# Circulating multimeric immune complexes contribute to immunopathology in COVID-19

**DOI:** 10.1038/s41467-022-32867-z

**Published:** 2022-09-26

**Authors:** Jakob Ankerhold, Sebastian Giese, Philipp Kolb, Andrea Maul-Pavicic, Reinhard E. Voll, Nathalie Göppert, Kevin Ciminski, Clemens Kreutz, Achim Lother, Ulrich Salzer, Wolfgang Bildl, Tim Welsink, Nils G. Morgenthaler, Andrea Busse Grawitz, Florian Emmerich, Daniel Steinmann, Daniela Huzly, Martin Schwemmle, Hartmut Hengel, Valeria Falcone

**Affiliations:** 1 Institute of Virology, Freiburg University Medical Center, Faculty of Medicine, Albert-Ludwigs-University of Freiburg, Freiburg, Germany; 2 Department of Rheumatology and Clinical Immunology, Freiburg University Medical Center, Faculty of Medicine, Albert-Ludwigs-University of Freiburg, Freiburg, Germany; 3 Center for Chronic Immunodeficiency (CCI), Freiburg University Medical Center, Faculty of Medicine, Albert-Ludwigs-University of Freiburg, Freiburg, Germany; 4 Institute of Medical Biometry and Statistics, Freiburg University Medical Center, Faculty of Medicine, Albert-Ludwigs-University of Freiburg, Freiburg, Germany; 5 Department of Cardiology and Angiology I, University Heart Center, Freiburg University Medical Center, Faculty of Medicine, Albert-Ludwigs-University of Freiburg, Freiburg, Germany; 6 Institute of Experimental and Clinical Pharmacology and Toxicology, Faculty of Medicine, Albert-Ludwigs-University of Freiburg, Freiburg, Germany; 7 Interdisciplinary Medical Intensive Care, Freiburg University Medical Center, Faculty of Medicine, Albert-Ludwigs-University of Freiburg, Freiburg, Germany; 8 Institute of Physiology II, Faculty of Medicine, Albert-Ludwigs-University of Freiburg, Freiburg, Germany; 9 InVivo BioTech Services GmbH, Hennigsdorf, Germany; 10 Institute of Clinical Chemistry and Laboratory Medicine, Faculty of Medicine, Albert-Ludwigs-University of Freiburg, Freiburg, Germany; 11 Institute for Transfusion Medicine and Gene Therapy, Freiburg University Medical Center, Faculty of Medicine, University of Freiburg, Freiburg, Germany; 12 Occupational Medical Service, Freiburg University Medical Center, Faculty of Medicine, Albert-Ludwigs-University of Freiburg, Freiburg, Germany

**Keywords:** SARS-CoV-2, Viral infection, Antibodies, Infection

## Abstract

A dysregulated immune response with high levels of SARS-CoV-2 specific IgG antibodies characterizes patients with severe or critical COVID-19. Although a robust IgG response is considered to be protective, excessive triggering of activating Fc-gamma-receptors (FcγRs) could be detrimental and cause immunopathology. Here, we document excessive FcγRIIIA/CD16A activation in patients developing severe or critical COVID-19 but not in those with mild disease. We identify two independent ligands mediating extreme FcγRIIIA/CD16A activation. Soluble circulating IgG immune complexes (sICs) are detected in about 80% of patients with severe and critical COVID-19 at levels comparable to active systemic lupus erythematosus (SLE) disease. FcγRIIIA/CD16A activation is further enhanced by afucosylation of SARS-CoV-2 specific IgG. Utilizing cell-based reporter systems we provide evidence that sICs can be formed prior to a specific humoral response against SARS-CoV-2. Our data suggest a cycle of immunopathology driven by an early formation of sICs in predisposed patients. These findings suggest a reason for the seemingly paradoxical findings of high antiviral IgG responses and systemic immune dysregulation in severe COVID-19. The involvement of circulating sICs in the promotion of immunopathology in predisposed patients opens new possibilities for intervention strategies to mitigate critical COVID-19 progression.

## Abstract (web summary)

During viral infections high levels of antibodies can form soluble immune complexes (sICs) with antigen and trigger Fcγ receptors (FcγR) leading to increased immunopathology. Here the authors measure FcγRs activation by sICs and consider how these may lead to excessive immunopathology during severe SARS-CoV-2 infection.

## Introduction

Since the emergence of SARS-CoV-2 in late December 2019^1^, more than 381 million laboratory confirmed infections (as of February 2nd, 2022) have been reported, with cases still on the rise^2^. Accordingly, rapid insights into the disease manifestations and pathogenesis have been globally obtained. A hallmark of the coronavirus disease 2019 (COVID-19) is a respiratory infection which can progress to an acute respiratory distress syndrome (ARDS) and multi-organ failure. Next to asymptomatic infections, COVID-19 symptoms differ widely according to the disease process and may comprise fever, coughing, pneumonia, dyspnea and hypoxia^3^. Pre-existing heterotypic immunity against circulating human coronaviruses may provide a partial explanation for the varying outcomes observed in SARS-CoV-2 infected individuals, yet this concept remains highly controversial^4–6^. While fever and coughing are common symptoms, pneumonia, hypoxia, dyspnea, certain organ manifestations like acute renal failure and lymphopenia indicate critical or fatal infections^3,7–9^. Pronounced dyspnea can eventually progress to ARDS, a severe complication frequently observed in critically ill patients^10,11^. Although overall disease severity, and breathing difficulties in particular, are related to viral load^12^, age^7,13–16^ and underlying medical conditions^7,14,15^, the delayed kinetics of respiratory failure and multi-organ dysfunction strongly suggest an essential function of the host immune response^3,14,17^. Typically, aggravation of disease occurs between 9-11 days after symptom onset^15^ and correlates with high levels of SARS-CoV-2 specific IgG antibodies and systemic effects of pro-inflammatory cytokines^3,18–20^. This cytokine release, mediated by myeloid cells such as macrophages and neutrophils^21,22^ or lymphoid T helper (T_H_) cells^18^, is either triggered by pattern recognition receptor (PRR) signaling in the context of innate immunity but can also occur by Fcγ receptor (FcγR) activation^23^. Stimulated by matrix- or cell-bound immune complexes (antibody-antigen complex), the cytokine release following FcγR activation represents a potent defense mechanism against invading pathogens. A prototypical activating FcγR in this regard is FcγRIII/CD16 expressed most notably by NK cells^24,25^ and monocyte-derived macrophages (CD16A)^26^. Moreover, FcγRIII/CD16^+^ expressing highly activated CD4^+^, CD8^+^, TCRαβ^+^ and TCRγδ^+^ T cell subpopulations with increased cytotoxic functions have been recently detected during severe COVID-19^27^. Besides matrix- or cell-bound immune complexes, FcγRIII/CD16 is able to sense circulating soluble immune complexes (sICs) as they are formed in particular autoimmune diseases such as systemic lupus erythematosus (SLE)^28–31^ and certain viral infections^32^. Overstimulation of activating FcγRs is associated with disease severity^32–34^ and thus an FcγR-driven overshooting inflammatory response^23^ might be an explanation for the pronounced immunopathology observed during severe courses of COVID-19. Consistently, hyper-inflammation in SARS-CoV-1 and MERS infected patients has been previously proposed as a possible pathogenic factor^35^ and could be recapitulated in mice and macaques infected with SARS-CoV-1^36,37^. Furthermore, N297-dependent glycan-modifications such as afucosylation within the constant region of IgG antibodies are known to enhance FcγR binding, in turn promoting inflammation. It has been shown that enhanced FcγRIII/CD16 activation by low-fucosylated anti-SARS-CoV-2-S IgG leads to excessive macrophage and monocyte activation, associated with severe COVID-19 disease progression^38,39^. Further, it has been proposed that uncleared antigen-antibody immune complexes (ICs) might be involved in the pathogenesis of severe disease involving systemic complement activation and tissue damage, neutrophil activation, cytokine storm, systemic vasculitis, microvascular thrombosis and organ failure^40–46^. However, comprehensive evidence that circulating sICs impact disease progression is still missing.

Our multiparametric analysis shows a marked correlation between FcγRIII/CD16 activation by patient SARS-CoV-2 specific IgG and severity of disease. Additionally, our data show that circulating FcγRIIIA/CD16A-triggering sICs are abundantly present in the serum of patients with critical and severe disease, but not in the serum of patients with a mild course of infection. sIC activation levels are comparable to those found in SLE patients with active disease. As sIC formation occasionally precedes a SARS-CoV-2 specific humoral response, we propose that a so far undisclosed predisposing condition divides patients into sIC-prone and non-sIC-prone individuals. In conclusion, here we show that patients generating sICs in response to SARS-CoV-2 infection develop enhanced disease manifestations due to systemic and uncontrolled FcγRIIIA/CD16A-mediated immune cell activation. Our findings suggest a cycle leading to immunopathology involving the early formation of sICs.

## Results

### Patients and clinical information

We retrospectively analyzed serial serum samples collected for routine diagnostic testing from 41 patients hospitalized at our tertiary care center between March and June 2020 with SARS-CoV-2 infection confirmed by real-time PCR. Based on the clinical course, we categorized patients as either severely diseased (hospitalized on regular wards with COVID-19 related pneumonia) versus critically diseased (COVID-19 related pneumonia and eventually in need of invasive mechanical ventilation). In total, 27 patients with critical and 14 with severe course of disease were grouped into separate cohorts. A cohort comprising 28 mild non-hospitalized COVID-19 cases served as control (Table 1). Most patients were older than 60 years with an overall mean age of 68 years (63 years and 76 years in the critically and severely diseased patients respectively). The majority of patients in both groups had comorbidities of different origin with cardiovascular diseases including hypertension representing the most frequent condition (35/41, 85%). Comparable with previous reports, high Interleukin 6 (IL-6), C-reactive protein (CRP) and lactate dehydrogenase (LDH) levels were associated with severity of disease (mean IL-6 levels: 1452.1 pg/ml in the critical group vs 46.1 pg/ml in the severe group, mean CRP levels: 162.2 mg/l vs 65.3 mg/l and mean LDH levels 1061.7 U/l vs 305.6 U/l 13-25 days post symptom onset respectively). Similarly, procalcitonin (PCT), a biomarker of microbial infection, was higher in critically diseased patients (mean value 9.9 ng/ml vs 0.17 ng/ml). Bacterial superinfection represented a further complication in 39% of the patients and was only slightly more frequent in patients with critical disease (11/27, 41% vs 5/14, 33%). Accordingly, and irrespective of the clinical presentation, PCT levels were significantly enhanced in patients suffering from bacterial superinfection (Supplementary Fig. 1a). More than half of the patients (59%) were treated with hydroxychloroquine/Lopinavir and Ritonavir (Kaletra), (18/27, 67% in the critical group vs 6/14, 43% in the severe group). Notably, at the time of serum acquisition, only one patient received steroid treatment, which was given due to underlying chronic obstructive pulmonary disease. Finally, the mortality rate was 37% (10/27) in critically and 7% (1/14) in severely diseased patients.

**Table 1:** Clinical characteristics of SARS-CoV-2-infected patients

	All patients: 69	%	Critical *n*: 27	%	Severe *n*: 14	%	Mild *n*: 28	%
Age [years]	61 (24–90)	-	63 (39–79)	-	76 (31–90)	-	45 (24–65)	-
Female	24	34.8	5	18.5	3	21.4	16	57.1
Male	45	65.2	22	81.5	11	78.6	12	42.9
**Comorbidities**								
Hypertension	21	51.2	12	44.4	9	64.3	n.a.	-
Cardiovascular disease	14	34.1	5	18.5	9	64.3	n.a.	-
Pulmonary disease	6	14.6	2	7.4	4	28.6	n.a.	-
Chronic kidney disease	6	14.6	1	3.7	5	35.7	n.a.	-
Diabetes	10	24.4	6	22.2	4	28.6	n.a.	-
Malignancy	8	19.5	4	14.8	4	28.6	n.a.	-
none	6	14.6	6	22.2	0	0	n.a.	-
**Diagnostic markers**								
Interleukin-6 [pg/ml]	1012.8	-	1452.1 (3774.6)	-	46.1 (26.8)	-	n.a.	-
Procalcitonin [ng/ml]	7	-	9.9 (21.9)	-	0.17 (0.11)	-	n.a.	-
C-reactive protein [mg/l]	128.1	-	162.2 (75.8)	-	65.3 (47.1)	-	n.a.	-
Lactate dehydrogenase [U/l]	800.97	-	1061.7 (1976.3)	-	305.6 (78.3)	-	n.a.	-
**Complications**								
Bacterial superinfection	16	39	11	40.7	5	35.7	n.a.	-
**Treatment**								
Hydroxychloroquine, Ritonavir + Lopinavir (Kaletra)	24	58.5	18	66.7	6	42.9	n.a.	-
**Fatal outcome**								
Total	11	26.8	10	37	1	7.1	n.a.	-

Patients were categorized as either severely (hospitalized on regular ward, requiring O_2_ supplementation, *n* = 14), critically (intensive care unit admission and in need of invasive mechanical ventilation, *n* = 27) or mildly diseased (non-hospitalized, *n* = 28). Diagnostic markers are depicted as mean and standard deviation (SD) (in brackets) of all analyzed laboratory parameters obtained 13–25 days post symptom onset for critically and severely diseased patients. Percentage [%] is indicated. n.a.: not available. Source data are provided as a Source Data file.

### Kinetics of IgG antibody responses following symptom onset across severe and critical courses of disease

**Fig. 1:**
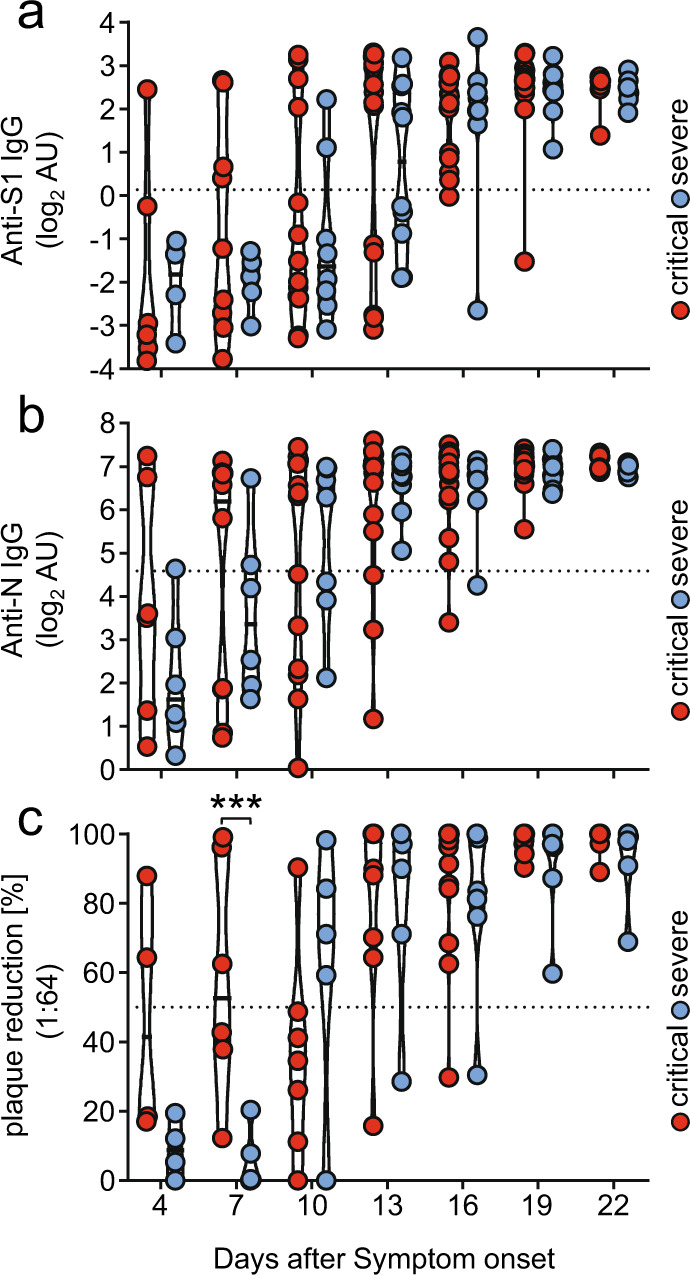
Antibody responses against SARS-CoV-2 across severe versus critical clinical course of disease. IgG antibody levels were analyzed in longitudinal serum samples from hospitalized SARS-CoV-2 infected individuals. Patients were categorized as critically diseased when in need of invasive mechanical ventilation (*n* = 27; red symbols) compared to severely diseased patients who did not require invasive ventilation (*n* = 14; blue symbols). Each dot represents the mean value obtained by the analysis of all samples, which were available at the indicated time points (+/− 1 day) following symptom onset. **a** IgG response against SARS-CoV-2 S1 –protein and **b** SARS-CoV-2 N-protein as determined by commercial ELISA assays. Dotted lines represent cut-off values for commercial S1- and N- specific ELISA assays. Each dot represents the mean value obtained by the analysis of all samples, which were available at the indicated time points (+/− 1 day) following symptom onset. **c** Serum neutralization capacity against SARS-CoV-2 measured by a plaque reduction assay. Sera were considered neutralizing at 50% plaque reduction (dotted line) at a 1:64 dilution. Solid black lines indicate the median. Significant differences were tested using a linear mixed effects model (one-sided, no adjustments) (^***^p < 0.001). Source data are provided as a Source Data file.

Elevated SARS-CoV-2 antibody titers are associated with disease severity^19,39^ and speculated to have a function not only in the clearance but also in the pathogenesis of SARS-CoV-2 infection^47,48^. We initially analyzed the levels and kinetics of SARS-CoV-2 specific IgG in serial serum samples from patients hospitalized with critical (*n* = 27) or severe (*n* = 14) illness, a setting we also used in the following experiments. A total of 125 (critically diseased) and 79 (severely diseased) serum samples, obtained from the aforementioned patients at different time points within 3–23 days following symptom onset were analyzed by commercially available S1- and N- specific ELISA-based assays. Assay specificity was confirmed analyzing healthy donor (HD) serum samples (*n* = 30) as negative control (Supplementary Fig. 1b, c). Most patients developed detectable SARS-CoV-2 specific IgG responses within 10–16 days after symptom onset. SARS-CoV-2 specific IgG gradually increased over time in both severely and critically diseased patients reaching a plateau at 18-20 days after symptom onset (Fig. 1a, b). Varying antibody response kinetics were observed for each individual patient (Supplementary Fig. 2a–d) with anti-N IgG titers rising significantly earlier than anti-S1 IgG (12.5 days ± 3.3 days vs 10.6 ± 3.8; *p* = 0.0091). A trend towards earlier seroconversion for anti-S1 IgG could be observed in critically diseased patients (mean time of seroconversion 11.4 ± 3.0 days in critically diseased patients vs 12.9 ± 3.8 days for severely diseased patients; *p* = 0.24), whereas time of seroconversion for anti-N IgG was similar in both groups (10.1 ± 3.2 and 10.4 ± 4.2 days for critically and severely diseased patients, respectively; *p* = 0.83). S1- and N-specific IgG levels at plateau did not significantly differ between the two groups. No significant difference between deceased and discharged patients was measured 13–25 days after symptom onset for anti- S1, N or RBD specific IgG antibodies (Supplementary Fig. 1d–f). Next, we evaluated and compared the neutralizing capacity of SARS-CoV-2 antibodies in either critically or severely diseased patients in a plaque-reduction assay (Fig. 1c). All patients mounted a robust neutralizing antibody response (96.5% ± 2% neutralization at a 1:64 serum dilution), with peaking titers at 18–20 days following symptom onset. Of note, two critically diseased patients developed a neutralizing antibody response already 4 days after symptom onset. In summary, we observed only minor differences in cohort wide kinetics of S1- or N- specific IgG levels between patients hospitalized with severe or critical clinical courses although in two critical patients earlier N-seroconversion and greater neutralizing capacity at very early stages could be detected.

### Patients with severe COVID 19 have enhanced FcγRIII/CD16 activation by S-specific IgG antibodies

**Fig. 2:**
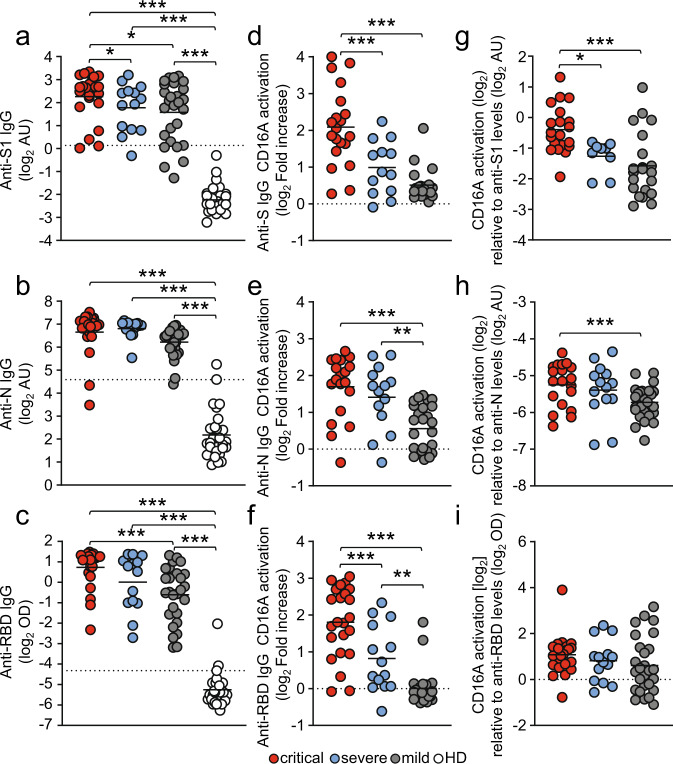
FcγRIIIA/CD16A activation by SARS-CoV-2 - specific IgG is enhanced in critically diseased patients. FcγRIIIA/CD16A activation of BW5147 reporter cells by SARS-CoV-2-specific IgG in serum samples obtained 13-25 days following symptom onset from 23 critically (red symbols) and 14 severely (blue symbols) diseased patients. Between 2 to 8 samples/patient were analyzed depending on the availability of sample material. Sera from 28 non-hospitalized patients with mild SARS-CoV-2 infection (grey symbols) and 30 healthy donors (open circles) served as reference. Each symbol represents the mean value of all available samples per patient. **a**–**c** ELISA levels for S1-, N- and RBD-specific IgG. Dotted lines represent cut-off values for commercial S1-, N- and RBD - specific ELISA assays. **d**–**f ** FcγRIIIA/CD16A activation by S-, N- and RBD-specific IgG expressed as log_2_ fold change relative to negative control. **g**–**i** FcγRIIIA/CD16A activation, expressed as log_2_ values relative to SARS-CoV-2-spcific IgG titers. Solid black lines indicate the mean. Significant differences over all three groups were tested by ANOVA and pairwise group comparison was made by Games-Howell post-hoc tests (^***^p < 0.001; ^**^p < 0.01, ^*^p < 0.05). Source data are provided as a Source Data file.

FcγRIIIA/CD16A triggering initiates multiple protective effector functions such as antibody-dependent cellular cytotoxicity (ADCC) by natural killer (NK) cells as well as cytokine and chemokine secretion by NK cells and macrophages^23,49^. However, excessive FcγR stimulation can have severe adverse effects such as uncontrolled cytokine release as observed in systemic autoimmune diseases or certain viral infections^23,50^. Therefore, we hypothesized that an exaggerated FcγR mediated activation triggered by SARS-CoV-2 specific IgG might contribute to the exacerbation of COVID-19 in critically compared to severely diseased patients. To address this, we analyzed the ability of SARS-CoV-2 specific antibodies to activate FcγRIIIA/CD16A (158 V) using a previously validated cell-based reporter system. The assay quantifies the capacity of virus-specific IgG to trigger FcγR signaling upon recognition of immobilized antigen^51–55^ (Supplementary Fig. 3a). Considering the typically late time point of health deterioration, we performed an analysis of FcγRIIIA/CD16A activation triggered by SARS-CoV-2 specific IgG with serum samples obtained 13-25 days following symptom onset (Fig. 2). Sera were analyzed at a 1:500 dilution to stay within the dynamic range of detection (Supplementary Fig. 4). Depending on the availability of sample material 2-8 samples/patient/time-point were included in this analysis. If available in sufficient quantity, sera were reanalyzed. Reproducibility was tested using available serum surplus (Supplementary Fig. 5). Sera from 28 patients with mild SARS-CoV-2 infection and 30 healthy blood donors were included for reference. Semi-quantitative assessment of IgG titers using antigen-specific ELISA revealed comparable levels between critically, severely and mildly diseased patient cohorts (Fig. 2a–c). In contrast, S- and RBD-specific but not N-specific IgG-mediated FcγRIIIA/CD16A activation was significantly increased in critically compared to severely diseased patients (Fig. 2d–f). Furthermore, normalizing FcγRIIIA/CD16A activation to antigen-specific IgG titers, revealed significantly stronger FcγRIIIA/CD16A activation by S- and N-specific IgG compared to mildly diseased patients (Fig. 2g–i). Intriguingly, we observed a heterogeneous CD16A activation pattern characterized by either high (OD_450_ > 1.2) or low (OD_450_ < 0.6) CD16A-activating sera irrespective of the clinical manifestation (Fig. 2d–f). Overall, a significant positive correlation could be determined between anti-SARS-CoV-2 antigen IgG titers and CD16A activation (Supplementary Fig. 6). Our data document a sustained FcγRIIIA/CD16A activation by SARS-CoV-2 specific antibodies particularly in patients suffering from critical COVID-19 disease. Based on these results we confirmed the notion that elevated FcγRIIIA/CD16A activation by S-specific IgG might contribute to disease severity of COVID-19.

### Enhanced Fcγ-afucosylation of S- and N-specific IgG in critically and severely diseased patients results in increased FcγRIIIA/CD16A activation

**Fig. 3:**
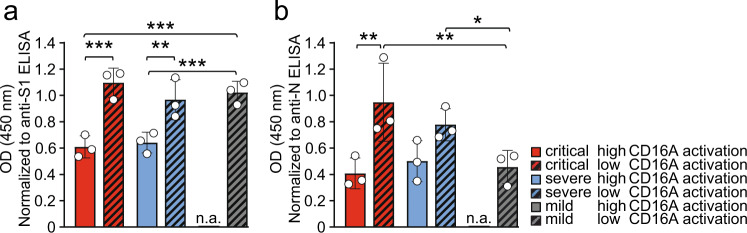
Anti SARS-CoV-2 IgG Fc core fucosylation in critical and severe COVID-19 cases. IgG-Fc core fucosylation levels of SARS-CoV-2–specific IgG in critically (red bars), severely (blue bars) and mildly (grey bars) diseased COVID-19 patients. Analysis was carried out on a pool of 5 different sera. Measured OD values (fucosylation) of the generated eluates were normalized to their respective IgG concentrations determined by antigen-specific S1 and N ELISA. **a** S-IgG-Fc-fucosylation and **b** N-IgG-Fc-fucosylation in critically and severely diseased patients characterized by either high (red) or low (patterned) CD16A-activation levels in the FcγRIIIA/CD16A reporter assay. Of note, for mildly diseased patients only sera displaying low CD16 activation levels were available and analyzed respectively. The mean and standard deviation (SD) of three independent experiments is depicted. Each dot represents one individual experiment. Statistical tests using a two-factorial linear model (one-sided, no adjustments) indicate three significant differences between the low and high categories as well as between patients of the different groups (^***^p < 0.001, ^**^p < 0.01, ^*^p < 0.05). n.a.: not available. Source data are provided as a Source Data file.

Based on the findings described above we reasoned that differences in Fcγ mediated effector functions might contribute to disease severity of COVID-19. We compared CD16A high (OD_450_ > 1.2) - versus CD16A low (OD_450_ < 0.6)-activating patient sera (based on Fig. 2d, e) regarding their SARS-CoV-2 specific IgG core fucosylation. Inspired by previous findings^38,56,57^ we focused on determining IgG core fucosylation of S- and N- specific SARS-CoV-2 IgG. To determine IgG core fucosylation we used a lectin-based ELISA preceded by antigen-specific antibody purification from immobilized SARS-CoV-2-antigen. Analysis of anti-S and anti-N IgG core fucosylation was performed on serum pools containing five sera of either critically or severely diseased patients obtained 13–25 days post symptom onset. A serum pool containing (anti-S *n* = 5/anti-N *n* = 4) sera of mildly diseased patients served as control. To stay within the dynamic detection range, relative fucosylation was analyzed at a dilution of 1:4. When analyzing serum pools from critically and severely diseased patients we determined a significantly lower level of core fucosylation among the high CD16A activators (Fig. 3, plain-colored bars) compared to the low CD16A activators (Fig. 3, shaded bars). This applied for both the S- (Fig. 3a) and N-specific antibodies (Fig. 3b). Only mildly diseased patients displaying low CD16 activation levels were available and, as expected, did not show decreased core fucosylation of SARS-CoV-2 S specific IgG. These results are in line with previously published findings regarding the effect of Fcγ-afucosylated IgG on FcγRIIIA/CD16A effector functions^56,58^ and recapitulate similar findings in the context of COVID-19^38,56,57^. However, we did not observe significant differences between critically and severely diseased patients.

### COVID-19 disease severity correlates with an increase in FcγRIIIA/CD16A-reactive soluble IgG complexes

**Fig. 4:**
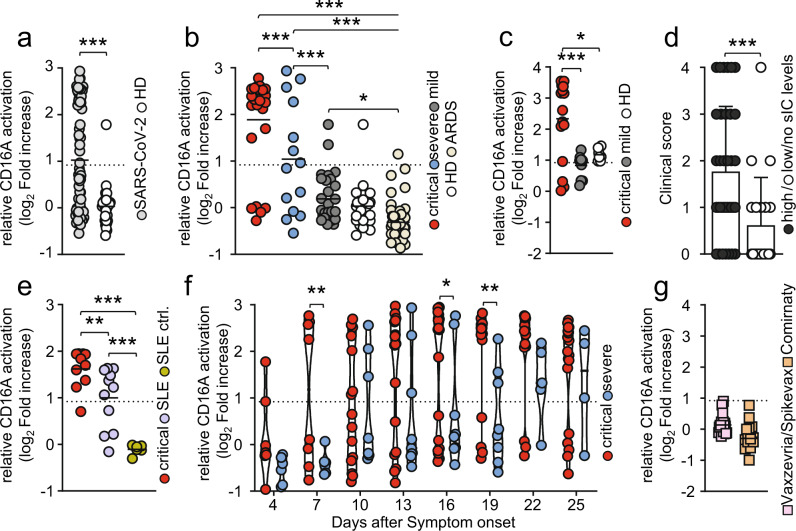
Severe COVID-19 disease coincides with high FcγRIIIA/CD16A activation by sICs. Serial serum samples obtained 13-25 days after onset of symptoms were analyzed in a cell-based reporter assay which is sensitive to sIC amount and size^31,59^. FcγRIIIA/CD16A activation is shown as log_2_ fold change relative to negative control. Dotted lines represent cut-off values. Each symbol represents the mean value obtained by the analysis of all samples available in the indicated time range for each individual patient. **a** Analysis of FcγRIIIA/CD16A activation by sICs in SARS-CoV-2-infected patients (*n* = 69) compared to healthy blood donors (*n* = 30) (*t*-test, unequal variances, one-sided). **b** Levels of sIC-mediated activation across critical (red, *n* = 27), severe (blue, *n* = 14) and mild (grey, *n* = 28) clinical courses of COVID-19 disease, in healthy donors (HD; white, *n* = 30) and in non-COVID-19 patients who developed acute respiratory distress syndrome (ARDS; beige, *n* = 47). Two-group comparisons with the linear model (linear model, one-sided, no adjustments) indicate significant differences between critical cases and all other groups, as well as between severe cases and all other groups (^***^p < 0.001, ^**^p < 0.01). No significant differences (*p* > 0.05) have been found for the comparisons mild vs. healthy and for HD vs. ARDS. **c** sIC-mediated FcγRIIIA/CD16A activation in 18 critically ill COVID-19 patients infected with either the alpha or delta SARS-CoV-2 variant. Mildly diseased patients (*n* = 11) and healthy donors (HD, *n* = 8) served as control (linear model, one-sided, no adjustments). **d** A clinical score displaying either high (>2 log_2_ fold increase) or low/no (<2 log_2_ fold increase) CD16A-activators including both critically (*n* = 42) and severely (*n* = 14) diseased patients analyzed in **b**) and **c**). The mean and SD is depicted. Statistical differences were calculated using a non-parametric Mann-Whitney test (^***^p = 0.0008). **e** Selected sera from critically diseased patients (red, *n* = 10) were compared to sera from SLE patients (purple, *n* = 11) with active disease regarding FcγRIIIA/CD16A activation. Sera from healthy donors (green, *n* = 6) served as SLE-negative control. Statistical differences were calculated using a linear model (one-sided, no adjustments) (^**^p < 0.01, ^***^p < 0.001). **f** Kinetics of sIC-mediated FcγRIIIA/CD16A activation in critically (red, *n* = 26) and severely (blue, *n* = 14) diseased patients. Days after symptom onset (+/− 1 day) are depicted. Solid black lines indicate the median. The linear mixed effects model (one-sided, no adjustments) indicates three time points with significant differences (^**^p < 0.01, ^*^p < 0.05). **g** Levels of sIC-mediated FcγRIIIA/CD16A activation in COVID-19 vaccinees either receiving heterologous (Vaxzevria/Spikevax; pink, *n* = 26) or homologous (Comirnaty; orange, *n* = 14) prime-boost vaccination. If not indicated otherwise, solid black lines indicate the mean. Source data are provided as a Source Data file.

Aside from afucosylated IgG, it has been proposed that uncleared antigen-antibody immune complexes (ICs) might be involved in the pathogenesis of severe COVID-19^40–42,44^. However, the actual presence of circulating, multimeric soluble ICs (sICs) in critically or severely diseased patients has not been shown yet. As extensive FcγR activation by sICs might contribute to the severe systemic inflammatory state occurring in some COVID-19 patients with prolonged disease we thus set out to characterize our patient cohort regarding the presence of sICs in serum samples taken at various time points during disease and after hospitalization. To this end, we deployed a cell-based reporter assay developed to quantify FcγRIIIA/CD16A (158 V) activation by IgG-containing sICs, measuring their bioactivity^31,59^. This assay does not react to monomeric IgG or small dimeric antigen-antibody complexes in solution, but specifically identifies multimeric sICs at nanomolar concentrations (Supplementary Fig. 3b) and has been successfully used to detect sICs in patients with systemic lupus erythematosus (SLE)^31^. Here, sICs are major drivers of inflammation^30^ and the assay finds sIC bioactivity to significantly correlate with SLE disease severity^31^. Analysis of serum samples, obtained 13–25 days after symptom onset, implicated the presence of highly FcγRIIIA/CD16A-reactive sICs in SARS-CoV-2 infected patients compared to healthy individuals (Fig. 4a). Next, we compared sIC-mediated FcγRIIIA/CD16A activation between COVID-19 patients with varying disease severity. We found that critically diseased patients show a striking increase in reactive sICs compared to patients with severe or mild disease (Fig. 4b). Only 5/26 patients with critical disease (19.2%) showed no sIC-mediated FcγRIIIA/CD16A activation. Of note, when we analyzed additional sera from COVID-19 patients hospitalized between April-December 2021, infected with either the alpha or delta SARS-CoV-2 variant, we found comparable levels of reactive sICs in the serum of critically diseased patients implying that sIC formation during COVID-19 pathogenesis is conserved across various SARS-CoV-2 strains (Fig. 4c). As depicted (Fig. 4b, c), we observed two subpopulations characterized by either high (>2 log_2_ fold increase) or none to low (<2 log_2_ fold increase) FcγRIIIA/CD16A-activation levels in both severely and critically ill patients. Highly reactive sICs were associated with increased mortality, higher frequency of extracorporeal membrane oxygenation (ECMO) therapy and acute kidney injury (AKI) (Table 2).

**Table 2:** Clinical characteristics associated with sIC-levels

	Total (*n*)	Deceased (*n*)	%	ECMO (*n*)	%	AKI (*n*)	%
sIC high	35	15	42.9	18	51.4	19	54.3
sIC no/low	21	2	9.5	4	19.0	5	23.8

Frequency of death, extracorporeal membrane oxygenation (ECMO) therapy and acute kidney injury (AKI) in patients (including critically and severely diseased patients) with high (> 2 log_2_ fold increase) or no /low (<2 log_2_ fold increase) reactive sIC levels. Percentage [%] is indicated. Based on these findings, we calculated a clinical score (death: 2 points, ECMO: 1 point, AKI: 1 point) which was significantly higher in patients displaying high sIC-mediated FcγRIIIA/CD16A activation (as shown in Fig. 4d). Fisher’s exact test: deceased (*p* = 0.0148), ECMO (*p* = 0.0237), AKI (*p* = 0.0301). Source data are provided as a Source Data file.

While we observed no direct correlation between sIC reactivity and either PCT (indicative of bacterial infection) or IL-6 levels (Supplementary Fig. 7a, b) sIC levels were directly associated with CRP and LDH, both undisputed correlates of severe COVID-19^11^ (Supplementary Fig. 7c, d). In addition, we observed a positive correlation between IL-12 and sIC reactivity (*p* = 0.04) and a similar tendency between IFN-γ cytokine levels and sIC levels (*p* = 0.07) (Supplementary Fig. 8a, b). Next, we compared sIC bioactivity between sera from critically diseased COVID-19 patients and sera from SLE patients with active disease (Fig. 4e). These data showed that sICs formed in COVID-19 are comparable to sICs formed during active SLE regarding their potential to drive FcγRIIIA/CD16A-mediated inflammation. As we did not detect sICs in the serum of 47 patients with acute respiratory distress syndrome (ARDS; mean age 57.5 years) in response to infections of different etiology including cytomegalovirus disease, HIV/AIDS, influenza or pulmonary tuberculosis, we conclude that the formation of multimeric sICs is selectively associated with severe SARS-CoV-2 disease (Fig. 4B and Supplementary Fig. 9). To verify that sICs and no other constituent represent the FcγRIIIA/CD16A-reactive component in the serum of COVID-19 patients, we analyzed serum-mediated FcγRIIIA/CD16A activation before and after PEG8000-precipitation. This treatment was previously shown to selectively precipitate large IgG complexes from solution^31,60^. For this analysis, pools of 8 sera, showing either high (sICs^+^) or no (sICs^-^) CD16A activation, were compared. Sera from healthy donors (HD) served as a negative control. Compatible with the hypothesis of serum-derived sICs driving FcγRIIIA/CD16A signaling, no activation was observed following incubation with 3.5% PEG8000 (Supplementary Fig. 10a). To ensure that sIC removal was selective and did not precipitate monomeric IgG, we tested the depleted sera for remaining S1- and N-specific IgG. As depicted S1- and N- specific IgG could still be detected at unchanged high levels in samples treated with 3.5% PEG8000 (Supplementary Fig. 10b). Remarkably, longitudinal analysis of reactive sICs in the serum of critically or severely diseased patients showed high FcγRIIIA/CD16A activation levels in 2 critically diseased patients already 4 days after symptom onset (Fig. 4f). Of note, 2 of 4 patients with an early increase of circulating reactive sICs eventually died. sIC-mediated FcγRIIIA/CD16A activation persisted in 14 of 19 critically diseased patients at high levels until day 25 after symptom onset. sIC-mediated FcγRIIIA/CD16A activation in severely diseased patients was slightly delayed compared to critically diseased patients and was first detected in 3 patients 10 days after symptom onset (Fig. 4f). Only 4 of 14 patients with severe disease showed detectable sIC-mediated FcγRIIIA/CD16A activation. When resolving sIC-mediated FcγRIIIA/CD16A activation over the entire time of hospitalization for selected patients (*n* = 27) of which sufficient samples at different time points were available, we observed that sIC reactivity precedes anti-S1 IgG in 22% (*n* = 6/27) of the cases whereas anti-S1 antibodies can be detected prior to sIC formation in about ~26% (*n* = 7/27) of the patients. Anti-S1 IgG and sICs appeared simultaneously in ~52% (*n* = 14/27) of the cases (Supplementary Fig. 11, Supplementary Fig. 12). In accordance with the very early emergence of sICs, we were not able to identify any SARS-CoV-2-derived antigens in PEG8000-precipitated sICs using extensive tandem mass spectrometry approaches or SARS–CoV-2 – RNA detection by RT-PCR. We did not detect bacterial antigens specific for patients with sICs versus patients without sICs (Supplementary note 1). To further exclude the formation of multimeric sICs formed from circulating S1 antigen^61^, we also specifically targeted S1 for precipitation from patient serum using biotinylated S1-specific monoclonal antibodies. Again we failed to identify S1 antigen in our samples (Supplementary Note 2). Consequently, no sIC-mediated FcγRIIIA/CD16A activation could be detected in the serum of COVID-19 Vaxzevria/Spikevax or Comirnaty vaccine recipients (Fig. 4g).

Previously, the function of neutrophil mediated intravascular NETosis was reported to have a critical function in thrombosis formation and subsequent organ damage observed in severe clinical forms of COVID-19^62–64^. Since these long extracellular traps can bind by strong electrostatic forces to negatively charged domains in immunoglobulins, thus facilitating the formation of aggregated IgG as a form of sICs^65^, we next tested whether Benzonase nuclease treatment of patient serum would dissolve reactive sICs. To this end, we analyzed sera from critically diseased COVID-19 patients or healthy individuals and compared FcγRIIIA/CD16A reactivity before and after nuclease treatment (Supplementary Fig. 13). Nuclease activity in diluted human serum was controlled using plasmid DNA for reference. This experiment indicated that nucleic acid was not involved in the formation of FcγRIIIA/CD16A-reactive sICs in critically diseased patients. Finally, we tested pooled patient sera for autoantibodies against a panel of prototypical autoantigens associated with autoimmune disease including anti-nuclear autoantibodies (ANA) by indirect immunofluorescence, dsDNA autoantibodies by ELISA and autoantibodies against the extractable nuclear antigens (nRNP/Sm, Sm, SS-A, Ro-52, SS-B, Scl-70, PM-Scl, Jo-1, CENP B, PCNA, nucleosomes, histones, ribosomal P-protein, AMA-M2, DFS70) by dot blot in case SARS-CoV-2 infection triggers autoantibody formation and possible sIC formation. However, no significant titers of free monomeric IgG autoantibodies with prototypical specificity could be detected in any serum pool (Supplementary Table 1).

Although we were not able to identify a singular origin for multimeric sICs, our data clearly show their presence in COVID-19 patients with an increase in FcγRIIIA/CD16A-reactive sICs corresponding with severity of disease reaching activation levels comparable to those observed in SLE patients with active disease. We conclude that circulating sICs are a contributing factor to COVID-19 disease severity.

## Discussion

We collected and analyzed data from 41 COVID-19 patients hospitalized at the University Hospital Freiburg during the first wave of the SARS-CoV-2 pandemic. Patients were categorized by severity of disease into severely (*n* = 14) and critically diseased patients (*n* = 27). Both groups were of comparable average age and had a similar male-to-female ratio. For comparison, we also analyzed 28 non-hospitalized mildly diseased and 30 healthy individuals. As key findings we identify de novo produced afucosylated SARS-CoV-2 IgG and the emergence of soluble circulating immune complexes (sICs) activating FcγRIIIA/CD16A as potential risk factors closely associated to COVID-19 severity. In contrast to afucosylated SARS-CoV-2 IgG which is targeting immune effector functions from FcγRIII/CD16^+^ cells and complement to virus-infected tissues upon antigen encounter circulating sICs trigger immune responses immediately and in a locally unlimited manner. For this reason the formation of sICs could be an accelerating event in the causal chain of COVID-19 immunopathogenesis, e.g. by linking prevalent autoantibody formation in prone individuals with systemic activation of various FcγRIIIA/CD16A-bearing immune cells contributing to subsequent disseminated tissue destruction and multi-organ disease as observed in severely ill COVID-19 patients (see below).

### Circulating sICs and their contribution to COVID-19 disease severity

Based on data from the study of SARS-CoV-1 and other respiratory viruses concerns were expressed that anti-SARS-CoV-2 IgG antibodies could exacerbate COVID-19 through antibody-dependent enhancement (ADE), i.e. through excessive antibody Fc-mediated effector functions or immune complex formation^66^. Using a ligand selective reporter cell activation assay^31,59^ we provide evidence of circulating IgG-containing multimeric sICs in the serum of COVID-19 patients and experimentally confirm previous hypotheses suggesting immune complexes as potential drivers of disease progression in COVID-19^40–42,44^. Our data confirm enhanced FcγRIIIA/CD16A triggering mediated by sICs in critically but not in mildly ill patients and  complete absence in COVID-19 vaccine recipients. In fundamental contrast to opsonized antigens decorating virus-infected cells in tissues, sICs become distributed systemically. Thus activation of constitutively FcγRIIIA/CD16A expressing monocytes, granulocytes and NK cells could readily explain systemic responses which potentiate local inflammation in virus-infected tissues intensifying organ damage and dysfunction, but may carry the disease process also across to uninfected tissues. Notably, a recent publication has shown that during SARS-CoV-2 infection, opsonizing antibodies may mediate abortive infection of monocytes via CD16/FcγRIIIA leading to inflammasome activation followed by pyropoptosis of infected cells^48^. Indeed, we show here that sIC reactivity correlates with LDH levels, a marker of pyropoptosis and one of the best correlates of severe COVID-19^11^. Strikingly, full-blown activated FcγRIII/CD16^+^ T cells with a very high cytotoxic potential including the ability to activate lung microvascular endothelial cells were recently detected in severe COVID-19^27^. Polyclonal differentiation of these T cells is promoted by the high inflammatory milieu generated by complement split products such as C3a, which is produced at high levels in severe COVID-19. The aberrantly FcγRIII/CD16 expressing T cells with a T cell receptor (TCR)-independent cytotoxic functionality were shown to persist beyond the acute phase of the infection and maintaining their cytotoxic phenotype^27^. Evidently, SARS-CoV-2-specific IgG with a proinflammatory phenotype together with abundant circulating sICs could greatly enhance the pathogenic potential of FcγRIII/CD16 expressing T cells in COVID-19 disease and readily explain the uncontrolled and disseminated clinical presentation of the SARS-CoV-2 induced disease complex. Simultaneous measurement of sICs and FcγRIII/CD16 expressing T cell numbers in patients is an obvious task for further COVID-19 research to assess the relative importance of both disease determinants. In addition to binding to FcγRIIIA/CD16A, sICs activate the classical pathway of the complement cascade leading to the membrane attack complex and cause tissue destruction in this way. While the size of sICs is highly variable, they can reach into the MDa range and thus cause massive vascular and organ injury^67^.

### Origin and composition of circulating sICs

Although the formation conditions of circulating immune complexes and the identity of the complexed antigens still remain elusive, we show that the presence of IgG-containing sICs during SARS-CoV-2 infection is directly responsible and sufficient for the observed dose-dependent FcγRIIIA/CD16A activation by patient serum. Detection of sICs in sera collected during various pandemic waves indicates a conserved feature across SARS-CoV-2-variants. Recent studies reported that viral antigens can be detected in the serum of patients^61,68^. However, as we were not able to detect any viral material including viral RNA within sICs and find sIC reactivity to often precede SARS-CoV-2-S specific IgG responses, we assume that circulating S or shed S1-antigens are unlikely to be involved in sIC formation. For the same reason the involvement of preexisting, cross-reactive IgG antibodies against circulating common cold human coronaviruses (HCoVs)^69,70^ in sIC formation seems also improbable. Likewise and despite considerable bacterial superinfections in critically and severely diseased COVID-19 patients, we did not detect any bacterial antigens within sICs making their contribution to sIC formation unlikely. sICs are commonly associated with immunopathology in autoimmunity^29,30,71^. When analyzing prototypical IgG autoantibodies as constituents of subsequently formed sICs we could not identify a distinct culprit self-antigen linked to sIC formation. However, several studies have described that acute SARS-CoV-2 infection triggers the de novo IgG production against a large variety of immunomodulatory proteins including cytokines, chemokines, complement components and cell-surface proteins^72–74^. Further, it has been shown that pre-existing neutralizing anti-type I interferon antibodies, which can be found in about 10% of patients with severe COVID-19 pneumonia, are related to the highest risk of developing life-threatening COVID-19 disease^75^. Therefore, the de novo induction of anti-cytokine auto-antibodies in a significant proportion of hospitalized COVID-19 patients, might indeed represent a source of heterogenous circulating sICs in COVID-19. In such a scenario, immune responses are initially hampered by an auto-antibody-driven immunodepletion of critical immunomodulatory components and subsequently exaggerated through the formation of persisting sICs. Lastly, it was speculated that anti-idiotypic autoantibodies could be elicited and resulting in transient sIC formation during the antiviral immune response^76^ in a similar process as characterized for coxsackievirus B3 in mice resulting in severe autoimmune myocarditis^77^.

As sIC formation is strongly reminiscent to SLE and sICs initiate a common terminal pathway of inflammation, we classified patients as sIC-prone or non-sIC-prone. Of note, besides sIC formation a range of additional phenotypical abnormalities shared between B cell populations in autoimmune disorders exemplified by active SLE and severe COVID-19 have been observed. This includes the pronounced engagement of extrafollicular B cell responses, associated with the activation of effector B cells lacking naïve (IgD) and memory markers (CD27) as well as class-switched antibody secreting cells^78^. In light of these massive changes in B cell development seen during COVID-19 we rather propose that disease severity is associated with a hidden predisposition in sIC-prone patients which initiates a strong early inflammatory response to SARS-CoV-2 infection and thereby triggers autoreactive B cells to undergo plasma blast formation followed by Ig production and autoantigen-sIC formation. Similar to SLE, this could promote subsequent changes in SARS-CoV-2-specific IgG glycan profiles^79^.

### sICs as a disease marker and potential target of therapeutic intervention

Notably, FcγRIIIA/CD16A activation levels in patients with critical disease were comparable to those measured in SLE patients, where circulating sICs are established to crucially contribute to tissue damage and disease manifestations^80,81^. Consistently we found that critically diseased COVID-19 patients exhibit significantly higher levels of reactive sICs compared to less severely diseased patients. Moreover, sICs in both critically and severely ill patients are associated with increased mortality, higher frequency of ECMO therapy and acute kidney injury (Table 2) thus providing evidence of the central function of sIC-mediated immunopathology. In addition, we provide evidence that sIC levels directly correlate with LDH, CRP, as well as IL-12 and to a certain extent IFN-γ serum levels. While CRP release can be triggered by various factors including IL-6^82^, IL-12 and IFN-γ can be indirect^83,84^ and direct^85,86^ indicators of FcγRIII activation. Overall, these findings strongly support a causal link between sICs and systemic inflammation in COVID-19 disease. Interestingly, we did not observe a significant correlation of sIC reactivity with PCT and IL-6 serum levels compatible with the notion that sIC formation acts independently of these factors and in particular of bacterial antigens.

**Fig. 5:**
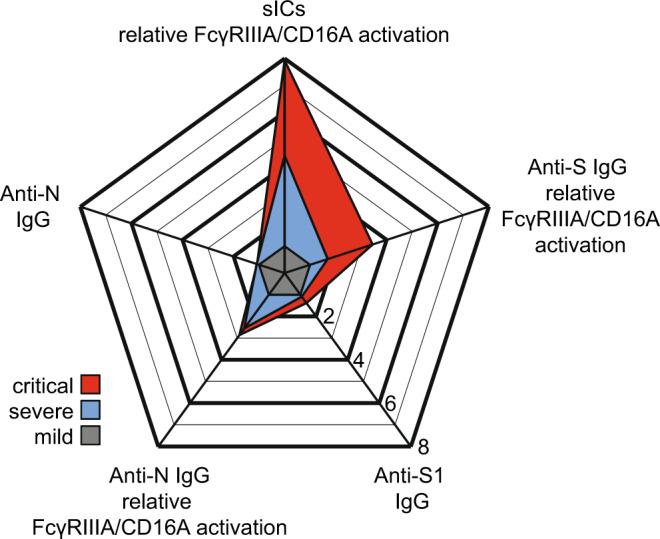
Summary of antibody features from SARS-CoV-2-infected patients with critical and severe disease. Relative multivariate antibody features illustrated as radar chart in critically (red) or severely (blue) diseased COVID-19 patients normalized to the corresponding features of patients with mild infection (grey). Each spoke represents one of the following variables: ELISA (S1-IgG, N-IgG,) and CD16 activation (S-IgG, N-IgG, multimeric sICs). Arithmetic mean values of log_2_ values were calculated for each group (days 13-25 post symptom onset), respectively. The fold change compared to mildly diseased patients is shown. Source data are provided as a Source Data file.

Based on these findings, together with the higher levels of afucosylated IgG, we conclude that FcγRIIIA/CD16A activation in COVID-19 disease is mainly governed by sIC formation (Fig. 5). Additionally, the decrease in core α-1-6 fucosylation, which has been associated to chronological aging^87^, might contribute to the higher frequency of severe disease observed in older adults^88^. Recognition by sICs is not restricted to FcγRIIIA/CD16A but also involves additional FcγRs such as CD32A genotype H, as recently shown by our group^31^. Bye et al. observed that immune complexes presented in suspension do not potently enhance platelet activation^89^. Still, CD32A might have an individual function in some patients as sIC sensitivity differs strongly between the H and R allelic forms of this receptor^31^. It has been reported that individuals show higher susceptibility to autoimmune diseases when they are homozygous for the R allelic form, presumably due to the lack of sIC clearance^90^ which could also impact SARS-CoV-2 immunopathogenesis.

**Fig. 6:**
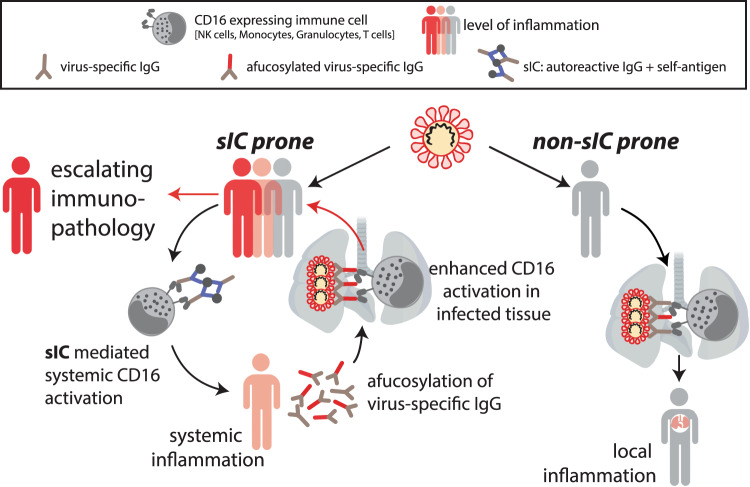
Model proposing a vicious cycle of immunopathology in COVID-19 patients driven by soluble multimeric immune complexes (sICs). SARS-CoV-2 infection triggers innate responses leading to autoreactive sIC formation in prone individuals. Activation of FcγRIIIA/CD16A expressing immune cells (NK cells, monocytes, T cells, granulocytes) by systemically emerging sICs leads to systemic inflammation. sIC-driven systemic inflammation, tissue inflammation mediated by virus infection and deposited ICs add to afucosylation of virus-specific IgG. This enhances FcγRIIIA/CD16A activation by opsonized targets in infected tissues. Resulting tissue damage intensifies autoantigen-release, further elevating sIC levels. sIC-mediated systemic immune cell activation ultimately leads to an escalating immunopathology. Created with Biorender.com.

As depicted (Fig. 6), sIC formation could be a crucial event in the causal chain of COVID-19 immunopathogenesis by linking prevalent autoantibody formation in prone individuals with systemic FcγRIII/CD16-mediated immune cell activation and subsequent disseminated tissue destruction and multi-organ disease. In our study we find that strong activation of FcγRIIIA/CD16A coincides with the presence of afucosylated virus-specific IgG. As Hoepel et al. show prolonged presence of afucosylated IgG in some individuals^38^ and as systemic inflammation in SLE has been linked to an increase in afucosylated IgG^79^, it is tempting to speculate that the formation of sICs in predisposed patients initiates a vicious cycle of FcγR-mediated inflammation leading to enhanced afucosylated IgG and FcγR activation by SARS-CoV-2-specific IgG, further contributing to inflammation and, conceivably, to *de-novo* sIC formation. Therefore, our findings provide an explanation for the sustained immunopathology following SARS-CoV-2 infection observed in a majority of severely diseased patients. Limitations of our study include the bounded number of retrospectively recruited patients in our cohorts that were amenable to us as well as the limited amounts of serum. As a result, some analyses could only be performed with pooled serum, which may be a reason why we have not yet been able to identify antigens within the sICs. Another restriction is due to the fact that only serum, but not immune cells from patients, was available to our study. Thus, we were not yet able to directly demonstrate which immune cell subpopulations were activated by sICs in vivo in a FcγRIIIA/CD16A-dependent manner. Extracorporeal therapies, such as plasma exchange or immunoadsorption have emerged to be valuable options in the treatment of multiple autoimmune disorders including SLE^91^. In the context of COVID-19, therapeutic plasma exchange effectively decreased circulating anti-type I interferon-antibodies, in four anti-type I interferon-antibody-positive, severely ill patients^92^ and in a child with autoimmune polyendocrinopathy syndrome type 1 suffering from severe COVID-19^93^. Moreover a German single center observational study recently provided promising clinical data for plasma exchange as a novel therapeutic strategy in a subset of critically ill COVID-19 patients by potentially reversing the complex immunopathology^94^. In light of our results such interventional approaches could be effective by removing sICs from the circulation. Future studies will be required to further establish sICs as a distinct biomarker in COVID-19 and confirm the rationale behind the approach of extracorporeal therapies. Eventually it remains to be shown whether soluble sICs may persist in reconvalescent patients and could contribute to the reported immunoglobulin signature predicting risks of a postacute COVID-19 syndrome^95^.

## Methods

### Ethical statement

The protocol of this study conforms to the ethical guidelines of the 1975 Declaration of Helsinki and was approved by the institutional ethical committee of the University of Freiburg (EK 153/20). Written informed consent was obtained from participants and the study was conducted according to federal guidelines, local ethics committee regulations (Albert-Ludwigs-Universität, Freiburg, Germany: No. F-2020-09-03-160428 and no. 322/20; No 507/16 and 624/14 for the SLE patients, No 20-1271_1 for the vaccinees). No compensation was given to research participants.

### Participants and specimens

Between March 2020 and April 2020, 41 patients with SARS-CoV-2 infection confirmed by real-time PCR were hospitalized at the University Medical Center, Freiburg. Serum samples were collected during hospitalization for routine laboratory testing. Clinical data were obtained from electronic medical records. A total of 27 patients necessitating invasive mechanical ventilation in intensive care unit were included in the critical group. Fourteen patients requiring O_2_ supplementation on regular ward were included in the severe group. Additionally, serum samples from 28 mild non-hospitalized COVID-19 cases and 30 plasma samples from anonymous healthy controls (HD) tested negative for SARS-CoV-2 IgG, were used as controls in this study. Serum samples collected between April 2021 and December 2021 from 16 patients with critical disease infected with either the alpha (B.1.1.7) or delta (B.1.617) SARS-Cov-2 variants served as a reference cohort. For the SLE patient control cohort, sera were obtained from the Immunologic, Rheumatologic Biobank (IR-B) of the Department of Rheumatology and Clinical Immunology. Vaccinees either receiving heterologous (Vaxzevria/Spikevax; *n* = 25) or homologous (Comirnaty; *n* = 10) prime-boost COVID-19 vaccination served as additional control group.

### Cell culture

African green monkey kidney Vero E6 cells (ATCC CRL-1586) were cultured at 37 °C in Dulbecco’s Modified Eagle Medium (DMEM) supplemented with 10% (vol/vol) fetal calf serum (FCS, Biochrom), sodium pyruvate (1×, Gibco) and 100 U/ml penicillin-Streptomycin (Gibco). BW5147 mouse thymoma cells (BW, obtained from ATCC: TIB-47) were stably transduced with human FcγR as previously described^51,52^. Cells were maintained at 3 × 10^5^ to 9 × 10^5^ cells/ml in Roswell Park Memorial Institute medium (RPMI GlutaMAX, Gibco) supplemented with 10% (vol/vol) FCS, sodium pyruvate (1×, Gibco), 100 U/ml penicillin-Streptomycin (Gibco) β-mercaptoethanol (0.1 mM, Gibco). Cells were cultured at 37 °C, 5% CO_2_. All cell lines were routinely tested for mycoplasma.

### Monitoring of antibody response to SARS-CoV-2 by ELISA

Serum IgG antibody titers targeting S1- and N-SARS-CoV-2 proteins were measured using commercial enzyme-linked immunosorbent assay (ELISA). Anti-S1- SARS-CoV-2 IgG was measured by the anti-SARS-CoV-2 ELISA (IgG) Euroimmune Kit (Euroimmune, Lübeck, Germany) according to manufacturer’s protocol. Results, expressed as arbitrary units (AU), were evaluated semi-quantitatively by calculation of the ratio of the extinction of the control or patient sample over the extinction of the calibrator. This ratio is interpreted as follows: <0.8 negative; ≥0.8 to <1.0 borderline; ≥1.1 positive. Anti-N SARS-CoV-2 IgG was detected using the recomWell SARS-CoV-2 IgG Kit (Mikrogen Diagnostik GmbH, Neuried, Germany) according to manufacturer’s protocol. The corresponding antibody activity expressed in AU/ml is calculated using the formula (absorbance of sample / absorbance of cut-off) ×20. Results are interpreted as follows: <20 negative; ≥20 to <24 borderline; >24 positive. IgG against the SARS-CoV-2 Spike Glycoprotein Receptor Binding Domain (RBD) were detected using SARS-CoV-2 IgG ELISA Reagent Set, kindly provided by InVivo (InVivo Biotech Services GmbH, Hennigsdorf, Germany) according to manufacturer’s protocol.

### Fcγ receptor activation assay

FcγRIIIA (CD16A, 158 V) activation was measured by a cell-based assay as previously described. Briefly, the assay detects antigen–antibody interactions, which initiate a FcγR activation cascade eventually leading to mIL-2 secretion^53^. For detection of anti-S and anti-RBD-specific FcγR activation, we utilized SARS-CoV-2-S- and RBD-coated plates (kindly provided by InVivo Biotech Services GmbH, Hennigsdorf, Germany). The recombinant (S)-protein was produced under serum-free conditions in mammalian cells and contains amino acid residues 1 to 1213 of the SARS-CoV-2 Wuhan-Hu-1-isolate (GenBank annotation QHD43416.1). The furin cleavage site was mutated, two mutations for protein stabilization were included, and the C-terminal domain was replaced by a T4 trimerization sequence and a C-terminal hexa-His-Tag^96^. The recombinant RBD-protein represented amino acids 319 to 541 of the (S)-protein mentioned before. Both recombinant proteins were purified using immobilized metal exchange chromatography (IMAC) and preparative SEC under standard conditions in a regulated environment. Microtiter plates were coated using 0.2 µg recombinant (S)-protein or RBD-protein per well. N-specific FcγR activation was determined using plates coated with SARS-CoV-2-N (Mikrogen Diagnostik GmbH, Neuried, Germany). Respective plates were subsequently incubated with serial dilutions of SARS-CoV-2 positive sera or control sera in RPMI 10% (v/v) FCS for 30 min at 37 °C. To remove non-immune IgG, all wells were thoroughly washed with RPMI 10% (v/v) before co-cultivation with 2 × 10^5^ BW:FcγR-ζ reporter cells for 16 h at 37 °C, 5% CO_2_. Afterwards, mouse IL-2 secretion was quantified by anti-IL-2 ELISA, using purified rat anti-mouse IL-2 (BD-Pharmingen, 1:500) and biotin rat anti-mouse IL-2 (BD-Pharmingen, 1:500)^53^. FcγRIIIA (CD16A) activation by multimeric sICs was measured by a recently developed cell-based assay^31,59^. Briefly, 2 × 10^5^ BW5147-CD16 reporter cells were incubated with SARS-CoV-2 sera in a total volume of 200 µl for 16 h at 37 °C, 5% CO_2_. Incubation was performed in a 96-well ELISA plate (Nunc Maxisorp) pre-treated with PBS containing10% FCS for 1 h at 4 °C to avoid direct binding of serum IgG to the plate. Reporter cell mIL-2 secretion was quantified via ELISA as described previously^53^. FcγRIIIA/CD16A activation by sICs is shown as log_2_ fold change relative to negative control. The cut-off for sIC reactivity was calculated with the following formula: log_2_ (mean OD_450 nm_ neg. control+3 SD / mean OD_450 nm_).

### Purification of SARS-CoV-2-S and –N specific antibodies from serum

SARS-CoV-2-specific antibodies were purified using SARS-CoV-2 spike protein (S)-coated plates (kindly provided by InVivo BioTech Services) and - nucleocapsid (N) - coated plates recomWell SARS-CoV-2 IgG (Mikrogen Diagnostik GmbH, Neuried, Germany). Patient sera were diluted 1:5 in 100 µl (two wells per serum sample) and incubated for one hour at 37 °C with the S- and N-precoated plates. After washing using PBS-T (0.05% Tween 20) 100 mM formic acid (30 µl/well) was added and incubated for 5 min on an orbital shaker at room temperature (RT) to elute bound IgG. Following pH neutralization using TRIS buffer (1 M), the eluates were either directly processed or stored at 4 °C.

### Quantitation of antigen-specific IgG concentration

In order to determine the relative S1- and N-SARS-CoV-2 specific IgG antibody concentration of the generated eluates, S1- and N-ELISA were performed by the anti-SARS-CoV-2 ELISA (IgG) Euroimmune Kit (Euroimmune, Lübeck, Germany; Cat # EI 2606-9601 G) and anti-N SARS-CoV-2 IgG ELISA (recomWell SARS-CoV-2 IgG Kit; Mikrogen Diagnostik GmbH, Neuried, Germany; Cat # 7304) as aformentioned.

### Analysis of antigen-specific IgG-Fc fucosylation

Fucosylation levels of S- and N-specific IgG were measured using a lectin-based ELISA assay. Briefly, 96-well Maxisorb plates (Nunc) were coated with 50 µl/well anti-human IgG-Fab fragment (MyBiosource, MBS674607) at a concentration of 2 µg/ml, diluted in PBS for one hour at 37 °C. After three washing steps with PBS-T (0.05% Tween20) unspecific binding sites were blocked adding 300 µl/well Carbo-free blocking solution (VectorLab, Inc., SP-5040,) for one hour at room temperature. After three further washing steps, eluted antibodies were serially diluted (2-fold) with PBS in a total volume of 30 µl/well and incubated for one hour at 37 °C and 5% CO_2_. After washing (3×) using PBS-T, 50 µl/well of 4 µg/ml biotinylated Aleuria Aurantia lectin (AAL, lectin, VectorLab, B-1395) diluted in lectin buffer (10 mM HEPES, 0.1 mM CaCl_2_, 0.15 M NaCl, 0.1% Tween20) was added and incubated for 45 min at room temperature (RT). Following another three washing steps using PBS-T, Streptavidin-Peroxidase Polymer (Sigma, S 2438), at 1 µg/ml final concentration diluted in LowCross-HRP-buffer (Candor) was added and incubated for one hour at RT. After washing five times with PBS-T, 50 µl/well of 1-Step™ Ultra TMB-ELISA Substrate Solution (ThermoFisher, 34028) was applied and the enzyme-substrate reaction was stopped after six minutes using 50 µl/well sulphuric acid (1 M H_2_SO_4_). Quantification of absorbance, OD_450nm_, was performed using a Tecan M2000. Relative fucosylation for each generated pool-eluate was calculated by normalizing OD_450nm_ (fucosylation) to its respective relative antigen-specific IgG amount.

### PEG precipitation

Sera pools, consisting of eight different sera per pool, were diluted with varying amounts of PEG8000, in order to reach a final PEG8000 concentration of 1, 2, 3.5, 5 and 7.5% respectively. Mixtures were vortexed and incubated overnight at 4 °C. For supernatant analysis, precipitates were sedimented via centrifugation at 16.089 *g* for 30 min at 4 °C. For Mass Spectrometry analysis, PEG8000-precipitated sICs were shortly run into 10% polyacrylamide gels. After over-night fixation (40% ethanol, 10% acetic acid, 50% water) and washing (3x), complete lanes were excised.

### Benzonase treatment of sera

Serum from six individual patients containing CD16-reactive soluble immune complexes, were treated with 50 Units/ml of Benzonase Nuclease (Sigma-Aldrich Chemie GmbH, Munich Germany) for 1 h at 4 °C. After treatment, sera were titrated in complete BW5147 culture medium and tested for CD16 reactivity. Non-treated sera served as control. To verify Benzonase activity in the presence of human serum, 3 µg of pIRES-eGFP plasmid DNA (Addgene) were digested with 50 Units/ml of Benzonase. Successful nucleic acid digestion was visualized using a 1% agarose gel stained with Midori Green.

### Immune precipitation

For mass spectrometry analysis of SARS-CoV-2-S specific precipitates, individual sera containing FcγRIII/CD16-reactive soluble immune complexes were subjected to immune precipitation (IP) using Pierce MS-compatible magnetic IP kit (ThermoFisher Scientific, Darmstadt, Germany) according to manufacturer’s protocol. Briefly 250 µl serum was incubated overnight at 4 °C with 5 µg of biotinylated anti-RBD-specific TRES-1-224.2.19 mouse monoclonal antibody (1:20) or TRES-II-480 (isotype control; (1:80) (kind gift of H.M. Jäck, Erlangen) before addition of streptavidin magnetic beads. Beads were subsequently collected via centrifugation and elution buffer was added to detach putative precipitated antigen. The elution was dried in a speed vacuum concentrator and shortly run into 10% polyacrylamide gels. After over-night fixation (40% ethanol, 10% acetic acid, 50% water) and washing (3x), complete lanes were excised. Antibody biotinylation was performed using a Pierce antibody biotinylation Kit for IP (ThermoFisher Scientific, Darmstadt, Germany) according to manufacturer’s protocol.

### Mass spectrometry

Proteins were in-gel digested with sequencing grade modified trypsin (Promega GmbH, Walldorf, Germany) similar to the procedure described by Pandey et al^97^. Vacuum-dried peptides were dissolved in 0.5% trifluoroacetic acid, loaded onto a trap column (C18 PepMap100, 5 µm particles, Thermo Fisher Scientific GmbH, Dreieich, Germany) with 0.05% trifluoroacetic acid (4 min, 10 µL/min) and separated on a C18 reversed phase column (SilicaTip emitter, 75 µm i.d., 8 µm tip, New Objective, Inc, Littleton, USA, manually packed 23 cm with ReproSil-Pur ODS-3, 3 µm particles, Dr. A. Maisch HPLC GmbH, Ammerbuch-Entringen, Germany; flow rate: 300 nL/min). For sample injection and multi-step gradient formation (eluent “A”: 0.5% acetic acid in water; eluent “B”: 0.5% acetic acid in 80% acetonitrile / 20% water; gradient length / acquisition time: 100 min or 175 min) an UltiMate 3000 RSLCnano system (Thermo Fisher Scientific GmbH, Dreieich, Germany) was used. Eluting peptides were electrosprayed at 2.3 kV via a Nanospray Flex ion source into a Q Exactive HF-X hybrid quadrupole-orbitrap mass spectrometer (both Thermo Fisher Scientific GmbH, Dreieich, Germany) and analyzed by data-dependent acquisition with HCD (higher energy collisional dissociation) fragmentation of doubly, triply and quadruply charged ions (loop count and dynamic exclusion dependent on the gradient length). Peak lists were generated with ProteoWizard msConvert (http://proteowizard.sourceforge.net/; version 3.0.11098), linear shift mass recalibrated (after a preliminary database search) using software developed in-house and searched against a database containing the SARS-CoV-2 UniProtKB reference proteome (proteome ID: UP000464024), all human UniProtKB/Swiss-Prot entries, and optionally (to reduce the number of incorrectly assigned matches) selected bacterial proteins (finally the Pseudomonas fluorescence (strain SBW25) reference proteome; proteome ID: UP000002332) with Mascot 2.6.2 (Matrix Science Ltd, London, UK; peptide mass tolerance: ± 5 ppm; fragment mass tolerance: ± 20 mmu; one missed trypsin cleavage and common variable modifications allowed).

### Cytokine measurements

Serum Interferon-gamma (IFN-γ) and interleukin-12p70 (IL-12) concentrations were measured using the Milliplex MAP human high sensitivity T cell magnetic bead panel (Merck Millipore) on a Luminex 200 analyzer (Luminex Corporation, Austin, Texas) according to the manufacturer’s recommendations. Data analysis was performed using the BioPlex manager software v 6.1 (Bio-Rad).

### Neutralization assay

Serum neutralization capacity was analyzed as previously described^98,99^. Briefly, VeroE6 cells were seeded in 12-well plates at a density of 4 × 10^5^ cells/well 24 h prior to infection. Serum samples were diluted at ratios of 1:16, 1:32 and 1:64 in 50 µL PBS total volume. Negative controls (PBS without serum) were included for each serum. Diluted sera and negative controls were subsequently mixed with 90 plaque forming units (PFU) of authentic SARS-CoV-2 (B.1) in 50 µl PBS (1600 PFU/mL) resulting in final sera dilution ratios of 1:32, 1:64, and 1:128. Following incubation at RT for 1 h, 400 µL PBS was added to each sample and the mixture was subsequently used to infect VeroE6 cells. After 1.5 h of incubation at RT, inoculum was removed and the cells were overlaid with 0.6% Oxoid-agar in DMEM, 20 mM HEPES (pH 7.4), 0.1% NaHCO_3_, 1% BSA and 0.01% DEAE-Dextran. Cells were fixed 48 h post-infection (4% formaldehyde for 30 min). Upon removal of the agar overlay, plaque neutralization was visualized using 1% crystal violet. PFU were counted manually. Plaques counted for serum-treated wells were compared to the average number of plaques counted for the untreated negative controls, which were set to 100%.

### Statistical analyses

Data were analyzed with GraphPad Prism 9.0.0 software and Matlab 9.8.0.1721703 (R2020a). Statistical analyses were performed using linear statistical models. I.e. the two-group comparisons were made based on the *t*-statistic of the estimated effects. Differences over more than two groups were tested by Analysis of Variance (ANOVA).We only report unadjusted *p*-values because the study was explorative and the hypotheses to be tested and to be considered with respect to multiple testing were not defined beforehand. For the time course data, patient differences were treated as random effects in a linear mixed effects model with time and clinical course (severe vs. critical) as fixed main and interaction effects. All analyses were performed at the log_2_ scale. Assumptions about variance heterogeneity and normal distribution were checked by visual inspection of diagnostic plots.

### Reporting summary

Further information on research design is available in the Nature Research Reporting Summary linked to this article.

## Supplementary information

Supplementary Information

Reporting Summary

## Data Availability

All other data are available in the article and its Supplementary files or from the corresponding author upon reasonable request. Source data are provided with this paper.

## Source data

Source Data
