# Supplementary material for: Circulating multimeric immune complexes contribute to immunopathology in COVID-19
Source: Nat Commun. 2022 Sep 26;13:5654. doi: 10.1038/s41467-022-32867-z (PMC9513013; doi:10.1038/s41467-022-32867-z)
Supplement: Supplementary file 1 — Supplementary Information [file 41467_2022_32867_MOESM1_ESM.pdf]

Supplementary Information

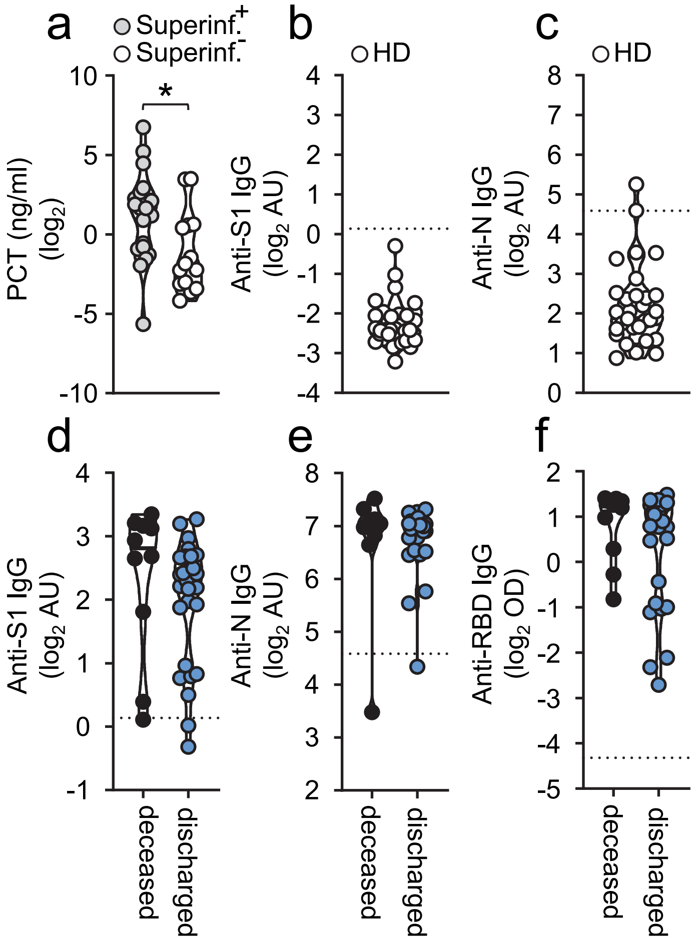

Supplementary Figure 1. Correlation between Procalcitonin levels and bacterial superinfections, together with SARS-CoV-2 specific IgG levels in seronegative patients and according to disease outcome.

a) Procalcitonin (PCT) levels in patients with (grey, n=20) or without (white, n=14) bacterial superinfection (*, $p<0.05$, t-test, equal variances, one-sided). b) S1- and c) N-specific IgG levels in 30 healthy donors. d) Cumulative S1-, e) N- and f) RBD-specific IgG levels measured 13-25 days after symptom onset in deceased (black, n= 10) and not deceased COVID-19 patients (blue, n=27). Each symbol represents the mean value obtained by the analysis of all samples available in the indicated time range for each individual patient. Dotted lines represent cut-off values for S1-, N- and RBD-specific ELISA assays. Solid black lines indicate the median. Source data are provided as a Source Data file.

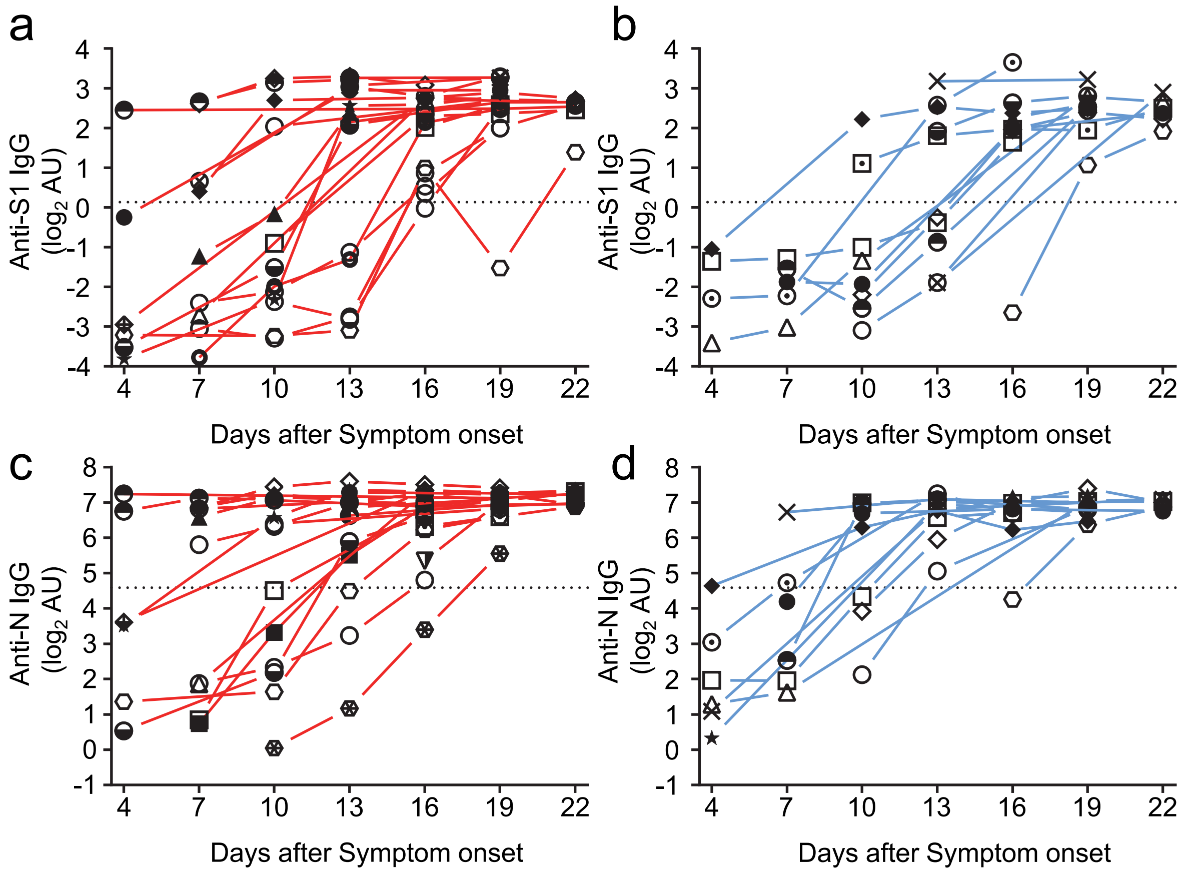

Supplementary Figure 2. Longitudinal changes in anti- SARS-CoV-2 IgG titers in severely and critically diseased patients.
Serial serum samples were collected from hospitalized COVID-19 patients and used for SARS-CoV-2–specific IgG measurement. IgG responses against SARS-CoV-2 S1- and N-protein in (a, c) critically (red lines) and (b, d) severely (blue lines) diseased patients. Days after symptom onset (+/- 1 day) are depicted. Dotted lines represent cut-off values for commercial S1- and N- specific ELISA assays. Each symbol represents the mean value of all samples which were available for each patient at the indicated time range after symptom onset. There are no significant t-tests (i.e. p>0.05 for all comparisons). Source data are provided as a Source Data file.

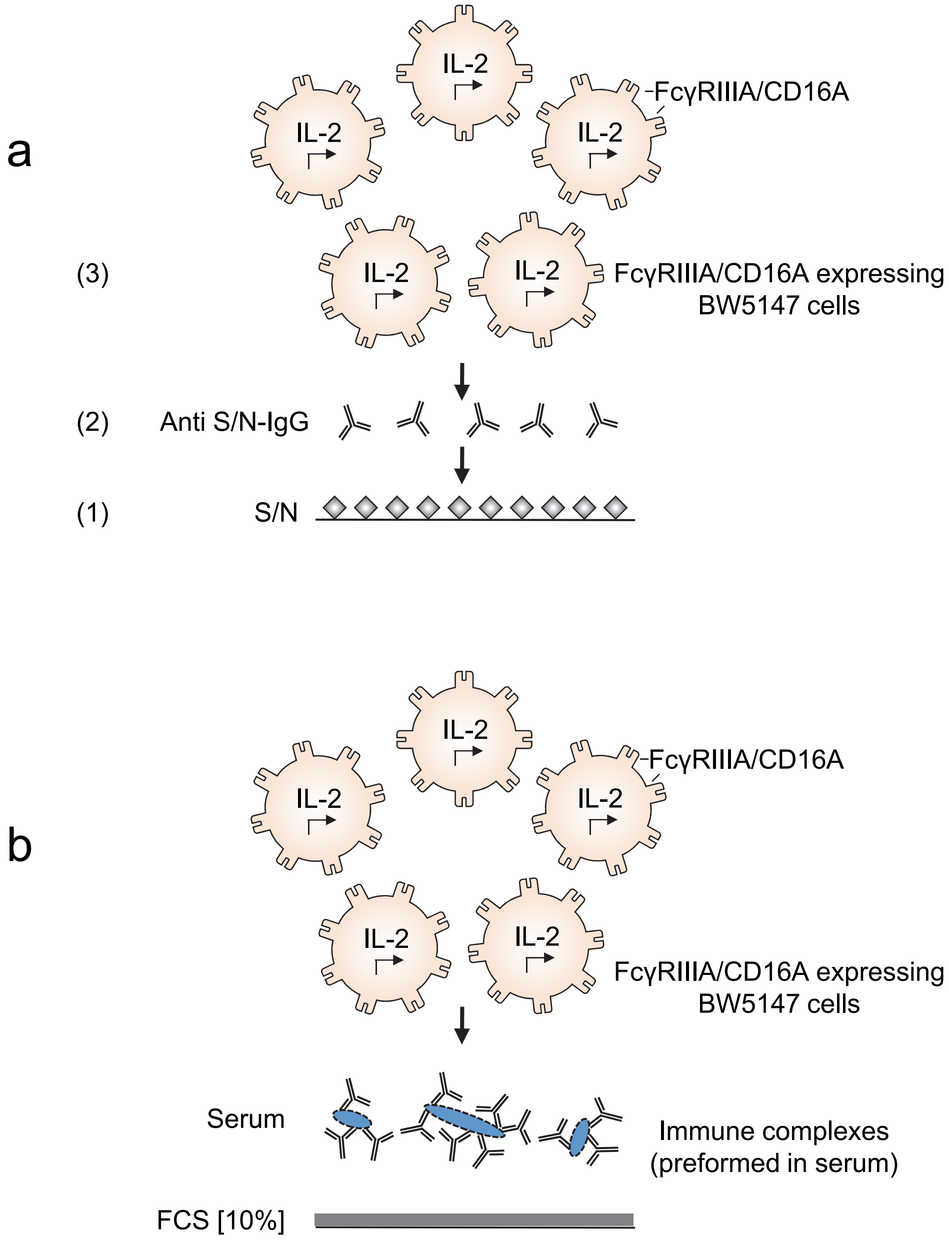

Supplementary Figure 3. Cell-based reporter assay measuring FcγRIIIA/CD16A activation in response to immobilized IgG and sICs.

BW5147 reporter cells expressing chimeric human FcγRIIIA/CD16A secrete IL-2 in response to FcγR activation by a) clustered viral specific IgG binding solid-phase antigen or b) soluble ICs. Solubility of sICs is achieved by pre-blocking an ELISA plate with PBS supplemented with 10% FCS as previously described[1,2].

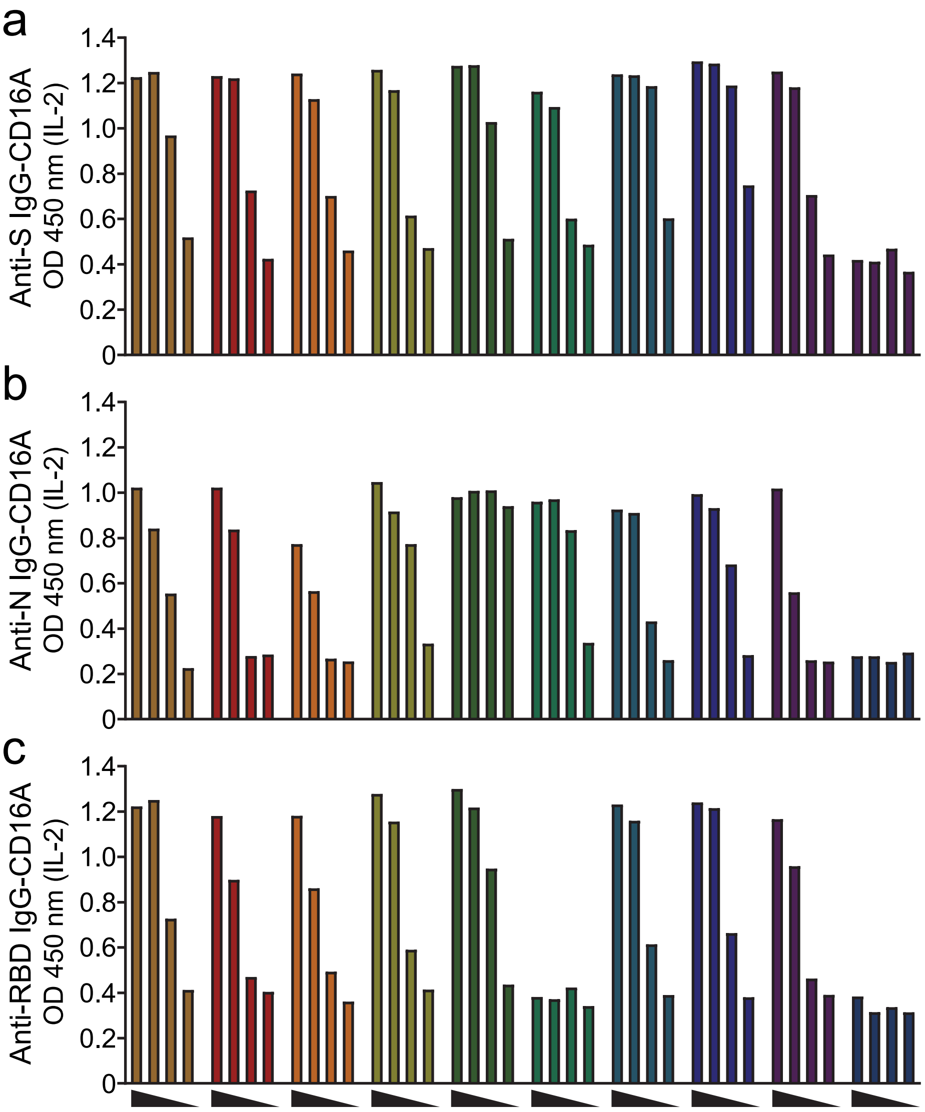

Supplementary Figure 4. Dose dependent FcγRIIIA/CD16A activation by SARS-CoV-2 specific IgG. FcγRIIIA/CD16A activation by a) S- , b) N- and c) RBD-specific IgG in 9 representatively selected serum samples and one SARS-CoV-2 negative serum (dark blue bars). Sera were serially diluted at 1:20, 1:100, 1:500 and 1:2500. FcγRIIIA/CD16A activation initiates IL-2 secretion by reporter cells, which is subsequently measured via ELISA (OD 450 nm). Based on this empirical pretesting all sera were thereafter tested at 1:100 and 1:500 dilutions to reach an optimal dynamic range of response. The OD values obtained by the 1:500 dilutions were used for subsequent data analysis. Source data are provided as a Source Data file.

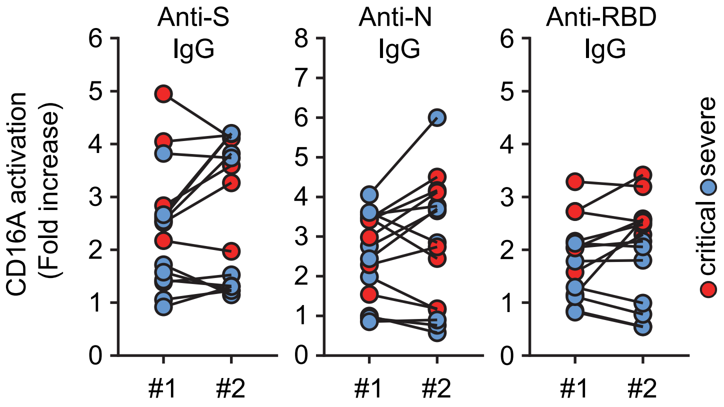

Supplementary Figure 5. Reproducibility of FcγRIIIA/CD16A activation measurements by SARS-CoV-2 specific IgG.
Selected sera which were available in sufficient amount from patients with critical (red symbols) or severe (blue symbols) SARS-CoV-2 infection were tested in two independent experiments to show reproducibility and consistency of results. FcγRIIIA/CD16A activation by S-, N- and RBD specific IgG is shown. Statistical tests using a Kolmogorov-Smirnov test indicate no significant differences. Source data are provided as a Source Data file.

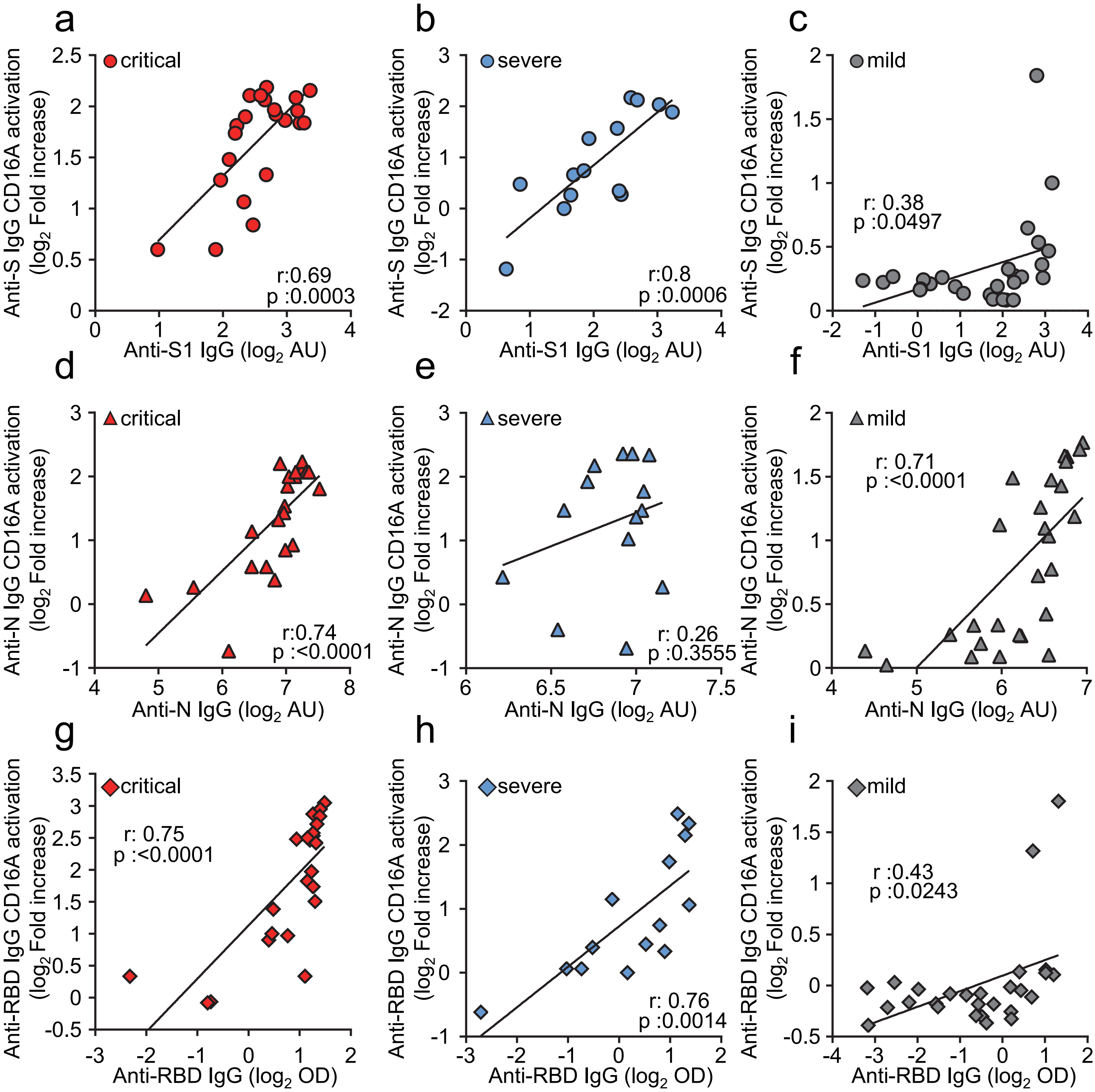

Supplementary Figure 6. Correlation of FcγRIIIA/CD16A activation by virus specific IgG and ELISA levels.

Pearson's correlation coefficient was used to assess the relationship between virus–specific IgG levels and their capability to trigger FcγRIIIA/CD16A activation on BW5147 reporter cells in 22-23 paired samples from patients with critical disease (red symbols), 14 paired samples from patients with severe disease (blue symbols) and 27 samples from patients with mild disease (grey symbols). Each dot represents the mean value obtained by the analysis of all samples available at the indicated time points. (a-c) anti-S IgG, (d-f) anti-N IgG and anti-RBD-IgG (g-i). Source data are provided as a Source Data file.

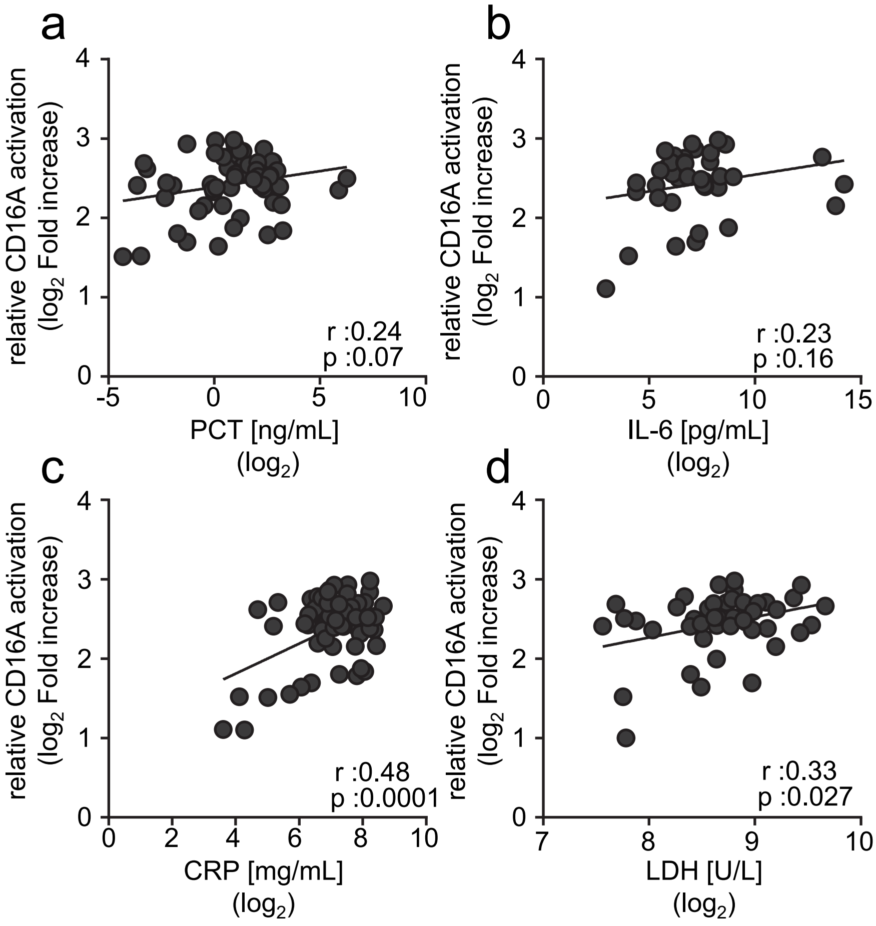

Supplementary Figure 7. Correlation of FcγRIIIA/CD16A activation by sICs and different diagnostic markers.

Serum samples obtained 13-25 days after onset of symptoms from critically and severely diseased patients were analyzed regarding sIC levels and diagnostic markers. Pearson's correlation coefficient was used to assess the relationship between FcγRIIIA/CD16A activation by sICs and a) PCT, b) IL-6, c) CRP and d) LDH. FcγRIIIA/CD16A activation is shown as log_2 fold change relative to negative control. Each symbol represents individual serum samples. Source data are provided as a Source Data file.

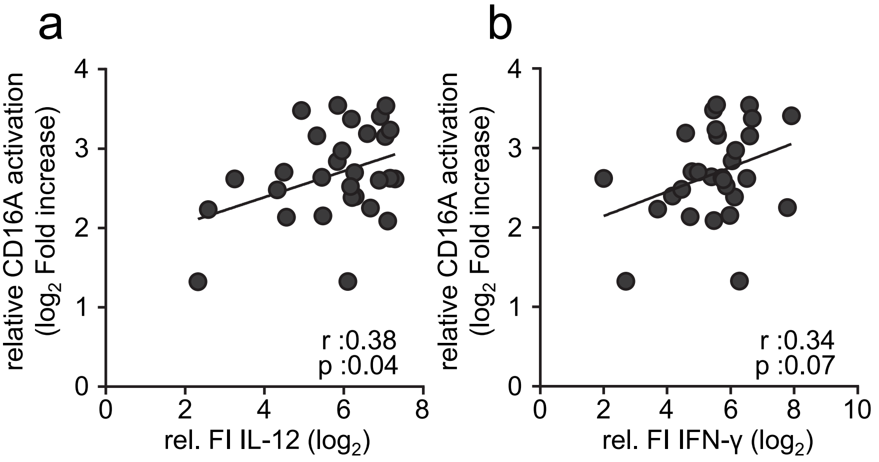

Supplementary Figure 8. IL-12 and IFN-γ levels in COVID-19 patient sera.
Analysis of sIC reactive serum samples (Figure 4.a, c) obtained between 13-25 days after symptom onset. Pearson's correlation coefficient was used to assess the relationship between FcγRIIIA/CD16A activation by sICs and a) IL-12 levels and b) IFN-γ levels. FcγRIIIA/CD16A activation is shown as $\log_2$ fold change relative to negative control. Cytokines levels are shown as relative fluorescence intensity. Each symbol represents individual serum samples. Source data are provided as a Source Data file.

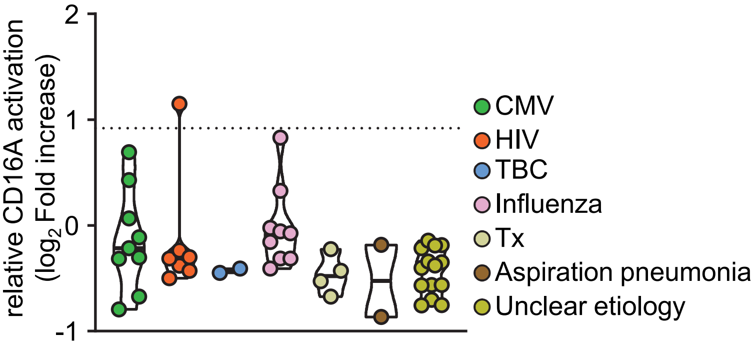

Supplementary Figure 9. FcγRIIIA/CD16A activation by sICs in non-COVID-19 patients with ARDS. Serum samples from 47 patients with ARDS in response to infections of different etiology were analyzed in a cell-based reporter assay which is sensitive to sIC amount and size[1,2]. FcγRIIIA/CD16A activation is shown as log_2 fold change relative to negative control. Each symbol represents one sample from one patient. CMV: Cytomegalovirus disease under immunosuppression; HIV: HIV infection; TBC: Mycobacterium tuberculosis infection; Influenza: influenza virus infection; TX: solid organ transplantation. Solid black lines indicate the median. Dashed line represents cut-off level for sIC activation. Source data are provided as a Source Data file.

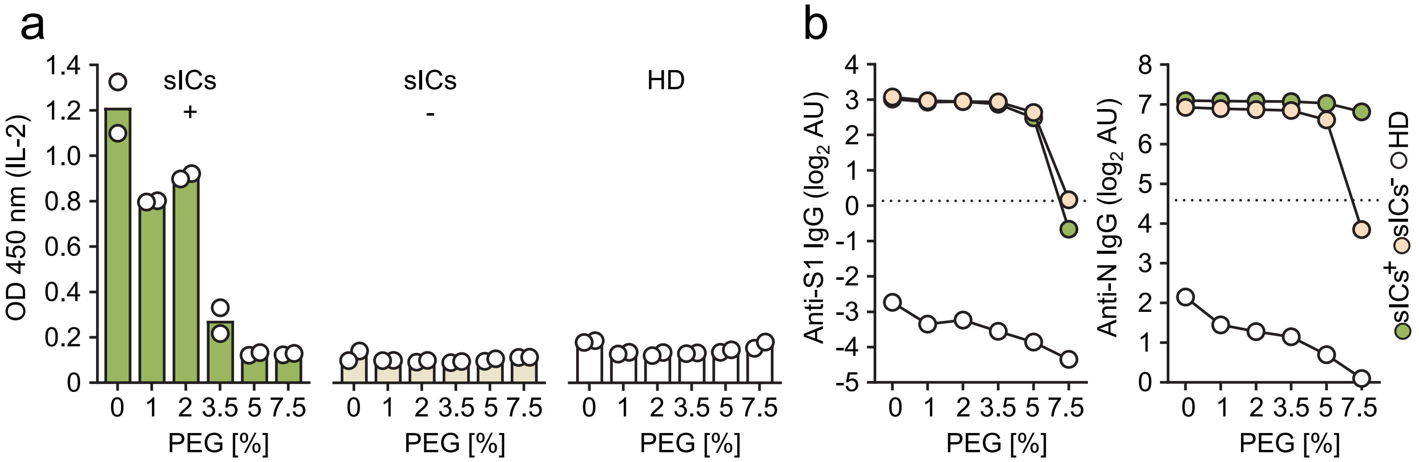

Supplementary Figure 10. PEG precipitation eliminates sIC-mediated FcγRIIIA/CD16A activation. Pools of 8 sera were incubated with equal volumes of PEG8000 to reach the indicated final PEG concentrations. a) FcγRIIIA/CD16A activation after PEG-precipitation in the pool supernatant, showing either high ($sICs^+$) or no ($sICs^-$) FcγRIIIA/CD16A activation. Sera from healthy donors (HD) were included as a negative control. Activation levels are expressed as IL-2 levels (OD 450 nm) released by reporter cells. Ech dot represents one independent experiment. b) Anti SARS-CoV-2 IgG levels against S1 (left panel) or N (right panel) IgG following PEG precipitation. The mean value of two independent experiments for both sIC^+ and sIC^- pools is depicted. HD (n=1) served as control. Dotted lines represent cut-off values for commercial S1- and N- specific ELISA assays. Source data are provided as a Source Data file.

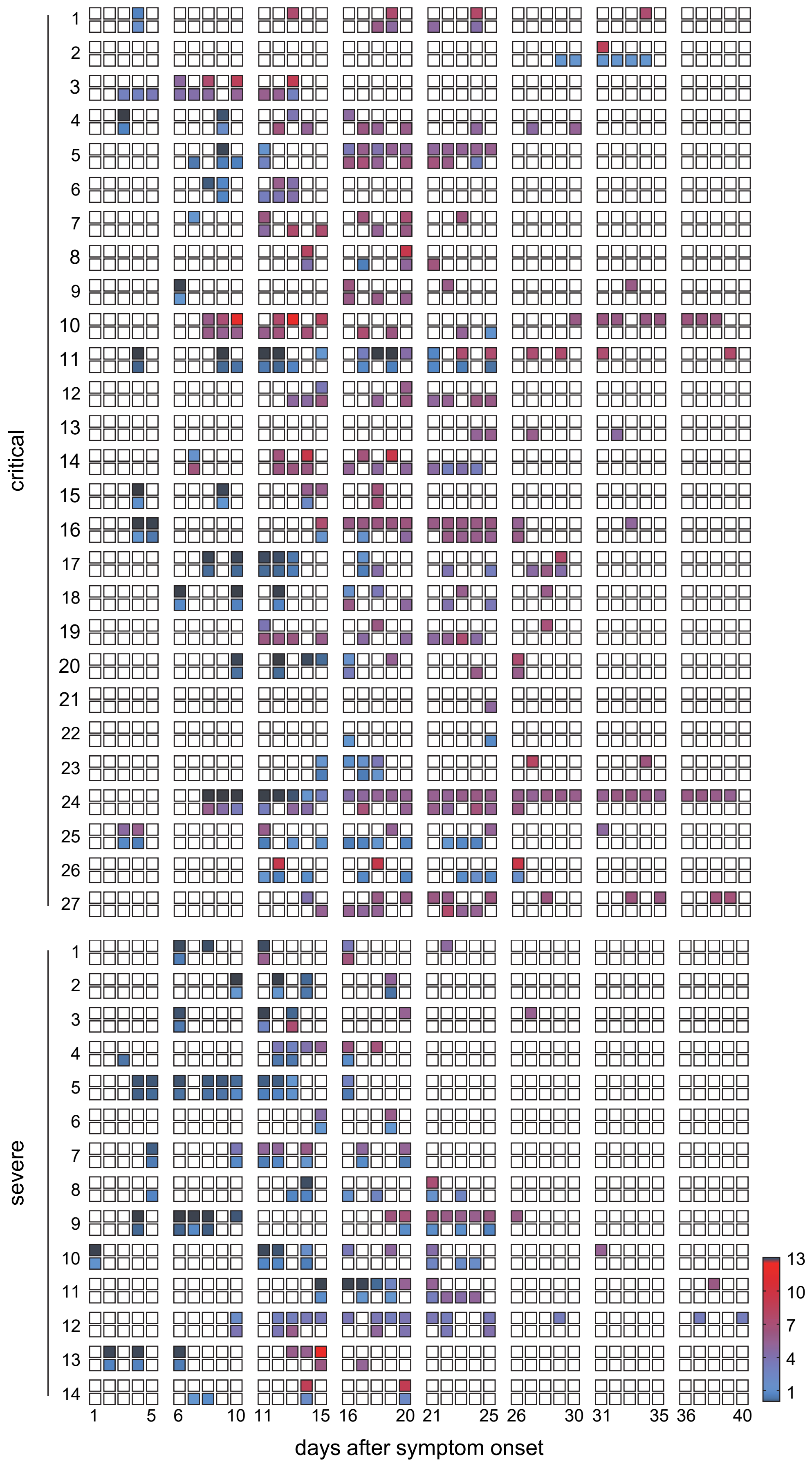

Supplementary Figure 11. Individual FcγRIIIA/CD16A activation by sICs and anti-S1 ELISA IgG kinetics post symptom onset.

Individual sera from either critically (n = 27) or severely (n = 14) diseased patients were analyzed via ELISA [AU] for anti S1-IgG (upper row) and for FcγRIIIA/CD16A activation by soluble immune complexes (lower row, relative FcγRIIIA/CD16A activation depicted as fold increase to the negative control) over time (1-40 days post symptom onset). White squares: not tested. Source data are provided as a Source Data file.

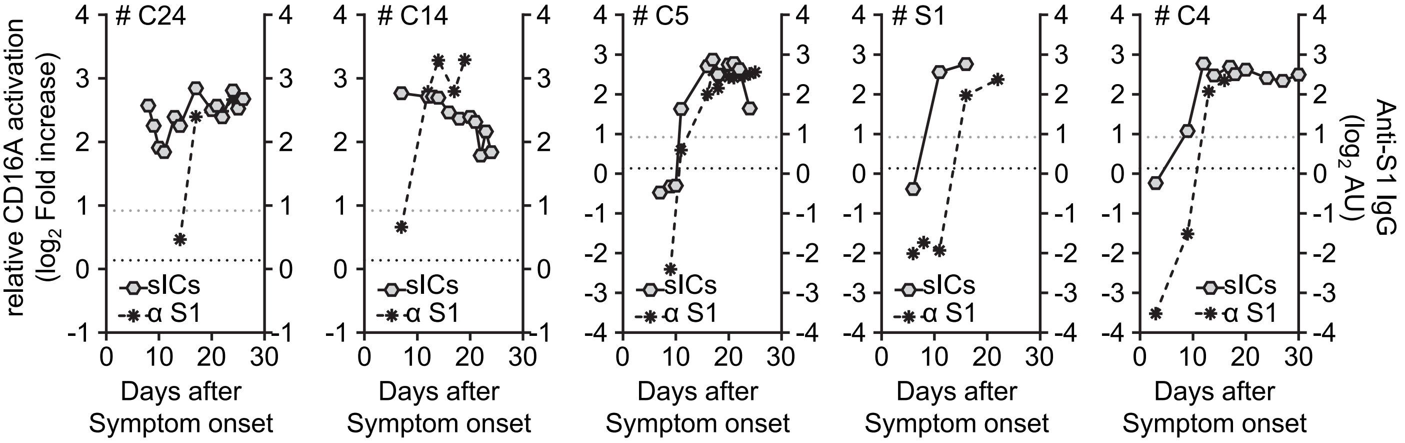

Supplementary Figure 12. Select patients showing sIC formation preceding SARS-CoV-2-IgG response.
Time course of sIC-mediated FcγRIIIA/CD16A activation (log_2 fold increase to negative control) vs. S1-ELISA in four individual critical patients (#C24, #C14, #C5, #C4) and one severe patient (#S1). Dashed line (black) represents commercial S1-ELISA-cut-off level, whereas dashed line (grey) represents cut-off levels for sIC activation. Individual longitudinal courses correspond to patients depicted in Supplementary Figure 11. Source data are provided as a Source Data file.

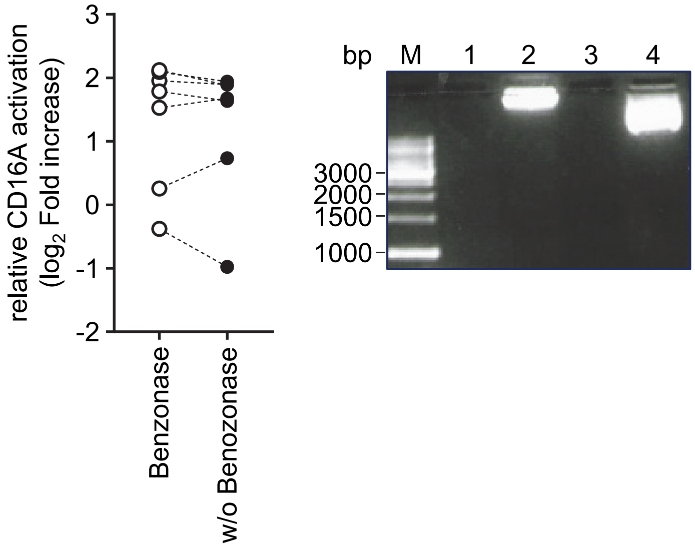

Supplementary Figure 13. Benzonase treatment of sIC-reactive sera does not abolish FcγRIIIA/CD16A activation.

Left panel: sIC-mediated FcγRIIIA/CD16A reactivity expressed as log_2 fold increase to the negative control, in serum of six individual patients before and after treatment with 50 U/ml of Benzonase Nuclease. A representative experiment is shown (n=2). Right panel: As positive control (n=1), 3 µg plasmid DNA was digested. M: 1kb DNA ladder, Lane 1: benzonase digestion in the presence of human serum, lane 2: plasmid DNA w/o benzonase in the presence of human serum, lane 3: benzonase digestion in medium only and lane 4: plasmid DNA w/o benzonase in medium only. Source data are provided as a Source Data file.

Supplementary Table 1.

Pooled patient sera were analyzed for a) Autoantibodies against extractable nuclear antigens (via dot blot), b) dsDNA autoantibodies (via ELISA, Cutoff: > 40IU/mL)) and c) anti-nuclear autoantibodies (ANA) by indirect immunofluorescence. HD: Healthy donors; sIC^{hi}: high sIC-mediated FcγRIIIA/CD16A activation; sIC^{low}: low sIC-mediated FcγRIIIA/CD16A activation in either critically or severely diseased patients

a

Group		ET	Ctrl.	DSF70	AMA-M2	RIB	HIS	NUC	PCNA	CENPB	Jo-1	PM-Scl	Scl-70	SS-B	Ro-52	SS-A	Sm	nRNP/Sm
HD	Intensity	-1	98	1	0	1	10	0	1	1	0	0	2	0	2	1	1	2
	Result	0	+++	0	0		(+)											
Critical sIC^{hi}	Intensity	-1	82	0	1	0	1	0	0	1	0	1	0	1	1	2	2	0
	Result	0	+++															
Critical sIC^{low}	Intensity	-1	101	0	1	1	0	0	0	0	1	0	3	1	1	1	0	0
	Result	0	+++	0	0	0	0	0	0	0	0	0	0	0	0	0	0	0
Severe sIC^{hi}	Intensity	-1	82	1	1	0	1	0	1	1	0	0	0	1	1	1	2	1
	Result	0	+++	0	0	0	0	0	0	0	0	0	0	0	0	0	0	0
Severe sIC^{low}	Intensity	-1	94	0	1	1	0	0	1	1	1	0	1	0	1	1	0	2
	Result	0	+++	0	0	0	0	0	0	0	0	0	0	0	0	0	0	0

b

Group	dsDNA (IU/mL)
HD	12
Critical sIC^{hi}	22
Critical sIC^{low}	7
Severe sIC^{hi}	17
Severe sIC^{low}	12

c

Group	Dilution	Nucleus	NKL	Chromo	Cyto
HD	50	negative	negative	negative	negative
Critical sIC^{hi}	50	negative	negative	negative	negative
Critical sIC^{low}	50	negative	negative	negative	negative
Severe sIC^{hi}	50	negative	negative	negative	negative
Severe sIC^{low}	50	negative	negative	negative	negative

Supplementary note 1

Details for MS_29012021 (whole lane cut) zip file: Mass spectrometry analysis of the whole lane of PEG8000- precipitated pooled sera.
sIC^{+}: sIC-mediated FcγRIIIA/CD16A activation; sIC^{-}: no sIC-mediated FcγRIIIA/CD16A.

Folder Name	**MS_29012021 (whole lane cut) .zip**	**Samples**
File name	QX20210214-01_Virologie(AG-Hengel)-20210129**_(V6)_**IC+_Lys_completelane_0k3v20_TRL_db_SA RS-COV-2+SP_H(F229058).pdf	Cell lysate of SARS-CoV-2-infected cells (pos control)
	QX20210214-01_Virologie(AG-Hengel)-20210129**_(V5)_**C-_ 7-5_completelane_0k3v20_TRL_db_SARS-COV-2+SP_H(F229078).pdf	SARS-CoV2 neg pooled sera after 7,5% PEG precipitation (neg ctrl)
	QX20210214-01_Virologie(AG-Hengel)-20210129**_(V4)_**IC-_3-5_completelane_0k3v20_TRL_db_SARS-COV- 2+SP_H(F229100).pdf	sIC-_critical pooled sera after 3,5% PEG precipitation
	QX20210214-01_Virologie(AG-Hengel)- 20210129**_(V3)_**IC+_3-5__completelane_0k3v20_TRL_db_SARS-COV- 2+SP_H(F229104).pdf	sIC+_critical pooled sera after 3,5% PEG precipitation
	QX20210214-01_Virologie(AG-Hengel)-20210129**_(V2)_**IC-_7-5_completelane_0k3v20_TRL_db_SARS-COV- 2+SP_H(F229120).pdf	sIC-_critical pooled sera after 7,5% PEG precipitation
	QX20210214-01_Virologie(AG-Hengel)- 20210129**_(V1)_**IC+_7-5_completelane_0k3v20_TRL_db_SARS-COV- 2+SP_H(F229151).pdf	sIC+_critical pooled sera after 7,5% PEG precipitation

Supplementary note 2

Details for MS_20210407 (immune precipitation) zip file: Mass spectrometry analysis of single sera from COVID19 critical patients after immune precipitation with anti-RBD- specific TRES-1-224.2.19 mouse monoclonal antibody or TRES-II-480 (isotype control).
sIC^{+}: sIC-mediated FcγRIIIA/CD16A activation; sIC^{-}: no sIC-mediated FcγRIIIA/CD16A.

Folder Name	**MS_20210407 (immune precipitation) .zip**	**Samples**
File name	QX20210616-01_Virologie(AG-Hengel)-20210407**_(V1)_**0k3v20_TRL_db_SC"+db_Pf+SP_H(F235773).pdf	sIC-_ critical COVID19_single serum
	QX20210616-02_Virologie(AG-Hengel)-20210407**_(V2)_**0k3v20_TRL_db_SC"+db_Pf+SP_H(F235783).pdf	sIC-_ critical COVID19_single serum
	QX20210616-03_Virologie(AG-Hengel)-20210407**_(V3)_**0k3v20_TRL_db_SC"+db_Pf+SP_H(F235788).pdf	sIC+_ critical COVID19_single serum
	QX20210616-04_Virologie(AG-Hengel)-20210407**_(V4)_**0k3v20_TRL_db_SC"+db_Pf+SP_H(F235795).pdf	sIC+_ critical COVID19_single serum
	QX20210616-05_Virologie(AG-Hengel)-20210407**_(V5)_**0k3v20_TRL_db_SC"+db_Pf+SP_H(F235800).pdf	sIC+_ critical COVID19_single serum
	QX20210616-06_Virologie(AG-Hengel)-20210407**_(V6)_**0k3v20_TRL_db_SC"+db_Pf+SP_H(F235806).pdf	sIC+_ critical COVID19_single serum
	QX20210616-09_Virologie(AG-Hengel)-20210407**_(V9)_**0k3v20_TRL_db_SC"+db_Pf+SP_H(F235822).pdf	sIC+_ critical COVID19_single serum
	QX20210616-10_Virologie(AG-Hengel)-20210407**_(V10)_**0k3v20_TRL_db_SC"+db_Pf+SP_H(F235831).pdf	sIC+_ critical COVID19_single serum
	QX20210616-11_Virologie(AG-Hengel)-20210407**_(V11)**_0k3v20_TRL_db_SC"+db_Pf+SP_H(F235838).pdf	sIC+_critical_ pool V5+V6_ Ctrl Isotype

Supplementary References

[1] Chen, H. *et al.* Detection and functional resolution of soluble immune complexes by an FcgammaR reporter cell panel. *EMBO Mol Med*, e14182, doi:10.15252/emmm.202114182 (2021).

[2] Zhao, S. *et al.* JAK inhibition prevents the induction of pro-inflammatory HLA-DR(+) CD90(+) RA synovial fibroblasts by IFN. *Arthritis Rheumatol*, doi:10.1002/art.41958 (2021).
