# Supplementary material for: Circulating multimeric immune complexes contribute to immunopathology in COVID-19
Source: Nat Commun. 2022 Sep 26;13:5654. doi: 10.1038/s41467-022-32867-z (PMC9513013; doi:10.1038/s41467-022-32867-z)
Supplement: Supplementary file 3 — Source Data [file 41467_2022_32867_MOESM3_ESM.zip › Source Data/MS_20210407 (immune precipitation).zip!/QX20210618-01_Virologie(AG-Hengel)-20210407_(V1)_0k3v20_TRL_db_SC2+db_Pf+SP_H(F235773).pdf]

MATRIX SCIENCE MASCOT Search Results

User : Wolfgang Bildl
E-mail : wolfgang.bildl@physiologie.uni-freiburg.de
Search title : QX 20210618 (db$ SARS-CoV-2+db$2 Pf+SP H$ 5 ppm 20 mmu), QX20210618-01_Virologie(AG-Hengel)-20210407_(V1)_0k3v20_TRL_shifted_-1.1.mgf
MS data file : D:\Xcalibur\Data\Wolfgang\Q-Exactive-HF-X_20210616\QX20210618-01_Virologie(AG-Hengel)-20210407_(V1)_0k3v20_TRL_shifted_-1.1.mgf
Databases : **1:** db$ 20210617 (17 sequences; 14,439 residues)
2: db$2 20210617 (6,388 sequences; 2,089,672 residues)
3: SwissProt_HUMAN$ 20201007 (20,389 sequences; 11,363,789 residues)
Timestamp : 21 Jun 2021 at 09:04:32 GMT

Not what you expected? Try **the select summary**.

Search parameters

Type of search : MS/MS Ion Search
Enzyme : Trypsin
Variable modifications : **Acetyl (Protein N-term)**, **Carbamidomethyl (C)**, **Formyl (Protein N-term)**, **Gln->pyro-Glu (N-term Q)**, **Oxidation (M)**
Mass values : Monoisotopic
Protein mass : Unrestricted
Peptide mass tolerance : ± 5 ppm
Fragment mass tolerance : ± 20 mmu
Max missed cleavages : 1
Instrument type : ESI-FTICR
Number of queries : 83,438

Score distribution

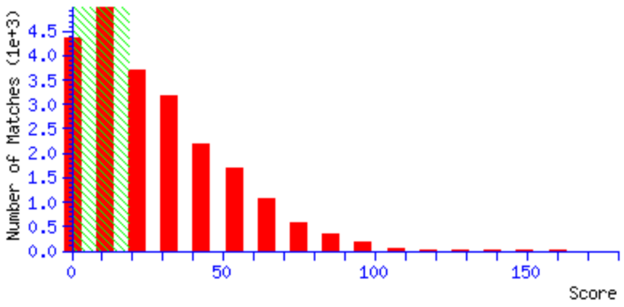

Peptide score distribution. Ions score is $-10\log(P)$, where P is the probability that the observed match is a random event.
There are **11,825** peptide matches above identity threshold and **12,583** matches above homology threshold for **83,438** queries.
On average, individual ions scores **> 19** (beyond green shading) indicate **identity or extensive homology** (p<0.05).

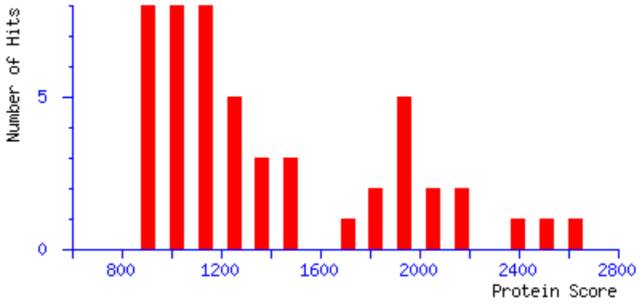

[Deprecated] Protein score distribution. Score distribution for family members in the first 50 proteins. Protein scores are derived from ions scores as a non-probabilistic basis for ranking protein families.

▼Modification statistics

Modification	Site	Above thr.
Oxidation	M	2264
Carbamidomethyl	C	1180
Gln->pyro-Glu	N-term	265
Formyl	Protein N-term	45
Acetyl	Protein N-term	15

▼Legend

Peptide columns and rows

Dupes	...	Expect	Rank	U	1	2	Peptide	
		**0.037**	▶**2**				**GAYSLSLR**	significant
		9	▶1				GFFLFVEGGR	top ranking
		**6.4e-005**	▶**1**				**GSSIFGLAPGK**	significant and top ranking
		**1.3e-006**	▶**1**		■		**SSGTSYPDVLK**	peptide is found in all proteins in family member 1
		**6.2e-007**	▶**1**			■	**VCNYVSWIK**	peptide is found in some but not all proteins in family member 2
		**6.4e-005**	▶**1**	**U**			**GSSIFGLAPGK**	unique
▶**2**		**5.7e-005**	▶**1**				**LNTLETEEWFFK**	peptide has two duplicates
		0.18	▶*1*				*LNTLETEEWFFK*	duplicate peptide

Right-facing triangle (▶) in the Dupes or Rank column indicates content that can be expanded by clicking on it. Down-facing triangle (▼) indicates the content is expanded and can be collapsed. For more details about particular columns, see **results format help**.

Protein quantitation ratios

	Score	...	114/113	115/113
**CFAH_HUMAN**	37559		*0.962*	**1.129**
**FHR2_HUMAN**	1330		**0.859**	1.128

When quantitation method is Reporter (e.g. iTRAQ) or Multiplex (e.g. IPTL), protein ratios are displayed when a family is expanded. Ratios in *italic* indicate that the peptide log-ratios do not appear to come from a normal distribution. **Bold** indicates that if you can assume peptide ratios are normally distributed, the protein ratio is significantly different from 1.0 (at significance level 0.05).

Note that lack of bold or italic can also mean that significance or normality testing has not been performed (for

example, if protein ratio type does not support it).

Protein Family Summary

Significance threshold p<	0.05	Max. number of families	AUTO
Ions score or expect cut-off	0.5	Dendrograms cut at	0
Show Percolator scores	☐		
Preferred taxonomy	All entries		

▼Sensitivity and FDR (reversed protein sequences)

			Target	Decoy	FDR		
PSMs	above	homology	12583	494	3.93%	Adjust to	1%

Decoy results are available in **the decoy report**.

Protein hits (2120 proteins)

▶Filters: (none)

Export as CSV

↑Family	M	DB	Accession	Score	Mass	Matches	Match(sig)	Sequences	Seq(sig)	emPAI	Description
1	1	db$2	2::C3K2Y3|RPOB_PSEFS	2626	150923	102	101	69	69	10.66	DNA-directed RNA poly
2	1	db$2	2::C3K2X8|EFTU_PSEFS	2466	43536	73	71	25	25	28.08	Elongation factor Tu O
3	1	db$2	2::C3K5G3|C3K5G3_PSEFS	2353	99192	77	71	36	32	6.83	Aconitate hydratase O:
4	1	db$2	2::C3K1A0|CH60_PSEFS	2205	56931	62	61	28	27	20.73	60 kDa chaperonin OS
5	1	db$2	2::C3K2X9|EFG_PSEFS	2165	77114	71	70	35	35	13.97	Elongation factor G OS
6	1	db$2	2::C3K1E6|ATPB_PSEFS	2050	49459	62	61	30	30	54.51	ATP synthase subunit I
7	1	db$2	2::C3K4N0|C3K4N0_PSEFS	2020	43479	62	59	26	24	35.14	Putative ornithine deca
8	1	db$2	2::C3KD82|C3KD82_PSEFS	1980	181591	74	71	57	55	3.94	Glutamate dehydroger
9	1	db$2	2::C3K2Y2|RPOC_PSEFS	1975	154528	93	85	67	63	8.17	DNA-directed RNA poly
10	1	db$2	2::C3K1E8|ATPA_PSEFS	1923	55245	64	62	28	28	27.28	ATP synthase subunit a
11	1	SwissProt_HUMAN$	3::P13645|K1C10_HUMAN	1897	58792	49	46	30	29	17.17	Keratin, type I cytoske
11	2	SwissProt_HUMAN$	3::P02533|K1C14_HUMAN	347	51529	15	12	12	9	1.51	Keratin, type I cytoske
11	3	SwissProt_HUMAN$	3::P08779|K1C16_HUMAN	228	51236	11	8	10	7	1.09	Keratin, type I cytoske
11	4	SwissProt_HUMAN$	3::P13646|K1C13_HUMAN	143	49557	5	5	4	4	0.61	Keratin, type I cytoske
11	5	SwissProt_HUMAN$	3::Q04695|K1C17_HUMAN	124	48076	10	6	8	5	0.64	Keratin, type I cytoske
11	6	SwissProt_HUMAN$	3::P05783|K1C18_HUMAN	40	48029	2	2	2	2	0.22	Keratin, type I cytoske
12	1	db$2	2::C3K255|PNP_PSEFS	1884	74889	58	57	32	32	12.41	Polyribonucleotide nuc
13	1	db$2	2::C3K4D5|C3K4D5_PSEFS	1867	34599	65	61	22	22	38.69	Phosphate-binding pro
14	1	db$2	2::C3K5Z6|EFTS_PSEFS	1826	30341	65	58	16	14	9.32	Elongation factor Ts O:
15	1	SwissProt_HUMAN$	3::P04264|K2C1_HUMAN	1671	65999	43	42	28	28	8.26	Keratin, type II cytosk
15	2	SwissProt_HUMAN$	3::P35908|K22E_HUMAN	983	65393	35	35	27	27	6.62	Keratin, type II cytosk

↑Family	M	DB	Accession	Score	Mass	Matches	Match(sig)	Sequences	Seq(sig)	emPAI	Description
15	3	SwissProt_HUMAN$	3::P13647|K2C5_HUMAN	194	62340	7	7	7	7	0.70	Keratin, type II cytosk
15	4	SwissProt_HUMAN$	3::P02538|K2C6A_HUMAN	172	60008	8	8	8	8	0.88	Keratin, type II cytosk
15	5	SwissProt_HUMAN$	3::P05787|K2C8_HUMAN	95	53671	5	5	5	5	0.55	Keratin, type II cytosk
15	6	SwissProt_HUMAN$	3::P08729|K2C7_HUMAN	75	51354	4	4	4	4	0.45	Keratin, type II cytosk
15	7	SwissProt_HUMAN$	3::Q14CN4|K2C72_HUMAN	58	55842	3	2	3	2	0.18	Keratin, type II cytosk
16	1	db$2	2::C3K6M5|C3K6M5_PSEFS	1536	63522	45	43	24	24	6.51	Succinate dehydrogen
17	1	db$2	2::C3KCP4|C3KCP4_PSEFS	1527	99394	67	63	43	40	12.22	Pyruvate dehydrogena
18	1	db$2	2::C3K6P2|C3K6P2_PSEFS	1424	42870	38	35	16	15	9.16	3-oxoacyl-[acyl-carrier
19	1	db$2	2::C3K0Q5|C3K0Q5_PSEFS	1412	107302	53	49	40	36	4.14	Ribonucleoside-diphos
20	1	db$2	2::C3KCJ5|C3KCJ5_PSEFS	1391	93957	53	51	40	38	6.95	Aconitate hydratase B
21	1	db$2	2::C3K275|DNAK_PSEFS	1348	68158	43	39	25	23	5.11	Chaperone protein Dn
22	1	db$2	2::C3K271|C3K271_PSEFS	1283	117563	47	45	35	33	3.46	Carbamoyl-phosphate
23	1	db$2	2::C3JYT1|SYDND_PSEFS	1237	66220	39	37	24	24	6.97	Aspartate--tRNA(Asp/
24	1	db$2	2::C3KBH1|C3KBH1_PSEFS	1215	161946	54	52	41	41	2.43	Glutamate synthase [
25	1	SwissProt_HUMAN$	3::P35527|K1C9_HUMAN	1213	62027	35	34	25	25	7.49	Keratin, type I cytoske
26	1	db$2	2::C3K6H4|PYRG_PSEFS	1202	59660	38	38	25	25	9.86	CTP synthase OS=Pse
27	1	db$2	2::C3K6N0|SUCC_PSEFS	1188	41262	32	31	18	17	11.42	Succinate--CoA ligase
28	1	db$2	2::C3K5J5|FABV_PSEFS	1164	42933	39	37	27	27	23.36	Enoyl-[acyl-carrier-pro
29	1	db$2	2::C3K7D1|C3K7D1_PSEFS	1160	67115	39	36	29	28	6.75	50S ribosomal subunit
30	1	db$2	2::C3K6M7|C3K6M7_PSEFS	1160	105930	47	43	31	30	3.60	Oxoglutarate dehydrog
31	1	db$2	2::C3K402|C3K402_PSEFS	1132	83101	49	47	28	26	4.87	Polyphosphate kinase
32	1	db$2	2::C3K0P3|C3K0P3_PSEFS	1116	118163	31	31	20	20	1.33	Ribonuclease E OS=Ps
33	1	db$2	2::C3K331|C3K331_PSEFS	1109	73691	46	41	33	32	8.51	AAA_PrkA domain-con
34	1	db$2	2::C3KEC5|C3KEC5_PSEFS	1092	97398	45	43	35	33	5.08	Phosphoenolpyruvate
35	1	db$2	2::C3JYU7|C3JYU7_PSEFS	1046	102460	40	39	27	26	3.21	Protein translocase su
36	1	db$2	2::C3K6N1|C3K6N1_PSEFS	1045	29928	31	30	15	14	22.26	Succinate--CoA ligase
37	1	db$2	2::C3KBY8|C3KBY8_PSEFS	1032	21919	45	42	17	17	72.43	Alkyl hydroperoxide re
38	1	db$2	2::C3K225|ILVC_PSEFS	1029	36188	40	38	21	20	16.67	Ketol-acid reductoisom
39	1	db$2	2::C3JYJ7|C3JYJ7_PSEFS	999	9057	57	50	8	8	260.84	DNA-binding protein H
40	1	db$2	2::C3K7D3|C3K7D3_PSEFS	977	91800	34	32	27	26	3.48	Alpha-1,4 glucan phos
41	1	db$2	2::C3K613|FADB_PSEFS	977	76938	38	35	28	26	3.97	Fatty acid oxidation co
42	1	db$2	2::C3K6M8|C3K6M8_PSEFS	966	42320	26	25	14	14	5.69	Dihydrolipoyllysine-res
43	1	db$2	2::C3K165|C3K165_PSEFS	955	54857	42	37	26	25	14.87	Fumarate hydratase cl
44	1	db$2	2::C3K259|IF2_PSEFS	947	90544	41	34	28	25	3.34	Translation initiation f
45	1	db$2	2::C3K1K1|C3K1K1_PSEFS	937	51928	40	34	23	21	9.70	Inosine-5'-monophosp
46	1	db$2	2::C3K3Z0|C3K3Z0_PSEFS	935	46925	32	30	20	18	8.18	Transcription terminat
47	1	db$2	2::C3K6K0|C3K6K0_PSEFS	930	62268	39	39	23	23	7.43	30S ribosomal protein
48	1	db$2	2::C3JY66|C3JY66_PSEFS	929	80156	37	35	25	23	4.25	Isocitrate dehydrogen
49	1	db$2	2::C3K3F3|C3K3F3_PSEFS	924	72324	44	39	28	26	5.27	Transketolase OS=Pse
50	1	db$2	2::C3KDP2|C3KDP2_PSEFS	909	68879	35	31	24	21	3.87	Multifunctional fusion

↑Family	M	DB	Accession	Score	Mass	Matches	Match(sig)	Sequences	Seq(sig)	emPAI	Description
51	1	db$2	2::C3K673|C3K673_PSEFS	907	47725	38	35	21	19	10.93	Citrate synthase OS=F
52	1	db$2	2::C3KBT0|C3KBT0_PSEFS	903	51607	34	32	17	16	8.04	Succinate-semialdehyc
53	1	db$2	2::C3K1L8|NDK_PSEFS	899	14928	26	25	8	8	15.84	Nucleoside diphosphat
54	1	db$2	2::C3KBT1|C3KBT1_PSEFS	890	44808	32	31	19	19	8.17	4-aminobutyrate amin
55	1	db$2	2::C3JYR1|GCSP_PSEFS	888	102176	36	34	22	21	1.91	Glycine dehydrogenase
56	1	db$2	2::C3K0T0|C3K0T0_PSEFS	858	34560	29	28	10	9	2.93	Putative outer membra
57	1	db$2	2::C3K7G0|C3K7G0_PSEFS	847	36980	24	24	16	16	8.98	Cell shape-determining
58	1	db$2	2::C3JYK1|TIG_PSEFS	847	48316	33	31	22	20	9.51	Trigger factor OS=Pse
59	1	db$2	2::C3KE86|C3KE86_PSEFS	845	95293	37	35	26	26	3.46	Chaperone protein Clp
60	1	db$2	2::C3K4D2|C3K4D2_PSEFS	840	30988	31	27	17	16	23.49	Phosphate import ATP-
61	1	db$2	2::C3KBJ2|C3KBJ2_PSEFS	831	24354	29	28	11	11	21.11	Carbonic anhydrase OS
62	1	db$2	2::C3KCB8|C3KCB8_PSEFS	830	36708	30	28	15	13	10.51	Phosphate-binding pro
63	1	db$2	2::C3K2V9|RS5_PSEFS	828	17675	24	24	11	11	68.71	30S ribosomal protein
64	1	db$2	2::C3K1J2|C3K1J2_PSEFS	827	140531	40	39	33	33	2.50	Phosphoribosylformylg
65	1	db$2	2::C3K367|C3K367_PSEFS	820	68142	33	32	22	22	4.69	Uncharacterized protei
66	1	db$2	2::C3K121|C3K121_PSEFS	817	86030	30	28	23	23	2.76	Phosphoenolpyruvate s
67	1	db$2	2::C3K6M9|C3K6M9_PSEFS	811	49948	26	26	14	14	4.51	Dihydrolipoyl dehydrog
68	1	db$2	2::C3K2D0|C3K2D0_PSEFS	804	61490	31	29	21	21	5.36	Energy-dependent trar
69	1	db$2	2::C3K6N7|HTPG_PSEFS	797	71589	34	34	24	24	4.60	Chaperone protein Htp
70	1	db$2	2::C3KE51|C3KE51_PSEFS	791	38026	31	30	22	21	14.34	Putative oxidoreductas
71	1	db$2	2::C3KAH7|C3KAH7_PSEFS	781	45018	23	22	17	16	6.35	Putative NADP-depend
72	1	db$2	2::C3K162|C3K162_PSEFS	773	52023	25	24	20	19	4.63	Pyruvate kinase OS=P
73	1	db$2	2::C3K0K6|C3K0K6_PSEFS	753	26352	24	24	13	13	19.96	Electron transfer flavo
74	1	db$2	2::C3KE67|RS6_PSEFS	739	16276	26	26	13	13	230.33	30S ribosomal protein
75	1	db$2	2::C3K3F1|PGK_PSEFS	738	40125	20	20	14	14	4.87	Phosphoglycerate kina
76	1	db$2	2::C3K603|C3K603_PSEFS	727	87560	29	27	23	22	2.48	Outer membrane prote
77	1	db$2	2::C3K6J4|C3K6J4_PSEFS	727	97893	36	32	28	25	2.70	DNA gyrase subunit A
78	1	db$2	2::C3K6H6|C3K6H6_PSEFS	724	45588	24	22	17	15	4.26	Enolase OS=Pseudomo
78	2	SwissProt_HUMAN$	3::P06733|ENOA_HUMAN	100	47139	2	2	2	2	0.22	Alpha-enolase OS=Hor
79	1	db$2	2::C3K1W9|C3K1W9_PSEFS	724	33929	17	15	9	8	3.01	Thioredoxin reductase
80	1	db$2	2::C3K3S2|C3K3S2_PSEFS	721	44244	25	25	18	18	5.84	2-oxoglutarate reducta
81	1	db$2	2::C3K1E2|C3K1E2_PSEFS	719	66300	25	23	15	15	2.38	Glutamine--fructose-6-
82	1	db$2	2::C3K4Y4|C3K4Y4_PSEFS	715	71842	26	25	21	21	3.88	Potassium-transporting
83	1	db$2	2::C3K2Y6|RL1_PSEFS	710	24061	22	22	12	12	14.39	50S ribosomal protein
84	1	db$2	2::C3JYS1|C3JYS1_PSEFS	709	17688	24	22	7	7	9.89	Peptidoglycan-associat
85	1	db$2	2::C3K3J7|C3K3J7_PSEFS	705	35008	27	26	13	13	16.05	Glutathione synthetase
86	1	db$2	2::C3K6G5|AMPA_PSEFS	704	52403	29	29	24	24	8.58	Probable cytosol aminc
87	1	db$2	2::C3K4J5|PCKA_PSEFS	703	55324	24	24	19	19	4.55	Phosphoenolpyruvate c
88	1	db$2	2::C3KCP3|C3KCP3_PSEFS	698	56221	24	24	14	14	2.54	Acetyltransferase com
89	1	db$2	2::C3K426|ARLY_PSEFS	694	51545	26	24	15	15	5.29	Argininosuccinate lyase

↑Family	M	DB	Accession	Score	Mass	Matches	Match(sig)	Sequences	Seq(sig)	emPAI	Description
90	1	db$2	2::C3KDU5|C3KDU5_PSEFS	693	90380	23	21	18	16	1.44	DNA gyrase subunit B
91	1	db$2	2::C3K2D3|C3K2D3_PSEFS	693	44901	23	22	15	14	4.39	Serine hydroxymethylt
92	1	db$2	2::C3K4D1|C3K4D1_PSEFS	692	28553	23	23	12	12	13.06	Phosphate-specific trai
93	1	db$2	2::C3K266|C3K266_PSEFS	689	69364	52	51	10	10	1.12	ATP-dependent zinc m
94	1	db$2	2::C3K178|C3K178_PSEFS	686	36139	18	18	11	11	5.22	Glyceraldehyde-3-phos
95	1	db$2	2::C3K2X5|RL4_PSEFS	685	21987	11	11	7	7	4.53	50S ribosomal protein
96	1	db$2	2::C3K4F6|C3K4F6_PSEFS	681	65489	33	29	22	19	4.29	Oxaloacetate decarbox
97	1	db$2	2::C3JXY1|C3JXY1_PSEFS	677	93962	28	24	23	19	1.75	Alanine--tRNA ligase C
98	1	db$2	2::C3JZS1|C3JZS1_PSEFS	676	55264	30	28	18	17	5.59	Amidophosphoribosyltı
99	1	db$2	2::C3KBX8|C3KBX8_PSEFS	671	26859	14	14	8	8	5.88	OmpA-like domain-cor
100	1	db$2	2::C3K1K0|GUAA_PSEFS	668	58398	28	25	24	21	4.96	GMP synthase [glutam
101	1	SwissProt_HUMAN$	3::P02751|FINC_HUMAN	664	272150	34	31	31	29	0.66	Fibronectin OS=Homo
102	1	db$2	2::C3KDW8|PURA_PSEFS	663	46535	25	25	16	16	5.91	Adenylosuccinate synt
103	1	db$2	2::C3JYT4|C3JYT4_PSEFS	650	17756	22	22	11	11	51.17	Putative ferritin DPS-fa
104	1	db$2	2::C3K1A7|C3K1A7_PSEFS	649	66070	26	24	20	19	3.85	Proline--tRNA ligase O
105	1	db$2	2::C3K1E7|ATPG_PSEFS	649	31374	19	17	14	12	6.04	ATP synthase gamma
106	1	db$2	2::C3KEC2|C3KEC2_PSEFS	637	28614	25	22	16	14	9.01	Putative lipoprotein OS
106	2	db$2	2::C3KAJ6|C3KAJ6_PSEFS	165	38502	8	6	6	4	0.85	Histidinol-phosphate a
107	1	db$2	2::C3K139|C3K139_PSEFS	630	48816	24	24	10	10	2.20	Uncharacterized protei
108	1	db$2	2::C3KBZ2|ASSY_PSEFS	626	45330	24	23	17	17	5.56	Argininosuccinate synt
109	1	db$2	2::C3JZN7|SYT_PSEFS	612	72513	26	21	19	18	2.47	Threonine--tRNA ligase
110	1	db$2	2::C3K4D4|C3K4D4_PSEFS	606	83859	32	30	21	21	3.36	Putative phosphate AB
111	1	db$2	2::C3JYS2|TOLB_PSEFS	605	47517	27	25	20	20	8.89	Tol-Pal system protein
112	1	db$2	2::C3JYV2|C3JYV2_PSEFS	603	52688	20	19	14	14	3.60	AAA domain-containin
113	1	db$2	2::C3JYJ9|CLPX_PSEFS	592	46924	27	25	17	15	5.79	ATP-dependent Clp pro
114	1	db$2	2::C3JZT6|C3JZT6_PSEFS	592	40569	17	16	12	11	3.56	Aspartate-semialdehyc
115	1	db$2	2::C3K6I0|C3K6I0_PSEFS	585	39056	20	19	10	10	4.44	Alcohol dehydrogenase
116	1	db$2	2::C3KDW5|C3KDW5_PSEFS	585	43265	24	22	17	16	6.15	Protein HflK OS=Pseuc
117	1	db$2	2::C3KBM0|GLPK_PSEFS	583	55508	30	28	18	16	4.06	Glycerol kinase OS=Ps
118	1	db$2	2::C3K2V2|RS4_PSEFS	582	23062	20	19	12	11	10.51	30S ribosomal protein
119	1	db$2	2::C3K9R0|C3K9R0_PSEFS	581	62265	18	18	14	14	2.13	Pyruvate dehydrogena
120	1	db$2	2::C3KA76|C3KA76_PSEFS	576	30860	17	17	9	9	3.61	UTP--glucose-1-phospl
121	1	db$2	2::C3K5E6|RS2_PSEFS	567	27078	22	21	15	14	15.16	30S ribosomal protein
122	1	db$2	2::C3K1N2|C3K1N2_PSEFS	567	67307	23	22	15	15	2.31	Protein translocase sul
123	1	db$2	2::C3K108|C3K108_PSEFS	566	83591	25	21	22	18	1.78	GTP pyrophosphokinas
124	1	db$2	2::C3JYQ6|C3JYQ6_PSEFS	565	37842	22	20	12	11	7.35	Ornithine carbamoyltra
125	1	db$2	2::C3KDG0|RECA_PSEFS	564	37499	23	21	16	14	7.51	Protein RecA OS=Pseu
126	1	db$2	2::C3KAK3|C3KAK3_PSEFS	557	54608	20	19	16	15	3.01	Probable malate:quino
127	1	db$2	2::C3JZ70|C3JZ70_PSEFS	556	54858	27	24	19	18	4.16	Glucose-6-phosphate :
127	2	SwissProt_HUMAN$	3::P11413|G6PD_HUMAN	35	59219	2	2	2	2	0.17	Glucose-6-phosphate :

↑Family	M	DB	Accession	Score	Mass	Matches	Match(sig)	Sequences	Seq(sig)	emPAI	Description
128	1	db$2	2::C3KDU7|C3KDU7_PSEFS	551	28504	22	20	14	13	7.56	Putative ABC transport
129	1	db$2	2::C3K2M9|PROA_PSEFS	551	45380	16	16	10	10	2.49	Gamma-glutamyl phos
130	1	db$2	2::C3K6W9|C3K6W9_PSEFS	547	69415	16	16	14	14	1.60	Putative methyl-accept
131	1	db$2	2::C3JYQ5|C3JYQ5_PSEFS	547	46409	22	21	17	16	5.27	Arginine deiminase OS
132	1	db$2	2::C3KDR8|OPGD_PSEFS	542	60485	22	20	16	15	2.50	Glucans biosynthesis p
133	1	db$2	2::C3K3E8|C3K3E8_PSEFS	542	38385	26	25	17	17	13.99	Fructose-1,6-bisphosp
134	1	db$2	2::C3K2W6|RL14_PSEFS	541	13401	17	17	9	9	63.09	50S ribosomal protein
135	1	db$2	2::C3KDD8|C3KDD8_PSEFS	538	44893	29	28	20	19	7.25	Cell division protein Ft
136	1	db$2	2::C3K7Y0|C3K7Y0_PSEFS	538	45268	21	20	15	14	4.91	Putative exported prot
137	1	db$2	2::C3K3G6|SAHH_PSEFS	538	51212	25	24	19	18	6.66	Adenosylhomocysteina
138	1	db$2	2::C3K244|C3K244_PSEFS	537	52276	16	16	14	14	2.55	Putative response regu
139	1	db$2	2::C3K2Y4|RL7_PSEFS	537	12456	14	14	2	2	5.48	50S ribosomal protein
140	1	db$2	2::C3K4C6|C3K4C6_PSEFS	532	25565	18	18	8	8	5.32	Phosphate regulon tra
141	1	db$2	2::C3KCS1|C3KCS1_PSEFS	530	34171	22	21	12	12	9.46	Ribose-phosphate pyro
142	1	db$2	2::C3JXL8|C3JXL8_PSEFS	528	112023	22	21	17	17	1.24	Putative efflux protein
143	1	db$2	2::C3K2V6|C3K2V6_PSEFS	527	47842	17	16	8	7	1.69	Protein translocase sul
144	1	db$2	2::C3K4F7|C3K4F7_PSEFS	525	51847	29	26	23	21	6.46	Biotin carboxylase OS=
144	2	db$2	2::C3K282|SSRP_PSEFS	60	18308	6	5	6	5	2.59	SsrA-binding protein C
145	1	db$2	2::C3K6G0|C3K6G0_PSEFS	523	9680	12	11	7	6	16.60	Putative DNA-binding p
146	1	db$2	2::C3KE40|SYGB_PSEFS	523	74833	28	27	19	18	2.79	Glycine--tRNA ligase b
147	1	db$2	2::C3K7D6|F16PA_PSEFS	522	37081	19	18	13	13	4.95	Fructose-1,6-bisphosp
148	1	db$2	2::C3K016|C3K016_PSEFS	522	85788	22	19	20	17	1.56	Ribosomal RNA large s
149	1	db$2	2::C3KDU3|C3KDU3_PSEFS	520	40518	22	19	16	14	4.12	Beta sliding clamp OS=
150	1	db$2	2::C3K914|C3K914_PSEFS	520	38509	16	16	11	11	3.93	Putative ABC transport
151	1	db$2	2::C3K1G5|C3K1G5_PSEFS	515	46195	16	15	13	13	2.79	Homoserine dehydroge
152	1	db$2	2::C3K2R9|C3K2R9_PSEFS	511	16440	11	10	7	6	6.33	6,7-dimethyl-8-ribityllu
153	1	db$2	2::C3K3F5|METK_PSEFS	509	42554	20	17	14	12	3.75	S-adenosylmethionine
154	1	db$2	2::C3K6H5|KDSA_PSEFS	508	30777	27	26	16	15	33.06	2-dehydro-3-deoxypho
155	1	db$2	2::C3K3P0|ILVD_PSEFS	506	65667	16	16	15	15	1.95	Dihydroxy-acid dehydr
156	1	db$2	2::C3K9R4|C3K9R4_PSEFS	506	62164	18	18	17	17	2.66	Putative sulfite reducta
157	1	db$2	2::C3K3R2|C3K3R2_PSEFS	500	83481	26	25	20	20	2.30	Phosphoenolpyruvate-
158	1	db$2	2::C3K6X4|C3K6X4_PSEFS	498	71161	17	17	16	16	1.91	Biosynthetic arginine c
159	1	db$2	2::C3JY65|C3JY65_PSEFS	497	45375	23	20	17	14	5.53	Isocitrate dehydrogena
160	1	db$2	2::C3JZV2|C3JZV2_PSEFS	497	43020	18	18	13	13	3.65	Aminotransferase OS=
161	1	db$2	2::C3JZN2|C3JZN2_PSEFS	496	86329	22	21	19	18	1.69	Phenylalanine--tRNA li
162	1	db$2	2::C3JZN3|SYFA_PSEFS	495	37892	16	16	13	13	4.74	Phenylalanine--tRNA li
163	1	db$2	2::C3K260|C3K260_PSEFS	493	54381	14	13	12	11	1.61	Transcription terminati
164	1	db$2	2::C3JYJ8|C3JYJ8_PSEFS	490	88851	24	22	23	21	2.07	Lon protease OS=Pseu
165	1	db$2	2::C3K2V1|C3K2V1_PSEFS	489	36623	29	23	22	19	12.19	DNA-directed RNA poly
166	1	db$2	2::C3K4C7|C3K4C7_PSEFS	489	50044	11	11	10	10	1.83	Phosphate regulon sen

↑Family	M	DB	Accession	Score	Mass	Matches	Match(sig)	Sequences	Seq(sig)	emPAI	Description
167	1	db$2	2::C3K1X6|C3K1X6_PSEFS	487	44711	13	12	11	10	1.88	GTPase Obg OS=Pseu
168	1	db$2	2::C3K865|C3K865_PSEFS	487	15847	11	11	5	5	6.85	Putative membrane pr
169	1	db$2	2::C3K6M2|C3K6M2_PSEFS	487	60584	17	16	15	14	2.23	ATP-dependent RNA he
170	1	db$2	2::C3K616|C3K616_PSEFS	478	97222	28	26	22	20	1.79	DNA topoisomerase 1
171	1	db$2	2::C3K6C2|C3K6C2_PSEFS	478	27720	14	14	10	10	5.47	Putative amino acid AE
172	1	db$2	2::C3KE65|C3KE65_PSEFS	477	97331	22	19	19	16	1.29	Ribonuclease R OS=Ps
173	1	db$2	2::C3JYA7|C3JYA7_PSEFS	475	59461	17	16	14	14	2.05	Putative NADP-depend
174	1	db$2	2::C3KE70|RL9_PSEFS	472	15488	15	15	8	8	13.96	50S ribosomal protein
175	1	db$2	2::C3K1M5|ISCS_PSEFS	471	44467	13	13	11	11	2.58	Cysteine desulfurase I
176	1	db$2	2::C3K6M6|C3K6M6_PSEFS	470	26063	21	18	12	10	9.45	Succinate dehydrogena
177	1	db$2	2::C3KBH2|C3KBH2_PSEFS	464	51951	14	13	10	9	1.49	Glutamate synthase [N
178	1	db$2	2::C3K4V9|C3K4V9_PSEFS	463	53185	16	16	14	14	3.16	Glyceraldehyde-3-phos
179	1	db$2	2::C3K4D3|C3K4D3_PSEFS	458	60551	18	17	14	14	2.23	Phosphate transport sy
180	1	SwissProt_HUMAN$	3::P00761|TRYP_PIG	458	24394	13	12	7	7	4.66	Trypsin OS=Sus scrofa
181	1	db$2	2::C3JY70|C3JY70_PSEFS	458	50668	20	19	14	14	3.47	Adenylosuccinate lyase
182	1	db$2	2::C3K341|C3K341_PSEFS	454	69250	23	22	16	16	2.67	RNA polymerase sigma
182	2	db$2	2::C3K708|C3K708_PSEFS	79	38066	3	3	3	3	0.45	RNA polymerase sigma
183	1	db$2	2::C3KDD9|C3KDD9_PSEFS	452	41969	13	13	13	13	3.33	Cell division protein Ft
184	1	db$2	2::C3K0D4|C3K0D4_PSEFS	451	29228	17	17	12	12	5.94	Cell division inhibitor M
185	1	db$2	2::C3K2X0|C3K2X0_PSEFS	450	25737	20	17	13	10	9.76	30S ribosomal protein
186	1	db$2	2::C3K3Z1|C3K3Z1_PSEFS	449	11808	13	13	6	6	22.48	Thioredoxin OS=Pseuc
187	1	db$2	2::C3JZV8|C3JZV8_PSEFS	448	38906	13	12	9	8	1.98	Neutral metalloprotein
188	1	db$2	2::C3K325|C3K325_PSEFS	446	49076	18	17	14	13	2.86	Chaperone SurA OS=P
189	1	db$2	2::C3K1B7|C3K1B7_PSEFS	445	51892	23	22	15	14	3.31	Threonine synthase OS
190	1	db$2	2::C3KEC8|C3KEC8_PSEFS	437	50623	19	15	11	8	1.55	Periplasmic serine end
191	1	db$2	2::C3K6E1|RL13_PSEFS	435	15695	16	14	9	8	13.68	50S ribosomal protein
192	1	db$2	2::C3K569|C3K569_PSEFS	434	44415	16	15	12	11	2.59	Tryptophan synthase b
193	1	db$2	2::C3K5E0|C3K5E0_PSEFS	434	36000	12	12	9	9	3.24	2,3,4,5-tetrahydropyri
194	1	db$2	2::C3K358|C3K358_PSEFS	434	65409	18	15	14	12	1.57	Putative dehydrogenas
194	2	db$2	2::C3K355|C3K355_PSEFS	367	63710	14	13	12	11	1.27	Putative dehydrogenas
195	1	db$2	2::C3KAA8|C3KAA8_PSEFS	434	132643	14	14	13	13	0.59	Histidine kinase OS=P
196	1	db$2	2::C3KEB4|C3KEB4_PSEFS	433	56978	18	15	16	13	2.21	Lysine--tRNA ligase OS
197	1	SwissProt_HUMAN$	3::P02768|ALBU_HUMAN	432	69321	23	22	19	18	2.42	Albumin OS=Homo sa
197	2	SwissProt_HUMAN$	3::P02769|ALBU_BOVIN	361	69248	16	16	15	15	1.79	Albumin OS=Bos tauru
198	1	db$2	2::C3K1F0|C3K1F0_PSEFS	430	16832	19	18	10	10	15.00	ATP synthase subunit
199	1	db$2	2::C3K1L4|ISPG_PSEFS	430	39660	21	19	17	16	7.56	4-hydroxy-3-methylbu
200	1	db$2	2::C3K2W1|RL6_PSEFS	430	19185	14	13	6	6	6.05	50S ribosomal protein
201	1	db$2	2::C3JXZ7|C3JXZ7_PSEFS	429	28415	17	16	9	9	4.25	Putative histidine-bind
202	1	db$2	2::C3KBN3|C3KBN3_PSEFS	428	112655	15	15	11	11	0.59	Efflux pump membran
202	2	db$2	2::C3KB18|C3KB18_PSEFS	88	115005	3	3	3	3	0.13	Efflux pump membran

↑Family	M	DB	Accession	Score	Mass	Matches	Match(sig)	Sequences	Seq(sig)	emPAI	Description
203	1	db$2	2::C3K3H0|C3K3H0_PSEFS	427	67796	17	15	13	11	1.16	ATP-dependent RNA he
204	1	SwissProt_HUMAN$	3::P60709|ACTB_HUMAN	426	41710	16	15	13	12	2.89	Actin, cytoplasmic 1 O
205	1	db$2	2::C3K2U7|C3K2U7_PSEFS	425	104087	22	18	20	17	1.17	UvrABC system proteir
206	1	db$2	2::C3K8B0|C3K8B0_PSEFS	424	25585	15	14	8	8	6.60	Peptidyl-prolyl cis-tran
207	1	db$2	2::C3JXY0|AK_PSEFS	419	44504	19	18	14	14	4.48	Aspartate kinase OS=I
208	1	db$2	2::C3K6W6|C3K6W6_PSEFS	418	49345	24	21	18	17	5.18	Biotin carboxylase OS=
209	1	db$2	2::C3K8X4|MURA_PSEFS	418	44975	23	19	16	14	4.98	UDP-N-acetylglucosam
210	1	db$2	2::C3K6H2|ACCA_PSEFS	417	34868	18	15	14	11	4.82	Acetyl-coenzyme A car
211	1	db$2	2::C3K6G2|C3K6G2_PSEFS	417	106936	23	21	21	19	1.33	Valine--tRNA ligase OS
212	1	db$2	2::C3K227|C3K227_PSEFS	416	62578	20	20	15	15	2.62	Acetolactate synthase
213	1	db$2	2::C3KCB5|C3KCB5_PSEFS	414	30173	23	20	12	10	4.57	Phosphate import ATP-
214	1	db$2	2::C3K484|C3K484_PSEFS	413	13502	14	14	10	10	42.95	Uncharacterized protei
215	1	db$2	2::C3KDP1|CYSD_PSEFS	412	35173	15	15	7	7	4.01	Sulfate adenylyltransfe
216	1	db$2	2::C3K7C9|C3K7C9_PSEFS	409	51641	20	17	15	12	2.96	Glutamine synthetase
217	1	db$2	2::C3K2Y7|RL11_PSEFS	407	14903	8	8	3	3	2.51	50S ribosomal protein
218	1	db$2	2::C3K2X6|RL3_PSEFS	404	22550	13	11	9	8	4.30	50S ribosomal protein
219	1	db$2	2::C3K3N4|COAD_PSEFS	401	17810	15	14	7	7	9.73	Phosphopantetheine a
220	1	db$2	2::C3K2M1|C3K2M1_PSEFS	401	42033	13	13	11	11	2.85	Serine-type D-Ala-D-A
221	1	db$2	2::C3KAD2|ARNA_PSEFS	400	74140	17	16	14	13	1.45	Bifunctional polymyxin
222	1	db$2	2::C3K1N3|C3K1N3_PSEFS	396	11888	8	8	5	5	6.06	Sec translocon accesso
223	1	db$2	2::C3KAL6|C3KAL6_PSEFS	395	52109	16	15	14	13	2.57	Putative aminotransfer
224	1	db$2	2::C3K7X9|C3K7X9_PSEFS	395	40858	14	13	10	10	3.01	Carboxynorspermidine
225	1	db$2	2::C3K2X3|RL2_PSEFS	389	29689	18	18	13	13	8.27	50S ribosomal protein
226	1	db$2	2::C3K2X1|RL22_PSEFS	388	11904	11	11	8	8	21.81	50S ribosomal protein
227	1	db$2	2::C3KE61|RIMK_PSEFS	387	32488	9	9	5	5	1.76	Probable alpha-L-gluta
228	1	db$2	2::C3K2V7|RL15_PSEFS	386	15215	12	12	8	8	28.29	50S ribosomal protein
229	1	db$2	2::C3K0N2|C3K0N2_PSEFS	383	43313	11	11	9	9	1.67	3-oxoacyl-[acyl-carrier
230	1	db$2	2::C3K3U2|C3K3U2_PSEFS	383	50985	18	15	13	12	2.05	Putative glutamine syr
231	1	db$2	2::C3KBL8|C3KBL8_PSEFS	383	56970	20	17	17	15	2.48	Glycerol-3-phosphate
232	1	db$2	2::C3JZS4|C3JZS4_PSEFS	379	33375	15	15	11	11	5.27	Acetyl-coenzyme A car
233	1	db$2	2::C3JZN6|IF3_PSEFS	377	20837	13	13	6	6	3.83	Translation initiation fa
234	1	db$2	2::C3JY74|C3JY74_PSEFS	377	48472	15	13	14	12	2.23	Isocitrase OS=Pseudor
235	1	SwissProt_HUMAN$	3::P07355|ANXA2_HUMAN	376	38580	13	12	13	12	3.34	Annexin A2 OS=Homo
236	1	db$2	2::C3K0H1|C3K0H1_PSEFS	376	17848	11	11	7	7	7.14	Chemotaxis protein Ch
237	1	db$2	2::C3K0Q7|C3K0Q7_PSEFS	375	45255	10	9	6	6	1.08	Flagellar hook protein
238	1	db$2	2::C3K8X5|HIS1_PSEFS	375	23098	15	15	10	10	10.51	ATP phosphoribosyltra
239	1	db$2	2::C3KA80|C3KA80_PSEFS	374	20438	12	12	8	8	6.91	Alkyl hydroperoxide re
240	1	db$2	2::C3K2C4|C3K2C4_PSEFS	374	89060	13	13	12	12	0.90	Endopeptidase La OS=
241	1	db$2	2::C3K5S8|C3K5S8_PSEFS	370	75646	15	15	12	12	1.41	Oligopeptidase A OS=I
242	1	db$2	2::C3KED7|PUR7_PSEFS	369	26862	17	15	14	12	7.20	Phosphoribosylaminoir

↑Family	M	DB	Accession	Score	Mass	Matches	Match(sig)	Sequences	Seq(sig)	emPAI	Description
243	1	db$2	2::C3K9U6|C3K9U6_PSEFS	368	35232	11	11	8	8	2.35	Putative 6-phosphoglu
244	1	db$2	2::C3K614|C3K614_PSEFS	367	41572	14	14	11	11	3.38	3-ketoacyl-CoA thiolas
245	1	db$2	2::C3K649|C3K649_PSEFS	367	8822	12	11	4	4	7.16	Major outer membrane
246	1	db$2	2::C3K5H9|C3K5H9_PSEFS	366	33776	14	12	12	10	3.66	Transaldolase OS=Pse
247	1	db$2	2::C3K6J9|KCY_PSEFS	366	24780	9	9	8	8	3.58	Cytidylate kinase OS=
248	1	db$2	2::C3K1F4|C3K1F4_PSEFS	364	31961	9	9	7	7	1.81	Probable chromosome-
249	1	db$2	2::C3K4W1|STHA_PSEFS	363	50963	23	18	19	15	3.42	Soluble pyridine nucle
250	1	db$2	2::C3K8X0|C3K8X0_PSEFS	363	16351	11	11	7	7	8.90	Putative ABC transport
251	1	db$2	2::C3K263|C3K263_PSEFS	361	26068	10	10	7	7	2.54	Triosephosphate isome
252	1	db$2	2::C3K2H6|C3K2H6_PSEFS	359	32512	8	8	4	4	1.07	Acyl-CoA thioesterase
253	1	db$2	2::C3KBN2|C3KBN2_PSEFS	358	52689	9	9	8	8	1.24	Outer membrane prote
254	1	db$2	2::C3KBY6|C3KBY6_PSEFS	354	17850	16	15	10	10	22.20	Bacterioferritin OS=Ps
255	1	db$2	2::C3JXZ0|C3JXZ0_PSEFS	354	43332	17	16	14	13	3.62	Acetylornithine aminot
256	1	db$2	2::C3K443|C3K443_PSEFS	353	12323	14	14	9	9	42.45	Nitrogen regulatory pr
257	1	db$2	2::C3K2W4|RL5_PSEFS	353	20490	16	14	9	9	8.83	50S ribosomal protein
258	1	db$2	2::C3K5Z7|C3K5Z7_PSEFS	351	26574	15	15	10	10	11.00	Uridylate kinase OS=P
259	1	db$2	2::C3K3V2|C3K3V2_PSEFS	350	49111	16	15	9	9	1.62	Putative outer membra
260	1	db$2	2::C3K226|C3K226_PSEFS	350	17820	9	9	7	7	5.26	Acetolactate synthase
261	1	db$2	2::C3K4K9|C3K4K9_PSEFS	349	27377	12	11	6	6	2.33	Putative amino acid ox
262	1	db$2	2::C3KCN5|C3KCN5_PSEFS	348	142346	15	13	15	13	0.54	Bifunctional protein Pu
263	1	db$2	2::C3K6R4|C3K6R4_PSEFS	348	18727	14	13	7	7	8.47	Uncharacterized protei
264	1	db$2	2::C3JY85|C3JY85_PSEFS	348	48759	16	14	14	12	2.21	NADH-quinone oxidore
265	1	db$2	2::C3K1Y2|C3K1Y2_PSEFS	343	21662	11	10	8	7	6.05	Peptidyl-prolyl cis-tran
266	1	db$2	2::C3K474|C3K474_PSEFS	340	50732	14	11	12	10	1.79	Putative phosphomanr
267	1	db$2	2::C3K1K7|LEU1_PSEFS	339	61997	19	17	17	15	2.15	2-isopropylmalate synt
268	1	db$2	2::C3KBX9|C3KBX9_PSEFS	339	20403	7	7	4	4	2.15	Putative phospholipid-l
269	1	db$2	2::C3K2J8|C3K2J8_PSEFS	339	54392	14	14	11	11	1.84	AMP nucleosidase OS=
270	1	db$2	2::C3KCB7|C3KCB7_PSEFS	337	34191	8	7	4	3	0.74	Phosphate transport sy
271	1	db$2	2::C3K476|PYRE_PSEFS	337	23079	7	7	6	6	2.39	Orotate phosphoribosy
272	1	db$2	2::C3K2T2|C3K2T2_PSEFS	336	24648	8	8	5	5	2.81	Putative ABC transport
273	1	db$2	2::C3KCR8|C3KCR8_PSEFS	336	39907	11	11	6	6	1.29	Ribosome-binding ATP
274	1	db$2	2::C3JY83|NUOCD_PSEFS	334	67660	27	22	21	19	3.35	NADH-quinone oxidore
275	1	db$2	2::C3JZV0|C3JZV0_PSEFS	334	56140	13	13	13	13	1.99	Glutamate--tRNA ligas
276	1	db$2	2::C3K2V0|RL17_PSEFS	333	14407	13	13	7	7	33.99	50S ribosomal protein
277	1	db$2	2::C3K1H6|PURT_PSEFS	330	42327	14	14	13	13	3.79	Formate-dependent ph
278	1	db$2	2::C3JZT7|C3JZT7_PSEFS	329	38793	12	12	10	10	2.83	3-isopropylmalate deh
279	1	db$2	2::C3KDL2|C3KDL2_PSEFS	329	52736	10	10	10	10	1.46	Putative outer membra
280	1	db$2	2::C3KBI6|C3KBI6_PSEFS	329	106629	15	13	14	12	0.71	Uncharacterized protei
281	1	db$2	2::C3K2W0|RL18_PSEFS	327	12651	11	11	6	6	12.10	50S ribosomal protein
282	1	db$2	2::C3K2Y5|RL10_PSEFS	327	17594	10	9	6	6	5.40	50S ribosomal protein

↑Family	M	DB	Accession	Score	Mass	Matches	Match(sig)	Sequences	Seq(sig)	emPAI	Description
283	1	db$2	2::C3JYD5|C3JYD5_PSEFS	326	18453	10	9	6	6	3.60	Peptidyl-prolyl cis-tran
284	1	db$2	2::C3K401|C3K401_PSEFS	325	56255	20	17	16	15	2.54	Exopolyphosphatase O
285	1	db$2	2::C3K1E9|ATPD_PSEFS	324	19246	16	15	9	8	6.05	ATP synthase subunit
286	1	db$2	2::C3K466|C3K466_PSEFS	324	52681	14	14	9	9	1.46	Putative aldehyde deh
287	1	db$2	2::C3K475|ARGB_PSEFS	324	31926	11	11	10	10	3.37	Acetylglutamate kinase
288	1	db$2	2::C3K638|C3K638_PSEFS	324	26394	7	7	3	3	1.44	Putative short chain de
289	1	db$2	2::C3K3V3|ARGA_PSEFS	323	47793	9	9	8	8	1.21	Amino-acid acetyltrans
290	1	db$2	2::C3KEA4|C3KEA4_PSEFS	323	51722	14	11	10	9	1.74	PINc domain-containin
291	1	db$2	2::C3JXZ8|C3JXZ8_PSEFS	323	71568	19	18	16	15	1.70	Acetyl-coenzyme A syr
292	1	db$2	2::C3K6D9|C3K6D9_PSEFS	321	45394	11	9	8	7	1.30	Phosphoribosylamine--
293	1	db$2	2::C3K8V3|HSLU_PSEFS	321	49607	17	15	13	12	2.14	ATP-dependent protea
294	1	db$2	2::C3KBN4|C3KBN4_PSEFS	321	40532	11	11	10	10	2.61	Acriflavine resistance
295	1	db$2	2::C3K3M4|C3K3M4_PSEFS	320	49883	8	8	8	8	1.14	Signal recognition part
296	1	db$2	2::C3JXL7|C3JXL7_PSEFS	320	39112	13	13	11	11	2.78	HlyD-family secretion
297	1	db$2	2::C3K2K3|GSA_PSEFS	318	45181	13	12	9	8	1.57	Glutamate-1-semialde
298	1	db$2	2::C3K123|C3K123_PSEFS	317	41542	10	10	8	8	1.49	Citrate synthase OS=F
299	1	db$2	2::C3JYW3|C3JYW3_PSEFS	316	36818	14	13	11	10	3.10	Branched-chain-amino
300	1	db$2	2::C3K0K5|C3K0K5_PSEFS	314	31365	11	10	6	6	2.32	Electron transfer flavo
301	1	db$2	2::C3K3A2|C3K3A2_PSEFS	314	38105	11	10	7	7	1.38	L-threonine aldolase O
302	1	db$2	2::C3K0N5|C3K0N5_PSEFS	313	32402	10	10	5	5	1.40	Malonyl CoA-acyl carri
303	1	db$2	2::C3JY86|C3JY86_PSEFS	312	98164	19	16	15	14	0.97	NADH-quinone oxidore
304	1	db$2	2::C3K7N8|RDGC_PSEFS	312	34160	16	15	12	12	5.02	Recombination-associa
305	1	db$2	2::C3K3K2|PYRB_PSEFS	311	36271	8	7	7	6	1.18	Aspartate carbamoyltr
306	1	db$2	2::C3K1L0|C3K1L0_PSEFS	311	55309	15	15	15	15	2.62	GTPase Der OS=Pseud
307	1	SwissProt_HUMAN$	3::P01871|IGHM_HUMAN	310	49409	13	12	9	9	1.61	Immunoglobulin heavy
308	1	db$2	2::C3K3Q2|C3K3Q2_PSEFS	309	37335	11	10	5	5	1.14	Putative cation ABC tra
309	1	db$2	2::C3K4V8|C3K4V8_PSEFS	308	128447	9	9	9	9	0.40	Transcription-repair-cc
310	1	db$2	2::C3K3P4|C3K3P4_PSEFS	307	39333	14	14	8	8	1.95	Uncharacterized protei
311	1	db$2	2::C3K2Y8|C3K2Y8_PSEFS	306	20023	13	12	9	8	7.27	Transcription terminati
312	1	db$2	2::C3K5Y0|C3K5Y0_PSEFS	303	38747	14	14	11	11	3.32	NADP(H)-dependt aldc
313	1	db$2	2::C3K907|C3K907_PSEFS	302	77648	16	13	16	13	1.21	Tail-specific protease (
314	1	db$2	2::C3K902|RAPA_PSEFS	302	106722	12	11	10	9	0.49	RNA polymerase-assoc
315	1	db$2	2::C3K734|C3K734_PSEFS	302	44623	9	9	6	6	1.34	Putative aminotransfer
316	1	db$2	2::C3K5I7|C3K5I7_PSEFS	302	59867	11	11	8	8	1.39	Probable malate:quino
317	1	db$2	2::C3K6J7|C3K6J7_PSEFS	302	39784	10	10	9	9	1.92	Histidinol-phosphate a
318	1	db$2	2::C3K5Z8|RRF_PSEFS	301	20297	12	12	7	7	7.00	Ribosome-recycling fac
319	1	db$2	2::C3K7L5|C3K7L5_PSEFS	300	19131	9	9	6	6	4.58	Molybdenum cofactor l
320	1	db$2	2::C3KDM2|C3KDM2_PSEFS	300	83508	14	14	14	14	1.22	DNA topoisomerase 4
321	1	db$2	2::C3K667|C3K667_PSEFS	300	48557	12	12	9	9	1.65	Conserved hypothetica
322	1	db$2	2::C3KCS2|RSMH_PSEFS	299	34344	11	11	9	9	2.45	Ribosomal RNA small s

↑Family	M	DB	Accession	Score	Mass	Matches	Match(sig)	Sequences	Seq(sig)	emPAI	Description
323	1	db$2	2::C3K1F2|ATP6_PSEFS	296	31626	8	8	3	3	0.56	ATP synthase subunit a
324	1	db$2	2::C3JZS0|C3JZS0_PSEFS	296	43144	16	16	14	14	4.17	O-succinylhomoserine
325	1	db$2	2::C3K403|C3K403_PSEFS	296	36964	10	10	7	7	1.78	Delta-aminolevulinic a
326	1	db$2	2::C3K0N4|C3K0N4_PSEFS	296	25612	8	7	6	6	2.61	3-oxoacyl-[acyl-carrier
327	1	db$2	2::C3JZ61|C3JZ61_PSEFS	294	61509	16	14	13	11	1.33	Acyl-CoA synthetase (I
328	1	db$2	2::C3K490|C3K490_PSEFS	294	33505	14	13	10	10	3.71	Putative oxidative stre
329	1	db$2	2::C3K2S6|C3K2S6_PSEFS	293	31662	8	8	7	7	1.84	Putative thioredoxin O
330	1	db$2	2::C3K7M6|C3K7M6_PSEFS	292	88918	10	9	8	7	0.45	Acyl-coenzyme A dehy
331	1	db$2	2::C3JYR3|C3JYR3_PSEFS	290	40135	14	14	10	10	2.65	Glycine cleavage syste
332	1	db$2	2::C3KAR0|C3KAR0_PSEFS	290	79563	11	9	8	6	0.52	Putative exported isoq
333	1	db$2	2::C3K7G9|C3K7G9_PSEFS	290	48266	11	9	8	8	1.19	Putative modulation of
334	1	db$2	2::C3K5A9|RF3_PSEFS	290	59584	12	10	9	7	0.89	Peptide chain release f
335	1	db$2	2::C3K645|C3K645_PSEFS	289	24522	9	8	7	6	2.16	Putative ABC transport
336	1	db$2	2::C3K0W7|C3K0W7_PSEFS	288	31068	11	11	8	8	2.37	Metallophos_2 domain
337	1	db$2	2::C3K142|C3K142_PSEFS	288	32099	10	10	8	8	2.76	2-methylisocitrate lyas
338	1	db$2	2::C3K7C1|SECB_PSEFS	288	17615	6	6	4	4	1.89	Protein-export protein
339	1	db$2	2::C3K604|C3K604_PSEFS	287	18933	18	16	14	13	30.73	Putative outer membra
340	1	db$2	2::C3KAI6|C3KAI6_PSEFS	285	24263	8	8	5	5	1.63	Putative dehydrogenas
341	1	db$2	2::C3K4M6|C3K4M6_PSEFS	284	57818	21	16	15	12	2.15	Putative exported alka
342	1	db$2	2::C3K187|C3K187_PSEFS	284	38556	14	13	11	10	2.86	Peptidase_M75 domair
343	1	db$2	2::C3K8U4|UBIE_PSEFS	284	28320	17	16	14	13	7.71	Ubiquinone/menaquinc
344	1	db$2	2::C3K1X9|C3K1X9_PSEFS	283	34909	12	12	10	10	2.88	Octaprenyl-diphosphat
345	1	db$2	2::C3KBQ2|C3KBQ2_PSEFS	283	37020	10	10	7	7	1.77	Putative quinone oxidc
346	1	db$2	2::C3JYQ7|C3JYQ7_PSEFS	282	32635	10	9	7	6	1.75	Carbamate kinase OS=
347	1	db$2	2::C3KDX4|SYI_PSEFS	282	105386	20	17	17	14	0.97	Isoleucine--tRNA ligas
348	1	db$2	2::C3K435|C3K435_PSEFS	281	45402	13	12	11	10	2.15	Diaminopimelate decai
349	1	db$2	2::C3K9T7|C3K9T7_PSEFS	281	99328	14	14	12	12	0.77	Aminopeptidase N OS=
350	1	SwissProt_HUMAN$	3::P02679|FIBG_HUMAN	281	51479	15	14	10	10	1.75	Fibrinogen gamma cha
351	1	db$2	2::C3JYS5|C3JYS5_PSEFS	281	25332	6	6	4	4	1.53	Tol-Pal system protein
352	1	db$2	2::C3K8X6|C3K8X6_PSEFS	280	47255	9	8	8	7	1.02	Histidinol dehydrogena
353	1	db$2	2::C3K2K9|C3K2K9_PSEFS	280	31417	13	12	9	9	2.86	Magnesium and cobalt
354	1	db$2	2::C3JY00|C3JY00_PSEFS	277	47211	9	9	8	8	1.23	Ribonucleoside-diphos
355	1	db$2	2::C3K6X1|C3K6X1_PSEFS	275	39290	9	8	7	6	1.06	Uncharacterized protei
356	1	db$2	2::C3K6H1|C3K6H1_PSEFS	273	131195	12	11	11	10	0.44	DNA polymerase III su
357	1	db$2	2::C3K6A2|C3K6A2_PSEFS	273	27847	13	11	10	9	3.58	Putative D-methionine
357	2	db$2	2::C3KD95|C3KD95_PSEFS	83	28020	5	5	4	4	0.96	Putative exported prot
358	1	db$2	2::C3KDE0|LPXC_PSEFS	271	33197	10	10	8	8	2.60	UDP-3-O-acyl-N-acety
359	1	db$2	2::C3KAC1|C3KAC1_PSEFS	269	27548	14	14	10	10	6.80	Putative phenylacetic a
360	1	db$2	2::C3K747|GPDA_PSEFS	269	36476	10	10	8	8	2.65	Glycerol-3-phosphate (
361	1	db$2	2::C3K9U7|C3K9U7_PSEFS	267	56147	9	9	9	9	1.14	Glucose-6-phosphate 1

↑Family	M	DB	Accession	Score	Mass	Matches	Match(sig)	Sequences	Seq(sig)	emPAI	Description
362	1	db$2	2::C3KDB1|C3KDB1_PSEFS	267	65565	16	15	14	13	1.56	Putative carbamoyltrar
363	1	SwissProt_HUMAN$	3::P01834|IGKC_HUMAN	267	11758	8	8	5	5	6.19	Immunoglobulin kappa
364	1	db$2	2::C3K1L3|SYH_PSEFS	266	47853	11	11	10	10	1.97	Histidine--tRNA ligase
365	1	db$2	2::C3K6E0|PUR9_PSEFS	265	57370	14	14	12	12	1.93	Bifunctional purine bio
366	1	db$2	2::C3JYT9|C3JYT9_PSEFS	264	46635	7	7	5	5	0.66	Putative family S43 no
367	1	db$2	2::C3K2N8|C3K2N8_PSEFS	263	23444	13	13	10	10	8.10	Semialdhyde_dh doma
368	1	db$2	2::C3K4V4|C3K4V4_PSEFS	263	32182	11	10	9	9	2.74	Putative ATP-binding p
369	1	db$2	2::C3K324|C3K324_PSEFS	262	105271	18	17	17	16	1.06	LPS-assembly protein
370	1	db$2	2::C3K091|C3K091_PSEFS	261	20089	8	8	6	6	3.06	GTP cyclohydrolase 1 (
370	2	db$2	2::C3K1W5|C3K1W5_PSEFS	73	20960	4	3	4	3	0.96	GTP cyclohydrolase 1 (
371	1	db$2	2::C3K2Q0|C3K2Q0_PSEFS	260	19348	9	7	7	6	3.29	Inorganic pyrophospha
372	1	db$2	2::C3K6F9|C3K6F9_PSEFS	260	37389	9	9	9	9	2.12	Nucleoid-associated pr
373	1	db$2	2::C3JZ71|C3JZ71_PSEFS	258	31565	8	6	7	5	1.11	Hex regulon repressor
374	1	db$2	2::C3K5J3|C3K5J3_PSEFS	258	50388	8	8	8	8	1.12	AMP nucleosidase OS=
375	1	db$2	2::C3K0K7|C3K0K7_PSEFS	257	60595	11	11	10	10	1.19	Electron transfer flavo
376	1	db$2	2::C3K310|C3K310_PSEFS	256	54246	12	10	12	10	1.39	Anthranilate synthase
377	1	db$2	2::C3K1N4|C3K1N4_PSEFS	256	41398	8	8	5	5	0.98	Queuine tRNA-ribosylt
378	1	db$2	2::C3K2Z7|ARGC_PSEFS	256	36612	11	11	7	7	2.19	N-acetyl-gamma-gluta
379	1	db$2	2::C3JZ68|C3JZ68_PSEFS	254	24197	11	11	9	9	5.99	2-dehydro-3-deoxy-ph
380	1	db$2	2::C3K422|C3K422_PSEFS	254	40250	9	8	8	7	1.27	Putative uroporphyrin-
381	1	db$2	2::C3K1X8|C3K1X8_PSEFS	253	11596	7	6	3	3	2.34	50S ribosomal protein
382	1	db$2	2::C3K4Q9|C3K4Q9_PSEFS	253	23656	6	6	5	5	1.70	Putative exported prot
383	1	db$2	2::C3KCT1|MURC_PSEFS	252	52132	9	9	6	6	0.72	UDP-N-acetylmuramat
384	1	db$2	2::C3K6J6|C3K6J6_PSEFS	252	40307	13	11	11	9	2.64	Bifunctional chorismat
385	1	db$2	2::C3K274|DNAJ_PSEFS	251	40315	10	10	9	9	1.87	Chaperone protein Dna
386	1	db$2	2::C3JZ88|C3JZ88_PSEFS	250	47232	8	8	5	5	0.82	Putative exported prot
387	1	db$2	2::C3K2E3|MSCL_PSEFS	249	14568	7	7	4	4	3.97	Large-conductance me
388	1	db$2	2::C3K4G1|C3K4G1_PSEFS	248	81186	11	11	10	10	0.79	DNA helicase OS=Pseu
389	1	db$2	2::C3K249|G6PI_PSEFS	248	61384	18	14	14	12	1.73	Glucose-6-phosphate i
390	1	db$2	2::C3K1X5|C3K1X5_PSEFS	247	39754	8	7	6	5	0.81	Glutamate 5-kinase OS
391	1	db$2	2::C3JZ29|C3JZ29_PSEFS	247	41047	10	9	8	8	1.51	Putative glycerophospl
392	1	db$2	2::C3K084|AROC_PSEFS	247	39094	10	10	8	8	1.97	Chorismate synthase (
393	1	db$2	2::C3K017|PYRD_PSEFS	247	35850	13	12	12	11	3.86	Dihydroorotate dehydr
394	1	db$2	2::C3K1E5|ATPE_PSEFS	247	14558	7	6	4	4	3.97	ATP synthase epsilon c
395	1	db$2	2::C3K2Y0|RS7_PSEFS	245	17636	16	15	11	11	17.50	30S ribosomal protein
396	1	db$2	2::C3K278|C3K278_PSEFS	245	15153	7	7	5	5	5.31	Ferric uptake regulatio
397	1	db$2	2::C3K2E9|C3K2E9_PSEFS	244	53251	7	7	4	4	0.70	Putative cytochrome o
398	1	db$2	2::C3K5D5|C3K5D5_PSEFS	244	41033	6	6	5	5	0.78	Succinyl-diaminopimel
399	1	db$2	2::C3JYR4|C3JYR4_PSEFS	243	7675	7	7	4	4	9.93	Major cold shock prote
400	1	db$2	2::C3K6G8|LEPA_PSEFS	242	66372	13	10	11	9	1.04	Elongation factor 4 OS

↑Family	M	DB	Accession	Score	Mass	Matches	Match(sig)	Sequences	Seq(sig)	emPAI	Description
401	1	db$2	2::C3KCT2|MUTS_PSEFS	241	95536	8	8	8	8	0.49	DNA mismatch repair p
402	1	db$2	2::C3KE41|SYGA_PSEFS	241	36196	10	10	9	9	2.24	Glycine--tRNA ligase a
403	1	db$2	2::C3KED5|C3KED5_PSEFS	240	40447	11	9	9	7	1.27	Putative lipoprotein OS
404	1	db$2	2::C3K1G8|RL19_PSEFS	240	12961	9	9	8	8	24.14	50S ribosomal protein
405	1	db$2	2::C3K6U8|C3K6U8_PSEFS	239	58654	7	7	5	5	0.62	Putative membrane pr
406	1	db$2	2::C3JYU1|Y4927_PSEFS	239	18225	10	10	7	7	9.13	UPF0234 protein PFLU_
407	1	db$2	2::C3K2X2|RS19_PSEFS	238	10313	10	7	7	5	8.39	30S ribosomal protein
408	1	db$2	2::C3KDU9|C3KDU9_PSEFS	235	34261	7	7	7	7	1.62	Serine O-acetyltransfe
409	1	db$2	2::C3K4H9|C3K4H9_PSEFS	235	34203	10	7	9	6	1.29	Quinone oxidoreductas
410	1	db$2	2::C3K3T9|C3K3T9_PSEFS	234	49785	9	9	7	7	1.14	Putative aminotransfer
411	1	db$2	2::C3KDW3|HFQ_PSEFS	233	9318	14	11	8	7	30.86	RNA-binding protein H
412	1	db$2	2::C3K2Z3|C3K2Z3_PSEFS	233	44017	11	8	11	8	1.36	Tyrosine--tRNA ligase
413	1	db$2	2::C3JXY7|ASTD_PSEFS	233	51479	5	5	5	5	0.58	N-succinylglutamate 5
414	1	db$2	2::C3JYN4|ZIPA_PSEFS	232	31818	9	7	6	5	1.82	Cell division protein Zi
415	1	db$2	2::C3K6R3|Y2135_PSEFS	232	24050	7	7	6	6	2.23	UPF0502 protein PFLU_
416	1	db$2	2::C3KAH3|SYR_PSEFS	232	63549	10	10	9	9	1.11	Arginine--tRNA ligase (
417	1	db$2	2::C3K469|RL28_PSEFS	232	8845	10	9	6	5	12.36	50S ribosomal protein
418	1	db$2	2::C3K104|C3K104_PSEFS	231	26102	9	8	6	5	1.95	Putative membrane pr
419	1	SwissProt_HUMAN$	3::P01876|IGHA1_HUMAN	231	37631	8	8	7	7	1.41	Immunoglobulin heavy
419	2	SwissProt_HUMAN$	3::P0DOX2|IGA2_HUMAN	127	48903	4	4	4	4	0.47	Immunoglobulin alpha
419	3	SwissProt_HUMAN$	3::P01764|HV323_HUMAN	36	12574	3	3	2	2	2.04	Immunoglobulin heavy
420	1	db$2	2::C3K6K6|C3K6K6_PSEFS	231	36089	11	10	10	9	2.25	HTH-type transcription
421	1	db$2	2::C3KBL1|C3KBL1_PSEFS	230	61934	10	9	9	8	0.99	Putative medium-chair
422	1	db$2	2::C3K9R8|C3K9R8_PSEFS	230	135461	12	8	11	8	0.32	Methionine synthase O
423	1	db$2	2::C3KBY9|C3KBY9_PSEFS	229	24532	5	5	4	4	1.15	Ribonuclease T OS=Ps
424	1	db$2	2::C3KEB7|C3KEB7_PSEFS	228	48036	10	10	9	9	1.68	Uncharacterized protei
425	1	db$2	2::C3K452|XPT_PSEFS	228	20267	10	10	9	9	9.07	Xanthine phosphoribos
426	1	db$2	2::C3KBQ3|C3KBQ3_PSEFS	228	33274	7	7	5	5	1.03	LysR-family transcripti
427	1	db$2	2::C3K245|C3K245_PSEFS	228	53442	14	13	13	12	2.17	Poly(A) polymerase I (
428	1	db$2	2::C3K2W5|RL24_PSEFS	227	11328	10	10	8	8	38.88	50S ribosomal protein
429	1	db$2	2::C3JY54|SYS_PSEFS	226	47115	12	9	10	8	1.24	Serine--tRNA ligase OS
430	1	db$2	2::C3K0T1|C3K0T1_PSEFS	225	94111	10	10	9	9	0.66	Aconitate hydratase O:
431	1	db$2	2::C3K9P3|C3K9P3_PSEFS	225	9882	7	7	5	5	9.36	Peptidyl-prolyl cis-tran
432	1	db$2	2::C3K839|Y879_PSEFS	225	32120	8	8	7	7	1.80	Nucleotide-binding pro
433	1	db$2	2::C3K6E2|RS9_PSEFS	225	14607	11	10	7	6	11.77	30S ribosomal protein
434	1	db$2	2::C3K2S0|C3K2S0_PSEFS	224	39319	7	6	6	5	0.82	3,4-dihydroxy-2-butan
435	1	db$2	2::C3K2U9|C3K2U9_PSEFS	223	53651	16	13	13	10	1.64	Catalase OS=Pseudom
436	1	db$2	2::C3K371|C3K371_PSEFS	223	78727	11	11	11	11	0.94	Malate synthase G OS:
437	1	db$2	2::C3K1L2|C3K1L2_PSEFS	222	22708	6	6	6	6	2.45	Ancillary SecYEG trans
438	1	db$2	2::C3KE79|C3KE79_PSEFS	221	29713	5	5	4	4	0.89	NH(3)-dependent NAD

↑Family	M	DB	Accession	Score	Mass	Matches	Match(sig)	Sequences	Seq(sig)	emPAI	Description
439	1	db$2	2::C3JYD4|C3JYD4_PSEFS	221	64707	12	11	10	9	0.93	Glutamine--tRNA ligas
440	1	db$2	2::C3K5N1|C3K5N1_PSEFS	221	36842	10	9	8	7	1.45	Sulfate ABC transport
441	1	db$2	2::C3K5X7|C3K5X7_PSEFS	221	42054	12	12	11	11	2.85	Cell division protein Za
442	1	db$2	2::C3JYI0|C3JYI0_PSEFS	220	25235	10	9	6	6	2.07	Probable transcriptiona
443	1	db$2	2::C3JZ69|C3JZ69_PSEFS	220	25612	7	7	5	5	1.50	6-phosphogluconolacto
444	1	db$2	2::C3JZQ0|C3JZQ0_PSEFS	219	38665	10	9	9	8	2.01	Putative L-asparaginas
445	1	db$2	2::C3K4G2|C3K4G2_PSEFS	219	30728	9	9	4	4	1.51	Putative exported prot
446	1	db$2	2::C3KDM0|C3KDM0_PSEFS	219	69594	10	10	9	9	0.85	DNA topoisomerase 4
447	1	db$2	2::C3K8K2|C3K8K2_PSEFS	219	418093	6	6	6	6	0.07	Haemagg_act domain-
448	1	db$2	2::C3K740|C3K740_PSEFS	218	33777	12	12	12	12	4.36	DUF815 domain-conta
449	1	db$2	2::C3K818|C3K818_PSEFS	218	29926	8	7	5	4	0.88	Putative ABC transport
450	1	db$2	2::C3K2L2|SYL_PSEFS	218	97066	12	10	12	10	0.63	Leucine--tRNA ligase C
451	1	db$2	2::C3KA84|C3KA84_PSEFS	217	13408	7	7	6	6	7.01	Putative regulatory pro
452	1	db$2	2::C3KAJ1|C3KAJ1_PSEFS	217	48585	12	10	11	9	1.65	Omega-amino acid--py
453	1	db$2	2::C3KAJ2|C3KAJ2_PSEFS	216	53335	10	10	9	9	1.23	Methylmalonate-semia
454	1	db$2	2::C3K1U7|C3K1U7_PSEFS	215	36908	7	7	2	2	1.15	Ubiquinol oxidase subu
455	1	db$2	2::C3KE71|C3KE71_PSEFS	215	51412	11	10	11	10	1.51	Replicative DNA helica
456	1	db$2	2::C3K1X2|C3K1X2_PSEFS	214	40166	8	8	8	8	1.56	Putative putative oxido
457	1	db$2	2::C3KDM7|C3KDM7_PSEFS	214	29519	10	10	8	8	3.21	Sulfurtransferase OS=
458	1	db$2	2::C3K2X7|RS10_PSEFS	213	11745	17	14	10	8	22.48	30S ribosomal protein
459	1	db$2	2::C3K5L3|C3K5L3_PSEFS	213	36401	8	7	7	6	1.18	Phosphonates ABC tra
460	1	db$2	2::C3KDW7|HISZ_PSEFS	213	42687	12	12	10	10	2.02	ATP phosphoribosyltra
461	1	db$2	2::C3JY88|C3JY88_PSEFS	212	20432	8	7	6	5	2.97	NADH-quinone oxidore
462	1	db$2	2::C3K8Z6|C3K8Z6_PSEFS	211	39611	11	10	7	7	1.60	Branched amino acid A
463	1	db$2	2::C3K581|C3K581_PSEFS	211	86479	8	7	8	7	0.47	Putative molybdopterin
464	1	db$2	2::C3KBP9|C3KBP9_PSEFS	210	58780	7	6	6	6	0.62	Phosphoglucomutase C
465	1	db$2	2::C3K4E4|C3K4E4_PSEFS	210	51389	10	10	10	10	1.51	Aspartate ammonia-lya
466	1	db$2	2::C3KAC7|C3KAC7_PSEFS	208	51379	10	10	9	9	1.51	UDP-glucose 6-dehydr
467	1	db$2	2::C3KDD6|C3KDD6_PSEFS	208	33869	5	5	5	5	1.01	D-alanine--D-alanine li
468	1	db$2	2::C3K102|PUR5_PSEFS	208	36950	13	11	9	8	3.08	Phosphoribosylformylg
469	1	SwissProt_HUMAN$	3::O00139|KIF2A_HUMAN	208	79905	24	23	1	1	0.06	Kinesin-like protein KI
470	1	db$2	2::C3K3M3|C3K3M3_PSEFS	207	24540	6	5	4	3	0.78	Cell division ATP-bindi
471	1	db$2	2::C3K5H5|C3K5H5_PSEFS	207	25721	8	8	5	5	1.99	Putative exported prot
472	1	db$2	2::C3K1A1|C3K1A1_PSEFS	207	10298	10	9	7	7	22.02	10 kDa chaperonin OS
473	1	db$2	2::C3K6Y3|GATB_PSEFS	206	52868	14	9	10	6	0.87	Aspartyl/glutamyl-tRN
474	1	db$2	2::C3KDW6|C3KDW6_PSEFS	204	33055	11	11	9	9	3.17	Protein HflC OS=Pseu
475	1	db$2	2::C3K0S7|Y4577_PSEFS	204	11900	5	5	3	3	6.06	Nucleoid-associated pr
476	1	db$2	2::C3K5X4|C3K5X4_PSEFS	203	16332	11	10	8	7	8.90	Putative membrane pr
477	1	db$2	2::C3K0S3|C3K0S3_PSEFS	202	67491	6	6	6	6	0.52	Putative ATP-binding c
478	1	db$2	2::C3KBL7|C3KBL7_PSEFS	202	33413	8	6	8	6	1.33	Glutamate/aspartate A

↑Family	M	DB	Accession	Score	Mass	Matches	Match(sig)	Sequences	Seq(sig)	emPAI	Description
479	1	db$2	2::C3K7G4|C3K7G4_PSEFS	202	54521	6	6	6	6	0.68	Ribonuclease G OS=Ps
480	1	db$2	2::C3JY62|C3JY62_PSEFS	201	83224	18	15	15	13	1.10	ATP-dependent Clp pro
480	2	db$2	2::C3K4B3|C3K4B3_PSEFS	80	97007	9	6	8	6	0.34	Chaperone OS=Pseudo
481	1	db$2	2::C3K1G2|YIDC_PSEFS	201	61977	12	9	12	9	0.99	Membrane protein inse
482	1	db$2	2::C3K262|C3K262_PSEFS	201	12956	8	6	4	3	1.93	Protein-export membra
483	1	db$2	2::C3K5V1|C3K5V1_PSEFS	199	47050	8	8	8	8	1.24	Outer membrane prote
484	1	db$2	2::C3K6Y4|GATA_PSEFS	199	51737	7	7	6	6	0.90	Glutamyl-tRNA(Gln) ar
485	1	db$2	2::C3K575|C3K575_PSEFS	199	14827	7	7	7	7	7.99	Osmotically inducible p
486	1	db$2	2::C3JXX2|C3JXX2_PSEFS	199	50328	7	7	7	7	0.93	ATP-dependent RNA he
487	1	db$2	2::C3K2I0|C3K2I0_PSEFS	198	48538	7	7	7	7	0.98	Putative ATP-depender
488	1	db$2	2::C3K733|HTPX_PSEFS	198	32382	8	7	5	4	1.07	Protease HtpX OS=Pse
489	1	db$2	2::C3JYE4|C3JYE4_PSEFS	198	30882	12	12	9	9	2.96	Exodeoxyribonuclease
490	1	db$2	2::C3JZ86|C3JZ86_PSEFS	198	21964	13	11	11	11	9.50	Superoxide dismutase
491	1	db$2	2::C3KBM6|C3KBM6_PSEFS	197	11934	7	6	4	3	5.94	Glutaredoxin OS=Pseu
492	1	db$2	2::C3K254|C3K254_PSEFS	196	21027	8	8	6	6	2.83	Osmotically inducible p
493	1	db$2	2::C3K472|C3K472_PSEFS	196	42804	9	9	8	8	1.70	Coenzyme A biosynthe
494	1	db$2	2::C3KBP1|C3KBP1_PSEFS	195	37670	9	7	7	6	1.12	Putative reductase OS
495	1	db$2	2::C3K5U6|C3K5U6_PSEFS	195	40174	6	6	5	5	0.80	dTDP-glucose 4,6-dehy
496	1	db$2	2::C3K606|FABZ_PSEFS	194	16691	8	8	6	6	4.39	3-hydroxyacyl-[acyl-ca
497	1	db$2	2::C3K3W3|C3K3W3_PSEFS	194	41294	5	5	4	4	0.77	Putative aminotransfer
498	1	db$2	2::C3K9Z4|C3K9Z4_PSEFS	194	58133	8	8	7	7	0.77	Putative ABC tranmspo
499	1	db$2	2::C3JZT0|C3JZT0_PSEFS	193	28122	5	5	5	5	1.31	Putative GntR-family tr
500	1	db$2	2::C3K417|C3K417_PSEFS	192	39396	4	4	3	3	0.43	Transcriptional regulat
501	1	db$2	2::C3KAK7|C3KAK7_PSEFS	192	20540	5	5	3	3	1.49	Putative lipoprotein OS
502	1	db$2	2::C3K177|C3K177_PSEFS	192	65030	12	10	11	9	0.93	Phosphogluconate deh
503	1	db$2	2::C3K1V6|C3K1V6_PSEFS	191	32555	4	4	4	4	0.79	Putative short-chain de
504	1	db$2	2::C3K152|C3K152_PSEFS	191	13999	8	8	7	7	13.38	Putative transcriptiona
505	1	db$2	2::C3JZS5|TRPF_PSEFS	191	21966	3	3	2	2	0.53	N-(5'-phosphoribosyl)a
506	1	db$2	2::C3KBK6|C3KBK6_PSEFS	191	29320	5	5	3	3	0.90	Putative exported pept
507	1	db$2	2::C3K4A8|C3K4A8_PSEFS	191	55832	10	9	8	8	0.97	Uncharacterized protei
508	1	db$2	2::C3K148|APT_PSEFS	190	20064	7	6	6	5	2.21	Adenine phosphoribosy
509	1	db$2	2::C3K8Y8|C3K8Y8_PSEFS	189	23915	7	7	5	5	1.68	Putative isomerase OS
510	1	db$2	2::C3KCP5|C3KCP5_PSEFS	188	109710	7	6	7	6	0.30	Bifunctional glutamine
511	1	db$2	2::C3K7M9|C3K7M9_PSEFS	188	34271	7	7	7	7	1.62	Putative ABC transport
512	1	db$2	2::C3K3P5|C3K3P5_PSEFS	188	48453	6	6	5	5	0.63	Putative symporter me
513	1	db$2	2::C3K6S8|C3K6S8_PSEFS	188	40355	7	7	7	7	1.27	Putative acetyl-CoA tra
514	1	db$2	2::C3K3W7|C3K3W7_PSEFS	188	36662	9	7	8	7	1.47	Putative iron (III) ABC
515	1	db$2	2::C3K859|C3K859_PSEFS	187	59064	8	7	7	6	0.62	Putative glutamate syr
516	1	db$2	2::C3K8W7|C3K8W7_PSEFS	187	34291	6	6	5	5	0.99	Arabinose 5-phosphate
517	1	db$2	2::C3K332|Y5583_PSEFS	187	48606	7	7	6	6	0.79	UPF0229 protein PFLU_

↑Family	M	DB	Accession	Score	Mass	Matches	Match(sig)	Sequences	Seq(sig)	emPAI	Description
518	1	db$2	2::C3KA77|C3KA77_PSEFS	187	49108	9	9	9	9	1.38	Glutathione reductase
519	1	db$2	2::C3K7I1|C3K7I1_PSEFS	187	85859	4	4	4	4	0.25	Quinoprotein glucose d
520	1	SwissProt_HUMAN$	3::P63104|1433Z_HUMAN	186	27728	7	7	7	7	2.28	14-3-3 protein zeta/de
520	2	SwissProt_HUMAN$	3::P27348|1433T_HUMAN	155	27747	4	4	4	4	0.97	14-3-3 protein theta O
521	1	db$2	2::C3KBL3|C3KBL3_PSEFS	186	50616	9	9	9	9	1.32	Putative exported prot
522	1	db$2	2::C3K1H1|RS16_PSEFS	186	9202	9	9	6	6	19.12	30S ribosomal protein
523	1	db$2	2::C3JYD3|SYC_PSEFS	186	51635	13	11	11	10	1.50	Cysteine--tRNA ligase
524	1	db$2	2::C3K368|C3K368_PSEFS	185	37353	6	5	5	5	0.88	Stress response kinase
525	1	db$2	2::C3K4T4|C3K4T4_PSEFS	185	21507	6	6	5	5	1.98	NAD(P)-bd_dom doma
526	1	db$2	2::C3K2S1|C3K2S1_PSEFS	185	23236	10	8	6	6	2.37	Riboflavin synthase alp
527	1	db$2	2::C3K3Q8|TYSY_PSEFS	184	31490	8	8	6	6	1.85	Thymidylate synthase
528	1	db$2	2::C3JYJ6|C3JYJ6_PSEFS	184	68407	10	9	9	9	0.87	Peptidyl-prolyl cis-tran
529	1	db$2	2::C3K3T6|C3K3T6_PSEFS	184	42390	9	9	7	7	1.73	Spermidine/putrescine
530	1	db$2	2::C3KBY3|C3KBY3_PSEFS	184	64998	7	5	6	5	0.44	Putative lipoprotein OS
531	1	db$2	2::C3K759|C3K759_PSEFS	184	35694	5	5	4	4	0.70	Putative dehydrogenas
531	2	db$2	2::C3K0S6|C3K0S6_PSEFS	76	36802	2	2	2	2	0.29	Putative oxidoreductas
532	1	db$2	2::B0B0A5|DNAA_PSEFS	183	56252	10	8	7	5	0.52	Chromosomal replicati
533	1	db$2	2::C3KAI8|C3KAI8_PSEFS	182	24497	7	6	6	5	2.16	Putative TetR-family re
534	1	db$2	2::C3K0V5|C3K0V5_PSEFS	182	33212	10	10	6	6	1.71	Putative keto/oxo acyl
535	1	db$2	2::C3K5E5|C3K5E5_PSEFS	182	28503	7	7	6	6	2.18	Methionine aminopepti
536	1	db$2	2::C3JXZ3|C3JXZ3_PSEFS	181	28123	7	7	6	6	1.74	Histidine transport ATF
537	1	db$2	2::C3K6L0|EFP_PSEFS	181	21303	5	5	4	4	1.41	Elongation factor P OS
538	1	db$2	2::C3K819|C3K819_PSEFS	181	35012	4	4	4	4	0.72	Putative ABC transport
539	1	db$2	2::C3KAC2|C3KAC2_PSEFS	181	41342	5	5	5	5	0.77	Acyl-CoA dehydrogena
540	1	db$2	2::C3K527|C3K527_PSEFS	180	65564	6	6	5	5	0.54	Putative peptidase OS=
541	1	db$2	2::C3K9M2|C3K9M2_PSEFS	180	37830	9	8	7	6	1.11	Putative peptidase OS=
542	1	SwissProt_HUMAN$	3::P62805|H4_HUMAN	180	11360	7	7	6	6	10.67	Histone H4 OS=Homo
543	1	db$2	2::C3K5H8|C3K5H8_PSEFS	180	17826	7	7	6	6	3.82	STAS domain-containi
544	1	db$2	2::C3K6U2|C3K6U2_PSEFS	179	47692	4	4	4	4	0.49	Probable proton/glutar
545	1	db$2	2::C3K272|C3K272_PSEFS	178	40774	9	8	7	6	1.25	Carbamoyl-phosphate
546	1	db$2	2::C3K264|GLMM_PSEFS	178	47719	10	8	9	7	1.21	Phosphoglucosamine n
547	1	db$2	2::C3JYU6|C3JYU6_PSEFS	178	42196	7	4	6	4	0.57	Arginine biosynthesis b
548	1	db$2	2::C3KE48|C3KE48_PSEFS	177	19341	6	6	5	5	2.37	Peptide deformylase O
549	1	db$2	2::C3K2W2|RS8_PSEFS	177	14083	8	8	4	4	4.22	30S ribosomal protein
550	1	db$2	2::C3K6M3|C3K6M3_PSEFS	177	13365	4	4	3	3	1.85	Succinate dehydrogena
551	1	db$2	2::C3K0I1|C3K0I1_PSEFS	177	29699	6	6	5	5	1.59	ParA family ATPase OS
552	1	db$2	2::C3K841|C3K841_PSEFS	176	11596	10	10	7	7	36.22	Putative sigma-54 mod
553	1	db$2	2::C3K7G7|C3K7G7_PSEFS	176	50136	8	7	7	6	0.76	Putative modulation of
554	1	db$2	2::C3K4Y3|KDPA_PSEFS	176	60181	8	8	4	4	0.48	Potassium-transportin
555	1	db$2	2::C3K116|PSRP_PSEFS	176	30838	7	7	7	7	1.92	Putative phosphoenolp

↑Family	M	DB	Accession	Score	Mass	Matches	Match(sig)	Sequences	Seq(sig)	emPAI	Description
556	1	db$2	2::C3K0T3|C3K0T3_PSEFS	175	28958	5	5	5	5	1.26	Putative membrane pr
557	1	db$2	2::C3JZV9|C3JZV9_PSEFS	175	34932	4	4	3	3	0.50	Putative DNA/RNA non
558	1	db$2	2::C3K8U2|UBIB_PSEFS	175	61616	8	7	8	7	0.71	Probable protein kinas
559	1	db$2	2::C3KE80|C3KE80_PSEFS	173	46502	7	7	7	7	1.04	Nicotinate phosphoribo
560	1	db$2	2::Q84CP9|DAPB_PSEFS	173	28276	11	11	7	7	2.21	4-hydroxy-tetrahydroc
561	1	db$2	2::C3K2L6|C3K2L6_PSEFS	171	47731	4	3	4	3	0.35	Putative lipoprotein OS
562	1	SwissProt_HUMAN$	3::Q5VTE0|EF1A3_HUMAN	171	50153	14	10	10	8	1.13	Putative elongation fac
563	1	db$2	2::C3K703|ISPF_PSEFS	171	16742	4	4	4	4	2.05	2-C-methyl-D-erythrito
564	1	db$2	2::C3K3X2|C3K3X2_PSEFS	171	19739	7	7	5	5	2.28	Uncharacterized protei
565	1	db$2	2::C3K3U0|C3K3U0_PSEFS	170	51001	8	7	7	7	0.92	Putative glutamine syr
566	1	db$2	2::C3JZU0|C3JZU0_PSEFS	169	50538	8	8	8	8	1.12	3-isopropylmalate deh
567	1	db$2	2::C3K4C0|C3K4C0_PSEFS	169	9802	4	4	4	4	5.62	DNA-binding protein H
568	1	db$2	2::C3K2W8|RL29_PSEFS	169	7168	6	6	5	5	22.70	50S ribosomal protein
569	1	db$2	2::C3K6V9|C3K6V9_PSEFS	169	54793	9	8	6	6	0.68	2,3-bisphosphoglycera
570	1	db$2	2::C3K1M1|HSCA_PSEFS	168	65896	8	8	8	8	0.78	Chaperone protein Hsc
571	1	db$2	2::C3K2C9|C3K2C9_PSEFS	168	48295	7	7	7	7	0.99	Glutamate dehydroger
572	1	db$2	2::C3KE99|C3KE99_PSEFS	168	30145	7	6	6	5	1.18	Quinolinate phosphorib
573	1	db$2	2::C3KDB7|C3KDB7_PSEFS	167	65793	7	6	7	6	0.54	ATP-dependent lipid A-
574	1	db$2	2::C3K611|C3K611_PSEFS	167	16065	7	7	4	4	3.27	Universal stress protei
575	1	db$2	2::C3K0P6|DCTA_PSEFS	167	46761	5	5	4	4	0.50	C4-dicarboxylate trans
576	1	db$2	2::C3KDX0|C3KDX0_PSEFS	167	16992	7	7	4	4	2.01	Peptidyl-prolyl cis-tran
577	1	db$2	2::C3K6K4|C3K6K4_PSEFS	166	32908	6	5	6	5	1.05	Putative 5'-nucleotidas
578	1	db$2	2::C3KAI4|C3KAI4_PSEFS	166	53827	7	7	6	6	0.85	SPOR domain-containi
579	1	db$2	2::C3JXZ2|C3JXZ2_PSEFS	165	36334	7	6	6	5	0.92	Putative AraC family re
580	1	db$2	2::C3K8U7|C3K8U7_PSEFS	165	30923	6	5	5	4	0.84	Transcriptional regulat
581	1	SwissProt_HUMAN$	3::P0CG04|IGLC1_HUMAN	165	11341	4	4	3	3	2.42	Immunoglobulin lambo
582	1	db$2	2::C3JYA9|C3JYA9_PSEFS	165	27795	5	5	5	5	1.34	Putative TetR-family re
583	1	db$2	2::C3K9M0|C3K9M0_PSEFS	165	11184	3	3	2	2	1.29	SCP2 domain-containir
584	1	db$2	2::C3K2W7|RS17_PSEFS	165	10090	6	6	5	5	8.85	30S ribosomal protein
585	1	db$2	2::C3K3Y8|UBID_PSEFS	164	54697	6	4	6	4	0.41	3-octaprenyl-4-hydrox
586	1	db$2	2::C3JYI3|PHNX_PSEFS	164	29973	5	5	4	4	1.20	Phosphonoacetaldehyc
587	1	db$2	2::C3K134|C3K134_PSEFS	163	52371	6	5	5	4	0.44	Coproporphyrinogen-II
588	1	db$2	2::C3K2M7|C3K2M7_PSEFS	163	18106	6	6	5	5	2.65	Ribosomal silencing fa
589	1	db$2	2::C3K480|C3K480_PSEFS	163	31946	6	5	5	4	0.80	Uncharacterized protei
590	1	db$2	2::C3K0D3|MINC_PSEFS	163	26249	11	11	9	9	5.03	Probable septum site-c
591	1	db$2	2::C3K2R1|DXS_PSEFS	163	68238	8	7	8	7	0.63	1-deoxy-D-xylulose-5-
592	1	db$2	2::C3K9S0|NFUA_PSEFS	163	20961	5	4	4	3	0.96	Fe/S biogenesis protei
593	1	db$2	2::C3K6A5|C3K6A5_PSEFS	163	78572	9	8	9	8	0.62	Catalase OS=Pseudom
594	1	db$2	2::C3K3G5|C3K3G5_PSEFS	162	15160	8	7	6	6	7.58	Putative thioesterase (
595	1	db$2	2::C3K4I0|HEM6_PSEFS	162	35313	8	7	7	6	1.23	Oxygen-dependent cop

↑Family	M	DB	Accession	Score	Mass	Matches	Match(sig)	Sequences	Seq(sig)	emPAI	Description
596	1	db$2	2::C3JY77|C3JY77_PSEFS	161	40451	5	5	4	4	0.60	Putative 3-oxoacyl-[ac
597	1	db$2	2::C3K5L8|C3K5L8_PSEFS	161	39360	6	6	6	6	1.05	Phosphorous compoun
598	1	db$2	2::C3K2V3|RS11_PSEFS	161	13607	4	4	3	3	1.78	30S ribosomal protein
599	1	db$2	2::C3K843|C3K843_PSEFS	160	26535	8	8	5	5	1.43	Lipopolysaccharide exp
600	1	db$2	2::C3K114|C3K114_PSEFS	160	74752	7	7	7	7	0.56	DNA polymerase III su
601	1	db$2	2::C3K4R1|PLSB_PSEFS	160	95327	7	7	7	7	0.42	Glycerol-3-phosphate
602	1	db$2	2::C3K5H7|C3K5H7_PSEFS	160	43348	4	4	3	3	0.39	Putative response regu
603	1	db$2	2::C3KDL6|C3KDL6_PSEFS	159	23139	6	4	6	4	1.26	ADP-ribose diphosphat
604	1	db$2	2::C3K309|C3K309_PSEFS	159	21661	3	3	3	3	0.92	Anthranilate synthase
605	1	db$2	2::C3JZN9|C3JZN9_PSEFS	158	7652	7	7	6	6	35.12	Major cold shock prote
606	1	db$2	2::C3K5U5|C3K5U5_PSEFS	157	32335	5	5	4	4	0.79	Glucose-1-phosphate t
607	1	db$2	2::C3K0G3|PPNP_PSEFS	157	10127	5	5	4	4	5.24	Pyrimidine/purine nucl
608	1	db$2	2::C3KE68|RS18_PSEFS	157	8912	7	7	4	4	12.36	30S ribosomal protein
609	1	db$2	2::C3K910|C3K910_PSEFS	157	36322	5	5	5	5	0.92	Putative ABC transport
610	1	db$2	2::C3K185|C3K185_PSEFS	156	20062	7	7	6	6	4.13	Putative NAD(P)H nitrc
611	1	db$2	2::C3K3D2|BETB_PSEFS	156	53123	8	7	5	5	0.56	Betaine aldehyde dehy
612	1	db$2	2::C3K421|C3K421_PSEFS	155	46244	7	7	7	7	1.05	Putative HemY domain
613	1	db$2	2::C3K483|C3K483_PSEFS	154	78630	11	11	11	11	0.94	Guanosine-3',5'-bis(Di
613	2	db$2	2::C3K1B1|C3K1B1_PSEFS	62	46678	3	3	3	3	0.36	Putative acid tolerance
614	1	db$2	2::C3K572|C3K572_PSEFS	154	7891	2	2	2	2	2.20	Uncharacterized protei
615	1	db$2	2::C3K743|FABA_PSEFS	153	18688	8	7	5	5	3.52	3-hydroxydecanoyl-[ac
616	1	db$2	2::C3K1K8|C3K1K8_PSEFS	153	31523	6	6	5	5	1.11	Putative hydrolase OS
617	1	db$2	2::C3K8W6|C3K8W6_PSEFS	152	18885	5	5	5	5	2.46	3-deoxy-D-manno-oct
618	1	db$2	2::C3K419|C3K419_PSEFS	152	17100	3	3	2	2	1.27	Transcriptional regulat
619	1	db$2	2::C3K5X2|C3K5X2_PSEFS	152	20559	6	5	5	4	1.49	Putative two-compone
620	1	db$2	2::C3K0N7|RL32_PSEFS	152	6752	5	5	3	3	6.53	50S ribosomal protein
621	1	db$2	2::C3K4E2|C3K4E2_PSEFS	151	16860	5	5	5	5	3.00	N5-carboxyaminoimida
622	1	db$2	2::C3K1E4|GLMU_PSEFS	151	48690	6	6	6	6	0.79	Bifunctional protein Gl
623	1	db$2	2::C3JY36|C3JY36_PSEFS	150	16095	6	5	5	4	2.19	Uncharacterized protei
624	1	db$2	2::C3KDK8|C3KDK8_PSEFS	150	41619	4	4	4	4	0.58	DAO domain-containin
625	1	db$2	2::C3K6T0|ENGB_PSEFS	150	23537	5	5	4	4	1.22	Probable GTP-binding
626	1	db$2	2::C3KAH1|HSLV_PSEFS	149	18731	4	4	3	3	1.72	ATP-dependent protea
627	1	db$2	2::C3K1M9|C3K1M9_PSEFS	149	29586	12	9	10	9	3.19	Inositol-1-monophospl
628	1	db$2	2::C3K1H2|C3K1H2_PSEFS	149	49205	8	7	7	6	0.78	Signal recognition part
629	1	db$2	2::C3K6J1|UBIG_PSEFS	149	26040	6	6	5	5	1.95	Ubiquinone biosynthes
630	1	db$2	2::C3JZV1|C3JZV1_PSEFS	149	76010	9	9	8	8	0.65	UvrABC system proteir
631	1	db$2	2::C3KBZ7|SYM_PSEFS	149	75525	10	10	9	9	0.87	Methionine--tRNA ligas
632	1	SwissProt_HUMAN$	3::Q9UBD5|ORC3_HUMAN	147	82201	24	21	1	1	0.06	Origin recognition com
633	1	SwissProt_HUMAN$	3::P0DOX5|IGG1_HUMAN	147	49298	7	6	6	6	0.78	Immunoglobulin gamn
633	2	SwissProt_HUMAN$	3::P01859|IGHG2_HUMAN	140	35878	6	6	6	6	1.20	Immunoglobulin heavy

↑Family	M	DB	Accession	Score	Mass	Matches	Match(sig)	Sequences	Seq(sig)	emPAI	Description
634	1	db$2	2::C3K7W0|C3K7W0_PSEFS	147	26786	4	4	4	4	1.02	Uncharacterized protei
635	1	db$2	2::C3JXY8|C3JXY8_PSEFS	147	37476	5	5	5	5	0.88	Arginine N-succinyltrar
636	1	db$2	2::C3KDG2|C3KDG2_PSEFS	147	41589	7	6	6	5	0.98	AMP nucleosidase OS=
637	1	db$2	2::C3K276|GRPE_PSEFS	146	20714	5	4	4	3	0.97	Protein GrpE OS=Pseu
638	1	db$2	2::C3JZ43|C3JZ43_PSEFS	146	44885	7	7	7	7	1.09	Putative aminotransfer
639	1	db$2	2::C3K6E3|C3K6E3_PSEFS	146	21087	7	6	4	4	2.04	Ubiquinol-cytochrome
640	1	db$2	2::C3KAV6|C3KAV6_PSEFS	145	17648	5	5	3	3	1.22	Thiol peroxidase OS=P
641	1	db$2	2::C3K4E1|C3K4E1_PSEFS	145	38799	6	6	6	6	1.07	N5-carboxyaminoimida
642	1	db$2	2::C3KBB4|OADC_PSEFS	145	31135	5	4	4	3	0.58	Oxaloacetate decarbox
643	1	db$2	2::C3K5E3|C3K5E3_PSEFS	144	43909	7	6	5	4	0.54	Putative aminotransfer
644	1	SwissProt_HUMAN$	3::P23528|COF1_HUMAN	144	18491	5	5	5	5	2.54	Cofilin-1 OS=Homo sa
645	1	db$2	2::C3JZT9|C3JZT9_PSEFS	144	24210	6	5	6	5	1.64	3-isopropylmalate deh
646	1	db$2	2::C3K2J9|C3K2J9_PSEFS	144	60860	4	3	3	2	0.26	Putative dehydrogenas
647	1	db$2	2::C3KCL0|C3KCL0_PSEFS	143	35424	6	6	5	5	0.95	Uncharacterized protei
648	1	db$2	2::C3K0H7|C3K0H7_PSEFS	142	77893	6	5	6	5	0.36	Histidine kinase OS=P
649	1	db$2	2::C3K2V4|RS13_PSEFS	142	13332	8	7	6	5	4.74	30S ribosomal protein
650	1	db$2	2::C3K2J5|C3K2J5_PSEFS	142	18057	4	4	4	4	1.82	Putative AsnC-family r
651	1	db$2	2::C3K6I1|C3K6I1_PSEFS	142	31224	7	6	6	6	1.48	S-formylglutathione hy
652	1	db$2	2::C3K2Z9|C3K2Z9_PSEFS	141	34289	5	5	3	3	0.73	NMO domain-containin
653	1	db$2	2::C3K1X7|RL27_PSEFS	141	9214	5	5	3	3	3.49	50S ribosomal protein
654	1	db$2	2::C3K6Z0|PDXJ_PSEFS	140	26758	6	6	5	5	1.41	Pyridoxine 5'-phosphat
655	1	db$2	2::C3JY82|NUOB_PSEFS	140	25341	7	6	5	5	1.53	NADH-quinone oxidore
656	1	db$2	2::C3KC10|C3KC10_PSEFS	140	23080	3	3	2	2	0.50	Uncharacterized protei
657	1	db$2	2::C3KE83|C3KE83_PSEFS	140	38700	9	8	7	6	1.08	Outer membrane prote
658	1	db$2	2::C3K268|C3K268_PSEFS	139	11552	6	5	5	4	6.46	CRM domain-containin
659	1	db$2	2::C3KDQ9|C3KDQ9_PSEFS	139	29676	9	7	9	7	2.05	Ferredoxin--NADP redu
660	1	db$2	2::C3K3S0|C3K3S0_PSEFS	139	23721	6	5	4	3	0.81	Putative hydrolase OS
661	1	db$2	2::C3K3L7|THIG_PSEFS	139	28373	6	6	5	5	1.29	Thiazole synthase OS=
662	1	db$2	2::C3K256|RS15_PSEFS	139	10112	4	4	3	3	2.95	30S ribosomal protein
663	1	db$2	2::C3K3L0|METXS_PSEFS	139	41664	5	5	4	4	0.76	Homoserine O-succiny
664	1	db$2	2::C3K2W9|RL16_PSEFS	139	15422	6	6	5	5	3.54	50S ribosomal protein
665	1	db$2	2::C3K307|TRPC_PSEFS	139	30252	8	8	8	8	2.47	Indole-3-glycerol phos
666	1	db$2	2::C3K6J5|SERC_PSEFS	137	39799	6	6	5	5	1.04	Phosphoserine aminotı
667	1	db$2	2::C3KED4|C3KED4_PSEFS	136	31038	4	4	4	4	0.84	4-hydroxy-tetrahydroc
668	1	db$2	2::C3K308|TRPD_PSEFS	136	36976	6	6	6	6	1.15	Anthranilate phosphori
669	1	db$2	2::C3K4C1|C3K4C1_PSEFS	135	40318	5	5	3	3	0.42	Putative nitric oxide de
670	1	db$2	2::C3K2U8|C3K2U8_PSEFS	135	18135	7	6	6	5	3.73	Bacterioferritin OS=Ps
671	1	db$2	2::C3K6W7|C3K6W7_PSEFS	135	16216	3	3	3	3	1.38	Biotin carboxyl carrier
672	1	db$2	2::C3K424|HEM3_PSEFS	134	33541	3	3	2	2	0.33	Porphobilinogen deami
673	1	db$2	2::C3JYK0|CLPP_PSEFS	134	23405	8	8	6	6	3.08	ATP-dependent Clp pro

↑Family	M	DB	Accession	Score	Mass	Matches	Match(sig)	Sequences	Seq(sig)	emPAI	Description
674	1	db$2	2::C3K0Q9|C3K0Q9_PSEFS	134	15688	4	3	3	3	1.45	Flagellar basal-body rc
675	1	db$2	2::C3KE43|C3KE43_PSEFS	134	32711	4	4	4	4	0.78	Lipid A biosynthesis lau
676	1	db$2	2::C3K5K3|C3K5K3_PSEFS	133	28692	3	3	2	2	0.64	CHAD domain-containi
677	1	db$2	2::C3KAJ8|UPP_PSEFS	133	22986	9	5	7	5	1.78	Uracil phosphoribosyltı
678	1	db$2	2::C3K8W8|C3K8W8_PSEFS	133	29386	5	5	4	4	0.90	Putative ABC transport
679	1	db$2	2::C3KBW8|C3KBW8_PSEFS	133	39031	7	6	7	6	1.07	D-alanine--D-alanine li
680	1	db$2	2::C3K610|C3K610_PSEFS	133	70283	9	7	9	7	0.60	ATP-binding protein Ui
681	1	db$2	2::C3JXP7|RECR_PSEFS	133	21528	6	6	6	6	2.70	Recombination protein
682	1	db$2	2::C3JXY5|C3JXY5_PSEFS	133	10610	4	4	3	3	2.68	Uncharacterized protei
683	1	db$2	2::C3K3M7|C3K3M7_PSEFS	133	22464	2	2	1	1	0.23	Ribosomal RNA small s
684	1	db$2	2::C3JYQ9|C3JYQ9_PSEFS	132	54767	3	3	3	3	0.30	Transcriptional regulat
685	1	db$2	2::C3JYS3|C3JYS3_PSEFS	132	38665	4	2	3	2	0.28	TolA colicin import me
686	1	db$2	2::C3K1L1|C3K1L1_PSEFS	132	40864	6	5	5	5	0.78	Outer membrane prote
687	1	db$2	2::C3JY78|C3JY78_PSEFS	132	22781	3	3	2	2	0.51	Putative cytochrome o
688	1	db$2	2::C3K6S9|C3K6S9_PSEFS	132	101727	9	9	9	9	0.52	DNA polymerase I OS=
689	1	db$2	2::C3K651|C3K651_PSEFS	131	39766	7	7	7	7	1.30	Phospho-2-dehydro-3-
690	1	db$2	2::C3K9F0|C3K9F0_PSEFS	131	40567	5	4	3	3	0.42	Putative transport-rela
691	1	db$2	2::C3KEC6|KAD_PSEFS	131	23251	7	6	6	6	2.37	Adenylate kinase OS=I
692	1	db$2	2::C3K075|C3K075_PSEFS	131	77370	8	7	8	7	0.54	Putative sulfatase OS=
693	1	db$2	2::C3JY53|CYSG_PSEFS	130	50259	5	5	5	5	0.60	Siroheme synthase OS
694	1	db$2	2::C3K5F4|C3K5F4_PSEFS	130	21753	4	4	4	4	1.37	Putative nitroreductase
695	1	db$2	2::C3K427|C3K427_PSEFS	130	23927	6	5	4	4	1.19	Putative glutathione S-
696	1	db$2	2::C3K2K2|THIE_PSEFS	130	22345	3	3	3	3	0.88	Thiamine-phosphate sy
697	1	db$2	2::C3K468|C3K468_PSEFS	129	6077	3	3	1	1	3.42	50S ribosomal protein
698	1	db$2	2::C3KAD3|ARNC_PSEFS	129	37700	3	3	3	3	0.46	Undecaprenyl-phospha
699	1	db$2	2::C3K494|C3K494_PSEFS	129	33626	3	3	3	3	0.52	Gly-zipper_YMGG dom
700	1	db$2	2::C3JXQ0|C3JXQ0_PSEFS	129	24880	7	6	6	5	1.58	Putative hydrolase OS
701	1	db$2	2::C3JZN1|IHFA_PSEFS	129	11469	5	5	4	4	4.07	Integration host factor
702	1	db$2	2::C3K6Z7|C3K6Z7_PSEFS	128	89469	9	9	9	9	0.61	Lon protease OS=Pseu
703	1	db$2	2::C3KCS8|C3KCS8_PSEFS	128	47767	5	5	5	5	0.64	UDP-N-acetylmuramoy
704	1	db$2	2::C3K231|C3K231_PSEFS	128	57678	6	5	5	5	0.51	APH domain-containin
705	1	db$2	2::C3K2R5|RIBA_PSEFS	127	22312	5	5	5	5	1.87	GTP cyclohydrolase-2
706	1	db$2	2::C3K5G1|C3K5G1_PSEFS	127	34097	5	5	4	4	1.00	Cysteine synthase OS=
707	1	db$2	2::C3JXY9|C3JXY9_PSEFS	126	37073	7	6	6	6	1.15	Putative arginine N-su
708	1	db$2	2::C3K6V2|HIS4_PSEFS	126	25679	7	7	7	7	2.61	1-(5-phosphoribosyl)-!
709	1	db$2	2::C3KEA8|C3KEA8_PSEFS	125	45495	4	4	3	3	0.37	ATP-dependent RNA he
710	1	db$2	2::C3K6E8|C3K6E8_PSEFS	125	27518	3	3	2	2	0.41	Putative GntR-family r
711	1	db$2	2::Q9ZEP8|PANB_PSEFS	125	27951	5	5	4	4	1.32	3-methyl-2-oxobutano
712	1	SwissProt_HUMAN$	3::P02671|FIBA_HUMAN	125	94914	3	2	3	2	0.11	Fibrinogen alpha chain
713	1	db$2	2::C3K4V5|C3K4V5_PSEFS	125	36313	8	5	7	4	0.92	Beta-hexosaminidase (

↑Family	M	DB	Accession	Score	Mass	Matches	Match(sig)	Sequences	Seq(sig)	emPAI	Description
714	1	db$2	2::C3JXX7|C3JXX7_PSEFS	124	56979	4	4	4	4	0.39	Conserved hypothetica
715	1	db$2	2::C3K3N8|C3K3N8_PSEFS	124	43920	5	5	5	5	0.71	PUA domain-containinı
716	1	db$2	2::C3K644|C3K644_PSEFS	124	89774	6	6	5	5	0.30	Putative ABC transport
717	1	db$2	2::C3KA90|C3KA90_PSEFS	124	36984	3	3	3	3	0.47	Putative aldo/keto redı
718	1	db$2	2::C3K479|RNPH_PSEFS	124	25645	6	5	5	4	1.08	Ribonuclease PH OS=F
719	1	db$2	2::C3KBM4|C3KBM4_PSEFS	123	34098	7	6	6	6	1.30	Ornithine carbamoyltra
720	1	db$2	2::C3K3L6|TRMB_PSEFS	123	26990	3	3	3	3	0.69	tRNA (guanine-N(7)-)-
721	1	db$2	2::C3K5L9|C3K5L9_PSEFS	123	31441	3	2	3	2	0.35	Alpha-D-ribose 1-meth
722	1	db$2	2::C3K763|C3K763_PSEFS	123	29996	3	3	2	2	0.37	Putative hydrolase OS:
723	1	db$2	2::C3KCR9|PTH_PSEFS	123	21041	4	3	4	3	0.95	Peptidyl-tRNA hydrolas
724	1	db$2	2::C3K0W5|PHS_PSEFS	123	13357	4	4	2	2	1.85	Putative pterin-4-alpha
725	1	db$2	2::C3JZN4|RL20_PSEFS	123	13348	8	6	7	5	4.74	50S ribosomal protein
726	1	db$2	2::C3K785|C3K785_PSEFS	122	24962	6	5	4	3	0.76	3-oxoadipate CoA-tran
727	1	db$2	2::C3KDM5|C3KDM5_PSEFS	122	43829	5	4	5	4	0.54	O-phosphoserine phos
728	1	db$2	2::C3K9G9|C3K9G9_PSEFS	122	16183	6	6	5	5	3.23	CMD domain-containin
729	1	db$2	2::C3K8W9|C3K8W9_PSEFS	122	28276	5	5	5	5	1.30	Intermembrane phosp
730	1	db$2	2::C3K796|C3K796_PSEFS	122	49835	6	5	5	5	0.61	Putative glutamine syr
731	1	db$2	2::C3KBK9|C3KBK9_PSEFS	122	34365	3	3	3	3	0.51	Glycerate dehydrogena
732	1	db$2	2::C3K571|C3K571_PSEFS	122	11755	4	4	3	3	2.27	Uncharacterized protei
733	1	db$2	2::C3K3V5|C3K3V5_PSEFS	121	52101	6	5	6	5	0.58	CYTH domain-containii
734	1	db$2	2::C3KBH3|DCUP_PSEFS	121	38973	6	5	6	5	0.83	Uroporphyrinogen deca
735	1	db$2	2::C3K0I0|C3K0I0_PSEFS	121	27472	6	5	5	4	0.99	RNA polymerase sigma
736	1	db$2	2::C3K6V7|C3K6V7_PSEFS	120	46336	7	5	6	5	0.67	Carboxy-terminal proc
737	1	db$2	2::C3K253|C3K253_PSEFS	120	106648	6	5	6	5	0.25	Putative membrane pr
738	1	db$2	2::C3JZ32|C3JZ32_PSEFS	120	38286	4	4	3	3	0.45	Putative cation transpo
739	1	db$2	2::C3K270|C3K270_PSEFS	119	17315	5	5	4	4	1.95	Transcription elongatic
740	1	db$2	2::C3KDQ1|C3KDQ1_PSEFS	119	35181	4	4	3	3	0.50	Putative LysR-family re
741	1	db$2	2::C3KDN5|C3KDN5_PSEFS	118	35878	3	3	3	3	0.48	Renalase OS=Pseudon
742	1	db$2	2::C3KDP4|C3KDP4_PSEFS	118	75338	8	7	7	6	0.46	Phosphate acetyltransf
743	1	db$2	2::C3JYV5|C3JYV5_PSEFS	118	45487	7	7	6	6	1.07	Putative oxidoreductas
744	1	db$2	2::C3K360|C3K360_PSEFS	118	23509	3	3	3	3	0.82	ATP-dependent dethiol
745	1	db$2	2::C3K240|C3K240_PSEFS	118	17616	7	7	5	5	3.91	RNA polymerase-bindiı
746	1	db$2	2::C3K1X0|CYSZ_PSEFS	117	28826	4	4	4	4	0.92	Sulfate transporter Cy:
747	1	db$2	2::C3JY91|C3JY91_PSEFS	117	66398	2	2	2	2	0.15	NADH-quinone oxidore
748	1	db$2	2::C3K458|C3K458_PSEFS	117	47152	3	3	2	2	0.22	D-amino acid dehydroı
749	1	db$2	2::C3K3L1|C3K3L1_PSEFS	117	23430	4	4	4	4	1.23	Uncharacterized protei
750	1	db$2	2::C3K1U9|C3K1U9_PSEFS	117	42728	6	6	4	4	0.74	Flavohemoglobin OS=I
751	1	db$2	2::C3K312|C3K312_PSEFS	117	24134	6	6	4	4	1.65	Ribulose-phosphate 3-
752	1	db$2	2::C3K4Z4|C3K4Z4_PSEFS	117	43117	4	4	4	4	0.55	Putative cytochrome C
753	1	db$2	2::C3K648|C3K648_PSEFS	117	33905	4	4	4	4	0.75	Conserved hypothetica

↑Family	M	DB	Accession	Score	Mass	Matches	Match(sig)	Sequences	Seq(sig)	emPAI	Description
754	1	db$2	2::C3K0V4|C3K0V4_PSEFS	116	48572	3	3	3	3	0.34	Flagellin OS=Pseudom
755	1	db$2	2::C3JYI6|C3JYI6_PSEFS	116	73112	5	4	5	4	0.30	Putative 2,4-dienoyl-C
756	1	SwissProt_HUMAN$	3::Q06830|PRDX1_HUMAN	116	22096	11	9	9	7	4.48	Peroxiredoxin-1 OS=H
757	1	db$2	2::C3K2V8|RL30_PSEFS	116	6521	4	3	3	2	3.01	50S ribosomal protein
758	1	db$2	2::C3JXZ5|C3JXZ5_PSEFS	116	25958	4	4	4	4	1.06	Putative histidine trans
759	1	db$2	2::C3K3G7|C3K3G7_PSEFS	116	31253	6	6	6	6	1.47	Methylenetetrahydrofo
760	1	db$2	2::C3K459|C3K459_PSEFS	115	18704	5	5	5	5	2.49	Leucine-responsive reg
761	1	db$2	2::C3K4U5|C3K4U5_PSEFS	115	75901	8	8	7	7	0.55	Putative protease OS=
762	1	db$2	2::C3K482|RPOZ_PSEFS	115	9821	4	3	3	2	3.13	DNA-directed RNA poly
763	1	db$2	2::C3K3H3|C3K3H3_PSEFS	115	52510	4	4	4	4	0.43	Adenosylmethionine-8
764	1	db$2	2::C3K5E4|GLND_PSEFS	115	102948	6	5	6	5	0.26	Bifunctional uridylyltra
765	1	db$2	2::C3KA81|C3KA81_PSEFS	114	55484	5	4	5	4	0.41	Alkyl hydroperoxide re
766	1	db$2	2::C3K3W5|C3K3W5_PSEFS	114	39645	7	6	5	5	0.82	Aminomethyltransfera
767	1	db$2	2::C3JYT6|C3JYT6_PSEFS	114	8291	7	6	6	6	26.39	Cold shock protein csp
768	1	SwissProt_HUMAN$	3::P02675|FIBB_HUMAN	113	55892	8	7	8	7	0.81	Fibrinogen beta chain (
769	1	db$2	2::C3K6S5|C3K6S5_PSEFS	113	35353	3	3	3	3	0.49	Delta-aminolevulinic a
770	1	db$2	2::C3JZN8|C3JZN8_PSEFS	113	10735	6	5	3	3	4.59	Uncharacterized protei
771	1	db$2	2::C3KDB8|HLDE_PSEFS	113	49913	8	7	8	7	0.94	Bifunctional protein Hl
772	1	db$2	2::C3KE08|C3KE08_PSEFS	113	25044	3	3	3	3	0.76	Conserved hypothetica
773	1	db$2	2::C3K477|C3K477_PSEFS	113	29836	5	4	4	4	0.88	Exodeoxyribonuclease
774	1	db$2	2::C3K1D1|C3K1D1_PSEFS	112	32188	3	3	3	3	0.55	Protein HflC OS=Pseuc
775	1	db$2	2::C3K7C5|C3K7C5_PSEFS	112	40067	3	2	3	2	0.27	Histidine kinase OS=P:
776	1	db$2	2::C3K7G1|C3K7G1_PSEFS	112	37563	5	5	5	5	0.88	Cell shape protein Mre
777	1	db$2	2::C3K1W7|C3K1W7_PSEFS	112	11247	4	3	3	2	1.29	DUF1244 domain-cont
778	1	db$2	2::C3K568|TRPA_PSEFS	112	28539	3	3	3	3	0.64	Tryptophan synthase a
779	1	db$2	2::C3K062|C3K062_PSEFS	111	48996	8	8	6	6	0.97	Fumarate hydratase cl
780	1	db$2	2::C3K2P1|C3K2P1_PSEFS	111	48286	4	3	4	3	0.34	UDP-N-acetylmuramat
781	1	db$2	2::C3K1G1|C3K1G1_PSEFS	111	48709	3	3	3	3	0.34	tRNA modification GTP
782	1	db$2	2::C3KAW2|C3KAW2_PSEFS	111	26230	3	3	3	3	0.71	Putative short chain de
783	1	db$2	2::C3K281|C3K281_PSEFS	111	15787	3	3	2	2	0.81	Ribosome association t
784	1	db$2	2::C3KAB1|C3KAB1_PSEFS	110	44397	5	4	4	3	0.38	Histidine kinase OS=P:
785	1	db$2	2::C3KDM4|C3KDM4_PSEFS	110	56715	7	5	7	5	0.52	Putative membrane pr
786	1	db$2	2::C3K2U6|C3K2U6_PSEFS	110	49484	5	5	3	3	0.47	Putative transport-rela
787	1	db$2	2::C3KD57|C3KD57_PSEFS	110	49686	4	4	4	4	0.46	Cystathionine beta-lya
788	1	db$2	2::C3K3L5|C3K3L5_PSEFS	109	11400	2	2	1	1	1.25	Putative membrane pr
789	1	db$2	2::C3K436|C3K436_PSEFS	109	30237	3	3	3	3	0.60	Diaminopimelate epim
790	1	db$2	2::C3K758|PYRF_PSEFS	109	24706	6	4	5	4	1.14	Orotidine 5'-phosphate
791	1	db$2	2::C3KDU8|C3KDU8_PSEFS	109	35029	7	7	5	5	1.24	D-cysteine desulfhydra
792	1	db$2	2::C3JYS8|RUVA_PSEFS	109	22218	3	3	3	3	0.89	Holliday junction ATP-(
793	1	db$2	2::C3KE31|C3KE31_PSEFS	109	22237	6	5	6	5	1.88	RNA polymerase sigma

↑Family	M	DB	Accession	Score	Mass	Matches	Match(sig)	Sequences	Seq(sig)	emPAI	Description
794	1	db$2	2::C3K6U9|HIS7_PSEFS	108	21830	6	4	5	3	0.90	Imidazoleglycerol-phos
795	1	db$2	2::C3K437|C3K437_PSEFS	108	26506	2	2	1	1	0.19	Uncharacterized protei
796	1	db$2	2::C3KAH8|C3KAH8_PSEFS	108	89028	7	7	7	7	0.45	DD-transpeptidase OS
797	1	db$2	2::C3K0W6|C3K0W6_PSEFS	108	42755	6	5	6	5	0.74	Aspartate aminotransf
798	1	db$2	2::C3K3S1|C3K3S1_PSEFS	107	51070	7	5	6	4	0.45	Putative oxidase OS=P
799	1	db$2	2::C3K7I9|C3K7I9_PSEFS	107	88241	7	6	7	6	0.38	Putative pinecillin acyl
800	1	db$2	2::C3KC07|C3KC07_PSEFS	107	21449	4	4	4	4	1.40	YceI domain-containin
801	1	db$2	2::C3KD21|C3KD21_PSEFS	106	81698	5	5	5	5	0.34	Putative glycogen debr
802	1	db$2	2::C3K6M4|C3K6M4_PSEFS	106	13463	4	4	2	2	3.00	Succinate dehydrogena
803	1	db$2	2::C3KCG1|C3KCG1_PSEFS	106	60384	2	2	2	2	0.17	Putative secreted amic
804	1	db$2	2::C3JZH3|C3JZH3_PSEFS	106	22428	3	2	3	2	0.52	Putative cytochrome o
805	1	SwissProt_HUMAN$	3::P0DJI8|SAA1_HUMAN	106	13524	2	2	1	1	0.41	Serum amyloid A-1 pro
806	1	SwissProt_HUMAN$	3::Q9BQE3|TBA1C_HUMAN	106	49863	5	4	5	4	0.46	Tubulin alpha-1C chair
807	1	db$2	2::C3KAS9|C3KAS9_PSEFS	106	109677	3	2	3	2	0.09	Efflux pump membran
808	1	db$2	2::C3K3K7|C3K3K7_PSEFS	106	24490	3	3	3	3	0.78	Pyridoxal phosphate h
809	1	db$2	2::C3KAC0|C3KAC0_PSEFS	105	40546	4	4	3	3	0.42	Butyryl-CoA dehydrog
810	1	db$2	2::C3KDX7|ISPH_PSEFS	105	34499	5	5	4	4	0.73	4-hydroxy-3-methylbu
811	1	db$2	2::C3KE82|C3KE82_PSEFS	105	53340	5	3	5	3	0.30	Aldehyde dehydrogena
812	1	db$2	2::C3K2U5|C3K2U5_PSEFS	105	18989	4	4	4	4	1.69	Single-stranded DNA-b
813	1	db$2	2::C3KBU0|C3KBU0_PSEFS	105	36726	5	4	5	4	0.67	Sulfate/thiosulfate imp
814	1	db$2	2::C3K709|C3K709_PSEFS	105	7722	4	4	2	2	2.25	Cold shock protein OS
815	1	db$2	2::C3K8W4|C3K8W4_PSEFS	105	18790	7	7	5	5	3.48	Lipopolysaccharide exp
816	1	db$2	2::C3K1A8|C3K1A8_PSEFS	104	35739	4	3	4	3	0.49	Putative lipoprotein OS
817	1	db$2	2::C3KBH8|C3KBH8_PSEFS	104	25745	4	4	4	4	1.08	3-oxoacyl-[acyl-carrier
818	1	db$2	2::C3KB19|C3KB19_PSEFS	104	44434	6	4	5	4	0.53	HlyD family secretion p
819	1	db$2	2::C3KDM3|C3KDM3_PSEFS	104	25575	2	2	2	2	0.45	Putative lipoprotein OS
820	1	db$2	2::C3K8K4|C3K8K4_PSEFS	104	58330	3	3	3	3	0.28	Putative outer membra
821	1	db$2	2::C3KE66|C3KE66_PSEFS	104	27011	4	4	4	4	1.01	23S rRNA (guanosine-
822	1	db$2	2::C3KCT0|MURG_PSEFS	104	37667	4	4	4	4	0.65	UDP-N-acetylglucosam
823	1	db$2	2::C3KDE9|C3KDE9_PSEFS	104	41781	3	3	3	3	0.40	Putative phage tail-rel
824	1	db$2	2::C3K650|C3K650_PSEFS	103	13083	5	5	4	4	3.14	N-acetyltransferase do
825	1	db$2	2::C3K5M0|C3K5M0_PSEFS	103	29300	4	4	4	4	0.90	Phosphonates ABC tra
826	1	db$2	2::C3JXQ1|C3JXQ1_PSEFS	103	24479	3	3	2	2	0.47	Putative enoyl-CoA hy
827	1	db$2	2::C3K7C4|C3K7C4_PSEFS	103	53137	11	7	10	6	0.71	DNA-binding transcript
828	1	db$2	2::C3K1F7|C3K1F7_PSEFS	103	69251	7	5	7	5	0.41	tRNA uridine 5-carbox
829	1	db$2	2::C3K2Y1|RS12_PSEFS	102	13716	3	3	2	2	0.97	30S ribosomal protein
830	1	db$2	2::C3K2S3|C3K2S3_PSEFS	102	17933	5	4	4	3	1.18	Transcriptional repress
831	1	db$2	2::C3K0D7|APEB_PSEFS	102	46914	5	5	5	5	0.66	Probable M18 family a
832	1	db$2	2::C3KAM2|C3KAM2_PSEFS	102	49640	2	2	2	2	0.21	Putative two-compone
833	1	db$2	2::C3K4J0|C3K4J0_PSEFS	102	17598	3	3	3	3	1.22	Zn_protease domain-c

↑Family	M	DB	Accession	Score	Mass	Matches	Match(sig)	Sequences	Seq(sig)	emPAI	Description
834	1	db$2	2::C3K486|C3K486_PSEFS	101	30593	4	3	4	3	0.59	Epimerase domain-cor
835	1	db$2	2::C3K061|C3K061_PSEFS	101	18792	4	4	4	4	1.72	DUF2059 domain-cont
836	1	db$2	2::C3JY71|C3JY71_PSEFS	101	43919	1	1	1	1	0.11	JmjC domain-containir
837	1	db$2	2::C3K3B7|C3K3B7_PSEFS	101	14934	2	2	2	2	0.86	Uncharacterized protei
838	1	db$2	2::C3K4W3|C3K4W3_PSEFS	101	21770	2	2	2	2	0.54	PilZ domain-containing
839	1	db$2	2::C3K3M6|C3K3M6_PSEFS	101	53012	4	3	4	3	0.31	Peptidase_M16_C dom
840	1	db$2	2::C3K5E8|C3K5E8_PSEFS	101	28997	3	3	3	3	0.63	Cyclohexadienyl dehyc
841	1	db$2	2::C3K197|C3K197_PSEFS	101	25331	4	3	3	3	0.75	Putative two-compone
842	1	db$2	2::C3K7Y8|C3K7Y8_PSEFS	101	53697	6	5	6	5	0.55	Putative hydrolase OS
843	1	db$2	2::C3K7E3|C3K7E3_PSEFS	101	178026	8	6	8	6	0.17	Alpha-2-macroglobulin
844	1	db$2	2::C3KBZ0|PYRC_PSEFS	100	38385	4	4	4	4	0.64	Dihydroorotase OS=Ps
845	1	db$2	2::C3KE20|C3KE20_PSEFS	100	33868	5	5	4	4	0.75	GCV_T domain-contair
846	1	db$2	2::C3K126|C3K126_PSEFS	100	54569	7	7	6	6	0.68	2-methylcitrate dehydı
847	1	db$2	2::C3K2E0|C3K2E0_PSEFS	100	48742	7	7	7	7	0.97	DNA repair protein Rac
848	1	db$2	2::C3K5K2|C3K5K2_PSEFS	100	9923	3	3	3	3	3.07	Putative lipoprotein OS
849	1	db$2	2::C3K6J2|C3K6J2_PSEFS	100	47817	3	3	3	3	0.35	Putative hydrolase OS
850	1	db$2	2::C3K2W3|C3K2W3_PSEFS	100	11427	7	6	6	6	10.41	30S ribosomal protein
851	1	db$2	2::C3JXH2|C3JXH2_PSEFS	100	91149	3	3	3	3	0.17	Putative excinuclease
852	1	SwissProt_HUMAN$	3::Q5QNW6|H2B2F_HUMAN	100	13912	4	4	3	3	2.83	Histone H2B type 2-F (
853	1	db$2	2::C3K9T0|NADK_PSEFS	100	32289	4	4	4	4	0.79	NAD kinase OS=Pseud
854	1	db$2	2::C3K2X4|RL23_PSEFS	99	10938	8	7	7	7	18.13	50S ribosomal protein
855	1	db$2	2::C3KAI3|AROB_PSEFS	99	39825	3	3	3	3	0.43	3-dehydroquinate synt
856	1	db$2	2::C3K8K8|C3K8K8_PSEFS	99	62203	6	6	6	6	0.58	Putative exported prot
857	1	db$2	2::C3K170|C3K170_PSEFS	99	22875	4	4	3	3	0.85	Flavin_Reduct domain-
858	1	db$2	2::C3KE58|C3KE58_PSEFS	99	84757	8	8	6	6	0.40	Putative S1 RNA-bindi
859	1	db$2	2::C3JXY4|ASTE_PSEFS	98	37404	5	4	4	4	0.66	Succinylglutamate des
860	1	db$2	2::C3JY61|C3JY61_PSEFS	98	8297	4	4	3	3	8.09	Translation initiation fa
861	1	db$2	2::C3JY84|C3JY84_PSEFS	98	18006	4	4	4	4	1.83	NADH dehydrogenase
862	1	SwissProt_HUMAN$	3::P02647|APOA1_HUMAN	98	30759	3	3	3	3	0.58	Apolipoprotein A-I OS=
863	1	db$2	2::C3KCS6|C3KCS6_PSEFS	98	47227	3	3	3	3	0.35	UDP-N-acetylmuramoy
864	1	db$2	2::C3KDA5|C3KDA5_PSEFS	97	54170	2	1	2	1	0.09	Uncharacterized protei
865	1	db$2	2::C3K6E5|C3K6E5_PSEFS	96	29069	4	3	3	3	0.63	Cytochrome c1 OS=Ps
866	1	db$2	2::C3K9P7|C3K9P7_PSEFS	96	69450	4	3	4	3	0.23	Putative substrate-bin
867	1	db$2	2::C3K5T5|C3K5T5_PSEFS	96	25405	4	4	2	2	0.45	Carbonic anhydrase OS
868	1	db$2	2::C3K487|C3K487_PSEFS	96	33600	2	2	2	2	0.32	Biopolymer transport p
869	1	SwissProt_HUMAN$	3::P0C0L4|CO4A_HUMAN	96	192664	2	2	2	2	0.05	Complement C4-A OS=
870	1	db$2	2::C3JXM0|C3JXM0_PSEFS	96	45141	3	2	3	2	0.23	Putative peptidase OS
871	1	db$2	2::C3K0P9|C3K0P9_PSEFS	95	25457	3	3	3	3	0.74	Two-component syster
872	1	db$2	2::C3K0R6|C3K0R6_PSEFS	95	28374	6	6	6	6	1.70	Uncharacterized protei
873	1	db$2	2::C3KDI2|C3KDI2_PSEFS	95	135789	2	2	2	2	0.07	Exonuclease OS=Pseu

↑Family	M	DB	Accession	Score	Mass	Matches	Match(sig)	Sequences	Seq(sig)	emPAI	Description
874	1	db$2	2::C3KDM6|PSD_PSEFS	95	31380	5	4	4	3	0.82	Phosphatidylserine dec
875	1	db$2	2::C3K1X3|C3K1X3_PSEFS	95	20670	5	5	4	4	1.48	Glutathione peroxidase
876	1	db$2	2::C3K3T8|C3K3T8_PSEFS	95	39499	3	3	3	3	0.43	Putrescine ABC transpo
877	1	db$2	2::C3KAX3|C3KAX3_PSEFS	94	30360	3	3	3	3	0.59	Conserved hypothetica
878	1	db$2	2::C3JXL6|C3JXL6_PSEFS	94	41978	2	2	1	1	0.12	Bcr/CflA family efflux t
879	1	db$2	2::C3K3K1|C3K3K1_PSEFS	94	18188	2	2	2	2	0.67	Bifunctional protein [p
880	1	db$2	2::C3K0T7|C3K0T7_PSEFS	94	76509	7	6	6	5	0.45	Putative fatty acid oxic
881	1	db$2	2::C3KAY1|C3KAY1_PSEFS	94	44657	5	5	5	5	0.70	3-phosphoshikimate 1-
882	1	db$2	2::C3K4W2|C3K4W2_PSEFS	94	26844	5	3	4	3	0.69	Putative glycerophosph
883	1	db$2	2::C3KBV0|C3KBV0_PSEFS	94	28603	5	4	4	3	0.64	Putative chromosome
884	1	SwissProt_HUMAN$	3::P23396|RS3_HUMAN	93	26671	4	4	4	4	1.03	40S ribosomal protein
885	1	db$2	2::C3KBJ5|C3KBJ5_PSEFS	93	62095	4	4	4	4	0.36	Phospholipase C OS=P
886	1	db$2	2::C3K304|C3K304_PSEFS	93	15054	3	3	3	3	1.53	Uncharacterized protei
887	1	db$2	2::C3K3K8|C3K3K8_PSEFS	93	27898	2	2	1	1	0.18	Pyrroline-5-carboxylat
888	1	db$2	2::C3K544|C3K544_PSEFS	93	74165	6	6	6	6	0.47	Uncharacterized protei
889	1	db$2	2::C3K7Z1|C3K7Z1_PSEFS	92	38822	6	6	6	6	1.07	NAD(P) transhydrogen
890	1	db$2	2::C3JZR8|C3JZR8_PSEFS	92	46446	9	7	7	6	0.84	Putative oxidoreductas
891	1	db$2	2::C3K1W6|C3K1W6_PSEFS	92	13390	4	4	4	4	3.00	D-erythro-7,8-dihydro
892	1	db$2	2::C3K6Y6|C3K6Y6_PSEFS	92	31839	9	6	7	5	1.10	Signal peptidase I OS=
893	1	db$2	2::C3K3J3|C3K3J3_PSEFS	92	72855	3	3	3	3	0.22	Type IV pilus biogenes
894	1	db$2	2::C3KDC4|C3KDC4_PSEFS	92	64677	3	3	3	3	0.25	Uncharacterized protei
895	1	db$2	2::C3KDP0|C3KDP0_PSEFS	92	27498	2	2	2	2	0.41	Uncharacterized protei
896	1	db$2	2::C3K9N1|C3K9N1_PSEFS	92	43216	5	5	4	4	0.55	D-ser_dehydrat domai
897	1	db$2	2::C3JZ77|C3JZ77_PSEFS	92	45611	3	3	2	2	0.23	Maltose-binding protei
898	1	db$2	2::C3K3X0|C3K3X0_PSEFS	91	42989	4	3	4	3	0.39	Putative ubiquinone bi
899	1	db$2	2::C3KDC6|RF1_PSEFS	91	39857	1	1	1	1	0.13	Peptide chain release f
900	1	db$2	2::C3K7C7|C3K7C7_PSEFS	91	19943	3	3	3	3	1.02	Chorismate mutase OS
901	1	db$2	2::C3KBI8|C3KBI8_PSEFS	91	48805	3	2	3	2	0.21	Uncharacterized protei
902	1	db$2	2::C3K6A4|C3K6A4_PSEFS	91	36459	4	3	4	3	0.48	Methionine import ATP
903	1	db$2	2::C3K617|C3K617_PSEFS	91	19384	5	3	4	2	0.62	Uncharacterized protei
904	1	db$2	2::C3K230|C3K230_PSEFS	90	85084	5	3	5	3	0.18	Penicillin-binding prote
905	1	db$2	2::C3KED2|C3KED2_PSEFS	90	17293	4	4	4	4	1.95	Thioredoxin peroxidase
906	1	db$2	2::C3KB73|C3KB73_PSEFS	90	9692	1	1	1	1	0.61	Putative lipoprotein OS
907	1	db$2	2::C3K103|C3K103_PSEFS	90	23527	5	4	4	3	0.82	Phosphoribosylglycinar
908	1	db$2	2::C3K868|C3K868_PSEFS	90	58117	5	4	5	4	0.39	Uncharacterized protei
909	1	db$2	2::C3KDC0|C3KDC0_PSEFS	90	36735	3	3	3	3	0.47	Uncharacterized protei
910	1	db$2	2::C3JYN9|C3JYN9_PSEFS	90	44994	4	3	4	3	0.37	Pseudouridine synthas
911	1	db$2	2::C3KE59|C3KE59_PSEFS	90	27398	8	6	8	6	1.80	DNA-binding response
912	1	db$2	2::C3KE56|C3KE56_PSEFS	90	59021	6	5	6	5	0.49	Glutamate--cysteine li
913	1	db$2	2::C3KEC3|C3KEC3_PSEFS	90	16145	4	3	3	2	0.79	Putative membrane pr

↑Family	M	DB	Accession	Score	Mass	Matches	Match(sig)	Sequences	Seq(sig)	emPAI	Description
914	1	db$2	2::C3K3R6|C3K3R6_PSEFS	89	54762	8	7	7	7	0.83	L-threonine dehydrata
915	1	db$2	2::C3K0K1|C3K0K1_PSEFS	88	53436	1	1	1	1	0.09	Putative GntR-family r
916	1	db$2	2::C3K3K9|C3K3K9_PSEFS	88	21575	4	4	2	2	0.55	Conserved hypothetica
917	1	db$2	2::C3KCS0|RL25_PSEFS	88	21502	8	7	4	4	1.98	50S ribosomal protein
918	1	db$2	2::C3K0H6|C3K0H6_PSEFS	88	40054	6	6	6	6	1.03	Protein-glutamate met
919	1	db$2	2::C3K0M6|C3K0M6_PSEFS	88	25497	6	5	6	5	1.51	Putative tetR-family re
920	1	db$2	2::C3K415|C3K415_PSEFS	88	70809	4	3	4	3	0.22	Putative ABC transport
921	1	db$2	2::C3K3S3|C3K3S3_PSEFS	88	15261	4	4	2	2	0.85	DUF4399 domain-cont
922	1	db$2	2::C3K087|C3K087_PSEFS	88	34228	3	2	3	2	0.32	50S ribosomal protein
923	1	db$2	2::C3K110|C3K110_PSEFS	88	32414	4	4	4	4	0.79	Cysteine synthase OS=
924	1	db$2	2::C3JY52|C3JY52_PSEFS	87	37777	3	3	3	3	0.46	GST C-terminal domai
925	1	db$2	2::C3K1N5|QUEA_PSEFS	87	38396	3	2	3	2	0.28	S-adenosylmethionine
926	1	db$2	2::C3K0U4|C3K0U4_PSEFS	87	36872	7	5	6	5	0.90	Flagellar motor switch
927	1	db$2	2::C3KDG5|C3KDG5_PSEFS	87	23599	5	5	5	5	1.70	Putative regulatory pro
928	1	db$2	2::C3K3E2|C3K3E2_PSEFS	87	17110	2	2	2	2	0.73	Putative acetyltransfer
929	1	db$2	2::C3KC04|C3KC04_PSEFS	86	39513	5	4	4	4	0.61	Putative hydrolase OS=
930	1	db$2	2::C3KAK2|C3KAK2_PSEFS	86	10989	6	5	5	4	4.40	Uncharacterized protei
931	1	db$2	2::C3K5X6|C3K5X6_PSEFS	86	49358	6	5	6	5	0.61	Tryptophan--tRNA liga
932	1	db$2	2::C3K015|C3K015_PSEFS	86	53791	5	4	5	4	0.42	Putative ferredoxin OS
933	1	db$2	2::C3K8P0|C3K8P0_PSEFS	86	38329	3	3	3	3	0.45	Putative nucleoside-se
934	1	db$2	2::C3KCP1|C3KCP1_PSEFS	86	23476	3	2	3	2	0.49	Peptide methionine sul
935	1	db$2	2::C3KDL3|C3KDL3_PSEFS	86	70148	7	6	6	5	0.40	Phosphomethylpyrimid
936	1	db$2	2::C3KE84|C3KE84_PSEFS	86	35425	5	3	4	3	0.49	Pseudouridine synthas
937	1	db$2	2::C3KBY4|C3KBY4_PSEFS	85	31613	3	3	3	3	0.56	Ribosomal RNA small s
938	1	db$2	2::C3JZH1|C3JZH1_PSEFS	85	34020	3	3	3	3	0.52	LysR-family transcripti
939	1	db$2	2::C3KAM8|C3KAM8_PSEFS	85	52747	4	4	4	4	0.43	Phosphate transporter
940	1	db$2	2::C3K1A3|C3K1A3_PSEFS	85	26906	1	1	1	1	0.19	Putative pyridoxamine
941	1	db$2	2::C3KBG3|C3KBG3_PSEFS	85	24033	4	4	4	4	1.18	Curli production asser
942	1	db$2	2::C3KCV6|C3KCV6_PSEFS	84	40154	5	5	5	5	0.80	Probable alcohol dehyc
943	1	db$2	2::C3K5G2|C3K5G2_PSEFS	84	35788	5	3	5	3	0.49	Putative membrane pr
944	1	SwissProt_HUMAN$	3::P62826|RAN_HUMAN	84	24408	3	3	3	3	0.78	GTP-binding nuclear pi
945	1	db$2	2::C3K7T1|C3K7T1_PSEFS	84	23131	6	5	6	5	1.77	Two-component syster
946	1	db$2	2::C3KDS5|C3KDS5_PSEFS	84	41122	3	3	3	3	0.41	Putative hydratase/iso
947	1	db$2	2::C3KE32|C3KE32_PSEFS	84	20912	1	1	1	1	0.25	Sigma factor negative
948	1	db$2	2::C3K303|COQ7_PSEFS	84	23866	3	3	3	3	0.81	3-demethoxyubiquinol
949	1	db$2	2::C3JXX3|C3JXX3_PSEFS	84	52842	3	2	3	2	0.20	Cytochrome-c oxidase
950	1	db$2	2::C3KC09|C3KC09_PSEFS	84	83087	3	3	3	3	0.19	Periplasmic beta-gluco
951	1	db$2	2::C3K470|C3K470_PSEFS	83	58011	5	5	5	5	0.50	Putative transport-rela
952	1	db$2	2::C3K9H7|C3K9H7_PSEFS	83	27490	2	2	2	2	0.41	Putative short-chain de
953	1	db$2	2::C3KEB0|C3KEB0_PSEFS	83	36597	4	4	2	2	0.29	Putative amino acid AE

↑Family	M	DB	Accession	Score	Mass	Matches	Match(sig)	Sequences	Seq(sig)	emPAI	Description
954	1	db$2	2::C3KBL9|C3KBL9_PSEFS	83	27726	5	5	5	5	1.34	Glycerol-3-phosphate
955	1	db$2	2::C3K968|C3K968_PSEFS	83	55197	4	4	4	4	0.41	Putative aldehyde deh
956	1	db$2	2::C3K481|C3K481_PSEFS	83	23434	3	3	3	3	0.83	Guanylate kinase OS=
957	1	db$2	2::C3K4D7|C3K4D7_PSEFS	83	14435	1	1	1	1	0.38	Putative thioesterase (
958	1	db$2	2::C3K705|SURE_PSEFS	82	26431	2	2	2	2	0.43	5'-nucleotidase SurE C
959	1	db$2	2::C3K0W1|C3K0W1_PSEFS	82	27483	4	4	4	4	0.98	Flagellar basal-body rc
960	1	db$2	2::C3JZ24|C3JZ24_PSEFS	82	24729	5	3	5	3	0.77	Putative GntR-family r
961	1	db$2	2::C3K1U6|C3K1U6_PSEFS	82	74730	5	5	5	5	0.37	Cytochrome bo(3) ubi
962	1	db$2	2::C3K257|C3K257_PSEFS	82	33362	6	6	4	4	0.76	tRNA pseudouridine sy
963	1	db$2	2::C3K4P8|C3K4P8_PSEFS	82	24008	1	1	1	1	0.22	t(6)A37 threonylcarba
964	1	db$2	2::C3KEC9|C3KEC9_PSEFS	81	53005	2	2	2	2	0.20	Putative beta-barrel as
965	1	db$2	2::C3KE89|C3KE89_PSEFS	81	24346	4	3	4	3	0.79	Uncharacterized protei
966	1	db$2	2::C3K0R0|C3K0R0_PSEFS	81	14751	2	2	2	2	0.88	Flagellar basal body ro
967	1	db$2	2::C3JXX6|DNLJ_PSEFS	81	85515	5	4	5	4	0.25	DNA ligase OS=Pseud
968	1	db$2	2::C3K5M1|C3K5M1_PSEFS	81	26375	4	2	3	2	0.43	Phosphonates ABC tra
969	1	db$2	2::C3KED3|C3KED3_PSEFS	81	20551	3	3	3	3	0.99	Glycine cleavage syste
970	1	db$2	2::C3K6Y8|C3K6Y8_PSEFS	80	34148	8	8	6	6	1.63	GTPase Era OS=Pseud
971	1	db$2	2::C3K0M7|C3K0M7_PSEFS	80	28601	2	2	2	2	0.39	Putative deoxyribonucl
972	1	db$2	2::C3K0X3|C3K0X3_PSEFS	80	16227	2	2	2	2	0.78	Transcriptional regulat
973	1	db$2	2::C3JZ82|C3JZ82_PSEFS	80	37007	2	2	2	2	0.29	Putative dehydrogenas
974	1	db$2	2::C3JXY2|C3JXY2_PSEFS	80	35731	2	2	2	2	0.30	L-allo-threonine aldola
975	1	db$2	2::C3K3V4|C3K3V4_PSEFS	80	41512	2	1	2	1	0.12	Acetylornithine deacet
976	1	db$2	2::C3JXJ5|C3JXJ5_PSEFS	80	22111	3	3	2	2	0.53	Putative SCO1/SenC fa
977	1	db$2	2::C3K0X7|C3K0X7_PSEFS	80	126663	1	1	1	1	0.04	Histidine kinase OS=P
978	1	db$2	2::C3KAH2|C3KAH2_PSEFS	80	24970	4	4	3	3	0.76	SPOR domain-containi
979	1	db$2	2::C3KAJ9|C3KAJ9_PSEFS	80	20836	4	3	4	3	0.96	Putative hypoxanthine
980	1	db$2	2::C3K0R1|C3K0R1_PSEFS	80	30842	2	2	2	2	0.36	Protein-glutamate O-m
981	1	db$2	2::C3K586|C3K586_PSEFS	80	21064	5	5	5	5	2.04	ADP compounds hydro
982	1	db$2	2::C3K754|C3K754_PSEFS	79	29479	1	1	1	1	0.17	Putative lipoprotein OS
983	1	db$2	2::C3K7B0|C3K7B0_PSEFS	79	28681	1	1	1	1	0.18	Putative ABC transport
984	1	db$2	2::C3JY38|C3JY38_PSEFS	79	16812	3	3	3	3	1.31	Putative thioredoxin-re
985	1	db$2	2::C3K0U9|C3K0U9_PSEFS	79	55465	4	3	3	2	0.19	Putative two-compone
986	1	db$2	2::C3KE17|C3KE17_PSEFS	79	24430	3	3	3	3	0.78	Two component syster
987	1	db$2	2::C3JYU5|C3JYU5_PSEFS	79	34090	3	3	3	3	0.52	Nudix hydrolase doma
988	1	db$2	2::C3KBY0|GMHA_PSEFS	78	21250	3	2	3	2	0.56	Phosphoheptose isome
989	1	db$2	2::C3KBM1|C3KBM1_PSEFS	78	29989	2	2	2	2	0.37	Glycerol uptake facilita
990	1	db$2	2::C3K349|C3K349_PSEFS	78	28994	4	4	4	4	0.92	Pyrroloquinoline-quino
991	1	SwissProt_HUMAN$	3::P62937|PPIA_HUMAN	78	18001	4	4	4	4	1.83	Peptidyl-prolyl cis-tran
992	1	db$2	2::C3KDN6|C3KDN6_PSEFS	78	32369	1	1	1	1	0.16	Uncharacterized protei
993	1	db$2	2::C3K7M5|C3K7M5_PSEFS	78	15789	2	2	2	2	0.81	Uncharacterized protei

↑Family	M	DB	Accession	Score	Mass	Matches	Match(sig)	Sequences	Seq(sig)	emPAI	Description
994	1	db$2	2::C3K3Q4|C3K3Q4_PSEFS	78	38576	4	4	3	3	0.44	Putative cation ABC tra
995	1	db$2	2::C3K1A4|C3K1A4_PSEFS	78	41199	1	1	1	1	0.12	Uncharacterized protei
996	1	SwissProt_HUMAN$	3::P62979|RS27A_HUMAN	77	17953	3	3	3	3	1.18	Ubiquitin-40S ribosom
997	1	db$2	2::C3K146|C3K146_PSEFS	77	27134	3	3	3	3	0.68	Fumarate and nitrate r
998	1	db$2	2::C3K605|LPXD_PSEFS	77	36124	2	2	2	2	0.30	UDP-3-O-acylglucosam
999	1	db$2	2::C3K1B3|C3K1B3_PSEFS	77	20726	3	2	3	2	0.57	Uncharacterized protei
1000	1	db$2	2::C3KCJ3|C3KCJ3_PSEFS	77	30975	5	4	3	2	0.36	Usp domain-containing
1001	1	db$2	2::C3K830|C3K830_PSEFS	77	47217	6	6	6	6	0.83	Putative short chain de
1002	1	db$2	2::C3K7J3|Y1319_PSEFS	77	16953	2	2	2	2	0.73	UPF0225 protein PFLU_
1003	1	db$2	2::C3KBT9|C3KBT9_PSEFS	77	31336	1	1	1	1	0.16	Sulfate ABC transport
1004	1	db$2	2::C3KE10|C3KE10_PSEFS	76	58823	3	3	3	3	0.27	Putative methyl-accept
1005	1	db$2	2::C3K838|C3K838_PSEFS	76	9715	2	2	2	2	1.57	Putative phosphocarrie
1006	1	db$2	2::C3K862|C3K862_PSEFS	76	39825	3	3	2	2	0.43	Putative outer membra
1007	1	db$2	2::C3K3N0|C3K3N0_PSEFS	76	25405	2	2	1	1	0.20	Putative TetR-family re
1008	1	db$2	2::C3K2K7|C3K2K7_PSEFS	76	37713	3	3	3	3	0.46	PhoH domain-containi
1009	1	db$2	2::C3K1L5|C3K1L5_PSEFS	75	34147	4	4	4	4	0.74	Putative DNA-binding
1010	1	db$2	2::C3JY31|C3JY31_PSEFS	75	22860	3	3	3	3	0.85	Putative biopolymer tr
1011	1	db$2	2::C3K666|C3K666_PSEFS	75	29461	2	2	2	2	0.38	Putative lipoprotein OS
1012	1	SwissProt_HUMAN$	3::Q71UM5|RS27L_HUMAN	75	9471	2	2	2	2	1.66	40S ribosomal protein
1013	1	db$2	2::C3K3D1|BETI_PSEFS	75	21992	1	1	1	1	0.24	HTH-type transcription
1014	1	db$2	2::C3JXY6|ASTB_PSEFS	74	48741	5	5	5	5	0.63	N-succinylarginine dihy
1015	1	db$2	2::C3K229|C3K229_PSEFS	74	24861	1	1	1	1	0.21	TPR_REGION domain-
1016	1	db$2	2::C3K440|C3K440_PSEFS	74	11389	4	4	3	3	4.07	Uncharacterized protei
1017	1	db$2	2::C3KCN6|C3KCN6_PSEFS	74	53164	2	2	2	2	0.20	Sodium/proline sympo
1018	1	db$2	2::C3K9Q9|C3K9Q9_PSEFS	74	19670	1	1	1	1	0.27	FMN_red domain-cont
1019	1	db$2	2::C3K863|PDXH_PSEFS	74	24884	3	3	3	3	0.76	Pyridoxine/pyridoxami
1020	1	SwissProt_HUMAN$	3::Q07020|RL18_HUMAN	74	21621	3	2	3	2	0.54	60S ribosomal protein
1021	1	db$2	2::C3K5X9|C3K5X9_PSEFS	74	41639	2	2	2	2	0.26	Putative acyl-CoA dehy
1022	1	db$2	2::C3K6V8|C3K6V8_PSEFS	74	47941	4	3	3	3	0.34	Putative peptidase OS
1023	1	db$2	2::C3K0Q8|C3K0Q8_PSEFS	74	24717	2	2	2	2	0.46	Basal-body rod modific
1024	1	db$2	2::C3K2D4|C3K2D4_PSEFS	73	35439	4	4	4	4	0.70	Putative cobalamin syr
1025	1	SwissProt_HUMAN$	3::Q86YZ3|HORN_HUMAN	73	282228	2	2	1	1	0.03	Hornerin OS=Homo sa
1026	1	db$2	2::Q9ZEP7|PANC_PSEFS	73	30696	2	2	2	2	0.36	Pantothenate syntheta
1027	1	SwissProt_HUMAN$	3::P15531|NDKA_HUMAN	73	17138	6	6	4	4	1.99	Nucleoside diphosphat
1028	1	db$2	2::C3K0C5|C3K0C5_PSEFS	73	27111	2	2	2	2	0.42	Putative amino transfe
1029	1	db$2	2::C3KDQ0|C3KDQ0_PSEFS	73	35105	2	2	2	2	0.31	2-ketogluconate reduc
1030	1	db$2	2::C3KE45|C3KE45_PSEFS	73	49639	7	5	7	5	0.61	Trk system potassium
1031	1	db$2	2::C3JYR2|C3JYR2_PSEFS	73	48934	4	4	4	4	0.47	L-serine dehydratase (
1031	2	db$2	2::C3K5Y8|C3K5Y8_PSEFS	61	48751	2	2	2	2	0.21	L-serine dehydratase (
1032	1	db$2	2::C3JYE6|C3JYE6_PSEFS	73	45437	3	3	3	3	0.37	Putative acyl-CoA dehy

↑Family	M	DB	Accession	Score	Mass	Matches	Match(sig)	Sequences	Seq(sig)	emPAI	Description
1033	1	db$2	2::C3K8W5|C3K8W5_PSEFS	73	21382	2	2	2	2	0.55	Lipopolysaccharide exp
1034	1	db$2	2::C3K0S0|C3K0S0_PSEFS	72	12378	4	4	4	4	3.46	Putative DNA-binding
1035	1	SwissProt_HUMAN$	3::P62249|RS16_HUMAN	72	16435	2	2	2	2	0.77	40S ribosomal protein
1036	1	db$2	2::C3K5B5|C3K5B5_PSEFS	72	37023	2	2	2	2	0.29	Dipeptide ABC transpo
1037	1	db$2	2::C3K6W8|C3K6W8_PSEFS	72	16611	2	2	2	2	0.75	3-dehydroquinate deh
1038	1	db$2	2::C3KC02|C3KC02_PSEFS	72	29258	3	3	3	3	0.62	Putative acyltransferas
1039	1	db$2	2::C3K4A7|C3K4A7_PSEFS	72	18391	3	2	3	2	0.66	Uncharacterized protei
1040	1	db$2	2::C3K998|C3K998_PSEFS	72	35021	4	3	4	3	0.50	Putative dehydrogenas
1041	1	db$2	2::C3KE78|C3KE78_PSEFS	72	15753	1	1	1	1	0.35	Azurin OS=Pseudomor
1042	1	db$2	2::C3K9B4|C3K9B4_PSEFS	72	16186	2	2	1	1	0.78	Type II secretion syste
1043	1	db$2	2::C3JY57|LOLA_PSEFS	71	22546	5	5	5	5	1.84	Outer-membrane lipop
1044	1	db$2	2::C3KDN7|HEMH_PSEFS	71	38415	5	3	5	3	0.45	Ferrochelatase OS=Ps
1045	1	db$2	2::C3K6W5|PRMA_PSEFS	71	31717	2	2	2	2	0.35	Ribosomal protein L11
1046	1	SwissProt_HUMAN$	3::P61604|CH10_HUMAN	71	10925	4	3	4	3	2.59	10 kDa heat shock pro
1047	1	db$2	2::C3K258|RBFA_PSEFS	71	14697	3	3	3	3	1.60	Ribosome-binding fact
1048	1	db$2	2::C3K621|C3K621_PSEFS	71	26068	4	4	4	4	1.06	Putative TetR-family re
1049	1	db$2	2::C3K0M3|C3K0M3_PSEFS	71	20567	4	4	4	4	1.49	Uncharacterized protei
1050	1	db$2	2::C3K2T3|C3K2T3_PSEFS	71	32553	4	4	4	4	0.79	dTDP-4-dehydrorhamr
1051	1	db$2	2::C3KDV5|C3KDV5_PSEFS	71	20461	5	3	4	3	0.99	Oligoribonuclease OS=
1052	1	db$2	2::C3KA82|C3KA82_PSEFS	71	19457	4	3	4	3	1.06	Lactoylglutathione lyas
1053	1	db$2	2::C3K3D8|C3K3D8_PSEFS	70	49942	5	5	4	4	0.46	ATP-independent RNA
1054	1	db$2	2::C3K092|C3K092_PSEFS	70	14085	4	4	4	4	2.75	Glutaredoxin domain-c
1055	1	db$2	2::C3KDV4|RSGA_PSEFS	70	37296	5	3	4	2	0.29	Small ribosomal subun
1056	1	db$2	2::C3K1C5|C3K1C5_PSEFS	70	44321	3	2	3	2	0.24	Putative cyclopropane-
1057	1	db$2	2::C3K328|APAG_PSEFS	70	13631	2	2	2	2	0.98	Protein ApaG OS=Pseu
1058	1	db$2	2::C3K364|C3K364_PSEFS	70	38824	3	3	3	3	0.44	Biotin synthase OS=Ps
1059	1	db$2	2::C3K6U7|C3K6U7_PSEFS	70	79141	6	4	6	4	0.27	Putative exported prot
1060	1	db$2	2::C3K5K4|C3K5K4_PSEFS	69	29062	3	2	3	2	0.38	Uncharacterized protei
1061	1	db$2	2::C3K1B0|C3K1B0_PSEFS	69	96490	1	1	1	1	0.05	Lysylphosphatidylglyce
1062	1	db$2	2::C3KB06|C3KB06_PSEFS	69	29724	1	1	1	1	0.17	Putative lipoprotein OS
1063	1	db$2	2::C3K5X5|C3K5X5_PSEFS	69	22739	2	2	2	2	0.51	Thioesterase domain-c
1064	1	db$2	2::C3KDX3|C3KDX3_PSEFS	69	34279	4	4	4	4	0.73	Riboflavin biosynthesis
1065	1	db$2	2::C3K6K2|IHFB_PSEFS	69	11021	4	4	3	3	2.54	Integration host factor
1066	1	db$2	2::C3JYE9|C3JYE9_PSEFS	69	31035	3	3	2	2	0.35	Putative hydroxymeth
1067	1	db$2	2::C3JY68|MNMA_PSEFS	69	41397	4	4	4	4	0.58	tRNA-specific 2-thiouri
1068	1	db$2	2::C3KD59|C3KD59_PSEFS	69	81779	4	4	4	4	0.26	YcaO domain-containir
1069	1	db$2	2::C3K1N0|C3K1N0_PSEFS	68	18655	2	2	2	2	0.65	Rick_17kDa_Anti dom
1070	1	db$2	2::C3K3P7|C3K3P7_PSEFS	68	49977	2	2	2	2	0.21	Putative membrane pr
1071	1	db$2	2::C3KEA2|C3KEA2_PSEFS	68	30824	2	2	2	2	0.36	Putative inner membra
1072	1	db$2	2::C3K6P0|C3K6P0_PSEFS	68	15266	2	2	2	2	0.84	Uncharacterized protei

↑Family	M	DB	Accession	Score	Mass	Matches	Match(sig)	Sequences	Seq(sig)	emPAI	Description
1073	1	db$2	2::C3K2P7|C3K2P7_PSEFS	68	17680	5	3	4	3	1.22	Putative acetyltransfer
1074	1	db$2	2::C3KDF0|C3KDF0_PSEFS	68	18221	3	2	3	2	0.67	Putative phage tail-rel
1075	1	db$2	2::C3K5K1|C3K5K1_PSEFS	67	38065	6	6	6	6	1.11	Putative phospholipase
1076	1	db$2	2::C3K1M4|C3K1M4_PSEFS	67	13705	4	3	3	3	1.78	Iron-sulfur cluster asse
1077	1	db$2	2::C3K3B8|C3K3B8_PSEFS	67	42763	1	1	1	1	0.12	Uncharacterized protei
1078	1	db$2	2::C3K3X4|C3K3X4_PSEFS	67	11561	3	3	2	2	2.34	Cell division protein Za
1079	1	db$2	2::C3K5K7|C3K5K7_PSEFS	67	52604	3	3	3	3	0.31	Putative methyl-accept
1080	1	db$2	2::C3K8W2|ADE_PSEFS	67	36048	3	3	3	3	0.48	Adenine deaminase OS
1081	1	db$2	2::C3K3M9|C3K3M9_PSEFS	67	30473	4	4	4	4	0.86	Putative sulfurtransfer
1082	1	db$2	2::C3K707|C3K707_PSEFS	67	29295	2	1	2	1	0.17	Putative lipoprotein OS
1083	1	db$2	2::C3K279|C3K279_PSEFS	67	19136	2	2	2	2	0.63	Outer membrane prote
1084	1	db$2	2::C3K491|C3K491_PSEFS	67	76137	2	1	2	1	0.06	ATP-dependent DNA h
1085	1	db$2	2::C3K4C8|C3K4C8_PSEFS	67	49694	3	3	3	3	0.33	Putative transport-rela
1086	1	SwissProt_HUMAN$	3::P62913|RL11_HUMAN	67	20240	2	2	2	2	0.59	60S ribosomal protein
1087	1	db$2	2::C3KBK8|C3KBK8_PSEFS	67	35740	2	2	2	2	0.30	Ribosomal RNA small s
1088	1	db$2	2::C3K3Y7|C3K3Y7_PSEFS	67	35706	4	3	4	3	0.49	Putative iron-sulfur bir
1089	1	db$2	2::C3K5U7|C3K5U7_PSEFS	67	105852	5	2	5	2	0.09	Putative aminotransfer
1090	1	db$2	2::C3K129|C3K129_PSEFS	67	42141	2	2	2	2	0.25	Putative acetyl CoA ac
1091	1	db$2	2::C3K5T7|C3K5T7_PSEFS	66	23769	3	3	2	2	0.49	Uncharacterized protei
1092	1	db$2	2::C3K2N1|C3K2N1_PSEFS	66	47217	1	1	1	1	0.11	Putative membrane pr
1093	1	db$2	2::C3JYR0|C3JYR0_PSEFS	66	13774	2	2	2	2	0.97	Glycine cleavage syste
1094	1	db$2	2::C3KAY5|C3KAY5_PSEFS	66	30881	2	2	2	2	0.36	NAD-capped RNA hydr
1095	1	db$2	2::C3JYK2|C3JYK2_PSEFS	66	30492	2	2	2	2	0.36	Bifunctional protein Fo
1096	1	db$2	2::C3K4J4|HSLO_PSEFS	66	33320	3	3	2	2	0.33	33 kDa chaperonin OS
1097	1	db$2	2::C3JZ46|C3JZ46_PSEFS	66	37091	3	3	3	3	0.47	Putative LacI family tr
1098	1	db$2	2::C3K3L2|C3K3L2_PSEFS	66	15596	3	3	3	3	1.45	DUF4426 domain-cont
1099	1	SwissProt_HUMAN$	3::Q9H0U4|RAB1B_HUMAN	65	22157	2	2	2	2	0.53	Ras-related protein Ra
1100	1	db$2	2::C3JYF7|C3JYF7_PSEFS	65	147681	4	3	4	3	0.10	ATP-dependent helicas
1101	1	db$2	2::C3JZR9|C3JZR9_PSEFS	65	27415	4	4	4	4	0.99	Putative dehydrogenas
1102	1	db$2	2::C3KDX1|RS20_PSEFS	65	10078	3	3	2	2	1.50	30S ribosomal protein
1103	1	db$2	2::C3K601|DXR_PSEFS	64	42341	3	3	3	3	0.40	1-deoxy-D-xylulose 5-
1104	1	db$2	2::C3K173|C3K173_PSEFS	64	34695	4	4	4	4	0.72	Putative AraC-family tı
1105	1	db$2	2::C3K068|C3K068_PSEFS	64	65454	5	5	5	5	0.44	Putative asparagine sy
1106	1	db$2	2::C3K333|C3K333_PSEFS	64	61021	1	1	1	1	0.08	Uncharacterized protei
1107	1	db$2	2::C3K221|C3K221_PSEFS	64	129628	2	2	1	1	0.04	RecBCD enzyme subur
1108	1	db$2	2::C3K1H0|RIMM_PSEFS	64	20004	3	3	3	3	1.02	Ribosome maturation f
1109	1	db$2	2::C3K300|C3K300_PSEFS	64	34122	1	1	1	1	0.15	Putative membrane pr
1110	1	db$2	2::C3K0J1|C3K0J1_PSEFS	63	36130	2	2	2	2	0.30	Flagellar motor switch
1111	1	db$2	2::C3K6Y7|RNC_PSEFS	63	25367	2	2	1	1	0.20	Ribonuclease 3 OS=Ps
1112	1	db$2	2::C3K815|C3K815_PSEFS	63	62777	4	3	4	3	0.25	Glucans biosynthesis p

↑Family	M	DB	Accession	Score	Mass	Matches	Match(sig)	Sequences	Seq(sig)	emPAI	Description
1113	1	db$2	2::C3K0R2|C3K0R2_PSEFS	63	34379	1	1	1	1	0.15	Chemotaxis protein OS
1114	1	db$2	2::C3JY56|C3JY56_PSEFS	63	48640	2	2	2	2	0.21	Replication-associated
1115	1	db$2	2::C3KCS4|C3KCS4_PSEFS	63	63231	4	3	4	3	0.25	Peptidoglycan D,D-tra
1116	1	db$2	2::C3K4Z0|CCME_PSEFS	63	16101	3	3	3	3	1.39	Cytochrome c-type bio
1117	1	db$2	2::C3K9R3|C3K9R3_PSEFS	63	18863	3	2	2	2	0.64	Uncharacterized protei
1118	1	db$2	2::C3KAA9|C3KAA9_PSEFS	62	31757	1	1	1	1	0.16	Putative chemotaxis pr
1119	1	db$2	2::C3K9M9|C3K9M9_PSEFS	62	14163	1	1	1	1	0.39	Putative endoribonucle
1120	1	db$2	2::C3JYN3|C3JYN3_PSEFS	62	52328	2	2	2	2	0.20	Family S13 unassignec
1121	1	db$2	2::C3KDM9|C3KDM9_PSEFS	62	28096	3	3	3	3	0.65	Putative oxidoreductas
1122	1	SwissProt_HUMAN$	3::Q6FI13|H2A2A_HUMAN	62	14087	3	3	3	3	1.70	Histone H2A type 2-A
1122	2	SwissProt_HUMAN$	3::P04908|H2A1B_HUMAN	60	14127	3	3	3	3	1.70	Histone H2A type 1-B/
1123	1	db$2	2::C3K584|C3K584_PSEFS	62	15399	3	3	3	3	1.48	Uncharacterized protei
1124	1	db$2	2::C3K2D6|C3K2D6_PSEFS	62	73930	3	3	3	3	0.21	Carbon starvation prot
1125	1	db$2	2::C3K737|C3K737_PSEFS	62	17727	4	2	3	2	0.69	Glutathione peroxidase
1126	1	db$2	2::C3KDR2|C3KDR2_PSEFS	62	26142	7	3	3	2	0.43	S4 RNA-binding domai
1127	1	db$2	2::C3K867|C3K867_PSEFS	62	39735	5	4	4	3	0.43	PALP domain-containir
1128	1	db$2	2::C3K3K3|C3K3K3_PSEFS	62	44101	4	4	4	4	0.54	Dihydroorotase-like pr
1129	1	db$2	2::C3K5B9|C3K5B9_PSEFS	62	60269	1	1	1	1	0.08	Dipeptide ABC transpo
1130	1	db$2	2::C3K1V9|C3K1V9_PSEFS	62	16219	2	2	2	2	0.78	Flavodoxin-like domair
1131	1	db$2	2::C3KDR1|C3KDR1_PSEFS	61	17628	3	3	3	3	1.22	Uncharacterized protei
1132	1	SwissProt_HUMAN$	3::P08865|RSSA_HUMAN	61	32833	1	1	1	1	0.15	40S ribosomal protein
1133	1	SwissProt_HUMAN$	3::P60174|TPIS_HUMAN	61	26653	3	2	3	2	0.42	Triosephosphate isome
1134	1	db$2	2::C3K216|C3K216_PSEFS	61	17384	2	2	2	2	0.71	Putative putative lipop
1135	1	db$2	2::C3K320|C3K320_PSEFS	60	34836	1	1	1	1	0.15	Uncharacterized protei
1136	1	db$2	2::C3KDR6|C3KDR6_PSEFS	60	40570	2	2	2	2	0.26	Putaive NADH oxidored
1137	1	db$2	2::C3K9Q2|C3K9Q2_PSEFS	60	16954	3	2	3	2	0.73	Ribonuclease H OS=Ps
1138	1	db$2	2::C3K0Y8|COBT_PSEFS	60	36066	1	1	1	1	0.14	Nicotinate-nucleotide--
1139	1	SwissProt_HUMAN$	3::P07900|HS90A_HUMAN	60	84607	3	3	3	3	0.18	Heat shock protein HS
1140	1	db$2	2::C3K710|DCD_PSEFS	60	21231	5	4	5	4	1.43	dCTP deaminase OS=F
1141	1	db$2	2::C3KCB9|C3KCB9_PSEFS	60	35987	4	4	2	2	0.48	Polyphosphate kinase
1142	1	db$2	2::C3KCS5|C3KCS5_PSEFS	60	51798	3	2	3	2	0.20	UDP-N-acetylmuramoy
1143	1	db$2	2::C3K5H2|QUEF_PSEFS	60	30808	4	4	4	4	0.84	NADPH-dependent 7-c
1144	1	db$2	2::C3JYV1|C3JYV1_PSEFS	60	32264	4	4	4	4	0.79	Formyltetrahydrofolate
1145	1	db$2	2::C3K224|C3K224_PSEFS	59	30473	2	2	1	1	0.17	CDP-diacylglycerol--se
1146	1	db$2	2::C3JYJ2|C3JYJ2_PSEFS	59	27205	2	2	2	2	0.41	Putative lipoprotein OS
1147	1	db$2	2::C3K6T7|C3K6T7_PSEFS	59	66001	1	1	1	1	0.07	Histidine kinase OS=P
1148	1	db$2	2::C3K0D5|MINE_PSEFS	59	9531	4	3	4	3	3.26	Cell division topologica
1149	1	SwissProt_HUMAN$	3::P46781|RS9_HUMAN	59	22578	4	3	4	3	0.87	40S ribosomal protein
1150	1	db$2	2::C3K7K5|C3K7K5_PSEFS	59	12541	1	1	1	1	0.45	Uncharacterized protei
1151	1	db$2	2::C3JY76|C3JY76_PSEFS	59	48249	3	3	3	3	0.34	Putative mechanosens

↑Family	M	DB	Accession	Score	Mass	Matches	Match(sig)	Sequences	Seq(sig)	emPAI	Description
1152	1	db$2	2::C3KDI4|C3KDI4_PSEFS	59	42151	3	3	3	3	0.40	Putative exported prot
1153	1	db$2	2::C3KDY6|C3KDY6_PSEFS	59	17128	1	1	1	1	0.31	Uncharacterized protei
1154	1	SwissProt_HUMAN$	3::P63244|RACK1_HUMAN	59	35055	2	2	2	2	0.31	Receptor of activated p
1155	1	db$2	2::C3K2V5|RL36_PSEFS	58	4431	1	1	1	1	1.73	50S ribosomal protein
1156	1	db$2	2::C3K753|C3K753_PSEFS	58	30652	1	1	1	1	0.17	Putative hydrolase OS
1157	1	db$2	2::C3K021|C3K021_PSEFS	58	27911	5	4	4	3	0.66	Phosphoadenosine pho
1158	1	db$2	2::C3K3Q3|C3K3Q3_PSEFS	58	61523	3	2	3	2	0.17	Putative cation ABC tra
1159	1	db$2	2::C3KB68|C3KB68_PSEFS	58	11363	2	2	2	2	1.27	DUF1883 domain-cont
1160	1	SwissProt_HUMAN$	3::Q32P51|RA1L2_HUMAN	58	34204	1	1	1	1	0.15	Heterogeneous nuclear
1161	1	db$2	2::C3K8Z2|C3K8Z2_PSEFS	58	25669	1	1	1	1	0.20	High-affinity branched-
1162	1	db$2	2::C3K2M0|Y5418_PSEFS	58	10305	1	1	1	1	0.57	UPF0250 protein PFLU_
1163	1	db$2	2::C3K0U5|C3K0U5_PSEFS	58	62992	1	1	1	1	0.08	Flagellar M-ring protei
1164	1	db$2	2::C3K4U6|C3K4U6_PSEFS	58	17513	2	2	1	1	0.31	Conserved hypothetica
1165	1	db$2	2::C3K0L5|C3K0L5_PSEFS	58	17844	2	2	2	2	0.69	Putative GNAT-family a
1166	1	db$2	2::C3KCH1|C3KCH1_PSEFS	58	32212	2	2	2	2	0.34	Putative carboxymethy
1167	1	SwissProt_HUMAN$	3::P04406|G3P_HUMAN	58	36030	4	3	4	3	0.48	Glyceraldehyde-3-phos
1168	1	db$2	2::C3K3T5|C3K3T5_PSEFS	58	34240	1	1	1	1	0.15	Putrescine ABC transp
1169	1	db$2	2::C3KDX6|C3KDX6_PSEFS	57	16394	2	2	1	1	0.33	Peptidyl-prolyl cis-tran
1170	1	db$2	2::C3KDC3|LOLB_PSEFS	57	23021	2	2	2	2	0.51	Outer-membrane lipop
1171	1	db$2	2::C3KBB9|C3KBB9_PSEFS	57	73354	3	2	2	2	0.14	Putative protease OS=
1172	1	db$2	2::C3K870|C3K870_PSEFS	57	20966	2	2	2	2	0.56	Uncharacterized protei
1173	1	db$2	2::C3KDB5|C3KDB5_PSEFS	57	33189	1	1	1	1	0.15	Putative glycosyl trans
1174	1	db$2	2::C3JY87|NUOH_PSEFS	57	37530	6	6	4	4	1.13	NADH-quinone oxidore
1175	1	db$2	2::C3K6B0|C3K6B0_PSEFS	57	33534	1	1	1	1	0.15	High-affinity zinc uptal
1176	1	db$2	2::C3K013|C3K013_PSEFS	57	20254	2	2	1	1	0.26	Peptidyl-prolyl cis-tran
1177	1	db$2	2::C3K5L2|C3K5L2_PSEFS	56	29854	2	2	2	2	0.37	Phosphonates import A
1178	1	db$2	2::C3JYP6|C3JYP6_PSEFS	56	10477	4	4	3	3	2.78	YCII domain-containin
1179	1	db$2	2::C3KB07|C3KB07_PSEFS	56	47911	1	1	1	1	0.10	Deferrochelatase/pero:
1180	1	db$2	2::C3K4V3|C3K4V3_PSEFS	56	45550	3	2	3	2	0.23	Uncharacterized protei
1181	1	db$2	2::C3K0T5|C3K0T5_PSEFS	56	37877	3	2	2	2	0.28	Zinc transport protein
1182	1	db$2	2::C3K5B3|C3K5B3_PSEFS	56	34937	2	2	2	2	0.31	Dipeptide ABC transpo
1183	1	db$2	2::C3K579|C3K579_PSEFS	56	15962	1	1	1	1	0.34	Putative thioesterase (
1184	1	db$2	2::C3JY81|C3JY81_PSEFS	55	15084	2	2	2	2	0.86	NADH-quinone oxidore
1185	1	db$2	2::C3K0N3|ACP_PSEFS	55	8637	2	2	2	2	1.89	Acyl carrier protein OS
1186	1	db$2	2::C3K820|C3K820_PSEFS	55	26990	2	2	2	2	0.42	Putative ABC transport
1187	1	db$2	2::C3K467|C3K467_PSEFS	55	13453	2	2	2	2	1.00	Cupin_3 domain-conta
1188	1	db$2	2::C3K098|C3K098_PSEFS	55	23000	1	1	1	1	0.23	Putative glutathione S-
1189	1	db$2	2::C3KBI7|C3KBI7_PSEFS	55	26715	2	2	2	2	0.42	Uncharacterized protei
1190	1	db$2	2::C3K8I3|C3K8I3_PSEFS	55	39921	2	2	1	1	0.13	3,4-dihydroxy-2-butan
1191	1	db$2	2::C3K7J4|C3K7J4_PSEFS	55	18330	2	2	1	1	0.29	Uncharacterized protei

↑Family	M	DB	Accession	Score	Mass	Matches	Match(sig)	Sequences	Seq(sig)	emPAI	Description
1192	1	db$2	2::C3K176|C3K176_PSEFS	55	33571	3	2	3	2	0.33	Glucokinase OS=Pseu
1193	1	db$2	2::C3K5E1|C3K5E1_PSEFS	55	13734	2	2	2	2	0.97	Uncharacterized protei
1194	1	db$2	2::C3K5J9|C3K5J9_PSEFS	55	96070	2	2	2	2	0.10	Histidine kinase OS=P
1195	1	db$2	2::C3K8H9|C3K8H9_PSEFS	55	41728	1	1	1	1	0.12	Putative polyamine AB
1196	1	SwissProt_HUMAN$	3::P62263|RS14_HUMAN	54	16263	2	2	1	1	0.33	40S ribosomal protein
1197	1	db$2	2::C3K2C2|C3K2C2_PSEFS	54	67328	1	1	1	1	0.07	Putative lipoprotein OS
1198	1	db$2	2::C3K2K4|C3K2K4_PSEFS	54	19926	1	1	1	1	0.26	Putative exported prot
1199	1	db$2	2::C3K5Y4|C3K5Y4_PSEFS	54	9690	1	1	1	1	0.61	Uncharacterized protei
1200	1	db$2	2::C3KAL0|C3KAL0_PSEFS	54	43962	2	2	2	2	0.24	PlsC domain-containin
1201	1	db$2	2::C3K158|C3K158_PSEFS	54	36421	2	2	2	2	0.30	Putative regulatory pr
1202	1	db$2	2::C3K261|RIMP_PSEFS	54	17653	3	2	3	2	0.70	Ribosome maturation f
1203	1	db$2	2::C3K2M6|RLMH_PSEFS	54	17669	2	2	2	2	0.70	Ribosomal RNA large s
1204	1	db$2	2::C3KDW1|MUTL_PSEFS	54	69574	4	3	4	3	0.23	DNA mismatch repair p
1205	1	db$2	2::C3KE85|C3KE85_PSEFS	54	26099	1	1	1	1	0.20	Purine nucleoside phos
1206	1	db$2	2::C3JY92|C3JY92_PSEFS	53	55851	3	3	3	3	0.29	NADH dehydrogenase
1207	1	db$2	2::C3KDJ8|C3KDJ8_PSEFS	53	23307	2	2	2	2	0.50	Methyltransf_25 doma
1208	1	db$2	2::C3K2S2|C3K2S2_PSEFS	53	38886	4	3	4	3	0.44	Riboflavin biosynthesis
1209	1	db$2	2::C3K1B4|C3K1B4_PSEFS	53	43355	2	2	2	2	0.24	Putative transferase O
1210	1	db$2	2::C3JYU3|C3JYU3_PSEFS	53	75598	2	2	2	2	0.13	Histidine kinase OS=P
1211	1	db$2	2::C3KBM3|C3KBM3_PSEFS	53	40194	2	2	2	2	0.27	Putative ABC transport
1212	1	db$2	2::C3K5Q0|C3K5Q0_PSEFS	53	53619	3	2	3	2	0.19	Mannose-1-phosphate
1213	1	db$2	2::C3KE16|C3KE16_PSEFS	53	37004	3	3	3	3	0.47	Putative chemotaxis-re
1214	1	db$2	2::C3K0H8|C3K0H8_PSEFS	53	29525	4	4	4	4	0.89	Protein phosphatase C
1215	1	db$2	2::C3JYS0|C3JYS0_PSEFS	53	29172	4	2	4	2	0.38	Cell division coordinat
1216	1	SwissProt_HUMAN$	3::P62750|RL23A_HUMAN	53	17684	1	1	1	1	0.30	60S ribosomal protein
1217	1	db$2	2::C3JZ44|C3JZ44_PSEFS	53	47432	2	2	2	2	0.22	Putative gluconate per
1218	1	db$2	2::C3JYL7|C3JYL7_PSEFS	53	12417	1	1	1	1	0.45	Putative copper resista
1219	1	SwissProt_HUMAN$	3::P46783|RS10_HUMAN	52	18886	2	1	2	1	0.28	40S ribosomal protein
1220	1	db$2	2::C3K5D6|C3K5D6_PSEFS	52	97394	2	2	2	2	0.10	Putative beta-(1-3)-glu
1221	1	db$2	2::C3K574|C3K574_PSEFS	52	36116	3	2	2	2	0.30	Putative oxidoreductas
1222	1	db$2	2::C3KDC7|C3KDC7_PSEFS	52	29930	2	2	2	2	0.37	Release factor glutami
1223	1	db$2	2::C3K132|C3K132_PSEFS	52	42760	2	2	2	2	0.25	Uncharacterized protei
1224	1	db$2	2::C3KDG3|C3KDG3_PSEFS	52	15637	3	3	2	2	0.82	Putative exported prot
1225	1	db$2	2::C3K8K1|C3K8K1_PSEFS	52	40207	2	2	1	1	0.12	Phosphate-binding pro
1226	1	SwissProt_HUMAN$	3::P62753|RS6_HUMAN	52	28663	1	1	1	1	0.18	40S ribosomal protein
1227	1	db$2	2::C3KBL5|C3KBL5_PSEFS	52	24580	2	2	2	2	0.47	Glutamate/aspartate A
1228	1	db$2	2::C3JY97|C3JY97_PSEFS	52	30357	1	1	1	1	0.17	N-hydroxyarylamine O
1229	1	db$2	2::C3K301|C3K301_PSEFS	52	28901	2	2	2	2	0.39	Putative dehydrogenas
1230	1	SwissProt_HUMAN$	3::P61978|HNRPK_HUMAN	52	50944	1	1	1	1	0.10	Heterogeneous nuclea
1231	1	db$2	2::C3K5K0|C3K5K0_PSEFS	52	25438	2	2	2	2	0.45	Two-component syster

↑Family	M	DB	Accession	Score	Mass	Matches	Match(sig)	Sequences	Seq(sig)	emPAI	Description
1232	1	db$2	2::C3K7L9|C3K7L9_PSEFS	52	28704	3	2	3	2	0.39	Putative membrane pr
1233	1	SwissProt_HUMAN$	3::Q2PPJ7|RGPA2_HUMAN	52	210636	2	2	2	2	0.05	Ral GTPase-activating
1233	2	SwissProt_HUMAN$	3::P61254|RL26_HUMAN	48	17248	3	2	3	2	0.72	60S ribosomal protein
1234	1	db$2	2::C3JY69|HFLD_PSEFS	52	23173	2	2	2	2	0.50	High frequency lysoge
1235	1	db$2	2::C3K150|C3K150_PSEFS	51	25194	3	3	3	3	0.75	Putative short-chain de
1236	1	db$2	2::C3K6T3|C3K6T3_PSEFS	51	23166	2	2	2	2	0.50	Thiol:disulfide intercha
1237	1	db$2	2::C3KDR4|C3KDR4_PSEFS	51	49531	4	4	4	4	0.47	Ribosomal protein S12
1238	1	db$2	2::C3K5I0|C3K5I0_PSEFS	51	36784	1	1	1	1	0.14	tRNA-dihydrouridine(2
1239	1	SwissProt_HUMAN$	3::Q5D862|FILA2_HUMAN	51	247928	1	1	1	1	0.02	Filaggrin-2 OS=Homo
1240	1	db$2	2::C3KDN9|C3KDN9_PSEFS	51	40608	1	1	1	1	0.12	Probable periplasmic s
1241	1	db$2	2::C3K6A1|C3K6A1_PSEFS	51	22990	1	1	1	1	0.23	Putative copper-bindin
1242	1	SwissProt_HUMAN$	3::P62847|RS24_HUMAN	51	15413	1	1	1	1	0.35	40S ribosomal protein
1243	1	db$2	2::C3K9Y7|C3K9Y7_PSEFS	51	30413	2	2	2	2	0.36	Undecaprenyl-diphosp
1244	1	db$2	2::C3JZ45|C3JZ45_PSEFS	51	19122	1	1	1	1	0.28	Gluconokinase OS=Pse
1245	1	db$2	2::C3KBN5|C3KBN5_PSEFS	51	24125	1	1	1	1	0.22	Putative TetR-family re
1246	1	db$2	2::C3K2Q9|EX7S_PSEFS	51	8900	3	3	3	3	3.74	Exodeoxyribonuclease
1247	1	db$2	2::C3K5F1|C3K5F1_PSEFS	51	42516	3	3	3	3	0.40	Ribonuclease D OS=Ps
1248	1	db$2	2::C3K2L9|LIPB_PSEFS	51	23473	2	1	2	1	0.22	Octanoyltransferase O
1249	1	db$2	2::C3K607|LPXA_PSEFS	51	28002	1	1	1	1	0.18	Acyl-[acyl-carrier-prot
1250	1	db$2	2::C3K5H4|C3K5H4_PSEFS	51	21385	1	1	1	1	0.25	Uncharacterized protei
1251	1	db$2	2::C3K302|C3K302_PSEFS	51	12723	4	4	2	2	1.07	Putative HIT domain-c
1252	1	db$2	2::C3K3T7|C3K3T7_PSEFS	51	39982	2	2	2	2	0.27	Putrescine ABC transp
1253	1	db$2	2::C3K0G4|C3K0G4_PSEFS	51	29541	1	1	1	1	0.17	MOSC domain-contain
1254	1	db$2	2::C3K326|C3K326_PSEFS	51	35169	2	1	2	1	0.14	4-hydroxythreonine-4-
1255	1	SwissProt_HUMAN$	3::P01591|IGJ_HUMAN	50	18087	2	1	2	1	0.30	Immunoglobulin J chai
1256	1	db$2	2::C3K3Q1|C3K3Q1_PSEFS	50	40925	1	1	1	1	0.12	Putative oxidoreductas
1257	1	db$2	2::C3JZ23|C3JZ23_PSEFS	50	131220	2	2	2	2	0.08	Chromosome partition
1258	1	SwissProt_HUMAN$	3::P62318|SMD3_HUMAN	50	13907	1	1	1	1	0.40	Small nuclear ribonucl
1259	1	SwissProt_HUMAN$	3::P60900|PSA6_HUMAN	50	27382	1	1	1	1	0.19	Proteasome subunit al
1260	1	db$2	2::C3KAW3|C3KAW3_PSEFS	50	52085	1	1	1	1	0.10	Putative GntR-family t
1261	1	db$2	2::C3K5G4|RLMM_PSEFS	50	40585	1	1	1	1	0.12	Ribosomal RNA large s
1262	1	db$2	2::C3K059|TRHO_PSEFS	50	35401	2	2	2	2	0.31	tRNA uridine(34) hydr
1263	1	db$2	2::C3JZU8|C3JZU8_PSEFS	50	30426	2	2	2	2	0.36	Hydrolase_4 domain-c
1264	1	SwissProt_HUMAN$	3::P46779|RL28_HUMAN	50	15738	1	1	1	1	0.35	60S ribosomal protein
1265	1	db$2	2::C3K6V0|C3K6V0_PSEFS	50	23354	3	2	2	2	0.50	Imidazole glycerol pho
1266	1	db$2	2::C3K136|C3K136_PSEFS	50	31053	2	2	2	2	0.35	Uncharacterized protei
1267	1	db$2	2::C3KCI1|C3KCI1_PSEFS	50	42811	1	1	1	1	0.12	Putative acyl-CoA deh
1268	1	db$2	2::C3K1R4|C3K1R4_PSEFS	49	17440	1	1	1	1	0.31	Putative plasmid-relate
1269	1	db$2	2::C3K9Q1|C3K9Q1_PSEFS	49	28823	1	1	1	1	0.18	Methyltransf_11 doma
1270	1	SwissProt_HUMAN$	3::Q6IS14|IF5AL_HUMAN	49	16762	1	1	1	1	0.32	Eukaryotic translation

↑Family	M	DB	Accession	Score	Mass	Matches	Match(sig)	Sequences	Seq(sig)	emPAI	Description
1271	1	db$2	2::C3K7I5|C3K7I5_PSEFS	49	28342	2	2	2	2	0.40	Putative iron utilisatior
1272	1	db$2	2::C3K2R8|NUSB_PSEFS	49	18764	3	2	2	1	0.28	Transcription antitermi
1273	1	db$2	2::C3K2L0|C3K2L0_PSEFS	49	55816	1	1	1	1	0.09	Apolipoprotein N-acylt
1274	1	db$2	2::C3K6G1|RLMF_PSEFS	49	38015	1	1	1	1	0.13	Ribosomal RNA large s
1275	1	db$2	2::C3K670|C3K670_PSEFS	49	30761	1	1	1	1	0.17	Putative glycine betain
1276	1	db$2	2::C3K0F5|C3K0F5_PSEFS	49	23098	1	1	1	1	0.23	Putative TetR-family tr
1277	1	db$2	2::C3K5B2|C3K5B2_PSEFS	49	35714	2	2	2	2	0.30	Dipeptide ABC transpo
1278	1	db$2	2::C3K6J0|C3K6J0_PSEFS	49	24596	1	1	1	1	0.21	Putative phosphoglyco
1279	1	SwissProt_HUMAN$	3::P07737|PROF1_HUMAN	49	15045	3	2	2	2	0.86	Profilin-1 OS=Homo sa
1280	1	db$2	2::C3JYE7|C3JYE7_PSEFS	49	35468	2	2	2	2	0.30	LysR family regulatory
1281	1	db$2	2::C3K4Z1|C3K4Z1_PSEFS	49	72318	2	2	2	2	0.14	Cytochrome C biogene
1282	1	db$2	2::C3K6L9|C3K6L9_PSEFS	48	49785	3	3	3	3	0.33	Phospho-2-dehydro-3-
1283	1	db$2	2::C3K5X1|C3K5X1_PSEFS	48	46611	1	1	1	1	0.11	Histidine kinase OS=P
1284	1	db$2	2::C3JZU7|C3JZU7_PSEFS	48	16433	2	2	2	2	0.77	Putative thioesterase (
1285	1	db$2	2::C3JZ30|C3JZ30_PSEFS	48	12225	2	1	2	1	0.46	DUF2025 domain-cont
1286	1	db$2	2::C3K453|C3K453_PSEFS	48	71119	1	1	1	1	0.07	AcetylCoA_hyd_C dom
1287	1	db$2	2::Q8VMR3|HUTH_PSEFS	48	54209	1	1	1	1	0.09	Histidine ammonia-lya
1288	1	db$2	2::C3KD16|C3KD16_PSEFS	48	34294	4	2	2	2	0.32	Putative 2-hydroxyacic
1289	1	db$2	2::C3K1N1|C3K1N1_PSEFS	48	33097	3	3	3	3	0.53	Protein-export membr
1290	1	db$2	2::C3K9E9|C3K9E9_PSEFS	48	21363	3	3	1	1	0.55	Putative TetR-family re
1291	1	db$2	2::C3JYU4|C3JYU4_PSEFS	48	20911	1	1	1	1	0.25	Corrinoid adenosyltran
1292	1	db$2	2::C3K4I1|C3K4I1_PSEFS	48	29333	3	2	3	2	0.38	Shikimate dehydrogen
1293	1	db$2	2::C3KDF9|C3KDF9_PSEFS	48	17633	3	3	3	3	1.22	CinA domain-containin
1294	1	db$2	2::C3K3Y2|C3K3Y2_PSEFS	48	34182	1	1	1	1	0.15	Putative sinc-binding c
1295	1	db$2	2::C3K1L7|RLMN_PSEFS	47	42342	1	1	1	1	0.12	Dual-specificity RNA m
1296	1	db$2	2::C3K3G8|C3K3G8_PSEFS	47	41555	2	2	2	2	0.26	Uncharacterized protei
1297	1	db$2	2::C3K591|C3K591_PSEFS	47	19872	2	2	2	2	0.60	Putative bacterioferriti
1298	1	db$2	2::C3K564|C3K564_PSEFS	47	54508	2	2	2	2	0.19	Methylmalonate-semia
1299	1	db$2	2::C3K0D2|C3K0D2_PSEFS	47	35834	2	2	2	2	0.30	Lipid A biosynthesis la
1300	1	db$2	2::C3K3D3|BETA_PSEFS	47	62642	2	2	2	2	0.16	Oxygen-dependent ch
1301	1	db$2	2::C3K0M9|KTHY_PSEFS	47	22917	2	2	2	2	0.51	Thymidylate kinase OS
1302	1	db$2	2::C3KAH5|RL31_PSEFS	47	8451	3	3	2	2	1.97	50S ribosomal protein
1303	1	db$2	2::C3KE39|C3KE39_PSEFS	47	18976	2	2	2	2	0.64	D,D-heptose 1,7-bisph
1304	1	db$2	2::C3K101|C3K101_PSEFS	47	37185	4	2	3	2	0.29	Uncharacterized protei
1305	1	db$2	2::C3K7A7|C3K7A7_PSEFS	47	43115	2	2	2	2	0.25	Conserved hypothetica
1306	1	db$2	2::C3K122|C3K122_PSEFS	47	33097	1	1	1	1	0.15	Putative O-acetylserine
1307	1	db$2	2::C3KCP6|C3KCP6_PSEFS	46	38240	3	2	3	2	0.28	ADP-heptose--LPS hep
1308	1	db$2	2::C3KBX7|C3KBX7_PSEFS	46	23636	1	1	1	1	0.22	Putative metallo-beta-
1309	1	db$2	2::C3K4A9|C3K4A9_PSEFS	46	17462	3	2	2	2	0.71	Uncharacterized protei
1310	1	db$2	2::C3K239|C3K239_PSEFS	46	42550	2	2	2	2	0.25	Aminotransferase OS=

↑Family	M	DB	Accession	Score	Mass	Matches	Match(sig)	Sequences	Seq(sig)	emPAI	Description
1311	1	db$2	2::C3K609|C3K609_PSEFS	46	73644	4	4	4	4	0.29	Putative lytic transglyc
1312	1	db$2	2::C3KAX0|C3KAX0_PSEFS	46	84238	3	3	3	3	0.18	1,4-alpha-glucan bran
1313	1	db$2	2::C3K493|C3K493_PSEFS	46	16495	2	2	2	2	0.76	Uncharacterized protei
1314	1	db$2	2::C3K1Y4|C3K1Y4_PSEFS	46	14766	1	1	1	1	0.37	Putative putative glyo
1315	1	db$2	2::C3K1W8|C3K1W8_PSEFS	46	11862	1	1	1	1	0.48	Uncharacterized protei
1316	1	db$2	2::C3K6V3|HIS6_PSEFS	46	27111	2	1	2	1	0.19	Imidazole glycerol pho
1317	1	SwissProt_HUMAN$	3::A6NJ78|MET15_HUMAN	46	46092	2	2	1	1	0.11	12S rRNA N4-methylcy
1318	1	db$2	2::C3K4W6|C3K4W6_PSEFS	45	44511	3	2	3	2	0.24	Putative lipoprotein rel
1319	1	db$2	2::C3K3E9|C3K3E9_PSEFS	45	12056	2	1	2	1	0.47	Putative lipoprotein OS
1320	1	db$2	2::C3JYQ1|C3JYQ1_PSEFS	45	48078	2	2	2	2	0.22	Putative CoA transfera
1321	1	db$2	2::C3K1A2|C3K1A2_PSEFS	45	17206	2	1	2	1	0.31	Putative membrane pr
1322	1	SwissProt_HUMAN$	3::Q02878|RL6_HUMAN	45	32708	1	1	1	1	0.16	60S ribosomal protein
1323	1	db$2	2::C3K1C9|C3K1C9_PSEFS	45	65670	2	1	2	1	0.07	Putative cation-transp
1324	1	db$2	2::C3K4U9|C3K4U9_PSEFS	45	34107	2	2	2	2	0.32	Putative LysR-family r
1325	1	SwissProt_HUMAN$	3::P31153|METK2_HUMAN	45	43633	1	1	1	1	0.11	S-adenosylmethionine
1326	1	SwissProt_HUMAN$	3::P07195|LDHB_HUMAN	45	36615	3	3	3	3	0.47	L-lactate dehydrogena
1327	1	db$2	2::C3KAB9|C3KAB9_PSEFS	45	40944	1	1	1	1	0.12	Putative acetyl-CoA ac
1328	1	db$2	2::C3K0J2|C3K0J2_PSEFS	45	17877	1	1	1	1	0.30	Flagellar protein FliL O
1329	1	db$2	2::C3KDB9|C3KDB9_PSEFS	45	32977	2	2	2	2	0.33	Putative lipopolysacch
1330	1	SwissProt_HUMAN$	3::P23284|PPIB_HUMAN	45	23728	1	1	1	1	0.22	Peptidyl-prolyl cis-tran
1331	1	db$2	2::C3K0S4|C3K0S4_PSEFS	45	65961	1	1	1	1	0.07	Isocitrate dehydrogen
1332	1	db$2	2::C3K047|C3K047_PSEFS	45	16614	2	2	2	2	0.75	Putative putative heat
1333	1	db$2	2::C3K6R7|C3K6R7_PSEFS	44	41832	2	1	2	1	0.12	Serine/threonine trans
1334	1	db$2	2::C3JXI0|C3JXI0_PSEFS	44	23030	1	1	1	1	0.23	Nudix hydrolase doma
1335	1	db$2	2::C3K127|C3K127_PSEFS	44	41295	2	2	2	2	0.26	Putative membrane pr
1336	1	SwissProt_HUMAN$	3::P62277|RS13_HUMAN	44	17212	2	2	2	2	0.72	40S ribosomal protein
1337	1	db$2	2::C3KE46|C3KE46_PSEFS	44	47174	3	2	3	2	0.22	16S rRNA m5C967 me
1338	1	db$2	2::C3K4R2|C3K4R2_PSEFS	44	7681	2	2	2	2	2.31	Major cold shock prote
1339	1	db$2	2::C3JZP0|C3JZP0_PSEFS	44	11251	2	1	2	1	0.51	Putative lipoprotein OS
1340	1	db$2	2::C3K0X2|C3K0X2_PSEFS	44	81009	2	2	2	2	0.12	Patatin-like phospholip
1341	1	db$2	2::C3JZP9|C3JZP9_PSEFS	44	33380	2	1	2	1	0.15	Putative ribose transp
1342	1	db$2	2::C3K646|C3K646_PSEFS	44	21182	1	1	1	1	0.25	Putative acyl-CoA thio
1343	1	db$2	2::C3KAD1|C3KAD1_PSEFS	44	32401	2	2	2	2	0.34	Probable 4-deoxy-4-fo
1344	1	db$2	2::C3K425|C3K425_PSEFS	44	27295	2	2	2	2	0.41	Positive alginate biosy
1345	1	db$2	2::C3K5Y3|C3K5Y3_PSEFS	44	35524	3	3	3	3	0.49	Putative leucine dehyd
1346	1	db$2	2::C3K6Y0|C3K6Y0_PSEFS	44	13973	1	1	1	1	0.40	4-carboxymuconolacto
1347	1	db$2	2::C3JY28|Y3771_PSEFS	44	6626	1	1	1	1	0.98	UPF0434 protein PFLU_
1348	1	db$2	2::C3K2U2|C3K2U2_PSEFS	44	20033	1	1	1	1	0.26	Putative hydrolase OS
1349	1	db$2	2::C3KDW4|C3KDW4_PSEFS	43	48912	2	2	2	2	0.21	GTPase HflX OS=Pseu
1350	1	db$2	2::C3KAR4|C3KAR4_PSEFS	43	11430	1	1	1	1	0.50	Uncharacterized protei

↑Family	M	DB	Accession	Score	Mass	Matches	Match(sig)	Sequences	Seq(sig)	emPAI	Description
1351	1	db$2	2::C3K353|C3K353_PSEFS	43	44910	1	1	1	1	0.11	Putative aminotransfer
1352	1	SwissProt_HUMAN$	3::P62888|RL30_HUMAN	43	12776	1	1	1	1	0.44	60S ribosomal protein
1353	1	db$2	2::C3K1F1|ATPL_PSEFS	43	8603	4	4	2	2	3.92	ATP synthase subunit
1354	1	db$2	2::C3K0T6|RRAAH_PSEFS	43	17479	2	2	1	1	0.31	Putative 4-hydroxy-4-
1355	1	db$2	2::C3K8U6|C3K8U6_PSEFS	43	15270	1	1	1	1	0.36	Putative polyhydroxyal
1356	1	db$2	2::C3K2L8|LIPA_PSEFS	43	38163	5	3	4	3	0.45	Lipoyl synthase OS=Ps
1357	1	db$2	2::C3KBL4|C3KBL4_PSEFS	43	26993	3	1	3	1	0.19	Glutamate/aspartate A
1358	1	SwissProt_HUMAN$	3::P61247|RS3A_HUMAN	43	29926	2	2	2	2	0.37	40S ribosomal protein
1359	1	db$2	2::C3K975|C3K975_PSEFS	42	21948	1	1	1	1	0.24	Putative glutathione S-
1360	1	db$2	2::C3K6E9|C3K6E9_PSEFS	42	41977	1	1	1	1	0.12	Phosphatidylcholine-hy
1361	1	db$2	2::C3K6E4|C3K6E4_PSEFS	42	46143	2	2	2	2	0.23	Cytochrome b OS=Pse
1362	1	db$2	2::C3K088|C3K088_PSEFS	42	20655	2	1	2	1	0.26	Putative isochorismata
1363	1	SwissProt_HUMAN$	3::P02747|C1QC_HUMAN	42	25757	2	1	2	1	0.20	Complement C1q subc
1364	1	db$2	2::C3K0B2|C3K0B2_PSEFS	42	20071	1	1	1	1	0.26	Putative membrane pr
1365	1	db$2	2::C3JYG6|C3JYG6_PSEFS	42	44622	3	1	3	1	0.11	Peptidase_M14 domair
1366	1	SwissProt_HUMAN$	3::P07437|TBB5_HUMAN	42	49639	2	2	2	2	0.21	Tubulin beta chain OS
1367	1	db$2	2::C3K4M2|C3K4M2_PSEFS	42	36363	2	1	2	1	0.14	Catabolite repressor/a
1368	1	db$2	2::C3K842|C3K842_PSEFS	42	56600	2	1	2	1	0.09	RNA polymerase sigma
1369	1	db$2	2::C3JYS6|C3JYS6_PSEFS	42	17060	2	2	2	2	0.73	Putative thioesterase (
1370	1	db$2	2::C3K816|OPGH_PSEFS	42	96236	5	5	5	5	0.28	Glucans biosynthesis g
1371	1	db$2	2::C3K485|C3K485_PSEFS	42	26565	1	1	1	1	0.19	Putative lipoprotein OS
1372	1	SwissProt_HUMAN$	3::P63220|RS21_HUMAN	42	9106	2	2	2	2	1.75	40S ribosomal protein
1373	1	db$2	2::C3JYP8|C3JYP8_PSEFS	42	24937	1	1	1	1	0.21	Putative two-compone
1374	1	db$2	2::C3K7W1|C3K7W1_PSEFS	42	12229	2	2	2	2	1.14	Putative lipoprotein OS
1375	1	SwissProt_HUMAN$	3::P62081|RS7_HUMAN	42	22113	1	1	1	1	0.24	40S ribosomal protein
1376	1	db$2	2::C3K654|C3K654_PSEFS	42	34290	1	1	1	1	0.15	2-dehydropantoate 2-r
1377	1	db$2	2::C3K8L0|C3K8L0_PSEFS	42	16885	1	1	1	1	0.32	Uncharacterized protei
1378	1	db$2	2::C3K4I9|C3K4I9_PSEFS	42	42202	1	1	1	1	0.12	Galactonate dehydrata
1379	1	SwissProt_HUMAN$	3::Q5JXB2|UE2NL_HUMAN	42	17366	1	1	1	1	0.31	Putative ubiquitin-conj
1380	1	db$2	2::C3K7N0|C3K7N0_PSEFS	41	28545	2	2	2	2	0.39	Transport permease pr
1381	1	db$2	2::C3K220|C3K220_PSEFS	41	136725	1	1	1	1	0.04	RecBCD enzyme subur
1382	1	db$2	2::C3KAF8|C3KAF8_PSEFS	41	74138	1	1	1	1	0.07	Putative lipoprotein OS
1383	1	db$2	2::C3K8V8|C3K8V8_PSEFS	41	36166	2	2	2	2	0.30	Zinc-type alcohol dehy
1384	1	db$2	2::C3K1V0|C3K1V0_PSEFS	41	56633	1	1	1	1	0.09	Nitric oxide reductase
1385	1	db$2	2::C3K3F9|C3K3F9_PSEFS	41	16338	1	1	1	1	0.33	Putative exported cyto
1386	1	db$2	2::C3JZA7|C3JZA7_PSEFS	41	51947	1	1	1	1	0.10	Putative succinate-sen
1387	1	db$2	2::C3KAM0|C3KAM0_PSEFS	41	50872	2	2	2	2	0.20	Putative two-compone
1388	1	db$2	2::C3JYS4|C3JYS4_PSEFS	41	16520	2	2	2	2	0.76	Tol-Pal system protein
1389	1	db$2	2::C3JZ62|C3JZ62_PSEFS	41	61473	2	2	2	2	0.17	Putative long-chain-fat
1390	1	db$2	2::C3K0N0|C3K0N0_PSEFS	41	41535	1	1	1	1	0.12	Endolytic murein trans

↑Family	M	DB	Accession	Score	Mass	Matches	Match(sig)	Sequences	Seq(sig)	emPAI	Description
1391	1	db$2	2::C3K5Q6|C3K5Q6_PSEFS	41	68754	1	1	1	1	0.07	Putative hydrolase OS=
1392	1	db$2	2::C3JZN5|RL35_PSEFS	41	7363	2	2	1	1	0.87	50S ribosomal protein
1393	1	db$2	2::C3JYQ4|C3JYQ4_PSEFS	41	52375	2	2	2	2	0.20	Arginine/ornithine anti
1394	1	db$2	2::C3K3M8|C3K3M8_PSEFS	40	36638	2	2	1	1	0.14	AB hydrolase-1 domai
1395	1	db$2	2::C3K0K4|C3K0K4_PSEFS	40	29536	2	2	2	2	0.38	PBPb domain-containir
1396	1	db$2	2::C3KDW0|C3KDW0_PSEFS	40	49939	3	3	3	3	0.33	N-acetylmuramoyl-L-a
1397	1	db$2	2::C3KE38|C3KE38_PSEFS	40	28697	3	3	3	3	0.64	Putative phospholipid l
1398	1	db$2	2::C3K1E3|C3K1E3_PSEFS	40	28123	1	1	1	1	0.18	Glucitol operon repres
1399	1	db$2	2::C3JY27|KDSB_PSEFS	40	27609	2	1	2	1	0.19	3-deoxy-manno-octulc
1400	1	db$2	2::C3K620|C3K620_PSEFS	40	22214	2	2	2	2	0.53	LexA repressor OS=Ps
1401	1	SwissProt_HUMAN$	3::P62899|RL31_HUMAN	40	14454	2	2	2	2	0.91	60S ribosomal protein
1402	1	db$2	2::C3K7S1|C3K7S1_PSEFS	40	25085	2	2	1	1	0.46	Protein MgtC OS=Pseu
1403	1	SwissProt_HUMAN$	3::Q8NHW5|RLA0L_HUMAN	40	34343	1	1	1	1	0.15	60S acidic ribosomal p
1404	1	db$2	2::C3K3M5|C3K3M5_PSEFS	39	50101	3	3	3	3	0.33	Putative peptidase OS=
1405	1	db$2	2::C3K7V7|C3K7V7_PSEFS	39	18111	1	1	1	1	0.30	Uncharacterized protei
1406	1	db$2	2::C3K8T8|C3K8T8_PSEFS	39	14354	3	1	2	1	0.38	Sec-independent prote
1407	1	db$2	2::C3KAX2|C3KAX2_PSEFS	39	79149	1	1	1	1	0.06	Histidine kinase OS=P:
1408	1	SwissProt_HUMAN$	3::P23246|SFPQ_HUMAN	39	76102	1	1	1	1	0.06	Splicing factor, proline
1409	1	SwissProt_HUMAN$	3::Q6IV72|ZN425_HUMAN	39	87663	1	1	1	1	0.06	Zinc finger protein 425
1410	1	db$2	2::C3K0U7|C3K0U7_PSEFS	39	49884	3	3	3	3	0.33	Two-component respo
1411	1	db$2	2::C3K637|C3K637_PSEFS	39	56441	2	2	2	2	0.18	Putative peptide ABC t
1412	1	db$2	2::C3K4N5|C3K4N5_PSEFS	39	28830	1	1	1	1	0.18	Putative amino acid AE
1413	1	db$2	2::C3K1D4|C3K1D4_PSEFS	39	64734	2	2	2	2	0.16	Uncharacterized protei
1414	1	db$2	2::C3KBI5|C3KBI5_PSEFS	39	21236	1	1	1	1	0.25	Putative membrane pr
1415	1	db$2	2::C3K334|CCA_PSEFS	39	45916	2	1	1	1	0.11	Multifunctional CCA pr
1416	1	db$2	2::C3K6T8|C3K6T8_PSEFS	39	49583	4	2	4	2	0.21	Alginate biosynthesis t
1417	1	db$2	2::C3KBH7|C3KBH7_PSEFS	39	44237	1	1	1	1	0.11	3-oxoacyl-[acyl-carrier
1418	1	db$2	2::C3K1K9|C3K1K9_PSEFS	39	42204	2	2	2	2	0.25	Putative aminotransfer
1419	1	db$2	2::C3KE30|C3KE30_PSEFS	39	59128	5	3	5	3	0.27	L-aspartate oxidase OS
1420	1	db$2	2::C3K6H7|C3K6H7_PSEFS	39	10540	1	1	1	1	0.55	Cell division protein Ft:
1421	1	db$2	2::C3K3W8|C3K3W8_PSEFS	39	44404	2	2	2	2	0.24	Putative ubiquinone bi
1422	1	db$2	2::C3K3V9|C3K3V9_PSEFS	38	33232	1	1	1	1	0.15	Protein QmcA OS=Pse
1423	1	db$2	2::C3KCK0|C3KCK0_PSEFS	38	26687	3	1	2	1	0.19	Putative short-chain de
1424	1	SwissProt_HUMAN$	3::P62304|RUXE_HUMAN	38	10797	1	1	1	1	0.54	Small nuclear ribonucl
1425	1	SwissProt_HUMAN$	3::P26373|RL13_HUMAN	38	24247	1	1	1	1	0.21	60S ribosomal protein
1426	1	db$2	2::C3K0K9|C3K0K9_PSEFS	38	37421	1	1	1	1	0.13	Putative NADP-depend
1427	1	db$2	2::C3K813|C3K813_PSEFS	38	36618	4	4	4	4	0.68	Proline iminopeptidase
1428	1	db$2	2::C3KDV8|C3KDV8_PSEFS	38	29265	3	3	3	3	0.62	ADP-dependent (S)-NA
1429	1	db$2	2::C3KBZ4|C3KBZ4_PSEFS	38	6850	1	1	1	1	0.94	Uncharacterized protei
1430	1	SwissProt_HUMAN$	3::Q8NBF4|YG006_HUMAN	38	16172	1	1	1	1	0.33	Putative uncharacteriz

↑Family	M	DB	Accession	Score	Mass	Matches	Match(sig)	Sequences	Seq(sig)	emPAI	Description
1431	1	db$2	2::C3K8L6|C3K8L6_PSEFS	38	28069	2	2	2	2	0.40	Precorrin-6A synthase
1432	1	db$2	2::C3K4U8|C3K4U8_PSEFS	38	20094	1	1	1	1	0.26	Conserved hypothetica
1433	1	SwissProt_HUMAN$	3::P31939|PUR9_HUMAN	38	64575	1	1	1	1	0.08	Bifunctional purine bio
1434	1	db$2	2::C3K386|C3K386_PSEFS	38	22957	1	1	1	1	0.23	Putative glutathione S-
1435	1	db$2	2::C3K5L0|C3K5L0_PSEFS	37	14786	1	1	1	1	0.37	Conserved hypothetica
1436	1	db$2	2::C3JYI2|C3JYI2_PSEFS	37	39477	1	1	1	1	0.13	2-aminoethylphosphor
1437	1	db$2	2::C3K3R7|RPIA_PSEFS	37	23281	2	2	2	2	0.50	Ribose-5-phosphate is
1438	1	db$2	2::C3K2E4|C3K2E4_PSEFS	37	29249	2	2	2	2	0.38	Ferredoxin--NADP redu
1439	1	db$2	2::C3K9N3|C3K9N3_PSEFS	37	10076	2	2	2	2	1.50	Peptidyl-prolyl cis-tran
1440	1	db$2	2::C3K1F5|C3K1F5_PSEFS	37	29048	1	1	1	1	0.18	Putative chromosome
1441	1	db$2	2::C3K4U7|C3K4U7_PSEFS	37	28919	1	1	1	1	0.18	Putative amidotransfer
1442	1	db$2	2::C3K6A6|C3K6A6_PSEFS	37	25667	1	1	1	1	0.20	Putative lipoprotein OS
1443	1	db$2	2::C3K8U0|HIS2_PSEFS	37	12007	2	2	2	2	1.17	Phosphoribosyl-ATP py
1444	1	SwissProt_HUMAN$	3::P33992|MCM5_HUMAN	37	82233	1	1	1	1	0.06	DNA replication licensi
1445	1	db$2	2::C3K8Z7|C3K8Z7_PSEFS	37	10906	2	2	2	2	1.34	Uncharacterized protei
1446	1	db$2	2::C3K6W0|C3K6W0_PSEFS	37	14786	2	2	2	2	0.88	Putative rhodanese do
1447	1	db$2	2::C3JY40|C3JY40_PSEFS	37	21505	1	1	1	1	0.24	Putative Trp repressor
1448	1	db$2	2::C3K832|C3K832_PSEFS	37	33163	2	2	2	2	0.33	Non-specific serine/thr
1449	1	SwissProt_HUMAN$	3::P01009|A1AT_HUMAN	37	46707	1	1	1	1	0.11	Alpha-1-antitrypsin OS
1450	1	db$2	2::C3K3H4|C3K3H4_PSEFS	37	26074	1	1	1	1	0.20	Ribosomal RNA small s
1451	1	SwissProt_HUMAN$	3::P19338|NUCL_HUMAN	37	76568	1	1	1	1	0.06	Nucleolin OS=Homo sa
1452	1	db$2	2::C3K9Y8|C3K9Y8_PSEFS	37	14463	1	1	1	1	0.38	Uncharacterized protei
1453	1	db$2	2::C3K8X1|C3K8X1_PSEFS	37	23435	2	2	2	2	0.49	Putative ABC transport
1454	1	db$2	2::C3K1G4|RL34_PSEFS	37	5064	2	2	2	2	4.81	50S ribosomal protein
1455	1	db$2	2::C3K2M3|C3K2M3_PSEFS	36	37518	2	1	2	1	0.13	Putative peptidoglycan
1456	1	db$2	2::C3K2U3|C3K2U3_PSEFS	36	27946	2	2	2	2	0.40	Trans-aconitate 2-met
1457	1	db$2	2::C3K672|C3K672_PSEFS	36	58670	2	2	2	2	0.18	Cation/acetate sympor
1458	1	db$2	2::C3K0L0|C3K0L0_PSEFS	36	29492	1	1	1	1	0.17	Putative IclR-family re
1459	1	db$2	2::C3K3R4|C3K3R4_PSEFS	36	24681	3	3	3	3	0.77	Putative hydrolase OS
1460	1	db$2	2::C3KDC8|C3KDC8_PSEFS	36	26520	1	1	1	1	0.19	Molybdopterin biosyntl
1461	1	db$2	2::C3KBZ8|C3KBZ8_PSEFS	36	38389	2	1	2	1	0.13	Iron-sulfur cluster carr
1462	1	db$2	2::C3K456|C3K456_PSEFS	36	38683	1	1	1	1	0.13	Alanine racemase OS=
1463	1	db$2	2::C3K2Q3|C3K2Q3_PSEFS	36	23107	1	1	1	1	0.23	Putative hydrolase OS
1464	1	db$2	2::C3K4R0|C3K4R0_PSEFS	36	14930	1	1	1	1	0.37	Putative lipoprotein OS
1465	1	db$2	2::C3KE42|C3KE42_PSEFS	36	20828	1	1	1	1	0.25	Putative DNA-3-methy
1466	1	db$2	2::C3KCD6|C3KCD6_PSEFS	36	27732	2	1	2	1	0.19	Short chain dehydroge
1467	1	db$2	2::C3K265|C3K265_PSEFS	36	29739	2	2	2	2	0.37	Dihydropteroate synth
1468	1	db$2	2::C3KBV6|C3KBV6_PSEFS	36	125347	1	1	1	1	0.04	Putative potassium eff
1469	1	SwissProt_HUMAN$	3::Q9BY11|PACN1_HUMAN	36	50934	4	1	3	1	0.10	Protein kinase C and c
1470	1	db$2	2::C3K406|C3K406_PSEFS	36	23424	2	2	2	2	0.49	Glyoxalase OS=Pseudo

↑Family	M	DB	Accession	Score	Mass	Matches	Match(sig)	Sequences	Seq(sig)	emPAI	Description
1471	1	db$2	2::C3K6C0|C3K6C0_PSEFS	36	25791	1	1	1	1	0.20	Putative amino acid AE
1472	1	db$2	2::C3KE75|C3KE75_PSEFS	36	123658	2	1	2	1	0.04	TGc domain-containing
1473	1	db$2	2::C3K451|C3K451_PSEFS	36	76463	2	2	2	2	0.13	ATP-dependent DNA h
1474	1	SwissProt_HUMAN$	3::P39019|RS19_HUMAN	35	16051	1	1	1	1	0.34	40S ribosomal protein
1475	1	db$2	2::C3K0N6|PLSX_PSEFS	35	35209	1	1	1	1	0.14	Phosphate acyltransfer
1476	1	SwissProt_HUMAN$	3::Q9BVV6|TALD3_HUMAN	35	169201	1	1	1	1	0.03	Protein TALPID3 OS=H
1477	1	db$2	2::C3K413|C3K413_PSEFS	35	22663	1	1	1	1	0.23	Putative ABC transport
1478	1	SwissProt_HUMAN$	3::P63173|RL38_HUMAN	35	8213	1	1	1	1	0.75	60S ribosomal protein
1479	1	SwissProt_HUMAN$	3::Q96T58|MINT_HUMAN	35	402004	1	1	1	1	0.01	Msx2-interacting prote
1480	1	db$2	2::C3K5Z0|C3K5Z0_PSEFS	35	15508	5	2	3	2	0.82	Uncharacterized protei
1481	1	db$2	2::C3K0Z5|C3K0Z5_PSEFS	35	22469	1	1	1	1	0.23	Corrinoid adenosyltran
1482	1	db$2	2::C3K4L1|C3K4L1_PSEFS	35	41720	2	2	1	1	0.12	Putative epimerase OS
1483	1	db$2	2::C3JZP3|C3JZP3_PSEFS	35	36685	2	2	2	2	0.29	Putative inosine-uridin
1484	1	db$2	2::C3K0Z9|C3K0Z9_PSEFS	35	26468	1	1	1	1	0.20	Bac_DnaA domain-con
1485	1	SwissProt_HUMAN$	3::Q9HCU5|PREB_HUMAN	35	45440	2	1	2	1	0.11	Prolactin regulatory ele
1486	1	SwissProt_HUMAN$	3::O75251|NDUS7_HUMAN	35	23548	1	1	1	1	0.22	NADH dehydrogenase
1487	1	db$2	2::C3K1R1|C3K1R1_PSEFS	35	13822	1	1	1	1	0.40	bPH_1 domain-contain
1488	1	db$2	2::C3K0Y2|C3K0Y2_PSEFS	35	46222	1	1	1	1	0.11	Putative fatty acid trar
1489	1	db$2	2::C3JY58|C3JY58_PSEFS	35	87640	4	2	3	2	0.11	DNA translocase FtsK (
1490	1	db$2	2::C3K3J8|C3K3J8_PSEFS	35	33397	1	1	1	1	0.15	TonB_C domain-contai
1491	1	db$2	2::C3K9J4|C3K9J4_PSEFS	34	15648	1	1	1	1	0.35	Putative DnaK suppres
1492	1	db$2	2::C3KCS7|MRAY_PSEFS	34	39243	2	2	2	2	0.27	Phospho-N-acetylmura
1493	1	db$2	2::C3JZ64|C3JZ64_PSEFS	34	17217	1	1	1	1	0.31	MaoC-like domain-con
1494	1	db$2	2::C3K6X3|C3K6X3_PSEFS	34	12829	1	1	1	1	0.44	Putative translation ini
1495	1	db$2	2::C3K704|TRUD_PSEFS	34	38571	3	3	2	2	0.28	tRNA pseudouridine sy
1496	1	db$2	2::C3K2Y9|C3K2Y9_PSEFS	34	13142	1	1	1	1	0.43	Protein translocase sul
1497	1	SwissProt_HUMAN$	3::P49207|RL34_HUMAN	34	13284	2	2	2	2	1.01	60S ribosomal protein
1498	1	SwissProt_HUMAN$	3::P62701|RS4X_HUMAN	34	29579	1	1	1	1	0.17	40S ribosomal protein
1499	1	db$2	2::C3K2Z1|COAX_PSEFS	34	26920	1	1	1	1	0.19	Type III pantothenate
1500	1	db$2	2::C3K7A9|C3K7A9_PSEFS	34	33658	1	1	1	1	0.15	Conserved hypothetica
1501	1	db$2	2::C3KAZ7|C3KAZ7_PSEFS	34	48577	1	1	1	1	0.10	Putative chain length c
1502	1	db$2	2::C3K7M0|C3K7M0_PSEFS	34	62717	1	1	1	1	0.08	Putative dipeptidase O
1503	1	SwissProt_HUMAN$	3::Q8IWU5|SULF2_HUMAN	34	100390	1	1	1	1	0.05	Extracellular sulfatase
1504	1	db$2	2::C3KE87|C3KE87_PSEFS	34	46792	3	3	3	3	0.35	NADH dehydrogenase
1505	1	db$2	2::C3K0Z1|C3K0Z1_PSEFS	34	36566	1	1	1	1	0.14	L-threonine-O-3-phosp
1506	1	db$2	2::C3K3X7|C3K3X7_PSEFS	34	16525	1	1	1	1	0.33	EVE domain-containing
1507	1	SwissProt_HUMAN$	3::P18627|LAG3_HUMAN	34	57413	1	1	1	1	0.09	Lymphocyte activation
1508	1	db$2	2::C3K6Z1|C3K6Z1_PSEFS	34	50711	2	2	2	2	0.21	Putative multicopper o
1509	1	db$2	2::C3K450|C3K450_PSEFS	34	61786	2	2	2	2	0.17	Putative EAL/GGDEF d
1510	1	SwissProt_HUMAN$	3::Q5SRN2|TSBP1_HUMAN	34	61588	1	1	1	1	0.08	Testis-expressed basic

↑Family	M	DB	Accession	Score	Mass	Matches	Match(sig)	Sequences	Seq(sig)	emPAI	Description
1511	1	db$2	2::C3K6Z6|C3K6Z6_PSEFS	34	13580	1	1	1	1	0.41	Putaitve lipoprotein OS
1512	1	SwissProt_HUMAN$	3::P37802|TAGL2_HUMAN	34	22377	1	1	1	1	0.23	Transgelin-2 OS=Hom
1513	1	SwissProt_HUMAN$	3::O75197|LRP5_HUMAN	34	179030	2	2	1	1	0.03	Low-density lipoprotei
1514	1	db$2	2::C3K5K8|C3K5K8_PSEFS	34	29952	1	1	1	1	0.17	Putative deoxyribonucl
1515	1	db$2	2::C3K0N8|C3K0N8_PSEFS	34	19586	1	1	1	1	0.27	23S rRNA accumulatio
1516	1	db$2	2::C3K6K7|C3K6K7_PSEFS	34	17395	1	1	1	1	0.31	GreA_GreB domain-co
1517	1	SwissProt_HUMAN$	3::O14827|RGRF2_HUMAN	33	140675	2	2	2	2	0.07	Ras-specific guanine n
1518	1	db$2	2::C3K330|GLPE_PSEFS	33	11747	1	1	1	1	0.48	Thiosulfate sulfurtrans
1519	1	db$2	2::C3KBM2|C3KBM2_PSEFS	33	16548	2	2	2	2	0.76	Cys-tRNA(Pro)/Cys-tRI
1520	1	db$2	2::C3K7G6|C3K7G6_PSEFS	33	30473	1	1	1	1	0.17	Putative hydrolase OS
1521	1	db$2	2::C3K6B1|C3K6B1_PSEFS	33	35098	2	2	2	2	0.31	Homoserine kinase OS
1522	1	db$2	2::C3K1M8|C3K1M8_PSEFS	33	28345	1	1	1	1	0.18	tRNA (cytidine/uridine
1523	1	db$2	2::C3JYL6|C3JYL6_PSEFS	33	25076	1	1	1	1	0.21	Putative TetR-family re
1524	1	SwissProt_HUMAN$	3::O94817|ATG12_HUMAN	33	15104	1	1	1	1	0.36	Ubiquitin-like protein A
1525	1	db$2	2::C3JZ52|C3JZ52_PSEFS	33	18715	1	1	1	1	0.28	Uncharacterized protei
1526	1	db$2	2::C3K0P2|C3K0P2_PSEFS	33	35618	3	2	3	2	0.30	Pseudouridine synthas
1527	1	db$2	2::C3K3W4|C3K3W4_PSEFS	33	13233	2	1	1	1	0.42	Glycine cleavage syste
1528	1	db$2	2::C3K3C7|C3K3C7_PSEFS	33	34452	2	1	2	1	0.15	Putative glycine betain
1529	1	db$2	2::C3K2P8|C3K2P8_PSEFS	33	24358	2	1	2	1	0.21	Membrane-related pro
1530	1	db$2	2::C3JYN0|C3JYN0_PSEFS	33	33944	2	2	2	2	0.32	Cbb3-type cytochrome
1530	2	db$2	2::C3JZS9|C3JZS9_PSEFS	23	35591	2	2	2	2	0.30	Cbb3-type cytochrome
1531	1	db$2	2::C3K8Z4|C3K8Z4_PSEFS	33	45613	3	2	3	2	0.23	High-affinity branched
1532	1	db$2	2::C3K7D7|C3K7D7_PSEFS	33	8979	1	1	1	1	0.67	Putative lipoprotein OS
1533	1	SwissProt_HUMAN$	3::Q4UJ75|A20A4_HUMAN	33	94090	2	1	1	1	0.05	Putative ankyrin repea
1534	1	db$2	2::C3KDN1|C3KDN1_PSEFS	33	54005	2	1	2	1	0.09	Deoxyribodipyrimidine
1535	1	db$2	2::C3JZ74|C3JZ74_PSEFS	33	42038	1	1	1	1	0.12	ABC transport system,
1536	1	db$2	2::C3K3M2|C3K3M2_PSEFS	32	37029	2	2	2	2	0.29	Cell division protein Ft
1537	1	db$2	2::C3K7J9|C3K7J9_PSEFS	32	83444	1	1	1	1	0.06	Putative exported prot
1538	1	db$2	2::C3K3P6|C3K3P6_PSEFS	32	19979	2	2	2	2	0.60	Dihydrofolate reductas
1539	1	db$2	2::C3K7K0|C3K7K0_PSEFS	32	41297	1	1	1	1	0.12	Esterase III OS=Pseuc
1540	1	SwissProt_HUMAN$	3::P57727|TMPS3_HUMAN	32	49373	1	1	1	1	0.10	Transmembrane prote
1541	1	db$2	2::C3JZ90|C3JZ90_PSEFS	32	52890	1	1	1	1	0.09	Trk system potassium
1542	1	db$2	2::C3K439|C3K439_PSEFS	32	25827	1	1	1	1	0.20	Putative hydrolase OS
1543	1	SwissProt_HUMAN$	3::Q14240|IF4A2_HUMAN	32	46373	2	2	2	2	0.23	Eukaryotic initiation fa
1544	1	SwissProt_HUMAN$	3::P06748|NPM_HUMAN	32	32555	2	1	2	1	0.16	Nucleophosmin OS=Hc
1545	1	SwissProt_HUMAN$	3::P62851|RS25_HUMAN	32	13734	2	1	2	1	0.40	40S ribosomal protein
1546	1	db$2	2::C3K0V2|C3K0V2_PSEFS	32	48301	2	2	2	2	0.22	Flagellar hook-associat
1547	1	SwissProt_HUMAN$	3::Q8IZ83|A16A1_HUMAN	32	85074	1	1	1	1	0.06	Aldehyde dehydrogena
1548	1	db$2	2::C3K911|C3K911_PSEFS	32	29460	1	1	1	1	0.17	Putative ABC transport
1549	1	db$2	2::C3K9T1|C3K9T1_PSEFS	32	36655	1	1	1	1	0.14	Putative phosphatase (

↑Family	M	DB	Accession	Score	Mass	Matches	Match(sig)	Sequences	Seq(sig)	emPAI	Description
1550	1	db$2	2::C3JXV4|C3JXV4_PSEFS	32	26256	1	1	1	1	0.20	Putative short-chain de
1551	1	db$2	2::C3K4Z5|C3K4Z5_PSEFS	32	14110	1	1	1	1	0.39	Putative lipoprotein OS
1552	1	db$2	2::C3K223|C3K223_PSEFS	32	37863	1	1	1	1	0.13	Protein-methionine-sul
1553	1	db$2	2::C3K663|C3K663_PSEFS	32	22341	2	1	2	1	0.23	Putative TetR-family re
1554	1	db$2	2::C3K3Q9|LGT_PSEFS	32	30444	3	3	2	2	0.59	Phosphatidylglycerol--p
1555	1	SwissProt_HUMAN$	3::Q6ZMT4|KDM7A_HUMAN	32	106489	2	1	2	1	0.05	Lysine-specific demeth
1556	1	db$2	2::C3KB05|C3KB05_PSEFS	32	50891	1	1	1	1	0.10	CDP-diacylglycerol-ser
1557	1	db$2	2::C3KAA5|C3KAA5_PSEFS	32	23984	3	3	2	2	0.48	Uncharacterized protei
1558	1	db$2	2::C3K457|C3K457_PSEFS	32	12769	2	2	2	2	1.07	Putative endoribonucle
1559	1	db$2	2::C3K5M4|C3K5M4_PSEFS	32	27564	1	1	1	1	0.19	Phosphorous compoun
1560	1	db$2	2::C3K8V7|C3K8V7_PSEFS	31	6725	1	1	1	1	0.96	Uncharacterized protei
1561	1	db$2	2::C3K8L9|C3K8L9_PSEFS	31	65348	3	1	2	1	0.08	Putative potassium-eff
1562	1	db$2	2::C3K267|RLME_PSEFS	31	24125	2	2	2	2	0.48	Ribosomal RNA large s
1563	1	db$2	2::C3K020|C3K020_PSEFS	31	23656	1	1	1	1	0.22	Putative hydrolase OS
1564	1	db$2	2::C3K4T5|C3K4T5_PSEFS	31	30974	2	2	2	2	0.36	Putative hydrolase OS
1565	1	SwissProt_HUMAN$	3::Q99618|CDCA3_HUMAN	31	28981	1	1	1	1	0.18	Cell division cycle-asso
1566	1	db$2	2::C3K4R7|C3K4R7_PSEFS	31	57887	1	1	1	1	0.09	Putative methyl-accept
1567	1	db$2	2::C3K4F9|C3K4F9_PSEFS	31	31102	1	1	1	1	0.16	Putative regulatory pro
1568	1	db$2	2::C3K8K6|C3K8K6_PSEFS	31	15117	1	1	1	1	0.36	Putative transport-rela
1569	1	SwissProt_HUMAN$	3::A6NJB7|PRR19_HUMAN	31	38692	1	1	1	1	0.13	Proline-rich protein 19
1570	1	db$2	2::C3K9Y9|C3K9Y9_PSEFS	31	13239	1	1	1	1	0.42	Uncharacterized protei
1571	1	db$2	2::C3KAN2|C3KAN2_PSEFS	31	30990	2	2	2	2	0.36	3-oxoadipate CoA-tran
1572	1	db$2	2::C3KAP0|C3KAP0_PSEFS	31	14934	1	1	1	1	0.37	4-carboxymuconolacto
1573	1	SwissProt_HUMAN$	3::P17480|UBF1_HUMAN	31	89350	2	1	2	1	0.05	Nucleolar transcription
1574	1	db$2	2::C3K3K0|YQGF_PSEFS	31	16158	1	1	1	1	0.33	Putative pre-16S rRNA
1575	1	SwissProt_HUMAN$	3::P26641|EF1G_HUMAN	31	50087	2	2	2	2	0.21	Elongation factor 1-ga
1576	1	SwissProt_HUMAN$	3::O00299|CLIC1_HUMAN	31	26906	1	1	1	1	0.19	Chloride intracellular c
1577	1	db$2	2::C3KDK6|C3KDK6_PSEFS	31	27451	1	1	1	1	0.19	Putative ABC transport
1578	1	db$2	2::C3K9N0|C3K9N0_PSEFS	31	28569	1	1	1	1	0.18	Putative transcriptiona
1579	1	db$2	2::C3K0A4|C3K0A4_PSEFS	31	12844	1	1	1	1	0.44	5-hydroxyisourate hyd
1580	1	db$2	2::C3KE19|C3KE19_PSEFS	30	30253	1	1	1	1	0.17	HDOD domain-contain
1581	1	db$2	2::C3K250|C3K250_PSEFS	30	71198	1	1	1	1	0.07	Acetyl-coenzyme A syn
1582	1	SwissProt_HUMAN$	3::Q9Y2G4|ANKR6_HUMAN	30	79922	1	1	1	1	0.06	Ankyrin repeat domain
1583	1	db$2	2::C3K0B1|C3K0B1_PSEFS	30	21506	1	1	1	1	0.24	Uncharacterized protei
1584	1	SwissProt_HUMAN$	3::Q6ZVD8|PHLP2_HUMAN	30	146658	2	1	2	1	0.03	PH domain leucine-rich
1585	1	db$2	2::C3K5I1|C3K5I1_PSEFS	30	43688	1	1	1	1	0.11	Putative phage integra
1586	1	db$2	2::C3K0Z0|COBQ_PSEFS	30	51487	1	1	1	1	0.10	Cobyric acid synthase
1587	1	db$2	2::C3K2K6|C3K2K6_PSEFS	30	49458	2	1	2	1	0.10	tRNA-2-methylthio-N(6
1588	1	SwissProt_HUMAN$	3::Q5VXU9|SHOC1_HUMAN	30	165097	1	1	1	1	0.03	Protein shortage in chi
1589	1	db$2	2::C3K340|C3K340_PSEFS	30	72802	2	2	2	2	0.14	DNA primase OS=Pseu

↑Family	M	DB	Accession	Score	Mass	Matches	Match(sig)	Sequences	Seq(sig)	emPAI	Description
1590	1	SwissProt_HUMAN$	3::Q96E52|OMA1_HUMAN	30	60081	1	1	1	1	0.08	Metalloendopeptidase
1591	1	db$2	2::C3K211|C3K211_PSEFS	30	35352	1	1	1	1	0.14	LysR-family transcripti
1592	1	SwissProt_HUMAN$	3::P42766|RL35_HUMAN	30	14543	1	1	1	1	0.38	60S ribosomal protein
1593	1	db$2	2::C3K3L3|C3K3L3_PSEFS	30	21091	4	3	3	2	0.56	dITP/XTP pyrophospha
1594	1	SwissProt_HUMAN$	3::Q6ZMY3|SPOC1_HUMAN	30	129945	2	2	1	1	0.04	SPOC domain-containi
1595	1	SwissProt_HUMAN$	3::Q9Y5B9|SP16H_HUMAN	30	119838	2	1	2	1	0.04	FACT complex subunit
1596	1	SwissProt_HUMAN$	3::Q8NA82|MARHA_HUMAN	30	90455	1	1	1	1	0.05	Probable E3 ubiquitin-(
1597	1	db$2	2::C3K7M1|C3K7M1_PSEFS	30	62712	1	1	1	1	0.08	Putative dipeptidase O
1598	1	SwissProt_HUMAN$	3::Q9NXE8|CWC25_HUMAN	30	49617	1	1	1	1	0.10	Pre-mRNA-splicing fact
1599	1	db$2	2::C3KE01|Y992_PSEFS	30	29383	3	3	3	3	0.62	UPF0246 protein PFLU_
1600	1	db$2	2::C3JY47|C3JY47_PSEFS	30	14227	1	1	1	1	0.39	DrsE domain-containin
1601	1	db$2	2::C3K065|C3K065_PSEFS	30	58554	3	3	3	3	0.27	Uncharacterized protei
1602	1	db$2	2::C3JXQ7|C3JXQ7_PSEFS	30	48832	3	2	2	1	0.10	UDP-glucose 6-dehydr
1603	1	SwissProt_HUMAN$	3::Q9Y265|RUVB1_HUMAN	30	50196	2	2	1	1	0.10	RuvB-like 1 OS=Homo
1604	1	SwissProt_HUMAN$	3::P35268|RL22_HUMAN	30	14778	1	1	1	1	0.37	60S ribosomal protein
1605	1	db$2	2::C3K1H9|C3K1H9_PSEFS	30	18692	2	2	2	2	0.65	Conserved hypothetica
1606	1	SwissProt_HUMAN$	3::Q9NR48|ASH1L_HUMAN	30	332582	1	1	1	1	0.01	Histone-lysine N-meth
1607	1	db$2	2::C3K4H8|C3K4H8_PSEFS	30	20657	2	1	2	1	0.26	Threonylcarbamoyl-AM
1608	1	SwissProt_HUMAN$	3::Q86TW2|ADCK1_HUMAN	30	60538	1	1	1	1	0.08	AarF domain-containin
1609	1	db$2	2::C3KBX2|C3KBX2_PSEFS	29	101201	1	1	1	1	0.05	Putative LuxR-family r
1610	1	SwissProt_HUMAN$	3::Q9UNA0|ATS5_HUMAN	29	101653	1	1	1	1	0.05	A disintegrin and meta
1611	1	db$2	2::C3K589|C3K589_PSEFS	29	50032	3	2	3	2	0.21	Transcriptional regulat
1612	1	db$2	2::C3K678|C3K678_PSEFS	29	45414	1	1	1	1	0.11	Cytosine deaminase O
1613	1	db$2	2::C3K0G1|C3K0G1_PSEFS	29	67986	1	1	1	1	0.07	Putative acetyl-/propic
1614	1	db$2	2::C3JXX9|C3JXX9_PSEFS	29	6925	3	2	2	2	2.76	Translational regulator
1615	1	SwissProt_HUMAN$	3::P61313|RL15_HUMAN	29	24131	1	1	1	1	0.22	60S ribosomal protein
1616	1	db$2	2::C3K3H5|C3K3H5_PSEFS	29	32718	1	1	1	1	0.16	Putative ABC transport
1617	1	db$2	2::C3K166|C3K166_PSEFS	29	65025	1	1	1	1	0.08	Histidine kinase OS=P
1618	1	db$2	2::C3K1G6|C3K1G6_PSEFS	29	25762	2	1	2	1	0.20	Thiol:disulfide intercha
1619	1	db$2	2::C3KB02|C3KB02_PSEFS	29	42163	1	1	1	1	0.12	Putative exported prot
1620	1	SwissProt_HUMAN$	3::Q8IV32|CCD71_HUMAN	29	49618	1	1	1	1	0.10	Coiled-coil domain-cor
1621	1	db$2	2::C3JYF1|C3JYF1_PSEFS	29	42707	1	1	1	1	0.12	Putative acyl-CoA deh
1622	1	db$2	2::C3KB17|C3KB17_PSEFS	29	51104	2	2	2	2	0.20	Outer membrane comp
1623	1	db$2	2::C3K327|C3K327_PSEFS	29	29832	2	2	2	2	0.37	Ribosomal RNA small s
1624	1	db$2	2::C3KAG2|C3KAG2_PSEFS	29	280833	1	1	1	1	0.02	Toxin CdiA OS=Pseudo
1625	1	db$2	2::C3JYT0|C3JYT0_PSEFS	29	26526	1	1	1	1	0.19	Probable transcriptiona
1626	1	SwissProt_HUMAN$	3::Q9P273|TEN3_HUMAN	28	300760	1	1	1	1	0.02	Teneurin-3 OS=Homo
1627	1	SwissProt_HUMAN$	3::P62280|RS11_HUMAN	28	18419	2	1	2	1	0.29	40S ribosomal protein
1628	1	SwissProt_HUMAN$	3::O15197|EPHB6_HUMAN	28	110630	1	1	1	1	0.04	Ephrin type-B receptor
1629	1	db$2	2::C3K8Z0|C3K8Z0_PSEFS	28	32439	1	1	1	1	0.16	UPF0276 protein PFLU_

↑Family	M	DB	Accession	Score	Mass	Matches	Match(sig)	Sequences	Seq(sig)	emPAI	Description
1630	1	SwissProt_HUMAN$	3::Q92963|RIT1_HUMAN	28	25129	1	1	1	1	0.21	GTP-binding protein Ri
1631	1	db$2	2::C3KCP9|C3KCP9_PSEFS	28	30268	1	1	1	1	0.17	Lipopolysaccharide cor
1632	1	db$2	2::C3JYG1|C3JYG1_PSEFS	28	30646	2	2	2	2	0.36	Putative LysR-family tr
1633	1	SwissProt_HUMAN$	3::Q9Y5A7|NUB1_HUMAN	28	70494	3	1	3	1	0.07	NEDD8 ultimate buste
1634	1	db$2	2::C3K6U5|FETP_PSEFS	28	10399	1	1	1	1	0.56	Probable Fe(2+)-traffi
1635	1	SwissProt_HUMAN$	3::P12111|CO6A3_HUMAN	28	343457	1	1	1	1	0.01	Collagen alpha-3(VI) c
1636	1	db$2	2::C3JYC8|C3JYC8_PSEFS	28	59331	1	1	1	1	0.08	Putative pH adaptatior
1637	1	db$2	2::C3K106|C3K106_PSEFS	28	20669	1	1	1	1	0.26	Uncharacterized protei
1638	1	db$2	2::C3KDC5|HEM1_PSEFS	28	46641	1	1	1	1	0.11	Glutamyl-tRNA reducta
1639	1	db$2	2::C3K660|C3K660_PSEFS	28	28970	1	1	1	1	0.18	Hydroxypyruvate isom
1640	1	SwissProt_HUMAN$	3::Q96GD3|SCMH1_HUMAN	28	73307	1	1	1	1	0.07	Polycomb protein SCM
1641	1	SwissProt_HUMAN$	3::Q9ULV4|COR1C_HUMAN	28	53215	1	1	1	1	0.09	Coronin-1C OS=Homo
1642	1	db$2	2::C3K909|C3K909_PSEFS	28	24537	1	1	1	1	0.21	Uncharacterized protei
1643	1	db$2	2::C3K2M4|C3K2M4_PSEFS	28	40052	1	1	1	1	0.13	Peptidoglycan glycosyl
1644	1	db$2	2::C3K1I0|C3K1I0_PSEFS	28	22456	2	1	2	1	0.23	Nudix hydrolase doma
1645	1	SwissProt_HUMAN$	3::O15270|SPTC2_HUMAN	28	62884	1	1	1	1	0.08	Serine palmitoyltransf
1646	1	db$2	2::C3K109|C3K109_PSEFS	28	49568	1	1	1	1	0.10	23S rRNA (uracil(1939
1647	1	SwissProt_HUMAN$	3::Q9BYK8|HELZ2_HUMAN	28	294465	1	1	1	1	0.02	Helicase with zinc fing
1648	1	db$2	2::C3JY07|C3JY07_PSEFS	28	19767	3	2	2	1	0.27	Putative RNA polymera
1649	1	db$2	2::C3K0N9|C3K0N9_PSEFS	28	21007	3	2	3	2	0.56	7-methyl-GTP pyropho
1650	1	db$2	2::C3K0X8|C3K0X8_PSEFS	28	55461	1	1	1	1	0.09	DNA recombination pr
1651	1	SwissProt_HUMAN$	3::Q7Z6Z7|HUWE1_HUMAN	28	481589	2	1	2	1	0.01	E3 ubiquitin-protein lig
1652	1	SwissProt_HUMAN$	3::P78549|NTH_HUMAN	28	34368	1	1	1	1	0.15	Endonuclease III-like p
1653	1	db$2	2::C3KAZ8|C3KAZ8_PSEFS	28	34711	1	1	1	1	0.15	Putative beta-hydroxyl
1654	1	db$2	2::C3K9P2|C3K9P2_PSEFS	28	69747	1	1	1	1	0.07	Putative substrate-bin
1655	1	db$2	2::C3KAJ7|C3KAJ7_PSEFS	28	44130	1	1	1	1	0.11	Uracil permease OS=P
1656	1	db$2	2::C3JXG8|C3JXG8_PSEFS	28	49590	1	1	1	1	0.10	Putative outer membra
1657	1	db$2	2::C3KDI3|C3KDI3_PSEFS	28	22379	1	1	1	1	0.23	Putative glutathione-S
1658	1	db$2	2::C3K9N7|C3K9N7_PSEFS	28	48751	1	1	1	1	0.10	Putative membrane pr
1659	1	db$2	2::C3JZT8|C3JZT8_PSEFS	28	28261	1	1	1	1	0.18	Putative methyltransfe
1660	1	db$2	2::C3KAT1|C3KAT1_PSEFS	28	27898	1	1	1	1	0.18	Response regulator pr
1661	1	SwissProt_HUMAN$	3::Q86VS8|HOOK3_HUMAN	27	83074	1	1	1	1	0.06	Protein Hook homolog
1662	1	db$2	2::C3JZ89|C3JZ89_PSEFS	27	157759	2	2	2	2	0.06	Uncharacterized protei
1663	1	SwissProt_HUMAN$	3::Q765P7|MTSS2_HUMAN	27	79880	2	1	2	1	0.06	Protein MTSS 2 OS=H
1664	1	db$2	2::C3K3P8|C3K3P8_PSEFS	27	49421	1	1	1	1	0.10	G domain-containing p
1665	1	db$2	2::C3KBB8|C3KBB8_PSEFS	27	13546	2	2	1	1	0.99	Uncharacterized protei
1666	1	db$2	2::C3JXW6|C3JXW6_PSEFS	27	64770	1	1	1	1	0.08	Putative exported prot
1667	1	db$2	2::C3K6L2|C3K6L2_PSEFS	27	14328	1	1	1	1	0.38	Organic hydroperoxide
1668	1	db$2	2::C3K391|C3K391_PSEFS	27	33913	2	2	2	2	0.32	Putative acyltransferas
1669	1	db$2	2::C3K6G9|LPXB_PSEFS	27	41468	1	1	1	1	0.12	Lipid-A-disaccharide sy

↑Family	M	DB	Accession	Score	Mass	Matches	Match(sig)	Sequences	Seq(sig)	emPAI	Description
1670	1	db$2	2::C3K8W0|C3K8W0_PSEFS	27	34311	1	1	1	1	0.15	Putative LysR-family re
1671	1	SwissProt_HUMAN$	3::Q86UK5|LBN_HUMAN	27	147856	1	1	1	1	0.03	Limbin OS=Homo sapi
1672	1	db$2	2::C3K1F6|C3K1F6_PSEFS	27	23787	2	2	2	2	0.49	Ribosomal RNA small s
1673	1	db$2	2::C3K629|C3K629_PSEFS	27	51787	1	1	1	1	0.10	Putative transport-rela
1674	1	db$2	2::C3JZ73|C3JZ73_PSEFS	27	49677	2	2	2	2	0.21	Porin OS=Pseudomona
1675	1	db$2	2::C3K7Z3|C3K7Z3_PSEFS	27	43142	1	1	1	1	0.12	Putative acyl-CoA dehy
1676	1	db$2	2::C3K811|C3K811_PSEFS	27	43409	1	1	1	1	0.12	Imidazolonepropionase
1677	1	db$2	2::C3KB44|C3KB44_PSEFS	27	23546	1	1	1	1	0.22	Putative membrane pr
1678	1	SwissProt_HUMAN$	3::P12883|MYH7_HUMAN	27	222959	1	1	1	1	0.02	Myosin-7 OS=Homo sa
1679	1	db$2	2::C3KAT0|C3KAT0_PSEFS	27	40804	1	1	1	1	0.12	Putative efflux system
1680	1	SwissProt_HUMAN$	3::Q7LBC6|KDM3B_HUMAN	27	191461	2	1	2	1	0.03	Lysine-specific demeth
1681	1	db$2	2::C3K6R2|C3K6R2_PSEFS	27	29309	1	1	1	1	0.17	Putative shikimate deh
1682	1	db$2	2::C3KAN8|C3KAN8_PSEFS	27	48228	1	1	1	1	0.10	3-carboxy-cis,cis-muco
1683	1	db$2	2::C3K659|C3K659_PSEFS	27	30767	2	2	2	2	0.36	2-hydroxy-3-oxopropio
1684	1	db$2	2::C3K0J0|C3K0J0_PSEFS	27	16514	1	1	1	1	0.33	Flagellar motor switch
1685	1	db$2	2::C3KBV2|C3KBV2_PSEFS	27	15686	1	1	1	1	0.35	Thioredoxin OS=Pseud
1686	1	SwissProt_HUMAN$	3::Q6PI48|SYDM_HUMAN	27	73516	1	1	1	1	0.07	Aspartate--tRNA ligase
1687	1	db$2	2::C3KB61|C3KB61_PSEFS	27	26669	1	1	1	1	0.19	Molybdate-binding per
1688	1	SwissProt_HUMAN$	3::Q9ULQ1|TPC1_HUMAN	27	94086	1	1	1	1	0.05	Two pore calcium char
1689	1	db$2	2::C3K7A1|C3K7A1_PSEFS	27	56003	2	2	1	1	0.09	Putative LuxR-family r
1690	1	db$2	2::C3JXL1|C3JXL1_PSEFS	27	51972	1	1	1	1	0.10	Putative transmembra
1691	1	SwissProt_HUMAN$	3::Q9Y6C2|EMIL1_HUMAN	27	106630	1	1	1	1	0.05	EMILIN-1 OS=Homo sa
1692	1	db$2	2::C3KCL5|C3KCL5_PSEFS	27	16185	1	1	1	1	0.33	Putative exported prot
1693	1	db$2	2::C3K125|C3K125_PSEFS	27	26783	2	2	1	1	0.19	Alanine--tRNA ligase C
1694	1	db$2	2::C3K8K5|C3K8K5_PSEFS	27	64377	1	1	1	1	0.08	Putative transport-rela
1695	1	db$2	2::C3K918|C3K918_PSEFS	27	26912	1	1	1	1	0.19	Putative ABC transport
1696	1	SwissProt_HUMAN$	3::Q9UQE7|SMC3_HUMAN	27	141454	1	1	1	1	0.03	Structural maintenanc
1697	1	db$2	2::C3KAF4|C3KAF4_PSEFS	27	50981	1	1	1	1	0.10	Putative lipoprotein OS
1698	1	db$2	2::C3K3J1|C3K3J1_PSEFS	27	16523	1	1	1	1	0.33	Putative chemotaxis-re
1699	1	db$2	2::C3K4K0|C3K4K0_PSEFS	27	42230	1	1	1	1	0.12	Hypothetical exported
1700	1	db$2	2::C3K473|DUT_PSEFS	27	16022	1	1	1	1	0.34	Deoxyuridine 5'-tripho
1701	1	db$2	2::C3K252|C3K252_PSEFS	26	37575	1	1	1	1	0.13	Uncharacterized protei
1702	1	db$2	2::C3K6S4|C3K6S4_PSEFS	26	30497	1	1	1	1	0.17	Putative antibiotic bios
1703	1	db$2	2::C3K746|C3K746_PSEFS	26	78906	1	1	1	1	0.06	Putative TonB-depende
1704	1	SwissProt_HUMAN$	3::Q02080|MEF2B_HUMAN	26	38615	1	1	1	1	0.13	Myocyte-specific enhar
1705	1	SwissProt_HUMAN$	3::O96017|CHK2_HUMAN	26	60877	2	1	2	1	0.08	Serine/threonine-prote
1706	1	SwissProt_HUMAN$	3::Q00526|CDK3_HUMAN	26	35024	1	1	1	1	0.14	Cyclin-dependent kina
1707	1	SwissProt_HUMAN$	3::Q9NQX4|MYO5C_HUMAN	26	202682	2	1	2	1	0.02	Unconventional myosir
1708	1	db$2	2::C3K6E6|C3K6E6_PSEFS	26	23384	1	1	1	1	0.22	Stringent starvation pr
1709	1	db$2	2::C3KCV5|C3KCV5_PSEFS	26	37705	2	2	2	2	0.28	Uncharacterized protei

↑Family	M	DB	Accession	Score	Mass	Matches	Match(sig)	Sequences	Seq(sig)	emPAI	Description
1710	1	db$2	2::C3K2E8|C3K2E8_PSEFS	26	37117	1	1	1	1	0.14	Putative cytochrome o
1711	1	SwissProt_HUMAN$	3::P59998|ARPC4_HUMAN	26	19654	1	1	1	1	0.27	Actin-related protein 2
1712	1	db$2	2::C3K9N4|C3K9N4_PSEFS	26	91939	2	1	2	1	0.05	Histidine kinase OS=P:
1713	1	SwissProt_HUMAN$	3::P62906|RL10A_HUMAN	26	24816	1	1	1	1	0.21	60S ribosomal protein
1714	1	db$2	2::C3K536|C3K536_PSEFS	26	22752	2	1	2	1	0.23	DUF4174 domain-cont
1715	1	db$2	2::C3KA92|C3KA92_PSEFS	26	24193	1	1	1	1	0.21	Putative ACP reductase
1716	1	db$2	2::C3KB65|C3KB65_PSEFS	26	37899	1	1	1	1	0.13	Methylated-DNA--[prot
1717	1	SwissProt_HUMAN$	3::Q16363|LAMA4_HUMAN	26	202397	1	1	1	1	0.02	Laminin subunit alpha-
1718	1	db$2	2::C3JYR6|C3JYR6_PSEFS	26	38174	3	2	3	2	0.28	Quinolinate synthase A
1719	1	SwissProt_HUMAN$	3::P54577|SYYC_HUMAN	26	59106	1	1	1	1	0.08	Tyrosine--tRNA ligase,
1720	1	db$2	2::C3JY26|C3JY26_PSEFS	26	17211	2	1	2	1	0.31	Protein-tyrosine-phosp
1721	1	db$2	2::C3K5C4|C3K5C4_PSEFS	26	17510	2	2	2	2	0.71	SnoaL-like domain-cor
1722	1	db$2	2::C3KAS1|C3KAS1_PSEFS	26	34986	1	1	1	1	0.14	Putative 2-hydroxyacic
1723	1	db$2	2::C3K9H5|C3K9H5_PSEFS	26	33119	1	1	1	1	0.15	Putative LysR-family tr
1724	1	db$2	2::C3K323|C3K323_PSEFS	26	38795	2	1	2	1	0.13	Putative phosphotrans
1725	1	SwissProt_HUMAN$	3::Q5VT06|CE350_HUMAN	26	350716	3	2	3	2	0.03	Centrosome-associated
1726	1	db$2	2::C3K7A2|C3K7A2_PSEFS	26	33841	1	1	1	1	0.15	Putative membrane pr
1727	1	SwissProt_HUMAN$	3::Q96AY4|TTC28_HUMAN	26	270715	1	1	1	1	0.02	Tetratricopeptide repea
1728	1	db$2	2::C3JZN0|C3JZN0_PSEFS	26	13917	1	1	1	1	0.40	MerR-family transcript
1729	1	db$2	2::C3KAN9|C3KAN9_PSEFS	26	28318	1	1	1	1	0.18	Putative aromatic-hydi
1730	1	SwissProt_HUMAN$	3::Q96AQ6|PBIP1_HUMAN	26	80594	1	1	1	1	0.06	Pre-B-cell leukemia tra
1731	1	SwissProt_HUMAN$	3::Q71RG4|TMUB2_HUMAN	26	33767	1	1	1	1	0.15	Transmembrane and u
1732	1	db$2	2::C3K0H9|C3K0H9_PSEFS	26	13855	2	2	2	2	0.96	Chemotaxis protein Ch
1733	1	SwissProt_HUMAN$	3::P62316|SMD2_HUMAN	26	13518	1	1	1	1	0.41	Small nuclear ribonucl
1734	1	SwissProt_HUMAN$	3::P04920|B3A2_HUMAN	25	136923	1	1	1	1	0.04	Anion exchange protei
1735	1	db$2	2::C3KDL9|C3KDL9_PSEFS	25	22175	1	1	1	1	0.24	Uncharacterized protei
1736	1	db$2	2::C3K454|C3K454_PSEFS	25	13613	2	1	1	1	0.41	Putative cytochrome c
1737	1	db$2	2::C3KBT7|C3KBT7_PSEFS	25	36676	4	3	3	3	0.47	Sulfate ABC transport
1738	1	db$2	2::C3K598|C3K598_PSEFS	25	34258	2	2	2	2	0.32	Curved DNA-binding p
1739	1	db$2	2::C3K602|C3K602_PSEFS	25	48346	2	1	2	1	0.10	Zinc metalloprotease (
1740	1	SwissProt_HUMAN$	3::Q969Y2|GTPB3_HUMAN	25	52026	1	1	1	1	0.10	tRNA modification GTP
1741	1	db$2	2::C3JY25|C3JY25_PSEFS	25	37002	1	1	1	1	0.14	UDP-N-acetylenolpyru
1742	1	db$2	2::C3KE60|C3KE60_PSEFS	25	49144	2	2	2	2	0.21	Histidine kinase OS=P:
1743	1	db$2	2::C3JXZ6|C3JXZ6_PSEFS	25	24822	1	1	1	1	0.21	Putative histidine trans
1744	1	SwissProt_HUMAN$	3::Q14746|COG2_HUMAN	25	83155	1	1	1	1	0.06	Conserved oligomeric (
1745	1	SwissProt_HUMAN$	3::Q75VX8|GARE2_HUMAN	25	92824	1	1	1	1	0.05	GRB2-associated and r
1746	1	db$2	2::C3K5T2|C3K5T2_PSEFS	25	65173	2	1	2	1	0.08	Putative dehydrogenas
1747	1	SwissProt_HUMAN$	3::Q3B7T1|EDRF1_HUMAN	25	138440	1	1	1	1	0.03	Erythroid differentiatio
1748	1	db$2	2::C3K9P4|C3K9P4_PSEFS	25	59261	4	2	3	2	0.17	Putative ATP-binding c
1749	1	db$2	2::C3KD58|C3KD58_PSEFS	25	41746	1	1	1	1	0.12	Putative cystathionine

↑Family	M	DB	Accession	Score	Mass	Matches	Match(sig)	Sequences	Seq(sig)	emPAI	Description
1750	1	SwissProt_HUMAN$	3::Q5JTZ9|SYAM_HUMAN	25	107273	1	1	1	1	0.05	Alanine--tRNA ligase, r
1751	1	db$2	2::C3K2L4|C3K2L4_PSEFS	25	37419	1	1	1	1	0.13	DNA polymerase III su
1752	1	db$2	2::C3K071|Y4323_PSEFS	25	8578	1	1	1	1	0.71	UPF0270 protein PFLU_
1753	1	SwissProt_HUMAN$	3::P98161|PKD1_HUMAN	25	462239	1	1	1	1	0.01	Polycystin-1 OS=Homo
1754	1	db$2	2::C3JXL2|C3JXL2_PSEFS	25	30967	1	1	1	1	0.16	Uncharacterized protei
1755	1	db$2	2::C3K3B4|C3K3B4_PSEFS	25	14608	1	1	1	1	0.37	Putative exported prot
1756	1	SwissProt_HUMAN$	3::O00231|PSD11_HUMAN	25	47434	1	1	1	1	0.11	26S proteasome non-A
1757	1	db$2	2::C3K6G7|C3K6G7_PSEFS	25	39119	1	1	1	1	0.13	Putative ABC transport
1758	1	SwissProt_HUMAN$	3::P81605|DCD_HUMAN	25	11277	1	1	1	1	0.51	Dermcidin OS=Homo s
1759	1	db$2	2::C3KCB4|C3KCB4_PSEFS	25	27191	1	1	1	1	0.19	Uncharacterized protei
1760	1	db$2	2::C3K4Y0|C3K4Y0_PSEFS	25	31989	1	1	1	1	0.16	DUF1989 domain-cont
1761	1	db$2	2::C3K286|C3K286_PSEFS	25	9470	1	1	1	1	0.63	Putative exported prot
1762	1	db$2	2::C3KDB4|C3KDB4_PSEFS	25	42765	1	1	1	1	0.12	Putative glycosyl trans
1763	1	SwissProt_HUMAN$	3::Q5K651|SAMD9_HUMAN	25	184165	1	1	1	1	0.03	Sterile alpha motif dor
1764	1	db$2	2::C3K0X5|C3K0X5_PSEFS	25	21842	2	1	2	1	0.24	Uncharacterized protei
1765	1	db$2	2::C3K5C9|C3K5C9_PSEFS	25	27544	1	1	1	1	0.19	Putative regulatory pro
1766	1	db$2	2::C3K0J8|C3K0J8_PSEFS	25	22091	2	2	2	2	0.53	START domain-contain
1767	1	SwissProt_HUMAN$	3::O00294|TULP1_HUMAN	25	60572	1	1	1	1	0.08	Tubby-related protein
1768	1	SwissProt_HUMAN$	3::P06312|KV401_HUMAN	25	13372	1	1	1	1	0.42	Immunoglobulin kappa
1769	1	db$2	2::C3K6N8|C3K6N8_PSEFS	25	25972	2	1	1	1	0.20	Putative hydrolase OS=
1770	1	db$2	2::C3K1H4|C3K1H4_PSEFS	25	45615	1	1	1	1	0.11	Putative membrane pr
1771	1	SwissProt_HUMAN$	3::Q6ZNX1|SHLD3_HUMAN	25	28824	1	1	1	1	0.18	Shieldin complex subu
1772	1	SwissProt_HUMAN$	3::O14917|PCD17_HUMAN	24	126151	1	1	1	1	0.04	Protocadherin-17 OS=
1773	1	SwissProt_HUMAN$	3::Q8NCM8|DYHC2_HUMAN	24	492311	3	2	3	2	0.02	Cytoplasmic dynein 2 l
1774	1	SwissProt_HUMAN$	3::P39687|AN32A_HUMAN	24	28568	1	1	1	1	0.18	Acidic leucine-rich nuc
1775	1	db$2	2::C3K7Q7|C3K7Q7_PSEFS	24	37715	1	1	1	1	0.13	Putative GntR-family r
1776	1	db$2	2::C3KDU6|C3KDU6_PSEFS	24	27726	1	1	1	1	0.19	Putative two-compone
1777	1	SwissProt_HUMAN$	3::Q9NRN5|OLFL3_HUMAN	24	45981	1	1	1	1	0.11	Olfactomedin-like prot
1778	1	db$2	2::C3KEB8|C3KEB8_PSEFS	24	34155	1	1	1	1	0.15	Putative lipoprotein OS
1779	1	SwissProt_HUMAN$	3::Q9BTV4|TMM43_HUMAN	24	44847	1	1	1	1	0.11	Transmembrane protei
1780	1	db$2	2::C3K1M6|C3K1M6_PSEFS	24	24782	1	1	1	1	0.21	Uncharacterized protei
1781	1	db$2	2::C3KDZ7|C3KDZ7_PSEFS	24	49934	2	2	1	1	0.10	Alginate biosynthesis p
1782	1	SwissProt_HUMAN$	3::P27708|PYR1_HUMAN	24	242829	1	1	1	1	0.02	CAD protein OS=Homo
1783	1	SwissProt_HUMAN$	3::P61353|RL27_HUMAN	24	15788	1	1	1	1	0.35	60S ribosomal protein
1784	1	SwissProt_HUMAN$	3::Q9C037|TRIM4_HUMAN	24	57424	1	1	1	1	0.09	E3 ubiquitin-protein lig
1785	1	db$2	2::C3K3T1|C3K3T1_PSEFS	24	86048	1	1	1	1	0.06	Putative penicillin amic
1786	1	SwissProt_HUMAN$	3::Q9P1Z2|CACO1_HUMAN	24	77289	2	1	2	1	0.06	Calcium-binding and c
1787	1	db$2	2::C3K4W4|C3K4W4_PSEFS	24	45211	3	3	3	3	0.37	Putative lipoprotein rel
1788	1	db$2	2::C3K0Y3|C3K0Y3_PSEFS	24	20085	1	1	1	1	0.26	Glutathione peroxidase
1789	1	db$2	2::C3KBT8|C3KBT8_PSEFS	24	29541	1	1	1	1	0.17	Sulfate transport syste

↑Family	M	DB	Accession	Score	Mass	Matches	Match(sig)	Sequences	Seq(sig)	emPAI	Description
1790	1	db$2	2::C3JY37|C3JY37_PSEFS	24	14829	1	1	1	1	0.37	Putative lipoprotein OS
1791	1	SwissProt_HUMAN$	3::P01024|CO3_HUMAN	24	187030	1	1	1	1	0.03	Complement C3 OS=H
1792	1	SwissProt_HUMAN$	3::O95153|RIMB1_HUMAN	24	199928	1	1	1	1	0.02	Peripheral-type benzoc
1793	1	SwissProt_HUMAN$	3::O43151|TET3_HUMAN	24	193583	2	2	2	2	0.05	Methylcytosine dioxyge
1794	1	SwissProt_HUMAN$	3::P62136|PP1A_HUMAN	24	37488	1	1	1	1	0.13	Serine/threonine-prote
1795	1	db$2	2::C3K348|PQQB_PSEFS	24	33093	1	1	1	1	0.15	Coenzyme PQQ synthe
1796	1	SwissProt_HUMAN$	3::Q96RL7|VP13A_HUMAN	24	360047	2	1	2	1	0.01	Vacuolar protein sortin
1797	1	db$2	2::C3K8T6|C3K8T6_PSEFS	24	26078	2	2	2	2	0.43	Ribosomal RNA small s
1798	1	SwissProt_HUMAN$	3::Q05BV3|EMAL5_HUMAN	24	219287	1	1	1	1	0.02	Echinoderm microtubu
1799	1	SwissProt_HUMAN$	3::Q7L0Q8|RHOU_HUMAN	24	28200	1	1	1	1	0.18	Rho-related GTP-bindi
1800	1	db$2	2::C3KE47|FMT_PSEFS	24	33416	1	1	1	1	0.15	Methionyl-tRNA formyl
1801	1	SwissProt_HUMAN$	3::Q6DHV5|C2D2B_HUMAN	24	166681	2	1	1	1	0.03	Protein CC2D2B OS=H
1802	1	db$2	2::C3K431|C3K431_PSEFS	24	14535	1	1	1	1	0.38	Regulator of nucleosid
1803	1	db$2	2::C3K4W5|C3K4W5_PSEFS	24	24663	1	1	1	1	0.21	Lipoprotein-releasing s
1804	1	db$2	2::C3JXK0|C3JXK0_PSEFS	24	17939	1	1	1	1	0.30	Putative acetyltransfer
1805	1	SwissProt_HUMAN$	3::P02741|CRP_HUMAN	24	25023	3	3	3	3	0.76	C-reactive protein OS=
1806	1	SwissProt_HUMAN$	3::O95819|M4K4_HUMAN	24	142013	1	1	1	1	0.03	Mitogen-activated prot
1807	1	SwissProt_HUMAN$	3::Q9Y2B5|VP9D1_HUMAN	24	68913	1	1	1	1	0.07	VPS9 domain-containir
1808	1	db$2	2::C3K6G6|C3K6G6_PSEFS	23	41185	1	1	1	1	0.12	Lipopolysaccharide exp
1809	1	SwissProt_HUMAN$	3::Q9HBJ7|UBP29_HUMAN	23	104090	1	1	1	1	0.05	Ubiquitin carboxyl-terr
1810	1	db$2	2::C3K1J1|C3K1J1_PSEFS	23	11312	1	1	1	1	0.51	Uncharacterized protei
1811	1	db$2	2::C3K3J6|C3K3J6_PSEFS	23	14927	1	1	1	1	0.37	Type IV pilus response
1812	1	db$2	2::C3KE72|C3KE72_PSEFS	23	85807	2	1	2	1	0.06	UPF0313 protein PFLU_
1813	1	SwissProt_HUMAN$	3::Q9H0A8|COMD4_HUMAN	23	21750	1	1	1	1	0.24	COMM domain-contain
1814	1	SwissProt_HUMAN$	3::Q6UX04|CWC27_HUMAN	23	53814	1	1	1	1	0.09	Spliceosome-associate
1815	1	SwissProt_HUMAN$	3::Q14246|AGRE1_HUMAN	23	97618	1	1	1	1	0.05	Adhesion G protein-cou
1816	1	db$2	2::C3K3V1|C3K3V1_PSEFS	23	23989	1	1	1	1	0.22	Alkyl hydroperoxide re
1817	1	SwissProt_HUMAN$	3::P27216|ANX13_HUMAN	23	35393	1	1	1	1	0.14	Annexin A13 OS=Hom
1818	1	db$2	2::C3K5B8|C3K5B8_PSEFS	23	59457	1	1	1	1	0.08	Dipeptide ABC transpo
1819	1	db$2	2::C3K2N6|C3K2N6_PSEFS	23	16796	1	1	1	1	0.32	MaoC-like domain-cont
1820	1	SwissProt_HUMAN$	3::Q6UWJ1|TMCO3_HUMAN	23	75549	1	1	1	1	0.06	Transmembrane and c
1821	1	SwissProt_HUMAN$	3::O94967|WDR47_HUMAN	23	101884	1	1	1	1	0.05	WD repeat-containing
1822	1	SwissProt_HUMAN$	3::P08397|HEM3_HUMAN	23	39306	1	1	1	1	0.13	Porphobilinogen deami
1823	1	db$2	2::C3K9S5|C3K9S5_PSEFS	23	38309	1	1	1	1	0.13	Putative cobalamin bio
1824	1	SwissProt_HUMAN$	3::P36507|MP2K2_HUMAN	23	44396	1	1	1	1	0.11	Dual specificity mitoge
1825	1	db$2	2::C3K5G7|PDXB_PSEFS	23	41465	1	1	1	1	0.12	Erythronate-4-phospha
1826	1	db$2	2::C3K2L3|C3K2L3_PSEFS	23	22416	1	1	1	1	0.23	LPS-assembly lipoprot
1827	1	SwissProt_HUMAN$	3::Q15075|EEA1_HUMAN	23	162367	1	1	1	1	0.03	Early endosome antige
1828	1	SwissProt_HUMAN$	3::Q05D60|DEUP1_HUMAN	23	70933	2	2	2	2	0.14	Deuterosome assembl
1829	1	SwissProt_HUMAN$	3::P00738|HPT_HUMAN	23	45177	1	1	1	1	0.11	Haptoglobin OS=Homo

↑Family	M	DB	Accession	Score	Mass	Matches	Match(sig)	Sequences	Seq(sig)	emPAI	Description
1830	1	SwissProt_HUMAN$	3::O60861|GAS7_HUMAN	23	54337	1	1	1	1	0.09	Growth arrest-specific
1831	1	SwissProt_HUMAN$	3::Q7Z5H4|VN1R5_HUMAN	23	40752	1	1	1	1	0.12	Vomeronasal type-1 re
1832	1	SwissProt_HUMAN$	3::P54821|PRRX1_HUMAN	23	27280	1	1	1	1	0.19	Paired mesoderm hom
1833	1	SwissProt_HUMAN$	3::Q96FC7|PHIPL_HUMAN	23	42459	1	1	1	1	0.12	Phytanoyl-CoA hydrox
1834	1	SwissProt_HUMAN$	3::Q9H2M9|RBGPR_HUMAN	23	155886	1	1	1	1	0.03	Rab3 GTPase-activatin
1835	1	SwissProt_HUMAN$	3::Q96ES6|MFSD3_HUMAN	23	42669	1	1	1	1	0.12	Major facilitator superf
1836	1	SwissProt_HUMAN$	3::Q03001|DYST_HUMAN	23	860127	2	1	2	1	0.01	Dystonin OS=Homo sa
1837	1	db$2	2::C3K188|C3K188_PSEFS	22	39897	1	1	1	1	0.13	Putative exported prot
1838	1	SwissProt_HUMAN$	3::Q96A44|SPSB4_HUMAN	22	30160	1	1	1	1	0.17	SPRY domain-containir
1839	1	SwissProt_HUMAN$	3::O94827|PKHG5_HUMAN	22	117377	2	1	2	1	0.04	Pleckstrin homology dc
1840	1	SwissProt_HUMAN$	3::A4UGR9|XIRP2_HUMAN	22	382063	1	1	1	1	0.01	Xin actin-binding repea
1841	1	db$2	2::C3JZ42|C3JZ42_PSEFS	22	19185	1	1	1	1	0.28	Putative AraC family tr
1842	1	db$2	2::C3K4L7|C3K4L7_PSEFS	22	23283	1	1	1	1	0.22	Putative TetR-family re
1843	1	db$2	2::C3K3D4|C3K3D4_PSEFS	22	52472	2	1	2	1	0.09	Uncharacterized protei
1844	1	db$2	2::C3K1V5|C3K1V5_PSEFS	22	64171	1	1	1	1	0.08	Uncharacterized protei
1845	1	SwissProt_HUMAN$	3::Q9UPN4|CP131_HUMAN	22	122075	1	1	1	1	0.04	Centrosomal protein of
1846	1	db$2	2::C3K732|TPMT_PSEFS	22	24819	1	1	1	1	0.21	Thiopurine S-methyltra
1847	1	SwissProt_HUMAN$	3::A6NM36|LRC30_HUMAN	22	34012	1	1	1	1	0.15	Leucine-rich repeat-co
1848	1	SwissProt_HUMAN$	3::P78395|PRAME_HUMAN	22	57852	2	1	2	1	0.09	Melanoma antigen pre
1849	1	SwissProt_HUMAN$	3::Q9H244|P2Y12_HUMAN	22	39413	1	1	1	1	0.13	P2Y purinoceptor 12 O
1850	1	SwissProt_HUMAN$	3::P52333|JAK3_HUMAN	22	125019	1	1	1	1	0.04	Tyrosine-protein kinas
1851	1	db$2	2::C3K3F2|C3K3F2_PSEFS	22	38228	1	1	1	1	0.13	D-erythrose-4-phosph
1852	1	SwissProt_HUMAN$	3::P05546|HEP2_HUMAN	22	57034	1	1	1	1	0.09	Heparin cofactor 2 OS
1853	1	db$2	2::C3JZP4|RBSD_PSEFS	22	14393	1	1	1	1	0.38	D-ribose pyranase OS
1854	1	db$2	2::C3K0I8|C3K0I8_PSEFS	22	26895	1	1	1	1	0.19	Flagellar biosynthetic p
1855	1	SwissProt_HUMAN$	3::P49961|ENTP1_HUMAN	22	57927	1	1	1	1	0.09	Ectonucleoside triphos
1856	1	SwissProt_HUMAN$	3::P18077|RL35A_HUMAN	22	12530	1	1	1	1	0.45	60S ribosomal protein
1857	1	db$2	2::A4V7G0|A4V7G0_PSEFS	22	59387	2	1	2	1	0.08	Putative RNA polymera
1858	1	db$2	2::C3K6S3|C3K6S3_PSEFS	22	25881	2	2	2	2	0.44	Putative glutathione S-
1859	1	SwissProt_HUMAN$	3::Q9H299|SH3L3_HUMAN	22	10431	1	1	1	1	0.56	SH3 domain-binding g
1860	1	SwissProt_HUMAN$	3::P02788|TRFL_HUMAN	22	78132	1	1	1	1	0.06	Lactotransferrin OS=H
1861	1	db$2	2::C3K029|C3K029_PSEFS	22	46068	1	1	1	1	0.11	Uncharacterized protei
1862	1	db$2	2::C3JXS4|C3JXS4_PSEFS	22	25108	1	1	1	1	0.21	Tol-Pal system protein
1863	1	db$2	2::C3JXZ4|C3JXZ4_PSEFS	22	40619	1	1	1	1	0.12	Uncharacterized protei
1864	1	db$2	2::C3KAI2|C3KAI2_PSEFS	21	19361	1	1	1	1	0.27	Shikimate kinase OS=
1865	1	SwissProt_HUMAN$	3::Q96PC2|IP6K3_HUMAN	21	46387	1	1	1	1	0.11	Inositol hexakisphosph
1866	1	db$2	2::C3JYS7|RUVB_PSEFS	21	38919	1	1	1	1	0.13	Holliday junction ATP-d
1867	1	db$2	2::C3K5L6|C3K5L6_PSEFS	21	16214	2	2	2	2	0.78	Putative phosphorous
1868	1	SwissProt_HUMAN$	3::Q9Y4B4|ARIP4_HUMAN	21	162667	1	1	1	1	0.03	Helicase ARIP4 OS=Hc
1869	1	SwissProt_HUMAN$	3::Q15382|RHEB_HUMAN	21	20485	1	1	1	1	0.26	GTP-binding protein Rł

↑Family	M	DB	Accession	Score	Mass	Matches	Match(sig)	Sequences	Seq(sig)	emPAI	Description
1870	1	SwissProt_HUMAN$	3::P0DKB5|TPBGL_HUMAN	21	40374	2	1	1	1	0.12	Trophoblast glycoprote
1871	1	db$2	2::C3JZR0|C3JZR0_PSEFS	21	25453	1	1	1	1	0.20	Response regulatory p
1872	1	SwissProt_HUMAN$	3::Q9H6P5|TASP1_HUMAN	21	44427	1	1	1	1	0.11	Threonine aspartase 1
1873	1	db$2	2::C3K0P0|C3K0P0_PSEFS	21	35982	1	1	1	1	0.14	Putative peptidase OS:
1874	1	SwissProt_HUMAN$	3::P62258|1433E_HUMAN	21	29155	1	1	1	1	0.18	14-3-3 protein epsilon
1875	1	SwissProt_HUMAN$	3::P55199|ELL_HUMAN	21	68223	1	1	1	1	0.07	RNA polymerase II elo
1876	1	db$2	2::C3K1S4|C3K1S4_PSEFS	21	23334	1	1	1	1	0.22	Putative membrane pr
1877	1	db$2	2::C3K9I7|C3K9I7_PSEFS	21	69543	1	1	1	1	0.07	Putative thiamine pyro
1878	1	SwissProt_HUMAN$	3::P02765|FETUA_HUMAN	21	39316	1	1	1	1	0.13	Alpha-2-HS-glycoprote
1879	1	db$2	2::C3K814|DTD_PSEFS	21	15391	1	1	1	1	0.35	D-aminoacyl-tRNA dea
1880	1	db$2	2::C3K1Q2|C3K1Q2_PSEFS	21	73203	1	1	1	1	0.07	Histidine kinase OS=P:
1881	1	db$2	2::C3KCP8|C3KCP8_PSEFS	21	42283	1	1	1	1	0.12	Lipopolysaccharide cor
1882	1	db$2	2::C3K757|C3K757_PSEFS	21	27432	2	1	2	1	0.19	Putative two-compone
1883	1	db$2	2::C3K883|C3K883_PSEFS	21	60772	1	1	1	1	0.08	Putative transposase s
1884	1	db$2	2::C3K5Y5|C3K5Y5_PSEFS	21	18019	1	1	1	1	0.30	Protein PsiE OS=Pseuc
1885	1	db$2	2::C3K4U2|C3K4U2_PSEFS	21	24386	1	1	1	1	0.21	Uncharacterized protei
1886	1	db$2	2::C3JXT0|C3JXT0_PSEFS	21	22445	1	1	1	1	0.23	Putative isochorismata
1887	1	SwissProt_HUMAN$	3::Q9BXL7|CAR11_HUMAN	21	133200	1	1	1	1	0.04	Caspase recruitment d
1888	1	SwissProt_HUMAN$	3::P04075|ALDOA_HUMAN	21	39395	1	1	1	1	0.13	Fructose-bisphosphate
1889	1	db$2	2::C3K321|C3K321_PSEFS	21	28336	1	1	1	1	0.18	Putative heat shock pr
1890	1	SwissProt_HUMAN$	3::Q9NQZ6|ZC4H2_HUMAN	21	26227	1	1	1	1	0.20	Zinc finger C4H2 doma
1891	1	db$2	2::C3K2M2|C3K2M2_PSEFS	21	35893	1	1	1	1	0.14	Endolytic peptidoglyca
1892	1	db$2	2::C3K2H9|C3K2H9_PSEFS	21	43500	1	1	1	1	0.11	Uncharacterized protei
1893	1	db$2	2::C3K800|C3K800_PSEFS	21	26032	1	1	1	1	0.20	Putative GntR-family r
1894	1	db$2	2::C3K1Y0|C3K1Y0_PSEFS	21	12222	1	1	1	1	0.46	Uncharacterized protei
1895	1	db$2	2::C3JZQ4|C3JZQ4_PSEFS	20	8106	1	1	1	1	0.76	Translational regulator
1896	1	SwissProt_HUMAN$	3::P31943|HNRH1_HUMAN	20	49198	1	1	1	1	0.10	Heterogeneous nuclea
1897	1	db$2	2::C3KCP0|C3KCP0_PSEFS	20	31742	1	1	1	1	0.16	Ribosomal RNA large s
1898	1	SwissProt_HUMAN$	3::A0A1B0GVD1|F237B_HUMAN	20	16320	1	1	1	1	0.33	Protein FAM237B OS=
1899	1	db$2	2::C3K0J3|C3K0J3_PSEFS	20	45387	2	1	2	1	0.11	Putative flagellar hook
1900	1	SwissProt_HUMAN$	3::Q7Z5L9|I2BP2_HUMAN	20	60987	1	1	1	1	0.08	Interferon regulatory f
1901	1	SwissProt_HUMAN$	3::Q53EZ4|CEP55_HUMAN	20	54145	1	1	1	1	0.09	Centrosomal protein of
1902	1	SwissProt_HUMAN$	3::A6NNT2|CP096_HUMAN	20	124963	1	1	1	1	0.04	Uncharacterized protei
1903	1	db$2	2::C3K5M2|C3K5M2_PSEFS	20	41151	1	1	1	1	0.12	Phosphorous compoun
1904	1	db$2	2::C3K3X9|C3K3X9_PSEFS	20	98433	3	1	3	1	0.05	Putative ABC transport
1905	1	SwissProt_HUMAN$	3::Q7Z410|TMPS9_HUMAN	20	113948	1	1	1	1	0.04	Transmembrane prote
1906	1	SwissProt_HUMAN$	3::P30041|PRDX6_HUMAN	20	25019	1	1	1	1	0.21	Peroxiredoxin-6 OS=H
1907	1	SwissProt_HUMAN$	3::Q8WXH0|SYNE2_HUMAN	20	795944	1	1	1	1	0.01	Nesprin-2 OS=Homo s
1908	1	db$2	2::C3JXS7|C3JXS7_PSEFS	20	34119	1	1	1	1	0.15	Putative GGDEF domai
1909	1	SwissProt_HUMAN$	3::O60671|RAD1_HUMAN	20	31807	1	1	1	1	0.16	Cell cycle checkpoint p

↑Family	M	DB	Accession	Score	Mass	Matches	Match(sig)	Sequences	Seq(sig)	emPAI	Description
1910	1	SwissProt_HUMAN$	3::Q9ULR3|PPM1H_HUMAN	20	56413	2	1	1	1	0.09	Protein phosphatase 1
1911	1	db$2	2::C3K5A1|C3K5A1_PSEFS	20	60517	1	1	1	1	0.08	Urease subunit alpha (
1912	1	db$2	2::C3K9Q3|C3K9Q3_PSEFS	20	26964	1	1	1	1	0.19	DNA polymerase III su
1913	1	db$2	2::C3K706|PIMT_PSEFS	20	23451	2	1	2	1	0.22	Protein-L-isoaspartate
1914	1	SwissProt_HUMAN$	3::Q13835|PKP1_HUMAN	20	82808	1	1	1	1	0.06	Plakophilin-1 OS=Hom
1915	1	SwissProt_HUMAN$	3::Q8IUR0|TPPC5_HUMAN	20	20770	1	1	1	1	0.25	Trafficking protein part
1916	1	db$2	2::C3K9H2|C3K9H2_PSEFS	20	39760	1	1	1	1	0.13	Putrescine ABC transp
1917	1	SwissProt_HUMAN$	3::P28072|PSB6_HUMAN	20	25341	1	1	1	1	0.20	Proteasome subunit be
1918	1	SwissProt_HUMAN$	3::Q9P0Z9|SOX_HUMAN	20	44038	1	1	1	1	0.11	Peroxisomal sarcosine
1919	1	SwissProt_HUMAN$	3::Q8ND30|LIPB2_HUMAN	20	98482	1	1	1	1	0.05	Liprin-beta-2 OS=Hom
1920	1	db$2	2::C3K3J5|C3K3J5_PSEFS	20	13219	1	1	1	1	0.42	Type IV pilus response
1921	1	SwissProt_HUMAN$	3::Q99928|GBRG3_HUMAN	20	54253	1	1	1	1	0.09	Gamma-aminobutyric
1922	1	db$2	2::C3KAF6|C3KAF6_PSEFS	20	52919	1	1	1	1	0.09	Putative membrane pr
1923	1	SwissProt_HUMAN$	3::P04275|VWF_HUMAN	20	309058	1	1	1	1	0.02	von Willebrand factor (
1924	1	db$2	2::C3K120|C3K120_PSEFS	20	69519	1	1	1	1	0.07	Putative sulfurylase OS
1925	1	SwissProt_HUMAN$	3::P46939|UTRO_HUMAN	20	394220	3	2	2	1	0.01	Utrophin OS=Homo sa
1926	1	SwissProt_HUMAN$	3::P34947|GRK5_HUMAN	20	67743	1	1	1	1	0.07	G protein-coupled rece
1927	1	db$2	2::C3KCG3|C3KCG3_PSEFS	20	16758	1	1	1	1	0.32	YjgF_endoribonc doma
1928	1	db$2	2::C3K2J0|C3K2J0_PSEFS	19	40846	2	1	2	1	0.12	Putative exported prot
1929	1	SwissProt_HUMAN$	3::Q5TGY3|AHDC1_HUMAN	19	168245	2	1	1	1	0.03	AT-hook DNA-binding
1930	1	db$2	2::C3KBV8|C3KBV8_PSEFS	19	32477	1	1	1	1	0.16	Cobalt-zinc-cadmium r
1931	1	db$2	2::C3K578|C3K578_PSEFS	19	39452	1	1	1	1	0.13	Putative lipoprotein OS
1932	1	SwissProt_HUMAN$	3::P11362|FGFR1_HUMAN	19	91809	1	1	1	1	0.05	Fibroblast growth facto
1933	1	SwissProt_HUMAN$	3::Q14118|DAG1_HUMAN	19	97381	1	1	1	1	0.05	Dystroglycan OS=Hom
1934	1	db$2	2::C3K492|C3K492_PSEFS	19	51080	1	1	1	1	0.10	HDOD domain-contain
1935	1	db$2	2::C3K3N1|C3K3N1_PSEFS	19	52088	1	1	1	1	0.10	Aldehyde dehydrogena
1936	1	SwissProt_HUMAN$	3::Q15334|L2GL1_HUMAN	19	115346	1	1	1	1	0.04	Lethal(2) giant larvae
1937	1	db$2	2::C3K869|C3K869_PSEFS	19	24016	1	1	1	1	0.22	Putative lipoprotein OS
1938	1	db$2	2::C3K4N6|C3K4N6_PSEFS	19	18219	1	1	1	1	0.29	Uncharacterized protei
1939	1	db$2	2::C3K597|C3K597_PSEFS	19	46587	2	2	2	2	0.23	Putative chaperone/he
1940	1	SwissProt_HUMAN$	3::O95347|SMC2_HUMAN	19	135572	1	1	1	1	0.04	Structural maintenanc
1941	1	SwissProt_HUMAN$	3::P08514|ITA2B_HUMAN	19	113306	1	1	1	1	0.04	Integrin alpha-IIb OS=
1942	1	SwissProt_HUMAN$	3::O75051|PLXA2_HUMAN	19	210969	2	1	2	1	0.02	Plexin-A2 OS=Homo s
1943	1	SwissProt_HUMAN$	3::O00635|TRI38_HUMAN	19	53381	1	1	1	1	0.09	E3 ubiquitin-protein lig
1944	1	SwissProt_HUMAN$	3::Q5VWM6|PRA13_HUMAN	19	54878	2	1	2	1	0.09	Putative PRAME family
1945	1	SwissProt_HUMAN$	3::Q71DI3|H32_HUMAN	19	15379	2	2	2	2	0.83	Histone H3.2 OS=Hom
1946	1	db$2	2::C3KAB6|C3KAB6_PSEFS	19	39151	1	1	1	1	0.13	Conserved hypothetica
1947	1	db$2	2::C3K908|C3K908_PSEFS	19	33577	2	1	2	1	0.15	Putative dehydrogenas
1948	1	db$2	2::C3KBN1|C3KBN1_PSEFS	19	62708	1	1	1	1	0.08	Chaperone OS=Pseud(
1949	1	db$2	2::C3KDS6|UNG_PSEFS	19	25820	2	1	2	1	0.20	Uracil-DNA glycosylase

↑Family	M	DB	Accession	Score	Mass	Matches	Match(sig)	Sequences	Seq(sig)	emPAI	Description
1950	1	SwissProt_HUMAN$	3::P60953|CDC42_HUMAN	19	21245	1	1	1	1	0.25	Cell division control pr
1951	1	db$2	2::C3K5C6|C3K5C6_PSEFS	19	33902	1	1	1	1	0.15	Uncharacterized protei
1952	1	db$2	2::C3K5D7|C3K5D7_PSEFS	19	29152	1	1	1	1	0.18	ThiF domain-containin
1953	1	SwissProt_HUMAN$	3::P61513|RL37A_HUMAN	19	10268	2	1	2	1	0.57	60S ribosomal protein
1954	1	SwissProt_HUMAN$	3::Q09666|AHNK_HUMAN	19	628699	1	1	1	1	0.01	Neuroblast differentiat
1955	1	SwissProt_HUMAN$	3::Q8IZ21|PHAR4_HUMAN	19	78163	1	1	1	1	0.06	Phosphatase and actin
1956	1	SwissProt_HUMAN$	3::O95696|BRD1_HUMAN	19	119444	1	1	1	1	0.04	Bromodomain-containi
1957	1	SwissProt_HUMAN$	3::Q4V348|Z658B_HUMAN	19	94270	1	1	1	1	0.05	Zinc finger protein 65
1958	1	SwissProt_HUMAN$	3::P22894|MMP8_HUMAN	19	53378	1	1	1	1	0.09	Neutrophil collagenase
1959	1	SwissProt_HUMAN$	3::Q5JNZ5|RS26L_HUMAN	19	12994	1	1	1	1	0.43	Putative 40S ribosoma
1960	1	SwissProt_HUMAN$	3::Q9H1D9|RPC6_HUMAN	19	35661	1	1	1	1	0.14	DNA-directed RNA poly
1961	1	db$2	2::C3KC05|C3KC05_PSEFS	19	11915	1	1	1	1	0.48	Conserved hypothetica
1962	1	SwissProt_HUMAN$	3::P62857|RS28_HUMAN	19	7836	2	2	2	2	2.20	40S ribosomal protein
1963	1	db$2	2::C3JZR3|PCP_PSEFS	19	22533	1	1	1	1	0.23	Pyrrolidone-carboxylat
1964	1	db$2	2::C3K2F9|C3K2F9_PSEFS	19	48516	1	1	1	1	0.10	Putative ABC transport
1965	1	db$2	2::C3K205|C3K205_PSEFS	19	51020	1	1	1	1	0.10	Aromatic amino acid tr
1966	1	SwissProt_HUMAN$	3::P53814|SMTN_HUMAN	19	98998	1	1	1	1	0.05	Smoothelin OS=Homo
1967	1	SwissProt_HUMAN$	3::Q9Y5I4|PCDC2_HUMAN	19	109383	2	1	2	1	0.04	Protocadherin alpha-C
1968	1	db$2	2::C3K713|C3K713_PSEFS	19	28108	1	1	1	1	0.18	Arginine/ornithine ABC
1969	1	SwissProt_HUMAN$	3::Q8NFC6|BD1L1_HUMAN	19	330266	1	1	1	1	0.01	Biorientation of chrom
1970	1	SwissProt_HUMAN$	3::Q9UHW9|S12A6_HUMAN	19	127534	1	1	1	1	0.04	Solute carrier family 1
1971	1	db$2	2::C3K546|C3K546_PSEFS	19	33665	1	1	1	1	0.15	Putative lipopolysacch
1972	1	SwissProt_HUMAN$	3::P62241|RS8_HUMAN	19	24190	1	1	1	1	0.21	40S ribosomal protein
1973	1	SwissProt_HUMAN$	3::P04080|CYTB_HUMAN	18	11133	1	1	1	1	0.52	Cystatin-B OS=Homo
1974	1	db$2	2::C3JYZ9|C3JYZ9_PSEFS	18	61887	1	1	1	1	0.08	Undecaprenyl phospha
1975	1	db$2	2::C3K7J6|C3K7J6_PSEFS	18	25687	1	1	1	1	0.20	Putative exported prot
1976	1	db$2	2::C3K8V1|C3K8V1_PSEFS	18	62361	1	1	1	1	0.08	Poly(3-hydroxyalkanoa
1977	1	SwissProt_HUMAN$	3::Q9BQ75|CMS1_HUMAN	18	31864	1	1	1	1	0.16	Protein CMSS1 OS=Ho
1978	1	SwissProt_HUMAN$	3::Q9P202|WHRN_HUMAN	18	96499	3	2	2	1	0.05	Whirlin OS=Homo sapi
1979	1	db$2	2::C3K2F6|TRMA_PSEFS	18	40867	2	2	2	2	0.26	tRNA/tmRNA (uracil-C
1980	1	SwissProt_HUMAN$	3::Q49MG5|MAP9_HUMAN	18	74189	1	1	1	1	0.07	Microtubule-associatec
1981	1	db$2	2::C3K2Z6|ERPA_PSEFS	18	12493	1	1	1	1	0.45	Iron-sulfur cluster inse
1982	1	db$2	2::C3KCB6|C3KCB6_PSEFS	18	31268	1	1	1	1	0.16	Phosphate transport sy
1983	1	SwissProt_HUMAN$	3::Q96P48|ARAP1_HUMAN	18	162089	1	1	1	1	0.03	Arf-GAP with Rho-GAP
1984	1	db$2	2::C3JYP1|C3JYP1_PSEFS	18	35226	2	2	2	2	0.31	Uncharacterized protei
1985	1	db$2	2::C3K5J0|C3K5J0_PSEFS	18	13887	1	1	1	1	0.40	Putative lipoprotein OS
1986	1	db$2	2::C3KE21|C3KE21_PSEFS	18	10230	1	1	1	1	0.57	FAD assembly factor S
1987	1	SwissProt_HUMAN$	3::Q8N8R5|CB069_HUMAN	18	43421	1	1	1	1	0.12	UPF0565 protein C2orf
1988	1	db$2	2::C3JY01|C3JY01_PSEFS	18	74110	1	1	1	1	0.07	AAA_15 domain-conta
1989	1	SwissProt_HUMAN$	3::Q9BXF9|TEKT3_HUMAN	18	56601	1	1	1	1	0.09	Tektin-3 OS=Homo sa

↑Family	M	DB	Accession	Score	Mass	Matches	Match(sig)	Sequences	Seq(sig)	emPAI	Description
1990	1	SwissProt_HUMAN$	3::Q9NRF8|PYRG2_HUMAN	18	65636	1	1	1	1	0.07	CTP synthase 2 OS=H
1991	1	db$2	2::C3K433|CYAY_PSEFS	18	12442	2	2	2	2	1.11	Iron-sulfur cluster asse
1992	1	SwissProt_HUMAN$	3::Q9NZI6|TF2L1_HUMAN	18	54593	1	1	1	1	0.09	Transcription factor CP
1993	1	db$2	2::C3K6C3|C3K6C3_PSEFS	18	28259	2	1	2	1	0.18	Putative amino acid AE
1994	1	SwissProt_HUMAN$	3::O14640|DVL1_HUMAN	18	75140	1	1	1	1	0.07	Segment polarity prote
1995	1	SwissProt_HUMAN$	3::Q14139|UBE4A_HUMAN	18	122482	2	1	1	1	0.04	Ubiquitin conjugation f
1996	1	SwissProt_HUMAN$	3::Q6TFL3|CC171_HUMAN	18	152714	2	1	2	1	0.03	Coiled-coil domain-con
1997	1	db$2	2::C3K9P8|C3K9P8_PSEFS	18	69666	1	1	1	1	0.07	Putative substrate-bin
1998	1	SwissProt_HUMAN$	3::P15924|DESP_HUMAN	18	331569	1	1	1	1	0.01	Desmoplakin OS=Hom
1999	1	db$2	2::C3K963|C3K963_PSEFS	18	78429	1	1	1	1	0.06	Putative TonB-depend
2000	1	db$2	2::C3KAU8|C3KAU8_PSEFS	18	52023	1	1	1	1	0.10	Xylulose kinase OS=P
2001	1	SwissProt_HUMAN$	3::Q9BYN0|SRXN1_HUMAN	18	14250	1	1	1	1	0.39	Sulfiredoxin-1 OS=Ho
2002	1	SwissProt_HUMAN$	3::P30876|RPB2_HUMAN	18	133811	1	1	1	1	0.04	DNA-directed RNA pol
2003	1	SwissProt_HUMAN$	3::P11142|HSP7C_HUMAN	18	70854	2	2	2	2	0.14	Heat shock cognate 71
2004	1	db$2	2::C3JZ98|C3JZ98_PSEFS	18	32170	1	1	1	1	0.16	Putative acid phosphat
2005	1	db$2	2::C3K749|C3K749_PSEFS	18	16397	1	1	1	1	0.33	Putative phosphohistid
2006	1	db$2	2::C3KDU2|C3KDU2_PSEFS	18	32215	1	1	1	1	0.16	Putative short chain de
2007	1	SwissProt_HUMAN$	3::O96028|NSD2_HUMAN	18	152160	1	1	1	1	0.03	Histone-lysine N-meth
2008	1	SwissProt_HUMAN$	3::O75155|CAND2_HUMAN	18	135171	1	1	1	1	0.04	Cullin-associated NEDI
2009	1	SwissProt_HUMAN$	3::Q5F1R6|DJC21_HUMAN	17	61989	1	1	1	1	0.08	DnaJ homolog subfami
2010	1	SwissProt_HUMAN$	3::Q13873|BMPR2_HUMAN	17	115129	1	1	1	1	0.04	Bone morphogenetic p
2011	1	SwissProt_HUMAN$	3::Q4KMP7|TB10B_HUMAN	17	87145	1	1	1	1	0.06	TBC1 domain family m
2012	1	SwissProt_HUMAN$	3::Q9H158|PCDC1_HUMAN	17	103877	1	1	1	1	0.05	Protocadherin alpha-C
2013	1	SwissProt_HUMAN$	3::P16885|PLCG2_HUMAN	17	147776	1	1	1	1	0.03	1-phosphatidylinositol
2014	1	db$2	2::C3K6J8|C3K6J8_PSEFS	17	78979	1	1	1	1	0.06	3-phosphoshikimate 1-
2015	1	db$2	2::C3K1W4|C3K1W4_PSEFS	17	25488	1	1	1	1	0.20	Probable short-chain d
2016	1	db$2	2::C3JYT5|C3JYT5_PSEFS	17	13226	1	1	1	1	0.42	Uncharacterized protei
2017	1	SwissProt_HUMAN$	3::Q9BQS8|FYCO1_HUMAN	17	166879	2	1	2	1	0.03	FYVE and coiled-coil d
2018	1	db$2	2::C3K444|C3K444_PSEFS	17	9491	1	1	1	1	0.62	Ubiquinone biosynthes
2019	1	SwissProt_HUMAN$	3::Q9BT17|MTG1_HUMAN	17	37213	1	1	1	1	0.14	Mitochondrial ribosome
2020	1	SwissProt_HUMAN$	3::Q8TEQ6|GEMI5_HUMAN	17	168483	1	1	1	1	0.03	Gem-associated protei
2021	1	SwissProt_HUMAN$	3::Q8N139|ABCA6_HUMAN	17	184167	1	1	1	1	0.03	ATP-binding cassette s
2022	1	db$2	2::C3JXY3|C3JXY3_PSEFS	17	18788	1	1	1	1	0.28	6,7-dimethyl-8-ribityll
2023	1	SwissProt_HUMAN$	3::O00444|PLK4_HUMAN	17	108903	1	1	1	1	0.04	Serine/threonine-prote
2024	1	db$2	2::C3K600|C3K600_PSEFS	17	28761	1	1	1	1	0.18	Phosphatidate cytidyly
2025	1	db$2	2::C3K9S7|C3K9S7_PSEFS	17	35611	1	1	1	1	0.14	Putative magnesium c
2026	1	SwissProt_HUMAN$	3::P58166|INHBE_HUMAN	17	38537	1	1	1	1	0.13	Inhibin beta E chain O
2027	1	SwissProt_HUMAN$	3::Q9BWT7|CAR10_HUMAN	17	115859	1	1	1	1	0.04	Caspase recruitment d
2028	1	db$2	2::C3K8R2|C3K8R2_PSEFS	17	22663	1	1	1	1	0.23	Uncharacterized protei
2029	1	db$2	2::A4V763|A4V763_PSEFS	16	28708	1	1	1	1	0.18	Uncharacterized protei

↑Family	M	DB	Accession	Score	Mass	Matches	Match(sig)	Sequences	Seq(sig)	emPAI	Description
2030	1	SwissProt_HUMAN$	3::Q9UKI8|TLK1_HUMAN	16	86646	2	1	1	1	0.06	Serine/threonine-prote
2031	1	SwissProt_HUMAN$	3::Q6MZW2|FSTL4_HUMAN	16	93037	1	1	1	1	0.05	Follistatin-related prot
2032	1	SwissProt_HUMAN$	3::Q7L1T6|NB5R4_HUMAN	16	59436	1	1	1	1	0.08	Cytochrome b5 reduct
2033	1	db$2	2::C3JY51|C3JY51_PSEFS	16	37030	2	2	1	1	0.14	Putative glycosyl trans
2034	1	db$2	2::C3K0B8|C3K0B8_PSEFS	16	24379	1	1	1	1	0.21	Uncharacterized protei
2035	1	db$2	2::C3K7T3|C3K7T3_PSEFS	16	20773	1	1	1	1	0.25	CDP-diacylglycerol--gly
2036	1	SwissProt_HUMAN$	3::Q9Y4K0|LOXL2_HUMAN	16	86668	2	1	2	1	0.06	Lysyl oxidase homolog
2037	1	db$2	2::C3KCA2|C3KCA2_PSEFS	16	48472	2	1	1	1	0.10	Putative family S43 no
2038	1	SwissProt_HUMAN$	3::Q8TEU7|RPGF6_HUMAN	16	179310	1	1	1	1	0.03	Rap guanine nucleotid
2039	1	SwissProt_HUMAN$	3::P02545|LMNA_HUMAN	16	74095	1	1	1	1	0.07	Prelamin-A/C OS=Hom
2040	1	db$2	2::C3JYM6|C3JYM6_PSEFS	16	40915	1	1	1	1	0.12	Putative HlyD-family s
2041	1	SwissProt_HUMAN$	3::Q6NVV1|R13P3_HUMAN	16	12127	1	1	1	1	0.47	Putative 60S ribosoma
2042	1	SwissProt_HUMAN$	3::P62269|RS18_HUMAN	16	17708	2	1	2	1	0.30	40S ribosomal protein
2043	1	SwissProt_HUMAN$	3::O15397|IPO8_HUMAN	16	119861	1	1	1	1	0.04	Importin-8 OS=Homo
2044	1	SwissProt_HUMAN$	3::O15050|TRNK1_HUMAN	16	336007	1	1	1	1	0.01	TPR and ankyrin repea
2045	1	SwissProt_HUMAN$	3::Q8IYW2|CFA46_HUMAN	16	303308	3	1	3	1	0.02	Cilia- and flagella-asso
2046	1	SwissProt_HUMAN$	3::P62266|RS23_HUMAN	15	15798	1	1	1	1	0.35	40S ribosomal protein
2047	1	SwissProt_HUMAN$	3::Q9UL62|TRPC5_HUMAN	15	111340	2	1	2	1	0.04	Short transient recept
2048	1	db$2	2::C3JY96|C3JY96_PSEFS	15	27408	1	1	1	1	0.19	OMP_b-brl domain-cor
2049	1	SwissProt_HUMAN$	3::P35227|PCGF2_HUMAN	15	37764	1	1	1	1	0.13	Polycomb group RING
2050	1	db$2	2::C3K3S9|C3K3S9_PSEFS	15	35576	1	1	1	1	0.14	Hydrolase_4 domain-c
2051	1	SwissProt_HUMAN$	3::D6REC4|CFA99_HUMAN	15	52261	1	1	1	1	0.09	Cilia- and flagella-asso
2052	1	SwissProt_HUMAN$	3::Q8IVF4|DYH10_HUMAN	15	514512	1	1	1	1	0.01	Dynein heavy chain 10
2053	1	db$2	2::C3K0X1|C3K0X1_PSEFS	15	10638	1	1	1	1	0.54	Uncharacterized protei
2054	1	SwissProt_HUMAN$	3::A6NE01|F186A_HUMAN	15	262595	1	1	1	1	0.02	Protein FAM186A OS=
2055	1	SwissProt_HUMAN$	3::Q70EL3|UBP50_HUMAN	15	38929	1	1	1	1	0.13	Inactive ubiquitin carb
2056	1	SwissProt_HUMAN$	3::Q9P1U1|ARP3B_HUMAN	15	47577	2	1	2	1	0.10	Actin-related protein 3
2057	1	db$2	2::C3JXH7|C3JXH7_PSEFS	15	21910	1	1	1	1	0.24	LexA repressor OS=Ps
2058	1	db$2	2::C3K9Z1|C3K9Z1_PSEFS	15	21192	1	1	1	1	0.25	FMN dependent NADH
2059	1	SwissProt_HUMAN$	3::Q5VSL9|STRP1_HUMAN	15	95515	1	1	1	1	0.05	Striatin-interacting pro
2060	1	SwissProt_HUMAN$	3::Q86VD1|MORC1_HUMAN	15	112810	1	1	1	1	0.04	MORC family CW-type
2061	1	db$2	2::C3K751|C3K751_PSEFS	15	15943	1	1	1	1	0.34	Putative thioesterase (
2062	1	SwissProt_HUMAN$	3::Q53GA4|PHLA2_HUMAN	15	17082	1	1	1	1	0.31	Pleckstrin homology-lil
2063	1	SwissProt_HUMAN$	3::P21127|CD11B_HUMAN	15	92563	2	1	2	1	0.05	Cyclin-dependent kina
2064	1	SwissProt_HUMAN$	3::Q86VN1|VPS36_HUMAN	15	43789	1	1	1	1	0.11	Vacuolar protein-sortir
2065	1	db$2	2::C3K8U1|C3K8U1_PSEFS	15	15020	1	1	1	1	0.37	Phosphoribosyl-AMP cy
2066	1	db$2	2::C3K0F1|C3K0F1_PSEFS	15	476575	2	1	2	1	0.01	Pyoverdine synthetase
2067	1	db$2	2::C3K0P5|C3K0P5_PSEFS	15	35572	1	1	1	1	0.14	Uncharacterized protei
2068	1	SwissProt_HUMAN$	3::Q9NUQ8|ABCF3_HUMAN	14	79695	1	1	1	1	0.06	ATP-binding cassette s
2069	1	SwissProt_HUMAN$	3::Q9BV86|NTM1A_HUMAN	14	25371	1	1	1	1	0.20	N-terminal Xaa-Pro-Ly

↑Family	M	DB	Accession	Score	Mass	Matches	Match(sig)	Sequences	Seq(sig)	emPAI	Description
2070	1	db$2	2::C3K2K1|C3K2K1_PSEFS	14	28157	1	1	1	1	0.18	Phos_pyr_kin domain-
2071	1	db$2	2::C3KAY4|C3KAY4_PSEFS	14	29479	1	1	1	1	0.17	Putative enoyl-CoA hy
2072	1	SwissProt_HUMAN$	3::Q13905|RPGF1_HUMAN	14	120472	1	1	1	1	0.04	Rap guanine nucleotid
2073	1	SwissProt_HUMAN$	3::Q5THR3|EFCB6_HUMAN	14	172820	1	1	1	1	0.03	EF-hand calcium-bindi
2074	1	SwissProt_HUMAN$	3::Q8WZ42|TITIN_HUMAN	14	3813652	3	1	3	1	0.00	Titin OS=Homo sapien
2075	1	SwissProt_HUMAN$	3::Q8IYB3|SRRM1_HUMAN	14	102274	1	1	1	1	0.05	Serine/arginine repetit
2076	1	SwissProt_HUMAN$	3::Q86WN2|IFNE_HUMAN	14	24399	1	1	1	1	0.21	Interferon epsilon OS=
2077	1	SwissProt_HUMAN$	3::Q8IUS5|EPHX4_HUMAN	14	42297	1	1	1	1	0.12	Epoxide hydrolase 4 O
2078	1	db$2	2::C3K9C4|C3K9C4_PSEFS	14	31700	1	1	1	1	0.16	Putative transposase f
2079	1	SwissProt_HUMAN$	3::P49321|NASP_HUMAN	14	85186	1	1	1	1	0.06	Nuclear autoantigenic
2080	1	SwissProt_HUMAN$	3::Q16512|PKN1_HUMAN	14	103868	1	1	1	1	0.05	Serine/threonine-prote
2081	1	db$2	2::C3K3D5|C3K3D5_PSEFS	14	47715	1	1	1	1	0.10	Uncharacterized protei
2082	1	SwissProt_HUMAN$	3::Q9P2E9|RRBP1_HUMAN	14	152365	1	1	1	1	0.03	Ribosome-binding prot
2083	1	SwissProt_HUMAN$	3::P30050|RL12_HUMAN	13	17808	2	1	2	1	0.30	60S ribosomal protein
2084	1	SwissProt_HUMAN$	3::Q9Y5G0|PCDGH_HUMAN	13	99813	1	1	1	1	0.05	Protocadherin gamma-
2085	1	db$2	2::C3KAN4|C3KAN4_PSEFS	13	41646	1	1	1	1	0.12	3-oxoadipyl-CoA thiola
2086	1	SwissProt_HUMAN$	3::Q7Z333|SETX_HUMAN	13	302689	1	1	1	1	0.02	Probable helicase sena
2087	1	SwissProt_HUMAN$	3::O43897|TLL1_HUMAN	13	114635	1	1	1	1	0.04	Tolloid-like protein 1 C
2088	1	db$2	2::A4V768|A4V768_PSEFS	13	12827	1	1	1	1	0.44	Uncharacterized protei

Export as CSV

Not what you expected? Try **the select summary**.

Mascot: http://www.matrixscience.com/
