# Supplementary material for: Circulating multimeric immune complexes contribute to immunopathology in COVID-19
Source: Nat Commun. 2022 Sep 26;13:5654. doi: 10.1038/s41467-022-32867-z (PMC9513013; doi:10.1038/s41467-022-32867-z)
Supplement: Supplementary file 3 — Source Data [file 41467_2022_32867_MOESM3_ESM.zip › Source Data/MS_20210407 (immune precipitation).zip!/QX20210618-02_Virologie(AG-Hengel)-20210407_(V2)_0k3v20_TRL_db_SC2+db_Pf+SP_H(F235783).pdf]

MATRIX SCIENCE MASCOT Search Results

**User**	: Wolfgang Bildl
**E-mail**	: wolfgang.bildl@physiologie.uni-freiburg.de
**Search title**	: QX 20210618 (db$ SARS-CoV-2+db$2 Pf+SP H$ 5 ppm 20 mmu), QX20210618-02_Virologie(AG-Hengel)-20210407_(V2)_0k3v20_TRL_shifted_-1.6.mgf
**MS data file**	: D:\Xcalibur\Data\Wolfgang\Q-Exactive-HF-X_20210616\QX20210618-02_Virologie(AG-Hengel)-20210407_(V2)_0k3v20_TRL_shifted_-1.6.mgf
**Databases**	: **1:** db$ 20210617 (17 sequences; 14,439 residues)<br>**2:** db$2 20210617 (6,388 sequences; 2,089,672 residues)<br>**3:** SwissProt_HUMAN$ 20201007 (20,389 sequences; 11,363,789 residues)
**Timestamp**	: 21 Jun 2021 at 09:36:00 GMT

Not what you expected? Try **the select summary**.

▼Search parameters

**Type of search**	: MS/MS Ion Search
**Enzyme**	: Trypsin
**Variable modifications**	: **Acetyl (Protein N-term)**, **Carbamidomethyl (C)**, **Formyl (Protein N-term)**, **Gln->pyro-Glu (N-term Q)**, **Oxidation (M)**
**Mass values**	: Monoisotopic
**Protein mass**	: Unrestricted
**Peptide mass tolerance**	: ± 5 ppm
**Fragment mass tolerance**	: ± 20 mmu
**Max missed cleavages**	: 1
**Instrument type**	: ESI-FTICR
**Number of queries**	: 85,533

▼Score distribution

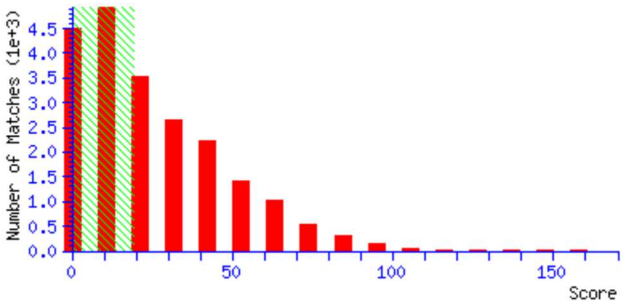

Peptide score distribution. Ions score is $-10\log(P)$, where P is the probability that the observed match is a random event.

There are **10,847** peptide matches above identity threshold and **11,639** matches above homology threshold for **85,533** queries. On average, individual ions scores **> 19** (beyond green shading) indicate **identity or extensive homology** (p<0.05).

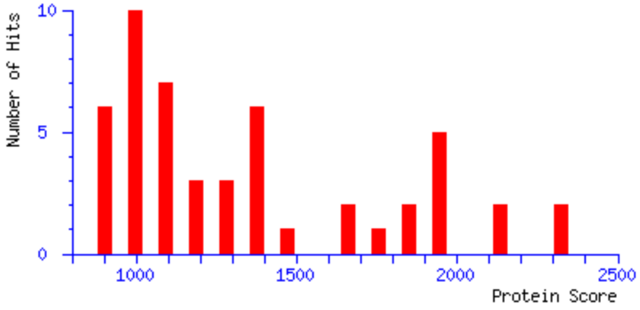

[Deprecated] Protein score distribution. Score distribution for family members in the first 50 proteins. Protein scores are derived from ions scores as a non-probabilistic basis for ranking protein families.

▼Modification statistics

Modification	Site	Above thr.
Oxidation	M	1988
Carbamidomethyl	C	1095
Gln->pyro-Glu	N-term	221
Formyl	Protein N-term	49
Acetyl	Protein N-term	26

▼Legend

Peptide columns and rows

Dupes	...	Expect	Rank	U	1	2	Peptide	
		**0.037**	▶**2**				**GAYSLSLR**	significant
		9	▶1				GFFLFVEGGR	top ranking
		**6.4e-005**	▶**1**				**GSSIFGLAPGK**	significant and top ranking
		**1.3e-006**	▶**1**		■		**SSGTSYPDVLK**	peptide is found in all proteins in family member 1
		**6.2e-007**	▶**1**			■	**VCNYVSWIK**	peptide is found in some but not all proteins in family member 2
		**6.4e-005**	▶**1**	**U**			**GSSIFGLAPGK**	unique
▶**2**		**5.7e-005**	▶**1**				**LNTLETEEWFFK**	peptide has two duplicates
		0.18	▶*1*				*LNTLETEEWFFK*	duplicate peptide

Right-facing triangle (▶) in the Dupes or Rank column indicates content that can be expanded by clicking on it. Down-facing triangle (▼) indicates the content is expanded and can be collapsed. For more details about particular columns, see **results format help**.

Protein quantitation ratios

	Score	...	114/113	115/113
**CFAH_HUMAN**	37559		*0.962*	**1.129**
**FHR2_HUMAN**	1330		**0.859**	1.128

When quantitation method is Reporter (e.g. iTRAQ) or Multiplex (e.g. IPTL), protein ratios are displayed when a family is expanded. Ratios in *italic* indicate that the peptide log-ratios do not appear to come from a normal distribution. **Bold** indicates that if you can assume peptide ratios are normally distributed, the protein ratio is significantly different from 1.0 (at significance level 0.05).

Note that lack of bold or italic can also mean that significance or normality testing has not been performed (for

example, if protein ratio type does not support it).

Protein Family Summary

Significance threshold p<	0.05	Max. number of families	AUTO
Ions score or expect cut-off	0.5	Dendrograms cut at	0
Show Percolator scores	☐		
Preferred taxonomy	All entries		

Sensitivity and FDR (reversed protein sequences)

			Target	Decoy	FDR		
PSMs	above	homology	11639	411	3.53%	Adjust to	1%

Decoy results are available in **the decoy report**.

Protein hits (2005 proteins)

Filters: (none)

Export as CSV

↑Family	M	DB	Accession	Score	Mass	Matches	Match(sig)	Sequences	Seq(sig)	emPAI	Description
1	1	db$2	2::C3K2X8|EFTU_PSEFS	2322	43536	79	71	25	24	31.32	Elongation factor Tu O
2	1	db$2	2::C3K2Y3|RPOB_PSEFS	2299	150923	94	88	66	62	7.49	DNA-directed RNA poly
3	1	db$2	2::C3K5Z6|EFTS_PSEFS	2171	30341	75	73	16	16	13.06	Elongation factor Ts O
4	1	db$2	2::C3K5G3|C3K5G3_PSEFS	2169	99192	78	71	34	32	6.81	Aconitate hydratase O
5	1	SwissProt_HUMAN$	3::P13645|K1C10_HUMAN	1979	58792	53	50	32	29	14.43	Keratin, type I cytoske
5	2	SwissProt_HUMAN$	3::P02533|K1C14_HUMAN	291	51529	17	15	16	14	2.62	Keratin, type I cytoske
5	3	SwissProt_HUMAN$	3::P08779|K1C16_HUMAN	256	51236	14	12	13	11	1.76	Keratin, type I cytoske
6	1	db$2	2::C3K1E6|ATPB_PSEFS	1970	49459	61	61	28	28	33.30	ATP synthase subunit I
7	1	db$2	2::C3K4D5|C3K4D5_PSEFS	1937	34599	66	61	23	22	44.34	Phosphate-binding pro
8	1	db$2	2::C3K4N0|C3K4N0_PSEFS	1918	43479	54	52	26	25	25.01	Putative ornithine deca
9	1	db$2	2::C3K1E8|ATPA_PSEFS	1915	55245	62	60	29	28	24.89	ATP synthase subunit a
10	1	db$2	2::C3KD82|C3KD82_PSEFS	1882	181591	64	60	50	47	2.90	Glutamate dehydroger
11	1	db$2	2::C3K1A0|CH60_PSEFS	1881	56931	58	57	30	30	29.22	60 kDa chaperonin OS
12	1	db$2	2::C3K2X9|EFG_PSEFS	1759	77114	70	66	35	35	13.05	Elongation factor G OS
13	1	db$2	2::C3K2Y2|RPOC_PSEFS	1688	154528	83	75	58	53	5.32	DNA-directed RNA poly
14	1	db$2	2::C3K255|PNP_PSEFS	1651	74889	54	51	32	30	10.07	Polyribonucleotide nuc
15	1	SwissProt_HUMAN$	3::P04264|K2C1_HUMAN	1459	65999	40	40	31	31	9.67	Keratin, type II cytosk
15	2	SwissProt_HUMAN$	3::P35908|K22E_HUMAN	1027	65393	42	38	34	33	9.92	Keratin, type II cytosk
15	3	SwissProt_HUMAN$	3::P13647|K2C5_HUMAN	252	62340	14	13	13	12	1.49	Keratin, type II cytosk
15	4	SwissProt_HUMAN$	3::P02538|K2C6A_HUMAN	238	60008	14	13	14	13	1.79	Keratin, type II cytosk
15	5	SwissProt_HUMAN$	3::P05787|K2C8_HUMAN	127	53671	6	6	6	6	0.70	Keratin, type II cytosk

↑Family	M	DB	Accession	Score	Mass	Matches	Match(sig)	Sequences	Seq(sig)	emPAI	Description
15	6	SwissProt_HUMAN$	3::P08729|K2C7_HUMAN	87	51354	5	5	5	5	0.58	Keratin, type II cytosk
16	1	db$2	2::C3K6P2|C3K6P2_PSEFS	1412	42870	31	31	14	14	8.08	3-oxoacyl-[acyl-carrier
17	1	db$2	2::C3K6M5|C3K6M5_PSEFS	1396	63522	39	37	23	22	5.46	Succinate dehydrogena
18	1	db$2	2::C3KCP4|C3KCP4_PSEFS	1385	99394	67	63	44	42	9.89	Pyruvate dehydrogena
19	1	db$2	2::C3KBY8|C3KBY8_PSEFS	1380	21919	48	46	16	15	58.04	Alkyl hydroperoxide re
20	1	db$2	2::C3K0Q5|C3K0Q5_PSEFS	1375	107302	59	51	35	31	4.13	Ribonucleoside-diphos
21	1	db$2	2::C3KCJ5|C3KCJ5_PSEFS	1344	93957	48	43	34	30	4.30	Aconitate hydratase B
22	1	db$2	2::C3K275|DNAK_PSEFS	1276	68158	39	37	23	21	4.69	Chaperone protein Dna
23	1	db$2	2::C3K402|C3K402_PSEFS	1254	83101	49	46	29	28	5.95	Polyphosphate kinase
24	1	db$2	2::C3K5J5|FABV_PSEFS	1242	42933	39	39	23	23	16.47	Enoyl-[acyl-carrier-pro
25	1	db$2	2::C3K6N0|SUCC_PSEFS	1202	41262	38	36	22	22	18.58	Succinate--CoA ligase
26	1	db$2	2::C3K0P3|C3K0P3_PSEFS	1195	118163	34	31	24	23	1.73	Ribonuclease E OS=Ps
27	1	db$2	2::C3K225|ILVC_PSEFS	1192	36188	43	40	21	20	21.88	Ketol-acid reductoisom
28	1	db$2	2::C3KDP2|C3KDP2_PSEFS	1124	68879	34	32	21	20	3.86	Multifunctional fusion p
29	1	db$2	2::C3K271|C3K271_PSEFS	1123	117563	41	36	32	29	2.64	Carbamoyl-phosphate
30	1	db$2	2::C3K7D1|C3K7D1_PSEFS	1107	67115	43	38	29	26	7.30	50S ribosomal subunit
31	1	SwissProt_HUMAN$	3::P35527|K1C9_HUMAN	1101	62027	31	30	21	20	4.36	Keratin, type I cytoske
32	1	db$2	2::C3K6M7|C3K6M7_PSEFS	1096	105930	49	42	35	30	3.59	Oxoglutarate dehydrog
33	1	db$2	2::C3JYJ7|C3JYJ7_PSEFS	1093	9057	63	56	8	8	260.08	DNA-binding protein H
34	1	db$2	2::C3JYU7|C3JYU7_PSEFS	1058	102460	40	37	25	23	2.49	Protein translocase sul
35	1	db$2	2::C3K7D3|C3K7D3_PSEFS	1044	91800	30	30	23	23	2.64	Alpha-1,4 glucan phos
36	1	db$2	2::C3KBH1|C3KBH1_PSEFS	1042	161946	52	48	42	39	2.43	Glutamate synthase [N
37	1	db$2	2::C3K6K0|C3K6K0_PSEFS	1040	62268	49	45	27	26	9.57	30S ribosomal protein
38	1	db$2	2::C3K259|IF2_PSEFS	1027	90544	40	35	32	27	3.81	Translation initiation fa
39	1	db$2	2::C3KBT0|C3KBT0_PSEFS	1007	51607	30	28	15	14	4.71	Succinate-semialdehyd
40	1	db$2	2::C3K331|C3K331_PSEFS	1006	73691	45	42	35	33	8.49	AAA_PrkA domain-con
41	1	db$2	2::C3KEC5|C3KEC5_PSEFS	984	97398	39	36	29	26	3.11	Phosphoenolpyruvate
42	1	db$2	2::C3KE86|C3KE86_PSEFS	978	95293	44	42	34	34	5.01	Chaperone protein Clp
43	1	db$2	2::C3JYK1|TIG_PSEFS	961	48316	37	34	25	22	13.08	Trigger factor OS=Pse
44	1	db$2	2::C3KCB8|C3KCB8_PSEFS	954	36708	31	25	15	14	9.10	Phosphate-binding pro
45	1	db$2	2::C3JYR1|GCSP_PSEFS	945	102176	39	39	24	24	2.34	Glycine dehydrogenase
46	1	db$2	2::C3K3Z0|C3K3Z0_PSEFS	940	46925	30	29	21	21	10.21	Transcription terminati
47	1	db$2	2::C3K6H4|PYRG_PSEFS	920	59660	39	32	24	23	6.89	CTP synthase OS=Pseu
48	1	db$2	2::C3K613|FADB_PSEFS	908	76938	35	32	25	23	3.13	Fatty acid oxidation co
49	1	db$2	2::C3K3F3|C3K3F3_PSEFS	905	72324	45	43	29	29	7.13	Transketolase OS=Pse
50	1	db$2	2::C3K0T0|C3K0T0_PSEFS	902	34560	33	31	10	9	2.92	Putative outer membra
51	1	db$2	2::C3JYT1|SYDND_PSEFS	902	66220	34	33	25	24	4.97	Aspartate--tRNA(Asp/A
52	1	db$2	2::C3JY66|C3JY66_PSEFS	897	80156	39	37	24	23	3.94	Isocitrate dehydrogena
53	1	db$2	2::C3JYJ8|C3JYJ8_PSEFS	882	88851	39	37	18	16	1.35	Lon protease OS=Pseu
54	1	db$2	2::C3K121|C3K121_PSEFS	875	86030	44	39	32	29	5.17	Phosphoenolpyruvate s

↑Family	M	DB	Accession	Score	Mass	Matches	Match(sig)	Sequences	Seq(sig)	emPAI	Description
55	1	db$2	2::C3K6M9|C3K6M9_PSEFS	874	49948	28	28	17	17	5.65	Dihydrolipoyl dehydrog
56	1	db$2	2::C3K1K1|C3K1K1_PSEFS	874	51928	33	30	19	17	5.77	Inosine-5'-monophosp
57	1	db$2	2::C3K6M8|C3K6M8_PSEFS	862	42320	26	24	15	14	5.68	Dihydrolipoyllysine-res
58	1	db$2	2::C3K367|C3K367_PSEFS	846	68142	33	32	20	20	4.30	Uncharacterized protei
59	1	db$2	2::C3K1L8|NDK_PSEFS	844	14928	24	23	9	8	11.28	Nucleoside diphosphat
60	1	db$2	2::C3K2V9|RS5_PSEFS	817	17675	24	24	11	11	52.31	30S ribosomal protein
61	1	db$2	2::C3K603|C3K603_PSEFS	813	87560	26	25	21	21	2.12	Outer membrane prote
61	2	db$2	2::C3K3R2|C3K3R2_PSEFS	352	83481	19	19	18	18	1.78	Phosphoenolpyruvate-
62	1	db$2	2::C3KBT1|C3KBT1_PSEFS	810	44808	32	32	19	19	8.15	4-aminobutyrate amin
63	1	db$2	2::C3K0K6|C3K0K6_PSEFS	806	26352	22	21	13	13	13.62	Electron transfer flavo
64	1	db$2	2::C3K1J2|C3K1J2_PSEFS	802	140531	31	31	29	29	1.66	Phosphoribosylformylg
65	1	db$2	2::C3K7G0|C3K7G0_PSEFS	801	36980	23	20	18	15	6.72	Cell shape-determining
66	1	db$2	2::C3K673|C3K673_PSEFS	791	47725	36	33	20	18	9.78	Citrate synthase OS=F
67	1	db$2	2::C3K6N1|C3K6N1_PSEFS	783	29928	28	26	15	14	13.47	Succinate--CoA ligase
68	1	db$2	2::C3K3J7|C3K3J7_PSEFS	778	35008	29	28	16	16	16.01	Glutathione synthetase
69	1	db$2	2::C3KBJ2|C3KBJ2_PSEFS	773	24354	31	25	14	12	21.06	Carbonic anhydrase OS
70	1	db$2	2::C3K1K0|GUAA_PSEFS	773	58398	29	29	23	23	5.46	GMP synthase [glutam
71	1	db$2	2::C3JZS1|C3JZS1_PSEFS	771	55264	29	27	17	17	4.09	Amidophosphoribosyltı
72	1	db$2	2::C3K162|C3K162_PSEFS	763	52023	26	25	20	19	5.74	Pyruvate kinase OS=P:
73	1	db$2	2::C3K2D0|C3K2D0_PSEFS	758	61490	27	26	22	21	4.88	Energy-dependent trar
74	1	db$2	2::C3K266|C3K266_PSEFS	754	69364	72	71	13	12	1.43	ATP-dependent zinc m
75	1	db$2	2::C3KDU5|C3KDU5_PSEFS	745	90380	23	23	20	20	1.86	DNA gyrase subunit B
76	1	db$2	2::C3KE51|C3KE51_PSEFS	745	38026	28	26	19	18	8.32	Putative oxidoreductas
77	1	db$2	2::C3KCS1|C3KCS1_PSEFS	732	34171	27	27	16	16	17.13	Ribose-phosphate pyrc
78	1	db$2	2::C3K2D3|C3K2D3_PSEFS	722	44901	26	25	16	15	5.64	Serine hydroxymethylt
79	1	db$2	2::C3KEC2|C3KEC2_PSEFS	719	28614	26	25	18	17	15.37	Putative lipoprotein OS
79	2	db$2	2::C3KAJ6|C3KAJ6_PSEFS	227	38502	8	7	5	4	0.85	Histidinol-phosphate a
80	1	db$2	2::C3K4D2|C3K4D2_PSEFS	716	30988	32	30	17	16	37.56	Phosphate import ATP-
81	1	db$2	2::C3K6N7|HTPG_PSEFS	715	71589	27	24	18	16	2.29	Chaperone protein Htp
82	1	db$2	2::C3KBZ2|ASSY_PSEFS	713	45330	27	26	17	17	6.27	Argininosuccinate synt
83	1	db$2	2::C3K4D1|C3K4D1_PSEFS	713	28553	26	25	14	14	18.51	Phosphate-specific trar
84	1	db$2	2::C3KBM0|GLPK_PSEFS	706	55508	26	24	18	16	3.64	Glycerol kinase OS=Ps
85	1	db$2	2::C3JZN7|SYT_PSEFS	695	72513	27	24	21	19	2.95	Threonine--tRNA ligase
86	1	db$2	2::C3K3S2|C3K3S2_PSEFS	674	44244	26	25	18	17	5.14	2-oxoglutarate reducta
87	1	db$2	2::C3K2X5|RL4_PSEFS	665	21987	13	12	8	8	5.84	50S ribosomal protein
88	1	db$2	2::C3K6J4|C3K6J4_PSEFS	663	97893	33	29	27	24	2.36	DNA gyrase subunit A
89	1	db$2	2::C3K2Y6|RL1_PSEFS	663	24061	22	22	12	12	21.69	50S ribosomal protein
90	1	db$2	2::C3K6G5|AMPA_PSEFS	651	52403	27	25	21	19	5.66	Probable cytosol aminc
91	1	db$2	2::C3K6H6|C3K6H6_PSEFS	645	45588	23	22	14	13	3.27	Enolase OS=Pseudomc
92	1	db$2	2::C3K139|C3K139_PSEFS	643	48816	25	23	13	12	2.88	Uncharacterized protei

↑Family	M	DB	Accession	Score	Mass	Matches	Match(sig)	Sequences	Seq(sig)	emPAI	Description
93	1	db$2	2::C3JYS1|C3JYS1_PSEFS	640	17688	22	21	8	8	17.46	Peptidoglycan-associat
94	1	db$2	2::C3K1A7|C3K1A7_PSEFS	640	66070	27	25	20	19	3.85	Proline--tRNA ligase O
95	1	db$2	2::C3K2V1|C3K2V1_PSEFS	634	36623	26	25	19	18	12.16	DNA-directed RNA poly
96	1	db$2	2::C3KE67|RS6_PSEFS	625	16276	23	22	13	13	96.61	30S ribosomal protein
97	1	db$2	2::C3K3G6|SAHH_PSEFS	624	51212	31	27	19	17	5.97	Adenosylhomocysteina
98	1	db$2	2::C3KE40|SYGB_PSEFS	620	74833	29	26	20	19	2.55	Glycine--tRNA ligase b
99	1	db$2	2::C3KDU7|C3KDU7_PSEFS	613	28504	23	22	16	15	10.89	Putative ABC transport
100	1	db$2	2::C3K2R9|C3K2R9_PSEFS	612	16440	13	13	6	6	8.73	6,7-dimethyl-8-ribityll
101	1	db$2	2::C3JY70|C3JY70_PSEFS	601	50668	17	16	12	11	2.07	Adenylosuccinate lyase
102	1	db$2	2::C3K1E7|ATPG_PSEFS	599	31374	19	18	13	12	7.17	ATP synthase gamma
103	1	db$2	2::C3K165|C3K165_PSEFS	599	54857	31	26	23	19	6.28	Fumarate hydratase cl
103	2	db$2	2::C3K604|C3K604_PSEFS	230	18933	14	14	10	10	14.10	Putative outer membra
104	1	db$2	2::C3K178|C3K178_PSEFS	596	36139	22	21	16	16	10.92	Glyceraldehyde-3-phos
105	1	db$2	2::C3K5E6|RS2_PSEFS	596	27078	25	25	16	16	26.15	30S ribosomal protein
106	1	db$2	2::C3K4F6|C3K4F6_PSEFS	595	65489	32	27	24	20	3.57	Oxaloacetate decarbox
107	1	db$2	2::C3K3P0|ILVD_PSEFS	591	65667	24	21	21	18	2.93	Dihydroxy-acid dehydr
108	1	db$2	2::C3K4J5|PCKA_PSEFS	591	55324	23	21	18	18	4.09	Phosphoenolpyruvate
109	1	db$2	2::C3KDD8|C3KDD8_PSEFS	590	44893	28	26	18	17	5.67	Cell division protein Ft
110	1	db$2	2::C3KDW5|C3KDW5_PSEFS	589	43265	26	23	20	19	8.90	Protein HflK OS=Pseu
111	1	db$2	2::C3JXY1|C3JXY1_PSEFS	587	93962	29	26	21	19	1.61	Alanine--tRNA ligase C
112	1	db$2	2::C3K426|ARLY_PSEFS	586	51545	24	22	17	16	4.73	Argininosuccinate lyas
113	1	db$2	2::C3K3F1|PGK_PSEFS	581	40125	16	16	13	13	4.21	Phosphoglycerate kina
114	1	db$2	2::C3K7C9|C3K7C9_PSEFS	576	51641	25	24	19	18	4.71	Glutamine synthetase
115	1	db$2	2::C3K8B0|C3K8B0_PSEFS	576	25585	18	18	9	9	8.12	Peptidyl-prolyl cis-tran
116	1	db$2	2::C3K4Y4|C3K4Y4_PSEFS	575	71842	21	20	18	18	2.50	Potassium-transportin
117	1	db$2	2::C3K6W6|C3K6W6_PSEFS	569	49345	30	25	20	18	5.17	Biotin carboxylase OS=
118	1	db$2	2::C3JYT4|C3JYT4_PSEFS	569	17756	21	19	10	9	22.60	Putative ferritin DPS-fa
119	1	db$2	2::C3K1B7|C3K1B7_PSEFS	568	51892	18	17	11	11	1.73	Threonine synthase OS
120	1	db$2	2::C3K7Y0|C3K7Y0_PSEFS	567	45268	20	18	13	12	2.88	Putative exported prot
121	1	db$2	2::C3K914|C3K914_PSEFS	566	38509	22	21	14	14	7.04	Putative ABC transport
122	1	db$2	2::C3K1W9|C3K1W9_PSEFS	564	33929	12	12	9	9	2.49	Thioredoxin reductase
123	1	db$2	2::C3KAH7|C3KAH7_PSEFS	560	45018	25	24	19	18	8.05	Putative NADP-depend
124	1	db$2	2::C3JYJ9|CLPX_PSEFS	551	46924	24	22	14	13	4.01	ATP-dependent Clp pro
125	1	db$2	2::C3K865|C3K865_PSEFS	541	15847	11	11	5	5	4.84	Putative membrane pr
126	1	db$2	2::C3K260|C3K260_PSEFS	539	54381	19	17	15	14	2.69	Transcription terminati
127	1	db$2	2::C3K1F0|C3K1F0_PSEFS	534	16832	18	18	10	10	14.97	ATP synthase subunit
128	1	db$2	2::C3KA76|C3KA76_PSEFS	527	30860	17	16	11	11	6.28	UTP--glucose-1-phosp
129	1	db$2	2::C3K6W9|C3K6W9_PSEFS	524	69415	16	14	13	12	1.27	Putative methyl-accep
130	1	db$2	2::C3K2V2|RS4_PSEFS	520	23062	20	20	14	14	20.15	30S ribosomal protein
131	1	db$2	2::C3K2Y4|RL7_PSEFS	518	12456	11	10	2	2	5.48	50S ribosomal protein

↑Family	M	DB	Accession	Score	Mass	Matches	Match(sig)	Sequences	Seq(sig)	emPAI	Description
132	1	db$2	2::C3K1K7|LEU1_PSEFS	517	61997	22	21	20	19	3.26	2-isopropylmalate synt
133	1	db$2	2::C3KCB5|C3KCB5_PSEFS	516	30173	24	21	12	11	6.60	Phosphate import ATP-
134	1	db$2	2::C3K1N2|C3K1N2_PSEFS	515	67307	23	23	15	15	2.55	Protein translocase sul
135	1	db$2	2::C3JXZ7|C3JXZ7_PSEFS	515	28415	16	14	9	8	3.44	Putative histidine-bind
136	1	db$2	2::C3K6I0|C3K6I0_PSEFS	514	39056	24	22	14	14	6.80	Alcohol dehydrogenase
137	1	db$2	2::C3JZT6|C3JZT6_PSEFS	512	40569	19	19	11	11	5.46	Aspartate-semialdehyc
138	1	db$2	2::C3KED7|PUR7_PSEFS	508	26862	24	21	14	13	12.84	Phosphoribosylaminoir
139	1	db$2	2::C3JYQ5|C3JYQ5_PSEFS	504	46409	22	22	17	17	5.26	Arginine deiminase OS
140	1	db$2	2::C3K6X4|C3K6X4_PSEFS	504	71161	22	21	17	16	2.32	Biosynthetic arginine c
141	1	db$2	2::C3K016|C3K016_PSEFS	501	85788	17	16	15	14	1.29	Ribosomal RNA large s
142	1	db$2	2::C3KDW8|PURA_PSEFS	493	46535	22	22	17	17	4.63	Adenylosuccinate syntl
143	1	db$2	2::C3K4V9|C3K4V9_PSEFS	493	53185	20	19	16	16	3.54	Glyceraldehyde-3-phos
144	1	db$2	2::C3JYS2|TOLB_PSEFS	493	47517	24	22	18	18	5.00	Tol-Pal system protein
145	1	db$2	2::C3KAD2|ARNA_PSEFS	490	74140	19	19	16	16	1.96	Bifunctional polymyxin
146	1	db$2	2::C3KDR8|OPGD_PSEFS	486	60485	16	14	14	12	1.56	Glucans biosynthesis p
147	1	db$2	2::C3K2V6|C3K2V6_PSEFS	483	47842	15	15	6	6	1.43	Protein translocase sul
148	1	db$2	2::C3K6M2|C3K6M2_PSEFS	480	60584	17	17	15	15	2.49	ATP-dependent RNA h
149	1	db$2	2::C3K6M6|C3K6M6_PSEFS	479	26063	19	17	14	13	16.91	Succinate dehydrogena
150	1	db$2	2::C3K7D6|F16PA_PSEFS	475	37081	22	21	13	12	5.75	Fructose-1,6-bisphosp
151	1	db$2	2::C3K3F5|METK_PSEFS	471	42554	17	16	12	11	2.80	S-adenosylmethionine
152	1	db$2	2::C3K108|C3K108_PSEFS	466	83591	21	19	19	17	1.62	GTP pyrophosphokinas
153	1	db$2	2::C3K5S8|C3K5S8_PSEFS	456	75646	17	17	15	15	1.73	Oligopeptidase A OS=I
154	1	db$2	2::C3K2X6|RL3_PSEFS	454	22550	13	12	9	9	7.04	50S ribosomal protein
155	1	db$2	2::C3JXL8|C3JXL8_PSEFS	448	112023	19	17	14	12	0.89	Putative efflux protein
156	1	db$2	2::C3JZV2|C3JZV2_PSEFS	448	43020	17	17	13	13	3.16	Aminotransferase OS=
157	1	db$2	2::C3KBY6|C3KBY6_PSEFS	448	17850	16	14	9	9	16.82	Bacterioferritin OS=Ps
158	1	db$2	2::C3K4D4|C3K4D4_PSEFS	447	83859	24	22	16	15	1.93	Putative phosphate AB
159	1	db$2	2::C3KCP3|C3KCP3_PSEFS	447	56221	19	19	14	14	2.54	Acetyltransferase com
160	1	db$2	2::C3K2H6|C3K2H6_PSEFS	445	32512	8	8	5	5	1.39	Acyl-CoA thioesterase
161	1	db$2	2::C3K6H5|KDSA_PSEFS	444	30777	27	22	16	12	17.39	2-dehydro-3-deoxyphc
162	1	db$2	2::C3K341|C3K341_PSEFS	440	69250	21	21	16	16	2.93	RNA polymerase sigma
162	2	db$2	2::C3K708|C3K708_PSEFS	123	38066	5	4	5	4	0.64	RNA polymerase sigma
163	1	db$2	2::C3K2X0|C3K2X0_PSEFS	440	25737	20	17	13	12	11.90	30S ribosomal protein
164	1	db$2	2::C3JXY0|AK_PSEFS	439	44504	20	19	16	15	5.08	Aspartate kinase OS=I
165	1	db$2	2::C3KBH2|C3KBH2_PSEFS	438	51951	16	14	15	13	2.27	Glutamate synthase [N
166	1	db$2	2::C3KDD9|C3KDD9_PSEFS	437	41969	16	14	15	14	3.84	Cell division protein Ft
167	1	db$2	2::C3KDW6|C3KDW6_PSEFS	436	33055	22	20	15	14	8.79	Protein HflC OS=Pseuc
168	1	db$2	2::C3K6C2|C3K6C2_PSEFS	431	27720	15	15	12	12	6.66	Putative amino acid AE
169	1	db$2	2::C3K3V2|C3K3V2_PSEFS	429	49111	21	18	13	11	1.89	Putative outer membra
170	1	db$2	2::C3K1E2|C3K1E2_PSEFS	428	66300	21	21	17	17	2.62	Glutamine--fructose-6-

↑Family	M	DB	Accession	Score	Mass	Matches	Match(sig)	Sequences	Seq(sig)	emPAI	Description
171	1	db$2	2::C3K484|C3K484_PSEFS	425	13502	13	13	9	9	30.09	Uncharacterized protei
172	1	db$2	2::C3KBL8|C3KBL8_PSEFS	424	56970	21	20	18	17	3.11	Glycerol-3-phosphate
173	1	db$2	2::C3K1M5|ISCS_PSEFS	420	44467	16	14	14	12	2.58	Cysteine desulfurase I
174	1	db$2	2::C3K8X4|MURA_PSEFS	420	44975	19	19	16	16	5.64	UDP-N-acetylglucosam
175	1	db$2	2::C3K5H9|C3K5H9_PSEFS	419	33776	18	18	12	12	7.14	Transaldolase OS=Pse
176	1	db$2	2::C3JYA7|C3JYA7_PSEFS	415	59461	16	15	11	11	1.81	Putative NADP-depend
177	1	db$2	2::C3KDP1|CYSD_PSEFS	414	35173	13	13	9	9	4.00	Sulfate adenylyltransfe
178	1	db$2	2::C3K1G5|C3K1G5_PSEFS	414	46195	13	13	10	10	1.78	Homoserine dehydroge
179	1	db$2	2::C3K0Q7|C3K0Q7_PSEFS	414	45255	10	10	7	7	1.30	Flagellar hook protein
180	1	db$2	2::C3JXL7|C3JXL7_PSEFS	411	39112	13	13	10	10	2.35	HlyD-family secretion
181	1	db$2	2::C3K4C6|C3K4C6_PSEFS	408	25565	13	13	9	9	6.59	Phosphate regulon tra
182	1	db$2	2::C3JYV2|C3JYV2_PSEFS	408	52688	17	17	14	14	2.84	AAA domain-containin
183	1	db$2	2::C3K8X5|HIS1_PSEFS	404	23098	16	16	12	12	16.26	ATP phosphoribosyltra
184	1	db$2	2::C3K3E8|C3K3E8_PSEFS	401	38385	19	19	15	15	8.14	Fructose-1,6-bisphosp
185	1	db$2	2::C3JY65|C3JY65_PSEFS	399	45375	25	19	19	17	5.52	Isocitrate dehydrogena
186	1	db$2	2::C3K4C7|C3K4C7_PSEFS	397	50044	13	12	12	11	2.11	Phosphate regulon sen
187	1	db$2	2::C3JZN2|C3JZN2_PSEFS	396	86329	19	18	18	17	1.54	Phenylalanine--tRNA li
188	1	db$2	2::C3K2Z7|ARGC_PSEFS	394	36612	17	17	11	11	5.07	N-acetyl-gamma-gluta
189	1	db$2	2::C3K358|C3K358_PSEFS	393	65409	16	16	11	11	1.39	Putative dehydrogenas
189	2	db$2	2::C3K355|C3K355_PSEFS	278	63710	13	12	10	9	0.95	Putative dehydrogenas
190	1	db$2	2::C3K2M9|PROA_PSEFS	393	45380	14	13	9	8	1.83	Gamma-glutamyl phos
191	1	db$2	2::C3K2W1|RL6_PSEFS	392	19185	17	15	10	9	10.47	50S ribosomal protein
192	1	db$2	2::C3K9R4|C3K9R4_PSEFS	392	62164	15	15	13	13	1.91	Putative sulfite reducta
193	1	db$2	2::C3KAL6|C3KAL6_PSEFS	391	52109	19	16	15	13	2.57	Putative aminotransfer
194	1	db$2	2::C3K649|C3K649_PSEFS	391	8822	12	12	5	5	12.77	Major outer membrane
195	1	db$2	2::C3KDL2|C3KDL2_PSEFS	390	52736	11	11	11	11	1.68	Putative outer membra
196	1	db$2	2::C3JZ71|C3JZ71_PSEFS	389	31565	10	8	9	7	1.85	Hex regulon repressor
197	1	db$2	2::C3K9R0|C3K9R0_PSEFS	389	62265	15	14	11	11	1.49	Pyruvate dehydrogena
198	1	db$2	2::C3K0H1|C3K0H1_PSEFS	388	17848	10	9	7	6	5.25	Chemotaxis protein Ch
199	1	db$2	2::C3K1Y2|C3K1Y2_PSEFS	387	21662	10	10	8	8	6.04	Peptidyl-prolyl cis-tran
200	1	db$2	2::C3K2W6|RL14_PSEFS	387	13401	13	13	8	8	30.97	50S ribosomal protein
201	1	db$2	2::C3JZN6|IF3_PSEFS	381	20837	14	14	7	7	5.04	Translation initiation fa
202	1	db$2	2::C3KDE0|LPXC_PSEFS	381	33197	12	12	10	10	3.14	UDP-3-O-acyl-N-acety
203	1	db$2	2::C3KE65|C3KE65_PSEFS	381	97331	23	22	22	21	1.78	Ribonuclease R OS=Ps
204	1	db$2	2::C3JZ68|C3JZ68_PSEFS	381	24197	13	13	10	10	7.47	2-dehydro-3-deoxy-ph
205	1	db$2	2::C3K3U2|C3K3U2_PSEFS	379	50985	12	12	10	10	1.53	Putative glutamine syr
206	1	db$2	2::C3K569|C3K569_PSEFS	378	44415	19	18	13	12	2.99	Tryptophan synthase b
207	1	db$2	2::C3JYD5|C3JYD5_PSEFS	376	18453	10	10	6	6	3.59	Peptidyl-prolyl cis-tran
208	1	db$2	2::C3K6D9|C3K6D9_PSEFS	376	45394	12	12	10	10	1.83	Phosphoribosylamine--
209	1	db$2	2::C3K2C4|C3K2C4_PSEFS	369	89060	16	16	15	15	1.22	Endopeptidase La OS=

↑Family	M	DB	Accession	Score	Mass	Matches	Match(sig)	Sequences	Seq(sig)	emPAI	Description
210	1	db$2	2::C3KCR8|C3KCR8_PSEFS	367	39907	14	14	9	9	2.67	Ribosome-binding ATP
211	1	db$2	2::C3JZN3|SYFA_PSEFS	366	37892	13	13	11	11	3.47	Phenylalanine--tRNA li
212	1	db$2	2::C3K1L4|ISPG_PSEFS	365	39660	15	15	13	13	4.30	4-hydroxy-3-methylbu
213	1	db$2	2::C3KDG0|RECA_PSEFS	365	37499	20	18	17	15	7.49	Protein RecA OS=Pseu
214	1	db$2	2::C3K2Y0|RS7_PSEFS	364	17636	16	15	12	12	23.07	30S ribosomal protein
215	1	db$2	2::C3JZV8|C3JZV8_PSEFS	359	38906	13	11	9	8	1.98	Neutral metalloprotein
216	1	db$2	2::C3K4D3|C3K4D3_PSEFS	359	60551	21	20	15	15	2.23	Phosphate transport sy
217	1	db$2	2::C3K4F7|C3K4F7_PSEFS	354	51847	18	18	16	16	3.31	Biotin carboxylase OS=
218	1	db$2	2::C3KEC8|C3KEC8_PSEFS	354	50623	13	12	10	9	1.55	Periplasmic serine end
219	1	SwissProt_HUMAN$	3::P60709|ACTB_HUMAN	353	41710	19	16	13	12	3.88	Actin, cytoplasmic 1 O
220	1	SwissProt_HUMAN$	3::P00761|TRYP_PIG	352	24394	11	9	6	6	3.66	Trypsin OS=Sus scrofa
221	1	db$2	2::C3K2U7|C3K2U7_PSEFS	352	104087	23	20	19	17	1.17	UvrABC system protein
222	1	db$2	2::C3K5E0|C3K5E0_PSEFS	352	36000	11	11	8	8	2.71	2,3,4,5-tetrahydropyri
223	1	db$2	2::C3KDU3|C3KDU3_PSEFS	351	40518	15	14	11	10	2.21	Beta sliding clamp OS=
224	1	db$2	2::C3K0D4|C3K0D4_PSEFS	350	29228	15	14	9	9	3.27	Cell division inhibitor M
225	1	db$2	2::C3JYW3|C3JYW3_PSEFS	350	36818	15	15	12	12	4.29	Branched-chain-amino
226	1	db$2	2::C3KCN5|C3KCN5_PSEFS	349	142346	14	13	12	11	0.44	Bifunctional protein Pu
227	1	db$2	2::C3JXZ0|C3JXZ0_PSEFS	348	43332	11	11	11	11	2.32	Acetylornithine aminot
228	1	db$2	2::C3K3V3|ARGA_PSEFS	347	47793	12	10	11	9	1.43	Amino-acid acetyltrans
229	1	db$2	2::C3KE61|RIMK_PSEFS	347	32488	9	9	6	6	1.76	Probable alpha-L-gluta
230	1	db$2	2::C3K4M6|C3K4M6_PSEFS	344	57818	19	17	15	14	2.41	Putative exported alka
231	1	db$2	2::C3K902|RAPA_PSEFS	339	106722	13	13	12	12	0.71	RNA polymerase-assoc
232	1	db$2	2::C3K6G2|C3K6G2_PSEFS	339	106936	20	19	19	18	1.22	Valine--tRNA ligase OS
233	1	db$2	2::C3K123|C3K123_PSEFS	338	41542	11	10	9	8	1.49	Citrate synthase OS=P
234	1	db$2	2::C3K2X3|RL2_PSEFS	337	29689	14	14	12	12	5.74	50S ribosomal protein
235	1	db$2	2::C3K7L5|C3K7L5_PSEFS	336	19131	11	11	10	10	10.63	Molybdenum cofactor I
236	1	db$2	2::C3K4W1|STHA_PSEFS	336	50963	19	18	14	13	3.03	Soluble pyridine nucle
237	1	db$2	2::C3K0N4|C3K0N4_PSEFS	335	25612	7	7	5	5	2.61	3-oxoacyl-[acyl-carrier
238	1	db$2	2::C3KAK3|C3KAK3_PSEFS	333	54608	16	15	12	11	1.83	Probable malate:quino
239	1	db$2	2::C3K2W0|RL18_PSEFS	333	12651	13	12	8	7	12.07	50S ribosomal protein
240	1	db$2	2::C3K3Z1|C3K3Z1_PSEFS	332	11808	12	12	6	6	22.43	Thioredoxin OS=Pseuc
241	1	db$2	2::C3KAA8|C3KAA8_PSEFS	331	132643	13	13	13	13	0.59	Histidine kinase OS=P:
242	1	db$2	2::C3K1G8|RL19_PSEFS	329	12961	11	11	7	7	16.54	50S ribosomal protein
243	1	db$2	2::C3K6E1|RL13_PSEFS	329	15695	13	13	7	7	9.87	50S ribosomal protein
244	1	db$2	2::C3K452|XPT_PSEFS	328	20267	12	10	10	8	6.98	Xanthine phosphoribos
245	1	db$2	2::C3K6J9|KCY_PSEFS	327	24780	8	7	7	6	2.13	Cytidylate kinase OS=
246	1	db$2	2::C3K1X6|C3K1X6_PSEFS	326	44711	9	8	8	7	1.10	GTPase Obg OS=Pseu
247	1	db$2	2::C3JZ70|C3JZ70_PSEFS	325	54858	20	19	18	17	3.34	Glucose-6-phosphate :
248	1	db$2	2::C3KE70|RL9_PSEFS	324	15488	10	10	6	6	7.19	50S ribosomal protein
249	1	SwissProt_HUMAN$	3::P02768|ALBU_HUMAN	324	69321	14	12	13	12	1.27	Albumin OS=Homo sa

↑Family	M	DB	Accession	Score	Mass	Matches	Match(sig)	Sequences	Seq(sig)	emPAI	Description
249	2	SwissProt_HUMAN$	3::P02769|ALBU_BOVIN	130	69248	5	5	5	5	0.41	Albumin OS=Bos tauru
250	1	db$2	2::C3JY85|C3JY85_PSEFS	324	48759	15	15	13	13	2.53	NADH-quinone oxidore
251	1	db$2	2::C3K614|C3K614_PSEFS	323	41572	17	17	11	11	3.37	3-ketoacyl-CoA thiolas
252	1	db$2	2::C3K1E9|ATPD_PSEFS	323	19246	13	13	7	7	7.99	ATP synthase subunit
253	1	db$2	2::C3K2M1|C3K2M1_PSEFS	322	42033	17	15	14	13	3.82	Serine-type D-Ala-D-A
254	1	db$2	2::C3KEB4|C3KEB4_PSEFS	320	56978	17	15	14	12	2.20	Lysine--tRNA ligase OS
255	1	db$2	2::C3KDX4|SYI_PSEFS	319	105386	17	14	16	13	0.80	Isoleucine--tRNA ligase
256	1	db$2	2::C3JY83|NUOCD_PSEFS	318	67660	24	21	20	18	3.35	NADH-quinone oxidore
257	1	db$2	2::C3JZS4|C3JZS4_PSEFS	317	33375	13	13	9	9	3.72	Acetyl-coenzyme A car
258	1	db$2	2::C3K8X0|C3K8X0_PSEFS	315	16351	10	9	8	7	6.42	Putative ABC transport
259	1	db$2	2::C3K6E0|PUR9_PSEFS	315	57370	15	15	12	12	2.17	Bifunctional purine bio
260	1	db$2	2::C3K324|C3K324_PSEFS	313	105271	18	16	15	14	0.88	LPS-assembly protein
261	1	db$2	2::C3K8U4|UBIE_PSEFS	313	28320	20	19	14	13	7.69	Ubiquinone/menaquinc
262	1	db$2	2::C3K6R4|C3K6R4_PSEFS	313	18727	13	12	5	4	3.47	Uncharacterized protei
263	1	db$2	2::C3JZ29|C3JZ29_PSEFS	313	41047	7	7	7	7	1.24	Putative glycerophospl
264	1	db$2	2::C3KBP1|C3KBP1_PSEFS	311	37670	13	12	9	8	2.09	Putative reductase OS
265	1	db$2	2::C3K8X6|C3K8X6_PSEFS	311	47255	12	11	10	10	1.72	Histidinol dehydrogena
266	1	db$2	2::C3K466|C3K466_PSEFS	311	52681	11	11	8	8	1.05	Putative aldehyde deh
267	1	db$2	2::C3K2X7|RS10_PSEFS	311	11745	18	14	9	8	22.43	30S ribosomal protein
268	1	db$2	2::C3KEA4|C3KEA4_PSEFS	310	51722	11	9	8	7	1.08	PINc domain-containin
269	1	db$2	2::C3JZV0|C3JZV0_PSEFS	310	56140	17	15	16	14	2.54	Glutamate--tRNA ligas
270	1	db$2	2::C3K2V7|RL15_PSEFS	310	15215	13	12	8	8	28.22	50S ribosomal protein
271	1	db$2	2::C3K616|C3K616_PSEFS	309	97222	17	16	15	15	1.18	DNA topoisomerase 1
272	1	db$2	2::C3K5Z7|C3K5Z7_PSEFS	309	26574	14	13	10	10	7.40	Uridylate kinase OS=P
273	1	db$2	2::C3KAC1|C3KAC1_PSEFS	308	27548	15	14	11	10	6.78	Putative phenylacetic a
274	1	db$2	2::C3K278|C3K278_PSEFS	306	15153	9	8	5	5	3.64	Ferric uptake regulatio
275	1	db$2	2::C3K2T2|C3K2T2_PSEFS	306	24648	8	8	5	5	2.80	Putative ABC transport
276	1	db$2	2::C3K0K5|C3K0K5_PSEFS	304	31365	14	14	7	7	2.86	Electron transfer flavo
277	1	db$2	2::C3JZS0|C3JZS0_PSEFS	302	43144	17	15	13	13	3.15	O-succinylhomoserine
278	1	db$2	2::C3K443|C3K443_PSEFS	302	12323	13	11	8	7	19.40	Nitrogen regulatory pr
279	1	db$2	2::C3K9U6|C3K9U6_PSEFS	300	35232	10	10	7	7	2.34	Putative 6-phosphoglu
280	1	db$2	2::C3KBI6|C3KBI6_PSEFS	297	106629	17	16	16	15	0.95	Uncharacterized protei
281	1	db$2	2::C3K2S0|C3K2S0_PSEFS	297	39319	8	7	6	5	0.82	3,4-dihydroxy-2-butan
282	1	db$2	2::C3K245|C3K245_PSEFS	296	53442	13	12	12	11	1.90	Poly(A) polymerase I (
283	1	db$2	2::C3K3N4|COAD_PSEFS	296	17810	14	13	9	9	12.94	Phosphopantetheine a
284	1	db$2	2::C3K474|C3K474_PSEFS	295	50732	14	12	12	10	1.79	Putative phosphomanr
285	1	db$2	2::C3K249|G6PI_PSEFS	295	61384	19	15	13	12	1.94	Glucose-6-phosphate i
286	1	db$2	2::C3K2S6|C3K2S6_PSEFS	295	31662	7	7	6	6	1.44	Putative thioredoxin O
287	1	db$2	2::C3K7X9|C3K7X9_PSEFS	294	40858	16	14	11	10	2.57	Carboxynorspermidine
288	1	db$2	2::C3K3M4|C3K3M4_PSEFS	294	49883	8	8	8	8	1.14	Signal recognition part

↑Family	M	DB	Accession	Score	Mass	Matches	Match(sig)	Sequences	Seq(sig)	emPAI	Description
289	1	db$2	2::C3K9T7|C3K9T7_PSEFS	292	99328	17	17	15	15	1.05	Aminopeptidase N OS=
290	1	db$2	2::C3K907|C3K907_PSEFS	292	77648	17	14	16	13	1.21	Tail-specific protease (
291	1	SwissProt_HUMAN$	3::P07355|ANXA2_HUMAN	290	38580	12	11	11	10	2.40	Annexin A2 OS=Homo
292	1	db$2	2::C3K476|PYRE_PSEFS	289	23079	7	7	6	6	2.39	Orotate phosphoribosy
293	1	db$2	2::C3JY74|C3JY74_PSEFS	286	48472	13	13	13	13	2.55	Isocitrase OS=Pseudor
294	1	db$2	2::C3JYU6|C3JYU6_PSEFS	286	42196	9	7	9	7	1.19	Arginine biosynthesis b
295	1	db$2	2::C3KA80|C3KA80_PSEFS	285	20438	14	13	10	9	11.50	Alkyl hydroperoxide re
296	1	db$2	2::C3K244|C3K244_PSEFS	282	52276	12	12	12	12	1.96	Putative response regu
297	1	db$2	2::C3K142|C3K142_PSEFS	282	32099	12	11	11	10	4.04	2-methylisocitrate lyas
298	1	db$2	2::C3K2X1|RL22_PSEFS	282	11904	11	7	8	6	9.42	50S ribosomal protein
299	1	db$2	2::C3K401|C3K401_PSEFS	281	56255	19	17	14	13	2.25	Exopolyphosphatase O
300	1	db$2	2::C3JYQ6|C3JYQ6_PSEFS	279	37842	14	13	11	10	3.47	Ornithine carbamoyltra
301	1	db$2	2::C3JY62|C3JY62_PSEFS	279	83224	12	11	10	9	0.67	ATP-dependent Clp pro
301	2	db$2	2::C3K4B3|C3K4B3_PSEFS	71	97007	6	5	6	5	0.28	Chaperone OS=Pseudo
302	1	db$2	2::C3K3Q2|C3K3Q2_PSEFS	279	37335	10	10	5	5	1.43	Putative cation ABC tra
303	1	db$2	2::C3K6G0|C3K6G0_PSEFS	278	9680	8	7	5	4	5.76	Putative DNA-binding p
304	1	db$2	2::C3K6J6|C3K6J6_PSEFS	278	40307	11	10	9	8	1.27	Bifunctional chorismate
305	1	db$2	2::C3K7C1|SECB_PSEFS	278	17615	5	5	2	2	0.70	Protein-export protein
306	1	db$2	2::C3JYS5|C3JYS5_PSEFS	277	25332	10	10	7	7	4.32	Tol-Pal system protein
307	1	db$2	2::C3KAJ2|C3KAJ2_PSEFS	277	53335	11	10	10	9	1.22	Methylmalonate-semia
308	1	db$2	2::C3KBX9|C3KBX9_PSEFS	276	20403	5	5	3	3	1.51	Putative phospholipid-l
309	1	db$2	2::C3K3W3|C3K3W3_PSEFS	276	41294	8	7	6	6	1.23	Putative aminotransfer
310	1	db$2	2::C3K7N8|RDGC_PSEFS	275	34160	16	16	13	13	6.92	Recombination-associa
311	1	db$2	2::C3K6A2|C3K6A2_PSEFS	275	27847	11	11	9	9	3.58	Putative D-methionine
311	2	db$2	2::C3KD95|C3KD95_PSEFS	197	28020	6	6	5	5	1.32	Putative exported prot
312	1	db$2	2::C3KCP5|C3KCP5_PSEFS	274	109710	9	8	9	8	0.41	Bifunctional glutamine
313	1	db$2	2::C3K5Y0|C3K5Y0_PSEFS	273	38747	13	12	11	10	2.38	NADP(H)-dependt aldo
314	1	db$2	2::C3K227|C3K227_PSEFS	272	62578	16	15	11	10	1.13	Acetolactate synthase
315	1	db$2	2::C3K490|C3K490_PSEFS	272	33505	13	12	9	9	3.08	Putative oxidative stre
316	1	db$2	2::C3KDB1|C3KDB1_PSEFS	272	65565	14	14	13	13	1.56	Putative carbamoyltran
317	1	db$2	2::C3KBX8|C3KBX8_PSEFS	272	26859	8	8	6	6	2.41	OmpA-like domain-con
318	1	db$2	2::C3K2J8|C3K2J8_PSEFS	269	54392	14	12	12	11	1.60	AMP nucleosidase OS=
319	1	db$2	2::C3K7M6|C3K7M6_PSEFS	267	88918	11	10	8	7	0.53	Acyl-coenzyme A dehy
320	1	db$2	2::C3KE79|C3KE79_PSEFS	266	29713	6	6	5	5	1.21	NH(3)-dependent NAD
321	1	db$2	2::C3KAR0|C3KAR0_PSEFS	264	79563	9	8	7	6	0.52	Putative exported isoq
322	1	db$2	2::C3KDU9|C3KDU9_PSEFS	264	34261	9	9	8	8	2.00	Serine O-acetyltransfe
323	1	db$2	2::C3K638|C3K638_PSEFS	264	26394	6	5	3	2	0.71	Putative short chain de
324	1	db$2	2::C3JY00|C3JY00_PSEFS	263	47211	9	8	7	6	0.82	Ribonucleoside-diphosp
325	1	db$2	2::C3K5I7|C3K5I7_PSEFS	263	59867	15	14	11	10	1.39	Probable malate:quino
326	1	db$2	2::C3K1X8|C3K1X8_PSEFS	262	11596	6	6	3	3	2.34	50S ribosomal protein

↑Family	M	DB	Accession	Score	Mass	Matches	Match(sig)	Sequences	Seq(sig)	emPAI	Description
327	1	db$2	2::C3K5Z8|RRF_PSEFS	262	20297	11	11	5	5	5.34	Ribosome-recycling fac
328	1	db$2	2::C3K0S7|Y4577_PSEFS	261	11900	5	5	3	3	3.77	Nucleoid-associated pr
329	1	db$2	2::C3K274|DNAJ_PSEFS	259	40315	11	10	9	9	1.87	Chaperone protein Dna
330	1	db$2	2::C3K2Y8|C3K2Y8_PSEFS	259	20023	10	9	7	7	5.53	Transcription terminati
331	1	db$2	2::C3KAI6|C3KAI6_PSEFS	258	24263	10	8	6	5	1.63	Putative dehydrogenas
332	1	db$2	2::C3K226|C3K226_PSEFS	256	17820	8	8	7	7	5.25	Acetolactate synthase
333	1	db$2	2::C3K734|C3K734_PSEFS	256	44623	13	12	9	8	2.21	Putative aminotransfer
334	1	db$2	2::C3K645|C3K645_PSEFS	255	24522	7	7	6	6	2.16	Putative ABC transport
335	1	db$2	2::C3K7G9|C3K7G9_PSEFS	255	48266	11	10	10	9	1.41	Putative modulation of
336	1	db$2	2::C3KAH3|SYR_PSEFS	253	63549	9	9	6	6	0.68	Arginine--tRNA ligase (
337	1	db$2	2::C3K3H0|C3K3H0_PSEFS	252	67796	13	11	12	10	1.01	ATP-dependent RNA he
338	1	db$2	2::C3K2Y5|RL10_PSEFS	251	17594	9	9	8	8	7.34	50S ribosomal protein
339	1	db$2	2::C3K5X7|C3K5X7_PSEFS	250	42054	11	10	10	9	1.75	Cell division protein Za
340	1	db$2	2::C3K6R3|Y2135_PSEFS	250	24050	10	8	8	6	2.22	UPF0502 protein PFLU_
341	1	db$2	2::C3K0W7|C3K0W7_PSEFS	249	31068	7	7	6	6	1.48	Metallophos_2 domain
342	1	db$2	2::C3K2U9|C3K2U9_PSEFS	249	53651	13	11	11	9	1.21	Catalase OS=Pseudom
343	1	db$2	2::C3K091|C3K091_PSEFS	249	20089	10	9	8	7	4.12	GTP cyclohydrolase 1 (
344	1	db$2	2::C3K2K9|C3K2K9_PSEFS	249	31417	8	8	7	7	1.86	Magnesium and cobalt
345	1	db$2	2::C3K2Z3|C3K2Z3_PSEFS	248	44017	12	10	10	9	1.92	Tyrosine--tRNA ligase
346	1	db$2	2::C3K102|PUR5_PSEFS	248	36950	11	11	7	7	2.59	Phosphoribosylformylg
347	1	db$2	2::C3K6E2|RS9_PSEFS	246	14607	8	8	6	6	8.27	30S ribosomal protein
348	1	db$2	2::C3JYQ7|C3JYQ7_PSEFS	246	32635	8	7	6	6	1.38	Carbamate kinase OS=
349	1	db$2	2::C3K0D3|MINC_PSEFS	246	26249	9	9	6	6	2.52	Probable septum site-(
350	1	db$2	2::C3K5A9|RF3_PSEFS	246	59584	10	8	8	6	0.74	Peptide chain release f
351	1	db$2	2::C3K8V3|HSLU_PSEFS	245	49607	16	14	13	11	1.85	ATP-dependent protea
352	1	db$2	2::C3K651|C3K651_PSEFS	245	39766	10	10	9	9	1.91	Phospho-2-dehydro-3-
353	1	db$2	2::C3JYT9|C3JYT9_PSEFS	245	46635	9	9	6	6	1.03	Putative family S43 no
354	1	db$2	2::C3K2Y7|RL11_PSEFS	245	14903	9	8	6	6	7.98	50S ribosomal protein
355	1	db$2	2::C3K310|C3K310_PSEFS	244	54246	9	8	9	8	1.01	Anthranilate synthase
356	1	db$2	2::C3K2K3|GSA_PSEFS	243	45181	7	7	5	5	0.87	Glutamate-1-semialde
357	1	db$2	2::C3K325|C3K325_PSEFS	242	49076	17	14	12	10	1.89	Chaperone SurA OS=P
358	1	db$2	2::C3K2W5|RL24_PSEFS	241	11328	10	10	7	7	25.43	50S ribosomal protein
359	1	db$2	2::C3K263|C3K263_PSEFS	241	26068	8	8	6	6	1.95	Triosephosphate isome
360	1	db$2	2::C3K5X4|C3K5X4_PSEFS	240	16332	12	11	8	8	8.88	Putative membrane pr
361	1	db$2	2::C3KAH1|HSLV_PSEFS	240	18731	5	5	4	4	2.48	ATP-dependent protea
362	1	db$2	2::C3K1X9|C3K1X9_PSEFS	240	34909	11	11	9	9	2.38	Octaprenyl-diphosphat
363	1	db$2	2::C3KCT2|MUTS_PSEFS	239	95536	11	9	10	8	0.49	DNA mismatch repair p
364	1	db$2	2::C3JZN9|C3JZN9_PSEFS	239	7652	8	8	6	6	35.06	Major cold shock prote
365	1	db$2	2::C3K1U7|C3K1U7_PSEFS	239	36908	6	6	2	2	1.15	Ubiquinol oxidase subu
366	1	db$2	2::C3K4K9|C3K4K9_PSEFS	238	27377	9	9	6	6	2.95	Putative amino acid ox

↑Family	M	DB	Accession	Score	Mass	Matches	Match(sig)	Sequences	Seq(sig)	emPAI	Description
367	1	db$2	2::C3K5V1|C3K5V1_PSEFS	238	47050	7	7	6	6	1.02	Outer membrane prote
368	1	db$2	2::C3KAK7|C3KAK7_PSEFS	237	20540	7	7	4	4	2.13	Putative lipoprotein OS
369	1	db$2	2::C3K1L1|C3K1L1_PSEFS	236	40864	13	10	10	10	2.18	Outer membrane prote
370	1	db$2	2::C3K8U2|UBIB_PSEFS	235	61616	9	9	8	8	0.85	Probable protein kinas
371	1	db$2	2::C3K818|C3K818_PSEFS	234	29926	8	8	5	5	1.19	Putative ABC transport
372	1	db$2	2::C3KBL1|C3KBL1_PSEFS	234	61934	10	7	8	6	0.58	Putative medium-chair
373	1	db$2	2::C3K2W4|RL5_PSEFS	233	20490	14	13	11	11	14.49	50S ribosomal protein
374	1	db$2	2::C3K6G8|LEPA_PSEFS	232	66372	8	8	7	7	0.65	Elongation factor 4 OS
375	1	db$2	2::C3K6H2|ACCA_PSEFS	231	34868	12	12	9	9	2.87	Acetyl-coenzyme A car
376	1	db$2	2::C3K8Y8|C3K8Y8_PSEFS	231	23915	6	6	5	5	1.68	Putative isomerase OS
377	1	db$2	2::C3KDM0|C3KDM0_PSEFS	230	69594	10	10	9	9	0.85	DNA topoisomerase 4
378	1	db$2	2::C3K2Q0|C3K2Q0_PSEFS	230	19348	10	10	8	8	7.87	Inorganic pyrophospha
379	1	db$2	2::C3JZT7|C3JZT7_PSEFS	230	38793	10	9	9	8	2.00	3-isopropylmalate deh
380	1	db$2	2::C3K1F2|ATP6_PSEFS	230	31626	6	6	3	3	0.56	ATP synthase subunit
381	1	db$2	2::C3K403|C3K403_PSEFS	229	36964	11	9	9	7	1.44	Delta-aminolevulinic a
382	1	db$2	2::C3KDD6|C3KDD6_PSEFS	228	33869	5	5	5	5	1.01	D-alanine--D-alanine l
383	1	db$2	2::C3KE41|SYGA_PSEFS	227	36196	8	8	5	5	0.92	Glycine--tRNA ligase a
384	1	db$2	2::C3K1X2|C3K1X2_PSEFS	226	40166	10	9	10	9	1.88	Putative putative oxidc
385	1	db$2	2::C3K667|C3K667_PSEFS	225	48557	8	8	8	8	1.18	Conserved hypothetica
386	1	db$2	2::C3K5D5|C3K5D5_PSEFS	224	41033	6	6	5	5	0.78	Succinyl-diaminopimel
387	1	db$2	2::C3K6H1|C3K6H1_PSEFS	222	131195	15	14	15	14	0.66	DNA polymerase III su
388	1	db$2	2::C3JZ61|C3JZ61_PSEFS	221	61509	12	10	10	8	0.85	Acyl-CoA synthetase (l
389	1	db$2	2::C3K1G2|YIDC_PSEFS	221	61977	14	10	12	10	1.15	Membrane protein inse
390	1	db$2	2::C3K422|C3K422_PSEFS	221	40250	9	7	8	6	1.02	Putative uroporphyrin-
391	1	db$2	2::C3K475|ARGB_PSEFS	220	31926	10	9	10	9	2.77	Acetylglutamate kinas
392	1	db$2	2::C3JZ69|C3JZ69_PSEFS	219	25612	8	8	5	5	1.50	6-phosphogluconolactc
393	1	db$2	2::C3JZT0|C3JZT0_PSEFS	219	28122	5	5	5	5	1.31	Putative GntR-family t
394	1	db$2	2::C3K4V4|C3K4V4_PSEFS	218	32182	7	6	6	6	1.41	Putative ATP-binding p
395	1	db$2	2::C3KDN5|C3KDN5_PSEFS	218	35878	5	5	3	3	0.48	Renalase OS=Pseudon
396	1	db$2	2::C3K0N2|C3K0N2_PSEFS	217	43313	7	5	7	5	0.73	3-oxoacyl-[acyl-carrier
397	1	db$2	2::C3K3K2|PYRB_PSEFS	216	36271	5	5	4	4	0.68	Aspartate carbamoyltr
398	1	SwissProt_HUMAN$	3::P02751|FINC_HUMAN	214	272150	15	14	14	13	0.25	Fibronectin OS=Homo
399	1	db$2	2::C3JY86|C3JY86_PSEFS	214	98164	16	13	15	12	0.79	NADH-quinone oxidore
400	1	db$2	2::C3KBN3|C3KBN3_PSEFS	214	112655	10	10	8	8	0.40	Efflux pump membran
401	1	db$2	2::C3K2V0|RL17_PSEFS	214	14407	10	9	6	6	8.59	50S ribosomal protein
402	1	db$2	2::C3K6S8|C3K6S8_PSEFS	214	40355	8	8	7	7	1.55	Putative acetyl-CoA tra
403	1	db$2	2::C3K3L7|THIG_PSEFS	214	28373	9	9	8	8	2.76	Thiazole synthase OS=
404	1	db$2	2::C3KDB7|C3KDB7_PSEFS	213	65793	6	6	6	6	0.54	ATP-dependent lipid A-
405	1	db$2	2::C3KDW3|HFQ_PSEFS	213	9318	12	12	8	8	51.14	RNA-binding protein H
406	1	db$2	2::C3K262|C3K262_PSEFS	213	12956	9	8	4	4	3.19	Protein-export membr

↑Family	M	DB	Accession	Score	Mass	Matches	Match(sig)	Sequences	Seq(sig)	emPAI	Description
407	1	db$2	2::C3JY54|SYS_PSEFS	213	47115	11	9	9	7	1.23	Serine--tRNA ligase OS
408	1	db$2	2::C3K1A1|C3K1A1_PSEFS	213	10298	9	9	7	7	21.98	10 kDa chaperonin OS
409	1	db$2	2::C3K3A2|C3K3A2_PSEFS	213	38105	10	9	7	7	1.38	L-threonine aldolase O
410	1	db$2	2::C3K1L2|C3K1L2_PSEFS	213	22708	6	6	6	6	2.45	Ancillary SecYEG trans
411	1	db$2	2::C3JZ88|C3JZ88_PSEFS	212	47232	5	5	4	4	0.65	Putative exported prot
412	1	db$2	2::C3K4E2|C3K4E2_PSEFS	211	16860	8	8	7	7	5.95	N5-carboxyaminoimida
413	1	db$2	2::C3KAC7|C3KAC7_PSEFS	211	51379	12	8	11	7	0.90	UDP-glucose 6-dehydr
414	1	db$2	2::C3KEB7|C3KEB7_PSEFS	210	48036	10	10	9	9	1.67	Uncharacterized protei
415	1	db$2	2::C3KBN2|C3KBN2_PSEFS	210	52689	7	6	6	6	0.71	Outer membrane prote
416	1	db$2	2::C3KEC6|KAD_PSEFS	210	23251	10	9	7	7	4.05	Adenylate kinase OS=I
417	1	db$2	2::C3JYR3|C3JYR3_PSEFS	210	40135	10	10	7	7	1.56	Glycine cleavage syste
418	1	db$2	2::C3KA77|C3KA77_PSEFS	209	49108	7	6	7	6	0.78	Glutathione reductase
419	1	db$2	2::C3K0G3|PPNP_PSEFS	209	10127	7	6	4	4	8.84	Pyrimidine/purine nucl
420	1	db$2	2::C3K3U0|C3K3U0_PSEFS	208	51001	9	7	6	5	0.59	Putative glutamine syr
421	1	db$2	2::C3KED4|C3KED4_PSEFS	207	31038	7	7	6	6	1.48	4-hydroxy-tetrahydroc
422	1	db$2	2::C3K104|C3K104_PSEFS	207	26102	9	9	6	6	2.53	Putative membrane pr
423	1	db$2	2::C3K4V8|C3K4V8_PSEFS	207	128447	8	8	8	8	0.34	Transcription-repair-cc
424	1	db$2	2::C3JXY7|ASTD_PSEFS	207	51479	4	4	4	4	0.44	N-succinylglutamate 5
425	1	db$2	2::C3JYR4|C3JYR4_PSEFS	207	7675	5	5	3	3	5.01	Major cold shock prote
426	1	db$2	2::C3K1L0|C3K1L0_PSEFS	206	55309	12	11	12	11	1.56	GTPase Der OS=Pseud
427	1	db$2	2::C3K1F4|C3K1F4_PSEFS	206	31961	7	7	6	6	1.42	Probable chromosome-
428	1	db$2	2::C3K5L8|C3K5L8_PSEFS	206	39360	5	5	4	4	0.82	Phosphorous compoun
429	1	db$2	2::C3K0Q9|C3K0Q9_PSEFS	206	15688	5	5	4	4	2.30	Flagellar basal-body rc
430	1	db$2	2::C3K3V5|C3K3V5_PSEFS	206	52101	10	9	9	8	1.07	CYTH domain-containi
431	1	db$2	2::C3KDM7|C3KDM7_PSEFS	206	29519	8	8	7	7	2.05	Sulfurtransferase OS=
432	1	db$2	2::C3KBL7|C3KBL7_PSEFS	205	33413	9	8	8	7	1.69	Glutamate/aspartate A
433	1	db$2	2::C3JYD4|C3JYD4_PSEFS	204	64707	8	6	7	5	0.44	Glutamine--tRNA ligas
434	1	db$2	2::C3KCT1|MURC_PSEFS	204	52132	8	7	6	5	0.57	UDP-N-acetylmuramat
435	1	db$2	2::C3K7G7|C3K7G7_PSEFS	203	50136	7	7	6	6	0.76	Putative modulation of
436	1	db$2	2::C3K6X1|C3K6X1_PSEFS	203	39290	8	7	7	6	1.06	Uncharacterized protei
437	1	db$2	2::C3K1N4|C3K1N4_PSEFS	202	41398	8	8	5	5	0.98	Queuine tRNA-ribosylt
438	1	db$2	2::C3K1H6|PURT_PSEFS	201	42327	10	9	9	8	1.44	Formate-dependent ph
439	1	db$2	2::C3K469|RL28_PSEFS	201	8845	7	6	4	3	3.73	50S ribosomal protein
440	1	db$2	2::C3K9S0|NFUA_PSEFS	201	20961	6	6	6	6	2.83	Fe/S biogenesis protei
441	1	db$2	2::C3K6U2|C3K6U2_PSEFS	199	47692	6	6	5	5	0.64	Probable proton/glutar
442	1	db$2	2::C3K231|C3K231_PSEFS	199	57678	8	6	5	5	0.51	APH domain-containin
443	1	db$2	2::C3K6K6|C3K6K6_PSEFS	198	36089	12	10	11	9	2.25	HTH-type transcription
444	1	db$2	2::C3K1L3|SYH_PSEFS	198	47853	10	10	9	9	1.69	Histidine--tRNA ligase
445	1	db$2	2::C3KBY3|C3KBY3_PSEFS	197	64998	4	4	4	4	0.34	Putative lipoprotein OS
446	1	db$2	2::C3K116|PSRP_PSEFS	197	30838	6	6	6	6	1.50	Putative phosphoenolp

↑Family	M	DB	Accession	Score	Mass	Matches	Match(sig)	Sequences	Seq(sig)	emPAI	Description
447	1	db$2	2::C3K2L2|SYL_PSEFS	196	97066	11	10	9	9	0.63	Leucine--tRNA ligase C
448	1	db$2	2::C3K5L3|C3K5L3_PSEFS	195	36401	6	5	5	4	0.68	Phosphonates ABC trai
449	1	db$2	2::C3K2E3|MSCL_PSEFS	193	14568	5	5	3	3	2.60	Large-conductance me
450	1	db$2	2::C3KCB7|C3KCB7_PSEFS	193	34191	5	4	3	2	0.51	Phosphate transport sy
451	1	db$2	2::C3KDM2|C3KDM2_PSEFS	192	83508	10	9	10	9	0.67	DNA topoisomerase 4 :
452	1	db$2	2::C3KE83|C3KE83_PSEFS	191	38700	9	8	7	6	1.08	Outer membrane prote
453	1	db$2	2::C3KE59|C3KE59_PSEFS	190	27398	14	13	10	10	5.61	DNA-binding response
454	1	db$2	2::C3K3T9|C3K3T9_PSEFS	189	49785	6	6	4	4	0.61	Putative aminotransfer
455	1	db$2	2::C3K264|GLMM_PSEFS	189	47719	10	10	8	8	1.69	Phosphoglucosamine n
456	1	db$2	2::C3JXZ8|C3JXZ8_PSEFS	188	71568	10	10	10	10	0.94	Acetyl-coenzyme A syr
457	1	db$2	2::C3K5J3|C3K5J3_PSEFS	188	50388	6	6	6	6	0.76	AMP nucleosidase OS=
458	1	db$2	2::C3JXQ0|C3JXQ0_PSEFS	184	24880	8	7	7	6	2.11	Putative hydrolase OS:
459	1	db$2	2::C3K270|C3K270_PSEFS	184	17315	10	9	7	6	5.62	Transcription elongatic
460	1	db$2	2::C3K610|C3K610_PSEFS	183	70283	17	14	16	14	1.57	ATP-binding protein Uu
461	1	db$2	2::C3K6Y3|GATB_PSEFS	183	52868	12	10	9	7	1.05	Aspartyl/glutamyl-tRN.
462	1	db$2	2::C3K187|C3K187_PSEFS	182	38556	10	9	9	8	2.01	Peptidase_M75 domair
463	1	db$2	2::C3JZQ0|C3JZQ0_PSEFS	182	38665	8	8	7	7	1.66	Putative L-asparaginas
464	1	db$2	2::C3K185|C3K185_PSEFS	182	20062	7	7	5	5	4.12	Putative NAD(P)H nitrc
465	1	db$2	2::C3K572|C3K572_PSEFS	182	7891	2	2	2	2	2.20	Uncharacterized protei
466	1	db$2	2::C3K9P3|C3K9P3_PSEFS	181	9882	7	7	4	4	5.48	Peptidyl-prolyl cis-tran
467	1	db$2	2::C3KAC2|C3KAC2_PSEFS	181	41342	5	5	5	5	0.77	Acyl-CoA dehydrogena
468	1	db$2	2::C3K2S1|C3K2S1_PSEFS	181	23236	8	7	6	5	1.75	Riboflavin synthase alp
469	1	db$2	2::C3K4G1|C3K4G1_PSEFS	181	81186	10	6	10	6	0.42	DNA helicase OS=Pseu
470	1	db$2	2::C3JYN4|ZIPA_PSEFS	181	31818	7	6	5	4	0.81	Cell division protein Zi
471	1	db$2	2::C3K8Z6|C3K8Z6_PSEFS	178	39611	8	7	6	5	1.04	Branched amino acid A
472	1	db$2	2::C3KCS2|RSMH_PSEFS	178	34344	8	8	8	8	2.00	Ribosomal RNA small s
473	1	db$2	2::C3K2K2|THIE_PSEFS	176	22345	4	4	3	3	0.88	Thiamine-phosphate sy
474	1	db$2	2::C3K3D2|BETB_PSEFS	176	53123	9	9	8	8	1.04	Betaine aldehyde dehy
475	1	db$2	2::C3K5H8|C3K5H8_PSEFS	176	17826	6	6	6	6	3.81	STAS domain-containi
476	1	db$2	2::C3K843|C3K843_PSEFS	176	26535	10	9	6	5	1.43	Lipopolysaccharide exp
477	1	db$2	2::C3KCL0|C3KCL0_PSEFS	174	35424	6	6	5	5	0.95	Uncharacterized protei
478	1	db$2	2::C3K839|Y879_PSEFS	174	32120	7	6	7	6	1.42	Nucleotide-binding pro
479	1	db$2	2::C3JYE4|C3JYE4_PSEFS	173	30882	16	15	12	11	4.36	Exodeoxyribonuclease
480	1	db$2	2::C3JXY8|C3JXY8_PSEFS	172	37476	7	6	7	6	1.13	Arginine N-succinyltrai
481	1	db$2	2::C3KCT0|MURG_PSEFS	172	37667	6	6	6	6	1.12	UDP-N-acetylglucosam
482	1	db$2	2::C3K0N5|C3K0N5_PSEFS	172	32402	7	7	5	5	1.07	Malonyl CoA-acyl carri
483	1	db$2	2::C3KA84|C3KA84_PSEFS	172	13408	7	6	5	5	4.65	Putative regulatory prc
484	1	db$2	2::C3JYJ6|C3JYJ6_PSEFS	171	68407	10	9	8	7	0.62	Peptidyl-prolyl cis-tran
485	1	db$2	2::C3KBL3|C3KBL3_PSEFS	171	50616	10	10	8	8	1.11	Putative exported prot
486	1	db$2	2::C3K437|C3K437_PSEFS	170	26506	2	2	1	1	0.19	Uncharacterized protei

↑Family	M	DB	Accession	Score	Mass	Matches	Match(sig)	Sequences	Seq(sig)	emPAI	Description
487	1	db$2	2::C3K017|PYRD_PSEFS	170	35850	9	9	8	8	2.27	Dihydroorotate dehydr
488	1	db$2	2::C3K3W7|C3K3W7_PSEFS	170	36662	10	7	9	6	1.17	Putative iron (III) ABC
489	1	db$2	2::C3K6M3|C3K6M3_PSEFS	170	13365	8	8	6	6	7.13	Succinate dehydrogena
490	1	db$2	2::C3JXX2|C3JXX2_PSEFS	170	50328	6	6	6	6	0.76	ATP-dependent RNA he
491	1	db$2	2::C3K3P4|C3K3P4_PSEFS	169	39333	7	7	6	6	1.06	Uncharacterized protei
492	1	db$2	2::C3K7M9|C3K7M9_PSEFS	168	34271	7	7	7	7	1.62	Putative ABC transport
493	1	db$2	2::C3JY82|NUOB_PSEFS	168	25341	7	6	7	6	2.05	NADH-quinone oxidore
494	1	db$2	2::C3K3M3|C3K3M3_PSEFS	168	24540	5	5	4	4	1.15	Cell division ATP-bindi
495	1	db$2	2::C3KE48|C3KE48_PSEFS	167	19341	5	5	4	4	1.64	Peptide deformylase O
496	1	db$2	2::C3JZU0|C3JZU0_PSEFS	167	50538	8	7	8	7	0.93	3-isopropylmalate deh
497	1	db$2	2::C3K3G5|C3K3G5_PSEFS	166	15160	6	6	5	5	5.30	Putative thioesterase (
498	1	db$2	2::C3K0T1|C3K0T1_PSEFS	165	94111	8	7	8	7	0.42	Aconitate hydratase O
499	1	db$2	2::C3K4H9|C3K4H9_PSEFS	165	34203	7	5	7	5	0.99	Quinone oxidoreductas
500	1	db$2	2::C3K581|C3K581_PSEFS	165	86479	9	8	9	8	0.55	Putative molybdopterir
501	1	db$2	2::C3KDL6|C3KDL6_PSEFS	165	23139	4	4	4	4	1.26	ADP-ribose diphosphat
502	1	db$2	2::C3K611|C3K611_PSEFS	165	16065	7	7	5	5	4.70	Universal stress protei
503	1	db$2	2::C3K6L0|EFP_PSEFS	164	21303	6	6	5	5	2.01	Elongation factor P OS
504	1	db$2	2::C3JZ86|C3JZ86_PSEFS	164	21964	10	7	8	7	3.46	Superoxide dismutase
505	1	db$2	2::C3K417|C3K417_PSEFS	164	39396	4	4	3	3	0.43	Transcriptional regulat
506	1	db$2	2::C3K5H7|C3K5H7_PSEFS	164	43348	5	4	5	4	0.55	Putative response regu
507	1	db$2	2::C3K3S3|C3K3S3_PSEFS	163	15261	6	5	2	2	0.85	DUF4399 domain-cont
508	1	db$2	2::C3JYV5|C3JYV5_PSEFS	163	45487	7	6	6	5	0.87	Putative oxidoreductas
509	1	db$2	2::C3K1M9|C3K1M9_PSEFS	161	29586	8	7	7	6	1.60	Inositol-1-monophospl
510	1	db$2	2::C3K6Z7|C3K6Z7_PSEFS	160	89469	9	6	9	6	0.37	Lon protease OS=Pseu
511	1	db$2	2::C3KBZ0|PYRC_PSEFS	160	38385	5	5	5	5	0.85	Dihydroorotase OS=Ps
512	1	db$2	2::C3K371|C3K371_PSEFS	160	78727	10	7	10	7	0.52	Malate synthase G OS
513	1	db$2	2::C3K084|AROC_PSEFS	159	39094	7	7	6	6	1.07	Chorismate synthase (
514	1	db$2	2::C3K859|C3K859_PSEFS	159	59064	6	6	6	6	0.62	Putative glutamate syr
515	1	db$2	2::C3JYU1|Y4927_PSEFS	159	18225	8	6	7	5	2.62	UPF0234 protein PFLU
516	1	db$2	2::C3K4Q9|C3K4Q9_PSEFS	159	23656	3	3	3	3	0.82	Putative exported prot
517	1	db$2	2::C3K5E5|C3K5E5_PSEFS	158	28503	8	8	7	7	2.75	Methionine aminopepti
518	1	db$2	2::C3K2V3|RS11_PSEFS	158	13607	5	5	4	4	2.91	30S ribosomal protein
519	1	SwissProt_HUMAN$	3::P62937|PPIA_HUMAN	156	18001	5	5	5	5	2.67	Peptidyl-prolyl cis-tran
520	1	SwissProt_HUMAN$	3::P62805|H4_HUMAN	155	11360	7	7	6	6	10.66	Histone H4 OS=Homo
521	1	db$2	2::C3K9Z4|C3K9Z4_PSEFS	155	58133	8	8	7	7	0.77	Putative ABC tranmspo
522	1	db$2	2::C3K758|PYRF_PSEFS	155	24706	7	5	7	5	1.58	Orotidine 5'-phosphate
523	1	db$2	2::C3JY91|C3JY91_PSEFS	154	66398	5	5	3	3	0.24	NADH-quinone oxidore
524	1	db$2	2::C3K5K4|C3K5K4_PSEFS	154	29062	5	5	4	4	0.91	Uncharacterized protei
525	1	db$2	2::C3K3M6|C3K3M6_PSEFS	154	53012	6	5	6	5	0.56	Peptidase_M16_C dom
526	1	db$2	2::C3K2X2|RS19_PSEFS	154	10313	7	7	5	5	8.38	30S ribosomal protein

↑Family	M	DB	Accession	Score	Mass	Matches	Match(sig)	Sequences	Seq(sig)	emPAI	Description
527	1	db$2	2::C3KAD3|ARNC_PSEFS	154	37700	4	4	4	4	0.65	Undecaprenyl-phospha
528	1	db$2	2::C3KBN4|C3KBN4_PSEFS	153	40532	8	8	8	8	1.54	Acriflavine resistance p
529	1	db$2	2::C3K1K8|C3K1K8_PSEFS	153	31523	7	6	5	5	1.11	Putative hydrolase OS=
530	1	db$2	2::C3K747|GPDA_PSEFS	153	36476	8	6	7	6	1.17	Glycerol-3-phosphate (
531	1	db$2	2::C3K606|FABZ_PSEFS	153	16691	6	6	6	6	4.39	3-hydroxyacyl-[acyl-ca
532	1	db$2	2::C3K6K4|C3K6K4_PSEFS	153	32908	6	6	6	6	1.37	Putative 5'-nucleotidas
533	1	db$2	2::C3JYS3|C3JYS3_PSEFS	153	38665	2	2	2	2	0.28	TolA colicin import me
534	1	db$2	2::C3K7I9|C3K7I9_PSEFS	152	88241	5	5	5	5	0.31	Putative pinecillin acyl
535	1	db$2	2::C3K2C9|C3K2C9_PSEFS	152	48295	9	9	8	8	1.19	Glutamate dehydroger
536	1	db$2	2::C3JYI3|PHNX_PSEFS	152	29973	7	5	5	4	1.19	Phosphonoacetaldehyc
537	1	db$2	2::C3K6F9|C3K6F9_PSEFS	152	37389	8	7	8	7	1.42	Nucleoid-associated pr
538	1	db$2	2::C3K467|C3K467_PSEFS	151	13453	3	3	2	2	1.00	Cupin_3 domain-conta
539	1	db$2	2::C3KDK8|C3KDK8_PSEFS	150	41619	4	4	4	4	0.57	DAO domain-containin
540	1	db$2	2::C3JYT6|C3JYT6_PSEFS	150	8291	8	8	6	6	46.47	Cold shock protein csp
541	1	db$2	2::C3K2W9|RL16_PSEFS	150	15422	6	5	6	5	3.54	50S ribosomal protein
542	1	db$2	2::C3K815|C3K815_PSEFS	150	62777	11	7	9	6	0.57	Glucans biosynthesis p
543	1	db$2	2::C3JZV9|C3JZV9_PSEFS	150	34932	4	4	3	3	0.50	Putative DNA/RNA non
544	1	db$2	2::C3K2U5|C3K2U5_PSEFS	150	18989	5	5	4	4	1.68	Single-stranded DNA-b
545	1	db$2	2::C3K2N8|C3K2N8_PSEFS	149	23444	10	9	8	7	3.98	Semialdhyde_dh doma
546	1	db$2	2::C3JZS5|TRPF_PSEFS	149	21966	3	3	3	3	0.90	N-(5'-phosphoribosyl)a
547	1	db$2	2::C3KDW7|HISZ_PSEFS	149	42687	9	7	8	6	0.94	ATP phosphoribosyltra
548	1	db$2	2::C3K740|C3K740_PSEFS	148	33777	12	9	11	8	2.06	DUF815 domain-conta
549	1	db$2	2::C3K2U6|C3K2U6_PSEFS	148	49484	3	3	2	2	0.33	Putative transport-rela
550	1	db$2	2::C3K170|C3K170_PSEFS	147	22875	5	5	4	4	1.27	Flavin_Reduct domain-
551	1	db$2	2::C3KED5|C3KED5_PSEFS	147	40447	6	6	6	6	1.02	Putative lipoprotein OS
552	1	db$2	2::C3K276|GRPE_PSEFS	147	20714	4	3	3	2	0.57	Protein GrpE OS=Pseu
553	1	db$2	2::C3JZT9|C3JZT9_PSEFS	146	24210	7	6	7	6	2.21	3-isopropylmalate deh
554	1	db$2	2::C3K0S3|C3K0S3_PSEFS	146	67491	7	7	7	7	0.63	Putative ATP-binding c
555	1	db$2	2::C3KAJ8|UPP_PSEFS	146	22986	8	7	8	7	3.18	Uracil phosphoribosyltı
556	1	db$2	2::C3KBZ7|SYM_PSEFS	146	75525	8	7	7	6	0.46	Methionine--tRNA ligas
557	1	db$2	2::C3K0T3|C3K0T3_PSEFS	146	28958	4	4	4	4	0.92	Putative membrane pr
558	1	db$2	2::C3K5X2|C3K5X2_PSEFS	145	20559	5	4	4	3	0.98	Putative two-compone
559	1	SwissProt_HUMAN$	3::P62979|RS27A_HUMAN	145	17953	4	4	4	4	1.83	Ubiquitin-40S ribosom
560	1	db$2	2::C3K9M2|C3K9M2_PSEFS	145	37830	6	6	5	5	0.87	Putative peptidase OS=
561	1	db$2	2::C3K177|C3K177_PSEFS	145	65030	9	9	9	9	0.93	Phosphogluconate deh
562	1	db$2	2::C3KDQ9|C3KDQ9_PSEFS	144	29676	10	10	10	10	3.90	Ferredoxin--NADP redu
563	1	db$2	2::C3K472|C3K472_PSEFS	144	42804	6	5	6	5	0.74	Coenzyme A biosynthe
564	1	db$2	2::C3K1X5|C3K1X5_PSEFS	143	39754	7	5	5	4	0.61	Glutamate 5-kinase OS
565	1	db$2	2::C3K6J7|C3K6J7_PSEFS	143	39784	8	6	8	6	1.04	Histidinol-phosphate a
566	1	db$2	2::C3K2S3|C3K2S3_PSEFS	143	17933	6	6	5	5	3.77	Transcriptional repress

↑Family	M	DB	Accession	Score	Mass	Matches	Match(sig)	Sequences	Seq(sig)	emPAI	Description
567	1	db$2	2::C3K8K5|C3K8K5_PSEFS	142	64377	2	2	2	2	0.16	Putative transport-rela
568	1	db$2	2::C3KAY1|C3KAY1_PSEFS	142	44657	5	5	5	5	0.70	3-phosphoshikimate 1-
569	1	db$2	2::C3K527|C3K527_PSEFS	142	65564	7	6	6	5	0.43	Putative peptidase OS=
570	1	db$2	2::C3K0N7|RL32_PSEFS	141	6752	4	4	2	2	2.84	50S ribosomal protein
571	1	db$2	2::C3K4Z4|C3K4Z4_PSEFS	141	43117	6	6	5	5	0.73	Putative cytochrome C
572	1	db$2	2::C3K6U8|C3K6U8_PSEFS	141	58654	5	4	3	3	0.27	Putative membrane pr
573	1	db$2	2::C3K575|C3K575_PSEFS	141	14827	4	3	4	3	1.56	Osmotically inducible p
574	1	db$2	2::C3K796|C3K796_PSEFS	141	49835	7	7	7	7	0.94	Putative glutamine syr
575	1	db$2	2::C3K1U9|C3K1U9_PSEFS	140	42728	5	5	4	4	0.74	Flavohemoglobin OS=I
576	1	db$2	2::C3KED3|C3KED3_PSEFS	140	20551	5	5	4	4	2.13	Glycine cleavage syste
577	1	db$2	2::C3K1V6|C3K1V6_PSEFS	139	32555	4	3	4	3	0.54	Putative short-chain de
578	1	db$2	2::C3KBP9|C3KBP9_PSEFS	139	58780	6	6	5	5	0.50	Phosphoglucomutase (
579	1	db$2	2::C3KAJ1|C3KAJ1_PSEFS	139	48585	6	6	6	6	0.79	Omega-amino acid--py
580	1	db$2	2::C3K5H5|C3K5H5_PSEFS	139	25721	7	7	5	5	1.99	Putative exported prot
581	1	db$2	2::C3K368|C3K368_PSEFS	139	37353	4	3	4	3	0.46	Stress response kinase
582	1	db$2	2::C3KD21|C3KD21_PSEFS	138	81698	5	5	4	4	0.26	Putative glycogen debr
583	1	db$2	2::C3JXZ2|C3JXZ2_PSEFS	138	36334	5	5	4	4	0.68	Putative AraC family re
584	1	db$2	2::C3K3M9|C3K3M9_PSEFS	138	30473	4	4	4	4	0.85	Putative sulfurtransfer
585	1	db$2	2::C3K309|C3K309_PSEFS	138	21661	2	2	2	2	0.54	Anthranilate synthase
586	1	db$2	2::C3K152|C3K152_PSEFS	138	13999	8	7	7	6	6.37	Putative transcriptiona
587	1	db$2	2::C3KAI4|C3KAI4_PSEFS	138	53827	5	5	4	4	0.55	SPOR domain-containi
588	1	SwissProt_HUMAN$	3::P23528|COF1_HUMAN	138	18491	5	5	5	5	2.54	Cofilin-1 OS=Homo sa
589	1	db$2	2::C3K4U5|C3K4U5_PSEFS	137	75901	7	6	6	5	0.37	Putative protease OS=
590	1	db$2	2::C3K9U7|C3K9U7_PSEFS	137	56147	6	6	6	6	0.66	Glucose-6-phosphate 1
591	1	db$2	2::C3K4G2|C3K4G2_PSEFS	137	30728	6	6	3	3	1.15	Putative exported prot
592	1	db$2	2::C3K2X4|RL23_PSEFS	136	10938	8	8	7	7	18.10	50S ribosomal protein
593	1	db$2	2::C3K6Z0|PDXJ_PSEFS	136	26758	5	5	4	4	1.02	Pyridoxine 5'-phosphat
594	1	db$2	2::C3JZ43|C3JZ43_PSEFS	136	44885	5	5	5	5	0.69	Putative aminotransfer
595	1	db$2	2::C3JY61|C3JY61_PSEFS	136	8297	4	4	4	4	8.08	Translation initiation fa
596	1	db$2	2::C3K272|C3K272_PSEFS	136	40774	7	5	6	5	0.79	Carbamoyl-phosphate
597	1	db$2	2::C3K5M0|C3K5M0_PSEFS	136	29300	6	5	5	5	1.23	Phosphonates ABC trai
598	1	db$2	2::C3K1H1|RS16_PSEFS	136	9202	7	7	5	5	11.18	30S ribosomal protein
599	1	db$2	2::C3KDC4|C3KDC4_PSEFS	136	64677	4	4	4	4	0.34	Uncharacterized protei
600	1	db$2	2::C3K868|C3K868_PSEFS	135	58117	7	7	6	6	0.63	Uncharacterized protei
601	1	db$2	2::C3K6V9|C3K6V9_PSEFS	135	54793	6	5	5	4	0.41	2,3-bisphosphoglycera
602	1	db$2	2::C3K8W7|C3K8W7_PSEFS	134	34291	4	4	4	4	0.73	Arabinose 5-phosphate
603	1	db$2	2::C3K7I1|C3K7I1_PSEFS	134	85859	7	7	6	6	0.47	Quinoprotein glucose c
604	1	db$2	2::C3K240|C3K240_PSEFS	134	17616	7	7	5	5	5.39	RNA polymerase-bindi
605	1	db$2	2::C3JYK0|CLPP_PSEFS	134	23405	8	8	6	6	3.07	ATP-dependent Clp pro
606	1	db$2	2::C3K256|RS15_PSEFS	133	10112	4	4	3	3	2.94	30S ribosomal protein

↑Family	M	DB	Accession	Score	Mass	Matches	Match(sig)	Sequences	Seq(sig)	emPAI	Description
607	1	db$2	2::C3KAH8|C3KAH8_PSEFS	133	89028	8	7	7	6	0.45	DD-transpeptidase OS
608	1	db$2	2::C3K5E8|C3K5E8_PSEFS	133	28997	4	4	4	4	0.92	Cyclohexadienyl dehyc
609	1	db$2	2::C3K1N3|C3K1N3_PSEFS	132	11888	5	5	3	3	2.23	Sec translocon accessc
610	1	db$2	2::C3K3S0|C3K3S0_PSEFS	132	23721	5	5	3	3	0.81	Putative hydrolase OS
611	1	db$2	2::C3KA90|C3KA90_PSEFS	132	36984	4	3	4	3	0.47	Putative aldo/keto redu
612	1	db$2	2::C3KE80|C3KE80_PSEFS	131	46502	6	6	6	6	0.84	Nicotinate phosphoribc
613	1	db$2	2::C3K2Y1|RS12_PSEFS	131	13716	3	3	2	2	0.97	30S ribosomal protein
614	1	db$2	2::C3KCR9|PTH_PSEFS	131	21041	4	4	4	4	1.43	Peptidyl-tRNA hydrolas
615	1	db$2	2::C3K480|C3K480_PSEFS	131	31946	2	2	1	1	0.16	Uncharacterized protei
616	1	db$2	2::C3K6Y4|GATA_PSEFS	131	51737	5	5	5	5	0.58	Glutamyl-tRNA(Gln) ar
617	1	db$2	2::C3K6E5|C3K6E5_PSEFS	130	29069	4	4	4	4	0.91	Cytochrome c1 OS=Ps
618	1	db$2	2::C3KBQ2|C3KBQ2_PSEFS	130	37020	6	4	5	4	0.66	Putative quinone oxidc
619	1	db$2	2::C3JXY9|C3JXY9_PSEFS	130	37073	5	5	4	4	0.66	Putative arginine N-su
620	1	db$2	2::C3K4C1|C3K4C1_PSEFS	129	40318	4	4	3	3	0.42	Putative nitric oxide de
621	1	db$2	2::C3K5N1|C3K5N1_PSEFS	129	36842	8	8	6	6	1.16	Sulfate ABC transport
622	1	db$2	2::Q84CP9|DAPB_PSEFS	129	28276	9	9	7	7	2.78	4-hydroxy-tetrahydroc
623	1	db$2	2::C3JYD3|SYC_PSEFS	129	51635	8	7	8	7	0.90	Cysteine--tRNA ligase
624	1	db$2	2::C3JY36|C3JY36_PSEFS	129	16095	6	6	5	5	3.27	Uncharacterized protei
625	1	db$2	2::C3K5F4|C3K5F4_PSEFS	128	21753	3	3	3	3	0.91	Putative nitroreductase
626	1	db$2	2::C3K2E9|C3K2E9_PSEFS	128	53251	2	2	2	2	0.19	Putative cytochrome o
627	1	db$2	2::C3JZ77|C3JZ77_PSEFS	128	45611	7	7	6	6	0.86	Maltose-binding protei
628	1	db$2	2::C3JXY4|ASTE_PSEFS	128	37404	9	8	7	7	1.42	Succinylglutamate des
629	1	db$2	2::C3K910|C3K910_PSEFS	128	36322	6	6	5	5	0.92	Putative ABC transport
630	1	db$2	2::C3JZV1|C3JZV1_PSEFS	128	76010	8	7	8	7	0.55	UvrABC system proteir
631	1	db$2	2::C3KED2|C3KED2_PSEFS	128	17293	4	4	4	4	1.95	Thioredoxin peroxidase
632	1	db$2	2::C3KEA8|C3KEA8_PSEFS	128	45495	5	5	4	4	0.52	ATP-dependent RNA he
633	1	db$2	2::C3K427|C3K427_PSEFS	127	23927	4	4	3	3	0.80	Putative glutathione S-
634	1	db$2	2::C3K813|C3K813_PSEFS	127	36618	6	5	6	5	0.90	Proline iminopeptidase
635	1	db$2	2::C3K9F0|C3K9F0_PSEFS	127	40567	5	5	4	4	0.59	Putative transport-rela
636	1	db$2	2::C3K1M1|HSCA_PSEFS	127	65896	7	7	6	6	0.54	Chaperone protein Hsc
637	1	db$2	2::C3KCP1|C3KCP1_PSEFS	127	23476	3	3	3	3	0.83	Peptide methionine sul
638	1	db$2	2::C3K494|C3K494_PSEFS	127	33626	4	3	4	3	0.52	Gly-zipper_YMGG dom
639	1	db$2	2::C3K254|C3K254_PSEFS	127	21027	9	9	6	6	2.83	Osmotically inducible p
640	1	db$2	2::C3JY53|CYSG_PSEFS	126	50259	5	5	5	5	0.60	Siroheme synthase OS
641	1	db$2	2::C3KC10|C3KC10_PSEFS	126	23080	4	4	3	3	0.84	Uncharacterized protei
642	1	db$2	2::C3K4I0|HEM6_PSEFS	126	35313	9	7	9	7	1.55	Oxygen-dependent cop
643	1	db$2	2::C3KE78|C3KE78_PSEFS	126	15753	3	3	2	2	0.81	Azurin OS=Pseudomor
644	1	db$2	2::C3K5G1|C3K5G1_PSEFS	125	34097	5	5	4	4	1.00	Cysteine synthase OS=
645	1	db$2	2::C3K307|TRPC_PSEFS	125	30252	9	9	8	8	2.47	Indole-3-glycerol phos
646	1	db$2	2::C3JYI6|C3JYI6_PSEFS	125	73112	4	4	4	4	0.30	Putative 2,4-dienoyl-C

↑Family	M	DB	Accession	Score	Mass	Matches	Match(sig)	Sequences	Seq(sig)	emPAI	Description
647	1	db$2	2::C3K435|C3K435_PSEFS	124	45402	9	7	9	7	1.07	Diaminopimelate decar
648	1	db$2	2::C3K4T4|C3K4T4_PSEFS	124	21507	3	3	2	2	0.55	NAD(P)-bd_dom doma
649	1	db$2	2::C3K281|C3K281_PSEFS	124	15787	3	3	2	2	0.81	Ribosome association t
650	1	db$2	2::C3JZN4|RL20_PSEFS	123	13348	4	4	3	3	1.85	50S ribosomal protein
651	1	db$2	2::C3K9H7|C3K9H7_PSEFS	123	27490	3	2	3	2	0.41	Putative short-chain de
652	1	db$2	2::C3KCS0|RL25_PSEFS	123	21502	7	7	3	3	2.70	50S ribosomal protein
653	1	db$2	2::C3K4A8|C3K4A8_PSEFS	123	55832	6	6	6	6	0.66	Uncharacterized protei
654	1	db$2	2::C3JXZ3|C3JXZ3_PSEFS	123	28123	6	6	6	6	1.73	Histidine transport ATF
655	1	db$2	2::C3K4E4|C3K4E4_PSEFS	123	51389	7	6	6	6	0.74	Aspartate ammonia-ly
656	1	db$2	2::C3KE31|C3KE31_PSEFS	123	22237	8	6	6	5	1.88	RNA polymerase sigma
657	1	db$2	2::C3K2V4|RS13_PSEFS	123	13332	6	6	5	5	4.73	30S ribosomal protein
658	1	db$2	2::C3K470|C3K470_PSEFS	123	58011	6	5	6	5	0.50	Putative transport-rela
659	1	db$2	2::C3K648|C3K648_PSEFS	122	33905	5	4	5	4	0.75	Conserved hypothetica
660	1	db$2	2::C3K9R8|C3K9R8_PSEFS	122	135461	9	9	8	8	0.32	Methionine synthase O
661	1	db$2	2::C3K2R5|RIBA_PSEFS	122	22312	3	3	3	3	0.88	GTP cyclohydrolase-2 (
662	1	db$2	2::C3K5E4|GLND_PSEFS	122	102948	6	5	6	5	0.26	Bifunctional uridylyltra
663	1	db$2	2::C3K7E3|C3K7E3_PSEFS	122	178026	7	7	7	7	0.21	Alpha-2-macroglobulin
664	1	db$2	2::C3KAB1|C3KAB1_PSEFS	122	44397	5	5	4	4	0.53	Histidine kinase OS=P
665	1	db$2	2::C3K5K3|C3K5K3_PSEFS	122	28692	3	3	2	2	0.64	CHAD domain-containi
666	1	db$2	2::C3K6I1|C3K6I1_PSEFS	122	31224	6	6	6	6	1.48	S-formylglutathione hy
667	1	SwissProt_HUMAN$	3::Q06830|PRDX1_HUMAN	121	22096	7	7	4	4	1.89	Peroxiredoxin-1 OS=H
668	1	db$2	2::C3K644|C3K644_PSEFS	121	89774	7	6	6	5	0.30	Putative ABC transport
669	1	db$2	2::C3K4Y3|KDPA_PSEFS	121	60181	6	5	3	3	0.37	Potassium-transporting
670	1	db$2	2::C3KBK9|C3KBK9_PSEFS	121	34365	4	4	4	4	0.73	Glycerate dehydrogena
671	1	db$2	2::C3K4P8|C3K4P8_PSEFS	120	24008	3	3	2	2	0.48	t(6)A37 threonylcarba
672	1	db$2	2::C3K1X7|RL27_PSEFS	120	9214	6	6	3	3	6.39	50S ribosomal protein
673	1	db$2	2::C3KDM4|C3KDM4_PSEFS	120	56715	6	4	6	4	0.40	Putative membrane pr
674	1	db$2	2::C3JXY5|C3JXY5_PSEFS	120	10610	4	3	2	2	1.38	Uncharacterized protei
675	1	db$2	2::C3KE87|C3KE87_PSEFS	119	46792	4	4	4	4	0.50	NADH dehydrogenase
676	1	db$2	2::C3KDE9|C3KDE9_PSEFS	119	41781	5	4	5	4	0.57	Putative phage tail-rel
677	1	db$2	2::C3K6E3|C3K6E3_PSEFS	119	21087	6	5	4	3	1.43	Ubiquinol-cytochrome
678	1	db$2	2::C3K568|TRPA_PSEFS	119	28539	5	4	3	3	0.64	Tryptophan synthase a
679	1	db$2	2::C3KAM2|C3KAM2_PSEFS	119	49640	2	2	2	2	0.21	Putative two-compone
680	1	db$2	2::C3KDU8|C3KDU8_PSEFS	118	35029	6	6	4	4	0.71	D-cysteine desulfhydra
681	1	db$2	2::C3JYN9|C3JYN9_PSEFS	118	44994	5	5	5	5	0.69	Pseudouridine synthas
682	1	db$2	2::C3K968|C3K968_PSEFS	118	55197	5	5	5	5	0.54	Putative aldehyde deh
683	1	db$2	2::C3JY57|LOLA_PSEFS	118	22546	7	5	7	5	1.83	Outer-membrane lipop
684	1	db$2	2::C3K0K7|C3K0K7_PSEFS	118	60595	10	9	9	9	1.02	Electron transfer flavo
685	1	db$2	2::C3JY78|C3JY78_PSEFS	118	22781	4	4	2	2	0.86	Putative cytochrome o
686	1	db$2	2::C3KBW8|C3KBW8_PSEFS	117	39031	7	6	7	6	1.07	D-alanine--D-alanine l

↑Family	M	DB	Accession	Score	Mass	Matches	Match(sig)	Sequences	Seq(sig)	emPAI	Description
687	1	db$2	2::C3KE68|RS18_PSEFS	117	8912	8	7	5	4	12.34	30S ribosomal protein
688	1	db$2	2::C3KBB8|C3KBB8_PSEFS	116	13546	5	4	4	3	2.95	Uncharacterized protei
689	1	SwissProt_HUMAN$	3::P62249|RS16_HUMAN	116	16435	5	5	4	4	2.12	40S ribosomal protein
690	1	db$2	2::C3K3J3|C3K3J3_PSEFS	116	72855	2	2	1	1	0.07	Type IV pilus biogenes
691	1	db$2	2::C3K6S9|C3K6S9_PSEFS	116	101727	6	5	5	5	0.26	DNA polymerase I OS=
692	1	db$2	2::C3JY88|C3JY88_PSEFS	116	20432	9	8	6	5	2.97	NADH-quinone oxidore
693	1	db$2	2::C3KE58|C3KE58_PSEFS	115	84757	7	6	6	6	0.40	Putative S1 RNA-bindi
694	1	db$2	2::C3K8K8|C3K8K8_PSEFS	115	62203	5	5	5	5	0.46	Putative exported prot
695	1	db$2	2::C3K4E1|C3K4E1_PSEFS	114	38799	5	5	5	5	0.84	N5-carboxyaminoimida
696	1	db$2	2::C3K3Q1|C3K3Q1_PSEFS	114	40925	4	4	4	4	0.59	Putative oxidoreductas
697	1	db$2	2::C3JY87|NUOH_PSEFS	114	37530	5	5	4	4	0.88	NADH-quinone oxidore
698	1	db$2	2::C3KDL3|C3KDL3_PSEFS	114	70148	7	5	6	4	0.31	Phosphomethylpyrimid
699	1	db$2	2::C3K197|C3K197_PSEFS	114	25331	4	4	3	3	0.75	Putative two-compone
700	1	db$2	2::C3K0V5|C3K0V5_PSEFS	114	33212	7	6	5	4	1.03	Putative keto/oxo acyl
701	1	db$2	2::C3K3M7|C3K3M7_PSEFS	113	22464	2	2	1	1	0.23	Ribosomal RNA small s
702	1	db$2	2::C3K3L5|C3K3L5_PSEFS	113	11400	3	3	2	2	2.37	Putative membrane pr
703	1	db$2	2::C3KDB8|HLDE_PSEFS	113	49913	5	5	5	5	0.61	Bifunctional protein Hl
704	1	db$2	2::C3K0I1|C3K0I1_PSEFS	113	29699	5	3	4	2	0.61	ParA family ATPase OS
705	1	db$2	2::C3KDB9|C3KDB9_PSEFS	112	32977	4	4	3	3	0.77	Putative lipopolysaccha
706	1	db$2	2::C3K302|C3K302_PSEFS	112	12723	7	6	4	4	3.29	Putative HIT domain-c
707	1	db$2	2::C3K5Y8|C3K5Y8_PSEFS	112	48751	5	5	5	5	0.62	L-serine dehydratase (
708	1	SwissProt_HUMAN$	3::P60174|TPIS_HUMAN	112	26653	5	4	5	4	1.03	Triosephosphate isome
709	1	db$2	2::C3K3W5|C3K3W5_PSEFS	112	39645	7	7	5	5	0.81	Aminomethyltransfera:
710	1	db$2	2::C3K0H6|C3K0H6_PSEFS	111	40054	5	4	5	4	0.60	Protein-glutamate met
711	1	db$2	2::C3K703|ISPF_PSEFS	111	16742	3	3	3	3	1.31	2-C-methyl-D-erythrit
712	1	db$2	2::C3KCS6|C3KCS6_PSEFS	111	47227	3	3	3	3	0.35	UDP-N-acetylmuramoy
713	1	db$2	2::C3KBH8|C3KBH8_PSEFS	110	25745	4	4	4	4	1.08	3-oxoacyl-[acyl-carrier
714	1	db$2	2::C3JYI0|C3JYI0_PSEFS	110	25235	5	4	4	3	0.75	Probable transcriptiona
715	1	db$2	2::C3KA81|C3KA81_PSEFS	110	55484	5	5	5	5	0.53	Alkyl hydroperoxide re
716	1	db$2	2::C3KBY9|C3KBY9_PSEFS	110	24532	4	4	4	4	1.15	Ribonuclease T OS=Ps
717	1	db$2	2::C3K6V2|HIS4_PSEFS	110	25679	6	6	6	6	2.01	1-(5-phosphoribosyl)-!
718	1	db$2	2::C3K3L1|C3K3L1_PSEFS	109	23430	3	3	3	3	0.83	Uncharacterized protei
719	1	db$2	2::C3JYE9|C3JYE9_PSEFS	109	31035	3	3	3	3	0.58	Putative hydroxymethy
720	1	db$2	2::C3KE99|C3KE99_PSEFS	109	30145	3	3	3	3	0.60	Quinolinate phosphorit
721	1	db$2	2::C3K0W5|PHS_PSEFS	109	13357	5	4	4	3	3.04	Putative pterin-4-alpha
722	1	db$2	2::C3K4A7|C3K4A7_PSEFS	109	18391	5	4	4	4	1.76	Uncharacterized protei
723	1	db$2	2::C3K7Z1|C3K7Z1_PSEFS	109	38822	5	5	5	5	0.84	NAD(P) transhydrogen
724	1	db$2	2::C3KBJ5|C3KBJ5_PSEFS	108	62095	3	3	3	3	0.26	Phospholipase C OS=P
725	1	db$2	2::C3KBV0|C3KBV0_PSEFS	108	28603	4	4	3	3	0.64	Putative chromosome
726	1	db$2	2::C3K8P0|C3K8P0_PSEFS	108	38329	5	5	4	4	0.64	Putative nucleoside-se

↑Family	M	DB	Accession	Score	Mass	Matches	Match(sig)	Sequences	Seq(sig)	emPAI	Description
727	1	db$2	2::C3JXY6|ASTB_PSEFS	107	48741	7	5	6	5	0.62	N-succinylarginine dihy
728	1	db$2	2::C3K4U6|C3K4U6_PSEFS	107	17513	2	2	1	1	0.31	Conserved hypothetica
729	1	db$2	2::C3K6T0|ENGB_PSEFS	107	23537	4	4	3	3	0.82	Probable GTP-binding
730	1	db$2	2::C3K709|C3K709_PSEFS	107	7722	4	4	3	3	4.86	Cold shock protein OS
731	1	db$2	2::C3KBB4|OADC_PSEFS	107	31135	3	3	2	2	0.35	Oxaloacetate decarbox
732	1	db$2	2::C3K424|HEM3_PSEFS	106	33541	3	3	3	3	0.53	Porphobilinogen deami
733	1	db$2	2::C3K785|C3K785_PSEFS	106	24962	5	5	5	5	1.56	3-oxoadipate CoA-tran
734	1	db$2	2::C3K617|C3K617_PSEFS	106	19384	5	4	4	4	1.62	Uncharacterized protei
735	1	db$2	2::C3K2M7|C3K2M7_PSEFS	105	18106	3	3	2	2	0.68	Ribosomal silencing fa
736	1	db$2	2::C3JXJ5|C3JXJ5_PSEFS	105	22111	4	4	3	3	0.89	Putative SCO1/SenC fa
737	1	db$2	2::C3K230|C3K230_PSEFS	105	85084	3	3	3	3	0.18	Penicillin-binding prote
738	1	db$2	2::C3KDN7|HEMH_PSEFS	105	38415	6	5	6	5	0.85	Ferrochelatase OS=Ps
739	1	db$2	2::C3K2I0|C3K2I0_PSEFS	105	48538	5	5	5	5	0.63	Putative ATP-depender
740	1	db$2	2::C3K2W2|RS8_PSEFS	105	14083	6	6	4	4	4.22	30S ribosomal protein
741	1	db$2	2::C3K6S5|C3K6S5_PSEFS	105	35353	5	3	4	3	0.49	Delta-aminolevulinic a
742	1	db$2	2::C3K2W8|RL29_PSEFS	104	7168	4	4	3	3	5.68	50S ribosomal protein
743	1	db$2	2::C3K253|C3K253_PSEFS	104	106648	4	4	4	4	0.19	Putative membrane pr
744	1	db$2	2::C3KCS8|C3KCS8_PSEFS	104	47767	4	4	4	4	0.49	UDP-N-acetylmuramoy
745	1	db$2	2::C3KAJ9|C3KAJ9_PSEFS	104	20836	3	3	3	3	0.96	Putative hypoxanthine
746	1	db$2	2::C3KDM6|PSD_PSEFS	103	31380	5	5	4	4	1.12	Phosphatidylserine dec
747	1	SwissProt_HUMAN$	3::P07195|LDHB_HUMAN	103	36615	4	4	4	4	0.67	L-lactate dehydrogena
747	2	SwissProt_HUMAN$	3::P00338|LDHA_HUMAN	90	36665	3	3	3	3	0.47	L-lactate dehydrogena
748	1	db$2	2::C3K332|Y5583_PSEFS	103	48606	3	3	3	3	0.34	UPF0229 protein PFLU_
749	1	db$2	2::C3JXX7|C3JXX7_PSEFS	103	56979	5	4	5	4	0.39	Conserved hypothetica
750	1	db$2	2::C3K6J2|C3K6J2_PSEFS	103	47817	3	3	3	3	0.35	Putative hydrolase OS
751	1	db$2	2::C3K609|C3K609_PSEFS	102	73644	4	4	4	4	0.29	Putative lytic transglyc
752	1	db$2	2::C3JYA9|C3JYA9_PSEFS	102	27795	3	3	3	3	0.66	Putative TetR-family re
753	1	db$2	2::C3JZN8|C3JZN8_PSEFS	102	10735	4	4	4	4	4.58	Uncharacterized protei
754	1	db$2	2::C3K6A5|C3K6A5_PSEFS	101	78572	6	6	6	6	0.44	Catalase OS=Pseudom
755	1	db$2	2::C3K1X0|CYSZ_PSEFS	101	28826	3	3	3	3	0.63	Sulfate transporter Cy
756	1	db$2	2::C3KAI3|AROB_PSEFS	101	39825	6	6	5	5	0.81	3-dehydroquinate synt
757	1	db$2	2::C3K479|RNPH_PSEFS	101	25645	3	3	3	3	0.73	Ribonuclease PH OS=F
758	1	db$2	2::C3K6E4|C3K6E4_PSEFS	101	46143	4	4	3	3	0.51	Cytochrome b OS=Pse
759	1	SwissProt_HUMAN$	3::P0DOX5|IGG1_HUMAN	100	49298	5	5	5	5	0.62	Immunoglobulin gamn
759	2	SwissProt_HUMAN$	3::P01859|IGHG2_HUMAN	47	35878	3	3	3	3	0.48	Immunoglobulin heavy
760	1	db$2	2::C3KAS9|C3KAS9_PSEFS	100	109677	2	2	2	2	0.09	Efflux pump membran
761	1	db$2	2::C3K2J9|C3K2J9_PSEFS	100	60860	2	2	2	2	0.17	Putative dehydrogenas
762	1	db$2	2::C3K5U6|C3K5U6_PSEFS	100	40174	4	4	3	3	0.42	dTDP-glucose 4,6-deh
763	1	db$2	2::C3KDI2|C3KDI2_PSEFS	99	135789	4	3	3	2	0.07	Exonuclease OS=Pseu
764	1	db$2	2::C3K8W6|C3K8W6_PSEFS	99	18885	4	4	3	3	1.11	3-deoxy-D-manno-oct

↑Family	M	DB	Accession	Score	Mass	Matches	Match(sig)	Sequences	Seq(sig)	emPAI	Description
765	1	db$2	2::C3K6N8|C3K6N8_PSEFS	99	25972	2	2	1	1	0.20	Putative hydrolase OS
766	1	SwissProt_HUMAN$	3::P07737|PROF1_HUMAN	99	15045	4	4	4	4	2.44	Profilin-1 OS=Homo sa
767	1	db$2	2::C3K360|C3K360_PSEFS	99	23509	3	3	3	3	0.82	ATP-dependent dethiol
768	1	db$2	2::C3K6A4|C3K6A4_PSEFS	98	36459	2	2	2	2	0.30	Methionine import ATP
769	1	db$2	2::C3KDQ0|C3KDQ0_PSEFS	98	35105	3	3	3	3	0.50	2-ketogluconate reduc
770	1	db$2	2::C3JXZ5|C3JXZ5_PSEFS	98	25958	5	4	4	4	1.06	Putative histidine trans
771	1	db$2	2::C3K0U9|C3K0U9_PSEFS	98	55465	4	4	3	3	0.29	Putative two-compone
772	1	db$2	2::C3K4W2|C3K4W2_PSEFS	98	26844	4	4	4	4	1.02	Putative glycerophospl
773	1	db$2	2::C3KE71|C3KE71_PSEFS	98	51412	8	8	7	7	1.09	Replicative DNA helica
774	1	db$2	2::C3K1A8|C3K1A8_PSEFS	97	35739	2	2	2	2	0.30	Putative lipoprotein OS
775	1	db$2	2::C3K2R8|NUSB_PSEFS	97	18764	4	4	2	2	1.11	Transcription antitermi
776	1	db$2	2::C3KC02|C3KC02_PSEFS	97	29258	4	3	4	3	0.62	Putative acyltransferas
777	1	db$2	2::C3KDM3|C3KDM3_PSEFS	97	25575	2	2	2	2	0.45	Putative lipoprotein OS
778	1	db$2	2::C3K5H2|QUEF_PSEFS	97	30808	5	5	5	5	1.15	NADPH-dependent 7-c
779	1	db$2	2::C3JYQ9|C3JYQ9_PSEFS	97	54767	2	2	2	2	0.19	Transcriptional regulat
780	1	db$2	2::C3K3T8|C3K3T8_PSEFS	97	39499	2	2	2	2	0.27	Putrescine ABC transp
781	1	db$2	2::C3K0S1|C3K0S1_PSEFS	97	53269	3	3	3	3	0.31	Magnesium transporte
782	1	db$2	2::C3K6W7|C3K6W7_PSEFS	97	16216	5	2	4	2	0.78	Biotin carboxyl carrier
783	1	db$2	2::C3KE66|C3KE66_PSEFS	97	27011	5	5	5	5	1.39	23S rRNA (guanosine-
784	1	db$2	2::C3K134|C3K134_PSEFS	96	52371	4	4	4	4	0.44	Coproporphyrinogen-II
785	1	db$2	2::C3KE43|C3KE43_PSEFS	96	32711	4	4	4	4	0.78	Lipid A biosynthesis la
786	1	db$2	2::C3JZH3|C3JZH3_PSEFS	96	22428	3	3	3	3	0.87	Putative cytochrome o
787	1	db$2	2::C3K3X2|C3K3X2_PSEFS	96	19739	3	3	3	3	1.04	Uncharacterized protei
788	1	db$2	2::C3K5K2|C3K5K2_PSEFS	96	9923	4	3	4	3	3.06	Putative lipoprotein OS
789	1	db$2	2::C3K3K1|C3K3K1_PSEFS	96	18188	2	2	2	2	0.67	Bifunctional protein [p
790	1	db$2	2::C3K1N5|QUEA_PSEFS	95	38396	4	4	4	4	0.64	S-adenosylmethionine
791	1	SwissProt_HUMAN$	3::P02679|FIBG_HUMAN	94	51479	8	6	5	3	0.32	Fibrinogen gamma cha
792	1	db$2	2::C3JXQ1|C3JXQ1_PSEFS	94	24479	3	3	2	2	0.47	Putative enoyl-CoA hy
793	1	db$2	2::C3KCG1|C3KCG1_PSEFS	94	60384	3	2	2	1	0.08	Putative secreted amic
794	1	SwissProt_HUMAN$	3::P06703|S10A6_HUMAN	94	10173	2	2	1	1	0.57	Protein S100-A6 OS=H
795	1	db$2	2::C3K2W3|C3K2W3_PSEFS	94	11427	4	3	4	3	2.37	30S ribosomal protein
796	1	db$2	2::C3K2P7|C3K2P7_PSEFS	94	17680	4	2	4	2	0.70	Putative acetyltransfer
797	1	db$2	2::C3KBQ3|C3KBQ3_PSEFS	94	33274	4	4	4	4	0.76	LysR-family transcripti
798	1	db$2	2::C3KDX7|ISPH_PSEFS	94	34499	4	4	3	3	0.51	4-hydroxy-3-methylbu
799	1	db$2	2::C3K754|C3K754_PSEFS	94	29479	2	2	2	2	0.38	Putative lipoprotein OS
800	1	db$2	2::C3K268|C3K268_PSEFS	94	11552	5	4	5	4	3.99	CRM domain-containin
801	1	db$2	2::C3K9R3|C3K9R3_PSEFS	93	18863	4	3	3	3	1.11	Uncharacterized protei
802	1	db$2	2::C3K0D7|APEB_PSEFS	93	46914	5	5	4	4	0.50	Probable M18 family a
803	1	db$2	2::C3K8W9|C3K8W9_PSEFS	93	28276	4	4	3	3	0.65	Intermembrane phosp
804	1	db$2	2::C3K7T1|C3K7T1_PSEFS	92	23131	7	5	7	5	1.77	Two-component syster

↑Family	M	DB	Accession	Score	Mass	Matches	Match(sig)	Sequences	Seq(sig)	emPAI	Description
805	1	db$2	2::C3KE84|C3KE84_PSEFS	92	35425	6	5	5	5	0.95	Pseudouridine synthas
806	1	db$2	2::C3K6Y8|C3K6Y8_PSEFS	92	34148	7	7	6	6	1.29	GTPase Era OS=Pseud
807	1	db$2	2::C3K3S1|C3K3S1_PSEFS	92	51070	8	5	6	4	0.45	Putative oxidase OS=F
808	1	db$2	2::C3K5Y4|C3K5Y4_PSEFS	91	9690	2	2	1	1	1.60	Uncharacterized protei
809	1	SwissProt_HUMAN$	3::P27348|1433T_HUMAN	91	27747	3	2	3	2	0.40	14-3-3 protein theta O
809	2	SwissProt_HUMAN$	3::P63104|1433Z_HUMAN	90	27728	6	4	6	4	0.97	14-3-3 protein zeta/de
810	1	db$2	2::C3K3P5|C3K3P5_PSEFS	91	48453	2	2	1	1	0.10	Putative symporter me
811	1	db$2	2::C3K0P0|C3K0P0_PSEFS	91	35982	4	4	4	4	0.69	Putative peptidase OS
812	1	db$2	2::C3K9M0|C3K9M0_PSEFS	90	11184	4	3	3	3	2.45	SCP2 domain-containi
813	1	db$2	2::C3K3R6|C3K3R6_PSEFS	90	54762	5	5	3	3	0.30	L-threonine dehydrata
814	1	db$2	2::C3KD59|C3KD59_PSEFS	90	81779	5	5	5	5	0.34	YcaO domain-containi
815	1	db$2	2::C3K3K8|C3K3K8_PSEFS	90	27898	2	2	1	1	0.40	Pyrroline-5-carboxylat
816	1	db$2	2::C3K6M4|C3K6M4_PSEFS	90	13463	4	4	2	2	3.00	Succinate dehydrogena
817	1	db$2	2::C3JYM6|C3JYM6_PSEFS	90	40915	2	2	2	2	0.26	Putative HlyD-family s
818	1	db$2	2::C3K8K4|C3K8K4_PSEFS	89	58330	3	3	3	3	0.28	Putative outer membra
819	1	db$2	2::C3K4D7|C3K4D7_PSEFS	89	14435	2	2	2	2	0.91	Putative thioesterase (
820	1	db$2	2::C3K8I3|C3K8I3_PSEFS	89	39921	2	2	1	1	0.13	3,4-dihydroxy-2-butan
821	1	db$2	2::C3K0W6|C3K0W6_PSEFS	89	42755	3	3	3	3	0.39	Aspartate aminotransf
822	1	db$2	2::C3K2J5|C3K2J5_PSEFS	89	18057	5	4	5	4	1.81	Putative AsnC-family r
823	1	db$2	2::C3K015|C3K015_PSEFS	89	53791	4	4	4	4	0.42	Putative ferredoxin OS
824	1	db$2	2::C3KB18|C3KB18_PSEFS	88	115005	4	3	4	3	0.13	Efflux pump membran
825	1	SwissProt_HUMAN$	3::P01834|IGKC_HUMAN	88	11758	4	2	4	2	1.20	Immunoglobulin kappa
826	1	db$2	2::C3K2L6|C3K2L6_PSEFS	88	47731	5	3	5	3	0.35	Putative lipoprotein OS
827	1	db$2	2::C3K2R1|DXS_PSEFS	88	68238	4	4	4	4	0.32	1-deoxy-D-xylulose-5-
828	1	db$2	2::C3K763|C3K763_PSEFS	88	29996	2	2	2	2	0.37	Putative hydrolase OS
829	1	db$2	2::C3K482|RPOZ_PSEFS	88	9821	5	3	3	2	3.13	DNA-directed RNA poly
830	1	db$2	2::C3K0R0|C3K0R0_PSEFS	88	14751	2	2	2	2	0.88	Flagellar basal body ro
831	1	db$2	2::C3KDR6|C3KDR6_PSEFS	87	40570	3	3	3	3	0.42	Putaive NADH oxidore
832	1	db$2	2::C3K3G7|C3K3G7_PSEFS	87	31253	4	4	4	4	0.83	Methylenetetrahydrofo
833	1	db$2	2::C3K9N0|C3K9N0_PSEFS	87	28569	2	2	2	2	0.39	Putative transcriptiona
834	1	db$2	2::C3KDF0|C3KDF0_PSEFS	87	18221	5	4	4	3	1.16	Putative phage tail-rel
835	1	db$2	2::C3K453|C3K453_PSEFS	87	71119	3	2	3	2	0.14	AcetylCoA_hyd_C dom
836	1	db$2	2::C3K2K6|C3K2K6_PSEFS	87	49458	4	3	4	3	0.33	tRNA-2-methylthio-N(
837	1	db$2	2::C3K1K9|C3K1K9_PSEFS	87	42204	4	4	4	4	0.57	Putative aminotransfer
838	1	db$2	2::C3K2E0|C3K2E0_PSEFS	86	48742	5	4	4	3	0.34	DNA repair protein Ra
839	1	db$2	2::C3K3L6|TRMB_PSEFS	86	26990	3	2	3	2	0.42	tRNA (guanine-N(7)-)-
840	1	db$2	2::C3K2W7|RS17_PSEFS	86	10090	3	3	3	3	2.94	30S ribosomal protein
841	1	db$2	2::C3JY84|C3JY84_PSEFS	86	18006	3	3	3	3	1.18	NADH dehydrogenase
842	1	db$2	2::C3KE30|C3KE30_PSEFS	86	59128	6	5	6	5	0.49	L-aspartate oxidase OS
843	1	db$2	2::C3K5K7|C3K5K7_PSEFS	86	52604	4	3	3	2	0.20	Putative methyl-accep

↑Family	M	DB	Accession	Score	Mass	Matches	Match(sig)	Sequences	Seq(sig)	emPAI	Description
844	1	db$2	2::C3K841|C3K841_PSEFS	86	11596	6	4	4	3	3.99	Putative sigma-54 mo(
845	1	db$2	2::C3K1N1|C3K1N1_PSEFS	86	33097	4	4	4	4	0.77	Protein-export membra
846	1	db$2	2::C3K3Y8|UBID_PSEFS	86	54697	5	5	5	5	0.54	3-octaprenyl-4-hydrox
847	1	db$2	2::C3K7W0|C3K7W0_PSEFS	86	26786	4	4	4	4	1.02	Uncharacterized protei
848	1	db$2	2::C3KE10|C3KE10_PSEFS	86	58823	5	4	5	4	0.38	Putative methyl-accep
849	1	db$2	2::C3K8K6|C3K8K6_PSEFS	86	15117	2	2	2	2	0.86	Putative transport-rela
850	1	db$2	2::C3K1E4|GLMU_PSEFS	85	48690	4	4	4	4	0.47	Bifunctional protein Gl
851	1	db$2	2::C3K862|C3K862_PSEFS	85	39825	4	3	4	3	0.43	Putative outer membra
852	1	db$2	2::C3KAM8|C3KAM8_PSEFS	85	52747	2	2	2	2	0.20	Phosphate transporter
853	1	db$2	2::C3K705|SURE_PSEFS	85	26431	2	2	2	2	0.43	5'-nucleotidase SurE C
854	1	db$2	2::C3K3L0|METXS_PSEFS	85	41664	2	2	2	2	0.25	Homoserine O-succiny
855	1	db$2	2::C3K013|C3K013_PSEFS	85	20254	3	3	2	2	0.59	Peptidyl-prolyl cis-tran
856	1	db$2	2::C3K3D4|C3K3D4_PSEFS	85	52472	3	3	3	3	0.31	Uncharacterized protei
857	1	db$2	2::C3K5X6|C3K5X6_PSEFS	85	49358	5	4	5	4	0.47	Tryptophan--tRNA liga
858	1	db$2	2::C3K0I0|C3K0I0_PSEFS	85	27472	3	3	2	2	0.41	RNA polymerase sigma
859	1	db$2	2::C3JYE7|C3JYE7_PSEFS	84	35468	3	3	3	3	0.49	LysR family regulatory
860	1	db$2	2::C3K6K2|IHFB_PSEFS	84	11021	4	2	4	2	1.32	Integration host factor
861	1	db$2	2::C3K3Q8|TYSY_PSEFS	84	31490	4	4	4	4	0.82	Thymidylate synthase
862	1	db$2	2::C3KDP4|C3KDP4_PSEFS	84	75338	6	5	6	5	0.37	Phosphate acetyltransf
863	1	db$2	2::C3KAJ5|C3KAJ5_PSEFS	84	83174	3	3	3	3	0.19	Putative paraquat-indu
864	1	db$2	2::C3K6U7|C3K6U7_PSEFS	84	79141	6	5	5	4	0.27	Putative exported prot
865	1	SwissProt_HUMAN$	3::Q5VTE0|EF1A3_HUMAN	84	50153	9	8	7	7	1.13	Putative elongation fac
866	1	db$2	2::C3K2D4|C3K2D4_PSEFS	83	35439	3	3	3	3	0.49	Putative cobalamin syr
867	1	db$2	2::C3K0H7|C3K0H7_PSEFS	83	77893	4	3	4	3	0.20	Histidine kinase OS=P:
868	1	db$2	2::C3KDM5|C3KDM5_PSEFS	83	43829	2	2	2	2	0.24	O-phosphoserine phos
869	1	db$2	2::C3K1U6|C3K1U6_PSEFS	82	74730	5	5	4	4	0.37	Cytochrome bo(3) ubi(
870	1	db$2	2::C3K743|FABA_PSEFS	82	18688	6	5	5	5	2.51	3-hydroxydecanoyl-[a(
871	1	db$2	2::C3KB69|C3KB69_PSEFS	82	12397	1	1	1	1	0.45	Putative lipoprotein OS
872	1	db$2	2::C3K0X3|C3K0X3_PSEFS	82	16227	2	2	2	2	0.78	Transcriptional regulat
873	1	db$2	2::C3KCS5|C3KCS5_PSEFS	82	51798	5	4	5	4	0.44	UDP-N-acetylmuramoy
874	1	db$2	2::C3K419|C3K419_PSEFS	82	17100	2	2	2	2	0.73	Transcriptional regulat
875	1	db$2	2::C3KDX0|C3KDX0_PSEFS	82	16992	4	4	3	3	1.28	Peptidyl-prolyl cis-tran
876	1	db$2	2::C3K1H2|C3K1H2_PSEFS	82	49205	5	4	5	4	0.47	Signal recognition part
877	1	db$2	2::C3K3N0|C3K3N0_PSEFS	81	25405	3	3	2	2	0.45	Putative TetR-family re
878	1	db$2	2::C3JY38|C3JY38_PSEFS	81	16812	2	1	2	1	0.32	Putative thioredoxin-re
879	1	db$2	2::B0B0A5|DNAA_PSEFS	81	56252	8	7	8	7	0.80	Chromosomal replicati
880	1	db$2	2::C3KDA5|C3KDA5_PSEFS	81	54170	2	1	2	1	0.09	Uncharacterized protei
881	1	db$2	2::C3K8U6|C3K8U6_PSEFS	81	15270	2	2	2	2	0.84	Putative polyhydroxyal
882	1	db$2	2::C3JY92|C3JY92_PSEFS	81	55851	5	4	4	3	0.29	NADH dehydrogenase
883	1	db$2	2::C3K436|C3K436_PSEFS	81	30237	3	3	3	3	0.60	Diaminopimelate epim

↑Family	M	DB	Accession	Score	Mass	Matches	Match(sig)	Sequences	Seq(sig)	emPAI	Description
884	1	db$2	2::C3K459|C3K459_PSEFS	81	18704	3	3	3	3	1.11	Leucine-responsive reg
885	1	db$2	2::C3K8U7|C3K8U7_PSEFS	80	30923	4	4	3	3	0.58	Transcriptional regulat
886	1	db$2	2::C3K224|C3K224_PSEFS	80	30473	2	2	1	1	0.17	CDP-diacylglycerol--se
887	1	db$2	2::C3K3H3|C3K3H3_PSEFS	80	52510	3	3	3	3	0.31	Adenosylmethionine-8
888	1	db$2	2::C3K6W5|PRMA_PSEFS	80	31717	4	4	3	3	0.56	Ribosomal protein L11
889	1	db$2	2::C3K1W7|C3K1W7_PSEFS	79	11247	3	3	2	2	1.29	DUF1244 domain-cont
890	1	db$2	2::C3KBM1|C3KBM1_PSEFS	79	29989	2	2	2	2	0.37	Glycerol uptake facilita
891	1	db$2	2::C3K126|C3K126_PSEFS	79	54569	7	7	6	6	0.68	2-methylcitrate dehydı
892	1	db$2	2::C3JYV1|C3JYV1_PSEFS	79	32264	5	3	5	3	0.55	Formyltetrahydrofolate
893	1	db$2	2::C3K148|APT_PSEFS	79	20064	4	4	4	4	1.54	Adenine phosphoribosy
894	1	db$2	2::C3K5L9|C3K5L9_PSEFS	78	31441	3	3	3	3	0.57	Alpha-D-ribose 1-meth
895	1	db$2	2::C3K819|C3K819_PSEFS	78	35012	2	2	2	2	0.31	Putative ABC transport
896	1	db$2	2::C3JY71|C3JY71_PSEFS	78	43919	2	2	2	2	0.24	JmjC domain-containir
897	1	db$2	2::C3JXH2|C3JXH2_PSEFS	78	91149	4	2	3	2	0.11	Putative excinuclease /
898	1	db$2	2::C3KBZ8|C3KBZ8_PSEFS	78	38389	2	2	2	2	0.28	Iron-sulfur cluster carr
899	1	db$2	2::Q9ZEP7|PANC_PSEFS	77	30696	2	2	2	2	0.36	Pantothenate syntheta
900	1	db$2	2::C3K1C9|C3K1C9_PSEFS	77	65670	2	2	2	2	0.16	Putative cation-transpo
901	1	db$2	2::C3K0M3|C3K0M3_PSEFS	77	20567	5	5	5	5	2.13	Uncharacterized protei
902	1	db$2	2::C3K114|C3K114_PSEFS	77	74752	3	3	3	3	0.21	DNA polymerase III su
903	1	db$2	2::C3KC07|C3KC07_PSEFS	77	21449	2	2	2	2	0.55	YceI domain-containin
904	1	db$2	2::C3KDG5|C3KDG5_PSEFS	77	23599	3	3	3	3	0.82	Putative regulatory pro
905	1	db$2	2::C3K4V5|C3K4V5_PSEFS	76	36313	4	4	4	4	0.68	Beta-hexosaminidase (
906	1	db$2	2::C3KBT9|C3KBT9_PSEFS	76	31336	1	1	1	1	0.16	Sulfate ABC transport
907	1	db$2	2::C3K3L2|C3K3L2_PSEFS	76	15596	2	2	2	2	0.82	DUF4426 domain-cont
908	1	db$2	2::C3K5X9|C3K5X9_PSEFS	76	41639	2	2	2	2	0.25	Putative acyl-CoA dehy
909	1	db$2	2::C3JZU7|C3JZU7_PSEFS	76	16433	3	3	2	2	0.77	Putative thioesterase (
910	1	db$2	2::C3K2U8|C3K2U8_PSEFS	76	18135	6	5	5	5	2.65	Bacterioferritin OS=Ps
911	1	db$2	2::C3K7Y8|C3K7Y8_PSEFS	76	53697	6	6	5	5	0.55	Putative hydrolase OS=
912	1	db$2	2::C3K487|C3K487_PSEFS	76	33600	3	2	2	2	0.32	Biopolymer transport p
913	1	db$2	2::C3K9G9|C3K9G9_PSEFS	76	16183	4	3	4	3	1.37	CMD domain-containin
914	1	db$2	2::C3K250|C3K250_PSEFS	76	71198	2	2	1	1	0.07	Acetyl-coenzyme A syı
915	1	db$2	2::C3JY31|C3JY31_PSEFS	75	22860	3	3	2	2	0.85	Putative biopolymer tr
916	1	db$2	2::C3JZQ4|C3JZQ4_PSEFS	75	8106	3	3	3	3	4.46	Translational regulator
917	1	db$2	2::C3K107|C3K107_PSEFS	75	31415	2	2	2	2	0.35	Uncharacterized protei
918	1	db$2	2::C3K816|OPGH_PSEFS	75	96236	7	7	5	5	0.34	Glucans biosynthesis g
919	1	db$2	2::C3K1H9|C3K1H9_PSEFS	75	18692	3	3	3	3	1.12	Conserved hypothetica
920	1	db$2	2::C3K0V4|C3K0V4_PSEFS	75	48572	3	3	3	3	0.34	Flagellin OS=Pseudom
921	1	db$2	2::C3K4A9|C3K4A9_PSEFS	75	17462	4	4	3	3	1.24	Uncharacterized protei
922	1	db$2	2::C3K481|C3K481_PSEFS	74	23434	3	3	3	3	0.83	Guanylate kinase OS=
923	1	db$2	2::C3K5T5|C3K5T5_PSEFS	74	25405	4	4	2	2	0.45	Carbonic anhydrase OS

↑Family	M	DB	Accession	Score	Mass	Matches	Match(sig)	Sequences	Seq(sig)	emPAI	Description
924	1	db$2	2::C3KCS7|MRAY_PSEFS	74	39243	2	2	2	2	0.27	Phospho-N-acetylmura
925	1	db$2	2::C3KE20|C3KE20_PSEFS	74	33868	5	4	5	4	0.75	GCV_T domain-contair
926	1	db$2	2::C3K483|C3K483_PSEFS	74	78630	5	4	5	4	0.27	Guanosine-3',5'-bis(Di
927	1	db$2	2::C3K4J0|C3K4J0_PSEFS	74	17598	3	3	3	3	1.21	Zn_protease domain-c
928	1	db$2	2::C3KB73|C3KB73_PSEFS	74	9692	1	1	1	1	0.61	Putative lipoprotein OS
929	1	db$2	2::C3K303|COQ7_PSEFS	74	23866	3	2	3	2	0.48	3-demethoxyubiquinol
930	1	db$2	2::C3K3N8|C3K3N8_PSEFS	74	43920	4	4	4	4	0.54	PUA domain-containin
931	1	db$2	2::C3K257|C3K257_PSEFS	74	33362	3	2	3	2	0.33	tRNA pseudouridine sy
932	1	db$2	2::C3K579|C3K579_PSEFS	74	15962	1	1	1	1	0.34	Putative thioesterase (
933	1	db$2	2::C3K176|C3K176_PSEFS	73	33571	5	4	5	4	0.76	Glucokinase OS=Pseuc
934	1	db$2	2::C3JXX6|DNLJ_PSEFS	73	85515	4	4	4	4	0.25	DNA ligase OS=Pseud
935	1	SwissProt_HUMAN$	3::O00139|KIF2A_HUMAN	73	79905	8	8	2	2	0.13	Kinesin-like protein KI
936	1	db$2	2::C3K5B9|C3K5B9_PSEFS	73	60269	1	1	1	1	0.08	Dipeptide ABC transpo
937	1	db$2	2::C3K6Y6|C3K6Y6_PSEFS	73	31839	7	5	5	4	0.81	Signal peptidase I OS=
938	1	db$2	2::C3K2D6|C3K2D6_PSEFS	73	73930	2	2	2	2	0.14	Carbon starvation prot
939	1	db$2	2::C3KAI8|C3KAI8_PSEFS	73	24497	4	3	3	2	0.78	Putative TetR-family re
940	1	db$2	2::C3K9P7|C3K9P7_PSEFS	73	69450	4	4	4	4	0.31	Putative substrate-bin
941	1	db$2	2::C3JYU3|C3JYU3_PSEFS	72	75598	2	2	2	2	0.13	Histidine kinase OS=P
942	1	db$2	2::C3JYR0|C3JYR0_PSEFS	72	13774	2	2	2	2	0.97	Glycine cleavage syste
943	1	db$2	2::C3K1B3|C3K1B3_PSEFS	72	20726	3	3	3	3	0.97	Uncharacterized protei
944	1	db$2	2::C3K6L9|C3K6L9_PSEFS	72	49785	7	6	7	6	0.77	Phospho-2-dehydro-3-
945	1	SwissProt_HUMAN$	3::Q5D862|FILA2_HUMAN	72	247928	1	1	1	1	0.02	Filaggrin-2 OS=Homo
946	1	SwissProt_HUMAN$	3::P62269|RS18_HUMAN	72	17708	1	1	1	1	0.30	40S ribosomal protein
947	1	db$2	2::C3JXP7|RECR_PSEFS	72	21528	4	3	3	3	0.92	Recombination protein
948	1	db$2	2::C3KD57|C3KD57_PSEFS	72	49686	5	4	5	4	0.46	Cystathionine beta-lya
949	1	db$2	2::C3K1B0|C3K1B0_PSEFS	72	96490	2	2	2	2	0.10	Lysylphosphatidylglyce
950	1	db$2	2::C3K075|C3K075_PSEFS	71	77370	7	4	7	4	0.28	Putative sulfatase OS=
951	1	db$2	2::C3K605|LPXD_PSEFS	71	36124	4	3	4	3	0.48	UDP-3-O-acylglucosam
952	1	db$2	2::C3K5U5|C3K5U5_PSEFS	71	32335	4	4	4	4	0.79	Glucose-1-phosphate t
953	1	db$2	2::C3K0Z1|C3K0Z1_PSEFS	71	36566	1	1	1	1	0.14	L-threonine-O-3-phosp
954	1	db$2	2::C3K707|C3K707_PSEFS	71	29295	2	2	2	2	0.38	Putative lipoprotein OS
955	1	db$2	2::C3K710|DCD_PSEFS	71	21231	5	5	5	5	2.02	dCTP deaminase OS=P
956	1	db$2	2::C3K863|PDXH_PSEFS	71	24884	3	3	2	2	0.46	Pyridoxine/pyridoxami
957	1	db$2	2::C3K433|CYAY_PSEFS	71	12442	2	2	1	1	0.45	Iron-sulfur cluster asse
958	1	db$2	2::C3K602|C3K602_PSEFS	71	48346	4	4	4	4	0.48	Zinc metalloprotease (
959	1	db$2	2::C3K0M7|C3K0M7_PSEFS	70	28601	2	2	2	2	0.39	Putative deoxyribonucl
960	1	db$2	2::C3K5G2|C3K5G2_PSEFS	70	35788	6	4	6	4	0.70	Putative membrane pr
961	1	SwissProt_HUMAN$	3::P23396|RS3_HUMAN	70	26671	2	2	2	2	0.42	40S ribosomal protein
962	1	db$2	2::C3K9I2|C3K9I2_PSEFS	70	33896	1	1	1	1	0.15	Putative transcriptiona
963	1	db$2	2::C3K0K1|C3K0K1_PSEFS	69	53436	1	1	1	1	0.09	Putative GntR-family r

↑Family	M	DB	Accession	Score	Mass	Matches	Match(sig)	Sequences	Seq(sig)	emPAI	Description
964	1	db$2	2::C3K5L2|C3K5L2_PSEFS	69	29854	3	3	3	3	0.61	Phosphonates import /
965	1	db$2	2::C3KEA2|C3KEA2_PSEFS	69	30824	2	2	2	2	0.36	Putative inner membra
966	1	db$2	2::C3K6J1|UBIG_PSEFS	69	26040	4	3	3	2	0.72	Ubiquinone biosynthes
967	1	SwissProt_HUMAN$	3::Q9BQE3|TBA1C_HUMAN	69	49863	3	3	3	3	0.33	Tubulin alpha-1C chair
968	1	db$2	2::C3K5X0|C3K5X0_PSEFS	69	25599	1	1	1	1	0.20	Putative exported prot
969	1	db$2	2::C3K7G1|C3K7G1_PSEFS	69	37563	2	2	2	2	0.29	Cell shape protein Mre
970	1	db$2	2::C3K3D1|BETI_PSEFS	69	21992	1	1	1	1	0.24	HTH-type transcription
971	1	db$2	2::C3K3D8|C3K3D8_PSEFS	68	49942	4	4	4	4	0.46	ATP-independent RNA
972	1	db$2	2::C3KBL4|C3KBL4_PSEFS	68	26993	2	2	1	1	0.19	Glutamate/aspartate A
973	1	db$2	2::C3KA82|C3KA82_PSEFS	68	19457	5	3	5	3	1.06	Lactoylglutathione lyas
974	1	db$2	2::C3K3R4|C3K3R4_PSEFS	68	24681	3	3	3	3	0.77	Putative hydrolase OS=
975	1	db$2	2::C3K328|APAG_PSEFS	68	13631	2	2	2	2	0.98	Protein ApaG OS=Pseu
976	1	db$2	2::C3KAC0|C3KAC0_PSEFS	68	40546	3	3	3	3	0.42	Butyryl-CoA dehydroge
977	1	db$2	2::C3JY77|C3JY77_PSEFS	68	40451	4	4	3	3	0.42	Putative 3-oxoacyl-[ac
978	1	db$2	2::C3K3B7|C3K3B7_PSEFS	68	14934	1	1	1	1	0.37	Uncharacterized protei
979	1	db$2	2::C3K6J5|SERC_PSEFS	68	39799	7	4	7	4	0.61	Phosphoserine aminotr
980	1	db$2	2::C3K0G9|C3K0G9_PSEFS	68	32198	2	2	2	2	0.34	Probable lipid kinase Y
981	1	db$2	2::C3K2P1|C3K2P1_PSEFS	68	48286	2	2	2	2	0.22	UDP-N-acetylmuramat
982	1	db$2	2::C3JZ62|C3JZ62_PSEFS	68	61473	1	1	1	1	0.08	Putative long-chain-fat
983	1	db$2	2::C3KDS5|C3KDS5_PSEFS	67	41122	3	3	2	2	0.41	Putative hydratase/iso
984	1	db$2	2::C3K3J5|C3K3J5_PSEFS	67	13219	4	4	3	3	3.09	Type IV pilus response
985	1	db$2	2::C3K062|C3K062_PSEFS	67	48996	6	6	6	6	0.79	Fumarate hydratase cl
986	1	db$2	2::C3K110|C3K110_PSEFS	67	32414	3	3	3	3	0.55	Cysteine synthase OS=
987	1	db$2	2::C3K9Q9|C3K9Q9_PSEFS	67	19670	1	1	1	1	0.27	FMN_red domain-conta
988	1	db$2	2::C3K1A4|C3K1A4_PSEFS	67	41199	1	1	1	1	0.12	Uncharacterized protei
989	1	db$2	2::C3K7M5|C3K7M5_PSEFS	67	15789	1	1	1	1	0.34	Uncharacterized protei
990	1	db$2	2::C3K9T0|NADK_PSEFS	67	32289	3	2	3	2	0.34	NAD kinase OS=Pseud
991	1	db$2	2::C3K312|C3K312_PSEFS	67	24134	3	2	3	2	0.48	Ribulose-phosphate 3-
992	1	SwissProt_HUMAN$	3::P62826|RAN_HUMAN	67	24408	1	1	1	1	0.21	GTP-binding nuclear pr
993	1	db$2	2::C3KAK2|C3KAK2_PSEFS	67	10989	3	3	3	3	2.54	Uncharacterized protei
994	1	db$2	2::C3K4U7|C3K4U7_PSEFS	67	28919	3	3	3	3	0.63	Putative amidotransfer
995	1	db$2	2::C3JZH1|C3JZH1_PSEFS	67	34020	2	2	2	2	0.32	LysR-family transcripti
996	1	db$2	2::C3K3Y7|C3K3Y7_PSEFS	67	35706	4	4	4	4	0.70	Putative iron-sulfur bir
997	1	db$2	2::C3JZN1|IHFA_PSEFS	67	11469	3	3	2	2	1.25	Integration host factor
998	1	SwissProt_HUMAN$	3::P04406|G3P_HUMAN	66	36030	2	2	2	2	0.30	Glyceraldehyde-3-phos
999	1	db$2	2::C3K6B0|C3K6B0_PSEFS	66	33534	1	1	1	1	0.15	High-affinity zinc uptal
1000	1	db$2	2::C3K6S4|C3K6S4_PSEFS	66	30497	1	1	1	1	0.17	Putative antibiotic bios
1001	1	db$2	2::C3KBK8|C3KBK8_PSEFS	66	35740	2	2	2	2	0.30	Ribosomal RNA small s
1002	1	db$2	2::C3KBM4|C3KBM4_PSEFS	66	34098	7	5	5	5	1.00	Ornithine carbamoyltra
1003	1	SwissProt_HUMAN$	3::P02671|FIBA_HUMAN	66	94914	1	1	1	1	0.05	Fibrinogen alpha chain

↑Family	M	DB	Accession	Score	Mass	Matches	Match(sig)	Sequences	Seq(sig)	emPAI	Description
1004	1	db$2	2::C3K6D7|C3K6D7_PSEFS	66	21411	1	1	1	1	0.24	UPF0056 inner membr
1005	1	db$2	2::C3K6A6|C3K6A6_PSEFS	66	25667	2	2	2	2	0.44	Putative lipoprotein OS
1006	1	db$2	2::C3K584|C3K584_PSEFS	65	15399	2	2	2	2	0.83	Uncharacterized protei
1007	1	db$2	2::C3KCI1|C3KCI1_PSEFS	65	42811	1	1	1	1	0.12	Putative acyl-CoA dehy
1008	1	db$2	2::C3K6Z1|C3K6Z1_PSEFS	65	50711	3	3	3	3	0.32	Putative multicopper o
1009	1	db$2	2::C3K6V7|C3K6V7_PSEFS	65	46336	5	5	4	4	0.50	Carboxy-terminal proc
1010	1	db$2	2::C3KDX3|C3KDX3_PSEFS	65	34279	3	3	3	3	0.51	Riboflavin biosynthesis
1011	1	db$2	2::C3K1C5|C3K1C5_PSEFS	65	44321	3	3	3	3	0.38	Putative cyclopropane-
1012	1	db$2	2::C3JYI2|C3JYI2_PSEFS	65	39477	2	2	2	2	0.27	2-aminoethylphosphor
1013	1	db$2	2::C3KDP0|C3KDP0_PSEFS	65	27498	2	2	2	2	0.41	Uncharacterized protei
1014	1	db$2	2::C3K221|C3K221_PSEFS	65	129628	1	1	1	1	0.04	RecBCD enzyme subur
1015	1	db$2	2::C3K0W1|C3K0W1_PSEFS	64	27483	3	3	3	3	0.67	Flagellar basal-body rc
1016	1	db$2	2::C3K261|RIMP_PSEFS	64	17653	2	2	2	2	0.70	Ribosome maturation f
1017	1	db$2	2::C3JY68|MNMA_PSEFS	64	41397	4	4	4	4	0.58	tRNA-specific 2-thiouri
1018	1	db$2	2::C3K0R2|C3K0R2_PSEFS	64	34379	3	3	1	1	0.15	Chemotaxis protein OS
1019	1	db$2	2::C3K304|C3K304_PSEFS	64	15054	4	4	2	2	2.44	Uncharacterized protei
1020	1	db$2	2::C3JYS8|RUVA_PSEFS	64	22218	3	2	3	2	0.53	Holliday junction ATP-(
1021	1	db$2	2::C3K9U9|C3K9U9_PSEFS	63	9554	1	1	1	1	0.62	Uncharacterized protei
1022	1	db$2	2::C3KC09|C3KC09_PSEFS	63	83087	2	2	2	2	0.12	Periplasmic beta-gluco
1023	1	db$2	2::C3KAF9|C3KAF9_PSEFS	63	50830	2	2	2	2	0.20	Poly-beta-1,6-N-acetyl
1024	1	db$2	2::C3K2Z9|C3K2Z9_PSEFS	63	34289	3	2	3	2	0.32	NMO domain-containin
1025	1	db$2	2::C3K1X4|C3K1X4_PSEFS	63	16845	3	3	3	3	1.30	Putative putative expo
1026	1	db$2	2::C3K415|C3K415_PSEFS	63	70809	2	2	2	2	0.14	Putative ABC transport
1027	1	db$2	2::C3K8W5|C3K8W5_PSEFS	63	21382	2	2	2	2	0.55	Lipopolysaccharide exp
1028	1	db$2	2::C3K8K2|C3K8K2_PSEFS	63	418093	3	3	3	3	0.03	Haemagg_act domain-
1029	1	SwissProt_HUMAN$	3::P08238|HS90B_HUMAN	63	83212	2	2	2	2	0.12	Heat shock protein HS
1030	1	db$2	2::C3JYE6|C3JYE6_PSEFS	63	45437	3	3	3	3	0.37	Putative acyl-CoA dehy
1031	1	db$2	2::C3K5E3|C3K5E3_PSEFS	63	43909	2	1	2	1	0.11	Putative aminotransfer
1032	1	db$2	2::C3K0N8|C3K0N8_PSEFS	63	19586	2	2	2	2	0.62	23S rRNA accumulatio
1033	1	db$2	2::C3K061|C3K061_PSEFS	62	18792	2	2	2	2	0.65	DUF2059 domain-cont
1034	1	db$2	2::C3K0T7|C3K0T7_PSEFS	62	76509	4	3	4	3	0.20	Putative fatty acid oxic
1035	1	db$2	2::C3K6U5|FETP_PSEFS	62	10399	2	2	2	2	1.43	Probable Fe(2+)-traffic
1036	1	SwissProt_HUMAN$	3::Q07020|RL18_HUMAN	62	21621	1	1	1	1	0.24	60S ribosomal protein
1037	1	db$2	2::C3K258|RBFA_PSEFS	62	14697	2	2	2	2	0.89	Ribosome-binding fact
1038	1	db$2	2::C3KDI4|C3KDI4_PSEFS	62	42151	3	3	3	3	0.40	Putative exported prot
1039	1	db$2	2::C3K2S2|C3K2S2_PSEFS	62	38886	4	4	4	4	0.63	Riboflavin biosynthesis
1040	1	db$2	2::C3K0L0|C3K0L0_PSEFS	62	29492	3	3	3	3	0.61	Putative IclR-family re
1041	1	db$2	2::C3KDR1|C3KDR1_PSEFS	62	17628	3	3	3	3	1.21	Uncharacterized protei
1042	1	db$2	2::C3K6Y7|RNC_PSEFS	62	25367	2	2	2	2	0.45	Ribonuclease 3 OS=Ps
1043	1	db$2	2::C3KE82|C3KE82_PSEFS	62	53340	5	4	5	4	0.43	Aldehyde dehydrogena

↑Family	M	DB	Accession	Score	Mass	Matches	Match(sig)	Sequences	Seq(sig)	emPAI	Description
1044	1	db$2	2::C3K998|C3K998_PSEFS	62	35021	4	3	3	3	0.50	Putative dehydrogenas
1045	1	db$2	2::C3K8H9|C3K8H9_PSEFS	62	41728	1	1	1	1	0.12	Putative polyamine AB
1046	1	db$2	2::C3KDM9|C3KDM9_PSEFS	61	28096	3	3	3	3	0.65	Putative oxidoreductas
1047	1	db$2	2::C3K0P9|C3K0P9_PSEFS	61	25457	2	2	2	2	0.45	Two-component syster
1048	1	db$2	2::C3K477|C3K477_PSEFS	61	29836	3	2	3	2	0.37	Exodeoxyribonuclease
1049	1	db$2	2::C3KDB5|C3KDB5_PSEFS	61	33189	1	1	1	1	0.15	Putative glycosyl trans
1050	1	db$2	2::C3KDX6|C3KDX6_PSEFS	61	16394	3	3	3	3	1.35	Peptidyl-prolyl cis-tran
1051	1	db$2	2::C3KBB9|C3KBB9_PSEFS	61	73354	3	2	3	2	0.14	Putative protease OS=
1052	1	db$2	2::C3K733|HTPX_PSEFS	61	32382	4	4	3	3	0.55	Protease HtpX OS=Pse
1053	1	db$2	2::C3K0R6|C3K0R6_PSEFS	61	28374	4	3	4	3	0.64	Uncharacterized protei
1054	1	db$2	2::C3K9M9|C3K9M9_PSEFS	61	14163	1	1	1	1	0.39	Putative endoribonucle
1055	1	db$2	2::C3K590|C3K590_PSEFS	61	74072	2	2	2	2	0.14	Putative membrane pr
1056	1	db$2	2::C3KDC0|C3KDC0_PSEFS	60	36735	1	1	1	1	0.14	Uncharacterized protei
1057	1	db$2	2::C3K3X0|C3K3X0_PSEFS	60	42989	1	1	1	1	0.12	Putative ubiquinone bi
1058	1	db$2	2::C3K456|C3K456_PSEFS	60	38683	1	1	1	1	0.13	Alanine racemase OS=
1059	1	db$2	2::C3K3M5|C3K3M5_PSEFS	60	50101	7	6	6	5	0.60	Putative peptidase OS
1060	1	db$2	2::C3JXL2|C3JXL2_PSEFS	60	30967	1	1	1	1	0.16	Uncharacterized protei
1061	1	db$2	2::C3JZR8|C3JZR8_PSEFS	60	46446	5	4	5	4	0.50	Putative oxidoreductas
1062	1	db$2	2::C3KE46|C3KE46_PSEFS	60	47174	5	5	4	4	0.49	16S rRNA m5C967 me
1063	1	db$2	2::C3K637|C3K637_PSEFS	60	56441	2	2	2	2	0.18	Putative peptide ABC t
1064	1	db$2	2::C3K3H4|C3K3H4_PSEFS	60	26074	1	1	1	1	0.20	Ribosomal RNA small s
1065	1	db$2	2::C3KDC3|LOLB_PSEFS	60	23021	4	4	3	3	0.85	Outer-membrane lipop
1066	1	db$2	2::C3JXL6|C3JXL6_PSEFS	60	41978	1	1	1	1	0.12	Bcr/CflA family efflux t
1067	1	db$2	2::C3JY26|C3JY26_PSEFS	60	17211	2	2	2	2	0.72	Protein-tyrosine-phosp
1068	1	db$2	2::C3K0C5|C3K0C5_PSEFS	60	27111	1	1	1	1	0.19	Putative amino transfe
1069	1	SwissProt_HUMAN$	3::P0DOX6|IGM_HUMAN	60	63446	3	3	3	3	0.25	Immunoglobulin mu he
1070	1	db$2	2::C3K6V8|C3K6V8_PSEFS	60	47941	3	3	3	3	0.34	Putative peptidase OS
1071	1	db$2	2::C3KE16|C3KE16_PSEFS	60	37004	4	4	3	3	0.47	Putative chemotaxis-re
1072	1	db$2	2::C3K1M6|C3K1M6_PSEFS	60	24782	3	3	3	3	0.77	Uncharacterized protei
1073	1	db$2	2::C3K8W4|C3K8W4_PSEFS	60	18790	7	5	5	4	1.71	Lipopolysaccharide exp
1074	1	db$2	2::C3K4C8|C3K4C8_PSEFS	59	49694	2	2	2	2	0.21	Putative transport-rela
1075	1	db$2	2::C3KBI7|C3KBI7_PSEFS	59	26715	2	2	2	2	0.42	Uncharacterized protei
1076	1	db$2	2::C3JYQ1|C3JYQ1_PSEFS	59	48078	1	1	1	1	0.10	Putative CoA transfera
1077	1	db$2	2::C3KE17|C3KE17_PSEFS	59	24430	2	2	2	2	0.47	Two component syster
1078	1	db$2	2::C3K5T7|C3K5T7_PSEFS	59	23769	2	2	2	2	0.49	Uncharacterized protei
1079	1	db$2	2::C3K0Q8|C3K0Q8_PSEFS	58	24717	1	1	1	1	0.21	Basal-body rod modific
1080	1	db$2	2::C3K1F7|C3K1F7_PSEFS	58	69251	2	1	2	1	0.07	tRNA uridine 5-carbox
1081	1	db$2	2::C3KCP6|C3KCP6_PSEFS	58	38240	2	1	2	1	0.13	ADP-heptose--LPS hep
1082	1	db$2	2::C3K4M2|C3K4M2_PSEFS	58	36363	5	3	5	3	0.48	Catabolite repressor/a
1083	1	db$2	2::C3K1E5|ATPE_PSEFS	58	14558	4	3	3	2	0.90	ATP synthase epsilon c

↑Family	M	DB	Accession	Score	Mass	Matches	Match(sig)	Sequences	Seq(sig)	emPAI	Description
1084	1	db$2	2::C3K1A3|C3K1A3_PSEFS	58	26906	2	2	2	2	0.42	Putative pyridoxamine
1085	1	db$2	2::C3K5Q0|C3K5Q0_PSEFS	57	53619	1	1	1	1	0.09	Mannose-1-phosphate
1086	1	db$2	2::C3K390|C3K390_PSEFS	57	18447	4	3	3	2	0.66	Zn_protease domain-c
1087	1	db$2	2::C3K088|C3K088_PSEFS	57	20655	2	1	2	1	0.26	Putative isochorismata
1088	1	db$2	2::C3KC04|C3KC04_PSEFS	57	39513	2	2	2	2	0.27	Putative hydrolase OS=
1089	1	SwissProt_HUMAN$	3::Q14240|IF4A2_HUMAN	57	46373	2	2	2	2	0.23	Eukaryotic initiation fa
1090	1	db$2	2::C3K4W6|C3K4W6_PSEFS	57	44511	3	2	3	2	0.24	Putative lipoprotein rel
1091	1	db$2	2::C3K0M6|C3K0M6_PSEFS	57	25497	2	2	2	2	0.45	Putative tetR-family re
1092	1	db$2	2::C3KE32|C3KE32_PSEFS	57	20912	2	2	1	1	0.25	Sigma factor negative
1093	1	db$2	2::C3K3T7|C3K3T7_PSEFS	57	39982	2	2	2	2	0.27	Putrescine ABC transp
1094	1	db$2	2::C3K0Z5|C3K0Z5_PSEFS	57	22469	1	1	1	1	0.23	Corrinoid adenosyltran
1095	1	db$2	2::C3K425|C3K425_PSEFS	57	27295	2	2	2	2	0.41	Positive alginate biosy
1096	1	db$2	2::C3K9N1|C3K9N1_PSEFS	57	43216	3	2	3	2	0.24	D-ser_dehydrat domai
1097	1	db$2	2::C3K468|C3K468_PSEFS	57	6077	2	1	1	1	1.10	50S ribosomal protein
1098	1	SwissProt_HUMAN$	3::Q5QNW6|H2B2F_HUMAN	57	13912	4	3	3	2	0.96	Histone H2B type 2-F (
1099	1	db$2	2::C3K308|TRPD_PSEFS	57	36976	3	3	3	3	0.47	Anthranilate phosphori
1100	1	SwissProt_HUMAN$	3::Q86YZ3|HORN_HUMAN	57	282228	3	3	1	1	0.03	Hornerin OS=Homo sa
1101	1	db$2	2::C3KCV6|C3KCV6_PSEFS	57	40154	4	3	4	3	0.42	Probable alcohol dehyc
1102	1	db$2	2::C3KDC6|RF1_PSEFS	57	39857	1	1	1	1	0.13	Peptide chain release f
1103	1	db$2	2::C3KBM6|C3KBM6_PSEFS	56	11934	3	3	1	1	1.17	Glutaredoxin OS=Pseu
1104	1	db$2	2::C3K589|C3K589_PSEFS	56	50032	3	3	3	3	0.33	Transcriptional regulat
1105	1	SwissProt_HUMAN$	3::P61604|CH10_HUMAN	56	10925	4	4	4	4	4.49	10 kDa heat shock pro
1106	1	SwissProt_HUMAN$	3::P46781|RS9_HUMAN	56	22578	3	3	3	3	0.87	40S ribosomal protein
1107	1	db$2	2::C3JXX3|C3JXX3_PSEFS	56	52842	1	1	1	1	0.09	Cytochrome-c oxidase
1108	1	db$2	2::C3KE89|C3KE89_PSEFS	56	24346	3	3	3	3	0.79	Uncharacterized protei
1109	1	db$2	2::C3JXW6|C3JXW6_PSEFS	56	64770	1	1	1	1	0.08	Putative exported prot
1110	1	db$2	2::C3K2V8|RL30_PSEFS	56	6521	3	3	2	2	3.01	50S ribosomal protein
1111	1	db$2	2::C3K5U7|C3K5U7_PSEFS	56	105852	3	3	3	3	0.14	Putative aminotransfer
1112	1	db$2	2::C3KBU0|C3KBU0_PSEFS	56	36726	4	4	4	4	0.67	Sulfate/thiosulfate imp
1113	1	db$2	2::C3KDE6|C3KDE6_PSEFS	55	25704	1	1	1	1	0.20	Tw-component system
1114	1	db$2	2::C3K3K9|C3K3K9_PSEFS	55	21575	3	3	2	2	0.55	Conserved hypothetica
1115	1	SwissProt_HUMAN$	3::P63244|RACK1_HUMAN	55	35055	2	2	2	2	0.31	Receptor of activated p
1116	1	db$2	2::C3JZS9|C3JZS9_PSEFS	55	35591	2	2	2	2	0.30	Cbb3-type cytochrome
1116	2	db$2	2::C3JYN0|C3JYN0_PSEFS	28	33944	3	3	3	3	0.52	Cbb3-type cytochrome
1117	1	db$2	2::C3K3W4|C3K3W4_PSEFS	55	13233	3	3	2	2	1.02	Glycine cleavage syste
1118	1	db$2	2::C3K0D5|MINE_PSEFS	55	9531	4	3	4	3	3.26	Cell division topologica
1119	1	db$2	2::C3KDG2|C3KDG2_PSEFS	55	41589	6	5	5	4	0.76	AMP nucleosidase OS=
1120	1	db$2	2::C3KAV6|C3KAV6_PSEFS	55	17648	3	3	3	3	1.21	Thiol peroxidase OS=F
1121	1	db$2	2::C3K6G1|RLMF_PSEFS	55	38015	3	3	3	3	0.45	Ribosomal RNA large s
1122	1	db$2	2::C3K146|C3K146_PSEFS	55	27134	3	3	3	3	0.68	Fumarate and nitrate r

↑Family	M	DB	Accession	Score	Mass	Matches	Match(sig)	Sequences	Seq(sig)	emPAI	Description
1123	1	db$2	2::C3JZU8|C3JZU8_PSEFS	55	30426	2	2	2	2	0.36	Hydrolase_4 domain-c
1124	1	db$2	2::C3K7K5|C3K7K5_PSEFS	54	12541	1	1	1	1	0.45	Uncharacterized protei
1125	1	db$2	2::C3K0S6|C3K0S6_PSEFS	54	36802	1	1	1	1	0.14	Putative oxidoreductas
1126	1	db$2	2::C3K0R1|C3K0R1_PSEFS	54	30842	1	1	1	1	0.16	Protein-glutamate O-m
1127	1	db$2	2::C3K8T8|C3K8T8_PSEFS	54	14354	2	1	2	1	0.38	Sec-independent prote
1128	1	db$2	2::C3K3V4|C3K3V4_PSEFS	54	41512	1	1	1	1	0.12	Acetylornithine deacet
1129	1	db$2	2::C3K0J1|C3K0J1_PSEFS	54	36130	2	2	2	2	0.30	Flagellar motor switch
1130	1	db$2	2::C3JYU4|C3JYU4_PSEFS	53	20911	3	1	2	1	0.25	Corrinoid adenosyltran
1131	1	db$2	2::C3KE08|C3KE08_PSEFS	53	25044	2	2	2	2	0.45	Conserved hypothetica
1132	1	db$2	2::C3K223|C3K223_PSEFS	53	37863	2	2	2	2	0.28	Protein-methionine-sul
1133	1	db$2	2::C3K8K1|C3K8K1_PSEFS	53	40207	2	1	1	1	0.12	Phosphate-binding pro
1134	1	db$2	2::C3JYS0|C3JYS0_PSEFS	53	29172	3	3	3	3	0.62	Cell division coordinato
1135	1	db$2	2::C3K450|C3K450_PSEFS	53	61786	2	2	2	2	0.17	Putative EAL/GGDEF d
1136	1	db$2	2::C3K2K7|C3K2K7_PSEFS	53	37713	2	2	2	2	0.28	PhoH domain-containir
1137	1	db$2	2::C3K650|C3K650_PSEFS	53	13083	3	3	3	3	1.90	N-acetyltransferase do
1138	1	db$2	2::C3K486|C3K486_PSEFS	53	30593	3	2	3	2	0.36	Epimerase domain-cor
1139	1	db$2	2::C3JYK2|C3JYK2_PSEFS	53	30492	3	2	3	2	0.36	Bifunctional protein Fo
1140	1	db$2	2::C3K103|C3K103_PSEFS	53	23527	2	2	2	2	0.49	Phosphoribosylglycinar
1141	1	db$2	2::C3K0X7|C3K0X7_PSEFS	53	126663	1	1	1	1	0.04	Histidine kinase OS=P:
1142	1	db$2	2::C3K870|C3K870_PSEFS	53	20966	3	3	2	2	0.56	Uncharacterized protei
1143	1	db$2	2::C3K4U9|C3K4U9_PSEFS	52	34107	1	1	1	1	0.15	Putative LysR-family re
1144	1	db$2	2::C3K3R7|RPIA_PSEFS	52	23281	4	2	4	2	0.50	Ribose-5-phosphate is
1145	1	db$2	2::C3K458|C3K458_PSEFS	52	47152	1	1	1	1	0.11	D-amino acid dehydro
1146	1	db$2	2::C3K6E8|C3K6E8_PSEFS	52	27518	1	1	1	1	0.19	Putative GntR-family r
1147	1	db$2	2::C3K5K1|C3K5K1_PSEFS	52	38065	3	3	3	3	0.45	Putative phospholipase
1148	1	db$2	2::C3JY97|C3JY97_PSEFS	52	30357	1	1	1	1	0.17	N-hydroxyarylamine O
1149	1	db$2	2::C3K4J4|HSLO_PSEFS	52	33320	2	2	2	2	0.33	33 kDa chaperonin OS
1150	1	db$2	2::C3K831|C3K831_PSEFS	52	45359	4	4	3	3	0.37	Putative thiolase OS=F
1151	1	db$2	2::C3K4U2|C3K4U2_PSEFS	52	24386	1	1	1	1	0.21	Uncharacterized protei
1152	1	db$2	2::C3KDC8|C3KDC8_PSEFS	52	26520	1	1	1	1	0.19	Molybdopterin biosyntl
1153	1	db$2	2::C3K8Z4|C3K8Z4_PSEFS	52	45613	2	2	2	2	0.23	High-affinity branched
1154	1	db$2	2::C3K9N3|C3K9N3_PSEFS	52	10076	1	1	1	1	0.58	Peptidyl-prolyl cis-tran
1155	1	db$2	2::C3K9Q2|C3K9Q2_PSEFS	52	16954	4	3	3	3	1.28	Ribonuclease H OS=Ps
1156	1	SwissProt_HUMAN$	3::P62701|RS4X_HUMAN	51	29579	2	2	2	2	0.38	40S ribosomal protein
1157	1	db$2	2::C3K7N0|C3K7N0_PSEFS	51	28545	1	1	1	1	0.18	Transport permease pr
1158	1	db$2	2::C3KE85|C3KE85_PSEFS	51	26099	1	1	1	1	0.20	Purine nucleoside phos
1159	1	db$2	2::C3KEC3|C3KEC3_PSEFS	51	16145	3	3	2	2	0.79	Putative membrane pr
1160	1	db$2	2::C3K8J9|C3K8J9_PSEFS	51	51515	1	1	1	1	0.10	Type II secretion syste
1161	1	db$2	2::C3KAL0|C3KAL0_PSEFS	51	43962	2	1	2	1	0.11	PlsC domain-containin
1162	1	SwissProt_HUMAN$	3::P46783|RS10_HUMAN	51	18886	1	1	1	1	0.28	40S ribosomal protein

↑Family	M	DB	Accession	Score	Mass	Matches	Match(sig)	Sequences	Seq(sig)	emPAI	Description
1163	1	db$2	2::C3K0U4|C3K0U4_PSEFS	50	36872	4	2	3	2	0.29	Flagellar motor switch
1164	1	db$2	2::C3KBY4|C3KBY4_PSEFS	50	31613	3	3	3	3	0.56	Ribosomal RNA small s
1165	1	db$2	2::C3K101|C3K101_PSEFS	50	37185	2	2	2	2	0.29	Uncharacterized protei
1166	1	db$2	2::C3KEC9|C3KEC9_PSEFS	50	53005	2	2	2	2	0.20	Putative beta-barrel as
1167	1	db$2	2::C3K421|C3K421_PSEFS	50	46244	4	2	4	2	0.23	Putative HemY domain
1168	1	db$2	2::C3KB19|C3KB19_PSEFS	50	44434	3	2	3	2	0.24	HlyD family secretion p
1169	1	db$2	2::C3KBL9|C3KBL9_PSEFS	50	27726	3	2	3	2	0.40	Glycerol-3-phosphate r
1170	1	db$2	2::C3JYL7|C3JYL7_PSEFS	50	12417	2	2	2	2	1.11	Putative copper resista
1171	1	db$2	2::C3KAF8|C3KAF8_PSEFS	50	74138	1	1	1	1	0.07	Putative lipoprotein OS
1172	1	db$2	2::C3K6S3|C3K6S3_PSEFS	50	25881	2	2	2	2	0.44	Putative glutathione S-
1173	1	db$2	2::C3JZ64|C3JZ64_PSEFS	50	17217	5	4	4	4	1.97	MaoC-like domain-cont
1174	1	db$2	2::C3K6G9|LPXB_PSEFS	50	41468	2	2	2	2	0.26	Lipid-A-disaccharide sy
1175	1	db$2	2::C3K820|C3K820_PSEFS	50	26990	2	2	2	2	0.42	Putative ABC transport
1176	1	db$2	2::C3K544|C3K544_PSEFS	50	74165	5	4	5	4	0.29	Uncharacterized protei
1177	1	db$2	2::C3K601|DXR_PSEFS	49	42341	2	2	2	2	0.25	1-deoxy-D-xylulose 5-
1178	1	db$2	2::C3K737|C3K737_PSEFS	49	17727	3	2	3	2	0.69	Glutathione peroxidase
1179	1	db$2	2::C3K5J9|C3K5J9_PSEFS	49	96070	2	2	2	2	0.10	Histidine kinase OS=Ps
1180	1	SwissProt_HUMAN$	3::P62318|SMD3_HUMAN	49	13907	1	1	1	1	0.40	Small nuclear ribonucl
1181	1	db$2	2::C3KDF9|C3KDF9_PSEFS	49	17633	2	2	2	2	0.70	CinA domain-containin
1182	1	db$2	2::C3K5G4|RLMM_PSEFS	49	40585	1	1	1	1	0.12	Ribosomal RNA large s
1183	1	db$2	2::C3K660|C3K660_PSEFS	49	28970	1	1	1	1	0.18	Hydroxypyruvate isom
1184	1	db$2	2::C3K3Q3|C3K3Q3_PSEFS	49	61523	3	3	3	3	0.26	Putative cation ABC tra
1185	1	db$2	2::C3K607|LPXA_PSEFS	49	28002	1	1	1	1	0.18	Acyl-[acyl-carrier-prot
1186	1	db$2	2::C3JXI0|C3JXI0_PSEFS	49	23030	1	1	1	1	0.23	Nudix hydrolase doma
1187	1	db$2	2::C3K9Y7|C3K9Y7_PSEFS	49	30413	1	1	1	1	0.17	Undecaprenyl-diphospl
1188	1	db$2	2::C3K6V0|C3K6V0_PSEFS	49	23354	1	1	1	1	0.22	Imidazole glycerol pho
1189	1	db$2	2::C3JY81|C3JY81_PSEFS	48	15084	2	2	2	2	0.86	NADH-quinone oxidore
1190	1	db$2	2::C3KB68|C3KB68_PSEFS	48	11363	1	1	1	1	0.51	DUF1883 domain-cont
1191	1	db$2	2::C3K685|C3K685_PSEFS	48	13635	2	2	2	2	0.98	6-carboxy-5,6,7,8-tetr
1192	1	db$2	2::C3JYS7|RUVB_PSEFS	48	38919	2	2	2	2	0.27	Holliday junction ATP-(
1193	1	db$2	2::C3K2T3|C3K2T3_PSEFS	48	32553	2	2	2	2	0.34	dTDP-4-dehydrorhamr
1194	1	db$2	2::C3K4W5|C3K4W5_PSEFS	48	24663	3	2	3	2	0.46	Lipoprotein-releasing s
1195	1	db$2	2::C3K439|C3K439_PSEFS	48	25827	3	3	3	3	0.73	Putative hydrolase OS=
1196	1	db$2	2::C3JZ23|C3JZ23_PSEFS	48	131220	2	1	2	1	0.04	Chromosome partition
1197	1	db$2	2::C3K7A7|C3K7A7_PSEFS	48	43115	2	2	2	2	0.25	Conserved hypothetica
1198	1	db$2	2::C3K911|C3K911_PSEFS	48	29460	1	1	1	1	0.17	Putative ABC transport
1199	1	db$2	2::C3K2U3|C3K2U3_PSEFS	48	27946	2	2	2	2	0.40	Trans-aconitate 2-met
1200	1	db$2	2::C3KDQ1|C3KDQ1_PSEFS	48	35181	2	2	2	2	0.31	Putative LysR-family re
1201	1	db$2	2::C3K7S1|C3K7S1_PSEFS	48	25085	2	2	1	1	0.45	Protein MgtC OS=Pseu
1202	1	SwissProt_HUMAN$	3::P39019|RS19_HUMAN	48	16051	2	2	2	2	0.79	40S ribosomal protein

↑Family	M	DB	Accession	Score	Mass	Matches	Match(sig)	Sequences	Seq(sig)	emPAI	Description
1203	1	db$2	2::C3K759|C3K759_PSEFS	48	35694	1	1	1	1	0.14	Putative dehydrogenas
1204	1	db$2	2::C3K0U5|C3K0U5_PSEFS	48	62992	2	2	2	2	0.16	Flagellar M-ring protei
1205	1	db$2	2::C3JYU5|C3JYU5_PSEFS	48	34090	3	3	3	3	0.51	Nudix hydrolase doma
1206	1	db$2	2::C3K1I0|C3K1I0_PSEFS	48	22456	2	1	2	1	0.23	Nudix hydrolase doma
1207	1	db$2	2::C3K1N0|C3K1N0_PSEFS	47	18655	1	1	1	1	0.29	Rick_17kDa_Anti dom
1208	1	db$2	2::C3K047|C3K047_PSEFS	47	16614	2	2	2	2	0.75	Putative putative heat
1209	1	db$2	2::C3K646|C3K646_PSEFS	47	21182	2	2	2	2	0.56	Putative acyl-CoA thio
1210	1	db$2	2::C3K1H0|RIMM_PSEFS	47	20004	3	3	3	3	1.02	Ribosome maturation f
1211	1	SwissProt_HUMAN$	3::P62888|RL30_HUMAN	47	12776	3	3	3	3	1.98	60S ribosomal protein
1212	1	db$2	2::C3K300|C3K300_PSEFS	47	34122	1	1	1	1	0.15	Putative membrane pr
1213	1	db$2	2::C3K068|C3K068_PSEFS	47	65454	4	4	4	4	0.34	Putative asparagine sy
1214	1	db$2	2::C3JYR2|C3JYR2_PSEFS	47	48934	2	2	2	2	0.21	L-serine dehydratase (
1215	1	db$2	2::C3KCB9|C3KCB9_PSEFS	47	35987	3	3	3	3	0.48	Polyphosphate kinase
1216	1	db$2	2::C3KAY5|C3KAY5_PSEFS	47	30881	1	1	1	1	0.16	NAD-capped RNA hydr
1217	1	db$2	2::C3K8R5|C3K8R5_PSEFS	47	48038	2	1	2	1	0.10	Putative methyl-accept
1218	1	db$2	2::C3K0H8|C3K0H8_PSEFS	47	29525	3	3	3	3	0.61	Protein phosphatase C
1219	1	db$2	2::C3JXY2|C3JXY2_PSEFS	47	35731	3	2	2	2	0.30	L-allo-threonine aldola
1220	1	db$2	2::C3K6V3|HIS6_PSEFS	47	27111	1	1	1	1	0.19	Imidazole glycerol pho
1221	1	db$2	2::C3K4V3|C3K4V3_PSEFS	46	45550	3	3	3	3	0.37	Uncharacterized protei
1222	1	db$2	2::C3K2M0|Y5418_PSEFS	46	10305	1	1	1	1	0.56	UPF0250 protein PFLU_
1223	1	db$2	2::C3K654|C3K654_PSEFS	46	34290	2	2	2	2	0.32	2-dehydropantoate 2-r
1224	1	db$2	2::C3K2Q3|C3K2Q3_PSEFS	46	23107	1	1	1	1	0.23	Putative hydrolase OS
1225	1	db$2	2::C3K1B1|C3K1B1_PSEFS	46	46678	1	1	1	1	0.11	Putative acid tolerance
1226	1	db$2	2::C3K386|C3K386_PSEFS	46	22957	2	2	2	2	0.51	Putative glutathione S-
1227	1	db$2	2::C3K7G4|C3K7G4_PSEFS	46	54521	4	4	4	4	0.42	Ribonuclease G OS=Ps
1228	1	db$2	2::C3K6W8|C3K6W8_PSEFS	46	16611	1	1	1	1	0.32	3-dehydroquinate deh
1229	1	db$2	2::C3K6U9|HIS7_PSEFS	46	21830	2	2	2	2	0.54	Imidazoleglycerol-pho
1230	1	db$2	2::C3K591|C3K591_PSEFS	46	19872	3	3	2	2	1.03	Putative bacterioferriti
1231	1	SwissProt_HUMAN$	3::P0CG04|IGLC1_HUMAN	46	11341	2	2	2	2	1.27	Immunoglobulin lamb
1232	1	db$2	2::C3KE56|C3KE56_PSEFS	46	59021	3	3	3	3	0.27	Glutamate--cysteine li
1233	1	SwissProt_HUMAN$	3::P07437|TBB5_HUMAN	46	49639	2	2	2	2	0.21	Tubulin beta chain OS
1234	1	db$2	2::C3K7L9|C3K7L9_PSEFS	46	28704	2	2	2	2	0.39	Putative membrane pr
1235	1	db$2	2::C3K5B2|C3K5B2_PSEFS	46	35714	2	2	2	2	0.30	Dipeptide ABC transpo
1236	1	db$2	2::C3K586|C3K586_PSEFS	46	21064	4	3	4	3	0.95	ADP compounds hydro
1237	1	db$2	2::C3K8W8|C3K8W8_PSEFS	45	29386	2	2	2	2	0.38	Putative ABC transport
1238	1	db$2	2::C3K838|C3K838_PSEFS	45	9715	2	2	2	2	1.57	Putative phosphocarrie
1239	1	db$2	2::C3KBP5|C3KBP5_PSEFS	45	30770	1	1	1	1	0.17	Putative AraC-family r
1240	1	db$2	2::C3K1R4|C3K1R4_PSEFS	45	17440	1	1	1	1	0.31	Putative plasmid-relate
1241	1	db$2	2::C3JZN0|C3JZN0_PSEFS	45	13917	2	2	2	2	0.96	MerR-family transcript
1242	1	SwissProt_HUMAN$	3::P62847|RS24_HUMAN	45	15413	1	1	1	1	0.35	40S ribosomal protein

↑Family	M	DB	Accession	Score	Mass	Matches	Match(sig)	Sequences	Seq(sig)	emPAI	Description
1243	1	db$2	2::C3KDN6|C3KDN6_PSEFS	45	32369	1	1	1	1	0.16	Uncharacterized protei
1244	1	db$2	2::C3JY69|HFLD_PSEFS	45	23173	2	2	2	2	0.50	High frequency lysoge
1245	1	db$2	2::C3K0D2|C3K0D2_PSEFS	45	35834	2	2	2	2	0.30	Lipid A biosynthesis la
1246	1	db$2	2::C3JZ24|C3JZ24_PSEFS	45	24729	4	3	4	3	0.77	Putative GntR-family r
1247	1	db$2	2::C3K0Y8|COBT_PSEFS	45	36066	1	1	1	1	0.14	Nicotinate-nucleotide--
1248	1	db$2	2::C3JZR9|C3JZR9_PSEFS	45	27415	2	1	2	1	0.19	Putative dehydrogenas
1249	1	db$2	2::C3K867|C3K867_PSEFS	45	39735	2	1	2	1	0.13	PALP domain-containir
1250	1	db$2	2::C3K353|C3K353_PSEFS	45	44910	1	1	1	1	0.11	Putative aminotransfer
1251	1	db$2	2::C3K0S4|C3K0S4_PSEFS	44	65961	1	1	1	1	0.07	Isocitrate dehydrogena
1252	1	db$2	2::C3K4N5|C3K4N5_PSEFS	44	28830	1	1	1	1	0.18	Putative amino acid AE
1253	1	db$2	2::C3K5X1|C3K5X1_PSEFS	44	46611	1	1	1	1	0.11	Histidine kinase OS=P:
1254	1	SwissProt_HUMAN$	3::P0C0S8|H2A1_HUMAN	44	14083	2	2	2	2	0.94	Histone H2A type 1 OS
1255	1	db$2	2::C3K391|C3K391_PSEFS	44	33913	1	1	1	1	0.15	Putative acyltransferas
1256	1	SwissProt_HUMAN$	3::Q6IS14|IF5AL_HUMAN	44	16762	1	1	1	1	0.32	Eukaryotic translation
1257	1	db$2	2::C3K0U7|C3K0U7_PSEFS	44	49884	3	3	3	3	0.33	Two-component respo
1258	1	SwissProt_HUMAN$	3::Q92805|GOGA1_HUMAN	44	88130	1	1	1	1	0.06	Golgin subfamily A me
1259	1	db$2	2::C3K536|C3K536_PSEFS	44	22752	1	1	1	1	0.23	DUF4174 domain-cont
1260	1	db$2	2::C3K574|C3K574_PSEFS	44	36116	2	2	2	2	0.30	Putative oxidoreductas
1261	1	db$2	2::C3K3J1|C3K3J1_PSEFS	44	16523	1	1	1	1	0.33	Putative chemotaxis-re
1262	1	db$2	2::C3K485|C3K485_PSEFS	44	26565	1	1	1	1	0.19	Putative lipoprotein OS
1263	1	db$2	2::C3K8U0|HIS2_PSEFS	44	12007	1	1	1	1	0.47	Phosphoribosyl-ATP py
1264	1	SwissProt_HUMAN$	3::A6NJ78|MET15_HUMAN	44	46092	1	1	1	1	0.11	12S rRNA N4-methylcy
1265	1	db$2	2::C3K2U2|C3K2U2_PSEFS	44	20033	2	2	2	2	0.60	Putative hydrolase OS:
1266	1	db$2	2::C3K1A2|C3K1A2_PSEFS	44	17206	1	1	1	1	0.31	Putative membrane pr
1267	1	SwissProt_HUMAN$	3::P18085|ARF4_HUMAN	44	20498	1	1	1	1	0.26	ADP-ribosylation factor
1268	1	db$2	2::C3KDW0|C3KDW0_PSEFS	43	49939	3	3	3	3	0.33	N-acetylmuramoyl-L-a
1269	1	db$2	2::C3K8L9|C3K8L9_PSEFS	43	65348	2	1	2	1	0.08	Putative potassium-eff
1270	1	db$2	2::C3K2L9|LIPB_PSEFS	43	23473	1	1	1	1	0.22	Octanoyltransferase O:
1271	1	db$2	2::C3K9N7|C3K9N7_PSEFS	43	48751	1	1	1	1	0.10	Putative membrane pr
1272	1	db$2	2::C3K1L7|RLMN_PSEFS	43	42342	4	4	4	4	0.56	Dual-specificity RNA m
1273	1	db$2	2::C3KDM8|C3KDM8_PSEFS	43	57105	3	2	3	2	0.18	HDOD domain-contain
1274	1	db$2	2::C3K735|MSRB_PSEFS	43	14471	1	1	1	1	0.38	Peptide methionine sul
1275	1	db$2	2::C3K092|C3K092_PSEFS	43	14085	3	2	3	2	0.94	Glutaredoxin domain-c
1276	1	db$2	2::C3K327|C3K327_PSEFS	43	29832	1	1	1	1	0.17	Ribosomal RNA small s
1277	1	db$2	2::C3K0L5|C3K0L5_PSEFS	43	17844	1	1	1	1	0.30	Putative GNAT-family a
1278	1	db$2	2::C3K3K7|C3K3K7_PSEFS	43	24490	3	3	3	3	0.78	Pyridoxal phosphate h
1279	1	SwissProt_HUMAN$	3::P19338|NUCL_HUMAN	43	76568	1	1	1	1	0.06	Nucleolin OS=Homo sa
1280	1	db$2	2::C3KAH2|C3KAH2_PSEFS	43	24970	4	4	3	3	0.76	SPOR domain-containi
1281	1	db$2	2::C3KAB9|C3KAB9_PSEFS	43	40944	1	1	1	1	0.12	Putative acetyl-CoA ac
1282	1	db$2	2::C3K6H7|C3K6H7_PSEFS	43	10540	1	1	1	1	0.55	Cell division protein Ft:

↑Family	M	DB	Accession	Score	Mass	Matches	Match(sig)	Sequences	Seq(sig)	emPAI	Description
1283	1	db$2	2::C3K6Y0|C3K6Y0_PSEFS	43	13973	1	1	1	1	0.40	4-carboxymuconolacto
1284	1	db$2	2::C3K6W0|C3K6W0_PSEFS	43	14786	3	3	2	2	0.88	Putative rhodanese do
1285	1	db$2	2::C3K129|C3K129_PSEFS	43	42141	1	1	1	1	0.12	Putative acetyl CoA ac
1286	1	db$2	2::C3JZ82|C3JZ82_PSEFS	42	37007	2	1	2	1	0.14	Putative dehydrogenas
1287	1	db$2	2::C3K1D4|C3K1D4_PSEFS	42	64734	3	2	3	2	0.16	Uncharacterized protei
1288	1	db$2	2::C3K5M2|C3K5M2_PSEFS	42	41151	1	1	1	1	0.12	Phosphorous compoun
1289	1	db$2	2::C3K3T5|C3K3T5_PSEFS	42	34240	1	1	1	1	0.15	Putrescine ABC transp
1290	1	db$2	2::C3K1W6|C3K1W6_PSEFS	42	13390	4	4	4	4	3.00	D-erythro-7,8-dihydro
1291	1	db$2	2::C3K158|C3K158_PSEFS	42	36421	1	1	1	1	0.14	Putative regulatory pr
1292	1	db$2	2::C3KB02|C3KB02_PSEFS	42	42163	2	1	2	1	0.12	Putative exported prot
1293	1	db$2	2::C3KB17|C3KB17_PSEFS	42	51104	4	2	4	2	0.20	Outer membrane comp
1294	1	db$2	2::C3KBY0|GMHA_PSEFS	42	21250	2	2	2	2	0.56	Phosphoheptose isome
1295	1	db$2	2::C3K6K7|C3K6K7_PSEFS	42	17395	1	1	1	1	0.31	GreA_GreB domain-co
1296	1	db$2	2::C3KDJ5|C3KDJ5_PSEFS	42	38272	1	1	1	1	0.13	Putative lipoprotein OS
1297	1	db$2	2::C3K132|C3K132_PSEFS	42	42760	2	2	2	2	0.25	Uncharacterized protei
1298	1	db$2	2::C3KCJ9|C3KCJ9_PSEFS	42	40399	1	1	1	1	0.12	Putative phosphotrans
1299	1	db$2	2::C3K0X8|C3K0X8_PSEFS	41	55461	2	2	2	2	0.19	DNA recombination pr
1300	1	SwissProt_HUMAN$	3::P62750|RL23A_HUMAN	41	17684	1	1	1	1	0.30	60S ribosomal protein
1301	1	SwissProt_HUMAN$	3::P15531|NDKA_HUMAN	41	17138	3	3	2	2	0.73	Nucleoside diphosphat
1302	1	db$2	2::C3JYN3|C3JYN3_PSEFS	41	52328	2	2	2	2	0.20	Family S13 unassignec
1303	1	SwissProt_HUMAN$	3::P02675|FIBB_HUMAN	41	55892	2	2	2	2	0.18	Fibrinogen beta chain
1304	1	db$2	2::C3K5K0|C3K5K0_PSEFS	41	25438	1	1	1	1	0.20	Two-component syster
1305	1	db$2	2::C3K8L0|C3K8L0_PSEFS	41	16885	1	1	1	1	0.32	Uncharacterized protei
1306	1	db$2	2::C3KDR2|C3KDR2_PSEFS	41	26142	3	2	3	2	0.43	S4 RNA-binding domai
1307	1	SwissProt_HUMAN$	3::O00189|AP4M1_HUMAN	41	49946	1	1	1	1	0.10	AP-4 complex subunit
1308	1	SwissProt_HUMAN$	3::Q9NRD0|FBX8_HUMAN	41	37045	1	1	1	1	0.14	F-box only protein 8 O
1309	1	SwissProt_HUMAN$	3::P05109|S10A8_HUMAN	41	10828	1	1	1	1	0.53	Protein S100-A8 OS=H
1310	1	db$2	2::C3K8L6|C3K8L6_PSEFS	41	28069	2	1	2	1	0.18	Precorrin-6A synthase
1311	1	db$2	2::C3K3W8|C3K3W8_PSEFS	41	44404	1	1	1	1	0.11	Putative ubiquinone bi
1312	1	SwissProt_HUMAN$	3::Q08828|ADCY1_HUMAN	41	123360	1	1	1	1	0.04	Adenylate cyclase type
1313	1	db$2	2::C3K4T5|C3K4T5_PSEFS	41	30974	2	2	2	2	0.36	Putative hydrolase OS
1314	1	db$2	2::C3K564|C3K564_PSEFS	40	54508	3	3	3	3	0.30	Methylmalonate-semia
1315	1	db$2	2::C3KAA6|C3KAA6_PSEFS	40	51931	2	1	2	1	0.10	Putative cell envelope
1316	1	db$2	2::C3KAX0|C3KAX0_PSEFS	40	84238	3	3	3	3	0.18	1,4-alpha-glucan bran
1317	1	db$2	2::C3KBL6|C3KBL6_PSEFS	40	27732	2	2	2	2	0.40	Glutamate/aspartate A
1318	1	db$2	2::C3K7V7|C3K7V7_PSEFS	40	18111	1	1	1	1	0.30	Uncharacterized protei
1319	1	db$2	2::C3K6A1|C3K6A1_PSEFS	40	22990	1	1	1	1	0.23	Putative copper-bindin
1320	1	db$2	2::C3K7J8|C3K7J8_PSEFS	40	78803	3	3	3	3	0.20	ATP-dependent DNA h
1321	1	db$2	2::C3KAZ7|C3KAZ7_PSEFS	40	48577	1	1	1	1	0.10	Putative chain length c
1322	1	db$2	2::C3KBL5|C3KBL5_PSEFS	40	24580	2	1	2	1	0.21	Glutamate/aspartate A

↑Family	M	DB	Accession	Score	Mass	Matches	Match(sig)	Sequences	Seq(sig)	emPAI	Description
1323	1	db$2	2::C3JY59|LFTR_PSEFS	39	25331	1	1	1	1	0.20	Leucyl/phenylalanyl-tR
1324	1	db$2	2::C3KE45|C3KE45_PSEFS	39	49639	3	2	2	1	0.10	Trk system potassium
1325	1	SwissProt_HUMAN$	3::P26641|EF1G_HUMAN	39	50087	1	1	1	1	0.10	Elongation factor 1-gai
1326	1	db$2	2::C3JZ32|C3JZ32_PSEFS	39	38286	2	2	2	2	0.28	Putative cation transpo
1327	1	db$2	2::C3JZP5|C3JZP5_PSEFS	39	30747	1	1	1	1	0.17	Ribokinase OS=Pseudo
1328	1	SwissProt_HUMAN$	3::P62913|RL11_HUMAN	39	20240	2	2	2	2	0.59	60S ribosomal protein
1329	1	db$2	2::C3K2P8|C3K2P8_PSEFS	39	24358	3	3	3	3	0.79	Membrane-related pro
1330	1	db$2	2::C3K3L3|C3K3L3_PSEFS	39	21091	3	3	3	3	0.95	dITP/XTP pyrophospha
1331	1	db$2	2::C3K8Z2|C3K8Z2_PSEFS	39	25669	1	1	1	1	0.20	High-affinity branched
1332	1	db$2	2::C3K282|SSRP_PSEFS	39	18308	2	2	2	2	0.67	SsrA-binding protein C
1333	1	db$2	2::C3K0V2|C3K0V2_PSEFS	39	48301	1	1	1	1	0.10	Flagellar hook-associat
1334	1	SwissProt_HUMAN$	3::P61353|RL27_HUMAN	39	15788	3	2	3	2	0.81	60S ribosomal protein
1335	1	db$2	2::C3K9Q1|C3K9Q1_PSEFS	39	28823	1	1	1	1	0.18	Methyltransf_11 doma
1336	1	SwissProt_HUMAN$	3::P62241|RS8_HUMAN	39	24190	1	1	1	1	0.21	40S ribosomal protein
1337	1	db$2	2::C3KEB0|C3KEB0_PSEFS	39	36597	3	3	2	2	0.29	Putative amino acid AE
1338	1	db$2	2::C3KDV4|RSGA_PSEFS	39	37296	1	1	1	1	0.13	Small ribosomal subun
1339	1	SwissProt_HUMAN$	3::P31939|PUR9_HUMAN	39	64575	1	1	1	1	0.08	Bifunctional purine bio
1340	1	SwissProt_HUMAN$	3::P42694|HELZ_HUMAN	39	218833	2	2	2	2	0.04	Probable helicase with
1341	1	SwissProt_HUMAN$	3::P33992|MCM5_HUMAN	38	82233	3	2	1	1	0.06	DNA replication licensi
1342	1	db$2	2::C3K5B3|C3K5B3_PSEFS	38	34937	1	1	1	1	0.14	Dipeptide ABC transpo
1343	1	db$2	2::C3K349|C3K349_PSEFS	38	28994	2	1	2	1	0.18	Pyrroloquinoline-quino
1344	1	db$2	2::C3K6P0|C3K6P0_PSEFS	38	15266	2	2	2	2	0.84	Uncharacterized protei
1345	1	db$2	2::C3KDP3|C3KDP3_PSEFS	38	34782	1	1	1	1	0.15	Putaive acyltransferase
1346	1	db$2	2::C3K0B2|C3K0B2_PSEFS	38	20071	1	1	1	1	0.26	Putative membrane pr
1347	1	db$2	2::C3K4I1|C3K4I1_PSEFS	38	29333	1	1	1	1	0.17	Shikimate dehydrogen
1348	1	db$2	2::C3KBX4|C3KBX4_PSEFS	38	59754	2	2	2	2	0.17	Putative membrane pr
1349	1	SwissProt_HUMAN$	3::Q6ZMT4|KDM7A_HUMAN	38	106489	3	1	3	1	0.05	Lysine-specific demeth
1350	1	db$2	2::C3K473|DUT_PSEFS	38	16022	1	1	1	1	0.34	Deoxyuridine 5'-tripho
1351	1	db$2	2::C3JY27|KDSB_PSEFS	38	27609	2	2	2	2	0.41	3-deoxy-manno-octulc
1352	1	db$2	2::C3JXX9|C3JXX9_PSEFS	38	6925	3	3	2	2	2.76	Translational regulator
1353	1	db$2	2::C3K8W2|ADE_PSEFS	38	36048	2	2	2	2	0.30	Adenine deaminase OS
1354	1	db$2	2::C3K3T6|C3K3T6_PSEFS	38	42390	6	4	5	3	0.56	Spermidine/putrescine
1355	1	db$2	2::C3KBM2|C3KBM2_PSEFS	38	16548	2	1	2	1	0.33	Cys-tRNA(Pro)/Cys-tRI
1356	1	db$2	2::C3K784|C3K784_PSEFS	38	23456	1	1	1	1	0.22	3-oxoadipate CoA-tran
1357	1	db$2	2::C3JZP7|C3JZP7_PSEFS	38	33454	1	1	1	1	0.15	Putative ribose transpo
1358	1	db$2	2::C3KE39|C3KE39_PSEFS	38	18976	4	2	4	2	0.64	D,D-heptose 1,7-bisph
1359	1	db$2	2::C3K4Z0|CCME_PSEFS	38	16101	1	1	1	1	0.34	Cytochrome c-type bio
1360	1	SwissProt_HUMAN$	3::Q70Z53|F10C1_HUMAN	38	37525	1	1	1	1	0.13	Protein FRA10AC1 OS=
1361	1	db$2	2::C3K830|C3K830_PSEFS	38	47217	4	4	4	4	0.49	Putative short chain de
1362	1	SwissProt_HUMAN$	3::P32969|RL9_HUMAN	38	21850	2	1	2	1	0.24	60S ribosomal protein

↑Family	M	DB	Accession	Score	Mass	Matches	Match(sig)	Sequences	Seq(sig)	emPAI	Description
1363	1	db$2	2::C3K4F9|C3K4F9_PSEFS	38	31102	1	1	1	1	0.16	Putative regulatory prc
1364	1	db$2	2::C3K741|C3K741_PSEFS	37	17249	1	1	1	1	0.31	GAF domain-containin
1365	1	db$2	2::C3KB07|C3KB07_PSEFS	37	47911	1	1	1	1	0.10	Deferrochelatase/pero:
1366	1	db$2	2::C3JZ73|C3JZ73_PSEFS	37	49677	2	2	2	2	0.21	Porin OS=Pseudomona
1367	1	db$2	2::C3K9Y8|C3K9Y8_PSEFS	37	14463	1	1	1	1	0.38	Uncharacterized protei
1368	1	SwissProt_HUMAN$	3::Q5JXB2|UE2NL_HUMAN	37	17366	1	1	1	1	0.31	Putative ubiquitin-conj
1369	1	db$2	2::C3K621|C3K621_PSEFS	37	26068	2	2	2	2	0.43	Putative TetR-family re
1370	1	db$2	2::C3K0Y2|C3K0Y2_PSEFS	37	46222	1	1	1	1	0.11	Putative fatty acid trar
1371	1	db$2	2::C3K1F5|C3K1F5_PSEFS	37	29048	1	1	1	1	0.18	Putative chromosome
1372	1	db$2	2::C3K546|C3K546_PSEFS	37	33665	1	1	1	1	0.15	Putative lipopolysaccha
1373	1	db$2	2::C3K6E9|C3K6E9_PSEFS	37	41977	1	1	1	1	0.12	Phosphatidylcholine-hy
1374	1	db$2	2::C3KDQ7|C3KDQ7_PSEFS	37	12961	1	1	1	1	0.43	Diacylglycerol kinase C
1375	1	db$2	2::C3K1M8|C3K1M8_PSEFS	37	28345	2	2	2	2	0.39	tRNA (cytidine/uridine-
1376	1	db$2	2::C3K2M6|RLMH_PSEFS	36	17669	1	1	1	1	0.30	Ribosomal RNA large s
1377	1	db$2	2::C3K2M3|C3K2M3_PSEFS	36	37518	2	1	2	1	0.13	Putative peptidoglycan
1378	1	SwissProt_HUMAN$	3::Q71UM5|RS27L_HUMAN	36	9471	1	1	1	1	0.63	40S ribosomal protein
1379	1	db$2	2::C3K2V5|RL36_PSEFS	36	4431	1	1	1	1	1.73	50S ribosomal protein
1380	1	db$2	2::C3K7C7|C3K7C7_PSEFS	36	19943	1	1	1	1	0.26	Chorismate mutase OS
1381	1	db$2	2::C3K5C4|C3K5C4_PSEFS	36	17510	2	2	2	2	0.70	SnoaL-like domain-cor
1382	1	db$2	2::C3K2Z2|C3K2Z2_PSEFS	36	34551	1	1	1	1	0.15	Bifunctional ligase/rep
1383	1	db$2	2::C3K1G1|C3K1G1_PSEFS	36	48709	1	1	1	1	0.10	tRNA modification GTP
1384	1	db$2	2::C3KBG3|C3KBG3_PSEFS	36	24033	3	3	3	3	0.80	Curli production asser
1385	1	db$2	2::C3K7B0|C3K7B0_PSEFS	36	28681	1	1	1	1	0.18	Putative ABC transport
1386	1	db$2	2::C3KBM3|C3KBM3_PSEFS	36	40194	2	2	2	2	0.27	Putative ABC transport
1387	1	SwissProt_HUMAN$	3::P10599|THIO_HUMAN	36	11730	1	1	1	1	0.48	Thioredoxin OS=Homo
1388	1	db$2	2::C3K6E6|C3K6E6_PSEFS	36	23384	1	1	1	1	0.22	Stringent starvation pr
1389	1	db$2	2::C3KBH3|DCUP_PSEFS	36	38973	2	2	2	2	0.27	Uroporphyrinogen dec
1390	1	db$2	2::C3KDW1|MUTL_PSEFS	36	69574	5	5	5	5	0.41	DNA mismatch repair p
1391	1	SwissProt_HUMAN$	3::Q2PPJ7|RGPA2_HUMAN	36	210636	1	1	1	1	0.02	Ral GTPase-activating
1392	1	db$2	2::C3JY67|C3JY67_PSEFS	36	16861	1	1	1	1	0.32	Phosphatase NudJ OS=
1393	1	db$2	2::C3KDN9|C3KDN9_PSEFS	36	40608	2	1	2	1	0.12	Probable periplasmic s
1394	1	db$2	2::C3K5L6|C3K5L6_PSEFS	36	16214	3	2	3	2	0.78	Putative phosphorous
1395	1	db$2	2::C3K098|C3K098_PSEFS	36	23000	1	1	1	1	0.23	Putative glutathione S-
1396	1	SwissProt_HUMAN$	3::Q7Z6Z7|HUWE1_HUMAN	36	481589	2	2	1	1	0.01	E3 ubiquitin-protein lig
1397	1	db$2	2::C3K905|C3K905_PSEFS	36	13812	1	1	1	1	0.40	Uncharacterized protei
1398	1	db$2	2::C3K172|C3K172_PSEFS	35	12407	1	1	1	1	0.45	ABM domain-containin
1399	1	db$2	2::C3K216|C3K216_PSEFS	35	17384	1	1	1	1	0.31	Putative putative lipop
1400	1	db$2	2::C3K5H4|C3K5H4_PSEFS	35	21385	1	1	1	1	0.24	Uncharacterized protei
1401	1	db$2	2::C3K5F1|C3K5F1_PSEFS	35	42516	1	1	1	1	0.12	Ribonuclease D OS=Ps
1402	1	db$2	2::C3K4W3|C3K4W3_PSEFS	35	21770	1	1	1	1	0.24	PilZ domain-containing

↑Family	M	DB	Accession	Score	Mass	Matches	Match(sig)	Sequences	Seq(sig)	emPAI	Description
1403	1	SwissProt_HUMAN$	3::Q9BVV6|TALD3_HUMAN	35	169201	1	1	1	1	0.03	Protein TALPID3 OS=H
1404	1	db$2	2::C3K0P2|C3K0P2_PSEFS	35	35618	1	1	1	1	0.14	Pseudouridine synthas
1405	1	db$2	2::C3KCB4|C3KCB4_PSEFS	35	27191	1	1	1	1	0.19	Uncharacterized protei
1406	1	db$2	2::C3K5S7|C3K5S7_PSEFS	35	19211	1	1	1	1	0.28	Uncharacterized protei
1407	1	SwissProt_HUMAN$	3::P04080|CYTB_HUMAN	35	11133	1	1	1	1	0.52	Cystatin-B OS=Homo s
1408	1	db$2	2::C3K3J8|C3K3J8_PSEFS	35	33397	2	2	2	2	0.33	TonB_C domain-contai
1409	1	SwissProt_HUMAN$	3::Q7RTV0|PHF5A_HUMAN	35	12397	1	1	1	1	0.45	PHD finger-like domair
1410	1	db$2	2::C3K334|CCA_PSEFS	35	45916	1	1	1	1	0.11	Multifunctional CCA pro
1411	1	db$2	2::C3K7W1|C3K7W1_PSEFS	35	12229	2	2	2	2	1.14	Putative lipoprotein OS
1412	1	db$2	2::C3K0S0|C3K0S0_PSEFS	35	12378	1	1	1	1	0.45	Putative DNA-binding p
1413	1	db$2	2::C3KE38|C3KE38_PSEFS	35	28697	4	3	3	2	0.39	Putative phospholipid b
1414	1	db$2	2::C3KE14|C3KE14_PSEFS	35	83238	1	1	1	1	0.06	Histidine kinase OS=Ps
1415	1	db$2	2::C3K5G7|PDXB_PSEFS	35	41465	2	2	2	2	0.26	Erythronate-4-phospha
1416	1	db$2	2::C3K7J6|C3K7J6_PSEFS	35	25687	1	1	1	1	0.20	Putative exported prot
1417	1	db$2	2::C3K7M1|C3K7M1_PSEFS	35	62712	2	2	2	2	0.16	Putative dipeptidase O
1418	1	SwissProt_HUMAN$	3::P48556|PSMD8_HUMAN	34	39587	2	1	2	1	0.13	26S proteasome non-A
1419	1	db$2	2::C3K3J2|C3K3J2_PSEFS	34	213513	1	1	1	1	0.02	Histidine kinase OS=Ps
1420	1	db$2	2::C3KA92|C3KA92_PSEFS	34	24193	1	1	1	1	0.21	Putative ACP reductase
1421	1	db$2	2::C3K241|C3K241_PSEFS	34	33217	1	1	1	1	0.15	Glutamyl-Q tRNA(Asp)
1422	1	db$2	2::C3KDV5|C3KDV5_PSEFS	34	20461	2	2	2	2	0.58	Oligoribonuclease OS=
1423	1	db$2	2::C3K2L8|LIPA_PSEFS	34	38163	5	4	4	3	0.45	Lipoyl synthase OS=Ps
1424	1	db$2	2::C3K1M2|HSCB_PSEFS	34	20288	1	1	1	1	0.26	Co-chaperone protein
1425	1	db$2	2::C3K7T3|C3K7T3_PSEFS	34	20773	1	1	1	1	0.25	CDP-diacylglycerol--gly
1426	1	db$2	2::C3K9N4|C3K9N4_PSEFS	34	91939	1	1	1	1	0.05	Histidine kinase OS=Ps
1427	1	db$2	2::C3K021|C3K021_PSEFS	34	27911	4	2	4	2	0.40	Phosphoadenosine pho
1428	1	db$2	2::C3KCP9|C3KCP9_PSEFS	34	30268	1	1	1	1	0.17	Lipopolysaccharide cor
1429	1	db$2	2::C3K6C0|C3K6C0_PSEFS	34	25791	1	1	1	1	0.20	Putative amino acid AE
1430	1	db$2	2::C3K0X5|C3K0X5_PSEFS	34	21842	2	2	1	1	0.54	Uncharacterized protei
1431	1	db$2	2::C3JY28|Y3771_PSEFS	34	6626	3	1	2	1	0.98	UPF0434 protein PFLU_
1432	1	db$2	2::C3KAA5|C3KAA5_PSEFS	34	23984	2	2	2	2	0.48	Uncharacterized protei
1433	1	SwissProt_HUMAN$	3::Q8NBF4|YG006_HUMAN	34	16172	1	1	1	1	0.33	Putative uncharacterize
1434	1	db$2	2::C3K5Y3|C3K5Y3_PSEFS	34	35524	1	1	1	1	0.14	Putative leucine dehyd
1435	1	db$2	2::C3KBK6|C3KBK6_PSEFS	34	29320	1	1	1	1	0.17	Putative exported pept
1436	1	db$2	2::C3K7K0|C3K7K0_PSEFS	33	41297	1	1	1	1	0.12	Esterase III OS=Pseuc
1437	1	db$2	2::C3KAW2|C3KAW2_PSEFS	33	26230	3	3	3	3	0.71	Putative short chain de
1438	1	db$2	2::C3JY43|TTCA_PSEFS	33	31052	1	1	1	1	0.16	tRNA-cytidine(32) 2-su
1439	1	db$2	2::C3JYL1|C3JYL1_PSEFS	33	44986	1	1	1	1	0.11	N-carbamoyl-L-amino
1440	1	db$2	2::C3K5K8|C3K5K8_PSEFS	33	29952	1	1	1	1	0.17	Putative deoxyribonucl
1441	1	SwissProt_HUMAN$	3::P27216|ANX13_HUMAN	33	35393	1	1	1	1	0.14	Annexin A13 OS=Hom
1442	1	db$2	2::C3KEB9|C3KEB9_PSEFS	33	11110	1	1	1	1	0.52	Putative lipoprotein OS

↑Family	M	DB	Accession	Score	Mass	Matches	Match(sig)	Sequences	Seq(sig)	emPAI	Description
1443	1	db$2	2::C3K2Y9|C3K2Y9_PSEFS	33	13142	2	2	2	2	1.03	Protein translocase sul
1444	1	SwissProt_HUMAN$	3::Q96E52|OMA1_HUMAN	33	60081	1	1	1	1	0.08	Metalloendopeptidase
1445	1	SwissProt_HUMAN$	3::O60262|GBG7_HUMAN	33	7517	3	2	1	1	0.83	Guanine nucleotide-bir
1446	1	db$2	2::C3K5Z0|C3K5Z0_PSEFS	33	15508	4	1	3	1	0.35	Uncharacterized protei
1447	1	db$2	2::C3K807|C3K807_PSEFS	33	30283	2	2	2	2	0.36	Putative histidine ABC
1448	1	db$2	2::C3K0P5|C3K0P5_PSEFS	33	35572	1	1	1	1	0.14	Uncharacterized protei
1449	1	db$2	2::C3K3M2|C3K3M2_PSEFS	33	37029	1	1	1	1	0.14	Cell division protein Ft
1450	1	SwissProt_HUMAN$	3::P62753|RS6_HUMAN	33	28663	2	2	2	2	0.39	40S ribosomal protein
1451	1	db$2	2::C3KDC2|ISPE_PSEFS	33	30632	1	1	1	1	0.17	4-diphosphocytidyl-2-(
1452	1	db$2	2::C3KDR5|KUP_PSEFS	33	68624	2	1	2	1	0.07	Probable potassium tra
1453	1	SwissProt_HUMAN$	3::Q14697|GANAB_HUMAN	33	106807	1	1	1	1	0.05	Neutral alpha-glucosid
1454	1	db$2	2::C3K6T8|C3K6T8_PSEFS	33	49583	4	4	4	4	0.46	Alginate biosynthesis t
1455	1	db$2	2::C3K4L7|C3K4L7_PSEFS	33	23283	2	1	2	1	0.22	Putative TetR-family re
1456	1	db$2	2::C3K265|C3K265_PSEFS	33	29739	2	2	2	2	0.37	Dihydropteroate synth
1457	1	db$2	2::C3JYS4|C3JYS4_PSEFS	32	16520	4	4	4	4	2.10	Tol-Pal system protein
1458	1	db$2	2::C3K0N3|ACP_PSEFS	32	8637	1	1	1	1	0.70	Acyl carrier protein OS
1459	1	db$2	2::C3K2L3|C3K2L3_PSEFS	32	22416	1	1	1	1	0.23	LPS-assembly lipoprot
1460	1	db$2	2::C3K1D1|C3K1D1_PSEFS	32	32188	2	2	2	2	0.34	Protein HflC OS=Pseuc
1461	1	db$2	2::C3KC05|C3KC05_PSEFS	32	11915	1	1	1	1	0.48	Conserved hypothetica
1462	1	db$2	2::C3K106|C3K106_PSEFS	32	20669	2	2	2	2	0.58	Uncharacterized protei
1463	1	db$2	2::C3K598|C3K598_PSEFS	32	34258	1	1	1	1	0.15	Curved DNA-binding p
1464	1	db$2	2::C3K150|C3K150_PSEFS	32	25194	2	2	2	2	0.45	Putative short-chain de
1465	1	db$2	2::C3K3P7|C3K3P7_PSEFS	32	49977	2	2	2	2	0.21	Putative membrane pr
1466	1	db$2	2::C3KAS1|C3KAS1_PSEFS	32	34986	2	2	1	1	0.14	Putative 2-hydroxyacic
1467	1	db$2	2::C3K330|GLPE_PSEFS	32	11747	1	1	1	1	0.48	Thiosulfate sulfurtrans
1468	1	db$2	2::C3K666|C3K666_PSEFS	32	29461	4	4	4	4	0.90	Putative lipoprotein OS
1469	1	db$2	2::C3K6R2|C3K6R2_PSEFS	32	29309	1	1	1	1	0.17	Putative shikimate deh
1470	1	db$2	2::C3K964|C3K964_PSEFS	32	26948	1	1	1	1	0.19	Putative GntR-family r
1471	1	SwissProt_HUMAN$	3::Q13472|TOP3A_HUMAN	32	112300	1	1	1	1	0.04	DNA topoisomerase 3-
1472	1	db$2	2::C3K1B6|C3K1B6_PSEFS	32	10582	1	1	1	1	0.55	Uncharacterized protei
1473	1	db$2	2::C3K0H5|C3K0H5_PSEFS	32	25946	1	1	1	1	0.20	Putative chemotaxis pı
1474	1	db$2	2::C3K0K9|C3K0K9_PSEFS	32	37421	2	2	1	1	0.13	Putative NADP-depend
1475	1	db$2	2::C3K1X3|C3K1X3_PSEFS	32	20670	3	2	3	2	0.58	Glutathione peroxidase
1476	1	SwissProt_HUMAN$	3::O94827|PKHG5_HUMAN	32	117377	2	2	2	2	0.08	Pleckstrin homology dc
1477	1	db$2	2::C3K7C4|C3K7C4_PSEFS	32	53137	3	2	3	2	0.19	DNA-binding transcript
1478	1	db$2	2::C3JYP6|C3JYP6_PSEFS	32	10477	3	3	3	3	2.78	YCII domain-containin
1479	1	db$2	2::C3JYL6|C3JYL6_PSEFS	32	25076	2	2	2	2	0.45	Putative TetR-family re
1480	1	db$2	2::C3KAN8|C3KAN8_PSEFS	32	48228	1	1	1	1	0.10	3-carboxy-cis,cis-mucc
1481	1	SwissProt_HUMAN$	3::P61247|RS3A_HUMAN	31	29926	1	1	1	1	0.17	40S ribosomal protein
1482	1	db$2	2::C3KAM0|C3KAM0_PSEFS	31	50872	3	2	3	2	0.20	Putative two-compone

↑Family	M	DB	Accession	Score	Mass	Matches	Match(sig)	Sequences	Seq(sig)	emPAI	Description
1483	1	SwissProt_HUMAN$	3::Q9UHN6|CEIP2_HUMAN	31	154276	3	2	3	2	0.06	Cell surface hyaluronic
1484	1	db$2	2::C3K0G4|C3K0G4_PSEFS	31	29541	1	1	1	1	0.17	MOSC domain-containi
1485	1	db$2	2::C3JY76|C3JY76_PSEFS	31	48249	2	2	2	2	0.22	Putative mechanosens
1486	1	db$2	2::C3K4T8|C3K4T8_PSEFS	31	33681	1	1	1	1	0.15	Putative AraC-family re
1487	1	db$2	2::C3K413|C3K413_PSEFS	31	22663	1	1	1	1	0.23	Putative ABC transport
1488	1	db$2	2::C3JYJ2|C3JYJ2_PSEFS	31	27205	1	1	1	1	0.19	Putative lipoprotein OS
1489	1	db$2	2::C3K9E9|C3K9E9_PSEFS	31	21363	2	2	1	1	0.25	Putative TetR-family re
1490	1	db$2	2::C3K0V8|C3K0V8_PSEFS	31	45209	1	1	1	1	0.11	Muramidase FlgJ OS=F
1491	1	db$2	2::C3K326|C3K326_PSEFS	31	35169	3	1	3	1	0.14	4-hydroxythreonine-4-
1492	1	SwissProt_HUMAN$	3::Q9P273|TEN3_HUMAN	31	300760	1	1	1	1	0.02	Teneurin-3 OS=Homo
1493	1	db$2	2::C3JZ52|C3JZ52_PSEFS	31	18715	1	1	1	1	0.28	Uncharacterized protei
1494	1	db$2	2::C3K3X4|C3K3X4_PSEFS	31	11561	2	2	1	1	1.23	Cell division protein Za
1495	1	db$2	2::C3K8Z3|C3K8Z3_PSEFS	31	28227	1	1	1	1	0.18	High-affinity branched-
1496	1	db$2	2::C3K4R7|C3K4R7_PSEFS	31	57887	1	1	1	1	0.09	Putative methyl-accept
1497	1	SwissProt_HUMAN$	3::Q96AQ6|PBIP1_HUMAN	31	80594	1	1	1	1	0.06	Pre-B-cell leukemia tra
1498	1	db$2	2::A4V6U2|A4V6U2_PSEFS	31	76547	1	1	1	1	0.06	Uncharacterized protei
1499	1	db$2	2::C3KAY4|C3KAY4_PSEFS	31	29479	3	2	3	2	0.38	Putative enoyl-CoA hy
1500	1	db$2	2::C3K6B1|C3K6B1_PSEFS	31	35098	1	1	1	1	0.14	Homoserine kinase OS
1501	1	db$2	2::C3K1Y4|C3K1Y4_PSEFS	30	14766	1	1	1	1	0.37	Putative putative glyox
1502	1	db$2	2::C3K8X1|C3K8X1_PSEFS	30	23435	3	3	2	2	0.83	Putative ABC transport
1503	1	SwissProt_HUMAN$	3::Q8WVY7|UBCP1_HUMAN	30	36781	2	1	1	1	0.14	Ubiquitin-like domain-
1504	1	db$2	2::C3K3X9|C3K3X9_PSEFS	30	98433	2	2	2	2	0.10	Putative ABC transport
1505	1	db$2	2::C3KAZ8|C3KAZ8_PSEFS	30	34711	2	2	2	2	0.31	Putative beta-hydroxyl
1506	1	db$2	2::C3K9P4|C3K9P4_PSEFS	30	59261	2	1	2	1	0.08	Putative ATP-binding c
1507	1	db$2	2::C3K0Y9|C3K0Y9_PSEFS	30	19041	1	1	1	1	0.28	Bifunctional adenosylc
1508	1	db$2	2::C3KCP8|C3KCP8_PSEFS	30	42283	1	1	1	1	0.12	Lipopolysaccharide cor
1509	1	db$2	2::C3JYG6|C3JYG6_PSEFS	30	44622	2	1	2	1	0.11	Peptidase_M14 domair
1510	1	db$2	2::C3K3G3|C3K3G3_PSEFS	30	32186	1	1	1	1	0.16	Putative membrane pr
1511	1	db$2	2::C3K7J9|C3K7J9_PSEFS	30	83444	1	1	1	1	0.06	Putative exported prot
1512	1	SwissProt_HUMAN$	3::O14640|DVL1_HUMAN	30	75140	1	1	1	1	0.07	Segment polarity prote
1513	1	SwissProt_HUMAN$	3::Q9ULQ1|TPC1_HUMAN	30	94086	1	1	1	1	0.05	Two pore calcium char
1514	1	SwissProt_HUMAN$	3::O94817|ATG12_HUMAN	30	15104	1	1	1	1	0.36	Ubiquitin-like protein A
1515	1	db$2	2::C3K2E2|C3K2E2_PSEFS	30	56906	1	1	1	1	0.09	Catalase OS=Pseudom
1516	1	db$2	2::C3K7I5|C3K7I5_PSEFS	30	28342	1	1	1	1	0.18	Putative iron utilisatior
1517	1	SwissProt_HUMAN$	3::P63220|RS21_HUMAN	30	9106	1	1	1	1	0.66	40S ribosomal protein
1518	1	db$2	2::C3K8T6|C3K8T6_PSEFS	30	26078	2	2	2	2	0.43	Ribosomal RNA small s
1519	1	db$2	2::C3K4R1|PLSB_PSEFS	30	95327	2	2	2	2	0.10	Glycerol-3-phosphate a
1520	1	SwissProt_HUMAN$	3::Q9Y231|FUT9_HUMAN	30	42044	1	1	1	1	0.12	4-galactosyl-N-acetylg
1521	1	db$2	2::C3K4K0|C3K4K0_PSEFS	30	42230	2	2	2	2	0.25	Hypothetical exported
1522	1	SwissProt_HUMAN$	3::Q5VT06|CE350_HUMAN	30	350716	2	1	2	1	0.01	Centrosome-associate

↑Family	M	DB	Accession	Score	Mass	Matches	Match(sig)	Sequences	Seq(sig)	emPAI	Description
1523	1	db$2	2::C3K1V5|C3K1V5_PSEFS	30	64171	1	1	1	1	0.08	Uncharacterized protei
1524	1	db$2	2::C3KBX7|C3KBX7_PSEFS	29	23636	1	1	1	1	0.22	Putative metallo-beta-
1525	1	db$2	2::Q8VMR3|HUTH_PSEFS	29	54209	1	1	1	1	0.09	Histidine ammonia-lya
1526	1	db$2	2::C3K4Z1|C3K4Z1_PSEFS	29	72318	1	1	1	1	0.07	Cytochrome C biogene
1527	1	db$2	2::C3K834|C3K834_PSEFS	29	76708	1	1	1	1	0.06	Copper-transporting P-
1528	1	SwissProt_HUMAN$	3::Q14789|GOGB1_HUMAN	29	375790	3	2	3	2	0.03	Golgin subfamily B me
1529	1	db$2	2::C3JY73|C3JY73_PSEFS	29	26430	1	1	1	1	0.19	Secretin_N domain-coı
1530	1	db$2	2::C3JYA8|C3JYA8_PSEFS	29	39123	1	1	1	1	0.13	Putative membrane pr
1531	1	db$2	2::C3K429|C3K429_PSEFS	29	8856	1	1	1	1	0.68	Uncharacterized protei
1532	1	SwissProt_HUMAN$	3::P62277|RS13_HUMAN	29	17212	1	1	1	1	0.31	40S ribosomal protein
1533	1	db$2	2::C3K2H7|C3K2H7_PSEFS	29	21527	1	1	1	1	0.24	Putative acetyltransfer
1534	1	db$2	2::C3KDN1|C3KDN1_PSEFS	29	54005	2	1	2	1	0.09	Deoxyribodipyrimidine
1535	1	SwissProt_HUMAN$	3::Q9Y399|RT02_HUMAN	29	33228	1	1	1	1	0.15	28S ribosomal protein
1536	1	db$2	2::C3KBN1|C3KBN1_PSEFS	29	62708	2	1	2	1	0.08	Chaperone OS=Pseudo
1537	1	db$2	2::C3K5W0|C3K5W0_PSEFS	29	170278	3	2	3	2	0.06	DUF4329 domain-cont
1538	1	db$2	2::C3JY37|C3JY37_PSEFS	29	14829	1	1	1	1	0.37	Putative lipoprotein OS
1539	1	SwissProt_HUMAN$	3::Q5K651|SAMD9_HUMAN	29	184165	1	1	1	1	0.03	Sterile alpha motif dor
1540	1	SwissProt_HUMAN$	3::P06727|APOA4_HUMAN	29	45344	1	1	1	1	0.11	Apolipoprotein A-IV OS
1541	1	db$2	2::C3K0Y3|C3K0Y3_PSEFS	29	20085	1	1	1	1	0.26	Glutathione peroxidase
1542	1	db$2	2::C3K406|C3K406_PSEFS	29	23424	2	2	2	2	0.49	Glyoxalase OS=Pseudo
1543	1	db$2	2::C3KDG3|C3KDG3_PSEFS	29	15637	1	1	1	1	0.35	Putative exported prot
1544	1	db$2	2::C3K3T4|C3K3T4_PSEFS	28	31991	1	1	1	1	0.16	Putrescine ABC transp
1545	1	db$2	2::C3KAG2|C3KAG2_PSEFS	28	280833	2	1	2	1	0.02	Toxin CdiA OS=Pseudo
1546	1	db$2	2::C3K6R7|C3K6R7_PSEFS	28	41832	2	2	2	2	0.25	Serine/threonine trans
1547	1	db$2	2::C3KDB4|C3KDB4_PSEFS	28	42765	1	1	1	1	0.12	Putative glycosyl trans
1548	1	db$2	2::C3K3P8|C3K3P8_PSEFS	28	49421	1	1	1	1	0.10	G domain-containing p
1549	1	db$2	2::C3K2Z1|COAX_PSEFS	28	26920	1	1	1	1	0.19	Type III pantothenate
1550	1	db$2	2::C3K1G4|RL34_PSEFS	28	5064	2	1	2	1	1.41	50S ribosomal protein
1551	1	db$2	2::C3K8W0|C3K8W0_PSEFS	28	34311	1	1	1	1	0.15	Putative LysR-family re
1552	1	SwissProt_HUMAN$	3::Q9Y2G4|ANKR6_HUMAN	28	79922	1	1	1	1	0.06	Ankyrin repeat domain
1553	1	SwissProt_HUMAN$	3::P05546|HEP2_HUMAN	28	57034	1	1	1	1	0.09	Heparin cofactor 2 OS=
1554	1	db$2	2::A4V7R9|A4V7R9_PSEFS	28	24067	1	1	1	1	0.22	Uncharacterized protei
1555	1	db$2	2::C3JZ89|C3JZ89_PSEFS	28	157759	2	2	2	2	0.06	Uncharacterized protei
1556	1	SwissProt_HUMAN$	3::Q99618|CDCA3_HUMAN	28	28981	1	1	1	1	0.18	Cell division cycle-asso
1557	1	db$2	2::Q9ZEP8|PANB_PSEFS	28	27951	2	1	2	1	0.18	3-methyl-2-oxobutano
1558	1	db$2	2::C3JZQ3|C3JZQ3_PSEFS	27	15084	1	1	1	1	0.36	Uncharacterized protei
1559	1	db$2	2::C3KD16|C3KD16_PSEFS	27	34294	2	1	2	1	0.15	Putative 2-hydroxyacid
1560	1	db$2	2::C3KDK6|C3KDK6_PSEFS	27	27451	2	1	2	1	0.19	Putative ABC transport
1561	1	SwissProt_HUMAN$	3::O15197|EPHB6_HUMAN	27	110630	1	1	1	1	0.04	Ephrin type-B receptor
1562	1	SwissProt_HUMAN$	3::P52179|MYOM1_HUMAN	27	187509	1	1	1	1	0.03	Myomesin-1 OS=Homo

↑Family	M	DB	Accession	Score	Mass	Matches	Match(sig)	Sequences	Seq(sig)	emPAI	Description
1563	1	db$2	2::C3K3N1|C3K3N1_PSEFS	27	52088	2	1	2	1	0.10	Aldehyde dehydrogena
1564	1	SwissProt_HUMAN$	3::Q9BYK8|HELZ2_HUMAN	27	294465	1	1	1	1	0.02	Helicase with zinc fing
1565	1	SwissProt_HUMAN$	3::P26373|RL13_HUMAN	27	24247	1	1	1	1	0.21	60S ribosomal protein
1566	1	db$2	2::C3KDI3|C3KDI3_PSEFS	27	22379	1	1	1	1	0.23	Putative glutathione-S
1567	1	db$2	2::C3K188|C3K188_PSEFS	27	39897	1	1	1	1	0.13	Putative exported prot
1568	1	db$2	2::C3K3S9|C3K3S9_PSEFS	27	35576	1	1	1	1	0.14	Hydrolase_4 domain-c
1569	1	SwissProt_HUMAN$	3::Q96P48|ARAP1_HUMAN	27	162089	1	1	1	1	0.03	Arf-GAP with Rho-GAP
1570	1	SwissProt_HUMAN$	3::P49411|EFTU_HUMAN	27	49510	1	1	1	1	0.10	Elongation factor Tu, r
1571	1	db$2	2::C3K267|RLME_PSEFS	27	24125	1	1	1	1	0.22	Ribosomal RNA large s
1572	1	SwissProt_HUMAN$	3::P05413|FABPH_HUMAN	27	14849	1	1	1	1	0.37	Fatty acid-binding prot
1573	1	db$2	2::C3JY93|C3JY93_PSEFS	27	51981	1	1	1	1	0.10	NADH-quinone oxidore
1574	1	db$2	2::C3K127|C3K127_PSEFS	27	41295	1	1	1	1	0.12	Putative membrane pr
1575	1	db$2	2::C3JXP2|C3JXP2_PSEFS	27	43336	1	1	1	1	0.12	Putative glycosyltransf
1576	1	db$2	2::C3KAR4|C3KAR4_PSEFS	27	11430	1	1	1	1	0.50	Uncharacterized protei
1577	1	SwissProt_HUMAN$	3::O75155|CAND2_HUMAN	27	135171	1	1	1	1	0.04	Cullin-associated NEDI
1578	1	SwissProt_HUMAN$	3::Q9UQE7|SMC3_HUMAN	27	141454	1	1	1	1	0.03	Structural maintenanc
1579	1	SwissProt_HUMAN$	3::Q13093|PAFA_HUMAN	27	50045	1	1	1	1	0.10	Platelet-activating fact
1580	1	db$2	2::C3K595|C3K595_PSEFS	27	10805	1	1	1	1	0.54	Phosphate starvation-i
1581	1	db$2	2::C3K3C7|C3K3C7_PSEFS	27	34452	1	1	1	1	0.15	Putative glycine betain
1582	1	SwissProt_HUMAN$	3::Q09MP3|R51A2_HUMAN	27	133822	1	1	1	1	0.04	RAD51-associated prot
1583	1	db$2	2::C3JY58|C3JY58_PSEFS	27	87640	4	3	4	3	0.18	DNA translocase FtsK (
1584	1	SwissProt_HUMAN$	3::Q9H2G2|SLK_HUMAN	27	142607	1	1	1	1	0.03	STE20-like serine/thre
1585	1	db$2	2::C3K0E2|C3K0E2_PSEFS	27	49920	1	1	1	1	0.10	Diaminobutyrate--2-ox
1586	1	db$2	2::C3K1M3|C3K1M3_PSEFS	27	11692	1	1	1	1	0.49	Iron-binding protein Is
1587	1	SwissProt_HUMAN$	3::Q9NU22|MDN1_HUMAN	27	632420	2	1	2	1	0.01	Midasin OS=Homo sap
1588	1	SwissProt_HUMAN$	3::Q8IZ83|A16A1_HUMAN	27	85074	1	1	1	1	0.06	Aldehyde dehydrogena
1589	1	db$2	2::C3K428|C3K428_PSEFS	27	10726	1	1	1	1	0.54	Uncharacterized protei
1590	1	db$2	2::C3JYS6|C3JYS6_PSEFS	27	17060	1	1	1	1	0.31	Putative thioesterase (
1591	1	SwissProt_HUMAN$	3::P62304|RUXE_HUMAN	27	10797	1	1	1	1	0.54	Small nuclear ribonucl
1592	1	SwissProt_HUMAN$	3::O75197|LRP5_HUMAN	26	179030	2	1	1	1	0.03	Low-density lipoprotei
1593	1	SwissProt_HUMAN$	3::O14917|PCD17_HUMAN	26	126151	1	1	1	1	0.04	Protocadherin-17 OS=
1594	1	db$2	2::C3KAH5|RL31_PSEFS	26	8451	3	1	2	1	0.72	50S ribosomal protein
1595	1	SwissProt_HUMAN$	3::Q13454|TUSC3_HUMAN	26	39650	1	1	1	1	0.13	Tumor suppressor can
1596	1	SwissProt_HUMAN$	3::P30041|PRDX6_HUMAN	26	25019	1	1	1	1	0.21	Peroxiredoxin-6 OS=H
1597	1	SwissProt_HUMAN$	3::P11532|DMD_HUMAN	26	426484	1	1	1	1	0.01	Dystrophin OS=Homo
1598	1	SwissProt_HUMAN$	3::P46020|KPB1_HUMAN	26	137225	2	2	2	2	0.07	Phosphorylase b kinas
1599	1	SwissProt_HUMAN$	3::P46779|RL28_HUMAN	26	15738	1	1	1	1	0.34	60S ribosomal protein
1600	1	db$2	2::C3K4R0|C3K4R0_PSEFS	26	14930	1	1	1	1	0.37	Putative lipoprotein OS
1601	1	SwissProt_HUMAN$	3::A6NJB7|PRR19_HUMAN	26	38692	1	1	1	1	0.13	Proline-rich protein 19
1602	1	db$2	2::C3K364|C3K364_PSEFS	26	38824	1	1	1	1	0.13	Biotin synthase OS=Ps

↑Family	M	DB	Accession	Score	Mass	Matches	Match(sig)	Sequences	Seq(sig)	emPAI	Description
1603	1	db$2	2::C3KDR4|C3KDR4_PSEFS	26	49531	2	2	2	2	0.21	Ribosomal protein S12
1604	1	db$2	2::C3K7B3|C3K7B3_PSEFS	26	51722	1	1	1	1	0.10	Putative membrane-bc
1605	1	db$2	2::C3K006|C3K006_PSEFS	26	41293	1	1	1	1	0.12	Acetylornithine deacet
1606	1	db$2	2::C3KCK0|C3KCK0_PSEFS	26	26687	3	1	2	1	0.19	Putative short-chain de
1607	1	db$2	2::C3KDT8|C3KDT8_PSEFS	26	91333	1	1	1	1	0.05	Acyl-coenzyme A dehy
1608	1	SwissProt_HUMAN$	3::P08865|RSSA_HUMAN	26	32833	1	1	1	1	0.15	40S ribosomal protein
1609	1	SwissProt_HUMAN$	3::Q75VX8|GARE2_HUMAN	26	92824	1	1	1	1	0.05	GRB2-associated and r
1610	1	SwissProt_HUMAN$	3::Q8TBY8|PMFBP_HUMAN	26	117420	1	1	1	1	0.04	Polyamine-modulated
1611	1	SwissProt_HUMAN$	3::P35268|RL22_HUMAN	26	14778	1	1	1	1	0.37	60S ribosomal protein
1612	1	db$2	2::C3K908|C3K908_PSEFS	26	33577	1	1	1	1	0.15	Putative dehydrogenas
1613	1	db$2	2::C3K4S7|C3K4S7_PSEFS	26	34300	1	1	1	1	0.15	Putative LysR-family re
1614	1	db$2	2::C3K294|C3K294_PSEFS	26	11674	1	1	1	1	0.49	Transposase for inserti
1615	1	SwissProt_HUMAN$	3::P39086|GRIK1_HUMAN	26	103914	1	1	1	1	0.05	Glutamate receptor ior
1616	1	SwissProt_HUMAN$	3::Q8N465|D2HDH_HUMAN	26	56380	1	1	1	1	0.09	D-2-hydroxyglutarate
1617	1	db$2	2::C3K672|C3K672_PSEFS	25	58670	2	2	2	2	0.17	Cation/acetate sympor
1618	1	SwissProt_HUMAN$	3::Q08170|SRSF4_HUMAN	25	56645	1	1	1	1	0.09	Serine/arginine-rich sp
1619	1	db$2	2::C3K7D7|C3K7D7_PSEFS	25	8979	1	1	1	1	0.67	Putative lipoprotein OS
1620	1	SwissProt_HUMAN$	3::Q14683|SMC1A_HUMAN	25	143144	1	1	1	1	0.03	Structural maintenanc
1621	1	db$2	2::C3K493|C3K493_PSEFS	25	16495	2	1	2	1	0.33	Uncharacterized protei
1622	1	db$2	2::C3JZ46|C3JZ46_PSEFS	25	37091	2	2	2	2	0.29	Putative LacI family tra
1623	1	db$2	2::C3K0E4|C3K0E4_PSEFS	25	48130	1	1	1	1	0.10	Sensor protein QseC C
1624	1	SwissProt_HUMAN$	3::O43927|CXL13_HUMAN	25	12656	1	1	1	1	0.44	C-X-C motif chemokine
1625	1	db$2	2::C3K2M2|C3K2M2_PSEFS	25	35893	2	2	2	2	0.30	Endolytic peptidoglyca
1626	1	db$2	2::C3K2K1|C3K2K1_PSEFS	25	28157	1	1	1	1	0.18	Phos_pyr_kin domain-
1627	1	db$2	2::C3JZP0|C3JZP0_PSEFS	25	11251	1	1	1	1	0.51	Putative lipoprotein OS
1628	1	db$2	2::C3JYT0|C3JYT0_PSEFS	25	26526	1	1	1	1	0.19	Probable transcriptiona
1629	1	db$2	2::C3K4L1|C3K4L1_PSEFS	25	41720	1	1	1	1	0.12	Putative epimerase OS
1630	1	db$2	2::C3K2F6|TRMA_PSEFS	25	40867	1	1	1	1	0.12	tRNA/tmRNA (uracil-C(
1631	1	SwissProt_HUMAN$	3::P54886|P5CS_HUMAN	25	87248	1	1	1	1	0.06	Delta-1-pyrroline-5-ca
1632	1	db$2	2::C3K0T6|RRAAH_PSEFS	25	17479	1	1	1	1	0.31	Putative 4-hydroxy-4-r
1633	1	SwissProt_HUMAN$	3::O00299|CLIC1_HUMAN	25	26906	1	1	1	1	0.19	Chloride intracellular c
1634	1	SwissProt_HUMAN$	3::Q9UBV2|SE1L1_HUMAN	25	88698	1	1	1	1	0.05	Protein sel-1 homolog
1635	1	db$2	2::C3K3T1|C3K3T1_PSEFS	25	86048	1	1	1	1	0.06	Putative penicillin amic
1636	1	SwissProt_HUMAN$	3::Q14DG7|T132B_HUMAN	25	119402	1	1	1	1	0.04	Transmembrane protei
1637	1	SwissProt_HUMAN$	3::Q9BTV4|TMM43_HUMAN	25	44847	1	1	1	1	0.11	Transmembrane protei
1638	1	db$2	2::C3K620|C3K620_PSEFS	25	22214	1	1	1	1	0.24	LexA repressor OS=Ps
1639	1	SwissProt_HUMAN$	3::Q92802|N42L2_HUMAN	25	67417	1	1	1	1	0.07	NEDD4-binding proteir
1640	1	SwissProt_HUMAN$	3::Q13219|PAPP1_HUMAN	24	180856	2	1	2	1	0.03	Pappalysin-1 OS=Hom
1641	1	db$2	2::C3K239|C3K239_PSEFS	24	42550	1	1	1	1	0.12	Aminotransferase OS=
1642	1	SwissProt_HUMAN$	3::O00159|MYO1C_HUMAN	24	121606	1	1	1	1	0.04	Unconventional myosir

↑Family	M	DB	Accession	Score	Mass	Matches	Match(sig)	Sequences	Seq(sig)	emPAI	Description
1643	1	SwissProt_HUMAN$	3::Q76L83|ASXL2_HUMAN	24	153725	1	1	1	1	0.03	Putative Polycomb gro
1644	1	SwissProt_HUMAN$	3::Q7Z7L7|ZER1_HUMAN	24	88113	2	2	1	1	0.11	Protein zer-1 homolog
1645	1	db$2	2::C3K9H5|C3K9H5_PSEFS	24	33119	1	1	1	1	0.15	Putative LysR-family tı
1646	1	SwissProt_HUMAN$	3::P68431|H31_HUMAN	24	15394	2	2	2	2	0.83	Histone H3.1 OS=Hom
1647	1	SwissProt_HUMAN$	3::Q8TD08|MK15_HUMAN	24	59795	1	1	1	1	0.08	Mitogen-activated prot
1648	1	db$2	2::C3KBI8|C3KBI8_PSEFS	24	48805	2	2	2	2	0.21	Uncharacterized protei
1649	1	db$2	2::C3K663|C3K663_PSEFS	24	22341	1	1	1	1	0.23	Putative TetR-family re
1650	1	SwissProt_HUMAN$	3::Q9NYY1|IL20_HUMAN	24	20059	1	1	1	1	0.26	Interleukin-20 OS=Hoı
1651	1	SwissProt_HUMAN$	3::Q96GD3|SCMH1_HUMAN	24	73307	1	1	1	1	0.07	Polycomb protein SCM
1652	1	db$2	2::C3JYA6|C3JYA6_PSEFS	24	31593	1	1	1	1	0.16	Uncharacterized protei
1653	1	SwissProt_HUMAN$	3::O95153|RIMB1_HUMAN	24	199928	2	1	1	1	0.02	Peripheral-type benzoc
1654	1	SwissProt_HUMAN$	3::P10909|CLUS_HUMAN	24	52461	2	1	2	1	0.09	Clusterin OS=Homo sa
1655	1	SwissProt_HUMAN$	3::P23276|KELL_HUMAN	24	82771	1	1	1	1	0.06	Kell blood group glyco
1656	1	db$2	2::C3JYC8|C3JYC8_PSEFS	24	59331	1	1	1	1	0.08	Putative pH adaptatior
1657	1	SwissProt_HUMAN$	3::Q96T58|MINT_HUMAN	24	402004	1	1	1	1	0.01	Msx2-interacting prote
1658	1	db$2	2::C3K832|C3K832_PSEFS	24	33163	1	1	1	1	0.15	Non-specific serine/thr
1659	1	SwissProt_HUMAN$	3::Q8NFC6|BD1L1_HUMAN	24	330266	1	1	1	1	0.01	Biorientation of chrom
1660	1	SwissProt_HUMAN$	3::Q3B7T1|EDRF1_HUMAN	24	138440	1	1	1	1	0.03	Erythroid differentiatio
1661	1	db$2	2::C3K8V8|C3K8V8_PSEFS	24	36166	2	2	2	2	0.30	Zinc-type alcohol dehy
1662	1	db$2	2::C3JZP3|C3JZP3_PSEFS	24	36685	1	1	1	1	0.14	Putative inosine-uridin
1663	1	SwissProt_HUMAN$	3::Q9Y2Q0|AT8A1_HUMAN	24	131285	2	1	2	1	0.04	Phospholipid-transport
1664	1	SwissProt_HUMAN$	3::Q15147|PLCB4_HUMAN	24	134378	2	1	1	1	0.04	1-phosphatidylinositol
1665	1	db$2	2::C3KDJ8|C3KDJ8_PSEFS	24	23307	1	1	1	1	0.22	Methyltransf_25 doma
1666	1	SwissProt_HUMAN$	3::Q8IWX8|CHERP_HUMAN	24	103637	1	1	1	1	0.05	Calcium homeostasis e
1667	1	db$2	2::C3KBJ0|C3KBJ0_PSEFS	24	16933	1	1	1	1	0.32	[Ribosomal protein S1
1668	1	db$2	2::C3JY48|C3JY48_PSEFS	24	12277	1	1	1	1	0.46	DrsE domain-containin
1669	1	db$2	2::C3K167|C3K167_PSEFS	24	48979	1	1	1	1	0.10	Putative two-compone
1670	1	db$2	2::C3KCJ3|C3KCJ3_PSEFS	24	30975	2	2	2	2	0.36	Usp domain-containing
1671	1	SwissProt_HUMAN$	3::O15270|SPTC2_HUMAN	24	62884	1	1	1	1	0.08	Serine palmitoyltransfe
1672	1	SwissProt_HUMAN$	3::P34059|GALNS_HUMAN	24	57989	1	1	1	1	0.09	N-acetylgalactosamine
1673	1	db$2	2::C3KCH1|C3KCH1_PSEFS	24	32212	1	1	1	1	0.16	Putative carboxymethy
1674	1	db$2	2::C3KD58|C3KD58_PSEFS	24	41746	1	1	1	1	0.12	Putative cystathionine
1675	1	SwissProt_HUMAN$	3::P62263|RS14_HUMAN	24	16263	2	1	2	1	0.33	40S ribosomal protein
1676	1	SwissProt_HUMAN$	3::Q9C037|TRIM4_HUMAN	24	57424	1	1	1	1	0.09	E3 ubiquitin-protein lig
1677	1	db$2	2::C3K020|C3K020_PSEFS	24	23656	2	1	2	1	0.22	Putative hydrolase OS
1678	1	SwissProt_HUMAN$	3::P62136|PP1A_HUMAN	24	37488	1	1	1	1	0.13	Serine/threonine-prote
1679	1	SwissProt_HUMAN$	3::Q8NHX9|TPC2_HUMAN	24	85188	1	1	1	1	0.06	Two pore calcium char
1680	1	SwissProt_HUMAN$	3::Q9NVN3|RIC8B_HUMAN	23	58787	1	1	1	1	0.08	Synembryn-B OS=Hor
1681	1	SwissProt_HUMAN$	3::P11413|G6PD_HUMAN	23	59219	1	1	1	1	0.08	Glucose-6-phosphate
1682	1	SwissProt_HUMAN$	3::Q8N8R5|CB069_HUMAN	23	43421	1	1	1	1	0.12	UPF0565 protein C2orf

↑Family	M	DB	Accession	Score	Mass	Matches	Match(sig)	Sequences	Seq(sig)	emPAI	Description
1683	1	SwissProt_HUMAN$	3::Q9Y265|RUVB1_HUMAN	23	50196	1	1	1	1	0.10	RuvB-like 1 OS=Homo
1684	1	SwissProt_HUMAN$	3::Q9H299|SH3L3_HUMAN	23	10431	1	1	1	1	0.56	SH3 domain-binding g
1685	1	SwissProt_HUMAN$	3::Q9H9E3|COG4_HUMAN	23	89026	2	1	2	1	0.05	Conserved oligomeric (
1686	1	db$2	2::C3K065|C3K065_PSEFS	23	58554	1	1	1	1	0.08	Uncharacterized protei
1687	1	db$2	2::C3K2E8|C3K2E8_PSEFS	23	37117	1	1	1	1	0.14	Putative cytochrome o
1688	1	db$2	2::C3K3N6|FPG_PSEFS	23	30025	2	1	1	1	0.17	Formamidopyrimidine-
1689	1	db$2	2::C3K6L2|C3K6L2_PSEFS	23	14328	1	1	1	1	0.38	Organic hydroperoxide
1690	1	db$2	2::C3K322|C3K322_PSEFS	23	23985	1	1	1	1	0.22	NTP_transferase doma
1691	1	db$2	2::C3JZ74|C3JZ74_PSEFS	23	42038	1	1	1	1	0.12	ABC transport system,
1692	1	SwissProt_HUMAN$	3::Q14129|DGCR6_HUMAN	23	24973	1	1	1	1	0.21	Protein DGCR6 OS=Ho
1693	1	db$2	2::C3JZN5|RL35_PSEFS	23	7363	2	1	1	1	0.87	50S ribosomal protein
1694	1	SwissProt_HUMAN$	3::O15212|PFD6_HUMAN	23	14574	1	1	1	1	0.38	Prefoldin subunit 6 OS
1695	1	SwissProt_HUMAN$	3::P36507|MP2K2_HUMAN	23	44396	1	1	1	1	0.11	Dual specificity mitoge
1696	1	SwissProt_HUMAN$	3::Q96RL7|VP13A_HUMAN	23	360047	3	1	3	1	0.01	Vacuolar protein sortin
1697	1	db$2	2::C3KE19|C3KE19_PSEFS	23	30253	1	1	1	1	0.17	HDOD domain-contain
1698	1	SwissProt_HUMAN$	3::Q9H0U4|RAB1B_HUMAN	23	22157	1	1	1	1	0.24	Ras-related protein Ra
1699	1	SwissProt_HUMAN$	3::Q9NTJ3|SMC4_HUMAN	23	147091	1	1	1	1	0.03	Structural maintenanc
1700	1	db$2	2::A4V7G0|A4V7G0_PSEFS	23	59387	2	1	2	1	0.08	Putative RNA polymera
1701	1	db$2	2::C3K5B8|C3K5B8_PSEFS	23	59457	1	1	1	1	0.08	Dipeptide ABC transpo
1702	1	SwissProt_HUMAN$	3::Q9UL62|TRPC5_HUMAN	23	111340	1	1	1	1	0.04	Short transient recepto
1703	1	db$2	2::C3K4U8|C3K4U8_PSEFS	23	20094	1	1	1	1	0.26	Conserved hypothetica
1704	1	SwissProt_HUMAN$	3::Q70UQ0|IKIP_HUMAN	23	39285	1	1	1	1	0.13	Inhibitor of nuclear fac
1705	1	SwissProt_HUMAN$	3::Q9ULG1|INO80_HUMAN	23	176643	1	1	1	1	0.03	Chromatin-remodeling
1706	1	db$2	2::C3KDC5|HEM1_PSEFS	23	46641	1	1	1	1	0.11	Glutamyl-tRNA reducta
1707	1	SwissProt_HUMAN$	3::Q7Z6K1|THAP5_HUMAN	23	45388	1	1	1	1	0.11	THAP domain-containi
1708	1	SwissProt_HUMAN$	3::O94967|WDR47_HUMAN	23	101884	1	1	1	1	0.05	WD repeat-containing
1709	1	db$2	2::C3K0N6|PLSX_PSEFS	23	35209	1	1	1	1	0.14	Phosphate acyltransfer
1710	1	db$2	2::C3KE47|FMT_PSEFS	23	33416	3	2	3	2	0.33	Methionyl-tRNA formyl
1711	1	db$2	2::C3K279|C3K279_PSEFS	23	19136	2	2	2	2	0.63	Outer membrane prote
1712	1	SwissProt_HUMAN$	3::P14618|KPYM_HUMAN	23	57900	1	1	1	1	0.09	Pyruvate kinase PKM C
1713	1	db$2	2::C3K9X2|C3K9X2_PSEFS	22	42284	1	1	1	1	0.12	Putative aminotransfer
1714	1	db$2	2::C3K7T7|C3K7T7_PSEFS	22	17319	1	1	1	1	0.31	Putative acetyltransfer
1715	1	db$2	2::C3KBT7|C3KBT7_PSEFS	22	36676	3	1	3	1	0.14	Sulfate ABC transport
1716	1	db$2	2::C3K3K3|C3K3K3_PSEFS	22	44101	2	2	2	2	0.24	Dihydroorotase-like pr
1717	1	db$2	2::C3KDK7|C3KDK7_PSEFS	22	24465	1	1	1	1	0.21	Putative ABC transport
1718	1	SwissProt_HUMAN$	3::Q7Z5H4|VN1R5_HUMAN	22	40752	1	1	1	1	0.12	Vomeronasal type-1 re
1719	1	SwissProt_HUMAN$	3::Q9H244|P2Y12_HUMAN	22	39413	1	1	1	1	0.13	P2Y purinoceptor 12 O
1720	1	SwissProt_HUMAN$	3::Q8WV24|PHLA1_HUMAN	22	44988	1	1	1	1	0.11	Pleckstrin homology-lil
1721	1	db$2	2::C3K992|C3K992_PSEFS	22	34600	1	1	1	1	0.15	Putative AraC-family r
1722	1	SwissProt_HUMAN$	3::Q9ULT8|HECD1_HUMAN	22	289203	1	1	1	1	0.02	E3 ubiquitin-protein lig

↑Family	M	DB	Accession	Score	Mass	Matches	Match(sig)	Sequences	Seq(sig)	emPAI	Description
1723	1	SwissProt_HUMAN$	3::P15924|DESP_HUMAN	22	331569	1	1	1	1	0.01	Desmoplakin OS=Hom
1724	1	SwissProt_HUMAN$	3::Q15075|EEA1_HUMAN	22	162367	1	1	1	1	0.03	Early endosome antige
1725	1	SwissProt_HUMAN$	3::Q8WZ42|TITIN_HUMAN	22	3813652	5	1	5	1	0.00	Titin OS=Homo sapien
1726	1	db$2	2::C3K1G6|C3K1G6_PSEFS	22	25762	2	2	1	1	0.20	Thiol:disulfide intercha
1727	1	SwissProt_HUMAN$	3::A0A1B0GVD1|F237B_HUMAN	22	16320	1	1	1	1	0.33	Protein FAM237B OS=
1728	1	SwissProt_HUMAN$	3::P06748|NPM_HUMAN	22	32555	1	1	1	1	0.16	Nucleophosmin OS=Hc
1729	1	SwissProt_HUMAN$	3::O43586|PPIP1_HUMAN	22	47562	2	2	1	1	0.10	Proline-serine-threonir
1730	1	SwissProt_HUMAN$	3::Q9HBJ7|UBP29_HUMAN	22	104090	2	1	2	1	0.05	Ubiquitin carboxyl-terr
1731	1	SwissProt_HUMAN$	3::Q9NRF8|PYRG2_HUMAN	22	65636	1	1	1	1	0.07	CTP synthase 2 OS=Hc
1732	1	db$2	2::C3K9J4|C3K9J4_PSEFS	22	15648	1	1	1	1	0.35	Putative DnaK suppres
1733	1	SwissProt_HUMAN$	3::P04920|B3A2_HUMAN	22	136923	1	1	1	1	0.04	Anion exchange protei
1734	1	db$2	2::C3K8V0|C3K8V0_PSEFS	22	31271	1	1	1	1	0.16	Poly(3-hydroxyalkanoa
1735	1	SwissProt_HUMAN$	3::Q9Y5I4|PCDC2_HUMAN	22	109383	1	1	1	1	0.04	Protocadherin alpha-C
1736	1	db$2	2::C3K1F1|ATPL_PSEFS	22	8603	1	1	1	1	0.70	ATP synthase subunit
1737	1	db$2	2::C3JYB1|C3JYB1_PSEFS	22	43274	1	1	1	1	0.12	Uncharacterized protei
1738	1	db$2	2::C3K3Q9|LGT_PSEFS	22	30444	2	2	2	2	0.36	Phosphatidylglycerol--
1739	1	db$2	2::C3JZA7|C3JZA7_PSEFS	22	51947	1	1	1	1	0.10	Putative succinate-sem
1740	1	db$2	2::C3KBB7|C3KBB7_PSEFS	22	43574	1	1	1	1	0.11	3-oxoacyl-[acyl-carrier
1741	1	db$2	2::C3K3Q4|C3K3Q4_PSEFS	22	38576	2	2	2	2	0.28	Putative cation ABC tra
1742	1	SwissProt_HUMAN$	3::O95819|M4K4_HUMAN	22	142013	2	1	2	1	0.03	Mitogen-activated prot
1743	1	db$2	2::C3K578|C3K578_PSEFS	22	39452	1	1	1	1	0.13	Putative lipoprotein OS
1744	1	SwissProt_HUMAN$	3::Q9BYT1|S17A9_HUMAN	22	47451	1	1	1	1	0.10	Solute carrier family 1
1745	1	SwissProt_HUMAN$	3::P0DOX2|IGA2_HUMAN	22	48903	1	1	1	1	0.10	Immunoglobulin alpha
1746	1	SwissProt_HUMAN$	3::Q9H6P5|TASP1_HUMAN	22	44427	1	1	1	1	0.11	Threonine aspartase 1
1747	1	SwissProt_HUMAN$	3::Q8IVE0|CROL2_HUMAN	22	33314	2	1	2	1	0.15	Putative ciliary rootlet
1748	1	SwissProt_HUMAN$	3::Q8IXV7|KLD8B_HUMAN	22	37652	1	1	1	1	0.13	Kelch domain-containi
1749	1	db$2	2::C3KC21|C3KC21_PSEFS	21	421641	3	2	3	2	0.02	Putative adhesin OS=F
1750	1	db$2	2::C3K348|PQQB_PSEFS	21	33093	1	1	1	1	0.15	Coenzyme PQQ synthe
1751	1	db$2	2::C3K9N9|C3K9N9_PSEFS	21	57231	1	1	1	1	0.09	Putative ABC transport
1752	1	db$2	2::C3K333|C3K333_PSEFS	21	61021	1	1	1	1	0.08	Uncharacterized protei
1753	1	SwissProt_HUMAN$	3::Q2VPJ9|LR75B_HUMAN	21	34718	1	1	1	1	0.15	Leucine-rich repeat-co
1754	1	db$2	2::C3K0A7|ALLC_PSEFS	21	36686	1	1	1	1	0.14	Probable allantoicase (
1755	1	db$2	2::C3K9N6|C3K9N6_PSEFS	21	26575	1	1	1	1	0.19	Putative oxidoreductas
1756	1	db$2	2::C3K8G1|C3K8G1_PSEFS	21	23138	1	1	1	1	0.23	Putative DNA-binding
1757	1	SwissProt_HUMAN$	3::Q9UJN7|ZN391_HUMAN	21	40634	1	1	1	1	0.12	Zinc finger protein 391
1758	1	db$2	2::C3JYF7|C3JYF7_PSEFS	21	147681	1	1	1	1	0.03	ATP-dependent helicas
1759	1	SwissProt_HUMAN$	3::P62857|RS28_HUMAN	21	7836	1	1	1	1	0.79	40S ribosomal protein
1760	1	db$2	2::C3K283|C3K283_PSEFS	21	28802	1	1	1	1	0.18	Pyruvate dehydrogena
1761	1	db$2	2::C3KAP0|C3KAP0_PSEFS	21	14934	1	1	1	1	0.37	4-carboxymuconolacto
1762	1	SwissProt_HUMAN$	3::Q9Y4G8|RPGF2_HUMAN	21	167313	2	2	2	2	0.06	Rap guanine nucleotid

↑*Family*	M	DB	Accession	Score	Mass	Matches	Match(sig)	Sequences	Seq(sig)	emPAI	Description
1763	1	db$2	2::C3JXS7|C3JXS7_PSEFS	21	34119	1	1	1	1	0.15	Putative GGDEF domai
1764	1	db$2	2::C3KAT0|C3KAT0_PSEFS	21	40804	1	1	1	1	0.12	Putative efflux system
1765	1	db$2	2::C3K4F4|C3K4F4_PSEFS	21	25363	1	1	1	1	0.20	Putative acetyltransfer
1766	1	db$2	2::C3KBV2|C3KBV2_PSEFS	21	15686	1	1	1	1	0.35	Thioredoxin OS=Pseuc
1767	1	db$2	2::C3KDX1|RS20_PSEFS	21	10078	1	1	1	1	0.58	30S ribosomal protein
1768	1	SwissProt_HUMAN$	3::Q9BWW9|APOL5_HUMAN	21	47014	1	1	1	1	0.11	Apolipoprotein L5 OS=
1769	1	db$2	2::C3KAD1|C3KAD1_PSEFS	21	32401	1	1	1	1	0.16	Probable 4-deoxy-4-fo
1770	1	db$2	2::C3K7A9|C3K7A9_PSEFS	21	33658	1	1	1	1	0.15	Conserved hypothetica
1771	1	db$2	2::C3K4U1|C3K4U1_PSEFS	21	34459	1	1	1	1	0.15	Putative lipoprotein OS
1772	1	SwissProt_HUMAN$	3::Q96T25|ZIC5_HUMAN	21	68404	1	1	1	1	0.07	Zinc finger protein ZIC
1773	1	SwissProt_HUMAN$	3::Q8WWY3|PRP31_HUMAN	21	55421	2	1	2	1	0.09	U4/U6 small nuclear ri
1774	1	db$2	2::C3K814|DTD_PSEFS	21	15391	1	1	1	1	0.35	D-aminoacyl-tRNA dea
1775	1	db$2	2::C3KB61|C3KB61_PSEFS	21	26669	3	1	3	1	0.19	Molybdate-binding per
1776	1	db$2	2::C3KAN4|C3KAN4_PSEFS	21	41646	1	1	1	1	0.12	3-oxoadipyl-CoA thiola
1777	1	db$2	2::C3KE01|Y992_PSEFS	21	29383	1	1	1	1	0.17	UPF0246 protein PFLU_
1778	1	SwissProt_HUMAN$	3::P46776|RL27A_HUMAN	21	16551	1	1	1	1	0.33	60S ribosomal protein
1779	1	SwissProt_HUMAN$	3::Q9BT17|MTG1_HUMAN	21	37213	1	1	1	1	0.14	Mitochondrial ribosome
1780	1	SwissProt_HUMAN$	3::Q9Y2J0|RP3A_HUMAN	21	76824	1	1	1	1	0.06	Rabphilin-3A OS=Hom
1781	1	SwissProt_HUMAN$	3::Q02080|MEF2B_HUMAN	21	38615	1	1	1	1	0.13	Myocyte-specific enhar
1782	1	SwissProt_HUMAN$	3::Q8N7X0|ADGB_HUMAN	21	189594	1	1	1	1	0.03	Androglobin OS=Homc
1783	1	db$2	2::C3K3K0|YQGF_PSEFS	20	16158	1	1	1	1	0.33	Putative pre-16S rRNA
1784	1	db$2	2::C3KDY6|C3KDY6_PSEFS	20	17128	1	1	1	1	0.31	Uncharacterized protei
1785	1	SwissProt_HUMAN$	3::Q8TCU5|NMD3A_HUMAN	20	125385	2	1	1	1	0.04	Glutamate receptor ior
1786	1	db$2	2::C3K2N6|C3K2N6_PSEFS	20	16796	1	1	1	1	0.32	MaoC-like domain-cont
1787	1	SwissProt_HUMAN$	3::P02545|LMNA_HUMAN	20	74095	2	1	2	1	0.07	Prelamin-A/C OS=Hom
1788	1	SwissProt_HUMAN$	3::P30050|RL12_HUMAN	20	17808	1	1	1	1	0.30	60S ribosomal protein
1789	1	db$2	2::C3K1S4|C3K1S4_PSEFS	20	23334	1	1	1	1	0.22	Putative membrane pr
1790	1	db$2	2::C3K7Z3|C3K7Z3_PSEFS	20	43142	1	1	1	1	0.12	Putative acyl-CoA deh
1791	1	SwissProt_HUMAN$	3::Q86VP1|TAXB1_HUMAN	20	90820	1	1	1	1	0.05	Tax1-binding protein 1
1792	1	SwissProt_HUMAN$	3::Q13422|IKZF1_HUMAN	20	57491	1	1	1	1	0.09	DNA-binding protein Ik
1793	1	SwissProt_HUMAN$	3::O75251|NDUS7_HUMAN	20	23548	1	1	1	1	0.22	NADH dehydrogenase
1794	1	db$2	2::C3K5C6|C3K5C6_PSEFS	20	33902	1	1	1	1	0.15	Uncharacterized protei
1795	1	SwissProt_HUMAN$	3::A6NNS2|DRS7C_HUMAN	20	34855	1	1	1	1	0.14	Dehydrogenase/reduct
1796	1	SwissProt_HUMAN$	3::Q8IZ21|PHAR4_HUMAN	20	78163	2	2	1	1	0.06	Phosphatase and actin
1797	1	SwissProt_HUMAN$	3::Q5TGY3|AHDC1_HUMAN	20	168245	2	1	1	1	0.03	AT-hook DNA-binding
1798	1	db$2	2::C3K6M0|C3K6M0_PSEFS	20	28018	1	1	1	1	0.18	PABS domain-containi
1799	1	db$2	2::C3JXG5|C3JXG5_PSEFS	20	36156	1	1	1	1	0.14	Putative GFO/IDH/Moc
1800	1	db$2	2::C3K3D3|BETA_PSEFS	20	62642	2	2	2	2	0.16	Oxygen-dependent chc
1801	1	db$2	2::C3KBM5|C3KBM5_PSEFS	20	75948	1	1	1	1	0.06	Putative formate dehy
1802	1	db$2	2::C3K1B4|C3K1B4_PSEFS	20	43355	1	1	1	1	0.12	Putative transferase O

↑Family	M	DB	Accession	Score	Mass	Matches	Match(sig)	Sequences	Seq(sig)	emPAI	Description
1803	1	SwissProt_HUMAN$	3::P62258|1433E_HUMAN	20	29155	3	1	3	1	0.18	14-3-3 protein epsilon
1804	1	db$2	2::C3KCJ8|C3KCJ8_PSEFS	20	33388	1	1	1	1	0.15	Putative 2-hydroxyacic
1805	1	SwissProt_HUMAN$	3::P34947|GRK5_HUMAN	20	67743	1	1	1	1	0.07	G protein-coupled rece
1806	1	db$2	2::C3K751|C3K751_PSEFS	20	15943	1	1	1	1	0.34	Putative thioesterase (
1807	1	SwissProt_HUMAN$	3::Q9NUQ8|ABCF3_HUMAN	20	79695	1	1	1	1	0.06	ATP-binding cassette s
1808	1	db$2	2::C3K2T4|C3K2T4_PSEFS	20	155869	1	1	1	1	0.03	Putative ATP-depender
1809	1	SwissProt_HUMAN$	3::Q99623|PHB2_HUMAN	20	33276	1	1	1	1	0.15	Prohibitin-2 OS=Homo
1810	1	db$	1::P0DTC1|R1A_SARS2	20	489672	1	1	1	1	0.01	Replicase polyprotein :
1811	1	SwissProt_HUMAN$	3::P08476|INHBA_HUMAN	20	47412	1	1	1	1	0.10	Inhibin beta A chain O
1812	1	db$2	2::C3K136|C3K136_PSEFS	20	31053	1	1	1	1	0.16	Uncharacterized protei
1813	1	db$2	2::C3K9H2|C3K9H2_PSEFS	20	39760	2	2	2	2	0.27	Putrescine ABC transp
1814	1	db$2	2::C3KAE4|C3KAE4_PSEFS	20	39865	1	1	1	1	0.13	Uncharacterized protei
1815	1	SwissProt_HUMAN$	3::Q8TEA8|DTD1_HUMAN	20	23409	1	1	1	1	0.22	D-aminoacyl-tRNA dea
1816	1	db$2	2::C3KAX3|C3KAX3_PSEFS	20	30360	1	1	1	1	0.17	Conserved hypothetica
1817	1	db$2	2::A4V6R3|A4V6R3_PSEFS	20	51478	1	1	1	1	0.10	Uncharacterized protei
1818	1	db$2	2::C3JXZ4|C3JXZ4_PSEFS	20	40619	1	1	1	1	0.12	Uncharacterized protei
1819	1	db$2	2::C3K3F2|C3K3F2_PSEFS	19	38228	1	1	1	1	0.13	D-erythrose-4-phosphi
1820	1	SwissProt_HUMAN$	3::P00738|HPT_HUMAN	19	45177	1	1	1	1	0.11	Haptoglobin OS=Homc
1821	1	db$2	2::C3K0X2|C3K0X2_PSEFS	19	81009	1	1	1	1	0.06	Patatin-like phospholip
1822	1	db$2	2::C3K125|C3K125_PSEFS	19	26783	1	1	1	1	0.19	Alanine--tRNA ligase C
1823	1	db$2	2::C3K5F3|C3K5F3_PSEFS	19	34108	1	1	1	1	0.15	Putative dehydrogenas
1824	1	db$2	2::C3K1Q2|C3K1Q2_PSEFS	19	73203	1	1	1	1	0.07	Histidine kinase OS=P:
1825	1	db$2	2::C3K301|C3K301_PSEFS	19	28901	1	1	1	1	0.18	Putative dehydrogenas
1826	1	SwissProt_HUMAN$	3::O96028|NSD2_HUMAN	19	152160	1	1	1	1	0.03	Histone-lysine N-meth
1827	1	SwissProt_HUMAN$	3::O15050|TRNK1_HUMAN	19	336007	3	2	3	2	0.03	TPR and ankyrin repea
1828	1	db$2	2::C3K9H9|C3K9H9_PSEFS	19	51634	1	1	1	1	0.10	Histidine kinase OS=P:
1829	1	db$2	2::C3KAI2|C3KAI2_PSEFS	19	19361	2	1	2	1	0.27	Shikimate kinase OS=
1830	1	db$2	2::C3KCG3|C3KCG3_PSEFS	19	16758	1	1	1	1	0.32	YjgF_endoribonc doma
1831	1	SwissProt_HUMAN$	3::Q9NR48|ASH1L_HUMAN	19	332582	1	1	1	1	0.01	Histone-lysine N-meth
1832	1	SwissProt_HUMAN$	3::P02741|CRP_HUMAN	19	25023	2	1	2	1	0.21	C-reactive protein OS=
1833	1	db$2	2::C3K7J3|Y1319_PSEFS	19	16953	1	1	1	1	0.32	UPF0225 protein PFLU_
1834	1	SwissProt_HUMAN$	3::Q9UHW9|S12A6_HUMAN	19	127534	1	1	1	1	0.04	Solute carrier family 1:
1835	1	SwissProt_HUMAN$	3::Q13835|PKP1_HUMAN	19	82808	1	1	1	1	0.06	Plakophilin-1 OS=Hom
1836	1	db$2	2::C3K9K0|C3K9K0_PSEFS	19	37025	1	1	1	1	0.14	Glycine betaine/L-proli
1837	1	db$2	2::C3K055|C3K055_PSEFS	19	30642	1	1	1	1	0.17	Uncharacterized protei
1838	1	SwissProt_HUMAN$	3::Q9Y5A7|NUB1_HUMAN	19	70494	2	1	2	1	0.07	NEDD8 ultimate buster
1839	1	db$2	2::C3JYG2|C3JYG2_PSEFS	19	36128	1	1	1	1	0.14	ATP-grasp domain-con
1840	1	SwissProt_HUMAN$	3::Q9BYV7|BCDO2_HUMAN	19	65632	1	1	1	1	0.07	Beta,beta-carotene 9',
1841	1	SwissProt_HUMAN$	3::Q7Z3T8|ZFY16_HUMAN	19	168797	1	1	1	1	0.03	Zinc finger FYVE doma
1842	1	SwissProt_HUMAN$	3::Q53EZ4|CEP55_HUMAN	19	54145	2	1	2	1	0.09	Centrosomal protein of

↑Family	M	DB	Accession	Score	Mass	Matches	Match(sig)	Sequences	Seq(sig)	emPAI	Description
1843	1	db$2	2::C3K7J4|C3K7J4_PSEFS	19	18330	1	1	1	1	0.29	Uncharacterized protei
1844	1	db$2	2::C3KBA7|C3KBA7_PSEFS	19	51338	2	1	2	1	0.10	Putative phenylacetald
1845	1	SwissProt_HUMAN$	3::Q8IWU5|SULF2_HUMAN	19	100390	1	1	1	1	0.05	Extracellular sulfatase
1846	1	SwissProt_HUMAN$	3::Q7Z5L9|I2BP2_HUMAN	19	60987	1	1	1	1	0.08	Interferon regulatory f
1847	1	SwissProt_HUMAN$	3::Q9UDT6|CLIP2_HUMAN	19	115767	1	1	1	1	0.04	CAP-Gly domain-conta
1848	1	db$2	2::C3K4R2|C3K4R2_PSEFS	19	7681	1	1	1	1	0.82	Major cold shock prote
1849	1	SwissProt_HUMAN$	3::Q9NZI6|TF2L1_HUMAN	19	54593	1	1	1	1	0.09	Transcription factor CP
1850	1	db$2	2::C3KE72|C3KE72_PSEFS	19	85807	1	1	1	1	0.06	UPF0313 protein PFLU_
1851	1	db$2	2::C3K979|C3K979_PSEFS	18	30391	1	1	1	1	0.17	Putative glutathione S-
1852	1	SwissProt_HUMAN$	3::P22894|MMP8_HUMAN	18	53378	1	1	1	1	0.09	Neutrophil collagenase
1853	1	db$2	2::C3K2F9|C3K2F9_PSEFS	18	48516	1	1	1	1	0.10	Putative ABC transport
1854	1	db$2	2::C3K5B7|C3K5B7_PSEFS	18	50883	1	1	1	1	0.10	Putative outer membra
1855	1	db$2	2::C3K757|C3K757_PSEFS	18	27432	3	1	3	1	0.19	Putative two-compone
1856	1	db$2	2::C3K8N0|C3K8N0_PSEFS	18	12883	1	1	1	1	0.43	Antitoxin OS=Pseudon
1857	1	db$2	2::C3K883|C3K883_PSEFS	18	60772	1	1	1	1	0.08	Putative transposase s
1858	1	SwissProt_HUMAN$	3::Q92541|RTF1_HUMAN	18	80265	1	1	1	1	0.06	RNA polymerase-assoc
1859	1	db$2	2::C3K8Z7|C3K8Z7_PSEFS	18	10906	2	1	2	1	0.53	Uncharacterized protei
1860	1	SwissProt_HUMAN$	3::Q9UPN3|MACF1_HUMAN	18	837787	2	1	2	1	0.01	Microtubule-actin cros:
1861	1	db$2	2::C3K1H4|C3K1H4_PSEFS	18	45615	1	1	1	1	0.11	Putative membrane pr
1862	1	SwissProt_HUMAN$	3::Q969X2|SIA7F_HUMAN	18	38043	1	1	1	1	0.13	Alpha-N-acetylgalactos
1863	1	SwissProt_HUMAN$	3::Q8IVF4|DYH10_HUMAN	18	514512	1	1	1	1	0.01	Dynein heavy chain 10
1864	1	SwissProt_HUMAN$	3::O14827|RGRF2_HUMAN	18	140675	1	1	1	1	0.03	Ras-specific guanine n
1865	1	SwissProt_HUMAN$	3::Q86XX4|FRAS1_HUMAN	18	442928	1	1	1	1	0.01	Extracellular matrix pr
1866	1	db$2	2::C3JY60|BPT_PSEFS	18	27663	1	1	1	1	0.19	Aspartate/glutamate le
1867	1	db$2	2::C3JZP4|RBSD_PSEFS	18	14393	1	1	1	1	0.38	D-ribose pyranase OS=
1868	1	db$2	2::C3K5F2|Y1517_PSEFS	18	11422	1	1	1	1	0.50	YcgL domain-containin
1869	1	db$2	2::C3K323|C3K323_PSEFS	18	38795	3	2	3	2	0.28	Putative phosphotrans
1870	1	db$2	2::C3KAG1|C3KAG1_PSEFS	18	64101	1	1	1	1	0.08	Putative exported adhe
1871	1	SwissProt_HUMAN$	3::Q2T9J0|TYSD1_HUMAN	18	59271	1	1	1	1	0.08	Peroxisomal leader pep
1872	1	SwissProt_HUMAN$	3::P08514|ITA2B_HUMAN	18	113306	1	1	1	1	0.04	Integrin alpha-IIb OS=
1873	1	SwissProt_HUMAN$	3::P42126|ECI1_HUMAN	18	32795	1	1	1	1	0.15	Enoyl-CoA delta isome
1874	1	db$2	2::C3KAX6|C3KAX6_PSEFS	18	25593	1	1	1	1	0.20	Putative acetyltransfer
1875	1	db$2	2::C3KDL9|C3KDL9_PSEFS	18	22175	1	1	1	1	0.24	Uncharacterized protei
1876	1	db$2	2::C3K670|C3K670_PSEFS	18	30761	1	1	1	1	0.17	Putative glycine betain
1877	1	db$2	2::C3K277|C3K277_PSEFS	18	60895	1	1	1	1	0.08	DNA repair protein Rec
1878	1	SwissProt_HUMAN$	3::P31943|HNRH1_HUMAN	18	49198	1	1	1	1	0.10	Heterogeneous nuclear
1879	1	db$2	2::C3K1J8|C3K1J8_PSEFS	17	26668	2	1	2	1	0.19	Trehalose operon trans
1880	1	SwissProt_HUMAN$	3::Q5F1R6|DJC21_HUMAN	17	61989	1	1	1	1	0.08	DnaJ homolog subfami
1881	1	SwissProt_HUMAN$	3::A8MV81|HIG1C_HUMAN	17	11072	1	1	1	1	0.52	HIG1 domain family m
1882	1	SwissProt_HUMAN$	3::P60903|S10AA_HUMAN	17	11196	1	1	1	1	0.51	Protein S100-A10 OS=

↑Family	M	DB	Accession	Score	Mass	Matches	Match(sig)	Sequences	Seq(sig)	emPAI	Description
1883	1	db$2	2::C3K4W4|C3K4W4_PSEFS	17	45211	2	1	2	1	0.11	Putative lipoprotein rel
1884	1	db$2	2::C3K9R9|C3K9R9_PSEFS	17	86327	1	1	1	1	0.06	Putative exported fatty
1885	1	SwissProt_HUMAN$	3::Q9BZE7|EVG1_HUMAN	17	24940	1	1	1	1	0.21	UPF0193 protein EVG1
1886	1	db$2	2::C3K2P5|C3K2P5_PSEFS	17	49850	1	1	1	1	0.10	Ethanolamine ammoni
1887	1	db$2	2::C3K2E4|C3K2E4_PSEFS	17	29249	1	1	1	1	0.17	Ferredoxin--NADP redu
1888	1	db$2	2::C3K5C9|C3K5C9_PSEFS	17	27544	1	1	1	1	0.19	Putative regulatory pro
1889	1	db$2	2::C3K842|C3K842_PSEFS	17	56600	1	1	1	1	0.09	RNA polymerase sigma
1890	1	SwissProt_HUMAN$	3::Q9NRJ2|GSAS1_HUMAN	17	17646	1	1	1	1	0.30	Putative uncharacteriz
1891	1	db$2	2::C3K000|C3K000_PSEFS	17	26225	1	1	1	1	0.20	Putative GntR-family t
1892	1	db$2	2::C3K5A1|C3K5A1_PSEFS	17	60517	1	1	1	1	0.08	Urease subunit alpha (
1893	1	db$2	2::C3K2K4|C3K2K4_PSEFS	17	19926	1	1	1	1	0.26	Putative exported prot
1894	1	db$2	2::C3KCA2|C3KCA2_PSEFS	17	48472	1	1	1	1	0.10	Putative family S43 no
1895	1	db$2	2::C3JZ44|C3JZ44_PSEFS	17	47432	1	1	1	1	0.10	Putative gluconate per
1896	1	SwissProt_HUMAN$	3::Q8NA19|LMBL4_HUMAN	17	71077	1	1	1	1	0.07	Lethal(3)malignant bra
1897	1	db$2	2::C3K2B9|C3K2B9_PSEFS	17	76650	1	1	1	1	0.06	Putative methyl-accept
1898	1	SwissProt_HUMAN$	3::Q92766|RREB1_HUMAN	17	181306	1	1	1	1	0.03	Ras-responsive elemen
1899	1	db$2	2::C3K0P6|DCTA_PSEFS	17	46761	1	1	1	1	0.11	C4-dicarboxylate trans
1900	1	db$2	2::C3K4V6|C3K4V6_PSEFS	17	101332	1	1	1	1	0.05	Putative helicase OS=I
1901	1	db$2	2::C3K6J8|C3K6J8_PSEFS	17	78979	1	1	1	1	0.06	3-phosphoshikimate 1-
1902	1	db$2	2::C3K1Z0|C3K1Z0_PSEFS	17	34051	1	1	1	1	0.15	Putative putative polys
1903	1	SwissProt_HUMAN$	3::O75437|ZN254_HUMAN	17	77110	1	1	1	1	0.06	Zinc finger protein 254
1904	1	SwissProt_HUMAN$	3::Q9UM11|FZR1_HUMAN	16	55145	1	1	1	1	0.09	Fizzy-related protein h
1905	1	SwissProt_HUMAN$	3::Q9H310|RHBG_HUMAN	16	47200	1	1	1	1	0.11	Ammonium transporte
1906	1	SwissProt_HUMAN$	3::Q86VD9|PIGZ_HUMAN	16	63430	1	1	1	1	0.08	GPI mannosyltransfera
1907	1	SwissProt_HUMAN$	3::P78527|PRKDC_HUMAN	16	468788	2	1	2	1	0.01	DNA-dependent protei
1908	1	SwissProt_HUMAN$	3::Q8N1H7|S6OS1_HUMAN	16	68123	1	1	1	1	0.07	Protein SIX6OS1 OS=I
1909	1	db$2	2::C3JXM0|C3JXM0_PSEFS	16	45141	2	1	2	1	0.11	Putative peptidase OS=
1910	1	SwissProt_HUMAN$	3::P42768|WASP_HUMAN	16	52880	1	1	1	1	0.09	Wiskott-Aldrich syndro
1911	1	db$2	2::C3KB05|C3KB05_PSEFS	16	50891	1	1	1	1	0.10	CDP-diacylglycerol-ser
1912	1	SwissProt_HUMAN$	3::Q9BT73|PSMG3_HUMAN	16	13096	1	1	1	1	0.43	Proteasome assembly
1913	1	SwissProt_HUMAN$	3::P42224|STAT1_HUMAN	16	87280	1	1	1	1	0.06	Signal transducer and
1914	1	SwissProt_HUMAN$	3::O15399|NMDE4_HUMAN	16	143661	1	1	1	1	0.03	Glutamate receptor ior
1915	1	db$2	2::C3K3H5|C3K3H5_PSEFS	16	32718	1	1	1	1	0.15	Putative ABC transport
1916	1	SwissProt_HUMAN$	3::Q9BV86|NTM1A_HUMAN	16	25371	1	1	1	1	0.20	N-terminal Xaa-Pro-Ly
1917	1	SwissProt_HUMAN$	3::Q96KP1|EXOC2_HUMAN	16	104000	1	1	1	1	0.05	Exocyst complex comp
1918	1	SwissProt_HUMAN$	3::Q3LIE5|ADPRM_HUMAN	16	39504	1	1	1	1	0.13	Manganese-dependent
1919	1	SwissProt_HUMAN$	3::Q9C098|DCLK3_HUMAN	16	73768	1	1	1	1	0.07	Serine/threonine-prote
1920	1	SwissProt_HUMAN$	3::Q8IYW2|CFA46_HUMAN	16	303308	2	1	2	1	0.02	Cilia- and flagella-asso
1921	1	db$2	2::C3KE75|C3KE75_PSEFS	16	123658	1	1	1	1	0.04	TGc domain-containing
1922	1	SwissProt_HUMAN$	3::Q7RTR0|NLRP9_HUMAN	16	113238	1	1	1	1	0.04	NACHT, LRR and PYD (

↑Family	M	DB	Accession	Score	Mass	Matches	Match(sig)	Sequences	Seq(sig)	emPAI	Description
1923	1	db$2	2::A4V819|A4V819_PSEFS	16	100853	1	1	1	1	0.05	PDDEXK_1 domain-cor
1924	1	SwissProt_HUMAN$	3::O43182|RHG06_HUMAN	16	105882	1	1	1	1	0.05	Rho GTPase-activating
1925	1	db$2	2::C3KBZ4|C3KBZ4_PSEFS	16	6850	1	1	1	1	0.94	Uncharacterized protei
1926	1	db$2	2::C3KCB6|C3KCB6_PSEFS	16	31268	1	1	1	1	0.16	Phosphate transport sy
1927	1	db$2	2::C3K933|C3K933_PSEFS	16	32686	1	1	1	1	0.16	Putative homocysteine
1928	1	SwissProt_HUMAN$	3::O15397|IPO8_HUMAN	16	119861	1	1	1	1	0.04	Importin-8 OS=Homo
1929	1	SwissProt_HUMAN$	3::Q9Y577|TRI17_HUMAN	15	54383	2	1	2	1	0.09	E3 ubiquitin-protein lig
1930	1	db$2	2::C3KCS4|C3KCS4_PSEFS	15	63231	1	1	1	1	0.08	Peptidoglycan D,D-trar
1931	1	SwissProt_HUMAN$	3::Q6PCB6|AB17C_HUMAN	15	35808	1	1	1	1	0.14	Alpha/beta hydrolase c
1932	1	SwissProt_HUMAN$	3::P52429|DGKE_HUMAN	15	63885	1	1	1	1	0.08	Diacylglycerol kinase e
1933	1	db$2	2::C3K577|C3K577_PSEFS	15	15787	1	1	1	1	0.34	Putative lipoprotein OS
1934	1	db$2	2::C3K5J0|C3K5J0_PSEFS	15	13887	1	1	1	1	0.40	Putative lipoprotein OS
1935	1	SwissProt_HUMAN$	3::Q05215|EGR4_HUMAN	15	61584	1	1	1	1	0.08	Early growth response
1936	1	db$2	2::C3K2L1|C3K2L1_PSEFS	15	28054	1	1	1	1	0.18	DUF218 domain-conta
1937	1	db$2	2::C3K915|C3K915_PSEFS	15	26912	1	1	1	1	0.19	Putative GntR-family r
1938	1	db$2	2::C3K1L5|C3K1L5_PSEFS	15	34147	3	1	3	1	0.15	Putative DNA-binding p
1939	1	db$2	2::C3JZI1|C3JZI1_PSEFS	15	33491	1	1	1	1	0.15	Putative glycine betain
1940	1	SwissProt_HUMAN$	3::P55285|CADH6_HUMAN	15	88254	1	1	1	1	0.06	Cadherin-6 OS=Homo
1941	1	db$2	2::C3K0M9|KTHY_PSEFS	15	22917	1	1	1	1	0.23	Thymidylate kinase OS
1942	1	SwissProt_HUMAN$	3::Q8TF21|ANR24_HUMAN	15	124111	2	1	2	1	0.04	Ankyrin repeat domain
1943	1	db$2	2::C3K2J0|C3K2J0_PSEFS	15	40846	1	1	1	1	0.12	Putative exported prot
1944	1	db$2	2::C3KB00|C3KB00_PSEFS	15	30565	1	1	1	1	0.17	Non-homologous end j
1945	1	SwissProt_HUMAN$	3::Q5VSL9|STRP1_HUMAN	15	95515	1	1	1	1	0.05	Striatin-interacting pro
1946	1	db$2	2::C3K5L4|C3K5L4_PSEFS	15	28633	1	1	1	1	0.18	Phosphonates ABC trar
1947	1	db$2	2::C3K805|C3K805_PSEFS	15	35193	1	1	1	1	0.14	Putative histidine trans
1948	1	db$2	2::C3K7D0|THII_PSEFS	15	54519	1	1	1	1	0.09	tRNA sulfurtransferase
1949	1	SwissProt_HUMAN$	3::Q8TC07|TBC15_HUMAN	15	79440	2	1	2	1	0.06	TBC1 domain family m
1950	1	db$2	2::C3KA88|C3KA88_PSEFS	15	40085	1	1	1	1	0.13	Putative 4-hydroxyphe
1951	1	SwissProt_HUMAN$	3::O75132|ZBED4_HUMAN	15	130239	1	1	1	1	0.04	Zinc finger BED domai
1952	1	SwissProt_HUMAN$	3::P11387|TOP1_HUMAN	15	90669	1	1	1	1	0.05	DNA topoisomerase 1
1953	1	db$2	2::C3KAB7|C3KAB7_PSEFS	15	33297	1	1	1	1	0.15	Putative membrane pr
1954	1	SwissProt_HUMAN$	3::P18077|RL35A_HUMAN	15	12530	1	1	1	1	0.45	60S ribosomal protein
1955	1	SwissProt_HUMAN$	3::A4UGR9|XIRP2_HUMAN	15	382063	1	1	1	1	0.01	Xin actin-binding repea
1956	1	SwissProt_HUMAN$	3::P22695|QCR2_HUMAN	15	48413	1	1	1	1	0.10	Cytochrome b-c1 comp
1957	1	SwissProt_HUMAN$	3::P58166|INHBE_HUMAN	14	38537	1	1	1	1	0.13	Inhibin beta E chain O:
1958	1	db$2	2::C3KBT8|C3KBT8_PSEFS	14	29541	1	1	1	1	0.17	Sulfate transport syste
1959	1	SwissProt_HUMAN$	3::P61024|CKS1_HUMAN	14	9654	1	1	1	1	0.61	Cyclin-dependent kina:
1960	1	SwissProt_HUMAN$	3::Q7Z4P5|GDF7_HUMAN	14	46921	1	1	1	1	0.11	Growth/differentiation
1961	1	db$2	2::C3K0E8|C3K0E8_PSEFS	14	27512	1	1	1	1	0.19	Thiol:disulfide intercha
1962	1	db$2	2::C3K3X1|C3K3X1_PSEFS	14	48834	1	1	1	1	0.10	Xaa-Pro aminopeptida:

↑Family	M	DB	Accession	Score	Mass	Matches	Match(sig)	Sequences	Seq(sig)	emPAI	Description
1963	1	SwissProt_HUMAN$	3::Q96GC6|ZN274_HUMAN	14	74131	3	2	1	1	0.07	Neurotrophin receptor-
1964	1	SwissProt_HUMAN$	3::Q6W2J9|BCOR_HUMAN	14	192069	1	1	1	1	0.03	BCL-6 corepressor OS=
1965	1	SwissProt_HUMAN$	3::O95297|MPZL1_HUMAN	14	29064	1	1	1	1	0.18	Myelin protein zero-lik
1966	1	SwissProt_HUMAN$	3::Q6I9Y2|THOC7_HUMAN	14	23728	1	1	1	1	0.22	THO complex subunit 7
1967	1	SwissProt_HUMAN$	3::Q92620|PRP16_HUMAN	14	140415	1	1	1	1	0.03	Pre-mRNA-splicing fact
1968	1	SwissProt_HUMAN$	3::Q8WXH0|SYNE2_HUMAN	14	795944	2	1	2	1	0.01	Nesprin-2 OS=Homo s
1969	1	SwissProt_HUMAN$	3::O00231|PSD11_HUMAN	14	47434	1	1	1	1	0.10	26S proteasome non-A
1970	1	db$2	2::C3K1M4|C3K1M4_PSEFS	14	13705	2	2	2	2	0.98	Iron-sulfur cluster asse
1971	1	SwissProt_HUMAN$	3::Q15652|JHD2C_HUMAN	14	284349	1	1	1	1	0.02	Probable JmjC domain
1972	1	SwissProt_HUMAN$	3::Q99797|MIPEP_HUMAN	14	80589	2	1	2	1	0.06	Mitochondrial intermed
1973	1	db$2	2::C3K0F1|C3K0F1_PSEFS	14	476575	2	1	2	1	0.01	Pyoverdine synthetase
1974	1	db$2	2::C3K869|C3K869_PSEFS	14	24016	1	1	1	1	0.22	Putative lipoprotein OS
1975	1	db$2	2::C3K3J6|C3K3J6_PSEFS	14	14927	1	1	1	1	0.37	Type IV pilus response
1976	1	SwissProt_HUMAN$	3::Q9Y446|PKP3_HUMAN	14	87029	1	1	1	1	0.06	Plakophilin-3 OS=Hom
1977	1	db$2	2::C3K6M1|C3K6M1_PSEFS	13	28266	1	1	1	1	0.18	Putative enoyl-CoA hy
1978	1	db$2	2::C3K6T3|C3K6T3_PSEFS	13	23166	1	1	1	1	0.22	Thiol:disulfide intercha
1979	1	SwissProt_HUMAN$	3::Q86VN1|VPS36_HUMAN	13	43789	1	1	1	1	0.11	Vacuolar protein-sortir
1980	1	SwissProt_HUMAN$	3::Q9Y6K1|DNM3A_HUMAN	13	101793	1	1	1	1	0.05	DNA (cytosine-5)-metl
1981	1	db$2	2::C3KCV5|C3KCV5_PSEFS	13	37705	1	1	1	1	0.13	Uncharacterized protei
1982	1	db$2	2::C3K8V2|C3K8V2_PSEFS	13	13938	1	1	1	1	0.40	GBBH-like_N domain-c
1983	1	SwissProt_HUMAN$	3::Q4KMP7|TB10B_HUMAN	13	87145	1	1	1	1	0.06	TBC1 domain family m
1984	1	db$2	2::C3K9C4|C3K9C4_PSEFS	13	31700	1	1	1	1	0.16	Putative transposase fo
1985	1	db$2	2::C3K2Z6|ERPA_PSEFS	13	12493	1	1	1	1	0.45	Iron-sulfur cluster inse
1986	1	SwissProt_HUMAN$	3::Q86XA9|HTR5A_HUMAN	13	221863	1	1	1	1	0.02	HEAT repeat-containin

Export as CSV

Not what you expected? Try **the select summary**.

Mascot: http://www.matrixscience.com/
