# Supplementary material for: Circulating multimeric immune complexes contribute to immunopathology in COVID-19
Source: Nat Commun. 2022 Sep 26;13:5654. doi: 10.1038/s41467-022-32867-z (PMC9513013; doi:10.1038/s41467-022-32867-z)
Supplement: Supplementary file 3 — Source Data [file 41467_2022_32867_MOESM3_ESM.zip › Source Data/MS_20210407 (immune precipitation).zip!/QX20210618-03_Virologie(AG-Hengel)-20210407_(V3)_0k3v20_TRL_db_SC2+db_Pf+SP_H(F235788).pdf]

MATRIX SCIENCE MASCOT Search Results

User : Wolfgang Bildl
E-mail : wolfgang.bildl@physiologie.uni-freiburg.de
Search title : QX 20210618 (db$ SARS-CoV-2+db$2 Pf+SP H$ 5 ppm 20 mmu), QX20210618-03_Virologie(AG-Hengel)-20210407_(V3)_0k3v20_TRL_shifted_-1.4.mgf
MS data file : D:\Xcalibur\Data\Wolfgang\Q-Exactive-HF-X_20210616\QX20210618-03_Virologie(AG-Hengel)-20210407_(V3)_0k3v20_TRL_shifted_-1.4.mgf
Databases : **1:** db$ 20210617 (17 sequences; 14,439 residues)
2: db$2 20210617 (6,388 sequences; 2,089,672 residues)
3: SwissProt_HUMAN$ 20201007 (20,389 sequences; 11,363,789 residues)
Timestamp : 21 Jun 2021 at 10:05:10 GMT

Not what you expected? Try **the select summary**.

Search parameters

Type of search : MS/MS Ion Search
Enzyme : Trypsin
Variable modifications : Acetyl (Protein N-term), Carbamidomethyl (C), Formyl (Protein N-term), Gln->pyro-Glu (N-term Q), Oxidation (M)
Mass values : Monoisotopic
Protein mass : Unrestricted
Peptide mass tolerance : ± 5 ppm
Fragment mass tolerance : ± 20 mmu
Max missed cleavages : 1
Instrument type : ESI-FTICR
Number of queries : 84,008

Score distribution

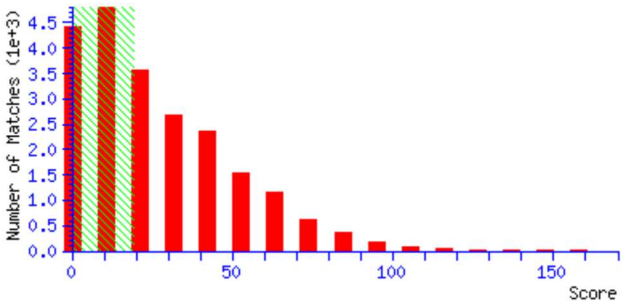

Peptide score distribution. Ions score is $-10\log(P)$, where P is the probability that the observed match is a random event.
There are **11,475** peptide matches above identity threshold and **12,235** matches above homology threshold for **84,008** queries.
On average, individual ions scores **> 19** (beyond green shading) indicate **identity or extensive homology** ($p<0.05$).

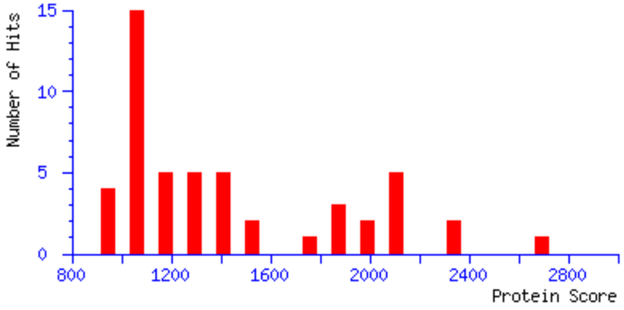

[Deprecated] Protein score distribution. Score distribution for family members in the first 50 proteins. Protein scores are derived from ions scores as a non-probabilistic basis for ranking protein families.

▼Modification statistics

Modification	Site	Above thr.
Oxidation	M	2200
Carbamidomethyl	C	1166
Gln->pyro-Glu	N-term	228
Formyl	Protein N-term	44
Acetyl	Protein N-term	15

▼Legend

Peptide columns and rows

Dupes	...	Expect	Rank	U	1	2	Peptide	
		**0.037**	▶**2**				**GAYSLSLR**	significant
		9	▶1				GFFLFVEGGR	top ranking
		**6.4e-005**	▶**1**				**GSSIFGLAPGK**	significant and top ranking
		**1.3e-006**	▶**1**		■		**SSGTSYPDVLK**	peptide is found in all proteins in family member 1
		**6.2e-007**	▶**1**			■	**VCNYVSWIK**	peptide is found in some but not all proteins in family member 2
		**6.4e-005**	▶**1**	**U**			**GSSIFGLAPGK**	unique
▶**2**		**5.7e-005**	▶**1**				**LNTLETEEWFFK**	peptide has two duplicates
		0.18	▶*1*				*LNTLETEEWFFK*	duplicate peptide

Right-facing triangle (▶) in the Dupes or Rank column indicates content that can be expanded by clicking on it. Down-facing triangle (▼) indicates the content is expanded and can be collapsed. For more details about particular columns, see **results format help**.

Protein quantitation ratios

	Score	...	114/113	115/113
**CFAH_HUMAN**	37559		*0.962*	**1.129**
**FHR2_HUMAN**	1330		**0.859**	1.128

When quantitation method is Reporter (e.g. iTRAQ) or Multiplex (e.g. IPTL), protein ratios are displayed when a family is expanded. Ratios in *italic* indicate that the peptide log-ratios do not appear to come from a normal distribution. **Bold** indicates that if you can assume peptide ratios are normally distributed, the protein ratio is significantly different from 1.0 (at significance level 0.05).

Note that lack of bold or italic can also mean that significance or normality testing has not been performed (for

example, if protein ratio type does not support it).

Protein Family Summary

Significance threshold p<	0.05	Max. number of families	AUTO
Ions score or expect cut-off	0.5	Dendrograms cut at	0
Show Percolator scores	☐		
Preferred taxonomy	All entries		

▼Sensitivity and FDR (reversed protein sequences)

			Target	Decoy	FDR		
PSMs	above	homology	12235	461	3.77%	Adjust to	1%

Decoy results are available in **the decoy report**.

Protein hits (2073 proteins)

▶Filters: (none)

Export as CSV

↑Family	M	DB	Accession	Score	Mass	Matches	Match(sig)	Sequences	Seq(sig)	emPAI	Description
1	1	db$2	2::C3K2Y3|RPOB_PSEFS	2692	150923	102	96	72	69	9.95	DNA-directed RNA poly
2	1	db$2	2::C3K5G3|C3K5G3_PSEFS	2366	99192	79	73	36	32	6.83	Aconitate hydratase OS
3	1	db$2	2::C3K2X9|EFG_PSEFS	2292	77114	79	76	35	35	13.97	Elongation factor G OS
4	1	db$2	2::C3K1E6|ATPB_PSEFS	2147	49459	64	63	30	30	44.84	ATP synthase subunit b
5	1	db$2	2::C3KD82|C3KD82_PSEFS	2115	181591	74	73	57	57	4.07	Glutamate dehydrogen
5	2	db$2	2::C3KAT2|C3KAT2_PSEFS	77	49009	2	2	2	2	0.21	Histidine kinase OS=Ps
6	1	SwissProt_HUMAN$	3::P13645|K1C10_HUMAN	2090	58792	52	47	30	27	12.17	Keratin, type I cytoske
6	2	SwissProt_HUMAN$	3::P02533|K1C14_HUMAN	361	51529	16	11	15	11	1.75	Keratin, type I cytoske
6	3	SwissProt_HUMAN$	3::P08779|K1C16_HUMAN	227	51236	11	7	11	7	0.91	Keratin, type I cytoske
6	4	SwissProt_HUMAN$	3::Q04695|K1C17_HUMAN	95	48076	10	5	9	5	0.64	Keratin, type I cytoske
6	5	SwissProt_HUMAN$	3::P08727|K1C19_HUMAN	43	44079	5	3	5	3	0.38	Keratin, type I cytoske
7	1	db$2	2::C3K2X8|EFTU_PSEFS	2082	43536	62	57	24	23	19.99	Elongation factor Tu OS
8	1	db$2	2::C3K4D5|C3K4D5_PSEFS	2055	34599	67	64	21	20	29.22	Phosphate-binding prot
9	1	db$2	2::C3K1A0|CH60_PSEFS	1987	56931	61	59	29	29	20.73	60 kDa chaperonin OS
10	1	db$2	2::C3K4N0|C3K4N0_PSEFS	1965	43479	51	49	28	27	35.14	Putative ornithine deca
11	1	db$2	2::C3K5Z6|EFTS_PSEFS	1884	30341	68	65	18	16	13.09	Elongation factor Ts OS
12	1	db$2	2::C3K1E8|ATPA_PSEFS	1854	55245	63	61	29	28	20.87	ATP synthase subunit a
13	1	db$2	2::C3K255|PNP_PSEFS	1817	74889	55	55	30	30	10.09	Polyribonucleotide nucl
14	1	db$2	2::C3K2Y2|RPOC_PSEFS	1811	154528	93	84	71	66	8.45	DNA-directed RNA poly
15	1	SwissProt_HUMAN$	3::P04264|K2C1_HUMAN	1474	65999	41	39	28	27	7.62	Keratin, type II cytosk
15	2	SwissProt_HUMAN$	3::P35908|K22E_HUMAN	1099	65393	31	28	25	23	4.70	Keratin, type II cytosk

↑Family	M	DB	Accession	Score	Mass	Matches	Match(sig)	Sequences	Seq(sig)	emPAI	Description
15	3	SwissProt_HUMAN$	3::P04259|K2C6B_HUMAN	231	60030	8	7	8	7	0.74	Keratin, type II cytosk
15	4	SwissProt_HUMAN$	3::P13647|K2C5_HUMAN	230	62340	10	9	10	9	0.98	Keratin, type II cytosk
15	5	SwissProt_HUMAN$	3::P05787|K2C8_HUMAN	154	53671	5	4	5	4	0.42	Keratin, type II cytosk
15	6	SwissProt_HUMAN$	3::P08729|K2C7_HUMAN	92	51354	4	4	4	4	0.45	Keratin, type II cytosk
16	1	db$2	2::C3KCP4|C3KCP4_PSEFS	1468	99394	70	67	43	42	13.54	Pyruvate dehydrogena
17	1	db$2	2::C3K275|DNAK_PSEFS	1458	68158	42	42	27	27	8.27	Chaperone protein Dna
18	1	db$2	2::C3K0Q5|C3K0Q5_PSEFS	1456	107302	56	51	38	36	5.14	Ribonucleoside-diphos
19	1	db$2	2::C3K271|C3K271_PSEFS	1383	117563	49	42	34	29	3.12	Carbamoyl-phosphate
20	1	db$2	2::C3K6P2|C3K6P2_PSEFS	1371	42870	35	33	16	16	10.34	3-oxoacyl-[acyl-carrier
21	1	db$2	2::C3KBH1|C3KBH1_PSEFS	1351	161946	55	50	42	39	2.53	Glutamate synthase [N
22	1	db$2	2::C3JYU7|C3JYU7_PSEFS	1314	102460	46	43	30	28	3.84	Protein translocase sub
23	1	db$2	2::C3KCJ5|C3KCJ5_PSEFS	1273	93957	49	45	36	33	5.17	Aconitate hydratase B
24	1	db$2	2::C3K6M5|C3K6M5_PSEFS	1257	63522	37	34	22	21	5.00	Succinate dehydrogena
25	1	db$2	2::C3KBY8|C3KBY8_PSEFS	1249	21919	57	51	18	18	90.02	Alkyl hydroperoxide re
26	1	db$2	2::C3K6N0|SUCC_PSEFS	1241	41262	37	34	22	21	16.51	Succinate--CoA ligase
27	1	db$2	2::C3K3Z0|C3K3Z0_PSEFS	1219	46925	40	39	24	24	17.60	Transcription terminati
28	1	SwissProt_HUMAN$	3::P35527|K1C9_HUMAN	1190	62027	32	30	22	21	4.80	Keratin, type I cytoske
29	1	db$2	2::C3K331|C3K331_PSEFS	1183	73691	49	45	34	33	9.15	AAA_PrkA domain-cont
30	1	db$2	2::C3K6K0|C3K6K0_PSEFS	1166	62268	42	40	25	25	8.09	30S ribosomal protein
31	1	db$2	2::C3K6M7|C3K6M7_PSEFS	1154	105930	50	45	34	32	3.81	Oxoglutarate dehydrog
32	1	db$2	2::C3K402|C3K402_PSEFS	1104	83101	52	44	33	27	4.87	Polyphosphate kinase
33	1	db$2	2::C3K225|ILVC_PSEFS	1100	36188	37	36	20	20	19.13	Ketol-acid reductoisom
34	1	db$2	2::C3K6N1|C3K6N1_PSEFS	1100	29928	34	31	14	14	22.26	Succinate--CoA ligase
35	1	db$2	2::C3K613|FADB_PSEFS	1096	76938	32	31	23	22	2.88	Fatty acid oxidation co
36	1	db$2	2::C3KDP2|C3KDP2_PSEFS	1091	68879	34	31	22	21	4.21	Multifunctional fusion p
37	1	db$2	2::C3JYT1|SYDND_PSEFS	1085	66220	38	35	28	25	6.42	Aspartate--tRNA(Asp/A
38	1	db$2	2::C3K0P3|C3K0P3_PSEFS	1065	118163	31	30	23	22	1.52	Ribonuclease E OS=Ps
39	1	db$2	2::C3K6H4|PYRG_PSEFS	1056	59660	38	34	24	23	7.56	CTP synthase OS=Pseu
40	1	db$2	2::C3JYJ7|C3JYJ7_PSEFS	1052	9057	58	52	9	9	433.37	DNA-binding protein H
41	1	db$2	2::C3JY66|C3JY66_PSEFS	1041	80156	38	35	25	23	3.39	Isocitrate dehydrogena
42	1	db$2	2::C3KEC5|C3KEC5_PSEFS	1040	97398	40	37	33	30	3.76	Phosphoenolpyruvate c
43	1	db$2	2::C3K7D1|C3K7D1_PSEFS	1039	67115	36	36	28	28	6.75	50S ribosomal subunit
44	1	db$2	2::C3K165|C3K165_PSEFS	1027	54857	47	38	28	25	13.56	Fumarate hydratase cl
45	1	db$2	2::C3JYK1|TIG_PSEFS	1014	48316	38	35	27	25	16.17	Trigger factor OS=Pseu
46	1	db$2	2::C3K5J5|FABV_PSEFS	1004	42933	34	33	23	22	11.58	Enoyl-[acyl-carrier-pro
47	1	db$2	2::C3KE86|C3KE86_PSEFS	1000	95293	39	36	28	27	3.46	Chaperone protein Clp
48	1	db$2	2::C3K6N7|HTPG_PSEFS	979	71589	36	36	24	24	4.98	Chaperone protein Htp
49	1	db$2	2::C3KCB8|C3KCB8_PSEFS	950	36708	36	31	14	13	6.82	Phosphate-binding pro
50	1	db$2	2::C3K6M8|C3K6M8_PSEFS	942	42320	28	28	14	14	6.48	Dihydrolipoyllysine-res
51	1	db$2	2::C3K6M9|C3K6M9_PSEFS	922	49948	27	26	16	16	5.66	Dihydrolipoyl dehydrog

↑Family	M	DB	Accession	Score	Mass	Matches	Match(sig)	Sequences	Seq(sig)	emPAI	Description
52	1	db$2	2::C3KBJ2|C3KBJ2_PSEFS	903	24354	27	27	11	11	14.02	Carbonic anhydrase OS
53	1	db$2	2::C3K367|C3K367_PSEFS	897	68142	36	31	22	20	4.31	Uncharacterized protei
54	1	db$2	2::C3K259|IF2_PSEFS	891	90544	34	31	27	25	3.12	Translation initiation fa
55	1	db$2	2::C3K2D3|C3K2D3_PSEFS	887	44901	28	26	17	16	5.65	Serine hydroxymethylt
56	1	db$2	2::C3KE51|C3KE51_PSEFS	885	38026	31	31	19	19	10.97	Putative oxidoreductas
57	1	db$2	2::C3K1L8|NDK_PSEFS	883	14928	25	25	7	7	15.84	Nucleoside diphosphate
58	1	db$2	2::C3K673|C3K673_PSEFS	882	47725	32	31	17	16	5.58	Citrate synthase OS=P
59	1	db$2	2::C3JZS1|C3JZS1_PSEFS	882	55264	28	26	17	17	4.10	Amidophosphoribosyltr
60	1	db$2	2::C3K3S2|C3K3S2_PSEFS	868	44244	29	28	23	23	10.67	2-oxoglutarate reducta
61	1	db$2	2::C3K162|C3K162_PSEFS	863	52023	31	30	22	21	7.10	Pyruvate kinase OS=Ps
62	1	db$2	2::C3K7D3|C3K7D3_PSEFS	861	91800	31	30	24	24	3.26	Alpha-1,4 glucan phos
63	1	db$2	2::C3K0T0|C3K0T0_PSEFS	859	34560	30	29	10	10	3.50	Putative outer membra
64	1	db$2	2::C3JYR1|GCSP_PSEFS	859	102176	39	37	23	23	2.35	Glycine dehydrogenase
65	1	db$2	2::C3K0K6|C3K0K6_PSEFS	826	26352	25	24	14	13	13.65	Electron transfer flavo
66	1	db$2	2::C3KBT0|C3KBT0_PSEFS	817	51607	28	21	15	13	4.21	Succinate-semialdehyd
67	1	db$2	2::C3KDW8|PURA_PSEFS	813	46535	30	30	18	18	7.47	Adenylosuccinate synth
68	1	db$2	2::C3K1J2|C3K1J2_PSEFS	810	140531	35	32	31	29	1.76	Phosphoribosylformylg
69	1	db$2	2::C3K426|ARLY_PSEFS	803	51545	26	25	15	14	5.29	Argininosuccinate lyase
70	1	db$2	2::C3K139|C3K139_PSEFS	792	48816	26	25	12	12	3.28	Uncharacterized protei
71	1	db$2	2::C3K1A7|C3K1A7_PSEFS	791	66070	26	25	19	18	3.85	Proline--tRNA ligase OS
72	1	db$2	2::C3K3G6|SAHH_PSEFS	788	51212	31	29	18	17	6.66	Adenosylhomocysteina
73	1	db$2	2::C3K7G0|C3K7G0_PSEFS	780	36980	24	23	17	16	8.98	Cell shape-determining
74	1	db$2	2::C3K3J7|C3K3J7_PSEFS	772	35008	28	27	15	15	13.89	Glutathione synthetase
75	1	db$2	2::C3K4D4|C3K4D4_PSEFS	771	83859	31	31	19	19	2.89	Putative phosphate AB
76	1	db$2	2::C3K4D1|C3K4D1_PSEFS	762	28553	23	22	12	12	10.92	Phosphate-specific trar
77	1	db$2	2::C3K2D0|C3K2D0_PSEFS	761	61490	30	28	24	22	6.42	Energy-dependent tran
78	1	db$2	2::C3K2V9|RS5_PSEFS	759	17675	24	24	11	11	52.46	30S ribosomal protein
79	1	db$2	2::C3K3F3|C3K3F3_PSEFS	757	72324	38	36	27	26	5.27	Transketolase OS=Pse
80	1	db$2	2::C3K121|C3K121_PSEFS	743	86030	33	32	27	26	3.95	Phosphoenolpyruvate s
81	1	db$2	2::C3K4D2|C3K4D2_PSEFS	738	30988	34	32	19	17	37.68	Phosphate import ATP-
82	1	db$2	2::C3JYS1|C3JYS1_PSEFS	736	17688	20	19	8	8	13.19	Peptidoglycan-associat
83	1	db$2	2::C3JXY1|C3JXY1_PSEFS	732	93962	32	28	25	22	2.20	Alanine--tRNA ligase O
84	1	db$2	2::C3K260|C3K260_PSEFS	725	54381	21	21	15	15	3.40	Transcription terminati
85	1	db$2	2::C3K2R9|C3K2R9_PSEFS	717	16440	14	13	7	7	11.96	6,7-dimethyl-8-ribitylli
86	1	db$2	2::C3KBT1|C3KBT1_PSEFS	717	44808	29	27	17	17	6.42	4-aminobutyrate amino
87	1	db$2	2::C3K1K0|GUAA_PSEFS	713	58398	30	28	23	22	5.47	GMP synthase [glutami
88	1	db$2	2::C3KA76|C3KA76_PSEFS	707	30860	20	20	13	13	8.90	UTP--glucose-1-phosph
89	1	db$2	2::C3K3F1|PGK_PSEFS	702	40125	19	19	12	12	3.64	Phosphoglycerate kinas
90	1	db$2	2::C3K4J5|PCKA_PSEFS	699	55324	23	22	17	16	3.29	Phosphoenolpyruvate c
91	1	db$2	2::C3KCP3|C3KCP3_PSEFS	696	56221	23	23	14	14	2.54	Acetyltransferase comp

↑Family	M	DB	Accession	Score	Mass	Matches	Match(sig)	Sequences	Seq(sig)	emPAI	Description
92	1	db$2	2::C3KE67|RS6_PSEFS	695	16276	26	23	13	12	172.70	30S ribosomal protein
93	1	db$2	2::C3K178|C3K178_PSEFS	690	36139	20	18	15	14	7.07	Glyceraldehyde-3-phos
94	1	db$2	2::C3K6G5|AMPA_PSEFS	687	52403	27	26	22	21	6.31	Probable cytosol aminc
95	1	db$2	2::C3KCS1|C3KCS1_PSEFS	673	34171	22	21	14	14	11.01	Ribose-phosphate pyro
96	1	db$2	2::C3K6H6|C3K6H6_PSEFS	667	45588	23	22	16	15	4.26	Enolase OS=Pseudomc
97	1	db$2	2::C3K7C9|C3K7C9_PSEFS	661	51641	31	29	18	18	5.87	Glutamine synthetase (
98	1	db$2	2::C3K8B0|C3K8B0_PSEFS	660	25585	19	18	10	10	8.14	Peptidyl-prolyl cis-tran:
99	1	db$2	2::C3K2V1|C3K2V1_PSEFS	660	36623	26	25	20	20	14.00	DNA-directed RNA poly
100	1	db$2	2::C3K1W9|C3K1W9_PSEFS	657	33929	15	14	10	9	3.01	Thioredoxin reductase
101	1	db$2	2::C3K1E7|ATPG_PSEFS	650	31374	21	17	15	12	7.19	ATP synthase gamma c
102	1	db$2	2::C3K1K1|C3K1K1_PSEFS	643	51928	33	30	21	19	7.14	Inosine-5'-monophospl
103	1	db$2	2::C3KDD8|C3KDD8_PSEFS	641	44893	25	25	20	20	8.17	Cell division protein Fts
104	1	db$2	2::C3KBZ2|ASSY_PSEFS	640	45330	27	24	19	17	6.28	Argininosuccinate syntl
105	1	db$2	2::C3KBM0|GLPK_PSEFS	633	55508	30	28	20	19	6.12	Glycerol kinase OS=Ps
106	1	db$2	2::C3K2Y6|RL1_PSEFS	627	24061	18	18	10	10	11.66	50S ribosomal protein
107	1	db$2	2::C3KE40|SYGB_PSEFS	626	74833	28	25	19	18	2.55	Glycine--tRNA ligase be
108	1	db$2	2::C3JYT4|C3JYT4_PSEFS	620	17756	23	23	10	10	66.91	Putative ferritin DPS-fa
109	1	db$2	2::C3K603|C3K603_PSEFS	614	87560	28	24	22	19	2.12	Outer membrane prote
110	1	db$2	2::C3K9R0|C3K9R0_PSEFS	613	62265	17	17	13	13	1.90	Pyruvate dehydrogena:
111	1	db$2	2::C3KAH7|C3KAH7_PSEFS	603	45018	25	24	18	17	7.16	Putative NADP-depend
112	1	db$2	2::C3K6M6|C3K6M6_PSEFS	598	26063	20	20	13	13	16.95	Succinate dehydrogena
113	1	db$2	2::C3K2X5|RL4_PSEFS	596	21987	11	11	7	7	4.53	50S ribosomal protein
114	1	db$2	2::C3K6X4|C3K6X4_PSEFS	595	71161	21	20	18	17	2.32	Biosynthetic arginine d
115	1	db$2	2::C3K6J4|C3K6J4_PSEFS	591	97893	31	28	23	21	2.20	DNA gyrase subunit A (
116	1	db$2	2::C3JZN7|SYT_PSEFS	590	72513	24	22	17	16	2.47	Threonine--tRNA ligase
117	1	db$2	2::C3KDW5|C3KDW5_PSEFS	588	43265	26	23	18	16	5.41	Protein HflK OS=Pseud
118	1	db$2	2::C3K4F6|C3K4F6_PSEFS	587	65489	33	30	26	24	6.06	Oxaloacetate decarbox
119	1	db$2	2::C3KE65|C3KE65_PSEFS	584	97331	23	22	21	20	1.65	Ribonuclease R OS=Ps
120	1	db$2	2::C3KDU7|C3KDU7_PSEFS	584	28504	23	21	15	14	9.10	Putative ABC transport
121	1	db$2	2::C3K5H9|C3K5H9_PSEFS	583	33776	16	14	13	11	5.16	Transaldolase OS=Pseu
122	1	db$2	2::C3K5E6|RS2_PSEFS	579	27078	23	21	15	13	15.16	30S ribosomal protein
123	1	db$2	2::C3K016|C3K016_PSEFS	574	85788	21	19	18	16	1.42	Ribosomal RNA large s
124	1	db$2	2::C3K2Y4|RL7_PSEFS	572	12456	12	12	2	2	5.48	50S ribosomal protein
125	1	db$2	2::C3JXL8|C3JXL8_PSEFS	571	112023	25	23	18	17	1.34	Putative efflux protein
126	1	db$2	2::C3K914|C3K914_PSEFS	567	38509	16	16	10	10	3.93	Putative ABC transport
127	1	db$2	2::C3JXZ0|C3JXZ0_PSEFS	567	43332	18	18	14	14	4.15	Acetylornithine aminot
128	1	db$2	2::C3JY65|C3JY65_PSEFS	566	45375	25	25	17	17	8.90	Isocitrate dehydrogena
129	1	db$2	2::C3KDR8|OPGD_PSEFS	565	60485	19	19	15	15	2.50	Glucans biosynthesis p
130	1	db$2	2::C3KDU5|C3KDU5_PSEFS	562	90380	24	20	18	15	1.44	DNA gyrase subunit B (
131	1	db$2	2::C3JZ70|C3JZ70_PSEFS	559	54858	24	24	18	18	3.74	Glucose-6-phosphate 1

↑Family	M	DB	Accession	Score	Mass	Matches	Match(sig)	Sequences	Seq(sig)	emPAI	Description
132	1	db$2	2::C3JYJ9|CLPX_PSEFS	559	46924	22	21	13	12	3.54	ATP-dependent Clp pro
133	1	db$2	2::C3KAD2|ARNA_PSEFS	555	74140	23	22	20	19	2.60	Bifunctional polymyxin
134	1	db$2	2::C3K266|C3K266_PSEFS	549	69364	20	18	15	14	1.79	ATP-dependent zinc me
135	1	db$2	2::C3KEC2|C3KEC2_PSEFS	548	28614	21	20	12	12	6.21	Putative lipoprotein OS
135	2	db$2	2::C3KAJ6|C3KAJ6_PSEFS	157	38502	9	8	7	6	1.36	Histidinol-phosphate ar
136	1	db$2	2::C3K2X0|C3K2X0_PSEFS	547	25737	21	19	14	12	14.52	30S ribosomal protein
137	1	db$2	2::C3K7D6|F16PA_PSEFS	545	37081	19	18	13	12	4.24	Fructose-1,6-bisphospl
138	1	db$2	2::C3K7Y0|C3K7Y0_PSEFS	544	45268	19	19	14	14	4.32	Putative exported prote
139	1	db$2	2::C3K4Y4|C3K4Y4_PSEFS	537	71842	26	23	20	19	3.28	Potassium-transporting
140	1	db$2	2::C3K6I0|C3K6I0_PSEFS	524	39056	21	20	14	14	6.82	Alcohol dehydrogenase
141	1	db$2	2::C3K1L4|ISPG_PSEFS	522	39660	21	19	16	15	6.60	4-hydroxy-3-methylbu
142	1	db$2	2::C3K2V6|C3K2V6_PSEFS	521	47842	17	15	7	6	1.44	Protein translocase sub
143	1	db$2	2::C3K6G0|C3K6G0_PSEFS	520	9680	11	10	6	5	9.91	Putative DNA-binding p
144	1	db$2	2::C3K0D4|C3K0D4_PSEFS	520	29228	18	18	11	11	4.91	Cell division inhibitor M
145	1	db$2	2::C3KCB5|C3KCB5_PSEFS	518	30173	19	18	10	10	4.57	Phosphate import ATP-
146	1	db$2	2::C3KE70|RL9_PSEFS	516	15488	14	13	7	7	13.96	50S ribosomal protein
147	1	db$2	2::C3K3E8|C3K3E8_PSEFS	516	38385	27	25	17	16	12.25	Fructose-1,6-bisphospl
148	1	db$2	2::C3K4F7|C3K4F7_PSEFS	515	51847	24	22	21	20	5.81	Biotin carboxylase OS=
148	2	db$2	2::C3K282|SSRP_PSEFS	55	18308	5	4	4	4	1.78	SsrA-binding protein O
149	1	db$2	2::C3K341|C3K341_PSEFS	514	69250	26	22	20	16	2.93	RNA polymerase sigma
149	2	db$2	2::C3K708|C3K708_PSEFS	122	38066	3	3	3	3	0.45	RNA polymerase sigma
150	1	db$2	2::C3K8X5|HIS1_PSEFS	513	23098	17	17	10	10	13.11	ATP phosphoribosyltrar
151	1	db$2	2::C3K1N2|C3K1N2_PSEFS	507	67307	22	22	17	17	2.82	Protein translocase sub
152	1	db$2	2::C3K4C6|C3K4C6_PSEFS	503	25565	18	18	10	10	8.14	Phosphate regulon trar
153	1	db$2	2::C3KDG0|RECA_PSEFS	503	37499	24	21	17	16	9.94	Protein RecA OS=Pseu
154	1	db$2	2::C3KED7|PUR7_PSEFS	502	26862	21	19	17	17	22.47	Phosphoribosylaminoin
155	1	db$2	2::C3KDU3|C3KDU3_PSEFS	502	40518	23	21	16	14	5.47	Beta sliding clamp OS=
156	1	SwissProt_HUMAN$	3::P02768|ALBU_HUMAN	497	69321	20	19	17	16	1.99	Albumin OS=Homo sap
156	2	SwissProt_HUMAN$	3::P02769|ALBU_BOVIN	288	69248	13	13	12	12	1.27	Albumin OS=Bos tauru
157	1	db$2	2::C3K649|C3K649_PSEFS	496	8822	15	15	5	5	12.79	Major outer membrane
158	1	db$2	2::C3K3V2|C3K3V2_PSEFS	492	49111	19	18	10	10	1.89	Putative outer membra
159	1	db$2	2::C3K2M9|PROA_PSEFS	488	45380	15	15	11	11	2.88	Gamma-glutamyl phos
160	1	db$2	2::C3K1E2|C3K1E2_PSEFS	486	66300	20	20	14	14	1.93	Glutamine--fructose-6-
161	1	db$2	2::C3JYQ6|C3JYQ6_PSEFS	479	37842	20	19	12	12	8.46	Ornithine carbamoyltra
162	1	db$2	2::C3JZN3|SYFA_PSEFS	477	37892	14	14	12	12	4.74	Phenylalanine--tRNA li
163	1	db$2	2::C3JZT6|C3JZT6_PSEFS	474	40569	15	15	10	10	3.06	Aspartate-semialdehyd
164	1	db$2	2::C3K569|C3K569_PSEFS	471	44415	21	19	12	10	3.00	Tryptophan synthase b
165	1	db$2	2::C3K4C7|C3K4C7_PSEFS	470	50044	13	13	11	11	2.11	Phosphate regulon sen
166	1	db$2	2::C3K2V2|RS4_PSEFS	470	23062	18	18	14	14	20.20	30S ribosomal protein
167	1	db$2	2::C3JZS0|C3JZS0_PSEFS	469	43144	21	19	15	14	4.77	O-succinylhomoserine

↑Family	M	DB	Accession	Score	Mass	Matches	Match(sig)	Sequences	Seq(sig)	emPAI	Description
168	1	db$2	2::C3JY86|C3JY86_PSEFS	466	98164	22	20	18	17	1.39	NADH-quinone oxidore
169	1	db$2	2::C3KDD9|C3KDD9_PSEFS	464	41969	17	14	16	13	3.33	Cell division protein Fts
170	1	db$2	2::C3K3P0|ILVD_PSEFS	460	65667	17	16	16	15	1.95	Dihydroxy-acid dehydr
171	1	db$2	2::C3KBY6|C3KBY6_PSEFS	459	17850	15	15	10	10	16.86	Bacterioferritin OS=Pse
172	1	db$2	2::C3JYS2|TOLB_PSEFS	458	47517	25	21	21	19	6.33	Tol-Pal system protein
173	1	db$2	2::C3K2W1|RL6_PSEFS	457	19185	17	16	10	9	10.49	50S ribosomal protein
174	1	db$2	2::C3K2W6|RL14_PSEFS	456	13401	15	15	8	8	63.09	50S ribosomal protein
175	1	db$2	2::C3K2H6|C3K2H6_PSEFS	456	32512	8	8	4	4	1.07	Acyl-CoA thioesterase
176	1	db$2	2::C3K108|C3K108_PSEFS	449	83591	22	20	19	17	1.63	GTP pyrophosphokinas
177	1	db$2	2::C3KBL8|C3KBL8_PSEFS	449	56970	20	20	17	17	3.11	Glycerol-3-phosphate c
178	1	db$2	2::C3K466|C3K466_PSEFS	446	52681	13	13	8	8	1.05	Putative aldehyde dehy
179	1	db$2	2::C3K1K7|LEU1_PSEFS	444	61997	19	19	17	17	2.66	2-isopropylmalate synt
180	1	db$2	2::C3JZV0|C3JZV0_PSEFS	443	56140	14	14	13	13	1.99	Glutamate--tRNA ligase
181	1	db$2	2::C3JYQ5|C3JYQ5_PSEFS	442	46409	20	18	14	13	3.62	Arginine deiminase OS
182	1	db$2	2::C3JZN2|C3JZN2_PSEFS	441	86329	18	18	15	15	1.28	Phenylalanine--tRNA lig
183	1	db$2	2::C3K6W6|C3K6W6_PSEFS	441	49345	26	26	19	19	8.06	Biotin carboxylase OS=
184	1	db$2	2::C3K6H2|ACCA_PSEFS	438	34868	18	17	12	12	6.63	Acetyl-coenzyme A car
185	1	db$2	2::C3K6H5|KDSA_PSEFS	437	30777	26	24	15	13	17.44	2-dehydro-3-deoxypho
186	1	db$2	2::C3JYA7|C3JYA7_PSEFS	437	59461	16	16	14	14	2.30	Putative NADP-depend
187	1	db$2	2::C3K9R4|C3K9R4_PSEFS	436	62164	16	15	15	14	1.91	Putative sulfite reducta
188	1	db$2	2::C3JYJ8|C3JYJ8_PSEFS	435	88851	17	15	16	14	1.11	Lon protease OS=Pseu
189	1	db$2	2::C3K2W0|RL18_PSEFS	434	12651	13	12	8	8	17.91	50S ribosomal protein
190	1	db$2	2::C3K1G5|C3K1G5_PSEFS	432	46195	18	17	13	13	3.20	Homoserine dehydroge
191	1	db$2	2::C3JXL7|C3JXL7_PSEFS	431	39112	12	12	10	10	2.78	HlyD-family secretion p
192	1	db$2	2::C3JYV2|C3JYV2_PSEFS	431	52688	15	15	12	12	2.21	AAA domain-containing
193	1	db$2	2::C3K1F0|C3K1F0_PSEFS	430	16832	14	14	11	11	20.11	ATP synthase subunit b
194	1	db$2	2::C3K3V3|ARGA_PSEFS	429	47793	12	12	11	11	1.97	Amino-acid acetyltrans
195	1	db$2	2::C3K6W9|C3K6W9_PSEFS	425	69415	12	12	10	10	0.98	Putative methyl-accept
196	1	SwissProt_HUMAN$	3::P00761|TRYP_PIG	424	24394	12	12	6	6	3.67	Trypsin OS=Sus scrofa
197	1	db$2	2::C3K2L2|SYL_PSEFS	424	97066	19	17	17	15	1.08	Leucine--tRNA ligase O
198	1	db$2	2::C3K4K9|C3K4K9_PSEFS	422	27377	13	13	7	7	2.95	Putative amino acid ox
199	1	db$2	2::C3K0Q7|C3K0Q7_PSEFS	421	45255	12	11	7	7	1.31	Flagellar hook protein I
200	1	db$2	2::C3K3Z1|C3K3Z1_PSEFS	420	11808	12	12	6	6	22.48	Thioredoxin OS=Pseud
201	1	db$2	2::C3JXY0|AK_PSEFS	418	44504	16	16	15	15	3.92	Aspartate kinase OS=P
202	1	db$2	2::C3K3R2|C3K3R2_PSEFS	417	83481	18	17	17	16	1.48	Phosphoenolpyruvate--
203	1	db$2	2::C3JXZ7|C3JXZ7_PSEFS	416	28415	14	13	8	8	2.77	Putative histidine-bindi
204	1	db$2	2::C3KEA4|C3KEA4_PSEFS	415	51722	14	14	10	10	2.00	PINc domain-containin
205	1	db$2	2::C3K2Z7|ARGC_PSEFS	415	36612	14	13	8	8	3.13	N-acetyl-gamma-gluta
206	1	db$2	2::C3K324|C3K324_PSEFS	414	105271	20	18	17	15	0.97	LPS-assembly protein L
207	1	db$2	2::C3K475|ARGB_PSEFS	414	31926	14	14	12	12	5.81	Acetylglutamate kinase

↑Family	M	DB	Accession	Score	Mass	Matches	Match(sig)	Sequences	Seq(sig)	emPAI	Description
208	1	db$2	2::C3JY70|C3JY70_PSEFS	411	50668	15	15	12	12	2.37	Adenylosuccinate lyase
209	1	db$2	2::C3KAA8|C3KAA8_PSEFS	411	132643	14	11	11	9	0.38	Histidine kinase OS=Ps
210	1	db$2	2::C3KCR8|C3KCR8_PSEFS	410	39907	14	14	9	9	2.26	Ribosome-binding ATPa
211	1	db$2	2::C3K227|C3K227_PSEFS	408	62578	23	23	17	17	2.91	Acetolactate synthase
212	1	db$2	2::C3K401|C3K401_PSEFS	408	56255	20	19	18	17	3.18	Exopolyphosphatase O
213	1	db$2	2::C3K4V9|C3K4V9_PSEFS	408	53185	20	19	15	15	3.55	Glyceraldehyde-3-phos
214	1	db$2	2::C3K310|C3K310_PSEFS	406	54246	14	14	10	10	2.11	Anthranilate synthase
215	1	db$2	2::C3KEB4|C3KEB4_PSEFS	405	56978	14	13	13	12	1.95	Lysine--tRNA ligase OS
216	1	db$2	2::C3K484|C3K484_PSEFS	404	13502	13	13	9	9	30.16	Uncharacterized protei
217	1	db$2	2::C3K443|C3K443_PSEFS	402	12323	16	16	9	9	42.45	Nitrogen regulatory pro
218	1	db$2	2::C3KDL2|C3KDL2_PSEFS	401	52736	12	11	10	9	1.24	Putative outer membra
219	1	db$2	2::C3JZV8|C3JZV8_PSEFS	401	38906	12	12	9	9	2.36	Neutral metalloproteina
220	1	db$2	2::C3K1L3|SYH_PSEFS	401	47853	17	17	15	15	3.87	Histidine--tRNA ligase
221	1	db$2	2::C3JY83|NUOCD_PSEFS	399	67660	22	18	19	17	2.53	NADH-quinone oxidore
222	1	db$2	2::C3KBI6|C3KBI6_PSEFS	399	106629	16	15	14	13	0.87	Uncharacterized protei
223	1	db$2	2::C3K274|DNAJ_PSEFS	394	40315	13	13	11	11	2.64	Chaperone protein Dna
224	1	db$2	2::C3K9T7|C3K9T7_PSEFS	393	99328	15	15	13	13	0.86	Aminopeptidase N OS=
225	1	db$2	2::C3K638|C3K638_PSEFS	391	26394	9	9	5	5	2.48	Putative short chain de
226	1	db$2	2::C3K226|C3K226_PSEFS	391	17820	9	9	6	6	3.82	Acetolactate synthase
227	1	db$2	2::C3K1M5|ISCS_PSEFS	389	44467	17	15	15	13	2.98	Cysteine desulfurase Is
228	1	db$2	2::C3K865|C3K865_PSEFS	389	15847	8	8	4	4	3.36	Putative membrane pro
229	1	db$2	2::C3K8X0|C3K8X0_PSEFS	389	16351	12	12	8	8	12.18	Putative ABC transport
230	1	db$2	2::C3K2X3|RL2_PSEFS	388	29689	17	17	11	11	4.75	50S ribosomal protein
231	1	db$2	2::C3K1X6|C3K1X6_PSEFS	387	44711	11	11	9	9	1.59	GTPase Obg OS=Pseuc
232	1	db$2	2::C3K123|C3K123_PSEFS	385	41542	11	10	9	8	1.49	Citrate synthase OS=P
233	1	db$2	2::C3K1B7|C3K1B7_PSEFS	385	51892	16	15	14	13	2.28	Threonine synthase OS
234	1	db$2	2::C3K3N4|COAD_PSEFS	383	17810	11	10	6	5	5.33	Phosphopantetheine ac
235	1	db$2	2::C3KDX4|SYI_PSEFS	379	105386	15	14	13	12	0.72	Isoleucine--tRNA ligase
236	1	db$2	2::C3K1X9|C3K1X9_PSEFS	379	34909	15	14	11	11	4.08	Octaprenyl-diphosphat
237	1	db$2	2::C3KAL6|C3KAL6_PSEFS	375	52109	17	16	14	14	2.91	Putative aminotransfer
238	1	db$2	2::C3K5Z8|RRF_PSEFS	374	20297	12	11	7	7	7.00	Ribosome-recycling fac
239	1	db$2	2::C3K3H0|C3K3H0_PSEFS	374	67796	14	14	12	12	1.48	ATP-dependent RNA he
240	1	db$2	2::C3K3F5|METK_PSEFS	364	42554	18	16	12	11	3.25	S-adenosylmethionine
241	1	db$2	2::C3K474|C3K474_PSEFS	364	50732	14	12	13	11	2.07	Putative phosphomann
242	1	db$2	2::C3KCN5|C3KCN5_PSEFS	364	142346	14	14	13	13	0.54	Bifunctional protein Pu
243	1	db$2	2::C3K358|C3K358_PSEFS	362	65409	16	12	14	11	1.22	Putative dehydrogenas
243	2	db$2	2::C3K355|C3K355_PSEFS	322	63710	13	12	12	11	1.27	Putative dehydrogenas
244	1	db$2	2::C3KBN3|C3KBN3_PSEFS	361	112655	17	17	13	13	0.80	Efflux pump membrane
244	2	db$2	2::C3KB18|C3KB18_PSEFS	122	115005	4	4	4	4	0.18	Efflux pump membrane
245	1	db$2	2::C3K249|G6PI_PSEFS	361	61384	21	18	14	12	1.95	Glucose-6-phosphate is

↑Family	M	DB	Accession	Score	Mass	Matches	Match(sig)	Sequences	Seq(sig)	emPAI	Description
246	1	db$2	2::C3JZV2|C3JZV2_PSEFS	360	43020	14	14	12	12	3.17	Aminotransferase OS=
247	1	db$2	2::C3KBX8|C3KBX8_PSEFS	360	26859	14	13	8	8	7.20	OmpA-like domain-con
248	1	db$2	2::C3K7X9|C3K7X9_PSEFS	359	40858	14	11	9	8	1.83	Carboxynorspermidine,
249	1	db$2	2::C3K2K3|GSA_PSEFS	359	45181	12	12	9	9	2.16	Glutamate-1-semialdel
250	1	db$2	2::C3K6R4|C3K6R4_PSEFS	359	18727	13	13	6	6	4.75	Uncharacterized protei
251	1	db$2	2::C3K1E9|ATPD_PSEFS	358	19246	14	14	7	7	6.05	ATP synthase subunit c
252	1	db$2	2::C3KBH2|C3KBH2_PSEFS	357	51951	14	13	13	12	1.99	Glutamate synthase [N
253	1	db$2	2::C3K6M2|C3K6M2_PSEFS	356	60584	13	13	12	12	1.76	ATP-dependent RNA he
254	1	db$2	2::C3K245|C3K245_PSEFS	355	53442	14	14	13	13	2.17	Poly(A) polymerase I C
255	1	db$2	2::C3K2X1|RL22_PSEFS	353	11904	11	10	8	7	14.43	50S ribosomal protein
256	1	db$2	2::C3K5S8|C3K5S8_PSEFS	352	75646	14	14	13	13	1.41	Oligopeptidase A OS=F
257	1	db$2	2::C3KCT2|MUTS_PSEFS	350	95536	10	10	10	10	0.64	DNA mismatch repair p
258	1	db$2	2::C3KCB7|C3KCB7_PSEFS	349	34191	7	6	4	3	0.74	Phosphate transport sy
259	1	db$2	2::C3JY85|C3JY85_PSEFS	346	48759	14	14	12	12	2.54	NADH-quinone oxidore
260	1	db$2	2::C3K263|C3K263_PSEFS	346	26068	8	8	6	6	1.95	Triosephosphate isome
261	1	db$2	2::C3KDE0|LPXC_PSEFS	346	33197	9	9	7	7	1.71	UDP-3-O-acyl-N-acetyl
262	1	db$2	2::C3K2X6|RL3_PSEFS	345	22550	11	8	10	8	4.30	50S ribosomal protein
263	1	db$2	2::C3K6K6|C3K6K6_PSEFS	345	36089	15	12	12	10	2.70	HTH-type transcription
264	1	db$2	2::C3JYD4|C3JYD4_PSEFS	345	64707	14	14	11	11	1.41	Glutamine--tRNA ligase
265	1	db$2	2::C3K8U4|UBIE_PSEFS	344	28320	16	15	12	12	7.71	Ubiquinone/menaquinc
266	1	db$2	2::C3K2Y8|C3K2Y8_PSEFS	342	20023	13	13	9	9	9.46	Transcription terminati
267	1	SwissProt_HUMAN$	3::P07355|ANXA2_HUMAN	342	38580	14	11	13	10	2.40	Annexin A2 OS=Homo
268	1	db$2	2::C3K1G8|RL19_PSEFS	341	12961	10	10	8	8	16.57	50S ribosomal protein
269	1	db$2	2::C3K2W4|RL5_PSEFS	340	20490	19	16	11	10	14.53	50S ribosomal protein
270	1	db$2	2::C3K2E3|MSCL_PSEFS	339	14568	7	7	4	4	3.97	Large-conductance me
271	1	db$2	2::C3K1G2|YIDC_PSEFS	339	61977	12	11	12	11	1.32	Membrane protein inse
272	1	db$2	2::C3K3P4|C3K3P4_PSEFS	338	39333	16	14	10	8	2.33	Uncharacterized protei
273	1	db$2	2::C3JYW3|C3JYW3_PSEFS	338	36818	14	14	12	12	4.29	Branched-chain-amino-
274	1	db$2	2::C3K6E1|RL13_PSEFS	338	15695	13	12	8	8	13.68	50S ribosomal protein
275	1	db$2	2::C3K902|RAPA_PSEFS	338	106722	14	12	13	11	0.63	RNA polymerase-assoc
276	1	db$2	2::C3K1H6|PURT_PSEFS	338	42327	14	14	14	14	3.79	Formate-dependent ph
277	1	db$2	2::C3K2U7|C3K2U7_PSEFS	337	104087	17	16	15	14	0.89	UvrABC system protein
278	1	db$2	2::C3K7G9|C3K7G9_PSEFS	336	48266	12	12	10	10	1.67	Putative modulation of
279	1	db$2	2::C3K0H1|C3K0H1_PSEFS	335	17848	8	8	7	7	5.26	Chemotaxis protein Ch
280	1	db$2	2::C3JZN6|IF3_PSEFS	335	20837	9	8	6	5	2.86	Translation initiation fa
281	1	db$2	2::C3JZS4|C3JZS4_PSEFS	334	33375	14	14	9	9	3.11	Acetyl-coenzyme A car
282	1	db$2	2::C3JYQ7|C3JYQ7_PSEFS	333	32635	9	9	7	7	1.75	Carbamate kinase OS=
283	1	db$2	2::C3K6D9|C3K6D9_PSEFS	333	45394	9	8	6	5	0.87	Phosphoribosylamine--
284	1	db$2	2::C3KE83|C3KE83_PSEFS	331	38700	11	10	8	7	1.35	Outer membrane prote
285	1	db$2	2::C3JY62|C3JY62_PSEFS	329	83224	21	19	17	17	1.64	ATP-dependent Clp pro

↑Family	M	DB	Accession	Score	Mass	Matches	Match(sig)	Sequences	Seq(sig)	emPAI	Description
285	2	db$2	2::C3K4B3|C3K4B3_PSEFS	74	97007	6	5	5	5	0.28	Chaperone OS=Pseudo
286	1	db$2	2::C3K6G2|C3K6G2_PSEFS	329	106936	18	14	17	13	0.78	Valine--tRNA ligase OS
287	1	db$2	2::C3K422|C3K422_PSEFS	325	40250	10	8	9	7	1.27	Putative uroporphyrin-
288	1	db$2	2::C3KAJ2|C3KAJ2_PSEFS	323	53335	14	13	13	12	1.91	Methylmalonate-semia
289	1	db$2	2::C3KAK3|C3KAK3_PSEFS	321	54608	16	16	11	11	1.83	Probable malate:quino
290	1	db$2	2::C3K2T2|C3K2T2_PSEFS	320	24648	9	9	8	8	4.58	Putative ABC transport
291	1	db$2	2::C3KDP1|CYSD_PSEFS	320	35173	11	11	7	7	1.93	Sulfate adenylyltransfe
292	1	db$2	2::C3K0K5|C3K0K5_PSEFS	320	31365	12	10	7	7	2.86	Electron transfer flavo
293	1	db$2	2::C3JZ61|C3JZ61_PSEFS	318	61509	12	12	9	9	1.00	Acyl-CoA synthetase (L
294	1	SwissProt_HUMAN$	3::P0DOX5|IGG1_HUMAN	318	49298	11	10	7	7	0.96	Immunoglobulin gamm
294	2	SwissProt_HUMAN$	3::P01859|IGHG2_HUMAN	72	35878	6	5	5	5	0.93	Immunoglobulin heavy
295	1	SwissProt_HUMAN$	3::P60709|ACTB_HUMAN	317	41710	15	14	12	12	3.36	Actin, cytoplasmic 1 OS
296	1	db$2	2::C3K2Y0|RS7_PSEFS	314	17636	16	16	12	12	23.12	30S ribosomal protein
297	1	db$2	2::C3K6J7|C3K6J7_PSEFS	313	39784	9	9	8	8	1.59	Histidinol-phosphate a
298	1	db$2	2::C3KCS2|RSMH_PSEFS	313	34344	12	12	9	9	2.45	Ribosomal RNA small s
299	1	db$2	2::C3KEC8|C3KEC8_PSEFS	311	50623	15	11	11	9	1.32	Periplasmic serine end
300	1	db$2	2::C3K6J9|KCY_PSEFS	310	24780	8	8	7	7	2.78	Cytidylate kinase OS=I
301	1	db$2	2::C3KCT1|MURC_PSEFS	307	52132	8	8	5	5	0.57	UDP-N-acetylmuramat
302	1	db$2	2::C3JXZ8|C3JXZ8_PSEFS	307	71568	14	13	13	12	1.21	Acetyl-coenzyme A syr
303	1	db$2	2::C3K1X5|C3K1X5_PSEFS	306	39754	10	10	8	8	1.59	Glutamate 5-kinase OS
304	1	db$2	2::C3K1X8|C3K1X8_PSEFS	306	11596	7	7	4	4	6.46	50S ribosomal protein
305	1	db$2	2::C3JZT7|C3JZT7_PSEFS	304	38793	13	13	10	10	2.39	3-isopropylmalate deh
306	1	db$2	2::C3JXY7|ASTD_PSEFS	304	51479	6	6	6	6	0.74	N-succinylglutamate 5-
307	1	db$2	2::C3K4W1|STHA_PSEFS	304	50963	18	16	14	12	2.35	Soluble pyridine nucleo
308	1	db$2	2::C3K1F2|ATP6_PSEFS	303	31626	8	8	3	3	0.56	ATP synthase subunit a
309	1	db$2	2::C3JYR3|C3JYR3_PSEFS	302	40135	11	11	9	9	2.24	Glycine cleavage syste
310	1	db$2	2::C3K907|C3K907_PSEFS	299	77648	18	15	18	15	1.50	Tail-specific protease C
311	1	db$2	2::C3K6C2|C3K6C2_PSEFS	298	27720	10	7	8	6	1.77	Putative amino acid AB
312	1	db$2	2::C3K2M1|C3K2M1_PSEFS	298	42033	12	11	10	9	1.75	Serine-type D-Ala-D-Al
313	1	db$2	2::C3K2S6|C3K2S6_PSEFS	297	31662	9	9	7	7	2.30	Putative thioredoxin OS
314	1	db$2	2::C3KBX9|C3KBX9_PSEFS	296	20403	6	6	4	4	1.51	Putative phospholipid-b
315	1	db$2	2::C3K8V3|HSLU_PSEFS	295	49607	13	13	10	10	1.60	ATP-dependent proteas
316	1	db$2	2::C3K5A9|RF3_PSEFS	295	59584	12	11	11	10	1.21	Peptide chain release f
317	1	db$2	2::C3K325|C3K325_PSEFS	294	49076	15	13	12	11	2.51	Chaperone SurA OS=P
318	1	db$2	2::C3K6A2|C3K6A2_PSEFS	294	27847	12	11	10	9	3.58	Putative D-methionine
318	2	db$2	2::C3KD95|C3KD95_PSEFS	216	28020	9	9	7	7	2.25	Putative exported prot
319	1	db$2	2::C3KDB1|C3KDB1_PSEFS	292	65565	14	12	14	12	1.38	Putative carbamoyltrar
320	1	db$2	2::C3K1Y2|C3K1Y2_PSEFS	292	21662	10	9	7	7	4.67	Peptidyl-prolyl cis-tran
321	1	db$2	2::C3K403|C3K403_PSEFS	292	36964	11	10	9	8	1.78	Delta-aminolevulinic ac
322	1	db$2	2::C3K0N5|C3K0N5_PSEFS	292	32402	7	7	5	5	1.07	Malonyl CoA-acyl carrie

↑Family	M	DB	Accession	Score	Mass	Matches	Match(sig)	Sequences	Seq(sig)	emPAI	Description
323	1	db$2	2::C3K6Z0|PDXJ_PSEFS	291	26758	8	8	6	6	1.88	Pyridoxine 5'-phosphat
324	1	db$2	2::C3K2Y7|RL11_PSEFS	291	14903	6	6	3	3	2.51	50S ribosomal protein
325	1	db$2	2::C3K1N3|C3K1N3_PSEFS	290	11888	8	7	5	4	3.78	Sec translocon accesso
326	1	SwissProt_HUMAN$	3::P01834|IGKC_HUMAN	290	11758	8	8	6	6	9.67	Immunoglobulin kappa
327	1	db$2	2::C3KE79|C3KE79_PSEFS	290	29713	6	6	5	5	1.21	NH(3)-dependent NAD
328	1	db$2	2::C3K6E0|PUR9_PSEFS	289	57370	13	13	11	11	1.48	Bifunctional purine bio
329	1	db$2	2::C3KDW6|C3KDW6_PSEFS	289	33055	16	15	13	12	6.37	Protein HflC OS=Pseud
330	1	db$2	2::C3KE41|SYGA_PSEFS	288	36196	14	12	12	11	3.20	Glycine--tRNA ligase al
331	1	db$2	2::C3K2V3|RS11_PSEFS	288	13607	7	7	5	5	4.51	30S ribosomal protein
332	1	db$2	2::C3K4D3|C3K4D3_PSEFS	288	60551	14	12	12	12	1.56	Phosphate transport sy
333	1	db$2	2::C3K3A2|C3K3A2_PSEFS	287	38105	9	9	7	7	1.38	L-threonine aldolase O
334	1	SwissProt_HUMAN$	3::P01871|IGHM_HUMAN	286	49409	11	11	9	9	1.61	Immunoglobulin heavy
335	1	db$2	2::C3K5E5|C3K5E5_PSEFS	286	28503	12	12	9	9	5.15	Methionine aminopepti
336	1	db$2	2::C3K2V7|RL15_PSEFS	286	15215	12	12	8	8	28.29	50S ribosomal protein
337	1	db$2	2::C3K5E0|C3K5E0_PSEFS	285	36000	6	6	5	5	1.20	2,3,4,5-tetrahydropyri
338	1	db$2	2::C3K6J6|C3K6J6_PSEFS	285	40307	13	10	10	8	1.87	Bifunctional chorismate
339	1	db$2	2::C3K5D5|C3K5D5_PSEFS	285	41033	6	6	5	5	0.78	Succinyl-diaminopimel
340	1	db$2	2::C3JYU6|C3JYU6_PSEFS	283	42196	8	8	6	6	0.96	Arginine biosynthesis b
341	1	db$2	2::C3KEC6|KAD_PSEFS	281	23251	13	12	6	6	4.06	Adenylate kinase OS=
342	1	db$2	2::C3K2V0|RL17_PSEFS	281	14407	11	11	7	7	17.33	50S ribosomal protein
343	1	db$2	2::C3K6E2|RS9_PSEFS	280	14607	9	9	7	7	11.77	30S ribosomal protein
344	1	db$2	2::C3K6X1|C3K6X1_PSEFS	279	39290	9	8	8	7	1.32	Uncharacterized protei
345	1	db$2	2::C3K616|C3K616_PSEFS	279	97222	12	11	11	10	0.63	DNA topoisomerase 1 (
346	1	db$2	2::C3KBZ7|SYM_PSEFS	279	75525	14	12	12	10	1.00	Methionine--tRNA ligas
347	1	db$2	2::C3K2Y5|RL10_PSEFS	278	17594	11	10	8	7	5.40	50S ribosomal protein
348	1	db$2	2::C3K8X4|MURA_PSEFS	278	44975	15	14	13	12	2.54	UDP-N-acetylglucosam
349	1	db$2	2::C3K1L0|C3K1L0_PSEFS	278	55309	17	15	14	12	1.80	GTPase Der OS=Pseud
350	1	db$2	2::C3KAC1|C3KAC1_PSEFS	278	27548	16	14	12	10	6.80	Putative phenylacetic a
351	1	db$2	2::C3K7C1|SECB_PSEFS	277	17615	5	5	3	3	1.22	Protein-export protein
352	1	db$2	2::C3K5Y0|C3K5Y0_PSEFS	277	38747	12	11	11	10	2.39	NADP(H)-dependt aldo
353	1	db$2	2::C3K818|C3K818_PSEFS	277	29926	12	10	9	7	2.52	Putative ABC transport
354	1	db$2	2::C3KDM2|C3KDM2_PSEFS	276	83508	16	14	15	13	1.09	DNA topoisomerase 4 s
355	1	db$2	2::C3JYU1|Y4927_PSEFS	275	18225	13	11	7	6	6.83	UPF0234 protein PFLU_
356	1	db$2	2::C3K142|C3K142_PSEFS	274	32099	13	13	10	10	4.04	2-methylisocitrate lyas
357	1	db$2	2::C3KBL1|C3KBL1_PSEFS	272	61934	13	13	12	12	1.70	Putative medium-chain
358	1	db$2	2::C3K4G1|C3K4G1_PSEFS	272	81186	12	8	12	8	0.60	DNA helicase OS=Pseu
359	1	db$2	2::C3K3U2|C3K3U2_PSEFS	270	50985	13	11	10	9	1.31	Putative glutamine syn
360	1	db$2	2::C3K102|PUR5_PSEFS	268	36950	12	10	9	9	2.59	Phosphoribosylformylgl
361	1	db$2	2::C3K2W5|RL24_PSEFS	267	11328	12	11	8	8	38.88	50S ribosomal protein
362	1	db$2	2::C3K264|GLMM_PSEFS	266	47719	12	12	10	10	1.98	Phosphoglucosamine m

↑Family	M	DB	Accession	Score	Mass	Matches	Match(sig)	Sequences	Seq(sig)	emPAI	Description
363	1	db$2	2::C3K187|C3K187_PSEFS	266	38556	13	12	10	10	2.41	Peptidase_M75 domain
364	1	db$2	2::C3K9U6|C3K9U6_PSEFS	266	35232	11	10	8	8	2.35	Putative 6-phosphoglu
365	1	db$2	2::C3KDL6|C3KDL6_PSEFS	266	23139	6	6	4	4	1.26	ADP-ribose diphosphat
366	1	db$2	2::C3KA80|C3KA80_PSEFS	266	20438	13	12	9	9	11.52	Alkyl hydroperoxide re
367	1	db$2	2::C3KCP5|C3KCP5_PSEFS	266	109710	8	8	8	8	0.41	Bifunctional glutamine
368	1	db$2	2::C3KDB7|C3KDB7_PSEFS	266	65793	8	8	7	7	0.66	ATP-dependent lipid A-
368	2	db$2	2::C3K608|C3K608_PSEFS	43	64881	2	2	2	2	0.16	Putative ABC transport
369	1	db$2	2::C3K476|PYRE_PSEFS	265	23079	8	8	6	6	2.39	Orotate phosphoribosy
370	1	db$2	2::C3K244|C3K244_PSEFS	265	52276	9	8	8	7	0.89	Putative response regu
371	1	db$2	2::C3K734|C3K734_PSEFS	265	44623	9	9	7	7	1.34	Putative aminotransfer
372	1	db$2	2::C3K278|C3K278_PSEFS	262	15153	6	5	4	4	2.41	Ferric uptake regulatio
373	1	db$2	2::C3K3K2|PYRB_PSEFS	262	36271	7	7	5	5	0.92	Aspartate carbamoyltra
374	1	db$2	2::C3K3Y8|UBID_PSEFS	261	54697	8	8	8	8	1.00	3-octaprenyl-4-hydrox
375	1	db$2	2::C3JZ69|C3JZ69_PSEFS	261	25612	7	7	6	6	2.01	6-phosphogluconolacto
376	1	db$2	2::C3K0N2|C3K0N2_PSEFS	260	43313	7	6	7	6	0.93	3-oxoacyl-[acyl-carrier
377	1	db$2	2::C3JZ88|C3JZ88_PSEFS	259	47232	7	7	5	5	1.02	Putative exported prot
378	1	db$2	2::C3JY54|SYS_PSEFS	258	47115	11	11	9	9	1.73	Serine--tRNA ligase OS
379	1	db$2	2::C3K1L2|C3K1L2_PSEFS	257	22708	9	9	8	8	4.22	Ancillary SecYEG transl
380	1	db$2	2::C3K614|C3K614_PSEFS	257	41572	12	11	8	8	1.78	3-ketoacyl-CoA thiolas
381	1	db$2	2::C3K3Q2|C3K3Q2_PSEFS	255	37335	9	9	4	4	1.14	Putative cation ABC tra
382	1	db$2	2::C3KBQ2|C3KBQ2_PSEFS	255	37020	11	11	8	8	2.15	Putative quinone oxido
383	1	db$2	2::C3JYD5|C3JYD5_PSEFS	254	18453	10	10	5	5	2.57	Peptidyl-prolyl cis-tran
384	1	db$2	2::C3K6U8|C3K6U8_PSEFS	254	58654	8	8	5	5	0.62	Putative membrane pr
385	1	db$2	2::C3JY74|C3JY74_PSEFS	253	48472	9	9	9	9	1.41	Isocitrase OS=Pseudor
386	1	db$2	2::C3KC10|C3KC10_PSEFS	252	23080	5	5	4	4	1.26	Uncharacterized protei
387	1	db$2	2::C3JYR4|C3JYR4_PSEFS	251	7675	5	5	3	3	5.01	Major cold shock prote
388	1	db$2	2::C3K5V1|C3K5V1_PSEFS	250	47050	11	10	9	9	1.47	Outer membrane prote
389	1	db$2	2::C3K2C9|C3K2C9_PSEFS	250	48295	8	8	8	8	1.19	Glutamate dehydrogen
390	1	db$2	2::C3K4A8|C3K4A8_PSEFS	249	55832	8	8	8	8	0.97	Uncharacterized protei
391	1	db$2	2::C3K667|C3K667_PSEFS	248	48557	10	10	10	10	1.65	Conserved hypothetica
392	1	db$2	2::C3K1X2|C3K1X2_PSEFS	248	40166	9	9	9	9	1.88	Putative putative oxido
393	1	db$2	2::C3K452|XPT_PSEFS	247	20267	9	9	8	8	7.00	Xanthine phosphoribos
394	1	db$2	2::C3K4E2|C3K4E2_PSEFS	246	16860	7	7	6	6	4.28	N5-carboxyaminoimida
395	1	db$2	2::C3KDW7|HISZ_PSEFS	246	42687	11	11	8	8	1.42	ATP phosphoribosyltrar
396	1	db$2	2::C3K645|C3K645_PSEFS	245	24522	8	8	6	6	2.16	Putative ABC transport
397	1	db$2	2::C3K1F4|C3K1F4_PSEFS	245	31961	8	8	7	7	1.81	Probable chromosome-
398	1	db$2	2::C3KAR0|C3KAR0_PSEFS	244	79563	9	8	7	6	0.52	Putative exported isoq
399	1	db$2	2::C3K7N8|RDGC_PSEFS	244	34160	15	14	11	11	4.24	Recombination-associa
400	1	db$2	2::C3KA77|C3KA77_PSEFS	243	49108	9	8	9	8	1.16	Glutathione reductase
401	1	db$2	2::C3K6Y3|GATB_PSEFS	243	52868	13	11	10	9	1.45	Aspartyl/glutamyl-tRN/

↑Family	M	DB	Accession	Score	Mass	Matches	Match(sig)	Sequences	Seq(sig)	emPAI	Description
402	1	db$2	2::C3KDW3|HFQ_PSEFS	242	9318	10	10	7	7	51.25	RNA-binding protein Hf
403	1	db$2	2::C3K8Z6|C3K8Z6_PSEFS	240	39611	10	10	7	7	1.60	Branched amino acid A
404	1	db$2	2::C3KAI6|C3KAI6_PSEFS	240	24263	9	8	7	7	2.87	Putative dehydrogenas
405	1	db$2	2::C3K2X2|RS19_PSEFS	240	10313	10	9	6	5	22.02	30S ribosomal protein
406	1	db$2	2::C3K5H5|C3K5H5_PSEFS	238	25721	11	11	6	6	3.32	Putative exported prot
407	1	db$2	2::C3K6R3|Y2135_PSEFS	236	24050	9	9	8	8	3.77	UPF0502 protein PFLU_
408	1	db$2	2::C3K3M4|C3K3M4_PSEFS	235	49883	7	7	7	7	0.94	Signal recognition part
409	1	db$2	2::C3K262|C3K262_PSEFS	235	12956	7	7	4	4	3.19	Protein-export membra
410	1	db$2	2::C3K2U9|C3K2U9_PSEFS	234	53651	16	13	13	10	1.42	Catalase OS=Pseudom
411	1	db$2	2::C3KBN4|C3KBN4_PSEFS	234	40532	9	9	8	8	1.86	Acriflavine resistance p
412	1	db$2	2::C3JXY9|C3JXY9_PSEFS	234	37073	9	9	8	8	1.77	Putative arginine N-suc
413	1	db$2	2::C3KDU9|C3KDU9_PSEFS	233	34261	14	11	11	9	2.96	Serine O-acetyltransfer
414	1	db$2	2::C3K2K9|C3K2K9_PSEFS	231	31417	10	8	8	7	1.86	Magnesium and cobalt
415	1	db$2	2::C3K5X4|C3K5X4_PSEFS	229	16332	9	9	7	7	6.43	Putative membrane pro
416	1	db$2	2::C3K0K7|C3K0K7_PSEFS	229	60595	10	10	10	10	1.19	Electron transfer flavop
417	1	db$2	2::C3K3W7|C3K3W7_PSEFS	228	36662	11	9	10	8	1.81	Putative iron (III) ABC
418	1	db$2	2::C3K2C4|C3K2C4_PSEFS	228	89060	9	9	8	8	0.53	Endopeptidase La OS=
419	1	db$2	2::C3K606|FABZ_PSEFS	227	16691	9	9	7	7	6.14	3-hydroxyacyl-[acyl-ca
420	1	db$2	2::C3K7L5|C3K7L5_PSEFS	227	19131	8	8	7	7	6.13	Molybdenum cofactor b
421	1	db$2	2::C3K1E5|ATPE_PSEFS	226	14558	5	5	3	3	2.61	ATP synthase epsilon c
422	1	db$2	2::C3K527|C3K527_PSEFS	225	65564	9	9	7	7	0.78	Putative peptidase OS=
423	1	db$2	2::C3KDD6|C3KDD6_PSEFS	225	33869	6	5	6	5	1.01	D-alanine--D-alanine li
424	1	db$2	2::C3JZN9|C3JZN9_PSEFS	224	7652	9	9	6	6	64.67	Major cold shock prote
425	1	db$2	2::C3JZT0|C3JZT0_PSEFS	224	28122	7	7	6	6	1.74	Putative GntR-family tr
426	1	db$2	2::C3K0S7|Y4577_PSEFS	224	11900	6	6	3	3	9.44	Nucleoid-associated pr
427	1	db$2	2::C3JYI0|C3JYI0_PSEFS	224	25235	9	9	8	8	3.46	Probable transcriptiona
428	1	db$2	2::C3K604|C3K604_PSEFS	223	18933	17	14	11	9	10.82	Putative outer membra
429	1	db$2	2::C3K5I7|C3K5I7_PSEFS	223	59867	13	12	9	8	1.21	Probable malate:quino
430	1	db$2	2::C3K575|C3K575_PSEFS	222	14827	7	6	6	5	3.80	Osmotically inducible p
431	1	db$2	2::C3JYT9|C3JYT9_PSEFS	218	46635	7	7	5	5	0.84	Putative family S43 no
432	1	db$2	2::C3K3G5|C3K3G5_PSEFS	217	15160	6	6	6	6	5.31	Putative thioesterase C
433	1	db$2	2::C3KAH1|HSLV_PSEFS	217	18731	6	6	4	4	2.49	ATP-dependent proteas
434	1	db$2	2::C3K469|RL28_PSEFS	217	8845	6	6	3	3	3.74	50S ribosomal protein
435	1	db$2	2::C3K0T3|C3K0T3_PSEFS	216	28958	6	6	5	5	1.65	Putative membrane pro
436	1	db$2	2::C3JYV5|C3JYV5_PSEFS	214	45487	9	9	8	8	1.55	Putative oxidoreductas
437	1	db$2	2::C3KED4|C3KED4_PSEFS	214	31038	7	7	5	5	1.14	4-hydroxy-tetrahydrod
438	1	db$2	2::C3K7G7|C3K7G7_PSEFS	213	50136	9	8	8	7	0.94	Putative modulation of
439	1	db$2	2::C3K0W7|C3K0W7_PSEFS	213	31068	8	8	6	6	1.49	Metallophos_2 domain-
440	1	db$2	2::C3JXZ2|C3JXZ2_PSEFS	213	36334	7	5	6	4	0.68	Putative AraC family re
441	1	db$2	2::C3K0T1|C3K0T1_PSEFS	212	94111	13	12	12	11	0.83	Aconitate hydratase OS

↑Family	M	DB	Accession	Score	Mass	Matches	Match(sig)	Sequences	Seq(sig)	emPAI	Description
442	1	db$2	2::C3K4M6|C3K4M6_PSEFS	212	57818	13	12	10	10	1.67	Putative exported alkal
443	1	db$2	2::C3JYS5|C3JYS5_PSEFS	211	25332	4	4	3	3	1.10	Tol-Pal system protein
444	1	db$2	2::C3KAH3|SYR_PSEFS	210	63549	11	10	9	8	0.96	Arginine--tRNA ligase (
445	1	db$2	2::C3K6L0|EFP_PSEFS	209	21303	7	7	6	6	2.75	Elongation factor P OS=
446	1	db$2	2::C3K6G8|LEPA_PSEFS	208	66372	10	10	10	10	1.04	Elongation factor 4 OS=
447	1	db$2	2::C3K6M3|C3K6M3_PSEFS	207	13365	7	7	5	5	4.74	Succinate dehydrogena
448	1	db$2	2::C3K2E9|C3K2E9_PSEFS	207	53251	5	5	4	4	0.56	Putative cytochrome ox
449	1	db$2	2::C3JZ29|C3JZ29_PSEFS	207	41047	6	6	6	6	1.00	Putative glycerophosph
450	1	db$2	2::C3K1N4|C3K1N4_PSEFS	207	41398	9	8	6	5	0.98	Queuine tRNA-ribosyltr
451	1	SwissProt_HUMAN$	3::P02679|FIBG_HUMAN	206	51479	11	9	8	7	1.09	Fibrinogen gamma cha
452	1	db$2	2::C3K6T0|ENGB_PSEFS	206	23537	6	6	4	4	1.22	Probable GTP-binding p
453	1	db$2	2::C3JY00|C3JY00_PSEFS	205	47211	9	9	8	8	1.23	Ribonucleoside-diphosp
454	1	db$2	2::C3K759|C3K759_PSEFS	205	35694	6	6	4	4	0.70	Putative dehydrogenas
454	2	db$2	2::C3K0S6|C3K0S6_PSEFS	98	36802	2	2	2	2	0.29	Putative oxidoreductas
455	1	db$2	2::C3K9U7|C3K9U7_PSEFS	204	56147	6	6	6	6	0.66	Glucose-6-phosphate 1
456	1	db$2	2::C3K4Q9|C3K4Q9_PSEFS	204	23656	8	6	6	5	1.70	Putative exported prote
457	1	SwissProt_HUMAN$	3::P62805|H4_HUMAN	204	11360	5	5	4	4	4.15	Histone H4 OS=Homo
458	1	db$2	2::C3JZ43|C3JZ43_PSEFS	204	44885	7	7	7	7	1.09	Putative aminotransfer
459	1	db$2	2::C3KBY3|C3KBY3_PSEFS	203	64998	6	5	6	5	0.44	Putative lipoprotein OS
460	1	db$2	2::C3K5X7|C3K5X7_PSEFS	202	42054	10	10	10	10	2.08	Cell division protein Za
461	1	db$2	2::C3KBP1|C3KBP1_PSEFS	202	37670	10	9	6	6	1.41	Putative reductase OS=
462	1	db$2	2::C3JZ71|C3JZ71_PSEFS	202	31565	11	8	10	8	2.31	Hex regulon repressor
463	1	db$2	2::C3K435|C3K435_PSEFS	202	45402	10	10	9	9	1.84	Diaminopimelate decar
464	1	db$2	2::C3KAM2|C3KAM2_PSEFS	200	49640	5	5	4	4	0.46	Putative two-componer
465	1	db$2	2::C3K3S1|C3K3S1_PSEFS	200	51070	10	8	10	8	1.10	Putative oxidase OS=P
466	1	db$2	2::C3K0D3|MINC_PSEFS	200	26249	12	12	9	9	5.03	Probable septum site-d
467	1	db$2	2::C3JYA9|C3JYA9_PSEFS	200	27795	7	7	6	6	1.77	Putative TetR-family re
468	1	db$2	2::C3KEB7|C3KEB7_PSEFS	200	48036	7	7	5	5	0.99	Uncharacterized protei
469	1	db$2	2::C3K104|C3K104_PSEFS	199	26102	10	10	7	7	3.24	Putative membrane pro
470	1	db$2	2::C3KBN2|C3KBN2_PSEFS	199	52689	8	8	8	8	1.05	Outer membrane prote
471	1	db$2	2::C3K8K2|C3K8K2_PSEFS	198	418093	4	4	4	4	0.05	Haemagg_act domain-
472	1	db$2	2::C3K0Q9|C3K0Q9_PSEFS	198	15688	7	6	4	4	3.45	Flagellar basal-body ro
473	1	db$2	2::Q84CP9|DAPB_PSEFS	197	28276	14	12	10	9	3.47	4-hydroxy-tetrahydrod
474	1	db$2	2::C3KAK7|C3KAK7_PSEFS	197	20540	4	4	2	2	0.99	Putative lipoprotein OS
475	1	db$2	2::C3KDM0|C3KDM0_PSEFS	197	69594	10	10	9	9	0.85	DNA topoisomerase 4 s
476	1	db$2	2::C3JY38|C3JY38_PSEFS	197	16812	5	5	5	5	3.04	Putative thioredoxin-re
477	1	db$2	2::C3K177|C3K177_PSEFS	196	65030	12	11	10	10	1.23	Phosphogluconate dehy
478	1	db$2	2::C3K4I0|HEM6_PSEFS	195	35313	9	9	9	9	2.33	Oxygen-dependent cop
479	1	db$2	2::C3KAH8|C3KAH8_PSEFS	195	89028	7	7	7	7	0.45	DD-transpeptidase OS=
480	1	db$2	2::C3K8U7|C3K8U7_PSEFS	195	30923	6	6	5	5	1.14	Transcriptional regulat

↑Family	M	DB	Accession	Score	Mass	Matches	Match(sig)	Sequences	Seq(sig)	emPAI	Description
481	1	db$2	2::C3KCT0|MURG_PSEFS	193	37667	8	8	8	8	1.73	UDP-N-acetylglucosam
482	1	db$2	2::C3K1L1|C3K1L1_PSEFS	193	40864	6	6	6	6	1.00	Outer membrane prote
483	1	db$2	2::C3K5H8|C3K5H8_PSEFS	193	17826	7	7	5	5	2.71	STAS domain-containir
484	1	db$2	2::C3K859|C3K859_PSEFS	192	59064	7	7	7	7	0.75	Putative glutamate syn
485	1	db$2	2::C3JZT9|C3JZT9_PSEFS	192	24210	8	8	8	8	3.74	3-isopropylmalate deh
486	1	db$2	2::C3K1U7|C3K1U7_PSEFS	192	36908	8	7	3	2	0.89	Ubiquinol oxidase subu
487	1	db$2	2::C3K7Z1|C3K7Z1_PSEFS	191	38822	8	7	7	6	1.07	NAD(P) transhydrogen
488	1	db$2	2::C3K6V2|HIS4_PSEFS	191	25679	10	10	7	7	3.34	1-(5-phosphoribosyl)-5
489	1	db$2	2::C3K371|C3K371_PSEFS	191	78727	11	10	11	10	0.83	Malate synthase G OS=
490	1	db$2	2::C3JZS5|TRPF_PSEFS	190	21966	3	3	2	2	0.53	N-(5'-phosphoribosyl)a
491	1	db$2	2::C3KE31|C3KE31_PSEFS	190	22237	9	8	8	7	3.40	RNA polymerase sigma
492	1	db$2	2::C3K091|C3K091_PSEFS	190	20089	6	6	5	5	2.21	GTP cyclohydrolase 1 (
492	2	db$2	2::C3K1W5|C3K1W5_PSEFS	44	20960	2	2	2	2	0.56	GTP cyclohydrolase 1 (
493	1	db$2	2::C3JZ68|C3JZ68_PSEFS	190	24197	9	9	8	8	3.74	2-dehydro-3-deoxy-ph
494	1	db$2	2::C3K6U2|C3K6U2_PSEFS	189	47692	8	6	6	5	0.64	Probable proton/glutan
495	1	db$2	2::C3K6J5|SERC_PSEFS	189	39799	9	8	7	6	1.58	Phosphoserine aminotr
496	1	db$2	2::C3K6Y4|GATA_PSEFS	189	51737	8	7	7	6	0.90	Glutamyl-tRNA(Gln) ar
497	1	db$2	2::C3K2S0|C3K2S0_PSEFS	188	39319	7	6	5	5	0.82	3,4-dihydroxy-2-butan
498	1	db$2	2::C3K3T9|C3K3T9_PSEFS	187	49785	6	6	6	6	0.77	Putative aminotransfer
499	1	db$2	2::C3K9P3|C3K9P3_PSEFS	187	9882	5	5	3	3	3.07	Peptidyl-prolyl cis-tran
500	1	db$2	2::C3K9S0|NFUA_PSEFS	187	20961	7	7	6	6	2.83	Fe/S biogenesis proteir
501	1	db$2	2::C3KE61|RIMK_PSEFS	187	32488	7	6	6	5	1.39	Probable alpha-L-gluta
502	1	db$2	2::C3K2N8|C3K2N8_PSEFS	187	23444	12	12	10	10	8.10	Semialdhyde_dh doma
503	1	db$2	2::C3JZ86|C3JZ86_PSEFS	186	21964	11	9	8	8	4.53	Superoxide dismutase
504	1	db$2	2::C3K8X6|C3K8X6_PSEFS	186	47255	11	8	10	7	1.02	Histidinol dehydrogena
505	1	db$2	2::C3K4R1|PLSB_PSEFS	185	95327	9	8	8	8	0.49	Glycerol-3-phosphate a
506	1	db$2	2::C3K2J8|C3K2J8_PSEFS	185	54392	12	8	11	8	1.01	AMP nucleosidase OS=
507	1	db$2	2::C3K490|C3K490_PSEFS	184	33505	10	9	9	8	2.08	Putative oxidative stre:
508	1	db$2	2::C3K0G3|PPNP_PSEFS	184	10127	7	7	4	4	8.85	Pyrimidine/purine nucl
509	1	db$2	2::C3K7M9|C3K7M9_PSEFS	184	34271	8	8	8	8	2.01	Putative ABC transport
510	1	db$2	2::C3K7I1|C3K7I1_PSEFS	184	85859	5	5	5	5	0.32	Quinoprotein glucose d
511	1	db$2	2::C3K4V8|C3K4V8_PSEFS	184	128447	7	7	7	7	0.30	Transcription-repair-co
512	1	db$2	2::C3K185|C3K185_PSEFS	184	20062	7	6	5	4	3.06	Putative NAD(P)H nitro
513	1	db$2	2::C3KBP9|C3KBP9_PSEFS	182	58780	8	7	8	7	0.76	Phosphoglucomutase C
514	1	db$2	2::C3K7M6|C3K7M6_PSEFS	182	88918	8	8	7	7	0.45	Acyl-coenzyme A dehy
515	1	db$2	2::C3K6F9|C3K6F9_PSEFS	182	37389	7	7	7	7	1.42	Nucleoid-associated pr
516	1	db$2	2::C3KDK8|C3KDK8_PSEFS	182	41619	4	4	4	4	0.58	DAO domain-containin
517	1	db$2	2::C3KAJ1|C3KAJ1_PSEFS	181	48585	9	8	8	7	1.18	Omega-amino acid--py
518	1	db$2	2::C3K4Y3|KDPA_PSEFS	181	60181	7	7	4	4	0.48	Potassium-transportin
519	1	db$2	2::C3K480|C3K480_PSEFS	181	31946	8	6	6	4	1.09	Uncharacterized protei

↑Family	M	DB	Accession	Score	Mass	Matches	Match(sig)	Sequences	Seq(sig)	emPAI	Description
520	1	db$2	2::C3K2W9|RL16_PSEFS	181	15422	8	8	7	7	7.32	50S ribosomal protein
521	1	db$2	2::C3K0N4|C3K0N4_PSEFS	180	25612	6	5	6	5	1.50	3-oxoacyl-[acyl-carrier
522	1	db$2	2::C3K3M3|C3K3M3_PSEFS	180	24540	4	4	3	3	0.78	Cell division ATP-bindir
523	1	db$2	2::C3JXQ1|C3JXQ1_PSEFS	179	24479	4	4	3	3	0.78	Putative enoyl-CoA hyc
524	1	db$2	2::C3JZV9|C3JZV9_PSEFS	178	34932	4	4	3	3	0.50	Putative DNA/RNA non
525	1	db$2	2::C3K6E3|C3K6E3_PSEFS	178	21087	8	7	6	5	2.80	Ubiquinol-cytochrome (
526	1	db$2	2::C3KBQ3|C3KBQ3_PSEFS	178	33274	5	5	5	5	1.03	LysR-family transcriptio
527	1	db$2	2::C3KAC7|C3KAC7_PSEFS	178	51379	11	9	9	8	1.09	UDP-glucose 6-dehydro
528	1	db$2	2::C3K8W9|C3K8W9_PSEFS	176	28276	6	6	6	6	1.72	Intermembrane phosph
529	1	db$2	2::C3K5N1|C3K5N1_PSEFS	176	36842	10	9	9	8	1.79	Sulfate ABC transport s
530	1	db$2	2::C3K0S3|C3K0S3_PSEFS	176	67491	7	6	7	6	0.52	Putative ATP-binding co
531	1	db$2	2::C3JXX2|C3JXX2_PSEFS	176	50328	7	7	7	7	0.93	ATP-dependent RNA he
532	1	db$2	2::C3K3D2|BETB_PSEFS	175	53123	9	8	8	7	0.87	Betaine aldehyde dehy
533	1	db$2	2::C3K1A1|C3K1A1_PSEFS	175	10298	7	7	5	5	8.39	10 kDa chaperonin OS:
534	1	db$2	2::C3JXY2|C3JXY2_PSEFS	175	35731	6	4	4	3	0.70	L-allo-threonine aldolas
535	1	db$2	2::C3K075|C3K075_PSEFS	174	77370	9	7	8	7	0.54	Putative sulfatase OS=
536	1	db$2	2::C3KDG2|C3KDG2_PSEFS	174	41589	9	7	7	5	0.98	AMP nucleosidase OS=
537	1	db$2	2::C3K5X2|C3K5X2_PSEFS	173	20559	7	6	5	5	2.14	Putative two-componer
538	1	db$2	2::C3K494|C3K494_PSEFS	173	33626	4	4	4	4	0.75	Gly-zipper_YMGG doma
539	1	db$2	2::C3KE68|RS18_PSEFS	173	8912	7	6	5	4	12.36	30S ribosomal protein
540	1	db$2	2::C3K2S1|C3K2S1_PSEFS	172	23236	10	8	7	6	3.13	Riboflavin synthase alp
541	1	db$2	2::C3K2J9|C3K2J9_PSEFS	172	60860	5	3	4	2	0.26	Putative dehydrogenas
542	1	db$2	2::C3K9R8|C3K9R8_PSEFS	172	135461	9	8	9	8	0.32	Methionine synthase O
543	1	db$2	2::C3K2V4|RS13_PSEFS	172	13332	8	7	5	5	4.74	30S ribosomal protein
544	1	db$2	2::C3K417|C3K417_PSEFS	171	39396	4	4	3	3	0.43	Transcriptional regulato
545	1	db$2	2::C3K270|C3K270_PSEFS	171	17315	12	11	7	7	7.69	Transcription elongatio
546	1	db$2	2::C3K5Z7|C3K5Z7_PSEFS	171	26574	10	9	8	8	3.14	Uridylate kinase OS=Ps
547	1	db$2	2::C3K4V4|C3K4V4_PSEFS	170	32182	10	8	7	6	1.79	Putative ATP-binding p
548	1	db$2	2::C3K3X2|C3K3X2_PSEFS	170	19739	5	5	5	5	2.28	Uncharacterized protei
549	1	db$2	2::C3K116|PSRP_PSEFS	170	30838	6	5	6	5	1.15	Putative phosphoenolp
550	1	db$2	2::C3K8U2|UBIB_PSEFS	170	61616	7	6	7	6	0.59	Probable protein kinase
551	1	db$2	2::C3K6V9|C3K6V9_PSEFS	170	54793	8	7	7	6	0.68	2,3-bisphosphoglycerat
552	1	db$2	2::C3KDN5|C3KDN5_PSEFS	170	35878	5	5	4	4	0.69	Renalase OS=Pseudom
553	1	db$2	2::C3KE71|C3KE71_PSEFS	170	51412	8	6	8	6	0.74	Replicative DNA helicas
554	1	db$2	2::C3JYS0|C3JYS0_PSEFS	169	29172	6	5	6	5	1.24	Cell division coordinato
555	1	db$2	2::C3K651|C3K651_PSEFS	169	39766	9	7	9	7	1.30	Phospho-2-dehydro-3-
556	1	db$2	2::C3K2Z3|C3K2Z3_PSEFS	168	44017	12	8	11	8	1.36	Tyrosine--tRNA ligase (
557	1	db$2	2::C3K3Q8|TYSY_PSEFS	167	31490	6	6	3	3	0.82	Thymidylate synthase (
558	1	db$2	2::C3KDM7|C3KDM7_PSEFS	167	29519	8	8	6	6	2.06	Sulfurtransferase OS=I
559	1	SwissProt_HUMAN$	3::Q06830|PRDX1_HUMAN	165	22096	10	9	7	6	3.43	Peroxiredoxin-1 OS=H(

↑Family	M	DB	Accession	Score	Mass	Matches	Match(sig)	Sequences	Seq(sig)	emPAI	Description
560	1	db$2	2::C3K421|C3K421_PSEFS	165	46244	8	7	8	7	1.05	Putative HemY domain
561	1	db$2	2::C3K0W5|PHS_PSEFS	165	13357	7	7	5	5	7.14	Putative pterin-4-alpha
562	1	db$2	2::C3KBL3|C3KBL3_PSEFS	165	50616	10	10	8	8	1.11	Putative exported prote
563	1	db$2	2::C3K3M7|C3K3M7_PSEFS	164	22464	2	2	1	1	0.23	Ribosomal RNA small s
564	1	SwissProt_HUMAN$	3::P23528|COF1_HUMAN	164	18491	6	5	6	5	2.54	Cofilin-1 OS=Homo sa
565	1	db$2	2::C3K839|Y879_PSEFS	164	32120	7	7	7	7	1.80	Nucleotide-binding pro
566	1	db$2	2::C3K5U6|C3K5U6_PSEFS	163	40174	6	6	5	5	0.80	dTDP-glucose 4,6-dehy
567	1	db$2	2::C3K084|AROC_PSEFS	162	39094	7	7	5	5	1.07	Chorismate synthase C
568	1	db$2	2::C3K5H7|C3K5H7_PSEFS	162	43348	6	5	4	4	0.55	Putative response regu
569	1	db$2	2::C3K268|C3K268_PSEFS	162	11552	7	7	7	7	15.66	CRM domain-containin
570	1	db$2	2::C3K8W7|C3K8W7_PSEFS	161	34291	5	5	5	5	0.99	Arabinose 5-phosphate
571	1	db$2	2::C3K1K9|C3K1K9_PSEFS	161	42204	6	5	5	4	0.57	Putative aminotransfer
572	1	db$2	2::C3KBM6|C3KBM6_PSEFS	161	11934	7	6	4	3	9.22	Glutaredoxin OS=Pseu
573	1	db$2	2::C3K152|C3K152_PSEFS	161	13999	8	7	7	7	9.31	Putative transcriptional
574	1	db$2	2::C3K968|C3K968_PSEFS	161	55197	8	7	8	7	0.82	Putative aldehyde dehy
575	1	db$2	2::C3KBZ0|PYRC_PSEFS	160	38385	5	5	4	4	0.64	Dihydroorotase OS=Ps
576	1	db$2	2::C3K5L3|C3K5L3_PSEFS	160	36401	5	5	4	4	0.68	Phosphonates ABC trar
577	1	db$2	2::C3JXQ0|C3JXQ0_PSEFS	160	24880	6	5	5	4	1.13	Putative hydrolase OS=
578	1	db$2	2::C3KA84|C3KA84_PSEFS	159	13408	7	7	7	7	10.32	Putative regulatory pro
579	1	db$2	2::C3K581|C3K581_PSEFS	159	86479	8	7	8	7	0.47	Putative molybdopterir
580	1	db$2	2::C3KAJ8|UPP_PSEFS	159	22986	8	7	6	5	2.41	Uracil phosphoribosyltr
581	1	db$2	2::C3JY36|C3JY36_PSEFS	159	16095	7	7	4	4	3.27	Uncharacterized protei
582	1	db$2	2::C3K6U9|HIS7_PSEFS	159	21830	9	9	6	6	3.50	Imidazoleglycerol-phos
583	1	db$2	2::C3K4H9|C3K4H9_PSEFS	159	34203	10	7	9	7	1.63	Quinone oxidoreductas
584	1	db$2	2::C3K910|C3K910_PSEFS	159	36322	5	5	5	5	0.92	Putative ABC transport
585	1	db$2	2::C3K5G1|C3K5G1_PSEFS	159	34097	5	5	4	4	1.00	Cysteine synthase OS=
586	1	db$2	2::C3K062|C3K062_PSEFS	158	48996	9	8	6	6	0.79	Fumarate hydratase cl
587	1	db$2	2::C3K703|ISPF_PSEFS	158	16742	3	3	3	3	1.31	2-C-methyl-D-erythrit
588	1	db$2	2::C3JY82|NUOB_PSEFS	158	25341	8	7	6	6	2.05	NADH-quinone oxidore
589	1	db$2	2::C3K6H1|C3K6H1_PSEFS	158	131195	10	8	10	8	0.34	DNA polymerase III su
590	1	db$2	2::C3JYK0|CLPP_PSEFS	157	23405	9	8	6	6	3.08	ATP-dependent Clp pro
591	1	db$2	2::C3K6K4|C3K6K4_PSEFS	157	32908	6	6	6	6	1.37	Putative 5'-nucleotidas
592	1	db$2	2::C3JZN1|IHFA_PSEFS	157	11469	6	6	5	5	6.60	Integration host factor
593	1	db$2	2::C3K4G2|C3K4G2_PSEFS	157	30728	9	9	4	4	1.93	Putative exported prote
594	1	db$2	2::C3JYI3|PHNX_PSEFS	157	29973	8	5	5	4	1.20	Phosphonoacetaldehyd
595	1	db$2	2::C3JZQ0|C3JZQ0_PSEFS	157	38665	9	8	7	6	1.35	Putative L-asparaginas
596	1	db$2	2::C3K1U9|C3K1U9_PSEFS	157	42728	7	4	5	3	0.56	Flavohemoglobin OS=F
597	1	SwissProt_HUMAN$	3::P0DOY2|IGLC2_HUMAN	157	11287	6	6	5	5	6.75	Immunoglobulin lambd
598	1	db$2	2::C3K5J3|C3K5J3_PSEFS	157	50388	9	7	9	7	0.93	AMP nucleosidase OS=
599	1	db$2	2::C3K2X4|RL23_PSEFS	157	10938	8	7	8	7	18.13	50S ribosomal protein

↑Family	M	DB	Accession	Score	Mass	Matches	Match(sig)	Sequences	Seq(sig)	emPAI	Description
600	1	db$2	2::C3K796|C3K796_PSEFS	156	49835	5	5	4	4	0.46	Putative glutamine syn
601	1	db$2	2::C3K5U5|C3K5U5_PSEFS	156	32335	6	5	5	5	1.08	Glucose-1-phosphate t
602	1	db$2	2::C3KBY9|C3KBY9_PSEFS	156	24532	4	4	4	4	1.15	Ribonuclease T OS=Ps
603	1	db$2	2::C3JYE4|C3JYE4_PSEFS	156	30882	11	10	10	9	2.96	Exodeoxyribonuclease
604	1	db$2	2::C3K8Y8|C3K8Y8_PSEFS	156	23915	3	3	3	3	0.81	Putative isomerase OS
605	1	SwissProt_HUMAN$	3::Q5QNW6|H2B2F_HUMAN	156	13912	6	6	4	4	4.36	Histone H2B type 2-F (
606	1	db$2	2::C3K479|RNPH_PSEFS	155	25645	5	4	4	3	0.73	Ribonuclease PH OS=P
607	1	db$2	2::C3JXP7|RECR_PSEFS	154	21528	6	6	5	5	2.70	Recombination protein
608	1	db$2	2::C3K332|Y5583_PSEFS	153	48606	6	5	6	5	0.63	UPF0229 protein PFLU_
609	1	db$2	2::C3K281|C3K281_PSEFS	153	15787	3	3	2	2	0.81	Ribosome association t
610	1	db$2	2::C3K843|C3K843_PSEFS	153	26535	7	7	5	5	1.43	Lipopolysaccharide exp
611	1	db$2	2::C3K9M2|C3K9M2_PSEFS	153	37830	6	6	5	5	0.87	Putative peptidase OS=
612	1	db$2	2::C3K4E4|C3K4E4_PSEFS	152	51389	8	7	8	7	0.91	Aspartate ammonia-lya
613	1	db$2	2::C3KDU8|C3KDU8_PSEFS	152	35029	7	7	6	6	1.24	D-cysteine desulfhydra
614	1	SwissProt_HUMAN$	3::P27348|1433T_HUMAN	152	27747	4	3	4	3	0.66	14-3-3 protein theta O
615	1	db$2	2::C3K1H2|C3K1H2_PSEFS	152	49205	7	5	6	4	0.47	Signal recognition part
616	1	db$2	2::C3KA81|C3KA81_PSEFS	152	55484	7	6	7	6	0.67	Alkyl hydroperoxide re
617	1	db$2	2::C3KBB4|OADC_PSEFS	151	31135	5	5	3	3	0.84	Oxaloacetate decarbox
618	1	db$2	2::C3K841|C3K841_PSEFS	151	11596	13	11	6	4	15.66	Putative sigma-54 moc
619	1	db$2	2::C3KAD3|ARNC_PSEFS	151	37700	4	4	4	4	0.65	Undecaprenyl-phospha
620	1	db$2	2::C3K572|C3K572_PSEFS	151	7891	2	2	2	2	2.20	Uncharacterized protei
621	1	db$2	2::C3KCL0|C3KCL0_PSEFS	151	35424	6	6	5	5	0.95	Uncharacterized protei
622	1	db$2	2::C3KBW8|C3KBW8_PSEFS	150	39031	8	7	8	7	1.33	D-alanine--D-alanine li
623	1	db$2	2::C3K472|C3K472_PSEFS	149	42804	6	6	5	5	0.94	Coenzyme A biosynthe
624	1	db$2	2::C3KAC2|C3KAC2_PSEFS	149	41342	4	4	4	4	0.58	Acyl-CoA dehydrogena
625	1	db$2	2::C3K368|C3K368_PSEFS	149	37353	6	6	6	6	1.13	Stress response kinase
626	1	db$2	2::C3K909|C3K909_PSEFS	149	24537	3	3	2	2	0.47	Uncharacterized protei
627	1	db$2	2::C3K9Z4|C3K9Z4_PSEFS	149	58133	7	7	7	7	0.77	Putative ABC tranmspo
628	1	db$2	2::C3KB19|C3KB19_PSEFS	149	44434	4	4	4	4	0.53	HlyD family secretion p
629	1	db$2	2::C3K4T4|C3K4T4_PSEFS	148	21507	5	5	5	5	1.98	NAD(P)-bd_dom domai
630	1	db$2	2::C3KED5|C3KED5_PSEFS	148	40447	5	5	5	5	0.80	Putative lipoprotein OS
631	1	db$2	2::C3K0I1|C3K0I1_PSEFS	148	29699	6	6	5	5	1.59	ParA family ATPase OS
632	1	db$2	2::C3K3T6|C3K3T6_PSEFS	148	42390	12	9	11	8	1.73	Spermidine/putrescine
633	1	db$2	2::C3JXY8|C3JXY8_PSEFS	147	37476	5	5	5	5	0.88	Arginine N-succinyltrar
634	1	db$2	2::C3KE32|C3KE32_PSEFS	146	20912	2	2	1	1	0.25	Sigma factor negative
635	1	db$2	2::C3K0V5|C3K0V5_PSEFS	146	33212	7	7	6	6	1.35	Putative keto/oxo acyl-
636	1	db$2	2::C3K747|GPDA_PSEFS	145	36476	6	5	5	5	0.91	Glycerol-3-phosphate c
637	1	db$2	2::C3K4U5|C3K4U5_PSEFS	145	75901	8	8	7	7	0.55	Putative protease OS=
638	1	db$2	2::C3KE48|C3KE48_PSEFS	145	19341	5	5	5	5	2.37	Peptide deformylase O:
639	1	db$2	2::C3K5K3|C3K5K3_PSEFS	144	28692	4	3	3	2	0.64	CHAD domain-containi

↑Family	M	DB	Accession	Score	Mass	Matches	Match(sig)	Sequences	Seq(sig)	emPAI	Description
640	1	db$2	2::C3JY91|C3JY91_PSEFS	144	66398	3	3	2	2	0.15	NADH-quinone oxidore
641	1	db$2	2::C3K733|HTPX_PSEFS	144	32382	6	6	4	4	1.07	Protease HtpX OS=Pse
642	1	db$2	2::C3K470|C3K470_PSEFS	143	58011	7	6	6	5	0.50	Putative transport-rela
643	1	db$2	2::C3JXY5|C3JXY5_PSEFS	142	10610	4	3	2	2	1.38	Uncharacterized protei
644	1	db$2	2::C3K437|C3K437_PSEFS	142	26506	3	3	2	2	0.43	Uncharacterized protei
645	1	db$2	2::C3JZV1|C3JZV1_PSEFS	142	76010	9	7	9	7	0.55	UvrABC system protein
646	1	db$2	2::C3KCG1|C3KCG1_PSEFS	142	60384	3	3	3	3	0.27	Putative secreted amid
647	1	db$2	2::C3K5B9|C3K5B9_PSEFS	142	60269	3	3	2	2	0.17	Dipeptide ABC transpo
648	1	db$2	2::C3K3L0|METXS_PSEFS	142	41664	5	5	5	5	0.76	Homoserine O-succinyl
649	1	db$2	2::C3JYQ9|C3JYQ9_PSEFS	141	54767	4	4	4	4	0.41	Transcriptional regulat
650	1	db$2	2::C3K3L7|THIG_PSEFS	141	28373	7	7	6	6	1.70	Thiazole synthase OS=
651	1	SwissProt_HUMAN$	3::P63104|1433Z_HUMAN	141	27728	6	5	5	4	0.97	14-3-3 protein zeta/de
652	1	db$2	2::C3K5L8|C3K5L8_PSEFS	141	39360	6	5	6	5	0.82	Phosphorous compoun
653	1	db$2	2::C3JZN8|C3JZN8_PSEFS	140	10735	5	5	3	3	4.59	Uncharacterized protei
654	1	db$2	2::C3K257|C3K257_PSEFS	140	33362	6	6	5	5	1.33	tRNA pseudouridine sy
655	1	db$2	2::C3K6I1|C3K6I1_PSEFS	140	31224	7	5	4	4	1.13	S-formylglutathione hy
656	1	db$2	2::C3K9F0|C3K9F0_PSEFS	140	40567	5	5	4	4	0.59	Putative transport-rela
657	1	db$2	2::C3K6S5|C3K6S5_PSEFS	140	35353	6	6	6	6	1.23	Delta-aminolevulinic ac
658	1	db$2	2::C3K2J5|C3K2J5_PSEFS	139	18057	4	4	4	4	1.82	Putative AsnC-family re
659	1	db$2	2::C3KEA8|C3KEA8_PSEFS	139	45495	4	4	3	3	0.37	ATP-dependent RNA he
660	1	SwissProt_HUMAN$	3::Q5VTE0|EF1A3_HUMAN	139	50153	6	6	5	5	0.60	Putative elongation fac
661	1	db$2	2::C3K3U0|C3K3U0_PSEFS	139	51001	6	5	6	5	0.59	Putative glutamine syn
662	1	db$2	2::C3JYD3|SYC_PSEFS	139	51635	11	11	10	10	1.50	Cysteine--tRNA ligase (
663	1	db$2	2::C3K424|HEM3_PSEFS	139	33541	2	2	1	1	0.15	Porphobilinogen deami
664	1	db$2	2::C3K2Q0|C3K2Q0_PSEFS	139	19348	11	10	9	9	7.89	Inorganic pyrophospha
665	1	db$2	2::C3KAI3|AROB_PSEFS	139	39825	6	6	5	5	0.81	3-dehydroquinate synt
666	1	db$2	2::C3K1D1|C3K1D1_PSEFS	139	32188	4	4	4	4	0.80	Protein HflC OS=Pseud
667	1	db$2	2::C3K2R1|DXS_PSEFS	137	68238	7	7	7	7	0.63	1-deoxy-D-xylulose-5-
668	1	db$2	2::C3K276|GRPE_PSEFS	137	20714	4	3	3	2	0.57	Protein GrpE OS=Pseu
669	1	db$2	2::C3JY78|C3JY78_PSEFS	137	22781	5	4	3	3	1.28	Putative cytochrome ox
670	1	db$2	2::C3JYS3|C3JYS3_PSEFS	137	38665	3	2	3	2	0.28	TolA colicin import mer
671	1	db$2	2::C3K017|PYRD_PSEFS	137	35850	10	9	8	8	2.27	Dihydroorotate dehydr
672	1	db$2	2::C3K617|C3K617_PSEFS	136	19384	4	4	3	3	1.06	Uncharacterized protei
673	1	db$2	2::C3K4E1|C3K4E1_PSEFS	136	38799	10	9	8	7	1.34	N5-carboxyaminoimida
674	1	db$2	2::C3K6E5|C3K6E5_PSEFS	136	29069	3	3	3	3	0.63	Cytochrome c1 OS=Ps
675	1	db$2	2::C3K1X7|RL27_PSEFS	136	9214	6	6	3	3	6.40	50S ribosomal protein
676	1	db$2	2::C3K061|C3K061_PSEFS	135	18792	3	3	3	3	1.12	DUF2059 domain-conta
677	1	db$2	2::C3K309|C3K309_PSEFS	134	21661	3	3	3	3	0.92	Anthranilate synthase (
678	1	db$2	2::C3K740|C3K740_PSEFS	134	33777	8	7	7	6	1.31	DUF815 domain-contai
679	1	db$2	2::C3K0P6|DCTA_PSEFS	134	46761	4	4	3	3	0.36	C4-dicarboxylate trans

↑Family	M	DB	Accession	Score	Mass	Matches	Match(sig)	Sequences	Seq(sig)	emPAI	Description
680	1	db$2	2::C3K611|C3K611_PSEFS	134	16065	5	5	4	4	3.27	Universal stress protei
681	1	db$2	2::C3KE99|C3KE99_PSEFS	134	30145	4	4	4	4	0.87	Quinolinate phosphorib
682	1	db$2	2::C3K610|C3K610_PSEFS	133	70283	11	8	11	8	0.72	ATP-binding protein Uu
683	1	SwissProt_HUMAN$	3::Q9BQE3|TBA1C_HUMAN	133	49863	4	4	4	4	0.46	Tubulin alpha-1C chain
684	1	db$2	2::C3JXX7|C3JXX7_PSEFS	133	56979	6	4	6	4	0.39	Conserved hypothetica
685	1	db$2	2::C3K6A5|C3K6A5_PSEFS	133	78572	6	6	6	6	0.44	Catalase OS=Pseudom
686	1	db$2	2::C3K1M1|HSCA_PSEFS	132	65896	6	6	5	5	0.43	Chaperone protein Hsc
687	1	db$2	2::C3KE59|C3KE59_PSEFS	132	27398	10	9	10	9	3.69	DNA-binding response
688	1	db$2	2::C3KAI4|C3KAI4_PSEFS	132	53827	5	4	5	4	0.42	SPOR domain-containi
689	1	db$2	2::C3K3S0|C3K3S0_PSEFS	131	23721	5	5	3	3	0.81	Putative hydrolase OS=
690	1	db$2	2::C3K3G7|C3K3G7_PSEFS	131	31253	6	6	6	6	1.47	Methylenetetrahydrofo
691	1	db$2	2::C3K254|C3K254_PSEFS	131	21027	8	7	7	6	2.83	Osmotically inducible p
692	1	db$2	2::C3K4W2|C3K4W2_PSEFS	130	26844	5	4	5	4	1.02	Putative glycerophosph
693	1	db$2	2::C3JYT6|C3JYT6_PSEFS	130	8291	7	6	6	5	14.78	Cold shock protein csp
694	1	db$2	2::C3KDB8|HLDE_PSEFS	130	49913	7	7	7	7	0.94	Bifunctional protein Hl
695	1	db$2	2::C3K2L6|C3K2L6_PSEFS	130	47731	5	4	5	4	0.49	Putative lipoprotein OS
696	1	db$2	2::C3K4V5|C3K4V5_PSEFS	130	36313	6	6	5	5	1.18	Beta-hexosaminidase (
697	1	db$2	2::C3K2I0|C3K2I0_PSEFS	129	48538	5	5	5	5	0.63	Putative ATP-depender
698	1	db$2	2::C3K785|C3K785_PSEFS	129	24962	4	4	4	4	1.12	3-oxoadipate CoA-tran
699	1	db$2	2::C3K8K8|C3K8K8_PSEFS	128	62203	6	6	6	6	0.58	Putative exported prot
700	1	db$2	2::C3K256|RS15_PSEFS	128	10112	4	4	3	3	2.95	30S ribosomal protein
701	1	db$2	2::C3K2K7|C3K2K7_PSEFS	128	37713	6	6	6	6	1.12	PhoH domain-containi
702	1	db$2	2::C3KBL7|C3KBL7_PSEFS	128	33413	5	4	4	3	0.53	Glutamate/aspartate A
703	1	db$2	2::C3K3W3|C3K3W3_PSEFS	128	41294	5	5	5	5	0.77	Putative aminotransfer
704	1	db$2	2::C3K2U8|C3K2U8_PSEFS	128	18135	5	5	5	5	2.65	Bacterioferritin OS=Ps
705	1	db$2	2::C3KDR6|C3KDR6_PSEFS	127	40570	3	3	3	3	0.42	Putaive NADH oxidore
706	1	db$2	2::C3JXZ3|C3JXZ3_PSEFS	127	28123	7	7	7	7	2.24	Histidine transport ATP
706	2	db$2	2::C3K713|C3K713_PSEFS	45	28108	2	2	2	2	0.40	Arginine/ornithine ABC
707	1	db$2	2::C3KCS5|C3KCS5_PSEFS	127	51798	6	6	6	6	0.73	UDP-N-acetylmuramoy
708	1	db$2	2::C3K2S3|C3K2S3_PSEFS	127	17933	6	6	3	3	1.18	Transcriptional repress
709	1	db$2	2::C3K0R0|C3K0R0_PSEFS	126	14751	2	2	2	2	0.88	Flagellar basal body ro
710	1	db$2	2::C3K7Z3|C3K7Z3_PSEFS	126	43142	5	5	4	4	0.55	Putative acyl-CoA dehy
711	1	db$2	2::C3KAW2|C3KAW2_PSEFS	126	26230	4	4	4	4	1.05	Putative short chain de
712	1	db$2	2::C3K568|TRPA_PSEFS	126	28539	4	3	4	3	0.64	Tryptophan synthase a
713	1	db$2	2::C3K1E4|GLMU_PSEFS	126	48690	6	6	6	6	0.79	Bifunctional protein Gl
714	1	db$2	2::C3KDE9|C3KDE9_PSEFS	126	41781	3	3	3	3	0.40	Putative phage tail-rela
715	1	db$2	2::C3K2W2|RS8_PSEFS	126	14083	7	7	5	5	6.27	30S ribosomal protein
716	1	db$2	2::C3KDX0|C3KDX0_PSEFS	126	16992	6	5	4	3	1.29	Peptidyl-prolyl cis-tran
717	1	db$2	2::C3K6S8|C3K6S8_PSEFS	125	40355	6	6	5	5	1.02	Putative acetyl-CoA tra
718	1	db$2	2::C3K709|C3K709_PSEFS	125	7722	7	7	6	6	33.38	Cold shock protein OS=

↑Family	M	DB	Accession	Score	Mass	Matches	Match(sig)	Sequences	Seq(sig)	emPAI	Description
719	1	db$2	2::C3K170|C3K170_PSEFS	125	22875	5	5	4	4	1.28	Flavin_Reduct domain-
720	1	db$2	2::C3K9M0|C3K9M0_PSEFS	125	11184	3	3	2	2	1.29	SCP2 domain-containir
721	1	db$2	2::C3K3N8|C3K3N8_PSEFS	124	43920	6	6	5	5	0.71	PUA domain-containing
722	1	db$2	2::C3JZ77|C3JZ77_PSEFS	124	45611	6	6	5	5	0.68	Maltose-binding proteir
723	1	db$2	2::C3K3V5|C3K3V5_PSEFS	124	52101	5	5	4	4	0.44	CYTH domain-containir
724	1	db$2	2::C3K6S9|C3K6S9_PSEFS	124	101727	9	7	8	7	0.39	DNA polymerase I OS=
725	1	db$2	2::C3K2K2|THIE_PSEFS	124	22345	3	3	3	3	0.88	Thiamine-phosphate sy
726	1	db$2	2::C3K815|C3K815_PSEFS	124	62777	8	8	7	7	0.70	Glucans biosynthesis p
727	1	db$2	2::C3K0T7|C3K0T7_PSEFS	123	76509	6	6	6	6	0.45	Putative fatty acid oxid
728	1	db$2	2::C3K467|C3K467_PSEFS	123	13453	3	3	2	2	1.00	Cupin_3 domain-conta
729	1	db$2	2::C3K114|C3K114_PSEFS	123	74752	4	4	4	4	0.29	DNA polymerase III su
730	1	db$2	2::C3KAB1|C3KAB1_PSEFS	123	44397	6	6	5	5	0.70	Histidine kinase OS=Ps
731	1	db$2	2::C3K272|C3K272_PSEFS	123	40774	8	6	7	5	0.79	Carbamoyl-phosphate
732	1	db$2	2::C3K3W5|C3K3W5_PSEFS	123	39645	6	6	5	5	0.82	Aminomethyltransferas
733	1	db$2	2::C3JZU0|C3JZU0_PSEFS	123	50538	6	6	6	6	0.76	3-isopropylmalate dehy
734	1	db$2	2::C3K7E3|C3K7E3_PSEFS	122	178026	9	8	9	8	0.24	Alpha-2-macroglobulin
735	1	db$2	2::C3K3L5|C3K3L5_PSEFS	122	11400	2	2	1	1	1.25	Putative membrane pro
736	1	db$2	2::C3K1X0|CYSZ_PSEFS	122	28826	4	4	4	4	0.92	Sulfate transporter Cys
737	1	db$2	2::C3KDX7|ISPH_PSEFS	122	34499	6	4	6	4	0.73	4-hydroxy-3-methylbu
738	1	db$2	2::C3KCS0|RL25_PSEFS	121	21502	9	9	5	5	3.60	50S ribosomal protein
739	1	db$2	2::C3K2U5|C3K2U5_PSEFS	121	18989	5	4	5	4	1.69	Single-stranded DNA-b
740	1	db$2	2::C3JYN4|ZIPA_PSEFS	121	31818	6	5	5	4	1.10	Cell division protein Zip
741	1	db$2	2::C3K240|C3K240_PSEFS	121	17616	8	7	6	5	3.91	RNA polymerase-bindir
742	1	db$2	2::C3KDM3|C3KDM3_PSEFS	121	25575	3	2	3	2	0.45	Putative lipoprotein OS
743	1	SwissProt_HUMAN$	3::P02675|FIBB_HUMAN	121	55892	7	5	7	5	0.53	Fibrinogen beta chain (
744	1	db$2	2::C3KDQ9|C3KDQ9_PSEFS	120	29676	9	8	7	7	2.05	Ferredoxin--NADP redu
745	1	db$2	2::C3KDM4|C3KDM4_PSEFS	120	56715	6	4	6	4	0.40	Putative membrane pro
746	1	db$2	2::C3K3L1|C3K3L1_PSEFS	120	23430	4	4	4	4	1.23	Uncharacterized protei
747	1	db$2	2::C3JZR8|C3JZR8_PSEFS	120	46446	7	6	6	5	0.67	Putative oxidoreductas
748	1	SwissProt_HUMAN$	3::P60174|TPIS_HUMAN	120	26653	8	8	7	7	2.45	Triosephosphate isome
749	1	db$2	2::C3K758|PYRF_PSEFS	120	24706	4	4	4	4	1.14	Orotidine 5'-phosphate
750	1	db$2	2::C3K1V6|C3K1V6_PSEFS	119	32555	2	2	2	2	0.34	Putative short-chain de
751	1	db$2	2::C3JY87|NUOH_PSEFS	119	37530	5	5	4	4	0.88	NADH-quinone oxidore
752	1	db$2	2::C3K586|C3K586_PSEFS	119	21064	7	7	5	5	2.80	ADP compounds hydrol
753	1	db$2	2::C3JYI6|C3JYI6_PSEFS	119	73112	6	4	6	4	0.30	Putative 2,4-dienoyl-Co
754	1	db$2	2::C3K304|C3K304_PSEFS	118	15054	4	4	3	3	2.44	Uncharacterized protei
755	1	db$2	2::C3KE66|C3KE66_PSEFS	118	27011	5	5	5	5	1.39	23S rRNA (guanosine-2
756	1	db$2	2::C3K4C1|C3K4C1_PSEFS	118	40318	5	5	4	4	0.60	Putative nitric oxide de
757	1	db$2	2::C3K015|C3K015_PSEFS	117	53791	4	4	3	3	0.30	Putative ferredoxin OS
758	1	db$2	2::C3K1M9|C3K1M9_PSEFS	116	29586	8	7	8	7	2.05	Inositol-1-monophosph

↑Family	M	DB	Accession	Score	Mass	Matches	Match(sig)	Sequences	Seq(sig)	emPAI	Description
759	1	db$2	2::C3KE08|C3KE08_PSEFS	116	25044	3	3	3	3	0.76	Conserved hypothetica
760	1	db$2	2::C3KE17|C3KE17_PSEFS	116	24430	4	4	4	4	1.16	Two component systen
761	1	db$2	2::C3K6V3|HIS6_PSEFS	116	27111	5	4	4	4	1.00	Imidazole glycerol pho:
762	1	db$2	2::C3KCP1|C3KCP1_PSEFS	115	23476	3	3	3	3	0.83	Peptide methionine sul
763	1	db$2	2::C3K3D8|C3K3D8_PSEFS	115	49942	5	5	5	5	0.61	ATP-independent RNA
764	1	db$2	2::C3K3B7|C3K3B7_PSEFS	115	14934	2	2	2	2	0.86	Uncharacterized protei
765	1	db$2	2::C3KBH3|DCUP_PSEFS	115	38973	6	6	6	6	1.07	Uroporphyrinogen deca
766	1	db$2	2::C3K1H1|RS16_PSEFS	115	9202	10	8	5	5	11.20	30S ribosomal protein
767	1	db$2	2::C3K8W6|C3K8W6_PSEFS	114	18885	3	3	3	3	1.11	3-deoxy-D-manno-octu
768	1	db$2	2::C3K126|C3K126_PSEFS	114	54569	9	7	5	5	0.54	2-methylcitrate dehydr
769	1	db$2	2::C3K2W8|RL29_PSEFS	114	7168	6	5	5	5	22.70	50S ribosomal protein
770	1	db$2	2::C3KAY1|C3KAY1_PSEFS	114	44657	7	5	6	5	0.70	3-phosphoshikimate 1-
771	1	db$2	2::C3K868|C3K868_PSEFS	114	58117	5	5	4	4	0.39	Uncharacterized protei
772	1	db$2	2::C3K3K1|C3K3K1_PSEFS	113	18188	2	2	2	2	0.67	Bifunctional protein [p)
773	1	db$2	2::C3K307|TRPC_PSEFS	113	30252	7	7	7	7	1.97	Indole-3-glycerol phos|
774	1	SwissProt_HUMAN$	3::P06703|S10A6_HUMAN	113	10173	2	2	1	1	0.57	Protein S100-A6 OS=H
775	1	db$2	2::C3K838|C3K838_PSEFS	113	9715	3	3	2	2	1.57	Putative phosphocarrie
776	1	db$2	2::C3KBU0|C3KBU0_PSEFS	112	36726	6	6	6	6	1.16	Sulfate/thiosulfate imp
777	1	db$2	2::C3KE43|C3KE43_PSEFS	112	32711	4	4	4	4	0.78	Lipid A biosynthesis lau
778	1	db$2	2::C3K979|C3K979_PSEFS	112	30391	3	3	2	2	0.36	Putative glutathione S-
779	1	db$2	2::C3K2R5|RIBA_PSEFS	112	22312	5	5	5	5	1.87	GTP cyclohydrolase-2 (
780	1	db$2	2::C3K7W0|C3K7W0_PSEFS	111	26786	3	3	3	3	0.70	Uncharacterized protei
781	1	db$2	2::C3K7T1|C3K7T1_PSEFS	111	23131	6	6	6	6	2.39	Two-component syster
782	1	db$2	2::C3KD57|C3KD57_PSEFS	111	49686	4	4	4	4	0.46	Cystathionine beta-lya:
783	1	db$2	2::C3K3P5|C3K3P5_PSEFS	111	48453	3	3	2	2	0.22	Putative symporter me
784	1	db$2	2::C3K302|C3K302_PSEFS	110	12723	6	5	4	3	1.98	Putative HIT domain-c
785	1	db$2	2::C3K231|C3K231_PSEFS	110	57678	4	4	4	4	0.39	APH domain-containing
786	1	db$2	2::C3K3M6|C3K3M6_PSEFS	109	53012	5	5	5	5	0.56	Peptidase_M16_C dom
787	1	db$2	2::C3K5K1|C3K5K1_PSEFS	108	38065	5	4	5	4	0.64	Putative phospholipase
788	1	db$2	2::C3K7C5|C3K7C5_PSEFS	108	40067	2	2	2	2	0.27	Histidine kinase OS=Ps
789	1	db$2	2::C3K2W7|RS17_PSEFS	107	10090	5	4	4	4	5.24	30S ribosomal protein
790	1	db$2	2::C3K197|C3K197_PSEFS	107	25331	4	4	4	4	1.10	Putative two-componer
791	1	db$2	2::C3K2X7|RS10_PSEFS	107	11745	12	11	7	7	14.82	30S ribosomal protein
792	1	db$2	2::C3K1C9|C3K1C9_PSEFS	107	65670	4	3	3	2	0.16	Putative cation-transpc
793	1	db$2	2::C3K3L6|TRMB_PSEFS	107	26990	2	2	2	2	0.42	tRNA (guanine-N(7)-)-
794	1	db$2	2::C3K8W4|C3K8W4_PSEFS	107	18790	7	6	5	5	3.48	Lipopolysaccharide exp
795	1	db$2	2::C3K605|LPXD_PSEFS	107	36124	3	3	3	3	0.48	UDP-3-O-acylglucosam
796	1	db$2	2::C3K148|APT_PSEFS	106	20064	6	6	5	5	2.21	Adenine phosphoribosy
797	1	db$2	2::C3JYN3|C3JYN3_PSEFS	106	52328	4	4	4	4	0.44	Family S13 unassigned
798	1	db$2	2::C3K0I0|C3K0I0_PSEFS	106	27472	5	5	4	4	0.99	RNA polymerase sigma

↑Family	M	DB	Accession	Score	Mass	Matches	Match(sig)	Sequences	Seq(sig)	emPAI	Description
799	1	db$2	2::C3KDM6|PSD_PSEFS	105	31380	3	3	3	3	0.57	Phosphatidylserine dec
800	1	db$2	2::C3K8W8|C3K8W8_PSEFS	105	29386	4	4	4	4	0.90	Putative ABC transport
801	1	db$2	2::C3K4J0|C3K4J0_PSEFS	105	17598	3	3	3	3	1.22	Zn_protease domain-c
802	1	db$2	2::C3K3Q4|C3K3Q4_PSEFS	105	38576	4	4	3	3	0.44	Putative cation ABC tra
803	1	db$2	2::C3K6J1|UBIG_PSEFS	105	26040	6	5	5	4	1.47	Ubiquinone biosynthesi
804	1	db$2	2::C3K7C4|C3K7C4_PSEFS	105	53137	10	7	9	6	0.87	DNA-binding transcript
805	1	db$2	2::C3K763|C3K763_PSEFS	104	29996	2	2	1	1	0.17	Putative hydrolase OS=
806	1	db$2	2::C3K110|C3K110_PSEFS	104	32414	5	5	5	5	1.07	Cysteine synthase OS=
807	1	db$2	2::C3JZN4|RL20_PSEFS	104	13348	8	6	7	5	4.74	50S ribosomal protein
808	1	db$2	2::C3JYF7|C3JYF7_PSEFS	104	147681	4	3	4	3	0.10	ATP-dependent helicas
809	1	db$2	2::C3KAI8|C3KAI8_PSEFS	104	24497	4	3	4	3	0.78	Putative TetR-family re
810	1	db$2	2::C3KDQ0|C3KDQ0_PSEFS	104	35105	4	4	4	4	0.71	2-ketogluconate reduct
811	1	db$2	2::C3K134|C3K134_PSEFS	103	52371	3	3	3	3	0.31	Coproporphyrinogen-II
812	1	db$2	2::C3K3T8|C3K3T8_PSEFS	103	39499	5	5	4	4	0.61	Putrescine ABC transpo
813	1	db$2	2::C3KBV0|C3KBV0_PSEFS	102	28603	5	5	4	4	0.93	Putative chromosome p
814	1	db$2	2::C3KE89|C3KE89_PSEFS	102	24346	5	5	5	5	1.63	Uncharacterized protei
815	1	db$2	2::C3K3Q1|C3K3Q1_PSEFS	102	40925	3	3	3	3	0.41	Putative oxidoreductas
816	1	db$2	2::C3KAV6|C3KAV6_PSEFS	102	17648	4	4	3	3	1.22	Thiol peroxidase OS=P
817	1	db$2	2::C3K9G9|C3K9G9_PSEFS	102	16183	4	3	4	3	1.38	CMD domain-containin
818	1	db$2	2::C3K3L2|C3K3L2_PSEFS	102	15596	3	3	3	3	1.45	DUF4426 domain-conta
819	1	db$2	2::C3KDC6|RF1_PSEFS	101	39857	3	3	3	3	0.43	Peptide chain release f
820	1	db$2	2::C3K9H7|C3K9H7_PSEFS	101	27490	4	2	3	2	0.41	Putative short-chain de
821	1	db$2	2::C3KBY4|C3KBY4_PSEFS	101	31613	2	2	2	2	0.35	Ribosomal RNA small s
822	1	db$2	2::C3K4U6|C3K4U6_PSEFS	101	17513	2	2	1	1	0.31	Conserved hypothetica
823	1	db$2	2::C3JYN9|C3JYN9_PSEFS	101	44994	5	4	5	4	0.52	Pseudouridine synthase
824	1	db$2	2::C3KE58|C3KE58_PSEFS	100	84757	10	9	10	9	0.65	Putative S1 RNA-bindir
825	1	db$2	2::C3K6E8|C3K6E8_PSEFS	100	27518	4	3	4	3	0.67	Putative GntR-family re
826	1	db$2	2::C3K5L9|C3K5L9_PSEFS	100	31441	2	2	2	2	0.35	Alpha-D-ribose 1-meth
827	1	db$2	2::C3K3K7|C3K3K7_PSEFS	100	24490	3	3	3	3	0.78	Pyridoxal phosphate hc
828	1	db$2	2::C3KDP0|C3KDP0_PSEFS	100	27498	3	2	3	2	0.41	Uncharacterized protei
829	1	db$2	2::C3JYJ6|C3JYJ6_PSEFS	100	68407	7	7	7	7	0.62	Peptidyl-prolyl cis-tran
830	1	db$2	2::C3K6L9|C3K6L9_PSEFS	100	49785	4	4	4	4	0.46	Phospho-2-dehydro-3-
831	1	db$2	2::C3K0J1|C3K0J1_PSEFS	100	36130	3	3	3	3	0.48	Flagellar motor switch
832	1	db$2	2::C3KBK6|C3KBK6_PSEFS	100	29320	3	3	2	2	0.62	Putative exported pept
833	1	db$2	2::C3K7S1|C3K7S1_PSEFS	100	25085	3	2	2	1	0.21	Protein MgtC OS=Pseu
834	1	db$2	2::C3K544|C3K544_PSEFS	100	74165	10	8	9	7	0.56	Uncharacterized protei
835	1	db$2	2::C3JXY4|ASTE_PSEFS	99	37404	8	6	7	6	1.13	Succinylglutamate desu
836	1	db$2	2::C3K5E8|C3K5E8_PSEFS	99	28997	3	3	3	3	0.63	Cyclohexadienyl dehyd
837	1	db$2	2::C3JYS8|RUVA_PSEFS	99	22218	3	3	3	3	0.89	Holliday junction ATP-c
838	1	db$2	2::C3K1B3|C3K1B3_PSEFS	99	20726	3	2	3	2	0.57	Uncharacterized protei

↑Family	M	DB	Accession	Score	Mass	Matches	Match(sig)	Sequences	Seq(sig)	emPAI	Description
839	1	db$2	2::C3KBK9|C3KBK9_PSEFS	99	34365	2	2	2	2	0.32	Glycerate dehydrogena
840	1	db$2	2::C3K621|C3K621_PSEFS	99	26068	3	3	3	3	0.72	Putative TetR-family re
841	1	db$2	2::C3K2P1|C3K2P1_PSEFS	99	48286	4	3	3	3	0.34	UDP-N-acetylmuramate
842	1	db$2	2::C3K644|C3K644_PSEFS	99	89774	6	4	5	3	0.17	Putative ABC transport
843	1	db$2	2::C3JY57|LOLA_PSEFS	98	22546	6	4	6	4	1.30	Outer-membrane lipop
844	1	SwissProt_HUMAN$	3::Q6FI13|H2A2A_HUMAN	98	14087	4	4	3	3	1.70	Histone H2A type 2-A (
844	2	SwissProt_HUMAN$	3::P0C0S5|H2AZ_HUMAN	42	13545	2	2	2	2	0.99	Histone H2A.Z OS=Hor
845	1	db$2	2::C3KC04|C3KC04_PSEFS	98	39513	4	4	4	4	0.61	Putative hydrolase OS=
846	1	db$2	2::C3KD21|C3KD21_PSEFS	97	81698	3	3	3	3	0.19	Putative glycogen debr
847	1	db$2	2::C3K4U7|C3K4U7_PSEFS	97	28919	3	3	2	2	0.38	Putative amidotransfer
848	1	db$2	2::C3K737|C3K737_PSEFS	97	17727	3	3	3	3	1.21	Glutathione peroxidase
849	1	db$2	2::C3JXX3|C3JXX3_PSEFS	97	52842	3	3	3	3	0.31	Cytochrome-c oxidase
850	1	db$2	2::C3K1K8|C3K1K8_PSEFS	97	31523	5	4	5	4	0.82	Putative hydrolase OS=
851	1	db$2	2::C3K862|C3K862_PSEFS	97	39825	4	3	4	3	0.43	Putative outer membra
852	1	db$2	2::C3KC02|C3KC02_PSEFS	97	29258	2	2	2	2	0.38	Putative acyltransferas
853	1	db$2	2::C3K8K4|C3K8K4_PSEFS	96	58330	4	3	4	3	0.28	Putative outer membra
854	1	db$2	2::C3K337|PLSY_PSEFS	96	20278	1	1	1	1	0.26	Glycerol-3-phosphate a
855	1	db$2	2::C3K5L2|C3K5L2_PSEFS	95	29854	4	3	3	3	0.61	Phosphonates import A
856	1	db$2	2::C3K5E4|GLND_PSEFS	95	102948	6	5	6	5	0.26	Bifunctional uridylyltra
857	1	db$2	2::C3K419|C3K419_PSEFS	95	17100	2	2	2	2	0.73	Transcriptional regulat
858	1	db$2	2::C3KDQ7|C3KDQ7_PSEFS	95	12961	2	2	1	1	0.43	Diacylglycerol kinase C
859	1	db$2	2::C3KAM8|C3KAM8_PSEFS	95	52747	3	3	3	3	0.31	Phosphate transporter
860	1	db$2	2::C3KDN6|C3KDN6_PSEFS	95	32369	2	2	2	2	0.34	Uncharacterized protei
861	1	db$2	2::C3K303|COQ7_PSEFS	95	23866	6	4	5	3	1.20	3-demethoxyubiquinol
862	1	db$2	2::C3KDQ1|C3KDQ1_PSEFS	95	35181	2	2	2	2	0.31	Putative LysR-family re
863	1	db$2	2::C3JY88|C3JY88_PSEFS	95	20432	7	6	7	6	2.97	NADH-quinone oxidore
864	1	db$2	2::C3KBH8|C3KBH8_PSEFS	95	25745	4	4	4	4	1.08	3-oxoacyl-[acyl-carrier
865	1	db$2	2::C3K0W6|C3K0W6_PSEFS	95	42755	4	4	3	3	0.39	Aspartate aminotransfe
866	1	db$2	2::B0B0A5|DNAA_PSEFS	95	56252	11	9	9	7	0.80	Chromosomal replicatio
867	1	db$2	2::C3KDI2|C3KDI2_PSEFS	94	135789	2	2	1	1	0.04	Exonuclease OS=Pseuc
868	1	db$2	2::C3K3X0|C3K3X0_PSEFS	94	42989	4	2	4	2	0.25	Putative ubiquinone bio
869	1	db$2	2::C3KEC3|C3KEC3_PSEFS	94	16145	3	2	2	2	0.79	Putative membrane pro
870	1	db$2	2::C3K427|C3K427_PSEFS	93	23927	5	5	4	4	1.67	Putative glutathione S-
871	1	db$2	2::C3JYV1|C3JYV1_PSEFS	93	32264	5	5	4	4	0.79	Formyltetrahydrofolate
872	1	db$2	2::C3JY31|C3JY31_PSEFS	93	22860	3	3	2	2	0.85	Putative biopolymer tra
873	1	db$2	2::Q9ZEP8|PANB_PSEFS	93	27951	2	2	2	2	0.40	3-methyl-2-oxobutano
874	1	db$2	2::C3K5K4|C3K5K4_PSEFS	93	29062	5	4	4	3	0.63	Uncharacterized protei
875	1	db$2	2::C3KE80|C3KE80_PSEFS	92	46502	5	4	5	4	0.50	Nicotinate phosphoribo
876	1	db$2	2::C3K2U6|C3K2U6_PSEFS	92	49484	2	2	2	2	0.21	Putative transport-rela
877	1	db$2	2::C3K607|LPXA_PSEFS	92	28002	3	3	2	2	0.40	Acyl-[acyl-carrier-prote

↑Family	M	DB	Accession	Score	Mass	Matches	Match(sig)	Sequences	Seq(sig)	emPAI	Description
878	1	db$2	2::C3K253|C3K253_PSEFS	92	106648	3	3	3	3	0.14	Putative membrane pro
879	1	db$2	2::C3JYP6|C3JYP6_PSEFS	92	10477	4	4	3	3	2.78	YCII domain-containing
880	1	db$2	2::C3K5F4|C3K5F4_PSEFS	92	21753	4	3	4	3	0.91	Putative nitroreductase
881	1	db$2	2::C3KDN7|HEMH_PSEFS	92	38415	5	4	5	4	0.64	Ferrochelatase OS=Pse
882	1	db$2	2::C3KBM4|C3KBM4_PSEFS	92	34098	5	5	5	5	1.00	Ornithine carbamoyltra
883	1	db$2	2::C3KDP4|C3KDP4_PSEFS	91	75338	7	5	5	4	0.29	Phosphate acetyltransf
884	1	db$2	2::C3K3S3|C3K3S3_PSEFS	91	15261	3	3	2	2	0.85	DUF4399 domain-conta
885	1	db$2	2::C3K650|C3K650_PSEFS	91	13083	4	4	4	4	3.14	N-acetyltransferase do
886	1	db$2	2::C3K0D7|APEB_PSEFS	90	46914	4	4	4	4	0.50	Probable M18 family ar
887	1	db$2	2::C3K468|C3K468_PSEFS	90	6077	3	2	1	1	1.10	50S ribosomal protein
888	1	db$2	2::C3K0H7|C3K0H7_PSEFS	90	77893	5	4	5	4	0.28	Histidine kinase OS=Ps
889	1	db$2	2::C3K485|C3K485_PSEFS	90	26565	2	2	2	2	0.43	Putative lipoprotein OS
890	1	db$2	2::C3K6Y6|C3K6Y6_PSEFS	90	31839	8	7	6	6	1.43	Signal peptidase I OS=
891	1	db$2	2::C3KD59|C3KD59_PSEFS	90	81779	3	3	3	3	0.19	YcaO domain-containin
892	1	db$2	2::C3K6V8|C3K6V8_PSEFS	90	47941	4	4	4	4	0.48	Putative peptidase OS=
893	1	db$2	2::C3KDC0|C3KDC0_PSEFS	90	36735	2	2	2	2	0.29	Uncharacterized protei
894	1	db$2	2::C3JXJ5|C3JXJ5_PSEFS	89	22111	4	4	3	3	0.89	Putative SCO1/SenC fa
895	1	db$2	2::C3K6V7|C3K6V7_PSEFS	89	46336	6	6	6	6	0.85	Carboxy-terminal proce
896	1	db$2	2::C3K4Z4|C3K4Z4_PSEFS	88	43117	3	3	3	3	0.39	Putative cytochrome C
897	1	db$2	2::C3K5M0|C3K5M0_PSEFS	88	29300	3	3	3	3	0.62	Phosphonates ABC trar
898	1	db$2	2::C3JYS6|C3JYS6_PSEFS	88	17060	2	2	2	2	0.73	Putative thioesterase C
899	1	db$2	2::C3K1C5|C3K1C5_PSEFS	87	44321	6	5	6	5	0.71	Putative cyclopropane-
900	1	db$2	2::C3KCV6|C3KCV6_PSEFS	87	40154	8	7	7	6	1.03	Probable alcohol dehyd
901	1	db$2	2::C3JY84|C3JY84_PSEFS	87	18006	2	2	2	2	0.68	NADH dehydrogenase I
902	1	db$2	2::C3K1M6|C3K1M6_PSEFS	87	24782	2	2	2	2	0.46	Uncharacterized protei
903	1	db$2	2::C3JYE9|C3JYE9_PSEFS	87	31035	3	2	3	2	0.35	Putative hydroxymethy
904	1	db$2	2::C3KBB9|C3KBB9_PSEFS	87	73354	4	3	3	2	0.14	Putative protease OS=
905	1	db$2	2::C3K103|C3K103_PSEFS	87	23527	3	3	3	3	0.82	Phosphoribosylglycinar
906	1	db$2	2::C3K2U3|C3K2U3_PSEFS	87	27946	3	3	3	3	0.66	Trans-aconitate 2-metl
907	1	db$2	2::C3K571|C3K571_PSEFS	86	11755	3	3	3	3	2.27	Uncharacterized protei
908	1	db$2	2::C3K1N0|C3K1N0_PSEFS	86	18655	2	1	2	1	0.29	Rick_17kDa_Anti doma
909	1	SwissProt_HUMAN$	3::A0A0B4J1X5|HV374_HUMAN	86	12831	3	3	2	2	1.96	Immunoglobulin heavy
910	1	db$2	2::C3K5H2|QUEF_PSEFS	86	30808	3	3	3	3	0.58	NADPH-dependent 7-cy
911	1	db$2	2::C3K4P8|C3K4P8_PSEFS	86	24008	2	2	2	2	0.48	t(6)A37 threonylcarbar
912	1	db$2	2::C3K9M9|C3K9M9_PSEFS	86	14163	2	2	2	2	0.93	Putative endoribonucle
913	1	db$2	2::C3K333|C3K333_PSEFS	86	61021	3	3	2	2	0.17	Uncharacterized protei
914	1	db$2	2::C3K5K2|C3K5K2_PSEFS	86	9923	3	3	3	3	3.07	Putative lipoprotein OS
915	1	db$2	2::C3K1W6|C3K1W6_PSEFS	86	13390	4	4	4	4	3.00	D-erythro-7,8-dihydror
916	1	db$2	2::C3K0N7|RL32_PSEFS	86	6752	4	4	3	3	6.53	50S ribosomal protein
917	1	db$2	2::C3KDY6|C3KDY6_PSEFS	85	17128	1	1	1	1	0.31	Uncharacterized protei

↑Family	M	DB	Accession	Score	Mass	Matches	Match(sig)	Sequences	Seq(sig)	emPAI	Description
918	1	db$2	2::C3K0P9|C3K0P9_PSEFS	85	25457	2	2	2	2	0.45	Two-component systen
919	1	db$2	2::C3KAS9|C3KAS9_PSEFS	85	109677	3	3	3	3	0.14	Efflux pump membrane
920	1	db$2	2::C3K0Q8|C3K0Q8_PSEFS	85	24717	2	2	2	2	0.46	Basal-body rod modific
921	1	db$2	2::C3K6J2|C3K6J2_PSEFS	84	47817	5	4	5	4	0.49	Putative hydrolase OS=
922	1	db$2	2::C3KBJ5|C3KBJ5_PSEFS	84	62095	2	2	2	2	0.17	Phospholipase C OS=P:
923	1	db$2	2::C3KAY5|C3KAY5_PSEFS	84	30881	2	2	1	1	0.36	NAD-capped RNA hydr
924	1	db$2	2::C3K360|C3K360_PSEFS	84	23509	2	2	2	2	0.49	ATP-dependent dethiob
925	1	db$2	2::C3K1V9|C3K1V9_PSEFS	83	16219	2	2	2	2	0.78	Flavodoxin-like domain
926	1	db$2	2::C3K5X6|C3K5X6_PSEFS	83	49358	5	4	4	3	0.33	Tryptophan--tRNA ligas
927	1	db$2	2::C3K5E3|C3K5E3_PSEFS	83	43909	4	3	4	3	0.38	Putative aminotransfer
928	1	db$2	2::C3KCS7|MRAY_PSEFS	83	39243	3	3	3	3	0.43	Phospho-N-acetylmura
929	1	db$2	2::C3KBL9|C3KBL9_PSEFS	83	27726	4	4	4	4	0.97	Glycerol-3-phosphate r
930	1	db$2	2::C3K7G1|C3K7G1_PSEFS	83	37563	4	4	4	4	0.65	Cell shape protein Mre(
931	1	db$2	2::C3K2D4|C3K2D4_PSEFS	83	35439	4	3	4	3	0.49	Putative cobalamin syn
932	1	db$2	2::C3KDS5|C3KDS5_PSEFS	83	41122	4	4	4	4	0.58	Putative hydratase/isor
933	1	db$2	2::C3JY52|C3JY52_PSEFS	83	37777	3	3	3	3	0.46	GST C-terminal domair
934	1	db$2	2::C3KE87|C3KE87_PSEFS	83	46792	5	3	4	3	0.35	NADH dehydrogenase I
935	1	db$2	2::C3K6Y8|C3K6Y8_PSEFS	82	34148	5	5	4	4	0.74	GTPase Era OS=Pseud(
936	1	db$2	2::C3K3J3|C3K3J3_PSEFS	82	72855	5	4	4	3	0.22	Type IV pilus biogenesi
937	1	db$2	2::C3K2W3|C3K2W3_PSEFS	81	11427	5	4	4	3	2.38	30S ribosomal protein
938	1	db$2	2::C3K830|C3K830_PSEFS	81	47217	8	8	8	8	1.23	Putative short chain de
939	1	db$2	2::C3KA90|C3KA90_PSEFS	81	36984	3	2	3	2	0.29	Putative aldo/keto redu
940	1	db$2	2::C3K0R6|C3K0R6_PSEFS	81	28374	7	7	6	6	1.70	Uncharacterized protei
941	1	db$2	2::C3K258|RBFA_PSEFS	81	14697	3	3	3	3	1.60	Ribosome-binding facto
942	1	SwissProt_HUMAN$	3::P02647|APOA1_HUMAN	80	30759	1	1	1	1	0.17	Apolipoprotein A-I OS=
943	1	db$2	2::C3K5J9|C3K5J9_PSEFS	80	96070	3	3	3	3	0.16	Histidine kinase OS=Ps
944	1	db$2	2::C3K0Z1|C3K0Z1_PSEFS	80	36566	2	2	2	2	0.30	L-threonine-O-3-phosp
945	1	db$2	2::C3K8P0|C3K8P0_PSEFS	80	38329	4	3	4	3	0.45	Putative nucleoside-se|
946	1	SwissProt_HUMAN$	3::P62979|RS27A_HUMAN	80	17953	3	3	3	3	1.18	Ubiquitin-40S ribosoma
947	1	db$2	2::C3K2M7|C3K2M7_PSEFS	80	18106	4	4	3	3	1.17	Ribosomal silencing fac
948	1	db$2	2::C3K998|C3K998_PSEFS	80	35021	5	5	5	5	0.96	Putative dehydrogenas
949	1	db$2	2::C3KEA2|C3KEA2_PSEFS	80	30824	2	2	2	2	0.36	Putative inner membra
950	1	db$2	2::C3KED3|C3KED3_PSEFS	80	20551	4	4	4	4	1.49	Glycine cleavage syste
951	1	db$2	2::C3K2Z9|C3K2Z9_PSEFS	80	34289	2	2	1	1	0.15	NMO domain-containin
952	1	db$2	2::C3K1N5|QUEA_PSEFS	79	38396	3	3	3	3	0.45	S-adenosylmethionine:
953	1	db$2	2::C3JY77|C3JY77_PSEFS	79	40451	2	2	2	2	0.26	Putative 3-oxoacyl-[ac
954	1	db$2	2::C3K0H6|C3K0H6_PSEFS	79	40054	5	5	5	5	0.80	Protein-glutamate metl
955	1	db$2	2::C3JXZ5|C3JXZ5_PSEFS	79	25958	3	3	3	3	0.72	Putative histidine trans
956	1	db$2	2::C3KAH2|C3KAH2_PSEFS	79	24970	4	4	3	3	0.76	SPOR domain-containir
957	1	db$2	2::C3JYI2|C3JYI2_PSEFS	79	39477	2	2	2	2	0.27	2-aminoethylphosphon

↑Family	M	DB	Accession	Score	Mass	Matches	Match(sig)	Sequences	Seq(sig)	emPAI	Description
958	1	db$2	2::C3K0M6|C3K0M6_PSEFS	79	25497	4	4	4	4	1.09	Putative tetR-family re
959	1	db$2	2::C3JYS7|RUVB_PSEFS	79	38919	1	1	1	1	0.13	Holliday junction ATP-c
960	1	SwissProt_HUMAN$	3::Q07020|RL18_HUMAN	79	21621	1	1	1	1	0.24	60S ribosomal protein
961	1	db$2	2::C3K2Y1|RS12_PSEFS	79	13716	4	4	4	4	2.87	30S ribosomal protein
962	1	db$2	2::C3KE84|C3KE84_PSEFS	78	35425	5	5	5	5	0.95	Pseudouridine synthase
963	1	db$2	2::C3K1N1|C3K1N1_PSEFS	78	33097	3	3	3	3	0.53	Protein-export membra
964	1	db$2	2::C3KAZ7|C3KAZ7_PSEFS	78	48577	2	2	2	2	0.21	Putative chain length d
965	1	db$2	2::C3KBT9|C3KBT9_PSEFS	78	31336	1	1	1	1	0.16	Sulfate ABC transport s
966	1	db$2	2::C3K4W3|C3K4W3_PSEFS	78	21770	2	2	2	2	0.54	PilZ domain-containing
967	1	db$2	2::C3K483|C3K483_PSEFS	78	78630	8	7	8	7	0.53	Guanosine-3',5'-bis(Dip
967	2	db$2	2::C3K1B1|C3K1B1_PSEFS	75	46678	3	3	3	3	0.36	Putative acid tolerance
968	1	SwissProt_HUMAN$	3::P02671|FIBA_HUMAN	78	94914	3	2	3	2	0.11	Fibrinogen alpha chain
969	1	db$2	2::C3K1A8|C3K1A8_PSEFS	78	35739	2	2	2	2	0.30	Putative lipoprotein OS
970	1	db$2	2::C3K229|C3K229_PSEFS	78	24861	1	1	1	1	0.21	TPR_REGION domain-c
971	1	db$2	2::C3JZZ4|C3JZZ4_PSEFS	78	23102	1	1	1	1	0.23	Putative glutathione S
972	1	db$2	2::C3KA82|C3KA82_PSEFS	78	19457	3	3	3	3	1.06	Lactoylglutathione lyas
973	1	db$2	2::C3K6W5|PRMA_PSEFS	78	31717	1	1	1	1	0.16	Ribosomal protein L11
974	1	db$2	2::C3K579|C3K579_PSEFS	77	15962	1	1	1	1	0.34	Putative thioesterase C
975	1	db$2	2::C3KBZ8|C3KBZ8_PSEFS	77	38389	2	2	2	2	0.28	Iron-sulfur cluster carr
976	1	db$2	2::C3K5G2|C3K5G2_PSEFS	77	35788	5	4	5	4	0.70	Putative membrane pro
977	1	db$2	2::C3K3Y7|C3K3Y7_PSEFS	77	35706	4	4	3	3	0.49	Putative iron-sulfur bin
978	1	db$2	2::C3K458|C3K458_PSEFS	76	47152	2	2	2	2	0.22	D-amino acid dehydrog
979	1	db$2	2::C3JY68|MNMA_PSEFS	76	41397	6	5	6	5	0.77	tRNA-specific 2-thiouri
980	1	db$2	2::C3K8X2|C3K8X2_PSEFS	76	10666	1	1	1	1	0.54	STAS domain-containir
981	1	db$2	2::C3K3R6|C3K3R6_PSEFS	76	54762	5	4	5	4	0.41	L-threonine dehydratas
982	1	db$2	2::C3JXH2|C3JXH2_PSEFS	76	91149	2	2	2	2	0.11	Putative excinuclease A
983	1	db$2	2::C3K6W8|C3K6W8_PSEFS	76	16611	2	2	2	2	0.75	3-dehydroquinate dehy
984	1	db$2	2::C3K436|C3K436_PSEFS	76	30237	3	3	3	3	0.60	Diaminopimelate epime
985	1	db$2	2::C3K5U7|C3K5U7_PSEFS	76	105852	3	3	2	2	0.09	Putative aminotransfer
986	1	db$2	2::C3K9U9|C3K9U9_PSEFS	76	9554	1	1	1	1	0.62	Uncharacterized protei
987	1	db$2	2::C3K3K3|C3K3K3_PSEFS	75	44101	5	5	5	5	0.71	Dihydroorotase-like pro
988	1	db$2	2::C3KDC4|C3KDC4_PSEFS	75	64677	2	2	2	2	0.16	Uncharacterized protei
989	1	db$2	2::C3K5X9|C3K5X9_PSEFS	75	41639	1	1	1	1	0.12	Putative acyl-CoA dehy
990	1	db$2	2::C3K9N0|C3K9N0_PSEFS	75	28569	2	2	2	2	0.39	Putative transcriptional
991	1	db$2	2::C3KAT0|C3KAT0_PSEFS	75	40804	2	2	2	2	0.26	Putative efflux system
992	1	db$2	2::C3KE56|C3KE56_PSEFS	75	59021	4	4	4	4	0.38	Glutamate--cysteine lig
993	1	db$2	2::C3K7I9|C3K7I9_PSEFS	75	88241	3	3	3	3	0.18	Putative pinecillin acyla
994	1	db$2	2::C3K146|C3K146_PSEFS	74	27134	4	4	4	4	1.00	Fumarate and nitrate r
995	1	db$2	2::C3K1G1|C3K1G1_PSEFS	74	48709	3	3	2	2	0.21	tRNA modification GTPa
996	1	db$2	2::C3KDR1|C3KDR1_PSEFS	74	17628	4	4	3	3	1.22	Uncharacterized protei

↑Family	M	DB	Accession	Score	Mass	Matches	Match(sig)	Sequences	Seq(sig)	emPAI	Description
997	1	db$2	2::C3K9N1|C3K9N1_PSEFS	73	43216	5	5	4	4	0.55	D-ser_dehydrat domai
998	1	db$2	2::C3JY81|C3JY81_PSEFS	73	15084	2	2	2	2	0.86	NADH-quinone oxidore
999	1	db$2	2::C3K230|C3K230_PSEFS	73	85084	2	2	2	2	0.12	Penicillin-binding prote
1000	1	db$2	2::C3K6P0|C3K6P0_PSEFS	73	15266	2	2	2	2	0.84	Uncharacterized protei
1001	1	db$2	2::C3K8X1|C3K8X1_PSEFS	73	23435	4	4	3	3	1.23	Putative ABC transport
1002	1	db$2	2::C3K3N0|C3K3N0_PSEFS	73	25405	2	2	2	2	0.45	Putative TetR-family re
1003	1	db$2	2::C3KDF0|C3KDF0_PSEFS	73	18221	2	2	2	2	0.67	Putative phage tail-rela
1004	1	SwissProt_HUMAN$	3::P62888|RL30_HUMAN	73	12776	2	2	2	2	1.07	60S ribosomal protein
1005	1	db$2	2::C3K2P7|C3K2P7_PSEFS	73	17680	5	3	4	2	0.70	Putative acetyltransfera
1006	1	db$2	2::C3K0U9|C3K0U9_PSEFS	73	55465	3	2	2	1	0.09	Putative two-componen
1007	1	db$2	2::C3KE30|C3KE30_PSEFS	73	59128	5	3	5	3	0.27	L-aspartate oxidase OS
1008	1	db$2	2::C3JZ82|C3JZ82_PSEFS	73	37007	2	2	2	2	0.29	Putative dehydrogenas
1009	1	db$2	2::C3K8W5|C3K8W5_PSEFS	72	21382	2	2	2	2	0.55	Lipopolysaccharide exp
1010	1	db$2	2::C3K3L3|C3K3L3_PSEFS	72	21091	4	3	3	3	0.95	dITP/XTP pyrophospha
1011	1	db$2	2::C3K1F7|C3K1F7_PSEFS	72	69251	3	2	3	2	0.15	tRNA uridine 5-carboxy
1012	1	db$2	2::C3K8W2|ADE_PSEFS	72	36048	3	3	3	3	0.48	Adenine deaminase OS
1013	1	SwissProt_HUMAN$	3::P00738|HPT_HUMAN	71	45177	5	5	5	5	0.69	Haptoglobin OS=Homo
1014	1	db$2	2::C3K0M3|C3K0M3_PSEFS	71	20567	3	3	3	3	0.99	Uncharacterized protei
1015	1	db$2	2::C3K6M4|C3K6M4_PSEFS	71	13463	3	3	2	2	1.83	Succinate dehydrogena
1016	1	SwissProt_HUMAN$	3::P62249|RS16_HUMAN	71	16435	2	2	2	2	0.77	40S ribosomal protein
1017	1	db$2	2::C3KB73|C3KB73_PSEFS	71	9692	2	2	2	2	1.60	Putative lipoprotein OS
1018	1	db$2	2::C3K743|FABA_PSEFS	71	18688	5	4	4	4	1.73	3-hydroxydecanoyl-[ac
1019	1	SwissProt_HUMAN$	3::P62937|PPIA_HUMAN	71	18001	3	3	2	2	0.68	Peptidyl-prolyl cis-tran
1020	1	db$2	2::C3K5Q0|C3K5Q0_PSEFS	71	53619	4	3	4	3	0.30	Mannose-1-phosphate
1021	1	db$2	2::C3KDW1|MUTL_PSEFS	71	69574	5	5	5	5	0.41	DNA mismatch repair p
1022	1	db$2	2::C3K3B8|C3K3B8_PSEFS	71	42763	1	1	1	1	0.12	Uncharacterized protei
1023	1	SwissProt_HUMAN$	3::P62913|RL11_HUMAN	71	20240	1	1	1	1	0.26	60S ribosomal protein
1024	1	db$2	2::C3K312|C3K312_PSEFS	71	24134	4	4	4	4	1.18	Ribulose-phosphate 3-
1025	1	db$2	2::C3K9T0|NADK_PSEFS	70	32289	3	2	3	2	0.34	NAD kinase OS=Pseud
1026	1	db$2	2::C3JXI0|C3JXI0_PSEFS	70	23030	2	2	2	2	0.51	Nudix hydrolase domai
1027	1	db$2	2::C3K221|C3K221_PSEFS	70	129628	3	3	2	2	0.08	RecBCD enzyme subun
1028	1	SwissProt_HUMAN$	3::Q86YZ3|HORN_HUMAN	70	282228	3	3	2	2	0.05	Hornerin OS=Homo sa
1029	1	db$2	2::C3K601|DXR_PSEFS	70	42341	3	3	3	3	0.40	1-deoxy-D-xylulose 5-
1030	1	db$2	2::C3K564|C3K564_PSEFS	70	54508	3	3	3	3	0.30	Methylmalonate-semia
1031	1	db$2	2::C3K013|C3K013_PSEFS	70	20254	3	3	2	2	0.59	Peptidyl-prolyl cis-tran
1032	1	db$2	2::C3KC09|C3KC09_PSEFS	70	83087	2	2	2	2	0.12	Periplasmic beta-gluco
1033	1	db$2	2::C3K5F1|C3K5F1_PSEFS	70	42516	3	2	3	2	0.25	Ribonuclease D OS=Ps
1034	1	db$2	2::C3K107|C3K107_PSEFS	69	31415	2	2	2	2	0.35	Uncharacterized protei
1035	1	db$2	2::C3K7Y8|C3K7Y8_PSEFS	69	53697	4	3	4	3	0.30	Putative hydrolase OS=
1036	1	db$2	2::C3KBV6|C3KBV6_PSEFS	69	125347	2	2	2	2	0.08	Putative potassium effl

↑Family	M	DB	Accession	Score	Mass	Matches	Match(sig)	Sequences	Seq(sig)	emPAI	Description
1037	1	db$2	2::C3K1M8|C3K1M8_PSEFS	69	28345	2	2	2	2	0.40	tRNA (cytidine/uridine-
1038	1	db$2	2::C3K6A4|C3K6A4_PSEFS	69	36459	2	2	2	2	0.30	Methionine import ATP-
1039	1	db$2	2::C3K1L7|RLMN_PSEFS	69	42342	2	2	2	2	0.25	Dual-specificity RNA m
1040	1	db$2	2::C3K602|C3K602_PSEFS	69	48346	3	3	3	3	0.34	Zinc metalloprotease C
1041	1	db$2	2::C3K415|C3K415_PSEFS	69	70809	3	2	3	2	0.14	Putative ABC transport
1042	1	db$2	2::C3K477|C3K477_PSEFS	68	29836	3	3	3	3	0.61	Exodeoxyribonuclease
1043	1	db$2	2::C3K2T3|C3K2T3_PSEFS	68	32553	3	2	3	2	0.34	dTDP-4-dehydrorhamn
1044	1	db$2	2::C3K391|C3K391_PSEFS	68	33913	1	1	1	1	0.15	Putative acyltransferas
1045	1	db$2	2::C3K8I3|C3K8I3_PSEFS	68	39921	2	2	1	1	0.13	3,4-dihydroxy-2-butan
1046	1	db$2	2::C3KED2|C3KED2_PSEFS	68	17293	2	2	2	2	0.72	Thioredoxin peroxidase
1047	1	db$2	2::C3K1L5|C3K1L5_PSEFS	68	34147	4	4	4	4	0.74	Putative DNA-binding p
1048	1	db$2	2::C3JZ64|C3JZ64_PSEFS	68	17217	3	3	3	3	1.26	MaoC-like domain-cont
1049	1	db$2	2::C3KAK2|C3KAK2_PSEFS	68	10989	6	5	5	4	4.40	Uncharacterized protei
1050	1	db$2	2::C3K349|C3K349_PSEFS	68	28994	4	4	4	4	0.92	Pyrroloquinoline-quinoi
1051	1	db$2	2::C3K326|C3K326_PSEFS	68	35169	2	1	2	1	0.14	4-hydroxythreonine-4-
1052	1	db$2	2::C3KDL3|C3KDL3_PSEFS	67	70148	5	4	5	4	0.31	Phosphomethylpyrimid
1053	1	db$2	2::C3K6T3|C3K6T3_PSEFS	67	23166	2	2	2	2	0.50	Thiol:disulfide intercha
1054	1	db$2	2::C3K1A4|C3K1A4_PSEFS	67	41199	2	2	2	2	0.26	Uncharacterized protei
1055	1	db$2	2::C3K7G4|C3K7G4_PSEFS	67	54521	3	3	3	3	0.30	Ribonuclease G OS=Ps
1056	1	db$2	2::C3K068|C3K068_PSEFS	67	65454	4	4	4	4	0.34	Putative asparagine sy
1057	1	db$2	2::C3KDX1|RS20_PSEFS	66	10078	3	3	2	2	1.50	30S ribosomal protein
1058	1	db$2	2::C3K710|DCD_PSEFS	66	21231	4	4	4	4	1.43	dCTP deaminase OS=P
1059	1	SwissProt_HUMAN$	3::P04406|G3P_HUMAN	66	36030	4	3	3	3	0.48	Glyceraldehyde-3-phos
1060	1	db$2	2::C3KCB9|C3KCB9_PSEFS	66	35987	3	3	2	2	0.30	Polyphosphate kinase (
1061	1	db$2	2::C3K3P7|C3K3P7_PSEFS	66	49977	2	2	2	2	0.21	Putative membrane pro
1062	1	db$2	2::C3K7A7|C3K7A7_PSEFS	66	43115	4	3	4	3	0.39	Conserved hypothetica
1063	1	db$2	2::C3KAJ9|C3KAJ9_PSEFS	66	20836	4	3	4	3	0.96	Putative hypoxanthine-
1064	1	db$2	2::C3K7X0|C3K7X0_PSEFS	66	85237	4	3	3	2	0.12	5-methyltetrahydropte
1065	1	db$2	2::C3KDV5|C3KDV5_PSEFS	66	20461	5	4	4	4	1.51	Oligoribonuclease OS=
1066	1	db$2	2::C3K0C5|C3K0C5_PSEFS	66	27111	1	1	1	1	0.19	Putative amino transfe
1067	1	db$2	2::C3K129|C3K129_PSEFS	66	42141	2	2	2	2	0.25	Putative acetyl CoA ace
1068	1	db$2	2::C3KDX3|C3KDX3_PSEFS	66	34279	3	3	3	3	0.51	Riboflavin biosynthesis
1069	1	db$2	2::C3K0M9|KTHY_PSEFS	65	22917	2	2	2	2	0.51	Thymidylate kinase OS
1070	1	db$2	2::C3K6W0|C3K6W0_PSEFS	65	14786	3	3	2	2	0.88	Putative rhodanese dor
1071	1	db$2	2::C3K4V3|C3K4V3_PSEFS	65	45550	2	2	2	2	0.23	Uncharacterized protei
1072	1	db$2	2::C3K4R2|C3K4R2_PSEFS	65	7681	3	3	3	3	5.01	Major cold shock prote
1073	1	db$2	2::C3KDG5|C3KDG5_PSEFS	65	23599	3	3	3	3	0.82	Putative regulatory pro
1074	1	db$2	2::C3K9Q9|C3K9Q9_PSEFS	65	19670	1	1	1	1	0.27	FMN_red domain-conta
1075	1	db$2	2::C3K1B4|C3K1B4_PSEFS	64	43355	2	2	2	2	0.24	Putative transferase OS
1076	1	db$2	2::C3KDM9|C3KDM9_PSEFS	64	28096	5	5	4	4	0.96	Putative oxidoreductas

↑Family	M	DB	Accession	Score	Mass	Matches	Match(sig)	Sequences	Seq(sig)	emPAI	Description
1077	1	db$2	2::C3K5B7|C3K5B7_PSEFS	64	50883	1	1	1	1	0.10	Putative outer membra
1078	1	db$2	2::C3K3V1|C3K3V1_PSEFS	64	23989	3	3	2	2	0.48	Alkyl hydroperoxide re
1079	1	db$2	2::C3K6E4|C3K6E4_PSEFS	64	46143	5	4	3	3	0.51	Cytochrome b OS=Pse
1080	1	db$2	2::C3KCR9|PTH_PSEFS	64	21041	2	2	2	2	0.56	Peptidyl-tRNA hydrolas
1081	1	SwissProt_HUMAN$	3::P08238|HS90B_HUMAN	64	83212	3	3	3	3	0.19	Heat shock protein HSI
1082	1	db$2	2::C3K0X7|C3K0X7_PSEFS	64	126663	1	1	1	1	0.04	Histidine kinase OS=Ps
1083	1	db$2	2::C3K4J4|HSLO_PSEFS	64	33320	2	2	2	2	0.33	33 kDa chaperonin OS
1084	1	db$2	2::C3K150|C3K150_PSEFS	64	25194	3	3	3	3	0.75	Putative short-chain de
1085	1	db$2	2::C3K6T8|C3K6T8_PSEFS	64	49583	2	2	2	2	0.21	Alginate biosynthesis t
1086	1	db$2	2::C3K3D1|BETI_PSEFS	63	21992	1	1	1	1	0.24	HTH-type transcription
1087	1	db$2	2::C3KDN9|C3KDN9_PSEFS	63	40608	1	1	1	1	0.12	Probable periplasmic se
1088	1	db$2	2::C3KCS8|C3KCS8_PSEFS	63	47767	3	3	3	3	0.35	UDP-N-acetylmuramoy
1089	1	db$2	2::C3K754|C3K754_PSEFS	63	29479	2	2	2	2	0.38	Putative lipoprotein OS
1090	1	db$2	2::C3K6A6|C3K6A6_PSEFS	63	25667	2	2	1	1	0.44	Putative lipoprotein OS
1091	1	db$2	2::C3KDR2|C3KDR2_PSEFS	63	26142	3	2	3	2	0.43	S4 RNA-binding domai
1092	1	db$2	2::C3K0Z5|C3K0Z5_PSEFS	63	22469	4	4	3	3	1.31	Corrinoid adenosyltran
1093	1	db$2	2::C3KE47|FMT_PSEFS	63	33416	3	2	2	1	0.15	Methionyl-tRNA formyl
1094	1	db$2	2::C3K1G6|C3K1G6_PSEFS	63	25762	3	3	2	2	0.44	Thiol:disulfide intercha
1095	1	db$2	2::C3K5Y8|C3K5Y8_PSEFS	62	48751	3	3	3	3	0.34	L-serine dehydratase C
1096	1	db$2	2::C3JY92|C3JY92_PSEFS	62	55851	2	2	2	2	0.18	NADH dehydrogenase I
1097	1	db$2	2::C3KEC9|C3KEC9_PSEFS	62	53005	3	3	2	2	0.20	Putative beta-barrel as
1098	1	db$2	2::C3K2E4|C3K2E4_PSEFS	62	29249	3	3	3	3	0.62	Ferredoxin--NADP redu
1099	1	db$2	2::C3K5X1|C3K5X1_PSEFS	62	46611	2	2	2	2	0.23	Histidine kinase OS=Ps
1100	1	db$2	2::C3K3R4|C3K3R4_PSEFS	62	24681	3	3	3	3	0.77	Putative hydrolase OS=
1101	1	db$2	2::C3KDX6|C3KDX6_PSEFS	62	16394	2	2	1	1	0.33	Peptidyl-prolyl cis-tran
1102	1	db$2	2::C3K3M9|C3K3M9_PSEFS	62	30473	4	3	4	3	0.59	Putative sulfurtransfera
1103	1	db$2	2::C3K6Y7|RNC_PSEFS	62	25367	2	2	2	2	0.45	Ribonuclease 3 OS=Ps
1104	1	db$2	2::C3K6G9|LPXB_PSEFS	62	41468	2	2	2	2	0.26	Lipid-A-disaccharide sy
1105	1	db$2	2::C3KB07|C3KB07_PSEFS	62	47911	1	1	1	1	0.10	Deferrochelatase/pero
1106	1	db$2	2::C3K0N3|ACP_PSEFS	62	8637	2	1	2	1	0.70	Acyl carrier protein OS
1107	1	db$2	2::C3K021|C3K021_PSEFS	62	27911	5	3	3	2	0.40	Phosphoadenosine pho
1108	1	db$2	2::C3K3J1|C3K3J1_PSEFS	62	16523	2	2	2	2	0.76	Putative chemotaxis-re
1109	1	db$2	2::C3K0K3|C3K0K3_PSEFS	62	12996	1	1	1	1	0.43	Putative lipoprotein OS
1110	1	db$2	2::C3K2K6|C3K2K6_PSEFS	62	49458	4	3	4	3	0.33	tRNA-2-methylthio-N(6
1111	1	db$2	2::C3JZP9|C3JZP9_PSEFS	61	33380	1	1	1	1	0.15	Putative ribose transpo
1112	1	db$2	2::C3JZP0|C3JZP0_PSEFS	61	11251	1	1	1	1	0.51	Putative lipoprotein OS
1113	1	db$2	2::C3K2V8|RL30_PSEFS	61	6521	4	2	3	2	3.01	50S ribosomal protein
1114	1	db$2	2::C3KBM3|C3KBM3_PSEFS	61	40194	2	2	2	2	0.27	Putative ABC transport
1115	1	db$2	2::C3K5M1|C3K5M1_PSEFS	61	26375	3	2	3	2	0.43	Phosphonates ABC trar
1116	1	db$2	2::C3K0V4|C3K0V4_PSEFS	61	48572	2	2	2	2	0.21	Flagellin OS=Pseudom

↑Family	M	DB	Accession	Score	Mass	Matches	Match(sig)	Sequences	Seq(sig)	emPAI	Description
1117	1	db$2	2::C3KDF9|C3KDF9_PSEFS	61	17633	3	3	2	2	1.22	CinA domain-containin
1118	1	db$2	2::C3JYE7|C3JYE7_PSEFS	61	35468	2	2	2	2	0.30	LysR family regulatory
1119	1	db$2	2::C3KBL4|C3KBL4_PSEFS	61	26993	4	4	3	3	0.69	Glutamate/aspartate A
1120	1	db$2	2::C3K3K9|C3K3K9_PSEFS	61	21575	3	3	2	2	0.55	Conserved hypothetica
1121	1	db$2	2::C3K820|C3K820_PSEFS	61	26990	2	2	2	2	0.42	Putative ABC transport
1122	1	db$2	2::C3KCJ3|C3KCJ3_PSEFS	61	30975	4	4	3	3	0.58	Usp domain-containing
1123	1	db$2	2::C3K1H9|C3K1H9_PSEFS	60	18692	4	4	3	3	1.13	Conserved hypothetica
1124	1	db$2	2::C3K3D3|BETA_PSEFS	60	62642	3	2	3	2	0.16	Oxygen-dependent chc
1125	1	db$2	2::C3K5K7|C3K5K7_PSEFS	60	52604	3	3	3	3	0.31	Putative methyl-accept
1126	1	db$2	2::C3K9P7|C3K9P7_PSEFS	60	69450	3	2	3	2	0.15	Putative substrate-binc
1127	1	db$2	2::C3K1X3|C3K1X3_PSEFS	60	20670	3	2	3	2	0.58	Glutathione peroxidase
1128	1	db$2	2::C3JZH3|C3JZH3_PSEFS	60	22428	1	1	1	1	0.23	Putative cytochrome o:
1129	1	db$2	2::C3K1D4|C3K1D4_PSEFS	60	64734	3	2	3	2	0.16	Uncharacterized protei
1130	1	db$2	2::C3JYK2|C3JYK2_PSEFS	60	30492	3	2	3	2	0.36	Bifunctional protein Fol
1131	1	db$2	2::C3K7K5|C3K7K5_PSEFS	60	12541	1	1	1	1	0.45	Uncharacterized protei
1132	1	db$2	2::C3KEB0|C3KEB0_PSEFS	60	36597	3	3	2	2	0.29	Putative amino acid AB
1133	1	db$2	2::C3K666|C3K666_PSEFS	60	29461	3	2	3	2	0.38	Putative lipoprotein OS
1134	1	db$2	2::C3KDV8|C3KDV8_PSEFS	60	29265	2	2	2	2	0.38	ADP-dependent (S)-NA
1135	1	db$2	2::C3K0W1|C3K0W1_PSEFS	60	27483	2	2	2	2	0.41	Flagellar basal-body ro
1136	1	db$2	2::C3K707|C3K707_PSEFS	59	29295	3	2	3	2	0.38	Putative lipoprotein OS
1137	1	db$2	2::C3K2E0|C3K2E0_PSEFS	59	48742	6	5	6	5	0.63	DNA repair protein Rac
1138	1	db$2	2::C3JZ30|C3JZ30_PSEFS	59	12225	1	1	1	1	0.46	DUF2025 domain-conta
1139	1	db$2	2::C3K5X0|C3K5X0_PSEFS	59	25599	2	1	2	1	0.20	Putative exported prote
1140	1	SwissProt_HUMAN$	3::P46783|RS10_HUMAN	59	18886	2	2	2	2	0.64	40S ribosomal protein
1141	1	db$2	2::C3K8K5|C3K8K5_PSEFS	59	64377	3	3	3	3	0.25	Putative transport-rela
1142	1	db$2	2::C3K433|CYAY_PSEFS	59	12442	2	2	1	1	0.45	Iron-sulfur cluster asse
1143	1	db$2	2::C3JZU7|C3JZU7_PSEFS	59	16433	2	2	2	2	0.77	Putative thioesterase C
1144	1	db$2	2::C3K6U5|FETP_PSEFS	59	10399	2	2	2	2	1.43	Probable Fe(2+)-traffic
1145	1	db$2	2::C3K459|C3K459_PSEFS	58	18704	3	3	3	3	1.12	Leucine-responsive reg
1146	1	db$2	2::C3K0X3|C3K0X3_PSEFS	58	16227	2	2	2	2	0.78	Transcriptional regulat
1147	1	db$2	2::C3K1H0|RIMM_PSEFS	58	20004	2	2	2	2	0.60	Ribosome maturation f
1148	1	db$2	2::C3KBM1|C3KBM1_PSEFS	58	29989	2	2	2	2	0.37	Glycerol uptake facilita
1149	1	db$2	2::C3JZQ4|C3JZQ4_PSEFS	58	8106	2	2	2	2	2.10	Translational regulator
1150	1	db$2	2::C3K867|C3K867_PSEFS	58	39735	3	3	2	2	0.27	PALP domain-containin
1151	1	db$2	2::C3K176|C3K176_PSEFS	58	33571	4	3	4	3	0.53	Glucokinase OS=Pseud
1152	1	SwissProt_HUMAN$	3::P46781|RS9_HUMAN	58	22578	3	3	2	2	0.52	40S ribosomal protein
1153	1	db$2	2::C3K3X4|C3K3X4_PSEFS	58	11561	2	2	1	1	1.23	Cell division protein Za
1154	1	db$2	2::C3KE46|C3KE46_PSEFS	58	47174	3	3	3	3	0.35	16S rRNA m5C967 met
1155	1	db$2	2::C3KE10|C3KE10_PSEFS	58	58823	2	1	2	1	0.08	Putative methyl-accept
1156	1	db$2	2::C3K7J3|Y1319_PSEFS	58	16953	1	1	1	1	0.32	UPF0225 protein PFLU_

↑Family	M	DB	Accession	Score	Mass	Matches	Match(sig)	Sequences	Seq(sig)	emPAI	Description
1157	1	db$2	2::C3JYG2|C3JYG2_PSEFS	58	36128	2	2	2	2	0.30	ATP-grasp domain-con
1158	1	db$2	2::C3K7M5|C3K7M5_PSEFS	58	15789	1	1	1	1	0.35	Uncharacterized protei
1159	1	db$2	2::C3K9Y7|C3K9Y7_PSEFS	57	30413	1	1	1	1	0.17	Undecaprenyl-diphosph
1160	1	db$2	2::C3KE20|C3KE20_PSEFS	57	33868	4	4	4	4	0.75	GCV_T domain-contain
1161	1	db$2	2::C3K2C2|C3K2C2_PSEFS	57	67328	1	1	1	1	0.07	Putative lipoprotein OS
1162	1	db$2	2::C3KE78|C3KE78_PSEFS	57	15753	2	2	2	2	0.81	Azurin OS=Pseudomon
1163	1	db$2	2::C3K819|C3K819_PSEFS	57	35012	1	1	1	1	0.14	Putative ABC transport
1164	1	db$2	2::C3JZU8|C3JZU8_PSEFS	57	30426	2	2	2	2	0.36	Hydrolase_4 domain-c
1165	1	db$2	2::C3K1A3|C3K1A3_PSEFS	57	26906	1	1	1	1	0.19	Putative pyridoxamine
1166	1	db$2	2::C3JZ44|C3JZ44_PSEFS	57	47432	2	2	2	2	0.22	Putative gluconate per
1167	1	SwissProt_HUMAN$	3::P07195|LDHB_HUMAN	57	36615	3	2	3	2	0.29	L-lactate dehydrogena
1168	1	db$2	2::C3KAW8|C3KAW8_PSEFS	57	75149	1	1	1	1	0.07	Alpha-1,4-glucan:malt
1169	1	db$2	2::C3K0S4|C3K0S4_PSEFS	56	65961	2	2	2	2	0.15	Isocitrate dehydrogena
1170	1	db$2	2::C3K364|C3K364_PSEFS	56	38824	2	2	2	2	0.28	Biotin synthase OS=Ps
1171	1	db$2	2::C3K6U7|C3K6U7_PSEFS	56	79141	6	4	6	4	0.27	Putative exported prot
1172	1	db$2	2::C3K1W7|C3K1W7_PSEFS	56	11247	3	2	3	2	1.29	DUF1244 domain-cont
1173	1	db$2	2::C3K425|C3K425_PSEFS	56	27295	3	2	2	2	0.41	Positive alginate biosy
1174	1	db$2	2::C3K101|C3K101_PSEFS	56	37185	3	3	3	3	0.46	Uncharacterized protei
1175	1	db$2	2::C3K5K0|C3K5K0_PSEFS	56	25438	4	4	4	4	1.10	Two-component syster
1176	1	db$2	2::C3KBM2|C3KBM2_PSEFS	56	16548	3	3	3	3	1.34	Cys-tRNA(Pro)/Cys-tRI
1177	1	db$2	2::C3K440|C3K440_PSEFS	56	11389	5	4	4	3	2.38	Uncharacterized protei
1178	1	db$2	2::C3KAX3|C3KAX3_PSEFS	56	30360	2	2	2	2	0.36	Conserved hypothetica
1179	1	db$2	2::C3K4M2|C3K4M2_PSEFS	55	36363	2	2	2	2	0.30	Catabolite repressor/a
1180	1	db$2	2::C3K491|C3K491_PSEFS	55	76137	1	1	1	1	0.06	ATP-dependent DNA he
1181	1	db$2	2::C3K3V4|C3K3V4_PSEFS	55	41512	1	1	1	1	0.12	Acetylornithine deacet
1182	1	db$2	2::C3K6B0|C3K6B0_PSEFS	55	33534	1	1	1	1	0.15	High-affinity zinc uptak
1183	1	db$2	2::C3KDC7|C3KDC7_PSEFS	55	29930	2	2	2	2	0.37	Release factor glutami
1184	1	db$2	2::C3KE45|C3KE45_PSEFS	55	49639	5	5	4	4	0.46	Trk system potassium
1185	1	db$2	2::C3K088|C3K088_PSEFS	55	20655	2	2	2	2	0.58	Putative isochorismata
1186	1	db$2	2::C3KDI4|C3KDI4_PSEFS	55	42151	4	4	4	4	0.57	Putative exported prot
1187	1	SwissProt_HUMAN$	3::P37802|TAGL2_HUMAN	55	22377	2	2	2	2	0.52	Transgelin-2 OS=Hom
1188	1	SwissProt_HUMAN$	3::P62263|RS14_HUMAN	55	16263	1	1	1	1	0.33	40S ribosomal protein
1189	1	db$2	2::C3K1M4|C3K1M4_PSEFS	54	13705	3	3	3	3	1.78	Iron-sulfur cluster asse
1190	1	db$2	2::C3JZP3|C3JZP3_PSEFS	54	36685	3	3	3	3	0.47	Putative inosine-uridin
1191	1	db$2	2::C3KDA5|C3KDA5_PSEFS	54	54170	1	1	1	1	0.09	Uncharacterized protei
1192	1	db$2	2::C3K591|C3K591_PSEFS	54	19872	3	3	3	3	1.03	Putative bacterioferriti
1193	1	db$2	2::C3K2S2|C3K2S2_PSEFS	54	38886	3	2	3	2	0.28	Riboflavin biosynthesis
1194	1	db$2	2::C3K3G8|C3K3G8_PSEFS	54	41555	1	1	1	1	0.12	Uncharacterized protei
1195	1	db$2	2::C3K0L5|C3K0L5_PSEFS	54	17844	1	1	1	1	0.30	Putative GNAT-family a
1196	1	db$2	2::C3JY53|CYSG_PSEFS	54	50259	4	4	4	4	0.46	Siroheme synthase OS

↑Family	M	DB	Accession	Score	Mass	Matches	Match(sig)	Sequences	Seq(sig)	emPAI	Description
1197	1	db$2	2::C3K481|C3K481_PSEFS	54	23434	3	2	3	2	0.49	Guanylate kinase OS=I
1198	1	db$2	2::C3KE85|C3KE85_PSEFS	53	26099	1	1	1	1	0.20	Purine nucleoside phos
1199	1	db$2	2::C3K9Y8|C3K9Y8_PSEFS	53	14463	1	1	1	1	0.38	Uncharacterized protei
1200	1	db$2	2::C3JY97|C3JY97_PSEFS	53	30357	1	1	1	1	0.17	N-hydroxyarylamine O-
1201	1	db$2	2::C3K6R2|C3K6R2_PSEFS	53	29309	2	2	2	2	0.38	Putative shikimate deh
1202	1	db$2	2::C3K6Z7|C3K6Z7_PSEFS	53	89469	8	5	8	5	0.30	Lon protease OS=Pseu
1203	1	db$2	2::C3K482|RPOZ_PSEFS	53	9821	4	3	3	2	3.13	DNA-directed RNA poly
1204	1	db$2	2::C3K1A2|C3K1A2_PSEFS	53	17206	1	1	1	1	0.31	Putative membrane pro
1205	1	SwissProt_HUMAN$	3::Q71UM5|RS27L_HUMAN	53	9471	1	1	1	1	0.63	40S ribosomal protein
1206	1	db$2	2::C3K6Z1|C3K6Z1_PSEFS	53	50711	3	2	3	2	0.21	Putative multicopper o:
1207	1	db$2	2::C3K595|C3K595_PSEFS	53	10805	2	2	2	2	1.36	Phosphate starvation-i
1208	1	db$2	2::C3JXX6|DNLJ_PSEFS	53	85515	4	4	4	4	0.25	DNA ligase OS=Pseudc
1209	1	db$2	2::C3K9Q2|C3K9Q2_PSEFS	52	16954	4	3	3	3	1.29	Ribonuclease H OS=Ps
1210	1	db$2	2::C3K2K4|C3K2K4_PSEFS	52	19926	1	1	1	1	0.26	Putative exported prote
1211	1	db$2	2::C3K2L9|LIPB_PSEFS	52	23473	1	1	1	1	0.22	Octanoyltransferase OS
1212	1	db$2	2::C3K5L0|C3K5L0_PSEFS	52	14786	1	1	1	1	0.37	Conserved hypothetica
1213	1	db$2	2::C3K609|C3K609_PSEFS	52	73644	3	2	3	2	0.14	Putative lytic transglyc
1214	1	db$2	2::C3K173|C3K173_PSEFS	52	34695	3	3	3	3	0.50	Putative AraC-family tr
1215	1	SwissProt_HUMAN$	3::Q02878|RL6_HUMAN	52	32708	1	1	1	1	0.16	60S ribosomal protein
1216	1	db$2	2::C3JZN0|C3JZN0_PSEFS	52	13917	2	2	2	2	0.96	MerR-family transcripti
1217	1	db$2	2::C3KDM5|C3KDM5_PSEFS	52	43829	5	4	4	3	0.38	O-phosphoserine phos
1218	1	db$2	2::C3JY28|Y3771_PSEFS	52	6626	2	2	1	1	0.98	UPF0434 protein PFLU_
1219	1	db$2	2::C3KDC8|C3KDC8_PSEFS	52	26520	1	1	1	1	0.19	Molybdopterin biosynth
1220	1	db$2	2::C3K7L3|C3K7L3_PSEFS	52	8069	2	1	1	1	0.76	Putative lipoprotein OS
1221	1	SwissProt_HUMAN$	3::Q2PPJ7|RGPA2_HUMAN	52	210636	2	2	2	2	0.05	Ral GTPase-activating
1221	2	SwissProt_HUMAN$	3::P61254|RL26_HUMAN	38	17248	2	2	2	2	0.72	60S ribosomal protein
1222	1	db$2	2::C3K0U7|C3K0U7_PSEFS	51	49884	3	3	3	3	0.33	Two-component respor
1223	1	SwissProt_HUMAN$	3::P61604|CH10_HUMAN	51	10925	4	3	4	3	2.59	10 kDa heat shock prot
1224	1	db$2	2::C3KB69|C3KB69_PSEFS	51	12397	1	1	1	1	0.45	Putative lipoprotein OS
1225	1	db$2	2::C3KBN5|C3KBN5_PSEFS	51	24125	1	1	1	1	0.22	Putative TetR-family re
1226	1	db$2	2::C3JXY6|ASTB_PSEFS	51	48741	4	4	4	4	0.47	N-succinylarginine dihy
1227	1	SwissProt_HUMAN$	3::P62847|RS24_HUMAN	51	15413	1	1	1	1	0.35	40S ribosomal protein
1228	1	db$2	2::C3K0K1|C3K0K1_PSEFS	51	53436	1	1	1	1	0.09	Putative GntR-family re
1229	1	SwissProt_HUMAN$	3::P08865|RSSA_HUMAN	51	32833	1	1	1	1	0.15	40S ribosomal protein
1230	1	db$2	2::C3K320|C3K320_PSEFS	51	34836	1	1	1	1	0.15	Uncharacterized protei
1231	1	db$2	2::C3K5D9|C3K5D9_PSEFS	51	43455	1	1	1	1	0.12	Putative cysteine desul
1232	1	db$2	2::C3KDB9|C3KDB9_PSEFS	51	32977	2	2	2	2	0.33	Putative lipopolysaccha
1233	1	db$2	2::C3K8H9|C3K8H9_PSEFS	51	41728	1	1	1	1	0.12	Putative polyamine AB(
1234	1	db$2	2::C3K8U0|HIS2_PSEFS	51	12007	2	2	2	2	1.17	Phosphoribosyl-ATP py
1235	1	db$2	2::C3K7N0|C3K7N0_PSEFS	51	28545	2	2	2	2	0.39	Transport permease pr

↑Family	M	DB	Accession	Score	Mass	Matches	Match(sig)	Sequences	Seq(sig)	emPAI	Description
1236	1	db$2	2::C3K328|APAG_PSEFS	51	13631	1	1	1	1	0.41	Protein ApaG OS=Pseu
1237	1	db$2	2::C3JYU4|C3JYU4_PSEFS	50	20911	1	1	1	1	0.25	Corrinoid adenosyltran
1238	1	db$2	2::C3K574|C3K574_PSEFS	50	36116	1	1	1	1	0.14	Putative oxidoreductas
1239	1	db$2	2::C3K5B2|C3K5B2_PSEFS	50	35714	2	2	2	2	0.30	Dipeptide ABC transpo
1240	1	db$2	2::C3KCP0|C3KCP0_PSEFS	50	31742	2	2	2	2	0.35	Ribosomal RNA large s
1241	1	db$2	2::C3KB17|C3KB17_PSEFS	50	51104	1	1	1	1	0.10	Outer membrane comp
1242	1	db$2	2::C3K098|C3K098_PSEFS	50	23000	1	1	1	1	0.23	Putative glutathione S-
1243	1	db$2	2::C3K224|C3K224_PSEFS	50	30473	2	2	1	1	0.17	CDP-diacylglycerol--se
1244	1	SwissProt_HUMAN$	3::P62851|RS25_HUMAN	50	13734	1	1	1	1	0.40	40S ribosomal protein
1245	1	db$2	2::C3KAB9|C3KAB9_PSEFS	50	40944	1	1	1	1	0.12	Putative acetyl-CoA ac
1246	1	SwissProt_HUMAN$	3::Q14240|IF4A2_HUMAN	49	46373	3	3	3	3	0.36	Eukaryotic initiation fa
1247	1	db$2	2::C3K735|MSRB_PSEFS	49	14471	2	1	2	1	0.38	Peptide methionine sul
1248	1	db$2	2::C3K0D5|MINE_PSEFS	49	9531	2	2	2	2	1.63	Cell division topologica
1249	1	db$2	2::C3JXL6|C3JXL6_PSEFS	49	41978	1	1	1	1	0.12	Bcr/CflA family efflux t
1250	1	db$2	2::C3K6Y0|C3K6Y0_PSEFS	49	13973	1	1	1	1	0.40	4-carboxymuconolacto
1251	1	db$2	2::C3K663|C3K663_PSEFS	49	22341	3	3	3	3	0.88	Putative TetR-family re
1252	1	SwissProt_HUMAN$	3::P63220|RS21_HUMAN	49	9106	2	2	2	2	1.75	40S ribosomal protein
1253	1	db$2	2::C3JYR0|C3JYR0_PSEFS	49	13774	2	2	2	2	0.97	Glycine cleavage syste
1254	1	db$2	2::C3K3Y9|C3K3Y9_PSEFS	49	73510	2	2	1	1	0.07	Putative membrane pr
1255	1	db$2	2::C3K261|RIMP_PSEFS	49	17653	3	2	3	2	0.70	Ribosome maturation f
1256	1	db$2	2::C3KCS6|C3KCS6_PSEFS	49	47227	2	2	2	2	0.22	UDP-N-acetylmuramoy
1257	1	db$2	2::C3K3U4|C3K3U4_PSEFS	49	23278	1	1	1	1	0.22	Putative TetR-family re
1258	1	SwissProt_HUMAN$	3::Q9H0U4|RAB1B_HUMAN	49	22157	1	1	1	1	0.24	Ras-related protein Ral
1259	1	db$2	2::C3KDJ8|C3KDJ8_PSEFS	49	23307	2	2	2	2	0.50	Methyltransf_25 domai
1260	1	db$2	2::C3K9R3|C3K9R3_PSEFS	48	18863	4	3	3	3	1.11	Uncharacterized protei
1261	1	db$2	2::C3KE82|C3KE82_PSEFS	48	53340	3	1	3	1	0.09	Aldehyde dehydrogena
1262	1	db$2	2::C3K2Y9|C3K2Y9_PSEFS	48	13142	2	2	2	2	1.04	Protein translocase sub
1263	1	db$2	2::C3KBK8|C3KBK8_PSEFS	48	35740	1	1	1	1	0.14	Ribosomal RNA small s
1264	1	db$2	2::C3JZ23|C3JZ23_PSEFS	48	131220	4	3	4	3	0.11	Chromosome partition
1265	1	db$2	2::C3K300|C3K300_PSEFS	48	34122	1	1	1	1	0.15	Putative membrane pr
1266	1	db$2	2::C3K6K2|IHFB_PSEFS	48	11021	4	3	3	3	2.54	Integration host factor
1267	1	db$2	2::C3K0P2|C3K0P2_PSEFS	48	35618	2	2	2	2	0.30	Pseudouridine synthase
1268	1	db$2	2::C3JYL7|C3JYL7_PSEFS	48	12417	1	1	1	1	0.45	Putative copper resista
1269	1	db$2	2::C3K5S7|C3K5S7_PSEFS	48	19211	1	1	1	1	0.28	Uncharacterized protei
1270	1	db$2	2::C3K0U4|C3K0U4_PSEFS	48	36872	7	4	6	4	0.67	Flagellar motor switch
1271	1	db$2	2::C3K047|C3K047_PSEFS	48	16614	2	2	2	2	0.75	Putative putative heat
1272	1	db$2	2::C3K8K1|C3K8K1_PSEFS	48	40207	1	1	1	1	0.12	Phosphate-binding prot
1273	1	db$2	2::C3JZ32|C3JZ32_PSEFS	48	38286	1	1	1	1	0.13	Putative cation transpo
1274	1	SwissProt_HUMAN$	3::P62277|RS13_HUMAN	48	17212	1	1	1	1	0.31	40S ribosomal protein
1275	1	db$2	2::C3JY69|HFLD_PSEFS	48	23173	1	1	1	1	0.22	High frequency lysoger

↑Family	M	DB	Accession	Score	Mass	Matches	Match(sig)	Sequences	Seq(sig)	emPAI	Description
1276	1	db$2	2::C3K301|C3K301_PSEFS	48	28901	3	3	3	3	0.63	Putative dehydrogenas
1277	1	db$2	2::C3K6A1|C3K6A1_PSEFS	47	22990	1	1	1	1	0.23	Putative copper-bindin
1278	1	db$2	2::C3K092|C3K092_PSEFS	47	14085	2	2	2	2	0.94	Glutaredoxin domain-c
1279	1	db$2	2::C3K1Y0|C3K1Y0_PSEFS	47	12222	1	1	1	1	0.46	Uncharacterized protei
1280	1	db$2	2::C3K5Y4|C3K5Y4_PSEFS	47	9690	1	1	1	1	0.61	Uncharacterized protei
1281	1	db$2	2::C3KDN1|C3KDN1_PSEFS	47	54005	4	3	4	3	0.30	Deoxyribodipyrimidine
1282	1	db$2	2::C3K456|C3K456_PSEFS	47	38683	1	1	1	1	0.13	Alanine racemase OS=
1283	1	db$2	2::C3K2V5|RL36_PSEFS	47	4431	1	1	1	1	1.73	50S ribosomal protein
1284	1	db$2	2::C3K5T5|C3K5T5_PSEFS	47	25405	1	1	1	1	0.20	Carbonic anhydrase OS
1285	1	SwissProt_HUMAN$	3::Q5VT06|CE350_HUMAN	47	350716	3	2	3	2	0.03	Centrosome-associatec
1286	1	db$2	2::C3KDV4|RSGA_PSEFS	47	37296	3	2	3	2	0.29	Small ribosomal subun
1287	1	SwissProt_HUMAN$	3::A6NJ78|MET15_HUMAN	47	46092	1	1	1	1	0.11	12S rRNA N4-methylcy
1288	1	SwissProt_HUMAN$	3::P06312|KV401_HUMAN	47	13372	1	1	1	1	0.42	Immunoglobulin kappa
1289	1	db$2	2::C3KE16|C3KE16_PSEFS	47	37004	3	3	3	3	0.47	Putative chemotaxis-re
1290	1	db$2	2::C3JYR2|C3JYR2_PSEFS	47	48934	1	1	1	1	0.10	L-serine dehydratase C
1291	1	SwissProt_HUMAN$	3::Q6IS14|IF5AL_HUMAN	47	16762	1	1	1	1	0.32	Eukaryotic translation i
1292	1	db$2	2::C3JXZ4|C3JXZ4_PSEFS	46	40619	1	1	1	1	0.12	Uncharacterized protei
1293	1	db$2	2::C3K453|C3K453_PSEFS	46	71119	3	2	3	2	0.14	AcetylCoA_hyd_C dom
1294	1	db$2	2::C3KC07|C3KC07_PSEFS	46	21449	1	1	1	1	0.25	YceI domain-containin
1295	1	db$2	2::C3KBY0|GMHA_PSEFS	46	21250	3	1	3	1	0.25	Phosphoheptose isome
1296	1	db$2	2::C3JXL2|C3JXL2_PSEFS	46	30967	1	1	1	1	0.16	Uncharacterized protei
1297	1	db$2	2::C3K250|C3K250_PSEFS	46	71198	1	1	1	1	0.07	Acetyl-coenzyme A syr
1298	1	db$2	2::C3KDC5|HEM1_PSEFS	46	46641	2	2	2	2	0.23	Glutamyl-tRNA reducta
1299	1	db$2	2::C3K4Z1|C3K4Z1_PSEFS	46	72318	3	3	2	2	0.14	Cytochrome C biogene
1300	1	db$2	2::C3KAR4|C3KAR4_PSEFS	46	11430	1	1	1	1	0.50	Uncharacterized protei
1301	1	db$2	2::C3K1W8|C3K1W8_PSEFS	46	11862	1	1	1	1	0.48	Uncharacterized protei
1302	1	db$2	2::C3JXZ6|C3JXZ6_PSEFS	46	24822	1	1	1	1	0.21	Putative histidine trans
1303	1	db$2	2::C3JZZ8|C3JZZ8_PSEFS	45	73265	3	3	2	2	0.14	Putative methyl-accept
1304	1	db$2	2::C3K0L0|C3K0L0_PSEFS	46	29492	2	2	2	2	0.38	Putative IclR-family reg
1305	1	db$2	2::C3K450|C3K450_PSEFS	45	61786	2	1	2	1	0.08	Putative EAL/GGDEF dc
1306	1	db$2	2::C3JYQ1|C3JYQ1_PSEFS	45	48078	1	1	1	1	0.10	Putative CoA transfera:
1307	1	db$2	2::C3K9N3|C3K9N3_PSEFS	45	10076	2	2	2	2	1.50	Peptidyl-prolyl cis-tran:
1308	1	db$2	2::C3K4U8|C3K4U8_PSEFS	45	20094	1	1	1	1	0.26	Conserved hypothetica
1309	1	db$2	2::C3K0T6|RRAAH_PSEFS	45	17479	1	1	1	1	0.31	Putative 4-hydroxy-4-r
1310	1	db$2	2::C3K6V0|C3K6V0_PSEFS	45	23354	2	2	2	2	0.50	Imidazole glycerol pho:
1311	1	db$2	2::C3KBZ4|C3KBZ4_PSEFS	45	6850	1	1	1	1	0.94	Uncharacterized protei
1312	1	db$2	2::C3KDC3|LOLB_PSEFS	45	23021	1	1	1	1	0.23	Outer-membrane lipop
1313	1	db$2	2::C3KAL0|C3KAL0_PSEFS	45	43962	1	1	1	1	0.11	PlsC domain-containin
1314	1	db$2	2::C3K223|C3K223_PSEFS	45	37863	2	2	2	2	0.28	Protein-methionine-sul
1315	1	db$2	2::C3K2P0|C3K2P0_PSEFS	45	22640	2	2	2	2	0.51	Flavin prenyltransferas

↑Family	M	DB	Accession	Score	Mass	Matches	Match(sig)	Sequences	Seq(sig)	emPAI	Description
1316	1	db$2	2::C3JY76|C3JY76_PSEFS	45	48249	4	3	4	3	0.34	Putative mechanosensi
1317	1	db$2	2::C3JY61|C3JY61_PSEFS	45	8297	4	3	3	3	4.23	Translation initiation fa
1318	1	db$2	2::C3K8T8|C3K8T8_PSEFS	45	14354	2	2	2	2	0.92	Sec-independent prote
1319	1	db$2	2::C3K7C7|C3K7C7_PSEFS	45	19943	1	1	1	1	0.26	Chorismate mutase OS
1320	1	SwissProt_HUMAN$	3::P61513|RL37A_HUMAN	45	10268	2	2	2	2	1.45	60S ribosomal protein
1321	1	db$2	2::C3K8U6|C3K8U6_PSEFS	45	15270	1	1	1	1	0.36	Putative polyhydroxyal
1322	1	db$2	2::C3K648|C3K648_PSEFS	44	33905	1	1	1	1	0.15	Conserved hypothetica
1323	1	db$2	2::C3KCK0|C3KCK0_PSEFS	44	26687	5	4	4	3	0.70	Putative short-chain de
1324	1	SwissProt_HUMAN$	3::P31153|METK2_HUMAN	44	43633	1	1	1	1	0.11	S-adenosylmethionine
1325	1	db$2	2::C3K0J2|C3K0J2_PSEFS	44	17877	1	1	1	1	0.30	Flagellar protein FliL O:
1326	1	db$2	2::C3K8Z2|C3K8Z2_PSEFS	44	25669	1	1	1	1	0.20	High-affinity branched-
1327	1	db$2	2::C3KDR4|C3KDR4_PSEFS	44	49531	3	2	3	2	0.21	Ribosomal protein S12
1327	2	SwissProt_HUMAN$	3::Q13439|GOGA4_HUMAN	30	260980	3	2	3	2	0.04	Golgin subfamily A me
1328	1	db$2	2::Q8VMR3|HUTH_PSEFS	44	54209	1	1	1	1	0.09	Histidine ammonia-lyas
1329	1	db$2	2::C3KC05|C3KC05_PSEFS	44	11915	1	1	1	1	0.48	Conserved hypothetica
1330	1	db$2	2::C3K2Z2|C3K2Z2_PSEFS	44	34551	1	1	1	1	0.15	Bifunctional ligase/repr
1331	1	db$2	2::C3K125|C3K125_PSEFS	44	26783	3	2	2	1	0.19	Alanine--tRNA ligase O
1332	1	db$2	2::C3K816|OPGH_PSEFS	44	96236	5	2	5	2	0.10	Glucans biosynthesis g
1333	1	db$2	2::C3K589|C3K589_PSEFS	44	50032	3	2	3	2	0.21	Transcriptional regulat
1334	1	db$2	2::C3K127|C3K127_PSEFS	44	41295	2	2	2	2	0.26	Putative membrane pr
1335	1	db$2	2::C3K386|C3K386_PSEFS	44	22957	2	2	2	2	0.51	Putative glutathione S-
1336	1	db$2	2::C3KAC0|C3KAC0_PSEFS	44	40546	2	1	2	1	0.12	Butyryl-CoA dehydroge
1337	1	db$2	2::C3K5B5|C3K5B5_PSEFS	44	37023	1	1	1	1	0.14	Dipeptide ABC transpo
1338	1	db$2	2::C3KBL6|C3KBL6_PSEFS	44	27732	2	2	2	2	0.40	Glutamate/aspartate A
1339	1	db$2	2::C3JXY3|C3JXY3_PSEFS	43	18788	2	2	2	2	0.65	6,7-dimethyl-8-ribityllu
1340	1	db$2	2::C3K5B3|C3K5B3_PSEFS	43	34937	2	2	2	2	0.31	Dipeptide ABC transpo
1341	1	db$2	2::C3K6G1|RLMF_PSEFS	43	38015	1	1	1	1	0.13	Ribosomal RNA large s
1342	1	db$2	2::C3K106|C3K106_PSEFS	43	20669	1	1	1	1	0.26	Uncharacterized protei
1343	1	SwissProt_HUMAN$	3::P62826|RAN_HUMAN	43	24408	2	2	2	2	0.47	GTP-binding nuclear pr
1344	1	db$2	2::C3K7T3|C3K7T3_PSEFS	43	20773	2	2	2	2	0.57	CDP-diacylglycerol--gly
1345	1	SwissProt_HUMAN$	3::P23284|PPIB_HUMAN	43	23728	1	1	1	1	0.22	Peptidyl-prolyl cis-tran:
1346	1	SwissProt_HUMAN$	3::P39019|RS19_HUMAN	43	16051	2	2	2	2	0.79	40S ribosomal protein
1347	1	db$2	2::C3KCV5|C3KCV5_PSEFS	43	37705	3	3	2	2	0.28	Uncharacterized protei
1348	1	db$2	2::C3K3W8|C3K3W8_PSEFS	43	44404	2	2	2	2	0.24	Putative ubiquinone bi
1349	1	db$2	2::C3KE76|C3KE76_PSEFS	43	92563	1	1	1	1	0.05	Alpha-E domain-contai
1350	1	db$2	2::C3KAA6|C3KAA6_PSEFS	43	51931	1	1	1	1	0.10	Putative cell envelope
1351	1	db$2	2::C3KCP6|C3KCP6_PSEFS	43	38240	3	2	3	2	0.28	ADP-heptose--LPS hep
1352	1	db$2	2::C3KAW3|C3KAW3_PSEFS	43	52085	1	1	1	1	0.10	Putative GntR-family tr
1353	1	db$2	2::C3JZS9|C3JZS9_PSEFS	43	35591	2	2	2	2	0.30	Cbb3-type cytochrome
1353	2	db$2	2::C3JYN0|C3JYN0_PSEFS	39	33944	3	3	2	2	0.32	Cbb3-type cytochrome

↑Family	M	DB	Accession	Score	Mass	Matches	Match(sig)	Sequences	Seq(sig)	emPAI	Description
1354	1	db$2	2::C3K6R7|C3K6R7_PSEFS	43	41832	1	1	1	1	0.12	Serine/threonine trans
1355	1	SwissProt_HUMAN$	3::P26373|RL13_HUMAN	43	24247	1	1	1	1	0.21	60S ribosomal protein
1356	1	db$2	2::C3K3H4|C3K3H4_PSEFS	42	26074	2	1	2	1	0.20	Ribosomal RNA small s
1357	1	SwissProt_HUMAN$	3::P62258|1433E_HUMAN	42	29155	3	3	2	2	0.62	14-3-3 protein epsilon
1358	1	db$2	2::C3K4A7|C3K4A7_PSEFS	42	18391	2	1	2	1	0.29	Uncharacterized protei
1359	1	db$2	2::C3K4I9|C3K4I9_PSEFS	42	42202	1	1	1	1	0.12	Galactonate dehydrata
1360	1	db$2	2::C3K664|C3K664_PSEFS	42	31550	1	1	1	1	0.16	Putative membrane pro
1361	1	db$2	2::C3K654|C3K654_PSEFS	42	34290	1	1	1	1	0.15	2-dehydropantoate 2-r
1362	1	db$2	2::C3K0Y8|COBT_PSEFS	42	36066	1	1	1	1	0.14	Nicotinate-nucleotide--
1363	1	db$2	2::C3K4L1|C3K4L1_PSEFS	42	41720	1	1	1	1	0.12	Putative epimerase OS
1364	1	db$2	2::C3K486|C3K486_PSEFS	42	30593	1	1	1	1	0.17	Epimerase domain-con
1365	1	db$2	2::C3KBX7|C3KBX7_PSEFS	42	23636	1	1	1	1	0.22	Putative metallo-beta-l
1366	1	db$2	2::C3K870|C3K870_PSEFS	42	20966	2	1	2	1	0.25	Uncharacterized protei
1367	1	SwissProt_HUMAN$	3::Q8NHW5|RLA0L_HUMAN	42	34343	1	1	1	1	0.15	60S acidic ribosomal p
1368	1	db$2	2::C3K3N1|C3K3N1_PSEFS	42	52088	2	2	2	2	0.20	Aldehyde dehydrogena
1369	1	db$2	2::C3K059|TRHO_PSEFS	42	35401	2	2	2	2	0.31	tRNA uridine(34) hydro
1370	1	db$2	2::C3K5L6|C3K5L6_PSEFS	42	16214	1	1	1	1	0.33	Putative phosphorous
1371	1	db$2	2::C3K4R0|C3K4R0_PSEFS	42	14930	1	1	1	1	0.37	Putative lipoprotein OS
1372	1	SwissProt_HUMAN$	3::Q5W0B1|OBI1_HUMAN	41	81066	1	1	1	1	0.06	ORC ubiquitin ligase 1
1373	1	db$2	2::C3K5G4|RLMM_PSEFS	41	40585	1	1	1	1	0.12	Ribosomal RNA large s
1374	1	db$2	2::C3K8U1|C3K8U1_PSEFS	41	15020	2	2	2	2	0.86	Phosphoribosyl-AMP cy
1375	1	db$2	2::C3K0U5|C3K0U5_PSEFS	41	62992	2	2	2	2	0.16	Flagellar M-ring protei
1376	1	db$2	2::C3K4K0|C3K4K0_PSEFS	41	42230	3	3	3	3	0.40	Hypothetical exported
1377	1	db$2	2::C3K2Q3|C3K2Q3_PSEFS	41	23107	1	1	1	1	0.23	Putative hydrolase OS=
1378	1	db$2	2::C3K0R1|C3K0R1_PSEFS	41	30842	2	2	2	2	0.36	Protein-glutamate O-m
1379	1	SwissProt_HUMAN$	3::P60900|PSA6_HUMAN	41	27382	1	1	1	1	0.19	Proteasome subunit alp
1380	1	db$2	2::C3K8L6|C3K8L6_PSEFS	41	28069	2	2	2	2	0.40	Precorrin-6A synthase
1381	1	db$2	2::C3K2M4|C3K2M4_PSEFS	41	40052	1	1	1	1	0.13	Peptidoglycan glycosylt
1382	1	db$2	2::C3KCS4|C3KCS4_PSEFS	41	63231	3	3	3	3	0.25	Peptidoglycan D,D-tran
1383	1	db$2	2::C3JYP8|C3JYP8_PSEFS	41	24937	1	1	1	1	0.21	Putative two-compone
1384	1	db$2	2::C3KBI7|C3KBI7_PSEFS	41	26715	2	2	2	2	0.42	Uncharacterized protei
1385	1	db$2	2::C3K8L0|C3K8L0_PSEFS	41	16885	1	1	1	1	0.32	Uncharacterized protei
1386	1	db$2	2::C3JYT2|C3JYT2_PSEFS	41	7867	1	1	1	1	0.79	CxxC_CXXC_SSSS don
1387	1	db$2	2::C3KBG3|C3KBG3_PSEFS	41	24033	4	4	4	4	1.18	Curli production assem
1388	1	db$2	2::C3K3R7|RPIA_PSEFS	41	23281	3	2	3	2	0.50	Ribose-5-phosphate isc
1389	1	db$2	2::C3K279|C3K279_PSEFS	40	19136	2	2	2	2	0.63	Outer membrane prote
1390	1	db$2	2::C3K8Z4|C3K8Z4_PSEFS	40	45613	2	2	2	2	0.23	High-affinity branched-
1391	1	SwissProt_HUMAN$	3::P15531|NDKA_HUMAN	40	17138	4	3	3	2	0.73	Nucleoside diphosphate
1392	1	db$2	2::C3K4A9|C3K4A9_PSEFS	40	17462	2	2	2	2	0.71	Uncharacterized protei
1393	1	db$2	2::C3K493|C3K493_PSEFS	40	16495	2	1	2	1	0.33	Uncharacterized protei

↑Family	M	DB	Accession	Score	Mass	Matches	Match(sig)	Sequences	Seq(sig)	emPAI	Description
1394	1	db$2	2::C3K3K0|YQGF_PSEFS	40	16158	1	1	1	1	0.33	Putative pre-16S rRNA
1395	1	db$2	2::C3K7W1|C3K7W1_PSEFS	40	12229	2	2	2	2	1.14	Putative lipoprotein OS
1396	1	db$2	2::C3K4U9|C3K4U9_PSEFS	40	34107	2	2	2	2	0.32	Putative LysR-family re
1397	1	SwissProt_HUMAN$	3::Q9HCU5|PREB_HUMAN	40	45440	2	2	2	2	0.23	Prolactin regulatory ele
1398	1	db$2	2::C3K166|C3K166_PSEFS	40	65025	1	1	1	1	0.08	Histidine kinase OS=Ps
1399	1	db$2	2::C3K4I1|C3K4I1_PSEFS	40	29333	2	2	2	2	0.38	Shikimate dehydrogena
1400	1	db$2	2::C3K2U2|C3K2U2_PSEFS	40	20033	3	3	3	3	1.02	Putative hydrolase OS=
1401	1	db$2	2::C3K2D6|C3K2D6_PSEFS	40	73930	4	3	4	3	0.21	Carbon starvation prot
1402	1	db$2	2::C3JYU5|C3JYU5_PSEFS	40	34090	1	1	1	1	0.15	Nudix hydrolase domai
1403	1	db$2	2::C3K2M6|RLMH_PSEFS	40	17669	2	2	2	2	0.70	Ribosomal RNA large s
1404	1	db$2	2::C3K7A2|C3K7A2_PSEFS	40	33841	1	1	1	1	0.15	Putative membrane pro
1405	1	SwissProt_HUMAN$	3::P05783|K1C18_HUMAN	40	48029	1	1	1	1	0.10	Keratin, type I cytoske
1406	1	db$2	2::C3K4W6|C3K4W6_PSEFS	40	44511	3	2	3	2	0.24	Putative lipoprotein rel
1407	1	SwissProt_HUMAN$	3::P62304|RUXE_HUMAN	39	10797	1	1	1	1	0.54	Small nuclear ribonucle
1408	1	db$2	2::C3K2M3|C3K2M3_PSEFS	39	37518	2	2	2	2	0.29	Putative peptidoglycan
1409	1	db$2	2::C3K4T5|C3K4T5_PSEFS	39	30974	2	2	2	2	0.36	Putative hydrolase OS=
1410	1	db$2	2::C3JZ52|C3JZ52_PSEFS	39	18715	1	1	1	1	0.28	Uncharacterized protei
1411	1	db$2	2::C3KBI5|C3KBI5_PSEFS	39	21236	1	1	1	1	0.25	Putative membrane pro
1412	1	db$2	2::C3K2F6|TRMA_PSEFS	39	40867	1	1	1	1	0.12	tRNA/tmRNA (uracil-C(
1413	1	db$2	2::C3K1G4|RL34_PSEFS	39	5064	1	1	1	1	1.41	50S ribosomal protein
1414	1	db$2	2::C3K6C3|C3K6C3_PSEFS	39	28259	1	1	1	1	0.18	Putative amino acid AB
1415	1	db$2	2::C3K413|C3K413_PSEFS	39	22663	1	1	1	1	0.23	Putative ABC transport
1416	1	db$2	2::C3JZN5|RL35_PSEFS	39	7363	2	2	1	1	0.87	50S ribosomal protein
1417	1	SwissProt_HUMAN$	3::P62318|SMD3_HUMAN	38	13907	1	1	1	1	0.40	Small nuclear ribonucle
1418	1	db$2	2::C3K3W4|C3K3W4_PSEFS	38	13233	2	2	2	2	1.02	Glycine cleavage syste
1419	1	db$2	2::C3KBB8|C3KBB8_PSEFS	38	13546	1	1	1	1	0.41	Uncharacterized protei
1420	1	db$2	2::C3K338|TSAD_PSEFS	38	36371	1	1	1	1	0.14	tRNA N6-adenosine thr
1421	1	SwissProt_HUMAN$	3::P62750|RL23A_HUMAN	38	17684	1	1	1	1	0.30	60S ribosomal protein
1422	1	db$2	2::C3K7M1|C3K7M1_PSEFS	38	62712	2	2	2	2	0.16	Putative dipeptidase OS
1423	1	db$2	2::C3K4U2|C3K4U2_PSEFS	38	24386	1	1	1	1	0.21	Uncharacterized protei
1424	1	SwissProt_HUMAN$	3::O14827|RGRF2_HUMAN	38	140675	2	2	2	2	0.07	Ras-specific guanine nu
1425	1	SwissProt_HUMAN$	3::Q8WYA0|IFT81_HUMAN	37	79696	1	1	1	1	0.06	Intraflagellar transport
1426	1	db$2	2::C3K0D2|C3K0D2_PSEFS	37	35834	3	3	2	2	0.30	Lipid A biosynthesis lau
1427	1	SwissProt_HUMAN$	3::P30050|RL12_HUMAN	37	17808	1	1	1	1	0.30	60S ribosomal protein
1428	1	db$2	2::C3K8K6|C3K8K6_PSEFS	37	15117	1	1	1	1	0.36	Putative transport-rela
1429	1	SwissProt_HUMAN$	3::P62753|RS6_HUMAN	37	28663	1	1	1	1	0.18	40S ribosomal protein
1430	1	SwissProt_HUMAN$	3::Q14697|GANAB_HUMAN	37	106807	2	2	2	2	0.09	Neutral alpha-glucosida
1431	1	db$2	2::C3K473|DUT_PSEFS	37	16022	1	1	1	1	0.34	Deoxyuridine 5'-tripho
1432	1	db$2	2::C3K308|TRPD_PSEFS	37	36976	2	2	2	2	0.29	Anthranilate phosphori
1433	1	db$2	2::C3JY27|KDSB_PSEFS	37	27609	3	2	3	2	0.41	3-deoxy-manno-octulo

↑Family	M	DB	Accession	Score	Mass	Matches	Match(sig)	Sequences	Seq(sig)	emPAI	Description
1434	1	db$2	2::C3K5H4|C3K5H4_PSEFS	37	21385	1	1	1	1	0.25	Uncharacterized protei
1435	1	db$2	2::C3K0S0|C3K0S0_PSEFS	37	12378	2	2	2	2	1.11	Putative DNA-binding p
1436	1	db$2	2::C3K9Z0|C3K9Z0_PSEFS	37	33445	1	1	1	1	0.15	LysR-family transcripti
1437	1	db$2	2::C3JXX9|C3JXX9_PSEFS	37	6925	3	2	3	2	2.76	Translational regulator
1438	1	SwissProt_HUMAN$	3::Q08379|GOGA2_HUMAN	37	113017	2	1	2	1	0.04	Golgin subfamily A mei
1439	1	SwissProt_HUMAN$	3::Q9NU22|MDN1_HUMAN	36	632420	3	2	2	1	0.01	Midasin OS=Homo sap
1440	1	db$2	2::C3K2Z1|COAX_PSEFS	36	26920	1	1	1	1	0.19	Type III pantothenate
1441	1	SwissProt_HUMAN$	3::P62269|RS18_HUMAN	36	17708	3	2	3	2	0.69	40S ribosomal protein
1442	1	SwissProt_HUMAN$	3::O94817|ATG12_HUMAN	36	15104	2	1	2	1	0.36	Ubiquitin-like protein A
1443	1	db$2	2::C3K7J9|C3K7J9_PSEFS	36	83444	2	1	2	1	0.06	Putative exported prot
1444	1	db$2	2::C3KE01|Y992_PSEFS	36	29383	4	4	4	4	0.90	UPF0246 protein PFLU_
1445	1	db$2	2::C3K2K1|C3K2K1_PSEFS	36	28157	3	3	3	3	0.65	Phos_pyr_kin domain-(
1446	1	SwissProt_HUMAN$	3::P07737|PROF1_HUMAN	36	15045	1	1	1	1	0.36	Profilin-1 OS=Homo sa
1447	1	db$2	2::C3KE42|C3KE42_PSEFS	36	20828	1	1	1	1	0.25	Putative DNA-3-methyl
1448	1	db$2	2::C3K753|C3K753_PSEFS	36	30652	1	1	1	1	0.17	Putative hydrolase OS=
1449	1	db$2	2::C3K7V7|C3K7V7_PSEFS	36	18111	1	1	1	1	0.30	Uncharacterized protei
1450	1	db$2	2::C3KBU2|C3KBU2_PSEFS	36	56055	3	1	3	1	0.09	Putative histidine amm
1451	1	db$2	2::C3KDM8|C3KDM8_PSEFS	36	57105	1	1	1	1	0.09	HDOD domain-containi
1452	1	db$2	2::C3K2S4|C3K2S4_PSEFS	36	15995	1	1	1	1	0.34	Putative lipoprotein OS
1453	1	db$2	2::C3KAX0|C3KAX0_PSEFS	36	84238	1	1	1	1	0.06	1,4-alpha-glucan branc
1454	1	SwissProt_HUMAN$	3::P31939|PUR9_HUMAN	36	64575	1	1	1	1	0.08	Bifunctional purine bio:
1455	1	db$2	2::C3K451|C3K451_PSEFS	36	76463	2	1	2	1	0.06	ATP-dependent DNA he
1456	1	db$2	2::C3K5B8|C3K5B8_PSEFS	34	59457	2	2	2	2	0.17	Dipeptide ABC transpoi
1457	1	SwissProt_HUMAN$	3::Q8NBF4|YG006_HUMAN	36	16172	1	1	1	1	0.33	Putative uncharacterize
1458	1	db$2	2::C3K4N5|C3K4N5_PSEFS	36	28830	1	1	1	1	0.18	Putative amino acid AB
1459	1	SwissProt_HUMAN$	3::Q7Z6Z7|HUWE1_HUMAN	35	481589	3	3	2	2	0.02	E3 ubiquitin-protein lig
1460	1	db$2	2::C3JZT8|C3JZT8_PSEFS	35	28261	1	1	1	1	0.18	Putative methyltransfe
1461	1	db$2	2::C3K537|C3K537_PSEFS	35	39684	1	1	1	1	0.13	Putative membrane pr(
1462	1	SwissProt_HUMAN$	3::Q5D862|FILA2_HUMAN	35	247928	1	1	1	1	0.02	Filaggrin-2 OS=Homo s
1463	1	SwissProt_HUMAN$	3::P63244|RACK1_HUMAN	35	35055	1	1	1	1	0.14	Receptor of activated p
1464	1	db$2	2::C3JY37|C3JY37_PSEFS	35	14829	2	2	2	2	0.87	Putative lipoprotein OS
1465	1	db$2	2::C3JXM0|C3JXM0_PSEFS	35	45141	2	1	2	1	0.11	Putative peptidase OS=
1466	1	SwissProt_HUMAN$	3::O75251|NDUS7_HUMAN	35	23548	1	1	1	1	0.22	NADH dehydrogenase |
1467	1	db$2	2::C3K2R8|NUSB_PSEFS	35	18764	2	2	2	2	0.65	Transcription antitermi
1468	1	db$2	2::C3JY58|C3JY58_PSEFS	35	87640	4	3	3	3	0.18	DNA translocase FtsK (
1469	1	db$2	2::Q9ZEP7|PANC_PSEFS	35	30696	1	1	1	1	0.17	Pantothenate syntheta:
1470	1	SwissProt_HUMAN$	3::P47900|P2RY1_HUMAN	35	42044	1	1	1	1	0.12	P2Y purinoceptor 1 OS
1471	1	db$2	2::C3KAN9|C3KAN9_PSEFS	35	28318	1	1	1	1	0.18	Putative aromatic-hydr
1472	1	SwissProt_HUMAN$	3::P26440|IVD_HUMAN	34	46621	1	1	1	1	0.11	Isovaleryl-CoA dehydrc
1473	1	SwissProt_HUMAN$	3::Q8IW45|NNRD_HUMAN	34	36553	1	1	1	1	0.14	ATP-dependent (S)-NA

↑Family	M	DB	Accession	Score	Mass	Matches	Match(sig)	Sequences	Seq(sig)	emPAI	Description
1474	1	db$2	2::C3K620|C3K620_PSEFS	34	22214	2	2	2	2	0.53	LexA repressor OS=Ps‹
1475	1	SwissProt_HUMAN$	3::P49207|RL34_HUMAN	34	13284	2	2	2	2	1.01	60S ribosomal protein
1476	1	db$2	2::C3K0B2|C3K0B2_PSEFS	34	20071	1	1	1	1	0.26	Putative membrane pr‹
1477	1	SwissProt_HUMAN$	3::P63173|RL38_HUMAN	34	8213	1	1	1	1	0.75	60S ribosomal protein
1478	1	db$2	2::C3KAA9|C3KAA9_PSEFS	34	31757	1	1	1	1	0.16	Putative chemotaxis pr
1479	1	db$2	2::C3KBB7|C3KBB7_PSEFS	34	43574	1	1	1	1	0.11	3-oxoacyl-[acyl-carrier
1480	1	SwissProt_HUMAN$	3::Q9UL62|TRPC5_HUMAN	34	111340	3	2	2	1	0.04	Short transient recepto
1481	1	db$2	2::C3K0H5|C3K0H5_PSEFS	34	25946	1	1	1	1	0.20	Putative chemotaxis pr
1482	1	db$2	2::C3K5Y3|C3K5Y3_PSEFS	34	35524	3	3	3	3	0.49	Putative leucine dehydı
1483	1	db$2	2::C3K3G3|C3K3G3_PSEFS	34	32186	1	1	1	1	0.16	Putative membrane pr‹
1484	1	db$2	2::C3KCP9|C3KCP9_PSEFS	34	30268	1	1	1	1	0.17	Lipopolysaccharide cor‹
1485	1	db$2	2::C3K7J8|C3K7J8_PSEFS	34	78803	2	2	2	2	0.13	ATP-dependent DNA he
1486	1	db$2	2::C3KDF1|C3KDF1_PSEFS	34	21060	1	1	1	1	0.25	Uncharacterized protei
1487	1	db$2	2::C3K0P5|C3K0P5_PSEFS	34	35572	2	2	2	2	0.30	Uncharacterized protei
1488	1	SwissProt_HUMAN$	3::P18627|LAG3_HUMAN	34	57413	1	1	1	1	0.09	Lymphocyte activation
1489	1	db$2	2::C3K0I8|C3K0I8_PSEFS	34	26895	1	1	1	1	0.19	Flagellar biosynthetic p
1490	1	db$2	2::C3K813|C3K813_PSEFS	33	36618	4	4	4	4	0.68	Proline iminopeptidase
1491	1	db$2	2::C3K3C7|C3K3C7_PSEFS	33	34452	2	2	2	2	0.32	Putative glycine betain
1492	1	db$2	2::C3K646|C3K646_PSEFS	33	21182	1	1	1	1	0.25	Putative acyl-CoA thio‹
1493	1	db$2	2::C3K6W7|C3K6W7_PSEFS	33	16216	2	1	2	1	0.33	Biotin carboxyl carrier
1494	1	SwissProt_HUMAN$	3::P62701|RS4X_HUMAN	33	29579	1	1	1	1	0.17	40S ribosomal protein
1495	1	SwissProt_HUMAN$	3::Q9HBJ7|UBP29_HUMAN	33	104090	1	1	1	1	0.05	Ubiquitin carboxyl-tern
1496	1	db$2	2::C3K3X9|C3K3X9_PSEFS	33	98433	3	2	3	2	0.10	Putative ABC transport
1497	1	db$2	2::C3KAM0|C3KAM0_PSEFS	33	50872	1	1	1	1	0.10	Putative two-compone‹
1498	1	db$2	2::C3K8W0|C3K8W0_PSEFS	33	34311	1	1	1	1	0.15	Putative LysR-family re
1499	1	SwissProt_HUMAN$	3::P27216|ANX13_HUMAN	33	35393	1	1	1	1	0.14	Annexin A13 OS=Hom‹
1500	1	db$2	2::C3JZH1|C3JZH1_PSEFS	33	34020	2	2	2	2	0.32	LysR-family transcripti
1501	1	db$2	2::C3JZA7|C3JZA7_PSEFS	33	51947	1	1	1	1	0.10	Putative succinate-sem
1502	1	db$2	2::C3KDQ6|C3KDQ6_PSEFS	33	14780	1	1	1	1	0.37	Uncharacterized protei
1503	1	SwissProt_HUMAN$	3::P06748|NPM_HUMAN	33	32555	1	1	1	1	0.16	Nucleophosmin OS=Hc
1504	1	db$2	2::C3K3H5|C3K3H5_PSEFS	33	32718	1	1	1	1	0.16	Putative ABC transport
1505	1	SwissProt_HUMAN$	3::P01876|IGHA1_HUMAN	33	37631	2	1	1	1	0.13	Immunoglobulin heavy
1506	1	db$2	2::C3K5Z0|C3K5Z0_PSEFS	33	15508	3	2	3	2	0.82	Uncharacterized protei
1507	1	db$2	2::C3K3F9|C3K3F9_PSEFS	33	16338	1	1	1	1	0.33	Putative exported cyto
1508	1	SwissProt_HUMAN$	3::P12883|MYH7_HUMAN	32	222959	1	1	1	1	0.02	Myosin-7 OS=Homo sa
1509	1	db$2	2::C3K7K0|C3K7K0_PSEFS	32	41297	2	1	2	1	0.12	Esterase III OS=Pseud
1510	1	SwissProt_HUMAN$	3::P57727|TMPS3_HUMAN	32	49373	1	1	1	1	0.10	Transmembrane protea
1511	1	db$2	2::C3K6S3|C3K6S3_PSEFS	32	25881	1	1	1	1	0.20	Putative glutathione S-
1512	1	db$2	2::C3JYE6|C3JYE6_PSEFS	32	45437	2	2	2	2	0.23	Putative acyl-CoA dehy
1513	1	db$2	2::C3K6Z6|C3K6Z6_PSEFS	32	13580	2	1	2	1	0.41	Putaitve lipoprotein OS

↑Family	M	DB	Accession	Score	Mass	Matches	Match(sig)	Sequences	Seq(sig)	emPAI	Description
1514	1	db$2	2::C3K6J0|C3K6J0_PSEFS	32	24596	1	1	1	1	0.21	Putative phosphoglycol
1515	1	db$2	2::C3KDW0|C3KDW0_PSEFS	32	49939	1	1	1	1	0.10	N-acetylmuramoyl-L-al
1516	1	SwissProt_HUMAN$	3::Q8IUR0|TPPC5_HUMAN	32	20770	1	1	1	1	0.25	Trafficking protein part
1517	1	SwissProt_HUMAN$	3::P17480|UBF1_HUMAN	32	89350	1	1	1	1	0.05	Nucleolar transcription
1518	1	db$2	2::C3KBE8|C3KBE8_PSEFS	32	8134	1	1	1	1	0.76	Putative lipoprotein OS
1519	1	db$2	2::C3K0H9|C3K0H9_PSEFS	32	13855	2	1	2	1	0.40	Chemotaxis protein Ch
1520	1	db$2	2::C3K6K7|C3K6K7_PSEFS	31	17395	1	1	1	1	0.31	GreA_GreB domain-cor
1521	1	SwissProt_HUMAN$	3::Q7Z624|CMKMT_HUMAN	31	36105	1	1	1	1	0.14	Calmodulin-lysine N-m
1522	1	db$2	2::C3K2P5|C3K2P5_PSEFS	31	49850	1	1	1	1	0.10	Ethanolamine ammonia
1523	1	db$2	2::C3K2P8|C3K2P8_PSEFS	31	24358	2	1	2	1	0.21	Membrane-related prot
1524	1	db$2	2::C3JYU3|C3JYU3_PSEFS	31	75598	1	1	1	1	0.06	Histidine kinase OS=Ps
1525	1	SwissProt_HUMAN$	3::Q92963|RIT1_HUMAN	31	25129	2	2	1	1	0.21	GTP-binding protein Rit
1526	1	db$2	2::C3JYG6|C3JYG6_PSEFS	31	44622	2	1	2	1	0.11	Peptidase_M14 domain
1527	1	SwissProt_HUMAN$	3::Q02413|DSG1_HUMAN	31	113676	1	1	1	1	0.04	Desmoglein-1 OS=Hon
1528	1	db$2	2::C3K916|C3K916_PSEFS	31	31897	1	1	1	1	0.16	Putative ABC transport
1529	1	db$2	2::C3JZ46|C3JZ46_PSEFS	31	37091	3	2	3	2	0.29	Putative LacI family tra
1530	1	db$2	2::C3KBZ5|C3KBZ5_PSEFS	31	23751	1	1	1	1	0.22	Endonuclease III OS=P
1531	1	db$2	2::C3K975|C3K975_PSEFS	31	21948	1	1	1	1	0.24	Putative glutathione S-
1532	1	SwissProt_HUMAN$	3::O43281|EFS_HUMAN	31	58779	1	1	1	1	0.08	Embryonal Fyn-associa
1533	1	db$2	2::A4V6U2|A4V6U2_PSEFS	31	76547	1	1	1	1	0.06	Uncharacterized protei
1534	1	db$2	2::C3K1Y4|C3K1Y4_PSEFS	31	14766	1	1	1	1	0.37	Putative putative glyox
1535	1	db$2	2::C3K200|C3K200_PSEFS	31	32052	1	1	1	1	0.16	Cat operon regulatory
1536	1	db$2	2::C3KE19|C3KE19_PSEFS	31	30253	1	1	1	1	0.17	HDOD domain-containi
1537	1	db$2	2::C3K5K8|C3K5K8_PSEFS	31	29952	1	1	1	1	0.17	Putative deoxyribonucl
1538	1	db$2	2::C3K5T7|C3K5T7_PSEFS	31	23769	3	2	2	2	0.49	Uncharacterized protei
1539	1	db$2	2::C3K5C4|C3K5C4_PSEFS	31	17510	1	1	1	1	0.31	SnoaL-like domain-con
1540	1	db$2	2::C3K4Z0|CCME_PSEFS	31	16101	1	1	1	1	0.34	Cytochrome c-type bio
1541	1	db$2	2::C3K9H2|C3K9H2_PSEFS	31	39760	3	3	3	3	0.43	Putrescine ABC transpo
1542	1	db$2	2::C3K1F5|C3K1F5_PSEFS	31	29048	1	1	1	1	0.18	Putative chromosome p
1543	1	db$2	2::C3KAF9|C3KAF9_PSEFS	31	50830	1	1	1	1	0.10	Poly-beta-1,6-N-acetyl
1544	1	SwissProt_HUMAN$	3::O14917|PCD17_HUMAN	31	126151	1	1	1	1	0.04	Protocadherin-17 OS=I
1545	1	db$2	2::C3K3T7|C3K3T7_PSEFS	30	39982	1	1	1	1	0.13	Putrescine ABC transpo
1546	1	db$2	2::C3KDB5|C3KDB5_PSEFS	30	33189	1	1	1	1	0.15	Putative glycosyl transf
1547	1	db$2	2::C3K1G3|C3K1G3_PSEFS	30	15162	1	1	1	1	0.36	Ribonuclease P protein
1548	1	SwissProt_HUMAN$	3::Q96E52|OMA1_HUMAN	30	60081	1	1	1	1	0.08	Metalloendopeptidase (
1549	1	db$2	2::C3K439|C3K439_PSEFS	30	25827	2	2	2	2	0.44	Putative hydrolase OS=
1550	1	db$2	2::C3K188|C3K188_PSEFS	30	39897	1	1	1	1	0.13	Putative exported prote
1551	1	db$2	2::C3K7C2|C3K7C2_PSEFS	30	16947	1	1	1	1	0.32	tRNA (cytidine(34)-2'-(
1552	1	SwissProt_HUMAN$	3::P26641|EF1G_HUMAN	30	50087	1	1	1	1	0.10	Elongation factor 1-gar
1553	1	db$2	2::C3K4C8|C3K4C8_PSEFS	30	49694	2	2	2	2	0.21	Putative transport-rela

↑Family	M	DB	Accession	Score	Mass	Matches	Match(sig)	Sequences	Seq(sig)	emPAI	Description
1554	1	db$2	2::C3K4C0|C3K4C0_PSEFS	30	9802	1	1	1	1	0.60	DNA-binding protein HU
1555	1	SwissProt_HUMAN$	3::O15078|CE290_HUMAN	30	290207	3	1	3	1	0.02	Centrosomal protein of
1556	1	SwissProt_HUMAN$	3::Q15365|PCBP1_HUMAN	30	37474	1	1	1	1	0.13	Poly(rC)-binding protei
1557	1	db$2	2::C3KB06|C3KB06_PSEFS	30	29724	1	1	1	1	0.17	Putative lipoprotein OS
1558	1	SwissProt_HUMAN$	3::Q5JXB2|UE2NL_HUMAN	30	17366	1	1	1	1	0.31	Putative ubiquitin-conj
1559	1	SwissProt_HUMAN$	3::Q8IWU5|SULF2_HUMAN	30	100390	1	1	1	1	0.05	Extracellular sulfatase
1560	1	SwissProt_HUMAN$	3::O00299|CLIC1_HUMAN	30	26906	1	1	1	1	0.19	Chloride intracellular cl
1561	1	SwissProt_HUMAN$	3::Q9ULQ1|TPC1_HUMAN	30	94086	1	1	1	1	0.05	Two pore calcium chan
1562	1	SwissProt_HUMAN$	3::Q7LBC6|KDM3B_HUMAN	30	191461	2	1	2	1	0.03	Lysine-specific demeth
1563	1	db$2	2::C3K9S5|C3K9S5_PSEFS	30	38309	1	1	1	1	0.13	Putative cobalamin bio
1564	1	db$2	2::C3K9Y9|C3K9Y9_PSEFS	30	13239	1	1	1	1	0.42	Uncharacterized protei
1565	1	SwissProt_HUMAN$	3::Q9Y265|RUVB1_HUMAN	30	50196	1	1	1	1	0.10	RuvB-like 1 OS=Homo
1566	1	db$2	2::C3KAF4|C3KAF4_PSEFS	30	50981	1	1	1	1	0.10	Putative lipoprotein OS
1567	1	db$2	2::C3K267|RLME_PSEFS	30	24125	2	2	2	2	0.48	Ribosomal RNA large s
1568	1	db$2	2::C3K6E9|C3K6E9_PSEFS	30	41977	2	2	2	2	0.25	Phosphatidylcholine-hy
1569	1	db$2	2::C3JZ74|C3JZ74_PSEFS	30	42038	1	1	1	1	0.12	ABC transport system,
1570	1	db$2	2::C3KDG3|C3KDG3_PSEFS	30	15637	2	1	2	1	0.35	Putative exported prote
1571	1	SwissProt_HUMAN$	3::P22626|ROA2_HUMAN	30	37407	2	2	2	2	0.29	Heterogeneous nuclear
1572	1	db$2	2::C3K3Y1|C3K3Y1_PSEFS	30	14748	1	1	1	1	0.37	Flagellar protein FliL O
1573	1	db$2	2::C3K1Q1|C3K1Q1_PSEFS	30	59090	1	1	1	1	0.08	Putative methyl accept
1574	1	db$2	2::C3K406|C3K406_PSEFS	29	23424	2	2	2	2	0.49	Glyoxalase OS=Pseudo
1575	1	db$2	2::C3K5E1|C3K5E1_PSEFS	29	13734	1	1	1	1	0.40	Uncharacterized protei
1576	1	SwissProt_HUMAN$	3::Q9NR48|ASH1L_HUMAN	29	332582	1	1	1	1	0.01	Histone-lysine N-methy
1577	1	db$2	2::C3JYS4|C3JYS4_PSEFS	29	16520	1	1	1	1	0.33	Tol-Pal system protein
1578	1	db$2	2::C3K732|TPMT_PSEFS	29	24819	1	1	1	1	0.21	Thiopurine S-methyltra
1579	1	db$2	2::C3K4H8|C3K4H8_PSEFS	29	20657	1	1	1	1	0.26	Threonylcarbamoyl-AM
1580	1	db$2	2::C3KE38|C3KE38_PSEFS	29	28697	3	2	3	2	0.39	Putative phospholipid b
1581	1	SwissProt_HUMAN$	3::Q5JTZ9|SYAM_HUMAN	29	107273	1	1	1	1	0.05	Alanine--tRNA ligase, r
1582	1	SwissProt_HUMAN$	3::Q6ZSJ9|SHSA6_HUMAN	29	55729	1	1	1	1	0.09	Protein shisa-6 OS=Ho
1583	1	SwissProt_HUMAN$	3::P28072|PSB6_HUMAN	29	25341	2	2	2	2	0.45	Proteasome subunit be
1584	1	db$2	2::C3KAI2|C3KAI2_PSEFS	29	19361	1	1	1	1	0.27	Shikimate kinase OS=P
1585	1	db$2	2::C3K8Z0|C3K8Z0_PSEFS	29	32439	1	1	1	1	0.16	UPF0276 protein PFLU_
1586	1	db$2	2::C3KAN8|C3KAN8_PSEFS	29	48228	1	1	1	1	0.10	3-carboxy-cis,cis-muco
1587	1	db$2	2::C3K4E5|C3K4E5_PSEFS	29	51748	1	1	1	1	0.10	Putative amino acid tra
1588	1	db$2	2::C3K6L2|C3K6L2_PSEFS	29	14328	1	1	1	1	0.38	Organic hydroperoxide
1589	1	SwissProt_HUMAN$	3::P10124|SRGN_HUMAN	29	17641	1	1	1	1	0.30	Serglycin OS=Homo sa
1590	1	SwissProt_HUMAN$	3::P10909|CLUS_HUMAN	29	52461	1	1	1	1	0.09	Clusterin OS=Homo sa
1591	1	SwissProt_HUMAN$	3::P35268|RL22_HUMAN	29	14778	1	1	1	1	0.37	60S ribosomal protein
1592	1	db$2	2::C3KBH6|C3KBH6_PSEFS	29	16609	1	1	1	1	0.32	Conserved hypothetica
1593	1	db$2	2::C3K3Q9|LGT_PSEFS	29	30444	3	2	2	2	0.36	Phosphatidylglycerol--p

↑Family	M	DB	Accession	Score	Mass	Matches	Match(sig)	Sequences	Seq(sig)	emPAI	Description
1594	1	SwissProt_HUMAN$	3::Q10713|MPPA_HUMAN	29	58216	1	1	1	1	0.08	Mitochondrial-processi
1595	1	db$2	2::C3KAH5|RL31_PSEFS	29	8451	2	1	1	1	0.72	50S ribosomal protein
1596	1	db$2	2::C3KDS6|UNG_PSEFS	29	25820	2	1	2	1	0.20	Uracil-DNA glycosylase
1597	1	SwissProt_HUMAN$	3::P25100|ADA1D_HUMAN	29	60424	2	1	2	1	0.08	Alpha-1D adrenergic re
1598	1	db$2	2::C3KBX4|C3KBX4_PSEFS	29	59754	1	1	1	1	0.08	Putative membrane pro
1599	1	db$2	2::C3K7L9|C3K7L9_PSEFS	29	28704	1	1	1	1	0.18	Putative membrane pro
1600	1	db$2	2::C3KBW9|C3KBW9_PSEFS	29	64127	1	1	1	1	0.08	Putative lipoprotein OS
1601	1	db$2	2::C3K2L3|C3K2L3_PSEFS	29	22416	1	1	1	1	0.23	LPS-assembly lipoprote
1602	1	db$2	2::C3KA92|C3KA92_PSEFS	28	24193	1	1	1	1	0.21	Putative ACP reductase
1603	1	SwissProt_HUMAN$	3::Q9BXF3|CECR2_HUMAN	28	164109	1	1	1	1	0.03	Cat eye syndrome criti
1604	1	db$2	2::C3KB02|C3KB02_PSEFS	28	42163	2	1	2	1	0.12	Putative exported prote
1605	1	db$2	2::C3KCP8|C3KCP8_PSEFS	28	42283	1	1	1	1	0.12	Lipopolysaccharide cor
1606	1	SwissProt_HUMAN$	3::P23396|RS3_HUMAN	28	26671	3	3	3	3	0.70	40S ribosomal protein
1607	1	db$2	2::C3K0P0|C3K0P0_PSEFS	28	35982	3	2	3	2	0.30	Putative peptidase OS=
1608	1	SwissProt_HUMAN$	3::Q96GD3|SCMH1_HUMAN	28	73307	1	1	1	1	0.07	Polycomb protein SCMI
1609	1	db$2	2::C3JXG8|C3JXG8_PSEFS	28	49590	1	1	1	1	0.10	Putative outer membra
1610	1	db$2	2::C3KCD6|C3KCD6_PSEFS	28	27732	1	1	1	1	0.19	Short chain dehydroge
1611	1	SwissProt_HUMAN$	3::P01009|A1AT_HUMAN	28	46707	2	1	2	1	0.11	Alpha-1-antitrypsin OS
1612	1	SwissProt_HUMAN$	3::Q5K651|SAMD9_HUMAN	28	184165	1	1	1	1	0.03	Sterile alpha motif dom
1613	1	SwissProt_HUMAN$	3::Q9Y5A7|NUB1_HUMAN	28	70494	3	1	3	1	0.07	NEDD8 ultimate buster
1614	1	db$2	2::C3K9P4|C3K9P4_PSEFS	28	59261	4	4	3	3	0.27	Putative ATP-binding c
1615	1	db$2	2::C3KAN3|C3KAN3_PSEFS	28	27088	1	1	1	1	0.19	3-oxoadipate CoA-tran
1616	1	db$2	2::C3K7A9|C3K7A9_PSEFS	28	33658	1	1	1	1	0.15	Conserved hypothetica
1617	1	db$2	2::C3KD16|C3KD16_PSEFS	28	34294	2	1	1	1	0.15	Putative 2-hydroxyacid
1618	1	SwissProt_HUMAN$	3::Q9UQE7|SMC3_HUMAN	28	141454	1	1	1	1	0.03	Structural maintenance
1619	1	db$2	2::C3K584|C3K584_PSEFS	28	15399	2	2	2	2	0.83	Uncharacterized protei
1620	1	db$2	2::C3K3H3|C3K3H3_PSEFS	28	52510	1	1	1	1	0.09	Adenosylmethionine-8-
1621	1	SwissProt_HUMAN$	3::Q8IZ83|A16A1_HUMAN	28	85074	1	1	1	1	0.06	Aldehyde dehydrogena
1622	1	db$2	2::C3K5D7|C3K5D7_PSEFS	28	29152	2	2	1	1	0.18	ThiF domain-containing
1623	1	SwissProt_HUMAN$	3::Q6ZVD8|PHLP2_HUMAN	28	146658	2	1	2	1	0.03	PH domain leucine-rich
1624	1	db$2	2::C3KDK6|C3KDK6_PSEFS	28	27451	1	1	1	1	0.19	Putative ABC transport
1625	1	db$2	2::C3K7D4|C3K7D4_PSEFS	28	125718	1	1	1	1	0.04	Putative membrane pro
1626	1	SwissProt_HUMAN$	3::P42694|HELZ_HUMAN	28	218833	3	1	3	1	0.02	Probable helicase with
1627	1	SwissProt_HUMAN$	3::Q71DI3|H32_HUMAN	27	15379	2	1	2	1	0.35	Histone H3.2 OS=Hom
1628	1	db$2	2::C3K5I1|C3K5I1_PSEFS	27	43688	1	1	1	1	0.11	Putative phage integra
1629	1	db$2	2::C3K7A1|C3K7A1_PSEFS	27	56003	3	2	2	2	0.18	Putative LuxR-family re
1630	1	db$2	2::C3K598|C3K598_PSEFS	27	34258	2	2	2	2	0.32	Curved DNA-binding pr
1631	1	db$2	2::C3K1Q2|C3K1Q2_PSEFS	27	73203	1	1	1	1	0.07	Histidine kinase OS=Ps
1632	1	SwissProt_HUMAN$	3::Q99996|AKAP9_HUMAN	27	452708	2	1	2	1	0.01	A-kinase anchor protei
1633	1	db$2	2::C3K5F2|Y1517_PSEFS	27	11422	1	1	1	1	0.50	YcgL domain-containin

↑Family	M	DB	Accession	Score	Mass	Matches	Match(sig)	Sequences	Seq(sig)	emPAI	Description
1634	1	db$2	2::C3K6B1|C3K6B1_PSEFS	27	35098	1	1	1	1	0.14	Homoserine kinase OS
1635	1	db$2	2::C3JYG1|C3JYG1_PSEFS	27	30646	2	2	2	2	0.36	Putative LysR-family tr
1636	1	db$2	2::C3K9B4|C3K9B4_PSEFS	27	16186	1	1	1	1	0.33	Type II secretion syste
1637	1	db$2	2::C3K4W5|C3K4W5_PSEFS	27	24663	2	1	2	1	0.21	Lipoprotein-releasing s
1638	1	db$2	2::C3K8L9|C3K8L9_PSEFS	27	65348	2	1	2	1	0.08	Putative potassium-effl
1639	1	db$2	2::C3K0G1|C3K0G1_PSEFS	27	67986	1	1	1	1	0.07	Putative acetyl-/propio
1640	1	SwissProt_HUMAN$	3::Q6PI48|SYDM_HUMAN	27	73516	1	1	1	1	0.07	Aspartate--tRNA ligase
1641	1	db$2	2::C3JYC8|C3JYC8_PSEFS	27	59331	1	1	1	1	0.08	Putative pH adaptation
1642	1	db$2	2::C3K3M5|C3K3M5_PSEFS	27	50101	2	2	2	2	0.21	Putative peptidase OS=
1643	1	SwissProt_HUMAN$	3::P05546|HEP2_HUMAN	27	57034	1	1	1	1	0.09	Heparin cofactor 2 OS=
1644	1	db$2	2::C3JYR6|C3JYR6_PSEFS	27	38174	1	1	1	1	0.13	Quinolinate synthase A
1645	1	db$2	2::C3JY90|NUOK_PSEFS	27	10944	1	1	1	1	0.52	NADH-quinone oxidore
1646	1	db$2	2::C3KDK7|C3KDK7_PSEFS	27	24465	2	1	2	1	0.21	Putative ABC transport
1647	1	db$2	2::C3K158|C3K158_PSEFS	26	36421	2	2	2	2	0.30	Putative regulatory pro
1648	1	SwissProt_HUMAN$	3::Q9BY11|PACN1_HUMAN	26	50934	3	2	2	1	0.10	Protein kinase C and ca
1649	1	SwissProt_HUMAN$	3::Q99618|CDCA3_HUMAN	26	28981	1	1	1	1	0.18	Cell division cycle-asso
1650	1	SwissProt_HUMAN$	3::Q9BQG2|NUD12_HUMAN	26	52043	1	1	1	1	0.10	Peroxisomal NADH pyr
1651	1	SwissProt_HUMAN$	3::Q02080|MEF2B_HUMAN	26	38615	1	1	1	1	0.13	Myocyte-specific enhar
1652	1	db$2	2::C3KCP7|C3KCP7_PSEFS	26	39236	1	1	1	1	0.13	Lipopolysaccharide hep
1653	1	db$2	2::C3K2G8|C3K2G8_PSEFS	26	27283	1	1	1	1	0.19	Putative TetR-family re
1654	1	SwissProt_HUMAN$	3::Q9C098|DCLK3_HUMAN	26	73768	1	1	1	1	0.07	Serine/threonine-prote
1655	1	db$2	2::C3K705|SURE_PSEFS	26	26431	1	1	1	1	0.20	5'-nucleotidase SurE O
1656	1	db$2	2::C3K7M8|C3K7M8_PSEFS	26	23309	2	1	2	1	0.22	Putative glutathione S-
1657	1	db$2	2::C3K746|C3K746_PSEFS	26	78906	2	1	1	1	0.06	Putative TonB-depende
1658	1	db$2	2::C3K9N4|C3K9N4_PSEFS	26	91939	1	1	1	1	0.05	Histidine kinase OS=Ps
1659	1	SwissProt_HUMAN$	3::Q9BYK8|HELZ2_HUMAN	26	294465	1	1	1	1	0.02	Helicase with zinc finge
1660	1	SwissProt_HUMAN$	3::P62266|RS23_HUMAN	26	15798	1	1	1	1	0.35	40S ribosomal protein
1661	1	db$2	2::C3KB65|C3KB65_PSEFS	26	37899	2	1	2	1	0.13	Methylated-DNA--[prot
1662	1	db$2	2::C3JY29|LPXK_PSEFS	26	37235	1	1	1	1	0.14	Tetraacyldisaccharide 4
1663	1	db$2	2::C3K3M8|C3K3M8_PSEFS	26	36638	1	1	1	1	0.14	AB hydrolase-1 domair
1664	1	db$2	2::C3K8T6|C3K8T6_PSEFS	26	26078	2	1	2	1	0.20	Ribosomal RNA small s
1665	1	db$2	2::C3KCS9|FTSW_PSEFS	26	44522	1	1	1	1	0.11	Probable peptidoglycar
1666	1	db$2	2::C3K8V8|C3K8V8_PSEFS	26	36166	2	1	2	1	0.14	Zinc-type alcohol dehy
1667	1	db$2	2::C3KCU6|C3KCU6_PSEFS	26	23028	2	1	1	1	0.23	Putative membrane prc
1668	1	db$2	2::C3K2L1|C3K2L1_PSEFS	26	28054	1	1	1	1	0.18	DUF218 domain-contai
1669	1	SwissProt_HUMAN$	3::Q75VX8|GARE2_HUMAN	26	92824	1	1	1	1	0.05	GRB2-associated and r
1670	1	SwissProt_HUMAN$	3::A6NJB7|PRR19_HUMAN	26	38692	1	1	1	1	0.13	Proline-rich protein 19
1671	1	db$2	2::C3KBV2|C3KBV2_PSEFS	26	15686	1	1	1	1	0.35	Thioredoxin OS=Pseud
1672	1	SwissProt_HUMAN$	3::O15270|SPTC2_HUMAN	26	62884	1	1	1	1	0.08	Serine palmitoyltransfe
1673	1	SwissProt_HUMAN$	3::O75197|LRP5_HUMAN	26	179030	2	1	1	1	0.03	Low-density lipoproteir

↑Family	M	DB	Accession	Score	Mass	Matches	Match(sig)	Sequences	Seq(sig)	emPAI	Description
1674	1	db$2	2::C3K660|C3K660_PSEFS	26	28970	1	1	1	1	0.18	Hydroxypyruvate isom
1675	1	db$2	2::C3K751|C3K751_PSEFS	26	15943	1	1	1	1	0.34	Putative thioesterase C
1676	1	db$2	2::C3JY59|LFTR_PSEFS	26	25331	1	1	1	1	0.20	Leucyl/phenylalanyl-tR
1677	1	db$2	2::C3KDB6|C3KDB6_PSEFS	26	73565	1	1	1	1	0.07	Putative membrane pr
1678	1	SwissProt_HUMAN$	3::P0DMR1|HNRC4_HUMAN	26	32010	1	1	1	1	0.16	Heterogeneous nuclear
1679	1	SwissProt_HUMAN$	3::P27708|PYR1_HUMAN	26	242829	1	1	1	1	0.02	CAD protein OS=Homo
1680	1	db$2	2::C3KB61|C3KB61_PSEFS	26	26669	1	1	1	1	0.19	Molybdate-binding peri
1681	1	db$2	2::C3K2E8|C3K2E8_PSEFS	26	37117	1	1	1	1	0.14	Putative cytochrome o
1682	1	db$2	2::C3K749|C3K749_PSEFS	26	16397	1	1	1	1	0.33	Putative phosphohistid
1683	1	SwissProt_HUMAN$	3::Q9Y5I4|PCDC2_HUMAN	26	109383	3	1	1	1	0.04	Protocadherin alpha-C
1684	1	SwissProt_HUMAN$	3::Q9Y2Q0|AT8A1_HUMAN	26	131285	2	1	2	1	0.04	Phospholipid-transport
1685	1	SwissProt_HUMAN$	3::P33992|MCM5_HUMAN	26	82233	2	1	1	1	0.06	DNA replication licensi
1686	1	db$2	2::C3KEB8|C3KEB8_PSEFS	25	34155	1	1	1	1	0.15	Putative lipoprotein OS
1687	1	db$2	2::C3K3J5|C3K3J5_PSEFS	25	13219	2	2	1	1	0.42	Type IV pilus response
1688	1	db$2	2::C3K9P2|C3K9P2_PSEFS	25	69747	2	1	2	1	0.07	Putative substrate-binc
1689	1	db$2	2::C3K3T1|C3K3T1_PSEFS	25	86048	2	1	2	1	0.06	Putative penicillin amid
1690	1	SwissProt_HUMAN$	3::Q9Y4G8|RPGF2_HUMAN	25	167313	1	1	1	1	0.03	Rap guanine nucleotide
1691	1	db$2	2::C3K265|C3K265_PSEFS	25	29739	4	4	3	3	0.61	Dihydropteroate synth
1692	1	SwissProt_HUMAN$	3::O75051|PLXA2_HUMAN	25	210969	2	1	2	1	0.02	Plexin-A2 OS=Homo sa
1693	1	SwissProt_HUMAN$	3::Q96AQ6|PBIP1_HUMAN	25	80594	1	1	1	1	0.06	Pre-B-cell leukemia tra
1694	1	SwissProt_HUMAN$	3::Q9NQX4|MYO5C_HUMAN	25	202682	1	1	1	1	0.02	Unconventional myosi
1695	1	SwissProt_HUMAN$	3::A6NM36|LRC30_HUMAN	25	34012	1	1	1	1	0.15	Leucine-rich repeat-co
1696	1	db$2	2::C3K637|C3K637_PSEFS	25	56441	1	1	1	1	0.09	Putative peptide ABC t
1697	1	SwissProt_HUMAN$	3::Q7Z6B0|CCD91_HUMAN	25	49941	3	1	3	1	0.10	Coiled-coil domain-con
1698	1	db$2	2::C3K0E4|C3K0E4_PSEFS	25	48130	1	1	1	1	0.10	Sensor protein QseC O
1699	1	db$2	2::C3K784|C3K784_PSEFS	25	23456	2	2	2	2	0.49	3-oxoadipate CoA-tran
1700	1	SwissProt_HUMAN$	3::P31946|1433B_HUMAN	25	28065	1	1	1	1	0.18	14-3-3 protein beta/al
1701	1	db$2	2::C3KBT8|C3KBT8_PSEFS	25	29541	1	1	1	1	0.17	Sulfate transport syste
1702	1	db$2	2::C3KDB4|C3KDB4_PSEFS	25	42765	1	1	1	1	0.12	Putative glycosyl trans
1703	1	SwissProt_HUMAN$	3::Q14746|COG2_HUMAN	25	83155	2	2	2	2	0.12	Conserved oligomeric
1704	1	db$2	2::C3K704|TRUD_PSEFS	25	38571	2	1	2	1	0.13	tRNA pseudouridine sy
1705	1	db$2	2::C3KAS1|C3KAS1_PSEFS	25	34986	1	1	1	1	0.14	Putative 2-hydroxyacid
1706	1	db$2	2::C3K1F1|ATPL_PSEFS	25	8603	3	3	1	1	1.89	ATP synthase subunit
1707	1	db$2	2::C3K5M2|C3K5M2_PSEFS	25	41151	1	1	1	1	0.12	Phosphorous compoun
1708	1	db$2	2::C3KB13|C3KB13_PSEFS	25	35564	1	1	1	1	0.14	Putative ThiF protein O
1709	1	SwissProt_HUMAN$	3::Q14129|DGCR6_HUMAN	25	24973	1	1	1	1	0.21	Protein DGCR6 OS=Ho
1710	1	db$2	2::C3K994|C3K994_PSEFS	25	23940	1	1	1	1	0.22	Putative regulatory pro
1711	1	db$2	2::C3KAD1|C3KAD1_PSEFS	25	32401	3	1	3	1	0.16	Probable 4-deoxy-4-fo
1712	1	db$2	2::C3KCQ3|C3KCQ3_PSEFS	25	36116	1	1	1	1	0.14	Putative type III secret
1713	1	db$2	2::C3KDU6|C3KDU6_PSEFS	24	27726	1	1	1	1	0.19	Putative two-compone

↑Family	M	DB	Accession	Score	Mass	Matches	Match(sig)	Sequences	Seq(sig)	emPAI	Description
1714	1	db$2	2::C3JXL1|C3JXL1_PSEFS	24	51972	1	1	1	1	0.10	Putative transmembrar
1715	1	db$2	2::C3KCB4|C3KCB4_PSEFS	24	27191	1	1	1	1	0.19	Uncharacterized protei
1716	1	db$2	2::C3KD58|C3KD58_PSEFS	24	41746	1	1	1	1	0.12	Putative cystathionine
1717	1	db$2	2::C3K323|C3K323_PSEFS	24	38795	2	2	2	2	0.28	Putative phosphotransf
1718	1	SwissProt_HUMAN$	3::O95153|RIMB1_HUMAN	24	199928	1	1	1	1	0.02	Peripheral-type benzod
1719	1	SwissProt_HUMAN$	3::P0DKB5|TPBGL_HUMAN	24	40374	1	1	1	1	0.12	Trophoblast glycoprote
1720	1	SwissProt_HUMAN$	3::Q5SRN2|TSBP1_HUMAN	24	61588	1	1	1	1	0.08	Testis-expressed basic
1721	1	db$2	2::C3K0N1|C3K0N1_PSEFS	24	29172	1	1	1	1	0.18	4-amino-4-deoxychoris
1722	1	db$2	2::C3KDL9|C3KDL9_PSEFS	24	22175	1	1	1	1	0.24	Uncharacterized protei
1723	1	db$2	2::C3KBL5|C3KBL5_PSEFS	24	24580	1	1	1	1	0.21	Glutamate/aspartate A
1724	1	db$2	2::C3K8Y2|C3K8Y2_PSEFS	24	49522	2	1	1	1	0.10	Putative two-componen
1725	1	db$2	2::C3K3D4|C3K3D4_PSEFS	24	52472	2	1	2	1	0.09	Uncharacterized protei
1726	1	SwissProt_HUMAN$	3::P81605|DCD_HUMAN	24	11277	1	1	1	1	0.51	Dermcidin OS=Homo s
1727	1	db$2	2::C3K8T9|TATA_PSEFS	24	10325	1	1	1	1	0.57	Sec-independent prote
1728	1	SwissProt_HUMAN$	3::P50607|TUB_HUMAN	24	55617	2	1	2	1	0.09	Tubby protein homolog
1729	1	db$2	2::C3K1S4|C3K1S4_PSEFS	24	23334	1	1	1	1	0.22	Putative membrane pro
1730	1	db$2	2::C3K136|C3K136_PSEFS	24	31053	2	2	2	2	0.35	Uncharacterized protei
1731	1	SwissProt_HUMAN$	3::P62136|PP1A_HUMAN	24	37488	1	1	1	1	0.13	Serine/threonine-prote
1732	1	SwissProt_HUMAN$	3::O43272|PROD_HUMAN	24	67959	1	1	1	1	0.07	Proline dehydrogenase
1733	1	db$2	2::C3K7I5|C3K7I5_PSEFS	24	28342	2	2	2	2	0.40	Putative iron utilisation
1734	1	SwissProt_HUMAN$	3::P32969|RL9_HUMAN	24	21850	1	1	1	1	0.24	60S ribosomal protein
1735	1	db$2	2::C3K802|C3K802_PSEFS	24	20330	1	1	1	1	0.26	Uncharacterized protei
1736	1	db$2	2::C3K0Y3|C3K0Y3_PSEFS	24	20085	1	1	1	1	0.26	Glutathione peroxidase
1737	1	db$2	2::C3K3F2|C3K3F2_PSEFS	24	38228	2	1	2	1	0.13	D-erythrose-4-phospha
1738	1	db$2	2::C3K2M2|C3K2M2_PSEFS	24	35893	1	1	1	1	0.14	Endolytic peptidoglycar
1739	1	db$2	2::C3KE39|C3KE39_PSEFS	24	18976	1	1	1	1	0.28	D,D-heptose 1,7-bisph
1740	1	db$2	2::C3KB05|C3KB05_PSEFS	24	50891	1	1	1	1	0.10	CDP-diacylglycerol-seri
1741	1	db$2	2::C3K672|C3K672_PSEFS	24	58670	2	2	2	2	0.18	Cation/acetate sympor
1742	1	db$2	2::C3K918|C3K918_PSEFS	23	26912	1	1	1	1	0.19	Putative ABC transport
1743	1	SwissProt_HUMAN$	3::Q9H9E3|COG4_HUMAN	23	89026	1	1	1	1	0.05	Conserved oligomeric (
1744	1	SwissProt_HUMAN$	3::O94827|PKHG5_HUMAN	23	117377	1	1	1	1	0.04	Pleckstrin homology dc
1745	1	db$2	2::C3K029|C3K029_PSEFS	23	46068	1	1	1	1	0.11	Uncharacterized protei
1746	1	SwissProt_HUMAN$	3::P60903|S10AA_HUMAN	23	11196	1	1	1	1	0.51	Protein S100-A10 OS=
1747	1	SwissProt_HUMAN$	3::O95819|M4K4_HUMAN	23	142013	2	1	2	1	0.03	Mitogen-activated prot
1748	1	SwissProt_HUMAN$	3::Q4KMP7|TB10B_HUMAN	23	87145	1	1	1	1	0.06	TBC1 domain family m
1749	1	db$2	2::C3JY42|C3JY42_PSEFS	23	25186	1	1	1	1	0.21	Uncharacterized protei
1750	1	SwissProt_HUMAN$	3::Q8N8R5|CB069_HUMAN	23	43421	1	1	1	1	0.12	UPF0565 protein C2orf
1751	1	db$2	2::C3K321|C3K321_PSEFS	23	28336	1	1	1	1	0.18	Putative heat shock pro
1752	1	db$2	2::C3KAA5|C3KAA5_PSEFS	23	23984	3	2	2	1	0.22	Uncharacterized protei
1753	1	db$2	2::C3KBB3|C3KBB3_PSEFS	23	202817	2	1	1	1	0.02	Helicase ATP-binding d

↑Family	M	DB	Accession	Score	Mass	Matches	Match(sig)	Sequences	Seq(sig)	emPAI	Description
1754	1	SwissProt_HUMAN$	3::Q6ZMT4|KDM7A_HUMAN	23	106489	1	1	1	1	0.05	Lysine-specific demeth
1755	1	SwissProt_HUMAN$	3::Q96AY4|TTC28_HUMAN	23	270715	2	1	2	1	0.02	Tetratricopeptide repea
1756	1	SwissProt_HUMAN$	3::Q9NZJ4|SACS_HUMAN	23	520795	2	1	2	1	0.01	Sacsin OS=Homo sapie
1757	1	SwissProt_HUMAN$	3::P53396|ACLY_HUMAN	23	120762	1	1	1	1	0.04	ATP-citrate synthase O
1758	1	SwissProt_HUMAN$	3::Q96JK2|DCAF5_HUMAN	23	103899	1	1	1	1	0.05	DDB1- and CUL4-assoc
1759	1	SwissProt_HUMAN$	3::P36507|MP2K2_HUMAN	23	44396	1	1	1	1	0.11	Dual specificity mitoge
1760	1	SwissProt_HUMAN$	3::Q9NTJ3|SMC4_HUMAN	23	147091	1	1	1	1	0.03	Structural maintenance
1761	1	SwissProt_HUMAN$	3::Q8TD08|MK15_HUMAN	23	59795	1	1	1	1	0.08	Mitogen-activated prot
1762	1	db$2	2::C3KE93|C3KE93_PSEFS	23	22964	1	1	1	1	0.23	Dephospho-CoA kinase
1763	1	db$2	2::C3KE21|C3KE21_PSEFS	23	10230	2	2	2	2	1.47	FAD assembly factor S
1764	1	SwissProt_HUMAN$	3::P16471|PRLR_HUMAN	23	69461	1	1	1	1	0.07	Prolactin receptor OS=
1765	1	SwissProt_HUMAN$	3::O94967|WDR47_HUMAN	23	101884	2	1	2	1	0.05	WD repeat-containing
1766	1	db$2	2::C3K600|C3K600_PSEFS	23	28761	1	1	1	1	0.18	Phosphatidate cytidylyl
1767	1	SwissProt_HUMAN$	3::Q6PCB6|AB17C_HUMAN	23	35808	1	1	1	1	0.14	Alpha/beta hydrolase d
1768	1	db$2	2::C3K7J4|C3K7J4_PSEFS	23	18330	2	2	1	1	0.29	Uncharacterized protei
1769	1	db$2	2::C3K4F9|C3K4F9_PSEFS	23	31102	1	1	1	1	0.16	Putative regulatory pro
1770	1	db$2	2::C3K685|C3K685_PSEFS	23	13635	1	1	1	1	0.41	6-carboxy-5,6,7,8-tetr
1771	1	db$2	2::C3K2L8|LIPA_PSEFS	23	38163	3	1	3	1	0.13	Lipoyl synthase OS=Ps
1772	1	db$2	2::C3JY43|TTCA_PSEFS	23	31052	1	1	1	1	0.16	tRNA-cytidine(32) 2-su
1773	1	db$2	2::C3K5X3|C3K5X3_PSEFS	23	64999	1	1	1	1	0.08	Putative ABC transport
1774	1	SwissProt_HUMAN$	3::P08397|HEM3_HUMAN	23	39306	1	1	1	1	0.13	Porphobilinogen deami
1775	1	SwissProt_HUMAN$	3::Q9Y2B5|VP9D1_HUMAN	23	68913	1	1	1	1	0.07	VPS9 domain-containir
1776	1	db$2	2::C3JXK0|C3JXK0_PSEFS	23	17939	1	1	1	1	0.30	Putative acetyltransfer
1777	1	SwissProt_HUMAN$	3::Q96JG8|MAGD4_HUMAN	23	81327	1	1	1	1	0.06	Melanoma-associated a
1778	1	db$2	2::C3K7G6|C3K7G6_PSEFS	22	30473	1	1	1	1	0.17	Putative hydrolase OS=
1779	1	db$2	2::C3K0N6|PLSX_PSEFS	22	35209	1	1	1	1	0.14	Phosphate acyltransfer
1780	1	db$2	2::C3JXS7|C3JXS7_PSEFS	22	34119	1	1	1	1	0.15	Putative GGDEF domai
1781	1	SwissProt_HUMAN$	3::Q7Z5H4|VN1R5_HUMAN	22	40752	1	1	1	1	0.12	Vomeronasal type-1 re
1782	1	db$2	2::C3K0Y2|C3K0Y2_PSEFS	22	46222	2	2	2	2	0.23	Putative fatty acid tran
1783	1	db$2	2::C3JXP2|C3JXP2_PSEFS	22	43336	1	1	1	1	0.12	Putative glycosyltransf
1784	1	db$2	2::C3K0X1|C3K0X1_PSEFS	22	10638	1	1	1	1	0.54	Uncharacterized protei
1785	1	SwissProt_HUMAN$	3::Q9H244|P2Y12_HUMAN	22	39413	1	1	1	1	0.13	P2Y purinoceptor 12 O
1786	1	db$2	2::C3K7D0|THII_PSEFS	22	54519	2	2	2	2	0.19	tRNA sulfurtransferase
1787	1	SwissProt_HUMAN$	3::Q9Y399|RT02_HUMAN	22	33228	1	1	1	1	0.15	28S ribosomal protein
1788	1	db$2	2::C3K546|C3K546_PSEFS	22	33665	1	1	1	1	0.15	Putative lipopolysaccha
1789	1	SwissProt_HUMAN$	3::Q8NDY3|ARHL1_HUMAN	22	40079	2	1	2	1	0.13	[Protein ADP-ribosylarg
1790	1	db$2	2::C3JXR0|C3JXR0_PSEFS	22	12414	1	1	1	1	0.45	Uncharacterized protei
1791	1	SwissProt_HUMAN$	3::Q86YF9|DZIP1_HUMAN	22	98602	1	1	1	1	0.05	Zinc finger protein DZI
1792	1	db$2	2::C3KCP2|C3KCP2_PSEFS	22	100291	1	1	1	1	0.05	Conserved GGDEF/EAL
1793	1	db$2	2::C3K3T5|C3K3T5_PSEFS	22	34240	2	1	2	1	0.15	Putrescine ABC transpo

↑Family	M	DB	Accession	Score	Mass	Matches	Match(sig)	Sequences	Seq(sig)	emPAI	Description
1794	1	db$2	2::C3K353|C3K353_PSEFS	22	44910	1	1	1	1	0.11	Putative aminotransfer
1795	1	SwissProt_HUMAN$	3::Q11130|FUT7_HUMAN	22	39214	1	1	1	1	0.13	Alpha-(1,3)-fucosyltrar
1796	1	SwissProt_HUMAN$	3::Q96A44|SPSB4_HUMAN	22	30160	1	1	1	1	0.17	SPRY domain-containir
1797	1	db$2	2::C3KAX6|C3KAX6_PSEFS	22	25593	1	1	1	1	0.20	Putative acetyltransfer
1798	1	db$2	2::C3K1F6|C3K1F6_PSEFS	22	23787	1	1	1	1	0.22	Ribosomal RNA small s
1799	1	db$2	2::C3KAJ5|C3KAJ5_PSEFS	22	83174	2	2	2	2	0.12	Putative paraquat-indu
1800	1	db$2	2::C3K1Z0|C3K1Z0_PSEFS	22	34051	1	1	1	1	0.15	Putative putative polys
1801	1	db$2	2::C3K0J8|C3K0J8_PSEFS	22	22091	1	1	1	1	0.24	START domain-contain
1802	1	db$2	2::C3K0X4|C3K0X4_PSEFS	22	79071	1	1	1	1	0.06	DNA helicase OS=Pseu
1803	1	db$2	2::C3K694|C3K694_PSEFS	22	48962	1	1	1	1	0.10	[utative peptidase OS=
1804	1	SwissProt_HUMAN$	3::Q53EZ4|CEP55_HUMAN	22	54145	1	1	1	1	0.09	Centrosomal protein of
1805	1	SwissProt_HUMAN$	3::P02741|CRP_HUMAN	22	25023	2	2	2	2	0.46	C-reactive protein OS=
1806	1	db$2	2::C3JZP4|RBSD_PSEFS	22	14393	1	1	1	1	0.38	D-ribose pyranase OS=
1807	1	SwissProt_HUMAN$	3::P61353|RL27_HUMAN	22	15788	2	1	2	1	0.35	60S ribosomal protein
1808	1	db$2	2::C3K1D6|C3K1D6_PSEFS	22	16178	1	1	1	1	0.33	Putative membrane pro
1809	1	SwissProt_HUMAN$	3::P29590|PML_HUMAN	22	97489	1	1	1	1	0.05	Protein PML OS=Homo
1810	1	db$2	2::C3KAP7|C3KAP7_PSEFS	22	26475	1	1	1	1	0.19	Putative short-chain de
1811	1	db$2	2::C3K678|C3K678_PSEFS	22	45414	1	1	1	1	0.11	Cytosine deaminase OS
1812	1	db$2	2::C3JYJ2|C3JYJ2_PSEFS	22	27205	1	1	1	1	0.19	Putative lipoprotein OS
1813	1	SwissProt_HUMAN$	3::P55199|ELL_HUMAN	22	68223	2	1	2	1	0.07	RNA polymerase II elor
1814	1	db$2	2::C3JY63|CLPS_PSEFS	22	13780	1	1	1	1	0.40	ATP-dependent Clp pro
1815	1	db$2	2::C3K5T8|C3K5T8_PSEFS	22	41768	1	1	1	1	0.12	Cytochrome c oxidase
1816	1	SwissProt_HUMAN$	3::Q17RB8|LONF1_HUMAN	22	86669	1	1	1	1	0.06	LON peptidase N-termi
1817	1	SwissProt_HUMAN$	3::P18077|RL35A_HUMAN	21	12530	1	1	1	1	0.45	60S ribosomal protein
1818	1	db$2	2::C3JXT0|C3JXT0_PSEFS	21	22445	1	1	1	1	0.23	Putative isochorismata
1819	1	db$2	2::C3K5M3|PHNN_PSEFS	21	20351	1	1	1	1	0.26	Ribose 1,5-bisphospha
1820	1	SwissProt_HUMAN$	3::Q14185|DOCK1_HUMAN	21	215209	1	1	1	1	0.02	Dedicator of cytokinesi
1821	1	db$2	2::C3KBT7|C3KBT7_PSEFS	21	36676	3	2	3	2	0.29	Sulfate ABC transport s
1822	1	db$2	2::C3K3P8|C3K3P8_PSEFS	21	49421	1	1	1	1	0.10	G domain-containing p
1823	1	db$2	2::C3K4T8|C3K4T8_PSEFS	21	33681	1	1	1	1	0.15	Putative AraC-family re
1824	1	db$2	2::C3KB68|C3KB68_PSEFS	21	11363	3	1	3	1	0.51	DUF1883 domain-conta
1825	1	db$2	2::C3K492|C3K492_PSEFS	21	51080	1	1	1	1	0.10	HDOD domain-containi
1826	1	db$2	2::C3KB08|C3KB08_PSEFS	21	43028	1	1	1	1	0.12	Putative exported prote
1827	1	SwissProt_HUMAN$	3::O95347|SMC2_HUMAN	21	135572	1	1	1	1	0.04	Structural maintenance
1828	1	SwissProt_HUMAN$	3::Q3SY89|ELB3B_HUMAN	21	59723	1	1	1	1	0.08	Elongin-A3 member B
1829	1	SwissProt_HUMAN$	3::Q674X7|KAZRN_HUMAN	21	86298	1	1	1	1	0.06	Kazrin OS=Homo sapie
1830	1	db$2	2::C3KBV8|C3KBV8_PSEFS	21	32477	1	1	1	1	0.16	Cobalt-zinc-cadmium r
1831	1	SwissProt_HUMAN$	3::Q9H6P5|TASP1_HUMAN	21	44427	1	1	1	1	0.11	Threonine aspartase 1
1832	1	db$2	2::C3K418|C3K418_PSEFS	21	24448	1	1	1	1	0.21	Peptidyl-prolyl cis-tran
1833	1	SwissProt_HUMAN$	3::P62244|RS15A_HUMAN	21	14830	2	1	2	1	0.37	40S ribosomal protein

↑Family	M	DB	Accession	Score	Mass	Matches	Match(sig)	Sequences	Seq(sig)	emPAI	Description
1834	1	SwissProt_HUMAN$	3::Q14139|UBE4A_HUMAN	21	122482	1	1	1	1	0.04	Ubiquitin conjugation fa
1835	1	SwissProt_HUMAN$	3::Q9BT17|MTG1_HUMAN	21	37213	1	1	1	1	0.14	Mitochondrial ribosome
1836	1	db$2	2::C3K7U0|C3K7U0_PSEFS	21	43041	1	1	1	1	0.12	Putative dehydrogenas
1837	1	SwissProt_HUMAN$	3::Q9BRZ2|TRI56_HUMAN	21	81436	1	1	1	1	0.06	E3 ubiquitin-protein lig
1838	1	db$2	2::C3K0H8|C3K0H8_PSEFS	21	29525	1	1	1	1	0.17	Protein phosphatase Cl
1839	1	db$2	2::C3K5C6|C3K5C6_PSEFS	21	33902	1	1	1	1	0.15	Uncharacterized protei
1840	1	SwissProt_HUMAN$	3::Q9UBN4|TRPC4_HUMAN	21	112030	1	1	1	1	0.04	Short transient recepto
1841	1	SwissProt_HUMAN$	3::Q9NSI6|BRWD1_HUMAN	21	262772	1	1	1	1	0.02	Bromodomain and WD
1842	1	db$2	2::C3K340|C3K340_PSEFS	21	72802	1	1	1	1	0.07	DNA primase OS=Pseu
1843	1	SwissProt_HUMAN$	3::P62280|RS11_HUMAN	21	18419	2	1	2	1	0.29	40S ribosomal protein
1844	1	db$2	2::C3K629|C3K629_PSEFS	21	51787	1	1	1	1	0.10	Putative transport-rela
1845	1	db$2	2::C3JYP1|C3JYP1_PSEFS	21	35226	1	1	1	1	0.14	Uncharacterized protei
1846	1	SwissProt_HUMAN$	3::P31943|HNRH1_HUMAN	21	49198	1	1	1	1	0.10	Heterogeneous nuclear
1847	1	db$2	2::C3K5T2|C3K5T2_PSEFS	20	65173	1	1	1	1	0.08	Putative dehydrogenas
1848	1	db$2	2::C3K1M3|C3K1M3_PSEFS	20	11692	1	1	1	1	0.49	Iron-binding protein Is
1849	1	SwissProt_HUMAN$	3::P08476|INHBA_HUMAN	20	47412	1	1	1	1	0.11	Inhibin beta A chain OS
1850	1	SwissProt_HUMAN$	3::Q8NEZ4|KMT2C_HUMAN	20	541028	1	1	1	1	0.01	Histone-lysine N-methy
1851	1	SwissProt_HUMAN$	3::Q8N7X0|ADGB_HUMAN	20	189594	1	1	1	1	0.03	Androglobin OS=Homo
1852	1	db$2	2::C3K963|C3K963_PSEFS	20	78429	1	1	1	1	0.06	Putative TonB-depende
1853	1	SwissProt_HUMAN$	3::Q9Y231|FUT9_HUMAN	20	42044	1	1	1	1	0.12	4-galactosyl-N-acetylgl
1854	1	db$2	2::C3K9R9|C3K9R9_PSEFS	20	86327	1	1	1	1	0.06	Putative exported fatty
1855	1	SwissProt_HUMAN$	3::Q9NZT2|OGFR_HUMAN	20	73280	1	1	1	1	0.07	Opioid growth factor re
1856	1	SwissProt_HUMAN$	3::Q9UBV2|SE1L1_HUMAN	20	88698	1	1	1	1	0.05	Protein sel-1 homolog
1857	1	db$2	2::C3K814|DTD_PSEFS	20	15391	1	1	1	1	0.35	D-aminoacyl-tRNA dea
1858	1	db$2	2::C3K1V0|C3K1V0_PSEFS	20	56633	1	1	1	1	0.09	Nitric oxide reductase s
1859	1	db$2	2::C3K1J8|C3K1J8_PSEFS	20	26668	1	1	1	1	0.19	Trehalose operon trans
1860	1	SwissProt_HUMAN$	3::P52333|JAK3_HUMAN	20	125019	1	1	1	1	0.04	Tyrosine-protein kinase
1861	1	SwissProt_HUMAN$	3::Q5T7B8|KIF24_HUMAN	20	151809	1	1	1	1	0.03	Kinesin-like protein KIF
1862	1	db$2	2::C3K5W0|C3K5W0_PSEFS	20	170278	2	2	2	2	0.06	DUF4329 domain-conta
1863	1	db$2	2::C3K109|C3K109_PSEFS	20	49568	1	1	1	1	0.10	23S rRNA (uracil(1939)
1864	1	SwissProt_HUMAN$	3::A6NNT2|CP096_HUMAN	20	124963	1	1	1	1	0.04	Uncharacterized protei
1865	1	SwissProt_HUMAN$	3::O95696|BRD1_HUMAN	20	119444	1	1	1	1	0.04	Bromodomain-containi
1866	1	SwissProt_HUMAN$	3::P62241|RS8_HUMAN	20	24190	1	1	1	1	0.21	40S ribosomal protein
1867	1	db$2	2::C3K4Y8|C3K4Y8_PSEFS	20	27802	1	1	1	1	0.19	Heme exporter protein
1868	1	db$2	2::C3K832|C3K832_PSEFS	20	33163	1	1	1	1	0.15	Non-specific serine/thr
1869	1	SwissProt_HUMAN$	3::Q86TG7|PEG10_HUMAN	20	80122	1	1	1	1	0.06	Retrotransposon-derive
1870	1	SwissProt_HUMAN$	3::Q9H1R3|MYLK2_HUMAN	20	64644	1	1	1	1	0.08	Myosin light chain kina
1871	1	SwissProt_HUMAN$	3::Q9Y3A2|UTP11_HUMAN	20	30428	1	1	1	1	0.17	Probable U3 small nucl
1872	1	db$2	2::C3K590|C3K590_PSEFS	20	74072	1	1	1	1	0.07	Putative membrane pro
1873	1	SwissProt_HUMAN$	3::P36956|SRBP1_HUMAN	20	121599	1	1	1	1	0.04	Sterol regulatory eleme

↑Family	M	DB	Accession	Score	Mass	Matches	Match(sig)	Sequences	Seq(sig)	emPAI	Description
1874	1	SwissProt_HUMAN$	3::Q8TBM8|DJB14_HUMAN	20	42489	1	1	1	1	0.12	DnaJ homolog subfami
1875	1	db$2	2::C3K3E2|C3K3E2_PSEFS	20	17110	1	1	1	1	0.31	Putative acetyltransfera
1876	1	SwissProt_HUMAN$	3::Q9P0Z9|SOX_HUMAN	20	44038	1	1	1	1	0.11	Peroxisomal sarcosine
1877	1	db$2	2::C3KAG2|C3KAG2_PSEFS	20	280833	1	1	1	1	0.02	Toxin CdiA OS=Pseudo
1878	1	db$2	2::C3K6J8|C3K6J8_PSEFS	20	78979	1	1	1	1	0.06	3-phosphoshikimate 1-
1879	1	db$2	2::C3K2Q9|EX7S_PSEFS	20	8900	2	1	2	1	0.68	Exodeoxyribonuclease
1880	1	db$2	2::C3K7B0|C3K7B0_PSEFS	20	28681	1	1	1	1	0.18	Putative ABC transport
1881	1	db$2	2::C3K908|C3K908_PSEFS	20	33577	1	1	1	1	0.15	Putative dehydrogenas
1882	1	SwissProt_HUMAN$	3::P62910|RL32_HUMAN	20	15850	1	1	1	1	0.34	60S ribosomal protein
1883	1	db$2	2::C3K0X8|C3K0X8_PSEFS	20	55461	1	1	1	1	0.09	DNA recombination pro
1884	1	SwissProt_HUMAN$	3::Q6P1X5|TAF2_HUMAN	20	136883	1	1	1	1	0.04	Transcription initiation
1885	1	SwissProt_HUMAN$	3::Q99623|PHB2_HUMAN	20	33276	1	1	1	1	0.15	Prohibitin-2 OS=Homo
1886	1	db$2	2::C3K0X5|C3K0X5_PSEFS	20	21842	1	1	1	1	0.24	Uncharacterized protei
1887	1	SwissProt_HUMAN$	3::Q13835|PKP1_HUMAN	20	82808	1	1	1	1	0.06	Plakophilin-1 OS=Hom
1888	1	SwissProt_HUMAN$	3::P04080|CYTB_HUMAN	20	11133	1	1	1	1	0.52	Cystatin-B OS=Homo s
1889	1	db$2	2::C3JZT3|C3JZT3_PSEFS	20	31726	1	1	1	1	0.16	tRNA pseudouridine sy
1890	1	SwissProt_HUMAN$	3::Q5TGY3|AHDC1_HUMAN	20	168245	2	1	1	1	0.03	AT-hook DNA-binding r
1891	1	db$2	2::C3K8V7|C3K8V7_PSEFS	20	6725	1	1	1	1	0.96	Uncharacterized protei
1892	1	SwissProt_HUMAN$	3::Q9P2D0|IBTK_HUMAN	20	150432	1	1	1	1	0.03	Inhibitor of Bruton tyro
1893	1	SwissProt_HUMAN$	3::Q8TCU5|NMD3A_HUMAN	19	125385	2	2	1	1	0.04	Glutamate receptor ion
1894	1	SwissProt_HUMAN$	3::A6NI79|CCD69_HUMAN	19	34775	3	1	3	1	0.15	Coiled-coil domain-con
1895	1	db$2	2::C3K8B2|C3K8B2_PSEFS	19	12313	1	1	1	1	0.46	HTH cro/C1-type doma
1896	1	db$2	2::C3K2H9|C3K2H9_PSEFS	19	43500	1	1	1	1	0.11	Uncharacterized protei
1897	1	SwissProt_HUMAN$	3::P49642|PRI1_HUMAN	19	49870	1	1	1	1	0.10	DNA primase small sub
1898	1	db$2	2::C3KBN1|C3KBN1_PSEFS	19	62708	2	1	2	1	0.08	Chaperone OS=Pseudo
1899	1	SwissProt_HUMAN$	3::Q86V20|SHLD2_HUMAN	19	93646	1	1	1	1	0.05	Shieldin complex subu
1900	1	SwissProt_HUMAN$	3::P53814|SMTN_HUMAN	19	98998	1	1	1	1	0.05	Smoothelin OS=Homo
1901	1	db$2	2::C3K597|C3K597_PSEFS	19	46587	1	1	1	1	0.11	Putative chaperone/hea
1902	1	db$2	2::C3K863|PDXH_PSEFS	19	24884	1	1	1	1	0.21	Pyridoxine/pyridoxamir
1903	1	SwissProt_HUMAN$	3::Q7Z7L1|SLN11_HUMAN	19	102770	1	1	1	1	0.05	Schlafen family membe
1904	1	SwissProt_HUMAN$	3::Q9H1Z9|TSN10_HUMAN	19	36475	1	1	1	1	0.14	Tetraspanin-10 OS=Ho
1905	1	db$2	2::C3KAB6|C3KAB6_PSEFS	19	39151	1	1	1	1	0.13	Conserved hypothetica
1906	1	db$2	2::C3K6U1|C3K6U1_PSEFS	19	17245	1	1	1	1	0.31	Conserved hypothetica
1907	1	SwissProt_HUMAN$	3::O00469|PLOD2_HUMAN	19	84632	1	1	1	1	0.06	Procollagen-lysine,2-ox
1908	1	db$2	2::C3K4W4|C3K4W4_PSEFS	19	45211	2	2	2	2	0.23	Putative lipoprotein rel
1909	1	db$2	2::C3KCN9|C3KCN9_PSEFS	19	45566	1	1	1	1	0.11	Putative homocysteine
1910	1	SwissProt_HUMAN$	3::P54886|P5CS_HUMAN	19	87248	1	1	1	1	0.06	Delta-1-pyrroline-5-car
1911	1	db$2	2::C3K327|C3K327_PSEFS	19	29832	1	1	1	1	0.17	Ribosomal RNA small s
1912	1	db$2	2::C3K2G9|C3K2G9_PSEFS	19	70101	1	1	1	1	0.07	3-dehydroshikimate de
1913	1	SwissProt_HUMAN$	3::Q09666|AHNK_HUMAN	19	628699	2	1	2	1	0.01	Neuroblast differentiati

↑Family	M	DB	Accession	Score	Mass	Matches	Match(sig)	Sequences	Seq(sig)	emPAI	Description
1914	1	db$2	2::C3K883|C3K883_PSEFS	19	60772	1	1	1	1	0.08	Putative transposase su
1915	1	db$2	2::C3K831|C3K831_PSEFS	19	45359	2	2	2	2	0.23	Putative thiolase OS=P
1916	1	SwissProt_HUMAN$	3::Q9Y4K0|LOXL2_HUMAN	19	86668	1	1	1	1	0.06	Lysyl oxidase homolog
1917	1	SwissProt_HUMAN$	3::Q8IVL0|NAV3_HUMAN	19	255491	2	1	2	1	0.02	Neuron navigator 3 OS
1918	1	db$2	2::C3K3M2|C3K3M2_PSEFS	19	37029	1	1	1	1	0.14	Cell division protein Fts
1919	1	SwissProt_HUMAN$	3::P06850|CRF_HUMAN	19	21408	1	1	1	1	0.25	Corticoliberin OS=Hom
1920	1	SwissProt_HUMAN$	3::P31040|SDHA_HUMAN	19	72645	1	1	1	1	0.07	Succinate dehydrogena
1921	1	SwissProt_HUMAN$	3::P04075|ALDOA_HUMAN	19	39395	1	1	1	1	0.13	Fructose-bisphosphate
1922	1	SwissProt_HUMAN$	3::Q8NFP9|NBEA_HUMAN	19	327614	1	1	1	1	0.01	Neurobeachin OS=Hom
1923	1	db$2	2::C3KAP1|C3KAP1_PSEFS	19	35319	1	1	1	1	0.14	Conserved hypothetica
1924	1	SwissProt_HUMAN$	3::A6NMS3|OR5K4_HUMAN	19	36686	1	1	1	1	0.14	Olfactory receptor 5K4
1925	1	db$2	2::C3K2F9|C3K2F9_PSEFS	19	48516	1	1	1	1	0.10	Putative ABC transport
1926	1	SwissProt_HUMAN$	3::Q6P0Q8|MAST2_HUMAN	19	196315	2	1	2	1	0.02	Microtubule-associated
1927	1	db$2	2::C3K3Q3|C3K3Q3_PSEFS	19	61523	2	1	2	1	0.08	Putative cation ABC tra
1928	1	db$2	2::C3K9E9|C3K9E9_PSEFS	19	21363	1	1	1	1	0.25	Putative TetR-family re
1929	1	db$2	2::C3K3J6|C3K3J6_PSEFS	19	14927	1	1	1	1	0.37	Type IV pilus response
1930	1	db$2	2::C3KDZ7|C3KDZ7_PSEFS	18	49934	2	1	2	1	0.10	Alginate biosynthesis p
1931	1	SwissProt_HUMAN$	3::Q9BXF9|TEKT3_HUMAN	18	56601	1	1	1	1	0.09	Tektin-3 OS=Homo sap
1932	1	SwissProt_HUMAN$	3::Q6UWJ1|TMCO3_HUMAN	18	75549	1	1	1	1	0.06	Transmembrane and co
1933	1	db$2	2::C3KB33|C3KB33_PSEFS	18	31594	1	1	1	1	0.16	Putative ABC transport
1934	1	SwissProt_HUMAN$	3::Q5F1R6|DJC21_HUMAN	18	61989	1	1	1	1	0.08	DnaJ homolog subfami
1935	1	db$2	2::C3K1K5|EX7L_PSEFS	18	51103	1	1	1	1	0.10	Exodeoxyribonuclease
1936	1	db$2	2::C3K7I3|C3K7I3_PSEFS	18	8950	1	1	1	1	0.67	Uncharacterized protei
1937	1	SwissProt_HUMAN$	3::Q7Z5L9|I2BP2_HUMAN	18	60987	1	1	1	1	0.08	Interferon regulatory fa
1938	1	SwissProt_HUMAN$	3::Q9BV81|EMC6_HUMAN	18	12010	1	1	1	1	0.47	ER membrane protein c
1939	1	SwissProt_HUMAN$	3::Q4V348|Z658B_HUMAN	18	94270	1	1	1	1	0.05	Zinc finger protein 658
1940	1	SwissProt_HUMAN$	3::Q96P48|ARAP1_HUMAN	18	162089	1	1	1	1	0.03	Arf-GAP with Rho-GAP
1941	1	SwissProt_HUMAN$	3::Q3T8J9|GON4L_HUMAN	18	248465	1	1	1	1	0.02	GON-4-like protein OS
1942	1	SwissProt_HUMAN$	3::O15397|IPO8_HUMAN	18	119861	1	1	1	1	0.04	Importin-8 OS=Homo s
1943	1	SwissProt_HUMAN$	3::P11413|G6PD_HUMAN	18	59219	1	1	1	1	0.08	Glucose-6-phosphate 1
1944	1	db$2	2::C3KE72|C3KE72_PSEFS	18	85807	2	1	2	1	0.06	UPF0313 protein PFLU_
1945	1	db$2	2::C3K216|C3K216_PSEFS	18	17384	1	1	1	1	0.31	Putative putative lipopr
1946	1	SwissProt_HUMAN$	3::Q9BWW9|APOL5_HUMAN	18	47014	1	1	1	1	0.11	Apolipoprotein L5 OS=
1947	1	db$2	2::C3JZ24|C3JZ24_PSEFS	18	24729	1	1	1	1	0.21	Putative GntR-family re
1948	1	db$2	2::C3JY01|C3JY01_PSEFS	18	74110	1	1	1	1	0.07	AAA_15 domain-contai
1949	1	SwissProt_HUMAN$	3::A4UGR9|XIRP2_HUMAN	18	382063	1	1	1	1	0.01	Xin actin-binding repea
1950	1	db$2	2::C3JYL6|C3JYL6_PSEFS	18	25076	2	1	2	1	0.21	Putative TetR-family re
1951	1	db$2	2::C3K277|C3K277_PSEFS	18	60895	1	1	1	1	0.08	DNA repair protein Rec
1952	1	SwissProt_HUMAN$	3::P36776|LONM_HUMAN	18	106422	1	1	1	1	0.05	Lon protease homolog,
1953	1	db$2	2::C3K906|C3K906_PSEFS	18	65595	1	1	1	1	0.07	Conserved GGDEF/EAL

↑Family	M	DB	Accession	Score	Mass	Matches	Match(sig)	Sequences	Seq(sig)	emPAI	Description
1954	1	SwissProt_HUMAN$	3::P08514|ITA2B_HUMAN	18	113306	1	1	1	1	0.04	Integrin alpha-IIb OS=
1955	1	SwissProt_HUMAN$	3::Q9NZ63|TLS1_HUMAN	18	33667	1	1	1	1	0.15	Telomere length and si
1956	1	SwissProt_HUMAN$	3::O14640|DVL1_HUMAN	18	75140	1	1	1	1	0.07	Segment polarity prote
1957	1	SwissProt_HUMAN$	3::O00139|KIF2A_HUMAN	18	79905	2	1	2	1	0.06	Kinesin-like protein KIF
1958	1	db$2	2::C3KBI8|C3KBI8_PSEFS	18	48805	1	1	1	1	0.10	Uncharacterized protei
1959	1	SwissProt_HUMAN$	3::P16885|PLCG2_HUMAN	18	147776	1	1	1	1	0.03	1-phosphatidylinositol
1960	1	db$2	2::C3K0N8|C3K0N8_PSEFS	18	19586	1	1	1	1	0.27	23S rRNA accumulatior
1961	1	db$2	2::C3K0E8|C3K0E8_PSEFS	18	27512	1	1	1	1	0.19	Thiol:disulfide intercha
1962	1	db$2	2::C3KAU8|C3KAU8_PSEFS	17	52023	1	1	1	1	0.10	Xylulose kinase OS=Ps
1963	1	db$2	2::C3K8R2|C3K8R2_PSEFS	17	22663	1	1	1	1	0.23	Uncharacterized protei
1964	1	db$2	2::C3K1H4|C3K1H4_PSEFS	17	45615	1	1	1	1	0.11	Putative membrane pro
1965	1	SwissProt_HUMAN$	3::Q9NRF8|PYRG2_HUMAN	17	65636	1	1	1	1	0.07	CTP synthase 2 OS=Hc
1966	1	db$2	2::C3K8B3|C3K8B3_PSEFS	17	48276	1	1	1	1	0.10	Putative transport-rela
1967	1	db$2	2::C3K2Z6|ERPA_PSEFS	17	12493	1	1	1	1	0.45	Iron-sulfur cluster inse
1968	1	SwissProt_HUMAN$	3::P61313|RL15_HUMAN	17	24131	1	1	1	1	0.22	60S ribosomal protein
1969	1	db$2	2::C3K4L7|C3K4L7_PSEFS	17	23283	1	1	1	1	0.22	Putative TetR-family re
1970	1	SwissProt_HUMAN$	3::P23526|SAHH_HUMAN	17	47685	1	1	1	1	0.10	Adenosylhomocysteina
1971	1	SwissProt_HUMAN$	3::P25398|RS12_HUMAN	17	14505	1	1	1	1	0.38	40S ribosomal protein
1972	1	SwissProt_HUMAN$	3::Q96P16|RPR1A_HUMAN	17	35698	1	1	1	1	0.14	Regulation of nuclear p
1973	1	db$2	2::C3KCT5|C3KCT5_PSEFS	17	27647	1	1	1	1	0.19	Putative regulatory pro
1974	1	SwissProt_HUMAN$	3::Q9H2K2|TNKS2_HUMAN	17	126838	1	1	1	1	0.04	Poly [ADP-ribose] poly
1975	1	db$2	2::C3JZ73|C3JZ73_PSEFS	17	49677	2	1	2	1	0.10	Porin OS=Pseudomona
1976	1	db$2	2::C3K457|C3K457_PSEFS	17	12769	1	1	1	1	0.44	Putative endoribonucle
1977	1	db$2	2::C3K670|C3K670_PSEFS	17	30761	1	1	1	1	0.17	Putative glycine betain
1978	1	SwissProt_HUMAN$	3::O95861|BPNT1_HUMAN	17	33371	1	1	1	1	0.15	3'(2'),5'-bisphosphate
1979	1	db$2	2::C3K2J0|C3K2J0_PSEFS	17	40846	1	1	1	1	0.12	Putative exported prote
1980	1	SwissProt_HUMAN$	3::Q8IZ21|PHAR4_HUMAN	17	78163	1	1	1	1	0.06	Phosphatase and actin
1981	1	db$2	2::C3KCV4|C3KCV4_PSEFS	17	19545	1	1	1	1	0.27	Putative DNA-binding r
1982	1	db$2	2::C3K3S9|C3K3S9_PSEFS	16	35576	1	1	1	1	0.14	Hydrolase_4 domain-co
1983	1	db$2	2::C3K0F1|C3K0F1_PSEFS	16	476575	2	1	2	1	0.01	Pyoverdine synthetase
1984	1	SwissProt_HUMAN$	3::Q5T011|SZT2_HUMAN	16	377791	1	1	1	1	0.01	KICSTOR complex prot
1985	1	db$2	2::A4V819|A4V819_PSEFS	16	100853	2	1	2	1	0.05	PDDEXK_1 domain-cor
1986	1	SwissProt_HUMAN$	3::Q8TD90|MAGE2_HUMAN	16	60339	1	1	1	1	0.08	Melanoma-associated a
1987	1	db$2	2::C3K0M7|C3K0M7_PSEFS	16	28601	1	1	1	1	0.18	Putative deoxyribonucl
1988	1	SwissProt_HUMAN$	3::P46020|KPB1_HUMAN	16	137225	1	1	1	1	0.04	Phosphorylase b kinase
1989	1	SwissProt_HUMAN$	3::P49321|NASP_HUMAN	16	85186	1	1	1	1	0.06	Nuclear autoantigenic s
1990	1	SwissProt_HUMAN$	3::O00231|PSD11_HUMAN	16	47434	1	1	1	1	0.11	26S proteasome non-A
1991	1	SwissProt_HUMAN$	3::O15050|TRNK1_HUMAN	16	336007	2	1	2	1	0.01	TPR and ankyrin repea
1992	1	SwissProt_HUMAN$	3::Q7RTR0|NLRP9_HUMAN	16	113238	1	1	1	1	0.04	NACHT, LRR and PYD d
1993	1	SwissProt_HUMAN$	3::P07437|TBB5_HUMAN	16	49639	1	1	1	1	0.10	Tubulin beta chain OS=

↑Family	M	DB	Accession	Score	Mass	Matches	Match(sig)	Sequences	Seq(sig)	emPAI	Description
1994	1	SwissProt_HUMAN$	3::Q8N1N0|CLC4F_HUMAN	16	65478	1	1	1	1	0.08	C-type lectin domain fa
1995	1	db$2	2::C3K3V9|C3K3V9_PSEFS	16	33232	1	1	1	1	0.15	Protein QmcA OS=Pseu
1996	1	SwissProt_HUMAN$	3::Q8IYW2|CFA46_HUMAN	16	303308	3	1	2	1	0.02	Cilia- and flagella-asso
1997	1	SwissProt_HUMAN$	3::P35222|CTNB1_HUMAN	15	85442	1	1	1	1	0.06	Catenin beta-1 OS=Ho
1998	1	SwissProt_HUMAN$	3::Q5VT40|FA78B_HUMAN	15	29815	1	1	1	1	0.17	Protein FAM78B OS=H
1999	1	SwissProt_HUMAN$	3::Q6ZRK6|CCD73_HUMAN	15	124077	2	1	2	1	0.04	Coiled-coil domain-con
2000	1	SwissProt_HUMAN$	3::Q969Y2|GTPB3_HUMAN	15	52026	1	1	1	1	0.10	tRNA modification GTP
2001	1	db$2	2::C3KCA2|C3KCA2_PSEFS	15	48472	2	1	1	1	0.10	Putative family S43 no
2002	1	SwissProt_HUMAN$	3::Q8IVF4|DYH10_HUMAN	15	514512	1	1	1	1	0.01	Dynein heavy chain 10
2003	1	SwissProt_HUMAN$	3::Q6ZUI0|TPRG1_HUMAN	15	31210	1	1	1	1	0.16	Tumor protein p63-reg
2004	1	db$2	2::C3K8Z7|C3K8Z7_PSEFS	15	10906	1	1	1	1	0.53	Uncharacterized protei
2005	1	db$2	2::C3K065|C3K065_PSEFS	15	58554	3	2	3	2	0.18	Uncharacterized protei
2006	1	SwissProt_HUMAN$	3::P60411|KR109_HUMAN	15	30014	1	1	1	1	0.17	Keratin-associated prot
2007	1	db$2	2::C3JYI7|C3JYI7_PSEFS	15	41303	1	1	1	1	0.12	Putative hydrolase OS=
2008	1	db$2	2::C3KBA7|C3KBA7_PSEFS	15	51338	1	1	1	1	0.10	Putative phenylacetald
2009	1	SwissProt_HUMAN$	3::O15230|LAMA5_HUMAN	15	399479	1	1	1	1	0.01	Laminin subunit alpha-
2010	1	SwissProt_HUMAN$	3::Q9BW66|CINP_HUMAN	15	24308	1	1	1	1	0.21	Cyclin-dependent kinas
2011	1	db$2	2::C3K8V2|C3K8V2_PSEFS	15	13938	1	1	1	1	0.40	GBBH-like_N domain-c
2012	1	db$2	2::C3KB03|C3KB03_PSEFS	15	18864	2	2	1	1	0.28	Uncharacterized protei
2013	1	db$2	2::C3K536|C3K536_PSEFS	15	22752	1	1	1	1	0.23	DUF4174 domain-conta
2014	1	db$2	2::C3KAN4|C3KAN4_PSEFS	15	41646	1	1	1	1	0.12	3-oxoadipyl-CoA thiola
2015	1	SwissProt_HUMAN$	3::Q9BV86|NTM1A_HUMAN	15	25371	1	1	1	1	0.20	N-terminal Xaa-Pro-Ly
2016	1	db$2	2::C3KAZ8|C3KAZ8_PSEFS	15	34711	1	1	1	1	0.15	Putative beta-hydroxyl
2017	1	db$2	2::C3KCB6|C3KCB6_PSEFS	15	31268	2	2	1	1	0.35	Phosphate transport sy
2018	1	db$2	2::C3K454|C3K454_PSEFS	15	13613	2	2	1	1	0.41	Putative cytochrome c5
2019	1	db$2	2::C3JXQ7|C3JXQ7_PSEFS	15	48832	1	1	1	1	0.10	UDP-glucose 6-dehydr
2020	1	SwissProt_HUMAN$	3::Q6WRX3|ZY11A_HUMAN	14	85752	1	1	1	1	0.06	Protein zyg-11 homolo
2021	1	SwissProt_HUMAN$	3::Q71H61|ILDR2_HUMAN	14	71155	2	1	2	1	0.07	Immunoglobulin-like d
2022	1	SwissProt_HUMAN$	3::Q5T9G4|ARM12_HUMAN	14	38607	1	1	1	1	0.13	Armadillo repeat-conta
2023	1	SwissProt_HUMAN$	3::Q13445|TMED1_HUMAN	14	25190	1	1	1	1	0.21	Transmembrane emp2
2024	1	SwissProt_HUMAN$	3::D6REC4|CFA99_HUMAN	14	52261	1	1	1	1	0.09	Cilia- and flagella-asso
2025	1	db$2	2::C3JZ63|C3JZ63_PSEFS	14	34729	1	1	1	1	0.15	Hydrolase_4 domain-c
2026	1	SwissProt_HUMAN$	3::Q8WXH0|SYNE2_HUMAN	14	795944	1	1	1	1	0.01	Nesprin-2 OS=Homo s
2027	1	db$2	2::C3K659|C3K659_PSEFS	14	30767	1	1	1	1	0.17	2-hydroxy-3-oxopropic
2028	1	SwissProt_HUMAN$	3::O96005|CLPT1_HUMAN	14	76048	1	1	1	1	0.06	Cleft lip and palate tra
2029	1	db$2	2::C3K9C4|C3K9C4_PSEFS	14	31700	1	1	1	1	0.16	Putative transposase f
2030	1	SwissProt_HUMAN$	3::P10599|THIO_HUMAN	14	11730	1	1	1	1	0.48	Thioredoxin OS=Homo
2031	1	SwissProt_HUMAN$	3::Q9C0C2|TB182_HUMAN	14	181685	1	1	1	1	0.03	182 kDa tankyrase-1-b
2032	1	SwissProt_HUMAN$	3::Q8IYB3|SRRM1_HUMAN	14	102274	1	1	1	1	0.05	Serine/arginine repetit
2033	1	db$2	2::C3KC46|C3KC46_PSEFS	14	40282	1	1	1	1	0.12	Multidrug resistance pr

↑Family	M	DB	Accession	Score	Mass	Matches	Match(sig)	Sequences	Seq(sig)	emPAI	Description
2034	1	SwissProt_HUMAN$	3::Q5TZA2|CROCC_HUMAN	14	228388	1	1	1	1	0.02	Rootletin OS=Homo sa
2035	1	SwissProt_HUMAN$	3::O94907|DKK1_HUMAN	13	28653	1	1	1	1	0.18	Dickkopf-related protei
2036	1	db$2	2::C3K869|C3K869_PSEFS	13	24016	1	1	1	1	0.22	Putative lipoprotein OS
2037	1	db$2	2::C3K9Z1|C3K9Z1_PSEFS	13	21192	1	1	1	1	0.25	FMN dependent NADH:
2038	1	SwissProt_HUMAN$	3::P60866|RS20_HUMAN	13	13364	1	1	1	1	0.42	40S ribosomal protein
2039	1	SwissProt_HUMAN$	3::Q8NF91|SYNE1_HUMAN	13	1010456	4	1	4	1	0.00	Nesprin-1 OS=Homo sa
2040	1	db$2	2::C3JY96|C3JY96_PSEFS	13	27408	1	1	1	1	0.19	OMP_b-brl domain-con
2041	1	db$2	2::C3JXJ1|C3JXJ1_PSEFS	13	47057	1	1	1	1	0.11	Putative oxygen-indepe
2042	1	SwissProt_HUMAN$	3::A6NE01|F186A_HUMAN	13	262595	1	1	1	1	0.02	Protein FAM186A OS=H
2043	1	db$2	2::C3K1V5|C3K1V5_PSEFS	13	64171	1	1	1	1	0.08	Uncharacterized protei
2044	1	SwissProt_HUMAN$	3::P11172|UMPS_HUMAN	13	52189	1	1	1	1	0.10	Uridine 5'-monophosph
2045	1	SwissProt_HUMAN$	3::P30041|PRDX6_HUMAN	13	25019	1	1	1	1	0.21	Peroxiredoxin-6 OS=Ho

Export as CSV

Not what you expected? Try **the select summary**.

Mascot: http://www.matrixscience.com/
