# Supplementary material for: Circulating multimeric immune complexes contribute to immunopathology in COVID-19
Source: Nat Commun. 2022 Sep 26;13:5654. doi: 10.1038/s41467-022-32867-z (PMC9513013; doi:10.1038/s41467-022-32867-z)
Supplement: Supplementary file 3 — Source Data [file 41467_2022_32867_MOESM3_ESM.zip › Source Data/MS_20210407 (immune precipitation).zip!/QX20210618-04_Virologie(AG-Hengel)-20210407_(V4)_0k3v20_TRL_db_SC2+db_Pf+SP_H(F235795).pdf]

MATRIX SCIENCE MASCOT Search Results

User : Wolfgang Bildl
E-mail : wolfgang.bildl@physiologie.uni-freiburg.de
Search title : QX 20210618 (db$ SARS-CoV-2+db$2 Pf+SP H$ 5 ppm 20 mmu), QX20210618-04_Virologie(AG-Hengel)-20210407_(V4)_0k3v20_TRL_shifted_-0.6.mgf
MS data file : D:\Xcalibur\Data\Wolfgang\Q-Exactive-HF-X_20210616\QX20210618-04_Virologie(AG-Hengel)-20210407_(V4)_0k3v20_TRL_shifted_-0.6.mgf
Databases : **1:** db$ 20210617 (17 sequences; 14,439 residues)
2: db$2 20210617 (6,388 sequences; 2,089,672 residues)
3: SwissProt_HUMAN$ 20201007 (20,389 sequences; 11,363,789 residues)
Timestamp : 21 Jun 2021 at 10:35:03 GMT

Not what you expected? Try **the select summary**.

Search parameters

Type of search : MS/MS Ion Search
Enzyme : Trypsin
Variable modifications : **Acetyl (Protein N-term)**, **Carbamidomethyl (C)**, **Formyl (Protein N-term)**, **Gln->pyro-Glu (N-term Q)**, **Oxidation (M)**
Mass values : Monoisotopic
Protein mass : Unrestricted
Peptide mass tolerance : ± 5 ppm
Fragment mass tolerance : ± 20 mmu
Max missed cleavages : 1
Instrument type : ESI-FTICR
Number of queries : 85,347

Score distribution

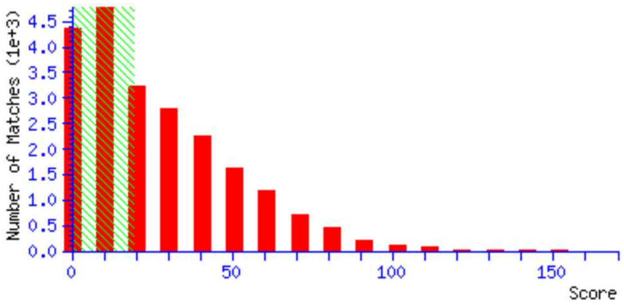

Peptide score distribution. Ions score is $-10\log(P)$, where P is the probability that the observed match is a random event.
There are **11,556** peptide matches above identity threshold and **12,310** matches above homology threshold for **85,347** queries.
On average, individual ions scores **> 19** (beyond green shading) indicate **identity or extensive homology** ($p<0.05$).

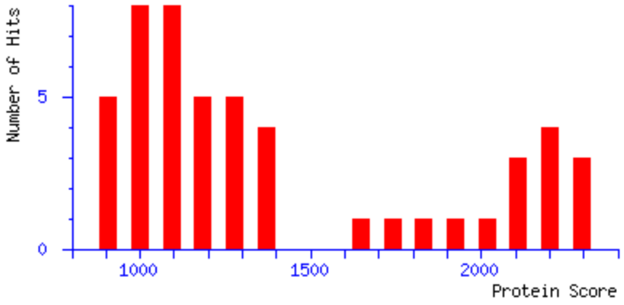

[Deprecated] Protein score distribution. Score distribution for family members in the first 50 proteins. Protein scores are derived from ions scores as a non-probabilistic basis for ranking protein families.

▼Modification statistics

Modification	Site	Above thr.
Oxidation	M	2132
Carbamidomethyl	C	1155
Gln->pyro-Glu	N-term	234
Formyl	Protein N-term	52
Acetyl	Protein N-term	14

▼Legend

Peptide columns and rows

Dupes	...	Expect	Rank	U	1	2	Peptide	
		**0.037**	▶**2**				**GAYSLSLR**	significant
		9	▶1				GFFLFVEGGR	top ranking
		**6.4e-005**	▶**1**				**GSSIFGLAPGK**	significant and top ranking
		**1.3e-006**	▶**1**		■		**SSGTSYPDVLK**	peptide is found in all proteins in family member 1
		**6.2e-007**	▶**1**			■	**VCNYVSWIK**	peptide is found in some but not all proteins in family member 2
		**6.4e-005**	▶**1**	**U**			**GSSIFGLAPGK**	unique
▶**2**		**5.7e-005**	▶**1**				**LNTLETEEWFFK**	peptide has two duplicates
		0.18	▶*1*				*LNTLETEEWFFK*	duplicate peptide

Right-facing triangle (▶) in the Dupes or Rank column indicates content that can be expanded by clicking on it. Down-facing triangle (▼) indicates the content is expanded and can be collapsed. For more details about particular columns, see **results format help**.

Protein quantitation ratios

	Score	...	114/113	115/113
**CFAH_HUMAN**	37559		*0.962*	**1.129**
**FHR2_HUMAN**	1330		**0.859**	1.128

When quantitation method is Reporter (e.g. iTRAQ) or Multiplex (e.g. IPTL), protein ratios are displayed when a family is expanded. Ratios in *italic* indicate that the peptide log-ratios do not appear to come from a normal distribution. **Bold** indicates that if you can assume peptide ratios are normally distributed, the protein ratio is significantly different from 1.0 (at significance level 0.05).

Note that lack of bold or italic can also mean that significance or normality testing has not been performed (for

example, if protein ratio type does not support it).

Protein Family Summary

Significance threshold p<	0.05	Max. number of families	AUTO
Ions score or expect cut-off	0.5	Dendrograms cut at	0
Show Percolator scores	☐		
Preferred taxonomy	All entries		

▼Sensitivity and FDR (reversed protein sequences)

			Target	Decoy	FDR		
PSMs	above	homology	12310	441	3.58%	Adjust to	1%

Decoy results are available in **the decoy report**.

Protein hits (2061 proteins)

▶Filters: (none)

Export as CSV

↑Family	M	DB	Accession	Score	Mass	Matches	Match(sig)	Sequences	Seq(sig)	emPAI	Description
1	1	db$2	2::C3K2Y3|RPOB_PSEFS	2294	150923	101	95	65	61	8.34	DNA-directed RNA poly
2	1	db$2	2::C3K1A0|CH60_PSEFS	2259	56931	65	63	31	31	29.22	60 kDa chaperonin OS
3	1	db$2	2::C3K1E6|ATPB_PSEFS	2254	49459	67	64	30	30	44.69	ATP synthase subunit b
4	1	db$2	2::C3K2Y2|RPOC_PSEFS	2207	154528	92	90	67	65	8.73	DNA-directed RNA poly
5	1	db$2	2::C3K4D5|C3K4D5_PSEFS	2190	34599	67	64	23	22	44.34	Phosphate-binding prot
6	1	db$2	2::C3K5G3|C3K5G3_PSEFS	2183	99192	73	67	36	33	6.45	Aconitate hydratase OS
7	1	db$2	2::C3K5Z6|EFTS_PSEFS	2183	30341	69	63	17	17	13.06	Elongation factor Ts OS
8	1	db$2	2::C3KD82|C3KD82_PSEFS	2134	181591	73	73	55	55	3.81	Glutamate dehydrogen
9	1	db$2	2::C3K2X9|EFG_PSEFS	2116	77114	68	65	34	33	12.21	Elongation factor G OS
10	1	db$2	2::C3K2X8|EFTU_PSEFS	2097	43536	71	66	24	22	15.84	Elongation factor Tu OS
11	1	SwissProt_HUMAN$	3::P13645|K1C10_HUMAN	2026	58792	48	45	29	28	14.43	Keratin, type I cytoske
11	2	SwissProt_HUMAN$	3::P35527|K1C9_HUMAN	806	62027	25	24	18	18	3.97	Keratin, type I cytoske
11	3	SwissProt_HUMAN$	3::P02533|K1C14_HUMAN	384	51529	18	15	16	13	2.62	Keratin, type I cytoske
11	4	SwissProt_HUMAN$	3::P08779|K1C16_HUMAN	355	51236	15	13	14	12	2.32	Keratin, type I cytoske
11	5	SwissProt_HUMAN$	3::Q04695|K1C17_HUMAN	200	48076	11	10	10	9	1.42	Keratin, type I cytoske
11	6	SwissProt_HUMAN$	3::P19012|K1C15_HUMAN	102	49181	7	7	6	6	0.78	Keratin, type I cytoske
11	7	SwissProt_HUMAN$	3::P08727|K1C19_HUMAN	85	44079	6	6	6	6	0.90	Keratin, type I cytoske
12	1	db$2	2::C3K4N0|C3K4N0_PSEFS	1910	43479	51	50	25	25	39.16	Putative ornithine deca
13	1	db$2	2::C3K1E8|ATPA_PSEFS	1822	55245	60	59	28	28	24.89	ATP synthase subunit a
14	1	db$2	2::C3K255|PNP_PSEFS	1706	74889	61	59	32	32	10.79	Polyribonucleotide nucl
15	1	db$2	2::C3K0Q5|C3K0Q5_PSEFS	1666	107302	56	53	37	35	4.86	Ribonucleoside-diphosp

↑Family	M	DB	Accession	Score	Mass	Matches	Match(sig)	Sequences	Seq(sig)	emPAI	Description
16	1	db$2	2::C3K6P2|C3K6P2_PSEFS	1390	42870	31	30	14	14	7.13	3-oxoacyl-[acyl-carrier
17	1	SwissProt_HUMAN$	3::P04264|K2C1_HUMAN	1388	65999	42	42	29	29	9.67	Keratin, type II cytosk
17	2	SwissProt_HUMAN$	3::P35908|K22E_HUMAN	918	65393	42	40	31	31	9.92	Keratin, type II cytosk
17	3	SwissProt_HUMAN$	3::P02538|K2C6A_HUMAN	256	60008	12	11	12	11	1.38	Keratin, type II cytosk
17	4	SwissProt_HUMAN$	3::P13647|K2C5_HUMAN	224	62340	13	11	13	11	1.31	Keratin, type II cytosk
17	5	SwissProt_HUMAN$	3::P05787|K2C8_HUMAN	188	53671	10	8	8	6	0.70	Keratin, type II cytosk
17	6	SwissProt_HUMAN$	3::O95678|K2C75_HUMAN	163	59524	8	8	8	8	0.89	Keratin, type II cytosk
17	7	SwissProt_HUMAN$	3::P08729|K2C7_HUMAN	129	51354	6	5	4	3	0.32	Keratin, type II cytosk
18	1	db$2	2::C3KCP4|C3KCP4_PSEFS	1360	99394	60	57	37	36	7.58	Pyruvate dehydrogena
19	1	db$2	2::C3K271|C3K271_PSEFS	1330	117563	44	41	32	29	2.79	Carbamoyl-phosphate
20	1	db$2	2::C3KCJ5|C3KCJ5_PSEFS	1322	93957	55	49	38	35	5.82	Aconitate hydratase B
21	1	db$2	2::C3JYU7|C3JYU7_PSEFS	1284	102460	41	35	29	25	2.66	Protein translocase sub
22	1	db$2	2::C3K0P3|C3K0P3_PSEFS	1283	118163	37	36	26	25	2.08	Ribonuclease E OS=Ps
23	1	db$2	2::C3KBH1|C3KBH1_PSEFS	1264	161946	49	46	41	39	2.33	Glutamate synthase [N
24	1	db$2	2::C3K6N0|SUCC_PSEFS	1239	41262	38	34	23	19	14.58	Succinate--CoA ligase
25	1	db$2	2::C3K331|C3K331_PSEFS	1228	73691	50	47	36	35	10.51	AAA_PrkA domain-cont
26	1	db$2	2::C3KBY8|C3KBY8_PSEFS	1218	21919	53	46	17	15	89.70	Alkyl hydroperoxide re
27	1	db$2	2::C3K275|DNAK_PSEFS	1205	68158	39	37	24	23	5.10	Chaperone protein Dna
28	1	db$2	2::C3K5J5|FABV_PSEFS	1183	42933	41	39	25	24	14.65	Enoyl-[acyl-carrier-pro
29	1	db$2	2::C3K6M5|C3K6M5_PSEFS	1161	63522	34	34	23	23	5.96	Succinate dehydrogena
30	1	db$2	2::C3K613|FADB_PSEFS	1114	76938	38	35	28	26	3.96	Fatty acid oxidation co
31	1	db$2	2::C3K6M8|C3K6M8_PSEFS	1096	42320	29	27	15	14	5.68	Dihydrolipoyllysine-res
32	1	db$2	2::C3K225|ILVC_PSEFS	1092	36188	40	40	22	22	28.69	Ketol-acid reductoisom
33	1	db$2	2::C3K3F3|C3K3F3_PSEFS	1092	72324	43	42	32	31	8.27	Transketolase OS=Pse
34	1	db$2	2::C3K6K0|C3K6K0_PSEFS	1079	62268	40	40	22	22	6.22	30S ribosomal protein
35	1	db$2	2::C3JYK1|TIG_PSEFS	1077	48316	40	40	26	26	19.83	Trigger factor OS=Pseu
36	1	db$2	2::C3KDP2|C3KDP2_PSEFS	1073	68879	33	32	21	20	4.21	Multifunctional fusion p
37	1	db$2	2::C3K7D1|C3K7D1_PSEFS	1062	67115	38	34	28	25	6.74	50S ribosomal subunit
38	1	db$2	2::C3K6M7|C3K6M7_PSEFS	1020	105930	52	46	36	35	4.49	Oxoglutarate dehydrog
39	1	db$2	2::C3K3Z0|C3K3Z0_PSEFS	1012	46925	33	31	23	21	10.21	Transcription terminati
40	1	db$2	2::C3K673|C3K673_PSEFS	1004	47725	46	44	22	22	18.54	Citrate synthase OS=P
41	1	db$2	2::C3K6H4|PYRG_PSEFS	1003	59660	39	36	24	23	8.25	CTP synthase OS=Pseu
42	1	db$2	2::C3K165|C3K165_PSEFS	991	54857	44	38	27	26	16.26	Fumarate hydratase cl
43	1	db$2	2::C3K0K6|C3K0K6_PSEFS	976	26352	26	25	14	13	13.62	Electron transfer flavo
44	1	db$2	2::C3K6N1|C3K6N1_PSEFS	972	29928	32	31	15	14	22.19	Succinate--CoA ligase
45	1	db$2	2::C3K402|C3K402_PSEFS	970	83101	46	43	30	29	6.79	Polyphosphate kinase (
46	1	db$2	2::C3K259|IF2_PSEFS	923	90544	35	31	27	24	3.11	Translation initiation fa
47	1	db$2	2::C3JY66|C3JY66_PSEFS	923	80156	34	34	20	20	2.90	Isocitrate dehydrogena
48	1	db$2	2::C3JYR1|GCSP_PSEFS	914	102176	39	38	25	24	2.19	Glycine dehydrogenase
49	1	db$2	2::C3K7G0|C3K7G0_PSEFS	909	36980	26	26	19	19	13.61	Cell shape-determining

↑Family	M	DB	Accession	Score	Mass	Matches	Match(sig)	Sequences	Seq(sig)	emPAI	Description
50	1	db$2	2::C3KEC5|C3KEC5_PSEFS	906	97398	36	33	28	25	2.73	Phosphoenolpyruvate c
51	1	db$2	2::C3KBJ2|C3KBJ2_PSEFS	906	24354	25	24	13	13	21.06	Carbonic anhydrase OS
52	1	db$2	2::C3K7D3|C3K7D3_PSEFS	906	91800	30	29	22	21	2.46	Alpha-1,4 glucan phos
53	1	db$2	2::C3K3S2|C3K3S2_PSEFS	894	44244	27	27	20	20	7.45	2-oxoglutarate reducta
54	1	db$2	2::C3JYJ7|C3JYJ7_PSEFS	891	9057	53	45	9	9	432.00	DNA-binding protein HI
55	1	db$2	2::C3K0T0|C3K0T0_PSEFS	886	34560	29	28	9	9	2.92	Putative outer membra
56	1	db$2	2::C3KDW8|PURA_PSEFS	884	46535	29	29	14	14	5.23	Adenylosuccinate synth
57	1	db$2	2::C3K1K1|C3K1K1_PSEFS	883	51928	34	34	21	21	9.68	Inosine-5'-monophospl
58	1	db$2	2::C3K2R9|C3K2R9_PSEFS	882	16440	17	17	8	8	16.19	6,7-dimethyl-8-ribitylu
59	1	db$2	2::C3K367|C3K367_PSEFS	881	68142	32	31	17	17	3.31	Uncharacterized protei
60	1	db$2	2::C3K4D2|C3K4D2_PSEFS	879	30988	38	32	20	20	43.90	Phosphate import ATP-
61	1	db$2	2::C3KE51|C3KE51_PSEFS	857	38026	32	31	25	24	18.61	Putative oxidoreductas
62	1	db$2	2::C3KBT0|C3KBT0_PSEFS	855	51607	30	26	16	16	5.85	Succinate-semialdehyd
63	1	db$2	2::C3K1A7|C3K1A7_PSEFS	852	66070	30	30	20	20	4.21	Proline--tRNA ligase OS
64	1	db$2	2::C3JYS1|C3JYS1_PSEFS	852	17688	22	21	9	8	13.16	Peptidoglycan-associat
65	1	db$2	2::C3K1K0|GUAA_PSEFS	834	58398	32	31	25	24	6.00	GMP synthase [glutami
66	1	db$2	2::C3JYT1|SYDND_PSEFS	834	66220	35	34	23	22	4.97	Aspartate--tRNA(Asp/A
67	1	db$2	2::C3KE86|C3KE86_PSEFS	827	95293	40	35	30	26	3.46	Chaperone protein ClpI
68	1	db$2	2::C3K6M9|C3K6M9_PSEFS	826	49948	26	25	16	16	5.05	Dihydrolipoyl dehydrog
69	1	db$2	2::C3K6N7|HTPG_PSEFS	814	71589	30	28	19	17	2.76	Chaperone protein Htp
70	1	db$2	2::C3KE67|RS6_PSEFS	811	16276	23	21	11	11	128.97	30S ribosomal protein
71	1	db$2	2::C3K1L8|NDK_PSEFS	810	14928	28	28	8	8	22.00	Nucleoside diphosphate
72	1	db$2	2::C3K1J2|C3K1J2_PSEFS	804	140531	38	35	35	32	2.05	Phosphoribosylformylg
73	1	db$2	2::C3K5E6|RS2_PSEFS	783	27078	26	26	17	17	31.30	30S ribosomal protein
74	1	db$2	2::C3K3J7|C3K3J7_PSEFS	781	35008	27	26	15	15	13.86	Glutathione synthetase
75	1	db$2	2::C3K162|C3K162_PSEFS	776	52023	26	25	19	18	4.13	Pyruvate kinase OS=Ps
76	1	db$2	2::C3K178|C3K178_PSEFS	766	36139	29	28	16	16	9.46	Glyceraldehyde-3-phos
77	1	db$2	2::C3KAD2|ARNA_PSEFS	751	74140	28	26	20	19	2.83	Bifunctional polymyxin
78	1	db$2	2::C3KE40|SYGB_PSEFS	737	74833	29	27	24	22	3.57	Glycine--tRNA ligase be
79	1	db$2	2::C3K139|C3K139_PSEFS	731	48816	22	22	13	13	3.71	Uncharacterized protei
80	1	db$2	2::C3KBT1|C3KBT1_PSEFS	726	44808	34	33	19	19	11.55	4-aminobutyrate amino
81	1	db$2	2::C3K4D1|C3K4D1_PSEFS	724	28553	25	22	13	11	9.08	Phosphate-specific trar
82	1	db$2	2::C3K6G5|AMPA_PSEFS	713	52403	31	31	21	21	6.98	Probable cytosol amino
83	1	db$2	2::C3K121|C3K121_PSEFS	712	86030	35	34	26	25	3.68	Phosphoenolpyruvate s
84	1	db$2	2::C3JXY1|C3JXY1_PSEFS	709	93962	31	26	24	20	1.89	Alanine--tRNA ligase O
85	1	db$2	2::C3K6J4|C3K6J4_PSEFS	708	97893	35	28	27	20	1.77	DNA gyrase subunit A (
86	1	db$2	2::C3KCB8|C3KCB8_PSEFS	692	36708	31	29	16	16	10.48	Phosphate-binding prot
87	1	db$2	2::C3K6H6|C3K6H6_PSEFS	692	45588	26	25	17	17	5.46	Enolase OS=Pseudomo
88	1	db$2	2::C3K3F1|PGK_PSEFS	686	40125	18	18	13	13	4.86	Phosphoglycerate kinas
89	1	db$2	2::C3K2D3|C3K2D3_PSEFS	684	44901	22	22	14	14	3.84	Serine hydroxymethylt

↑Family	M	DB	Accession	Score	Mass	Matches	Match(sig)	Sequences	Seq(sig)	emPAI	Description
90	1	db$2	2::C3KAH7|C3KAH7_PSEFS	673	45018	26	24	20	19	8.05	Putative NADP-depend
91	1	db$2	2::C3K3G6|SAHH_PSEFS	670	51212	35	32	22	20	10.07	Adenosylhomocysteina
92	1	db$2	2::C3K2D0|C3K2D0_PSEFS	668	61490	24	24	20	20	4.44	Energy-dependent trar
93	1	db$2	2::C3K4D4|C3K4D4_PSEFS	662	83859	29	29	17	17	2.67	Putative phosphate AB
94	1	db$2	2::C3JYA7|C3JYA7_PSEFS	657	59461	21	21	16	16	2.57	Putative NADP-depend
95	1	db$2	2::C3KDD8|C3KDD8_PSEFS	655	44893	32	32	22	22	12.95	Cell division protein Fts
96	1	db$2	2::C3K426|ARLY_PSEFS	648	51545	22	21	17	16	4.73	Argininosuccinate lyase
97	1	db$2	2::C3KDW5|C3KDW5_PSEFS	645	43265	27	23	19	18	6.14	Protein HflK OS=Pseud
98	1	db$2	2::C3K4F6|C3K4F6_PSEFS	637	65489	36	31	26	24	5.56	Oxaloacetate decarbox
99	1	db$2	2::C3JZV2|C3JZV2_PSEFS	633	43020	19	19	16	16	5.46	Aminotransferase OS=
100	1	db$2	2::C3JYT4|C3JYT4_PSEFS	630	17756	22	21	12	11	38.97	Putative ferritin DPS-fa
101	1	db$2	2::C3K6I0|C3K6I0_PSEFS	628	39056	17	17	10	10	3.81	Alcohol dehydrogenase
102	1	db$2	2::C3JZ70|C3JZ70_PSEFS	622	54858	23	21	16	14	2.65	Glucose-6-phosphate 1
103	1	db$2	2::C3JYQ5|C3JYQ5_PSEFS	619	46409	25	24	16	16	4.65	Arginine deiminase OS
104	1	db$2	2::C3K8B0|C3K8B0_PSEFS	619	25585	19	18	9	9	6.59	Peptidyl-prolyl cis-tran
105	1	db$2	2::C3JYJ9|CLPX_PSEFS	614	46924	25	24	16	15	4.54	ATP-dependent Clp pro
106	1	db$2	2::C3K7C9|C3K7C9_PSEFS	613	51641	28	26	17	16	5.25	Glutamine synthetase (
107	1	db$2	2::C3K1E2|C3K1E2_PSEFS	613	66300	21	21	17	17	2.62	Glutamine--fructose-6-
108	1	db$2	2::C3JZN2|C3JZN2_PSEFS	611	86329	23	20	20	17	1.54	Phenylalanine--tRNA li
109	1	db$2	2::C3JYS2|TOLB_PSEFS	608	47517	22	22	19	19	6.32	Tol-Pal system protein
110	1	db$2	2::C3KED7|PUR7_PSEFS	604	26862	23	22	16	16	18.65	Phosphoribosylaminoin
111	1	db$2	2::C3K2V9|RS5_PSEFS	599	17675	20	19	10	9	39.89	30S ribosomal protein
112	1	db$2	2::C3K6W9|C3K6W9_PSEFS	595	69415	16	16	14	14	1.60	Putative methyl-accept
112	2	db$2	2::C3JZ31|C3JZ31_PSEFS	95	58204	3	3	2	2	0.18	Putative methyl-accept
112	3	db$2	2::C3K5K7|C3K5K7_PSEFS	61	52604	3	2	3	2	0.20	Putative methyl-accept
113	1	db$2	2::C3JZN7|SYT_PSEFS	590	72513	24	23	18	17	2.46	Threonine--tRNA ligase
114	1	db$2	2::C3KCB5|C3KCB5_PSEFS	585	30173	23	21	14	12	9.39	Phosphate import ATP-
115	1	db$2	2::C3K2V6|C3K2V6_PSEFS	583	47842	22	22	10	10	2.99	Protein translocase sub
116	1	db$2	2::C3JZS1|C3JZS1_PSEFS	578	55264	20	17	14	12	1.79	Amidophosphoribosyltr
117	1	db$2	2::C3K5H9|C3K5H9_PSEFS	578	33776	16	16	13	13	7.14	Transaldolase OS=Pseu
118	1	db$2	2::C3K266|C3K266_PSEFS	577	69364	21	20	17	16	2.19	ATP-dependent zinc me
119	1	db$2	2::C3K108|C3K108_PSEFS	569	83591	24	24	20	20	2.11	GTP pyrophosphokinas
120	1	db$2	2::C3KBZ2|ASSY_PSEFS	567	45330	20	18	13	12	3.79	Argininosuccinate synt
121	1	db$2	2::C3KEC2|C3KEC2_PSEFS	562	28614	16	16	13	13	7.48	Putative lipoprotein OS
121	2	db$2	2::C3KAJ6|C3KAJ6_PSEFS	182	38502	9	7	7	5	1.09	Histidinol-phosphate ar
122	1	db$2	2::C3K1B7|C3K1B7_PSEFS	555	51892	20	18	13	12	2.28	Threonine synthase OS
123	1	db$2	2::C3K6X4|C3K6X4_PSEFS	555	71161	23	20	19	18	2.32	Biosynthetic arginine d
124	1	db$2	2::C3K1W9|C3K1W9_PSEFS	555	33929	13	13	8	8	3.01	Thioredoxin reductase
125	1	db$2	2::C3K914|C3K914_PSEFS	554	38509	17	17	9	9	3.92	Putative ABC transport
126	1	db$2	2::C3K2V1|C3K2V1_PSEFS	554	36623	23	20	17	15	7.94	DNA-directed RNA poly

↑Family	M	DB	Accession	Score	Mass	Matches	Match(sig)	Sequences	Seq(sig)	emPAI	Description
127	1	db$2	2::C3K2X5|RL4_PSEFS	550	21987	11	11	7	7	4.52	50S ribosomal protein
128	1	db$2	2::C3K6M6|C3K6M6_PSEFS	549	26063	22	19	14	13	16.91	Succinate dehydrogena
129	1	db$2	2::C3KCP3|C3KCP3_PSEFS	546	56221	21	21	13	13	2.54	Acetyltransferase comp
130	1	db$2	2::C3K4Y4|C3K4Y4_PSEFS	546	71842	22	22	18	18	2.74	Potassium-transporting
131	1	db$2	2::C3K6H5|KDSA_PSEFS	544	30777	27	26	16	15	20.44	2-dehydro-3-deoxypho
132	1	db$2	2::C3KE65|C3KE65_PSEFS	544	97331	28	27	22	21	1.92	Ribonuclease R OS=Ps
133	1	db$2	2::C3JZT6|C3JZT6_PSEFS	544	40569	18	17	14	13	5.46	Aspartate-semialdehyd
134	1	db$2	2::C3KDU5|C3KDU5_PSEFS	544	90380	19	17	17	15	1.20	DNA gyrase subunit B
135	1	db$2	2::C3K1N2|C3K1N2_PSEFS	542	67307	26	25	20	20	3.39	Protein translocase sub
136	1	db$2	2::C3K4F7|C3K4F7_PSEFS	541	51847	24	22	22	21	5.80	Biotin carboxylase OS=
136	2	db$2	2::C3K282|SSRP_PSEFS	46	18308	3	2	3	2	0.67	SsrA-binding protein O
137	1	db$2	2::C3K260|C3K260_PSEFS	539	54381	19	19	15	15	3.02	Transcription terminati
138	1	db$2	2::C3K2U7|C3K2U7_PSEFS	539	104087	23	22	19	18	1.27	UvrABC system protein
139	1	db$2	2::C3KAL6|C3KAL6_PSEFS	535	52109	20	18	15	15	2.91	Putative aminotransfer
140	1	db$2	2::C3KDG0|RECA_PSEFS	534	37499	20	19	14	13	5.60	Protein RecA OS=Pseu
141	1	db$2	2::C3K6H2|ACCA_PSEFS	534	34868	20	18	13	13	7.72	Acetyl-coenzyme A car
142	1	db$2	2::C3JYV2|C3JYV2_PSEFS	530	52688	17	14	14	11	1.94	AAA domain-containing
143	1	db$2	2::C3KDX4|SYI_PSEFS	526	105386	23	23	18	18	1.25	Isoleucine--tRNA ligase
144	1	db$2	2::C3K1K7|LEU1_PSEFS	522	61997	22	22	20	20	3.60	2-isopropylmalate synt
145	1	db$2	2::C3K6W6|C3K6W6_PSEFS	521	49345	23	22	16	15	4.09	Biotin carboxylase OS=
146	1	db$2	2::C3K4V9|C3K4V9_PSEFS	517	53185	18	17	15	14	3.15	Glyceraldehyde-3-phos
147	1	db$2	2::C3KDU7|C3KDU7_PSEFS	517	28504	21	18	14	13	6.25	Putative ABC transport
148	1	db$2	2::C3KBX8|C3KBX8_PSEFS	516	26859	11	11	7	7	3.84	OmpA-like domain-con
149	1	db$2	2::C3K1L4|ISPG_PSEFS	515	39660	20	18	16	16	6.58	4-hydroxy-3-methylbu
150	1	db$2	2::C3KCS1|C3KCS1_PSEFS	514	34171	16	16	11	11	5.90	Ribose-phosphate pyro
151	1	db$2	2::C3JY65|C3JY65_PSEFS	512	45375	28	27	20	19	11.17	Isocitrate dehydrogena
152	1	db$2	2::C3K4J5|PCKA_PSEFS	510	55324	24	22	18	17	3.29	Phosphoenolpyruvate c
153	1	db$2	2::C3K341|C3K341_PSEFS	510	69250	22	22	19	19	3.21	RNA polymerase sigma
153	2	db$2	2::C3K708|C3K708_PSEFS	107	38066	4	4	4	4	0.64	RNA polymerase sigma
154	1	db$2	2::C3K3R2|C3K3R2_PSEFS	509	83481	24	22	20	18	2.11	Phosphoenolpyruvate--
155	1	db$2	2::C3KDR8|OPGD_PSEFS	509	60485	18	17	15	15	2.24	Glucans biosynthesis p
156	1	db$2	2::C3K2Y4|RL7_PSEFS	508	12456	13	13	3	3	8.41	50S ribosomal protein
157	1	SwissProt_HUMAN$	3::P02768|ALBU_HUMAN	503	69321	20	18	17	15	1.79	Albumin OS=Homo sap
157	2	SwissProt_HUMAN$	3::P02769|ALBU_BOVIN	341	69248	15	13	13	11	1.12	Albumin OS=Bos tauru
158	1	db$2	2::C3K6M2|C3K6M2_PSEFS	494	60584	18	17	16	15	2.77	ATP-dependent RNA he
159	1	db$2	2::C3K9T7|C3K9T7_PSEFS	490	99328	19	17	15	13	0.86	Aminopeptidase N OS=
160	1	db$2	2::C3JZV8|C3JZV8_PSEFS	489	38906	13	13	7	7	1.64	Neutral metalloprotein
161	1	db$2	2::C3K7Y0|C3K7Y0_PSEFS	482	45268	17	16	12	11	2.88	Putative exported prot
162	1	db$2	2::C3JZS0|C3JZS0_PSEFS	482	43144	23	21	18	17	6.17	O-succinylhomoserine
163	1	db$2	2::C3K2X0|C3K2X0_PSEFS	482	25737	17	16	12	11	9.74	30S ribosomal protein

↑Family	M	DB	Accession	Score	Mass	Matches	Match(sig)	Sequences	Seq(sig)	emPAI	Description
164	1	db$2	2::C3K3E8|C3K3E8_PSEFS	481	38385	18	18	14	14	6.15	Fructose-1,6-bisphospl
165	1	db$2	2::C3K1G5|C3K1G5_PSEFS	479	46195	13	13	11	11	2.08	Homoserine dehydroge
166	1	db$2	2::C3JXL7|C3JXL7_PSEFS	477	39112	16	16	11	11	3.26	HlyD-family secretion p
167	1	db$2	2::C3K016|C3K016_PSEFS	474	85788	17	16	16	15	1.29	Ribosomal RNA large s
168	1	db$2	2::C3KBM0|GLPK_PSEFS	468	55508	24	21	15	14	3.26	Glycerol kinase OS=Ps
169	1	db$2	2::C3K4C6|C3K4C6_PSEFS	467	25565	17	16	10	10	8.12	Phosphate regulon trar
170	1	db$2	2::C3JY86|C3JY86_PSEFS	463	98164	21	21	15	15	1.17	NADH-quinone oxidore
171	1	db$2	2::C3K2H6|C3K2H6_PSEFS	462	32512	13	13	7	7	2.19	Acyl-CoA thioesterase
172	1	db$2	2::C3K2X3|RL2_PSEFS	461	29689	17	17	11	11	4.75	50S ribosomal protein
173	1	db$2	2::C3KBH2|C3KBH2_PSEFS	461	51951	15	13	12	10	1.72	Glutamate synthase [N
174	1	db$2	2::C3K2M9|PROA_PSEFS	460	45380	15	15	13	13	3.30	Gamma-glutamyl phos
175	1	db$2	2::C3K6G0|C3K6G0_PSEFS	459	9680	12	11	8	7	27.33	Putative DNA-binding p
176	1	db$2	2::C3K2Y6|RL1_PSEFS	458	24061	16	15	11	11	7.55	50S ribosomal protein
177	1	db$2	2::C3K569|C3K569_PSEFS	457	44415	18	17	12	12	3.44	Tryptophan synthase b
178	1	db$2	2::C3K227|C3K227_PSEFS	457	62578	25	23	19	17	2.90	Acetolactate synthase
179	1	db$2	2::C3K9R0|C3K9R0_PSEFS	456	62265	13	13	12	12	1.49	Pyruvate dehydrogenas
180	1	db$2	2::C3K2W0|RL18_PSEFS	455	12651	16	15	9	9	38.35	50S ribosomal protein
181	1	db$2	2::C3K1G8|RL19_PSEFS	450	12961	16	16	9	9	50.34	50S ribosomal protein
182	1	db$2	2::C3KDE0|LPXC_PSEFS	449	33197	14	14	10	10	3.14	UDP-3-O-acyl-N-acetyl
183	1	db$2	2::C3K1F0|C3K1F0_PSEFS	446	16832	15	15	10	10	14.97	ATP synthase subunit b
184	1	db$2	2::C3JXZ0|C3JXZ0_PSEFS	446	43332	18	16	15	13	3.61	Acetylornithine aminot
185	1	db$2	2::C3KEC8|C3KEC8_PSEFS	446	50623	20	20	12	12	2.70	Periplasmic serine end
186	1	db$2	2::C3K1E7|ATPG_PSEFS	445	31374	18	16	14	12	7.17	ATP synthase gamma c
187	1	db$2	2::C3JXY7|ASTD_PSEFS	444	51479	10	8	7	6	0.90	N-succinylglutamate 5-
188	1	db$2	2::C3KA76|C3KA76_PSEFS	443	30860	15	15	11	11	5.25	UTP--glucose-1-phospl
189	1	db$2	2::C3K649|C3K649_PSEFS	443	8822	14	12	4	4	7.15	Major outer membrane
190	1	db$2	2::C3K4D3|C3K4D3_PSEFS	440	60551	20	17	14	13	1.76	Phosphate transport sy
191	1	db$2	2::C3KBY6|C3KBY6_PSEFS	440	17850	15	13	8	6	12.71	Bacterioferritin OS=Pse
192	1	db$2	2::C3K902|RAPA_PSEFS	436	106722	17	16	14	14	0.86	RNA polymerase-assoc
193	1	db$2	2::C3JXY0|AK_PSEFS	435	44504	19	18	16	15	5.08	Aspartate kinase OS=P
194	1	db$2	2::C3K5E0|C3K5E0_PSEFS	435	36000	13	13	8	8	3.24	2,3,4,5-tetrahydropyric
195	1	db$2	2::C3K466|C3K466_PSEFS	433	52681	14	13	8	7	1.05	Putative aldehyde dehy
196	1	db$2	2::C3K603|C3K603_PSEFS	433	87560	21	19	16	14	1.38	Outer membrane prote
197	1	db$2	2::C3KE70|RL9_PSEFS	430	15488	15	14	9	8	19.16	50S ribosomal protein
198	1	db$2	2::C3K7D6|F16PA_PSEFS	429	37081	15	15	9	9	3.06	Fructose-1,6-bisphospl
199	1	db$2	2::C3JYQ6|C3JYQ6_PSEFS	428	37842	17	16	12	11	5.49	Ornithine carbamoyltra
200	1	db$2	2::C3K4K9|C3K4K9_PSEFS	428	27377	12	11	8	8	3.69	Putative amino acid ox
201	1	db$2	2::C3K0K5|C3K0K5_PSEFS	424	31365	15	15	8	8	3.48	Electron transfer flavop
202	1	db$2	2::C3JYJ8|C3JYJ8_PSEFS	422	88851	16	16	16	16	1.35	Lon protease OS=Pseu
203	1	db$2	2::C3K1E9|ATPD_PSEFS	421	19246	19	18	9	9	13.64	ATP synthase subunit c

↑Family	M	DB	Accession	Score	Mass	Matches	Match(sig)	Sequences	Seq(sig)	emPAI	Description
204	1	db$2	2::C3K226|C3K226_PSEFS	421	17820	10	9	6	6	3.81	Acetolactate synthase (
205	1	db$2	2::C3K1X6|C3K1X6_PSEFS	420	44711	13	13	10	10	1.88	GTPase Obg OS=Pseuc
206	1	db$2	2::C3K616|C3K616_PSEFS	416	97222	21	20	19	18	1.41	DNA topoisomerase 1 (
207	1	db$2	2::C3K2L2|SYL_PSEFS	411	97066	20	18	17	15	1.19	Leucine--tRNA ligase O
208	1	db$2	2::C3K907|C3K907_PSEFS	409	77648	20	19	19	18	2.00	Tail-specific protease C
209	1	db$2	2::C3KCB7|C3KCB7_PSEFS	408	34191	7	7	3	3	0.74	Phosphate transport sy
210	1	db$2	2::C3JY74|C3JY74_PSEFS	406	48472	18	17	16	15	3.32	Isocitrase OS=Pseudor
211	1	db$2	2::C3K263|C3K263_PSEFS	405	26068	9	8	7	6	1.95	Triosephosphate isome
212	1	db$2	2::C3K3V2|C3K3V2_PSEFS	405	49111	21	16	11	9	1.62	Putative outer membra
213	1	db$2	2::C3K8X4|MURA_PSEFS	405	44975	17	14	13	10	2.92	UDP-N-acetylglucosam
214	1	db$2	2::C3K484|C3K484_PSEFS	404	13502	10	10	6	6	6.86	Uncharacterized protei
215	1	db$2	2::C3K4W1|STHA_PSEFS	401	50963	19	18	16	15	3.42	Soluble pyridine nucleo
216	1	db$2	2::C3K2K3|GSA_PSEFS	400	45181	15	14	9	8	2.16	Glutamate-1-semialdeh
217	1	db$2	2::C3K244|C3K244_PSEFS	400	52276	11	9	9	8	1.06	Putative response regu
218	1	db$2	2::C3K1M5|ISCS_PSEFS	398	44467	17	16	13	13	3.42	Cysteine desulfurase Is
219	1	db$2	2::C3K5S8|C3K5S8_PSEFS	396	75646	11	11	9	9	0.87	Oligopeptidase A OS=F
220	1	db$2	2::C3K6Y3|GATB_PSEFS	395	52868	16	14	10	9	1.68	Aspartyl/glutamyl-tRNA
221	1	db$2	2::C3KA80|C3KA80_PSEFS	394	20438	16	16	10	10	14.72	Alkyl hydroperoxide re
222	1	db$2	2::C3K3F5|METK_PSEFS	393	42554	17	16	14	14	4.30	S-adenosylmethionine
223	1	db$2	2::C3K7G9|C3K7G9_PSEFS	392	48266	10	10	8	8	1.19	Putative modulation of
224	1	db$2	2::C3JXZ8|C3JXZ8_PSEFS	392	71568	16	16	12	12	1.21	Acetyl-coenzyme A syr
225	1	db$2	2::C3JXL8|C3JXL8_PSEFS	391	112023	18	17	15	14	0.89	Putative efflux protein
226	1	db$2	2::C3K0D4|C3K0D4_PSEFS	385	29228	12	12	8	8	3.27	Cell division inhibitor M
227	1	db$2	2::C3K422|C3K422_PSEFS	385	40250	9	9	7	7	1.27	Putative uroporphyrin-I
228	1	db$2	2::C3JZN6|IF3_PSEFS	385	20837	13	13	6	6	3.82	Translation initiation fa
229	1	db$2	2::C3K2X1|RL22_PSEFS	384	11904	9	9	6	6	9.42	50S ribosomal protein
230	1	db$2	2::C3JZ68|C3JZ68_PSEFS	383	24197	11	11	8	8	4.74	2-dehydro-3-deoxy-ph
231	1	db$2	2::C3K1L0|C3K1L0_PSEFS	380	55309	18	18	16	16	3.29	GTPase Der OS=Pseud
232	1	db$2	2::C3K9R4|C3K9R4_PSEFS	380	62164	22	20	17	16	2.65	Putative sulfite reducta
233	1	SwissProt_HUMAN$	3::P60709|ACTB_HUMAN	378	41710	14	14	13	13	3.88	Actin, cytoplasmic 1 OS
234	1	db$2	2::C3JXZ7|C3JXZ7_PSEFS	377	28415	12	12	8	8	3.44	Putative histidine-bindi
235	1	db$2	2::C3K0N2|C3K0N2_PSEFS	377	43313	7	7	6	6	1.15	3-oxoacyl-[acyl-carrier
236	1	db$2	2::C3KDD9|C3KDD9_PSEFS	376	41969	14	11	13	11	2.45	Cell division protein Fts
237	1	db$2	2::C3K4C7|C3K4C7_PSEFS	376	50044	14	14	11	11	2.11	Phosphate regulon sen
238	1	db$2	2::C3KAK3|C3KAK3_PSEFS	373	54608	13	13	10	10	1.38	Probable malate:quinoi
239	1	db$2	2::C3K401|C3K401_PSEFS	373	56255	16	15	16	15	2.53	Exopolyphosphatase O
240	1	db$2	2::C3K2C4|C3K2C4_PSEFS	371	89060	17	15	16	15	1.22	Endopeptidase La OS=
241	1	db$2	2::C3K2W4|RL5_PSEFS	370	20490	17	17	12	12	23.46	50S ribosomal protein
242	1	db$2	2::C3K3Z1|C3K3Z1_PSEFS	369	11808	11	11	5	5	14.80	Thioredoxin OS=Pseud
243	1	db$2	2::C3K3P4|C3K3P4_PSEFS	369	39333	15	13	8	7	1.95	Uncharacterized protei

↑Family	M	DB	Accession	Score	Mass	Matches	Match(sig)	Sequences	Seq(sig)	emPAI	Description
244	1	db$2	2::C3JYD5|C3JYD5_PSEFS	368	18453	11	11	7	7	6.63	Peptidyl-prolyl cis-tran:
245	1	db$2	2::C3K4G1|C3K4G1_PSEFS	367	81186	14	11	12	10	0.79	DNA helicase OS=Pseu
246	1	db$2	2::C3K475|ARGB_PSEFS	366	31926	13	13	12	12	5.80	Acetylglutamate kinase
247	1	db$2	2::C3K7N8|RDGC_PSEFS	366	34160	16	16	11	11	4.24	Recombination-associa
248	1	db$2	2::C3K6J6|C3K6J6_PSEFS	366	40307	13	12	10	9	1.87	Bifunctional chorismate
249	1	db$2	2::C3KBN3|C3KBN3_PSEFS	365	112655	15	15	13	13	0.73	Efflux pump membrane
249	2	db$2	2::C3KB18|C3KB18_PSEFS	160	115005	7	6	7	6	0.28	Efflux pump membrane
250	1	db$2	2::C3K6U8|C3K6U8_PSEFS	365	58654	10	10	6	6	0.76	Putative membrane pro
251	1	db$2	2::C3K8X5|HIS1_PSEFS	364	23098	14	14	10	10	10.49	ATP phosphoribosyltrar
252	1	db$2	2::C3K123|C3K123_PSEFS	363	41542	11	11	9	9	1.78	Citrate synthase OS=P
253	1	db$2	2::C3K865|C3K865_PSEFS	360	15847	10	8	5	5	3.35	Putative membrane pro
254	1	db$2	2::C3K474|C3K474_PSEFS	360	50732	14	13	12	11	2.06	Putative phosphomann
255	1	db$2	2::C3K2T2|C3K2T2_PSEFS	358	24648	9	9	7	7	4.57	Putative ABC transport
256	1	db$2	2::C3K2J8|C3K2J8_PSEFS	357	54392	13	11	11	10	1.60	AMP nucleosidase OS=
257	1	db$2	2::C3K274|DNAJ_PSEFS	357	40315	11	11	10	10	2.23	Chaperone protein Dna
258	1	db$2	2::C3K6A2|C3K6A2_PSEFS	357	27847	15	12	11	10	4.42	Putative D-methionine
258	2	db$2	2::C3KD95|C3KD95_PSEFS	230	28020	10	10	7	7	2.25	Putative exported prote
259	1	db$2	2::C3K7L5|C3K7L5_PSEFS	355	19131	10	10	7	7	6.12	Molybdenum cofactor b
260	1	db$2	2::C3K2W1|RL6_PSEFS	353	19185	16	15	9	8	7.99	50S ribosomal protein
261	1	db$2	2::C3K443|C3K443_PSEFS	352	12323	14	14	9	9	42.35	Nitrogen regulatory pro
262	1	db$2	2::C3K1X8|C3K1X8_PSEFS	349	11596	10	10	5	5	10.13	50S ribosomal protein
263	1	db$2	2::C3K638|C3K638_PSEFS	348	26394	9	9	6	6	2.48	Putative short chain de
264	1	db$2	2::C3K2W6|RL14_PSEFS	348	13401	12	11	9	8	21.61	50S ribosomal protein
265	1	db$2	2::C3K2Y5|RL10_PSEFS	347	17594	10	9	6	6	5.39	50S ribosomal protein
266	1	db$2	2::C3KEB4|C3KEB4_PSEFS	345	56978	17	15	17	15	2.48	Lysine--tRNA ligase OS
267	1	db$2	2::C3K6J9|KCY_PSEFS	344	24780	8	7	7	6	2.13	Cytidylate kinase OS=I
268	1	db$2	2::C3KDP1|CYSD_PSEFS	343	35173	13	13	10	10	4.00	Sulfate adenylyltransfe
269	1	db$2	2::C3K6K6|C3K6K6_PSEFS	342	36089	15	14	11	11	3.22	HTH-type transcription
270	1	db$2	2::C3KEA4|C3KEA4_PSEFS	341	51722	10	10	9	9	1.50	PINc domain-containin
271	1	db$2	2::C3K6G2|C3K6G2_PSEFS	340	106936	20	18	19	18	1.22	Valine--tRNA ligase OS
272	1	db$2	2::C3JZ71|C3JZ71_PSEFS	340	31565	11	10	10	9	2.84	Hex regulon repressor
273	1	db$2	2::C3KDU3|C3KDU3_PSEFS	339	40518	12	11	10	9	1.86	Beta sliding clamp OS=
274	1	db$2	2::C3K3U2|C3K3U2_PSEFS	339	50985	13	13	12	12	2.05	Putative glutamine syn
275	1	db$2	2::C3JYD4|C3JYD4_PSEFS	339	64707	12	12	10	10	1.24	Glutamine--tRNA ligase
276	1	db$2	2::C3JYU6|C3JYU6_PSEFS	339	42196	10	9	8	7	1.19	Arginine biosynthesis b
277	1	db$2	2::C3K8U4|UBIE_PSEFS	335	28320	17	14	12	10	5.23	Ubiquinone/menaquinc
278	1	db$2	2::C3K6E1|RL13_PSEFS	334	15695	14	12	8	7	9.87	50S ribosomal protein
279	1	db$2	2::C3K3P0|ILVD_PSEFS	334	65667	14	14	13	13	1.55	Dihydroxy-acid dehydr
280	1	db$2	2::C3K9U6|C3K9U6_PSEFS	334	35232	11	11	8	8	2.34	Putative 6-phosphoglu
281	1	db$2	2::C3K3V3|ARGA_PSEFS	332	47793	13	12	12	11	1.97	Amino-acid acetyltrans

↑Family	M	DB	Accession	Score	Mass	Matches	Match(sig)	Sequences	Seq(sig)	emPAI	Description
282	1	db$2	2::C3KDL2|C3KDL2_PSEFS	331	52736	10	9	10	9	1.24	Putative outer membra
283	1	db$2	2::C3K187|C3K187_PSEFS	331	38556	13	12	9	9	2.41	Peptidase_M75 domain
284	1	db$2	2::C3K2V2|RS4_PSEFS	330	23062	14	13	11	10	8.37	30S ribosomal protein
285	1	db$2	2::C3KE59|C3KE59_PSEFS	329	27398	14	11	12	10	4.57	DNA-binding response
286	1	db$2	2::C3K2Y0|RS7_PSEFS	329	17636	15	14	12	11	17.46	30S ribosomal protein
287	1	db$2	2::C3K734|C3K734_PSEFS	327	44623	12	12	8	8	1.88	Putative aminotransfer
288	1	db$2	2::C3JZT0|C3JZT0_PSEFS	326	28122	8	8	6	6	1.73	Putative GntR-family tr
289	1	db$2	2::C3K2X6|RL3_PSEFS	326	22550	11	9	8	7	3.30	50S ribosomal protein
290	1	SwissProt_HUMAN$	3::P00761|TRYP_PIG	326	24394	11	11	6	6	3.66	Trypsin OS=Sus scrofa
291	1	db$2	2::C3JY70|C3JY70_PSEFS	324	50668	12	12	11	11	1.80	Adenylosuccinate lyase
292	1	db$2	2::C3JZ61|C3JZ61_PSEFS	323	61509	13	12	10	9	1.16	Acyl-CoA synthetase (L
293	1	db$2	2::C3KDW3|HFQ_PSEFS	323	9318	15	15	8	8	84.46	RNA-binding protein Hf
294	1	db$2	2::C3K358|C3K358_PSEFS	322	65409	12	11	10	9	1.06	Putative dehydrogenas
294	2	db$2	2::C3K355|C3K355_PSEFS	321	63710	10	10	9	9	0.95	Putative dehydrogenas
295	1	db$2	2::C3KBI6|C3KBI6_PSEFS	322	106629	13	13	12	12	0.78	Uncharacterized protei
296	1	db$2	2::C3K0W7|C3K0W7_PSEFS	321	31068	11	10	8	7	1.89	Metallophos_2 domain-
297	1	db$2	2::C3K4M6|C3K4M6_PSEFS	321	57818	15	14	10	10	1.46	Putative exported alkal
298	1	db$2	2::C3KDW6|C3KDW6_PSEFS	321	33055	17	15	13	12	6.36	Protein HflC OS=Pseud
299	1	db$2	2::C3K325|C3K325_PSEFS	320	49076	16	13	13	11	1.89	Chaperone SurA OS=P
300	1	db$2	2::C3K7C1|SECB_PSEFS	320	17615	8	7	5	4	2.76	Protein-export protein
301	1	db$2	2::C3KCR8|C3KCR8_PSEFS	319	39907	16	15	9	9	2.67	Ribosome-binding ATPa
302	1	db$2	2::C3K2Y7|RL11_PSEFS	319	14903	7	7	4	4	3.80	50S ribosomal protein
303	1	db$2	2::C3K2S0|C3K2S0_PSEFS	319	39319	9	9	6	6	1.32	3,4-dihydroxy-2-butan
304	1	db$2	2::C3K5Z8|RRF_PSEFS	319	20297	12	11	6	6	5.34	Ribosome-recycling fac
305	1	db$2	2::C3K2V7|RL15_PSEFS	319	15215	13	11	8	8	20.50	50S ribosomal protein
306	1	db$2	2::C3K278|C3K278_PSEFS	318	15153	8	8	5	5	7.56	Ferric uptake regulatio
307	1	db$2	2::C3JY83|NUOCD_PSEFS	317	67660	25	22	22	20	3.66	NADH-quinone oxidore
308	1	db$2	2::C3K8X0|C3K8X0_PSEFS	317	16351	11	11	8	8	12.15	Putative ABC transport
309	1	db$2	2::C3K1F2|ATP6_PSEFS	317	31626	8	8	3	3	0.56	ATP synthase subunit a
310	1	db$2	2::C3JY62|C3JY62_PSEFS	317	83224	20	18	16	15	1.49	ATP-dependent Clp pro
310	2	db$2	2::C3K4B3|C3K4B3_PSEFS	76	97007	8	7	7	6	0.34	Chaperone OS=Pseudo
311	1	db$2	2::C3K2W5|RL24_PSEFS	316	11328	13	12	7	7	25.43	50S ribosomal protein
312	1	db$2	2::C3K3N4|COAD_PSEFS	315	17810	16	14	10	9	17.14	Phosphopantetheine ac
313	1	db$2	2::C3K1Y2|C3K1Y2_PSEFS	315	21662	9	9	7	7	4.67	Peptidyl-prolyl cis-tran
314	1	db$2	2::C3KAK7|C3KAK7_PSEFS	314	20540	7	7	4	4	2.13	Putative lipoprotein OS
315	1	db$2	2::C3K5Y0|C3K5Y0_PSEFS	313	38747	11	11	9	9	2.00	NADP(H)-dependt aldo
316	1	db$2	2::C3K0N4|C3K0N4_PSEFS	313	25612	9	8	6	5	2.01	3-oxoacyl-[acyl-carrier
317	1	db$2	2::C3K3K2|PYRB_PSEFS	311	36271	8	8	6	6	1.18	Aspartate carbamoyltra
318	1	db$2	2::C3K0H1|C3K0H1_PSEFS	311	17848	10	9	8	7	7.12	Chemotaxis protein Ch
319	1	db$2	2::C3K1L3|SYH_PSEFS	310	47853	14	14	12	12	2.99	Histidine--tRNA ligase (

↑Family	M	DB	Accession	Score	Mass	Matches	Match(sig)	Sequences	Seq(sig)	emPAI	Description
320	1	db$2	2::C3K2S6|C3K2S6_PSEFS	309	31662	10	9	7	6	1.84	Putative thioredoxin OS
321	1	db$2	2::C3JYS5|C3JYS5_PSEFS	307	25332	7	7	5	5	2.05	Tol-Pal system protein
322	1	db$2	2::C3K6C2|C3K6C2_PSEFS	307	27720	9	9	8	8	2.88	Putative amino acid AB
323	1	db$2	2::C3KCT2|MUTS_PSEFS	306	95536	11	11	10	10	0.64	DNA mismatch repair p
324	1	db$2	2::C3K8V3|HSLU_PSEFS	306	49607	16	15	12	11	1.85	ATP-dependent proteas
325	1	db$2	2::C3K5Z7|C3K5Z7_PSEFS	305	26574	14	13	9	8	7.40	Uridylate kinase OS=P
326	1	db$2	2::C3KE61|RIMK_PSEFS	305	32488	11	9	8	7	2.19	Probable alpha-L-gluta
327	1	db$2	2::C3K2Y8|C3K2Y8_PSEFS	304	20023	12	9	9	7	5.53	Transcription terminati
328	1	db$2	2::C3K1G2|YIDC_PSEFS	304	61977	13	13	12	12	1.50	Membrane protein inse
329	1	db$2	2::C3K614|C3K614_PSEFS	302	41572	13	13	10	10	2.49	3-ketoacyl-CoA thiolas
330	1	db$2	2::C3K5J3|C3K5J3_PSEFS	302	50388	9	9	9	9	1.33	AMP nucleosidase OS=
331	1	db$2	2::C3KAC1|C3KAC1_PSEFS	301	27548	16	15	11	10	6.78	Putative phenylacetic a
332	1	db$2	2::C3KDL6|C3KDL6_PSEFS	300	23139	8	8	5	5	1.77	ADP-ribose diphosphat
333	1	db$2	2::C3KBL1|C3KBL1_PSEFS	300	61934	12	11	11	11	1.32	Putative medium-chain
334	1	db$2	2::C3K645|C3K645_PSEFS	298	24522	7	6	7	6	2.16	Putative ABC transport
335	1	db$2	2::C3KDM2|C3KDM2_PSEFS	296	83508	15	15	13	13	1.09	DNA topoisomerase 4 s
336	1	db$2	2::C3K2X7|RS10_PSEFS	296	11745	19	15	8	6	14.80	30S ribosomal protein
337	1	db$2	2::C3K7X9|C3K7X9_PSEFS	294	40858	12	12	8	8	2.18	Carboxynorspermidine,
338	1	db$2	2::C3JZV0|C3JZV0_PSEFS	293	56140	12	11	12	11	1.53	Glutamate--tRNA ligase
339	1	db$2	2::C3KBL8|C3KBL8_PSEFS	293	56970	17	17	16	16	2.78	Glycerol-3-phosphate d
340	1	db$2	2::C3JZN3|SYFA_PSEFS	292	37892	11	11	9	9	2.94	Phenylalanine--tRNA lig
341	1	db$2	2::C3KE79|C3KE79_PSEFS	290	29713	4	4	3	3	0.61	NH(3)-dependent NAD
342	1	db$2	2::C3K6X1|C3K6X1_PSEFS	290	39290	12	11	11	10	2.33	Uncharacterized protei
343	1	db$2	2::C3K2V0|RL17_PSEFS	290	14407	13	12	7	7	33.91	50S ribosomal protein
344	1	db$2	2::C3K2Z3|C3K2Z3_PSEFS	290	44017	13	13	11	11	3.03	Tyrosine--tRNA ligase (
345	1	db$2	2::C3JYW3|C3JYW3_PSEFS	289	36818	14	13	11	10	2.60	Branched-chain-amino-
346	1	db$2	2::C3JZS4|C3JZS4_PSEFS	289	33375	11	11	7	7	2.09	Acetyl-coenzyme A car
347	1	db$2	2::C3K3V5|C3K3V5_PSEFS	289	52101	10	10	8	8	1.07	CYTH domain-containir
348	1	db$2	2::C3K604|C3K604_PSEFS	287	18933	18	17	12	12	23.73	Putative outer membra
349	1	db$2	2::C3KAH3|SYR_PSEFS	286	63549	10	10	8	8	0.95	Arginine--tRNA ligase (
350	1	db$2	2::C3KAR0|C3KAR0_PSEFS	286	79563	13	11	11	9	0.81	Putative exported isoqu
351	1	db$2	2::C3K2Q0|C3K2Q0_PSEFS	286	19348	11	11	8	8	7.87	Inorganic pyrophospha
352	1	db$2	2::C3K6G8|LEPA_PSEFS	284	66372	8	8	7	7	0.65	Elongation factor 4 OS
353	1	db$2	2::C3K3M4|C3K3M4_PSEFS	282	49883	8	8	8	8	1.14	Signal recognition part
354	1	db$2	2::C3K6M3|C3K6M3_PSEFS	281	13365	10	8	7	7	10.53	Succinate dehydrogena
355	1	db$2	2::C3KCS2|RSMH_PSEFS	280	34344	12	12	10	10	2.96	Ribosomal RNA small s
356	1	db$2	2::C3K3W7|C3K3W7_PSEFS	279	36662	11	10	9	8	2.19	Putative iron (III) ABC
357	1	db$2	2::C3K6E2|RS9_PSEFS	279	14607	10	10	6	6	11.75	30S ribosomal protein
358	1	db$2	2::C3KAJ2|C3KAJ2_PSEFS	279	53335	12	11	11	10	1.43	Methylmalonate-semia
359	1	db$2	2::C3K610|C3K610_PSEFS	276	70283	13	12	12	11	1.10	ATP-binding protein Uu

↑Family	M	DB	Accession	Score	Mass	Matches	Match(sig)	Sequences	Seq(sig)	emPAI	Description
360	1	db$2	2::C3KDU9|C3KDU9_PSEFS	276	34261	11	11	8	8	2.00	Serine O-acetyltransfe
361	1	db$2	2::C3K5H8|C3K5H8_PSEFS	275	17826	7	7	6	6	3.81	STAS domain-containir
362	1	db$2	2::C3K4H9|C3K4H9_PSEFS	275	34203	10	8	7	6	1.29	Quinone oxidoreductas
363	1	SwissProt_HUMAN$	3::P07355|ANXA2_HUMAN	275	38580	13	10	13	10	2.40	Annexin A2 OS=Homo
364	1	db$2	2::C3K4A8|C3K4A8_PSEFS	275	55832	10	8	9	8	0.97	Uncharacterized protei
365	1	db$2	2::C3KE41|SYGA_PSEFS	274	36196	15	13	13	11	3.20	Glycine--tRNA ligase al
366	1	db$2	2::C3K3H0|C3K3H0_PSEFS	273	67796	10	10	10	10	1.01	ATP-dependent RNA he
367	1	db$2	2::C3K6S8|C3K6S8_PSEFS	272	40355	10	10	9	9	2.22	Putative acetyl-CoA tra
368	1	db$2	2::C3K435|C3K435_PSEFS	271	45402	12	12	11	11	2.14	Diaminopimelate decar
369	1	db$2	2::C3KCN5|C3KCN5_PSEFS	271	142346	14	11	13	11	0.44	Bifunctional protein Pu
370	1	db$2	2::C3JZ29|C3JZ29_PSEFS	271	41047	8	8	7	7	1.24	Putative glycerophosph
371	1	db$2	2::C3K476|PYRE_PSEFS	269	23079	5	5	4	4	1.26	Orotate phosphoribosy
372	1	db$2	2::C3JYQ7|C3JYQ7_PSEFS	268	32635	9	9	7	7	1.75	Carbamate kinase OS=
373	1	db$2	2::C3K3D2|BETB_PSEFS	267	53123	11	10	9	9	1.23	Betaine aldehyde dehy
374	1	db$2	2::C3K667|C3K667_PSEFS	266	48557	13	12	11	11	2.22	Conserved hypothetica
375	1	db$2	2::C3K5V1|C3K5V1_PSEFS	264	47050	9	9	8	8	1.23	Outer membrane prote
376	1	db$2	2::C3K403|C3K403_PSEFS	264	36964	11	10	7	7	1.78	Delta-aminolevulinic ac
377	1	db$2	2::C3K116|PSRP_PSEFS	261	30838	7	6	7	6	1.50	Putative phosphoenolp
378	1	db$2	2::C3K091|C3K091_PSEFS	260	20089	9	9	6	6	3.06	GTP cyclohydrolase 1 C
378	2	db$2	2::C3K1W5|C3K1W5_PSEFS	100	20960	5	5	4	4	1.45	GTP cyclohydrolase 1 C
379	1	db$2	2::C3K6L0|EFP_PSEFS	260	21303	9	8	8	7	3.67	Elongation factor P OS
380	1	db$2	2::C3K0T1|C3K0T1_PSEFS	260	94111	10	10	9	9	0.57	Aconitate hydratase OS
381	1	db$2	2::C3JYR3|C3JYR3_PSEFS	260	40135	11	11	9	9	1.88	Glycine cleavage syste
382	1	db$2	2::C3JXZ3|C3JXZ3_PSEFS	258	28123	11	10	9	8	2.82	Histidine transport ATP
382	2	db$2	2::C3K713|C3K713_PSEFS	25	28108	2	2	2	2	0.40	Arginine/ornithine ABC
383	1	db$2	2::C3K1M9|C3K1M9_PSEFS	257	29586	11	11	9	9	3.18	Inositol-1-monophosph
384	1	db$2	2::C3JZ86|C3JZ86_PSEFS	257	21964	16	14	13	12	11.97	Superoxide dismutase
385	1	db$2	2::C3K8X6|C3K8X6_PSEFS	257	47255	11	9	10	8	1.23	Histidinol dehydrogena
386	1	db$2	2::C3KBQ2|C3KBQ2_PSEFS	257	37020	11	11	9	9	2.57	Putative quinone oxido
387	1	db$2	2::C3KCT1|MURC_PSEFS	256	52132	7	7	6	6	0.72	UDP-N-acetylmuramat
388	1	db$2	2::C3K3Q2|C3K3Q2_PSEFS	255	37335	11	10	6	6	1.75	Putative cation ABC tra
389	1	db$2	2::C3K469|RL28_PSEFS	255	8845	10	9	5	5	12.34	50S ribosomal protein
390	1	db$2	2::C3KE31|C3KE31_PSEFS	255	22237	10	8	9	8	4.43	RNA polymerase sigma
391	1	db$2	2::C3K3T9|C3K3T9_PSEFS	254	49785	11	10	10	9	1.35	Putative aminotransfer
392	1	db$2	2::C3K6D9|C3K6D9_PSEFS	250	45394	11	9	9	7	1.07	Phosphoribosylamine--
393	1	db$2	2::C3KDM7|C3KDM7_PSEFS	250	29519	8	7	5	5	1.22	Sulfurtransferase OS=I
394	1	db$2	2::C3K1N3|C3K1N3_PSEFS	249	11888	8	6	5	3	2.23	Sec translocon accesso
395	1	db$2	2::C3K4V4|C3K4V4_PSEFS	249	32182	11	10	10	9	2.74	Putative ATP-binding p
396	1	db$2	2::C3K6E0|PUR9_PSEFS	248	57370	11	11	8	8	1.10	Bifunctional purine bios
397	1	db$2	2::C3K0Q9|C3K0Q9_PSEFS	247	15688	6	6	4	4	3.44	Flagellar basal-body ro

↑Family	M	DB	Accession	Score	Mass	Matches	Match(sig)	Sequences	Seq(sig)	emPAI	Description
398	1	db$2	2::C3K0Q7|C3K0Q7_PSEFS	247	45255	8	8	5	5	0.87	Flagellar hook protein I
399	1	db$2	2::C3K3T6|C3K3T6_PSEFS	247	42390	10	9	8	7	1.44	Spermidine/putrescine
400	1	db$2	2::C3K1X2|C3K1X2_PSEFS	246	40166	9	8	9	8	1.56	Putative putative oxido
401	1	db$2	2::C3K1H6|PURT_PSEFS	246	42327	13	12	13	12	2.82	Formate-dependent ph
402	1	db$2	2::C3KDB7|C3KDB7_PSEFS	246	65793	7	7	7	7	0.65	ATP-dependent lipid A-
402	2	db$2	2::C3K608|C3K608_PSEFS	51	64881	2	2	2	2	0.16	Putative ABC transport
403	1	db$2	2::C3K2Z7|ARGC_PSEFS	245	36612	12	11	9	8	2.19	N-acetyl-gamma-gluta
404	1	db$2	2::C3K6V2|HIS4_PSEFS	245	25679	10	8	8	6	2.61	1-(5-phosphoribosyl)-5
405	1	db$2	2::C3K6R4|C3K6R4_PSEFS	245	18727	12	10	6	5	4.74	Uncharacterized protei
406	1	db$2	2::C3K075|C3K075_PSEFS	244	77370	9	9	8	8	0.63	Putative sulfatase OS=
407	1	db$2	2::C3KDM6|PSD_PSEFS	241	31380	7	7	5	5	1.46	Phosphatidylserine dec
408	1	db$2	2::C3K6S9|C3K6S9_PSEFS	241	101727	10	9	10	9	0.52	DNA polymerase I OS=
409	1	db$2	2::C3K1X5|C3K1X5_PSEFS	241	39754	9	9	8	8	1.59	Glutamate 5-kinase OS
410	1	db$2	2::C3K245|C3K245_PSEFS	241	53442	13	12	12	11	1.65	Poly(A) polymerase I C
411	1	db$2	2::C3JZ88|C3JZ88_PSEFS	240	47232	7	7	5	5	1.01	Putative exported prote
412	1	db$2	2::C3JY00|C3JY00_PSEFS	240	47211	9	9	8	8	1.23	Ribonucleoside-diphosp
413	1	db$2	2::C3K6Z0|PDXJ_PSEFS	240	26758	8	8	7	7	2.43	Pyridoxine 5'-phosphat
414	1	db$2	2::C3K0K7|C3K0K7_PSEFS	238	60595	14	12	11	9	1.02	Electron transfer flavop
415	1	db$2	2::C3K759|C3K759_PSEFS	237	35694	5	5	4	4	0.70	Putative dehydrogenas
415	2	db$2	2::C3K0S6|C3K0S6_PSEFS	115	36802	2	2	2	2	0.29	Putative oxidoreductas
416	1	db$2	2::C3K1H2|C3K1H2_PSEFS	237	49205	7	6	7	6	0.78	Signal recognition part
417	1	db$2	2::C3KDL3|C3KDL3_PSEFS	236	70148	10	9	10	9	0.84	Phosphomethylpyrimid
418	1	db$2	2::C3JZT7|C3JZT7_PSEFS	236	38793	11	10	8	8	2.00	3-isopropylmalate dehy
419	1	db$2	2::C3K249|G6PI_PSEFS	235	61384	16	14	13	12	1.52	Glucose-6-phosphate is
420	1	db$2	2::C3K1F4|C3K1F4_PSEFS	235	31961	8	7	7	6	1.42	Probable chromosome-
421	1	db$2	2::C3K264|GLMM_PSEFS	233	47719	13	12	12	11	2.28	Phosphoglucosamine m
422	1	db$2	2::C3K185|C3K185_PSEFS	233	20062	8	8	5	5	4.12	Putative NAD(P)H nitro
423	1	db$2	2::C3KEC6|KAD_PSEFS	232	23251	11	11	7	7	5.19	Adenylate kinase OS=P
424	1	db$2	2::C3JYV5|C3JYV5_PSEFS	230	45487	8	8	7	7	1.30	Putative oxidoreductas
425	1	db$2	2::C3K2E3|MSCL_PSEFS	230	14568	5	5	2	2	1.62	Large-conductance me
426	1	db$2	2::C3KDC6|RF1_PSEFS	229	39857	4	4	2	2	0.27	Peptide chain release f
427	1	db$2	2::C3K859|C3K859_PSEFS	229	59064	7	6	6	5	0.49	Putative glutamate syn
428	1	db$2	2::C3K0N5|C3K0N5_PSEFS	228	32402	7	6	5	5	1.07	Malonyl CoA-acyl carrie
429	1	db$2	2::C3KAA8|C3KAA8_PSEFS	227	132643	14	12	12	11	0.48	Histidine kinase OS=Ps
430	1	db$2	2::C3K4V8|C3K4V8_PSEFS	227	128447	9	8	9	8	0.34	Transcription-repair-co
431	1	db$2	2::C3K5A9|RF3_PSEFS	226	59584	13	11	10	8	0.89	Peptide chain release f
432	1	db$2	2::C3K2W9|RL16_PSEFS	226	15422	7	7	6	6	5.14	50S ribosomal protein
433	1	db$2	2::C3K5E5|C3K5E5_PSEFS	225	28503	8	8	6	6	2.18	Methionine aminopepti
434	1	db$2	2::C3K0H6|C3K0H6_PSEFS	225	40054	6	6	6	6	1.03	Protein-glutamate met
435	1	db$2	2::C3K1U7|C3K1U7_PSEFS	225	36908	8	8	3	3	1.44	Ubiquinol oxidase subu

↑Family	M	DB	Accession	Score	Mass	Matches	Match(sig)	Sequences	Seq(sig)	emPAI	Description
436	1	db$2	2::C3KAJ8|UPP_PSEFS	224	22986	14	11	9	8	4.13	Uracil phosphoribosyltr
437	1	db$2	2::C3K2K9|C3K2K9_PSEFS	224	31417	12	8	10	7	1.86	Magnesium and cobalt
438	1	db$2	2::C3K527|C3K527_PSEFS	224	65564	8	8	6	6	0.66	Putative peptidase OS=
439	1	db$2	2::C3K9R8|C3K9R8_PSEFS	224	135461	11	9	9	8	0.32	Methionine synthase O
440	1	db$2	2::C3KE43|C3KE43_PSEFS	223	32711	7	6	6	5	1.05	Lipid A biosynthesis lau
441	1	db$2	2::C3KBY9|C3KBY9_PSEFS	223	24532	5	5	5	5	1.61	Ribonuclease T OS=Ps
442	1	db$2	2::C3K324|C3K324_PSEFS	223	105271	13	13	11	11	0.64	LPS-assembly protein l
443	1	db$2	2::C3K6F9|C3K6F9_PSEFS	222	37389	11	10	9	8	1.74	Nucleoid-associated pr
443	2	db$2	2::A4V7R1|A4V7R1_PSEFS	28	37718	2	2	2	2	0.28	Putative nucleoid-asso
444	1	db$2	2::C3K2I0|C3K2I0_PSEFS	222	48538	8	8	8	8	1.18	Putative ATP-depender
445	1	db$2	2::C3KBQ3|C3KBQ3_PSEFS	220	33274	7	7	6	6	1.34	LysR-family transcripti
446	1	db$2	2::C3KBL3|C3KBL3_PSEFS	220	50616	10	10	8	8	1.32	Putative exported prot
447	1	db$2	2::C3K5X7|C3K5X7_PSEFS	219	42054	10	10	10	10	2.07	Cell division protein Za
448	1	db$2	2::C3K2M1|C3K2M1_PSEFS	219	42033	15	14	12	12	3.31	Serine-type D-Ala-D-A
449	1	db$2	2::C3JYU1|Y4927_PSEFS	218	18225	9	9	6	6	6.82	UPF0234 protein PFLU_
450	1	db$2	2::C3JYR4|C3JYR4_PSEFS	218	7675	5	5	3	3	5.01	Major cold shock prote
451	1	db$2	2::C3K1A1|C3K1A1_PSEFS	217	10298	10	9	7	7	21.98	10 kDa chaperonin OS
452	1	db$2	2::C3K177|C3K177_PSEFS	217	65030	13	13	12	12	1.40	Phosphogluconate deh
453	1	db$2	2::C3KAH1|HSLV_PSEFS	216	18731	6	6	4	4	2.48	ATP-dependent proteas
454	1	db$2	2::C3K651|C3K651_PSEFS	216	39766	13	9	11	8	1.59	Phospho-2-dehydro-3-
455	1	db$2	2::C3K152|C3K152_PSEFS	216	13999	10	9	8	7	9.29	Putative transcriptional
456	1	db$2	2::C3K2S1|C3K2S1_PSEFS	216	23236	7	7	4	4	1.75	Riboflavin synthase alp
457	1	db$2	2::C3K310|C3K310_PSEFS	215	54246	10	9	7	6	1.19	Anthranilate synthase
458	1	db$2	2::C3K368|C3K368_PSEFS	215	37353	5	5	5	5	0.88	Stress response kinase
459	1	db$2	2::C3JXY9|C3JXY9_PSEFS	214	37073	8	8	7	7	1.44	Putative arginine N-su
460	1	db$2	2::C3JZ69|C3JZ69_PSEFS	214	25612	5	5	5	5	1.50	6-phosphogluconolacto
461	1	db$2	2::C3KEB7|C3KEB7_PSEFS	214	48036	9	9	8	8	1.42	Uncharacterized protei
462	1	db$2	2::C3K2U9|C3K2U9_PSEFS	214	53651	14	12	11	9	1.21	Catalase OS=Pseudom
463	1	db$2	2::C3K3T8|C3K3T8_PSEFS	214	39499	8	8	6	6	1.31	Putrescine ABC transp
464	1	db$2	2::C3K3S1|C3K3S1_PSEFS	213	51070	10	9	9	8	1.30	Putative oxidase OS=P
465	1	db$2	2::C3K3P5|C3K3P5_PSEFS	213	48453	6	6	4	4	0.48	Putative symporter me
466	1	db$2	2::C3JY82|NUOB_PSEFS	213	25341	8	8	6	6	2.67	NADH-quinone oxidore
467	1	db$2	2::C3K6Y4|GATA_PSEFS	212	51737	7	7	6	6	0.90	Glutamyl-tRNA(Gln) an
468	1	db$2	2::C3K7M6|C3K7M6_PSEFS	212	88918	9	8	6	5	0.38	Acyl-coenzyme A dehy
469	1	db$2	2::C3JYT9|C3JYT9_PSEFS	212	46635	8	8	5	5	0.84	Putative family S43 no
470	1	db$2	2::C3K490|C3K490_PSEFS	211	33505	13	13	10	10	3.08	Putative oxidative stre
471	1	db$2	2::C3K6V9|C3K6V9_PSEFS	210	54793	11	11	8	8	1.17	2,3-bisphosphoglycera
472	1	db$2	2::C3K421|C3K421_PSEFS	210	46244	12	11	12	11	2.08	Putative HemY domain
473	1	db$2	2::C3KE80|C3KE80_PSEFS	210	46502	10	9	10	9	1.50	Nicotinate phosphoribo
474	1	db$2	2::C3KED5|C3KED5_PSEFS	209	40447	9	9	8	8	1.55	Putative lipoprotein OS

↑Family	M	DB	Accession	Score	Mass	Matches	Match(sig)	Sequences	Seq(sig)	emPAI	Description
475	1	SwissProt_HUMAN$	3::P01834|IGKC_HUMAN	209	11758	7	5	5	4	3.84	Immunoglobulin kappa
476	1	db$2	2::C3KBN2|C3KBN2_PSEFS	208	52689	8	8	5	5	0.87	Outer membrane prote
477	1	db$2	2::C3JY85|C3JY85_PSEFS	208	48759	15	15	13	13	2.89	NADH-quinone oxidore
478	1	db$2	2::C3K5I7|C3K5I7_PSEFS	207	59867	13	12	9	9	1.39	Probable malate:quino
479	1	db$2	2::C3K0S7|Y4577_PSEFS	205	11900	4	4	3	3	3.77	Nucleoid-associated pr
480	1	db$2	2::C3K841|C3K841_PSEFS	205	11596	12	12	6	6	36.14	Putative sigma-54 moc
481	1	db$2	2::C3K8U2|UBIB_PSEFS	204	61616	5	5	5	5	0.47	Probable protein kinase
482	1	db$2	2::C3K9M2|C3K9M2_PSEFS	204	37830	9	9	7	7	1.39	Putative peptidase OS=
483	1	db$2	2::C3KED4|C3KED4_PSEFS	204	31038	5	5	5	5	1.13	4-hydroxy-tetrahydrod
484	1	db$2	2::C3K2X2|RS19_PSEFS	203	10313	7	7	5	5	8.38	30S ribosomal protein
485	1	db$2	2::C3KBX9|C3KBX9_PSEFS	203	20403	5	5	3	3	1.51	Putative phospholipid-t
486	1	db$2	2::C3K102|PUR5_PSEFS	203	36950	11	8	8	6	1.44	Phosphoribosylformylgl
487	1	db$2	2::C3KE83|C3KE83_PSEFS	203	38700	6	6	5	5	0.84	Outer membrane prote
488	1	db$2	2::C3KCL0|C3KCL0_PSEFS	203	35424	8	7	7	6	1.22	Uncharacterized protei
489	1	db$2	2::C3JYJ6|C3JYJ6_PSEFS	201	68407	10	9	9	8	0.87	Peptidyl-prolyl cis-tran
490	1	db$2	2::C3K8Z6|C3K8Z6_PSEFS	201	39611	10	9	8	8	1.59	Branched amino acid A
491	1	db$2	2::C3K8W9|C3K8W9_PSEFS	201	28276	6	6	6	6	1.71	Intermembrane phospl
492	1	db$2	2::C3K2V3|RS11_PSEFS	201	13607	5	5	4	4	2.91	30S ribosomal protein
493	1	db$2	2::C3K2E9|C3K2E9_PSEFS	201	53251	5	5	3	3	0.43	Putative cytochrome o
494	1	db$2	2::C3K0W5|PHS_PSEFS	201	13357	7	7	5	5	7.13	Putative pterin-4-alpha
495	1	db$2	2::C3K9U7|C3K9U7_PSEFS	201	56147	7	6	7	6	0.66	Glucose-6-phosphate 1
496	1	db$2	2::Q84CP9|DAPB_PSEFS	199	28276	11	10	9	8	2.78	4-hydroxy-tetrahydrod
497	1	db$2	2::C3K1L2|C3K1L2_PSEFS	199	22708	6	6	6	6	2.45	Ancillary SecYEG transl
498	1	db$2	2::C3K4G2|C3K4G2_PSEFS	199	30728	7	7	4	4	1.92	Putative exported prote
499	1	db$2	2::C3K0G3|PPNP_PSEFS	198	10127	7	7	3	3	5.23	Pyrimidine/purine nucl
500	1	db$2	2::C3KC10|C3KC10_PSEFS	197	23080	4	4	3	3	0.84	Uncharacterized protei
501	1	db$2	2::C3KDU8|C3KDU8_PSEFS	197	35029	8	8	6	6	1.24	D-cysteine desulfhydra
502	1	db$2	2::C3KCP5|C3KCP5_PSEFS	196	109710	9	8	9	8	0.41	Bifunctional glutamine
503	1	db$2	2::C3KBZ7|SYM_PSEFS	195	75525	16	14	14	12	1.26	Methionine--tRNA ligas
504	1	db$2	2::C3K796|C3K796_PSEFS	195	49835	5	5	5	5	0.61	Putative glutamine syn
505	1	db$2	2::C3K254|C3K254_PSEFS	195	21027	7	7	5	5	2.06	Osmotically inducible p
506	1	db$2	2::C3KAI6|C3KAI6_PSEFS	195	24263	8	8	7	7	2.87	Putative dehydrogenas
507	1	db$2	2::C3KBP9|C3KBP9_PSEFS	195	58780	8	7	7	7	0.76	Phosphoglucomutase C
508	1	db$2	2::C3K5H7|C3K5H7_PSEFS	195	43348	7	6	6	5	0.72	Putative response regu
509	1	db$2	2::C3KDX7|ISPH_PSEFS	194	34499	8	7	7	6	1.27	4-hydroxy-3-methylbu
510	1	db$2	2::C3K3Y8|UBID_PSEFS	194	54697	7	6	7	6	0.68	3-octaprenyl-4-hydrox
511	1	db$2	2::C3K6A5|C3K6A5_PSEFS	193	78572	9	9	8	8	0.62	Catalase OS=Pseudom
512	1	db$2	2::C3JZS5|TRPF_PSEFS	193	21966	3	3	2	2	0.53	N-(5'-phosphoribosyl)a
513	1	db$2	2::C3K142|C3K142_PSEFS	193	32099	10	10	8	8	2.75	2-methylisocitrate lyas
514	1	db$2	2::C3K1E4|GLMU_PSEFS	192	48690	5	5	5	5	0.62	Bifunctional protein Glr

↑Family	M	DB	Accession	Score	Mass	Matches	Match(sig)	Sequences	Seq(sig)	emPAI	Description
515	1	db$2	2::C3KBZ0|PYRC_PSEFS	192	38385	6	6	5	5	0.85	Dihydroorotase OS=Ps
516	1	db$2	2::C3JXY2|C3JXY2_PSEFS	192	35731	6	6	3	3	0.70	L-allo-threonine aldola:
517	1	db$2	2::C3K2N8|C3K2N8_PSEFS	192	23444	8	8	7	7	3.07	Semialdhyde_dh doma
518	1	db$2	2::C3K5H5|C3K5H5_PSEFS	190	25721	8	8	5	5	1.99	Putative exported prot
519	1	db$2	2::C3K4E4|C3K4E4_PSEFS	190	51389	7	7	7	7	0.90	Aspartate ammonia-lya
520	1	db$2	2::C3JZU0|C3JZU0_PSEFS	190	50538	12	10	11	9	1.32	3-isopropylmalate deh
521	1	db$2	2::C3K452|XPT_PSEFS	190	20267	7	7	6	6	4.03	Xanthine phosphoribos
522	1	db$2	2::C3KAI4|C3KAI4_PSEFS	189	53827	4	4	3	3	0.30	SPOR domain-containir
523	1	db$2	2::C3K1L1|C3K1L1_PSEFS	186	40864	8	8	6	6	1.00	Outer membrane prote
524	1	db$2	2::C3K360|C3K360_PSEFS	183	23509	6	6	5	5	1.71	ATP-dependent dethiol
525	1	db$2	2::C3K611|C3K611_PSEFS	183	16065	6	6	3	3	2.19	Universal stress proteir
526	1	db$2	2::C3K3L7|THIG_PSEFS	183	28373	10	9	8	8	2.76	Thiazole synthase OS=
527	1	db$2	2::C3KDX0|C3KDX0_PSEFS	183	16992	5	5	3	3	1.28	Peptidyl-prolyl cis-tran:
528	1	db$2	2::C3K4I0|HEM6_PSEFS	182	35313	8	8	8	8	1.91	Oxygen-dependent cop
529	1	db$2	2::C3JXQ1|C3JXQ1_PSEFS	182	24479	5	5	3	3	0.78	Putative enoyl-CoA hyc
530	1	db$2	2::C3K2U5|C3K2U5_PSEFS	181	18989	7	7	6	6	3.40	Single-stranded DNA-b
531	1	db$2	2::C3KBM6|C3KBM6_PSEFS	181	11934	5	5	3	3	5.93	Glutaredoxin OS=Pseu
532	1	db$2	2::C3K703|ISPF_PSEFS	181	16742	5	5	4	4	2.05	2-C-methyl-D-erythrito
533	1	db$2	2::C3K371|C3K371_PSEFS	180	78727	9	8	9	8	0.62	Malate synthase G OS=
534	1	db$2	2::C3JXX2|C3JXX2_PSEFS	180	50328	8	8	7	7	0.93	ATP-dependent RNA he
535	1	SwissProt_HUMAN$	3::P0DOX5|IGG1_HUMAN	180	49298	12	11	8	7	1.15	Immunoglobulin gamm
535	2	SwissProt_HUMAN$	3::P01859|IGHG2_HUMAN	81	35878	7	7	4	4	0.93	Immunoglobulin heavy
536	1	db$2	2::C3JYT6|C3JYT6_PSEFS	179	8291	6	5	4	3	8.08	Cold shock protein csp
537	1	db$2	2::C3K1N4|C3K1N4_PSEFS	179	41398	6	6	5	5	0.77	Queuine tRNA-ribosyltr
538	1	db$2	2::C3K6J7|C3K6J7_PSEFS	179	39784	7	7	7	7	1.30	Histidinol-phosphate ar
539	1	db$2	2::C3K7G7|C3K7G7_PSEFS	179	50136	7	4	7	4	0.46	Putative modulation of
540	1	db$2	2::C3K6U2|C3K6U2_PSEFS	178	47692	4	4	3	3	0.35	Probable proton/glutan
541	1	db$2	2::C3K276|GRPE_PSEFS	177	20714	3	3	2	2	0.57	Protein GrpE OS=Pseu
542	1	db$2	2::C3K6J5|SERC_PSEFS	177	39799	7	7	7	7	1.29	Phosphoserine aminotr
543	1	db$2	2::C3K8U7|C3K8U7_PSEFS	177	30923	6	6	5	5	1.14	Transcriptional regulat
544	1	db$2	2::C3K818|C3K818_PSEFS	177	29926	10	7	8	5	1.19	Putative ABC transport
545	1	db$2	2::C3K424|HEM3_PSEFS	176	33541	5	4	4	3	0.53	Porphobilinogen deami
546	1	db$2	2::C3KE68|RS18_PSEFS	176	8912	9	8	5	5	12.34	30S ribosomal protein
547	1	SwissProt_HUMAN$	3::Q5VTE0|EF1A3_HUMAN	175	50153	9	9	8	8	1.34	Putative elongation fac
548	1	db$2	2::C3KAC7|C3KAC7_PSEFS	175	51379	10	10	9	9	1.29	UDP-glucose 6-dehydr
549	1	db$2	2::C3KAJ1|C3KAJ1_PSEFS	175	48585	8	6	7	6	0.79	Omega-amino acid--py
550	1	db$2	2::C3K3U0|C3K3U0_PSEFS	174	51001	8	7	7	6	0.75	Putative glutamine syn
551	1	db$2	2::C3KA84|C3KA84_PSEFS	174	13408	6	6	6	6	6.99	Putative regulatory pro
552	1	db$2	2::C3K5X4|C3K5X4_PSEFS	173	16332	7	7	5	5	3.19	Putative membrane pr
553	1	db$2	2::C3K9M0|C3K9M0_PSEFS	172	11184	3	3	2	2	1.29	SCP2 domain-containir

↑Family	M	DB	Accession	Score	Mass	Matches	Match(sig)	Sequences	Seq(sig)	emPAI	Description
554	1	db$2	2::C3K470|C3K470_PSEFS	172	58011	6	6	5	5	0.50	Putative transport-rela
555	1	db$2	2::C3JZN9|C3JZN9_PSEFS	171	7652	9	8	5	4	9.92	Major cold shock prote
556	1	db$2	2::C3K4E2|C3K4E2_PSEFS	171	16860	7	7	6	6	5.95	N5-carboxyaminoimida
557	1	db$2	2::C3K1K8|C3K1K8_PSEFS	171	31523	7	7	5	5	1.11	Putative hydrolase OS=
558	1	db$2	2::C3K104|C3K104_PSEFS	171	26102	8	8	5	5	1.95	Putative membrane pro
559	1	db$2	2::C3JY91|C3JY91_PSEFS	170	66398	4	4	3	3	0.24	NADH-quinone oxidore
560	1	db$2	2::C3KDB1|C3KDB1_PSEFS	170	65565	9	7	9	7	0.66	Putative carbamoyltrar
561	1	db$2	2::C3K8W7|C3K8W7_PSEFS	170	34291	4	4	4	4	0.73	Arabinose 5-phosphate
562	1	db$2	2::C3K5X2|C3K5X2_PSEFS	170	20559	7	7	5	5	2.13	Putative two-componer
563	1	db$2	2::C3K0N7|RL32_PSEFS	170	6752	5	5	3	3	6.52	50S ribosomal protein
564	1	db$2	2::C3K3G5|C3K3G5_PSEFS	170	15160	7	7	5	5	5.30	Putative thioesterase C
565	1	db$2	2::C3K2C9|C3K2C9_PSEFS	169	48295	8	5	8	5	0.63	Glutamate dehydrogen
566	1	db$2	2::C3K262|C3K262_PSEFS	169	12956	6	5	3	3	1.93	Protein-export membra
567	1	db$2	2::C3K758|PYRF_PSEFS	168	24706	7	7	6	6	2.78	Orotidine 5'-phosphate
568	1	db$2	2::C3K3L0|METXS_PSEFS	168	41664	7	6	6	5	0.98	Homoserine O-succinyl
569	1	db$2	2::C3KA77|C3KA77_PSEFS	168	49108	9	8	8	7	0.96	Glutathione reductase
570	1	db$2	2::C3K281|C3K281_PSEFS	168	15787	5	5	4	4	2.27	Ribosome association t
571	1	db$2	2::C3KBN4|C3KBN4_PSEFS	168	40532	7	6	6	5	1.01	Acriflavine resistance p
572	1	db$2	2::C3K9P3|C3K9P3_PSEFS	167	9882	7	6	4	3	3.06	Peptidyl-prolyl cis-tran
573	1	db$2	2::C3JZT9|C3JZT9_PSEFS	167	24210	7	6	7	6	2.21	3-isopropylmalate dehy
574	1	db$2	2::C3K1M1|HSCA_PSEFS	167	65896	10	8	8	8	0.78	Chaperone protein Hsc
575	1	db$2	2::C3KAH8|C3KAH8_PSEFS	166	89028	9	9	8	8	0.53	DD-transpeptidase OS=
576	1	db$2	2::C3K3W3|C3K3W3_PSEFS	166	41294	4	4	4	4	0.58	Putative aminotransfer
577	1	db$2	2::C3K0I0|C3K0I0_PSEFS	166	27472	7	7	6	6	1.80	RNA polymerase sigma
578	1	db$2	2::C3K5L8|C3K5L8_PSEFS	166	39360	4	3	3	2	0.43	Phosphorous compoun
579	1	db$2	2::C3K6J1|UBIG_PSEFS	166	26040	8	8	6	6	2.53	Ubiquinone biosynthesi
580	1	db$2	2::C3K968|C3K968_PSEFS	165	55197	8	7	7	6	0.67	Putative aldehyde dehy
581	1	db$2	2::C3KDN5|C3KDN5_PSEFS	165	35878	4	4	3	3	0.48	Renalase OS=Pseudom
582	1	db$2	2::C3KDQ9|C3KDQ9_PSEFS	165	29676	10	8	8	6	1.60	Ferredoxin--NADP redu
583	1	db$2	2::C3K7M9|C3K7M9_PSEFS	164	34271	5	5	5	5	0.99	Putative ABC transport
584	1	db$2	2::C3K6R3|Y2135_PSEFS	164	24050	8	7	7	6	2.22	UPF0502 protein PFLU_
585	1	db$2	2::C3KBV0|C3KBV0_PSEFS	164	28603	6	5	5	4	0.93	Putative chromosome p
586	1	db$2	2::C3K148|APT_PSEFS	163	20064	7	7	6	6	3.06	Adenine phosphoribosy
587	1	db$2	2::C3K0U9|C3K0U9_PSEFS	163	55465	9	6	7	5	0.53	Putative two-componer
588	1	db$2	2::C3K472|C3K472_PSEFS	162	42804	6	6	4	4	0.74	Coenzyme A biosynthe
589	1	SwissProt_HUMAN$	3::P0CG04|IGLC1_HUMAN	162	11341	3	3	2	2	1.27	Immunoglobulin lambd
590	1	db$2	2::C3K9Z4|C3K9Z4_PSEFS	162	58133	10	9	7	7	0.77	Putative ABC tranmspo
591	1	db$2	2::C3K843|C3K843_PSEFS	162	26535	9	9	6	6	1.90	Lipopolysaccharide exp
592	1	db$2	2::C3K1N1|C3K1N1_PSEFS	161	33097	4	4	4	4	0.77	Protein-export membra
593	1	db$2	2::C3K3R6|C3K3R6_PSEFS	161	54762	7	6	6	5	0.54	L-threonine dehydratas

↑Family	M	DB	Accession	Score	Mass	Matches	Match(sig)	Sequences	Seq(sig)	emPAI	Description
594	1	db$2	2::B0B0A5|DNAA_PSEFS	161	56252	11	9	10	8	0.96	Chromosomal replicatic
595	1	db$2	2::C3K1X7|RL27_PSEFS	161	9214	8	8	4	4	11.18	50S ribosomal protein
596	1	db$2	2::C3K332|Y5583_PSEFS	161	48606	6	6	6	6	0.79	UPF0229 protein PFLU_
597	1	db$2	2::C3K5G1|C3K5G1_PSEFS	160	34097	6	6	4	4	1.00	Cysteine synthase OS=
598	1	db$2	2::C3JY38|C3JY38_PSEFS	160	16812	4	4	4	4	2.05	Putative thioredoxin-re
599	1	db$2	2::C3K7Z1|C3K7Z1_PSEFS	158	38822	7	6	7	6	1.07	NAD(P) transhydrogen:
600	1	db$2	2::C3K581|C3K581_PSEFS	158	86479	5	5	5	5	0.32	Putative molybdopterir
601	1	db$2	2::C3KDW7|HISZ_PSEFS	157	42687	8	8	6	6	0.94	ATP phosphoribosyltrar
602	1	db$2	2::C3K7I1|C3K7I1_PSEFS	157	85859	4	4	4	4	0.25	Quinoprotein glucose d
603	1	db$2	2::C3KBW8|C3KBW8_PSEFS	157	39031	8	8	7	7	1.33	D-alanine--D-alanine li
604	1	db$2	2::C3JY54|SYS_PSEFS	156	47115	8	8	7	7	1.02	Serine--tRNA ligase OS
605	1	db$2	2::C3K601|DXR_PSEFS	156	42341	3	3	3	3	0.40	1-deoxy-D-xylulose 5-|
606	1	db$2	2::C3K0P6|DCTA_PSEFS	156	46761	4	4	4	4	0.50	C4-dicarboxylate trans
607	1	db$2	2::C3JXY5|C3JXY5_PSEFS	155	10610	3	3	2	2	1.38	Uncharacterized protei
608	1	db$2	2::C3KDM0|C3KDM0_PSEFS	155	69594	8	7	7	7	0.61	DNA topoisomerase 4 s
609	1	db$2	2::C3K1N5|QUEA_PSEFS	154	38396	3	3	3	3	0.45	S-adenosylmethionine:
610	1	db$2	2::C3K170|C3K170_PSEFS	153	22875	5	5	4	4	1.27	Flavin_Reduct domain-
611	1	db$2	2::C3KDM5|C3KDM5_PSEFS	152	43829	5	5	5	5	0.71	O-phosphoserine phos|
612	1	db$2	2::C3K417|C3K417_PSEFS	151	39396	4	4	3	3	0.43	Transcriptional regulat
613	1	db$2	2::C3K0I1|C3K0I1_PSEFS	151	29699	8	7	6	5	1.59	ParA family ATPase OS
614	1	db$2	2::C3K5X6|C3K5X6_PSEFS	151	49358	8	8	6	6	0.95	Tryptophan--tRNA liga:
615	1	db$2	2::C3K0D3|MINC_PSEFS	151	26249	7	7	6	6	1.94	Probable septum site-c
616	1	db$2	2::C3K3Q8|TYSY_PSEFS	151	31490	7	7	4	4	1.11	Thymidylate synthase (
617	1	db$2	2::C3K868|C3K868_PSEFS	151	58117	7	7	7	7	0.77	Uncharacterized protei
618	1	db$2	2::C3K240|C3K240_PSEFS	151	17616	7	7	6	6	3.91	RNA polymerase-bindir
619	1	db$2	2::C3K0T3|C3K0T3_PSEFS	150	28958	6	5	6	5	1.25	Putative membrane prc
620	1	db$2	2::C3JXY4|ASTE_PSEFS	150	37404	7	6	6	6	1.13	Succinylglutamate desu
621	1	db$2	2::C3K2R1|DXS_PSEFS	150	68238	7	5	7	5	0.41	1-deoxy-D-xylulose-5-
622	1	SwissProt_HUMAN$	3::Q9BQE3|TBA1C_HUMAN	150	49863	6	5	6	5	0.61	Tubulin alpha-1C chain
623	1	db$2	2::C3K910|C3K910_PSEFS	149	36322	6	6	6	6	1.18	Putative ABC transport
624	1	db$2	2::C3K419|C3K419_PSEFS	149	17100	3	3	2	2	1.27	Transcriptional regulat
625	1	db$2	2::C3K3S0|C3K3S0_PSEFS	149	23721	6	6	4	4	1.21	Putative hydrolase OS=
626	1	db$2	2::C3JZN8|C3JZN8_PSEFS	149	10735	4	4	3	3	4.58	Uncharacterized protei
627	1	db$2	2::C3K1V6|C3K1V6_PSEFS	148	32555	5	3	4	3	0.54	Putative short-chain de
628	1	db$2	2::C3K103|C3K103_PSEFS	148	23527	4	3	3	2	0.49	Phosphoribosylglycinar
629	1	db$2	2::C3K575|C3K575_PSEFS	148	14827	5	5	5	5	3.80	Osmotically inducible p
630	1	SwissProt_HUMAN$	3::P0DOX6|IGM_HUMAN	148	63446	7	3	6	3	0.25	Immunoglobulin mu he
631	1	SwissProt_HUMAN$	3::P62805|H4_HUMAN	147	11360	5	5	5	5	6.74	Histone H4 OS=Homo
632	1	db$2	2::C3K709|C3K709_PSEFS	146	7722	6	6	4	4	9.56	Cold shock protein OS=
633	1	db$2	2::C3K2V8|RL30_PSEFS	146	6521	7	7	3	3	15.08	50S ribosomal protein

↑Family	M	DB	Accession	Score	Mass	Matches	Match(sig)	Sequences	Seq(sig)	emPAI	Description
634	1	db$2	2::C3JYD3|SYC_PSEFS	146	51635	9	8	9	8	1.08	Cysteine--tRNA ligase (
635	1	db$2	2::C3K2W2|RS8_PSEFS	146	14083	6	6	5	5	6.26	30S ribosomal protein
636	1	db$2	2::C3K3A2|C3K3A2_PSEFS	146	38105	7	6	6	5	0.86	L-threonine aldolase O:
637	1	db$2	2::C3JYE4|C3JYE4_PSEFS	145	30882	12	11	10	9	2.95	Exodeoxyribonuclease
638	1	db$2	2::C3K733|HTPX_PSEFS	145	32382	7	6	5	4	1.07	Protease HtpX OS=Pse
639	1	db$2	2::C3K437|C3K437_PSEFS	145	26506	3	3	2	2	0.43	Uncharacterized protei
640	1	db$2	2::C3JZN1|IHFA_PSEFS	145	11469	5	5	4	4	4.06	Integration host factor
641	1	db$2	2::C3KE99|C3KE99_PSEFS	145	30145	5	4	5	4	0.87	Quinolinate phosphorib
642	1	db$2	2::C3K1H1|RS16_PSEFS	143	9202	8	6	5	5	11.18	30S ribosomal protein
643	1	db$2	2::C3K747|GPDA_PSEFS	143	36476	8	7	8	7	1.47	Glycerol-3-phosphate c
644	1	db$2	2::C3K9F0|C3K9F0_PSEFS	143	40567	6	6	4	4	0.59	Putative transport-rela
645	1	db$2	2::C3K0S3|C3K0S3_PSEFS	143	67491	8	6	8	6	0.52	Putative ATP-binding c
646	1	db$2	2::C3K272|C3K272_PSEFS	142	40774	7	7	6	6	1.25	Carbamoyl-phosphate
647	1	db$2	2::C3K494|C3K494_PSEFS	141	33626	4	4	4	4	0.75	Gly-zipper_YMGG dom
648	1	db$2	2::C3K3M7|C3K3M7_PSEFS	140	22464	2	2	1	1	0.23	Ribosomal RNA small s
649	1	SwissProt_HUMAN$	3::P27348|1433T_HUMAN	140	27747	2	2	2	2	0.40	14-3-3 protein theta O
650	1	db$2	2::C3K4U5|C3K4U5_PSEFS	139	75901	7	6	7	6	0.45	Putative protease OS=
651	1	db$2	2::C3K5G2|C3K5G2_PSEFS	139	35788	4	4	4	4	0.70	Putative membrane pro
652	1	db$2	2::C3JXZ2|C3JXZ2_PSEFS	139	36334	5	5	4	4	0.68	Putative AraC family re
653	1	db$2	2::C3K2U8|C3K2U8_PSEFS	138	18135	4	4	4	4	1.81	Bacterioferritin OS=Pse
654	1	db$2	2::C3K126|C3K126_PSEFS	138	54569	10	10	7	7	0.83	2-methylcitrate dehydr
655	1	db$2	2::C3K4E1|C3K4E1_PSEFS	138	38799	9	7	8	7	1.34	N5-carboxyaminoimida
656	1	db$2	2::C3K2M7|C3K2M7_PSEFS	138	18106	4	4	4	4	1.81	Ribosomal silencing fac
657	1	db$2	2::C3K763|C3K763_PSEFS	138	29996	3	3	2	2	0.37	Putative hydrolase OS=
658	1	db$2	2::C3JYS3|C3JYS3_PSEFS	138	38665	5	3	4	3	0.44	TolA colicin import mer
659	1	db$2	2::C3JY61|C3JY61_PSEFS	137	8297	5	5	4	4	8.08	Translation initiation fa
660	1	db$2	2::C3JZ43|C3JZ43_PSEFS	137	44885	5	5	5	5	0.69	Putative aminotransfer
661	1	db$2	2::C3K5M0|C3K5M0_PSEFS	137	29300	5	4	3	3	0.62	Phosphonates ABC trar
662	1	db$2	2::C3K5L9|C3K5L9_PSEFS	136	31441	3	2	3	2	0.35	Alpha-D-ribose 1-meth
663	1	db$2	2::C3JY88|C3JY88_PSEFS	136	20432	9	8	7	6	3.99	NADH-quinone oxidore
664	1	db$2	2::C3K3N8|C3K3N8_PSEFS	135	43920	8	7	7	6	0.91	PUA domain-containing
665	1	db$2	2::C3K2W7|RS17_PSEFS	134	10090	6	6	5	5	8.84	30S ribosomal protein
666	1	db$2	2::C3KBL4|C3KBL4_PSEFS	134	26993	5	4	4	3	0.69	Glutamate/aspartate A
667	1	db$2	2::C3K0T7|C3K0T7_PSEFS	134	76509	6	6	6	6	0.45	Putative fatty acid oxid
668	1	db$2	2::C3K2L6|C3K2L6_PSEFS	134	47731	6	5	6	5	0.64	Putative lipoprotein OS
669	1	db$2	2::C3K4C1|C3K4C1_PSEFS	134	40318	4	4	4	4	0.60	Putative nitric oxide de
670	1	db$2	2::C3KBJ5|C3KBJ5_PSEFS	134	62095	4	4	4	4	0.36	Phospholipase C OS=P
671	1	db$2	2::C3KBL7|C3KBL7_PSEFS	133	33413	7	7	5	5	1.03	Glutamate/aspartate A
672	1	db$2	2::C3K3X2|C3K3X2_PSEFS	133	19739	5	5	4	4	1.58	Uncharacterized protei
673	1	SwissProt_HUMAN$	3::P62937|PPIA_HUMAN	133	18001	5	5	4	4	1.83	Peptidyl-prolyl cis-tran

↑Family	M	DB	Accession	Score	Mass	Matches	Match(sig)	Sequences	Seq(sig)	emPAI	Description
674	1	db$2	2::C3K8Y8|C3K8Y8_PSEFS	132	23915	4	4	4	4	1.20	Putative isomerase OS
675	1	db$2	2::C3K9S0|NFUA_PSEFS	132	20961	5	5	5	5	2.06	Fe/S biogenesis protei
676	1	db$2	2::C3JZR8|C3JZR8_PSEFS	132	46446	7	7	6	6	0.84	Putative oxidoreductas
677	1	db$2	2::C3K2R5|RIBA_PSEFS	132	22312	4	4	3	3	1.32	GTP cyclohydrolase-2 (
678	1	db$2	2::C3KAB1|C3KAB1_PSEFS	132	44397	4	4	3	3	0.38	Histidine kinase OS=Ps
679	1	db$2	2::C3KDC0|C3KDC0_PSEFS	132	36735	4	4	4	4	0.67	Uncharacterized protei
680	1	db$2	2::C3K3L5|C3K3L5_PSEFS	132	11400	2	2	1	1	1.25	Putative membrane pr
681	1	db$2	2::C3KAV6|C3KAV6_PSEFS	131	17648	5	5	3	3	1.21	Thiol peroxidase OS=P
682	1	db$2	2::C3KAI8|C3KAI8_PSEFS	131	24497	4	4	3	3	1.15	Putative TetR-family re
683	1	db$2	2::C3K084|AROC_PSEFS	131	39094	8	7	6	6	1.07	Chorismate synthase C
684	1	db$2	2::C3KAY1|C3KAY1_PSEFS	131	44657	6	5	6	5	0.70	3-phosphoshikimate 1-
685	1	db$2	2::C3K015|C3K015_PSEFS	130	53791	5	5	4	4	0.42	Putative ferredoxin OS
686	1	db$2	2::C3K061|C3K061_PSEFS	129	18792	4	4	4	4	1.71	DUF2059 domain-cont
687	1	db$2	2::C3K427|C3K427_PSEFS	129	23927	5	5	4	4	1.66	Putative glutathione S-
688	1	db$2	2::C3K6V7|C3K6V7_PSEFS	129	46336	5	5	5	5	0.67	Carboxy-terminal proc
689	1	db$2	2::C3K1E5|ATPE_PSEFS	128	14558	5	5	3	3	2.60	ATP synthase epsilon c
690	1	db$2	2::C3JXY8|C3JXY8_PSEFS	128	37476	5	5	5	5	0.88	Arginine N-succinyltrar
691	1	db$2	2::C3K1X9|C3K1X9_PSEFS	128	34909	8	8	7	7	1.58	Octaprenyl-diphosphat
692	1	db$2	2::C3KBU0|C3KBU0_PSEFS	128	36726	4	3	4	3	0.47	Sulfate/thiosulfate imp
693	1	db$2	2::C3K7I9|C3K7I9_PSEFS	128	88241	7	7	7	7	0.46	Putative pinecillin acyla
694	1	db$2	2::C3JYI0|C3JYI0_PSEFS	127	25235	6	6	6	6	2.06	Probable transcriptiona
695	1	db$2	2::C3K4T4|C3K4T4_PSEFS	127	21507	4	4	4	4	1.39	NAD(P)-bd_dom doma
696	1	db$2	2::C3K4D7|C3K4D7_PSEFS	127	14435	3	3	2	2	0.91	Putative thioesterase C
697	1	db$2	2::C3K8W4|C3K8W4_PSEFS	127	18790	7	7	6	6	4.74	Lipopolysaccharide exp
698	1	db$2	2::C3K2J9|C3K2J9_PSEFS	127	60860	2	2	2	2	0.17	Putative dehydrogenas
699	1	db$2	2::C3JY57|LOLA_PSEFS	127	22546	7	6	5	5	1.83	Outer-membrane lipop
700	1	db$2	2::C3K101|C3K101_PSEFS	127	37185	4	4	3	3	0.46	Uncharacterized protei
701	1	db$2	2::C3K231|C3K231_PSEFS	127	57678	5	5	5	5	0.51	APH domain-containing
702	1	db$2	2::C3KDG2|C3KDG2_PSEFS	126	41589	7	7	5	5	0.98	AMP nucleosidase OS=
703	1	db$2	2::C3K467|C3K467_PSEFS	126	13453	2	2	1	1	0.41	Cupin_3 domain-conta
704	1	db$2	2::C3K5U5|C3K5U5_PSEFS	125	32335	7	6	5	5	1.07	Glucose-1-phosphate t
705	1	db$2	2::C3JYI3|PHNX_PSEFS	125	29973	7	5	6	5	1.19	Phosphonoacetaldehyd
706	1	db$2	2::C3KE71|C3KE71_PSEFS	124	51412	9	8	9	8	1.09	Replicative DNA helica
707	1	db$2	2::C3K4Y3|KDPA_PSEFS	124	60181	5	5	3	3	0.37	Potassium-transporting
708	1	db$2	2::C3K6K4|C3K6K4_PSEFS	123	32908	5	5	5	5	1.05	Putative 5'-nucleotidas
709	1	db$2	2::C3KE48|C3KE48_PSEFS	123	19341	6	6	5	5	2.36	Peptide deformylase O
710	1	db$2	2::C3K3Q4|C3K3Q4_PSEFS	123	38576	5	4	4	3	0.44	Putative cation ABC tra
711	1	db$2	2::C3K6J2|C3K6J2_PSEFS	123	47817	4	4	3	3	0.35	Putative hydrolase OS=
712	1	db$2	2::C3K754|C3K754_PSEFS	122	29479	4	4	4	4	0.89	Putative lipoprotein OS
713	1	db$2	2::C3K7E3|C3K7E3_PSEFS	122	178026	7	7	7	7	0.21	Alpha-2-macroglobulin

↑Family	M	DB	Accession	Score	Mass	Matches	Match(sig)	Sequences	Seq(sig)	emPAI	Description
714	1	db$2	2::C3K1B3|C3K1B3_PSEFS	122	20726	3	3	3	3	0.97	Uncharacterized protei
715	1	SwissProt_HUMAN$	3::P63104|1433Z_HUMAN	122	27728	7	6	6	5	1.34	14-3-3 protein zeta/de
716	1	db$2	2::C3K606|FABZ_PSEFS	122	16691	5	5	5	5	3.07	3-hydroxyacyl-[acyl-ca
717	1	db$2	2::C3K544|C3K544_PSEFS	122	74165	9	7	9	7	0.56	Uncharacterized protei
718	1	db$2	2::C3K568|TRPA_PSEFS	122	28539	4	4	3	3	0.64	Tryptophan synthase a
719	1	db$2	2::C3K6Z7|C3K6Z7_PSEFS	121	89469	11	9	10	9	0.61	Lon protease OS=Pseu
720	1	db$2	2::C3K2X4|RL23_PSEFS	121	10938	7	6	7	6	11.53	50S ribosomal protein
721	1	db$2	2::C3K2S3|C3K2S3_PSEFS	121	17933	6	6	5	5	3.77	Transcriptional repress
722	1	db$2	2::C3KCG1|C3KCG1_PSEFS	121	60384	1	1	1	1	0.08	Putative secreted amid
723	1	db$2	2::C3KE58|C3KE58_PSEFS	120	84757	10	9	9	9	0.65	Putative S1 RNA-bindir
724	1	db$2	2::C3JZV9|C3JZV9_PSEFS	120	34932	3	3	3	3	0.50	Putative DNA/RNA non
725	1	db$2	2::C3K0V4|C3K0V4_PSEFS	119	48572	4	4	4	4	0.48	Flagellin OS=Pseudom
726	1	db$2	2::C3KBP1|C3KBP1_PSEFS	119	37670	8	6	6	5	0.87	Putative reductase OS=
727	1	db$2	2::C3K197|C3K197_PSEFS	119	25331	7	6	5	5	1.53	Putative two-componen
728	1	db$2	2::C3K8K4|C3K8K4_PSEFS	119	58330	5	5	5	5	0.50	Putative outer membra
729	1	db$2	2::C3KAD3|ARNC_PSEFS	119	37700	4	4	4	4	0.65	Undecaprenyl-phospha
730	1	db$2	2::C3K0H7|C3K0H7_PSEFS	119	77893	6	6	6	6	0.44	Histidine kinase OS=Ps
731	1	db$2	2::C3KBH8|C3KBH8_PSEFS	118	25745	4	4	4	4	1.08	3-oxoacyl-[acyl-carrier
732	1	db$2	2::C3KAW2|C3KAW2_PSEFS	118	26230	5	3	4	3	0.71	Putative short chain de
733	1	db$2	2::C3KEA8|C3KEA8_PSEFS	118	45495	4	4	4	4	0.52	ATP-dependent RNA he
734	1	db$2	2::C3K1B0|C3K1B0_PSEFS	118	96490	3	3	1	1	0.10	Lysylphosphatidylglyce
735	1	db$2	2::C3JYQ9|C3JYQ9_PSEFS	118	54767	3	3	3	3	0.30	Transcriptional regulat
736	1	db$2	2::C3K617|C3K617_PSEFS	118	19384	5	4	4	3	1.06	Uncharacterized protei
737	1	db$2	2::C3JYR2|C3JYR2_PSEFS	117	48934	6	6	5	5	0.79	L-serine dehydratase C
737	2	db$2	2::C3K5Y8|C3K5Y8_PSEFS	94	48751	5	5	4	4	0.47	L-serine dehydratase C
738	1	db$2	2::C3K468|C3K468_PSEFS	117	6077	2	2	1	1	3.41	50S ribosomal protein
739	1	db$2	2::C3K6Y6|C3K6Y6_PSEFS	117	31839	7	7	7	7	1.82	Signal peptidase I OS=
740	1	db$2	2::C3K740|C3K740_PSEFS	117	33777	10	9	9	9	2.52	DUF815 domain-contai
741	1	db$2	2::C3K5D5|C3K5D5_PSEFS	116	41033	2	2	2	2	0.26	Succinyl-diaminopimela
742	1	db$2	2::C3K7C4|C3K7C4_PSEFS	116	53137	10	9	10	9	1.23	DNA-binding transcript
743	1	SwissProt_HUMAN$	3::P62979|RS27A_HUMAN	116	17953	3	3	3	3	1.18	Ubiquitin-40S ribosoma
744	1	db$2	2::C3K1U9|C3K1U9_PSEFS	116	42728	4	4	2	2	0.39	Flavohemoglobin OS=F
745	1	db$2	2::C3K3D4|C3K3D4_PSEFS	116	52472	3	3	3	3	0.31	Uncharacterized protei
746	1	db$2	2::C3K839|Y879_PSEFS	115	32120	3	3	3	3	0.55	Nucleotide-binding pro
747	1	db$2	2::C3JYK0|CLPP_PSEFS	115	23405	9	8	7	7	3.98	ATP-dependent Clp pro
748	1	db$2	2::C3JYA9|C3JYA9_PSEFS	115	27795	4	4	4	4	0.97	Putative TetR-family re
749	1	db$2	2::C3JXZ5|C3JXZ5_PSEFS	115	25958	4	4	4	4	1.06	Putative histidine trans
750	1	db$2	2::C3K6T0|ENGB_PSEFS	115	23537	3	3	3	3	0.82	Probable GTP-binding p
751	1	db$2	2::C3K644|C3K644_PSEFS	115	89774	7	7	6	6	0.37	Putative ABC transport
752	1	db$2	2::C3K609|C3K609_PSEFS	114	73644	4	4	4	4	0.29	Putative lytic transglyc

↑Family	M	DB	Accession	Score	Mass	Matches	Match(sig)	Sequences	Seq(sig)	emPAI	Description
753	1	db$2	2::C3K480|C3K480_PSEFS	114	31946	5	2	4	1	0.16	Uncharacterized protei
754	1	db$2	2::C3K7M5|C3K7M5_PSEFS	114	15789	2	2	2	2	0.81	Uncharacterized protei
755	1	db$2	2::C3K4Q9|C3K4Q9_PSEFS	113	23656	4	3	4	3	0.82	Putative exported prot
756	1	db$2	2::C3KBM2|C3KBM2_PSEFS	113	16548	4	4	3	3	1.33	Cys-tRNA(Pro)/Cys-tR
757	1	db$2	2::C3K5K3|C3K5K3_PSEFS	112	28692	3	3	1	1	0.39	CHAD domain-containi
758	1	db$2	2::C3K862|C3K862_PSEFS	112	39825	6	5	5	4	0.81	Putative outer membra
759	1	db$2	2::C3KDN7|HEMH_PSEFS	112	38415	6	5	5	5	0.85	Ferrochelatase OS=Pse
760	1	db$2	2::C3JZ24|C3JZ24_PSEFS	112	24729	6	6	6	6	2.13	Putative GntR-family re
761	1	db$2	2::C3JY71|C3JY71_PSEFS	112	43919	2	1	2	1	0.11	JmjC domain-containin
762	1	db$2	2::C3KA81|C3KA81_PSEFS	112	55484	7	5	7	5	0.53	Alkyl hydroperoxide re
763	1	db$2	2::C3K5E4|GLND_PSEFS	112	102948	8	7	8	7	0.38	Bifunctional uridylyltra
764	1	db$2	2::C3K3W8|C3K3W8_PSEFS	112	44404	5	5	5	5	0.70	Putative ubiquinone bi
765	1	db$2	2::C3K5N1|C3K5N1_PSEFS	112	36842	8	8	6	6	1.45	Sulfate ABC transport
766	1	db$2	2::C3K6E5|C3K6E5_PSEFS	111	29069	2	2	2	2	0.38	Cytochrome c1 OS=Ps
767	1	db$2	2::C3K0W1|C3K0W1_PSEFS	111	27483	5	3	5	3	0.67	Flagellar basal-body ro
768	1	db$2	2::C3K5U6|C3K5U6_PSEFS	111	40174	4	4	4	4	0.60	dTDP-glucose 4,6-dehy
769	1	db$2	2::C3K308|TRPD_PSEFS	111	36976	5	4	5	4	0.67	Anthranilate phosphori
770	1	db$2	2::C3KA90|C3KA90_PSEFS	111	36984	3	3	2	2	0.29	Putative aldo/keto redu
771	1	db$2	2::C3K4C0|C3K4C0_PSEFS	111	9802	3	3	3	3	3.13	DNA-binding protein H
772	1	db$2	2::C3K5U7|C3K5U7_PSEFS	110	105852	4	3	4	3	0.14	Putative aminotransfer
773	1	db$2	2::C3KDF0|C3KDF0_PSEFS	110	18221	4	4	4	4	1.80	Putative phage tail-rela
774	1	db$2	2::C3JZV1|C3JZV1_PSEFS	110	76010	7	6	6	6	0.45	UvrABC system protein
775	1	db$2	2::C3K5L3|C3K5L3_PSEFS	110	36401	7	4	6	3	0.48	Phosphonates ABC tran
776	1	db$2	2::C3K9N1|C3K9N1_PSEFS	110	43216	5	5	4	4	0.55	D-ser_dehydrat domai
777	1	db$2	2::C3JZQ0|C3JZQ0_PSEFS	110	38665	7	6	7	6	1.08	Putative L-asparaginas
778	1	db$2	2::C3KDE9|C3KDE9_PSEFS	110	41781	4	4	4	4	0.57	Putative phage tail-rela
779	1	db$2	2::C3KE17|C3KE17_PSEFS	110	24430	3	3	3	3	0.78	Two component systen
780	1	db$2	2::C3K3L6|TRMB_PSEFS	110	26990	2	2	1	1	0.19	tRNA (guanine-N(7)-)-
781	1	db$2	2::C3K0D7|APEB_PSEFS	109	46914	5	4	5	4	0.50	Probable M18 family ar
782	1	db$2	2::C3K621|C3K621_PSEFS	109	26068	2	2	2	2	0.43	Putative TetR-family re
783	1	db$2	2::C3K2W3|C3K2W3_PSEFS	109	11427	6	6	5	5	6.59	30S ribosomal protein
784	1	db$2	2::C3K3G7|C3K3G7_PSEFS	109	31253	6	6	6	6	1.47	Methylenetetrahydrofo
785	1	db$2	2::C3K017|PYRD_PSEFS	109	35850	7	7	6	6	1.51	Dihydroorotate dehydr
786	1	db$2	2::C3K2J5|C3K2J5_PSEFS	109	18057	4	4	4	4	1.81	Putative AsnC-family re
787	1	db$2	2::C3KCS7|MRAY_PSEFS	108	39243	3	3	2	2	0.43	Phospho-N-acetylmura
788	1	db$2	2::C3K816|OPGH_PSEFS	108	96236	4	3	4	3	0.16	Glucans biosynthesis g
789	1	db$2	2::C3JY36|C3JY36_PSEFS	108	16095	7	7	5	5	4.70	Uncharacterized protei
790	1	db$2	2::C3KE08|C3KE08_PSEFS	108	25044	3	3	3	3	0.75	Conserved hypothetica
791	1	db$2	2::C3K6S5|C3K6S5_PSEFS	108	35353	7	7	7	7	1.55	Delta-aminolevulinic ac
792	1	db$2	2::C3KDM3|C3KDM3_PSEFS	108	25575	2	2	2	2	0.45	Putative lipoprotein OS

↑Family	M	DB	Accession	Score	Mass	Matches	Match(sig)	Sequences	Seq(sig)	emPAI	Description
793	1	db$2	2::C3JY84|C3JY84_PSEFS	108	18006	2	2	2	2	0.68	NADH dehydrogenase I
794	1	db$2	2::C3K5H2|QUEF_PSEFS	107	30808	5	5	5	5	1.15	NADPH-dependent 7-cy
795	1	db$2	2::C3K5B2|C3K5B2_PSEFS	107	35714	3	3	3	3	0.49	Dipeptide ABC transpoi
796	1	db$2	2::C3K6W5|PRMA_PSEFS	107	31717	4	3	4	3	0.56	Ribosomal protein L11
797	1	db$2	2::C3KBB4|OADC_PSEFS	106	31135	3	3	2	2	0.58	Oxaloacetate decarbox
798	1	db$2	2::C3JXQ0|C3JXQ0_PSEFS	106	24880	8	7	6	5	2.11	Putative hydrolase OS=
799	1	db$2	2::C3K309|C3K309_PSEFS	106	21661	3	2	3	2	0.54	Anthranilate synthase (
800	1	db$2	2::C3KE78|C3KE78_PSEFS	106	15753	3	3	3	3	1.43	Azurin OS=Pseudomon
801	1	db$2	2::C3K110|C3K110_PSEFS	106	32414	5	5	5	5	1.07	Cysteine synthase OS=
802	1	db$2	2::C3K482|RPOZ_PSEFS	106	9821	3	3	2	2	3.13	DNA-directed RNA poly
803	1	db$2	2::C3JY53|CYSG_PSEFS	105	50259	4	4	4	4	0.46	Siroheme synthase OS
804	1	db$2	2::C3K9G9|C3K9G9_PSEFS	105	16183	3	3	3	3	1.37	CMD domain-containin
805	1	db$2	2::C3K1M6|C3K1M6_PSEFS	105	24782	4	4	3	3	1.14	Uncharacterized protei
806	1	db$2	2::C3K4R0|C3K4R0_PSEFS	105	14930	2	2	2	2	0.86	Putative lipoprotein OS
807	1	db$2	2::C3K261|RIMP_PSEFS	105	17653	3	3	3	3	1.21	Ribosome maturation f
808	1	db$2	2::C3K3M3|C3K3M3_PSEFS	104	24540	6	5	5	4	1.15	Cell division ATP-bindir
809	1	db$2	2::C3K3M5|C3K3M5_PSEFS	104	50101	7	6	7	6	0.76	Putative peptidase OS=
810	1	SwissProt_HUMAN$	3::Q5QNW6|H2B2F_HUMAN	104	13912	5	4	3	3	2.83	Histone H2B type 2-F (
811	1	db$2	2::C3K3H3|C3K3H3_PSEFS	104	52510	2	2	2	2	0.20	Adenosylmethionine-8-
812	1	db$2	2::C3KCP1|C3KCP1_PSEFS	104	23476	4	4	3	3	1.23	Peptide methionine sul
813	1	db$2	2::C3JY78|C3JY78_PSEFS	104	22781	5	3	4	3	0.86	Putative cytochrome ox
813	2	db$2	2::C3JZH3|C3JZH3_PSEFS	32	22428	3	2	3	2	0.52	Putative cytochrome ox
814	1	db$2	2::C3K8W6|C3K8W6_PSEFS	104	18885	5	3	4	3	1.11	3-deoxy-D-manno-octu
815	1	db$2	2::C3K1X0|CYSZ_PSEFS	103	28826	4	3	4	3	0.63	Sulfate transporter Cys
816	1	db$2	2::C3K268|C3K268_PSEFS	103	11552	5	5	4	4	6.45	CRM domain-containin
817	1	db$2	2::C3KBM3|C3KBM3_PSEFS	103	40194	2	2	2	2	0.27	Putative ABC transport
818	1	db$2	2::C3KDA5|C3KDA5_PSEFS	103	54170	2	2	2	2	0.19	Uncharacterized protei
819	1	db$2	2::C3K3W5|C3K3W5_PSEFS	103	39645	7	7	6	6	1.04	Aminomethyltransferas
820	1	db$2	2::C3K0V5|C3K0V5_PSEFS	103	33212	5	5	5	5	1.03	Putative keto/oxo acyl-
821	1	SwissProt_HUMAN$	3::P01876|IGHA1_HUMAN	102	37631	6	6	6	6	1.12	Immunoglobulin heavy
821	2	SwissProt_HUMAN$	3::P0DOX2|IGA2_HUMAN	58	48903	4	4	4	4	0.47	Immunoglobulin alpha-
821	3	SwissProt_HUMAN$	3::A0A0B4J1X5|HV374_HUMAN	38	12831	2	2	2	2	1.06	Immunoglobulin heavy
822	1	db$2	2::C3K9R3|C3K9R3_PSEFS	102	18863	3	2	3	2	0.64	Uncharacterized protei
823	1	db$2	2::C3K1D1|C3K1D1_PSEFS	101	32188	2	2	2	2	0.34	Protein HflC OS=Pseud
824	1	db$2	2::C3K785|C3K785_PSEFS	101	24962	3	3	3	3	0.76	3-oxoadipate CoA-tran
825	1	db$2	2::C3KE20|C3KE20_PSEFS	101	33868	4	4	4	4	0.75	GCV_T domain-contain
826	1	db$2	2::C3K3L2|C3K3L2_PSEFS	101	15596	3	3	3	3	1.45	DUF4426 domain-conta
827	1	db$2	2::C3K8K8|C3K8K8_PSEFS	101	62203	6	6	6	6	0.58	Putative exported prot
828	1	db$2	2::C3JYS0|C3JYS0_PSEFS	101	29172	4	4	3	3	0.62	Cell division coordinatc
829	1	db$2	2::C3JYI6|C3JYI6_PSEFS	101	73112	3	2	3	2	0.14	Putative 2,4-dienoyl-C

↑Family	M	DB	Accession	Score	Mass	Matches	Match(sig)	Sequences	Seq(sig)	emPAI	Description
830	1	db$2	2::C3K5E8|C3K5E8_PSEFS	101	28997	3	3	3	3	0.63	Cyclohexadienyl dehyd
831	1	db$2	2::C3K6A4|C3K6A4_PSEFS	101	36459	3	3	3	3	0.48	Methionine import ATP
832	1	db$2	2::C3K8K2|C3K8K2_PSEFS	101	418093	5	5	5	5	0.06	Haemagg_act domain-
833	1	db$2	2::C3KB06|C3KB06_PSEFS	101	29724	2	2	2	2	0.37	Putative lipoprotein OS
834	1	db$2	2::C3KBY3|C3KBY3_PSEFS	100	64998	8	6	7	6	0.55	Putative lipoprotein OS
835	1	db$2	2::C3KBK9|C3KBK9_PSEFS	100	34365	4	4	4	4	0.73	Glycerate dehydrogena
836	1	db$2	2::C3JYN3|C3JYN3_PSEFS	100	52328	4	3	4	3	0.31	Family S13 unassigned
837	1	db$2	2::C3JXX7|C3JXX7_PSEFS	100	56979	4	4	4	4	0.39	Conserved hypothetica
838	1	SwissProt_HUMAN$	3::P07737|PROF1_HUMAN	100	15045	4	3	3	3	1.53	Profilin-1 OS=Homo sa
839	1	db$2	2::C3K743|FABA_PSEFS	100	18688	7	6	6	5	2.51	3-hydroxydecanoyl-[ac
840	1	db$2	2::C3K572|C3K572_PSEFS	100	7891	1	1	1	1	0.79	Uncharacterized protei
841	1	db$2	2::C3K3M9|C3K3M9_PSEFS	99	30473	4	4	4	4	0.85	Putative sulfurtransfera
842	1	db$2	2::C3K8W8|C3K8W8_PSEFS	99	29386	4	4	3	3	0.62	Putative ABC transport
843	1	db$2	2::C3K3Q1|C3K3Q1_PSEFS	98	40925	3	2	3	2	0.26	Putative oxidoreductas
844	1	db$2	2::C3K6E8|C3K6E8_PSEFS	98	27518	2	2	1	1	0.19	Putative GntR-family re
845	1	db$2	2::C3KD59|C3KD59_PSEFS	98	81779	4	4	4	4	0.26	YcaO domain-containin
846	1	db$2	2::C3K1M4|C3K1M4_PSEFS	98	13705	6	3	3	3	1.78	Iron-sulfur cluster asse
847	1	db$2	2::C3KE84|C3KE84_PSEFS	98	35425	4	4	4	4	0.70	Pseudouridine synthase
848	1	db$2	2::C3K648|C3K648_PSEFS	97	33905	4	4	4	4	0.75	Conserved hypothetica
849	1	db$2	2::C3K3D8|C3K3D8_PSEFS	97	49942	6	6	6	6	0.77	ATP-independent RNA
850	1	db$2	2::C3K333|C3K333_PSEFS	97	61021	3	3	2	2	0.17	Uncharacterized protei
851	1	db$2	2::C3KDD6|C3KDD6_PSEFS	97	33869	4	4	4	4	0.75	D-alanine--D-alanine li
852	1	db$2	2::C3KD21|C3KD21_PSEFS	97	81698	3	3	3	3	0.19	Putative glycogen debr
853	1	db$2	2::C3K312|C3K312_PSEFS	97	24134	4	4	4	4	1.18	Ribulose-phosphate 3-
853	2	db$2	2::C3K906|C3K906_PSEFS	30	65595	2	2	2	2	0.16	Conserved GGDEF/EAL
854	1	db$2	2::C3K1G6|C3K1G6_PSEFS	96	25762	7	6	5	4	1.08	Thiol:disulfide intercha
855	1	db$2	2::C3KC04|C3KC04_PSEFS	96	39513	3	3	3	3	0.43	Putative hydrolase OS=
856	1	SwissProt_HUMAN$	3::P23528|COF1_HUMAN	96	18491	4	3	4	3	1.13	Cofilin-1 OS=Homo sa
857	1	db$2	2::C3K3S3|C3K3S3_PSEFS	96	15261	2	2	1	1	0.36	DUF4399 domain-cont
858	1	db$2	2::C3K5B9|C3K5B9_PSEFS	96	60269	4	3	3	2	0.17	Dipeptide ABC transpo
859	1	db$2	2::C3K605|LPXD_PSEFS	96	36124	2	2	2	2	0.30	UDP-3-O-acylglucosam
860	1	db$2	2::C3K6M4|C3K6M4_PSEFS	96	13463	4	4	2	2	3.00	Succinate dehydrogena
861	1	db$2	2::C3KD57|C3KD57_PSEFS	96	49686	3	3	3	3	0.33	Cystathionine beta-lya
862	1	db$2	2::C3K9N0|C3K9N0_PSEFS	95	28569	2	2	2	2	0.39	Putative transcriptional
863	1	db$2	2::C3K8Y2|C3K8Y2_PSEFS	95	49522	1	1	1	1	0.10	Putative two-compone
864	1	db$2	2::C3K487|C3K487_PSEFS	95	33600	2	1	2	1	0.15	Biopolymer transport p
865	1	db$2	2::C3K6W7|C3K6W7_PSEFS	95	16216	4	4	3	3	2.17	Biotin carboxyl carrier
866	1	db$2	2::C3K1K9|C3K1K9_PSEFS	94	42204	3	3	3	3	0.40	Putative aminotransfer
867	1	db$2	2::C3K3N0|C3K3N0_PSEFS	94	25405	1	1	1	1	0.20	Putative TetR-family re
868	1	db$2	2::C3K4V5|C3K4V5_PSEFS	94	36313	7	6	4	3	0.68	Beta-hexosaminidase (

↑Family	M	DB	Accession	Score	Mass	Matches	Match(sig)	Sequences	Seq(sig)	emPAI	Description
869	1	db$2	2::C3K586|C3K586_PSEFS	94	21064	5	4	4	3	0.95	ADP compounds hydro
870	1	db$2	2::C3KCS0|RL25_PSEFS	94	21502	10	7	4	4	2.70	50S ribosomal protein
871	1	db$2	2::Q9ZEP8|PANB_PSEFS	93	27951	3	2	3	2	0.40	3-methyl-2-oxobutano
872	1	db$2	2::C3K134|C3K134_PSEFS	93	52371	3	3	3	3	0.31	Coproporphyrinogen-II
873	1	db$2	2::C3JY77|C3JY77_PSEFS	93	40451	5	5	3	3	0.60	Putative 3-oxoacyl-[ac
874	1	db$2	2::C3K3R4|C3K3R4_PSEFS	93	24681	3	3	3	3	0.77	Putative hydrolase OS=
875	1	db$2	2::C3JXJ5|C3JXJ5_PSEFS	93	22111	4	4	3	3	0.89	Putative SCO1/SenC fa
876	1	db$2	2::C3K7S1|C3K7S1_PSEFS	93	25085	2	2	1	1	0.21	Protein MgtC OS=Pseu
877	1	db$2	2::C3KDM4|C3KDM4_PSEFS	93	56715	6	4	6	4	0.40	Putative membrane pro
878	1	db$2	2::C3K2U6|C3K2U6_PSEFS	93	49484	2	2	2	2	0.21	Putative transport-rela
879	1	db$2	2::C3KC02|C3KC02_PSEFS	92	29258	3	3	3	3	0.62	Putative acyltransferas
880	1	db$2	2::C3KDB8|HLDE_PSEFS	91	49913	6	5	6	5	0.61	Bifunctional protein Hl
881	1	db$2	2::C3K909|C3K909_PSEFS	91	24537	3	3	2	2	0.47	Uncharacterized protei
882	1	db$2	2::C3K2K2|THIE_PSEFS	90	22345	2	2	2	2	0.52	Thiamine-phosphate sy
883	1	db$2	2::C3K5F4|C3K5F4_PSEFS	90	21753	3	3	3	3	0.91	Putative nitroreductase
884	1	SwissProt_HUMAN$	3::Q07020|RL18_HUMAN	90	21621	2	2	2	2	0.54	60S ribosomal protein
885	1	db$2	2::C3KB73|C3KB73_PSEFS	90	9692	1	1	1	1	0.61	Putative lipoprotein OS
886	1	db$2	2::C3JZ82|C3JZ82_PSEFS	90	37007	2	2	2	2	0.29	Putative dehydrogenas
887	1	db$2	2::C3K1B1|C3K1B1_PSEFS	90	46678	2	2	2	2	0.22	Putative acid tolerance
888	1	db$2	2::C3KEB0|C3KEB0_PSEFS	90	36597	3	3	2	2	0.29	Putative amino acid AB
889	1	db$2	2::C3K3X0|C3K3X0_PSEFS	90	42989	3	3	3	3	0.39	Putative ubiquinone bi
890	1	db$2	2::C3K2L0|C3K2L0_PSEFS	89	55816	2	2	2	2	0.18	Apolipoprotein N-acyltr
891	1	db$2	2::C3K4Z4|C3K4Z4_PSEFS	89	43117	5	5	5	5	0.73	Putative cytochrome C
892	1	db$2	2::C3K3K3|C3K3K3_PSEFS	89	44101	8	7	7	6	0.90	Dihydroorotase-like pro
893	1	db$2	2::C3K3J3|C3K3J3_PSEFS	89	72855	3	3	3	3	0.22	Type IV pilus biogenesi
894	1	db$2	2::C3KDR6|C3KDR6_PSEFS	89	40570	3	3	3	3	0.42	Putaive NADH oxidorec
895	1	db$2	2::C3K4J4|HSLO_PSEFS	88	33320	2	2	2	2	0.33	33 kDa chaperonin OS
896	1	db$2	2::C3JYN4|ZIPA_PSEFS	88	31818	6	5	4	4	0.81	Cell division protein Zi
897	1	db$2	2::C3K0Q8|C3K0Q8_PSEFS	88	24717	2	2	2	2	0.46	Basal-body rod modific
898	1	db$2	2::C3K819|C3K819_PSEFS	87	35012	3	3	3	3	0.50	Putative ABC transport
899	1	db$2	2::C3K9H7|C3K9H7_PSEFS	87	27490	3	2	3	2	0.41	Putative short-chain de
900	1	db$2	2::C3K6U9|HIS7_PSEFS	87	21830	3	3	3	3	0.90	Imidazoleglycerol-phos
901	1	db$2	2::C3K479|RNPH_PSEFS	87	25645	3	3	3	3	0.73	Ribonuclease PH OS=P
902	1	db$2	2::C3K7C7|C3K7C7_PSEFS	86	19943	3	3	3	3	1.02	Chorismate mutase OS
903	1	db$2	2::C3K3M6|C3K3M6_PSEFS	86	53012	6	5	6	5	0.56	Peptidase_M16_C dom
904	1	db$2	2::C3K4J0|C3K4J0_PSEFS	86	17598	4	4	4	4	1.89	Zn_protease domain-c
905	1	db$2	2::C3KDY6|C3KDY6_PSEFS	86	17128	1	1	1	1	0.31	Uncharacterized protei
906	1	db$2	2::C3JYU5|C3JYU5_PSEFS	86	34090	2	2	2	2	0.32	Nudix hydrolase domai
907	1	SwissProt_HUMAN$	3::P61978|HNRPK_HUMAN	86	50944	1	1	1	1	0.10	Heterogeneous nuclear
908	1	db$2	2::C3K4W3|C3K4W3_PSEFS	86	21770	3	3	2	2	0.91	PilZ domain-containing

↑Family	M	DB	Accession	Score	Mass	Matches	Match(sig)	Sequences	Seq(sig)	emPAI	Description
909	1	db$2	2::C3K415|C3K415_PSEFS	85	70809	3	3	3	3	0.22	Putative ABC transport
910	1	db$2	2::C3K1A8|C3K1A8_PSEFS	85	35739	3	2	2	2	0.30	Putative lipoprotein OS
911	1	db$2	2::C3K4U6|C3K4U6_PSEFS	85	17513	2	2	1	1	0.31	Conserved hypothetica
912	1	db$2	2::C3K1A3|C3K1A3_PSEFS	85	26906	2	2	2	2	0.42	Putative pyridoxamine
913	1	db$2	2::C3K838|C3K838_PSEFS	85	9715	2	2	2	2	1.57	Putative phosphocarrie
914	1	db$2	2::C3KCV6|C3KCV6_PSEFS	85	40154	7	6	7	6	1.02	Probable alcohol dehyd
915	1	SwissProt_HUMAN$	3::P60174|TPIS_HUMAN	85	26653	5	5	4	4	1.03	Triosephosphate isome
916	1	db$2	2::C3JYU3|C3JYU3_PSEFS	85	75598	3	3	3	3	0.21	Histidine kinase OS=Ps
917	1	db$2	2::C3K481|C3K481_PSEFS	85	23434	2	2	2	2	0.49	Guanylate kinase OS=I
918	1	db$2	2::C3KCT0|MURG_PSEFS	85	37667	5	4	5	4	0.65	UDP-N-acetylglucosam
919	1	db$2	2::C3KAI3|AROB_PSEFS	85	39825	5	5	5	5	0.81	3-dehydroquinate synt
920	1	db$2	2::C3K2P1|C3K2P1_PSEFS	84	48286	4	4	3	3	0.34	UDP-N-acetylmuramat
921	1	db$2	2::C3K092|C3K092_PSEFS	84	14085	4	4	3	3	1.69	Glutaredoxin domain-c
922	1	db$2	2::C3K4R1|PLSB_PSEFS	84	95327	3	3	3	3	0.16	Glycerol-3-phosphate a
923	1	db$2	2::C3JXX6|DNLJ_PSEFS	84	85515	4	4	4	4	0.25	DNA ligase OS=Pseudc
924	1	db$2	2::C3K0N8|C3K0N8_PSEFS	84	19586	3	3	3	3	1.05	23S rRNA accumulatior
925	1	db$2	2::C3KE89|C3KE89_PSEFS	84	24346	4	4	4	4	1.17	Uncharacterized protei
926	1	db$2	2::C3JZN4|RL20_PSEFS	84	13348	5	4	4	4	3.04	50S ribosomal protein
927	1	db$2	2::C3KB19|C3KB19_PSEFS	83	44434	3	2	3	2	0.24	HlyD family secretion p
928	1	db$2	2::C3K0X3|C3K0X3_PSEFS	83	16227	3	3	3	3	1.37	Transcriptional regulat
929	1	db$2	2::C3K2D4|C3K2D4_PSEFS	83	35439	4	4	4	4	0.70	Putative cobalamin syn
930	1	db$2	2::C3K4Z0|CCME_PSEFS	83	16101	4	3	3	3	1.39	Cytochrome c-type bio
931	1	db$2	2::C3KE87|C3KE87_PSEFS	83	46792	4	3	4	3	0.35	NADH dehydrogenase I
932	1	db$2	2::C3K815|C3K815_PSEFS	82	62777	9	6	7	5	0.57	Glucans biosynthesis p
933	1	db$2	2::C3JYS8|RUVA_PSEFS	82	22218	4	3	4	3	0.89	Holliday junction ATP-c
934	1	db$2	2::C3K5K2|C3K5K2_PSEFS	82	9923	3	3	3	3	3.06	Putative lipoprotein OS
935	1	db$2	2::C3K0D5|MINE_PSEFS	82	9531	4	4	4	4	5.91	Cell division topologica
936	1	db$2	2::C3K1W6|C3K1W6_PSEFS	82	13390	3	3	3	3	1.83	D-erythro-7,8-dihydror
937	1	db$2	2::C3K7A7|C3K7A7_PSEFS	81	43115	4	4	4	4	0.55	Conserved hypothetica
938	1	db$2	2::C3KE56|C3KE56_PSEFS	81	59021	3	3	3	3	0.27	Glutamate--cysteine lig
939	1	db$2	2::C3KBM4|C3KBM4_PSEFS	80	34098	4	3	3	3	0.51	Ornithine carbamoyltra
940	1	db$2	2::C3KAC2|C3KAC2_PSEFS	80	41342	4	4	4	4	0.58	Acyl-CoA dehydrogena
941	1	db$2	2::C3KAL0|C3KAL0_PSEFS	80	43962	3	2	3	2	0.24	PlsC domain-containing
942	1	SwissProt_HUMAN$	3::Q6FI13|H2A2A_HUMAN	80	14087	3	3	3	3	1.69	Histone H2A type 2-A (
943	1	db$2	2::C3K3Y7|C3K3Y7_PSEFS	80	35706	3	3	3	3	0.49	Putative iron-sulfur bin
944	1	SwissProt_HUMAN$	3::Q02878|RL6_HUMAN	80	32708	1	1	1	1	0.15	60S ribosomal protein
945	1	db$2	2::C3KCS8|C3KCS8_PSEFS	80	47767	4	4	4	4	0.49	UDP-N-acetylmuramoy
946	1	db$2	2::C3K2Z9|C3K2Z9_PSEFS	80	34289	2	2	2	2	0.32	NMO domain-containin
947	1	db$2	2::C3KBZ8|C3KBZ8_PSEFS	80	38389	3	3	3	3	0.45	Iron-sulfur cluster carr
948	1	db$2	2::C3K257|C3K257_PSEFS	80	33362	6	6	5	5	1.03	tRNA pseudouridine sy

↑Family	M	DB	Accession	Score	Mass	Matches	Match(sig)	Sequences	Seq(sig)	emPAI	Description
949	1	db$2	2::C3K1H0|RIMM_PSEFS	80	20004	3	3	3	3	1.02	Ribosome maturation f
950	1	db$2	2::C3K6Y8|C3K6Y8_PSEFS	80	34148	5	4	5	4	0.74	GTPase Era OS=Pseud
951	1	db$2	2::C3K1C9|C3K1C9_PSEFS	80	65670	3	3	2	2	0.16	Putative cation-transpo
952	1	db$2	2::C3KDC3|LOLB_PSEFS	80	23021	2	1	2	1	0.23	Outer-membrane lipop
953	1	db$2	2::C3JZH1|C3JZH1_PSEFS	80	34020	2	2	2	2	0.32	LysR-family transcripti
954	1	db$2	2::C3K813|C3K813_PSEFS	80	36618	8	7	6	6	1.46	Proline iminopeptidase
955	1	db$2	2::C3K5L2|C3K5L2_PSEFS	79	29854	2	2	2	2	0.37	Phosphonates import A
956	1	db$2	2::C3KDS5|C3KDS5_PSEFS	79	41122	5	4	5	4	0.58	Putative hydratase/isor
957	1	db$2	2::C3KBB8|C3KBB8_PSEFS	79	13546	3	3	3	3	1.80	Uncharacterized protei
958	1	db$2	2::C3K8W5|C3K8W5_PSEFS	79	21382	2	2	2	2	0.55	Lipopolysaccharide exp
959	1	db$2	2::C3K8W2|ADE_PSEFS	79	36048	3	3	3	3	0.48	Adenine deaminase OS
960	1	db$2	2::C3K707|C3K707_PSEFS	79	29295	2	1	2	1	0.17	Putative lipoprotein OS
961	1	db$2	2::C3K3K1|C3K3K1_PSEFS	79	18188	1	1	1	1	0.29	Bifunctional protein [py
962	1	db$2	2::C3KAH2|C3KAH2_PSEFS	79	24970	4	4	3	3	0.76	SPOR domain-containir
963	1	db$2	2::C3KAS9|C3KAS9_PSEFS	79	109677	3	3	3	3	0.14	Efflux pump membrane
964	1	db$2	2::C3K9M9|C3K9M9_PSEFS	79	14163	3	3	3	3	1.67	Putative endoribonucle
965	1	db$2	2::C3JYE6|C3JYE6_PSEFS	78	45437	2	2	2	2	0.23	Putative acyl-CoA dehy
966	1	SwissProt_HUMAN$	3::Q06830|PRDX1_HUMAN	78	22096	8	6	8	6	2.58	Peroxiredoxin-1 OS=H
967	1	db$2	2::C3KDG5|C3KDG5_PSEFS	78	23599	4	4	4	4	1.21	Putative regulatory pro
968	1	db$2	2::C3K9Q9|C3K9Q9_PSEFS	78	19670	2	2	2	2	0.61	FMN_red domain-conta
969	1	db$2	2::C3KDI4|C3KDI4_PSEFS	78	42151	4	4	4	4	0.57	Putative exported prot
970	1	db$2	2::C3KBL6|C3KBL6_PSEFS	78	27732	2	2	2	2	0.40	Glutamate/aspartate A
971	1	db$2	2::C3K579|C3K579_PSEFS	77	15962	1	1	1	1	0.34	Putative thioesterase C
972	1	db$2	2::C3KDW0|C3KDW0_PSEFS	77	49939	3	3	3	3	0.33	N-acetylmuramoyl-L-al
973	1	db$2	2::C3K2U3|C3K2U3_PSEFS	77	27946	3	3	2	2	0.40	Trans-aconitate 2-metl
974	1	db$2	2::C3K6P0|C3K6P0_PSEFS	77	15266	3	3	2	2	0.84	Uncharacterized protei
975	1	db$2	2::C3KDX6|C3KDX6_PSEFS	77	16394	1	1	1	1	0.33	Peptidyl-prolyl cis-tran
976	1	db$2	2::C3K5T5|C3K5T5_PSEFS	77	25405	3	2	2	1	0.20	Carbonic anhydrase OS
977	1	db$2	2::C3K088|C3K088_PSEFS	77	20655	7	6	4	4	2.11	Putative isochorismata
978	1	db$2	2::C3JXX3|C3JXX3_PSEFS	77	52842	4	4	4	4	0.43	Cytochrome-c oxidase
979	1	db$2	2::C3KAM8|C3KAM8_PSEFS	77	52747	5	5	4	4	0.43	Phosphate transporter
980	1	db$2	2::C3JYE7|C3JYE7_PSEFS	76	35468	2	2	2	2	0.30	LysR family regulatory
981	1	db$2	2::C3K666|C3K666_PSEFS	76	29461	2	2	2	2	0.38	Putative lipoprotein OS
982	1	db$2	2::C3K221|C3K221_PSEFS	76	129628	1	1	1	1	0.04	RecBCD enzyme subun
983	1	db$2	2::C3K1G1|C3K1G1_PSEFS	76	48709	3	3	3	3	0.34	tRNA modification GTP
984	1	db$2	2::C3K224|C3K224_PSEFS	76	30473	2	2	1	1	0.17	CDP-diacylglycerol--se
985	1	SwissProt_HUMAN$	3::P05413|FABPH_HUMAN	76	14849	2	2	2	2	0.87	Fatty acid-binding prot
986	1	db$2	2::C3K062|C3K062_PSEFS	76	48996	4	4	3	3	0.34	Fumarate hydratase cla
987	1	db$2	2::C3K574|C3K574_PSEFS	76	36116	2	2	2	2	0.30	Putative oxidoreductas
988	1	db$2	2::C3KEC9|C3KEC9_PSEFS	76	53005	2	2	2	2	0.20	Putative beta-barrel as

↑Family	M	DB	Accession	Score	Mass	Matches	Match(sig)	Sequences	Seq(sig)	emPAI	Description
989	1	db$2	2::C3K303|COQ7_PSEFS	76	23866	4	2	3	2	0.48	3-demethoxyubiquinol
990	1	db$2	2::C3KBG3|C3KBG3_PSEFS	76	24033	3	3	3	3	0.80	Curli production assem
991	1	db$2	2::C3K0R6|C3K0R6_PSEFS	76	28374	3	3	3	3	0.64	Uncharacterized protei
992	1	db$2	2::C3JYN0|C3JYN0_PSEFS	75	33944	2	2	2	2	0.32	Cbb3-type cytochrome
992	2	db$2	2::C3JZS9|C3JZS9_PSEFS	53	35591	2	2	2	2	0.30	Cbb3-type cytochrome
993	1	db$2	2::C3K0C5|C3K0C5_PSEFS	75	27111	1	1	1	1	0.19	Putative amino transfe
994	1	db$2	2::C3K0P9|C3K0P9_PSEFS	75	25457	3	3	3	3	0.74	Two-component syster
995	1	db$2	2::C3JZZ4|C3JZZ4_PSEFS	75	23102	3	2	3	2	0.50	Putative glutathione S
996	1	db$2	2::C3K5F1|C3K5F1_PSEFS	75	42516	4	3	4	3	0.40	Ribonuclease D OS=Ps
997	1	SwissProt_HUMAN$	3::P62249|RS16_HUMAN	75	16435	2	2	2	2	0.77	40S ribosomal protein
998	1	db$2	2::C3KBL9|C3KBL9_PSEFS	75	27726	3	3	2	2	0.40	Glycerol-3-phosphate r
999	1	db$2	2::C3K0K1|C3K0K1_PSEFS	75	53436	1	1	1	1	0.09	Putative GntR-family re
1000	1	db$2	2::C3JZ77|C3JZ77_PSEFS	75	45611	2	2	1	1	0.11	Maltose-binding protei
1001	1	db$2	2::C3JYF7|C3JYF7_PSEFS	75	147681	4	3	4	3	0.10	ATP-dependent helicas
1002	1	db$2	2::C3K327|C3K327_PSEFS	75	29832	2	2	2	2	0.37	Ribosomal RNA small s
1003	1	db$2	2::Q9ZEP7|PANC_PSEFS	74	30696	2	2	2	2	0.36	Pantothenate syntheta
1004	1	db$2	2::C3K458|C3K458_PSEFS	74	47152	1	1	1	1	0.11	D-amino acid dehydrog
1005	1	db$2	2::C3K6N8|C3K6N8_PSEFS	74	25972	1	1	1	1	0.20	Putative hydrolase OS=
1006	1	db$2	2::C3KAM2|C3KAM2_PSEFS	74	49640	1	1	1	1	0.10	Putative two-componer
1007	1	db$2	2::C3K737|C3K737_PSEFS	74	17727	3	3	3	3	1.20	Glutathione peroxidase
1008	1	db$2	2::C3KC07|C3KC07_PSEFS	74	21449	2	2	2	2	0.55	YceI domain-containinc
1009	1	db$2	2::C3K477|C3K477_PSEFS	73	29836	4	4	4	4	0.88	Exodeoxyribonuclease
1010	1	db$2	2::C3K1C5|C3K1C5_PSEFS	73	44321	5	3	5	3	0.38	Putative cyclopropane-
1011	1	db$2	2::C3K301|C3K301_PSEFS	73	28901	4	3	4	3	0.63	Putative dehydrogenas
1012	1	db$2	2::C3K2Y1|RS12_PSEFS	73	13716	4	4	3	3	1.76	30S ribosomal protein
1013	1	db$2	2::C3K7W0|C3K7W0_PSEFS	73	26786	3	3	3	3	0.70	Uncharacterized protei
1014	1	db$2	2::C3KCP2|C3KCP2_PSEFS	73	100291	1	1	1	1	0.05	Conserved GGDEF/EAL
1015	1	db$2	2::C3KE46|C3KE46_PSEFS	73	47174	4	4	3	3	0.35	16S rRNA m5C967 met
1016	1	db$2	2::C3JZ62|C3JZ62_PSEFS	73	61473	1	1	1	1	0.08	Putative long-chain-fat
1017	1	db$2	2::C3JZP9|C3JZP9_PSEFS	73	33380	1	1	1	1	0.15	Putative ribose transpc
1018	1	db$2	2::C3K0M3|C3K0M3_PSEFS	73	20567	3	3	3	3	0.98	Uncharacterized protei
1019	1	SwissProt_HUMAN$	3::P61604|CH10_HUMAN	72	10925	4	4	4	4	4.49	10 kDa heat shock prot
1020	1	db$2	2::C3K650|C3K650_PSEFS	72	13083	4	4	4	4	3.14	N-acetyltransferase do
1021	1	db$2	2::C3K6Z2|C3K6Z2_PSEFS	72	17868	2	2	2	2	0.69	tRNA-specific adenosin
1022	1	db$2	2::C3K5J9|C3K5J9_PSEFS	72	96070	3	2	3	2	0.10	Histidine kinase OS=Ps
1023	1	db$2	2::C3KDR1|C3KDR1_PSEFS	71	17628	4	4	3	3	1.21	Uncharacterized protei
1024	1	db$2	2::C3KDV5|C3KDV5_PSEFS	71	20461	5	5	4	4	1.51	Oligoribonuclease OS=
1025	1	db$2	2::C3K710|DCD_PSEFS	71	21231	4	4	4	4	1.42	dCTP deaminase OS=P
1026	1	SwissProt_HUMAN$	3::P07195|LDHB_HUMAN	71	36615	4	3	4	3	0.47	L-lactate dehydrogenas
1027	1	db$2	2::C3K486|C3K486_PSEFS	71	30593	4	3	4	3	0.59	Epimerase domain-con

↑Family	M	DB	Accession	Score	Mass	Matches	Match(sig)	Sequences	Seq(sig)	emPAI	Description
1028	1	db$2	2::C3K584|C3K584_PSEFS	71	15399	2	2	2	2	0.83	Uncharacterized protei
1029	1	db$2	2::C3K2E4|C3K2E4_PSEFS	71	29249	3	3	2	2	0.38	Ferredoxin--NADP redu
1030	1	db$2	2::C3JY87|NUOH_PSEFS	70	37530	4	4	3	3	0.46	NADH-quinone oxidore
1031	1	db$2	2::C3K270|C3K270_PSEFS	70	17315	7	4	6	4	1.95	Transcription elongatio
1032	1	db$2	2::C3JXP7|RECR_PSEFS	70	21528	3	2	3	2	0.55	Recombination protein
1033	1	db$2	2::C3KBY0|GMHA_PSEFS	70	21250	2	2	2	2	0.56	Phosphoheptose isome
1034	1	db$2	2::C3K0W6|C3K0W6_PSEFS	70	42755	3	3	3	3	0.39	Aspartate aminotransfe
1035	1	db$2	2::C3K5E3|C3K5E3_PSEFS	70	43909	3	3	3	3	0.38	Putative aminotransfer
1036	1	db$2	2::C3K307|TRPC_PSEFS	70	30252	6	6	6	6	1.54	Indole-3-glycerol phos
1037	1	db$2	2::C3K591|C3K591_PSEFS	70	19872	3	3	2	2	1.03	Putative bacterioferriti
1038	1	db$2	2::C3K867|C3K867_PSEFS	70	39735	3	3	3	3	0.43	PALP domain-containin
1039	1	db$2	2::C3K8P0|C3K8P0_PSEFS	69	38329	4	2	4	2	0.28	Putative nucleoside-se
1040	1	db$2	2::C3K483|C3K483_PSEFS	69	78630	4	4	4	4	0.27	Guanosine-3',5'-bis(Di
1041	1	db$2	2::C3K571|C3K571_PSEFS	69	11755	2	2	2	2	1.20	Uncharacterized protei
1042	1	db$2	2::C3K1F7|C3K1F7_PSEFS	69	69251	3	2	3	2	0.15	tRNA uridine 5-carboxy
1043	1	db$2	2::C3K9T0|NADK_PSEFS	69	32289	3	2	3	2	0.34	NAD kinase OS=Pseud
1044	1	db$2	2::C3K6H1|C3K6H1_PSEFS	69	131195	7	5	7	5	0.20	DNA polymerase III su
1045	1	db$2	2::C3KAY5|C3KAY5_PSEFS	69	30881	3	3	3	3	0.58	NAD-capped RNA hydr
1046	1	db$2	2::C3KDC4|C3KDC4_PSEFS	69	64677	4	3	4	3	0.25	Uncharacterized protei
1047	1	db$2	2::C3K3L3|C3K3L3_PSEFS	69	21091	3	3	3	3	0.95	dITP/XTP pyrophospha
1048	1	db$2	2::C3KDX3|C3KDX3_PSEFS	68	34279	4	4	4	4	0.73	Riboflavin biosynthesis
1049	1	db$2	2::C3K2K7|C3K2K7_PSEFS	68	37713	5	4	5	4	0.65	PhoH domain-containir
1050	1	db$2	2::C3K0Z1|C3K0Z1_PSEFS	68	36566	3	2	3	2	0.29	L-threonine-O-3-phosp
1051	1	db$2	2::C3K4V3|C3K4V3_PSEFS	68	45550	4	2	3	2	0.23	Uncharacterized protei
1052	1	db$2	2::C3K2E0|C3K2E0_PSEFS	68	48742	6	5	6	5	0.62	DNA repair protein Rac
1053	1	db$2	2::C3KCS5|C3KCS5_PSEFS	68	51798	5	5	5	5	0.58	UDP-N-acetylmuramoy
1054	1	db$2	2::C3KDP0|C3KDP0_PSEFS	68	27498	3	3	3	3	0.67	Uncharacterized protei
1055	1	db$2	2::C3K0R2|C3K0R2_PSEFS	68	34379	3	3	2	2	0.32	Chemotaxis protein OS
1056	1	db$2	2::C3KE30|C3KE30_PSEFS	68	59128	5	4	5	4	0.38	L-aspartate oxidase OS
1057	1	db$2	2::C3K013|C3K013_PSEFS	68	20254	2	2	1	1	0.26	Peptidyl-prolyl cis-tran
1058	1	db$2	2::C3KA82|C3KA82_PSEFS	68	19457	5	4	5	4	1.62	Lactoylglutathione lyas
1059	1	db$2	2::C3K1K5|EX7L_PSEFS	68	51103	2	2	2	2	0.20	Exodeoxyribonuclease
1060	1	SwissProt_HUMAN$	3::P08238|HS90B_HUMAN	68	83212	2	2	2	2	0.12	Heat shock protein HSI
1061	1	db$2	2::C3KE45|C3KE45_PSEFS	68	49639	3	3	3	3	0.33	Trk system potassium
1062	1	db$2	2::C3K150|C3K150_PSEFS	68	25194	3	3	3	3	0.75	Putative short-chain de
1063	1	db$2	2::C3K0U4|C3K0U4_PSEFS	67	36872	4	4	4	4	0.67	Flagellar motor switch
1064	1	db$2	2::C3JYR0|C3JYR0_PSEFS	67	13774	2	2	2	2	0.97	Glycine cleavage syste
1065	1	db$2	2::C3K5K1|C3K5K1_PSEFS	67	38065	4	3	4	3	0.45	Putative phospholipase
1066	1	SwissProt_HUMAN$	3::P62701|RS4X_HUMAN	67	29579	1	1	1	1	0.17	40S ribosomal protein
1067	1	db$2	2::C3KA92|C3KA92_PSEFS	67	24193	1	1	1	1	0.21	Putative ACP reductase

↑Family	M	DB	Accession	Score	Mass	Matches	Match(sig)	Sequences	Seq(sig)	emPAI	Description
1068	1	db$2	2::C3K0M6|C3K0M6_PSEFS	66	25497	4	3	4	3	0.74	Putative tetR-family re
1069	1	db$2	2::C3K6W0|C3K6W0_PSEFS	66	14786	4	2	3	2	0.88	Putative rhodanese dor
1070	1	db$2	2::C3K7Y8|C3K7Y8_PSEFS	66	53697	4	4	3	3	0.42	Putative hydrolase OS=
1071	1	db$2	2::C3K4U7|C3K4U7_PSEFS	66	28919	2	2	2	2	0.38	Putative amidotransfer
1072	1	db$2	2::C3K6Z1|C3K6Z1_PSEFS	66	50711	2	2	2	2	0.21	Putative multicopper o:
1073	1	db$2	2::C3JXH2|C3JXH2_PSEFS	66	91149	3	3	3	3	0.17	Putative excinuclease A
1074	1	db$2	2::C3K459|C3K459_PSEFS	66	18704	2	2	2	2	0.65	Leucine-responsive reg
1075	1	db$2	2::C3JXL6|C3JXL6_PSEFS	66	41978	1	1	1	1	0.12	Bcr/CflA family efflux t
1076	1	db$2	2::C3K5X3|C3K5X3_PSEFS	65	64999	2	2	2	2	0.16	Putative ABC transport
1077	1	db$2	2::C3K3V1|C3K3V1_PSEFS	65	23989	3	2	3	2	0.48	Alkyl hydroperoxide re
1078	1	db$2	2::C3K6E3|C3K6E3_PSEFS	65	21087	6	4	5	4	1.43	Ubiquinol-cytochrome
1079	1	db$2	2::C3K1A4|C3K1A4_PSEFS	65	41199	2	2	2	2	0.26	Uncharacterized protei
1080	1	db$2	2::C3KC09|C3KC09_PSEFS	65	83087	2	2	2	2	0.12	Periplasmic beta-gluco:
1081	1	db$2	2::C3K620|C3K620_PSEFS	65	22214	4	2	4	2	0.53	LexA repressor OS=Ps
1082	1	db$2	2::C3K0J1|C3K0J1_PSEFS	65	36130	4	4	4	4	0.69	Flagellar motor switch
1083	1	db$2	2::C3K2V4|RS13_PSEFS	65	13332	4	4	3	3	1.85	30S ribosomal protein
1084	1	db$2	2::C3K4M2|C3K4M2_PSEFS	65	36363	3	2	3	2	0.30	Catabolite repressor/ac
1085	1	db$2	2::C3K664|C3K664_PSEFS	65	31550	1	1	1	1	0.16	Putative membrane pro
1086	1	db$2	2::C3KCJ3|C3KCJ3_PSEFS	65	30975	4	4	3	3	0.58	Usp domain-containing
1087	1	db$2	2::C3K6L9|C3K6L9_PSEFS	64	49785	4	4	4	4	0.46	Phospho-2-dehydro-3-
1088	1	db$2	2::C3K9I2|C3K9I2_PSEFS	64	33896	2	2	2	2	0.32	Putative transcriptional
1089	1	db$2	2::C3K4R2|C3K4R2_PSEFS	64	7681	3	3	3	3	5.01	Major cold shock prote
1090	1	db$2	2::C3KBM1|C3KBM1_PSEFS	64	29989	2	2	2	2	0.37	Glycerol uptake facilita
1091	1	db$2	2::C3JXS4|C3JXS4_PSEFS	64	25108	1	1	1	1	0.21	Tol-Pal system protein
1092	1	db$2	2::C3K098|C3K098_PSEFS	64	23000	1	1	1	1	0.23	Putative glutathione S-
1093	1	db$2	2::C3KE38|C3KE38_PSEFS	64	28697	4	3	4	3	0.63	Putative phospholipid b
1094	1	SwissProt_HUMAN$	3::P61513|RL37A_HUMAN	64	10268	1	1	1	1	0.56	60S ribosomal protein
1095	1	db$2	2::C3KCP6|C3KCP6_PSEFS	64	38240	2	2	2	2	0.28	ADP-heptose--LPS hep
1096	1	db$2	2::C3K6R7|C3K6R7_PSEFS	64	41832	2	2	2	2	0.25	Serine/threonine trans
1097	1	db$2	2::C3K3E9|C3K3E9_PSEFS	63	12056	1	1	1	1	0.47	Putative lipoprotein OS
1098	1	db$2	2::C3K7T1|C3K7T1_PSEFS	63	23131	4	3	4	3	0.84	Two-component syster
1099	1	db$2	2::C3K706|PIMT_PSEFS	63	23451	1	1	1	1	0.22	Protein-L-isoaspartate
1100	1	db$2	2::C3K830|C3K830_PSEFS	63	47217	6	4	6	4	0.49	Putative short chain de
1101	1	SwissProt_HUMAN$	3::Q71DI3|H32_HUMAN	63	15379	4	3	3	2	0.83	Histone H3.2 OS=Hom
1102	1	db$2	2::C3K2P7|C3K2P7_PSEFS	63	17680	3	3	3	3	1.21	Putative acetyltransfer
1103	1	db$2	2::C3JY93|C3JY93_PSEFS	63	51981	2	1	2	1	0.10	NADH-quinone oxidore
1104	1	db$2	2::C3K8T9|TATA_PSEFS	63	10325	2	1	2	1	0.56	Sec-independent prote
1105	1	db$2	2::C3K2K6|C3K2K6_PSEFS	63	49458	3	3	3	3	0.33	tRNA-2-methylthio-N(6
1106	1	db$2	2::C3K6T3|C3K6T3_PSEFS	63	23166	1	1	1	1	0.22	Thiol:disulfide intercha
1107	1	db$2	2::C3KED3|C3KED3_PSEFS	62	20551	4	3	4	3	0.98	Glycine cleavage syste

↑Family	M	DB	Accession	Score	Mass	Matches	Match(sig)	Sequences	Seq(sig)	emPAI	Description
1108	1	db$2	2::C3K219|C3K219_PSEFS	62	76943	1	1	1	1	0.06	RecBCD enzyme subun
1109	1	db$2	2::C3K7K0|C3K7K0_PSEFS	62	41297	2	2	2	2	0.26	Esterase III OS=Pseud
1110	1	db$2	2::C3K2R8|NUSB_PSEFS	62	18764	2	2	2	2	0.65	Transcription antitermi
1111	1	db$2	2::C3K9B4|C3K9B4_PSEFS	62	16186	1	1	1	1	0.33	Type II secretion syste
1112	1	db$2	2::C3KDC7|C3KDC7_PSEFS	62	29930	1	1	1	1	0.17	Release factor glutamir
1113	1	db$2	2::C3K3D1|BETI_PSEFS	62	21992	1	1	1	1	0.24	HTH-type transcription
1114	1	db$2	2::C3K8L0|C3K8L0_PSEFS	62	16885	1	1	1	1	0.32	Uncharacterized protei
1115	1	db$2	2::C3K654|C3K654_PSEFS	62	34290	4	4	2	2	0.32	2-dehydropantoate 2-r
1116	1	db$2	2::C3K258|RBFA_PSEFS	62	14697	4	3	4	3	1.60	Ribosome-binding facto
1117	1	db$2	2::C3JYS4|C3JYS4_PSEFS	62	16520	3	3	3	3	1.33	Tol-Pal system protein
1118	1	db$2	2::C3K021|C3K021_PSEFS	62	27911	5	4	4	3	0.66	Phosphoadenosine pho
1119	1	db$2	2::C3KDP4|C3KDP4_PSEFS	62	75338	4	4	4	4	0.29	Phosphate acetyltransf
1120	1	db$2	2::C3K7G1|C3K7G1_PSEFS	61	37563	3	3	3	3	0.46	Cell shape protein Mre(
1121	1	db$2	2::C3K0Z5|C3K0Z5_PSEFS	61	22469	3	3	3	3	0.87	Corrinoid adenosyltran:
1122	1	db$2	2::C3JZ64|C3JZ64_PSEFS	61	17217	3	3	3	3	1.26	MaoC-like domain-cont
1123	1	db$2	2::C3KBT9|C3KBT9_PSEFS	61	31336	1	1	1	1	0.16	Sulfate ABC transport s
1124	1	db$2	2::C3K0K9|C3K0K9_PSEFS	61	37421	2	2	1	1	0.13	Putative NADP-depend
1125	1	db$2	2::C3K453|C3K453_PSEFS	61	71119	2	1	2	1	0.07	AcetylCoA_hyd_C dom
1126	1	db$2	2::C3K256|RS15_PSEFS	61	10112	5	5	4	4	5.23	30S ribosomal protein
1127	1	db$2	2::C3K2S2|C3K2S2_PSEFS	61	38886	6	3	6	3	0.44	Riboflavin biosynthesis
1128	1	SwissProt_HUMAN$	3::Q86YZ3|HORN_HUMAN	61	282228	2	2	2	2	0.03	Hornerin OS=Homo sa
1129	1	db$2	2::C3K3B8|C3K3B8_PSEFS	61	42763	3	3	3	3	0.39	Uncharacterized protei
1130	1	db$2	2::C3KDX1|RS20_PSEFS	61	10078	2	2	2	2	1.50	30S ribosomal protein
1131	1	db$2	2::C3KE16|C3KE16_PSEFS	60	37004	4	2	3	2	0.29	Putative chemotaxis-re
1132	1	db$2	2::C3K6W8|C3K6W8_PSEFS	60	16611	1	1	1	1	0.32	3-dehydroquinate dehy
1133	1	db$2	2::C3K0X7|C3K0X7_PSEFS	60	126663	1	1	1	1	0.04	Histidine kinase OS=Ps
1134	1	db$2	2::C3K663|C3K663_PSEFS	60	22341	4	2	4	2	0.52	Putative TetR-family re
1135	1	db$2	2::C3JXZ6|C3JXZ6_PSEFS	60	24822	1	1	1	1	0.21	Putative histidine trans
1136	1	db$2	2::C3K493|C3K493_PSEFS	60	16495	3	3	3	3	1.33	Uncharacterized protei
1137	1	db$2	2::C3K107|C3K107_PSEFS	59	31415	3	3	3	3	0.57	Uncharacterized protei
1138	1	db$2	2::C3KDN1|C3KDN1_PSEFS	59	54005	4	3	3	2	0.19	Deoxyribodipyrimidine
1139	1	db$2	2::C3K068|C3K068_PSEFS	59	65454	4	4	4	4	0.34	Putative asparagine sy
1140	1	db$2	2::C3KAR4|C3KAR4_PSEFS	59	11430	1	1	1	1	0.50	Uncharacterized protei
1141	1	db$2	2::C3K7W1|C3K7W1_PSEFS	59	12229	3	2	3	2	1.14	Putative lipoprotein OS
1142	1	db$2	2::C3K6Y0|C3K6Y0_PSEFS	59	13973	1	1	1	1	0.40	4-carboxymuconolacto
1143	1	db$2	2::C3KDN6|C3KDN6_PSEFS	59	32369	2	2	2	2	0.34	Uncharacterized protei
1144	1	db$2	2::C3KDI2|C3KDI2_PSEFS	59	135789	2	1	2	1	0.04	Exonuclease OS=Pseuc
1145	1	db$2	2::C3K9Q2|C3K9Q2_PSEFS	59	16954	3	2	3	2	0.73	Ribonuclease H OS=Ps
1146	1	db$2	2::C3JY31|C3JY31_PSEFS	59	22860	1	1	1	1	0.23	Putative biopolymer tra
1147	1	db$2	2::C3K8I3|C3K8I3_PSEFS	59	39921	1	1	1	1	0.13	3,4-dihydroxy-2-butan

↑Family	M	DB	Accession	Score	Mass	Matches	Match(sig)	Sequences	Seq(sig)	emPAI	Description
1148	1	db$2	2::C3K1W7|C3K1W7_PSEFS	59	11247	2	2	2	2	1.29	DUF1244 domain-conta
1149	1	db$2	2::C3KBB9|C3KBB9_PSEFS	58	73354	2	2	2	2	0.14	Putative protease OS=
1150	1	db$2	2::C3K9M1|C3K9M1_PSEFS	58	26106	1	1	1	1	0.20	Putative phosphoglycer
1151	1	db$2	2::C3K3H4|C3K3H4_PSEFS	58	26074	2	2	2	2	0.43	Ribosomal RNA small s
1152	1	db$2	2::C3K9K0|C3K9K0_PSEFS	58	37025	1	1	1	1	0.14	Glycine betaine/L-proli
1153	1	db$2	2::C3K2G8|C3K2G8_PSEFS	58	27283	1	1	1	1	0.19	Putative TetR-family re
1154	1	db$2	2::C3K598|C3K598_PSEFS	58	34258	2	2	2	2	0.32	Curved DNA-binding pr
1155	1	db$2	2::C3KE10|C3KE10_PSEFS	58	58823	2	1	2	1	0.08	Putative methyl-accept
1156	1	db$2	2::C3K473|DUT_PSEFS	58	16022	1	1	1	1	0.34	Deoxyuridine 5'-tripho
1157	1	db$2	2::C3K3K7|C3K3K7_PSEFS	57	24490	3	3	2	2	0.47	Pyridoxal phosphate hc
1158	1	db$2	2::C3K265|C3K265_PSEFS	57	29739	4	4	3	3	0.61	Dihydropteroate syntha
1159	1	db$2	2::C3JYG2|C3JYG2_PSEFS	57	36128	2	2	2	2	0.30	ATP-grasp domain-con
1160	1	db$2	2::C3K6R2|C3K6R2_PSEFS	57	29309	2	2	2	2	0.38	Putative shikimate deh
1161	1	db$2	2::C3KAA6|C3KAA6_PSEFS	57	51931	2	2	2	2	0.20	Putative cell envelope
1162	1	db$2	2::C3K7K5|C3K7K5_PSEFS	57	12541	1	1	1	1	0.45	Uncharacterized protei
1163	1	db$2	2::C3K7A2|C3K7A2_PSEFS	57	33841	1	1	1	1	0.15	Putative membrane pro
1164	1	db$2	2::C3K6E4|C3K6E4_PSEFS	56	46143	4	3	3	2	0.36	Cytochrome b OS=Pse
1165	1	db$2	2::C3JXL2|C3JXL2_PSEFS	56	30967	1	1	1	1	0.16	Uncharacterized protei
1166	1	db$2	2::C3KED2|C3KED2_PSEFS	56	17293	3	3	2	2	0.72	Thioredoxin peroxidase
1167	1	db$2	2::C3JZU8|C3JZU8_PSEFS	56	30426	2	2	2	2	0.36	Hydrolase_4 domain-c
1168	1	db$2	2::C3K5B5|C3K5B5_PSEFS	56	37023	1	1	1	1	0.14	Dipeptide ABC transpo
1169	1	db$2	2::C3K106|C3K106_PSEFS	56	20669	2	2	2	2	0.58	Uncharacterized protei
1170	1	db$2	2::C3KEC3|C3KEC3_PSEFS	56	16145	4	3	3	2	0.79	Putative membrane pro
1171	1	db$2	2::C3K2M0|Y5418_PSEFS	56	10305	1	1	1	1	0.56	UPF0250 protein PFLU_
1172	1	db$2	2::C3KE66|C3KE66_PSEFS	56	27011	2	2	2	2	0.42	23S rRNA (guanosine-2
1173	1	db$2	2::C3K3J5|C3K3J5_PSEFS	55	13219	1	1	1	1	0.42	Type IV pilus response
1174	1	db$2	2::C3KAD1|C3KAD1_PSEFS	55	32401	3	3	3	3	0.55	Probable 4-deoxy-4-fo
1175	1	db$2	2::C3KBH3|DCUP_PSEFS	55	38973	4	4	4	4	0.62	Uroporphyrinogen deca
1176	1	db$2	2::C3K9U9|C3K9U9_PSEFS	55	9554	1	1	1	1	0.62	Uncharacterized protei
1177	1	SwissProt_HUMAN$	3::P15531|NDKA_HUMAN	55	17138	4	3	3	3	1.27	Nucleoside diphosphate
1178	1	db$2	2::C3KDJ8|C3KDJ8_PSEFS	55	23307	3	2	3	2	0.50	Methyltransf_25 domai
1179	1	db$2	2::C3K391|C3K391_PSEFS	55	33913	1	1	1	1	0.15	Putative acyltransferas
1180	1	db$2	2::C3K3V4|C3K3V4_PSEFS	55	41512	1	1	1	1	0.12	Acetylornithine deacety
1181	1	db$2	2::C3JY68|MNMA_PSEFS	55	41397	2	2	2	2	0.26	tRNA-specific 2-thiouri
1182	1	db$2	2::C3KCS6|C3KCS6_PSEFS	55	47227	1	1	1	1	0.11	UDP-N-acetylmuramoy
1183	1	db$2	2::C3KDR4|C3KDR4_PSEFS	55	49531	4	3	2	2	0.21	Ribosomal protein S12
1184	1	db$2	2::C3KDB5|C3KDB5_PSEFS	55	33189	2	1	2	1	0.15	Putative glycosyl trans
1185	1	db$2	2::C3K5L6|C3K5L6_PSEFS	55	16214	2	2	2	2	0.78	Putative phosphorous c
1186	1	SwissProt_HUMAN$	3::P06727|APOA4_HUMAN	55	45344	2	2	1	1	0.11	Apolipoprotein A-IV OS
1187	1	db$2	2::C3K3K9|C3K3K9_PSEFS	54	21575	3	3	2	2	0.55	Conserved hypothetica

↑Family	M	DB	Accession	Score	Mass	Matches	Match(sig)	Sequences	Seq(sig)	emPAI	Description
1188	1	db$2	2::C3K9P4|C3K9P4_PSEFS	54	59261	4	4	3	3	0.27	Putative ATP-binding c
1189	1	db$2	2::C3K1V9|C3K1V9_PSEFS	54	16219	1	1	1	1	0.33	Flavodoxin-like domain
1190	1	db$2	2::C3K0B8|C3K0B8_PSEFS	54	24379	1	1	1	1	0.21	Uncharacterized protei
1191	1	db$2	2::C3K320|C3K320_PSEFS	54	34836	2	2	2	2	0.31	Uncharacterized protei
1192	1	db$2	2::C3K6S3|C3K6S3_PSEFS	54	25881	2	2	2	2	0.44	Putative glutathione S-
1193	1	db$2	2::C3JXY3|C3JXY3_PSEFS	54	18788	2	2	2	2	0.65	6,7-dimethyl-8-ribityllu
1194	1	db$2	2::C3K784|C3K784_PSEFS	54	23456	2	2	2	2	0.49	3-oxoadipate CoA-tran
1195	1	db$2	2::C3K4A9|C3K4A9_PSEFS	54	17462	3	2	2	2	0.71	Uncharacterized protei
1196	1	db$2	2::C3K0M9|KTHY_PSEFS	54	22917	2	2	2	2	0.51	Thymidylate kinase OS
1197	1	db$2	2::C3K3P7|C3K3P7_PSEFS	54	49977	2	2	2	2	0.21	Putative membrane pro
1198	1	db$2	2::C3K2L8|LIPA_PSEFS	54	38163	4	4	4	4	0.64	Lipoyl synthase OS=Ps
1199	1	db$2	2::C3K2N1|C3K2N1_PSEFS	54	47217	1	1	1	1	0.11	Putative membrane pro
1200	1	db$2	2::C3KBK6|C3KBK6_PSEFS	54	29320	3	3	1	1	0.38	Putative exported pept
1201	1	SwissProt_HUMAN$	3::P63244|RACK1_HUMAN	54	35055	1	1	1	1	0.14	Receptor of activated p
1202	1	SwissProt_HUMAN$	3::P62266|RS23_HUMAN	54	15798	2	2	2	2	0.81	40S ribosomal protein
1203	1	db$2	2::C3KE32|C3KE32_PSEFS	54	20912	1	1	1	1	0.25	Sigma factor negative
1204	1	db$2	2::C3JZ52|C3JZ52_PSEFS	54	18715	2	2	1	1	0.28	Uncharacterized protei
1205	1	db$2	2::C3JY37|C3JY37_PSEFS	54	14829	1	1	1	1	0.37	Putative lipoprotein OS
1206	1	SwissProt_HUMAN$	3::P04406|G3P_HUMAN	54	36030	1	1	1	1	0.14	Glyceraldehyde-3-phos
1207	1	db$2	2::C3KCJ9|C3KCJ9_PSEFS	53	40399	1	1	1	1	0.12	Putative phosphotransf
1208	1	db$2	2::C3KAI2|C3KAI2_PSEFS	53	19361	2	2	2	2	0.62	Shikimate kinase OS=P
1209	1	db$2	2::C3K4W2|C3K4W2_PSEFS	53	26844	4	3	4	3	0.69	Putative glycerophosph
1210	1	db$2	2::C3K3B7|C3K3B7_PSEFS	53	14934	1	1	1	1	0.37	Uncharacterized protei
1211	1	db$2	2::C3KDB9|C3KDB9_PSEFS	53	32977	2	2	2	2	0.33	Putative lipopolysaccha
1212	1	db$2	2::C3JY81|C3JY81_PSEFS	53	15084	2	2	2	2	0.86	NADH-quinone oxidore
1213	1	db$2	2::C3K4Z1|C3K4Z1_PSEFS	53	72318	4	4	3	3	0.22	Cytochrome C biogene:
1214	1	db$2	2::C3K1U6|C3K1U6_PSEFS	53	74730	3	1	3	1	0.07	Cytochrome bo(3) ubic
1215	1	db$2	2::C3K9P7|C3K9P7_PSEFS	53	69450	3	2	3	2	0.15	Putative substrate-binc
1216	1	db$2	2::C3K8K6|C3K8K6_PSEFS	53	15117	1	1	1	1	0.36	Putative transport-rela
1217	1	db$2	2::C3JYE9|C3JYE9_PSEFS	53	31035	2	2	2	2	0.35	Putative hydroxymethy
1218	1	db$2	2::C3JY97|C3JY97_PSEFS	53	30357	1	1	1	1	0.17	N-hydroxyarylamine O-
1219	1	db$2	2::C3JYK2|C3JYK2_PSEFS	52	30492	2	2	2	2	0.36	Bifunctional protein Fol
1220	1	db$2	2::C3K6G1|RLMF_PSEFS	52	38015	1	1	1	1	0.13	Ribosomal RNA large s
1221	1	db$2	2::C3K0H8|C3K0H8_PSEFS	52	29525	3	3	2	2	0.38	Protein phosphatase Cl
1222	1	db$2	2::C3JY69|HFLD_PSEFS	52	23173	3	3	3	3	0.84	High frequency lysoger
1223	1	SwissProt_HUMAN$	3::Q2PPJ7|RGPA2_HUMAN	52	210636	2	2	2	2	0.05	Ral GTPase-activating p
1223	2	SwissProt_HUMAN$	3::P61254|RL26_HUMAN	45	17248	2	2	2	2	0.72	60S ribosomal protein
1224	1	db$2	2::C3K5G4|RLMM_PSEFS	52	40585	2	1	2	1	0.12	Ribosomal RNA large s
1225	1	db$2	2::C3KAN8|C3KAN8_PSEFS	52	48228	2	2	2	2	0.22	3-carboxy-cis,cis-mucc
1226	1	db$2	2::C3K6I1|C3K6I1_PSEFS	52	31224	4	4	2	2	0.57	S-formylglutathione hy

↑Family	M	DB	Accession	Score	Mass	Matches	Match(sig)	Sequences	Seq(sig)	emPAI	Description
1227	1	db$2	2::C3KBK8|C3KBK8_PSEFS	52	35740	1	1	1	1	0.14	Ribosomal RNA small s
1228	1	db$2	2::C3KDC8|C3KDC8_PSEFS	52	26520	1	1	1	1	0.19	Molybdopterin biosynth
1229	1	db$2	2::C3K6T8|C3K6T8_PSEFS	52	49583	3	3	3	3	0.33	Alginate biosynthesis t
1230	1	db$2	2::C3K3T7|C3K3T7_PSEFS	52	39982	3	3	3	3	0.43	Putrescine ABC transpo
1231	1	db$2	2::C3K0N3|ACP_PSEFS	52	8637	2	2	2	2	1.89	Acyl carrier protein OS
1232	1	db$2	2::C3KDD0|C3KDD0_PSEFS	51	19011	1	1	1	1	0.28	Lipid A deacylase OS=I
1233	1	db$2	2::C3K059|TRHO_PSEFS	51	35401	2	1	2	1	0.14	tRNA uridine(34) hydro
1234	1	db$2	2::C3K7X0|C3K7X0_PSEFS	51	85237	2	2	2	2	0.12	5-methyltetrahydropte
1235	1	db$2	2::C3KCP9|C3KCP9_PSEFS	51	30268	2	2	2	2	0.36	Lipopolysaccharide core
1236	1	db$2	2::C3KAU7|C3KAU7_PSEFS	51	33821	1	1	1	1	0.15	Fructokinase OS=Pseu
1237	1	db$2	2::C3KDQ0|C3KDQ0_PSEFS	51	35105	1	1	1	1	0.14	2-ketogluconate reduct
1238	1	db$2	2::C3K0U5|C3K0U5_PSEFS	51	62992	2	2	1	1	0.08	Flagellar M-ring protein
1239	1	db$2	2::C3K3X4|C3K3X4_PSEFS	51	11561	2	2	1	1	1.23	Cell division protein Za
1240	1	db$2	2::C3K146|C3K146_PSEFS	51	27134	2	2	2	2	0.42	Fumarate and nitrate r
1241	1	db$2	2::C3K0B2|C3K0B2_PSEFS	51	20071	1	1	1	1	0.26	Putative membrane pro
1242	1	db$2	2::C3K820|C3K820_PSEFS	50	26990	2	2	2	2	0.42	Putative ABC transport
1243	1	db$2	2::C3K0L0|C3K0L0_PSEFS	50	29492	2	2	2	2	0.38	Putative IclR-family reg
1244	1	db$2	2::C3K672|C3K672_PSEFS	50	58670	3	3	3	3	0.27	Cation/acetate sympor
1245	1	db$2	2::C3K6B0|C3K6B0_PSEFS	50	33534	1	1	1	1	0.15	High-affinity zinc uptak
1246	1	db$2	2::C3KDV4|RSGA_PSEFS	50	37296	2	2	2	2	0.29	Small ribosomal subun
1247	1	SwissProt_HUMAN$	3::Q5D862|FILA2_HUMAN	50	247928	1	1	1	1	0.02	Filaggrin-2 OS=Homo s
1248	1	SwissProt_HUMAN$	3::P62857|RS28_HUMAN	50	7836	1	1	1	1	0.79	40S ribosomal protein
1249	1	SwissProt_HUMAN$	3::P46781|RS9_HUMAN	50	22578	3	2	3	2	0.52	40S ribosomal protein
1250	1	db$2	2::C3K1R4|C3K1R4_PSEFS	50	17440	1	1	1	1	0.31	Putative plasmid-relate
1251	1	db$2	2::C3K4P8|C3K4P8_PSEFS	50	24008	3	3	2	2	0.48	t(6)A37 threonylcarbar
1252	1	db$2	2::C3KDN9|C3KDN9_PSEFS	49	40608	3	2	3	2	0.26	Probable periplasmic se
1253	1	db$2	2::C3K564|C3K564_PSEFS	49	54508	3	3	2	2	0.19	Methylmalonate-semia
1254	1	db$2	2::C3K9N4|C3K9N4_PSEFS	49	91939	2	1	2	1	0.05	Histidine kinase OS=Ps
1255	1	db$2	2::C3JZ46|C3JZ46_PSEFS	49	37091	1	1	1	1	0.14	Putative LacI family tra
1256	1	db$2	2::C3JYP6|C3JYP6_PSEFS	49	10477	3	3	3	3	2.78	YCII domain-containing
1257	1	db$2	2::C3K6G9|LPXB_PSEFS	49	41468	3	3	3	3	0.41	Lipid-A-disaccharide sy
1258	1	db$2	2::C3K047|C3K047_PSEFS	49	16614	2	2	2	2	0.75	Putative putative heat
1259	1	db$2	2::C3K9S5|C3K9S5_PSEFS	49	38309	2	2	2	2	0.28	Putative cobalamin bio
1260	1	db$2	2::C3JY40|C3JY40_PSEFS	49	21505	2	2	2	2	0.55	Putative Trp repressor
1261	1	SwissProt_HUMAN$	3::P60900|PSA6_HUMAN	49	27382	1	1	1	1	0.19	Proteasome subunit alp
1262	1	SwissProt_HUMAN$	3::Q5VT06|CE350_HUMAN	49	350716	3	2	3	2	0.03	Centrosome-associated
1263	1	db$2	2::C3K386|C3K386_PSEFS	49	22957	2	2	2	2	0.51	Putative glutathione S-
1264	1	db$2	2::C3K1X4|C3K1X4_PSEFS	49	16845	3	3	3	3	1.30	Putative putative expo
1265	1	db$2	2::C3JXS9|C3JXS9_PSEFS	49	32539	1	1	1	1	0.16	Putative LysR-family tr
1266	1	db$2	2::C3KDW1|MUTL_PSEFS	49	69574	4	3	4	3	0.23	DNA mismatch repair p

↑Family	M	DB	Accession	Score	Mass	Matches	Match(sig)	Sequences	Seq(sig)	emPAI	Description
1267	1	db$2	2::C3K3L1|C3K3L1_PSEFS	48	23430	2	2	2	2	0.49	Uncharacterized protei
1268	1	db$2	2::C3KB17|C3KB17_PSEFS	48	51104	5	2	4	2	0.20	Outer membrane comp
1269	1	db$2	2::C3K253|C3K253_PSEFS	48	106648	2	2	2	2	0.09	Putative membrane pro
1270	1	db$2	2::C3K6V8|C3K6V8_PSEFS	48	47941	3	2	3	2	0.22	Putative peptidase OS=
1271	1	db$2	2::C3K436|C3K436_PSEFS	48	30237	1	1	1	1	0.17	Diaminopimelate epime
1272	1	db$2	2::C3K433|CYAY_PSEFS	48	12442	2	2	1	1	0.45	Iron-sulfur cluster asse
1273	1	db$2	2::C3KDM9|C3KDM9_PSEFS	48	28096	2	2	2	2	0.40	Putative oxidoreductas
1274	1	db$2	2::C3KCB9|C3KCB9_PSEFS	48	35987	4	4	3	3	0.48	Polyphosphate kinase (
1275	1	SwissProt_HUMAN$	3::Q13472|TOP3A_HUMAN	48	112300	1	1	1	1	0.04	DNA topoisomerase 3-a
1276	1	db$2	2::C3KDQ1|C3KDQ1_PSEFS	48	35181	2	2	1	1	0.14	Putative LysR-family re
1277	1	db$2	2::C3JZU7|C3JZU7_PSEFS	48	16433	1	1	1	1	0.33	Putative thioesterase C
1278	1	db$2	2::C3K911|C3K911_PSEFS	48	29460	1	1	1	1	0.17	Putative ABC transport
1279	1	db$2	2::C3KAN3|C3KAN3_PSEFS	47	27088	1	1	1	1	0.19	3-oxoadipate CoA-tran
1280	1	db$2	2::C3K6V3|HIS6_PSEFS	47	27111	2	2	2	2	0.42	Imidazole glycerol pho:
1281	1	db$2	2::C3K250|C3K250_PSEFS	47	71198	1	1	1	1	0.07	Acetyl-coenzyme A syr
1282	1	db$2	2::C3JXI0|C3JXI0_PSEFS	47	23030	1	1	1	1	0.23	Nudix hydrolase domai
1283	1	db$2	2::C3K5Q0|C3K5Q0_PSEFS	47	53619	4	2	4	2	0.19	Mannose-1-phosphate
1284	1	db$2	2::Q8VMR3|HUTH_PSEFS	47	54209	2	1	2	1	0.09	Histidine ammonia-lyas
1285	1	db$2	2::C3K3G8|C3K3G8_PSEFS	47	41555	2	2	2	2	0.26	Uncharacterized protei
1286	1	db$2	2::C3K300|C3K300_PSEFS	47	34122	1	1	1	1	0.15	Putative membrane pro
1287	1	db$2	2::C3K5X1|C3K5X1_PSEFS	47	46611	1	1	1	1	0.11	Histidine kinase OS=Ps
1288	1	db$2	2::C3KDF9|C3KDF9_PSEFS	47	17633	2	2	2	2	0.70	CinA domain-containin
1289	1	db$2	2::C3K440|C3K440_PSEFS	47	11389	3	2	2	2	1.25	Uncharacterized protei
1290	1	db$2	2::C3K3J6|C3K3J6_PSEFS	47	14927	2	2	2	2	0.87	Type IV pilus response
1291	1	db$2	2::C3K0D2|C3K0D2_PSEFS	47	35834	5	3	4	3	0.48	Lipid A biosynthesis lau
1292	1	SwissProt_HUMAN$	3::P62826|RAN_HUMAN	47	24408	1	1	1	1	0.21	GTP-binding nuclear pr
1293	1	db$2	2::C3K6U7|C3K6U7_PSEFS	47	79141	6	4	6	4	0.27	Putative exported prote
1294	1	db$2	2::C3KE01|Y992_PSEFS	47	29383	2	2	2	2	0.38	UPF0246 protein PFLU_
1295	1	db$2	2::C3K3D3|BETA_PSEFS	47	62642	2	2	2	2	0.16	Oxygen-dependent cho
1296	1	db$2	2::C3K1D4|C3K1D4_PSEFS	47	64734	3	2	3	2	0.16	Uncharacterized protei
1297	1	db$2	2::C3K454|C3K454_PSEFS	47	13613	3	3	2	2	0.98	Putative cytochrome c5
1298	1	SwissProt_HUMAN$	3::Q6IS14|IF5AL_HUMAN	47	16762	1	1	1	1	0.32	Eukaryotic translation i
1299	1	db$2	2::C3K685|C3K685_PSEFS	46	13635	2	2	2	2	0.98	6-carboxy-5,6,7,8-tetra
1300	1	db$2	2::C3K600|C3K600_PSEFS	46	28761	3	3	3	3	0.63	Phosphatidate cytidylyl
1301	1	db$2	2::C3K2L3|C3K2L3_PSEFS	46	22416	2	2	1	1	0.23	LPS-assembly lipoprote
1302	1	db$2	2::C3K9Q0|GLO2_PSEFS	46	28360	1	1	1	1	0.18	Hydroxyacylglutathione
1303	1	db$2	2::C3K8V8|C3K8V8_PSEFS	46	36166	3	2	3	2	0.30	Zinc-type alcohol dehy
1304	1	db$2	2::C3K114|C3K114_PSEFS	46	74752	5	4	5	4	0.29	DNA polymerase III su
1305	1	db$2	2::C3K8Z4|C3K8Z4_PSEFS	46	45613	2	1	2	1	0.11	High-affinity branched-
1306	1	SwissProt_HUMAN$	3::P46783|RS10_HUMAN	46	18886	1	1	1	1	0.28	40S ribosomal protein

↑Family	M	DB	Accession	Score	Mass	Matches	Match(sig)	Sequences	Seq(sig)	emPAI	Description
1307	1	db$2	2::C3K2L9|LIPB_PSEFS	46	23473	1	1	1	1	0.22	Octanoyltransferase O[illegible]
1308	1	SwissProt_HUMAN$	3::P61353|RL27_HUMAN	46	15788	1	1	1	1	0.34	60S ribosomal protein
1309	1	db$2	2::C3JYV1|C3JYV1_PSEFS	46	32264	3	3	3	3	0.55	Formyltetrahydrofolate
1310	1	SwissProt_HUMAN$	3::P50914|RL14_HUMAN	46	23417	1	1	1	1	0.22	60S ribosomal protein
1311	1	db$2	2::C3K3N1|C3K3N1_PSEFS	46	52088	1	1	1	1	0.10	Aldehyde dehydrogena
1312	1	db$2	2::C3JYI2|C3JYI2_PSEFS	45	39477	2	2	2	2	0.27	2-aminoethylphosphon
1313	1	db$2	2::C3K6Y7|RNC_PSEFS	45	25367	1	1	1	1	0.20	Ribonuclease 3 OS=Ps[illegible]
1314	1	db$2	2::C3KE39|C3KE39_PSEFS	45	18976	2	2	2	2	0.64	D,D-heptose 1,7-bisph
1315	1	db$2	2::C3K129|C3K129_PSEFS	45	42141	2	2	2	2	0.25	Putative acetyl CoA ac[illegible]
1316	1	SwissProt_HUMAN$	3::Q14240|IF4A2_HUMAN	45	46373	2	2	2	2	0.23	Eukaryotic initiation fa[illegible]
1317	1	db$2	2::C3K0T6|RRAAH_PSEFS	45	17479	2	1	2	1	0.31	Putative 4-hydroxy-4-r
1318	1	db$2	2::C3K125|C3K125_PSEFS	45	26783	2	2	1	1	0.19	Alanine--tRNA ligase O
1319	1	db$2	2::C3KBZ4|C3KBZ4_PSEFS	45	6850	1	1	1	1	0.94	Uncharacterized protei[illegible]
1320	1	SwissProt_HUMAN$	3::P10599|THIO_HUMAN	45	11730	1	1	1	1	0.48	Thioredoxin OS=Homo
1321	1	db$2	2::C3KEA2|C3KEA2_PSEFS	45	30824	1	1	1	1	0.16	Putative inner membra
1322	1	db$2	2::C3K585|C3K585_PSEFS	45	25105	1	1	1	1	0.21	Putative hydrolase OS=
1323	1	db$2	2::C3K5X9|C3K5X9_PSEFS	45	41639	2	1	2	1	0.12	Putative acyl-CoA dehy
1324	1	db$2	2::C3KBE8|C3KBE8_PSEFS	45	8134	2	2	2	2	2.10	Putative lipoprotein OS
1325	1	db$2	2::C3K2M3|C3K2M3_PSEFS	45	37518	3	2	3	2	0.29	Putative peptidoglycan
1326	1	db$2	2::C3K173|C3K173_PSEFS	45	34695	4	4	4	4	0.72	Putative AraC-family tr
1327	1	db$2	2::C3K6Z6|C3K6Z6_PSEFS	45	13580	2	2	2	2	0.99	Putaitve lipoprotein OS
1328	1	db$2	2::C3K2L4|C3K2L4_PSEFS	45	37419	1	1	1	1	0.13	DNA polymerase III su
1329	1	db$2	2::C3K7J8|C3K7J8_PSEFS	44	78803	2	2	2	2	0.13	ATP-dependent DNA he
1330	1	db$2	2::C3K8U1|C3K8U1_PSEFS	44	15020	2	2	2	2	0.86	Phosphoribosyl-AMP cy
1331	1	db$2	2::C3KAH5|RL31_PSEFS	44	8451	2	1	2	1	0.72	50S ribosomal protein
1332	1	db$2	2::C3K442|C3K442_PSEFS	44	46322	1	1	1	1	0.11	Ammonium transporte[illegible]
1333	1	db$2	2::C3K0S0|C3K0S0_PSEFS	44	12378	7	3	2	2	1.11	Putative DNA-binding [illegible]
1334	1	db$2	2::C3K1H9|C3K1H9_PSEFS	44	18692	2	2	2	2	0.65	Conserved hypothetica
1335	1	db$2	2::C3K6A6|C3K6A6_PSEFS	44	25667	1	1	1	1	0.20	Putative lipoprotein OS
1336	1	db$2	2::C3JYJ2|C3JYJ2_PSEFS	44	27205	2	2	2	2	0.41	Putative lipoprotein OS
1337	1	db$2	2::C3K456|C3K456_PSEFS	44	38683	3	2	3	2	0.28	Alanine racemase OS=
1338	1	db$2	2::C3JY27|KDSB_PSEFS	44	27609	3	3	3	3	0.67	3-deoxy-manno-octulo
1339	1	db$2	2::C3K6A1|C3K6A1_PSEFS	44	22990	2	1	2	1	0.23	Putative copper-bindin[illegible]
1340	1	db$2	2::C3K7B3|C3K7B3_PSEFS	44	51722	1	1	1	1	0.10	Putative membrane-bo
1341	1	db$2	2::C3K390|C3K390_PSEFS	44	18447	2	2	1	1	0.29	Zn_protease domain-c[illegible]
1342	1	db$2	2::C3K1X3|C3K1X3_PSEFS	44	20670	2	2	2	2	0.58	Glutathione peroxidase
1343	1	db$2	2::C3K6J8|C3K6J8_PSEFS	44	78979	1	1	1	1	0.06	3-phosphoshikimate 1-
1344	1	db$2	2::C3K660|C3K660_PSEFS	43	28970	1	1	1	1	0.18	Hydroxypyruvate isom[illegible]
1345	1	SwissProt_HUMAN$	3::P62280|RS11_HUMAN	43	18419	3	1	3	1	0.29	40S ribosomal protein
1346	1	db$2	2::C3K1U1|C3K1U1_PSEFS	43	26390	1	1	1	1	0.19	Uncharacterized protei[illegible]

↑Family	M	DB	Accession	Score	Mass	Matches	Match(sig)	Sequences	Seq(sig)	emPAI	Description
1347	1	db$2	2::C3KAW3|C3KAW3_PSEFS	43	52085	1	1	1	1	0.10	Putative GntR-family tr
1348	1	db$2	2::C3K5B4|C3K5B4_PSEFS	43	32752	2	1	1	1	0.15	Dipeptide ABC transpo
1349	1	db$2	2::C3KBY4|C3KBY4_PSEFS	43	31613	2	2	2	2	0.35	Ribosomal RNA small s
1350	1	db$2	2::C3KAZ7|C3KAZ7_PSEFS	43	48577	1	1	1	1	0.10	Putative chain length d
1351	1	db$2	2::C3K8U6|C3K8U6_PSEFS	43	15270	2	2	2	2	0.84	Putative polyhydroxyal
1352	1	db$2	2::C3K2Q3|C3K2Q3_PSEFS	43	23107	1	1	1	1	0.23	Putative hydrolase OS=
1353	1	db$2	2::C3K6H7|C3K6H7_PSEFS	43	10540	1	1	1	1	0.55	Cell division protein Fts
1354	1	SwissProt_HUMAN$	3::P62851|RS25_HUMAN	43	13734	2	2	2	2	0.97	40S ribosomal protein
1355	1	db$2	2::C3K3Q6|C3K3Q6_PSEFS	43	79554	1	1	1	1	0.06	Putative heavy metal A
1356	1	db$2	2::C3KAK0|C3KAK0_PSEFS	42	16987	1	1	1	1	0.32	Uncharacterized protei
1357	1	db$2	2::C3JYF1|C3JYF1_PSEFS	42	42707	1	1	1	1	0.12	Putative acyl-CoA dehy
1358	1	db$2	2::C3K9Y8|C3K9Y8_PSEFS	42	14463	1	1	1	1	0.38	Uncharacterized protei
1359	1	db$2	2::C3K607|LPXA_PSEFS	42	28002	1	1	1	1	0.18	Acyl-[acyl-carrier-prote
1360	1	db$2	2::C3KE14|C3KE14_PSEFS	42	83238	1	1	1	1	0.06	Histidine kinase OS=Ps
1361	1	SwissProt_HUMAN$	3::P23284|PPIB_HUMAN	42	23728	1	1	1	1	0.22	Peptidyl-prolyl cis-tran
1362	1	db$2	2::C3KCR9|PTH_PSEFS	42	21041	2	2	2	2	0.56	Peptidyl-tRNA hydrolas
1363	1	db$2	2::C3K9M3|C3K9M3_PSEFS	42	57514	1	1	1	1	0.09	Putative membrane pro
1364	1	db$2	2::C3K870|C3K870_PSEFS	42	20966	2	2	2	2	0.56	Uncharacterized protei
1365	1	db$2	2::C3K1L5|C3K1L5_PSEFS	42	34147	3	1	3	1	0.15	Putative DNA-binding p
1366	1	db$2	2::C3K0V2|C3K0V2_PSEFS	42	48301	2	1	2	1	0.10	Flagellar hook-associat
1367	1	db$2	2::C3K8U0|HIS2_PSEFS	42	12007	3	3	2	2	1.17	Phosphoribosyl-ATP py
1368	1	db$2	2::C3KE82|C3KE82_PSEFS	42	53340	2	1	2	1	0.09	Aldehyde dehydrogena
1369	1	db$2	2::C3KBU2|C3KBU2_PSEFS	41	56055	1	1	1	1	0.09	Putative histidine amm
1370	1	db$2	2::C3K0M7|C3K0M7_PSEFS	41	28601	2	2	2	2	0.39	Putative deoxyribonucl
1371	1	db$2	2::C3K132|C3K132_PSEFS	41	42760	3	3	3	3	0.39	Uncharacterized protei
1372	1	db$2	2::C3JYU4|C3JYU4_PSEFS	41	20911	2	2	2	2	0.57	Corrinoid adenosyltran
1373	1	db$2	2::C3K5Q6|C3K5Q6_PSEFS	41	68754	2	2	2	2	0.15	Putative hydrolase OS=
1374	1	db$2	2::C3K6K2|IHFB_PSEFS	41	11021	3	2	3	2	1.32	Integration host factor
1375	1	db$2	2::C3K998|C3K998_PSEFS	41	35021	4	3	3	3	0.50	Putative dehydrogenas
1376	1	db$2	2::C3K4C8|C3K4C8_PSEFS	41	49694	2	2	2	2	0.21	Putative transport-rela
1377	1	SwissProt_HUMAN$	3::Q9UQE7|SMC3_HUMAN	41	141454	1	1	1	1	0.03	Structural maintenance
1378	1	SwissProt_HUMAN$	3::P62244|RS15A_HUMAN	41	14830	1	1	1	1	0.37	40S ribosomal protein
1379	1	db$2	2::C3K492|C3K492_PSEFS	41	51080	3	2	3	2	0.20	HDOD domain-containi
1380	1	db$2	2::C3K425|C3K425_PSEFS	41	27295	2	2	2	2	0.41	Positive alginate biosyr
1381	1	db$2	2::C3KBX4|C3KBX4_PSEFS	41	59754	3	2	3	2	0.17	Putative membrane pro
1382	1	db$2	2::C3K2Q9|EX7S_PSEFS	40	8900	3	2	3	2	1.82	Exodeoxyribonuclease
1383	1	db$2	2::C3K8L6|C3K8L6_PSEFS	40	28069	1	1	1	1	0.18	Precorrin-6A synthase
1384	1	SwissProt_HUMAN$	3::P62888|RL30_HUMAN	40	12776	1	1	1	1	0.44	60S ribosomal protein
1385	1	SwissProt_HUMAN$	3::Q71UM5|RS27L_HUMAN	40	9471	1	1	1	1	0.63	40S ribosomal protein
1386	1	SwissProt_HUMAN$	3::Q8NHW5|RLA0L_HUMAN	40	34343	1	1	1	1	0.15	60S acidic ribosomal pr

↑Family	M	DB	Accession	Score	Mass	Matches	Match(sig)	Sequences	Seq(sig)	emPAI	Description
1387	1	SwissProt_HUMAN$	3::P31939|PUR9_HUMAN	40	64575	1	1	1	1	0.08	Bifunctional purine bios
1388	1	SwissProt_HUMAN$	3::A6NJ78|MET15_HUMAN	40	46092	1	1	1	1	0.11	12S rRNA N4-methylcy
1389	1	db$2	2::C3K3M2|C3K3M2_PSEFS	40	37029	3	3	2	2	0.29	Cell division protein Fts
1390	1	db$2	2::C3K5C4|C3K5C4_PSEFS	40	17510	1	1	1	1	0.31	SnoaL-like domain-con
1391	1	db$2	2::C3K8Z7|C3K8Z7_PSEFS	40	10906	3	3	3	3	2.58	Uncharacterized protei
1392	1	SwissProt_HUMAN$	3::Q14168|MPP2_HUMAN	40	64540	1	1	1	1	0.08	MAGUK p55 subfamily
1393	1	db$2	2::C3K6E9|C3K6E9_PSEFS	40	41977	2	2	2	2	0.25	Phosphatidylcholine-hy
1394	1	db$2	2::C3JZ44|C3JZ44_PSEFS	40	47432	2	2	2	2	0.22	Putative gluconate peri
1395	1	db$2	2::C3K1M7|C3K1M7_PSEFS	40	27957	1	1	1	1	0.18	Serine O-acetyltransfer
1396	1	db$2	2::C3K8K1|C3K8K1_PSEFS	40	40207	2	1	1	1	0.12	Phosphate-binding prot
1397	1	SwissProt_HUMAN$	3::Q7Z7L7|ZER1_HUMAN	40	88113	2	2	1	1	0.11	Protein zer-1 homolog
1398	1	db$2	2::C3K590|C3K590_PSEFS	40	74072	2	2	2	2	0.14	Putative membrane pro
1399	1	db$2	2::C3K7Z3|C3K7Z3_PSEFS	40	43142	2	2	2	2	0.24	Putative acyl-CoA dehy
1400	1	db$2	2::C3K4W5|C3K4W5_PSEFS	40	24663	3	1	3	1	0.21	Lipoprotein-releasing s
1401	1	db$2	2::C3K0J8|C3K0J8_PSEFS	40	22091	2	2	2	2	0.53	START domain-contain
1402	1	db$2	2::A4V6U2|A4V6U2_PSEFS	39	76547	2	2	1	1	0.06	Uncharacterized protei
1403	1	SwissProt_HUMAN$	3::P61247|RS3A_HUMAN	39	29926	4	3	4	3	0.60	40S ribosomal protein
1404	1	db$2	2::C3JZ32|C3JZ32_PSEFS	39	38286	2	2	2	2	0.28	Putative cation transpo
1405	1	db$2	2::C3K9N7|C3K9N7_PSEFS	39	48751	1	1	1	1	0.10	Putative membrane pro
1406	1	db$2	2::C3JZ81|C3JZ81_PSEFS	39	25240	2	1	2	1	0.21	Putative exported prote
1407	1	db$2	2::C3K277|C3K277_PSEFS	39	60895	2	1	2	1	0.08	DNA repair protein Rec
1408	1	db$2	2::C3K602|C3K602_PSEFS	39	48346	3	3	3	3	0.34	Zinc metalloprotease C
1409	1	db$2	2::C3K451|C3K451_PSEFS	39	76463	4	1	3	1	0.06	ATP-dependent DNA he
1410	1	db$2	2::C3K304|C3K304_PSEFS	39	15054	3	3	3	3	1.53	Uncharacterized protei
1411	1	db$2	2::C3K2U2|C3K2U2_PSEFS	39	20033	5	5	5	5	2.23	Putative hydrolase OS=
1412	1	db$2	2::C3K4T5|C3K4T5_PSEFS	39	30974	2	2	2	2	0.36	Putative hydrolase OS=
1413	1	db$2	2::C3K3F2|C3K3F2_PSEFS	39	38228	2	1	2	1	0.13	D-erythrose-4-phospha
1414	1	db$2	2::C3KDG3|C3KDG3_PSEFS	39	15637	1	1	1	1	0.35	Putative exported prote
1415	1	db$2	2::C3K302|C3K302_PSEFS	39	12723	3	3	1	1	0.44	Putative HIT domain-co
1416	1	db$2	2::C3K1B4|C3K1B4_PSEFS	38	43355	2	2	2	2	0.24	Putative transferase OS
1417	1	db$2	2::C3JY76|C3JY76_PSEFS	38	48249	2	2	2	2	0.22	Putative mechanosensi
1418	1	db$2	2::C3KAX3|C3KAX3_PSEFS	38	30360	1	1	1	1	0.17	Conserved hypothetica
1419	1	db$2	2::C3KBJ0|C3KBJ0_PSEFS	38	16933	2	2	2	2	0.73	[Ribosomal protein S18
1420	1	db$2	2::C3K4N5|C3K4N5_PSEFS	38	28830	1	1	1	1	0.18	Putative amino acid AB
1421	1	db$2	2::C3K7C5|C3K7C5_PSEFS	38	40067	2	2	1	1	0.13	Histidine kinase OS=Ps
1422	1	SwissProt_HUMAN$	3::P04003|C4BPA_HUMAN	38	66989	1	1	1	1	0.07	C4b-binding protein alp
1423	1	db$2	2::C3KBB7|C3KBB7_PSEFS	38	43574	1	1	1	1	0.11	3-oxoacyl-[acyl-carrier
1424	1	db$2	2::C3K020|C3K020_PSEFS	38	23656	3	2	2	2	0.49	Putative hydrolase OS=
1425	1	SwissProt_HUMAN$	3::P60903|S10AA_HUMAN	38	11196	1	1	1	1	0.51	Protein S100-A10 OS=
1426	1	db$2	2::C3K2F6|TRMA_PSEFS	38	40867	1	1	1	1	0.12	tRNA/tmRNA (uracil-C(

↑Family	M	DB	Accession	Score	Mass	Matches	Match(sig)	Sequences	Seq(sig)	emPAI	Description
1427	1	SwissProt_HUMAN$	3::Q7Z6Z7|HUWE1_HUMAN	38	481589	6	3	4	2	0.02	E3 ubiquitin-protein lig
1428	1	db$2	2::C3K1M0|C3K1M0_PSEFS	38	12311	1	1	1	1	0.46	2Fe-2S ferredoxin OS=
1429	1	db$2	2::C3K2Z1|COAX_PSEFS	38	26920	1	1	1	1	0.19	Type III pantothenate
1430	1	db$2	2::C3K413|C3K413_PSEFS	38	22663	1	1	1	1	0.23	Putative ABC transport
1431	1	db$2	2::C3K5B3|C3K5B3_PSEFS	38	34937	2	1	2	1	0.14	Dipeptide ABC transpo
1432	1	db$2	2::C3K406|C3K406_PSEFS	38	23424	2	2	2	2	0.49	Glyoxalase OS=Pseudc
1433	1	db$2	2::C3JY52|C3JY52_PSEFS	38	37777	2	2	2	2	0.28	GST C-terminal domair
1434	1	db$2	2::C3K5G7|PDXB_PSEFS	38	41465	1	1	1	1	0.12	Erythronate-4-phospha
1435	1	db$2	2::C3KBL5|C3KBL5_PSEFS	38	24580	2	2	2	2	0.47	Glutamate/aspartate A
1436	1	SwissProt_HUMAN$	3::Q9H0U4|RAB1B_HUMAN	38	22157	1	1	1	1	0.24	Ras-related protein Ral
1437	1	SwissProt_HUMAN$	3::P0DKB5|TPBGL_HUMAN	37	40374	1	1	1	1	0.12	Trophoblast glycoprote
1438	1	db$2	2::C3JXG5|C3JXG5_PSEFS	37	36156	2	2	2	2	0.30	Putative GFO/IDH/Moc.
1439	1	SwissProt_HUMAN$	3::P33992|MCM5_HUMAN	37	82233	2	1	1	1	0.06	DNA replication licensir
1440	1	SwissProt_HUMAN$	3::P36507|MP2K2_HUMAN	37	44396	1	1	1	1	0.11	Dual specificity mitoge
1441	1	db$2	2::C3K8J9|C3K8J9_PSEFS	37	51515	1	1	1	1	0.10	Type II secretion syste
1442	1	db$2	2::C3K1N0|C3K1N0_PSEFS	37	18655	3	1	3	1	0.29	Rick_17kDa_Anti doma
1443	1	db$2	2::C3KBT8|C3KBT8_PSEFS	37	29541	2	1	2	1	0.17	Sulfate transport syste
1444	1	db$2	2::C3K6W4|C3K6W4_PSEFS	37	45779	1	1	1	1	0.11	Znf/thioredoxin_put dc
1445	1	db$2	2::C3K0Y2|C3K0Y2_PSEFS	37	46222	2	2	2	2	0.23	Putative fatty acid tran
1446	1	db$2	2::C3KBI7|C3KBI7_PSEFS	37	26715	1	1	1	1	0.19	Uncharacterized protei
1447	1	db$2	2::C3KAL9|C3KAL9_PSEFS	37	64520	1	1	1	1	0.08	Histidine kinase OS=Ps
1448	1	SwissProt_HUMAN$	3::O75251|NDUS7_HUMAN	37	23548	1	1	1	1	0.22	NADH dehydrogenase |
1449	1	db$2	2::C3K4I1|C3K4I1_PSEFS	37	29333	2	1	2	1	0.17	Shikimate dehydrogen
1450	1	SwissProt_HUMAN$	3::P62277|RS13_HUMAN	37	17212	2	2	2	2	0.72	40S ribosomal protein
1451	1	db$2	2::C3KDV8|C3KDV8_PSEFS	37	29265	1	1	1	1	0.17	ADP-dependent (S)-NA
1452	1	db$2	2::C3K589|C3K589_PSEFS	37	50032	2	2	2	2	0.21	Transcriptional regulat
1453	1	db$2	2::C3KD16|C3KD16_PSEFS	36	34294	2	2	2	2	0.32	Putative 2-hydroxyacid
1454	1	db$2	2::C3K0I2|C3K0I2_PSEFS	36	47755	1	1	1	1	0.10	Flagella-associated GTI
1455	1	db$2	2::C3K439|C3K439_PSEFS	36	25827	1	1	1	1	0.20	Putative hydrolase OS=
1456	1	db$2	2::C3K0R0|C3K0R0_PSEFS	36	14751	1	1	1	1	0.37	Flagellar basal body ro
1457	1	db$2	2::C3K3Q3|C3K3Q3_PSEFS	36	61523	2	2	2	2	0.17	Putative cation ABC tra
1458	1	SwissProt_HUMAN$	3::Q14147|DHX34_HUMAN	36	128040	3	2	1	1	0.04	Probable ATP-depende
1459	1	SwissProt_HUMAN$	3::Q13304|GPR17_HUMAN	36	40963	1	1	1	1	0.12	Uracil nucleotide/cyste
1460	1	db$2	2::C3JZR9|C3JZR9_PSEFS	36	27415	2	2	2	2	0.41	Putative dehydrogenas
1461	1	db$2	2::C3JY26|C3JY26_PSEFS	36	17211	2	1	2	1	0.31	Protein-tyrosine-phosp
1462	1	db$2	2::C3K2L1|C3K2L1_PSEFS	36	28054	1	1	1	1	0.18	DUF218 domain-contai
1463	1	db$2	2::C3K5Y3|C3K5Y3_PSEFS	36	35524	4	4	3	3	0.49	Putative leucine dehyd
1464	1	db$2	2::C3K8Z2|C3K8Z2_PSEFS	36	25669	1	1	1	1	0.20	High-affinity branched-
1465	1	SwissProt_HUMAN$	3::Q9H1K0|RBNS5_HUMAN	36	88815	2	1	2	1	0.05	Rabenosyn-5 OS=Hom
1466	1	db$2	2::C3KDR2|C3KDR2_PSEFS	36	26142	2	2	2	2	0.43	S4 RNA-binding domai

↑Family	M	DB	Accession	Score	Mass	Matches	Match(sig)	Sequences	Seq(sig)	emPAI	Description
1467	1	db$2	2::C3K109|C3K109_PSEFS	36	49568	1	1	1	1	0.10	23S rRNA (uracil(1939
1468	1	db$2	2::C3KE42|C3KE42_PSEFS	36	20828	1	1	1	1	0.25	Putative DNA-3-methyl
1469	1	db$2	2::C3K223|C3K223_PSEFS	36	37863	1	1	1	1	0.13	Protein-methionine-sul
1470	1	db$2	2::C3JYL6|C3JYL6_PSEFS	36	25076	2	2	2	2	0.45	Putative TetR-family re
1471	1	db$2	2::C3JZ73|C3JZ73_PSEFS	36	49677	2	2	2	2	0.21	Porin OS=Pseudomona
1472	1	db$2	2::C3K3Y2|C3K3Y2_PSEFS	36	34182	1	1	1	1	0.15	Putative sinc-binding o
1473	1	SwissProt_HUMAN$	3::Q9BVV6|TALD3_HUMAN	35	169201	1	1	1	1	0.03	Protein TALPID3 OS=H
1474	1	db$2	2::C3KBN5|C3KBN5_PSEFS	35	24125	1	1	1	1	0.22	Putative TetR-family re
1475	1	db$2	2::C3K7L9|C3K7L9_PSEFS	35	28704	1	1	1	1	0.18	Putative membrane pr
1476	1	db$2	2::C3JY73|C3JY73_PSEFS	35	26430	1	1	1	1	0.19	Secretin_N domain-cor
1477	1	SwissProt_HUMAN$	3::Q6IV72|ZN425_HUMAN	35	87663	1	1	1	1	0.06	Zinc finger protein 425
1478	1	db$2	2::C3K537|C3K537_PSEFS	35	39684	1	1	1	1	0.13	Putative membrane pr
1479	1	db$2	2::C3K6M1|C3K6M1_PSEFS	35	28266	2	1	2	1	0.18	Putative enoyl-CoA hy
1480	1	db$2	2::C3K0H9|C3K0H9_PSEFS	35	13855	2	2	2	2	0.96	Chemotaxis protein Ch
1481	1	db$2	2::C3K6C3|C3K6C3_PSEFS	35	28259	3	3	3	3	0.65	Putative amino acid AB
1482	1	db$2	2::C3KAJ9|C3KAJ9_PSEFS	35	20836	2	1	2	1	0.25	Putative hypoxanthine-
1483	1	SwissProt_HUMAN$	3::Q5J8M3|EMC4_HUMAN	35	20073	1	1	1	1	0.26	ER membrane protein
1484	1	SwissProt_HUMAN$	3::Q5JTZ9|SYAM_HUMAN	35	107273	1	1	1	1	0.05	Alanine--tRNA ligase, r
1485	1	db$2	2::C3K127|C3K127_PSEFS	35	41295	1	1	1	1	0.12	Putative membrane pr
1486	1	SwissProt_HUMAN$	3::Q6ZVD8|PHLP2_HUMAN	35	146658	2	1	2	1	0.03	PH domain leucine-rich
1487	1	db$2	2::C3K4A7|C3K4A7_PSEFS	35	18391	1	1	1	1	0.29	Uncharacterized protei
1488	1	db$2	2::C3KDK8|C3KDK8_PSEFS	35	41619	1	1	1	1	0.12	DAO domain-containin
1489	1	db$2	2::C3K5T7|C3K5T7_PSEFS	35	23769	2	2	2	2	0.49	Uncharacterized protei
1490	1	db$2	2::C3K2D6|C3K2D6_PSEFS	35	73930	1	1	1	1	0.07	Carbon starvation prot
1491	1	db$2	2::C3K646|C3K646_PSEFS	34	21182	1	1	1	1	0.25	Putative acyl-CoA thioe
1492	1	db$2	2::C3KB68|C3KB68_PSEFS	34	11363	4	1	3	1	0.51	DUF1883 domain-conta
1493	1	db$2	2::C3K757|C3K757_PSEFS	34	27432	4	1	3	1	0.19	Putative two-compone
1494	1	db$2	2::C3K5Y4|C3K5Y4_PSEFS	34	9690	1	1	1	1	0.61	Uncharacterized protei
1495	1	db$2	2::C3JYN9|C3JYN9_PSEFS	34	44994	2	2	2	2	0.23	Pseudouridine synthas
1496	1	db$2	2::C3K349|C3K349_PSEFS	34	28994	2	2	2	2	0.38	Pyrroloquinoline-quino
1497	1	SwissProt_HUMAN$	3::A6NCI4|VWA3A_HUMAN	34	133935	1	1	1	1	0.04	von Willebrand factor
1498	1	db$2	2::C3K2M6|RLMH_PSEFS	34	17669	1	1	1	1	0.30	Ribosomal RNA large s
1499	1	db$2	2::C3KAJ5|C3KAJ5_PSEFS	34	83174	2	2	2	2	0.12	Putative paraquat-indu
1500	1	db$2	2::C3KCN6|C3KCN6_PSEFS	34	53164	2	1	2	1	0.09	Sodium/proline sympo
1501	1	SwissProt_HUMAN$	3::P30050|RL12_HUMAN	34	17808	2	1	2	1	0.30	60S ribosomal protein
1502	1	db$2	2::C3K595|C3K595_PSEFS	34	10805	1	1	1	1	0.54	Phosphate starvation-i
1503	1	db$2	2::C3K8V7|C3K8V7_PSEFS	34	6725	1	1	1	1	0.96	Uncharacterized protei
1504	1	SwissProt_HUMAN$	3::P46776|RL27A_HUMAN	34	16551	1	1	1	1	0.33	60S ribosomal protein
1505	1	db$2	2::C3JY47|C3JY47_PSEFS	34	14227	1	1	1	1	0.39	DrsE domain-containin
1506	1	db$2	2::C3K172|C3K172_PSEFS	34	12407	1	1	1	1	0.45	ABM domain-containin

↑Family	M	DB	Accession	Score	Mass	Matches	Match(sig)	Sequences	Seq(sig)	emPAI	Description
1507	1	db$2	2::C3KAP0|C3KAP0_PSEFS	33	14934	1	1	1	1	0.37	4-carboxymuconolacto
1508	1	SwissProt_HUMAN$	3::P37802|TAGL2_HUMAN	33	22377	1	1	1	1	0.23	Transgelin-2 OS=Homo
1509	1	db$2	2::C3K2W8|RL29_PSEFS	33	7168	4	3	3	3	5.68	50S ribosomal protein
1510	1	db$2	2::C3K0Y8|COBT_PSEFS	33	36066	1	1	1	1	0.14	Nicotinate-nucleotide--
1511	1	db$2	2::C3K0J0|C3K0J0_PSEFS	33	16514	1	1	1	1	0.33	Flagellar motor switch
1512	1	db$2	2::C3KCD6|C3KCD6_PSEFS	33	27732	1	1	1	1	0.18	Short chain dehydroge
1513	1	db$2	2::C3K7G4|C3K7G4_PSEFS	33	54521	2	2	2	2	0.19	Ribonuclease G OS=Ps
1514	1	db$2	2::C3K0X1|C3K0X1_PSEFS	33	10638	1	1	1	1	0.54	Uncharacterized protei
1515	1	SwissProt_HUMAN$	3::Q96QU8|XPO6_HUMAN	33	128801	1	1	1	1	0.04	Exportin-6 OS=Homo s
1516	1	db$2	2::C3JZP3|C3JZP3_PSEFS	33	36685	2	2	2	2	0.29	Putative inosine-uridine
1517	1	db$2	2::C3K0J3|C3K0J3_PSEFS	33	45387	1	1	1	1	0.11	Putative flagellar hook-
1518	1	db$2	2::C3K323|C3K323_PSEFS	33	38795	3	3	3	3	0.44	Putative phosphotransf
1519	1	db$2	2::C3K0P0|C3K0P0_PSEFS	33	35982	2	2	2	2	0.30	Putative peptidase OS=
1520	1	SwissProt_HUMAN$	3::P30566|PUR8_HUMAN	33	54854	1	1	1	1	0.09	Adenylosuccinate lyase
1521	1	db$2	2::C3K8H9|C3K8H9_PSEFS	33	41728	1	1	1	1	0.12	Putative polyamine AB(
1522	1	db$2	2::C3KAH4|C3KAH4_PSEFS	33	82035	1	1	1	1	0.06	Primosomal protein N'
1523	1	db$2	2::C3KCK0|C3KCK0_PSEFS	33	26687	4	1	2	1	0.19	Putative short-chain de
1524	1	db$2	2::C3K328|APAG_PSEFS	33	13631	2	2	2	2	0.98	Protein ApaG OS=Pseu
1525	1	SwissProt_HUMAN$	3::P26440|IVD_HUMAN	33	46621	1	1	1	1	0.11	Isovaleryl-CoA dehydro
1526	1	db$2	2::C3JY58|C3JY58_PSEFS	32	87640	3	3	3	3	0.18	DNA translocase FtsK (
1527	1	db$2	2::C3K3W4|C3K3W4_PSEFS	32	13233	1	1	1	1	0.42	Glycine cleavage syste
1528	1	db$2	2::C3K5M1|C3K5M1_PSEFS	32	26375	2	1	2	1	0.19	Phosphonates ABC trar
1529	1	db$2	2::C3KE21|C3KE21_PSEFS	32	10230	1	1	1	1	0.57	FAD assembly factor S(
1530	1	SwissProt_HUMAN$	3::P39019|RS19_HUMAN	32	16051	2	2	2	2	0.79	40S ribosomal protein
1531	1	db$2	2::C3K5K0|C3K5K0_PSEFS	32	25438	2	2	2	2	0.45	Two-component syster
1532	1	db$2	2::C3K7J4|C3K7J4_PSEFS	32	18330	1	1	1	1	0.29	Uncharacterized protei
1533	1	SwissProt_HUMAN$	3::P62304|RUXE_HUMAN	32	10797	1	1	1	1	0.54	Small nuclear ribonucle
1534	1	SwissProt_HUMAN$	3::P35268|RL22_HUMAN	32	14778	1	1	1	1	0.37	60S ribosomal protein
1535	1	SwissProt_HUMAN$	3::Q8NBF4|YG006_HUMAN	32	16172	1	1	1	1	0.33	Putative characterize
1536	1	db$2	2::C3K4Y0|C3K4Y0_PSEFS	32	31989	1	1	1	1	0.16	DUF1989 domain-conta
1537	1	SwissProt_HUMAN$	3::P04080|CYTB_HUMAN	32	11133	1	1	1	1	0.52	Cystatin-B OS=Homo s
1538	1	db$2	2::C3KAF9|C3KAF9_PSEFS	32	50830	1	1	1	1	0.10	Poly-beta-1,6-N-acetyl
1539	1	db$2	2::C3JYP8|C3JYP8_PSEFS	32	24937	1	1	1	1	0.21	Putative two-componer
1540	1	db$2	2::C3KBW9|C3KBW9_PSEFS	32	64127	1	1	1	1	0.08	Putative lipoprotein OS
1541	1	db$2	2::C3K065|C3K065_PSEFS	32	58554	2	2	2	2	0.18	Uncharacterized protei
1542	1	SwissProt_HUMAN$	3::O00459|P85B_HUMAN	32	81495	3	2	2	1	0.06	Phosphatidylinositol 3-
1543	1	db$2	2::C3KDL9|C3KDL9_PSEFS	31	22175	1	1	1	1	0.24	Uncharacterized protei
1544	1	SwissProt_HUMAN$	3::Q9UDT6|CLIP2_HUMAN	31	115767	2	2	1	1	0.04	CAP-Gly domain-contai
1545	1	db$2	2::C3K2P8|C3K2P8_PSEFS	31	24358	1	1	1	1	0.21	Membrane-related prot
1546	1	db$2	2::C3K364|C3K364_PSEFS	31	38824	2	2	2	2	0.28	Biotin synthase OS=Ps

↑Family	M	DB	Accession	Score	Mass	Matches	Match(sig)	Sequences	Seq(sig)	emPAI	Description
1547	1	SwissProt_HUMAN$	3::P62899|RL31_HUMAN	31	14454	1	1	1	1	0.38	60S ribosomal protein
1548	1	SwissProt_HUMAN$	3::Q8TAT2|FGFP3_HUMAN	31	27573	1	1	1	1	0.19	Fibroblast growth facto
1549	1	db$2	2::C3K6C0|C3K6C0_PSEFS	31	25791	1	1	1	1	0.20	Putative amino acid AB
1550	1	db$2	2::C3K1Q2|C3K1Q2_PSEFS	31	73203	1	1	1	1	0.07	Histidine kinase OS=Ps
1551	1	db$2	2::C3JY51|C3JY51_PSEFS	31	37030	2	2	2	2	0.29	Putative glycosyl transf
1552	1	SwissProt_HUMAN$	3::Q96F81|DISP1_HUMAN	31	170822	1	1	1	1	0.03	Protein dispatched hom
1553	1	db$2	2::C3K0X8|C3K0X8_PSEFS	31	55461	2	2	2	2	0.19	DNA recombination pro
1554	1	db$2	2::C3K6D3|C3K6D3_PSEFS	31	21795	1	1	1	1	0.24	Precorrin-8X methylmu
1555	1	db$2	2::C3K279|C3K279_PSEFS	31	19136	3	3	2	2	0.63	Outer membrane prote
1556	1	SwissProt_HUMAN$	3::P48556|PSMD8_HUMAN	31	39587	3	1	2	1	0.13	26S proteasome non-A
1557	1	db$2	2::C3K9P8|C3K9P8_PSEFS	31	69666	1	1	1	1	0.07	Putative substrate-binc
1558	1	db$2	2::C3K0X2|C3K0X2_PSEFS	31	81009	1	1	1	1	0.06	Patatin-like phospholip
1559	1	db$2	2::C3K216|C3K216_PSEFS	31	17384	1	1	1	1	0.31	Putative putative lipopr
1560	1	db$2	2::C3KDJ3|C3KDJ3_PSEFS	31	42677	1	1	1	1	0.12	Uncharacterized protei
1561	1	db$2	2::C3K7L3|C3K7L3_PSEFS	31	8069	1	1	1	1	0.76	Putative lipoprotein OS
1562	1	db$2	2::C3KAK2|C3KAK2_PSEFS	31	10989	2	2	2	2	1.32	Uncharacterized protei
1563	1	db$2	2::C3K340|C3K340_PSEFS	31	72802	1	1	1	1	0.07	DNA primase OS=Pseu
1564	1	db$2	2::C3K136|C3K136_PSEFS	31	31053	2	2	2	2	0.35	Uncharacterized protei
1565	1	SwissProt_HUMAN$	3::P17480|UBF1_HUMAN	31	89350	1	1	1	1	0.05	Nucleolar transcription
1566	1	db$2	2::C3K0G1|C3K0G1_PSEFS	31	67986	1	1	1	1	0.07	Putative acetyl-/propio
1567	1	db$2	2::C3K7N0|C3K7N0_PSEFS	31	28545	1	1	1	1	0.18	Transport permease pr
1568	1	db$2	2::C3K9N3|C3K9N3_PSEFS	31	10076	2	2	2	2	1.50	Peptidyl-prolyl cis-trans
1569	1	db$2	2::C3K348|PQQB_PSEFS	30	33093	1	1	1	1	0.15	Coenzyme PQQ synthe
1570	1	db$2	2::C3K1I1|C3K1I1_PSEFS	30	24836	1	1	1	1	0.21	Nudix hydrolase domai
1571	1	SwissProt_HUMAN$	3::P06748|NPM_HUMAN	30	32555	1	1	1	1	0.16	Nucleophosmin OS=Hc
1572	1	db$2	2::C3K0N0|C3K0N0_PSEFS	30	41535	1	1	1	1	0.12	Endolytic murein trans
1573	1	db$2	2::C3K1Y4|C3K1Y4_PSEFS	30	14766	1	1	1	1	0.37	Putative putative glyox
1574	1	db$2	2::C3K3T5|C3K3T5_PSEFS	30	34240	1	1	1	1	0.15	Putrescine ABC transpc
1575	1	db$2	2::C3K732|TPMT_PSEFS	30	24819	1	1	1	1	0.21	Thiopurine S-methyltra
1576	1	SwissProt_HUMAN$	3::Q69YQ0|CYTSA_HUMAN	30	124525	2	1	1	1	0.04	Cytospin-A OS=Homo
1577	1	db$2	2::C3K457|C3K457_PSEFS	30	12769	1	1	1	1	0.44	Putative endoribonucle
1578	1	SwissProt_HUMAN$	3::Q96E52|OMA1_HUMAN	30	60081	1	1	1	1	0.08	Metalloendopeptidase (
1579	1	db$2	2::C3K488|C3K488_PSEFS	30	15239	2	2	2	2	0.85	Biopolymer transport p
1580	1	db$2	2::C3K4W6|C3K4W6_PSEFS	30	44511	2	2	2	2	0.24	Putative lipoprotein rel
1581	1	db$2	2::C3K0K2|C3K0K2_PSEFS	30	29783	2	2	2	2	0.37	Putative outer membra
1582	1	db$2	2::C3K0R1|C3K0R1_PSEFS	30	30842	1	1	1	1	0.16	Protein-glutamate O-m
1583	1	db$2	2::C3K6B1|C3K6B1_PSEFS	30	35098	2	2	2	2	0.31	Homoserine kinase OS
1584	1	db$2	2::C3K5Z0|C3K5Z0_PSEFS	30	15508	2	2	2	2	0.82	Uncharacterized protei
1585	1	db$2	2::C3K0A4|C3K0A4_PSEFS	30	12844	1	1	1	1	0.43	5-hydroxyisourate hyd
1586	1	db$2	2::C3K5K8|C3K5K8_PSEFS	30	29952	1	1	1	1	0.17	Putative deoxyribonucl

↑Family	M	DB	Accession	Score	Mass	Matches	Match(sig)	Sequences	Seq(sig)	emPAI	Description
1587	1	SwissProt_HUMAN$	3::O15078|CE290_HUMAN	30	290207	3	1	3	1	0.02	Centrosomal protein of
1588	1	db$2	2::C3K0P5|C3K0P5_PSEFS	30	35572	2	1	2	1	0.14	Uncharacterized protei
1589	1	db$2	2::C3K1S4|C3K1S4_PSEFS	30	23334	1	1	1	1	0.22	Putative membrane pro
1590	1	SwissProt_HUMAN$	3::Q2M3C7|SPKAP_HUMAN	30	186339	2	1	2	1	0.03	A-kinase anchor protei
1591	1	db$2	2::C3JYG6|C3JYG6_PSEFS	29	44622	3	2	3	2	0.24	Peptidase_M14 domain
1592	1	db$2	2::C3K3M8|C3K3M8_PSEFS	29	36638	3	1	1	1	0.14	AB hydrolase-1 domair
1593	1	db$2	2::C3K7A1|C3K7A1_PSEFS	29	56003	3	2	3	2	0.18	Putative LuxR-family re
1594	1	SwissProt_HUMAN$	3::Q9UHN6|CEIP2_HUMAN	29	154276	4	1	4	1	0.03	Cell surface hyaluronid
1595	1	db$2	2::C3KAB8|C3KAB8_PSEFS	29	25944	1	1	1	1	0.20	Putative short-chain de
1596	1	db$2	2::C3K1Z7|C3K1Z7_PSEFS	29	34470	1	1	1	1	0.15	Catechol 1,2-dioxygena
1597	1	SwissProt_HUMAN$	3::Q9NQI0|DDX4_HUMAN	29	79258	2	1	2	1	0.06	Probable ATP-depender
1598	1	db$2	2::C3K6V0|C3K6V0_PSEFS	29	23354	2	2	2	2	0.50	Imidazole glycerol phos
1599	1	SwissProt_HUMAN$	3::Q96AY4|TTC28_HUMAN	29	270715	1	1	1	1	0.02	Tetratricopeptide repea
1600	1	db$2	2::C3JZN5|RL35_PSEFS	29	7363	1	1	1	1	0.87	50S ribosomal protein
1601	1	db$2	2::C3K2S4|C3K2S4_PSEFS	29	15995	1	1	1	1	0.34	Putative lipoprotein OS
1602	1	db$2	2::C3K5A3|C3K5A3_PSEFS	29	19531	1	1	1	1	0.27	Putative acetyltransfera
1603	1	SwissProt_HUMAN$	3::O15270|SPTC2_HUMAN	29	62884	1	1	1	1	0.08	Serine palmitoyltransfe
1604	1	db$2	2::C3K2E6|C3K2E6_PSEFS	29	40463	2	2	2	2	0.26	Ribosomal RNA large s
1605	1	db$2	2::C3JY67|C3JY67_PSEFS	29	16861	1	1	1	1	0.32	Phosphatase NudJ OS=
1606	1	db$2	2::C3JXG8|C3JXG8_PSEFS	29	49590	1	1	1	1	0.10	Putative outer membra
1607	1	db$2	2::C3K338|TSAD_PSEFS	29	36371	1	1	1	1	0.14	tRNA N6-adenosine thr
1608	1	db$2	2::C3K8W1|C3K8W1_PSEFS	29	24415	1	1	1	1	0.21	Putative ArsR-family re
1609	1	db$2	2::C3K8T6|C3K8T6_PSEFS	29	26078	2	1	2	1	0.20	Ribosomal RNA small s
1610	1	db$2	2::C3K3R7|RPIA_PSEFS	29	23281	2	2	2	2	0.50	Ribose-5-phosphate isc
1611	1	db$2	2::C3KE76|C3KE76_PSEFS	29	92563	1	1	1	1	0.05	Alpha-E domain-contai
1612	1	db$2	2::C3JYC8|C3JYC8_PSEFS	29	59331	1	1	1	1	0.08	Putative pH adaptation
1613	1	db$2	2::C3KBI8|C3KBI8_PSEFS	29	48805	1	1	1	1	0.10	Uncharacterized protei
1614	1	db$2	2::C3K1H4|C3K1H4_PSEFS	29	45615	1	1	1	1	0.11	Putative membrane pro
1615	1	db$2	2::C3K1Y0|C3K1Y0_PSEFS	29	12222	1	1	1	1	0.46	Uncharacterized protei
1616	1	db$2	2::C3K4H8|C3K4H8_PSEFS	28	20657	4	2	4	2	0.58	Threonylcarbamoyl-AM
1617	1	db$2	2::C3K7B1|C3K7B1_PSEFS	28	40323	1	1	1	1	0.12	Intermembrane phospl
1618	1	db$2	2::C3K3P6|C3K3P6_PSEFS	28	19979	1	1	1	1	0.26	Dihydrofolate reductas
1619	1	db$2	2::C3JY07|C3JY07_PSEFS	28	19767	1	1	1	1	0.27	Putative RNA polymera
1620	1	db$2	2::C3K239|C3K239_PSEFS	28	42550	2	2	2	2	0.25	Aminotransferase OS=
1621	1	SwissProt_HUMAN$	3::Q9Y231|FUT9_HUMAN	28	42044	1	1	1	1	0.12	4-galactosyl-N-acetylgl
1622	1	db$2	2::C3JYR6|C3JYR6_PSEFS	28	38174	5	4	4	3	0.45	Quinolinate synthase A
1623	1	db$2	2::C3K353|C3K353_PSEFS	28	44910	1	1	1	1	0.11	Putative aminotransfer
1624	1	db$2	2::C3K975|C3K975_PSEFS	28	21948	1	1	1	1	0.24	Putative glutathione S-
1625	1	SwissProt_HUMAN$	3::Q6ZMT4|KDM7A_HUMAN	28	106489	1	1	1	1	0.05	Lysine-specific demeth
1626	1	db$2	2::C3K0P1|C3K0P1_PSEFS	28	24214	1	1	1	1	0.21	Putative hydrolase OS=

↑Family	M	DB	Accession	Score	Mass	Matches	Match(sig)	Sequences	Seq(sig)	emPAI	Description
1627	1	db$2	2::C3K3J2|C3K3J2_PSEFS	28	213513	1	1	1	1	0.02	Histidine kinase OS=Ps
1628	1	SwissProt_HUMAN$	3::Q99618|CDCA3_HUMAN	28	28981	1	1	1	1	0.18	Cell division cycle-asso
1629	1	db$2	2::C3KCP7|C3KCP7_PSEFS	28	39236	1	1	1	1	0.13	Lipopolysaccharide hep
1630	1	SwissProt_HUMAN$	3::A0A494C0N9|F246B_HUMAN	28	24337	2	1	1	1	0.21	Protein FAM246B OS=H
1631	1	db$2	2::C3K869|C3K869_PSEFS	28	24016	2	2	2	2	0.48	Putative lipoprotein OS
1632	1	db$2	2::C3K3S9|C3K3S9_PSEFS	28	35576	1	1	1	1	0.14	Hydrolase_4 domain-co
1633	1	SwissProt_HUMAN$	3::Q8IZ83|A16A1_HUMAN	28	85074	1	1	1	1	0.06	Aldehyde dehydrogena
1634	1	db$2	2::C3K8X2|C3K8X2_PSEFS	28	10666	1	1	1	1	0.54	STAS domain-containir
1635	1	SwissProt_HUMAN$	3::P56749|CLD12_HUMAN	28	27092	1	1	1	1	0.19	Claudin-12 OS=Homo s
1636	1	db$2	2::C3K1F1|ATPL_PSEFS	28	8603	4	3	2	1	1.89	ATP synthase subunit c
1637	1	SwissProt_HUMAN$	3::P47900|P2RY1_HUMAN	28	42044	1	1	1	1	0.12	P2Y purinoceptor 1 OS
1638	1	db$2	2::C3KA94|C3KA94_PSEFS	28	41012	1	1	1	1	0.12	Uncharacterized protei
1639	1	db$2	2::C3K694|C3K694_PSEFS	28	48962	1	1	1	1	0.10	[utative peptidase OS=
1640	1	db$2	2::C3K670|C3K670_PSEFS	28	30761	2	1	2	1	0.17	Putative glycine betain
1641	1	db$2	2::C3K6T6|C3K6T6_PSEFS	28	30009	1	1	1	1	0.17	Putative membrane-bo
1642	1	db$2	2::C3JZN0|C3JZN0_PSEFS	28	13917	1	1	1	1	0.40	MerR-family transcripti
1643	1	SwissProt_HUMAN$	3::Q75VX8|GARE2_HUMAN	28	92824	1	1	1	1	0.05	GRB2-associated and r
1644	1	SwissProt_HUMAN$	3::Q2M3G4|SHRM1_HUMAN	28	90730	1	1	1	1	0.05	Protein Shroom1 OS=H
1645	1	db$2	2::C3K678|C3K678_PSEFS	27	45414	1	1	1	1	0.11	Cytosine deaminase OS
1646	1	SwissProt_HUMAN$	3::Q92625|ANS1A_HUMAN	27	123032	1	1	1	1	0.04	Ankyrin repeat and SAI
1647	1	db$2	2::C3K230|C3K230_PSEFS	27	85084	1	1	1	1	0.06	Penicillin-binding prote
1648	1	db$2	2::C3KAT0|C3KAT0_PSEFS	27	40804	2	1	2	1	0.12	Putative efflux system
1649	1	db$2	2::C3K5J0|C3K5J0_PSEFS	27	13887	1	1	1	1	0.40	Putative lipoprotein OS
1650	1	db$2	2::C3KDB4|C3KDB4_PSEFS	27	42765	2	2	2	2	0.25	Putative glycosyl transf
1651	1	db$2	2::C3K5I1|C3K5I1_PSEFS	27	43688	1	1	1	1	0.11	Putative phage integra
1652	1	SwissProt_HUMAN$	3::Q6WCQ1|MPRIP_HUMAN	27	116461	1	1	1	1	0.04	Myosin phosphatase Rh
1653	1	SwissProt_HUMAN$	3::Q3B7T1|EDRF1_HUMAN	27	138440	1	1	1	1	0.03	Erythroid differentiatio
1654	1	db$2	2::C3JXX9|C3JXX9_PSEFS	27	6925	3	2	2	2	2.76	Translational regulator
1655	1	SwissProt_HUMAN$	3::Q14129|DGCR6_HUMAN	27	24973	1	1	1	1	0.21	Protein DGCR6 OS=Ho
1656	1	SwissProt_HUMAN$	3::Q9Y6C2|EMIL1_HUMAN	27	106630	1	1	1	1	0.05	EMILIN-1 OS=Homo sa
1657	1	SwissProt_HUMAN$	3::P12883|MYH7_HUMAN	27	222959	1	1	1	1	0.02	Myosin-7 OS=Homo sa
1658	1	db$2	2::C3KB02|C3KB02_PSEFS	27	42163	1	1	1	1	0.12	Putative exported prote
1659	1	db$2	2::C3K4E5|C3K4E5_PSEFS	27	51748	1	1	1	1	0.10	Putative amino acid tra
1660	1	SwissProt_HUMAN$	3::Q8NA82|MARHA_HUMAN	27	90455	1	1	1	1	0.05	Probable E3 ubiquitin-p
1661	1	db$2	2::C3KCL5|C3KCL5_PSEFS	27	16185	1	1	1	1	0.33	Putative exported prote
1662	1	SwissProt_HUMAN$	3::O95153|RIMB1_HUMAN	27	199928	1	1	1	1	0.02	Peripheral-type benzod
1663	1	SwissProt_HUMAN$	3::P46060|RAGP1_HUMAN	27	63502	1	1	1	1	0.08	Ran GTPase-activating
1664	1	db$2	2::C3K8Z3|C3K8Z3_PSEFS	27	28227	2	1	2	1	0.18	High-affinity branched-
1665	1	SwissProt_HUMAN$	3::Q00526|CDK3_HUMAN	27	35024	1	1	1	1	0.14	Cyclin-dependent kinas
1666	1	db$2	2::C3K5M2|C3K5M2_PSEFS	27	41151	1	1	1	1	0.12	Phosphorous compoun

↑Family	M	DB	Accession	Score	Mass	Matches	Match(sig)	Sequences	Seq(sig)	emPAI	Description
1667	1	SwissProt_HUMAN$	3::Q8NG92|O13H1_HUMAN	27	34547	1	1	1	1	0.15	Olfactory receptor 13H
1668	1	db$2	2::C3K2C2|C3K2C2_PSEFS	27	67328	1	1	1	1	0.07	Putative lipoprotein OS
1669	1	db$2	2::C3K8T8|C3K8T8_PSEFS	27	14354	1	1	1	1	0.38	Sec-independent prote
1670	1	db$2	2::C3KAF8|C3KAF8_PSEFS	27	74138	1	1	1	1	0.07	Putative lipoprotein OS
1671	1	SwissProt_HUMAN$	3::O60237|MYPT2_HUMAN	27	110337	1	1	1	1	0.04	Protein phosphatase 1
1672	1	db$2	2::C3K444|C3K444_PSEFS	27	9491	1	1	1	1	0.62	Ubiquinone biosynthesi
1673	1	SwissProt_HUMAN$	3::Q5TB80|CE162_HUMAN	27	161843	3	1	2	1	0.03	Centrosomal protein of
1674	1	SwissProt_HUMAN$	3::O00299|CLIC1_HUMAN	27	26906	1	1	1	1	0.19	Chloride intracellular cl
1675	1	db$2	2::C3K3N6|FPG_PSEFS	26	30025	2	2	2	2	0.37	Formamidopyrimidine-
1676	1	SwissProt_HUMAN$	3::Q9BYK8|HELZ2_HUMAN	26	294465	1	1	1	1	0.02	Helicase with zinc finge
1677	1	SwissProt_HUMAN$	3::O94817|ATG12_HUMAN	26	15104	1	1	1	1	0.36	Ubiquitin-like protein A
1678	1	SwissProt_HUMAN$	3::P49207|RL34_HUMAN	26	13284	1	1	1	1	0.42	60S ribosomal protein
1679	1	SwissProt_HUMAN$	3::A6NJB7|PRR19_HUMAN	26	38692	1	1	1	1	0.13	Proline-rich protein 19
1680	1	SwissProt_HUMAN$	3::P05546|HEP2_HUMAN	26	57034	1	1	1	1	0.09	Heparin cofactor 2 OS=
1681	1	db$2	2::C3KE60|C3KE60_PSEFS	26	49144	1	1	1	1	0.10	Histidine kinase OS=Ps
1682	1	SwissProt_HUMAN$	3::P29590|PML_HUMAN	26	97489	2	1	2	1	0.05	Protein PML OS=Homo
1683	1	db$2	2::C3JZ14|C3JZ14_PSEFS	26	26480	1	1	1	1	0.19	Putative short-chain de
1684	1	db$2	2::C3JZP7|C3JZP7_PSEFS	26	33454	1	1	1	1	0.15	Putative ribose transpc
1685	1	SwissProt_HUMAN$	3::Q9C098|DCLK3_HUMAN	26	73768	1	1	1	1	0.07	Serine/threonine-prote
1686	1	db$2	2::C3K536|C3K536_PSEFS	26	22752	1	1	1	1	0.23	DUF4174 domain-conta
1687	1	SwissProt_HUMAN$	3::Q9UKR0|KLK12_HUMAN	26	26716	1	1	1	1	0.19	Kallikrein-12 OS=Hom
1688	1	SwissProt_HUMAN$	3::Q92887|MRP2_HUMAN	26	174096	1	1	1	1	0.03	Canalicular multispecifi
1689	1	SwissProt_HUMAN$	3::Q9UBQ5|EIF3K_HUMAN	26	25043	1	1	1	1	0.21	Eukaryotic translation i
1690	1	SwissProt_HUMAN$	3::O14917|PCD17_HUMAN	26	126151	1	1	1	1	0.04	Protocadherin-17 OS=I
1691	1	db$2	2::C3K0N9|C3K0N9_PSEFS	26	21007	2	1	2	1	0.25	7-methyl-GTP pyropho
1692	1	db$2	2::C3JXM0|C3JXM0_PSEFS	26	45141	1	1	1	1	0.11	Putative peptidase OS=
1693	1	db$2	2::C3K6G7|C3K6G7_PSEFS	26	39119	1	1	1	1	0.13	Putative ABC transport
1694	1	SwissProt_HUMAN$	3::Q9H223|EHD4_HUMAN	26	61137	1	1	1	1	0.08	EH domain-containing
1695	1	db$2	2::C3K404|C3K404_PSEFS	26	23654	1	1	1	1	0.22	Putative membrane pr
1696	1	db$2	2::C3K705|SURE_PSEFS	26	26431	1	1	1	1	0.19	5'-nucleotidase SurE O
1697	1	SwissProt_HUMAN$	3::Q5TH69|BIG3_HUMAN	26	240498	2	2	1	1	0.04	Brefeldin A-inhibited gu
1698	1	SwissProt_HUMAN$	3::Q9Y265|RUVB1_HUMAN	26	50196	1	1	1	1	0.10	RuvB-like 1 OS=Homo
1699	1	db$2	2::C3K7T3|C3K7T3_PSEFS	26	20773	2	2	2	2	0.57	CDP-diacylglycerol--gly
1700	1	db$2	2::C3K0S1|C3K0S1_PSEFS	26	53269	1	1	1	1	0.09	Magnesium transporter
1701	1	db$2	2::C3KBH6|C3KBH6_PSEFS	26	16609	1	1	1	1	0.32	Conserved hypothetica
1702	1	db$2	2::C3K5L4|C3K5L4_PSEFS	26	28633	1	1	1	1	0.18	Phosphonates ABC trar
1703	1	db$2	2::C3K1L7|RLMN_PSEFS	26	42342	1	1	1	1	0.12	Dual-specificity RNA m
1704	1	db$2	2::C3K5D6|C3K5D6_PSEFS	26	97394	1	1	1	1	0.05	Putative beta-(1-3)-glu
1705	1	db$2	2::C3K4L1|C3K4L1_PSEFS	26	41720	1	1	1	1	0.12	Putative epimerase OS
1706	1	db$2	2::C3K431|C3K431_PSEFS	26	14535	1	1	1	1	0.38	Regulator of nucleoside

↑Family	M	DB	Accession	Score	Mass	Matches	Match(sig)	Sequences	Seq(sig)	emPAI	Description
1707	1	SwissProt_HUMAN$	3::Q14392|LRC32_HUMAN	26	71934	1	1	1	1	0.07	Transforming growth fa
1708	1	db$2	2::C3KAN4|C3KAN4_PSEFS	25	41646	1	1	1	1	0.12	3-oxoadipyl-CoA thiola
1709	1	SwissProt_HUMAN$	3::P27708|PYR1_HUMAN	25	242829	1	1	1	1	0.02	CAD protein OS=Homo
1710	1	db$2	2::C3K429|C3K429_PSEFS	25	8856	1	1	1	1	0.68	Uncharacterized protei
1711	1	db$2	2::C3K5Y5|C3K5Y5_PSEFS	25	18019	1	1	1	1	0.30	Protein PsiE OS=Pseud
1712	1	SwissProt_HUMAN$	3::O00159|MYO1C_HUMAN	25	121606	1	1	1	1	0.04	Unconventional myosir
1713	1	db$2	2::C3JZP5|C3JZP5_PSEFS	25	30747	1	1	1	1	0.17	Ribokinase OS=Pseudc
1714	1	SwissProt_HUMAN$	3::Q14DG7|T132B_HUMAN	25	119402	2	1	2	1	0.04	Transmembrane protei
1715	1	db$2	2::C3K7E0|C3K7E0_PSEFS	25	27725	1	1	1	1	0.18	Histidine utilization rep
1716	1	db$2	2::C3K8K5|C3K8K5_PSEFS	25	64377	1	1	1	1	0.08	Putative transport-rela
1717	1	SwissProt_HUMAN$	3::Q8TF21|ANR24_HUMAN	25	124111	2	2	2	2	0.08	Ankyrin repeat domain
1718	1	SwissProt_HUMAN$	3::O95819|M4K4_HUMAN	25	142013	2	2	2	2	0.07	Mitogen-activated prot
1719	1	db$2	2::C3K2N6|C3K2N6_PSEFS	25	16796	3	2	2	1	0.32	MaoC-like domain-cont
1720	1	db$2	2::C3K4U9|C3K4U9_PSEFS	25	34107	1	1	1	1	0.15	Putative LysR-family re
1721	1	SwissProt_HUMAN$	3::Q7L1V2|MON1B_HUMAN	25	59180	1	1	1	1	0.08	Vacuolar fusion protein
1722	1	db$2	2::C3JY28|Y3771_PSEFS	25	6626	2	2	1	1	0.98	UPF0434 protein PFLU_
1723	1	SwissProt_HUMAN$	3::O00515|LAD1_HUMAN	25	57097	1	1	1	1	0.09	Ladinin-1 OS=Homo sa
1724	1	SwissProt_HUMAN$	3::P0DN79|CBSL_HUMAN	25	60548	2	1	1	1	0.08	Cystathionine beta-syn
1725	1	SwissProt_HUMAN$	3::Q92543|SNX19_HUMAN	25	108530	1	1	1	1	0.04	Sorting nexin-19 OS=H
1726	1	db$2	2::C3K8L9|C3K8L9_PSEFS	25	65348	1	1	1	1	0.08	Putative potassium-effl
1727	1	SwissProt_HUMAN$	3::Q96AQ6|PBIP1_HUMAN	25	80594	1	1	1	1	0.06	Pre-B-cell leukemia tra
1728	1	db$2	2::C3KD58|C3KD58_PSEFS	25	41746	1	1	1	1	0.12	Putative cystathionine
1729	1	SwissProt_HUMAN$	3::O15111|IKKA_HUMAN	25	84585	1	1	1	1	0.06	Inhibitor of nuclear fac
1730	1	db$2	2::C3K5F5|Y1520_PSEFS	25	17238	1	1	1	1	0.31	UPF0260 protein PFLU_
1731	1	SwissProt_HUMAN$	3::Q08945|SSRP1_HUMAN	25	81024	1	1	1	1	0.06	FACT complex subunit
1732	1	db$2	2::C3K0L5|C3K0L5_PSEFS	25	17844	1	1	1	1	0.30	Putative GNAT-family a
1733	1	db$2	2::C3JY92|C3JY92_PSEFS	25	55851	1	1	1	1	0.09	NADH dehydrogenase I
1734	1	SwissProt_HUMAN$	3::Q5K651|SAMD9_HUMAN	25	184165	1	1	1	1	0.03	Sterile alpha motif dom
1735	1	db$2	2::C3K1Z0|C3K1Z0_PSEFS	25	34051	1	1	1	1	0.15	Putative putative polys
1736	1	SwissProt_HUMAN$	3::Q9Y263|PLAP_HUMAN	25	87101	1	1	1	1	0.06	Phospholipase A-2-acti
1737	1	db$2	2::C3K336|C3K336_PSEFS	24	13078	1	1	1	1	0.43	7,8-dihydroneopterin a
1738	1	SwissProt_HUMAN$	3::Q92805|GOGA1_HUMAN	24	88130	3	1	2	1	0.06	Golgin subfamily A mei
1739	1	SwissProt_HUMAN$	3::Q9NZY2|FA30A_HUMAN	24	14616	1	1	1	1	0.37	Putative uncharacterize
1740	1	SwissProt_HUMAN$	3::O00469|PLOD2_HUMAN	24	84632	1	1	1	1	0.06	Procollagen-lysine,2-ox
1741	1	db$2	2::C3K9Y7|C3K9Y7_PSEFS	24	30413	1	1	1	1	0.17	Undecaprenyl-diphospl
1742	1	SwissProt_HUMAN$	3::Q9H244|P2Y12_HUMAN	24	39413	1	1	1	1	0.13	P2Y purinoceptor 12 O:
1743	1	SwissProt_HUMAN$	3::Q9UBN4|TRPC4_HUMAN	24	112030	1	1	1	1	0.04	Short transient recepto
1744	1	SwissProt_HUMAN$	3::Q8NE79|POPD1_HUMAN	24	41424	2	1	2	1	0.12	Blood vessel epicardial
1745	1	SwissProt_HUMAN$	3::Q969W8|ZN566_HUMAN	24	49188	1	1	1	1	0.10	Zinc finger protein 566
1746	1	SwissProt_HUMAN$	3::O75155|CAND2_HUMAN	24	135171	2	2	1	1	0.04	Cullin-associated NEDD

↑Family	M	DB	Accession	Score	Mass	Matches	Match(sig)	Sequences	Seq(sig)	emPAI	Description
1747	1	db$2	2::C3KDS6|UNG_PSEFS	24	25820	1	1	1	1	0.20	Uracil-DNA glycosylase
1748	1	SwissProt_HUMAN$	3::O43182|RHG06_HUMAN	24	105882	1	1	1	1	0.05	Rho GTPase-activating
1749	1	SwissProt_HUMAN$	3::P01009|A1AT_HUMAN	24	46707	1	1	1	1	0.11	Alpha-1-antitrypsin OS
1750	1	SwissProt_HUMAN$	3::P62263|RS14_HUMAN	24	16263	1	1	1	1	0.33	40S ribosomal protein
1751	1	db$2	2::C3KEB8|C3KEB8_PSEFS	24	34155	1	1	1	1	0.15	Putative lipoprotein OS
1752	1	SwissProt_HUMAN$	3::Q96GD3|SCMH1_HUMAN	24	73307	1	1	1	1	0.07	Polycomb protein SCMI
1753	1	db$2	2::C3K982|C3K982_PSEFS	24	35465	1	1	1	1	0.14	Putative oxidoreductas
1754	1	SwissProt_HUMAN$	3::Q93075|TATD2_HUMAN	24	84970	1	1	1	1	0.06	Putative deoxyribonucl
1755	1	db$2	2::C3K2E8|C3K2E8_PSEFS	24	37117	1	1	1	1	0.14	Putative cytochrome o›
1756	1	db$2	2::C3JY42|C3JY42_PSEFS	24	25186	1	1	1	1	0.21	Uncharacterized protei
1757	1	SwissProt_HUMAN$	3::Q6P1X5|TAF2_HUMAN	24	136883	1	1	1	1	0.04	Transcription initiation
1758	1	db$2	2::C3K6U5|FETP_PSEFS	24	10399	1	1	1	1	0.56	Probable Fe(2+)-traffic
1759	1	SwissProt_HUMAN$	3::Q9C037|TRIM4_HUMAN	24	57424	1	1	1	1	0.09	E3 ubiquitin-protein lig
1760	1	db$2	2::C3JZP8|C3JZP8_PSEFS	24	55398	1	1	1	1	0.09	Putative ribose transpc
1761	1	db$2	2::C3K8N0|C3K8N0_PSEFS	24	12883	1	1	1	1	0.43	Antitoxin OS=Pseudorr
1762	1	db$2	2::C3K7C2|C3K7C2_PSEFS	23	16947	1	1	1	1	0.32	tRNA (cytidine(34)-2'-(
1763	1	SwissProt_HUMAN$	3::Q4KMP7|TB10B_HUMAN	23	87145	1	1	1	1	0.06	TBC1 domain family m
1764	1	SwissProt_HUMAN$	3::P08476|INHBA_HUMAN	23	47412	1	1	1	1	0.10	Inhibin beta A chain OS
1765	1	db$2	2::C3K5M3|PHNN_PSEFS	23	20351	2	2	2	2	0.59	Ribose 1,5-bisphospha
1766	1	db$2	2::C3K267|RLME_PSEFS	23	24125	1	1	1	1	0.22	Ribosomal RNA large s
1767	1	db$2	2::C3KAM0|C3KAM0_PSEFS	23	50872	3	2	3	2	0.20	Putative two-componer
1768	1	db$2	2::C3K2Y9|C3K2Y9_PSEFS	23	13142	1	1	1	1	0.43	Protein translocase sut
1769	1	db$2	2::C3K8Q6|C3K8Q6_PSEFS	23	41029	1	1	1	1	0.12	Uncharacterized protei
1770	1	SwissProt_HUMAN$	3::O75197|LRP5_HUMAN	23	179030	1	1	1	1	0.03	Low-density lipoproteir
1771	1	SwissProt_HUMAN$	3::Q9NZT2|OGFR_HUMAN	23	73280	1	1	1	1	0.07	Opioid growth factor re
1772	1	SwissProt_HUMAN$	3::Q92538|GBF1_HUMAN	23	206315	2	1	2	1	0.02	Golgi-specific brefeldin
1773	1	SwissProt_HUMAN$	3::Q8IVE3|PKHH2_HUMAN	23	168124	1	1	1	1	0.03	Pleckstrin homology dc
1774	1	db$2	2::C3K4G7|PDXY_PSEFS	23	31241	1	1	1	1	0.16	Pyridoxal kinase PdxY (
1775	1	SwissProt_HUMAN$	3::P12110|CO6A2_HUMAN	23	108512	1	1	1	1	0.04	Collagen alpha-2(VI) cl
1776	1	SwissProt_HUMAN$	3::P60866|RS20_HUMAN	23	13364	1	1	1	1	0.42	40S ribosomal protein
1777	1	db$2	2::C3KAU8|C3KAU8_PSEFS	23	52023	1	1	1	1	0.10	Xylulose kinase OS=Ps
1778	1	SwissProt_HUMAN$	3::P04920|B3A2_HUMAN	23	136923	1	1	1	1	0.04	Anion exchange proteir
1779	1	SwissProt_HUMAN$	3::P59536|T2R41_HUMAN	23	35872	1	1	1	1	0.14	Taste receptor type 2 r
1780	1	SwissProt_HUMAN$	3::P28072|PSB6_HUMAN	23	25341	1	1	1	1	0.20	Proteasome subunit be
1781	1	SwissProt_HUMAN$	3::Q8TB72|PUM2_HUMAN	23	114145	1	1	1	1	0.04	Pumilio homolog 2 OS=
1782	1	SwissProt_HUMAN$	3::Q9ULX9|MAFF_HUMAN	23	17749	1	1	1	1	0.30	Transcription factor Ma
1783	1	db$2	2::C3K8R2|C3K8R2_PSEFS	23	22663	1	1	1	1	0.23	Uncharacterized protei
1784	1	SwissProt_HUMAN$	3::Q14966|ZN638_HUMAN	23	220488	1	1	1	1	0.02	Zinc finger protein 638
1785	1	db$2	2::C3K306|C3K306_PSEFS	23	24918	1	1	1	1	0.21	BPL/LPL catalytic doma
1786	1	SwissProt_HUMAN$	3::P62753|RS6_HUMAN	23	28663	1	1	1	1	0.18	40S ribosomal protein

↑Family	M	DB	Accession	Score	Mass	Matches	Match(sig)	Sequences	Seq(sig)	emPAI	Description
1787	1	db$2	2::C3K7I5|C3K7I5_PSEFS	23	28342	2	2	2	2	0.39	Putative iron utilisation
1788	1	SwissProt_HUMAN$	3::P27216|ANX13_HUMAN	23	35393	1	1	1	1	0.14	Annexin A13 OS=Homo
1789	1	db$2	2::C3KBV2|C3KBV2_PSEFS	22	15686	1	1	1	1	0.35	Thioredoxin OS=Pseud
1790	1	SwissProt_HUMAN$	3::Q9UBV2|SE1L1_HUMAN	22	88698	1	1	1	1	0.05	Protein sel-1 homolog
1791	1	SwissProt_HUMAN$	3::Q9NRF8|PYRG2_HUMAN	22	65636	1	1	1	1	0.07	CTP synthase 2 OS=Ho
1792	1	SwissProt_HUMAN$	3::P54886|P5CS_HUMAN	22	87248	1	1	1	1	0.06	Delta-1-pyrroline-5-car
1793	1	SwissProt_HUMAN$	3::P11532|DMD_HUMAN	22	426484	1	1	1	1	0.01	Dystrophin OS=Homo
1794	1	SwissProt_HUMAN$	3::Q14679|TTLL4_HUMAN	22	133294	3	2	1	1	0.04	Tubulin polyglutamylas
1795	1	db$2	2::C3JXT0|C3JXT0_PSEFS	22	22445	1	1	1	1	0.23	Putative isochorismata
1796	1	db$2	2::C3K1D6|C3K1D6_PSEFS	22	16178	1	1	1	1	0.33	Putative membrane pro
1797	1	SwissProt_HUMAN$	3::P62136|PP1A_HUMAN	22	37488	1	1	1	1	0.13	Serine/threonine-prote
1798	1	SwissProt_HUMAN$	3::O94967|WDR47_HUMAN	22	101884	1	1	1	1	0.05	WD repeat-containing
1799	1	db$2	2::A4V7G0|A4V7G0_PSEFS	22	59387	1	1	1	1	0.08	Putative RNA polymera
1800	1	db$2	2::C3K6X3|C3K6X3_PSEFS	22	12829	1	1	1	1	0.43	Putative translation init
1801	1	db$2	2::C3K4S7|C3K4S7_PSEFS	22	34300	2	2	1	1	0.15	Putative LysR-family re
1802	1	db$2	2::C3KCU6|C3KCU6_PSEFS	22	23028	2	2	2	2	0.51	Putative membrane pro
1803	1	db$2	2::C3K3H6|C3K3H6_PSEFS	22	12460	1	1	1	1	0.45	Putative endoribonucle
1804	1	db$2	2::C3K0S4|C3K0S4_PSEFS	22	65961	2	2	2	2	0.15	Isocitrate dehydrogena
1805	1	SwissProt_HUMAN$	3::O15397|IPO8_HUMAN	22	119861	1	1	1	1	0.04	Importin-8 OS=Homo
1806	1	db$2	2::C3K5B7|C3K5B7_PSEFS	22	50883	1	1	1	1	0.10	Putative outer membra
1807	1	SwissProt_HUMAN$	3::Q7Z5H4|VN1R5_HUMAN	22	40752	1	1	1	1	0.12	Vomeronasal type-1 re
1808	1	db$2	2::C3K4M3|C3K4M3_PSEFS	22	101390	1	1	1	1	0.05	Phosphocarrier protein
1809	1	SwissProt_HUMAN$	3::P62913|RL11_HUMAN	22	20240	1	1	1	1	0.26	60S ribosomal protein
1810	1	db$2	2::C3K2T4|C3K2T4_PSEFS	22	155869	1	1	1	1	0.03	Putative ATP-depender
1811	1	db$2	2::C3K211|C3K211_PSEFS	22	35352	1	1	1	1	0.14	LysR-family transcripti
1812	1	SwissProt_HUMAN$	3::A6NE01|F186A_HUMAN	22	262595	1	1	1	1	0.02	Protein FAM186A OS=H
1813	1	db$2	2::C3K4Q6|C3K4Q6_PSEFS	22	39864	1	1	1	1	0.13	Putative lipoprotein OS
1814	1	SwissProt_HUMAN$	3::P35611|ADDA_HUMAN	21	80905	1	1	1	1	0.06	Alpha-adducin OS=Hor
1815	1	db$2	2::C3JYV6|C3JYV6_PSEFS	21	31643	1	1	1	1	0.16	Metallo-beta-lactamase
1816	1	SwissProt_HUMAN$	3::A4UGR9|XIRP2_HUMAN	21	382063	1	1	1	1	0.01	Xin actin-binding repea
1817	1	SwissProt_HUMAN$	3::Q8WUI4|HDAC7_HUMAN	21	102863	1	1	1	1	0.05	Histone deacetylase 7
1818	1	SwissProt_HUMAN$	3::P63173|RL38_HUMAN	21	8213	1	1	1	1	0.75	60S ribosomal protein
1819	1	SwissProt_HUMAN$	3::Q9UL62|TRPC5_HUMAN	21	111340	1	1	1	1	0.04	Short transient recepto
1820	1	db$2	2::C3KCB4|C3KCB4_PSEFS	21	27191	2	1	2	1	0.19	Uncharacterized protei
1821	1	SwissProt_HUMAN$	3::Q8WVP7|LMBR1_HUMAN	21	55062	1	1	1	1	0.09	Limb region 1 protein h
1822	1	db$2	2::C3K080|C3K080_PSEFS	21	10402	1	1	1	1	0.56	Uncharacterized protei
1823	1	db$2	2::C3K4V6|C3K4V6_PSEFS	21	101332	1	1	1	1	0.05	Putative helicase OS=P
1824	1	SwissProt_HUMAN$	3::Q99623|PHB2_HUMAN	21	33276	1	1	1	1	0.15	Prohibitin-2 OS=Homo
1825	1	db$2	2::C3K831|C3K831_PSEFS	21	45359	1	1	1	1	0.11	Putative thiolase OS=P
1826	1	db$2	2::C3KE47|FMT_PSEFS	21	33416	1	1	1	1	0.15	Methionyl-tRNA formyl

↑Family	M	DB	Accession	Score	Mass	Matches	Match(sig)	Sequences	Seq(sig)	emPAI	Description
1827	1	SwissProt_HUMAN$	3::P81605|DCD_HUMAN	21	11277	1	1	1	1	0.51	Dermcidin OS=Homo s
1828	1	db$2	2::C3K330|GLPE_PSEFS	21	11747	1	1	1	1	0.48	Thiosulfate sulfurtransf
1829	1	SwissProt_HUMAN$	3::Q86TG7|PEG10_HUMAN	21	80122	1	1	1	1	0.06	Retrotransposon-derive
1830	1	db$2	2::C3K7Y9|C3K7Y9_PSEFS	21	49907	1	1	1	1	0.10	NAD(P) transhydrogena
1831	1	db$2	2::C3K329|APAH_PSEFS	21	31643	1	1	1	1	0.16	Bis(5'-nucleosyl)-tetra
1832	1	db$2	2::C3JYM3|C3JYM3_PSEFS	21	19231	1	1	1	1	0.28	Putative C4-dicarboxyla
1833	1	SwissProt_HUMAN$	3::Q8NFP9|NBEA_HUMAN	21	327614	2	1	2	1	0.01	Neurobeachin OS=Hon
1834	1	SwissProt_HUMAN$	3::Q14118|DAG1_HUMAN	21	97381	1	1	1	1	0.05	Dystroglycan OS=Hom
1835	1	db$2	2::C3K7D0|THII_PSEFS	21	54519	1	1	1	1	0.09	tRNA sulfurtransferase
1836	1	SwissProt_HUMAN$	3::Q02080|MEF2B_HUMAN	21	38615	1	1	1	1	0.13	Myocyte-specific enhar
1837	1	db$2	2::C3JZQ4|C3JZQ4_PSEFS	21	8106	2	2	2	2	2.10	Translational regulator
1838	1	db$2	2::C3K176|C3K176_PSEFS	21	33571	1	1	1	1	0.15	Glucokinase OS=Pseud
1839	1	SwissProt_HUMAN$	3::P16885|PLCG2_HUMAN	21	147776	1	1	1	1	0.03	1-phosphatidylinositol
1840	1	SwissProt_HUMAN$	3::Q8NEZ4|KMT2C_HUMAN	21	541028	1	1	1	1	0.01	Histone-lysine N-methy
1841	1	db$2	2::C3K637|C3K637_PSEFS	21	56441	2	1	2	1	0.09	Putative peptide ABC tr
1842	1	SwissProt_HUMAN$	3::Q8N5H7|SH2D3_HUMAN	21	94352	1	1	1	1	0.05	SH2 domain-containing
1843	1	SwissProt_HUMAN$	3::Q14789|GOGB1_HUMAN	21	375790	1	1	1	1	0.01	Golgin subfamily B mei
1844	1	SwissProt_HUMAN$	3::P26641|EF1G_HUMAN	21	50087	2	1	2	1	0.10	Elongation factor 1-gar
1845	1	db$2	2::C3KAJ0|C3KAJ0_PSEFS	21	34086	2	1	1	1	0.15	Putative LysR-family re
1846	1	db$2	2::C3K3P8|C3K3P8_PSEFS	21	49421	2	1	2	1	0.10	G domain-containing p
1847	1	db$2	2::C3KB13|C3KB13_PSEFS	21	35564	1	1	1	1	0.14	Putative ThiF protein O
1848	1	SwissProt_HUMAN$	3::O94827|PKHG5_HUMAN	21	117377	1	1	1	1	0.04	Pleckstrin homology dc
1849	1	db$2	2::C3KDM8|C3KDM8_PSEFS	21	57105	1	1	1	1	0.09	HDOD domain-containi
1850	1	SwissProt_HUMAN$	3::A6NNS2|DRS7C_HUMAN	21	34855	1	1	1	1	0.14	Dehydrogenase/reduct
1851	1	db$2	2::C3K0V8|C3K0V8_PSEFS	21	45209	1	1	1	1	0.11	Muramidase FlgJ OS=P
1852	1	db$2	2::C3KE72|C3KE72_PSEFS	21	85807	2	1	2	1	0.06	UPF0313 protein PFLU_
1853	1	SwissProt_HUMAN$	3::Q96SQ7|ATOH8_HUMAN	20	34622	1	1	1	1	0.15	Protein atonal homolog
1854	1	db$2	2::C3K883|C3K883_PSEFS	20	60772	1	1	1	1	0.08	Putative transposase su
1855	1	SwissProt_HUMAN$	3::P15880|RS2_HUMAN	20	31305	1	1	1	1	0.16	40S ribosomal protein
1856	1	db$2	2::C3K3K0|YQGF_PSEFS	20	16158	1	1	1	1	0.33	Putative pre-16S rRNA
1857	1	SwissProt_HUMAN$	3::P62269|RS18_HUMAN	20	17708	1	1	1	1	0.30	40S ribosomal protein
1858	1	SwissProt_HUMAN$	3::Q8TEU7|RPGF6_HUMAN	20	179310	1	1	1	1	0.03	Rap guanine nucleotide
1859	1	SwissProt_HUMAN$	3::P15924|DESP_HUMAN	20	331569	1	1	1	1	0.01	Desmoplakin OS=Hom
1860	1	db$2	2::C3K5A1|C3K5A1_PSEFS	20	60517	1	1	1	1	0.08	Urease subunit alpha C
1861	1	SwissProt_HUMAN$	3::P47869|GBRA2_HUMAN	20	51293	1	1	1	1	0.10	Gamma-aminobutyric a
1862	1	db$2	2::C3KDK6|C3KDK6_PSEFS	20	27451	2	1	2	1	0.19	Putative ABC transport
1863	1	SwissProt_HUMAN$	3::Q8WV24|PHLA1_HUMAN	20	44988	1	1	1	1	0.11	Pleckstrin homology-lik
1864	1	db$2	2::C3K0D9|C3K0D9_PSEFS	20	79601	1	1	1	1	0.06	Putative transport-rela
1865	1	SwissProt_HUMAN$	3::Q9HCH0|NCK5L_HUMAN	20	139345	1	1	1	1	0.03	Nck-associated protein
1866	1	SwissProt_HUMAN$	3::P23396|RS3_HUMAN	20	26671	3	2	3	2	0.42	40S ribosomal protein

↑Family	M	DB	Accession	Score	Mass	Matches	Match(sig)	Sequences	Seq(sig)	emPAI	Description
1867	1	db$2	2::C3K428|C3K428_PSEFS	20	10726	2	1	2	1	0.54	Uncharacterized protei
1868	1	db$2	2::C3K4F9|C3K4F9_PSEFS	20	31102	1	1	1	1	0.16	Putative regulatory pro
1869	1	SwissProt_HUMAN$	3::Q14315|FLNC_HUMAN	20	290841	1	1	1	1	0.02	Filamin-C OS=Homo sa
1870	1	SwissProt_HUMAN$	3::O95347|SMC2_HUMAN	20	135572	1	1	1	1	0.04	Structural maintenance
1871	1	db$2	2::C3JZP4|RBSD_PSEFS	20	14393	1	1	1	1	0.38	D-ribose pyranase OS=
1872	1	SwissProt_HUMAN$	3::Q8TEQ6|GEMI5_HUMAN	20	168483	2	1	2	1	0.03	Gem-associated protei
1873	1	db$2	2::C3K863|PDXH_PSEFS	20	24884	1	1	1	1	0.21	Pyridoxine/pyridoxamir
1874	1	db$2	2::C3KBI5|C3KBI5_PSEFS	20	21236	1	1	1	1	0.25	Putative membrane pro
1875	1	SwissProt_HUMAN$	3::Q8N9B5|JMY_HUMAN	20	111376	2	1	2	1	0.04	Junction-mediating anc
1876	1	db$2	2::C3JYI7|C3JYI7_PSEFS	20	41303	1	1	1	1	0.12	Putative hydrolase OS=
1877	1	SwissProt_HUMAN$	3::Q08378|GOGA3_HUMAN	20	167252	1	1	1	1	0.03	Golgin subfamily A mei
1878	1	db$2	2::C3K058|C3K058_PSEFS	20	21557	1	1	1	1	0.24	DSBA domain-containii
1879	1	db$2	2::C3KAF7|C3KAF7_PSEFS	20	91635	3	1	3	1	0.05	TPR_REGION domain-c
1880	1	SwissProt_HUMAN$	3::Q6RI45|BRWD3_HUMAN	20	203471	1	1	1	1	0.02	Bromodomain and WD
1881	1	db$2	2::C3K4U8|C3K4U8_PSEFS	20	20094	1	1	1	1	0.26	Conserved hypothetica
1882	1	db$2	2::C3KCQ3|C3KCQ3_PSEFS	19	36116	1	1	1	1	0.14	Putative type III secret
1883	1	db$2	2::C3K0U7|C3K0U7_PSEFS	19	49884	2	2	2	2	0.21	Two-component respor
1884	1	SwissProt_HUMAN$	3::O14640|DVL1_HUMAN	19	75140	1	1	1	1	0.07	Segment polarity prote
1885	1	db$2	2::C3KDV1|C3KDV1_PSEFS	19	30184	1	1	1	1	0.17	Chemotaxis MotA prote
1886	1	db$2	2::C3K814|DTD_PSEFS	19	15391	1	1	1	1	0.35	D-aminoacyl-tRNA dea
1887	1	db$2	2::C3K1J8|C3K1J8_PSEFS	19	26668	1	1	1	1	0.19	Trehalose operon trans
1888	1	SwissProt_HUMAN$	3::Q93074|MED12_HUMAN	19	242928	2	1	2	1	0.02	Mediator of RNA polym
1889	1	SwissProt_HUMAN$	3::Q6NSW7|NANP8_HUMAN	19	34650	1	1	1	1	0.15	Homeobox protein NAN
1890	1	db$2	2::C3KAE4|C3KAE4_PSEFS	19	39865	1	1	1	1	0.13	Uncharacterized protei
1891	1	db$2	2::C3KCG3|C3KCG3_PSEFS	19	16758	1	1	1	1	0.32	YjgF_endoribonc doma
1892	1	SwissProt_HUMAN$	3::Q13275|SEM3F_HUMAN	19	88325	1	1	1	1	0.06	Semaphorin-3F OS=Hc
1893	1	db$2	2::C3KBT7|C3KBT7_PSEFS	19	36676	2	1	2	1	0.14	Sulfate ABC transport s
1894	1	SwissProt_HUMAN$	3::O15457|MSH4_HUMAN	19	104689	1	1	1	1	0.05	MutS protein homolog
1895	1	db$2	2::C3K3Q9|LGT_PSEFS	19	30444	1	1	1	1	0.17	Phosphatidylglycerol--p
1896	1	db$2	2::C3K8X1|C3K8X1_PSEFS	19	23435	2	2	2	2	0.49	Putative ABC transport
1897	1	db$2	2::C3K0A7|ALLC_PSEFS	19	36686	1	1	1	1	0.14	Probable allantoicase C
1898	1	SwissProt_HUMAN$	3::Q8WXF8|DEDD2_HUMAN	19	36157	1	1	1	1	0.14	DNA-binding death effe
1899	1	SwissProt_HUMAN$	3::Q13835|PKP1_HUMAN	19	82808	2	1	2	1	0.06	Plakophilin-1 OS=Hom
1900	1	SwissProt_HUMAN$	3::Q92802|N42L2_HUMAN	19	67417	1	1	1	1	0.07	NEDD4-binding protein
1901	1	SwissProt_HUMAN$	3::Q5TGY3|AHDC1_HUMAN	19	168245	2	1	1	1	0.03	AT-hook DNA-binding r
1902	1	SwissProt_HUMAN$	3::Q96P48|ARAP1_HUMAN	19	162089	1	1	1	1	0.03	Arf-GAP with Rho-GAP
1903	1	db$2	2::C3K979|C3K979_PSEFS	19	30391	1	1	1	1	0.17	Putative glutathione S-
1904	1	db$2	2::C3K1V5|C3K1V5_PSEFS	19	64171	1	1	1	1	0.08	Uncharacterized protei
1905	1	SwissProt_HUMAN$	3::Q8NCM8|DYHC2_HUMAN	19	492311	1	1	1	1	0.01	Cytoplasmic dynein 2 h
1906	1	SwissProt_HUMAN$	3::P23526|SAHH_HUMAN	19	47685	1	1	1	1	0.10	Adenosylhomocysteina

↑Family	M	DB	Accession	Score	Mass	Matches	Match(sig)	Sequences	Seq(sig)	emPAI	Description
1907	1	SwissProt_HUMAN$	3::P17987|TCPA_HUMAN	19	60306	1	1	1	1	0.08	T-complex protein 1 su
1908	1	SwissProt_HUMAN$	3::Q13613|MTMR1_HUMAN	19	74631	3	1	2	1	0.07	Myotubularin-related p
1909	1	db$2	2::C3JZG7|C3JZG7_PSEFS	19	24981	1	1	1	1	0.21	Putative GntR-family tr
1910	1	SwissProt_HUMAN$	3::Q9HCC9|LST2_HUMAN	19	96429	1	1	1	1	0.05	Lateral signaling target
1911	1	db$2	2::C3K2F9|C3K2F9_PSEFS	19	48516	1	1	1	1	0.10	Putative ABC transport
1912	1	db$2	2::C3K326|C3K326_PSEFS	19	35169	2	1	2	1	0.14	4-hydroxythreonine-4-
1913	1	SwissProt_HUMAN$	3::Q9P2D0|IBTK_HUMAN	19	150432	1	1	1	1	0.03	Inhibitor of Bruton tyrc
1914	1	db$2	2::C3K8V0|C3K8V0_PSEFS	19	31271	1	1	1	1	0.16	Poly(3-hydroxyalkanoa
1915	1	db$2	2::C3KAA5|C3KAA5_PSEFS	19	23984	2	1	2	1	0.22	Uncharacterized protei
1916	1	SwissProt_HUMAN$	3::Q09MP3|R51A2_HUMAN	19	133822	2	1	2	1	0.04	RAD51-associated prot
1917	1	SwissProt_HUMAN$	3::Q8N1H7|S6OS1_HUMAN	19	68123	2	1	2	1	0.07	Protein SIX6OS1 OS=H
1918	1	SwissProt_HUMAN$	3::Q9GZP7|VN1R1_HUMAN	19	39994	1	1	1	1	0.13	Vomeronasal type-1 re
1919	1	SwissProt_HUMAN$	3::Q7Z7L1|SLN11_HUMAN	19	102770	2	1	2	1	0.05	Schlafen family membe
1920	1	db$2	2::C3K9X2|C3K9X2_PSEFS	19	42284	1	1	1	1	0.12	Putative aminotransfer
1921	1	db$2	2::C3K1F6|C3K1F6_PSEFS	18	23787	1	1	1	1	0.22	Ribosomal RNA small s
1922	1	SwissProt_HUMAN$	3::P16471|PRLR_HUMAN	18	69461	1	1	1	1	0.07	Prolactin receptor OS=
1923	1	db$2	2::C3K0Z0|COBQ_PSEFS	18	51487	1	1	1	1	0.10	Cobyric acid synthase (
1924	1	SwissProt_HUMAN$	3::A6NE52|WDR97_HUMAN	18	180192	2	1	2	1	0.03	WD repeat-containing
1925	1	db$2	2::C3KAC0|C3KAC0_PSEFS	18	40546	1	1	1	1	0.12	Butyryl-CoA dehydroge
1926	1	db$2	2::C3K9H2|C3K9H2_PSEFS	18	39760	1	1	1	1	0.13	Putrescine ABC transpc
1927	1	db$2	2::C3K5T8|C3K5T8_PSEFS	18	41768	1	1	1	1	0.12	Cytochrome c oxidase
1928	1	SwissProt_HUMAN$	3::Q6UWJ1|TMCO3_HUMAN	18	75549	1	1	1	1	0.06	Transmembrane and co
1929	1	SwissProt_HUMAN$	3::Q9H0L4|CSTFT_HUMAN	18	64396	1	1	1	1	0.08	Cleavage stimulation fa
1930	1	db$2	2::C3K1M8|C3K1M8_PSEFS	18	28345	1	1	1	1	0.18	tRNA (cytidine/uridine-
1931	1	SwissProt_HUMAN$	3::P42126|ECI1_HUMAN	18	32795	1	1	1	1	0.15	Enoyl-CoA delta isomer
1932	1	SwissProt_HUMAN$	3::Q96AQ8|MCUR1_HUMAN	18	39669	1	1	1	1	0.13	Mitochondrial calcium u
1933	1	SwissProt_HUMAN$	3::P04114|APOB_HUMAN	18	515283	1	1	1	1	0.01	Apolipoprotein B-100 C
1934	1	db$2	2::C3K3W9|C3K3W9_PSEFS	18	18528	1	1	1	1	0.29	Uncharacterized protei
1935	1	SwissProt_HUMAN$	3::Q8IWX8|CHERP_HUMAN	18	103637	1	1	1	1	0.05	Calcium homeostasis e
1936	1	SwissProt_HUMAN$	3::O00635|TRI38_HUMAN	18	53381	1	1	1	1	0.09	E3 ubiquitin-protein lig
1937	1	SwissProt_HUMAN$	3::O95297|MPZL1_HUMAN	18	29064	1	1	1	1	0.18	Myelin protein zero-like
1938	1	SwissProt_HUMAN$	3::Q7RTR0|NLRP9_HUMAN	18	113238	1	1	1	1	0.04	NACHT, LRR and PYD c
1939	1	SwissProt_HUMAN$	3::P04083|ANXA1_HUMAN	18	38690	1	1	1	1	0.13	Annexin A1 OS=Homo
1940	1	db$2	2::C3KAN2|C3KAN2_PSEFS	18	30990	2	1	2	1	0.16	3-oxoadipate CoA-tran
1941	1	SwissProt_HUMAN$	3::P10745|RET3_HUMAN	18	135278	2	1	2	1	0.04	Retinol-binding protein
1942	1	db$2	2::C3K751|C3K751_PSEFS	18	15943	1	1	1	1	0.34	Putative thioesterase C
1943	1	db$2	2::C3JY60|BPT_PSEFS	18	27663	1	1	1	1	0.19	Aspartate/glutamate le
1944	1	db$2	2::C3K3R1|C3K3R1_PSEFS	18	26777	1	1	1	1	0.19	Uncharacterized protei
1945	1	db$2	2::C3JXY6|ASTB_PSEFS	18	48741	3	2	3	2	0.21	N-succinylarginine dihy
1946	1	db$2	2::C3K0E8|C3K0E8_PSEFS	17	27512	3	1	2	1	0.19	Thiol:disulfide intercha

↑Family	M	DB	Accession	Score	Mass	Matches	Match(sig)	Sequences	Seq(sig)	emPAI	Description
1947	1	SwissProt_HUMAN$	3::Q9BWT7|CAR10_HUMAN	17	115859	1	1	1	1	0.04	Caspase recruitment d
1948	1	SwissProt_HUMAN$	3::Q14204|DYHC1_HUMAN	17	532072	3	1	3	1	0.01	Cytoplasmic dynein 1 h
1949	1	db$2	2::A4V7A1|A4V7A1_PSEFS	17	43655	1	1	1	1	0.11	Putative partitioning pr
1950	1	SwissProt_HUMAN$	3::Q9P2F6|RHG20_HUMAN	17	132525	1	1	1	1	0.04	Rho GTPase-activating
1951	1	db$2	2::C3K3T1|C3K3T1_PSEFS	17	86048	1	1	1	1	0.06	Putative penicillin amid
1952	1	SwissProt_HUMAN$	3::P26373|RL13_HUMAN	17	24247	1	1	1	1	0.21	60S ribosomal protein
1953	1	db$2	2::C3K1R1|C3K1R1_PSEFS	17	13822	1	1	1	1	0.40	bPH_1 domain-contain
1954	1	SwissProt_HUMAN$	3::P31943|HNRH1_HUMAN	17	49198	1	1	1	1	0.10	Heterogeneous nuclear
1955	1	db$2	2::C3K7L6|C3K7L6_PSEFS	17	44010	1	1	1	1	0.11	Molybdopterin molybde
1956	1	db$2	2::C3JY96|C3JY96_PSEFS	17	27408	1	1	1	1	0.19	OMP_b-brl domain-con
1957	1	db$2	2::C3K175|C3K175_PSEFS	17	27428	1	1	1	1	0.19	Putative two-componer
1958	1	db$2	2::C3K5S7|C3K5S7_PSEFS	17	19211	1	1	1	1	0.28	Uncharacterized protei
1959	1	SwissProt_HUMAN$	3::Q16798|MAON_HUMAN	17	67026	1	1	1	1	0.07	NADP-dependent malic
1960	1	db$2	2::C3K659|C3K659_PSEFS	17	30767	1	1	1	1	0.17	2-hydroxy-3-oxopropic
1961	1	db$2	2::C3KBV7|NHAP2_PSEFS	17	61387	1	1	1	1	0.08	K(+)/H(+) antiporter N
1962	1	SwissProt_HUMAN$	3::Q92820|GGH_HUMAN	17	35941	1	1	1	1	0.14	Gamma-glutamyl hydr
1963	1	SwissProt_HUMAN$	3::Q9UI33|SCNBA_HUMAN	17	204788	1	1	1	1	0.02	Sodium channel protei
1964	1	db$2	2::C3K9J9|C3K9J9_PSEFS	17	17304	1	1	1	1	0.31	Uncharacterized protei
1965	1	db$2	2::C3KCA2|C3KCA2_PSEFS	17	48472	1	1	1	1	0.10	Putative family S43 no
1966	1	db$2	2::C3K5T2|C3K5T2_PSEFS	17	65173	1	1	1	1	0.08	Putative dehydrogenas
1967	1	SwissProt_HUMAN$	3::Q5THJ4|VP13D_HUMAN	17	491606	1	1	1	1	0.01	Vacuolar protein sortin
1968	1	SwissProt_HUMAN$	3::Q96P16|RPR1A_HUMAN	16	35698	1	1	1	1	0.14	Regulation of nuclear p
1969	1	db$2	2::C3K6K7|C3K6K7_PSEFS	16	17395	1	1	1	1	0.31	GreA_GreB domain-cor
1970	1	SwissProt_HUMAN$	3::O00155|GPR25_HUMAN	16	38754	1	1	1	1	0.13	Probable G-protein cou
1971	1	db$2	2::C3K832|C3K832_PSEFS	16	33163	1	1	1	1	0.15	Non-specific serine/thr
1972	1	SwissProt_HUMAN$	3::P46020|KPB1_HUMAN	16	137225	2	1	2	1	0.04	Phosphorylase b kinase
1973	1	db$2	2::C3K842|C3K842_PSEFS	16	56600	1	1	1	1	0.09	RNA polymerase sigma
1974	1	db$2	2::C3K2E2|C3K2E2_PSEFS	16	56906	1	1	1	1	0.09	Catalase OS=Pseudom
1975	1	db$2	2::C3KCV5|C3KCV5_PSEFS	16	37705	1	1	1	1	0.13	Uncharacterized protei
1976	1	db$2	2::C3K5I5|C3K5I5_PSEFS	16	25298	1	1	1	1	0.20	Putative fimbria-relate
1977	1	SwissProt_HUMAN$	3::O75717|WDHD1_HUMAN	16	125888	1	1	1	1	0.04	WD repeat and HMG-b
1978	1	SwissProt_HUMAN$	3::P30041|PRDX6_HUMAN	16	25019	1	1	1	1	0.21	Peroxiredoxin-6 OS=H
1979	1	db$2	2::C3JYS6|C3JYS6_PSEFS	16	17060	1	1	1	1	0.31	Putative thioesterase C
1980	1	SwissProt_HUMAN$	3::Q9BYV7|BCDO2_HUMAN	16	65632	1	1	1	1	0.07	Beta,beta-carotene 9',
1981	1	db$2	2::C3KB69|C3KB69_PSEFS	16	12397	1	1	1	1	0.45	Putative lipoprotein OS
1982	1	SwissProt_HUMAN$	3::Q9Y2G4|ANKR6_HUMAN	16	79922	1	1	1	1	0.06	Ankyrin repeat domain
1983	1	db$2	2::C3K461|C3K461_PSEFS	15	46893	1	1	1	1	0.11	Putative oxidoreductas
1984	1	SwissProt_HUMAN$	3::Q16629|SRSF7_HUMAN	15	27350	1	1	1	1	0.19	Serine/arginine-rich sp
1985	1	SwissProt_HUMAN$	3::Q13219|PAPP1_HUMAN	15	180856	1	1	1	1	0.03	Pappalysin-1 OS=Hom
1986	1	db$2	2::C3K158|C3K158_PSEFS	15	36421	1	1	1	1	0.14	Putative regulatory pro

↑Family	M	DB	Accession	Score	Mass	Matches	Match(sig)	Sequences	Seq(sig)	emPAI	Description
1987	1	SwissProt_HUMAN$	3::Q5VSL9|STRP1_HUMAN	15	95515	1	1	1	1	0.05	Striatin-interacting pro
1988	1	db$2	2::C3KCS4|C3KCS4_PSEFS	15	63231	1	1	1	1	0.08	Peptidoglycan D,D-trar
1989	1	db$2	2::C3K1G3|C3K1G3_PSEFS	15	15162	1	1	1	1	0.36	Ribonuclease P protein
1990	1	db$2	2::C3K9S7|C3K9S7_PSEFS	15	35611	1	1	1	1	0.14	Putative magnesium ch
1991	1	db$2	2::C3KB03|C3KB03_PSEFS	15	18864	1	1	1	1	0.28	Uncharacterized protei
1992	1	db$2	2::C3K284|C3K284_PSEFS	15	59644	1	1	1	1	0.08	L-lactate permease OS
1993	1	SwissProt_HUMAN$	3::P24386|RAE1_HUMAN	15	73429	1	1	1	1	0.07	Rab proteins geranylge
1994	1	db$2	2::C3K6L2|C3K6L2_PSEFS	15	14328	1	1	1	1	0.38	Organic hydroperoxide
1995	1	db$2	2::C3KAN9|C3KAN9_PSEFS	15	28318	1	1	1	1	0.18	Putative aromatic-hydr
1996	1	SwissProt_HUMAN$	3::Q02153|GCYB1_HUMAN	14	70470	2	1	2	1	0.07	Guanylate cyclase solu
1997	1	SwissProt_HUMAN$	3::P01178|NEU1_HUMAN	14	12713	1	1	1	1	0.44	Oxytocin-neurophysin
1998	1	db$2	2::C3K0E6|C3K0E6_PSEFS	14	61696	1	1	1	1	0.08	Thiol:disulfide intercha
1999	1	SwissProt_HUMAN$	3::A6NNT2|CP096_HUMAN	14	124963	1	1	1	1	0.04	Uncharacterized protei
2000	1	SwissProt_HUMAN$	3::Q4G0U5|PCDP1_HUMAN	14	96829	2	1	2	1	0.05	Cilia- and flagella-asso
2001	1	SwissProt_HUMAN$	3::Q14573|ITPR3_HUMAN	14	303912	1	1	1	1	0.02	Inositol 1,4,5-trisphosp
2002	1	SwissProt_HUMAN$	3::Q8NF91|SYNE1_HUMAN	14	1010456	2	1	2	1	0.00	Nesprin-1 OS=Homo sa
2003	1	SwissProt_HUMAN$	3::Q9H819|DJC18_HUMAN	14	41525	1	1	1	1	0.12	DnaJ homolog subfami
2004	1	db$2	2::C3K1I4|C3K1I4_PSEFS	14	39250	1	1	1	1	0.13	Putative N-acetylglucos
2005	1	db$2	2::C3K0F1|C3K0F1_PSEFS	14	476575	1	1	1	1	0.01	Pyoverdine synthetase
2006	1	db$2	2::C3K3X1|C3K3X1_PSEFS	14	48834	1	1	1	1	0.10	Xaa-Pro aminopeptidas
2007	1	SwissProt_HUMAN$	3::P11362|FGFR1_HUMAN	14	91809	1	1	1	1	0.05	Fibroblast growth facto
2008	1	db$2	2::C3K3V9|C3K3V9_PSEFS	14	33232	1	1	1	1	0.15	Protein QmcA OS=Pseu
2009	1	SwissProt_HUMAN$	3::P62633|CNBP_HUMAN	14	19450	1	1	1	1	0.27	Cellular nucleic acid-bi
2010	1	db$2	2::C3KCP0|C3KCP0_PSEFS	14	31742	1	1	1	1	0.16	Ribosomal RNA large s
2011	1	db$2	2::C3K7J3|Y1319_PSEFS	14	16953	1	1	1	1	0.32	UPF0225 protein PFLU_
2012	1	SwissProt_HUMAN$	3::Q9ULU8|CAPS1_HUMAN	14	152690	2	1	2	1	0.03	Calcium-dependent sec
2013	1	SwissProt_HUMAN$	3::Q96Q89|KI20B_HUMAN	14	210500	1	1	1	1	0.02	Kinesin-like protein KIF
2014	1	SwissProt_HUMAN$	3::P05181|CP2E1_HUMAN	14	56812	1	1	1	1	0.09	Cytochrome P450 2E1
2015	1	SwissProt_HUMAN$	3::Q49MG5|MAP9_HUMAN	14	74189	1	1	1	1	0.07	Microtubule-associated
2016	1	SwissProt_HUMAN$	3::P19338|NUCL_HUMAN	14	76568	1	1	1	1	0.06	Nucleolin OS=Homo sa
2017	1	SwissProt_HUMAN$	3::Q7Z4P5|GDF7_HUMAN	14	46921	2	2	1	1	0.11	Growth/differentiation
2018	1	SwissProt_HUMAN$	3::Q8NG08|HELB_HUMAN	14	123175	1	1	1	1	0.04	DNA helicase B OS=Ho
2019	1	SwissProt_HUMAN$	3::C9JTQ0|ANR63_HUMAN	13	39596	1	1	1	1	0.13	Ankyrin repeat domain
2020	1	SwissProt_HUMAN$	3::Q9H254|SPTN4_HUMAN	13	288808	2	1	2	1	0.02	Spectrin beta chain, nc
2021	1	db$2	2::C3JZG9|C3JZG9_PSEFS	13	47015	1	1	1	1	0.11	Histidine kinase OS=Ps
2022	1	SwissProt_HUMAN$	3::Q08211|DHX9_HUMAN	13	140869	1	1	1	1	0.03	ATP-dependent RNA he
2023	1	db$2	2::C3K1I0|C3K1I0_PSEFS	13	22456	1	1	1	1	0.23	Nudix hydrolase domai
2024	1	SwissProt_HUMAN$	3::Q96N23|CFA54_HUMAN	13	351744	1	1	1	1	0.01	Cilia- and flagella-asso
2025	1	db$2	2::C3K0F8|C3K0F8_PSEFS	13	57361	1	1	1	1	0.09	Acetyl-CoA carboxylase
2026	1	SwissProt_HUMAN$	3::P02741|CRP_HUMAN	13	25023	1	1	1	1	0.21	C-reactive protein OS=

Export as CSV

Not what you expected? Try **the select summary**.

Mascot: http://www.matrixscience.com/
