# Supplementary material for: Circulating multimeric immune complexes contribute to immunopathology in COVID-19
Source: Nat Commun. 2022 Sep 26;13:5654. doi: 10.1038/s41467-022-32867-z (PMC9513013; doi:10.1038/s41467-022-32867-z)
Supplement: Supplementary file 3 — Source Data [file 41467_2022_32867_MOESM3_ESM.zip › Source Data/MS_20210407 (immune precipitation).zip!/QX20210618-05_Virologie(AG-Hengel)-20210407_(V5)_0k3v20_TRL_db_SC2+db_Pf+SP_H(F235800).pdf]

MATRIX SCIENCE MASCOT Search Results

User : Wolfgang Bildl
E-mail : wolfgang.bildl@physiologie.uni-freiburg.de
Search title : QX 20210618 (db$ SARS-CoV-2+db$2 Pf+SP H$ 5 ppm 20 mmu), QX20210618-05_Virologie(AG-Hengel)-20210407_(V5)_0k3v20_TRL_shifted_0.3.mgf
MS data file : D:\Xcalibur\Data\Wolfgang\Q-Exactive-HF-X_20210616\QX20210618-05_Virologie(AG-Hengel)-20210407_(V5)_0k3v20_TRL_shifted_0.3.mgf
Databases : **1:** db$ 20210617 (17 sequences; 14,439 residues)
2: db$2 20210617 (6,388 sequences; 2,089,672 residues)
3: SwissProt_HUMAN$ 20201007 (20,389 sequences; 11,363,789 residues)
Timestamp : 21 Jun 2021 at 11:04:24 GMT

Not what you expected? Try **the select summary**.

Search parameters

Type of search : MS/MS Ion Search
Enzyme : Trypsin
Variable modifications : **Acetyl (Protein N-term)**, **Carbamidomethyl (C)**, **Formyl (Protein N-term)**, **Gln->pyro-Glu (N-term Q)**, **Oxidation (M)**
Mass values : Monoisotopic
Protein mass : Unrestricted
Peptide mass tolerance : ± 5 ppm
Fragment mass tolerance : ± 20 mmu
Max missed cleavages : 1
Instrument type : ESI-FTICR
Number of queries : 85,387

Score distribution

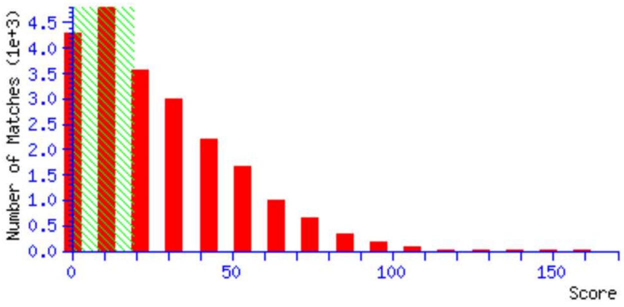

Peptide score distribution. Ions score is $-10\log(P)$, where P is the probability that the observed match is a random event.
There are **11,604** peptide matches above identity threshold and **12,332** matches above homology threshold for **85,387** queries.
On average, individual ions scores **> 19** (beyond green shading) indicate **identity or extensive homology** (p<0.05).

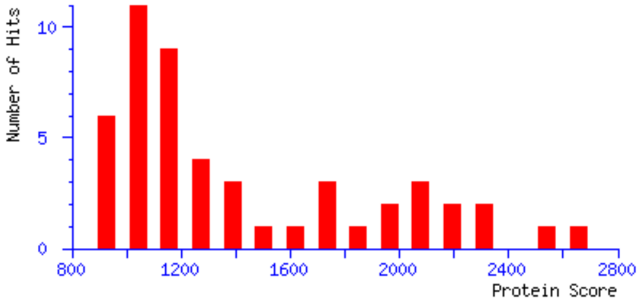

[Deprecated] Protein score distribution. Score distribution for family members in the first 50 proteins. Protein scores are derived from ions scores as a non-probabilistic basis for ranking protein families.

▼Modification statistics

Modification	Site	Above thr.
Oxidation	M	2167
Carbamidomethyl	C	1196
Gln->pyro-Glu	N-term	235
Formyl	Protein N-term	44
Acetyl	Protein N-term	13

▼Legend

Peptide columns and rows

Dupes	...	Expect	Rank	U	1	2	Peptide	
		**0.037**	▶**2**				**GAYSLSLR**	significant
		9	▶1				GFFLFVEGGR	top ranking
		**6.4e-005**	▶**1**				**GSSIFGLAPGK**	significant and top ranking
		**1.3e-006**	▶**1**		■		**SSGTSYPDVLK**	peptide is found in all proteins in family member 1
		**6.2e-007**	▶**1**			■	**VCNYVSWIK**	peptide is found in some but not all proteins in family member 2
		**6.4e-005**	▶**1**	**U**			**GSSIFGLAPGK**	unique
▶**2**		**5.7e-005**	▶**1**				**LNTLETEEWFFK**	peptide has two duplicates
		0.18	▶*1*				*LNTLETEEWFFK*	duplicate peptide

Right-facing triangle (▶) in the Dupes or Rank column indicates content that can be expanded by clicking on it. Down-facing triangle (▼) indicates the content is expanded and can be collapsed. For more details about particular columns, see **results format help**.

Protein quantitation ratios

	Score	...	114/113	115/113
**CFAH_HUMAN**	37559		*0.962*	**1.129**
**FHR2_HUMAN**	1330		**0.859**	1.128

When quantitation method is Reporter (e.g. iTRAQ) or Multiplex (e.g. IPTL), protein ratios are displayed when a family is expanded. Ratios in *italic* indicate that the peptide log-ratios do not appear to come from a normal distribution. **Bold** indicates that if you can assume peptide ratios are normally distributed, the protein ratio is significantly different from 1.0 (at significance level 0.05).

Note that lack of bold or italic can also mean that significance or normality testing has not been performed (for

example, if protein ratio type does not support it).

Protein Family Summary

Significance threshold p<	0.05	Max. number of families	AUTO
Ions score or expect cut-off	0.5	Dendrograms cut at	0
Show Percolator scores	☐		
Preferred taxonomy	All entries		

Sensitivity and FDR (reversed protein sequences)

			Target	Decoy	FDR		
PSMs	above	homology	12332	467	3.79%	Adjust to	1%

Decoy results are available in **the decoy report**.

Protein hits (2093 proteins)

Filters: (none)

Export as CSV

↑Family	M	DB	Accession	Score	Mass	Matches	Match(sig)	Sequences	Seq(sig)	emPAI	Description
1	1	db$2	2::C3K5G3|C3K5G3_PSEFS	2654	99192	82	78	38	35	8.02	Aconitate hydratase OS=
2	1	db$2	2::C3K2X8|EFTU_PSEFS	2488	43536	72	67	24	24	25.01	Elongation factor Tu OS=
3	1	db$2	2::C3K2Y3|RPOB_PSEFS	2302	150923	100	96	72	69	9.93	DNA-directed RNA polyn
4	1	db$2	2::C3K1A0|CH60_PSEFS	2280	56931	63	62	29	29	31.84	60 kDa chaperonin OS=
5	1	db$2	2::C3K2Y2|RPOC_PSEFS	2205	154528	92	88	64	63	7.60	DNA-directed RNA polyn
6	1	db$2	2::C3K1E6|ATPB_PSEFS	2150	49459	65	64	30	30	44.69	ATP synthase subunit be
7	1	db$2	2::C3K5Z6|EFTS_PSEFS	2104	30341	83	78	16	16	11.03	Elongation factor Ts OS=
8	1	db$2	2::C3K4N0|C3K4N0_PSEFS	2060	43479	55	55	27	27	39.16	Putative ornithine decarl
9	1	db$2	2::C3K4D5|C3K4D5_PSEFS	2058	34599	68	66	22	22	44.34	Phosphate-binding prote
10	1	db$2	2::C3K2X9|EFG_PSEFS	1974	77114	72	69	34	34	11.42	Elongation factor G OS=
11	1	db$2	2::C3K1E8|ATPA_PSEFS	1954	55245	65	62	31	30	35.46	ATP synthase subunit al
12	1	db$2	2::C3K0Q5|C3K0Q5_PSEFS	1887	107302	61	60	41	40	6.64	Ribonucleoside-diphospl
13	1	db$2	2::C3K255|PNP_PSEFS	1786	74889	57	53	32	30	10.07	Polyribonucleotide nucle
14	1	db$2	2::C3KD82|C3KD82_PSEFS	1757	181591	65	63	51	51	3.22	Glutamate dehydrogena
15	1	SwissProt_HUMAN$	3::P13645|K1C10_HUMAN	1691	58792	44	41	29	26	9.32	Keratin, type I cytoskele
15	2	SwissProt_HUMAN$	3::P02533|K1C14_HUMAN	331	51529	16	13	14	12	2.01	Keratin, type I cytoskele
15	3	SwissProt_HUMAN$	3::P08779|K1C16_HUMAN	242	51236	12	9	11	9	1.29	Keratin, type I cytoskele
15	4	SwissProt_HUMAN$	3::Q04695|K1C17_HUMAN	112	48076	11	8	9	7	0.99	Keratin, type I cytoskele
16	1	db$2	2::C3KCP4|C3KCP4_PSEFS	1564	99394	66	63	40	39	10.42	Pyruvate dehydrogenase
17	1	db$2	2::C3K6P2|C3K6P2_PSEFS	1508	42870	41	41	16	16	11.64	3-oxoacyl-[acyl-carrier-|
18	1	db$2	2::C3JYU7|C3JYU7_PSEFS	1414	102460	48	47	27	27	3.61	Protein translocase subu

↑Family	M	DB	Accession	Score	Mass	Matches	Match(sig)	Sequences	Seq(sig)	emPAI	Description
19	1	db$2	2::C3KBH1|C3KBH1_PSEFS	1366	161946	54	52	41	39	2.23	Glutamate synthase [NA
20	1	db$2	2::C3K275|DNAK_PSEFS	1345	68158	40	38	24	23	5.10	Chaperone protein Dnak
21	1	db$2	2::C3K0P3|C3K0P3_PSEFS	1322	118163	40	39	28	27	2.47	Ribonuclease E OS=Pseu
22	1	db$2	2::C3K5J5|FABV_PSEFS	1276	42933	42	42	26	26	20.76	Enoyl-[acyl-carrier-prote
23	1	SwissProt_HUMAN$	3::P35908|K22E_HUMAN	1269	65393	37	36	29	29	7.79	Keratin, type II cytoskel
23	2	SwissProt_HUMAN$	3::P04264|K2C1_HUMAN	1211	65999	37	36	26	26	7.01	Keratin, type II cytoskel
23	3	SwissProt_HUMAN$	3::P13647|K2C5_HUMAN	245	62340	10	7	10	7	0.70	Keratin, type II cytoskel
23	4	SwissProt_HUMAN$	3::P02538|K2C6A_HUMAN	230	60008	7	6	7	6	0.60	Keratin, type II cytoskel
23	5	SwissProt_HUMAN$	3::P05787|K2C8_HUMAN	141	53671	6	4	5	3	0.30	Keratin, type II cytoskel
23	6	SwissProt_HUMAN$	3::P08729|K2C7_HUMAN	86	51354	4	3	3	2	0.20	Keratin, type II cytoskel
24	1	db$2	2::C3K6M5|C3K6M5_PSEFS	1223	63522	38	36	20	19	3.79	Succinate dehydrogenas
25	1	db$2	2::C3KCJ5|C3KCJ5_PSEFS	1193	93957	49	46	35	32	4.86	Aconitate hydratase B O
26	1	db$2	2::C3K331|C3K331_PSEFS	1182	73691	47	43	33	31	8.49	AAA_PrkA domain-conta
27	1	db$2	2::C3K225|ILVC_PSEFS	1163	36188	37	37	21	21	25.06	Ketol-acid reductoisome
28	1	db$2	2::C3JYT1|SYDND_PSEFS	1157	66220	42	41	28	27	8.85	Aspartate--tRNA(Asp/As
29	1	db$2	2::C3K6M7|C3K6M7_PSEFS	1152	105930	48	46	34	32	4.02	Oxoglutarate dehydroge
30	1	db$2	2::C3K259|IF2_PSEFS	1136	90544	39	34	29	26	3.11	Translation initiation fac
31	1	db$2	2::C3KBY8|C3KBY8_PSEFS	1127	21919	47	45	18	18	89.70	Alkyl hydroperoxide redu
32	1	db$2	2::C3K271|C3K271_PSEFS	1123	117563	41	39	32	30	3.11	Carbamoyl-phosphate sy
33	1	db$2	2::C3JYJ7|C3JYJ7_PSEFS	1110	9057	57	55	9	9	432.00	DNA-binding protein HU
34	1	db$2	2::C3JYK1|TIG_PSEFS	1094	48316	37	34	24	24	16.12	Trigger factor OS=Pseuc
35	1	db$2	2::C3K6M8|C3K6M8_PSEFS	1073	42320	29	28	15	14	5.68	Dihydrolipoyllysine-resic
36	1	db$2	2::C3K6H4|PYRG_PSEFS	1062	59660	37	33	22	21	5.73	CTP synthase OS=Pseuc
37	1	db$2	2::C3K6N1|C3K6N1_PSEFS	1059	29928	31	28	15	13	15.94	Succinate--CoA ligase [/
38	1	db$2	2::C3K613|FADB_PSEFS	1046	76938	36	33	28	26	3.96	Fatty acid oxidation com
39	1	db$2	2::C3KDP2|C3KDP2_PSEFS	1042	68879	32	29	23	20	3.86	Multifunctional fusion pr
40	1	db$2	2::C3K6N0|SUCC_PSEFS	1030	41262	32	31	22	21	12.89	Succinate--CoA ligase [/
41	1	db$2	2::C3K3Z0|C3K3Z0_PSEFS	1025	46925	30	30	22	22	10.21	Transcription terminatio
42	1	db$2	2::C3K7D3|C3K7D3_PSEFS	1021	91800	34	32	25	25	3.04	Alpha-1,4 glucan phosph
43	1	db$2	2::C3K165|C3K165_PSEFS	1013	54857	45	39	27	25	14.83	Fumarate hydratase clas
44	1	SwissProt_HUMAN$	3::P35527|K1C9_HUMAN	1013	62027	30	29	21	20	4.36	Keratin, type I cytoskele
45	1	db$2	2::C3K6K0|C3K6K0_PSEFS	977	62268	37	35	22	21	6.22	30S ribosomal protein S
46	1	db$2	2::C3K121|C3K121_PSEFS	976	86030	39	36	29	27	3.68	Phosphoenolpyruvate sy
47	1	db$2	2::C3K7G0|C3K7G0_PSEFS	973	36980	32	28	20	17	11.86	Cell shape-determining |
48	1	db$2	2::C3K7D1|C3K7D1_PSEFS	968	67115	39	38	28	28	8.56	50S ribosomal subunit a
49	1	db$2	2::C3JYR1|GCSP_PSEFS	958	102176	44	38	27	23	2.19	Glycine dehydrogenase
50	1	db$2	2::C3K367|C3K367_PSEFS	927	68142	36	34	19	19	4.30	Uncharacterized protein
51	1	db$2	2::C3KE51|C3KE51_PSEFS	916	38026	33	30	22	20	10.94	Putative oxidoreductase
52	1	db$2	2::C3JY66|C3JY66_PSEFS	915	80156	37	34	24	23	3.39	Isocitrate dehydrogenas
53	1	db$2	2::C3K3F3|C3K3F3_PSEFS	914	72324	44	44	30	30	7.68	Transketolase OS=Pseu

↑Family	M	DB	Accession	Score	Mass	Matches	Match(sig)	Sequences	Seq(sig)	emPAI	Description
54	1	db$2	2::C3K1J2|C3K1J2_PSEFS	894	140531	36	33	32	29	1.95	Phosphoribosylformylgly
55	1	db$2	2::C3KE86|C3KE86_PSEFS	878	95293	38	34	29	26	2.84	Chaperone protein ClpB
56	1	db$2	2::C3K1L8|NDK_PSEFS	874	14928	28	28	9	9	30.46	Nucleoside diphosphate
57	1	db$2	2::C3KEC5|C3KEC5_PSEFS	870	97398	39	35	30	28	3.31	Phosphoenolpyruvate ca
58	1	db$2	2::C3KDW8|PURA_PSEFS	858	46535	30	30	18	18	7.46	Adenylosuccinate synthe
59	1	db$2	2::C3K1K0|GUAA_PSEFS	853	58398	34	32	26	24	6.59	GMP synthase [glutamin
60	1	db$2	2::C3KBJ2|C3KBJ2_PSEFS	852	24354	27	25	14	13	25.76	Carbonic anhydrase OS=
61	1	db$2	2::C3K3S2|C3K3S2_PSEFS	847	44244	31	30	23	22	9.46	2-oxoglutarate reductas
62	1	db$2	2::C3K0K6|C3K0K6_PSEFS	842	26352	25	23	12	11	11.23	Electron transfer flavopr
63	1	db$2	2::C3K402|C3K402_PSEFS	839	83101	40	37	27	25	4.23	Polyphosphate kinase O:
64	1	db$2	2::C3K673|C3K673_PSEFS	835	47725	34	33	20	20	13.51	Citrate synthase OS=Ps
65	1	db$2	2::C3K426|ARLY_PSEFS	833	51545	28	27	18	18	6.55	Argininosuccinate lyase
66	1	db$2	2::C3KBT1|C3KBT1_PSEFS	828	44808	34	31	19	18	7.23	4-aminobutyrate aminot
67	1	db$2	2::C3KE67|RS6_PSEFS	825	16276	25	25	11	11	128.97	30S ribosomal protein S
68	1	db$2	2::C3KDW5|C3KDW5_PSEFS	820	43265	29	27	20	19	8.90	Protein HflK OS=Pseudo
69	1	db$2	2::C3K2D3|C3K2D3_PSEFS	817	44901	30	29	18	17	6.37	Serine hydroxymethyltra
70	1	db$2	2::C3KCB8|C3KCB8_PSEFS	812	36708	34	33	16	16	12.06	Phosphate-binding prote
71	1	db$2	2::C3K2R9|C3K2R9_PSEFS	802	16440	15	15	7	7	11.93	6,7-dimethyl-8-ribityllur
72	1	db$2	2::C3K6M9|C3K6M9_PSEFS	794	49948	25	24	15	15	4.50	Dihydrolipoyl dehydroge
73	1	db$2	2::C3K4D1|C3K4D1_PSEFS	792	28553	25	23	14	13	13.03	Phosphate-specific trans
74	1	db$2	2::C3K0T0|C3K0T0_PSEFS	788	34560	30	30	9	9	2.92	Putative outer membran
75	1	db$2	2::C3K6N7|HTPG_PSEFS	787	71589	33	31	25	23	4.23	Chaperone protein HtpG
76	1	db$2	2::C3KBT0|C3KBT0_PSEFS	769	51607	25	22	15	15	4.71	Succinate-semialdehyde
77	1	db$2	2::C3K2D0|C3K2D0_PSEFS	762	61490	27	27	22	22	5.86	Energy-dependent trans
78	1	db$2	2::C3JYS1|C3JYS1_PSEFS	751	17688	22	21	9	8	13.16	Peptidoglycan-associate
79	1	db$2	2::C3K1W9|C3K1W9_PSEFS	749	33929	17	17	9	9	5.08	Thioredoxin reductase O
80	1	db$2	2::C3K162|C3K162_PSEFS	745	52023	29	26	20	19	5.16	Pyruvate kinase OS=Pse
81	1	db$2	2::C3K6G5|AMPA_PSEFS	744	52403	25	25	20	20	6.29	Probable cytosol aminop
82	1	db$2	2::C3K1E2|C3K1E2_PSEFS	739	66300	23	22	18	17	2.62	Glutamine--fructose-6-p
83	1	db$2	2::C3K1A7|C3K1A7_PSEFS	733	66070	26	24	19	17	3.20	Proline--tRNA ligase OS:
84	1	db$2	2::C3K3J7|C3K3J7_PSEFS	729	35008	25	24	16	16	13.86	Glutathione synthetase (
85	1	db$2	2::C3K2V1|C3K2V1_PSEFS	722	36623	29	28	22	22	21.03	DNA-directed RNA polyn
86	1	db$2	2::C3K3F1|PGK_PSEFS	707	40125	20	18	15	15	5.59	Phosphoglycerate kinase
87	1	db$2	2::C3K6H6|C3K6H6_PSEFS	707	45588	25	24	16	15	4.25	Enolase OS=Pseudomon
88	1	db$2	2::C3KCP3|C3KCP3_PSEFS	697	56221	21	21	13	13	2.54	Acetyltransferase compo
89	1	db$2	2::C3K2V9|RS5_PSEFS	695	17675	21	21	10	10	39.89	30S ribosomal protein S
90	1	db$2	2::C3KDD8|C3KDD8_PSEFS	684	44893	29	29	21	21	11.55	Cell division protein FtsA
91	1	db$2	2::C3JZS1|C3JZS1_PSEFS	682	55264	25	23	18	16	3.29	Amidophosphoribosyltra
92	1	db$2	2::C3K4J5|PCKA_PSEFS	677	55324	23	22	17	17	4.09	Phosphoenolpyruvate ca
93	1	db$2	2::C3K1K1|C3K1K1_PSEFS	668	51928	33	31	23	22	9.68	Inosine-5'-monophospha

↑Family	M	DB	Accession	Score	Mass	Matches	Match(sig)	Sequences	Seq(sig)	emPAI	Description
94	1	db$2	2::C3JXY1|C3JXY1_PSEFS	664	93962	25	24	21	20	1.89	Alanine--tRNA ligase OS
95	1	db$2	2::C3JZN7|SYT_PSEFS	664	72513	21	18	16	14	1.67	Threonine--tRNA ligase
96	1	db$2	2::C3K603|C3K603_PSEFS	662	87560	30	27	21	19	2.12	Outer membrane protei
97	1	db$2	2::C3K3E8|C3K3E8_PSEFS	658	38385	24	24	16	16	10.69	Fructose-1,6-bisphospha
98	1	db$2	2::C3KAH7|C3KAH7_PSEFS	658	45018	27	26	17	16	5.61	Putative NADP-depende
99	1	db$2	2::C3JY65|C3JY65_PSEFS	635	45375	25	22	16	14	6.23	Isocitrate dehydrogenas
100	1	db$2	2::C3K6X4|C3K6X4_PSEFS	633	71161	22	21	18	18	2.54	Biosynthetic arginine de
101	1	db$2	2::C3K7C9|C3K7C9_PSEFS	632	51641	28	27	17	17	4.71	Glutamine synthetase O
102	1	db$2	2::C3K2V6|C3K2V6_PSEFS	630	47842	21	19	9	8	2.27	Protein translocase subu
103	1	db$2	2::C3KE40|SYGB_PSEFS	624	74833	30	26	25	21	3.29	Glycine--tRNA ligase bet
104	1	db$2	2::C3KDU5|C3KDU5_PSEFS	618	90380	24	23	18	17	1.71	DNA gyrase subunit B O
105	1	db$2	2::C3KDR8|OPGD_PSEFS	616	60485	20	20	16	16	2.50	Glucans biosynthesis pr
106	1	db$2	2::C3KCS1|C3KCS1_PSEFS	614	34171	17	17	12	12	8.09	Ribose-phosphate pyrop
107	1	db$2	2::C3K8B0|C3K8B0_PSEFS	612	25585	18	18	10	10	8.12	Peptidyl-prolyl cis-trans
108	1	db$2	2::C3K6W9|C3K6W9_PSEFS	612	69415	17	16	14	13	1.43	Putative methyl-accepti
108	2	db$2	2::C3JZ31|C3JZ31_PSEFS	115	58204	3	3	2	2	0.18	Putative methyl-accepti
108	3	db$2	2::C3K5K7|C3K5K7_PSEFS	87	52604	5	4	5	4	0.43	Putative methyl-accepti
108	4	db$2	2::C3KD60|C3KD60_PSEFS	50	55774	2	2	2	2	0.19	Methyl-accepting chemo
109	1	db$2	2::C3KDG0|RECA_PSEFS	612	37499	25	24	16	15	8.63	Protein RecA OS=Pseud
110	1	db$2	2::C3K914|C3K914_PSEFS	610	38509	19	18	11	10	5.29	Putative ABC transport
111	1	db$2	2::C3K3G6|SAHH_PSEFS	609	51212	31	29	19	19	7.38	Adenosylhomocysteinas
112	1	db$2	2::C3KAD2|ARNA_PSEFS	602	74140	23	21	19	17	2.16	Bifunctional polymyxin r
113	1	db$2	2::C3K4D2|C3K4D2_PSEFS	602	30988	29	27	20	19	27.44	Phosphate import ATP-b
114	1	db$2	2::C3KEC2|C3KEC2_PSEFS	598	28614	19	18	13	12	6.19	Putative lipoprotein OS=
114	2	db$2	2::C3KAJ6|C3KAJ6_PSEFS	195	38502	10	8	8	6	1.36	Histidinol-phosphate am
115	1	db$2	2::C3K6J4|C3K6J4_PSEFS	597	97893	28	28	21	21	1.90	DNA gyrase subunit A O
116	1	db$2	2::C3K016|C3K016_PSEFS	597	85788	21	20	19	18	1.70	Ribosomal RNA large su
117	1	db$2	2::C3K6G0|C3K6G0_PSEFS	595	9680	13	12	8	7	27.33	Putative DNA-binding pr
118	1	db$2	2::C3K2X5|RL4_PSEFS	590	21987	12	12	7	7	4.52	50S ribosomal protein L
119	1	db$2	2::C3K139|C3K139_PSEFS	589	48816	20	19	9	9	1.90	Uncharacterized protein
120	1	db$2	2::C3K2X0|C3K2X0_PSEFS	584	25737	19	17	13	11	14.48	30S ribosomal protein S
121	1	db$2	2::C3K5E6|RS2_PSEFS	580	27078	24	23	15	14	18.18	30S ribosomal protein S
122	1	db$2	2::C3K4F6|C3K4F6_PSEFS	579	65489	34	26	24	20	4.28	Oxaloacetate decarboxy
123	1	db$2	2::C3KE70|RL9_PSEFS	578	15488	15	15	7	7	10.05	50S ribosomal protein L
124	1	db$2	2::C3K6I0|C3K6I0_PSEFS	574	39056	19	19	12	12	5.13	Alcohol dehydrogenase
125	1	db$2	2::C3K6C2|C3K6C2_PSEFS	572	27720	15	15	9	9	3.60	Putative amino acid ABC
126	1	db$2	2::C3JYJ9|CLPX_PSEFS	569	46924	25	25	16	16	5.13	ATP-dependent Clp prot
127	1	db$2	2::C3K266|C3K266_PSEFS	562	69364	34	34	13	13	1.60	ATP-dependent zinc met
128	1	db$2	2::C3KBZ2|ASSY_PSEFS	561	45330	24	24	16	16	5.55	Argininosuccinate synth
129	1	db$2	2::C3K1N2|C3K1N2_PSEFS	557	67307	26	23	21	18	3.09	Protein translocase subu

↑Family	M	DB	Accession	Score	Mass	Matches	Match(sig)	Sequences	Seq(sig)	emPAI	Description
130	1	db$2	2::C3K260|C3K260_PSEFS	556	54381	18	17	13	12	2.38	Transcription terminatio
131	1	db$2	2::C3K2M9|PROA_PSEFS	555	45380	16	16	10	10	2.49	Gamma-glutamyl phosp
132	1	db$2	2::C3JZN2|C3JZN2_PSEFS	554	86329	24	23	20	19	1.84	Phenylalanine--tRNA liga
133	1	db$2	2::C3K6M6|C3K6M6_PSEFS	554	26063	20	20	13	13	16.91	Succinate dehydrogenas
134	1	db$2	2::C3KCB5|C3KCB5_PSEFS	551	30173	23	21	13	12	7.89	Phosphate import ATP-b
135	1	db$2	2::C3K2Y6|RL1_PSEFS	546	24061	18	17	11	11	11.63	50S ribosomal protein L
136	1	db$2	2::C3K178|C3K178_PSEFS	546	36139	20	17	14	12	5.21	Glyceraldehyde-3-phosp
137	1	db$2	2::C3K3V3|ARGA_PSEFS	539	47793	16	16	13	13	2.61	Amino-acid acetyltransfe
138	1	db$2	2::C3KBM0|GLPK_PSEFS	538	55508	31	26	21	18	5.53	Glycerol kinase OS=Pse
139	1	db$2	2::C3K5H9|C3K5H9_PSEFS	538	33776	19	18	14	13	7.14	Transaldolase OS=Pseu
140	1	db$2	2::C3K9R0|C3K9R0_PSEFS	533	62265	17	16	13	13	1.69	Pyruvate dehydrogenase
141	1	db$2	2::C3K2Y4|RL7_PSEFS	531	12456	13	13	2	2	5.48	50S ribosomal protein L
142	1	db$2	2::C3K1B7|C3K1B7_PSEFS	531	51892	19	18	13	12	2.28	Threonine synthase OS=
143	1	db$2	2::C3JZS0|C3JZS0_PSEFS	527	43144	23	22	17	16	6.17	O-succinylhomoserine su
144	1	db$2	2::C3KBX8|C3KBX8_PSEFS	522	26859	17	17	9	9	10.62	OmpA-like domain-conta
145	1	db$2	2::C3JXY0|AK_PSEFS	521	44504	21	19	17	15	5.08	Aspartate kinase OS=Ps
146	1	db$2	2::C3K341|C3K341_PSEFS	520	69250	27	25	21	19	3.50	RNA polymerase sigma f
146	2	db$2	2::C3K708|C3K708_PSEFS	126	38066	3	3	3	3	0.45	RNA polymerase sigma f
147	1	db$2	2::C3JYS2|TOLB_PSEFS	518	47517	27	25	20	19	7.09	Tol-Pal system protein T
148	1	db$2	2::C3JYT4|C3JYT4_PSEFS	516	17756	20	18	10	9	22.60	Putative ferritin DPS-fan
149	1	db$2	2::C3K0D4|C3K0D4_PSEFS	516	29228	17	16	10	10	4.90	Cell division inhibitor Mi
150	1	db$2	2::C3JY70|C3JY70_PSEFS	513	50668	37	33	14	14	3.06	Adenylosuccinate lyase (
151	1	db$2	2::C3K4F7|C3K4F7_PSEFS	511	51847	26	23	21	19	5.21	Biotin carboxylase OS=F
151	2	db$2	2::C3K282|SSRP_PSEFS	83	18308	4	4	3	3	1.15	SsrA-binding protein OS
152	1	db$2	2::C3JZ70|C3JZ70_PSEFS	505	54858	27	23	19	15	2.98	Glucose-6-phosphate 1-
153	1	db$2	2::C3K123|C3K123_PSEFS	501	41542	13	12	10	9	1.78	Citrate synthase OS=Ps
154	1	db$2	2::C3JXZ0|C3JXZ0_PSEFS	500	43332	17	16	14	14	3.61	Acetylornithine aminotra
155	1	db$2	2::C3K3F5|METK_PSEFS	499	42554	20	19	14	14	4.30	S-adenosylmethionine s
156	1	db$2	2::C3K4D4|C3K4D4_PSEFS	495	83859	25	25	17	17	2.10	Putative phosphate ABC
157	1	db$2	2::C3K7D6|F16PA_PSEFS	494	37081	16	15	10	9	3.06	Fructose-1,6-bisphospha
158	1	db$2	2::C3JXL8|C3JXL8_PSEFS	493	112023	23	22	17	16	1.14	Putative efflux protein O
159	1	db$2	2::C3KBI6|C3KBI6_PSEFS	491	106629	15	15	13	13	0.86	Uncharacterized protein
160	1	db$2	2::C3K108|C3K108_PSEFS	491	83591	24	21	21	18	1.78	GTP pyrophosphokinase
161	1	SwissProt_HUMAN$	3::P02768|ALBU_HUMAN	490	69321	20	19	17	17	2.19	Albumin OS=Homo sapi
161	2	SwissProt_HUMAN$	3::P02769|ALBU_BOVIN	289	69248	13	13	12	12	1.27	Albumin OS=Bos taurus
162	1	SwissProt_HUMAN$	3::P07355|ANXA2_HUMAN	483	38580	19	18	16	15	5.26	Annexin A2 OS=Homo s
163	1	db$2	2::C3KAK3|C3KAK3_PSEFS	481	54608	16	16	12	12	1.83	Probable malate:quinon
164	1	db$2	2::C3JZT6|C3JZT6_PSEFS	480	40569	15	15	11	11	4.12	Aspartate-semialdehyde
165	1	db$2	2::C3JXL7|C3JXL7_PSEFS	478	39112	15	15	11	11	3.26	HlyD-family secretion pr
166	1	db$2	2::C3KDD9|C3KDD9_PSEFS	478	41969	16	14	14	12	3.32	Cell division protein FtsZ

↑Family	M	DB	Accession	Score	Mass	Matches	Match(sig)	Sequences	Seq(sig)	emPAI	Description
167	1	db$2	2::C3K3P0|ILVD_PSEFS	477	65667	19	18	17	16	2.17	Dihydroxy-acid dehydra
168	1	db$2	2::C3K6M2|C3K6M2_PSEFS	475	60584	17	17	15	15	2.49	ATP-dependent RNA heli
169	1	db$2	2::C3K3V2|C3K3V2_PSEFS	475	49111	18	15	8	8	1.38	Putative outer membran
170	1	db$2	2::C3K4V9|C3K4V9_PSEFS	474	53185	21	18	15	14	2.80	Glyceraldehyde-3-phosp
171	1	db$2	2::C3KA76|C3KA76_PSEFS	473	30860	16	15	11	11	5.25	UTP--glucose-1-phospha
172	1	db$2	2::C3K865|C3K865_PSEFS	472	15847	10	10	5	5	4.84	Putative membrane prot
173	1	db$2	2::C3KCR8|C3KCR8_PSEFS	469	39907	16	14	9	8	2.26	Ribosome-binding ATPas
174	1	db$2	2::C3JYV2|C3JYV2_PSEFS	468	52688	17	17	15	15	2.84	AAA domain-containing
175	1	db$2	2::C3K616|C3K616_PSEFS	465	97222	24	21	20	17	1.29	DNA topoisomerase 1 O
176	1	db$2	2::C3K649|C3K649_PSEFS	464	8822	13	13	5	5	12.77	Major outer membrane l
177	1	db$2	2::C3K244|C3K244_PSEFS	463	52276	15	14	13	12	1.96	Putative response regul
178	1	db$2	2::C3KED7|PUR7_PSEFS	459	26862	20	18	15	13	15.49	Phosphoribosylaminoimi
179	1	db$2	2::C3K4Y4|C3K4Y4_PSEFS	457	71842	20	20	15	15	2.28	Potassium-transporting
180	1	db$2	2::C3K1G8|RL19_PSEFS	454	12961	14	13	9	9	34.89	50S ribosomal protein L
181	1	db$2	2::C3KDX4|SYI_PSEFS	449	105386	20	18	19	17	1.15	Isoleucine--tRNA ligase
182	1	db$2	2::C3K6H5|KDSA_PSEFS	449	30777	28	27	17	16	32.96	2-dehydro-3-deoxyphos
183	1	db$2	2::C3K569|C3K569_PSEFS	446	44415	17	17	11	11	2.99	Tryptophan synthase be
184	1	db$2	2::C3K6G2|C3K6G2_PSEFS	446	106936	23	20	21	18	1.32	Valine--tRNA ligase OS=
185	1	db$2	2::C3K9U6|C3K9U6_PSEFS	443	35232	13	13	9	9	3.37	Putative 6-phosphogluc
186	1	db$2	2::C3JYQ6|C3JYQ6_PSEFS	442	37842	19	16	11	9	4.73	Ornithine carbamoyltran
187	1	db$2	2::C3KDP1|CYSD_PSEFS	441	35173	14	14	9	9	3.37	Sulfate adenylyltransfer
188	1	db$2	2::C3K7Y0|C3K7Y0_PSEFS	439	45268	17	17	13	13	3.31	Putative exported protei
189	1	db$2	2::C3K6W6|C3K6W6_PSEFS	439	49345	21	19	16	15	4.09	Biotin carboxylase OS=I
190	1	db$2	2::C3K4W1|STHA_PSEFS	438	50963	20	20	16	16	3.85	Soluble pyridine nucleot
191	1	db$2	2::C3KDE0|LPXC_PSEFS	436	33197	13	13	8	8	2.59	UDP-3-O-acyl-N-acetylg
192	1	db$2	2::C3K4C6|C3K4C6_PSEFS	436	25565	16	16	9	9	6.59	Phosphate regulon trans
193	1	db$2	2::C3K4K9|C3K4K9_PSEFS	434	27377	14	14	8	8	3.69	Putative amino acid oxic
194	1	db$2	2::C3K6H2|ACCA_PSEFS	434	34868	21	21	11	11	7.72	Acetyl-coenzyme A carb
195	1	db$2	2::C3KBY6|C3KBY6_PSEFS	433	17850	12	12	9	9	12.71	Bacterioferritin OS=Pseu
196	1	db$2	2::C3K0K5|C3K0K5_PSEFS	426	31365	13	13	7	7	2.32	Electron transfer flavopr
197	1	db$2	2::C3K1K7|LEU1_PSEFS	425	61997	20	20	17	17	2.95	2-isopropylmalate synth
198	1	db$2	2::C3K2W1|RL6_PSEFS	423	19185	19	18	10	10	17.68	50S ribosomal protein L
199	1	db$2	2::C3K6D9|C3K6D9_PSEFS	422	45394	13	13	10	10	2.49	Phosphoribosylamine--g
200	1	db$2	2::C3JYU6|C3JYU6_PSEFS	421	42196	10	10	8	8	1.45	Arginine biosynthesis bi
201	1	db$2	2::C3K3Z1|C3K3Z1_PSEFS	419	11808	12	12	6	6	22.43	Thioredoxin OS=Pseudo
202	1	db$2	2::C3JZV2|C3JZV2_PSEFS	419	43020	18	17	13	12	3.65	Aminotransferase OS=P
203	1	db$2	2::C3K1M5|ISCS_PSEFS	413	44467	15	14	13	12	2.58	Cysteine desulfurase Isc
204	1	db$2	2::C3K1L3|SYH_PSEFS	413	47853	16	15	13	13	3.40	Histidine--tRNA ligase O
205	1	db$2	2::C3K8X0|C3K8X0_PSEFS	413	16351	12	12	9	9	12.15	Putative ABC transport s
206	1	db$2	2::C3JY83|NUOCD_PSEFS	412	67660	29	23	22	18	3.05	NADH-quinone oxidored

↑Family	M	DB	Accession	Score	Mass	Matches	Match(sig)	Sequences	Seq(sig)	emPAI	Description
207	1	db$2	2::C3K4D3|C3K4D3_PSEFS	411	60551	18	17	13	13	1.76	Phosphate transport sys
208	1	db$2	2::C3K1L4|ISPG_PSEFS	409	39660	20	20	15	15	5.73	4-hydroxy-3-methylbut-
209	1	db$2	2::C3K734|C3K734_PSEFS	408	44623	13	13	9	9	2.21	Putative aminotransfera
210	1	db$2	2::C3K1F0|C3K1F0_PSEFS	407	16832	16	13	10	10	14.97	ATP synthase subunit b
211	1	db$2	2::C3K263|C3K263_PSEFS	407	26068	8	8	7	7	2.53	Triosephosphate isomera
212	1	db$2	2::C3K1L0|C3K1L0_PSEFS	406	55309	20	19	18	17	3.29	GTPase Der OS=Pseudo
213	1	db$2	2::C3KDU7|C3KDU7_PSEFS	403	28504	20	18	15	14	9.08	Putative ABC transport s
214	1	db$2	2::C3JYQ5|C3JYQ5_PSEFS	403	46409	20	18	13	13	2.76	Arginine deiminase OS=
215	1	db$2	2::C3K6J9|KCY_PSEFS	401	24780	9	9	8	8	3.57	Cytidylate kinase OS=Ps
216	1	db$2	2::C3K2X1|RL22_PSEFS	400	11904	11	9	7	6	9.42	50S ribosomal protein L
217	1	db$2	2::C3JYJ8|C3JYJ8_PSEFS	399	88851	14	14	14	14	1.11	Lon protease OS=Pseud
218	1	db$2	2::C3K2Z7|ARGC_PSEFS	398	36612	13	13	6	6	1.80	N-acetyl-gamma-glutam
219	1	db$2	2::C3JYA7|C3JYA7_PSEFS	396	59461	11	11	9	9	1.22	Putative NADP-depende
220	1	db$2	2::C3JZN6|IF3_PSEFS	395	20837	14	14	8	8	6.56	Translation initiation fac
221	1	db$2	2::C3K3N4|COAD_PSEFS	394	17810	14	13	8	7	9.71	Phosphopantetheine ade
222	1	db$2	2::C3K4C7|C3K4C7_PSEFS	394	50044	11	11	9	9	1.57	Phosphate regulon sens
223	1	db$2	2::C3K3R2|C3K3R2_PSEFS	393	83481	21	18	17	15	1.34	Phosphoenolpyruvate--p
224	1	db$2	2::C3KEA4|C3KEA4_PSEFS	392	51722	13	12	10	10	1.74	PINc domain-containing
225	1	db$2	2::C3K274|DNAJ_PSEFS	388	40315	15	15	13	13	3.59	Chaperone protein DnaJ
226	1	db$2	2::C3JZN3|SYFA_PSEFS	388	37892	14	14	11	11	4.06	Phenylalanine--tRNA liga
227	1	db$2	2::C3KAL6|C3KAL6_PSEFS	388	52109	17	17	14	14	2.57	Putative aminotransfera
228	1	db$2	2::C3K6J6|C3K6J6_PSEFS	387	40307	13	12	10	9	1.87	Bifunctional chorismate
229	1	db$2	2::C3K1E7|ATPG_PSEFS	384	31374	17	16	15	14	8.49	ATP synthase gamma ch
230	1	db$2	2::C3K466|C3K466_PSEFS	380	52681	12	12	8	8	1.05	Putative aldehyde dehyd
231	1	db$2	2::C3JZV0|C3JZV0_PSEFS	378	56140	15	14	15	14	2.25	Glutamate--tRNA ligase
232	1	db$2	2::C3K227|C3K227_PSEFS	377	62578	18	17	14	13	1.88	Acetolactate synthase O
233	1	db$2	2::C3K2X3|RL2_PSEFS	373	29689	16	16	13	13	6.90	50S ribosomal protein L
234	1	db$2	2::C3K2X6|RL3_PSEFS	372	22550	13	12	11	10	8.90	50S ribosomal protein L
235	1	db$2	2::C3K2L2|SYL_PSEFS	372	97066	18	14	17	14	0.98	Leucine--tRNA ligase OS
236	1	db$2	2::C3K7G9|C3K7G9_PSEFS	372	48266	12	11	10	9	1.41	Putative modulation of D
237	1	db$2	2::C3K2V2|RS4_PSEFS	372	23062	17	15	11	11	10.49	30S ribosomal protein S
238	1	db$2	2::C3K3U2|C3K3U2_PSEFS	370	50985	13	13	10	10	1.53	Putative glutamine synth
239	1	db$2	2::C3K2W0|RL18_PSEFS	368	12651	12	11	8	8	26.25	50S ribosomal protein L
240	1	db$2	2::C3K1G5|C3K1G5_PSEFS	365	46195	12	12	8	8	1.51	Homoserine dehydrogen
241	1	db$2	2::C3K6K6|C3K6K6_PSEFS	362	36089	15	15	13	13	4.48	HTH-type transcriptiona
242	1	db$2	2::C3KCS2|RSMH_PSEFS	361	34344	14	13	11	11	4.21	Ribosomal RNA small su
243	1	db$2	2::C3K2J8|C3K2J8_PSEFS	360	54392	14	14	11	11	1.84	AMP nucleosidase OS=P
244	1	db$2	2::C3KBH2|C3KBH2_PSEFS	359	51951	10	10	8	8	1.07	Glutamate synthase [NA
245	1	db$2	2::C3K8X5|HIS1_PSEFS	359	23098	15	15	10	10	8.37	ATP phosphoribosyltrans
246	1	db$2	2::C3KBL8|C3KBL8_PSEFS	358	56970	21	21	17	17	3.11	Glycerol-3-phosphate de

↑Family	M	DB	Accession	Score	Mass	Matches	Match(sig)	Sequences	Seq(sig)	emPAI	Description
247	1	db$2	2::C3KCB7|C3KCB7_PSEFS	357	34191	8	8	4	4	0.99	Phosphate transport sys
248	1	db$2	2::C3K2C4|C3K2C4_PSEFS	356	89060	14	13	13	13	1.00	Endopeptidase La OS=P
249	1	db$2	2::C3K2Y8|C3K2Y8_PSEFS	356	20023	14	13	11	10	12.21	Transcription terminatio
250	1	db$2	2::C3KDM2|C3KDM2_PSEFS	355	83508	17	16	14	13	1.09	DNA topoisomerase 4 su
251	1	db$2	2::C3KEC8|C3KEC8_PSEFS	353	50623	17	15	9	7	1.32	Periplasmic serine endo
252	1	db$2	2::C3K8X6|C3K8X6_PSEFS	353	47255	10	10	8	8	1.23	Histidinol dehydrogenas
253	1	db$2	2::C3K1H6|PURT_PSEFS	352	42327	10	10	10	10	2.06	Formate-dependent pho
254	1	db$2	2::C3K2U7|C3K2U7_PSEFS	352	104087	18	17	16	15	0.98	UvrABC system protein
255	1	db$2	2::C3K1X8|C3K1X8_PSEFS	351	11596	8	7	4	3	3.99	50S ribosomal protein L
256	1	db$2	2::C3K1E9|ATPD_PSEFS	350	19246	18	18	9	9	10.47	ATP synthase subunit de
257	1	db$2	2::C3K358|C3K358_PSEFS	350	65409	15	14	10	10	1.22	Putative dehydrogenase
257	2	db$2	2::C3K355|C3K355_PSEFS	315	63710	11	11	10	10	1.10	Putative dehydrogenase
258	1	db$2	2::C3K5I7|C3K5I7_PSEFS	349	59867	15	14	9	9	1.21	Probable malate:quinon
259	1	db$2	2::C3K435|C3K435_PSEFS	349	45402	13	13	9	9	1.55	Diaminopimelate decarb
260	1	db$2	2::C3JZV8|C3JZV8_PSEFS	348	38906	13	12	10	9	2.36	Neutral metalloproteina
261	1	db$2	2::C3K0Q7|C3K0Q7_PSEFS	347	45255	8	8	5	5	0.87	Flagellar hook protein Fl
262	1	db$2	2::C3K9T7|C3K9T7_PSEFS	347	99328	18	17	14	14	0.95	Aminopeptidase N OS=P
263	1	db$2	2::C3K902|RAPA_PSEFS	346	106722	14	13	12	11	0.63	RNA polymerase-associa
264	1	db$2	2::C3KDU3|C3KDU3_PSEFS	346	40518	17	16	13	12	3.55	Beta sliding clamp OS=P
265	1	db$2	2::C3KE59|C3KE59_PSEFS	346	27398	16	16	10	10	6.85	DNA-binding response r
266	1	db$2	2::C3JY86|C3JY86_PSEFS	345	98164	17	16	15	14	0.97	NADH-quinone oxidored
267	1	db$2	2::C3K614|C3K614_PSEFS	343	41572	14	14	9	9	2.49	3-ketoacyl-CoA thiolase
268	1	db$2	2::C3JYW3|C3JYW3_PSEFS	343	36818	12	12	9	9	2.60	Branched-chain-amino-a
269	1	db$2	2::C3JZT0|C3JZT0_PSEFS	342	28122	8	8	6	6	1.73	Putative GntR-family tra
270	1	db$2	2::C3K484|C3K484_PSEFS	341	13502	11	11	6	6	10.09	Uncharacterized protein
271	1	db$2	2::C3K9R4|C3K9R4_PSEFS	337	62164	20	19	17	16	2.65	Putative sulfite reductas
272	1	db$2	2::C3K6E2|RS9_PSEFS	337	14607	11	11	7	7	23.09	30S ribosomal protein S
273	1	db$2	2::C3KE65|C3KE65_PSEFS	336	97331	19	17	17	15	1.08	Ribonuclease R OS=Pse
274	1	db$2	2::C3KBP1|C3KBP1_PSEFS	335	37670	12	11	8	8	2.09	Putative reductase OS=
275	1	db$2	2::C3K5Y0|C3K5Y0_PSEFS	334	38747	14	13	11	11	2.82	NADP(H)-dependt aldo-
276	1	db$2	2::C3K226|C3K226_PSEFS	334	17820	10	10	6	6	3.81	Acetolactate synthase O
277	1	db$2	2::C3K4M6|C3K4M6_PSEFS	334	57818	15	15	10	10	1.46	Putative exported alkali
278	1	db$2	2::C3K2T2|C3K2T2_PSEFS	331	24648	12	11	9	9	7.15	Putative ABC transport s
279	1	db$2	2::C3K3Q2|C3K3Q2_PSEFS	330	37335	16	14	8	7	2.12	Putative cation ABC tran
280	1	db$2	2::C3KDU9|C3KDU9_PSEFS	330	34261	11	11	8	8	2.00	Serine O-acetyltransfera
281	1	SwissProt_HUMAN$	3::P60709|ACTB_HUMAN	330	41710	16	15	15	14	3.88	Actin, cytoplasmic 1 OS
282	1	db$2	2::C3K2Y0|RS7_PSEFS	329	17636	19	17	12	11	17.46	30S ribosomal protein S
283	1	db$2	2::C3KDL2|C3KDL2_PSEFS	329	52736	10	9	10	9	1.24	Putative outer membran
284	1	db$2	2::C3K7N8|RDGC_PSEFS	329	34160	13	13	11	11	4.24	Recombination-associat
285	1	db$2	2::C3K5V1|C3K5V1_PSEFS	327	47050	9	9	8	8	1.23	Outer membrane protei

↑Family	M	DB	Accession	Score	Mass	Matches	Match(sig)	Sequences	Seq(sig)	emPAI	Description
286	1	db$2	2::C3K6E0|PUR9_PSEFS	327	57370	15	15	12	12	1.92	Bifunctional purine biosy
287	1	db$2	2::C3K474|C3K474_PSEFS	326	50732	10	10	9	9	1.54	Putative phosphomanno
288	1	db$2	2::Q84CP9|DAPB_PSEFS	324	28276	13	13	8	8	3.47	4-hydroxy-tetrahydrodip
289	1	db$2	2::C3K278|C3K278_PSEFS	323	15153	10	10	5	5	7.56	Ferric uptake regulation
290	1	db$2	2::C3K2E3|MSCL_PSEFS	321	14568	7	7	4	4	3.97	Large-conductance mecl
291	1	db$2	2::C3K907|C3K907_PSEFS	319	77648	17	16	17	16	1.65	Tail-specific protease OS
292	1	db$2	2::C3K075|C3K075_PSEFS	319	77370	12	12	9	9	0.74	Putative sulfatase OS=P
293	1	db$2	2::C3JYR4|C3JYR4_PSEFS	318	7675	7	7	3	3	5.01	Major cold shock proteir
294	1	db$2	2::C3K6V2|HIS4_PSEFS	318	25679	14	14	9	9	5.26	1-(5-phosphoribosyl)-5-
295	1	db$2	2::C3K6U8|C3K6U8_PSEFS	317	58654	12	10	7	6	0.76	Putative membrane prot
296	1	db$2	2::C3KBN2|C3KBN2_PSEFS	314	52689	7	7	6	6	0.87	Outer membrane proteir
297	1	db$2	2::C3K1G2|YIDC_PSEFS	313	61977	10	9	9	9	0.99	Membrane protein insert
298	1	db$2	2::C3KAJ2|C3KAJ2_PSEFS	313	53335	14	14	13	13	2.17	Methylmalonate-semialc
299	1	db$2	2::C3K2V0|RL17_PSEFS	312	14407	13	12	8	7	24.27	50S ribosomal protein L
300	1	db$2	2::C3KA80|C3KA80_PSEFS	312	20438	14	13	11	11	14.72	Alkyl hydroperoxide redu
301	1	db$2	2::C3K5Z8|RRF_PSEFS	311	20297	12	11	6	6	5.34	Ribosome-recycling fact
302	1	db$2	2::C3K5E0|C3K5E0_PSEFS	310	36000	10	10	7	7	2.26	2,3,4,5-tetrahydropyridi
303	1	db$2	2::C3JY74|C3JY74_PSEFS	309	48472	17	14	16	13	2.55	Isocitrase OS=Pseudom
304	1	db$2	2::C3K2Y7|RL11_PSEFS	308	14903	8	7	4	3	1.56	50S ribosomal protein L
305	1	db$2	2::C3K2M1|C3K2M1_PSEFS	307	42033	12	11	11	10	2.07	Serine-type D-Ala-D-Ala
306	1	db$2	2::C3KAC1|C3KAC1_PSEFS	306	27548	17	14	13	11	6.78	Putative phenylacetic ac
307	1	db$2	2::C3K1X9|C3K1X9_PSEFS	305	34909	11	11	9	9	2.87	Octaprenyl-diphosphate
308	1	db$2	2::C3K2S0|C3K2S0_PSEFS	305	39319	9	7	6	5	0.82	3,4-dihydroxy-2-butano
309	1	db$2	2::C3K3P4|C3K3P4_PSEFS	304	39333	15	14	7	6	1.32	Uncharacterized protein
310	1	db$2	2::C3K3H0|C3K3H0_PSEFS	304	67796	12	11	11	10	1.01	ATP-dependent RNA heli
311	1	db$2	2::C3K325|C3K325_PSEFS	303	49076	15	14	13	12	2.18	Chaperone SurA OS=Ps
312	1	db$2	2::C3K401|C3K401_PSEFS	303	56255	18	16	15	13	1.98	Exopolyphosphatase OS
313	1	db$2	2::C3JY85|C3JY85_PSEFS	301	48759	16	16	13	13	2.53	NADH-quinone oxidored
314	1	db$2	2::C3K0H1|C3K0H1_PSEFS	301	17848	9	9	8	8	7.12	Chemotaxis protein Che
315	1	db$2	2::C3KBQ2|C3KBQ2_PSEFS	300	37020	13	13	9	9	2.57	Putative quinone oxidor
316	1	db$2	2::C3K324|C3K324_PSEFS	300	105271	14	10	13	9	0.50	LPS-assembly protein Lp
317	1	db$2	2::C3K5Z7|C3K5Z7_PSEFS	299	26574	15	15	8	8	7.40	Uridylate kinase OS=Pse
318	1	db$2	2::C3K1X6|C3K1X6_PSEFS	299	44711	10	10	8	8	1.33	GTPase Obg OS=Pseudo
319	1	db$2	2::C3JXZ8|C3JXZ8_PSEFS	299	71568	14	13	12	11	1.07	Acetyl-coenzyme A synt
320	1	db$2	2::C3K2V7|RL15_PSEFS	298	15215	12	11	7	7	10.64	50S ribosomal protein L
321	1	db$2	2::C3K6A2|C3K6A2_PSEFS	297	27847	12	12	9	9	3.58	Putative D-methionine A
321	2	db$2	2::C3KD95|C3KD95_PSEFS	194	28020	8	8	7	7	2.25	Putative exported protei
322	1	db$2	2::C3KCN5|C3KCN5_PSEFS	295	142346	16	12	16	12	0.49	Bifunctional protein PutA
323	1	db$2	2::C3K2W4|RL5_PSEFS	294	20490	14	14	10	10	11.33	50S ribosomal protein L
324	1	db$2	2::C3K3K2|PYRB_PSEFS	294	36271	8	8	6	6	1.18	Aspartate carbamoyltrar

↑Family	M	DB	Accession	Score	Mass	Matches	Match(sig)	Sequences	Seq(sig)	emPAI	Description
325	1	db$2	2::C3KE83|C3KE83_PSEFS	290	38700	8	8	5	5	0.84	Outer membrane protei
326	1	db$2	2::C3K8X4|MURA_PSEFS	290	44975	20	16	15	12	3.84	UDP-N-acetylglucosamir
327	1	db$2	2::C3K310|C3K310_PSEFS	289	54246	12	11	10	9	1.61	Anthranilate synthase cc
328	1	db$2	2::C3JZ69|C3JZ69_PSEFS	288	25612	6	6	4	4	1.08	6-phosphogluconolacton
329	1	db$2	2::C3K422|C3K422_PSEFS	288	40250	10	10	8	8	1.55	Putative uroporphyrin-II
330	1	SwissProt_HUMAN$	3::P00761|TRYP_PIG	287	24394	10	10	6	6	2.85	Trypsin OS=Sus scrofa (
331	1	db$2	2::C3K1F2|ATP6_PSEFS	287	31626	8	8	4	4	0.81	ATP synthase subunit a
332	1	db$2	2::C3K4V8|C3K4V8_PSEFS	286	128447	11	9	10	8	0.34	Transcription-repair-cou
333	1	db$2	2::C3JXY7|ASTD_PSEFS	285	51479	8	7	8	7	0.90	N-succinylglutamate 5-s
334	1	db$2	2::C3K841|C3K841_PSEFS	284	11596	15	13	6	5	36.14	Putative sigma-54 modu
335	1	db$2	2::C3KE61|RIMK_PSEFS	284	32488	10	8	6	5	1.76	Probable alpha-L-glutam
336	1	db$2	2::C3K8V3|HSLU_PSEFS	283	49607	14	12	12	10	1.59	ATP-dependent protease
337	1	db$2	2::C3K116|PSRP_PSEFS	282	30838	10	7	10	7	1.91	Putative phosphoenolpyı
338	1	db$2	2::C3K0N2|C3K0N2_PSEFS	281	43313	7	7	6	6	1.15	3-oxoacyl-[acyl-carrier-ı
339	1	db$2	2::C3JY54|SYS_PSEFS	281	47115	12	11	12	11	2.02	Serine--tRNA ligase OS=
340	1	db$2	2::C3K527|C3K527_PSEFS	280	65564	12	10	10	8	0.92	Putative peptidase OS=I
341	1	db$2	2::C3K2S6|C3K2S6_PSEFS	280	31662	10	10	7	7	2.29	Putative thioredoxin OS
342	1	db$2	2::C3K2K3|GSA_PSEFS	278	45181	10	10	7	7	1.56	Glutamate-1-semialdehy
343	1	db$2	2::C3K2H6|C3K2H6_PSEFS	277	32512	8	8	6	6	1.39	Acyl-CoA thioesterase II
344	1	db$2	2::C3K476|PYRE_PSEFS	276	23079	6	6	5	5	1.77	Orotate phosphoribosylt
345	1	db$2	2::C3K6R3|Y2135_PSEFS	276	24050	10	10	9	9	4.79	UPF0502 protein PFLU_2
346	1	db$2	2::C3JZ68|C3JZ68_PSEFS	274	24197	11	11	8	8	4.74	2-dehydro-3-deoxy-pho
347	1	db$2	2::C3K747|GPDA_PSEFS	273	36476	7	6	5	5	1.17	Glycerol-3-phosphate de
348	1	db$2	2::C3K7C1|SECB_PSEFS	273	17615	6	5	3	3	1.21	Protein-export protein S
349	1	db$2	2::C3K0N4|C3K0N4_PSEFS	272	25612	9	7	7	6	2.61	3-oxoacyl-[acyl-carrier-ı
350	1	db$2	2::C3KAJ1|C3KAJ1_PSEFS	272	48585	12	10	10	10	1.65	Omega-amino acid--pyr
351	1	db$2	2::C3K1Y2|C3K1Y2_PSEFS	270	21662	7	7	7	7	3.56	Peptidyl-prolyl cis-trans
352	1	db$2	2::C3K8U4|UBIE_PSEFS	270	28320	16	15	13	12	6.36	Ubiquinone/menaquinon
353	1	db$2	2::C3JYV5|C3JYV5_PSEFS	269	45487	9	9	8	8	1.55	Putative oxidoreductase
354	1	db$2	2::C3K249|G6PI_PSEFS	269	61384	17	16	13	12	1.73	Glucose-6-phosphate isc
355	1	db$2	2::C3JZ61|C3JZ61_PSEFS	268	61509	12	11	8	7	0.85	Acyl-CoA synthetase (Lc
356	1	db$2	2::C3K5S8|C3K5S8_PSEFS	268	75646	15	13	12	10	0.99	Oligopeptidase A OS=Ps
357	1	db$2	2::C3K859|C3K859_PSEFS	267	59064	8	8	7	7	0.75	Putative glutamate syntl
358	1	db$2	2::C3K0G3|PPNP_PSEFS	267	10127	9	8	5	5	14.55	Pyrimidine/purine nucle
359	1	db$2	2::C3KBP9|C3KBP9_PSEFS	267	58780	8	8	7	7	0.76	Phosphoglucomutase OS
360	1	db$2	2::C3KBZ7|SYM_PSEFS	266	75525	16	13	14	11	1.12	Methionine--tRNA ligase
361	1	db$2	2::C3K638|C3K638_PSEFS	266	26394	6	6	3	3	1.04	Putative short chain deh
362	1	db$2	2::C3K6Y3|GATB_PSEFS	264	52868	11	11	8	8	1.24	Aspartyl/glutamyl-tRNA(
363	1	db$2	2::C3JYR3|C3JYR3_PSEFS	264	40135	14	13	9	9	2.64	Glycine cleavage system
364	1	db$2	2::C3KAH3|SYR_PSEFS	263	63549	8	8	8	8	0.81	Arginine--tRNA ligase OS

↑Family	M	DB	Accession	Score	Mass	Matches	Match(sig)	Sequences	Seq(sig)	emPAI	Description
365	1	db$2	2::C3K102|PUR5_PSEFS	263	36950	9	9	8	8	2.16	Phosphoribosylformylgly
366	1	db$2	2::C3K2W9|RL16_PSEFS	262	15422	7	7	6	6	5.14	50S ribosomal protein L
367	1	db$2	2::C3K7L5|C3K7L5_PSEFS	262	19131	7	6	5	5	2.41	Molybdenum cofactor bi
368	1	db$2	2::C3JZS4|C3JZS4_PSEFS	262	33375	12	11	10	10	3.10	Acetyl-coenzyme A carb
369	1	db$2	2::C3K2W6|RL14_PSEFS	261	13401	13	12	9	8	30.97	50S ribosomal protein L
370	1	db$2	2::C3K452|XPT_PSEFS	261	20267	6	6	5	5	2.99	Xanthine phosphoribosy
371	1	db$2	2::C3JZT7|C3JZT7_PSEFS	261	38793	13	9	12	9	2.00	3-isopropylmalate dehy
372	1	db$2	2::C3K0N5|C3K0N5_PSEFS	259	32402	8	8	5	5	1.39	Malonyl CoA-acyl carrier
373	1	db$2	2::C3JYD5|C3JYD5_PSEFS	259	18453	8	8	5	5	2.56	Peptidyl-prolyl cis-trans
374	1	db$2	2::C3K2Z3|C3K2Z3_PSEFS	256	44017	11	10	9	8	1.92	Tyrosine--tRNA ligase O
375	1	db$2	2::C3K2K9|C3K2K9_PSEFS	255	31417	10	9	9	8	2.32	Magnesium and cobalt e
376	1	db$2	2::C3K7X9|C3K7X9_PSEFS	254	40858	10	7	9	6	1.00	Carboxynorspermidine/c
377	1	db$2	2::C3KBX9|C3KBX9_PSEFS	253	20403	5	5	3	3	1.51	Putative phospholipid-bi
378	1	db$2	2::C3KBN3|C3KBN3_PSEFS	252	112655	14	13	11	10	0.59	Efflux pump membrane
378	2	db$2	2::C3KB18|C3KB18_PSEFS	78	115005	2	2	2	2	0.09	Efflux pump membrane
379	1	db$2	2::C3K443|C3K443_PSEFS	252	12323	11	11	8	8	28.73	Nitrogen regulatory prot
380	1	db$2	2::C3K475|ARGB_PSEFS	251	31926	9	8	9	8	2.25	Acetylglutamate kinase
381	1	db$2	2::C3K667|C3K667_PSEFS	250	48557	12	11	11	10	1.65	Conserved hypothetical
382	1	db$2	2::C3K6X1|C3K6X1_PSEFS	247	39290	10	9	9	8	1.62	Uncharacterized protein
383	1	db$2	2::C3K2X7|RS10_PSEFS	247	11745	17	16	8	8	22.43	30S ribosomal protein S
384	1	db$2	2::C3K0T1|C3K0T1_PSEFS	246	94111	8	8	6	6	0.42	Aconitate hydratase OS=
385	1	db$2	2::C3K3L7|THIG_PSEFS	246	28373	12	11	9	9	3.44	Thiazole synthase OS=P
386	1	db$2	2::C3K604|C3K604_PSEFS	245	18933	16	14	13	12	23.73	Putative outer membran
387	1	db$2	2::C3KAR0|C3KAR0_PSEFS	244	79563	7	4	6	4	0.27	Putative exported isoqui
388	1	db$2	2::C3K2Y5|RL10_PSEFS	243	17594	7	6	5	4	1.89	50S ribosomal protein L
389	1	db$2	2::C3K7M6|C3K7M6_PSEFS	243	88918	9	8	7	6	0.45	Acyl-coenzyme A dehyd
390	1	db$2	2::C3KEB4|C3KEB4_PSEFS	243	56978	12	11	12	11	1.50	Lysine--tRNA ligase OS=
391	1	db$2	2::C3KEB7|C3KEB7_PSEFS	243	48036	12	12	9	9	1.95	Uncharacterized protein
392	1	db$2	2::C3JY00|C3JY00_PSEFS	242	47211	10	10	8	8	1.23	Ribonucleoside-diphosp
393	1	db$2	2::C3KCT1|MURC_PSEFS	242	52132	8	8	6	6	0.72	UDP-N-acetylmuramate-
394	1	db$2	2::C3JZ29|C3JZ29_PSEFS	242	41047	9	8	7	7	1.24	Putative glycerophosph
395	1	db$2	2::C3K0T3|C3K0T3_PSEFS	242	28958	7	7	7	7	2.12	Putative membrane prot
396	1	db$2	2::C3KDD6|C3KDD6_PSEFS	241	33869	5	5	5	5	1.01	D-alanine--D-alanine lig
397	1	db$2	2::C3K1F4|C3K1F4_PSEFS	241	31961	8	8	7	7	1.81	Probable chromosome-p
398	1	db$2	2::C3K3M3|C3K3M3_PSEFS	239	24540	6	6	4	4	1.15	Cell division ATP-bindin
399	1	db$2	2::C3K6E1|RL13_PSEFS	239	15695	11	11	7	7	9.87	50S ribosomal protein L
400	1	db$2	2::C3JZ86|C3JZ86_PSEFS	237	21964	14	11	11	9	7.46	Superoxide dismutase O
401	1	db$2	2::C3K5H8|C3K5H8_PSEFS	237	17826	9	9	6	6	3.81	STAS domain-containin
402	1	db$2	2::C3K262|C3K262_PSEFS	236	12956	7	6	3	3	1.93	Protein-export membra
403	1	db$2	2::C3KDW3|HFQ_PSEFS	236	9318	12	11	7	7	51.14	RNA-binding protein Hfq

↑Family	M	DB	Accession	Score	Mass	Matches	Match(sig)	Sequences	Seq(sig)	emPAI	Description
404	1	db$2	2::C3K6R4|C3K6R4_PSEFS	234	18727	11	9	6	4	2.48	Uncharacterized protein
405	1	db$2	2::C3KE41|SYGA_PSEFS	234	36196	14	11	10	9	2.23	Glycine--tRNA ligase alp
406	1	db$2	2::C3K4H9|C3K4H9_PSEFS	233	34203	11	7	10	7	1.63	Quinone oxidoreductase
407	1	db$2	2::C3K2W5|RL24_PSEFS	232	11328	10	10	7	7	25.43	50S ribosomal protein L
408	1	db$2	2::C3K6Y4|GATA_PSEFS	231	51737	8	8	6	6	0.90	Glutamyl-tRNA(Gln) ami
409	1	db$2	2::C3K6S9|C3K6S9_PSEFS	231	101727	12	12	11	11	0.75	DNA polymerase I OS=F
410	1	db$2	2::C3KBL1|C3KBL1_PSEFS	231	61934	11	9	10	9	0.99	Putative medium-chain-
411	1	db$2	2::C3KDW6|C3KDW6_PSEFS	230	33055	19	15	16	12	6.36	Protein HflC OS=Pseudo
412	1	db$2	2::C3KBZ0|PYRC_PSEFS	228	38385	6	6	6	6	1.09	Dihydroorotase OS=Pse
413	1	db$2	2::C3K8Z6|C3K8Z6_PSEFS	228	39611	8	8	6	6	1.30	Branched amino acid AB
414	1	db$2	2::C3KED5|C3KED5_PSEFS	226	40447	10	9	10	9	1.86	Putative lipoprotein OS=
415	1	db$2	2::C3K3Y8|UBID_PSEFS	225	54697	6	6	6	6	0.68	3-octaprenyl-4-hydroxyl
416	1	db$2	2::C3KAK7|C3KAK7_PSEFS	225	20540	7	6	5	4	2.13	Putative lipoprotein OS=
417	1	db$2	2::C3K5J3|C3K5J3_PSEFS	225	50388	7	5	7	5	0.60	AMP nucleosidase OS=P
418	1	db$2	2::C3K6M3|C3K6M3_PSEFS	224	13365	5	4	4	3	1.85	Succinate dehydrogenas
419	1	db$2	2::C3K490|C3K490_PSEFS	224	33505	13	11	10	9	2.55	Putative oxidative stress
420	1	db$2	2::C3K469|RL28_PSEFS	223	8845	8	8	5	5	12.34	50S ribosomal protein L
421	1	db$2	2::C3K0W7|C3K0W7_PSEFS	222	31068	8	7	7	6	1.48	Metallophos_2 domain-c
422	1	SwissProt_HUMAN$	3::P0DOX7|IGK_HUMAN	221	23364	5	4	4	4	1.24	Immunoglobulin kappa l
423	1	db$2	2::C3KCT2|MUTS_PSEFS	221	95536	8	8	8	8	0.49	DNA mismatch repair pr
424	1	db$2	2::C3K2W8|RL29_PSEFS	221	7168	8	8	6	6	43.57	50S ribosomal protein L
425	1	db$2	2::C3KBY9|C3KBY9_PSEFS	221	24532	4	4	4	4	1.15	Ribonuclease T OS=Pseu
426	1	db$2	2::C3K759|C3K759_PSEFS	221	35694	6	6	5	5	0.94	Putative dehydrogenase
426	2	db$2	2::C3K0S6|C3K0S6_PSEFS	68	36802	2	2	2	2	0.29	Putative oxidoreductase
427	1	db$2	2::C3K4E1|C3K4E1_PSEFS	220	38799	8	7	7	6	1.07	N5-carboxyaminoimidaz
428	1	db$2	2::C3K4E2|C3K4E2_PSEFS	220	16860	7	7	6	6	5.95	N5-carboxyaminoimidaz
429	1	db$2	2::C3K0S3|C3K0S3_PSEFS	220	67491	8	8	8	8	0.75	Putative ATP-binding co
430	1	db$2	2::C3JZN9|C3JZN9_PSEFS	219	7652	9	9	6	6	64.55	Major cold shock protein
431	1	db$2	2::C3K1X5|C3K1X5_PSEFS	219	39754	11	10	9	9	1.91	Glutamate 5-kinase OS=
432	1	db$2	2::C3K4Q9|C3K4Q9_PSEFS	218	23656	7	7	6	6	2.29	Putative exported protei
433	1	db$2	2::C3KE31|C3KE31_PSEFS	218	22237	8	7	8	7	3.39	RNA polymerase sigma f
434	1	db$2	2::C3K1M9|C3K1M9_PSEFS	218	29586	10	10	9	9	3.18	Inositol-1-monophospha
435	1	db$2	2::C3K5N1|C3K5N1_PSEFS	218	36842	10	9	8	7	1.45	Sulfate ABC transport sy
436	1	db$2	2::C3K245|C3K245_PSEFS	217	53442	13	12	11	10	1.65	Poly(A) polymerase I OS
437	1	db$2	2::C3JYQ7|C3JYQ7_PSEFS	217	32635	7	6	7	6	1.38	Carbamate kinase OS=F
438	1	db$2	2::C3K6J7|C3K6J7_PSEFS	217	39784	10	10	9	9	1.91	Histidinol-phosphate am
439	1	db$2	2::C3JXZ7|C3JXZ7_PSEFS	216	28415	8	6	7	5	1.29	Putative histidine-bindin
440	1	db$2	2::C3K1H2|C3K1H2_PSEFS	215	49205	9	7	8	6	0.78	Signal recognition partic
441	1	db$2	2::C3K581|C3K581_PSEFS	215	86479	10	10	10	10	0.73	Putative molybdopterin
442	1	db$2	2::C3JZS5|TRPF_PSEFS	215	21966	3	3	2	2	0.53	N-(5'-phosphoribosyl)an

↑Family	M	DB	Accession	Score	Mass	Matches	Match(sig)	Sequences	Seq(sig)	emPAI	Description
443	1	db$2	2::C3JYT9|C3JYT9_PSEFS	215	46635	8	8	6	6	0.84	Putative family S43 non
444	1	db$2	2::C3KAA8|C3KAA8_PSEFS	215	132643	8	8	8	8	0.33	Histidine kinase OS=Pse
445	1	db$2	2::C3JXZ2|C3JXZ2_PSEFS	214	36334	8	7	6	6	1.18	Putative AraC family reg
446	1	db$2	2::C3KBL3|C3KBL3_PSEFS	214	50616	11	9	9	8	1.11	Putative exported protei
447	1	db$2	2::C3K3T9|C3K3T9_PSEFS	213	49785	8	7	7	6	0.77	Putative aminotransfera
448	1	db$2	2::C3K610|C3K610_PSEFS	213	70283	13	13	13	13	1.40	ATP-binding protein Uup
449	1	db$2	2::C3JYI0|C3JYI0_PSEFS	212	25235	8	8	7	7	2.69	Probable transcriptional
450	1	db$2	2::C3JY62|C3JY62_PSEFS	212	83224	16	16	14	14	1.22	ATP-dependent Clp prot
450	2	db$2	2::C3K4B3|C3K4B3_PSEFS	78	97007	7	6	7	6	0.34	Chaperone OS=Pseudon
451	1	db$2	2::C3K0I1|C3K0I1_PSEFS	212	29699	6	6	6	6	1.59	ParA family ATPase OS=
452	1	db$2	2::C3K5L8|C3K5L8_PSEFS	211	39360	6	6	5	5	1.05	Phosphorous compound:
453	1	db$2	2::C3KDB7|C3KDB7_PSEFS	211	65793	6	6	6	6	0.54	ATP-dependent lipid A-c
454	1	db$2	2::C3K4V4|C3K4V4_PSEFS	211	32182	13	10	11	9	3.33	Putative ATP-binding pr
455	1	db$2	2::C3K5X7|C3K5X7_PSEFS	211	42054	9	9	9	9	1.75	Cell division protein Zap
456	1	db$2	2::C3K5X4|C3K5X4_PSEFS	209	16332	8	8	8	8	8.88	Putative membrane prot
457	1	db$2	2::C3K187|C3K187_PSEFS	209	38556	12	11	9	8	1.67	Peptidase_M75 domain-
458	1	db$2	2::C3JZ71|C3JZ71_PSEFS	209	31565	9	7	9	7	1.85	Hex regulon repressor C
459	1	db$2	2::C3K0P6|DCTA_PSEFS	209	46761	5	5	4	4	0.50	C4-dicarboxylate transp
460	1	db$2	2::C3K0Q9|C3K0Q9_PSEFS	208	15688	7	5	4	4	2.30	Flagellar basal-body rod
461	1	db$2	2::C3KA77|C3KA77_PSEFS	207	49108	10	8	9	8	1.16	Glutathione reductase O
462	1	db$2	2::C3K3X2|C3K3X2_PSEFS	207	19739	9	9	6	6	3.15	Uncharacterized protein
463	1	db$2	2::C3K1L1|C3K1L1_PSEFS	205	40864	8	8	7	7	1.25	Outer membrane proteir
464	1	db$2	2::C3KDM0|C3KDM0_PSEFS	205	69594	10	10	10	10	0.98	DNA topoisomerase 4 su
465	1	db$2	2::C3K0K7|C3K0K7_PSEFS	205	60595	12	11	11	10	1.18	Electron transfer flavopr
466	1	db$2	2::C3K9P3|C3K9P3_PSEFS	204	9882	7	7	5	5	9.34	Peptidyl-prolyl cis-trans
467	1	db$2	2::C3KBQ3|C3KBQ3_PSEFS	204	33274	7	7	6	6	1.34	LysR-family transcriptio
468	1	db$2	2::C3K3U0|C3K3U0_PSEFS	203	51001	9	9	8	8	1.10	Putative glutamine syntl
469	1	db$2	2::C3K3D2|BETB_PSEFS	202	53123	10	10	9	9	1.23	Betaine aldehyde dehyd
470	1	db$2	2::C3KAY1|C3KAY1_PSEFS	200	44657	8	6	7	5	0.89	3-phosphoshikimate 1-c
471	1	db$2	2::C3K3L0|METXS_PSEFS	200	41664	7	6	6	5	0.98	Homoserine O-succinyltı
472	1	db$2	2::C3K7I1|C3K7I1_PSEFS	199	85859	6	5	5	5	0.32	Quinoprotein glucose de
473	1	db$2	2::C3K6L0|EFP_PSEFS	199	21303	8	8	6	6	2.75	Elongation factor P OS=
474	1	db$2	2::C3K017|PYRD_PSEFS	199	35850	11	10	9	9	2.73	Dihydroorotate dehydro
475	1	db$2	2::C3JXY9|C3JXY9_PSEFS	199	37073	7	7	6	6	1.44	Putative arginine N-succ
476	1	db$2	2::C3K3W7|C3K3W7_PSEFS	198	36662	9	9	7	7	1.80	Putative iron (III) ABC t
477	1	db$2	2::C3K5H5|C3K5H5_PSEFS	198	25721	9	7	6	4	1.49	Putative exported protei
478	1	db$2	2::C3K6Z0|PDXJ_PSEFS	198	26758	6	6	5	5	1.41	Pyridoxine 5'-phosphate
479	1	db$2	2::C3K2U9|C3K2U9_PSEFS	198	53651	13	8	12	7	0.85	Catalase OS=Pseudomo
480	1	db$2	2::C3K2S1|C3K2S1_PSEFS	197	23236	8	7	7	6	2.37	Riboflavin synthase alph
481	1	db$2	2::C3K2X2|RS19_PSEFS	197	10313	7	7	5	5	8.38	30S ribosomal protein S

↑Family	M	DB	Accession	Score	Mass	Matches	Match(sig)	Sequences	Seq(sig)	emPAI	Description
482	1	db$2	2::C3K6U9|HIS7_PSEFS	197	21830	8	7	5	5	2.63	Imidazoleglycerol-phosp
483	1	db$2	2::C3K8K2|C3K8K2_PSEFS	197	418093	6	5	6	5	0.06	Haemagg_act domain-co
484	1	db$2	2::C3K403|C3K403_PSEFS	195	36964	7	7	5	5	1.15	Delta-aminolevulinic aci
485	1	db$2	2::C3K091|C3K091_PSEFS	195	20089	8	8	7	7	4.12	GTP cyclohydrolase 1 OS
485	2	db$2	2::C3K1W5|C3K1W5_PSEFS	79	20960	4	4	4	4	1.45	GTP cyclohydrolase 1 OS
486	1	db$2	2::C3K0S7|Y4577_PSEFS	195	11900	5	5	3	3	3.77	Nucleoid-associated prot
487	1	db$2	2::C3K240|C3K240_PSEFS	195	17616	9	9	6	6	5.39	RNA polymerase-binding
488	1	db$2	2::C3JY91|C3JY91_PSEFS	194	66398	4	4	3	3	0.24	NADH-quinone oxidored
489	1	db$2	2::C3K6S5|C3K6S5_PSEFS	194	35353	6	6	6	6	1.23	Delta-aminolevulinic aci
490	1	db$2	2::C3KB19|C3KB19_PSEFS	194	44434	7	7	7	7	1.11	HlyD family secretion pr
491	1	db$2	2::C3K0D3|MINC_PSEFS	194	26249	10	9	7	6	2.52	Probable septum site-de
492	1	db$2	2::C3K5A9|RF3_PSEFS	193	59584	8	7	6	5	0.49	Peptide chain release fa
493	1	db$2	2::C3JYE4|C3JYE4_PSEFS	193	30882	14	13	11	11	4.36	Exodeoxyribonuclease II
494	1	db$2	2::C3KAH1|HSLV_PSEFS	193	18731	5	5	4	4	2.48	ATP-dependent protease
495	1	db$2	2::C3K818|C3K818_PSEFS	193	29926	10	10	9	9	3.12	Putative ABC transport s
496	1	db$2	2::C3KC10|C3KC10_PSEFS	192	23080	6	6	4	4	1.26	Uncharacterized protein
497	1	db$2	2::C3K4A8|C3K4A8_PSEFS	192	55832	9	9	8	8	1.14	Uncharacterized protein
498	1	db$2	2::C3K4G1|C3K4G1_PSEFS	192	81186	7	6	7	6	0.42	DNA helicase OS=Pseud
498	2	db$2	2::C3K5Z0|C3K5Z0_PSEFS	64	15508	5	4	4	3	1.46	Uncharacterized protein
499	1	db$2	2::C3K276|GRPE_PSEFS	191	20714	4	3	3	2	0.57	Protein GrpE OS=Pseud
500	1	db$2	2::C3JYT6|C3JYT6_PSEFS	190	8291	7	7	5	5	26.35	Cold shock protein cspc
501	1	db$2	2::C3K254|C3K254_PSEFS	190	21027	8	7	6	5	2.06	Osmotically inducible pr
502	1	db$2	2::C3KE79|C3KE79_PSEFS	190	29713	3	3	2	2	0.37	NH(3)-dependent NAD(-
503	1	db$2	2::C3KDW7|HISZ_PSEFS	189	42687	13	12	10	10	2.02	ATP phosphoribosyltrans
504	1	db$2	2::C3K4E4|C3K4E4_PSEFS	187	51389	8	7	8	7	0.90	Aspartate ammonia-lyas
505	1	db$2	2::C3K862|C3K862_PSEFS	187	39825	6	6	5	5	0.81	Putative outer membran
506	1	db$2	2::C3K7M9|C3K7M9_PSEFS	186	34271	6	6	6	6	1.28	Putative ABC transport s
507	1	db$2	2::C3K368|C3K368_PSEFS	186	37353	6	6	5	5	0.88	Stress response kinase /
508	1	db$2	2::C3K104|C3K104_PSEFS	186	26102	8	8	6	6	2.53	Putative membrane prot
509	1	db$2	2::C3KDC6|RF1_PSEFS	185	39857	4	4	4	4	0.61	Peptide chain release fa
510	1	db$2	2::C3K7G7|C3K7G7_PSEFS	185	50136	8	7	8	7	0.93	Putative modulation of D
511	1	db$2	2::C3K177|C3K177_PSEFS	185	65030	13	12	10	9	1.07	Phosphogluconate dehyd
512	1	db$2	2::C3JXZ3|C3JXZ3_PSEFS	185	28123	7	6	7	6	1.73	Histidine transport ATP-l
513	1	db$2	2::C3K2C9|C3K2C9_PSEFS	185	48295	8	6	8	6	0.80	Glutamate dehydrogena
514	1	db$2	2::C3KAJ8|UPP_PSEFS	184	22986	10	10	8	8	5.29	Uracil phosphoribosyltra
515	1	db$2	2::C3JYU1|Y4927_PSEFS	184	18225	8	7	5	5	2.62	UPF0234 protein PFLU_4
516	1	db$2	2::C3KCL0|C3KCL0_PSEFS	183	35424	7	7	6	6	1.22	Uncharacterized protein
517	1	db$2	2::C3K264|GLMM_PSEFS	183	47719	13	11	12	10	1.69	Phosphoglucosamine mu
518	1	db$2	2::C3JZ43|C3JZ43_PSEFS	183	44885	6	6	6	6	0.88	Putative aminotransfera
519	1	db$2	2::C3K142|C3K142_PSEFS	182	32099	9	8	7	6	1.80	2-methylisocitrate lyase

↑Family	M	DB	Accession	Score	Mass	Matches	Match(sig)	Sequences	Seq(sig)	emPAI	Description
520	1	db$2	2::C3KE68|RS18_PSEFS	182	8912	7	7	5	5	12.34	30S ribosomal protein S
521	1	db$2	2::C3K6G8|LEPA_PSEFS	182	66372	9	8	7	6	0.53	Elongation factor 4 OS=
522	1	db$2	2::C3K4C1|C3K4C1_PSEFS	182	40318	5	5	3	3	0.42	Putative nitric oxide det
523	1	db$2	2::C3KCP5|C3KCP5_PSEFS	182	109710	8	7	8	7	0.35	Bifunctional glutamine s
524	1	db$2	2::C3JZT9|C3JZT9_PSEFS	181	24210	7	7	7	7	2.89	3-isopropylmalate dehyd
525	1	db$2	2::C3KDL6|C3KDL6_PSEFS	181	23139	6	6	4	4	1.26	ADP-ribose diphosphata
526	1	db$2	2::C3KDB1|C3KDB1_PSEFS	180	65565	13	12	12	12	1.38	Putative carbamoyltrans
527	1	db$2	2::C3K8W7|C3K8W7_PSEFS	180	34291	4	4	4	4	0.73	Arabinose 5-phosphate i
528	1	db$2	2::C3KAC7|C3KAC7_PSEFS	180	51379	10	7	8	5	0.74	UDP-glucose 6-dehydrog
529	1	db$2	2::C3K4P8|C3K4P8_PSEFS	177	24008	4	4	2	2	0.80	t(6)A37 threonylcarbam
530	1	db$2	2::C3K740|C3K740_PSEFS	177	33777	15	11	13	11	3.65	DUF815 domain-contain
531	1	db$2	2::C3K8Y8|C3K8Y8_PSEFS	177	23915	6	6	5	5	1.68	Putative isomerase OS=
532	1	db$2	2::C3K3A2|C3K3A2_PSEFS	177	38105	8	7	5	5	0.86	L-threonine aldolase OS
533	1	db$2	2::C3KBN4|C3KBN4_PSEFS	176	40532	6	6	5	5	1.01	Acriflavine resistance pr
534	1	db$2	2::C3K472|C3K472_PSEFS	176	42804	7	7	6	6	1.16	Coenzyme A biosynthesi
535	1	db$2	2::C3K307|TRPC_PSEFS	176	30252	8	8	8	8	2.47	Indole-3-glycerol phospl
536	1	db$2	2::C3K2E9|C3K2E9_PSEFS	176	53251	6	4	4	3	0.43	Putative cytochrome oxi
537	1	SwissProt_HUMAN$	3::P0DOX6|IGM_HUMAN	176	63446	7	7	7	7	0.69	Immunoglobulin mu hea
538	1	db$2	2::C3K2V3|RS11_PSEFS	175	13607	6	5	3	3	1.78	30S ribosomal protein S
539	1	db$2	2::C3K3Q8|TYSY_PSEFS	174	31490	6	6	4	4	1.11	Thymidylate synthase O
540	1	db$2	2::C3K843|C3K843_PSEFS	174	26535	8	8	5	5	1.43	Lipopolysaccharide expo
541	1	db$2	2::C3JZU0|C3JZU0_PSEFS	173	50538	6	6	6	6	0.75	3-isopropylmalate dehyd
542	1	db$2	2::C3K8U2|UBIB_PSEFS	173	61616	6	6	6	6	0.59	Probable protein kinase
543	1	db$2	2::C3K3S0|C3K3S0_PSEFS	171	23721	6	6	4	4	1.21	Putative hydrolase OS=I
544	1	db$2	2::C3K645|C3K645_PSEFS	171	24522	6	6	6	6	2.16	Putative ABC transports
545	1	db$2	2::C3K651|C3K651_PSEFS	171	39766	10	9	9	9	1.91	Phospho-2-dehydro-3-d
546	1	db$2	2::C3K796|C3K796_PSEFS	171	49835	5	5	5	5	0.61	Putative glutamine syntl
547	1	db$2	2::C3JYJ6|C3JYJ6_PSEFS	170	68407	9	9	8	8	0.74	Peptidyl-prolyl cis-trans
548	1	db$2	2::C3K308|TRPD_PSEFS	170	36976	7	7	6	6	1.15	Anthranilate phosphorib
549	1	db$2	2::C3JZQ0|C3JZQ0_PSEFS	169	38665	8	8	8	8	1.66	Putative L-asparaginase
550	1	db$2	2::C3K148|APT_PSEFS	169	20064	6	6	5	5	2.21	Adenine phosphoribosylt
551	1	db$2	2::C3K968|C3K968_PSEFS	168	55197	8	8	7	7	0.82	Putative aldehyde dehyd
552	1	db$2	2::C3K575|C3K575_PSEFS	168	14827	4	3	4	3	1.56	Osmotically inducible pr
553	1	db$2	2::C3KBH8|C3KBH8_PSEFS	167	25745	5	5	4	4	1.08	3-oxoacyl-[acyl-carrier-|
554	1	db$2	2::C3K733|HTPX_PSEFS	166	32382	7	6	5	4	0.79	Protease HtpX OS=Pseu
555	1	db$2	2::C3K084|AROC_PSEFS	166	39094	7	6	5	4	0.83	Chorismate synthase OS
556	1	db$2	2::C3K1U7|C3K1U7_PSEFS	166	36908	4	4	2	2	0.47	Ubiquinol oxidase subun
557	1	db$2	2::C3K703|ISPF_PSEFS	166	16742	5	4	4	3	1.31	2-C-methyl-D-erythritol
558	1	db$2	2::C3K9M0|C3K9M0_PSEFS	165	11184	4	3	3	2	1.29	SCP2 domain-containing
559	1	db$2	2::C3K9S0|NFUA_PSEFS	165	20961	5	5	5	5	2.06	Fe/S biogenesis protein

↑Family	M	DB	Accession	Score	Mass	Matches	Match(sig)	Sequences	Seq(sig)	emPAI	Description
560	1	db$2	2::C3K611|C3K611_PSEFS	165	16065	5	5	3	3	2.19	Universal stress protein
561	1	db$2	2::C3JXQ1|C3JXQ1_PSEFS	164	24479	4	4	3	3	0.78	Putative enoyl-CoA hydr
562	1	db$2	2::C3K1E4|GLMU_PSEFS	164	48690	7	7	7	7	0.97	Bifunctional protein Glm
563	1	db$2	2::C3KCT0|MURG_PSEFS	163	37667	8	7	7	6	1.12	UDP-N-acetylglucosamir
564	1	db$2	2::C3K5T5|C3K5T5_PSEFS	162	25405	4	4	2	2	0.45	Carbonic anhydrase OS=
565	1	db$2	2::C3K6T0|ENGB_PSEFS	162	23537	5	5	4	4	1.22	Probable GTP-binding pr
566	1	db$2	2::C3KE99|C3KE99_PSEFS	162	30145	5	4	5	4	0.87	Quinolinate phosphoribo
567	1	db$2	2::C3K185|C3K185_PSEFS	161	20062	6	6	4	4	3.06	Putative NAD(P)H nitror
568	1	db$2	2::C3K281|C3K281_PSEFS	161	15787	4	4	3	3	1.43	Ribosome association to
569	1	db$2	2::C3K2Q0|C3K2Q0_PSEFS	161	19348	8	6	6	5	3.29	Inorganic pyrophosphat
570	1	db$2	2::C3K480|C3K480_PSEFS	161	31946	6	4	5	3	0.56	Uncharacterized protein
571	1	db$2	2::C3KEC6|KAD_PSEFS	161	23251	8	8	5	5	2.37	Adenylate kinase OS=Ps
572	1	db$2	2::C3KAM2|C3KAM2_PSEFS	160	49640	6	6	5	5	0.61	Putative two-component
573	1	db$2	2::C3JYN4|ZIPA_PSEFS	160	31818	5	5	4	4	0.81	Cell division protein Zip/
574	1	db$2	2::C3K1V6|C3K1V6_PSEFS	160	32555	6	5	6	5	1.06	Putative short-chain deh
575	1	db$2	2::C3KDU8|C3KDU8_PSEFS	160	35029	7	7	6	6	1.24	D-cysteine desulfhydras
576	1	db$2	2::C3K1X7|RL27_PSEFS	159	9214	7	7	4	4	6.39	50S ribosomal protein L
577	1	db$2	2::C3K4V5|C3K4V5_PSEFS	159	36313	5	5	4	4	0.68	Beta-hexosaminidase O
578	1	db$2	2::C3KDE9|C3KDE9_PSEFS	159	41781	4	4	4	4	0.57	Putative phage tail-relat
579	1	db$2	2::C3K6K4|C3K6K4_PSEFS	159	32908	6	6	6	6	1.37	Putative 5'-nucleotidase
580	1	db$2	2::C3KED4|C3KED4_PSEFS	159	31038	7	6	6	5	1.13	4-hydroxy-tetrahydrodip
581	1	db$2	2::C3JY87|NUOH_PSEFS	159	37530	8	8	4	4	1.13	NADH-quinone oxidored
582	1	db$2	2::C3K2L6|C3K2L6_PSEFS	159	47731	5	5	5	5	0.64	Putative lipoprotein OS=
583	1	db$2	2::C3K3M4|C3K3M4_PSEFS	159	49883	7	7	7	7	0.94	Signal recognition partic
584	1	db$2	2::C3K470|C3K470_PSEFS	158	58011	6	6	5	5	0.50	Putative transport-relate
585	1	db$2	2::C3K6F9|C3K6F9_PSEFS	158	37389	8	7	8	7	1.42	Nucleoid-associated prot
586	1	db$2	2::C3KBM6|C3KBM6_PSEFS	158	11934	7	6	5	4	5.93	Glutaredoxin OS=Pseud
587	1	db$2	2::C3K0W5|PHS_PSEFS	157	13357	6	6	4	4	4.73	Putative pterin-4-alpha-
588	1	db$2	2::C3K868|C3K868_PSEFS	157	58117	8	7	8	7	0.77	Uncharacterized protein
589	1	db$2	2::C3JYQ9|C3JYQ9_PSEFS	157	54767	5	4	5	4	0.41	Transcriptional regulato
590	1	db$2	2::C3K785|C3K785_PSEFS	157	24962	5	5	4	4	1.12	3-oxoadipate CoA-trans
591	1	db$2	2::C3K6J5|SERC_PSEFS	157	39799	8	7	8	7	1.29	Phosphoserine aminotra
592	1	db$2	2::C3K4I0|HEM6_PSEFS	156	35313	9	8	9	8	1.91	Oxygen-dependent copr
593	1	db$2	2::C3KBK9|C3KBK9_PSEFS	156	34365	5	5	5	5	0.98	Glycerate dehydrogenas
594	1	db$2	2::C3K606|FABZ_PSEFS	155	16691	8	8	7	7	6.13	3-hydroxyacyl-[acyl-car
595	1	db$2	2::C3K4T4|C3K4T4_PSEFS	155	21507	4	4	4	4	1.39	NAD(P)-bd_dom domain
596	1	db$2	2::C3JYD4|C3JYD4_PSEFS	155	64707	11	10	10	9	0.93	Glutamine--tRNA ligase
597	1	db$2	2::C3K0V5|C3K0V5_PSEFS	155	33212	6	6	5	5	1.35	Putative keto/oxo acyl-A
598	1	db$2	2::C3KE48|C3KE48_PSEFS	155	19341	6	6	6	6	3.29	Peptide deformylase OS
599	1	db$2	2::C3JYU5|C3JYU5_PSEFS	155	34090	5	5	4	4	0.74	Nudix hydrolase domain

↑Family	M	DB	Accession	Score	Mass	Matches	Match(sig)	Sequences	Seq(sig)	emPAI	Description
600	1	db$2	2::C3K6I1|C3K6I1_PSEFS	154	31224	5	5	4	4	1.13	S-formylglutathione hyd
601	1	db$2	2::C3K5E5|C3K5E5_PSEFS	154	28503	9	9	7	7	2.75	Methionine aminopeptid
602	1	db$2	2::C3K8W6|C3K8W6_PSEFS	154	18885	5	4	5	4	1.70	3-deoxy-D-manno-octul
603	1	db$2	2::C3K5H7|C3K5H7_PSEFS	154	43348	7	7	5	5	0.92	Putative response regula
604	1	db$2	2::C3K1A1|C3K1A1_PSEFS	154	10298	6	6	5	5	8.38	10 kDa chaperonin OS=
605	1	db$2	2::C3K4G2|C3K4G2_PSEFS	154	30728	9	8	5	5	1.92	Putative exported protei
606	1	db$2	2::C3K3G7|C3K3G7_PSEFS	153	31253	7	7	7	7	1.87	Methylenetetrahydrofola
607	1	db$2	2::C3K2I0|C3K2I0_PSEFS	153	48538	8	8	8	8	1.18	Putative ATP-dependent
608	1	db$2	2::C3KE80|C3KE80_PSEFS	153	46502	7	7	7	7	1.04	Nicotinate phosphoribos
609	1	db$2	2::C3K2J9|C3K2J9_PSEFS	153	60860	3	3	2	2	0.26	Putative dehydrogenase
610	1	db$2	2::C3K6E3|C3K6E3_PSEFS	152	21087	6	5	4	3	0.95	Ubiquinol-cytochrome c
611	1	db$2	2::C3KDX7|ISPH_PSEFS	152	34499	8	7	7	6	1.27	4-hydroxy-3-methylbut-
612	1	db$2	2::C3K3R6|C3K3R6_PSEFS	152	54762	9	8	8	7	0.83	L-threonine dehydratase
613	1	db$2	2::C3K134|C3K134_PSEFS	152	52371	5	5	4	4	0.44	Coproporphyrinogen-III
614	1	db$2	2::C3KBW8|C3KBW8_PSEFS	151	39031	7	5	7	5	0.83	D-alanine--D-alanine lig
615	1	db$2	2::C3KDR6|C3KDR6_PSEFS	151	40570	5	4	5	4	0.59	Putaive NADH oxidoredu
616	1	SwissProt_HUMAN$	3::Q5VTE0|EF1A3_HUMAN	151	50153	7	6	7	6	0.76	Putative elongation facto
617	1	db$2	2::C3K062|C3K062_PSEFS	151	48996	9	7	8	6	0.79	Fumarate hydratase clas
618	1	SwissProt_HUMAN$	3::P62805|H4_HUMAN	150	11360	5	5	5	5	6.74	Histone H4 OS=Homo sa
619	1	db$2	2::C3JZ88|C3JZ88_PSEFS	150	47232	6	5	4	3	0.65	Putative exported protei
620	1	SwissProt_HUMAN$	3::P23528|COF1_HUMAN	150	18491	7	7	6	6	3.55	Cofilin-1 OS=Homo sapi
621	1	db$2	2::C3K3W3|C3K3W3_PSEFS	150	41294	3	3	3	3	0.41	Putative aminotransfera
622	1	db$2	2::C3KDG2|C3KDG2_PSEFS	149	41589	7	6	6	5	0.98	AMP nucleosidase OS=P
623	1	db$2	2::C3K1N4|C3K1N4_PSEFS	149	41398	5	5	3	3	0.58	Queuine tRNA-ribosyltra
624	1	db$2	2::C3K6Z7|C3K6Z7_PSEFS	149	89469	8	8	8	8	0.53	Lon protease OS=Pseud
625	1	db$2	2::C3K3V5|C3K3V5_PSEFS	149	52101	6	6	4	4	0.44	CYTH domain-containing
626	1	db$2	2::C3K5Y8|C3K5Y8_PSEFS	148	48751	4	4	4	4	0.47	L-serine dehydratase OS
627	1	db$2	2::C3K6S8|C3K6S8_PSEFS	148	40355	8	8	8	8	1.55	Putative acetyl-CoA tran
628	1	db$2	2::C3K5U5|C3K5U5_PSEFS	147	32335	6	6	5	5	1.07	Glucose-1-phosphate th
629	1	db$2	2::C3K371|C3K371_PSEFS	147	78727	8	7	8	7	0.52	Malate synthase G OS=I
630	1	db$2	2::C3K3S3|C3K3S3_PSEFS	147	15261	3	3	2	2	0.85	DUF4399 domain-contai
631	1	db$2	2::C3JXP7|RECR_PSEFS	147	21528	8	7	6	6	2.70	Recombination protein F
632	1	db$2	2::C3K1H1|RS16_PSEFS	146	9202	7	6	5	4	6.39	30S ribosomal protein S
633	1	db$2	2::C3JYS3|C3JYS3_PSEFS	146	38665	4	3	4	3	0.44	TolA colicin import mem
634	1	db$2	2::C3K605|LPXD_PSEFS	146	36124	4	4	4	4	0.69	UDP-3-O-acylglucosamir
635	1	db$2	2::C3K3S1|C3K3S1_PSEFS	145	51070	6	5	6	5	0.59	Putative oxidase OS=Ps
636	1	db$2	2::C3K2U8|C3K2U8_PSEFS	145	18135	6	5	6	5	2.65	Bacterioferritin OS=Pseu
637	1	db$2	2::C3KDM7|C3KDM7_PSEFS	145	29519	7	6	7	6	1.60	Sulfurtransferase OS=Ps
638	1	db$2	2::C3KAC2|C3KAC2_PSEFS	144	41342	4	4	4	4	0.58	Acyl-CoA dehydrogenase
639	1	SwissProt_HUMAN$	3::Q5QNW6|H2B2F_HUMAN	144	13912	7	7	5	5	6.49	Histone H2B type 2-F OS

↑Family	M	DB	Accession	Score	Mass	Matches	Match(sig)	Sequences	Seq(sig)	emPAI	Description
640	1	db$2	2::C3K3T6|C3K3T6_PSEFS	144	42390	7	5	6	5	0.75	Spermidine/putrescine i
641	1	db$2	2::C3K3K3|C3K3K3_PSEFS	144	44101	7	7	5	5	0.71	Dihydroorotase-like prot
642	1	db$2	2::C3K568|TRPA_PSEFS	143	28539	4	4	4	4	0.94	Tryptophan synthase alp
643	1	db$2	2::C3JXY2|C3JXY2_PSEFS	143	35731	5	4	4	3	0.70	L-allo-threonine aldolase
644	1	db$2	2::C3JYI3|PHNX_PSEFS	142	29973	6	4	4	3	0.88	Phosphonoacetaldehyde
645	1	db$2	2::C3K9U7|C3K9U7_PSEFS	142	56147	7	7	7	7	0.80	Glucose-6-phosphate 1-
646	1	db$2	2::C3K1C9|C3K1C9_PSEFS	142	65670	3	2	2	1	0.07	Putative cation-transpor
647	1	SwissProt_HUMAN$	3::P62937|PPIA_HUMAN	142	18001	5	5	4	4	1.83	Peptidyl-prolyl cis-trans
648	1	db$2	2::C3JYS5|C3JYS5_PSEFS	142	25332	5	5	5	5	1.53	Tol-Pal system protein T
649	1	db$2	2::C3JZN1|IHFA_PSEFS	142	11469	6	6	5	5	6.59	Integration host factor s
650	1	db$2	2::C3KDB9|C3KDB9_PSEFS	141	32977	3	3	3	3	0.54	Putative lipopolysacchar
651	1	SwissProt_HUMAN$	3::P0DOX5|IGG1_HUMAN	141	49298	7	6	5	5	0.62	Immunoglobulin gamma
651	2	SwissProt_HUMAN$	3::P01860|IGHG3_HUMAN	120	41260	5	4	4	4	0.58	Immunoglobulin heavy c
652	1	db$2	2::C3K304|C3K304_PSEFS	141	15054	3	3	3	3	1.53	Uncharacterized protein
653	1	db$2	2::C3KDX0|C3KDX0_PSEFS	141	16992	5	5	4	4	2.01	Peptidyl-prolyl cis-trans
654	1	SwissProt_HUMAN$	3::P27348|1433T_HUMAN	141	27747	2	2	2	2	0.40	14-3-3 protein theta OS
655	1	db$2	2::C3K3P5|C3K3P5_PSEFS	140	48453	4	4	3	3	0.34	Putative symporter mem
656	1	db$2	2::B0B0A5|DNAA_PSEFS	140	56252	13	9	10	7	0.80	Chromosomal replicatior
657	1	db$2	2::C3K3M9|C3K3M9_PSEFS	140	30473	4	4	4	4	0.85	Putative sulfurtransferas
658	1	db$2	2::C3K3L5|C3K3L5_PSEFS	139	11400	3	2	2	1	1.25	Putative membrane prot
659	1	db$2	2::C3K5E4|GLND_PSEFS	138	102948	9	7	8	7	0.38	Bifunctional uridylyltrans
660	1	db$2	2::C3K8U7|C3K8U7_PSEFS	138	30923	4	4	3	3	0.58	Transcriptional regulatoı
661	1	db$2	2::C3JY88|C3JY88_PSEFS	138	20432	8	8	7	7	3.99	NADH-quinone oxidored
662	1	db$2	2::C3K6J1|UBIG_PSEFS	137	26040	5	5	4	4	1.46	Ubiquinone biosynthesis
663	1	db$2	2::C3KAI3|AROB_PSEFS	137	39825	6	6	5	5	0.81	3-dehydroquinate synth
664	1	db$2	2::C3K5G2|C3K5G2_PSEFS	137	35788	4	3	4	3	0.49	Putative membrane prot
665	1	db$2	2::C3KDF0|C3KDF0_PSEFS	137	18221	4	4	4	4	1.80	Putative phage tail-relat
666	1	db$2	2::C3K2N8|C3K2N8_PSEFS	136	23444	6	6	6	6	2.33	Semialdhyde_dh domair
667	1	db$2	2::C3K6A5|C3K6A5_PSEFS	136	78572	6	6	6	6	0.44	Catalase OS=Pseudomo
668	1	db$2	2::C3JYI6|C3JYI6_PSEFS	136	73112	5	4	5	4	0.30	Putative 2,4-dienoyl-Co/
669	1	db$2	2::C3KDQ9|C3KDQ9_PSEFS	136	29676	8	7	7	6	1.60	Ferredoxin--NADP reduc
670	1	db$2	2::C3K3L3|C3K3L3_PSEFS	136	21091	5	4	4	3	0.95	dITP/XTP pyrophosphata
671	1	db$2	2::C3K9N1|C3K9N1_PSEFS	136	43216	8	8	6	6	0.93	D-ser_dehydrat domain-
672	1	db$2	2::C3K437|C3K437_PSEFS	136	26506	2	2	1	1	0.19	Uncharacterized protein
673	1	db$2	2::C3K839|Y879_PSEFS	136	32120	6	6	6	6	1.42	Nucleotide-binding prote
674	1	db$2	2::C3K419|C3K419_PSEFS	135	17100	4	3	3	2	1.27	Transcriptional regulatoı
675	1	SwissProt_HUMAN$	3::P07195|LDHB_HUMAN	135	36615	5	3	3	2	0.29	L-lactate dehydrogenase
675	2	SwissProt_HUMAN$	3::P00338|LDHA_HUMAN	110	36665	3	3	2	2	0.29	L-lactate dehydrogenase
676	1	db$2	2::C3K5X2|C3K5X2_PSEFS	135	20559	6	6	4	4	1.49	Putative two-component
677	1	db$2	2::C3KDG5|C3KDG5_PSEFS	135	23599	5	5	4	4	1.21	Putative regulatory prot

↑Family	M	DB	Accession	Score	Mass	Matches	Match(sig)	Sequences	Seq(sig)	emPAI	Description
678	1	db$2	2::C3KDN5|C3KDN5_PSEFS	135	35878	4	3	4	3	0.48	Renalase OS=Pseudomc
679	1	db$2	2::C3JZV1|C3JZV1_PSEFS	135	76010	8	7	8	7	0.55	UvrABC system protein I
680	1	db$2	2::C3KBL7|C3KBL7_PSEFS	135	33413	7	7	7	7	1.69	Glutamate/aspartate AB
681	1	db$2	2::C3K9R8|C3K9R8_PSEFS	134	135461	6	5	6	5	0.19	Methionine synthase OS
682	1	db$2	2::C3K6U2|C3K6U2_PSEFS	134	47692	6	5	6	5	0.64	Probable proton/glutama
683	1	db$2	2::C3KA90|C3KA90_PSEFS	134	36984	4	3	4	3	0.47	Putative aldo/keto reduc
684	1	db$2	2::C3K601|DXR_PSEFS	134	42341	3	3	3	3	0.40	1-deoxy-D-xylulose 5-pl
685	1	db$2	2::C3K231|C3K231_PSEFS	133	57678	5	4	5	4	0.39	APH domain-containing
686	1	db$2	2::C3K2V4|RS13_PSEFS	133	13332	8	5	5	4	3.04	30S ribosomal protein S
687	1	db$2	2::C3JXX2|C3JXX2_PSEFS	133	50328	8	8	8	8	1.12	ATP-dependent RNA heli
688	1	db$2	2::C3JY53|CYSG_PSEFS	132	50259	5	5	5	5	0.60	Siroheme synthase OS=
689	1	db$2	2::C3K2U3|C3K2U3_PSEFS	132	27946	4	3	3	2	0.40	Trans-aconitate 2-methy
690	1	db$2	2::C3K1B1|C3K1B1_PSEFS	132	46678	3	3	3	3	0.35	Putative acid tolerance p
690	2	db$2	2::C3K483|C3K483_PSEFS	83	78630	5	5	5	5	0.35	Guanosine-3',5'-bis(Dipl
691	1	db$2	2::C3K2J5|C3K2J5_PSEFS	132	18057	4	4	4	4	1.81	Putative AsnC-family reg
692	1	SwissProt_HUMAN$	3::P02751|FINC_HUMAN	132	272150	7	7	7	7	0.13	Fibronectin OS=Homo sa
693	1	db$2	2::C3JXQ0|C3JXQ0_PSEFS	132	24880	5	5	4	4	1.13	Putative hydrolase OS=I
694	1	db$2	2::C3KBV0|C3KBV0_PSEFS	132	28603	4	4	3	3	0.64	Putative chromosome pa
695	1	db$2	2::C3KAI6|C3KAI6_PSEFS	131	24263	5	4	5	4	1.17	Putative dehydrogenase
696	1	db$2	2::C3K2K2|THIE_PSEFS	131	22345	3	3	3	3	0.88	Thiamine-phosphate syr
697	1	db$2	2::C3K0T7|C3K0T7_PSEFS	130	76509	9	8	8	7	0.54	Putative fatty acid oxida
698	1	SwissProt_HUMAN$	3::Q06830|PRDX1_HUMAN	130	22096	8	7	6	5	1.89	Peroxiredoxin-1 OS=Hoı
699	1	db$2	2::C3KAI4|C3KAI4_PSEFS	130	53827	4	4	4	4	0.42	SPOR domain-containing
700	1	db$2	2::C3K494|C3K494_PSEFS	130	33626	3	3	2	2	0.32	Gly-zipper_YMGG domai
701	1	db$2	2::C3JXZ5|C3JXZ5_PSEFS	130	25958	3	3	3	3	0.72	Putative histidine transp
702	1	db$2	2::C3K479|RNPH_PSEFS	129	25645	5	5	4	4	1.08	Ribonuclease PH OS=Ps
703	1	db$2	2::C3K1L2|C3K1L2_PSEFS	129	22708	4	3	4	3	0.86	Ancillary SecYEG translo
704	1	db$2	2::C3K0I0|C3K0I0_PSEFS	129	27472	6	6	5	5	1.36	RNA polymerase sigma f
705	1	db$2	2::C3KEA8|C3KEA8_PSEFS	128	45495	4	4	3	3	0.37	ATP-dependent RNA heli
706	1	db$2	2::C3K015|C3K015_PSEFS	128	53791	5	5	5	5	0.55	Putative ferredoxin OS=
707	1	db$2	2::C3KCP1|C3KCP1_PSEFS	127	23476	5	3	5	3	0.83	Peptide methionine sulfo
708	1	db$2	2::C3K644|C3K644_PSEFS	127	89774	9	7	7	6	0.37	Putative ABC transport s
709	1	db$2	2::C3JYS0|C3JYS0_PSEFS	127	29172	4	4	4	4	0.91	Cell division coordinator
710	1	db$2	2::C3JXY4|ASTE_PSEFS	127	37404	10	7	7	6	1.13	Succinylglutamate desu
711	1	db$2	2::C3K5K1|C3K5K1_PSEFS	126	38065	6	6	6	6	1.10	Putative phospholipase (
712	1	db$2	2::C3K3N8|C3K3N8_PSEFS	126	43920	5	4	5	4	0.54	PUA domain-containing
713	1	db$2	2::C3K7Z1|C3K7Z1_PSEFS	126	38822	8	7	7	6	1.07	NAD(P) transhydrogenas
714	1	db$2	2::C3K5B9|C3K5B9_PSEFS	125	60269	2	2	2	2	0.17	Dipeptide ABC transport
715	1	db$2	2::C3K1M1|HSCA_PSEFS	125	65896	5	5	4	4	0.33	Chaperone protein HscA
716	1	db$2	2::C3K0U9|C3K0U9_PSEFS	125	55465	5	5	4	4	0.41	Putative two-component

↑Family	M	DB	Accession	Score	Mass	Matches	Match(sig)	Sequences	Seq(sig)	emPAI	Description
717	1	db$2	2::C3JYA9|C3JYA9_PSEFS	125	27795	5	5	5	5	1.34	Putative TetR-family reg
718	1	db$2	2::C3K910|C3K910_PSEFS	125	36322	5	5	5	5	0.92	Putative ABC transport s
719	1	db$2	2::C3K3Q1|C3K3Q1_PSEFS	124	40925	5	5	4	4	0.59	Putative oxidoreductase
720	1	db$2	2::C3K3T8|C3K3T8_PSEFS	124	39499	7	7	5	5	0.82	Putrescine ABC transpor
721	1	db$2	2::C3JYD3|SYC_PSEFS	124	51635	8	8	8	8	1.08	Cysteine--tRNA ligase O
722	1	db$2	2::C3K9M2|C3K9M2_PSEFS	124	37830	5	5	4	4	0.65	Putative peptidase OS=I
723	1	db$2	2::C3K0H7|C3K0H7_PSEFS	124	77893	5	4	5	4	0.28	Histidine kinase OS=Pse
724	1	SwissProt_HUMAN$	3::P63104|1433Z_HUMAN	124	27728	3	3	3	3	0.66	14-3-3 protein zeta/delt
725	1	db$2	2::C3K1X0|CYSZ_PSEFS	123	28826	5	5	5	5	1.26	Sulfate transporter CysZ
726	1	db$2	2::C3K6L9|C3K6L9_PSEFS	123	49785	5	5	4	4	0.46	Phospho-2-dehydro-3-d
727	1	db$2	2::C3K1B3|C3K1B3_PSEFS	123	20726	4	3	4	3	0.97	Uncharacterized protein
728	1	db$2	2::C3K9Z4|C3K9Z4_PSEFS	123	58133	9	7	8	6	0.63	Putative ABC tranmspor
729	1	db$2	2::C3K197|C3K197_PSEFS	123	25331	7	5	5	5	1.53	Putative two-component
730	1	db$2	2::C3K909|C3K909_PSEFS	123	24537	3	3	2	2	0.47	Uncharacterized protein
731	1	db$2	2::C3K1X2|C3K1X2_PSEFS	123	40166	6	6	6	6	1.02	Putative putative oxidor
732	1	db$2	2::C3KA84|C3KA84_PSEFS	122	13408	5	5	5	5	4.65	Putative regulatory prot
733	1	db$2	2::C3K2S3|C3K2S3_PSEFS	122	17933	7	6	5	5	3.77	Transcriptional represso
734	1	db$2	2::C3K2U6|C3K2U6_PSEFS	122	49484	3	3	2	2	0.33	Putative transport-relate
735	1	db$2	2::C3K2U5|C3K2U5_PSEFS	122	18989	6	6	5	5	2.43	Single-stranded DNA-bir
736	1	db$2	2::C3KDN7|HEMH_PSEFS	122	38415	6	5	6	5	0.85	Ferrochelatase OS=Pseu
737	1	db$2	2::C3JXY5|C3JXY5_PSEFS	121	10610	3	3	2	2	1.38	Uncharacterized protein
738	1	db$2	2::C3K0D7|APEB_PSEFS	121	46914	6	6	5	5	0.65	Probable M18 family am
739	1	db$2	2::C3K544|C3K544_PSEFS	121	74165	8	7	8	7	0.56	Uncharacterized protein
740	1	db$2	2::C3K270|C3K270_PSEFS	121	17315	6	5	4	3	1.25	Transcription elongation
741	1	db$2	2::C3K6A4|C3K6A4_PSEFS	121	36459	3	3	3	3	0.48	Methionine import ATP-b
742	1	db$2	2::C3K3E9|C3K3E9_PSEFS	120	12056	3	3	3	3	2.16	Putative lipoprotein OS=
743	1	SwissProt_HUMAN$	3::P60174|TPIS_HUMAN	120	26653	5	5	5	5	1.42	Triosephosphate isomera
744	1	db$2	2::C3K8W9|C3K8W9_PSEFS	120	28276	3	3	3	3	0.65	Intermembrane phospho
745	1	db$2	2::C3K5E8|C3K5E8_PSEFS	119	28997	4	4	4	4	0.92	Cyclohexadienyl dehydra
746	1	db$2	2::C3K763|C3K763_PSEFS	119	29996	3	3	2	2	0.37	Putative hydrolase OS=I
747	1	db$2	2::C3K0R0|C3K0R0_PSEFS	119	14751	4	4	2	2	0.88	Flagellar basal body rod
748	1	db$2	2::C3K256|RS15_PSEFS	118	10112	6	6	4	4	5.23	30S ribosomal protein S
749	1	db$2	2::C3K1G1|C3K1G1_PSEFS	118	48709	3	3	3	3	0.34	tRNA modification GTPa
750	1	db$2	2::C3K0R1|C3K0R1_PSEFS	118	30842	4	3	3	2	0.36	Protein-glutamate O-me
751	1	db$2	2::C3K6V9|C3K6V9_PSEFS	118	54793	9	8	7	6	0.68	2,3-bisphosphoglycerate
752	1	db$2	2::C3K2R5|RIBA_PSEFS	118	22312	3	3	3	3	0.88	GTP cyclohydrolase-2 OS
753	1	db$2	2::C3KDM6|PSD_PSEFS	118	31380	6	6	4	4	1.46	Phosphatidylserine deca
754	1	db$2	2::C3K3M6|C3K3M6_PSEFS	117	53012	5	5	5	5	0.56	Peptidase_M16_C doma
755	1	SwissProt_HUMAN$	3::P07737|PROF1_HUMAN	116	15045	4	4	4	4	2.44	Profilin-1 OS=Homo sap
756	1	db$2	2::C3K1E5|ATPE_PSEFS	116	14558	4	4	3	3	2.60	ATP synthase epsilon ch

↑Family	M	DB	Accession	Score	Mass	Matches	Match(sig)	Sequences	Seq(sig)	emPAI	Description
757	1	db$2	2::C3JXJ5|C3JXJ5_PSEFS	116	22111	4	4	3	3	0.89	Putative SCO1/SenC fan
758	1	db$2	2::C3K2W3|C3K2W3_PSEFS	115	11427	6	5	5	4	4.06	30S ribosomal protein S
759	1	db$2	2::C3K8K8|C3K8K8_PSEFS	115	62203	5	5	5	5	0.46	Putative exported protei
760	1	db$2	2::C3K126|C3K126_PSEFS	115	54569	8	7	5	5	0.54	2-methylcitrate dehydra
761	1	db$2	2::C3KED2|C3KED2_PSEFS	114	17293	4	4	4	4	1.95	Thioredoxin peroxidase
762	1	db$2	2::C3JYS6|C3JYS6_PSEFS	114	17060	2	2	2	2	0.73	Putative thioesterase OS
763	1	db$2	2::C3KAW2|C3KAW2_PSEFS	114	26230	4	4	4	4	1.05	Putative short chain deh
764	1	db$2	2::C3K257|C3K257_PSEFS	114	33362	7	7	5	5	1.03	tRNA pseudouridine synt
765	1	db$2	2::C3JZR8|C3JZR8_PSEFS	113	46446	6	5	5	4	0.50	Putative oxidoreductase
766	1	db$2	2::C3KBM4|C3KBM4_PSEFS	113	34098	9	7	7	5	1.29	Ornithine carbamoyltran
767	1	db$2	2::C3KAB1|C3KAB1_PSEFS	113	44397	5	4	4	3	0.38	Histidine kinase OS=Pse
768	1	db$2	2::C3K0W1|C3K0W1_PSEFS	112	27483	4	4	4	4	0.98	Flagellar basal-body rod
769	1	db$2	2::C3KDB8|HLDE_PSEFS	112	49913	6	5	6	5	0.61	Bifunctional protein HldE
770	1	db$2	2::C3K2D6|C3K2D6_PSEFS	112	73930	4	3	3	3	0.21	Carbon starvation protei
771	1	db$2	2::C3K3G5|C3K3G5_PSEFS	112	15160	5	5	4	4	2.41	Putative thioesterase OS
772	1	db$2	2::C3K8K4|C3K8K4_PSEFS	112	58330	3	2	3	2	0.18	Putative outer membran
773	1	SwissProt_HUMAN$	3::Q9BQE3|TBA1C_HUMAN	111	49863	5	5	5	5	0.61	Tubulin alpha-1C chain (
774	1	db$2	2::C3JXX3|C3JXX3_PSEFS	111	52842	2	2	2	2	0.20	Cytochrome-c oxidase C
775	1	db$2	2::C3K1D1|C3K1D1_PSEFS	111	32188	2	2	2	2	0.34	Protein HflC OS=Pseudo
776	1	db$2	2::C3K1N3|C3K1N3_PSEFS	111	11888	6	5	4	3	2.23	Sec translocon accessor
777	1	db$2	2::C3K5E3|C3K5E3_PSEFS	110	43909	5	5	4	4	0.54	Putative aminotransfera
778	1	db$2	2::C3K6J2|C3K6J2_PSEFS	110	47817	4	4	3	3	0.35	Putative hydrolase OS=I
779	1	db$2	2::C3KDQ1|C3KDQ1_PSEFS	109	35181	3	3	2	2	0.31	Putative LysR-family reg
780	1	db$2	2::C3K2W7|RS17_PSEFS	109	10090	4	4	4	4	5.23	30S ribosomal protein S
781	1	db$2	2::C3KBM2|C3KBM2_PSEFS	109	16548	3	3	3	3	1.33	Cys-tRNA(Pro)/Cys-tRNA
782	1	db$2	2::C3K9H7|C3K9H7_PSEFS	109	27490	4	3	4	3	0.67	Putative short-chain deh
783	1	db$2	2::C3JYN9|C3JYN9_PSEFS	109	44994	5	4	4	3	0.37	Pseudouridine synthase
784	1	db$2	2::C3K6V7|C3K6V7_PSEFS	108	46336	5	5	5	5	0.67	Carboxy-terminal proces
785	1	db$2	2::C3K5K3|C3K5K3_PSEFS	108	28692	3	3	2	2	0.64	CHAD domain-containin
786	1	db$2	2::C3K617|C3K617_PSEFS	108	19384	3	3	2	2	0.62	Uncharacterized protein
787	1	db$2	2::C3K2P7|C3K2P7_PSEFS	107	17680	3	3	3	3	1.21	Putative acetyltransferas
788	1	db$2	2::C3K3K1|C3K3K1_PSEFS	107	18188	1	1	1	1	0.29	Bifunctional protein [pyr
789	1	db$2	2::C3KBL9|C3KBL9_PSEFS	107	27726	6	5	5	4	0.97	Glycerol-3-phosphate re
790	1	db$2	2::C3KBH3|DCUP_PSEFS	107	38973	4	4	4	4	0.62	Uroporphyrinogen decar
791	1	db$2	2::C3K1M6|C3K1M6_PSEFS	107	24782	3	3	3	3	0.77	Uncharacterized protein
792	1	db$2	2::C3JY31|C3JY31_PSEFS	107	22860	3	3	3	3	0.85	Putative biopolymer trar
793	1	db$2	2::C3K7I9|C3K7I9_PSEFS	107	88241	6	6	6	6	0.38	Putative pinecillin acylas
794	1	db$2	2::C3K8W4|C3K8W4_PSEFS	106	18790	7	7	5	5	2.48	Lipopolysaccharide expo
795	1	db$2	2::C3K0P9|C3K0P9_PSEFS	106	25457	4	4	3	3	0.74	Two-component system,
796	1	db$2	2::C3K309|C3K309_PSEFS	106	21661	2	2	2	2	0.54	Anthranilate synthase co

↑Family	M	DB	Accession	Score	Mass	Matches	Match(sig)	Sequences	Seq(sig)	emPAI	Description
797	1	db$2	2::C3K268|C3K268_PSEFS	106	11552	6	4	5	4	3.99	CRM domain-containing
798	1	db$2	2::C3K5G1|C3K5G1_PSEFS	106	34097	7	6	5	4	1.00	Cysteine synthase OS=P
799	1	db$2	2::C3KA82|C3KA82_PSEFS	105	19457	4	4	3	3	1.06	Lactoylglutathione lyase
800	1	db$2	2::C3KE66|C3KE66_PSEFS	105	27011	5	4	5	4	1.01	23S rRNA (guanosine-2'
801	1	db$2	2::C3K6E8|C3K6E8_PSEFS	104	27518	2	2	2	2	0.41	Putative GntR-family reg
802	1	db$2	2::C3JZN8|C3JZN8_PSEFS	104	10735	5	5	4	4	4.58	Uncharacterized protein
803	1	SwissProt_HUMAN$	3::P02647|APOA1_HUMAN	104	30759	4	3	4	3	0.58	Apolipoprotein A-I OS=H
804	1	db$2	2::C3K0N3|ACP_PSEFS	104	8637	3	3	2	2	1.89	Acyl carrier protein OS=
805	1	db$2	2::C3JY78|C3JY78_PSEFS	104	22781	3	2	3	2	0.51	Putative cytochrome oxi
806	1	db$2	2::C3K621|C3K621_PSEFS	103	26068	4	4	4	4	1.06	Putative TetR-family reg
807	1	db$2	2::C3K0N7|RL32_PSEFS	103	6752	3	3	3	3	6.52	50S ribosomal protein L
808	1	db$2	2::C3KAV6|C3KAV6_PSEFS	103	17648	4	4	3	3	1.21	Thiol peroxidase OS=Ps
809	1	db$2	2::Q9ZEP8|PANB_PSEFS	103	27951	3	3	3	3	0.66	3-methyl-2-oxobutanoat
810	1	db$2	2::C3K4C0|C3K4C0_PSEFS	103	9802	2	2	2	2	1.57	DNA-binding protein HU
811	1	db$2	2::C3K3X0|C3K3X0_PSEFS	102	42989	5	3	5	3	0.39	Putative ubiquinone bios
812	1	db$2	2::C3K5L9|C3K5L9_PSEFS	102	31441	3	3	3	3	0.57	Alpha-D-ribose 1-methy
813	1	db$2	2::C3K101|C3K101_PSEFS	102	37185	4	4	3	3	0.46	Uncharacterized protein
814	1	db$2	2::C3KB06|C3KB06_PSEFS	102	29724	2	2	2	2	0.37	Putative lipoprotein OS=
815	1	db$2	2::C3K424|HEM3_PSEFS	102	33541	5	4	4	3	0.53	Porphobilinogen deamin
816	1	db$2	2::C3K4W2|C3K4W2_PSEFS	101	26844	5	5	4	4	1.02	Putative glycerophospho
817	1	db$2	2::C3K6W7|C3K6W7_PSEFS	101	16216	5	5	4	4	3.23	Biotin carboxyl carrier p
818	1	db$2	2::C3KBK6|C3KBK6_PSEFS	101	29320	4	4	3	3	0.90	Putative exported peptic
819	1	db$2	2::C3KDA5|C3KDA5_PSEFS	101	54170	2	2	2	2	0.19	Uncharacterized protein
820	1	db$2	2::C3K5M0|C3K5M0_PSEFS	101	29300	3	3	3	3	0.62	Phosphonates ABC trans
821	1	db$2	2::C3K2D4|C3K2D4_PSEFS	100	35439	3	3	3	3	0.49	Putative cobalamin synt
822	1	db$2	2::C3JY84|C3JY84_PSEFS	100	18006	1	1	1	1	0.30	NADH dehydrogenase I
823	1	db$2	2::C3K6E5|C3K6E5_PSEFS	100	29069	3	2	3	2	0.38	Cytochrome c1 OS=Pseu
824	1	db$2	2::C3K110|C3K110_PSEFS	100	32414	5	5	5	5	1.07	Cysteine synthase OS=P
825	1	db$2	2::C3KE56|C3KE56_PSEFS	100	59021	5	5	4	4	0.38	Glutamate--cysteine liga
826	1	db$2	2::C3K572|C3K572_PSEFS	100	7891	1	1	1	1	0.79	Uncharacterized protein
827	1	db$2	2::C3JY82|NUOB_PSEFS	99	25341	7	5	7	5	1.53	NADH-quinone oxidored
828	1	db$2	2::C3KEA2|C3KEA2_PSEFS	99	30824	3	3	3	3	0.58	Putative inner membran
829	1	db$2	2::C3JYK0|CLPP_PSEFS	99	23405	10	6	7	5	1.73	ATP-dependent Clp prot
830	1	db$2	2::C3K1U9|C3K1U9_PSEFS	99	42728	4	4	3	3	0.39	Flavohemoglobin OS=Ps
831	1	db$2	2::C3K5L2|C3K5L2_PSEFS	99	29854	2	2	2	2	0.37	Phosphonates import AT
832	1	db$2	2::C3KDK8|C3KDK8_PSEFS	99	41619	3	3	3	3	0.41	DAO domain-containing
833	1	db$2	2::C3K2M7|C3K2M7_PSEFS	99	18106	5	4	4	3	1.17	Ribosomal silencing fact
834	1	db$2	2::C3K9F0|C3K9F0_PSEFS	98	40567	5	5	4	4	0.79	Putative transport-relate
835	1	db$2	2::C3K754|C3K754_PSEFS	98	29479	4	3	4	3	0.61	Putative lipoprotein OS=
836	1	db$2	2::C3KCG1|C3KCG1_PSEFS	98	60384	3	3	3	3	0.27	Putative secreted amida

↑Family	M	DB	Accession	Score	Mass	Matches	Match(sig)	Sequences	Seq(sig)	emPAI	Description
837	1	db$2	2::C3JXX6|DNLJ_PSEFS	98	85515	6	5	6	5	0.32	DNA ligase OS=Pseudon
838	1	db$2	2::C3KDM3|C3KDM3_PSEFS	97	25575	2	2	2	2	0.45	Putative lipoprotein OS=
839	1	db$2	2::C3K061|C3K061_PSEFS	97	18792	3	3	3	3	1.11	DUF2059 domain-contai
840	1	db$2	2::C3KAM8|C3KAM8_PSEFS	96	52747	4	4	3	3	0.31	Phosphate transporter C
841	1	db$2	2::C3KE78|C3KE78_PSEFS	96	15753	3	3	3	3	1.43	Azurin OS=Pseudomona
842	1	db$2	2::C3K1M4|C3K1M4_PSEFS	96	13705	5	4	3	3	1.78	Iron-sulfur cluster asser
843	1	db$2	2::C3K4Z4|C3K4Z4_PSEFS	96	43117	2	2	2	2	0.25	Putative cytochrome C b
844	1	db$2	2::C3KD59|C3KD59_PSEFS	95	81779	3	3	3	3	0.19	YcaO domain-containing
845	1	db$2	2::C3K7E3|C3K7E3_PSEFS	95	178026	8	6	8	6	0.17	Alpha-2-macroglobulin (
846	1	db$2	2::C3KD21|C3KD21_PSEFS	95	81698	4	4	4	4	0.26	Putative glycogen debra
847	1	db$2	2::C3JXY8|C3JXY8_PSEFS	95	37476	4	4	4	4	0.65	Arginine N-succinyltrans
848	1	db$2	2::C3K5X6|C3K5X6_PSEFS	95	49358	6	6	5	5	0.78	Tryptophan--tRNA ligase
849	1	db$2	2::C3KBB4|OADC_PSEFS	95	31135	5	5	3	3	0.83	Oxaloacetate decarboxy
850	1	db$2	2::C3KBZ8|C3KBZ8_PSEFS	95	38389	5	4	4	4	0.64	Iron-sulfur cluster carrie
851	1	db$2	2::C3KBL4|C3KBL4_PSEFS	94	26993	5	5	4	4	1.01	Glutamate/aspartate AB
852	1	db$2	2::C3KBY3|C3KBY3_PSEFS	94	64998	4	4	4	4	0.34	Putative lipoprotein OS=
853	1	SwissProt_HUMAN$	3::P62979|RS27A_HUMAN	94	17953	2	2	2	2	0.68	Ubiquitin-40S ribosomal
854	1	db$2	2::C3KDC4|C3KDC4_PSEFS	93	64677	4	4	4	4	0.34	Uncharacterized protein
855	1	db$2	2::C3K3N0|C3K3N0_PSEFS	93	25405	2	2	1	1	0.20	Putative TetR-family reg
856	1	db$2	2::C3K3L2|C3K3L2_PSEFS	93	15596	3	3	3	3	1.45	DUF4426 domain-contai
857	1	db$2	2::C3K7Z3|C3K7Z3_PSEFS	93	43142	3	3	3	3	0.39	Putative acyl-CoA dehyd
858	1	db$2	2::C3KBY0|GMHA_PSEFS	93	21250	4	3	4	3	0.94	Phosphoheptose isomer
859	1	db$2	2::C3K7C4|C3K7C4_PSEFS	93	53137	8	6	8	6	0.71	DNA-binding transcriptic
860	1	db$2	2::C3JY71|C3JY71_PSEFS	93	43919	3	2	3	2	0.24	JmjC domain-containing
861	1	db$2	2::C3K4U5|C3K4U5_PSEFS	92	75901	7	6	7	6	0.45	Putative protease OS=P
862	1	db$2	2::C3K4W3|C3K4W3_PSEFS	92	21770	2	2	2	2	0.54	PilZ domain-containing p
863	1	db$2	2::C3K272|C3K272_PSEFS	92	40774	8	6	7	6	1.01	Carbamoyl-phosphate sy
864	1	db$2	2::C3KC02|C3KC02_PSEFS	92	29258	3	2	3	2	0.38	Putative acyltransferase
865	1	db$2	2::C3K7M5|C3K7M5_PSEFS	92	15789	2	2	2	2	0.81	Uncharacterized protein
866	1	db$2	2::C3K4R1|PLSB_PSEFS	91	95327	3	3	3	3	0.16	Glycerol-3-phosphate ac
867	1	db$2	2::C3K1C5|C3K1C5_PSEFS	91	44321	4	4	3	3	0.38	Putative cyclopropane-fa
868	1	db$2	2::C3K3J3|C3K3J3_PSEFS	91	72855	3	3	3	3	0.22	Type IV pilus biogenesis
869	1	db$2	2::C3KDQ7|C3KDQ7_PSEFS	91	12961	2	2	1	1	0.43	Diacylglycerol kinase OS
870	1	db$2	2::C3K579|C3K579_PSEFS	91	15962	1	1	1	1	0.34	Putative thioesterase OS
871	1	db$2	2::C3K312|C3K312_PSEFS	91	24134	3	3	3	3	0.80	Ribulose-phosphate 3-ep
872	1	db$2	2::C3K979|C3K979_PSEFS	91	30391	3	3	2	2	0.36	Putative glutathione S-tı
873	1	db$2	2::C3K258|RBFA_PSEFS	91	14697	3	3	3	3	1.60	Ribosome-binding factor
874	1	db$2	2::C3K421|C3K421_PSEFS	90	46244	4	4	4	4	0.50	Putative HemY domain p
875	1	db$2	2::C3K360|C3K360_PSEFS	90	23509	4	4	4	4	1.22	ATP-dependent dethiobi
876	1	db$2	2::C3JZQ4|C3JZQ4_PSEFS	90	8106	2	2	2	2	2.10	Translational regulator (

↑Family	M	DB	Accession	Score	Mass	Matches	Match(sig)	Sequences	Seq(sig)	emPAI	Description
877	1	SwissProt_HUMAN$	3::P10909|CLUS_HUMAN	90	52461	6	4	5	3	0.31	Clusterin OS=Homo sap
878	1	db$2	2::C3K3D8|C3K3D8_PSEFS	90	49942	6	5	6	5	0.61	ATP-independent RNA h
879	1	db$2	2::C3KCP6|C3KCP6_PSEFS	90	38240	3	3	3	3	0.45	ADP-heptose--LPS hepto
880	1	db$2	2::C3KDP0|C3KDP0_PSEFS	90	27498	2	2	2	2	0.41	Uncharacterized protein
881	1	db$2	2::C3KB73|C3KB73_PSEFS	90	9692	1	1	1	1	0.61	Putative lipoprotein OS=
882	1	db$2	2::C3JZV9|C3JZV9_PSEFS	90	34932	3	3	2	2	0.31	Putative DNA/RNA non-s
883	1	db$2	2::C3KBJ5|C3KBJ5_PSEFS	90	62095	4	3	3	3	0.26	Phospholipase C OS=Pse
884	1	db$2	2::C3K2V8|RL30_PSEFS	90	6521	4	4	3	3	7.03	50S ribosomal protein L
885	1	db$2	2::C3K0J1|C3K0J1_PSEFS	89	36130	5	5	5	5	0.92	Flagellar motor switch p
886	1	db$2	2::C3KDM5|C3KDM5_PSEFS	89	43829	4	4	4	4	0.54	O-phosphoserine phosph
887	1	db$2	2::C3K2W2|RS8_PSEFS	89	14083	7	7	4	4	4.22	30S ribosomal protein S
888	1	db$2	2::C3K3R7|RPIA_PSEFS	89	23281	4	4	3	3	0.83	Ribose-5-phosphate isor
889	1	db$2	2::C3KE87|C3KE87_PSEFS	89	46792	2	2	2	2	0.22	NADH dehydrogenase II
890	1	db$2	2::C3K758|PYRF_PSEFS	88	24706	5	5	4	4	1.58	Orotidine 5'-phosphate (
891	1	db$2	2::C3K303|COQ7_PSEFS	88	23866	6	5	6	5	1.68	3-demethoxyubiquinol 3
892	1	db$2	2::C3JYS8|RUVA_PSEFS	88	22218	4	4	4	4	1.33	Holliday junction ATP-de
893	1	db$2	2::C3K4Y3|KDPA_PSEFS	88	60181	4	4	3	3	0.27	Potassium-transporting
894	1	db$2	2::C3JXX7|C3JXX7_PSEFS	88	56979	2	2	2	2	0.18	Conserved hypothetical
895	1	db$2	2::C3JYF7|C3JYF7_PSEFS	88	147681	4	3	3	2	0.07	ATP-dependent helicase
896	1	db$2	2::C3K417|C3K417_PSEFS	87	39396	3	3	3	3	0.43	Transcriptional regulator
897	1	db$2	2::C3K482|RPOZ_PSEFS	87	9821	5	4	3	3	5.62	DNA-directed RNA polyn
898	1	db$2	2::C3JY57|LOLA_PSEFS	87	22546	6	5	6	5	1.83	Outer-membrane lipopro
899	1	db$2	2::C3K9M9|C3K9M9_PSEFS	87	14163	2	2	2	2	0.93	Putative endoribonuclea
900	1	db$2	2::C3KAH2|C3KAH2_PSEFS	87	24970	5	5	4	4	1.12	SPOR domain-containing
901	1	db$2	2::C3KDQ0|C3KDQ0_PSEFS	87	35105	4	4	4	4	0.71	2-ketogluconate reducta
902	1	SwissProt_HUMAN$	3::P0C0L4|CO4A_HUMAN	87	192664	2	2	2	2	0.05	Complement C4-A OS=H
903	1	db$2	2::C3JZ62|C3JZ62_PSEFS	86	61473	3	3	3	3	0.26	Putative long-chain-fatty
904	1	db$2	2::C3K6E4|C3K6E4_PSEFS	86	46143	8	6	5	4	0.51	Cytochrome b OS=Pseu
905	1	db$2	2::C3K3W5|C3K3W5_PSEFS	86	39645	5	5	4	4	0.61	Aminomethyltransferase
906	1	db$2	2::C3K1B4|C3K1B4_PSEFS	86	43355	2	2	2	2	0.24	Putative transferase OS
907	1	db$2	2::C3K3L1|C3K3L1_PSEFS	86	23430	2	2	2	2	0.49	Uncharacterized protein
908	1	db$2	2::C3JY61|C3JY61_PSEFS	86	8297	5	4	3	3	4.23	Translation initiation fac
909	1	db$2	2::C3JY77|C3JY77_PSEFS	86	40451	5	5	4	4	0.60	Putative 3-oxoacyl-[acyl
910	1	db$2	2::C3K088|C3K088_PSEFS	86	20655	5	4	4	4	1.48	Putative isochorismatase
911	1	db$2	2::C3K586|C3K586_PSEFS	86	21064	5	3	5	3	0.95	ADP compounds hydrola
912	1	db$2	2::C3K7T1|C3K7T1_PSEFS	85	23131	3	3	3	3	0.84	Two-component system
913	1	db$2	2::C3KCR9|PTH_PSEFS	85	21041	3	3	3	3	0.95	Peptidyl-tRNA hydrolase
914	1	db$2	2::C3K253|C3K253_PSEFS	85	106648	2	2	2	2	0.09	Putative membrane prot
915	1	db$2	2::C3KBU0|C3KBU0_PSEFS	85	36726	3	3	3	3	0.47	Sulfate/thiosulfate impo
916	1	db$2	2::C3KAD3|ARNC_PSEFS	85	37700	3	3	3	3	0.46	Undecaprenyl-phosphate

↑Family	M	DB	Accession	Score	Mass	Matches	Match(sig)	Sequences	Seq(sig)	emPAI	Description
917	1	db$2	2::C3K9M1|C3K9M1_PSEFS	85	26106	1	1	1	1	0.20	Putative phosphoglycera
918	1	db$2	2::C3K648|C3K648_PSEFS	85	33905	3	3	3	3	0.52	Conserved hypothetical
919	1	db$2	2::C3JYU3|C3JYU3_PSEFS	84	75598	4	2	4	2	0.13	Histidine kinase OS=Pse
920	1	db$2	2::C3K427|C3K427_PSEFS	84	23927	3	3	3	3	0.80	Putative glutathione S-tı
921	1	db$2	2::C3JZ32|C3JZ32_PSEFS	84	38286	4	3	3	3	0.45	Putative cation transpor
922	1	db$2	2::C3K9R3|C3K9R3_PSEFS	84	18863	3	3	3	3	1.11	Uncharacterized protein
923	1	db$2	2::C3K114|C3K114_PSEFS	84	74752	3	3	3	3	0.21	DNA polymerase III sub
924	1	db$2	2::C3K415|C3K415_PSEFS	84	70809	3	3	3	3	0.22	Putative ABC transport s
925	1	db$2	2::C3K3Q4|C3K3Q4_PSEFS	84	38576	3	3	2	2	0.28	Putative cation ABC tran
926	1	db$2	2::C3K7S1|C3K7S1_PSEFS	84	25085	3	3	1	1	0.45	Protein MgtC OS=Pseud
927	1	db$2	2::C3K4J0|C3K4J0_PSEFS	84	17598	3	3	3	3	1.21	Zn_protease domain-coı
928	1	db$2	2::C3KBG3|C3KBG3_PSEFS	84	24033	3	3	3	3	0.80	Curli production assemb
929	1	db$2	2::C3K1N5|QUEA_PSEFS	83	38396	3	3	3	3	0.45	S-adenosylmethionine:t
930	1	db$2	2::C3K813|C3K813_PSEFS	83	36618	4	4	4	4	0.67	Proline iminopeptidase (
931	1	db$2	2::C3KDV5|C3KDV5_PSEFS	83	20461	5	5	5	5	2.15	Oligoribonuclease OS=P
932	1	db$2	2::C3KE71|C3KE71_PSEFS	83	51412	9	7	8	7	0.90	Replicative DNA helicase
933	1	db$2	2::C3K327|C3K327_PSEFS	83	29832	2	2	2	2	0.37	Ribosomal RNA small su
934	1	db$2	2::C3K650|C3K650_PSEFS	83	13083	5	5	5	5	4.90	N-acetyltransferase dom
935	1	db$2	2::C3K5L3|C3K5L3_PSEFS	83	36401	6	5	6	5	0.91	Phosphonates ABC trans
936	1	db$2	2::C3JY36|C3JY36_PSEFS	83	16095	5	5	3	3	2.19	Uncharacterized protein
937	1	db$2	2::C3K0U4|C3K0U4_PSEFS	83	36872	5	4	5	4	0.67	Flagellar motor switch p
938	1	db$2	2::C3K1N1|C3K1N1_PSEFS	82	33097	4	4	4	4	0.77	Protein-export membrar
939	1	db$2	2::C3K9Y7|C3K9Y7_PSEFS	82	30413	1	1	1	1	0.17	Undecaprenyl-diphospha
940	1	db$2	2::C3K436|C3K436_PSEFS	82	30237	2	2	2	2	0.37	Diaminopimelate epimer
941	1	db$2	2::C3KE89|C3KE89_PSEFS	82	24346	3	3	3	3	0.79	Uncharacterized protein
942	1	db$2	2::C3K2R1|DXS_PSEFS	82	68238	6	6	6	6	0.52	1-deoxy-D-xylulose-5-p
943	1	db$2	2::C3K9B4|C3K9B4_PSEFS	82	16186	2	2	2	2	0.78	Type II secretion systen
944	1	db$2	2::C3KC04|C3KC04_PSEFS	82	39513	3	3	3	3	0.43	Putative hydrolase OS=I
945	1	db$2	2::C3K838|C3K838_PSEFS	82	9715	2	2	2	2	1.57	Putative phosphocarrier
946	1	db$2	2::C3KCS6|C3KCS6_PSEFS	81	47227	3	3	3	3	0.35	UDP-N-acetylmuramoyl-
947	1	db$2	2::C3K706|PIMT_PSEFS	81	23451	1	1	1	1	0.22	Protein-L-isoaspartate O
948	1	db$2	2::C3K5B2|C3K5B2_PSEFS	81	35714	2	2	2	2	0.30	Dipeptide ABC transport
949	1	db$2	2::C3KDN9|C3KDN9_PSEFS	81	40608	3	2	3	2	0.26	Probable periplasmic ser
950	1	db$2	2::C3KE43|C3KE43_PSEFS	81	32711	3	3	3	3	0.54	Lipid A biosynthesis laur
951	1	db$2	2::C3K6H1|C3K6H1_PSEFS	81	131195	5	5	5	5	0.20	DNA polymerase III sub
952	1	db$2	2::C3K5U6|C3K5U6_PSEFS	81	40174	4	4	2	2	0.27	dTDP-glucose 4,6-dehyc
953	1	db$2	2::C3KE82|C3KE82_PSEFS	81	53340	3	3	3	3	0.30	Aldehyde dehydrogenas
954	1	db$2	2::C3K487|C3K487_PSEFS	81	33600	1	1	1	1	0.15	Biopolymer transport pr
955	1	db$2	2::C3K1N0|C3K1N0_PSEFS	81	18655	3	2	3	2	0.65	Rick_17kDa_Anti domai
956	1	db$2	2::C3JYE7|C3JYE7_PSEFS	81	35468	3	3	3	3	0.49	LysR family regulatory p

↑Family	M	DB	Accession	Score	Mass	Matches	Match(sig)	Sequences	Seq(sig)	emPAI	Description
957	1	db$2	2::C3K2K7|C3K2K7_PSEFS	81	37713	5	4	5	4	0.65	PhoH domain-containing
958	1	db$2	2::C3K9G9|C3K9G9_PSEFS	80	16183	2	2	2	2	0.78	CMD domain-containing
959	1	db$2	2::C3K0M6|C3K0M6_PSEFS	80	25497	3	3	3	3	0.74	Putative tetR-family reg
960	1	db$2	2::C3JYE9|C3JYE9_PSEFS	80	31035	3	2	3	2	0.35	Putative hydroxymethyl
961	1	db$2	2::C3K3P7|C3K3P7_PSEFS	80	49977	2	2	2	2	0.21	Putative membrane prot
962	1	db$2	2::C3K867|C3K867_PSEFS	80	39735	4	4	4	4	0.61	PALP domain-containing
963	1	db$2	2::C3KAI8|C3KAI8_PSEFS	80	24497	2	2	2	2	0.47	Putative TetR-family reg
964	1	db$2	2::C3KE84|C3KE84_PSEFS	80	35425	4	3	4	3	0.49	Pseudouridine synthase
965	1	db$2	2::C3KDL3|C3KDL3_PSEFS	80	70148	6	5	6	5	0.40	Phosphomethylpyrimidir
966	1	db$2	2::C3K1W7|C3K1W7_PSEFS	80	11247	3	2	2	1	0.51	DUF1244 domain-contai
967	1	SwissProt_HUMAN$	3::P61604|CH10_HUMAN	80	10925	5	4	5	4	4.49	10 kDa heat shock prote
968	1	db$2	2::C3K607|LPXA_PSEFS	79	28002	2	2	2	2	0.40	Acyl-[acyl-carrier-protei
969	1	db$2	2::C3K863|PDXH_PSEFS	79	24884	3	2	3	2	0.46	Pyridoxine/pyridoxamine
970	1	db$2	2::C3K229|C3K229_PSEFS	79	24861	1	1	1	1	0.21	TPR_REGION domain-co
971	1	db$2	2::C3K6N8|C3K6N8_PSEFS	79	25972	2	2	2	2	0.44	Putative hydrolase OS=I
972	1	db$2	2::C3KC09|C3KC09_PSEFS	78	83087	3	3	3	3	0.19	Periplasmic beta-glucosi
973	1	db$2	2::C3K491|C3K491_PSEFS	78	76137	3	2	3	2	0.13	ATP-dependent DNA hel
974	1	db$2	2::C3K337|PLSY_PSEFS	78	20278	1	1	1	1	0.26	Glycerol-3-phosphate ac
975	1	SwissProt_HUMAN$	3::P23396|RS3_HUMAN	77	26671	3	3	3	3	0.70	40S ribosomal protein S
976	1	db$2	2::C3K4J4|HSLO_PSEFS	76	33320	2	2	2	2	0.33	33 kDa chaperonin OS=
977	1	db$2	2::C3KDC3|LOLB_PSEFS	76	23021	2	2	2	2	0.51	Outer-membrane lipopr
978	1	db$2	2::C3K477|C3K477_PSEFS	76	29836	3	3	3	3	0.61	Exodeoxyribonuclease II
979	1	db$2	2::C3JY38|C3JY38_PSEFS	76	16812	3	3	3	3	1.31	Putative thioredoxin-rela
980	1	SwissProt_HUMAN$	3::P62249|RS16_HUMAN	76	16435	3	2	3	2	0.77	40S ribosomal protein S
981	1	db$2	2::C3K3X4|C3K3X4_PSEFS	76	11561	2	2	1	1	1.23	Cell division protein Zap
982	1	db$2	2::C3JY58|C3JY58_PSEFS	76	87640	4	4	4	4	0.24	DNA translocase FtsK OS
983	1	db$2	2::C3K170|C3K170_PSEFS	75	22875	3	3	2	2	0.51	Flavin_Reduct domain-c
984	1	db$2	2::C3K5D5|C3K5D5_PSEFS	75	41033	2	2	2	2	0.26	Succinyl-diaminopimelat
985	1	db$2	2::C3KEC9|C3KEC9_PSEFS	75	53005	3	3	2	2	0.20	Putative beta-barrel ass
986	1	db$2	2::C3K3B8|C3K3B8_PSEFS	75	42763	1	1	1	1	0.12	Uncharacterized protein
987	1	db$2	2::C3K2X4|RL23_PSEFS	75	10938	4	4	4	4	4.40	50S ribosomal protein L
988	1	db$2	2::C3K3Y7|C3K3Y7_PSEFS	75	35706	4	2	4	2	0.30	Putative iron-sulfur bind
989	1	db$2	2::C3K709|C3K709_PSEFS	74	7722	3	3	3	3	4.86	Cold shock protein OS=I
990	1	db$2	2::C3KAA9|C3KAA9_PSEFS	74	31757	1	1	1	1	0.16	Putative chemotaxis pro
991	1	db$2	2::C3K336|C3K336_PSEFS	74	13078	1	1	1	1	0.43	7,8-dihydroneopterin ald
992	1	db$2	2::C3JYV1|C3JYV1_PSEFS	74	32264	4	4	4	4	0.79	Formyltetrahydrofolate d
993	1	db$2	2::C3K6G9|LPXB_PSEFS	74	41468	2	2	2	2	0.26	Lipid-A-disaccharide syn
994	1	db$2	2::C3K8W8|C3K8W8_PSEFS	73	29386	3	2	2	2	0.38	Putative ABC transport s
995	1	db$2	2::C3K6G1|RLMF_PSEFS	73	38015	2	2	2	2	0.28	Ribosomal RNA large sul
996	1	db$2	2::C3K3K8|C3K3K8_PSEFS	73	27898	1	1	1	1	0.18	Pyrroline-5-carboxylate

↑Family	M	DB	Accession	Score	Mass	Matches	Match(sig)	Sequences	Seq(sig)	emPAI	Description
997	1	db$2	2::C3K4U6|C3K4U6_PSEFS	73	17513	2	2	1	1	0.31	Conserved hypothetical
998	1	db$2	2::C3K4A7|C3K4A7_PSEFS	73	18391	3	2	3	2	0.66	Uncharacterized protein
999	1	SwissProt_HUMAN$	3::P02671|FIBA_HUMAN	73	94914	2	2	2	2	0.11	Fibrinogen alpha chain (
1000	1	db$2	2::C3KB17|C3KB17_PSEFS	73	51104	3	3	3	3	0.32	Outer membrane compc
1001	1	db$2	2::C3K0J3|C3K0J3_PSEFS	72	45387	4	4	4	4	0.52	Putative flagellar hook-l
1002	1	db$2	2::C3K0Z1|C3K0Z1_PSEFS	72	36566	3	3	3	3	0.47	L-threonine-O-3-phosph
1003	1	db$2	2::C3KDC0|C3KDC0_PSEFS	72	36735	3	3	3	3	0.47	Uncharacterized protein
1004	1	db$2	2::C3JZN4|RL20_PSEFS	72	13348	4	3	3	2	1.01	50S ribosomal protein L
1005	1	db$2	2::C3K2T3|C3K2T3_PSEFS	72	32553	2	2	2	2	0.34	dTDP-4-dehydrorhamno
1006	1	db$2	2::C3K1G6|C3K1G6_PSEFS	72	25762	3	3	3	3	0.73	Thiol:disulfide interchan
1007	1	db$2	2::C3K5X9|C3K5X9_PSEFS	72	41639	1	1	1	1	0.12	Putative acyl-CoA dehyd
1008	1	db$2	2::C3K3L6|TRMB_PSEFS	72	26990	1	1	1	1	0.19	tRNA (guanine-N(7)-)-m
1009	1	db$2	2::C3K707|C3K707_PSEFS	72	29295	2	1	2	1	0.17	Putative lipoprotein OS=
1010	1	db$2	2::C3K2E0|C3K2E0_PSEFS	72	48742	6	5	5	5	0.62	DNA repair protein RadA
1011	1	db$2	2::C3K0H6|C3K0H6_PSEFS	72	40054	5	5	5	5	0.80	Protein-glutamate meth
1012	1	db$2	2::C3K3M7|C3K3M7_PSEFS	72	22464	1	1	1	1	0.23	Ribosomal RNA small su
1013	1	db$2	2::C3KCB9|C3KCB9_PSEFS	71	35987	5	4	3	2	0.48	Polyphosphate kinase O
1014	1	db$2	2::C3K591|C3K591_PSEFS	71	19872	3	3	2	2	1.03	Putative bacterioferritin
1015	1	db$2	2::C3KA81|C3KA81_PSEFS	71	55484	6	6	6	6	0.67	Alkyl hydroperoxide redu
1016	1	db$2	2::C3K6A1|C3K6A1_PSEFS	71	22990	2	2	2	2	0.51	Putative copper-binding
1017	1	db$2	2::C3K127|C3K127_PSEFS	71	41295	3	3	3	3	0.41	Putative membrane prot
1018	1	db$2	2::C3K6S3|C3K6S3_PSEFS	70	25881	3	3	3	3	0.73	Putative glutathione S-t
1019	1	db$2	2::C3K6T3|C3K6T3_PSEFS	70	23166	2	2	2	2	0.50	Thiol:disulfide interchan
1020	1	db$2	2::C3KED3|C3KED3_PSEFS	70	20551	2	2	2	2	0.58	Glycine cleavage system
1021	1	db$2	2::C3K7G4|C3K7G4_PSEFS	70	54521	3	3	3	3	0.30	Ribonuclease G OS=Pse
1022	1	db$2	2::C3K5F4|C3K5F4_PSEFS	70	21753	3	3	3	3	0.91	Putative nitroreductase (
1023	1	db$2	2::C3K098|C3K098_PSEFS	70	23000	1	1	1	1	0.23	Putative glutathione S-t
1024	1	db$2	2::C3JXH2|C3JXH2_PSEFS	70	91149	2	2	2	2	0.11	Putative excinuclease AE
1025	1	db$2	2::C3KDM4|C3KDM4_PSEFS	70	56715	6	3	6	3	0.28	Putative membrane prot
1026	1	db$2	2::C3K0C5|C3K0C5_PSEFS	70	27111	1	1	1	1	0.19	Putative amino transfera
1027	1	db$2	2::C3K6Z2|C3K6Z2_PSEFS	70	17868	2	2	2	2	0.69	tRNA-specific adenosine
1028	1	db$2	2::C3K1X3|C3K1X3_PSEFS	69	20670	3	3	3	3	0.98	Glutathione peroxidase (
1029	1	db$2	2::C3K0L5|C3K0L5_PSEFS	69	17844	3	2	2	2	0.69	Putative GNAT-family ac
1030	1	db$2	2::C3K8P0|C3K8P0_PSEFS	69	38329	3	3	3	3	0.45	Putative nucleoside-sepa
1031	1	db$2	2::C3K332|Y5583_PSEFS	69	48606	4	4	4	4	0.48	UPF0229 protein PFLU_5
1032	1	db$2	2::C3K221|C3K221_PSEFS	69	129628	1	1	1	1	0.04	RecBCD enzyme subunit
1033	1	db$2	2::C3JZP9|C3JZP9_PSEFS	68	33380	1	1	1	1	0.15	Putative ribose transpor
1034	1	db$2	2::C3K107|C3K107_PSEFS	68	31415	3	1	3	1	0.16	Uncharacterized protein
1035	1	db$2	2::C3KCS5|C3KCS5_PSEFS	68	51798	5	5	5	5	0.58	UDP-N-acetylmuramoyl-
1036	1	db$2	2::C3K152|C3K152_PSEFS	68	13999	8	5	8	5	4.29	Putative transcriptional r

↑Family	M	DB	Accession	Score	Mass	Matches	Match(sig)	Sequences	Seq(sig)	emPAI	Description
1037	1	db$2	2::C3KC07|C3KC07_PSEFS	68	21449	2	2	2	2	0.55	YceI domain-containing
1038	1	db$2	2::C3KDV8|C3KDV8_PSEFS	68	29265	2	2	2	2	0.38	ADP-dependent (S)-NAD
1039	1	db$2	2::C3JXL6|C3JXL6_PSEFS	68	41978	1	1	1	1	0.12	Bcr/CflA family efflux tra
1040	1	db$2	2::C3K8K6|C3K8K6_PSEFS	68	15117	2	2	2	2	0.86	Putative transport-relate
1041	1	db$2	2::C3KCS0|RL25_PSEFS	68	21502	6	6	3	3	1.97	50S ribosomal protein L
1042	1	db$2	2::C3KBM3|C3KBM3_PSEFS	68	40194	2	2	2	2	0.27	Putative ABC transport s
1043	1	db$2	2::C3K1F7|C3K1F7_PSEFS	68	69251	2	2	2	2	0.15	tRNA uridine 5-carboxyr
1044	1	db$2	2::C3K6M4|C3K6M4_PSEFS	67	13463	4	4	2	2	3.00	Succinate dehydrogenas
1045	1	db$2	2::C3KE20|C3KE20_PSEFS	67	33868	2	2	1	1	0.15	GCV_T domain-containir
1046	1	db$2	2::C3KEC3|C3KEC3_PSEFS	67	16145	4	3	3	2	0.79	Putative membrane prot
1047	1	db$2	2::C3K6Y8|C3K6Y8_PSEFS	67	34148	7	5	5	4	0.74	GTPase Era OS=Pseudo
1048	1	db$2	2::C3K3D4|C3K3D4_PSEFS	67	52472	2	2	2	2	0.20	Uncharacterized protein
1049	1	db$2	2::C3K1K8|C3K1K8_PSEFS	67	31523	5	5	5	5	1.11	Putative hydrolase OS=I
1050	1	db$2	2::C3K5K2|C3K5K2_PSEFS	67	9923	4	3	3	2	1.55	Putative lipoprotein OS=
1051	1	db$2	2::C3K1A4|C3K1A4_PSEFS	67	41199	1	1	1	1	0.12	Uncharacterized protein
1052	1	db$2	2::C3K320|C3K320_PSEFS	67	34836	2	2	2	2	0.31	Uncharacterized protein
1053	1	db$2	2::C3K326|C3K326_PSEFS	67	35169	3	1	3	1	0.14	4-hydroxythreonine-4-p
1054	1	db$2	2::C3KD57|C3KD57_PSEFS	67	49686	1	1	1	1	0.10	Cystathionine beta-lyase
1055	1	db$2	2::C3K3B7|C3K3B7_PSEFS	67	14934	1	1	1	1	0.37	Uncharacterized protein
1056	1	db$2	2::C3K439|C3K439_PSEFS	67	25827	3	3	3	3	0.73	Putative hydrolase OS=I
1057	1	db$2	2::C3K173|C3K173_PSEFS	67	34695	5	5	5	5	0.97	Putative AraC-family tra
1058	1	db$2	2::C3K5H2|QUEF_PSEFS	67	30808	7	6	7	6	1.50	NADPH-dependent 7-cya
1059	1	db$2	2::C3K6Z1|C3K6Z1_PSEFS	67	50711	2	2	2	2	0.21	Putative multicopper oxi
1060	1	db$2	2::C3K705|SURE_PSEFS	66	26431	2	2	2	2	0.43	5'-nucleotidase SurE OS
1061	1	db$2	2::C3KDP4|C3KDP4_PSEFS	66	75338	3	3	3	3	0.21	Phosphate acetyltransfe
1062	1	db$2	2::C3KE17|C3KE17_PSEFS	66	24430	3	3	3	3	0.78	Two component system,
1063	1	db$2	2::C3KE16|C3KE16_PSEFS	66	37004	3	3	3	3	0.47	Putative chemotaxis-rela
1064	1	db$2	2::C3JY76|C3JY76_PSEFS	66	48249	3	3	3	3	0.34	Putative mechanosensiti
1065	1	SwissProt_HUMAN$	3::Q14240|IF4A2_HUMAN	66	46373	2	2	2	2	0.23	Eukaryotic initiation fact
1066	1	db$2	2::C3KE58|C3KE58_PSEFS	65	84757	6	5	6	5	0.32	Putative S1 RNA-binding
1067	1	db$2	2::C3K7C7|C3K7C7_PSEFS	65	19943	3	3	3	3	1.02	Chorismate mutase OS=
1068	1	db$2	2::C3K830|C3K830_PSEFS	65	47217	6	6	6	6	0.82	Putative short chain deh
1069	1	db$2	2::C3KDN6|C3KDN6_PSEFS	65	32369	2	2	2	2	0.34	Uncharacterized protein
1070	1	db$2	2::C3K3J5|C3K3J5_PSEFS	65	13219	2	2	2	2	1.02	Type IV pilus response r
1071	1	db$2	2::C3K0M3|C3K0M3_PSEFS	65	20567	3	3	3	3	0.98	Uncharacterized protein
1072	1	db$2	2::C3K3T7|C3K3T7_PSEFS	65	39982	2	2	2	2	0.27	Putrescine ABC transpor
1073	1	db$2	2::C3KDI4|C3KDI4_PSEFS	65	42151	3	3	3	3	0.40	Putative exported protei
1074	1	db$2	2::C3K7W0|C3K7W0_PSEFS	65	26786	2	1	2	1	0.19	Uncharacterized protein
1075	1	db$2	2::C3KBT9|C3KBT9_PSEFS	65	31336	1	1	1	1	0.16	Sulfate ABC transport sy
1076	1	db$2	2::C3K0R6|C3K0R6_PSEFS	65	28374	4	4	4	4	0.94	Uncharacterized protein

↑Family	M	DB	Accession	Score	Mass	Matches	Match(sig)	Sequences	Seq(sig)	emPAI	Description
1077	1	db$2	2::C3K7J8|C3K7J8_PSEFS	64	78803	4	3	3	3	0.20	ATP-dependent DNA hel
1078	1	db$2	2::C3K0K1|C3K0K1_PSEFS	64	53436	1	1	1	1	0.09	Putative GntR-family reg
1079	1	db$2	2::C3K9T0|NADK_PSEFS	64	32289	3	3	3	3	0.55	NAD kinase OS=Pseudo
1080	1	SwissProt_HUMAN$	3::P06703|S10A6_HUMAN	64	10173	2	2	1	1	0.57	Protein S100-A6 OS=Ho
1081	1	db$2	2::C3K3D1|BETI_PSEFS	64	21992	1	1	1	1	0.24	HTH-type transcriptiona
1082	1	SwissProt_HUMAN$	3::P08519|APOA_HUMAN	64	500995	3	3	3	3	0.03	Apolipoprotein(a) OS=H
1083	1	db$2	2::C3K224|C3K224_PSEFS	64	30473	3	3	2	2	0.36	CDP-diacylglycerol--seri
1084	1	db$2	2::C3K9Y8|C3K9Y8_PSEFS	64	14463	1	1	1	1	0.38	Uncharacterized protein
1085	1	db$2	2::C3K7G1|C3K7G1_PSEFS	64	37563	5	3	5	3	0.46	Cell shape protein MreC
1086	1	db$2	2::C3K0H8|C3K0H8_PSEFS	63	29525	4	4	3	3	0.61	Protein phosphatase Che
1087	1	db$2	2::C3K1W6|C3K1W6_PSEFS	63	13390	4	3	3	3	1.83	D-erythro-7,8-dihydrone
1088	1	db$2	2::C3K2Q9|EX7S_PSEFS	63	8900	2	2	2	2	1.82	Exodeoxyribonuclease 7
1089	1	db$2	2::C3K6K2|IHFB_PSEFS	63	11021	4	4	3	3	2.54	Integration host factor s
1090	1	db$2	2::C3K663|C3K663_PSEFS	63	22341	4	2	4	2	0.52	Putative TetR-family reg
1091	1	db$2	2::C3JZ82|C3JZ82_PSEFS	63	37007	3	3	3	3	0.47	Putative dehydrogenase
1092	1	db$2	2::C3K9U9|C3K9U9_PSEFS	63	9554	1	1	1	1	0.62	Uncharacterized protein
1093	1	db$2	2::C3KBB9|C3KBB9_PSEFS	63	73354	2	2	2	2	0.14	Putative protease OS=P
1094	1	db$2	2::C3JYS7|RUVB_PSEFS	63	38919	2	2	2	2	0.27	Holliday junction ATP-de
1095	1	db$2	2::C3K7W1|C3K7W1_PSEFS	63	12229	2	2	2	2	1.14	Putative lipoprotein OS=
1096	1	db$2	2::C3K5Y4|C3K5Y4_PSEFS	63	9690	1	1	1	1	0.61	Uncharacterized protein
1097	1	db$2	2::C3JYI2|C3JYI2_PSEFS	63	39477	3	3	3	3	0.43	2-aminoethylphosphona
1098	1	db$2	2::C3KAR4|C3KAR4_PSEFS	63	11430	1	1	1	1	0.50	Uncharacterized protein
1099	1	db$2	2::C3JYN3|C3JYN3_PSEFS	62	52328	2	2	2	2	0.20	Family S13 unassigned
1100	1	SwissProt_HUMAN$	3::P62277|RS13_HUMAN	62	17212	2	2	2	2	0.72	40S ribosomal protein S
1101	1	db$2	2::C3K815|C3K815_PSEFS	62	62777	8	6	7	6	0.57	Glucans biosynthesis pr
1102	1	db$2	2::C3KBB8|C3KBB8_PSEFS	62	13546	3	3	3	3	1.80	Uncharacterized protein
1103	1	db$2	2::C3K486|C3K486_PSEFS	62	30593	2	2	2	2	0.36	Epimerase domain-conta
1104	1	db$2	2::C3K743|FABA_PSEFS	62	18688	3	3	3	3	1.12	3-hydroxydecanoyl-[acy
1105	1	db$2	2::C3K4R0|C3K4R0_PSEFS	62	14930	2	2	2	2	0.86	Putative lipoprotein OS=
1106	1	SwissProt_HUMAN$	3::A0A0B4J1X5|HV374_HUMAN	62	12831	3	3	2	2	1.95	Immunoglobulin heavy
1106	2	SwissProt_HUMAN$	3::P0DOX2|IGA2_HUMAN	35	48903	3	3	3	3	0.34	Immunoglobulin alpha-2
1106	3	SwissProt_HUMAN$	3::P01876|IGHA1_HUMAN	31	37631	3	3	3	3	0.46	Immunoglobulin heavy
1107	1	db$2	2::C3K6W0|C3K6W0_PSEFS	62	14786	4	3	3	2	0.88	Putative rhodanese dom
1108	1	db$2	2::C3K0K9|C3K0K9_PSEFS	62	37421	2	2	1	1	0.13	Putative NADP-depende
1109	1	db$2	2::C3KDX1|RS20_PSEFS	62	10078	3	3	2	2	1.50	30S ribosomal protein S
1110	1	db$2	2::C3KCJ3|C3KCJ3_PSEFS	62	30975	4	4	3	3	0.58	Usp domain-containing
1111	1	SwissProt_HUMAN$	3::P02679|FIBG_HUMAN	62	51479	5	3	5	3	0.32	Fibrinogen gamma chair
1112	1	db$2	2::C3K8W5|C3K8W5_PSEFS	62	21382	1	1	1	1	0.24	Lipopolysaccharide expo
1113	1	db$2	2::C3K5U7|C3K5U7_PSEFS	61	105852	2	1	2	1	0.05	Putative aminotransfera
1114	1	db$2	2::C3K8U1|C3K8U1_PSEFS	61	15020	3	3	3	3	1.54	Phosphoribosyl-AMP cyc

↑Family	M	DB	Accession	Score	Mass	Matches	Match(sig)	Sequences	Seq(sig)	emPAI	Description
1115	1	db$2	2::C3K0D5|MINE_PSEFS	61	9531	3	3	3	3	3.26	Cell division topological
1116	1	db$2	2::C3K7Y8|C3K7Y8_PSEFS	61	53697	4	4	4	4	0.42	Putative hydrolase OS=I
1117	1	db$2	2::C3K0Z5|C3K0Z5_PSEFS	61	22469	4	2	4	2	0.52	Corrinoid adenosyltransf
1118	1	db$2	2::C3K0X3|C3K0X3_PSEFS	61	16227	3	3	3	3	1.37	Transcriptional regulator
1119	1	db$2	2::C3K6W8|C3K6W8_PSEFS	60	16611	2	2	2	2	0.75	3-dehydroquinate dehyc
1120	1	db$2	2::C3JZU7|C3JZU7_PSEFS	60	16433	2	2	2	2	0.77	Putative thioesterase OS
1121	1	db$2	2::C3KBE8|C3KBE8_PSEFS	60	8134	2	2	1	1	0.76	Putative lipoprotein OS=
1122	1	db$2	2::C3K7K0|C3K7K0_PSEFS	60	41297	2	2	2	2	0.26	Esterase III OS=Pseudo
1123	1	db$2	2::C3K485|C3K485_PSEFS	60	26565	2	1	2	1	0.19	Putative lipoprotein OS=
1124	1	db$2	2::C3KAC0|C3KAC0_PSEFS	60	40546	2	1	2	1	0.12	Butyryl-CoA dehydroger
1125	1	db$2	2::C3JY28|Y3771_PSEFS	60	6626	4	4	2	2	2.92	UPF0434 protein PFLU_3
1126	1	db$2	2::C3K459|C3K459_PSEFS	60	18704	3	2	3	2	0.65	Leucine-responsive regu
1127	1	db$2	2::C3K440|C3K440_PSEFS	60	11389	4	3	4	3	2.37	Uncharacterized protein
1128	1	db$2	2::C3K1K9|C3K1K9_PSEFS	59	42204	2	2	2	2	0.25	Putative aminotransfera
1129	1	db$2	2::C3JYE6|C3JYE6_PSEFS	59	45437	2	2	2	2	0.23	Putative acyl-CoA dehyd
1130	1	db$2	2::C3K5I0|C3K5I0_PSEFS	59	36784	2	2	2	2	0.29	tRNA-dihydrouridine(20/
1131	1	db$2	2::C3KA92|C3KA92_PSEFS	59	24193	1	1	1	1	0.21	Putative ACP reductase (
1132	1	db$2	2::C3K2M6|RLMH_PSEFS	59	17669	2	2	2	2	0.70	Ribosomal RNA large sul
1133	1	db$2	2::C3K5F1|C3K5F1_PSEFS	59	42516	4	4	4	4	0.56	Ribonuclease D OS=Pse
1134	1	db$2	2::C3KAX3|C3KAX3_PSEFS	59	30360	2	2	2	2	0.36	Conserved hypothetical
1135	1	db$2	2::C3K831|C3K831_PSEFS	59	45359	4	4	3	3	0.37	Putative thiolase OS=Ps
1136	1	db$2	2::C3JYN0|C3JYN0_PSEFS	59	33944	2	2	2	2	0.32	Cbb3-type cytochrome c
1136	2	db$2	2::C3JZS9|C3JZS9_PSEFS	21	35591	2	2	2	2	0.30	Cbb3-type cytochrome c
1137	1	db$2	2::C3K5S6|C3K5S6_PSEFS	59	24824	1	1	1	1	0.21	Putative hydrolase OS=I
1138	1	db$2	2::C3K468|C3K468_PSEFS	59	6077	2	2	1	1	1.10	50S ribosomal protein L
1139	1	db$2	2::C3KBN5|C3KBN5_PSEFS	58	24125	1	1	1	1	0.22	Putative TetR-family reg
1140	1	db$2	2::C3K751|C3K751_PSEFS	58	15943	1	1	1	1	0.34	Putative thioesterase OS
1141	1	db$2	2::C3K6P0|C3K6P0_PSEFS	58	15266	3	2	2	2	0.84	Uncharacterized protein
1142	1	db$2	2::C3K602|C3K602_PSEFS	58	48346	5	5	5	5	0.63	Zinc metalloprotease OS
1143	1	db$2	2::C3K7K5|C3K7K5_PSEFS	57	12541	1	1	1	1	0.45	Uncharacterized protein
1144	1	db$2	2::C3KAA6|C3KAA6_PSEFS	57	51931	2	2	2	2	0.20	Putative cell envelope pı
1145	1	db$2	2::C3KAY5|C3KAY5_PSEFS	57	30881	1	1	1	1	0.16	NAD-capped RNA hydrol
1146	1	db$2	2::C3K7J3|Y1319_PSEFS	57	16953	1	1	1	1	0.32	UPF0225 protein PFLU_1
1147	1	db$2	2::C3KDF9|C3KDF9_PSEFS	57	17633	2	2	2	2	0.70	CinA domain-containing
1148	1	db$2	2::C3K1M8|C3K1M8_PSEFS	57	28345	2	2	2	2	0.39	tRNA (cytidine/uridine-2
1149	1	db$2	2::C3K2L9|LIPB_PSEFS	57	23473	1	1	1	1	0.22	Octanoyltransferase OS=
1150	1	db$2	2::C3K349|C3K349_PSEFS	57	28994	2	2	2	2	0.38	Pyrroloquinoline-quinone
1151	1	db$2	2::C3K666|C3K666_PSEFS	57	29461	2	2	2	2	0.38	Putative lipoprotein OS=
1152	1	db$2	2::C3K0K3|C3K0K3_PSEFS	57	12996	1	1	1	1	0.43	Putative lipoprotein OS=
1153	1	db$2	2::C3KEB0|C3KEB0_PSEFS	57	36597	4	3	3	2	0.29	Putative amino acid ABC

↑Family	M	DB	Accession	Score	Mass	Matches	Match(sig)	Sequences	Seq(sig)	emPAI	Description
1154	1	db$2	2::C3KDY6|C3KDY6_PSEFS	57	17128	1	1	1	1	0.31	Uncharacterized protein
1155	1	db$2	2::C3K068|C3K068_PSEFS	56	65454	3	3	3	3	0.24	Putative asparagine synt
1156	1	db$2	2::C3KB44|C3KB44_PSEFS	56	23546	1	1	1	1	0.22	Putative membrane prot
1157	1	SwissProt_HUMAN$	3::Q71DI3|H32_HUMAN	56	15379	3	3	3	3	1.48	Histone H3.2 OS=Homo
1158	1	db$2	2::C3K7A7|C3K7A7_PSEFS	56	43115	2	2	2	2	0.25	Conserved hypothetical
1159	1	db$2	2::C3K5L6|C3K5L6_PSEFS	56	16214	3	3	3	3	1.37	Putative phosphorous cc
1160	1	db$2	2::C3JXL2|C3JXL2_PSEFS	56	30967	1	1	1	1	0.16	Uncharacterized protein
1161	1	db$2	2::C3KAH5|RL31_PSEFS	56	8451	2	2	2	2	1.97	50S ribosomal protein L
1162	1	db$2	2::C3KBM1|C3KBM1_PSEFS	56	29989	1	1	1	1	0.17	Glycerol uptake facilitat
1163	1	db$2	2::C3KE38|C3KE38_PSEFS	56	28697	1	1	1	1	0.18	Putative phospholipid bi
1164	1	db$2	2::C3KAJ9|C3KAJ9_PSEFS	55	20836	3	3	3	3	0.96	Putative hypoxanthine-g
1165	1	db$2	2::C3K0U5|C3K0U5_PSEFS	55	62992	1	1	1	1	0.08	Flagellar M-ring protein
1166	1	db$2	2::C3KAN3|C3KAN3_PSEFS	55	27088	1	1	1	1	0.19	3-oxoadipate CoA-transf
1167	1	db$2	2::C3KAS9|C3KAS9_PSEFS	55	109677	3	3	3	3	0.14	Efflux pump membrane
1168	1	db$2	2::C3K5B7|C3K5B7_PSEFS	55	50883	1	1	1	1	0.10	Putative outer membran
1169	1	db$2	2::C3K1V9|C3K1V9_PSEFS	55	16219	2	1	2	1	0.33	Flavodoxin-like domain-
1170	1	db$2	2::C3K820|C3K820_PSEFS	55	26990	2	2	2	2	0.42	Putative ABC transport s
1171	1	db$2	2::C3K6B0|C3K6B0_PSEFS	55	33534	1	1	1	1	0.15	High-affinity zinc uptake
1172	1	db$2	2::C3K1X4|C3K1X4_PSEFS	55	16845	1	1	1	1	0.32	Putative putative export
1173	1	db$2	2::C3K2N1|C3K2N1_PSEFS	55	47217	1	1	1	1	0.11	Putative membrane prot
1174	1	db$2	2::C3KAL0|C3KAL0_PSEFS	55	43962	1	1	1	1	0.11	PlsC domain-containing
1175	1	db$2	2::C3KAK2|C3KAK2_PSEFS	55	10989	5	3	4	3	2.54	Uncharacterized protein
1176	1	db$2	2::C3K2Y1|RS12_PSEFS	55	13716	1	1	1	1	0.40	30S ribosomal protein S
1177	1	db$2	2::C3K564|C3K564_PSEFS	55	54508	2	2	2	2	0.19	Methylmalonate-semialc
1178	1	db$2	2::C3K6Z6|C3K6Z6_PSEFS	54	13580	2	2	2	2	0.99	Putaitve lipoprotein OS=
1179	1	db$2	2::C3K3G8|C3K3G8_PSEFS	54	41555	2	2	2	2	0.26	Uncharacterized protein
1180	1	db$2	2::C3K5X5|C3K5X5_PSEFS	54	22739	2	1	2	1	0.23	Thioesterase domain-co
1181	1	SwissProt_HUMAN$	3::P62913|RL11_HUMAN	54	20240	1	1	1	1	0.26	60S ribosomal protein L
1182	1	db$2	2::C3K425|C3K425_PSEFS	54	27295	2	2	2	2	0.41	Positive alginate biosynt
1183	1	SwissProt_HUMAN$	3::P23284|PPIB_HUMAN	54	23728	1	1	1	1	0.22	Peptidyl-prolyl cis-trans
1184	1	SwissProt_HUMAN$	3::P46781|RS9_HUMAN	54	22578	3	3	3	3	0.87	40S ribosomal protein S
1185	1	db$2	2::C3K3K9|C3K3K9_PSEFS	54	21575	4	4	2	2	0.55	Conserved hypothetical
1186	1	db$2	2::C3KDX3|C3KDX3_PSEFS	53	34279	3	3	3	3	0.51	Riboflavin biosynthesis p
1187	1	db$2	2::C3K3V1|C3K3V1_PSEFS	53	23989	2	2	2	2	0.48	Alkyl hydroperoxide redu
1188	1	db$2	2::C3JY37|C3JY37_PSEFS	53	14829	1	1	1	1	0.37	Putative lipoprotein OS=
1189	1	db$2	2::C3K7C5|C3K7C5_PSEFS	53	40067	2	2	1	1	0.13	Histidine kinase OS=Pse
1190	1	db$2	2::C3KE85|C3KE85_PSEFS	53	26099	1	1	1	1	0.20	Purine nucleoside phosp
1191	1	db$2	2::C3JZ64|C3JZ64_PSEFS	53	17217	3	3	3	3	1.26	MaoC-like domain-conta
1192	1	db$2	2::C3KE32|C3KE32_PSEFS	53	20912	1	1	1	1	0.25	Sigma factor negative re
1193	1	db$2	2::C3JXY3|C3JXY3_PSEFS	53	18788	3	3	2	2	0.65	6,7-dimethyl-8-ribityllur

↑Family	M	DB	Accession	Score	Mass	Matches	Match(sig)	Sequences	Seq(sig)	emPAI	Description
1194	1	db$2	2::C3K4A9|C3K4A9_PSEFS	53	17462	3	3	3	3	1.24	Uncharacterized protein
1195	1	db$2	2::C3K8K1|C3K8K1_PSEFS	53	40207	2	2	1	1	0.12	Phosphate-binding prote
1196	1	db$2	2::C3K4Z0|CCME_PSEFS	53	16101	2	2	2	2	0.79	Cytochrome c-type biog
1197	1	db$2	2::C3JY97|C3JY97_PSEFS	53	30357	1	1	1	1	0.17	N-hydroxyarylamine O-a
1198	1	db$2	2::C3KDV4|RSGA_PSEFS	53	37296	5	3	4	3	0.46	Small ribosomal subunit
1199	1	db$2	2::C3K0M7|C3K0M7_PSEFS	53	28601	1	1	1	1	0.18	Putative deoxyribonucle
1200	1	db$2	2::C3K646|C3K646_PSEFS	53	21182	2	2	1	1	0.25	Putative acyl-CoA thioes
1201	1	db$2	2::C3K5L0|C3K5L0_PSEFS	53	14786	1	1	1	1	0.37	Conserved hypothetical
1202	1	SwissProt_HUMAN$	3::P04406|G3P_HUMAN	53	36030	1	1	1	1	0.14	Glyceraldehyde-3-phosp
1203	1	db$2	2::C3KDS5|C3KDS5_PSEFS	52	41122	2	2	2	2	0.26	Putative hydratase/isom
1204	1	db$2	2::C3K132|C3K132_PSEFS	52	42760	2	1	2	1	0.12	Uncharacterized protein
1205	1	db$2	2::C3K7N0|C3K7N0_PSEFS	52	28545	2	2	2	2	0.39	Transport permease pro
1206	1	SwissProt_HUMAN$	3::P0CF74|IGLC6_HUMAN	52	11270	2	2	2	2	1.27	Immunoglobulin lambda
1207	1	db$2	2::C3JZ52|C3JZ52_PSEFS	52	18715	2	2	1	1	0.28	Uncharacterized protein
1208	1	db$2	2::C3K3W8|C3K3W8_PSEFS	52	44404	2	2	2	2	0.24	Putative ubiquinone bios
1209	1	db$2	2::C3K4D7|C3K4D7_PSEFS	52	14435	1	1	1	1	0.38	Putative thioesterase OS
1210	1	SwissProt_HUMAN$	3::Q5D862|FILA2_HUMAN	52	247928	1	1	1	1	0.02	Filaggrin-2 OS=Homo sa
1211	1	db$2	2::C3K4C8|C3K4C8_PSEFS	52	49694	1	1	1	1	0.10	Putative transport-relate
1212	1	db$2	2::C3JYG6|C3JYG6_PSEFS	52	44622	3	2	3	2	0.24	Peptidase_M14 domain-
1213	1	db$2	2::C3K6V3|HIS6_PSEFS	51	27111	4	3	4	3	0.68	Imidazole glycerol phos
1214	1	db$2	2::C3K5J9|C3K5J9_PSEFS	51	96070	2	2	2	2	0.10	Histidine kinase OS=Pse
1215	1	db$2	2::C3KDN1|C3KDN1_PSEFS	51	54005	3	2	3	2	0.19	Deoxyribodipyrimidine p
1216	1	db$2	2::C3JYR2|C3JYR2_PSEFS	51	48934	3	3	3	3	0.34	L-serine dehydratase OS
1217	1	db$2	2::C3K4T5|C3K4T5_PSEFS	51	30974	3	3	3	3	0.58	Putative hydrolase OS=I
1218	1	db$2	2::C3K261|RIMP_PSEFS	51	17653	2	2	2	2	0.70	Ribosome maturation fa
1219	1	db$2	2::C3K8I3|C3K8I3_PSEFS	51	39921	1	1	1	1	0.13	3,4-dihydroxy-2-butano
1220	1	db$2	2::C3KAX0|C3KAX0_PSEFS	51	84238	2	2	2	2	0.12	1,4-alpha-glucan branch
1221	1	db$2	2::C3K6W5|PRMA_PSEFS	51	31717	1	1	1	1	0.16	Ribosomal protein L11 n
1222	1	db$2	2::C3K6Y0|C3K6Y0_PSEFS	51	13973	1	1	1	1	0.40	4-carboxymuconolacton
1223	1	db$2	2::C3JYR6|C3JYR6_PSEFS	50	38174	4	4	4	4	0.64	Quinolinate synthase A (
1224	1	db$2	2::C3KE47|FMT_PSEFS	50	33416	2	2	2	2	0.33	Methionyl-tRNA formyltr
1225	1	db$2	2::C3KBL5|C3KBL5_PSEFS	50	24580	3	3	3	3	0.78	Glutamate/aspartate AB
1226	1	db$2	2::C3K300|C3K300_PSEFS	50	34122	1	1	1	1	0.15	Putative membrane prot
1227	1	SwissProt_HUMAN$	3::A6NJ78|MET15_HUMAN	50	46092	1	1	1	1	0.11	12S rRNA N4-methylcyti
1228	1	db$2	2::C3JZU8|C3JZU8_PSEFS	50	30426	2	2	2	2	0.36	Hydrolase_4 domain-co
1229	1	SwissProt_HUMAN$	3::P62750|RL23A_HUMAN	50	17684	1	1	1	1	0.30	60S ribosomal protein L
1230	1	db$2	2::C3JY81|C3JY81_PSEFS	50	15084	2	2	2	2	0.86	NADH-quinone oxidored
1231	1	db$2	2::C3K103|C3K103_PSEFS	50	23527	2	2	2	2	0.49	Phosphoribosylglycinami
1232	1	db$2	2::C3K6R7|C3K6R7_PSEFS	50	41832	2	2	2	2	0.25	Serine/threonine transp
1233	1	db$2	2::C3KE14|C3KE14_PSEFS	50	83238	1	1	1	1	0.06	Histidine kinase OS=Pse

↑Family	M	DB	Accession	Score	Mass	Matches	Match(sig)	Sequences	Seq(sig)	emPAI	Description
1234	1	db$2	2::C3KE30|C3KE30_PSEFS	50	59128	3	2	3	2	0.17	L-aspartate oxidase OS=
1235	1	SwissProt_HUMAN$	3::Q02878|RL6_HUMAN	50	32708	1	1	1	1	0.15	60S ribosomal protein L
1236	1	db$2	2::C3K250|C3K250_PSEFS	50	71198	1	1	1	1	0.07	Acetyl-coenzyme A synt
1237	1	db$2	2::C3KCH1|C3KCH1_PSEFS	50	32212	1	1	1	1	0.16	Putative carboxymethyle
1238	1	db$2	2::C3KBK8|C3KBK8_PSEFS	50	35740	2	1	2	1	0.14	Ribosomal RNA small su
1239	1	db$2	2::C3K5B3|C3K5B3_PSEFS	50	34937	2	1	2	1	0.14	Dipeptide ABC transport
1240	1	db$2	2::C3K654|C3K654_PSEFS	50	34290	3	3	2	2	0.32	2-dehydropantoate 2-re
1241	1	db$2	2::C3K386|C3K386_PSEFS	50	22957	1	1	1	1	0.23	Putative glutathione S-t
1242	1	db$2	2::C3K6R2|C3K6R2_PSEFS	49	29309	2	1	2	1	0.17	Putative shikimate dehy
1243	1	db$2	2::C3K4V3|C3K4V3_PSEFS	49	45550	2	1	2	1	0.11	Uncharacterized protein
1244	1	db$2	2::C3K333|C3K333_PSEFS	49	61021	2	2	2	2	0.17	Uncharacterized protein
1245	1	db$2	2::C3K047|C3K047_PSEFS	49	16614	2	2	2	2	0.75	Putative putative heat sl
1246	1	db$2	2::C3K302|C3K302_PSEFS	49	12723	4	3	2	2	1.07	Putative HIT domain-cor
1247	1	db$2	2::C3K4G7|PDXY_PSEFS	49	31241	1	1	1	1	0.16	Pyridoxal kinase PdxY O
1248	1	db$2	2::C3JZN0|C3JZN0_PSEFS	49	13917	1	1	1	1	0.40	MerR-family transcriptio
1249	1	db$2	2::C3JY69|HFLD_PSEFS	49	23173	2	1	2	1	0.22	High frequency lysogeni
1250	1	db$2	2::C3KDK6|C3KDK6_PSEFS	49	27451	2	2	2	2	0.41	Putative ABC transport s
1251	1	db$2	2::C3K1L7|RLMN_PSEFS	49	42342	1	1	1	1	0.12	Dual-specificity RNA me
1252	1	SwissProt_HUMAN$	3::Q13472|TOP3A_HUMAN	49	112300	2	1	2	1	0.04	DNA topoisomerase 3-al
1253	1	db$2	2::C3K1Y0|C3K1Y0_PSEFS	49	12222	1	1	1	1	0.46	Uncharacterized protein
1254	1	db$2	2::C3JYG2|C3JYG2_PSEFS	49	36128	1	1	1	1	0.14	ATP-grasp domain-conta
1255	1	db$2	2::C3K660|C3K660_PSEFS	49	28970	1	1	1	1	0.18	Hydroxypyruvate isomei
1256	1	db$2	2::C3KE39|C3KE39_PSEFS	49	18976	2	1	2	1	0.28	D,D-heptose 1,7-bispho:
1257	1	db$2	2::C3JZH1|C3JZH1_PSEFS	49	34020	3	3	3	3	0.52	LysR-family transcriptioi
1258	1	db$2	2::C3KDX6|C3KDX6_PSEFS	49	16394	2	2	2	2	0.77	Peptidyl-prolyl cis-trans
1259	1	SwissProt_HUMAN$	3::P62701|RS4X_HUMAN	48	29579	2	1	2	1	0.17	40S ribosomal protein S
1260	1	db$2	2::C3K8J9|C3K8J9_PSEFS	48	51515	1	1	1	1	0.10	Type II secretion systen
1261	1	db$2	2::C3K456|C3K456_PSEFS	48	38683	2	2	2	2	0.28	Alanine racemase OS=P
1262	1	db$2	2::C3KDC8|C3KDC8_PSEFS	48	26520	1	1	1	1	0.19	Molybdopterin biosynthe
1263	1	db$2	2::C3KDR1|C3KDR1_PSEFS	48	17628	2	2	2	2	0.70	Uncharacterized protein
1264	1	db$2	2::C3KE46|C3KE46_PSEFS	48	47174	3	3	2	2	0.22	16S rRNA m5C967 meth
1265	1	db$2	2::C3JXI0|C3JXI0_PSEFS	48	23030	1	1	1	1	0.23	Nudix hydrolase domain
1266	1	db$2	2::C3K6V0|C3K6V0_PSEFS	48	23354	4	3	4	3	0.83	Imidazole glycerol phos
1267	1	db$2	2::C3K5Y3|C3K5Y3_PSEFS	48	35524	3	3	2	2	0.30	Putative leucine dehydrc
1268	1	db$2	2::C3K106|C3K106_PSEFS	48	20669	2	2	2	2	0.58	Uncharacterized protein
1269	1	db$2	2::C3JYS4|C3JYS4_PSEFS	48	16520	3	3	3	3	1.33	Tol-Pal system protein T
1270	1	db$2	2::C3KDC7|C3KDC7_PSEFS	48	29930	1	1	1	1	0.17	Release factor glutamine
1271	1	SwissProt_HUMAN$	3::P46783|RS10_HUMAN	48	18886	1	1	1	1	0.28	40S ribosomal protein S
1272	1	db$2	2::C3K5E1|C3K5E1_PSEFS	47	13734	2	2	2	2	0.97	Uncharacterized protein
1273	1	SwissProt_HUMAN$	3::Q58FF8|H90B2_HUMAN	47	44321	1	1	1	1	0.11	Putative heat shock prot

↑Family	M	DB	Accession	Score	Mass	Matches	Match(sig)	Sequences	Seq(sig)	emPAI	Description
1274	1	db$2	2::C3K092|C3K092_PSEFS	47	14085	4	3	4	3	1.69	Glutaredoxin domain-co
1275	1	db$2	2::C3K3H4|C3K3H4_PSEFS	47	26074	2	2	2	2	0.43	Ribosomal RNA small su
1276	1	db$2	2::C3KAH8|C3KAH8_PSEFS	47	89028	4	3	4	3	0.17	DD-transpeptidase OS=
1277	1	db$2	2::C3K589|C3K589_PSEFS	47	50032	3	3	3	3	0.33	Transcriptional regulator
1278	1	db$2	2::C3K5Q0|C3K5Q0_PSEFS	47	53619	4	3	4	3	0.30	Mannose-1-phosphate g
1279	1	db$2	2::C3K364|C3K364_PSEFS	47	38824	2	1	2	1	0.13	Biotin synthase OS=Pse
1280	1	db$2	2::C3JZZ4|C3JZZ4_PSEFS	47	23102	1	1	1	1	0.23	Putative glutathione S tr
1281	1	db$2	2::C3K1B0|C3K1B0_PSEFS	47	96490	1	1	1	1	0.05	Lysylphosphatidylglycer
1282	1	db$2	2::C3KDW1|MUTL_PSEFS	47	69574	7	5	7	5	0.41	DNA mismatch repair pr
1283	1	db$2	2::C3K3J2|C3K3J2_PSEFS	47	213513	1	1	1	1	0.02	Histidine kinase OS=Pse
1284	1	SwissProt_HUMAN$	3::Q5VT06|CE350_HUMAN	46	350716	2	2	2	2	0.03	Centrosome-associated
1285	1	db$2	2::C3KBI7|C3KBI7_PSEFS	46	26715	1	1	1	1	0.19	Uncharacterized protein
1286	1	db$2	2::C3JY47|C3JY47_PSEFS	46	14227	1	1	1	1	0.39	DrsE domain-containing
1287	1	db$2	2::C3JZ77|C3JZ77_PSEFS	46	45611	3	3	2	2	0.23	Maltose-binding protein
1288	1	db$2	2::C3K2Z9|C3K2Z9_PSEFS	46	34289	4	4	4	4	0.73	NMO domain-containing
1289	1	db$2	2::C3K685|C3K685_PSEFS	46	13635	4	3	4	3	1.78	6-carboxy-5,6,7,8-tetral
1290	1	db$2	2::C3K122|C3K122_PSEFS	46	33097	1	1	1	1	0.15	Putative O-acetylserine
1291	1	db$2	2::C3JZP3|C3JZP3_PSEFS	46	36685	2	2	2	2	0.29	Putative inosine-uridine
1292	1	db$2	2::C3KAR1|C3KAR1_PSEFS	46	16023	1	1	1	1	0.34	Putative isoquinoline 1-(
1293	1	db$2	2::C3K609|C3K609_PSEFS	46	73644	5	3	4	3	0.21	Putative lytic transglyco:
1294	1	db$2	2::C3K5X3|C3K5X3_PSEFS	46	64999	1	1	1	1	0.08	Putative ABC transport s
1295	1	db$2	2::C3K0P5|C3K0P5_PSEFS	46	35572	2	2	2	2	0.30	Uncharacterized protein
1296	1	db$2	2::C3K150|C3K150_PSEFS	46	25194	2	2	2	2	0.45	Putative short-chain deh
1297	1	db$2	2::C3K8U6|C3K8U6_PSEFS	46	15270	2	2	2	2	0.84	Putative polyhydroxyalk
1298	1	db$2	2::C3K3M5|C3K3M5_PSEFS	46	50101	5	5	5	5	0.60	Putative peptidase OS=I
1299	1	db$2	2::C3K4R2|C3K4R2_PSEFS	46	7681	3	3	3	3	5.01	Major cold shock proteir
1300	1	db$2	2::C3K0W6|C3K0W6_PSEFS	46	42755	3	2	3	2	0.25	Aspartate aminotransfer
1301	1	db$2	2::Q8VMR3|HUTH_PSEFS	46	54209	1	1	1	1	0.09	Histidine ammonia-lyase
1302	1	db$2	2::C3K3N1|C3K3N1_PSEFS	46	52088	2	1	2	1	0.10	Aldehyde dehydrogenas
1303	1	db$2	2::C3K013|C3K013_PSEFS	46	20254	1	1	1	1	0.26	Peptidyl-prolyl cis-trans
1304	1	db$2	2::C3K2Z1|COAX_PSEFS	46	26920	1	1	1	1	0.19	Type III pantothenate ki
1305	1	db$2	2::C3K9P4|C3K9P4_PSEFS	45	59261	5	2	5	2	0.17	Putative ATP-binding cor
1306	1	db$2	2::C3K737|C3K737_PSEFS	45	17727	3	3	3	3	1.20	Glutathione peroxidase (
1307	1	db$2	2::C3KBX4|C3KBX4_PSEFS	45	59754	3	2	2	2	0.17	Putative membrane prot
1308	1	db$2	2::C3K8Z4|C3K8Z4_PSEFS	45	45613	2	2	2	2	0.23	High-affinity branched-c
1309	1	SwissProt_HUMAN$	3::P63220|RS21_HUMAN	45	9106	2	2	2	2	1.75	40S ribosomal protein S
1310	1	db$2	2::C3K4M2|C3K4M2_PSEFS	45	36363	3	2	3	2	0.30	Catabolite repressor/act
1311	1	db$2	2::C3K5X1|C3K5X1_PSEFS	45	46611	1	1	1	1	0.11	Histidine kinase OS=Pse
1312	1	db$2	2::C3K1M3|C3K1M3_PSEFS	45	11692	1	1	1	1	0.49	Iron-binding protein IscA
1313	1	db$2	2::C3K3Y1|C3K3Y1_PSEFS	45	14748	1	1	1	1	0.37	Flagellar protein FliL OS

↑Family	M	DB	Accession	Score	Mass	Matches	Match(sig)	Sequences	Seq(sig)	emPAI	Description
1314	1	db$2	2::C3K710|DCD_PSEFS	45	21231	3	3	3	3	0.94	dCTP deaminase OS=Ps
1315	1	db$2	2::C3JYL7|C3JYL7_PSEFS	45	12417	2	2	2	2	1.11	Putative copper resistan
1316	1	db$2	2::C3KAD1|C3KAD1_PSEFS	45	32401	3	2	3	2	0.34	Probable 4-deoxy-4-forr
1317	1	db$2	2::C3K0X7|C3K0X7_PSEFS	45	126663	1	1	1	1	0.04	Histidine kinase OS=Pse
1318	1	db$2	2::C3K9S5|C3K9S5_PSEFS	45	38309	3	2	3	2	0.28	Putative cobalamin biosy
1319	1	db$2	2::C3KAL9|C3KAL9_PSEFS	45	64520	2	2	2	2	0.16	Histidine kinase OS=Pse
1320	1	db$2	2::C3KCV5|C3KCV5_PSEFS	45	37705	2	2	2	2	0.28	Uncharacterized protein
1321	1	db$2	2::C3K1M0|C3K1M0_PSEFS	45	12311	1	1	1	1	0.46	2Fe-2S ferredoxin OS=F
1322	1	db$2	2::C3K8Z2|C3K8Z2_PSEFS	45	25669	1	1	1	1	0.20	High-affinity branched-c
1323	1	db$2	2::C3K2S2|C3K2S2_PSEFS	44	38886	2	1	2	1	0.13	Riboflavin biosynthesis p
1324	1	db$2	2::C3K176|C3K176_PSEFS	44	33571	2	2	2	2	0.32	Glucokinase OS=Pseudo
1325	1	db$2	2::C3KDE6|C3KDE6_PSEFS	44	25704	2	2	2	2	0.44	Tw-component system,
1326	1	db$2	2::C3KB68|C3KB68_PSEFS	44	11363	3	2	3	2	1.27	DUF1883 domain-contai
1327	1	db$2	2::C3K4Q4|RSMJ_PSEFS	44	27840	1	1	1	1	0.18	Ribosomal RNA small su
1328	1	SwissProt_HUMAN$	3::P61254|RL26_HUMAN	44	17248	2	2	2	2	0.72	60S ribosomal protein L
1328	2	SwissProt_HUMAN$	3::Q2PPJ7|RGPA2_HUMAN	40	210636	2	2	2	2	0.05	Ral GTPase-activating pr
1329	1	SwissProt_HUMAN$	3::O15078|CE290_HUMAN	44	290207	3	1	2	1	0.02	Centrosomal protein of 2
1330	1	db$2	2::C3K0S4|C3K0S4_PSEFS	44	65961	1	1	1	1	0.07	Isocitrate dehydrogenas
1331	1	db$2	2::C3K3M8|C3K3M8_PSEFS	44	36638	3	2	1	1	0.14	AB hydrolase-1 domain-
1332	1	db$2	2::C3KE45|C3KE45_PSEFS	44	49639	4	4	4	4	0.46	Trk system potassium u
1333	1	db$2	2::C3KCV6|C3KCV6_PSEFS	44	40154	4	2	4	2	0.27	Probable alcohol dehydr
1334	1	db$2	2::C3KDW0|C3KDW0_PSEFS	44	49939	2	2	2	2	0.21	N-acetylmuramoyl-L-ala
1335	1	db$2	2::C3JZ24|C3JZ24_PSEFS	44	24729	3	2	3	2	0.46	Putative GntR-family reg
1336	1	db$2	2::C3K753|C3K753_PSEFS	44	30652	1	1	1	1	0.17	Putative hydrolase OS=I
1337	1	db$2	2::C3K265|C3K265_PSEFS	44	29739	3	3	2	2	0.37	Dihydropteroate synthas
1338	1	db$2	2::C3K832|C3K832_PSEFS	44	33163	2	2	2	2	0.33	Non-specific serine/thre
1339	1	db$2	2::C3JZR0|C3JZR0_PSEFS	44	25453	1	1	1	1	0.20	Response regulatory prc
1340	1	db$2	2::C3K2P1|C3K2P1_PSEFS	43	48286	3	2	3	2	0.22	UDP-N-acetylmuramate-
1341	1	db$2	2::C3K9N0|C3K9N0_PSEFS	43	28569	2	2	2	2	0.39	Putative transcriptional r
1342	1	db$2	2::C3KAP0|C3KAP0_PSEFS	43	14934	2	2	2	2	0.86	4-carboxymuconolacton
1343	1	db$2	2::C3K450|C3K450_PSEFS	43	61786	1	1	1	1	0.08	Putative EAL/GGDEF dor
1344	1	db$2	2::C3JXT0|C3JXT0_PSEFS	43	22445	2	2	2	2	0.52	Putative isochorismatase
1345	1	db$2	2::C3JZW1|C3JZW1_PSEFS	43	36968	1	1	1	1	0.14	Putative cobalamin-bind
1346	1	db$2	2::C3KDG3|C3KDG3_PSEFS	43	15637	1	1	1	1	0.35	Putative exported protei
1347	1	db$2	2::C3K166|C3K166_PSEFS	43	65025	1	1	1	1	0.08	Histidine kinase OS=Pse
1348	1	db$2	2::C3K0S0|C3K0S0_PSEFS	43	12378	2	2	2	2	1.11	Putative DNA-binding pr
1349	1	db$2	2::C3K620|C3K620_PSEFS	43	22214	2	2	2	2	0.53	LexA repressor OS=Pseu
1350	1	db$2	2::C3KDM9|C3KDM9_PSEFS	43	28096	3	2	3	2	0.40	Putative oxidoreductase
1351	1	db$2	2::C3K0H9|C3K0H9_PSEFS	43	13855	1	1	1	1	0.40	Chemotaxis protein Che
1352	1	db$2	2::C3KDJ8|C3KDJ8_PSEFS	43	23307	2	2	2	2	0.50	Methyltransf_25 domain

↑Family	M	DB	Accession	Score	Mass	Matches	Match(sig)	Sequences	Seq(sig)	emPAI	Description
1353	1	db$2	2::C3KEB8|C3KEB8_PSEFS	42	34155	2	2	2	2	0.32	Putative lipoprotein OS=
1354	1	db$2	2::C3K129|C3K129_PSEFS	42	42141	1	1	1	1	0.12	Putative acetyl CoA acet
1355	1	db$2	2::C3K4U7|C3K4U7_PSEFS	42	28919	2	2	2	2	0.38	Putative amidotransfera
1356	1	db$2	2::C3K279|C3K279_PSEFS	42	19136	3	3	2	2	1.09	Outer membrane protei
1357	1	db$2	2::C3KAT0|C3KAT0_PSEFS	42	40804	2	2	2	2	0.26	Putative efflux system ir
1358	1	db$2	2::C3K6T6|C3K6T6_PSEFS	42	30009	1	1	1	1	0.17	Putative membrane-bou
1359	1	SwissProt_HUMAN$	3::P62280|RS11_HUMAN	42	18419	2	1	2	1	0.29	40S ribosomal protein S
1360	1	SwissProt_HUMAN$	3::Q86YZ3|HORN_HUMAN	42	282228	2	2	1	1	0.03	Hornerin OS=Homo sapi
1361	1	db$2	2::C3K2M3|C3K2M3_PSEFS	42	37518	2	2	2	2	0.29	Putative peptidoglycan-r
1362	1	db$2	2::C3KBZ4|C3KBZ4_PSEFS	42	6850	1	1	1	1	0.94	Uncharacterized protein
1363	1	db$2	2::C3K0Y8|COBT_PSEFS	42	36066	1	1	1	1	0.14	Nicotinate-nucleotide--d
1364	1	db$2	2::C3K8Z7|C3K8Z7_PSEFS	42	10906	2	2	2	2	1.34	Uncharacterized protein
1365	1	db$2	2::C3K4L1|C3K4L1_PSEFS	42	41720	1	1	1	1	0.12	Putative epimerase OS=
1366	1	db$2	2::C3K454|C3K454_PSEFS	42	13613	4	4	3	3	1.78	Putative cytochrome c5
1367	1	db$2	2::C3KCK0|C3KCK0_PSEFS	42	26687	5	2	4	2	0.42	Putative short-chain deh
1368	1	db$2	2::C3K8L6|C3K8L6_PSEFS	42	28069	1	1	1	1	0.18	Precorrin-6A synthase O
1369	1	db$2	2::C3K9N9|C3K9N9_PSEFS	42	57231	2	1	2	1	0.09	Putative ABC transport s
1370	1	db$2	2::C3K735|MSRB_PSEFS	42	14471	2	2	2	2	0.91	Peptide methionine sulfc
1371	1	db$2	2::C3JYR0|C3JYR0_PSEFS	42	13774	3	3	2	2	0.97	Glycine cleavage system
1372	1	db$2	2::C3KDC1|C3KDC1_PSEFS	41	29143	1	1	1	1	0.18	Putative oxidoreductase
1373	1	db$2	2::C3K8L0|C3K8L0_PSEFS	41	16885	1	1	1	1	0.32	Uncharacterized protein
1374	1	db$2	2::C3K1G3|C3K1G3_PSEFS	41	15162	2	2	1	1	0.36	Ribonuclease P protein c
1375	1	db$2	2::C3K4A6|C3K4A6_PSEFS	41	36556	1	1	1	1	0.14	ImpA_N domain-contain
1376	1	SwissProt_HUMAN$	3::P62244|RS15A_HUMAN	41	14830	3	1	3	1	0.37	40S ribosomal protein S
1377	1	db$2	2::C3K5G4|RLMM_PSEFS	41	40585	1	1	1	1	0.12	Ribosomal RNA large su
1378	1	SwissProt_HUMAN$	3::P15531|NDKA_HUMAN	41	17138	3	3	3	3	1.27	Nucleoside diphosphate
1379	1	db$2	2::C3K1V0|C3K1V0_PSEFS	41	56633	1	1	1	1	0.09	Nitric oxide reductase si
1380	1	SwissProt_HUMAN$	3::Q6FI13|H2A2A_HUMAN	41	14087	2	2	2	2	0.94	Histone H2A type 2-A O
1381	1	db$2	2::C3K4K0|C3K4K0_PSEFS	41	42230	1	1	1	1	0.12	Hypothetical exported p
1382	1	db$2	2::C3K6T8|C3K6T8_PSEFS	41	49583	3	3	3	3	0.33	Alginate biosynthesis tra
1383	1	SwissProt_HUMAN$	3::P33992|MCM5_HUMAN	41	82233	5	2	2	1	0.06	DNA replication licensin
1384	1	db$2	2::C3K2Q3|C3K2Q3_PSEFS	41	23107	1	1	1	1	0.23	Putative hydrolase OS=I
1385	1	db$2	2::C3K8V8|C3K8V8_PSEFS	41	36166	2	1	2	1	0.14	Zinc-type alcohol dehyd
1386	1	db$2	2::C3K3J1|C3K3J1_PSEFS	41	16523	1	1	1	1	0.33	Putative chemotaxis-rela
1387	1	db$2	2::C3K453|C3K453_PSEFS	41	71119	4	2	4	2	0.14	AcetylCoA_hyd_C domai
1388	1	SwissProt_HUMAN$	3::P61513|RL37A_HUMAN	41	10268	2	2	2	2	1.45	60S ribosomal protein L
1389	1	db$2	2::C3KDD0|C3KDD0_PSEFS	40	19011	1	1	1	1	0.28	Lipid A deacylase OS=P
1390	1	db$2	2::C3K3N6|FPG_PSEFS	40	30025	2	1	2	1	0.17	Formamidopyrimidine-D
1391	1	SwissProt_HUMAN$	3::Q6IS14|IF5AL_HUMAN	40	16762	1	1	1	1	0.32	Eukaryotic translation in
1392	1	db$2	2::C3K3X9|C3K3X9_PSEFS	40	98433	2	2	2	2	0.10	Putative ABC transport s

↑Family	M	DB	Accession	Score	Mass	Matches	Match(sig)	Sequences	Seq(sig)	emPAI	Description
1393	1	db$2	2::C3K4I9|C3K4I9_PSEFS	40	42202	1	1	1	1	0.12	Galactonate dehydratase
1394	1	SwissProt_HUMAN$	3::P62888|RL30_HUMAN	40	12776	1	1	1	1	0.44	60S ribosomal protein L
1395	1	db$2	2::C3KCS7|MRAY_PSEFS	40	39243	3	2	3	2	0.27	Phospho-N-acetylmuram
1396	1	SwissProt_HUMAN$	3::P31939|PUR9_HUMAN	40	64575	1	1	1	1	0.08	Bifunctional purine biosy
1397	1	db$2	2::C3K6U7|C3K6U7_PSEFS	40	79141	6	5	5	5	0.35	Putative exported protei
1398	1	db$2	2::C3K8T8|C3K8T8_PSEFS	40	14354	2	2	2	2	0.92	Sec-independent proteir
1399	1	db$2	2::C3K451|C3K451_PSEFS	40	76463	2	1	2	1	0.06	ATP-dependent DNA hel
1400	1	db$2	2::C3JYK2|C3JYK2_PSEFS	40	30492	1	1	1	1	0.17	Bifunctional protein FolD
1401	1	db$2	2::C3K536|C3K536_PSEFS	40	22752	2	2	2	2	0.51	DUF4174 domain-contai
1402	1	db$2	2::C3JYU4|C3JYU4_PSEFS	40	20911	1	1	1	1	0.25	Corrinoid adenosyltransf
1403	1	SwissProt_HUMAN$	3::P0DKB5|TPBGL_HUMAN	40	40374	1	1	1	1	0.12	Trophoblast glycoproteir
1404	1	db$2	2::C3K7A2|C3K7A2_PSEFS	39	33841	1	1	1	1	0.15	Putative membrane prot
1405	1	db$2	2::C3KDC2|ISPE_PSEFS	39	30632	1	1	1	1	0.17	4-diphosphocytidyl-2-C-
1406	1	db$2	2::C3KE08|C3KE08_PSEFS	39	25044	3	3	3	3	0.75	Conserved hypothetical
1407	1	SwissProt_HUMAN$	3::O94817|ATG12_HUMAN	39	15104	1	1	1	1	0.36	Ubiquitin-like protein AT
1408	1	SwissProt_HUMAN$	3::Q71UM5|RS27L_HUMAN	39	9471	2	1	2	1	0.63	40S ribosomal protein S
1409	1	db$2	2::C3KBZ5|C3KBZ5_PSEFS	39	23751	1	1	1	1	0.22	Endonuclease III OS=Ps
1410	1	db$2	2::C3JXX9|C3JXX9_PSEFS	39	6925	3	3	2	2	2.76	Translational regulator C
1411	1	db$2	2::C3K600|C3K600_PSEFS	39	28761	3	3	2	2	0.39	Phosphatidate cytidylyltı
1412	1	db$2	2::C3K598|C3K598_PSEFS	39	34258	1	1	1	1	0.15	Curved DNA-binding pro
1413	1	db$2	2::C3JYG1|C3JYG1_PSEFS	39	30646	2	2	2	2	0.36	Putative LysR-family tra
1414	1	db$2	2::C3K8L9|C3K8L9_PSEFS	39	65348	1	1	1	1	0.08	Putative potassium-efflu
1415	1	db$2	2::C3K7V7|C3K7V7_PSEFS	39	18111	1	1	1	1	0.30	Uncharacterized protein
1416	1	db$2	2::C3JXZ4|C3JXZ4_PSEFS	39	40619	2	2	2	2	0.26	Uncharacterized protein
1417	1	db$2	2::C3KBJ0|C3KBJ0_PSEFS	39	16933	1	1	1	1	0.32	[Ribosomal protein S18]
1418	1	db$2	2::C3K5B5|C3K5B5_PSEFS	39	37023	1	1	1	1	0.14	Dipeptide ABC transport
1419	1	db$2	2::C3K6C0|C3K6C0_PSEFS	39	25791	2	1	2	1	0.20	Putative amino acid ABC
1420	1	db$2	2::C3K0L0|C3K0L0_PSEFS	39	29492	2	1	2	1	0.17	Putative IclR-family regu
1421	1	db$2	2::C3KDR4|C3KDR4_PSEFS	39	49531	4	2	4	2	0.21	Ribosomal protein S12 r
1422	1	db$2	2::C3K5T7|C3K5T7_PSEFS	39	23769	1	1	1	1	0.22	Uncharacterized protein
1423	1	db$2	2::C3K2K6|C3K2K6_PSEFS	39	49458	3	3	3	3	0.33	tRNA-2-methylthio-N(6)
1424	1	db$2	2::C3JY56|C3JY56_PSEFS	39	48640	2	2	2	2	0.21	Replication-associated r
1425	1	db$2	2::C3K6T7|C3K6T7_PSEFS	39	66001	1	1	1	1	0.07	Histidine kinase OS=Pse
1426	1	SwissProt_HUMAN$	3::P30050|RL12_HUMAN	38	17808	1	1	1	1	0.30	60S ribosomal protein L
1427	1	db$2	2::C3K7C2|C3K7C2_PSEFS	38	16947	1	1	1	1	0.32	tRNA (cytidine(34)-2'-O
1428	1	db$2	2::C3K200|C3K200_PSEFS	38	32052	1	1	1	1	0.16	Cat operon regulatory p
1429	1	db$2	2::C3K429|C3K429_PSEFS	38	8856	2	2	2	2	1.82	Uncharacterized protein
1430	1	db$2	2::C3JY51|C3JY51_PSEFS	38	37030	1	1	1	1	0.14	Putative glycosyl transfe
1431	1	db$2	2::C3K2P0|C3K2P0_PSEFS	38	22640	2	2	2	2	0.51	Flavin prenyltransferase
1432	1	SwissProt_HUMAN$	3::P60866|RS20_HUMAN	38	13364	1	1	1	1	0.42	40S ribosomal protein S

↑Family	M	DB	Accession	Score	Mass	Matches	Match(sig)	Sequences	Seq(sig)	emPAI	Description
1433	1	SwissProt_HUMAN$	3::O75251|NDUS7_HUMAN	38	23548	1	1	1	1	0.22	NADH dehydrogenase [u
1434	1	db$2	2::C3KBI9|C3KBI9_PSEFS	38	29043	1	1	1	1	0.18	Uncharacterized protein
1435	1	db$2	2::C3K0P0|C3K0P0_PSEFS	38	35982	2	2	2	2	0.30	Putative peptidase OS=I
1436	1	SwissProt_HUMAN$	3::P04003|C4BPA_HUMAN	38	66989	1	1	1	1	0.07	C4b-binding protein alpl
1437	1	db$2	2::C3K637|C3K637_PSEFS	38	56441	1	1	1	1	0.09	Putative peptide ABC tra
1438	1	db$2	2::C3K0B2|C3K0B2_PSEFS	38	20071	1	1	1	1	0.26	Putative membrane prot
1439	1	db$2	2::C3K1Y4|C3K1Y4_PSEFS	38	14766	2	2	2	2	0.88	Putative putative glyoxa
1440	1	db$2	2::C3K0D2|C3K0D2_PSEFS	37	35834	2	1	2	1	0.14	Lipid A biosynthesis laur
1441	1	db$2	2::C3JY25|C3JY25_PSEFS	37	37002	2	1	2	1	0.14	UDP-N-acetylenolpyruvc
1442	1	SwissProt_HUMAN$	3::Q15075|EEA1_HUMAN	37	162367	3	2	2	1	0.03	Early endosome antigen
1443	1	db$2	2::C3JZP0|C3JZP0_PSEFS	37	11251	1	1	1	1	0.51	Putative lipoprotein OS=
1444	1	db$2	2::C3K0M9|KTHY_PSEFS	37	22917	3	2	3	2	0.51	Thymidylate kinase OS=
1445	1	db$2	2::C3K7J4|C3K7J4_PSEFS	37	18330	2	2	1	1	0.29	Uncharacterized protein
1446	1	db$2	2::C3KA94|C3KA94_PSEFS	37	41012	1	1	1	1	0.12	Uncharacterized protein
1447	1	SwissProt_HUMAN$	3::Q6IV72|ZN425_HUMAN	37	87663	1	1	1	1	0.06	Zinc finger protein 425 (
1448	1	SwissProt_HUMAN$	3::P46779|RL28_HUMAN	37	15738	1	1	1	1	0.34	60S ribosomal protein L
1449	1	db$2	2::C3KBI8|C3KBI8_PSEFS	37	48805	2	2	2	2	0.21	Uncharacterized protein
1450	1	db$2	2::C3K1H9|C3K1H9_PSEFS	37	18692	2	2	2	2	0.65	Conserved hypothetical
1451	1	db$2	2::C3K8W2|ADE_PSEFS	37	36048	2	2	2	2	0.30	Adenine deaminase OS=
1452	1	db$2	2::C3K7D7|C3K7D7_PSEFS	37	8979	1	1	1	1	0.67	Putative lipoprotein OS=
1453	1	db$2	2::C3K8U0|HIS2_PSEFS	37	12007	3	3	2	2	1.17	Phosphoribosyl-ATP pyrc
1454	1	db$2	2::C3K6Y6|C3K6Y6_PSEFS	37	31839	6	3	5	3	0.56	Signal peptidase I OS=F
1455	1	db$2	2::C3K5D9|C3K5D9_PSEFS	37	43455	1	1	1	1	0.12	Putative cysteine desulfu
1456	1	db$2	2::C3K6C3|C3K6C3_PSEFS	37	28259	1	1	1	1	0.18	Putative amino acid ABC
1457	1	db$2	2::C3K4I1|C3K4I1_PSEFS	37	29333	1	1	1	1	0.17	Shikimate dehydrogenas
1458	1	SwissProt_HUMAN$	3::P0DMR1|HNRC4_HUMAN	37	32010	1	1	1	1	0.16	Heterogeneous nuclear r
1459	1	db$2	2::C3JY27|KDSB_PSEFS	36	27609	1	1	1	1	0.19	3-deoxy-manno-octulos
1460	1	SwissProt_HUMAN$	3::P48059|LIMS1_HUMAN	36	37226	2	1	2	1	0.14	LIM and senescent cell a
1461	1	db$2	2::C3KE01|Y992_PSEFS	36	29383	4	3	4	3	0.62	UPF0246 protein PFLU_(
1462	1	SwissProt_HUMAN$	3::Q8NHW5|RLA0L_HUMAN	36	34343	1	1	1	1	0.15	60S acidic ribosomal prc
1463	1	db$2	2::C3JYP6|C3JYP6_PSEFS	36	10477	3	2	2	2	1.43	YCII domain-containing
1464	1	db$2	2::C3KE42|C3KE42_PSEFS	36	20828	1	1	1	1	0.25	Putative DNA-3-methyla
1465	1	SwissProt_HUMAN$	3::Q96T58|MINT_HUMAN	36	402004	1	1	1	1	0.01	Msx2-interacting proteir
1466	1	db$2	2::C3KDR2|C3KDR2_PSEFS	36	26142	2	1	2	1	0.20	S4 RNA-binding domain-
1467	1	db$2	2::C3K223|C3K223_PSEFS	36	37863	2	2	2	2	0.28	Protein-methionine-sulfc
1468	1	SwissProt_HUMAN$	3::Q9H1K0|RBNS5_HUMAN	36	88815	1	1	1	1	0.05	Rabenosyn-5 OS=Homo
1469	1	db$2	2::C3KCP9|C3KCP9_PSEFS	36	30268	2	2	2	2	0.36	Lipopolysaccharide core
1470	1	db$2	2::C3K2E4|C3K2E4_PSEFS	36	29249	3	2	3	2	0.38	Ferredoxin--NADP reduc
1471	1	db$2	2::C3K0I8|C3K0I8_PSEFS	36	26895	1	1	1	1	0.19	Flagellar biosynthetic pr
1472	1	db$2	2::C3KE72|C3KE72_PSEFS	36	85807	3	2	3	2	0.12	UPF0313 protein PFLU_(

↑Family	M	DB	Accession	Score	Mass	Matches	Match(sig)	Sequences	Seq(sig)	emPAI	Description
1473	1	db$2	2::C3K1D4|C3K1D4_PSEFS	36	64734	1	1	1	1	0.08	Uncharacterized protein
1474	1	db$2	2::C3K2S4|C3K2S4_PSEFS	36	15995	1	1	1	1	0.34	Putative lipoprotein OS=
1475	1	db$2	2::C3K2V5|RL36_PSEFS	36	4431	1	1	1	1	1.73	50S ribosomal protein L
1476	1	db$2	2::C3K2F6|TRMA_PSEFS	36	40867	1	1	1	1	0.12	tRNA/tmRNA (uracil-C(5
1477	1	SwissProt_HUMAN$	3::P04075|ALDOA_HUMAN	36	39395	2	2	2	2	0.27	Fructose-bisphosphate a
1478	1	SwissProt_HUMAN$	3::P39019|RS19_HUMAN	36	16051	2	2	2	2	0.79	40S ribosomal protein S
1479	1	db$2	2::C3K390|C3K390_PSEFS	36	18447	3	1	2	1	0.29	Zn_protease domain-coi
1480	1	SwissProt_HUMAN$	3::P63173|RL38_HUMAN	36	8213	1	1	1	1	0.75	60S ribosomal protein L
1481	1	SwissProt_HUMAN$	3::Q9BVV6|TALD3_HUMAN	36	169201	1	1	1	1	0.03	Protein TALPID3 OS=Ho
1482	1	SwissProt_HUMAN$	3::Q9ULV4|COR1C_HUMAN	35	53215	1	1	1	1	0.09	Coronin-1C OS=Homo s
1483	1	SwissProt_HUMAN$	3::P62318|SMD3_HUMAN	35	13907	1	1	1	1	0.40	Small nuclear ribonuclec
1484	1	db$2	2::C3K3R4|C3K3R4_PSEFS	35	24681	3	3	3	3	0.77	Putative hydrolase OS=I
1485	1	db$2	2::C3K694|C3K694_PSEFS	35	48962	1	1	1	1	0.10	[utative peptidase OS=F
1486	1	db$2	2::C3K2L8|LIPA_PSEFS	35	38163	4	2	4	2	0.28	Lipoyl synthase OS=Pse
1487	1	db$2	2::C3K9Q2|C3K9Q2_PSEFS	35	16954	4	2	3	2	0.73	Ribonuclease H OS=Pse
1488	1	db$2	2::C3JZ73|C3JZ73_PSEFS	35	49677	2	2	2	2	0.21	Porin OS=Pseudomonas
1489	1	db$2	2::C3K1F1|ATPL_PSEFS	35	8603	6	5	2	2	7.36	ATP synthase subunit c
1490	1	db$2	2::C3K467|C3K467_PSEFS	35	13453	1	1	1	1	0.41	Cupin_3 domain-contair
1491	1	db$2	2::C3KAX2|C3KAX2_PSEFS	35	79149	1	1	1	1	0.06	Histidine kinase OS=Pse
1492	1	SwissProt_HUMAN$	3::P62847|RS24_HUMAN	35	15413	1	1	1	1	0.35	40S ribosomal protein S
1493	1	db$2	2::C3K9P7|C3K9P7_PSEFS	35	69450	2	2	2	2	0.15	Putative substrate-bindi
1494	1	db$2	2::C3K4N5|C3K4N5_PSEFS	35	28830	1	1	1	1	0.18	Putative amino acid ABC
1495	1	db$2	2::C3K0P1|C3K0P1_PSEFS	35	24214	1	1	1	1	0.21	Putative hydrolase OS=I
1496	1	db$2	2::C3K413|C3K413_PSEFS	35	22663	1	1	1	1	0.23	Putative ABC transport s
1497	1	db$2	2::C3KDM8|C3KDM8_PSEFS	35	57105	1	1	1	1	0.09	HDOD domain-containin
1498	1	db$2	2::C3K0P2|C3K0P2_PSEFS	35	35618	2	2	2	2	0.30	Pseudouridine synthase
1499	1	db$2	2::C3K998|C3K998_PSEFS	35	35021	3	2	3	2	0.31	Putative dehydrogenase
1500	1	db$2	2::C3K6H7|C3K6H7_PSEFS	35	10540	1	1	1	1	0.55	Cell division protein FtsE
1501	1	SwissProt_HUMAN$	3::Q9ULM0|PKHH1_HUMAN	35	151137	2	2	2	2	0.06	Pleckstrin homology dor
1502	1	db$2	2::C3KAW1|C3KAW1_PSEFS	35	75166	1	1	1	1	0.07	Putative EAL/GGDEF dor
1503	1	SwissProt_HUMAN$	3::P10599|THIO_HUMAN	35	11730	1	1	1	1	0.48	Thioredoxin OS=Homo s
1504	1	db$2	2::C3JZ90|C3JZ90_PSEFS	35	52890	1	1	1	1	0.09	Trk system potassium u
1505	1	db$2	2::C3K0Q8|C3K0Q8_PSEFS	34	24717	1	1	1	1	0.21	Basal-body rod modifica
1506	1	SwissProt_HUMAN$	3::Q9UKE5|TNIK_HUMAN	34	154848	1	1	1	1	0.03	TRAF2 and NCK-interact
1507	1	db$2	2::C3K2Y9|C3K2Y9_PSEFS	34	13142	2	2	2	2	1.03	Protein translocase subu
1508	1	db$2	2::C3K493|C3K493_PSEFS	34	16495	2	1	2	1	0.33	Uncharacterized protein
1509	1	SwissProt_HUMAN$	3::Q9H0U4|RAB1B_HUMAN	34	22157	1	1	1	1	0.24	Ras-related protein Rab-
1510	1	db$2	2::C3K4W6|C3K4W6_PSEFS	34	44511	3	2	3	2	0.24	Putative lipoprotein rele
1511	1	SwissProt_HUMAN$	3::P01009|A1AT_HUMAN	34	46707	1	1	1	1	0.11	Alpha-1-antitrypsin OS=
1512	1	SwissProt_HUMAN$	3::Q6ZSJ9|SHSA6_HUMAN	34	55729	1	1	1	1	0.09	Protein shisa-6 OS=Hon

↑Family	M	DB	Accession	Score	Mass	Matches	Match(sig)	Sequences	Seq(sig)	emPAI	Description
1513	1	SwissProt_HUMAN$	3::A6NCI4|VWA3A_HUMAN	34	133935	2	1	2	1	0.04	von Willebrand factor A
1514	1	SwissProt_HUMAN$	3::P46776|RL27A_HUMAN	34	16551	1	1	1	1	0.33	60S ribosomal protein L
1515	1	db$2	2::C3K2R8|NUSB_PSEFS	34	18764	1	1	1	1	0.28	Transcription antitermin
1516	1	SwissProt_HUMAN$	3::P42694|HELZ_HUMAN	34	218833	3	2	2	1	0.02	Probable helicase with z
1517	1	SwissProt_HUMAN$	3::P02741|CRP_HUMAN	34	25023	2	2	2	2	0.46	C-reactive protein OS=H
1518	1	db$2	2::C3K9Z1|C3K9Z1_PSEFS	34	21192	1	1	1	1	0.25	FMN dependent NADH:q
1519	1	db$2	2::C3K8V7|C3K8V7_PSEFS	34	6725	1	1	1	1	0.96	Uncharacterized protein
1520	1	SwissProt_HUMAN$	3::P32969|RL9_HUMAN	34	21850	1	1	1	1	0.24	60S ribosomal protein L
1521	1	SwissProt_HUMAN$	3::P62263|RS14_HUMAN	34	16263	1	1	1	1	0.33	40S ribosomal protein S
1522	1	db$2	2::C3K7L9|C3K7L9_PSEFS	34	28704	1	1	1	1	0.18	Putative membrane prot
1523	1	db$2	2::C3JZ46|C3JZ46_PSEFS	34	37091	1	1	1	1	0.14	Putative LacI family trar
1524	1	SwissProt_HUMAN$	3::P04350|TBB4A_HUMAN	34	49554	1	1	1	1	0.10	Tubulin beta-4A chain O
1525	1	db$2	2::C3K975|C3K975_PSEFS	34	21948	1	1	1	1	0.24	Putative glutathione S-t
1526	1	db$2	2::C3K3W4|C3K3W4_PSEFS	33	13233	2	2	2	2	1.02	Glycine cleavage system
1527	1	db$2	2::C3K2K1|C3K2K1_PSEFS	33	28157	1	1	1	1	0.18	Phos_pyr_kin domain-cc
1528	1	db$2	2::C3K2M4|C3K2M4_PSEFS	33	40052	1	1	1	1	0.13	Peptidoglycan glycosyltr
1529	1	db$2	2::C3K6Y7|RNC_PSEFS	33	25367	1	1	1	1	0.20	Ribonuclease 3 OS=Pseu
1530	1	db$2	2::C3K4Z1|C3K4Z1_PSEFS	33	72318	2	2	2	2	0.14	Cytochrome C biogenesi
1531	1	db$2	2::C3K4H8|C3K4H8_PSEFS	33	20657	1	1	1	1	0.26	Threonylcarbamoyl-AMP
1532	1	db$2	2::C3JXS4|C3JXS4_PSEFS	33	25108	1	1	1	1	0.21	Tol-Pal system protein T
1533	1	db$2	2::C3KBT8|C3KBT8_PSEFS	33	29541	2	2	2	2	0.38	Sulfate transport system
1534	1	db$2	2::C3KAZ7|C3KAZ7_PSEFS	33	48577	1	1	1	1	0.10	Putative chain length de
1535	1	db$2	2::C3K216|C3K216_PSEFS	33	17384	1	1	1	1	0.31	Putative putative lipopro
1536	1	db$2	2::C3JY92|C3JY92_PSEFS	33	55851	2	2	2	2	0.18	NADH dehydrogenase I
1537	1	db$2	2::A4V6U2|A4V6U2_PSEFS	33	76547	2	2	1	1	0.06	Uncharacterized protein
1538	1	db$2	2::C3K7D0|THII_PSEFS	33	54519	1	1	1	1	0.09	tRNA sulfurtransferase C
1539	1	SwissProt_HUMAN$	3::P02675|FIBB_HUMAN	33	55892	3	1	3	1	0.09	Fibrinogen beta chain OS
1540	1	db$2	2::C3KD16|C3KD16_PSEFS	33	34294	2	1	2	1	0.15	Putative 2-hydroxyacid c
1541	1	SwissProt_HUMAN$	3::Q8NBF4|YG006_HUMAN	33	16172	1	1	1	1	0.33	Putative uncharacterized
1542	1	db$2	2::C3K3M2|C3K3M2_PSEFS	33	37029	2	2	2	2	0.29	Cell division protein FtsX
1543	1	db$2	2::C3K458|C3K458_PSEFS	33	47152	2	2	2	2	0.22	D-amino acid dehydroge
1544	1	db$2	2::C3K5D6|C3K5D6_PSEFS	33	97394	2	2	2	2	0.10	Putative beta-(1-3)-gluc
1545	1	db$2	2::C3K6A6|C3K6A6_PSEFS	33	25667	1	1	1	1	0.20	Putative lipoprotein OS=
1546	1	SwissProt_HUMAN$	3::Q13304|GPR17_HUMAN	33	40963	1	1	1	1	0.12	Uracil nucleotide/cysteir
1547	1	db$2	2::C3K1H0|RIMM_PSEFS	33	20004	2	2	2	2	0.60	Ribosome maturation fa
1548	1	db$2	2::C3K328|APAG_PSEFS	33	13631	2	2	2	2	0.98	Protein ApaG OS=Pseud
1549	1	db$2	2::C3K574|C3K574_PSEFS	33	36116	2	1	2	1	0.14	Putative oxidoreductase
1550	1	db$2	2::C3K9Y9|C3K9Y9_PSEFS	32	13239	1	1	1	1	0.42	Uncharacterized protein
1551	1	SwissProt_HUMAN$	3::Q06546|GABPA_HUMAN	32	51263	1	1	1	1	0.10	GA-binding protein alpha
1552	1	db$2	2::C3KBL6|C3KBL6_PSEFS	32	27732	1	1	1	1	0.18	Glutamate/aspartate AB

↑Family	M	DB	Accession	Score	Mass	Matches	Match(sig)	Sequences	Seq(sig)	emPAI	Description
1553	1	db$2	2::C3KBV2|C3KBV2_PSEFS	32	15686	2	2	2	2	0.82	Thioredoxin OS=Pseudo
1554	1	db$2	2::C3K585|C3K585_PSEFS	32	25105	1	1	1	1	0.21	Putative hydrolase OS=I
1555	1	db$2	2::C3K4W5|C3K4W5_PSEFS	32	24663	4	3	4	3	0.77	Lipoprotein-releasing sy
1556	1	db$2	2::C3K0B8|C3K0B8_PSEFS	32	24379	1	1	1	1	0.21	Uncharacterized protein
1557	1	db$2	2::C3JXG5|C3JXG5_PSEFS	32	36156	1	1	1	1	0.14	Putative GFO/IDH/MocA
1558	1	SwissProt_HUMAN$	3::Q7LBC6|KDM3B_HUMAN	32	191461	1	1	1	1	0.03	Lysine-specific demethy
1559	1	db$2	2::C3JXM0|C3JXM0_PSEFS	32	45141	2	1	2	1	0.11	Putative peptidase OS=I
1560	1	db$2	2::C3K915|C3K915_PSEFS	32	26912	1	1	1	1	0.19	Putative GntR-family reg
1561	1	db$2	2::C3K6V8|C3K6V8_PSEFS	32	47941	3	1	3	1	0.10	Putative peptidase OS=I
1562	1	db$2	2::C3K7B3|C3K7B3_PSEFS	32	51722	1	1	1	1	0.10	Putative membrane-bou
1563	1	db$2	2::C3K2U2|C3K2U2_PSEFS	32	20033	3	3	3	3	1.02	Putative hydrolase OS=I
1564	1	db$2	2::C3K1A2|C3K1A2_PSEFS	32	17206	2	2	2	2	0.72	Putative membrane prot
1565	1	SwissProt_HUMAN$	3::O00299|CLIC1_HUMAN	32	26906	1	1	1	1	0.19	Chloride intracellular cha
1566	1	db$2	2::C3K811|C3K811_PSEFS	32	43409	1	1	1	1	0.12	Imidazolonepropionase (
1567	1	db$2	2::C3K784|C3K784_PSEFS	32	23456	2	2	2	2	0.49	3-oxoadipate CoA-transf
1568	1	db$2	2::C3K7A1|C3K7A1_PSEFS	32	56003	2	2	2	2	0.18	Putative LuxR-family reg
1569	1	db$2	2::C3K146|C3K146_PSEFS	32	27134	2	2	2	2	0.42	Fumarate and nitrate re
1570	1	db$2	2::C3KE21|C3KE21_PSEFS	32	10230	2	1	2	1	0.57	FAD assembly factor Sdl
1571	1	db$2	2::C3KCN6|C3KCN6_PSEFS	32	53164	1	1	1	1	0.09	Sodium/proline symport
1572	1	db$2	2::C3K4Q6|C3K4Q6_PSEFS	32	39864	1	1	1	1	0.13	Putative lipoprotein OS=
1573	1	SwissProt_HUMAN$	3::Q8WXD0|RXFP2_HUMAN	32	86396	1	1	1	1	0.06	Relaxin receptor 2 OS=H
1574	1	db$2	2::C3JZN5|RL35_PSEFS	32	7363	1	1	1	1	0.87	50S ribosomal protein L
1575	1	db$2	2::C3JYQ1|C3JYQ1_PSEFS	32	48078	2	2	2	2	0.22	Putative CoA transferase
1576	1	db$2	2::C3JZ44|C3JZ44_PSEFS	32	47432	1	1	1	1	0.10	Putative gluconate perm
1577	1	SwissProt_HUMAN$	3::Q8IW45|NNRD_HUMAN	32	36553	1	1	1	1	0.14	ATP-dependent (S)-NAD
1578	1	db$2	2::C3K5W0|C3K5W0_PSEFS	31	170278	3	3	3	3	0.09	DUF4329 domain-contai
1579	1	SwissProt_HUMAN$	3::Q14147|DHX34_HUMAN	31	128040	1	1	1	1	0.04	Probable ATP-dependent
1580	1	db$2	2::C3JYJ2|C3JYJ2_PSEFS	31	27205	2	2	2	2	0.41	Putative lipoprotein OS=
1581	1	db$2	2::C3K672|C3K672_PSEFS	31	58670	1	1	1	1	0.08	Cation/acetate symporte
1582	1	SwissProt_HUMAN$	3::P17480|UBF1_HUMAN	31	89350	1	1	1	1	0.05	Nucleolar transcription f
1583	1	SwissProt_HUMAN$	3::Q99618|CDCA3_HUMAN	31	28981	1	1	1	1	0.18	Cell division cycle-assoc
1584	1	SwissProt_HUMAN$	3::Q8IUI8|CRLF3_HUMAN	31	49735	1	1	1	1	0.10	Cytokine receptor-like fa
1585	1	db$2	2::C3K0N8|C3K0N8_PSEFS	31	19586	2	2	2	2	0.62	23S rRNA accumulation
1586	1	SwissProt_HUMAN$	3::P62269|RS18_HUMAN	31	17708	3	2	3	2	0.69	40S ribosomal protein S
1587	1	db$2	2::C3K4F4|C3K4F4_PSEFS	31	25363	1	1	1	1	0.20	Putative acetyltransferas
1588	1	db$2	2::C3K021|C3K021_PSEFS	31	27911	3	2	3	2	0.40	Phosphoadenosine phos
1589	1	SwissProt_HUMAN$	3::P61247|RS3A_HUMAN	31	29926	1	1	1	1	0.17	40S ribosomal protein S
1590	1	db$2	2::C3K353|C3K353_PSEFS	31	44910	1	1	1	1	0.11	Putative aminotransfera
1591	1	db$2	2::C3K7E9|C3K7E9_PSEFS	31	20872	1	1	1	1	0.25	Putative lipoprotein OS=
1592	1	db$2	2::C3K6W1|C3K6W1_PSEFS	31	9231	1	1	1	1	0.65	Glutaredoxin OS=Pseud

↑Family	M	DB	Accession	Score	Mass	Matches	Match(sig)	Sequences	Seq(sig)	emPAI	Description
1593	1	db$2	2::C3K3K7|C3K3K7_PSEFS	31	24490	2	2	1	1	0.21	Pyridoxal phosphate hor
1594	1	db$2	2::C3KB69|C3KB69_PSEFS	31	12397	1	1	1	1	0.45	Putative lipoprotein OS=
1595	1	db$2	2::C3K277|C3K277_PSEFS	31	60895	3	3	3	3	0.26	DNA repair protein RecN
1596	1	db$2	2::C3K340|C3K340_PSEFS	31	72802	1	1	1	1	0.07	DNA primase OS=Pseud
1597	1	db$2	2::C3K391|C3K391_PSEFS	31	33913	1	1	1	1	0.15	Putative acyltransferase
1598	1	SwissProt_HUMAN$	3::P26641|EF1G_HUMAN	31	50087	1	1	1	1	0.10	Elongation factor 1-gam
1599	1	SwissProt_HUMAN$	3::O14917|PCD17_HUMAN	31	126151	1	1	1	1	0.04	Protocadherin-17 OS=H
1600	1	db$2	2::C3K1I1|C3K1I1_PSEFS	31	24836	1	1	1	1	0.21	Nudix hydrolase domain
1601	1	SwissProt_HUMAN$	3::P08397|HEM3_HUMAN	30	39306	1	1	1	1	0.13	Porphobilinogen deamin
1602	1	db$2	2::C3KAM0|C3KAM0_PSEFS	30	50872	2	1	2	1	0.10	Putative two-component
1603	1	db$2	2::C3K732|TPMT_PSEFS	30	24819	1	1	1	1	0.21	Thiopurine S-methyltrar
1604	1	SwissProt_HUMAN$	3::Q69YQ0|CYTSA_HUMAN	30	124525	2	1	1	1	0.04	Cytospin-A OS=Homo sa
1605	1	SwissProt_HUMAN$	3::Q9ULQ1|TPC1_HUMAN	30	94086	1	1	1	1	0.05	Two pore calcium chann
1606	1	db$2	2::C3K2P8|C3K2P8_PSEFS	30	24358	1	1	1	1	0.21	Membrane-related prote
1607	1	db$2	2::C3K0F5|C3K0F5_PSEFS	30	23098	1	1	1	1	0.23	Putative TetR-family trai
1608	1	db$2	2::C3K0N0|C3K0N0_PSEFS	30	41535	1	1	1	1	0.12	Endolytic murein transgl
1609	1	db$2	2::C3K5M1|C3K5M1_PSEFS	30	26375	1	1	1	1	0.19	Phosphonates ABC trans
1610	1	db$2	2::C3K0J8|C3K0J8_PSEFS	30	22091	1	1	1	1	0.24	START domain-containir
1611	1	db$2	2::C3K1U6|C3K1U6_PSEFS	30	74730	2	2	2	2	0.14	Cytochrome bo(3) ubiqu
1612	1	db$2	2::C3K5I1|C3K5I1_PSEFS	30	43688	1	1	1	1	0.11	Putative phage integrase
1613	1	db$2	2::C3K717|C3K717_PSEFS	30	27466	1	1	1	1	0.19	Putative amino acid trar
1614	1	db$2	2::C3KCP7|C3KCP7_PSEFS	30	39236	1	1	1	1	0.13	Lipopolysaccharide hept
1615	1	SwissProt_HUMAN$	3::Q9UQE7|SMC3_HUMAN	30	141454	1	1	1	1	0.03	Structural maintenance
1616	1	SwissProt_HUMAN$	3::A6NNS2|DRS7C_HUMAN	30	34855	1	1	1	1	0.14	Dehydrogenase/reducta
1617	1	SwissProt_HUMAN$	3::Q96E52|OMA1_HUMAN	30	60081	1	1	1	1	0.08	Metalloendopeptidase O
1618	1	db$2	2::C3K6B1|C3K6B1_PSEFS	30	35098	1	1	1	1	0.14	Homoserine kinase OS=
1619	1	db$2	2::C3KCS4|C3KCS4_PSEFS	30	63231	1	1	1	1	0.08	Peptidoglycan D,D-trans
1620	1	db$2	2::C3KE76|C3KE76_PSEFS	30	92563	1	1	1	1	0.05	Alpha-E domain-contain
1621	1	db$2	2::C3K2L0|C3K2L0_PSEFS	30	55816	2	1	2	1	0.09	Apolipoprotein N-acyltra
1622	1	db$2	2::C3K9N3|C3K9N3_PSEFS	30	10076	1	1	1	1	0.58	Peptidyl-prolyl cis-trans
1623	1	SwissProt_HUMAN$	3::Q7Z624|CMKMT_HUMAN	30	36105	1	1	1	1	0.14	Calmodulin-lysine N-me
1624	1	db$2	2::C3K5F6|C3K5F6_PSEFS	30	45088	1	1	1	1	0.11	Uncharacterized protein
1625	1	SwissProt_HUMAN$	3::Q9UL62|TRPC5_HUMAN	30	111340	2	2	1	1	0.04	Short transient receptor
1626	1	db$2	2::C3K8X1|C3K8X1_PSEFS	30	23435	2	2	2	2	0.49	Putative ABC transport s
1627	1	db$2	2::C3K3X7|C3K3X7_PSEFS	30	16525	1	1	1	1	0.33	EVE domain-containing
1628	1	SwissProt_HUMAN$	3::P62258|1433E_HUMAN	30	29155	2	1	2	1	0.18	14-3-3 protein epsilon C
1629	1	db$2	2::C3K243|C3K243_PSEFS	30	108098	1	1	1	1	0.04	Histidine kinase OS=Pse
1630	1	db$2	2::C3JY68|MNMA_PSEFS	30	41397	5	3	5	3	0.41	tRNA-specific 2-thiourid
1631	1	db$2	2::C3K757|C3K757_PSEFS	29	27432	2	2	2	2	0.41	Putative two-component
1632	1	SwissProt_HUMAN$	3::P00738|HPT_HUMAN	30	45177	1	1	1	1	0.11	Haptoglobin OS=Homo s

↑Family	M	DB	Accession	Score	Mass	Matches	Match(sig)	Sequences	Seq(sig)	emPAI	Description
1633	1	db$2	2::C3K125|C3K125_PSEFS	29	26783	2	1	1	1	0.19	Alanine--tRNA ligase OS
1634	1	db$2	2::C3KAA5|C3KAA5_PSEFS	29	23984	2	1	1	1	0.22	Uncharacterized protein
1635	1	SwissProt_HUMAN$	3::P26440|IVD_HUMAN	29	46621	1	1	1	1	0.11	Isovaleryl-CoA dehydrog
1636	1	db$2	2::C3K9N4|C3K9N4_PSEFS	29	91939	2	2	2	2	0.11	Histidine kinase OS=Pse
1637	1	db$2	2::C3KCV4|C3KCV4_PSEFS	29	19545	1	1	1	1	0.27	Putative DNA-binding re
1638	1	SwissProt_HUMAN$	3::P06748|NPM_HUMAN	29	32555	1	1	1	1	0.16	Nucleophosmin OS=Hom
1639	1	SwissProt_HUMAN$	3::Q9P273|TEN3_HUMAN	29	300760	1	1	1	1	0.02	Teneurin-3 OS=Homo sa
1640	1	SwissProt_HUMAN$	3::Q5JTZ9|SYAM_HUMAN	29	107273	1	1	1	1	0.05	Alanine--tRNA ligase, m
1641	1	SwissProt_HUMAN$	3::Q3SY89|ELB3B_HUMAN	29	59723	1	1	1	1	0.08	Elongin-A3 member B O
1642	1	SwissProt_HUMAN$	3::Q96AY4|TTC28_HUMAN	29	270715	1	1	1	1	0.02	Tetratricopeptide repeat
1643	1	db$2	2::C3K945|C3K945_PSEFS	29	100224	1	1	1	1	0.05	Putative transferase/cys
1644	1	db$2	2::C3KCB4|C3KCB4_PSEFS	29	27191	1	1	1	1	0.19	Uncharacterized protein
1645	1	db$2	2::C3KBX7|C3KBX7_PSEFS	29	23636	1	1	1	1	0.22	Putative metallo-beta-la
1646	1	db$2	2::C3K9H2|C3K9H2_PSEFS	29	39760	3	2	3	2	0.27	Putrescine ABC transpor
1647	1	db$2	2::C3K3H3|C3K3H3_PSEFS	29	52510	1	1	1	1	0.09	Adenosylmethionine-8-a
1648	1	db$2	2::C3KDB5|C3KDB5_PSEFS	29	33189	1	1	1	1	0.15	Putative glycosyl transfe
1649	1	db$2	2::C3K3D9|C3K3D9_PSEFS	29	42615	1	1	1	1	0.12	Uncharacterized protein
1650	1	db$2	2::C3KDS6|UNG_PSEFS	29	25820	1	1	1	1	0.20	Uracil-DNA glycosylase (
1651	1	SwissProt_HUMAN$	3::Q7Z6Z7|HUWE1_HUMAN	28	481589	3	2	2	2	0.02	E3 ubiquitin-protein liga
1652	1	SwissProt_HUMAN$	3::Q9BYK8|HELZ2_HUMAN	28	294465	1	1	1	1	0.02	Helicase with zinc finger
1653	1	SwissProt_HUMAN$	3::P12883|MYH7_HUMAN	28	222959	1	1	1	1	0.02	Myosin-7 OS=Homo sap
1654	1	db$2	2::C3K1M7|C3K1M7_PSEFS	28	27957	1	1	1	1	0.18	Serine O-acetyltransfera
1655	1	db$2	2::C3K5G7|PDXB_PSEFS	28	41465	1	1	1	1	0.12	Erythronate-4-phosphat
1656	1	SwissProt_HUMAN$	3::P37802|TAGL2_HUMAN	28	22377	1	1	1	1	0.23	Transgelin-2 OS=Homo
1657	1	db$2	2::C3K428|C3K428_PSEFS	28	10726	2	2	2	2	1.36	Uncharacterized protein
1658	1	db$2	2::C3K821|C3K821_PSEFS	28	68185	1	1	1	1	0.07	Putative methyl-acceptin
1659	1	db$2	2::C3K7I5|C3K7I5_PSEFS	28	28342	2	2	2	2	0.39	Putative iron utilisation p
1660	1	SwissProt_HUMAN$	3::P62081|RS7_HUMAN	28	22113	1	1	1	1	0.24	40S ribosomal protein S
1661	1	db$2	2::C3K670|C3K670_PSEFS	28	30761	1	1	1	1	0.17	Putative glycine betaine-
1662	1	SwissProt_HUMAN$	3::Q13474|DRP2_HUMAN	28	107894	1	1	1	1	0.04	Dystrophin-related prote
1663	1	db$2	2::C3K2M2|C3K2M2_PSEFS	28	35893	1	1	1	1	0.14	Endolytic peptidoglycan
1664	1	db$2	2::C3KBW9|C3KBW9_PSEFS	28	64127	1	1	1	1	0.08	Putative lipoprotein OS=
1665	1	SwissProt_HUMAN$	3::P61353|RL27_HUMAN	28	15788	2	2	2	2	0.81	60S ribosomal protein L
1666	1	db$2	2::C3K0U7|C3K0U7_PSEFS	28	49884	3	3	3	3	0.33	Two-component respons
1667	1	SwissProt_HUMAN$	3::Q96AQ6|PBIP1_HUMAN	28	80594	1	1	1	1	0.06	Pre-B-cell leukemia tran
1668	1	db$2	2::C3KDB4|C3KDB4_PSEFS	28	42765	1	1	1	1	0.12	Putative glycosyl transfe
1669	1	SwissProt_HUMAN$	3::P47900|P2RY1_HUMAN	28	42044	1	1	1	1	0.12	P2Y purinoceptor 1 OS=
1670	1	db$2	2::C3K406|C3K406_PSEFS	28	23424	2	2	2	2	0.49	Glyoxalase OS=Pseudon
1671	1	db$2	2::C3K816|OPGH_PSEFS	28	96236	2	2	2	2	0.10	Glucans biosynthesis glu
1672	1	db$2	2::C3KAI2|C3KAI2_PSEFS	28	19361	1	1	1	1	0.27	Shikimate kinase OS=Ps

↑Family	M	DB	Accession	Score	Mass	Matches	Match(sig)	Sequences	Seq(sig)	emPAI	Description
1673	1	db$2	2::C3K4U9|C3K4U9_PSEFS	28	34107	1	1	1	1	0.15	Putative LysR-family reg
1674	1	db$2	2::C3JY07|C3JY07_PSEFS	28	19767	2	1	1	1	0.27	Putative RNA polymeras
1675	1	db$2	2::C3K0H5|C3K0H5_PSEFS	28	25946	1	1	1	1	0.20	Putative chemotaxis pro
1676	1	SwissProt_HUMAN$	3::Q6UWR7|ENPP6_HUMAN	28	50208	1	1	1	1	0.10	Glycerophosphocholine (
1677	1	SwissProt_HUMAN$	3::O43272|PROD_HUMAN	28	67959	1	1	1	1	0.07	Proline dehydrogenase 1
1678	1	db$2	2::C3K7X0|C3K7X0_PSEFS	27	85237	3	2	3	2	0.12	5-methyltetrahydropter(
1679	1	SwissProt_HUMAN$	3::Q5S007|LRRK2_HUMAN	27	285919	1	1	1	1	0.02	Leucine-rich repeat serir
1680	1	db$2	2::C3K220|C3K220_PSEFS	27	136725	1	1	1	1	0.04	RecBCD enzyme subunit
1681	1	SwissProt_HUMAN$	3::Q9NQI0|DDX4_HUMAN	27	79258	1	1	1	1	0.06	Probable ATP-dependent
1682	1	SwissProt_HUMAN$	3::Q7Z7L7|ZER1_HUMAN	27	88113	2	2	1	1	0.11	Protein zer-1 homolog C
1683	1	db$2	2::C3K1L5|C3K1L5_PSEFS	27	34147	1	1	1	1	0.15	Putative DNA-binding pr
1684	1	db$2	2::C3KAN8|C3KAN8_PSEFS	27	48228	1	1	1	1	0.10	3-carboxy-cis,cis-mucor
1685	1	SwissProt_HUMAN$	3::P36507|MP2K2_HUMAN	27	44396	1	1	1	1	0.11	Dual specificity mitogen-
1686	1	db$2	2::C3K5A1|C3K5A1_PSEFS	27	60517	3	3	3	3	0.26	Urease subunit alpha OS
1687	1	db$2	2::C3K0T5|C3K0T5_PSEFS	27	37877	1	1	1	1	0.13	Zinc transport protein O
1688	1	db$2	2::C3JZR9|C3JZR9_PSEFS	27	27415	3	2	3	2	0.41	Putative dehydrogenase,
1689	1	SwissProt_HUMAN$	3::Q9Y265|RUVB1_HUMAN	27	50196	1	1	1	1	0.10	RuvB-like 1 OS=Homo s
1690	1	db$2	2::C3K1Z0|C3K1Z0_PSEFS	27	34051	1	1	1	1	0.15	Putative putative polysa
1691	1	db$2	2::C3K750|C3K750_PSEFS	27	60230	1	1	1	1	0.08	Putative AMP-binding pr
1692	1	SwissProt_HUMAN$	3::Q9Y399|RT02_HUMAN	27	33228	1	1	1	1	0.15	28S ribosomal protein S
1693	1	db$2	2::C3KB61|C3KB61_PSEFS	27	26669	1	1	1	1	0.19	Molybdate-binding perip
1694	1	SwissProt_HUMAN$	3::Q8N0Y7|PGAM4_HUMAN	27	28759	1	1	1	1	0.18	Probable phosphoglycera
1695	1	db$2	2::C3K3T4|C3K3T4_PSEFS	27	31991	1	1	1	1	0.16	Putrescine ABC transpor
1696	1	db$2	2::C3K2T4|C3K2T4_PSEFS	27	155869	1	1	1	1	0.03	Putative ATP-dependent
1697	1	db$2	2::C3KAU8|C3KAU8_PSEFS	27	52023	1	1	1	1	0.10	Xylulose kinase OS=Pse
1698	1	SwissProt_HUMAN$	3::O15197|EPHB6_HUMAN	27	110630	1	1	1	1	0.04	Ephrin type-B receptor 6
1699	1	SwissProt_HUMAN$	3::Q15147|PLCB4_HUMAN	27	134378	1	1	1	1	0.04	1-phosphatidylinositol 4
1700	1	db$2	2::C3K0E8|C3K0E8_PSEFS	27	27512	3	2	3	2	0.41	Thiol:disulfide interchan
1701	1	SwissProt_HUMAN$	3::Q9Y6D9|MD1L1_HUMAN	27	83016	2	1	2	1	0.06	Mitotic spindle assembly
1702	1	SwissProt_HUMAN$	3::O00515|LAD1_HUMAN	27	57097	1	1	1	1	0.09	Ladinin-1 OS=Homo sap
1703	1	SwissProt_HUMAN$	3::Q8TF21|ANR24_HUMAN	27	124111	3	3	2	2	0.08	Ankyrin repeat domain-(
1704	1	db$2	2::C3JZP7|C3JZP7_PSEFS	27	33454	1	1	1	1	0.15	Putative ribose transpor
1705	1	SwissProt_HUMAN$	3::Q14129|DGCR6_HUMAN	27	24973	1	1	1	1	0.21	Protein DGCR6 OS=Hom
1706	1	db$2	2::C3K9C4|C3K9C4_PSEFS	27	31700	1	1	1	1	0.16	Putative transposase for
1707	1	SwissProt_HUMAN$	3::A6NJB7|PRR19_HUMAN	27	38692	1	1	1	1	0.13	Proline-rich protein 19 C
1708	1	SwissProt_HUMAN$	3::P15880|RS2_HUMAN	27	31305	1	1	1	1	0.16	40S ribosomal protein S
1709	1	SwissProt_HUMAN$	3::Q149M9|NWD1_HUMAN	27	174441	1	1	1	1	0.03	NACHT domain- and WD
1710	1	SwissProt_HUMAN$	3::Q6PI48|SYDM_HUMAN	27	73516	1	1	1	1	0.07	Aspartate--tRNA ligase,
1711	1	SwissProt_HUMAN$	3::Q9C098|DCLK3_HUMAN	27	73768	1	1	1	1	0.07	Serine/threonine-proteir
1712	1	db$2	2::C3K0Z9|C3K0Z9_PSEFS	27	26468	1	1	1	1	0.19	Bac_DnaA domain-conta

↑Family	M	DB	Accession	Score	Mass	Matches	Match(sig)	Sequences	Seq(sig)	emPAI	Description
1713	1	db$2	2::C3KD58|C3KD58_PSEFS	26	41746	1	1	1	1	0.12	Putative cystathionine g
1714	1	db$2	2::C3K3F2|C3K3F2_PSEFS	26	38228	2	2	2	2	0.28	D-erythrose-4-phosphat
1715	1	db$2	2::C3K5C4|C3K5C4_PSEFS	26	17510	2	2	2	2	0.70	SnoaL-like domain-conta
1716	1	db$2	2::C3K9Q0|GLO2_PSEFS	26	28360	1	1	1	1	0.18	Hydroxyacylglutathione
1717	1	db$2	2::C3K7J9|C3K7J9_PSEFS	26	83444	1	1	1	1	0.06	Putative exported protei
1718	1	SwissProt_HUMAN$	3::Q92887|MRP2_HUMAN	26	174096	1	1	1	1	0.03	Canalicular multispecific
1719	1	db$2	2::C3KE10|C3KE10_PSEFS	26	58823	2	2	2	2	0.17	Putative methyl-acceptir
1720	1	db$2	2::C3K595|C3K595_PSEFS	26	10805	1	1	1	1	0.54	Phosphate starvation-inc
1721	1	db$2	2::C3K1Q2|C3K1Q2_PSEFS	26	73203	1	1	1	1	0.07	Histidine kinase OS=Pse
1722	1	db$2	2::C3K3D3|BETA_PSEFS	26	62642	4	2	4	2	0.16	Oxygen-dependent choli
1723	1	db$2	2::C3KCU6|C3KCU6_PSEFS	26	23028	1	1	1	1	0.23	Putative membrane prot
1724	1	db$2	2::C3K3Y9|C3K3Y9_PSEFS	26	73510	1	1	1	1	0.07	Putative membrane prot
1725	1	db$2	2::C3KAN4|C3KAN4_PSEFS	26	41646	2	2	1	1	0.25	3-oxoadipyl-CoA thiolase
1726	1	SwissProt_HUMAN$	3::O75197|LRP5_HUMAN	26	179030	3	1	2	1	0.03	Low-density lipoprotein
1727	1	db$2	2::C3K2M0|Y5418_PSEFS	26	10305	1	1	1	1	0.56	UPF0250 protein PFLU_5
1728	1	SwissProt_HUMAN$	3::Q6PKH6|DR4L2_HUMAN	26	24570	1	1	1	1	0.21	Dehydrogenase/reducta
1729	1	db$2	2::C3JXY6|ASTB_PSEFS	26	48741	3	2	3	2	0.21	N-succinylarginine dihyc
1730	1	SwissProt_HUMAN$	3::Q9BSW2|EFC4B_HUMAN	26	45564	1	1	1	1	0.11	EF-hand calcium-binding
1731	1	SwissProt_HUMAN$	3::O75155|CAND2_HUMAN	26	135171	2	2	2	2	0.07	Cullin-associated NEDD8
1732	1	SwissProt_HUMAN$	3::P27708|PYR1_HUMAN	26	242829	1	1	1	1	0.02	CAD protein OS=Homo s
1733	1	SwissProt_HUMAN$	3::P56373|P2RX3_HUMAN	26	44260	1	1	1	1	0.11	P2X purinoceptor 3 OS=
1734	1	SwissProt_HUMAN$	3::P62266|RS23_HUMAN	26	15798	1	1	1	1	0.34	40S ribosomal protein S
1735	1	db$2	2::C3K8R2|C3K8R2_PSEFS	26	22663	1	1	1	1	0.23	Uncharacterized protein
1736	1	SwissProt_HUMAN$	3::O60237|MYPT2_HUMAN	25	110337	1	1	1	1	0.04	Protein phosphatase 1 r
1737	1	SwissProt_HUMAN$	3::Q02080|MEF2B_HUMAN	25	38615	1	1	1	1	0.13	Myocyte-specific enhanc
1738	1	SwissProt_HUMAN$	3::O94827|PKHG5_HUMAN	25	117377	2	1	2	1	0.04	Pleckstrin homology dor
1739	1	db$2	2::C3K4E5|C3K4E5_PSEFS	25	51748	1	1	1	1	0.10	Putative amino acid tran
1740	1	SwissProt_HUMAN$	3::Q8NA82|MARHA_HUMAN	25	90455	1	1	1	1	0.05	Probable E3 ubiquitin-pr
1741	1	SwissProt_HUMAN$	3::P81605|DCD_HUMAN	25	11277	1	1	1	1	0.51	Dermcidin OS=Homo sa
1742	1	SwissProt_HUMAN$	3::Q92817|EVPL_HUMAN	25	231463	3	1	3	1	0.02	Envoplakin OS=Homo sa
1743	1	SwissProt_HUMAN$	3::Q5K651|SAMD9_HUMAN	25	184165	1	1	1	1	0.03	Sterile alpha motif doma
1744	1	db$2	2::C3K294|C3K294_PSEFS	25	11674	2	1	2	1	0.49	Transposase for insertio
1745	1	db$2	2::C3K2P3|C3K2P3_PSEFS	25	54945	1	1	1	1	0.09	Putative aldehyde dehyc
1746	1	db$2	2::C3JZP4|RBSD_PSEFS	25	14393	1	1	1	1	0.38	D-ribose pyranase OS=F
1747	1	db$2	2::C3K918|C3K918_PSEFS	25	26912	1	1	1	1	0.19	Putative ABC transport s
1748	1	SwissProt_HUMAN$	3::Q96RL7|VP13A_HUMAN	25	360047	1	1	1	1	0.01	Vacuolar protein sorting
1749	1	db$2	2::C3KAY4|C3KAY4_PSEFS	25	29479	2	1	2	1	0.17	Putative enoyl-CoA hydr
1750	1	SwissProt_HUMAN$	3::P12110|CO6A2_HUMAN	25	108512	1	1	1	1	0.04	Collagen alpha-2(VI) cha
1751	1	db$2	2::C3K0G4|C3K0G4_PSEFS	25	29541	1	1	1	1	0.17	MOSC domain-containin
1752	1	db$2	2::C3K870|C3K870_PSEFS	25	20966	1	1	1	1	0.25	Uncharacterized protein

↑Family	M	DB	Accession	Score	Mass	Matches	Match(sig)	Sequences	Seq(sig)	emPAI	Description
1753	1	db$2	2::C3K158|C3K158_PSEFS	25	36421	1	1	1	1	0.14	Putative regulatory prot
1754	1	SwissProt_HUMAN$	3::Q00536|CDK16_HUMAN	25	55681	1	1	1	1	0.09	Cyclin-dependent kinase
1755	1	db$2	2::C3K9Q1|C3K9Q1_PSEFS	25	28823	1	1	1	1	0.18	Methyltransf_11 domain
1756	1	SwissProt_HUMAN$	3::P62136|PP1A_HUMAN	25	37488	1	1	1	1	0.13	Serine/threonine-proteir
1757	1	SwissProt_HUMAN$	3::O00139|KIF2A_HUMAN	25	79905	2	2	2	2	0.13	Kinesin-like protein KIF2
1758	1	db$2	2::C3K0X5|C3K0X5_PSEFS	25	21842	2	2	2	2	0.54	Uncharacterized protein
1759	1	SwissProt_HUMAN$	3::Q96S38|KS6C1_HUMAN	25	118609	1	1	1	1	0.04	Ribosomal protein S6 ki
1760	1	db$2	2::C3K267|RLME_PSEFS	25	24125	3	2	3	2	0.48	Ribosomal RNA large su
1761	1	SwissProt_HUMAN$	3::Q9BTV4|TMM43_HUMAN	24	44847	1	1	1	1	0.11	Transmembrane protein
1762	1	db$2	2::C3K7M0|C3K7M0_PSEFS	24	62717	1	1	1	1	0.08	Putative dipeptidase OS
1763	1	SwissProt_HUMAN$	3::Q93075|TATD2_HUMAN	24	84970	1	1	1	1	0.06	Putative deoxyribonucle
1764	1	db$2	2::C3K7M8|C3K7M8_PSEFS	24	23309	1	1	1	1	0.22	Putative glutathione S-t
1765	1	db$2	2::C3K2L3|C3K2L3_PSEFS	24	22416	1	1	1	1	0.23	LPS-assembly lipoprotei
1766	1	SwissProt_HUMAN$	3::A6NE01|F186A_HUMAN	24	262595	1	1	1	1	0.02	Protein FAM186A OS=H
1767	1	db$2	2::C3JYC8|C3JYC8_PSEFS	24	59331	1	1	1	1	0.08	Putative pH adaptation
1768	1	db$2	2::C3K3U4|C3K3U4_PSEFS	24	23278	1	1	1	1	0.22	Putative TetR-family reg
1769	1	SwissProt_HUMAN$	3::Q96AA8|JKIP2_HUMAN	24	94875	2	1	2	1	0.05	Janus kinase and microt
1770	1	db$2	2::C3K6U5|FETP_PSEFS	24	10399	1	1	1	1	0.56	Probable Fe(2+)-traffick
1771	1	SwissProt_HUMAN$	3::Q8NFZ0|FBH1_HUMAN	24	117611	1	1	1	1	0.04	F-box DNA helicase 1 O
1772	1	db$2	2::C3KCS8|C3KCS8_PSEFS	24	47767	2	2	2	2	0.22	UDP-N-acetylmuramoyla
1773	1	SwissProt_HUMAN$	3::Q8NE79|POPD1_HUMAN	24	41424	1	1	1	1	0.12	Blood vessel epicardial s
1774	1	db$2	2::C3K3V9|C3K3V9_PSEFS	24	33232	1	1	1	1	0.15	Protein QmcA OS=Pseu
1775	1	SwissProt_HUMAN$	3::O95153|RIMB1_HUMAN	24	199928	1	1	1	1	0.02	Peripheral-type benzodi
1776	1	SwissProt_HUMAN$	3::P52333|JAK3_HUMAN	24	125019	1	1	1	1	0.04	Tyrosine-protein kinase
1777	1	SwissProt_HUMAN$	3::P05546|HEP2_HUMAN	24	57034	1	1	1	1	0.09	Heparin cofactor 2 OS=
1778	1	SwissProt_HUMAN$	3::P27216|ANX13_HUMAN	24	35393	1	1	1	1	0.14	Annexin A13 OS=Homo
1779	1	SwissProt_HUMAN$	3::Q99584|S10AD_HUMAN	24	11464	1	1	1	1	0.50	Protein S100-A13 OS=H
1780	1	SwissProt_HUMAN$	3::Q9ULX9|MAFF_HUMAN	24	17749	2	1	2	1	0.30	Transcription factor Maf
1781	1	db$2	2::C3K7Y9|C3K7Y9_PSEFS	24	49907	1	1	1	1	0.10	NAD(P) transhydrogena
1782	1	SwissProt_HUMAN$	3::Q7Z6I6|RHG30_HUMAN	24	118509	2	1	1	1	0.04	Rho GTPase-activating p
1783	1	db$2	2::C3KC11|C3KC11_PSEFS	24	29623	1	1	1	1	0.17	Putative GerE familyreg
1784	1	db$2	2::C3K3P8|C3K3P8_PSEFS	24	49421	1	1	1	1	0.10	G domain-containing pr
1785	1	SwissProt_HUMAN$	3::O94804|STK10_HUMAN	24	112065	1	1	1	1	0.04	Serine/threonine-proteir
1786	1	db$2	2::C3K1R1|C3K1R1_PSEFS	24	13822	1	1	1	1	0.40	bPH_1 domain-containir
1787	1	SwissProt_HUMAN$	3::Q9UDT6|CLIP2_HUMAN	24	115767	2	2	1	1	0.04	CAP-Gly domain-contain
1788	1	SwissProt_HUMAN$	3::P02747|C1QC_HUMAN	24	25757	1	1	1	1	0.20	Complement C1q subcor
1789	1	SwissProt_HUMAN$	3::Q969W8|ZN566_HUMAN	24	49188	1	1	1	1	0.10	Zinc finger protein 566 (
1790	1	SwissProt_HUMAN$	3::P62857|RS28_HUMAN	24	7836	1	1	1	1	0.79	40S ribosomal protein S
1791	1	db$2	2::C3K3X1|C3K3X1_PSEFS	23	48834	1	1	1	1	0.10	Xaa-Pro aminopeptidase
1792	1	SwissProt_HUMAN$	3::P27540|ARNT_HUMAN	23	86582	1	1	1	1	0.06	Aryl hydrocarbon recept

↑Family	M	DB	Accession	Score	Mass	Matches	Match(sig)	Sequences	Seq(sig)	emPAI	Description
1793	1	db$2	2::C3K5X0|C3K5X0_PSEFS	23	25599	2	2	2	2	0.44	Putative exported protei
1794	1	SwissProt_HUMAN$	3::Q08828|ADCY1_HUMAN	23	123360	1	1	1	1	0.04	Adenylate cyclase type :
1795	1	SwissProt_HUMAN$	3::Q14246|AGRE1_HUMAN	23	97618	1	1	1	1	0.05	Adhesion G protein-coup
1796	1	SwissProt_HUMAN$	3::Q14789|GOGB1_HUMAN	23	375790	3	2	3	2	0.03	Golgin subfamily B mem
1797	1	SwissProt_HUMAN$	3::O15270|SPTC2_HUMAN	23	62884	1	1	1	1	0.08	Serine palmitoyltransfer
1798	1	db$2	2::C3K492|C3K492_PSEFS	23	51080	2	1	2	1	0.10	HDOD domain-containin
1799	1	db$2	2::C3K6W4|C3K6W4_PSEFS	23	45779	1	1	1	1	0.11	Znf/thioredoxin_put don
1800	1	SwissProt_HUMAN$	3::Q8N8R5|CB069_HUMAN	23	43421	1	1	1	1	0.12	UPF0565 protein C2orf6
1801	1	db$2	2::C3K4T8|C3K4T8_PSEFS	23	33681	1	1	1	1	0.15	Putative AraC-family reg
1802	1	SwissProt_HUMAN$	3::P54577|SYYC_HUMAN	23	59106	1	1	1	1	0.08	Tyrosine--tRNA ligase, c
1803	1	db$2	2::C3KE19|C3KE19_PSEFS	23	30253	1	1	1	1	0.17	HDOD domain-containin
1804	1	SwissProt_HUMAN$	3::Q9UKR0|KLK12_HUMAN	23	26716	1	1	1	1	0.19	Kallikrein-12 OS=Homo
1805	1	db$2	2::C3K3B4|C3K3B4_PSEFS	23	14608	1	1	1	1	0.37	Putative exported protei
1806	1	db$2	2::C3K0S1|C3K0S1_PSEFS	23	53269	1	1	1	1	0.09	Magnesium transporter
1807	1	db$2	2::C3K5B8|C3K5B8_PSEFS	23	59457	1	1	1	1	0.08	Dipeptide ABC transport
1808	1	db$2	2::C3KAZ8|C3KAZ8_PSEFS	23	34711	1	1	1	1	0.15	Putative beta-hydroxyla
1809	1	db$2	2::C3K4S7|C3K4S7_PSEFS	23	34300	1	1	1	1	0.15	Putative LysR-family reg
1810	1	SwissProt_HUMAN$	3::P23276|KELL_HUMAN	23	82771	1	1	1	1	0.06	Kell blood group glycopr
1811	1	SwissProt_HUMAN$	3::Q7Z5H4|VN1R5_HUMAN	23	40752	1	1	1	1	0.12	Vomeronasal type-1 rec
1812	1	db$2	2::C3KCF3|C3KCF3_PSEFS	23	94057	3	2	3	2	0.11	Putative signalling-relate
1813	1	SwissProt_HUMAN$	3::Q9ULD4|BRPF3_HUMAN	23	135660	1	1	1	1	0.04	Bromodomain and PHD '
1814	1	SwissProt_HUMAN$	3::Q8TCU5|NMD3A_HUMAN	23	125385	2	1	1	1	0.04	Glutamate receptor iono
1815	1	SwissProt_HUMAN$	3::P00519|ABL1_HUMAN	23	122796	1	1	1	1	0.04	Tyrosine-protein kinase
1816	1	SwissProt_HUMAN$	3::O94967|WDR47_HUMAN	23	101884	2	1	2	1	0.05	WD repeat-containing pi
1817	1	SwissProt_HUMAN$	3::P49207|RL34_HUMAN	23	13284	1	1	1	1	0.42	60S ribosomal protein L
1818	1	SwissProt_HUMAN$	3::P08476|INHBA_HUMAN	23	47412	1	1	1	1	0.10	Inhibin beta A chain OS:
1819	1	SwissProt_HUMAN$	3::Q8TD26|CHD6_HUMAN	23	305220	1	1	1	1	0.02	Chromodomain-helicase
1820	1	db$2	2::C3K5K4|C3K5K4_PSEFS	23	29062	1	1	1	1	0.18	Uncharacterized protein
1821	1	db$2	2::C3K992|C3K992_PSEFS	22	34600	1	1	1	1	0.15	Putative AraC-family reg
1822	1	db$2	2::C3K6K7|C3K6K7_PSEFS	22	17395	1	1	1	1	0.31	GreA_GreB domain-cont
1823	1	SwissProt_HUMAN$	3::P23508|CRCM_HUMAN	22	92970	1	1	1	1	0.05	Colorectal mutant cance
1824	1	db$2	2::C3K9J9|C3K9J9_PSEFS	22	17304	1	1	1	1	0.31	Uncharacterized protein
1825	1	SwissProt_HUMAN$	3::Q96P48|ARAP1_HUMAN	22	162089	1	1	1	1	0.03	Arf-GAP with Rho-GAP d
1826	1	db$2	2::C3K9E9|C3K9E9_PSEFS	22	21363	3	2	2	1	0.55	Putative TetR-family reg
1827	1	SwissProt_HUMAN$	3::Q9BYW2|SETD2_HUMAN	22	287418	1	1	1	1	0.02	Histone-lysine N-methyl
1828	1	db$2	2::C3K5J0|C3K5J0_PSEFS	22	13887	1	1	1	1	0.40	Putative lipoprotein OS=
1829	1	SwissProt_HUMAN$	3::Q9H244|P2Y12_HUMAN	22	39413	1	1	1	1	0.13	P2Y purinoceptor 12 OS
1830	1	db$2	2::C3K115|C3K115_PSEFS	22	24088	1	1	1	1	0.22	DsbD_2 domain-contain
1831	1	SwissProt_HUMAN$	3::P25391|LAMA1_HUMAN	22	336867	1	1	1	1	0.01	Laminin subunit alpha-1
1832	1	db$2	2::C3KAX6|C3KAX6_PSEFS	22	25593	1	1	1	1	0.20	Putative acetyltransfera:

↑Family	M	DB	Accession	Score	Mass	Matches	Match(sig)	Sequences	Seq(sig)	emPAI	Description
1833	1	SwissProt_HUMAN$	3::O95347|SMC2_HUMAN	22	135572	1	1	1	1	0.04	Structural maintenance
1834	1	db$2	2::C3K0N6|PLSX_PSEFS	22	35209	2	1	1	1	0.14	Phosphate acyltransfera
1835	1	db$2	2::C3KBB6|C3KBB6_PSEFS	22	18242	1	1	1	1	0.29	Putative transcriptional
1836	1	SwissProt_HUMAN$	3::Q8WX94|NALP7_HUMAN	22	111735	1	1	1	1	0.04	NACHT, LRR and PYD dc
1837	1	SwissProt_HUMAN$	3::Q9H1K4|GHC2_HUMAN	22	33826	1	1	1	1	0.15	Mitochondrial glutamate
1838	1	db$2	2::C3KCQ3|C3KCQ3_PSEFS	22	36116	1	1	1	1	0.14	Putative type III secreti
1839	1	SwissProt_HUMAN$	3::Q6Y7W6|GGYF2_HUMAN	22	149978	1	1	1	1	0.03	GRB10-interacting GYF p
1840	1	db$2	2::C3JXS9|C3JXS9_PSEFS	22	32539	1	1	1	1	0.16	Putative LysR-family tra
1841	1	db$2	2::C3KAB6|C3KAB6_PSEFS	22	39151	1	1	1	1	0.13	Conserved hypothetical
1842	1	db$2	2::C3KBZ9|C3KBZ9_PSEFS	22	12125	1	1	1	1	0.47	Ferredoxin OS=Pseudon
1843	1	SwissProt_HUMAN$	3::Q56UQ5|TPT1L_HUMAN	22	15747	1	1	1	1	0.34	TPT1-like protein OS=Ho
1844	1	db$2	2::C3KDR5|KUP_PSEFS	22	68624	1	1	1	1	0.07	Probable potassium tran
1845	1	db$2	2::C3JY93|C3JY93_PSEFS	22	51981	1	1	1	1	0.10	NADH-quinone oxidored
1846	1	db$2	2::C3K065|C3K065_PSEFS	22	58554	1	1	1	1	0.08	Uncharacterized protein
1847	1	SwissProt_HUMAN$	3::Q8TBY8|PMFBP_HUMAN	22	117420	1	1	1	1	0.04	Polyamine-modulated fa
1848	1	SwissProt_HUMAN$	3::P62316|SMD2_HUMAN	22	13518	1	1	1	1	0.41	Small nuclear ribonucle
1849	1	SwissProt_HUMAN$	3::Q96GD3|SCMH1_HUMAN	22	73307	1	1	1	1	0.07	Polycomb protein SCMH
1850	1	SwissProt_HUMAN$	3::Q13972|RGRF1_HUMAN	22	145142	1	1	1	1	0.03	Ras-specific guanine nu
1851	1	db$2	2::C3K4F9|C3K4F9_PSEFS	22	31102	1	1	1	1	0.16	Putative regulatory prot
1852	1	db$2	2::C3K814|DTD_PSEFS	22	15391	1	1	1	1	0.35	D-aminoacyl-tRNA deac
1853	1	SwissProt_HUMAN$	3::Q9C0K3|ARP3C_HUMAN	22	23697	1	1	1	1	0.22	Actin-related protein 3C
1854	1	db$2	2::C3K7T3|C3K7T3_PSEFS	21	20773	1	1	1	1	0.25	CDP-diacylglycerol--glyc
1855	1	SwissProt_HUMAN$	3::Q9H9E3|COG4_HUMAN	21	89026	1	1	1	1	0.05	Conserved oligomeric G
1856	1	SwissProt_HUMAN$	3::P04080|CYTB_HUMAN	21	11133	1	1	1	1	0.52	Cystatin-B OS=Homo sa
1857	1	SwissProt_HUMAN$	3::Q7Z410|TMPS9_HUMAN	21	113948	1	1	1	1	0.04	Transmembrane proteas
1858	1	SwissProt_HUMAN$	3::Q8NFP9|NBEA_HUMAN	21	327614	1	1	1	1	0.01	Neurobeachin OS=Homo
1859	1	db$2	2::C3JY63|CLPS_PSEFS	21	13780	1	1	1	1	0.40	ATP-dependent Clp prot
1860	1	SwissProt_HUMAN$	3::P62851|RS25_HUMAN	21	13734	1	1	1	1	0.40	40S ribosomal protein S
1861	1	SwissProt_HUMAN$	3::Q15811|ITSN1_HUMAN	21	195300	2	1	2	1	0.02	Intersectin-1 OS=Homo
1862	1	SwissProt_HUMAN$	3::Q6ZMQ8|LMTK1_HUMAN	21	144479	2	1	1	1	0.03	Serine/threonine-protein
1863	1	db$2	2::C3K9I3|C3K9I3_PSEFS	21	71292	1	1	1	1	0.07	Putative carbohydrate ki
1864	1	SwissProt_HUMAN$	3::O00155|GPR25_HUMAN	21	38754	1	1	1	1	0.13	Probable G-protein coup
1865	1	SwissProt_HUMAN$	3::A6NCM1|IQCAL_HUMAN	21	95001	3	1	3	1	0.05	IQ and AAA domain-con
1866	1	db$2	2::C3K7R8|C3K7R8_PSEFS	21	12576	1	1	1	1	0.45	Peptidyl-prolyl cis-trans
1867	1	db$2	2::C3K4W4|C3K4W4_PSEFS	21	45211	2	2	2	2	0.23	Putative lipoprotein rele
1868	1	SwissProt_HUMAN$	3::Q7Z5L9|I2BP2_HUMAN	21	60987	1	1	1	1	0.08	Interferon regulatory fa
1869	1	db$2	2::C3K1J1|C3K1J1_PSEFS	21	11312	1	1	1	1	0.51	Uncharacterized protein
1870	1	SwissProt_HUMAN$	3::Q96N23|CFA54_HUMAN	21	351744	1	1	1	1	0.01	Cilia- and flagella-associ
1871	1	SwissProt_HUMAN$	3::P18627|LAG3_HUMAN	21	57413	1	1	1	1	0.09	Lymphocyte activation g
1872	1	db$2	2::C3K3Q9|LGT_PSEFS	21	30444	1	1	1	1	0.17	Phosphatidylglycerol--pr

↑Family	M	DB	Accession	Score	Mass	Matches	Match(sig)	Sequences	Seq(sig)	emPAI	Description
1873	1	db$2	2::C3K334|CCA_PSEFS	21	45916	1	1	1	1	0.11	Multifunctional CCA prot
1874	1	SwissProt_HUMAN$	3::P35611|ADDA_HUMAN	21	80905	1	1	1	1	0.06	Alpha-adducin OS=Hom
1875	1	SwissProt_HUMAN$	3::Q96PC2|IP6K3_HUMAN	21	46387	1	1	1	1	0.11	Inositol hexakisphospha
1876	1	SwissProt_HUMAN$	3::O43255|SIAH2_HUMAN	21	34592	1	1	1	1	0.15	E3 ubiquitin-protein liga
1877	1	db$2	2::C3JXG8|C3JXG8_PSEFS	21	49590	1	1	1	1	0.10	Putative outer membran
1878	1	SwissProt_HUMAN$	3::Q92625|ANS1A_HUMAN	21	123032	1	1	1	1	0.04	Ankyrin repeat and SAM
1879	1	db$2	2::C3KB02|C3KB02_PSEFS	21	42163	1	1	1	1	0.12	Putative exported protei
1880	1	db$2	2::C3KAB2|C3KAB2_PSEFS	21	13873	1	1	1	1	0.40	Putative two-component
1881	1	SwissProt_HUMAN$	3::Q49MG5|MAP9_HUMAN	21	74189	1	1	1	1	0.07	Microtubule-associated p
1882	1	SwissProt_HUMAN$	3::P62899|RL31_HUMAN	21	14454	1	1	1	1	0.38	60S ribosomal protein L
1883	1	db$2	2::C3K321|C3K321_PSEFS	21	28336	1	1	1	1	0.18	Putative heat shock prot
1884	1	SwissProt_HUMAN$	3::Q03001|DYST_HUMAN	20	860127	2	1	2	1	0.01	Dystonin OS=Homo sap
1885	1	db$2	2::C3JZP8|C3JZP8_PSEFS	20	55398	1	1	1	1	0.09	Putative ribose transpor
1886	1	db$2	2::C3KCP8|C3KCP8_PSEFS	20	42283	1	1	1	1	0.12	Lipopolysaccharide core
1887	1	SwissProt_HUMAN$	3::P31943|HNRH1_HUMAN	20	49198	1	1	1	1	0.10	Heterogeneous nuclear r
1888	1	SwissProt_HUMAN$	3::Q9BWW9|APOL5_HUMAN	20	47014	1	1	1	1	0.11	Apolipoprotein L5 OS=H
1889	1	db$2	2::C3K2R0|C3K2R0_PSEFS	20	31322	1	1	1	1	0.16	Geranyltranstransferase
1890	1	db$2	2::C3K1U1|C3K1U1_PSEFS	20	26390	1	1	1	1	0.19	Uncharacterized protein
1891	1	SwissProt_HUMAN$	3::A4D0S4|LAMB4_HUMAN	20	193413	1	1	1	1	0.02	Laminin subunit beta-4 (
1892	1	db$2	2::C3K1S4|C3K1S4_PSEFS	20	23334	1	1	1	1	0.22	Putative membrane prot
1893	1	SwissProt_HUMAN$	3::Q9C037|TRIM4_HUMAN	20	57424	1	1	1	1	0.09	E3 ubiquitin-protein liga
1894	1	db$2	2::C3JY67|C3JY67_PSEFS	20	16861	1	1	1	1	0.32	Phosphatase NudJ OS=P
1895	1	db$2	2::C3K906|C3K906_PSEFS	20	65595	1	1	1	1	0.07	Conserved GGDEF/EAL c
1896	1	db$2	2::C3KDD7|C3KDD7_PSEFS	20	32365	1	1	1	1	0.16	Cell division protein FtsC
1897	1	SwissProt_HUMAN$	3::O43776|SYNC_HUMAN	20	62903	1	1	1	1	0.08	Asparagine--tRNA ligase
1898	1	SwissProt_HUMAN$	3::P38405|GNAL_HUMAN	20	44280	1	1	1	1	0.11	Guanine nucleotide-bind
1899	1	SwissProt_HUMAN$	3::Q6UWN5|LYPD5_HUMAN	20	26918	1	1	1	1	0.19	Ly6/PLAUR domain-cont
1900	1	db$2	2::C3K5F2|Y1517_PSEFS	20	11422	1	1	1	1	0.50	YcgL domain-containing
1901	1	SwissProt_HUMAN$	3::P11362|FGFR1_HUMAN	20	91809	1	1	1	1	0.05	Fibroblast growth factor
1902	1	db$2	2::C3K9P8|C3K9P8_PSEFS	20	69666	1	1	1	1	0.07	Putative substrate-bindi
1903	1	SwissProt_HUMAN$	3::P16471|PRLR_HUMAN	20	69461	1	1	1	1	0.07	Prolactin receptor OS=H
1904	1	SwissProt_HUMAN$	3::P61978|HNRPK_HUMAN	20	50944	1	1	1	1	0.10	Heterogeneous nuclear r
1905	1	db$2	2::C3K2E2|C3K2E2_PSEFS	20	56906	1	1	1	1	0.09	Catalase OS=Pseudomo
1906	1	db$2	2::C3KBV8|C3KBV8_PSEFS	20	32477	1	1	1	1	0.16	Cobalt-zinc-cadmium re
1907	1	SwissProt_HUMAN$	3::Q9BXF9|TEKT3_HUMAN	20	56601	1	1	1	1	0.09	Tektin-3 OS=Homo sapi
1908	1	db$2	2::C3K5K0|C3K5K0_PSEFS	20	25438	1	1	1	1	0.20	Two-component system,
1909	1	SwissProt_HUMAN$	3::Q9NZT2|OGFR_HUMAN	20	73280	1	1	1	1	0.07	Opioid growth factor rec
1910	1	db$2	2::C3K0Z0|COBQ_PSEFS	20	51487	1	1	1	1	0.10	Cobyric acid synthase O
1911	1	db$2	2::C3K596|C3K596_PSEFS	20	36487	1	1	1	1	0.14	Putative membrane prot
1912	1	db$2	2::C3KBY4|C3KBY4_PSEFS	20	31613	1	1	1	1	0.16	Ribosomal RNA small su

↑Family	M	DB	Accession	Score	Mass	Matches	Match(sig)	Sequences	Seq(sig)	emPAI	Description
1913	1	SwissProt_HUMAN$	3::P57727|TMPS3_HUMAN	20	49373	1	1	1	1	0.10	Transmembrane proteas
1914	1	SwissProt_HUMAN$	3::Q9H6P5|TASP1_HUMAN	20	44427	1	1	1	1	0.11	Threonine aspartase 1 C
1915	1	SwissProt_HUMAN$	3::P11532|DMD_HUMAN	20	426484	2	1	2	1	0.01	Dystrophin OS=Homo sa
1916	1	SwissProt_HUMAN$	3::Q05D60|DEUP1_HUMAN	20	70933	2	1	2	1	0.07	Deuterosome assembly
1917	1	db$2	2::C3K4Y8|C3K4Y8_PSEFS	20	27802	1	1	1	1	0.18	Heme exporter protein C
1918	1	SwissProt_HUMAN$	3::Q9ULR3|PPM1H_HUMAN	20	56413	1	1	1	1	0.09	Protein phosphatase 1H
1919	1	SwissProt_HUMAN$	3::P60903|S10AA_HUMAN	19	11196	1	1	1	1	0.51	Protein S100-A10 OS=H
1920	1	db$2	2::C3K2P5|C3K2P5_PSEFS	19	49850	1	1	1	1	0.10	Ethanolamine ammonia-
1921	1	db$2	2::C3K3T5|C3K3T5_PSEFS	19	34240	1	1	1	1	0.15	Putrescine ABC transpor
1922	1	db$2	2::C3K323|C3K323_PSEFS	19	38795	1	1	1	1	0.13	Putative phosphotransfe
1923	1	db$2	2::C3K0Y2|C3K0Y2_PSEFS	19	46222	1	1	1	1	0.11	Putative fatty acid trans
1924	1	db$2	2::C3K807|C3K807_PSEFS	19	30283	1	1	1	1	0.17	Putative histidine ABC tr
1925	1	db$2	2::C3JZK2|C3JZK2_PSEFS	19	18051	1	1	1	1	0.30	Putative AsnC-family tra
1926	1	db$2	2::C3K6A9|C3K6A9_PSEFS	19	17428	1	1	1	1	0.31	Ferric uptake regulation
1927	1	SwissProt_HUMAN$	3::Q9BVJ7|DUS23_HUMAN	19	16578	1	1	1	1	0.33	Dual specificity protein p
1928	1	db$2	2::C3K058|C3K058_PSEFS	19	21557	1	1	1	1	0.24	DSBA domain-containing
1929	1	SwissProt_HUMAN$	3::Q9Y3A2|UTP11_HUMAN	19	30428	1	1	1	1	0.17	Probable U3 small nucle
1930	1	db$2	2::C3JZT3|C3JZT3_PSEFS	19	31726	1	1	1	1	0.16	tRNA pseudouridine synt
1931	1	db$2	2::C3K8T6|C3K8T6_PSEFS	19	26078	3	2	3	2	0.43	Ribosomal RNA small su
1932	1	SwissProt_HUMAN$	3::Q13835|PKP1_HUMAN	19	82808	3	1	2	1	0.06	Plakophilin-1 OS=Homo
1933	1	db$2	2::C3K9R9|C3K9R9_PSEFS	19	86327	1	1	1	1	0.06	Putative exported fatty a
1934	1	db$2	2::C3K834|C3K834_PSEFS	19	76708	1	1	1	1	0.06	Copper-transporting P-ty
1935	1	db$2	2::C3KCG3|C3KCG3_PSEFS	19	16758	1	1	1	1	0.32	YjgF_endoribonc domain
1936	1	db$2	2::C3K0V2|C3K0V2_PSEFS	19	48301	1	1	1	1	0.10	Flagellar hook-associate
1937	1	db$2	2::C3K348|PQQB_PSEFS	19	33093	1	1	1	1	0.15	Coenzyme PQQ synthesi
1938	1	SwissProt_HUMAN$	3::P54886|P5CS_HUMAN	19	87248	3	2	3	2	0.11	Delta-1-pyrroline-5-carb
1939	1	db$2	2::C3K109|C3K109_PSEFS	19	49568	1	1	1	1	0.10	23S rRNA (uracil(1939)-
1940	1	db$2	2::C3KAF6|C3KAF6_PSEFS	19	52919	1	1	1	1	0.09	Putative membrane prot
1941	1	db$2	2::C3K883|C3K883_PSEFS	19	60772	1	1	1	1	0.08	Putative transposase sul
1942	1	db$2	2::C3K2F9|C3K2F9_PSEFS	19	48516	1	1	1	1	0.10	Putative ABC transport s
1943	1	db$2	2::Q9ZEP7|PANC_PSEFS	19	30696	2	1	2	1	0.17	Pantothenate synthetase
1944	1	db$2	2::C3K1B2|C3K1B2_PSEFS	19	62071	1	1	1	1	0.08	Single-stranded-DNA-sp
1945	1	db$2	2::C3JZT5|C3JZT5_PSEFS	19	35785	1	1	1	1	0.14	Putative aspartate-semia
1946	1	db$2	2::C3K571|C3K571_PSEFS	19	11755	1	1	1	1	0.48	Uncharacterized protein
1947	1	SwissProt_HUMAN$	3::Q9Y5R8|TPPC1_HUMAN	19	16821	1	1	1	1	0.32	Trafficking protein partic
1948	1	SwissProt_HUMAN$	3::O15397|IPO8_HUMAN	19	119861	1	1	1	1	0.04	Importin-8 OS=Homo sa
1949	1	SwissProt_HUMAN$	3::P0DPD5|ZN723_HUMAN	19	59113	1	1	1	1	0.08	Zinc finger protein 723 (
1950	1	db$2	2::C3K5B4|C3K5B4_PSEFS	19	32752	1	1	1	1	0.15	Dipeptide ABC transport
1951	1	SwissProt_HUMAN$	3::Q7Z7L1|SLN11_HUMAN	19	102770	1	1	1	1	0.05	Schlafen family member
1952	1	db$2	2::C3K136|C3K136_PSEFS	19	31053	3	2	3	2	0.35	Uncharacterized protein

↑Family	M	DB	Accession	Score	Mass	Matches	Match(sig)	Sequences	Seq(sig)	emPAI	Description
1953	1	db$2	2::C3K7I3|C3K7I3_PSEFS	19	8950	1	1	1	1	0.67	Uncharacterized protein
1954	1	SwissProt_HUMAN$	3::Q8ND30|LIPB2_HUMAN	18	98482	1	1	1	1	0.05	Liprin-beta-2 OS=Homo
1955	1	SwissProt_HUMAN$	3::P41225|SOX3_HUMAN	18	45182	1	1	1	1	0.11	Transcription factor SOX
1956	1	SwissProt_HUMAN$	3::Q6WRX3|ZY11A_HUMAN	18	85752	2	1	2	1	0.06	Protein zyg-11 homolog
1957	1	SwissProt_HUMAN$	3::Q5JXB2|UE2NL_HUMAN	18	17366	1	1	1	1	0.31	Putative ubiquitin-conju
1958	1	SwissProt_HUMAN$	3::Q6UWJ1|TMCO3_HUMAN	18	75549	1	1	1	1	0.06	Transmembrane and coi
1959	1	SwissProt_HUMAN$	3::Q8IZF0|NALCN_HUMAN	18	200201	2	1	1	1	0.02	Sodium leak channel no
1960	1	db$2	2::C3KCP0|C3KCP0_PSEFS	18	31742	1	1	1	1	0.16	Ribosomal RNA large su
1961	1	db$2	2::C3K842|C3K842_PSEFS	18	56600	1	1	1	1	0.09	RNA polymerase sigma-
1962	1	db$2	2::C3K3K0|YQGF_PSEFS	18	16158	1	1	1	1	0.33	Putative pre-16S rRNA r
1963	1	SwissProt_HUMAN$	3::Q7Z7G8|VP13B_HUMAN	18	448378	2	2	2	2	0.02	Vacuolar protein sorting
1964	1	db$2	2::C3K055|C3K055_PSEFS	18	30642	1	1	1	1	0.17	Uncharacterized protein
1965	1	SwissProt_HUMAN$	3::Q9H0L4|CSTFT_HUMAN	18	64396	1	1	1	1	0.08	Cleavage stimulation fac
1966	1	SwissProt_HUMAN$	3::Q5VWM6|PRA13_HUMAN	18	54878	2	1	2	1	0.09	Putative PRAME family n
1967	1	SwissProt_HUMAN$	3::Q96M60|F227B_HUMAN	18	59918	1	1	1	1	0.08	Protein FAM227B OS=H
1968	1	SwissProt_HUMAN$	3::Q96P16|RPR1A_HUMAN	18	35698	1	1	1	1	0.14	Regulation of nuclear pr
1969	1	SwissProt_HUMAN$	3::O95782|AP2A1_HUMAN	18	107478	1	1	1	1	0.05	AP-2 complex subunit al
1970	1	SwissProt_HUMAN$	3::Q4KMP7|TB10B_HUMAN	18	87145	1	1	1	1	0.06	TBC1 domain family me
1971	1	SwissProt_HUMAN$	3::Q9NZI6|TF2L1_HUMAN	17	54593	1	1	1	1	0.09	Transcription factor CP2
1972	1	SwissProt_HUMAN$	3::Q96M02|CJ090_HUMAN	17	77862	1	1	1	1	0.06	(E2-independent) E3 ub
1973	1	SwissProt_HUMAN$	3::Q5F1R6|DJC21_HUMAN	17	61989	1	1	1	1	0.08	DnaJ homolog subfamily
1974	1	db$2	2::C3JY26|C3JY26_PSEFS	17	17211	1	1	1	1	0.31	Protein-tyrosine-phosph
1975	1	db$2	2::A4V819|A4V819_PSEFS	17	100853	2	1	2	1	0.05	PDDEXK_1 domain-cont
1976	1	db$2	2::C3K153|C3K153_PSEFS	17	54699	2	1	2	1	0.09	Exodeoxyribonuclease I
1977	1	SwissProt_HUMAN$	3::Q6P995|F171B_HUMAN	17	92124	1	1	1	1	0.05	Protein FAM171B OS=H
1978	1	db$2	2::C3JY52|C3JY52_PSEFS	17	37777	1	1	1	1	0.13	GST C-terminal domain-
1979	1	db$2	2::C3K3Q3|C3K3Q3_PSEFS	17	61523	1	1	1	1	0.08	Putative cation ABC tran
1980	1	db$2	2::C3K457|C3K457_PSEFS	17	12769	1	1	1	1	0.44	Putative endoribonuclea
1981	1	db$2	2::C3KAJ5|C3KAJ5_PSEFS	17	83174	1	1	1	1	0.06	Putative paraquat-induc
1982	1	db$2	2::C3K704|TRUD_PSEFS	17	38571	2	1	2	1	0.13	tRNA pseudouridine syn
1983	1	db$2	2::C3K1H4|C3K1H4_PSEFS	17	45615	1	1	1	1	0.11	Putative membrane prot
1984	1	SwissProt_HUMAN$	3::P01040|CYTA_HUMAN	17	11000	1	1	1	1	0.52	Cystatin-A OS=Homo sa
1985	1	SwissProt_HUMAN$	3::Q9P202|WHRN_HUMAN	17	96499	2	2	1	1	0.05	Whirlin OS=Homo sapie
1986	1	SwissProt_HUMAN$	3::P16403|H12_HUMAN	17	21352	1	1	1	1	0.25	Histone H1.2 OS=Homo
1987	1	db$2	2::C3KDV1|C3KDV1_PSEFS	17	30184	1	1	1	1	0.17	Chemotaxis MotA protei
1988	1	SwissProt_HUMAN$	3::Q8IZ21|PHAR4_HUMAN	17	78163	1	1	1	1	0.06	Phosphatase and actin r
1989	1	SwissProt_HUMAN$	3::Q8IWI9|MGAP_HUMAN	17	335951	1	1	1	1	0.01	MAX gene-associated pr
1990	1	db$2	2::C3K9I2|C3K9I2_PSEFS	16	33896	1	1	1	1	0.15	Putative transcriptional
1991	1	db$2	2::C3K659|C3K659_PSEFS	16	30767	1	1	1	1	0.17	2-hydroxy-3-oxopropion
1992	1	SwissProt_HUMAN$	3::Q9BV86|NTM1A_HUMAN	16	25371	1	1	1	1	0.20	N-terminal Xaa-Pro-Lys

↑Family	M	DB	Accession	Score	Mass	Matches	Match(sig)	Sequences	Seq(sig)	emPAI	Description
1993	1	SwissProt_HUMAN$	3::P63244|RACK1_HUMAN	16	35055	1	1	1	1	0.14	Receptor of activated pr
1994	1	db$2	2::C3KDK7|C3KDK7_PSEFS	16	24465	1	1	1	1	0.21	Putative ABC transport s
1995	1	SwissProt_HUMAN$	3::Q9NRF8|PYRG2_HUMAN	16	65636	1	1	1	1	0.07	CTP synthase 2 OS=Hor
1996	1	SwissProt_HUMAN$	3::Q9H1R3|MYLK2_HUMAN	16	64644	1	1	1	1	0.08	Myosin light chain kinas
1997	1	SwissProt_HUMAN$	3::O00294|TULP1_HUMAN	16	60572	1	1	1	1	0.08	Tubby-related protein 1
1998	1	SwissProt_HUMAN$	3::P23526|SAHH_HUMAN	16	47685	1	1	1	1	0.10	Adenosylhomocysteinas
1999	1	SwissProt_HUMAN$	3::Q93074|MED12_HUMAN	16	242928	2	1	2	1	0.02	Mediator of RNA polyme
2000	1	db$2	2::C3K744|C3K744_PSEFS	16	69999	1	1	1	1	0.07	Histidine kinase OS=Pse
2001	1	db$2	2::C3K8K5|C3K8K5_PSEFS	16	64377	1	1	1	1	0.08	Putative transport-relate
2002	1	db$2	2::C3K431|C3K431_PSEFS	16	14535	2	1	2	1	0.38	Regulator of nucleoside
2003	1	SwissProt_HUMAN$	3::Q7L576|CYFP1_HUMAN	16	145089	1	1	1	1	0.03	Cytoplasmic FMR1-inter
2004	1	SwissProt_HUMAN$	3::P42566|EPS15_HUMAN	16	98595	1	1	1	1	0.05	Epidermal growth factor
2005	1	db$2	2::C3KE60|C3KE60_PSEFS	16	49144	1	1	1	1	0.10	Histidine kinase OS=Pse
2006	1	db$2	2::C3KDU2|C3KDU2_PSEFS	16	32215	2	1	1	1	0.16	Putative short chain deh
2007	1	db$2	2::C3K4K4|C3K4K4_PSEFS	16	39850	1	1	1	1	0.13	Putative branched amin
2008	1	SwissProt_HUMAN$	3::A4UGR9|XIRP2_HUMAN	16	382063	1	1	1	1	0.01	Xin actin-binding repeat
2009	1	SwissProt_HUMAN$	3::Q7RTR0|NLRP9_HUMAN	16	113238	1	1	1	1	0.04	NACHT, LRR and PYD dc
2010	1	db$2	2::C3K0Y3|C3K0Y3_PSEFS	16	20085	1	1	1	1	0.26	Glutathione peroxidase (
2011	1	SwissProt_HUMAN$	3::Q13206|DDX10_HUMAN	16	100825	2	1	1	1	0.05	Probable ATP-dependent
2012	1	SwissProt_HUMAN$	3::Q9NZ63|TLS1_HUMAN	16	33667	1	1	1	1	0.15	Telomere length and sile
2013	1	SwissProt_HUMAN$	3::Q13724|MOGS_HUMAN	16	91861	1	1	1	1	0.05	Mannosyl-oligosaccharid
2014	1	db$2	2::C3JYZ6|C3JYZ6_PSEFS	16	88576	1	1	1	1	0.06	Putative ABC transport s
2015	1	SwissProt_HUMAN$	3::C9JTQ0|ANR63_HUMAN	16	39596	1	1	1	1	0.13	Ankyrin repeat domain-(
2016	1	db$2	2::C3KC79|C3KC79_PSEFS	15	13177	1	1	1	1	0.42	Cupin_3 domain-contair
2017	1	db$2	2::C3K819|C3K819_PSEFS	15	35012	1	1	1	1	0.14	Putative ABC transport s
2018	1	SwissProt_HUMAN$	3::Q5T011|SZT2_HUMAN	15	377791	1	1	1	1	0.01	KICSTOR complex prote
2019	1	SwissProt_HUMAN$	3::Q71H61|ILDR2_HUMAN	15	71155	2	1	2	1	0.07	Immunoglobulin-like do
2020	1	SwissProt_HUMAN$	3::Q8IYW2|CFA46_HUMAN	15	303308	3	1	3	1	0.02	Cilia- and flagella-associ
2021	1	SwissProt_HUMAN$	3::P30041|PRDX6_HUMAN	15	25019	1	1	1	1	0.21	Peroxiredoxin-6 OS=Ho
2022	1	SwissProt_HUMAN$	3::Q6ZSZ6|TSH1_HUMAN	15	117843	1	1	1	1	0.04	Teashirt homolog 1 OS=
2023	1	SwissProt_HUMAN$	3::P0DMV8|HS71A_HUMAN	15	70009	1	1	1	1	0.07	Heat shock 70 kDa prote
2024	1	db$2	2::C3K230|C3K230_PSEFS	15	85084	1	1	1	1	0.06	Penicillin-binding protei
2025	1	SwissProt_HUMAN$	3::Q8WXH0|SYNE2_HUMAN	15	795944	1	1	1	1	0.01	Nesprin-2 OS=Homo sa
2026	1	db$2	2::C3KBH6|C3KBH6_PSEFS	15	16609	1	1	1	1	0.32	Conserved hypothetical
2027	1	db$2	2::C3K3J6|C3K3J6_PSEFS	15	14927	1	1	1	1	0.37	Type IV pilus response r
2028	1	db$2	2::A4V768|A4V768_PSEFS	15	12827	1	1	1	1	0.43	Uncharacterized protein
2029	1	db$2	2::C3K9S7|C3K9S7_PSEFS	14	35611	1	1	1	1	0.14	Putative magnesium che
2030	1	db$2	2::C3JXQ7|C3JXQ7_PSEFS	14	48832	1	1	1	1	0.10	UDP-glucose 6-dehydro
2031	1	db$2	2::C3K0N1|C3K0N1_PSEFS	14	29172	1	1	1	1	0.18	4-amino-4-deoxychorisr
2032	1	SwissProt_HUMAN$	3::P49321|NASP_HUMAN	14	85186	1	1	1	1	0.06	Nuclear autoantigenic sp

↑Family	M	DB	Accession	Score	Mass	Matches	Match(sig)	Sequences	Seq(sig)	emPAI	Description
2033	1	db$2	2::C3KB03|C3KB03_PSEFS	14	18864	2	1	2	1	0.28	Uncharacterized protein
2034	1	db$2	2::C3K239|C3K239_PSEFS	14	42550	1	1	1	1	0.12	Aminotransferase OS=P
2035	1	SwissProt_HUMAN$	3::Q6ZVD7|STOX1_HUMAN	14	110893	1	1	1	1	0.04	Storkhead-box protein 1
2036	1	db$2	2::C3K855|C3K855_PSEFS	14	192299	1	1	1	1	0.03	Uncharacterized protein
2037	1	SwissProt_HUMAN$	3::O75116|ROCK2_HUMAN	14	160799	2	1	2	1	0.03	Rho-associated protein k
2038	1	db$2	2::C3K0K2|C3K0K2_PSEFS	14	29783	1	1	1	1	0.17	Putative outer membran
2039	1	SwissProt_HUMAN$	3::Q8N468|MFD4A_HUMAN	14	56228	1	1	1	1	0.09	Major facilitator superfa
2040	1	SwissProt_HUMAN$	3::Q7L592|NDUF7_HUMAN	14	49206	1	1	1	1	0.10	Protein arginine methylt
2041	1	SwissProt_HUMAN$	3::Q8IVF4|DYH10_HUMAN	14	514512	1	1	1	1	0.01	Dynein heavy chain 10,
2042	1	SwissProt_HUMAN$	3::Q00994|BEX3_HUMAN	14	12950	1	1	1	1	0.43	Protein BEX3 OS=Homo
2043	1	SwissProt_HUMAN$	3::P51608|MECP2_HUMAN	14	52409	1	1	1	1	0.09	Methyl-CpG-binding prot
2044	1	SwissProt_HUMAN$	3::P08842|STS_HUMAN	14	65450	1	1	1	1	0.08	Steryl-sulfatase OS=Hor
2045	1	db$2	2::C3K908|C3K908_PSEFS	14	33577	2	1	2	1	0.15	Putative dehydrogenase
2046	1	db$2	2::C3K0I5|C3K0I5_PSEFS	14	41628	1	1	1	1	0.12	Flagellar biosynthetic pr
2047	1	SwissProt_HUMAN$	3::Q8IXJ9|ASXL1_HUMAN	14	165329	1	1	1	1	0.03	Polycomb group protein
2048	1	SwissProt_HUMAN$	3::A6NNT2|CP096_HUMAN	14	124963	2	1	2	1	0.04	Uncharacterized protein
2049	1	db$2	2::C3K0W2|C3K0W2_PSEFS	14	26131	1	1	1	1	0.20	Flagellar basal body prot
2050	1	db$2	2::C3K2Z6|ERPA_PSEFS	14	12493	1	1	1	1	0.45	Iron-sulfur cluster insert
2051	1	db$2	2::C3KCT9|C3KCT9_PSEFS	14	12132	1	1	1	1	0.47	Hypothetical phage-relat
2052	1	SwissProt_HUMAN$	3::Q9P2J5|SYLC_HUMAN	14	134379	1	1	1	1	0.04	Leucine--tRNA ligase, cy
2053	1	db$2	2::C3K1F5|C3K1F5_PSEFS	14	29048	1	1	1	1	0.18	Putative chromosome pa
2054	1	SwissProt_HUMAN$	3::Q9NZQ0|DJC27_HUMAN	14	30836	1	1	1	1	0.16	DnaJ homolog subfamily
2055	1	SwissProt_HUMAN$	3::Q2NL98|VMAC_HUMAN	13	18337	1	1	1	1	0.29	Vimentin-type intermedi
2056	1	SwissProt_HUMAN$	3::Q7KZF4|SND1_HUMAN	13	101934	1	1	1	1	0.05	Staphylococcal nuclease
2057	1	db$2	2::C3KC46|C3KC46_PSEFS	13	40282	2	1	2	1	0.12	Multidrug resistance pro
2058	1	SwissProt_HUMAN$	3::Q14146|URB2_HUMAN	13	170435	1	1	1	1	0.03	Unhealthy ribosome bio
2059	1	db$2	2::C3K0Z2|C3K0Z2_PSEFS	13	32838	1	1	1	1	0.15	Cobalamin biosynthesis
2060	1	SwissProt_HUMAN$	3::O43897|TLL1_HUMAN	13	114635	1	1	1	1	0.04	Tolloid-like protein 1 OS
2061	1	SwissProt_HUMAN$	3::Q96GC6|ZN274_HUMAN	13	74131	1	1	1	1	0.07	Neurotrophin receptor-ir
2062	1	SwissProt_HUMAN$	3::Q8N5G0|SIM20_HUMAN	13	7697	1	1	1	1	0.82	Small integral membran
2063	1	SwissProt_HUMAN$	3::Q13275|SEM3F_HUMAN	13	88325	1	1	1	1	0.06	Semaphorin-3F OS=Hor
2064	1	db$2	2::C3K418|C3K418_PSEFS	13	24448	1	1	1	1	0.21	Peptidyl-prolyl cis-trans

Export as CSV

Not what you expected? Try **the select summary**.

Mascot: http://www.matrixscience.com/
