# Supplementary material for: Circulating multimeric immune complexes contribute to immunopathology in COVID-19
Source: Nat Commun. 2022 Sep 26;13:5654. doi: 10.1038/s41467-022-32867-z (PMC9513013; doi:10.1038/s41467-022-32867-z)
Supplement: Supplementary file 3 — Source Data [file 41467_2022_32867_MOESM3_ESM.zip › Source Data/MS_20210407 (immune precipitation).zip!/QX20210618-06_Virologie(AG-Hengel)-20210407_(V6)_0k3v20_TRL_db_SC2+db_Pf+SP_H(F235806).pdf]

MATRIX SCIENCE MASCOT Search Results

User : Wolfgang Bildl
E-mail : wolfgang.bildl@physiologie.uni-freiburg.de
Search title : QX 20210618 (db$ SARS-CoV-2+db$2 Pf+SP H$ 5 ppm 20 mmu), QX20210618-06_Virologie(AG-Hengel)-20210407_(V6)_0k3v20_TRL_shifted_-0.4.mgf
MS data file : D:\Xcalibur\Data\Wolfgang\Q-Exactive-HF-X_20210616\QX20210618-06_Virologie(AG-Hengel)-20210407_(V6)_0k3v20_TRL_shifted_-0.4.mgf
Databases : **1:** db$ 20210617 (17 sequences; 14,439 residues)
2: db$2 20210617 (6,388 sequences; 2,089,672 residues)
3: SwissProt_HUMAN$ 20201007 (20,389 sequences; 11,363,789 residues)
Timestamp : 21 Jun 2021 at 11:33:04 GMT

Not what you expected? Try **the select summary**.

Search parameters

Type of search : MS/MS Ion Search
Enzyme : Trypsin
Variable modifications : **Acetyl (Protein N-term)**, **Carbamidomethyl (C)**, **Formyl (Protein N-term)**, **Gln->pyro-Glu (N-term Q)**, **Oxidation (M)**
Mass values : Monoisotopic
Protein mass : Unrestricted
Peptide mass tolerance : ± 5 ppm
Fragment mass tolerance : ± 20 mmu
Max missed cleavages : 1
Instrument type : ESI-FTICR
Number of queries : 86,530

Score distribution

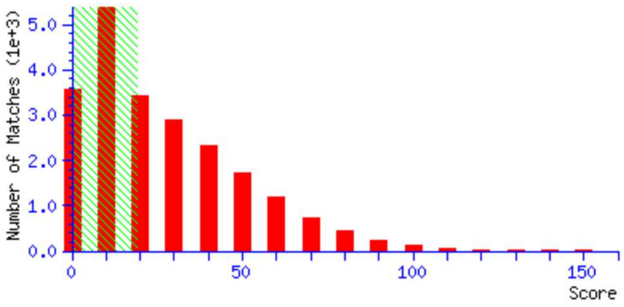

Peptide score distribution. Ions score is $-10\log(P)$, where P is the probability that the observed match is a random event.
There are **11,588** peptide matches above identity threshold and **12,404** matches above homology threshold for **86,530** queries.
On average, individual ions scores **> 19** (beyond green shading) indicate **identity or extensive homology** (p<0.05).

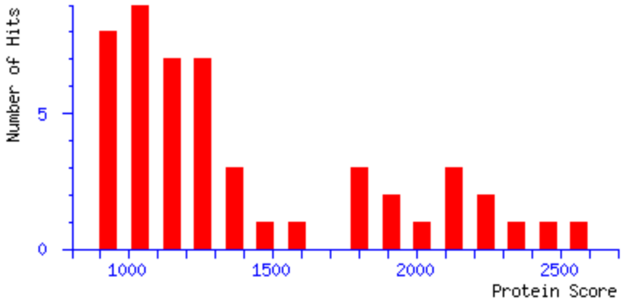

[Deprecated] Protein score distribution. Score distribution for family members in the first 50 proteins. Protein scores are derived from ions scores as a non-probabilistic basis for ranking protein families.

▼Modification statistics

Modification	Site	Above thr.
Oxidation	M	2167
Carbamidomethyl	C	1196
Gln->pyro-Glu	N-term	268
Formyl	Protein N-term	46
Acetyl	Protein N-term	16

▼Legend

Peptide columns and rows

Dupes	...	Expect	Rank	U	1	2	Peptide	
		**0.037**	▶**2**				**GAYSLSLR**	significant
		9	▶1				GFFLFVEGGR	top ranking
		**6.4e-005**	▶**1**				**GSSIFGLAPGK**	significant and top ranking
		**1.3e-006**	▶**1**		■		**SSGTSYPDVLK**	peptide is found in all proteins in family member 1
		**6.2e-007**	▶**1**			■	**VCNYVSWIK**	peptide is found in some but not all proteins in family member 2
		**6.4e-005**	▶**1**	**U**			**GSSIFGLAPGK**	unique
▶**2**		**5.7e-005**	▶**1**				**LNTLETEEWFFK**	peptide has two duplicates
		0.18	▶*1*				*LNTLETEEWFFK*	duplicate peptide

Right-facing triangle (▶) in the Dupes or Rank column indicates content that can be expanded by clicking on it. Down-facing triangle (▼) indicates the content is expanded and can be collapsed. For more details about particular columns, see **results format help**.

Protein quantitation ratios

	Score	...	114/113	115/113
**CFAH_HUMAN**	37559		*0.962*	**1.129**
**FHR2_HUMAN**	1330		**0.859**	1.128

When quantitation method is Reporter (e.g. iTRAQ) or Multiplex (e.g. IPTL), protein ratios are displayed when a family is expanded. Ratios in *italic* indicate that the peptide log-ratios do not appear to come from a normal distribution. **Bold** indicates that if you can assume peptide ratios are normally distributed, the protein ratio is significantly different from 1.0 (at significance level 0.05).

Note that lack of bold or italic can also mean that significance or normality testing has not been performed (for

example, if protein ratio type does not support it).

Protein Family Summary

Significance threshold p<	0.05	Max. number of families	AUTO
Ions score or expect cut-off	0.5	Dendrograms cut at	0
Show Percolator scores	☐		
Preferred taxonomy	All entries		

▼Sensitivity and FDR (reversed protein sequences)

			Target	Decoy	FDR		
PSMs	above	homology	12404	432	3.48%	Adjust to	1%

Decoy results are available in **the decoy report**.

Protein hits (2103 proteins)

▶Filters: (none)

Export as CSV

↑Family	M	DB	Accession	Score	Mass	Matches	Match(sig)	Sequences	Seq(sig)	emPAI	Description
1	1	db$2	2::C3K5Z6|EFTS_PSEFS	2565	30341	84	82	13	13	7.81	Elongation factor Ts OS
2	1	db$2	2::C3K5G3|C3K5G3_PSEFS	2506	99192	81	77	36	34	7.59	Aconitate hydratase OS
3	1	db$2	2::C3K2Y3|RPOB_PSEFS	2338	150923	99	92	69	64	8.63	DNA-directed RNA poly
4	1	db$2	2::C3K2X8|EFTU_PSEFS	2251	43536	73	68	25	24	24.98	Elongation factor Tu OS
5	1	db$2	2::C3K1E6|ATPB_PSEFS	2233	49459	69	68	28	28	36.69	ATP synthase subunit b
6	1	db$2	2::C3K4N0|C3K4N0_PSEFS	2181	43479	60	58	25	23	24.98	Putative ornithine deca
7	1	db$2	2::C3K1A0|CH60_PSEFS	2115	56931	62	60	28	28	26.77	60 kDa chaperonin OS=
8	1	db$2	2::C3K4D5|C3K4D5_PSEFS	2077	34599	65	63	23	22	38.51	Phosphate-binding prot
9	1	db$2	2::C3KD82|C3KD82_PSEFS	2003	181591	72	69	54	52	3.56	Glutamate dehydrogena
10	1	db$2	2::C3K2X9|EFG_PSEFS	1945	77114	68	64	34	31	11.41	Elongation factor G OS=
11	1	SwissProt_HUMAN$	3::P13645|K1C10_HUMAN	1934	58792	48	44	32	30	13.22	Keratin, type I cytoskel
11	2	SwissProt_HUMAN$	3::P35527|K1C9_HUMAN	947	62027	26	25	22	21	3.96	Keratin, type I cytoskel
11	3	SwissProt_HUMAN$	3::P02533|K1C14_HUMAN	319	51529	18	15	17	14	2.62	Keratin, type I cytoskel
11	4	SwissProt_HUMAN$	3::P08779|K1C16_HUMAN	222	51236	13	10	13	10	1.51	Keratin, type I cytoskel
11	5	SwissProt_HUMAN$	3::Q04695|K1C17_HUMAN	118	48076	11	7	10	6	0.80	Keratin, type I cytoskel
11	6	SwissProt_HUMAN$	3::P08727|K1C19_HUMAN	65	44079	6	4	6	4	0.54	Keratin, type I cytoskel
11	7	SwissProt_HUMAN$	3::P05783|K1C18_HUMAN	59	48029	4	3	4	3	0.34	Keratin, type I cytoskel
12	1	db$2	2::C3K2Y2|RPOC_PSEFS	1826	154528	85	80	59	56	6.15	DNA-directed RNA poly
13	1	db$2	2::C3K1E8|ATPA_PSEFS	1797	55245	60	59	31	31	32.42	ATP synthase subunit a
14	1	db$2	2::C3K255|PNP_PSEFS	1770	74889	61	58	30	29	10.78	Polyribonucleotide nucl
15	1	db$2	2::C3K0Q5|C3K0Q5_PSEFS	1559	107302	55	50	38	35	5.12	Ribonucleoside-diphosp

↑Family	M	DB	Accession	Score	Mass	Matches	Match(sig)	Sequences	Seq(sig)	emPAI	Description
16	1	SwissProt_HUMAN$	3::P04264|K2C1_HUMAN	1510	65999	43	42	28	28	8.23	Keratin, type II cytoske
16	2	SwissProt_HUMAN$	3::P35908|K22E_HUMAN	1044	65393	37	35	31	29	7.78	Keratin, type II cytoske
16	3	SwissProt_HUMAN$	3::P04259|K2C6B_HUMAN	262	60030	12	11	12	11	1.38	Keratin, type II cytoske
16	4	SwissProt_HUMAN$	3::P13647|K2C5_HUMAN	221	62340	10	10	10	10	1.14	Keratin, type II cytoske
16	5	SwissProt_HUMAN$	3::P05787|K2C8_HUMAN	144	53671	7	7	5	5	0.55	Keratin, type II cytoske
16	6	SwissProt_HUMAN$	3::P08729|K2C7_HUMAN	126	51354	6	6	4	4	0.44	Keratin, type II cytoske
16	7	SwissProt_HUMAN$	3::Q14CN4|K2C72_HUMAN	55	55842	3	3	3	3	0.29	Keratin, type II cytoske
17	1	db$2	2::C3KCP4|C3KCP4_PSEFS	1401	99394	64	61	39	37	9.88	Pyruvate dehydrogenas
18	1	db$2	2::C3K275|DNAK_PSEFS	1342	68158	42	42	23	23	4.68	Chaperone protein Dna
19	1	db$2	2::C3K6P2|C3K6P2_PSEFS	1334	42870	31	30	13	13	6.28	3-oxoacyl-[acyl-carrier-
20	1	db$2	2::C3K6N0|SUCC_PSEFS	1288	41262	38	38	21	21	18.56	Succinate--CoA ligase [
21	1	db$2	2::C3K6M5|C3K6M5_PSEFS	1246	63522	34	34	20	20	4.56	Succinate dehydrogena
22	1	db$2	2::C3KCJ5|C3KCJ5_PSEFS	1240	93957	49	45	38	35	5.48	Aconitate hydratase B (
23	1	db$2	2::C3K271|C3K271_PSEFS	1226	117563	49	46	35	33	3.45	Carbamoyl-phosphate s
24	1	db$2	2::C3K0P3|C3K0P3_PSEFS	1208	118163	34	30	22	19	1.42	Ribonuclease E OS=Pse
25	1	db$2	2::C3K5J5|FABV_PSEFS	1204	42933	39	39	23	23	13.00	Enoyl-[acyl-carrier-prot
26	1	db$2	2::C3K6M7|C3K6M7_PSEFS	1201	105930	46	43	32	29	3.39	Oxoglutarate dehydrog
27	1	db$2	2::C3K402|C3K402_PSEFS	1193	83101	57	51	37	32	7.73	Polyphosphate kinase C
28	1	db$2	2::C3JYU7|C3JYU7_PSEFS	1184	102460	39	38	26	25	2.83	Protein translocase sub
29	1	db$2	2::C3K331|C3K331_PSEFS	1161	73691	47	42	35	32	7.90	AAA_PrkA domain-cont
30	1	db$2	2::C3KBH1|C3KBH1_PSEFS	1151	161946	49	48	41	40	2.33	Glutamate synthase [N
31	1	db$2	2::C3KBY8|C3KBY8_PSEFS	1116	21919	50	47	16	16	72.07	Alkyl hydroperoxide rec
32	1	db$2	2::C3K7D3|C3K7D3_PSEFS	1095	91800	34	34	24	24	3.03	Alpha-1,4 glucan phosp
33	1	db$2	2::C3K613|FADB_PSEFS	1095	76938	35	33	24	22	2.88	Fatty acid oxidation cor
34	1	db$2	2::C3K225|ILVC_PSEFS	1084	36188	47	45	24	23	32.79	Ketol-acid reductoisome
35	1	db$2	2::C3JY66|C3JY66_PSEFS	1058	80156	37	35	22	21	3.65	Isocitrate dehydrogena
36	1	db$2	2::C3JYK1|TIG_PSEFS	1044	48316	43	36	26	21	13.06	Trigger factor OS=Pseu
37	1	db$2	2::C3K6H4|PYRG_PSEFS	1039	59660	40	35	24	22	7.53	CTP synthase OS=Pseu
38	1	db$2	2::C3K259|IF2_PSEFS	1030	90544	40	39	27	26	3.81	Translation initiation fa
39	1	db$2	2::C3K1L8|NDK_PSEFS	1030	14928	30	28	9	9	21.97	Nucleoside diphosphate
40	1	db$2	2::C3K673|C3K673_PSEFS	1024	47725	42	39	23	22	15.01	Citrate synthase OS=Ps
41	1	db$2	2::C3K367|C3K367_PSEFS	1015	68142	38	36	21	19	3.61	Uncharacterized protei
42	1	db$2	2::C3K6N1|C3K6N1_PSEFS	1000	29928	26	26	12	12	15.92	Succinate--CoA ligase [
43	1	db$2	2::C3JYJ7|C3JYJ7_PSEFS	976	9057	60	50	8	8	156.26	DNA-binding protein HU
44	1	db$2	2::C3JYT1|SYDND_PSEFS	975	66220	38	38	25	25	6.40	Aspartate--tRNA(Asp/A
45	1	db$2	2::C3KEC5|C3KEC5_PSEFS	955	97398	36	34	28	27	3.31	Phosphoenolpyruvate c
46	1	db$2	2::C3K3Z0|C3K3Z0_PSEFS	954	46925	32	32	21	21	9.13	Transcription terminati
47	1	db$2	2::C3K266|C3K266_PSEFS	953	69364	75	74	14	13	1.60	ATP-dependent zinc me
48	1	db$2	2::C3KE86|C3KE86_PSEFS	935	95293	42	39	33	32	4.71	Chaperone protein ClpB
49	1	SwissProt_HUMAN$	3::P02751|FINC_HUMAN	928	272150	49	46	42	40	1.01	Fibronectin OS=Homo s

↑Family	M	DB	Accession	Score	Mass	Matches	Match(sig)	Sequences	Seq(sig)	emPAI	Description
50	1	db$2	2::C3K1J2|C3K1J2_PSEFS	927	140531	42	34	33	26	1.57	Phosphoribosylformylgl
51	1	db$2	2::C3K6K0|C3K6K0_PSEFS	925	62268	39	37	22	20	5.69	30S ribosomal protein S
52	1	db$2	2::C3K0K6|C3K0K6_PSEFS	918	26352	25	25	15	15	16.47	Electron transfer flavop
53	1	db$2	2::C3JYR1|GCSP_PSEFS	908	102176	49	42	28	25	2.50	Glycine dehydrogenase
54	1	db$2	2::C3K7D1|C3K7D1_PSEFS	891	67115	39	35	25	24	7.90	50S ribosomal subunit a
55	1	db$2	2::C3K121|C3K121_PSEFS	880	86030	41	37	29	28	4.52	Phosphoenolpyruvate sy
56	1	db$2	2::C3JYS1|C3JYS1_PSEFS	878	17688	23	21	8	8	13.15	Peptidoglycan-associate
57	1	db$2	2::C3K6N7|HTPG_PSEFS	862	71589	29	27	20	18	2.76	Chaperone protein HtpG
58	1	db$2	2::C3K3F3|C3K3F3_PSEFS	861	72324	38	37	25	25	5.67	Transketolase OS=Pseu
59	1	db$2	2::C3K6M9|C3K6M9_PSEFS	860	49948	28	27	15	14	3.55	Dihydrolipoyl dehydrog
60	1	db$2	2::C3KE67|RS6_PSEFS	857	16276	28	26	14	13	229.04	30S ribosomal protein S
61	1	db$2	2::C3K7G0|C3K7G0_PSEFS	851	36980	28	26	18	18	11.85	Cell shape-determining
62	1	db$2	2::C3K1K1|C3K1K1_PSEFS	840	51928	38	35	20	18	7.12	Inosine-5'-monophosph
63	1	db$2	2::C3KBT1|C3KBT1_PSEFS	840	44808	31	31	20	20	9.16	4-aminobutyrate amino
64	1	db$2	2::C3K3S2|C3K3S2_PSEFS	830	44244	27	26	18	17	5.13	2-oxoglutarate reductas
65	1	db$2	2::C3K2D0|C3K2D0_PSEFS	828	61490	31	27	21	20	5.34	Energy-dependent trans
66	1	db$2	2::C3K0T0|C3K0T0_PSEFS	824	34560	30	29	7	7	2.42	Putative outer membran
67	1	db$2	2::C3K4D2|C3K4D2_PSEFS	804	30988	36	33	21	20	69.77	Phosphate import ATP-b
68	1	db$2	2::C3K165|C3K165_PSEFS	796	54857	37	33	24	22	10.20	Fumarate hydratase cla
68	2	db$2	2::C3K604|C3K604_PSEFS	306	18933	17	16	11	10	10.79	Putative outer membran
69	1	db$2	2::C3K6G5|AMPA_PSEFS	787	52403	32	30	25	25	9.46	Probable cytosol amino
70	1	db$2	2::C3K6M8|C3K6M8_PSEFS	779	42320	24	22	13	12	4.34	Dihydrolipoyllysine-resi
71	1	db$2	2::C3K1K0|GUAA_PSEFS	775	58398	32	28	23	20	4.95	GMP synthase [glutami
72	1	db$2	2::C3KBJ2|C3KBJ2_PSEFS	774	24354	22	21	13	13	21.03	Carbonic anhydrase OS
73	1	db$2	2::C3KCB8|C3KCB8_PSEFS	774	36708	30	27	12	11	6.80	Phosphate-binding prot
74	1	db$2	2::C3K6J4|C3K6J4_PSEFS	771	97893	29	28	21	20	1.77	DNA gyrase subunit A O
75	1	db$2	2::C3KBT0|C3KBT0_PSEFS	763	51607	27	26	14	14	4.20	Succinate-semialdehyde
76	1	db$2	2::C3KAD2|ARNA_PSEFS	743	74140	23	21	19	17	2.16	Bifunctional polymyxin
77	1	db$2	2::C3KDP2|C3KDP2_PSEFS	741	68879	26	24	20	18	2.95	Multifunctional fusion p
78	1	db$2	2::C3K1A7|C3K1A7_PSEFS	736	66070	27	25	21	19	3.84	Proline--tRNA ligase OS
79	1	db$2	2::C3JXY1|C3JXY1_PSEFS	734	93962	29	25	23	21	2.03	Alanine--tRNA ligase OS
80	1	db$2	2::C3K162|C3K162_PSEFS	733	52023	28	27	20	19	5.15	Pyruvate kinase OS=Ps
81	1	db$2	2::C3K2V9|RS5_PSEFS	718	17675	22	21	11	11	52.24	30S ribosomal protein S
82	1	db$2	2::C3KEC2|C3KEC2_PSEFS	710	28614	24	24	16	16	12.87	Putative lipoprotein OS=
82	2	db$2	2::C3KAJ6|C3KAJ6_PSEFS	227	38502	8	7	5	4	0.85	Histidinol-phosphate an
83	1	db$2	2::C3K139|C3K139_PSEFS	707	48816	21	21	11	11	2.88	Uncharacterized protein
84	1	db$2	2::C3JY70|C3JY70_PSEFS	707	50668	41	23	16	16	4.37	Adenylosuccinate lyase
85	1	db$2	2::C3K4D1|C3K4D1_PSEFS	706	28553	24	22	14	13	13.01	Phosphate-specific tran
86	1	db$2	2::C3K4J5|PCKA_PSEFS	704	55324	26	25	19	18	4.54	Phosphoenolpyruvate c
87	1	db$2	2::C3K426|ARLY_PSEFS	702	51545	27	26	16	15	5.88	Argininosuccinate lyase

↑Family	M	DB	Accession	Score	Mass	Matches	Match(sig)	Sequences	Seq(sig)	emPAI	Description
88	1	db$2	2::C3JYJ9|CLPX_PSEFS	699	46924	26	24	17	15	5.12	ATP-dependent Clp prot
89	1	db$2	2::C3K3J7|C3K3J7_PSEFS	692	35008	26	25	14	14	11.97	Glutathione synthetase
90	1	db$2	2::C3K3F1|PGK_PSEFS	679	40125	17	17	12	12	3.63	Phosphoglycerate kinas
91	1	db$2	2::C3KE51|C3KE51_PSEFS	678	38026	27	25	19	19	9.54	Putative oxidoreductase
92	1	db$2	2::C3JZN7|SYT_PSEFS	677	72513	22	20	16	15	2.04	Threonine--tRNA ligase
93	1	db$2	2::C3KA76|C3KA76_PSEFS	674	30860	19	18	13	12	7.48	UTP--glucose-1-phosph
94	1	db$2	2::C3KDU5|C3KDU5_PSEFS	674	90380	22	20	18	17	1.57	DNA gyrase subunit B C
95	1	db$2	2::C3K6H6|C3K6H6_PSEFS	672	45588	26	23	16	14	3.73	Enolase OS=Pseudomo
96	1	db$2	2::C3K2R9|C3K2R9_PSEFS	662	16440	15	15	7	7	8.72	6,7-dimethyl-8-ribityllu
97	1	db$2	2::C3KAH7|C3KAH7_PSEFS	660	45018	24	23	16	15	5.60	Putative NADP-depende
98	1	db$2	2::C3K178|C3K178_PSEFS	655	36139	21	18	12	12	5.20	Glyceraldehyde-3-phos
99	1	db$2	2::C3K2D3|C3K2D3_PSEFS	653	44901	23	21	16	15	4.38	Serine hydroxymethyltr
100	1	db$2	2::C3JYT4|C3JYT4_PSEFS	649	17756	24	23	12	11	86.98	Putative ferritin DPS-fa
101	1	db$2	2::C3K603|C3K603_PSEFS	648	87560	27	25	19	18	1.65	Outer membrane protei
101	2	db$2	2::C3K3R2|C3K3R2_PSEFS	457	83481	21	18	19	17	1.63	Phosphoenolpyruvate--
102	1	db$2	2::C3K1W9|C3K1W9_PSEFS	647	33929	14	14	9	9	3.60	Thioredoxin reductase (
103	1	db$2	2::C3KCS1|C3KCS1_PSEFS	647	34171	22	21	14	14	14.78	Ribose-phosphate pyro
104	1	db$2	2::C3KCP3|C3KCP3_PSEFS	639	56221	23	23	15	15	2.54	Acetyltransferase comp
105	1	db$2	2::C3K4Y4|C3K4Y4_PSEFS	638	71842	22	21	17	17	2.74	Potassium-transporting
106	1	db$2	2::C3KDD8|C3KDD8_PSEFS	636	44893	29	24	21	18	7.23	Cell division protein Fts
107	1	db$2	2::C3K7C9|C3K7C9_PSEFS	634	51641	27	24	18	16	4.20	Glutamine synthetase C
108	1	db$2	2::C3KDG0|RECA_PSEFS	631	37499	24	23	16	15	8.62	Protein RecA OS=Pseuc
109	1	db$2	2::C3K1E7|ATPG_PSEFS	630	31374	18	17	12	12	7.17	ATP synthase gamma c
110	1	db$2	2::C3JZS1|C3JZS1_PSEFS	626	55264	23	23	14	14	2.61	Amidophosphoribosyltra
111	1	db$2	2::C3KE40|SYGB_PSEFS	626	74833	32	29	23	20	3.03	Glycine--tRNA ligase be
112	1	db$2	2::C3K2X5|RL4_PSEFS	624	21987	14	14	9	9	7.46	50S ribosomal protein L
113	1	db$2	2::C3JYS2|TOLB_PSEFS	623	47517	22	22	18	18	6.32	Tol-Pal system protein
114	1	db$2	2::C3JY65|C3JY65_PSEFS	611	45375	28	27	16	15	7.02	Isocitrate dehydrogena
115	1	db$2	2::C3JYV2|C3JYV2_PSEFS	609	52688	18	18	15	15	3.20	AAA domain-containing
116	1	db$2	2::C3KDW5|C3KDW5_PSEFS	608	43265	23	22	17	16	5.39	Protein HflK OS=Pseud
117	1	db$2	2::C3K341|C3K341_PSEFS	603	69250	24	23	19	18	3.20	RNA polymerase sigma
117	2	db$2	2::C3K708|C3K708_PSEFS	119	38066	3	3	3	3	0.45	RNA polymerase sigma
118	1	db$2	2::C3KBM0|GLPK_PSEFS	601	55508	30	25	20	16	4.05	Glycerol kinase OS=Pse
119	1	SwissProt_HUMAN$	3::P02675|FIBB_HUMAN	599	55892	24	21	21	19	3.99	Fibrinogen beta chain C
120	1	db$2	2::C3K7Y0|C3K7Y0_PSEFS	596	45268	20	19	16	15	4.31	Putative exported prote
121	1	db$2	2::C3K3G6|SAHH_PSEFS	596	51212	28	25	20	18	5.96	Adenosylhomocysteinas
122	1	db$2	2::C3K1E2|C3K1E2_PSEFS	593	66300	24	23	18	17	3.18	Glutamine--fructose-6-
123	1	db$2	2::C3KDD9|C3KDD9_PSEFS	592	41969	19	16	17	15	5.06	Cell division protein Fts
124	1	db$2	2::C3K8B0|C3K8B0_PSEFS	585	25585	19	18	11	10	9.96	Peptidyl-prolyl cis-trans
125	1	db$2	2::C3K4F6|C3K4F6_PSEFS	584	65489	33	29	23	22	4.67	Oxaloacetate decarbox

↑Family	M	DB	Accession	Score	Mass	Matches	Match(sig)	Sequences	Seq(sig)	emPAI	Description
126	1	db$2	2::C3KDW8|PURA_PSEFS	578	46535	27	27	17	17	5.90	Adenylosuccinate synth
127	1	db$2	2::C3K6H5|KDSA_PSEFS	575	30777	26	25	14	13	23.96	2-dehydro-3-deoxypho:
128	1	db$2	2::C3K4D4|C3K4D4_PSEFS	575	83859	23	22	17	16	2.10	Putative phosphate ABC
129	1	db$2	2::C3K1K7|LEU1_PSEFS	571	61997	22	22	18	18	3.26	2-isopropylmalate syntl
130	1	db$2	2::C3K6I0|C3K6I0_PSEFS	565	39056	20	18	11	11	3.81	Alcohol dehydrogenase
131	1	db$2	2::C3K2Y6|RL1_PSEFS	564	24061	18	17	11	11	11.62	50S ribosomal protein l
132	1	db$2	2::C3K914|C3K914_PSEFS	558	38509	18	18	12	12	5.28	Putative ABC transport
133	1	db$2	2::C3K2V1|C3K2V1_PSEFS	555	36623	24	21	16	14	5.90	DNA-directed RNA poly
134	1	db$2	2::C3KED7|PUR7_PSEFS	554	26862	20	20	14	14	15.48	Phosphoribosylaminoim
135	1	db$2	2::C3JZV8|C3JZV8_PSEFS	553	38906	11	11	7	7	1.64	Neutral metalloproteina
136	1	db$2	2::C3K5E6|RS2_PSEFS	551	27078	27	26	16	15	18.16	30S ribosomal protein S
137	1	db$2	2::C3K2V2|RS4_PSEFS	547	23062	23	20	12	12	13.07	30S ribosomal protein S
138	1	db$2	2::C3K1G5|C3K1G5_PSEFS	540	46195	21	19	14	13	3.19	Homoserine dehydroge
139	1	db$2	2::C3JZT6|C3JZT6_PSEFS	536	40569	16	16	11	11	3.55	Aspartate-semialdehyde
140	1	db$2	2::C3KBH2|C3KBH2_PSEFS	533	51951	15	13	13	11	1.72	Glutamate synthase [N
141	1	db$2	2::C3K6W9|C3K6W9_PSEFS	532	69415	15	15	13	13	1.43	Putative methyl-accepti
141	2	db$2	2::C3JZ31|C3JZ31_PSEFS	74	58204	2	2	2	2	0.18	Putative methyl-accepti
141	3	db$2	2::C3K4R7|C3K4R7_PSEFS	68	57887	2	2	2	2	0.18	Putative methyl-accepti
141	4	db$2	2::C3KD60|C3KD60_PSEFS	62	55774	2	2	2	2	0.19	Methyl-accepting chem
141	5	db$2	2::C3K5K7|C3K5K7_PSEFS	54	52604	4	2	4	2	0.20	Putative methyl-accepti
142	1	db$2	2::C3K3P0|ILVD_PSEFS	526	65667	21	20	20	19	2.93	Dihydroxy-acid dehydra
143	1	db$2	2::C3K5H9|C3K5H9_PSEFS	525	33776	17	14	12	9	3.65	Transaldolase OS=Pseu
144	1	db$2	2::C3KBZ2|ASSY_PSEFS	525	45330	22	21	17	16	4.89	Argininosuccinate synth
145	1	db$2	2::C3K6X4|C3K6X4_PSEFS	523	71161	20	20	18	18	2.31	Biosynthetic arginine de
146	1	db$2	2::C3K2H6|C3K2H6_PSEFS	518	32512	12	11	7	6	2.19	Acyl-CoA thioesterase I
147	1	db$2	2::C3JZV2|C3JZV2_PSEFS	515	43020	18	18	15	15	4.78	Aminotransferase OS=F
148	1	db$2	2::C3K1B7|C3K1B7_PSEFS	512	51892	17	16	13	12	2.27	Threonine synthase OS
149	1	db$2	2::C3K2X0|C3K2X0_PSEFS	505	25737	23	21	14	12	17.57	30S ribosomal protein S
150	1	db$2	2::C3K2V6|C3K2V6_PSEFS	502	47842	17	16	8	7	1.69	Protein translocase sub
151	1	db$2	2::C3JXL8|C3JXL8_PSEFS	498	112023	23	21	18	16	1.24	Putative efflux protein (
152	1	db$2	2::C3JZN3|SYFA_PSEFS	497	37892	17	15	15	13	4.73	Phenylalanine--tRNA lig
153	1	db$2	2::C3K016|C3K016_PSEFS	492	85788	22	21	20	19	1.86	Ribosomal RNA large su
154	1	db$2	2::C3K1N2|C3K1N2_PSEFS	492	67307	23	22	16	16	2.81	Protein translocase sub
155	1	db$2	2::C3K2Y4|RL7_PSEFS	491	12456	11	10	3	2	5.47	50S ribosomal protein l
156	1	db$2	2::C3K649|C3K649_PSEFS	485	8822	14	14	4	4	7.15	Major outer membrane
157	1	db$2	2::C3KDX4|SYI_PSEFS	484	105386	24	21	21	18	1.25	Isoleucine--tRNA ligase
158	1	db$2	2::C3KEC8|C3KEC8_PSEFS	483	50623	18	16	11	10	1.79	Periplasmic serine endo
159	1	db$2	2::C3K9R4|C3K9R4_PSEFS	482	62164	22	22	17	17	3.25	Putative sulfite reducta:
160	1	db$2	2::C3K2X6|RL3_PSEFS	482	22550	14	13	9	9	5.52	50S ribosomal protein l
161	1	db$2	2::C3K260|C3K260_PSEFS	481	54381	17	15	13	11	1.84	Transcription terminatic

↑Family	M	DB	Accession	Score	Mass	Matches	Match(sig)	Sequences	Seq(sig)	emPAI	Description
162	1	db$2	2::C3JYJ8|C3JYJ8_PSEFS	477	88851	29	28	15	14	1.11	Lon protease OS=Pseu
163	1	db$2	2::C3K4F7|C3K4F7_PSEFS	476	51847	24	23	21	21	5.79	Biotin carboxylase OS=
164	1	SwissProt_HUMAN$	3::P02679|FIBG_HUMAN	474	51479	20	17	16	13	2.62	Fibrinogen gamma chai
165	1	db$2	2::C3K6M2|C3K6M2_PSEFS	471	60584	19	19	17	17	3.08	ATP-dependent RNA he
166	1	db$2	2::C3K6C2|C3K6C2_PSEFS	470	27720	14	13	10	9	3.60	Putative amino acid AB
167	1	db$2	2::C3KDR8|OPGD_PSEFS	466	60485	18	18	14	14	2.23	Glucans biosynthesis pr
168	1	db$2	2::C3K2W6|RL14_PSEFS	464	13401	16	15	10	9	62.84	50S ribosomal protein L
169	1	db$2	2::C3JZ70|C3JZ70_PSEFS	464	54858	22	18	15	12	2.07	Glucose-6-phosphate 1
170	1	db$2	2::C3KA80|C3KA80_PSEFS	457	20438	20	19	12	12	23.86	Alkyl hydroperoxide rec
171	1	db$2	2::C3K244|C3K244_PSEFS	456	52276	14	12	13	11	1.71	Putative response regul
172	1	db$2	2::C3K1L4|ISPG_PSEFS	453	39660	18	17	16	15	5.73	4-hydroxy-3-methylbut
173	1	db$2	2::C3KAK3|C3KAK3_PSEFS	451	54608	15	14	11	11	1.83	Probable malate:quinor
174	1	db$2	2::C3K9R0|C3K9R0_PSEFS	451	62265	15	15	14	14	2.13	Pyruvate dehydrogenas
175	1	db$2	2::C3K3V3|ARGA_PSEFS	450	47793	13	12	11	11	1.97	Amino-acid acetyltransf
176	1	db$2	2::C3K4D3|C3K4D3_PSEFS	449	60551	19	19	15	15	2.77	Phosphate transport sy
177	1	db$2	2::C3JXY0|AK_PSEFS	447	44504	18	18	16	16	5.76	Aspartate kinase OS=P
178	1	db$2	2::C3K4V9|C3K4V9_PSEFS	447	53185	20	19	15	15	3.15	Glyceraldehyde-3-phos
179	1	db$2	2::C3K1F0|C3K1F0_PSEFS	444	16832	16	14	9	9	11.09	ATP synthase subunit b
180	1	db$2	2::C3K2X7|RS10_PSEFS	442	11745	20	19	10	10	50.50	30S ribosomal protein S
181	1	db$2	2::C3K2M9|PROA_PSEFS	440	45380	11	10	7	6	1.07	Gamma-glutamyl phosp
182	1	db$2	2::C3K6G0|C3K6G0_PSEFS	438	9680	12	11	7	6	16.56	Putative DNA-binding p
183	1	db$2	2::C3K2W1|RL6_PSEFS	437	19185	18	16	8	7	7.98	50S ribosomal protein L
184	1	db$2	2::C3K358|C3K358_PSEFS	437	65409	17	15	12	10	1.38	Putative dehydrogenase
184	2	db$2	2::C3K355|C3K355_PSEFS	319	63710	14	11	11	8	0.81	Putative dehydrogenase
185	1	db$2	2::C3JXL7|C3JXL7_PSEFS	435	39112	13	13	11	11	3.26	HlyD-family secretion p
186	1	db$2	2::C3K6M6|C3K6M6_PSEFS	432	26063	24	22	13	13	11.48	Succinate dehydrogena
187	1	db$2	2::C3K1M5|ISCS_PSEFS	432	44467	15	13	14	12	2.57	Cysteine desulfurase Is
188	1	db$2	2::C3K2W0|RL18_PSEFS	432	12651	16	15	9	8	26.23	50S ribosomal protein L
189	1	db$2	2::C3KDU7|C3KDU7_PSEFS	431	28504	21	18	14	13	7.54	Putative ABC transport
190	1	SwissProt_HUMAN$	3::P60709|ACTB_HUMAN	430	41710	20	16	16	14	4.46	Actin, cytoplasmic 1 OS
191	1	db$2	2::C3JYQ5|C3JYQ5_PSEFS	427	46409	19	18	14	14	3.61	Arginine deiminase OS=
192	1	db$2	2::C3K3V2|C3K3V2_PSEFS	426	49111	18	14	9	6	0.78	Putative outer membra
193	1	db$2	2::C3K2T2|C3K2T2_PSEFS	422	24648	11	11	7	7	4.56	Putative ABC transport
194	1	db$2	2::C3KCB5|C3KCB5_PSEFS	421	30173	21	18	12	9	4.56	Phosphate import ATP-l
195	1	db$2	2::C3KBY6|C3KBY6_PSEFS	421	17850	18	13	11	9	12.70	Bacterioferritin OS=Pse
196	1	db$2	2::C3JZS4|C3JZS4_PSEFS	420	33375	16	15	10	9	3.10	Acetyl-coenzyme A carb
197	1	db$2	2::C3K108|C3K108_PSEFS	418	83591	18	17	17	16	1.48	GTP pyrophosphokinase
198	1	db$2	2::C3K5S8|C3K5S8_PSEFS	416	75646	18	18	18	18	2.09	Oligopeptidase A OS=P
199	1	db$2	2::C3KBL8|C3KBL8_PSEFS	415	56970	20	19	15	14	2.20	Glycerol-3-phosphate d
200	1	db$2	2::C3K4C6|C3K4C6_PSEFS	414	25565	13	13	6	6	3.36	Phosphate regulon tran

↑Family	M	DB	Accession	Score	Mass	Matches	Match(sig)	Sequences	Seq(sig)	emPAI	Description
201	1	db$2	2::C3K3U2|C3K3U2_PSEFS	414	50985	14	12	10	9	1.31	Putative glutamine synt
202	1	db$2	2::C3KE70|RL9_PSEFS	411	15488	15	14	6	5	5.06	50S ribosomal protein L
203	1	db$2	2::C3K5J3|C3K5J3_PSEFS	410	50388	11	10	11	10	1.56	AMP nucleosidase OS=I
204	1	db$2	2::C3KAA8|C3KAA8_PSEFS	404	132643	12	12	12	12	0.54	Histidine kinase OS=Ps
205	1	db$2	2::C3K4C7|C3K4C7_PSEFS	404	50044	13	12	11	10	1.83	Phosphate regulon sens
206	1	db$2	2::C3K476|PYRE_PSEFS	402	23079	8	8	7	7	3.15	Orotate phosphoribosyl
207	1	db$2	2::C3KDP1|CYSD_PSEFS	402	35173	14	13	9	9	4.00	Sulfate adenylyltransfer
208	1	db$2	2::C3K278|C3K278_PSEFS	402	15153	11	10	5	5	5.30	Ferric uptake regulatior
209	1	db$2	2::C3K7D6|F16PA_PSEFS	401	37081	17	17	9	9	3.61	Fructose-1,6-bisphosph
210	1	db$2	2::C3K2V7|RL15_PSEFS	397	15215	13	13	9	9	28.19	50S ribosomal protein L
211	1	db$2	2::C3K902|RAPA_PSEFS	397	106722	19	18	17	17	1.13	RNA polymerase-associ
212	1	db$2	2::C3JYA7|C3JYA7_PSEFS	394	59461	16	15	12	12	1.81	Putative NADP-depende
213	1	db$2	2::C3K2Y0|RS7_PSEFS	392	17636	16	16	11	11	17.45	30S ribosomal protein S
214	1	db$2	2::C3K0Q7|C3K0Q7_PSEFS	392	45255	11	11	7	7	1.56	Flagellar hook protein F
215	1	db$2	2::C3KE65|C3KE65_PSEFS	392	97331	22	21	20	19	1.52	Ribonuclease R OS=Pse
216	1	db$2	2::C3K2Y7|RL11_PSEFS	392	14903	10	9	4	4	3.79	50S ribosomal protein L
217	1	db$2	2::C3K6W6|C3K6W6_PSEFS	391	49345	22	20	18	17	5.16	Biotin carboxylase OS=
218	1	db$2	2::C3JXZ0|C3JXZ0_PSEFS	391	43332	15	15	13	13	3.13	Acetylornithine aminotr
219	1	db$2	2::C3K4K9|C3K4K9_PSEFS	389	27377	15	15	9	9	5.60	Putative amino acid oxi
220	1	db$2	2::C3JZN2|C3JZN2_PSEFS	388	86329	19	16	17	15	1.28	Phenylalanine--tRNA lig
221	1	db$2	2::C3JXZ7|C3JXZ7_PSEFS	387	28415	15	13	9	8	3.44	Putative histidine-bindi
222	1	db$2	2::C3K3F5|METK_PSEFS	385	42554	17	17	13	13	3.74	S-adenosylmethionine s
223	1	db$2	2::C3K3E8|C3K3E8_PSEFS	382	38385	20	19	14	13	6.14	Fructose-1,6-bisphosph
224	1	db$2	2::C3K865|C3K865_PSEFS	380	15847	9	9	5	5	3.35	Putative membrane pro
225	1	db$2	2::C3JYQ6|C3JYQ6_PSEFS	378	37842	17	12	12	9	2.94	Ornithine carbamoyltra
226	1	db$2	2::C3K4W1|STHA_PSEFS	377	50963	20	17	17	14	3.02	Soluble pyridine nucleo
227	1	db$2	2::C3K123|C3K123_PSEFS	377	41542	13	11	10	8	1.48	Citrate synthase OS=Ps
228	1	db$2	2::C3JZS0|C3JZS0_PSEFS	377	43144	19	17	14	13	3.63	O-succinylhomoserine s
229	1	db$2	2::C3K6H2|ACCA_PSEFS	376	34868	19	18	11	11	5.65	Acetyl-coenzyme A carb
230	1	db$2	2::C3KAI6|C3KAI6_PSEFS	374	24263	10	9	8	7	2.87	Putative dehydrogenase
231	1	db$2	2::C3K2C4|C3K2C4_PSEFS	373	89060	11	11	10	10	0.70	Endopeptidase La OS=I
232	1	db$2	2::C3KEB4|C3KEB4_PSEFS	373	56978	18	14	16	12	2.20	Lysine--tRNA ligase OS
233	1	db$2	2::C3KBX8|C3KBX8_PSEFS	371	26859	8	8	6	6	2.41	OmpA-like domain-cont
234	1	db$2	2::C3KBP1|C3KBP1_PSEFS	369	37670	11	11	8	8	2.09	Putative reductase OS=
235	1	db$2	2::C3K6U8|C3K6U8_PSEFS	368	58654	9	9	5	5	0.62	Putative membrane pro
236	1	db$2	2::C3K0D4|C3K0D4_PSEFS	368	29228	16	15	11	10	4.02	Cell division inhibitor M
237	1	db$2	2::C3K5E0|C3K5E0_PSEFS	367	36000	12	11	9	8	2.71	2,3,4,5-tetrahydropyrid
238	1	db$2	2::C3K5Z8|RRF_PSEFS	366	20297	14	14	7	7	6.98	Ribosome-recycling fact
239	1	db$2	2::C3KCN5|C3KCN5_PSEFS	365	142346	14	14	14	14	0.60	Bifunctional protein Put
240	1	db$2	2::C3K4M6|C3K4M6_PSEFS	364	57818	18	17	14	13	2.14	Putative exported alkali

↑Family	M	DB	Accession	Score	Mass	Matches	Match(sig)	Sequences	Seq(sig)	emPAI	Description
241	1	db$2	2::C3JYW3|C3JYW3_PSEFS	364	36818	18	16	15	14	5.82	Branched-chain-amino-
242	1	db$2	2::C3K667|C3K667_PSEFS	364	48557	12	12	11	11	2.22	Conserved hypothetical
243	1	db$2	2::C3K8X0|C3K8X0_PSEFS	364	16351	9	9	6	6	4.57	Putative ABC transport
244	1	db$2	2::C3K475|ARGB_PSEFS	363	31926	15	15	12	12	4.86	Acetylglutamate kinase
245	1	db$2	2::C3JY86|C3JY86_PSEFS	361	98164	21	18	17	15	1.06	NADH-quinone oxidored
246	1	db$2	2::C3JZ29|C3JZ29_PSEFS	361	41047	10	10	8	8	1.51	Putative glycerophosph
247	1	db$2	2::C3K102|PUR5_PSEFS	361	36950	14	13	10	9	3.07	Phosphoribosylformylgl
248	1	db$2	2::C3K263|C3K263_PSEFS	359	26068	8	8	7	7	2.53	Triosephosphate isomeı
249	1	db$2	2::C3KE41|SYGA_PSEFS	359	36196	14	13	11	10	2.68	Glycine--tRNA ligase al
250	1	db$2	2::C3K1X8|C3K1X8_PSEFS	358	11596	12	11	6	5	10.13	50S ribosomal protein L
251	1	db$2	2::C3K7L5|C3K7L5_PSEFS	358	19131	11	11	7	7	8.09	Molybdenum cofactor b
252	1	db$2	2::C3K0W7|C3K0W7_PSEFS	358	31068	10	10	7	7	1.89	Metallophos_2 domain-
253	1	db$2	2::C3K6D9|C3K6D9_PSEFS	357	45394	14	13	11	10	2.14	Phosphoribosylamine--
254	1	db$2	2::C3KDE0|LPXC_PSEFS	356	33197	10	10	9	9	2.59	UDP-3-O-acyl-N-acetyl
255	1	db$2	2::C3KBN2|C3KBN2_PSEFS	355	52689	12	11	11	10	1.68	Outer membrane protei
256	1	db$2	2::C3K6J9|KCY_PSEFS	354	24780	8	8	6	6	2.78	Cytidylate kinase OS=P
257	1	db$2	2::C3K2J8|C3K2J8_PSEFS	352	54392	13	13	11	11	1.60	AMP nucleosidase OS=
258	1	db$2	2::C3JXY7|ASTD_PSEFS	351	51479	7	6	5	4	0.58	N-succinylglutamate 5-
259	1	db$2	2::C3K484|C3K484_PSEFS	348	13502	11	11	8	8	14.62	Uncharacterized proteir
260	1	db$2	2::C3KAC1|C3KAC1_PSEFS	347	27548	18	17	13	12	9.95	Putative phenylacetic a
261	1	db$2	2::C3JYR3|C3JYR3_PSEFS	346	40135	16	16	11	11	3.10	Glycine cleavage syster
262	1	db$2	2::C3KDU3|C3KDU3_PSEFS	346	40518	11	11	9	9	1.85	Beta sliding clamp OS=
263	1	db$2	2::C3KAJ8|UPP_PSEFS	346	22986	13	13	9	9	6.72	Uracil phosphoribosyltr
264	1	db$2	2::C3K616|C3K616_PSEFS	345	97222	20	19	18	17	1.41	DNA topoisomerase 1 C
265	1	db$2	2::C3K2X3|RL2_PSEFS	344	29689	21	16	14	12	5.73	50S ribosomal protein L
266	1	db$2	2::C3K2Z7|ARGC_PSEFS	344	36612	14	14	9	9	3.69	N-acetyl-gamma-glutar
267	1	db$2	2::C3K6R4|C3K6R4_PSEFS	344	18727	14	13	6	6	4.74	Uncharacterized proteir
268	1	db$2	2::C3K2Q0|C3K2Q0_PSEFS	343	19348	14	14	9	9	10.30	Inorganic pyrophosphat
269	1	db$2	2::C3K2X1|RL22_PSEFS	342	11904	10	10	6	6	9.42	50S ribosomal protein L
270	1	db$2	2::C3K6G2|C3K6G2_PSEFS	341	106936	20	17	19	16	1.03	Valine--tRNA ligase OS
271	1	db$2	2::C3K3M4|C3K3M4_PSEFS	340	49883	8	8	8	8	1.14	Signal recognition parti
272	1	db$2	2::C3K907|C3K907_PSEFS	339	77648	15	12	15	12	1.08	Tail-specific protease O
273	1	db$2	2::C3K6E0|PUR9_PSEFS	339	57370	15	15	14	14	2.44	Bifunctional purine bios
274	1	db$2	2::C3K1Y2|C3K1Y2_PSEFS	338	21662	10	10	8	8	6.03	Peptidyl-prolyl cis-trans
275	1	db$2	2::C3K5I7|C3K5I7_PSEFS	338	59867	14	14	10	10	1.58	Probable malate:quinor
276	1	db$2	2::C3K569|C3K569_PSEFS	337	44415	13	13	11	11	2.59	Tryptophan synthase be
277	1	db$2	2::C3KEA4|C3KEA4_PSEFS	336	51722	12	11	7	7	1.28	PINc domain-containing
278	1	db$2	2::C3JYU6|C3JYU6_PSEFS	335	42196	9	9	8	8	1.45	Arginine biosynthesis bi
279	1	db$2	2::C3KCR8|C3KCR8_PSEFS	335	39907	13	13	7	7	1.90	Ribosome-binding ATPa
280	1	db$2	2::C3K325|C3K325_PSEFS	333	49076	20	18	14	14	3.24	Chaperone SurA OS=Ps

↑Family	M	DB	Accession	Score	Mass	Matches	Match(sig)	Sequences	Seq(sig)	emPAI	Description
281	1	db$2	2::C3JYD5|C3JYD5_PSEFS	333	18453	11	10	6	6	4.92	Peptidyl-prolyl cis-trans
282	1	db$2	2::C3K645|C3K645_PSEFS	333	24522	8	8	7	7	2.82	Putative ABC transports
283	1	db$2	2::C3K8X5|HIS1_PSEFS	333	23098	16	13	12	10	8.37	ATP phosphoribosyltran
284	1	db$2	2::C3JZN6|IF3_PSEFS	333	20837	15	13	9	8	6.56	Translation initiation fa
285	1	db$2	2::C3JZ71|C3JZ71_PSEFS	332	31565	10	9	8	7	1.85	Hex regulon repressor (
286	1	db$2	2::C3KBN3|C3KBN3_PSEFS	329	112655	13	13	10	10	0.52	Efflux pump membrane
286	2	db$2	2::C3KB18|C3KB18_PSEFS	69	115005	3	3	3	3	0.13	Efflux pump membrane
287	1	db$2	2::C3JZV0|C3JZV0_PSEFS	328	56140	16	14	14	12	1.99	Glutamate--tRNA ligase
288	1	db$2	2::C3KDL2|C3KDL2_PSEFS	326	52736	11	9	11	9	1.24	Putative outer membra
289	1	db$2	2::C3KAJ2|C3KAJ2_PSEFS	326	53335	12	12	11	11	1.66	Methylmalonate-semial
290	1	db$2	2::C3K249|G6PI_PSEFS	324	61384	23	19	16	14	2.71	Glucose-6-phosphate is
291	1	db$2	2::C3K2K3|GSA_PSEFS	324	45181	11	11	8	8	1.56	Glutamate-1-semialdeh
292	1	db$2	2::C3JZ61|C3JZ61_PSEFS	322	61509	14	13	11	10	1.16	Acyl-CoA synthetase (L
293	1	db$2	2::C3K1H6|PURT_PSEFS	322	42327	15	15	14	14	4.34	Formate-dependent pho
294	1	db$2	2::C3KAH3|SYR_PSEFS	321	63549	12	12	9	9	1.11	Arginine--tRNA ligase C
295	1	db$2	2::C3JY85|C3JY85_PSEFS	321	48759	12	11	11	10	1.64	NADH-quinone oxidore
296	1	db$2	2::C3JY62|C3JY62_PSEFS	320	83224	21	19	17	16	1.63	ATP-dependent Clp prot
296	2	db$2	2::C3K4B3|C3K4B3_PSEFS	90	97007	10	8	9	8	0.48	Chaperone OS=Pseudo
297	1	db$2	2::C3JYD4|C3JYD4_PSEFS	319	64707	14	13	12	11	1.24	Glutamine--tRNA ligase
298	1	db$2	2::C3K4H9|C3K4H9_PSEFS	319	34203	11	9	9	8	2.01	Quinone oxidoreductase
299	1	db$2	2::C3K1M9|C3K1M9_PSEFS	319	29586	11	9	10	8	2.56	Inositol-1-monophosph
300	1	db$2	2::C3K0H1|C3K0H1_PSEFS	318	17848	9	9	6	6	5.25	Chemotaxis protein Che
301	1	db$2	2::C3K7G9|C3K7G9_PSEFS	318	48266	9	9	8	8	1.19	Putative modulation of
302	1	db$2	2::C3K226|C3K226_PSEFS	317	17820	11	10	8	7	5.25	Acetolactate synthase (
303	1	db$2	2::C3K2S6|C3K2S6_PSEFS	316	31662	9	8	7	6	1.44	Putative thioredoxin OS
304	1	db$2	2::C3K9U6|C3K9U6_PSEFS	316	35232	9	9	7	7	1.92	Putative 6-phosphogluc
305	1	db$2	2::C3KBX9|C3KBX9_PSEFS	316	20403	8	7	5	4	2.15	Putative phospholipid-b
306	1	db$2	2::C3K5A9|RF3_PSEFS	316	59584	12	11	8	7	1.04	Peptide chain release fa
307	1	db$2	2::C3K1G2|YIDC_PSEFS	315	61977	15	13	13	12	1.50	Membrane protein inse
308	1	db$2	2::C3K466|C3K466_PSEFS	314	52681	10	10	7	7	0.87	Putative aldehyde dehy
309	1	db$2	2::C3K7C1|SECB_PSEFS	314	17615	7	7	4	4	2.76	Protein-export protein S
310	1	db$2	2::C3KAL6|C3KAL6_PSEFS	310	52109	14	12	11	9	1.48	Putative aminotransfera
311	1	db$2	2::C3K6R3|Y2135_PSEFS	309	24050	15	15	11	11	7.55	UPF0502 protein PFLU_
312	1	db$2	2::C3KEB7|C3KEB7_PSEFS	308	48036	14	13	11	10	1.95	Uncharacterized protei
313	1	db$2	2::C3K2M1|C3K2M1_PSEFS	308	42033	14	14	12	12	3.30	Serine-type D-Ala-D-Al
314	1	db$2	2::C3K0N5|C3K0N5_PSEFS	307	32402	8	8	5	5	1.39	Malonyl CoA-acyl carrie
315	1	db$2	2::C3K8X6|C3K8X6_PSEFS	306	47255	13	12	12	11	2.00	Histidinol dehydrogena
316	1	db$2	2::C3KDW3|HFQ_PSEFS	305	9318	14	13	8	7	30.78	RNA-binding protein Hf
317	1	db$2	2::C3K7X9|C3K7X9_PSEFS	304	40858	11	8	9	6	1.25	Carboxynorspermidine/
318	1	db$2	2::C3K0K5|C3K0K5_PSEFS	304	31365	13	13	7	7	3.48	Electron transfer flavop

↑Family	M	DB	Accession	Score	Mass	Matches	Match(sig)	Sequences	Seq(sig)	emPAI	Description
319	1	db$2	2::C3JXZ8|C3JXZ8_PSEFS	304	71568	14	13	14	13	1.36	Acetyl-coenzyme A syn
320	1	db$2	2::C3K227|C3K227_PSEFS	303	62578	18	17	15	14	1.88	Acetolactate synthase (
321	1	db$2	2::C3K0N4|C3K0N4_PSEFS	302	25612	8	7	7	6	2.61	3-oxoacyl-[acyl-carrier-
322	1	db$2	2::C3K274|DNAJ_PSEFS	300	40315	10	10	8	8	1.55	Chaperone protein Dna
323	1	db$2	2::C3K6E1|RL13_PSEFS	299	15695	13	11	8	7	9.86	50S ribosomal protein L
324	1	db$2	2::C3KBI6|C3KBI6_PSEFS	299	106629	14	13	13	12	0.78	Uncharacterized proteir
325	1	db$2	2::C3KBP9|C3KBP9_PSEFS	298	58780	10	10	9	9	1.06	Phosphoglucomutase O
326	1	db$2	2::C3K3Q2|C3K3Q2_PSEFS	298	37335	11	10	5	5	1.42	Putative cation ABC trai
327	1	db$2	2::C3K324|C3K324_PSEFS	297	105271	17	15	16	14	0.88	LPS-assembly protein L
328	1	db$2	2::C3K6A2|C3K6A2_PSEFS	297	27847	10	9	8	7	2.26	Putative D-methionine /
328	2	db$2	2::C3KD95|C3KD95_PSEFS	220	28020	12	10	9	8	2.84	Putative exported prote
329	1	SwissProt_HUMAN$	3::P01871|IGHM_HUMAN	296	49409	12	10	9	7	1.15	Immunoglobulin heavy
330	1	db$2	2::C3K1X5|C3K1X5_PSEFS	296	39754	11	10	8	8	1.59	Glutamate 5-kinase OS
331	1	db$2	2::C3K474|C3K474_PSEFS	292	50732	11	11	9	9	1.54	Putative phosphomanno
332	1	SwissProt_HUMAN$	3::P00761|TRYP_PIG	291	24394	8	8	6	6	2.17	Trypsin OS=Sus scrofa
333	1	db$2	2::C3K7M6|C3K7M6_PSEFS	288	88918	9	9	6	6	0.45	Acyl-coenzyme A dehyc
334	1	db$2	2::C3JZT7|C3JZT7_PSEFS	288	38793	10	9	8	7	1.65	3-isopropylmalate dehy
335	1	db$2	2::C3K8V3|HSLU_PSEFS	286	49607	18	16	15	14	2.80	ATP-dependent proteas
336	1	db$2	2::C3JZ68|C3JZ68_PSEFS	285	24197	10	10	9	9	4.74	2-dehydro-3-deoxy-phc
337	1	db$2	2::C3KAK7|C3KAK7_PSEFS	284	20540	7	6	4	4	2.13	Putative lipoprotein OS
338	1	db$2	2::C3KDW6|C3KDW6_PSEFS	284	33055	15	13	12	10	3.16	Protein HflC OS=Pseudo
339	1	db$2	2::C3K3U0|C3K3U0_PSEFS	283	51001	9	9	7	7	0.92	Putative glutamine synt
340	1	db$2	2::C3K3A2|C3K3A2_PSEFS	282	38105	9	9	5	5	0.86	L-threonine aldolase OS
341	1	db$2	2::C3JY74|C3JY74_PSEFS	282	48472	12	10	10	8	1.18	Isocitrase OS=Pseudom
342	1	db$2	2::C3JZ86|C3JZ86_PSEFS	281	21964	19	16	12	12	11.96	Superoxide dismutase (
343	1	db$2	2::C3JZN9|C3JZN9_PSEFS	281	7652	12	12	5	5	35.04	Major cold shock protei
344	1	SwissProt_HUMAN$	3::P02671|FIBA_HUMAN	280	94914	13	11	12	10	0.65	Fibrinogen alpha chain
345	1	db$2	2::C3KCS2|RSMH_PSEFS	280	34344	13	12	11	11	3.54	Ribosomal RNA small su
346	1	db$2	2::C3K2Y8|C3K2Y8_PSEFS	280	20023	11	10	8	8	7.26	Transcription terminatic
347	1	db$2	2::C3K3Z1|C3K3Z1_PSEFS	279	11808	11	11	6	6	14.78	Thioredoxin OS=Pseudo
348	1	db$2	2::C3KDB1|C3KDB1_PSEFS	279	65565	15	14	15	14	1.75	Putative carbamoyltran
349	1	db$2	2::C3K9T7|C3K9T7_PSEFS	277	99328	13	12	11	10	0.61	Aminopeptidase N OS=
350	1	db$2	2::C3K841|C3K841_PSEFS	274	11596	10	10	5	5	23.84	Putative sigma-54 mod
351	1	db$2	2::C3K5N1|C3K5N1_PSEFS	273	36842	11	11	9	9	2.17	Sulfate ABC transport s
352	1	db$2	2::C3KDU9|C3KDU9_PSEFS	273	34261	10	9	9	8	2.00	Serine O-acetyltransfer
353	1	db$2	2::C3K734|C3K734_PSEFS	272	44623	12	10	8	7	1.88	Putative aminotransfera
354	1	db$2	2::C3K185|C3K185_PSEFS	271	20062	10	9	6	5	4.12	Putative NAD(P)H nitror
355	1	db$2	2::C3K091|C3K091_PSEFS	271	20089	11	11	7	7	5.46	GTP cyclohydrolase 1 O
355	2	db$2	2::C3K1W5|C3K1W5_PSEFS	50	20960	4	3	3	2	0.56	GTP cyclohydrolase 1 O
356	1	db$2	2::C3K2L2|SYL_PSEFS	269	97066	12	11	11	10	0.63	Leucine--tRNA ligase OS

↑Family	M	DB	Accession	Score	Mass	Matches	Match(sig)	Sequences	Seq(sig)	emPAI	Description
357	1	db$2	2::C3K3K2|PYRB_PSEFS	269	36271	9	7	7	5	0.92	Aspartate carbamoyltra
358	1	db$2	2::C3K5V1|C3K5V1_PSEFS	268	47050	9	9	9	9	1.47	Outer membrane protei
359	1	db$2	2::C3K6Y4|GATA_PSEFS	268	51737	10	9	7	6	1.08	Glutamyl-tRNA(Gln) am
360	1	db$2	2::C3K651|C3K651_PSEFS	267	39766	10	10	9	9	1.91	Phospho-2-dehydro-3-c
361	1	db$2	2::C3K2V0|RL17_PSEFS	267	14407	8	8	5	5	8.58	50S ribosomal protein L
362	1	db$2	2::C3JYR4|C3JYR4_PSEFS	266	7675	5	5	3	3	5.00	Major cold shock protei
363	1	db$2	2::C3K6G8|LEPA_PSEFS	266	66372	13	11	10	9	1.04	Elongation factor 4 OS=
364	1	db$2	2::C3JY00|C3JY00_PSEFS	265	47211	10	8	8	7	1.02	Ribonucleoside-diphosp
365	1	db$2	2::C3K403|C3K403_PSEFS	263	36964	10	10	7	7	1.78	Delta-aminolevulinic ac
366	1	db$2	2::C3K1F4|C3K1F4_PSEFS	263	31961	8	7	7	6	1.42	Probable chromosome-p
367	1	db$2	2::C3K6M3|C3K6M3_PSEFS	263	13365	6	6	5	5	4.73	Succinate dehydrogena
368	1	db$2	2::C3K614|C3K614_PSEFS	262	41572	14	13	10	9	2.11	3-ketoacyl-CoA thiolase
369	1	db$2	2::C3K2U7|C3K2U7_PSEFS	260	104087	18	17	15	14	0.89	UvrABC system protein
370	1	db$2	2::C3KCT1|MURC_PSEFS	260	52132	10	9	8	7	0.89	UDP-N-acetylmuramate
371	1	db$2	2::C3K2S0|C3K2S0_PSEFS	259	39319	7	7	5	5	1.06	3,4-dihydroxy-2-butanc
372	1	db$2	2::C3K401|C3K401_PSEFS	259	56255	14	12	13	12	1.74	Exopolyphosphatase OS
373	1	db$2	2::C3K638|C3K638_PSEFS	259	26394	5	5	3	3	1.04	Putative short chain del
374	1	SwissProt_HUMAN$	3::P01834|IGKC_HUMAN	258	11758	7	7	5	5	6.18	Immunoglobulin kappa
375	1	db$2	2::C3KE61|RIMK_PSEFS	257	32488	9	7	6	5	1.39	Probable alpha-L-glutar
376	1	db$2	2::C3K422|C3K422_PSEFS	257	40250	8	6	7	6	1.02	Putative uroporphyrin-I
377	1	db$2	2::C3K8X4|MURA_PSEFS	257	44975	17	15	14	12	2.92	UDP-N-acetylglucosami
378	1	db$2	2::C3K472|C3K472_PSEFS	256	42804	9	8	7	6	1.16	Coenzyme A biosynthes
379	1	db$2	2::C3K9S0|NFUA_PSEFS	254	20961	9	8	8	7	3.78	Fe/S biogenesis protein
380	1	db$2	2::C3K3N4|COAD_PSEFS	254	17810	12	11	8	7	7.22	Phosphopantetheine ad
381	1	db$2	2::C3KBQ2|C3KBQ2_PSEFS	253	37020	13	13	10	10	3.06	Putative quinone oxidor
382	1	db$2	2::C3JY83|NUOCD_PSEFS	250	67660	19	18	15	14	2.06	NADH-quinone oxidored
383	1	db$2	2::C3K7N8|RDGC_PSEFS	249	34160	15	15	13	13	5.90	Recombination-associat
384	1	db$2	2::C3K5H8|C3K5H8_PSEFS	248	17826	8	8	6	6	3.81	STAS domain-containin
385	1	db$2	2::C3K4G1|C3K4G1_PSEFS	247	81186	14	9	13	8	0.60	DNA helicase OS=Pseu
385	2	db$2	2::C3K5Z0|C3K5Z0_PSEFS	42	15508	4	3	2	2	0.82	Uncharacterized protein
386	1	db$2	2::C3K0Q9|C3K0Q9_PSEFS	247	15688	5	5	4	4	2.30	Flagellar basal-body roc
387	1	db$2	2::C3K8U4|UBIE_PSEFS	247	28320	17	14	14	12	6.36	Ubiquinone/menaquinon
388	1	db$2	2::C3KE79|C3KE79_PSEFS	245	29713	5	5	4	4	0.88	NH(3)-dependent NAD(
389	1	db$2	2::C3K371|C3K371_PSEFS	245	78727	11	11	11	11	0.94	Malate synthase G OS=
390	1	db$2	2::C3K1E9|ATPD_PSEFS	244	19246	14	11	8	7	6.04	ATP synthase subunit d
391	1	db$2	2::C3K3H0|C3K3H0_PSEFS	243	67796	12	11	12	11	1.16	ATP-dependent RNA he
392	1	db$2	2::C3K2Y5|RL10_PSEFS	243	17594	7	6	5	5	2.76	50S ribosomal protein L
393	1	db$2	2::C3K2E3|MSCL_PSEFS	242	14568	6	6	3	3	2.60	Large-conductance mec
394	1	db$2	2::C3K5Z7|C3K5Z7_PSEFS	242	26574	11	10	7	7	3.93	Uridylate kinase OS=Ps
395	1	db$2	2::C3K469|RL28_PSEFS	241	8845	9	9	4	4	6.94	50S ribosomal protein L

↑Family	M	DB	Accession	Score	Mass	Matches	Match(sig)	Sequences	Seq(sig)	emPAI	Description
396	1	db$2	2::C3K1F2|ATP6_PSEFS	241	31626	6	6	3	3	0.56	ATP synthase subunit a
397	1	SwissProt_HUMAN$	3::P02768|ALBU_HUMAN	240	69321	15	13	14	12	1.27	Albumin OS=Homo sap
397	2	SwissProt_HUMAN$	3::P02769|ALBU_BOVIN	235	69248	11	10	11	10	0.98	Albumin OS=Bos taurus
398	1	db$2	2::C3K452|XPT_PSEFS	239	20267	8	8	7	7	5.33	Xanthine phosphoribosy
399	1	db$2	2::C3K3D2|BETB_PSEFS	238	53123	9	8	8	7	0.86	Betaine aldehyde dehyc
400	1	db$2	2::C3JYT9|C3JYT9_PSEFS	238	46635	9	7	6	5	0.84	Putative family S43 nor
401	1	db$2	2::C3KAC7|C3KAC7_PSEFS	238	51379	12	12	10	10	1.75	UDP-glucose 6-dehydro
402	1	db$2	2::C3K1X9|C3K1X9_PSEFS	238	34909	12	10	11	9	2.38	Octaprenyl-diphosphate
403	1	db$2	2::C3KBL3|C3KBL3_PSEFS	237	50616	10	10	8	8	1.11	Putative exported prote
404	1	db$2	2::C3K1L2|C3K1L2_PSEFS	236	22708	8	8	6	6	2.45	Ancillary SecYEG transl
405	1	db$2	2::C3K818|C3K818_PSEFS	236	29926	11	11	8	8	2.52	Putative ABC transport
406	1	db$2	2::C3K4V4|C3K4V4_PSEFS	236	32182	10	10	7	7	2.23	Putative ATP-binding pr
407	1	db$2	2::C3K270|C3K270_PSEFS	234	17315	12	11	8	7	7.67	Transcription elongatior
408	1	db$2	2::C3KDD6|C3KDD6_PSEFS	234	33869	7	6	7	6	1.31	D-alanine--D-alanine lic
409	1	db$2	2::C3K9P3|C3K9P3_PSEFS	233	9882	8	7	5	4	5.48	Peptidyl-prolyl cis-trans
410	1	db$2	2::C3KBL1|C3KBL1_PSEFS	232	61934	9	8	8	8	0.84	Putative medium-chain-
411	1	db$2	2::C3KE71|C3KE71_PSEFS	232	51412	11	10	9	8	1.51	Replicative DNA helicas
412	1	db$2	2::C3KDM2|C3KDM2_PSEFS	232	83508	12	11	12	11	0.87	DNA topoisomerase 4 s
413	1	db$2	2::C3K435|C3K435_PSEFS	231	45402	10	9	10	9	1.55	Diaminopimelate decarl
414	1	db$2	2::C3K3W3|C3K3W3_PSEFS	230	41294	4	4	4	4	0.58	Putative aminotransfera
415	1	db$2	2::C3K6V2|HIS4_PSEFS	229	25679	11	10	8	8	4.21	1-(5-phosphoribosyl)-5
416	1	db$2	2::C3K1A1|C3K1A1_PSEFS	228	10298	9	8	6	6	13.67	10 kDa chaperonin OS=
417	1	db$2	2::C3K2W5|RL24_PSEFS	227	11328	8	8	7	7	16.54	50S ribosomal protein L
418	1	db$2	2::C3KDK8|C3KDK8_PSEFS	226	41619	5	5	4	4	0.57	DAO domain-containing
419	1	SwissProt_HUMAN$	3::P07355|ANXA2_HUMAN	225	38580	8	7	8	7	1.35	Annexin A2 OS=Homo :
420	1	db$2	2::C3K5D5|C3K5D5_PSEFS	225	41033	4	4	3	3	0.41	Succinyl-diaminopimela
421	1	db$2	2::C3K2V3|RS11_PSEFS	225	13607	7	7	5	5	4.50	30S ribosomal protein S
422	1	db$2	2::C3K1G8|RL19_PSEFS	224	12961	9	7	6	6	7.56	50S ribosomal protein L
423	1	db$2	2::C3K1L3|SYH_PSEFS	222	47853	16	12	13	11	2.27	Histidine--tRNA ligase C
424	1	db$2	2::C3JZT0|C3JZT0_PSEFS	222	28122	7	6	7	6	1.73	Putative GntR-family tra
425	1	db$2	2::C3KDM7|C3KDM7_PSEFS	222	29519	10	10	9	9	3.20	Sulfurtransferase OS=P
426	1	db$2	2::C3K8Z6|C3K8Z6_PSEFS	221	39611	10	8	7	6	1.30	Branched amino acid AI
427	1	db$2	2::C3K3X2|C3K3X2_PSEFS	221	19739	8	8	6	6	3.15	Uncharacterized proteir
428	1	db$2	2::C3K5E5|C3K5E5_PSEFS	221	28503	8	8	7	7	2.74	Methionine aminopeptic
429	1	db$2	2::C3K1X6|C3K1X6_PSEFS	220	44711	9	9	8	8	1.33	GTPase Obg OS=Pseud
430	1	db$2	2::C3KEC6|KAD_PSEFS	219	23251	12	11	9	8	5.18	Adenylate kinase OS=P
431	1	db$2	2::C3KA77|C3KA77_PSEFS	219	49108	8	7	8	7	0.96	Glutathione reductase C
432	1	db$2	2::C3K703|ISPF_PSEFS	219	16742	5	5	4	4	2.05	2-C-methyl-D-erythrito
433	1	db$2	2::C3K490|C3K490_PSEFS	217	33505	11	11	8	8	2.55	Putative oxidative stres
434	1	db$2	2::C3KDM0|C3KDM0_PSEFS	217	69594	11	11	10	10	0.98	DNA topoisomerase 4 s

↑Family	M	DB	Accession	Score	Mass	Matches	Match(sig)	Sequences	Seq(sig)	emPAI	Description
435	1	db$2	2::C3K6Y3|GATB_PSEFS	217	52868	14	14	10	10	1.93	Aspartyl/glutamyl-tRNA
436	1	db$2	2::C3JYS5|C3JYS5_PSEFS	217	25332	5	5	4	4	1.53	Tol-Pal system protein
437	1	db$2	2::C3K759|C3K759_PSEFS	214	35694	5	5	4	4	0.70	Putative dehydrogenase
437	2	db$2	2::C3K0S6|C3K0S6_PSEFS	100	36802	2	2	2	2	0.29	Putative oxidoreductase
438	1	db$2	2::C3K1L1|C3K1L1_PSEFS	213	40864	10	7	8	7	1.25	Outer membrane protei
439	1	db$2	2::C3KBZ0|PYRC_PSEFS	213	38385	7	7	7	7	1.36	Dihydroorotase OS=Pse
440	1	db$2	2::C3K6L0|EFP_PSEFS	212	21303	9	8	7	6	2.75	Elongation factor P OS=
441	1	db$2	2::C3K4Z4|C3K4Z4_PSEFS	212	43117	9	9	7	7	1.16	Putative cytochrome C
442	1	db$2	2::C3K610|C3K610_PSEFS	212	70283	12	11	12	11	1.10	ATP-binding protein Uu
443	1	db$2	2::C3KCB7|C3KCB7_PSEFS	212	34191	5	4	3	2	0.51	Phosphate transport sy
444	1	db$2	2::C3K1L0|C3K1L0_PSEFS	211	55309	11	10	11	10	1.35	GTPase Der OS=Pseudo
445	1	db$2	2::C3K307|TRPC_PSEFS	210	30252	11	10	10	10	3.73	Indole-3-glycerol phosp
446	1	db$2	2::C3KBZ7|SYM_PSEFS	210	75525	12	10	10	8	0.65	Methionine--tRNA ligase
447	1	db$2	2::C3K2Z3|C3K2Z3_PSEFS	210	44017	12	11	10	9	1.92	Tyrosine--tRNA ligase C
448	1	db$2	2::C3K575|C3K575_PSEFS	209	14827	4	4	4	4	2.50	Osmotically inducible pr
449	1	db$2	2::C3K142|C3K142_PSEFS	208	32099	9	9	7	7	2.24	2-methylisocitrate lyase
450	1	db$2	2::C3K4Q9|C3K4Q9_PSEFS	208	23656	5	4	5	4	1.21	Putative exported prote
451	1	db$2	2::C3K4V8|C3K4V8_PSEFS	208	128447	10	8	10	8	0.34	Transcription-repair-cou
452	1	db$2	2::C3KCL0|C3KCL0_PSEFS	208	35424	5	5	4	4	0.70	Uncharacterized protein
453	1	db$2	2::C3K4Y3|KDPA_PSEFS	207	60181	6	5	2	1	0.17	Potassium-transporting
454	1	db$2	2::C3K5X7|C3K5X7_PSEFS	207	42054	12	12	12	12	2.85	Cell division protein Zap
455	1	db$2	2::C3JYE4|C3JYE4_PSEFS	206	30882	15	15	13	13	6.28	Exodeoxyribonuclease I
456	1	db$2	2::C3KAM2|C3KAM2_PSEFS	206	49640	5	5	5	5	0.61	Putative two-componen
457	1	db$2	2::C3K245|C3K245_PSEFS	205	53442	10	10	9	9	1.22	Poly(A) polymerase I O
458	1	db$2	2::C3K6X1|C3K6X1_PSEFS	204	39290	6	6	5	5	0.82	Uncharacterized protein
459	1	db$2	2::C3KBQ3|C3KBQ3_PSEFS	204	33274	7	7	6	6	1.34	LysR-family transcriptic
460	1	db$2	2::C3JYN4|ZIPA_PSEFS	204	31818	8	8	4	4	1.43	Cell division protein Zip
461	1	db$2	2::C3K3T6|C3K3T6_PSEFS	204	42390	6	6	6	6	0.95	Spermidine/putrescine
462	1	db$2	2::C3JZV9|C3JZV9_PSEFS	204	34932	4	4	3	3	0.50	Putative DNA/RNA non-
463	1	db$2	2::C3K262|C3K262_PSEFS	203	12956	7	7	4	4	3.19	Protein-export membra
464	1	db$2	2::C3K443|C3K443_PSEFS	203	12323	13	13	7	7	19.38	Nitrogen regulatory pro
465	1	db$2	2::C3K8Y8|C3K8Y8_PSEFS	202	23915	6	6	5	5	1.68	Putative isomerase OS=
466	1	db$2	2::C3JXY8|C3JXY8_PSEFS	202	37476	6	6	6	6	1.13	Arginine N-succinyltran
467	1	db$2	2::C3K0G3|PPNP_PSEFS	200	10127	7	5	4	3	5.23	Pyrimidine/purine nucle
468	1	db$2	2::C3K6J6|C3K6J6_PSEFS	199	40307	8	7	7	6	1.02	Bifunctional chorismate
469	1	db$2	2::C3JXZ2|C3JXZ2_PSEFS	198	36334	7	7	7	7	1.48	Putative AraC family re
470	1	db$2	2::Q84CP9|DAPB_PSEFS	198	28276	14	11	10	9	3.47	4-hydroxy-tetrahydrodi
471	1	db$2	2::C3K6I1|C3K6I1_PSEFS	198	31224	10	6	5	3	1.13	S-formylglutathione hy
472	1	db$2	2::C3K6U2|C3K6U2_PSEFS	198	47692	6	5	5	4	0.49	Probable proton/glutam
473	1	db$2	2::C3K5L8|C3K5L8_PSEFS	197	39360	7	7	6	6	1.31	Phosphorous compounc

↑Family	M	DB	Accession	Score	Mass	Matches	Match(sig)	Sequences	Seq(sig)	emPAI	Description
474	1	db$2	2::C3K3P4|C3K3P4_PSEFS	197	39333	9	8	6	6	1.32	Uncharacterized proteir
475	1	db$2	2::C3KDN5|C3KDN5_PSEFS	196	35878	5	5	4	4	0.69	Renalase OS=Pseudom
476	1	SwissProt_HUMAN$	3::P23528|COF1_HUMAN	196	18491	7	7	6	6	3.55	Cofilin-1 OS=Homo sap
477	1	db$2	2::C3K2S1|C3K2S1_PSEFS	195	23236	11	11	6	6	2.37	Riboflavin synthase alpl
478	1	db$2	2::C3K7I1|C3K7I1_PSEFS	195	85859	8	8	8	8	0.55	Quinoprotein glucose de
479	1	db$2	2::C3JZQ0|C3JZQ0_PSEFS	195	38665	8	8	7	7	1.66	Putative L-asparaginase
480	1	db$2	2::C3K6K6|C3K6K6_PSEFS	194	36089	8	8	8	8	1.85	HTH-type transcriptiona
481	1	db$2	2::C3K4T4|C3K4T4_PSEFS	194	21507	5	5	4	4	1.39	NAD(P)-bd_dom domai
482	1	db$2	2::C3K9M2|C3K9M2_PSEFS	194	37830	6	6	5	5	0.87	Putative peptidase OS=
483	1	db$2	2::C3K177|C3K177_PSEFS	194	65030	8	8	8	8	0.79	Phosphogluconate dehy
484	1	db$2	2::C3K4E4|C3K4E4_PSEFS	193	51389	8	8	8	8	1.09	Aspartate ammonia-lya
485	1	db$2	2::C3K6J7|C3K6J7_PSEFS	192	39784	9	8	8	8	1.59	Histidinol-phosphate an
486	1	db$2	2::C3KAR0|C3KAR0_PSEFS	192	79563	6	6	5	5	0.35	Putative exported isoqu
487	1	db$2	2::C3K581|C3K581_PSEFS	192	86479	8	7	7	6	0.39	Putative molybdopterin
488	1	db$2	2::C3K2N8|C3K2N8_PSEFS	192	23444	8	8	7	7	3.97	Semialdhyde_dh domai
489	1	db$2	2::C3K5Y0|C3K5Y0_PSEFS	192	38747	9	8	7	6	1.08	NADP(H)-dependt aldo-
490	1	db$2	2::C3KDB7|C3KDB7_PSEFS	192	65793	7	6	7	6	0.54	ATP-dependent lipid A-(
491	1	db$2	2::C3K6S8|C3K6S8_PSEFS	191	40355	7	7	6	6	1.27	Putative acetyl-CoA trai
492	1	db$2	2::C3K6E3|C3K6E3_PSEFS	191	21087	10	9	8	7	4.92	Ubiquinol-cytochrome c
493	1	db$2	2::C3K8W7|C3K8W7_PSEFS	190	34291	7	6	6	5	0.99	Arabinose 5-phosphate
494	1	db$2	2::C3K3Q8|TYSY_PSEFS	190	31490	8	8	6	6	1.85	Thymidylate synthase (
495	1	db$2	2::C3KE80|C3KE80_PSEFS	190	46502	9	9	8	8	1.25	Nicotinate phosphoribos
496	1	db$2	2::C3KDX0|C3KDX0_PSEFS	189	16992	7	7	4	4	2.01	Peptidyl-prolyl cis-trans
497	1	db$2	2::C3K3Y8|UBID_PSEFS	188	54697	9	9	8	8	1.00	3-octaprenyl-4-hydroxy
498	1	db$2	2::C3JYS3|C3JYS3_PSEFS	188	38665	5	4	4	4	0.63	TolA colicin import men
499	1	db$2	2::C3K4G2|C3K4G2_PSEFS	188	30728	8	8	5	5	1.92	Putative exported prote
500	1	db$2	2::C3K5X4|C3K5X4_PSEFS	186	16332	8	8	6	6	6.42	Putative membrane pro
501	1	db$2	2::C3K3W7|C3K3W7_PSEFS	186	36662	8	6	7	5	0.90	Putative iron (III) ABC !
502	1	db$2	2::C3K2E9|C3K2E9_PSEFS	186	53251	4	4	3	3	0.43	Putative cytochrome ox
503	1	db$2	2::B0B0A5|DNAA_PSEFS	185	56252	10	7	8	6	0.66	Chromosomal replicatio
504	1	db$2	2::C3JZ88|C3JZ88_PSEFS	185	47232	5	4	4	3	0.49	Putative exported prote
505	1	db$2	2::C3JZT9|C3JZT9_PSEFS	184	24210	9	7	8	7	2.89	3-isopropylmalate dehy
506	1	db$2	2::C3K0S7|Y4577_PSEFS	184	11900	5	4	3	3	3.77	Nucleoid-associated prc
507	1	db$2	2::C3K0T3|C3K0T3_PSEFS	183	28958	4	4	4	4	0.92	Putative membrane pro
508	1	db$2	2::C3K527|C3K527_PSEFS	183	65564	10	9	6	6	0.66	Putative peptidase OS=
509	1	db$2	2::C3JYI0|C3JYI0_PSEFS	183	25235	7	7	5	5	1.54	Probable transcriptional
510	1	db$2	2::C3KBY9|C3KBY9_PSEFS	183	24532	5	4	4	4	1.15	Ribonuclease T OS=Pse
511	1	db$2	2::C3JYU1|Y4927_PSEFS	182	18225	8	7	6	5	3.68	UPF0234 protein PFLU_
512	1	db$2	2::C3K2U9|C3K2U9_PSEFS	182	53651	12	10	10	9	1.21	Catalase OS=Pseudomc
513	1	db$2	2::C3K2U5|C3K2U5_PSEFS	181	18989	5	5	4	4	1.68	Single-stranded DNA-bi

↑Family	M	DB	Accession	Score	Mass	Matches	Match(sig)	Sequences	Seq(sig)	emPAI	Description
514	1	db$2	2::C3JXQ0|C3JXQ0_PSEFS	181	24880	8	8	5	5	2.11	Putative hydrolase OS=
515	1	db$2	2::C3K6Z0|PDXJ_PSEFS	181	26758	6	6	5	5	1.41	Pyridoxine 5'-phosphate
516	1	db$2	2::C3KDM6|PSD_PSEFS	180	31380	7	6	5	4	1.46	Phosphatidylserine deca
517	1	db$2	2::C3K494|C3K494_PSEFS	180	33626	4	4	4	4	0.75	Gly-zipper_YMGG doma
518	1	db$2	2::C3KCS8|C3KCS8_PSEFS	179	47767	5	5	5	5	0.64	UDP-N-acetylmuramoyl
519	1	db$2	2::C3K0N2|C3K0N2_PSEFS	179	43313	6	6	5	5	0.73	3-oxoacyl-[acyl-carrier-
520	1	db$2	2::C3K3T9|C3K3T9_PSEFS	179	49785	9	9	8	8	1.35	Putative aminotransfera
521	1	db$2	2::C3K1H2|C3K1H2_PSEFS	179	49205	11	9	7	7	1.16	Signal recognition parti
522	1	db$2	2::C3K3S0|C3K3S0_PSEFS	177	23721	6	6	4	4	1.21	Putative hydrolase OS=
523	1	db$2	2::C3K2W8|RL29_PSEFS	176	7168	7	6	6	5	22.66	50S ribosomal protein L
524	1	db$2	2::C3K6V9|C3K6V9_PSEFS	176	54793	9	8	7	7	0.83	2,3-bisphosphoglycerat
525	1	db$2	2::C3K6T0|ENGB_PSEFS	176	23537	5	5	3	3	0.82	Probable GTP-binding p
526	1	db$2	2::C3K9M0|C3K9M0_PSEFS	176	11184	3	3	2	2	1.28	SCP2 domain-containin
527	1	db$2	2::C3K1N3|C3K1N3_PSEFS	176	11888	6	6	3	3	2.23	Sec translocon accessor
528	1	db$2	2::C3K8U2|UBIB_PSEFS	174	61616	6	6	6	6	0.59	Probable protein kinase
529	1	db$2	2::C3JXZ3|C3JXZ3_PSEFS	174	28123	10	9	8	7	2.23	Histidine transport ATP-
530	1	db$2	2::C3KDL6|C3KDL6_PSEFS	174	23139	4	4	4	4	1.26	ADP-ribose diphosphata
531	1	db$2	2::C3KBN4|C3KBN4_PSEFS	174	40532	8	8	7	7	1.54	Acriflavine resistance pi
532	1	db$2	2::C3K7E3|C3K7E3_PSEFS	174	178026	9	8	9	8	0.24	Alpha-2-macroglobulin
533	1	db$2	2::C3K015|C3K015_PSEFS	173	53791	6	6	4	4	0.42	Putative ferredoxin OS=
534	1	db$2	2::C3K1U9|C3K1U9_PSEFS	172	42728	6	6	4	4	0.74	Flavohemoglobin OS=P
535	1	db$2	2::C3K9Z4|C3K9Z4_PSEFS	172	58133	9	9	9	9	1.08	Putative ABC tranmspo
536	1	db$2	2::C3K310|C3K310_PSEFS	172	54246	7	6	7	6	0.69	Anthranilate synthase c
537	1	db$2	2::C3JXP7|RECR_PSEFS	171	21528	6	6	5	5	2.70	Recombination protein
538	1	db$2	2::C3K6W5|PRMA_PSEFS	171	31717	3	3	2	2	0.35	Ribosomal protein L11 r
539	1	db$2	2::C3JZS5|TRPF_PSEFS	171	21966	4	3	3	2	0.53	N-(5'-phosphoribosyl)a
540	1	db$2	2::C3KE83|C3KE83_PSEFS	170	38700	7	7	6	6	1.08	Outer membrane protei
541	1	db$2	2::C3K1E5|ATPE_PSEFS	170	14558	5	5	3	3	2.60	ATP synthase epsilon ch
542	1	db$2	2::C3K9U7|C3K9U7_PSEFS	170	56147	8	8	7	7	0.80	Glucose-6-phosphate 1-
543	1	db$2	2::C3K2K9|C3K2K9_PSEFS	170	31417	9	8	8	7	2.32	Magnesium and cobalt e
544	1	db$2	2::C3KAD3|ARNC_PSEFS	169	37700	5	5	5	5	0.87	Undecaprenyl-phosphat
545	1	db$2	2::C3K3T8|C3K3T8_PSEFS	169	39499	6	6	5	5	0.82	Putrescine ABC transpo
546	1	db$2	2::C3JY54|SYS_PSEFS	169	47115	9	8	8	7	1.23	Serine--tRNA ligase OS
547	1	db$2	2::C3K276|GRPE_PSEFS	169	20714	3	3	2	2	0.57	Protein GrpE OS=Pseuc
548	1	db$2	2::C3K1X2|C3K1X2_PSEFS	168	40166	10	10	10	10	2.24	Putative putative oxidoı
549	1	db$2	2::C3K368|C3K368_PSEFS	168	37353	7	7	6	6	1.13	Stress response kinase
550	1	db$2	2::C3K2C9|C3K2C9_PSEFS	168	48295	7	7	6	6	0.80	Glutamate dehydrogena
551	1	db$2	2::C3K7G7|C3K7G7_PSEFS	168	50136	6	5	6	5	0.60	Putative modulation of
552	1	db$2	2::C3K5L3|C3K5L3_PSEFS	168	36401	6	4	6	4	0.68	Phosphonates ABC tran
553	1	db$2	2::C3K0N7|RL32_PSEFS	168	6752	5	5	3	3	6.52	50S ribosomal protein L

↑Family	M	DB	Accession	Score	Mass	Matches	Match(sig)	Sequences	Seq(sig)	emPAI	Description
554	1	db$2	2::C3K6E2|RS9_PSEFS	168	14607	6	5	4	3	2.57	30S ribosomal protein S
555	1	db$2	2::C3JYD3|SYC_PSEFS	167	51635	11	11	11	11	1.74	Cysteine--tRNA ligase C
556	1	db$2	2::C3JYQ7|C3JYQ7_PSEFS	167	32635	5	5	4	4	0.78	Carbamate kinase OS=
557	1	db$2	2::C3K2J9|C3K2J9_PSEFS	166	60860	4	4	3	3	0.37	Putative dehydrogenase
558	1	db$2	2::C3JXX2|C3JXX2_PSEFS	165	50328	7	7	7	7	0.93	ATP-dependent RNA he
559	1	db$2	2::C3K2W2|RS8_PSEFS	165	14083	7	6	5	4	4.22	30S ribosomal protein S
560	1	db$2	2::C3K5U6|C3K5U6_PSEFS	165	40174	8	8	6	6	1.02	dTDP-glucose 4,6-dehy
561	1	db$2	2::C3K170|C3K170_PSEFS	163	22875	5	5	4	4	1.27	Flavin_Reduct domain-c
562	1	db$2	2::C3KC10|C3KC10_PSEFS	163	23080	5	5	3	3	0.84	Uncharacterized protein
563	1	db$2	2::C3KED4|C3KED4_PSEFS	163	31038	5	5	5	5	1.13	4-hydroxy-tetrahydrodi
564	1	db$2	2::C3K254|C3K254_PSEFS	163	21027	9	9	6	6	2.82	Osmotically inducible pr
565	1	db$2	2::C3K1U7|C3K1U7_PSEFS	163	36908	4	4	2	2	0.67	Ubiquinol oxidase subur
566	1	db$2	2::C3KE48|C3KE48_PSEFS	162	19341	5	5	4	4	1.64	Peptide deformylase OS
567	1	db$2	2::C3K968|C3K968_PSEFS	162	55197	8	7	6	5	0.54	Putative aldehyde dehy
568	1	db$2	2::C3K6K4|C3K6K4_PSEFS	162	32908	6	6	6	6	1.37	Putative 5'-nucleotidase
569	1	db$2	2::C3KAV6|C3KAV6_PSEFS	162	17648	6	6	3	3	1.21	Thiol peroxidase OS=Ps
570	1	db$2	2::C3K7M9|C3K7M9_PSEFS	161	34271	5	5	5	5	0.99	Putative ABC transport
571	1	SwissProt_HUMAN$	3::P01876|IGHA1_HUMAN	161	37631	7	7	7	7	1.41	Immunoglobulin heavy
571	2	SwissProt_HUMAN$	3::P0DOX2|IGA2_HUMAN	80	48903	4	4	4	4	0.47	Immunoglobulin alpha-
571	3	SwissProt_HUMAN$	3::A0A0B4J1X5|HV374_HUMAN	64	12831	3	3	2	2	1.95	Immunoglobulin heavy
572	1	db$2	2::C3K0D3|MINC_PSEFS	161	26249	10	8	9	7	2.51	Probable septum site-d
573	1	db$2	2::C3K0U9|C3K0U9_PSEFS	161	55465	6	6	5	5	0.53	Putative two-componen
574	1	db$2	2::C3K417|C3K417_PSEFS	161	39396	4	4	3	3	0.43	Transcriptional regulato
575	1	db$2	2::C3K6H1|C3K6H1_PSEFS	161	131195	12	10	12	10	0.44	DNA polymerase III sub
576	1	db$2	2::C3K0K7|C3K0K7_PSEFS	160	60595	13	9	11	9	1.02	Electron transfer flavop
577	1	db$2	2::C3K1M1|HSCA_PSEFS	160	65896	8	8	7	7	0.65	Chaperone protein HscA
578	1	SwissProt_HUMAN$	3::P62805|H4_HUMAN	160	11360	7	7	6	6	10.65	Histone H4 OS=Homo s
579	1	db$2	2::C3K3M6|C3K3M6_PSEFS	158	53012	5	5	5	5	0.56	Peptidase_M16_C doma
580	1	db$2	2::C3K6F9|C3K6F9_PSEFS	158	37389	8	8	8	8	1.74	Nucleoid-associated pro
581	1	db$2	2::C3JY82|NUOB_PSEFS	158	25341	11	9	8	7	4.32	NADH-quinone oxidored
582	1	db$2	2::C3K606|FABZ_PSEFS	158	16691	8	8	8	8	8.44	3-hydroxyacyl-[acyl-car
583	1	db$2	2::C3K868|C3K868_PSEFS	157	58117	7	6	6	5	0.50	Uncharacterized protein
584	1	db$2	2::C3K424|HEM3_PSEFS	157	33541	4	4	3	3	0.52	Porphobilinogen deamir
585	1	db$2	2::C3K2D6|C3K2D6_PSEFS	156	73930	5	5	5	5	0.38	Carbon starvation prote
586	1	db$2	2::C3K1V6|C3K1V6_PSEFS	156	32555	4	3	4	3	0.54	Putative short-chain de
587	1	db$2	2::C3K2I0|C3K2I0_PSEFS	156	48538	5	5	5	5	0.63	Putative ATP-dependent
588	1	db$2	2::C3K3J3|C3K3J3_PSEFS	156	72855	5	5	4	4	0.30	Type IV pilus biogenesis
589	1	db$2	2::C3KAH1|HSLV_PSEFS	155	18731	4	4	3	3	1.11	ATP-dependent proteas
590	1	db$2	2::C3K8K2|C3K8K2_PSEFS	155	418093	4	4	4	4	0.05	Haemagg_act domain-c
591	1	db$2	2::C3K740|C3K740_PSEFS	155	33777	9	9	8	8	2.06	DUF815 domain-contair

↑Family	M	DB	Accession	Score	Mass	Matches	Match(sig)	Sequences	Seq(sig)	emPAI	Description
592	1	db$2	2::C3K483|C3K483_PSEFS	155	78630	11	9	11	9	0.72	Guanosine-3',5'-bis(Dip
592	2	db$2	2::C3K1B1|C3K1B1_PSEFS	84	46678	3	3	3	3	0.35	Putative acid tolerance
593	1	db$2	2::C3JYI3|PHNX_PSEFS	155	29973	5	5	4	4	1.19	Phosphonoacetaldehyde
594	1	db$2	2::C3KA81|C3KA81_PSEFS	155	55484	6	6	6	6	0.67	Alkyl hydroperoxide rec
595	1	db$2	2::C3KDG5|C3KDG5_PSEFS	154	23599	4	4	4	4	1.21	Putative regulatory prot
596	1	db$2	2::C3KBY3|C3KBY3_PSEFS	154	64998	6	6	6	6	0.55	Putative lipoprotein OS=
597	1	db$2	2::C3K785|C3K785_PSEFS	153	24962	4	4	4	4	1.12	3-oxoadipate CoA-trans
598	1	db$2	2::C3K3G7|C3K3G7_PSEFS	153	31253	8	8	8	8	2.33	Methylenetetrahydrofol
599	1	db$2	2::C3KED3|C3KED3_PSEFS	153	20551	5	5	4	4	2.13	Glycine cleavage syster
600	1	db$2	2::C3KDW7|HISZ_PSEFS	153	42687	11	9	8	7	1.17	ATP phosphoribosyltran
601	1	db$2	2::C3JXY4|ASTE_PSEFS	153	37404	9	8	7	7	1.42	Succinylglutamate desu
602	1	db$2	2::C3K859|C3K859_PSEFS	152	59064	4	4	4	4	0.38	Putative glutamate synt
603	1	db$2	2::C3K480|C3K480_PSEFS	151	31946	8	7	7	6	1.42	Uncharacterized proteir
604	1	db$2	2::C3JYJ6|C3JYJ6_PSEFS	151	68407	7	6	6	5	0.52	Peptidyl-prolyl cis-trans
605	1	db$2	2::C3K5H5|C3K5H5_PSEFS	151	25721	5	5	3	3	0.73	Putative exported prote
606	1	db$2	2::C3JZ32|C3JZ32_PSEFS	151	38286	4	4	3	3	0.45	Putative cation transpo
607	1	db$2	2::C3JXQ1|C3JXQ1_PSEFS	151	24479	4	4	2	2	0.47	Putative enoyl-CoA hyd
608	1	db$2	2::C3KDX7|ISPH_PSEFS	151	34499	8	6	6	5	0.98	4-hydroxy-3-methylbut
609	1	db$2	2::C3K116|PSRP_PSEFS	151	30838	5	5	5	5	1.15	Putative phosphoenolpy
610	1	SwissProt_HUMAN$	3::Q06830|PRDX1_HUMAN	150	22096	10	9	7	7	3.42	Peroxiredoxin-1 OS=Hc
611	1	db$2	2::C3JXY2|C3JXY2_PSEFS	150	35731	4	3	3	2	0.49	L-allo-threonine aldolas
612	1	db$2	2::C3K2W9|RL16_PSEFS	150	15422	5	4	5	4	2.35	50S ribosomal protein L
613	1	db$2	2::C3JYV5|C3JYV5_PSEFS	149	45487	8	7	7	6	1.07	Putative oxidoreductase
614	1	db$2	2::C3K3P5|C3K3P5_PSEFS	149	48453	4	4	3	3	0.34	Putative symporter mer
615	1	SwissProt_HUMAN$	3::Q5QNW6|H2B2F_HUMAN	149	13912	6	6	4	4	4.35	Histone H2B type 2-F O
616	1	db$2	2::C3K839|Y879_PSEFS	149	32120	6	6	6	6	1.41	Nucleotide-binding prot
617	1	db$2	2::C3K6Z7|C3K6Z7_PSEFS	149	89469	9	8	9	8	0.53	Lon protease OS=Pseu
618	1	db$2	2::C3K611|C3K611_PSEFS	148	16065	4	4	3	3	1.39	Universal stress protein
619	1	db$2	2::C3KAJ1|C3KAJ1_PSEFS	148	48585	7	6	7	6	0.79	Omega-amino acid--pyi
620	1	db$2	2::C3K0W1|C3K0W1_PSEFS	148	27483	6	6	5	5	1.35	Flagellar basal-body roc
621	1	db$2	2::C3K4E1|C3K4E1_PSEFS	148	38799	6	6	6	6	1.07	N5-carboxyaminoimida:
622	1	db$2	2::C3K862|C3K862_PSEFS	148	39825	7	7	6	6	1.29	Putative outer membra
623	1	db$2	2::C3K4R1|PLSB_PSEFS	148	95327	8	7	8	7	0.42	Glycerol-3-phosphate a
624	1	db$2	2::C3K477|C3K477_PSEFS	147	29836	4	4	4	4	0.88	Exodeoxyribonuclease I
625	1	db$2	2::C3K6J5|SERC_PSEFS	147	39799	9	8	7	6	1.29	Phosphoserine aminotr
626	1	db$2	2::C3K3M9|C3K3M9_PSEFS	147	30473	5	5	5	5	1.16	Putative sulfurtransfera
627	1	db$2	2::C3K126|C3K126_PSEFS	147	54569	9	7	7	6	0.68	2-methylcitrate dehydr
628	1	db$2	2::C3K485|C3K485_PSEFS	146	26565	3	3	2	2	0.43	Putative lipoprotein OS=
629	1	db$2	2::C3K309|C3K309_PSEFS	145	21661	4	4	3	3	0.92	Anthranilate synthase c
630	1	db$2	2::C3K0T1|C3K0T1_PSEFS	145	94111	8	7	8	7	0.42	Aconitate hydratase OS

↑Family	M	DB	Accession	Score	Mass	Matches	Match(sig)	Sequences	Seq(sig)	emPAI	Description
631	1	db$2	2::C3JZV1|C3JZV1_PSEFS	145	76010	11	10	10	10	0.86	UvrABC system protein
632	1	db$2	2::C3K6E5|C3K6E5_PSEFS	144	29069	4	4	4	4	0.91	Cytochrome c1 OS=Pse
633	1	db$2	2::C3KDQ0|C3KDQ0_PSEFS	144	35105	4	4	4	4	0.71	2-ketogluconate reduct
634	1	db$2	2::C3JZN8|C3JZN8_PSEFS	144	10735	4	4	3	3	4.58	Uncharacterized proteir
635	1	db$2	2::C3K062|C3K062_PSEFS	144	48996	8	7	7	6	0.79	Fumarate hydratase cla
636	1	db$2	2::C3K360|C3K360_PSEFS	143	23509	4	4	4	4	1.22	ATP-dependent dethiob
637	1	db$2	2::C3K5G1|C3K5G1_PSEFS	143	34097	6	6	5	5	1.29	Cysteine synthase OS=
638	1	db$2	2::C3K0I1|C3K0I1_PSEFS	143	29699	5	4	5	4	0.88	ParA family ATPase OS:
639	1	SwissProt_HUMAN$	3::P62979|RS27A_HUMAN	142	17953	4	4	4	4	1.83	Ubiquitin-40S ribosoma
640	1	db$2	2::C3KBV0|C3KBV0_PSEFS	142	28603	4	4	3	3	0.64	Putative chromosome p
641	1	db$2	2::C3KE99|C3KE99_PSEFS	142	30145	6	5	5	5	1.18	Quinolinate phosphoribo
642	1	db$2	2::C3K187|C3K187_PSEFS	142	38556	11	10	8	8	2.41	Peptidase_M75 domain-
643	1	db$2	2::C3KDB8|HLDE_PSEFS	141	49913	7	7	7	7	0.94	Bifunctional protein Hld
644	1	db$2	2::C3K0S3|C3K0S3_PSEFS	141	67491	5	4	5	4	0.32	Putative ATP-binding cc
645	1	db$2	2::C3K813|C3K813_PSEFS	140	36618	8	8	7	7	1.80	Proline iminopeptidase
646	1	db$2	2::C3KC02|C3KC02_PSEFS	140	29258	4	4	3	3	0.62	Putative acyltransferase
647	1	db$2	2::C3K017|PYRD_PSEFS	139	35850	11	11	10	10	3.25	Dihydroorotate dehydro
648	1	db$2	2::C3K075|C3K075_PSEFS	138	77370	6	5	6	5	0.36	Putative sulfatase OS=I
649	1	db$2	2::C3KED2|C3KED2_PSEFS	138	17293	4	4	4	4	1.94	Thioredoxin peroxidase
650	1	db$2	2::C3K544|C3K544_PSEFS	138	74165	9	8	8	8	0.67	Uncharacterized proteir
651	1	db$2	2::C3JY88|C3JY88_PSEFS	138	20432	8	8	7	7	5.27	NADH-quinone oxidorec
652	1	db$2	2::C3JYT6|C3JYT6_PSEFS	138	8291	9	8	6	6	46.43	Cold shock protein cspc
653	1	db$2	2::C3JY36|C3JY36_PSEFS	138	16095	6	6	5	5	3.27	Uncharacterized proteir
654	1	db$2	2::C3K467|C3K467_PSEFS	138	13453	3	3	2	2	1.00	Cupin_3 domain-contai
655	1	db$2	2::C3K264|GLMM_PSEFS	137	47719	9	7	7	6	0.81	Phosphoglucosamine m
656	1	db$2	2::C3KBK9|C3KBK9_PSEFS	137	34365	4	4	4	4	0.73	Glycerate dehydrogena:
657	1	db$2	2::C3K308|TRPD_PSEFS	136	36976	4	4	4	4	0.67	Anthranilate phosphorib
658	1	db$2	2::C3K3L7|THIG_PSEFS	136	28373	8	8	8	8	2.76	Thiazole synthase OS=I
659	1	db$2	2::C3K152|C3K152_PSEFS	136	13999	8	8	6	6	9.28	Putative transcriptional
660	1	db$2	2::C3JZ69|C3JZ69_PSEFS	136	25612	7	7	6	6	2.00	6-phosphogluconolactor
661	1	db$2	2::C3K2W4|RL5_PSEFS	136	20490	10	9	9	9	6.80	50S ribosomal protein L
662	1	db$2	2::C3K1A8|C3K1A8_PSEFS	135	35739	3	3	3	3	0.49	Putative lipoprotein OS:
663	1	db$2	2::C3K4P8|C3K4P8_PSEFS	135	24008	2	2	2	2	0.48	t(6)A37 threonylcarbam
664	1	db$2	2::C3K0U4|C3K0U4_PSEFS	134	36872	5	3	5	3	0.47	Flagellar motor switch p
665	1	db$2	2::C3KAI3|AROB_PSEFS	134	39825	4	4	4	4	0.61	3-dehydroquinate synth
666	1	db$2	2::C3K4E2|C3K4E2_PSEFS	134	16860	8	8	7	7	5.95	N5-carboxyaminoimida:
667	1	db$2	2::C3K468|C3K468_PSEFS	133	6077	2	2	1	1	1.10	50S ribosomal protein L
668	1	db$2	2::C3K0H7|C3K0H7_PSEFS	133	77893	7	6	7	6	0.44	Histidine kinase OS=Ps
669	1	db$2	2::C3K148|APT_PSEFS	133	20064	6	6	5	5	2.21	Adenine phosphoribosy
670	1	db$2	2::C3K4I0|HEM6_PSEFS	133	35313	7	6	7	6	1.23	Oxygen-dependent cop

↑Family	M	DB	Accession	Score	Mass	Matches	Match(sig)	Sequences	Seq(sig)	emPAI	Description
671	1	db$2	2::C3K1N4|C3K1N4_PSEFS	133	41398	7	7	5	5	0.98	Queuine tRNA-ribosyltra
672	1	db$2	2::C3K419|C3K419_PSEFS	133	17100	3	3	2	2	1.27	Transcriptional regulato
673	1	db$2	2::C3K5K3|C3K5K3_PSEFS	133	28692	4	4	2	2	0.64	CHAD domain-containir
674	1	db$2	2::C3K843|C3K843_PSEFS	132	26535	9	8	5	4	1.03	Lipopolysaccharide exp
675	1	db$2	2::C3K9F0|C3K9F0_PSEFS	132	40567	4	4	3	3	0.42	Putative transport-relat
676	1	db$2	2::C3K4C1|C3K4C1_PSEFS	132	40318	6	4	4	3	0.42	Putative nitric oxide de
677	1	db$2	2::C3K303|COQ7_PSEFS	132	23866	4	4	4	4	1.20	3-demethoxyubiquinol
678	1	db$2	2::C3K733|HTPX_PSEFS	132	32382	4	4	2	2	0.55	Protease HtpX OS=Pseu
679	1	db$2	2::C3K6L9|C3K6L9_PSEFS	131	49785	5	5	5	5	0.61	Phospho-2-dehydro-3-c
680	1	db$2	2::C3K134|C3K134_PSEFS	131	52371	6	5	5	4	0.43	Coproporphyrinogen-III
681	1	db$2	2::C3K3Q1|C3K3Q1_PSEFS	131	40925	4	4	4	4	0.59	Putative oxidoreductase
682	1	db$2	2::C3K1B4|C3K1B4_PSEFS	131	43355	3	3	3	3	0.39	Putative transferase OS
683	1	db$2	2::C3K1X7|RL27_PSEFS	131	9214	6	5	3	3	3.48	50S ribosomal protein L
684	1	db$2	2::C3K0R1|C3K0R1_PSEFS	131	30842	3	3	3	3	0.58	Protein-glutamate O-m
685	1	db$2	2::C3K3M3|C3K3M3_PSEFS	131	24540	4	4	3	3	0.78	Cell division ATP-bindin
686	1	db$2	2::C3K281|C3K281_PSEFS	130	15787	3	3	2	2	0.81	Ribosome association t
687	1	db$2	2::C3JXY9|C3JXY9_PSEFS	130	37073	5	5	5	5	0.89	Putative arginine N-suc
688	1	db$2	2::C3K3S1|C3K3S1_PSEFS	130	51070	7	7	7	7	0.91	Putative oxidase OS=Ps
689	1	db$2	2::C3K1B0|C3K1B0_PSEFS	130	96490	3	3	2	2	0.16	Lysylphosphatidylglycer
690	1	db$2	2::C3K421|C3K421_PSEFS	130	46244	9	7	9	7	1.04	Putative HemY domain
691	1	db$2	2::C3K6V7|C3K6V7_PSEFS	130	46336	4	4	4	4	0.50	Carboxy-terminal proce
692	1	db$2	2::C3K0W6|C3K0W6_PSEFS	130	42755	4	4	4	4	0.56	Aspartate aminotransfe
693	1	db$2	2::C3K7I9|C3K7I9_PSEFS	130	88241	8	7	8	7	0.46	Putative pinecillin acyla
694	1	db$2	2::C3K104|C3K104_PSEFS	129	26102	6	6	5	5	1.95	Putative membrane pro
695	1	SwissProt_HUMAN$	3::P06703|S10A6_HUMAN	129	10173	2	2	1	1	0.57	Protein S100-A6 OS=H
696	1	db$2	2::C3KBB4|OADC_PSEFS	129	31135	5	4	4	3	0.58	Oxaloacetate decarboxy
697	1	db$2	2::C3KAB1|C3KAB1_PSEFS	129	44397	4	4	3	3	0.38	Histidine kinase OS=Ps
698	1	db$2	2::C3K910|C3K910_PSEFS	129	36322	5	5	5	5	0.92	Putative ABC transport
699	1	db$2	2::C3K0V5|C3K0V5_PSEFS	128	33212	8	8	6	6	1.70	Putative keto/oxo acyl-
700	1	db$2	2::C3K5M0|C3K5M0_PSEFS	128	29300	4	4	4	4	0.90	Phosphonates ABC tran
701	1	db$2	2::C3JYI6|C3JYI6_PSEFS	128	73112	4	4	4	4	0.30	Putative 2,4-dienoyl-Co
702	1	db$2	2::C3K0W5|PHS_PSEFS	128	13357	3	3	2	2	1.85	Putative pterin-4-alpha
703	1	db$2	2::C3JXY6|ASTB_PSEFS	128	48741	7	6	6	5	0.79	N-succinylarginine dihy
704	1	db$2	2::C3KCP1|C3KCP1_PSEFS	127	23476	3	3	3	3	0.82	Peptide methionine sulf
705	1	db$2	2::C3K4A8|C3K4A8_PSEFS	127	55832	6	4	6	4	0.40	Uncharacterized proteir
706	1	db$2	2::C3K2W7|RS17_PSEFS	127	10090	6	5	4	4	5.23	30S ribosomal protein S
707	1	db$2	2::C3K3G5|C3K3G5_PSEFS	126	15160	4	4	3	3	2.41	Putative thioesterase O
708	1	db$2	2::C3K268|C3K268_PSEFS	126	11552	6	6	6	6	10.13	CRM domain-containing
709	1	db$2	2::C3JYA9|C3JYA9_PSEFS	126	27795	7	7	7	7	2.28	Putative TetR-family re
710	1	db$2	2::C3K6W7|C3K6W7_PSEFS	126	16216	4	4	4	4	2.17	Biotin carboxyl carrier p

↑Family	M	DB	Accession	Score	Mass	Matches	Match(sig)	Sequences	Seq(sig)	emPAI	Description
711	1	SwissProt_HUMAN$	3::Q5VTE0|EF1A3_HUMAN	126	50153	8	5	8	5	0.60	Putative elongation fact
712	1	db$2	2::C3JZ43|C3JZ43_PSEFS	125	44885	7	6	7	6	0.88	Putative aminotransfera
713	1	db$2	2::C3K5E3|C3K5E3_PSEFS	125	43909	5	5	3	3	0.38	Putative aminotransfera
714	1	db$2	2::C3K253|C3K253_PSEFS	125	106648	3	3	3	3	0.14	Putative membrane pro
715	1	db$2	2::C3K3V5|C3K3V5_PSEFS	125	52101	7	7	6	6	0.72	CYTH domain-containin
716	1	SwissProt_HUMAN$	3::P0DOX5|IGG1_HUMAN	124	49298	8	8	5	5	0.62	Immunoglobulin gamm
716	2	SwissProt_HUMAN$	3::P01860|IGHG3_HUMAN	104	41260	5	5	4	4	0.58	Immunoglobulin heavy
716	3	SwissProt_HUMAN$	3::P01859|IGHG2_HUMAN	66	35878	5	5	4	4	0.69	Immunoglobulin heavy
717	1	db$2	2::C3K0D7|APEB_PSEFS	124	46914	6	5	6	5	0.65	Probable M18 family an
718	1	db$2	2::C3KAC2|C3KAC2_PSEFS	124	41342	4	4	4	4	0.58	Acyl-CoA dehydrogenas
719	1	SwissProt_HUMAN$	3::Q9BQE3|TBA1C_HUMAN	123	49863	5	5	5	5	0.61	Tubulin alpha-1C chain
720	1	db$2	2::C3K9G9|C3K9G9_PSEFS	123	16183	6	5	6	5	3.22	CMD domain-containing
721	1	db$2	2::C3KDP4|C3KDP4_PSEFS	123	75338	9	7	9	7	0.55	Phosphate acetyltransfe
722	1	db$2	2::C3JYL7|C3JYL7_PSEFS	122	12417	3	3	2	2	1.11	Putative copper resistar
723	1	db$2	2::C3KDN7|HEMH_PSEFS	122	38415	7	6	7	6	1.09	Ferrochelatase OS=Pse
724	1	SwissProt_HUMAN$	3::P27348|1433T_HUMAN	122	27747	4	4	4	4	0.97	14-3-3 protein theta OS
724	2	SwissProt_HUMAN$	3::P63104|1433Z_HUMAN	110	27728	6	6	6	6	1.77	14-3-3 protein zeta/del
724	3	SwissProt_HUMAN$	3::P31946|1433B_HUMAN	58	28065	4	4	4	4	0.95	14-3-3 protein beta/alp
725	1	db$2	2::C3KEA8|C3KEA8_PSEFS	122	45495	4	4	3	3	0.37	ATP-dependent RNA he
726	1	db$2	2::C3KE58|C3KE58_PSEFS	121	84757	11	9	10	9	0.65	Putative S1 RNA-bindin
727	1	db$2	2::C3KBL7|C3KBL7_PSEFS	121	33413	6	4	6	4	0.76	Glutamate/aspartate AE
728	1	db$2	2::C3JYE9|C3JYE9_PSEFS	121	31035	4	4	4	4	0.83	Putative hydroxymethy
729	1	db$2	2::C3K2R5|RIBA_PSEFS	121	22312	3	3	3	3	0.88	GTP cyclohydrolase-2 C
730	1	db$2	2::C3KA84|C3KA84_PSEFS	121	13408	6	5	5	4	3.00	Putative regulatory prot
731	1	db$2	2::C3K240|C3K240_PSEFS	120	17616	7	7	5	5	5.39	RNA polymerase-bindin
732	1	db$2	2::C3KCP5|C3KCP5_PSEFS	120	109710	5	5	5	5	0.24	Bifunctional glutamine s
733	1	db$2	2::C3K758|PYRF_PSEFS	120	24706	5	5	4	4	1.58	Orotidine 5'-phosphate
733	2	db$2	2::C3K1G3|C3K1G3_PSEFS	44	15162	3	3	3	3	1.51	Ribonuclease P protein
734	1	db$2	2::C3K3M7|C3K3M7_PSEFS	119	22464	3	3	2	2	0.52	Ribosomal RNA small su
735	1	db$2	2::C3KBG3|C3KBG3_PSEFS	119	24033	4	4	4	4	1.18	Curli production asseml
736	1	db$2	2::C3KE32|C3KE32_PSEFS	119	20912	2	2	1	1	0.25	Sigma factor negative r
737	1	db$2	2::C3JZN4|RL20_PSEFS	119	13348	5	5	4	4	3.04	50S ribosomal protein L
738	1	db$2	2::C3K754|C3K754_PSEFS	119	29479	3	3	3	3	0.61	Putative lipoprotein OS=
739	1	db$2	2::C3JZU0|C3JZU0_PSEFS	118	50538	6	6	6	6	0.75	3-isopropylmalate dehy
740	1	db$2	2::C3KDB9|C3KDB9_PSEFS	118	32977	3	3	3	3	0.54	Putative lipopolysaccha
741	1	db$2	2::C3KDQ9|C3KDQ9_PSEFS	118	29676	6	6	6	6	1.59	Ferredoxin--NADP redu
742	1	db$2	2::C3K2Z9|C3K2Z9_PSEFS	118	34289	4	3	3	2	0.32	NMO domain-containing
743	1	db$2	2::C3K176|C3K176_PSEFS	118	33571	5	4	5	4	0.76	Glucokinase OS=Pseud
744	1	db$2	2::C3KBW8|C3KBW8_PSEFS	117	39031	7	6	7	6	1.06	D-alanine--D-alanine lig
745	1	db$2	2::C3KE84|C3KE84_PSEFS	117	35425	4	4	4	4	0.70	Pseudouridine synthase

↑Family	M	DB	Accession	Score	Mass	Matches	Match(sig)	Sequences	Seq(sig)	emPAI	Description
746	1	db$2	2::C3JY78|C3JY78_PSEFS	117	22781	4	4	3	3	0.86	Putative cytochrome ox
747	1	db$2	2::C3KDU8|C3KDU8_PSEFS	117	35029	5	5	5	5	0.96	D-cysteine desulfhydra
748	1	db$2	2::C3K427|C3K427_PSEFS	117	23927	4	4	3	3	0.80	Putative glutathione S-t
749	1	db$2	2::C3KDM3|C3KDM3_PSEFS	116	25575	3	2	3	2	0.45	Putative lipoprotein OS
750	1	db$2	2::C3K2S3|C3K2S3_PSEFS	116	17933	5	5	5	5	2.67	Transcriptional repressc
751	1	db$2	2::C3JYN9|C3JYN9_PSEFS	116	44994	5	4	5	4	0.52	Pseudouridine synthase
752	1	db$2	2::C3K482|RPOZ_PSEFS	116	9821	6	4	3	2	3.12	DNA-directed RNA poly
753	1	db$2	2::C3KBM6|C3KBM6_PSEFS	116	11934	8	7	5	5	9.20	Glutaredoxin OS=Pseuc
754	1	db$2	2::C3KCS5|C3KCS5_PSEFS	116	51798	5	5	5	5	0.58	UDP-N-acetylmuramoyl
755	1	db$2	2::C3KAY1|C3KAY1_PSEFS	116	44657	4	4	4	4	0.53	3-phosphoshikimate 1-
756	1	db$2	2::C3KDM4|C3KDM4_PSEFS	115	56715	6	5	6	5	0.52	Putative membrane pro
757	1	db$2	2::C3K6U9|HIS7_PSEFS	115	21830	9	7	7	6	2.62	Imidazoleglycerol-phos
758	1	db$2	2::C3KE59|C3KE59_PSEFS	115	27398	11	8	8	6	2.32	DNA-binding response r
759	1	SwissProt_HUMAN$	3::P60174|TPIS_HUMAN	115	26653	4	4	4	4	1.03	Triosephosphate isomer
760	1	db$2	2::C3K6E4|C3K6E4_PSEFS	115	46143	6	6	4	4	0.67	Cytochrome b OS=Pseu
761	1	db$2	2::C3K830|C3K830_PSEFS	114	47217	6	6	6	6	0.82	Putative short chain del
762	1	db$2	2::C3KA90|C3KA90_PSEFS	114	36984	2	2	2	2	0.29	Putative aldo/keto redu
763	1	db$2	2::C3K2X4|RL23_PSEFS	113	10938	7	6	7	6	11.52	50S ribosomal protein L
764	1	db$2	2::C3K332|Y5583_PSEFS	113	48606	5	5	5	5	0.63	UPF0229 protein PFLU_
765	1	db$2	2::C3K2D4|C3K2D4_PSEFS	113	35439	3	3	3	3	0.49	Putative cobalamin synt
766	1	db$2	2::C3K2J5|C3K2J5_PSEFS	113	18057	5	4	5	4	1.81	Putative AsnC-family re
767	1	db$2	2::C3K6S9|C3K6S9_PSEFS	113	101727	8	7	7	6	0.39	DNA polymerase I OS=
768	1	db$2	2::C3K6N8|C3K6N8_PSEFS	113	25972	2	2	1	1	0.20	Putative hydrolase OS=
769	1	db$2	2::C3K7Z1|C3K7Z1_PSEFS	112	38822	7	6	7	6	1.07	NAD(P) transhydrogena
770	1	db$2	2::C3KAS9|C3KAS9_PSEFS	112	109677	2	2	2	2	0.09	Efflux pump membrane
771	1	db$2	2::C3JY91|C3JY91_PSEFS	112	66398	4	4	3	3	0.24	NADH-quinone oxidored
772	1	db$2	2::C3K5H2|QUEF_PSEFS	112	30808	5	5	5	5	1.15	NADPH-dependent 7-cy
773	1	db$2	2::C3KCS7|MRAY_PSEFS	111	39243	4	4	3	3	0.62	Phospho-N-acetylmurar
774	1	db$2	2::C3K763|C3K763_PSEFS	111	29996	3	3	2	2	0.37	Putative hydrolase OS=
775	1	db$2	2::C3K572|C3K572_PSEFS	111	7891	2	2	2	2	2.20	Uncharacterized proteir
776	1	db$2	2::C3K272|C3K272_PSEFS	110	40774	5	4	4	4	0.59	Carbamoyl-phosphate s
777	1	db$2	2::C3K2X2|RS19_PSEFS	110	10313	7	7	5	5	13.67	30S ribosomal protein S
778	1	db$2	2::C3K5F4|C3K5F4_PSEFS	110	21753	3	3	3	3	0.91	Putative nitroreductase
779	1	db$2	2::C3K8U7|C3K8U7_PSEFS	110	30923	4	4	3	3	0.58	Transcriptional regulatc
780	1	db$2	2::C3K1W7|C3K1W7_PSEFS	109	11247	5	4	4	3	2.45	DUF1244 domain-conta
781	1	db$2	2::C3K8I3|C3K8I3_PSEFS	109	39921	3	3	1	1	0.13	3,4-dihydroxy-2-butanc
782	1	db$2	2::C3K4V5|C3K4V5_PSEFS	109	36313	6	5	6	5	0.92	Beta-hexosaminidase O
783	1	db$2	2::C3K5U5|C3K5U5_PSEFS	109	32335	5	5	4	4	0.79	Glucose-1-phosphate th
784	1	db$2	2::C3K256|RS15_PSEFS	109	10112	4	4	3	3	2.94	30S ribosomal protein S
785	1	db$2	2::C3KE68|RS18_PSEFS	108	8912	5	4	4	3	3.73	30S ribosomal protein S

↑Family	M	DB	Accession	Score	Mass	Matches	Match(sig)	Sequences	Seq(sig)	emPAI	Description
786	1	db$2	2::C3K0Q8|C3K0Q8_PSEFS	108	24717	2	2	2	2	0.46	Basal-body rod modifica
787	1	db$2	2::C3K2K2|THIE_PSEFS	108	22345	2	2	2	2	0.52	Thiamine-phosphate sy
788	1	db$2	2::C3K2P7|C3K2P7_PSEFS	108	17680	4	3	4	3	1.21	Putative acetyltransfera
789	1	db$2	2::C3K1H1|RS16_PSEFS	107	9202	9	7	5	5	11.18	30S ribosomal protein S
790	1	db$2	2::C3KDC0|C3KDC0_PSEFS	107	36735	2	2	2	2	0.29	Uncharacterized proteir
791	1	db$2	2::C3KDC3|LOLB_PSEFS	107	23021	3	3	3	3	0.85	Outer-membrane lipopr
792	1	db$2	2::C3K1K8|C3K1K8_PSEFS	107	31523	5	5	4	4	0.82	Putative hydrolase OS=
793	1	db$2	2::C3JYQ9|C3JYQ9_PSEFS	107	54767	2	2	2	2	0.19	Transcriptional regulatc
794	1	db$2	2::C3K231|C3K231_PSEFS	107	57678	6	6	5	5	0.64	APH domain-containing
795	1	db$2	2::C3K7G4|C3K7G4_PSEFS	107	54521	6	5	6	5	0.54	Ribonuclease G OS=Pse
796	1	db$2	2::C3JZR8|C3JZR8_PSEFS	107	46446	8	7	8	7	1.04	Putative oxidoreductase
797	1	db$2	2::C3K2L6|C3K2L6_PSEFS	106	47731	5	5	5	5	0.64	Putative lipoprotein OS
798	1	db$2	2::C3K9H7|C3K9H7_PSEFS	106	27490	4	3	4	3	0.67	Putative short-chain de
799	1	db$2	2::C3K4C8|C3K4C8_PSEFS	106	49694	5	5	5	5	0.61	Putative transport-relat
800	1	db$2	2::C3K3J5|C3K3J5_PSEFS	106	13219	4	3	3	2	1.02	Type IV pilus response
801	1	db$2	2::C3KBY0|GMHA_PSEFS	105	21250	4	4	4	4	1.42	Phosphoheptose isomer
802	1	db$2	2::C3K3L5|C3K3L5_PSEFS	105	11400	2	2	1	1	1.25	Putative membrane pro
803	1	db$2	2::C3JZH3|C3JZH3_PSEFS	105	22428	2	2	2	2	0.52	Putative cytochrome ox
804	1	db$2	2::C3KCG1|C3KCG1_PSEFS	105	60384	3	3	2	2	0.17	Putative secreted amid
805	1	db$2	2::C3K617|C3K617_PSEFS	105	19384	5	5	4	4	1.62	Uncharacterized proteir
806	1	db$2	2::C3JZH1|C3JZH1_PSEFS	105	34020	3	3	3	3	0.52	LysR-family transcriptic
807	1	db$2	2::C3K4U5|C3K4U5_PSEFS	104	75901	8	7	8	7	0.55	Putative protease OS=I
808	1	db$2	2::C3K1X0|CYSZ_PSEFS	104	28826	5	5	5	5	1.26	Sulfate transporter Cys
809	1	db$2	2::C3K3L3|C3K3L3_PSEFS	104	21091	5	5	4	4	1.43	dITP/XTP pyrophosphat
810	1	db$2	2::C3K491|C3K491_PSEFS	104	76137	2	2	2	2	0.13	ATP-dependent DNA he
811	1	db$2	2::C3K737|C3K737_PSEFS	104	17727	5	5	4	4	1.87	Glutathione peroxidase
812	1	db$2	2::C3K3X4|C3K3X4_PSEFS	103	11561	3	3	1	1	1.23	Cell division protein Za
813	1	db$2	2::C3KDA5|C3KDA5_PSEFS	103	54170	3	2	3	2	0.19	Uncharacterized proteir
814	1	db$2	2::C3K8W9|C3K8W9_PSEFS	103	28276	4	4	4	4	0.94	Intermembrane phosph
815	1	db$2	2::C3K5X2|C3K5X2_PSEFS	103	20559	5	5	4	4	1.49	Putative two-componen
816	1	db$2	2::C3K8P0|C3K8P0_PSEFS	102	38329	4	4	4	4	0.64	Putative nucleoside-sep
817	1	SwissProt_HUMAN$	3::P0C0S8|H2A1_HUMAN	102	14083	4	3	4	3	1.69	Histone H2A type 1 OS
817	2	SwissProt_HUMAN$	3::Q16777|H2A2C_HUMAN	54	13980	4	3	4	3	1.71	Histone H2A type 2-C C
818	1	db$2	2::C3KD59|C3KD59_PSEFS	102	81779	6	5	6	5	0.34	YcaO domain-containin
819	1	SwissProt_HUMAN$	3::P10599|THIO_HUMAN	102	11730	3	2	2	1	0.48	Thioredoxin OS=Homo
820	1	db$2	2::C3K061|C3K061_PSEFS	102	18792	3	3	3	3	1.11	DUF2059 domain-conta
821	1	db$2	2::C3K3B7|C3K3B7_PSEFS	101	14934	2	2	2	2	0.86	Uncharacterized proteir
822	1	db$2	2::C3K3Y7|C3K3Y7_PSEFS	101	35706	3	3	3	3	0.49	Putative iron-sulfur binc
823	1	db$2	2::C3KE30|C3KE30_PSEFS	100	59128	6	5	6	5	0.49	L-aspartate oxidase OS
824	1	db$2	2::C3KDQ1|C3KDQ1_PSEFS	100	35181	4	4	3	3	0.49	Putative LysR-family re

↑Family	M	DB	Accession	Score	Mass	Matches	Match(sig)	Sequences	Seq(sig)	emPAI	Description
825	1	db$2	2::C3K261|RIMP_PSEFS	100	17653	2	2	2	2	0.70	Ribosome maturation fa
826	1	db$2	2::C3K7C4|C3K7C4_PSEFS	100	53137	10	8	9	7	0.86	DNA-binding transcripti
827	1	db$2	2::C3K9N0|C3K9N0_PSEFS	100	28569	2	2	2	2	0.39	Putative transcriptional
828	1	db$2	2::C3KAK2|C3KAK2_PSEFS	100	10989	6	5	4	4	7.22	Uncharacterized proteir
829	1	db$2	2::C3KCT2|MUTS_PSEFS	100	95536	5	4	5	4	0.22	DNA mismatch repair p
830	1	db$2	2::C3K6Y6|C3K6Y6_PSEFS	99	31839	6	5	5	5	1.10	Signal peptidase I OS=
831	1	db$2	2::C3K1K9|C3K1K9_PSEFS	99	42204	4	4	4	4	0.57	Putative aminotransfera
832	1	db$2	2::C3K1B3|C3K1B3_PSEFS	99	20726	3	3	3	3	0.97	Uncharacterized proteir
833	1	db$2	2::C3KBZ8|C3KBZ8_PSEFS	99	38389	3	2	3	2	0.28	Iron-sulfur cluster carri
834	1	db$2	2::C3K644|C3K644_PSEFS	99	89774	7	4	5	3	0.17	Putative ABC transport
835	1	db$2	2::C3K3S3|C3K3S3_PSEFS	99	15261	4	3	2	1	0.36	DUF4399 domain-conta
836	1	db$2	2::C3K0R0|C3K0R0_PSEFS	99	14751	3	2	2	2	0.88	Flagellar basal body roc
837	1	db$2	2::C3K3X0|C3K3X0_PSEFS	98	42989	3	3	3	3	0.39	Putative ubiquinone bio
838	1	db$2	2::C3KE66|C3KE66_PSEFS	98	27011	6	6	6	6	1.85	23S rRNA (guanosine-2
839	1	db$2	2::C3K3N8|C3K3N8_PSEFS	98	43920	5	4	5	4	0.54	PUA domain-containing
840	1	db$2	2::C3K2Y1|RS12_PSEFS	98	13716	4	4	3	3	1.76	30S ribosomal protein S
841	1	db$2	2::C3K4W5|C3K4W5_PSEFS	97	24663	5	4	5	4	1.14	Lipoprotein-releasing sy
842	1	db$2	2::C3KE43|C3KE43_PSEFS	97	32711	4	4	4	4	0.78	Lipid A biosynthesis lau
843	1	db$2	2::C3K4W3|C3K4W3_PSEFS	97	21770	2	2	2	2	0.54	PilZ domain-containing
844	1	db$2	2::C3K2E0|C3K2E0_PSEFS	97	48742	5	4	5	4	0.47	DNA repair protein Rad
845	1	db$2	2::C3K084|AROC_PSEFS	97	39094	5	5	5	5	0.83	Chorismate synthase O
846	1	db$2	2::C3KE87|C3KE87_PSEFS	97	46792	4	2	3	2	0.22	NADH dehydrogenase I
847	1	db$2	2::C3K819|C3K819_PSEFS	97	35012	3	3	3	3	0.50	Putative ABC transport
848	1	db$2	2::C3K3L6|TRMB_PSEFS	97	26990	3	3	3	3	0.69	tRNA (guanine-N(7)-)-r
849	1	db$2	2::C3KDX6|C3KDX6_PSEFS	96	16394	3	3	2	2	0.77	Peptidyl-prolyl cis-trans
850	1	db$2	2::C3K8K4|C3K8K4_PSEFS	96	58330	3	3	3	3	0.28	Putative outer membrai
851	1	db$2	2::C3K1C9|C3K1C9_PSEFS	96	65670	2	2	1	1	0.07	Putative cation-transpo
852	1	db$2	2::C3KED5|C3KED5_PSEFS	96	40447	8	6	8	6	1.02	Putative lipoprotein OS
853	1	db$2	2::C3JY57|LOLA_PSEFS	96	22546	5	3	5	3	0.87	Outer-membrane lipopr
854	1	db$2	2::C3KE08|C3KE08_PSEFS	96	25044	3	3	3	3	0.75	Conserved hypothetical
855	1	db$2	2::C3K1E4|GLMU_PSEFS	96	48690	4	4	4	4	0.47	Bifunctional protein Glr
856	1	db$2	2::C3K3R6|C3K3R6_PSEFS	96	54762	6	6	4	4	0.41	L-threonine dehydratas
857	1	db$2	2::C3KDR6|C3KDR6_PSEFS	96	40570	2	2	2	2	0.26	Putaive NADH oxidored
858	1	db$2	2::C3K9R3|C3K9R3_PSEFS	96	18863	4	3	3	3	1.11	Uncharacterized proteir
859	1	db$2	2::C3KE89|C3KE89_PSEFS	96	24346	5	4	5	4	1.17	Uncharacterized proteir
860	1	db$2	2::C3KBH8|C3KBH8_PSEFS	95	25745	5	5	5	5	1.49	3-oxoacyl-[acyl-carrier-
861	1	db$2	2::C3K9P7|C3K9P7_PSEFS	95	69450	5	5	5	5	0.41	Putative substrate-bind
862	1	db$2	2::C3K230|C3K230_PSEFS	95	85084	3	3	3	3	0.18	Penicillin-binding protei
863	1	db$2	2::C3KAM8|C3KAM8_PSEFS	95	52747	3	3	3	3	0.31	Phosphate transporter (
864	1	db$2	2::C3K0T7|C3K0T7_PSEFS	95	76509	5	4	5	4	0.28	Putative fatty acid oxida

↑Family	M	DB	Accession	Score	Mass	Matches	Match(sig)	Sequences	Seq(sig)	emPAI	Description
865	1	db$2	2::C3KCT0|MURG_PSEFS	95	37667	4	4	4	4	0.65	UDP-N-acetylglucosami
866	1	db$2	2::C3KCS0|RL25_PSEFS	95	21502	9	7	4	4	1.97	50S ribosomal protein L
867	1	db$2	2::C3KE31|C3KE31_PSEFS	95	22237	6	5	6	5	1.88	RNA polymerase sigma
868	1	SwissProt_HUMAN$	3::P0CG04|IGLC1_HUMAN	94	11341	3	3	3	3	2.41	Immunoglobulin lambda
869	1	SwissProt_HUMAN$	3::A0A0C4DH25|KVD20_HUMAN	94	12507	1	1	1	1	0.45	Immunoglobulin kappa
870	1	db$2	2::C3K796|C3K796_PSEFS	94	49835	3	3	3	3	0.33	Putative glutamine synt
871	1	db$2	2::C3JXY5|C3JXY5_PSEFS	94	10610	2	2	2	2	1.38	Uncharacterized protein
872	1	db$2	2::C3K5K2|C3K5K2_PSEFS	94	9923	4	4	4	4	5.48	Putative lipoprotein OS
873	1	db$2	2::C3JXJ5|C3JXJ5_PSEFS	94	22111	4	3	3	2	0.53	Putative SCO1/SenC fa
874	1	db$2	2::C3K4R2|C3K4R2_PSEFS	94	7681	3	3	3	3	5.00	Major cold shock protei
875	1	db$2	2::C3KDS5|C3KDS5_PSEFS	93	41122	3	3	3	3	0.41	Putative hydratase/ison
876	1	db$2	2::C3K8W8|C3K8W8_PSEFS	93	29386	4	4	4	4	0.90	Putative ABC transport
877	1	db$2	2::C3K0X3|C3K0X3_PSEFS	93	16227	3	2	3	2	0.78	Transcriptional regulato
878	1	db$2	2::C3K5E4|GLND_PSEFS	93	102948	6	4	6	4	0.20	Bifunctional uridylyltran
879	1	db$2	2::C3K648|C3K648_PSEFS	92	33905	4	4	4	4	0.75	Conserved hypothetical
880	1	db$2	2::C3K7Y8|C3K7Y8_PSEFS	92	53697	5	5	5	5	0.55	Putative hydrolase OS=
881	1	db$2	2::C3K568|TRPA_PSEFS	92	28539	4	3	3	3	0.64	Tryptophan synthase al
882	1	db$2	2::C3K747|GPDA_PSEFS	92	36476	3	3	2	2	0.47	Glycerol-3-phosphate d
883	1	db$2	2::C3K3K3|C3K3K3_PSEFS	92	44101	5	5	4	4	0.54	Dihydroorotase-like pro
884	1	db$2	2::C3KDP0|C3KDP0_PSEFS	92	27498	3	3	3	3	0.67	Uncharacterized protein
885	1	db$2	2::C3K0U7|C3K0U7_PSEFS	91	49884	3	3	3	3	0.33	Two-component respon
886	1	db$2	2::C3K2V8|RL30_PSEFS	91	6521	4	4	3	3	7.03	50S ribosomal protein L
887	1	db$2	2::C3K605|LPXD_PSEFS	91	36124	4	3	4	3	0.48	UDP-3-O-acylglucosami
888	1	db$2	2::C3JYK0|CLPP_PSEFS	91	23405	6	5	5	4	1.23	ATP-dependent Clp prot
889	1	db$2	2::C3KA82|C3KA82_PSEFS	90	19457	4	3	4	3	1.06	Lactoylglutathione lyase
890	1	db$2	2::C3K479|RNPH_PSEFS	90	25645	3	2	3	2	0.44	Ribonuclease PH OS=Ps
891	1	db$2	2::C3KC09|C3KC09_PSEFS	90	83087	3	3	3	3	0.19	Periplasmic beta-glucos
892	1	db$2	2::C3K2K6|C3K2K6_PSEFS	90	49458	2	2	2	2	0.21	tRNA-2-methylthio-N(6
893	1	SwissProt_HUMAN$	3::P07737|PROF1_HUMAN	90	15045	4	4	4	4	2.44	Profilin-1 OS=Homo sa
894	1	db$2	2::C3K437|C3K437_PSEFS	90	26506	2	2	2	2	0.43	Uncharacterized protein
895	1	db$2	2::C3JYS0|C3JYS0_PSEFS	90	29172	5	3	3	3	0.62	Cell division coordinato
896	1	SwissProt_HUMAN$	3::P62937|PPIA_HUMAN	90	18001	5	5	4	4	1.83	Peptidyl-prolyl cis-trans
897	1	db$2	2::C3K2U6|C3K2U6_PSEFS	89	49484	2	2	2	2	0.21	Putative transport-relat
898	1	db$2	2::C3KDM5|C3KDM5_PSEFS	89	43829	6	6	5	5	0.71	O-phosphoserine phosp
899	1	db$2	2::C3K4U6|C3K4U6_PSEFS	89	17513	2	2	1	1	0.31	Conserved hypothetical
900	1	db$2	2::C3K0P6|DCTA_PSEFS	89	46761	4	4	3	3	0.35	C4-dicarboxylate transp
901	1	db$2	2::C3KBY4|C3KBY4_PSEFS	89	31613	2	2	2	2	0.35	Ribosomal RNA small su
902	1	db$2	2::C3K979|C3K979_PSEFS	88	30391	3	2	2	1	0.17	Putative glutathione S-t
903	1	db$2	2::C3K5X6|C3K5X6_PSEFS	88	49358	5	5	5	5	0.61	Tryptophan--tRNA ligas
904	1	db$2	2::C3K6A5|C3K6A5_PSEFS	88	78572	5	5	5	5	0.35	Catalase OS=Pseudomo

↑Family	M	DB	Accession	Score	Mass	Matches	Match(sig)	Sequences	Seq(sig)	emPAI	Description
905	1	db$2	2::C3KDL3|C3KDL3_PSEFS	88	70148	5	3	5	3	0.22	Phosphomethylpyrimidi
906	1	db$2	2::C3K0H9|C3K0H9_PSEFS	88	13855	3	3	2	2	1.74	Chemotaxis protein Che
907	1	db$2	2::C3K257|C3K257_PSEFS	87	33362	4	4	3	3	0.53	tRNA pseudouridine syr
908	1	db$2	2::C3KB69|C3KB69_PSEFS	87	12397	2	2	2	2	1.11	Putative lipoprotein OS
909	1	db$2	2::C3KAJ9|C3KAJ9_PSEFS	87	20836	2	2	2	2	0.57	Putative hypoxanthine-
910	1	db$2	2::C3K7W0|C3K7W0_PSEFS	86	26786	3	3	3	3	0.69	Uncharacterized proteir
911	1	db$2	2::C3KCV6|C3KCV6_PSEFS	86	40154	4	4	4	4	0.60	Probable alcohol dehydı
912	1	SwissProt_HUMAN$	3::P68371|TBB4B_HUMAN	86	49799	2	2	2	2	0.21	Tubulin beta-4B chain (
912	2	SwissProt_HUMAN$	3::P07437|TBB5_HUMAN	52	49639	2	2	2	2	0.21	Tubulin beta chain OS=
913	1	db$2	2::C3KB19|C3KB19_PSEFS	86	44434	4	4	4	4	0.53	HlyD family secretion p
914	1	db$2	2::C3K5E8|C3K5E8_PSEFS	86	28997	3	3	3	3	0.63	Cyclohexadienyl dehydı
915	1	db$2	2::C3K9N1|C3K9N1_PSEFS	86	43216	4	4	4	4	0.55	D-ser_dehydrat domair
916	1	db$2	2::C3K1F7|C3K1F7_PSEFS	85	69251	4	4	4	4	0.31	tRNA uridine 5-carboxy
917	1	db$2	2::C3JZN0|C3JZN0_PSEFS	85	13917	1	1	1	1	0.40	MerR-family transcriptic
918	1	db$2	2::C3K9M1|C3K9M1_PSEFS	85	26106	1	1	1	1	0.20	Putative phosphoglycer
919	1	db$2	2::C3K816|OPGH_PSEFS	85	96236	4	4	4	4	0.22	Glucans biosynthesis gl
920	1	db$2	2::C3K327|C3K327_PSEFS	85	29832	3	3	3	3	0.61	Ribosomal RNA small sı
921	1	db$2	2::C3K4W2|C3K4W2_PSEFS	84	26844	4	4	4	4	1.01	Putative glycerophosph
922	1	db$2	2::C3KC07|C3KC07_PSEFS	84	21449	2	2	2	2	0.55	YceI domain-containing
923	1	db$2	2::C3KCS6|C3KCS6_PSEFS	84	47227	3	3	3	3	0.35	UDP-N-acetylmuramoyl
924	1	db$2	2::C3KDF0|C3KDF0_PSEFS	84	18221	2	2	2	2	0.67	Putative phage tail-rela
925	1	db$2	2::C3K2S2|C3K2S2_PSEFS	84	38886	6	5	6	5	0.84	Riboflavin biosynthesis
926	1	db$2	2::C3KBB8|C3KBB8_PSEFS	84	13546	3	3	3	3	1.80	Uncharacterized proteir
927	1	SwissProt_HUMAN$	3::P62249|RS16_HUMAN	84	16435	3	2	3	2	0.77	40S ribosomal protein S
928	1	db$2	2::C3K2U8|C3K2U8_PSEFS	83	18135	5	5	5	5	2.64	Bacterioferritin OS=Pse
929	1	db$2	2::C3KAI8|C3KAI8_PSEFS	83	24497	3	3	2	2	0.78	Putative TetR-family reı
930	1	db$2	2::C3JYS6|C3JYS6_PSEFS	83	17060	2	2	2	2	0.73	Putative thioesterase O
931	1	db$2	2::C3K279|C3K279_PSEFS	83	19136	4	4	3	3	1.67	Outer membrane protei
932	1	db$2	2::C3JZT8|C3JZT8_PSEFS	83	28261	1	1	1	1	0.18	Putative methyltransfer
933	1	db$2	2::C3KD21|C3KD21_PSEFS	83	81698	3	3	3	3	0.19	Putative glycogen debra
934	1	db$2	2::C3K6M4|C3K6M4_PSEFS	83	13463	3	3	2	2	1.83	Succinate dehydrogena
935	1	db$2	2::C3K013|C3K013_PSEFS	83	20254	3	3	2	2	0.59	Peptidyl-prolyl cis-trans
936	1	db$2	2::C3JXX6|DNLJ_PSEFS	83	85515	4	4	4	4	0.25	DNA ligase OS=Pseudoı
937	1	db$2	2::C3JYS8|RUVA_PSEFS	83	22218	3	3	3	3	0.89	Holliday junction ATP-d
938	1	db$2	2::C3K9R8|C3K9R8_PSEFS	83	135461	5	5	5	5	0.19	Methionine synthase OS
939	1	SwissProt_HUMAN$	3::P04275|VWF_HUMAN	82	309058	4	4	4	4	0.06	von Willebrand factor O
940	1	db$2	2::C3K5Y8|C3K5Y8_PSEFS	82	48751	3	3	3	3	0.34	L-serine dehydratase O
941	1	db$2	2::C3JYN3|C3JYN3_PSEFS	82	52328	3	3	3	3	0.31	Family S13 unassigned
942	1	db$2	2::C3K5K4|C3K5K4_PSEFS	82	29062	4	4	4	4	0.91	Uncharacterized proteir
943	1	db$2	2::C3K5B2|C3K5B2_PSEFS	82	35714	3	3	3	3	0.49	Dipeptide ABC transpor

↑Family	M	DB	Accession	Score	Mass	Matches	Match(sig)	Sequences	Seq(sig)	emPAI	Description
944	1	db$2	2::C3JZN1|IHFA_PSEFS	81	11469	3	2	2	2	1.25	Integration host factor
945	1	db$2	2::C3KBJ5|C3KBJ5_PSEFS	81	62095	3	3	3	3	0.26	Phospholipase C OS=Ps
946	1	db$2	2::C3K2T3|C3K2T3_PSEFS	81	32553	3	3	3	3	0.54	dTDP-4-dehydrorhamn
947	1	db$2	2::C3K2R1|DXS_PSEFS	81	68238	6	6	6	6	0.52	1-deoxy-D-xylulose-5-p
948	1	db$2	2::C3K3D8|C3K3D8_PSEFS	81	49942	4	4	4	4	0.46	ATP-independent RNA h
949	1	db$2	2::C3K7T1|C3K7T1_PSEFS	81	23131	5	4	5	4	1.26	Two-component system
950	1	db$2	2::C3KEC9|C3KEC9_PSEFS	81	53005	2	2	2	2	0.20	Putative beta-barrel ass
951	1	db$2	2::C3KEC3|C3KEC3_PSEFS	81	16145	4	3	3	2	0.79	Putative membrane pro
952	1	db$2	2::C3K8U6|C3K8U6_PSEFS	81	15270	2	2	2	2	0.84	Putative polyhydroxyalk
953	1	db$2	2::C3KBH3|DCUP_PSEFS	81	38973	4	4	4	4	0.62	Uroporphyrinogen deca
954	1	db$2	2::C3JXH2|C3JXH2_PSEFS	80	91149	3	3	3	3	0.17	Putative excinuclease A
955	1	db$2	2::C3K7J8|C3K7J8_PSEFS	80	78803	5	4	5	4	0.27	ATP-dependent DNA he
956	1	db$2	2::C3KC04|C3KC04_PSEFS	80	39513	3	2	3	2	0.27	Putative hydrolase OS=
957	1	db$2	2::C3K304|C3K304_PSEFS	80	15054	2	2	2	2	0.85	Uncharacterized protein
958	1	db$2	2::C3K7B0|C3K7B0_PSEFS	79	28681	1	1	1	1	0.18	Putative ABC transport
959	1	db$2	2::C3K9B4|C3K9B4_PSEFS	79	16186	2	2	2	2	0.78	Type II secretion syster
960	1	db$2	2::C3KAW2|C3KAW2_PSEFS	79	26230	3	3	3	3	0.71	Putative short chain del
961	1	db$2	2::C3K114|C3K114_PSEFS	79	74752	5	5	5	5	0.37	DNA polymerase III sub
962	1	db$2	2::C3KBT9|C3KBT9_PSEFS	78	31336	1	1	1	1	0.16	Sulfate ABC transport s
963	1	db$2	2::C3K258|RBFA_PSEFS	78	14697	3	3	3	3	1.60	Ribosome-binding facto
964	1	db$2	2::C3KDR1|C3KDR1_PSEFS	78	17628	4	4	4	4	1.89	Uncharacterized protein
965	1	db$2	2::C3K3W5|C3K3W5_PSEFS	77	39645	4	4	3	3	0.43	Aminomethyltransferas
966	1	db$2	2::C3JZ77|C3JZ77_PSEFS	77	45611	10	8	8	6	0.86	Maltose-binding protein
967	1	db$2	2::C3K8K8|C3K8K8_PSEFS	77	62203	4	4	4	4	0.36	Putative exported prote
968	1	db$2	2::C3K5X9|C3K5X9_PSEFS	77	41639	2	2	2	2	0.25	Putative acyl-CoA dehy
969	1	db$2	2::C3K709|C3K709_PSEFS	77	7722	4	4	2	2	2.25	Cold shock protein OS=
970	1	db$2	2::C3K1C5|C3K1C5_PSEFS	76	44321	4	3	4	3	0.38	Putative cyclopropane-f
971	1	db$2	2::C3K302|C3K302_PSEFS	76	12723	5	5	2	2	1.98	Putative HIT domain-co
972	1	db$2	2::C3JY68|MNMA_PSEFS	76	41397	6	5	5	4	0.58	tRNA-specific 2-thiouric
973	1	db$2	2::C3KAF9|C3KAF9_PSEFS	76	50830	2	2	2	2	0.20	Poly-beta-1,6-N-acetyl-
974	1	db$2	2::C3KD57|C3KD57_PSEFS	76	49686	4	3	4	3	0.33	Cystathionine beta-lyas
975	1	db$2	2::C3JYV1|C3JYV1_PSEFS	76	32264	6	4	6	4	0.79	Formyltetrahydrofolate
976	1	db$2	2::C3K333|C3K333_PSEFS	76	61021	3	3	2	2	0.17	Uncharacterized protein
977	1	db$2	2::C3K3N0|C3K3N0_PSEFS	75	25405	2	2	2	2	0.45	Putative TetR-family re
978	1	db$2	2::C3K6J2|C3K6J2_PSEFS	75	47817	3	3	3	3	0.35	Putative hydrolase OS=
979	1	db$2	2::C3K8W4|C3K8W4_PSEFS	75	18790	6	6	4	4	1.71	Lipopolysaccharide exp
980	1	db$2	2::C3JYR0|C3JYR0_PSEFS	75	13774	3	2	2	2	0.97	Glycine cleavage syster
981	1	db$2	2::C3KE46|C3KE46_PSEFS	75	47174	4	4	3	3	0.35	16S rRNA m5C967 met
982	1	db$2	2::C3K4A9|C3K4A9_PSEFS	75	17462	3	3	3	3	1.24	Uncharacterized protein
983	1	db$2	2::C3K0H6|C3K0H6_PSEFS	75	40054	4	4	4	4	0.60	Protein-glutamate meth

↑Family	M	DB	Accession	Score	Mass	Matches	Match(sig)	Sequences	Seq(sig)	emPAI	Description
984	1	db$2	2::C3K1G6|C3K1G6_PSEFS	75	25762	4	4	3	3	0.73	Thiol:disulfide interchar
985	1	db$2	2::C3K6S3|C3K6S3_PSEFS	75	25881	2	2	2	2	0.44	Putative glutathione S-t
986	1	db$2	2::C3K2M7|C3K2M7_PSEFS	75	18106	4	2	4	2	0.68	Ribosomal silencing fac
987	1	db$2	2::C3KAH5|RL31_PSEFS	75	8451	3	3	3	3	4.12	50S ribosomal protein L
988	1	db$2	2::C3KBL4|C3KBL4_PSEFS	74	26993	5	4	4	3	0.69	Glutamate/aspartate AE
989	1	db$2	2::C3K092|C3K092_PSEFS	74	14085	4	4	4	4	2.75	Glutaredoxin domain-cc
990	1	db$2	2::C3K5K0|C3K5K0_PSEFS	74	25438	2	2	2	2	0.45	Two-component system
991	1	db$2	2::C3K3Q4|C3K3Q4_PSEFS	74	38576	4	4	2	2	0.28	Putative cation ABC trai
992	1	db$2	2::C3K470|C3K470_PSEFS	74	58011	2	2	2	2	0.18	Putative transport-relat
993	1	SwissProt_HUMAN$	3::P01009|A1AT_HUMAN	74	46707	3	3	3	3	0.35	Alpha-1-antitrypsin OS=
994	1	db$2	2::C3K815|C3K815_PSEFS	74	62777	7	5	7	5	0.46	Glucans biosynthesis pr
995	1	db$2	2::C3K3E2|C3K3E2_PSEFS	73	17110	1	1	1	1	0.31	Putative acetyltransfera
996	1	db$2	2::C3JZ62|C3JZ62_PSEFS	73	61473	1	1	1	1	0.08	Putative long-chain-fatt
997	1	db$2	2::C3KDV5|C3KDV5_PSEFS	73	20461	6	6	4	4	1.50	Oligoribonuclease OS=F
998	1	db$2	2::C3K0R2|C3K0R2_PSEFS	73	34379	2	2	1	1	0.15	Chemotaxis protein OS=
999	1	db$2	2::C3JY53|CYSG_PSEFS	73	50259	3	3	3	3	0.33	Siroheme synthase OS=
1000	1	db$2	2::C3JY31|C3JY31_PSEFS	73	22860	3	2	3	2	0.51	Putative biopolymer tra
1001	1	db$2	2::C3K2V4|RS13_PSEFS	73	13332	5	5	5	5	4.73	30S ribosomal protein S
1002	1	db$2	2::C3K487|C3K487_PSEFS	73	33600	3	2	3	2	0.32	Biopolymer transport pr
1003	1	db$2	2::C3K4A7|C3K4A7_PSEFS	73	18391	4	3	4	3	1.14	Uncharacterized proteir
1004	1	db$2	2::C3KE20|C3KE20_PSEFS	73	33868	2	2	2	2	0.32	GCV_T domain-containi
1005	1	db$2	2::C3KAY5|C3KAY5_PSEFS	73	30881	2	2	1	1	0.16	NAD-capped RNA hydrc
1006	1	db$2	2::C3KDC4|C3KDC4_PSEFS	73	64677	5	3	5	3	0.25	Uncharacterized proteir
1007	1	db$2	2::C3KBU0|C3KBU0_PSEFS	73	36726	4	4	4	4	0.67	Sulfate/thiosulfate impc
1008	1	db$2	2::C3K2P1|C3K2P1_PSEFS	73	48286	3	2	3	2	0.22	UDP-N-acetylmuramate
1009	1	db$2	2::C3K3M2|C3K3M2_PSEFS	73	37029	3	3	3	3	0.46	Cell division protein Fts
1010	1	db$2	2::C3KAI4|C3KAI4_PSEFS	73	53827	4	4	4	4	0.42	SPOR domain-containin
1011	1	db$2	2::C3K312|C3K312_PSEFS	72	24134	4	3	3	2	0.48	Ribulose-phosphate 3-e
1012	1	db$2	2::C3K4J0|C3K4J0_PSEFS	72	17598	4	4	4	4	1.89	Zn_protease domain-cc
1013	1	db$2	2::C3K173|C3K173_PSEFS	72	34695	3	3	3	3	0.50	Putative AraC-family tra
1014	1	db$2	2::C3K2K4|C3K2K4_PSEFS	72	19926	3	3	2	2	0.60	Putative exported prote
1015	1	db$2	2::C3JY71|C3JY71_PSEFS	72	43919	1	1	1	1	0.11	JmjC domain-containinc
1016	1	db$2	2::C3KBK6|C3KBK6_PSEFS	71	29320	2	2	1	1	0.38	Putative exported pepti
1017	1	db$2	2::C3JY97|C3JY97_PSEFS	71	30357	1	1	1	1	0.17	N-hydroxyarylamine O-
1018	1	db$2	2::C3KBL6|C3KBL6_PSEFS	71	27732	2	2	2	2	0.40	Glutamate/aspartate AE
1019	1	db$2	2::C3JXM0|C3JXM0_PSEFS	71	45141	3	2	3	2	0.23	Putative peptidase OS=
1020	1	db$2	2::C3K7Z3|C3K7Z3_PSEFS	71	43142	2	2	2	2	0.24	Putative acyl-CoA dehy
1021	1	db$2	2::C3K2W3|C3K2W3_PSEFS	71	11427	6	3	5	3	2.37	30S ribosomal protein S
1022	1	db$2	2::C3JY77|C3JY77_PSEFS	70	40451	3	3	3	3	0.42	Putative 3-oxoacyl-[acy
1023	1	db$2	2::C3K0J1|C3K0J1_PSEFS	70	36130	5	4	5	4	0.69	Flagellar motor switch p

↑Family	M	DB	Accession	Score	Mass	Matches	Match(sig)	Sequences	Seq(sig)	emPAI	Description
1024	1	db$2	2::C3K564|C3K564_PSEFS	70	54508	3	3	3	3	0.30	Methylmalonate-semial
1025	1	db$2	2::C3K1N0|C3K1N0_PSEFS	70	18655	1	1	1	1	0.29	Rick_17kDa_Anti doma
1026	1	db$2	2::C3K8W5|C3K8W5_PSEFS	70	21382	3	2	3	2	0.55	Lipopolysaccharide exp
1027	1	db$2	2::C3K1X3|C3K1X3_PSEFS	70	20670	3	2	3	2	0.57	Glutathione peroxidase
1028	1	db$2	2::C3K349|C3K349_PSEFS	69	28994	3	3	3	3	0.63	Pyrroloquinoline-quinor
1029	1	db$2	2::C3K4G7|PDXY_PSEFS	69	31241	1	1	1	1	0.16	Pyridoxal kinase PdxY C
1030	1	db$2	2::C3KDC5|HEM1_PSEFS	69	46641	3	2	3	2	0.22	Glutamyl-tRNA reducta:
1031	1	db$2	2::C3K0M3|C3K0M3_PSEFS	69	20567	4	4	4	4	1.49	Uncharacterized protein
1032	1	db$2	2::C3K579|C3K579_PSEFS	69	15962	1	1	1	1	0.34	Putative thioesterase O
1033	1	db$2	2::C3JYR2|C3JYR2_PSEFS	69	48934	2	2	2	2	0.21	L-serine dehydratase O
1034	1	db$2	2::C3K0K1|C3K0K1_PSEFS	69	53436	1	1	1	1	0.09	Putative GntR-family re
1035	1	db$2	2::C3JZP9|C3JZP9_PSEFS	68	33380	2	2	2	2	0.33	Putative ribose transpo
1036	1	db$2	2::C3JXZ5|C3JXZ5_PSEFS	68	25958	3	3	3	3	0.72	Putative histidine trans
1037	1	db$2	2::C3KBB9|C3KBB9_PSEFS	68	73354	5	4	4	3	0.21	Putative protease OS=F
1038	1	db$2	2::C3KE10|C3KE10_PSEFS	68	58823	2	2	2	2	0.17	Putative methyl-accepti
1039	1	db$2	2::C3K3L2|C3K3L2_PSEFS	68	15596	2	2	2	2	0.82	DUF4426 domain-conta
1040	1	db$2	2::C3K591|C3K591_PSEFS	68	19872	3	2	3	2	0.60	Putative bacterioferritin
1041	1	db$2	2::C3K3D3|BETA_PSEFS	68	62642	2	2	2	2	0.16	Oxygen-dependent cho
1042	1	db$2	2::C3K440|C3K440_PSEFS	67	11389	4	3	3	3	2.37	Uncharacterized protein
1043	1	db$2	2::C3K0V4|C3K0V4_PSEFS	67	48572	2	2	2	2	0.21	Flagellin OS=Pseudomo
1044	1	db$2	2::C3K3Q3|C3K3Q3_PSEFS	67	61523	3	2	3	2	0.17	Putative cation ABC tra
1045	1	db$2	2::C3KE78|C3KE78_PSEFS	67	15753	2	2	1	1	0.34	Azurin OS=Pseudomona
1046	1	SwissProt_HUMAN$	3::P23396|RS3_HUMAN	67	26671	2	2	2	2	0.42	40S ribosomal protein S
1047	1	db$2	2::C3K1B2|C3K1B2_PSEFS	67	62071	2	1	2	1	0.08	Single-stranded-DNA-s
1048	1	db$2	2::C3K6Z2|C3K6Z2_PSEFS	67	17868	2	2	2	2	0.69	tRNA-specific adenosine
1049	1	db$2	2::C3K5T7|C3K5T7_PSEFS	67	23769	3	3	2	2	0.49	Uncharacterized protein
1050	1	db$2	2::C3KDC6|RF1_PSEFS	67	39857	3	2	3	2	0.27	Peptide chain release fa
1051	1	db$2	2::C3JXX7|C3JXX7_PSEFS	67	56979	5	4	5	4	0.39	Conserved hypothetical
1052	1	db$2	2::C3K584|C3K584_PSEFS	67	15399	2	2	2	2	0.83	Uncharacterized protein
1053	1	db$2	2::C3K1H9|C3K1H9_PSEFS	67	18692	2	2	2	2	0.65	Conserved hypothetical
1054	1	SwissProt_HUMAN$	3::P07195|LDHB_HUMAN	63	36615	3	3	3	3	0.47	L-lactate dehydrogenas
1055	1	db$2	2::C3K707|C3K707_PSEFS	67	29295	2	2	2	2	0.38	Putative lipoprotein OS
1056	1	db$2	2::C3K6B0|C3K6B0_PSEFS	66	33534	1	1	1	1	0.15	High-affinity zinc uptak
1057	1	db$2	2::C3K863|PDXH_PSEFS	66	24884	3	3	3	3	0.76	Pyridoxine/pyridoxamin
1058	1	db$2	2::C3KBI8|C3KBI8_PSEFS	66	48805	3	2	3	2	0.21	Uncharacterized protein
1059	1	SwissProt_HUMAN$	3::P04406|G3P_HUMAN	66	36030	2	2	2	2	0.30	Glyceraldehyde-3-phos
1060	1	db$2	2::C3K9Q2|C3K9Q2_PSEFS	66	16954	5	4	4	4	2.01	Ribonuclease H OS=Pse
1061	1	db$2	2::C3K705|SURE_PSEFS	66	26431	1	1	1	1	0.19	5'-nucleotidase SurE OS
1062	1	db$2	2::C3K8L9|C3K8L9_PSEFS	66	65348	2	1	2	1	0.08	Putative potassium-efflu
1063	1	db$2	2::C3K243|C3K243_PSEFS	65	108098	1	1	1	1	0.04	Histidine kinase OS=Ps

↑Family	M	DB	Accession	Score	Mass	Matches	Match(sig)	Sequences	Seq(sig)	emPAI	Description
1064	1	db$2	2::C3KBK8|C3KBK8_PSEFS	65	35740	2	1	2	1	0.14	Ribosomal RNA small su
1065	1	db$2	2::C3K6V8|C3K6V8_PSEFS	65	47941	4	4	4	4	0.48	Putative peptidase OS=
1066	1	db$2	2::C3K6J1|UBIG_PSEFS	65	26040	5	4	5	4	1.06	Ubiquinone biosynthesi
1067	1	db$2	2::C3JYU5|C3JYU5_PSEFS	65	34090	3	3	3	3	0.51	Nudix hydrolase domair
1068	1	db$2	2::C3K4J4|HSLO_PSEFS	65	33320	3	3	3	3	0.53	33 kDa chaperonin OS=
1069	1	db$2	2::C3K459|C3K459_PSEFS	64	18704	3	3	3	3	1.11	Leucine-responsive regı
1070	1	db$2	2::C3KDX1|RS20_PSEFS	64	10078	2	2	1	1	0.58	30S ribosomal protein S
1071	1	db$2	2::C3K364|C3K364_PSEFS	64	38824	2	2	2	2	0.28	Biotin synthase OS=Pse
1072	1	db$2	2::C3K481|C3K481_PSEFS	64	23434	4	4	4	4	1.23	Guanylate kinase OS=P
1073	1	db$2	2::C3K998|C3K998_PSEFS	64	35021	4	4	4	4	0.71	Putative dehydrogenase
1074	1	db$2	2::C3K1X4|C3K1X4_PSEFS	64	16845	2	2	2	2	0.74	Putative putative expor
1075	1	SwissProt_HUMAN$	3::Q07020|RL18_HUMAN	64	21621	1	1	1	1	0.24	60S ribosomal protein L
1076	1	db$2	2::C3K0C5|C3K0C5_PSEFS	64	27111	1	1	1	1	0.19	Putative amino transfer
1077	1	db$2	2::C3K0R6|C3K0R6_PSEFS	64	28374	5	3	5	3	0.64	Uncharacterized proteir
1078	1	db$2	2::C3K146|C3K146_PSEFS	64	27134	4	4	4	4	1.00	Fumarate and nitrate re
1079	1	db$2	2::C3K0M6|C3K0M6_PSEFS	64	25497	4	2	3	1	0.45	Putative tetR-family reg
1080	1	db$2	2::C3K107|C3K107_PSEFS	64	31415	3	2	2	2	0.35	Uncharacterized proteir
1081	1	db$2	2::C3K8W6|C3K8W6_PSEFS	64	18885	2	2	2	2	0.64	3-deoxy-D-manno-octu
1082	1	db$2	2::C3K3B8|C3K3B8_PSEFS	64	42763	1	1	1	1	0.12	Uncharacterized proteir
1083	1	db$2	2::C3K2U3|C3K2U3_PSEFS	63	27946	2	2	2	2	0.40	Trans-aconitate 2-meth
1084	1	db$2	2::C3K743|FABA_PSEFS	63	18688	4	4	4	4	1.73	3-hydroxydecanoyl-[acy
1085	1	db$2	2::C3K9Q9|C3K9Q9_PSEFS	63	19670	1	1	1	1	0.27	FMN_red domain-conta
1086	1	db$2	2::C3K150|C3K150_PSEFS	63	25194	4	3	4	3	0.75	Putative short-chain de
1087	1	db$2	2::C3K6W0|C3K6W0_PSEFS	63	14786	3	2	2	1	0.37	Putative rhodanese don
1088	1	db$2	2::C3K9U9|C3K9U9_PSEFS	62	9554	1	1	1	1	0.62	Uncharacterized proteir
1089	1	db$2	2::C3K386|C3K386_PSEFS	62	22957	1	1	1	1	0.23	Putative glutathione S-t
1090	1	db$2	2::C3K6A1|C3K6A1_PSEFS	62	22990	1	1	1	1	0.23	Putative copper-binding
1091	1	db$2	2::C3K0S4|C3K0S4_PSEFS	62	65961	2	2	2	2	0.15	Isocitrate dehydrogena:
1092	1	db$2	2::C3K3K7|C3K3K7_PSEFS	62	24490	2	2	2	2	0.47	Pyridoxal phosphate ho
1093	1	db$2	2::C3K571|C3K571_PSEFS	62	11755	2	2	2	2	1.20	Uncharacterized proteir
1094	1	db$2	2::C3KB06|C3KB06_PSEFS	62	29724	1	1	1	1	0.17	Putative lipoprotein OS:
1095	1	db$2	2::C3K3D1|BETI_PSEFS	62	21992	1	1	1	1	0.24	HTH-type transcriptiona
1096	1	SwissProt_HUMAN$	3::P01023|A2MG_HUMAN	62	163188	3	3	3	3	0.09	Alpha-2-macroglobulin
1097	1	db$2	2::C3JXX3|C3JXX3_PSEFS	62	52842	1	1	1	1	0.09	Cytochrome-c oxidase (
1098	1	db$2	2::C3K221|C3K221_PSEFS	62	129628	1	1	1	1	0.04	RecBCD enzyme subuni
1099	1	db$2	2::C3JY84|C3JY84_PSEFS	62	18006	2	2	2	2	0.68	NADH dehydrogenase I
1100	1	db$2	2::C3K6Z1|C3K6Z1_PSEFS	62	50711	2	2	2	2	0.20	Putative multicopper ox
1101	1	db$2	2::C3K109|C3K109_PSEFS	61	49568	1	1	1	1	0.10	23S rRNA (uracil(1939)
1102	1	db$2	2::C3K1M3|C3K1M3_PSEFS	61	11692	2	2	2	2	1.22	Iron-binding protein Isc
1103	1	db$2	2::C3K6S5|C3K6S5_PSEFS	61	35353	7	6	6	5	0.95	Delta-aminolevulinic ac

↑Family	M	DB	Accession	Score	Mass	Matches	Match(sig)	Sequences	Seq(sig)	emPAI	Description
1104	1	db$2	2::C3KE17|C3KE17_PSEFS	61	24430	2	2	2	2	0.47	Two component system
1105	1	db$2	2::C3K101|C3K101_PSEFS	61	37185	2	2	2	2	0.29	Uncharacterized proteir
1106	1	db$2	2::C3JY61|C3JY61_PSEFS	61	8297	2	2	2	2	2.01	Translation initiation fac
1107	1	db$2	2::C3K7I5|C3K7I5_PSEFS	61	28342	3	3	3	3	0.65	Putative iron utilisation
1108	1	db$2	2::C3KCK0|C3KCK0_PSEFS	61	26687	6	3	4	3	0.70	Putative short-chain de
1109	1	db$2	2::C3JYS9|RUVC_PSEFS	61	18321	2	2	2	2	0.67	Crossover junction endo
1110	1	db$2	2::C3KDG2|C3KDG2_PSEFS	60	41589	4	4	3	3	0.57	AMP nucleosidase OS=P
1111	1	db$2	2::C3K334|CCA_PSEFS	60	45916	2	1	1	1	0.11	Multifunctional CCA pro
1112	1	db$2	2::C3K666|C3K666_PSEFS	60	29461	2	2	2	2	0.38	Putative lipoprotein OS=
1113	1	db$2	2::C3K0P9|C3K0P9_PSEFS	60	25457	3	2	3	2	0.45	Two-component systen
1114	1	db$2	2::C3K5H7|C3K5H7_PSEFS	60	43348	2	2	2	2	0.24	Putative response regul
1115	1	db$2	2::C3K5J9|C3K5J9_PSEFS	60	96070	3	3	3	3	0.16	Histidine kinase OS=Ps
1116	1	db$2	2::C3K2H3|C3K2H3_PSEFS	60	31916	2	1	2	1	0.16	Putative metallopeptida
1117	1	db$2	2::C3K6D7|C3K6D7_PSEFS	60	21411	1	1	1	1	0.24	UPF0056 inner membra
1118	1	db$2	2::C3K158|C3K158_PSEFS	60	36421	2	2	2	2	0.30	Putative regulatory prot
1119	1	db$2	2::C3JY76|C3JY76_PSEFS	60	48249	4	4	4	4	0.48	Putative mechanosensit
1120	1	db$2	2::C3KBM2|C3KBM2_PSEFS	60	16548	2	2	2	2	0.76	Cys-tRNA(Pro)/Cys-tRN
1121	1	db$2	2::C3K415|C3K415_PSEFS	60	70809	3	3	3	3	0.22	Putative ABC transport
1122	1	db$2	2::C3JZ23|C3JZ23_PSEFS	60	131220	4	3	4	3	0.11	Chromosome partition p
1123	1	db$2	2::C3K1A3|C3K1A3_PSEFS	59	26906	1	1	1	1	0.19	Putative pyridoxamine (
1124	1	db$2	2::C3JYI2|C3JYI2_PSEFS	59	39477	3	3	3	3	0.43	2-aminoethylphosphona
1125	1	db$2	2::C3K602|C3K602_PSEFS	59	48346	5	4	5	4	0.48	Zinc metalloprotease O
1126	1	db$2	2::C3K9T0|NADK_PSEFS	59	32289	2	1	2	1	0.16	NAD kinase OS=Pseudo
1127	1	db$2	2::C3K7C7|C3K7C7_PSEFS	59	19943	2	2	2	2	0.60	Chorismate mutase OS=
1128	1	db$2	2::C3K751|C3K751_PSEFS	59	15943	2	2	2	2	0.79	Putative thioesterase O
1129	1	db$2	2::C3K241|C3K241_PSEFS	59	33217	2	2	2	2	0.33	Glutamyl-Q tRNA(Asp)
1130	1	db$2	2::C3K2L9|LIPB_PSEFS	59	23473	1	1	1	1	0.22	Octanoyltransferase OS
1131	1	db$2	2::C3JY92|C3JY92_PSEFS	59	55851	2	2	2	2	0.18	NADH dehydrogenase I
1132	1	db$2	2::C3KDW0|C3KDW0_PSEFS	59	49939	2	2	2	2	0.21	N-acetylmuramoyl-L-ala
1133	1	db$2	2::C3K1M4|C3K1M4_PSEFS	59	13705	5	5	4	4	2.91	Iron-sulfur cluster asse
1134	1	db$2	2::C3KBM4|C3KBM4_PSEFS	59	34098	7	6	6	5	1.29	Ornithine carbamoyltra
1135	1	SwissProt_HUMAN$	3::P05109|S10A8_HUMAN	58	10828	1	1	1	1	0.53	Protein S100-A8 OS=H
1136	1	db$2	2::C3KDR2|C3KDR2_PSEFS	58	26142	3	3	3	3	0.71	S4 RNA-binding domair
1137	1	db$2	2::C3K021|C3K021_PSEFS	58	27911	5	5	4	4	0.97	Phosphoadenosine phos
1138	1	db$2	2::C3K0U5|C3K0U5_PSEFS	58	62992	2	2	2	2	0.16	Flagellar M-ring protein
1139	1	db$2	2::C3K6S4|C3K6S4_PSEFS	58	30497	1	1	1	1	0.17	Putative antibiotic biosy
1140	1	db$2	2::C3KE82|C3KE82_PSEFS	58	53340	4	3	4	3	0.30	Aldehyde dehydrogenas
1141	1	db$2	2::C3JYE7|C3JYE7_PSEFS	58	35468	2	2	2	2	0.30	LysR family regulatory p
1142	1	db$2	2::C3K0K3|C3K0K3_PSEFS	57	12996	1	1	1	1	0.43	Putative lipoprotein OS=
1143	1	db$2	2::C3K3F2|C3K3F2_PSEFS	57	38228	2	2	1	1	0.13	D-erythrose-4-phospha

↑Family	M	DB	Accession	Score	Mass	Matches	Match(sig)	Sequences	Seq(sig)	emPAI	Description
1144	1	db$2	2::C3K3K8|C3K3K8_PSEFS	57	27898	1	1	1	1	0.18	Pyrroline-5-carboxylate
1145	1	SwissProt_HUMAN$	3::P61604|CH10_HUMAN	57	10925	4	4	4	4	4.48	10 kDa heat shock prot
1146	1	db$2	2::C3K601|DXR_PSEFS	57	42341	3	3	3	3	0.40	1-deoxy-D-xylulose 5-p
1147	1	db$2	2::C3K3V1|C3K3V1_PSEFS	57	23989	2	2	2	2	0.48	Alkyl hydroperoxide red
1148	1	db$2	2::C3K5G7|PDXB_PSEFS	57	41465	2	2	2	2	0.26	Erythronate-4-phospha
1149	1	db$2	2::C3K436|C3K436_PSEFS	57	30237	2	2	2	2	0.37	Diaminopimelate epime
1150	1	db$2	2::C3K5Q0|C3K5Q0_PSEFS	57	53619	4	1	4	1	0.09	Mannose-1-phosphate g
1151	1	db$2	2::C3K0K4|C3K0K4_PSEFS	57	29536	2	2	2	2	0.38	PBPb domain-containin
1152	1	db$2	2::C3K1H0|RIMM_PSEFS	57	20004	3	3	3	3	1.02	Ribosome maturation fa
1153	1	db$2	2::C3KDN9|C3KDN9_PSEFS	57	40608	1	1	1	1	0.12	Probable periplasmic se
1154	1	db$2	2::C3K9Y7|C3K9Y7_PSEFS	56	30413	2	2	2	2	0.36	Undecaprenyl-diphosph
1155	1	db$2	2::C3KDM9|C3KDM9_PSEFS	56	28096	3	3	3	3	0.65	Putative oxidoreductase
1156	1	db$2	2::C3K6T8|C3K6T8_PSEFS	56	49583	4	3	4	3	0.33	Alginate biosynthesis tr
1157	1	db$2	2::C3KAB6|C3KAB6_PSEFS	56	39151	2	2	2	2	0.27	Conserved hypothetical
1158	1	db$2	2::C3KB73|C3KB73_PSEFS	56	9692	1	1	1	1	0.61	Putative lipoprotein OS
1159	1	db$2	2::C3KAH8|C3KAH8_PSEFS	56	89028	3	3	3	3	0.17	DD-transpeptidase OS=
1160	1	db$2	2::C3K1N5|QUEA_PSEFS	56	38396	6	3	6	3	0.45	S-adenosylmethionine:
1161	1	db$2	2::C3K7L9|C3K7L9_PSEFS	56	28704	2	2	2	2	0.39	Putative membrane pro
1162	1	db$2	2::C3K224|C3K224_PSEFS	56	30473	2	2	1	1	0.17	CDP-diacylglycerol--ser
1163	1	db$2	2::C3K197|C3K197_PSEFS	56	25331	2	2	2	2	0.45	Putative two-componen
1164	1	db$2	2::C3JYE6|C3JYE6_PSEFS	56	45437	2	2	2	2	0.23	Putative acyl-CoA dehy
1165	1	db$2	2::C3K7R8|C3K7R8_PSEFS	56	12576	2	2	2	2	1.10	Peptidyl-prolyl cis-trans
1166	1	db$2	2::C3K4L1|C3K4L1_PSEFS	56	41720	2	2	1	1	0.12	Putative epimerase OS=
1167	1	db$2	2::C3K6A4|C3K6A4_PSEFS	56	36459	1	1	1	1	0.14	Methionine import ATP-
1168	1	SwissProt_HUMAN$	3::P08865|RSSA_HUMAN	55	32833	2	2	2	2	0.33	40S ribosomal protein S
1169	1	SwissProt_HUMAN$	3::P39019|RS19_HUMAN	55	16051	2	2	2	2	0.79	40S ribosomal protein S
1170	1	db$2	2::C3JZU7|C3JZU7_PSEFS	55	16433	2	2	2	2	0.77	Putative thioesterase O
1171	1	db$2	2::C3K5K1|C3K5K1_PSEFS	55	38065	2	2	2	2	0.28	Putative phospholipase
1172	1	db$2	2::C3K9Y8|C3K9Y8_PSEFS	55	14463	1	1	1	1	0.38	Uncharacterized protein
1173	1	db$2	2::C3K3H4|C3K3H4_PSEFS	55	26074	1	1	1	1	0.20	Ribosomal RNA small su
1174	1	db$2	2::C3KDE9|C3KDE9_PSEFS	55	41781	4	4	4	4	0.57	Putative phage tail-rela
1175	1	db$2	2::C3KAH2|C3KAH2_PSEFS	55	24970	3	3	2	2	0.46	SPOR domain-containin
1176	1	db$2	2::C3K300|C3K300_PSEFS	55	34122	1	1	1	1	0.15	Putative membrane pro
1177	1	db$2	2::C3K216|C3K216_PSEFS	55	17384	2	2	2	2	0.71	Putative putative lipopr
1178	1	SwissProt_HUMAN$	3::Q5D862|FILA2_HUMAN	55	247928	1	1	1	1	0.02	Filaggrin-2 OS=Homo s
1179	1	db$2	2::C3K5B3|C3K5B3_PSEFS	55	34937	3	3	3	3	0.50	Dipeptide ABC transpor
1180	1	db$2	2::C3K2N1|C3K2N1_PSEFS	55	47217	1	1	1	1	0.11	Putative membrane pro
1181	1	SwissProt_HUMAN$	3::P15531|NDKA_HUMAN	55	17138	4	3	3	2	0.73	Nucleoside diphosphate
1182	1	SwissProt_HUMAN$	3::P60900|PSA6_HUMAN	55	27382	1	1	1	1	0.19	Proteasome subunit alp
1183	1	db$2	2::C3JZQ4|C3JZQ4_PSEFS	54	8106	2	2	2	2	2.10	Translational regulator

↑Family	M	DB	Accession	Score	Mass	Matches	Match(sig)	Sequences	Seq(sig)	emPAI	Description
1184	1	db$2	2::C3K250|C3K250_PSEFS	54	71198	2	2	1	1	0.07	Acetyl-coenzyme A syn
1185	1	db$2	2::C3KBE8|C3KBE8_PSEFS	54	8134	2	2	2	2	2.10	Putative lipoprotein OS
1186	1	db$2	2::C3JY69|HFLD_PSEFS	54	23173	2	2	2	2	0.50	High frequency lysogen
1187	1	db$2	2::C3KDB5|C3KDB5_PSEFS	54	33189	1	1	1	1	0.15	Putative glycosyl transf
1188	1	db$2	2::C3K5D8|C3K5D8_PSEFS	54	15101	1	1	1	1	0.36	Putative post-transcript
1189	1	db$2	2::C3K4I1|C3K4I1_PSEFS	54	29333	3	3	3	3	0.62	Shikimate dehydrogena
1190	1	db$2	2::C3K0S1|C3K0S1_PSEFS	54	53269	2	1	2	1	0.09	Magnesium transporter
1191	1	db$2	2::C3K425|C3K425_PSEFS	54	27295	3	3	2	2	0.41	Positive alginate biosyn
1192	1	db$2	2::C3KAX3|C3KAX3_PSEFS	54	30360	2	2	2	2	0.36	Conserved hypothetical
1193	1	db$2	2::C3K5F1|C3K5F1_PSEFS	54	42516	5	3	5	3	0.40	Ribonuclease D OS=Pse
1194	1	db$2	2::C3K607|LPXA_PSEFS	53	28002	1	1	1	1	0.18	Acyl-[acyl-carrier-prote
1195	1	db$2	2::C3K5X1|C3K5X1_PSEFS	53	46611	1	1	1	1	0.11	Histidine kinase OS=Ps
1196	1	db$2	2::C3KDR4|C3KDR4_PSEFS	53	49531	4	4	4	4	0.47	Ribosomal protein S12
1197	1	db$2	2::C3K4K0|C3K4K0_PSEFS	53	42230	2	2	2	2	0.25	Hypothetical exported p
1198	1	db$2	2::C3KC05|C3KC05_PSEFS	53	11915	1	1	1	1	0.48	Conserved hypothetical
1199	1	db$2	2::C3K456|C3K456_PSEFS	53	38683	2	2	2	2	0.28	Alanine racemase OS=I
1200	1	db$2	2::C3K239|C3K239_PSEFS	53	42550	2	2	2	2	0.25	Aminotransferase OS=I
1201	1	db$2	2::C3K132|C3K132_PSEFS	53	42760	3	3	3	3	0.39	Uncharacterized protein
1202	1	db$2	2::C3K3E9|C3K3E9_PSEFS	53	12056	1	1	1	1	0.47	Putative lipoprotein OS
1203	1	db$2	2::C3KBM3|C3KBM3_PSEFS	53	40194	2	2	2	2	0.26	Putative ABC transport
1204	1	db$2	2::C3K5L2|C3K5L2_PSEFS	53	29854	3	2	3	2	0.37	Phosphonates import A
1205	1	SwissProt_HUMAN$	3::P46783|RS10_HUMAN	53	18886	2	1	2	1	0.28	40S ribosomal protein S
1206	1	db$2	2::C3K4W6|C3K4W6_PSEFS	52	44511	3	2	3	2	0.24	Putative lipoprotein rele
1207	1	SwissProt_HUMAN$	3::Q5VT06|CE350_HUMAN	52	350716	3	3	3	3	0.04	Centrosome-associated
1208	1	db$2	2::C3JZU8|C3JZU8_PSEFS	52	30426	2	2	2	2	0.36	Hydrolase_4 domain-co
1209	1	db$2	2::C3K2Y9|C3K2Y9_PSEFS	52	13142	3	2	3	2	1.03	Protein translocase sub
1210	1	db$2	2::C3KAA6|C3KAA6_PSEFS	52	51931	1	1	1	1	0.10	Putative cell envelope p
1211	1	db$2	2::C3K596|C3K596_PSEFS	52	36487	2	2	2	2	0.29	Putative membrane pro
1212	1	db$2	2::C3JYF7|C3JYF7_PSEFS	52	147681	3	2	3	2	0.07	ATP-dependent helicase
1213	1	db$2	2::C3KDX3|C3KDX3_PSEFS	52	34279	2	2	2	2	0.32	Riboflavin biosynthesis
1214	1	db$2	2::C3K8L6|C3K8L6_PSEFS	52	28069	2	2	2	2	0.40	Precorrin-6A synthase (
1215	1	db$2	2::C3KCB9|C3KCB9_PSEFS	52	35987	4	3	3	2	0.30	Polyphosphate kinase C
1216	1	db$2	2::C3K0I0|C3K0I0_PSEFS	51	27472	3	2	2	1	0.19	RNA polymerase sigma
1217	1	db$2	2::C3KDW1|MUTL_PSEFS	51	69574	6	6	6	6	0.50	DNA mismatch repair p
1218	1	db$2	2::C3K454|C3K454_PSEFS	51	13613	2	2	1	1	0.41	Putative cytochrome c5
1219	1	SwissProt_HUMAN$	3::Q86YZ3|HORN_HUMAN	51	282228	2	2	1	1	0.03	Hornerin OS=Homo sap
1220	1	db$2	2::C3JYQ1|C3JYQ1_PSEFS	51	48078	2	1	2	1	0.10	Putative CoA transferas
1221	1	db$2	2::C3K0V2|C3K0V2_PSEFS	51	48301	1	1	1	1	0.10	Flagellar hook-associate
1222	1	db$2	2::C3K9J9|C3K9J9_PSEFS	51	17304	2	2	2	2	0.72	Uncharacterized protein
1223	1	db$2	2::C3K7A7|C3K7A7_PSEFS	51	43115	2	2	2	2	0.25	Conserved hypothetical

↑Family	M	DB	Accession	Score	Mass	Matches	Match(sig)	Sequences	Seq(sig)	emPAI	Description
1224	1	db$2	2::C3KAT0|C3KAT0_PSEFS	51	40804	2	2	2	2	0.26	Putative efflux system i
1225	1	db$2	2::C3K0L0|C3K0L0_PSEFS	51	29492	2	2	2	2	0.38	Putative IclR-family reg
1226	1	db$2	2::C3K8U3|C3K8U3_PSEFS	51	23009	1	1	1	1	0.23	Ubiquinone biosynthesi
1227	1	db$2	2::C3JY81|C3JY81_PSEFS	51	15084	1	1	1	1	0.36	NADH-quinone oxidore
1228	1	db$2	2::C3K2M0|Y5418_PSEFS	51	10305	1	1	1	1	0.56	UPF0250 protein PFLU_
1229	1	db$2	2::C3JY87|NUOH_PSEFS	51	37530	3	3	3	3	0.46	NADH-quinone oxidore
1230	1	db$2	2::C3K486|C3K486_PSEFS	51	30593	1	1	1	1	0.17	Epimerase domain-cont
1231	1	SwissProt_HUMAN$	3::P62913|RL11_HUMAN	50	20240	1	1	1	1	0.26	60S ribosomal protein L
1232	1	db$2	2::C3K6A6|C3K6A6_PSEFS	50	25667	2	2	1	1	0.44	Putative lipoprotein OS
1233	1	SwissProt_HUMAN$	3::O60262|GBG7_HUMAN	50	7517	2	1	1	1	0.83	Guanine nucleotide-bin
1234	1	db$2	2::C3K660|C3K660_PSEFS	50	28970	1	1	1	1	0.18	Hydroxypyruvate isome
1235	1	db$2	2::C3K4M2|C3K4M2_PSEFS	50	36363	3	3	3	3	0.48	Catabolite repressor/ac
1236	1	db$2	2::C3KD16|C3KD16_PSEFS	50	34294	5	4	3	3	0.51	Putative 2-hydroxyacid
1237	1	db$2	2::C3KDV8|C3KDV8_PSEFS	50	29265	2	2	2	2	0.38	ADP-dependent (S)-NA
1238	1	db$2	2::C3K3H3|C3K3H3_PSEFS	50	52510	3	3	3	3	0.31	Adenosylmethionine-8-
1239	1	db$2	2::C3JYU0|C3JYU0_PSEFS	50	30784	2	1	2	1	0.17	Putative transmembran
1240	1	db$2	2::C3K1M8|C3K1M8_PSEFS	50	28345	3	1	2	1	0.18	tRNA (cytidine/uridine-
1241	1	db$2	2::C3K5G4|RLMM_PSEFS	50	40585	1	1	1	1	0.12	Ribosomal RNA large su
1242	1	db$2	2::C3KCD6|C3KCD6_PSEFS	50	27732	1	1	1	1	0.18	Short chain dehydroger
1243	1	db$2	2::C3KBL9|C3KBL9_PSEFS	50	27726	2	2	2	2	0.40	Glycerol-3-phosphate r
1244	1	db$2	2::C3JYU4|C3JYU4_PSEFS	50	20911	1	1	1	1	0.25	Corrinoid adenosyltrans
1245	1	db$2	2::C3K0M9|KTHY_PSEFS	50	22917	4	3	4	3	0.85	Thymidylate kinase OS
1246	1	SwissProt_HUMAN$	3::Q6IS14|IF5AL_HUMAN	49	16762	1	1	1	1	0.32	Eukaryotic translation i
1247	1	db$2	2::C3K3W8|C3K3W8_PSEFS	49	44404	3	3	3	3	0.38	Putative ubiquinone bio
1248	1	SwissProt_HUMAN$	3::P62318|SMD3_HUMAN	49	13907	1	1	1	1	0.40	Small nuclear ribonucle
1249	1	db$2	2::C3KAZ8|C3KAZ8_PSEFS	49	34711	2	2	2	2	0.31	Putative beta-hydroxyla
1250	1	db$2	2::C3KAF8|C3KAF8_PSEFS	49	74138	2	2	2	2	0.14	Putative lipoprotein OS
1251	1	db$2	2::C3K1R4|C3K1R4_PSEFS	49	17440	1	1	1	1	0.31	Putative plasmid-relate
1252	1	db$2	2::C3JXI0|C3JXI0_PSEFS	49	23030	2	2	2	2	0.50	Nudix hydrolase domai
1253	1	db$2	2::C3JZ64|C3JZ64_PSEFS	49	17217	3	3	3	3	1.26	MaoC-like domain-cont
1254	1	SwissProt_HUMAN$	3::P46776|RL27A_HUMAN	49	16551	1	1	1	1	0.33	60S ribosomal protein L
1255	1	db$2	2::C3K110|C3K110_PSEFS	49	32414	3	2	3	2	0.34	Cysteine synthase OS=
1256	1	db$2	2::C3K5I0|C3K5I0_PSEFS	49	36784	1	1	1	1	0.14	tRNA-dihydrouridine(2
1257	1	db$2	2::C3K7K0|C3K7K0_PSEFS	49	41297	1	1	1	1	0.12	Esterase III OS=Pseud
1258	1	db$2	2::C3K6H7|C3K6H7_PSEFS	49	10540	1	1	1	1	0.55	Cell division protein Fts
1259	1	db$2	2::C3JXX9|C3JXX9_PSEFS	49	6925	5	4	3	3	6.29	Translational regulator
1260	1	db$2	2::C3K4L7|C3K4L7_PSEFS	48	23283	2	2	2	2	0.50	Putative TetR-family re
1261	1	db$2	2::C3K3L8|C3K3L8_PSEFS	48	7061	1	1	1	1	0.90	Putative thiamine biosy
1262	1	db$2	2::C3K4R0|C3K4R0_PSEFS	48	14930	2	2	2	2	0.86	Putative lipoprotein OS
1263	1	db$2	2::C3K6V0|C3K6V0_PSEFS	48	23354	1	1	1	1	0.22	Imidazole glycerol phos

↑Family	M	DB	Accession	Score	Mass	Matches	Match(sig)	Sequences	Seq(sig)	emPAI	Description
1264	1	SwissProt_HUMAN$	3::Q2PPJ7|RGPA2_HUMAN	48	210636	2	2	2	2	0.05	Ral GTPase-activating p
1264	2	SwissProt_HUMAN$	3::P61254|RL26_HUMAN	39	17248	2	2	2	2	0.72	60S ribosomal protein L
1265	1	db$2	2::C3KDC8|C3KDC8_PSEFS	48	26520	1	1	1	1	0.19	Molybdopterin biosynth
1266	1	db$2	2::C3K8X1|C3K8X1_PSEFS	48	23435	2	2	2	2	0.49	Putative ABC transport
1267	1	db$2	2::C3K4Z0|CCME_PSEFS	48	16101	4	2	4	2	0.79	Cytochrome c-type biog
1268	1	db$2	2::C3JYN0|C3JYN0_PSEFS	48	33944	2	2	2	2	0.32	Cbb3-type cytochrome
1269	1	db$2	2::C3K5G2|C3K5G2_PSEFS	48	35788	3	2	3	2	0.30	Putative membrane pro
1270	1	SwissProt_HUMAN$	3::P62847|RS24_HUMAN	48	15413	1	1	1	1	0.35	40S ribosomal protein S
1271	1	db$2	2::C3K6K7|C3K6K7_PSEFS	48	17395	1	1	1	1	0.31	GreA_GreB domain-con
1272	1	db$2	2::C3K7N0|C3K7N0_PSEFS	48	28545	1	1	1	1	0.18	Transport permease pro
1273	1	db$2	2::C3KCN6|C3KCN6_PSEFS	48	53164	1	1	1	1	0.09	Sodium/proline sympor
1274	1	db$2	2::C3K5B5|C3K5B5_PSEFS	48	37023	1	1	1	1	0.14	Dipeptide ABC transpor
1275	1	db$2	2::C3K589|C3K589_PSEFS	48	50032	3	3	3	3	0.33	Transcriptional regulato
1276	1	db$2	2::C3K6Y0|C3K6Y0_PSEFS	48	13973	1	1	1	1	0.40	4-carboxymuconolactor
1277	1	SwissProt_HUMAN$	3::P62750|RL23A_HUMAN	48	17684	1	1	1	1	0.30	60S ribosomal protein L
1278	1	db$2	2::C3K0Y9|C3K0Y9_PSEFS	48	19041	1	1	1	1	0.28	Bifunctional adenosylco
1279	1	db$2	2::C3K1W6|C3K1W6_PSEFS	47	13390	4	3	3	3	1.83	D-erythro-7,8-dihydron
1280	1	db$2	2::C3K129|C3K129_PSEFS	47	42141	2	2	2	2	0.25	Putative acetyl CoA ace
1281	1	db$2	2::C3K4N5|C3K4N5_PSEFS	47	28830	1	1	1	1	0.18	Putative amino acid AB(
1282	1	db$2	2::C3K7Y7|C3K7Y7_PSEFS	47	8356	1	1	1	1	0.74	Uncharacterized proteir
1283	1	db$2	2::C3K838|C3K838_PSEFS	47	9715	2	2	2	2	1.57	Putative phosphocarrier
1284	1	db$2	2::C3K1B6|C3K1B6_PSEFS	47	10582	1	1	1	1	0.55	Uncharacterized proteir
1285	1	db$2	2::C3JYP8|C3JYP8_PSEFS	47	24937	1	1	1	1	0.21	Putative two-componen
1286	1	SwissProt_HUMAN$	3::P62701|RS4X_HUMAN	47	29579	3	1	3	1	0.17	40S ribosomal protein S
1287	1	db$2	2::C3K5L9|C3K5L9_PSEFS	47	31441	3	2	3	2	0.35	Alpha-D-ribose 1-methy
1288	1	db$2	2::C3K125|C3K125_PSEFS	47	26783	2	2	1	1	0.19	Alanine--tRNA ligase OS
1289	1	db$2	2::C3K7S1|C3K7S1_PSEFS	47	25085	2	2	1	1	0.21	Protein MgtC OS=Pseud
1290	1	db$2	2::C3K6E6|C3K6E6_PSEFS	46	23384	2	2	2	2	0.49	Stringent starvation pro
1291	1	db$2	2::C3K0T5|C3K0T5_PSEFS	46	37877	1	1	1	1	0.13	Zinc transport protein C
1292	1	db$2	2::C3JY28|Y3771_PSEFS	46	6626	4	3	2	2	2.92	UPF0434 protein PFLU_
1293	1	db$2	2::C3JYM6|C3JYM6_PSEFS	46	40915	1	1	1	1	0.12	Putative HlyD-family se
1294	1	db$2	2::C3K0X7|C3K0X7_PSEFS	46	126663	2	2	2	2	0.08	Histidine kinase OS=Ps
1295	1	db$2	2::C3K1A2|C3K1A2_PSEFS	46	17206	2	1	2	1	0.31	Putative membrane pro
1296	1	db$2	2::C3K8K1|C3K8K1_PSEFS	46	40207	1	1	1	1	0.12	Phosphate-binding prot
1297	1	db$2	2::C3K0J8|C3K0J8_PSEFS	46	22091	2	2	2	2	0.53	START domain-containi
1298	1	db$2	2::C3K6Y7|RNC_PSEFS	46	25367	1	1	1	1	0.20	Ribonuclease 3 OS=Pse
1299	1	db$2	2::C3K5Y3|C3K5Y3_PSEFS	46	35524	2	2	2	2	0.30	Putative leucine dehydr
1300	1	db$2	2::C3K7X0|C3K7X0_PSEFS	46	85237	2	1	2	1	0.06	5-methyltetrahydropter
1301	1	db$2	2::Q9ZEP7|PANC_PSEFS	46	30696	2	2	2	2	0.36	Pantothenate synthetas
1302	1	db$2	2::C3K650|C3K650_PSEFS	46	13083	3	3	3	3	1.90	N-acetyltransferase dor

↑Family	M	DB	Accession	Score	Mass	Matches	Match(sig)	Sequences	Seq(sig)	emPAI	Description
1303	1	db$2	2::C3K2C2|C3K2C2_PSEFS	46	67328	1	1	1	1	0.07	Putative lipoprotein OS=
1304	1	SwissProt_HUMAN$	3::O00299|CLIC1_HUMAN	46	26906	2	2	2	2	0.42	Chloride intracellular ch
1305	1	db$2	2::C3K328|APAG_PSEFS	46	13631	3	2	3	2	0.98	Protein ApaG OS=Pseu
1306	1	db$2	2::C3K2Z1|COAX_PSEFS	45	26920	1	1	1	1	0.19	Type III pantothenate k
1307	1	db$2	2::C3K8Z4|C3K8Z4_PSEFS	45	45613	3	2	3	2	0.23	High-affinity branched-
1308	1	db$2	2::C3K5U7|C3K5U7_PSEFS	45	105852	2	1	2	1	0.05	Putative aminotransfera
1309	1	db$2	2::C3KAX0|C3KAX0_PSEFS	45	84238	3	3	3	3	0.18	1,4-alpha-glucan branc
1310	1	db$2	2::C3K282|SSRP_PSEFS	45	18308	5	3	4	3	1.15	SsrA-binding protein OS
1311	1	db$2	2::C3K8L0|C3K8L0_PSEFS	45	16885	1	1	1	1	0.32	Uncharacterized proteir
1312	1	db$2	2::C3K8H9|C3K8H9_PSEFS	45	41728	1	1	1	1	0.12	Putative polyamine ABC
1313	1	db$2	2::C3K0P0|C3K0P0_PSEFS	45	35982	3	3	3	3	0.48	Putative peptidase OS=
1314	1	db$2	2::C3K870|C3K870_PSEFS	45	20966	2	2	2	2	0.56	Uncharacterized proteir
1315	1	db$2	2::C3K5M2|C3K5M2_PSEFS	45	41151	3	2	2	1	0.12	Phosphorous compounc
1316	1	db$2	2::C3K229|C3K229_PSEFS	45	24861	1	1	1	1	0.21	TPR_REGION domain-c
1317	1	db$2	2::C3K3J1|C3K3J1_PSEFS	45	16523	1	1	1	1	0.33	Putative chemotaxis-rel
1318	1	db$2	2::C3K5S7|C3K5S7_PSEFS	45	19211	1	1	1	1	0.28	Uncharacterized proteir
1319	1	db$2	2::C3K646|C3K646_PSEFS	44	21182	2	2	2	2	0.56	Putative acyl-CoA thioe
1320	1	db$2	2::C3K4I9|C3K4I9_PSEFS	44	42202	1	1	1	1	0.12	Galactonate dehydratas
1321	1	db$2	2::C3KBL5|C3KBL5_PSEFS	44	24580	2	2	2	2	0.47	Glutamate/aspartate AE
1322	1	db$2	2::C3K390|C3K390_PSEFS	44	18447	2	1	2	1	0.29	Zn_protease domain-cc
1323	1	db$2	2::C3JY27|KDSB_PSEFS	44	27609	3	2	3	2	0.41	3-deoxy-manno-octulos
1324	1	db$2	2::C3K047|C3K047_PSEFS	44	16614	2	1	2	1	0.32	Putative putative heat s
1325	1	db$2	2::C3JYP6|C3JYP6_PSEFS	44	10477	3	3	2	2	1.43	YCII domain-containing
1326	1	db$2	2::C3K220|C3K220_PSEFS	44	136725	1	1	1	1	0.04	RecBCD enzyme subuni
1327	1	db$2	2::C3K7G1|C3K7G1_PSEFS	44	37563	2	2	2	2	0.29	Cell shape protein MreC
1328	1	db$2	2::C3KB11|C3KB11_PSEFS	44	38044	2	2	2	2	0.28	Putative AraC family re
1329	1	db$2	2::C3KCR9|PTH_PSEFS	44	21041	2	2	2	2	0.56	Peptidyl-tRNA hydrolase
1330	1	db$2	2::C3K574|C3K574_PSEFS	43	36116	2	2	2	2	0.30	Putative oxidoreductase
1331	1	db$2	2::C3K088|C3K088_PSEFS	43	20655	4	2	3	2	0.57	Putative isochorismatas
1332	1	db$2	2::C3K8T8|C3K8T8_PSEFS	43	14354	2	1	2	1	0.38	Sec-independent protei
1333	1	db$2	2::C3K586|C3K586_PSEFS	43	21064	4	4	4	4	1.43	ADP compounds hydrol
1334	1	db$2	2::C3JZ24|C3JZ24_PSEFS	43	24729	3	3	3	3	0.77	Putative GntR-family re
1335	1	db$2	2::C3K3L1|C3K3L1_PSEFS	43	23430	1	1	1	1	0.22	Uncharacterized proteir
1336	1	db$2	2::C3JYQ4|C3JYQ4_PSEFS	43	52375	2	2	1	1	0.09	Arginine/ornithine antip
1337	1	db$2	2::C3K353|C3K353_PSEFS	43	44910	1	1	1	1	0.11	Putative aminotransfera
1338	1	db$2	2::C3K0T6|RRAAH_PSEFS	43	17479	2	2	1	1	0.31	Putative 4-hydroxy-4-m
1339	1	db$2	2::C3K735|MSRB_PSEFS	43	14471	1	1	1	1	0.38	Peptide methionine sulf
1340	1	db$2	2::C3KBJ0|C3KBJ0_PSEFS	43	16933	3	3	3	3	1.28	[Ribosomal protein S18
1341	1	db$2	2::C3K301|C3K301_PSEFS	43	28901	3	2	3	2	0.39	Putative dehydrogenase
1342	1	db$2	2::C3K0P2|C3K0P2_PSEFS	43	35618	3	2	3	2	0.30	Pseudouridine synthase

↑Family	M	DB	Accession	Score	Mass	Matches	Match(sig)	Sequences	Seq(sig)	emPAI	Description
1343	1	db$2	2::C3K277|C3K277_PSEFS	43	60895	2	2	2	2	0.17	DNA repair protein Recl
1344	1	db$2	2::C3K7W1|C3K7W1_PSEFS	43	12229	2	2	2	2	1.14	Putative lipoprotein OS
1345	1	db$2	2::C3KBM1|C3KBM1_PSEFS	43	29989	2	2	2	2	0.37	Glycerol uptake facilitat
1346	1	db$2	2::C3K3L0|METXS_PSEFS	43	41664	2	1	2	1	0.12	Homoserine O-succinylt
1347	1	db$2	2::C3JZW1|C3JZW1_PSEFS	43	36968	1	1	1	1	0.14	Putative cobalamin-binc
1348	1	db$2	2::C3K098|C3K098_PSEFS	42	23000	1	1	1	1	0.23	Putative glutathione S-t
1349	1	db$2	2::C3KAD1|C3KAD1_PSEFS	42	32401	1	1	1	1	0.16	Probable 4-deoxy-4-for
1350	1	db$2	2::C3K1N1|C3K1N1_PSEFS	42	33097	3	3	3	3	0.53	Protein-export membra
1351	1	db$2	2::C3K2L0|C3K2L0_PSEFS	42	55816	2	1	2	1	0.09	Apolipoprotein N-acyltra
1352	1	SwissProt_HUMAN$	3::P62263|RS14_HUMAN	42	16263	1	1	1	1	0.33	40S ribosomal protein S
1353	1	db$2	2::C3K1G1|C3K1G1_PSEFS	42	48709	1	1	1	1	0.10	tRNA modification GTPa
1354	1	db$2	2::C3KAR4|C3KAR4_PSEFS	42	11430	1	1	1	1	0.50	Uncharacterized proteir
1355	1	db$2	2::C3KDK7|C3KDK7_PSEFS	42	24465	3	1	3	1	0.21	Putative ABC transport
1356	1	db$2	2::C3K7M1|C3K7M1_PSEFS	42	62712	2	2	2	2	0.16	Putative dipeptidase OS
1357	1	db$2	2::C3KBX7|C3KBX7_PSEFS	42	23636	1	1	1	1	0.22	Putative metallo-beta-la
1358	1	db$2	2::C3K2M3|C3K2M3_PSEFS	42	37518	3	2	3	2	0.29	Putative peptidoglycan-
1359	1	db$2	2::C3K7K5|C3K7K5_PSEFS	42	12541	1	1	1	1	0.45	Uncharacterized proteir
1360	1	db$2	2::C3K0J2|C3K0J2_PSEFS	42	17877	1	1	1	1	0.30	Flagellar protein FliL OS
1361	1	db$2	2::C3K6E8|C3K6E8_PSEFS	42	27518	1	1	1	1	0.19	Putative GntR-family re
1362	1	db$2	2::C3KE16|C3KE16_PSEFS	42	37004	4	4	4	4	0.67	Putative chemotaxis-rel
1363	1	db$2	2::C3KB02|C3KB02_PSEFS	42	42163	2	1	2	1	0.12	Putative exported prote
1364	1	db$2	2::C3K4T5|C3K4T5_PSEFS	42	30974	2	2	2	2	0.36	Putative hydrolase OS=
1365	1	SwissProt_HUMAN$	3::P46781|RS9_HUMAN	41	22578	3	2	3	2	0.52	40S ribosomal protein S
1366	1	db$2	2::C3K9E9|C3K9E9_PSEFS	41	21363	3	2	1	1	0.25	Putative TetR-family re
1367	1	SwissProt_HUMAN$	3::Q8NHW5|RLA0L_HUMAN	41	34343	1	1	1	1	0.15	60S acidic ribosomal pr
1368	1	db$2	2::C3K3K9|C3K3K9_PSEFS	41	21575	2	2	1	1	0.24	Conserved hypothetical
1369	1	SwissProt_HUMAN$	3::P62826|RAN_HUMAN	41	24408	1	1	1	1	0.21	GTP-binding nuclear pro
1370	1	db$2	2::C3K1L7|RLMN_PSEFS	41	42342	4	3	4	3	0.40	Dual-specificity RNA me
1371	1	db$2	2::C3JZ60|C3JZ60_PSEFS	41	14055	1	1	1	1	0.39	Uncharacterized proteir
1372	1	db$2	2::C3K5Q6|C3K5Q6_PSEFS	41	68754	1	1	1	1	0.07	Putative hydrolase OS=
1373	1	db$2	2::C3K7M5|C3K7M5_PSEFS	41	15789	1	1	1	1	0.34	Uncharacterized proteir
1374	1	db$2	2::C3K068|C3K068_PSEFS	41	65454	3	3	3	3	0.24	Putative asparagine syr
1375	1	SwissProt_HUMAN$	3::Q8TCB0|IFI44_HUMAN	41	50459	1	1	1	1	0.10	Interferon-induced prot
1376	1	db$2	2::C3KDC7|C3KDC7_PSEFS	40	29930	1	1	1	1	0.17	Release factor glutamin
1377	1	db$2	2::C3K326|C3K326_PSEFS	40	35169	3	1	3	1	0.14	4-hydroxythreonine-4-p
1378	1	db$2	2::C3K6V3|HIS6_PSEFS	40	27111	2	2	2	2	0.42	Imidazole glycerol phos
1379	1	db$2	2::C3K1M2|HSCB_PSEFS	40	20288	1	1	1	1	0.26	Co-chaperone protein H
1380	1	db$2	2::C3K6T3|C3K6T3_PSEFS	40	23166	2	1	2	1	0.22	Thiol:disulfide interchar
1381	1	SwissProt_HUMAN$	3::P62888|RL30_HUMAN	40	12776	3	3	3	3	1.98	60S ribosomal protein L
1382	1	db$2	2::C3K6R7|C3K6R7_PSEFS	40	41832	2	2	2	2	0.25	Serine/threonine transp

↑Family	M	DB	Accession	Score	Mass	Matches	Match(sig)	Sequences	Seq(sig)	emPAI	Description
1383	1	db$2	2::C3K0N0|C3K0N0_PSEFS	40	41535	1	1	1	1	0.12	Endolytic murein transg
1384	1	db$2	2::C3K7V7|C3K7V7_PSEFS	40	18111	1	1	1	1	0.30	Uncharacterized proteir
1385	1	db$2	2::C3K0M7|C3K0M7_PSEFS	40	28601	2	2	2	2	0.39	Putative deoxyribonucle
1386	1	db$2	2::C3JZ82|C3JZ82_PSEFS	40	37007	1	1	1	1	0.14	Putative dehydrogenase
1387	1	db$2	2::C3K103|C3K103_PSEFS	40	23527	1	1	1	1	0.22	Phosphoribosylglycinam
1388	1	db$2	2::C3JZN5|RL35_PSEFS	40	7363	2	2	1	1	0.87	50S ribosomal protein L
1389	1	db$2	2::C3KDC1|C3KDC1_PSEFS	40	29143	1	1	1	1	0.18	Putative oxidoreductase
1390	1	db$2	2::C3K6P0|C3K6P0_PSEFS	40	15266	2	2	2	2	0.84	Uncharacterized proteir
1391	1	db$2	2::C3K4C0|C3K4C0_PSEFS	39	9802	1	1	1	1	0.60	DNA-binding protein HU
1392	1	SwissProt_HUMAN$	3::P63220|RS21_HUMAN	39	9106	1	1	1	1	0.66	40S ribosomal protein S
1393	1	db$2	2::C3K0S0|C3K0S0_PSEFS	39	12378	2	1	2	1	0.45	Putative DNA-binding p
1394	1	db$2	2::C3JXG7|C3JXG7_PSEFS	39	101527	2	1	2	1	0.05	Uncharacterized proteir
1395	1	db$2	2::C3K8Z7|C3K8Z7_PSEFS	39	10906	3	3	3	3	2.58	Uncharacterized proteir
1396	1	db$2	2::C3KAL0|C3KAL0_PSEFS	39	43962	1	1	1	1	0.11	PlsC domain-containing
1397	1	SwissProt_HUMAN$	3::O14827|RGRF2_HUMAN	39	140675	3	2	2	2	0.07	Ras-specific guanine nu
1398	1	SwissProt_HUMAN$	3::P31153|METK2_HUMAN	39	43633	1	1	1	1	0.11	S-adenosylmethionine s
1399	1	db$2	2::C3K320|C3K320_PSEFS	39	34836	2	2	2	2	0.31	Uncharacterized proteir
1400	1	SwissProt_HUMAN$	3::Q13136|LIPA1_HUMAN	39	135695	1	1	1	1	0.04	Liprin-alpha-1 OS=Hom
1401	1	db$2	2::C3KE39|C3KE39_PSEFS	39	18976	3	3	3	3	1.10	D,D-heptose 1,7-bispho
1402	1	SwissProt_HUMAN$	3::P62277|RS13_HUMAN	39	17212	2	2	2	2	0.72	40S ribosomal protein S
1403	1	db$2	2::C3K820|C3K820_PSEFS	39	26990	2	2	2	2	0.42	Putative ABC transport
1404	1	db$2	2::C3K2Z2|C3K2Z2_PSEFS	39	34551	1	1	1	1	0.15	Bifunctional ligase/repr
1405	1	db$2	2::C3K6E9|C3K6E9_PSEFS	39	41977	2	2	2	2	0.25	Phosphatidylcholine-hy
1406	1	db$2	2::C3K0X1|C3K0X1_PSEFS	39	10638	1	1	1	1	0.54	Uncharacterized proteir
1407	1	SwissProt_HUMAN$	3::A6NJ78|MET15_HUMAN	39	46092	1	1	1	1	0.11	12S rRNA N4-methylcyt
1408	1	db$2	2::C3K1U6|C3K1U6_PSEFS	38	74730	1	1	1	1	0.07	Cytochrome bo(3) ubiq
1409	1	db$2	2::C3K710|DCD_PSEFS	38	21231	4	4	4	4	1.42	dCTP deaminase OS=Ps
1410	1	db$2	2::C3K2P8|C3K2P8_PSEFS	38	24358	2	2	2	2	0.47	Membrane-related prot
1411	1	db$2	2::C3K654|C3K654_PSEFS	38	34290	2	1	2	1	0.15	2-dehydropantoate 2-re
1412	1	db$2	2::C3JZZ4|C3JZZ4_PSEFS	38	23102	1	1	1	1	0.23	Putative glutathione S t
1413	1	db$2	2::C3KAL9|C3KAL9_PSEFS	38	64520	1	1	1	1	0.08	Histidine kinase OS=Ps
1414	1	db$2	2::C3K4U7|C3K4U7_PSEFS	38	28919	2	2	2	2	0.38	Putative amidotransfera
1415	1	db$2	2::C3K1M7|C3K1M7_PSEFS	38	27957	1	1	1	1	0.18	Serine O-acetyltransfer
1416	1	db$2	2::C3K4T8|C3K4T8_PSEFS	38	33681	2	2	2	2	0.32	Putative AraC-family re
1417	1	db$2	2::C3K911|C3K911_PSEFS	38	29460	1	1	1	1	0.17	Putative ABC transport
1418	1	db$2	2::C3K6Y8|C3K6Y8_PSEFS	38	34148	5	5	5	5	0.99	GTPase Era OS=Pseudo
1419	1	db$2	2::C3KE56|C3KE56_PSEFS	38	59021	3	2	3	2	0.17	Glutamate--cysteine lig
1420	1	db$2	2::C3K413|C3K413_PSEFS	38	22663	1	1	1	1	0.23	Putative ABC transport
1421	1	SwissProt_HUMAN$	3::Q14168|MPP2_HUMAN	38	64540	1	1	1	1	0.08	MAGUK p55 subfamily r
1422	1	db$2	2::C3K3U4|C3K3U4_PSEFS	38	23278	1	1	1	1	0.22	Putative TetR-family re

↑Family	M	DB	Accession	Score	Mass	Matches	Match(sig)	Sequences	Seq(sig)	emPAI	Description
1423	1	db$2	2::C3K706|PIMT_PSEFS	37	23451	3	1	2	1	0.22	Protein-L-isoaspartate (
1424	1	db$2	2::C3K9P4|C3K9P4_PSEFS	37	59261	3	2	2	2	0.17	Putative ATP-binding cc
1425	1	db$2	2::C3K473|DUT_PSEFS	37	16022	1	1	1	1	0.34	Deoxyuridine 5'-triphos
1426	1	db$2	2::C3K6K2|IHFB_PSEFS	37	11021	3	2	2	2	1.32	Integration host factor
1427	1	SwissProt_HUMAN$	3::P33992|MCM5_HUMAN	37	82233	1	1	1	1	0.06	DNA replication licensin
1428	1	SwissProt_HUMAN$	3::Q9H1K0|RBNS5_HUMAN	37	88815	2	1	2	1	0.05	Rabenosyn-5 OS=Homo
1429	1	SwissProt_HUMAN$	3::Q6ZSJ9|SHSA6_HUMAN	37	55729	1	1	1	1	0.09	Protein shisa-6 OS=Hor
1430	1	db$2	2::C3KDJ8|C3KDJ8_PSEFS	37	23307	2	2	2	2	0.50	Methyltransf_25 domai
1431	1	SwissProt_HUMAN$	3::P26641|EF1G_HUMAN	37	50087	2	2	2	2	0.21	Elongation factor 1-gan
1432	1	db$2	2::C3K637|C3K637_PSEFS	37	56441	1	1	1	1	0.09	Putative peptide ABC tr
1433	1	db$2	2::C3K3T7|C3K3T7_PSEFS	37	39982	1	1	1	1	0.13	Putrescine ABC transpo
1434	1	db$2	2::C3KE45|C3KE45_PSEFS	37	49639	4	3	4	3	0.33	Trk system potassium u
1435	1	db$2	2::C3JYH9|C3JYH9_PSEFS	37	47184	1	1	1	1	0.11	Protein kinase domain-
1436	1	db$2	2::C3JXY3|C3JXY3_PSEFS	37	18788	1	1	1	1	0.28	6,7-dimethyl-8-ribityllu
1437	1	SwissProt_HUMAN$	3::Q58FF8|H90B2_HUMAN	37	44321	1	1	1	1	0.11	Putative heat shock pro
1438	1	SwissProt_HUMAN$	3::P06748|NPM_HUMAN	37	32555	2	1	2	1	0.16	Nucleophosmin OS=Hor
1439	1	db$2	2::C3K4F9|C3K4F9_PSEFS	37	31102	1	1	1	1	0.16	Putative regulatory prot
1440	1	db$2	2::C3K9H2|C3K9H2_PSEFS	37	39760	4	4	3	3	0.43	Putrescine ABC transpo
1441	1	SwissProt_HUMAN$	3::Q7Z6Z7|HUWE1_HUMAN	37	481589	3	2	2	1	0.01	E3 ubiquitin-protein liga
1442	1	db$2	2::C3K3R7|RPIA_PSEFS	37	23281	3	3	3	3	0.83	Ribose-5-phosphate iso
1443	1	db$2	2::C3K7A2|C3K7A2_PSEFS	37	33841	1	1	1	1	0.15	Putative membrane pro
1444	1	db$2	2::C3K5L6|C3K5L6_PSEFS	37	16214	5	4	4	3	1.37	Putative phosphorous c
1445	1	db$2	2::C3KEB0|C3KEB0_PSEFS	36	36597	3	2	2	1	0.14	Putative amino acid AB
1446	1	db$2	2::C3K6W8|C3K6W8_PSEFS	36	16611	1	1	1	1	0.32	3-dehydroquinate dehy
1447	1	db$2	2::C3K0D5|MINE_PSEFS	36	9531	2	2	2	2	1.63	Cell division topological
1448	1	db$2	2::C3K2M6|RLMH_PSEFS	36	17669	1	1	1	1	0.30	Ribosomal RNA large su
1449	1	db$2	2::C3JZ34|C3JZ34_PSEFS	36	26312	1	1	1	1	0.20	Probable short chain de
1450	1	SwissProt_HUMAN$	3::Q86YF9|DZIP1_HUMAN	36	98602	1	1	1	1	0.05	Zinc finger protein DZII
1451	1	SwissProt_HUMAN$	3::P62753|RS6_HUMAN	36	28663	1	1	1	1	0.18	40S ribosomal protein S
1452	1	db$2	2::C3JZS9|C3JZS9_PSEFS	36	35591	1	1	1	1	0.14	Cbb3-type cytochrome
1453	1	db$2	2::C3K2K7|C3K2K7_PSEFS	36	37713	1	1	1	1	0.13	PhoH domain-containin
1454	1	db$2	2::C3K172|C3K172_PSEFS	36	12407	1	1	1	1	0.45	ABM domain-containing
1455	1	db$2	2::C3KEA2|C3KEA2_PSEFS	36	30824	1	1	1	1	0.16	Putative inner membran
1456	1	db$2	2::C3K678|C3K678_PSEFS	36	45414	1	1	1	1	0.11	Cytosine deaminase OS
1457	1	db$2	2::C3JYU3|C3JYU3_PSEFS	36	75598	3	2	3	2	0.13	Histidine kinase OS=Ps
1458	1	db$2	2::C3KAC0|C3KAC0_PSEFS	35	40546	2	2	2	2	0.26	Butyryl-CoA dehydroge
1459	1	db$2	2::C3K2Q3|C3K2Q3_PSEFS	35	23107	1	1	1	1	0.23	Putative hydrolase OS=
1460	1	db$2	2::C3K0K9|C3K0K9_PSEFS	35	37421	2	1	1	1	0.13	Putative NADP-depende
1461	1	db$2	2::C3K537|C3K537_PSEFS	35	39684	1	1	1	1	0.13	Putative membrane pro
1462	1	SwissProt_HUMAN$	3::Q5JTZ9|SYAM_HUMAN	35	107273	2	2	1	1	0.05	Alanine--tRNA ligase, m

↑Family	M	DB	Accession	Score	Mass	Matches	Match(sig)	Sequences	Seq(sig)	emPAI	Description
1463	1	SwissProt_HUMAN$	3::Q7RTV0|PHF5A_HUMAN	35	12397	1	1	1	1	0.45	PHD finger-like domain-
1464	1	db$2	2::C3K598|C3K598_PSEFS	35	34258	2	2	2	2	0.32	Curved DNA-binding pr
1465	1	db$2	2::C3K2R8|NUSB_PSEFS	35	18764	1	1	1	1	0.28	Transcription antitermi
1466	1	db$2	2::C3KAM0|C3KAM0_PSEFS	35	50872	2	1	2	1	0.10	Putative two-componen
1467	1	db$2	2::C3K3S9|C3K3S9_PSEFS	35	35576	1	1	1	1	0.14	Hydrolase_4 domain-co
1468	1	db$2	2::C3KDF9|C3KDF9_PSEFS	35	17633	2	2	2	2	0.70	CinA domain-containing
1469	1	db$2	2::C3K3M5|C3K3M5_PSEFS	35	50101	4	4	4	4	0.46	Putative peptidase OS=
1470	1	db$2	2::C3K6R2|C3K6R2_PSEFS	35	29309	1	1	1	1	0.17	Putative shikimate deh
1471	1	db$2	2::C3K3E3|C3K3E3_PSEFS	35	10928	1	1	1	1	0.53	Uncharacterized protei
1472	1	db$2	2::C3K428|C3K428_PSEFS	35	10726	1	1	1	1	0.54	Uncharacterized protei
1473	1	db$2	2::C3K4Q6|C3K4Q6_PSEFS	35	39864	1	1	1	1	0.13	Putative lipoprotein OS
1474	1	SwissProt_HUMAN$	3::Q70Z53|F10C1_HUMAN	35	37525	1	1	1	1	0.13	Protein FRA10AC1 OS=
1475	1	db$2	2::C3K3N1|C3K3N1_PSEFS	35	52088	2	2	2	2	0.20	Aldehyde dehydrogenas
1476	1	db$2	2::C3K0P5|C3K0P5_PSEFS	35	35572	2	2	2	2	0.30	Uncharacterized protei
1477	1	db$2	2::C3KAX2|C3KAX2_PSEFS	34	79149	1	1	1	1	0.06	Histidine kinase OS=Ps
1478	1	db$2	2::C3K6X3|C3K6X3_PSEFS	34	12829	1	1	1	1	0.43	Putative translation init
1479	1	db$2	2::C3K1R1|C3K1R1_PSEFS	34	13822	2	1	2	1	0.40	bPH_1 domain-containi
1480	1	db$2	2::C3KAB2|C3KAB2_PSEFS	34	13873	1	1	1	1	0.40	Putative two-componen
1481	1	db$2	2::C3K265|C3K265_PSEFS	34	29739	2	2	2	2	0.37	Dihydropteroate syntha
1482	1	SwissProt_HUMAN$	3::O96017|CHK2_HUMAN	34	60877	2	2	2	2	0.17	Serine/threonine-protei
1483	1	db$2	2::C3KCJ3|C3KCJ3_PSEFS	34	30975	2	2	1	1	0.16	Usp domain-containing
1484	1	db$2	2::C3K536|C3K536_PSEFS	34	22752	1	1	1	1	0.23	DUF4174 domain-conta
1485	1	db$2	2::C3JZ30|C3JZ30_PSEFS	34	12225	1	1	1	1	0.46	DUF2025 domain-conta
1486	1	db$2	2::C3JXL6|C3JXL6_PSEFS	34	41978	1	1	1	1	0.12	Bcr/CflA family efflux tr
1487	1	db$2	2::C3K1F1|ATPL_PSEFS	34	8603	3	2	2	1	1.89	ATP synthase subunit c
1488	1	db$2	2::C3K620|C3K620_PSEFS	34	22214	1	1	1	1	0.24	LexA repressor OS=Pse
1489	1	db$2	2::C3JZ52|C3JZ52_PSEFS	34	18715	1	1	1	1	0.28	Uncharacterized protei
1490	1	SwissProt_HUMAN$	3::P28072|PSB6_HUMAN	34	25341	1	1	1	1	0.20	Proteasome subunit bet
1491	1	SwissProt_HUMAN$	3::Q6ZMT4|KDM7A_HUMAN	33	106489	2	1	2	1	0.05	Lysine-specific demeth
1492	1	db$2	2::C3K493|C3K493_PSEFS	33	16495	2	2	2	2	0.76	Uncharacterized protei
1493	1	db$2	2::C3KBX4|C3KBX4_PSEFS	33	59754	2	2	2	2	0.17	Putative membrane pro
1494	1	db$2	2::C3KEB9|C3KEB9_PSEFS	33	11110	1	1	1	1	0.52	Putative lipoprotein OS
1495	1	SwissProt_HUMAN$	3::P61247|RS3A_HUMAN	33	29926	2	2	2	2	0.37	40S ribosomal protein S
1496	1	db$2	2::C3K340|C3K340_PSEFS	33	72802	1	1	1	1	0.07	DNA primase OS=Pseu
1497	1	db$2	2::C3K059|TRHO_PSEFS	33	35401	4	2	4	2	0.31	tRNA uridine(34) hydro
1498	1	SwissProt_HUMAN$	3::P27216|ANX13_HUMAN	33	35393	1	1	1	1	0.14	Annexin A13 OS=Homo
1499	1	db$2	2::C3K9H5|C3K9H5_PSEFS	33	33119	1	1	1	1	0.15	Putative LysR-family tra
1500	1	db$2	2::C3K1F5|C3K1F5_PSEFS	33	29048	1	1	1	1	0.18	Putative chromosome p
1501	1	db$2	2::C3K1G4|RL34_PSEFS	33	5064	1	1	1	1	1.41	50S ribosomal protein L
1502	1	SwissProt_HUMAN$	3::Q15075|EEA1_HUMAN	33	162367	1	1	1	1	0.03	Early endosome antiger

↑Family	M	DB	Accession	Score	Mass	Matches	Match(sig)	Sequences	Seq(sig)	emPAI	Description
1503	1	db$2	2::C3K488|C3K488_PSEFS	33	15239	1	1	1	1	0.36	Biopolymer transport pı
1504	1	SwissProt_HUMAN$	3::Q96RL7|VP13A_HUMAN	33	360047	2	2	2	2	0.03	Vacuolar protein sorting
1505	1	db$2	2::C3K1D4|C3K1D4_PSEFS	33	64734	2	2	2	2	0.16	Uncharacterized protein
1506	1	db$2	2::C3K8Z2|C3K8Z2_PSEFS	33	25669	1	1	1	1	0.20	High-affinity branched-(
1507	1	db$2	2::C3K200|C3K200_PSEFS	33	32052	1	1	1	1	0.16	Cat operon regulatory p
1508	1	db$2	2::C3K439|C3K439_PSEFS	33	25827	2	2	2	2	0.44	Putative hydrolase OS=
1509	1	SwissProt_HUMAN$	3::O94817|ATG12_HUMAN	33	15104	1	1	1	1	0.36	Ubiquitin-like protein A'
1510	1	SwissProt_HUMAN$	3::P19338|NUCL_HUMAN	33	76568	1	1	1	1	0.06	Nucleolin OS=Homo sap
1511	1	db$2	2::C3K8K6|C3K8K6_PSEFS	33	15117	1	1	1	1	0.36	Putative transport-relat
1512	1	db$2	2::C3K753|C3K753_PSEFS	32	30652	3	3	2	2	0.36	Putative hydrolase OS=
1513	1	SwissProt_HUMAN$	3::P62258|1433E_HUMAN	32	29155	2	2	2	2	0.38	14-3-3 protein epsilon (
1514	1	db$2	2::C3KAB9|C3KAB9_PSEFS	32	40944	1	1	1	1	0.12	Putative acetyl-CoA ace
1515	1	db$2	2::C3KDM8|C3KDM8_PSEFS	32	57105	1	1	1	1	0.09	HDOD domain-containir
1516	1	SwissProt_HUMAN$	3::P63244|RACK1_HUMAN	32	35055	1	1	1	1	0.14	Receptor of activated p
1517	1	db$2	2::C3K8Z0|C3K8Z0_PSEFS	32	32439	1	1	1	1	0.16	UPF0276 protein PFLU_
1518	1	db$2	2::C3K451|C3K451_PSEFS	32	76463	3	2	3	2	0.13	ATP-dependent DNA he
1519	1	db$2	2::C3KDJ5|C3KDJ5_PSEFS	32	38272	1	1	1	1	0.13	Putative lipoprotein OS=
1520	1	db$2	2::C3K0X2|C3K0X2_PSEFS	32	81009	1	1	1	1	0.06	Patatin-like phospholipa
1521	1	db$2	2::C3K223|C3K223_PSEFS	32	37863	1	1	1	1	0.13	Protein-methionine-sulf
1522	1	db$2	2::C3K6Z6|C3K6Z6_PSEFS	32	13580	1	1	1	1	0.41	Putaitve lipoprotein OS=
1523	1	SwissProt_HUMAN$	3::Q8NBF4|YG006_HUMAN	32	16172	2	1	1	1	0.33	Putative uncharacterize
1524	1	db$2	2::C3K0H5|C3K0H5_PSEFS	32	25946	1	1	1	1	0.20	Putative chemotaxis pro
1525	1	db$2	2::C3JY26|C3JY26_PSEFS	32	17211	2	1	2	1	0.31	Protein-tyrosine-phospł
1526	1	SwissProt_HUMAN$	3::Q13454|TUSC3_HUMAN	32	39650	1	1	1	1	0.13	Tumor suppressor cand
1527	1	db$2	2::C3JY34|C3JY34_PSEFS	31	100208	1	1	1	1	0.05	Histidine kinase OS=Ps
1528	1	db$2	2::C3K0B2|C3K0B2_PSEFS	31	20071	1	1	1	1	0.26	Putative membrane pro
1529	1	db$2	2::C3K8Z3|C3K8Z3_PSEFS	31	28227	1	1	1	1	0.18	High-affinity branched-(
1530	1	db$2	2::C3KCP9|C3KCP9_PSEFS	31	30268	1	1	1	1	0.17	Lipopolysaccharide core
1531	1	SwissProt_HUMAN$	3::P04080|CYTB_HUMAN	31	11133	1	1	1	1	0.52	Cystatin-B OS=Homo s
1532	1	db$2	2::C3K9Q1|C3K9Q1_PSEFS	31	28823	1	1	1	1	0.18	Methyltransf_11 domair
1533	1	db$2	2::C3KDY6|C3KDY6_PSEFS	31	17128	1	1	1	1	0.31	Uncharacterized protein
1534	1	db$2	2::C3KAN9|C3KAN9_PSEFS	31	28318	1	1	1	1	0.18	Putative aromatic-hydro
1535	1	db$2	2::C3KDG3|C3KDG3_PSEFS	31	15637	1	1	1	1	0.35	Putative exported prote
1536	1	SwissProt_HUMAN$	3::O75197|LRP5_HUMAN	31	179030	3	1	1	1	0.03	Low-density lipoprotein
1537	1	db$2	2::C3JZR9|C3JZR9_PSEFS	31	27415	2	2	2	2	0.41	Putative dehydrogenase
1538	1	SwissProt_HUMAN$	3::O75251|NDUS7_HUMAN	31	23548	1	1	1	1	0.22	NADH dehydrogenase [
1539	1	db$2	2::C3K0G4|C3K0G4_PSEFS	31	29541	1	1	1	1	0.17	MOSC domain-containir
1540	1	db$2	2::C3K1M6|C3K1M6_PSEFS	31	24782	1	1	1	1	0.21	Uncharacterized protein
1541	1	db$2	2::C3KCP6|C3KCP6_PSEFS	31	38240	1	1	1	1	0.13	ADP-heptose--LPS hept
1542	1	db$2	2::C3JYS4|C3JYS4_PSEFS	31	16520	2	2	2	2	0.76	Tol-Pal system protein

↑Family	M	DB	Accession	Score	Mass	Matches	Match(sig)	Sequences	Seq(sig)	emPAI	Description
1543	1	db$2	2::C3K5B9|C3K5B9_PSEFS	31	60269	1	1	1	1	0.08	Dipeptide ABC transpor
1544	1	db$2	2::C3K136|C3K136_PSEFS	31	31053	3	3	3	3	0.58	Uncharacterized protein
1545	1	SwissProt_HUMAN$	3::P17480|UBF1_HUMAN	31	89350	2	1	2	1	0.05	Nucleolar transcription
1546	1	db$2	2::C3KDV4|RSGA_PSEFS	31	37296	2	1	2	1	0.13	Small ribosomal subuni
1547	1	db$2	2::C3K7C5|C3K7C5_PSEFS	31	40067	3	2	3	2	0.27	Histidine kinase OS=Ps
1548	1	db$2	2::Q8VMR3|HUTH_PSEFS	31	54209	1	1	1	1	0.09	Histidine ammonia-lyas
1549	1	db$2	2::C3K5A2|URE2_PSEFS	31	11072	1	1	1	1	0.52	Urease subunit beta OS
1550	1	SwissProt_HUMAN$	3::Q9H0U4|RAB1B_HUMAN	31	22157	1	1	1	1	0.24	Ras-related protein Rab
1551	1	SwissProt_HUMAN$	3::P02741|CRP_HUMAN	31	25023	1	1	1	1	0.21	C-reactive protein OS=
1552	1	db$2	2::C3JY67|C3JY67_PSEFS	31	16861	1	1	1	1	0.32	Phosphatase NudJ OS=
1553	1	db$2	2::C3K1D1|C3K1D1_PSEFS	31	32188	2	2	2	2	0.34	Protein HflC OS=Pseud
1554	1	db$2	2::C3K0A7|ALLC_PSEFS	31	36686	1	1	1	1	0.14	Probable allantoicase O
1555	1	SwissProt_HUMAN$	3::P63173|RL38_HUMAN	31	8213	3	3	2	2	2.06	60S ribosomal protein L
1556	1	SwissProt_HUMAN$	3::P08397|HEM3_HUMAN	30	39306	1	1	1	1	0.13	Porphobilinogen deamir
1557	1	db$2	2::C3KBI7|C3KBI7_PSEFS	30	26715	2	2	2	2	0.42	Uncharacterized protein
1558	1	SwissProt_HUMAN$	3::O15078|CE290_HUMAN	30	290207	2	1	2	1	0.02	Centrosomal protein of
1559	1	SwissProt_HUMAN$	3::Q69YQ0|CYTSA_HUMAN	30	124525	2	1	1	1	0.04	Cytospin-A OS=Homo s
1560	1	db$2	2::C3K2E8|C3K2E8_PSEFS	30	37117	1	1	1	1	0.14	Putative cytochrome ox
1561	1	db$2	2::C3K3Y1|C3K3Y1_PSEFS	30	14748	1	1	1	1	0.37	Flagellar protein FliL OS
1562	1	db$2	2::C3KB17|C3KB17_PSEFS	30	51104	1	1	1	1	0.10	Outer membrane comp
1563	1	db$2	2::C3KBU2|C3KBU2_PSEFS	30	56055	2	1	2	1	0.09	Putative histidine amm
1564	1	db$2	2::C3JY38|C3JY38_PSEFS	30	16812	3	2	3	2	0.75	Putative thioredoxin-rel
1565	1	db$2	2::C3K5I1|C3K5I1_PSEFS	30	43688	1	1	1	1	0.11	Putative phage integras
1566	1	SwissProt_HUMAN$	3::Q9Y265|RUVB1_HUMAN	30	50196	1	1	1	1	0.10	RuvB-like 1 OS=Homo
1567	1	SwissProt_HUMAN$	3::Q8N9V3|WSDU1_HUMAN	30	52783	1	1	1	1	0.09	WD repeat, SAM and U
1568	1	SwissProt_HUMAN$	3::Q14240|IF4A2_HUMAN	30	46373	1	1	1	1	0.11	Eukaryotic initiation fac
1569	1	db$2	2::C3KBZ5|C3KBZ5_PSEFS	30	23751	2	1	2	1	0.22	Endonuclease III OS=P
1570	1	db$2	2::C3K3J8|C3K3J8_PSEFS	30	33397	2	1	2	1	0.15	TonB_C domain-contair
1571	1	db$2	2::C3K0N9|C3K0N9_PSEFS	30	21007	2	1	2	1	0.25	7-methyl-GTP pyrophos
1572	1	db$2	2::C3K8U1|C3K8U1_PSEFS	30	15020	1	1	1	1	0.37	Phosphoribosyl-AMP cy
1573	1	db$2	2::C3JY56|C3JY56_PSEFS	30	48640	1	1	1	1	0.10	Replication-associated
1574	1	SwissProt_HUMAN$	3::Q96E52|OMA1_HUMAN	30	60081	1	1	1	1	0.08	Metalloendopeptidase C
1575	1	SwissProt_HUMAN$	3::O60716|CTND1_HUMAN	30	108103	1	1	1	1	0.04	Catenin delta-1 OS=Ho
1576	1	db$2	2::C3K8U0|HIS2_PSEFS	30	12007	2	2	2	2	1.17	Phosphoribosyl-ATP pyr
1577	1	db$2	2::C3K7D7|C3K7D7_PSEFS	30	8979	1	1	1	1	0.67	Putative lipoprotein OS
1578	1	db$2	2::C3K0J0|C3K0J0_PSEFS	30	16514	1	1	1	1	0.33	Flagellar motor switch p
1579	1	db$2	2::C3K1Q1|C3K1Q1_PSEFS	30	59090	1	1	1	1	0.08	Putative methyl accepti
1580	1	db$2	2::C3JYJ2|C3JYJ2_PSEFS	30	27205	1	1	1	1	0.19	Putative lipoprotein OS
1581	1	db$2	2::C3K0H8|C3K0H8_PSEFS	29	29525	2	2	2	2	0.38	Protein phosphatase Ch
1582	1	db$2	2::C3KAS1|C3KAS1_PSEFS	29	34986	2	1	1	1	0.14	Putative 2-hydroxyacid

↑Family	M	DB	Accession	Score	Mass	Matches	Match(sig)	Sequences	Seq(sig)	emPAI	Description
1583	1	db$2	2::C3K458|C3K458_PSEFS	29	47152	1	1	1	1	0.11	D-amino acid dehydrog
1584	1	db$2	2::C3KAX5|C3KAX5_PSEFS	29	33022	1	1	1	1	0.15	Putative LysR family tra
1585	1	db$2	2::C3KB68|C3KB68_PSEFS	29	11363	3	1	3	1	0.51	DUF1883 domain-conta
1586	1	db$2	2::C3K6U7|C3K6U7_PSEFS	29	79141	3	2	3	2	0.13	Putative exported prote
1587	1	db$2	2::C3K595|C3K595_PSEFS	29	10805	1	1	1	1	0.54	Phosphate starvation-ir
1588	1	db$2	2::C3K1L5|C3K1L5_PSEFS	29	34147	3	1	3	1	0.15	Putative DNA-binding p
1589	1	SwissProt_HUMAN$	3::Q9Y231|FUT9_HUMAN	29	42044	1	1	1	1	0.12	4-galactosyl-N-acetylgl
1590	1	db$2	2::C3K7D4|C3K7D4_PSEFS	29	125718	1	1	1	1	0.04	Putative membrane pro
1591	1	db$2	2::C3KAN8|C3KAN8_PSEFS	29	48228	1	1	1	1	0.10	3-carboxy-cis,cis-muco
1592	1	db$2	2::C3K330|GLPE_PSEFS	29	11747	1	1	1	1	0.48	Thiosulfate sulfurtransf
1593	1	db$2	2::C3JZR0|C3JZR0_PSEFS	29	25453	1	1	1	1	0.20	Response regulatory pr
1594	1	db$2	2::C3K4Z1|C3K4Z1_PSEFS	29	72318	1	1	1	1	0.07	Cytochrome C biogenes
1595	1	SwissProt_HUMAN$	3::O60637|TSN3_HUMAN	29	27999	1	1	1	1	0.18	Tetraspanin-3 OS=Hor
1596	1	db$2	2::C3K0N6|PLSX_PSEFS	29	35209	1	1	1	1	0.14	Phosphate acyltransfera
1597	1	db$2	2::C3K020|C3K020_PSEFS	29	23656	2	2	2	2	0.49	Putative hydrolase OS=
1598	1	db$2	2::C3K663|C3K663_PSEFS	29	22341	1	1	1	1	0.23	Putative TetR-family re
1599	1	db$2	2::C3K3Y2|C3K3Y2_PSEFS	29	34182	1	1	1	1	0.15	Putative sinc-binding o
1600	1	db$2	2::C3K621|C3K621_PSEFS	29	26068	2	2	2	2	0.43	Putative TetR-family re
1601	1	db$2	2::C3JXZ4|C3JXZ4_PSEFS	29	40619	1	1	1	1	0.12	Uncharacterized proteir
1602	1	SwissProt_HUMAN$	3::P26373|RL13_HUMAN	29	24247	1	1	1	1	0.21	60S ribosomal protein L
1603	1	db$2	2::C3K0N8|C3K0N8_PSEFS	29	19586	1	1	1	1	0.27	23S rRNA accumulation
1604	1	db$2	2::C3K5C4|C3K5C4_PSEFS	28	17510	1	1	1	1	0.31	SnoaL-like domain-cont
1605	1	db$2	2::C3K9N7|C3K9N7_PSEFS	28	48751	1	1	1	1	0.10	Putative membrane pro
1606	1	db$2	2::C3K7A1|C3K7A1_PSEFS	28	56003	1	1	1	1	0.09	Putative LuxR-family re
1607	1	SwissProt_HUMAN$	3::Q96P48|ARAP1_HUMAN	28	162089	1	1	1	1	0.03	Arf-GAP with Rho-GAP
1608	1	db$2	2::C3K5W0|C3K5W0_PSEFS	28	170278	2	2	2	2	0.06	DUF4329 domain-conta
1609	1	db$2	2::C3JYG6|C3JYG6_PSEFS	28	44622	2	1	2	1	0.11	Peptidase_M14 domain
1610	1	SwissProt_HUMAN$	3::Q9P273|TEN3_HUMAN	28	300760	1	1	1	1	0.02	Teneurin-3 OS=Homo s
1611	1	SwissProt_HUMAN$	3::P54886|P5CS_HUMAN	28	87248	1	1	1	1	0.06	Delta-1-pyrroline-5-car
1612	1	SwissProt_HUMAN$	3::Q9UL62|TRPC5_HUMAN	28	111340	2	2	1	1	0.04	Short transient recepto
1613	1	db$2	2::C3K3K0|YQGF_PSEFS	28	16158	1	1	1	1	0.33	Putative pre-16S rRNA
1614	1	SwissProt_HUMAN$	3::Q9BYK8|HELZ2_HUMAN	28	294465	1	1	1	1	0.02	Helicase with zinc finge
1615	1	db$2	2::C3K609|C3K609_PSEFS	28	73644	2	1	2	1	0.07	Putative lytic transglyc
1616	1	db$2	2::C3K590|C3K590_PSEFS	28	74072	1	1	1	1	0.07	Putative membrane pro
1617	1	db$2	2::C3KD06|C3KD06_PSEFS	28	82856	1	1	1	1	0.06	Putative amidase OS=P
1618	1	db$2	2::C3K6G9|LPXB_PSEFS	28	41468	1	1	1	1	0.12	Lipid-A-disaccharide sy
1619	1	SwissProt_HUMAN$	3::Q96AY4|TTC28_HUMAN	28	270715	2	2	2	2	0.04	Tetratricopeptide repea
1620	1	SwissProt_HUMAN$	3::P10909|CLUS_HUMAN	28	52461	1	1	1	1	0.09	Clusterin OS=Homo sa
1621	1	db$2	2::C3K3R4|C3K3R4_PSEFS	28	24681	3	2	3	2	0.46	Putative hydrolase OS=
1622	1	db$2	2::A4V7R9|A4V7R9_PSEFS	28	24067	1	1	1	1	0.22	Uncharacterized proteir

↑Family	M	DB	Accession	Score	Mass	Matches	Match(sig)	Sequences	Seq(sig)	emPAI	Description
1623	1	db$2	2::C3K286|C3K286_PSEFS	28	9470	1	1	1	1	0.63	Putative exported prote
1624	1	db$2	2::C3K166|C3K166_PSEFS	28	65025	1	1	1	1	0.08	Histidine kinase OS=Ps
1625	1	db$2	2::C3K1Y4|C3K1Y4_PSEFS	28	14766	1	1	1	1	0.37	Putative putative glyox
1626	1	SwissProt_HUMAN$	3::P61353|RL27_HUMAN	28	15788	1	1	1	1	0.34	60S ribosomal protein L
1627	1	SwissProt_HUMAN$	3::Q9H6P5|TASP1_HUMAN	28	44427	1	1	1	1	0.11	Threonine aspartase 1 (
1628	1	db$2	2::C3JYC8|C3JYC8_PSEFS	28	59331	1	1	1	1	0.08	Putative pH adaptation
1629	1	SwissProt_HUMAN$	3::Q99618|CDCA3_HUMAN	28	28981	1	1	1	1	0.18	Cell division cycle-asso
1630	1	db$2	2::C3K4U9|C3K4U9_PSEFS	27	34107	1	1	1	1	0.15	Putative LysR-family re
1631	1	db$2	2::C3KDK6|C3KDK6_PSEFS	27	27451	1	1	1	1	0.19	Putative ABC transport
1632	1	db$2	2::C3JY63|CLPS_PSEFS	27	13780	1	1	1	1	0.40	ATP-dependent Clp prot
1633	1	db$2	2::C3K4F4|C3K4F4_PSEFS	27	25363	1	1	1	1	0.20	Putative acetyltransfera
1634	1	SwissProt_HUMAN$	3::Q5SW79|CE170_HUMAN	27	175187	1	1	1	1	0.03	Centrosomal protein of
1635	1	db$2	2::C3K3M8|C3K3M8_PSEFS	27	36638	1	1	1	1	0.14	AB hydrolase-1 domain
1636	1	db$2	2::C3KE21|C3KE21_PSEFS	27	10230	1	1	1	1	0.57	FAD assembly factor Sc
1637	1	SwissProt_HUMAN$	3::P08476|INHBA_HUMAN	27	47412	1	1	1	1	0.10	Inhibin beta A chain OS
1638	1	db$2	2::C3K975|C3K975_PSEFS	27	21948	1	1	1	1	0.24	Putative glutathione S-t
1639	1	db$2	2::C3JXG5|C3JXG5_PSEFS	27	36156	1	1	1	1	0.14	Putative GFO/IDH/MocA
1640	1	db$2	2::C3K406|C3K406_PSEFS	27	23424	2	2	2	2	0.49	Glyoxalase OS=Pseudo
1641	1	SwissProt_HUMAN$	3::Q8NA82|MARHA_HUMAN	27	90455	1	1	1	1	0.05	Probable E3 ubiquitin-p
1642	1	SwissProt_HUMAN$	3::Q99996|AKAP9_HUMAN	27	452708	3	1	3	1	0.01	A-kinase anchor protein
1643	1	db$2	2::C3KAK0|C3KAK0_PSEFS	27	16987	1	1	1	1	0.32	Uncharacterized protein
1644	1	SwissProt_HUMAN$	3::Q9UPN3|MACF1_HUMAN	27	837787	4	2	4	2	0.01	Microtubule-actin cross-
1645	1	SwissProt_HUMAN$	3::Q9ULQ1|TPC1_HUMAN	27	94086	1	1	1	1	0.05	Two pore calcium chann
1646	1	db$2	2::C3K3P6|C3K3P6_PSEFS	27	19979	1	1	1	1	0.26	Dihydrofolate reductase
1647	1	db$2	2::C3K348|PQQB_PSEFS	27	33093	1	1	1	1	0.15	Coenzyme PQQ synthes
1648	1	SwissProt_HUMAN$	3::Q71RG4|TMUB2_HUMAN	27	33767	1	1	1	1	0.15	Transmembrane and ub
1649	1	SwissProt_HUMAN$	3::Q13077|TRAF1_HUMAN	27	46134	1	1	1	1	0.11	TNF receptor-associated
1650	1	SwissProt_HUMAN$	3::O95153|RIMB1_HUMAN	27	199928	1	1	1	1	0.02	Peripheral-type benzodi
1651	1	db$2	2::C3K0E8|C3K0E8_PSEFS	27	27512	2	2	2	2	0.41	Thiol:disulfide interchar
1652	1	SwissProt_HUMAN$	3::Q6P587|FAHD1_HUMAN	27	24827	1	1	1	1	0.21	Acylpyruvase FAHD1, m
1653	1	db$2	2::C3K3W4|C3K3W4_PSEFS	27	13233	2	1	1	1	0.42	Glycine cleavage syster
1654	1	db$2	2::C3K8K5|C3K8K5_PSEFS	27	64377	1	1	1	1	0.08	Putative transport-relat
1655	1	SwissProt_HUMAN$	3::Q6PI48|SYDM_HUMAN	27	73516	1	1	1	1	0.07	Aspartate--tRNA ligase,
1656	1	SwissProt_HUMAN$	3::Q96N76|HUTU_HUMAN	27	74783	1	1	1	1	0.07	Urocanate hydratase OS
1657	1	SwissProt_HUMAN$	3::Q9NQZ6|ZC4H2_HUMAN	27	26227	1	1	1	1	0.20	Zinc finger C4H2 domai
1658	1	SwissProt_HUMAN$	3::P56749|CLD12_HUMAN	26	27092	1	1	1	1	0.19	Claudin-12 OS=Homo s
1659	1	SwissProt_HUMAN$	3::Q6XUX3|DUSTY_HUMAN	26	105139	1	1	1	1	0.05	Dual serine/threonine a
1660	1	SwissProt_HUMAN$	3::P62906|RL10A_HUMAN	26	24816	2	2	2	2	0.46	60S ribosomal protein L
1661	1	db$2	2::C3KAF4|C3KAF4_PSEFS	26	50981	1	1	1	1	0.10	Putative lipoprotein OS=
1662	1	db$2	2::C3K391|C3K391_PSEFS	26	33913	1	1	1	1	0.15	Putative acyltransferase

↑Family	M	DB	Accession	Score	Mass	Matches	Match(sig)	Sequences	Seq(sig)	emPAI	Description
1663	1	db$2	2::C3KBI5|C3KBI5_PSEFS	26	21236	1	1	1	1	0.25	Putative membrane pro
1664	1	SwissProt_HUMAN$	3::Q14789|GOGB1_HUMAN	26	375790	3	2	3	2	0.03	Golgin subfamily B mer
1665	1	SwissProt_HUMAN$	3::O14917|PCD17_HUMAN	26	126151	1	1	1	1	0.04	Protocadherin-17 OS=H
1666	1	db$2	2::C3K0G1|C3K0G1_PSEFS	26	67986	1	1	1	1	0.07	Putative acetyl-/propior
1667	1	SwissProt_HUMAN$	3::P23508|CRCM_HUMAN	26	92970	1	1	1	1	0.05	Colorectal mutant canc
1668	1	db$2	2::C3K5T2|C3K5T2_PSEFS	26	65173	3	2	3	2	0.16	Putative dehydrogenase
1669	1	db$2	2::C3K4S7|C3K4S7_PSEFS	26	34300	1	1	1	1	0.15	Putative LysR-family re
1670	1	db$2	2::C3K713|C3K713_PSEFS	26	28108	1	1	1	1	0.18	Arginine/ornithine ABC
1671	1	SwissProt_HUMAN$	3::P16885|PLCG2_HUMAN	26	147776	1	1	1	1	0.03	1-phosphatidylinositol 4
1672	1	db$2	2::C3K9J4|C3K9J4_PSEFS	26	15648	1	1	1	1	0.35	Putative DnaK suppress
1673	1	db$2	2::C3K8T6|C3K8T6_PSEFS	26	26078	2	1	2	1	0.20	Ribosomal RNA small su
1674	1	db$2	2::C3K5K8|C3K5K8_PSEFS	26	29952	1	1	1	1	0.17	Putative deoxyribonucle
1675	1	db$2	2::C3K6U5|FETP_PSEFS	26	10399	1	1	1	1	0.56	Probable Fe(2+)-traffic
1676	1	SwissProt_HUMAN$	3::A6NJB7|PRR19_HUMAN	26	38692	1	1	1	1	0.13	Proline-rich protein 19 (
1677	1	SwissProt_HUMAN$	3::Q8N465|D2HDH_HUMAN	26	56380	1	1	1	1	0.09	D-2-hydroxyglutarate d
1678	1	db$2	2::C3KAP0|C3KAP0_PSEFS	26	14934	1	1	1	1	0.37	4-carboxymuconolactor
1679	1	db$2	2::C3K1Y0|C3K1Y0_PSEFS	26	12222	1	1	1	1	0.46	Uncharacterized proteir
1680	1	db$2	2::C3K0Y8|COBT_PSEFS	26	36066	1	1	1	1	0.14	Nicotinate-nucleotide--(
1681	1	SwissProt_HUMAN$	3::P35579|MYH9_HUMAN	26	226392	1	1	1	1	0.02	Myosin-9 OS=Homo sa
1682	1	db$2	2::C3JXW6|C3JXW6_PSEFS	26	64770	1	1	1	1	0.08	Putative exported prote
1683	1	db$2	2::C3K5T5|C3K5T5_PSEFS	26	25405	1	1	1	1	0.20	Carbonic anhydrase OS
1684	1	SwissProt_HUMAN$	3::O00321|ETV2_HUMAN	26	36609	1	1	1	1	0.14	ETS translocation variar
1685	1	SwissProt_HUMAN$	3::Q9P1V8|SAM15_HUMAN	26	77103	2	1	1	1	0.06	Sterile alpha motif dom
1686	1	db$2	2::C3K7A9|C3K7A9_PSEFS	26	33658	1	1	1	1	0.15	Conserved hypothetical
1687	1	SwissProt_HUMAN$	3::P04920|B3A2_HUMAN	25	136923	1	1	1	1	0.04	Anion exchange protein
1688	1	db$2	2::C3K994|C3K994_PSEFS	25	23940	1	1	1	1	0.22	Putative regulatory prot
1689	1	SwissProt_HUMAN$	3::Q9P202|WHRN_HUMAN	25	96499	3	2	2	2	0.10	Whirlin OS=Homo sapie
1690	1	SwissProt_HUMAN$	3::P05546|HEP2_HUMAN	25	57034	1	1	1	1	0.09	Heparin cofactor 2 OS=
1691	1	db$2	2::C3K127|C3K127_PSEFS	25	41295	2	1	2	1	0.12	Putative membrane pro
1692	1	SwissProt_HUMAN$	3::O00159|MYO1C_HUMAN	25	121606	1	1	1	1	0.04	Unconventional myosin
1693	1	db$2	2::C3K9S5|C3K9S5_PSEFS	25	38309	3	2	3	2	0.28	Putative cobalamin bios
1694	1	SwissProt_HUMAN$	3::Q5K651|SAMD9_HUMAN	25	184165	1	1	1	1	0.03	Sterile alpha motif dom
1695	1	db$2	2::C3K3Q9|LGT_PSEFS	25	30444	1	1	1	1	0.17	Phosphatidylglycerol--p
1696	1	db$2	2::C3K0V8|C3K0V8_PSEFS	25	45209	1	1	1	1	0.11	Muramidase FlgJ OS=Ps
1697	1	db$2	2::C3K1D8|C3K1D8_PSEFS	25	34344	1	1	1	1	0.15	Putative phosphatidate
1698	1	SwissProt_HUMAN$	3::P28356|HXD9_HUMAN	25	36472	1	1	1	1	0.14	Homeobox protein Hox-
1699	1	db$2	2::C3KDW2|MIAA_PSEFS	25	35360	1	1	1	1	0.14	tRNA dimethylallyltrans
1700	1	db$2	2::C3K2L8|LIPA_PSEFS	25	38163	5	3	5	3	0.45	Lipoyl synthase OS=Pse
1701	1	SwissProt_HUMAN$	3::Q7Z7L7|ZER1_HUMAN	25	88113	2	2	1	1	0.11	Protein zer-1 homolog (
1702	1	db$2	2::C3K6B1|C3K6B1_PSEFS	25	35098	2	2	2	2	0.31	Homoserine kinase OS=

↑Family	M	DB	Accession	Score	Mass	Matches	Match(sig)	Sequences	Seq(sig)	emPAI	Description
1703	1	db$2	2::A4V6U2|A4V6U2_PSEFS	25	76547	2	1	1	1	0.06	Uncharacterized proteir
1704	1	db$2	2::C3K7J9|C3K7J9_PSEFS	25	83444	1	1	1	1	0.06	Putative exported prote
1705	1	db$2	2::C3JZ63|C3JZ63_PSEFS	25	34729	1	1	1	1	0.15	Hydrolase_4 domain-co
1706	1	db$2	2::C3K1Z0|C3K1Z0_PSEFS	25	34051	1	1	1	1	0.15	Putative putative polysa
1707	1	db$2	2::C3K600|C3K600_PSEFS	25	28761	1	1	1	1	0.18	Phosphatidate cytidylyl
1708	1	db$2	2::C3JZ46|C3JZ46_PSEFS	25	37091	3	2	3	2	0.29	Putative LacI family tra
1709	1	db$2	2::C3KB05|C3KB05_PSEFS	25	50891	2	2	2	2	0.20	CDP-diacylglycerol-seri
1710	1	db$2	2::C3K8W0|C3K8W0_PSEFS	25	34311	1	1	1	1	0.15	Putative LysR-family re
1711	1	SwissProt_HUMAN$	3::P98161|PKD1_HUMAN	25	462239	1	1	1	1	0.01	Polycystin-1 OS=Homo
1712	1	db$2	2::C3KBV2|C3KBV2_PSEFS	25	15686	1	1	1	1	0.35	Thioredoxin OS=Pseudo
1713	1	SwissProt_HUMAN$	3::Q8TD08|MK15_HUMAN	25	59795	1	1	1	1	0.08	Mitogen-activated prote
1714	1	db$2	2::C3K6L2|C3K6L2_PSEFS	25	14328	1	1	1	1	0.38	Organic hydroperoxide
1715	1	SwissProt_HUMAN$	3::Q9Y4G8|RPGF2_HUMAN	25	167313	2	2	2	2	0.06	Rap guanine nucleotide
1716	1	db$2	2::C3K9Y9|C3K9Y9_PSEFS	25	13239	1	1	1	1	0.42	Uncharacterized proteir
1717	1	db$2	2::C3K211|C3K211_PSEFS	24	35352	1	1	1	1	0.14	LysR-family transcriptic
1718	1	SwissProt_HUMAN$	3::Q9UQE7|SMC3_HUMAN	24	141454	2	1	2	1	0.03	Structural maintenance
1719	1	SwissProt_HUMAN$	3::Q71DI3|H32_HUMAN	24	15379	2	2	2	2	0.83	Histone H3.2 OS=Homo
1720	1	db$2	2::C3K8V0|C3K8V0_PSEFS	24	31271	1	1	1	1	0.16	Poly(3-hydroxyalkanoat
1721	1	SwissProt_HUMAN$	3::P37802|TAGL2_HUMAN	24	22377	2	2	2	2	0.52	Transgelin-2 OS=Homo
1722	1	db$2	2::C3KE19|C3KE19_PSEFS	24	30253	1	1	1	1	0.17	HDOD domain-containir
1723	1	SwissProt_HUMAN$	3::Q9UBV2|SE1L1_HUMAN	24	88698	1	1	1	1	0.05	Protein sel-1 homolog 1
1724	1	SwissProt_HUMAN$	3::P23284|PPIB_HUMAN	24	23728	1	1	1	1	0.22	Peptidyl-prolyl cis-trans
1725	1	db$2	2::C3JYP1|C3JYP1_PSEFS	24	35226	2	1	2	1	0.14	Uncharacterized proteir
1726	1	SwissProt_HUMAN$	3::Q3SXY7|LRIT3_HUMAN	24	74706	1	1	1	1	0.07	Leucine-rich repeat, im
1727	1	SwissProt_HUMAN$	3::P36507|MP2K2_HUMAN	24	44396	1	1	1	1	0.11	Dual specificity mitoger
1728	1	SwissProt_HUMAN$	3::Q9Y6N6|LAMC3_HUMAN	24	171117	1	1	1	1	0.03	Laminin subunit gamma
1729	1	SwissProt_HUMAN$	3::P49207|RL34_HUMAN	24	13284	1	1	1	1	0.42	60S ribosomal protein L
1730	1	SwissProt_HUMAN$	3::P27708|PYR1_HUMAN	24	242829	1	1	1	1	0.02	CAD protein OS=Homo
1731	1	SwissProt_HUMAN$	3::P30041|PRDX6_HUMAN	24	25019	1	1	1	1	0.21	Peroxiredoxin-6 OS=Hc
1732	1	SwissProt_HUMAN$	3::Q16610|ECM1_HUMAN	24	60635	1	1	1	1	0.08	Extracellular matrix pro
1733	1	db$2	2::C3KDN6|C3KDN6_PSEFS	24	32369	1	1	1	1	0.16	Uncharacterized proteir
1734	1	db$2	2::C3K3P7|C3K3P7_PSEFS	24	49977	1	1	1	1	0.10	Putative membrane pro
1735	1	db$2	2::C3KAJ5|C3KAJ5_PSEFS	24	83174	3	3	3	3	0.19	Putative paraquat-induc
1736	1	db$2	2::C3KED0|C3KED0_PSEFS	24	8582	1	1	1	1	0.71	Putative SirA-like prote
1737	1	SwissProt_HUMAN$	3::P54577|SYYC_HUMAN	24	59106	1	1	1	1	0.08	Tyrosine--tRNA ligase,
1738	1	db$2	2::C3K7J4|C3K7J4_PSEFS	24	18330	1	1	1	1	0.29	Uncharacterized proteir
1739	1	SwissProt_HUMAN$	3::P62136|PP1A_HUMAN	24	37488	1	1	1	1	0.13	Serine/threonine-protei
1740	1	db$2	2::C3K4Z5|C3K4Z5_PSEFS	24	14110	1	1	1	1	0.39	Putative lipoprotein OS
1741	1	SwissProt_HUMAN$	3::O95819|M4K4_HUMAN	24	142013	1	1	1	1	0.03	Mitogen-activated prote
1742	1	db$2	2::C3JXL1|C3JXL1_PSEFS	24	51972	1	1	1	1	0.10	Putative transmembran

↑Family	M	DB	Accession	Score	Mass	Matches	Match(sig)	Sequences	Seq(sig)	emPAI	Description
1743	1	db$2	2::C3JZ73|C3JZ73_PSEFS	24	49677	3	2	3	2	0.21	Porin OS=Pseudomonas
1744	1	SwissProt_HUMAN$	3::Q8TF62|AT8B4_HUMAN	24	135782	3	1	2	1	0.04	Probable phospholipid-t
1745	1	db$2	2::C3K8V8|C3K8V8_PSEFS	24	36166	2	1	2	1	0.14	Zinc-type alcohol dehyc
1746	1	db$2	2::C3KAR1|C3KAR1_PSEFS	24	16023	1	1	1	1	0.34	Putative isoquinoline 1-
1747	1	SwissProt_HUMAN$	3::Q8TAY7|F110D_HUMAN	24	28693	1	1	1	1	0.18	Protein FAM110D OS=H
1748	1	SwissProt_HUMAN$	3::Q9C037|TRIM4_HUMAN	24	57424	1	1	1	1	0.09	E3 ubiquitin-protein liga
1749	1	db$2	2::C3K0N3|ACP_PSEFS	24	8637	1	1	1	1	0.70	Acyl carrier protein OS=
1750	1	db$2	2::C3K122|C3K122_PSEFS	24	33097	1	1	1	1	0.15	Putative O-acetylserine
1751	1	db$2	2::C3K2L3|C3K2L3_PSEFS	24	22416	1	1	1	1	0.23	LPS-assembly lipoprote
1752	1	db$2	2::C3K8C0|C3K8C0_PSEFS	24	26199	1	1	1	1	0.20	Putative dehydrogenase
1753	1	SwissProt_HUMAN$	3::Q8N8R5|CB069_HUMAN	24	43421	1	1	1	1	0.11	UPF0565 protein C2orf6
1754	1	SwissProt_HUMAN$	3::Q96N23|CFA54_HUMAN	24	351744	1	1	1	1	0.01	Cilia- and flagella-assoc
1755	1	db$2	2::C3K2S4|C3K2S4_PSEFS	24	15995	1	1	1	1	0.34	Putative lipoprotein OS=
1756	1	SwissProt_HUMAN$	3::Q8WX39|LCN9_HUMAN	24	20272	1	1	1	1	0.26	Epididymal-specific lipo
1757	1	db$2	2::C3K0Z5|C3K0Z5_PSEFS	24	22469	2	1	2	1	0.23	Corrinoid adenosyltrans
1758	1	SwissProt_HUMAN$	3::Q9H173|SIL1_HUMAN	24	52052	2	1	1	1	0.10	Nucleotide exchange fa
1759	1	SwissProt_HUMAN$	3::O15270|SPTC2_HUMAN	24	62884	1	1	1	1	0.08	Serine palmitoyltransfe
1760	1	SwissProt_HUMAN$	3::P15924|DESP_HUMAN	23	331569	1	1	1	1	0.01	Desmoplakin OS=Homo
1761	1	db$2	2::C3JZP4|RBSD_PSEFS	23	14393	1	1	1	1	0.38	D-ribose pyranase OS=
1762	1	db$2	2::C3KD58|C3KD58_PSEFS	23	41746	1	1	1	1	0.12	Putative cystathionine g
1763	1	db$2	2::C3K433|CYAY_PSEFS	23	12442	1	1	1	1	0.45	Iron-sulfur cluster asse
1764	1	SwissProt_HUMAN$	3::P02747|C1QC_HUMAN	23	25757	2	2	2	2	0.44	Complement C1q subcc
1765	1	db$2	2::C3KDI2|C3KDI2_PSEFS	23	135789	1	1	1	1	0.04	Exonuclease OS=Pseud
1766	1	SwissProt_HUMAN$	3::Q8NER1|TRPV1_HUMAN	23	94895	1	1	1	1	0.05	Transient receptor pote
1767	1	SwissProt_HUMAN$	3::Q9Y5I4|PCDC2_HUMAN	23	109383	2	1	1	1	0.04	Protocadherin alpha-C2
1768	1	db$2	2::C3KAI2|C3KAI2_PSEFS	23	19361	1	1	1	1	0.27	Shikimate kinase OS=P
1769	1	SwissProt_HUMAN$	3::Q08828|ADCY1_HUMAN	23	123360	1	1	1	1	0.04	Adenylate cyclase type
1770	1	SwissProt_HUMAN$	3::Q14129|DGCR6_HUMAN	23	24973	2	1	1	1	0.21	Protein DGCR6 OS=Hor
1771	1	db$2	2::C3JZ48|C3JZ48_PSEFS	23	11531	1	1	1	1	0.49	Uncharacterized proteir
1772	1	db$2	2::C3JYK2|C3JYK2_PSEFS	23	30492	5	2	3	2	0.36	Bifunctional protein Foll
1773	1	db$2	2::C3JYF1|C3JYF1_PSEFS	23	42707	1	1	1	1	0.12	Putative acyl-CoA dehy
1774	1	SwissProt_HUMAN$	3::Q86WI3|NLRC5_HUMAN	23	204464	1	1	1	1	0.02	Protein NLRC5 OS=Hon
1775	1	db$2	2::C3K7T3|C3K7T3_PSEFS	23	20773	1	1	1	1	0.25	CDP-diacylglycerol--gly
1776	1	db$2	2::C3KCB4|C3KCB4_PSEFS	23	27191	1	1	1	1	0.19	Uncharacterized proteir
1777	1	SwissProt_HUMAN$	3::Q9H9E3|COG4_HUMAN	23	89026	1	1	1	1	0.05	Conserved oligomeric G
1778	1	db$2	2::C3K3D5|C3K3D5_PSEFS	23	47715	1	1	1	1	0.10	Uncharacterized proteir
1779	1	db$2	2::C3K5C6|C3K5C6_PSEFS	23	33902	1	1	1	1	0.15	Uncharacterized proteir
1780	1	db$2	2::C3K546|C3K546_PSEFS	23	33665	1	1	1	1	0.15	Putative lipopolysaccha
1781	1	SwissProt_HUMAN$	3::O00294|TULP1_HUMAN	23	60572	1	1	1	1	0.08	Tubby-related protein 1
1782	1	SwissProt_HUMAN$	3::Q8N565|MREG_HUMAN	23	24912	1	1	1	1	0.21	Melanoregulin OS=Hom

↑Family	M	DB	Accession	Score	Mass	Matches	Match(sig)	Sequences	Seq(sig)	emPAI	Description
1783	1	SwissProt_HUMAN$	3::Q8NDA8|MROH1_HUMAN	23	181134	1	1	1	1	0.03	Maestro heat-like repea
1784	1	SwissProt_HUMAN$	3::Q969W8|ZN566_HUMAN	23	49188	1	1	1	1	0.10	Zinc finger protein 566
1785	1	SwissProt_HUMAN$	3::Q8IWX8|CHERP_HUMAN	23	103637	1	1	1	1	0.05	Calcium homeostasis er
1786	1	db$2	2::C3K071|Y4323_PSEFS	23	8578	1	1	1	1	0.71	UPF0270 protein PFLU_
1787	1	db$2	2::C3K807|C3K807_PSEFS	23	30283	1	1	1	1	0.17	Putative histidine ABC t
1788	1	SwissProt_HUMAN$	3::Q7Z5H4|VN1R5_HUMAN	23	40752	1	1	1	1	0.12	Vomeronasal type-1 rec
1789	1	SwissProt_HUMAN$	3::Q2TAL5|SMTL2_HUMAN	23	50165	1	1	1	1	0.10	Smoothelin-like protein
1790	1	db$2	2::C3K065|C3K065_PSEFS	23	58554	1	1	1	1	0.08	Uncharacterized proteir
1791	1	db$2	2::C3JY47|C3JY47_PSEFS	23	14227	1	1	1	1	0.39	DrsE domain-containinc
1792	1	db$2	2::C3JZ42|C3JZ42_PSEFS	23	19185	1	1	1	1	0.28	Putative AraC family tra
1793	1	db$2	2::C3K2H7|C3K2H7_PSEFS	22	21527	1	1	1	1	0.24	Putative acetyltransfera
1794	1	db$2	2::C3KBB7|C3KBB7_PSEFS	22	43574	1	1	1	1	0.11	3-oxoacyl-[acyl-carrier-
1795	1	SwissProt_HUMAN$	3::Q9ULT8|HECD1_HUMAN	22	289203	1	1	1	1	0.02	E3 ubiquitin-protein liga
1796	1	db$2	2::C3KCP2|C3KCP2_PSEFS	22	100291	1	1	1	1	0.05	Conserved GGDEF/EAL
1797	1	SwissProt_HUMAN$	3::Q05397|FAK1_HUMAN	22	119157	1	1	1	1	0.04	Focal adhesion kinase 1
1798	1	SwissProt_HUMAN$	3::O94967|WDR47_HUMAN	22	101884	2	1	2	1	0.05	WD repeat-containing p
1799	1	db$2	2::C3K006|C3K006_PSEFS	22	41293	1	1	1	1	0.12	Acetylornithine deacety
1800	1	SwissProt_HUMAN$	3::Q9H244|P2Y12_HUMAN	22	39413	1	1	1	1	0.13	P2Y purinoceptor 12 OS
1801	1	db$2	2::C3K3Y9|C3K3Y9_PSEFS	22	73510	2	1	2	1	0.07	Putative membrane pro
1802	1	db$2	2::C3K834|C3K834_PSEFS	22	76708	1	1	1	1	0.06	Copper-transporting P-t
1803	1	SwissProt_HUMAN$	3::P62899|RL31_HUMAN	22	14454	1	1	1	1	0.38	60S ribosomal protein L
1804	1	db$2	2::C3KA95|C3KA95_PSEFS	22	39629	1	1	1	1	0.13	Glycerate kinase OS=P:
1805	1	db$2	2::C3K2J0|C3K2J0_PSEFS	22	40846	2	2	2	2	0.26	Putative exported prote
1806	1	SwissProt_HUMAN$	3::O94827|PKHG5_HUMAN	22	117377	2	1	2	1	0.04	Pleckstrin homology do
1807	1	db$2	2::C3K867|C3K867_PSEFS	22	39735	1	1	1	1	0.13	PALP domain-containinc
1808	1	db$2	2::C3K831|C3K831_PSEFS	22	45359	2	2	2	2	0.23	Putative thiolase OS=P:
1809	1	db$2	2::C3K1H4|C3K1H4_PSEFS	22	45615	1	1	1	1	0.11	Putative membrane pro
1810	1	SwissProt_HUMAN$	3::P25391|LAMA1_HUMAN	22	336867	1	1	1	1	0.01	Laminin subunit alpha-:
1811	1	db$2	2::C3K5E1|C3K5E1_PSEFS	22	13734	2	1	2	1	0.40	Uncharacterized proteir
1812	1	db$2	2::C3K3H5|C3K3H5_PSEFS	22	32718	1	1	1	1	0.15	Putative ABC transport
1813	1	db$2	2::C3JY58|C3JY58_PSEFS	22	87640	3	2	2	2	0.11	DNA translocase FtsK O
1814	1	db$2	2::C3KDI4|C3KDI4_PSEFS	22	42151	1	1	1	1	0.12	Putative exported prote
1815	1	db$2	2::C3K992|C3K992_PSEFS	22	34600	1	1	1	1	0.15	Putative AraC-family re
1816	1	SwissProt_HUMAN$	3::Q8N7X0|ADGB_HUMAN	22	189594	1	1	1	1	0.03	Androglobin OS=Homo
1817	1	db$2	2::C3JXR0|C3JXR0_PSEFS	22	12414	1	1	1	1	0.45	Uncharacterized proteir
1818	1	db$2	2::C3K811|C3K811_PSEFS	22	43409	1	1	1	1	0.11	Imidazolonepropionase
1819	1	db$2	2::C3K578|C3K578_PSEFS	22	39452	1	1	1	1	0.13	Putative lipoprotein OS:
1820	1	db$2	2::C3K585|C3K585_PSEFS	22	25105	1	1	1	1	0.21	Putative hydrolase OS=
1821	1	SwissProt_HUMAN$	3::Q9Y239|NOD1_HUMAN	22	107622	1	1	1	1	0.05	Nucleotide-binding oligo
1822	1	db$2	2::C3K3J6|C3K3J6_PSEFS	22	14927	1	1	1	1	0.37	Type IV pilus response

↑Family	M	DB	Accession	Score	Mass	Matches	Match(sig)	Sequences	Seq(sig)	emPAI	Description
1823	1	db$2	2::C3KAA5|C3KAA5_PSEFS	22	23984	2	2	2	2	0.48	Uncharacterized protein
1824	1	SwissProt_HUMAN$	3::Q8NFP9|NBEA_HUMAN	22	327614	1	1	1	1	0.01	Neurobeachin OS=Hom
1825	1	db$2	2::C3K5A4|C3K5A4_PSEFS	22	19103	1	1	1	1	0.28	Putative acetyltransfera
1826	1	db$2	2::C3KE47|FMT_PSEFS	22	33416	1	1	1	1	0.15	Methionyl-tRNA formylt
1827	1	db$2	2::C3KE38|C3KE38_PSEFS	21	28697	3	2	3	2	0.39	Putative phospholipid b
1828	1	db$2	2::C3JY43|TTCA_PSEFS	21	31052	1	1	1	1	0.16	tRNA-cytidine(32) 2-sul
1829	1	SwissProt_HUMAN$	3::Q149M9|NWD1_HUMAN	21	174441	1	1	1	1	0.03	NACHT domain- and WI
1830	1	db$2	2::C3K4H8|C3K4H8_PSEFS	21	20657	1	1	1	1	0.25	Threonylcarbamoyl-AMI
1831	1	db$2	2::C3K5F3|C3K5F3_PSEFS	21	34108	1	1	1	1	0.15	Putative dehydrogenase
1832	1	SwissProt_HUMAN$	3::Q9Y2B5|VP9D1_HUMAN	21	68913	1	1	1	1	0.07	VPS9 domain-containin
1833	1	db$2	2::C3K9N9|C3K9N9_PSEFS	21	57231	2	2	2	2	0.18	Putative ABC transport
1834	1	SwissProt_HUMAN$	3::Q15046|SYK_HUMAN	21	68005	2	1	2	1	0.07	Lysine--tRNA ligase OS
1835	1	db$2	2::C3K8Y5|C3K8Y5_PSEFS	21	34001	1	1	1	1	0.15	HTH-type transcriptiona
1836	1	db$2	2::C3K167|C3K167_PSEFS	21	48979	1	1	1	1	0.10	Putative two-componen
1837	1	SwissProt_HUMAN$	3::Q9BT17|MTG1_HUMAN	21	37213	2	1	2	1	0.14	Mitochondrial ribosome
1838	1	db$2	2::C3KBZ4|C3KBZ4_PSEFS	21	6850	2	1	1	1	0.94	Uncharacterized protein
1839	1	SwissProt_HUMAN$	3::P16471|PRLR_HUMAN	21	69461	1	1	1	1	0.07	Prolactin receptor OS=H
1840	1	db$2	2::C3K2E6|C3K2E6_PSEFS	21	40463	1	1	1	1	0.12	Ribosomal RNA large su
1841	1	db$2	2::C3K3C7|C3K3C7_PSEFS	21	34452	2	1	2	1	0.15	Putative glycine betaine
1842	1	SwissProt_HUMAN$	3::P57727|TMPS3_HUMAN	21	49373	1	1	1	1	0.10	Transmembrane protea
1843	1	db$2	2::C3JXP2|C3JXP2_PSEFS	21	43336	2	1	1	1	0.12	Putative glycosyltransfe
1844	1	db$2	2::C3K321|C3K321_PSEFS	21	28336	1	1	1	1	0.18	Putative heat shock pro
1845	1	SwissProt_HUMAN$	3::Q7Z5L9|I2BP2_HUMAN	21	60987	1	1	1	1	0.08	Interferon regulatory fa
1846	1	db$2	2::C3KAW9|C3KAW9_PSEFS	21	125598	1	1	1	1	0.04	Maltokinase OS=Pseudo
1847	1	SwissProt_HUMAN$	3::Q9Y263|PLAP_HUMAN	21	87101	1	1	1	1	0.06	Phospholipase A-2-activ
1848	1	SwissProt_HUMAN$	3::Q9NRF8|PYRG2_HUMAN	21	65636	1	1	1	1	0.07	CTP synthase 2 OS=Ho
1849	1	SwissProt_HUMAN$	3::Q13563|PKD2_HUMAN	21	109622	1	1	1	1	0.04	Polycystin-2 OS=Homo
1850	1	SwissProt_HUMAN$	3::Q8N1K5|THMS1_HUMAN	21	73406	1	1	1	1	0.07	Protein THEMIS OS=Ho
1851	1	db$2	2::C3JZP0|C3JZP0_PSEFS	21	11251	1	1	1	1	0.51	Putative lipoprotein OS=
1852	1	SwissProt_HUMAN$	3::Q96GD3|SCMH1_HUMAN	21	73307	1	1	1	1	0.07	Polycomb protein SCMH
1853	1	db$2	2::C3KE72|C3KE72_PSEFS	21	85807	1	1	1	1	0.06	UPF0313 protein PFLU_
1854	1	SwissProt_HUMAN$	3::Q49MG5|MAP9_HUMAN	21	74189	1	1	1	1	0.07	Microtubule-associated
1855	1	db$2	2::C3K5B8|C3K5B8_PSEFS	21	59457	1	1	1	1	0.08	Dipeptide ABC transpor
1856	1	db$2	2::C3K1S4|C3K1S4_PSEFS	21	23334	1	1	1	1	0.22	Putative membrane pro
1857	1	db$2	2::C3K0B8|C3K0B8_PSEFS	21	24379	1	1	1	1	0.21	Uncharacterized protein
1858	1	db$2	2::C3K883|C3K883_PSEFS	21	60772	2	1	2	1	0.08	Putative transposase su
1859	1	db$2	2::C3KCS4|C3KCS4_PSEFS	21	63231	2	1	2	1	0.08	Peptidoglycan D,D-tran
1860	1	SwissProt_HUMAN$	3::P08842|STS_HUMAN	20	65450	1	1	1	1	0.07	Steryl-sulfatase OS=Ho
1861	1	SwissProt_HUMAN$	3::Q5TGY3|AHDC1_HUMAN	20	168245	2	1	1	1	0.03	AT-hook DNA-binding n
1862	1	db$2	2::C3K153|C3K153_PSEFS	20	54699	1	1	1	1	0.09	Exodeoxyribonuclease I

↑Family	M	DB	Accession	Score	Mass	Matches	Match(sig)	Sequences	Seq(sig)	emPAI	Description
1863	1	db$2	2::C3K9N3|C3K9N3_PSEFS	20	10076	1	1	1	1	0.58	Peptidyl-prolyl cis-trans
1864	1	SwissProt_HUMAN$	3::A0A1B0GVD1|F237B_HUMAN	20	16320	1	1	1	1	0.33	Protein FAM237B OS=H
1865	1	SwissProt_HUMAN$	3::Q86TG7|PEG10_HUMAN	20	80122	1	1	1	1	0.06	Retrotransposon-derive
1866	1	SwissProt_HUMAN$	3::Q8TCU5|NMD3A_HUMAN	20	125385	2	1	1	1	0.04	Glutamate receptor ion
1867	1	db$2	2::C3KBT8|C3KBT8_PSEFS	20	29541	1	1	1	1	0.17	Sulfate transport syster
1868	1	SwissProt_HUMAN$	3::Q9H299|SH3L3_HUMAN	20	10431	1	1	1	1	0.56	SH3 domain-binding glu
1869	1	db$2	2::C3JY52|C3JY52_PSEFS	20	37777	1	1	1	1	0.13	GST C-terminal domain
1870	1	SwissProt_HUMAN$	3::P60903|S10AA_HUMAN	20	11196	1	1	1	1	0.51	Protein S100-A10 OS=I
1871	1	db$2	2::C3K7J3|Y1319_PSEFS	20	16953	1	1	1	1	0.32	UPF0225 protein PFLU_
1872	1	db$2	2::C3KDB4|C3KDB4_PSEFS	20	42765	1	1	1	1	0.12	Putative glycosyl transf
1873	1	db$2	2::C3K789|C3K789_PSEFS	20	16242	1	1	1	1	0.33	Uncharacterized proteir
1874	1	SwissProt_HUMAN$	3::P78357|CNTP1_HUMAN	20	156167	2	1	2	1	0.03	Contactin-associated pr
1875	1	db$2	2::C3K7K3|C3K7K3_PSEFS	20	49881	1	1	1	1	0.10	Putative deoxyguanosir
1876	1	SwissProt_HUMAN$	3::P81274|GPSM2_HUMAN	20	76614	1	1	1	1	0.06	G-protein-signaling mo
1877	1	SwissProt_HUMAN$	3::Q02080|MEF2B_HUMAN	20	38615	1	1	1	1	0.13	Myocyte-specific enhan
1878	1	db$2	2::C3K4E3|C3K4E3_PSEFS	20	32989	2	1	2	1	0.15	Putative LysR-family re
1879	1	db$2	2::C3JYL6|C3JYL6_PSEFS	20	25076	1	1	1	1	0.21	Putative TetR-family re
1880	1	db$2	2::C3K4Y8|C3K4Y8_PSEFS	20	27802	1	1	1	1	0.18	Heme exporter protein
1881	1	db$2	2::C3JY42|C3JY42_PSEFS	20	25186	1	1	1	1	0.21	Uncharacterized proteir
1882	1	db$2	2::C3K404|C3K404_PSEFS	20	23654	1	1	1	1	0.22	Putative membrane pro
1883	1	db$2	2::C3K3G8|C3K3G8_PSEFS	20	41555	1	1	1	1	0.12	Uncharacterized proteir
1884	1	SwissProt_HUMAN$	3::Q9ULR3|PPM1H_HUMAN	20	56413	1	1	1	1	0.09	Protein phosphatase 1H
1885	1	SwissProt_HUMAN$	3::Q86UA1|PRP39_HUMAN	20	78381	1	1	1	1	0.06	Pre-mRNA-processing f
1886	1	SwissProt_HUMAN$	3::Q06828|FMOD_HUMAN	20	43152	1	1	1	1	0.12	Fibromodulin OS=Homo
1887	1	SwissProt_HUMAN$	3::Q99623|PHB2_HUMAN	20	33276	1	1	1	1	0.15	Prohibitin-2 OS=Homo
1888	1	db$2	2::C3JYG2|C3JYG2_PSEFS	20	36128	1	1	1	1	0.14	ATP-grasp domain-cont
1889	1	db$2	2::C3K0D2|C3K0D2_PSEFS	20	35834	1	1	1	1	0.14	Lipid A biosynthesis lau
1890	1	SwissProt_HUMAN$	3::Q3MIP1|IPIL2_HUMAN	20	58409	2	1	2	1	0.08	Inositol 1,4,5-trisphosp
1891	1	SwissProt_HUMAN$	3::P00738|HPT_HUMAN	20	45177	1	1	1	1	0.11	Haptoglobin OS=Homo
1892	1	db$2	2::C3K2Q9|EX7S_PSEFS	20	8900	1	1	1	1	0.68	Exodeoxyribonuclease
1893	1	SwissProt_HUMAN$	3::P18627|LAG3_HUMAN	20	57413	1	1	1	1	0.09	Lymphocyte activation
1894	1	db$2	2::C3K267|RLME_PSEFS	20	24125	1	1	1	1	0.22	Ribosomal RNA large su
1895	1	db$2	2::C3K5F2|Y1517_PSEFS	20	11422	1	1	1	1	0.50	YcgL domain-containing
1896	1	SwissProt_HUMAN$	3::Q8WXH0|SYNE2_HUMAN	20	795944	2	1	2	1	0.01	Nesprin-2 OS=Homo sa
1897	1	db$2	2::C3K9N4|C3K9N4_PSEFS	20	91939	2	1	2	1	0.05	Histidine kinase OS=Ps
1898	1	db$2	2::C3KAE4|C3KAE4_PSEFS	20	39865	1	1	1	1	0.13	Uncharacterized proteir
1899	1	db$2	2::C3JXK0|C3JXK0_PSEFS	20	17939	1	1	1	1	0.30	Putative acetyltransfera
1900	1	db$2	2::C3KCG3|C3KCG3_PSEFS	19	16758	1	1	1	1	0.32	YjgF_endoribonc domai
1901	1	db$2	2::C3KDZ7|C3KDZ7_PSEFS	19	49934	1	1	1	1	0.10	Alginate biosynthesis pr
1902	1	SwissProt_HUMAN$	3::P35227|PCGF2_HUMAN	19	37764	1	1	1	1	0.13	Polycomb group RING f

↑Family	M	DB	Accession	Score	Mass	Matches	Match(sig)	Sequences	Seq(sig)	emPAI	Description
1903	1	db$2	2::C3K1V9|C3K1V9_PSEFS	19	16219	1	1	1	1	0.33	Flavodoxin-like domain-
1904	1	db$2	2::C3JYN8|C3JYN8_PSEFS	19	39948	2	1	1	1	0.13	Putative noncomposite
1905	1	db$2	2::C3JXS7|C3JXS7_PSEFS	19	34119	1	1	1	1	0.15	Putative GGDEF domair
1906	1	SwissProt_HUMAN$	3::Q9NR48|ASH1L_HUMAN	19	332582	1	1	1	1	0.01	Histone-lysine N-methy
1907	1	SwissProt_HUMAN$	3::Q76N89|HECW1_HUMAN	19	179442	1	1	1	1	0.03	E3 ubiquitin-protein liga
1908	1	SwissProt_HUMAN$	3::P62942|FKB1A_HUMAN	19	11943	1	1	1	1	0.47	Peptidyl-prolyl cis-trans
1909	1	SwissProt_HUMAN$	3::Q96PE3|INP4A_HUMAN	19	109885	1	1	1	1	0.04	Inositol polyphosphate-
1910	1	SwissProt_HUMAN$	3::P0DKB5|TPBGL_HUMAN	19	40374	1	1	1	1	0.12	Trophoblast glycoprotei
1911	1	db$2	2::C3JYS7|RUVB_PSEFS	19	38919	1	1	1	1	0.13	Holliday junction ATP-d
1912	1	db$2	2::C3K672|C3K672_PSEFS	19	58670	1	1	1	1	0.08	Cation/acetate symport
1913	1	SwissProt_HUMAN$	3::P19838|NFKB1_HUMAN	19	105290	1	1	1	1	0.05	Nuclear factor NF-kappa
1914	1	db$2	2::C3K0Y2|C3K0Y2_PSEFS	19	46222	1	1	1	1	0.11	Putative fatty acid trans
1915	1	db$2	2::C3JY93|C3JY93_PSEFS	19	51981	1	1	1	1	0.10	NADH-quinone oxidorec
1916	1	SwissProt_HUMAN$	3::A6NM36|LRC30_HUMAN	19	34012	1	1	1	1	0.15	Leucine-rich repeat-con
1917	1	db$2	2::C3K749|C3K749_PSEFS	19	16397	1	1	1	1	0.33	Putative phosphohistidi
1918	1	db$2	2::C3K3D4|C3K3D4_PSEFS	19	52472	1	1	1	1	0.09	Uncharacterized proteir
1919	1	SwissProt_HUMAN$	3::O60671|RAD1_HUMAN	19	31807	1	1	1	1	0.16	Cell cycle checkpoint pr
1920	1	db$2	2::C3KBG6|C3KBG6_PSEFS	19	59843	2	1	1	1	0.08	VWFA domain-containir
1921	1	db$2	2::C3K431|C3K431_PSEFS	19	14535	1	1	1	1	0.38	Regulator of nucleoside
1922	1	db$2	2::C3K7M0|C3K7M0_PSEFS	19	62717	1	1	1	1	0.08	Putative dipeptidase OS
1923	1	db$2	2::C3K1W1|C3K1W1_PSEFS	19	22921	2	2	1	1	0.23	Uncharacterized proteir
1924	1	db$2	2::C3KAR9|C3KAR9_PSEFS	19	50146	1	1	1	1	0.10	Putative transmembran
1925	1	SwissProt_HUMAN$	3::Q99932|SPAG8_HUMAN	19	51106	1	1	1	1	0.10	Sperm-associated antig
1926	1	SwissProt_HUMAN$	3::P31943|HNRH1_HUMAN	19	49198	1	1	1	1	0.10	Heterogeneous nuclear
1927	1	db$2	2::C3KAU8|C3KAU8_PSEFS	19	52023	1	1	1	1	0.10	Xylulose kinase OS=Pse
1928	1	SwissProt_HUMAN$	3::P61513|RL37A_HUMAN	19	10268	2	1	2	1	0.56	60S ribosomal protein L
1929	1	SwissProt_HUMAN$	3::Q8NCM8|DYHC2_HUMAN	19	492311	2	1	2	1	0.01	Cytoplasmic dynein 2 h
1930	1	db$2	2::C3K188|C3K188_PSEFS	19	39897	1	1	1	1	0.13	Putative exported prote
1931	1	db$2	2::C3KE75|C3KE75_PSEFS	19	123658	1	1	1	1	0.04	TGc domain-containing
1932	1	db$2	2::C3K3Z3|C3K3Z3_PSEFS	19	27990	1	1	1	1	0.18	Putative ABC transport
1933	1	SwissProt_HUMAN$	3::Q8IVF4|DYH10_HUMAN	19	514512	1	1	1	1	0.01	Dynein heavy chain 10,
1934	1	SwissProt_HUMAN$	3::Q4V348|Z658B_HUMAN	19	94270	1	1	1	1	0.05	Zinc finger protein 658I
1935	1	SwissProt_HUMAN$	3::Q09MP3|R51A2_HUMAN	19	133822	1	1	1	1	0.04	RAD51-associated prote
1936	1	db$2	2::C3K9P8|C3K9P8_PSEFS	19	69666	1	1	1	1	0.07	Putative substrate-bind
1937	1	SwissProt_HUMAN$	3::Q08378|GOGA3_HUMAN	18	167252	1	1	1	1	0.03	Golgin subfamily A mer
1938	1	SwissProt_HUMAN$	3::P00488|F13A_HUMAN	18	83215	1	1	1	1	0.06	Coagulation factor XIII
1939	1	SwissProt_HUMAN$	3::Q9NZI6|TF2L1_HUMAN	18	54593	1	1	1	1	0.09	Transcription factor CP2
1940	1	db$2	2::C3KAN4|C3KAN4_PSEFS	18	41646	1	1	1	1	0.12	3-oxoadipyl-CoA thiolas
1941	1	SwissProt_HUMAN$	3::Q9Y3A2|UTP11_HUMAN	18	30428	1	1	1	1	0.17	Probable U3 small nucle
1942	1	db$2	2::C3K9P2|C3K9P2_PSEFS	18	69747	1	1	1	1	0.07	Putative substrate-bind

↑Family	M	DB	Accession	Score	Mass	Matches	Match(sig)	Sequences	Seq(sig)	emPAI	Description
1943	1	SwissProt_HUMAN$	3::P61978|HNRPK_HUMAN	18	50944	1	1	1	1	0.10	Heterogeneous nuclear
1944	1	SwissProt_HUMAN$	3::Q6TFL3|CC171_HUMAN	18	152714	1	1	1	1	0.03	Coiled-coil domain-cont
1945	1	SwissProt_HUMAN$	3::Q6P0Q8|MAST2_HUMAN	18	196315	3	1	2	1	0.02	Microtubule-associated
1946	1	db$2	2::C3JXQ7|C3JXQ7_PSEFS	18	48832	1	1	1	1	0.10	UDP-glucose 6-dehydro
1947	1	SwissProt_HUMAN$	3::Q969X2|SIA7F_HUMAN	18	38043	1	1	1	1	0.13	Alpha-N-acetylgalactos
1948	1	db$2	2::C3KE01|Y992_PSEFS	18	29383	2	2	2	2	0.38	UPF0246 protein PFLU_
1949	1	SwissProt_HUMAN$	3::O43151|TET3_HUMAN	18	193583	2	1	2	1	0.02	Methylcytosine dioxyge
1950	1	SwissProt_HUMAN$	3::Q6UWJ1|TMCO3_HUMAN	18	75549	1	1	1	1	0.06	Transmembrane and co
1951	1	db$2	2::C3JZS2|C3JZS2_PSEFS	18	19776	1	1	1	1	0.27	Putative colicin V produ
1952	1	db$2	2::C3K5M1|C3K5M1_PSEFS	18	26375	2	1	2	1	0.19	Phosphonates ABC tran
1953	1	db$2	2::A4V7Q1|A4V7Q1_PSEFS	18	42078	2	1	2	1	0.12	Uncharacterized protei
1954	1	db$2	2::C3KDL9|C3KDL9_PSEFS	18	22175	2	2	2	2	0.53	Uncharacterized protei
1955	1	db$2	2::C3KDU2|C3KDU2_PSEFS	18	32215	1	1	1	1	0.16	Putative short chain de
1956	1	SwissProt_HUMAN$	3::Q9Y2G4|ANKR6_HUMAN	18	79922	1	1	1	1	0.06	Ankyrin repeat domain-
1957	1	SwissProt_HUMAN$	3::P54855|UDB15_HUMAN	18	60996	1	1	1	1	0.08	UDP-glucuronosyltransf
1958	1	db$2	2::C3KEB8|C3KEB8_PSEFS	18	34155	1	1	1	1	0.15	Putative lipoprotein OS
1959	1	SwissProt_HUMAN$	3::Q92802|N42L2_HUMAN	18	67417	1	1	1	1	0.07	NEDD4-binding protein
1960	1	db$2	2::C3K2E4|C3K2E4_PSEFS	18	29249	2	2	2	2	0.38	Ferredoxin--NADP redu
1961	1	SwissProt_HUMAN$	3::P62269|RS18_HUMAN	18	17708	1	1	1	1	0.30	40S ribosomal protein
1962	1	db$2	2::C3K329|APAH_PSEFS	18	31643	1	1	1	1	0.16	Bis(5'-nucleosyl)-tetrap
1963	1	SwissProt_HUMAN$	3::Q86VQ0|LCA5_HUMAN	18	80505	1	1	1	1	0.06	Lebercilin OS=Homo sa
1964	1	db$2	2::C3KAZ7|C3KAZ7_PSEFS	18	48577	1	1	1	1	0.10	Putative chain length d
1965	1	SwissProt_HUMAN$	3::P62304|RUXE_HUMAN	18	10797	1	1	1	1	0.54	Small nuclear ribonucle
1966	1	SwissProt_HUMAN$	3::O75155|CAND2_HUMAN	18	135171	1	1	1	1	0.04	Cullin-associated NEDD
1967	1	SwissProt_HUMAN$	3::P42126|ECI1_HUMAN	18	32795	1	1	1	1	0.15	Enoyl-CoA delta isomer
1968	1	db$2	2::C3JZ35|C3JZ35_PSEFS	18	31443	1	1	1	1	0.16	Putative LysR-family tr
1969	1	SwissProt_HUMAN$	3::Q9BZH6|WDR11_HUMAN	18	136598	1	1	1	1	0.04	WD repeat-containing
1970	1	db$2	2::C3K8B2|C3K8B2_PSEFS	18	12313	1	1	1	1	0.46	HTH cro/C1-type domai
1971	1	SwissProt_HUMAN$	3::B2RPK0|HGB1A_HUMAN	17	24223	1	1	1	1	0.21	Putative high mobility
1972	1	SwissProt_HUMAN$	3::Q14683|SMC1A_HUMAN	17	143144	1	1	1	1	0.03	Structural maintenance
1973	1	SwissProt_HUMAN$	3::O15397|IPO8_HUMAN	17	119861	1	1	1	1	0.04	Importin-8 OS=Homo s
1974	1	db$2	2::C3K000|C3K000_PSEFS	17	26225	1	1	1	1	0.20	Putative GntR-family tr
1975	1	SwissProt_HUMAN$	3::P18077|RL35A_HUMAN	17	12530	2	1	2	1	0.45	60S ribosomal protein
1976	1	db$2	2::C3JY48|C3JY48_PSEFS	17	12277	1	1	1	1	0.46	DrsE domain-containin
1977	1	SwissProt_HUMAN$	3::P02686|MBP_HUMAN	17	33097	1	1	1	1	0.15	Myelin basic protein OS
1978	1	db$2	2::C3K0J3|C3K0J3_PSEFS	17	45387	2	2	1	1	0.11	Putative flagellar hook-
1979	1	db$2	2::C3K2Z6|ERPA_PSEFS	17	12493	1	1	1	1	0.45	Iron-sulfur cluster inse
1980	1	SwissProt_HUMAN$	3::Q96P16|RPR1A_HUMAN	17	35698	1	1	1	1	0.14	Regulation of nuclear p
1981	1	SwissProt_HUMAN$	3::O14511|NRG2_HUMAN	17	91622	1	1	1	1	0.05	Pro-neuregulin-2, mem
1982	1	SwissProt_HUMAN$	3::P42224|STAT1_HUMAN	17	87280	1	1	1	1	0.06	Signal transducer and a

↑Family	M	DB	Accession	Score	Mass	Matches	Match(sig)	Sequences	Seq(sig)	emPAI	Description
1983	1	db$2	2::C3K2E2|C3K2E2_PSEFS	17	56906	1	1	1	1	0.09	Catalase OS=Pseudomo
1984	1	SwissProt_HUMAN$	3::Q9UNA0|ATS5_HUMAN	17	101653	1	1	1	1	0.05	A disintegrin and metal
1985	1	db$2	2::C3K1I1|C3K1I1_PSEFS	17	24836	1	1	1	1	0.21	Nudix hydrolase domair
1986	1	SwissProt_HUMAN$	3::Q9BSF4|TIM29_HUMAN	17	29215	1	1	1	1	0.18	Mitochondrial import in
1987	1	SwissProt_HUMAN$	3::Q6I9Y2|THOC7_HUMAN	17	23728	1	1	1	1	0.22	THO complex subunit 7
1988	1	db$2	2::C3K963|C3K963_PSEFS	17	78429	1	1	1	1	0.06	Putative TonB-depende
1989	1	db$2	2::C3KA85|C3KA85_PSEFS	17	57956	1	1	1	1	0.09	Cyclic-guanylate-specifi
1990	1	SwissProt_HUMAN$	3::Q8IWU5|SULF2_HUMAN	17	100390	1	1	1	1	0.05	Extracellular sulfatase S
1991	1	db$2	2::C3KAJ7|C3KAJ7_PSEFS	17	44130	1	1	1	1	0.11	Uracil permease OS=Ps
1992	1	SwissProt_HUMAN$	3::O00231|PSD11_HUMAN	17	47434	1	1	1	1	0.10	26S proteasome non-AT
1993	1	SwissProt_HUMAN$	3::P11362|FGFR1_HUMAN	17	91809	1	1	1	1	0.05	Fibroblast growth factor
1994	1	db$2	2::C3K908|C3K908_PSEFS	17	33577	1	1	1	1	0.15	Putative dehydrogenase
1995	1	SwissProt_HUMAN$	3::Q14847|LASP1_HUMAN	17	29698	1	1	1	1	0.17	LIM and SH3 domain pr
1996	1	SwissProt_HUMAN$	3::Q9UHW9|S12A6_HUMAN	17	127534	1	1	1	1	0.04	Solute carrier family 12
1997	1	SwissProt_HUMAN$	3::Q13867|BLMH_HUMAN	17	52528	1	1	1	1	0.09	Bleomycin hydrolase OS
1998	1	db$2	2::C3K5A1|C3K5A1_PSEFS	17	60517	1	1	1	1	0.08	Urease subunit alpha O
1999	1	SwissProt_HUMAN$	3::O75746|CMC1_HUMAN	17	74715	1	1	1	1	0.07	Calcium-binding mitoch
2000	1	db$2	2::C3JY73|C3JY73_PSEFS	17	26430	1	1	1	1	0.19	Secretin_N domain-con
2001	1	db$2	2::C3JZP3|C3JZP3_PSEFS	17	36685	1	1	1	1	0.14	Putative inosine-uridine
2002	1	db$2	2::C3K832|C3K832_PSEFS	17	33163	1	1	1	1	0.15	Non-specific serine/thre
2003	1	SwissProt_HUMAN$	3::P42696|RBM34_HUMAN	17	48535	1	1	1	1	0.10	RNA-binding protein 34
2004	1	SwissProt_HUMAN$	3::A8MXV6|CD15L_HUMAN	17	30081	1	1	1	1	0.17	CMT1A duplicated regio
2005	1	SwissProt_HUMAN$	3::Q9BT73|PSMG3_HUMAN	17	13096	2	2	2	2	1.03	Proteasome assembly c
2006	1	db$2	2::C3KCA2|C3KCA2_PSEFS	17	48472	1	1	1	1	0.10	Putative family S43 nor
2007	1	db$2	2::C3K0X8|C3K0X8_PSEFS	17	55461	1	1	1	1	0.09	DNA recombination pro
2008	1	SwissProt_HUMAN$	3::P46020|KPB1_HUMAN	17	137225	2	1	2	1	0.04	Phosphorylase b kinase
2009	1	db$2	2::C3K4U1|C3K4U1_PSEFS	17	34459	1	1	1	1	0.15	Putative lipoprotein OS
2010	1	SwissProt_HUMAN$	3::O94832|MYO1D_HUMAN	17	116129	1	1	1	1	0.04	Unconventional myosin
2011	1	db$2	2::C3K3N7|C3K3N7_PSEFS	17	31800	2	2	2	2	0.35	HDOD domain-containi
2012	1	db$2	2::C3K4V6|C3K4V6_PSEFS	16	101332	1	1	1	1	0.05	Putative helicase OS=P
2013	1	SwissProt_HUMAN$	3::Q8TC07|TBC15_HUMAN	16	79440	1	1	1	1	0.06	TBC1 domain family me
2014	1	SwissProt_HUMAN$	3::Q5VSL9|STRP1_HUMAN	16	95515	1	1	1	1	0.05	Striatin-interacting prot
2015	1	SwissProt_HUMAN$	3::Q7L1T6|NB5R4_HUMAN	16	59436	1	1	1	1	0.08	Cytochrome b5 reducta
2016	1	SwissProt_HUMAN$	3::Q71H61|ILDR2_HUMAN	16	71155	2	1	2	1	0.07	Immunoglobulin-like dc
2017	1	SwissProt_HUMAN$	3::Q9P278|FNIP2_HUMAN	16	122038	1	1	1	1	0.04	Folliculin-interacting pr
2018	1	db$2	2::C3KBH7|C3KBH7_PSEFS	16	44237	1	1	1	1	0.11	3-oxoacyl-[acyl-carrier-
2019	1	SwissProt_HUMAN$	3::Q8IWY9|CDAN1_HUMAN	16	134035	1	1	1	1	0.04	Codanin-1 OS=Homo sa
2020	1	SwissProt_HUMAN$	3::P19174|PLCG1_HUMAN	16	148438	1	1	1	1	0.03	1-phosphatidylinositol 4
2021	1	SwissProt_HUMAN$	3::Q8IZ21|PHAR4_HUMAN	16	78163	1	1	1	1	0.06	Phosphatase and actin
2022	1	SwissProt_HUMAN$	3::Q96EG3|ZN837_HUMAN	16	58041	1	1	1	1	0.08	Zinc finger protein 837

↑Family	M	DB	Accession	Score	Mass	Matches	Match(sig)	Sequences	Seq(sig)	emPAI	Description
2023	1	db$2	2::C3KCV5|C3KCV5_PSEFS	16	37705	1	1	1	1	0.13	Uncharacterized protein
2024	1	db$2	2::C3K055|C3K055_PSEFS	16	30642	1	1	1	1	0.17	Uncharacterized protein
2025	1	SwissProt_HUMAN$	3::O15050|TRNK1_HUMAN	16	336007	1	1	1	1	0.01	TPR and ankyrin repeat
2026	1	db$2	2::C3K964|C3K964_PSEFS	16	26948	1	1	1	1	0.19	Putative GntR-family re
2027	1	db$2	2::C3JYY4|C3JYY4_PSEFS	16	32072	1	1	1	1	0.16	Putative LysR-family tra
2028	1	SwissProt_HUMAN$	3::P54821|PRRX1_HUMAN	16	27280	1	1	1	1	0.19	Paired mesoderm home
2029	1	SwissProt_HUMAN$	3::Q6PD62|CTR9_HUMAN	16	133420	1	1	1	1	0.04	RNA polymerase-associ
2030	1	db$2	2::C3K6G1|RLMF_PSEFS	15	38015	1	1	1	1	0.13	Ribosomal RNA large su
2031	1	db$2	2::C3KAT1|C3KAT1_PSEFS	15	27898	1	1	1	1	0.18	Response regulator pro
2032	1	SwissProt_HUMAN$	3::Q14185|DOCK1_HUMAN	15	215209	1	1	1	1	0.02	Dedicator of cytokinesis
2033	1	SwissProt_HUMAN$	3::Q13219|PAPP1_HUMAN	15	180856	1	1	1	1	0.03	Pappalysin-1 OS=Homo
2034	1	db$2	2::C3K732|TPMT_PSEFS	15	24819	1	1	1	1	0.21	Thiopurine S-methyltra
2035	1	db$2	2::C3K2T4|C3K2T4_PSEFS	15	155869	1	1	1	1	0.03	Putative ATP-dependen
2036	1	db$2	2::C3K5L4|C3K5L4_PSEFS	15	28633	1	1	1	1	0.18	Phosphonates ABC tran
2037	1	SwissProt_HUMAN$	3::P01303|NPY_HUMAN	15	10845	1	1	1	1	0.53	Pro-neuropeptide Y OS=
2038	1	db$2	2::C3KCH1|C3KCH1_PSEFS	15	32212	1	1	1	1	0.16	Putative carboxymethyl
2039	1	SwissProt_HUMAN$	3::Q05D60|DEUP1_HUMAN	15	70933	1	1	1	1	0.07	Deuterosome assembly
2040	1	db$2	2::C3K323|C3K323_PSEFS	15	38795	1	1	1	1	0.13	Putative phosphotransf
2041	1	db$2	2::C3K1I0|C3K1I0_PSEFS	15	22456	1	1	1	1	0.23	Nudix hydrolase domain
2042	1	db$2	2::C3KDD0|C3KDD0_PSEFS	14	19011	1	1	1	1	0.28	Lipid A deacylase OS=P
2043	1	db$2	2::C3KCU6|C3KCU6_PSEFS	14	23028	2	1	2	1	0.23	Putative membrane pro
2044	1	SwissProt_HUMAN$	3::Q8NHQ9|DDX55_HUMAN	14	68503	1	1	1	1	0.07	ATP-dependent RNA he
2045	1	SwissProt_HUMAN$	3::Q7L590|MCM10_HUMAN	14	98121	1	1	1	1	0.05	Protein MCM10 homolog
2046	1	SwissProt_HUMAN$	3::P14649|MYL6B_HUMAN	14	22750	1	1	1	1	0.23	Myosin light chain 6B O
2047	1	db$2	2::C3K5J0|C3K5J0_PSEFS	14	13887	1	1	1	1	0.40	Putative lipoprotein OS=
2048	1	SwissProt_HUMAN$	3::Q15652|JHD2C_HUMAN	14	284349	1	1	1	1	0.02	Probable JmjC domain-
2049	1	db$2	2::C3JZ45|C3JZ45_PSEFS	14	19122	1	1	1	1	0.28	Gluconokinase OS=Pseu
2050	1	SwissProt_HUMAN$	3::O00139|KIF2A_HUMAN	14	79905	2	1	2	1	0.06	Kinesin-like protein KIF
2051	1	db$2	2::C3K4W4|C3K4W4_PSEFS	14	45211	1	1	1	1	0.11	Putative lipoprotein rele
2052	1	db$2	2::C3JZI1|C3JZI1_PSEFS	14	33491	1	1	1	1	0.15	Putative glycine betaine
2053	1	SwissProt_HUMAN$	3::Q9BXU3|TX13A_HUMAN	14	45554	1	1	1	1	0.11	Testis-expressed protei
2054	1	db$2	2::C3K869|C3K869_PSEFS	14	24016	1	1	1	1	0.22	Putative lipoprotein OS=
2055	1	SwissProt_HUMAN$	3::O43166|SI1L1_HUMAN	14	199904	1	1	1	1	0.02	Signal-induced prolifera
2056	1	db$2	2::C3K1F6|C3K1F6_PSEFS	13	23787	2	1	2	1	0.22	Ribosomal RNA small su
2057	1	SwissProt_HUMAN$	3::O43897|TLL1_HUMAN	13	114635	1	1	1	1	0.04	Tolloid-like protein 1 OS
2058	1	db$2	2::C3K558|C3K558_PSEFS	13	65740	1	1	1	1	0.07	Exo-poly-alpha-D-galac
2059	1	db$2	2::C3K6C3|C3K6C3_PSEFS	13	28259	1	1	1	1	0.18	Putative amino acid AB(
2060	1	SwissProt_HUMAN$	3::Q3SY89|ELB3B_HUMAN	13	59723	1	1	1	1	0.08	Elongin-A3 member B (
2061	1	SwissProt_HUMAN$	3::Q86VN1|VPS36_HUMAN	13	43789	1	1	1	1	0.11	Vacuolar protein-sorting
2062	1	db$2	2::C3KC11|C3KC11_PSEFS	13	29623	1	1	1	1	0.17	Putative GerE familyreg

↑Family	M	DB	Accession	Score	Mass	Matches	Match(sig)	Sequences	Seq(sig)	emPAI	Description
2063	1	SwissProt_HUMAN$	3::Q9ULU8|CAPS1_HUMAN	13	152690	1	1	1	1	0.03	Calcium-dependent sec
2064	1	SwissProt_HUMAN$	3::Q96GC6|ZN274_HUMAN	13	74131	1	1	1	1	0.07	Neurotrophin receptor-i

Export as CSV

Not what you expected? Try **the select summary**.

Mascot: http://www.matrixscience.com/
