# Supplementary material for: Circulating multimeric immune complexes contribute to immunopathology in COVID-19
Source: Nat Commun. 2022 Sep 26;13:5654. doi: 10.1038/s41467-022-32867-z (PMC9513013; doi:10.1038/s41467-022-32867-z)
Supplement: Supplementary file 3 — Source Data [file 41467_2022_32867_MOESM3_ESM.zip › Source Data/MS_20210407 (immune precipitation).zip!/QX20210618-09_Virologie(AG-Hengel)-20210407_(V9)_0k3v20_TRL_db_SC2+db_Pf+SP_H(F235822).pdf]

MATRIX SCIENCE MASCOT Search Results

**User**	: Wolfgang Bildl
**E-mail**	: wolfgang.bildl@physiologie.uni-freiburg.de
**Search title**	: QX 20210618 (db$ SARS-CoV-2+db$2 Pf+SP H$ 5 ppm 20 mmu), QX20210618-09_Virologie(AG-Hengel)-20210407_(V9)_0k3v20_TRL_shifted_-1.0.mgf
**MS data file**	: D:\Xcalibur\Data\Wolfgang\Q-Exactive-HF-X_20210616\QX20210618-09_Virologie(AG-Hengel)-20210407_(V9)_0k3v20_TRL_shifted_-1.0.mgf
**Databases**	: **1:** db$ 20210617 (17 sequences; 14,439 residues)<br>**2:** db$2 20210617 (6,388 sequences; 2,089,672 residues)<br>**3:** SwissProt_HUMAN$ 20201007 (20,389 sequences; 11,363,789 residues)
**Timestamp**	: 21 Jun 2021 at 13:06:35 GMT

Not what you expected? Try **the select summary**.

Search parameters

**Type of search**	: MS/MS Ion Search
**Enzyme**	: Trypsin
**Variable modifications**	: **Acetyl (Protein N-term)**, **Carbamidomethyl (C)**, **Formyl (Protein N-term)**, **Gln->pyro-Glu (N-term Q)**, **Oxidation (M)**
**Mass values**	: Monoisotopic
**Protein mass**	: Unrestricted
**Peptide mass tolerance**	: ± 5 ppm
**Fragment mass tolerance**	: ± 20 mmu
**Max missed cleavages**	: 1
**Instrument type**	: ESI-FTICR
**Number of queries**	: 84,099

Score distribution

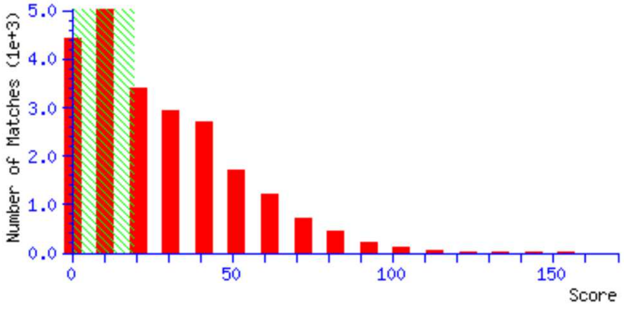

Peptide score distribution. Ions score is $-10\log(P)$, where P is the probability that the observed match is a random event.
There are **12,348** peptide matches above identity threshold and **13,123** matches above homology threshold for **84,099** queries.
On average, individual ions scores **> 19** (beyond green shading) indicate **identity or extensive homology** (p<0.05).

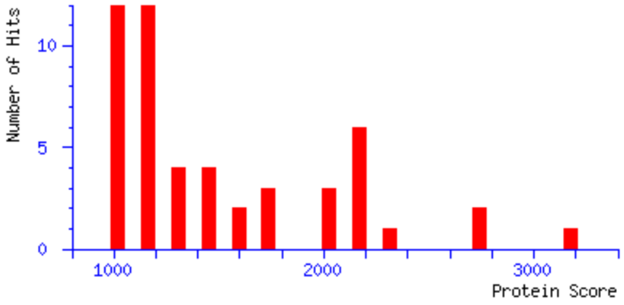

[Deprecated] Protein score distribution. Score distribution for family members in the first 50 proteins. Protein scores are derived from ions scores as a non-probabilistic basis for ranking protein families.

▼Modification statistics

Modification	Site	Above thr.
Oxidation	M	2430
Carbamidomethyl	C	1328
Gln->pyro-Glu	N-term	275
Formyl	Protein N-term	37
Acetyl	Protein N-term	14

▼Legend

Peptide columns and rows

Dupes	...	Expect	Rank	U	1	2	Peptide	
		**0.037**	▶**2**				**GAYSLSLR**	significant
		9	▶1				GFFLFVEGGR	top ranking
		**6.4e-005**	▶**1**				**GSSIFGLAPGK**	significant and top ranking
		**1.3e-006**	▶**1**		■		**SSGTSYPDVLK**	peptide is found in all proteins in family member 1
		**6.2e-007**	▶**1**			■	**VCNYVSWIK**	peptide is found in some but not all proteins in family member 2
		**6.4e-005**	▶**1**	**U**			**GSSIFGLAPGK**	unique
▶**2**		**5.7e-005**	▶**1**				**LNTLETEEWFFK**	peptide has two duplicates
		0.18	▶*1*				*LNTLETEEWFFK*	duplicate peptide

Right-facing triangle (▶) in the Dupes or Rank column indicates content that can be expanded by clicking on it. Down-facing triangle (▼) indicates the content is expanded and can be collapsed. For more details about particular columns, see **results format help**.

Protein quantitation ratios

	Score	...	114/113	115/113
**CFAH_HUMAN**	37559		*0.962*	**1.129**
**FHR2_HUMAN**	1330		**0.859**	1.128

When quantitation method is Reporter (e.g. iTRAQ) or Multiplex (e.g. IPTL), protein ratios are displayed when a family is expanded. Ratios in *italic* indicate that the peptide log-ratios do not appear to come from a normal distribution. **Bold** indicates that if you can assume peptide ratios are normally distributed, the protein ratio is significantly different from 1.0 (at significance level 0.05).

Note that lack of bold or italic can also mean that significance or normality testing has not been performed (for

example, if protein ratio type does not support it).

Protein Family Summary

Significance threshold p<	0.05	Max. number of families	AUTO
Ions score or expect cut-off	0.5	Dendrograms cut at	0
Show Percolator scores	☐		
Preferred taxonomy	All entries		

Sensitivity and FDR (reversed protein sequences)

			Target	Decoy	FDR		
PSMs	above	homology	13123	449	3.42%	Adjust to	1%

Decoy results are available in **the decoy report**.

Protein hits (2118 proteins)

Filters: (none)

Export as CSV

↑Family	M	DB	Accession	Score	Mass	Matches	Match(sig)	Sequences	Seq(sig)	emPAI	Description
1	1	db$2	2::C3K2Y3|RPOB_PSEFS	3174	150923	121	117	75	74	12.62	DNA-directed RNA poly
2	1	db$2	2::C3K2X8|EFTU_PSEFS	2758	43536	86	80	28	27	43.77	Elongation factor Tu O
2	2	SwissProt_HUMAN$	3::Q96SB8|SMC6_HUMAN	38	126246	3	2	3	2	0.08	Structural maintenanc
2	3	SwissProt_HUMAN$	3::Q5TB80|CE162_HUMAN	32	161843	2	2	2	2	0.06	Centrosomal protein o
3	1	db$2	2::C3K5G3|C3K5G3_PSEFS	2711	99192	85	81	37	37	9.92	Aconitate hydratase O
4	1	db$2	2::C3K2Y2|RPOC_PSEFS	2373	154528	101	97	71	68	10.34	DNA-directed RNA poly
5	1	db$2	2::C3K4N0|C3K4N0_PSEFS	2190	43479	57	57	25	25	39.16	Putative ornithine deca
6	1	SwissProt_HUMAN$	3::P13645|K1C10_HUMAN	2181	58792	57	53	32	30	15.72	Keratin, type I cytoske
6	2	SwissProt_HUMAN$	3::P02533|K1C14_HUMAN	531	51529	21	18	17	14	2.97	Keratin, type I cytoske
6	3	db$2	2::C3K610|C3K610_PSEFS	302	70283	20	18	17	16	1.94	ATP-binding protein U
6	4	SwissProt_HUMAN$	3::P08779|K1C16_HUMAN	275	51236	15	13	11	9	1.52	Keratin, type I cytoske
6	5	SwissProt_HUMAN$	3::P13646|K1C13_HUMAN	159	49557	11	9	8	7	0.95	Keratin, type I cytoske
6	6	SwissProt_HUMAN$	3::P05783|K1C18_HUMAN	32	48029	4	4	4	4	0.48	Keratin, type I cytoske
7	1	db$2	2::C3K5Z6|EFTS_PSEFS	2150	30341	79	73	16	16	11.03	Elongation factor Ts O
8	1	db$2	2::C3K1E6|ATPB_PSEFS	2133	49459	68	66	29	29	36.74	ATP synthase subunit
9	1	db$2	2::C3K1A0|CH60_PSEFS	2113	56931	69	65	30	29	26.81	60 kDa chaperonin OS
10	1	db$2	2::C3K4D5|C3K4D5_PSEFS	2097	34599	73	69	22	21	44.34	Phosphate-binding pro
11	1	db$2	2::C3KD82|C3KD82_PSEFS	2058	181591	71	67	56	52	3.81	Glutamate dehydroger
12	1	db$2	2::C3K1E8|ATPA_PSEFS	1998	55245	64	61	29	28	24.89	ATP synthase subunit
13	1	db$2	2::C3K2X9|EFG_PSEFS	1994	77114	73	70	31	31	12.21	Elongation factor G OS
14	1	db$2	2::C3K0Q5|C3K0Q5_PSEFS	1722	107302	63	58	43	39	6.99	Ribonucleoside-diphos

↑Family	M	DB	Accession	Score	Mass	Matches	Match(sig)	Sequences	Seq(sig)	emPAI	Description
15	1	db$2	2::C3KCJ5|C3KCJ5_PSEFS	1708	93957	58	56	35	35	6.54	Aconitate hydratase B
16	1	SwissProt_HUMAN$	3::P04264|K2C1_HUMAN	1704	65999	53	51	33	33	13.21	Keratin, type II cytosk
16	2	SwissProt_HUMAN$	3::P35908|K22E_HUMAN	1262	65393	51	47	38	38	17.14	Keratin, type II cytosk
16	3	SwissProt_HUMAN$	3::P02538|K2C6A_HUMAN	367	60008	17	16	17	16	2.53	Keratin, type II cytosk
16	4	SwissProt_HUMAN$	3::P13647|K2C5_HUMAN	366	62340	16	16	15	15	2.12	Keratin, type II cytosk
16	5	SwissProt_HUMAN$	3::P05787|K2C8_HUMAN	176	53671	7	7	6	6	0.70	Keratin, type II cytosk
16	6	SwissProt_HUMAN$	3::P08729|K2C7_HUMAN	126	51354	6	6	5	5	0.58	Keratin, type II cytosk
16	7	SwissProt_HUMAN$	3::Q7RTS7|K2C74_HUMAN	98	57830	6	6	4	4	0.39	Keratin, type II cytosk
17	1	db$2	2::C3K255|PNP_PSEFS	1593	74889	55	51	32	29	10.79	Polyribonucleotide nuc
18	1	db$2	2::C3K6M5|C3K6M5_PSEFS	1543	63522	39	37	22	21	4.99	Succinate dehydrogena
19	1	db$2	2::C3K275|DNAK_PSEFS	1498	68158	43	42	25	25	6.51	Chaperone protein Dna
20	1	db$2	2::C3KCP4|C3KCP4_PSEFS	1490	99394	74	67	44	41	9.89	Pyruvate dehydrogena
21	1	db$2	2::C3KBH1|C3KBH1_PSEFS	1476	161946	55	55	40	40	2.74	Glutamate synthase [N
22	1	db$2	2::C3K0P3|C3K0P3_PSEFS	1424	118163	38	34	24	21	1.52	Ribonuclease E OS=Ps
23	1	db$2	2::C3K6N0|SUCC_PSEFS	1293	41262	42	41	25	24	20.96	Succinate--CoA ligase
24	1	db$2	2::C3JYU7|C3JYU7_PSEFS	1287	102460	37	34	25	24	2.66	Protein translocase sul
25	1	db$2	2::C3K331|C3K331_PSEFS	1272	73691	47	43	32	31	7.35	AAA_PrkA domain-con
26	1	db$2	2::C3K225|ILVC_PSEFS	1265	36188	43	43	24	24	32.83	Ketol-acid reductoisom
27	1	db$2	2::C3K121|C3K121_PSEFS	1216	86030	48	46	31	31	5.89	Phosphoenolpyruvate s
28	1	db$2	2::C3K402|C3K402_PSEFS	1215	83101	49	47	31	29	6.79	Polyphosphate kinase
29	1	db$2	2::C3K271|C3K271_PSEFS	1205	117563	41	37	30	27	2.64	Carbamoyl-phosphate
30	1	db$2	2::C3K613|FADB_PSEFS	1192	76938	43	42	27	26	4.62	Fatty acid oxidation co
31	1	db$2	2::C3K5J5|FABV_PSEFS	1188	42933	47	44	25	22	14.65	Enoyl-[acyl-carrier-pro
32	1	db$2	2::C3K7D1|C3K7D1_PSEFS	1171	67115	43	42	28	27	8.56	50S ribosomal subunit
33	1	db$2	2::C3K6P2|C3K6P2_PSEFS	1151	42870	34	33	15	15	9.14	3-oxoacyl-[acyl-carrier
34	1	db$2	2::C3KBY8|C3KBY8_PSEFS	1143	21919	44	41	16	16	72.18	Alkyl hydroperoxide re
35	1	db$2	2::C3K259|IF2_PSEFS	1143	90544	46	43	31	28	4.93	Translation initiation fa
36	1	db$2	2::C3K3Z0|C3K3Z0_PSEFS	1140	46925	34	33	24	23	12.71	Transcription terminati
37	1	db$2	2::C3K7D3|C3K7D3_PSEFS	1137	91800	37	37	27	27	3.96	Alpha-1,4 glucan phos
38	1	SwissProt_HUMAN$	3::P35527|K1C9_HUMAN	1119	62027	31	30	22	21	5.25	Keratin, type I cytoske
39	1	db$2	2::C3JYK1|TIG_PSEFS	1090	48316	40	36	23	20	11.76	Trigger factor OS=Pse
40	1	db$2	2::C3K6M7|C3K6M7_PSEFS	1088	105930	47	43	34	32	4.02	Oxoglutarate dehydrog
41	1	db$2	2::C3K3F3|C3K3F3_PSEFS	1077	72324	46	46	30	30	8.89	Transketolase OS=Pse
42	1	db$2	2::C3K673|C3K673_PSEFS	1074	47725	39	36	20	19	12.14	Citrate synthase OS=F
43	1	db$2	2::C3KEC5|C3KEC5_PSEFS	1071	97398	44	40	33	31	3.99	Phosphoenolpyruvate
44	1	db$2	2::C3K6H4|PYRG_PSEFS	1070	59660	35	31	21	19	4.74	CTP synthase OS=Pse
45	1	db$2	2::C3K0T0|C3K0T0_PSEFS	1068	34560	37	37	10	10	4.15	Putative outer membra
46	1	db$2	2::C3K162|C3K162_PSEFS	1055	52023	33	33	21	21	7.86	Pyruvate kinase OS=P
47	1	db$2	2::C3K6K0|C3K6K0_PSEFS	1051	62268	46	42	29	25	8.08	30S ribosomal protein
48	1	db$2	2::C3JYT1|SYDND_PSEFS	1048	66220	37	33	23	20	4.18	Aspartate--tRNA(Asp/

↑Family	M	DB	Accession	Score	Mass	Matches	Match(sig)	Sequences	Seq(sig)	emPAI	Description
49	1	db$2	2::C3JYR1|GCSP_PSEFS	1033	102176	45	43	28	27	2.84	Glycine dehydrogenase
50	1	db$2	2::C3JYJ7|C3JYJ7_PSEFS	1019	9057	58	48	9	9	156.42	DNA-binding protein H
51	1	db$2	2::C3KE86|C3KE86_PSEFS	977	95293	44	40	36	32	4.44	Chaperone protein Clp
52	1	db$2	2::C3K6N1|C3K6N1_PSEFS	963	29928	27	25	14	13	15.94	Succinate--CoA ligase
53	1	db$2	2::C3K6M8|C3K6M8_PSEFS	962	42320	25	24	14	14	5.68	Dihydrolipoyllysine-res
54	1	db$2	2::C3KBJ2|C3KBJ2_PSEFS	960	24354	36	35	13	13	31.47	Carbonic anhydrase OS
55	1	db$2	2::C3KBT1|C3KBT1_PSEFS	959	44808	37	36	21	20	12.95	4-aminobutyrate amin
56	1	db$2	2::C3K7G0|C3K7G0_PSEFS	952	36980	29	28	18	18	11.86	Cell shape-determining
57	1	db$2	2::C3KBT0|C3KBT0_PSEFS	928	51607	34	33	19	18	8.89	Succinate-semialdehyd
58	1	db$2	2::C3K139|C3K139_PSEFS	925	48816	27	26	10	10	2.20	Uncharacterized protei
59	1	SwissProt_HUMAN$	3::P02768|ALBU_HUMAN	923	69321	42	39	29	28	7.90	Albumin OS=Homo sa
59	2	SwissProt_HUMAN$	3::P02769|ALBU_BOVIN	253	69248	16	14	13	12	1.27	Albumin OS=Bos tauru
60	1	db$2	2::C3K6N7|HTPG_PSEFS	923	71589	35	32	23	22	3.90	Chaperone protein Htp
61	1	db$2	2::C3JYS1|C3JYS1_PSEFS	921	17688	24	23	8	7	9.87	Peptidoglycan-associat
62	1	db$2	2::C3KDP2|C3KDP2_PSEFS	920	68879	34	32	24	24	4.58	Multifunctional fusion p
63	1	db$2	2::C3K1J2|C3K1J2_PSEFS	919	140531	44	40	32	28	2.05	Phosphoribosylformylg
64	1	db$2	2::C3K1L8|NDK_PSEFS	915	14928	30	28	8	7	11.28	Nucleoside diphosphat
65	1	db$2	2::C3K1K0|GUAA_PSEFS	904	58398	34	32	27	26	7.93	GMP synthase [glutam
66	1	db$2	2::C3K3S2|C3K3S2_PSEFS	897	44244	30	25	19	16	4.52	2-oxoglutarate reducta
67	1	db$2	2::C3JY65|C3JY65_PSEFS	896	45375	34	32	19	18	15.64	Isocitrate dehydrogena
68	1	db$2	2::C3JY66|C3JY66_PSEFS	885	80156	37	30	23	20	2.90	Isocitrate dehydrogena
69	1	db$2	2::C3K6J4|C3K6J4_PSEFS	881	97893	39	38	30	29	3.49	DNA gyrase subunit A
70	1	db$2	2::C3KCS1|C3KCS1_PSEFS	874	34171	28	28	16	16	14.79	Ribose-phosphate pyro
71	1	db$2	2::C3K165|C3K165_PSEFS	869	54857	38	34	24	23	10.21	Fumarate hydratase cl
71	2	db$2	2::C3K604|C3K604_PSEFS	353	18933	18	17	12	11	18.32	Putative outer membra
72	1	db$2	2::C3K4D2|C3K4D2_PSEFS	864	30988	38	35	20	19	51.28	Phosphate import ATP-
73	1	db$2	2::C3K6M9|C3K6M9_PSEFS	862	49948	27	26	13	13	3.55	Dihydrolipoyl dehydrog
74	1	db$2	2::C3KCB8|C3KCB8_PSEFS	848	36708	32	30	16	15	10.48	Phosphate-binding pro
75	1	db$2	2::C3K426|ARLY_PSEFS	847	51545	27	26	15	15	5.28	Argininosuccinate lyase
76	1	db$2	2::C3K1A7|C3K1A7_PSEFS	845	66070	33	28	24	21	4.21	Proline--tRNA ligase O
77	1	db$2	2::C3K2V9|RS5_PSEFS	820	17675	25	23	13	11	52.31	30S ribosomal protein
78	1	db$2	2::C3K4J5|PCKA_PSEFS	805	55324	31	27	21	18	4.54	Phosphoenolpyruvate c
79	1	db$2	2::C3JYJ9|CLPX_PSEFS	804	46924	26	25	18	17	6.49	ATP-dependent Clp pro
80	1	db$2	2::C3K0K6|C3K0K6_PSEFS	803	26352	22	21	12	11	11.23	Electron transfer flavo
81	1	db$2	2::C3K1K1|C3K1K1_PSEFS	790	51928	38	36	24	22	10.69	Inosine-5'-monophosp
82	1	db$2	2::C3KE67|RS6_PSEFS	786	16276	25	22	14	13	96.61	30S ribosomal protein
83	1	db$2	2::C3KDD8|C3KDD8_PSEFS	782	44893	29	29	21	21	11.55	Cell division protein Fts
84	1	db$2	2::C3KE40|SYGB_PSEFS	777	74833	33	32	24	24	4.53	Glycine--tRNA ligase b
85	1	db$2	2::C3KDW8|PURA_PSEFS	763	46535	32	31	19	18	8.36	Adenylosuccinate synth
86	1	db$2	2::C3K2Y6|RL1_PSEFS	759	24061	23	22	13	13	21.69	50S ribosomal protein

↑Family	M	DB	Accession	Score	Mass	Matches	Match(sig)	Sequences	Seq(sig)	emPAI	Description
87	1	db$2	2::C3KE51|C3KE51_PSEFS	756	38026	31	30	21	20	12.52	Putative oxidoreductas
88	1	db$2	2::C3K2R9|C3K2R9_PSEFS	743	16440	16	16	6	6	8.73	6,7-dimethyl-8-ribitylli
89	1	db$2	2::C3K2D3|C3K2D3_PSEFS	741	44901	27	26	17	17	6.37	Serine hydroxymethylt
90	1	db$2	2::C3K1E7|ATPG_PSEFS	740	31374	21	21	12	12	10.03	ATP synthase gamma
91	1	db$2	2::C3K603|C3K603_PSEFS	734	87560	31	31	23	23	3.09	Outer membrane prote
91	2	db$2	2::C3K3R2|C3K3R2_PSEFS	567	83481	26	26	20	20	2.11	Phosphoenolpyruvate-
91	3	SwissProt_HUMAN$	3::Q5K651|SAMD9_HUMAN	26	184165	2	2	2	2	0.05	Sterile alpha motif dor
92	1	db$2	2::C3K6H6|C3K6H6_PSEFS	732	45588	25	23	16	14	3.73	Enolase OS=Pseudomo
92	2	SwissProt_HUMAN$	3::P06733|ENOA_HUMAN	54	47139	2	2	2	2	0.22	Alpha-enolase OS=Hor
93	1	db$2	2::C3KDG0|RECA_PSEFS	731	37499	22	22	14	14	9.92	Protein RecA OS=Pseu
94	1	db$2	2::C3K6G5|AMPA_PSEFS	731	52403	34	32	23	22	7.74	Probable cytosol amino
95	1	db$2	2::C3JYT4|C3JYT4_PSEFS	720	17756	26	24	10	9	51.02	Putative ferritin DPS-fa
96	1	db$2	2::C3K569|C3K569_PSEFS	718	44415	31	30	12	11	2.59	Tryptophan synthase b
97	1	db$2	2::C3K3J7|C3K3J7_PSEFS	715	35008	31	30	17	17	16.01	Glutathione synthetase
98	1	db$2	2::C3KBM0|GLPK_PSEFS	704	55508	32	30	21	20	5.53	Glycerol kinase OS=Ps
99	1	db$2	2::C3JXY1|C3JXY1_PSEFS	701	93962	28	27	22	21	2.03	Alanine--tRNA ligase C
100	1	db$2	2::C3JY70|C3JY70_PSEFS	699	50668	23	23	16	16	4.38	Adenylosuccinate lyase
101	1	db$2	2::C3K367|C3K367_PSEFS	695	68142	30	29	19	19	3.02	Uncharacterized protei
102	1	db$2	2::C3K178|C3K178_PSEFS	692	36139	20	19	13	13	7.06	Glyceraldehyde-3-phos
103	1	db$2	2::C3K5E6|RS2_PSEFS	687	27078	25	22	17	15	18.18	30S ribosomal protein
104	1	db$2	2::C3K4D1|C3K4D1_PSEFS	685	28553	25	24	15	14	18.51	Phosphate-specific trai
105	1	db$2	2::C3K1W9|C3K1W9_PSEFS	660	33929	16	16	9	9	4.29	Thioredoxin reductase
106	1	db$2	2::C3K108|C3K108_PSEFS	656	83591	30	27	24	22	2.69	GTP pyrophosphokinas
107	1	db$2	2::C3KDW5|C3KDW5_PSEFS	653	43265	27	25	20	19	8.90	Protein HflK OS=Pseuc
108	1	db$2	2::C3K7C9|C3K7C9_PSEFS	652	51641	30	27	18	17	4.71	Glutamine synthetase
109	1	db$2	2::C3K6I0|C3K6I0_PSEFS	651	39056	27	26	15	15	10.21	Alcohol dehydrogenase
110	1	db$2	2::C3JZN7|SYT_PSEFS	650	72513	22	20	18	17	2.46	Threonine--tRNA ligase
111	1	db$2	2::C3KCP3|C3KCP3_PSEFS	648	56221	21	21	11	11	1.53	Acetyltransferase com
112	1	db$2	2::C3K2D0|C3K2D0_PSEFS	648	61490	23	21	18	18	3.00	Energy-dependent trai
113	1	db$2	2::C3K6H5|KDSA_PSEFS	647	30777	27	26	11	10	12.54	2-dehydro-3-deoxypho
114	1	db$2	2::C3K260|C3K260_PSEFS	644	54381	21	20	14	14	3.79	Transcription terminati
115	1	db$2	2::C3KDR8|OPGD_PSEFS	640	60485	22	21	16	16	2.50	Glucans biosynthesis p
116	1	db$2	2::C3KDU5|C3KDU5_PSEFS	633	90380	23	22	19	19	2.01	DNA gyrase subunit B
117	1	db$2	2::C3K1E2|C3K1E2_PSEFS	632	66300	20	20	14	14	1.92	Glutamine--fructose-6-
118	1	db$2	2::C3K4F6|C3K4F6_PSEFS	624	65489	36	34	25	24	6.05	Oxaloacetate decarbox
119	1	db$2	2::C3K016|C3K016_PSEFS	621	85788	23	21	18	16	1.56	Ribosomal RNA large s
120	1	db$2	2::C3K3G6|SAHH_PSEFS	613	51212	29	27	20	18	8.20	Adenosylhomocysteina
121	1	db$2	2::C3JYQ5|C3JYQ5_PSEFS	610	46409	24	22	17	17	5.26	Arginine deiminase OS
122	1	db$2	2::C3K6W6|C3K6W6_PSEFS	607	49345	31	25	21	18	6.47	Biotin carboxylase OS=
123	1	db$2	2::C3K7Y0|C3K7Y0_PSEFS	596	45268	22	22	14	14	5.55	Putative exported prot

↑Family	M	DB	Accession	Score	Mass	Matches	Match(sig)	Sequences	Seq(sig)	emPAI	Description
124	1	db$2	2::C3K2X5|RL4_PSEFS	593	21987	14	14	9	9	7.46	50S ribosomal protein
125	1	db$2	2::C3KAH7|C3KAH7_PSEFS	592	45018	28	27	18	18	8.05	Putative NADP-depend
126	1	db$2	2::C3JZN3|SYFA_PSEFS	592	37892	15	14	12	12	4.73	Phenylalanine--tRNA li
127	1	db$2	2::C3KBZ2|ASSY_PSEFS	590	45330	30	22	19	16	4.90	Argininosuccinate synt
128	1	db$2	2::C3K4D4|C3K4D4_PSEFS	589	83859	32	27	20	17	2.67	Putative phosphate AB
129	1	db$2	2::C3KA76|C3KA76_PSEFS	589	30860	18	16	12	12	6.28	UTP--glucose-1-phospl
130	1	db$2	2::C3K8B0|C3K8B0_PSEFS	587	25585	18	18	10	10	8.12	Peptidyl-prolyl cis-tran
131	1	db$2	2::C3JZT6|C3JZT6_PSEFS	586	40569	17	15	13	11	3.55	Aspartate-semialdehyc
132	1	db$2	2::C3JZS1|C3JZS1_PSEFS	582	55264	26	22	18	16	3.67	Amidophosphoribosylti
133	1	db$2	2::C3KEC8|C3KEC8_PSEFS	580	50623	18	17	12	11	2.37	Periplasmic serine end
134	1	db$2	2::C3K2V1|C3K2V1_PSEFS	575	36623	26	26	20	20	13.97	DNA-directed RNA poly
135	1	db$2	2::C3KEC2|C3KEC2_PSEFS	570	28614	21	20	14	13	7.48	Putative lipoprotein OS
135	2	db$2	2::C3KAJ6|C3KAJ6_PSEFS	172	38502	7	6	5	4	0.85	Histidinol-phosphate a
136	1	db$2	2::C3KAD2|ARNA_PSEFS	569	74140	20	19	17	17	1.96	Bifunctional polymyxin
137	1	db$2	2::C3KBH2|C3KBH2_PSEFS	565	51951	18	17	15	14	2.58	Glutamate synthase [N
138	1	db$2	2::C3K6M2|C3K6M2_PSEFS	564	60584	20	20	16	16	3.41	ATP-dependent RNA he
139	1	db$2	2::C3K484|C3K484_PSEFS	553	13502	13	12	9	8	21.05	Uncharacterized protei
140	1	db$2	2::C3K6H2|ACCA_PSEFS	549	34868	21	20	15	14	6.62	Acetyl-coenzyme A car
141	1	db$2	2::C3K2V6|C3K2V6_PSEFS	549	47842	17	17	8	8	1.97	Protein translocase sul
142	1	db$2	2::C3K4V9|C3K4V9_PSEFS	549	53185	26	22	18	16	3.96	Glyceraldehyde-3-phos
143	1	db$2	2::C3K2X0|C3K2X0_PSEFS	543	25737	21	20	14	14	17.58	30S ribosomal protein
144	1	db$2	2::C3K4C6|C3K4C6_PSEFS	543	25565	17	17	11	11	12.18	Phosphate regulon trai
145	1	db$2	2::C3JZV8|C3JZV8_PSEFS	539	38906	15	15	9	9	2.79	Neutral metalloprotein
146	1	db$2	2::C3K4Y4|C3K4Y4_PSEFS	539	71842	20	19	17	17	2.28	Potassium-transporting
147	1	db$2	2::C3KBL8|C3KBL8_PSEFS	538	56970	21	20	15	15	2.78	Glycerol-3-phosphate (
148	1	db$2	2::C3K266|C3K266_PSEFS	536	69364	16	16	12	12	1.43	ATP-dependent zinc m
149	1	db$2	2::C3JYS2|TOLB_PSEFS	533	47517	22	18	17	17	5.00	Tol-Pal system protein
150	1	db$2	2::C3JYV2|C3JYV2_PSEFS	532	52688	17	15	14	12	2.21	AAA domain-containing
151	1	db$2	2::C3JZV2|C3JZV2_PSEFS	531	43020	22	20	16	15	5.46	Aminotransferase OS=
152	1	db$2	2::C3K3P0|ILVD_PSEFS	530	65667	20	17	19	16	2.17	Dihydroxy-acid dehydr
153	1	db$2	2::C3K3F1|PGK_PSEFS	530	40125	17	16	13	12	3.63	Phosphoglycerate kina
154	1	db$2	2::C3K2W1|RL6_PSEFS	529	19185	17	16	8	8	10.47	50S ribosomal protein
155	1	db$2	2::C3K2M9|PROA_PSEFS	521	45380	18	18	12	12	3.30	Gamma-glutamyl phos
156	1	db$2	2::C3K649|C3K649_PSEFS	518	8822	15	15	4	4	7.15	Major outer membrane
157	1	db$2	2::C3JXL7|C3JXL7_PSEFS	517	39112	15	14	11	10	2.78	HlyD-family secretion (
158	1	db$2	2::C3K341|C3K341_PSEFS	513	69250	29	26	22	20	4.16	RNA polymerase sigma
158	2	db$2	2::C3K708|C3K708_PSEFS	262	38066	5	5	4	4	0.64	RNA polymerase sigma
159	1	db$2	2::C3K6W9|C3K6W9_PSEFS	503	69415	15	15	12	12	1.27	Putative methyl-accept
159	2	db$2	2::C3K5K7|C3K5K7_PSEFS	85	52604	4	3	3	2	0.20	Putative methyl-accept
159	3	db$2	2::C3JZ31|C3JZ31_PSEFS	77	58204	3	3	2	2	0.18	Putative methyl-accept

↑Family	M	DB	Accession	Score	Mass	Matches	Match(sig)	Sequences	Seq(sig)	emPAI	Description
159	4	db$2	2::C3K7Z5|C3K7Z5_PSEFS	47	66997	3	2	3	2	0.15	Putative methyl-accept
160	1	db$2	2::C3JZS4|C3JZS4_PSEFS	498	33375	16	15	12	11	4.44	Acetyl-coenzyme A car
161	1	db$2	2::C3KED7|PUR7_PSEFS	498	26862	21	20	15	14	12.84	Phosphoribosylaminoir
162	1	db$2	2::C3K2W6|RL14_PSEFS	496	13401	14	14	7	7	44.20	50S ribosomal protein
163	1	db$2	2::C3JZN6|IF3_PSEFS	496	20837	17	17	6	6	5.04	Translation initiation fa
164	1	db$2	2::C3K1N2|C3K1N2_PSEFS	493	67307	19	18	14	13	1.88	Protein translocase sul
165	1	db$2	2::C3K1L4|ISPG_PSEFS	490	39660	18	15	15	12	3.71	4-hydroxy-3-methylbu
166	1	db$2	2::C3JXY0|AK_PSEFS	488	44504	20	16	16	14	4.47	Aspartate kinase OS=I
167	1	db$2	2::C3KCB5|C3KCB5_PSEFS	487	30173	23	21	12	10	5.51	Phosphate import ATP-
168	1	db$2	2::C3KDU7|C3KDU7_PSEFS	486	28504	23	22	14	13	7.55	Putative ABC transport
169	1	db$2	2::C3K6C2|C3K6C2_PSEFS	484	27720	13	13	10	10	4.45	Putative amino acid AE
170	1	db$2	2::C3K9R0|C3K9R0_PSEFS	482	62265	16	15	13	13	1.90	Pyruvate dehydrogena
171	1	SwissProt_HUMAN$	3::P00761|TRYP_PIG	477	24394	11	11	6	6	2.85	Trypsin OS=Sus scrofa
172	1	db$2	2::C3K6X4|C3K6X4_PSEFS	477	71161	26	21	21	18	2.54	Biosynthetic arginine c
173	1	db$2	2::C3JY83|NUOCD_PSEFS	476	67660	26	24	19	18	4.36	NADH-quinone oxidore
174	1	db$2	2::C3K6G0|C3K6G0_PSEFS	476	9680	10	10	7	7	27.33	Putative DNA-binding
175	1	db$2	2::C3K4K9|C3K4K9_PSEFS	476	27377	14	13	9	8	4.57	Putative amino acid ox
176	1	db$2	2::C3K5E0|C3K5E0_PSEFS	475	36000	15	15	9	9	4.51	2,3,4,5-tetrahydropyri
177	1	db$2	2::C3JZ70|C3JZ70_PSEFS	473	54858	22	21	17	16	2.98	Glucose-6-phosphate
178	1	db$2	2::C3K1G5|C3K1G5_PSEFS	473	46195	15	15	12	12	2.79	Homoserine dehydroge
179	1	db$2	2::C3K865|C3K865_PSEFS	469	15847	10	10	5	5	4.84	Putative membrane pr
180	1	db$2	2::C3K4D3|C3K4D3_PSEFS	467	60551	18	17	14	14	2.23	Phosphate transport sy
181	1	db$2	2::C3KE70|RL9_PSEFS	467	15488	17	16	8	8	13.93	50S ribosomal protein
182	1	db$2	2::C3K9R4|C3K9R4_PSEFS	464	62164	16	16	14	14	1.91	Putative sulfite reducta
183	1	db$2	2::C3K123|C3K123_PSEFS	463	41542	11	11	8	8	1.49	Citrate synthase OS=F
184	1	db$2	2::C3K8X4|MURA_PSEFS	463	44975	22	21	16	15	4.97	UDP-N-acetylglucosam
185	1	db$2	2::C3K3F5|METK_PSEFS	462	42554	17	14	14	11	3.74	S-adenosylmethionine
186	1	db$2	2::C3K244|C3K244_PSEFS	460	52276	16	14	14	12	1.96	Putative response regu
187	1	db$2	2::C3K2Y7|RL11_PSEFS	458	14903	10	10	5	5	5.56	50S ribosomal protein
188	1	db$2	2::C3K1K7|LEU1_PSEFS	454	61997	23	21	19	17	2.95	2-isopropylmalate synt
189	1	db$2	2::C3KDE0|LPXC_PSEFS	453	33197	16	15	12	12	4.50	UDP-3-O-acyl-N-acety
190	1	db$2	2::C3K3U2|C3K3U2_PSEFS	452	50985	17	16	13	12	2.05	Putative glutamine syr
191	1	db$2	2::C3K358|C3K358_PSEFS	451	65409	17	16	14	13	1.76	Putative dehydrogenas
191	2	db$2	2::C3K355|C3K355_PSEFS	337	63710	12	12	11	11	1.27	Putative dehydrogenas
192	1	db$2	2::C3K7D6|F16PA_PSEFS	451	37081	19	18	13	12	4.23	Fructose-1,6-bisphosp
193	1	db$2	2::C3KAK3|C3KAK3_PSEFS	450	54608	19	16	16	14	2.36	Probable malate:quino
194	1	db$2	2::C3KE65|C3KE65_PSEFS	449	97331	23	23	20	20	1.78	Ribonuclease R OS=Ps
195	1	db$2	2::C3KBN3|C3KBN3_PSEFS	449	112655	17	17	11	11	0.73	Efflux pump membran
195	2	db$2	2::C3KB18|C3KB18_PSEFS	67	115005	4	4	4	4	0.18	Efflux pump membran
196	1	db$2	2::C3K2C4|C3K2C4_PSEFS	448	89060	17	16	16	15	1.22	Endopeptidase La OS=

↑Family	M	DB	Accession	Score	Mass	Matches	Match(sig)	Sequences	Seq(sig)	emPAI	Description
197	1	db$2	2::C3JXZ0|C3JXZ0_PSEFS	448	43332	16	15	11	11	2.32	Acetylornithine aminot
198	1	db$2	2::C3JZV0|C3JZV0_PSEFS	447	56140	16	15	16	15	2.54	Glutamate--tRNA ligas
199	1	db$2	2::C3K5Z7|C3K5Z7_PSEFS	447	26574	16	15	9	9	7.40	Uridylate kinase OS=P
200	1	db$2	2::C3K1X8|C3K1X8_PSEFS	446	11596	11	11	5	5	10.13	50S ribosomal protein
201	1	db$2	2::C3K6J6|C3K6J6_PSEFS	444	40307	17	16	12	11	4.16	Bifunctional chorismat
202	1	db$2	2::C3KEB4|C3KEB4_PSEFS	443	56978	21	18	17	15	2.78	Lysine--tRNA ligase OS
203	1	db$2	2::C3K3V3|ARGA_PSEFS	440	47793	13	11	11	9	1.43	Amino-acid acetyltrans
204	1	db$2	2::C3JY86|C3JY86_PSEFS	439	98164	20	18	17	15	1.07	NADH-quinone oxidore
205	1	db$2	2::C3JXL8|C3JXL8_PSEFS	438	112023	22	20	17	15	1.06	Putative efflux protein
206	1	db$2	2::C3JZN2|C3JZN2_PSEFS	433	86329	19	18	17	16	1.41	Phenylalanine--tRNA li
207	1	db$2	2::C3K324|C3K324_PSEFS	433	105271	20	20	18	18	1.25	LPS-assembly protein
208	1	db$2	2::C3K907|C3K907_PSEFS	432	77648	19	19	17	17	2.00	Tail-specific protease (
209	1	db$2	2::C3K914|C3K914_PSEFS	428	38509	17	16	10	9	3.35	Putative ABC transport
210	1	db$2	2::C3K2W0|RL18_PSEFS	424	12651	10	10	9	9	26.25	50S ribosomal protein
211	1	db$2	2::C3KBI6|C3KBI6_PSEFS	424	106629	20	17	17	14	0.95	Uncharacterized protei
212	1	db$2	2::C3K1L3|SYH_PSEFS	424	47853	19	17	14	12	3.86	Histidine--tRNA ligase
213	1	db$2	2::C3K0D4|C3K0D4_PSEFS	423	29228	16	16	11	11	5.93	Cell division inhibitor M
214	1	db$2	2::C3K616|C3K616_PSEFS	421	97222	23	22	22	21	1.79	DNA topoisomerase 1
215	1	db$2	2::C3K227|C3K227_PSEFS	421	62578	24	21	18	17	3.21	Acetolactate synthase
216	1	db$2	2::C3KAL6|C3KAL6_PSEFS	420	52109	16	16	13	13	2.57	Putative aminotransfer
217	1	db$2	2::C3K4C7|C3K4C7_PSEFS	420	50044	13	13	12	12	2.42	Phosphate regulon sen
218	1	db$2	2::C3K1G8|RL19_PSEFS	419	12961	15	14	9	9	50.34	50S ribosomal protein
219	1	db$2	2::C3K8X0|C3K8X0_PSEFS	419	16351	9	9	5	5	4.57	Putative ABC transport
220	1	db$2	2::C3K3N4|COAD_PSEFS	417	17810	16	16	8	8	12.94	Phosphopantetheine a
221	1	db$2	2::C3K325|C3K325_PSEFS	416	49076	17	17	14	14	2.86	Chaperone SurA OS=P
222	1	db$2	2::C3KDL2|C3KDL2_PSEFS	416	52736	15	14	13	12	1.94	Putative outer membra
223	1	db$2	2::C3K8X5|HIS1_PSEFS	416	23098	17	16	12	12	13.08	ATP phosphoribosyltra
224	1	db$2	2::C3K6D9|C3K6D9_PSEFS	415	45394	12	12	8	8	1.55	Phosphoribosylamine--
225	1	db$2	2::C3K0K5|C3K0K5_PSEFS	413	31365	15	15	8	8	3.48	Electron transfer flavo
226	1	db$2	2::C3KDU3|C3KDU3_PSEFS	411	40518	16	13	12	11	3.05	Beta sliding clamp OS=
227	1	db$2	2::C3JZS0|C3JZS0_PSEFS	411	43144	17	17	12	12	3.63	O-succinylhomoserine
228	1	db$2	2::C3K102|PUR5_PSEFS	407	36950	15	14	11	10	3.63	Phosphoribosylformylg
229	1	db$2	2::C3K6E0|PUR9_PSEFS	406	57370	16	15	14	14	2.45	Bifunctional purine bio
230	1	db$2	2::C3K3E8|C3K3E8_PSEFS	406	38385	24	22	16	15	9.34	Fructose-1,6-bisphosp
231	1	db$2	2::C3K2Z7|ARGC_PSEFS	404	36612	18	18	9	9	4.34	N-acetyl-gamma-gluta
232	1	db$2	2::C3K4W1|STHA_PSEFS	403	50963	18	17	14	13	3.03	Soluble pyridine nucle
233	1	db$2	2::C3K278|C3K278_PSEFS	403	15153	9	9	5	5	5.30	Ferric uptake regulatio
234	1	db$2	2::C3K5H9|C3K5H9_PSEFS	403	33776	16	14	13	11	4.35	Transaldolase OS=Pse
235	1	db$2	2::C3KDX4|SYI_PSEFS	401	105386	17	15	15	13	0.80	Isoleucine--tRNA ligas
236	1	db$2	2::C3K2V2|RS4_PSEFS	401	23062	19	15	12	11	10.49	30S ribosomal protein

↑Family	M	DB	Accession	Score	Mass	Matches	Match(sig)	Sequences	Seq(sig)	emPAI	Description
237	1	db$2	2::C3K1B7|C3K1B7_PSEFS	400	51892	16	15	12	11	1.99	Threonine synthase OS
238	1	db$2	2::C3K2Y4|RL7_PSEFS	400	12456	10	9	3	3	5.48	50S ribosomal protein
239	1	db$2	2::C3K1E9|ATPD_PSEFS	398	19246	16	16	9	9	10.47	ATP synthase subunit
240	1	db$2	2::C3K1M5|ISCS_PSEFS	398	44467	12	12	9	9	1.89	Cysteine desulfurase I
241	1	db$2	2::C3KE79|C3KE79_PSEFS	398	29713	9	9	7	7	2.03	NH(3)-dependent NAD
242	1	db$2	2::C3K0Q7|C3K0Q7_PSEFS	396	45255	8	8	7	7	1.08	Flagellar hook protein
243	1	db$2	2::C3K0H1|C3K0H1_PSEFS	396	17848	10	10	7	7	9.55	Chemotaxis protein Ch
244	1	db$2	2::C3K2X6|RL3_PSEFS	390	22550	13	12	8	8	4.30	50S ribosomal protein
245	1	db$2	2::C3K6J9|KCY_PSEFS	388	24780	9	9	7	7	2.78	Cytidylate kinase OS=
246	1	db$2	2::C3K2J8|C3K2J8_PSEFS	387	54392	16	14	14	12	2.10	AMP nucleosidase OS=
247	1	db$2	2::C3KDW6|C3KDW6_PSEFS	386	33055	22	18	15	11	6.36	Protein HflC OS=Pseuc
248	1	db$2	2::C3JYQ6|C3JYQ6_PSEFS	386	37842	22	19	14	12	5.49	Ornithine carbamoyltra
249	1	db$2	2::C3K2V0|RL17_PSEFS	385	14407	14	13	6	5	24.27	50S ribosomal protein
250	1	db$2	2::C3K3V2|C3K3V2_PSEFS	385	49111	15	14	9	8	1.16	Putative outer membra
251	1	db$2	2::C3K475|ARGB_PSEFS	384	31926	15	13	13	11	4.06	Acetylglutamate kinase
252	1	db$2	2::C3K2X3|RL2_PSEFS	383	29689	17	17	10	10	3.90	50S ribosomal protein
253	1	db$2	2::C3K6M6|C3K6M6_PSEFS	380	26063	15	15	12	12	9.43	Succinate dehydrogena
254	1	db$2	2::C3JYA7|C3JYA7_PSEFS	377	59461	16	16	11	11	1.40	Putative NADP-depend
255	1	db$2	2::C3K2K3|GSA_PSEFS	377	45181	14	12	11	10	2.51	Glutamate-1-semialde
256	1	db$2	2::C3KCR8|C3KCR8_PSEFS	375	39907	11	11	8	8	1.90	Ribosome-binding ATP
257	1	db$2	2::C3K645|C3K645_PSEFS	374	24522	10	10	8	8	3.63	Putative ABC transport
258	1	db$2	2::C3K274|DNAJ_PSEFS	373	40315	14	14	11	11	2.63	Chaperone protein Dna
259	1	db$2	2::C3K6M3|C3K6M3_PSEFS	373	13365	10	8	8	6	7.13	Succinate dehydrogena
260	1	db$2	2::C3JYU6|C3JYU6_PSEFS	373	42196	9	9	8	8	1.74	Arginine biosynthesis b
261	1	db$2	2::C3K6E1|RL13_PSEFS	371	15695	14	13	8	8	13.65	50S ribosomal protein
262	1	db$2	2::C3K902|RAPA_PSEFS	371	106722	16	15	15	14	0.86	RNA polymerase-assoc
263	1	db$2	2::C3KDP1|CYSD_PSEFS	370	35173	12	10	8	8	2.82	Sulfate adenylyltransfe
264	1	db$2	2::C3KCB7|C3KCB7_PSEFS	368	34191	7	6	4	3	0.74	Phosphate transport sy
265	1	db$2	2::C3K4F7|C3K4F7_PSEFS	368	51847	19	18	16	15	2.93	Biotin carboxylase OS=
266	1	db$2	2::C3K5A9|RF3_PSEFS	367	59584	15	13	12	10	1.40	Peptide chain release f
267	1	db$2	2::C3KDD9|C3KDD9_PSEFS	366	41969	13	13	13	13	3.32	Cell division protein Ft
268	1	db$2	2::C3K1F0|C3K1F0_PSEFS	365	16832	16	16	9	9	11.10	ATP synthase subunit I
269	1	db$2	2::C3KBQ2|C3KBQ2_PSEFS	364	37020	11	11	8	8	2.15	Putative quinone oxidc
270	1	db$2	2::C3K1X6|C3K1X6_PSEFS	363	44711	10	8	8	7	1.10	GTPase Obg OS=Pseuc
271	1	db$2	2::C3K401|C3K401_PSEFS	362	56255	19	18	17	16	2.84	Exopolyphosphatase O
272	1	db$2	2::C3K2L2|SYL_PSEFS	361	97066	22	19	19	18	1.53	Leucine--tRNA ligase C
273	1	db$2	2::C3KBX8|C3KBX8_PSEFS	361	26859	12	11	8	8	4.76	OmpA-like domain-cor
274	1	db$2	2::C3JYD4|C3JYD4_PSEFS	361	64707	16	15	14	13	1.59	Glutamine--tRNA ligas
275	1	db$2	2::C3JXZ7|C3JXZ7_PSEFS	360	28415	13	13	8	8	3.44	Putative histidine-bind
276	1	db$2	2::C3K142|C3K142_PSEFS	359	32099	11	11	9	9	3.35	2-methylisocitrate lyas

↑Family	M	DB	Accession	Score	Mass	Matches	Match(sig)	Sequences	Seq(sig)	emPAI	Description
277	1	db$2	2::C3KBY6|C3KBY6_PSEFS	359	17850	11	11	8	8	9.55	Bacterioferritin OS=Ps
278	1	db$2	2::C3KAC1|C3KAC1_PSEFS	357	27548	15	13	11	9	6.78	Putative phenylacetic a
279	1	db$2	2::C3JYW3|C3JYW3_PSEFS	357	36818	13	13	11	11	3.09	Branched-chain-amino
280	1	db$2	2::C3K403|C3K403_PSEFS	355	36964	12	12	8	8	1.78	Delta-aminolevulinic a
281	1	db$2	2::C3K4V4|C3K4V4_PSEFS	355	32182	11	10	8	8	2.74	Putative ATP-binding p
282	1	db$2	2::C3K7L5|C3K7L5_PSEFS	355	19131	10	10	7	7	6.12	Molybdenum cofactor l
283	1	db$2	2::C3JYJ8|C3JYJ8_PSEFS	354	88851	18	15	18	15	1.23	Lon protease OS=Pseu
284	1	db$2	2::C3K2S0|C3K2S0_PSEFS	353	39319	8	7	5	5	0.82	3,4-dihydroxy-2-butan
285	1	db$2	2::C3K6A2|C3K6A2_PSEFS	352	27847	13	12	10	9	3.58	Putative D-methionine
285	2	db$2	2::C3KD95|C3KD95_PSEFS	213	28020	7	6	7	6	1.75	Putative exported prot
286	1	SwissProt_HUMAN$	3::P07355|ANXA2_HUMAN	351	38580	13	13	12	12	3.34	Annexin A2 OS=Homo
287	1	db$2	2::C3K5I7|C3K5I7_PSEFS	350	59867	18	16	13	12	2.03	Probable malate:quino
288	1	db$2	2::C3K0W7|C3K0W7_PSEFS	347	31068	14	12	9	8	2.36	Metallophos_2 domain
289	1	db$2	2::C3K2V7|RL15_PSEFS	346	15215	12	12	8	8	20.50	50S ribosomal protein
290	1	db$2	2::C3K249|G6PI_PSEFS	346	61384	20	18	14	14	2.71	Glucose-6-phosphate i
291	1	db$2	2::C3KA80|C3KA80_PSEFS	346	20438	14	14	8	8	8.93	Alkyl hydroperoxide re
292	1	db$2	2::C3K2Y5|RL10_PSEFS	342	17594	12	12	8	8	9.87	50S ribosomal protein
293	1	db$2	2::C3JXX2|C3JXX2_PSEFS	340	50328	14	12	12	11	1.81	ATP-dependent RNA h
294	1	db$2	2::C3K8X6|C3K8X6_PSEFS	339	47255	14	14	11	11	2.00	Histidinol dehydrogena
295	1	db$2	2::C3KDM2|C3KDM2_PSEFS	339	83508	17	17	16	16	1.48	DNA topoisomerase 4
296	1	db$2	2::C3K9U6|C3K9U6_PSEFS	338	35232	10	10	7	7	1.92	Putative 6-phosphoglu
297	1	db$2	2::C3KAR0|C3KAR0_PSEFS	337	79563	10	9	7	7	0.61	Putative exported isoq
298	1	db$2	2::C3K2W5|RL24_PSEFS	336	11328	14	14	8	8	38.80	50S ribosomal protein
299	1	db$2	2::C3K7G9|C3K7G9_PSEFS	336	48266	11	10	9	9	1.41	Putative modulation of
300	1	db$2	2::C3K1N3|C3K1N3_PSEFS	336	11888	9	9	4	4	3.77	Sec translocon accesso
301	1	db$2	2::C3K8V3|HSLU_PSEFS	335	49607	15	15	11	11	1.85	ATP-dependent protea
302	1	db$2	2::C3KBN2|C3KBN2_PSEFS	335	52689	9	8	8	7	1.05	Outer membrane prote
303	1	db$2	2::C3K5Y0|C3K5Y0_PSEFS	334	38747	13	12	11	10	2.82	NADP(H)-dependt aldo
304	1	db$2	2::C3K5Z8|RRF_PSEFS	333	20297	16	15	8	8	11.67	Ribosome-recycling fa
305	1	db$2	2::C3K2X7|RS10_PSEFS	331	11745	13	11	6	5	6.18	30S ribosomal protein
306	1	db$2	2::C3K818|C3K818_PSEFS	330	29926	13	12	10	9	3.82	Putative ABC transport
307	1	db$2	2::C3K2U7|C3K2U7_PSEFS	330	104087	18	16	17	15	0.98	UvrABC system protein
308	1	db$2	2::C3K2H6|C3K2H6_PSEFS	329	32512	9	7	7	5	1.39	Acyl-CoA thioesterase
309	1	db$2	2::C3K1U7|C3K1U7_PSEFS	327	36908	9	7	3	2	0.89	Ubiquinol oxidase subu
310	1	db$2	2::C3JZ68|C3JZ68_PSEFS	327	24197	13	13	10	10	7.47	2-dehydro-3-deoxy-ph
311	1	db$2	2::C3K8U4|UBIE_PSEFS	327	28320	17	13	13	9	3.47	Ubiquinone/menaquino
312	1	db$2	2::C3KBN4|C3KBN4_PSEFS	325	40532	12	11	9	8	2.21	Acriflavine resistance p
313	1	db$2	2::C3K226|C3K226_PSEFS	324	17820	9	9	7	7	5.25	Acetolactate synthase
314	1	db$2	2::C3JYD5|C3JYD5_PSEFS	324	18453	10	10	6	6	4.92	Peptidyl-prolyl cis-tran
315	1	db$2	2::C3KE61|RIMK_PSEFS	323	32488	10	9	7	7	2.19	Probable alpha-L-gluta

↑Family	M	DB	Accession	Score	Mass	Matches	Match(sig)	Sequences	Seq(sig)	emPAI	Description
316	1	db$2	2::C3K2M1|C3K2M1_PSEFS	323	42033	13	13	12	12	3.31	Serine-type D-Ala-D-A
317	1	db$2	2::C3K6K6|C3K6K6_PSEFS	322	36089	14	13	11	10	2.70	HTH-type transcription
318	1	db$2	2::C3K614|C3K614_PSEFS	321	41572	16	16	10	10	2.90	3-ketoacyl-CoA thiolas
319	1	db$2	2::C3K310|C3K310_PSEFS	321	54246	11	11	9	9	1.39	Anthranilate synthase
320	1	db$2	2::C3KCN5|C3KCN5_PSEFS	320	142346	16	15	16	15	0.65	Bifunctional protein Pu
321	1	db$2	2::C3KE41|SYGA_PSEFS	318	36196	13	12	10	9	2.23	Glycine--tRNA ligase a
322	1	db$2	2::C3JYR3|C3JYR3_PSEFS	316	40135	13	12	9	9	2.24	Glycine cleavage syste
323	1	db$2	2::C3K7M6|C3K7M6_PSEFS	314	88918	13	13	10	10	0.80	Acyl-coenzyme A dehy
324	1	db$2	2::C3K3U0|C3K3U0_PSEFS	314	51001	9	9	7	7	0.92	Putative glutamine syr
325	1	db$2	2::C3K2Y0|RS7_PSEFS	313	17636	17	16	11	11	17.46	30S ribosomal protein
326	1	db$2	2::C3K1H6|PURT_PSEFS	313	42327	13	12	13	12	2.82	Formate-dependent ph
327	1	db$2	2::C3K476|PYRE_PSEFS	312	23079	7	7	7	7	3.15	Orotate phosphoribosy
328	1	db$2	2::C3K490|C3K490_PSEFS	311	33505	18	17	12	12	5.23	Putative oxidative stre
329	1	db$2	2::C3K2T2|C3K2T2_PSEFS	311	24648	14	13	7	7	5.74	Putative ABC transport
330	1	db$2	2::C3K2X1|RL22_PSEFS	311	11904	10	8	6	5	6.05	50S ribosomal protein
331	1	db$2	2::C3K435|C3K435_PSEFS	310	45402	14	13	12	11	2.49	Diaminopimelate decar
332	1	db$2	2::C3KAI6|C3KAI6_PSEFS	310	24263	10	10	8	8	3.70	Putative dehydrogenas
333	1	db$2	2::C3K472|C3K472_PSEFS	310	42804	9	9	8	8	1.70	Coenzyme A biosynthe
334	1	db$2	2::C3JY54|SYS_PSEFS	309	47115	12	11	11	10	1.73	Serine--tRNA ligase OS
335	1	db$2	2::C3K466|C3K466_PSEFS	305	52681	14	12	10	9	1.45	Putative aldehyde deh
336	1	db$2	2::C3K264|GLMM_PSEFS	304	47719	15	15	13	13	3.42	Phosphoglucosamine n
337	1	db$2	2::C3K3Q2|C3K3Q2_PSEFS	303	37335	11	9	6	4	1.14	Putative cation ABC tra
338	1	db$2	2::C3K2Y8|C3K2Y8_PSEFS	301	20023	15	11	8	7	5.53	Transcription terminati
339	1	db$2	2::C3JXY7|ASTD_PSEFS	301	51479	7	7	6	6	0.74	N-succinylglutamate 5
340	1	db$2	2::C3K1F4|C3K1F4_PSEFS	301	31961	9	8	8	7	1.81	Probable chromosome-
341	1	db$2	2::C3K667|C3K667_PSEFS	301	48557	15	14	12	12	2.22	Conserved hypothetica
342	1	db$2	2::C3JZ29|C3JZ29_PSEFS	301	41047	10	8	9	7	1.51	Putative glycerophospl
343	1	db$2	2::C3JYT9|C3JYT9_PSEFS	301	46635	9	9	4	4	0.84	Putative family S43 no
344	1	db$2	2::C3KDB1|C3KDB1_PSEFS	300	65565	16	14	15	13	1.56	Putative carbamoyltrar
345	1	db$2	2::C3KED5|C3KED5_PSEFS	299	40447	12	10	12	10	2.22	Putative lipoprotein OS
346	1	db$2	2::C3K5S8|C3K5S8_PSEFS	299	75646	12	11	11	10	0.87	Oligopeptidase A OS=I
347	1	db$2	2::C3K7C1|SECB_PSEFS	299	17615	6	6	3	3	1.89	Protein-export protein
348	1	db$2	2::C3K6R4|C3K6R4_PSEFS	298	18727	12	11	5	5	3.47	Uncharacterized protei
349	1	db$2	2::C3K6G2|C3K6G2_PSEFS	297	106936	17	15	16	14	0.86	Valine--tRNA ligase OS
350	1	SwissProt_HUMAN$	3::P01871|IGHM_HUMAN	295	49409	12	11	10	10	1.61	Immunoglobulin heavy
351	1	db$2	2::C3K263|C3K263_PSEFS	294	26068	8	7	6	5	1.46	Triosephosphate isome
352	1	db$2	2::C3K3Z1|C3K3Z1_PSEFS	293	11808	9	9	6	6	14.80	Thioredoxin OS=Pseuc
353	1	db$2	2::C3K527|C3K527_PSEFS	292	65564	13	12	10	9	1.06	Putative peptidase OS
354	1	db$2	2::C3K4V8|C3K4V8_PSEFS	292	128447	14	12	14	12	0.56	Transcription-repair-cc
355	1	db$2	2::C3K0N4|C3K0N4_PSEFS	291	25612	9	8	6	6	3.34	3-oxoacyl-[acyl-carrier

↑Family	M	DB	Accession	Score	Mass	Matches	Match(sig)	Sequences	Seq(sig)	emPAI	Description
356	1	db$2	2::C3K1L0|C3K1L0_PSEFS	290	55309	17	17	16	16	3.29	GTPase Der OS=Pseud
357	1	db$2	2::C3K0N2|C3K0N2_PSEFS	289	43313	9	9	8	8	1.39	3-oxoacyl-[acyl-carrier
358	1	db$2	2::C3KEB7|C3KEB7_PSEFS	289	48036	11	10	10	9	1.67	Uncharacterized protei
359	1	db$2	2::C3K859|C3K859_PSEFS	287	59064	9	8	7	7	0.75	Putative glutamate syr
360	1	db$2	2::C3K3D2|BETB_PSEFS	286	53123	11	11	10	10	1.44	Betaine aldehyde dehy
361	1	db$2	2::C3K7X9|C3K7X9_PSEFS	286	40858	13	10	11	8	1.52	Carboxynorspermidine
362	1	db$2	2::C3K8Z6|C3K8Z6_PSEFS	285	39611	10	9	7	6	1.30	Branched amino acid A
363	1	db$2	2::C3K3S1|C3K3S1_PSEFS	284	51070	9	8	9	8	1.10	Putative oxidase OS=P
364	1	db$2	2::C3KCS2|RSMH_PSEFS	284	34344	11	11	10	10	2.96	Ribosomal RNA small s
365	1	db$2	2::C3JZ86|C3JZ86_PSEFS	283	21964	18	16	11	11	11.97	Superoxide dismutase
366	1	db$2	2::C3K6U8|C3K6U8_PSEFS	283	58654	7	7	6	6	0.62	Putative membrane pr
367	1	db$2	2::C3KEA4|C3KEA4_PSEFS	282	51722	13	13	10	10	1.74	PINc domain-containin
368	1	db$2	2::C3K4M6|C3K4M6_PSEFS	281	57818	18	17	12	12	1.90	Putative exported alka
369	1	db$2	2::C3K581|C3K581_PSEFS	280	86479	12	10	12	10	0.73	Putative molybdopterir
370	1	db$2	2::C3K2X2|RS19_PSEFS	280	10313	12	11	8	7	34.95	30S ribosomal protein
371	1	db$2	2::C3K2V3|RS11_PSEFS	278	13607	8	8	6	6	6.73	30S ribosomal protein
372	1	SwissProt_HUMAN$	3::P60709|ACTB_HUMAN	278	41710	16	12	12	9	2.47	Actin, cytoplasmic 1 O
373	1	db$2	2::C3K3T9|C3K3T9_PSEFS	277	49785	9	9	7	7	1.14	Putative aminotransfer
374	1	db$2	2::C3KAA8|C3KAA8_PSEFS	276	132643	13	12	13	12	0.54	Histidine kinase OS=P:
375	1	db$2	2::C3K1F2|ATP6_PSEFS	275	31626	8	7	4	3	0.56	ATP synthase subunit a
376	1	db$2	2::C3K1X5|C3K1X5_PSEFS	275	39754	11	9	8	7	1.30	Glutamate 5-kinase OS
377	1	db$2	2::C3K2U9|C3K2U9_PSEFS	271	53651	17	15	13	11	1.64	Catalase OS=Pseudom
378	1	db$2	2::C3JZN9|C3JZN9_PSEFS	271	7652	11	10	5	5	35.06	Major cold shock prote
379	1	db$2	2::C3K3W3|C3K3W3_PSEFS	270	41294	7	7	6	6	1.23	Putative aminotransfer
380	1	db$2	2::C3JZ88|C3JZ88_PSEFS	270	47232	7	6	4	3	0.65	Putative exported prot
381	1	db$2	2::C3K7N8|RDGC_PSEFS	270	34160	16	14	14	12	5.01	Recombination-associa
382	1	db$2	2::C3K0N5|C3K0N5_PSEFS	269	32402	9	9	5	5	1.39	Malonyl CoA-acyl carri
383	1	db$2	2::C3KA77|C3KA77_PSEFS	269	49108	10	8	10	8	1.16	Glutathione reductase
384	1	db$2	2::C3K4A8|C3K4A8_PSEFS	268	55832	11	11	11	11	1.54	Uncharacterized protei
385	1	db$2	2::C3KE83|C3KE83_PSEFS	267	38700	8	8	7	7	1.35	Outer membrane prote
386	1	db$2	2::C3K2S6|C3K2S6_PSEFS	267	31662	9	9	7	7	2.29	Putative thioredoxin O
387	1	db$2	2::C3K5X4|C3K5X4_PSEFS	267	16332	10	10	5	5	3.19	Putative membrane pr
388	1	db$2	2::C3K2Z3|C3K2Z3_PSEFS	266	44017	13	11	11	10	1.92	Tyrosine--tRNA ligase
389	1	db$2	2::C3JYU1|Y4927_PSEFS	264	18225	7	7	5	5	3.68	UPF0234 protein PFLU_
390	1	db$2	2::C3K443|C3K443_PSEFS	264	12323	11	10	9	9	42.35	Nitrogen regulatory pr
391	1	db$2	2::C3K187|C3K187_PSEFS	264	38556	10	10	8	8	2.41	Peptidase_M75 domair
392	1	db$2	2::C3K2S1|C3K2S1_PSEFS	264	23236	11	10	8	7	4.05	Riboflavin synthase alp
392	2	db$2	2::C3K5X7|C3K5X7_PSEFS	195	42054	12	12	12	12	2.85	Cell division protein Za
393	1	db$2	2::C3JZT7|C3JZT7_PSEFS	263	38793	11	10	9	8	1.65	3-isopropylmalate deh
394	1	db$2	2::C3JYV5|C3JYV5_PSEFS	262	45487	9	8	9	8	1.30	Putative oxidoreductas

↑Family	M	DB	Accession	Score	Mass	Matches	Match(sig)	Sequences	Seq(sig)	emPAI	Description
395	1	db$2	2::C3K6V9|C3K6V9_PSEFS	261	54793	11	11	8	8	1.17	2,3-bisphosphoglycera
396	1	db$2	2::C3K9T7|C3K9T7_PSEFS	261	99328	17	15	15	13	0.86	Aminopeptidase N OS=
397	1	db$2	2::C3KE31|C3KE31_PSEFS	260	22237	9	9	8	8	4.43	RNA polymerase sigma
398	1	db$2	2::C3JY74|C3JY74_PSEFS	260	48472	15	13	13	12	2.22	Isocitrase OS=Pseudo
399	1	db$2	2::C3K1L2|C3K1L2_PSEFS	260	22708	7	7	6	6	2.45	Ancillary SecYEG trans
400	1	db$2	2::C3K1M9|C3K1M9_PSEFS	259	29586	10	9	8	7	2.04	Inositol-1-monophospl
401	1	db$2	2::C3K4G2|C3K4G2_PSEFS	259	30728	9	9	4	4	1.92	Putative exported prot
402	1	db$2	2::C3KAH3|SYR_PSEFS	257	63549	14	14	10	10	1.11	Arginine--tRNA ligase (
403	1	db$2	2::C3K9S0|NFUA_PSEFS	257	20961	8	7	6	5	2.06	Fe/S biogenesis protei
404	1	db$2	2::C3K5J3|C3K5J3_PSEFS	256	50388	13	12	11	11	2.08	AMP nucleosidase OS=
405	1	db$2	2::C3K6F9|C3K6F9_PSEFS	256	37389	9	9	9	9	2.11	Nucleoid-associated pr
406	1	db$2	2::C3K6Z0|PDXJ_PSEFS	256	26758	9	8	7	6	1.87	Pyridoxine 5'-phospha
407	1	db$2	2::C3JY85|C3JY85_PSEFS	255	48759	12	9	9	7	0.97	NADH-quinone oxidore
408	1	db$2	2::C3KBL1|C3KBL1_PSEFS	253	61934	11	7	9	6	0.58	Putative medium-chair
409	1	db$2	2::C3K4E4|C3K4E4_PSEFS	252	51389	13	12	11	10	1.75	Aspartate ammonia-ly
410	1	db$2	2::C3K1Y2|C3K1Y2_PSEFS	252	21662	8	7	7	6	2.67	Peptidyl-prolyl cis-tran
411	1	db$2	2::C3K2J9|C3K2J9_PSEFS	252	60860	7	5	6	4	0.48	Putative dehydrogenas
412	1	db$2	2::C3KDU9|C3KDU9_PSEFS	251	34261	9	9	8	8	2.00	Serine O-acetyltransfe
413	1	db$2	2::C3K0D3|MINC_PSEFS	249	26249	11	11	9	9	5.02	Probable septum site-c
414	1	db$2	2::C3KE99|C3KE99_PSEFS	249	30145	7	6	7	6	1.55	Quinolinate phosphorit
415	1	db$2	2::C3K734|C3K734_PSEFS	248	44623	10	10	8	8	1.88	Putative aminotransfer
416	1	db$2	2::C3JZT0|C3JZT0_PSEFS	247	28122	5	5	5	5	1.31	Putative GntR-family t
417	1	db$2	2::C3K3X2|C3K3X2_PSEFS	246	19739	8	8	6	6	3.15	Uncharacterized protei
418	1	db$2	2::C3K091|C3K091_PSEFS	245	20089	8	7	7	6	3.06	GTP cyclohydrolase 1 (
418	2	db$2	2::C3K1W5|C3K1W5_PSEFS	94	20960	5	5	5	5	2.06	GTP cyclohydrolase 1 (
419	1	db$2	2::C3KBP1|C3KBP1_PSEFS	244	37670	10	9	9	8	1.73	Putative reductase OS
420	1	db$2	2::C3K2Q0|C3K2Q0_PSEFS	243	19348	11	11	9	9	7.87	Inorganic pyrophospha
421	1	db$2	2::C3K6T0|ENGB_PSEFS	242	23537	5	5	4	4	1.22	Probable GTP-binding
422	1	db$2	2::C3K3V5|C3K3V5_PSEFS	242	52101	12	10	9	7	1.07	CYTH domain-containi
423	1	db$2	2::C3K5G2|C3K5G2_PSEFS	241	35788	11	11	9	9	2.28	Putative membrane pr
424	1	db$2	2::C3K469|RL28_PSEFS	241	8845	7	7	3	3	3.73	50S ribosomal protein
425	1	db$2	2::C3K474|C3K474_PSEFS	241	50732	12	11	11	10	1.79	Putative phosphomanr
426	1	db$2	2::C3K452|XPT_PSEFS	241	20267	11	9	10	8	6.98	Xanthine phosphoribos
427	1	db$2	2::C3K2E3|MSCL_PSEFS	240	14568	5	5	3	3	2.60	Large-conductance me
428	1	db$2	2::C3KCT1|MURC_PSEFS	240	52132	9	9	6	6	0.72	UDP-N-acetylmuramat
429	1	db$2	2::C3K5U5|C3K5U5_PSEFS	240	32335	7	7	6	6	1.40	Glucose-1-phosphate t
430	1	db$2	2::C3K6R3|Y2135_PSEFS	239	24050	11	10	8	7	2.92	UPF0502 protein PFLU_
431	1	db$2	2::C3K6Y4|GATA_PSEFS	239	51737	7	6	5	4	0.58	Glutamyl-tRNA(Gln) ar
432	1	db$2	2::C3JYR4|C3JYR4_PSEFS	239	7675	5	5	3	3	5.01	Major cold shock prote
433	1	db$2	2::C3JZU0|C3JZU0_PSEFS	239	50538	11	11	10	10	1.55	3-isopropylmalate deh

↑Family	M	DB	Accession	Score	Mass	Matches	Match(sig)	Sequences	Seq(sig)	emPAI	Description
434	1	db$2	2::C3K6S9|C3K6S9_PSEFS	238	101727	11	10	11	10	0.59	DNA polymerase I OS=
435	1	db$2	2::C3KAJ2|C3KAJ2_PSEFS	238	53335	11	11	10	10	1.43	Methylmalonate-semia
436	1	db$2	2::C3K6Y3|GATB_PSEFS	238	52868	11	11	7	7	1.24	Aspartyl/glutamyl-tRN.
437	1	db$2	2::C3K5D5|C3K5D5_PSEFS	235	41033	4	4	3	3	0.41	Succinyl-diaminopimel
438	1	db$2	2::C3K839|Y879_PSEFS	235	32120	8	7	8	7	1.80	Nucleotide-binding pro
439	1	db$2	2::C3JZ61|C3JZ61_PSEFS	235	61509	17	17	11	11	1.94	Acyl-CoA synthetase (I
440	1	db$2	2::C3JYI3|PHNX_PSEFS	234	29973	8	7	6	6	1.57	Phosphonoacetaldehyc
441	1	db$2	2::C3K4G1|C3K4G1_PSEFS	234	81186	11	10	10	9	0.69	DNA helicase OS=Pseu
441	2	db$2	2::C3K5Z0|C3K5Z0_PSEFS	61	15508	6	4	5	4	2.32	Uncharacterized protei
442	1	db$2	2::C3K0S7|Y4577_PSEFS	234	11900	6	6	3	3	3.77	Nucleoid-associated pr
443	1	db$2	2::C3KEC6|KAD_PSEFS	234	23251	12	11	8	8	5.19	Adenylate kinase OS=I
444	1	db$2	2::C3JY62|C3JY62_PSEFS	233	83224	15	12	13	11	0.87	ATP-dependent Clp prc
444	2	db$2	2::C3K4B3|C3K4B3_PSEFS	115	97007	9	7	8	7	0.41	Chaperone OS=Pseudc
445	1	db$2	2::C3K6J7|C3K6J7_PSEFS	233	39784	11	11	10	10	2.28	Histidinol-phosphate a
446	1	db$2	2::C3K017|PYRD_PSEFS	232	35850	9	9	9	9	2.27	Dihydroorotate dehydr
447	1	db$2	2::C3K0Q9|C3K0Q9_PSEFS	228	15688	5	5	3	3	2.30	Flagellar basal-body rc
448	1	db$2	2::C3K1G2|YIDC_PSEFS	227	61977	13	11	11	10	1.15	Membrane protein inse
449	1	db$2	2::C3K245|C3K245_PSEFS	227	53442	12	11	11	11	1.65	Poly(A) polymerase I (
450	1	SwissProt_HUMAN$	3::P01834|IGKC_HUMAN	226	11758	8	7	5	5	6.18	Immunoglobulin kappa
451	1	db$2	2::C3KBY9|C3KBY9_PSEFS	226	24532	5	5	4	4	1.15	Ribonuclease T OS=Ps
452	1	db$2	2::C3KDB7|C3KDB7_PSEFS	226	65793	9	9	8	8	0.78	ATP-dependent lipid A-
452	2	db$2	2::C3K608|C3K608_PSEFS	43	64881	2	2	2	2	0.16	Putative ABC transport
453	1	db$2	2::C3K3H0|C3K3H0_PSEFS	225	67796	12	11	11	10	1.01	ATP-dependent RNA he
454	1	db$2	2::C3KAI4|C3KAI4_PSEFS	225	53827	6	6	5	5	0.69	SPOR domain-containi
455	1	db$2	2::C3K6U2|C3K6U2_PSEFS	225	47692	6	5	5	4	0.49	Probable proton/glutar
456	1	db$2	2::C3KCP5|C3KCP5_PSEFS	225	109710	8	8	8	8	0.41	Bifunctional glutamine
457	1	db$2	2::C3KBZ0|PYRC_PSEFS	224	38385	8	7	8	7	1.36	Dihydroorotase OS=Ps
458	1	db$2	2::C3K3M4|C3K3M4_PSEFS	224	49883	7	7	6	6	0.77	Signal recognition part
459	1	db$2	2::C3KBZ7|SYM_PSEFS	224	75525	11	10	10	10	0.87	Methionine--tRNA ligas
460	1	db$2	2::C3K747|GPDA_PSEFS	223	36476	9	9	7	7	2.20	Glycerol-3-phosphate (
461	1	db$2	2::C3JXZ8|C3JXZ8_PSEFS	222	71568	13	10	12	10	0.94	Acetyl-coenzyme A syr
462	1	db$2	2::C3KCL0|C3KCL0_PSEFS	221	35424	5	5	4	4	0.70	Uncharacterized protei
463	1	db$2	2::C3KBP9|C3KBP9_PSEFS	220	58780	9	9	8	8	0.90	Phosphoglucomutase (
464	1	db$2	2::C3K0K7|C3K0K7_PSEFS	220	60595	13	12	11	10	1.18	Electron transfer flavo
465	1	db$2	2::C3K104|C3K104_PSEFS	219	26102	10	9	7	6	3.23	Putative membrane pr
466	1	db$2	2::C3K733|HTPX_PSEFS	218	32382	7	7	4	4	1.07	Protease HtpX OS=Pse
467	1	db$2	2::C3K1L1|C3K1L1_PSEFS	218	40864	9	8	7	6	1.00	Outer membrane prote
468	1	db$2	2::C3KEA8|C3KEA8_PSEFS	218	45495	6	6	5	5	0.68	ATP-dependent RNA he
469	1	db$2	2::C3K3W7|C3K3W7_PSEFS	217	36662	12	12	11	11	3.13	Putative iron (III) ABC
470	1	db$2	2::C3K1M1|HSCA_PSEFS	217	65896	7	7	6	6	0.54	Chaperone protein Hsc

↑Family	M	DB	Accession	Score	Mass	Matches	Match(sig)	Sequences	Seq(sig)	emPAI	Description
471	1	db$2	2::C3K6V7|C3K6V7_PSEFS	215	46336	9	8	9	8	1.26	Carboxy-terminal proc
472	1	db$2	2::C3K1X2|C3K1X2_PSEFS	215	40166	7	6	7	6	1.02	Putative putative oxidc
473	1	db$2	2::C3K1N4|C3K1N4_PSEFS	215	41398	12	11	7	6	1.49	Queuine tRNA-ribosylti
474	1	db$2	2::C3K368|C3K368_PSEFS	213	37353	8	7	7	6	1.13	Stress response kinase
475	1	db$2	2::C3K177|C3K177_PSEFS	213	65030	13	11	10	10	1.23	Phosphogluconate deh
476	1	db$2	2::C3K6E2|RS9_PSEFS	212	14607	12	11	7	7	11.75	30S ribosomal protein
477	1	db$2	2::C3K7G7|C3K7G7_PSEFS	212	50136	6	6	6	6	0.76	Putative modulation of
478	1	db$2	2::C3K3K2|PYRB_PSEFS	212	36271	10	9	9	8	1.83	Aspartate carbamoyltra
479	1	db$2	2::C3K9R8|C3K9R8_PSEFS	212	135461	10	10	9	9	0.37	Methionine synthase O
480	1	db$2	2::C3KAC7|C3KAC7_PSEFS	211	51379	12	9	10	7	1.09	UDP-glucose 6-dehydr
480	2	db$2	2::C3K2K9|C3K2K9_PSEFS	183	31417	12	12	9	9	2.86	Magnesium and cobalt
480	3	db$2	2::C3KAL9|C3KAL9_PSEFS	28	64520	2	2	2	2	0.16	Histidine kinase OS=Ps
481	1	db$2	2::C3K3P4|C3K3P4_PSEFS	210	39333	14	12	9	8	1.95	Uncharacterized protei
482	1	db$2	2::C3KDD6|C3KDD6_PSEFS	210	33869	6	6	6	6	1.31	D-alanine--D-alanine li
483	1	db$2	2::C3K4I0|HEM6_PSEFS	210	35313	8	8	7	7	1.55	Oxygen-dependent cop
484	1	db$2	2::C3K1X9|C3K1X9_PSEFS	209	34909	8	8	7	7	1.58	Octaprenyl-diphosphat
485	1	db$2	2::C3K5V1|C3K5V1_PSEFS	209	47050	7	7	7	7	1.02	Outer membrane prote
486	1	db$2	2::C3K9Z4|C3K9Z4_PSEFS	208	58133	10	10	9	9	1.08	Putative ABC tranmspc
487	1	db$2	2::C3KDW3|HFQ_PSEFS	208	9318	8	8	5	5	18.40	RNA-binding protein H
488	1	db$2	2::C3K110|C3K110_PSEFS	207	32414	7	7	7	7	1.77	Cysteine synthase OS=
489	1	db$2	2::C3KBV0|C3KBV0_PSEFS	207	28603	7	7	4	4	0.93	Putative chromosome
490	1	db$2	2::C3K1E5|ATPE_PSEFS	206	14558	6	5	5	4	3.97	ATP synthase epsilon c
491	1	db$2	2::C3KE59|C3KE59_PSEFS	206	27398	10	10	9	9	3.69	DNA-binding response
492	1	SwissProt_HUMAN$	3::P00738|HPT_HUMAN	205	45177	12	11	8	7	1.56	Haptoglobin OS=Homc
493	1	db$2	2::C3K3Y8|UBID_PSEFS	204	54697	9	8	8	8	1.00	3-octaprenyl-4-hydrox
494	1	db$2	2::C3JZ71|C3JZ71_PSEFS	204	31565	8	7	8	7	1.85	Hex regulon repressor
495	1	db$2	2::C3KAM2|C3KAM2_PSEFS	204	49640	6	6	5	5	0.61	Putative two-compone
496	1	db$2	2::C3K7C4|C3K7C4_PSEFS	203	53137	13	11	12	10	1.44	DNA-binding transcript
497	1	db$2	2::C3K644|C3K644_PSEFS	203	89774	9	8	7	7	0.45	Putative ABC transport
498	1	db$2	2::C3KDP4|C3KDP4_PSEFS	203	75338	13	10	11	9	0.76	Phosphate acetyltransf
499	1	db$2	2::C3JYT6|C3JYT6_PSEFS	202	8291	7	6	6	5	14.76	Cold shock protein csp
500	1	db$2	2::Q84CP9|DAPB_PSEFS	202	28276	10	9	9	8	2.78	4-hydroxy-tetrahydroc
501	1	db$2	2::C3K5L8|C3K5L8_PSEFS	202	39360	9	9	7	7	1.61	Phosphorous compoun
502	1	db$2	2::C3KED4|C3KED4_PSEFS	202	31038	6	5	6	5	1.13	4-hydroxy-tetrahydroc
503	1	db$2	2::C3K116|PSRP_PSEFS	202	30838	7	7	7	7	1.91	Putative phosphoenolp
504	1	db$2	2::C3K5L3|C3K5L3_PSEFS	202	36401	5	5	5	5	0.91	Phosphonates ABC trai
505	1	db$2	2::C3K705|SURE_PSEFS	201	26431	2	2	1	1	0.19	5'-nucleotidase SurE C
506	1	db$2	2::C3K3P5|C3K3P5_PSEFS	200	48453	5	5	4	4	0.48	Putative symporter me
507	1	db$2	2::C3K1A1|C3K1A1_PSEFS	200	10298	8	7	6	5	8.38	10 kDa chaperonin OS
508	1	db$2	2::C3JZ69|C3JZ69_PSEFS	199	25612	5	5	4	4	1.08	6-phosphogluconolactc

↑Family	M	DB	Accession	Score	Mass	Matches	Match(sig)	Sequences	Seq(sig)	emPAI	Description
509	1	db$2	2::C3JYE4|C3JYE4_PSEFS	199	30882	12	12	11	11	4.36	Exodeoxyribonuclease
510	1	db$2	2::C3JYD3|SYC_PSEFS	198	51635	11	10	8	7	0.90	Cysteine--tRNA ligase
511	1	db$2	2::C3K1K8|C3K1K8_PSEFS	197	31523	9	6	7	5	1.11	Putative hydrolase OS=
512	1	db$2	2::C3K5E5|C3K5E5_PSEFS	197	28503	8	7	6	6	2.18	Methionine aminopepti
513	1	db$2	2::C3JYS0|C3JYS0_PSEFS	196	29172	7	7	6	6	1.63	Cell division coordinato
514	1	db$2	2::C3KE80|C3KE80_PSEFS	194	46502	8	7	8	7	1.04	Nicotinate phosphoribo
515	1	db$2	2::C3KC10|C3KC10_PSEFS	194	23080	5	5	4	4	1.26	Uncharacterized protei
516	1	db$2	2::C3K307|TRPC_PSEFS	193	30252	11	11	10	10	3.73	Indole-3-glycerol phos
517	1	db$2	2::C3K2W4|RL5_PSEFS	193	20490	15	12	11	9	8.81	50S ribosomal protein
518	1	db$2	2::C3K7Z1|C3K7Z1_PSEFS	192	38822	5	5	4	4	0.63	NAD(P) transhydrogen
519	1	db$2	2::C3K4E2|C3K4E2_PSEFS	192	16860	9	8	7	6	4.27	N5-carboxyaminoimida
520	1	db$2	2::C3K651|C3K651_PSEFS	191	39766	10	10	10	10	2.28	Phospho-2-dehydro-3-
521	1	db$2	2::C3KBL7|C3KBL7_PSEFS	191	33413	7	6	6	5	1.03	Glutamate/aspartate A
522	1	db$2	2::C3JYQ7|C3JYQ7_PSEFS	191	32635	7	5	7	5	1.06	Carbamate kinase OS=
523	1	db$2	2::B0B0A5|DNAA_PSEFS	191	56252	12	11	10	9	1.13	Chromosomal replicati
524	1	db$2	2::C3K152|C3K152_PSEFS	191	13999	8	8	7	7	9.29	Putative transcriptiona
525	1	db$2	2::C3K1E4|GLMU_PSEFS	191	48690	6	6	5	5	0.62	Bifunctional protein Gl
526	1	db$2	2::C3K0I1|C3K0I1_PSEFS	190	29699	10	9	9	8	3.16	ParA family ATPase OS
527	1	db$2	2::C3K3L7|THIG_PSEFS	189	28373	9	7	8	6	2.19	Thiazole synthase OS=
528	1	db$2	2::C3K1H2|C3K1H2_PSEFS	189	49205	9	7	7	5	0.62	Signal recognition part
529	1	db$2	2::C3K703|ISPF_PSEFS	188	16742	5	5	5	5	3.03	2-C-methyl-D-erythrit
530	1	db$2	2::C3JZ43|C3JZ43_PSEFS	188	44885	8	8	8	8	1.32	Putative aminotransfer
531	1	db$2	2::C3K4T4|C3K4T4_PSEFS	188	21507	5	5	4	4	1.39	NAD(P)-bd_dom doma
532	1	db$2	2::C3K3Q8|TYSY_PSEFS	188	31490	8	8	5	5	1.45	Thymidylate synthase
533	1	db$2	2::C3KAK7|C3KAK7_PSEFS	188	20540	7	5	5	4	2.13	Putative lipoprotein OS
534	1	db$2	2::C3K2E9|C3K2E9_PSEFS	187	53251	5	5	4	4	0.56	Putative cytochrome o
535	1	db$2	2::C3JZV9|C3JZV9_PSEFS	186	34932	3	3	2	2	0.31	Putative DNA/RNA non
536	1	db$2	2::C3K6S8|C3K6S8_PSEFS	186	40355	11	11	9	9	2.22	Putative acetyl-CoA tra
537	1	db$2	2::C3K6L0|EFP_PSEFS	184	21303	9	8	7	7	3.67	Elongation factor P OS
538	1	db$2	2::C3K3J3|C3K3J3_PSEFS	184	72855	6	6	5	5	0.38	Type IV pilus biogenes
539	1	db$2	2::C3KE43|C3KE43_PSEFS	184	32711	6	6	5	5	1.05	Lipid A biosynthesis la
540	1	db$2	2::C3K5N1|C3K5N1_PSEFS	183	36842	9	8	6	5	0.90	Sulfate ABC transport
541	1	db$2	2::C3K3A2|C3K3A2_PSEFS	182	38105	8	8	7	7	1.38	L-threonine aldolase O
542	1	db$2	2::C3K8Y8|C3K8Y8_PSEFS	181	23915	3	3	3	3	0.81	Putative isomerase OS
543	1	db$2	2::C3K1A8|C3K1A8_PSEFS	180	35739	4	4	3	3	0.49	Putative lipoprotein OS
544	1	db$2	2::C3K2S2|C3K2S2_PSEFS	180	38886	5	5	5	5	0.84	Riboflavin biosynthesis
545	1	db$2	2::C3KAH1|HSLV_PSEFS	180	18731	5	5	5	5	2.48	ATP-dependent protea
546	1	db$2	2::C3K477|C3K477_PSEFS	180	29836	6	5	5	5	1.20	Exodeoxyribonuclease
547	1	db$2	2::C3K2C9|C3K2C9_PSEFS	179	48295	10	10	9	9	1.41	Glutamate dehydroger
548	1	db$2	2::C3K868|C3K868_PSEFS	179	58117	6	5	6	5	0.50	Uncharacterized protei

↑Family	M	DB	Accession	Score	Mass	Matches	Match(sig)	Sequences	Seq(sig)	emPAI	Description
549	1	db$2	2::C3KCT0|MURG_PSEFS	179	37667	6	6	6	6	1.12	UDP-N-acetylglucosam
550	1	db$2	2::C3K9P3|C3K9P3_PSEFS	179	9882	6	6	4	4	5.48	Peptidyl-prolyl cis-tran
551	1	db$2	2::C3K606|FABZ_PSEFS	179	16691	9	8	7	6	4.39	3-hydroxyacyl-[acyl-ca
552	1	db$2	2::C3KDM5|C3KDM5_PSEFS	178	43829	11	8	9	7	1.37	O-phosphoserine phos
553	1	db$2	2::C3K3K3|C3K3K3_PSEFS	178	44101	7	7	6	6	0.90	Dihydroorotase-like pr
554	1	db$2	2::C3KAJ8|UPP_PSEFS	178	22986	10	9	7	6	3.18	Uracil phosphoribosylt
555	1	db$2	2::C3K262|C3K262_PSEFS	177	12956	7	7	3	3	1.93	Protein-export membr
556	1	db$2	2::C3KBB4|OADC_PSEFS	177	31135	5	4	3	3	0.58	Oxaloacetate decarbox
557	1	db$2	2::C3KE68|RS18_PSEFS	176	8912	7	7	5	5	12.34	30S ribosomal protein
558	1	db$2	2::C3KBX9|C3KBX9_PSEFS	176	20403	4	4	3	3	0.99	Putative phospholipid-l
559	1	db$2	2::C3K5M0|C3K5M0_PSEFS	176	29300	6	6	5	5	1.23	Phosphonates ABC trai
560	1	db$2	2::C3K8U2|UBIB_PSEFS	176	61616	6	6	6	6	0.59	Probable protein kinas
561	1	db$2	2::C3K4Q9|C3K4Q9_PSEFS	176	23656	6	5	5	5	1.70	Putative exported prot
562	1	db$2	2::C3K5H8|C3K5H8_PSEFS	175	17826	8	8	6	6	3.81	STAS domain-containi
563	1	db$2	2::C3K9M2|C3K9M2_PSEFS	175	37830	8	8	6	6	1.11	Putative peptidase OS
564	1	db$2	2::C3KDM0|C3KDM0_PSEFS	175	69594	11	10	10	10	0.98	DNA topoisomerase 4
565	1	db$2	2::C3K6K4|C3K6K4_PSEFS	175	32908	7	6	7	6	1.37	Putative 5'-nucleotidas
566	1	db$2	2::C3JYJ6|C3JYJ6_PSEFS	175	68407	10	10	9	9	1.00	Peptidyl-prolyl cis-tran
567	1	db$2	2::C3K2N8|C3K2N8_PSEFS	173	23444	9	9	7	7	3.07	Semialdhyde_dh doma
568	1	db$2	2::C3JZN4|RL20_PSEFS	173	13348	8	8	7	7	10.53	50S ribosomal protein
569	1	db$2	2::C3KAD3|ARNC_PSEFS	173	37700	4	4	4	4	0.65	Undecaprenyl-phospha
570	1	db$2	2::C3K5H5|C3K5H5_PSEFS	172	25721	9	7	6	5	1.99	Putative exported prot
571	1	db$2	2::C3JYS5|C3JYS5_PSEFS	172	25332	7	7	7	7	2.67	Tol-Pal system protein
572	1	db$2	2::C3JY00|C3JY00_PSEFS	172	47211	4	4	3	3	0.35	Ribonucleoside-diphos
573	1	db$2	2::C3KAJ1|C3KAJ1_PSEFS	172	48585	11	11	9	9	1.92	Omega-amino acid--py
574	1	db$2	2::C3K0H6|C3K0H6_PSEFS	170	40054	4	4	4	4	0.60	Protein-glutamate met
575	1	db$2	2::C3K5K3|C3K5K3_PSEFS	170	28692	4	4	2	2	0.64	CHAD domain-containi
576	1	db$2	2::C3K2U5|C3K2U5_PSEFS	169	18989	6	6	4	4	1.68	Single-stranded DNA-b
577	1	db$2	2::C3K5G1|C3K5G1_PSEFS	169	34097	6	6	5	5	1.29	Cysteine synthase OS=
578	1	db$2	2::C3K2W9|RL16_PSEFS	169	15422	7	7	6	6	5.14	50S ribosomal protein
579	1	db$2	2::C3K084|AROC_PSEFS	169	39094	7	7	6	6	1.33	Chorismate synthase (
580	1	db$2	2::C3K0G3|PPNP_PSEFS	169	10127	4	4	4	4	5.23	Pyrimidine/purine nucl
581	1	db$2	2::C3KDU8|C3KDU8_PSEFS	169	35029	10	10	6	6	1.56	D-cysteine desulfhydra
582	1	db$2	2::C3K7M9|C3K7M9_PSEFS	169	34271	7	7	7	7	1.62	Putative ABC transport
583	1	db$2	2::C3KAY1|C3KAY1_PSEFS	168	44657	5	5	4	4	0.53	3-phosphoshikimate 1-
584	1	db$2	2::C3K6G8|LEPA_PSEFS	167	66372	8	8	7	7	0.65	Elongation factor 4 OS
585	1	db$2	2::C3KE71|C3KE71_PSEFS	167	51412	10	10	9	9	1.51	Replicative DNA helica
586	1	db$2	2::C3KCS8|C3KCS8_PSEFS	167	47767	5	5	5	5	0.64	UDP-N-acetylmuramoy
587	1	db$2	2::C3K9F0|C3K9F0_PSEFS	166	40567	6	6	4	4	0.79	Putative transport-rela
588	1	db$2	2::C3JXY4|ASTE_PSEFS	165	37404	8	7	6	6	1.13	Succinylglutamate des

↑Family	M	DB	Accession	Score	Mass	Matches	Match(sig)	Sequences	Seq(sig)	emPAI	Description
589	1	db$2	2::C3JZS5|TRPF_PSEFS	165	21966	4	4	3	3	0.90	N-(5'-phosphoribosyl)a
590	1	db$2	2::C3KDL6|C3KDL6_PSEFS	164	23139	5	5	5	5	1.77	ADP-ribose diphosphat
591	1	SwissProt_HUMAN$	3::P62805|H4_HUMAN	164	11360	7	7	6	6	10.66	Histone H4 OS=Homo
592	1	db$2	2::C3K185|C3K185_PSEFS	163	20062	7	7	6	6	4.12	Putative NAD(P)H nitrc
593	1	db$2	2::C3KCT2|MUTS_PSEFS	163	95536	6	6	6	6	0.35	DNA mismatch repair p
594	1	db$2	2::C3K3S0|C3K3S0_PSEFS	163	23721	6	6	3	3	0.81	Putative hydrolase OS:
595	1	SwissProt_HUMAN$	3::P60174|TPIS_HUMAN	163	26653	6	6	6	6	1.89	Triosephosphate isome
596	1	db$2	2::C3K254|C3K254_PSEFS	162	21027	7	5	6	5	2.06	Osmotically inducible p
597	1	db$2	2::C3K371|C3K371_PSEFS	162	78727	9	8	8	8	0.62	Malate synthase G OS:
598	1	db$2	2::C3KDM7|C3KDM7_PSEFS	162	29519	8	8	8	8	2.58	Sulfurtransferase OS=
599	1	db$2	2::C3KDG2|C3KDG2_PSEFS	161	41589	9	7	8	6	1.21	AMP nucleosidase OS=
600	1	db$2	2::C3K0T7|C3K0T7_PSEFS	161	76509	10	9	10	9	0.75	Putative fatty acid oxic
601	1	db$2	2::C3K638|C3K638_PSEFS	160	26394	6	6	3	3	1.04	Putative short chain de
602	1	db$2	2::C3K568|TRPA_PSEFS	159	28539	4	4	4	4	0.94	Tryptophan synthase a
603	1	db$2	2::C3JZN1|IHFA_PSEFS	159	11469	5	5	5	5	6.59	Integration host factor
604	1	db$2	2::C3JXZ3|C3JXZ3_PSEFS	159	28123	8	7	7	6	1.73	Histidine transport ATF
605	1	SwissProt_HUMAN$	3::P23528|COF1_HUMAN	158	18491	6	5	6	5	2.54	Cofilin-1 OS=Homo sa
606	1	db$2	2::C3JZN8|C3JZN8_PSEFS	157	10735	5	5	4	4	7.58	Uncharacterized protei
607	1	db$2	2::C3K6J1|UBIG_PSEFS	157	26040	6	6	5	5	1.95	Ubiquinone biosynthes
608	1	db$2	2::C3KBY3|C3KBY3_PSEFS	156	64998	5	5	5	5	0.44	Putative lipoprotein OS
609	1	db$2	2::C3K5U6|C3K5U6_PSEFS	156	40174	8	7	6	5	0.80	dTDP-glucose 4,6-deh
610	1	db$2	2::C3K6I1|C3K6I1_PSEFS	156	31224	9	7	6	5	1.13	S-formylglutathione hy
611	1	db$2	2::C3KED3|C3KED3_PSEFS	156	20551	5	5	4	4	2.13	Glycine cleavage syste
612	1	db$2	2::C3KAC2|C3KAC2_PSEFS	155	41342	4	4	4	4	0.58	Acyl-CoA dehydrogena
613	1	db$2	2::C3K572|C3K572_PSEFS	155	7891	2	2	2	2	2.20	Uncharacterized protei
614	1	db$2	2::C3K843|C3K843_PSEFS	155	26535	7	7	5	5	1.43	Lipopolysaccharide exp
615	1	db$2	2::C3K332|Y5583_PSEFS	155	48606	6	6	6	6	0.79	UPF0229 protein PFLU_
616	1	db$2	2::C3JZV1|C3JZV1_PSEFS	155	76010	10	8	10	8	0.65	UvrABC system proteir
617	1	db$2	2::C3K6H1|C3K6H1_PSEFS	154	131195	12	11	12	11	0.49	DNA polymerase III su
618	1	db$2	2::C3JYN4|ZIPA_PSEFS	153	31818	7	6	5	4	1.10	Cell division protein Zi
619	1	db$2	2::C3K075|C3K075_PSEFS	153	77370	10	8	10	8	0.63	Putative sulfatase OS=
620	1	db$2	2::C3K419|C3K419_PSEFS	153	17100	3	3	2	2	1.27	Transcriptional regulat
621	1	db$2	2::Q9ZEP7|PANC_PSEFS	152	30696	3	3	2	2	0.36	Pantothenate syntheta
622	1	db$2	2::C3KE48|C3KE48_PSEFS	152	19341	5	5	4	4	1.64	Peptide deformylase O
623	1	db$2	2::C3JXY2|C3JXY2_PSEFS	152	35731	4	4	3	3	0.70	L-allo-threonine aldola
624	1	db$2	2::C3JXZ2|C3JXZ2_PSEFS	150	36334	8	6	7	5	0.92	Putative AraC family re
625	1	db$2	2::C3K5X2|C3K5X2_PSEFS	150	20559	6	5	5	4	1.49	Putative two-compone
626	1	db$2	2::C3K0N7|RL32_PSEFS	150	6752	5	5	3	3	6.52	50S ribosomal protein
627	1	db$2	2::C3K2W2|RS8_PSEFS	150	14083	7	7	6	6	9.11	30S ribosomal protein
628	1	db$2	2::C3K7I1|C3K7I1_PSEFS	149	85859	7	6	6	5	0.32	Quinoprotein glucose c

↑Family	M	DB	Accession	Score	Mass	Matches	Match(sig)	Sequences	Seq(sig)	emPAI	Description
629	1	db$2	2::C3K3M3|C3K3M3_PSEFS	149	24540	5	4	3	3	0.78	Cell division ATP-bindi
630	1	db$2	2::C3K611|C3K611_PSEFS	148	16065	7	6	4	4	3.27	Universal stress protei
631	1	db$2	2::C3JZT9|C3JZT9_PSEFS	148	24210	6	6	5	5	1.64	3-isopropylmalate deh
632	1	db$2	2::C3KDN5|C3KDN5_PSEFS	148	35878	4	4	3	3	0.48	Renalase OS=Pseudon
633	1	db$2	2::C3K2D6|C3K2D6_PSEFS	148	73930	5	4	5	4	0.29	Carbon starvation prot
634	1	db$2	2::C3K480|C3K480_PSEFS	148	31946	7	6	6	5	1.09	Uncharacterized protei
635	1	db$2	2::C3K6Z7|C3K6Z7_PSEFS	148	89469	9	8	8	8	0.53	Lon protease OS=Pseu
636	1	db$2	2::C3K4Z4|C3K4Z4_PSEFS	147	43117	5	5	4	4	0.55	Putative cytochrome C
637	1	db$2	2::C3K422|C3K422_PSEFS	147	40250	4	3	4	3	0.42	Putative uroporphyrin-
638	1	db$2	2::C3K479|RNPH_PSEFS	147	25645	4	4	3	3	0.73	Ribonuclease PH OS=F
639	1	db$2	2::C3K4U5|C3K4U5_PSEFS	147	75901	9	8	8	7	0.55	Putative protease OS=
640	1	db$2	2::C3K3M9|C3K3M9_PSEFS	146	30473	4	4	4	4	0.85	Putative sulfurtransfer
641	1	db$2	2::C3K0T1|C3K0T1_PSEFS	146	94111	10	8	10	8	0.50	Aconitate hydratase O
642	1	db$2	2::C3K743|FABA_PSEFS	145	18688	7	6	6	5	2.51	3-hydroxydecanoyl-[a
643	1	db$2	2::C3KE66|C3KE66_PSEFS	145	27011	8	7	7	6	1.85	23S rRNA (guanosine-
644	1	db$2	2::C3KCS6|C3KCS6_PSEFS	144	47227	4	4	4	4	0.49	UDP-N-acetylmuramoy
645	1	db$2	2::C3K417|C3K417_PSEFS	144	39396	4	4	3	3	0.43	Transcriptional regulat
646	1	db$2	2::C3K5E4|GLND_PSEFS	144	102948	8	6	8	6	0.32	Bifunctional uridylyltra
647	1	db$2	2::C3KAW2|C3KAW2_PSEFS	144	26230	8	7	7	6	1.94	Putative short chain de
648	1	db$2	2::C3K1C9|C3K1C9_PSEFS	143	65670	3	3	2	2	0.16	Putative cation-transp
649	1	db$2	2::C3K0T3|C3K0T3_PSEFS	143	28958	4	4	4	4	0.92	Putative membrane pr
650	1	SwissProt_HUMAN$	3::P0CG04|IGLC1_HUMAN	143	11341	3	3	3	3	2.41	Immunoglobulin lambc
651	1	db$2	2::C3K709|C3K709_PSEFS	143	7722	4	4	3	3	4.86	Cold shock protein OS
652	1	db$2	2::C3K8W7|C3K8W7_PSEFS	142	34291	5	5	5	5	0.99	Arabinose 5-phosphate
653	1	db$2	2::C3K0W1|C3K0W1_PSEFS	142	27483	5	5	5	5	1.35	Flagellar basal-body rc
654	1	db$2	2::C3K2R5|RIBA_PSEFS	142	22312	8	8	6	6	3.36	GTP cyclohydrolase-2
655	1	db$2	2::C3K1K9|C3K1K9_PSEFS	142	42204	6	6	5	5	0.75	Putative aminotransfer
656	1	db$2	2::C3JYK0|CLPP_PSEFS	142	23405	8	6	6	4	2.33	ATP-dependent Clp prc
657	1	db$2	2::C3JY88|C3JY88_PSEFS	142	20432	8	7	6	5	3.99	NADH-quinone oxidore
658	1	db$2	2::C3K759|C3K759_PSEFS	142	35694	3	3	3	3	0.49	Putative dehydrogenas
658	2	db$2	2::C3K0S6|C3K0S6_PSEFS	94	36802	2	2	2	2	0.29	Putative oxidoreductas
659	1	db$2	2::C3K3L6|TRMB_PSEFS	142	26990	3	3	3	3	0.69	tRNA (guanine-N(7)-)-
660	1	db$2	2::C3K796|C3K796_PSEFS	142	49835	6	5	5	4	0.46	Putative glutamine syr
661	1	db$2	2::C3K617|C3K617_PSEFS	141	19384	5	5	4	4	1.62	Uncharacterized protei
662	1	SwissProt_HUMAN$	3::P27348|1433T_HUMAN	141	27747	2	2	2	2	0.40	14-3-3 protein theta O
663	1	db$2	2::C3K910|C3K910_PSEFS	141	36322	5	5	5	5	0.92	Putative ABC transport
664	1	db$2	2::C3JXQ0|C3JXQ0_PSEFS	140	24880	7	6	5	4	1.13	Putative hydrolase OS
665	1	db$2	2::C3KB68|C3KB68_PSEFS	139	11363	2	2	2	2	1.27	DUF1883 domain-cont
666	1	db$2	2::C3K5B9|C3K5B9_PSEFS	139	60269	4	3	3	3	0.27	Dipeptide ABC transpo
667	1	db$2	2::C3K0S3|C3K0S3_PSEFS	139	67491	7	6	7	6	0.52	Putative ATP-binding c

↑Family	M	DB	Accession	Score	Mass	Matches	Match(sig)	Sequences	Seq(sig)	emPAI	Description
668	1	db$2	2::C3K3R6|C3K3R6_PSEFS	139	54762	11	9	9	7	0.83	L-threonine dehydrata
669	1	db$2	2::C3K1X7|RL27_PSEFS	139	9214	6	6	3	3	6.39	50S ribosomal protein
670	1	db$2	2::C3KAI8|C3KAI8_PSEFS	138	24497	5	5	3	3	1.15	Putative TetR-family re
671	1	db$2	2::C3K2R1|DXS_PSEFS	137	68238	9	9	9	9	0.87	1-deoxy-D-xylulose-5-
672	1	db$2	2::C3KB69|C3KB69_PSEFS	137	12397	2	2	1	1	0.45	Putative lipoprotein OS
673	1	db$2	2::C3JXY9|C3JXY9_PSEFS	136	37073	4	4	4	4	0.66	Putative arginine N-su
674	1	db$2	2::C3K4W2|C3K4W2_PSEFS	136	26844	7	5	5	4	1.02	Putative glycerophospl
675	1	db$2	2::C3K061|C3K061_PSEFS	136	18792	4	4	4	4	1.71	DUF2059 domain-cont
676	1	db$2	2::C3KAZ7|C3KAZ7_PSEFS	136	48577	2	2	2	2	0.21	Putative chain length c
677	1	db$2	2::C3K3L0|METXS_PSEFS	136	41664	5	4	5	4	0.57	Homoserine O-succiny
678	1	db$2	2::C3K5L2|C3K5L2_PSEFS	135	29854	3	3	3	3	0.61	Phosphonates import A
679	1	db$2	2::C3K240|C3K240_PSEFS	135	17616	5	5	4	4	2.76	RNA polymerase-bindi
680	1	db$2	2::C3K1D1|C3K1D1_PSEFS	135	32188	4	4	4	4	0.80	Protein HflC OS=Pseuc
681	1	db$2	2::C3K3G5|C3K3G5_PSEFS	134	15160	5	4	4	3	1.51	Putative thioesterase (
682	1	db$2	2::C3K197|C3K197_PSEFS	134	25331	5	5	5	5	1.53	Putative two-compone
683	1	db$2	2::C3KE16|C3KE16_PSEFS	134	37004	4	3	4	3	0.47	Putative chemotaxis-re
684	1	db$2	2::C3JZQ0|C3JZQ0_PSEFS	134	38665	8	7	6	5	1.08	Putative L-asparaginas
685	1	db$2	2::C3K276|GRPE_PSEFS	133	20714	5	2	3	1	0.25	Protein GrpE OS=Pseu
686	1	db$2	2::C3K421|C3K421_PSEFS	133	46244	4	3	4	3	0.36	Putative HemY domain
687	1	db$2	2::C3K134|C3K134_PSEFS	133	52371	6	6	5	5	0.57	Coproporphyrinogen-II
688	1	db$2	2::C3JY91|C3JY91_PSEFS	133	66398	6	6	4	4	0.33	NADH-quinone oxidore
689	1	db$2	2::C3KBM6|C3KBM6_PSEFS	133	11934	3	3	1	1	1.17	Glutaredoxin OS=Pseu
690	1	db$2	2::C3K6X1|C3K6X1_PSEFS	133	39290	6	4	6	4	0.62	Uncharacterized protei
691	1	db$2	2::C3K6A5|C3K6A5_PSEFS	133	78572	7	7	7	7	0.53	Catalase OS=Pseudom
692	1	db$2	2::C3JYA9|C3JYA9_PSEFS	133	27795	8	8	7	7	2.28	Putative TetR-family re
693	1	db$2	2::C3KDW7|HISZ_PSEFS	133	42687	8	6	7	5	0.74	ATP phosphoribosyltra
694	1	db$2	2::C3KDG5|C3KDG5_PSEFS	133	23599	4	3	3	3	0.82	Putative regulatory pro
695	1	db$2	2::C3K3K8|C3K3K8_PSEFS	133	27898	3	3	1	1	0.40	Pyrroline-5-carboxylat
696	1	db$2	2::C3K308|TRPD_PSEFS	132	36976	7	7	7	7	1.44	Anthranilate phosphori
697	1	db$2	2::C3K9R3|C3K9R3_PSEFS	132	18863	3	3	3	3	1.11	Uncharacterized protei
698	1	db$2	2::C3JYI0|C3JYI0_PSEFS	132	25235	6	6	5	5	1.54	Probable transcriptiona
699	1	db$2	2::C3KDQ9|C3KDQ9_PSEFS	132	29676	10	9	9	8	2.57	Ferredoxin--NADP redu
700	1	db$2	2::C3K7I9|C3K7I9_PSEFS	132	88241	6	5	6	5	0.31	Putative pinecillin acyl
701	1	db$2	2::C3KBK9|C3KBK9_PSEFS	132	34365	3	2	3	2	0.32	Glycerate dehydrogena
702	1	db$2	2::C3JXQ1|C3JXQ1_PSEFS	131	24479	4	4	3	3	0.78	Putative enoyl-CoA hy
703	1	SwissProt_HUMAN$	3::P01859|IGHG2_HUMAN	131	35878	5	5	5	5	0.93	Immunoglobulin heavy
703	2	SwissProt_HUMAN$	3::P01857|IGHG1_HUMAN	106	36083	7	6	6	5	0.92	Immunoglobulin heavy
703	3	SwissProt_HUMAN$	3::P01861|IGHG4_HUMAN	90	35918	5	5	5	5	0.93	Immunoglobulin heavy
704	1	db$2	2::C3K8K4|C3K8K4_PSEFS	131	58330	3	3	3	3	0.28	Putative outer membra
705	1	db$2	2::C3K2P7|C3K2P7_PSEFS	131	17680	6	5	6	5	2.76	Putative acetyltransfer

↑Family	M	DB	Accession	Score	Mass	Matches	Match(sig)	Sequences	Seq(sig)	emPAI	Description
706	1	db$2	2::C3K3Q3|C3K3Q3_PSEFS	131	61523	3	3	3	3	0.26	Putative cation ABC tra
707	1	db$2	2::C3K4V5|C3K4V5_PSEFS	131	36313	5	5	5	5	0.92	Beta-hexosaminidase (
708	1	db$2	2::C3K494|C3K494_PSEFS	131	33626	4	4	4	4	0.75	Gly-zipper_YMGG dom
709	1	db$2	2::C3K3T6|C3K3T6_PSEFS	130	42390	7	6	7	6	0.95	Spermidine/putrescine
710	1	db$2	2::C3K3M6|C3K3M6_PSEFS	129	53012	5	5	5	5	0.56	Peptidase_M16_C dom
711	1	db$2	2::Q9ZEP8|PANB_PSEFS	129	27951	6	6	4	4	1.32	3-methyl-2-oxobutano
712	1	SwissProt_HUMAN$	3::Q5QNW6|H2B2F_HUMAN	129	13912	5	5	4	4	2.83	Histone H2B type 2-F (
713	1	db$2	2::C3K737|C3K737_PSEFS	129	17727	5	5	5	5	2.73	Glutathione peroxidase
714	1	db$2	2::C3KE20|C3KE20_PSEFS	129	33868	6	6	5	5	1.01	GCV_T domain-contair
715	1	db$2	2::C3JZ77|C3JZ77_PSEFS	129	45611	7	6	7	6	0.86	Maltose-binding protei
716	1	db$2	2::C3KDC4|C3KDC4_PSEFS	129	64677	4	4	4	4	0.34	Uncharacterized protei
717	1	db$2	2::C3K0H7|C3K0H7_PSEFS	128	77893	5	5	5	5	0.36	Histidine kinase OS=P:
718	1	db$2	2::C3KDK8|C3KDK8_PSEFS	128	41619	3	3	3	3	0.41	DAO domain-containin
719	1	db$2	2::C3KE58|C3KE58_PSEFS	128	84757	11	10	9	9	0.65	Putative S1 RNA-bindi
720	1	db$2	2::C3K3G7|C3K3G7_PSEFS	128	31253	6	5	6	5	1.12	Methylenetetrahydrofo
721	1	db$2	2::C3JXY8|C3JXY8_PSEFS	128	37476	5	5	5	5	0.88	Arginine N-succinyltrai
722	1	db$2	2::C3JYI6|C3JYI6_PSEFS	128	73112	7	6	7	6	0.48	Putative 2,4-dienoyl-C
723	1	db$2	2::C3K0D7|APEB_PSEFS	128	46914	4	4	4	4	0.50	Probable M18 family ai
724	1	db$2	2::C3JYS6|C3JYS6_PSEFS	128	17060	3	3	2	2	0.73	Putative thioesterase (
725	1	db$2	2::C3K8W9|C3K8W9_PSEFS	128	28276	4	4	3	3	0.65	Intermembrane phosp
726	1	db$2	2::C3K7E3|C3K7E3_PSEFS	128	178026	8	8	8	8	0.24	Alpha-2-macroglobulin
727	1	db$2	2::C3K270|C3K270_PSEFS	127	17315	7	6	6	5	2.86	Transcription elongatic
728	1	db$2	2::C3K5K2|C3K5K2_PSEFS	127	9923	6	5	4	4	5.48	Putative lipoprotein OS
729	1	SwissProt_HUMAN$	3::P63104|1433Z_HUMAN	127	27728	4	4	4	4	0.97	14-3-3 protein zeta/de
730	1	db$2	2::C3K648|C3K648_PSEFS	127	33905	5	5	5	5	1.01	Conserved hypothetica
731	1	db$2	2::C3K9H7|C3K9H7_PSEFS	127	27490	3	3	3	3	0.67	Putative short-chain de
732	1	db$2	2::C3JYE9|C3JYE9_PSEFS	127	31035	4	4	3	3	0.58	Putative hydroxymethy
733	1	db$2	2::C3K309|C3K309_PSEFS	127	21661	5	4	4	4	1.38	Anthranilate synthase
734	1	db$2	2::C3K6J5|SERC_PSEFS	126	39799	8	7	7	6	1.04	Phosphoserine aminotı
735	1	SwissProt_HUMAN$	3::Q5VTE0|EF1A3_HUMAN	126	50153	8	7	7	7	0.93	Putative elongation fac
736	1	db$2	2::C3KDB8|HLDE_PSEFS	126	49913	8	7	8	7	0.94	Bifunctional protein Hl
737	1	db$2	2::C3K6J2|C3K6J2_PSEFS	126	47817	4	4	3	3	0.35	Putative hydrolase OS:
738	1	db$2	2::C3KAJ9|C3KAJ9_PSEFS	125	20836	7	7	6	6	2.85	Putative hypoxanthine
739	1	db$2	2::C3K3M7|C3K3M7_PSEFS	125	22464	2	2	1	1	0.23	Ribosomal RNA small s
740	1	db$2	2::C3K8W4|C3K8W4_PSEFS	125	18790	7	7	5	5	3.47	Lipopolysaccharide exp
741	1	db$2	2::C3JY77|C3JY77_PSEFS	125	40451	5	5	4	4	0.60	Putative 3-oxoacyl-[ac
742	1	db$2	2::C3K0W5|PHS_PSEFS	124	13357	6	6	4	4	3.04	Putative pterin-4-alpha
743	1	db$2	2::C3K7T1|C3K7T1_PSEFS	124	23131	5	4	5	4	1.26	Two-component syster
744	1	db$2	2::C3K2V4|RS13_PSEFS	124	13332	9	6	6	3	1.85	30S ribosomal protein
745	1	db$2	2::C3K3M2|C3K3M2_PSEFS	124	37029	4	4	4	4	0.66	Cell division protein Ft:

↑Family	M	DB	Accession	Score	Mass	Matches	Match(sig)	Sequences	Seq(sig)	emPAI	Description
746	1	db$2	2::C3K3W8|C3K3W8_PSEFS	124	44404	4	4	4	4	0.53	Putative ubiquinone bi
747	1	db$2	2::C3KAI3|AROB_PSEFS	123	39825	4	4	4	4	0.61	3-dehydroquinate synt
748	1	db$2	2::C3K8W6|C3K8W6_PSEFS	123	18885	6	6	6	6	3.43	3-deoxy-D-manno-oct
749	1	db$2	2::C3KE32|C3KE32_PSEFS	123	20912	2	2	1	1	0.25	Sigma factor negative
750	1	db$2	2::C3K483|C3K483_PSEFS	122	78630	10	8	9	7	0.53	Guanosine-3',5'-bis(Di
750	2	db$2	2::C3K1B1|C3K1B1_PSEFS	119	46678	3	3	3	3	0.35	Putative acid tolerance
751	1	db$2	2::C3K8K2|C3K8K2_PSEFS	122	418093	6	4	6	4	0.05	Haemagg_act domain-
752	1	db$2	2::C3KDC0|C3KDC0_PSEFS	122	36735	3	3	3	3	0.47	Uncharacterized protei
753	1	db$2	2::C3KED2|C3KED2_PSEFS	122	17293	5	4	5	4	1.95	Thioredoxin peroxidase
754	1	db$2	2::C3KE89|C3KE89_PSEFS	122	24346	5	4	5	4	1.17	Uncharacterized protei
755	1	db$2	2::C3K6V2|HIS4_PSEFS	121	25679	5	4	4	3	1.08	1-(5-phosphoribosyl)-!
756	1	db$2	2::C3JXH2|C3JXH2_PSEFS	121	91149	3	3	3	3	0.17	Putative excinuclease /
757	1	db$2	2::C3K4C8|C3K4C8_PSEFS	121	49694	6	5	6	5	0.61	Putative transport-rela
758	1	db$2	2::C3K272|C3K272_PSEFS	120	40774	7	4	5	4	0.59	Carbamoyl-phosphate
759	1	db$2	2::C3K253|C3K253_PSEFS	120	106648	4	4	4	4	0.19	Putative membrane pr
760	1	db$2	2::C3K3S3|C3K3S3_PSEFS	120	15261	5	5	3	3	1.51	DUF4399 domain-cont
761	1	db$2	2::C3K427|C3K427_PSEFS	120	23927	3	3	2	2	0.48	Putative glutathione S-
762	1	db$2	2::C3KCP1|C3KCP1_PSEFS	119	23476	5	5	5	5	1.73	Peptide methionine sul
763	1	db$2	2::C3K229|C3K229_PSEFS	119	24861	1	1	1	1	0.21	TPR_REGION domain-(
764	1	db$2	2::C3K5H2|QUEF_PSEFS	119	30808	5	5	5	5	1.15	NADPH-dependent 7-c
765	1	db$2	2::C3JYV1|C3JYV1_PSEFS	119	32264	6	6	6	6	1.40	Formyltetrahydrofolate
766	1	db$2	2::C3KBM4|C3KBM4_PSEFS	119	34098	9	8	8	8	2.03	Ornithine carbamoyltra
767	1	db$2	2::C3KA90|C3KA90_PSEFS	119	36984	4	4	4	4	0.67	Putative aldo/keto red
768	1	db$2	2::C3JYS8|RUVA_PSEFS	119	22218	3	3	3	3	0.89	Holliday junction ATP-(
769	1	db$2	2::C3K2X4|RL23_PSEFS	118	10938	8	7	6	6	11.53	50S ribosomal protein
770	1	db$2	2::C3K2K2|THIE_PSEFS	118	22345	3	3	2	2	0.52	Thiamine-phosphate s
771	1	db$2	2::C3K231|C3K231_PSEFS	118	57678	6	5	6	5	0.51	APH domain-containin
772	1	db$2	2::C3KB44|C3KB44_PSEFS	117	23546	2	2	1	1	0.49	Putative membrane pr
773	1	db$2	2::C3K7Y8|C3K7Y8_PSEFS	117	53697	6	5	4	4	0.55	Putative hydrolase OS
774	1	db$2	2::C3K0I0|C3K0I0_PSEFS	117	27472	5	5	5	5	1.36	RNA polymerase sigma
775	1	db$2	2::C3KAS9|C3KAS9_PSEFS	117	109677	3	3	3	3	0.14	Efflux pump membran
776	1	db$2	2::C3K9U7|C3K9U7_PSEFS	117	56147	8	7	7	6	0.80	Glucose-6-phosphate :
777	1	db$2	2::C3K740|C3K740_PSEFS	117	33777	12	9	11	9	2.52	DUF815 domain-conta
778	1	db$2	2::C3K0N3|ACP_PSEFS	116	8637	3	3	2	2	1.89	Acyl carrier protein OS
779	1	db$2	2::C3KDX7|ISPH_PSEFS	116	34499	6	6	5	5	0.98	4-hydroxy-3-methylbu
780	1	db$2	2::C3K5X6|C3K5X6_PSEFS	116	49358	5	4	5	4	0.47	Tryptophan--tRNA liga
781	1	db$2	2::C3K4E1|C3K4E1_PSEFS	116	38799	8	6	6	5	0.84	N5-carboxyaminoimida
782	1	db$2	2::C3K1W6|C3K1W6_PSEFS	116	13390	5	5	5	5	4.65	D-erythro-7,8-dihydro
783	1	db$2	2::C3K785|C3K785_PSEFS	116	24962	6	5	5	5	1.56	3-oxoadipate CoA-tran
784	1	db$2	2::C3K2I0|C3K2I0_PSEFS	115	48538	5	5	5	5	0.63	Putative ATP-depender

↑Family	M	DB	Accession	Score	Mass	Matches	Match(sig)	Sequences	Seq(sig)	emPAI	Description
785	1	db$2	2::C3KAK2|C3KAK2_PSEFS	115	10989	6	5	4	4	7.22	Uncharacterized protei
786	1	db$2	2::C3JYK2|C3JYK2_PSEFS	115	30492	6	6	4	4	0.85	Bifunctional protein Fo
787	1	db$2	2::C3K754|C3K754_PSEFS	114	29479	3	3	3	3	0.61	Putative lipoprotein OS
788	1	db$2	2::C3K1X0|CYSZ_PSEFS	114	28826	4	4	4	4	0.92	Sulfate transporter Cy:
789	1	db$2	2::C3K0U5|C3K0U5_PSEFS	114	62992	4	3	4	3	0.25	Flagellar M-ring protei
790	1	db$2	2::C3K148|APT_PSEFS	114	20064	6	6	4	4	1.54	Adenine phosphoribos
791	1	db$2	2::C3K320|C3K320_PSEFS	114	34836	2	2	2	2	0.31	Uncharacterized protei
792	1	db$2	2::C3K2T3|C3K2T3_PSEFS	113	32553	4	4	4	4	0.79	dTDP-4-dehydrorhamr
793	1	db$2	2::C3KDX6|C3KDX6_PSEFS	113	16394	2	2	2	2	0.77	Peptidyl-prolyl cis-tran
794	1	db$2	2::C3K3T8|C3K3T8_PSEFS	113	39499	5	4	4	3	0.43	Putrescine ABC transp
795	1	db$2	2::C3K9P7|C3K9P7_PSEFS	113	69450	4	4	4	4	0.31	Putative substrate-bin
796	1	db$2	2::C3K2W8|RL29_PSEFS	113	7168	6	4	5	3	5.68	50S ribosomal protein
797	1	db$2	2::C3KDM6|PSD_PSEFS	113	31380	7	5	6	4	1.12	Phosphatidylserine dec
798	1	db$2	2::C3K6Y8|C3K6Y8_PSEFS	112	34148	7	7	6	6	1.29	GTPase Era OS=Pseud
799	1	db$2	2::C3K486|C3K486_PSEFS	112	30593	3	3	3	3	0.59	Epimerase domain-cor
800	1	db$2	2::C3KD59|C3KD59_PSEFS	111	81779	5	5	5	5	0.34	YcaO domain-containir
801	1	db$2	2::C3K3L3|C3K3L3_PSEFS	111	21091	5	2	4	2	0.56	dITP/XTP pyrophospha
802	1	db$2	2::C3K3M5|C3K3M5_PSEFS	111	50101	7	7	7	7	0.94	Putative peptidase OS:
803	1	db$2	2::C3JZH3|C3JZH3_PSEFS	111	22428	5	4	4	3	1.31	Putative cytochrome o
803	2	db$2	2::C3JY78|C3JY78_PSEFS	111	22781	4	4	3	3	1.28	Putative cytochrome o
804	1	db$2	2::C3KA81|C3KA81_PSEFS	111	55484	7	6	7	6	0.67	Alkyl hydroperoxide re
805	1	db$2	2::C3K1W7|C3K1W7_PSEFS	111	11247	4	3	3	2	1.29	DUF1244 domain-cont
806	1	db$2	2::C3KE84|C3KE84_PSEFS	110	35425	5	5	5	5	0.95	Pseudouridine synthas
807	1	db$2	2::C3K9G9|C3K9G9_PSEFS	110	16183	4	4	4	4	2.17	CMD domain-containin
808	1	db$2	2::C3KE45|C3KE45_PSEFS	110	49639	6	5	5	5	0.61	Trk system potassium
809	1	db$2	2::C3JY36|C3JY36_PSEFS	110	16095	4	4	3	3	1.39	Uncharacterized protei
810	1	SwissProt_HUMAN$	3::P07737|PROF1_HUMAN	110	15045	5	5	4	4	3.69	Profilin-1 OS=Homo sa
811	1	db$2	2::C3KBI8|C3KBI8_PSEFS	109	48805	4	4	4	4	0.47	Uncharacterized protei
812	1	db$2	2::C3K2U8|C3K2U8_PSEFS	109	18135	6	5	6	5	2.65	Bacterioferritin OS=Ps
813	1	db$2	2::C3JXP7|RECR_PSEFS	109	21528	5	5	5	5	1.97	Recombination protein
814	1	db$2	2::C3KBL3|C3KBL3_PSEFS	109	50616	8	7	7	6	0.75	Putative exported prot
815	1	db$2	2::C3KD57|C3KD57_PSEFS	109	49686	5	3	5	3	0.33	Cystathionine beta-lya
816	1	db$2	2::C3K3K7|C3K3K7_PSEFS	109	24490	5	5	4	4	1.15	Pyridoxal phosphate h
817	1	SwissProt_HUMAN$	3::P01876|IGHA1_HUMAN	109	37631	5	4	4	3	0.46	Immunoglobulin heavy
817	2	SwissProt_HUMAN$	3::P0DOX2|IGA2_HUMAN	104	48903	4	4	4	4	0.47	Immunoglobulin alpha
817	3	SwissProt_HUMAN$	3::P01764|HV323_HUMAN	58	12574	4	4	2	2	2.04	Immunoglobulin heavy
818	1	db$2	2::C3K0V5|C3K0V5_PSEFS	109	33212	8	7	7	6	1.70	Putative keto/oxo acyl
819	1	db$2	2::C3K0P6|DCTA_PSEFS	109	46761	3	3	3	3	0.35	C4-dicarboxylate trans
820	1	db$2	2::C3K2L6|C3K2L6_PSEFS	108	47731	6	5	6	5	0.64	Putative lipoprotein OS
821	1	SwissProt_HUMAN$	3::P68363|TBA1B_HUMAN	108	50120	6	4	6	4	0.46	Tubulin alpha-1B chair

↑Family	M	DB	Accession	Score	Mass	Matches	Match(sig)	Sequences	Seq(sig)	emPAI	Description
822	1	db$2	2::C3JY87|NUOH_PSEFS	108	37530	4	4	4	4	0.65	NADH-quinone oxidore
823	1	db$2	2::C3K1H1|RS16_PSEFS	108	9202	5	5	3	3	3.48	30S ribosomal protein
824	1	db$2	2::C3K5K1|C3K5K1_PSEFS	108	38065	6	6	5	5	0.86	Putative phospholipase
825	1	db$2	2::C3K710|DCD_PSEFS	108	21231	4	4	4	4	1.42	dCTP deaminase OS=F
826	1	db$2	2::C3JZH1|C3JZH1_PSEFS	108	34020	4	4	3	3	0.52	LysR-family transcripti
827	1	db$2	2::C3K5B2|C3K5B2_PSEFS	108	35714	3	3	3	3	0.49	Dipeptide ABC transpo
828	1	db$2	2::C3KDF0|C3KDF0_PSEFS	107	18221	5	3	5	3	1.16	Putative phage tail-rel
829	1	db$2	2::C3K4C1|C3K4C1_PSEFS	107	40318	5	5	2	2	0.26	Putative nitric oxide de
830	1	db$2	2::C3KDX0|C3KDX0_PSEFS	107	16992	6	5	4	3	1.28	Peptidyl-prolyl cis-tran
831	1	db$2	2::C3K6U9|HIS7_PSEFS	107	21830	8	6	6	5	1.92	Imidazoleglycerol-pho
832	1	db$2	2::C3KDS5|C3KDS5_PSEFS	107	41122	4	4	3	3	0.58	Putative hydratase/iso
833	1	db$2	2::C3K2W7|RS17_PSEFS	106	10090	4	4	4	4	5.23	30S ribosomal protein
834	1	SwissProt_HUMAN$	3::P01009|A1AT_HUMAN	106	46707	4	4	4	4	0.50	Alpha-1-antitrypsin OS
835	1	db$2	2::C3K5E8|C3K5E8_PSEFS	106	28997	3	3	3	3	0.63	Cyclohexadienyl dehyc
836	1	db$2	2::C3K3N8|C3K3N8_PSEFS	106	43920	5	4	5	4	0.54	PUA domain-containin
837	1	db$2	2::C3KCV6|C3KCV6_PSEFS	106	40154	5	5	4	4	0.80	Probable alcohol dehyc
838	1	db$2	2::C3K256|RS15_PSEFS	105	10112	4	4	3	3	2.94	30S ribosomal protein
839	1	db$2	2::C3KBU0|C3KBU0_PSEFS	105	36726	6	4	6	4	0.67	Sulfate/thiosulfate imp
840	1	SwissProt_HUMAN$	3::P62979|RS27A_HUMAN	105	17953	4	4	3	3	1.18	Ubiquitin-40S ribosom
841	1	db$2	2::C3JY53|CYSG_PSEFS	105	50259	4	4	4	4	0.46	Siroheme synthase OS
842	1	db$2	2::C3JY82|NUOB_PSEFS	105	25341	6	4	6	4	1.10	NADH-quinone oxidore
843	1	db$2	2::C3KBK6|C3KBK6_PSEFS	105	29320	3	3	2	2	0.62	Putative exported pept
844	1	db$2	2::C3K349|C3K349_PSEFS	104	28994	4	4	4	4	0.92	Pyrroloquinoline-quino
845	1	db$2	2::C3K4H9|C3K4H9_PSEFS	104	34203	8	6	7	6	1.29	Quinone oxidoreducta
846	1	db$2	2::C3K170|C3K170_PSEFS	104	22875	5	5	4	4	1.27	Flavin_Reduct domain-
847	1	db$2	2::C3K2U6|C3K2U6_PSEFS	104	49484	2	2	1	1	0.21	Putative transport-rela
848	1	db$2	2::C3K2Z9|C3K2Z9_PSEFS	104	34289	5	5	4	4	0.73	NMO domain-containin
849	1	db$2	2::C3K0U9|C3K0U9_PSEFS	103	55465	6	4	5	3	0.29	Putative two-compone
850	1	db$2	2::C3KAB1|C3KAB1_PSEFS	103	44397	6	5	5	4	0.53	Histidine kinase OS=P
851	1	db$2	2::C3K2K6|C3K2K6_PSEFS	103	49458	4	4	4	4	0.47	tRNA-2-methylthio-N(
852	1	db$2	2::C3K230|C3K230_PSEFS	103	85084	2	2	2	2	0.12	Penicillin-binding prote
853	1	db$2	2::C3K107|C3K107_PSEFS	103	31415	5	4	5	4	0.82	Uncharacterized protei
854	1	db$2	2::C3K301|C3K301_PSEFS	103	28901	3	3	3	3	0.63	Putative dehydrogenas
855	1	db$2	2::C3K1H0|RIMM_PSEFS	103	20004	3	3	3	3	1.02	Ribosome maturation f
856	1	db$2	2::C3JZ82|C3JZ82_PSEFS	103	37007	2	2	2	2	0.29	Putative dehydrogenas
857	1	db$2	2::C3K816|OPGH_PSEFS	103	96236	5	4	5	4	0.22	Glucans biosynthesis g
858	1	db$2	2::C3K268|C3K268_PSEFS	102	11552	6	5	6	5	6.45	CRM domain-containin
859	1	db$2	2::C3JYQ9|C3JYQ9_PSEFS	102	54767	4	2	4	2	0.19	Transcriptional regulat
860	1	db$2	2::C3K062|C3K062_PSEFS	102	48996	7	5	5	4	0.47	Fumarate hydratase cl
861	1	db$2	2::C3K2P1|C3K2P1_PSEFS	102	48286	4	4	4	4	0.48	UDP-N-acetylmuramat

↑Family	M	DB	Accession	Score	Mass	Matches	Match(sig)	Sequences	Seq(sig)	emPAI	Description
862	1	db$2	2::C3K1U9|C3K1U9_PSEFS	102	42728	4	3	3	3	0.39	Flavohemoglobin OS=I
863	1	SwissProt_HUMAN$	3::Q06830|PRDX1_HUMAN	102	22096	6	6	6	6	2.58	Peroxiredoxin-1 OS=H
864	1	db$2	2::C3K2W3|C3K2W3_PSEFS	102	11427	6	3	5	3	2.37	30S ribosomal protein
865	1	db$2	2::C3K360|C3K360_PSEFS	102	23509	3	3	3	3	0.82	ATP-dependent dethiol
866	1	db$2	2::C3K437|C3K437_PSEFS	101	26506	2	2	2	2	0.43	Uncharacterized protei
867	1	db$2	2::C3K3K1|C3K3K1_PSEFS	101	18188	1	1	1	1	0.29	Bifunctional protein [p
868	1	db$2	2::C3K862|C3K862_PSEFS	101	39825	5	3	5	3	0.43	Putative outer membra
869	1	db$2	2::C3K8K5|C3K8K5_PSEFS	101	64377	2	2	2	2	0.16	Putative transport-rela
870	1	db$2	2::C3K303|COQ7_PSEFS	101	23866	4	4	4	4	1.20	3-demethoxyubiquinol
871	1	db$2	2::C3KDQ0|C3KDQ0_PSEFS	101	35105	5	5	5	5	0.96	2-ketogluconate reduc
872	1	db$2	2::C3K2K7|C3K2K7_PSEFS	101	37713	4	3	4	3	0.46	PhoH domain-containir
873	1	db$2	2::C3K467|C3K467_PSEFS	101	13453	2	2	2	2	1.00	Cupin_3 domain-conta
874	1	db$2	2::C3K2C2|C3K2C2_PSEFS	101	67328	3	3	3	3	0.23	Putative lipoprotein OS
875	1	db$2	2::C3K8W8|C3K8W8_PSEFS	101	29386	4	4	3	3	0.62	Putative ABC transport
876	1	db$2	2::C3KDB9|C3KDB9_PSEFS	100	32977	3	3	3	3	0.54	Putative lipopolysaccha
877	1	db$2	2::C3K8K8|C3K8K8_PSEFS	100	62203	7	7	6	6	0.58	Putative exported prot
878	1	db$2	2::C3JYS4|C3JYS4_PSEFS	100	16520	5	5	5	5	3.11	Tol-Pal system protein
879	1	db$2	2::C3KEA2|C3KEA2_PSEFS	100	30824	3	3	2	2	0.36	Putative inner membra
880	1	db$2	2::C3K3L5|C3K3L5_PSEFS	100	11400	2	2	2	2	1.25	Putative membrane pr
881	1	db$2	2::C3K4W3|C3K4W3_PSEFS	100	21770	3	3	3	3	0.91	PilZ domain-containinc
882	1	db$2	2::C3K2D4|C3K2D4_PSEFS	100	35439	4	3	3	2	0.31	Putative cobalamin syr
883	1	db$2	2::C3K3L1|C3K3L1_PSEFS	100	23430	3	3	2	2	0.49	Uncharacterized protei
884	1	db$2	2::C3KAH8|C3KAH8_PSEFS	99	89028	5	5	5	5	0.31	DD-transpeptidase OS
885	1	db$2	2::C3K250|C3K250_PSEFS	99	71198	3	3	2	2	0.14	Acetyl-coenzyme A syr
886	1	db$2	2::C3K4Y3|KDPA_PSEFS	99	60181	3	3	3	3	0.27	Potassium-transportin
887	1	db$2	2::C3K813|C3K813_PSEFS	99	36618	8	5	7	4	0.67	Proline iminopeptidase
888	1	db$2	2::C3KDC6|RF1_PSEFS	98	39857	3	3	3	3	0.43	Peptide chain release f
889	1	SwissProt_HUMAN$	3::P10909|CLUS_HUMAN	98	52461	4	4	4	4	0.43	Clusterin OS=Homo sa
890	1	SwissProt_HUMAN$	3::P04279|SEMG1_HUMAN	98	52100	2	2	2	2	0.20	Semenogelin-1 OS=Hc
891	1	db$2	2::C3JXL6|C3JXL6_PSEFS	98	41978	2	2	1	1	0.12	Bcr/CflA family efflux t
892	1	db$2	2::C3K6E8|C3K6E8_PSEFS	98	27518	2	2	2	2	0.41	Putative GntR-family r
893	1	db$2	2::C3K302|C3K302_PSEFS	98	12723	6	5	3	3	3.29	Putative HIT domain-c
894	1	db$2	2::C3K424|HEM3_PSEFS	98	33541	3	2	2	1	0.15	Porphobilinogen deami
895	1	db$2	2::C3K261|RIMP_PSEFS	98	17653	4	3	4	3	1.21	Ribosome maturation f
896	1	db$2	2::C3K4J0|C3K4J0_PSEFS	98	17598	5	4	5	4	1.89	Zn_protease domain-c
897	1	db$2	2::C3K312|C3K312_PSEFS	98	24134	6	4	4	4	1.18	Ribulose-phosphate 3-
898	1	db$2	2::C3K1N5|QUEA_PSEFS	98	38396	5	5	5	5	0.85	S-adenosylmethionine
899	1	db$2	2::C3KBM1|C3KBM1_PSEFS	97	29989	2	2	2	2	0.37	Glycerol uptake facilita
900	1	db$2	2::C3K6M4|C3K6M4_PSEFS	97	13463	4	4	2	2	3.00	Succinate dehydrogena
901	1	db$2	2::C3K830|C3K830_PSEFS	97	47217	6	6	6	6	0.82	Putative short chain de

↑Family	M	DB	Accession	Score	Mass	Matches	Match(sig)	Sequences	Seq(sig)	emPAI	Description
902	1	db$2	2::C3K6Y6|C3K6Y6_PSEFS	96	31839	7	7	5	5	1.10	Signal peptidase I OS=
903	1	db$2	2::C3K1F7|C3K1F7_PSEFS	96	69251	3	2	3	2	0.15	tRNA uridine 5-carboxy
904	1	db$2	2::C3K468|C3K468_PSEFS	96	6077	2	2	1	1	3.41	50S ribosomal protein
905	1	db$2	2::C3KCP6|C3KCP6_PSEFS	96	38240	4	4	4	4	0.64	ADP-heptose--LPS hep
906	1	db$2	2::C3K5T5|C3K5T5_PSEFS	96	25405	2	2	1	1	0.20	Carbonic anhydrase OS
907	1	db$2	2::C3K0S1|C3K0S1_PSEFS	96	53269	2	2	2	2	0.19	Magnesium transporte
908	1	db$2	2::C3KD21|C3KD21_PSEFS	95	81698	4	4	4	4	0.26	Putative glycogen debr
909	1	db$2	2::C3K5H7|C3K5H7_PSEFS	95	43348	3	3	3	3	0.39	Putative response regu
910	1	db$2	2::C3K3X0|C3K3X0_PSEFS	95	42989	5	4	4	3	0.39	Putative ubiquinone bi
911	1	db$2	2::C3K8U7|C3K8U7_PSEFS	95	30923	5	4	5	4	0.84	Transcriptional regulat
912	1	db$2	2::C3K4Z0|CCME_PSEFS	95	16101	3	3	3	3	1.39	Cytochrome c-type bio
913	1	db$2	2::C3KBW8|C3KBW8_PSEFS	95	39031	6	6	6	6	1.07	D-alanine--D-alanine l
914	1	db$2	2::C3K575|C3K575_PSEFS	95	14827	1	1	1	1	0.37	Osmotically inducible p
915	1	db$2	2::C3KBL4|C3KBL4_PSEFS	94	26993	5	4	4	3	0.69	Glutamate/aspartate A
916	1	db$2	2::C3KC09|C3KC09_PSEFS	94	83087	3	3	3	3	0.19	Periplasmic beta-gluco
917	1	db$2	2::C3K1L7|RLMN_PSEFS	94	42342	5	5	5	5	0.75	Dual-specificity RNA m
918	1	db$2	2::C3K544|C3K544_PSEFS	94	74165	4	3	4	3	0.21	Uncharacterized protei
919	1	db$2	2::C3K6W7|C3K6W7_PSEFS	93	16216	4	3	4	3	1.37	Biotin carboxyl carrier
920	1	db$2	2::C3K815|C3K815_PSEFS	93	62777	8	7	7	6	0.57	Glucans biosynthesis p
921	1	db$2	2::C3K3Q1|C3K3Q1_PSEFS	93	40925	5	3	4	3	0.41	Putative oxidoreductas
922	1	db$2	2::C3K015|C3K015_PSEFS	93	53791	3	3	3	3	0.30	Putative ferredoxin OS
923	1	db$2	2::C3K4U7|C3K4U7_PSEFS	92	28919	2	2	2	2	0.38	Putative amidotransfer
924	1	db$2	2::C3K3N0|C3K3N0_PSEFS	92	25405	5	5	3	3	0.74	Putative TetR-family re
925	1	db$2	2::C3K257|C3K257_PSEFS	92	33362	5	4	4	3	0.53	tRNA pseudouridine sy
926	1	db$2	2::C3K106|C3K106_PSEFS	92	20669	4	2	4	2	0.58	Uncharacterized protei
927	1	SwissProt_HUMAN$	3::P62937|PPIA_HUMAN	91	18001	4	4	4	4	1.83	Peptidyl-prolyl cis-tran
928	1	db$2	2::C3JY84|C3JY84_PSEFS	91	18006	2	2	2	2	0.68	NADH dehydrogenase
929	1	db$2	2::C3KAR1|C3KAR1_PSEFS	91	16023	1	1	1	1	0.34	Putative isoquinoline 1
930	1	db$2	2::C3K103|C3K103_PSEFS	91	23527	3	3	3	3	0.82	Phosphoribosylglycinar
931	1	db$2	2::C3K6E4|C3K6E4_PSEFS	91	46143	5	5	4	4	0.51	Cytochrome b OS=Pse
932	1	db$2	2::C3K1V6|C3K1V6_PSEFS	90	32555	2	2	2	2	0.34	Putative short-chain de
933	1	db$2	2::C3K2Y1|RS12_PSEFS	90	13716	4	4	3	3	1.76	30S ribosomal protein
934	1	db$2	2::C3K6E3|C3K6E3_PSEFS	90	21087	5	5	3	3	1.43	Ubiquinol-cytochrome
935	1	db$2	2::C3K9Q2|C3K9Q2_PSEFS	90	16954	3	2	3	2	0.73	Ribonuclease H OS=Ps
936	1	db$2	2::C3K0Q8|C3K0Q8_PSEFS	89	24717	2	2	2	2	0.46	Basal-body rod modific
937	1	db$2	2::C3K909|C3K909_PSEFS	89	24537	2	2	2	2	0.47	Uncharacterized protei
938	1	db$2	2::C3K1G1|C3K1G1_PSEFS	89	48709	3	3	3	3	0.34	tRNA modification GTP
939	1	db$2	2::C3KAT0|C3KAT0_PSEFS	89	40804	2	2	2	2	0.26	Putative efflux system
940	1	db$2	2::C3KAH5|RL31_PSEFS	88	8451	4	3	3	3	4.12	50S ribosomal protein
941	1	db$2	2::C3K6A4|C3K6A4_PSEFS	88	36459	4	3	3	3	0.48	Methionine import ATP

↑Family	M	DB	Accession	Score	Mass	Matches	Match(sig)	Sequences	Seq(sig)	emPAI	Description
942	1	db$2	2::C3K2Q9|EX7S_PSEFS	88	8900	3	2	3	2	1.82	Exodeoxyribonuclease
943	1	db$2	2::C3KBJ5|C3KBJ5_PSEFS	88	62095	3	3	3	3	0.26	Phospholipase C OS=P
944	1	db$2	2::C3K3Q4|C3K3Q4_PSEFS	88	38576	3	2	3	2	0.28	Putative cation ABC tra
945	1	db$2	2::C3K6K2|IHFB_PSEFS	88	11021	3	3	3	3	2.54	Integration host factor
946	1	db$2	2::C3K470|C3K470_PSEFS	88	58011	3	3	3	3	0.28	Putative transport-rela
947	1	db$2	2::C3JXX6|DNLJ_PSEFS	88	85515	4	4	4	4	0.25	DNA ligase OS=Pseudo
948	1	db$2	2::C3K3B7|C3K3B7_PSEFS	88	14934	2	2	2	2	0.86	Uncharacterized protei
949	1	db$2	2::C3K2J5|C3K2J5_PSEFS	88	18057	4	3	4	3	1.17	Putative AsnC-family r
950	1	db$2	2::C3KCS0|RL25_PSEFS	88	21502	8	8	3	3	2.70	50S ribosomal protein
951	1	db$2	2::C3KDM3|C3KDM3_PSEFS	87	25575	1	1	1	1	0.20	Putative lipoprotein OS
952	1	db$2	2::C3K7C5|C3K7C5_PSEFS	87	40067	3	3	3	3	0.42	Histidine kinase OS=Ps
953	1	db$2	2::C3JZR8|C3JZR8_PSEFS	87	46446	9	8	8	7	1.04	Putative oxidoreductas
954	1	db$2	2::C3K9M0|C3K9M0_PSEFS	87	11184	2	2	2	2	1.29	SCP2 domain-containi
955	1	db$2	2::C3K0W6|C3K0W6_PSEFS	86	42755	4	4	3	3	0.39	Aspartate aminotransf
956	1	db$2	2::C3KAM8|C3KAM8_PSEFS	86	52747	3	3	2	2	0.20	Phosphate transporter
957	1	db$2	2::C3K6E5|C3K6E5_PSEFS	86	29069	4	3	4	3	0.63	Cytochrome c1 OS=Ps
958	1	db$2	2::C3K337|PLSY_PSEFS	86	20278	1	1	1	1	0.26	Glycerol-3-phosphate a
959	1	SwissProt_HUMAN$	3::P02647|APOA1_HUMAN	85	30759	2	2	2	2	0.36	Apolipoprotein A-I OS=
960	1	db$2	2::C3KBY0|GMHA_PSEFS	85	21250	2	2	2	2	0.56	Phosphoheptose isome
961	1	db$2	2::C3JY57|LOLA_PSEFS	85	22546	4	4	4	4	1.30	Outer-membrane lipop
962	1	db$2	2::C3K482|RPOZ_PSEFS	85	9821	4	4	3	3	5.62	DNA-directed RNA poly
963	1	db$2	2::C3KDC3|LOLB_PSEFS	84	23021	3	2	3	2	0.51	Outer-membrane lipop
964	1	db$2	2::C3K9N1|C3K9N1_PSEFS	84	43216	4	3	4	3	0.39	D-ser_dehydrat domai
965	1	db$2	2::C3KC02|C3KC02_PSEFS	84	29258	3	3	3	3	0.62	Putative acyltransferas
966	1	db$2	2::C3K4R1|PLSB_PSEFS	84	95327	5	5	5	5	0.28	Glycerol-3-phosphate a
967	1	db$2	2::C3K220|C3K220_PSEFS	84	136725	2	2	2	2	0.07	RecBCD enzyme subur
968	1	db$2	2::C3JZN0|C3JZN0_PSEFS	84	13917	2	2	2	2	0.96	MerR-family transcript
969	1	db$2	2::C3KE87|C3KE87_PSEFS	83	46792	5	3	4	3	0.35	NADH dehydrogenase
970	1	db$2	2::C3K9U9|C3K9U9_PSEFS	83	9554	1	1	1	1	0.62	Uncharacterized protei
971	1	db$2	2::C3KCS5|C3KCS5_PSEFS	83	51798	3	2	3	2	0.20	UDP-N-acetylmuramoy
972	1	db$2	2::C3K1L5|C3K1L5_PSEFS	83	34147	5	4	3	3	0.51	Putative DNA-binding p
973	1	db$2	2::C3K1V9|C3K1V9_PSEFS	82	16219	1	1	1	1	0.33	Flavodoxin-like domair
974	1	db$2	2::C3K5F4|C3K5F4_PSEFS	82	21753	3	3	3	3	0.91	Putative nitroreductase
975	1	db$2	2::C3KDR6|C3KDR6_PSEFS	82	40570	3	2	3	2	0.26	Putaive NADH oxidored
976	1	db$2	2::C3K487|C3K487_PSEFS	82	33600	3	1	3	1	0.15	Biopolymer transport p
977	1	db$2	2::C3K9I2|C3K9I2_PSEFS	82	33896	2	2	2	2	0.32	Putative transcriptiona
978	1	db$2	2::C3KAC0|C3KAC0_PSEFS	82	40546	3	3	3	3	0.42	Butyryl-CoA dehydroge
979	1	db$2	2::C3K3R4|C3K3R4_PSEFS	82	24681	3	3	3	3	0.77	Putative hydrolase OS=
980	1	db$2	2::C3JXJ5|C3JXJ5_PSEFS	82	22111	3	3	3	3	0.89	Putative SCO1/SenC fa
981	1	db$2	2::C3K485|C3K485_PSEFS	81	26565	2	2	2	2	0.43	Putative lipoprotein OS

↑Family	M	DB	Accession	Score	Mass	Matches	Match(sig)	Sequences	Seq(sig)	emPAI	Description
982	1	db$2	2::C3K0Z1|C3K0Z1_PSEFS	81	36566	2	2	2	2	0.29	L-threonine-O-3-phosp
983	1	db$2	2::C3K7G4|C3K7G4_PSEFS	81	54521	5	4	5	4	0.42	Ribonuclease G OS=Ps
984	1	db$2	2::C3JYE7|C3JYE7_PSEFS	81	35468	3	3	2	2	0.30	LysR family regulatory
985	1	db$2	2::C3K3V1|C3K3V1_PSEFS	80	23989	3	2	3	2	0.48	Alkyl hydroperoxide re
986	1	db$2	2::C3K2V8|RL30_PSEFS	80	6521	5	5	3	3	15.08	50S ribosomal protein
987	1	db$2	2::C3K968|C3K968_PSEFS	80	55197	3	3	3	3	0.29	Putative aldehyde deh
988	1	db$2	2::C3K3B8|C3K3B8_PSEFS	80	42763	3	3	3	3	0.39	Uncharacterized protei
989	1	db$2	2::C3KDN7|HEMH_PSEFS	80	38415	8	5	8	5	0.85	Ferrochelatase OS=Ps
990	1	db$2	2::C3K7S1|C3K7S1_PSEFS	80	25085	2	2	1	1	0.21	Protein MgtC OS=Pseu
991	1	db$2	2::C3JYN9|C3JYN9_PSEFS	80	44994	7	5	7	5	0.69	Pseudouridine synthas
992	1	db$2	2::C3K415|C3K415_PSEFS	80	70809	3	2	3	2	0.14	Putative ABC transport
993	1	db$2	2::C3K068|C3K068_PSEFS	79	65454	5	4	5	4	0.34	Putative asparagine sy
994	1	db$2	2::C3K605|LPXD_PSEFS	79	36124	3	3	3	3	0.48	UDP-3-O-acylglucosam
995	1	db$2	2::C3K491|C3K491_PSEFS	79	76137	2	2	2	2	0.13	ATP-dependent DNA h
996	1	db$2	2::C3K1B2|C3K1B2_PSEFS	79	62071	3	2	3	2	0.16	Single-stranded-DNA-s
997	1	SwissProt_HUMAN$	3::P07195|LDHB_HUMAN	79	36615	4	4	4	4	0.67	L-lactate dehydrogena
998	1	db$2	2::C3K2K4|C3K2K4_PSEFS	79	19926	2	2	2	2	0.60	Putative exported prot
999	1	db$2	2::C3K5Y8|C3K5Y8_PSEFS	79	48751	3	3	3	3	0.34	L-serine dehydratase (
999	2	db$2	2::C3JYR2|C3JYR2_PSEFS	50	48934	2	2	2	2	0.21	L-serine dehydratase (
1000	1	db$2	2::C3K364|C3K364_PSEFS	79	38824	2	2	2	2	0.28	Biotin synthase OS=Ps
1001	1	db$2	2::C3K819|C3K819_PSEFS	78	35012	2	2	2	2	0.31	Putative ABC transport
1002	1	db$2	2::C3KAH2|C3KAH2_PSEFS	78	24970	6	4	6	4	1.12	SPOR domain-containi
1003	1	db$2	2::C3K1X3|C3K1X3_PSEFS	78	20670	4	2	4	2	0.58	Glutathione peroxidase
1004	1	db$2	2::C3KDV8|C3KDV8_PSEFS	78	29265	2	2	2	2	0.38	ADP-dependent (S)-NA
1005	1	db$2	2::C3KDX3|C3KDX3_PSEFS	78	34279	5	4	5	4	0.73	Riboflavin biosynthesis
1006	1	db$2	2::C3KBK8|C3KBK8_PSEFS	78	35740	3	3	3	3	0.49	Ribosomal RNA small s
1007	1	db$2	2::C3KDE9|C3KDE9_PSEFS	78	41781	4	4	4	4	0.57	Putative phage tail-rela
1008	1	db$2	2::C3K7N0|C3K7N0_PSEFS	78	28545	2	2	2	2	0.39	Transport permease pr
1009	1	db$2	2::C3K571|C3K571_PSEFS	78	11755	3	3	2	2	1.20	Uncharacterized protei
1010	1	db$2	2::C3JZ32|C3JZ32_PSEFS	78	38286	4	4	3	3	0.45	Putative cation transpo
1011	1	SwissProt_HUMAN$	3::P62269|RS18_HUMAN	77	17708	3	2	3	2	0.69	40S ribosomal protein
1012	1	db$2	2::C3KBH8|C3KBH8_PSEFS	77	25745	2	2	2	2	0.44	3-oxoacyl-[acyl-carrier
1013	1	db$2	2::C3K6S5|C3K6S5_PSEFS	77	35353	5	4	4	3	0.71	Delta-aminolevulinic a
1014	1	db$2	2::C3K3D1|BETI_PSEFS	77	21992	2	2	2	2	0.53	HTH-type transcription
1015	1	db$2	2::C3KCS7|MRAY_PSEFS	77	39243	4	4	3	3	0.62	Phospho-N-acetylmura
1016	1	db$2	2::C3KDW1|MUTL_PSEFS	77	69574	7	6	6	5	0.41	DNA mismatch repair p
1017	1	db$2	2::C3K5F1|C3K5F1_PSEFS	77	42516	3	3	3	3	0.40	Ribonuclease D OS=Ps
1018	1	db$2	2::C3K3E2|C3K3E2_PSEFS	76	17110	1	1	1	1	0.31	Putative acetyltransfer
1019	1	db$2	2::C3KDG3|C3KDG3_PSEFS	76	15637	2	2	2	2	0.82	Putative exported prot
1020	1	db$2	2::C3JY71|C3JY71_PSEFS	76	43919	1	1	1	1	0.11	JmjC domain-containir

↑Family	M	DB	Accession	Score	Mass	Matches	Match(sig)	Sequences	Seq(sig)	emPAI	Description
1021	1	db$2	2::C3K6U5|FETP_PSEFS	76	10399	2	2	2	2	1.43	Probable Fe(2+)-traffic
1022	1	db$2	2::C3K5X1|C3K5X1_PSEFS	76	46611	3	3	3	3	0.36	Histidine kinase OS=Ps
1023	1	db$2	2::C3KBL9|C3KBL9_PSEFS	76	27726	3	3	3	3	0.66	Glycerol-3-phosphate
1024	1	db$2	2::C3K4A9|C3K4A9_PSEFS	76	17462	4	4	3	3	1.24	Uncharacterized protei
1025	1	db$2	2::C3K0P9|C3K0P9_PSEFS	76	25457	3	3	3	3	0.74	Two-component syster
1026	1	db$2	2::C3K8I3|C3K8I3_PSEFS	75	39921	2	2	1	1	0.13	3,4-dihydroxy-2-butan
1027	1	db$2	2::C3KB06|C3KB06_PSEFS	75	29724	1	1	1	1	0.17	Putative lipoprotein OS
1028	1	db$2	2::C3K279|C3K279_PSEFS	75	19136	4	4	3	3	1.67	Outer membrane prote
1029	1	db$2	2::C3K088|C3K088_PSEFS	75	20655	5	4	3	3	1.48	Putative isochorismata
1030	1	SwissProt_HUMAN$	3::P04406|G3P_HUMAN	75	36030	3	2	3	2	0.30	Glyceraldehyde-3-phos
1031	1	db$2	2::C3K433|CYAY_PSEFS	75	12442	2	2	1	1	0.45	Iron-sulfur cluster asse
1032	1	db$2	2::C3K1B4|C3K1B4_PSEFS	75	43355	3	3	3	3	0.39	Putative transferase O
1033	1	db$2	2::C3K0J1|C3K0J1_PSEFS	75	36130	3	3	3	3	0.48	Flagellar motor switch
1034	1	db$2	2::C3KA84|C3KA84_PSEFS	75	13408	5	4	5	4	3.00	Putative regulatory pro
1035	1	db$2	2::C3K1A4|C3K1A4_PSEFS	75	41199	2	2	2	2	0.26	Uncharacterized protei
1036	1	db$2	2::C3JY68|MNMA_PSEFS	74	41397	5	5	4	4	0.58	tRNA-specific 2-thiouri
1037	1	db$2	2::C3KDA5|C3KDA5_PSEFS	74	54170	2	2	2	2	0.19	Uncharacterized protei
1038	1	db$2	2::C3K2L0|C3K2L0_PSEFS	74	55816	3	3	2	2	0.29	Apolipoprotein N-acylti
1039	1	db$2	2::C3JY31|C3JY31_PSEFS	74	22860	4	4	4	4	1.27	Putative biopolymer tra
1040	1	db$2	2::C3JY39|C3JY39_PSEFS	74	12732	1	1	1	1	0.44	Arsenate reductase OS
1041	1	db$2	2::C3K390|C3K390_PSEFS	74	18447	5	5	4	4	1.76	Zn_protease domain-c
1042	1	db$2	2::C3K5K0|C3K5K0_PSEFS	74	25438	2	2	2	2	0.45	Two-component syster
1043	1	db$2	2::C3K841|C3K841_PSEFS	73	11596	6	5	3	3	6.45	Putative sigma-54 moc
1044	1	db$2	2::C3KAX0|C3KAX0_PSEFS	73	84238	4	3	4	3	0.18	1,4-alpha-glucan bran
1045	1	db$2	2::C3KCG1|C3KCG1_PSEFS	73	60384	3	2	3	2	0.17	Putative secreted amic
1046	1	db$2	2::C3K6J0|C3K6J0_PSEFS	73	24596	2	2	2	2	0.46	Putative phosphoglyco
1047	1	db$2	2::C3JYR0|C3JYR0_PSEFS	73	13774	2	2	2	2	0.97	Glycine cleavage syste
1048	1	db$2	2::C3JYN3|C3JYN3_PSEFS	73	52328	3	3	3	3	0.31	Family S13 unassignec
1049	1	db$2	2::C3K0Y8|COBT_PSEFS	73	36066	1	1	1	1	0.14	Nicotinate-nucleotide--
1050	1	db$2	2::C3JXY6|ASTB_PSEFS	72	48741	5	4	4	4	0.47	N-succinylarginine dihy
1051	1	db$2	2::C3K459|C3K459_PSEFS	72	18704	4	3	4	3	1.11	Leucine-responsive reg
1052	1	db$2	2::C3K114|C3K114_PSEFS	72	74752	4	4	4	4	0.29	DNA polymerase III su
1053	1	SwissProt_HUMAN$	3::P07437|TBB5_HUMAN	70	49639	3	3	3	3	0.33	Tubulin beta chain OS=
1054	1	db$2	2::C3KDN6|C3KDN6_PSEFS	72	32369	1	1	1	1	0.16	Uncharacterized protei
1055	1	db$2	2::C3JZ44|C3JZ44_PSEFS	71	47432	3	3	3	3	0.35	Putative gluconate per
1056	1	db$2	2::C3K2M7|C3K2M7_PSEFS	71	18106	3	3	3	3	1.17	Ribosomal silencing fa
1057	1	db$2	2::C3K0V2|C3K0V2_PSEFS	71	48301	2	2	2	2	0.22	Flagellar hook-associat
1058	1	db$2	2::C3JYU4|C3JYU4_PSEFS	71	20911	2	2	2	2	0.57	Corrinoid adenosyltran
1059	1	db$2	2::C3K6U7|C3K6U7_PSEFS	71	79141	5	4	5	4	0.27	Putative exported prot
1060	1	db$2	2::C3K2S3|C3K2S3_PSEFS	71	17933	5	4	4	3	1.18	Transcriptional repress

↑Family	M	DB	Accession	Score	Mass	Matches	Match(sig)	Sequences	Seq(sig)	emPAI	Description
1061	1	db$2	2::C3KDV5|C3KDV5_PSEFS	71	20461	4	3	4	3	0.99	Oligoribonuclease OS=
1062	1	db$2	2::C3KE78|C3KE78_PSEFS	70	15753	2	2	1	1	0.81	Azurin OS=Pseudomor
1063	1	db$2	2::C3KDS6|UNG_PSEFS	70	25820	3	3	3	3	0.73	Uracil-DNA glycosylase
1064	1	db$2	2::C3K453|C3K453_PSEFS	70	71119	3	3	3	3	0.22	AcetylCoA_hyd_C dom
1065	1	db$2	2::C3JYP6|C3JYP6_PSEFS	70	10477	3	3	2	2	2.78	YCII domain-containin
1066	1	db$2	2::C3K1C5|C3K1C5_PSEFS	70	44321	4	3	4	3	0.38	Putative cyclopropane-
1067	1	db$2	2::C3KE14|C3KE14_PSEFS	70	83238	2	2	2	2	0.12	Histidine kinase OS=P
1068	1	db$2	2::C3KE82|C3KE82_PSEFS	69	53340	4	4	4	4	0.43	Aldehyde dehydrogena
1069	1	db$2	2::C3K1Y0|C3K1Y0_PSEFS	69	12222	1	1	1	1	0.46	Uncharacterized protei
1070	1	db$2	2::C3K0P0|C3K0P0_PSEFS	69	35982	4	3	4	3	0.48	Putative peptidase OS
1071	1	db$2	2::C3KDM9|C3KDM9_PSEFS	69	28096	4	3	4	3	0.65	Putative oxidoreductas
1072	1	db$2	2::C3KA82|C3KA82_PSEFS	69	19457	3	3	3	3	1.06	Lactoylglutathione lyas
1073	1	db$2	2::C3JZ24|C3JZ24_PSEFS	69	24729	4	4	4	4	1.14	Putative GntR-family r
1074	1	db$2	2::C3KAV6|C3KAV6_PSEFS	69	17648	6	6	3	3	1.21	Thiol peroxidase OS=F
1075	1	db$2	2::C3K654|C3K654_PSEFS	69	34290	2	2	2	2	0.32	2-dehydropantoate 2-r
1076	1	db$2	2::C3K784|C3K784_PSEFS	69	23456	2	2	2	2	0.49	3-oxoadipate CoA-tran
1077	1	db$2	2::C3K6W0|C3K6W0_PSEFS	69	14786	4	4	4	4	2.54	Putative rhodanese do
1078	1	db$2	2::C3K650|C3K650_PSEFS	69	13083	2	2	2	2	1.03	N-acetyltransferase do
1079	1	db$2	2::C3K758|PYRF_PSEFS	69	24706	4	2	4	2	0.46	Orotidine 5'-phosphate
1080	1	db$2	2::C3K3P7|C3K3P7_PSEFS	69	49977	2	2	2	2	0.21	Putative membrane pr
1081	1	db$2	2::C3K2U2|C3K2U2_PSEFS	68	20033	3	3	2	2	0.60	Putative hydrolase OS
1082	1	db$2	2::C3K0M7|C3K0M7_PSEFS	68	28601	3	3	3	3	0.64	Putative deoxyribonucl
1083	1	db$2	2::C3K4D7|C3K4D7_PSEFS	68	14435	1	1	1	1	0.38	Putative thioesterase (
1084	1	db$2	2::C3K666|C3K666_PSEFS	68	29461	4	4	4	4	0.90	Putative lipoprotein OS
1085	1	db$2	2::C3KCB9|C3KCB9_PSEFS	68	35987	4	4	3	3	0.48	Polyphosphate kinase
1086	1	db$2	2::C3KAJ5|C3KAJ5_PSEFS	68	83174	1	1	1	1	0.06	Putative paraquat-indu
1087	1	db$2	2::C3K6S3|C3K6S3_PSEFS	68	25881	2	2	2	2	0.44	Putative glutathione S-
1088	1	db$2	2::C3K1M6|C3K1M6_PSEFS	68	24782	3	3	3	3	0.77	Uncharacterized protei
1089	1	db$2	2::C3K4A6|C3K4A6_PSEFS	68	36556	1	1	1	1	0.14	ImpA_N domain-conta
1090	1	db$2	2::C3KAB9|C3KAB9_PSEFS	68	40944	1	1	1	1	0.12	Putative acetyl-CoA ac
1091	1	db$2	2::C3JYS3|C3JYS3_PSEFS	68	38665	2	2	2	2	0.28	TolA colicin import me
1092	1	db$2	2::C3KDW0|C3KDW0_PSEFS	67	49939	4	3	4	3	0.33	N-acetylmuramoyl-L-a
1093	1	db$2	2::C3K7C7|C3K7C7_PSEFS	67	19943	4	3	4	3	1.02	Chorismate mutase OS
1094	1	db$2	2::C3K0U4|C3K0U4_PSEFS	67	36872	4	2	4	2	0.29	Flagellar motor switch
1095	1	db$2	2::C3KBT9|C3KBT9_PSEFS	67	31336	1	1	1	1	0.16	Sulfate ABC transport
1096	1	SwissProt_HUMAN$	3::P07900|HS90A_HUMAN	67	84607	2	2	2	2	0.12	Heat shock protein HS
1097	1	db$2	2::C3KBZ8|C3KBZ8_PSEFS	67	38389	2	2	2	2	0.28	Iron-sulfur cluster carr
1098	1	db$2	2::C3KE30|C3KE30_PSEFS	67	59128	3	2	3	2	0.17	L-aspartate oxidase OS
1099	1	db$2	2::C3K4J4|HSLO_PSEFS	67	33320	2	2	2	2	0.33	33 kDa chaperonin OS
1100	1	db$2	2::C3KBM3|C3KBM3_PSEFS	67	40194	3	3	3	3	0.42	Putative ABC transport

↑Family	M	DB	Accession	Score	Mass	Matches	Match(sig)	Sequences	Seq(sig)	emPAI	Description
1101	1	SwissProt_HUMAN$	3::Q5D862|FILA2_HUMAN	67	247928	1	1	1	1	0.02	Filaggrin-2 OS=Homo
1102	1	db$2	2::C3K7J3|Y1319_PSEFS	67	16953	2	2	2	2	0.73	UPF0225 protein PFLU
1103	1	db$2	2::C3KBT7|C3KBT7_PSEFS	67	36676	4	3	4	3	0.47	Sulfate ABC transport
1104	1	db$2	2::C3K6G1|RLMF_PSEFS	67	38015	1	1	1	1	0.13	Ribosomal RNA large s
1105	1	db$2	2::C3JYI2|C3JYI2_PSEFS	67	39477	4	3	4	3	0.43	2-aminoethylphosphor
1106	1	db$2	2::C3K2L4|C3K2L4_PSEFS	66	37419	1	1	1	1	0.13	DNA polymerase III su
1107	1	db$2	2::C3K136|C3K136_PSEFS	66	31053	2	2	2	2	0.35	Uncharacterized protei
1108	1	db$2	2::C3K304|C3K304_PSEFS	66	15054	4	3	3	3	1.53	Uncharacterized protei
1109	1	db$2	2::C3K0R6|C3K0R6_PSEFS	66	28374	4	3	4	3	0.64	Uncharacterized protei
1110	1	db$2	2::C3K6B0|C3K6B0_PSEFS	66	33534	1	1	1	1	0.15	High-affinity zinc uptal
1111	1	db$2	2::C3K481|C3K481_PSEFS	66	23434	4	4	4	4	1.23	Guanylate kinase OS=
1112	1	SwissProt_HUMAN$	3::Q6FI13|H2A2A_HUMAN	66	14087	3	3	3	3	1.69	Histone H2A type 2-A
1113	1	db$2	2::C3K0V8|C3K0V8_PSEFS	66	45209	1	1	1	1	0.11	Muramidase FlgJ OS=F
1114	1	SwissProt_HUMAN$	3::Q14240|IF4A2_HUMAN	66	46373	2	2	2	2	0.23	Eukaryotic initiation fa
1115	1	db$2	2::C3K1N0|C3K1N0_PSEFS	65	18655	2	1	2	1	0.29	Rick_17kDa_Anti dom
1116	1	db$2	2::C3K3U4|C3K3U4_PSEFS	65	23278	2	2	2	2	0.50	Putative TetR-family re
1117	1	db$2	2::C3K3D3|BETA_PSEFS	65	62642	4	3	4	3	0.25	Oxygen-dependent ch
1118	1	db$2	2::C3KAY5|C3KAY5_PSEFS	65	30881	2	2	1	1	0.16	NAD-capped RNA hydr
1119	1	db$2	2::C3K9E9|C3K9E9_PSEFS	65	21363	3	2	2	1	0.25	Putative TetR-family re
1120	1	db$2	2::C3KBN5|C3KBN5_PSEFS	65	24125	1	1	1	1	0.22	Putative TetR-family re
1121	1	db$2	2::C3KDM4|C3KDM4_PSEFS	65	56715	5	3	5	3	0.28	Putative membrane pr
1122	1	db$2	2::C3K4I1|C3K4I1_PSEFS	65	29333	3	3	3	3	0.62	Shikimate dehydrogen
1123	1	SwissProt_HUMAN$	3::P62249|RS16_HUMAN	65	16435	4	3	4	3	1.35	40S ribosomal protein
1124	1	db$2	2::C3KDL9|C3KDL9_PSEFS	65	22175	2	2	2	2	0.53	Uncharacterized protei
1125	1	db$2	2::C3K223|C3K223_PSEFS	65	37863	3	3	3	3	0.45	Protein-methionine-su
1126	1	db$2	2::C3JXS4|C3JXS4_PSEFS	65	25108	1	1	1	1	0.21	Tol-Pal system protein
1127	1	db$2	2::C3JY28|Y3771_PSEFS	65	6626	3	3	2	2	2.92	UPF0434 protein PFLU
1128	1	db$2	2::C3K3L2|C3K3L2_PSEFS	64	15596	2	2	2	2	0.82	DUF4426 domain-cont
1129	1	db$2	2::C3K224|C3K224_PSEFS	64	30473	2	2	1	1	0.17	CDP-diacylglycerol--se
1130	1	db$2	2::C3K126|C3K126_PSEFS	64	54569	4	4	3	3	0.30	2-methylcitrate dehyd
1131	1	db$2	2::C3JXY3|C3JXY3_PSEFS	64	18788	3	3	3	3	1.11	6,7-dimethyl-8-ribityll
1132	1	SwissProt_HUMAN$	3::P22626|ROA2_HUMAN	64	37407	1	1	1	1	0.13	Heterogeneous nuclea
1133	1	db$2	2::C3K1B0|C3K1B0_PSEFS	64	96490	2	2	2	2	0.10	Lysylphosphatidylglyce
1134	1	db$2	2::C3JXX3|C3JXX3_PSEFS	64	52842	2	2	2	2	0.20	Cytochrome-c oxidase
1135	1	db$2	2::C3K0H8|C3K0H8_PSEFS	64	29525	3	3	3	3	0.61	Protein phosphatase C
1136	1	db$2	2::C3JY69|HFLD_PSEFS	64	23173	2	2	2	2	0.50	High frequency lysoge
1137	1	db$2	2::C3K832|C3K832_PSEFS	64	33163	2	2	1	1	0.15	Non-specific serine/thr
1138	1	SwissProt_HUMAN$	3::P15531|NDKA_HUMAN	64	17138	5	5	4	4	1.99	Nucleoside diphosphat
1139	1	db$2	2::C3K9M9|C3K9M9_PSEFS	64	14163	1	1	1	1	0.39	Putative endoribonucle
1140	1	db$2	2::C3K0K1|C3K0K1_PSEFS	64	53436	1	1	1	1	0.09	Putative GntR-family r

↑Family	M	DB	Accession	Score	Mass	Matches	Match(sig)	Sequences	Seq(sig)	emPAI	Description
1141	1	db$2	2::C3K0R1|C3K0R1_PSEFS	64	30842	2	2	2	2	0.36	Protein-glutamate O-m
1142	1	db$2	2::C3K1A2|C3K1A2_PSEFS	63	17206	1	1	1	1	0.31	Putative membrane pr
1143	1	db$2	2::C3K0M3|C3K0M3_PSEFS	63	20567	4	4	4	4	1.49	Uncharacterized protei
1144	1	db$2	2::C3KC04|C3KC04_PSEFS	63	39513	3	2	3	2	0.27	Putative hydrolase OS
1145	1	db$2	2::C3K0T5|C3K0T5_PSEFS	63	37877	2	2	2	2	0.28	Zinc transport protein
1146	1	SwissProt_HUMAN$	3::Q86YZ3|HORN_HUMAN	63	282228	3	2	2	2	0.03	Hornerin OS=Homo sa
1147	1	db$2	2::C3K4W6|C3K4W6_PSEFS	63	44511	3	3	2	2	0.24	Putative lipoprotein rel
1148	1	db$2	2::C3K282|SSRP_PSEFS	63	18308	4	3	4	3	1.15	SsrA-binding protein C
1149	1	db$2	2::C3K9M1|C3K9M1_PSEFS	62	26106	1	1	1	1	0.20	Putative phosphoglyce
1150	1	db$2	2::C3K4U9|C3K4U9_PSEFS	62	34107	1	1	1	1	0.15	Putative LysR-family re
1151	1	db$2	2::C3K2E0|C3K2E0_PSEFS	62	48742	3	3	3	3	0.34	DNA repair protein Rad
1152	1	db$2	2::C3KCI1|C3KCI1_PSEFS	62	42811	1	1	1	1	0.12	Putative acyl-CoA dehy
1153	1	db$2	2::C3JY52|C3JY52_PSEFS	62	37777	2	2	2	2	0.28	GST C-terminal domai
1154	1	db$2	2::C3K591|C3K591_PSEFS	62	19872	2	2	2	2	0.60	Putative bacterioferriti
1155	1	db$2	2::C3K8L0|C3K8L0_PSEFS	62	16885	2	1	2	1	0.32	Uncharacterized protei
1156	1	db$2	2::C3K3D8|C3K3D8_PSEFS	62	49942	3	2	3	2	0.21	ATP-independent RNA
1157	1	db$2	2::C3KBI7|C3KBI7_PSEFS	61	26715	2	2	2	2	0.42	Uncharacterized protei
1158	1	db$2	2::C3KE39|C3KE39_PSEFS	61	18976	7	4	5	4	1.68	D,D-heptose 1,7-bisph
1159	1	db$2	2::C3K7W0|C3K7W0_PSEFS	61	26786	2	2	2	2	0.42	Uncharacterized protei
1160	1	db$2	2::C3K6D7|C3K6D7_PSEFS	61	21411	1	1	1	1	0.24	UPF0056 inner membr
1161	1	db$2	2::C3KBQ3|C3KBQ3_PSEFS	61	33274	4	4	4	4	0.76	LysR-family transcripti
1162	1	db$2	2::C3K2M0|Y5418_PSEFS	61	10305	1	1	1	1	0.56	UPF0250 protein PFLU
1163	1	db$2	2::C3K6V0|C3K6V0_PSEFS	61	23354	4	3	4	3	0.83	Imidazole glycerol pho
1164	1	db$2	2::C3JYN0|C3JYN0_PSEFS	61	33944	3	3	3	3	0.52	Cbb3-type cytochrome
1164	2	db$2	2::C3JZS9|C3JZS9_PSEFS	43	35591	2	2	2	2	0.30	Cbb3-type cytochrome
1165	1	db$2	2::C3K4K0|C3K4K0_PSEFS	61	42230	3	3	3	3	0.40	Hypothetical exported
1166	1	db$2	2::C3K1N1|C3K1N1_PSEFS	61	33097	3	3	3	3	0.53	Protein-export membra
1167	1	db$2	2::C3K8Z4|C3K8Z4_PSEFS	61	45613	2	2	2	2	0.23	High-affinity branched
1168	1	db$2	2::C3K047|C3K047_PSEFS	61	16614	2	2	2	2	0.75	Putative putative heat
1169	1	db$2	2::C3K867|C3K867_PSEFS	61	39735	4	4	3	3	0.43	PALP domain-containir
1170	1	db$2	2::C3KAR4|C3KAR4_PSEFS	61	11430	1	1	1	1	0.50	Uncharacterized protei
1171	1	db$2	2::C3K0V4|C3K0V4_PSEFS	60	48572	2	2	2	2	0.21	Flagellin OS=Pseudom
1172	1	db$2	2::C3K574|C3K574_PSEFS	60	36116	3	2	3	2	0.30	Putative oxidoreductas
1173	1	db$2	2::C3K3K9|C3K3K9_PSEFS	60	21575	4	4	3	3	0.92	Conserved hypothetica
1174	1	db$2	2::C3K1A3|C3K1A3_PSEFS	60	26906	1	1	1	1	0.19	Putative pyridoxamine
1175	1	SwissProt_HUMAN$	3::P02741|CRP_HUMAN	60	25023	3	3	3	3	0.76	C-reactive protein OS=
1176	1	db$2	2::C3K6W8|C3K6W8_PSEFS	60	16611	2	2	2	2	0.75	3-dehydroquinate dehy
1177	1	db$2	2::C3K0D5|MINE_PSEFS	60	9531	5	3	5	3	3.26	Cell division topologica
1178	1	db$2	2::C3K300|C3K300_PSEFS	60	34122	1	1	1	1	0.15	Putative membrane pr
1179	1	db$2	2::C3K7A2|C3K7A2_PSEFS	60	33841	1	1	1	1	0.15	Putative membrane pr

↑Family	M	DB	Accession	Score	Mass	Matches	Match(sig)	Sequences	Seq(sig)	emPAI	Description
1180	1	db$2	2::C3JZP0|C3JZP0_PSEFS	60	11251	2	2	2	2	1.29	Putative lipoprotein OS
1181	1	db$2	2::C3K158|C3K158_PSEFS	60	36421	3	3	3	3	0.48	Putative regulatory pro
1182	1	db$2	2::C3K1G6|C3K1G6_PSEFS	60	25762	8	7	5	4	1.49	Thiol:disulfide intercha
1183	1	db$2	2::C3JZP3|C3JZP3_PSEFS	60	36685	3	3	2	2	0.29	Putative inosine-uridin
1184	1	db$2	2::C3K1V5|C3K1V5_PSEFS	60	64171	2	2	1	1	0.08	Uncharacterized protei
1185	1	db$2	2::C3K9I1|C3K9I1_PSEFS	60	31064	1	1	1	1	0.16	Putative transmembrai
1186	1	db$2	2::C3K7K5|C3K7K5_PSEFS	59	12541	1	1	1	1	0.45	Uncharacterized protei
1187	1	db$2	2::C3K869|C3K869_PSEFS	59	24016	2	2	2	2	0.48	Putative lipoprotein OS
1188	1	db$2	2::C3K0C5|C3K0C5_PSEFS	59	27111	1	1	1	1	0.19	Putative amino transfe
1189	1	db$2	2::C3K221|C3K221_PSEFS	59	129628	1	1	1	1	0.04	RecBCD enzyme subur
1190	1	db$2	2::C3K013|C3K013_PSEFS	59	20254	2	2	1	1	0.26	Peptidyl-prolyl cis-tran
1191	1	db$2	2::C3KDX1|RS20_PSEFS	59	10078	2	2	1	1	0.58	30S ribosomal protein
1192	1	db$2	2::C3KAL0|C3KAL0_PSEFS	59	43962	3	2	3	2	0.24	PlsC domain-containin
1193	1	db$2	2::C3K3W5|C3K3W5_PSEFS	59	39645	7	5	5	4	0.61	Aminomethyltransfera
1194	1	db$2	2::C3JYQ1|C3JYQ1_PSEFS	59	48078	2	1	2	1	0.10	Putative CoA transfera
1195	1	db$2	2::C3K5S6|C3K5S6_PSEFS	59	24824	2	1	2	1	0.21	Putative hydrolase OS
1196	1	db$2	2::C3K998|C3K998_PSEFS	59	35021	3	3	3	3	0.50	Putative dehydrogenas
1197	1	db$2	2::C3K098|C3K098_PSEFS	58	23000	1	1	1	1	0.23	Putative glutathione S-
1198	1	db$2	2::C3KDR4|C3KDR4_PSEFS	58	49531	4	4	4	4	0.47	Ribosomal protein S12
1199	1	db$2	2::C3K789|C3K789_PSEFS	58	16242	1	1	1	1	0.33	Uncharacterized protei
1200	1	db$2	2::C3K101|C3K101_PSEFS	58	37185	3	3	3	3	0.46	Uncharacterized protei
1201	1	db$2	2::C3JXM0|C3JXM0_PSEFS	58	45141	2	2	2	2	0.23	Putative peptidase OS
1202	1	db$2	2::C3JYL7|C3JYL7_PSEFS	58	12417	2	2	2	2	1.11	Putative copper resista
1203	1	db$2	2::C3K0S4|C3K0S4_PSEFS	58	65961	5	4	5	4	0.33	Isocitrate dehydrogena
1204	1	db$2	2::C3K5B3|C3K5B3_PSEFS	58	34937	3	2	3	2	0.31	Dipeptide ABC transpo
1205	1	db$2	2::C3K5Y3|C3K5Y3_PSEFS	58	35524	3	3	3	3	0.49	Putative leucine dehyd
1206	1	db$2	2::C3K6P0|C3K6P0_PSEFS	57	15266	3	2	2	2	0.84	Uncharacterized protei
1207	1	db$2	2::C3K8W5|C3K8W5_PSEFS	57	21382	1	1	1	1	0.24	Lipopolysaccharide exp
1208	1	db$2	2::C3KE47|FMT_PSEFS	57	33416	3	2	3	2	0.33	Methionyl-tRNA formyl
1209	1	db$2	2::C3K5U7|C3K5U7_PSEFS	57	105852	3	3	3	3	0.14	Putative aminotransfer
1210	1	db$2	2::C3JZ64|C3JZ64_PSEFS	57	17217	3	3	3	3	1.26	MaoC-like domain-cont
1211	1	db$2	2::C3K2H3|C3K2H3_PSEFS	57	31916	2	2	2	2	0.34	Putative metallopeptid
1212	1	db$2	2::C3K4C0|C3K4C0_PSEFS	57	9802	4	4	3	3	3.13	DNA-binding protein H
1213	1	db$2	2::C3K0X7|C3K0X7_PSEFS	57	126663	2	2	2	2	0.08	Histidine kinase OS=P
1214	1	db$2	2::C3K706|PIMT_PSEFS	57	23451	3	1	3	1	0.22	Protein-L-isoaspartate
1215	1	db$2	2::C3K7M5|C3K7M5_PSEFS	57	15789	2	2	2	2	0.81	Uncharacterized protei
1216	1	db$2	2::C3K9K0|C3K9K0_PSEFS	57	37025	1	1	1	1	0.14	Glycine betaine/L-proli
1217	1	db$2	2::C3K7I5|C3K7I5_PSEFS	56	28342	2	2	2	2	0.39	Putative iron utilisatior
1218	1	db$2	2::C3K602|C3K602_PSEFS	56	48346	5	4	4	3	0.34	Zinc metalloprotease (
1219	1	db$2	2::C3JXT1|C3JXT1_PSEFS	56	67768	1	1	1	1	0.07	Amidohydro_3 domain

↑Family	M	DB	Accession	Score	Mass	Matches	Match(sig)	Sequences	Seq(sig)	emPAI	Description
1220	1	db$2	2::C3KAF9|C3KAF9_PSEFS	56	50830	1	1	1	1	0.10	Poly-beta-1,6-N-acetyl
1221	1	db$2	2::C3K6Z6|C3K6Z6_PSEFS	56	13580	2	2	2	2	0.99	Putaitve lipoprotein OS
1222	1	db$2	2::C3KB73|C3KB73_PSEFS	55	9692	1	1	1	1	0.61	Putative lipoprotein OS
1223	1	db$2	2::C3K601|DXR_PSEFS	55	42341	4	4	4	4	0.56	1-deoxy-D-xylulose 5-
1224	1	db$2	2::C3K735|MSRB_PSEFS	55	14471	1	1	1	1	0.38	Peptide methionine sul
1225	1	db$2	2::C3K6S4|C3K6S4_PSEFS	55	30497	1	1	1	1	0.17	Putative antibiotic bios
1226	1	db$2	2::C3K265|C3K265_PSEFS	55	29739	2	2	2	2	0.37	Dihydropteroate synth
1227	1	db$2	2::C3K146|C3K146_PSEFS	55	27134	2	2	2	2	0.42	Fumarate and nitrate r
1228	1	db$2	2::C3JY61|C3JY61_PSEFS	55	8297	2	2	2	2	2.01	Translation initiation fa
1229	1	db$2	2::C3K1F6|C3K1F6_PSEFS	55	23787	2	2	2	2	0.49	Ribosomal RNA small s
1230	1	db$2	2::C3KAY4|C3KAY4_PSEFS	54	29479	1	1	1	1	0.17	Putative enoyl-CoA hy
1231	1	db$2	2::C3JY27|KDSB_PSEFS	54	27609	3	3	3	3	0.67	3-deoxy-manno-octulc
1232	1	db$2	2::C3K741|C3K741_PSEFS	54	17249	2	2	1	1	0.31	GAF domain-containin
1233	1	db$2	2::C3K9Q9|C3K9Q9_PSEFS	54	19670	1	1	1	1	0.27	FMN_red domain-conta
1234	1	db$2	2::C3JY38|C3JY38_PSEFS	54	16812	4	3	4	3	1.31	Putative thioredoxin-re
1235	1	db$2	2::C3K0Z9|C3K0Z9_PSEFS	54	26468	1	1	1	1	0.19	Bac_DnaA domain-con
1236	1	db$2	2::C3KDI2|C3KDI2_PSEFS	54	135789	2	2	2	2	0.07	Exonuclease OS=Pseu
1237	1	SwissProt_HUMAN$	3::P61247|RS3A_HUMAN	54	29926	2	2	2	2	0.37	40S ribosomal protein
1238	1	db$2	2::C3K4M4|C3K4M4_PSEFS	54	31652	1	1	1	1	0.16	Phosphofructokinase C
1239	1	db$2	2::C3K5T2|C3K5T2_PSEFS	54	65173	3	3	3	3	0.24	Putative dehydrogenas
1240	1	db$2	2::C3K6B1|C3K6B1_PSEFS	54	35098	3	3	3	3	0.50	Homoserine kinase OS
1241	1	db$2	2::C3K8Y2|C3K8Y2_PSEFS	54	49522	1	1	1	1	0.10	Putative two-compone
1242	1	db$2	2::C3JZU8|C3JZU8_PSEFS	54	30426	2	2	2	2	0.36	Hydrolase_4 domain-c
1243	1	db$2	2::C3K092|C3K092_PSEFS	54	14085	2	2	2	2	0.94	Glutaredoxin domain-c
1244	1	SwissProt_HUMAN$	3::P46781|RS9_HUMAN	54	22578	3	3	3	3	0.87	40S ribosomal protein
1245	1	db$2	2::C3K5E1|C3K5E1_PSEFS	54	13734	4	4	3	3	1.76	Uncharacterized protei
1246	1	db$2	2::C3JZU7|C3JZU7_PSEFS	53	16433	2	2	2	2	0.77	Putative thioesterase (
1247	1	db$2	2::C3K763|C3K763_PSEFS	53	29996	2	2	2	2	0.37	Putative hydrolase OS
1248	1	SwissProt_HUMAN$	3::Q07020|RL18_HUMAN	53	21621	1	1	1	1	0.24	60S ribosomal protein
1249	1	db$2	2::C3K607|LPXA_PSEFS	53	28002	1	1	1	1	0.18	Acyl-[acyl-carrier-prot
1250	1	db$2	2::C3KDB5|C3KDB5_PSEFS	53	33189	1	1	1	1	0.15	Putative glycosyl trans
1251	1	db$2	2::C3JYU5|C3JYU5_PSEFS	53	34090	2	2	2	2	0.32	Nudix hydrolase doma
1252	1	db$2	2::C3K0L0|C3K0L0_PSEFS	53	29492	1	1	1	1	0.17	Putative IclR-family re
1253	1	db$2	2::C3K595|C3K595_PSEFS	53	10805	2	2	2	2	1.36	Phosphate starvation-i
1254	1	db$2	2::C3K4U8|C3K4U8_PSEFS	53	20094	1	1	1	1	0.26	Conserved hypothetica
1255	1	db$2	2::C3K0I8|C3K0I8_PSEFS	53	26895	1	1	1	1	0.19	Flagellar biosynthetic p
1256	1	db$2	2::C3KE08|C3KE08_PSEFS	52	25044	2	2	2	2	0.45	Conserved hypothetica
1257	1	SwissProt_HUMAN$	3::Q6IS14|IF5AL_HUMAN	52	16762	1	1	1	1	0.32	Eukaryotic translation
1258	1	db$2	2::C3KAA5|C3KAA5_PSEFS	52	23984	3	3	2	2	0.48	Uncharacterized protei
1259	1	db$2	2::C3K586|C3K586_PSEFS	52	21064	4	3	4	3	0.95	ADP compounds hydro

↑Family	M	DB	Accession	Score	Mass	Matches	Match(sig)	Sequences	Seq(sig)	emPAI	Description
1260	1	db$2	2::C3K0X3|C3K0X3_PSEFS	52	16227	2	1	2	1	0.33	Transcriptional regulat
1261	1	db$2	2::C3JZT8|C3JZT8_PSEFS	52	28261	1	1	1	1	0.18	Putative methyltransfe
1262	1	db$2	2::C3JXI0|C3JXI0_PSEFS	52	23030	1	1	1	1	0.23	Nudix hydrolase doma
1263	1	db$2	2::C3K664|C3K664_PSEFS	52	31550	1	1	1	1	0.16	Putative membrane pr
1264	1	db$2	2::C3KBY5|MRAZ_PSEFS	52	17051	1	1	1	1	0.31	Transcriptional regulat
1265	1	db$2	2::C3K6J8|C3K6J8_PSEFS	52	78979	3	2	3	2	0.13	3-phosphoshikimate 1-
1266	1	db$2	2::C3K132|C3K132_PSEFS	52	42760	2	2	2	2	0.25	Uncharacterized protei
1267	1	db$2	2::C3JYG2|C3JYG2_PSEFS	52	36128	1	1	1	1	0.14	ATP-grasp domain-con
1268	1	db$2	2::C3KDC8|C3KDC8_PSEFS	52	26520	1	1	1	1	0.19	Molybdopterin biosyntl
1269	1	db$2	2::C3JY81|C3JY81_PSEFS	52	15084	1	1	1	1	0.36	NADH-quinone oxidore
1270	1	db$2	2::C3K1R1|C3K1R1_PSEFS	52	13822	2	2	2	2	0.96	bPH_1 domain-contain
1271	1	db$2	2::C3KAA9|C3KAA9_PSEFS	52	31757	3	3	2	2	0.35	Putative chemotaxis pı
1272	1	db$2	2::C3KC05|C3KC05_PSEFS	52	11915	2	2	2	2	1.18	Conserved hypothetica
1273	1	db$2	2::C3KB02|C3KB02_PSEFS	52	42163	2	2	2	2	0.25	Putative exported prot
1274	1	db$2	2::C3K258|RBFA_PSEFS	52	14697	2	2	2	2	0.89	Ribosome-binding fact
1275	1	db$2	2::C3K3X4|C3K3X4_PSEFS	51	11561	3	2	2	1	1.23	Cell division protein Za
1276	1	db$2	2::C3JXX7|C3JXX7_PSEFS	51	56979	2	1	2	1	0.09	Conserved hypothetica
1277	1	db$2	2::C3K0L5|C3K0L5_PSEFS	51	17844	2	2	2	2	0.69	Putative GNAT-family a
1278	1	db$2	2::C3JZR9|C3JZR9_PSEFS	51	27415	2	2	2	2	0.41	Putative dehydrogenas
1279	1	db$2	2::C3K4V3|C3K4V3_PSEFS	51	45550	3	2	3	2	0.23	Uncharacterized protei
1280	1	db$2	2::C3KDJ8|C3KDJ8_PSEFS	51	23307	3	3	2	2	0.50	Methyltransf_25 doma
1281	1	db$2	2::C3K286|C3K286_PSEFS	51	9470	2	2	2	2	1.66	Putative exported prot
1282	1	db$2	2::C3K7X0|C3K7X0_PSEFS	51	85237	2	2	2	2	0.12	5-methyltetrahydropte
1283	1	db$2	2::C3K3F9|C3K3F9_PSEFS	51	16338	1	1	1	1	0.33	Putative exported cyto
1284	1	db$2	2::C3K473|DUT_PSEFS	51	16022	1	1	1	1	0.34	Deoxyuridine 5'-tripho
1285	1	db$2	2::C3K436|C3K436_PSEFS	51	30237	2	2	2	2	0.37	Diaminopimelate epim
1286	1	db$2	2::C3K7W1|C3K7W1_PSEFS	51	12229	2	2	2	2	1.14	Putative lipoprotein OS
1287	1	db$2	2::C3KCR9|PTH_PSEFS	51	21041	3	3	3	3	0.95	Peptidyl-tRNA hydrolas
1288	1	SwissProt_HUMAN$	3::Q8NHW5|RLA0L_HUMAN	51	34343	1	1	1	1	0.15	60S acidic ribosomal p
1289	1	db$2	2::C3K3P8|C3K3P8_PSEFS	51	49421	2	2	2	2	0.21	G domain-containing p
1290	1	db$2	2::C3JXL2|C3JXL2_PSEFS	51	30967	1	1	1	1	0.16	Uncharacterized protei
1291	1	db$2	2::C3K8U6|C3K8U6_PSEFS	51	15270	1	1	1	1	0.36	Putative polyhydroxyal
1292	1	db$2	2::C3K1U6|C3K1U6_PSEFS	50	74730	3	2	3	2	0.14	Cytochrome bo(3) ubi(
1293	1	db$2	2::C3KBB8|C3KBB8_PSEFS	50	13546	3	3	3	3	1.80	Uncharacterized protei
1294	1	SwissProt_HUMAN$	3::P46783|RS10_HUMAN	50	18886	1	1	1	1	0.28	40S ribosomal protein
1295	1	db$2	2::C3KDR1|C3KDR1_PSEFS	50	17628	2	2	2	2	0.70	Uncharacterized protei
1296	1	db$2	2::C3JYS7|RUVB_PSEFS	50	38919	2	1	2	1	0.13	Holliday junction ATP-(
1297	1	db$2	2::C3K6A1|C3K6A1_PSEFS	50	22990	1	1	1	1	0.23	Putative copper-bindin
1298	1	db$2	2::C3KAF8|C3KAF8_PSEFS	50	74138	2	1	2	1	0.07	Putative lipoprotein OS
1299	1	db$2	2::C3K598|C3K598_PSEFS	50	34258	2	2	2	2	0.32	Curved DNA-binding p

↑Family	M	DB	Accession	Score	Mass	Matches	Match(sig)	Sequences	Seq(sig)	emPAI	Description
1300	1	db$2	2::C3K3E9|C3K3E9_PSEFS	50	12056	1	1	1	1	0.47	Putative lipoprotein OS
1301	1	SwissProt_HUMAN$	3::Q9H0U4|RAB1B_HUMAN	50	22157	2	2	2	2	0.53	Ras-related protein Ra
1302	1	db$2	2::C3K3H4|C3K3H4_PSEFS	50	26074	1	1	1	1	0.20	Ribosomal RNA small s
1303	1	db$2	2::C3KAF7|C3KAF7_PSEFS	50	91635	2	2	2	2	0.11	TPR_REGION domain-c
1304	1	SwissProt_HUMAN$	3::Q71UM5|RS27L_HUMAN	50	9471	2	1	2	1	0.63	40S ribosomal protein
1305	1	db$2	2::C3K0K3|C3K0K3_PSEFS	49	12996	1	1	1	1	0.43	Putative lipoprotein OS
1306	1	db$2	2::C3K637|C3K637_PSEFS	49	56441	2	2	2	2	0.18	Putative peptide ABC t
1307	1	db$2	2::C3K4W5|C3K4W5_PSEFS	49	24663	3	2	3	2	0.46	Lipoprotein-releasing s
1308	1	db$2	2::C3KB17|C3KB17_PSEFS	49	51104	4	3	4	3	0.32	Outer membrane comp
1309	1	db$2	2::C3KE64|C3KE64_PSEFS	49	37016	1	1	1	1	0.14	Putative iron-binding p
1310	1	db$2	2::C3K9P4|C3K9P4_PSEFS	49	59261	4	4	4	4	0.38	Putative ATP-binding c
1311	1	db$2	2::C3K3H3|C3K3H3_PSEFS	49	52510	2	2	2	2	0.20	Adenosylmethionine-8
1312	1	db$2	2::C3K564|C3K564_PSEFS	49	54508	2	2	2	2	0.19	Methylmalonate-semia
1313	1	db$2	2::C3K493|C3K493_PSEFS	49	16495	3	2	3	2	0.76	Uncharacterized protei
1314	1	db$2	2::C3K5J9|C3K5J9_PSEFS	49	96070	2	1	2	1	0.05	Histidine kinase OS=Ps
1315	1	db$2	2::C3K0M9|KTHY_PSEFS	49	22917	3	3	3	3	0.85	Thymidylate kinase OS
1316	1	db$2	2::C3KBU2|C3KBU2_PSEFS	49	56055	1	1	1	1	0.09	Putative histidine amm
1317	1	db$2	2::C3K2Z1|COAX_PSEFS	49	26920	1	1	1	1	0.19	Type III pantothenate
1318	1	db$2	2::C3K8T8|C3K8T8_PSEFS	48	14354	2	1	2	1	0.38	Sec-independent prote
1319	1	db$2	2::C3K8V8|C3K8V8_PSEFS	48	36166	3	3	3	3	0.48	Zinc-type alcohol dehy
1320	1	db$2	2::C3K7A7|C3K7A7_PSEFS	48	43115	2	2	2	2	0.25	Conserved hypothetica
1321	1	db$2	2::C3K4N5|C3K4N5_PSEFS	48	28830	1	1	1	1	0.18	Putative amino acid AE
1322	1	db$2	2::C3KBB9|C3KBB9_PSEFS	48	73354	3	2	2	2	0.14	Putative protease OS=
1323	1	db$2	2::C3K2P5|C3K2P5_PSEFS	48	49850	1	1	1	1	0.10	Ethanolamine ammoni
1324	1	SwissProt_HUMAN$	3::P62750|RL23A_HUMAN	48	17684	1	1	1	1	0.30	60S ribosomal protein
1325	1	db$2	2::C3K6R7|C3K6R7_PSEFS	48	41832	3	3	2	2	0.25	Serine/threonine trans
1326	1	db$2	2::C3K0E8|C3K0E8_PSEFS	48	27512	3	3	3	3	0.67	Thiol:disulfide intercha
1327	1	db$2	2::C3K5G4|RLMM_PSEFS	48	40585	1	1	1	1	0.12	Ribosomal RNA large s
1328	1	db$2	2::C3K685|C3K685_PSEFS	48	13635	3	2	3	2	0.98	6-carboxy-5,6,7,8-tetr
1329	1	SwissProt_HUMAN$	3::P62258|1433E_HUMAN	48	29155	3	3	3	3	0.62	14-3-3 protein epsilon
1330	1	db$2	2::C3K1R4|C3K1R4_PSEFS	48	17440	1	1	1	1	0.31	Putative plasmid-relate
1331	1	db$2	2::C3JZ46|C3JZ46_PSEFS	48	37091	2	2	2	2	0.29	Putative LacI family tra
1332	1	db$2	2::C3K7M0|C3K7M0_PSEFS	48	62717	1	1	1	1	0.08	Putative dipeptidase O
1333	1	db$2	2::C3K6U1|C3K6U1_PSEFS	48	17245	2	2	2	2	0.72	Conserved hypothetica
1334	1	db$2	2::C3K663|C3K663_PSEFS	48	22341	2	2	2	2	0.52	Putative TetR-family re
1335	1	db$2	2::C3K8X1|C3K8X1_PSEFS	47	23435	2	2	2	2	0.49	Putative ABC transport
1336	1	SwissProt_HUMAN$	3::P19338|NUCL_HUMAN	47	76568	2	2	2	2	0.13	Nucleolin OS=Homo sa
1337	1	db$2	2::C3K456|C3K456_PSEFS	47	38683	2	1	2	1	0.13	Alanine racemase OS=
1338	1	SwissProt_HUMAN$	3::P62913|RL11_HUMAN	47	20240	2	2	2	2	0.59	60S ribosomal protein
1339	1	db$2	2::C3KBX7|C3KBX7_PSEFS	47	23636	1	1	1	1	0.22	Putative metallo-beta-

↑Family	M	DB	Accession	Score	Mass	Matches	Match(sig)	Sequences	Seq(sig)	emPAI	Description
1340	1	db$2	2::C3K8P0|C3K8P0_PSEFS	47	38329	6	3	6	3	0.45	Putative nucleoside-se
1341	1	db$2	2::C3JYG1|C3JYG1_PSEFS	47	30646	2	2	2	2	0.36	Putative LysR-family tr
1342	1	db$2	2::C3K7L9|C3K7L9_PSEFS	47	28704	2	2	2	2	0.39	Putative membrane pr
1343	1	db$2	2::C3K751|C3K751_PSEFS	47	15943	1	1	1	1	0.34	Putative thioesterase (
1344	1	db$2	2::C3JXX9|C3JXX9_PSEFS	47	6925	4	3	3	3	6.29	Translational regulator
1345	1	db$2	2::C3K5G7|PDXB_PSEFS	47	41465	1	1	1	1	0.12	Erythronate-4-phospha
1346	1	db$2	2::C3K8L6|C3K8L6_PSEFS	47	28069	1	1	1	1	0.18	Precorrin-6A synthase
1347	1	db$2	2::C3K5K4|C3K5K4_PSEFS	46	29062	1	1	1	1	0.18	Uncharacterized protei
1348	1	SwissProt_HUMAN$	3::P62851|RS25_HUMAN	46	13734	2	2	1	1	0.40	40S ribosomal protein
1349	1	db$2	2::C3KBP5|C3KBP5_PSEFS	46	30770	1	1	1	1	0.17	Putative AraC-family re
1350	1	db$2	2::C3K0F5|C3K0F5_PSEFS	46	23098	1	1	1	1	0.23	Putative TetR-family tr
1351	1	SwissProt_HUMAN$	3::P63244|RACK1_HUMAN	46	35055	2	2	2	2	0.31	Receptor of activated p
1352	1	db$2	2::C3JYM6|C3JYM6_PSEFS	46	40915	2	1	2	1	0.12	Putative HlyD-family s
1353	1	db$2	2::C3K6L9|C3K6L9_PSEFS	46	49785	2	2	2	2	0.21	Phospho-2-dehydro-3-
1354	1	SwissProt_HUMAN$	3::P62847|RS24_HUMAN	46	15413	1	1	1	1	0.35	40S ribosomal protein
1355	1	db$2	2::C3KE10|C3KE10_PSEFS	46	58823	3	2	3	2	0.17	Putative methyl-accept
1356	1	db$2	2::C3JZ73|C3JZ73_PSEFS	46	49677	4	4	4	4	0.46	Porin OS=Pseudomona
1357	1	db$2	2::C3KEC3|C3KEC3_PSEFS	45	16145	2	2	2	2	0.79	Putative membrane pr
1358	1	db$2	2::C3JY92|C3JY92_PSEFS	45	55851	4	3	4	3	0.29	NADH dehydrogenase
1359	1	db$2	2::C3K4R2|C3K4R2_PSEFS	45	7681	1	1	1	1	0.82	Major cold shock prote
1360	1	db$2	2::C3K4H8|C3K4H8_PSEFS	45	20657	1	1	1	1	0.26	Threonylcarbamoyl-AM
1361	1	db$2	2::C3K1X4|C3K1X4_PSEFS	45	16845	1	1	1	1	0.32	Putative putative expo
1362	1	db$2	2::C3JZZ4|C3JZZ4_PSEFS	45	23102	2	1	2	1	0.23	Putative glutathione S
1363	1	db$2	2::C3K979|C3K979_PSEFS	45	30391	2	2	2	2	0.36	Putative glutathione S-
1364	1	db$2	2::C3K6N8|C3K6N8_PSEFS	45	25972	3	2	3	2	0.44	Putative hydrolase OS=
1365	1	db$2	2::C3KDC2|ISPE_PSEFS	45	30632	1	1	1	1	0.17	4-diphosphocytidyl-2-(
1366	1	db$2	2::C3K0N8|C3K0N8_PSEFS	45	19586	2	1	2	1	0.27	23S rRNA accumulatio
1367	1	db$2	2::C3KAG2|C3KAG2_PSEFS	45	280833	1	1	1	1	0.02	Toxin CdiA OS=Pseudo
1368	1	db$2	2::C3KDL3|C3KDL3_PSEFS	45	70148	3	2	3	2	0.14	Phosphomethylpyrimid
1369	1	db$2	2::C3K6Y0|C3K6Y0_PSEFS	45	13973	1	1	1	1	0.40	4-carboxymuconolacto
1370	1	db$2	2::C3KE17|C3KE17_PSEFS	45	24430	2	2	2	2	0.47	Two component syster
1371	1	db$2	2::C3K9H2|C3K9H2_PSEFS	45	39760	3	3	3	3	0.43	Putrescine ABC transp
1372	1	db$2	2::C3K3J1|C3K3J1_PSEFS	44	16523	1	1	1	1	0.33	Putative chemotaxis-re
1373	1	db$2	2::C3K6Z1|C3K6Z1_PSEFS	44	50711	4	4	3	3	0.32	Putative multicopper o
1374	1	db$2	2::C3KBG3|C3KBG3_PSEFS	44	24033	3	2	3	2	0.48	Curli production asser
1375	1	db$2	2::C3K1K5|EX7L_PSEFS	44	51103	3	2	3	2	0.20	Exodeoxyribonuclease
1376	1	db$2	2::C3K7V7|C3K7V7_PSEFS	44	18111	1	1	1	1	0.30	Uncharacterized protei
1377	1	SwissProt_HUMAN$	3::P61604|CH10_HUMAN	44	10925	4	3	4	3	2.58	10 kDa heat shock pro
1378	1	db$2	2::C3K911|C3K911_PSEFS	44	29460	1	1	1	1	0.17	Putative ABC transport
1379	1	db$2	2::C3K6T6|C3K6T6_PSEFS	44	30009	1	1	1	1	0.17	Putative membrane-bc

↑Family	M	DB	Accession	Score	Mass	Matches	Match(sig)	Sequences	Seq(sig)	emPAI	Description
1380	1	db$2	2::C3K587|C3K587_PSEFS	44	29705	1	1	1	1	0.17	3'(2'),5'-bisphosphate
1381	1	db$2	2::C3K328|APAG_PSEFS	44	13631	2	2	2	2	0.98	Protein ApaG OS=Pseu
1382	1	db$2	2::C3K3D4|C3K3D4_PSEFS	44	52472	2	2	2	2	0.20	Uncharacterized protei
1383	1	db$2	2::C3K8K1|C3K8K1_PSEFS	44	40207	1	1	1	1	0.12	Phosphate-binding pro
1384	1	db$2	2::C3KB07|C3KB07_PSEFS	44	47911	1	1	1	1	0.10	Deferrochelatase/pero:
1385	1	db$2	2::C3K1F5|C3K1F5_PSEFS	44	29048	1	1	1	1	0.18	Putative chromosome
1386	1	db$2	2::C3KE01|Y992_PSEFS	44	29383	3	3	3	3	0.62	UPF0246 protein PFLU_
1387	1	db$2	2::C3K0P5|C3K0P5_PSEFS	44	35572	2	2	2	2	0.30	Uncharacterized protei
1388	1	SwissProt_HUMAN$	3::Q8NG92|O13H1_HUMAN	44	34547	1	1	1	1	0.15	Olfactory receptor 13H
1389	1	db$2	2::C3K021|C3K021_PSEFS	44	27911	2	2	2	2	0.40	Phosphoadenosine phc
1390	1	SwissProt_HUMAN$	3::P62277|RS13_HUMAN	44	17212	1	1	1	1	0.31	40S ribosomal protein
1391	1	db$2	2::C3KDN9|C3KDN9_PSEFS	43	40608	2	2	2	2	0.26	Probable periplasmic s
1392	1	db$2	2::C3KE46|C3KE46_PSEFS	43	47174	3	3	2	2	0.22	16S rRNA m5C967 me
1393	1	SwissProt_HUMAN$	3::P62753|RS6_HUMAN	43	28663	1	1	1	1	0.18	40S ribosomal protein
1394	1	db$2	2::C3K7L3|C3K7L3_PSEFS	43	8069	1	1	1	1	0.76	Putative lipoprotein OS
1395	1	db$2	2::C3K2Q3|C3K2Q3_PSEFS	43	23107	1	1	1	1	0.23	Putative hydrolase OS:
1396	1	db$2	2::C3K3G8|C3K3G8_PSEFS	43	41555	1	1	1	1	0.12	Uncharacterized protei
1397	1	db$2	2::C3K353|C3K353_PSEFS	43	44910	1	1	1	1	0.11	Putative aminotransfer
1398	1	db$2	2::C3K127|C3K127_PSEFS	43	41295	2	2	2	2	0.26	Putative membrane pr
1399	1	db$2	2::C3KCP8|C3KCP8_PSEFS	43	42283	2	2	2	2	0.25	Lipopolysaccharide cor
1400	1	db$2	2::C3KCS4|C3KCS4_PSEFS	43	63231	3	3	3	3	0.25	Peptidoglycan D,D-trai
1401	1	db$2	2::C3K333|C3K333_PSEFS	43	61021	2	2	2	2	0.17	Uncharacterized protei
1402	1	SwissProt_HUMAN$	3::O00189|AP4M1_HUMAN	43	49946	1	1	1	1	0.10	AP-4 complex subunit
1403	1	db$2	2::C3K327|C3K327_PSEFS	43	29832	2	2	2	2	0.37	Ribosomal RNA small s
1404	1	db$2	2::C3K9N4|C3K9N4_PSEFS	43	91939	2	2	2	2	0.11	Histidine kinase OS=P:
1405	1	SwissProt_HUMAN$	3::P62888|RL30_HUMAN	43	12776	2	2	2	2	1.07	60S ribosomal protein
1406	1	db$2	2::C3K4A7|C3K4A7_PSEFS	43	18391	3	3	3	3	1.14	Uncharacterized protei
1407	1	db$2	2::C3K6Y7|RNC_PSEFS	42	25367	2	2	2	2	0.45	Ribonuclease 3 OS=Ps
1408	1	db$2	2::C3K620|C3K620_PSEFS	42	22214	3	2	3	2	0.53	LexA repressor OS=Ps
1409	1	db$2	2::C3K4I9|C3K4I9_PSEFS	42	42202	1	1	1	1	0.12	Galactonate dehydrata
1410	1	db$2	2::C3KEB0|C3KEB0_PSEFS	42	36597	2	2	1	1	0.14	Putative amino acid AE
1411	1	db$2	2::C3KBE8|C3KBE8_PSEFS	42	8134	2	2	1	1	0.76	Putative lipoprotein OS
1412	1	db$2	2::C3K425|C3K425_PSEFS	42	27295	1	1	1	1	0.19	Positive alginate biosyi
1413	1	SwissProt_HUMAN$	3::P26641|EF1G_HUMAN	42	50087	3	2	3	2	0.21	Elongation factor 1-gai
1414	1	SwissProt_HUMAN$	3::P62424|RL7A_HUMAN	42	29977	1	1	1	1	0.17	60S ribosomal protein
1415	1	db$2	2::C3K3Y7|C3K3Y7_PSEFS	42	35706	2	2	2	2	0.30	Putative iron-sulfur bir
1416	1	SwissProt_HUMAN$	3::P05109|S10A8_HUMAN	42	10828	1	1	1	1	0.53	Protein S100-A8 OS=H
1417	1	db$2	2::C3K458|C3K458_PSEFS	42	47152	2	2	2	2	0.22	D-amino acid dehydro(
1418	1	db$2	2::C3KCV5|C3KCV5_PSEFS	42	37705	2	2	2	2	0.28	Uncharacterized protei
1419	1	db$2	2::C3K1F1|ATPL_PSEFS	41	8603	3	3	2	2	3.92	ATP synthase subunit (

↑Family	M	DB	Accession	Score	Mass	Matches	Match(sig)	Sequences	Seq(sig)	emPAI	Description
1420	1	db$2	2::C3K6H7|C3K6H7_PSEFS	41	10540	1	1	1	1	0.55	Cell division protein Ft
1421	1	db$2	2::C3JZP9|C3JZP9_PSEFS	41	33380	2	2	2	2	0.33	Putative ribose transpo
1422	1	db$2	2::C3K2L9|LIPB_PSEFS	41	23473	1	1	1	1	0.22	Octanoyltransferase O
1423	1	db$2	2::C3JZQ4|C3JZQ4_PSEFS	41	8106	3	2	3	2	2.10	Translational regulator
1424	1	db$2	2::C3K820|C3K820_PSEFS	41	26990	1	1	1	1	0.19	Putative ABC transport
1425	1	db$2	2::C3JYE6|C3JYE6_PSEFS	41	45437	2	2	2	2	0.23	Putative acyl-CoA dehy
1426	1	db$2	2::C3K0S0|C3K0S0_PSEFS	41	12378	2	2	1	1	0.45	Putative DNA-binding
1427	1	db$2	2::C3KBY4|C3KBY4_PSEFS	41	31613	1	1	1	1	0.16	Ribosomal RNA small s
1428	1	db$2	2::Q8VMR3|HUTH_PSEFS	41	54209	1	1	1	1	0.09	Histidine ammonia-lya
1429	1	db$2	2::C3K4P8|C3K4P8_PSEFS	41	24008	3	3	3	3	0.80	t(6)A37 threonylcarba
1430	1	db$2	2::C3KBV6|C3KBV6_PSEFS	40	125347	1	1	1	1	0.04	Putative potassium eff
1431	1	db$2	2::C3K686|C3K686_PSEFS	40	39658	1	1	1	1	0.13	Uncharacterized protei
1432	1	db$2	2::C3JY97|C3JY97_PSEFS	40	30357	1	1	1	1	0.17	N-hydroxyarylamine O
1433	1	SwissProt_HUMAN$	3::P14618|KPYM_HUMAN	40	57900	1	1	1	1	0.09	Pyruvate kinase PKM C
1434	1	db$2	2::C3KBX4|C3KBX4_PSEFS	40	59754	2	2	2	2	0.17	Putative membrane pr
1435	1	db$2	2::C3K7G1|C3K7G1_PSEFS	40	37563	2	2	2	2	0.29	Cell shape protein Mre
1436	1	db$2	2::C3K0N9|C3K0N9_PSEFS	40	21007	3	2	3	2	0.56	7-methyl-GTP pyropho
1437	1	SwissProt_HUMAN$	3::Q96GW7|PGCB_HUMAN	40	99056	1	1	1	1	0.05	Brevican core protein (
1438	1	db$2	2::C3KAX2|C3KAX2_PSEFS	40	79149	1	1	1	1	0.06	Histidine kinase OS=P
1439	1	db$2	2::C3K241|C3K241_PSEFS	40	33217	1	1	1	1	0.15	Glutamyl-Q tRNA(Asp)
1440	1	db$2	2::C3K277|C3K277_PSEFS	40	60895	1	1	1	1	0.08	DNA repair protein Re
1441	1	db$2	2::C3K596|C3K596_PSEFS	40	36487	2	2	2	2	0.29	Putative membrane pr
1442	1	db$2	2::C3K9T0|NADK_PSEFS	39	32289	3	3	3	3	0.55	NAD kinase OS=Pseud
1443	1	db$2	2::C3K0B8|C3K0B8_PSEFS	39	24379	1	1	1	1	0.21	Uncharacterized protei
1444	1	SwissProt_HUMAN$	3::P05413|FABPH_HUMAN	39	14849	2	2	2	2	0.87	Fatty acid-binding prot
1445	1	db$2	2::C3K0T6|RRAAH_PSEFS	39	17479	1	1	1	1	0.31	Putative 4-hydroxy-4-
1446	1	db$2	2::C3K1M4|C3K1M4_PSEFS	39	13705	1	1	1	1	0.41	Iron-sulfur cluster asse
1447	1	db$2	2::C3K6W5|PRMA_PSEFS	39	31717	2	1	2	1	0.16	Ribosomal protein L11
1448	1	db$2	2::C3KAX3|C3KAX3_PSEFS	39	30360	3	3	3	3	0.59	Conserved hypothetica
1449	1	db$2	2::C3K5X0|C3K5X0_PSEFS	39	25599	2	2	2	2	0.44	Putative exported prot
1450	1	db$2	2::C3K8K6|C3K8K6_PSEFS	39	15117	1	1	1	1	0.36	Putative transport-rela
1451	1	db$2	2::C3JZ30|C3JZ30_PSEFS	39	12225	1	1	1	1	0.46	DUF2025 domain-cont
1452	1	db$2	2::C3K5L0|C3K5L0_PSEFS	39	14786	2	1	2	1	0.37	Conserved hypothetica
1453	1	db$2	2::C3K3J5|C3K3J5_PSEFS	39	13219	2	1	2	1	0.42	Type IV pilus response
1454	1	db$2	2::C3K4M2|C3K4M2_PSEFS	39	36363	2	1	2	1	0.14	Catabolite repressor/a
1455	1	db$2	2::C3KDP3|C3KDP3_PSEFS	39	34782	1	1	1	1	0.15	Putaive acyltransferase
1456	1	db$2	2::C3KDK7|C3KDK7_PSEFS	39	24465	1	1	1	1	0.21	Putative ABC transport
1457	1	db$2	2::C3K2L3|C3K2L3_PSEFS	39	22416	3	2	2	2	0.52	LPS-assembly lipoprot
1458	1	db$2	2::C3K9Y8|C3K9Y8_PSEFS	39	14463	1	1	1	1	0.38	Uncharacterized protei
1459	1	SwissProt_HUMAN$	3::A6NJ78|MET15_HUMAN	39	46092	1	1	1	1	0.11	12S rRNA N4-methylcy

↑Family	M	DB	Accession	Score	Mass	Matches	Match(sig)	Sequences	Seq(sig)	emPAI	Description
1460	1	db$2	2::C3K1V0|C3K1V0_PSEFS	39	56633	1	1	1	1	0.09	Nitric oxide reductase
1461	1	db$2	2::C3K3S9|C3K3S9_PSEFS	39	35576	1	1	1	1	0.14	Hydrolase_4 domain-c
1462	1	db$2	2::C3K964|C3K964_PSEFS	38	26948	2	2	2	2	0.42	Putative GntR-family r
1463	1	db$2	2::C3JXW6|C3JXW6_PSEFS	38	64770	1	1	1	1	0.08	Putative exported prot
1464	1	db$2	2::C3JZN5|RL35_PSEFS	38	7363	1	1	1	1	0.87	50S ribosomal protein
1465	1	SwissProt_HUMAN$	3::P33992|MCM5_HUMAN	38	82233	2	2	1	1	0.06	DNA replication licensi
1466	1	db$2	2::C3K0K2|C3K0K2_PSEFS	38	29783	1	1	1	1	0.17	Putative outer membra
1467	1	SwissProt_HUMAN$	3::P62701|RS4X_HUMAN	38	29579	3	2	2	2	0.38	40S ribosomal protein
1468	1	db$2	2::C3K6V3|HIS6_PSEFS	38	27111	2	1	2	1	0.19	Imidazole glycerol pho
1469	1	db$2	2::C3KDQ1|C3KDQ1_PSEFS	38	35181	4	3	4	3	0.50	Putative LysR-family re
1470	1	db$2	2::C3K0J2|C3K0J2_PSEFS	38	17877	1	1	1	1	0.30	Flagellar protein FliL O
1471	1	db$2	2::C3K1B3|C3K1B3_PSEFS	38	20726	4	3	4	3	0.97	Uncharacterized protei
1472	1	db$2	2::C3K2M3|C3K2M3_PSEFS	38	37518	3	2	3	2	0.29	Putative peptidoglycan
1473	1	db$2	2::C3KD16|C3KD16_PSEFS	38	34294	3	2	3	2	0.32	Putative 2-hydroxyacic
1474	1	db$2	2::C3JY37|C3JY37_PSEFS	38	14829	2	2	2	2	0.87	Putative lipoprotein OS
1475	1	db$2	2::C3K2H7|C3K2H7_PSEFS	38	21527	1	1	1	1	0.24	Putative acetyltransfer
1476	1	db$2	2::C3JZ63|C3JZ63_PSEFS	38	34729	1	1	1	1	0.15	Hydrolase_4 domain-c
1477	1	db$2	2::C3K5M2|C3K5M2_PSEFS	38	41151	3	2	3	2	0.26	Phosphorous compoun
1478	1	SwissProt_HUMAN$	3::Q2PPJ7|RGPA2_HUMAN	38	210636	2	1	2	1	0.02	Ral GTPase-activating
1479	1	db$2	2::C3K3T7|C3K3T7_PSEFS	38	39982	1	1	1	1	0.13	Putrescine ABC transp
1480	1	SwissProt_HUMAN$	3::P61254|RL26_HUMAN	38	17248	2	1	2	1	0.31	60S ribosomal protein
1481	1	db$2	2::C3K492|C3K492_PSEFS	38	51080	1	1	1	1	0.10	HDOD domain-contain
1482	1	db$2	2::C3K2P8|C3K2P8_PSEFS	37	24358	3	2	3	2	0.47	Membrane-related pro
1483	1	db$2	2::C3JZ52|C3JZ52_PSEFS	37	18715	1	1	1	1	0.28	Uncharacterized protei
1484	1	db$2	2::C3KE72|C3KE72_PSEFS	37	85807	3	2	3	2	0.12	UPF0313 protein PFLU_
1485	1	db$2	2::C3K150|C3K150_PSEFS	37	25194	2	2	2	2	0.45	Putative short-chain de
1486	1	db$2	2::C3K071|Y4323_PSEFS	37	8578	1	1	1	1	0.71	UPF0270 protein PFLU_
1487	1	db$2	2::A4V7S8|A4V7S8_PSEFS	37	15780	1	1	1	1	0.34	Mercuric resistance op
1488	1	db$2	2::C3JY67|C3JY67_PSEFS	37	16861	1	1	1	1	0.32	Phosphatase NudJ OS=
1489	1	db$2	2::C3K5E3|C3K5E3_PSEFS	37	43909	3	2	3	2	0.24	Putative aminotransfer
1490	1	db$2	2::C3K0P2|C3K0P2_PSEFS	37	35618	2	2	2	2	0.30	Pseudouridine synthas
1491	1	SwissProt_HUMAN$	3::P62136|PP1A_HUMAN	37	37488	1	1	1	1	0.13	Serine/threonine-prote
1492	1	SwissProt_HUMAN$	3::P26440|IVD_HUMAN	37	46621	1	1	1	1	0.11	Isovaleryl-CoA dehydr
1493	1	SwissProt_HUMAN$	3::P46779|RL28_HUMAN	37	15738	1	1	1	1	0.34	60S ribosomal protein
1494	1	db$2	2::C3K1S9|C3K1S9_PSEFS	37	82606	1	1	1	1	0.06	Putative phytochrome/
1495	1	db$2	2::C3K646|C3K646_PSEFS	36	21182	1	1	1	1	0.25	Putative acyl-CoA thio
1496	1	db$2	2::C3KDF9|C3KDF9_PSEFS	36	17633	2	2	2	2	0.70	CinA domain-containin
1497	1	db$2	2::C3K3R7|RPIA_PSEFS	36	23281	3	2	3	2	0.50	Ribose-5-phosphate is
1498	1	db$2	2::C3K6V8|C3K6V8_PSEFS	36	47941	2	2	2	2	0.22	Putative peptidase OS
1499	1	db$2	2::C3K3V4|C3K3V4_PSEFS	36	41512	2	2	2	2	0.26	Acetylornithine deacet

↑Family	M	DB	Accession	Score	Mass	Matches	Match(sig)	Sequences	Seq(sig)	emPAI	Description
1500	1	db$2	2::C3K0G4|C3K0G4_PSEFS	36	29541	2	2	2	2	0.38	MOSC domain-containi
1501	1	db$2	2::C3JY43|TTCA_PSEFS	36	31052	3	3	3	3	0.58	tRNA-cytidine(32) 2-su
1502	1	db$2	2::C3KE38|C3KE38_PSEFS	36	28697	4	2	3	1	0.18	Putative phospholipid I
1503	1	db$2	2::C3JYF7|C3JYF7_PSEFS	36	147681	3	1	2	1	0.03	ATP-dependent helicas
1504	1	db$2	2::C3K870|C3K870_PSEFS	36	20966	2	2	2	2	0.56	Uncharacterized protei
1505	1	db$2	2::C3KCK0|C3KCK0_PSEFS	36	26687	2	2	2	2	0.42	Putative short-chain de
1506	1	db$2	2::C3K2M6|RLMH_PSEFS	36	17669	2	2	2	2	0.70	Ribosomal RNA large s
1507	1	db$2	2::C3K8Z7|C3K8Z7_PSEFS	36	10906	1	1	1	1	0.53	Uncharacterized protei
1508	1	db$2	2::C3K0J8|C3K0J8_PSEFS	36	22091	1	1	1	1	0.24	START domain-contain
1509	1	db$2	2::C3K1M7|C3K1M7_PSEFS	36	27957	1	1	1	1	0.18	Serine O-acetyltransfe
1510	1	db$2	2::C3KA94|C3KA94_PSEFS	36	41012	1	1	1	1	0.12	Uncharacterized protei
1511	1	SwissProt_HUMAN$	3::P02675|FIBB_HUMAN	36	55892	1	1	1	1	0.09	Fibrinogen beta chain (
1512	1	SwissProt_HUMAN$	3::Q8WYA0|IFT81_HUMAN	36	79696	1	1	1	1	0.06	Intraflagellar transport
1513	1	db$2	2::C3KA88|C3KA88_PSEFS	36	40085	1	1	1	1	0.13	Putative 4-hydroxyphe
1514	1	db$2	2::C3K4F9|C3K4F9_PSEFS	36	31102	2	2	2	2	0.35	Putative regulatory pro
1515	1	db$2	2::C3K842|C3K842_PSEFS	36	56600	2	2	2	2	0.18	RNA polymerase sigma
1516	1	db$2	2::C3K413|C3K413_PSEFS	35	22663	1	1	1	1	0.23	Putative ABC transport
1517	1	db$2	2::C3K0Z5|C3K0Z5_PSEFS	35	22469	2	2	2	2	0.52	Corrinoid adenosyltran
1518	1	db$2	2::C3K6T8|C3K6T8_PSEFS	35	49583	2	2	2	2	0.21	Alginate biosynthesis t
1519	1	db$2	2::C3K442|C3K442_PSEFS	35	46322	1	1	1	1	0.11	Ammonium transporte
1520	1	db$2	2::C3K2F6|TRMA_PSEFS	35	40867	2	2	2	2	0.26	tRNA/tmRNA (uracil-C(
1521	1	db$2	2::C3K3X7|C3K3X7_PSEFS	35	16525	1	1	1	1	0.33	EVE domain-containing
1522	1	db$2	2::C3K5H4|C3K5H4_PSEFS	35	21385	1	1	1	1	0.24	Uncharacterized protei
1523	1	SwissProt_HUMAN$	3::Q9NSI6|BRWD1_HUMAN	35	262772	1	1	1	1	0.02	Bromodomain and WD
1524	1	db$2	2::C3K834|C3K834_PSEFS	35	76708	1	1	1	1	0.06	Copper-transporting P-
1525	1	SwissProt_HUMAN$	3::Q9BVV6|TALD3_HUMAN	35	169201	1	1	1	1	0.03	Protein TALPID3 OS=H
1526	1	db$2	2::C3K5M3|PHNN_PSEFS	35	20351	2	2	2	2	0.59	Ribose 1,5-bisphospha
1527	1	db$2	2::C3K0B2|C3K0B2_PSEFS	35	20071	2	2	1	1	0.26	Putative membrane pr
1528	1	SwissProt_HUMAN$	3::Q9UKE5|TNIK_HUMAN	35	154848	1	1	1	1	0.03	TRAF2 and NCK-intera
1529	1	db$2	2::C3K4Q6|C3K4Q6_PSEFS	35	39864	2	1	2	1	0.13	Putative lipoprotein OS
1530	1	SwissProt_HUMAN$	3::P06312|KV401_HUMAN	35	13372	1	1	1	1	0.42	Immunoglobulin kappa
1531	1	SwissProt_HUMAN$	3::Q765P7|MTSS2_HUMAN	35	79880	1	1	1	1	0.06	Protein MTSS 2 OS=Ho
1532	1	db$2	2::C3K2Y9|C3K2Y9_PSEFS	35	13142	1	1	1	1	0.43	Protein translocase sul
1533	1	db$2	2::C3K2U3|C3K2U3_PSEFS	34	27946	2	1	2	1	0.18	Trans-aconitate 2-met
1534	1	db$2	2::C3K078|C3K078_PSEFS	34	17799	1	1	1	1	0.30	Putative ankyrin-like p
1535	1	db$2	2::C3K546|C3K546_PSEFS	34	33665	1	1	1	1	0.15	Putative lipopolysaccha
1536	1	SwissProt_HUMAN$	3::Q6DHV5|C2D2B_HUMAN	34	166681	1	1	1	1	0.03	Protein CC2D2B OS=H
1537	1	db$2	2::C3K4Z1|C3K4Z1_PSEFS	34	72318	1	1	1	1	0.07	Cytochrome C biogene
1538	1	SwissProt_HUMAN$	3::P62826|RAN_HUMAN	34	24408	3	3	3	3	0.78	GTP-binding nuclear pr
1539	1	db$2	2::C3K7K0|C3K7K0_PSEFS	34	41297	1	1	1	1	0.12	Esterase III OS=Pseud

↑Family	M	DB	Accession	Score	Mass	Matches	Match(sig)	Sequences	Seq(sig)	emPAI	Description
1540	1	db$2	2::C3K2E2|C3K2E2_PSEFS	34	56906	1	1	1	1	0.09	Catalase OS=Pseudom
1541	1	db$2	2::C3K9A0|C3K9A0_PSEFS	34	31788	3	3	2	2	0.35	Putative LysR-family re
1542	1	db$2	2::C3K1Q1|C3K1Q1_PSEFS	34	59090	1	1	1	1	0.08	Putative methyl accept
1543	1	db$2	2::C3K5C4|C3K5C4_PSEFS	34	17510	1	1	1	1	0.31	SnoaL-like domain-cor
1544	1	db$2	2::C3K1G4|RL34_PSEFS	34	5064	2	2	2	2	4.81	50S ribosomal protein
1545	1	db$2	2::C3K219|C3K219_PSEFS	34	76943	2	2	2	2	0.13	RecBCD enzyme subur
1546	1	db$2	2::C3K8Z2|C3K8Z2_PSEFS	34	25669	1	1	1	1	0.20	High-affinity branched
1547	1	db$2	2::C3KCB4|C3KCB4_PSEFS	34	27191	1	1	1	1	0.19	Uncharacterized protei
1548	1	db$2	2::C3K9N0|C3K9N0_PSEFS	34	28569	2	1	2	1	0.18	Putative transcriptiona
1549	1	db$2	2::C3K3W4|C3K3W4_PSEFS	34	13233	2	1	1	1	0.42	Glycine cleavage syste
1550	1	db$2	2::C3K1H4|C3K1H4_PSEFS	34	45615	1	1	1	1	0.11	Putative membrane pr
1551	1	db$2	2::C3K5S7|C3K5S7_PSEFS	33	19211	1	1	1	1	0.28	Uncharacterized protei
1552	1	db$2	2::C3K6A9|C3K6A9_PSEFS	33	17428	1	1	1	1	0.31	Ferric uptake regulatio
1553	1	db$2	2::C3JXZ5|C3JXZ5_PSEFS	33	25958	1	1	1	1	0.20	Putative histidine trans
1554	1	db$2	2::C3K450|C3K450_PSEFS	33	61786	1	1	1	1	0.08	Putative EAL/GGDEF d
1555	1	db$2	2::C3K0R0|C3K0R0_PSEFS	33	14751	1	1	1	1	0.37	Flagellar basal body ro
1556	1	db$2	2::C3KD58|C3KD58_PSEFS	33	41746	1	1	1	1	0.12	Putative cystathionine
1557	1	SwissProt_HUMAN$	3::Q7Z6Z7|HUWE1_HUMAN	33	481589	3	3	2	2	0.02	E3 ubiquitin-protein lig
1558	1	db$2	2::C3K7A1|C3K7A1_PSEFS	33	56003	1	1	1	1	0.09	Putative LuxR-family r
1559	1	db$2	2::C3KBZ5|C3KBZ5_PSEFS	33	23751	2	1	2	1	0.22	Endonuclease III OS=I
1560	1	db$2	2::C3KDI4|C3KDI4_PSEFS	33	42151	2	2	2	2	0.25	Putative exported prot
1561	1	db$2	2::C3K2M2|C3K2M2_PSEFS	33	35893	2	2	2	2	0.30	Endolytic peptidoglyca
1562	1	db$2	2::C3KE21|C3KE21_PSEFS	33	10230	1	1	1	1	0.57	FAD assembly factor S
1563	1	db$2	2::C3JXT0|C3JXT0_PSEFS	33	22445	1	1	1	1	0.23	Putative isochorismata
1564	1	SwissProt_HUMAN$	3::P81605|DCD_HUMAN	33	11277	2	1	1	1	0.51	Dermcidin OS=Homo s
1565	1	db$2	2::C3KBL5|C3KBL5_PSEFS	33	24580	1	1	1	1	0.21	Glutamate/aspartate A
1566	1	db$2	2::C3K454|C3K454_PSEFS	33	13613	2	2	1	1	0.41	Putative cytochrome c
1567	1	db$2	2::C3K6W4|C3K6W4_PSEFS	33	45779	1	1	1	1	0.11	Znf/thioredoxin_put dc
1568	1	db$2	2::C3K176|C3K176_PSEFS	32	33571	2	1	2	1	0.15	Glucokinase OS=Pseuc
1569	1	db$2	2::C3K129|C3K129_PSEFS	32	42141	1	1	1	1	0.12	Putative acetyl CoA ac
1570	1	db$2	2::C3KAP0|C3KAP0_PSEFS	32	14934	1	1	1	1	0.37	4-carboxymuconolacto
1571	1	db$2	2::C3KCP9|C3KCP9_PSEFS	32	30268	1	1	1	1	0.17	Lipopolysaccharide cor
1572	1	db$2	2::C3K8H9|C3K8H9_PSEFS	32	41728	2	1	2	1	0.12	Putative polyamine AB
1573	1	db$2	2::C3K537|C3K537_PSEFS	32	39684	1	1	1	1	0.13	Putative membrane pr
1574	1	SwissProt_HUMAN$	3::Q13472|TOP3A_HUMAN	32	112300	1	1	1	1	0.04	DNA topoisomerase 3-
1575	1	db$2	2::C3K5C6|C3K5C6_PSEFS	32	33902	1	1	1	1	0.15	Uncharacterized protei
1576	1	SwissProt_HUMAN$	3::Q96M32|KAD7_HUMAN	32	82607	2	1	2	1	0.06	Adenylate kinase 7 OS
1577	1	SwissProt_HUMAN$	3::P56749|CLD12_HUMAN	32	27092	1	1	1	1	0.19	Claudin-12 OS=Homo
1578	1	SwissProt_HUMAN$	3::P32969|RL9_HUMAN	32	21850	1	1	1	1	0.24	60S ribosomal protein
1579	1	db$2	2::C3K838|C3K838_PSEFS	32	9715	1	1	1	1	0.60	Putative phosphocarrie

↑Family	M	DB	Accession	Score	Mass	Matches	Match(sig)	Sequences	Seq(sig)	emPAI	Description
1580	1	db$2	2::C3K707|C3K707_PSEFS	32	29295	4	2	4	2	0.38	Putative lipoprotein OS
1581	1	db$2	2::C3JZW1|C3JZW1_PSEFS	32	36968	1	1	1	1	0.14	Putative cobalamin-bir
1582	1	SwissProt_HUMAN$	3::P01011|AACT_HUMAN	32	47621	1	1	1	1	0.10	Alpha-1-antichymotryp
1583	1	db$2	2::C3KBJ0|C3KBJ0_PSEFS	32	16933	3	3	3	3	1.28	[Ribosomal protein S1
1584	1	SwissProt_HUMAN$	3::Q13422|IKZF1_HUMAN	32	57491	2	1	2	1	0.09	DNA-binding protein Ik
1585	1	db$2	2::C3K125|C3K125_PSEFS	32	26783	2	2	1	1	0.19	Alanine--tRNA ligase C
1586	1	db$2	2::C3K0H5|C3K0H5_PSEFS	32	25946	1	1	1	1	0.20	Putative chemotaxis pr
1587	1	db$2	2::C3JY76|C3JY76_PSEFS	32	48249	2	2	2	2	0.22	Putative mechanosens
1588	1	SwissProt_HUMAN$	3::P23396|RS3_HUMAN	32	26671	1	1	1	1	0.19	40S ribosomal protein
1589	1	db$2	2::C3KBH3|DCUP_PSEFS	32	38973	3	2	3	2	0.27	Uroporphyrinogen deca
1590	1	db$2	2::C3K6E9|C3K6E9_PSEFS	32	41977	2	2	2	2	0.25	Phosphatidylcholine-hy
1591	1	SwissProt_HUMAN$	3::P62318|SMD3_HUMAN	32	13907	1	1	1	1	0.40	Small nuclear ribonucl
1592	1	db$2	2::C3KB05|C3KB05_PSEFS	31	50891	1	1	1	1	0.10	CDP-diacylglycerol-ser
1593	1	db$2	2::C3K5T7|C3K5T7_PSEFS	31	23769	1	1	1	1	0.22	Uncharacterized protei
1594	1	db$2	2::C3KEB9|C3KEB9_PSEFS	31	11110	1	1	1	1	0.52	Putative lipoprotein OS
1595	1	db$2	2::C3JYB1|C3JYB1_PSEFS	31	43274	3	1	3	1	0.12	Uncharacterized protei
1596	1	SwissProt_HUMAN$	3::B2RPK0|HGB1A_HUMAN	31	24223	1	1	1	1	0.21	Putative high mobility
1597	1	SwissProt_HUMAN$	3::P0DME0|SETLP_HUMAN	31	34861	1	1	1	1	0.14	Protein SETSIP OS=Hc
1598	1	SwissProt_HUMAN$	3::P01024|CO3_HUMAN	31	187030	2	1	2	1	0.03	Complement C3 OS=H
1599	1	db$2	2::C3K281|C3K281_PSEFS	31	15787	1	1	1	1	0.34	Ribosome association t
1600	1	db$2	2::C3K4U6|C3K4U6_PSEFS	31	17513	1	1	1	1	0.31	Conserved hypothetica
1601	1	SwissProt_HUMAN$	3::P28072|PSB6_HUMAN	31	25341	1	1	1	1	0.20	Proteasome subunit be
1602	1	db$2	2::C3K440|C3K440_PSEFS	31	11389	5	3	3	3	2.37	Uncharacterized protei
1603	1	db$2	2::C3K5I1|C3K5I1_PSEFS	31	43688	1	1	1	1	0.11	Putative phage integra
1604	1	db$2	2::C3K3N1|C3K3N1_PSEFS	31	52088	1	1	1	1	0.10	Aldehyde dehydrogena
1605	1	SwissProt_HUMAN$	3::Q8TBY8|PMFBP_HUMAN	31	117420	1	1	1	1	0.04	Polyamine-modulated
1606	1	db$2	2::C3JZ62|C3JZ62_PSEFS	31	61473	1	1	1	1	0.08	Putative long-chain-fat
1607	1	db$2	2::C3JYJ2|C3JYJ2_PSEFS	31	27205	2	1	2	1	0.19	Putative lipoprotein OS
1608	1	db$2	2::C3K8K3|C3K8K3_PSEFS	31	22975	1	1	1	1	0.23	Putative transposase C
1609	1	db$2	2::C3JYG6|C3JYG6_PSEFS	31	44622	2	1	2	1	0.11	Peptidase_M14 domair
1610	1	db$2	2::C3KE75|C3KE75_PSEFS	31	123658	2	2	2	2	0.08	TGc domain-containing
1611	1	db$2	2::C3K1W1|C3K1W1_PSEFS	31	22921	2	2	1	1	0.23	Uncharacterized protei
1612	1	SwissProt_HUMAN$	3::Q9NU22|MDN1_HUMAN	31	632420	3	1	3	1	0.01	Midasin OS=Homo sap
1613	1	SwissProt_HUMAN$	3::Q96P48|ARAP1_HUMAN	31	162089	1	1	1	1	0.03	Arf-GAP with Rho-GAP
1614	1	SwissProt_HUMAN$	3::Q5SRN2|TSBP1_HUMAN	31	61588	1	1	1	1	0.08	Testis-expressed basic
1615	1	SwissProt_HUMAN$	3::P37802|TAGL2_HUMAN	31	22377	1	1	1	1	0.23	Transgelin-2 OS=Hom
1616	1	db$2	2::C3K5L6|C3K5L6_PSEFS	31	16214	3	3	3	3	1.37	Putative phosphorous
1617	1	SwissProt_HUMAN$	3::Q8NBF4|YG006_HUMAN	31	16172	2	1	1	1	0.33	Putative characteriz
1618	1	SwissProt_HUMAN$	3::O75197|LRP5_HUMAN	31	179030	4	1	1	1	0.03	Low-density lipoprotei
1619	1	SwissProt_HUMAN$	3::Q4UJ75|A20A4_HUMAN	31	94090	2	1	1	1	0.05	Putative ankyrin repea

↑Family	M	DB	Accession	Score	Mass	Matches	Match(sig)	Sequences	Seq(sig)	emPAI	Description
1620	1	db$2	2::C3JYF1|C3JYF1_PSEFS	31	42707	2	1	2	1	0.12	Putative acyl-CoA dehy
1621	1	db$2	2::C3KCP0|C3KCP0_PSEFS	30	31742	1	1	1	1	0.16	Ribosomal RNA large s
1622	1	SwissProt_HUMAN$	3::O94817|ATG12_HUMAN	30	15104	1	1	1	1	0.36	Ubiquitin-like protein A
1623	1	db$2	2::C3KCE1|C3KCE1_PSEFS	30	45800	1	1	1	1	0.11	Putative exported prot
1624	1	db$2	2::C3K6K7|C3K6K7_PSEFS	30	17395	1	1	1	1	0.31	GreA_GreB domain-co
1625	1	db$2	2::C3KDN1|C3KDN1_PSEFS	30	54005	2	1	2	1	0.09	Deoxyribodipyrimidine
1626	1	SwissProt_HUMAN$	3::Q96E52|OMA1_HUMAN	30	60081	1	1	1	1	0.08	Metalloendopeptidase
1627	1	db$2	2::C3JZP7|C3JZP7_PSEFS	30	33454	1	1	1	1	0.15	Putative ribose transpo
1628	1	SwissProt_HUMAN$	3::O43281|EFS_HUMAN	30	58779	1	1	1	1	0.08	Embryonal Fyn-associa
1629	1	SwissProt_HUMAN$	3::O00459|P85B_HUMAN	30	81495	3	2	2	2	0.12	Phosphatidylinositol 3-
1630	1	db$2	2::C3K600|C3K600_PSEFS	30	28761	2	2	2	2	0.39	Phosphatidate cytidyly
1631	1	db$2	2::C3K9N3|C3K9N3_PSEFS	30	10076	2	1	2	1	0.58	Peptidyl-prolyl cis-tran
1632	1	SwissProt_HUMAN$	3::Q9Y265|RUVB1_HUMAN	30	50196	1	1	1	1	0.10	RuvB-like 1 OS=Homo
1633	1	db$2	2::C3K0G1|C3K0G1_PSEFS	30	67986	1	1	1	1	0.07	Putative acetyl-/propic
1634	1	db$2	2::C3K8T6|C3K8T6_PSEFS	30	26078	1	1	1	1	0.20	Ribosomal RNA small s
1635	1	db$2	2::C3K9N7|C3K9N7_PSEFS	29	48751	1	1	1	1	0.10	Putative membrane pr
1636	1	db$2	2::C3K3J2|C3K3J2_PSEFS	29	213513	1	1	1	1	0.02	Histidine kinase OS=Ps
1637	1	db$2	2::C3K243|C3K243_PSEFS	29	108098	1	1	1	1	0.04	Histidine kinase OS=Ps
1638	1	db$2	2::C3KDM8|C3KDM8_PSEFS	29	57105	1	1	1	1	0.09	HDOD domain-contain
1639	1	db$2	2::C3K1Y4|C3K1Y4_PSEFS	29	14766	1	1	1	1	0.37	Putative putative glyox
1640	1	db$2	2::C3K386|C3K386_PSEFS	29	22957	1	1	1	1	0.23	Putative glutathione S-
1641	1	db$2	2::C3K808|C3K808_PSEFS	29	53666	1	1	1	1	0.09	Histidine ammonia-lya
1642	1	db$2	2::C3KE56|C3KE56_PSEFS	29	59021	3	3	3	3	0.27	Glutamate--cysteine li
1643	1	db$2	2::C3KC07|C3KC07_PSEFS	29	21449	1	1	1	1	0.24	YceI domain-containin
1644	1	db$2	2::C3K3H6|C3K3H6_PSEFS	29	12460	1	1	1	1	0.45	Putative endoribonucle
1645	1	db$2	2::C3K6X0|C3K6X0_PSEFS	29	62906	1	1	1	1	0.08	Putative GGDEF domai
1646	1	db$2	2::C3K3K0|YQGF_PSEFS	29	16158	1	1	1	1	0.33	Putative pre-16S rRNA
1647	1	db$2	2::C3JY58|C3JY58_PSEFS	29	87640	5	4	4	4	0.24	DNA translocase FtsK (
1648	1	db$2	2::C3JYL6|C3JYL6_PSEFS	29	25076	1	1	1	1	0.21	Putative TetR-family re
1649	1	SwissProt_HUMAN$	3::Q5VT06|CE350_HUMAN	29	350716	1	1	1	1	0.01	Centrosome-associated
1650	1	db$2	2::C3K1J8|C3K1J8_PSEFS	29	26668	1	1	1	1	0.19	Trehalose operon trans
1651	1	db$2	2::C3KDP0|C3KDP0_PSEFS	29	27498	2	2	2	2	0.41	Uncharacterized protei
1652	1	db$2	2::C3K8U1|C3K8U1_PSEFS	29	15020	1	1	1	1	0.37	Phosphoribosyl-AMP cy
1653	1	db$2	2::C3K5Y5|C3K5Y5_PSEFS	29	18019	1	1	1	1	0.30	Protein PsiE OS=Pseuc
1654	1	db$2	2::C3K5A1|C3K5A1_PSEFS	29	60517	1	1	1	1	0.08	Urease subunit alpha (
1655	1	db$2	2::C3K659|C3K659_PSEFS	29	30767	2	1	2	1	0.17	2-hydroxy-3-oxopropic
1656	1	SwissProt_HUMAN$	3::Q96PX9|PKH4B_HUMAN	29	139580	2	1	2	1	0.03	Pleckstrin homology do
1657	1	db$2	2::C3K428|C3K428_PSEFS	28	10726	1	1	1	1	0.54	Uncharacterized protei
1658	1	db$2	2::C3K340|C3K340_PSEFS	28	72802	1	1	1	1	0.07	DNA primase OS=Pseu
1659	1	db$2	2::C3KDY6|C3KDY6_PSEFS	28	17128	1	1	1	1	0.31	Uncharacterized protei

↑Family	M	DB	Accession	Score	Mass	Matches	Match(sig)	Sequences	Seq(sig)	emPAI	Description
1660	1	SwissProt_HUMAN$	3::Q8TDI7|TMC2_HUMAN	28	102544	1	1	1	1	0.05	Transmembrane chanr
1661	1	SwissProt_HUMAN$	3::O15049|N4BP3_HUMAN	28	60433	2	1	1	1	0.08	NEDD4-binding proteir
1662	1	db$2	2::C3K7M1|C3K7M1_PSEFS	28	62712	2	2	2	2	0.16	Putative dipeptidase O
1663	1	db$2	2::C3K7A9|C3K7A9_PSEFS	28	33658	1	1	1	1	0.15	Conserved hypothetica
1664	1	db$2	2::C3KD14|C3KD14_PSEFS	28	16704	1	1	1	1	0.32	Putative transmembra
1665	1	db$2	2::C3K6T3|C3K6T3_PSEFS	28	23166	2	2	1	1	0.22	Thiol:disulfide intercha
1666	1	SwissProt_HUMAN$	3::Q8TDR2|STK35_HUMAN	28	58015	1	1	1	1	0.08	Serine/threonine-prote
1667	1	db$2	2::C3K9Q1|C3K9Q1_PSEFS	28	28823	1	1	1	1	0.18	Methyltransf_11 doma
1668	1	db$2	2::C3K5U8|AGUA_PSEFS	28	40372	2	1	2	1	0.12	Agmatine deiminase O
1669	1	SwissProt_HUMAN$	3::O00231|PSD11_HUMAN	28	47434	1	1	1	1	0.10	26S proteasome non-A
1670	1	SwissProt_HUMAN$	3::Q8IV33|K0825_HUMAN	28	147640	1	1	1	1	0.03	Uncharacterized protei
1671	1	SwissProt_HUMAN$	3::A6NJB7|PRR19_HUMAN	28	38692	1	1	1	1	0.13	Proline-rich protein 19
1672	1	SwissProt_HUMAN$	3::Q6ZNX1|SHLD3_HUMAN	28	28824	1	1	1	1	0.18	Shieldin complex subu
1673	1	SwissProt_HUMAN$	3::Q9HBJ7|UBP29_HUMAN	28	104090	2	2	2	2	0.10	Ubiquitin carboxyl-terr
1674	1	db$2	2::C3KBV2|C3KBV2_PSEFS	28	15686	1	1	1	1	0.35	Thioredoxin OS=Pseuc
1675	1	db$2	2::C3K0R2|C3K0R2_PSEFS	28	34379	2	2	2	2	0.32	Chemotaxis protein OS
1676	1	db$2	2::C3KDC5|HEM1_PSEFS	28	46641	2	2	2	2	0.22	Glutamyl-tRNA reducta
1677	1	SwissProt_HUMAN$	3::P31946|1433B_HUMAN	28	28065	1	1	1	1	0.18	14-3-3 protein beta/al
1678	1	db$2	2::C3K4T5|C3K4T5_PSEFS	28	30974	2	1	2	1	0.16	Putative hydrolase OS
1679	1	db$2	2::C3KDK6|C3KDK6_PSEFS	28	27451	1	1	1	1	0.19	Putative ABC transport
1680	1	SwissProt_HUMAN$	3::Q8IWF6|DEN6A_HUMAN	28	69530	1	1	1	1	0.07	Protein DENND6A OS=
1681	1	db$2	2::C3K1Z0|C3K1Z0_PSEFS	28	34051	1	1	1	1	0.15	Putative putative polys
1682	1	SwissProt_HUMAN$	3::Q02224|CENPE_HUMAN	28	316219	3	1	3	1	0.02	Centromere-associatec
1683	1	SwissProt_HUMAN$	3::Q9H6P5|TASP1_HUMAN	28	44427	1	1	1	1	0.11	Threonine aspartase 1
1684	1	db$2	2::C3KAP7|C3KAP7_PSEFS	28	26475	1	1	1	1	0.19	Putative short-chain de
1685	1	db$2	2::C3JZI1|C3JZI1_PSEFS	28	33491	1	1	1	1	0.15	Putative glycine betain
1686	1	SwissProt_HUMAN$	3::O75155|CAND2_HUMAN	28	135171	1	1	1	1	0.04	Cullin-associated NEDI
1687	1	db$2	2::C3K4F4|C3K4F4_PSEFS	28	25363	1	1	1	1	0.20	Putative acetyltransfer
1688	1	db$2	2::C3JXP4|C3JXP4_PSEFS	28	23087	1	1	1	1	0.23	Putative putative ECF-
1689	1	db$2	2::C3K3P6|C3K3P6_PSEFS	28	19979	1	1	1	1	0.26	Dihydrofolate reductas
1690	1	db$2	2::C3KAF4|C3KAF4_PSEFS	28	50981	1	1	1	1	0.10	Putative lipoprotein OS
1691	1	db$2	2::C3K3J8|C3K3J8_PSEFS	28	33397	1	1	1	1	0.15	TonB_C domain-contai
1692	1	db$2	2::C3K7J4|C3K7J4_PSEFS	27	18330	1	1	1	1	0.29	Uncharacterized protei
1693	1	db$2	2::C3K7J6|C3K7J6_PSEFS	27	25687	1	1	1	1	0.20	Putative exported prot
1694	1	db$2	2::C3K9R9|C3K9R9_PSEFS	27	86327	1	1	1	1	0.06	Putative exported fatty
1695	1	SwissProt_HUMAN$	3::P15880|RS2_HUMAN	27	31305	2	1	2	1	0.16	40S ribosomal protein
1696	1	SwissProt_HUMAN$	3::Q96AQ6|PBIP1_HUMAN	27	80594	1	1	1	1	0.06	Pre-B-cell leukemia tra
1697	1	SwissProt_HUMAN$	3::P02747|C1QC_HUMAN	27	25757	2	2	2	2	0.44	Complement C1q subc
1698	1	db$2	2::C3K334|CCA_PSEFS	27	45916	1	1	1	1	0.11	Multifunctional CCA pr
1699	1	SwissProt_HUMAN$	3::P00519|ABL1_HUMAN	27	122796	1	1	1	1	0.04	Tyrosine-protein kinas

↑Family	M	DB	Accession	Score	Mass	Matches	Match(sig)	Sequences	Seq(sig)	emPAI	Description
1700	1	SwissProt_HUMAN$	3::Q7Z624|CMKMT_HUMAN	27	36105	1	1	1	1	0.14	Calmodulin-lysine N-m
1701	1	db$2	2::C3K5B4|C3K5B4_PSEFS	27	32752	1	1	1	1	0.15	Dipeptide ABC transpo
1702	1	SwissProt_HUMAN$	3::Q8NFV4|ABHDB_HUMAN	27	34668	1	1	1	1	0.15	Protein ABHD11 OS=H
1703	1	SwissProt_HUMAN$	3::Q07283|TRHY_HUMAN	27	253777	1	1	1	1	0.02	Trichohyalin OS=Homo
1704	1	SwissProt_HUMAN$	3::Q9Y5B9|SP16H_HUMAN	27	119838	2	2	2	2	0.08	FACT complex subunit
1705	1	db$2	2::C3K915|C3K915_PSEFS	27	26912	1	1	1	1	0.19	Putative GntR-family r
1706	1	db$2	2::C3KD06|C3KD06_PSEFS	27	82856	1	1	1	1	0.06	Putative amidase OS=
1707	1	SwissProt_HUMAN$	3::P04080|CYTB_HUMAN	27	11133	1	1	1	1	0.52	Cystatin-B OS=Homo s
1708	1	SwissProt_HUMAN$	3::O14917|PCD17_HUMAN	27	126151	1	1	1	1	0.04	Protocadherin-17 OS=
1709	1	SwissProt_HUMAN$	3::P27216|ANX13_HUMAN	27	35393	1	1	1	1	0.14	Annexin A13 OS=Hom
1710	1	db$2	2::C3KDR2|C3KDR2_PSEFS	27	26142	2	1	2	1	0.20	S4 RNA-binding domai
1711	1	db$2	2::C3K267|RLME_PSEFS	27	24125	1	1	1	1	0.22	Ribosomal RNA large s
1712	1	SwissProt_HUMAN$	3::Q96SK2|TM209_HUMAN	27	62881	1	1	1	1	0.08	Transmembrane protei
1713	1	SwissProt_HUMAN$	3::P54821|PRRX1_HUMAN	27	27280	1	1	1	1	0.19	Paired mesoderm hom
1714	1	db$2	2::C3K1Q2|C3K1Q2_PSEFS	27	73203	1	1	1	1	0.07	Histidine kinase OS=P:
1715	1	db$2	2::C3K660|C3K660_PSEFS	27	28970	1	1	1	1	0.18	Hydroxypyruvate isom
1716	1	db$2	2::C3K0E2|C3K0E2_PSEFS	27	49920	1	1	1	1	0.10	Diaminobutyrate--2-ox
1717	1	SwissProt_HUMAN$	3::O95153|RIMB1_HUMAN	27	199928	1	1	1	1	0.02	Peripheral-type benzo
1718	1	db$2	2::C3K7Z3|C3K7Z3_PSEFS	27	43142	1	1	1	1	0.12	Putative acyl-CoA deh
1719	1	SwissProt_HUMAN$	3::Q6UWJ1|TMCO3_HUMAN	27	75549	1	1	1	1	0.06	Transmembrane and c
1720	1	db$2	2::C3JY26|C3JY26_PSEFS	27	17211	1	1	1	1	0.31	Protein-tyrosine-phosp
1721	1	db$2	2::C3K0D9|C3K0D9_PSEFS	27	79601	1	1	1	1	0.06	Putative transport-rela
1722	1	db$2	2::C3JXY5|C3JXY5_PSEFS	27	10610	1	1	1	1	0.54	Uncharacterized protei
1723	1	db$2	2::C3K963|C3K963_PSEFS	27	78429	1	1	1	1	0.06	Putative TonB-depend
1724	1	db$2	2::C3K746|C3K746_PSEFS	27	78906	2	1	1	1	0.06	Putative TonB-depend
1725	1	db$2	2::C3K536|C3K536_PSEFS	27	22752	2	2	2	2	0.51	DUF4174 domain-cont
1726	1	SwissProt_HUMAN$	3::Q6NUI2|GPAT2_HUMAN	26	87779	1	1	1	1	0.06	Glycerol-3-phosphate
1727	1	db$2	2::C3K629|C3K629_PSEFS	26	51787	2	1	1	1	0.10	Putative transport-rela
1728	1	db$2	2::C3K8J9|C3K8J9_PSEFS	26	51515	1	1	1	1	0.10	Type II secretion syste
1729	1	SwissProt_HUMAN$	3::O15270|SPTC2_HUMAN	26	62884	1	1	1	1	0.08	Serine palmitoyltransf
1730	1	db$2	2::C3K6Z2|C3K6Z2_PSEFS	26	17868	1	1	1	1	0.30	tRNA-specific adenosir
1731	1	db$2	2::C3KB19|C3KB19_PSEFS	26	44434	1	1	1	1	0.11	HlyD family secretion p
1732	1	db$2	2::A4V7U9|A4V7U9_PSEFS	26	58926	1	1	1	1	0.08	Mercuric reductase OS
1733	1	SwissProt_HUMAN$	3::P46776|RL27A_HUMAN	26	16551	1	1	1	1	0.33	60S ribosomal protein
1734	1	SwissProt_HUMAN$	3::O00299|CLIC1_HUMAN	26	26906	1	1	1	1	0.19	Chloride intracellular c
1735	1	SwissProt_HUMAN$	3::P11309|PIM1_HUMAN	26	35663	1	1	1	1	0.14	Serine/threonine-prote
1736	1	db$2	2::C3KBH6|C3KBH6_PSEFS	26	16609	1	1	1	1	0.32	Conserved hypothetica
1737	1	SwissProt_HUMAN$	3::Q9NRG4|SMYD2_HUMAN	26	49656	1	1	1	1	0.10	N-lysine methyltransfe
1738	1	db$2	2::C3KDV4|RSGA_PSEFS	26	37296	2	2	2	2	0.29	Small ribosomal subun
1739	1	db$2	2::C3KAI2|C3KAI2_PSEFS	26	19361	2	2	2	2	0.62	Shikimate kinase OS=

↑Family	M	DB	Accession	Score	Mass	Matches	Match(sig)	Sequences	Seq(sig)	emPAI	Description
1740	1	db$2	2::C3K6R2|C3K6R2_PSEFS	26	29309	1	1	1	1	0.17	Putative shikimate deh
1741	1	db$2	2::C3K9N8|C3K9N8_PSEFS	26	51066	1	1	1	1	0.10	Putative sigma-54 dep
1742	1	SwissProt_HUMAN$	3::Q00526|CDK3_HUMAN	26	35024	1	1	1	1	0.14	Cyclin-dependent kina:
1743	1	SwissProt_HUMAN$	3::Q8N9W6|BOLL_HUMAN	26	31282	1	1	1	1	0.16	Protein boule-like OS=
1744	1	SwissProt_HUMAN$	3::Q8WZ42|TITIN_HUMAN	26	3813652	5	2	5	2	0.00	Titin OS=Homo sapien
1745	1	SwissProt_HUMAN$	3::Q99618|CDCA3_HUMAN	26	28981	1	1	1	1	0.18	Cell division cycle-assc
1746	1	SwissProt_HUMAN$	3::Q9H1K0|RBNS5_HUMAN	26	88815	1	1	1	1	0.05	Rabenosyn-5 OS=Hom
1747	1	SwissProt_HUMAN$	3::Q6PI48|SYDM_HUMAN	26	73516	1	1	1	1	0.07	Aspartate--tRNA ligase
1748	1	SwissProt_HUMAN$	3::P06748|NPM_HUMAN	25	32555	1	1	1	1	0.16	Nucleophosmin OS=Hc
1749	1	db$2	2::C3K2M4|C3K2M4_PSEFS	25	40052	1	1	1	1	0.13	Peptidoglycan glycosyl
1750	1	db$2	2::C3K6E6|C3K6E6_PSEFS	25	23384	2	2	2	2	0.49	Stringent starvation pr
1751	1	db$2	2::C3JXZ4|C3JXZ4_PSEFS	25	40619	2	1	1	1	0.12	Uncharacterized protei
1752	1	SwissProt_HUMAN$	3::P23284|PPIB_HUMAN	25	23728	1	1	1	1	0.22	Peptidyl-prolyl cis-tran
1753	1	db$2	2::C3K831|C3K831_PSEFS	25	45359	4	3	4	3	0.37	Putative thiolase OS=F
1754	1	SwissProt_HUMAN$	3::P48165|CXA8_HUMAN	25	48199	1	1	1	1	0.10	Gap junction alpha-8 p
1755	1	SwissProt_HUMAN$	3::Q06455|MTG8_HUMAN	25	67524	2	1	2	1	0.07	Protein CBFA2T1 OS=I
1756	1	db$2	2::C3K4V6|C3K4V6_PSEFS	25	101332	1	1	1	1	0.05	Putative helicase OS=I
1757	1	db$2	2::C3K444|C3K444_PSEFS	25	9491	1	1	1	1	0.62	Ubiquinone biosynthes
1758	1	db$2	2::C3K908|C3K908_PSEFS	25	33577	1	1	1	1	0.15	Putative dehydrogenas
1759	1	db$2	2::C3K1M8|C3K1M8_PSEFS	25	28345	3	2	2	2	0.39	tRNA (cytidine/uridine-
1760	1	SwissProt_HUMAN$	3::Q9Y5A7|NUB1_HUMAN	25	70494	2	1	2	1	0.07	NEDD8 ultimate buster
1761	1	SwissProt_HUMAN$	3::P36507|MP2K2_HUMAN	25	44396	1	1	1	1	0.11	Dual specificity mitoge
1762	1	db$2	2::C3K5B8|C3K5B8_PSEFS	25	59457	2	2	2	2	0.17	Dipeptide ABC transpo
1763	1	db$2	2::C3K8Z3|C3K8Z3_PSEFS	25	28227	2	1	2	1	0.18	High-affinity branched-
1764	1	db$2	2::C3K4T8|C3K4T8_PSEFS	25	33681	1	1	1	1	0.15	Putative AraC-family re
1765	1	db$2	2::C3K4R0|C3K4R0_PSEFS	25	14930	1	1	1	1	0.37	Putative lipoprotein OS
1766	1	SwissProt_HUMAN$	3::P31939|PUR9_HUMAN	25	64575	1	1	1	1	0.08	Bifunctional purine bio
1767	1	SwissProt_HUMAN$	3::Q14DG7|T132B_HUMAN	25	119402	1	1	1	1	0.04	Transmembrane protei
1768	1	db$2	2::C3K5J0|C3K5J0_PSEFS	25	13887	1	1	1	1	0.40	Putative lipoprotein OS
1769	1	SwissProt_HUMAN$	3::O00139|KIF2A_HUMAN	25	79905	5	2	1	1	0.06	Kinesin-like protein KII
1770	1	db$2	2::C3KBZ4|C3KBZ4_PSEFS	25	6850	2	2	1	1	0.94	Uncharacterized protei
1771	1	db$2	2::C3KCB6|C3KCB6_PSEFS	25	31268	3	2	2	1	0.35	Phosphate transport sy
1772	1	SwissProt_HUMAN$	3::Q9UHN6|CEIP2_HUMAN	25	154276	2	1	2	1	0.03	Cell surface hyaluronic
1773	1	SwissProt_HUMAN$	3::Q6PKH6|DR4L2_HUMAN	25	24570	1	1	1	1	0.21	Dehydrogenase/reduct
1774	1	SwissProt_HUMAN$	3::Q96GD3|SCMH1_HUMAN	25	73307	1	1	1	1	0.07	Polycomb protein SCM
1775	1	SwissProt_HUMAN$	3::Q15075|EEA1_HUMAN	25	162367	1	1	1	1	0.03	Early endosome antige
1776	1	db$2	2::C3K9P2|C3K9P2_PSEFS	24	69747	1	1	1	1	0.07	Putative substrate-bin
1777	1	db$2	2::C3JZP4|RBSD_PSEFS	24	14393	1	1	1	1	0.38	D-ribose pyranase OS=
1778	1	db$2	2::C3K059|TRHO_PSEFS	24	35401	1	1	1	1	0.14	tRNA uridine(34) hydr
1779	1	SwissProt_HUMAN$	3::P09622|DLDH_HUMAN	24	54143	1	1	1	1	0.09	Dihydrolipoyl dehydrog

↑Family	M	DB	Accession	Score	Mass	Matches	Match(sig)	Sequences	Seq(sig)	emPAI	Description
1780	1	db$2	2::C3K3C7|C3K3C7_PSEFS	24	34452	2	1	2	1	0.15	Putative glycine betain
1781	1	db$2	2::C3K9G3|C3K9G3_PSEFS	24	409423	2	1	2	1	0.01	Putative non-ribosoma
1782	1	db$2	2::C3K5Q0|C3K5Q0_PSEFS	24	53619	1	1	1	1	0.09	Mannose-1-phosphate
1783	1	db$2	2::C3K0X4|C3K0X4_PSEFS	24	79071	1	1	1	1	0.06	DNA helicase OS=Pseu
1784	1	SwissProt_HUMAN$	3::O43707|ACTN4_HUMAN	24	104788	2	1	1	1	0.05	Alpha-actinin-4 OS=Hc
1785	1	db$2	2::C3K992|C3K992_PSEFS	24	34600	2	2	2	2	0.31	Putative AraC-family re
1786	1	db$2	2::C3K8L9|C3K8L9_PSEFS	24	65348	2	1	2	1	0.08	Putative potassium-eff
1787	1	SwissProt_HUMAN$	3::Q8TD08|MK15_HUMAN	24	59795	1	1	1	1	0.08	Mitogen-activated prot
1788	1	SwissProt_HUMAN$	3::P54886|P5CS_HUMAN	24	87248	1	1	1	1	0.06	Delta-1-pyrroline-5-ca
1789	1	db$2	2::C3K8U0|HIS2_PSEFS	24	12007	1	1	1	1	0.47	Phosphoribosyl-ATP py
1790	1	db$2	2::C3K457|C3K457_PSEFS	24	12769	1	1	1	1	0.44	Putative endoribonucle
1791	1	db$2	2::C3K330|GLPE_PSEFS	24	11747	1	1	1	1	0.48	Thiosulfate sulfurtrans
1792	1	SwissProt_HUMAN$	3::Q9UL62|TRPC5_HUMAN	24	111340	1	1	1	1	0.04	Short transient recepto
1793	1	SwissProt_HUMAN$	3::O00159|MYO1C_HUMAN	24	121606	1	1	1	1	0.04	Unconventional myosir
1794	1	SwissProt_HUMAN$	3::P17480|UBF1_HUMAN	24	89350	1	1	1	1	0.05	Nucleolar transcription
1795	1	db$2	2::C3K1M3|C3K1M3_PSEFS	24	11692	1	1	1	1	0.49	Iron-binding protein Is
1796	1	SwissProt_HUMAN$	3::P05546|HEP2_HUMAN	24	57034	1	1	1	1	0.09	Heparin cofactor 2 OS=
1797	1	SwissProt_HUMAN$	3::P27708|PYR1_HUMAN	24	242829	1	1	1	1	0.02	CAD protein OS=Homo
1798	1	db$2	2::C3K9I0|C3K9I0_PSEFS	24	25315	1	1	1	1	0.20	Putative two-compone
1799	1	db$2	2::C3KCV4|C3KCV4_PSEFS	24	19545	1	1	1	1	0.27	Putative DNA-binding r
1800	1	SwissProt_HUMAN$	3::Q7Z7L7|ZER1_HUMAN	24	88113	2	2	1	1	0.11	Protein zer-1 homolog
1801	1	SwissProt_HUMAN$	3::Q9H6U8|ALG9_HUMAN	24	69818	1	1	1	1	0.07	Alpha-1,2-mannosyltra
1802	1	db$2	2::C3K7D7|C3K7D7_PSEFS	24	8979	1	1	1	1	0.67	Putative lipoprotein OS
1803	1	db$2	2::C3JXG5|C3JXG5_PSEFS	24	36156	2	1	2	1	0.14	Putative GFO/IDH/Moc
1804	1	SwissProt_HUMAN$	3::Q9H244|P2Y12_HUMAN	24	39413	1	1	1	1	0.13	P2Y purinoceptor 12 O
1805	1	SwissProt_HUMAN$	3::Q4G0P3|HYDIN_HUMAN	24	575528	1	1	1	1	0.01	Hydrocephalus-inducin
1806	1	SwissProt_HUMAN$	3::Q9BYX4|IFIH1_HUMAN	24	116615	2	2	2	2	0.08	Interferon-induced hel
1807	1	SwissProt_HUMAN$	3::O43586|PPIP1_HUMAN	24	47562	1	1	1	1	0.10	Proline-serine-threonir
1808	1	db$2	2::C3K9X2|C3K9X2_PSEFS	24	42284	2	2	2	2	0.25	Putative aminotransfer
1809	1	db$2	2::C3K5X3|C3K5X3_PSEFS	24	64999	1	1	1	1	0.08	Putative ABC transport
1810	1	db$2	2::C3KB65|C3KB65_PSEFS	24	37899	1	1	1	1	0.13	Methylated-DNA--[prot
1811	1	SwissProt_HUMAN$	3::A0A1B0GVD1|F237B_HUMAN	24	16320	1	1	1	1	0.33	Protein FAM237B OS=
1812	1	SwissProt_HUMAN$	3::Q53EZ4|CEP55_HUMAN	24	54145	1	1	1	1	0.09	Centrosomal protein of
1813	1	db$2	2::C3KDS1|C3KDS1_PSEFS	24	26155	2	1	1	1	0.20	Pseudouridine synthas
1814	1	SwissProt_HUMAN$	3::Q6ZUM4|RHG27_HUMAN	23	98335	1	1	1	1	0.05	Rho GTPase-activating
1815	1	SwissProt_HUMAN$	3::A8MX19|F90AC_HUMAN	23	49755	1	1	1	1	0.10	Putative protein FAM9(
1816	1	SwissProt_HUMAN$	3::Q14683|SMC1A_HUMAN	23	143144	2	1	2	1	0.03	Structural maintenanc
1817	1	SwissProt_HUMAN$	3::P04083|ANXA1_HUMAN	23	38690	1	1	1	1	0.13	Annexin A1 OS=Homo
1818	1	SwissProt_HUMAN$	3::Q9NRF8|PYRG2_HUMAN	23	65636	1	1	1	1	0.07	CTP synthase 2 OS=Hc
1819	1	SwissProt_HUMAN$	3::Q92805|GOGA1_HUMAN	23	88130	1	1	1	1	0.06	Golgin subfamily A me

↑Family	M	DB	Accession	Score	Mass	Matches	Match(sig)	Sequences	Seq(sig)	emPAI	Description
1820	1	SwissProt_HUMAN$	3::Q8TDN4|CABL1_HUMAN	23	67557	1	1	1	1	0.07	CDK5 and ABL1 enzym
1821	1	db$2	2::A4V7G0|A4V7G0_PSEFS	23	59387	2	1	2	1	0.08	Putative RNA polymera
1822	1	db$2	2::C3K7D0|THII_PSEFS	23	54519	1	1	1	1	0.09	tRNA sulfurtransferase
1823	1	SwissProt_HUMAN$	3::Q9P2E3|ZNFX1_HUMAN	23	220084	1	1	1	1	0.02	NFX1-type zinc finger-
1824	1	SwissProt_HUMAN$	3::O94967|WDR47_HUMAN	23	101884	1	1	1	1	0.05	WD repeat-containing
1825	1	db$2	2::C3KCJ3|C3KCJ3_PSEFS	23	30975	2	2	1	1	0.16	Usp domain-containing
1826	1	SwissProt_HUMAN$	3::P49207|RL34_HUMAN	23	13284	2	2	2	2	1.01	60S ribosomal protein
1827	1	db$2	2::C3KEC9|C3KEC9_PSEFS	23	53005	1	1	1	1	0.09	Putative beta-barrel as
1828	1	SwissProt_HUMAN$	3::P11532|DMD_HUMAN	23	426484	3	2	3	2	0.02	Dystrophin OS=Homo
1829	1	SwissProt_HUMAN$	3::P55283|CADH4_HUMAN	23	100218	1	1	1	1	0.05	Cadherin-4 OS=Homo
1830	1	db$2	2::C3KE19|C3KE19_PSEFS	23	30253	1	1	1	1	0.17	HDOD domain-contain
1831	1	SwissProt_HUMAN$	3::A6NGH7|CC160_HUMAN	23	38253	1	1	1	1	0.13	Coiled-coil domain-con
1832	1	SwissProt_HUMAN$	3::Q92538|GBF1_HUMAN	23	206315	2	1	2	1	0.02	Golgi-specific brefeldin
1833	1	SwissProt_HUMAN$	3::P15924|DESP_HUMAN	23	331569	2	1	2	1	0.01	Desmoplakin OS=Hom
1834	1	db$2	2::C3K5C9|C3K5C9_PSEFS	23	27544	1	1	1	1	0.19	Putative regulatory prc
1835	1	db$2	2::C3K0E5|C3K0E5_PSEFS	23	24553	1	1	1	1	0.21	Two-component syster
1836	1	db$2	2::C3K6T5|C3K6T5_PSEFS	23	75466	1	1	1	1	0.06	Conserved GGDEF don
1837	1	SwissProt_HUMAN$	3::Q96FN5|KIF12_HUMAN	23	70615	3	1	1	1	0.07	Kinesin-like protein KII
1838	1	SwissProt_HUMAN$	3::P04920|B3A2_HUMAN	23	136923	1	1	1	1	0.04	Anion exchange protei
1839	1	db$2	2::C3K0X1|C3K0X1_PSEFS	23	10638	1	1	1	1	0.54	Uncharacterized protei
1840	1	db$2	2::C3JZ42|C3JZ42_PSEFS	23	19185	1	1	1	1	0.28	Putative AraC family tr
1841	1	SwissProt_HUMAN$	3::P23526|SAHH_HUMAN	23	47685	1	1	1	1	0.10	Adenosylhomocysteina
1842	1	SwissProt_HUMAN$	3::Q9Y399|RT02_HUMAN	23	33228	1	1	1	1	0.15	28S ribosomal protein
1843	1	SwissProt_HUMAN$	3::Q96D53|COQ8B_HUMAN	23	60031	1	1	1	1	0.08	Atypical kinase COQ8E
1844	1	db$2	2::C3JXP2|C3JXP2_PSEFS	23	43336	1	1	1	1	0.12	Putative glycosyltransf
1845	1	db$2	2::C3K3Q9|LGT_PSEFS	23	30444	1	1	1	1	0.17	Phosphatidylglycerol--
1846	1	SwissProt_HUMAN$	3::A6NM36|LRC30_HUMAN	23	34012	1	1	1	1	0.15	Leucine-rich repeat-co
1847	1	SwissProt_HUMAN$	3::P18627|LAG3_HUMAN	23	57413	1	1	1	1	0.09	Lymphocyte activation
1848	1	db$2	2::C3K2R0|C3K2R0_PSEFS	22	31322	1	1	1	1	0.16	Geranyltranstransferas
1849	1	SwissProt_HUMAN$	3::A8K8P3|SFI1_HUMAN	22	147571	1	1	1	1	0.03	Protein SFI1 homolog
1850	1	SwissProt_HUMAN$	3::P53814|SMTN_HUMAN	22	98998	2	2	2	2	0.10	Smoothelin OS=Homo
1851	1	SwissProt_HUMAN$	3::Q8NFX7|STXB6_HUMAN	22	23539	1	1	1	1	0.22	Syntaxin-binding prote
1852	1	db$2	2::C3JZ48|C3JZ48_PSEFS	22	11531	1	1	1	1	0.49	Uncharacterized protei
1853	1	db$2	2::C3JZ23|C3JZ23_PSEFS	22	131220	2	1	2	1	0.04	Chromosome partition
1854	1	SwissProt_HUMAN$	3::P18077|RL35A_HUMAN	22	12530	1	1	1	1	0.45	60S ribosomal protein
1855	1	db$2	2::C3K0N6|PLSX_PSEFS	22	35209	2	1	1	1	0.14	Phosphate acyltransfer
1856	1	SwissProt_HUMAN$	3::Q7Z5H4|VN1R5_HUMAN	22	40752	1	1	1	1	0.12	Vomeronasal type-1 re
1857	1	db$2	2::C3K713|C3K713_PSEFS	22	28108	1	1	1	1	0.18	Arginine/ornithine ABC
1858	1	db$2	2::C3K585|C3K585_PSEFS	22	25105	1	1	1	1	0.21	Putative hydrolase OS
1859	1	db$2	2::C3K3J6|C3K3J6_PSEFS	22	14927	1	1	1	1	0.37	Type IV pilus response

↑Family	M	DB	Accession	Score	Mass	Matches	Match(sig)	Sequences	Seq(sig)	emPAI	Description
1860	1	SwissProt_HUMAN$	3::Q9ULT8|HECD1_HUMAN	22	289203	1	1	1	1	0.02	E3 ubiquitin-protein lig
1861	1	db$2	2::C3K0Y2|C3K0Y2_PSEFS	22	46222	1	1	1	1	0.11	Putative fatty acid trar
1862	1	SwissProt_HUMAN$	3::Q5TC12|ATPF1_HUMAN	22	36414	1	1	1	1	0.14	ATP synthase mitochoı
1863	1	db$2	2::C3K323|C3K323_PSEFS	22	38795	2	2	2	2	0.28	Putative phosphotrans
1864	1	db$2	2::C3K5B6|C3K5B6_PSEFS	22	58846	1	1	1	1	0.08	Dipeptide ABC transpo
1865	1	db$2	2::C3K020|C3K020_PSEFS	22	23656	1	1	1	1	0.22	Putative hydrolase OS
1866	1	db$2	2::C3K4J1|C3K4J1_PSEFS	22	29005	1	1	1	1	0.18	Conserved EAL domair
1867	1	SwissProt_HUMAN$	3::P29590|PML_HUMAN	22	97489	1	1	1	1	0.05	Protein PML OS=Homc
1868	1	SwissProt_HUMAN$	3::Q8WV24|PHLA1_HUMAN	22	44988	2	1	2	1	0.11	Pleckstrin homology-lil
1869	1	db$2	2::C3JYI7|C3JYI7_PSEFS	22	41303	1	1	1	1	0.12	Putative hydrolase OS
1870	1	SwissProt_HUMAN$	3::Q8IW75|SPA12_HUMAN	22	47145	2	1	2	1	0.11	Serpin A12 OS=Homo
1871	1	db$2	2::C3JZ02|C3JZ02_PSEFS	22	231164	2	1	1	1	0.02	Putative non-ribosoma
1872	1	db$2	2::C3K2N6|C3K2N6_PSEFS	22	16796	1	1	1	1	0.32	MaoC-like domain-con
1873	1	db$2	2::C3K1H9|C3K1H9_PSEFS	22	18692	3	1	3	1	0.29	Conserved hypothetica
1874	1	SwissProt_HUMAN$	3::Q8WXH0|SYNE2_HUMAN	22	795944	1	1	1	1	0.01	Nesprin-2 OS=Homo s
1875	1	db$2	2::C3K2L8|LIPA_PSEFS	22	38163	4	2	3	2	0.28	Lipoyl synthase OS=Ps
1876	1	db$2	2::C3K166|C3K166_PSEFS	22	65025	1	1	1	1	0.08	Histidine kinase OS=P
1877	1	SwissProt_HUMAN$	3::Q15645|PCH2_HUMAN	22	48520	1	1	1	1	0.10	Pachytene checkpoint
1878	1	db$2	2::C3JZ81|C3JZ81_PSEFS	22	25240	1	1	1	1	0.21	Putative exported prot
1879	1	SwissProt_HUMAN$	3::A6NE01|F186A_HUMAN	22	262595	1	1	1	1	0.02	Protein FAM186A OS=
1880	1	SwissProt_HUMAN$	3::P11362|FGFR1_HUMAN	22	91809	1	1	1	1	0.05	Fibroblast growth facto
1881	1	db$2	2::C3KAT1|C3KAT1_PSEFS	22	27898	2	1	2	1	0.18	Response regulator pr
1882	1	SwissProt_HUMAN$	3::P82094|TMF1_HUMAN	22	122767	1	1	1	1	0.04	TATA element modulat
1883	1	db$2	2::C3K0D2|C3K0D2_PSEFS	22	35834	1	1	1	1	0.14	Lipid A biosynthesis la
1884	1	db$2	2::C3K6L2|C3K6L2_PSEFS	21	14328	1	1	1	1	0.38	Organic hydroperoxide
1885	1	db$2	2::C3KDC7|C3KDC7_PSEFS	21	29930	1	1	1	1	0.17	Release factor glutami
1886	1	db$2	2::C3JXQ7|C3JXQ7_PSEFS	21	48832	2	1	2	1	0.10	UDP-glucose 6-dehydr
1887	1	db$2	2::C3KAJ0|C3KAJ0_PSEFS	21	34086	1	1	1	1	0.15	Putative LysR-family re
1888	1	SwissProt_HUMAN$	3::O15197|EPHB6_HUMAN	21	110630	1	1	1	1	0.04	Ephrin type-B receptor
1889	1	db$2	2::C3K672|C3K672_PSEFS	21	58670	1	1	1	1	0.08	Cation/acetate sympor
1890	1	SwissProt_HUMAN$	3::Q9BWW9|APOL5_HUMAN	21	47014	1	1	1	1	0.11	Apolipoprotein L5 OS=
1891	1	SwissProt_HUMAN$	3::Q14103|HNRPD_HUMAN	21	38410	2	2	1	1	0.13	Heterogeneous nuclea
1892	1	SwissProt_HUMAN$	3::Q9NQI0|DDX4_HUMAN	21	79258	3	1	2	1	0.06	Probable ATP-depende
1893	1	SwissProt_HUMAN$	3::Q9NTJ3|SMC4_HUMAN	21	147091	1	1	1	1	0.03	Structural maintenanc
1894	1	db$2	2::C3K4K4|C3K4K4_PSEFS	21	39850	1	1	1	1	0.13	Putative branched ami
1895	1	SwissProt_HUMAN$	3::P30041|PRDX6_HUMAN	21	25019	2	1	2	1	0.21	Peroxiredoxin-6 OS=H
1896	1	db$2	2::C3K807|C3K807_PSEFS	21	30283	1	1	1	1	0.17	Putative histidine ABC
1897	1	db$2	2::C3K1U3|C3K1U3_PSEFS	21	32198	1	1	1	1	0.16	Protoheme IX farnesyl
1898	1	SwissProt_HUMAN$	3::P52333|JAK3_HUMAN	21	125019	1	1	1	1	0.04	Tyrosine-protein kinas
1899	1	db$2	2::C3K4M3|C3K4M3_PSEFS	21	101390	3	2	3	2	0.10	Phosphocarrier protein

↑Family	M	DB	Accession	Score	Mass	Matches	Match(sig)	Sequences	Seq(sig)	emPAI	Description
1900	1	SwissProt_HUMAN$	3::Q5JNZ5|RS26L_HUMAN	21	12994	1	1	1	1	0.43	Putative 40S ribosoma
1901	1	db$2	2::C3K5X5|C3K5X5_PSEFS	21	22739	1	1	1	1	0.23	Thioesterase domain-c
1902	1	db$2	2::C3K3T1|C3K3T1_PSEFS	21	86048	2	1	2	1	0.06	Putative penicillin amic
1903	1	SwissProt_HUMAN$	3::P62280|RS11_HUMAN	21	18419	1	1	1	1	0.29	40S ribosomal protein
1904	1	db$2	2::C3JY56|C3JY56_PSEFS	21	48640	1	1	1	1	0.10	Replication-associated
1905	1	SwissProt_HUMAN$	3::O95819|M4K4_HUMAN	21	142013	1	1	1	1	0.03	Mitogen-activated prot
1906	1	db$2	2::C3K883|C3K883_PSEFS	21	60772	1	1	1	1	0.08	Putative transposase s
1907	1	SwissProt_HUMAN$	3::Q9BYE7|PCGF6_HUMAN	21	39023	1	1	1	1	0.13	Polycomb group RING
1908	1	SwissProt_HUMAN$	3::P63173|RL38_HUMAN	20	8213	2	1	2	1	0.75	60S ribosomal protein
1909	1	db$2	2::C3K5Q7|C3K5Q7_PSEFS	20	27879	1	1	1	1	0.18	Putative dehydrogenas
1910	1	SwissProt_HUMAN$	3::A0A2R8YFL7|OSP4A_HUMAN	20	21026	1	1	1	1	0.25	Oocyte-secreted prote
1911	1	db$2	2::C3K9S5|C3K9S5_PSEFS	20	38309	2	2	2	2	0.28	Putative cobalamin bio
1912	1	db$2	2::C3KAE4|C3KAE4_PSEFS	20	39865	1	1	1	1	0.13	Uncharacterized protei
1913	1	db$2	2::C3K8G1|C3K8G1_PSEFS	20	23138	1	1	1	1	0.23	Putative DNA-binding
1914	1	db$2	2::C3K609|C3K609_PSEFS	20	73644	2	1	2	1	0.07	Putative lytic transglyc
1915	1	SwissProt_HUMAN$	3::O94966|UBP19_HUMAN	20	145559	1	1	1	1	0.03	Ubiquitin carboxyl-terr
1916	1	db$2	2::C3K326|C3K326_PSEFS	20	35169	1	1	1	1	0.14	4-hydroxythreonine-4-
1917	1	db$2	2::C3K9S7|C3K9S7_PSEFS	20	35611	1	1	1	1	0.14	Putative magnesium cl
1918	1	SwissProt_HUMAN$	3::O00294|TULP1_HUMAN	20	60572	1	1	1	1	0.08	Tubby-related protein
1919	1	db$2	2::C3K1S4|C3K1S4_PSEFS	20	23334	1	1	1	1	0.22	Putative membrane pr
1920	1	db$2	2::C3K2R8|NUSB_PSEFS	20	18764	1	1	1	1	0.28	Transcription antitermi
1921	1	db$2	2::C3KB11|C3KB11_PSEFS	20	38044	1	1	1	1	0.13	Putative AraC family re
1922	1	SwissProt_HUMAN$	3::Q5JXB2|UE2NL_HUMAN	20	17366	1	1	1	1	0.31	Putative ubiquitin-conj
1923	1	SwissProt_HUMAN$	3::Q8WXW3|PIBF1_HUMAN	20	89750	1	1	1	1	0.05	Progesterone-induced-
1924	1	SwissProt_HUMAN$	3::Q96RL7|VP13A_HUMAN	20	360047	1	1	1	1	0.01	Vacuolar protein sortin
1925	1	db$2	2::C3K7G5|C3K7G5_PSEFS	20	138643	1	1	1	1	0.03	Putative conserved ex
1926	1	db$2	2::C3KCT5|C3KCT5_PSEFS	20	27647	1	1	1	1	0.19	Putative regulatory pro
1927	1	db$2	2::C3K7T7|C3K7T7_PSEFS	20	17319	1	1	1	1	0.31	Putative acetyltransfer
1928	1	SwissProt_HUMAN$	3::P08514|ITA2B_HUMAN	20	113306	1	1	1	1	0.04	Integrin alpha-IIb OS=
1929	1	db$2	2::C3K0M6|C3K0M6_PSEFS	20	25497	4	2	4	2	0.45	Putative tetR-family re
1930	1	SwissProt_HUMAN$	3::Q6ZSB9|ZBT49_HUMAN	20	85007	1	1	1	1	0.06	Zinc finger and BTB dc
1931	1	SwissProt_HUMAN$	3::P42766|RL35_HUMAN	20	14543	2	1	2	1	0.38	60S ribosomal protein
1932	1	db$2	2::C3K8Y1|C3K8Y1_PSEFS	20	70024	1	1	1	1	0.07	Conserved GGDEF dom
1933	1	db$2	2::C3KBT8|C3KBT8_PSEFS	20	29541	1	1	1	1	0.17	Sulfate transport syste
1934	1	SwissProt_HUMAN$	3::Q9Y2G4|ANKR6_HUMAN	20	79922	1	1	1	1	0.06	Ankyrin repeat domain
1935	1	SwissProt_HUMAN$	3::B7Z8K6|TRDC_HUMAN	20	17074	1	1	1	1	0.31	T cell receptor delta cc
1936	1	SwissProt_HUMAN$	3::Q6ZW33|MICLK_HUMAN	20	77230	1	1	1	1	0.06	MICAL C-terminal-like
1937	1	SwissProt_HUMAN$	3::P42224|STAT1_HUMAN	19	87280	1	1	1	1	0.06	Signal transducer and
1938	1	db$2	2::C3KDE3|C3KDE3_PSEFS	19	55008	1	1	1	1	0.09	Putaive GGDEF domain
1939	1	db$2	2::C3K000|C3K000_PSEFS	19	26225	1	1	1	1	0.20	Putative GntR-family tı

↑Family	M	DB	Accession	Score	Mass	Matches	Match(sig)	Sequences	Seq(sig)	emPAI	Description
1940	1	SwissProt_HUMAN$	3::P49454|CENPF_HUMAN	19	357306	2	1	2	1	0.01	Centromere protein F (
1941	1	db$2	2::C3JXL1|C3JXL1_PSEFS	19	51972	1	1	1	1	0.10	Putative transmembra
1942	1	db$2	2::C3KCG3|C3KCG3_PSEFS	19	16758	1	1	1	1	0.32	YjgF_endoribonc doma
1943	1	SwissProt_HUMAN$	3::O14640|DVL1_HUMAN	19	75140	1	1	1	1	0.07	Segment polarity prote
1944	1	db$2	2::C3JZA7|C3JZA7_PSEFS	19	51947	1	1	1	1	0.10	Putative succinate-sem
1945	1	SwissProt_HUMAN$	3::Q5TH69|BIG3_HUMAN	19	240498	1	1	1	1	0.02	Brefeldin A-inhibited g
1946	1	SwissProt_HUMAN$	3::P15169|CBPN_HUMAN	19	52253	1	1	1	1	0.09	Carboxypeptidase N ca
1947	1	SwissProt_HUMAN$	3::Q9NR48|ASH1L_HUMAN	19	332582	1	1	1	1	0.01	Histone-lysine N-meth
1948	1	SwissProt_HUMAN$	3::Q03001|DYST_HUMAN	19	860127	3	1	3	1	0.01	Dystonin OS=Homo sa
1949	1	db$2	2::C3K4L1|C3K4L1_PSEFS	19	41720	1	1	1	1	0.12	Putative epimerase OS
1950	1	db$2	2::C3K8Q6|C3K8Q6_PSEFS	19	41029	1	1	1	1	0.12	Uncharacterized protei
1951	1	SwissProt_HUMAN$	3::O95932|TGM3L_HUMAN	19	79263	1	1	1	1	0.06	Protein-glutamine gam
1952	1	db$2	2::C3K1T8|C3K1T8_PSEFS	19	11622	1	1	1	1	0.49	Antitoxin OS=Pseudon
1953	1	db$2	2::C3JY40|C3JY40_PSEFS	19	21505	1	1	1	1	0.24	Putative Trp repressor
1954	1	SwissProt_HUMAN$	3::Q9BWT7|CAR10_HUMAN	19	115859	1	1	1	1	0.04	Caspase recruitment d
1955	1	db$2	2::C3K055|C3K055_PSEFS	19	30642	1	1	1	1	0.17	Uncharacterized protei
1956	1	SwissProt_HUMAN$	3::Q13459|MYO9B_HUMAN	19	243249	1	1	1	1	0.02	Unconventional myosir
1957	1	db$	1::P0DTD1|R1AB_SARS2	19	793544	2	1	2	1	0.01	Replicase polyprotein :
1958	1	db$2	2::A4V7I2|A4V7I2_PSEFS	19	194095	1	1	1	1	0.02	Uncharacterized protei
1959	1	SwissProt_HUMAN$	3::O15397|IPO8_HUMAN	18	119861	1	1	1	1	0.04	Importin-8 OS=Homo
1960	1	SwissProt_HUMAN$	3::P62266|RS23_HUMAN	18	15798	1	1	1	1	0.34	40S ribosomal protein
1961	1	SwissProt_HUMAN$	3::Q9BYV7|BCDO2_HUMAN	18	65632	1	1	1	1	0.07	Beta,beta-carotene 9',
1962	1	db$2	2::C3KEB8|C3KEB8_PSEFS	18	34155	2	1	2	1	0.15	Putative lipoprotein OS
1963	1	db$2	2::C3JYC7|C3JYC7_PSEFS	18	12317	1	1	1	1	0.46	Putative pH adaptatior
1964	1	db$2	2::C3JYR6|C3JYR6_PSEFS	18	38174	5	1	4	1	0.13	Quinolinate synthase A
1965	1	db$2	2::C3KB61|C3KB61_PSEFS	18	26669	2	1	2	1	0.19	Molybdate-binding per
1966	1	db$2	2::C3K2E4|C3K2E4_PSEFS	18	29249	1	1	1	1	0.17	Ferredoxin--NADP redu
1967	1	SwissProt_HUMAN$	3::Q92802|N42L2_HUMAN	18	67417	1	1	1	1	0.07	NEDD4-binding proteir
1968	1	SwissProt_HUMAN$	3::P25398|RS12_HUMAN	18	14505	1	1	1	1	0.38	40S ribosomal protein
1969	1	db$2	2::C3K8V0|C3K8V0_PSEFS	18	31271	1	1	1	1	0.16	Poly(3-hydroxyalkanoa
1970	1	db$2	2::C3KCA2|C3KCA2_PSEFS	18	48472	1	1	1	1	0.10	Putative family S43 no
1971	1	SwissProt_HUMAN$	3::Q8N8R5|CB069_HUMAN	18	43421	1	1	1	1	0.12	UPF0565 protein C2orf
1972	1	SwissProt_HUMAN$	3::P30050|RL12_HUMAN	18	17808	1	1	1	1	0.30	60S ribosomal protein
1973	1	db$2	2::C3K2I7|C3K2I7_PSEFS	18	17463	1	1	1	1	0.31	NYN domain-containin
1974	1	SwissProt_HUMAN$	3::Q5VU65|P210L_HUMAN	18	210471	1	1	1	1	0.02	Nuclear pore membrar
1975	1	SwissProt_HUMAN$	3::P31943|HNRH1_HUMAN	18	49198	1	1	1	1	0.10	Heterogeneous nuclea
1976	1	SwissProt_HUMAN$	3::P58166|INHBE_HUMAN	18	38537	1	1	1	1	0.13	Inhibin beta E chain O:
1977	1	db$2	2::C3JY93|C3JY93_PSEFS	18	51981	1	1	1	1	0.10	NADH-quinone oxidore
1978	1	SwissProt_HUMAN$	3::Q5F1R6|DJC21_HUMAN	18	61989	1	1	1	1	0.08	DnaJ homolog subfami
1979	1	SwissProt_HUMAN$	3::Q12899|TRI26_HUMAN	18	62127	1	1	1	1	0.08	Tripartite motif-contair

↑Family	M	DB	Accession	Score	Mass	Matches	Match(sig)	Sequences	Seq(sig)	emPAI	Description
1980	1	db$2	2::C3K2K1|C3K2K1_PSEFS	18	28157	2	1	2	1	0.18	Phos_pyr_kin domain-
1981	1	db$2	2::C3KAZ8|C3KAZ8_PSEFS	18	34711	1	1	1	1	0.15	Putative beta-hydroxyl
1982	1	SwissProt_HUMAN$	3::O15050|TRNK1_HUMAN	18	336007	2	1	2	1	0.01	TPR and ankyrin repea
1983	1	db$2	2::C3JY42|C3JY42_PSEFS	18	25186	1	1	1	1	0.21	Uncharacterized protei
1984	1	SwissProt_HUMAN$	3::A0A075B6P5|KV228_HUMAN	18	12948	1	1	1	1	0.43	Immunoglobulin kappa
1985	1	db$2	2::C3KAA6|C3KAA6_PSEFS	18	51931	1	1	1	1	0.10	Putative cell envelope
1986	1	db$2	2::C3K863|PDXH_PSEFS	18	24884	1	1	1	1	0.21	Pyridoxine/pyridoxami
1987	1	SwissProt_HUMAN$	3::O95297|MPZL1_HUMAN	18	29064	2	2	2	2	0.38	Myelin protein zero-lik
1988	1	SwissProt_HUMAN$	3::Q9NYQ6|CELR1_HUMAN	18	329278	2	1	2	1	0.01	Cadherin EGF LAG sev
1989	1	SwissProt_HUMAN$	3::P59910|DJB13_HUMAN	18	36096	1	1	1	1	0.14	DnaJ homolog subfami
1990	1	SwissProt_HUMAN$	3::Q14204|DYHC1_HUMAN	17	532072	2	1	2	1	0.01	Cytoplasmic dynein 1 l
1991	1	db$2	2::C3JXS7|C3JXS7_PSEFS	17	34119	1	1	1	1	0.15	Putative GGDEF domai
1992	1	SwissProt_HUMAN$	3::Q8TEU7|RPGF6_HUMAN	17	179310	1	1	1	1	0.03	Rap guanine nucleotid
1993	1	SwissProt_HUMAN$	3::Q9C037|TRIM4_HUMAN	17	57424	1	1	1	1	0.09	E3 ubiquitin-protein lig
1994	1	db$2	2::C3K5L9|C3K5L9_PSEFS	17	31441	1	1	1	1	0.16	Alpha-D-ribose 1-meth
1995	1	db$2	2::C3KCP7|C3KCP7_PSEFS	17	39236	1	1	1	1	0.13	Lipopolysaccharide hep
1996	1	SwissProt_HUMAN$	3::Q13835|PKP1_HUMAN	17	82808	2	2	2	2	0.12	Plakophilin-1 OS=Hom
1997	1	db$2	2::C3KAB6|C3KAB6_PSEFS	17	39151	1	1	1	1	0.13	Conserved hypothetica
1998	1	db$2	2::C3KAD9|C3KAD9_PSEFS	17	13242	1	1	1	1	0.42	HTH hxlR-type domain
1999	1	SwissProt_HUMAN$	3::Q58FF3|ENPLL_HUMAN	17	45830	1	1	1	1	0.11	Putative endoplasmin-
2000	1	SwissProt_HUMAN$	3::P39019|RS19_HUMAN	17	16051	2	1	2	1	0.34	40S ribosomal protein
2001	1	SwissProt_HUMAN$	3::Q86VQ0|LCA5_HUMAN	17	80505	1	1	1	1	0.06	Lebercilin OS=Homo s
2002	1	db$2	2::C3K2Z6|ERPA_PSEFS	17	12493	1	1	1	1	0.45	Iron-sulfur cluster inse
2003	1	db$2	2::C3K4A3|C3K4A3_PSEFS	17	48421	1	1	1	1	0.10	Uncharacterized protei
2004	1	db$2	2::C3K0P1|C3K0P1_PSEFS	17	24214	1	1	1	1	0.21	Putative hydrolase OS
2005	1	SwissProt_HUMAN$	3::Q8NBF6|AVL9_HUMAN	17	71902	1	1	1	1	0.07	Late secretory pathwa
2006	1	SwissProt_HUMAN$	3::Q9C098|DCLK3_HUMAN	17	73768	1	1	1	1	0.07	Serine/threonine-prote
2007	1	SwissProt_HUMAN$	3::Q9ULQ1|TPC1_HUMAN	17	94086	1	1	1	1	0.05	Two pore calcium char
2008	1	db$2	2::C3K4X6|C3K4X6_PSEFS	16	73857	1	1	1	1	0.07	Putative polysaccharid
2009	1	SwissProt_HUMAN$	3::P62906|RL10A_HUMAN	16	24816	1	1	1	1	0.21	60S ribosomal protein
2010	1	db$2	2::C3K173|C3K173_PSEFS	16	34695	2	2	2	2	0.31	Putative AraC-family tı
2011	1	SwissProt_HUMAN$	3::Q7L592|NDUF7_HUMAN	16	49206	1	1	1	1	0.10	Protein arginine methy
2012	1	SwissProt_HUMAN$	3::Q9BSF4|TIM29_HUMAN	16	29215	1	1	1	1	0.18	Mitochondrial import ir
2013	1	db$2	2::C3JZ35|C3JZ35_PSEFS	16	31443	1	1	1	1	0.16	Putative LysR-family tı
2014	1	db$2	2::C3K590|C3K590_PSEFS	16	74072	1	1	1	1	0.07	Putative membrane pr
2015	1	SwissProt_HUMAN$	3::Q8IWU5|SULF2_HUMAN	16	100390	1	1	1	1	0.05	Extracellular sulfatase
2016	1	db$2	2::C3K0U7|C3K0U7_PSEFS	16	49884	1	1	1	1	0.10	Two-component respo
2017	1	db$2	2::C3JY01|C3JY01_PSEFS	16	74110	1	1	1	1	0.07	AAA_15 domain-conta
2018	1	db$2	2::C3K3M0|MTGA_PSEFS	16	27247	1	1	1	1	0.19	Biosynthetic peptidogl
2019	1	db$2	2::C3K0J3|C3K0J3_PSEFS	16	45387	2	1	2	1	0.11	Putative flagellar hook

↑Family	M	DB	Accession	Score	Mass	Matches	Match(sig)	Sequences	Seq(sig)	emPAI	Description
2020	1	SwissProt_HUMAN$	3::Q9P202|WHRN_HUMAN	16	96499	1	1	1	1	0.05	Whirlin OS=Homo sapi
2021	1	SwissProt_HUMAN$	3::Q9Y4D8|HECD4_HUMAN	16	439065	2	1	2	1	0.01	Probable E3 ubiquitin-|
2022	1	SwissProt_HUMAN$	3::Q9BT73|PSMG3_HUMAN	16	13096	1	1	1	1	0.43	Proteasome assembly
2023	1	db$2	2::C3KE69|C3KE69_PSEFS	16	32125	2	1	2	1	0.16	Putative membrane pr
2024	1	SwissProt_HUMAN$	3::Q9NUQ8|ABCF3_HUMAN	16	79695	1	1	1	1	0.06	ATP-binding cassette s
2025	1	db$2	2::C3K577|C3K577_PSEFS	16	15787	1	1	1	1	0.34	Putative lipoprotein OS
2026	1	db$2	2::C3KA78|C3KA78_PSEFS	16	37650	1	1	1	1	0.13	Putative phage cointeg
2027	1	SwissProt_HUMAN$	3::Q8TEQ6|GEMI5_HUMAN	16	168483	1	1	1	1	0.03	Gem-associated protei
2028	1	db$2	2::C3JY51|C3JY51_PSEFS	16	37030	2	2	1	1	0.14	Putative glycosyl trans
2029	1	SwissProt_HUMAN$	3::Q8TAE8|G45IP_HUMAN	16	25368	1	1	1	1	0.20	Growth arrest and DN/
2030	1	SwissProt_HUMAN$	3::P46020|KPB1_HUMAN	16	137225	1	1	1	1	0.04	Phosphorylase b kinas
2031	1	db$2	2::C3K3N6|FPG_PSEFS	16	30025	3	1	3	1	0.17	Formamidopyrimidine-
2032	1	db$2	2::C3KDV1|C3KDV1_PSEFS	16	30184	1	1	1	1	0.17	Chemotaxis MotA prot
2033	1	SwissProt_HUMAN$	3::Q149M9|NWD1_HUMAN	16	174441	1	1	1	1	0.03	NACHT domain- and W
2034	1	SwissProt_HUMAN$	3::P61353|RL27_HUMAN	16	15788	2	1	2	1	0.34	60S ribosomal protein
2035	1	db$2	2::C3KDU4|RECF_PSEFS	15	41521	1	1	1	1	0.12	DNA replication and re
2036	1	db$2	2::C3K216|C3K216_PSEFS	15	17384	1	1	1	1	0.31	Putative putative lipop
2037	1	SwissProt_HUMAN$	3::Q9P215|POGK_HUMAN	15	69399	1	1	1	1	0.07	Pogo transposable eler
2038	1	db$2	2::C3K2L1|C3K2L1_PSEFS	15	28054	1	1	1	1	0.18	DUF218 domain-conta
2039	1	SwissProt_HUMAN$	3::Q7Z6J6|FRMD5_HUMAN	15	65023	1	1	1	1	0.08	FERM domain-containi
2040	1	SwissProt_HUMAN$	3::Q9UPN4|CP131_HUMAN	15	122075	2	1	2	1	0.04	Centrosomal protein of
2041	1	SwissProt_HUMAN$	3::P13693|TCTP_HUMAN	15	19583	1	1	1	1	0.27	Translationally-control
2042	1	SwissProt_HUMAN$	3::Q6NXR0|IIGP5_HUMAN	15	50257	1	1	1	1	0.10	Interferon-inducible G
2043	1	db$2	2::C3KB13|C3KB13_PSEFS	15	35564	1	1	1	1	0.14	Putative ThiF protein C
2044	1	SwissProt_HUMAN$	3::Q5VSL9|STRP1_HUMAN	15	95515	1	1	1	1	0.05	Striatin-interacting pro
2045	1	SwissProt_HUMAN$	3::A6H8Y1|BDP1_HUMAN	15	293705	2	1	2	1	0.02	Transcription factor TF
2046	1	SwissProt_HUMAN$	3::P60866|RS20_HUMAN	15	13364	1	1	1	1	0.42	40S ribosomal protein
2047	1	SwissProt_HUMAN$	3::P50454|SERPH_HUMAN	15	46411	1	1	1	1	0.11	Serpin H1 OS=Homo s
2048	1	SwissProt_HUMAN$	3::Q9NPE6|SPAG4_HUMAN	15	48135	1	1	1	1	0.10	Sperm-associated anti
2049	1	db$2	2::C3JY96|C3JY96_PSEFS	15	27408	1	1	1	1	0.19	OMP_b-brl domain-cor
2050	1	SwissProt_HUMAN$	3::Q8WXI2|CNKR2_HUMAN	15	117461	1	1	1	1	0.04	Connector enhancer of
2051	1	SwissProt_HUMAN$	3::Q8TF21|ANR24_HUMAN	15	124111	2	1	2	1	0.04	Ankyrin repeat domain
2052	1	SwissProt_HUMAN$	3::O94827|PKHG5_HUMAN	15	117377	1	1	1	1	0.04	Pleckstrin homology do
2053	1	SwissProt_HUMAN$	3::A0A1B0GV85|RELD1_HUMAN	14	56801	1	1	1	1	0.09	Reelin domain-contain
2054	1	SwissProt_HUMAN$	3::Q5T9G4|ARM12_HUMAN	14	38607	1	1	1	1	0.13	Armadillo repeat-conta
2055	1	SwissProt_HUMAN$	3::O75717|WDHD1_HUMAN	14	125888	1	1	1	1	0.04	WD repeat and HMG-b
2056	1	db$2	2::C3K913|C3K913_PSEFS	14	30998	1	1	1	1	0.16	Uncharacterized protei
2057	1	db$2	2::C3KAN4|C3KAN4_PSEFS	14	41646	1	1	1	1	0.12	3-oxoadipyl-CoA thiola
2058	1	SwissProt_HUMAN$	3::Q15768|EFNB3_HUMAN	14	35812	1	1	1	1	0.14	Ephrin-B3 OS=Homo s
2059	1	db$2	2::C3K7M8|C3K7M8_PSEFS	14	23309	2	1	2	1	0.22	Putative glutathione S-

↑Family	M	DB	Accession	Score	Mass	Matches	Match(sig)	Sequences	Seq(sig)	emPAI	Description
2060	1	SwissProt_HUMAN$	3::O00339|MATN2_HUMAN	14	106767	1	1	1	1	0.05	Matrilin-2 OS=Homo s
2061	1	SwissProt_HUMAN$	3::P02746|C1QB_HUMAN	14	26704	2	1	2	1	0.19	Complement C1q subc
2062	1	SwissProt_HUMAN$	3::Q16512|PKN1_HUMAN	14	103868	2	1	2	1	0.05	Serine/threonine-prote
2063	1	db$2	2::C3K7S0|C3K7S0_PSEFS	14	99580	1	1	1	1	0.05	Magnesium-transportir
2064	1	db$2	2::C3JZD7|C3JZD7_PSEFS	14	86003	1	1	1	1	0.06	Putative outer membra
2065	1	db$2	2::C3K439|C3K439_PSEFS	13	25827	1	1	1	1	0.20	Putative hydrolase OS
2066	1	SwissProt_HUMAN$	3::Q8IVF4|DYH10_HUMAN	13	514512	1	1	1	1	0.01	Dynein heavy chain 10
2067	1	SwissProt_HUMAN$	3::Q12870|TCF15_HUMAN	13	20804	1	1	1	1	0.25	Transcription factor 15
2068	1	db$2	2::C3JXF2|C3JXF2_PSEFS	13	13982	1	1	1	1	0.40	Uncharacterized protei
2069	1	SwissProt_HUMAN$	3::Q9UBS9|SUCO_HUMAN	13	139343	1	1	1	1	0.03	SUN domain-containin
2070	1	db$2	2::C3K0F1|C3K0F1_PSEFS	13	476575	2	1	2	1	0.01	Pyoverdine synthetase
2071	1	SwissProt_HUMAN$	3::Q13219|PAPP1_HUMAN	13	180856	1	1	1	1	0.03	Pappalysin-1 OS=Hom
2072	1	SwissProt_HUMAN$	3::Q14980|NUMA1_HUMAN	13	238115	3	1	3	1	0.02	Nuclear mitotic appara
2073	1	SwissProt_HUMAN$	3::O43897|TLL1_HUMAN	13	114635	1	1	1	1	0.04	Tolloid-like protein 1 C
2074	1	db$2	2::C3K2J7|C3K2J7_PSEFS	13	23861	1	1	1	1	0.22	Pseudouridine synthas
2075	1	db$2	2::C3K0Y9|C3K0Y9_PSEFS	13	19041	1	1	1	1	0.28	Bifunctional adenosylc
2076	1	SwissProt_HUMAN$	3::O60487|MPZL2_HUMAN	13	24469	1	1	1	1	0.21	Myelin protein zero-lik

Export as CSV

Not what you expected? Try **the select summary**.

Mascot: http://www.matrixscience.com/
