# Supplementary material for: Circulating multimeric immune complexes contribute to immunopathology in COVID-19
Source: Nat Commun. 2022 Sep 26;13:5654. doi: 10.1038/s41467-022-32867-z (PMC9513013; doi:10.1038/s41467-022-32867-z)
Supplement: Supplementary file 3 — Source Data [file 41467_2022_32867_MOESM3_ESM.zip › Source Data/MS_20210407 (immune precipitation).zip!/QX20210618-10_Virologie(AG-Hengel)-20210407_(V10)_0k3v20_TRL_db_SC2+db_Pf+SP_H(F235831).pdf]

MASCOT Search Results

User : Wolfgang Bildl
E-mail : wolfgang.bildl@physiologie.uni-freiburg.de
Search title : QX 20210618 (db$ SARS-CoV-2+db$2 Pf+SP H$ 5 ppm 20 mmu), QX20210618-10_Virologie(AG-Hengel)-20210407_(V10)_0k3v20_TRL_shifted_-1.3.mgf
MS data file : D:\Xcalibur\Data\Wolfgang\Q-Exactive-HF-X_20210616\QX20210618-10_Virologie(AG-Hengel)-20210407_(V10)_0k3v20_TRL_shifted_-1.3.mgf
Databases : **1:** db$ 20210617 (17 sequences; 14,439 residues)
2: db$2 20210617 (6,388 sequences; 2,089,672 residues)
3: SwissProt_HUMAN$ 20201007 (20,389 sequences; 11,363,789 residues)
Timestamp : 21 Jun 2021 at 13:40:36 GMT

Not what you expected? Try **the select summary**.

Search parameters

Type of search : MS/MS Ion Search
Enzyme : Trypsin
Variable modifications : **Acetyl (Protein N-term)**, **Carbamidomethyl (C)**, **Formyl (Protein N-term)**, **Gln->pyro-Glu (N-term Q)**, **Oxidation (M)**
Mass values : Monoisotopic
Protein mass : Unrestricted
Peptide mass tolerance : ± 5 ppm
Fragment mass tolerance : ± 20 mmu
Max missed cleavages : 1
Instrument type : ESI-FTICR
Number of queries : 83,518

Score distribution

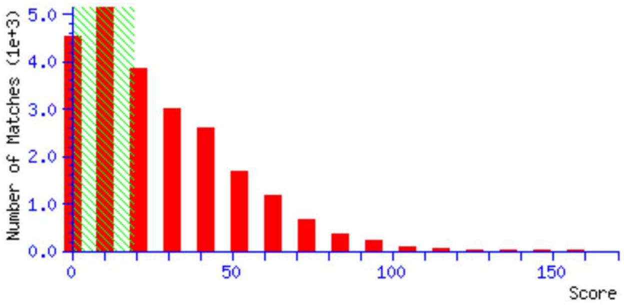

Peptide score distribution. Ions score is $-10\log(P)$, where P is the probability that the observed match is a random event.
There are **12,425** peptide matches above identity threshold and **13,290** matches above homology threshold for **83,518** queries.
On average, individual ions scores **> 19** (beyond green shading) indicate **identity or extensive homology** (p<0.05).

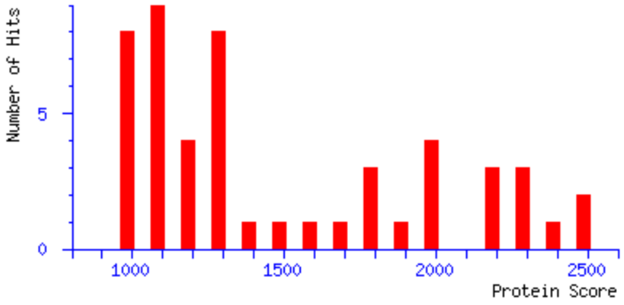

[Deprecated] Protein score distribution. Score distribution for family members in the first 50 proteins. Protein scores are derived from ions scores as a non-probabilistic basis for ranking protein families.

▼Modification statistics

**Modification**	**Site**	**Above thr.**
Oxidation	M	2477
Carbamidomethyl	C	1301
Gln->pyro-Glu	N-term	248
Formyl	Protein N-term	43
Acetyl	Protein N-term	13

▼Legend

Peptide columns and rows

Dupes	...	Expect	Rank	U	1	2	Peptide	
		**0.037**	▶**2**				**GAYSLSLR**	significant
		9	▶1				GFFLFVEGGR	top ranking
		**6.4e-005**	▶**1**				**GSSIFGLAPGK**	significant and top ranking
		**1.3e-006**	▶**1**		■		**SSGTSYPDVLK**	peptide is found in all proteins in family member 1
		**6.2e-007**	▶**1**			■	**VCNYVSWIK**	peptide is found in some but not all proteins in family member 2
		**6.4e-005**	▶**1**	**U**			**GSSIFGLAPGK**	unique
▶**2**		**5.7e-005**	▶**1**				**LNTLETEEWFFK**	peptide has two duplicates
		0.18	▶*1*				*LNTLETEEWFFK*	duplicate peptide

Right-facing triangle (▶) in the Dupes or Rank column indicates content that can be expanded by clicking on it. Down-facing triangle (▼) indicates the content is expanded and can be collapsed. For more details about particular columns, see **results format help**.

Protein quantitation ratios

	**Score**	...	**114/113**	**115/113**
**CFAH_HUMAN**	37559		*0.962*	**1.129**
**FHR2_HUMAN**	1330		**0.859**	1.128

When quantitation method is Reporter (e.g. iTRAQ) or Multiplex (e.g. IPTL), protein ratios are displayed when a family is expanded. Ratios in *italic* indicate that the peptide log-ratios do not appear to come from a normal distribution. **Bold** indicates that if you can assume peptide ratios are normally distributed, the protein ratio is significantly different from 1.0 (at significance level 0.05).

Note that lack of bold or italic can also mean that significance or normality testing has not been performed (for

example, if protein ratio type does not support it).

Protein Family Summary

Significance threshold p<	0.05	Max. number of families	AUTO
Ions score or expect cut-off	0.5	Dendrograms cut at	0
Show Percolator scores	☐		
Preferred taxonomy	All entries		

▼Sensitivity and FDR (reversed protein sequences)

			Target	Decoy	FDR		
PSMs	above	homology	13290	473	3.56%	Adjust to	1%

Decoy results are available in **the decoy report**.

Protein hits (2184 proteins)

▶Filters: (none)

Export as CSV

↑Family	M	DB	Accession	Score	Mass	Matches	Match(sig)	Sequences	Seq(sig)	emPAI	Description
1	1	db$2	2::C3K2X8|EFTU_PSEFS	2488	43536	79	74	23	23	28.08	Elongation factor Tu O
1	2	SwissProt_HUMAN$	3::Q8IWZ3|ANKH1_HUMAN	52	269291	2	2	2	2	0.04	Ankyrin repeat and KH
2	1	db$2	2::C3K2Y3|RPOB_PSEFS	2463	150923	103	97	72	67	9.61	DNA-directed RNA pol
3	1	db$2	2::C3K1E6|ATPB_PSEFS	2428	49459	71	70	31	31	36.86	ATP synthase subunit
4	1	db$2	2::C3K2Y2|RPOC_PSEFS	2272	154528	100	97	73	70	10.37	DNA-directed RNA pol
5	1	db$2	2::C3K5G3|C3K5G3_PSEFS	2262	99192	80	74	40	38	8.95	Aconitate hydratase O
6	1	db$2	2::C3K4N0|C3K4N0_PSEFS	2240	43479	65	63	28	28	68.39	Putative ornithine dec
7	1	db$2	2::C3K4D5|C3K4D5_PSEFS	2182	34599	71	64	21	20	29.22	Phosphate-binding pro
8	1	SwissProt_HUMAN$	3::P13645|K1C10_HUMAN	2178	58792	51	49	30	28	13.27	Keratin, type I cytoske
8	2	SwissProt_HUMAN$	3::P02533|K1C14_HUMAN	584	51529	21	20	19	18	4.23	Keratin, type I cytoske
8	3	SwissProt_HUMAN$	3::P08779|K1C16_HUMAN	338	51236	13	12	12	11	1.76	Keratin, type I cytoske
8	4	SwissProt_HUMAN$	3::P13646|K1C13_HUMAN	166	49557	9	9	7	7	1.36	Keratin, type I cytoske
8	5	SwissProt_HUMAN$	3::P08727|K1C19_HUMAN	94	44079	7	6	7	6	0.90	Keratin, type I cytoske
9	1	db$2	2::C3K5Z6|EFTS_PSEFS	2162	30341	70	66	17	16	11.06	Elongation factor Ts O
10	1	db$2	2::C3KD82|C3KD82_PSEFS	2013	181591	67	67	51	51	3.45	Glutamate dehydroger
11	1	db$2	2::C3K2X9|EFG_PSEFS	2003	77114	77	72	32	30	12.24	Elongation factor G OS
12	1	db$2	2::C3K1A0|CH60_PSEFS	1952	56931	63	59	32	29	34.80	60 kDa chaperonin OS
13	1	db$2	2::C3K0Q5|C3K0Q5_PSEFS	1949	107302	64	60	42	39	8.14	Ribonucleoside-diphos
14	1	db$2	2::C3K1E8|ATPA_PSEFS	1866	55245	60	58	27	27	22.83	ATP synthase subunit
15	1	db$2	2::C3K255|PNP_PSEFS	1769	74889	59	57	32	32	13.29	Polyribonucleotide nuc
16	1	SwissProt_HUMAN$	3::P04264|K2C1_HUMAN	1761	65999	55	52	35	32	14.31	Keratin, type II cytosk

↑Family	M	DB	Accession	Score	Mass	Matches	Match(sig)	Sequences	Seq(sig)	emPAI	Description
16	2	SwissProt_HUMAN$	3::P35908|K22E_HUMAN	1148	65393	45	41	35	33	12.61	Keratin, type II cytosk
16	3	SwissProt_HUMAN$	3::P13647|K2C5_HUMAN	406	62340	18	18	16	16	2.64	Keratin, type II cytosk
16	4	SwissProt_HUMAN$	3::P04259|K2C6B_HUMAN	380	60030	17	16	14	14	2.27	Keratin, type II cytosk
16	5	SwissProt_HUMAN$	3::P05787|K2C8_HUMAN	234	53671	7	7	6	6	0.70	Keratin, type II cytosk
16	6	SwissProt_HUMAN$	3::P08729|K2C7_HUMAN	175	51354	6	6	5	5	0.59	Keratin, type II cytosk
16	7	SwissProt_HUMAN$	3::Q14CN4|K2C72_HUMAN	112	55842	6	6	4	4	0.40	Keratin, type II cytosk
17	1	db$2	2::C3KCP4|C3KCP4_PSEFS	1755	99394	73	69	45	41	12.22	Pyruvate dehydrogena
18	1	db$2	2::C3KCJ5|C3KCJ5_PSEFS	1713	93957	55	51	38	35	6.18	Aconitate hydratase B
19	1	db$2	2::C3K6M5|C3K6M5_PSEFS	1601	63522	37	37	21	21	5.00	Succinate dehydrogen
20	1	db$2	2::C3K275|DNAK_PSEFS	1443	68158	45	42	21	21	4.69	Chaperone protein Dn
21	1	SwissProt_HUMAN$	3::P35527|K1C9_HUMAN	1384	62027	42	41	29	28	9.68	Keratin, type I cytoske
22	1	db$2	2::C3K0P3|C3K0P3_PSEFS	1322	118163	37	35	26	25	2.08	Ribonuclease E OS=Ps
23	1	db$2	2::C3K6P2|C3K6P2_PSEFS	1314	42870	34	32	17	17	11.67	3-oxoacyl-[acyl-carrie
24	1	db$2	2::C3K225|ILVC_PSEFS	1312	36188	52	50	24	24	37.66	Ketol-acid reductoison
25	1	db$2	2::C3KBH1|C3KBH1_PSEFS	1283	161946	52	51	41	40	2.53	Glutamate synthase [I
26	1	db$2	2::C3K6N0|SUCC_PSEFS	1275	41262	44	43	25	25	30.04	Succinate--CoA ligase
27	1	db$2	2::C3K271|C3K271_PSEFS	1255	117563	41	38	28	26	2.50	Carbamoyl-phosphate
28	1	db$2	2::C3K331|C3K331_PSEFS	1255	73691	46	44	32	30	7.92	AAA_PrkA domain-con
29	1	db$2	2::C3K402|C3K402_PSEFS	1245	83101	53	52	36	35	8.81	Polyphosphate kinase
30	1	db$2	2::C3K5J5|FABV_PSEFS	1224	42933	41	38	24	22	13.05	Enoyl-[acyl-carrier-pro
31	1	db$2	2::C3KBY8|C3KBY8_PSEFS	1212	21919	54	50	17	17	90.02	Alkyl hydroperoxide re
32	1	db$2	2::C3K7D1|C3K7D1_PSEFS	1163	67115	46	40	31	26	7.93	50S ribosomal subunit
33	1	db$2	2::C3JYU7|C3JYU7_PSEFS	1142	102460	49	41	33	26	3.21	Protein translocase su
34	1	db$2	2::C3K6M7|C3K6M7_PSEFS	1128	105930	50	47	38	37	5.01	Oxoglutarate dehydro
35	1	db$2	2::C3JYR1|GCSP_PSEFS	1121	102176	45	40	28	25	2.51	Glycine dehydrogenas
36	1	db$2	2::C3K613|FADB_PSEFS	1115	76938	42	41	30	30	5.77	Fatty acid oxidation cc
37	1	db$2	2::C3KCB8|C3KCB8_PSEFS	1099	36708	34	30	15	12	7.90	Phosphate-binding prc
38	1	db$2	2::C3JYJ7|C3JYJ7_PSEFS	1091	9057	54	50	8	8	260.84	DNA-binding protein H
39	1	db$2	2::C3KEC5|C3KEC5_PSEFS	1070	97398	39	37	31	30	4.00	Phosphoenolpyruvate
40	1	db$2	2::C3K6N7|HTPG_PSEFS	1049	71589	33	33	22	22	4.60	Chaperone protein Htp
41	1	db$2	2::C3K1K1|C3K1K1_PSEFS	1047	51928	39	36	19	17	7.14	Inosine-5'-monophosp
42	1	db$2	2::C3K3F3|C3K3F3_PSEFS	1043	72324	44	44	26	26	6.63	Transketolase OS=Pse
43	1	db$2	2::C3KBT0|C3KBT0_PSEFS	1016	51607	31	30	17	17	8.91	Succinate-semialdehy
44	1	db$2	2::C3K0K6|C3K0K6_PSEFS	1016	26352	26	25	14	13	24.07	Electron transfer flavo
45	1	db$2	2::C3KDP2|C3KDP2_PSEFS	1016	68879	33	33	23	23	4.98	Multifunctional fusion
46	1	db$2	2::C3K259|IF2_PSEFS	1007	90544	45	40	30	28	4.35	Translation initiation fa
47	1	db$2	2::C3K6H4|PYRG_PSEFS	1005	59660	43	39	26	25	9.03	CTP synthase OS=Pse
48	1	db$2	2::C3JYK1|TIG_PSEFS	998	48316	40	37	27	26	16.17	Trigger factor OS=Pse
49	1	db$2	2::C3K121|C3K121_PSEFS	986	86030	41	39	29	27	4.85	Phosphoenolpyruvate
50	1	db$2	2::C3K1L8|NDK_PSEFS	982	14928	35	34	9	9	22.05	Nucleoside diphosphat

↑Family	M	DB	Accession	Score	Mass	Matches	Match(sig)	Sequences	Seq(sig)	emPAI	Description
51	1	db$2	2::C3K162|C3K162_PSEFS	956	52023	33	33	21	21	8.72	Pyruvate kinase OS=P
52	1	db$2	2::C3K6N1|C3K6N1_PSEFS	955	29928	27	27	12	12	15.98	Succinate--CoA ligase
53	1	db$2	2::C3K6M9|C3K6M9_PSEFS	951	49948	27	26	14	14	4.01	Dihydrolipoyl dehydro
54	1	db$2	2::C3K367|C3K367_PSEFS	947	68142	35	33	21	20	3.96	Uncharacterized prote
55	1	db$2	2::C3K673|C3K673_PSEFS	942	47725	40	39	21	20	13.55	Citrate synthase OS=F
56	1	db$2	2::C3K6M8|C3K6M8_PSEFS	942	42320	26	25	14	14	5.69	Dihydrolipoyllysine-res
57	1	db$2	2::C3K0T0|C3K0T0_PSEFS	941	34560	38	34	12	10	3.50	Putative outer membra
58	1	db$2	2::C3K7D3|C3K7D3_PSEFS	911	91800	30	30	23	23	2.64	Alpha-1,4 glucan phos
59	1	db$2	2::C3JYS1|C3JYS1_PSEFS	906	17688	26	24	9	9	17.50	Peptidoglycan-associa
60	1	db$2	2::C3KE86|C3KE86_PSEFS	899	95293	42	40	35	33	5.02	Chaperone protein Clp
61	1	db$2	2::C3JY66|C3JY66_PSEFS	896	80156	36	33	22	20	3.14	Isocitrate dehydrogen
62	1	db$2	2::C3K7G0|C3K7G0_PSEFS	893	36980	25	25	15	15	7.78	Cell shape-determinin
63	1	db$2	2::C3JYT1|SYDND_PSEFS	893	66220	35	31	23	20	4.18	Aspartate--tRNA(Asp/
64	1	db$2	2::C3K5E6|RS2_PSEFS	883	27078	29	29	17	17	31.40	30S ribosomal protein
65	1	db$2	2::C3K1J2|C3K1J2_PSEFS	881	140531	39	37	32	30	2.16	Phosphoribosylformylg
66	1	db$2	2::C3K6K0|C3K6K0_PSEFS	881	62268	43	39	27	25	8.81	30S ribosomal protein
67	1	db$2	2::C3KCS1|C3KCS1_PSEFS	876	34171	27	26	15	15	22.95	Ribose-phosphate pyr
68	1	db$2	2::C3K139|C3K139_PSEFS	869	48816	25	25	11	11	2.88	Uncharacterized prote
69	1	SwissProt_HUMAN$	3::P02768|ALBU_HUMAN	867	69321	41	39	29	28	7.33	Albumin OS=Homo sa
69	2	SwissProt_HUMAN$	3::P02769|ALBU_BOVIN	380	69248	20	19	15	15	1.79	Albumin OS=Bos taur
70	1	db$2	2::C3K3Z0|C3K3Z0_PSEFS	859	46925	28	27	20	19	7.30	Transcription terminat
71	1	db$2	2::C3K3S2|C3K3S2_PSEFS	857	44244	28	27	17	17	5.15	2-oxoglutarate reduct
72	1	db$2	2::C3K165|C3K165_PSEFS	849	54857	41	37	23	23	11.25	Fumarate hydratase c
73	1	db$2	2::C3KDW8|PURA_PSEFS	847	46535	31	30	19	18	8.38	Adenylosuccinate synt
74	1	db$2	2::C3K2V9|RS5_PSEFS	846	17675	23	23	9	9	30.45	30S ribosomal protein
75	1	db$2	2::C3K4J5|PCKA_PSEFS	817	55324	31	29	23	22	5.59	Phosphoenolpyruvate
76	1	db$2	2::C3KBJ2|C3KBJ2_PSEFS	812	24354	32	29	15	15	38.51	Carbonic anhydrase O
77	1	db$2	2::C3K2D3|C3K2D3_PSEFS	799	44901	29	28	17	16	5.65	Serine hydroxymethyl
78	1	db$2	2::C3K2R9|C3K2R9_PSEFS	791	16440	18	16	8	7	8.75	6,7-dimethyl-8-ribityl
79	1	db$2	2::C3JYJ9|CLPX_PSEFS	791	46924	29	28	19	18	7.30	ATP-dependent Clp pr
80	1	db$2	2::C3K1K0|GUAA_PSEFS	779	58398	36	34	29	27	8.70	GMP synthase [glutam
81	1	db$2	2::C3K6J4|C3K6J4_PSEFS	764	97893	33	31	26	24	2.53	DNA gyrase subunit A
82	1	db$2	2::C3KE67|RS6_PSEFS	764	16276	23	21	13	12	129.43	30S ribosomal protein
83	1	db$2	2::C3K1W9|C3K1W9_PSEFS	760	33929	16	16	8	8	3.61	Thioredoxin reductase
84	1	db$2	2::C3K6H6|C3K6H6_PSEFS	759	45588	27	25	18	16	4.83	Enolase OS=Pseudom
84	2	SwissProt_HUMAN$	3::P06733|ENOA_HUMAN	81	47139	3	3	2	2	0.22	Alpha-enolase OS=Ho
85	1	db$2	2::C3K3F1|PGK_PSEFS	748	40125	22	22	15	15	6.43	Phosphoglycerate kina
86	1	db$2	2::C3K6G5|AMPA_PSEFS	747	52403	28	26	21	20	6.31	Probable cytosol amin
87	1	db$2	2::C3K4D2|C3K4D2_PSEFS	732	30988	33	32	19	19	60.08	Phosphate import ATP
88	1	db$2	2::C3KE51|C3KE51_PSEFS	731	38026	29	28	18	17	8.34	Putative oxidoreducta

↑Family	M	DB	Accession	Score	Mass	Matches	Match(sig)	Sequences	Seq(sig)	emPAI	Description
89	1	db$2	2::C3JZT6|C3JZT6_PSEFS	729	40569	22	20	13	12	6.27	Aspartate-semialdehy
90	1	db$2	2::C3K603|C3K603_PSEFS	722	87560	35	32	25	23	3.09	Outer membrane prot
91	1	db$2	2::C3K426|ARLY_PSEFS	709	51545	26	24	16	15	5.90	Argininosuccinate lyas
92	1	db$2	2::C3K1E2|C3K1E2_PSEFS	706	66300	26	25	18	17	2.89	Glutamine--fructose-6
93	1	db$2	2::C3K108|C3K108_PSEFS	704	83591	28	27	24	24	3.14	GTP pyrophosphokina
94	1	db$2	2::C3KBT1|C3KBT1_PSEFS	703	44808	27	26	17	17	7.25	4-aminobutyrate amin
95	1	db$2	2::C3JY65|C3JY65_PSEFS	699	45375	33	32	19	19	14.03	Isocitrate dehydrogen
96	1	db$2	2::C3K2Y6|RL1_PSEFS	696	24061	20	19	13	12	14.39	50S ribosomal protein
97	1	db$2	2::C3KDG0|RECA_PSEFS	689	37499	23	23	14	14	9.94	Protein RecA OS=Pseu
98	1	db$2	2::C3KBM0|GLPK_PSEFS	686	55508	29	28	20	19	5.54	Glycerol kinase OS=Ps
99	1	db$2	2::C3KDD8|C3KDD8_PSEFS	682	44893	29	28	19	19	10.32	Cell division protein Ft
100	1	db$2	2::C3K4F6|C3K4F6_PSEFS	675	65489	41	36	27	23	6.59	Oxaloacetate decarbo
101	1	db$2	2::C3K4D1|C3K4D1_PSEFS	675	28553	22	21	14	13	13.06	Phosphate-specific tra
102	1	db$2	2::C3K2D0|C3K2D0_PSEFS	670	61490	25	22	21	20	3.67	Energy-dependent tra
103	1	db$2	2::C3KDU5|C3KDU5_PSEFS	670	90380	24	23	22	21	2.01	DNA gyrase subunit B
104	1	db$2	2::C3K1A7|C3K1A7_PSEFS	669	66070	26	22	20	17	2.64	Proline--tRNA ligase O
105	1	db$2	2::C3K2V6|C3K2V6_PSEFS	666	47842	19	17	9	7	1.69	Protein translocase su
106	1	db$2	2::C3JYT4|C3JYT4_PSEFS	657	17756	20	20	9	9	22.66	Putative ferritin DPS-f
107	1	db$2	2::C3KAD2|ARNA_PSEFS	656	74140	23	22	18	18	2.16	Bifunctional polymyxir
108	1	db$2	2::C3KA76|C3KA76_PSEFS	655	30860	20	19	13	13	8.90	UTP--glucose-1-phosp
109	1	db$2	2::C3K2X5|RL4_PSEFS	651	21987	14	12	9	7	4.53	50S ribosomal protein
110	1	db$2	2::C3K1E7|ATPG_PSEFS	651	31374	22	20	13	13	10.05	ATP synthase gamma
111	1	db$2	2::C3K4C6|C3K4C6_PSEFS	648	25565	19	19	11	11	14.89	Phosphate regulon tra
112	1	db$2	2::C3KE40|SYGB_PSEFS	643	74833	26	25	21	20	2.79	Glycine--tRNA ligase b
113	1	db$2	2::C3K3J7|C3K3J7_PSEFS	641	35008	33	28	16	15	16.05	Glutathione synthetas
114	1	db$2	2::C3KDW5|C3KDW5_PSEFS	629	43265	28	24	19	16	6.15	Protein HflK OS=Pseu
115	1	db$2	2::C3KAH7|C3KAH7_PSEFS	625	45018	33	30	22	20	10.19	Putative NADP-depenc
116	1	db$2	2::C3K341|C3K341_PSEFS	625	69250	33	28	22	19	4.54	RNA polymerase sigm
116	2	db$2	2::C3K708|C3K708_PSEFS	191	38066	4	4	4	4	0.64	RNA polymerase sigm
117	1	db$2	2::C3JXY1|C3JXY1_PSEFS	618	93962	27	25	22	21	2.04	Alanine--tRNA ligase (
118	1	db$2	2::C3K7C9|C3K7C9_PSEFS	617	51641	28	25	17	14	3.76	Glutamine synthetase
119	1	db$2	2::C3KDU7|C3KDU7_PSEFS	615	28504	24	22	16	15	10.92	Putative ABC transpor
120	1	db$2	2::C3K649|C3K649_PSEFS	612	8822	18	18	5	5	12.79	Major outer membran
121	1	db$2	2::C3K6W6|C3K6W6_PSEFS	609	49345	35	30	20	18	7.24	Biotin carboxylase OS
122	1	db$2	2::C3JYQ5|C3JYQ5_PSEFS	608	46409	23	22	16	16	4.66	Arginine deiminase OS
123	1	db$2	2::C3JZN7|SYT_PSEFS	607	72513	29	25	21	20	3.22	Threonine--tRNA ligas
124	1	db$2	2::C3K7Y0|C3K7Y0_PSEFS	605	45268	23	20	14	13	4.32	Putative exported prot
125	1	db$2	2::C3JZS1|C3JZS1_PSEFS	599	55264	29	28	18	18	5.05	Amidophosphoribosylt
126	1	db$2	2::C3KAK3|C3KAK3_PSEFS	587	54608	20	18	14	12	2.09	Probable malate:quinc
127	1	db$2	2::C3K4Y4|C3K4Y4_PSEFS	582	71842	21	21	17	17	2.75	Potassium-transportin

↑Family	M	DB	Accession	Score	Mass	Matches	Match(sig)	Sequences	Seq(sig)	emPAI	Description
128	1	db$2	2::C3K3V2|C3K3V2_PSEFS	581	49111	20	19	12	11	2.18	Putative outer membr
129	1	db$2	2::C3K8B0|C3K8B0_PSEFS	580	25585	18	18	10	10	6.60	Peptidyl-prolyl cis-trar
130	1	db$2	2::C3JZN3|SYFA_PSEFS	577	37892	15	14	13	12	4.74	Phenylalanine--tRNA li
131	1	db$2	2::C3K3E8|C3K3E8_PSEFS	571	38385	25	24	18	17	13.99	Fructose-1,6-bisphosp
132	1	db$2	2::C3K914|C3K914_PSEFS	571	38509	17	17	11	11	3.93	Putative ABC transpor
133	1	db$2	2::C3KE65|C3KE65_PSEFS	566	97331	28	27	24	23	2.39	Ribonuclease R OS=Ps
134	1	db$2	2::C3K5H9|C3K5H9_PSEFS	563	33776	19	19	15	15	9.79	Transaldolase OS=Pse
135	1	db$2	2::C3K6C2|C3K6C2_PSEFS	563	27720	14	14	10	10	4.46	Putative amino acid AI
136	1	db$2	2::C3K2M9|PROA_PSEFS	562	45380	18	18	13	13	3.77	Gamma-glutamyl phos
137	1	db$2	2::C3KBZ2|ASSY_PSEFS	561	45330	23	21	17	16	5.56	Argininosuccinate synt
138	1	db$2	2::C3K3G6|SAHH_PSEFS	561	51212	32	30	21	20	10.09	Adenosylhomocysteina
139	1	db$2	2::C3KDR8|OPGD_PSEFS	558	60485	20	20	16	16	2.50	Glucans biosynthesis p
140	1	db$2	2::C3K6W9|C3K6W9_PSEFS	555	69415	15	15	14	14	1.60	Putative methyl-accep
140	2	db$2	2::C3K7Z5|C3K7Z5_PSEFS	110	66997	3	3	3	3	0.24	Putative methyl-accep
140	3	db$2	2::C3K5K7|C3K5K7_PSEFS	90	52604	4	3	4	3	0.31	Putative methyl-accep
140	4	db$2	2::C3JZ31|C3JZ31_PSEFS	70	58204	2	2	2	2	0.18	Putative methyl-accep
141	1	db$2	2::C3KED7|PUR7_PSEFS	553	26862	21	19	12	11	7.20	Phosphoribosylaminoir
142	1	db$2	2::C3KCB5|C3KCB5_PSEFS	550	30173	22	20	11	9	4.57	Phosphate import ATP
143	1	db$2	2::C3K6I0|C3K6I0_PSEFS	550	39056	21	18	14	13	5.14	Alcohol dehydrogenas
144	1	db$2	2::C3K1K7|LEU1_PSEFS	549	61997	25	24	22	21	4.37	2-isopropylmalate syn
145	1	db$2	2::C3K1N2|C3K1N2_PSEFS	549	67307	24	21	19	16	2.31	Protein translocase su
146	1	db$2	2::C3JZV2|C3JZV2_PSEFS	547	43020	19	19	14	14	4.80	Aminotransferase OS=
147	1	db$2	2::C3KEC2|C3KEC2_PSEFS	546	28614	20	18	12	12	6.21	Putative lipoprotein OS
147	2	db$2	2::C3KAJ6|C3KAJ6_PSEFS	213	38502	9	8	6	5	1.09	Histidinol-phosphate a
148	1	db$2	2::C3JZV8|C3JZV8_PSEFS	543	38906	13	13	8	8	2.36	Neutral metalloprotein
149	1	db$2	2::C3JYA7|C3JYA7_PSEFS	540	59461	17	17	12	12	2.05	Putative NADP-depenc
150	1	db$2	2::C3K7D6|F16PA_PSEFS	536	37081	17	17	11	11	4.24	Fructose-1,6-bisphosp
151	1	db$2	2::C3JZ70|C3JZ70_PSEFS	535	54858	25	23	19	18	4.16	Glucose-6-phosphate
152	1	db$2	2::C3JXL8|C3JXL8_PSEFS	533	112023	25	21	18	15	1.06	Putative efflux protein
153	1	db$2	2::C3K2V1|C3K2V1_PSEFS	533	36623	28	26	21	20	14.00	DNA-directed RNA pol
154	1	db$2	2::C3K3R2|C3K3R2_PSEFS	532	83481	24	23	21	21	2.49	Phosphoenolpyruvate-
155	1	db$2	2::C3K3P0|ILVD_PSEFS	531	65667	19	17	17	15	1.95	Dihydroxy-acid dehydr
156	1	db$2	2::C3K2Y4|RL7_PSEFS	530	12456	15	14	3	3	8.42	50S ribosomal protein
157	1	db$2	2::C3K260|C3K260_PSEFS	528	54381	18	18	14	14	3.03	Transcription terminat
158	1	db$2	2::C3JXY0|AK_PSEFS	526	44504	21	21	17	17	5.77	Aspartate kinase OS=
159	1	db$2	2::C3K484|C3K484_PSEFS	522	13502	16	16	9	9	30.16	Uncharacterized prote
160	1	db$2	2::C3K2W1|RL6_PSEFS	518	19185	17	16	10	9	13.67	50S ribosomal protein
161	1	db$2	2::C3KE70|RL9_PSEFS	517	15488	21	19	8	7	10.07	50S ribosomal protein
162	1	db$2	2::C3KA80|C3KA80_PSEFS	509	20438	17	17	10	10	14.76	Alkyl hydroperoxide re
163	1	db$2	2::C3JXL7|C3JXL7_PSEFS	502	39112	17	17	10	10	2.78	HlyD-family secretion

↑Family	M	DB	Accession	Score	Mass	Matches	Match(sig)	Sequences	Seq(sig)	emPAI	Description
164	1	db$2	2::C3K9R0|C3K9R0_PSEFS	501	62265	16	15	13	13	1.90	Pyruvate dehydrogena
165	1	db$2	2::C3JXZ7|C3JXZ7_PSEFS	500	28415	15	15	8	8	3.45	Putative histidine-bind
166	1	db$2	2::C3K9R4|C3K9R4_PSEFS	499	62164	21	19	17	17	2.95	Putative sulfite reduct
167	1	db$2	2::C3K2X6|RL3_PSEFS	499	22550	17	15	10	9	5.53	50S ribosomal protein
168	1	db$2	2::C3K1E9|ATPD_PSEFS	498	19246	19	18	8	8	10.49	ATP synthase subunit
169	1	db$2	2::C3K178|C3K178_PSEFS	497	36139	21	19	14	13	7.07	Glyceraldehyde-3-pho
170	1	db$2	2::C3KDD9|C3KDD9_PSEFS	495	41969	17	15	15	13	3.84	Cell division protein Ft
171	1	db$2	2::C3K6M2|C3K6M2_PSEFS	493	60584	16	16	14	14	2.23	ATP-dependent RNA h
172	1	db$2	2::C3JY70|C3JY70_PSEFS	483	50668	20	20	15	15	3.91	Adenylosuccinate lyas
173	1	db$2	2::C3JYV2|C3JYV2_PSEFS	480	52688	15	15	10	10	1.69	AAA domain-containin
174	1	db$2	2::C3K4V9|C3K4V9_PSEFS	480	53185	19	18	16	15	3.16	Glyceraldehyde-3-pho
175	1	db$2	2::C3KBH2|C3KBH2_PSEFS	478	51951	17	15	15	13	2.27	Glutamate synthase [I
176	1	db$2	2::C3JZN6|IF3_PSEFS	471	20837	17	17	9	9	10.87	Translation initiation f
177	1	db$2	2::C3JZN2|C3JZN2_PSEFS	470	86329	23	21	20	19	1.84	Phenylalanine--tRNA li
178	1	db$2	2::C3K8X5|HIS1_PSEFS	468	23098	18	16	13	13	16.30	ATP phosphoribosyltra
179	1	db$2	2::C3K4D4|C3K4D4_PSEFS	468	83859	20	19	14	14	1.62	Putative phosphate AE
180	1	db$2	2::C3K016|C3K016_PSEFS	468	85788	20	18	18	16	1.56	Ribosomal RNA large s
181	1	db$2	2::C3JYJ8|C3JYJ8_PSEFS	467	88851	18	17	17	16	1.35	Lon protease OS=Pseu
182	1	db$2	2::C3KEC8|C3KEC8_PSEFS	466	50623	16	14	11	9	1.80	Periplasmic serine end
183	1	db$2	2::C3K3V3|ARGA_PSEFS	464	47793	14	14	12	12	2.28	Amino-acid acetyltran
184	1	db$2	2::C3K2X0|C3K2X0_PSEFS	463	25737	18	17	12	11	9.76	30S ribosomal protein
185	1	db$2	2::C3JYS2|TOLB_PSEFS	462	47517	25	23	19	17	5.01	Tol-Pal system protein
186	1	db$2	2::C3K0Q7|C3K0Q7_PSEFS	462	45255	10	10	7	7	1.31	Flagellar hook protein
187	1	db$2	2::C3K3N4|COAD_PSEFS	461	17810	16	15	9	9	17.17	Phosphopantetheine a
188	1	db$2	2::C3K266|C3K266_PSEFS	455	69364	17	16	13	13	1.60	ATP-dependent zinc m
189	1	db$2	2::C3K2W6|RL14_PSEFS	453	13401	13	13	9	9	63.09	50S ribosomal protein
190	1	db$2	2::C3K907|C3K907_PSEFS	452	77648	23	20	21	18	2.19	Tail-specific protease (
191	1	db$2	2::C3K865|C3K865_PSEFS	452	15847	12	12	6	6	4.85	Putative membrane pr
192	1	db$2	2::C3K614|C3K614_PSEFS	450	41572	23	21	10	10	3.91	3-ketoacyl-CoA thiolas
193	1	db$2	2::C3K6E1|RL13_PSEFS	448	15695	16	16	10	10	25.66	50S ribosomal protein
194	1	db$2	2::C3K263|C3K263_PSEFS	446	26068	10	10	7	7	3.24	Triosephosphate isom
195	1	db$2	2::C3K4F7|C3K4F7_PSEFS	445	51847	18	17	15	14	2.59	Biotin carboxylase OS
196	1	db$2	2::C3K6H5|KDSA_PSEFS	443	30777	22	20	11	10	8.98	2-dehydro-3-deoxyph
197	1	db$2	2::C3K0D4|C3K0D4_PSEFS	442	29228	18	17	11	11	5.94	Cell division inhibitor N
198	1	db$2	2::C3K5A9|RF3_PSEFS	440	59584	16	15	14	13	2.30	Peptide chain release
199	1	db$2	2::C3KE83|C3KE83_PSEFS	440	38700	11	11	8	8	2.00	Outer membrane prot
200	1	db$2	2::C3K6M6|C3K6M6_PSEFS	437	26063	17	16	14	13	13.99	Succinate dehydrogen
201	1	db$2	2::C3K581|C3K581_PSEFS	436	86479	15	15	13	13	1.04	Putative molybdopteri
202	1	db$2	2::C3JZS0|C3JZS0_PSEFS	436	43144	17	16	13	12	3.15	O-succinylhomoserine
203	1	db$2	2::C3K2X3|RL2_PSEFS	435	29689	16	16	11	11	4.75	50S ribosomal protein

↑Family	M	DB	Accession	Score	Mass	Matches	Match(sig)	Sequences	Seq(sig)	emPAI	Description
204	1	db$2	2::C3JZS4|C3JZS4_PSEFS	434	33375	17	14	12	10	3.73	Acetyl-coenzyme A ca
205	1	db$2	2::C3KDX4|SYI_PSEFS	433	105386	18	17	15	15	0.97	Isoleucine--tRNA ligas
206	1	db$2	2::C3K5Z7|C3K5Z7_PSEFS	432	26574	18	17	9	8	11.00	Uridylate kinase OS=F
207	1	db$2	2::C3K3U2|C3K3U2_PSEFS	432	50985	14	14	11	11	2.05	Putative glutamine syı
208	1	db$2	2::C3K8X0|C3K8X0_PSEFS	431	16351	12	12	7	7	8.90	Putative ABC transpor
209	1	db$2	2::C3K6X4|C3K6X4_PSEFS	431	71161	20	18	18	17	2.11	Biosynthetic arginine (
210	1	db$2	2::C3K123|C3K123_PSEFS	430	41542	16	13	12	10	2.12	Citrate synthase OS=F
211	1	db$2	2::C3K278|C3K278_PSEFS	426	15153	9	9	5	5	5.31	Ferric uptake regulatic
212	1	db$2	2::C3K4C7|C3K4C7_PSEFS	424	50044	13	12	11	10	1.83	Phosphate regulon ser
213	1	db$2	2::C3K1L4|ISPG_PSEFS	421	39660	15	15	12	12	4.31	4-hydroxy-3-methylbu
214	1	db$2	2::C3K9U6|C3K9U6_PSEFS	421	35232	11	11	8	8	2.35	Putative 6-phosphoglu
215	1	SwissProt_HUMAN$	3::P07355|ANXA2_HUMAN	418	38580	22	18	17	14	4.54	Annexin A2 OS=Homc
216	1	db$2	2::C3K2T2|C3K2T2_PSEFS	417	24648	12	12	7	7	4.58	Putative ABC transpor
217	1	db$2	2::C3K4W1|STHA_PSEFS	416	50963	22	20	17	15	4.33	Soluble pyridine nucle
218	1	db$2	2::C3KEA4|C3KEA4_PSEFS	415	51722	15	14	11	11	2.29	PINc domain-containir
219	1	db$2	2::C3K358|C3K358_PSEFS	413	65409	18	18	15	15	2.43	Putative dehydrogena:
219	2	db$2	2::C3K355|C3K355_PSEFS	273	63710	11	11	9	9	0.95	Putative dehydrogena:
220	1	db$2	2::C3K2Z7|ARGC_PSEFS	411	36612	19	18	12	11	5.08	N-acetyl-gamma-gluta
221	1	db$2	2::C3KCP3|C3KCP3_PSEFS	409	56221	19	18	12	11	1.75	Acetyltransferase com
222	1	db$2	2::C3K6J6|C3K6J6_PSEFS	408	40307	18	17	13	13	4.17	Bifunctional chorismat
223	1	db$2	2::C3K6H2|ACCA_PSEFS	408	34868	17	16	11	10	5.66	Acetyl-coenzyme A ca
224	1	db$2	2::C3KAA8|C3KAA8_PSEFS	408	132643	14	13	14	13	0.59	Histidine kinase OS=P
225	1	db$2	2::C3K1G5|C3K1G5_PSEFS	407	46195	14	14	9	9	1.79	Homoserine dehydrog
226	1	db$2	2::C3K5I7|C3K5I7_PSEFS	407	59867	17	17	13	13	2.55	Probable malate:quinc
227	1	db$2	2::C3K1X8|C3K1X8_PSEFS	406	11596	9	9	4	4	6.46	50S ribosomal protein
228	1	db$2	2::C3K8X4|MURA_PSEFS	406	44975	21	19	15	14	3.85	UDP-N-acetylglucosan
229	1	db$2	2::C3K4K9|C3K4K9_PSEFS	405	27377	13	11	7	6	2.95	Putative amino acid o›
230	1	db$2	2::C3K325|C3K325_PSEFS	404	49076	18	18	14	14	2.86	Chaperone SurA OS=F
231	1	db$2	2::C3KDP1|CYSD_PSEFS	404	35173	15	13	9	8	3.38	Sulfate adenylyltransf
232	1	db$2	2::C3K3F5|METK_PSEFS	400	42554	15	15	12	12	3.75	S-adenosylmethionine
233	1	db$2	2::C3JY86|C3JY86_PSEFS	398	98164	22	20	20	18	1.39	NADH-quinone oxidore
234	1	db$2	2::C3K616|C3K616_PSEFS	397	97222	18	18	16	16	1.18	DNA topoisomerase 1
235	1	db$2	2::C3K2Y7|RL11_PSEFS	396	14903	9	8	5	4	3.80	50S ribosomal protein
236	1	db$2	2::C3K3P4|C3K3P4_PSEFS	394	39333	15	14	9	8	2.33	Uncharacterized prote
237	1	db$2	2::C3K2L2|SYL_PSEFS	394	97066	19	19	16	16	1.30	Leucine--tRNA ligase (
238	1	db$2	2::C3K6A2|C3K6A2_PSEFS	393	27847	14	13	10	10	4.43	Putative D-methionine
238	2	db$2	2::C3KD95|C3KD95_PSEFS	289	28020	9	9	8	8	2.85	Putative exported prot
239	1	db$2	2::C3K2H6|C3K2H6_PSEFS	392	32512	11	10	6	5	1.39	Acyl-CoA thioesterase
240	1	db$2	2::C3KBN3|C3KBN3_PSEFS	391	112655	17	16	11	10	0.66	Efflux pump membran
241	1	db$2	2::C3K6E0|PUR9_PSEFS	391	57370	18	17	14	14	2.75	Bifunctional purine bic

↑Family	M	DB	Accession	Score	Mass	Matches	Match(sig)	Sequences	Seq(sig)	emPAI	Description
242	1	db$2	2::C3K102|PUR5_PSEFS	389	36950	12	12	7	7	2.59	Phosphoribosylformylg
243	1	db$2	2::C3JYT9|C3JYT9_PSEFS	387	46635	11	11	6	6	1.25	Putative family S43 nc
244	1	db$2	2::C3JZ69|C3JZ69_PSEFS	387	25612	9	9	6	6	2.01	6-phosphogluconolact
245	1	db$2	2::C3K324|C3K324_PSEFS	387	105271	17	17	16	16	1.06	LPS-assembly protein
246	1	db$2	2::C3K1B7|C3K1B7_PSEFS	387	51892	18	16	14	13	2.59	Threonine synthase O!
247	1	db$2	2::C3KDE0|LPXC_PSEFS	386	33197	14	12	11	10	3.15	UDP-3-O-acyl-N-acety
248	1	db$2	2::C3K244|C3K244_PSEFS	385	52276	14	13	12	11	1.71	Putative response regu
249	1	db$2	2::C3K7L5|C3K7L5_PSEFS	382	19131	11	11	6	6	6.13	Molybdenum cofactor
250	1	db$2	2::C3JY83|NUOCD_PSEFS	381	67660	27	25	23	21	4.76	NADH-quinone oxidore
251	1	db$2	2::C3K1G8|RL19_PSEFS	379	12961	16	13	8	8	34.97	50S ribosomal protein
252	1	db$2	2::C3K2U9|C3K2U9_PSEFS	378	53651	19	18	15	14	2.76	Catalase OS=Pseudon
253	1	db$2	2::C3JYQ6|C3JYQ6_PSEFS	378	37842	22	19	14	13	6.37	Ornithine carbamoyltr
254	1	db$2	2::C3K7C1|SECB_PSEFS	376	17615	9	8	4	4	2.77	Protein-export protein
255	1	db$2	2::C3K4D3|C3K4D3_PSEFS	376	60551	18	15	13	12	1.76	Phosphate transport s
256	1	db$2	2::C3JYD4|C3JYD4_PSEFS	375	64707	15	15	13	13	1.59	Glutamine--tRNA ligas
257	1	db$2	2::C3K8X6|C3K8X6_PSEFS	374	47255	13	12	11	10	2.01	Histidinol dehydrogena
258	1	db$2	2::C3K9T7|C3K9T7_PSEFS	372	99328	20	17	16	13	0.95	Aminopeptidase N OS=
259	1	db$2	2::C3K3Z1|C3K3Z1_PSEFS	372	11808	11	11	6	6	14.82	Thioredoxin OS=Pseuc
260	1	db$2	2::C3KDW6|C3KDW6_PSEFS	371	33055	23	19	15	12	7.50	Protein HflC OS=Pseu
261	1	db$2	2::C3K401|C3K401_PSEFS	371	56255	17	17	16	16	2.85	Exopolyphosphatase C
262	1	db$2	2::C3K6G0|C3K6G0_PSEFS	370	9680	10	9	7	6	16.60	Putative DNA-binding
263	1	db$2	2::C3K2C4|C3K2C4_PSEFS	369	89060	18	16	16	14	1.23	Endopeptidase La OS=
264	1	db$2	2::C3K2V7|RL15_PSEFS	369	15215	15	15	9	9	38.81	50S ribosomal protein
265	1	db$2	2::C3K5S8|C3K5S8_PSEFS	368	75646	16	14	12	10	1.12	Oligopeptidase A OS=
266	1	db$2	2::C3K7G9|C3K7G9_PSEFS	368	48266	10	10	9	9	1.42	Putative modulation of
267	1	db$2	2::C3K1U7|C3K1U7_PSEFS	368	36908	8	8	2	2	1.15	Ubiquinol oxidase subu
268	1	db$2	2::C3K6R4|C3K6R4_PSEFS	366	18727	13	13	7	7	8.47	Uncharacterized prote
269	1	db$2	2::C3K466|C3K466_PSEFS	366	52681	18	17	10	9	1.69	Putative aldehyde deh
270	1	db$2	2::C3K1M5|ISCS_PSEFS	365	44467	16	15	14	13	2.98	Cysteine desulfurase I
271	1	db$2	2::C3K227|C3K227_PSEFS	364	62578	21	18	16	13	2.11	Acetolactate synthase
272	1	db$2	2::C3K1F0|C3K1F0_PSEFS	363	16832	15	14	8	8	8.19	ATP synthase subunit
273	1	db$2	2::C3JXZ8|C3JXZ8_PSEFS	362	71568	15	14	13	12	1.21	Acetyl-coenzyme A sy
274	1	db$2	2::C3JZ68|C3JZ68_PSEFS	361	24197	14	13	12	11	9.30	2-dehydro-3-deoxy-ph
275	1	db$2	2::C3KBN2|C3KBN2_PSEFS	360	52689	10	10	9	9	1.46	Outer membrane prot
276	1	db$2	2::C3KAL6|C3KAL6_PSEFS	359	52109	17	16	13	12	2.26	Putative aminotransfe
277	1	db$2	2::C3KBQ2|C3KBQ2_PSEFS	357	37020	12	12	8	8	2.15	Putative quinone oxid
278	1	SwissProt_HUMAN$	3::P00761|TRYP_PIG	357	24394	10	10	6	6	2.85	Trypsin OS=Sus scrof
279	1	db$2	2::C3K2W0|RL18_PSEFS	356	12651	10	10	6	6	8.07	50S ribosomal protein
280	1	db$2	2::C3K6D9|C3K6D9_PSEFS	356	45394	13	12	10	10	2.15	Phosphoribosylamine-
281	1	db$2	2::C3K2K3|GSA_PSEFS	355	45181	16	15	10	10	2.90	Glutamate-1-semialde

↑Family	M	DB	Accession	Score	Mass	Matches	Match(sig)	Sequences	Seq(sig)	emPAI	Description
282	1	db$2	2::C3K2X1|RL22_PSEFS	353	11904	9	9	6	6	9.44	50S ribosomal protein
283	1	db$2	2::C3K1H6|PURT_PSEFS	353	42327	15	15	13	13	3.28	Formate-dependent pl
284	1	db$2	2::C3K5Z8|RRF_PSEFS	352	20297	11	11	7	7	7.00	Ribosome-recycling fa
285	1	db$2	2::C3K2V0|RL17_PSEFS	351	14407	14	13	7	7	24.33	50S ribosomal protein
286	1	db$2	2::C3K2U7|C3K2U7_PSEFS	350	104087	24	20	22	19	1.38	UvrABC system protei
287	1	db$2	2::C3K7X9|C3K7X9_PSEFS	349	40858	15	14	11	10	3.01	Carboxynorspermidine
288	1	db$2	2::C3K5Y0|C3K5Y0_PSEFS	346	38747	14	14	10	10	2.39	NADP(H)-dependt aldo
289	1	db$2	2::C3K0Q9|C3K0Q9_PSEFS	344	15688	8	8	4	4	7.08	Flagellar basal-body ro
290	1	db$2	2::C3KCR8|C3KCR8_PSEFS	344	39907	14	12	7	7	1.58	Ribosome-binding ATP
291	1	db$2	2::C3KAJ2|C3KAJ2_PSEFS	343	53335	14	14	13	13	2.18	Methylmalonate-semia
292	1	db$2	2::C3JYU6|C3JYU6_PSEFS	341	42196	12	11	11	10	2.43	Arginine biosynthesis
293	1	db$2	2::C3K6U8|C3K6U8_PSEFS	340	58654	8	7	5	5	0.50	Putative membrane pr
294	1	db$2	2::C3KBL8|C3KBL8_PSEFS	339	56970	22	19	18	15	2.79	Glycerol-3-phosphate
295	1	db$2	2::C3KBI6|C3KBI6_PSEFS	339	106629	16	16	14	14	0.95	Uncharacterized prote
296	1	db$2	2::C3JYS5|C3JYS5_PSEFS	337	25332	9	9	8	8	4.33	Tol-Pal system protein
297	1	db$2	2::C3K2Y5|RL10_PSEFS	337	17594	14	14	8	8	9.89	50S ribosomal protein
298	1	db$2	2::C3KBX8|C3KBX8_PSEFS	336	26859	9	9	6	6	3.07	OmpA-like domain-cor
299	1	db$2	2::C3K1Y2|C3K1Y2_PSEFS	335	21662	12	12	7	7	7.76	Peptidyl-prolyl cis-trar
300	1	db$2	2::C3KAH3|SYR_PSEFS	333	63549	12	12	9	9	1.11	Arginine--tRNA ligase
301	1	db$2	2::C3K4M6|C3K4M6_PSEFS	332	57818	14	13	10	10	1.67	Putative exported alka
302	1	db$2	2::C3KE79|C3KE79_PSEFS	331	29713	7	7	5	5	1.21	NH(3)-dependent NAD
303	1	db$2	2::C3K734|C3K734_PSEFS	330	44623	15	15	11	11	2.97	Putative aminotransfe
304	1	db$2	2::C3K476|PYRE_PSEFS	330	23079	7	7	6	6	2.39	Orotate phosphoribosy
305	1	db$2	2::C3K142|C3K142_PSEFS	324	32099	10	10	9	9	3.35	2-methylisocitrate lya
306	1	db$2	2::C3JYD5|C3JYD5_PSEFS	322	18453	11	10	7	7	6.65	Peptidyl-prolyl cis-trar
307	1	db$2	2::C3KDL2|C3KDL2_PSEFS	321	52736	13	13	12	12	1.94	Putative outer membra
308	1	db$2	2::C3JXZ0|C3JXZ0_PSEFS	319	43332	16	15	11	11	2.33	Acetylornithine aminot
309	1	db$2	2::C3K2J8|C3K2J8_PSEFS	318	54392	16	16	13	13	2.10	AMP nucleosidase OS=
310	1	db$2	2::C3KEB4|C3KEB4_PSEFS	318	56978	18	16	15	13	2.21	Lysine--tRNA ligase OS
311	1	db$2	2::C3K2Y8|C3K2Y8_PSEFS	316	20023	14	10	9	7	5.54	Transcription terminat
312	1	db$2	2::C3K443|C3K443_PSEFS	316	12323	14	13	10	10	62.36	Nitrogen regulatory pr
313	1	db$2	2::C3KCS2|RSMH_PSEFS	315	34344	12	12	10	10	2.96	Ribosomal RNA small
314	1	db$2	2::C3K0N2|C3K0N2_PSEFS	313	43313	8	7	7	6	0.93	3-oxoacyl-[acyl-carrie
315	1	db$2	2::C3JZ61|C3JZ61_PSEFS	311	61509	15	14	11	10	1.52	Acyl-CoA synthetase (
316	1	db$2	2::C3KE41|SYGA_PSEFS	310	36196	13	12	10	9	2.24	Glycine--tRNA ligase a
317	1	db$2	2::C3KAC1|C3KAC1_PSEFS	310	27548	12	12	9	9	5.57	Putative phenylacetic
318	1	db$2	2::C3K818|C3K818_PSEFS	310	29926	10	10	7	7	2.01	Putative ABC transpor
319	1	db$2	2::C3JYQ7|C3JYQ7_PSEFS	309	32635	9	8	7	6	1.75	Carbamate kinase OS=
320	1	db$2	2::C3KDU3|C3KDU3_PSEFS	309	40518	16	15	12	12	3.56	Beta sliding clamp OS
321	1	db$2	2::C3K1N3|C3K1N3_PSEFS	308	11888	8	8	5	5	6.06	Sec translocon access

↑Family	M	DB	Accession	Score	Mass	Matches	Match(sig)	Sequences	Seq(sig)	emPAI	Description
322	1	db$2	2::C3K3S1|C3K3S1_PSEFS	308	51070	10	10	8	8	1.30	Putative oxidase OS=I
323	1	db$2	2::C3K6Y4|GATA_PSEFS	304	51737	10	10	7	7	1.28	Glutamyl-tRNA(Gln) a
324	1	db$2	2::C3KDB1|C3KDB1_PSEFS	303	65565	12	12	12	12	1.38	Putative carbamoyltra
325	1	db$2	2::C3KDM2|C3KDM2_PSEFS	303	83508	18	16	17	15	1.35	DNA topoisomerase 4
326	1	db$2	2::C3JYR3|C3JYR3_PSEFS	302	40135	13	13	9	9	2.24	Glycine cleavage syste
327	1	db$2	2::C3K4H9|C3K4H9_PSEFS	301	34203	8	7	7	6	1.29	Quinone oxidoreducta
328	1	db$2	2::C3K6I1|C3K6I1_PSEFS	301	31224	7	7	5	5	1.88	S-formylglutathione h
329	1	db$2	2::C3K274|DNAJ_PSEFS	300	40315	11	11	10	10	2.64	Chaperone protein Dn
330	1	db$2	2::C3K667|C3K667_PSEFS	300	48557	10	9	8	8	1.18	Conserved hypothetica
331	1	db$2	2::C3K6M3|C3K6M3_PSEFS	299	13365	8	6	7	5	4.74	Succinate dehydrogen
332	1	db$2	2::C3JYU1|Y4927_PSEFS	299	18225	10	9	6	6	6.83	UPF0234 protein PFLU
333	1	db$2	2::C3K604|C3K604_PSEFS	298	18933	18	17	11	10	14.13	Putative outer membr
334	1	db$2	2::C3K0H1|C3K0H1_PSEFS	297	17848	10	10	6	6	5.26	Chemotaxis protein Ch
335	1	db$2	2::C3K403|C3K403_PSEFS	295	36964	12	11	7	7	1.78	Delta-aminolevulinic a
336	1	db$2	2::C3K474|C3K474_PSEFS	294	50732	14	14	12	12	2.37	Putative phosphomanr
337	1	db$2	2::Q84CP9|DAPB_PSEFS	293	28276	16	13	11	9	4.28	4-hydroxy-tetrahydro
338	1	db$2	2::C3KEB7|C3KEB7_PSEFS	292	48036	13	13	10	10	1.95	Uncharacterized prote
339	1	db$2	2::C3KED5|C3KED5_PSEFS	291	40447	11	9	11	9	1.87	Putative lipoprotein O
340	1	SwissProt_HUMAN$	3::P60709|ACTB_HUMAN	291	41710	19	17	13	12	3.89	Actin, cytoplasmic 1 O
341	1	db$2	2::C3K0W7|C3K0W7_PSEFS	291	31068	10	9	9	8	2.37	Metallophos_2 domain
342	1	db$2	2::C3K6J9|KCY_PSEFS	291	24780	6	6	5	5	1.59	Cytidylate kinase OS=
343	1	db$2	2::C3K1F2|ATP6_PSEFS	290	31626	9	8	4	3	0.56	ATP synthase subunit
344	1	db$2	2::C3K3Q2|C3K3Q2_PSEFS	290	37335	11	10	6	5	1.43	Putative cation ABC tr
345	1	db$2	2::C3K6X1|C3K6X1_PSEFS	289	39290	10	9	9	8	1.62	Uncharacterized prote
346	1	db$2	2::C3KE61|RIMK_PSEFS	287	32488	11	9	7	7	2.19	Probable alpha-L-gluta
347	1	db$2	2::C3KBY6|C3KBY6_PSEFS	287	17850	11	11	7	7	9.57	Bacterioferritin OS=Ps
348	1	db$2	2::C3KA77|C3KA77_PSEFS	285	49108	9	8	9	8	1.16	Glutathione reductase
349	1	db$2	2::C3K1F4|C3K1F4_PSEFS	285	31961	9	8	8	7	1.81	Probable chromosome
350	1	db$2	2::C3K475|ARGB_PSEFS	284	31926	14	12	12	11	4.07	Acetylglutamate kinas
351	1	db$2	2::C3KAR0|C3KAR0_PSEFS	284	79563	8	8	6	6	0.52	Putative exported isoq
352	1	db$2	2::C3JYV5|C3JYV5_PSEFS	284	45487	10	9	9	8	1.55	Putative oxidoreducta
353	1	db$2	2::C3KDU9|C3KDU9_PSEFS	283	34261	11	11	9	9	2.96	Serine O-acetyltransfe
354	1	db$2	2::C3K2K9|C3K2K9_PSEFS	283	31417	12	11	10	9	2.86	Magnesium and cobalt
355	1	db$2	2::C3K1X9|C3K1X9_PSEFS	283	34909	12	11	9	9	2.38	Octaprenyl-diphospha
356	1	db$2	2::C3KBZ7|SYM_PSEFS	282	75525	16	15	14	13	1.26	Methionine--tRNA liga
357	1	db$2	2::C3JZ71|C3JZ71_PSEFS	282	31565	8	8	6	6	1.45	Hex regulon repressor
358	1	db$2	2::C3K3D2|BETB_PSEFS	281	53123	9	9	7	7	0.87	Betaine aldehyde deh
359	1	db$2	2::C3K651|C3K651_PSEFS	280	39766	10	10	9	9	1.92	Phospho-2-dehydro-3-
360	1	db$2	2::C3K1L3|SYH_PSEFS	279	47853	19	15	14	11	3.41	Histidine--tRNA ligase
361	1	db$2	2::C3K0S7|Y4577_PSEFS	279	11900	6	6	3	3	6.06	Nucleoid-associated p

↑Family	M	DB	Accession	Score	Mass	Matches	Match(sig)	Sequences	Seq(sig)	emPAI	Description
362	1	db$2	2::C3K472|C3K472_PSEFS	279	42804	9	9	7	7	1.42	Coenzyme A biosynthe
363	1	db$2	2::C3K2W4|RL5_PSEFS	278	20490	18	16	11	10	14.53	50S ribosomal protein
364	1	db$2	2::C3KAI4|C3KAI4_PSEFS	278	53827	8	8	6	6	0.85	SPOR domain-containi
365	1	db$2	2::C3JZ88|C3JZ88_PSEFS	277	47232	10	8	6	5	1.02	Putative exported prot
366	1	db$2	2::C3K645|C3K645_PSEFS	276	24522	8	8	6	6	2.16	Putative ABC transpor
367	1	db$2	2::C3K2E3|MSCL_PSEFS	276	14568	5	5	2	2	1.62	Large-conductance me
368	1	db$2	2::C3KCN5|C3KCN5_PSEFS	275	142346	14	14	14	14	0.60	Bifunctional protein Pu
369	1	db$2	2::C3K2M1|C3K2M1_PSEFS	275	42033	19	17	14	14	4.40	Serine-type D-Ala-D-A
370	1	db$2	2::C3K3H0|C3K3H0_PSEFS	274	67796	14	11	11	8	0.75	ATP-dependent RNA h
371	1	db$2	2::C3K2W5|RL24_PSEFS	274	11328	11	11	7	7	25.48	50S ribosomal protein
372	1	db$2	2::C3K0K5|C3K0K5_PSEFS	274	31365	13	12	8	7	2.86	Electron transfer flavo
373	1	db$2	2::C3K5X4|C3K5X4_PSEFS	274	16332	10	10	8	8	8.90	Putative membrane pr
374	1	db$2	2::C3K469|RL28_PSEFS	274	8845	10	10	5	5	12.36	50S ribosomal protein
375	1	db$2	2::C3K5H8|C3K5H8_PSEFS	272	17826	10	10	8	8	7.14	STAS domain-containi
376	1	db$2	2::C3K6K6|C3K6K6_PSEFS	272	36089	14	11	11	9	2.25	HTH-type transcriptior
377	1	db$2	2::C3K249|G6PI_PSEFS	269	61384	20	16	14	12	2.44	Glucose-6-phosphate
378	1	db$2	2::C3JXY7|ASTD_PSEFS	268	51479	6	6	6	6	0.74	N-succinylglutamate 5
379	1	db$2	2::C3K7M6|C3K7M6_PSEFS	268	88918	9	9	8	8	0.53	Acyl-coenzyme A dehy
380	1	db$2	2::C3K264|GLMM_PSEFS	268	47719	15	13	12	11	2.63	Phosphoglucosamine r
381	1	db$2	2::C3K6Z0|PDXJ_PSEFS	267	26758	9	9	7	7	2.43	Pyridoxine 5'-phospha
382	1	db$2	2::C3K7N8|RDGC_PSEFS	267	34160	18	18	14	14	6.94	Recombination-associ
383	1	db$2	2::C3K8V3|HSLU_PSEFS	267	49607	16	15	14	13	2.46	ATP-dependent protea
384	1	db$2	2::C3K6G2|C3K6G2_PSEFS	267	106936	14	9	14	9	0.49	Valine--tRNA ligase OS
385	1	db$2	2::C3K2S0|C3K2S0_PSEFS	266	39319	7	7	5	5	1.06	3,4-dihydroxy-2-butar
386	1	db$2	2::C3KCT2|MUTS_PSEFS	265	95536	10	10	9	9	0.56	DNA mismatch repair
387	1	db$2	2::C3K310|C3K310_PSEFS	265	54246	13	13	10	10	1.85	Anthranilate synthase
388	1	db$2	2::C3K1L0|C3K1L0_PSEFS	264	55309	14	13	14	13	2.05	GTPase Der OS=Pseuc
389	1	db$2	2::C3K4V8|C3K4V8_PSEFS	264	128447	12	11	12	11	0.50	Transcription-repair-co
390	1	db$2	2::C3KBN4|C3KBN4_PSEFS	263	40532	9	9	8	8	1.86	Acriflavine resistance
391	1	db$2	2::C3K5E0|C3K5E0_PSEFS	263	36000	11	8	10	7	1.86	2,3,4,5-tetrahydropyri
392	1	db$2	2::C3JZT7|C3JZT7_PSEFS	263	38793	11	11	10	10	2.39	3-isopropylmalate deh
393	1	db$2	2::C3K0N4|C3K0N4_PSEFS	262	25612	10	10	8	8	5.27	3-oxoacyl-[acyl-carrie
394	1	db$2	2::C3KBL1|C3KBL1_PSEFS	262	61934	10	8	9	8	0.84	Putative medium-chair
395	1	db$2	2::C3K902|RAPA_PSEFS	261	106722	14	11	13	10	0.56	RNA polymerase-asso
396	1	db$2	2::C3K2S6|C3K2S6_PSEFS	261	31662	9	9	7	7	2.30	Putative thioredoxin O
397	1	db$2	2::C3KDL6|C3KDL6_PSEFS	261	23139	7	7	5	5	1.77	ADP-ribose diphospha
398	1	db$2	2::C3K1X5|C3K1X5_PSEFS	260	39754	10	8	9	7	1.30	Glutamate 5-kinase O
399	1	db$2	2::C3K2C9|C3K2C9_PSEFS	260	48295	15	13	13	11	2.24	Glutamate dehydroger
400	1	db$2	2::C3JZN9|C3JZN9_PSEFS	260	7652	11	10	5	5	35.12	Major cold shock prote
401	1	db$2	2::C3K569|C3K569_PSEFS	260	44415	13	13	12	12	2.59	Tryptophan synthase

↑Family	M	DB	Accession	Score	Mass	Matches	Match(sig)	Sequences	Seq(sig)	emPAI	Description
402	1	db$2	2::C3K3W7|C3K3W7_PSEFS	259	36662	9	9	8	8	1.81	Putative iron (III) ABC
403	1	db$2	2::C3JZV0|C3JZV0_PSEFS	259	56140	13	13	12	12	1.99	Glutamate--tRNA ligas
404	1	db$2	2::C3K759|C3K759_PSEFS	258	35694	5	5	4	4	0.70	Putative dehydrogena:
404	2	db$2	2::C3K0S6|C3K0S6_PSEFS	79	36802	2	2	2	2	0.29	Putative oxidoreducta:
405	1	db$2	2::C3K2N8|C3K2N8_PSEFS	257	23444	12	12	8	8	5.09	Semialdhyde_dh doma
406	1	db$2	2::C3K226|C3K226_PSEFS	257	17820	8	8	6	6	3.82	Acetolactate synthase
407	1	db$2	2::C3K1X6|C3K1X6_PSEFS	254	44711	8	8	7	7	1.33	GTPase Obg OS=Pseu
408	1	db$2	2::C3K2Y0|RS7_PSEFS	253	17636	15	12	11	10	13.19	30S ribosomal protein
409	1	db$2	2::C3K3K2|PYRB_PSEFS	253	36271	10	10	9	9	2.23	Aspartate carbamoyltr
410	1	db$2	2::C3K7Z1|C3K7Z1_PSEFS	252	38822	8	8	7	7	1.34	NAD(P) transhydroger
411	1	db$2	2::C3K610|C3K610_PSEFS	251	70283	15	13	15	13	1.40	ATP-binding protein U
412	1	db$2	2::C3K859|C3K859_PSEFS	250	59064	8	7	8	7	0.75	Putative glutamate syı
413	1	db$2	2::C3KE59|C3KE59_PSEFS	250	27398	13	12	10	9	4.57	DNA-binding response
414	1	db$2	2::C3JY54|SYS_PSEFS	250	47115	12	9	11	8	1.24	Serine--tRNA ligase O:
415	1	db$2	2::C3K839|Y879_PSEFS	250	32120	7	7	6	6	1.42	Nucleotide-binding prc
416	1	db$2	2::C3K1X2|C3K1X2_PSEFS	249	40166	9	9	9	9	1.88	Putative putative oxid
417	1	db$2	2::C3K2X7|RS10_PSEFS	249	11745	12	11	5	5	6.19	30S ribosomal protein
418	1	db$2	2::C3KBP1|C3KBP1_PSEFS	248	37670	11	11	7	7	1.73	Putative reductase OS
419	1	db$2	2::C3KE31|C3KE31_PSEFS	248	22237	11	10	8	8	5.72	RNA polymerase sigma
420	1	db$2	2::C3JY74|C3JY74_PSEFS	248	48472	12	9	11	8	1.18	Isocitrase OS=Pseudo
421	1	db$2	2::C3K1L1|C3K1L1_PSEFS	247	40864	11	11	9	9	1.83	Outer membrane prot
422	1	db$2	2::C3K6G8|LEPA_PSEFS	245	66372	12	11	10	10	1.04	Elongation factor 4 OS
423	1	db$2	2::C3K5L3|C3K5L3_PSEFS	245	36401	7	7	7	7	1.48	Phosphonates ABC tra
424	1	db$2	2::C3JYW3|C3JYW3_PSEFS	245	36818	13	12	13	12	3.66	Branched-chain-aminc
425	1	db$2	2::C3K1G2|YIDC_PSEFS	244	61977	15	11	12	9	0.99	Membrane protein ins
426	1	db$2	2::C3K0N5|C3K0N5_PSEFS	243	32402	8	8	5	5	1.40	Malonyl CoA-acyl carri
427	1	db$2	2::C3K309|C3K309_PSEFS	243	21661	6	5	4	3	0.92	Anthranilate synthase
428	1	db$2	2::C3K3W3|C3K3W3_PSEFS	242	41294	7	7	7	7	1.23	Putative aminotransfe
429	1	db$2	2::C3JYR4|C3JYR4_PSEFS	242	7675	6	6	4	4	9.93	Major cold shock prote
430	1	db$2	2::C3K2Q0|C3K2Q0_PSEFS	242	19348	11	11	9	9	7.89	Inorganic pyrophosphi
431	1	db$2	2::C3K644|C3K644_PSEFS	242	89774	10	9	9	9	0.61	Putative ABC transpor
432	1	db$2	2::C3K270|C3K270_PSEFS	241	17315	9	8	7	6	4.06	Transcription elongatic
433	1	db$2	2::C3K4G1|C3K4G1_PSEFS	241	81186	11	9	9	8	0.60	DNA helicase OS=Pseu
434	1	db$2	2::C3JY62|C3JY62_PSEFS	240	83224	16	12	13	11	0.87	ATP-dependent Clp pr
434	2	db$2	2::C3K4B3|C3K4B3_PSEFS	67	97007	7	5	6	5	0.28	Chaperone OS=Pseud
435	1	db$2	2::C3K8U4|UBIE_PSEFS	239	28320	18	17	12	11	9.29	Ubiquinone/menaquin
436	1	db$2	2::C3KCB7|C3KCB7_PSEFS	238	34191	5	5	4	4	0.74	Phosphate transport s
437	1	db$2	2::C3K3U0|C3K3U0_PSEFS	238	51001	9	8	7	7	0.92	Putative glutamine syı
438	1	db$2	2::C3K6F9|C3K6F9_PSEFS	238	37389	9	8	9	8	1.75	Nucleoid-associated pı
439	1	SwissProt_HUMAN$	3::P01871|IGHM_HUMAN	237	49409	10	9	9	9	1.37	Immunoglobulin heavy

↑Family	M	DB	Accession	Score	Mass	Matches	Match(sig)	Sequences	Seq(sig)	emPAI	Description
440	1	db$2	2::C3K245|C3K245_PSEFS	237	53442	13	12	12	11	1.65	Poly(A) polymerase I (
441	1	db$2	2::C3K2V3|RS11_PSEFS	236	13607	9	8	7	7	9.89	30S ribosomal protein
442	1	db$2	2::C3K4E2|C3K4E2_PSEFS	236	16860	11	10	9	8	11.13	N5-carboxyaminoimid:
443	1	db$2	2::C3K4Q9|C3K4Q9_PSEFS	236	23656	8	6	6	5	1.70	Putative exported prot
444	1	db$2	2::B0B0A5|DNAA_PSEFS	236	56252	12	11	9	8	0.96	Chromosomal replicati
445	1	db$2	2::C3K6E2|RS9_PSEFS	236	14607	10	9	6	6	8.29	30S ribosomal protein
446	1	db$2	2::C3K4A8|C3K4A8_PSEFS	234	55832	11	10	10	9	1.34	Uncharacterized prote
447	1	db$2	2::C3JY00|C3JY00_PSEFS	234	47211	7	6	6	5	0.65	Ribonucleoside-diphos
448	1	SwissProt_HUMAN$	3::P01834|IGKC_HUMAN	233	11758	8	8	6	6	9.67	Immunoglobulin kappa
449	1	db$2	2::C3K747|GPDA_PSEFS	233	36476	9	9	7	7	2.20	Glycerol-3-phosphate
450	1	db$2	2::C3KAH1|HSLV_PSEFS	232	18731	6	6	5	5	2.49	ATP-dependent protea
451	1	db$2	2::C3KAI6|C3KAI6_PSEFS	232	24263	10	9	8	7	3.70	Putative dehydrogena:
452	1	db$2	2::C3KC10|C3KC10_PSEFS	232	23080	5	5	4	4	1.26	Uncharacterized prote
453	1	db$2	2::C3JY85|C3JY85_PSEFS	232	48759	17	15	15	13	2.54	NADH-quinone oxidore
454	1	db$2	2::C3K2V2|RS4_PSEFS	231	23062	14	11	7	5	2.39	30S ribosomal protein
455	1	db$2	2::C3K6J7|C3K6J7_PSEFS	231	39784	11	10	10	9	1.92	Histidinol-phosphate a
456	1	db$2	2::C3K1M9|C3K1M9_PSEFS	231	29586	10	9	9	8	2.57	Inositol-1-monophosp
457	1	db$2	2::C3K2Z3|C3K2Z3_PSEFS	230	44017	13	13	11	11	2.63	Tyrosine--tRNA ligase
458	1	db$2	2::C3K733|HTPX_PSEFS	228	32382	9	9	5	5	1.40	Protease HtpX OS=Pse
459	1	db$2	2::C3K8Z6|C3K8Z6_PSEFS	227	39611	8	7	6	6	1.05	Branched amino acid /
460	1	db$2	2::C3JZ29|C3JZ29_PSEFS	227	41047	9	8	7	6	1.24	Putative glycerophosp
461	1	db$2	2::C3K6V9|C3K6V9_PSEFS	226	54793	12	10	10	9	1.18	2,3-bisphosphoglycera
462	1	db$2	2::C3K611|C3K611_PSEFS	225	16065	8	8	5	5	6.64	Universal stress protei
463	1	db$2	2::C3K815|C3K815_PSEFS	225	62777	9	9	5	5	0.70	Glucans biosynthesis p
464	1	db$2	2::C3K4G2|C3K4G2_PSEFS	224	30728	9	9	4	4	1.93	Putative exported prot
465	1	db$2	2::C3KDM7|C3KDM7_PSEFS	222	29519	12	11	10	9	3.94	Sulfurtransferase OS=
466	1	db$2	2::C3K104|C3K104_PSEFS	221	26102	14	13	8	7	5.08	Putative membrane pr
467	1	db$2	2::C3K304|C3K304_PSEFS	221	15054	8	7	4	4	3.69	Uncharacterized prote
468	1	db$2	2::C3KCL0|C3KCL0_PSEFS	220	35424	7	7	6	6	1.23	Uncharacterized prote
469	1	db$2	2::C3KBY9|C3KBY9_PSEFS	220	24532	5	5	4	4	1.15	Ribonuclease T OS=Ps
470	1	db$2	2::C3K2X2|RS19_PSEFS	218	10313	11	9	7	6	13.71	30S ribosomal protein
471	1	db$2	2::C3K5V1|C3K5V1_PSEFS	217	47050	10	10	10	10	1.73	Outer membrane prot
472	1	db$2	2::C3K6T0|ENGB_PSEFS	214	23537	6	6	5	5	1.72	Probable GTP-binding
473	1	db$2	2::C3K1E5|ATPE_PSEFS	213	14558	5	5	4	4	3.97	ATP synthase epsilon (
474	1	db$2	2::C3KAJ8|UPP_PSEFS	213	22986	11	10	9	8	5.30	Uracil phosphoribosylt
475	1	db$2	2::C3K1H1|RS16_PSEFS	212	9202	8	7	6	5	11.20	30S ribosomal protein
476	1	db$2	2::C3K6S9|C3K6S9_PSEFS	212	101727	12	11	12	11	0.67	DNA polymerase I OS:
477	1	db$2	2::C3K116|PSRP_PSEFS	211	30838	6	5	6	5	1.15	Putative phosphoenolp
478	1	db$2	2::C3K3M4|C3K3M4_PSEFS	211	49883	9	9	9	9	1.35	Signal recognition part
479	1	db$2	2::C3KEC6|KAD_PSEFS	211	23251	12	10	8	6	4.06	Adenylate kinase OS=

↑Family	M	DB	Accession	Score	Mass	Matches	Match(sig)	Sequences	Seq(sig)	emPAI	Description
480	1	db$2	2::C3K910|C3K910_PSEFS	210	36322	8	8	8	8	1.83	Putative ABC transpor
481	1	db$2	2::C3K1N4|C3K1N4_PSEFS	210	41398	11	11	7	7	1.80	Queuine tRNA-ribosylt
482	1	db$2	2::C3K1L2|C3K1L2_PSEFS	210	22708	9	8	7	7	3.25	Ancillary SecYEG trans
483	1	db$2	2::C3K490|C3K490_PSEFS	209	33505	16	16	12	12	4.42	Putative oxidative stre
484	1	db$2	2::C3K152|C3K152_PSEFS	209	13999	7	7	5	5	4.29	Putative transcriptiona
485	1	db$2	2::C3KDB7|C3KDB7_PSEFS	209	65793	8	8	7	7	0.66	ATP-dependent lipid A
486	1	db$2	2::C3K0T3|C3K0T3_PSEFS	209	28958	5	5	5	5	1.26	Putative membrane pr
487	1	db$2	2::C3K5D5|C3K5D5_PSEFS	209	41033	5	5	4	4	0.58	Succinyl-diaminopimel
488	1	db$2	2::C3K9U7|C3K9U7_PSEFS	208	56147	11	10	10	9	1.32	Glucose-6-phosphate
489	1	db$2	2::C3K307|TRPC_PSEFS	208	30252	12	11	10	10	3.74	Indole-3-glycerol phos
490	1	db$2	2::C3K5J3|C3K5J3_PSEFS	207	50388	8	8	7	7	0.93	AMP nucleosidase OS=
491	1	db$2	2::C3K3A2|C3K3A2_PSEFS	207	38105	11	10	8	7	1.38	L-threonine aldolase C
492	1	db$2	2::C3K3P5|C3K3P5_PSEFS	207	48453	6	6	5	5	0.63	Putative symporter me
493	1	db$2	2::C3KBZ0|PYRC_PSEFS	207	38385	7	6	7	6	1.09	Dihydroorotase OS=Ps
494	1	db$2	2::C3KDQ9|C3KDQ9_PSEFS	206	29676	14	11	12	9	3.19	Ferredoxin--NADP red
495	1	db$2	2::C3K187|C3K187_PSEFS	204	38556	15	12	11	10	2.86	Peptidase_M75 domai
496	1	db$2	2::C3K5L8|C3K5L8_PSEFS	204	39360	7	7	6	6	1.32	Phosphorous compour
497	1	db$2	2::C3K6R3|Y2135_PSEFS	204	24050	8	8	7	7	2.92	UPF0502 protein PFLU
498	1	db$2	2::C3K110|C3K110_PSEFS	203	32414	7	7	7	7	1.77	Cysteine synthase OS
499	1	db$2	2::C3K4E4|C3K4E4_PSEFS	203	51389	12	10	11	10	1.51	Aspartate ammonia-ly
500	1	db$2	2::C3K3M3|C3K3M3_PSEFS	203	24540	8	7	5	4	1.15	Cell division ATP-bindi
501	1	db$2	2::C3K0T1|C3K0T1_PSEFS	203	94111	10	9	9	8	0.50	Aconitate hydratase O
502	1	db$2	2::C3KBL7|C3KBL7_PSEFS	203	33413	8	8	8	8	2.10	Glutamate/aspartate /
503	1	db$2	2::C3K8Y8|C3K8Y8_PSEFS	202	23915	5	5	4	4	1.20	Putative isomerase OS
504	1	db$2	2::C3K262|C3K262_PSEFS	202	12956	8	8	4	4	3.19	Protein-export membr
505	1	db$2	2::C3K0I1|C3K0I1_PSEFS	202	29699	7	6	5	4	1.21	ParA family ATPase OS
506	1	db$2	2::C3K0W1|C3K0W1_PSEFS	201	27483	7	7	7	7	2.31	Flagellar basal-body r
507	1	db$2	2::C3JYD3|SYC_PSEFS	200	51635	9	9	7	7	0.90	Cysteine--tRNA ligase
508	1	db$2	2::C3K435|C3K435_PSEFS	200	45402	11	11	11	11	2.15	Diaminopimelate deca
509	1	db$2	2::C3KED4|C3KED4_PSEFS	200	31038	6	6	5	5	1.14	4-hydroxy-tetrahydro
510	1	db$2	2::C3JYI3|PHNX_PSEFS	200	29973	6	5	5	4	1.20	Phosphonoacetaldehy
511	1	db$2	2::C3KDW3|HFQ_PSEFS	199	9318	11	11	7	7	30.86	RNA-binding protein H
512	1	db$2	2::C3K177|C3K177_PSEFS	198	65030	12	11	12	11	1.23	Phosphogluconate deh
513	1	db$2	2::C3KDM5|C3KDM5_PSEFS	198	43829	9	8	7	6	1.13	O-phosphoserine phos
514	1	db$2	2::C3K6V7|C3K6V7_PSEFS	197	46336	8	8	7	7	1.05	Carboxy-terminal proc
515	1	db$2	2::C3K6J5|SERC_PSEFS	197	39799	11	10	8	8	1.91	Phosphoserine aminot
516	1	db$2	2::C3KA84|C3KA84_PSEFS	197	13408	7	7	6	6	7.01	Putative regulatory pr
517	1	db$2	2::C3JYI0|C3JYI0_PSEFS	196	25235	6	6	5	5	1.54	Probable transcription
518	1	db$2	2::C3K527|C3K527_PSEFS	195	65564	8	8	7	7	0.78	Putative peptidase OS
519	1	db$2	2::C3K421|C3K421_PSEFS	195	46244	9	8	7	7	1.05	Putative HemY domair

↑Family	M	DB	Accession	Score	Mass	Matches	Match(sig)	Sequences	Seq(sig)	emPAI	Description
520	1	db$2	2::C3K452|XPT_PSEFS	195	20267	9	7	9	7	4.04	Xanthine phosphoribo:
521	1	db$2	2::C3K1M1|HSCA_PSEFS	194	65896	7	6	6	5	0.43	Chaperone protein Hs
522	1	db$2	2::C3K4T4|C3K4T4_PSEFS	194	21507	5	4	4	3	0.92	NAD(P)-bd_dom doma
523	1	db$2	2::C3K2S1|C3K2S1_PSEFS	194	23236	9	9	6	6	3.13	Riboflavin synthase al
524	1	db$2	2::C3JYK0|CLPP_PSEFS	194	23405	9	7	7	6	3.08	ATP-dependent Clp pr
525	1	db$2	2::C3K638|C3K638_PSEFS	194	26394	6	5	3	2	0.71	Putative short chain d
526	1	db$2	2::C3JYS0|C3JYS0_PSEFS	194	29172	7	7	5	5	1.24	Cell division coordinat
527	1	db$2	2::C3JYN4|ZIPA_PSEFS	193	31818	7	7	4	4	1.10	Cell division protein Zi
528	1	db$2	2::C3K7G7|C3K7G7_PSEFS	193	50136	7	6	7	6	0.76	Putative modulation o
529	1	db$2	2::C3K9M2|C3K9M2_PSEFS	192	37830	8	8	7	7	1.40	Putative peptidase OS
530	1	db$2	2::C3KBY3|C3KBY3_PSEFS	192	64998	5	5	5	5	0.44	Putative lipoprotein O:
531	1	db$2	2::C3K709|C3K709_PSEFS	191	7722	6	6	5	5	18.07	Cold shock protein OS
532	1	db$2	2::C3K6Y3|GATB_PSEFS	191	52868	10	9	9	8	1.05	Aspartyl/glutamyl-tRN
533	1	db$2	2::C3K868|C3K868_PSEFS	191	58117	5	4	5	4	0.39	Uncharacterized prote
534	1	db$2	2::C3JZ86|C3JZ86_PSEFS	191	21964	12	10	8	7	3.46	Superoxide dismutase
535	1	db$2	2::C3K1K8|C3K1K8_PSEFS	191	31523	8	6	6	4	1.11	Putative hydrolase OS
536	1	db$2	2::C3K3J3|C3K3J3_PSEFS	189	72855	6	6	5	5	0.39	Type IV pilus biogenes
537	1	db$2	2::C3KE99|C3KE99_PSEFS	189	30145	5	5	5	5	1.18	Quinolinate phosphoril
538	1	db$2	2::C3KDX0|C3KDX0_PSEFS	189	16992	6	5	3	2	0.73	Peptidyl-prolyl cis-trar
539	1	db$2	2::C3K2E9|C3K2E9_PSEFS	188	53251	5	4	3	2	0.31	Putative cytochrome c
540	1	db$2	2::C3K483|C3K483_PSEFS	188	78630	10	8	9	7	0.53	Guanosine-3',5'-bis(Di
540	2	db$2	2::C3K1B1|C3K1B1_PSEFS	90	46678	3	3	3	3	0.36	Putative acid toleranc
541	1	db$2	2::C3K4I0|HEM6_PSEFS	188	35313	8	7	8	7	1.55	Oxygen-dependent co
542	1	db$2	2::C3K843|C3K843_PSEFS	188	26535	9	8	6	5	1.43	Lipopolysaccharide ex
543	1	db$2	2::C3JZS5|TRPF_PSEFS	187	21966	3	3	2	2	0.53	N-(5'-phosphoribosyl)
544	1	db$2	2::C3K6S8|C3K6S8_PSEFS	187	40355	11	11	9	9	2.22	Putative acetyl-CoA tr
545	1	db$2	2::C3K7M9|C3K7M9_PSEFS	187	34271	8	8	8	8	2.01	Putative ABC transpor
546	1	db$2	2::C3JZN1|IHFA_PSEFS	186	11469	9	8	7	6	10.41	Integration host facto
547	1	db$2	2::C3JZN8|C3JZN8_PSEFS	186	10735	4	4	3	3	4.59	Uncharacterized prote
548	1	db$2	2::C3KCS6|C3KCS6_PSEFS	186	47227	6	5	6	5	0.65	UDP-N-acetylmuramo
549	1	db$2	2::C3JXX2|C3JXX2_PSEFS	184	50328	10	10	9	9	1.33	ATP-dependent RNA h
550	1	db$2	2::C3K3Q8|TYSY_PSEFS	184	31490	10	9	6	5	1.45	Thymidylate synthase
551	1	db$2	2::C3K1H2|C3K1H2_PSEFS	184	49205	11	8	9	7	0.96	Signal recognition par
552	1	db$2	2::C3KDW7|HISZ_PSEFS	184	42687	12	10	10	8	1.42	ATP phosphoribosyltra
553	1	db$2	2::C3KDC4|C3KDC4_PSEFS	184	64677	6	6	6	6	0.55	Uncharacterized prote
554	1	db$2	2::C3JXY2|C3JXY2_PSEFS	183	35731	5	4	4	3	0.49	L-allo-threonine aldola
555	1	db$2	2::C3K487|C3K487_PSEFS	183	33600	3	2	2	1	0.15	Biopolymer transport
556	1	db$2	2::C3K4U5|C3K4U5_PSEFS	183	75901	11	10	10	9	0.75	Putative protease OS=
557	1	db$2	2::C3K5N1|C3K5N1_PSEFS	183	36842	7	6	6	6	1.16	Sulfate ABC transport
558	1	db$2	2::C3K9R8|C3K9R8_PSEFS	182	135461	9	9	8	8	0.32	Methionine synthase C

↑Family	M	DB	Accession	Score	Mass	Matches	Match(sig)	Sequences	Seq(sig)	emPAI	Description
559	1	db$2	2::C3JZU0|C3JZU0_PSEFS	182	50538	9	8	8	8	1.12	3-isopropylmalate deh
560	1	db$2	2::C3KA81|C3KA81_PSEFS	181	55484	11	11	11	11	1.56	Alkyl hydroperoxide re
561	1	db$2	2::C3K703|ISPF_PSEFS	180	16742	4	4	4	4	2.05	2-C-methyl-D-erythrit
562	1	db$2	2::C3KDP4|C3KDP4_PSEFS	180	75338	9	8	9	8	0.65	Phosphate acetyltrans
563	1	db$2	2::C3K1A1|C3K1A1_PSEFS	180	10298	8	7	7	7	22.02	10 kDa chaperonin OS
564	1	db$2	2::C3KBP9|C3KBP9_PSEFS	179	58780	8	8	7	7	0.76	Phosphoglucomutase (
565	1	db$2	2::C3JXY8|C3JXY8_PSEFS	179	37476	6	6	6	6	1.13	Arginine N-succinyltra
566	1	db$2	2::C3K494|C3K494_PSEFS	178	33626	4	4	4	4	0.75	Gly-zipper_YMGG dor
567	1	db$2	2::C3KAK7|C3KAK7_PSEFS	178	20540	6	6	5	5	2.94	Putative lipoprotein OS
568	1	db$2	2::C3K479|RNPH_PSEFS	177	25645	5	4	5	4	1.08	Ribonuclease PH OS=I
569	1	db$2	2::C3KAM2|C3KAM2_PSEFS	177	49640	5	5	5	5	0.61	Putative two-compone
570	1	db$2	2::C3KD59|C3KD59_PSEFS	176	81779	7	7	6	6	0.42	YcaO domain-containi
571	1	db$2	2::C3JYT6|C3JYT6_PSEFS	176	8291	6	6	5	5	14.78	Cold shock protein csp
572	1	db$2	2::C3K240|C3K240_PSEFS	176	17616	9	9	6	6	7.35	RNA polymerase-bindi
573	1	db$2	2::C3K9Z4|C3K9Z4_PSEFS	176	58133	11	11	11	11	1.45	Putative ABC tranmsp
574	1	db$2	2::C3K2U5|C3K2U5_PSEFS	176	18989	6	6	4	4	1.69	Single-stranded DNA-I
575	1	db$2	2::C3KE71|C3KE71_PSEFS	175	51412	9	8	9	8	1.09	Replicative DNA helica
576	1	db$2	2::C3K8W7|C3K8W7_PSEFS	175	34291	6	6	5	5	0.99	Arabinose 5-phosphat
577	1	db$2	2::C3JZT9|C3JZT9_PSEFS	174	24210	6	6	5	5	1.64	3-isopropylmalate deh
578	1	db$2	2::C3K968|C3K968_PSEFS	174	55197	8	8	6	6	0.67	Putative aldehyde deh
579	1	db$2	2::C3K422|C3K422_PSEFS	174	40250	6	5	6	5	0.80	Putative uroporphyrin-
580	1	db$2	2::C3KBK6|C3KBK6_PSEFS	173	29320	5	5	4	4	1.24	Putative exported pep
581	1	db$2	2::C3KDN5|C3KDN5_PSEFS	173	35878	6	5	4	3	0.48	Renalase OS=Pseudor
582	1	db$2	2::C3K9P3|C3K9P3_PSEFS	173	9882	5	5	3	3	3.07	Peptidyl-prolyl cis-trar
583	1	SwissProt_HUMAN$	3::P68363|TBA1B_HUMAN	172	50120	7	6	7	6	0.76	Tubulin alpha-1B chai
584	1	db$2	2::C3KBX9|C3KBX9_PSEFS	172	20403	5	4	4	3	0.99	Putative phospholipid-
585	1	db$2	2::C3KBM6|C3KBM6_PSEFS	172	11934	5	5	3	3	3.71	Glutaredoxin OS=Pseu
586	1	db$2	2::C3JYE9|C3JYE9_PSEFS	172	31035	5	4	4	3	0.58	Putative hydroxymeth
587	1	db$2	2::C3K758|PYRF_PSEFS	172	24706	4	3	3	3	0.77	Orotidine 5'-phosphat
588	1	db$2	2::C3K185|C3K185_PSEFS	171	20062	5	5	4	4	2.21	Putative NAD(P)H nitr
589	1	db$2	2::C3K3X2|C3K3X2_PSEFS	171	19739	6	6	5	5	2.28	Uncharacterized prote
590	1	db$2	2::C3K2R1|DXS_PSEFS	171	68238	7	7	6	6	0.52	1-deoxy-D-xylulose-5-
591	1	db$2	2::C3KEA8|C3KEA8_PSEFS	171	45495	6	6	5	5	0.68	ATP-dependent RNA h
592	1	db$2	2::C3K0T7|C3K0T7_PSEFS	170	76509	9	9	8	8	0.75	Putative fatty acid oxi
593	1	db$2	2::C3KE48|C3KE48_PSEFS	169	19341	6	5	5	4	1.64	Peptide deformylase C
594	1	SwissProt_HUMAN$	3::P60174|TPIS_HUMAN	169	26653	6	6	6	6	1.89	Triosephosphate isom
595	1	db$2	2::C3K303|COQ7_PSEFS	169	23866	5	5	5	5	1.68	3-demethoxyubiquinol
596	1	db$2	2::C3JZN4|RL20_PSEFS	168	13348	5	5	5	5	4.74	50S ribosomal protein
597	1	db$2	2::C3KDR6|C3KDR6_PSEFS	168	40570	6	4	5	4	0.59	Putaive NADH oxidore
598	1	db$2	2::C3KAB1|C3KAB1_PSEFS	168	44397	5	5	3	3	0.38	Histidine kinase OS=P

↑Family	M	DB	Accession	Score	Mass	Matches	Match(sig)	Sequences	Seq(sig)	emPAI	Description
599	1	db$2	2::C3K743|FABA_PSEFS	168	18688	6	6	4	4	2.51	3-hydroxydecanoyl-[a
600	1	db$2	2::C3K2W9|RL16_PSEFS	168	15422	7	6	6	5	3.54	50S ribosomal protein
601	1	db$2	2::C3K7I1|C3K7I1_PSEFS	168	85859	7	6	6	5	0.32	Quinoprotein glucose (
602	1	db$2	2::C3K606|FABZ_PSEFS	167	16691	8	6	6	5	3.07	3-hydroxyacyl-[acyl-ca
603	1	db$2	2::C3K4Y3|KDPA_PSEFS	167	60181	6	5	3	2	0.27	Potassium-transportin
604	1	db$2	2::C3K084|AROC_PSEFS	167	39094	8	8	7	7	1.33	Chorismate synthase (
605	1	db$2	2::C3KAI8|C3KAI8_PSEFS	166	24497	5	5	4	4	1.61	Putative TetR-family r
606	1	db$2	2::C3K480|C3K480_PSEFS	165	31946	7	6	5	5	1.09	Uncharacterized prote
607	1	SwissProt_HUMAN$	3::P0DOX5|IGG1_HUMAN	165	49298	8	8	6	6	0.78	Immunoglobulin gamr
607	2	SwissProt_HUMAN$	3::P01859|IGHG2_HUMAN	106	35878	4	4	4	4	0.69	Immunoglobulin heavy
607	3	SwissProt_HUMAN$	3::P01861|IGHG4_HUMAN	73	35918	5	5	5	5	0.93	Immunoglobulin heavy
608	1	db$2	2::C3K5H5|C3K5H5_PSEFS	165	25721	8	8	6	6	2.60	Putative exported prot
609	1	db$2	2::C3K015|C3K015_PSEFS	165	53791	6	6	5	5	0.55	Putative ferredoxin OS
610	1	db$2	2::C3K5E5|C3K5E5_PSEFS	164	28503	8	8	7	7	2.75	Methionine aminopept
611	1	db$2	2::C3K2K6|C3K2K6_PSEFS	164	49458	6	5	6	5	0.61	tRNA-2-methylthio-N(
612	1	db$2	2::C3K4V4|C3K4V4_PSEFS	163	32182	9	9	6	6	1.79	Putative ATP-binding p
613	1	db$2	2::C3KDK8|C3KDK8_PSEFS	163	41619	4	4	4	4	0.58	DAO domain-containir
614	1	db$2	2::C3KCT0|MURG_PSEFS	163	37667	7	7	6	6	1.12	UDP-N-acetylglucosam
615	1	db$2	2::C3KDU8|C3KDU8_PSEFS	162	35029	5	5	5	5	0.96	D-cysteine desulfhydra
616	1	db$2	2::C3JYS3|C3JYS3_PSEFS	161	38665	3	3	3	3	0.44	TolA colicin import me
617	1	db$2	2::C3K3V5|C3K3V5_PSEFS	161	52101	9	7	7	6	0.73	CYTH domain-containi
618	1	db$2	2::C3KBV0|C3KBV0_PSEFS	161	28603	5	5	4	4	0.93	Putative chromosome
619	1	db$2	2::C3K0R1|C3K0R1_PSEFS	161	30842	4	4	3	3	0.58	Protein-glutamate O-n
620	1	db$2	2::C3K5G1|C3K5G1_PSEFS	161	34097	5	5	5	5	1.00	Cysteine synthase OS=
621	1	db$2	2::C3K2J9|C3K2J9_PSEFS	160	60860	5	4	3	2	0.26	Putative dehydrogenas
622	1	db$2	2::C3JXQ0|C3JXQ0_PSEFS	160	24880	8	7	6	5	1.58	Putative hydrolase OS
623	1	db$2	2::C3KCS5|C3KCS5_PSEFS	160	51798	8	7	6	5	0.73	UDP-N-acetylmuramoy
624	1	db$2	2::C3K5U6|C3K5U6_PSEFS	160	40174	10	8	6	5	0.80	dTDP-glucose 4,6-deh
625	1	db$2	2::C3K3Y8|UBID_PSEFS	160	54697	9	7	8	7	0.83	3-octaprenyl-4-hydrox
626	1	db$2	2::C3JXY4|ASTE_PSEFS	159	37404	8	7	7	7	1.42	Succinylglutamate des
627	1	db$2	2::C3JXP7|RECR_PSEFS	159	21528	7	6	6	5	1.98	Recombination proteir
628	1	db$2	2::C3KDD6|C3KDD6_PSEFS	159	33869	5	5	5	5	1.01	D-alanine--D-alanine l
629	1	db$2	2::C3K740|C3K740_PSEFS	159	33777	11	9	11	9	2.52	DUF815 domain-conta
630	1	db$2	2::C3K2L6|C3K2L6_PSEFS	159	47731	4	4	4	4	0.49	Putative lipoprotein OS
631	1	db$2	2::C3JZT0|C3JZT0_PSEFS	159	28122	5	5	5	5	1.31	Putative GntR-family t
632	1	db$2	2::C3K437|C3K437_PSEFS	159	26506	3	2	2	1	0.19	Uncharacterized prote
633	1	db$2	2::C3K3N8|C3K3N8_PSEFS	158	43920	5	5	4	4	0.54	PUA domain-containin
634	1	db$2	2::C3K5X7|C3K5X7_PSEFS	158	42054	9	8	8	7	1.20	Cell division protein Za
635	1	db$2	2::C3K2X4|RL23_PSEFS	158	10938	6	6	5	5	7.23	50S ribosomal protein
636	1	db$2	2::C3K2D6|C3K2D6_PSEFS	157	73930	6	6	6	6	0.47	Carbon starvation prot

↑Family	M	DB	Accession	Score	Mass	Matches	Match(sig)	Sequences	Seq(sig)	emPAI	Description
637	1	db$2	2::C3K6U2|C3K6U2_PSEFS	157	47692	5	5	4	4	0.49	Probable proton/glutai
638	1	db$2	2::C3K3Q1|C3K3Q1_PSEFS	156	40925	8	6	6	5	0.78	Putative oxidoreducta:
639	1	db$2	2::C3K2R5|RIBA_PSEFS	156	22312	6	5	5	4	1.87	GTP cyclohydrolase-2
640	1	db$2	2::C3K3T9|C3K3T9_PSEFS	156	49785	6	6	6	6	0.77	Putative aminotransfe
641	1	db$2	2::C3K368|C3K368_PSEFS	155	37353	5	5	5	5	0.88	Stress response kinas
642	1	db$2	2::C3K419|C3K419_PSEFS	155	17100	3	3	2	2	1.27	Transcriptional regulat
643	1	db$2	2::C3K568|TRPA_PSEFS	155	28539	4	4	4	4	0.94	Tryptophan synthase a
644	1	db$2	2::C3K3S3|C3K3S3_PSEFS	155	15261	5	5	3	3	1.51	DUF4399 domain-cont
645	1	db$2	2::C3KAC7|C3KAC7_PSEFS	155	51379	10	8	9	7	1.09	UDP-glucose 6-dehydr
646	1	db$2	2::C3KE58|C3KE58_PSEFS	154	84757	12	10	11	9	0.65	Putative S1 RNA-bindi
647	1	db$2	2::C3JYQ9|C3JYQ9_PSEFS	153	54767	4	4	4	4	0.41	Transcriptional regulat
648	1	db$2	2::C3K0M6|C3K0M6_PSEFS	153	25497	5	4	4	3	1.09	Putative tetR-family re
649	1	db$2	2::C3K763|C3K763_PSEFS	153	29996	4	4	2	2	0.37	Putative hydrolase OS
650	1	db$2	2::C3JZ77|C3JZ77_PSEFS	153	45611	8	6	7	5	0.68	Maltose-binding protei
651	1	db$2	2::C3KBB4|OADC_PSEFS	152	31135	5	5	3	3	0.58	Oxaloacetate decarbo:
652	1	db$2	2::C3K3M6|C3K3M6_PSEFS	152	53012	5	5	5	5	0.56	Peptidase_M16_C dom
653	1	db$2	2::C3K075|C3K075_PSEFS	152	77370	10	7	10	7	0.54	Putative sulfatase OS=
654	1	db$2	2::C3K3L7|THIG_PSEFS	152	28373	8	7	7	6	1.70	Thiazole synthase OS=
655	1	db$2	2::C3K254|C3K254_PSEFS	151	21027	7	6	5	4	1.45	Osmotically inducible
656	1	db$2	2::C3K2W8|RL29_PSEFS	151	7168	3	3	3	3	5.68	50S ribosomal protein
657	1	db$2	2::C3K5U5|C3K5U5_PSEFS	151	32335	6	5	5	4	0.79	Glucose-1-phosphate
658	1	db$2	2::C3KAY1|C3KAY1_PSEFS	149	44657	4	4	4	4	0.53	3-phosphoshikimate 1
659	1	db$2	2::C3K360|C3K360_PSEFS	149	23509	4	4	4	4	1.22	ATP-dependent dethio
660	1	db$2	2::C3K0D3|MINC_PSEFS	148	26249	11	9	10	8	4.04	Probable septum site-
661	1	db$2	2::C3K4R1|PLSB_PSEFS	148	95327	7	7	7	7	0.42	Glycerol-3-phosphate
662	1	db$2	2::C3K3M5|C3K3M5_PSEFS	148	50101	6	6	6	6	0.76	Putative peptidase OS
663	1	SwissProt_HUMAN$	3::Q5VTE0|EF1A3_HUMAN	148	50153	9	7	7	5	0.60	Putative elongation fa
664	1	db$2	2::C3JZ43|C3JZ43_PSEFS	148	44885	7	7	6	6	1.09	Putative aminotransfe
665	1	db$2	2::C3JY88|C3JY88_PSEFS	147	20432	9	9	7	7	6.91	NADH-quinone oxidore
666	1	db$2	2::C3KBQ3|C3KBQ3_PSEFS	146	33274	5	5	4	4	0.76	LysR-family transcript
667	1	db$2	2::C3JXZ2|C3JXZ2_PSEFS	146	36334	3	3	3	3	0.48	Putative AraC family r
668	1	SwissProt_HUMAN$	3::P62805|H4_HUMAN	145	11360	7	7	6	6	10.67	Histone H4 OS=Homo
669	1	db$2	2::C3K3G5|C3K3G5_PSEFS	145	15160	5	5	5	5	3.64	Putative thioesterase
670	1	db$2	2::C3K9S0|NFUA_PSEFS	144	20961	5	5	4	4	1.45	Fe/S biogenesis protei
671	1	db$2	2::C3K3S0|C3K3S0_PSEFS	144	23721	5	5	3	3	0.81	Putative hydrolase OS
672	1	db$2	2::C3K276|GRPE_PSEFS	143	20714	5	4	4	3	0.97	Protein GrpE OS=Pseu
673	1	db$2	2::C3JY77|C3JY77_PSEFS	143	40451	7	7	6	6	1.02	Putative 3-oxoacyl-[ac
674	1	db$2	2::C3K0V5|C3K0V5_PSEFS	143	33212	8	7	6	6	1.71	Putative keto/oxo acyl
675	1	db$2	2::C3K0G3|PPNP_PSEFS	142	10127	4	4	3	3	5.24	Pyrimidine/purine nuc
676	1	db$2	2::C3KCS8|C3KCS8_PSEFS	142	47767	5	5	5	5	0.64	UDP-N-acetylmuramoy

↑Family	M	DB	Accession	Score	Mass	Matches	Match(sig)	Sequences	Seq(sig)	emPAI	Description
677	1	db$2	2::C3K1W6|C3K1W6_PSEFS	142	13390	6	6	5	5	4.66	D-erythro-7,8-dihydro
678	1	db$2	2::C3K3K8|C3K3K8_PSEFS	142	27898	3	3	1	1	0.40	Pyrroline-5-carboxylat
679	1	db$2	2::C3K0W5|PHS_PSEFS	141	13357	6	6	4	4	3.05	Putative pterin-4-alph
680	1	db$2	2::C3KE68|RS18_PSEFS	141	8912	8	8	5	5	21.43	30S ribosomal protein
681	1	db$2	2::C3KED3|C3KED3_PSEFS	141	20551	5	4	4	4	1.49	Glycine cleavage syste
682	1	db$2	2::C3K6E5|C3K6E5_PSEFS	140	29069	5	4	4	4	0.91	Cytochrome c1 OS=Ps
683	1	db$2	2::C3K5E8|C3K5E8_PSEFS	140	28997	4	4	4	4	0.92	Cyclohexadienyl dehy
684	1	db$2	2::C3KBW8|C3KBW8_PSEFS	140	39031	8	7	8	7	1.33	D-alanine--D-alanine l
685	1	db$2	2::C3K017|PYRD_PSEFS	140	35850	8	8	8	8	1.87	Dihydroorotate dehydı
686	1	db$2	2::C3K2W2|RS8_PSEFS	140	14083	9	9	7	7	13.09	30S ribosomal protein
687	1	db$2	2::C3JY53|CYSG_PSEFS	139	50259	6	6	6	6	0.76	Siroheme synthase OS
688	1	db$2	2::C3KDC0|C3KDC0_PSEFS	139	36735	4	3	4	3	0.47	Uncharacterized prote
689	1	db$2	2::C3K6H1|C3K6H1_PSEFS	139	131195	12	12	12	12	0.54	DNA polymerase III su
690	1	db$2	2::C3KDM0|C3KDM0_PSEFS	138	69594	9	9	8	8	0.72	DNA topoisomerase 4
691	1	db$2	2::C3KCT1|MURC_PSEFS	137	52132	7	6	6	5	0.57	UDP-N-acetylmuramat
692	1	db$2	2::C3K8U2|UBIB_PSEFS	137	61616	7	7	7	7	0.71	Probable protein kinas
693	1	db$2	2::C3JXY9|C3JXY9_PSEFS	137	37073	4	4	4	4	0.66	Putative arginine N-su
694	1	db$2	2::C3JZV9|C3JZV9_PSEFS	137	34932	4	4	3	3	0.50	Putative DNA/RNA nor
695	1	db$2	2::C3KAD3|ARNC_PSEFS	137	37700	4	3	4	3	0.46	Undecaprenyl-phospha
696	1	db$2	2::C3K4V5|C3K4V5_PSEFS	137	36313	6	5	5	5	0.92	Beta-hexosaminidase
697	1	db$2	2::C3K1N1|C3K1N1_PSEFS	137	33097	5	5	5	5	1.04	Protein-export membr
698	1	db$2	2::C3K0N7|RL32_PSEFS	136	6752	5	5	3	3	6.53	50S ribosomal protein
699	1	db$2	2::C3K6Y8|C3K6Y8_PSEFS	136	34148	8	8	6	6	1.63	GTPase Era OS=Pseuc
700	1	db$2	2::C3K6A5|C3K6A5_PSEFS	136	78572	6	6	5	5	0.44	Catalase OS=Pseudon
701	1	db$2	2::C3KCV6|C3KCV6_PSEFS	136	40154	9	7	8	6	1.03	Probable alcohol dehy
702	1	db$2	2::C3KC02|C3KC02_PSEFS	136	29258	3	3	3	3	0.62	Putative acyltransfera
703	1	db$2	2::C3KDG2|C3KDG2_PSEFS	135	41589	7	6	6	5	0.98	AMP nucleosidase OS=
704	1	db$2	2::C3K5X2|C3K5X2_PSEFS	135	20559	6	5	5	5	2.14	Putative two-compone
705	1	db$2	2::C3K3T6|C3K3T6_PSEFS	135	42390	9	8	7	6	1.18	Spermidine/putrescine
706	1	db$2	2::C3K2D4|C3K2D4_PSEFS	135	35439	4	4	3	3	0.49	Putative cobalamin sy
707	1	db$2	2::C3K3Q3|C3K3Q3_PSEFS	135	61523	3	3	3	3	0.26	Putative cation ABC tr
708	1	db$2	2::C3K0I0|C3K0I0_PSEFS	134	27472	5	4	4	4	0.99	RNA polymerase sigm
709	1	db$2	2::C3KED2|C3KED2_PSEFS	134	17293	4	4	4	4	1.95	Thioredoxin peroxidas
710	1	db$2	2::C3KCG1|C3KCG1_PSEFS	134	60384	3	3	3	3	0.27	Putative secreted amic
711	1	db$2	2::C3K1D1|C3K1D1_PSEFS	134	32188	5	4	5	4	0.80	Protein HflC OS=Pseu
712	1	db$2	2::C3K062|C3K062_PSEFS	134	48996	8	7	6	6	0.97	Fumarate hydratase cl
713	1	db$2	2::C3JYA9|C3JYA9_PSEFS	133	27795	6	5	6	5	1.34	Putative TetR-family r
714	1	db$2	2::C3K371|C3K371_PSEFS	133	78727	12	11	11	10	0.83	Malate synthase G OS
715	1	db$2	2::C3K3T8|C3K3T8_PSEFS	133	39499	7	6	5	5	0.82	Putrescine ABC transp
716	1	db$2	2::C3KDM6|PSD_PSEFS	133	31380	9	6	7	4	1.46	Phosphatidylserine de

↑Family	M	DB	Accession	Score	Mass	Matches	Match(sig)	Sequences	Seq(sig)	emPAI	Description
717	1	db$2	2::C3K0H7|C3K0H7_PSEFS	133	77893	6	5	6	5	0.36	Histidine kinase OS=P
718	1	db$2	2::C3K5B9|C3K5B9_PSEFS	132	60269	3	3	2	2	0.27	Dipeptide ABC transpc
719	1	db$2	2::C3JXM0|C3JXM0_PSEFS	132	45141	3	3	3	3	0.37	Putative peptidase OS
720	1	db$2	2::C3KE43|C3KE43_PSEFS	132	32711	4	4	4	4	0.78	Lipid A biosynthesis la
721	1	db$2	2::C3K572|C3K572_PSEFS	132	7891	2	2	2	2	2.20	Uncharacterized prote
722	1	SwissProt_HUMAN$	3::P62937|PPIA_HUMAN	131	18001	5	5	5	5	2.68	Peptidyl-prolyl cis-trar
723	1	db$2	2::C3KB44|C3KB44_PSEFS	131	23546	2	2	1	1	0.49	Putative membrane pr
724	1	db$2	2::C3JY87|NUOH_PSEFS	131	37530	6	6	4	4	1.13	NADH-quinone oxidore
725	1	db$2	2::C3KCS7|MRAY_PSEFS	131	39243	3	3	3	3	0.43	Phospho-N-acetylmura
726	1	db$2	2::C3JXJ5|C3JXJ5_PSEFS	130	22111	4	4	3	3	0.89	Putative SCO1/SenC fa
727	1	db$2	2::C3K5U7|C3K5U7_PSEFS	130	105852	4	3	4	3	0.14	Putative aminotransfe
728	1	db$2	2::C3K8W6|C3K8W6_PSEFS	130	18885	5	5	5	5	2.46	3-deoxy-D-manno-oct
729	1	SwissProt_HUMAN$	3::P02679|FIBG_HUMAN	130	51479	7	6	7	6	0.74	Fibrinogen gamma cha
730	1	db$2	2::C3KAI3|AROB_PSEFS	129	39825	6	5	6	5	0.81	3-dehydroquinate synt
731	1	db$2	2::C3K648|C3K648_PSEFS	129	33905	7	7	7	7	1.65	Conserved hypothetica
732	1	db$2	2::C3K4P8|C3K4P8_PSEFS	129	24008	3	3	2	2	0.48	t(6)A37 threonylcarba
733	1	db$2	2::C3K6W7|C3K6W7_PSEFS	128	16216	5	5	4	4	3.23	Biotin carboxyl carrier
734	1	db$2	2::C3K5M0|C3K5M0_PSEFS	128	29300	7	5	6	5	1.24	Phosphonates ABC tra
735	1	db$2	2::C3KAJ9|C3KAJ9_PSEFS	128	20836	7	7	7	7	3.83	Putative hypoxanthine
736	1	db$2	2::C3K6Z7|C3K6Z7_PSEFS	128	89469	11	9	11	9	0.61	Lon protease OS=Pseu
737	1	db$2	2::C3K3L5|C3K3L5_PSEFS	128	11400	4	3	2	2	2.38	Putative membrane pr
738	1	db$2	2::C3K862|C3K862_PSEFS	128	39825	6	5	5	4	0.81	Putative outer membra
739	1	db$2	2::C3K417|C3K417_PSEFS	128	39396	4	4	3	3	0.43	Transcriptional regulat
740	1	db$2	2::C3KBM4|C3KBM4_PSEFS	128	34098	8	8	7	7	1.64	Ornithine carbamoyltra
741	1	db$2	2::C3K2P7|C3K2P7_PSEFS	127	17680	4	4	4	4	1.89	Putative acetyltransfer
742	1	db$2	2::C3K8K4|C3K8K4_PSEFS	127	58330	4	4	4	4	0.38	Putative outer membra
743	1	db$2	2::C3K6J1|UBIG_PSEFS	127	26040	5	5	3	3	1.06	Ubiquinone biosynthes
744	1	db$2	2::C3K424|HEM3_PSEFS	127	33541	4	3	3	2	0.33	Porphobilinogen deam
745	1	db$2	2::C3JXQ1|C3JXQ1_PSEFS	127	24479	5	4	3	2	0.47	Putative enoyl-CoA hy
746	1	db$2	2::C3K197|C3K197_PSEFS	127	25331	5	5	5	5	1.53	Putative two-compone
747	1	db$2	2::C3JYE4|C3JYE4_PSEFS	125	30882	10	10	9	9	2.96	Exodeoxyribonuclease
748	1	db$2	2::C3K6K4|C3K6K4_PSEFS	125	32908	3	3	3	3	0.54	Putative 5'-nucleotidas
749	1	db$2	2::Q9ZEP8|PANB_PSEFS	125	27951	4	4	3	3	0.96	3-methyl-2-oxobutanc
750	1	SwissProt_HUMAN$	3::P0DOY2|IGLC2_HUMAN	124	11287	4	4	4	4	4.15	Immunoglobulin lamb
751	1	db$2	2::C3K8W4|C3K8W4_PSEFS	125	18790	10	10	7	7	6.38	Lipopolysaccharide ex
752	1	db$2	2::C3KE78|C3KE78_PSEFS	125	15753	3	3	2	2	0.81	Azurin OS=Pseudomo
753	1	db$2	2::C3K5G2|C3K5G2_PSEFS	124	35788	8	6	8	6	1.21	Putative membrane pr
754	1	db$2	2::C3K134|C3K134_PSEFS	124	52371	4	4	3	3	0.31	Coproporphyrinogen-I
755	1	db$2	2::C3K126|C3K126_PSEFS	124	54569	6	5	5	5	0.54	2-methylcitrate dehyd
756	1	db$2	2::C3K0V4|C3K0V4_PSEFS	124	48572	5	5	5	5	0.63	Flagellin OS=Pseudom

↑Family	M	DB	Accession	Score	Mass	Matches	Match(sig)	Sequences	Seq(sig)	emPAI	Description
757	1	db$2	2::C3K4E1|C3K4E1_PSEFS	124	38799	8	8	7	7	1.34	N5-carboxyaminoimid
758	1	db$2	2::C3KE32|C3KE32_PSEFS	123	20912	2	2	1	1	0.25	Sigma factor negative
759	1	db$2	2::C3K9M1|C3K9M1_PSEFS	123	26106	2	2	2	2	0.43	Putative phosphoglyce
760	1	db$2	2::C3K7T1|C3K7T1_PSEFS	123	23131	4	4	4	4	1.26	Two-component syste
761	1	db$2	2::C3JZH3|C3JZH3_PSEFS	123	22428	6	5	6	5	1.85	Putative cytochrome c
761	2	db$2	2::C3JY78|C3JY78_PSEFS	49	22781	3	3	3	3	0.86	Putative cytochrome c
762	1	db$2	2::C3K467|C3K467_PSEFS	123	13453	3	3	2	2	1.00	Cupin_3 domain-conta
763	1	db$2	2::C3KBK9|C3KBK9_PSEFS	122	34365	4	4	4	4	0.73	Glycerate dehydrogen
764	1	db$2	2::C3K1E4|GLMU_PSEFS	122	48690	7	6	7	6	0.79	Bifunctional protein Gl
765	1	db$2	2::C3K1U9|C3K1U9_PSEFS	122	42728	4	4	3	3	0.56	Flavohemoglobin OS=
766	1	SwissProt_HUMAN$	3::P0C0S8|H2A1_HUMAN	122	14083	4	4	3	3	1.70	Histone H2A type 1 OS
766	2	SwissProt_HUMAN$	3::Q16777|H2A2C_HUMAN	70	13980	3	3	3	3	1.72	Histone H2A type 2-C
767	1	db$2	2::C3K3L6|TRMB_PSEFS	122	26990	3	3	3	3	0.69	tRNA (guanine-N(7)-)-
768	1	db$2	2::C3KE80|C3KE80_PSEFS	121	46502	6	5	6	5	0.66	Nicotinate phosphoribo
769	1	db$2	2::C3K8K2|C3K8K2_PSEFS	121	418093	4	4	4	4	0.05	Haemagg_act domain-
770	1	db$2	2::C3K7G4|C3K7G4_PSEFS	121	54521	9	8	9	8	1.00	Ribonuclease G OS=Ps
771	1	db$2	2::C3K3R6|C3K3R6_PSEFS	121	54762	10	7	8	6	0.83	L-threonine dehydrata
772	1	db$2	2::C3K6L0|EFP_PSEFS	121	21303	8	8	8	8	4.83	Elongation factor P OS
773	1	db$2	2::C3KAW2|C3KAW2_PSEFS	121	26230	5	5	5	5	1.46	Putative short chain de
774	1	db$2	2::C3K6A4|C3K6A4_PSEFS	121	36459	5	4	4	4	0.68	Methionine import ATP
775	1	db$2	2::C3K5F4|C3K5F4_PSEFS	120	21753	4	4	4	4	1.37	Putative nitroreductas
776	1	db$2	2::C3JYJ6|C3JYJ6_PSEFS	120	68407	11	10	11	10	1.00	Peptidyl-prolyl cis-trar
777	1	db$2	2::C3K229|C3K229_PSEFS	119	24861	2	1	2	1	0.21	TPR_REGION domain-
778	1	db$2	2::C3K308|TRPD_PSEFS	119	36976	6	6	6	6	1.15	Anthranilate phosphor
779	1	db$2	2::C3JZV1|C3JZV1_PSEFS	118	76010	10	8	9	8	0.65	UvrABC system protei
780	1	db$2	2::C3K2I0|C3K2I0_PSEFS	118	48538	4	4	4	4	0.48	Putative ATP-depende
781	1	db$2	2::C3JYK2|C3JYK2_PSEFS	118	30492	4	4	3	3	0.59	Bifunctional protein Fo
782	1	db$2	2::C3K481|C3K481_PSEFS	118	23434	5	5	5	5	1.73	Guanylate kinase OS=
783	1	db$2	2::C3K256|RS15_PSEFS	118	10112	4	4	3	3	2.95	30S ribosomal protein
784	1	db$2	2::C3JZQ0|C3JZQ0_PSEFS	117	38665	5	5	4	4	0.63	Putative L-asparagina
785	1	SwissProt_HUMAN$	3::P23528|COF1_HUMAN	117	18491	4	3	4	3	1.13	Cofilin-1 OS=Homo sa
786	1	db$2	2::C3JY31|C3JY31_PSEFS	117	22860	6	5	5	4	1.79	Putative biopolymer tr
787	1	db$2	2::C3K710|DCD_PSEFS	117	21231	4	4	4	4	1.43	dCTP deaminase OS=I
788	1	SwissProt_HUMAN$	3::P62979|RS27A_HUMAN	117	17953	5	5	4	4	1.83	Ubiquitin-40S ribosom
789	1	db$2	2::C3K7C4|C3K7C4_PSEFS	117	53137	10	7	9	6	0.87	DNA-binding transcrip
790	1	SwissProt_HUMAN$	3::Q5QNW6|H2B2F_HUMAN	117	13912	5	5	5	5	4.36	Histone H2B type 2-F
791	1	db$2	2::C3JYI6|C3JYI6_PSEFS	117	73112	7	5	7	5	0.38	Putative 2,4-dienoyl-C
792	1	db$2	2::C3KDS5|C3KDS5_PSEFS	117	41122	5	5	4	4	0.78	Putative hydratase/isc
793	1	db$2	2::C3K272|C3K272_PSEFS	116	40774	8	6	6	4	0.79	Carbamoyl-phosphate
794	1	db$2	2::C3K5H2|QUEF_PSEFS	116	30808	6	6	6	6	1.50	NADPH-dependent 7-c

↑Family	M	DB	Accession	Score	Mass	Matches	Match(sig)	Sequences	Seq(sig)	emPAI	Description
795	1	db$2	2::C3K253|C3K253_PSEFS	116	106648	7	6	7	6	0.31	Putative membrane pr
796	1	db$2	2::C3K091|C3K091_PSEFS	116	20089	8	7	6	5	2.21	GTP cyclohydrolase 1
796	2	db$2	2::C3K1W5|C3K1W5_PSEFS	97	20960	6	6	5	5	2.06	GTP cyclohydrolase 1
797	1	db$2	2::C3K0R0|C3K0R0_PSEFS	115	14751	3	3	2	2	0.88	Flagellar basal body rc
798	1	db$2	2::C3JXZ3|C3JXZ3_PSEFS	115	28123	6	5	5	4	0.96	Histidine transport ATI
799	1	db$2	2::C3KDG5|C3KDG5_PSEFS	115	23599	3	3	2	2	0.49	Putative regulatory pr
800	1	db$2	2::C3JY36|C3JY36_PSEFS	114	16095	6	5	6	5	3.27	Uncharacterized prote
801	1	SwissProt_HUMAN$	3::P06703|S10A6_HUMAN	114	10173	2	2	1	1	0.57	Protein S100-A6 OS=I
802	1	db$2	2::C3K257|C3K257_PSEFS	114	33362	7	7	6	6	1.33	tRNA pseudouridine sy
803	1	db$2	2::C3K114|C3K114_PSEFS	114	74752	6	5	6	5	0.37	DNA polymerase III su
804	1	db$2	2::C3K813|C3K813_PSEFS	113	36618	4	3	4	3	0.47	Proline iminopeptidase
805	1	db$2	2::C3KAY5|C3KAY5_PSEFS	113	30881	3	3	3	3	0.58	NAD-capped RNA hydr
806	1	SwissProt_HUMAN$	3::P07737|PROF1_HUMAN	113	15045	5	5	4	4	3.69	Profilin-1 OS=Homo sa
807	1	db$2	2::C3K9M0|C3K9M0_PSEFS	113	11184	3	2	3	2	1.29	SCP2 domain-containi
808	1	db$2	2::C3KE10|C3KE10_PSEFS	113	58823	4	3	4	3	0.27	Putative methyl-accep
809	1	db$2	2::C3KDL3|C3KDL3_PSEFS	113	70148	5	5	5	5	0.40	Phosphomethylpyrimic
810	1	db$2	2::C3K9G9|C3K9G9_PSEFS	113	16183	3	3	3	3	1.38	CMD domain-containir
811	1	db$2	2::C3K427|C3K427_PSEFS	113	23927	3	3	2	2	0.48	Putative glutathione S
812	1	db$2	2::C3K9Y7|C3K9Y7_PSEFS	113	30413	3	3	2	2	0.59	Undecaprenyl-diphosp
813	1	db$2	2::C3KD57|C3KD57_PSEFS	113	49686	4	4	4	4	0.46	Cystathionine beta-lya
814	1	db$2	2::C3K796|C3K796_PSEFS	113	49835	4	4	4	4	0.46	Putative glutamine syı
815	1	db$2	2::C3K1X7|RL27_PSEFS	112	9214	6	5	3	3	3.49	50S ribosomal protein
816	1	SwissProt_HUMAN$	3::P63104|1433Z_HUMAN	112	27728	6	4	6	4	0.97	14-3-3 protein zeta/de
817	1	db$2	2::C3KAH8|C3KAH8_PSEFS	112	89028	6	6	6	6	0.38	DD-transpeptidase OS
818	1	db$2	2::C3K6M4|C3K6M4_PSEFS	112	13463	4	4	2	2	3.00	Succinate dehydrogen
819	1	db$2	2::C3K9F0|C3K9F0_PSEFS	111	40567	6	6	4	4	0.79	Putative transport-rela
820	1	db$2	2::C3KAV6|C3KAV6_PSEFS	111	17648	6	6	3	3	1.22	Thiol peroxidase OS=F
821	1	db$2	2::C3KCP5|C3KCP5_PSEFS	111	109710	4	4	4	4	0.19	Bifunctional glutamine
822	1	db$2	2::C3K332|Y5583_PSEFS	110	48606	5	5	5	5	0.63	UPF0229 protein PFLU
823	1	db$2	2::C3K4C1|C3K4C1_PSEFS	110	40318	5	5	4	4	0.80	Putative nitric oxide de
824	1	db$2	2::C3K485|C3K485_PSEFS	109	26565	2	2	2	2	0.43	Putative lipoprotein OS
825	1	db$2	2::C3K2J5|C3K2J5_PSEFS	109	18057	5	4	5	4	1.82	Putative AsnC-family r
826	1	db$2	2::C3K2Y1|RS12_PSEFS	109	13716	4	4	3	3	1.76	30S ribosomal protein
827	1	db$2	2::C3K302|C3K302_PSEFS	109	12723	5	5	3	3	3.29	Putative HIT domain-c
828	1	db$2	2::C3K0U9|C3K0U9_PSEFS	108	55465	7	7	6	6	0.67	Putative two-compone
829	1	db$2	2::C3K5L2|C3K5L2_PSEFS	108	29854	4	3	4	3	0.61	Phosphonates import /
830	1	SwissProt_HUMAN$	3::P27348|1433T_HUMAN	108	27747	2	2	2	2	0.40	14-3-3 protein theta C
831	1	db$2	2::C3K337|PLSY_PSEFS	108	20278	1	1	1	1	0.26	Glycerol-3-phosphate
832	1	db$2	2::C3KBZ8|C3KBZ8_PSEFS	108	38389	4	3	3	2	0.28	Iron-sulfur cluster carı
833	1	db$2	2::C3JY82|NUOB_PSEFS	108	25341	7	7	6	6	2.68	NADH-quinone oxidore

↑Family	M	DB	Accession	Score	Mass	Matches	Match(sig)	Sequences	Seq(sig)	emPAI	Description
834	1	db$2	2::C3K0K7|C3K0K7_PSEFS	108	60595	6	6	6	6	0.60	Electron transfer flavo
835	1	db$2	2::C3K1X0|CYSZ_PSEFS	108	28826	5	4	5	4	0.92	Sulfate transporter Cy
836	1	db$2	2::C3KBX4|C3KBX4_PSEFS	108	59754	5	5	4	4	0.37	Putative membrane pr
837	1	db$2	2::C3K8U7|C3K8U7_PSEFS	108	30923	6	4	6	4	0.84	Transcriptional regulat
838	1	db$2	2::C3K3W8|C3K3W8_PSEFS	108	44404	5	5	5	5	0.70	Putative ubiquinone bi
839	1	db$2	2::C3KDM4|C3KDM4_PSEFS	107	56715	8	5	8	5	0.52	Putative membrane pr
840	1	db$2	2::C3KCS0|RL25_PSEFS	107	21502	7	7	3	3	2.70	50S ribosomal protein
841	1	db$2	2::C3K3X0|C3K3X0_PSEFS	107	42989	3	3	2	2	0.25	Putative ubiquinone bi
842	1	db$2	2::C3K3B7|C3K3B7_PSEFS	106	14934	2	2	2	2	0.86	Uncharacterized prote
843	1	db$2	2::C3K068|C3K068_PSEFS	106	65454	5	5	5	5	0.44	Putative asparagine sy
844	1	db$2	2::C3KAC2|C3KAC2_PSEFS	106	41342	4	4	4	4	0.58	Acyl-CoA dehydrogena
845	1	db$2	2::C3K5K3|C3K5K3_PSEFS	106	28692	3	3	2	2	0.64	CHAD domain-contain
846	1	db$2	2::C3K1K9|C3K1K9_PSEFS	105	42204	5	5	5	5	0.75	Putative aminotransfe
847	1	db$2	2::C3K3K3|C3K3K3_PSEFS	105	44101	6	4	5	3	0.38	Dihydroorotase-like pr
848	1	db$2	2::C3JXY6|ASTB_PSEFS	105	48741	7	6	6	5	0.79	N-succinylarginine dih
849	1	db$2	2::C3JYU5|C3JYU5_PSEFS	105	34090	4	4	4	4	0.74	Nudix hydrolase doma
850	1	db$2	2::C3K617|C3K617_PSEFS	104	19384	7	7	5	5	2.34	Uncharacterized prote
851	1	db$2	2::C3K4W2|C3K4W2_PSEFS	104	26844	4	4	4	4	1.02	Putative glycerophosp
852	1	db$2	2::C3K9H7|C3K9H7_PSEFS	104	27490	3	3	3	3	0.67	Putative short-chain d
853	1	db$2	2::C3KD21|C3KD21_PSEFS	103	81698	5	4	5	4	0.26	Putative glycogen deb
854	1	SwissProt_HUMAN$	3::P07195|LDHB_HUMAN	103	36615	5	4	5	4	0.68	L-lactate dehydrogena
854	2	SwissProt_HUMAN$	3::P00338|LDHA_HUMAN	89	36665	4	4	4	4	0.68	L-lactate dehydrogena
855	1	db$2	2::C3KBI8|C3KBI8_PSEFS	103	48805	5	3	5	3	0.34	Uncharacterized prote
856	1	db$2	2::C3KAR1|C3KAR1_PSEFS	103	16023	1	1	1	1	0.34	Putative isoquinoline 1
857	1	db$2	2::C3JYN3|C3JYN3_PSEFS	102	52328	3	3	3	3	0.31	Family S13 unassigne
858	1	db$2	2::C3KBG3|C3KBG3_PSEFS	102	24033	4	4	4	4	1.18	Curli production assen
859	1	db$2	2::C3K0M7|C3K0M7_PSEFS	102	28601	5	5	4	4	0.93	Putative deoxyribonuc
860	1	db$2	2::C3K8W9|C3K8W9_PSEFS	102	28276	3	3	2	2	0.40	Intermembrane phosp
861	1	SwissProt_HUMAN$	3::P02647|APOA1_HUMAN	102	30759	7	5	6	5	1.15	Apolipoprotein A-I OS
862	1	db$2	2::C3K2U8|C3K2U8_PSEFS	101	18135	8	8	6	6	3.73	Bacterioferritin OS=Ps
863	1	db$2	2::C3JYS6|C3JYS6_PSEFS	101	17060	2	2	2	2	0.73	Putative thioesterase (
864	1	db$2	2::C3K6W0|C3K6W0_PSEFS	100	14786	5	5	4	4	2.54	Putative rhodanese do
865	1	db$2	2::C3JYP6|C3JYP6_PSEFS	100	10477	2	2	2	2	1.43	YCII domain-containin
866	1	db$2	2::C3K3M7|C3K3M7_PSEFS	100	22464	1	1	1	1	0.23	Ribosomal RNA small
867	1	db$2	2::C3JY84|C3JY84_PSEFS	100	18006	2	2	2	2	0.68	NADH dehydrogenase
868	1	db$2	2::C3K281|C3K281_PSEFS	100	15787	3	3	2	2	0.81	Ribosome association
869	1	db$2	2::C3KBL3|C3KBL3_PSEFS	100	50616	8	8	8	8	1.11	Putative exported prot
870	1	db$2	2::C3K2K7|C3K2K7_PSEFS	99	37713	6	5	6	5	0.87	PhoH domain-containi
871	1	db$2	2::C3K1X3|C3K1X3_PSEFS	99	20670	5	5	5	5	2.12	Glutathione peroxidas
872	1	db$2	2::C3K7Y8|C3K7Y8_PSEFS	99	53697	6	6	5	5	0.55	Putative hydrolase OS

↑Family	M	DB	Accession	Score	Mass	Matches	Match(sig)	Sequences	Seq(sig)	emPAI	Description
873	1	db$2	2::C3K5K2|C3K5K2_PSEFS	99	9923	3	3	3	3	3.07	Putative lipoprotein O
874	1	db$2	2::C3K2E0|C3K2E0_PSEFS	99	48742	3	3	3	3	0.34	DNA repair protein Ra
875	1	db$2	2::C3K1H0|RIMM_PSEFS	99	20004	2	2	2	2	0.60	Ribosome maturation
876	1	db$2	2::C3KDF0|C3KDF0_PSEFS	99	18221	2	2	2	2	0.67	Putative phage tail-rel
877	1	db$2	2::C3K754|C3K754_PSEFS	98	29479	3	3	3	3	0.61	Putative lipoprotein O
878	1	db$2	2::C3K5T5|C3K5T5_PSEFS	98	25405	3	3	2	2	0.45	Carbonic anhydrase O
879	1	db$2	2::C3KEC3|C3KEC3_PSEFS	98	16145	3	3	2	2	0.79	Putative membrane pr
880	1	db$2	2::C3K0W6|C3K0W6_PSEFS	98	42755	5	5	4	4	0.74	Aspartate aminotransf
881	1	db$2	2::C3K4C8|C3K4C8_PSEFS	98	49694	3	3	3	3	0.33	Putative transport-rela
882	1	db$2	2::C3KDM3|C3KDM3_PSEFS	97	25575	2	2	2	2	0.45	Putative lipoprotein O
883	1	SwissProt_HUMAN$	3::A0A0C4DH25|KVD20_HUMAN	97	12507	1	1	1	1	0.45	Immunoglobulin kappa
884	1	db$2	2::C3K3M9|C3K3M9_PSEFS	97	30473	3	3	3	3	0.59	Putative sulfurtransfer
885	1	db$2	2::C3K021|C3K021_PSEFS	97	27911	5	5	5	5	1.33	Phosphoadenosine ph
886	1	db$2	2::C3KEA2|C3KEA2_PSEFS	97	30824	3	3	2	2	0.36	Putative inner membra
887	1	db$2	2::C3K0S1|C3K0S1_PSEFS	97	53269	2	2	2	2	0.19	Magnesium transporte
888	1	db$2	2::C3K312|C3K312_PSEFS	97	24134	4	4	3	3	0.80	Ribulose-phosphate 3-
889	1	db$2	2::C3KDB8|HLDE_PSEFS	97	49913	6	6	6	6	0.77	Bifunctional protein Hl
890	1	db$2	2::C3JZ82|C3JZ82_PSEFS	97	37007	3	2	3	2	0.29	Putative dehydrogena
891	1	db$2	2::C3K0H6|C3K0H6_PSEFS	97	40054	4	2	4	2	0.27	Protein-glutamate me
892	1	db$2	2::C3K7I9|C3K7I9_PSEFS	96	88241	6	5	6	5	0.31	Putative pinecillin acyl
893	1	db$2	2::C3K785|C3K785_PSEFS	96	24962	5	5	4	4	1.12	3-oxoadipate CoA-tra
894	1	db$2	2::C3K579|C3K579_PSEFS	96	15962	1	1	1	1	0.34	Putative thioesterase
895	1	db$2	2::C3K3K1|C3K3K1_PSEFS	96	18188	1	1	1	1	0.29	Bifunctional protein [p
896	1	db$2	2::C3K6T3|C3K6T3_PSEFS	96	23166	3	3	2	2	0.50	Thiol:disulfide intercha
897	1	db$2	2::C3K1F7|C3K1F7_PSEFS	96	69251	5	4	5	4	0.32	tRNA uridine 5-carbox
898	1	db$2	2::C3K061|C3K061_PSEFS	95	18792	4	4	4	4	1.72	DUF2059 domain-cont
899	1	db$2	2::C3KAT0|C3KAT0_PSEFS	95	40804	3	3	2	2	0.26	Putative efflux system
900	1	db$2	2::C3KBM1|C3KBM1_PSEFS	95	29989	3	3	2	2	0.60	Glycerol uptake facilita
901	1	db$2	2::C3K459|C3K459_PSEFS	95	18704	3	3	3	3	1.12	Leucine-responsive re
902	1	db$2	2::C3K107|C3K107_PSEFS	95	31415	4	4	4	4	0.82	Uncharacterized prote
903	1	db$2	2::C3K2W7|RS17_PSEFS	95	10090	3	3	3	3	2.95	30S ribosomal protein
904	1	SwissProt_HUMAN$	3::P62269|RS18_HUMAN	94	17708	2	2	2	2	0.69	40S ribosomal protein
905	1	db$2	2::C3JYN9|C3JYN9_PSEFS	94	44994	7	6	7	6	0.88	Pseudouridine synthas
906	1	db$2	2::C3KE89|C3KE89_PSEFS	94	24346	4	4	4	4	1.17	Uncharacterized prote
907	1	db$2	2::C3K8I3|C3K8I3_PSEFS	94	39921	2	2	1	1	0.13	3,4-dihydroxy-2-buta
908	1	db$2	2::C3JYS8|RUVA_PSEFS	94	22218	2	2	2	2	0.53	Holliday junction ATP-
909	1	db$2	2::C3KDB9|C3KDB9_PSEFS	94	32977	4	4	3	3	0.77	Putative lipopolysacch
910	1	db$2	2::C3K0S3|C3K0S3_PSEFS	94	67491	4	2	4	2	0.15	Putative ATP-binding
911	1	db$2	2::C3K6Y6|C3K6Y6_PSEFS	94	31839	3	3	3	3	0.56	Signal peptidase I OS
912	1	db$2	2::C3K544|C3K544_PSEFS	94	74165	6	4	5	4	0.29	Uncharacterized prote

↑Family	M	DB	Accession	Score	Mass	Matches	Match(sig)	Sequences	Seq(sig)	emPAI	Description
913	1	db$2	2::C3K5B2|C3K5B2_PSEFS	93	35714	3	3	3	3	0.49	Dipeptide ABC transpc
914	1	db$2	2::C3K6J2|C3K6J2_PSEFS	93	47817	3	3	3	3	0.35	Putative hydrolase OS
915	1	db$2	2::C3K3G7|C3K3G7_PSEFS	93	31253	8	7	7	6	1.47	Methylenetetrahydrofc
916	1	db$2	2::C3K4A7|C3K4A7_PSEFS	93	18391	4	3	3	2	1.14	Uncharacterized prote
917	1	db$2	2::C3KE20|C3KE20_PSEFS	93	33868	5	5	4	4	0.75	GCV_T domain-contai
918	1	db$2	2::C3K1A8|C3K1A8_PSEFS	93	35739	2	2	2	2	0.30	Putative lipoprotein OS
919	1	db$2	2::C3KDX7|ISPH_PSEFS	92	34499	6	6	6	6	1.27	4-hydroxy-3-methylbu
920	1	db$2	2::C3K2K4|C3K2K4_PSEFS	92	19926	2	2	2	2	0.60	Putative exported prot
921	1	db$2	2::C3K268|C3K268_PSEFS	92	11552	6	4	6	4	3.99	CRM domain-containir
922	1	db$2	2::C3K477|C3K477_PSEFS	92	29836	5	5	4	4	1.20	Exodeoxyribonuclease
923	1	db$2	2::C3K5K0|C3K5K0_PSEFS	92	25438	4	4	4	4	1.10	Two-component syste
924	1	db$2	2::C3KCB9|C3KCB9_PSEFS	92	35987	5	4	3	2	0.48	Polyphosphate kinase
925	1	db$2	2::C3K3P7|C3K3P7_PSEFS	92	49977	2	2	2	2	0.21	Putative membrane pr
926	1	db$2	2::C3K1C9|C3K1C9_PSEFS	92	65670	2	2	2	2	0.16	Putative cation-transp
927	1	SwissProt_HUMAN$	3::Q8NHW5|RLA0L_HUMAN	91	34343	2	2	2	2	0.32	60S acidic ribosomal p
928	1	db$2	2::C3JY91|C3JY91_PSEFS	91	66398	4	4	2	2	0.15	NADH-quinone oxidore
929	1	db$2	2::C3K841|C3K841_PSEFS	91	11596	7	5	6	4	6.46	Putative sigma-54 mo
930	1	db$2	2::C3K6K2|IHFB_PSEFS	90	11021	5	4	4	3	2.54	Integration host factor
931	1	db$2	2::C3K4R2|C3K4R2_PSEFS	90	7681	2	2	2	2	2.31	Major cold shock prote
932	1	db$2	2::C3K3D1|BETI_PSEFS	90	21992	2	2	2	2	0.53	HTH-type transcriptior
933	1	SwissProt_HUMAN$	3::P60842|IF4A1_HUMAN	90	46125	4	4	4	4	0.51	Eukaryotic initiation fa
934	1	db$2	2::C3K575|C3K575_PSEFS	90	14827	2	2	2	2	0.87	Osmotically inducible p
935	1	db$2	2::C3JXX6|DNLJ_PSEFS	90	85515	5	5	5	5	0.32	DNA ligase OS=Pseud
936	1	db$2	2::C3KDE9|C3KDE9_PSEFS	90	41781	3	3	3	3	0.40	Putative phage tail-rel
937	1	db$2	2::C3K468|C3K468_PSEFS	89	6077	1	1	1	1	1.10	50S ribosomal protein
938	1	db$2	2::C3K5H7|C3K5H7_PSEFS	89	43348	3	3	3	3	0.39	Putative response regu
939	1	db$2	2::C3KBB9|C3KBB9_PSEFS	89	73354	4	3	4	3	0.21	Putative protease OS=
940	1	db$2	2::C3KAJ1|C3KAJ1_PSEFS	89	48585	9	8	9	8	1.18	Omega-amino acid--py
941	1	db$2	2::C3KA90|C3KA90_PSEFS	88	36984	4	3	4	3	0.47	Putative aldo/keto red
942	1	db$2	2::C3K3D8|C3K3D8_PSEFS	88	49942	6	4	6	4	0.46	ATP-independent RNA
943	1	db$2	2::C3K6V2|HIS4_PSEFS	88	25679	6	5	4	3	1.08	1-(5-phosphoribosyl)-
944	1	db$2	2::C3KAC0|C3KAC0_PSEFS	88	40546	3	3	3	3	0.42	Butyryl-CoA dehydrog
945	1	db$2	2::C3K0S0|C3K0S0_PSEFS	87	12378	5	5	4	4	3.46	Putative DNA-binding
946	1	db$2	2::C3KDQ0|C3KDQ0_PSEFS	87	35105	4	4	4	4	0.71	2-ketogluconate reduc
947	1	db$2	2::C3K491|C3K491_PSEFS	87	76137	2	2	2	2	0.13	ATP-dependent DNA h
948	1	db$2	2::C3KAH2|C3KAH2_PSEFS	87	24970	5	3	5	3	0.76	SPOR domain-containi
949	1	db$2	2::C3K0L5|C3K0L5_PSEFS	87	17844	4	4	4	4	1.85	Putative GNAT-family
950	1	db$2	2::C3KDC7|C3KDC7_PSEFS	87	29930	2	2	2	2	0.37	Release factor glutami
951	1	db$2	2::C3K1B0|C3K1B0_PSEFS	87	96490	3	2	3	2	0.10	Lysylphosphatidylglyce
952	1	db$2	2::C3K6E4|C3K6E4_PSEFS	87	46143	6	5	4	4	0.51	Cytochrome b OS=Pse

↑Family	M	DB	Accession	Score	Mass	Matches	Match(sig)	Sequences	Seq(sig)	emPAI	Description
953	1	db$2	2::C3K6U9|HIS7_PSEFS	87	21830	5	4	5	4	1.36	Imidazoleglycerol-pho
954	1	db$2	2::C3JYV1|C3JYV1_PSEFS	87	32264	5	4	5	4	0.79	Formyltetrahydrofolate
955	1	db$2	2::C3K1B2|C3K1B2_PSEFS	87	62071	1	1	1	1	0.08	Single-stranded-DNA-
956	1	db$2	2::C3K4J0|C3K4J0_PSEFS	87	17598	4	3	4	3	1.22	Zn_protease domain-c
957	1	db$2	2::C3K1A4|C3K1A4_PSEFS	86	41199	4	4	3	3	0.41	Uncharacterized prote
958	1	db$2	2::C3K5E4|GLND_PSEFS	86	102948	3	3	3	3	0.15	Bifunctional uridylyltra
959	1	db$2	2::C3K2K2|THIE_PSEFS	86	22345	2	2	2	2	0.52	Thiamine-phosphate s
960	1	db$2	2::C3KDC3|LOLB_PSEFS	86	23021	3	3	3	3	0.85	Outer-membrane lipop
961	1	db$2	2::C3K2W3|C3K2W3_PSEFS	86	11427	4	2	4	2	1.25	30S ribosomal protein
962	1	db$2	2::C3JZN0|C3JZN0_PSEFS	86	13917	1	1	1	1	0.40	MerR-family transcript
963	1	db$2	2::C3K7E3|C3K7E3_PSEFS	85	178026	6	5	6	5	0.14	Alpha-2-macroglobulir
964	1	db$2	2::C3K1C5|C3K1C5_PSEFS	85	44321	5	5	5	5	0.71	Putative cyclopropane
965	1	db$2	2::C3KDX6|C3KDX6_PSEFS	85	16394	3	3	2	2	0.77	Peptidyl-prolyl cis-trar
966	1	db$2	2::C3K2V4|RS13_PSEFS	85	13332	13	9	6	4	3.05	30S ribosomal protein
967	1	db$2	2::C3K1W7|C3K1W7_PSEFS	85	11247	3	3	2	2	1.29	DUF1244 domain-cont
968	1	db$2	2::C3K0R6|C3K0R6_PSEFS	85	28374	4	4	4	4	0.94	Uncharacterized prote
969	1	db$2	2::C3KDR1|C3KDR1_PSEFS	84	17628	4	3	4	3	1.22	Uncharacterized prote
970	1	db$2	2::C3K148|APT_PSEFS	84	20064	4	4	4	4	1.54	Adenine phosphoribos
971	1	db$2	2::C3KBM3|C3KBM3_PSEFS	84	40194	2	2	2	2	0.27	Putative ABC transpor
972	1	db$2	2::C3K9N1|C3K9N1_PSEFS	84	43216	7	4	6	4	0.55	D-ser_dehydrat doma
973	1	SwissProt_HUMAN$	3::Q86YZ3|HORN_HUMAN	84	282228	2	2	2	2	0.03	Hornerin OS=Homo sa
974	1	db$2	2::C3K5K4|C3K5K4_PSEFS	84	29062	5	4	4	4	0.91	Uncharacterized prote
975	1	SwissProt_HUMAN$	3::P02747|C1QC_HUMAN	84	25757	3	2	3	2	0.44	Complement C1q subc
976	1	db$2	2::C3K1H9|C3K1H9_PSEFS	83	18692	3	3	2	2	0.65	Conserved hypothetica
977	1	db$2	2::C3K2P1|C3K2P1_PSEFS	83	48286	3	3	3	3	0.34	UDP-N-acetylmuramat
978	1	db$2	2::C3KDN7|HEMH_PSEFS	83	38415	6	3	6	3	0.45	Ferrochelatase OS=Ps
979	1	db$2	2::C3JY38|C3JY38_PSEFS	83	16812	5	5	4	4	2.05	Putative thioredoxin-re
980	1	db$2	2::C3K9U9|C3K9U9_PSEFS	83	9554	1	1	1	1	0.62	Uncharacterized prote
981	1	db$2	2::C3K486|C3K486_PSEFS	83	30593	2	2	2	2	0.36	Epimerase domain-cor
982	1	db$2	2::C3K830|C3K830_PSEFS	83	47217	6	6	6	6	0.83	Putative short chain de
983	1	db$2	2::C3K9Q2|C3K9Q2_PSEFS	83	16954	4	3	3	3	1.29	Ribonuclease H OS=Ps
984	1	db$2	2::C3K282|SSRP_PSEFS	83	18308	5	5	4	4	1.78	SsrA-binding protein C
985	1	db$2	2::C3K3D3|BETA_PSEFS	82	62642	4	3	3	2	0.16	Oxygen-dependent ch
986	1	db$2	2::C3K8K8|C3K8K8_PSEFS	82	62203	6	6	6	6	0.58	Putative exported prot
987	1	db$2	2::C3K0D2|C3K0D2_PSEFS	82	35834	4	4	4	4	0.69	Lipid A biosynthesis la
988	1	db$2	2::C3JY57|LOLA_PSEFS	82	22546	5	5	5	5	1.84	Outer-membrane lipop
989	1	db$2	2::C3K4W5|C3K4W5_PSEFS	82	24663	4	4	4	4	1.15	Lipoprotein-releasing s
990	1	db$2	2::C3KAS9|C3KAS9_PSEFS	82	109677	3	3	3	3	0.14	Efflux pump membran
991	1	db$2	2::C3K1G1|C3K1G1_PSEFS	81	48709	3	3	3	3	0.34	tRNA modification GTF
992	1	db$2	2::C3K170|C3K170_PSEFS	81	22875	4	3	4	3	0.85	Flavin_Reduct domain

↑Family	M	DB	Accession	Score	Mass	Matches	Match(sig)	Sequences	Seq(sig)	emPAI	Description
993	1	db$2	2::C3KBY0|GMHA_PSEFS	81	21250	2	2	2	2	0.56	Phosphoheptose isom
994	1	db$2	2::C3JXX3|C3JXX3_PSEFS	81	52842	2	2	2	2	0.20	Cytochrome-c oxidase
995	1	db$2	2::C3K6U7|C3K6U7_PSEFS	81	79141	6	5	6	5	0.35	Putative exported prot
996	1	db$2	2::C3K605|LPXD_PSEFS	81	36124	3	3	3	3	0.48	UDP-3-O-acylglucosan
997	1	db$2	2::C3K751|C3K751_PSEFS	81	15943	2	2	2	2	0.79	Putative thioesterase (
998	1	db$2	2::C3KDV8|C3KDV8_PSEFS	80	29265	2	2	2	2	0.38	ADP-dependent (S)-N/
999	1	db$2	2::C3K216|C3K216_PSEFS	80	17384	2	2	2	2	0.71	Putative putative lipop
1000	1	db$2	2::C3K609|C3K609_PSEFS	80	73644	4	4	4	4	0.29	Putative lytic transgly
1001	1	db$2	2::C3K4Z4|C3K4Z4_PSEFS	80	43117	3	3	3	3	0.39	Putative cytochrome C
1002	1	db$2	2::C3KBE8|C3KBE8_PSEFS	80	8134	2	2	2	2	2.10	Putative lipoprotein OS
1003	1	db$2	2::C3K1V6|C3K1V6_PSEFS	80	32555	4	3	4	3	0.55	Putative short-chain d
1004	1	db$2	2::C3K4J4|HSLO_PSEFS	80	33320	3	3	3	3	0.53	33 kDa chaperonin OS
1005	1	db$2	2::C3K9R3|C3K9R3_PSEFS	79	18863	2	2	2	2	0.64	Uncharacterized prote
1006	1	SwissProt_HUMAN$	3::Q06830|PRDX1_HUMAN	79	22096	7	7	6	6	2.58	Peroxiredoxin-1 OS=H
1007	1	db$2	2::C3K106|C3K106_PSEFS	79	20669	3	2	3	2	0.58	Uncharacterized prote
1008	1	db$2	2::C3K2L0|C3K2L0_PSEFS	79	55816	4	3	4	3	0.29	Apolipoprotein N-acylt
1009	1	db$2	2::C3K7G1|C3K7G1_PSEFS	78	37563	4	3	4	3	0.46	Cell shape protein Mre
1010	1	db$2	2::C3K6V3|HIS6_PSEFS	78	27111	5	5	5	5	1.39	Imidazole glycerol pho
1011	1	db$2	2::C3JXH2|C3JXH2_PSEFS	78	91149	2	2	2	2	0.11	Putative excinuclease
1012	1	db$2	2::C3K0C5|C3K0C5_PSEFS	78	27111	1	1	1	1	0.19	Putative amino transfe
1013	1	db$2	2::C3KAM8|C3KAM8_PSEFS	78	52747	2	2	2	2	0.20	Phosphate transporter
1014	1	db$2	2::C3K2M7|C3K2M7_PSEFS	77	18106	5	4	5	4	1.82	Ribosomal silencing fa
1015	1	db$2	2::C3JY39|C3JY39_PSEFS	77	12732	2	2	2	2	1.07	Arsenate reductase OS
1016	1	db$2	2::C3K2R8|NUSB_PSEFS	77	18764	2	2	2	2	0.65	Transcription antiterm
1017	1	db$2	2::C3KA82|C3KA82_PSEFS	77	19457	3	3	3	3	1.06	Lactoylglutathione lya
1018	1	db$2	2::C3K364|C3K364_PSEFS	77	38824	2	2	2	2	0.28	Biotin synthase OS=P
1019	1	db$2	2::C3K3H3|C3K3H3_PSEFS	77	52510	4	4	3	3	0.31	Adenosylmethionine-8
1020	1	db$2	2::C3K5E3|C3K5E3_PSEFS	76	43909	3	2	3	2	0.24	Putative aminotransfe
1021	1	db$2	2::C3KAH5|RL31_PSEFS	76	8451	3	3	2	2	1.97	50S ribosomal protein
1022	1	db$2	2::C3K9T0|NADK_PSEFS	76	32289	4	4	4	4	0.79	NAD kinase OS=Pseu
1023	1	db$2	2::C3K5J9|C3K5J9_PSEFS	76	96070	2	2	2	2	0.10	Histidine kinase OS=P
1024	1	db$2	2::C3K5F1|C3K5F1_PSEFS	76	42516	4	3	4	3	0.40	Ribonuclease D OS=P
1025	1	db$2	2::C3K574|C3K574_PSEFS	76	36116	2	2	2	2	0.30	Putative oxidoreducta
1026	1	db$2	2::C3K8P0|C3K8P0_PSEFS	76	38329	4	4	4	4	0.64	Putative nucleoside-se
1027	1	db$2	2::C3K4U2|C3K4U2_PSEFS	76	24386	1	1	1	1	0.21	Uncharacterized prote
1028	1	db$2	2::C3K9P7|C3K9P7_PSEFS	76	69450	4	4	4	4	0.31	Putative substrate-bin
1029	1	db$2	2::C3K132|C3K132_PSEFS	76	42760	2	2	2	2	0.25	Uncharacterized prote
1030	1	SwissProt_HUMAN$	3::O00139|KIF2A_HUMAN	76	79905	33	18	1	1	0.06	Kinesin-like protein KI
1031	1	db$2	2::C3K0J1|C3K0J1_PSEFS	76	36130	3	3	3	3	0.48	Flagellar motor switch
1032	1	db$2	2::C3K3Q4|C3K3Q4_PSEFS	75	38576	3	2	3	2	0.28	Putative cation ABC tr

↑Family	M	DB	Accession	Score	Mass	Matches	Match(sig)	Sequences	Seq(sig)	emPAI	Description
1033	1	db$2	2::C3KDV5|C3KDV5_PSEFS	75	20461	3	3	3	3	0.99	Oligoribonuclease OS=
1034	1	db$2	2::C3K819|C3K819_PSEFS	75	35012	1	1	1	1	0.14	Putative ABC transpor
1035	1	db$2	2::C3K8U6|C3K8U6_PSEFS	75	15270	2	2	2	2	0.84	Putative polyhydroxya
1036	1	db$2	2::C3K221|C3K221_PSEFS	75	129628	1	1	1	1	0.04	RecBCD enzyme subui
1037	1	db$2	2::C3K0P0|C3K0P0_PSEFS	75	35982	3	3	3	3	0.48	Putative peptidase OS
1038	1	db$2	2::C3KBL4|C3KBL4_PSEFS	75	26993	4	4	3	3	0.69	Glutamate/aspartate /
1039	1	db$2	2::C3K8V8|C3K8V8_PSEFS	75	36166	4	4	4	4	0.69	Zinc-type alcohol dehy
1040	1	db$2	2::C3JYL7|C3JYL7_PSEFS	75	12417	3	3	2	2	1.11	Putative copper resista
1041	1	db$2	2::C3JYU4|C3JYU4_PSEFS	75	20911	3	3	2	2	0.57	Corrinoid adenosyltrar
1042	1	db$2	2::C3K250|C3K250_PSEFS	74	71198	2	2	1	1	0.07	Acetyl-coenzyme A sy
1043	1	db$2	2::C3KDC5|HEM1_PSEFS	74	46641	4	4	4	4	0.50	Glutamyl-tRNA reducta
1044	1	db$2	2::C3KDK7|C3KDK7_PSEFS	74	24465	3	3	3	3	0.78	Putative ABC transpor
1045	1	db$2	2::C3KDR4|C3KDR4_PSEFS	74	49531	5	4	5	4	0.47	Ribosomal protein S12
1046	1	db$2	2::C3K816|OPGH_PSEFS	74	96236	5	5	5	5	0.28	Glucans biosynthesis g
1047	1	db$2	2::C3KB18|C3KB18_PSEFS	74	115005	3	3	3	3	0.13	Efflux pump membran
1048	1	db$2	2::C3KE45|C3KE45_PSEFS	73	49639	3	3	3	3	0.33	Trk system potassium
1049	1	db$2	2::C3K3V1|C3K3V1_PSEFS	73	23989	2	2	2	2	0.48	Alkyl hydroperoxide re
1050	1	db$2	2::C3JYE7|C3JYE7_PSEFS	73	35468	2	2	2	2	0.30	LysR family regulatory
1051	1	db$2	2::C3K2U6|C3K2U6_PSEFS	73	49484	2	1	2	1	0.10	Putative transport-rela
1052	1	db$2	2::C3JZ64|C3JZ64_PSEFS	73	17217	4	3	3	3	1.26	MaoC-like domain-con
1053	1	db$2	2::C3K3N0|C3K3N0_PSEFS	73	25405	2	2	2	2	0.45	Putative TetR-family r
1054	1	db$2	2::C3JY61|C3JY61_PSEFS	72	8297	4	4	4	4	8.09	Translation initiation fa
1055	1	db$2	2::C3K0Z1|C3K0Z1_PSEFS	72	36566	2	1	2	1	0.14	L-threonine-O-3-phos
1056	1	db$2	2::C3KDX3|C3KDX3_PSEFS	72	34279	5	5	5	5	0.99	Riboflavin biosynthesis
1057	1	db$2	2::C3K6T8|C3K6T8_PSEFS	72	49583	4	4	4	4	0.46	Alginate biosynthesis t
1058	1	db$2	2::C3K9N0|C3K9N0_PSEFS	72	28569	2	2	2	2	0.39	Putative transcriptiona
1059	1	db$2	2::C3KE47|FMT_PSEFS	72	33416	4	3	4	3	0.53	Methionyl-tRNA formy
1060	1	db$2	2::C3JZR8|C3JZR8_PSEFS	72	46446	6	6	5	5	0.67	Putative oxidoreductas
1061	1	db$2	2::C3K8X1|C3K8X1_PSEFS	71	23435	3	3	3	3	0.83	Putative ABC transpor
1062	1	db$2	2::C3KE30|C3KE30_PSEFS	71	59128	4	2	4	2	0.17	L-aspartate oxidase O
1063	1	db$2	2::C3K737|C3K737_PSEFS	71	17727	5	4	5	4	1.87	Glutathione peroxidas
1064	1	db$2	2::C3K9P4|C3K9P4_PSEFS	71	59261	5	5	5	5	0.49	Putative ATP-binding c
1065	1	db$2	2::C3K9M9|C3K9M9_PSEFS	71	14163	2	2	2	2	0.93	Putative endoribonucle
1066	1	db$2	2::C3K265|C3K265_PSEFS	71	29739	2	2	2	2	0.37	Dihydropteroate synth
1067	1	db$2	2::C3K5Y8|C3K5Y8_PSEFS	71	48751	5	5	4	4	0.63	L-serine dehydratase (
1067	2	db$2	2::C3JYR2|C3JYR2_PSEFS	50	48934	2	2	2	2	0.21	L-serine dehydratase (
1068	1	db$2	2::C3K7C7|C3K7C7_PSEFS	71	19943	3	3	3	3	1.02	Chorismate mutase OS
1069	1	db$2	2::C3K2T3|C3K2T3_PSEFS	71	32553	3	3	3	3	0.55	dTDP-4-dehydrorhamr
1070	1	db$2	2::C3K5Y3|C3K5Y3_PSEFS	71	35524	3	3	3	3	0.49	Putative leucine dehyc
1071	1	db$2	2::C3KB17|C3KB17_PSEFS	70	51104	2	2	2	2	0.20	Outer membrane com

↑Family	M	DB	Accession	Score	Mass	Matches	Match(sig)	Sequences	Seq(sig)	emPAI	Description
1072	1	db$2	2::C3JY69|HFLD_PSEFS	70	23173	3	3	3	3	0.84	High frequency lysoge
1073	1	db$2	2::C3K1M3|C3K1M3_PSEFS	70	11692	3	3	3	3	2.30	Iron-binding protein I:
1074	1	db$2	2::C3KB07|C3KB07_PSEFS	70	47911	2	2	2	2	0.22	Deferrochelatase/pero
1075	1	db$2	2::C3K867|C3K867_PSEFS	70	39735	3	3	3	3	0.43	PALP domain-containi
1076	1	db$2	2::C3KDW1|MUTL_PSEFS	70	69574	5	4	5	4	0.31	DNA mismatch repair
1077	1	db$2	2::C3K087|C3K087_PSEFS	70	34228	3	2	2	2	0.32	50S ribosomal protein
1078	1	db$2	2::C3K5L4|C3K5L4_PSEFS	69	28633	1	1	1	1	0.18	Phosphonates ABC tra
1079	1	db$2	2::C3K1X4|C3K1X4_PSEFS	69	16845	4	4	4	4	2.03	Putative putative expc
1080	1	db$2	2::C3KE39|C3KE39_PSEFS	69	18976	7	4	4	3	1.10	D,D-heptose 1,7-bispł
1081	1	db$2	2::C3KDM9|C3KDM9_PSEFS	69	28096	3	3	3	3	0.65	Putative oxidoreducta:
1082	1	db$2	2::C3K4C0|C3K4C0_PSEFS	69	9802	3	3	3	3	3.13	DNA-binding protein H
1083	1	db$2	2::C3KC05|C3KC05_PSEFS	69	11915	2	2	2	2	1.19	Conserved hypothetica
1084	1	db$2	2::C3K349|C3K349_PSEFS	69	28994	4	3	4	3	0.63	Pyrroloquinoline-quinc
1085	1	db$2	2::C3K146|C3K146_PSEFS	69	27134	1	1	1	1	0.19	Fumarate and nitrate
1086	1	SwissProt_HUMAN$	3::Q07020|RL18_HUMAN	69	21621	1	1	1	1	0.24	60S ribosomal protein
1087	1	db$2	2::C3K4D7|C3K4D7_PSEFS	69	14435	2	2	2	2	0.91	Putative thioesterase (
1088	1	db$2	2::C3K749|C3K749_PSEFS	69	16397	2	2	1	1	0.33	Putative phosphohistic
1089	1	db$2	2::C3K3B8|C3K3B8_PSEFS	69	42763	2	2	2	2	0.25	Uncharacterized prote
1090	1	db$2	2::C3K3L1|C3K3L1_PSEFS	68	23430	4	3	4	3	0.83	Uncharacterized prote
1091	1	db$2	2::C3K150|C3K150_PSEFS	68	25194	3	3	3	3	0.75	Putative short-chain d
1092	1	db$2	2::C3K470|C3K470_PSEFS	68	58011	6	4	6	4	0.39	Putative transport-rela
1093	1	db$2	2::C3K6E3|C3K6E3_PSEFS	68	21087	3	3	2	2	0.56	Ubiquinol-cytochrome
1094	1	db$2	2::C3K3K7|C3K3K7_PSEFS	68	24490	4	4	4	4	1.15	Pyridoxal phosphate h
1095	1	db$2	2::C3K2C2|C3K2C2_PSEFS	68	67328	3	2	3	2	0.15	Putative lipoprotein O:
1096	1	db$2	2::C3KEC7|C3KEC7_PSEFS	68	35636	1	1	1	1	0.14	Sigma factor regulator
1097	1	db$2	2::C3KAX0|C3KAX0_PSEFS	68	84238	4	2	4	2	0.12	1,4-alpha-glucan bran
1098	1	db$2	2::C3KDS6|UNG_PSEFS	68	25820	3	3	3	3	0.73	Uracil-DNA glycosylas
1099	1	db$2	2::C3K6B0|C3K6B0_PSEFS	68	33534	1	1	1	1	0.15	High-affinity zinc upta
1100	1	db$2	2::C3KCP6|C3KCP6_PSEFS	68	38240	3	3	3	3	0.45	ADP-heptose--LPS hep
1101	1	db$2	2::C3K415|C3K415_PSEFS	68	70809	2	2	2	2	0.14	Putative ABC transpor
1102	1	db$2	2::C3K5Y4|C3K5Y4_PSEFS	68	9690	1	1	1	1	0.61	Uncharacterized prote
1103	1	db$2	2::C3K4A6|C3K4A6_PSEFS	68	36556	1	1	1	1	0.14	ImpA_N domain-conta
1104	1	db$2	2::C3K3L2|C3K3L2_PSEFS	68	15596	3	3	3	3	1.45	DUF4426 domain-cont
1105	1	db$2	2::C3K4W3|C3K4W3_PSEFS	68	21770	3	2	3	2	0.54	PilZ domain-containin
1106	1	db$2	2::C3JZ24|C3JZ24_PSEFS	67	24729	4	4	4	4	1.14	Putative GntR-family r
1107	1	db$2	2::C3K8L0|C3K8L0_PSEFS	67	16885	2	2	2	2	0.74	Uncharacterized prote
1108	1	db$2	2::C3K3E2|C3K3E2_PSEFS	67	17110	1	1	1	1	0.31	Putative acetyltransfer
1109	1	db$2	2::C3K3E9|C3K3E9_PSEFS	67	12056	3	2	3	2	1.16	Putative lipoprotein O:
1110	1	db$2	2::C3KE17|C3KE17_PSEFS	67	24430	3	3	3	3	0.78	Two component syster
1111	1	db$2	2::C3K1N5|QUEA_PSEFS	66	38396	3	3	3	3	0.45	S-adenosylmethionine

↑Family	M	DB	Accession	Score	Mass	Matches	Match(sig)	Sequences	Seq(sig)	emPAI	Description
1112	1	db$2	2::C3KCP1|C3KCP1_PSEFS	66	23476	3	3	3	3	0.83	Peptide methionine su
1113	1	db$2	2::C3KBL5|C3KBL5_PSEFS	66	24580	2	2	2	2	0.47	Glutamate/aspartate /
1114	1	db$2	2::C3KBB8|C3KBB8_PSEFS	66	13546	4	4	4	4	2.96	Uncharacterized prote
1115	1	db$2	2::C3K7W1|C3K7W1_PSEFS	66	12229	2	2	2	2	1.14	Putative lipoprotein OS
1116	1	db$2	2::C3K4K0|C3K4K0_PSEFS	66	42230	2	2	2	2	0.25	Hypothetical exported
1117	1	SwissProt_HUMAN$	3::P61247|RS3A_HUMAN	66	29926	4	4	4	4	0.88	40S ribosomal protein
1118	1	db$2	2::C3K0U5|C3K0U5_PSEFS	66	62992	1	1	1	1	0.08	Flagellar M-ring protei
1119	1	db$2	2::C3K3K9|C3K3K9_PSEFS	66	21575	3	3	2	2	0.55	Conserved hypothetica
1120	1	SwissProt_HUMAN$	3::P0DP03|HVC05_HUMAN	66	12938	3	3	2	2	1.93	Immunoglobulin heavy
1120	2	SwissProt_HUMAN$	3::P0DOX2|IGA2_HUMAN	22	48903	2	2	2	2	0.21	Immunoglobulin alpha
1121	1	db$2	2::C3K301|C3K301_PSEFS	66	28901	3	3	3	3	0.63	Putative dehydrogenas
1122	1	db$2	2::C3K453|C3K453_PSEFS	66	71119	2	1	2	1	0.07	AcetylCoA_hyd_C dom
1123	1	db$2	2::C3KBJ5|C3KBJ5_PSEFS	66	62095	2	2	2	2	0.17	Phospholipase C OS=F
1124	1	db$2	2::C3K2H3|C3K2H3_PSEFS	66	31916	1	1	1	1	0.16	Putative metallopeptid
1125	1	db$2	2::C3JY71|C3JY71_PSEFS	65	43919	1	1	1	1	0.11	JmjC domain-containi
1126	1	db$2	2::C3K261|RIMP_PSEFS	65	17653	2	2	2	2	0.70	Ribosome maturation
1127	1	db$2	2::C3KE66|C3KE66_PSEFS	65	27011	4	3	4	3	0.69	23S rRNA (guanosine-
1128	1	db$2	2::C3KDG3|C3KDG3_PSEFS	65	15637	1	1	1	1	0.35	Putative exported prot
1129	1	db$2	2::C3KAB9|C3KAB9_PSEFS	65	40944	1	1	1	1	0.12	Putative acetyl-CoA ac
1130	1	SwissProt_HUMAN$	3::P02675|FIBB_HUMAN	65	55892	5	5	5	5	0.53	Fibrinogen beta chain
1131	1	db$2	2::C3K654|C3K654_PSEFS	65	34290	2	2	2	2	0.32	2-dehydropantoate 2-
1132	1	db$2	2::C3K1M6|C3K1M6_PSEFS	65	24782	2	2	2	2	0.46	Uncharacterized prote
1133	1	db$2	2::C3K6S4|C3K6S4_PSEFS	65	30497	1	1	1	1	0.17	Putative antibiotic bios
1134	1	db$2	2::C3K0M9|KTHY_PSEFS	64	22917	4	4	4	4	1.28	Thymidylate kinase OS
1135	1	db$2	2::C3JZ32|C3JZ32_PSEFS	64	38286	2	2	2	2	0.28	Putative cation transp
1136	1	db$2	2::C3K0M3|C3K0M3_PSEFS	64	20567	4	4	4	4	1.49	Uncharacterized prote
1137	1	db$2	2::C3KDB5|C3KDB5_PSEFS	64	33189	1	1	1	1	0.15	Putative glycosyl trans
1138	1	SwissProt_HUMAN$	3::P08238|HS90B_HUMAN	64	83212	3	3	3	3	0.19	Heat shock protein HS
1139	1	db$2	2::C3K433|CYAY_PSEFS	64	12442	3	3	2	2	1.11	Iron-sulfur cluster ass
1140	1	db$2	2::C3K6E9|C3K6E9_PSEFS	64	41977	3	3	3	3	0.40	Phosphatidylcholine-h
1141	1	db$2	2::C3K7J3|Y1319_PSEFS	64	16953	2	2	2	2	0.73	UPF0225 protein PFLU
1142	1	db$2	2::C3K7K5|C3K7K5_PSEFS	64	12541	1	1	1	1	0.45	Uncharacterized prote
1143	1	db$2	2::C3K5X1|C3K5X1_PSEFS	64	46611	2	2	2	2	0.23	Histidine kinase OS=P
1144	1	db$2	2::C3K320|C3K320_PSEFS	64	34836	1	1	1	1	0.15	Uncharacterized prote
1145	1	db$2	2::Q9ZEP7|PANC_PSEFS	64	30696	1	1	1	1	0.17	Pantothenate syntheta
1146	1	db$2	2::C3K0U7|C3K0U7_PSEFS	64	49884	2	1	2	1	0.10	Two-component respo
1147	1	db$2	2::C3K230|C3K230_PSEFS	63	85084	4	4	4	4	0.25	Penicillin-binding prot
1148	1	db$2	2::C3K101|C3K101_PSEFS	63	37185	2	2	2	2	0.29	Uncharacterized prote
1149	1	db$2	2::C3JZ23|C3JZ23_PSEFS	63	131220	3	2	3	2	0.08	Chromosome partition
1150	1	db$2	2::C3KBL9|C3KBL9_PSEFS	63	27726	4	4	4	4	0.97	Glycerol-3-phosphate

↑Family	M	DB	Accession	Score	Mass	Matches	Match(sig)	Sequences	Seq(sig)	emPAI	Description
1151	1	db$2	2::C3K598|C3K598_PSEFS	63	34258	4	4	4	4	0.73	Curved DNA-binding p
1152	1	db$2	2::C3KBT9|C3KBT9_PSEFS	63	31336	1	1	1	1	0.16	Sulfate ABC transport
1153	1	db$2	2::C3K9I2|C3K9I2_PSEFS	63	33896	2	2	2	2	0.32	Putative transcriptiona
1154	1	db$2	2::C3K863|PDXH_PSEFS	63	24884	3	3	3	3	0.76	Pyridoxine/pyridoxami
1155	1	db$2	2::C3JYR0|C3JYR0_PSEFS	63	13774	3	2	2	2	0.97	Glycine cleavage syste
1156	1	db$2	2::C3JYQ1|C3JYQ1_PSEFS	63	48078	1	1	1	1	0.10	Putative CoA transfera
1157	1	db$2	2::C3K6E8|C3K6E8_PSEFS	63	27518	2	1	1	1	0.19	Putative GntR-family r
1158	1	db$2	2::C3KB69|C3KB69_PSEFS	63	12397	1	1	1	1	0.45	Putative lipoprotein OS
1159	1	db$2	2::C3K047|C3K047_PSEFS	63	16614	2	2	2	2	0.75	Putative putative heat
1160	1	db$2	2::C3K0D7|APEB_PSEFS	63	46914	3	2	3	2	0.22	Probable M18 family a
1161	1	db$2	2::C3K231|C3K231_PSEFS	62	57678	3	3	3	3	0.28	APH domain-containin
1162	1	SwissProt_HUMAN$	3::P31946|1433B_HUMAN	62	28065	2	2	2	2	0.40	14-3-3 protein beta/al
1163	1	db$2	2::C3JYJ2|C3JYJ2_PSEFS	62	27205	2	2	2	2	0.41	Putative lipoprotein OS
1164	1	SwissProt_HUMAN$	3::Q32P51|RA1L2_HUMAN	62	34204	1	1	1	1	0.15	Heterogeneous nuclea
1165	1	db$2	2::C3K7N0|C3K7N0_PSEFS	62	28545	2	1	2	1	0.18	Transport permease p
1166	1	db$2	2::C3KAJ5|C3KAJ5_PSEFS	62	83174	3	2	3	2	0.12	Putative paraquat-indu
1167	1	db$2	2::C3K2Z9|C3K2Z9_PSEFS	62	34289	6	5	5	4	0.73	NMO domain-containir
1168	1	db$2	2::C3K838|C3K838_PSEFS	62	9715	2	2	2	2	1.57	Putative phosphocarrie
1169	1	db$2	2::C3KE87|C3KE87_PSEFS	62	46792	3	3	3	3	0.35	NADH dehydrogenase
1170	1	db$2	2::C3K0I8|C3K0I8_PSEFS	62	26895	1	1	1	1	0.19	Flagellar biosynthetic
1171	1	db$2	2::C3JZS9|C3JZS9_PSEFS	62	35591	3	2	3	2	0.30	Cbb3-type cytochrome
1171	2	db$2	2::C3JYN0|C3JYN0_PSEFS	58	33944	2	2	2	2	0.32	Cbb3-type cytochrome
1172	1	db$2	2::C3K279|C3K279_PSEFS	62	19136	5	4	2	2	1.67	Outer membrane prot
1173	1	db$2	2::C3K258|RBFA_PSEFS	62	14697	2	2	1	1	0.37	Ribosome-binding fact
1174	1	db$2	2::C3KCK0|C3KCK0_PSEFS	62	26687	6	4	5	4	1.03	Putative short-chain d
1175	1	db$2	2::C3K1L5|C3K1L5_PSEFS	61	34147	3	2	3	2	0.32	Putative DNA-binding
1176	1	db$2	2::C3KEB0|C3KEB0_PSEFS	61	36597	2	2	1	1	0.14	Putative amino acid AI
1177	1	db$2	2::C3KBI7|C3KBI7_PSEFS	61	26715	2	2	2	2	0.42	Uncharacterized prote
1178	1	SwissProt_HUMAN$	3::P62750|RL23A_HUMAN	61	17684	1	1	1	1	0.30	60S ribosomal protein
1179	1	db$2	2::C3K456|C3K456_PSEFS	61	38683	4	2	4	2	0.28	Alanine racemase OS=
1180	1	SwissProt_HUMAN$	3::P62249|RS16_HUMAN	61	16435	4	4	4	4	2.12	40S ribosomal protein
1181	1	db$2	2::C3K6P0|C3K6P0_PSEFS	61	15266	3	3	2	2	0.84	Uncharacterized prote
1182	1	db$2	2::C3K0P9|C3K0P9_PSEFS	61	25457	5	4	5	4	1.10	Two-component syste
1183	1	db$2	2::C3K7M5|C3K7M5_PSEFS	61	15789	1	1	1	1	0.35	Uncharacterized prote
1184	1	db$2	2::C3JYI2|C3JYI2_PSEFS	61	39477	3	3	3	3	0.43	2-aminoethylphosphor
1185	1	db$2	2::C3K2S3|C3K2S3_PSEFS	60	17933	2	1	2	1	0.30	Transcriptional repres
1186	1	db$2	2::C3K601|DXR_PSEFS	60	42341	3	3	3	3	0.40	1-deoxy-D-xylulose 5-
1187	1	db$2	2::C3K6J0|C3K6J0_PSEFS	60	24596	1	1	1	1	0.21	Putative phosphoglycc
1188	1	db$2	2::C3JXS4|C3JXS4_PSEFS	60	25108	1	1	1	1	0.21	Tol-Pal system protein
1189	1	db$2	2::C3K0X7|C3K0X7_PSEFS	60	126663	1	1	1	1	0.04	Histidine kinase OS=P

↑Family	M	DB	Accession	Score	Mass	Matches	Match(sig)	Sequences	Seq(sig)	emPAI	Description
1190	1	db$2	2::C3KC04|C3KC04_PSEFS	60	39513	3	3	3	3	0.43	Putative hydrolase OS
1191	1	db$2	2::C3K3L3|C3K3L3_PSEFS	59	21091	3	2	3	2	0.56	dITP/XTP pyrophospha
1192	1	SwissProt_HUMAN$	3::P02787|TRFE_HUMAN	59	77014	4	3	4	3	0.20	Serotransferrin OS=H
1193	1	db$2	2::C3K6D7|C3K6D7_PSEFS	59	21411	1	1	1	1	0.25	UPF0056 inner membr
1194	1	SwissProt_HUMAN$	3::O00299|CLIC1_HUMAN	59	26906	3	3	3	3	0.69	Chloride intracellular c
1195	1	db$2	2::C3K0S4|C3K0S4_PSEFS	59	65961	3	3	3	3	0.24	Isocitrate dehydrogen
1196	1	db$2	2::C3K2U2|C3K2U2_PSEFS	59	20033	3	2	2	1	0.26	Putative hydrolase OS
1197	1	db$2	2::C3JY92|C3JY92_PSEFS	59	55851	4	4	3	3	0.40	NADH dehydrogenase
1198	1	SwissProt_HUMAN$	3::Q9H0U4|RAB1B_HUMAN	59	22157	1	1	1	1	0.24	Ras-related protein Ra
1199	1	db$2	2::C3K440|C3K440_PSEFS	59	11389	5	4	3	3	2.38	Uncharacterized prote
1200	1	db$2	2::C3K6W5|PRMA_PSEFS	59	31717	1	1	1	1	0.16	Ribosomal protein L11
1201	1	db$2	2::C3K4I1|C3K4I1_PSEFS	59	29333	4	4	3	3	0.62	Shikimate dehydroger
1202	1	db$2	2::C3KAH4|C3KAH4_PSEFS	59	82035	2	1	2	1	0.06	Primosomal protein N'
1203	1	db$2	2::C3KBH8|C3KBH8_PSEFS	59	25745	1	1	1	1	0.20	3-oxoacyl-[acyl-carrie
1204	1	db$2	2::C3KAR4|C3KAR4_PSEFS	59	11430	1	1	1	1	0.50	Uncharacterized prote
1205	1	SwissProt_HUMAN$	3::Q9GZV4|IF5A2_HUMAN	58	16782	2	2	2	2	0.75	Eukaryotic translation
1206	1	db$2	2::C3K4A9|C3K4A9_PSEFS	58	17462	4	3	2	2	1.24	Uncharacterized prote
1207	1	db$2	2::C3K3H4|C3K3H4_PSEFS	58	26074	1	1	1	1	0.20	Ribosomal RNA small s
1208	1	db$2	2::C3K3W4|C3K3W4_PSEFS	58	13233	4	4	2	2	1.02	Glycine cleavage syste
1209	1	db$2	2::C3JZN5|RL35_PSEFS	58	7363	2	2	1	1	0.87	50S ribosomal protein
1210	1	db$2	2::C3K6S5|C3K6S5_PSEFS	58	35353	7	4	6	4	0.71	Delta-aminolevulinic a
1211	1	db$2	2::C3K454|C3K454_PSEFS	58	13613	3	2	3	2	0.98	Putative cytochrome c
1212	1	db$2	2::C3K3U4|C3K3U4_PSEFS	58	23278	1	1	1	1	0.22	Putative TetR-family r
1213	1	db$2	2::C3K1Y0|C3K1Y0_PSEFS	58	12222	1	1	1	1	0.46	Uncharacterized prote
1214	1	db$2	2::C3JY27|KDSB_PSEFS	58	27609	3	2	3	2	0.41	3-deoxy-manno-octulo
1215	1	db$2	2::C3K6U5|FETP_PSEFS	58	10399	2	2	2	2	1.43	Probable Fe(2+)-traffi
1216	1	db$2	2::C3KDF9|C3KDF9_PSEFS	58	17633	3	3	3	3	1.22	CinA domain-containir
1217	1	db$2	2::C3JYS4|C3JYS4_PSEFS	57	16520	3	3	3	3	1.34	Tol-Pal system protein
1218	1	db$2	2::C3JXL6|C3JXL6_PSEFS	57	41978	1	1	1	1	0.12	Bcr/CflA family efflux t
1219	1	db$2	2::C3KDN9|C3KDN9_PSEFS	57	40608	2	1	2	1	0.12	Probable periplasmic s
1220	1	SwissProt_HUMAN$	3::P46781|RS9_HUMAN	57	22578	2	2	2	2	0.52	40S ribosomal protein
1221	1	db$2	2::C3K7W0|C3K7W0_PSEFS	57	26786	2	2	2	2	0.42	Uncharacterized prote
1222	1	db$2	2::C3KB73|C3KB73_PSEFS	56	9692	1	1	1	1	0.61	Putative lipoprotein OS
1223	1	db$2	2::C3K0Q8|C3K0Q8_PSEFS	56	24717	1	1	1	1	0.21	Basal-body rod modifi
1224	1	db$2	2::C3JZT8|C3JZT8_PSEFS	56	28261	1	1	1	1	0.18	Putative methyltransfe
1225	1	db$2	2::C3K6U1|C3K6U1_PSEFS	56	17245	2	2	2	2	0.72	Conserved hypothetica
1226	1	db$2	2::C3K334|CCA_PSEFS	56	45916	2	2	1	1	0.11	Multifunctional CCA pr
1227	1	db$2	2::C3KDP0|C3KDP0_PSEFS	56	27498	2	2	2	2	0.41	Uncharacterized prote
1228	1	db$2	2::C3K6C3|C3K6C3_PSEFS	56	28259	1	1	1	1	0.18	Putative amino acid AI
1229	1	db$2	2::C3K5E1|C3K5E1_PSEFS	56	13734	5	3	4	3	1.76	Uncharacterized prote

↑Family	M	DB	Accession	Score	Mass	Matches	Match(sig)	Sequences	Seq(sig)	emPAI	Description
1230	1	SwissProt_HUMAN$	3::P62913|RL11_HUMAN	56	20240	2	2	2	2	0.59	60S ribosomal protein
1231	1	db$2	2::C3JZU8|C3JZU8_PSEFS	56	30426	2	2	2	2	0.36	Hydrolase_4 domain-c
1232	1	db$2	2::C3JXZ6|C3JXZ6_PSEFS	56	24822	1	1	1	1	0.21	Putative histidine tran
1233	1	db$2	2::C3K591|C3K591_PSEFS	56	19872	2	1	2	1	0.27	Putative bacterioferriti
1234	1	db$2	2::C3KAG2|C3KAG2_PSEFS	55	280833	2	2	2	2	0.03	Toxin CdiA OS=Pseud
1235	1	db$2	2::C3K7A2|C3K7A2_PSEFS	55	33841	1	1	1	1	0.15	Putative membrane pr
1236	1	SwissProt_HUMAN$	3::P01011|AACT_HUMAN	55	47621	2	2	2	2	0.22	Alpha-1-antichymotry
1237	1	db$2	2::C3K6Z2|C3K6Z2_PSEFS	55	17868	2	2	2	2	0.69	tRNA-specific adenosir
1238	1	SwissProt_HUMAN$	3::P04406|G3P_HUMAN	55	36030	1	1	1	1	0.14	Glyceraldehyde-3-pho
1239	1	db$2	2::C3K0Y8|COBT_PSEFS	55	36066	1	1	1	1	0.14	Nicotinate-nucleotide-
1240	1	db$2	2::C3K4M2|C3K4M2_PSEFS	55	36363	3	2	3	2	0.30	Catabolite repressor/a
1241	1	db$2	2::C3KDN1|C3KDN1_PSEFS	55	54005	2	2	2	2	0.19	Deoxyribodipyrimidine
1242	1	db$2	2::C3K9Q9|C3K9Q9_PSEFS	55	19670	1	1	1	1	0.27	FMN_red domain-cont
1243	1	SwissProt_HUMAN$	3::A6NJ78|MET15_HUMAN	55	46092	2	2	1	1	0.11	12S rRNA N4-methylc
1244	1	db$2	2::C3KDN6|C3KDN6_PSEFS	55	32369	1	1	1	1	0.16	Uncharacterized prote
1245	1	db$2	2::C3K1G6|C3K1G6_PSEFS	55	25762	6	5	5	4	1.08	Thiol:disulfide intercha
1246	1	SwissProt_HUMAN$	3::P62826|RAN_HUMAN	55	24408	2	1	2	1	0.21	GTP-binding nuclear p
1247	1	db$2	2::C3K5Z0|C3K5Z0_PSEFS	55	15508	5	3	5	3	1.46	Uncharacterized prote
1248	1	db$2	2::C3K0P5|C3K0P5_PSEFS	55	35572	3	3	3	3	0.49	Uncharacterized prote
1249	1	db$2	2::C3K092|C3K092_PSEFS	55	14085	2	2	2	2	0.94	Glutaredoxin domain-(
1250	1	db$2	2::C3K103|C3K103_PSEFS	54	23527	5	4	5	4	1.22	Phosphoribosylglycina
1251	1	db$2	2::C3K2L4|C3K2L4_PSEFS	54	37419	1	1	1	1	0.13	DNA polymerase III su
1252	1	db$2	2::C3K1A2|C3K1A2_PSEFS	54	17206	1	1	1	1	0.31	Putative membrane pr
1253	1	db$2	2::C3K584|C3K584_PSEFS	54	15399	2	1	2	1	0.35	Uncharacterized prote
1254	1	db$2	2::C3K2M0|Y5418_PSEFS	54	10305	1	1	1	1	0.57	UPF0250 protein PFLU
1255	1	db$2	2::C3KAL0|C3KAL0_PSEFS	54	43962	2	2	2	2	0.24	PlsC domain-containin
1256	1	db$2	2::C3K0K4|C3K0K4_PSEFS	54	29536	1	1	1	1	0.17	PBPb domain-containi
1257	1	db$2	2::C3K9N4|C3K9N4_PSEFS	54	91939	3	2	3	2	0.11	Histidine kinase OS=P
1258	1	SwissProt_HUMAN$	3::P62424|RL7A_HUMAN	54	29977	1	1	1	1	0.17	60S ribosomal protein
1259	1	db$2	2::C3JXS3|C3JXS3_PSEFS	54	15974	1	1	1	1	0.34	Tol-Pal system protein
1260	1	SwissProt_HUMAN$	3::P23396|RS3_HUMAN	54	26671	3	3	3	3	0.70	40S ribosomal protein
1261	1	db$2	2::C3JY68|MNMA_PSEFS	54	41397	3	3	3	3	0.41	tRNA-specific 2-thiour
1262	1	db$2	2::C3K979|C3K979_PSEFS	54	30391	4	3	3	2	0.36	Putative glutathione S
1263	1	db$2	2::C3K8K6|C3K8K6_PSEFS	54	15117	1	1	1	1	0.36	Putative transport-rela
1264	1	db$2	2::C3K4Z0|CCME_PSEFS	54	16101	2	2	2	2	0.79	Cytochrome c-type bic
1265	1	db$2	2::C3K595|C3K595_PSEFS	54	10805	1	1	1	1	0.54	Phosphate starvation-
1266	1	db$2	2::C3JXY3|C3JXY3_PSEFS	53	18788	2	1	2	1	0.28	6,7-dimethyl-8-ribityll
1267	1	db$2	2::C3K6A6|C3K6A6_PSEFS	53	25667	2	2	2	2	0.44	Putative lipoprotein OS
1268	1	db$2	2::C3KB68|C3KB68_PSEFS	53	11363	1	1	1	1	0.51	DUF1883 domain-cont
1269	1	db$2	2::C3K4U6|C3K4U6_PSEFS	53	17513	1	1	1	1	0.31	Conserved hypothetica

↑Family	M	DB	Accession	Score	Mass	Matches	Match(sig)	Sequences	Seq(sig)	emPAI	Description
1270	1	db$2	2::C3KB19|C3KB19_PSEFS	53	44434	5	4	5	4	0.53	HlyD family secretion
1271	1	db$2	2::C3K6V0|C3K6V0_PSEFS	53	23354	1	1	1	1	0.22	Imidazole glycerol pho
1272	1	db$2	2::C3K0N3|ACP_PSEFS	53	8637	2	1	2	1	0.70	Acyl carrier protein OS
1273	1	db$2	2::C3K6Y0|C3K6Y0_PSEFS	53	13973	1	1	1	1	0.40	4-carboxymuconolacto
1274	1	db$2	2::C3JZ63|C3JZ63_PSEFS	53	34729	2	2	2	2	0.31	Hydrolase_4 domain-c
1275	1	SwissProt_HUMAN$	3::P61604|CH10_HUMAN	53	10925	5	4	5	4	4.49	10 kDa heat shock pro
1276	1	db$2	2::C3KCI1|C3KCI1_PSEFS	53	42811	1	1	1	1	0.12	Putative acyl-CoA deh
1277	1	db$2	2::C3KCJ9|C3KCJ9_PSEFS	53	40399	1	1	1	1	0.12	Putative phosphotrans
1278	1	db$2	2::C3K0P2|C3K0P2_PSEFS	53	35618	1	1	1	1	0.14	Pseudouridine synthas
1279	1	db$2	2::C3JZ44|C3JZ44_PSEFS	53	47432	3	3	3	3	0.35	Putative gluconate per
1280	1	db$2	2::C3K831|C3K831_PSEFS	53	45359	5	4	5	4	0.52	Putative thiolase OS=I
1281	1	db$2	2::C3K098|C3K098_PSEFS	53	23000	1	1	1	1	0.23	Putative glutathione S
1282	1	db$2	2::C3KBU0|C3KBU0_PSEFS	53	36726	4	3	4	3	0.47	Sulfate/thiosulfate imp
1283	1	db$2	2::C3K241|C3K241_PSEFS	53	33217	1	1	1	1	0.15	Glutamyl-Q tRNA(Asp)
1284	1	db$2	2::C3K482|RPOZ_PSEFS	52	9821	3	3	3	3	3.13	DNA-directed RNA pol
1285	1	db$2	2::C3K4H8|C3K4H8_PSEFS	52	20657	2	2	2	2	0.58	Threonylcarbamoyl-AN
1286	1	db$2	2::C3JXX9|C3JXX9_PSEFS	52	6925	5	5	3	3	6.30	Translational regulator
1287	1	db$2	2::C3KAF8|C3KAF8_PSEFS	52	74138	2	1	2	1	0.07	Putative lipoprotein OS
1288	1	db$2	2::C3K173|C3K173_PSEFS	52	34695	3	3	3	3	0.50	Putative AraC-family t
1289	1	db$2	2::C3K621|C3K621_PSEFS	52	26068	3	3	3	3	0.72	Putative TetR-family r
1290	1	db$2	2::C3K391|C3K391_PSEFS	52	33913	2	1	2	1	0.15	Putative acyltransferas
1291	1	db$2	2::C3K789|C3K789_PSEFS	52	16242	1	1	1	1	0.33	Uncharacterized prote
1292	1	db$2	2::C3K6S3|C3K6S3_PSEFS	52	25881	3	2	3	2	0.44	Putative glutathione S
1293	1	db$2	2::C3K5G7|PDXB_PSEFS	52	41465	2	2	2	2	0.26	Erythronate-4-phosph
1294	1	db$2	2::C3K590|C3K590_PSEFS	52	74072	2	2	2	2	0.14	Putative membrane pr
1295	1	db$2	2::C3K3G3|C3K3G3_PSEFS	52	32186	1	1	1	1	0.16	Putative membrane pr
1296	1	db$2	2::C3K666|C3K666_PSEFS	52	29461	2	2	2	2	0.38	Putative lipoprotein OS
1297	1	db$2	2::C3KDI2|C3KDI2_PSEFS	52	135789	2	2	2	2	0.07	Exonuclease OS=Pseu
1298	1	db$2	2::C3K3X4|C3K3X4_PSEFS	52	11561	2	2	1	1	1.23	Cell division protein Za
1299	1	db$2	2::C3K2E4|C3K2E4_PSEFS	52	29249	1	1	1	1	0.17	Ferredoxin--NADP red
1300	1	db$2	2::C3K425|C3K425_PSEFS	52	27295	2	2	2	2	0.41	Positive alginate biosy
1301	1	SwissProt_HUMAN$	3::P62258|1433E_HUMAN	52	29155	3	2	3	2	0.38	14-3-3 protein epsilon
1302	1	db$2	2::C3K586|C3K586_PSEFS	51	21064	5	4	5	4	1.44	ADP compounds hydro
1303	1	db$2	2::C3K3J5|C3K3J5_PSEFS	51	13219	3	3	3	3	1.88	Type IV pilus response
1304	1	db$2	2::C3K0V2|C3K0V2_PSEFS	51	48301	1	1	1	1	0.10	Flagellar hook-associa
1305	1	db$2	2::C3JZP0|C3JZP0_PSEFS	51	11251	2	2	2	2	1.29	Putative lipoprotein OS
1306	1	db$2	2::C3KC09|C3KC09_PSEFS	51	83087	3	2	3	2	0.12	Periplasmic beta-gluco
1307	1	db$2	2::C3JZH1|C3JZH1_PSEFS	51	34020	2	2	2	2	0.32	LysR-family transcript
1308	1	db$2	2::C3JXX7|C3JXX7_PSEFS	51	56979	4	3	4	3	0.28	Conserved hypothetica
1309	1	db$2	2::C3K5G4|RLMM_PSEFS	51	40585	1	1	1	1	0.12	Ribosomal RNA large s

↑Family	M	DB	Accession	Score	Mass	Matches	Match(sig)	Sequences	Seq(sig)	emPAI	Description
1310	1	db$2	2::C3K0J0|C3K0J0_PSEFS	51	16514	1	1	1	1	0.33	Flagellar motor switch
1311	1	db$2	2::C3K4U7|C3K4U7_PSEFS	51	28919	2	2	2	2	0.38	Putative amidotransfe
1312	1	db$2	2::C3K5X6|C3K5X6_PSEFS	51	49358	2	2	2	2	0.21	Tryptophan--tRNA liga
1313	1	db$2	2::C3KAF9|C3KAF9_PSEFS	51	50830	1	1	1	1	0.10	Poly-beta-1,6-N-acety
1314	1	db$2	2::C3K650|C3K650_PSEFS	51	13083	3	3	3	3	1.90	N-acetyltransferase do
1315	1	db$2	2::C3KCR9|PTH_PSEFS	51	21041	2	2	2	2	0.56	Peptidyl-tRNA hydrola
1316	1	db$2	2::C3K998|C3K998_PSEFS	51	35021	2	2	2	2	0.31	Putative dehydrogena
1317	1	db$2	2::C3K3R4|C3K3R4_PSEFS	51	24681	3	3	3	3	0.77	Putative hydrolase OS
1318	1	db$2	2::C3K300|C3K300_PSEFS	51	34122	1	1	1	1	0.15	Putative membrane pr
1319	1	SwissProt_HUMAN$	3::Q5D862|FILA2_HUMAN	50	247928	1	1	1	1	0.02	Filaggrin-2 OS=Homo
1320	1	db$2	2::C3K0L0|C3K0L0_PSEFS	50	29492	1	1	1	1	0.17	Putative IclR-family re
1321	1	db$2	2::C3K473|DUT_PSEFS	50	16022	1	1	1	1	0.34	Deoxyuridine 5'-tripho
1322	1	db$2	2::C3KE82|C3KE82_PSEFS	50	53340	3	3	3	3	0.30	Aldehyde dehydrogena
1323	1	db$2	2::C3K0U4|C3K0U4_PSEFS	50	36872	4	1	4	1	0.14	Flagellar motor switch
1324	1	db$2	2::C3K1B6|C3K1B6_PSEFS	50	10582	1	1	1	1	0.55	Uncharacterized prote
1325	1	db$2	2::C3JZP9|C3JZP9_PSEFS	50	33380	2	2	2	2	0.33	Putative ribose transp
1326	1	db$2	2::C3K0Z9|C3K0Z9_PSEFS	50	26468	1	1	1	1	0.20	Bac_DnaA domain-cor
1327	1	db$2	2::C3KB11|C3KB11_PSEFS	50	38044	1	1	1	1	0.13	Putative AraC family r
1328	1	db$2	2::C3JZ45|C3JZ45_PSEFS	50	19122	1	1	1	1	0.28	Gluconokinase OS=Ps
1329	1	db$2	2::C3K6L9|C3K6L9_PSEFS	50	49785	3	3	3	3	0.33	Phospho-2-dehydro-3-
1330	1	db$2	2::C3K7X0|C3K7X0_PSEFS	50	85237	2	1	2	1	0.06	5-methyltetrahydropte
1331	1	db$2	2::C3KAM0|C3KAM0_PSEFS	50	50872	2	2	2	2	0.20	Putative two-compone
1332	1	db$2	2::C3K6G1|RLMF_PSEFS	49	38015	2	2	2	2	0.28	Ribosomal RNA large s
1333	1	db$2	2::C3K5K1|C3K5K1_PSEFS	49	38065	3	3	3	3	0.45	Putative phospholipas
1334	1	db$2	2::C3K5T2|C3K5T2_PSEFS	49	65173	3	2	3	2	0.16	Putative dehydrogena
1335	1	db$2	2::C3JYU0|C3JYU0_PSEFS	49	30784	1	1	1	1	0.17	Putative transmembra
1336	1	SwissProt_HUMAN$	3::P62701|RS4X_HUMAN	49	29579	3	2	3	2	0.38	40S ribosomal protein
1337	1	db$2	2::C3KDA5|C3KDA5_PSEFS	49	54170	2	1	2	1	0.09	Uncharacterized prote
1338	1	db$2	2::C3K5B3|C3K5B3_PSEFS	49	34937	3	2	3	2	0.31	Dipeptide ABC transpo
1339	1	db$2	2::C3K0P6|DCTA_PSEFS	49	46761	1	1	1	1	0.11	C4-dicarboxylate trans
1340	1	db$2	2::C3JZQ4|C3JZQ4_PSEFS	49	8106	3	3	3	3	4.47	Translational regulato
1341	1	db$2	2::C3K1M4|C3K1M4_PSEFS	49	13705	3	2	2	2	0.98	Iron-sulfur cluster ass
1342	1	db$2	2::C3JY76|C3JY76_PSEFS	49	48249	3	3	3	3	0.34	Putative mechanosens
1343	1	db$2	2::C3K450|C3K450_PSEFS	49	61786	2	2	2	2	0.17	Putative EAL/GGDEF d
1344	1	db$2	2::C3K8T8|C3K8T8_PSEFS	49	14354	1	1	1	1	0.38	Sec-independent prote
1345	1	db$2	2::C3KE08|C3KE08_PSEFS	49	25044	2	2	2	2	0.46	Conserved hypothetica
1346	1	db$2	2::C3KAZ7|C3KAZ7_PSEFS	49	48577	1	1	1	1	0.10	Putative chain length
1347	1	db$2	2::C3K2L3|C3K2L3_PSEFS	48	22416	2	2	1	1	0.23	LPS-assembly lipoprot
1348	1	db$2	2::C3JZU7|C3JZU7_PSEFS	48	16433	1	1	1	1	0.33	Putative thioesterase
1349	1	db$2	2::C3JY81|C3JY81_PSEFS	48	15084	1	1	1	1	0.36	NADH-quinone oxidore

↑Family	M	DB	Accession	Score	Mass	Matches	Match(sig)	Sequences	Seq(sig)	emPAI	Description
1350	1	db$2	2::C3K088|C3K088_PSEFS	48	20655	2	2	2	2	0.58	Putative isochorismata
1351	1	db$2	2::C3K1R4|C3K1R4_PSEFS	48	17440	1	1	1	1	0.31	Putative plasmid-relat
1352	1	db$2	2::C3KAK2|C3KAK2_PSEFS	48	10989	4	4	3	3	2.54	Uncharacterized prote
1353	1	SwissProt_HUMAN$	3::P46783|RS10_HUMAN	48	18886	1	1	1	1	0.28	40S ribosomal protein
1354	1	db$2	2::C3K223|C3K223_PSEFS	48	37863	2	2	2	2	0.28	Protein-methionine-su
1355	1	db$2	2::C3KBT7|C3KBT7_PSEFS	48	36676	4	3	3	2	0.29	Sulfate ABC transport
1356	1	SwissProt_HUMAN$	3::P15531|NDKA_HUMAN	48	17138	4	4	3	3	1.27	Nucleoside diphosphat
1357	1	db$2	2::C3K5X9|C3K5X9_PSEFS	48	41639	1	1	1	1	0.12	Putative acyl-CoA deh
1358	1	db$2	2::C3K8K1|C3K8K1_PSEFS	48	40207	1	1	1	1	0.12	Phosphate-binding prc
1359	1	db$2	2::C3K0K3|C3K0K3_PSEFS	47	12996	1	1	1	1	0.43	Putative lipoprotein O!
1360	1	db$2	2::C3K607|LPXA_PSEFS	47	28002	2	2	2	2	0.40	Acyl-[acyl-carrier-prot
1361	1	db$2	2::C3K158|C3K158_PSEFS	47	36421	2	1	2	1	0.14	Putative regulatory pr
1362	1	db$2	2::C3K8H9|C3K8H9_PSEFS	47	41728	1	1	1	1	0.12	Putative polyamine AE
1363	1	db$2	2::C3K0Z5|C3K0Z5_PSEFS	47	22469	2	2	2	2	0.52	Corrinoid adenosyltrar
1364	1	db$2	2::C3K0D5|MINE_PSEFS	47	9531	5	4	5	4	5.91	Cell division topologica
1365	1	db$2	2::C3JYH9|C3JYH9_PSEFS	47	47184	2	1	2	1	0.11	Protein kinase domain
1366	1	db$2	2::C3KE46|C3KE46_PSEFS	47	47174	2	2	2	2	0.22	16S rRNA m5C967 me
1367	1	db$2	2::C3KE64|C3KE64_PSEFS	47	37016	1	1	1	1	0.14	Putative iron-binding p
1368	1	db$2	2::C3K5M2|C3K5M2_PSEFS	47	41151	4	3	3	2	0.26	Phosphorous compour
1369	1	SwissProt_HUMAN$	3::P12273|PIP_HUMAN	47	16562	2	2	2	2	0.76	Prolactin-inducible pro
1370	1	db$2	2::C3JY52|C3JY52_PSEFS	47	37777	2	2	2	2	0.28	GST C-terminal domai
1371	1	db$2	2::C3K0X3|C3K0X3_PSEFS	47	16227	2	2	2	2	0.78	Transcriptional regulat
1372	1	db$2	2::C3K1V0|C3K1V0_PSEFS	46	56633	1	1	1	1	0.09	Nitric oxide reductase
1373	1	SwissProt_HUMAN$	3::P56749|CLD12_HUMAN	46	27092	1	1	1	1	0.19	Claudin-12 OS=Homo
1374	1	SwissProt_HUMAN$	3::P01009|A1AT_HUMAN	46	46707	3	3	3	3	0.36	Alpha-1-antitrypsin OS
1375	1	db$2	2::C3K9H2|C3K9H2_PSEFS	46	39760	3	3	2	2	0.27	Putrescine ABC transp
1376	1	db$2	2::C3K0K1|C3K0K1_PSEFS	46	53436	1	1	1	1	0.09	Putative GntR-family r
1377	1	SwissProt_HUMAN$	3::P62277|RS13_HUMAN	46	17212	1	1	1	1	0.31	40S ribosomal protein
1378	1	db$2	2::C3K6W8|C3K6W8_PSEFS	46	16611	1	1	1	1	0.32	3-dehydroquinate deh
1379	1	db$2	2::C3K3T7|C3K3T7_PSEFS	46	39982	1	1	1	1	0.13	Putrescine ABC transp
1380	1	db$2	2::C3KC07|C3KC07_PSEFS	46	21449	1	1	1	1	0.25	YceI domain-containin
1381	1	db$2	2::C3K2Q3|C3K2Q3_PSEFS	46	23107	1	1	1	1	0.23	Putative hydrolase OS
1382	1	db$2	2::C3K2V5|RL36_PSEFS	46	4431	1	1	1	1	1.73	50S ribosomal protein
1383	1	db$2	2::C3K8V7|C3K8V7_PSEFS	46	6725	1	1	1	1	0.96	Uncharacterized prote
1384	1	db$2	2::C3K0T6|RRAAH_PSEFS	46	17479	2	2	2	2	0.71	Putative 4-hydroxy-4-
1385	1	SwissProt_HUMAN$	3::P37802|TAGL2_HUMAN	46	22377	2	2	2	2	0.52	Transgelin-2 OS=Hom
1386	1	SwissProt_HUMAN$	3::P19338|NUCL_HUMAN	46	76568	1	1	1	1	0.06	Nucleolin OS=Homo s
1387	1	db$2	2::C3K224|C3K224_PSEFS	45	30473	2	2	1	1	0.17	CDP-diacylglycerol--se
1388	1	db$2	2::C3K2Z1|COAX_PSEFS	45	26920	1	1	1	1	0.19	Type III pantothenate
1389	1	db$2	2::C3K390|C3K390_PSEFS	45	18447	3	2	3	2	0.66	Zn_protease domain-c

↑Family	M	DB	Accession	Score	Mass	Matches	Match(sig)	Sequences	Seq(sig)	emPAI	Description
1390	1	db$2	2::C3K333|C3K333_PSEFS	45	61021	3	3	3	3	0.26	Uncharacterized prote
1391	1	SwissProt_HUMAN$	3::P31939|PUR9_HUMAN	45	64575	1	1	1	1	0.08	Bifunctional purine bic
1392	1	db$2	2::C3K9Q1|C3K9Q1_PSEFS	45	28823	1	1	1	1	0.18	Methyltransf_11 doma
1393	1	db$2	2::C3K564|C3K564_PSEFS	45	54508	2	2	2	2	0.19	Methylmalonate-semia
1394	1	db$2	2::C3K353|C3K353_PSEFS	45	44910	1	1	1	1	0.11	Putative aminotransfe
1395	1	db$2	2::C3K911|C3K911_PSEFS	45	29460	1	1	1	1	0.17	Putative ABC transpor
1396	1	db$2	2::C3K7A7|C3K7A7_PSEFS	45	43115	3	2	3	2	0.25	Conserved hypothetica
1397	1	db$2	2::C3K4W6|C3K4W6_PSEFS	45	44511	2	2	2	2	0.24	Putative lipoprotein re
1398	1	db$2	2::C3K3Y7|C3K3Y7_PSEFS	45	35706	1	1	1	1	0.14	Putative iron-sulfur bi
1399	1	db$2	2::C3K3J1|C3K3J1_PSEFS	45	16523	1	1	1	1	0.33	Putative chemotaxis-r
1400	1	db$2	2::C3KBL6|C3KBL6_PSEFS	45	27732	3	3	3	3	0.66	Glutamate/aspartate A
1401	1	db$2	2::C3K1G3|C3K1G3_PSEFS	45	15162	2	2	2	2	0.85	Ribonuclease P protein
1402	1	db$2	2::C3K7V7|C3K7V7_PSEFS	44	18111	2	1	1	1	0.30	Uncharacterized prote
1403	1	SwissProt_HUMAN$	3::P46776|RL27A_HUMAN	44	16551	1	1	1	1	0.33	60S ribosomal protein
1404	1	db$2	2::C3KDC1|C3KDC1_PSEFS	44	29143	1	1	1	1	0.18	Putative oxidoreducta
1405	1	db$2	2::C3KDW4|C3KDW4_PSEFS	44	48912	2	2	2	2	0.21	GTPase HflX OS=Pseu
1406	1	db$2	2::C3K1J8|C3K1J8_PSEFS	44	26668	1	1	1	1	0.19	Trehalose operon tran
1407	1	db$2	2::C3K2L9|LIPB_PSEFS	44	23473	1	1	1	1	0.22	Octanoyltransferase O
1408	1	db$2	2::C3K1B4|C3K1B4_PSEFS	44	43355	2	2	2	2	0.24	Putative transferase O
1409	1	db$2	2::C3K660|C3K660_PSEFS	44	28970	1	1	1	1	0.18	Hydroxypyruvate isom
1410	1	db$2	2::C3JYF7|C3JYF7_PSEFS	44	147681	4	2	4	2	0.07	ATP-dependent helica
1411	1	db$2	2::C3KCT5|C3KCT5_PSEFS	44	27647	2	2	2	2	0.41	Putative regulatory pr
1412	1	db$2	2::C3K8U1|C3K8U1_PSEFS	44	15020	2	2	2	2	0.86	Phosphoribosyl-AMP c
1413	1	db$2	2::C3K243|C3K243_PSEFS	44	108098	1	1	1	1	0.04	Histidine kinase OS=P
1414	1	db$2	2::C3KCS4|C3KCS4_PSEFS	43	63231	2	2	2	2	0.16	Peptidoglycan D,D-tra
1415	1	db$2	2::C3K5T7|C3K5T7_PSEFS	43	23769	2	2	2	2	0.49	Uncharacterized prote
1416	1	db$2	2::C3KDQ1|C3KDQ1_PSEFS	43	35181	4	2	4	2	0.31	Putative LysR-family r
1417	1	db$2	2::C3K800|C3K800_PSEFS	43	26032	1	1	1	1	0.20	Putative GntR-family r
1418	1	SwissProt_HUMAN$	3::P05413|FABPH_HUMAN	43	14849	1	1	1	1	0.37	Fatty acid-binding pro
1419	1	db$2	2::C3K1R1|C3K1R1_PSEFS	43	13822	2	2	2	2	0.96	bPH_1 domain-contair
1420	1	db$2	2::C3K3G8|C3K3G8_PSEFS	43	41555	1	1	1	1	0.12	Uncharacterized prote
1421	1	db$2	2::C3K5L6|C3K5L6_PSEFS	43	16214	4	4	3	3	1.38	Putative phosphorous
1422	1	db$2	2::C3K328|APAG_PSEFS	43	13631	2	2	2	2	0.98	Protein ApaG OS=Pseu
1423	1	SwissProt_HUMAN$	3::P61626|LYSC_HUMAN	43	16526	1	1	1	1	0.33	Lysozyme C OS=Hom
1424	1	db$2	2::C3K663|C3K663_PSEFS	43	22341	3	1	2	1	0.23	Putative TetR-family r
1425	1	db$2	2::C3K735|MSRB_PSEFS	42	14471	1	1	1	1	0.38	Peptide methionine su
1426	1	db$2	2::C3K2V8|RL30_PSEFS	42	6521	3	3	1	1	3.01	50S ribosomal protein
1427	1	db$2	2::C3K0B8|C3K0B8_PSEFS	42	24379	1	1	1	1	0.21	Uncharacterized prote
1428	1	db$2	2::C3JZP3|C3JZP3_PSEFS	42	36685	4	3	3	2	0.29	Putative inosine-uridir
1429	1	db$2	2::C3JZR0|C3JZR0_PSEFS	42	25453	1	1	1	1	0.20	Response regulatory p

↑Family	M	DB	Accession	Score	Mass	Matches	Match(sig)	Sequences	Seq(sig)	emPAI	Description
1430	1	db$2	2::C3K6H7|C3K6H7_PSEFS	42	10540	1	1	1	1	0.55	Cell division protein Ft
1431	1	db$2	2::C3K6Z1|C3K6Z1_PSEFS	42	50711	3	3	2	2	0.21	Putative multicopper c
1432	1	db$2	2::C3K9K0|C3K9K0_PSEFS	42	37025	1	1	1	1	0.14	Glycine betaine/L-prol
1433	1	db$2	2::C3K9E9|C3K9E9_PSEFS	42	21363	2	2	1	1	0.25	Putative TetR-family r
1434	1	db$2	2::C3K2E2|C3K2E2_PSEFS	42	56906	3	2	3	2	0.18	Catalase OS=Pseudon
1435	1	db$2	2::C3KDC8|C3KDC8_PSEFS	42	26520	1	1	1	1	0.19	Molybdopterin biosynt
1436	1	db$2	2::C3K0J2|C3K0J2_PSEFS	42	17877	1	1	1	1	0.30	Flagellar protein FliL C
1437	1	db$2	2::C3JYB1|C3JYB1_PSEFS	42	43274	3	1	3	1	0.12	Uncharacterized prote
1438	1	db$2	2::C3KE01|Y992_PSEFS	41	29383	3	3	3	3	0.62	UPF0246 protein PFLU
1439	1	db$2	2::C3K1L7|RLMN_PSEFS	41	42342	3	3	3	3	0.40	Dual-specificity RNA m
1440	1	db$2	2::C3K8W2|ADE_PSEFS	41	36048	2	2	2	2	0.30	Adenine deaminase OS
1441	1	SwissProt_HUMAN$	3::P46779|RL28_HUMAN	41	15738	1	1	1	1	0.35	60S ribosomal protein
1442	1	db$2	2::C3KDJ8|C3KDJ8_PSEFS	41	23307	2	2	2	2	0.50	Methyltransf_25 doma
1443	1	db$2	2::C3K0B2|C3K0B2_PSEFS	41	20071	1	1	1	1	0.26	Putative membrane pr
1444	1	db$2	2::C3K9Y8|C3K9Y8_PSEFS	41	14463	1	1	1	1	0.38	Uncharacterized prote
1445	1	db$2	2::C3JZ52|C3JZ52_PSEFS	41	18715	1	1	1	1	0.28	Uncharacterized prote
1446	1	db$2	2::C3K2D2|C3K2D2_PSEFS	41	145573	1	1	1	1	0.03	Putative putative sens
1447	1	db$2	2::C3JYP8|C3JYP8_PSEFS	41	24937	1	1	1	1	0.21	Putative two-compone
1448	1	SwissProt_HUMAN$	3::O75818|RPP40_HUMAN	41	41807	1	1	1	1	0.12	Ribonuclease P proteir
1449	1	db$2	2::C3K7I5|C3K7I5_PSEFS	41	28342	2	2	2	2	0.40	Putative iron utilisatio
1450	1	SwissProt_HUMAN$	3::O75197|LRP5_HUMAN	41	179030	3	2	1	1	0.03	Low-density lipoprotei
1451	1	db$2	2::C3K6N8|C3K6N8_PSEFS	40	25972	2	2	2	2	0.44	Putative hydrolase OS
1452	1	SwissProt_HUMAN$	3::Q8NG92|O13H1_HUMAN	40	34547	1	1	1	1	0.15	Olfactory receptor 13H
1453	1	db$2	2::C3KAW3|C3KAW3_PSEFS	40	52085	1	1	1	1	0.10	Putative GntR-family t
1454	1	SwissProt_HUMAN$	3::P61088|UBE2N_HUMAN	40	17127	2	2	2	2	0.73	Ubiquitin-conjugating
1455	1	db$2	2::C3K964|C3K964_PSEFS	40	26948	2	2	2	2	0.42	Putative GntR-family r
1456	1	db$2	2::C3K5H4|C3K5H4_PSEFS	40	21385	1	1	1	1	0.25	Uncharacterized prote
1457	1	db$2	2::C3K832|C3K832_PSEFS	40	33163	1	1	1	1	0.15	Non-specific serine/thi
1458	1	db$2	2::C3K4V3|C3K4V3_PSEFS	40	45550	2	1	2	1	0.11	Uncharacterized prote
1459	1	db$2	2::C3JYT0|C3JYT0_PSEFS	40	26526	2	2	2	2	0.43	Probable transcription
1460	1	SwissProt_HUMAN$	3::P62888|RL30_HUMAN	40	12776	2	2	2	2	1.07	60S ribosomal protein
1461	1	db$2	2::C3K820|C3K820_PSEFS	40	26990	1	1	1	1	0.19	Putative ABC transpor
1462	1	db$2	2::C3K8L6|C3K8L6_PSEFS	40	28069	2	1	2	1	0.18	Precorrin-6A synthase
1463	1	SwissProt_HUMAN$	3::P42694|HELZ_HUMAN	40	218833	1	1	1	1	0.02	Probable helicase with
1464	1	db$2	2::C3K9S7|C3K9S7_PSEFS	40	35611	1	1	1	1	0.14	Putative magnesium c
1465	1	SwissProt_HUMAN$	3::P62851|RS25_HUMAN	39	13734	3	3	2	2	0.97	40S ribosomal protein
1466	1	db$2	2::C3JY43|TTCA_PSEFS	39	31052	2	1	2	1	0.16	tRNA-cytidine(32) 2-s
1467	1	db$2	2::C3K602|C3K602_PSEFS	39	48346	2	2	2	2	0.22	Zinc metalloprotease (
1468	1	db$2	2::C3K2M6|RLMH_PSEFS	39	17669	2	2	2	2	0.70	Ribosomal RNA large s
1469	1	db$2	2::C3K5I0|C3K5I0_PSEFS	39	36784	1	1	1	1	0.14	tRNA-dihydrouridine(2

↑Family	M	DB	Accession	Score	Mass	Matches	Match(sig)	Sequences	Seq(sig)	emPAI	Description
1470	1	db$2	2::C3K059|TRHO_PSEFS	39	35401	1	1	1	1	0.14	tRNA uridine(34) hydr
1471	1	db$2	2::C3JXI0|C3JXI0_PSEFS	39	23030	1	1	1	1	0.23	Nudix hydrolase doma
1472	1	db$2	2::C3KCB4|C3KCB4_PSEFS	39	27191	1	1	1	1	0.19	Uncharacterized prote
1473	1	db$2	2::C3K220|C3K220_PSEFS	39	136725	1	1	1	1	0.04	RecBCD enzyme subu
1474	1	db$2	2::C3K870|C3K870_PSEFS	39	20966	3	2	3	2	0.56	Uncharacterized prote
1475	1	db$2	2::C3K0J8|C3K0J8_PSEFS	39	22091	2	2	2	2	0.53	START domain-contai
1476	1	db$2	2::C3K2U3|C3K2U3_PSEFS	39	27946	3	2	3	2	0.40	Trans-aconitate 2-met
1477	1	db$2	2::C3K4S7|C3K4S7_PSEFS	39	34300	1	1	1	1	0.15	Putative LysR-family r
1478	1	db$2	2::C3K3S9|C3K3S9_PSEFS	38	35576	1	1	1	1	0.14	Hydrolase_4 domain-c
1479	1	db$2	2::C3JY97|C3JY97_PSEFS	38	30357	1	1	1	1	0.17	N-hydroxyarylamine C
1480	1	db$2	2::C3K2M3|C3K2M3_PSEFS	38	37518	2	2	2	2	0.29	Putative peptidoglycar
1481	1	db$2	2::C3KDP3|C3KDP3_PSEFS	38	34782	1	1	1	1	0.15	Putaive acyltransferas
1482	1	db$2	2::C3K7L3|C3K7L3_PSEFS	38	8069	1	1	1	1	0.76	Putative lipoprotein O
1483	1	db$2	2::C3KAX2|C3KAX2_PSEFS	38	79149	1	1	1	1	0.06	Histidine kinase OS=P
1484	1	db$2	2::C3KE16|C3KE16_PSEFS	38	37004	1	1	1	1	0.14	Putative chemotaxis-r
1485	1	db$2	2::C3K4G5|C3K4G5_PSEFS	38	18193	1	1	1	1	0.29	Putative thioesterase (
1486	1	db$2	2::C3K3R7|RPIA_PSEFS	38	23281	1	1	1	1	0.22	Ribose-5-phosphate is
1487	1	db$2	2::C3K013|C3K013_PSEFS	38	20254	3	3	2	2	0.59	Peptidyl-prolyl cis-trar
1488	1	SwissProt_HUMAN$	3::P33992|MCM5_HUMAN	38	82233	3	2	1	1	0.06	DNA replication licensi
1489	1	db$2	2::C3JXZ5|C3JXZ5_PSEFS	38	25958	1	1	1	1	0.20	Putative histidine tran
1490	1	db$2	2::C3K4F4|C3K4F4_PSEFS	38	25363	1	1	1	1	0.20	Putative acetyltransfer
1491	1	db$2	2::C3K975|C3K975_PSEFS	38	21948	1	1	1	1	0.24	Putative glutathione S
1492	1	db$2	2::C3K637|C3K637_PSEFS	38	56441	2	2	2	2	0.18	Putative peptide ABC t
1493	1	db$2	2::C3K753|C3K753_PSEFS	38	30652	1	1	1	1	0.17	Putative hydrolase OS
1494	1	SwissProt_HUMAN$	3::P61254|RL26_HUMAN	37	17248	1	1	1	1	0.31	60S ribosomal protein
1495	1	db$2	2::C3K5C4|C3K5C4_PSEFS	37	17510	1	1	1	1	0.31	SnoaL-like domain-co
1496	1	SwissProt_HUMAN$	3::Q9HCU5|PREB_HUMAN	37	45440	2	2	2	2	0.23	Prolactin regulatory el
1497	1	SwissProt_HUMAN$	3::P10909|CLUS_HUMAN	37	52461	2	2	2	2	0.20	Clusterin OS=Homo sa
1498	1	db$2	2::C3K3M2|C3K3M2_PSEFS	37	37029	2	1	2	1	0.14	Cell division protein Ft
1499	1	db$2	2::C3K0N9|C3K0N9_PSEFS	37	21007	2	2	2	2	0.56	7-methyl-GTP pyropho
1500	1	SwissProt_HUMAN$	3::P62906|RL10A_HUMAN	37	24816	1	1	1	1	0.21	60S ribosomal protein
1501	1	db$2	2::C3K6Y7|RNC_PSEFS	37	25367	1	1	1	1	0.20	Ribonuclease 3 OS=Ps
1502	1	db$2	2::C3K7L9|C3K7L9_PSEFS	37	28704	1	1	1	1	0.18	Putative membrane pr
1503	1	SwissProt_HUMAN$	3::P62136|PP1A_HUMAN	37	37488	1	1	1	1	0.13	Serine/threonine-prot
1504	1	db$2	2::C3K4R0|C3K4R0_PSEFS	37	14930	2	2	2	2	0.86	Putative lipoprotein O
1505	1	SwissProt_HUMAN$	3::P00519|ABL1_HUMAN	37	122796	1	1	1	1	0.04	Tyrosine-protein kinas
1506	1	db$2	2::C3K909|C3K909_PSEFS	36	24537	1	1	1	1	0.21	Uncharacterized prote
1507	1	db$2	2::C3JYL6|C3JYL6_PSEFS	36	25076	1	1	1	1	0.21	Putative TetR-family r
1508	1	SwissProt_HUMAN$	3::P61353|RL27_HUMAN	36	15788	3	2	3	2	0.81	60S ribosomal protein
1509	1	SwissProt_HUMAN$	3::Q71UM5|RS27L_HUMAN	36	9471	2	1	2	1	0.63	40S ribosomal protein

↑Family	M	DB	Accession	Score	Mass	Matches	Match(sig)	Sequences	Seq(sig)	emPAI	Description
1510	1	db$2	2::C3KAA6|C3KAA6_PSEFS	36	51931	1	1	1	1	0.10	Putative cell envelope
1511	1	db$2	2::C3K5D9|C3K5D9_PSEFS	36	43455	1	1	1	1	0.12	Putative cysteine desu
1512	1	db$2	2::C3KE84|C3KE84_PSEFS	36	35425	1	1	1	1	0.14	Pseudouridine synthas
1513	1	db$2	2::C3K327|C3K327_PSEFS	36	29832	2	2	2	2	0.37	Ribosomal RNA small :
1514	1	SwissProt_HUMAN$	3::P01024|CO3_HUMAN	36	187030	3	3	3	3	0.08	Complement C3 OS=H
1515	1	db$2	2::C3K9B4|C3K9B4_PSEFS	36	16186	1	1	1	1	0.33	Type II secretion syste
1516	1	db$2	2::C3K0Y3|C3K0Y3_PSEFS	36	20085	1	1	1	1	0.26	Glutathione peroxidas
1517	1	db$2	2::C3K3X7|C3K3X7_PSEFS	36	16525	1	1	1	1	0.33	EVE domain-containin
1518	1	db$2	2::C3K2S2|C3K2S2_PSEFS	36	38886	3	3	3	3	0.44	Riboflavin biosynthesis
1519	1	db$2	2::C3KCV5|C3KCV5_PSEFS	36	37705	2	2	2	2	0.28	Uncharacterized prote
1520	1	db$2	2::C3K1D4|C3K1D4_PSEFS	36	64734	3	2	3	2	0.16	Uncharacterized prote
1521	1	db$2	2::C3K4M3|C3K4M3_PSEFS	36	101390	2	2	2	2	0.10	Phosphocarrier proteir
1522	1	db$2	2::C3K0H8|C3K0H8_PSEFS	35	29525	2	2	2	2	0.38	Protein phosphatase C
1523	1	SwissProt_HUMAN$	3::P23284|PPIB_HUMAN	35	23728	1	1	1	1	0.22	Peptidyl-prolyl cis-trar
1524	1	db$2	2::C3K3K0|YQGF_PSEFS	35	16158	2	2	2	2	0.78	Putative pre-16S rRNA
1525	1	db$2	2::C3JYE8|C3JYE8_PSEFS	35	14928	1	1	1	1	0.37	MerR-family transcript
1526	1	db$2	2::C3KE91|C3KE91_PSEFS	35	8193	1	1	1	1	0.75	Uncharacterized prote
1527	1	db$2	2::C3K7A1|C3K7A1_PSEFS	35	56003	2	1	2	1	0.09	Putative LuxR-family r
1528	1	db$2	2::C3K127|C3K127_PSEFS	35	41295	3	2	3	2	0.26	Putative membrane pr
1529	1	SwissProt_HUMAN$	3::Q9BVV6|TALD3_HUMAN	35	169201	1	1	1	1	0.03	Protein TALPID3 OS=H
1530	1	db$2	2::C3JYE6|C3JYE6_PSEFS	35	45437	1	1	1	1	0.11	Putative acyl-CoA deh
1531	1	SwissProt_HUMAN$	3::Q96QU8|XPO6_HUMAN	35	128801	1	1	1	1	0.04	Exportin-6 OS=Homo
1532	1	db$2	2::C3K4T8|C3K4T8_PSEFS	35	33681	1	1	1	1	0.15	Putative AraC-family r
1533	1	db$2	2::C3K7K0|C3K7K0_PSEFS	35	41297	1	1	1	1	0.12	Esterase III OS=Pseud
1534	1	db$2	2::C3JY37|C3JY37_PSEFS	35	14829	2	2	2	2	0.87	Putative lipoprotein OS
1535	1	db$2	2::C3KBV6|C3KBV6_PSEFS	35	125347	1	1	1	1	0.04	Putative potassium eff
1536	1	db$2	2::C3KD58|C3KD58_PSEFS	35	41746	1	1	1	1	0.12	Putative cystathionine
1537	1	db$2	2::C3JYS7|RUVB_PSEFS	35	38919	1	1	1	1	0.13	Holliday junction ATP-
1538	1	db$2	2::C3K330|GLPE_PSEFS	35	11747	1	1	1	1	0.48	Thiosulfate sulfurtrans
1539	1	SwissProt_HUMAN$	3::P06748|NPM_HUMAN	35	32555	2	2	2	2	0.34	Nucleophosmin OS=H
1540	1	SwissProt_HUMAN$	3::Q4UJ75|A20A4_HUMAN	35	94090	1	1	1	1	0.05	Putative ankyrin repea
1541	1	db$2	2::C3KDI4|C3KDI4_PSEFS	34	42151	2	2	2	2	0.25	Putative exported prot
1542	1	db$2	2::C3KE56|C3KE56_PSEFS	34	59021	3	3	3	3	0.27	Glutamate--cysteine li
1543	1	db$2	2::C3JYF1|C3JYF1_PSEFS	34	42707	2	1	2	1	0.12	Putative acyl-CoA deh
1544	1	db$2	2::C3K3T1|C3K3T1_PSEFS	34	86048	1	1	1	1	0.06	Putative penicillin ami
1545	1	db$2	2::C3K3D4|C3K3D4_PSEFS	34	52472	3	1	3	1	0.09	Uncharacterized prote
1546	1	db$2	2::C3JZR9|C3JZR9_PSEFS	34	27415	3	3	3	3	0.67	Putative dehydrogenas
1547	1	db$2	2::C3K6B1|C3K6B1_PSEFS	34	35098	2	2	2	2	0.31	Homoserine kinase OS
1548	1	db$2	2::C3K6G9|LPXB_PSEFS	34	41468	1	1	1	1	0.12	Lipid-A-disaccharide s
1549	1	db$2	2::C3K1Q1|C3K1Q1_PSEFS	34	59090	1	1	1	1	0.08	Putative methyl accep

↑Family	M	DB	Accession	Score	Mass	Matches	Match(sig)	Sequences	Seq(sig)	emPAI	Description
1550	1	db$2	2::C3K7S1|C3K7S1_PSEFS	34	25085	3	3	2	2	0.76	Protein MgtC OS=Pseu
1551	1	db$2	2::C3KBP5|C3KBP5_PSEFS	34	30770	1	1	1	1	0.17	Putative AraC-family r
1552	1	db$2	2::C3K4N5|C3K4N5_PSEFS	34	28830	1	1	1	1	0.18	Putative amino acid AI
1553	1	SwissProt_HUMAN$	3::P61978|HNRPK_HUMAN	34	50944	1	1	1	1	0.10	Heterogeneous nuclea
1554	1	SwissProt_HUMAN$	3::P28072|PSB6_HUMAN	34	25341	1	1	1	1	0.20	Proteasome subunit be
1555	1	db$2	2::C3K546|C3K546_PSEFS	34	33665	1	1	1	1	0.15	Putative lipopolysacch
1556	1	db$2	2::C3K620|C3K620_PSEFS	34	22214	1	1	1	1	0.24	LexA repressor OS=Ps
1557	1	db$2	2::C3K685|C3K685_PSEFS	34	13635	2	2	2	2	0.98	6-carboxy-5,6,7,8-tetı
1558	1	db$2	2::C3K600|C3K600_PSEFS	34	28761	2	2	2	2	0.39	Phosphatidate cytidyly
1559	1	db$2	2::C3JZZ4|C3JZZ4_PSEFS	34	23102	1	1	1	1	0.23	Putative glutathione S
1560	1	db$2	2::C3K8T9|TATA_PSEFS	34	10325	3	3	3	3	2.83	Sec-independent prote
1561	1	db$2	2::C3K5B6|C3K5B6_PSEFS	34	58846	4	4	3	3	0.27	Dipeptide ABC transpo
1562	1	db$2	2::C3K8L9|C3K8L9_PSEFS	34	65348	2	2	2	2	0.16	Putative potassium-efl
1563	1	db$2	2::C3K8T6|C3K8T6_PSEFS	34	26078	2	1	2	1	0.20	Ribosomal RNA small s
1564	1	db$2	2::C3K0X4|C3K0X4_PSEFS	34	79071	1	1	1	1	0.06	DNA helicase OS=Pseu
1565	1	db$2	2::C3K1H4|C3K1H4_PSEFS	34	45615	1	1	1	1	0.11	Putative membrane pr
1566	1	db$2	2::C3K7M1|C3K7M1_PSEFS	33	62712	1	1	1	1	0.08	Putative dipeptidase C
1567	1	db$2	2::C3K8V0|C3K8V0_PSEFS	33	31271	1	1	1	1	0.16	Poly(3-hydroxyalkanoa
1568	1	db$2	2::C3K239|C3K239_PSEFS	33	42550	1	1	1	1	0.12	Aminotransferase OS=
1569	1	db$2	2::C3K3J2|C3K3J2_PSEFS	33	213513	1	1	1	1	0.02	Histidine kinase OS=P
1570	1	db$2	2::C3K7C2|C3K7C2_PSEFS	33	16947	2	1	2	1	0.32	tRNA (cytidine(34)-2'-
1571	1	db$2	2::C3K3W5|C3K3W5_PSEFS	33	39645	4	2	3	2	0.27	Aminomethyltransfera
1572	1	db$2	2::C3KDC2|ISPE_PSEFS	33	30632	2	1	2	1	0.17	4-diphosphocytidyl-2-
1573	1	SwissProt_HUMAN$	3::Q02413|DSG1_HUMAN	33	113676	1	1	1	1	0.04	Desmoglein-1 OS=Hor
1574	1	SwissProt_HUMAN$	3::P00738|HPT_HUMAN	33	45177	2	2	2	2	0.23	Haptoglobin OS=Homo
1575	1	db$2	2::C3K488|C3K488_PSEFS	33	15239	2	1	2	1	0.36	Biopolymer transport p
1576	1	db$2	2::C3JYP3|C3JYP3_PSEFS	33	22876	1	1	1	1	0.23	YrdC-like domain-cont
1577	1	db$2	2::C3KD16|C3KD16_PSEFS	33	34294	3	2	3	2	0.32	Putative 2-hydroxyaci
1578	1	SwissProt_HUMAN$	3::P14618|KPYM_HUMAN	32	57900	1	1	1	1	0.09	Pyruvate kinase PKM (
1579	1	SwissProt_HUMAN$	3::Q96P48|ARAP1_HUMAN	32	162089	1	1	1	1	0.03	Arf-GAP with Rho-GAP
1580	1	SwissProt_HUMAN$	3::P62753|RS6_HUMAN	32	28663	1	1	1	1	0.18	40S ribosomal protein
1581	1	db$2	2::C3K2T0|C3K2T0_PSEFS	32	46040	1	1	1	1	0.11	Putative ABC transpor
1582	1	db$2	2::C3KBZ4|C3KBZ4_PSEFS	32	6850	1	1	1	1	0.94	Uncharacterized prote
1583	1	SwissProt_HUMAN$	3::Q14683|SMC1A_HUMAN	32	143144	2	1	2	1	0.03	Structural maintenanc
1584	1	db$2	2::C3K413|C3K413_PSEFS	32	22663	1	1	1	1	0.23	Putative ABC transpor
1585	1	db$2	2::C3JY58|C3JY58_PSEFS	32	87640	2	2	2	2	0.11	DNA translocase FtsK
1586	1	db$2	2::C3K136|C3K136_PSEFS	32	31053	2	1	2	1	0.16	Uncharacterized prote
1587	1	db$2	2::C3K741|C3K741_PSEFS	32	17249	1	1	1	1	0.31	GAF domain-containin
1588	1	db$2	2::C3K2P5|C3K2P5_PSEFS	32	49850	1	1	1	1	0.10	Ethanolamine ammoni
1589	1	db$2	2::C3KAT1|C3KAT1_PSEFS	32	27898	2	2	2	2	0.40	Response regulator pr

↑Family	M	DB	Accession	Score	Mass	Matches	Match(sig)	Sequences	Seq(sig)	emPAI	Description
1590	1	db$2	2::C3K4E5|C3K4E5_PSEFS	32	51748	1	1	1	1	0.10	Putative amino acid tr
1591	1	db$2	2::C3JZ89|C3JZ89_PSEFS	32	157759	2	2	2	2	0.06	Uncharacterized prote
1592	1	db$2	2::C3K0H5|C3K0H5_PSEFS	32	25946	1	1	1	1	0.20	Putative chemotaxis p
1593	1	db$2	2::C3K1I1|C3K1I1_PSEFS	32	24836	1	1	1	1	0.21	Nudix hydrolase doma
1594	1	db$2	2::C3K537|C3K537_PSEFS	32	39684	1	1	1	1	0.13	Putative membrane pr
1595	1	SwissProt_HUMAN$	3::Q9UKE5|TNIK_HUMAN	32	154848	2	1	2	1	0.03	TRAF2 and NCK-intera
1596	1	db$2	2::C3K8W8|C3K8W8_PSEFS	32	29386	1	1	1	1	0.17	Putative ABC transpor
1597	1	db$2	2::C3K2P8|C3K2P8_PSEFS	31	24358	2	1	2	1	0.21	Membrane-related pro
1598	1	db$2	2::C3JXP4|C3JXP4_PSEFS	31	23087	1	1	1	1	0.23	Putative putative ECF-
1599	1	SwissProt_HUMAN$	3::P07437|TBB5_HUMAN	31	49639	2	1	2	1	0.10	Tubulin beta chain OS
1600	1	db$2	2::C3JZP7|C3JZP7_PSEFS	31	33454	1	1	1	1	0.15	Putative ribose transp
1601	1	db$2	2::C3KAX3|C3KAX3_PSEFS	31	30360	1	1	1	1	0.17	Conserved hypothetica
1602	1	SwissProt_HUMAN$	3::Q96F81|DISP1_HUMAN	31	170822	1	1	1	1	0.03	Protein dispatched hor
1603	1	SwissProt_HUMAN$	3::P04080|CYTB_HUMAN	31	11133	1	1	1	1	0.52	Cystatin-B OS=Homo
1604	1	db$2	2::C3K915|C3K915_PSEFS	31	26912	1	1	1	1	0.19	Putative GntR-family r
1605	1	db$2	2::C3KDS1|C3KDS1_PSEFS	31	26155	2	1	1	1	0.20	Pseudouridine synthas
1606	1	db$2	2::C3KDY6|C3KDY6_PSEFS	31	17128	1	1	1	1	0.31	Uncharacterized prote
1607	1	db$2	2::C3K7J4|C3K7J4_PSEFS	31	18330	1	1	1	1	0.29	Uncharacterized prote
1608	1	SwissProt_HUMAN$	3::Q8TBY8|PMFBP_HUMAN	31	117420	1	1	1	1	0.04	Polyamine-modulated
1609	1	SwissProt_HUMAN$	3::Q15147|PLCB4_HUMAN	31	134378	2	1	2	1	0.04	1-phosphatidylinositol
1610	1	db$2	2::C3K0A4|C3K0A4_PSEFS	31	12844	1	1	1	1	0.44	5-hydroxyisourate hyc
1611	1	db$2	2::A4V6U2|A4V6U2_PSEFS	31	76547	3	1	1	1	0.06	Uncharacterized prote
1612	1	SwissProt_HUMAN$	3::Q8NBF4|YG006_HUMAN	31	16172	2	1	1	1	0.33	Putative uncharacteriz
1613	1	db$2	2::C3KDK6|C3KDK6_PSEFS	31	27451	1	1	1	1	0.19	Putative ABC transpor
1614	1	SwissProt_HUMAN$	3::P62304|RUXE_HUMAN	31	10797	1	1	1	1	0.54	Small nuclear ribonucl
1615	1	db$2	2::C3K4K4|C3K4K4_PSEFS	31	39850	1	1	1	1	0.13	Putative branched ami
1616	1	db$2	2::C3JY28|Y3771_PSEFS	31	6626	3	1	1	1	0.98	UPF0434 protein PFLU
1617	1	db$2	2::C3K834|C3K834_PSEFS	31	76708	1	1	1	1	0.06	Copper-transporting P
1618	1	db$2	2::C3KCP0|C3KCP0_PSEFS	31	31742	1	1	1	1	0.16	Ribosomal RNA large s
1619	1	db$2	2::C3K2Y9|C3K2Y9_PSEFS	31	13142	2	2	2	2	1.04	Protein translocase su
1620	1	SwissProt_HUMAN$	3::P26440|IVD_HUMAN	31	46621	1	1	1	1	0.11	Isovaleryl-CoA dehydr
1621	1	db$2	2::C3K428|C3K428_PSEFS	31	10726	1	1	1	1	0.54	Uncharacterized prote
1622	1	db$2	2::A4V7U9|A4V7U9_PSEFS	31	58926	1	1	1	1	0.08	Mercuric reductase OS
1623	1	db$2	2::C3K1U6|C3K1U6_PSEFS	31	74730	2	1	2	1	0.07	Cytochrome bo(3) ubi
1624	1	db$2	2::C3K842|C3K842_PSEFS	31	56600	1	1	1	1	0.09	RNA polymerase sigma
1625	1	SwissProt_HUMAN$	3::O14640|DVL1_HUMAN	31	75140	1	1	1	1	0.07	Segment polarity prot
1626	1	db$2	2::C3KE60|C3KE60_PSEFS	31	49144	1	1	1	1	0.10	Histidine kinase OS=P
1627	1	SwissProt_HUMAN$	3::Q13472|TOP3A_HUMAN	31	112300	1	1	1	1	0.04	DNA topoisomerase 3-
1628	1	db$2	2::C3K0J3|C3K0J3_PSEFS	31	45387	1	1	1	1	0.11	Putative flagellar hook
1629	1	db$2	2::C3K1F6|C3K1F6_PSEFS	31	23787	2	2	2	2	0.49	Ribosomal RNA small s

↑Family	M	DB	Accession	Score	Mass	Matches	Match(sig)	Sequences	Seq(sig)	emPAI	Description
1630	1	db$2	2::C3KBK8|C3KBK8_PSEFS	30	35740	1	1	1	1	0.14	Ribosomal RNA small :
1631	1	SwissProt_HUMAN$	3::Q96AQ6|PBIP1_HUMAN	30	80594	1	1	1	1	0.06	Pre-B-cell leukemia tra
1632	1	db$2	2::C3K3P6|C3K3P6_PSEFS	30	19979	1	1	1	1	0.26	Dihydrofolate reductas
1633	1	db$2	2::C3KBU2|C3KBU2_PSEFS	30	56055	1	1	1	1	0.09	Putative histidine amn
1634	1	db$2	2::C3K4F9|C3K4F9_PSEFS	30	31102	1	1	1	1	0.16	Putative regulatory pr
1635	1	SwissProt_HUMAN$	3::Q00526|CDK3_HUMAN	30	35024	2	2	1	1	0.14	Cyclin-dependent kina
1636	1	db$2	2::C3KBM2|C3KBM2_PSEFS	30	16548	1	1	1	1	0.33	Cys-tRNA(Pro)/Cys-tR
1637	1	db$2	2::C3K2L8|LIPA_PSEFS	30	38163	5	3	4	3	0.45	Lipoyl synthase OS=P:
1638	1	db$2	2::C3K5A2|URE2_PSEFS	30	11072	1	1	1	1	0.52	Urease subunit beta O
1639	1	db$2	2::C3K3L0|METXS_PSEFS	30	41664	2	2	2	2	0.26	Homoserine O-succiny
1640	1	db$2	2::C3KBV2|C3KBV2_PSEFS	30	15686	1	1	1	1	0.35	Thioredoxin OS=Pseu
1641	1	db$2	2::C3K908|C3K908_PSEFS	30	33577	1	1	1	1	0.15	Putative dehydrogena:
1642	1	db$2	2::C3K9H0|C3K9H0_PSEFS	30	78680	1	1	1	1	0.06	Putative ferric siderop
1643	1	db$2	2::C3K6D8|C3K6D8_PSEFS	30	104098	2	2	2	2	0.10	Putative sensor kinase
1644	1	SwissProt_HUMAN$	3::Q96E52|OMA1_HUMAN	30	60081	1	1	1	1	0.08	Metalloendopeptidase
1645	1	db$2	2::C3KBH3|DCUP_PSEFS	30	38973	4	2	4	2	0.27	Uroporphyrinogen dec
1646	1	SwissProt_HUMAN$	3::Q9UQL6|HDAC5_HUMAN	30	121902	1	1	1	1	0.04	Histone deacetylase 5
1647	1	db$2	2::C3K672|C3K672_PSEFS	30	58670	2	2	2	2	0.18	Cation/acetate sympo
1648	1	SwissProt_HUMAN$	3::Q7Z624|CMKMT_HUMAN	30	36105	1	1	1	1	0.14	Calmodulin-lysine N-m
1649	1	db$2	2::C3K129|C3K129_PSEFS	30	42141	1	1	1	1	0.12	Putative acetyl CoA ac
1650	1	db$2	2::C3K4Y9|C3K4Y9_PSEFS	30	6637	1	1	1	1	0.98	Heme exporter proteir
1651	1	db$2	2::C3JYM6|C3JYM6_PSEFS	30	40915	2	2	2	2	0.26	Putative HlyD-family s
1652	1	db$2	2::C3K571|C3K571_PSEFS	30	11755	2	1	2	1	0.48	Uncharacterized prote
1653	1	db$2	2::C3K0F5|C3K0F5_PSEFS	30	23098	2	1	2	1	0.23	Putative TetR-family ti
1654	1	db$2	2::C3K0T5|C3K0T5_PSEFS	30	37877	2	1	2	1	0.13	Zinc transport protein
1655	1	db$2	2::C3K6X0|C3K6X0_PSEFS	30	62906	1	1	1	1	0.08	Putative GGDEF doma
1656	1	db$2	2::C3K5K8|C3K5K8_PSEFS	30	29952	1	1	1	1	0.17	Putative deoxyribonuc
1657	1	db$2	2::C3KDX1|RS20_PSEFS	30	10078	1	1	1	1	0.58	30S ribosomal protein
1658	1	db$2	2::C3K2R0|C3K2R0_PSEFS	30	31322	1	1	1	1	0.16	Geranyltransferas
1659	1	db$2	2::C3K1I0|C3K1I0_PSEFS	29	22456	1	1	1	1	0.23	Nudix hydrolase doma
1660	1	db$2	2::C3K5Y5|C3K5Y5_PSEFS	29	18019	1	1	1	1	0.30	Protein PsiE OS=Pseu
1661	1	db$2	2::C3K7A9|C3K7A9_PSEFS	29	33658	1	1	1	1	0.15	Conserved hypothetica
1662	1	db$2	2::C3K4U9|C3K4U9_PSEFS	29	34107	2	2	2	2	0.32	Putative LysR-family r
1663	1	db$2	2::C3K1Q2|C3K1Q2_PSEFS	29	73203	1	1	1	1	0.07	Histidine kinase OS=P
1664	1	SwissProt_HUMAN$	3::O94817|ATG12_HUMAN	29	15104	1	1	1	1	0.36	Ubiquitin-like protein /
1665	1	db$2	2::C3K0N8|C3K0N8_PSEFS	29	19586	2	1	2	1	0.27	23S rRNA accumulatio
1666	1	db$2	2::C3K2P0|C3K2P0_PSEFS	29	22640	3	3	3	3	0.86	Flavin prenyltransfera:
1667	1	db$2	2::C3KBX7|C3KBX7_PSEFS	29	23636	1	1	1	1	0.22	Putative metallo-beta-
1668	1	SwissProt_HUMAN$	3::P27216|ANX13_HUMAN	29	35393	1	1	1	1	0.14	Annexin A13 OS=Hom
1669	1	SwissProt_HUMAN$	3::Q15366|PCBP2_HUMAN	29	38556	1	1	1	1	0.13	Poly(rC)-binding prote

↑Family	M	DB	Accession	Score	Mass	Matches	Match(sig)	Sequences	Seq(sig)	emPAI	Description
1670	1	db$2	2::C3JXZ4|C3JXZ4_PSEFS	29	40619	1	1	1	1	0.12	Uncharacterized prote
1671	1	db$2	2::C3JYA8|C3JYA8_PSEFS	29	39123	1	1	1	1	0.13	Putative membrane pr
1672	1	db$2	2::C3K6R2|C3K6R2_PSEFS	29	29309	1	1	1	1	0.17	Putative shikimate del
1673	1	SwissProt_HUMAN$	3::Q96MV8|ZDH15_HUMAN	29	39305	1	1	1	1	0.13	Palmitoyltransferase Z
1674	1	db$2	2::C3K2S9|C3K2S9_PSEFS	29	25730	2	2	2	2	0.44	Putative ABC transpor
1675	1	SwissProt_HUMAN$	3::Q8IYG6|LRC56_HUMAN	29	58697	1	1	1	1	0.08	Leucine-rich repeat-cc
1676	1	db$2	2::C3K5L9|C3K5L9_PSEFS	29	31441	3	3	3	3	0.57	Alpha-D-ribose 1-metl
1677	1	db$2	2::C3K157|C3K157_PSEFS	29	43413	1	1	1	1	0.12	Putative membrane pr
1678	1	db$2	2::C3K444|C3K444_PSEFS	29	9491	1	1	1	1	0.62	Ubiquinone biosynthes
1679	1	db$2	2::C3K3H6|C3K3H6_PSEFS	29	12460	1	1	1	1	0.45	Putative endoribonucle
1680	1	SwissProt_HUMAN$	3::B2RPK0|HGB1A_HUMAN	29	24223	1	1	1	1	0.21	Putative high mobility
1681	1	SwissProt_HUMAN$	3::Q96CM3|RUSD4_HUMAN	29	42178	1	1	1	1	0.12	Mitochondrial RNA pse
1682	1	db$2	2::C3K3J8|C3K3J8_PSEFS	29	33397	2	1	2	1	0.15	TonB_C domain-conta
1683	1	SwissProt_HUMAN$	3::Q9Y4K0|LOXL2_HUMAN	29	86668	2	2	1	1	0.06	Lysyl oxidase homolog
1684	1	db$2	2::C3K6L2|C3K6L2_PSEFS	29	14328	1	1	1	1	0.38	Organic hydroperoxide
1685	1	db$2	2::C3KDV4|RSGA_PSEFS	29	37296	1	1	1	1	0.14	Small ribosomal subur
1686	1	db$2	2::C3K706|PIMT_PSEFS	29	23451	2	1	2	1	0.22	Protein-L-isoaspartate
1687	1	SwissProt_HUMAN$	3::Q92805|GOGA1_HUMAN	29	88130	1	1	1	1	0.06	Golgin subfamily A me
1688	1	SwissProt_HUMAN$	3::Q9P2M7|CING_HUMAN	29	136304	2	1	2	1	0.04	Cingulin OS=Homo sa
1689	1	SwissProt_HUMAN$	3::Q7Z6Z7|HUWE1_HUMAN	29	481589	3	2	2	1	0.01	E3 ubiquitin-protein lig
1690	1	db$2	2::C3K9N9|C3K9N9_PSEFS	29	57231	2	2	2	2	0.18	Putative ABC transpor
1691	1	db$2	2::C3K029|C3K029_PSEFS	29	46068	1	1	1	1	0.11	Uncharacterized prote
1692	1	SwissProt_HUMAN$	3::Q7Z442|PK1L2_HUMAN	29	272340	1	1	1	1	0.02	Polycystic kidney disea
1693	1	db$2	2::C3K6A9|C3K6A9_PSEFS	28	17428	1	1	1	1	0.31	Ferric uptake regulatic
1694	1	db$2	2::C3KAF4|C3KAF4_PSEFS	28	50981	1	1	1	1	0.10	Putative lipoprotein OS
1695	1	SwissProt_HUMAN$	3::Q9Y6C2|EMIL1_HUMAN	28	106630	1	1	1	1	0.05	EMILIN-1 OS=Homo s
1696	1	db$2	2::C3JY34|C3JY34_PSEFS	28	100208	1	1	1	1	0.05	Histidine kinase OS=P
1697	1	SwissProt_HUMAN$	3::P62318|SMD3_HUMAN	28	13907	1	1	1	1	0.40	Small nuclear ribonucl
1698	1	SwissProt_HUMAN$	3::P22626|ROA2_HUMAN	28	37407	1	1	1	1	0.13	Heterogeneous nuclea
1699	1	db$2	2::C3KCF3|C3KCF3_PSEFS	28	94057	3	1	2	1	0.05	Putative signalling-rela
1700	1	db$2	2::C3K8K5|C3K8K5_PSEFS	28	64377	1	1	1	1	0.08	Putative transport-rela
1701	1	db$2	2::C3KE38|C3KE38_PSEFS	28	28697	1	1	1	1	0.18	Putative phospholipid
1702	1	db$2	2::C3K5B7|C3K5B7_PSEFS	28	50883	1	1	1	1	0.10	Putative outer membra
1703	1	SwissProt_HUMAN$	3::O00231|PSD11_HUMAN	28	47434	1	1	1	1	0.11	26S proteasome non-/
1704	1	db$2	2::C3K5C6|C3K5C6_PSEFS	28	33902	1	1	1	1	0.15	Uncharacterized prote
1705	1	SwissProt_HUMAN$	3::Q9H6P5|TASP1_HUMAN	28	44427	1	1	1	1	0.11	Threonine aspartase 1
1706	1	db$2	2::C3KEA7|C3KEA7_PSEFS	28	16450	1	1	1	1	0.33	MPT synthase subunit
1707	1	db$2	2::C3K9P2|C3K9P2_PSEFS	28	69747	1	1	1	1	0.07	Putative substrate-bin
1708	1	db$2	2::C3K9Q3|C3K9Q3_PSEFS	28	26964	1	1	1	1	0.19	DNA polymerase III su
1709	1	db$2	2::C3K5U8|AGUA_PSEFS	28	40372	1	1	1	1	0.12	Agmatine deiminase C

↑Family	M	DB	Accession	Score	Mass	Matches	Match(sig)	Sequences	Seq(sig)	emPAI	Description
1710	1	SwissProt_HUMAN$	3::Q9BYK8|HELZ2_HUMAN	28	294465	1	1	1	1	0.02	Helicase with zinc fing
1711	1	SwissProt_HUMAN$	3::Q99618|CDCA3_HUMAN	28	28981	1	1	1	1	0.18	Cell division cycle-ass
1712	1	SwissProt_HUMAN$	3::O75155|CAND2_HUMAN	28	135171	2	1	2	1	0.04	Cullin-associated NEDI
1713	1	db$2	2::C3JYR6|C3JYR6_PSEFS	28	38174	5	3	4	2	0.28	Quinolinate synthase /
1714	1	db$2	2::C3KAD1|C3KAD1_PSEFS	28	32401	2	1	2	1	0.16	Probable 4-deoxy-4-fc
1715	1	SwissProt_HUMAN$	3::Q9P273|TEN3_HUMAN	28	300760	1	1	1	1	0.02	Teneurin-3 OS=Homo
1716	1	SwissProt_HUMAN$	3::Q5SRN2|TSBP1_HUMAN	27	61588	1	1	1	1	0.08	Testis-expressed basic
1717	1	db$2	2::C3KCN6|C3KCN6_PSEFS	27	53164	2	2	2	2	0.20	Sodium/proline sympc
1718	1	db$2	2::C3K211|C3K211_PSEFS	27	35352	1	1	1	1	0.14	LysR-family transcript
1719	1	db$2	2::C3JXY5|C3JXY5_PSEFS	27	10610	1	1	1	1	0.54	Uncharacterized prote
1720	1	db$2	2::C3K5T8|C3K5T8_PSEFS	27	41768	1	1	1	1	0.12	Cytochrome c oxidase
1721	1	db$2	2::C3K589|C3K589_PSEFS	27	50032	2	2	2	2	0.21	Transcriptional regulat
1722	1	SwissProt_HUMAN$	3::P49207|RL34_HUMAN	27	13284	1	1	1	1	0.42	60S ribosomal protein
1723	1	SwissProt_HUMAN$	3::O15197|EPHB6_HUMAN	27	110630	1	1	1	1	0.04	Ephrin type-B recepto
1724	1	db$2	2::C3K4Z1|C3K4Z1_PSEFS	27	72318	1	1	1	1	0.07	Cytochrome C biogene
1725	1	db$2	2::C3KCP9|C3KCP9_PSEFS	27	30268	1	1	1	1	0.17	Lipopolysaccharide co
1726	1	db$2	2::C3K5I1|C3K5I1_PSEFS	27	43688	1	1	1	1	0.11	Putative phage integra
1727	1	db$2	2::C3K643|C3K643_PSEFS	27	18796	1	1	1	1	0.28	Transcription elongatio
1728	1	db$2	2::C3JXP2|C3JXP2_PSEFS	27	43336	1	1	1	1	0.12	Putative glycosyltrans
1729	1	db$2	2::C3JZ42|C3JZ42_PSEFS	27	19185	1	1	1	1	0.28	Putative AraC family t
1730	1	db$2	2::C3K0Y2|C3K0Y2_PSEFS	27	46222	1	1	1	1	0.11	Putative fatty acid tra
1731	1	db$2	2::C3K065|C3K065_PSEFS	27	58554	1	1	1	1	0.08	Uncharacterized prote
1732	1	db$2	2::C3KDM8|C3KDM8_PSEFS	27	57105	1	1	1	1	0.09	HDOD domain-contain
1733	1	db$2	2::C3K0E2|C3K0E2_PSEFS	27	49920	1	1	1	1	0.10	Diaminobutyrate--2-o
1734	1	db$2	2::C3K807|C3K807_PSEFS	27	30283	1	1	1	1	0.17	Putative histidine ABC
1735	1	SwissProt_HUMAN$	3::Q13093|PAFA_HUMAN	27	50045	2	1	2	1	0.10	Platelet-activating fact
1736	1	db$2	2::C3K3V4|C3K3V4_PSEFS	27	41512	1	1	1	1	0.12	Acetylornithine deacet
1737	1	SwissProt_HUMAN$	3::Q6PI48|SYDM_HUMAN	27	73516	1	1	1	1	0.07	Aspartate--tRNA ligase
1738	1	SwissProt_HUMAN$	3::O43678|NDUA2_HUMAN	27	10915	1	1	1	1	0.53	NADH dehydrogenase
1739	1	db$2	2::C3K3J6|C3K3J6_PSEFS	27	14927	3	2	3	2	0.87	Type IV pilus response
1740	1	db$2	2::C3K629|C3K629_PSEFS	27	51787	2	1	1	1	0.10	Putative transport-rela
1741	1	SwissProt_HUMAN$	3::Q8WX94|NALP7_HUMAN	26	111735	1	1	1	1	0.04	NACHT, LRR and PYD
1742	1	SwissProt_HUMAN$	3::P11532|DMD_HUMAN	26	426484	2	1	2	1	0.01	Dystrophin OS=Homo
1743	1	db$2	2::C3K267|RLME_PSEFS	26	24125	1	1	1	1	0.22	Ribosomal RNA large s
1744	1	SwissProt_HUMAN$	3::A0A1B0GVD1|F237B_HUMAN	26	16320	1	1	1	1	0.33	Protein FAM237B OS=
1745	1	SwissProt_HUMAN$	3::Q6UWR7|ENPP6_HUMAN	26	50208	1	1	1	1	0.10	Glycerophosphocholin
1746	1	SwissProt_HUMAN$	3::P01023|A2MG_HUMAN	26	163188	1	1	1	1	0.03	Alpha-2-macroglobulir
1747	1	SwissProt_HUMAN$	3::O43586|PPIP1_HUMAN	26	47562	1	1	1	1	0.10	Proline-serine-threonir
1748	1	db$2	2::C3K0G1|C3K0G1_PSEFS	26	67986	1	1	1	1	0.07	Putative acetyl-/propic
1749	1	db$2	2::C3K3F2|C3K3F2_PSEFS	26	38228	2	2	2	2	0.28	D-erythrose-4-phosph

↑Family	M	DB	Accession	Score	Mass	Matches	Match(sig)	Sequences	Seq(sig)	emPAI	Description
1750	1	db$2	2::C3KDQ6|C3KDQ6_PSEFS	26	14780	1	1	1	1	0.37	Uncharacterized prote
1751	1	db$2	2::C3KDT1|C3KDT1_PSEFS	26	36973	1	1	1	1	0.14	Putative membrane-as
1752	1	db$2	2::C3JY46|C3JY46_PSEFS	26	42076	2	2	2	2	0.25	Putative racemase OS
1753	1	db$2	2::C3K1M8|C3K1M8_PSEFS	26	28345	2	2	2	2	0.40	tRNA (cytidine/uridine
1754	1	db$2	2::C3JY26|C3JY26_PSEFS	26	17211	1	1	1	1	0.31	Protein-tyrosine-phos
1755	1	db$2	2::C3KDC6|RF1_PSEFS	26	39857	2	2	2	2	0.27	Peptide chain release
1756	1	SwissProt_HUMAN$	3::O15270|SPTC2_HUMAN	26	62884	1	1	1	1	0.08	Serine palmitoyltransf
1757	1	db$2	2::C3JZ73|C3JZ73_PSEFS	26	49677	1	1	1	1	0.10	Porin OS=Pseudomon
1758	1	SwissProt_HUMAN$	3::P30050|RL12_HUMAN	26	17808	2	1	2	1	0.30	60S ribosomal protein
1759	1	db$2	2::C3K1K5|EX7L_PSEFS	26	51103	1	1	1	1	0.10	Exodeoxyribonuclease
1760	1	SwissProt_HUMAN$	3::P48165|CXA8_HUMAN	26	48199	1	1	1	1	0.10	Gap junction alpha-8
1761	1	SwissProt_HUMAN$	3::Q5K651|SAMD9_HUMAN	26	184165	1	1	1	1	0.03	Sterile alpha motif do
1762	1	db$2	2::C3K429|C3K429_PSEFS	26	8856	1	1	1	1	0.68	Uncharacterized prote
1763	1	SwissProt_HUMAN$	3::Q8N9W6|BOLL_HUMAN	26	31282	1	1	1	1	0.16	Protein boule-like OS=
1764	1	SwissProt_HUMAN$	3::Q15365|PCBP1_HUMAN	26	37474	1	1	1	1	0.13	Poly(rC)-binding prote
1765	1	db$2	2::C3K1G4|RL34_PSEFS	26	5064	1	1	1	1	1.41	50S ribosomal protein
1766	1	db$2	2::C3K919|C3K919_PSEFS	26	33229	2	1	2	1	0.15	Putative exported prot
1767	1	db$2	2::C3K1V5|C3K1V5_PSEFS	26	64171	1	1	1	1	0.08	Uncharacterized prote
1768	1	db$2	2::C3K4V6|C3K4V6_PSEFS	26	101332	1	1	1	1	0.05	Putative helicase OS=
1769	1	db$2	2::C3K9E1|C3K9E1_PSEFS	26	24133	1	1	1	1	0.22	Putative LuxR-family r
1770	1	SwissProt_HUMAN$	3::P05546|HEP2_HUMAN	26	57034	1	1	1	1	0.09	Heparin cofactor 2 OS
1771	1	SwissProt_HUMAN$	3::P54821|PRRX1_HUMAN	26	27280	1	1	1	1	0.19	Paired mesoderm hor
1772	1	SwissProt_HUMAN$	3::Q96GD3|SCMH1_HUMAN	26	73307	1	1	1	1	0.07	Polycomb protein SCM
1773	1	db$2	2::C3KCB6|C3KCB6_PSEFS	26	31268	1	1	1	1	0.16	Phosphate transport s
1774	1	db$2	2::C3K176|C3K176_PSEFS	25	33571	1	1	1	1	0.15	Glucokinase OS=Pseu
1775	1	SwissProt_HUMAN$	3::P58557|YBEY_HUMAN	25	19286	1	1	1	1	0.27	Endoribonuclease Ybe
1776	1	SwissProt_HUMAN$	3::Q96GC6|ZN274_HUMAN	25	74131	1	1	1	1	0.07	Neurotrophin receptor
1777	1	SwissProt_HUMAN$	3::P32969|RL9_HUMAN	25	21850	1	1	1	1	0.24	60S ribosomal protein
1778	1	db$2	2::C3K6V8|C3K6V8_PSEFS	25	47941	3	3	3	3	0.34	Putative peptidase OS
1779	1	SwissProt_HUMAN$	3::Q5VT97|SYDE2_HUMAN	25	133146	1	1	1	1	0.04	Rho GTPase-activating
1780	1	db$2	2::C3KDR2|C3KDR2_PSEFS	25	26142	1	1	1	1	0.20	S4 RNA-binding doma
1781	1	db$2	2::C3K7C5|C3K7C5_PSEFS	25	40067	2	1	2	1	0.13	Histidine kinase OS=P
1782	1	SwissProt_HUMAN$	3::Q5S007|LRRK2_HUMAN	25	285919	1	1	1	1	0.02	Leucine-rich repeat se
1783	1	db$2	2::C3JY63|CLPS_PSEFS	25	13780	1	1	1	1	0.40	ATP-dependent Clp pr
1784	1	db$2	2::C3K6E6|C3K6E6_PSEFS	25	23384	1	1	1	1	0.22	Stringent starvation p
1785	1	SwissProt_HUMAN$	3::P36507|MP2K2_HUMAN	25	44396	1	1	1	1	0.11	Dual specificity mitoge
1786	1	db$2	2::C3K2H7|C3K2H7_PSEFS	25	21527	1	1	1	1	0.24	Putative acetyltransfe
1787	1	db$2	2::C3KAY4|C3KAY4_PSEFS	25	29479	3	2	3	2	0.38	Putative enoyl-CoA hy
1788	1	SwissProt_HUMAN$	3::P81605|DCD_HUMAN	25	11277	1	1	1	1	0.51	Dermcidin OS=Homo
1789	1	db$2	2::C3JXG8|C3JXG8_PSEFS	25	49590	1	1	1	1	0.10	Putative outer membr

↑Family	M	DB	Accession	Score	Mass	Matches	Match(sig)	Sequences	Seq(sig)	emPAI	Description
1790	1	SwissProt_HUMAN$	3::P62266|RS23_HUMAN	25	15798	1	1	1	1	0.35	40S ribosomal protein
1791	1	db$2	2::C3JXQ7|C3JXQ7_PSEFS	25	48832	2	1	2	1	0.10	UDP-glucose 6-dehydr
1792	1	SwissProt_HUMAN$	3::Q14DG7|T132B_HUMAN	25	119402	1	1	1	1	0.04	Transmembrane prote
1793	1	SwissProt_HUMAN$	3::P12883|MYH7_HUMAN	25	222959	1	1	1	1	0.02	Myosin-7 OS=Homo s
1794	1	SwissProt_HUMAN$	3::Q5VZ66|JKIP3_HUMAN	25	98469	1	1	1	1	0.05	Janus kinase and micr
1795	1	db$2	2::C3K0N6|PLSX_PSEFS	25	35209	3	2	2	2	0.31	Phosphate acyltransfe
1796	1	SwissProt_HUMAN$	3::Q9H158|PCDC1_HUMAN	25	103877	1	1	1	1	0.05	Protocadherin alpha-C
1797	1	db$2	2::C3K4T5|C3K4T5_PSEFS	25	30974	1	1	1	1	0.16	Putative hydrolase OS
1798	1	db$2	2::C3K6J8|C3K6J8_PSEFS	25	78979	3	1	3	1	0.06	3-phosphoshikimate 1
1799	1	db$2	2::C3K1Z0|C3K1Z0_PSEFS	24	34051	1	1	1	1	0.15	Putative putative poly
1800	1	db$2	2::C3K493|C3K493_PSEFS	24	16495	1	1	1	1	0.33	Uncharacterized prote
1801	1	db$2	2::C3JY90|NUOK_PSEFS	24	10944	1	1	1	1	0.52	NADH-quinone oxidore
1802	1	db$2	2::C3JZV4|C3JZV4_PSEFS	24	49113	1	1	1	1	0.10	Putative aldehyde deh
1803	1	db$2	2::C3K305|C3K305_PSEFS	24	24275	1	1	1	1	0.21	Catabolite gene activa
1804	1	SwissProt_HUMAN$	3::Q9UL62|TRPC5_HUMAN	24	111340	1	1	1	1	0.04	Short transient recept
1805	1	SwissProt_HUMAN$	3::O43707|ACTN4_HUMAN	24	104788	2	1	1	1	0.05	Alpha-actinin-4 OS=H
1806	1	db$2	2::C3KAA5|C3KAA5_PSEFS	24	23984	3	2	2	2	0.48	Uncharacterized prote
1807	1	SwissProt_HUMAN$	3::P62241|RS8_HUMAN	24	24190	1	1	1	1	0.21	40S ribosomal protein
1808	1	db$2	2::C3K436|C3K436_PSEFS	24	30237	1	1	1	1	0.17	Diaminopimelate epim
1809	1	SwissProt_HUMAN$	3::P27708|PYR1_HUMAN	24	242829	1	1	1	1	0.02	CAD protein OS=Hom
1810	1	db$2	2::C3K4Q6|C3K4Q6_PSEFS	24	39864	1	1	1	1	0.13	Putative lipoprotein O
1811	1	db$2	2::C3K8Z2|C3K8Z2_PSEFS	24	25669	1	1	1	1	0.20	High-affinity branched
1812	1	SwissProt_HUMAN$	3::Q6NUI2|GPAT2_HUMAN	24	87779	1	1	1	1	0.06	Glycerol-3-phosphate
1813	1	SwissProt_HUMAN$	3::P35268|RL22_HUMAN	24	14778	1	1	1	1	0.37	60S ribosomal protein
1814	1	db$2	2::C3K2S4|C3K2S4_PSEFS	24	15995	1	1	1	1	0.34	Putative lipoprotein O
1815	1	db$2	2::C3KAB2|C3KAB2_PSEFS	24	13873	1	1	1	1	0.40	Putative two-compone
1816	1	db$2	2::C3KBZ5|C3KBZ5_PSEFS	24	23751	2	2	2	2	0.49	Endonuclease III OS=
1817	1	db$2	2::C3K323|C3K323_PSEFS	24	38795	2	2	2	2	0.28	Putative phosphotrans
1818	1	SwissProt_HUMAN$	3::Q96MR6|CFA57_HUMAN	24	144870	2	1	2	1	0.03	Cilia- and flagella-ass
1819	1	SwissProt_HUMAN$	3::Q9UPN3|MACF1_HUMAN	24	837787	3	1	3	1	0.01	Microtubule-actin cros
1820	1	db$2	2::C3K7D0|THII_PSEFS	24	54519	1	1	1	1	0.09	tRNA sulfurtransferase
1821	1	SwissProt_HUMAN$	3::Q765P7|MTSS2_HUMAN	24	79880	2	1	2	1	0.06	Protein MTSS 2 OS=H
1822	1	SwissProt_HUMAN$	3::Q9H244|P2Y12_HUMAN	24	39413	1	1	1	1	0.13	P2Y purinoceptor 12 C
1823	1	SwissProt_HUMAN$	3::Q8TD08|MK15_HUMAN	24	59795	1	1	1	1	0.08	Mitogen-activated pro
1824	1	SwissProt_HUMAN$	3::Q70Z53|F10C1_HUMAN	24	37525	1	1	1	1	0.13	Protein FRA10AC1 OS
1825	1	db$2	2::C3KB47|C3KB47_PSEFS	24	100225	1	1	1	1	0.05	Magnesium-transporti
1826	1	SwissProt_HUMAN$	3::Q5VT06|CE350_HUMAN	24	350716	1	1	1	1	0.01	Centrosome-associate
1827	1	SwissProt_HUMAN$	3::Q15075|EEA1_HUMAN	24	162367	1	1	1	1	0.03	Early endosome antige
1828	1	SwissProt_HUMAN$	3::Q9Y265|RUVB1_HUMAN	24	50196	1	1	1	1	0.10	RuvB-like 1 OS=Homo
1829	1	SwissProt_HUMAN$	3::O00159|MYO1C_HUMAN	24	121606	1	1	1	1	0.04	Unconventional myosi

↑Family	M	DB	Accession	Score	Mass	Matches	Match(sig)	Sequences	Seq(sig)	emPAI	Description
1830	1	SwissProt_HUMAN$	3::O95153|RIMB1_HUMAN	24	199928	1	1	1	1	0.02	Peripheral-type benzo
1831	1	db$2	2::C3K9S5|C3K9S5_PSEFS	24	38309	1	1	1	1	0.13	Putative cobalamin bic
1832	1	SwissProt_HUMAN$	3::P62263|RS14_HUMAN	24	16263	1	1	1	1	0.33	40S ribosomal protein
1833	1	SwissProt_HUMAN$	3::Q5TAX3|TUT4_HUMAN	24	185048	3	1	2	1	0.03	Terminal uridylyltrans
1834	1	db$2	2::C3K167|C3K167_PSEFS	24	48979	1	1	1	1	0.10	Putative two-compone
1835	1	db$2	2::C3K3Q6|C3K3Q6_PSEFS	24	79554	1	1	1	1	0.06	Putative heavy metal
1836	1	db$2	2::C3K1F1|ATPL_PSEFS	24	8603	3	2	1	1	1.89	ATP synthase subunit
1837	1	SwissProt_HUMAN$	3::Q5TH69|BIG3_HUMAN	23	240498	2	1	2	1	0.02	Brefeldin A-inhibited g
1838	1	db$2	2::C3K492|C3K492_PSEFS	23	51080	1	1	1	1	0.10	HDOD domain-contain
1839	1	db$2	2::C3KE75|C3KE75_PSEFS	23	123658	1	1	1	1	0.04	TGc domain-containin
1840	1	db$2	2::C3K6W4|C3K6W4_PSEFS	23	45779	1	1	1	1	0.11	Znf/thioredoxin_put d
1841	1	db$2	2::C3K1A6|C3K1A6_PSEFS	23	56046	2	1	2	1	0.09	Beta-lactamase induct
1842	1	db$2	2::C3KEB9|C3KEB9_PSEFS	23	11110	1	1	1	1	0.52	Putative lipoprotein O
1843	1	SwissProt_HUMAN$	3::Q8TF62|AT8B4_HUMAN	23	135782	1	1	1	1	0.04	Probable phospholipid
1844	1	db$2	2::C3K125|C3K125_PSEFS	23	26783	1	1	1	1	0.19	Alanine--tRNA ligase (
1845	1	SwissProt_HUMAN$	3::P62917|RL8_HUMAN	23	28007	2	1	2	1	0.18	60S ribosomal protein
1846	1	SwissProt_HUMAN$	3::O00459|P85B_HUMAN	23	81495	2	1	1	1	0.06	Phosphatidylinositol 3-
1847	1	db$2	2::C3K6K7|C3K6K7_PSEFS	23	17395	1	1	1	1	0.31	GreA_GreB domain-co
1848	1	db$2	2::C3K0D1|C3K0D1_PSEFS	23	40054	1	1	1	1	0.13	PNPLA domain-contair
1849	1	SwissProt_HUMAN$	3::Q96SK2|TM209_HUMAN	23	62881	1	1	1	1	0.08	Transmembrane prote
1850	1	db$2	2::C3KCJ3|C3KCJ3_PSEFS	23	30975	2	2	2	2	0.36	Usp domain-containin
1851	1	SwissProt_HUMAN$	3::P52333|JAK3_HUMAN	23	125019	1	1	1	1	0.04	Tyrosine-protein kinas
1852	1	SwissProt_HUMAN$	3::Q8NB16|MLKL_HUMAN	23	54445	1	1	1	1	0.09	Mixed lineage kinase c
1853	1	SwissProt_HUMAN$	3::P08476|INHBA_HUMAN	23	47412	1	1	1	1	0.11	Inhibin beta A chain O
1854	1	SwissProt_HUMAN$	3::Q8N302|AGGF1_HUMAN	23	80928	1	1	1	1	0.06	Angiogenic factor with
1855	1	db$2	2::C3K5M3|PHNN_PSEFS	23	20351	1	1	1	1	0.26	Ribose 1,5-bisphospha
1856	1	db$2	2::C3KBT8|C3KBT8_PSEFS	23	29541	1	1	1	1	0.17	Sulfate transport syste
1857	1	db$2	2::C3K6R7|C3K6R7_PSEFS	23	41832	2	2	2	2	0.25	Serine/threonine trans
1858	1	db$2	2::C3K5X3|C3K5X3_PSEFS	23	64999	1	1	1	1	0.08	Putative ABC transpor
1859	1	SwissProt_HUMAN$	3::Q8N8R5|CB069_HUMAN	23	43421	1	1	1	1	0.12	UPF0565 protein C2or
1860	1	db$2	2::C3K7J6|C3K7J6_PSEFS	23	25687	1	1	1	1	0.20	Putative exported prot
1861	1	SwissProt_HUMAN$	3::Q9C037|TRIM4_HUMAN	23	57424	1	1	1	1	0.09	E3 ubiquitin-protein li
1862	1	SwissProt_HUMAN$	3::P15924|DESP_HUMAN	23	331569	1	1	1	1	0.01	Desmoplakin OS=Hom
1863	1	SwissProt_HUMAN$	3::Q9UJN7|ZN391_HUMAN	23	40634	1	1	1	1	0.12	Zinc finger protein 39
1864	1	SwissProt_HUMAN$	3::Q8IX18|DHX40_HUMAN	23	88504	1	1	1	1	0.06	Probable ATP-depende
1865	1	db$2	2::C3K6T7|C3K6T7_PSEFS	23	66001	2	1	2	1	0.07	Histidine kinase OS=P
1866	1	SwissProt_HUMAN$	3::P60900|PSA6_HUMAN	23	27382	1	1	1	1	0.19	Proteasome subunit al
1867	1	db$2	2::C3JYG6|C3JYG6_PSEFS	23	44622	1	1	1	1	0.11	Peptidase_M14 domai
1868	1	SwissProt_HUMAN$	3::P11362|FGFR1_HUMAN	23	91809	1	1	1	1	0.05	Fibroblast growth fact
1869	1	SwissProt_HUMAN$	3::A6NM36|LRC30_HUMAN	23	34012	1	1	1	1	0.15	Leucine-rich repeat-cc

↑Family	M	DB	Accession	Score	Mass	Matches	Match(sig)	Sequences	Seq(sig)	emPAI	Description
1870	1	db$2	2::C3K458|C3K458_PSEFS	23	47152	2	2	2	2	0.22	D-amino acid dehydro
1871	1	db$2	2::C3K5Q0|C3K5Q0_PSEFS	23	53619	4	1	4	1	0.09	Mannose-1-phosphate
1872	1	SwissProt_HUMAN$	3::Q8TDR2|STK35_HUMAN	23	58015	1	1	1	1	0.09	Serine/threonine-prote
1873	1	db$2	2::C3K577|C3K577_PSEFS	23	15787	2	1	2	1	0.35	Putative lipoprotein OS
1874	1	SwissProt_HUMAN$	3::Q9UBV2|SE1L1_HUMAN	23	88698	1	1	1	1	0.05	Protein sel-1 homolog
1875	1	SwissProt_HUMAN$	3::P08514|ITA2B_HUMAN	23	113306	1	1	1	1	0.04	Integrin alpha-IIb OS=
1876	1	SwissProt_HUMAN$	3::O94967|WDR47_HUMAN	23	101884	1	1	1	1	0.05	WD repeat-containing
1877	1	db$2	2::C3K386|C3K386_PSEFS	23	22957	1	1	1	1	0.23	Putative glutathione S
1878	1	SwissProt_HUMAN$	3::Q5TZA2|CROCC_HUMAN	23	228388	1	1	1	1	0.02	Rootletin OS=Homo sa
1879	1	db$2	2::C3K5A1|C3K5A1_PSEFS	23	60517	1	1	1	1	0.08	Urease subunit alpha (
1880	1	SwissProt_HUMAN$	3::Q7Z5H4|VN1R5_HUMAN	23	40752	1	1	1	1	0.12	Vomeronasal type-1 re
1881	1	db$2	2::C3KA94|C3KA94_PSEFS	23	41012	1	1	1	1	0.12	Uncharacterized prote
1882	1	SwissProt_HUMAN$	3::Q15645|PCH2_HUMAN	23	48520	1	1	1	1	0.10	Pachytene checkpoint
1883	1	SwissProt_HUMAN$	3::O14776|TCRG1_HUMAN	22	123823	2	2	2	2	0.08	Transcription elongatio
1884	1	db$2	2::C3K3Q9|LGT_PSEFS	22	30444	1	1	1	1	0.17	Phosphatidylglycerol--
1885	1	SwissProt_HUMAN$	3::Q11130|FUT7_HUMAN	22	39214	1	1	1	1	0.13	Alpha-(1,3)-fucosyltra
1886	1	SwissProt_HUMAN$	3::O95835|LATS1_HUMAN	22	126790	1	1	1	1	0.04	Serine/threonine-prote
1887	1	SwissProt_HUMAN$	3::P61313|RL15_HUMAN	22	24131	1	1	1	1	0.22	60S ribosomal protein
1888	1	SwissProt_HUMAN$	3::O94827|PKHG5_HUMAN	22	117377	1	1	1	1	0.04	Pleckstrin homology d
1889	1	db$2	2::C3JY48|C3JY48_PSEFS	22	12277	1	1	1	1	0.46	DrsE domain-containin
1890	1	db$2	2::C3K5K6|C3K5K6_PSEFS	22	177143	1	1	1	1	0.03	Putative RHS repeat-li
1891	1	SwissProt_HUMAN$	3::Q86YF9|DZIP1_HUMAN	22	98602	1	1	1	1	0.05	Zinc finger protein DZ
1892	1	SwissProt_HUMAN$	3::Q99623|PHB2_HUMAN	22	33276	1	1	1	1	0.15	Prohibitin-2 OS=Homo
1893	1	SwissProt_HUMAN$	3::Q9BWW9|APOL5_HUMAN	22	47014	1	1	1	1	0.11	Apolipoprotein L5 OS=
1894	1	SwissProt_HUMAN$	3::Q9Y5J3|HEY1_HUMAN	22	32593	1	1	1	1	0.16	Hairy/enhancer-of-spli
1895	1	SwissProt_HUMAN$	3::Q96BI1|S22AI_HUMAN	22	44816	1	1	1	1	0.11	Solute carrier family 2
1896	1	SwissProt_HUMAN$	3::Q7RTV0|PHF5A_HUMAN	22	12397	1	1	1	1	0.45	PHD finger-like domai
1897	1	SwissProt_HUMAN$	3::Q9NSI6|BRWD1_HUMAN	22	262772	1	1	1	1	0.02	Bromodomain and WD
1898	1	SwissProt_HUMAN$	3::P35579|MYH9_HUMAN	22	226392	1	1	1	1	0.02	Myosin-9 OS=Homo sa
1899	1	db$2	2::C3K5X0|C3K5X0_PSEFS	22	25599	2	2	2	2	0.44	Putative exported prot
1900	1	db$2	2::C3K2P3|C3K2P3_PSEFS	22	54945	2	2	1	1	0.09	Putative aldehyde deh
1901	1	db$2	2::C3K406|C3K406_PSEFS	22	23424	1	1	1	1	0.22	Glyoxalase OS=Pseud
1902	1	db$2	2::C3KE69|C3KE69_PSEFS	22	32125	1	1	1	1	0.16	Putative membrane pr
1903	1	SwissProt_HUMAN$	3::Q9UHC6|CNTP2_HUMAN	22	148073	1	1	1	1	0.03	Contactin-associated p
1904	1	db$2	2::C3K659|C3K659_PSEFS	22	30767	1	1	1	1	0.17	2-hydroxy-3-oxopropi
1905	1	SwissProt_HUMAN$	3::P82094|TMF1_HUMAN	22	122767	1	1	1	1	0.04	TATA element modula
1906	1	db$2	2::C3K2N6|C3K2N6_PSEFS	22	16796	1	1	1	1	0.32	MaoC-like domain-con
1907	1	SwissProt_HUMAN$	3::A6NE01|F186A_HUMAN	22	262595	1	1	1	1	0.02	Protein FAM186A OS=
1908	1	db$2	2::C3K431|C3K431_PSEFS	22	14535	1	1	1	1	0.38	Regulator of nucleosid
1909	1	db$2	2::C3K6Z6|C3K6Z6_PSEFS	22	13580	1	1	1	1	0.41	Putaitve lipoprotein OS

↑Family	M	DB	Accession	Score	Mass	Matches	Match(sig)	Sequences	Seq(sig)	emPAI	Description
1910	1	db$2	2::C3K8Z7|C3K8Z7_PSEFS	22	10906	1	1	1	1	0.53	Uncharacterized prote
1911	1	SwissProt_HUMAN$	3::P29590|PML_HUMAN	22	97489	2	1	2	1	0.05	Protein PML OS=Homo
1912	1	SwissProt_HUMAN$	3::Q9H7V2|SYNG1_HUMAN	22	28533	1	1	1	1	0.18	Synapse differentiatio
1913	1	SwissProt_HUMAN$	3::Q4V328|GRAP1_HUMAN	22	95947	1	1	1	1	0.05	GRIP1-associated prot
1914	1	db$2	2::C3K8Z4|C3K8Z4_PSEFS	22	45613	1	1	1	1	0.11	High-affinity branched
1915	1	SwissProt_HUMAN$	3::Q9Y2G4|ANKR6_HUMAN	22	79922	1	1	1	1	0.06	Ankyrin repeat domair
1916	1	SwissProt_HUMAN$	3::Q9ULI4|KI26A_HUMAN	22	194468	1	1	1	1	0.02	Kinesin-like protein KI
1917	1	SwissProt_HUMAN$	3::P47756|CAPZB_HUMAN	22	31331	1	1	1	1	0.16	F-actin-capping protei
1918	1	SwissProt_HUMAN$	3::Q14588|ZN234_HUMAN	22	80507	1	1	1	1	0.06	Zinc finger protein 23
1919	1	db$2	2::C3K1D6|C3K1D6_PSEFS	22	16178	1	1	1	1	0.33	Putative membrane pr
1920	1	SwissProt_HUMAN$	3::Q9H9E3|COG4_HUMAN	22	89026	2	1	2	1	0.05	Conserved oligomeric
1921	1	SwissProt_HUMAN$	3::Q8TDI7|TMC2_HUMAN	22	102544	1	1	1	1	0.05	Transmembrane chan
1922	1	SwissProt_HUMAN$	3::P18077|RL35A_HUMAN	21	12530	1	1	1	1	0.45	60S ribosomal protein
1923	1	db$2	2::C3K277|C3K277_PSEFS	21	60895	1	1	1	1	0.08	DNA repair protein Re
1924	1	SwissProt_HUMAN$	3::P15169|CBPN_HUMAN	21	52253	1	1	1	1	0.09	Carboxypeptidase N c
1925	1	SwissProt_HUMAN$	3::P83731|RL24_HUMAN	21	17768	1	1	1	1	0.30	60S ribosomal protein
1926	1	db$2	2::C3KCP8|C3KCP8_PSEFS	21	42283	1	1	1	1	0.12	Lipopolysaccharide co
1927	1	db$2	2::C3K0R2|C3K0R2_PSEFS	21	34379	1	1	1	1	0.15	Chemotaxis protein O
1928	1	db$2	2::C3K585|C3K585_PSEFS	21	25105	1	1	1	1	0.21	Putative hydrolase OS
1929	1	SwissProt_HUMAN$	3::Q13304|GPR17_HUMAN	21	40963	1	1	1	1	0.12	Uracil nucleotide/cyste
1930	1	SwissProt_HUMAN$	3::Q8WXW3|PIBF1_HUMAN	21	89750	1	1	1	1	0.05	Progesterone-induced
1931	1	SwissProt_HUMAN$	3::Q53EZ4|CEP55_HUMAN	21	54145	2	1	2	1	0.09	Centrosomal protein o
1932	1	db$2	2::C3K7T7|C3K7T7_PSEFS	21	17319	1	1	1	1	0.31	Putative acetyltransfe
1933	1	SwissProt_HUMAN$	3::O95819|M4K4_HUMAN	21	142013	2	1	2	1	0.03	Mitogen-activated pro
1934	1	db$2	2::C3KB02|C3KB02_PSEFS	21	42163	2	2	2	2	0.25	Putative exported prot
1935	1	SwissProt_HUMAN$	3::A8MPX8|PP2D1_HUMAN	21	71597	1	1	1	1	0.07	Protein phosphatase 2
1936	1	db$2	2::C3K0E8|C3K0E8_PSEFS	21	27512	1	1	1	1	0.19	Thiol:disulfide intercha
1937	1	db$2	2::C3K236|C3K236_PSEFS	21	30467	1	1	1	1	0.17	Putative putative hem
1938	1	db$2	2::C3JY42|C3JY42_PSEFS	21	25186	1	1	1	1	0.21	Uncharacterized prote
1939	1	db$2	2::C3JZP4|RBSD_PSEFS	21	14393	1	1	1	1	0.38	D-ribose pyranase OS
1940	1	SwissProt_HUMAN$	3::Q13422|IKZF1_HUMAN	21	57491	2	1	2	1	0.09	DNA-binding protein I
1941	1	db$2	2::C3K1V9|C3K1V9_PSEFS	21	16219	1	1	1	1	0.33	Flavodoxin-like domai
1942	1	SwissProt_HUMAN$	3::Q4G0N8|SL9C1_HUMAN	21	135118	1	1	1	1	0.04	Sodium/hydrogen exc
1943	1	SwissProt_HUMAN$	3::Q8WZ42|TITIN_HUMAN	21	3813652	4	1	4	1	0.00	Titin OS=Homo sapier
1944	1	SwissProt_HUMAN$	3::Q96RT6|CTGE2_HUMAN	21	85228	1	1	1	1	0.06	cTAGE family member
1945	1	db$2	2::C3KE19|C3KE19_PSEFS	21	30253	1	1	1	1	0.17	HDOD domain-contain
1946	1	SwissProt_HUMAN$	3::Q4G0P3|HYDIN_HUMAN	21	575528	1	1	1	1	0.01	Hydrocephalus-inducir
1947	1	db$2	2::C3JY93|C3JY93_PSEFS	21	51981	2	2	2	2	0.20	NADH-quinone oxidore
1948	1	db$2	2::C3JY56|C3JY56_PSEFS	21	48640	1	1	1	1	0.10	Replication-associated
1949	1	db$2	2::C3JYC8|C3JYC8_PSEFS	21	59331	2	1	2	1	0.08	Putative pH adaptatior

↑Family	M	DB	Accession	Score	Mass	Matches	Match(sig)	Sequences	Seq(sig)	emPAI	Description
1950	1	SwissProt_HUMAN$	3::A8MT33|SYC1L_HUMAN	21	27380	1	1	1	1	0.19	Synaptonemal comple
1951	1	db$2	2::C3K992|C3K992_PSEFS	21	34600	1	1	1	1	0.15	Putative AraC-family r
1952	1	SwissProt_HUMAN$	3::O95347|SMC2_HUMAN	21	135572	1	1	1	1	0.04	Structural maintenanc
1953	1	db$2	2::C3K1S4|C3K1S4_PSEFS	21	23334	1	1	1	1	0.22	Putative membrane pr
1954	1	SwissProt_HUMAN$	3::P35611|ADDA_HUMAN	21	80905	1	1	1	1	0.06	Alpha-adducin OS=Ho
1955	1	db$2	2::C3KB13|C3KB13_PSEFS	21	35564	1	1	1	1	0.14	Putative ThiF protein (
1956	1	SwissProt_HUMAN$	3::Q49MG5|MAP9_HUMAN	21	74189	1	1	1	1	0.07	Microtubule-associatec
1957	1	db$2	2::C3KBJ0|C3KBJ0_PSEFS	21	16933	2	2	2	2	0.73	[Ribosomal protein S1
1958	1	db$2	2::C3JZI1|C3JZI1_PSEFS	21	33491	1	1	1	1	0.15	Putative glycine betair
1959	1	SwissProt_HUMAN$	3::Q8NEM8|CBPC3_HUMAN	21	115938	1	1	1	1	0.04	Cytosolic carboxypepti
1960	1	SwissProt_HUMAN$	3::Q8NHX9|TPC2_HUMAN	21	85188	1	1	1	1	0.06	Two pore calcium char
1961	1	SwissProt_HUMAN$	3::P31943|HNRH1_HUMAN	21	49198	1	1	1	1	0.10	Heterogeneous nuclea
1962	1	SwissProt_HUMAN$	3::P62244|RS15A_HUMAN	20	14830	1	1	1	1	0.37	40S ribosomal protein
1963	1	SwissProt_HUMAN$	3::Q7Z5L9|I2BP2_HUMAN	20	60987	1	1	1	1	0.08	Interferon regulatory f
1964	1	db$2	2::C3K4L7|C3K4L7_PSEFS	20	23283	1	1	1	1	0.22	Putative TetR-family r
1965	1	db$2	2::C3KAE4|C3KAE4_PSEFS	20	39865	1	1	1	1	0.13	Uncharacterized prote
1966	1	SwissProt_HUMAN$	3::Q9NZM1|MYOF_HUMAN	20	234561	1	1	1	1	0.02	Myoferlin OS=Homo s
1967	1	SwissProt_HUMAN$	3::Q06455|MTG8_HUMAN	20	67524	2	1	2	1	0.07	Protein CBFA2T1 OS=
1968	1	SwissProt_HUMAN$	3::P31040|SDHA_HUMAN	20	72645	1	1	1	1	0.07	Succinate dehydrogen
1969	1	SwissProt_HUMAN$	3::Q8N7X0|ADGB_HUMAN	20	189594	1	1	1	1	0.03	Androglobin OS=Hom
1970	1	db$2	2::C3K0K9|C3K0K9_PSEFS	20	37421	2	1	1	1	0.13	Putative NADP-depenc
1971	1	db$2	2::C3K2Q9|EX7S_PSEFS	20	8900	1	1	1	1	0.68	Exodeoxyribonuclease
1972	1	db$2	2::C3KA92|C3KA92_PSEFS	20	24193	1	1	1	1	0.21	Putative ACP reductas
1973	1	SwissProt_HUMAN$	3::Q5TGY3|AHDC1_HUMAN	20	168245	1	1	1	1	0.03	AT-hook DNA-binding
1974	1	SwissProt_HUMAN$	3::Q6P280|ZN529_HUMAN	20	65822	1	1	1	1	0.07	Zinc finger protein 52
1975	1	SwissProt_HUMAN$	3::Q08828|ADCY1_HUMAN	20	123360	1	1	1	1	0.04	Adenylate cyclase type
1976	1	db$2	2::C3KCD6|C3KCD6_PSEFS	20	27732	1	1	1	1	0.19	Short chain dehydroge
1977	1	db$2	2::C3K8Z3|C3K8Z3_PSEFS	20	28227	2	1	2	1	0.18	High-affinity branched
1978	1	db$2	2::C3K2I7|C3K2I7_PSEFS	20	17463	1	1	1	1	0.31	NYN domain-containin
1979	1	SwissProt_HUMAN$	3::A6NI79|CCD69_HUMAN	20	34775	2	1	2	1	0.15	Coiled-coil domain-cor
1980	1	SwissProt_HUMAN$	3::P36776|LONM_HUMAN	20	106422	2	1	2	1	0.05	Lon protease homolog
1981	1	db$2	2::C3JZ98|C3JZ98_PSEFS	20	32170	1	1	1	1	0.16	Putative acid phospha
1982	1	db$2	2::C3K7T3|C3K7T3_PSEFS	20	20773	1	1	1	1	0.25	CDP-diacylglycerol--gl
1983	1	SwissProt_HUMAN$	3::Q9NQS7|INCE_HUMAN	20	105365	1	1	1	1	0.05	Inner centromere prot
1984	1	SwissProt_HUMAN$	3::O15397|IPO8_HUMAN	20	119861	1	1	1	1	0.04	Importin-8 OS=Homo
1985	1	db$2	2::C3K232|C3K232_PSEFS	20	12849	1	1	1	1	0.44	Uncharacterized prote
1986	1	db$2	2::C3KBH7|C3KBH7_PSEFS	20	44237	1	1	1	1	0.11	3-oxoacyl-[acyl-carrie
1987	1	SwissProt_HUMAN$	3::Q96PF2|TSSK2_HUMAN	20	40913	1	1	1	1	0.12	Testis-specific serine/t
1988	1	db$2	2::C3JZ46|C3JZ46_PSEFS	20	37091	1	1	1	1	0.14	Putative LacI family tr
1989	1	db$2	2::C3K2K1|C3K2K1_PSEFS	19	28157	2	2	2	2	0.40	Phos_pyr_kin domain-

↑Family	M	DB	Accession	Score	Mass	Matches	Match(sig)	Sequences	Seq(sig)	emPAI	Description
1990	1	db$2	2::C3K340|C3K340_PSEFS	19	72802	1	1	1	1	0.07	DNA primase OS=Pseu
1991	1	db$2	2::C3K6A1|C3K6A1_PSEFS	19	22990	1	1	1	1	0.23	Putative copper-bindir
1992	1	db$2	2::C3KDW0|C3KDW0_PSEFS	19	49939	1	1	1	1	0.10	N-acetylmuramoyl-L-a
1993	1	db$2	2::C3K3Y2|C3K3Y2_PSEFS	19	34182	1	1	1	1	0.15	Putative sinc-binding c
1994	1	SwissProt_HUMAN$	3::P30041|PRDX6_HUMAN	19	25019	1	1	1	1	0.21	Peroxiredoxin-6 OS=H
1995	1	db$2	2::C3K707|C3K707_PSEFS	19	29295	1	1	1	1	0.17	Putative lipoprotein OS
1996	1	db$2	2::C3K0G4|C3K0G4_PSEFS	19	29541	2	2	2	2	0.38	MOSC domain-contain
1997	1	SwissProt_HUMAN$	3::Q9H299|SH3L3_HUMAN	19	10431	1	1	1	1	0.56	SH3 domain-binding g
1998	1	db$2	2::C3JZ74|C3JZ74_PSEFS	19	42038	1	1	1	1	0.12	ABC transport system,
1999	1	SwissProt_HUMAN$	3::P08865|RSSA_HUMAN	19	32833	1	1	1	1	0.15	40S ribosomal protein
2000	1	db$2	2::C3JZQ3|C3JZQ3_PSEFS	19	15084	2	1	2	1	0.36	Uncharacterized prote
2001	1	db$2	2::C3KBV8|C3KBV8_PSEFS	19	32477	1	1	1	1	0.16	Cobalt-zinc-cadmium r
2002	1	db$2	2::C3KCE1|C3KCE1_PSEFS	19	45800	1	1	1	1	0.11	Putative exported prot
2003	1	SwissProt_HUMAN$	3::Q9Y5A7|NUB1_HUMAN	19	70494	1	1	1	1	0.07	NEDD8 ultimate buste
2004	1	SwissProt_HUMAN$	3::Q9BSJ5|CQ080_HUMAN	19	67272	1	1	1	1	0.07	Uncharacterized prote
2005	1	SwissProt_HUMAN$	3::Q9UL33|TPC2L_HUMAN	19	16135	1	1	1	1	0.34	Trafficking protein par
2006	1	SwissProt_HUMAN$	3::Q92625|ANS1A_HUMAN	19	123032	1	1	1	1	0.04	Ankyrin repeat and SA
2007	1	db$2	2::C3K326|C3K326_PSEFS	19	35169	1	1	1	1	0.14	4-hydroxythreonine-4
2008	1	SwissProt_HUMAN$	3::Q16649|NFIL3_HUMAN	19	51440	1	1	1	1	0.10	Nuclear factor interleu
2009	1	SwissProt_HUMAN$	3::Q16629|SRSF7_HUMAN	19	27350	1	1	1	1	0.19	Serine/arginine-rich sp
2010	1	db$2	2::C3K686|C3K686_PSEFS	19	39658	1	1	1	1	0.13	Uncharacterized prote
2011	1	db$2	2::C3K8Q1|C3K8Q1_PSEFS	19	34464	1	1	1	1	0.15	Putative oxidoreductas
2012	1	db$2	2::C3K0W2|C3K0W2_PSEFS	19	26131	1	1	1	1	0.20	Flagellar basal body pr
2013	1	SwissProt_HUMAN$	3::P26641|EF1G_HUMAN	19	50087	2	2	2	2	0.21	Elongation factor 1-ga
2014	1	SwissProt_HUMAN$	3::Q9NR48|ASH1L_HUMAN	19	332582	1	1	1	1	0.01	Histone-lysine N-meth
2015	1	db$2	2::C3K5X5|C3K5X5_PSEFS	19	22739	1	1	1	1	0.23	Thioesterase domain-c
2016	1	SwissProt_HUMAN$	3::P63173|RL38_HUMAN	19	8213	1	1	1	1	0.75	60S ribosomal protein
2017	1	SwissProt_HUMAN$	3::P02749|APOH_HUMAN	19	38273	1	1	1	1	0.13	Beta-2-glycoprotein 1
2018	1	db$2	2::C3KB08|C3KB08_PSEFS	19	43028	1	1	1	1	0.12	Putative exported prot
2019	1	SwissProt_HUMAN$	3::Q13613|MTMR1_HUMAN	19	74631	1	1	1	1	0.07	Myotubularin-related p
2020	1	db$2	2::C3K2F9|C3K2F9_PSEFS	19	48516	1	1	1	1	0.10	Putative ABC transpor
2021	1	SwissProt_HUMAN$	3::Q9GZP7|VN1R1_HUMAN	19	39994	1	1	1	1	0.13	Vomeronasal type-1 re
2022	1	SwissProt_HUMAN$	3::P62829|RL23_HUMAN	19	14856	1	1	1	1	0.37	60S ribosomal protein
2023	1	db$2	2::C3K7M8|C3K7M8_PSEFS	19	23309	2	1	2	1	0.22	Putative glutathione S
2024	1	db$2	2::C3K811|C3K811_PSEFS	18	43409	1	1	1	1	0.12	Imidazolonepropionas
2025	1	db$2	2::C3K883|C3K883_PSEFS	18	60772	1	1	1	1	0.08	Putative transposase s
2026	1	db$2	2::C3KCH1|C3KCH1_PSEFS	18	32212	1	1	1	1	0.16	Putative carboxymethy
2027	1	SwissProt_HUMAN$	3::Q8N7X2|STPG3_HUMAN	18	42470	1	1	1	1	0.12	Protein STPG3 OS=Ho
2028	1	db$2	2::C3KE72|C3KE72_PSEFS	18	85807	2	2	2	2	0.12	UPF0313 protein PFLU
2029	1	db$2	2::C3K7G5|C3K7G5_PSEFS	18	138643	1	1	1	1	0.03	Putative conserved ex

↑Family	M	DB	Accession	Score	Mass	Matches	Match(sig)	Sequences	Seq(sig)	emPAI	Description
2030	1	SwissProt_HUMAN$	3::P0DME0|SETLP_HUMAN	18	34861	1	1	1	1	0.15	Protein SETSIP OS=Hc
2031	1	db$2	2::C3K219|C3K219_PSEFS	18	76943	2	1	1	1	0.06	RecBCD enzyme subu
2032	1	db$2	2::C3K0Y9|C3K0Y9_PSEFS	18	19041	1	1	1	1	0.28	Bifunctional adenosylc
2033	1	db$2	2::C3JY73|C3JY73_PSEFS	18	26430	1	1	1	1	0.20	Secretin_N domain-co
2034	1	SwissProt_HUMAN$	3::Q12899|TRI26_HUMAN	18	62127	1	1	1	1	0.08	Tripartite motif-contai
2035	1	SwissProt_HUMAN$	3::Q9Y446|PKP3_HUMAN	18	87029	1	1	1	1	0.06	Plakophilin-3 OS=Hom
2036	1	SwissProt_HUMAN$	3::Q9HCE3|ZN532_HUMAN	18	141606	1	1	1	1	0.03	Zinc finger protein 53:
2037	1	SwissProt_HUMAN$	3::P16471|PRLR_HUMAN	18	69461	1	1	1	1	0.07	Prolactin receptor OS=
2038	1	SwissProt_HUMAN$	3::A6NJB7|PRR19_HUMAN	18	38692	1	1	1	1	0.13	Proline-rich protein 19
2039	1	db$2	2::C3K2Z6|ERPA_PSEFS	18	12493	1	1	1	1	0.45	Iron-sulfur cluster inse
2040	1	SwissProt_HUMAN$	3::Q5JNZ5|RS26L_HUMAN	18	12994	1	1	1	1	0.43	Putative 40S ribosoma
2041	1	SwissProt_HUMAN$	3::Q13216|ERCC8_HUMAN	18	44027	1	1	1	1	0.11	DNA excision repair pr
2042	1	SwissProt_HUMAN$	3::Q9NUQ8|ABCF3_HUMAN	18	79695	1	1	1	1	0.06	ATP-binding cassette s
2043	1	SwissProt_HUMAN$	3::Q9Y4G6|TLN2_HUMAN	18	271443	1	1	1	1	0.02	Talin-2 OS=Homo sap
2044	1	db$2	2::C3K9J4|C3K9J4_PSEFS	18	15648	1	1	1	1	0.35	Putative DnaK suppres
2045	1	db$2	2::C3K596|C3K596_PSEFS	18	36487	1	1	1	1	0.14	Putative membrane pr
2046	1	SwissProt_HUMAN$	3::Q9Y530|OARD1_HUMAN	18	17014	1	1	1	1	0.32	ADP-ribose glycohydrc
2047	1	db$2	2::C3K0P1|C3K0P1_PSEFS	18	24214	1	1	1	1	0.21	Putative hydrolase OS
2048	1	db$2	2::C3KBU4|C3KBU4_PSEFS	18	61261	1	1	1	1	0.08	Uncharacterized prote
2049	1	SwissProt_HUMAN$	3::P58166|INHBE_HUMAN	17	38537	1	1	1	1	0.13	Inhibin beta E chain O
2050	1	SwissProt_HUMAN$	3::Q5F1R6|DJC21_HUMAN	17	61989	1	1	1	1	0.08	DnaJ homolog subfam
2051	1	SwissProt_HUMAN$	3::Q9BZE7|EVG1_HUMAN	17	24940	1	1	1	1	0.21	UPF0193 protein EVG1
2052	1	db$2	2::C3K8U0|HIS2_PSEFS	17	12007	1	1	1	1	0.47	Phosphoribosyl-ATP py
2053	1	SwissProt_HUMAN$	3::Q00341|VIGLN_HUMAN	17	141368	2	1	1	1	0.03	Vigilin OS=Homo sapi
2054	1	SwissProt_HUMAN$	3::Q5VWN6|TASO2_HUMAN	17	268675	2	1	1	1	0.02	Protein TASOR 2 OS=I
2055	1	SwissProt_HUMAN$	3::P54886|P5CS_HUMAN	17	87248	1	1	1	1	0.06	Delta-1-pyrroline-5-ca
2056	1	SwissProt_HUMAN$	3::Q9Y4G8|RPGF2_HUMAN	17	167313	1	1	1	1	0.03	Rap guanine nucleotid
2057	1	SwissProt_HUMAN$	3::Q8TD90|MAGE2_HUMAN	17	60339	1	1	1	1	0.08	Melanoma-associated
2058	1	db$2	2::C3K1F5|C3K1F5_PSEFS	17	29048	1	1	1	1	0.18	Putative chromosome
2059	1	SwissProt_HUMAN$	3::Q13873|BMPR2_HUMAN	17	115129	1	1	1	1	0.04	Bone morphogenetic p
2060	1	db$2	2::C3KEC9|C3KEC9_PSEFS	17	53005	1	1	1	1	0.09	Putative beta-barrel a:
2061	1	db$2	2::C3K3X1|C3K3X1_PSEFS	17	48834	1	1	1	1	0.10	Xaa-Pro aminopeptida
2062	1	db$2	2::C3K1Y4|C3K1Y4_PSEFS	17	14766	2	1	1	1	0.37	Putative putative glyo:
2063	1	db$2	2::C3K8W5|C3K8W5_PSEFS	17	21382	2	1	2	1	0.25	Lipopolysaccharide ex
2064	1	SwissProt_HUMAN$	3::Q9BSF4|TIM29_HUMAN	17	29215	1	1	1	1	0.18	Mitochondrial import ir
2065	1	db$2	2::C3K3H5|C3K3H5_PSEFS	17	32718	1	1	1	1	0.16	Putative ABC transpor
2066	1	SwissProt_HUMAN$	3::D6RIA3|CD054_HUMAN	17	189958	1	1	1	1	0.03	Uncharacterized prote
2067	1	db$2	2::C3K3G0|C3K3G0_PSEFS	17	42161	4	1	3	1	0.12	Membrane-bound lytic
2068	1	SwissProt_HUMAN$	3::Q7L592|NDUF7_HUMAN	17	49206	1	1	1	1	0.10	Protein arginine methy
2069	1	SwissProt_HUMAN$	3::Q9NU22|MDN1_HUMAN	17	632420	1	1	1	1	0.01	Midasin OS=Homo sap

↑Family	M	DB	Accession	Score	Mass	Matches	Match(sig)	Sequences	Seq(sig)	emPAI	Description
2070	1	SwissProt_HUMAN$	3::Q9BRP8|PYM1_HUMAN	17	22642	1	1	1	1	0.23	Partner of Y14 and ma
2071	1	SwissProt_HUMAN$	3::Q9ULQ1|TPC1_HUMAN	17	94086	1	1	1	1	0.05	Two pore calcium chai
2072	1	db$2	2::C3K0X2|C3K0X2_PSEFS	16	81009	1	1	1	1	0.06	Patatin-like phospholi
2073	1	SwissProt_HUMAN$	3::Q14997|PSME4_HUMAN	16	211199	1	1	1	1	0.02	Proteasome activator
2074	1	SwissProt_HUMAN$	3::Q8NDA2|HMCN2_HUMAN	16	541639	1	1	1	1	0.01	Hemicentin-2 OS=Hor
2075	1	db$2	2::C3KAI2|C3KAI2_PSEFS	16	19361	1	1	1	1	0.27	Shikimate kinase OS=
2076	1	SwissProt_HUMAN$	3::O75437|ZN254_HUMAN	16	77110	1	1	1	1	0.06	Zinc finger protein 25
2077	1	db$2	2::C3K5F5|Y1520_PSEFS	16	17238	1	1	1	1	0.31	UPF0260 protein PFLU
2078	1	db$2	2::C3K526|C3K526_PSEFS	16	40471	1	1	1	1	0.12	Cysteine desulfurase (
2079	1	db$2	2::C3JXF2|C3JXF2_PSEFS	16	13982	1	1	1	1	0.40	Uncharacterized prote
2080	1	db$2	2::C3K0X8|C3K0X8_PSEFS	16	55461	1	1	1	1	0.09	DNA recombination pr
2081	1	db$2	2::C3KCP2|C3KCP2_PSEFS	16	100291	2	1	2	1	0.05	Conserved GGDEF/EAI
2082	1	SwissProt_HUMAN$	3::O60229|KALRN_HUMAN	16	340047	1	1	1	1	0.01	Kalirin OS=Homo sapi
2083	1	SwissProt_HUMAN$	3::Q8IWU5|SULF2_HUMAN	16	100390	1	1	1	1	0.05	Extracellular sulfatase
2084	1	SwissProt_HUMAN$	3::P78371|TCPB_HUMAN	16	57452	1	1	1	1	0.09	T-complex protein 1 s
2085	1	db$2	2::C3K2L1|C3K2L1_PSEFS	16	28054	1	1	1	1	0.18	DUF218 domain-conta
2086	1	SwissProt_HUMAN$	3::P46020|KPB1_HUMAN	16	137225	3	1	2	1	0.04	Phosphorylase b kinas
2087	1	db$2	2::C3K0X1|C3K0X1_PSEFS	16	10638	1	1	1	1	0.54	Uncharacterized prote
2088	1	db$2	2::C3K3N6|FPG_PSEFS	16	30025	2	1	2	1	0.17	Formamidopyrimidine-
2089	1	SwissProt_HUMAN$	3::Q9BT73|PSMG3_HUMAN	16	13096	1	1	1	1	0.43	Proteasome assembly
2090	1	SwissProt_HUMAN$	3::O96017|CHK2_HUMAN	16	60877	2	1	2	1	0.08	Serine/threonine-prot
2091	1	SwissProt_HUMAN$	3::Q6NXP2|F71F2_HUMAN	16	34494	1	1	1	1	0.15	Protein FAM71F2 OS=
2092	1	db$2	2::C3K994|C3K994_PSEFS	16	23940	1	1	1	1	0.22	Putative regulatory pr
2093	1	db$2	2::C3JYX2|C3JYX2_PSEFS	16	42003	1	1	1	1	0.12	Putative periplasmic p
2094	1	db$2	2::C3K457|C3K457_PSEFS	16	12769	1	1	1	1	0.44	Putative endoribonucle
2095	1	db$2	2::C3K4X6|C3K4X6_PSEFS	16	73857	1	1	1	1	0.07	Putative polysaccharid
2096	1	db$2	2::C3K439|C3K439_PSEFS	15	25827	1	1	1	1	0.20	Putative hydrolase OS
2097	1	SwissProt_HUMAN$	3::P42768|WASP_HUMAN	15	52880	1	1	1	1	0.09	Wiskott-Aldrich syndro
2098	1	SwissProt_HUMAN$	3::Q13219|PAPP1_HUMAN	15	180856	1	1	1	1	0.03	Pappalysin-1 OS=Hom
2099	1	SwissProt_HUMAN$	3::P55285|CADH6_HUMAN	15	88254	1	1	1	1	0.06	Cadherin-6 OS=Homo
2100	1	db$2	2::C3K122|C3K122_PSEFS	15	33097	1	1	1	1	0.15	Putative O-acetylserin
2101	1	SwissProt_HUMAN$	3::Q8TEQ6|GEMI5_HUMAN	15	168483	1	1	1	1	0.03	Gem-associated prote
2102	1	SwissProt_HUMAN$	3::P67936|TPM4_HUMAN	15	28504	2	1	2	1	0.18	Tropomyosin alpha-4
2103	1	SwissProt_HUMAN$	3::Q9BYV7|BCDO2_HUMAN	15	65632	1	1	1	1	0.07	Beta,beta-carotene 9',
2104	1	SwissProt_HUMAN$	3::Q7RTR0|NLRP9_HUMAN	15	113238	1	1	1	1	0.04	NACHT, LRR and PYD
2105	1	SwissProt_HUMAN$	3::Q5THR3|EFCB6_HUMAN	15	172820	1	1	1	1	0.03	EF-hand calcium-bindi
2106	1	db$2	2::C3K5Q6|C3K5Q6_PSEFS	15	68754	2	1	2	1	0.07	Putative hydrolase OS
2107	1	db$2	2::C3K1W1|C3K1W1_PSEFS	15	22921	1	1	1	1	0.23	Uncharacterized prote
2108	1	SwissProt_HUMAN$	3::P11172|UMPS_HUMAN	15	52189	1	1	1	1	0.10	Uridine 5'-monophosp
2109	1	SwissProt_HUMAN$	3::Q13835|PKP1_HUMAN	15	82808	1	1	1	1	0.06	Plakophilin-1 OS=Hom

↑Family	M	DB	Accession	Score	Mass	Matches	Match(sig)	Sequences	Seq(sig)	emPAI	Description
2110	1	SwissProt_HUMAN$	3::Q9Y4D8|HECD4_HUMAN	15	439065	2	2	1	1	0.01	Probable E3 ubiquitin-
2111	1	SwissProt_HUMAN$	3::Q6NXR0|IIGP5_HUMAN	15	50257	1	1	1	1	0.10	Interferon-inducible G
2112	1	SwissProt_HUMAN$	3::Q5VSL9|STRP1_HUMAN	15	95515	1	1	1	1	0.05	Striatin-interacting pro
2113	1	SwissProt_HUMAN$	3::Q9H173|SIL1_HUMAN	15	52052	2	1	1	1	0.10	Nucleotide exchange f
2114	1	SwissProt_HUMAN$	3::Q07002|CDK18_HUMAN	15	54390	1	1	1	1	0.09	Cyclin-dependent kina
2115	1	db$2	2::C3K020|C3K020_PSEFS	15	23656	1	1	1	1	0.22	Putative hydrolase OS
2116	1	SwissProt_HUMAN$	3::P24386|RAE1_HUMAN	15	73429	1	1	1	1	0.07	Rab proteins geranylg
2117	1	SwissProt_HUMAN$	3::Q8ND24|RN214_HUMAN	15	77619	1	1	1	1	0.06	RING finger protein 21
2118	1	SwissProt_HUMAN$	3::Q4KMP7|TB10B_HUMAN	15	87145	1	1	1	1	0.06	TBC1 domain family m
2119	1	SwissProt_HUMAN$	3::P04075|ALDOA_HUMAN	15	39395	1	1	1	1	0.13	Fructose-bisphosphate
2120	1	SwissProt_HUMAN$	3::P63244|RACK1_HUMAN	15	35055	1	1	1	1	0.14	Receptor of activated
2121	1	db$2	2::C3K7D7|C3K7D7_PSEFS	15	8979	1	1	1	1	0.67	Putative lipoprotein OS
2122	1	db$2	2::C3K0T2|C3K0T2_PSEFS	15	23233	1	1	1	1	0.22	Putative RNA polymera
2123	1	SwissProt_HUMAN$	3::Q6TFL3|CC171_HUMAN	15	152714	1	1	1	1	0.03	Coiled-coil domain-con
2124	1	SwissProt_HUMAN$	3::Q9H5Z1|DHX35_HUMAN	15	78860	1	1	1	1	0.06	Probable ATP-depende
2125	1	SwissProt_HUMAN$	3::P32321|DCTD_HUMAN	15	20003	1	1	1	1	0.26	Deoxycytidylate deam
2126	1	SwissProt_HUMAN$	3::Q12870|TCF15_HUMAN	15	20804	1	1	1	1	0.25	Transcription factor 15
2127	1	SwissProt_HUMAN$	3::Q7Z6J6|FRMD5_HUMAN	15	65023	1	1	1	1	0.08	FERM domain-containi
2128	1	SwissProt_HUMAN$	3::P50454|SERPH_HUMAN	15	46411	1	1	1	1	0.11	Serpin H1 OS=Homo s
2129	1	db$2	2::C3KBW9|C3KBW9_PSEFS	15	64127	2	1	2	1	0.08	Putative lipoprotein OS
2130	1	db$2	2::C3JY51|C3JY51_PSEFS	15	37030	1	1	1	1	0.14	Putative glycosyl trans
2131	1	db$2	2::C3K3C9|C3K3C9_PSEFS	14	43507	1	1	1	1	0.11	Putative glycine betair
2132	1	db$2	2::C3K6M1|C3K6M1_PSEFS	14	28266	1	1	1	1	0.18	Putative enoyl-CoA hy
2133	1	db$2	2::C3K4U0|C3K4U0_PSEFS	14	54029	1	1	1	1	0.09	Putative transport rela
2134	1	db$2	2::C3K1B3|C3K1B3_PSEFS	14	20726	2	1	2	1	0.25	Uncharacterized prote
2135	1	SwissProt_HUMAN$	3::O60237|MYPT2_HUMAN	14	110337	1	1	1	1	0.04	Protein phosphatase 1
2136	1	db$2	2::C3KDB4|C3KDB4_PSEFS	14	42765	1	1	1	1	0.12	Putative glycosyl trans
2137	1	SwissProt_HUMAN$	3::Q9P202|WHRN_HUMAN	14	96499	1	1	1	1	0.05	Whirlin OS=Homo sap
2138	1	SwissProt_HUMAN$	3::Q8NEN9|PDZD8_HUMAN	14	128483	1	1	1	1	0.04	PDZ domain-containin
2139	1	SwissProt_HUMAN$	3::Q8WU90|ZC3HF_HUMAN	14	48573	1	1	1	1	0.10	Zinc finger CCCH dom
2140	1	SwissProt_HUMAN$	3::Q9UBF2|COPG2_HUMAN	14	97560	1	1	1	1	0.05	Coatomer subunit gan
2141	1	SwissProt_HUMAN$	3::Q8IUL8|CILP2_HUMAN	14	126211	1	1	1	1	0.04	Cartilage intermediate
2142	1	SwissProt_HUMAN$	3::Q16512|PKN1_HUMAN	14	103868	1	1	1	1	0.05	Serine/threonine-prote
2143	1	SwissProt_HUMAN$	3::Q96KR4|LMLN_HUMAN	14	73521	1	1	1	1	0.07	Leishmanolysin-like pe
2144	1	SwissProt_HUMAN$	3::Q9BZB8|CPEB1_HUMAN	14	62555	1	1	1	1	0.08	Cytoplasmic polyaden
2145	1	db$2	2::C3K8R0|C3K8R0_PSEFS	13	13570	1	1	1	1	0.41	Uncharacterized prote
2146	1	db$2	2::C3KCK7|C3KCK7_PSEFS	13	25535	1	1	1	1	0.20	Putative GntR-family r
2147	1	db$2	2::C3KBV7|NHAP2_PSEFS	13	61387	1	1	1	1	0.08	K(+)/H(+) antiporter
2148	1	SwissProt_HUMAN$	3::Q7Z6B0|CCD91_HUMAN	13	49941	3	1	3	1	0.10	Coiled-coil domain-con
2149	1	SwissProt_HUMAN$	3::Q9Y5G7|PCDG6_HUMAN	13	100809	1	1	1	1	0.05	Protocadherin gamma

↑Family	M	DB	Accession	Score	Mass	Matches	Match(sig)	Sequences	Seq(sig)	emPAI	Description
2150	1	db$2	2::C3JYZ6|C3JYZ6_PSEFS	13	88576	1	1	1	1	0.06	Putative ABC transpor
2151	1	SwissProt_HUMAN$	3::O00635|TRI38_HUMAN	13	53381	1	1	1	1	0.09	E3 ubiquitin-protein li
2152	1	db$2	2::C3K7G6|C3K7G6_PSEFS	13	30473	1	1	1	1	0.17	Putative hydrolase OS

Export as CSV

Not what you expected? Try the select summary.

Mascot: http://www.matrixscience.com/
