# Supplementary material for: Circulating multimeric immune complexes contribute to immunopathology in COVID-19
Source: Nat Commun. 2022 Sep 26;13:5654. doi: 10.1038/s41467-022-32867-z (PMC9513013; doi:10.1038/s41467-022-32867-z)
Supplement: Supplementary file 3 — Source Data [file 41467_2022_32867_MOESM3_ESM.zip › Source Data/MS_20210407 (immune precipitation).zip!/QX20210618-11_Virologie(AG-Hengel)-20210407_(V11)_0k3v20_TRL_db_SC2+db_Pf+SP_H(F235838).pdf]

MASCOT Search Results

**User**	: Wolfgang Bildl
**E-mail**	: wolfgang.bildl@physiologie.uni-freiburg.de
**Search title**	: QX 20210618 (db$ SARS-CoV-2+db$2 Pf+SP H$ 5 ppm 20 mmu), QX20210618-11_Virologie(AG-Hengel)-20210407_(V11)_0k3v20_TRL_shifted_-1.3.mgf
**MS data file**	: D:\Xcalibur\Data\Wolfgang\Q-Exactive-HF-X_20210616\QX20210618-11_Virologie(AG-Hengel)-20210407_(V11)_0k3v20_TRL_shifted_-1.3.mgf
**Databases**	: **1:** db$ 20210617 (17 sequences; 14,439 residues)<br>**2:** db$2 20210617 (6,388 sequences; 2,089,672 residues)<br>**3:** SwissProt_HUMAN$ 20201007 (20,389 sequences; 11,363,789 residues)
**Timestamp**	: 21 Jun 2021 at 14:11:43 GMT

Not what you expected? Try **the select summary**.

Search parameters

**Type of search**	: MS/MS Ion Search
**Enzyme**	: Trypsin
**Variable modifications**	: **Acetyl (Protein N-term)**, **Carbamidomethyl (C)**, **Formyl (Protein N-term)**, **Gln->pyro-Glu (N-term Q)**, **Oxidation (M)**
**Mass values**	: Monoisotopic
**Protein mass**	: Unrestricted
**Peptide mass tolerance**	: ± 5 ppm
**Fragment mass tolerance**	: ± 20 mmu
**Max missed cleavages**	: 1
**Instrument type**	: ESI-FTICR
**Number of queries**	: 83,553

Score distribution

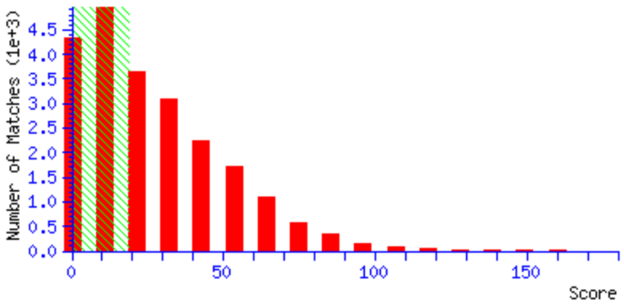

Peptide score distribution. Ions score is $-10\log(P)$, where P is the probability that the observed match is a random event.
There are **11,976** peptide matches above identity threshold and **12,720** matches above homology threshold for **83,553** queries.
On average, individual ions scores **> 19** (beyond green shading) indicate **identity or extensive homology** (p<0.05).

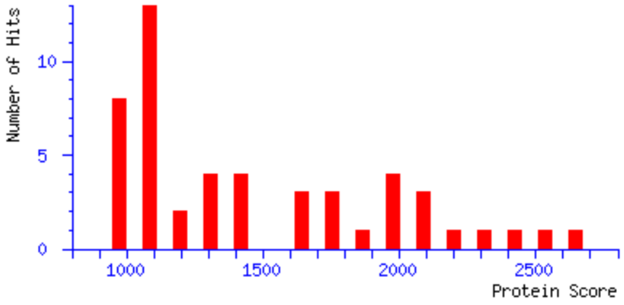

[Deprecated] Protein score distribution. Score distribution for family members in the first 50 proteins. Protein scores are derived from ions scores as a non-probabilistic basis for ranking protein families.

▼Modification statistics

Modification	Site	Above thr.
Oxidation	M	2380
Carbamidomethyl	C	1211
Gln->pyro-Glu	N-term	266
Formyl	Protein N-term	29
Acetyl	Protein N-term	15

▼Legend

Peptide columns and rows

Dupes	...	Expect	Rank	U	1	2	Peptide	
		**0.037**	▶**2**				**GAYSLSLR**	significant
		9	▶1				GFFLFVEGGR	top ranking
		**6.4e-005**	▶**1**				**GSSIFGLAPGK**	significant and top ranking
		**1.3e-006**	▶**1**		■		**SSGTSYPDVLK**	peptide is found in all proteins in family member 1
		**6.2e-007**	▶**1**			■	**VCNYVSWIK**	peptide is found in some but not all proteins in family member 2
		**6.4e-005**	▶**1**	**U**			**GSSIFGLAPGK**	unique
▶**2**		**5.7e-005**	▶**1**				**LNTLETEEWFFK**	peptide has two duplicates
		0.18	▶*1*				*LNTLETEEWFFK*	duplicate peptide

Right-facing triangle (▶) in the Dupes or Rank column indicates content that can be expanded by clicking on it. Down-facing triangle (▼) indicates the content is expanded and can be collapsed. For more details about particular columns, see **results format help**.

Protein quantitation ratios

	Score	...	114/113	115/113
**CFAH_HUMAN**	37559		*0.962*	**1.129**
**FHR2_HUMAN**	1330		**0.859**	1.128

When quantitation method is Reporter (e.g. iTRAQ) or Multiplex (e.g. IPTL), protein ratios are displayed when a family is expanded. Ratios in *italic* indicate that the peptide log-ratios do not appear to come from a normal distribution. **Bold** indicates that if you can assume peptide ratios are normally distributed, the protein ratio is significantly different from 1.0 (at significance level 0.05).

Note that lack of bold or italic can also mean that significance or normality testing has not been performed (for

example, if protein ratio type does not support it).

Protein Family Summary

Significance threshold p<	0.05	Max. number of families	AUTO
Ions score or expect cut-off	0.5	Dendrograms cut at	0
Show Percolator scores	☐		
Preferred taxonomy	All entries		

▼Sensitivity and FDR (reversed protein sequences)

			Target	Decoy	FDR		
PSMs	above	homology	12720	426	3.35%	Adjust to	1%

Decoy results are available in **the decoy report**.

Protein hits (2108 proteins)

▶Filters: (none)

Export as CSV

↑Family	M	DB	Accession	Score	Mass	Matches	Match(sig)	Sequences	Seq(sig)	emPAI	Description
1	1	db$2	2::C3K2Y3|RPOB_PSEFS	2642	150923	105	99	66	61	8.07	DNA-directed RNA poly
2	1	db$2	2::C3K2X8|EFTU_PSEFS	2506	43536	81	76	24	23	39.29	Elongation factor Tu O
3	1	db$2	2::C3K4N0|C3K4N0_PSEFS	2394	43479	60	58	24	22	31.42	Putative ornithine deca
4	1	db$2	2::C3K5G3|C3K5G3_PSEFS	2254	99192	77	73	38	37	8.48	Aconitate hydratase O
5	1	db$2	2::C3K1E6|ATPB_PSEFS	2180	49459	69	67	30	30	36.86	ATP synthase subunit I
6	1	db$2	2::C3K1A0|CH60_PSEFS	2061	56931	64	62	33	32	29.31	60 kDa chaperonin OS
7	1	db$2	2::C3KD82|C3KD82_PSEFS	2060	181591	69	69	54	54	3.69	Glutamate dehydroger
7	2	db$2	2::C3KAT2|C3KAT2_PSEFS	37	49009	2	2	2	2	0.21	Histidine kinase OS=P
8	1	SwissProt_HUMAN$	3::P13645|K1C10_HUMAN	2043	58792	49	48	28	27	12.17	Keratin, type I cytoske
8	2	SwissProt_HUMAN$	3::P02533|K1C14_HUMAN	529	51529	22	19	18	16	3.77	Keratin, type I cytoske
8	3	SwissProt_HUMAN$	3::P08779|K1C16_HUMAN	367	51236	15	14	12	12	2.32	Keratin, type I cytoske
8	4	SwissProt_HUMAN$	3::Q04695|K1C17_HUMAN	184	48076	11	10	9	9	1.43	Keratin, type I cytoske
8	5	SwissProt_HUMAN$	3::P13646|K1C13_HUMAN	115	49557	7	6	6	6	0.77	Keratin, type I cytoske
8	6	SwissProt_HUMAN$	3::P08727|K1C19_HUMAN	94	44079	6	6	6	6	0.90	Keratin, type I cytoske
9	1	db$2	2::C3K2Y2|RPOC_PSEFS	2029	154528	92	86	65	59	7.10	DNA-directed RNA poly
10	1	db$2	2::C3K2X9|EFG_PSEFS	2013	77114	75	71	34	33	13.97	Elongation factor G OS
11	1	db$2	2::C3K4D5|C3K4D5_PSEFS	1996	34599	65	61	19	19	29.22	Phosphate-binding pro
12	1	db$2	2::C3K1E8|ATPA_PSEFS	1957	55245	66	65	30	30	32.57	ATP synthase subunit a
13	1	db$2	2::C3K5Z6|EFTS_PSEFS	1865	30341	69	62	16	15	9.32	Elongation factor Ts O
14	1	db$2	2::C3KCJ5|C3KCJ5_PSEFS	1733	93957	53	49	37	34	5.83	Aconitate hydratase B
15	1	db$2	2::C3K255|PNP_PSEFS	1718	74889	55	55	33	33	12.41	Polyribonucleotide nuc

↑Family	M	DB	Accession	Score	Mass	Matches	Match(sig)	Sequences	Seq(sig)	emPAI	Description
16	1	db$2	2::C3K0Q5|C3K0Q5_PSEFS	1717	107302	65	61	43	41	8.14	Ribonucleoside-diphos
17	1	SwissProt_HUMAN$	3::P04264|K2C1_HUMAN	1675	65999	49	48	33	32	13.24	Keratin, type II cytosk
17	2	SwissProt_HUMAN$	3::P35908|K22E_HUMAN	1320	65393	44	42	37	37	13.63	Keratin, type II cytosk
17	3	SwissProt_HUMAN$	3::P02538|K2C6A_HUMAN	460	60008	20	17	18	16	2.54	Keratin, type II cytosk
17	4	SwissProt_HUMAN$	3::P13647|K2C5_HUMAN	437	62340	17	17	16	16	2.37	Keratin, type II cytosk
17	5	SwissProt_HUMAN$	3::P05787|K2C8_HUMAN	204	53671	10	9	8	7	0.85	Keratin, type II cytosk
17	6	SwissProt_HUMAN$	3::P08729|K2C7_HUMAN	138	51354	7	7	5	5	0.59	Keratin, type II cytosk
17	7	SwissProt_HUMAN$	3::Q14CN4|K2C72_HUMAN	85	55842	5	5	3	3	0.29	Keratin, type II cytosk
18	1	db$2	2::C3KCP4|C3KCP4_PSEFS	1620	99394	66	60	44	39	9.41	Pyruvate dehydrogena
19	1	db$2	2::C3K275|DNAK_PSEFS	1604	68158	47	46	26	25	6.52	Chaperone protein Dna
20	1	db$2	2::C3K6M5|C3K6M5_PSEFS	1460	63522	37	36	21	20	5.00	Succinate dehydrogena
21	1	db$2	2::C3K5J5|FABV_PSEFS	1419	42933	59	58	25	24	16.51	Enoyl-[acyl-carrier-prc
22	1	SwissProt_HUMAN$	3::P35527|K1C9_HUMAN	1405	62027	38	36	23	23	6.87	Keratin, type I cytoske
23	1	db$2	2::C3K0P3|C3K0P3_PSEFS	1397	118163	41	39	26	25	1.96	Ribonuclease E OS=Ps
24	1	db$2	2::C3KBH1|C3KBH1_PSEFS	1354	161946	54	49	43	41	2.43	Glutamate synthase [N
25	1	db$2	2::C3K331|C3K331_PSEFS	1296	73691	51	50	35	35	11.31	AAA_PrkA domain-con
26	1	db$2	2::C3JYU7|C3JYU7_PSEFS	1288	102460	40	37	25	25	2.50	Protein translocase sul
27	1	db$2	2::C3JYK1|TIG_PSEFS	1264	48316	42	39	25	24	17.93	Trigger factor OS=Psei
28	1	db$2	2::C3K6H4|PYRG_PSEFS	1243	59660	42	39	24	23	7.56	CTP synthase OS=Pseu
29	1	db$2	2::C3K225|ILVC_PSEFS	1235	36188	47	45	25	23	32.93	Ketol-acid reductoisom
30	1	db$2	2::C3K271|C3K271_PSEFS	1129	117563	41	37	28	25	2.23	Carbamoyl-phosphate
31	1	db$2	2::C3K6N0|SUCC_PSEFS	1119	41262	36	35	19	19	12.93	Succinate--CoA ligase
32	1	db$2	2::C3K6P2|C3K6P2_PSEFS	1113	42870	29	28	12	12	6.29	3-oxoacyl-[acyl-carrier
33	1	db$2	2::C3K402|C3K402_PSEFS	1111	83101	44	43	30	30	6.37	Polyphosphate kinase
34	1	db$2	2::C3KEC5|C3KEC5_PSEFS	1090	97398	41	40	30	30	4.25	Phosphoenolpyruvate
35	1	db$2	2::C3K7D3|C3K7D3_PSEFS	1071	91800	29	28	21	21	2.84	Alpha-1,4 glucan phos
36	1	db$2	2::C3K7D1|C3K7D1_PSEFS	1070	67115	46	40	30	26	7.32	50S ribosomal subunit
37	1	db$2	2::C3K1K1|C3K1K1_PSEFS	1064	51928	40	38	21	19	8.77	Inosine-5'-monophosp
37	2	db$2	2::C3KAJ8|UPP_PSEFS	113	22986	9	7	7	6	2.41	Uracil phosphoribosylti
38	1	db$2	2::C3K6K0|C3K6K0_PSEFS	1057	62268	41	39	24	22	6.81	30S ribosomal protein
39	1	db$2	2::C3K6M8|C3K6M8_PSEFS	1050	42320	30	29	17	16	8.36	Dihydrolipoyllysine-res
40	1	db$2	2::C3K6M7|C3K6M7_PSEFS	1048	105930	50	45	35	33	4.03	Oxoglutarate dehydrog
41	1	db$2	2::C3K613|FADB_PSEFS	1040	76938	41	39	30	29	5.77	Fatty acid oxidation co
42	1	db$2	2::C3JYR1|GCSP_PSEFS	1027	102176	37	34	24	23	2.05	Glycine dehydrogenase
43	1	db$2	2::C3JYT1|SYDND_PSEFS	1022	66220	36	35	23	23	5.90	Aspartate--tRNA(Asp/A
44	1	db$2	2::C3KBY8|C3KBY8_PSEFS	1013	21919	43	38	16	16	46.78	Alkyl hydroperoxide re
45	1	db$2	2::C3K4D2|C3K4D2_PSEFS	1012	30988	40	37	20	20	70.13	Phosphate import ATP-
46	1	db$2	2::C3K259|IF2_PSEFS	1007	90544	39	37	27	25	3.82	Translation initiation fa
47	1	db$2	2::C3KBT0|C3KBT0_PSEFS	999	51607	32	31	17	16	6.53	Succinate-semialdehyc
48	1	db$2	2::C3K121|C3K121_PSEFS	990	86030	44	41	34	32	5.90	Phosphoenolpyruvate s

↑Family	M	DB	Accession	Score	Mass	Matches	Match(sig)	Sequences	Seq(sig)	emPAI	Description
49	1	db$2	2::C3KE86|C3KE86_PSEFS	979	95293	45	43	33	31	4.73	Chaperone protein Clp
50	1	db$2	2::C3K3F3|C3K3F3_PSEFS	971	72324	39	36	24	21	4.87	Transketolase OS=Pse
51	1	db$2	2::C3K162|C3K162_PSEFS	961	52023	34	34	20	20	7.10	Pyruvate kinase OS=P
52	1	db$2	2::C3K6N7|HTPG_PSEFS	956	71589	32	32	23	23	5.39	Chaperone protein Htp
53	1	db$2	2::C3K673|C3K673_PSEFS	942	47725	36	35	18	17	10.93	Citrate synthase OS=F
54	1	db$2	2::C3K139|C3K139_PSEFS	941	48816	26	26	12	12	3.28	Uncharacterized protei
55	1	db$2	2::C3K3Z0|C3K3Z0_PSEFS	940	46925	36	33	23	22	10.24	Transcription terminati
56	1	db$2	2::C3K1J2|C3K1J2_PSEFS	927	140531	45	40	33	30	2.16	Phosphoribosylformylg
57	1	db$2	2::C3JYS1|C3JYS1_PSEFS	905	17688	23	22	7	7	9.89	Peptidoglycan-associat
58	1	db$2	2::C3K0T0|C3K0T0_PSEFS	904	34560	35	34	10	10	4.16	Putative outer membra
59	1	db$2	2::C3K0K6|C3K0K6_PSEFS	893	26352	27	26	14	13	19.96	Electron transfer flavo
60	1	db$2	2::C3JYJ7|C3JYJ7_PSEFS	882	9057	51	47	8	8	156.84	DNA-binding protein H
61	1	db$2	2::C3K165|C3K165_PSEFS	873	54857	38	35	23	22	11.25	Fumarate hydratase cl
61	2	db$2	2::C3K604|C3K604_PSEFS	225	18933	12	12	9	9	8.23	Putative outer membra
62	1	db$2	2::C3KBJ2|C3KBJ2_PSEFS	866	24354	30	27	13	13	25.83	Carbonic anhydrase O
63	1	db$2	2::C3K6N1|C3K6N1_PSEFS	864	29928	29	26	15	14	18.87	Succinate--CoA ligase
64	1	db$2	2::C3KCB8|C3KCB8_PSEFS	837	36708	30	26	16	14	7.90	Phosphate-binding pro
65	1	db$2	2::C3K6M9|C3K6M9_PSEFS	834	49948	24	24	14	14	4.01	Dihydrolipoyl dehydro
66	1	db$2	2::C3K7G0|C3K7G0_PSEFS	829	36980	25	22	16	15	7.78	Cell shape-determinin
67	1	db$2	2::C3KE67|RS6_PSEFS	823	16276	22	21	13	12	96.93	30S ribosomal protein
68	1	db$2	2::C3K603|C3K603_PSEFS	808	87560	34	30	24	21	2.48	Outer membrane prote
69	1	db$2	2::C3K367|C3K367_PSEFS	806	68142	33	32	20	20	3.96	Uncharacterized protei
70	1	db$2	2::C3K1K0|GUAA_PSEFS	784	58398	37	35	29	27	9.52	GMP synthase [glutam
71	1	db$2	2::C3KE51|C3KE51_PSEFS	783	38026	28	27	20	20	12.55	Putative oxidoreductas
72	1	db$2	2::C3KDP2|C3KDP2_PSEFS	782	68879	30	30	21	21	3.87	Multifunctional fusion
73	1	db$2	2::C3K4J5|PCKA_PSEFS	780	55324	26	25	20	19	4.55	Phosphoenolpyruvate
74	1	db$2	2::C3KCS1|C3KCS1_PSEFS	775	34171	28	28	17	17	26.50	Ribose-phosphate pyr
75	1	db$2	2::C3KBT1|C3KBT1_PSEFS	773	44808	29	26	17	17	5.68	4-aminobutyrate amin
76	1	db$2	2::C3K2Y6|RL1_PSEFS	770	24061	27	25	14	13	21.74	50S ribosomal protein
77	1	db$2	2::C3K4D1|C3K4D1_PSEFS	765	28553	21	19	13	13	10.92	Phosphate-specific tra
78	1	db$2	2::C3K2V9|RS5_PSEFS	764	17675	18	18	8	8	23.12	30S ribosomal protein
79	1	db$2	2::C3K2R9|C3K2R9_PSEFS	760	16440	18	18	8	8	16.22	6,7-dimethyl-8-ribityll
80	1	db$2	2::C3JY66|C3JY66_PSEFS	757	80156	38	33	22	19	3.14	Isocitrate dehydrogena
81	1	db$2	2::C3K6J4|C3K6J4_PSEFS	756	97893	37	35	29	27	2.89	DNA gyrase subunit A
82	1	db$2	2::C3K2D3|C3K2D3_PSEFS	747	44901	24	24	14	14	4.39	Serine hydroxymethylt
83	1	db$2	2::C3KAD2|ARNA_PSEFS	745	74140	22	20	19	18	2.16	Bifunctional polymyxin
84	1	db$2	2::C3K1A7|C3K1A7_PSEFS	740	66070	28	25	20	18	3.85	Proline--tRNA ligase O
85	1	db$2	2::C3KDW8|PURA_PSEFS	739	46535	27	27	19	19	7.47	Adenylosuccinate synt
86	1	db$2	2::C3KBM0|GLPK_PSEFS	731	55508	30	29	19	18	5.54	Glycerol kinase OS=Ps
87	1	db$2	2::C3JYT4|C3JYT4_PSEFS	713	17756	23	22	12	11	51.17	Putative ferritin DPS-fa

↑Family	M	DB	Accession	Score	Mass	Matches	Match(sig)	Sequences	Seq(sig)	emPAI	Description
88	1	db$2	2::C3KDD8|C3KDD8_PSEFS	711	44893	33	32	24	24	18.18	Cell division protein Ft
89	1	db$2	2::C3K3J7|C3K3J7_PSEFS	704	35008	29	28	16	15	16.05	Glutathione synthetase
90	1	db$2	2::C3K2V6|C3K2V6_PSEFS	700	47842	20	20	10	10	2.62	Protein translocase sul
91	1	db$2	2::C3KA76|C3KA76_PSEFS	699	30860	21	20	13	13	10.54	UTP--glucose-1-phospl
92	1	db$2	2::C3K2D0|C3K2D0_PSEFS	693	61490	23	22	18	17	3.00	Energy-dependent trar
93	1	db$2	2::C3K6G5|AMPA_PSEFS	692	52403	32	29	23	21	7.00	Probable cytosol aminc
94	1	db$2	2::C3JZS1|C3JZS1_PSEFS	682	55264	29	26	18	16	4.10	Amidophosphoribosyltı
95	1	db$2	2::C3JYJ9|CLPX_PSEFS	679	46924	29	26	19	17	6.51	ATP-dependent Clp prc
96	1	db$2	2::C3K6H6|C3K6H6_PSEFS	677	45588	21	21	15	15	4.26	Enolase OS=Pseudomc
96	2	SwissProt_HUMAN$	3::P06733|ENOA_HUMAN	35	47139	4	4	4	4	0.49	Alpha-enolase OS=Hor
97	1	db$2	2::C3KDU5|C3KDU5_PSEFS	676	90380	21	21	18	18	1.86	DNA gyrase subunit B
98	1	db$2	2::C3K178|C3K178_PSEFS	672	36139	20	18	14	13	7.07	Glyceraldehyde-3-phos
99	1	db$2	2::C3K4F6|C3K4F6_PSEFS	672	65489	31	28	21	20	3.92	Oxaloacetate decarbox
100	1	db$2	2::C3K3S2|C3K3S2_PSEFS	667	44244	22	21	16	15	3.96	2-oxoglutarate reducta
101	1	db$2	2::C3KDG0|RECA_PSEFS	666	37499	21	20	13	13	5.61	Protein RecA OS=Pseu
102	1	db$2	2::C3K7Y0|C3K7Y0_PSEFS	656	45268	23	22	16	16	4.91	Putative exported prot
103	1	db$2	2::C3JZN7|SYT_PSEFS	653	72513	26	22	21	18	2.47	Threonine--tRNA ligase
104	1	db$2	2::C3K8B0|C3K8B0_PSEFS	646	25585	20	20	10	10	8.14	Peptidyl-prolyl cis-tran
105	1	db$2	2::C3JXY1|C3JXY1_PSEFS	646	93962	28	25	23	21	2.04	Alanine--tRNA ligase C
106	1	db$2	2::C3K1E7|ATPG_PSEFS	643	31374	19	19	14	14	8.51	ATP synthase gamma
107	1	db$2	2::C3K5E6|RS2_PSEFS	637	27078	24	23	15	14	18.23	30S ribosomal protein
108	1	db$2	2::C3K1L8|NDK_PSEFS	635	14928	23	20	7	6	5.57	Nucleoside diphosphat
109	1	db$2	2::C3JY65|C3JY65_PSEFS	634	45375	30	28	17	17	9.99	Isocitrate dehydrogena
110	1	db$2	2::C3KAH7|C3KAH7_PSEFS	633	45018	30	29	22	21	10.19	Putative NADP-depend
111	1	db$2	2::C3K1E2|C3K1E2_PSEFS	632	66300	20	20	14	14	1.93	Glutamine--fructose-6
112	1	db$2	2::C3K1W9|C3K1W9_PSEFS	632	33929	14	14	8	8	3.01	Thioredoxin reductase
113	1	db$2	2::C3K4V9|C3K4V9_PSEFS	628	53185	23	22	17	16	3.97	Glyceraldehyde-3-phos
114	1	db$2	2::C3KE40|SYGB_PSEFS	628	74833	29	26	23	20	3.04	Glycine--tRNA ligase b
115	1	db$2	2::C3K108|C3K108_PSEFS	623	83591	30	28	27	25	3.38	GTP pyrophosphokinas
116	1	db$2	2::C3K7C9|C3K7C9_PSEFS	614	51641	28	27	18	17	5.26	Glutamine synthetase
117	1	db$2	2::C3KDW5|C3KDW5_PSEFS	614	43265	25	22	19	17	6.97	Protein HflK OS=Pseuc
118	1	db$2	2::C3K6W9|C3K6W9_PSEFS	612	69415	16	15	13	13	1.43	Putative methyl-accep
118	2	db$2	2::C3K5K7|C3K5K7_PSEFS	102	52604	3	3	2	2	0.20	Putative methyl-accep
118	3	db$2	2::C3JZ31|C3JZ31_PSEFS	47	58204	2	2	2	2	0.18	Putative methyl-accep
118	4	db$2	2::C3K7Z5|C3K7Z5_PSEFS	43	66997	2	2	2	2	0.15	Putative methyl-accep
118	5	db$2	2::C3KD60|C3KD60_PSEFS	34	55774	2	2	2	2	0.19	Methyl-accepting chen
119	1	db$2	2::C3K1K7|LEU1_PSEFS	608	61997	23	22	19	18	3.27	2-isopropylmalate synt
120	1	db$2	2::C3JZT6|C3JZT6_PSEFS	608	40569	23	23	15	15	9.32	Aspartate-semialdehyc
121	1	db$2	2::C3K2X5|RL4_PSEFS	606	21987	12	10	8	6	3.46	50S ribosomal protein
122	1	db$2	2::C3K341|C3K341_PSEFS	602	69250	27	26	21	20	3.83	RNA polymerase sigma

↑Family	M	DB	Accession	Score	Mass	Matches	Match(sig)	Sequences	Seq(sig)	emPAI	Description
122	2	db$2	2::C3K708|C3K708_PSEFS	165	38066	4	4	4	4	0.64	RNA polymerase sigma
123	1	db$2	2::C3JYV2|C3JYV2_PSEFS	602	52688	20	19	16	15	3.21	AAA domain-containing
124	1	db$2	2::C3KBZ2|ASSY_PSEFS	589	45330	27	24	19	18	7.08	Argininosuccinate synt
125	1	db$2	2::C3K6I0|C3K6I0_PSEFS	588	39056	22	21	12	12	5.93	Alcohol dehydrogenase
126	1	db$2	2::C3K3G6|SAHH_PSEFS	587	51212	33	30	19	17	5.98	Adenosylhomocysteina
127	1	db$2	2::C3KCB5|C3KCB5_PSEFS	583	30173	20	19	11	10	4.57	Phosphate import ATP-
128	1	db$2	2::C3K3F1|PGK_PSEFS	581	40125	18	18	15	15	5.60	Phosphoglycerate kina
129	1	db$2	2::C3K4C6|C3K4C6_PSEFS	572	25565	22	22	12	12	12.21	Phosphate regulon trai
130	1	db$2	2::C3KDD9|C3KDD9_PSEFS	572	41969	15	15	13	13	3.33	Cell division protein Ft:
131	1	db$2	2::C3K649|C3K649_PSEFS	570	8822	16	16	5	5	12.79	Major outer membrane
132	1	db$2	2::C3K6W6|C3K6W6_PSEFS	564	49345	28	27	17	16	5.80	Biotin carboxylase OS=
133	1	db$2	2::C3K2V1|C3K2V1_PSEFS	561	36623	27	25	19	18	9.19	DNA-directed RNA poly
134	1	db$2	2::C3K426|ARLY_PSEFS	560	51545	20	19	12	12	3.35	Argininosuccinate lyase
135	1	db$2	2::C3KAK3|C3KAK3_PSEFS	549	54608	16	16	10	10	1.60	Probable malate:quino
136	1	db$2	2::C3K2M9|PROA_PSEFS	547	45380	15	15	9	9	2.15	Gamma-glutamyl phos
137	1	db$2	2::C3K123|C3K123_PSEFS	542	41542	13	13	10	10	2.12	Citrate synthase OS=P
138	1	db$2	2::C3JY70|C3JY70_PSEFS	537	50668	20	20	15	15	3.47	Adenylosuccinate lyase
139	1	db$2	2::C3K016|C3K016_PSEFS	536	85788	18	16	16	14	1.17	Ribosomal RNA large s
140	1	db$2	2::C3K3V2|C3K3V2_PSEFS	535	49111	23	20	12	10	1.62	Putative outer membra
141	1	db$2	2::C3KE65|C3KE65_PSEFS	533	97331	29	27	24	23	2.22	Ribonuclease R OS=Ps
142	1	db$2	2::C3K6X4|C3K6X4_PSEFS	529	71161	21	21	21	21	3.06	Biosynthetic arginine c
143	1	db$2	2::C3JXZ7|C3JXZ7_PSEFS	528	28415	15	15	9	9	3.45	Putative histidine-bind
144	1	db$2	2::C3K9R0|C3K9R0_PSEFS	526	62265	16	16	13	13	1.90	Pyruvate dehydrogena
145	1	db$2	2::C3KE70|RL9_PSEFS	519	15488	14	13	6	6	7.20	50S ribosomal protein
146	1	db$2	2::C3JXY0|AK_PSEFS	518	44504	19	18	15	15	4.48	Aspartate kinase OS=F
147	1	db$2	2::C3JZN6|IF3_PSEFS	512	20837	16	16	7	7	6.57	Translation initiation fa
148	1	db$2	2::C3K4Y4|C3K4Y4_PSEFS	511	71842	19	17	16	14	1.88	Potassium-transporting
149	1	db$2	2::C3KBN3|C3KBN3_PSEFS	509	112655	21	21	13	13	0.88	Efflux pump membrane
149	2	db$2	2::C3KB18|C3KB18_PSEFS	128	115005	4	4	4	4	0.18	Efflux pump membrane
150	1	db$2	2::C3K3P0|ILVD_PSEFS	506	65667	19	16	15	12	1.55	Dihydroxy-acid dehydr
151	1	db$2	2::C3K4D4|C3K4D4_PSEFS	506	83859	23	23	15	15	2.10	Putative phosphate AB
152	1	db$2	2::C3K6H2|ACCA_PSEFS	500	34868	16	16	11	11	4.82	Acetyl-coenzyme A car
153	1	db$2	2::C3K260|C3K260_PSEFS	498	54381	18	17	14	13	2.69	Transcription terminati
154	1	db$2	2::C3KDU7|C3KDU7_PSEFS	497	28504	21	20	15	14	9.10	Putative ABC transport
155	1	db$2	2::C3K6H5|KDSA_PSEFS	496	30777	25	23	13	11	17.44	2-dehydro-3-deoxypho
156	1	db$2	2::C3K5H9|C3K5H9_PSEFS	495	33776	16	16	13	13	7.15	Transaldolase OS=Pse
156	2	SwissProt_HUMAN$	3::P37837|TALDO_HUMAN	75	37516	3	3	2	2	0.29	Transaldolase OS=Hor
157	1	db$2	2::C3K6M2|C3K6M2_PSEFS	495	60584	18	18	16	16	2.78	ATP-dependent RNA he
158	1	db$2	2::C3JYA7|C3JYA7_PSEFS	494	59461	18	18	14	14	2.30	Putative NADP-depend
159	1	db$2	2::C3JYJ8|C3JYJ8_PSEFS	491	88851	17	16	17	16	1.35	Lon protease OS=Pseu

↑Family	M	DB	Accession	Score	Mass	Matches	Match(sig)	Sequences	Seq(sig)	emPAI	Description
160	1	db$2	2::C3K3E8|C3K3E8_PSEFS	490	38385	25	23	17	16	10.72	Fructose-1,6-bisphosp
161	1	db$2	2::C3K3N4|COAD_PSEFS	487	17810	16	15	9	9	17.17	Phosphopantetheine a
162	1	db$2	2::C3K1B7|C3K1B7_PSEFS	486	51892	21	20	15	14	2.94	Threonine synthase OS
163	1	db$2	2::C3K4F7|C3K4F7_PSEFS	483	51847	23	23	18	18	4.18	Biotin carboxylase OS=
164	1	db$2	2::C3KEC2|C3KEC2_PSEFS	483	28614	24	22	15	14	9.01	Putative lipoprotein OS
164	2	db$2	2::C3KAJ6|C3KAJ6_PSEFS	218	38502	7	5	5	3	0.63	Histidinol-phosphate a
165	1	db$2	2::C3JXL8|C3JXL8_PSEFS	481	112023	31	24	22	18	1.44	Putative efflux protein
166	1	db$2	2::C3K2X0|C3K2X0_PSEFS	480	25737	21	19	14	12	14.52	30S ribosomal protein
167	1	db$2	2::C3JZV8|C3JZV8_PSEFS	480	38906	13	13	8	8	1.98	Neutral metalloprotein
168	1	db$2	2::C3KCR8|C3KCR8_PSEFS	480	39907	14	14	7	7	1.58	Ribosome-binding ATP
169	1	db$2	2::C3JYQ5|C3JYQ5_PSEFS	478	46409	21	21	16	16	4.66	Arginine deiminase OS
170	1	db$2	2::C3K266|C3K266_PSEFS	477	69364	16	16	13	13	1.60	ATP-dependent zinc m
171	1	db$2	2::C3K2H6|C3K2H6_PSEFS	477	32512	12	11	7	6	1.76	Acyl-CoA thioesterase
172	1	db$2	2::C3K2W6|RL14_PSEFS	475	13401	15	14	7	7	31.04	50S ribosomal protein
173	1	db$2	2::C3K914|C3K914_PSEFS	473	38509	18	18	11	11	4.57	Putative ABC transport
174	1	SwissProt_HUMAN$	3::P02768|ALBU_HUMAN	473	69321	19	17	17	16	1.99	Albumin OS=Homo sa
174	2	SwissProt_HUMAN$	3::P02769|ALBU_BOVIN	306	69248	12	12	11	11	1.12	Albumin OS=Bos tauru
175	1	db$2	2::C3K278|C3K278_PSEFS	472	15153	13	13	5	5	10.66	Ferric uptake regulatio
176	1	db$2	2::C3JY83|NUOCD_PSEFS	469	67660	29	27	22	21	5.18	NADH-quinone oxidore
177	1	db$2	2::C3KED7|PUR7_PSEFS	463	26862	19	17	13	11	8.77	Phosphoribosylaminoir
178	1	db$2	2::C3K9R4|C3K9R4_PSEFS	462	62164	19	18	16	15	2.39	Putative sulfite reducta
179	1	db$2	2::C3K3F5|METK_PSEFS	462	42554	16	15	12	11	3.75	S-adenosylmethionine
180	1	db$2	2::C3KDR8|OPGD_PSEFS	461	60485	18	18	16	16	2.50	Glucans biosynthesis p
181	1	db$2	2::C3K102|PUR5_PSEFS	460	36950	17	17	11	11	4.27	Phosphoribosylformylg
182	1	db$2	2::C3K7D6|F16PA_PSEFS	458	37081	15	14	10	9	3.06	Fructose-1,6-bisphosp
183	1	db$2	2::C3K0H1|C3K0H1_PSEFS	457	17848	12	12	7	7	7.14	Chemotaxis protein Ch
184	1	db$2	2::C3K244|C3K244_PSEFS	457	52276	13	13	13	13	2.25	Putative response regu
185	1	db$2	2::C3K2Y4|RL7_PSEFS	455	12456	11	9	3	2	3.46	50S ribosomal protein
186	1	db$2	2::C3K484|C3K484_PSEFS	455	13502	13	13	8	8	21.09	Uncharacterized protei
187	1	db$2	2::C3K2X3|RL2_PSEFS	453	29689	20	19	13	12	6.91	50S ribosomal protein
188	1	db$2	2::C3K2X6|RL3_PSEFS	450	22550	12	12	8	8	4.30	50S ribosomal protein
189	1	db$2	2::C3JYQ6|C3JYQ6_PSEFS	447	37842	23	20	14	12	5.50	Ornithine carbamoyltra
190	1	db$2	2::C3KBX8|C3KBX8_PSEFS	446	26859	13	12	7	7	4.77	OmpA-like domain-cor
191	1	db$2	2::C3K1N2|C3K1N2_PSEFS	445	67307	21	21	16	16	2.56	Protein translocase sul
192	1	db$2	2::C3K2Y7|RL11_PSEFS	443	14903	10	10	5	5	5.57	50S ribosomal protein
193	1	db$2	2::C3JZ70|C3JZ70_PSEFS	441	54858	25	23	19	18	4.16	Glucose-6-phosphate :
194	1	db$2	2::C3KBL8|C3KBL8_PSEFS	438	56970	21	19	17	15	2.79	Glycerol-3-phosphate (
195	1	db$2	2::C3JZN3|SYFA_PSEFS	436	37892	14	13	12	11	3.47	Phenylalanine--tRNA li
196	1	db$2	2::C3JYS2|TOLB_PSEFS	434	47517	20	16	17	15	3.92	Tol-Pal system protein
197	1	db$2	2::C3K4C7|C3K4C7_PSEFS	433	50044	13	13	12	12	2.42	Phosphate regulon sen

↑Family	M	DB	Accession	Score	Mass	Matches	Match(sig)	Sequences	Seq(sig)	emPAI	Description
198	1	db$2	2::C3K1G5|C3K1G5_PSEFS	427	46195	15	13	11	9	1.79	Homoserine dehydroge
199	1	db$2	2::C3K1L0|C3K1L0_PSEFS	425	55309	16	15	14	13	2.32	GTPase Der OS=Pseud
200	1	db$2	2::C3JZV2|C3JZV2_PSEFS	423	43020	16	13	12	10	2.35	Aminotransferase OS=
201	1	db$2	2::C3JXL7|C3JXL7_PSEFS	420	39112	13	13	11	11	3.27	HlyD-family secretion
202	1	db$2	2::C3KEC8|C3KEC8_PSEFS	420	50623	15	14	9	8	1.55	Periplasmic serine end
203	1	db$2	2::C3K8X4|MURA_PSEFS	418	44975	20	18	14	13	4.39	UDP-N-acetylglucosam
204	1	db$2	2::C3K3U2|C3K3U2_PSEFS	416	50985	14	13	10	9	1.53	Putative glutamine syr
205	1	db$2	2::C3K6D9|C3K6D9_PSEFS	413	45394	12	12	10	10	2.15	Phosphoribosylamine--
206	1	db$2	2::C3KAL6|C3KAL6_PSEFS	413	52109	18	15	14	12	2.26	Putative aminotransfer
207	1	db$2	2::C3K6C2|C3K6C2_PSEFS	412	27720	13	11	9	8	2.89	Putative amino acid AE
208	1	db$2	2::C3K1M5|ISCS_PSEFS	408	44467	13	11	11	10	1.89	Cysteine desulfurase I
209	1	db$2	2::C3K3R2|C3K3R2_PSEFS	407	83481	21	19	20	19	1.95	Phosphoenolpyruvate-
210	1	db$2	2::C3KBI6|C3KBI6_PSEFS	404	106629	19	19	17	17	1.23	Uncharacterized protei
211	1	db$2	2::C3KBH2|C3KBH2_PSEFS	404	51951	17	15	15	13	2.27	Glutamate synthase [N
212	1	db$2	2::C3K6G0|C3K6G0_PSEFS	403	9680	9	9	5	5	9.91	Putative DNA-binding
213	1	db$2	2::C3K2K3|GSA_PSEFS	401	45181	15	14	11	10	2.16	Glutamate-1-semialde
214	1	db$2	2::C3K3V3|ARGA_PSEFS	401	47793	13	12	11	10	1.69	Amino-acid acetyltrans
215	1	db$2	2::C3K3Z1|C3K3Z1_PSEFS	401	11808	11	11	6	6	22.48	Thioredoxin OS=Pseuc
216	1	db$2	2::C3K0D4|C3K0D4_PSEFS	393	29228	16	14	11	10	4.91	Cell division inhibitor M
217	1	db$2	2::C3JZS0|C3JZS0_PSEFS	391	43144	16	15	12	11	3.15	O-succinylhomoserine
218	1	db$2	2::C3K902|RAPA_PSEFS	388	106722	14	13	12	12	0.71	RNA polymerase-assoc
219	1	db$2	2::C3KDU3|C3KDU3_PSEFS	386	40518	16	15	13	12	3.56	Beta sliding clamp OS=
220	1	db$2	2::C3K5E0|C3K5E0_PSEFS	385	36000	11	11	9	9	2.26	2,3,4,5-tetrahydropyri
221	1	db$2	2::C3K2Z7|ARGC_PSEFS	383	36612	17	17	11	11	5.08	N-acetyl-gamma-gluta
222	1	db$2	2::C3K1L4|ISPG_PSEFS	381	39660	16	15	13	12	3.71	4-hydroxy-3-methylbu
223	1	db$2	2::C3K8X5|HIS1_PSEFS	381	23098	18	17	13	12	13.11	ATP phosphoribosyltra
224	1	db$2	2::C3K4W1|STHA_PSEFS	381	50963	19	17	16	15	3.03	Soluble pyridine nucle
225	1	db$2	2::C3JZS4|C3JZS4_PSEFS	380	33375	11	11	8	8	2.57	Acetyl-coenzyme A car
226	1	db$2	2::C3K6M6|C3K6M6_PSEFS	379	26063	20	19	12	11	11.51	Succinate dehydrogena
227	1	db$2	2::C3K7X9|C3K7X9_PSEFS	378	40858	16	14	11	10	3.01	Carboxynorspermidine
228	1	db$2	2::C3K401|C3K401_PSEFS	377	56255	19	16	16	14	2.25	Exopolyphosphatase O
229	1	db$2	2::C3JY86|C3JY86_PSEFS	377	98164	18	15	15	12	0.88	NADH-quinone oxidore
230	1	db$2	2::C3KBY6|C3KBY6_PSEFS	374	17850	14	13	8	8	12.74	Bacterioferritin OS=Ps
231	1	db$2	2::C3K4M6|C3K4M6_PSEFS	373	57818	19	18	12	12	1.90	Putative exported alka
232	1	db$2	2::C3K907|C3K907_PSEFS	372	77648	27	19	25	18	2.00	Tail-specific protease (
233	1	db$2	2::C3K5S8|C3K5S8_PSEFS	371	75646	17	17	16	16	1.73	Oligopeptidase A OS=I
234	1	db$2	2::C3KA80|C3KA80_PSEFS	371	20438	13	13	9	9	11.52	Alkyl hydroperoxide re
235	1	db$2	2::C3K0Q7|C3K0Q7_PSEFS	370	45255	9	8	6	6	1.08	Flagellar hook protein
236	1	db$2	2::C3K2V2|RS4_PSEFS	370	23062	19	15	12	9	6.66	30S ribosomal protein
237	1	db$2	2::C3K475|ARGB_PSEFS	369	31926	12	12	10	10	3.37	Acetylglutamate kinase

↑Family	M	DB	Accession	Score	Mass	Matches	Match(sig)	Sequences	Seq(sig)	emPAI	Description
238	1	db$2	2::C3KCP3|C3KCP3_PSEFS	369	56221	13	12	8	7	0.80	Acetyltransferase com
239	1	SwissProt_HUMAN$	3::P07355|ANXA2_HUMAN	367	38580	17	15	14	12	3.34	Annexin A2 OS=Homo
240	1	db$2	2::C3JZ68|C3JZ68_PSEFS	366	24197	11	11	9	9	5.99	2-dehydro-3-deoxy-ph
241	1	SwissProt_HUMAN$	3::P00761|TRYP_PIG	366	24394	11	11	6	6	3.67	Trypsin OS=Sus scrofa
242	1	db$2	2::C3K4K9|C3K4K9_PSEFS	364	27377	13	12	8	8	3.69	Putative amino acid ox
243	1	db$2	2::C3KEB4|C3KEB4_PSEFS	362	56978	19	16	15	13	2.21	Lysine--tRNA ligase OS
244	1	db$2	2::C3K9T7|C3K9T7_PSEFS	362	99328	17	15	14	12	0.77	Aminopeptidase N OS=
245	1	db$2	2::C3K6R4|C3K6R4_PSEFS	361	18727	12	12	6	6	4.75	Uncharacterized protei
246	1	db$2	2::C3K6J9|KCY_PSEFS	360	24780	7	7	6	6	2.13	Cytidylate kinase OS=
247	1	db$2	2::C3KDL2|C3KDL2_PSEFS	360	52736	13	12	12	11	1.69	Putative outer membra
248	1	db$2	2::C3K734|C3K734_PSEFS	356	44623	11	11	9	9	2.21	Putative aminotransfer
249	1	db$2	2::C3K1F0|C3K1F0_PSEFS	356	16832	19	19	11	11	20.11	ATP synthase subunit I
250	1	db$2	2::C3K2W4|RL5_PSEFS	356	20490	18	17	12	12	23.52	50S ribosomal protein
251	1	db$2	2::C3K358|C3K358_PSEFS	356	65409	16	15	12	12	1.57	Putative dehydrogenas
251	2	db$2	2::C3K355|C3K355_PSEFS	351	63710	13	13	12	12	1.44	Putative dehydrogenas
252	1	db$2	2::C3KAH3|SYR_PSEFS	356	63549	14	13	11	10	1.27	Arginine--tRNA ligase
253	1	db$2	2::C3K5Y0|C3K5Y0_PSEFS	354	38747	17	17	12	12	3.88	NADP(H)-dependt aldo
254	1	db$2	2::C3K466|C3K466_PSEFS	352	52681	18	18	10	10	1.69	Putative aldehyde deh
255	1	db$2	2::C3K2Y0|RS7_PSEFS	352	17636	17	16	12	12	23.12	30S ribosomal protein
256	1	db$2	2::C3K865|C3K865_PSEFS	352	15847	13	13	6	6	4.85	Putative membrane pr
257	1	db$2	2::C3KBP1|C3KBP1_PSEFS	349	37670	13	12	9	8	1.73	Putative reductase OS
258	1	db$2	2::C3K8X6|C3K8X6_PSEFS	348	47255	14	13	12	11	2.01	Histidinol dehydrogena
259	1	db$2	2::C3K6E0|PUR9_PSEFS	347	57370	13	12	11	10	1.48	Bifunctional purine bio
260	1	db$2	2::C3K9U6|C3K9U6_PSEFS	347	35232	10	10	7	7	2.35	Putative 6-phosphoglu
261	1	db$2	2::C3K5I7|C3K5I7_PSEFS	346	59867	17	17	11	11	1.80	Probable malate:quino
262	1	db$2	2::C3KAA8|C3KAA8_PSEFS	345	132643	12	12	12	12	0.54	Histidine kinase OS=P
263	1	db$2	2::C3K4V8|C3K4V8_PSEFS	344	128447	17	15	17	15	0.74	Transcription-repair-co
264	1	db$2	2::C3KEA4|C3KEA4_PSEFS	344	51722	13	12	9	9	1.74	PINc domain-containin
265	1	db$2	2::C3JYW3|C3JYW3_PSEFS	343	36818	18	18	13	13	4.29	Branched-chain-amino
266	1	db$2	2::C3K0Q9|C3K0Q9_PSEFS	343	15688	7	7	5	5	4.99	Flagellar basal-body ro
267	1	db$2	2::C3JYT9|C3JYT9_PSEFS	343	46635	13	12	7	7	1.49	Putative family S43 no
268	1	db$2	2::C3JY85|C3JY85_PSEFS	341	48759	16	15	14	13	2.54	NADH-quinone oxidore
269	1	db$2	2::C3K2W1|RL6_PSEFS	339	19185	14	13	9	9	10.49	50S ribosomal protein
270	1	db$2	2::C3K1H6|PURT_PSEFS	338	42327	14	14	13	13	3.28	Formate-dependent ph
271	1	db$2	2::C3JXZ0|C3JXZ0_PSEFS	338	43332	12	12	9	9	1.67	Acetylornithine aminot
272	1	db$2	2::C3K403|C3K403_PSEFS	337	36964	13	11	9	8	2.16	Delta-aminolevulinic a
273	1	db$2	2::C3K616|C3K616_PSEFS	337	97222	22	20	20	19	1.53	DNA topoisomerase 1
274	1	db$2	2::C3K818|C3K818_PSEFS	336	29926	12	10	9	7	2.01	Putative ABC transport
275	1	db$2	2::C3K0K5|C3K0K5_PSEFS	336	31365	13	12	7	7	2.86	Electron transfer flavo
276	1	db$2	2::C3K8X0|C3K8X0_PSEFS	335	16351	11	11	8	8	8.90	Putative ABC transport

↑Family	M	DB	Accession	Score	Mass	Matches	Match(sig)	Sequences	Seq(sig)	emPAI	Description
277	1	db$2	2::C3K1X8|C3K1X8_PSEFS	333	11596	9	9	4	4	3.99	50S ribosomal protein
278	1	db$2	2::C3K7M6|C3K7M6_PSEFS	332	88918	14	11	10	8	0.62	Acyl-coenzyme A dehy
279	1	db$2	2::C3K3K2|PYRB_PSEFS	330	36271	8	8	7	7	1.49	Aspartate carbamoyltr
280	1	db$2	2::C3K0N2|C3K0N2_PSEFS	330	43313	10	10	7	7	1.15	3-oxoacyl-[acyl-carrier
281	1	db$2	2::C3K2X1|RL22_PSEFS	329	11904	10	10	6	6	9.44	50S ribosomal protein
282	1	db$2	2::C3K2Q0|C3K2Q0_PSEFS	329	19348	15	14	11	10	13.45	Inorganic pyrophospha
283	1	db$2	2::C3K4D3|C3K4D3_PSEFS	327	60551	18	16	13	11	1.76	Phosphate transport sy
284	1	db$2	2::C3JZN2|C3JZN2_PSEFS	326	86329	21	19	19	17	1.55	Phenylalanine--tRNA li
285	1	db$2	2::C3K6G2|C3K6G2_PSEFS	326	106936	19	14	19	14	0.86	Valine--tRNA ligase OS
286	1	db$2	2::C3KDE0|LPXC_PSEFS	326	33197	12	10	9	8	2.12	UDP-3-O-acyl-N-acety
287	1	db$2	2::C3JZ69|C3JZ69_PSEFS	326	25612	9	9	6	6	2.01	6-phosphogluconolacto
288	1	db$2	2::C3K1F2|ATP6_PSEFS	325	31626	9	9	4	4	0.82	ATP synthase subunit a
289	1	db$2	2::C3KCN5|C3KCN5_PSEFS	324	142346	17	16	16	15	0.65	Bifunctional protein Pu
290	1	db$2	2::C3K4G1|C3K4G1_PSEFS	323	81186	13	12	11	10	0.79	DNA helicase OS=Pseu
291	1	db$2	2::C3K2Z3|C3K2Z3_PSEFS	322	44017	17	16	14	14	3.49	Tyrosine--tRNA ligase
292	1	db$2	2::C3K227|C3K227_PSEFS	320	62578	20	19	14	13	2.11	Acetolactate synthase
293	1	db$2	2::C3K6E1|RL13_PSEFS	320	15695	14	12	10	9	18.78	50S ribosomal protein
294	1	db$2	2::C3KCT2|MUTS_PSEFS	319	95536	11	11	9	9	0.56	DNA mismatch repair p
295	1	db$2	2::C3K263|C3K263_PSEFS	319	26068	9	9	7	7	2.54	Triosephosphate isome
296	1	db$2	2::C3K1M9|C3K1M9_PSEFS	318	29586	12	10	10	8	2.57	Inositol-1-monophospl
297	1	db$2	2::C3JYU6|C3JYU6_PSEFS	318	42196	8	8	8	8	1.45	Arginine biosynthesis b
298	1	db$2	2::C3K0W7|C3K0W7_PSEFS	317	31068	11	11	8	8	2.37	Metallophos_2 domain
299	1	db$2	2::C3K2J8|C3K2J8_PSEFS	317	54392	15	14	11	10	1.84	AMP nucleosidase OS=
300	1	db$2	2::C3K2W0|RL18_PSEFS	316	12651	11	11	7	7	12.10	50S ribosomal protein
301	1	db$2	2::C3K6E2|RS9_PSEFS	315	14607	11	11	7	7	11.77	30S ribosomal protein
302	1	db$2	2::C3KBQ2|C3KBQ2_PSEFS	315	37020	11	11	7	7	1.77	Putative quinone oxido
303	1	db$2	2::C3KDW6|C3KDW6_PSEFS	314	33055	17	16	13	12	5.39	Protein HflC OS=Pseuc
304	1	db$2	2::C3JY74|C3JY74_PSEFS	311	48472	16	12	14	10	1.65	Isocitrase OS=Pseudor
305	1	db$2	2::C3KBN2|C3KBN2_PSEFS	311	52689	9	9	8	8	1.24	Outer membrane prote
306	1	db$2	2::C3K1X6|C3K1X6_PSEFS	309	44711	10	10	9	9	1.59	GTPase Obg OS=Pseuc
307	1	db$2	2::C3KAJ2|C3KAJ2_PSEFS	308	53335	13	12	11	10	1.43	Methylmalonate-semia
308	1	db$2	2::C3K2S6|C3K2S6_PSEFS	308	31662	10	9	7	6	1.84	Putative thioredoxin O
309	1	db$2	2::C3K5J3|C3K5J3_PSEFS	308	50388	11	10	11	10	1.56	AMP nucleosidase OS=
310	1	db$2	2::C3K5Z7|C3K5Z7_PSEFS	307	26574	18	14	10	9	9.05	Uridylate kinase OS=P
311	1	db$2	2::C3K5Z8|RRF_PSEFS	307	20297	13	13	8	8	9.07	Ribosome-recycling fac
312	1	db$2	2::C3JXZ8|C3JXZ8_PSEFS	307	71568	15	15	13	13	1.37	Acetyl-coenzyme A syr
313	1	db$2	2::C3K6A2|C3K6A2_PSEFS	306	27847	11	9	9	7	2.27	Putative D-methionine
313	2	db$2	2::C3KD95|C3KD95_PSEFS	184	28020	9	7	8	7	2.25	Putative exported prot
314	1	db$2	2::C3K1G8|RL19_PSEFS	306	12961	12	11	8	8	24.14	50S ribosomal protein
315	1	db$2	2::C3K8U4|UBIE_PSEFS	305	28320	15	14	12	11	6.37	Ubiquinone/menaquino

↑Family	M	DB	Accession	Score	Mass	Matches	Match(sig)	Sequences	Seq(sig)	emPAI	Description
316	1	db$2	2::C3JZV0|C3JZV0_PSEFS	304	56140	13	13	12	12	1.99	Glutamate--tRNA ligas
317	1	db$2	2::C3JYD4|C3JYD4_PSEFS	303	64707	16	16	14	14	1.79	Glutamine--tRNA ligas
318	1	db$2	2::C3K651|C3K651_PSEFS	303	39766	11	10	10	9	1.92	Phospho-2-dehydro-3-
319	1	db$2	2::C3K6V7|C3K6V7_PSEFS	301	46336	10	10	8	8	1.27	Carboxy-terminal proc
320	1	db$2	2::C3K274|DNAJ_PSEFS	298	40315	12	11	10	9	2.23	Chaperone protein Dna
321	1	db$2	2::C3K6U8|C3K6U8_PSEFS	297	58654	6	6	4	4	0.50	Putative membrane pr
322	1	db$2	2::C3K2U7|C3K2U7_PSEFS	297	104087	18	16	17	15	0.98	UvrABC system protein
323	1	db$2	2::C3K249|G6PI_PSEFS	296	61384	14	13	12	11	1.73	Glucose-6-phosphate i
324	1	db$2	2::C3K3M4|C3K3M4_PSEFS	296	49883	7	7	7	7	0.94	Signal recognition part
325	1	db$2	2::C3KDX4|SYI_PSEFS	296	105386	13	12	12	11	0.64	Isoleucine--tRNA ligase
326	1	db$2	2::C3K2X7|RS10_PSEFS	295	11745	13	12	7	7	14.82	30S ribosomal protein
327	1	db$2	2::C3K324|C3K324_PSEFS	294	105271	16	15	14	13	0.80	LPS-assembly protein
328	1	db$2	2::C3KCB7|C3KCB7_PSEFS	294	34191	7	6	4	3	0.74	Phosphate transport sy
329	1	db$2	2::C3K270|C3K270_PSEFS	293	17315	10	9	8	7	5.63	Transcription elongatic
330	1	db$2	2::C3K474|C3K474_PSEFS	293	50732	11	9	9	7	1.11	Putative phosphomanr
331	1	db$2	2::C3K5A9|RF3_PSEFS	293	59584	11	11	9	9	1.21	Peptide chain release f
332	1	db$2	2::C3KE41|SYGA_PSEFS	292	36196	13	12	10	10	2.69	Glycine--tRNA ligase a
333	1	db$2	2::C3K2M1|C3K2M1_PSEFS	292	42033	15	14	13	12	3.31	Serine-type D-Ala-D-A
334	1	db$2	2::C3K2C4|C3K2C4_PSEFS	292	89060	14	13	13	12	0.90	Endopeptidase La OS=
335	1	db$2	2::C3K2Y5|RL10_PSEFS	292	17594	11	10	7	7	7.35	50S ribosomal protein
336	1	db$2	2::C3K6Y3|GATB_PSEFS	291	52868	14	11	10	7	1.24	Aspartyl/glutamyl-tRN.
337	1	db$2	2::C3K1G2|YIDC_PSEFS	290	61977	14	11	11	9	0.99	Membrane protein inse
338	1	db$2	2::C3K1Y2|C3K1Y2_PSEFS	289	21662	10	10	6	6	6.05	Peptidyl-prolyl cis-tran
339	1	db$2	2::C3K3P4|C3K3P4_PSEFS	289	39333	13	12	7	7	1.62	Uncharacterized protei
340	1	db$2	2::C3KBZ0|PYRC_PSEFS	288	38385	8	8	7	7	1.37	Dihydroorotase OS=Ps
341	1	db$2	2::C3K3D2|BETB_PSEFS	287	53123	10	10	8	8	1.04	Betaine aldehyde dehy
342	1	db$2	2::C3JYD5|C3JYD5_PSEFS	285	18453	9	8	6	6	3.60	Peptidyl-prolyl cis-tran
343	1	db$2	2::C3K2E3|MSCL_PSEFS	285	14568	7	6	4	3	2.61	Large-conductance me
344	1	db$2	2::C3K3Q2|C3K3Q2_PSEFS	284	37335	10	10	4	4	1.14	Putative cation ABC tra
345	1	db$2	2::C3JZT7|C3JZT7_PSEFS	283	38793	12	9	9	7	1.65	3-isopropylmalate deh
346	1	db$2	2::C3K5X4|C3K5X4_PSEFS	283	16332	11	10	8	8	8.90	Putative membrane pr
347	1	db$2	2::C3K310|C3K310_PSEFS	282	54246	11	11	9	9	1.39	Anthranilate synthase
348	1	db$2	2::C3K645|C3K645_PSEFS	282	24522	8	8	7	7	2.83	Putative ABC transport
349	1	db$2	2::C3KDB1|C3KDB1_PSEFS	282	65565	12	12	12	12	1.38	Putative carbamoyltrar
350	1	db$2	2::C3K569|C3K569_PSEFS	282	44415	14	13	10	10	2.23	Tryptophan synthase b
351	1	db$2	2::C3KE83|C3KE83_PSEFS	280	38700	11	11	8	8	1.65	Outer membrane prote
352	1	db$2	2::C3K7C1|SECB_PSEFS	280	17615	7	5	3	3	1.22	Protein-export protein
353	1	db$2	2::C3K4A8|C3K4A8_PSEFS	279	55832	12	12	11	11	1.54	Uncharacterized protei
354	1	db$2	2::C3JYS5|C3JYS5_PSEFS	278	25332	9	9	8	8	4.33	Tol-Pal system protein
355	1	db$2	2::C3JZN9|C3JZN9_PSEFS	278	7652	11	10	5	5	35.12	Major cold shock prote

↑Family	M	DB	Accession	Score	Mass	Matches	Match(sig)	Sequences	Seq(sig)	emPAI	Description
356	1	db$2	2::C3K226|C3K226_PSEFS	278	17820	11	10	7	6	3.82	Acetolactate synthase
357	1	db$2	2::C3K452|XPT_PSEFS	278	20267	10	9	8	7	5.35	Xanthine phosphoribos
358	1	db$2	2::C3KAI4|C3KAI4_PSEFS	274	53827	8	7	6	5	0.70	SPOR domain-containi
359	1	db$2	2::C3KDP1|CYSD_PSEFS	273	35173	12	12	9	9	3.38	Sulfate adenylyltransfe
360	1	db$2	2::C3K3W7|C3K3W7_PSEFS	272	36662	11	11	9	9	2.19	Putative iron (III) ABC
361	1	db$2	2::C3K7G9|C3K7G9_PSEFS	272	48266	10	9	9	8	1.19	Putative modulation of
362	1	db$2	2::C3K1X5|C3K1X5_PSEFS	271	39754	9	8	7	7	1.30	Glutamate 5-kinase OS
363	1	db$2	2::C3KDL6|C3KDL6_PSEFS	271	23139	8	8	5	5	1.77	ADP-ribose diphosphat
364	1	db$2	2::C3JY00|C3JY00_PSEFS	271	47211	11	10	8	8	1.23	Ribonucleoside-diphos
365	1	db$2	2::C3K2V0|RL17_PSEFS	270	14407	11	11	6	6	17.33	50S ribosomal protein
366	1	db$2	2::C3KAI8|C3KAI8_PSEFS	269	24497	7	7	4	4	1.61	Putative TetR-family re
367	1	db$2	2::C3K6Y4|GATA_PSEFS	269	51737	10	9	8	7	1.08	Glutamyl-tRNA(Gln) ar
368	1	db$2	2::C3K1F4|C3K1F4_PSEFS	268	31961	7	7	6	6	1.42	Probable chromosome-
369	1	db$2	2::C3K2L2|SYL_PSEFS	268	97066	14	13	14	13	0.89	Leucine--tRNA ligase C
370	1	db$2	2::C3K7N8|RDGC_PSEFS	267	34160	15	14	12	11	4.24	Recombination-associa
371	1	db$2	2::C3KDW7|HISZ_PSEFS	267	42687	11	8	8	7	1.17	ATP phosphoribosyltra
372	1	db$2	2::C3K476|PYRE_PSEFS	266	23079	6	6	6	6	2.39	Orotate phosphoribosy
373	1	db$2	2::C3K422|C3K422_PSEFS	263	40250	10	8	9	7	1.27	Putative uroporphyrin-
374	1	db$2	2::C3JYI0|C3JYI0_PSEFS	262	25235	6	6	5	5	1.54	Probable transcriptiona
375	1	db$2	2::C3KAC1|C3KAC1_PSEFS	262	27548	14	14	11	11	8.25	Putative phenylacetic a
376	1	db$2	2::C3K7L5|C3K7L5_PSEFS	261	19131	10	10	8	8	6.13	Molybdenum cofactor I
377	1	db$2	2::C3K2S0|C3K2S0_PSEFS	261	39319	6	6	4	4	0.62	3,4-dihydroxy-2-butan
378	1	db$2	2::C3K5D5|C3K5D5_PSEFS	261	41033	5	5	4	4	0.58	Succinyl-diaminopimel
379	1	db$2	2::C3JZ61|C3JZ61_PSEFS	259	61509	15	14	12	11	1.52	Acyl-CoA synthetase (I
380	1	db$2	2::C3K8Y8|C3K8Y8_PSEFS	258	23915	6	6	5	5	1.68	Putative isomerase OS
381	1	db$2	2::C3K6H1|C3K6H1_PSEFS	258	131195	15	12	15	12	0.54	DNA polymerase III su
382	1	db$2	2::C3JY54|SYS_PSEFS	258	47115	13	13	11	11	2.34	Serine--tRNA ligase OS
383	1	db$2	2::C3K6J6|C3K6J6_PSEFS	257	40307	12	10	10	8	1.56	Bifunctional chorismate
384	1	db$2	2::C3K3M3|C3K3M3_PSEFS	257	24540	8	7	5	5	1.61	Cell division ATP-bindi
385	1	db$2	2::C3K4V4|C3K4V4_PSEFS	255	32182	11	9	8	7	1.41	Putative ATP-binding p
386	1	db$2	2::C3K7Z1|C3K7Z1_PSEFS	255	38822	8	8	7	7	1.34	NAD(P) transhydrogen
387	1	db$2	2::C3K2J9|C3K2J9_PSEFS	253	60860	4	4	3	3	0.37	Putative dehydrogenas
388	1	db$2	2::C3JY62|C3JY62_PSEFS	252	83224	10	10	9	9	0.67	ATP-dependent Clp pro
388	2	db$2	2::C3K4B3|C3K4B3_PSEFS	57	97007	5	5	5	5	0.28	Chaperone OS=Pseudo
389	1	db$2	2::C3K606|FABZ_PSEFS	252	16691	9	9	7	7	6.14	3-hydroxyacyl-[acyl-ca
390	1	db$2	2::C3K6K6|C3K6K6_PSEFS	251	36089	9	9	8	8	1.85	HTH-type transcription
391	1	db$2	2::C3KDU9|C3KDU9_PSEFS	251	34261	11	11	9	9	2.96	Serine O-acetyltransfe
392	1	db$2	2::C3K6L0|EFP_PSEFS	251	21303	10	8	8	6	2.75	Elongation factor P OS
393	1	db$2	2::C3K8Z6|C3K8Z6_PSEFS	250	39611	11	11	10	10	2.30	Branched amino acid A
394	1	db$2	2::C3K614|C3K614_PSEFS	250	41572	17	16	10	10	3.38	3-ketoacyl-CoA thiolas

↑Family	M	DB	Accession	Score	Mass	Matches	Match(sig)	Sequences	Seq(sig)	emPAI	Description
395	1	db$2	2::C3K667|C3K667_PSEFS	250	48557	13	13	12	12	2.56	Conserved hypothetica
396	1	db$2	2::C3KCS2|RSMH_PSEFS	249	34344	10	10	10	10	2.96	Ribosomal RNA small s
397	1	db$2	2::C3K2V7|RL15_PSEFS	249	15215	11	10	8	7	14.85	50S ribosomal protein
398	1	db$2	2::C3K177|C3K177_PSEFS	248	65030	13	12	9	9	1.07	Phosphogluconate deh
399	1	db$2	2::C3K2T2|C3K2T2_PSEFS	247	24648	10	9	7	6	3.61	Putative ABC transport
400	1	SwissProt_HUMAN$	3::P60709|ACTB_HUMAN	247	41710	18	16	12	11	3.36	Actin, cytoplasmic 1 O
401	1	db$2	2::C3K759|C3K759_PSEFS	246	35694	5	5	4	4	0.70	Putative dehydrogenas
401	2	db$2	2::C3K0S6|C3K0S6_PSEFS	107	36802	2	2	2	2	0.29	Putative oxidoreductas
402	1	db$2	2::C3JZ29|C3JZ29_PSEFS	246	41047	8	7	8	7	1.24	Putative glycerophospl
403	1	db$2	2::C3K264|GLMM_PSEFS	245	47719	18	17	14	13	3.43	Phosphoglucosamine n
404	1	db$2	2::C3JYR4|C3JYR4_PSEFS	245	7675	5	5	3	3	5.01	Major cold shock prote
405	1	db$2	2::C3K1U7|C3K1U7_PSEFS	245	36908	7	7	2	2	0.89	Ubiquinol oxidase subu
406	1	db$2	2::C3KE79|C3KE79_PSEFS	245	29713	8	8	7	7	2.03	NH(3)-dependent NAD
407	1	db$2	2::C3K421|C3K421_PSEFS	244	46244	9	8	9	8	1.27	Putative HemY domain
408	1	db$2	2::C3K0S7|Y4577_PSEFS	244	11900	6	6	3	3	6.06	Nucleoid-associated pr
409	1	db$2	2::C3K4T4|C3K4T4_PSEFS	244	21507	6	6	4	4	1.39	NAD(P)-bd_dom doma
410	1	db$2	2::C3K272|C3K272_PSEFS	243	40774	9	9	6	6	1.25	Carbamoyl-phosphate
411	1	db$2	2::C3KEB7|C3KEB7_PSEFS	242	48036	12	11	10	9	1.68	Uncharacterized protei
412	1	db$2	2::C3JYT6|C3JYT6_PSEFS	241	8291	9	9	6	6	46.56	Cold shock protein csp
413	1	db$2	2::C3KDM2|C3KDM2_PSEFS	240	83508	16	13	14	11	0.87	DNA topoisomerase 4 :
414	1	db$2	2::C3KBY9|C3KBY9_PSEFS	240	24532	7	6	6	5	1.61	Ribonuclease T OS=Ps
415	1	db$2	2::C3K091|C3K091_PSEFS	240	20089	9	9	6	6	3.06	GTP cyclohydrolase 1 (
416	1	db$2	2::C3K1X9|C3K1X9_PSEFS	239	34909	10	10	7	7	1.96	Octaprenyl-diphosphat
417	1	db$2	2::C3K6R3|Y2135_PSEFS	239	24050	10	9	8	7	2.92	UPF0502 protein PFLU_
418	1	db$2	2::C3KDK8|C3KDK8_PSEFS	238	41619	4	4	3	3	0.41	DAO domain-containin
419	1	db$2	2::C3KA77|C3KA77_PSEFS	238	49108	10	9	10	9	1.38	Glutathione reductase
420	1	db$2	2::C3K3L7|THIG_PSEFS	237	28373	11	11	8	8	2.77	Thiazole synthase OS=
421	1	db$2	2::C3K2U9|C3K2U9_PSEFS	237	53651	14	11	12	9	1.21	Catalase OS=Pseudom
422	1	db$2	2::C3K262|C3K262_PSEFS	236	12956	7	7	3	3	1.93	Protein-export membra
423	1	db$2	2::C3K4E2|C3K4E2_PSEFS	236	16860	8	8	6	6	5.96	N5-carboxyaminoimida
424	1	db$2	2::C3K644|C3K644_PSEFS	235	89774	9	8	8	7	0.45	Putative ABC transport
425	1	db$2	2::C3K6F9|C3K6F9_PSEFS	234	37389	8	8	8	8	1.75	Nucleoid-associated pr
426	1	db$2	2::C3K705|SURE_PSEFS	234	26431	3	3	2	2	0.43	5'-nucleotidase SurE C
427	1	db$2	2::C3K325|C3K325_PSEFS	234	49076	14	14	10	10	1.89	Chaperone SurA OS=P
428	1	db$2	2::C3K2V3|RS11_PSEFS	234	13607	8	7	6	5	4.51	30S ribosomal protein
429	1	db$2	2::C3KAI6|C3KAI6_PSEFS	233	24263	11	9	7	6	2.19	Putative dehydrogenas
430	1	db$2	2::C3K6G8|LEPA_PSEFS	233	66372	8	8	7	7	0.65	Elongation factor 4 OS
431	1	db$2	2::C3JYR3|C3JYR3_PSEFS	233	40135	11	11	7	7	1.56	Glycine cleavage syste
432	1	db$2	2::C3K8V3|HSLU_PSEFS	233	49607	14	13	11	10	1.60	ATP-dependent protea
433	1	db$2	2::C3K084|AROC_PSEFS	232	39094	10	10	8	8	1.97	Chorismate synthase (

↑Family	M	DB	Accession	Score	Mass	Matches	Match(sig)	Sequences	Seq(sig)	emPAI	Description
434	1	db$2	2::C3K0T1|C3K0T1_PSEFS	232	94111	8	8	7	7	0.42	Aconitate hydratase O
435	1	db$2	2::C3KBX9|C3KBX9_PSEFS	231	20403	4	4	3	3	0.99	Putative phospholipid-
436	1	db$2	2::C3KCP5|C3KCP5_PSEFS	231	109710	6	6	6	6	0.30	Bifunctional glutamine
437	1	db$2	2::C3K1N3|C3K1N3_PSEFS	230	11888	5	5	3	3	2.23	Sec translocon access
438	1	db$2	2::C3K2E9|C3K2E9_PSEFS	230	53251	6	6	3	3	0.56	Putative cytochrome o
439	1	db$2	2::C3JYQ7|C3JYQ7_PSEFS	229	32635	6	6	4	4	0.78	Carbamate kinase OS=
440	1	db$2	2::C3K6J7|C3K6J7_PSEFS	227	39784	11	10	10	9	1.92	Histidinol-phosphate a
441	1	db$2	2::C3KCT1|MURC_PSEFS	226	52132	11	11	9	9	1.26	UDP-N-acetylmuramat
442	1	db$2	2::C3K2K9|C3K2K9_PSEFS	225	31417	11	11	9	9	2.86	Magnesium and cobalt
443	1	db$2	2::C3K490|C3K490_PSEFS	225	33505	11	11	9	9	2.55	Putative oxidative stre
444	1	db$2	2::C3K9U7|C3K9U7_PSEFS	224	56147	7	7	7	7	0.80	Glucose-6-phosphate 1
445	1	db$2	2::C3K2C9|C3K2C9_PSEFS	224	48295	11	10	11	10	1.67	Glutamate dehydroger
446	1	db$2	2::C3K4Q9|C3K4Q9_PSEFS	224	23656	5	5	4	4	1.22	Putative exported prot
447	1	db$2	2::C3K2S1|C3K2S1_PSEFS	223	23236	10	7	7	5	2.37	Riboflavin synthase alp
448	1	db$2	2::C3K5H8|C3K5H8_PSEFS	223	17826	8	8	6	6	3.82	STAS domain-containi
449	1	db$2	2::C3KBP9|C3KBP9_PSEFS	222	58780	9	9	9	9	1.06	Phosphoglucomutase (
450	1	db$2	2::C3KEC6|KAD_PSEFS	220	23251	11	11	7	7	4.06	Adenylate kinase OS=
451	1	db$2	2::C3K843|C3K843_PSEFS	220	26535	11	11	7	7	2.46	Lipopolysaccharide exp
452	1	db$2	2::C3K6T0|ENGB_PSEFS	220	23537	6	6	5	5	1.72	Probable GTP-binding
453	1	db$2	2::C3K1E9|ATPD_PSEFS	220	19246	12	11	8	7	4.52	ATP synthase subunit
454	1	db$2	2::C3K469|RL28_PSEFS	219	8845	7	7	3	3	3.74	50S ribosomal protein
455	1	db$2	2::C3K9R8|C3K9R8_PSEFS	219	135461	9	9	8	8	0.32	Methionine synthase O
456	1	db$2	2::C3JZU0|C3JZU0_PSEFS	218	50538	8	8	7	7	0.93	3-isopropylmalate deh
457	1	db$2	2::C3K5V1|C3K5V1_PSEFS	218	47050	8	8	8	8	1.24	Outer membrane prote
458	1	db$2	2::C3JY82|NUOB_PSEFS	218	25341	11	9	8	7	3.43	NADH-quinone oxidore
459	1	db$2	2::C3K3W3|C3K3W3_PSEFS	218	41294	7	7	6	6	0.99	Putative aminotransfer
460	1	db$2	2::C3K443|C3K443_PSEFS	218	12323	9	9	6	6	13.02	Nitrogen regulatory pr
461	1	db$2	2::C3K3T9|C3K3T9_PSEFS	217	49785	7	7	6	6	0.94	Putative aminotransfer
462	1	db$2	2::C3K2W5|RL24_PSEFS	217	11328	10	9	7	6	10.67	50S ribosomal protein
463	1	db$2	2::C3K0H6|C3K0H6_PSEFS	217	40054	7	6	6	5	0.80	Protein-glutamate met
464	1	db$2	2::C3K2Y8|C3K2Y8_PSEFS	216	20023	13	9	8	7	5.54	Transcription terminati
465	1	db$2	2::C3K3S0|C3K3S0_PSEFS	216	23721	7	7	3	3	0.81	Putative hydrolase OS
466	1	db$2	2::C3K581|C3K581_PSEFS	215	86479	9	8	9	8	0.55	Putative molybdopteri
467	1	db$2	2::C3KCT0|MURG_PSEFS	215	37667	6	6	6	6	1.12	UDP-N-acetylglucosam
468	1	db$2	2::C3JYU1|Y4927_PSEFS	214	18225	7	7	5	5	3.68	UPF0234 protein PFLU_
469	1	db$2	2::C3K5L8|C3K5L8_PSEFS	213	39360	8	7	7	6	1.32	Phosphorous compoun
470	1	db$2	2::C3K6X1|C3K6X1_PSEFS	212	39290	9	7	8	6	1.06	Uncharacterized protei
471	1	db$2	2::C3KE31|C3KE31_PSEFS	211	22237	10	10	9	9	5.72	RNA polymerase sigma
472	1	db$2	2::C3K9P3|C3K9P3_PSEFS	211	9882	7	7	5	5	9.36	Peptidyl-prolyl cis-tran
473	1	db$2	2::C3K2X2|RS19_PSEFS	211	10313	9	9	6	6	13.71	30S ribosomal protein

↑Family	M	DB	Accession	Score	Mass	Matches	Match(sig)	Sequences	Seq(sig)	emPAI	Description
474	1	db$2	2::C3K6M3|C3K6M3_PSEFS	211	13365	7	7	6	6	7.14	Succinate dehydrogen
475	1	db$2	2::C3K815|C3K815_PSEFS	210	62777	10	10	7	7	0.97	Glucans biosynthesis p
476	1	db$2	2::C3K0D3|MINC_PSEFS	210	26249	8	8	6	6	2.52	Probable septum site-(
477	1	db$2	2::C3K638|C3K638_PSEFS	210	26394	6	5	4	4	1.44	Putative short chain de
478	1	db$2	2::C3K062|C3K062_PSEFS	210	48996	9	9	7	7	1.17	Fumarate hydratase cl
479	1	db$2	2::C3KAJ1|C3KAJ1_PSEFS	209	48585	10	10	9	9	1.65	Omega-amino acid--py
480	1	db$2	2::C3K3P5|C3K3P5_PSEFS	209	48453	5	5	4	4	0.48	Putative symporter me
481	1	db$2	2::C3JZ43|C3JZ43_PSEFS	208	44885	7	7	7	7	1.09	Putative aminotransfer
482	1	db$2	2::C3K187|C3K187_PSEFS	208	38556	12	11	10	9	2.41	Peptidase_M75 domair
483	1	db$2	2::C3K0N4|C3K0N4_PSEFS	207	25612	11	9	9	7	3.34	3-oxoacyl-[acyl-carrier
484	1	db$2	2::C3KBZ7|SYM_PSEFS	206	75525	11	10	10	9	0.76	Methionine--tRNA ligas
485	1	db$2	2::C3K3S1|C3K3S1_PSEFS	206	51070	8	5	8	5	0.59	Putative oxidase OS=P
486	1	db$2	2::C3K8W7|C3K8W7_PSEFS	206	34291	7	7	5	5	0.99	Arabinose 5-phosphate
487	1	db$2	2::C3K4H9|C3K4H9_PSEFS	205	34203	7	6	7	6	1.29	Quinone oxidoreductas
488	1	db$2	2::C3KED5|C3KED5_PSEFS	205	40447	9	8	9	8	1.55	Putative lipoprotein OS
489	1	db$2	2::C3K2W8|RL29_PSEFS	204	7168	6	5	4	4	11.58	50S ribosomal protein
490	1	db$2	2::C3K968|C3K968_PSEFS	204	55197	9	8	7	6	0.67	Putative aldehyde deh
491	1	db$2	2::C3K4G2|C3K4G2_PSEFS	202	30728	7	7	3	3	1.51	Putative exported prot
492	1	db$2	2::C3JZ71|C3JZ71_PSEFS	202	31565	7	7	7	7	1.85	Hex regulon repressor
493	1	db$2	2::C3K5N1|C3K5N1_PSEFS	202	36842	8	6	6	4	0.67	Sulfate ABC transport
494	1	db$2	2::C3KE99|C3KE99_PSEFS	201	30145	6	6	5	5	1.18	Quinolinate phosphorit
495	1	db$2	2::C3JZV9|C3JZV9_PSEFS	201	34932	4	4	3	3	0.50	Putative DNA/RNA non
496	1	db$2	2::C3KDQ9|C3KDQ9_PSEFS	200	29676	10	9	10	9	3.19	Ferredoxin--NADP redu
497	1	db$2	2::C3K1L3|SYH_PSEFS	200	47853	13	9	11	8	1.44	Histidine--tRNA ligase
498	1	db$2	2::C3K1L2|C3K1L2_PSEFS	200	22708	7	7	7	7	3.25	Ancillary SecYEG trans
499	1	db$2	2::C3KE61|RIMK_PSEFS	200	32488	8	5	5	4	1.07	Probable alpha-L-gluta
500	1	db$2	2::C3KBM6|C3KBM6_PSEFS	199	11934	6	6	2	2	3.71	Glutaredoxin OS=Pseu
501	1	db$2	2::C3K6J5|SERC_PSEFS	198	39799	11	9	7	5	1.04	Phosphoserine aminotr
502	1	db$2	2::C3K6Z0|PDXJ_PSEFS	198	26758	7	7	6	6	1.88	Pyridoxine 5'-phosphat
503	1	db$2	2::C3JZ88|C3JZ88_PSEFS	198	47232	6	6	4	4	0.65	Putative exported prot
504	1	db$2	2::C3K142|C3K142_PSEFS	198	32099	7	6	6	5	1.42	2-methylisocitrate lyas
505	1	db$2	2::C3K4E4|C3K4E4_PSEFS	197	51389	10	9	10	9	1.29	Aspartate ammonia-ly
506	1	db$2	2::C3K2U5|C3K2U5_PSEFS	197	18989	9	8	5	5	2.44	Single-stranded DNA-t
507	1	db$2	2::C3KDM0|C3KDM0_PSEFS	196	69594	9	9	7	7	0.61	DNA topoisomerase 4
508	1	db$2	2::C3K1X2|C3K1X2_PSEFS	195	40166	7	7	7	7	1.28	Putative putative oxid
509	1	db$2	2::C3K3G7|C3K3G7_PSEFS	195	31253	7	7	6	6	1.47	Methylenetetrahydrofo
510	1	db$2	2::C3KAR0|C3KAR0_PSEFS	195	79563	6	5	5	4	0.35	Putative exported isoq
511	1	db$2	2::C3JYJ6|C3JYJ6_PSEFS	195	68407	11	11	11	11	1.14	Peptidyl-prolyl cis-tran
512	1	db$2	2::C3K2U8|C3K2U8_PSEFS	195	18135	8	7	5	5	3.73	Bacterioferritin OS=Ps
513	1	db$2	2::C3KDM5|C3KDM5_PSEFS	194	43829	7	6	7	6	0.91	O-phosphoserine phos

↑Family	M	DB	Accession	Score	Mass	Matches	Match(sig)	Sequences	Seq(sig)	emPAI	Description
514	1	db$2	2::C3KC10|C3KC10_PSEFS	194	23080	5	5	3	3	0.84	Uncharacterized protei
515	1	db$2	2::C3KBL1|C3KBL1_PSEFS	193	61934	9	9	8	8	0.84	Putative medium-chair
516	1	db$2	2::C3K8W6|C3K8W6_PSEFS	193	18885	6	6	5	5	2.46	3-deoxy-D-manno-octu
517	1	db$2	2::C3K472|C3K472_PSEFS	192	42804	8	8	7	7	1.17	Coenzyme A biosynthe
518	1	db$2	2::C3K245|C3K245_PSEFS	192	53442	12	12	11	11	1.65	Poly(A) polymerase I (
519	1	db$2	2::C3KE48|C3KE48_PSEFS	191	19341	7	7	6	6	3.29	Peptide deformylase O
520	1	db$2	2::C3JXY4|ASTE_PSEFS	191	37404	10	9	7	7	1.42	Succinylglutamate des
521	1	db$2	2::C3K1E5|ATPE_PSEFS	191	14558	6	6	4	4	3.97	ATP synthase epsilon c
522	1	db$2	2::C3JXY8|C3JXY8_PSEFS	191	37476	6	5	6	5	0.88	Arginine N-succinyltrar
523	1	db$2	2::C3K368|C3K368_PSEFS	190	37353	7	7	7	7	1.42	Stress response kinase
524	1	db$2	2::C3JXZ2|C3JXZ2_PSEFS	190	36334	7	7	5	5	0.92	Putative AraC family re
525	1	db$2	2::C3K0N5|C3K0N5_PSEFS	190	32402	5	5	4	4	0.79	Malonyl CoA-acyl carri
526	1	db$2	2::C3K152|C3K152_PSEFS	189	13999	8	8	6	6	6.38	Putative transcriptiona
527	1	db$2	2::C3K9Z4|C3K9Z4_PSEFS	189	58133	10	10	9	9	1.08	Putative ABC tranmspo
528	1	db$2	2::C3KDM7|C3KDM7_PSEFS	188	29519	10	9	8	7	2.59	Sulfurtransferase OS=
529	1	db$2	2::C3K3A2|C3K3A2_PSEFS	188	38105	9	8	6	5	1.11	L-threonine aldolase O
530	1	db$2	2::C3KBB4|OADC_PSEFS	188	31135	6	5	5	4	0.84	Oxaloacetate decarbox
531	1	db$2	2::C3JYV5|C3JYV5_PSEFS	188	45487	9	9	8	8	1.55	Putative oxidoreductas
532	1	db$2	2::C3KE71|C3KE71_PSEFS	187	51412	9	8	9	8	1.09	Replicative DNA helica:
533	1	db$2	2::C3K3G5|C3K3G5_PSEFS	187	15160	7	6	7	6	5.31	Putative thioesterase (
534	1	db$2	2::C3K0W1|C3K0W1_PSEFS	186	27483	6	6	6	6	1.79	Flagellar basal-body rc
535	1	db$2	2::C3K3U0|C3K3U0_PSEFS	186	51001	9	9	8	8	1.10	Putative glutamine syr
536	1	db$2	2::C3KBN4|C3KBN4_PSEFS	185	40532	6	5	6	5	0.79	Acriflavine resistance p
537	1	db$2	2::C3KBQ3|C3KBQ3_PSEFS	185	33274	5	5	5	5	1.03	LysR-family transcripti
538	1	db$2	2::C3JZN8|C3JZN8_PSEFS	185	10735	5	5	4	4	7.59	Uncharacterized protei
539	1	db$2	2::C3K7I1|C3K7I1_PSEFS	184	85859	6	6	6	6	0.39	Quinoprotein glucose c
540	1	db$2	2::C3K268|C3K268_PSEFS	184	11552	8	8	7	7	23.91	CRM domain-containin
541	1	db$2	2::C3K1L1|C3K1L1_PSEFS	183	40864	9	8	6	5	0.78	Outer membrane prote
542	1	db$2	2::C3K3X2|C3K3X2_PSEFS	183	19739	6	5	6	5	2.28	Uncharacterized protei
543	1	db$2	2::B0B0A5|DNAA_PSEFS	183	56252	14	11	12	9	1.13	Chromosomal replicati
544	1	db$2	2::C3K2V4|RS13_PSEFS	183	13332	9	8	6	5	4.74	30S ribosomal protein
545	1	db$2	2::C3K116|PSRP_PSEFS	182	30838	7	6	6	6	1.50	Putative phosphoenolp
546	1	db$2	2::C3K568|TRPA_PSEFS	182	28539	5	4	4	4	0.94	Tryptophan synthase a
547	1	db$2	2::C3K7M9|C3K7M9_PSEFS	182	34271	8	8	8	8	2.01	Putative ABC transport
548	1	db$2	2::C3K015|C3K015_PSEFS	182	53791	6	6	4	4	0.42	Putative ferredoxin OS
549	1	db$2	2::C3K7T1|C3K7T1_PSEFS	181	23131	6	6	6	6	2.39	Two-component syster
550	1	db$2	2::C3KAC7|C3KAC7_PSEFS	180	51379	9	9	8	8	1.29	UDP-glucose 6-dehydr
551	1	db$2	2::C3K0G3|PPNP_PSEFS	180	10127	5	5	4	4	8.85	Pyrimidine/purine nucl
552	1	db$2	2::C3K572|C3K572_PSEFS	180	7891	2	2	2	2	2.20	Uncharacterized protei
553	1	db$2	2::C3K610|C3K610_PSEFS	180	70283	11	10	11	10	0.96	ATP-binding protein Ut

↑Family	M	DB	Accession	Score	Mass	Matches	Match(sig)	Sequences	Seq(sig)	emPAI	Description
554	1	db$2	2::C3KDX0|C3KDX0_PSEFS	179	16992	7	6	3	3	1.29	Peptidyl-prolyl cis-tran
555	1	db$2	2::C3JY53|CYSG_PSEFS	179	50259	7	6	7	6	0.76	Siroheme synthase OS
556	1	db$2	2::C3K5H5|C3K5H5_PSEFS	179	25721	9	8	7	6	2.60	Putative exported prot
557	1	db$2	2::C3JYS3|C3JYS3_PSEFS	179	38665	3	2	3	2	0.28	TolA colicin import me
558	1	db$2	2::C3K8U2|UBIB_PSEFS	177	61616	6	5	6	5	0.47	Probable protein kinas
559	1	db$2	2::C3K733|HTPX_PSEFS	176	32382	8	8	5	5	1.40	Protease HtpX OS=Pse
560	1	db$2	2::C3KE80|C3KE80_PSEFS	176	46502	7	6	7	6	0.84	Nicotinate phosphoribo
561	1	db$2	2::C3K5E8|C3K5E8_PSEFS	176	28997	4	4	3	3	0.63	Cyclohexadienyl dehyc
562	1	db$2	2::C3K7G7|C3K7G7_PSEFS	176	50136	7	7	7	7	0.94	Putative modulation of
563	1	db$2	2::C3K611|C3K611_PSEFS	175	16065	5	5	5	5	3.27	Universal stress protei
564	1	db$2	2::C3K6U9|HIS7_PSEFS	175	21830	8	8	8	8	4.58	Imidazoleglycerol-phos
565	1	db$2	2::C3K868|C3K868_PSEFS	175	58117	6	6	6	6	0.63	Uncharacterized protei
566	1	db$2	2::C3K747|GPDA_PSEFS	175	36476	8	8	6	6	1.81	Glycerol-3-phosphate
567	1	db$2	2::C3K2L6|C3K2L6_PSEFS	175	47731	5	5	5	5	0.64	Putative lipoprotein OS
568	1	db$2	2::C3K703|ISPF_PSEFS	174	16742	4	4	4	4	2.05	2-C-methyl-D-erythrit
569	1	db$2	2::C3K6V9|C3K6V9_PSEFS	173	54793	9	8	8	7	1.00	2,3-bisphosphoglycera
570	1	db$2	2::C3KDB8|HLDE_PSEFS	173	49913	6	6	6	6	0.77	Bifunctional protein Hl
571	1	db$2	2::C3K859|C3K859_PSEFS	172	59064	6	6	5	5	0.49	Putative glutamate syr
572	1	db$2	2::C3K6Z7|C3K6Z7_PSEFS	172	89469	10	9	10	9	0.61	Lon protease OS=Pseu
573	1	db$2	2::C3JXY7|ASTD_PSEFS	171	51479	4	4	4	4	0.44	N-succinylglutamate 5
574	1	db$2	2::C3K6S8|C3K6S8_PSEFS	171	40355	9	9	8	8	1.87	Putative acetyl-CoA tra
575	1	db$2	2::C3K307|TRPC_PSEFS	171	30252	9	9	8	8	2.47	Indole-3-glycerol phos
576	1	db$2	2::C3K281|C3K281_PSEFS	170	15787	3	3	2	2	0.81	Ribosome association t
577	1	db$2	2::C3K740|C3K740_PSEFS	170	33777	12	10	11	10	3.05	DUF815 domain-conta
578	1	db$2	2::C3KDC3|LOLB_PSEFS	169	23021	4	4	4	4	1.27	Outer-membrane lipop
579	1	db$2	2::C3JZS5|TRPF_PSEFS	168	21966	4	4	3	3	0.90	N-(5'-phosphoribosyl)a
580	1	db$2	2::C3KDW3|HFQ_PSEFS	168	9318	8	7	5	5	10.85	RNA-binding protein H
581	1	db$2	2::C3JXX2|C3JXX2_PSEFS	168	50328	8	8	7	7	0.93	ATP-dependent RNA he
582	1	db$2	2::C3K3L0|METXS_PSEFS	167	41664	5	5	4	4	0.76	Homoserine O-succiny
583	1	db$2	2::C3KDD6|C3KDD6_PSEFS	167	33869	5	5	5	5	1.01	D-alanine--D-alanine li
584	1	db$2	2::C3K2W9|RL16_PSEFS	166	15422	6	6	5	5	3.54	50S ribosomal protein
585	1	db$2	2::C3K1A8|C3K1A8_PSEFS	166	35739	4	4	3	3	0.49	Putative lipoprotein OS
586	1	db$2	2::C3KDA5|C3KDA5_PSEFS	166	54170	4	4	3	3	0.30	Uncharacterized protei
587	1	db$2	2::C3KBK6|C3KBK6_PSEFS	166	29320	4	4	3	3	0.90	Putative exported pept
588	1	db$2	2::C3K7C4|C3K7C4_PSEFS	164	53137	11	8	9	7	0.87	DNA-binding transcript
589	1	db$2	2::C3K4Y3|KDPA_PSEFS	164	60181	6	5	3	2	0.27	Potassium-transporting
590	1	db$2	2::C3K2R1|DXS_PSEFS	164	68238	6	6	6	6	0.52	1-deoxy-D-xylulose-5-
591	1	db$2	2::C3K2I0|C3K2I0_PSEFS	163	48538	5	5	5	5	0.63	Putative ATP-depender
592	1	db$2	2::C3KA90|C3KA90_PSEFS	163	36984	5	4	5	4	0.67	Putative aldo/keto redu
593	1	db$2	2::C3JZ86|C3JZ86_PSEFS	163	21964	10	8	7	6	2.61	Superoxide dismutase

↑Family	M	DB	Accession	Score	Mass	Matches	Match(sig)	Sequences	Seq(sig)	emPAI	Description
594	1	db$2	2::C3K5G1|C3K5G1_PSEFS	162	34097	6	6	5	5	1.30	Cysteine synthase OS=
595	1	db$2	2::C3K1A1|C3K1A1_PSEFS	162	10298	6	6	5	5	8.39	10 kDa chaperonin OS
596	1	db$2	2::C3K494|C3K494_PSEFS	161	33626	4	4	4	4	0.75	Gly-zipper_YMGG dom
597	1	db$2	2::C3JYD3|SYC_PSEFS	161	51635	11	9	10	8	1.08	Cysteine--tRNA ligase
598	1	db$2	2::C3K839|Y879_PSEFS	160	32120	7	6	7	6	1.42	Nucleotide-binding pro
599	1	db$2	2::C3K4I0|HEM6_PSEFS	159	35313	8	7	8	7	1.55	Oxygen-dependent cop
600	1	db$2	2::C3K5B9|C3K5B9_PSEFS	159	60269	3	3	2	2	0.17	Dipeptide ABC transpo
601	1	db$2	2::C3KBL7|C3KBL7_PSEFS	159	33413	6	5	6	5	1.03	Glutamate/aspartate A
602	1	db$2	2::C3K1N4|C3K1N4_PSEFS	159	41398	8	8	7	7	1.49	Queuine tRNA-ribosylt
603	1	db$2	2::C3K1M1|HSCA_PSEFS	159	65896	7	6	6	5	0.43	Chaperone protein Hsc
604	1	db$2	2::C3K4V5|C3K4V5_PSEFS	159	36313	5	5	5	5	0.92	Beta-hexosaminidase (
605	1	db$2	2::C3K6W5|PRMA_PSEFS	158	31717	3	3	2	2	0.35	Ribosomal protein L11
606	1	db$2	2::C3K4U5|C3K4U5_PSEFS	158	75901	10	9	10	9	0.75	Putative protease OS=
607	1	db$2	2::C3KAW2|C3KAW2_PSEFS	158	26230	7	7	6	6	1.94	Putative short chain de
608	1	db$2	2::C3K1X7|RL27_PSEFS	157	9214	8	7	4	3	6.40	50S ribosomal protein
609	1	db$2	2::C3KED4|C3KED4_PSEFS	157	31038	6	6	6	6	1.49	4-hydroxy-tetrahydroc
610	1	db$2	2::C3KD21|C3KD21_PSEFS	156	81698	6	6	6	6	0.42	Putative glycogen debr
611	1	db$2	2::C3K3Q8|TYSY_PSEFS	156	31490	7	7	6	6	1.45	Thymidylate synthase
612	1	db$2	2::C3K3K8|C3K3K8_PSEFS	156	27898	3	3	1	1	0.40	Pyrroline-5-carboxylat
613	1	db$2	2::C3K197|C3K197_PSEFS	156	25331	6	6	5	5	1.53	Putative two-compone
614	1	db$2	2::C3K3H0|C3K3H0_PSEFS	156	67796	11	9	9	7	0.63	ATP-dependent RNA he
615	1	db$2	2::C3K332|Y5583_PSEFS	155	48606	6	5	6	5	0.63	UPF0229 protein PFLU_
616	1	db$2	2::C3JZN1|IHFA_PSEFS	155	11469	7	7	6	6	10.41	Integration host factor
617	1	db$2	2::C3K253|C3K253_PSEFS	155	106648	6	6	6	6	0.31	Putative membrane pr
618	1	db$2	2::C3K1X3|C3K1X3_PSEFS	154	20670	5	5	3	3	0.98	Glutathione peroxidase
619	1	SwissProt_HUMAN$	3::P27348|1433T_HUMAN	154	27747	4	4	4	4	0.97	14-3-3 protein theta O
619	2	SwissProt_HUMAN$	3::P63104|1433Z_HUMAN	154	27728	6	6	5	5	1.34	14-3-3 protein zeta/de
619	3	SwissProt_HUMAN$	3::P31946|1433B_HUMAN	31	28065	2	2	2	2	0.40	14-3-3 protein beta/al
620	1	db$2	2::C3JYN4|ZIPA_PSEFS	154	31818	7	6	5	4	1.10	Cell division protein Zi
621	1	db$2	2::C3K3Q3|C3K3Q3_PSEFS	154	61523	3	3	3	3	0.26	Putative cation ABC tra
622	1	db$2	2::C3K527|C3K527_PSEFS	154	65564	7	7	7	7	0.66	Putative peptidase OS=
623	1	db$2	2::C3KAB1|C3KAB1_PSEFS	153	44397	5	5	4	4	0.53	Histidine kinase OS=Ps
624	1	db$2	2::C3K5X2|C3K5X2_PSEFS	152	20559	5	5	5	5	2.14	Putative two-compone
625	1	db$2	2::C3KAH1|HSLV_PSEFS	151	18731	4	4	3	3	1.12	ATP-dependent protea
626	1	db$2	2::Q84CP9|DAPB_PSEFS	151	28276	10	9	8	7	2.21	4-hydroxy-tetrahydroc
627	1	db$2	2::C3K5X7|C3K5X7_PSEFS	151	42054	7	6	7	6	0.96	Cell division protein Za
628	1	db$2	2::C3K9S0|NFUA_PSEFS	151	20961	5	5	4	4	1.45	Fe/S biogenesis protei
629	1	SwissProt_HUMAN$	3::Q9BQE3|TBA1C_HUMAN	151	49863	5	5	5	5	0.61	Tubulin alpha-1C chain
630	1	db$2	2::C3JYE4|C3JYE4_PSEFS	150	30882	14	12	12	10	3.61	Exodeoxyribonuclease
631	1	db$2	2::C3JYV1|C3JYV1_PSEFS	150	32264	5	5	4	4	0.79	Formyltetrahydrofolate

↑Family	M	DB	Accession	Score	Mass	Matches	Match(sig)	Sequences	Seq(sig)	emPAI	Description
632	1	db$2	2::C3K5H7|C3K5H7_PSEFS	150	43348	4	4	3	3	0.39	Putative response regu
633	1	db$2	2::C3K2W2|RS8_PSEFS	150	14083	9	8	5	5	6.27	30S ribosomal protein
634	1	db$2	2::C3K480|C3K480_PSEFS	150	31946	5	4	4	3	0.56	Uncharacterized protei
635	1	db$2	2::C3K479|RNPH_PSEFS	149	25645	4	4	4	4	1.08	Ribonuclease PH OS=F
635	2	SwissProt_HUMAN$	3::O75051|PLXA2_HUMAN	36	210969	2	2	2	2	0.05	Plexin-A2 OS=Homo sa
636	1	db$2	2::C3K424|HEM3_PSEFS	149	33541	5	3	4	2	0.33	Porphobilinogen deami
637	1	db$2	2::C3K6S9|C3K6S9_PSEFS	148	101727	7	6	7	6	0.32	DNA polymerase I OS=
638	1	db$2	2::C3K435|C3K435_PSEFS	147	45402	8	7	8	7	1.07	Diaminopimelate decar
639	1	db$2	2::C3K3M6|C3K3M6_PSEFS	146	53012	5	5	5	5	0.56	Peptidase_M16_C dom
640	1	db$2	2::C3JY88|C3JY88_PSEFS	146	20432	9	9	6	6	5.28	NADH-quinone oxidore
641	1	db$2	2::C3K8K5|C3K8K5_PSEFS	146	64377	3	3	3	3	0.25	Putative transport-rela
642	1	db$2	2::C3K104|C3K104_PSEFS	146	26102	7	5	5	5	1.47	Putative membrane pr
643	1	db$2	2::C3JYP6|C3JYP6_PSEFS	145	10477	3	3	2	2	2.78	YCII domain-containin
644	1	db$2	2::C3KAC0|C3KAC0_PSEFS	145	40546	6	6	5	5	0.79	Butyryl-CoA dehydroge
645	1	db$2	2::C3K134|C3K134_PSEFS	145	52371	5	5	4	4	0.44	Coproporphyrinogen-II
646	1	SwissProt_HUMAN$	3::P62805|H4_HUMAN	145	11360	8	8	6	6	10.67	Histone H4 OS=Homo
647	1	db$2	2::C3K4P8|C3K4P8_PSEFS	145	24008	4	4	3	3	0.80	t(6)A37 threonylcarba
648	1	db$2	2::C3K9F0|C3K9F0_PSEFS	145	40567	6	6	4	4	0.79	Putative transport-rela
649	1	db$2	2::C3K5U5|C3K5U5_PSEFS	144	32335	4	4	3	3	0.55	Glucose-1-phosphate t
650	1	db$2	2::C3JZ77|C3JZ77_PSEFS	144	45611	6	6	5	5	0.68	Maltose-binding protei
651	1	db$2	2::C3K3S3|C3K3S3_PSEFS	144	15261	4	3	3	2	0.85	DUF4399 domain-cont
652	1	db$2	2::C3KAK2|C3KAK2_PSEFS	144	10989	6	6	4	4	7.23	Uncharacterized protei
653	1	db$2	2::C3K6I1|C3K6I1_PSEFS	144	31224	7	6	6	5	1.13	S-formylglutathione hy
654	1	db$2	2::C3K276|GRPE_PSEFS	144	20714	3	3	2	2	0.57	Protein GrpE OS=Pseu
655	1	db$2	2::C3JYK2|C3JYK2_PSEFS	143	30492	6	6	5	5	1.17	Bifunctional protein Fo
656	1	db$2	2::C3JZT9|C3JZT9_PSEFS	143	24210	8	8	7	7	2.90	3-isopropylmalate deh
657	1	db$2	2::C3K3L5|C3K3L5_PSEFS	143	11400	3	3	2	2	2.38	Putative membrane pr
658	1	db$2	2::C3K419|C3K419_PSEFS	142	17100	3	3	2	2	1.27	Transcriptional regulat
659	1	db$2	2::C3KEA8|C3KEA8_PSEFS	142	45495	5	5	4	4	0.52	ATP-dependent RNA he
660	1	db$2	2::C3K7I9|C3K7I9_PSEFS	141	88241	8	7	7	6	0.38	Putative pinecillin acyl
661	1	db$2	2::C3KDU8|C3KDU8_PSEFS	141	35029	7	7	6	6	1.57	D-cysteine desulfhydra
662	1	db$2	2::C3JYQ9|C3JYQ9_PSEFS	141	54767	3	3	3	3	0.30	Transcriptional regulat
663	1	db$2	2::C3KCS5|C3KCS5_PSEFS	140	51798	6	4	6	4	0.44	UDP-N-acetylmuramoy
664	1	db$2	2::C3K709|C3K709_PSEFS	140	7722	6	6	4	4	9.57	Cold shock protein OS
665	1	db$2	2::C3K256|RS15_PSEFS	140	10112	5	5	4	4	5.24	30S ribosomal protein
666	1	db$2	2::C3K240|C3K240_PSEFS	140	17616	5	5	4	4	2.77	RNA polymerase-bindi
667	1	db$2	2::C3K0T3|C3K0T3_PSEFS	140	28958	4	4	4	4	0.92	Putative membrane pr
668	1	db$2	2::C3K1K8|C3K1K8_PSEFS	139	31523	7	6	5	4	0.82	Putative hydrolase OS
669	1	db$2	2::C3KCS6|C3KCS6_PSEFS	139	47227	3	3	3	3	0.35	UDP-N-acetylmuramoy
670	1	db$2	2::C3K758|PYRF_PSEFS	139	24706	6	5	6	5	1.59	Orotidine 5'-phosphate

↑Family	M	DB	Accession	Score	Mass	Matches	Match(sig)	Sequences	Seq(sig)	emPAI	Description
671	1	db$2	2::C3K2R5|RIBA_PSEFS	139	22312	4	4	4	4	1.32	GTP cyclohydrolase-2 (
672	1	db$2	2::C3JXP7|RECR_PSEFS	138	21528	6	6	6	6	2.70	Recombination protein
673	1	db$2	2::C3KDP4|C3KDP4_PSEFS	137	75338	7	6	6	6	0.46	Phosphate acetyltransf
674	1	db$2	2::C3K483|C3K483_PSEFS	137	78630	9	9	9	9	0.72	Guanosine-3',5'-bis(Di
674	2	db$2	2::C3K1B1|C3K1B1_PSEFS	84	46678	3	3	3	3	0.36	Putative acid tolerance
675	1	db$2	2::C3K4E1|C3K4E1_PSEFS	137	38799	8	8	6	6	1.07	N5-carboxyaminoimida
676	1	db$2	2::C3K417|C3K417_PSEFS	137	39396	4	4	3	3	0.43	Transcriptional regulat
677	1	db$2	2::C3K017|PYRD_PSEFS	136	35850	7	6	7	6	1.20	Dihydroorotate dehydr
678	1	db$2	2::C3K0U9|C3K0U9_PSEFS	136	55465	5	5	4	4	0.41	Putative two-compone
679	1	db$2	2::C3K3M7|C3K3M7_PSEFS	136	22464	2	2	1	1	0.23	Ribosomal RNA small s
680	1	db$2	2::C3K4R1|PLSB_PSEFS	136	95327	5	5	5	5	0.28	Glycerol-3-phosphate a
681	1	db$2	2::C3K6E4|C3K6E4_PSEFS	135	46143	10	8	5	5	0.67	Cytochrome b OS=Pse
682	1	db$2	2::C3K862|C3K862_PSEFS	135	39825	5	5	5	5	0.81	Putative outer membra
683	1	db$2	2::C3K5K2|C3K5K2_PSEFS	134	9923	4	4	4	4	5.49	Putative lipoprotein OS
684	1	SwissProt_HUMAN$	3::P60174|TPIS_HUMAN	134	26653	7	7	6	6	1.89	Triosephosphate isome
685	1	db$2	2::C3K0D7|APEB_PSEFS	134	46914	5	5	5	5	0.66	Probable M18 family ar
686	1	db$2	2::C3K9M2|C3K9M2_PSEFS	134	37830	5	4	4	3	0.45	Putative peptidase OS=
687	1	db$2	2::C3JYS8|RUVA_PSEFS	134	22218	3	3	3	3	0.89	Holliday junction ATP-c
688	1	db$2	2::C3JYS0|C3JYS0_PSEFS	133	29172	6	6	5	5	1.24	Cell division coordinato
689	1	SwissProt_HUMAN$	3::Q5VTE0|EF1A3_HUMAN	133	50153	7	6	6	5	0.60	Putative elongation fac
690	1	db$2	2::C3K3Y8|UBID_PSEFS	133	54697	6	5	6	5	0.54	3-octaprenyl-4-hydrox
691	1	SwissProt_HUMAN$	3::Q5QNW6|H2B2F_HUMAN	132	13912	4	4	4	4	2.83	Histone H2B type 2-F (
692	1	db$2	2::C3K2E0|C3K2E0_PSEFS	132	48742	5	5	4	4	0.47	DNA repair protein Rad
693	1	db$2	2::C3K0I1|C3K0I1_PSEFS	130	29699	3	3	3	3	0.61	ParA family ATPase OS
694	1	db$2	2::C3KAC2|C3KAC2_PSEFS	130	41342	4	3	4	3	0.41	Acyl-CoA dehydrogena
695	1	db$2	2::C3JY78|C3JY78_PSEFS	130	22781	6	6	4	4	2.45	Putative cytochrome o
695	2	db$2	2::C3JZH3|C3JZH3_PSEFS	105	22428	5	5	5	5	1.85	Putative cytochrome o
696	1	db$2	2::C3K0U4|C3K0U4_PSEFS	130	36872	8	4	6	4	0.67	Flagellar motor switch
697	1	db$2	2::C3K6E5|C3K6E5_PSEFS	130	29069	4	4	3	3	0.63	Cytochrome c1 OS=Ps
698	1	SwissProt_HUMAN$	3::P62937|PPIA_HUMAN	130	18001	5	5	5	5	2.68	Peptidyl-prolyl cis-tran
699	1	db$2	2::C3KE58|C3KE58_PSEFS	129	84757	7	7	6	6	0.40	Putative S1 RNA-bindi
700	1	db$2	2::C3JZQ0|C3JZQ0_PSEFS	129	38665	6	4	5	3	0.44	Putative L-asparaginas
701	1	db$2	2::C3K8K2|C3K8K2_PSEFS	129	418093	4	3	4	3	0.03	Haemagg_act domain-
702	1	db$2	2::C3KED2|C3KED2_PSEFS	128	17293	5	5	5	5	2.86	Thioredoxin peroxidase
703	1	db$2	2::C3K5E5|C3K5E5_PSEFS	128	28503	7	7	5	5	1.69	Methionine aminopepti
704	1	db$2	2::C3K1H1|RS16_PSEFS	128	9202	5	5	5	5	11.20	30S ribosomal protein
705	1	db$2	2::C3K185|C3K185_PSEFS	128	20062	6	5	5	4	2.21	Putative NAD(P)H nitro
706	1	SwissProt_HUMAN$	3::P0DOX7|IGK_HUMAN	128	23364	3	3	3	3	0.83	Immunoglobulin kappa
707	1	db$2	2::C3K841|C3K841_PSEFS	128	11596	9	6	6	4	6.46	Putative sigma-54 mod
708	1	db$2	2::C3KA84|C3KA84_PSEFS	128	13408	4	4	4	4	3.00	Putative regulatory pro

↑Family	M	DB	Accession	Score	Mass	Matches	Match(sig)	Sequences	Seq(sig)	emPAI	Description
709	1	db$2	2::C3K0R1|C3K0R1_PSEFS	128	30842	3	3	3	3	0.58	Protein-glutamate O-m
710	1	db$2	2::C3KAD3|ARNC_PSEFS	127	37700	3	3	3	3	0.46	Undecaprenyl-phospha
711	1	db$2	2::C3KE59|C3KE59_PSEFS	127	27398	7	6	7	6	1.80	DNA-binding response
712	1	db$2	2::C3KE68|RS18_PSEFS	127	8912	9	8	4	4	12.36	30S ribosomal protein
713	1	db$2	2::C3K2N8|C3K2N8_PSEFS	127	23444	10	9	7	6	3.08	Semialdhyde_dh doma
714	1	db$2	2::C3K2U6|C3K2U6_PSEFS	126	49484	3	2	2	1	0.21	Putative transport-rela
715	1	db$2	2::C3K0V5|C3K0V5_PSEFS	126	33212	9	9	6	6	1.71	Putative keto/oxo acyl
716	1	db$2	2::C3JXY2|C3JXY2_PSEFS	126	35731	3	3	2	2	0.49	L-allo-threonine aldola
717	1	db$2	2::C3K4Z4|C3K4Z4_PSEFS	126	43117	5	5	4	4	0.55	Putative cytochrome C
718	1	db$2	2::C3KDB7|C3KDB7_PSEFS	126	65793	8	7	6	5	0.43	ATP-dependent lipid A-
719	1	db$2	2::C3KDN5|C3KDN5_PSEFS	125	35878	4	4	4	4	0.69	Renalase OS=Pseudon
720	1	db$2	2::C3KE45|C3KE45_PSEFS	125	49639	7	7	6	6	0.77	Trk system potassium
721	1	db$2	2::C3K8W9|C3K8W9_PSEFS	125	28276	4	4	4	4	0.95	Intermembrane phosp
722	1	db$2	2::C3K743|FABA_PSEFS	125	18688	9	7	7	6	3.52	3-hydroxydecanoyl-[ac
723	1	db$2	2::C3K9H7|C3K9H7_PSEFS	125	27490	5	3	5	3	0.67	Putative short-chain de
724	1	db$2	2::C3KAK7|C3KAK7_PSEFS	125	20540	5	5	4	4	2.14	Putative lipoprotein OS
725	1	db$2	2::C3K3T6|C3K3T6_PSEFS	125	42390	6	6	6	6	0.95	Spermidine/putrescine
726	1	db$2	2::C3K5E4|GLND_PSEFS	125	102948	6	6	6	6	0.32	Bifunctional uridylyltra
727	1	db$2	2::C3K371|C3K371_PSEFS	124	78727	11	8	11	8	0.62	Malate synthase G OS=
728	1	db$2	2::C3K9Q2|C3K9Q2_PSEFS	124	16954	4	3	4	3	1.29	Ribonuclease H OS=Ps
729	1	db$2	2::C3K2X4|RL23_PSEFS	124	10938	7	7	6	6	11.55	50S ribosomal protein
730	1	db$2	2::C3JYE9|C3JYE9_PSEFS	124	31035	2	2	2	2	0.35	Putative hydroxymethy
731	1	db$2	2::C3K6Y8|C3K6Y8_PSEFS	124	34148	6	6	5	5	0.99	GTPase Era OS=Pseud
732	1	db$2	2::C3KDG2|C3KDG2_PSEFS	124	41589	6	5	5	4	0.76	AMP nucleosidase OS=
733	1	db$2	2::C3K6U2|C3K6U2_PSEFS	124	47692	5	5	5	5	0.64	Probable proton/glutar
734	1	db$2	2::C3JXQ0|C3JXQ0_PSEFS	122	24880	7	6	5	4	1.13	Putative hydrolase OS=
735	1	db$2	2::C3K6V2|HIS4_PSEFS	122	25679	5	4	4	3	1.08	1-(5-phosphoribosyl)-5
736	1	db$2	2::C3K5U6|C3K5U6_PSEFS	122	40174	7	6	6	5	0.80	dTDP-glucose 4,6-dehy
737	1	db$2	2::C3KA81|C3KA81_PSEFS	122	55484	5	5	5	5	0.53	Alkyl hydroperoxide re
738	1	db$2	2::C3K0H7|C3K0H7_PSEFS	122	77893	4	4	4	4	0.28	Histidine kinase OS=Ps
739	1	SwissProt_HUMAN$	3::P62979|RS27A_HUMAN	122	17953	3	3	3	3	1.18	Ubiquitin-40S ribosom
740	1	db$2	2::C3JYN9|C3JYN9_PSEFS	122	44994	7	6	7	6	0.88	Pseudouridine synthas
741	1	db$2	2::C3K6A5|C3K6A5_PSEFS	121	78572	6	6	5	5	0.35	Catalase OS=Pseudom
742	1	db$2	2::C3K2K2|THIE_PSEFS	121	22345	3	3	3	3	0.88	Thiamine-phosphate sy
743	1	db$2	2::C3JXY9|C3JXY9_PSEFS	121	37073	5	5	5	5	0.89	Putative arginine N-suc
744	1	db$2	2::C3KBU0|C3KBU0_PSEFS	120	36726	6	5	6	5	0.90	Sulfate/thiosulfate imp
745	1	db$2	2::C3JZV1|C3JZV1_PSEFS	120	76010	6	6	6	6	0.45	UvrABC system protein
746	1	db$2	2::C3JXZ3|C3JXZ3_PSEFS	120	28123	8	6	7	6	1.74	Histidine transport ATP
747	1	db$2	2::C3K650|C3K650_PSEFS	119	13083	3	3	3	3	1.90	N-acetyltransferase do
748	1	db$2	2::C3JZT0|C3JZT0_PSEFS	119	28122	3	3	3	3	0.65	Putative GntR-family tr

↑Family	M	DB	Accession	Score	Mass	Matches	Match(sig)	Sequences	Seq(sig)	emPAI	Description
749	1	db$2	2::C3K785|C3K785_PSEFS	119	24962	4	4	3	3	0.76	3-oxoadipate CoA-tran
750	1	SwissProt_HUMAN$	3::Q06830|PRDX1_HUMAN	119	22096	5	5	4	4	1.34	Peroxiredoxin-1 OS=H
751	1	db$2	2::C3K075|C3K075_PSEFS	119	77370	8	7	8	7	0.54	Putative sulfatase OS=
752	1	db$2	2::C3K6K4|C3K6K4_PSEFS	119	32908	4	3	4	3	0.54	Putative 5'-nucleotidas
753	1	db$2	2::C3K5M0|C3K5M0_PSEFS	119	29300	5	4	4	4	0.90	Phosphonates ABC trai
754	1	db$2	2::C3K230|C3K230_PSEFS	119	85084	3	2	3	2	0.12	Penicillin-binding prote
755	1	db$2	2::C3K0N7|RL32_PSEFS	118	6752	5	5	2	2	2.84	50S ribosomal protein
756	1	db$2	2::C3JYI6|C3JYI6_PSEFS	118	73112	6	4	6	4	0.30	Putative 2,4-dienoyl-C
757	1	db$2	2::C3K6J2|C3K6J2_PSEFS	117	47817	5	5	4	4	0.49	Putative hydrolase OS=
758	1	db$2	2::C3K3J3|C3K3J3_PSEFS	117	72855	5	5	4	4	0.30	Type IV pilus biogenes
759	1	db$2	2::C3K1X0|CYSZ_PSEFS	117	28826	5	3	5	3	0.63	Sulfate transporter Cy:
760	1	db$2	2::C3K360|C3K360_PSEFS	117	23509	4	4	3	3	0.82	ATP-dependent dethiol
761	1	db$2	2::C3K0S3|C3K0S3_PSEFS	117	67491	7	5	7	5	0.42	Putative ATP-binding c
762	1	db$2	2::C3KAI3|AROB_PSEFS	117	39825	4	4	3	3	0.43	3-dehydroquinate synt
763	1	db$2	2::C3KDC4|C3KDC4_PSEFS	117	64677	3	3	3	3	0.25	Uncharacterized protei
764	1	db$2	2::C3KBK9|C3KBK9_PSEFS	117	34365	2	2	2	2	0.32	Glycerate dehydrogena
765	1	db$2	2::C3K3H3|C3K3H3_PSEFS	116	52510	3	3	3	3	0.31	Adenosylmethionine-8
766	1	db$2	2::C3K3L3|C3K3L3_PSEFS	116	21091	6	4	5	4	1.44	dITP/XTP pyrophospha
767	1	db$2	2::C3JXX7|C3JXX7_PSEFS	116	56979	4	3	4	3	0.28	Conserved hypothetica
768	1	db$2	2::C3K0P6|DCTA_PSEFS	115	46761	3	3	3	3	0.36	C4-dicarboxylate trans
769	1	db$2	2::C3K5K3|C3K5K3_PSEFS	115	28692	4	4	2	2	0.64	CHAD domain-containi
770	1	db$2	2::C3K3R6|C3K3R6_PSEFS	114	54762	7	6	6	6	0.68	L-threonine dehydrata
771	1	db$2	2::C3K2K7|C3K2K7_PSEFS	114	37713	5	5	5	5	0.87	PhoH domain-containir
772	1	SwissProt_HUMAN$	3::P23528|COF1_HUMAN	114	18491	5	5	5	5	2.54	Cofilin-1 OS=Homo sa
773	1	db$2	2::C3K5T5|C3K5T5_PSEFS	113	25405	2	2	1	1	0.20	Carbonic anhydrase O:
774	1	db$2	2::C3K5B2|C3K5B2_PSEFS	113	35714	3	3	3	3	0.49	Dipeptide ABC transpo
775	1	db$2	2::C3K126|C3K126_PSEFS	113	54569	10	7	6	6	0.68	2-methylcitrate dehydı
776	1	db$2	2::C3JYK0|CLPP_PSEFS	112	23405	8	8	7	7	3.08	ATP-dependent Clp prc
777	1	db$2	2::C3JZN0|C3JZN0_PSEFS	112	13917	2	2	2	2	0.96	MerR-family transcript
778	1	db$2	2::C3K816|OPGH_PSEFS	112	96236	4	4	4	4	0.22	Glucans biosynthesis g
779	1	db$2	2::C3K302|C3K302_PSEFS	112	12723	6	6	3	3	3.29	Putative HIT domain-c
780	1	db$2	2::C3K0W6|C3K0W6_PSEFS	112	42755	4	4	4	4	0.56	Aspartate aminotransf
781	1	db$2	2::C3KCL0|C3KCL0_PSEFS	112	35424	4	4	4	4	0.70	Uncharacterized protei
782	1	db$2	2::C3K3L6|TRMB_PSEFS	111	26990	3	3	3	3	0.69	tRNA (guanine-N(7)-)-
783	1	db$2	2::C3K0R6|C3K0R6_PSEFS	111	28374	6	6	6	6	1.70	Uncharacterized protei
784	1	db$2	2::C3K0R0|C3K0R0_PSEFS	110	14751	2	2	2	2	0.88	Flagellar basal body ro
785	1	db$2	2::C3K6E3|C3K6E3_PSEFS	110	21087	6	5	5	4	1.44	Ubiquinol-cytochrome
786	1	db$2	2::C3K6J1|UBIG_PSEFS	110	26040	5	4	5	4	1.06	Ubiquinone biosynthes
787	1	db$2	2::C3K2P1|C3K2P1_PSEFS	110	48286	4	3	3	3	0.34	UDP-N-acetylmuramat
788	1	db$2	2::C3K114|C3K114_PSEFS	110	74752	8	6	8	6	0.46	DNA polymerase III su

↑Family	M	DB	Accession	Score	Mass	Matches	Match(sig)	Sequences	Seq(sig)	emPAI	Description
789	1	db$2	2::C3K754|C3K754_PSEFS	110	29479	3	3	3	3	0.61	Putative lipoprotein OS
790	1	db$2	2::C3K3T8|C3K3T8_PSEFS	109	39499	6	5	5	4	0.61	Putrescine ABC transp
791	1	db$2	2::C3K304|C3K304_PSEFS	109	15054	4	4	3	3	1.53	Uncharacterized protei
792	1	db$2	2::C3JYI3|PHNX_PSEFS	109	29973	5	5	5	5	1.20	Phosphonoacetaldehyc
793	1	db$2	2::C3K349|C3K349_PSEFS	109	28994	5	5	4	4	0.92	Pyrroloquinoline-quino
794	1	db$2	2::C3JXZ5|C3JXZ5_PSEFS	109	25958	3	3	2	2	0.44	Putative histidine trans
795	1	db$2	2::C3KE89|C3KE89_PSEFS	108	24346	4	4	4	4	1.17	Uncharacterized protei
796	1	db$2	2::C3K3V5|C3K3V5_PSEFS	108	52101	10	9	8	7	0.89	CYTH domain-containi
797	1	db$2	2::C3K5K4|C3K5K4_PSEFS	108	29062	4	4	3	3	0.63	Uncharacterized protei
798	1	db$2	2::C3KDM6|PSD_PSEFS	108	31380	6	6	5	5	1.46	Phosphatidylserine dec
799	1	db$2	2::C3K1U9|C3K1U9_PSEFS	107	42728	4	3	3	3	0.39	Flavohemoglobin OS=I
800	1	db$2	2::C3K5L3|C3K5L3_PSEFS	107	36401	6	5	6	5	0.91	Phosphonates ABC trai
801	1	db$2	2::C3KBH8|C3KBH8_PSEFS	107	25745	3	3	3	3	0.73	3-oxoacyl-[acyl-carrier
802	1	db$2	2::C3KDR6|C3KDR6_PSEFS	107	40570	4	4	3	3	0.42	Putaive NADH oxidore
803	1	db$2	2::C3K648|C3K648_PSEFS	107	33905	4	4	4	4	0.75	Conserved hypothetica
804	1	db$2	2::C3K1D1|C3K1D1_PSEFS	106	32188	4	4	4	4	0.80	Protein HflC OS=Pseuc
805	1	db$2	2::C3K910|C3K910_PSEFS	106	36322	5	3	5	3	0.48	Putative ABC transport
806	1	db$2	2::C3K867|C3K867_PSEFS	106	39735	5	5	4	4	0.61	PALP domain-containir
807	1	db$2	2::C3K231|C3K231_PSEFS	106	57678	5	4	5	4	0.39	APH domain-containing
808	1	db$2	2::C3K2Y1|RS12_PSEFS	106	13716	3	3	2	2	0.97	30S ribosomal protein
809	1	db$2	2::C3K8K8|C3K8K8_PSEFS	106	62203	8	8	6	6	0.58	Putative exported prot
810	1	db$2	2::C3K1N5|QUEA_PSEFS	106	38396	5	5	5	5	0.85	S-adenosylmethionine
811	1	db$2	2::C3K468|C3K468_PSEFS	105	6077	2	2	1	1	3.42	50S ribosomal protein
812	1	db$2	2::C3JZ32|C3JZ32_PSEFS	105	38286	3	3	3	3	0.45	Putative cation transpo
813	1	db$2	2::C3KDN7|HEMH_PSEFS	105	38415	6	5	6	5	0.85	Ferrochelatase OS=Ps
814	1	db$2	2::C3KDX7|ISPH_PSEFS	104	34499	6	5	6	5	0.98	4-hydroxy-3-methylbu
815	1	db$2	2::C3KAV6|C3KAV6_PSEFS	104	17648	5	5	3	3	1.22	Thiol peroxidase OS=F
816	1	db$2	2::C3K6W7|C3K6W7_PSEFS	104	16216	4	2	3	2	0.78	Biotin carboxyl carrier
817	1	db$2	2::C3K257|C3K257_PSEFS	104	33362	4	4	4	4	0.76	tRNA pseudouridine sy
818	1	db$2	2::C3KAY1|C3KAY1_PSEFS	103	44657	4	4	4	4	0.53	3-phosphoshikimate 1-
819	1	db$2	2::C3KDI2|C3KDI2_PSEFS	103	135789	3	3	2	2	0.07	Exonuclease OS=Pseu
820	1	db$2	2::C3K2V8|RL30_PSEFS	103	6521	5	5	3	3	15.09	50S ribosomal protein
821	1	db$2	2::C3K6S5|C3K6S5_PSEFS	102	35353	7	6	5	4	1.23	Delta-aminolevulinic a
822	1	db$2	2::C3K467|C3K467_PSEFS	102	13453	3	3	3	3	1.83	Cupin_3 domain-conta
823	1	db$2	2::C3K5G2|C3K5G2_PSEFS	102	35788	7	6	7	6	1.21	Putative membrane pr
824	1	db$2	2::C3K3Q1|C3K3Q1_PSEFS	102	40925	4	4	4	4	0.59	Putative oxidoreductas
825	1	db$2	2::C3K1H9|C3K1H9_PSEFS	102	18692	3	3	3	3	1.13	Conserved hypothetica
826	1	db$2	2::C3JY87|NUOH_PSEFS	102	37530	5	5	3	3	0.65	NADH-quinone oxidore
827	1	db$2	2::C3K3M9|C3K3M9_PSEFS	102	30473	3	3	3	3	0.59	Putative sulfurtransfer
828	1	db$2	2::C3K9R3|C3K9R3_PSEFS	101	18863	3	3	3	3	1.11	Uncharacterized protei

↑Family	M	DB	Accession	Score	Mass	Matches	Match(sig)	Sequences	Seq(sig)	emPAI	Description
829	1	db$2	2::C3K1U6|C3K1U6_PSEFS	101	74730	3	3	3	3	0.21	Cytochrome bo(3) ubic
830	1	db$2	2::C3K254|C3K254_PSEFS	101	21027	4	4	4	4	1.45	Osmotically inducible p
831	1	db$2	2::C3JY91|C3JY91_PSEFS	101	66398	3	3	2	2	0.15	NADH-quinone oxidore
832	1	db$2	2::C3K6Y6|C3K6Y6_PSEFS	101	31839	5	5	5	5	1.10	Signal peptidase I OS=
833	1	db$2	2::C3K1V9|C3K1V9_PSEFS	100	16219	2	2	2	2	0.78	Flavodoxin-like domair
834	1	db$2	2::C3K309|C3K309_PSEFS	99	21661	2	2	2	2	0.54	Anthranilate synthase
835	1	db$2	2::C3JXQ1|C3JXQ1_PSEFS	99	24479	3	3	2	2	0.47	Putative enoyl-CoA hy
836	1	db$2	2::C3K221|C3K221_PSEFS	99	129628	2	2	2	2	0.08	RecBCD enzyme subur
837	1	db$2	2::C3K0V2|C3K0V2_PSEFS	99	48301	3	3	2	2	0.22	Flagellar hook-associat
838	1	db$2	2::C3KE30|C3KE30_PSEFS	99	59128	4	3	4	3	0.27	L-aspartate oxidase OS
839	1	db$2	2::C3K5X6|C3K5X6_PSEFS	98	49358	6	5	6	5	0.61	Tryptophan--tRNA liga
840	1	db$2	2::C3K4D7|C3K4D7_PSEFS	98	14435	3	3	3	3	1.64	Putative thioesterase (
841	1	db$2	2::C3K2W7|RS17_PSEFS	98	10090	4	3	4	3	2.95	30S ribosomal protein
842	1	db$2	2::C3KCS7|MRAY_PSEFS	98	39243	3	3	3	3	0.43	Phospho-N-acetylmura
843	1	db$2	2::C3K9G9|C3K9G9_PSEFS	98	16183	4	4	4	4	2.17	CMD domain-containin
844	1	db$2	2::C3K8U7|C3K8U7_PSEFS	98	30923	5	4	4	3	0.58	Transcriptional regulat
845	1	db$2	2::C3JY71|C3JY71_PSEFS	98	43919	2	2	2	2	0.24	JmjC domain-containir
846	1	db$2	2::C3KBG3|C3KBG3_PSEFS	98	24033	5	4	4	3	0.80	Curli production asser
847	1	db$2	2::C3K3M2|C3K3M2_PSEFS	97	37029	6	4	5	3	0.47	Cell division protein Ft
848	1	db$2	2::C3KBU2|C3KBU2_PSEFS	97	56055	3	3	3	3	0.29	Putative histidine amm
849	1	db$2	2::C3K4J0|C3K4J0_PSEFS	97	17598	3	3	3	3	1.22	Zn_protease domain-c
850	1	db$2	2::C3K6W0|C3K6W0_PSEFS	97	14786	5	4	4	3	1.58	Putative rhodanese do
851	1	db$2	2::C3K106|C3K106_PSEFS	97	20669	2	2	2	2	0.58	Uncharacterized protei
852	1	db$2	2::C3K0X7|C3K0X7_PSEFS	97	126663	2	2	2	2	0.08	Histidine kinase OS=P
853	1	db$2	2::C3KAS9|C3KAS9_PSEFS	97	109677	3	3	3	3	0.14	Efflux pump membran
854	1	db$2	2::C3KBJ5|C3KBJ5_PSEFS	96	62095	3	3	3	3	0.26	Phospholipase C OS=P
855	1	db$2	2::C3K6L9|C3K6L9_PSEFS	96	49785	3	3	3	3	0.33	Phospho-2-dehydro-3-
856	1	db$2	2::C3JXH2|C3JXH2_PSEFS	96	91149	4	4	3	3	0.17	Putative excinuclease
857	1	db$2	2::C3K103|C3K103_PSEFS	96	23527	5	4	4	3	0.82	Phosphoribosylglycinar
858	1	db$2	2::C3KE78|C3KE78_PSEFS	96	15753	4	4	3	3	1.43	Azurin OS=Pseudomor
859	1	db$2	2::C3K813|C3K813_PSEFS	96	36618	6	5	6	5	0.91	Proline iminopeptidase
860	1	db$2	2::C3K796|C3K796_PSEFS	96	49835	4	4	4	4	0.46	Putative glutamine syr
861	1	db$2	2::C3K491|C3K491_PSEFS	96	76137	3	3	3	3	0.21	ATP-dependent DNA h
862	1	db$2	2::C3K7M5|C3K7M5_PSEFS	96	15789	3	3	3	3	1.43	Uncharacterized protei
863	1	db$2	2::C3K2K6|C3K2K6_PSEFS	96	49458	5	5	5	5	0.61	tRNA-2-methylthio-N(
864	1	db$2	2::C3K9M0|C3K9M0_PSEFS	95	11184	2	2	2	2	1.29	SCP2 domain-containir
865	1	db$2	2::C3KDF0|C3KDF0_PSEFS	95	18221	5	3	5	3	1.16	Putative phage tail-rel
866	1	db$2	2::C3K617|C3K617_PSEFS	95	19384	4	4	3	3	1.06	Uncharacterized protei
867	1	db$2	2::C3KBZ8|C3KBZ8_PSEFS	95	38389	2	2	2	2	0.28	Iron-sulfur cluster carr
868	1	SwissProt_HUMAN$	3::O00139|KIF2A_HUMAN	95	79905	15	14	1	1	0.06	Kinesin-like protein KI

↑Family	M	DB	Accession	Score	Mass	Matches	Match(sig)	Sequences	Seq(sig)	emPAI	Description
869	1	db$2	2::C3K1A4|C3K1A4_PSEFS	95	41199	1	1	1	1	0.12	Uncharacterized protei
870	1	db$2	2::C3K1V6|C3K1V6_PSEFS	95	32555	3	3	3	3	0.55	Putative short-chain de
871	1	db$2	2::C3K1E4|GLMU_PSEFS	95	48690	4	3	4	3	0.34	Bifunctional protein Gl
872	1	db$2	2::C3KD57|C3KD57_PSEFS	95	49686	5	4	5	4	0.46	Cystathionine beta-lya
873	1	db$2	2::C3K148|APT_PSEFS	94	20064	6	6	4	4	1.54	Adenine phosphoribosy
874	1	db$2	2::C3KE66|C3KE66_PSEFS	94	27011	5	5	5	5	1.39	23S rRNA (guanosine-
875	1	db$2	2::C3KDC0|C3KDC0_PSEFS	94	36735	2	2	2	2	0.29	Uncharacterized protei
876	1	db$2	2::C3JYU5|C3JYU5_PSEFS	94	34090	5	5	4	4	0.74	Nudix hydrolase doma
877	1	db$2	2::C3K0Y2|C3K0Y2_PSEFS	94	46222	2	2	2	2	0.23	Putative fatty acid trar
878	1	db$2	2::C3KD59|C3KD59_PSEFS	94	81779	6	5	6	5	0.34	YcaO domain-containir
879	1	db$2	2::C3K470|C3K470_PSEFS	94	58011	5	4	5	4	0.39	Putative transport-rela
880	1	db$2	2::C3K110|C3K110_PSEFS	94	32414	5	5	4	4	1.07	Cysteine synthase OS=
881	1	db$2	2::C3JY61|C3JY61_PSEFS	94	8297	4	4	3	3	8.09	Translation initiation fa
882	1	db$2	2::C3K0Q8|C3K0Q8_PSEFS	93	24717	2	2	2	2	0.46	Basal-body rod modific
883	1	db$2	2::C3KCS0|RL25_PSEFS	93	21502	7	6	4	4	1.39	50S ribosomal protein
884	1	SwissProt_HUMAN$	3::P07737|PROF1_HUMAN	93	15045	4	4	4	4	2.44	Profilin-1 OS=Homo sa
885	1	db$2	2::C3K061|C3K061_PSEFS	93	18792	3	3	3	3	1.12	DUF2059 domain-cont
886	1	db$2	2::C3K2J5|C3K2J5_PSEFS	93	18057	4	4	4	4	1.82	Putative AsnC-family r
887	1	SwissProt_HUMAN$	3::P07437|TBB5_HUMAN	93	49639	3	2	3	2	0.21	Tubulin beta chain OS=
888	1	db$2	2::C3K3Q4|C3K3Q4_PSEFS	92	38576	5	4	5	4	0.63	Putative cation ABC tra
889	1	db$2	2::C3KDM4|C3KDM4_PSEFS	92	56715	5	4	5	4	0.40	Putative membrane pr
890	1	db$2	2::C3K3L1|C3K3L1_PSEFS	92	23430	2	2	2	2	0.49	Uncharacterized protei
891	1	db$2	2::C3K3D3|BETA_PSEFS	92	62642	3	3	2	2	0.16	Oxygen-dependent cho
892	1	db$2	2::Q9ZEP7|PANC_PSEFS	92	30696	2	2	2	2	0.36	Pantothenate syntheta
893	1	db$2	2::C3K5F1|C3K5F1_PSEFS	91	42516	5	4	5	4	0.56	Ribonuclease D OS=Ps
894	1	db$2	2::C3K3N8|C3K3N8_PSEFS	91	43920	6	5	6	5	0.71	PUA domain-containing
895	1	db$2	2::C3K261|RIMP_PSEFS	91	17653	3	2	3	2	0.70	Ribosome maturation f
896	1	db$2	2::C3K2P7|C3K2P7_PSEFS	91	17680	6	4	5	3	1.22	Putative acetyltransfer
897	1	db$2	2::C3JYS4|C3JYS4_PSEFS	91	16520	3	3	3	3	1.34	Tol-Pal system protein
898	1	db$2	2::C3KB69|C3KB69_PSEFS	91	12397	1	1	1	1	0.45	Putative lipoprotein OS
899	1	db$2	2::C3JZN4|RL20_PSEFS	91	13348	7	5	6	5	4.74	50S ribosomal protein
900	1	db$2	2::C3K4C1|C3K4C1_PSEFS	91	40318	3	3	3	3	0.42	Putative nitric oxide de
901	1	db$2	2::C3KBY3|C3KBY3_PSEFS	91	64998	3	3	3	3	0.24	Putative lipoprotein OS
902	1	db$2	2::C3K0T7|C3K0T7_PSEFS	91	76509	7	7	7	7	0.54	Putative fatty acid oxic
903	1	db$2	2::C3K450|C3K450_PSEFS	91	61786	2	2	2	2	0.17	Putative EAL/GGDEF d
904	1	db$2	2::C3K0U5|C3K0U5_PSEFS	91	62992	3	3	3	3	0.25	Flagellar M-ring protei
905	1	db$2	2::C3K8W2|ADE_PSEFS	90	36048	3	3	3	3	0.48	Adenine deaminase OS
906	1	db$2	2::C3K437|C3K437_PSEFS	90	26506	2	2	2	2	0.43	Uncharacterized protei
907	1	db$2	2::C3JYE7|C3JYE7_PSEFS	90	35468	5	5	4	4	0.70	LysR family regulatory
908	1	db$2	2::C3K8W4|C3K8W4_PSEFS	90	18790	5	4	4	3	1.12	Lipopolysaccharide exp

↑Family	M	DB	Accession	Score	Mass	Matches	Match(sig)	Sequences	Seq(sig)	emPAI	Description
909	1	db$2	2::C3JYR2|C3JYR2_PSEFS	90	48934	4	4	4	4	0.47	L-serine dehydratase (
909	2	db$2	2::C3K5Y8|C3K5Y8_PSEFS	62	48751	3	3	3	3	0.34	L-serine dehydratase (
910	1	db$2	2::C3K303|COQ7_PSEFS	90	23866	4	3	4	3	0.81	3-demethoxyubiquinol
911	1	db$2	2::C3K544|C3K544_PSEFS	90	74165	5	3	5	3	0.21	Uncharacterized protei
912	1	db$2	2::C3K3M5|C3K3M5_PSEFS	90	50101	6	6	6	6	0.76	Putative peptidase OS=
913	1	db$2	2::C3K1N1|C3K1N1_PSEFS	90	33097	3	3	3	3	0.53	Protein-export membra
914	1	db$2	2::C3KCP1|C3KCP1_PSEFS	90	23476	4	3	4	3	0.83	Peptide methionine sul
915	1	db$2	2::C3KB73|C3KB73_PSEFS	89	9692	1	1	1	1	0.61	Putative lipoprotein OS
916	1	db$2	2::C3JYI2|C3JYI2_PSEFS	89	39477	4	4	4	4	0.61	2-aminoethylphosphor
917	1	db$2	2::C3K3D4|C3K3D4_PSEFS	89	52472	4	3	4	3	0.31	Uncharacterized protei
918	1	db$2	2::C3K3X0|C3K3X0_PSEFS	88	42989	3	2	3	2	0.25	Putative ubiquinone bi
919	1	db$2	2::C3K1F7|C3K1F7_PSEFS	88	69251	6	5	6	5	0.41	tRNA uridine 5-carboxy
920	1	db$2	2::C3KBL6|C3KBL6_PSEFS	88	27732	5	5	2	2	0.40	Glutamate/aspartate A
921	1	db$2	2::C3K0W5|PHS_PSEFS	88	13357	9	8	5	4	4.74	Putative pterin-4-alpha
922	1	db$2	2::C3K087|C3K087_PSEFS	88	34228	1	1	1	1	0.15	50S ribosomal protein
923	1	db$2	2::C3JZR8|C3JZR8_PSEFS	88	46446	8	5	6	5	0.67	Putative oxidoreductas
924	1	SwissProt_HUMAN$	3::P06703|S10A6_HUMAN	88	10173	2	2	1	1	0.57	Protein S100-A6 OS=H
925	1	db$2	2::C3K591|C3K591_PSEFS	88	19872	3	3	3	3	1.03	Putative bacterioferriti
926	1	db$2	2::C3K8I3|C3K8I3_PSEFS	88	39921	2	2	1	1	0.13	3,4-dihydroxy-2-butan
927	1	db$2	2::C3K2Z9|C3K2Z9_PSEFS	87	34289	5	3	4	2	0.32	NMO domain-containin
928	1	db$2	2::C3K763|C3K763_PSEFS	87	29996	3	3	2	2	0.37	Putative hydrolase OS=
929	1	db$2	2::C3JY38|C3JY38_PSEFS	87	16812	4	3	3	2	1.31	Putative thioredoxin-re
930	1	db$2	2::C3K312|C3K312_PSEFS	87	24134	4	4	4	4	1.18	Ribulose-phosphate 3-
930	2	db$2	2::C3K906|C3K906_PSEFS	41	65595	2	2	2	2	0.16	Conserved GGDEF/EAL
931	1	db$2	2::C3K575|C3K575_PSEFS	87	14827	3	3	3	3	1.56	Osmotically inducible p
932	1	db$2	2::C3K485|C3K485_PSEFS	87	26565	2	2	2	2	0.43	Putative lipoprotein OS
933	1	db$2	2::C3K265|C3K265_PSEFS	87	29739	2	2	2	2	0.37	Dihydropteroate synth
934	1	db$2	2::C3KBL9|C3KBL9_PSEFS	87	27726	4	4	4	4	0.97	Glycerol-3-phosphate r
935	1	db$2	2::C3K0U7|C3K0U7_PSEFS	87	49884	4	4	4	4	0.46	Two-component respo
936	1	db$2	2::C3K0J0|C3K0J0_PSEFS	86	16514	1	1	1	1	0.33	Flagellar motor switch
937	1	db$2	2::C3K5K0|C3K5K0_PSEFS	86	25438	5	5	5	5	1.52	Two-component syster
938	1	db$2	2::C3K0S1|C3K0S1_PSEFS	86	53269	2	2	2	2	0.19	Magnesium transporte
939	1	db$2	2::C3K3L2|C3K3L2_PSEFS	86	15596	3	3	3	3	1.45	DUF4426 domain-cont
940	1	db$2	2::C3K2M7|C3K2M7_PSEFS	86	18106	4	4	4	4	1.82	Ribosomal silencing fa
941	1	db$2	2::C3K3W8|C3K3W8_PSEFS	86	44404	3	3	3	3	0.38	Putative ubiquinone bi
942	1	db$2	2::C3K176|C3K176_PSEFS	86	33571	4	2	4	2	0.33	Glucokinase OS=Pseuc
943	1	db$2	2::C3KBW8|C3KBW8_PSEFS	86	39031	7	7	7	7	1.33	D-alanine--D-alanine li
944	1	db$2	2::C3K1X4|C3K1X4_PSEFS	85	16845	4	4	4	4	2.03	Putative putative expo
945	1	db$2	2::C3K415|C3K415_PSEFS	85	70809	3	3	3	3	0.22	Putative ABC transport
946	1	db$2	2::C3JY57|LOLA_PSEFS	85	22546	5	5	4	4	1.30	Outer-membrane lipop

↑Family	M	DB	Accession	Score	Mass	Matches	Match(sig)	Sequences	Seq(sig)	emPAI	Description
947	1	db$2	2::C3KDQ0|C3KDQ0_PSEFS	85	35105	3	3	3	3	0.50	2-ketogluconate reduc
948	1	db$2	2::C3K1W6|C3K1W6_PSEFS	85	13390	5	5	5	5	4.66	D-erythro-7,8-dihydro
949	1	db$2	2::C3KDL3|C3KDL3_PSEFS	85	70148	7	6	7	6	0.50	Phosphomethylpyrimid
950	1	db$2	2::C3JZP3|C3JZP3_PSEFS	85	36685	5	5	4	4	0.68	Putative inosine-uridin
951	1	db$2	2::C3KDR4|C3KDR4_PSEFS	85	49531	4	4	4	4	0.47	Ribosomal protein S12
952	1	db$2	2::C3K337|PLSY_PSEFS	85	20278	1	1	1	1	0.26	Glycerol-3-phosphate a
953	1	db$2	2::C3K170|C3K170_PSEFS	85	22875	3	3	3	3	0.85	Flavin_Reduct domain-
954	1	db$2	2::C3KBL3|C3KBL3_PSEFS	84	50616	9	8	8	7	0.92	Putative exported prot
955	1	db$2	2::C3KCR9|PTH_PSEFS	84	21041	3	3	3	3	0.95	Peptidyl-tRNA hydrolas
956	1	db$2	2::C3K1K9|C3K1K9_PSEFS	84	42204	4	4	3	3	0.40	Putative aminotransfer
957	1	db$2	2::C3KAM8|C3KAM8_PSEFS	84	52747	4	4	3	3	0.31	Phosphate transporter
958	1	db$2	2::C3K3D1|BETI_PSEFS	84	21992	2	2	2	2	0.53	HTH-type transcription
959	1	db$2	2::C3K6V3|HIS6_PSEFS	84	27111	5	5	5	5	1.39	Imidazole glycerol pho
960	1	db$2	2::C3K4A7|C3K4A7_PSEFS	84	18391	5	5	3	3	1.77	Uncharacterized protei
961	1	db$2	2::C3K6E8|C3K6E8_PSEFS	83	27518	2	2	2	2	0.41	Putative GntR-family r
962	1	db$2	2::C3K7N0|C3K7N0_PSEFS	83	28545	3	3	3	3	0.64	Transport permease pr
963	1	db$2	2::C3JZT8|C3JZT8_PSEFS	83	28261	1	1	1	1	0.18	Putative methyltransfe
964	1	db$2	2::C3K9P7|C3K9P7_PSEFS	83	69450	6	3	6	3	0.23	Putative substrate-bin
965	1	db$2	2::C3K6V8|C3K6V8_PSEFS	83	47941	5	5	5	5	0.64	Putative peptidase OS
966	1	db$2	2::C3K436|C3K436_PSEFS	83	30237	3	3	3	3	0.60	Diaminopimelate epim
967	1	db$2	2::C3JZ44|C3JZ44_PSEFS	83	47432	3	3	3	3	0.35	Putative gluconate per
968	1	db$2	2::C3K9U9|C3K9U9_PSEFS	83	9554	1	1	1	1	0.62	Uncharacterized protei
969	1	db$2	2::C3JXL6|C3JXL6_PSEFS	83	41978	3	3	1	1	0.12	Bcr/CflA family efflux t
970	1	db$2	2::C3KE43|C3KE43_PSEFS	83	32711	4	4	4	4	0.78	Lipid A biosynthesis la
971	1	db$2	2::C3KBV0|C3KBV0_PSEFS	83	28603	4	4	3	3	0.64	Putative chromosome
972	1	db$2	2::C3K0K7|C3K0K7_PSEFS	82	60595	8	5	8	5	0.48	Electron transfer flavo
973	1	SwissProt_HUMAN$	3::P62269|RS18_HUMAN	82	17708	1	1	1	1	0.30	40S ribosomal protein
974	1	db$2	2::C3K737|C3K737_PSEFS	82	17727	4	2	4	2	0.69	Glutathione peroxidase
975	1	db$2	2::C3K364|C3K364_PSEFS	82	38824	4	4	4	4	0.63	Biotin synthase OS=Ps
976	1	db$2	2::C3KCP6|C3KCP6_PSEFS	82	38240	2	2	2	2	0.28	ADP-heptose--LPS hep
977	1	db$2	2::C3K2S3|C3K2S3_PSEFS	82	17933	2	2	2	2	0.68	Transcriptional repress
978	1	db$2	2::C3K4U6|C3K4U6_PSEFS	82	17513	2	2	1	1	0.31	Conserved hypothetica
979	1	db$2	2::C3KCG1|C3KCG1_PSEFS	82	60384	2	2	2	2	0.17	Putative secreted amic
980	1	db$2	2::C3KDS5|C3KDS5_PSEFS	81	41122	4	3	4	3	0.41	Putative hydratase/iso
981	1	db$2	2::C3K487|C3K487_PSEFS	81	33600	2	1	2	1	0.15	Biopolymer transport p
982	1	db$2	2::C3K4R2|C3K4R2_PSEFS	81	7681	2	2	2	2	2.31	Major cold shock prote
983	1	db$2	2::C3K5X0|C3K5X0_PSEFS	81	25599	2	2	2	2	0.44	Putative exported prot
984	1	db$2	2::C3K2K4|C3K2K4_PSEFS	81	19926	2	2	2	2	0.60	Putative exported prot
985	1	db$2	2::C3K5H2|QUEF_PSEFS	81	30808	5	5	5	5	1.15	NADPH-dependent 7-c
986	1	db$2	2::C3K229|C3K229_PSEFS	80	24861	1	1	1	1	0.21	TPR_REGION domain-

↑Family	M	DB	Accession	Score	Mass	Matches	Match(sig)	Sequences	Seq(sig)	emPAI	Description
987	1	SwissProt_HUMAN$	3::P01859|IGHG2_HUMAN	80	35878	4	3	4	3	0.48	Immunoglobulin heavy
987	2	SwissProt_HUMAN$	3::P01857|IGHG1_HUMAN	32	36083	4	3	4	3	0.48	Immunoglobulin heavy
988	1	db$2	2::C3K0M6|C3K0M6_PSEFS	80	25497	3	3	3	3	0.74	Putative tetR-family re
989	1	db$2	2::C3KC02|C3KC02_PSEFS	80	29258	2	2	2	2	0.38	Putative acyltransferas
990	1	db$2	2::C3JXY6|ASTB_PSEFS	80	48741	4	3	4	3	0.34	N-succinylarginine dihy
991	1	db$2	2::C3KAH5|RL31_PSEFS	79	8451	4	4	3	3	4.12	50S ribosomal protein
992	1	db$2	2::C3K0M9|KTHY_PSEFS	79	22917	5	4	4	4	1.28	Thymidylate kinase OS
993	1	db$2	2::C3KE10|C3KE10_PSEFS	79	58823	2	2	2	2	0.17	Putative methyl-accept
994	1	db$2	2::C3K8K4|C3K8K4_PSEFS	79	58330	2	2	2	2	0.18	Putative outer membra
995	1	db$2	2::C3KBT9|C3KBT9_PSEFS	79	31336	1	1	1	1	0.16	Sulfate ABC transport
996	1	db$2	2::C3K5K1|C3K5K1_PSEFS	79	38065	3	3	3	3	0.45	Putative phospholipase
997	1	db$2	2::C3KDM3|C3KDM3_PSEFS	79	25575	1	1	1	1	0.20	Putative lipoprotein OS
998	1	db$2	2::C3KCS8|C3KCS8_PSEFS	79	47767	3	3	3	3	0.35	UDP-N-acetylmuramoy
999	1	db$2	2::C3K1W7|C3K1W7_PSEFS	79	11247	2	2	1	1	0.51	DUF1244 domain-cont
1000	1	db$2	2::C3KDX3|C3KDX3_PSEFS	79	34279	8	7	8	7	1.62	Riboflavin biosynthesis
1001	1	db$2	2::C3K0P0|C3K0P0_PSEFS	79	35982	3	3	3	3	0.48	Putative peptidase OS=
1002	1	db$2	2::C3K308|TRPD_PSEFS	79	36976	5	5	5	5	0.89	Anthranilate phosphori
1003	1	db$2	2::C3K068|C3K068_PSEFS	78	65454	3	3	3	3	0.24	Putative asparagine sy
1004	1	db$2	2::C3K7G4|C3K7G4_PSEFS	78	54521	4	3	4	3	0.30	Ribonuclease G OS=Ps
1005	1	db$2	2::C3K250|C3K250_PSEFS	78	71198	2	2	1	1	0.07	Acetyl-coenzyme A syn
1006	1	db$2	2::C3KDC5|HEM1_PSEFS	78	46641	3	3	3	3	0.36	Glutamyl-tRNA reducta
1007	1	db$2	2::C3K3K3|C3K3K3_PSEFS	78	44101	4	4	4	4	0.54	Dihydroorotase-like pr
1008	1	db$2	2::C3JY68|MNMA_PSEFS	78	41397	5	5	5	5	0.77	tRNA-specific 2-thiouri
1009	1	db$2	2::C3K7E8|C3K7E8_PSEFS	77	21596	1	1	1	1	0.24	Putative lipoprotein OS
1010	1	db$2	2::C3K132|C3K132_PSEFS	77	42760	3	3	3	3	0.39	Uncharacterized protei
1011	1	db$2	2::C3K0N3|ACP_PSEFS	77	8637	1	1	1	1	0.70	Acyl carrier protein OS
1012	1	db$2	2::C3K4J4|HSLO_PSEFS	77	33320	2	2	2	2	0.33	33 kDa chaperonin OS
1013	1	db$2	2::C3K0B8|C3K0B8_PSEFS	77	24379	2	2	2	2	0.47	Uncharacterized protei
1014	1	db$2	2::C3K7S1|C3K7S1_PSEFS	77	25085	3	3	1	1	0.46	Protein MgtC OS=Pseu
1015	1	db$2	2::C3KBH3|DCUP_PSEFS	76	38973	4	4	4	4	0.62	Uroporphyrinogen deca
1016	1	db$2	2::C3K486|C3K486_PSEFS	76	30593	3	2	3	2	0.36	Epimerase domain-cor
1017	1	db$2	2::C3KDG3|C3KDG3_PSEFS	76	15637	2	2	2	2	0.82	Putative exported prot
1018	1	db$2	2::C3K5F4|C3K5F4_PSEFS	76	21753	4	3	4	3	0.91	Putative nitroreductase
1019	1	db$2	2::C3K9N0|C3K9N0_PSEFS	76	28569	2	2	2	2	0.39	Putative transcriptiona
1020	1	db$2	2::C3K0N8|C3K0N8_PSEFS	76	19586	3	3	3	3	1.05	23S rRNA accumulatio
1021	1	db$2	2::C3KAJ9|C3KAJ9_PSEFS	76	20836	4	3	4	3	0.96	Putative hypoxanthine
1022	1	db$2	2::C3K3E9|C3K3E9_PSEFS	76	12056	2	2	2	2	1.16	Putative lipoprotein OS
1023	1	db$2	2::C3K620|C3K620_PSEFS	76	22214	3	2	3	2	0.53	LexA repressor OS=Ps
1024	1	db$2	2::C3KAH8|C3KAH8_PSEFS	76	89028	5	5	5	5	0.31	DD-transpeptidase OS
1025	1	db$2	2::C3K3X4|C3K3X4_PSEFS	76	11561	3	3	2	2	2.34	Cell division protein Za

↑Family	M	DB	Accession	Score	Mass	Matches	Match(sig)	Sequences	Seq(sig)	emPAI	Description
1026	1	db$2	2::C3KDL9|C3KDL9_PSEFS	75	22175	2	1	2	1	0.24	Uncharacterized protei
1027	1	db$2	2::C3JYA9|C3JYA9_PSEFS	75	27795	4	3	4	3	0.66	Putative TetR-family re
1028	1	db$2	2::C3JY77|C3JY77_PSEFS	75	40451	4	4	3	3	0.60	Putative 3-oxoacyl-[ac
1029	1	db$2	2::C3K2W3|C3K2W3_PSEFS	75	11427	5	3	4	3	2.38	30S ribosomal protein
1030	1	db$2	2::C3K8U0|HIS2_PSEFS	75	12007	2	2	2	2	1.17	Phosphoribosyl-ATP py
1031	1	db$2	2::C3K8P0|C3K8P0_PSEFS	75	38329	4	4	4	4	0.64	Putative nucleoside-se
1032	1	db$2	2::C3JZH1|C3JZH1_PSEFS	75	34020	5	5	5	5	1.00	LysR-family transcripti
1033	1	db$2	2::C3K3J5|C3K3J5_PSEFS	75	13219	3	2	3	2	1.02	Type IV pilus response
1034	1	db$2	2::C3K595|C3K595_PSEFS	75	10805	3	3	2	2	1.36	Phosphate starvation-i
1035	1	db$2	2::C3KBB8|C3KBB8_PSEFS	74	13546	4	3	4	3	1.81	Uncharacterized protei
1036	1	db$2	2::C3KDR1|C3KDR1_PSEFS	74	17628	4	3	4	3	1.22	Uncharacterized protei
1037	1	db$2	2::C3JXX3|C3JXX3_PSEFS	74	52842	1	1	1	1	0.09	Cytochrome-c oxidase
1038	1	db$2	2::C3K4W5|C3K4W5_PSEFS	74	24663	5	4	4	3	0.77	Lipoprotein-releasing s
1039	1	db$2	2::C3JY39|C3JY39_PSEFS	74	12732	2	1	2	1	0.44	Arsenate reductase OS
1040	1	db$2	2::C3K101|C3K101_PSEFS	74	37185	4	4	4	4	0.66	Uncharacterized protei
1041	1	db$2	2::C3K0P9|C3K0P9_PSEFS	73	25457	4	4	4	4	1.10	Two-component syster
1042	1	db$2	2::C3K2S2|C3K2S2_PSEFS	73	38886	4	4	4	4	0.63	Riboflavin biosynthesis
1043	1	db$2	2::C3K7W1|C3K7W1_PSEFS	73	12229	2	2	2	2	1.14	Putative lipoprotein OS
1044	1	SwissProt_HUMAN$	3::P07900|HS90A_HUMAN	73	84607	2	2	2	2	0.12	Heat shock protein HS
1045	1	db$2	2::C3K1H2|C3K1H2_PSEFS	73	49205	7	6	7	6	0.78	Signal recognition part
1046	1	SwissProt_HUMAN$	3::Q86YZ3|HORN_HUMAN	73	282228	4	3	3	3	0.05	Hornerin OS=Homo sa
1047	1	db$2	2::C3K605|LPXD_PSEFS	73	36124	2	2	2	2	0.30	UDP-3-O-acylglucosan
1048	1	db$2	2::C3K0I0|C3K0I0_PSEFS	72	27472	4	4	4	4	0.99	RNA polymerase sigma
1049	1	db$2	2::C3K258|RBFA_PSEFS	72	14697	2	2	2	2	0.89	Ribosome-binding fact
1050	1	SwissProt_HUMAN$	3::Q6FI13|H2A2A_HUMAN	72	14087	3	3	3	3	1.70	Histone H2A type 2-A
1051	1	db$2	2::C3K8X1|C3K8X1_PSEFS	72	23435	3	3	3	3	0.83	Putative ABC transport
1052	1	db$2	2::C3K0V4|C3K0V4_PSEFS	72	48572	3	2	3	2	0.21	Flagellin OS=Pseudom
1053	1	db$2	2::C3K7W0|C3K7W0_PSEFS	72	26786	2	2	2	2	0.42	Uncharacterized protei
1054	1	db$2	2::C3K6Z1|C3K6Z1_PSEFS	71	50711	3	3	3	3	0.32	Putative multicopper o
1055	1	SwissProt_HUMAN$	3::P04406|G3P_HUMAN	71	36030	3	2	3	2	0.30	Glyceraldehyde-3-phos
1056	1	db$2	2::C3K427|C3K427_PSEFS	71	23927	3	3	2	2	0.48	Putative glutathione S-
1057	1	db$2	2::C3K6U7|C3K6U7_PSEFS	71	79141	7	6	7	6	0.43	Putative exported prot
1058	1	db$2	2::C3KAM2|C3KAM2_PSEFS	71	49640	2	2	2	2	0.21	Putative two-compone
1059	1	db$2	2::C3K1B0|C3K1B0_PSEFS	71	96490	2	1	2	1	0.05	Lysylphosphatidylglyce
1060	1	db$2	2::C3K1G6|C3K1G6_PSEFS	70	25762	6	5	3	3	1.08	Thiol:disulfide intercha
1061	1	db$2	2::C3KDW1|MUTL_PSEFS	70	69574	4	3	4	3	0.23	DNA mismatch repair p
1062	1	db$2	2::C3K6B0|C3K6B0_PSEFS	70	33534	1	1	1	1	0.15	High-affinity zinc uptal
1063	1	db$2	2::C3K6S4|C3K6S4_PSEFS	70	30497	1	1	1	1	0.17	Putative antibiotic bios
1064	1	db$2	2::C3JY81|C3JY81_PSEFS	70	15084	2	2	2	2	0.86	NADH-quinone oxidore
1065	1	db$2	2::C3JYJ2|C3JYJ2_PSEFS	70	27205	2	2	2	2	0.41	Putative lipoprotein OS

↑Family	M	DB	Accession	Score	Mass	Matches	Match(sig)	Sequences	Seq(sig)	emPAI	Description
1066	1	db$2	2::C3K0M3|C3K0M3_PSEFS	70	20567	4	4	4	4	1.49	Uncharacterized protei
1067	1	db$2	2::C3KDM9|C3KDM9_PSEFS	69	28096	3	3	3	3	0.65	Putative oxidoreductas
1068	1	db$2	2::C3KDK7|C3KDK7_PSEFS	69	24465	3	3	3	3	0.78	Putative ABC transport
1069	1	db$2	2::C3K220|C3K220_PSEFS	69	136725	1	1	1	1	0.04	RecBCD enzyme subur
1070	1	db$2	2::C3K3K7|C3K3K7_PSEFS	69	24490	3	3	3	3	0.78	Pyridoxal phosphate h
1071	1	db$2	2::C3JZ73|C3JZ73_PSEFS	69	49677	3	3	3	3	0.33	Porin OS=Pseudomona
1072	1	db$2	2::C3K0C5|C3K0C5_PSEFS	69	27111	2	2	2	2	0.42	Putative amino transfe
1073	1	db$2	2::C3K4U7|C3K4U7_PSEFS	69	28919	3	3	3	3	0.63	Putative amidotransfer
1074	1	db$2	2::C3KE82|C3KE82_PSEFS	69	53340	4	3	4	3	0.30	Aldehyde dehydrogena
1075	1	db$2	2::C3K586|C3K586_PSEFS	69	21064	5	4	5	4	1.44	ADP compounds hydro
1076	1	db$2	2::C3KC05|C3KC05_PSEFS	69	11915	1	1	1	1	0.48	Conserved hypothetica
1077	1	db$2	2::C3K6M4|C3K6M4_PSEFS	68	13463	4	4	2	2	3.00	Succinate dehydrogena
1078	1	db$2	2::C3K146|C3K146_PSEFS	68	27134	4	4	4	4	1.00	Fumarate and nitrate r
1079	1	db$2	2::C3KAT0|C3KAT0_PSEFS	68	40804	2	2	2	2	0.26	Putative efflux system
1080	1	db$2	2::C3KE20|C3KE20_PSEFS	68	33868	4	3	4	3	0.52	GCV_T domain-contair
1081	1	db$2	2::C3K7Y8|C3K7Y8_PSEFS	68	53697	3	3	2	2	0.19	Putative hydrolase OS
1082	1	db$2	2::C3K9I2|C3K9I2_PSEFS	68	33896	2	2	2	2	0.32	Putative transcriptiona
1083	1	db$2	2::C3K6Z2|C3K6Z2_PSEFS	68	17868	1	1	1	1	0.30	tRNA-specific adenosir
1084	1	db$2	2::C3KE84|C3KE84_PSEFS	68	35425	2	2	2	2	0.31	Pseudouridine synthas
1085	1	db$2	2::C3JXS4|C3JXS4_PSEFS	68	25108	1	1	1	1	0.21	Tol-Pal system protein
1086	1	SwissProt_HUMAN$	3::Q5D862|FILA2_HUMAN	67	247928	1	1	1	1	0.02	Filaggrin-2 OS=Homo
1087	1	db$2	2::C3JZR9|C3JZR9_PSEFS	67	27415	2	1	2	1	0.19	Putative dehydrogenas
1088	1	db$2	2::C3K5L9|C3K5L9_PSEFS	67	31441	3	3	3	3	0.57	Alpha-D-ribose 1-meth
1089	1	db$2	2::C3K6E6|C3K6E6_PSEFS	67	23384	3	3	3	3	0.83	Stringent starvation pr
1090	1	db$2	2::C3K5L2|C3K5L2_PSEFS	67	29854	3	2	3	2	0.37	Phosphonates import /
1091	1	db$2	2::C3K3B7|C3K3B7_PSEFS	67	14934	1	1	1	1	0.37	Uncharacterized protei
1092	1	db$2	2::C3K7E3|C3K7E3_PSEFS	67	178026	4	3	4	3	0.08	Alpha-2-macroglobulin
1093	1	SwissProt_HUMAN$	3::P62258|1433E_HUMAN	67	29155	3	3	3	3	0.62	14-3-3 protein epsilon
1094	1	db$2	2::C3K6A1|C3K6A1_PSEFS	67	22990	1	1	1	1	0.23	Putative copper-bindin
1095	1	db$2	2::C3KBM4|C3KBM4_PSEFS	67	34098	5	4	5	4	0.74	Ornithine carbamoyltra
1096	1	db$2	2::C3K5Y4|C3K5Y4_PSEFS	67	9690	1	1	1	1	0.61	Uncharacterized protei
1097	1	db$2	2::C3JYU3|C3JYU3_PSEFS	67	75598	2	2	2	2	0.13	Histidine kinase OS=P
1098	1	db$2	2::C3K390|C3K390_PSEFS	66	18447	3	3	2	2	0.66	Zn_protease domain-c
1099	1	db$2	2::C3K8L0|C3K8L0_PSEFS	66	16885	2	2	2	2	0.74	Uncharacterized protei
1100	1	db$2	2::C3JY69|HFLD_PSEFS	66	23173	3	3	3	3	0.84	High frequency lysoge
1101	1	SwissProt_HUMAN$	3::P62888|RL30_HUMAN	66	12776	3	3	3	3	1.98	60S ribosomal protein
1102	1	db$2	2::C3KBB9|C3KBB9_PSEFS	66	73354	3	2	3	2	0.14	Putative protease OS=
1103	1	db$2	2::C3JYL7|C3JYL7_PSEFS	66	12417	3	3	3	3	2.07	Putative copper resista
1104	1	db$2	2::C3KB68|C3KB68_PSEFS	66	11363	1	1	1	1	0.51	DUF1883 domain-cont
1105	1	db$2	2::C3K1L7|RLMN_PSEFS	66	42342	4	3	4	3	0.40	Dual-specificity RNA m

↑Family	M	DB	Accession	Score	Mass	Matches	Match(sig)	Sequences	Seq(sig)	emPAI	Description
1106	1	SwissProt_HUMAN$	3::Q07020|RL18_HUMAN	66	21621	1	1	1	1	0.24	60S ribosomal protein
1107	1	db$2	2::C3K0S4|C3K0S4_PSEFS	66	65961	3	3	3	3	0.24	Isocitrate dehydrogena
1108	1	db$2	2::C3K482|RPOZ_PSEFS	65	9821	3	2	2	1	1.57	DNA-directed RNA poly
1109	1	db$2	2::C3KE16|C3KE16_PSEFS	65	37004	1	1	1	1	0.14	Putative chemotaxis-re
1110	1	db$2	2::C3K6A4|C3K6A4_PSEFS	65	36459	3	2	2	2	0.30	Methionine import ATP
1111	1	db$2	2::C3K574|C3K574_PSEFS	65	36116	2	2	2	2	0.30	Putative oxidoreductas
1112	1	db$2	2::C3KDC7|C3KDC7_PSEFS	65	29930	1	1	1	1	0.17	Release factor glutami
1113	1	db$2	2::C3JYN3|C3JYN3_PSEFS	65	52328	2	2	2	2	0.20	Family S13 unassignec
1114	1	db$2	2::C3JZZ4|C3JZZ4_PSEFS	65	23102	2	2	2	2	0.50	Putative glutathione S
1115	1	db$2	2::C3K5J9|C3K5J9_PSEFS	65	96070	3	2	3	2	0.10	Histidine kinase OS=P:
1116	1	db$2	2::C3JXJ5|C3JXJ5_PSEFS	65	22111	4	4	3	3	0.89	Putative SCO1/SenC fa
1117	1	db$2	2::C3K2D4|C3K2D4_PSEFS	65	35439	2	2	2	2	0.31	Putative cobalamin syr
1118	1	SwissProt_HUMAN$	3::P62249|RS16_HUMAN	64	16435	1	1	1	1	0.33	40S ribosomal protein
1119	1	db$2	2::C3JYS6|C3JYS6_PSEFS	64	17060	2	2	2	2	0.73	Putative thioesterase (
1120	1	db$2	2::C3K9P4|C3K9P4_PSEFS	64	59261	6	6	5	5	0.49	Putative ATP-binding c
1121	1	db$2	2::C3K6T3|C3K6T3_PSEFS	64	23166	3	3	2	2	0.50	Thiol:disulfide intercha
1122	1	db$2	2::C3K7X0|C3K7X0_PSEFS	64	85237	2	2	2	2	0.12	5-methyltetrahydropte
1123	1	db$2	2::C3KBY4|C3KBY4_PSEFS	64	31613	2	2	2	2	0.35	Ribosomal RNA small s
1124	1	db$2	2::C3K098|C3K098_PSEFS	64	23000	2	2	2	2	0.51	Putative glutathione S-
1125	1	db$2	2::C3K6P0|C3K6P0_PSEFS	63	15266	2	2	2	2	0.84	Uncharacterized protei
1126	1	db$2	2::C3K0H9|C3K0H9_PSEFS	63	13855	1	1	1	1	0.40	Chemotaxis protein Ch
1127	1	db$2	2::C3K2T3|C3K2T3_PSEFS	63	32553	3	3	3	3	0.55	dTDP-4-dehydrorhamr
1128	1	db$2	2::C3K6W8|C3K6W8_PSEFS	63	16611	3	3	3	3	1.32	3-dehydroquinate deh\
1129	1	SwissProt_HUMAN$	3::P30086|PEBP1_HUMAN	63	21044	2	1	2	1	0.25	Phosphatidylethanolan
1130	1	db$2	2::C3KDX1|RS20_PSEFS	63	10078	2	2	1	1	0.58	30S ribosomal protein
1131	1	db$2	2::C3K9M1|C3K9M1_PSEFS	63	26106	1	1	1	1	0.20	Putative phosphoglyce
1132	1	db$2	2::C3K710|DCD_PSEFS	63	21231	3	3	3	3	0.94	dCTP deaminase OS=F
1133	1	db$2	2::C3K279|C3K279_PSEFS	63	19136	4	3	2	2	1.09	Outer membrane prote
1134	1	db$2	2::C3K5Y3|C3K5Y3_PSEFS	63	35524	4	4	4	4	0.70	Putative leucine dehyd
1135	1	db$2	2::C3K579|C3K579_PSEFS	62	15962	1	1	1	1	0.34	Putative thioesterase (
1136	1	db$2	2::C3K7G1|C3K7G1_PSEFS	62	37563	2	2	2	2	0.29	Cell shape protein Mre
1137	1	db$2	2::C3KDX6|C3KDX6_PSEFS	62	16394	1	1	1	1	0.33	Peptidyl-prolyl cis-tran
1138	1	db$2	2::C3K2U3|C3K2U3_PSEFS	62	27946	3	3	3	3	0.66	Trans-aconitate 2-met
1139	1	db$2	2::C3JYN0|C3JYN0_PSEFS	62	33944	3	3	3	3	0.52	Cbb3-type cytochrome
1139	2	db$2	2::C3JZS9|C3JZS9_PSEFS	57	35591	2	2	2	2	0.30	Cbb3-type cytochrome
1140	1	db$2	2::C3K869|C3K869_PSEFS	62	24016	1	1	1	1	0.22	Putative lipoprotein OS
1141	1	db$2	2::C3K706|PIMT_PSEFS	62	23451	3	2	3	2	0.49	Protein-L-isoaspartate
1142	1	db$2	2::C3K6U5|FETP_PSEFS	62	10399	1	1	1	1	0.56	Probable Fe(2+)-traffic
1143	1	db$2	2::C3JYL6|C3JYL6_PSEFS	62	25076	2	2	2	2	0.46	Putative TetR-family re
1144	1	db$2	2::C3KED3|C3KED3_PSEFS	61	20551	3	3	3	3	0.99	Glycine cleavage syste

↑Family	M	DB	Accession	Score	Mass	Matches	Match(sig)	Sequences	Seq(sig)	emPAI	Description
1145	1	db$2	2::C3K0Z5|C3K0Z5_PSEFS	61	22469	4	4	3	3	0.88	Corrinoid adenosyltran
1146	1	db$2	2::C3JY76|C3JY76_PSEFS	61	48249	3	3	3	3	0.34	Putative mechanosens
1147	1	db$2	2::C3KBK8|C3KBK8_PSEFS	61	35740	3	2	3	2	0.30	Ribosomal RNA small s
1148	1	SwissProt_HUMAN$	3::Q8NHW5|RLA0L_HUMAN	61	34343	1	1	1	1	0.15	60S acidic ribosomal p
1149	1	db$2	2::C3K4W3|C3K4W3_PSEFS	61	21770	3	3	3	3	0.91	PilZ domain-containing
1150	1	db$2	2::C3K5U7|C3K5U7_PSEFS	61	105852	4	3	4	3	0.14	Putative aminotransfer
1151	1	db$2	2::C3KDW0|C3KDW0_PSEFS	61	49939	2	2	2	2	0.21	N-acetylmuramoyl-L-a
1152	1	db$2	2::C3K607|LPXA_PSEFS	60	28002	3	3	3	3	0.66	Acyl-[acyl-carrier-prot
1153	1	db$2	2::C3K819|C3K819_PSEFS	60	35012	2	1	2	1	0.14	Putative ABC transport
1154	1	db$2	2::C3K1A3|C3K1A3_PSEFS	60	26906	2	2	2	2	0.42	Putative pyridoxamine
1155	1	SwissProt_HUMAN$	3::P61247|RS3A_HUMAN	60	29926	2	2	2	2	0.37	40S ribosomal protein
1156	1	db$2	2::C3KE76|C3KE76_PSEFS	60	92563	1	1	1	1	0.05	Alpha-E domain-contai
1157	1	db$2	2::C3K9Y8|C3K9Y8_PSEFS	60	14463	1	1	1	1	0.38	Uncharacterized protei
1158	1	db$2	2::C3K459|C3K459_PSEFS	60	18704	2	2	2	2	0.65	Leucine-responsive reg
1159	1	db$2	2::C3K1K5|EX7L_PSEFS	60	51103	2	2	2	2	0.20	Exodeoxyribonuclease
1160	1	db$2	2::C3KDF9|C3KDF9_PSEFS	60	17633	3	3	3	3	1.22	CinA domain-containin
1161	1	db$2	2::C3K5B3|C3K5B3_PSEFS	60	34937	2	2	2	2	0.31	Dipeptide ABC transpo
1162	1	db$2	2::C3JY27|KDSB_PSEFS	60	27609	4	2	4	2	0.41	3-deoxy-manno-octulc
1163	1	db$2	2::C3K2Q9|EX7S_PSEFS	59	8900	3	1	2	1	0.68	Exodeoxyribonuclease
1164	1	db$2	2::C3K0Y8|COBT_PSEFS	59	36066	1	1	1	1	0.14	Nicotinate-nucleotide--
1165	1	db$2	2::C3KE87|C3KE87_PSEFS	59	46792	3	2	3	2	0.22	NADH dehydrogenase
1166	1	db$2	2::C3K808|C3K808_PSEFS	59	53666	1	1	1	1	0.09	Histidine ammonia-lya
1167	1	db$2	2::C3K2D6|C3K2D6_PSEFS	59	73930	2	2	2	2	0.14	Carbon starvation prot
1168	1	db$2	2::C3K0Z9|C3K0Z9_PSEFS	59	26468	2	2	1	1	0.43	Bac_DnaA domain-con
1169	1	db$2	2::C3K1M4|C3K1M4_PSEFS	59	13705	4	3	3	3	1.78	Iron-sulfur cluster asse
1170	1	db$2	2::C3K5E1|C3K5E1_PSEFS	59	13734	5	5	3	3	1.76	Uncharacterized protei
1171	1	db$2	2::C3K8V8|C3K8V8_PSEFS	59	36166	4	4	4	4	0.69	Zinc-type alcohol dehy
1172	1	db$2	2::C3K7J3|Y1319_PSEFS	58	16953	2	2	2	2	0.73	UPF0225 protein PFLU_
1173	1	db$2	2::C3K663|C3K663_PSEFS	58	22341	2	2	2	2	0.52	Putative TetR-family re
1174	1	db$2	2::C3JZ45|C3JZ45_PSEFS	58	19122	1	1	1	1	0.28	Gluconokinase OS=Pse
1175	1	db$2	2::C3K666|C3K666_PSEFS	58	29461	2	2	2	2	0.38	Putative lipoprotein OS
1176	1	db$2	2::C3KE01|Y992_PSEFS	58	29383	4	4	4	4	0.90	UPF0246 protein PFLU_
1177	1	SwissProt_HUMAN$	3::P15531|NDKA_HUMAN	58	17138	4	4	3	3	1.27	Nucleoside diphosphat
1178	1	db$2	2::C3K8W8|C3K8W8_PSEFS	58	29386	2	2	2	2	0.38	Putative ABC transport
1179	1	db$2	2::C3K088|C3K088_PSEFS	58	20655	3	2	2	2	0.58	Putative isochorismata
1180	1	db$2	2::C3K601|DXR_PSEFS	58	42341	3	3	3	3	0.40	1-deoxy-D-xylulose 5-
1181	1	db$2	2::C3KCV6|C3KCV6_PSEFS	58	40154	5	3	5	3	0.42	Probable alcohol dehyc
1182	1	db$2	2::C3K6V0|C3K6V0_PSEFS	58	23354	2	2	2	2	0.50	Imidazole glycerol pho
1183	1	db$2	2::C3JYQ1|C3JYQ1_PSEFS	57	48078	1	1	1	1	0.10	Putative CoA transfera
1184	1	db$2	2::C3K4K0|C3K4K0_PSEFS	57	42230	2	2	2	2	0.25	Hypothetical exported

↑Family	M	DB	Accession	Score	Mass	Matches	Match(sig)	Sequences	Seq(sig)	emPAI	Description
1185	1	db$2	2::C3K7C7|C3K7C7_PSEFS	57	19943	3	2	3	2	0.60	Chorismate mutase OS
1186	1	db$2	2::C3KDE6|C3KDE6_PSEFS	57	25704	1	1	1	1	0.20	Tw-component system
1187	1	db$2	2::C3K3N0|C3K3N0_PSEFS	57	25405	2	2	2	2	0.45	Putative TetR-family re
1188	1	db$2	2::C3K065|C3K065_PSEFS	57	58554	3	2	3	2	0.18	Uncharacterized protei
1189	1	db$2	2::C3KAH2|C3KAH2_PSEFS	57	24970	3	3	3	3	0.76	SPOR domain-containi
1190	1	db$2	2::C3K1G1|C3K1G1_PSEFS	57	48709	2	2	2	2	0.21	tRNA modification GTP
1191	1	db$2	2::C3K493|C3K493_PSEFS	57	16495	3	3	3	3	1.34	Uncharacterized protei
1192	1	db$2	2::C3KBM3|C3KBM3_PSEFS	57	40194	3	3	3	3	0.42	Putative ABC transport
1193	1	db$2	2::C3KAA9|C3KAA9_PSEFS	57	31757	1	1	1	1	0.16	Putative chemotaxis pı
1194	1	db$2	2::C3K059|TRHO_PSEFS	56	35401	1	1	1	1	0.14	tRNA uridine(34) hydr
1195	1	db$2	2::C3K621|C3K621_PSEFS	56	26068	3	3	3	3	0.72	Putative TetR-family re
1196	1	db$2	2::C3K3W5|C3K3W5_PSEFS	56	39645	5	3	3	3	0.43	Aminomethyltransfera
1197	1	db$2	2::C3JYU4|C3JYU4_PSEFS	56	20911	2	2	2	2	0.57	Corrinoid adenosyltran
1198	1	db$2	2::C3KDV8|C3KDV8_PSEFS	56	29265	2	2	2	2	0.38	ADP-dependent (S)-NA
1199	1	db$2	2::C3JZ82|C3JZ82_PSEFS	56	37007	1	1	1	1	0.14	Putative dehydrogenas
1200	1	db$2	2::C3JZI1|C3JZI1_PSEFS	56	33491	1	1	1	1	0.15	Putative glycine betain
1201	1	db$2	2::C3KE08|C3KE08_PSEFS	55	25044	2	2	2	2	0.46	Conserved hypothetica
1202	1	db$2	2::C3JY36|C3JY36_PSEFS	55	16095	4	3	4	3	1.39	Uncharacterized protei
1203	1	db$2	2::C3K0K9|C3K0K9_PSEFS	55	37421	2	2	1	1	0.13	Putative NADP-depend
1204	1	db$2	2::C3K1M6|C3K1M6_PSEFS	55	24782	2	2	2	2	0.46	Uncharacterized protei
1205	1	db$2	2::C3K838|C3K838_PSEFS	55	9715	3	3	2	2	1.57	Putative phosphocarrie
1206	1	db$2	2::C3KAJ5|C3KAJ5_PSEFS	55	83174	4	3	4	3	0.19	Putative paraquat-indu
1207	1	db$2	2::C3KBL5|C3KBL5_PSEFS	55	24580	2	2	2	2	0.47	Glutamate/aspartate A
1208	1	SwissProt_HUMAN$	3::P62826|RAN_HUMAN	55	24408	1	1	1	1	0.21	GTP-binding nuclear pı
1209	1	db$2	2::C3JY31|C3JY31_PSEFS	55	22860	4	3	4	3	0.85	Putative biopolymer tra
1210	1	db$2	2::C3K0J1|C3K0J1_PSEFS	55	36130	5	4	5	4	0.69	Flagellar motor switch
1211	1	db$2	2::C3KAJ0|C3KAJ0_PSEFS	55	34086	1	1	1	1	0.15	Putative LysR-family re
1212	1	db$2	2::C3K282|SSRP_PSEFS	55	18308	5	5	4	4	1.78	SsrA-binding protein C
1213	1	db$2	2::C3K9H2|C3K9H2_PSEFS	55	39760	2	2	2	2	0.27	Putrescine ABC transpo
1214	1	db$2	2::C3K9M9|C3K9M9_PSEFS	55	14163	1	1	1	1	0.39	Putative endoribonucle
1215	1	db$2	2::C3K0Z1|C3K0Z1_PSEFS	55	36566	1	1	1	1	0.14	L-threonine-O-3-phosp
1216	1	db$2	2::C3K433|CYAY_PSEFS	54	12442	2	2	2	2	1.11	Iron-sulfur cluster asse
1217	1	db$2	2::C3KDQ1|C3KDQ1_PSEFS	54	35181	3	2	3	2	0.31	Putative LysR-family re
1218	1	db$2	2::C3K654|C3K654_PSEFS	54	34290	3	2	2	2	0.32	2-dehydropantoate 2-r
1219	1	db$2	2::C3K4Z1|C3K4Z1_PSEFS	54	72318	2	2	2	2	0.14	Cytochrome C biogene
1220	1	db$2	2::C3K7A7|C3K7A7_PSEFS	54	43115	4	3	4	3	0.39	Conserved hypothetica
1221	1	db$2	2::C3KDN6|C3KDN6_PSEFS	54	32369	1	1	1	1	0.16	Uncharacterized protei
1222	1	db$2	2::C3K660|C3K660_PSEFS	54	28970	1	1	1	1	0.18	Hydroxypyruvate isom
1223	1	db$2	2::C3JY84|C3JY84_PSEFS	54	18006	3	3	3	3	1.18	NADH dehydrogenase
1224	1	db$2	2::C3JY28|Y3771_PSEFS	54	6626	4	3	2	2	2.92	UPF0434 protein PFLU

↑Family	M	DB	Accession	Score	Mass	Matches	Match(sig)	Sequences	Seq(sig)	emPAI	Description
1225	1	db$2	2::C3K2M6|RLMH_PSEFS	53	17669	2	2	2	2	0.70	Ribosomal RNA large s
1226	1	db$2	2::C3JZP0|C3JZP0_PSEFS	53	11251	2	1	2	1	0.51	Putative lipoprotein OS
1227	1	db$2	2::C3JZ30|C3JZ30_PSEFS	53	12225	3	3	3	3	2.13	DUF2025 domain-cont
1228	1	db$2	2::C3K7K5|C3K7K5_PSEFS	53	12541	1	1	1	1	0.45	Uncharacterized protei
1229	1	db$2	2::C3KAB9|C3KAB9_PSEFS	53	40944	1	1	1	1	0.12	Putative acetyl-CoA ac
1230	1	SwissProt_HUMAN$	3::P0DJI8|SAA1_HUMAN	53	13524	1	1	1	1	0.41	Serum amyloid A-1 pr
1231	1	db$2	2::C3K3H4|C3K3H4_PSEFS	53	26074	1	1	1	1	0.20	Ribosomal RNA small s
1232	1	db$2	2::C3KC09|C3KC09_PSEFS	53	83087	2	2	2	2	0.12	Periplasmic beta-gluco
1233	1	db$2	2::C3K6D7|C3K6D7_PSEFS	53	21411	1	1	1	1	0.25	UPF0056 inner membr
1234	1	db$2	2::C3K6U1|C3K6U1_PSEFS	53	17245	2	1	2	1	0.31	Conserved hypothetica
1235	1	db$2	2::C3K1L5|C3K1L5_PSEFS	53	34147	4	3	3	3	0.51	Putative DNA-binding
1236	1	db$2	2::C3JZU8|C3JZU8_PSEFS	53	30426	2	2	2	2	0.36	Hydrolase_4 domain-c
1237	1	db$2	2::C3K1H0|RIMM_PSEFS	52	20004	3	2	3	2	0.60	Ribosome maturation f
1238	1	db$2	2::C3K1V0|C3K1V0_PSEFS	52	56633	1	1	1	1	0.09	Nitric oxide reductase
1239	1	db$2	2::C3K686|C3K686_PSEFS	52	39658	1	1	1	1	0.13	Uncharacterized protei
1240	1	db$2	2::C3KDV5|C3KDV5_PSEFS	52	20461	2	2	2	2	0.58	Oligoribonuclease OS=
1241	1	db$2	2::C3K391|C3K391_PSEFS	52	33913	1	1	1	1	0.15	Putative acyltransferas
1242	1	db$2	2::C3K013|C3K013_PSEFS	52	20254	3	3	2	2	0.59	Peptidyl-prolyl cis-tran
1243	1	db$2	2::C3K9Q9|C3K9Q9_PSEFS	52	19670	1	1	1	1	0.27	FMN_red domain-conta
1244	1	SwissProt_HUMAN$	3::P18085|ARF4_HUMAN	52	20498	1	1	1	1	0.26	ADP-ribosylation factor
1245	1	db$2	2::C3KDR2|C3KDR2_PSEFS	52	26142	2	2	2	2	0.43	S4 RNA-binding domai
1246	1	db$2	2::C3K8W5|C3K8W5_PSEFS	52	21382	1	1	1	1	0.25	Lipopolysaccharide exp
1247	1	SwissProt_HUMAN$	3::P46783|RS10_HUMAN	52	18886	1	1	1	1	0.28	40S ribosomal protein
1248	1	db$2	2::C3KDC8|C3KDC8_PSEFS	52	26520	1	1	1	1	0.19	Molybdopterin biosynt
1249	1	db$2	2::C3KBT7|C3KBT7_PSEFS	52	36676	3	2	3	2	0.29	Sulfate ABC transport
1250	1	db$2	2::C3K998|C3K998_PSEFS	52	35021	4	3	4	3	0.50	Putative dehydrogenas
1251	1	db$2	2::C3K300|C3K300_PSEFS	52	34122	1	1	1	1	0.15	Putative membrane pr
1252	1	db$2	2::C3K602|C3K602_PSEFS	51	48346	3	3	3	3	0.34	Zinc metalloprotease (
1253	1	db$2	2::C3JZ23|C3JZ23_PSEFS	51	131220	2	2	2	2	0.08	Chromosome partition
1254	1	db$2	2::C3K3Y7|C3K3Y7_PSEFS	51	35706	2	1	2	1	0.14	Putative iron-sulfur bir
1255	1	db$2	2::C3K4I1|C3K4I1_PSEFS	51	29333	3	3	3	3	0.62	Shikimate dehydrogen
1256	1	db$2	2::C3K047|C3K047_PSEFS	51	16614	2	2	2	2	0.75	Putative putative heat
1257	1	db$2	2::C3K915|C3K915_PSEFS	51	26912	2	2	2	2	0.42	Putative GntR-family r
1258	1	db$2	2::C3K870|C3K870_PSEFS	51	20966	2	2	2	2	0.56	Uncharacterized protei
1259	1	db$2	2::C3KEB0|C3KEB0_PSEFS	51	36597	2	2	1	1	0.14	Putative amino acid AE
1260	1	db$2	2::C3K150|C3K150_PSEFS	51	25194	2	2	2	2	0.45	Putative short-chain de
1261	1	SwissProt_HUMAN$	3::P62913|RL11_HUMAN	51	20240	1	1	1	1	0.26	60S ribosomal protein
1262	1	db$2	2::C3K6G1|RLMF_PSEFS	51	38015	2	2	2	2	0.28	Ribosomal RNA large s
1263	1	db$2	2::C3KE14|C3KE14_PSEFS	51	83238	3	3	3	3	0.19	Histidine kinase OS=P
1264	1	db$2	2::C3K473|DUT_PSEFS	51	16022	1	1	1	1	0.34	Deoxyuridine 5'-tripho

↑Family	M	DB	Accession	Score	Mass	Matches	Match(sig)	Sequences	Seq(sig)	emPAI	Description
1265	1	db$2	2::C3K4U8|C3K4U8_PSEFS	51	20094	1	1	1	1	0.26	Conserved hypothetica
1266	1	db$2	2::C3K0L0|C3K0L0_PSEFS	51	29492	1	1	1	1	0.17	Putative IclR-family re
1267	1	db$2	2::C3KCB9|C3KCB9_PSEFS	50	35987	3	2	3	2	0.30	Polyphosphate kinase
1268	1	SwissProt_HUMAN$	3::Q9H0U4|RAB1B_HUMAN	50	22157	1	1	1	1	0.24	Ras-related protein Ra
1269	1	db$2	2::C3K2L0|C3K2L0_PSEFS	50	55816	3	3	3	3	0.29	Apolipoprotein N-acylt
1270	1	db$2	2::C3KAF8|C3KAF8_PSEFS	50	74138	1	1	1	1	0.07	Putative lipoprotein OS
1271	1	db$2	2::C3K1C5|C3K1C5_PSEFS	50	44321	3	2	3	2	0.24	Putative cyclopropane-
1272	1	db$2	2::C3K021|C3K021_PSEFS	50	27911	4	3	4	3	0.66	Phosphoadenosine pho
1273	1	SwissProt_HUMAN$	3::Q14240|IF4A2_HUMAN	50	46373	2	2	2	2	0.23	Eukaryotic initiation fa
1274	1	db$2	2::C3K646|C3K646_PSEFS	50	21182	2	2	2	2	0.56	Putative acyl-CoA thio
1275	1	db$2	2::C3K909|C3K909_PSEFS	50	24537	1	1	1	1	0.21	Uncharacterized protei
1276	1	db$2	2::C3K2N1|C3K2N1_PSEFS	50	47217	1	1	1	1	0.11	Putative membrane pr
1277	1	db$2	2::C3JZ24|C3JZ24_PSEFS	50	24729	3	3	3	3	0.77	Putative GntR-family r
1278	1	db$2	2::C3K5G4|RLMM_PSEFS	49	40585	1	1	1	1	0.12	Ribosomal RNA large s
1279	1	db$2	2::C3K8H9|C3K8H9_PSEFS	49	41728	1	1	1	1	0.12	Putative polyamine AB
1280	1	db$2	2::C3KBY5|MRAZ_PSEFS	49	17051	1	1	1	1	0.31	Transcriptional regulat
1281	1	db$2	2::C3KAL0|C3KAL0_PSEFS	49	43962	3	2	3	2	0.24	PlsC domain-containin
1282	1	db$2	2::C3KE46|C3KE46_PSEFS	49	47174	1	1	1	1	0.11	16S rRNA m5C967 me
1283	1	SwissProt_HUMAN$	3::Q6IS14|IF5AL_HUMAN	49	16762	1	1	1	1	0.32	Eukaryotic translation
1284	1	db$2	2::C3K4N5|C3K4N5_PSEFS	49	28830	1	1	1	1	0.18	Putative amino acid AE
1285	1	db$2	2::C3K5L6|C3K5L6_PSEFS	49	16214	4	3	4	3	1.38	Putative phosphorous
1286	1	db$2	2::C3K831|C3K831_PSEFS	49	45359	3	3	3	3	0.37	Putative thiolase OS=F
1287	1	SwissProt_HUMAN$	3::P07195|LDHB_HUMAN	49	36615	3	3	3	3	0.47	L-lactate dehydrogena
1287	2	SwissProt_HUMAN$	3::P00338|LDHA_HUMAN	33	36665	2	2	2	2	0.29	L-lactate dehydrogena
1288	1	db$2	2::C3KD16|C3KD16_PSEFS	49	34294	2	1	2	1	0.15	Putative 2-hydroxyacic
1289	1	db$2	2::C3K125|C3K125_PSEFS	49	26783	3	3	1	1	0.19	Alanine--tRNA ligase C
1290	1	db$2	2::C3K1A2|C3K1A2_PSEFS	49	17206	1	1	1	1	0.31	Putative membrane pr
1291	1	db$2	2::C3K751|C3K751_PSEFS	49	15943	1	1	1	1	0.34	Putative thioesterase (
1292	1	db$2	2::C3KBV6|C3KBV6_PSEFS	48	125347	3	3	3	3	0.12	Putative potassium eff
1292	2	SwissProt_HUMAN$	3::Q9NZM1|MYOF_HUMAN	21	234561	2	2	2	2	0.04	Myoferlin OS=Homo sa
1293	1	SwissProt_HUMAN$	3::P62701|RS4X_HUMAN	48	29579	2	2	2	2	0.38	40S ribosomal protein
1294	1	db$2	2::C3K685|C3K685_PSEFS	48	13635	2	2	2	2	0.98	6-carboxy-5,6,7,8-tetr
1295	1	db$2	2::C3KC07|C3KC07_PSEFS	48	21449	2	2	2	2	0.55	YceI domain-containin
1296	1	db$2	2::C3K8L6|C3K8L6_PSEFS	48	28069	2	2	2	2	0.40	Precorrin-6A synthase
1297	1	SwissProt_HUMAN$	3::P22626|ROA2_HUMAN	48	37407	1	1	1	1	0.13	Heterogeneous nuclea
1298	1	SwissProt_HUMAN$	3::P46781|RS9_HUMAN	48	22578	3	2	3	2	0.52	40S ribosomal protein
1299	1	db$2	2::C3KAX0|C3KAX0_PSEFS	48	84238	2	1	2	1	0.06	1,4-alpha-glucan bran
1300	1	db$2	2::C3KB19|C3KB19_PSEFS	48	44434	3	1	3	1	0.11	HlyD family secretion p
1301	1	db$2	2::C3JY92|C3JY92_PSEFS	48	55851	3	3	3	3	0.29	NADH dehydrogenase
1302	1	db$2	2::C3JXI0|C3JXI0_PSEFS	48	23030	1	1	1	1	0.23	Nudix hydrolase doma

↑Family	M	DB	Accession	Score	Mass	Matches	Match(sig)	Sequences	Seq(sig)	emPAI	Description
1303	1	db$2	2::C3K1Y0|C3K1Y0_PSEFS	47	12222	2	1	2	1	0.46	Uncharacterized protei
1304	1	db$2	2::C3K6E9|C3K6E9_PSEFS	47	41977	3	3	3	3	0.40	Phosphatidylcholine-hy
1305	1	db$2	2::C3K267|RLME_PSEFS	47	24125	2	2	2	2	0.48	Ribosomal RNA large s
1306	1	db$2	2::C3JZ64|C3JZ64_PSEFS	47	17217	2	2	2	2	0.72	MaoC-like domain-cont
1307	1	db$2	2::C3K7C5|C3K7C5_PSEFS	47	40067	2	2	2	2	0.27	Histidine kinase OS=Ps
1308	1	db$2	2::C3K735|MSRB_PSEFS	47	14471	2	2	2	2	0.91	Peptide methionine sul
1309	1	db$2	2::C3KCP9|C3KCP9_PSEFS	47	30268	2	2	2	2	0.37	Lipopolysaccharide cor
1310	1	db$2	2::C3K172|C3K172_PSEFS	47	12407	1	1	1	1	0.45	ABM domain-containin
1311	1	db$2	2::C3K784|C3K784_PSEFS	47	23456	2	2	2	2	0.49	3-oxoadipate CoA-tran
1312	1	db$2	2::C3KDE9|C3KDE9_PSEFS	47	41781	3	3	3	3	0.40	Putative phage tail-rel
1313	1	db$2	2::C3K3B8|C3K3B8_PSEFS	47	42763	1	1	1	1	0.12	Uncharacterized protei
1314	1	db$2	2::C3K224|C3K224_PSEFS	47	30473	1	1	1	1	0.17	CDP-diacylglycerol--se
1315	1	db$2	2::C3K9T0|NADK_PSEFS	47	32289	5	3	5	3	0.55	NAD kinase OS=Pseud
1316	1	db$2	2::C3K6C3|C3K6C3_PSEFS	47	28259	3	1	3	1	0.18	Putative amino acid AE
1317	1	db$2	2::C3KDS6|UNG_PSEFS	47	25820	2	2	2	2	0.44	Uracil-DNA glycosylase
1318	1	SwissProt_HUMAN$	3::P05413|FABPH_HUMAN	47	14849	2	2	2	2	0.87	Fatty acid-binding prot
1319	1	db$2	2::C3K0T5|C3K0T5_PSEFS	47	37877	2	2	2	2	0.28	Zinc transport protein
1320	1	db$2	2::C3K4M3|C3K4M3_PSEFS	47	101390	3	3	3	3	0.15	Phosphocarrier protein
1321	1	db$2	2::C3KCI1|C3KCI1_PSEFS	47	42811	1	1	1	1	0.12	Putative acyl-CoA dehy
1322	1	db$2	2::C3K9N1|C3K9N1_PSEFS	47	43216	4	3	3	3	0.39	D-ser_dehydrat domai
1323	1	db$2	2::C3KDP0|C3KDP0_PSEFS	47	27498	3	3	2	2	0.41	Uncharacterized protei
1324	1	db$2	2::C3JZP9|C3JZP9_PSEFS	46	33380	2	2	2	2	0.33	Putative ribose transpo
1325	1	db$2	2::C3K4W2|C3K4W2_PSEFS	46	26844	2	2	2	2	0.42	Putative glycerophosph
1326	1	db$2	2::C3K5E3|C3K5E3_PSEFS	46	43909	2	1	2	1	0.11	Putative aminotransfer
1327	1	db$2	2::C3K1B4|C3K1B4_PSEFS	46	43355	4	2	4	2	0.24	Putative transferase O
1328	1	db$2	2::C3K1F1|ATPL_PSEFS	46	8603	4	4	2	2	7.37	ATP synthase subunit c
1329	1	db$2	2::C3KAW8|C3KAW8_PSEFS	46	75149	1	1	1	1	0.07	Alpha-1,4-glucan:malt
1330	1	db$2	2::C3K353|C3K353_PSEFS	46	44910	1	1	1	1	0.11	Putative aminotransfer
1331	1	db$2	2::C3K975|C3K975_PSEFS	46	21948	1	1	1	1	0.24	Putative glutathione S-
1332	1	db$2	2::C3K6Y0|C3K6Y0_PSEFS	46	13973	2	1	2	1	0.40	4-carboxymuconolacto
1333	1	db$2	2::C3JYG6|C3JYG6_PSEFS	46	44622	2	2	2	2	0.24	Peptidase_M14 domair
1334	1	db$2	2::C3K0M7|C3K0M7_PSEFS	46	28601	2	2	2	2	0.39	Putative deoxyribonucl
1335	1	SwissProt_HUMAN$	3::P63244|RACK1_HUMAN	46	35055	3	3	3	3	0.50	Receptor of activated p
1336	1	db$2	2::C3K4M4|C3K4M4_PSEFS	46	31652	1	1	1	1	0.16	Phosphofructokinase C
1337	1	db$2	2::C3KB11|C3KB11_PSEFS	46	38044	2	1	2	1	0.13	Putative AraC family re
1338	1	db$2	2::C3K3W4|C3K3W4_PSEFS	45	13233	4	4	2	2	1.02	Glycine cleavage syste
1339	1	db$2	2::C3KBX4|C3KBX4_PSEFS	45	59754	2	2	2	2	0.17	Putative membrane pr
1340	1	db$2	2::C3K456|C3K456_PSEFS	45	38683	1	1	1	1	0.13	Alanine racemase OS=
1341	1	db$2	2::C3K0J8|C3K0J8_PSEFS	45	22091	2	2	2	2	0.53	START domain-contain
1342	1	db$2	2::C3KAY4|C3KAY4_PSEFS	45	29479	3	3	3	3	0.61	Putative enoyl-CoA hy

↑Family	M	DB	Accession	Score	Mass	Matches	Match(sig)	Sequences	Seq(sig)	emPAI	Description
1343	1	db$2	2::C3K5T2|C3K5T2_PSEFS	45	65173	2	2	2	2	0.16	Putative dehydrogenas
1344	1	db$2	2::C3JXL2|C3JXL2_PSEFS	45	30967	1	1	1	1	0.16	Uncharacterized protei
1345	1	db$2	2::C3K5X1|C3K5X1_PSEFS	45	46611	1	1	1	1	0.11	Histidine kinase OS=P:
1346	1	db$2	2::C3K5B5|C3K5B5_PSEFS	45	37023	2	2	2	2	0.29	Dipeptide ABC transpo
1347	1	db$2	2::C3K911|C3K911_PSEFS	45	29460	1	1	1	1	0.17	Putative ABC transport
1348	1	db$2	2::C3JZP5|C3JZP5_PSEFS	45	30747	1	1	1	1	0.17	Ribokinase OS=Pseudo
1349	1	SwissProt_HUMAN$	3::P62857|RS28_HUMAN	45	7836	1	1	1	1	0.79	40S ribosomal protein
1350	1	db$2	2::C3KEC9|C3KEC9_PSEFS	44	53005	3	3	3	3	0.31	Putative beta-barrel as
1351	1	db$2	2::C3K4C4|UBIA_PSEFS	44	33068	1	1	1	1	0.15	4-hydroxybenzoate oc
1352	1	db$2	2::C3KBN5|C3KBN5_PSEFS	44	24125	1	1	1	1	0.22	Putative TetR-family re
1353	1	db$2	2::C3K7V7|C3K7V7_PSEFS	44	18111	1	1	1	1	0.30	Uncharacterized protei
1354	1	SwissProt_HUMAN$	3::Q8NG92|O13H1_HUMAN	44	34547	1	1	1	1	0.15	Olfactory receptor 13H
1355	1	db$2	2::C3K6H7|C3K6H7_PSEFS	44	10540	1	1	1	1	0.55	Cell division protein Ft
1356	1	db$2	2::C3KAU7|C3KAU7_PSEFS	44	33821	1	1	1	1	0.15	Fructokinase OS=Pseu
1357	1	db$2	2::C3KEA2|C3KEA2_PSEFS	44	30824	2	2	2	2	0.36	Putative inner membra
1358	1	SwissProt_HUMAN$	3::A6NJ78|MET15_HUMAN	44	46092	1	1	1	1	0.11	12S rRNA N4-methylcy
1359	1	SwissProt_HUMAN$	3::Q96FN5|KIF12_HUMAN	44	70615	3	3	1	1	0.07	Kinesin-like protein KI
1360	1	db$2	2::C3KCK0|C3KCK0_PSEFS	44	26687	6	4	4	4	1.03	Putative short-chain de
1361	1	db$2	2::C3K3R7|RPIA_PSEFS	44	23281	2	2	2	2	0.50	Ribose-5-phosphate is
1362	1	db$2	2::C3JYE6|C3JYE6_PSEFS	44	45437	4	2	3	2	0.23	Putative acyl-CoA dehy
1363	1	db$2	2::C3K598|C3K598_PSEFS	44	34258	3	3	3	3	0.51	Curved DNA-binding p
1364	1	db$2	2::C3K0X3|C3K0X3_PSEFS	44	16227	1	1	1	1	0.33	Transcriptional regulat
1365	1	db$2	2::Q9ZEP8|PANB_PSEFS	43	27951	1	1	1	1	0.18	3-methyl-2-oxobutano
1366	1	db$2	2::C3KBM2|C3KBM2_PSEFS	43	16548	3	3	3	3	1.34	Cys-tRNA(Pro)/Cys-tR
1367	1	db$2	2::C3JXM0|C3JXM0_PSEFS	43	45141	2	2	2	2	0.23	Putative peptidase OS=
1368	1	db$2	2::C3K3S9|C3K3S9_PSEFS	43	35576	1	1	1	1	0.14	Hydrolase_4 domain-c
1369	1	SwissProt_HUMAN$	3::Q02543|RL18A_HUMAN	43	20749	1	1	1	1	0.25	60S ribosomal protein
1370	1	SwissProt_HUMAN$	3::P62277|RS13_HUMAN	43	17212	1	1	1	1	0.31	40S ribosomal protein
1371	1	db$2	2::C3K4W6|C3K4W6_PSEFS	43	44511	3	2	3	2	0.24	Putative lipoprotein rel
1372	1	db$2	2::C3K9B4|C3K9B4_PSEFS	43	16186	2	2	2	2	0.78	Type II secretion syste
1373	1	SwissProt_HUMAN$	3::P14618|KPYM_HUMAN	43	57900	1	1	1	1	0.09	Pyruvate kinase PKM C
1374	1	db$2	2::C3K5I0|C3K5I0_PSEFS	43	36784	1	1	1	1	0.14	tRNA-dihydrouridine(2
1375	1	db$2	2::C3K8Z4|C3K8Z4_PSEFS	43	45613	2	2	2	2	0.23	High-affinity branched
1376	1	db$2	2::C3K2L4|C3K2L4_PSEFS	43	37419	1	1	1	1	0.13	DNA polymerase III su
1377	1	db$2	2::C3K704|TRUD_PSEFS	43	38571	1	1	1	1	0.13	tRNA pseudouridine sy
1378	1	db$2	2::C3K5T7|C3K5T7_PSEFS	43	23769	2	2	2	2	0.49	Uncharacterized protei
1379	1	db$2	2::C3K3T5|C3K3T5_PSEFS	43	34240	1	1	1	1	0.15	Putrescine ABC transp
1380	1	db$2	2::C3KAW3|C3KAW3_PSEFS	43	52085	1	1	1	1	0.10	Putative GntR-family t
1381	1	db$2	2::C3K7K0|C3K7K0_PSEFS	42	41297	2	2	1	1	0.12	Esterase III OS=Pseuc
1382	1	db$2	2::C3K3P8|C3K3P8_PSEFS	42	49421	2	2	1	1	0.10	G domain-containing p

↑Family	M	DB	Accession	Score	Mass	Matches	Match(sig)	Sequences	Seq(sig)	emPAI	Description
1383	1	db$2	2::C3KAX2|C3KAX2_PSEFS	42	79149	2	2	2	2	0.13	Histidine kinase OS=Ps
1384	1	db$2	2::C3K4S7|C3K4S7_PSEFS	42	34300	2	2	1	1	0.15	Putative LysR-family re
1385	1	SwissProt_HUMAN$	3::P23396|RS3_HUMAN	42	26671	3	2	3	2	0.42	40S ribosomal protein
1386	1	db$2	2::C3KBI8|C3KBI8_PSEFS	42	48805	3	2	3	2	0.21	Uncharacterized protei
1387	1	db$2	2::C3K1F5|C3K1F5_PSEFS	42	29048	3	2	3	2	0.38	Putative chromosome
1388	1	db$2	2::C3KB06|C3KB06_PSEFS	42	29724	1	1	1	1	0.17	Putative lipoprotein OS
1389	1	db$2	2::C3K6T8|C3K6T8_PSEFS	42	49583	5	3	5	3	0.33	Alginate biosynthesis t
1390	1	db$2	2::C3K0I8|C3K0I8_PSEFS	42	26895	1	1	1	1	0.19	Flagellar biosynthetic p
1391	1	db$2	2::C3K9E9|C3K9E9_PSEFS	42	21363	2	2	1	1	0.25	Putative TetR-family re
1392	1	db$2	2::C3KCJ9|C3KCJ9_PSEFS	42	40399	1	1	1	1	0.12	Putative phosphotrans
1393	1	db$2	2::C3K4V3|C3K4V3_PSEFS	42	45550	2	1	2	1	0.11	Uncharacterized protei
1394	1	db$2	2::C3KE75|C3KE75_PSEFS	42	123658	2	2	2	2	0.08	TGc domain-containing
1395	1	db$2	2::C3KC04|C3KC04_PSEFS	42	39513	3	2	3	2	0.27	Putative hydrolase OS
1396	1	db$2	2::C3K8U1|C3K8U1_PSEFS	42	15020	2	2	2	2	0.86	Phosphoribosyl-AMP cy
1397	1	db$2	2::C3K9Q1|C3K9Q1_PSEFS	42	28823	1	1	1	1	0.18	Methyltransf_11 doma
1398	1	db$2	2::C3JZ63|C3JZ63_PSEFS	42	34729	1	1	1	1	0.15	Hydrolase_4 domain-c
1399	1	db$2	2::C3K7Z3|C3K7Z3_PSEFS	42	43142	3	2	3	2	0.24	Putative acyl-CoA dehy
1400	1	db$2	2::C3K863|PDXH_PSEFS	42	24884	4	3	3	3	0.76	Pyridoxine/pyridoxami
1401	1	db$2	2::C3JY37|C3JY37_PSEFS	42	14829	2	2	2	2	0.87	Putative lipoprotein OS
1402	1	db$2	2::C3K2L9|LIPB_PSEFS	42	23473	1	1	1	1	0.22	Octanoyltransferase O
1403	1	db$2	2::C3K5H4|C3K5H4_PSEFS	42	21385	1	1	1	1	0.25	Uncharacterized protei
1404	1	db$2	2::C3KDG5|C3KDG5_PSEFS	41	23599	3	3	3	3	0.82	Putative regulatory pro
1405	1	db$2	2::C3KAG2|C3KAG2_PSEFS	41	280833	2	1	2	1	0.02	Toxin CdiA OS=Pseudo
1406	1	db$2	2::C3K0P2|C3K0P2_PSEFS	41	35618	2	1	2	1	0.14	Pseudouridine synthas
1407	1	db$2	2::C3K3K9|C3K3K9_PSEFS	41	21575	2	2	2	2	0.55	Conserved hypothetica
1408	1	db$2	2::C3K3T7|C3K3T7_PSEFS	41	39982	1	1	1	1	0.13	Putrescine ABC transp
1409	1	db$2	2::C3JY52|C3JY52_PSEFS	41	37777	1	1	1	1	0.13	GST C-terminal domai
1410	1	db$2	2::C3K6Z6|C3K6Z6_PSEFS	41	13580	1	1	1	1	0.41	Putaitve lipoprotein OS
1411	1	db$2	2::C3K6K2|IHFB_PSEFS	41	11021	4	3	3	2	1.32	Integration host factor
1412	1	db$2	2::C3K243|C3K243_PSEFS	41	108098	1	1	1	1	0.04	Histidine kinase OS=Ps
1413	1	db$2	2::C3KE32|C3KE32_PSEFS	41	20912	1	1	1	1	0.25	Sigma factor negative
1414	1	db$2	2::C3K600|C3K600_PSEFS	41	28761	2	2	2	2	0.39	Phosphatidate cytidyly
1415	1	db$2	2::C3K8T8|C3K8T8_PSEFS	41	14354	1	1	1	1	0.38	Sec-independent prote
1416	1	db$2	2::C3K4R0|C3K4R0_PSEFS	41	14930	1	1	1	1	0.37	Putative lipoprotein OS
1417	1	db$2	2::C3K979|C3K979_PSEFS	41	30391	2	2	2	2	0.36	Putative glutathione S-
1418	1	db$2	2::C3JXW6|C3JXW6_PSEFS	41	64770	1	1	1	1	0.08	Putative exported prot
1419	1	db$2	2::C3K832|C3K832_PSEFS	40	33163	1	1	1	1	0.15	Non-specific serine/thr
1420	1	SwissProt_HUMAN$	3::P12273|PIP_HUMAN	40	16562	2	2	2	2	0.76	Prolactin-inducible pro
1421	1	db$2	2::C3K0N9|C3K0N9_PSEFS	40	21007	3	3	3	3	0.96	7-methyl-GTP pyropho
1422	1	db$2	2::C3KCN6|C3KCN6_PSEFS	40	53164	3	3	3	3	0.31	Sodium/proline sympo

↑Family	M	DB	Accession	Score	Mass	Matches	Match(sig)	Sequences	Seq(sig)	emPAI	Description
1423	1	db$2	2::C3K5C9|C3K5C9_PSEFS	40	27544	1	1	1	1	0.19	Putative regulatory prc
1424	1	db$2	2::C3KBL4|C3KBL4_PSEFS	40	26993	4	2	3	2	0.42	Glutamate/aspartate A
1425	1	db$2	2::C3K071|Y4323_PSEFS	40	8578	1	1	1	1	0.71	UPF0270 protein PFLU_
1426	1	db$2	2::C3K3U4|C3K3U4_PSEFS	40	23278	1	1	1	1	0.22	Putative TetR-family re
1427	1	db$2	2::C3KE17|C3KE17_PSEFS	40	24430	1	1	1	1	0.21	Two component syster
1428	1	db$2	2::C3K6R2|C3K6R2_PSEFS	40	29309	1	1	1	1	0.17	Putative shikimate deh
1429	1	db$2	2::C3KBP5|C3KBP5_PSEFS	40	30770	1	1	1	1	0.17	Putative AraC-family re
1430	1	db$2	2::C3K5F3|C3K5F3_PSEFS	40	34108	1	1	1	1	0.15	Putative dehydrogenas
1431	1	db$2	2::C3K0D5|MINE_PSEFS	40	9531	3	2	3	2	1.63	Cell division topologica
1432	1	db$2	2::C3K0T6|RRAAH_PSEFS	40	17479	1	1	1	1	0.31	Putative 4-hydroxy-4-r
1433	1	db$2	2::C3KB05|C3KB05_PSEFS	40	50891	2	2	2	2	0.20	CDP-diacylglycerol-ser
1434	1	db$2	2::C3K109|C3K109_PSEFS	40	49568	1	1	1	1	0.10	23S rRNA (uracil(1939
1435	1	db$2	2::C3K9J9|C3K9J9_PSEFS	40	17304	1	1	1	1	0.31	Uncharacterized protei
1436	1	db$2	2::C3KBE8|C3KBE8_PSEFS	40	8134	2	2	2	2	2.10	Putative lipoprotein OS
1437	1	db$2	2::C3KA82|C3KA82_PSEFS	40	19457	2	2	2	2	0.62	Lactoylglutathione lyas
1438	1	db$2	2::C3K0B2|C3K0B2_PSEFS	39	20071	2	2	1	1	0.26	Putative membrane pr
1439	1	db$2	2::C3K4T8|C3K4T8_PSEFS	39	33681	1	1	1	1	0.15	Putative AraC-family re
1440	1	db$2	2::C3K0N6|PLSX_PSEFS	39	35209	3	2	2	2	0.31	Phosphate acyltransfer
1441	1	db$2	2::C3KE47|FMT_PSEFS	39	33416	2	1	2	1	0.15	Methionyl-tRNA formyl
1442	1	db$2	2::C3K453|C3K453_PSEFS	39	71119	2	2	2	2	0.14	AcetylCoA_hyd_C dom
1443	1	SwissProt_HUMAN$	3::P19338|NUCL_HUMAN	39	76568	1	1	1	1	0.06	Nucleolin OS=Homo sa
1444	1	db$2	2::C3K4Y9|C3K4Y9_PSEFS	39	6637	2	2	1	1	0.98	Heme exporter protein
1445	1	db$2	2::C3KDB9|C3KDB9_PSEFS	39	32977	2	2	2	2	0.33	Putative lipopolysacchа
1446	1	SwissProt_HUMAN$	3::P10809|CH60_HUMAN	39	61016	2	2	2	2	0.17	60 kDa heat shock pro
1447	1	db$2	2::C3JZN5|RL35_PSEFS	39	7363	1	1	1	1	0.87	50S ribosomal protein
1448	1	db$2	2::C3K5X9|C3K5X9_PSEFS	39	41639	2	2	2	2	0.26	Putative acyl-CoA deh
1449	1	db$2	2::C3K3P7|C3K3P7_PSEFS	39	49977	2	2	2	2	0.21	Putative membrane pr
1450	1	db$2	2::C3KAZ7|C3KAZ7_PSEFS	39	48577	1	1	1	1	0.10	Putative chain length c
1451	1	db$2	2::C3K5B6|C3K5B6_PSEFS	39	58846	4	4	4	4	0.38	Dipeptide ABC transpo
1452	1	db$2	2::C3K107|C3K107_PSEFS	39	31415	2	2	2	2	0.35	Uncharacterized protei
1453	1	db$2	2::C3K7L9|C3K7L9_PSEFS	39	28704	1	1	1	1	0.18	Putative membrane pr
1454	1	db$2	2::C3K333|C3K333_PSEFS	39	61021	2	2	2	2	0.17	Uncharacterized protei
1455	1	db$2	2::C3K6Y7|RNC_PSEFS	38	25367	2	2	2	2	0.45	Ribonuclease 3 OS=Ps
1456	1	db$2	2::C3KAJ7|C3KAJ7_PSEFS	38	44130	1	1	1	1	0.11	Uracil permease OS=P
1457	1	db$2	2::C3K092|C3K092_PSEFS	38	14085	1	1	1	1	0.39	Glutaredoxin domain-c
1458	1	SwissProt_HUMAN$	3::P32969|RL9_HUMAN	38	21850	1	1	1	1	0.24	60S ribosomal protein
1459	1	db$2	2::C3K1C9|C3K1C9_PSEFS	38	65670	2	2	2	2	0.16	Putative cation-transpo
1460	1	db$2	2::C3K5C4|C3K5C4_PSEFS	38	17510	2	2	2	2	0.71	SnoaL-like domain-cor
1461	1	db$2	2::C3JXY5|C3JXY5_PSEFS	38	10610	2	2	1	1	0.54	Uncharacterized protei
1462	1	db$2	2::C3JZQ4|C3JZQ4_PSEFS	38	8106	3	3	3	3	4.47	Translational regulator

↑Family	M	DB	Accession	Score	Mass	Matches	Match(sig)	Sequences	Seq(sig)	emPAI	Description
1463	1	db$2	2::C3K4C8|C3K4C8_PSEFS	38	49694	2	2	2	2	0.21	Putative transport-rela
1464	1	db$2	2::C3K2U2|C3K2U2_PSEFS	38	20033	2	2	2	2	0.60	Putative hydrolase OS
1465	1	db$2	2::C3K3V1|C3K3V1_PSEFS	38	23989	1	1	1	1	0.22	Alkyl hydroperoxide re
1466	1	SwissProt_HUMAN$	3::P62753|RS6_HUMAN	38	28663	2	2	2	2	0.39	40S ribosomal protein
1467	1	db$2	2::C3K7L3|C3K7L3_PSEFS	38	8069	1	1	1	1	0.76	Putative lipoprotein OS
1468	1	db$2	2::C3JZ52|C3JZ52_PSEFS	38	18715	1	1	1	1	0.28	Uncharacterized protei
1469	1	db$2	2::C3K587|C3K587_PSEFS	38	29705	1	1	1	1	0.17	3'(2'),5'-bisphosphate
1470	1	db$2	2::C3K3Q7|C3K3Q7_PSEFS	37	16308	1	1	1	1	0.33	Putative MerR-family r
1471	1	db$2	2::C3K7M8|C3K7M8_PSEFS	37	23309	3	2	3	2	0.50	Putative glutathione S-
1472	1	db$2	2::C3K327|C3K327_PSEFS	37	29832	1	1	1	1	0.17	Ribosomal RNA small s
1473	1	db$2	2::C3KDI4|C3KDI4_PSEFS	37	42151	1	1	1	1	0.12	Putative exported prot
1474	1	db$2	2::C3JY97|C3JY97_PSEFS	37	30357	1	1	1	1	0.17	N-hydroxyarylamine O
1475	1	db$2	2::C3K830|C3K830_PSEFS	37	47217	5	5	5	5	0.65	Putative short chain de
1476	1	SwissProt_HUMAN$	3::P26641|EF1G_HUMAN	37	50087	2	2	2	2	0.21	Elongation factor 1-ga
1477	1	db$2	2::C3K9N4|C3K9N4_PSEFS	37	91939	3	1	2	1	0.05	Histidine kinase OS=P
1478	1	db$2	2::C3K6J0|C3K6J0_PSEFS	37	24596	1	1	1	1	0.21	Putative phosphoglyco
1479	1	db$2	2::C3KBX7|C3KBX7_PSEFS	37	23636	1	1	1	1	0.22	Putative metallo-beta-
1480	1	db$2	2::C3KAM0|C3KAM0_PSEFS	37	50872	2	2	2	2	0.20	Putative two-compone
1481	1	db$2	2::C3K749|C3K749_PSEFS	37	16397	2	2	1	1	0.33	Putative phosphohistid
1482	1	db$2	2::C3K2F6|TRMA_PSEFS	36	40867	2	2	2	2	0.26	tRNA/tmRNA (uracil-C
1483	1	db$2	2::C3K8K1|C3K8K1_PSEFS	36	40207	1	1	1	1	0.12	Phosphate-binding pro
1484	1	SwissProt_HUMAN$	3::Q8WYA0|IFT81_HUMAN	36	79696	1	1	1	1	0.06	Intraflagellar transport
1485	1	db$2	2::C3K241|C3K241_PSEFS	36	33217	1	1	1	1	0.15	Glutamyl-Q tRNA(Asp)
1486	1	db$2	2::C3KDB5|C3KDB5_PSEFS	36	33189	1	1	1	1	0.15	Putative glycosyl trans
1487	1	SwissProt_HUMAN$	3::P46776|RL27A_HUMAN	36	16551	1	1	1	1	0.33	60S ribosomal protein
1488	1	db$2	2::C3K5B7|C3K5B7_PSEFS	36	50883	2	2	2	2	0.20	Putative outer membra
1489	1	SwissProt_HUMAN$	3::P62136|PP1A_HUMAN	36	37488	1	1	1	1	0.13	Serine/threonine-prote
1490	1	SwissProt_HUMAN$	3::Q7Z6Z7|HUWE1_HUMAN	36	481589	4	2	3	1	0.01	E3 ubiquitin-protein lig
1491	1	db$2	2::C3K0F5|C3K0F5_PSEFS	36	23098	1	1	1	1	0.23	Putative TetR-family tr
1492	1	SwissProt_HUMAN$	3::P46779|RL28_HUMAN	36	15738	1	1	1	1	0.35	60S ribosomal protein
1493	1	db$2	2::C3K6G9|LPXB_PSEFS	36	41468	4	3	3	3	0.41	Lipid-A-disaccharide sy
1494	1	db$2	2::C3K0X8|C3K0X8_PSEFS	36	55461	2	2	2	2	0.19	DNA recombination pr
1495	1	db$2	2::C3K2Q3|C3K2Q3_PSEFS	36	23107	1	1	1	1	0.23	Putative hydrolase OS
1496	1	SwissProt_HUMAN$	3::P61254|RL26_HUMAN	36	17248	2	2	2	2	0.72	60S ribosomal protein
1497	1	db$2	2::C3KBV8|C3KBV8_PSEFS	36	32477	1	1	1	1	0.16	Cobalt-zinc-cadmium r
1498	1	db$2	2::C3KDS1|C3KDS1_PSEFS	36	26155	2	1	1	1	0.20	Pseudouridine synthas
1499	1	SwissProt_HUMAN$	3::Q2PPJ7|RGPA2_HUMAN	36	210636	1	1	1	1	0.02	Ral GTPase-activating
1500	1	db$2	2::C3K0H8|C3K0H8_PSEFS	35	29525	2	2	2	2	0.38	Protein phosphatase C
1501	1	db$2	2::C3K1H4|C3K1H4_PSEFS	35	45615	1	1	1	1	0.11	Putative membrane pr
1502	1	db$2	2::C3K753|C3K753_PSEFS	35	30652	2	2	2	2	0.36	Putative hydrolase OS

↑Family	M	DB	Accession	Score	Mass	Matches	Match(sig)	Sequences	Seq(sig)	emPAI	Description
1503	1	db$2	2::C3K6R7|C3K6R7_PSEFS	35	41832	1	1	1	1	0.12	Serine/threonine trans
1504	1	SwissProt_HUMAN$	3::Q9BVV6|TALD3_HUMAN	35	169201	1	1	1	1	0.03	Protein TALPID3 OS=H
1505	1	SwissProt_HUMAN$	3::P08865|RSSA_HUMAN	35	32833	1	1	1	1	0.15	40S ribosomal protein
1506	1	db$2	2::C3K537|C3K537_PSEFS	35	39684	1	1	1	1	0.13	Putative membrane pr
1507	1	db$2	2::C3K9Y7|C3K9Y7_PSEFS	35	30413	1	1	1	1	0.17	Undecaprenyl-diphospl
1508	1	SwissProt_HUMAN$	3::P23284|PPIB_HUMAN	35	23728	1	1	1	1	0.22	Peptidyl-prolyl cis-tran
1509	1	db$2	2::C3K3K0|YQGF_PSEFS	35	16158	2	2	2	2	0.78	Putative pre-16S rRNA
1510	1	db$2	2::C3K158|C3K158_PSEFS	35	36421	3	3	3	3	0.48	Putative regulatory prc
1511	1	db$2	2::C3K1M2|HSCB_PSEFS	35	20288	1	1	1	1	0.26	Co-chaperone protein
1512	1	SwissProt_HUMAN$	3::P61604|CH10_HUMAN	35	10925	4	2	4	2	1.34	10 kDa heat shock pro
1513	1	db$2	2::C3KDJ8|C3KDJ8_PSEFS	35	23307	4	3	4	3	0.83	Methyltransf_25 doma
1514	1	db$2	2::C3K0D1|C3K0D1_PSEFS	35	40054	1	1	1	1	0.13	PNPLA domain-contain
1515	1	db$2	2::C3K1E3|C3K1E3_PSEFS	35	28123	1	1	1	1	0.18	Glucitol operon repres
1516	1	SwissProt_HUMAN$	3::P61353|RL27_HUMAN	35	15788	2	1	2	1	0.35	60S ribosomal protein
1517	1	db$2	2::C3K477|C3K477_PSEFS	35	29836	5	2	3	2	0.37	Exodeoxyribonuclease
1518	1	db$2	2::C3KBM1|C3KBM1_PSEFS	35	29989	1	1	1	1	0.17	Glycerol uptake facilita
1519	1	db$2	2::C3K326|C3K326_PSEFS	35	35169	1	1	1	1	0.14	4-hydroxythreonine-4-
1520	1	SwissProt_HUMAN$	3::O94817|ATG12_HUMAN	35	15104	1	1	1	1	0.36	Ubiquitin-like protein A
1521	1	db$2	2::C3KCD6|C3KCD6_PSEFS	35	27732	2	2	2	2	0.40	Short chain dehydroge
1522	1	db$2	2::C3K3D8|C3K3D8_PSEFS	35	49942	3	3	3	3	0.33	ATP-independent RNA
1523	1	db$2	2::C3KCV5|C3KCV5_PSEFS	34	37705	2	2	2	2	0.28	Uncharacterized protei
1524	1	db$2	2::C3KDY6|C3KDY6_PSEFS	34	17128	1	1	1	1	0.31	Uncharacterized protei
1525	1	db$2	2::C3K127|C3K127_PSEFS	34	41295	3	3	3	3	0.41	Putative membrane pr
1526	1	db$2	2::C3K0P5|C3K0P5_PSEFS	34	35572	2	2	2	2	0.30	Uncharacterized protei
1527	1	db$2	2::C3K219|C3K219_PSEFS	34	76943	2	1	1	1	0.06	RecBCD enzyme subur
1528	1	SwissProt_HUMAN$	3::Q96E52|OMA1_HUMAN	34	60081	1	1	1	1	0.08	Metalloendopeptidase
1529	1	db$2	2::C3K406|C3K406_PSEFS	34	23424	3	3	3	3	0.83	Glyoxalase OS=Pseudo
1530	1	db$2	2::C3K2M3|C3K2M3_PSEFS	34	37518	3	2	3	2	0.29	Putative peptidoglycan
1531	1	db$2	2::C3JY43|TTCA_PSEFS	34	31052	2	2	2	2	0.35	tRNA-cytidine(32) 2-su
1532	1	SwissProt_HUMAN$	3::P63220|RS21_HUMAN	34	9106	1	1	1	1	0.66	40S ribosomal protein
1533	1	SwissProt_HUMAN$	3::Q6DHV5|C2D2B_HUMAN	34	166681	1	1	1	1	0.03	Protein CC2D2B OS=H
1534	1	db$2	2::C3K2Y9|C3K2Y9_PSEFS	34	13142	1	1	1	1	0.43	Protein translocase sul
1535	1	db$2	2::C3JXX9|C3JXX9_PSEFS	34	6925	3	2	3	2	2.76	Translational regulator
1536	1	db$2	2::C3K707|C3K707_PSEFS	34	29295	2	2	2	2	0.38	Putative lipoprotein OS
1537	1	db$2	2::C3K0D2|C3K0D2_PSEFS	34	35834	3	3	3	3	0.48	Lipid A biosynthesis lau
1538	1	db$2	2::C3K1Q1|C3K1Q1_PSEFS	34	59090	1	1	1	1	0.08	Putative methyl accept
1539	1	db$2	2::C3KE60|C3KE60_PSEFS	34	49144	1	1	1	1	0.10	Histidine kinase OS=Ps
1540	1	db$2	2::C3K2H3|C3K2H3_PSEFS	34	31916	1	1	1	1	0.16	Putative metallopeptid
1541	1	SwissProt_HUMAN$	3::Q9P273|TEN3_HUMAN	34	300760	1	1	1	1	0.02	Teneurin-3 OS=Homo
1542	1	db$2	2::C3K2R8|NUSB_PSEFS	34	18764	1	1	1	1	0.28	Transcription antitermi

↑Family	M	DB	Accession	Score	Mass	Matches	Match(sig)	Sequences	Seq(sig)	emPAI	Description
1543	1	SwissProt_HUMAN$	3::P62847|RS24_HUMAN	34	15413	1	1	1	1	0.35	40S ribosomal protein
1544	1	db$2	2::C3K3G3|C3K3G3_PSEFS	34	32186	1	1	1	1	0.16	Putative membrane pr
1545	1	SwissProt_HUMAN$	3::O14640|DVL1_HUMAN	34	75140	1	1	1	1	0.07	Segment polarity prote
1546	1	db$2	2::C3K239|C3K239_PSEFS	34	42550	1	1	1	1	0.12	Aminotransferase OS=
1547	1	SwissProt_HUMAN$	3::P0CG04|IGLC1_HUMAN	34	11341	1	1	1	1	0.51	Immunoglobulin lambc
1548	1	SwissProt_HUMAN$	3::P42694|HELZ_HUMAN	33	218833	2	1	2	1	0.02	Probable helicase with
1549	1	db$2	2::C3KBV2|C3KBV2_PSEFS	33	15686	1	1	1	1	0.35	Thioredoxin OS=Pseuc
1550	1	db$2	2::C3K8T6|C3K8T6_PSEFS	33	26078	1	1	1	1	0.20	Ribosomal RNA small s
1551	1	db$2	2::C3K1F6|C3K1F6_PSEFS	33	23787	2	2	2	2	0.49	Ribosomal RNA small s
1552	1	db$2	2::C3K425|C3K425_PSEFS	33	27295	1	1	1	1	0.19	Positive alginate biosy
1553	1	SwissProt_HUMAN$	3::Q5VT06|CE350_HUMAN	33	350716	1	1	1	1	0.01	Centrosome-associate
1554	1	db$2	2::C3K1B3|C3K1B3_PSEFS	33	20726	3	3	3	3	0.97	Uncharacterized protei
1555	1	db$2	2::C3K742|C3K742_PSEFS	33	135455	1	1	1	1	0.04	Histidine kinase OS=P
1556	1	db$2	2::C3K136|C3K136_PSEFS	33	31053	3	2	3	2	0.35	Uncharacterized protei
1557	1	db$2	2::C3K7M1|C3K7M1_PSEFS	33	62712	1	1	1	1	0.08	Putative dipeptidase O
1558	1	db$2	2::C3KCK4|C3KCK4_PSEFS	33	14419	1	1	1	1	0.38	Putative exported prot
1559	1	db$2	2::C3K440|C3K440_PSEFS	33	11389	2	1	2	1	0.50	Uncharacterized protei
1560	1	db$2	2::C3K637|C3K637_PSEFS	33	56441	2	2	2	2	0.18	Putative peptide ABC t
1561	1	db$2	2::Q8VMR3|HUTH_PSEFS	33	54209	1	1	1	1	0.09	Histidine ammonia-lya
1562	1	db$2	2::C3K1D4|C3K1D4_PSEFS	33	64734	3	2	3	2	0.16	Uncharacterized protei
1563	1	db$2	2::C3K2C2|C3K2C2_PSEFS	33	67328	1	1	1	1	0.07	Putative lipoprotein OS
1564	1	SwissProt_HUMAN$	3::P56749|CLD12_HUMAN	33	27092	1	1	1	1	0.19	Claudin-12 OS=Homo
1565	1	db$2	2::C3K5Z0|C3K5Z0_PSEFS	33	15508	3	1	3	1	0.35	Uncharacterized protei
1566	1	db$2	2::C3KE39|C3KE39_PSEFS	33	18976	5	3	3	2	0.64	D,D-heptose 1,7-bisph
1567	1	SwissProt_HUMAN$	3::Q13472|TOP3A_HUMAN	33	112300	1	1	1	1	0.04	DNA topoisomerase 3-
1568	1	db$2	2::C3K571|C3K571_PSEFS	33	11755	1	1	1	1	0.48	Uncharacterized protei
1569	1	db$2	2::C3K481|C3K481_PSEFS	32	23434	2	2	2	2	0.49	Guanylate kinase OS=
1570	1	db$2	2::C3KAT1|C3KAT1_PSEFS	32	27898	1	1	1	1	0.18	Response regulator pro
1571	1	db$2	2::C3JYM6|C3JYM6_PSEFS	32	40915	2	1	2	1	0.12	Putative HlyD-family s
1572	1	db$2	2::C3KE56|C3KE56_PSEFS	32	59021	2	1	2	1	0.08	Glutamate--cysteine li
1573	1	db$2	2::C3K6S3|C3K6S3_PSEFS	32	25881	2	2	2	2	0.44	Putative glutathione S-
1574	1	db$2	2::C3K129|C3K129_PSEFS	32	42141	1	1	1	1	0.12	Putative acetyl CoA ac
1575	1	db$2	2::C3KBU6|C3KBU6_PSEFS	32	9578	1	1	1	1	0.62	Putative acyl carrier pr
1576	1	db$2	2::C3K0Y9|C3K0Y9_PSEFS	32	19041	1	1	1	1	0.28	Bifunctional adenosylc
1577	1	db$2	2::C3K386|C3K386_PSEFS	32	22957	1	1	1	1	0.23	Putative glutathione S-
1578	1	db$2	2::C3K536|C3K536_PSEFS	32	22752	3	2	3	2	0.51	DUF4174 domain-cont
1579	1	db$2	2::C3K8T9|TATA_PSEFS	32	10325	1	1	1	1	0.57	Sec-independent prote
1580	1	SwissProt_HUMAN$	3::P06748|NPM_HUMAN	32	32555	2	2	2	2	0.34	Nucleophosmin OS=Hc
1581	1	db$2	2::C3KAU1|C3KAU1_PSEFS	32	56168	1	1	1	1	0.09	ATP-grasp domain-con
1582	1	db$2	2::C3K223|C3K223_PSEFS	32	37863	2	1	2	1	0.13	Protein-methionine-sul

↑Family	M	DB	Accession	Score	Mass	Matches	Match(sig)	Sequences	Seq(sig)	emPAI	Description
1583	1	db$2	2::C3K1W1|C3K1W1_PSEFS	32	22921	2	2	1	1	0.23	Uncharacterized protei
1584	1	SwissProt_HUMAN$	3::P04080|CYTB_HUMAN	32	11133	1	1	1	1	0.52	Cystatin-B OS=Homo s
1585	1	db$2	2::C3K020|C3K020_PSEFS	32	23656	1	1	1	1	0.22	Putative hydrolase OS:
1586	1	SwissProt_HUMAN$	3::Q6ZVD8|PHLP2_HUMAN	32	146658	1	1	1	1	0.03	PH domain leucine-rich
1587	1	SwissProt_HUMAN$	3::P28072|PSB6_HUMAN	32	25341	2	1	2	1	0.20	Proteasome subunit be
1588	1	db$2	2::C3K2P5|C3K2P5_PSEFS	31	49850	1	1	1	1	0.10	Ethanolamine ammoni
1589	1	db$2	2::C3KBI5|C3KBI5_PSEFS	31	21236	1	1	1	1	0.25	Putative membrane pr
1590	1	db$2	2::C3K5M3|PHNN_PSEFS	31	20351	1	1	1	1	0.26	Ribose 1,5-bisphospha
1591	1	SwissProt_HUMAN$	3::P57727|TMPS3_HUMAN	31	49373	1	1	1	1	0.10	Transmembrane prote
1592	1	SwissProt_HUMAN$	3::Q9NQS7|INCE_HUMAN	31	105365	1	1	1	1	0.05	Inner centromere prot
1593	1	db$2	2::C3KCB4|C3KCB4_PSEFS	31	27191	1	1	1	1	0.19	Uncharacterized protei
1594	1	db$2	2::C3K3J1|C3K3J1_PSEFS	31	16523	1	1	1	1	0.33	Putative chemotaxis-re
1595	1	db$2	2::C3K4Z0|CCME_PSEFS	31	16101	2	1	2	1	0.34	Cytochrome c-type bio
1596	1	db$2	2::A4V6U2|A4V6U2_PSEFS	31	76547	1	1	1	1	0.06	Uncharacterized protei
1597	1	SwissProt_HUMAN$	3::Q9NU22|MDN1_HUMAN	31	632420	2	1	2	1	0.01	Midasin OS=Homo sap
1598	1	db$2	2::C3K4F9|C3K4F9_PSEFS	31	31102	1	1	1	1	0.16	Putative regulatory pro
1599	1	db$2	2::C3K8K6|C3K8K6_PSEFS	31	15117	1	1	1	1	0.36	Putative transport-rela
1600	1	db$2	2::C3JYA8|C3JYA8_PSEFS	31	39123	1	1	1	1	0.13	Putative membrane pr
1601	1	db$2	2::C3KDK6|C3KDK6_PSEFS	31	27451	1	1	1	1	0.19	Putative ABC transport
1602	1	SwissProt_HUMAN$	3::Q96P48|ARAP1_HUMAN	31	162089	1	1	1	1	0.03	Arf-GAP with Rho-GAP
1603	1	db$2	2::A4V7U9|A4V7U9_PSEFS	31	58926	1	1	1	1	0.08	Mercuric reductase OS
1604	1	SwissProt_HUMAN$	3::O15078|CE290_HUMAN	31	290207	4	1	3	1	0.02	Centrosomal protein of
1605	1	SwissProt_HUMAN$	3::Q13422|IKZF1_HUMAN	31	57491	2	1	2	1	0.09	DNA-binding protein Ik
1606	1	SwissProt_HUMAN$	3::P15880|RS2_HUMAN	31	31305	2	2	2	2	0.35	40S ribosomal protein
1607	1	db$2	2::C3K340|C3K340_PSEFS	30	72802	1	1	1	1	0.07	DNA primase OS=Pseu
1608	1	db$2	2::C3K2P0|C3K2P0_PSEFS	30	22640	2	2	2	2	0.51	Flavin prenyltransferas
1609	1	db$2	2::C3K1S9|C3K1S9_PSEFS	30	82606	1	1	1	1	0.06	Putative phytochrome/
1610	1	db$2	2::C3KBZ5|C3KBZ5_PSEFS	30	23751	2	2	2	2	0.49	Endonuclease III OS=I
1611	1	db$2	2::C3K2E2|C3K2E2_PSEFS	30	56906	1	1	1	1	0.09	Catalase OS=Pseudom
1612	1	SwissProt_HUMAN$	3::Q8NB16|MLKL_HUMAN	30	54445	1	1	1	1	0.09	Mixed lineage kinase d
1613	1	db$2	2::C3K590|C3K590_PSEFS	30	74072	1	1	1	1	0.07	Putative membrane pr
1614	1	db$2	2::C3KDN1|C3KDN1_PSEFS	30	54005	2	1	2	1	0.09	Deoxyribodipyrimidine
1615	1	db$2	2::C3KDM8|C3KDM8_PSEFS	30	57105	1	1	1	1	0.09	HDOD domain-contain
1616	1	SwissProt_HUMAN$	3::P81605|DCD_HUMAN	30	11277	1	1	1	1	0.51	Dermcidin OS=Homo s
1617	1	db$2	2::C3K8N0|C3K8N0_PSEFS	30	12883	2	2	1	1	0.44	Antitoxin OS=Pseudom
1618	1	db$2	2::C3KB07|C3KB07_PSEFS	30	47911	1	1	1	1	0.10	Deferrochelatase/pero:
1619	1	SwissProt_HUMAN$	3::Q9ULQ1|TPC1_HUMAN	30	94086	1	1	1	1	0.05	Two pore calcium char
1620	1	db$2	2::C3K5X3|C3K5X3_PSEFS	30	64999	2	2	2	2	0.16	Putative ABC transport
1621	1	SwissProt_HUMAN$	3::Q7Z624|CMKMT_HUMAN	30	36105	1	1	1	1	0.14	Calmodulin-lysine N-m
1622	1	db$2	2::C3K834|C3K834_PSEFS	30	76708	1	1	1	1	0.06	Copper-transporting P-

↑Family	M	DB	Accession	Score	Mass	Matches	Match(sig)	Sequences	Seq(sig)	emPAI	Description
1623	1	db$2	2::C3KCS4|C3KCS4_PSEFS	30	63231	2	2	2	2	0.16	Peptidoglycan D,D-trar
1624	1	db$2	2::C3K3C7|C3K3C7_PSEFS	30	34452	1	1	1	1	0.15	Putative glycine betain
1625	1	db$2	2::C3JYF1|C3JYF1_PSEFS	30	42707	1	1	1	1	0.12	Putative acyl-CoA deh
1626	1	SwissProt_HUMAN$	3::P37802|TAGL2_HUMAN	30	22377	1	1	1	1	0.23	Transgelin-2 OS=Hom
1627	1	db$2	2::C3K546|C3K546_PSEFS	30	33665	1	1	1	1	0.15	Putative lipopolysacch
1628	1	db$2	2::C3K2L3|C3K2L3_PSEFS	30	22416	1	1	1	1	0.23	LPS-assembly lipoprot
1629	1	SwissProt_HUMAN$	3::O75197|LRP5_HUMAN	30	179030	3	2	1	1	0.03	Low-density lipoprotei
1630	1	db$2	2::C3K7J7|C3K7J7_PSEFS	29	16971	1	1	1	1	0.32	Putative membrane pr
1631	1	db$2	2::C3K0E2|C3K0E2_PSEFS	29	49920	1	1	1	1	0.10	Diaminobutyrate--2-o
1632	1	db$2	2::C3K1R4|C3K1R4_PSEFS	29	17440	1	1	1	1	0.31	Putative plasmid-relat
1633	1	db$2	2::C3K2P3|C3K2P3_PSEFS	29	54945	1	1	1	1	0.09	Putative aldehyde deh
1634	1	db$2	2::C3KDL5|C3KDL5_PSEFS	29	27487	2	2	1	1	0.19	Putative lipoprotein OS
1635	1	db$2	2::C3K6B1|C3K6B1_PSEFS	29	35098	2	2	2	2	0.31	Homoserine kinase OS
1636	1	db$2	2::C3K4A1|C3K4A1_PSEFS	29	128725	1	1	1	1	0.04	Putative membrane pr
1637	1	db$2	2::C3K532|C3K532_PSEFS	29	17901	1	1	1	1	0.30	Uncharacterized protei
1638	1	db$2	2::C3K488|C3K488_PSEFS	29	15239	2	2	2	2	0.85	Biopolymer transport
1639	1	db$2	2::C3K0G1|C3K0G1_PSEFS	29	67986	1	1	1	1	0.07	Putative acetyl-/propic
1640	1	db$2	2::C3KDW4|C3KDW4_PSEFS	29	48912	2	2	2	2	0.21	GTPase HflX OS=Pseu
1641	1	db$2	2::C3K458|C3K458_PSEFS	29	47152	1	1	1	1	0.11	D-amino acid dehydro
1642	1	db$2	2::C3JY46|C3JY46_PSEFS	29	42076	1	1	1	1	0.12	Putative racemase OS
1643	1	db$2	2::C3K678|C3K678_PSEFS	29	45414	1	1	1	1	0.11	Cytosine deaminase O
1644	1	db$2	2::C3KDQ7|C3KDQ7_PSEFS	29	12961	1	1	1	1	0.43	Diacylglycerol kinase
1645	1	db$2	2::C3JZ07|C3JZ07_PSEFS	29	24563	1	1	1	1	0.21	Putative ABC transport
1646	1	db$2	2::C3K2R0|C3K2R0_PSEFS	29	31322	1	1	1	1	0.16	Geranyltranstransferas
1647	1	db$2	2::C3K2P8|C3K2P8_PSEFS	29	24358	2	1	2	1	0.21	Membrane-related pro
1648	1	db$2	2::C3K741|C3K741_PSEFS	29	17249	1	1	1	1	0.31	GAF domain-containin
1649	1	SwissProt_HUMAN$	3::P30050|RL12_HUMAN	29	17808	2	1	2	1	0.30	60S ribosomal protein
1650	1	db$2	2::C3K451|C3K451_PSEFS	28	76463	3	2	3	2	0.13	ATP-dependent DNA h
1651	1	SwissProt_HUMAN$	3::Q96AQ6|PBIP1_HUMAN	28	80594	1	1	1	1	0.06	Pre-B-cell leukemia tra
1652	1	db$2	2::C3K789|C3K789_PSEFS	28	16242	1	1	1	1	0.33	Uncharacterized protei
1653	1	db$2	2::C3K2S4|C3K2S4_PSEFS	28	15995	1	1	1	1	0.34	Putative lipoprotein OS
1654	1	db$2	2::C3K9A0|C3K9A0_PSEFS	28	31788	4	3	3	3	0.56	Putative LysR-family r
1655	1	db$2	2::C3K4M2|C3K4M2_PSEFS	28	36363	1	1	1	1	0.14	Catabolite repressor/a
1656	1	SwissProt_HUMAN$	3::Q6ZSZ5|ARHGI_HUMAN	28	151549	2	1	1	1	0.03	Rho guanine nucleotid
1657	1	SwissProt_HUMAN$	3::Q9BYK8|HELZ2_HUMAN	28	294465	1	1	1	1	0.02	Helicase with zinc fing
1658	1	db$2	2::C3K2Z1|COAX_PSEFS	28	26920	1	1	1	1	0.19	Type III pantothenate
1659	1	db$2	2::C3K157|C3K157_PSEFS	28	43413	1	1	1	1	0.12	Putative membrane pr
1660	1	db$2	2::C3KAB2|C3KAB2_PSEFS	28	13873	2	2	1	1	0.40	Putative two-compone
1661	1	db$2	2::C3K454|C3K454_PSEFS	28	13613	2	2	2	2	0.98	Putative cytochrome c
1662	1	db$2	2::C3K8U6|C3K8U6_PSEFS	28	15270	1	1	1	1	0.36	Putative polyhydroxya

↑Family	M	DB	Accession	Score	Mass	Matches	Match(sig)	Sequences	Seq(sig)	emPAI	Description
1663	1	SwissProt_HUMAN$	3::Q2KJY2|KI26B_HUMAN	28	223744	2	1	2	1	0.02	Kinesin-like protein KI
1664	1	db$2	2::C3JY59|LFTR_PSEFS	28	25331	1	1	1	1	0.20	Leucyl/phenylalanyl-tR
1665	1	SwissProt_HUMAN$	3::O15197|EPHB6_HUMAN	28	110630	1	1	1	1	0.04	Ephrin type-B receptor
1666	1	db$2	2::C3K0R2|C3K0R2_PSEFS	28	34379	1	1	1	1	0.15	Chemotaxis protein OS
1667	1	SwissProt_HUMAN$	3::Q2M3G4|SHRM1_HUMAN	28	90730	1	1	1	1	0.05	Protein Shroom1 OS=I
1668	1	db$2	2::C3KAF4|C3KAF4_PSEFS	28	50981	1	1	1	1	0.10	Putative lipoprotein OS
1669	1	db$2	2::C3K5S6|C3K5S6_PSEFS	28	24824	1	1	1	1	0.21	Putative hydrolase OS
1670	1	SwissProt_HUMAN$	3::O75155|CAND2_HUMAN	28	135171	2	1	2	1	0.04	Cullin-associated NEDI
1671	1	SwissProt_HUMAN$	3::Q9H6P5|TASP1_HUMAN	28	44427	1	1	1	1	0.11	Threonine aspartase 1
1672	1	db$2	2::C3K9S5|C3K9S5_PSEFS	28	38309	1	1	1	1	0.13	Putative cobalamin bio
1673	1	SwissProt_HUMAN$	3::Q8N9V3|WSDU1_HUMAN	28	52783	2	1	1	1	0.09	WD repeat, SAM and U
1674	1	db$2	2::C3K4G7|PDXY_PSEFS	27	31241	1	1	1	1	0.16	Pyridoxal kinase PdxY
1675	1	db$2	2::C3K7A1|C3K7A1_PSEFS	27	56003	1	1	1	1	0.09	Putative LuxR-family r
1676	1	SwissProt_HUMAN$	3::Q6UWJ1|TMCO3_HUMAN	27	75549	1	1	1	1	0.06	Transmembrane and c
1677	1	db$2	2::C3K5U8|AGUA_PSEFS	27	40372	1	1	1	1	0.12	Agmatine deiminase O
1678	1	db$2	2::C3K7C2|C3K7C2_PSEFS	27	16947	2	1	2	1	0.32	tRNA (cytidine(34)-2'-
1679	1	db$2	2::C3K672|C3K672_PSEFS	27	58670	2	2	2	2	0.18	Cation/acetate sympor
1680	1	db$2	2::C3K173|C3K173_PSEFS	27	34695	2	2	2	2	0.31	Putative AraC-family t
1681	1	SwissProt_HUMAN$	3::O14917|PCD17_HUMAN	27	126151	1	1	1	1	0.04	Protocadherin-17 OS=
1682	1	db$2	2::C3JXP2|C3JXP2_PSEFS	27	43336	1	1	1	1	0.12	Putative glycosyltransf
1683	1	SwissProt_HUMAN$	3::Q8NA82|MARHA_HUMAN	27	90455	1	1	1	1	0.05	Probable E3 ubiquitin-
1684	1	SwissProt_HUMAN$	3::Q14185|DOCK1_HUMAN	27	215209	1	1	1	1	0.02	Dedicator of cytokinesi
1685	1	SwissProt_HUMAN$	3::Q9UK58|CCNL1_HUMAN	27	59597	2	1	2	1	0.08	Cyclin-L1 OS=Homo sa
1686	1	db$2	2::C3K3T1|C3K3T1_PSEFS	27	86048	1	1	1	1	0.06	Putative penicillin amic
1687	1	SwissProt_HUMAN$	3::P23526|SAHH_HUMAN	27	47685	1	1	1	1	0.10	Adenosylhomocysteina
1688	1	db$2	2::C3KDC6|RF1_PSEFS	27	39857	2	1	2	1	0.13	Peptide chain release f
1689	1	SwissProt_HUMAN$	3::P12883|MYH7_HUMAN	27	222959	1	1	1	1	0.02	Myosin-7 OS=Homo sa
1690	1	SwissProt_HUMAN$	3::P62244|RS15A_HUMAN	27	14830	2	1	2	1	0.37	40S ribosomal protein
1691	1	db$2	2::C3K4T5|C3K4T5_PSEFS	27	30974	2	2	2	2	0.36	Putative hydrolase OS
1692	1	SwissProt_HUMAN$	3::P08514|ITA2B_HUMAN	27	113306	1	1	1	1	0.04	Integrin alpha-IIb OS=
1693	1	SwissProt_HUMAN$	3::Q75VX8|GARE2_HUMAN	27	92824	1	1	1	1	0.05	GRB2-associated and r
1694	1	db$2	2::C3JZ60|C3JZ60_PSEFS	27	14055	1	1	1	1	0.39	Uncharacterized protei
1695	1	SwissProt_HUMAN$	3::Q9H814|PHAX_HUMAN	27	44375	1	1	1	1	0.11	Phosphorylated adapte
1696	1	db$2	2::C3K7J8|C3K7J8_PSEFS	27	78803	2	1	2	1	0.06	ATP-dependent DNA h
1697	1	SwissProt_HUMAN$	3::A0A1B0GVD1|F237B_HUMAN	27	16320	1	1	1	1	0.33	Protein FAM237B OS=
1698	1	SwissProt_HUMAN$	3::Q96N76|HUTU_HUMAN	27	74783	1	1	1	1	0.07	Urocanate hydratase C
1699	1	db$2	2::C3K7A9|C3K7A9_PSEFS	26	33658	1	1	1	1	0.15	Conserved hypothetica
1700	1	SwissProt_HUMAN$	3::P05546|HEP2_HUMAN	26	57034	1	1	1	1	0.09	Heparin cofactor 2 OS
1701	1	db$2	2::C3K9P2|C3K9P2_PSEFS	26	69747	1	1	1	1	0.07	Putative substrate-bin
1702	1	SwissProt_HUMAN$	3::O15270|SPTC2_HUMAN	26	62884	1	1	1	1	0.08	Serine palmitoyltransfe

↑Family	M	DB	Accession	Score	Mass	Matches	Match(sig)	Sequences	Seq(sig)	emPAI	Description
1703	1	SwissProt_HUMAN$	3::Q9H0A9|SPC1L_HUMAN	26	37589	1	1	1	1	0.13	Speriolin-like protein C
1704	1	SwissProt_HUMAN$	3::Q9H930|SP14L_HUMAN	26	66962	1	1	1	1	0.07	Nuclear body protein S
1705	1	db$2	2::C3JY34|C3JY34_PSEFS	26	100208	1	1	1	1	0.05	Histidine kinase OS=Ps
1706	1	db$2	2::C3JXZ4|C3JXZ4_PSEFS	26	40619	2	1	1	1	0.12	Uncharacterized protei
1707	1	db$2	2::C3K1Q2|C3K1Q2_PSEFS	26	73203	1	1	1	1	0.07	Histidine kinase OS=Ps
1708	1	db$2	2::C3K3N6|FPG_PSEFS	26	30025	2	2	2	2	0.37	Formamidopyrimidine-
1709	1	db$2	2::C3K2N6|C3K2N6_PSEFS	26	16796	2	2	2	2	0.75	MaoC-like domain-cont
1710	1	SwissProt_HUMAN$	3::Q8N9W6|BOLL_HUMAN	26	31282	1	1	1	1	0.16	Protein boule-like OS=
1711	1	db$2	2::C3KEC3|C3KEC3_PSEFS	26	16145	2	2	2	2	0.79	Putative membrane pr
1712	1	SwissProt_HUMAN$	3::Q9BRG2|SH23A_HUMAN	26	63054	2	2	2	2	0.16	SH2 domain-containin
1713	1	SwissProt_HUMAN$	3::Q13219|PAPP1_HUMAN	26	180856	1	1	1	1	0.03	Pappalysin-1 OS=Hom
1714	1	db$2	2::C3K994|C3K994_PSEFS	26	23940	2	1	1	1	0.22	Putative regulatory pro
1715	1	SwissProt_HUMAN$	3::Q9P2P5|HECW2_HUMAN	26	175660	1	1	1	1	0.03	E3 ubiquitin-protein lig
1716	1	SwissProt_HUMAN$	3::B5MCY1|TDR15_HUMAN	26	221580	2	2	2	2	0.04	Tudor domain-containi
1717	1	SwissProt_HUMAN$	3::P48165|CXA8_HUMAN	26	48199	1	1	1	1	0.10	Gap junction alpha-8 p
1718	1	db$2	2::C3K4F4|C3K4F4_PSEFS	26	25363	1	1	1	1	0.20	Putative acetyltransfer
1719	1	SwissProt_HUMAN$	3::Q14697|GANAB_HUMAN	26	106807	2	1	2	1	0.05	Neutral alpha-glucosid
1720	1	SwissProt_HUMAN$	3::Q99618|CDCA3_HUMAN	26	28981	1	1	1	1	0.18	Cell division cycle-asso
1721	1	SwissProt_HUMAN$	3::P17480|UBF1_HUMAN	26	89350	1	1	1	1	0.05	Nucleolar transcription
1722	1	db$2	2::C3K334|CCA_PSEFS	26	45916	1	1	1	1	0.11	Multifunctional CCA pro
1723	1	SwissProt_HUMAN$	3::P11532|DMD_HUMAN	26	426484	2	1	2	1	0.01	Dystrophin OS=Homo
1724	1	db$2	2::C3K3H6|C3K3H6_PSEFS	26	12460	1	1	1	1	0.45	Putative endoribonucle
1725	1	SwissProt_HUMAN$	3::Q9C037|TRIM4_HUMAN	26	57424	1	1	1	1	0.09	E3 ubiquitin-protein lig
1726	1	db$2	2::C3KE18|C3KE18_PSEFS	26	50306	1	1	1	1	0.10	Sensor protein QseC O
1727	1	SwissProt_HUMAN$	3::P49207|RL34_HUMAN	26	13284	1	1	1	1	0.42	60S ribosomal protein
1728	1	SwissProt_HUMAN$	3::Q9Y2G4|ANKR6_HUMAN	26	79922	1	1	1	1	0.06	Ankyrin repeat domain
1729	1	db$2	2::C3K5L1|C3K5L1_PSEFS	26	14502	1	1	1	1	0.38	Uncharacterized protei
1730	1	SwissProt_HUMAN$	3::Q5JXB2|UE2NL_HUMAN	25	17366	1	1	1	1	0.31	Putative ubiquitin-conj
1731	1	db$2	2::C3K2E4|C3K2E4_PSEFS	25	29249	2	2	1	1	0.17	Ferredoxin--NADP redu
1732	1	SwissProt_HUMAN$	3::Q8NBF4|YG006_HUMAN	25	16172	2	1	1	1	0.33	Putative characteriz
1733	1	SwissProt_HUMAN$	3::Q5SRN2|TSBP1_HUMAN	25	61588	1	1	1	1	0.08	Testis-expressed basic
1734	1	db$2	2::C3KB08|C3KB08_PSEFS	25	43028	1	1	1	1	0.12	Putative exported prot
1735	1	SwissProt_HUMAN$	3::Q00526|CDK3_HUMAN	25	35024	1	1	1	1	0.14	Cyclin-dependent kina
1736	1	db$2	2::C3K7J6|C3K7J6_PSEFS	25	25687	1	1	1	1	0.20	Putative exported prot
1737	1	db$2	2::C3K5Y5|C3K5Y5_PSEFS	25	18019	1	1	1	1	0.30	Protein PsiE OS=Pseuc
1738	1	db$2	2::C3JY93|C3JY93_PSEFS	25	51981	2	2	2	2	0.20	NADH-quinone oxidore
1739	1	SwissProt_HUMAN$	3::Q9H9E3|COG4_HUMAN	25	89026	2	2	1	1	0.05	Conserved oligomeric
1740	1	SwissProt_HUMAN$	3::Q8NFV4|ABHDB_HUMAN	25	34668	1	1	1	1	0.15	Protein ABHD11 OS=H
1741	1	db$2	2::C3JY90|NUOK_PSEFS	25	10944	1	1	1	1	0.52	NADH-quinone oxidore
1742	1	SwissProt_HUMAN$	3::Q7Z7L7|ZER1_HUMAN	25	88113	2	2	1	1	0.11	Protein zer-1 homolog

↑Family	M	DB	Accession	Score	Mass	Matches	Match(sig)	Sequences	Seq(sig)	emPAI	Description
1743	1	SwissProt_HUMAN$	3::Q14789|GOGB1_HUMAN	25	375790	3	2	3	2	0.03	Golgin subfamily B me
1744	1	SwissProt_HUMAN$	3::P29144|TPP2_HUMAN	25	138263	1	1	1	1	0.03	Tripeptidyl-peptidase 2
1745	1	db$2	2::C3K5Q0|C3K5Q0_PSEFS	25	53619	3	2	3	2	0.19	Mannose-1-phosphate
1746	1	SwissProt_HUMAN$	3::P36507|MP2K2_HUMAN	25	44396	1	1	1	1	0.11	Dual specificity mitoge
1747	1	db$2	2::C3K6W1|C3K6W1_PSEFS	25	9231	1	1	1	1	0.65	Glutaredoxin OS=Pseu
1748	1	SwissProt_HUMAN$	3::Q96AC6|KIFC2_HUMAN	25	90092	1	1	1	1	0.05	Kinesin-like protein KI
1749	1	SwissProt_HUMAN$	3::Q6UVW9|CLC2A_HUMAN	25	19959	1	1	1	1	0.26	C-type lectin domain fa
1750	1	SwissProt_HUMAN$	3::O00159|MYO1C_HUMAN	25	121606	1	1	1	1	0.04	Unconventional myosir
1751	1	db$2	2::C3KAP7|C3KAP7_PSEFS	25	26475	1	1	1	1	0.19	Putative short-chain de
1752	1	db$2	2::C3KA92|C3KA92_PSEFS	25	24193	1	1	1	1	0.21	Putative ACP reductase
1753	1	SwissProt_HUMAN$	3::P62851|RS25_HUMAN	25	13734	2	1	2	1	0.40	40S ribosomal protein
1754	1	db$2	2::C3K5I1|C3K5I1_PSEFS	25	43688	1	1	1	1	0.11	Putative phage integra
1755	1	SwissProt_HUMAN$	3::Q9HBJ7|UBP29_HUMAN	25	104090	1	1	1	1	0.05	Ubiquitin carboxyl-terr
1756	1	SwissProt_HUMAN$	3::Q9BRX2|PELO_HUMAN	25	43332	2	1	2	1	0.12	Protein pelota homolog
1757	1	db$2	2::C3KCB6|C3KCB6_PSEFS	25	31268	2	2	1	1	0.35	Phosphate transport sy
1758	1	db$2	2::C3KAL9|C3KAL9_PSEFS	25	64520	1	1	1	1	0.08	Histidine kinase OS=P
1759	1	SwissProt_HUMAN$	3::P13637|AT1A3_HUMAN	24	111677	1	1	1	1	0.04	Sodium/potassium-tra
1760	1	db$2	2::C3K9R9|C3K9R9_PSEFS	24	86327	1	1	1	1	0.06	Putative exported fatty
1761	1	db$2	2::C3KE72|C3KE72_PSEFS	24	85807	2	1	2	1	0.06	UPF0313 protein PFLU_
1762	1	SwissProt_HUMAN$	3::Q9UL62|TRPC5_HUMAN	24	111340	1	1	1	1	0.04	Short transient recepto
1763	1	db$2	2::C3K5F5|Y1520_PSEFS	24	17238	1	1	1	1	0.31	UPF0260 protein PFLU_
1764	1	db$2	2::C3K3J8|C3K3J8_PSEFS	24	33397	1	1	1	1	0.15	TonB_C domain-contai
1765	1	SwissProt_HUMAN$	3::O95153|RIMB1_HUMAN	24	199928	1	1	1	1	0.02	Peripheral-type benzod
1766	1	SwissProt_HUMAN$	3::Q96MR6|CFA57_HUMAN	24	144870	1	1	1	1	0.03	Cilia- and flagella-asso
1767	1	SwissProt_HUMAN$	3::P27708|PYR1_HUMAN	24	242829	2	1	2	1	0.02	CAD protein OS=Homo
1768	1	SwissProt_HUMAN$	3::Q17RB8|LONF1_HUMAN	24	86669	1	1	1	1	0.06	LON peptidase N-term
1769	1	SwissProt_HUMAN$	3::O00299|CLIC1_HUMAN	24	26906	1	1	1	1	0.19	Chloride intracellular c
1770	1	SwissProt_HUMAN$	3::Q9H244|P2Y12_HUMAN	24	39413	1	1	1	1	0.13	P2Y purinoceptor 12 O
1771	1	SwissProt_HUMAN$	3::Q96GD3|SCMH1_HUMAN	24	73307	1	1	1	1	0.07	Polycomb protein SCM
1772	1	db$2	2::C3K429|C3K429_PSEFS	24	8856	1	1	1	1	0.68	Uncharacterized protei
1773	1	db$2	2::C3K0E5|C3K0E5_PSEFS	24	24553	1	1	1	1	0.21	Two-component syster
1774	1	db$2	2::C3K1G4|RL34_PSEFS	24	5064	1	1	1	1	1.41	50S ribosomal protein
1775	1	db$2	2::C3KCT5|C3KCT5_PSEFS	24	27647	1	1	1	1	0.19	Putative regulatory pro
1776	1	db$2	2::C3JY58|C3JY58_PSEFS	24	87640	2	1	2	1	0.06	DNA translocase FtsK (
1777	1	db$2	2::C3K6M1|C3K6M1_PSEFS	24	28266	1	1	1	1	0.18	Putative enoyl-CoA hy
1778	1	SwissProt_HUMAN$	3::Q92805|GOGA1_HUMAN	24	88130	1	1	1	1	0.06	Golgin subfamily A me
1779	1	db$2	2::C3KAX3|C3KAX3_PSEFS	24	30360	1	1	1	1	0.17	Conserved hypothetica
1780	1	db$2	2::C3KEB9|C3KEB9_PSEFS	24	11110	1	1	1	1	0.52	Putative lipoprotein OS
1781	1	SwissProt_HUMAN$	3::Q8TD08|MK15_HUMAN	24	59795	1	1	1	1	0.08	Mitogen-activated prot
1782	1	db$2	2::C3K439|C3K439_PSEFS	24	25827	1	1	1	1	0.20	Putative hydrolase OS

↑Family	M	DB	Accession	Score	Mass	Matches	Match(sig)	Sequences	Seq(sig)	emPAI	Description
1783	1	db$2	2::C3K5M9|C3K5M9_PSEFS	24	42011	1	1	1	1	0.12	Utative transport-relat
1784	1	db$2	2::C3K659|C3K659_PSEFS	23	30767	1	1	1	1	0.17	2-hydroxy-3-oxopropic
1785	1	db$2	2::C3K9N7|C3K9N7_PSEFS	23	48751	1	1	1	1	0.10	Putative membrane pr
1786	1	db$2	2::C3K4A9|C3K4A9_PSEFS	23	17462	1	1	1	1	0.31	Uncharacterized protei
1787	1	SwissProt_HUMAN$	3::Q14746|COG2_HUMAN	23	83155	2	2	2	2	0.12	Conserved oligomeric (
1788	1	db$2	2::C3KAA6|C3KAA6_PSEFS	23	51931	1	1	1	1	0.10	Putative cell envelope
1789	1	db$2	2::C3K5B8|C3K5B8_PSEFS	23	59457	1	1	1	1	0.08	Dipeptide ABC transpo
1790	1	db$2	2::C3K2M4|C3K2M4_PSEFS	23	40052	1	1	1	1	0.13	Peptidoglycan glycosyl
1791	1	SwissProt_HUMAN$	3::Q96D53|COQ8B_HUMAN	23	60031	1	1	1	1	0.08	Atypical kinase COQ8E
1792	1	SwissProt_HUMAN$	3::P15924|DESP_HUMAN	23	331569	2	1	2	1	0.01	Desmoplakin OS=Hom
1793	1	db$2	2::C3K2T4|C3K2T4_PSEFS	23	155869	2	1	2	1	0.03	Putative ATP-depender
1794	1	SwissProt_HUMAN$	3::P55283|CADH4_HUMAN	23	100218	1	1	1	1	0.05	Cadherin-4 OS=Homo
1795	1	SwissProt_HUMAN$	3::P62304|RUXE_HUMAN	23	10797	1	1	1	1	0.54	Small nuclear ribonucl
1796	1	db$2	2::C3K842|C3K842_PSEFS	23	56600	1	1	1	1	0.09	RNA polymerase sigma
1797	1	SwissProt_HUMAN$	3::P35268|RL22_HUMAN	23	14778	2	1	2	1	0.37	60S ribosomal protein
1798	1	SwissProt_HUMAN$	3::O94967|WDR47_HUMAN	23	101884	1	1	1	1	0.05	WD repeat-containing
1799	1	db$2	2::C3JZT4|C3JZT4_PSEFS	23	90038	1	1	1	1	0.05	Putative membrane pr
1800	1	db$2	2::C3JZ81|C3JZ81_PSEFS	23	25240	2	1	2	1	0.21	Putative exported prot
1801	1	SwissProt_HUMAN$	3::O75962|TRIO_HUMAN	23	346683	3	1	2	1	0.01	Triple functional doma
1802	1	SwissProt_HUMAN$	3::A6NM36|LRC30_HUMAN	23	34012	1	1	1	1	0.15	Leucine-rich repeat-co
1803	1	db$2	2::C3K301|C3K301_PSEFS	23	28901	1	1	1	1	0.18	Putative dehydrogenas
1804	1	db$2	2::C3KBY0|GMHA_PSEFS	23	21250	1	1	1	1	0.25	Phosphoheptose isome
1805	1	SwissProt_HUMAN$	3::P10909|CLUS_HUMAN	23	52461	1	1	1	1	0.09	Clusterin OS=Homo sa
1806	1	db$2	2::C3K3X1|C3K3X1_PSEFS	23	48834	1	1	1	1	0.10	Xaa-Pro aminopeptida:
1807	1	SwissProt_HUMAN$	3::P53814|SMTN_HUMAN	23	98998	2	2	2	2	0.10	Smoothelin OS=Homo
1808	1	SwissProt_HUMAN$	3::P04920|B3A2_HUMAN	23	136923	1	1	1	1	0.04	Anion exchange protei
1809	1	db$2	2::C3KE19|C3KE19_PSEFS	23	30253	1	1	1	1	0.17	HDOD domain-contain
1810	1	db$2	2::C3K1S4|C3K1S4_PSEFS	23	23334	1	1	1	1	0.22	Putative membrane pr
1811	1	db$2	2::C3KDV4|RSGA_PSEFS	23	37296	3	1	3	1	0.14	Small ribosomal subun
1812	1	SwissProt_HUMAN$	3::Q7Z5H4|VN1R5_HUMAN	23	40752	1	1	1	1	0.12	Vomeronasal type-1 re
1813	1	SwissProt_HUMAN$	3::P04083|ANXA1_HUMAN	23	38690	1	1	1	1	0.13	Annexin A1 OS=Homo
1814	1	db$2	2::C3KAA5|C3KAA5_PSEFS	22	23984	2	1	1	1	0.22	Uncharacterized protei
1815	1	db$2	2::C3K0Y3|C3K0Y3_PSEFS	22	20085	1	1	1	1	0.26	Glutathione peroxidase
1816	1	SwissProt_HUMAN$	3::Q5TZA2|CROCC_HUMAN	22	228388	1	1	1	1	0.02	Rootletin OS=Homo sa
1817	1	db$2	2::C3K5C7|C3K5C7_PSEFS	22	87287	1	1	1	1	0.06	Putative ferric sideropl
1818	1	db$2	2::C3K908|C3K908_PSEFS	22	33577	2	1	2	1	0.15	Putative dehydrogenas
1819	1	SwissProt_HUMAN$	3::Q8WV24|PHLA1_HUMAN	22	44988	1	1	1	1	0.11	Pleckstrin homology-lil
1820	1	db$2	2::C3K0X1|C3K0X1_PSEFS	22	10638	1	1	1	1	0.54	Uncharacterized protei
1821	1	SwissProt_HUMAN$	3::Q96BI1|S22AI_HUMAN	22	44816	1	1	1	1	0.11	Solute carrier family 2.
1822	1	db$2	2::C3KAB8|C3KAB8_PSEFS	22	25944	1	1	1	1	0.20	Putative short-chain de

↑Family	M	DB	Accession	Score	Mass	Matches	Match(sig)	Sequences	Seq(sig)	emPAI	Description
1823	1	SwissProt_HUMAN$	3::Q5TC12|ATPF1_HUMAN	22	36414	1	1	1	1	0.14	ATP synthase mitochor
1824	1	SwissProt_HUMAN$	3::Q92625|ANS1A_HUMAN	22	123032	1	1	1	1	0.04	Ankyrin repeat and SA
1825	1	SwissProt_HUMAN$	3::P62633|CNBP_HUMAN	22	19450	1	1	1	1	0.27	Cellular nucleic acid-bi
1826	1	db$2	2::C3KEB8|C3KEB8_PSEFS	22	34155	1	1	1	1	0.15	Putative lipoprotein OS
1827	1	SwissProt_HUMAN$	3::Q9BY77|PDIP3_HUMAN	22	46060	1	1	1	1	0.11	Polymerase delta-inter
1828	1	db$2	2::C3K006|C3K006_PSEFS	22	41293	1	1	1	1	0.12	Acetylornithine deacet
1829	1	SwissProt_HUMAN$	3::P62906|RL10A_HUMAN	22	24816	1	1	1	1	0.21	60S ribosomal protein
1830	1	db$2	2::C3K2L7|C3K2L7_PSEFS	22	32785	1	1	1	1	0.16	Putative muramoyltetr
1831	1	db$2	2::C3KBE6|C3KBE6_PSEFS	22	84273	1	1	1	1	0.06	Uncharacterized protei
1832	1	db$2	2::C3K0Z2|C3K0Z2_PSEFS	22	32838	1	1	1	1	0.15	Cobalamin biosynthesi
1833	1	SwissProt_HUMAN$	3::Q9NRC6|SPTN5_HUMAN	22	416493	1	1	1	1	0.01	Spectrin beta chain, no
1834	1	db$2	2::C3K1M3|C3K1M3_PSEFS	22	11692	1	1	1	1	0.49	Iron-binding protein Is
1835	1	SwissProt_HUMAN$	3::Q70Z53|F10C1_HUMAN	22	37525	1	1	1	1	0.13	Protein FRA10AC1 OS=
1836	1	SwissProt_HUMAN$	3::Q7RTV0|PHF5A_HUMAN	22	12397	1	1	1	1	0.45	PHD finger-like domair
1837	1	SwissProt_HUMAN$	3::Q9NY15|STAB1_HUMAN	22	275300	1	1	1	1	0.02	Stabilin-1 OS=Homo s
1838	1	SwissProt_HUMAN$	3::P18077|RL35A_HUMAN	22	12530	1	1	1	1	0.45	60S ribosomal protein
1839	1	SwissProt_HUMAN$	3::P28356|HXD9_HUMAN	22	36472	1	1	1	1	0.14	Homeobox protein Hox
1840	1	db$2	2::C3KBJ0|C3KBJ0_PSEFS	22	16933	1	1	1	1	0.32	[Ribosomal protein S1
1841	1	SwissProt_HUMAN$	3::P06727|APOA4_HUMAN	22	45344	2	1	2	1	0.11	Apolipoprotein A-IV OS
1842	1	SwissProt_HUMAN$	3::P35580|MYH10_HUMAN	22	228858	1	1	1	1	0.02	Myosin-10 OS=Homo s
1843	1	db$2	2::C3K3R4|C3K3R4_PSEFS	22	24681	1	1	1	1	0.21	Putative hydrolase OS=
1844	1	db$2	2::C3K4C0|C3K4C0_PSEFS	21	9802	1	1	1	1	0.60	DNA-binding protein H
1845	1	SwissProt_HUMAN$	3::Q13304|GPR17_HUMAN	21	40963	1	1	1	1	0.12	Uracil nucleotide/cyste
1846	1	SwissProt_HUMAN$	3::Q5K651|SAMD9_HUMAN	21	184165	2	1	1	1	0.03	Sterile alpha motif dor
1847	1	SwissProt_HUMAN$	3::O60296|TRAK2_HUMAN	21	101356	1	1	1	1	0.05	Trafficking kinesin-bind
1848	1	SwissProt_HUMAN$	3::Q8WX94|NALP7_HUMAN	21	111735	1	1	1	1	0.04	NACHT, LRR and PYD d
1849	1	db$2	2::C3K3F2|C3K3F2_PSEFS	21	38228	1	1	1	1	0.13	D-erythrose-4-phospha
1850	1	SwissProt_HUMAN$	3::P47756|CAPZB_HUMAN	21	31331	1	1	1	1	0.16	F-actin-capping proteir
1851	1	SwissProt_HUMAN$	3::Q15075|EEA1_HUMAN	21	162367	1	1	1	1	0.03	Early endosome antige
1852	1	SwissProt_HUMAN$	3::P52333|JAK3_HUMAN	21	125019	1	1	1	1	0.04	Tyrosine-protein kinas
1853	1	SwissProt_HUMAN$	3::Q8WXW3|PIBF1_HUMAN	21	89750	1	1	1	1	0.05	Progesterone-induced-
1854	1	db$2	2::C3K596|C3K596_PSEFS	21	36487	1	1	1	1	0.14	Putative membrane pr
1855	1	db$2	2::C3K992|C3K992_PSEFS	21	34600	2	1	2	1	0.15	Putative AraC-family re
1856	1	db$2	2::C3KCP8|C3KCP8_PSEFS	21	42283	1	1	1	1	0.12	Lipopolysaccharide cor
1857	1	SwissProt_HUMAN$	3::P62241|RS8_HUMAN	21	24190	1	1	1	1	0.21	40S ribosomal protein
1858	1	SwissProt_HUMAN$	3::Q9Y265|RUVB1_HUMAN	21	50196	1	1	1	1	0.10	RuvB-like 1 OS=Homo
1859	1	SwissProt_HUMAN$	3::Q92547|TOPB1_HUMAN	21	170571	1	1	1	1	0.03	DNA topoisomerase 2-
1860	1	db$2	2::C3JZP4|RBSD_PSEFS	21	14393	1	1	1	1	0.38	D-ribose pyranase OS=
1861	1	db$2	2::C3K2F9|C3K2F9_PSEFS	21	48516	1	1	1	1	0.10	Putative ABC transport
1862	1	db$2	2::C3K9G3|C3K9G3_PSEFS	21	409423	2	1	2	1	0.01	Putative non-ribosoma

↑Family	M	DB	Accession	Score	Mass	Matches	Match(sig)	Sequences	Seq(sig)	emPAI	Description
1863	1	db$2	2::C3K5Q7|C3K5Q7_PSEFS	21	27879	1	1	1	1	0.18	Putative dehydrogenas
1864	1	SwissProt_HUMAN$	3::Q9UPN3|MACF1_HUMAN	21	837787	3	1	3	1	0.01	Microtubule-actin cross
1865	1	db$2	2::C3K6A6|C3K6A6_PSEFS	21	25667	1	1	1	1	0.20	Putative lipoprotein OS
1866	1	db$2	2::C3K5U3|C3K5U3_PSEFS	21	31637	1	1	1	1	0.16	dTDP-4-dehydrorhamr
1867	1	db$2	2::C3K5F2|Y1517_PSEFS	21	11422	1	1	1	1	0.50	YcgL domain-containin
1868	1	db$2	2::C3KAN9|C3KAN9_PSEFS	21	28318	1	1	1	1	0.18	Putative aromatic-hydı
1869	1	SwissProt_HUMAN$	3::P63173|RL38_HUMAN	21	8213	1	1	1	1	0.75	60S ribosomal protein
1870	1	db$2	2::C3K2I7|C3K2I7_PSEFS	21	17463	1	1	1	1	0.31	NYN domain-containin
1871	1	SwissProt_HUMAN$	3::Q9NRF8|PYRG2_HUMAN	21	65636	1	1	1	1	0.07	CTP synthase 2 OS=H
1872	1	db$2	2::C3K7D0|THII_PSEFS	21	54519	1	1	1	1	0.09	tRNA sulfurtransferase
1873	1	SwissProt_HUMAN$	3::Q8WZ42|TITIN_HUMAN	21	3813652	3	2	3	2	0.00	Titin OS=Homo sapien
1874	1	db$2	2::C3KDQ6|C3KDQ6_PSEFS	21	14780	1	1	1	1	0.37	Uncharacterized protei
1875	1	db$2	2::C3K5T8|C3K5T8_PSEFS	21	41768	1	1	1	1	0.12	Cytochrome c oxidase
1876	1	SwissProt_HUMAN$	3::P31943|HNRH1_HUMAN	20	49198	1	1	1	1	0.10	Heterogeneous nuclea
1877	1	SwissProt_HUMAN$	3::P58557|YBEY_HUMAN	20	19286	1	1	1	1	0.27	Endoribonuclease Ybe
1878	1	db$2	2::C3KAZ8|C3KAZ8_PSEFS	20	34711	1	1	1	1	0.15	Putative beta-hydroxyl
1879	1	SwissProt_HUMAN$	3::O43586|PPIP1_HUMAN	20	47562	1	1	1	1	0.10	Proline-serine-threonir
1880	1	SwissProt_HUMAN$	3::Q96PH1|NOX5_HUMAN	20	86383	1	1	1	1	0.06	NADPH oxidase 5 OS=
1881	1	db$2	2::C3K9U8|C3K9U8_PSEFS	20	28987	1	1	1	1	0.18	Putative haloacid deha
1882	1	db$2	2::A4V6S3|A4V6S3_PSEFS	20	39347	1	1	1	1	0.13	ALP_N domain-contain
1883	1	SwissProt_HUMAN$	3::A6NNS2|DRS7C_HUMAN	20	34855	1	1	1	1	0.15	Dehydrogenase/reduct
1884	1	db$2	2::C3K564|C3K564_PSEFS	20	54508	2	1	2	1	0.09	Methylmalonate-semia
1885	1	db$2	2::C3K8Z3|C3K8Z3_PSEFS	20	28227	2	1	2	1	0.18	High-affinity branched
1886	1	db$2	2::C3K3G8|C3K3G8_PSEFS	20	41555	1	1	1	1	0.12	Uncharacterized protei
1887	1	SwissProt_HUMAN$	3::Q8NBP5|MFSD9_HUMAN	20	50586	1	1	1	1	0.10	Major facilitator superf
1888	1	SwissProt_HUMAN$	3::P62917|RL8_HUMAN	20	28007	1	1	1	1	0.18	60S ribosomal protein
1889	1	db$2	2::C3K0D6|C3K0D6_PSEFS	20	24032	1	1	1	1	0.22	Ribosomal large subun
1890	1	SwissProt_HUMAN$	3::Q53EZ4|CEP55_HUMAN	20	54145	2	1	2	1	0.09	Centrosomal protein o
1891	1	SwissProt_HUMAN$	3::Q70UQ0|IKIP_HUMAN	20	39285	1	1	1	1	0.13	Inhibitor of nuclear fac
1892	1	db$2	2::C3JXX6|DNLJ_PSEFS	20	85515	2	1	2	1	0.06	DNA ligase OS=Pseud
1893	1	db$2	2::C3KB03|C3KB03_PSEFS	20	18864	1	1	1	1	0.28	Uncharacterized protei
1894	1	SwissProt_HUMAN$	3::Q5S007|LRRK2_HUMAN	20	285919	2	1	2	1	0.02	Leucine-rich repeat se
1895	1	db$2	2::C3K0W2|C3K0W2_PSEFS	20	26131	1	1	1	1	0.20	Flagellar basal body pr
1896	1	db$2	2::C3JY42|C3JY42_PSEFS	20	25186	1	1	1	1	0.21	Uncharacterized protei
1897	1	SwissProt_HUMAN$	3::P53396|ACLY_HUMAN	20	120762	1	1	1	1	0.04	ATP-citrate synthase C
1898	1	SwissProt_HUMAN$	3::O95613|PCNT_HUMAN	20	377806	2	1	2	1	0.01	Pericentrin OS=Homo
1899	1	db$2	2::C3K7J4|C3K7J4_PSEFS	20	18330	1	1	1	1	0.29	Uncharacterized protei
1900	1	db$2	2::C3K609|C3K609_PSEFS	20	73644	1	1	1	1	0.07	Putative lytic transglyc
1901	1	db$2	2::C3K8V0|C3K8V0_PSEFS	20	31271	1	1	1	1	0.16	Poly(3-hydroxyalkanoa
1902	1	db$2	2::C3K5G7|PDXB_PSEFS	20	41465	1	1	1	1	0.12	Erythronate-4-phospha

↑Family	M	DB	Accession	Score	Mass	Matches	Match(sig)	Sequences	Seq(sig)	emPAI	Description
1903	1	db$2	2::C3KAD1|C3KAD1_PSEFS	20	32401	2	1	2	1	0.16	Probable 4-deoxy-4-fo
1904	1	db$2	2::C3K9X2|C3K9X2_PSEFS	20	42284	2	1	2	1	0.12	Putative aminotransfer
1905	1	SwissProt_HUMAN$	3::Q7Z7L1|SLN11_HUMAN	19	102770	1	1	1	1	0.05	Schlafen family memb
1906	1	db$2	2::C3K2H7|C3K2H7_PSEFS	19	21527	1	1	1	1	0.24	Putative acetyltransfer
1907	1	SwissProt_HUMAN$	3::O96028|NSD2_HUMAN	19	152160	1	1	1	1	0.03	Histone-lysine N-meth
1908	1	db$2	2::C3K7T7|C3K7T7_PSEFS	19	17319	1	1	1	1	0.31	Putative acetyltransfer
1909	1	SwissProt_HUMAN$	3::P16471|PRLR_HUMAN	19	69461	1	1	1	1	0.07	Prolactin receptor OS=
1910	1	SwissProt_HUMAN$	3::Q9BRZ2|TRI56_HUMAN	19	81436	1	1	1	1	0.06	E3 ubiquitin-protein lig
1911	1	db$2	2::C3KAR1|C3KAR1_PSEFS	19	16023	1	1	1	1	0.34	Putative isoquinoline 1
1912	1	SwissProt_HUMAN$	3::P26373|RL13_HUMAN	19	24247	1	1	1	1	0.21	60S ribosomal protein
1913	1	db$2	2::C3JZ90|C3JZ90_PSEFS	19	52890	1	1	1	1	0.09	Trk system potassium
1914	1	db$2	2::C3K2L1|C3K2L1_PSEFS	19	28054	1	1	1	1	0.18	DUF218 domain-conta
1915	1	SwissProt_HUMAN$	3::Q9BWW9|APOL5_HUMAN	19	47014	1	1	1	1	0.11	Apolipoprotein L5 OS=
1916	1	db$2	2::C3K3H5|C3K3H5_PSEFS	19	32718	1	1	1	1	0.16	Putative ABC transport
1917	1	SwissProt_HUMAN$	3::Q8N8R5|CB069_HUMAN	19	43421	1	1	1	1	0.12	UPF0565 protein C2orf
1918	1	SwissProt_HUMAN$	3::Q9NR48|ASH1L_HUMAN	19	332582	1	1	1	1	0.01	Histone-lysine N-meth
1919	1	SwissProt_HUMAN$	3::Q9ULV3|CIZ1_HUMAN	19	99983	1	1	1	1	0.05	Cip1-interacting zinc fi
1920	1	SwissProt_HUMAN$	3::Q13085|ACACA_HUMAN	19	265385	2	1	2	1	0.02	Acetyl-CoA carboxylas
1921	1	SwissProt_HUMAN$	3::P17014|ZNF12_HUMAN	19	81149	1	1	1	1	0.06	Zinc finger protein 12
1922	1	db$2	2::C3K4W4|C3K4W4_PSEFS	19	45211	2	1	2	1	0.11	Putative lipoprotein rel
1923	1	db$2	2::C3KAE4|C3KAE4_PSEFS	19	39865	1	1	1	1	0.13	Uncharacterized protei
1924	1	db$2	2::C3KCG3|C3KCG3_PSEFS	19	16758	1	1	1	1	0.32	YjgF_endoribonc doma
1925	1	SwissProt_HUMAN$	3::P01009|A1AT_HUMAN	19	46707	1	1	1	1	0.11	Alpha-1-antitrypsin OS
1926	1	db$2	2::C3K7Y9|C3K7Y9_PSEFS	19	49907	1	1	1	1	0.10	NAD(P) transhydrogen
1927	1	db$2	2::C3K4U1|C3K4U1_PSEFS	19	34459	1	1	1	1	0.15	Putative lipoprotein OS
1928	1	db$2	2::C3K820|C3K820_PSEFS	19	26990	1	1	1	1	0.19	Putative ABC transport
1929	1	SwissProt_HUMAN$	3::O15397|IPO8_HUMAN	19	119861	1	1	1	1	0.04	Importin-8 OS=Homo
1930	1	SwissProt_HUMAN$	3::P62280|RS11_HUMAN	19	18419	1	1	1	1	0.29	40S ribosomal protein
1931	1	db$2	2::C3K3V4|C3K3V4_PSEFS	19	41512	1	1	1	1	0.12	Acetylornithine deacet
1932	1	db$2	2::C3K7J2|C3K7J2_PSEFS	19	18572	1	1	1	1	0.29	Putative membrane pr
1933	1	SwissProt_HUMAN$	3::Q14683|SMC1A_HUMAN	19	143144	2	1	2	1	0.03	Structural maintenanc
1934	1	db$2	2::C3K526|C3K526_PSEFS	19	40471	1	1	1	1	0.12	Cysteine desulfurase C
1935	1	db$2	2::C3KDW2|MIAA_PSEFS	19	35360	1	1	1	1	0.14	tRNA dimethylallyltran
1936	1	SwissProt_HUMAN$	3::Q9BSJ5|CQ080_HUMAN	19	67272	1	1	1	1	0.07	Uncharacterized protei
1937	1	db$2	2::C3K6L2|C3K6L2_PSEFS	19	14328	1	1	1	1	0.38	Organic hydroperoxide
1938	1	SwissProt_HUMAN$	3::Q8TEQ6|GEMI5_HUMAN	19	168483	1	1	1	1	0.03	Gem-associated protei
1939	1	db$2	2::C3KBE4|C3KBE4_PSEFS	18	35935	1	1	1	1	0.14	Alpha-E domain-contai
1940	1	SwissProt_HUMAN$	3::Q9NWS8|RMND1_HUMAN	18	51571	1	1	1	1	0.10	Required for meiotic n
1941	1	SwissProt_HUMAN$	3::Q92802|N42L2_HUMAN	18	67417	1	1	1	1	0.07	NEDD4-binding proteir
1942	1	db$2	2::C3KDP3|C3KDP3_PSEFS	18	34782	1	1	1	1	0.15	Putaive acyltransferas

↑Family	M	DB	Accession	Score	Mass	Matches	Match(sig)	Sequences	Seq(sig)	emPAI	Description
1943	1	db$2	2::C3KE69|C3KE69_PSEFS	18	32125	1	1	1	1	0.16	Putative membrane pr
1944	1	SwissProt_HUMAN$	3::Q9BYV7|BCDO2_HUMAN	18	65632	1	1	1	1	0.07	Beta,beta-carotene 9',
1945	1	db$2	2::C3JZA7|C3JZA7_PSEFS	18	51947	1	1	1	1	0.10	Putative succinate-sem
1946	1	SwissProt_HUMAN$	3::Q96F81|DISP1_HUMAN	18	170822	1	1	1	1	0.03	Protein dispatched hor
1947	1	db$2	2::C3JYR6|C3JYR6_PSEFS	18	38174	4	1	4	1	0.13	Quinolinate synthase A
1948	1	db$2	2::C3K0P1|C3K0P1_PSEFS	18	24214	1	1	1	1	0.21	Putative hydrolase OS=
1949	1	db$2	2::C3KAY5|C3KAY5_PSEFS	18	30881	3	1	3	1	0.17	NAD-capped RNA hydr
1950	1	SwissProt_HUMAN$	3::Q9BWT7|CAR10_HUMAN	18	115859	1	1	1	1	0.04	Caspase recruitment d
1951	1	db$2	2::C3JYG1|C3JYG1_PSEFS	18	30646	2	2	2	2	0.36	Putative LysR-family tr
1952	1	SwissProt_HUMAN$	3::Q9NSQ0|RRP7B_HUMAN	18	12568	1	1	1	1	0.45	Putative ribosomal RNA
1953	1	db$2	2::C3JYF7|C3JYF7_PSEFS	18	147681	1	1	1	1	0.03	ATP-dependent helicas
1954	1	db$2	2::C3JY01|C3JY01_PSEFS	18	74110	1	1	1	1	0.07	AAA_15 domain-conta
1955	1	db$2	2::C3K8Z7|C3K8Z7_PSEFS	18	10906	3	2	3	2	1.34	Uncharacterized protei
1956	1	SwissProt_HUMAN$	3::Q9H7M9|VISTA_HUMAN	18	33887	1	1	1	1	0.15	V-type immunoglobulir
1957	1	SwissProt_HUMAN$	3::Q13610|PWP1_HUMAN	18	55793	1	1	1	1	0.09	Periodic tryptophan pr
1958	1	db$2	2::C3KCV4|C3KCV4_PSEFS	18	19545	1	1	1	1	0.27	Putative DNA-binding r
1959	1	SwissProt_HUMAN$	3::Q13873|BMPR2_HUMAN	18	115129	1	1	1	1	0.04	Bone morphogenetic p
1960	1	db$2	2::C3KD58|C3KD58_PSEFS	18	41746	1	1	1	1	0.12	Putative cystathionine
1961	1	SwissProt_HUMAN$	3::P00558|PGK1_HUMAN	18	44586	2	2	1	1	0.11	Phosphoglycerate kina
1962	1	SwissProt_HUMAN$	3::P62266|RS23_HUMAN	18	15798	1	1	1	1	0.35	40S ribosomal protein
1963	1	db$2	2::C3KCJ3|C3KCJ3_PSEFS	18	30975	1	1	1	1	0.16	Usp domain-containing
1964	1	SwissProt_HUMAN$	3::Q8TEU7|RPGF6_HUMAN	18	179310	1	1	1	1	0.03	Rap guanine nucleotide
1965	1	SwissProt_HUMAN$	3::O95782|AP2A1_HUMAN	18	107478	1	1	1	1	0.05	AP-2 complex subunit
1966	1	db$2	2::C3K0E8|C3K0E8_PSEFS	18	27512	2	1	2	1	0.19	Thiol:disulfide intercha
1967	1	db$2	2::C3K670|C3K670_PSEFS	17	30761	1	1	1	1	0.17	Putative glycine betain
1968	1	db$2	2::C3K2L8|LIPA_PSEFS	17	38163	2	1	2	1	0.13	Lipoyl synthase OS=Ps
1969	1	db$2	2::C3K277|C3K277_PSEFS	17	60895	1	1	1	1	0.08	DNA repair protein Rec
1970	1	db$2	2::C3K597|C3K597_PSEFS	17	46587	1	1	1	1	0.11	Putative chaperone/he
1971	1	SwissProt_HUMAN$	3::Q5JV73|FRPD3_HUMAN	17	199085	2	1	2	1	0.02	FERM and PDZ domain
1972	1	db$2	2::C3KDV1|C3KDV1_PSEFS	17	30184	1	1	1	1	0.17	Chemotaxis MotA prote
1973	1	SwissProt_HUMAN$	3::Q9NP72|RAB18_HUMAN	17	22963	1	1	1	1	0.23	Ras-related protein Ra
1974	1	SwissProt_HUMAN$	3::P02647|APOA1_HUMAN	17	30759	1	1	1	1	0.17	Apolipoprotein A-I OS=
1975	1	db$2	2::C3K6P6|C3K6P6_PSEFS	17	34930	1	1	1	1	0.14	Putative ornithine cycl
1976	1	db$2	2::C3K323|C3K323_PSEFS	17	38795	1	1	1	1	0.13	Putative phosphotrans
1977	1	db$2	2::C3KAD9|C3KAD9_PSEFS	17	13242	2	1	2	1	0.42	HTH hxlR-type domain
1978	1	SwissProt_HUMAN$	3::P58166|INHBE_HUMAN	17	38537	1	1	1	1	0.13	Inhibin beta E chain O:
1979	1	SwissProt_HUMAN$	3::Q86TB3|ALPK2_HUMAN	17	236865	1	1	1	1	0.02	Alpha-protein kinase 2
1980	1	SwissProt_HUMAN$	3::P0DMQ9|CH089_HUMAN	17	18149	2	1	2	1	0.29	Putative uncharacteriz
1981	1	SwissProt_HUMAN$	3::Q7Z6J6|FRMD5_HUMAN	17	65023	1	1	1	1	0.08	FERM domain-containi
1982	1	SwissProt_HUMAN$	3::Q9NUQ8|ABCF3_HUMAN	16	79695	1	1	1	1	0.06	ATP-binding cassette s

↑Family	M	DB	Accession	Score	Mass	Matches	Match(sig)	Sequences	Seq(sig)	emPAI	Description
1983	1	db$2	2::C3K444|C3K444_PSEFS	16	9491	1	1	1	1	0.62	Ubiquinone biosynthes
1984	1	db$2	2::C3JY73|C3JY73_PSEFS	16	26430	1	1	1	1	0.20	Secretin_N domain-coı
1985	1	SwissProt_HUMAN$	3::Q9C098|DCLK3_HUMAN	16	73768	1	1	1	1	0.07	Serine/threonine-prote
1986	1	SwissProt_HUMAN$	3::P55285|CADH6_HUMAN	16	88254	1	1	1	1	0.06	Cadherin-6 OS=Homo
1987	1	SwissProt_HUMAN$	3::O75437|ZN254_HUMAN	16	77110	1	1	1	1	0.06	Zinc finger protein 254
1988	1	SwissProt_HUMAN$	3::Q9BXA5|SUCR1_HUMAN	16	38672	1	1	1	1	0.13	Succinate receptor 1 C
1989	1	db$2	2::C3JZ46|C3JZ46_PSEFS	16	37091	1	1	1	1	0.14	Putative LacI family tra
1990	1	SwissProt_HUMAN$	3::Q7L592|NDUF7_HUMAN	16	49206	1	1	1	1	0.10	Protein arginine methy
1991	1	SwissProt_HUMAN$	3::Q6P280|ZN529_HUMAN	16	65822	1	1	1	1	0.07	Zinc finger protein 529
1992	1	db$2	2::C3K8Z2|C3K8Z2_PSEFS	16	25669	1	1	1	1	0.20	High-affinity branched·
1993	1	db$2	2::C3K4V6|C3K4V6_PSEFS	16	101332	1	1	1	1	0.05	Putative helicase OS=I
1994	1	SwissProt_HUMAN$	3::P11362|FGFR1_HUMAN	16	91809	1	1	1	1	0.05	Fibroblast growth facto
1995	1	SwissProt_HUMAN$	3::Q7Z2T5|TRM1L_HUMAN	16	81695	1	1	1	1	0.06	TRMT1-like protein OS
1996	1	SwissProt_HUMAN$	3::Q9H427|KCNKF_HUMAN	16	36107	1	1	1	1	0.14	Potassium channel sub
1997	1	SwissProt_HUMAN$	3::Q6ZMU5|TRI72_HUMAN	16	52697	1	1	1	1	0.09	Tripartite motif-contair
1998	1	db$2	2::C3K577|C3K577_PSEFS	16	15787	1	1	1	1	0.35	Putative lipoprotein OS
1999	1	SwissProt_HUMAN$	3::P46020|KPB1_HUMAN	16	137225	1	1	1	1	0.04	Phosphorylase b kinas
2000	1	db$2	2::C3K3M0|MTGA_PSEFS	16	27247	1	1	1	1	0.19	Biosynthetic peptidogly
2001	1	db$2	2::C3K0S0|C3K0S0_PSEFS	16	12378	1	1	1	1	0.45	Putative DNA-binding p
2002	1	db$2	2::C3K589|C3K589_PSEFS	16	50032	2	1	2	1	0.10	Transcriptional regulat
2003	1	SwissProt_HUMAN$	3::Q9BT73|PSMG3_HUMAN	16	13096	1	1	1	1	0.43	Proteasome assembly
2004	1	SwissProt_HUMAN$	3::P67936|TPM4_HUMAN	16	28504	2	1	2	1	0.18	Tropomyosin alpha-4 c
2005	1	SwissProt_HUMAN$	3::Q7Z5N4|SDK1_HUMAN	16	241962	1	1	1	1	0.02	Protein sidekick-1 OS=
2006	1	SwissProt_HUMAN$	3::Q6PI48|SYDM_HUMAN	16	73516	1	1	1	1	0.07	Aspartate--tRNA ligase
2007	1	db$2	2::C3K9Q8|C3K9Q8_PSEFS	16	9438	1	1	1	1	0.63	Uncharacterized protei
2008	1	SwissProt_HUMAN$	3::Q8N1K5|THMS1_HUMAN	16	73406	1	1	1	1	0.07	Protein THEMIS OS=H
2009	1	SwissProt_HUMAN$	3::P98160|PGBM_HUMAN	16	468532	2	1	2	1	0.01	Basement membrane-:
2010	1	db$2	2::C3K1M8|C3K1M8_PSEFS	16	28345	1	1	1	1	0.18	tRNA (cytidine/uridine·
2011	1	db$2	2::C3KBW9|C3KBW9_PSEFS	15	64127	2	2	2	2	0.16	Putative lipoprotein OS
2012	1	SwissProt_HUMAN$	3::Q9NY46|SCN3A_HUMAN	15	226147	1	1	1	1	0.02	Sodium channel protei
2013	1	db$2	2::C3K7D7|C3K7D7_PSEFS	15	8979	1	1	1	1	0.67	Putative lipoprotein OS
2014	1	db$2	2::C3K0X5|C3K0X5_PSEFS	15	21842	1	1	1	1	0.24	Uncharacterized protei
2015	1	SwissProt_HUMAN$	3::P35611|ADDA_HUMAN	15	80905	1	1	1	1	0.06	Alpha-adducin OS=Hoı
2016	1	SwissProt_HUMAN$	3::Q4KMP7|TB10B_HUMAN	15	87145	1	1	1	1	0.06	TBC1 domain family m
2017	1	db$2	2::C3KA85|C3KA85_PSEFS	15	57956	1	1	1	1	0.09	Cyclic-guanylate-speci
2018	1	SwissProt_HUMAN$	3::Q02779|M3K10_HUMAN	15	103630	1	1	1	1	0.05	Mitogen-activated prot
2019	1	SwissProt_HUMAN$	3::Q9ULQ0|STRP2_HUMAN	15	95299	2	1	2	1	0.05	Striatin-interacting prc
2020	1	db$2	2::C3K5W0|C3K5W0_PSEFS	15	170278	2	1	2	1	0.03	DUF4329 domain-cont
2021	1	SwissProt_HUMAN$	3::Q9BQ69|MACD1_HUMAN	15	35483	1	1	1	1	0.14	ADP-ribose glycohydro
2022	1	SwissProt_HUMAN$	3::Q8N0W3|FCSK_HUMAN	15	117548	1	1	1	1	0.04	L-fucose kinase OS=H

↑Family	M	DB	Accession	Score	Mass	Matches	Match(sig)	Sequences	Seq(sig)	emPAI	Description
2023	1	db$2	2::C3JYV9|C3JYV9_PSEFS	15	44973	1	1	1	1	0.11	2-oxoisovalerate dehy
2024	1	db$2	2::C3K913|C3K913_PSEFS	15	30998	1	1	1	1	0.16	Uncharacterized protei
2025	1	SwissProt_HUMAN$	3::Q9Y6Z4|KIAS1_HUMAN	15	19398	1	1	1	1	0.27	Putative uncharacteriz
2026	1	SwissProt_HUMAN$	3::P08519|APOA_HUMAN	15	500995	1	1	1	1	0.01	Apolipoprotein(a) OS=
2027	1	db$2	2::C3K4L6|C3K4L6_PSEFS	15	17954	1	1	1	1	0.30	Putative exported prot
2028	1	SwissProt_HUMAN$	3::Q9UKR0|KLK12_HUMAN	15	26716	1	1	1	1	0.19	Kallikrein-12 OS=Hom
2029	1	db$2	2::C3K0H5|C3K0H5_PSEFS	15	25946	1	1	1	1	0.20	Putative chemotaxis pı
2030	1	db$2	2::C3K8R0|C3K8R0_PSEFS	15	13570	1	1	1	1	0.41	Uncharacterized protei
2031	1	SwissProt_HUMAN$	3::Q5JNZ5|RS26L_HUMAN	15	12994	1	1	1	1	0.43	Putative 40S ribosoma
2032	1	SwissProt_HUMAN$	3::P42224|STAT1_HUMAN	15	87280	1	1	1	1	0.06	Signal transducer and
2033	1	db$2	2::C3K2S9|C3K2S9_PSEFS	15	25730	2	1	2	1	0.20	Putative ABC transport
2034	1	SwissProt_HUMAN$	3::Q8IYT3|CC170_HUMAN	15	82226	1	1	1	1	0.06	Coiled-coil domain-cor
2035	1	SwissProt_HUMAN$	3::P39019|RS19_HUMAN	15	16051	1	1	1	1	0.34	40S ribosomal protein
2036	1	db$2	2::C3K404|C3K404_PSEFS	15	23654	1	1	1	1	0.22	Putative membrane pr
2037	1	SwissProt_HUMAN$	3::Q8TF21|ANR24_HUMAN	15	124111	2	1	2	1	0.04	Ankyrin repeat domair
2038	1	SwissProt_HUMAN$	3::Q14703|MBTP1_HUMAN	14	117674	1	1	1	1	0.04	Membrane-bound tran
2039	1	SwissProt_HUMAN$	3::Q7L945|ZN627_HUMAN	14	52819	1	1	1	1	0.09	Zinc finger protein 627
2040	1	db$2	2::C3JY26|C3JY26_PSEFS	14	17211	1	1	1	1	0.31	Protein-tyrosine-phosp
2041	1	db$2	2::C3K6K7|C3K6K7_PSEFS	14	17395	1	1	1	1	0.31	GreA_GreB domain-co
2042	1	db$2	2::C3K498|C3K498_PSEFS	14	106734	1	1	1	1	0.05	Non-specific serine/thr
2043	1	SwissProt_HUMAN$	3::Q5M9Q1|NKAPL_HUMAN	14	46284	2	1	1	1	0.11	NKAP-like protein OS=
2044	1	SwissProt_HUMAN$	3::P0C854|CECR9_HUMAN	14	23587	1	1	1	1	0.22	Putative cat eye syndr
2045	1	db$2	2::C3K5D7|C3K5D7_PSEFS	14	29152	1	1	1	1	0.18	ThiF domain-containin
2046	1	SwissProt_HUMAN$	3::O94827|PKHG5_HUMAN	14	117377	2	1	2	1	0.04	Pleckstrin homology d
2047	1	db$2	2::C3K0Q2|C3K0Q2_PSEFS	14	59785	1	1	1	1	0.08	Histidine kinase OS=P
2048	1	db$2	2::C3KBV7|NHAP2_PSEFS	14	61387	1	1	1	1	0.08	K(+)/H(+) antiporter I
2049	1	db$2	2::C3K954|C3K954_PSEFS	14	11787	1	1	1	1	0.48	Uncharacterized protei
2050	1	SwissProt_HUMAN$	3::Q6PCB6|AB17C_HUMAN	14	35808	1	1	1	1	0.14	Alpha/beta hydrolase d
2051	1	SwissProt_HUMAN$	3::Q9BYW2|SETD2_HUMAN	14	287418	1	1	1	1	0.02	Histone-lysine N-meth
2052	1	SwissProt_HUMAN$	3::Q00341|VIGLN_HUMAN	14	141368	2	1	2	1	0.03	Vigilin OS=Homo sapie
2053	1	SwissProt_HUMAN$	3::P02549|SPTA1_HUMAN	14	279842	1	1	1	1	0.02	Spectrin alpha chain, e
2054	1	SwissProt_HUMAN$	3::A6NE01|F186A_HUMAN	14	262595	1	1	1	1	0.02	Protein FAM186A OS=
2055	1	db$2	2::C3K457|C3K457_PSEFS	14	12769	1	1	1	1	0.44	Putative endoribonucle
2056	1	SwissProt_HUMAN$	3::Q96GC6|ZN274_HUMAN	14	74131	2	1	2	1	0.07	Neurotrophin receptor-
2057	1	db$2	2::C3K3J6|C3K3J6_PSEFS	13	14927	1	1	1	1	0.37	Type IV pilus response
2058	1	db$2	2::C3K0F1|C3K0F1_PSEFS	13	476575	3	1	3	1	0.01	Pyoverdine synthetase
2059	1	db$2	2::C3KC46|C3KC46_PSEFS	13	40282	1	1	1	1	0.12	Multidrug resistance pı
2060	1	SwissProt_HUMAN$	3::Q16512|PKN1_HUMAN	13	103868	1	1	1	1	0.05	Serine/threonine-prote
2061	1	SwissProt_HUMAN$	3::Q9UBS9|SUCO_HUMAN	13	139343	1	1	1	1	0.03	SUN domain-containin
2062	1	db$2	2::C3K009|C3K009_PSEFS	13	29965	1	1	1	1	0.17	Putative glutamine-bin

↑Family	M	DB	Accession	Score	Mass	Matches	Match(sig)	Sequences	Seq(sig)	emPAI	Description
2063	1	db$2	2::C3K694|C3K694_PSEFS	13	48962	1	1	1	1	0.10	[utative peptidase OS=
2064	1	SwissProt_HUMAN$	3::O60487|MPZL2_HUMAN	13	24469	1	1	1	1	0.21	Myelin protein zero-lik
2065	1	SwissProt_HUMAN$	3::Q96Q05|TPPC9_HUMAN	13	128449	1	1	1	1	0.04	Trafficking protein part
2066	1	db$2	2::C3KDN9|C3KDN9_PSEFS	13	40608	1	1	1	1	0.12	Probable periplasmic s
2067	1	SwissProt_HUMAN$	3::O75717|WDHD1_HUMAN	13	125888	1	1	1	1	0.04	WD repeat and HMG-b
2068	1	SwissProt_HUMAN$	3::Q6MZP7|LIN54_HUMAN	13	79444	1	1	1	1	0.06	Protein lin-54 homolog
2069	1	SwissProt_HUMAN$	3::O43897|TLL1_HUMAN	13	114635	1	1	1	1	0.04	Tolloid-like protein 1 C

Export as CSV

Not what you expected? Try **the select summary**.

Mascot: http://www.matrixscience.com/
