# Supplementary material for: Circulating multimeric immune complexes contribute to immunopathology in COVID-19
Source: Nat Commun. 2022 Sep 26;13:5654. doi: 10.1038/s41467-022-32867-z (PMC9513013; doi:10.1038/s41467-022-32867-z)
Supplement: Supplementary file 3 — Source Data [file 41467_2022_32867_MOESM3_ESM.zip › Source Data/MS_29012021 (whole lane cut).zip!/QX20210214-01_Virologie(AG-Hengel)-20210129_(V6)_IC+_Lys_complete-lane_0k3v20_TRL_db_SARS-CoV-2+SP_H(F229058).pdf]

MATRIX SCIENCE MASCOT Search Results

User : Wolfgang Bildl
E-mail : wolfgang.bildl@physiologie.uni-freiburg.de
Search title : QX 20210212 (db$ SARS-CoV-2+SwissProt HUMAN$ 5 ppm 20 mmu), QX20210214-01_Virologie(AG-Hengel)-20210129_(V6)_IC+_Lys_complete-lane_0k3v20_TRL_shifted_-4.0.mgf
MS data file : D:\Xcalibur\Data\Wolfgang\Q-Exactive-HF-X_20210212\QX20210214-01_Virologie(AG-Hengel)-20210129_(V6)_IC+_Lys_complete-lane_0k3v20_TRL_shifted_-4.0.mgf
Databases : **1:** db$ 20210114 (14 sequences; 14,319 residues)
2: SwissProt_HUMAN$ 20201007 (20,389 sequences; 11,363,789 residues)
Timestamp : 14 Feb 2021 at 09:44:07 GMT

Not what you expected? Try **the select summary**.

Search parameters

Type of search : MS/MS Ion Search
Enzyme : Trypsin
Variable modifications : **Acetyl (Protein N-term)**, **Carbamidomethyl (C)**, **Gln->pyro-Glu (N-term Q)**, **Oxidation (M)**
Mass values : Monoisotopic
Protein mass : Unrestricted
Peptide mass tolerance : ± 5 ppm
Fragment mass tolerance : ± 20 mmu
Max missed cleavages : 1
Instrument type : ESI-FTICR
Number of queries : 95,763

Score distribution

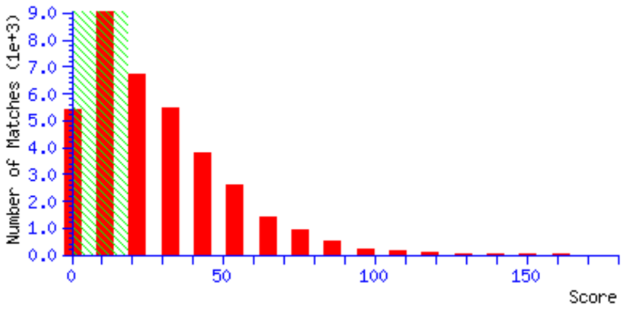

Peptide score distribution. Ions score is $-10\log(P)$, where P is the probability that the observed match is a random event.
There are **21,019** peptide matches above identity threshold and **21,841** matches above homology threshold for **95,763** queries.
On average, individual ions scores **> 18** (beyond green shading) indicate **identity or extensive homology** (p<0.05).

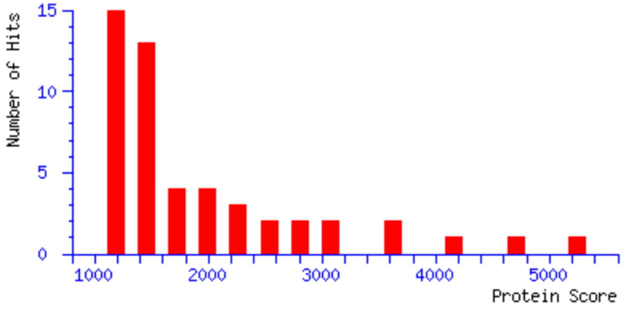

[Deprecated] Protein score distribution. Score distribution for family members in the first 50 proteins. Protein scores are derived from ions scores as a non-probabilistic basis for ranking protein families.

▼Modification statistics

Modification	Site	Above thr.
Carbamidomethyl	C	4160
Gln->pyro-Glu	N-term	1014
Oxidation	M	667
Acetyl	Protein N-term	390

▼Legend

Peptide columns and rows

Dupes	...	Expect	Rank	U	1	2	Peptide	
		**0.037**	▶**2**				**GAYSLSLR**	significant
		9	▶1				GFFLFVEGGR	top ranking
		**6.4e-005**	▶**1**				**GSSIFGLAPGK**	significant and top ranking
		**1.3e-006**	▶**1**		■		**SSGTSYPDVLK**	peptide is found in all proteins in family member 1
		**6.2e-007**	▶**1**			■	**VCNYVSWIK**	peptide is found in some but not all proteins in family member 2
		**6.4e-005**	▶**1**	**U**			**GSSIFGLAPGK**	unique
▶**2**		**5.7e-005**	▶**1**				**LNTLETEEWFFK**	peptide has two duplicates
		0.18	▶*1*				*LNTLETEEWFFK*	duplicate peptide

Right-facing triangle (▶) in the Dupes or Rank column indicates content that can be expanded by clicking on it. Down-facing triangle (▼) indicates the content is expanded and can be collapsed. For more details about particular columns, see **results format help**.

Protein quantitation ratios

	Score	...	114/113	115/113
**CFAH_HUMAN**	37559		*0.962*	**1.129**
**FHR2_HUMAN**	1330		**0.859**	1.128

When quantitation method is Reporter (e.g. iTRAQ) or Multiplex (e.g. IPTL), protein ratios are displayed when a family is expanded. Ratios in *italic* indicate that the peptide log-ratios do not appear to come from a normal distribution. **Bold** indicates that if you can assume peptide ratios are normally distributed, the protein ratio is significantly different from 1.0 (at significance level 0.05).

Note that lack of bold or italic can also mean that significance or normality testing has not been performed (for example, if protein ratio type does not support it).

Protein Family Summary

Significance threshold p<	0.05	Max. number of families	AUTO
Ions score or expect cut-off	0.5	Dendrograms cut at	0
Show Percolator scores	☐		
Preferred taxonomy	All entries		

Sensitivity and FDR (reversed protein sequences)

			Target	Decoy	FDR		
PSMs	above	homology	21841	325	1.49%	Adjust to	1%

Decoy results are available in **the decoy report**.

Protein hits (3224 proteins)

Filters: (none)

Export as CSV

↑Family	M	DB	Accession	Score	Mass	Matches	Match(sig)	Sequences	Seq(sig)	emPAI	Description
1	1	SwissProt_HUMAN$	2::P05787|K2C8_HUMAN	5243	53671	208	193	54	53	473.15	Keratin, type II cytosk
1	2	SwissProt_HUMAN$	2::P04264|K2C1_HUMAN	1396	65999	46	43	18	18	2.63	Keratin, type II cytosk
1	3	SwissProt_HUMAN$	2::P08729|K2C7_HUMAN	991	51354	48	43	9	8	1.09	Keratin, type II cytosk
1	4	SwissProt_HUMAN$	2::P35908|K22E_HUMAN	210	65393	12	12	9	9	0.92	Keratin, type II cytosk
1	5	SwissProt_HUMAN$	2::P13647|K2C5_HUMAN	136	62340	10	9	7	7	0.70	Keratin, type II cytosk
1	6	SwissProt_HUMAN$	2::O43790|KRT86_HUMAN	30	53466	3	2	2	2	0.19	Keratin, type II cuticu
2	1	SwissProt_HUMAN$	2::P02769|ALBU_BOVIN	4620	69248	177	170	66	65	110.81	Albumin OS=Bos tauri
2	2	SwissProt_HUMAN$	2::P02768|ALBU_HUMAN	595	69321	32	30	22	21	3.49	Albumin OS=Homo sa
3	1	SwissProt_HUMAN$	2::P05783|K1C18_HUMAN	4245	48029	150	129	40	35	100.62	Keratin, type I cytosk
3	2	SwissProt_HUMAN$	2::P08727|K1C19_HUMAN	2534	44079	99	91	40	38	137.42	Keratin, type I cytosk
3	3	SwissProt_HUMAN$	2::P13645|K1C10_HUMAN	736	58792	25	24	19	19	3.61	Keratin, type I cytosk
3	4	SwissProt_HUMAN$	2::Q04695|K1C17_HUMAN	337	48076	17	17	12	12	2.25	Keratin, type I cytosk
3	5	SwissProt_HUMAN$	2::P35527|K1C9_HUMAN	174	62027	6	6	6	6	0.58	Keratin, type I cytosk
3	6	SwissProt_HUMAN$	2::P19012|K1C15_HUMAN	159	49181	16	15	9	9	1.61	Keratin, type I cytosk
4	1	SwissProt_HUMAN$	2::P49327|FAS_HUMAN	3670	273254	153	142	107	100	5.20	Fatty acid synthase O:
4	2	SwissProt_HUMAN$	2::P28340|DPOD1_HUMAN	72	123553	4	3	3	2	0.08	DNA polymerase delta
5	1	SwissProt_HUMAN$	2::P10809|CH60_HUMAN	3559	61016	100	98	38	37	27.02	60 kDa heat shock prc
6	1	SwissProt_HUMAN$	2::P08238|HS90B_HUMAN	3211	83212	145	128	49	47	21.88	Heat shock protein HS
6	2	SwissProt_HUMAN$	2::P07900|HS90A_HUMAN	2805	84607	139	122	50	45	18.48	Heat shock protein HS
6	3	SwissProt_HUMAN$	2::P14625|ENPL_HUMAN	1405	92411	53	46	38	35	5.33	Endoplasmin OS=Hom
6	4	SwissProt_HUMAN$	2::Q12931|TRAP1_HUMAN	735	80060	31	30	21	21	2.47	Heat shock protein 75
6	5	SwissProt_HUMAN$	2::Q9NQH7|XPP3_HUMAN	59	56997	5	5	3	3	0.28	Xaa-Pro aminopeptida
7	1	SwissProt_HUMAN$	2::P07437|TBB5_HUMAN	3007	49639	102	100	32	31	59.12	Tubulin beta chain OS

↑Family	M	DB	Accession	Score	Mass	Matches	Match(sig)	Sequences	Seq(sig)	emPAI	Description
7	2	SwissProt_HUMAN$	2::P68371|TBB4B_HUMAN	2729	49799	100	96	32	31	52.70	Tubulin beta-4B chain
7	3	SwissProt_HUMAN$	2::Q9BVA1|TBB2B_HUMAN	2366	49921	86	84	29	28	26.44	Tubulin beta-2B chain
7	4	SwissProt_HUMAN$	2::Q13885|TBB2A_HUMAN	2282	49875	84	82	28	27	24.14	Tubulin beta-2A chain
7	5	SwissProt_HUMAN$	2::P04350|TBB4A_HUMAN	2226	49554	85	82	25	24	24.69	Tubulin beta-4A chain
7	6	SwissProt_HUMAN$	2::Q13509|TBB3_HUMAN	1287	50400	48	44	18	15	5.53	Tubulin beta-3 chain (
7	7	SwissProt_HUMAN$	2::Q9BUF5|TBB6_HUMAN	839	49825	34	32	12	12	3.15	Tubulin beta-6 chain (
8	1	SwissProt_HUMAN$	2::Q00610|CLH1_HUMAN	2870	191493	110	102	75	72	5.42	Clathrin heavy chain 1
9	1	SwissProt_HUMAN$	2::P06733|ENOA_HUMAN	2765	47139	83	79	32	31	32.31	Alpha-enolase OS=Ho
10	1	SwissProt_HUMAN$	2::P60709|ACTB_HUMAN	2664	41710	95	90	27	27	91.29	Actin, cytoplasmic 1 C
10	2	SwissProt_HUMAN$	2::P63261|ACTG_HUMAN	2639	41766	95	90	27	27	91.29	Actin, cytoplasmic 2 C
10	3	SwissProt_HUMAN$	2::P68032|ACTC_HUMAN	869	41992	47	43	17	16	8.49	Actin, alpha cardiac m
10	4	SwissProt_HUMAN$	2::Q562R1|ACTBL_HUMAN	500	41976	25	24	9	9	2.45	Beta-actin-like proteir
11	1	SwissProt_HUMAN$	2::P68104|EF1A1_HUMAN	2416	50109	114	106	27	26	21.56	Elongation factor 1-al
12	1	SwissProt_HUMAN$	2::P13639|EF2_HUMAN	2277	95277	88	80	49	46	12.31	Elongation factor 2 OS
13	1	SwissProt_HUMAN$	2::P11142|HSP7C_HUMAN	2269	70854	75	70	39	37	16.73	Heat shock cognate 7
13	2	SwissProt_HUMAN$	2::P0DMV8|HS71A_HUMAN	1451	70009	54	51	34	32	9.66	Heat shock 70 kDa pr
13	3	SwissProt_HUMAN$	2::P11021|BIP_HUMAN	1357	72288	50	47	29	27	5.27	Endoplasmic reticulum
14	1	SwissProt_HUMAN$	2::P68363|TBA1B_HUMAN	2215	50120	67	65	25	23	15.89	Tubulin alpha-1B chai
14	2	SwissProt_HUMAN$	2::Q9BQE3|TBA1C_HUMAN	2155	49863	67	65	26	24	17.92	Tubulin alpha-1C chai
14	3	SwissProt_HUMAN$	2::P68366|TBA4A_HUMAN	1520	49892	46	44	18	16	6.33	Tubulin alpha-4A chai
15	1	SwissProt_HUMAN$	2::P14618|KPYM_HUMAN	2055	57900	76	71	44	42	53.64	Pyruvate kinase PKM (
16	1	db$	1::P0DTC9|NCAP_SARS2	1985	45598	69	67	31	31	50.16	Nucleoprotein OS=Se
17	1	SwissProt_HUMAN$	2::P21333|FLNA_HUMAN	1914	280564	77	73	63	60	1.76	Filamin-A OS=Homo s
17	2	SwissProt_HUMAN$	2::O75369|FLNB_HUMAN	1490	277990	68	62	53	50	1.35	Filamin-B OS=Homo s
17	3	SwissProt_HUMAN$	2::Q14315|FLNC_HUMAN	614	290841	40	31	35	27	0.55	Filamin-C OS=Homo s
18	1	SwissProt_HUMAN$	2::P42704|LPPRC_HUMAN	1885	157805	77	71	58	56	4.39	Leucine-rich PPR moti
18	2	SwissProt_HUMAN$	2::Q13057|COASY_HUMAN	25	62290	2	2	2	2	0.16	Bifunctional coenzyme
19	1	SwissProt_HUMAN$	2::O43707|ACTN4_HUMAN	1798	104788	75	68	54	48	9.05	Alpha-actinin-4 OS=H
19	2	SwissProt_HUMAN$	2::P12814|ACTN1_HUMAN	960	102993	42	38	32	29	2.98	Alpha-actinin-1 OS=H
20	1	SwissProt_HUMAN$	2::P38646|GRP75_HUMAN	1752	73635	59	56	36	35	9.82	Stress-70 protein, mit
21	1	SwissProt_HUMAN$	2::P22314|UBA1_HUMAN	1601	117774	52	48	34	33	2.93	Ubiquitin-like modifier
22	1	SwissProt_HUMAN$	2::P55060|XPO2_HUMAN	1595	110346	72	64	42	39	5.35	Exportin-2 OS=Homo
22	2	SwissProt_HUMAN$	2::O60814|H2B1K_HUMAN	786	13882	35	34	12	12	152.17	Histone H2B type 1-K
22	3	SwissProt_HUMAN$	2::Q8N257|H2B3B_HUMAN	763	13900	36	34	12	12	152.17	Histone H2B type 3-B
23	1	SwissProt_HUMAN$	2::Q13813|SPTN1_HUMAN	1582	284364	69	66	58	55	1.59	Spectrin alpha chain,
24	1	SwissProt_HUMAN$	2::P04406|G3P_HUMAN	1503	36030	68	61	24	21	64.58	Glyceraldehyde-3-pho
25	1	SwissProt_HUMAN$	2::P19338|NUCL_HUMAN	1501	76568	50	45	30	28	5.39	Nucleolin OS=Homo s
26	1	db$	1::P0DTC2|SPIKE_SARS2	1487	141088	47	44	35	33	2.25	Spike glycoprotein OS
27	1	SwissProt_HUMAN$	2::Q9Y490|TLN1_HUMAN	1455	269599	59	53	47	41	1.06	Talin-1 OS=Homo sap
27	2	SwissProt_HUMAN$	2::Q9Y4G6|TLN2_HUMAN	100	271443	5	5	4	4	0.07	Talin-2 OS=Homo sap

↑Family	M	DB	Accession	Score	Mass	Matches	Match(sig)	Sequences	Seq(sig)	emPAI	Description
28	1	SwissProt_HUMAN$	2::P13010|XRCC5_HUMAN	1435	82652	52	50	36	34	9.48	X-ray repair cross-con
28	2	SwissProt_HUMAN$	2::Q13617|CUL2_HUMAN	64	86927	5	5	5	5	0.31	Cullin-2 OS=Homo sa
29	1	SwissProt_HUMAN$	2::P09327|VILI_HUMAN	1432	92637	51	49	33	32	4.41	Villin-1 OS=Homo sap
30	1	SwissProt_HUMAN$	2::P55072|TERA_HUMAN	1382	89266	58	50	39	34	5.77	Transitional endoplasr
31	1	SwissProt_HUMAN$	2::Q14974|IMB1_HUMAN	1369	97108	56	51	38	34	5.39	Importin subunit beta
32	1	SwissProt_HUMAN$	2::P60174|TPIS_HUMAN	1366	26653	44	41	21	20	39.72	Triosephosphate isom
33	1	SwissProt_HUMAN$	2::O00410|IPO5_HUMAN	1340	123550	44	41	33	32	2.42	Importin-5 OS=Homo
34	1	SwissProt_HUMAN$	2::P25705|ATPA_HUMAN	1318	59714	42	41	27	27	8.19	ATP synthase subunit
35	1	SwissProt_HUMAN$	2::P48163|MAOX_HUMAN	1317	64109	31	30	20	19	4.07	NADP-dependent mali
36	1	SwissProt_HUMAN$	2::Q01082|SPTB2_HUMAN	1314	274439	69	55	58	48	1.30	Spectrin beta chain, n
37	1	SwissProt_HUMAN$	2::P07814|SYEP_HUMAN	1291	170483	52	49	41	40	2.05	Bifunctional glutamate
38	1	SwissProt_HUMAN$	2::Q08211|DHX9_HUMAN	1287	140869	60	53	47	40	3.12	ATP-dependent RNA h
39	1	SwissProt_HUMAN$	2::P07355|ANXA2_HUMAN	1275	38580	50	49	29	29	43.11	Annexin A2 OS=Homo
39	2	SwissProt_HUMAN$	2::P07910|HNRPC_HUMAN	448	33650	25	23	14	13	7.18	Heterogeneous nuclea
39	3	SwissProt_HUMAN$	2::Q9UKM9|RALY_HUMAN	241	32444	12	12	10	10	3.28	RNA-binding protein R
40	1	SwissProt_HUMAN$	2::P31939|PUR9_HUMAN	1261	64575	47	44	35	33	11.08	Bifunctional purine bic
41	1	SwissProt_HUMAN$	2::Q92616|GCN1_HUMAN	1258	292572	49	48	40	39	0.88	eIF-2-alpha kinase act
42	1	SwissProt_HUMAN$	2::P78371|TCPB_HUMAN	1253	57452	48	44	30	28	10.80	T-complex protein 1 s
43	1	SwissProt_HUMAN$	2::P04075|ALDOA_HUMAN	1247	39395	41	40	24	24	24.37	Fructose-bisphosphate
43	2	SwissProt_HUMAN$	2::P09972|ALDOC_HUMAN	662	39431	16	16	9	9	1.94	Fructose-bisphosphate
44	1	SwissProt_HUMAN$	2::P09211|GSTP1_HUMAN	1244	23341	36	34	14	13	24.09	Glutathione S-transfer
45	1	SwissProt_HUMAN$	2::P62937|PPIA_HUMAN	1235	18001	44	39	15	13	106.95	Peptidyl-prolyl cis-trar
46	1	SwissProt_HUMAN$	2::Q9NR30|DDX21_HUMAN	1228	87290	54	47	36	33	5.00	Nucleolar RNA helicase
46	2	SwissProt_HUMAN$	2::Q9BQ39|DDX50_HUMAN	141	82514	7	6	6	5	0.33	ATP-dependent RNA h
47	1	SwissProt_HUMAN$	2::P35579|MYH9_HUMAN	1223	226392	55	45	45	38	1.22	Myosin-9 OS=Homo s
47	2	SwissProt_HUMAN$	2::P35580|MYH10_HUMAN	615	228858	28	24	25	22	0.58	Myosin-10 OS=Homo
47	3	SwissProt_HUMAN$	2::Q7Z406|MYH14_HUMAN	208	227732	12	11	11	10	0.23	Myosin-14 OS=Homo
48	1	SwissProt_HUMAN$	2::P00338|LDHA_HUMAN	1220	36665	43	39	25	23	31.35	L-lactate dehydrogena
48	2	SwissProt_HUMAN$	2::P07195|LDHB_HUMAN	1209	36615	40	40	22	22	24.00	L-lactate dehydrogena
49	1	SwissProt_HUMAN$	2::P02545|LMNA_HUMAN	1198	74095	46	41	37	33	7.77	Prelamin-A/C OS=Hor
49	2	SwissProt_HUMAN$	2::P20700|LMNB1_HUMAN	1059	66368	42	40	33	32	8.80	Lamin-B1 OS=Homo s
49	3	SwissProt_HUMAN$	2::Q03252|LMNB2_HUMAN	483	69906	27	24	24	22	3.44	Lamin-B2 OS=Homo s
50	1	SwissProt_HUMAN$	2::P26599|PTBP1_HUMAN	1180	57186	39	38	23	23	6.29	Polypyrimidine tract-b
50	2	SwissProt_HUMAN$	2::O95758|PTBP3_HUMAN	123	59652	7	6	6	5	0.61	Polypyrimidine tract-b
51	1	SwissProt_HUMAN$	2::O14980|XPO1_HUMAN	1153	123306	49	44	37	35	3.15	Exportin-1 OS=Homo
52	1	SwissProt_HUMAN$	2::P22626|ROA2_HUMAN	1152	37407	42	40	22	21	22.40	Heterogeneous nuclea
52	2	SwissProt_HUMAN$	2::P09651|ROA1_HUMAN	703	38723	28	26	15	13	4.50	Heterogeneous nuclea
52	3	SwissProt_HUMAN$	2::Q13151|ROA0_HUMAN	443	30822	18	16	12	12	5.24	Heterogeneous nuclea
52	4	SwissProt_HUMAN$	2::P51991|ROA3_HUMAN	380	39571	18	18	14	14	4.32	Heterogeneous nuclea
53	1	SwissProt_HUMAN$	2::P15924|DESP_HUMAN	1149	331569	64	51	57	45	0.91	Desmoplakin OS=Hon

↑Family	M	DB	Accession	Score	Mass	Matches	Match(sig)	Sequences	Seq(sig)	emPAI	Description
54	1	SwissProt_HUMAN$	2::P33176|KINH_HUMAN	1148	109617	34	33	27	26	2.21	Kinesin-1 heavy chain
54	2	SwissProt_HUMAN$	2::Q15046|SYK_HUMAN	377	68005	26	24	21	20	3.02	Lysine--tRNA ligase O
54	3	SwissProt_HUMAN$	2::P56937|DHB7_HUMAN	107	38182	4	4	4	4	0.64	3-keto-steroid reducta
54	4	SwissProt_HUMAN$	2::Q96MT7|CFA44_HUMAN	23	213731	3	2	3	2	0.05	Cilia- and flagella-asso
55	1	SwissProt_HUMAN$	2::P40227|TCPZ_HUMAN	1144	57988	38	35	21	20	5.03	T-complex protein 1 s
56	1	SwissProt_HUMAN$	2::P21796|VDAC1_HUMAN	1136	30754	29	27	19	18	14.75	Voltage-dependent an
56	2	SwissProt_HUMAN$	2::Q9Y277|VDAC3_HUMAN	279	30639	10	10	7	7	1.93	Voltage-dependent an
57	1	SwissProt_HUMAN$	2::Q13200|PSMD2_HUMAN	1129	100136	39	36	26	24	2.12	26S proteasome non-/
58	1	SwissProt_HUMAN$	2::P07237|PDIA1_HUMAN	1128	57081	42	39	30	29	13.20	Protein disulfide-isome
59	1	SwissProt_HUMAN$	2::P50395|GDIB_HUMAN	1125	50631	36	36	25	25	9.33	Rab GDP dissociation
60	1	SwissProt_HUMAN$	2::O75643|U520_HUMAN	1103	244353	50	45	42	37	1.05	U5 small nuclear ribor
61	1	SwissProt_HUMAN$	2::P53396|ACLY_HUMAN	1099	120762	47	43	34	32	2.80	ATP-citrate synthase (
62	1	SwissProt_HUMAN$	2::P14923|PLAK_HUMAN	1084	81693	37	36	27	26	3.79	Junction plakoglobin C
62	2	SwissProt_HUMAN$	2::P35222|CTNB1_HUMAN	191	85442	13	12	13	12	0.95	Catenin beta-1 OS=H
63	1	SwissProt_HUMAN$	2::P17987|TCPA_HUMAN	1081	60306	39	37	22	21	4.62	T-complex protein 1 s
64	1	SwissProt_HUMAN$	2::P34932|HSP74_HUMAN	1075	94271	39	37	33	32	4.26	Heat shock 70 kDa pro
64	2	SwissProt_HUMAN$	2::Q92598|HS105_HUMAN	820	96804	41	38	32	30	3.35	Heat shock protein 10
64	3	SwissProt_HUMAN$	2::O95757|HS74L_HUMAN	397	94453	17	17	16	16	1.23	Heat shock 70 kDa pro
65	1	SwissProt_HUMAN$	2::Q15029|U5S1_HUMAN	1050	109366	40	39	33	32	3.01	116 kDa U5 small nuc
66	1	SwissProt_HUMAN$	2::O00571|DDX3X_HUMAN	1040	73198	40	37	26	25	4.74	ATP-dependent RNA h
66	2	SwissProt_HUMAN$	2::P36776|LONM_HUMAN	314	106422	21	16	18	14	0.87	Lon protease homolog
67	1	SwissProt_HUMAN$	2::Q99832|TCPH_HUMAN	1012	59329	38	36	29	27	9.96	T-complex protein 1 s
68	1	SwissProt_HUMAN$	2::P09874|PARP1_HUMAN	1002	113012	43	39	32	30	2.67	Poly [ADP-ribose] poly
69	1	SwissProt_HUMAN$	2::O75533|SF3B1_HUMAN	999	145738	44	42	36	35	2.23	Splicing factor 3B sub
70	1	SwissProt_HUMAN$	2::P35221|CTNA1_HUMAN	987	100009	37	33	27	23	1.98	Catenin alpha-1 OS=H
71	1	SwissProt_HUMAN$	2::P50991|TCPD_HUMAN	983	57888	42	40	26	26	10.58	T-complex protein 1 s
71	2	SwissProt_HUMAN$	2::P48643|TCPE_HUMAN	586	59633	30	26	21	20	3.89	T-complex protein 1 s
72	1	SwissProt_HUMAN$	2::Q86VP6|CAND1_HUMAN	982	136289	48	43	31	29	1.84	Cullin-associated NED
73	1	SwissProt_HUMAN$	2::P40939|ECHA_HUMAN	973	82947	35	33	27	26	3.68	Trifunctional enzyme s
74	1	SwissProt_HUMAN$	2::P13489|RINI_HUMAN	958	49941	21	19	17	16	3.55	Ribonuclease inhibitor
75	1	SwissProt_HUMAN$	2::P00558|PGK1_HUMAN	954	44586	29	29	21	21	11.69	Phosphoglycerate kina
76	1	SwissProt_HUMAN$	2::P06576|ATPB_HUMAN	953	56525	31	31	20	20	5.86	ATP synthase subunit
77	1	SwissProt_HUMAN$	2::P50990|TCPQ_HUMAN	944	59583	43	36	32	28	9.82	T-complex protein 1 s
78	1	SwissProt_HUMAN$	2::P62805|H4_HUMAN	934	11360	56	50	14	14	1047.29	Histone H4 OS=Homo
79	1	SwissProt_HUMAN$	2::P61978|HNRPK_HUMAN	929	50944	43	41	28	27	13.74	Heterogeneous nuclea
80	1	SwissProt_HUMAN$	2::O60701|UGDH_HUMAN	924	54989	30	30	23	23	6.24	UDP-glucose 6-dehydr
81	1	SwissProt_HUMAN$	2::Q16555|DPYL2_HUMAN	922	62255	32	30	26	24	5.20	Dihydropyrimidinase-r
82	1	SwissProt_HUMAN$	2::P40926|MDHM_HUMAN	921	35481	33	30	19	18	11.46	Malate dehydrogenase
83	1	SwissProt_HUMAN$	2::P05023|AT1A1_HUMAN	916	112824	32	30	23	22	1.74	Sodium/potassium-tra
84	1	SwissProt_HUMAN$	2::P54886|P5CS_HUMAN	916	87248	48	41	31	29	4.69	Delta-1-pyrroline-5-ca

↑Family	M	DB	Accession	Score	Mass	Matches	Match(sig)	Sequences	Seq(sig)	emPAI	Description
85	1	SwissProt_HUMAN$	2::P43243|MATR3_HUMAN	899	94565	46	41	27	25	2.87	Matrin-3 OS=Homo sa
86	1	SwissProt_HUMAN$	2::P52272|HNRPM_HUMAN	873	77464	42	38	32	30	5.65	Heterogeneous nuclea
87	1	SwissProt_HUMAN$	2::P45974|UBP5_HUMAN	867	95725	33	30	25	22	2.28	Ubiquitin carboxyl-ter
87	2	SwissProt_HUMAN$	2::Q92995|UBP13_HUMAN	63	97265	3	3	3	3	0.16	Ubiquitin carboxyl-ter
88	1	SwissProt_HUMAN$	2::P30153|2AAA_HUMAN	860	65267	30	29	19	18	2.97	Serine/threonine-prot
88	2	SwissProt_HUMAN$	2::P30154|2AAB_HUMAN	710	66171	25	24	17	17	2.63	Serine/threonine-prot
89	1	SwissProt_HUMAN$	2::O00299|CLIC1_HUMAN	860	26906	28	27	16	16	18.61	Chloride intracellular c
90	1	SwissProt_HUMAN$	2::P04844|RPN2_HUMAN	858	69241	27	26	18	17	2.20	Dolichyl-diphosphoolig
91	1	SwissProt_HUMAN$	2::Q14697|GANAB_HUMAN	857	106807	41	40	30	29	2.62	Neutral alpha-glucosic
92	1	SwissProt_HUMAN$	2::P63104|1433Z_HUMAN	847	27728	33	29	22	20	24.05	14-3-3 protein zeta/d
92	2	SwissProt_HUMAN$	2::P62258|1433E_HUMAN	794	29155	28	25	18	16	17.21	14-3-3 protein epsilon
92	3	SwissProt_HUMAN$	2::P27348|1433T_HUMAN	756	27747	28	27	18	18	24.05	14-3-3 protein theta (
92	4	SwissProt_HUMAN$	2::P31946|1433B_HUMAN	604	28065	23	22	16	16	16.25	14-3-3 protein beta/a
92	5	SwissProt_HUMAN$	2::P61981|1433G_HUMAN	396	28285	19	17	15	13	7.68	14-3-3 protein gamma
92	6	SwissProt_HUMAN$	2::Q04917|1433F_HUMAN	222	28201	11	9	10	9	3.49	14-3-3 protein eta OS
92	7	SwissProt_HUMAN$	2::P31947|1433S_HUMAN	205	27757	8	7	7	6	1.77	14-3-3 protein sigma
93	1	SwissProt_HUMAN$	2::P12956|XRCC6_HUMAN	844	69799	40	35	21	20	3.45	X-ray repair cross-con
93	2	SwissProt_HUMAN$	2::Q9BXJ9|NAA15_HUMAN	344	101208	25	20	21	18	1.32	N-alpha-acetyltransfer
94	1	SwissProt_HUMAN$	2::Q15084|PDIA6_HUMAN	840	48091	33	26	18	17	4.32	Protein disulfide-isom
95	1	SwissProt_HUMAN$	2::Q00839|HNRPU_HUMAN	840	90528	41	33	28	24	2.90	Heterogeneous nuclea
96	1	SwissProt_HUMAN$	2::P29401|TKT_HUMAN	838	67835	40	37	28	26	6.06	Transketolase OS=Ho
97	1	SwissProt_HUMAN$	2::P36578|RL4_HUMAN	828	47667	38	36	23	22	10.95	60S ribosomal protein
98	1	SwissProt_HUMAN$	2::Q9NZI8|IF2B1_HUMAN	820	63441	36	33	27	25	6.48	Insulin-like growth fac
98	2	SwissProt_HUMAN$	2::O00425|IF2B3_HUMAN	601	63666	22	19	15	14	1.83	Insulin-like growth fac
98	3	SwissProt_HUMAN$	2::Q9Y6M1|IF2B2_HUMAN	520	66081	29	25	20	18	2.90	Insulin-like growth fac
99	1	SwissProt_HUMAN$	2::P02786|TFR1_HUMAN	813	84818	38	34	28	24	2.82	Transferrin receptor p
100	1	SwissProt_HUMAN$	2::P27708|PYR1_HUMAN	807	242829	38	34	32	29	0.76	CAD protein OS=Hom
101	1	SwissProt_HUMAN$	2::P78527|PRKDC_HUMAN	800	468788	40	37	38	35	0.43	DNA-dependent protei
102	1	SwissProt_HUMAN$	2::P26640|SYVC_HUMAN	799	140387	38	34	29	28	1.58	Valine--tRNA ligase O
102	2	SwissProt_HUMAN$	2::O75475|PSIP1_HUMAN	38	60067	2	2	2	2	0.17	PC4 and SFRS1-intera
103	1	SwissProt_HUMAN$	2::P23526|SAHH_HUMAN	788	47685	32	30	23	23	8.75	Adenosylhomocysteina
103	2	SwissProt_HUMAN$	2::O43865|SAHH2_HUMAN	77	58913	5	5	4	4	0.38	S-adenosylhomocyste
104	1	SwissProt_HUMAN$	2::P60842|IF4A1_HUMAN	787	46125	30	28	19	17	5.99	Eukaryotic initiation fa
104	2	SwissProt_HUMAN$	2::P38919|IF4A3_HUMAN	504	46841	19	16	16	13	2.71	Eukaryotic initiation fa
104	3	SwissProt_HUMAN$	2::Q14240|IF4A2_HUMAN	500	46373	18	16	13	11	2.07	Eukaryotic initiation fa
105	1	SwissProt_HUMAN$	2::P63244|RACK1_HUMAN	787	35055	25	21	15	14	5.57	Receptor of activated
106	1	SwissProt_HUMAN$	2::P18669|PGAM1_HUMAN	786	28786	27	27	13	13	12.72	Phosphoglycerate mut
107	1	SwissProt_HUMAN$	2::P18206|VINC_HUMAN	785	123722	38	35	30	28	2.04	Vinculin OS=Homo sa
108	1	SwissProt_HUMAN$	2::O60610|DIAP1_HUMAN	784	141258	35	28	30	25	1.32	Protein diaphanous ho
109	1	SwissProt_HUMAN$	2::Q13263|TIF1B_HUMAN	774	88493	36	29	27	25	2.82	Transcription intermed

↑Family	M	DB	Accession	Score	Mass	Matches	Match(sig)	Sequences	Seq(sig)	emPAI	Description
110	1	SwissProt_HUMAN$	2::P49588|SYAC_HUMAN	773	106743	34	32	30	29	2.62	Alanine--tRNA ligase,
111	1	SwissProt_HUMAN$	2::P49736|MCM2_HUMAN	768	101832	29	27	21	19	1.66	DNA replication licensi
112	1	SwissProt_HUMAN$	2::O60716|CTND1_HUMAN	764	108103	29	28	23	22	1.74	Catenin delta-1 OS=H
113	1	SwissProt_HUMAN$	2::P13796|PLSL_HUMAN	764	70244	29	27	21	19	2.60	Plastin-2 OS=Homo sa
113	2	SwissProt_HUMAN$	2::P13797|PLST_HUMAN	435	70766	17	16	14	13	1.39	Plastin-3 OS=Homo sa
113	3	SwissProt_HUMAN$	2::Q14651|PLSI_HUMAN	303	70209	10	10	6	6	0.50	Plastin-1 OS=Homo sa
114	1	SwissProt_HUMAN$	2::P11586|C1TC_HUMAN	761	101495	37	33	33	29	2.88	C-1-tetrahydrofolate s
115	1	SwissProt_HUMAN$	2::O43175|SERA_HUMAN	758	56614	29	27	18	18	4.32	D-3-phosphoglycerate
116	1	SwissProt_HUMAN$	2::P61247|RS3A_HUMAN	758	29926	27	24	19	17	15.91	40S ribosomal protein
117	1	SwissProt_HUMAN$	2::P35232|PHB_HUMAN	757	29786	27	25	21	19	22.69	Prohibitin OS=Homo s
118	1	SwissProt_HUMAN$	2::P62979|RS27A_HUMAN	756	17953	34	30	10	10	21.67	Ubiquitin-40S ribosom
119	1	SwissProt_HUMAN$	2::O95373|IPO7_HUMAN	754	119440	32	29	26	23	1.49	Importin-7 OS=Homo
119	2	SwissProt_HUMAN$	2::O15397|IPO8_HUMAN	187	119861	8	8	7	7	0.32	Importin-8 OS=Homo
120	1	SwissProt_HUMAN$	2::P12270|TPR_HUMAN	750	267131	32	29	28	26	0.59	Nucleoprotein TPR OS
121	1	SwissProt_HUMAN$	2::Q7KZF4|SND1_HUMAN	744	101934	34	28	31	26	2.35	Staphylococcal nuclea
122	1	SwissProt_HUMAN$	2::P22102|PUR2_HUMAN	741	107699	40	35	32	28	2.59	Trifunctional purine bi
123	1	SwissProt_HUMAN$	2::P56192|SYMC_HUMAN	736	101052	25	24	21	20	1.56	Methionine--tRNA liga
124	1	SwissProt_HUMAN$	2::Q15365|PCBP1_HUMAN	724	37474	20	19	13	12	5.59	Poly(rC)-binding prote
124	2	SwissProt_HUMAN$	2::Q15366|PCBP2_HUMAN	451	38556	15	13	10	8	2.40	Poly(rC)-binding prote
124	3	SwissProt_HUMAN$	2::P57721|PCBP3_HUMAN	247	39440	8	7	5	4	1.05	Poly(rC)-binding prote
125	1	SwissProt_HUMAN$	2::P49411|EFTU_HUMAN	724	49510	28	25	21	19	5.14	Elongation factor Tu, r
126	1	SwissProt_HUMAN$	2::P52292|IMA1_HUMAN	718	57826	25	23	16	14	2.70	Importin subunit alpha
127	1	SwissProt_HUMAN$	2::Q6P2Q9|PRP8_HUMAN	718	273427	50	39	45	34	0.81	Pre-mRNA-processing
128	1	SwissProt_HUMAN$	2::Q9Y4L1|HYOU1_HUMAN	710	111266	28	26	22	20	1.56	Hypoxia up-regulated
129	1	SwissProt_HUMAN$	2::P23396|RS3_HUMAN	693	26671	35	32	20	18	27.61	40S ribosomal protein
130	1	SwissProt_HUMAN$	2::Q92621|NU205_HUMAN	693	227776	32	29	29	26	0.72	Nuclear pore complex
131	1	SwissProt_HUMAN$	2::P30101|PDIA3_HUMAN	689	56747	32	28	25	22	5.26	Protein disulfide-isome
132	1	SwissProt_HUMAN$	2::P15311|EZRI_HUMAN	689	69370	40	34	29	24	5.31	Ezrin OS=Homo sapie
132	2	SwissProt_HUMAN$	2::P35241|RADI_HUMAN	492	68521	22	19	19	16	2.02	Radixin OS=Homo sap
133	1	SwissProt_HUMAN$	2::Q9Y262|EIF3L_HUMAN	682	66684	27	26	21	21	3.76	Eukaryotic translation
134	1	SwissProt_HUMAN$	2::P54136|SYRC_HUMAN	675	75331	36	34	29	27	4.81	Arginine--tRNA ligase,
135	1	SwissProt_HUMAN$	2::P08195|4F2_HUMAN	673	67952	25	25	20	20	3.03	4F2 cell-surface antige
136	1	SwissProt_HUMAN$	2::Q14980|NUMA1_HUMAN	669	238115	47	40	45	38	1.13	Nuclear mitotic appara
137	1	SwissProt_HUMAN$	2::P08865|RSSA_HUMAN	665	32833	22	19	13	12	5.45	40S ribosomal protein
138	1	SwissProt_HUMAN$	2::Q9UQ80|PA2G4_HUMAN	663	43759	21	20	15	15	4.05	Proliferation-associate
139	1	SwissProt_HUMAN$	2::P55884|EIF3B_HUMAN	662	92424	29	26	22	20	1.93	Eukaryotic translation
140	1	SwissProt_HUMAN$	2::P31943|HNRH1_HUMAN	660	49198	22	21	16	15	3.23	Heterogeneous nuclea
140	2	SwissProt_HUMAN$	2::P52597|HNRPF_HUMAN	498	45643	35	34	12	12	2.46	Heterogeneous nuclea
140	3	SwissProt_HUMAN$	2::P31942|HNRH3_HUMAN	343	36903	12	10	8	7	1.78	Heterogeneous nuclea
141	1	SwissProt_HUMAN$	2::P50454|SERPH_HUMAN	651	46411	22	21	13	13	2.76	Serpin H1 OS=Homo s

↑Family	M	DB	Accession	Score	Mass	Matches	Match(sig)	Sequences	Seq(sig)	emPAI	Description
142	1	SwissProt_HUMAN$	2::P22234|PUR6_HUMAN	651	47049	27	27	23	23	10.13	Multifunctional protein
143	1	SwissProt_HUMAN$	2::P25205|MCM3_HUMAN	649	90924	27	25	22	20	1.84	DNA replication licensi
144	1	SwissProt_HUMAN$	2::Q92945|FUBP2_HUMAN	648	73070	23	21	20	18	2.21	Far upstream element
145	1	SwissProt_HUMAN$	2::P49368|TCPG_HUMAN	647	60495	34	30	24	21	5.04	T-complex protein 1 s
146	1	SwissProt_HUMAN$	2::P62701|RS4X_HUMAN	645	29579	31	30	25	24	52.83	40S ribosomal protein
147	1	SwissProt_HUMAN$	2::P41250|GARS_HUMAN	645	83113	25	21	17	14	1.22	Glycine--tRNA ligase (
148	1	SwissProt_HUMAN$	2::P05141|ADT2_HUMAN	641	32831	24	23	17	16	12.22	ADP/ATP translocase 2
148	2	SwissProt_HUMAN$	2::P12235|ADT1_HUMAN	549	33043	17	16	12	12	4.53	ADP/ATP translocase 1
148	3	SwissProt_HUMAN$	2::P12236|ADT3_HUMAN	543	32845	22	20	17	16	8.92	ADP/ATP translocase 3
149	1	SwissProt_HUMAN$	2::P53621|COPA_HUMAN	640	138258	38	33	30	26	1.53	Coatomer subunit alpl
150	1	SwissProt_HUMAN$	2::Q15436|SC23A_HUMAN	628	86105	21	19	18	17	1.55	Protein transport prot
150	2	SwissProt_HUMAN$	2::Q15437|SC23B_HUMAN	91	86424	4	4	4	4	0.25	Protein transport prot
151	1	SwissProt_HUMAN$	2::P21964|COMT_HUMAN	624	30018	18	17	11	11	4.63	Catechol O-methyltrar
152	1	SwissProt_HUMAN$	2::P04843|RPN1_HUMAN	623	68527	30	30	23	23	3.90	Dolichyl-diphosphoolig
153	1	SwissProt_HUMAN$	2::P00367|DHE3_HUMAN	622	61359	29	25	20	18	3.01	Glutamate dehydroger
154	1	SwissProt_HUMAN$	2::Q04637|IF4G1_HUMAN	619	175382	32	30	28	26	1.02	Eukaryotic translation
154	2	SwissProt_HUMAN$	2::P26368|U2AF2_HUMAN	358	53467	15	14	10	9	1.22	Splicing factor U2AF 6
154	3	SwissProt_HUMAN$	2::O43432|IF4G3_HUMAN	136	176542	6	5	5	4	0.11	Eukaryotic translation
154	4	SwissProt_HUMAN$	2::Q8TEA8|DTD1_HUMAN	130	23409	5	4	4	3	0.82	D-aminoacyl-tRNA dea
154	5	SwissProt_HUMAN$	2::Q8WZ42|TITIN_HUMAN	19	3813652	5	2	4	2	0.00	Titin OS=Homo sapier
155	1	SwissProt_HUMAN$	2::Q12906|ILF3_HUMAN	612	95279	33	26	21	18	1.45	Interleukin enhancer-l
156	1	SwissProt_HUMAN$	2::O15371|EIF3D_HUMAN	608	63932	20	18	15	14	1.82	Eukaryotic translation
157	1	SwissProt_HUMAN$	2::Q8TEX9|IPO4_HUMAN	607	118640	23	21	20	18	1.14	Importin-4 OS=Homo
158	1	SwissProt_HUMAN$	2::P29692|EF1D_HUMAN	601	31103	19	16	11	9	3.55	Elongation factor 1-de
158	2	SwissProt_HUMAN$	2::P24534|EF1B_HUMAN	217	24748	7	7	7	7	2.78	Elongation factor 1-be
159	1	SwissProt_HUMAN$	2::O43390|HNRPR_HUMAN	600	70899	30	28	20	20	2.80	Heterogeneous nuclea
159	2	SwissProt_HUMAN$	2::O60506|HNRPQ_HUMAN	465	69560	23	21	17	17	2.18	Heterogeneous nuclea
160	1	SwissProt_HUMAN$	2::Q99714|HCD2_HUMAN	597	26906	17	17	13	13	8.74	3-hydroxyacyl-CoA de
161	1	SwissProt_HUMAN$	2::Q9P2M7|CING_HUMAN	597	136304	27	22	25	21	1.08	Cingulin OS=Homo sa
162	1	SwissProt_HUMAN$	2::Q96P70|IPO9_HUMAN	596	115889	20	20	17	17	1.01	Importin-9 OS=Homo
163	1	SwissProt_HUMAN$	2::P13804|ETFA_HUMAN	595	35058	23	22	16	16	11.86	Electron transfer flavo
164	1	SwissProt_HUMAN$	2::O43143|DHX15_HUMAN	594	90875	29	27	21	19	1.84	Pre-mRNA-splicing fac
165	1	SwissProt_HUMAN$	2::O76021|RL1D1_HUMAN	591	54939	21	20	15	14	2.64	Ribosomal L1 domain-
166	1	SwissProt_HUMAN$	2::P52209|6PGD_HUMAN	587	53106	23	23	16	16	3.55	6-phosphogluconate d
167	1	SwissProt_HUMAN$	2::Q9Y678|COPG1_HUMAN	587	97655	25	24	21	20	1.64	Coatomer subunit gan
167	2	SwissProt_HUMAN$	2::Q9UBF2|COPG2_HUMAN	174	97560	6	6	5	5	0.27	Coatomer subunit gan
168	1	SwissProt_HUMAN$	2::P26641|EF1G_HUMAN	584	50087	25	23	20	19	5.61	Elongation factor 1-ga
169	1	SwissProt_HUMAN$	2::P68431|H31_HUMAN	583	15394	21	19	9	8	19.55	Histone H3.1 OS=Hon
169	2	SwissProt_HUMAN$	2::Q71DI3|H32_HUMAN	515	15379	21	18	9	8	19.55	Histone H3.2 OS=Hon
169	3	SwissProt_HUMAN$	2::P84243|H33_HUMAN	371	15318	17	15	9	8	10.42	Histone H3.3 OS=Hon

↑Family	M	DB	Accession	Score	Mass	Matches	Match(sig)	Sequences	Seq(sig)	emPAI	Description
170	1	SwissProt_HUMAN$	2::Q14204|DYHC1_HUMAN	581	532072	38	33	36	33	0.34	Cytoplasmic dynein 1
171	1	SwissProt_HUMAN$	2::P06744|G6PI_HUMAN	578	63107	28	27	20	19	3.48	Glucose-6-phosphate
172	1	SwissProt_HUMAN$	2::Q96T76|MMS19_HUMAN	576	113217	23	21	19	17	1.13	MMS19 nucleotide exc
173	1	SwissProt_HUMAN$	2::P51659|DHB4_HUMAN	575	79636	26	22	22	19	2.10	Peroxisomal multifunc
174	1	SwissProt_HUMAN$	2::Q99623|PHB2_HUMAN	574	33276	19	16	16	13	6.25	Prohibitin-2 OS=Homo
175	1	SwissProt_HUMAN$	2::P06748|NPM_HUMAN	571	32555	25	23	13	11	7.79	Nucleophosmin OS=H
176	1	SwissProt_HUMAN$	2::P49915|GUAA_HUMAN	568	76667	28	23	21	19	2.23	GMP synthase [glutam
177	1	SwissProt_HUMAN$	2::P23246|SFPQ_HUMAN	564	76102	28	23	19	16	1.88	Splicing factor, proline
177	2	SwissProt_HUMAN$	2::Q15233|NONO_HUMAN	457	54197	58	24	14	14	3.04	Non-POU domain-cont
177	3	SwissProt_HUMAN$	2::Q8WXF1|PSPC1_HUMAN	372	58706	50	15	10	9	1.07	Paraspeckle componei
178	1	SwissProt_HUMAN$	2::Q16891|MIC60_HUMAN	561	83626	32	28	25	22	2.68	MICOS complex subur
179	1	SwissProt_HUMAN$	2::Q96AG4|LRC59_HUMAN	559	34909	22	20	16	14	5.64	Leucine-rich repeat-cc
180	1	SwissProt_HUMAN$	2::P47897|SYQ_HUMAN	556	87743	27	27	25	25	2.86	Glutamine--tRNA ligas
181	1	SwissProt_HUMAN$	2::P46782|RS5_HUMAN	555	22862	18	17	12	11	8.56	40S ribosomal protein
182	1	SwissProt_HUMAN$	2::P05388|RLA0_HUMAN	552	34252	19	19	12	12	4.20	60S acidic ribosomal p
183	1	SwissProt_HUMAN$	2::Q9H0A0|NAT10_HUMAN	552	115657	38	34	28	26	1.90	RNA cytidine acetyltra
183	2	SwissProt_HUMAN$	2::E9PRG8|CK098_HUMAN	52	14225	6	4	4	3	1.67	Uncharacterized prote
183	3	SwissProt_HUMAN$	2::Q96CN9|GCC1_HUMAN	27	87757	3	2	2	2	0.11	GRIP and coiled-coil d
184	1	SwissProt_HUMAN$	2::Q9UNM6|PSD13_HUMAN	550	42918	20	19	14	13	3.17	26S proteasome non-/
185	1	SwissProt_HUMAN$	2::P14866|HNRPL_HUMAN	550	64092	27	25	19	18	3.07	Heterogeneous nuclea
186	1	SwissProt_HUMAN$	2::P17655|CAN2_HUMAN	548	79945	23	21	19	18	1.91	Calpain-2 catalytic sul
187	1	SwissProt_HUMAN$	2::Q9HAV4|XPO5_HUMAN	545	136222	23	21	18	16	0.75	Exportin-5 OS=Homo
188	1	SwissProt_HUMAN$	2::Q9P2E9|RRBP1_HUMAN	544	152365	21	20	20	19	0.81	Ribosome-binding pro
189	1	SwissProt_HUMAN$	2::Q12905|ILF2_HUMAN	544	43035	22	17	16	13	3.16	Interleukin enhancer-l
190	1	db$	1::P0DTC1|R1A_SARS2	543	489672	29	24	28	23	0.25	Replicase polyprotein
191	1	SwissProt_HUMAN$	2::Q9NY33|DPP3_HUMAN	541	82538	21	20	17	16	1.50	Dipeptidyl peptidase 3
192	1	SwissProt_HUMAN$	2::Q01518|CAP1_HUMAN	541	51869	25	25	19	19	4.66	Adenylyl cyclase-asso
192	2	SwissProt_HUMAN$	2::P40123|CAP2_HUMAN	27	52791	2	2	2	2	0.20	Adenylyl cyclase-asso
193	1	SwissProt_HUMAN$	2::P16435|NCPR_HUMAN	538	76641	19	19	17	17	1.86	NADPH--cytochrome P
194	1	SwissProt_HUMAN$	2::O00429|DNM1L_HUMAN	536	81826	19	18	16	15	1.38	Dynamin-1-like protei
195	1	SwissProt_HUMAN$	2::P27797|CALR_HUMAN	535	48112	21	20	14	13	2.59	Calreticulin OS=Homo
196	1	SwissProt_HUMAN$	2::P37802|TAGL2_HUMAN	534	22377	25	21	16	14	22.08	Transgelin-2 OS=Hom
197	1	SwissProt_HUMAN$	2::Q14152|EIF3A_HUMAN	533	166468	44	30	35	25	1.04	Eukaryotic translation
198	1	SwissProt_HUMAN$	2::Q06210|GFPT1_HUMAN	533	78756	19	16	16	14	1.46	Glutamine--fructose-6
199	1	SwissProt_HUMAN$	2::P12268|IMDH2_HUMAN	532	55770	18	18	15	15	2.57	Inosine-5'-monophosp
199	2	SwissProt_HUMAN$	2::P20839|IMDH1_HUMAN	32	55370	5	5	5	5	0.53	Inosine-5'-monophosp
200	1	SwissProt_HUMAN$	2::P13667|PDIA4_HUMAN	530	72887	23	20	20	18	2.22	Protein disulfide-isome
201	1	SwissProt_HUMAN$	2::P31948|STIP1_HUMAN	526	62599	27	25	22	20	3.54	Stress-induced-phospl
202	1	SwissProt_HUMAN$	2::Q9UJS0|CMC2_HUMAN	521	74129	23	23	15	15	1.61	Calcium-binding mitoc
202	2	SwissProt_HUMAN$	2::O75746|CMC1_HUMAN	142	74715	10	10	9	9	0.77	Calcium-binding mitoc

↑Family	M	DB	Accession	Score	Mass	Matches	Match(sig)	Sequences	Seq(sig)	emPAI	Description
203	1	SwissProt_HUMAN$	2::P53618|COPB_HUMAN	521	107074	29	24	23	19	1.32	Coatomer subunit beta
204	1	SwissProt_HUMAN$	2::P11940|PABP1_HUMAN	520	70626	25	23	20	18	2.58	Polyadenylate-binding
204	2	SwissProt_HUMAN$	2::Q13310|PABP4_HUMAN	382	70738	14	14	11	11	1.23	Polyadenylate-binding
205	1	SwissProt_HUMAN$	2::P25787|PSA2_HUMAN	520	25882	15	12	11	8	3.28	Proteasome subunit al
206	1	SwissProt_HUMAN$	2::O60664|PLIN3_HUMAN	520	47046	14	14	13	13	2.69	Perilipin-3 OS=Homo
207	1	SwissProt_HUMAN$	2::Q9H4A4|AMPB_HUMAN	519	72549	21	19	19	18	2.24	Aminopeptidase B OS=
208	1	SwissProt_HUMAN$	2::Q02878|RL6_HUMAN	519	32708	26	23	17	15	9.00	60S ribosomal protein
209	1	SwissProt_HUMAN$	2::Q02790|FKBP4_HUMAN	513	51772	28	25	20	19	5.22	Peptidyl-prolyl cis-trar
210	1	SwissProt_HUMAN$	2::P33993|MCM7_HUMAN	512	81257	24	22	18	16	1.69	DNA replication licensi
211	1	SwissProt_HUMAN$	2::P12277|KCRB_HUMAN	510	42617	22	20	16	15	4.27	Creatine kinase B-type
211	2	SwissProt_HUMAN$	2::P68400|CSK21_HUMAN	166	45115	9	8	7	7	1.08	Casein kinase II subur
211	3	SwissProt_HUMAN$	2::P19784|CSK22_HUMAN	58	41187	5	4	4	4	0.58	Casein kinase II subur
211	4	SwissProt_HUMAN$	2::P06732|KCRM_HUMAN	23	43074	2	2	2	2	0.25	Creatine kinase M-typ
212	1	SwissProt_HUMAN$	2::Q6FI13|H2A2A_HUMAN	509	14087	17	15	7	7	13.04	Histone H2A type 2-A
212	2	SwissProt_HUMAN$	2::P04908|H2A1B_HUMAN	469	14127	17	15	7	7	9.09	Histone H2A type 1-B/
212	3	SwissProt_HUMAN$	2::P16104|H2AX_HUMAN	464	15135	13	11	6	6	5.38	Histone H2AX OS=Hoi
212	4	SwissProt_HUMAN$	2::P0C0S5|H2AZ_HUMAN	219	13545	9	8	5	5	4.57	Histone H2A.Z OS=Hc
213	1	SwissProt_HUMAN$	2::P55786|PSA_HUMAN	508	103211	25	24	20	19	1.39	Puromycin-sensitive a
214	1	SwissProt_HUMAN$	2::O75874|IDHC_HUMAN	506	46630	20	20	15	15	3.57	Isocitrate dehydrogen
215	1	SwissProt_HUMAN$	2::P62241|RS8_HUMAN	503	24190	23	21	15	14	14.14	40S ribosomal protein
216	1	SwissProt_HUMAN$	2::Q15691|MARE1_HUMAN	503	29980	17	15	13	12	6.71	Microtubule-associated
217	1	SwissProt_HUMAN$	2::O00567|NOP56_HUMAN	501	66009	24	22	19	17	2.38	Nucleolar protein 56 C
218	1	SwissProt_HUMAN$	2::Q9UL46|PSME2_HUMAN	500	27384	14	14	10	10	5.60	Proteasome activator
219	1	SwissProt_HUMAN$	2::P12004|PCNA_HUMAN	499	28750	19	19	12	12	10.65	Proliferating cell nucle
220	1	SwissProt_HUMAN$	2::P16615|AT2A2_HUMAN	499	114683	21	20	18	17	1.02	Sarcoplasmic/endopla
221	1	SwissProt_HUMAN$	2::P27824|CALX_HUMAN	498	67526	24	24	17	17	2.53	Calnexin OS=Homo sa
222	1	SwissProt_HUMAN$	2::Q08945|SSRP1_HUMAN	497	81024	20	17	17	15	1.40	FACT complex subunit
223	1	SwissProt_HUMAN$	2::P33991|MCM4_HUMAN	497	96498	26	24	24	22	1.95	DNA replication licensi
224	1	SwissProt_HUMAN$	2::P46940|IQGA1_HUMAN	494	189134	26	23	23	22	0.74	Ras GTPase-activating
225	1	SwissProt_HUMAN$	2::P62820|RAB1A_HUMAN	493	22663	18	18	12	12	11.02	Ras-related protein Ra
225	2	SwissProt_HUMAN$	2::Q9H0U4|RAB1B_HUMAN	424	22157	15	15	10	10	9.22	Ras-related protein Ra
225	3	SwissProt_HUMAN$	2::P61026|RAB10_HUMAN	238	22527	11	11	9	9	5.52	Ras-related protein Ra
225	4	SwissProt_HUMAN$	2::P61006|RAB8A_HUMAN	216	23653	8	8	6	6	2.29	Ras-related protein Ra
225	5	SwissProt_HUMAN$	2::P61106|RAB14_HUMAN	197	23882	8	7	6	5	1.67	Ras-related protein Ra
225	6	SwissProt_HUMAN$	2::Q15286|RAB35_HUMAN	150	23011	6	6	4	4	1.26	Ras-related protein Ra
225	7	SwissProt_HUMAN$	2::P20340|RAB6A_HUMAN	97	23578	7	6	5	4	1.22	Ras-related protein Ra
225	8	SwissProt_HUMAN$	2::O95716|RAB3D_HUMAN	86	24252	5	5	3	3	0.79	Ras-related protein Ra
225	9	SwissProt_HUMAN$	2::P51153|RAB13_HUMAN	50	22760	2	2	2	2	0.51	Ras-related protein Ra
226	1	SwissProt_HUMAN$	2::P63241|IF5A1_HUMAN	489	16821	14	14	10	10	26.74	Eukaryotic translation
227	1	SwissProt_HUMAN$	2::P17844|DDX5_HUMAN	487	69105	28	23	23	20	3.51	Probable ATP-depende

↑Family	M	DB	Accession	Score	Mass	Matches	Match(sig)	Sequences	Seq(sig)	emPAI	Description
227	2	SwissProt_HUMAN$	2::Q92841|DDX17_HUMAN	473	80222	28	26	23	21	2.89	Probable ATP-depende
228	1	SwissProt_HUMAN$	2::Q99613|EIF3C_HUMAN	485	105278	23	21	19	17	1.15	Eukaryotic translation
229	1	SwissProt_HUMAN$	2::Q8WUM4|PDC6I_HUMAN	484	95963	21	19	19	17	1.31	Programmed cell deat
230	1	SwissProt_HUMAN$	2::P28074|PSB5_HUMAN	483	28462	15	15	11	11	5.18	Proteasome subunit b
231	1	SwissProt_HUMAN$	2::P04181|OAT_HUMAN	483	48504	23	22	17	17	4.24	Ornithine aminotransf
232	1	SwissProt_HUMAN$	2::O00159|MYO1C_HUMAN	482	121606	24	22	22	21	1.27	Unconventional myosi
233	1	SwissProt_HUMAN$	2::P39023|RL3_HUMAN	478	46080	30	26	19	16	6.02	60S ribosomal protein
234	1	SwissProt_HUMAN$	2::P23921|RIR1_HUMAN	477	90013	18	17	14	13	0.98	Ribonucleoside-diphos
235	1	SwissProt_HUMAN$	2::P54578|UBP14_HUMAN	474	56033	18	18	14	14	2.26	Ubiquitin carboxyl-ter
236	1	SwissProt_HUMAN$	2::Q96HE7|ERO1A_HUMAN	472	54358	21	19	16	15	3.02	ERO1-like protein alph
237	1	SwissProt_HUMAN$	2::Q9H583|HEAT1_HUMAN	471	242215	23	22	22	21	0.51	HEAT repeat-containir
238	1	SwissProt_HUMAN$	2::P07741|APT_HUMAN	470	19595	15	14	11	10	9.85	Adenine phosphoribos
239	1	SwissProt_HUMAN$	2::P07384|CAN1_HUMAN	470	81838	22	19	18	15	1.38	Calpain-1 catalytic sul
240	1	SwissProt_HUMAN$	2::P36873|PP1G_HUMAN	469	36960	17	15	12	11	3.07	Serine/threonine-prot
240	2	SwissProt_HUMAN$	2::P62136|PP1A_HUMAN	436	37488	17	15	12	11	2.99	Serine/threonine-prot
240	3	SwissProt_HUMAN$	2::P62140|PP1B_HUMAN	423	37163	17	15	12	11	3.04	Serine/threonine-prot
241	1	SwissProt_HUMAN$	2::Q9BWD1|THIC_HUMAN	468	41324	15	15	11	11	2.52	Acetyl-CoA acetyltrans
242	1	SwissProt_HUMAN$	2::Q9BQG0|MBB1A_HUMAN	467	148762	20	16	18	14	0.56	Myb-binding protein 1
243	1	SwissProt_HUMAN$	2::Q06830|PRDX1_HUMAN	466	22096	30	25	19	15	35.99	Peroxiredoxin-1 OS=H
243	2	SwissProt_HUMAN$	2::Q13162|PRDX4_HUMAN	404	30521	17	16	11	10	4.46	Peroxiredoxin-4 OS=H
243	3	SwissProt_HUMAN$	2::P32119|PRDX2_HUMAN	382	21878	20	19	12	11	12.12	Peroxiredoxin-2 OS=H
244	1	SwissProt_HUMAN$	2::P14868|SYDC_HUMAN	466	57100	24	23	20	20	4.70	Aspartate--tRNA ligas
245	1	SwissProt_HUMAN$	2::Q9UJZ1|STML2_HUMAN	465	38510	12	11	9	9	2.01	Stomatin-like protein
246	1	SwissProt_HUMAN$	2::P12429|ANXA3_HUMAN	460	36353	16	16	14	14	5.13	Annexin A3 OS=Homc
247	1	SwissProt_HUMAN$	2::Q7L1Q6|BZW1_HUMAN	459	48013	19	19	15	15	3.37	Basic leucine zipper ar
247	2	SwissProt_HUMAN$	2::Q9Y6E2|BZW2_HUMAN	424	48132	16	15	12	11	2.25	Basic leucine zipper ar
248	1	SwissProt_HUMAN$	2::P48637|GSHB_HUMAN	459	52352	15	14	13	12	1.95	Glutathione synthetas
249	1	SwissProt_HUMAN$	2::Q86V81|THOC4_HUMAN	459	26872	13	12	8	7	3.83	THO complex subunit
250	1	SwissProt_HUMAN$	2::Q9UHB6|LIMA1_HUMAN	458	85173	28	24	25	22	2.40	LIM domain and actin-
251	1	SwissProt_HUMAN$	2::P15880|RS2_HUMAN	458	31305	21	20	14	14	8.55	40S ribosomal protein
252	1	SwissProt_HUMAN$	2::Q9Y265|RUVB1_HUMAN	457	50196	15	15	10	10	1.82	RuvB-like 1 OS=Homc
253	1	SwissProt_HUMAN$	2::P25398|RS12_HUMAN	456	14505	14	14	6	6	11.98	40S ribosomal protein
254	1	SwissProt_HUMAN$	2::Q13838|DX39B_HUMAN	454	48960	16	15	13	12	2.51	Spliceosome RNA helic
254	2	SwissProt_HUMAN$	2::O00148|DX39A_HUMAN	262	49098	14	14	12	12	2.18	ATP-dependent RNA h
255	1	SwissProt_HUMAN$	2::Q9BSJ8|ESYT1_HUMAN	452	122780	21	19	17	15	0.79	Extended synaptotagn
256	1	SwissProt_HUMAN$	2::P38606|VATA_HUMAN	451	68260	18	18	15	15	1.83	V-type proton ATPase
257	1	SwissProt_HUMAN$	2::Q99460|PSMD1_HUMAN	450	105769	19	18	19	18	1.24	26S proteasome non-/
258	1	SwissProt_HUMAN$	2::Q9Y230|RUVB2_HUMAN	448	51125	17	17	15	15	3.00	RuvB-like 2 OS=Homc
259	1	SwissProt_HUMAN$	2::Q9Y446|PKP3_HUMAN	447	87029	17	13	13	11	0.82	Plakophilin-3 OS=Hon
260	1	SwissProt_HUMAN$	2::Q9Y3F4|STRAP_HUMAN	446	38414	16	14	14	12	3.37	Serine-threonine kinas

↑Family	M	DB	Accession	Score	Mass	Matches	Match(sig)	Sequences	Seq(sig)	emPAI	Description
261	1	SwissProt_HUMAN$	2::P49720|PSB3_HUMAN	445	22933	14	13	10	9	8.46	Proteasome subunit be
262	1	SwissProt_HUMAN$	2::Q92973|TNPO1_HUMAN	443	102289	23	19	16	14	0.91	Transportin-1 OS=Hor
262	2	SwissProt_HUMAN$	2::O14787|TNPO2_HUMAN	76	101322	8	6	5	5	0.26	Transportin-2 OS=Hor
263	1	SwissProt_HUMAN$	2::P39656|OST48_HUMAN	442	50769	16	15	12	11	1.79	Dolichyl-diphosphoolig
264	1	SwissProt_HUMAN$	2::P31040|SDHA_HUMAN	441	72645	15	12	11	8	0.69	Succinate dehydrogen
265	1	SwissProt_HUMAN$	2::O43795|MYO1B_HUMAN	440	131902	21	19	17	15	0.72	Unconventional myosi
266	1	SwissProt_HUMAN$	2::O75694|NU155_HUMAN	440	155100	17	16	17	16	0.63	Nuclear pore complex
267	1	SwissProt_HUMAN$	2::P37837|TALDO_HUMAN	439	37516	18	18	15	15	5.59	Transaldolase OS=Hor
268	1	SwissProt_HUMAN$	2::Q9NSE4|SYIM_HUMAN	438	113719	22	19	19	16	1.03	Isoleucine--tRNA ligas
269	1	SwissProt_HUMAN$	2::P42166|LAP2A_HUMAN	437	75446	15	15	12	12	1.26	Lamina-associated pol
269	2	SwissProt_HUMAN$	2::P42167|LAP2B_HUMAN	413	50639	12	12	8	8	1.32	Lamina-associated pol
270	1	SwissProt_HUMAN$	2::P43304|GPDM_HUMAN	436	80802	21	19	18	17	1.71	Glycerol-3-phosphate
271	1	SwissProt_HUMAN$	2::O94832|MYO1D_HUMAN	433	116129	25	23	22	20	1.26	Unconventional myosi
272	1	SwissProt_HUMAN$	2::P45880|VDAC2_HUMAN	433	31547	16	16	12	12	5.01	Voltage-dependent an
273	1	SwissProt_HUMAN$	2::P49321|NASP_HUMAN	432	85186	13	12	11	10	0.74	Nuclear autoantigenic
274	1	SwissProt_HUMAN$	2::P62826|RAN_HUMAN	429	24408	18	18	13	13	11.19	GTP-binding nuclear p
275	1	SwissProt_HUMAN$	2::P34897|GLYM_HUMAN	426	55958	21	20	19	18	3.58	Serine hydroxymethyl
276	1	SwissProt_HUMAN$	2::Q96PK6|RBM14_HUMAN	425	69449	17	17	14	14	1.60	RNA-binding protein 1
277	1	SwissProt_HUMAN$	2::Q14247|SRC8_HUMAN	423	61549	25	23	19	18	3.00	Src substrate cortactir
278	1	SwissProt_HUMAN$	2::P35637|FUS_HUMAN	422	53394	9	9	7	7	0.86	RNA-binding protein F
278	2	SwissProt_HUMAN$	2::Q92804|RBP56_HUMAN	169	61793	6	5	4	4	0.36	TATA-binding protein-
279	1	SwissProt_HUMAN$	2::P60228|EIF3E_HUMAN	419	52187	16	14	12	11	1.97	Eukaryotic translation
280	1	SwissProt_HUMAN$	2::P08237|PFKAM_HUMAN	417	85128	18	16	15	15	1.30	ATP-dependent 6-pho:
280	2	SwissProt_HUMAN$	2::P17858|PFKAL_HUMAN	385	84964	15	13	13	11	0.85	ATP-dependent 6-pho:
280	3	SwissProt_HUMAN$	2::Q01813|PFKAP_HUMAN	343	85542	15	15	13	13	1.05	ATP-dependent 6-pho:
281	1	SwissProt_HUMAN$	2::P04083|ANXA1_HUMAN	415	38690	14	13	12	11	2.82	Annexin A1 OS=Homc
282	1	SwissProt_HUMAN$	2::P12955|PEPD_HUMAN	415	54513	14	13	13	12	1.83	Xaa-Pro dipeptidase O
283	1	SwissProt_HUMAN$	2::Q9UMS4|PRP19_HUMAN	414	55146	21	21	14	14	2.94	Pre-mRNA-processing
284	1	SwissProt_HUMAN$	2::P26639|SYTC_HUMAN	413	83382	26	23	22	20	2.12	Threonine--tRNA ligas
285	1	SwissProt_HUMAN$	2::O75083|WDR1_HUMAN	413	66152	18	17	15	14	1.72	WD repeat-containing
286	1	SwissProt_HUMAN$	2::Q96QK1|VPS35_HUMAN	412	91649	17	17	12	12	0.86	Vacuolar protein sortir
287	1	SwissProt_HUMAN$	2::P16152|CBR1_HUMAN	411	30356	14	12	13	12	5.45	Carbonyl reductase [N
288	1	SwissProt_HUMAN$	2::P00352|AL1A1_HUMAN	407	54827	18	16	14	12	1.82	Retinal dehydrogenase
288	2	SwissProt_HUMAN$	2::P05091|ALDH2_HUMAN	321	56346	17	15	14	12	1.74	Aldehyde dehydrogena
289	1	SwissProt_HUMAN$	2::P62195|PRS8_HUMAN	407	45597	16	14	14	13	2.84	26S proteasome regul
289	2	SwissProt_HUMAN$	2::P62191|PRS4_HUMAN	236	49154	17	13	15	11	1.88	26S proteasome regul
290	1	SwissProt_HUMAN$	2::P99999|CYC_HUMAN	406	11741	13	12	10	9	50.45	Cytochrome c OS=Hor
291	1	SwissProt_HUMAN$	2::O75367|H2AY_HUMAN	406	39592	17	16	12	12	3.18	Core histone macro-H
292	1	SwissProt_HUMAN$	2::Q92499|DDX1_HUMAN	406	82380	22	21	21	20	2.16	ATP-dependent RNA h
293	1	SwissProt_HUMAN$	2::O00232|PSD12_HUMAN	404	52871	18	18	17	17	3.58	26S proteasome non-/

↑Family	M	DB	Accession	Score	Mass	Matches	Match(sig)	Sequences	Seq(sig)	emPAI	Description
294	1	SwissProt_HUMAN$	2::Q12792|TWF1_HUMAN	402	40258	12	10	10	9	1.87	Twinfilin-1 OS=Homo
294	2	SwissProt_HUMAN$	2::Q6IBS0|TWF2_HUMAN	47	39523	4	3	4	3	0.43	Twinfilin-2 OS=Homo
295	1	SwissProt_HUMAN$	2::Q01581|HMCS1_HUMAN	402	57257	13	13	9	9	1.10	Hydroxymethylglutary
295	2	SwissProt_HUMAN$	2::P54868|HMCS2_HUMAN	199	56599	9	7	8	6	0.65	Hydroxymethylglutary
296	1	SwissProt_HUMAN$	2::P07737|PROF1_HUMAN	402	15045	20	20	10	10	54.42	Profilin-1 OS=Homo s
297	1	SwissProt_HUMAN$	2::P23528|COF1_HUMAN	402	18491	13	13	10	10	11.48	Cofilin-1 OS=Homo sa
297	2	SwissProt_HUMAN$	2::Q9Y281|COF2_HUMAN	177	18725	7	7	6	6	3.47	Cofilin-2 OS=Homo sa
297	3	SwissProt_HUMAN$	2::P60981|DEST_HUMAN	142	18493	7	7	6	6	3.55	Destrin OS=Homo sap
298	1	SwissProt_HUMAN$	2::P32969|RL9_HUMAN	401	21850	12	12	8	8	4.56	60S ribosomal protein
299	1	SwissProt_HUMAN$	2::Q9UNZ2|NSF1C_HUMAN	397	40548	12	12	10	10	2.21	NSFL1 cofactor p47 O
300	1	SwissProt_HUMAN$	2::P49419|AL7A1_HUMAN	396	58450	20	13	16	11	1.43	Alpha-aminoadipic ser
301	1	SwissProt_HUMAN$	2::Q9UIA9|XPO7_HUMAN	395	123828	20	18	16	14	0.71	Exportin-7 OS=Homo
302	1	SwissProt_HUMAN$	2::Q9UBE0|SAE1_HUMAN	394	38426	13	13	11	11	2.86	SUMO-activating enzy
303	1	SwissProt_HUMAN$	2::P31689|DNJA1_HUMAN	394	44839	18	17	15	15	3.86	DnaJ homolog subfam
304	1	SwissProt_HUMAN$	2::P42285|MTREX_HUMAN	394	117729	18	15	17	14	0.76	Exosome RNA helicase
305	1	SwissProt_HUMAN$	2::P63010|AP2B1_HUMAN	393	104486	18	16	16	14	0.89	AP-2 complex subunit
305	2	SwissProt_HUMAN$	2::Q10567|AP1B1_HUMAN	278	104570	12	9	10	7	0.37	AP-1 complex subunit
306	1	SwissProt_HUMAN$	2::Q9BZZ5|API5_HUMAN	392	58968	14	12	11	9	1.06	Apoptosis inhibitor 5 (
307	1	SwissProt_HUMAN$	2::P09525|ANXA4_HUMAN	392	35860	12	12	11	11	3.25	Annexin A4 OS=Homo
308	1	SwissProt_HUMAN$	2::O94973|AP2A2_HUMAN	390	103895	17	16	13	12	0.73	AP-2 complex subunit
308	2	SwissProt_HUMAN$	2::O95782|AP2A1_HUMAN	333	107478	15	13	13	11	0.62	AP-2 complex subunit
309	1	SwissProt_HUMAN$	2::Q9ULV4|COR1C_HUMAN	389	53215	15	13	10	10	1.43	Coronin-1C OS=Homo
310	1	SwissProt_HUMAN$	2::P67936|TPM4_HUMAN	387	28504	16	14	13	12	6.24	Tropomyosin alpha-4
310	2	SwissProt_HUMAN$	2::P06753|TPM3_HUMAN	264	32930	13	11	10	9	2.62	Tropomyosin alpha-3
311	1	SwissProt_HUMAN$	2::Q96AE4|FUBP1_HUMAN	387	67518	14	13	12	12	1.32	Far upstream element
312	1	SwissProt_HUMAN$	2::Q16576|RBBP7_HUMAN	385	47790	14	14	12	12	2.27	Histone-binding protei
312	2	SwissProt_HUMAN$	2::Q09028|RBBP4_HUMAN	286	47626	9	9	7	7	1.00	Histone-binding protei
313	1	SwissProt_HUMAN$	2::P62424|RL7A_HUMAN	384	29977	22	20	16	14	8.02	60S ribosomal protein
314	1	SwissProt_HUMAN$	2::Q99959|PKP2_HUMAN	383	97355	20	18	20	18	1.40	Plakophilin-2 OS=Hon
315	1	SwissProt_HUMAN$	2::Q9UHG3|PCYOX_HUMAN	382	56604	18	13	15	11	1.51	Prenylcysteine oxidase
316	1	SwissProt_HUMAN$	2::P50995|ANX11_HUMAN	381	54355	16	14	13	12	2.10	Annexin A11 OS=Hon
316	2	SwissProt_HUMAN$	2::P20073|ANXA7_HUMAN	320	52706	11	9	9	7	0.87	Annexin A7 OS=Homo
317	1	SwissProt_HUMAN$	2::P08708|RS17_HUMAN	381	15540	15	15	8	8	13.90	40S ribosomal protein
318	1	SwissProt_HUMAN$	2::P15531|NDKA_HUMAN	379	17138	17	16	10	10	25.59	Nucleoside diphosphat
318	2	SwissProt_HUMAN$	2::P22392|NDKB_HUMAN	279	17287	15	11	10	8	10.35	Nucleoside diphosphat
319	1	SwissProt_HUMAN$	2::Q9Y2X3|NOP58_HUMAN	377	59541	14	12	11	9	1.05	Nucleolar protein 58 C
320	1	SwissProt_HUMAN$	2::P62495|ERF1_HUMAN	377	49000	12	12	10	10	1.63	Eukaryotic peptide cha
321	1	SwissProt_HUMAN$	2::P16403|H12_HUMAN	375	21352	16	15	11	10	10.25	Histone H1.2 OS=Hon
321	2	SwissProt_HUMAN$	2::P10412|H14_HUMAN	371	21852	14	13	9	8	5.89	Histone H1.4 OS=Hon
321	3	SwissProt_HUMAN$	2::P16401|H15_HUMAN	326	22566	13	12	10	9	5.52	Histone H1.5 OS=Hon

↑Family	M	DB	Accession	Score	Mass	Matches	Match(sig)	Sequences	Seq(sig)	emPAI	Description
321	4	SwissProt_HUMAN$	2::P16402|H13_HUMAN	257	22336	10	9	8	7	3.36	Histone H1.3 OS=Hon
322	1	SwissProt_HUMAN$	2::Q9P2R7|SUCB1_HUMAN	374	50285	12	12	11	11	1.81	Succinate--CoA ligase
323	1	SwissProt_HUMAN$	2::P30084|ECHM_HUMAN	373	31367	14	12	8	7	1.86	Enoyl-CoA hydratase,
324	1	SwissProt_HUMAN$	2::P52948|NUP98_HUMAN	372	197457	18	17	17	16	0.47	Nuclear pore complex
325	1	SwissProt_HUMAN$	2::P84077|ARF1_HUMAN	372	20684	14	14	10	10	8.68	ADP-ribosylation facto
325	2	SwissProt_HUMAN$	2::P61204|ARF3_HUMAN	310	20588	13	13	9	9	6.80	ADP-ribosylation facto
325	3	SwissProt_HUMAN$	2::P18085|ARF4_HUMAN	282	20498	8	8	7	7	3.94	ADP-ribosylation facto
325	4	SwissProt_HUMAN$	2::P84085|ARF5_HUMAN	257	20517	8	7	7	6	2.93	ADP-ribosylation facto
326	1	SwissProt_HUMAN$	2::P06493|CDK1_HUMAN	372	34074	17	15	13	11	3.58	Cyclin-dependent kina
326	2	SwissProt_HUMAN$	2::Q00535|CDK5_HUMAN	161	33283	6	6	5	5	1.03	Cyclin-dependent-like
326	3	SwissProt_HUMAN$	2::P07947|YES_HUMAN	145	60763	8	8	7	7	0.73	Tyrosine-protein kinas
326	4	SwissProt_HUMAN$	2::P50750|CDK9_HUMAN	109	42750	4	4	3	3	0.39	Cyclin-dependent kina
326	5	SwissProt_HUMAN$	2::Q00534|CDK6_HUMAN	104	36915	4	4	3	3	0.47	Cyclin-dependent kina
326	6	SwissProt_HUMAN$	2::P24941|CDK2_HUMAN	95	33908	4	4	3	3	0.52	Cyclin-dependent kina
326	7	SwissProt_HUMAN$	2::Q14004|CDK13_HUMAN	73	164823	3	3	2	2	0.06	Cyclin-dependent kina
326	8	SwissProt_HUMAN$	2::P12931|SRC_HUMAN	65	59797	7	6	7	6	0.61	Proto-oncogene tyrosi
327	1	SwissProt_HUMAN$	2::Q16401|PSMD5_HUMAN	368	56160	13	12	12	11	1.53	26S proteasome non-/
328	1	SwissProt_HUMAN$	2::Q9ULC5|ACSL5_HUMAN	367	75942	16	15	13	13	1.25	Long-chain-fatty-acid-
329	1	SwissProt_HUMAN$	2::P07148|FABPL_HUMAN	366	14199	13	12	7	7	18.10	Fatty acid-binding pro
330	1	SwissProt_HUMAN$	2::Q99497|PARK7_HUMAN	366	19878	15	15	10	10	9.57	Parkinson disease pro
331	1	SwissProt_HUMAN$	2::Q8N1F7|NUP93_HUMAN	365	93430	17	15	13	12	0.93	Nuclear pore complex
332	1	SwissProt_HUMAN$	2::Q08J23|NSUN2_HUMAN	365	86416	17	16	15	15	1.28	RNA cytosine C(5)-me
333	1	SwissProt_HUMAN$	2::Q13347|EIF3I_HUMAN	365	36479	17	15	13	12	3.71	Eukaryotic translation
334	1	SwissProt_HUMAN$	2::P67809|YBOX1_HUMAN	364	35903	11	11	8	8	2.27	Y-box-binding protein
334	2	SwissProt_HUMAN$	2::P16989|YBOX3_HUMAN	74	40066	3	3	3	3	0.42	Y-box-binding protein
335	1	SwissProt_HUMAN$	2::O75153|CLU_HUMAN	363	146577	28	26	26	24	1.17	Clustered mitochondri
336	1	SwissProt_HUMAN$	2::P61221|ABCE1_HUMAN	362	67271	15	14	13	12	1.33	ATP-binding cassette s
337	1	SwissProt_HUMAN$	2::P30041|PRDX6_HUMAN	362	25019	19	18	13	12	10.54	Peroxiredoxin-6 OS=H
338	1	SwissProt_HUMAN$	2::P46776|RL27A_HUMAN	361	16551	10	9	6	6	4.44	60S ribosomal protein
339	1	SwissProt_HUMAN$	2::P62753|RS6_HUMAN	359	28663	15	11	12	8	2.72	40S ribosomal protein
340	1	SwissProt_HUMAN$	2::P01834|IGKC_HUMAN	357	11758	10	10	5	5	6.17	Immunoglobulin kapp
341	1	SwissProt_HUMAN$	2::Q16181|SEPT7_HUMAN	355	50648	14	14	9	9	1.32	Septin-7 OS=Homo sa
341	2	SwissProt_HUMAN$	2::Q9NVA2|SEP11_HUMAN	337	49367	12	11	9	8	1.15	Septin-11 OS=Homo s
341	3	SwissProt_HUMAN$	2::Q9P0V9|SEP10_HUMAN	157	52560	3	3	3	3	0.31	Septin-10 OS=Homo s
341	4	SwissProt_HUMAN$	2::Q92599|SEPT8_HUMAN	115	55721	7	6	5	4	0.40	Septin-8 OS=Homo sa
342	1	SwissProt_HUMAN$	2::Q15758|AAAT_HUMAN	355	56562	13	12	11	11	1.51	Neutral amino acid tra
343	1	SwissProt_HUMAN$	2::P46060|RAGP1_HUMAN	354	63502	53	14	15	14	1.84	Ran GTPase-activating
344	1	SwissProt_HUMAN$	2::Q15393|SF3B3_HUMAN	353	135492	17	14	14	12	0.52	Splicing factor 3B sub
345	1	SwissProt_HUMAN$	2::P05455|LA_HUMAN	352	46808	18	15	17	14	3.10	Lupus La protein OS=
346	1	SwissProt_HUMAN$	2::Q14103|HNRPD_HUMAN	351	38410	14	12	11	10	2.42	Heterogeneous nuclea

↑Family	M	DB	Accession	Score	Mass	Matches	Match(sig)	Sequences	Seq(sig)	emPAI	Description
346	2	SwissProt_HUMAN$	2::Q99729|ROAA_HUMAN	241	36202	10	9	10	9	2.23	Heterogeneous nuclea
346	3	SwissProt_HUMAN$	2::O14979|HNRDL_HUMAN	108	46409	8	8	7	7	1.04	Heterogeneous nuclea
347	1	SwissProt_HUMAN$	2::P35573|GDE_HUMAN	351	174652	16	15	15	14	0.46	Glycogen debranching
348	1	SwissProt_HUMAN$	2::Q9Y224|RTRAF_HUMAN	349	28051	16	14	13	12	6.47	RNA transcription, tra
349	1	SwissProt_HUMAN$	2::P30566|PUR8_HUMAN	348	54854	15	13	12	10	1.58	Adenylosuccinate lyas
350	1	SwissProt_HUMAN$	2::Q15813|TBCE_HUMAN	346	59309	11	11	10	10	1.22	Tubulin-specific chape
351	1	SwissProt_HUMAN$	2::P35270|SPRE_HUMAN	345	28031	8	7	6	5	1.31	Sepiapterin reductase
352	1	SwissProt_HUMAN$	2::P35606|COPB2_HUMAN	344	102422	22	15	18	14	0.91	Coatomer subunit bet
353	1	SwissProt_HUMAN$	2::P08758|ANXA5_HUMAN	344	35914	18	17	16	15	6.15	Annexin A5 OS=Homo
354	1	SwissProt_HUMAN$	2::P27695|APEX1_HUMAN	343	35532	9	9	8	8	1.89	DNA-(apurinic or apyr
355	1	SwissProt_HUMAN$	2::P22087|FBRL_HUMAN	340	33763	17	15	11	10	3.04	rRNA 2'-O-methyltran
356	1	SwissProt_HUMAN$	2::P11387|TOP1_HUMAN	340	90669	21	17	19	16	1.31	DNA topoisomerase 1
357	1	SwissProt_HUMAN$	2::O76094|SRP72_HUMAN	339	74560	16	14	16	14	1.43	Signal recognition par
358	1	SwissProt_HUMAN$	2::P38117|ETFB_HUMAN	338	27826	14	12	11	9	3.57	Electron transfer flavo
359	1	SwissProt_HUMAN$	2::P00505|AATM_HUMAN	338	47487	17	14	11	10	1.70	Aspartate aminotransf
360	1	SwissProt_HUMAN$	2::P27635|RL10_HUMAN	338	24588	16	16	10	10	10.92	60S ribosomal protein
361	1	SwissProt_HUMAN$	2::P62277|RS13_HUMAN	336	17212	14	14	8	8	7.79	40S ribosomal protein
362	1	SwissProt_HUMAN$	2::P62333|PRS10_HUMAN	336	44145	17	14	13	11	2.24	26S proteasome regul
363	1	SwissProt_HUMAN$	2::Q09666|AHNK_HUMAN	336	628699	19	16	19	16	0.13	Neuroblast differentia
364	1	SwissProt_HUMAN$	2::P31930|QCR1_HUMAN	335	52612	10	10	10	10	1.46	Cytochrome b-c1 com
365	1	SwissProt_HUMAN$	2::Q07065|CKAP4_HUMAN	334	65983	17	16	15	14	1.73	Cytoskeleton-associat
366	1	SwissProt_HUMAN$	2::O94925|GLSK_HUMAN	334	73414	15	15	13	13	1.31	Glutaminase kidney is
367	1	SwissProt_HUMAN$	2::Q96CM8|ACSF2_HUMAN	333	68081	13	12	10	9	0.87	Medium-chain acyl-Co
368	1	SwissProt_HUMAN$	2::P61289|PSME3_HUMAN	333	29488	13	13	9	9	3.20	Proteasome activator
369	1	SwissProt_HUMAN$	2::Q9Y285|SYFA_HUMAN	332	57528	11	10	9	8	0.93	Phenylalanine--tRNA l
370	1	SwissProt_HUMAN$	2::P17980|PRS6A_HUMAN	332	49172	15	15	13	13	2.84	26S proteasome regul
371	1	SwissProt_HUMAN$	2::P25789|PSA4_HUMAN	330	29465	13	13	9	9	3.22	Proteasome subunit al
372	1	SwissProt_HUMAN$	2::P40925|MDHC_HUMAN	330	36403	13	13	12	12	3.73	Malate dehydrogenase
373	1	SwissProt_HUMAN$	2::Q9BT78|CSN4_HUMAN	329	46240	11	11	8	8	1.26	COP9 signalosome cor
374	1	SwissProt_HUMAN$	2::Q9HB71|CYBP_HUMAN	328	26194	19	16	13	12	7.62	Calcyclin-binding prot
375	1	SwissProt_HUMAN$	2::Q9HCC0|MCCB_HUMAN	328	61294	18	14	15	11	1.34	Methylcrotonoyl-CoA c
376	1	SwissProt_HUMAN$	2::P46781|RS9_HUMAN	326	22578	19	17	16	14	21.73	40S ribosomal protein
377	1	SwissProt_HUMAN$	2::P07954|FUMH_HUMAN	325	54602	10	10	9	9	1.18	Fumarate hydratase, r
378	1	SwissProt_HUMAN$	2::Q16531|DDB1_HUMAN	325	126887	17	14	15	13	0.63	DNA damage-binding
379	1	SwissProt_HUMAN$	2::P35998|PRS7_HUMAN	325	48603	18	15	15	13	2.54	26S proteasome regul
380	1	SwissProt_HUMAN$	2::O00505|IMA4_HUMAN	324	57775	14	11	11	8	0.93	Importin subunit alph
380	2	SwissProt_HUMAN$	2::O00629|IMA3_HUMAN	234	57851	9	8	7	6	0.63	Importin subunit alph
381	1	SwissProt_HUMAN$	2::Q14566|MCM6_HUMAN	324	92831	19	18	15	15	1.15	DNA replication licensi
382	1	SwissProt_HUMAN$	2::P51665|PSMD7_HUMAN	323	37002	14	13	8	8	1.78	26S proteasome non-/
383	1	SwissProt_HUMAN$	2::O43242|PSMD3_HUMAN	323	60939	17	13	16	12	1.54	26S proteasome non-/

↑Family	M	DB	Accession	Score	Mass	Matches	Match(sig)	Sequences	Seq(sig)	emPAI	Description
384	1	SwissProt_HUMAN$	2::Q9Y295|DRG1_HUMAN	323	40517	14	10	13	9	1.85	Developmentally-regu
385	1	SwissProt_HUMAN$	2::P54577|SYYC_HUMAN	322	59106	20	15	18	13	1.83	Tyrosine--tRNA ligase,
386	1	SwissProt_HUMAN$	2::Q9Y2Z4|SYYM_HUMAN	322	53166	14	13	13	12	1.91	Tyrosine--tRNA ligase,
387	1	SwissProt_HUMAN$	2::P62249|RS16_HUMAN	321	16435	15	13	11	10	16.16	40S ribosomal protein
388	1	SwissProt_HUMAN$	2::P33992|MCM5_HUMAN	318	82233	16	16	13	13	1.11	DNA replication licensi
389	1	SwissProt_HUMAN$	2::O00764|PDXK_HUMAN	317	35080	7	7	5	5	0.96	Pyridoxal kinase OS=H
390	1	SwissProt_HUMAN$	2::P43246|MSH2_HUMAN	317	104677	16	12	14	10	0.57	DNA mismatch repair
391	1	SwissProt_HUMAN$	2::Q9Y5B9|SP16H_HUMAN	317	119838	15	11	11	9	0.43	FACT complex subunit
392	1	SwissProt_HUMAN$	2::Q15021|CND1_HUMAN	314	157082	17	15	17	15	0.57	Condensin complex su
393	1	SwissProt_HUMAN$	2::Q9NVP1|DDX18_HUMAN	314	75359	12	11	11	10	0.87	ATP-dependent RNA h
394	1	SwissProt_HUMAN$	2::O95573|ACSL3_HUMAN	313	80368	12	11	11	11	0.91	Long-chain-fatty-acid-
394	2	SwissProt_HUMAN$	2::O60488|ACSL4_HUMAN	86	79137	7	6	6	6	0.43	Long-chain-fatty-acid-
395	1	SwissProt_HUMAN$	2::P27694|RFA1_HUMAN	313	68095	11	8	10	8	0.74	Replication protein A 7
396	1	SwissProt_HUMAN$	2::Q15155|NOMO1_HUMAN	311	134239	15	15	15	15	0.70	Nodal modulator 1 OS
396	2	SwissProt_HUMAN$	2::Q5JPE7|NOMO2_HUMAN	296	139351	16	16	16	16	0.72	Nodal modulator 2 OS
397	1	SwissProt_HUMAN$	2::Q99733|NP1L4_HUMAN	311	42797	13	11	11	9	2.01	Nucleosome assembly
397	2	SwissProt_HUMAN$	2::P55209|NP1L1_HUMAN	150	45346	6	6	5	5	0.68	Nucleosome assembly
398	1	SwissProt_HUMAN$	2::P35268|RL22_HUMAN	311	14778	11	9	6	5	5.65	60S ribosomal protein
399	1	SwissProt_HUMAN$	2::Q15019|SEPT2_HUMAN	311	41461	9	9	9	9	1.78	Septin-2 OS=Homo sa
400	1	SwissProt_HUMAN$	2::P60900|PSA6_HUMAN	310	27382	12	10	9	8	2.95	Proteasome subunit al
401	1	SwissProt_HUMAN$	2::O94826|TOM70_HUMAN	310	67412	12	11	9	8	0.75	Mitochondrial import r
402	1	SwissProt_HUMAN$	2::P62081|RS7_HUMAN	310	22113	18	16	12	10	9.34	40S ribosomal protein
403	1	SwissProt_HUMAN$	2::Q92878|RAD50_HUMAN	308	153797	24	18	19	16	0.64	DNA repair protein RA
404	1	SwissProt_HUMAN$	2::O43765|SGTA_HUMAN	308	34042	10	10	8	8	2.02	Small glutamine-rich t
405	1	SwissProt_HUMAN$	2::Q9BXP5|SRRT_HUMAN	308	100604	21	18	19	17	1.23	Serrate RNA effector r
406	1	SwissProt_HUMAN$	2::P21281|VATB2_HUMAN	307	56465	18	16	15	14	2.23	V-type proton ATPase
407	1	SwissProt_HUMAN$	2::P20290|BTF3_HUMAN	307	22154	7	7	5	5	1.88	Transcription factor BT
408	1	SwissProt_HUMAN$	2::O95232|LC7L3_HUMAN	307	51435	14	13	8	8	1.09	Luc7-like protein 3 OS
409	1	SwissProt_HUMAN$	2::Q9Y3I0|RTCB_HUMAN	306	55175	12	12	8	8	1.16	RNA-splicing ligase Rt
410	1	SwissProt_HUMAN$	2::O95831|AIFM1_HUMAN	306	66859	12	11	11	10	1.03	Apoptosis-inducing fac
411	1	SwissProt_HUMAN$	2::Q13492|PICAL_HUMAN	305	70710	15	14	13	12	1.23	Phosphatidylinositol-b
412	1	SwissProt_HUMAN$	2::Q12788|TBL3_HUMAN	304	88978	13	12	12	11	0.80	Transducin beta-like p
413	1	SwissProt_HUMAN$	2::Q10713|MPPA_HUMAN	303	58216	9	8	7	6	0.63	Mitochondrial-process
414	1	SwissProt_HUMAN$	2::O60763|USO1_HUMAN	302	107828	14	11	13	10	0.55	General vesicular tran
415	1	SwissProt_HUMAN$	2::P39019|RS19_HUMAN	302	16051	16	14	12	11	23.29	40S ribosomal protein
416	1	SwissProt_HUMAN$	2::P18124|RL7_HUMAN	301	29207	19	17	14	13	8.56	60S ribosomal protein
416	2	SwissProt_HUMAN$	2::P57678|GEMI4_HUMAN	120	119960	7	7	6	6	0.27	Gem-associated prote
417	1	SwissProt_HUMAN$	2::Q13177|PAK2_HUMAN	301	58006	13	12	12	11	1.45	Serine/threonine-prot
417	2	SwissProt_HUMAN$	2::Q13153|PAK1_HUMAN	108	60609	4	3	4	3	0.26	Serine/threonine-prot
418	1	SwissProt_HUMAN$	2::O43747|AP1G1_HUMAN	298	91293	15	14	11	10	0.68	AP-1 complex subunit

↑Family	M	DB	Accession	Score	Mass	Matches	Match(sig)	Sequences	Seq(sig)	emPAI	Description
419	1	SwissProt_HUMAN$	2::O60684|IMA7_HUMAN	298	59991	10	8	8	6	0.61	Importin subunit alpha
419	2	SwissProt_HUMAN$	2::O15131|IMA6_HUMAN	197	60311	6	5	5	4	0.37	Importin subunit alpha
419	3	SwissProt_HUMAN$	2::P52294|IMA5_HUMAN	190	60184	8	7	7	6	0.60	Importin subunit alpha
420	1	SwissProt_HUMAN$	2::O00515|LAD1_HUMAN	297	57097	13	12	13	12	1.70	Ladinin-1 OS=Homo s
421	1	SwissProt_HUMAN$	2::P30740|ILEU_HUMAN	296	42715	14	13	10	9	1.70	Leukocyte elastase inh
422	1	SwissProt_HUMAN$	2::Q15459|SF3A1_HUMAN	296	88831	15	11	13	9	0.62	Splicing factor 3A sub
423	1	SwissProt_HUMAN$	2::P62917|RL8_HUMAN	295	28007	17	16	12	11	5.36	60S ribosomal protein
424	1	SwissProt_HUMAN$	2::P24752|THIL_HUMAN	295	45171	13	13	11	11	2.16	Acetyl-CoA acetyltrans
425	1	SwissProt_HUMAN$	2::O95470|SGPL1_HUMAN	295	63483	10	7	9	6	0.56	Sphingosine-1-phosph
426	1	SwissProt_HUMAN$	2::Q5JTH9|RRP12_HUMAN	294	143611	17	15	15	13	0.54	RRP12-like protein OS
427	1	SwissProt_HUMAN$	2::Q8N163|CCAR2_HUMAN	294	102838	22	20	19	17	1.29	Cell cycle and apoptos
428	1	SwissProt_HUMAN$	2::P23193|TCEA1_HUMAN	294	33948	8	7	6	5	1.00	Transcription elongatio
429	1	SwissProt_HUMAN$	2::P46087|NOP2_HUMAN	294	89247	18	14	17	14	1.10	Probable 28S rRNA (cy
430	1	SwissProt_HUMAN$	2::Q8NBS9|TXND5_HUMAN	293	47599	14	13	12	11	1.98	Thioredoxin domain-co
431	1	SwissProt_HUMAN$	2::P49748|ACADV_HUMAN	293	70345	14	12	12	11	1.10	Very long-chain specif
432	1	SwissProt_HUMAN$	2::P46379|BAG6_HUMAN	293	119334	11	11	9	9	0.43	Large proline-rich prot
433	1	SwissProt_HUMAN$	2::Q9NQR4|NIT2_HUMAN	293	30589	12	12	9	9	2.99	Omega-amidase NIT2
434	1	SwissProt_HUMAN$	2::Q99873|ANM1_HUMAN	292	42434	12	12	10	10	2.05	Protein arginine N-me
435	1	SwissProt_HUMAN$	2::P51148|RAB5C_HUMAN	292	23468	10	9	8	7	3.97	Ras-related protein Ra
435	2	SwissProt_HUMAN$	2::P61020|RAB5B_HUMAN	37	23692	5	3	5	3	0.81	Ras-related protein Ra
436	1	SwissProt_HUMAN$	2::P00390|GSHR_HUMAN	292	56221	12	11	11	10	1.32	Glutathione reductase
437	1	SwissProt_HUMAN$	2::P61586|RHOA_HUMAN	292	21754	13	12	8	8	5.96	Transforming protein
437	2	SwissProt_HUMAN$	2::P08134|RHOC_HUMAN	160	21992	11	9	7	6	2.60	Rho-related GTP-bindi
438	1	SwissProt_HUMAN$	2::P60660|MYL6_HUMAN	291	16919	7	7	5	5	4.27	Myosin light polypepti
439	1	SwissProt_HUMAN$	2::Q13501|SQSTM_HUMAN	290	47657	9	9	7	7	1.00	Sequestosome-1 OS=
440	1	SwissProt_HUMAN$	2::Q13724|MOGS_HUMAN	290	91861	14	13	11	11	0.76	Mannosyl-oligosacchar
441	1	SwissProt_HUMAN$	2::P62906|RL10A_HUMAN	289	24816	17	17	11	11	8.66	60S ribosomal protein
442	1	SwissProt_HUMAN$	2::P26373|RL13_HUMAN	287	24247	11	10	10	9	4.74	60S ribosomal protein
443	1	SwissProt_HUMAN$	2::Q9BY44|EIF2A_HUMAN	287	64949	15	13	14	12	1.40	Eukaryotic translation
444	1	SwissProt_HUMAN$	2::O60264|SMCA5_HUMAN	287	121828	20	18	19	17	0.94	SWI/SNF-related matr
444	2	SwissProt_HUMAN$	2::P28370|SMCA1_HUMAN	172	122527	13	12	12	11	0.53	Probable global transc
445	1	SwissProt_HUMAN$	2::P62888|RL30_HUMAN	287	12776	14	13	8	7	17.38	60S ribosomal protein
446	1	SwissProt_HUMAN$	2::Q7L576|CYFP1_HUMAN	287	145089	14	13	14	13	0.53	Cytoplasmic FMR1-inte
447	1	SwissProt_HUMAN$	2::Q9Y5M8|SRPRB_HUMAN	287	29684	13	13	10	10	3.90	Signal recognition par
448	1	SwissProt_HUMAN$	2::P43686|PRS6B_HUMAN	287	47337	15	14	12	11	2.00	26S proteasome regul
449	1	SwissProt_HUMAN$	2::Q14203|DCTN1_HUMAN	286	141607	9	8	8	8	0.31	Dynactin subunit 1 OS
450	1	SwissProt_HUMAN$	2::P78347|GTF2I_HUMAN	286	112346	22	18	19	15	0.88	General transcription f
451	1	SwissProt_HUMAN$	2::P61313|RL15_HUMAN	286	24131	13	13	10	10	6.03	60S ribosomal protein
452	1	SwissProt_HUMAN$	2::P12081|HARS1_HUMAN	286	57374	18	13	16	13	1.92	Histidine--tRNA ligase
452	2	SwissProt_HUMAN$	2::P49590|SYHM_HUMAN	56	56852	3	2	3	2	0.18	Histidine--tRNA ligase

↑Family	M	DB	Accession	Score	Mass	Matches	Match(sig)	Sequences	Seq(sig)	emPAI	Description
453	1	SwissProt_HUMAN$	2::P42765|THIM_HUMAN	285	41898	12	11	12	11	2.46	3-ketoacyl-CoA thiolas
454	1	SwissProt_HUMAN$	2::P46777|RL5_HUMAN	285	34341	21	17	18	15	6.86	60S ribosomal protein
455	1	SwissProt_HUMAN$	2::Q9H0S4|DDX47_HUMAN	285	50615	10	9	8	7	0.92	Probable ATP-depende
456	1	SwissProt_HUMAN$	2::P30419|NMT1_HUMAN	284	56770	12	12	9	9	1.12	Glycylpeptide N-tetrac
457	1	SwissProt_HUMAN$	2::Q01105|SET_HUMAN	284	33469	11	10	9	8	2.08	Protein SET OS=Homo
458	1	SwissProt_HUMAN$	2::O15067|PUR4_HUMAN	283	144643	12	12	10	10	0.39	Phosphoribosylformylg
459	1	SwissProt_HUMAN$	2::Q09161|NCBP1_HUMAN	283	91781	16	14	15	13	0.96	Nuclear cap-binding p
460	1	SwissProt_HUMAN$	2::P52788|SPSY_HUMAN	283	41242	13	11	12	10	2.14	Spermine synthase OS
461	1	SwissProt_HUMAN$	2::Q9UBT2|SAE2_HUMAN	282	71179	19	15	17	14	1.54	SUMO-activating enzy
462	1	SwissProt_HUMAN$	2::O96019|ACL6A_HUMAN	282	47430	12	10	10	8	1.22	Actin-like protein 6A C
463	1	SwissProt_HUMAN$	2::P49189|AL9A1_HUMAN	281	53767	12	11	10	10	1.41	4-trimethylaminobuty
464	1	SwissProt_HUMAN$	2::P14324|FPPS_HUMAN	281	48245	16	14	11	10	1.66	Farnesyl pyrophospha
465	1	SwissProt_HUMAN$	2::O75534|CSDE1_HUMAN	281	88829	17	16	15	14	1.11	Cold shock domain-co
466	1	SwissProt_HUMAN$	2::P37108|SRP14_HUMAN	281	14561	9	9	7	7	8.42	Signal recognition par
467	1	SwissProt_HUMAN$	2::P23588|IF4B_HUMAN	280	69110	13	11	9	7	0.61	Eukaryotic translation
468	1	SwissProt_HUMAN$	2::Q6PKG0|LARP1_HUMAN	279	123434	14	13	13	12	0.59	La-related protein 1 O
469	1	SwissProt_HUMAN$	2::P30086|PEBP1_HUMAN	279	21044	10	8	7	6	2.80	Phosphatidylethanolar
470	1	SwissProt_HUMAN$	2::Q02543|RL18A_HUMAN	279	20749	16	12	13	9	6.63	60S ribosomal protein
471	1	SwissProt_HUMAN$	2::Q9UHB9|SRP68_HUMAN	278	70686	10	10	10	10	0.95	Signal recognition par
472	1	SwissProt_HUMAN$	2::P11413|G6PD_HUMAN	277	59219	14	12	10	9	1.05	Glucose-6-phosphate
473	1	SwissProt_HUMAN$	2::Q07020|RL18_HUMAN	276	21621	11	11	8	8	4.66	60S ribosomal protein
474	1	SwissProt_HUMAN$	2::Q06323|PSME1_HUMAN	276	28705	9	8	7	6	1.67	Proteasome activator
475	1	SwissProt_HUMAN$	2::P83731|RL24_HUMAN	275	17768	15	13	10	9	12.91	60S ribosomal protein
476	1	SwissProt_HUMAN$	2::P38159|RBMX_HUMAN	273	42306	21	15	16	11	2.41	RNA-binding motif pro
477	1	SwissProt_HUMAN$	2::Q14258|TRI25_HUMAN	273	70928	15	14	14	13	1.38	E3 ubiquitin/ISG15 lig
478	1	SwissProt_HUMAN$	2::Q13616|CUL1_HUMAN	272	89622	16	13	16	13	0.99	Cullin-1 OS=Homo sa
479	1	SwissProt_HUMAN$	2::Q8IZ83|A16A1_HUMAN	271	85074	9	9	7	7	0.48	Aldehyde dehydrogena
480	1	SwissProt_HUMAN$	2::O95347|SMC2_HUMAN	270	135572	22	18	18	15	0.69	Structural maintenanc
481	1	SwissProt_HUMAN$	2::P17174|AATC_HUMAN	270	46219	12	12	11	11	2.08	Aspartate aminotransf
482	1	SwissProt_HUMAN$	2::Q99536|VAT1_HUMAN	270	41893	5	5	4	4	0.57	Synaptic vesicle meml
483	1	SwissProt_HUMAN$	2::Q9Y2Z0|SGT1_HUMAN	270	40998	11	9	10	8	1.51	Protein SGT1 homolog
484	1	SwissProt_HUMAN$	2::P25786|PSA1_HUMAN	269	29537	18	17	17	16	11.82	Proteasome subunit al
485	1	SwissProt_HUMAN$	2::P30050|RL12_HUMAN	269	17808	11	9	8	7	7.22	60S ribosomal protein
486	1	SwissProt_HUMAN$	2::O43592|XPOT_HUMAN	269	109893	15	14	11	10	0.54	Exportin-T OS=Homo
487	1	SwissProt_HUMAN$	2::P78344|IF4G2_HUMAN	269	102297	14	12	14	12	0.74	Eukaryotic translation
488	1	SwissProt_HUMAN$	2::P21399|ACOC_HUMAN	269	98337	19	15	18	14	0.96	Cytoplasmic aconitate
489	1	SwissProt_HUMAN$	2::P34896|GLYC_HUMAN	268	53049	11	9	11	9	1.23	Serine hydroxymethyl
490	1	SwissProt_HUMAN$	2::Q16851|UGPA_HUMAN	268	56905	10	9	8	7	0.79	UTP--glucose-1-phosp
491	1	SwissProt_HUMAN$	2::Q13620|CUL4B_HUMAN	267	103916	15	13	14	12	0.73	Cullin-4B OS=Homo s
491	2	SwissProt_HUMAN$	2::Q13619|CUL4A_HUMAN	206	87624	12	12	11	11	0.81	Cullin-4A OS=Homo s

↑Family	M	DB	Accession	Score	Mass	Matches	Match(sig)	Sequences	Seq(sig)	emPAI	Description
492	1	SwissProt_HUMAN$	2::Q6XQN6|PNCB_HUMAN	267	57542	8	8	7	7	0.78	Nicotinate phosphorib
493	1	SwissProt_HUMAN$	2::P29144|TPP2_HUMAN	267	138263	20	17	18	15	0.67	Tripeptidyl-peptidase
494	1	SwissProt_HUMAN$	2::P55957|BID_HUMAN	266	21981	7	7	5	5	1.91	BH3-interacting doma
495	1	SwissProt_HUMAN$	2::O14579|COPE_HUMAN	265	34460	9	9	7	7	1.99	Coatomer subunit eps
496	1	SwissProt_HUMAN$	2::O94906|PRP6_HUMAN	264	106858	11	10	10	9	0.49	Pre-mRNA-processing
497	1	SwissProt_HUMAN$	2::Q6YN16|HSDL2_HUMAN	264	45366	8	8	6	6	0.87	Hydroxysteroid dehyd
498	1	SwissProt_HUMAN$	2::Q12769|NU160_HUMAN	264	162017	16	14	12	11	0.38	Nuclear pore complex
499	1	SwissProt_HUMAN$	2::Q9BR76|COR1B_HUMAN	264	54200	10	7	9	6	0.69	Coronin-1B OS=Homo
500	1	SwissProt_HUMAN$	2::P62244|RS15A_HUMAN	263	14830	13	11	8	6	7.97	40S ribosomal protein
501	1	SwissProt_HUMAN$	2::P06454|PTMA_HUMAN	263	12196	4	4	3	3	2.13	Prothymosin alpha OS
502	1	SwissProt_HUMAN$	2::P41252|SYIC_HUMAN	263	144406	19	17	15	13	0.58	Isoleucine--tRNA ligas
503	1	SwissProt_HUMAN$	2::Q13185|CBX3_HUMAN	263	20798	9	9	7	7	3.86	Chromobox protein hc
503	2	SwissProt_HUMAN$	2::P45973|CBX5_HUMAN	33	22211	3	3	3	3	0.89	Chromobox protein hc
504	1	SwissProt_HUMAN$	2::Q6P996|PDXD1_HUMAN	262	86652	12	11	12	11	0.82	Pyridoxal-dependent c
505	1	SwissProt_HUMAN$	2::Q13148|TADBP_HUMAN	262	44711	8	6	6	5	0.70	TAR DNA-binding prot
506	1	SwissProt_HUMAN$	2::P48047|ATPO_HUMAN	262	23263	8	8	6	6	2.35	ATP synthase subunit
507	1	SwissProt_HUMAN$	2::P55265|DSRAD_HUMAN	262	135981	14	10	11	8	0.32	Double-stranded RNA-
508	1	SwissProt_HUMAN$	2::P11216|PYGB_HUMAN	262	96635	15	13	15	13	0.89	Glycogen phosphoryla
508	2	SwissProt_HUMAN$	2::P06737|PYGL_HUMAN	256	97087	14	14	12	12	0.80	Glycogen phosphoryla
509	1	SwissProt_HUMAN$	2::P54819|KAD2_HUMAN	260	26461	10	9	8	7	2.48	Adenylate kinase 2, m
510	1	SwissProt_HUMAN$	2::P26583|HMGB2_HUMAN	260	24019	12	10	6	6	2.94	High mobility group pı
510	2	SwissProt_HUMAN$	2::P09429|HMGB1_HUMAN	196	24878	18	13	9	9	5.62	High mobility group pı
511	1	SwissProt_HUMAN$	2::Q15738|NSDHL_HUMAN	259	41874	8	8	7	7	1.20	Sterol-4-alpha-carbox
512	1	SwissProt_HUMAN$	2::P20618|PSB1_HUMAN	259	26472	14	14	10	10	7.46	Proteasome subunit b
513	1	SwissProt_HUMAN$	2::P28838|AMPL_HUMAN	259	56131	14	13	13	12	1.75	Cytosol aminopeptidas
514	1	SwissProt_HUMAN$	2::P55263|ADK_HUMAN	259	40520	11	10	9	8	1.54	Adenosine kinase OS=
515	1	db$	1::P0DTC5|VME1_SARS2	259	25130	13	12	10	9	5.51	Membrane protein OS
516	1	SwissProt_HUMAN$	2::P39687|AN32A_HUMAN	258	28568	8	8	6	6	1.69	Acidic leucine-rich nuc
516	2	SwissProt_HUMAN$	2::Q92688|AN32B_HUMAN	252	28770	11	11	8	8	3.36	Acidic leucine-rich nuc
517	1	SwissProt_HUMAN$	2::Q9BUJ2|HNRL1_HUMAN	258	95679	11	10	10	9	0.56	Heterogeneous nuclea
518	1	SwissProt_HUMAN$	2::Q14008|CKAP5_HUMAN	258	225352	17	14	16	13	0.32	Cytoskeleton-associat
519	1	SwissProt_HUMAN$	2::O15355|PPM1G_HUMAN	258	59235	11	11	9	9	1.05	Protein phosphatase 1
520	1	SwissProt_HUMAN$	2::Q7L2H7|EIF3M_HUMAN	257	42476	14	14	11	11	2.39	Eukaryotic translation
521	1	SwissProt_HUMAN$	2::Q00688|FKBP3_HUMAN	257	25161	9	8	7	6	2.06	Peptidyl-prolyl cis-trar
522	1	SwissProt_HUMAN$	2::P30046|DOPD_HUMAN	257	12704	9	8	6	6	8.05	D-dopachrome decarb
523	1	SwissProt_HUMAN$	2::P56537|IF6_HUMAN	257	26582	8	8	5	5	1.90	Eukaryotic translation
524	1	SwissProt_HUMAN$	2::P08754|GNAI3_HUMAN	256	40506	12	10	10	8	1.54	Guanine nucleotide-bi
524	2	SwissProt_HUMAN$	2::P63092|GNAS2_HUMAN	181	45636	7	7	6	6	0.86	Guanine nucleotide-bi
524	3	SwissProt_HUMAN$	2::Q14344|GNA13_HUMAN	102	44022	4	4	3	3	0.38	Guanine nucleotide-bi
525	1	SwissProt_HUMAN$	2::O96008|TOM40_HUMAN	255	37869	8	8	7	7	1.39	Mitochondrial import r

↑Family	M	DB	Accession	Score	Mass	Matches	Match(sig)	Sequences	Seq(sig)	emPAI	Description
526	1	SwissProt_HUMAN$	2::Q14690|RRP5_HUMAN	254	208570	15	14	14	13	0.34	Protein RRP5 homolog
527	1	SwissProt_HUMAN$	2::P16422|EPCAM_HUMAN	254	34910	8	8	6	6	1.25	Epithelial cell adhesior
528	1	SwissProt_HUMAN$	2::E9PAV3|NACAM_HUMAN	254	205295	8	7	6	6	0.15	Nascent polypeptide-a
529	1	SwissProt_HUMAN$	2::P27816|MAP4_HUMAN	254	120930	8	8	8	8	0.37	Microtubule-associate
530	1	SwissProt_HUMAN$	2::Q1KMD3|HNRL2_HUMAN	253	85052	13	11	12	10	0.75	Heterogeneous nuclea
531	1	SwissProt_HUMAN$	2::O95994|AGR2_HUMAN	252	19967	13	13	9	9	7.25	Anterior gradient prot
532	1	SwissProt_HUMAN$	2::Q9UL25|RAB21_HUMAN	252	24332	9	9	7	7	2.87	Ras-related protein Ra
533	1	SwissProt_HUMAN$	2::Q00341|VIGLN_HUMAN	251	141368	16	12	16	12	0.50	Vigilin OS=Homo sapi
534	1	SwissProt_HUMAN$	2::O75390|CISY_HUMAN	251	51680	12	12	9	9	1.28	Citrate synthase, mito
535	1	SwissProt_HUMAN$	2::P09960|LKHA4_HUMAN	250	69241	15	13	13	11	1.12	Leukotriene A-4 hydro
536	1	SwissProt_HUMAN$	2::Q9H9B4|SFXN1_HUMAN	250	35596	10	10	9	9	2.29	Sideroflexin-1 OS=Ho
536	2	SwissProt_HUMAN$	2::Q9BWM7|SFXN3_HUMAN	118	35481	3	3	3	3	0.49	Sideroflexin-3 OS=Ho
537	1	SwissProt_HUMAN$	2::O75131|CPNE3_HUMAN	249	60092	15	13	7	6	0.60	Copine-3 OS=Homo s
538	1	SwissProt_HUMAN$	2::Q9NVI7|ATD3A_HUMAN	249	71325	18	15	17	14	1.53	ATPase family AAA do
539	1	SwissProt_HUMAN$	2::O95202|LETM1_HUMAN	249	83302	12	10	10	8	0.58	Mitochondrial proton/
540	1	SwissProt_HUMAN$	2::Q15003|CND2_HUMAN	248	82511	9	9	8	8	0.58	Condensin complex su
541	1	SwissProt_HUMAN$	2::Q9UBM7|DHCR7_HUMAN	248	54454	8	6	6	6	0.68	7-dehydrocholesterol
542	1	SwissProt_HUMAN$	2::P22695|QCR2_HUMAN	247	48413	12	11	9	9	1.41	Cytochrome b-c1 com
543	1	SwissProt_HUMAN$	2::Q9H3P7|GCP60_HUMAN	247	60556	4	4	3	3	0.26	Golgi resident protein
544	1	SwissProt_HUMAN$	2::P23284|PPIB_HUMAN	247	23728	12	10	10	8	3.86	Peptidyl-prolyl cis-trar
545	1	SwissProt_HUMAN$	2::O43776|SYNC_HUMAN	247	62903	9	9	8	8	0.97	Asparagine--tRNA liga
546	1	SwissProt_HUMAN$	2::P49591|SYSC_HUMAN	247	58740	17	16	13	13	1.85	Serine--tRNA ligase, c
547	1	SwissProt_HUMAN$	2::P05387|RLA2_HUMAN	246	11658	8	8	5	5	6.31	60S acidic ribosomal p
548	1	SwissProt_HUMAN$	2::P62913|RL11_HUMAN	245	20240	10	10	7	7	5.39	60S ribosomal protein
549	1	SwissProt_HUMAN$	2::O15260|SURF4_HUMAN	244	30374	8	8	5	5	1.17	Surfeit locus protein 4
550	1	SwissProt_HUMAN$	2::Q9NZM1|MYOF_HUMAN	244	234561	16	14	16	14	0.33	Myoferlin OS=Homo s
551	1	SwissProt_HUMAN$	2::Q6NUK1|SCMC1_HUMAN	244	53320	8	8	6	6	0.70	Calcium-binding mitoc
552	1	SwissProt_HUMAN$	2::Q9HC35|EMAL4_HUMAN	242	108848	18	15	17	14	0.84	Echinoderm microtubu
553	1	SwissProt_HUMAN$	2::O76003|GLRX3_HUMAN	242	37408	11	10	10	9	2.11	Glutaredoxin-3 OS=H
554	1	SwissProt_HUMAN$	2::P0DN76|U2AF5_HUMAN	241	27854	6	6	5	5	1.33	Splicing factor U2AF 3
555	1	SwissProt_HUMAN$	2::Q9Y365|STA10_HUMAN	241	33028	11	9	7	6	1.71	START domain-contair
556	1	SwissProt_HUMAN$	2::P61163|ACTZ_HUMAN	241	42587	12	11	10	10	2.03	Alpha-centractin OS=I
557	1	SwissProt_HUMAN$	2::P62879|GBB2_HUMAN	241	37307	8	8	8	8	1.75	Guanine nucleotide-bi
557	2	SwissProt_HUMAN$	2::P62873|GBB1_HUMAN	240	37353	9	8	8	8	1.74	Guanine nucleotide-bi
558	1	SwissProt_HUMAN$	2::P48444|COPD_HUMAN	241	57174	16	14	15	13	1.93	Coatomer subunit delt
559	1	SwissProt_HUMAN$	2::Q9Y2S7|PDIP2_HUMAN	240	42007	7	7	6	6	0.96	Polymerase delta-inte
560	1	SwissProt_HUMAN$	2::Q92900|RENT1_HUMAN	239	124267	20	19	18	17	0.91	Regulator of nonsense
561	1	SwissProt_HUMAN$	2::O00116|ADAS_HUMAN	239	72866	11	11	10	10	0.92	Alkyldihydroxyacetone
562	1	SwissProt_HUMAN$	2::Q86XP3|DDX42_HUMAN	238	102912	13	11	11	10	0.59	ATP-dependent RNA h
563	1	SwissProt_HUMAN$	2::Q12904|AIMP1_HUMAN	236	34331	10	8	8	7	1.62	Aminoacyl tRNA synth

↑Family	M	DB	Accession	Score	Mass	Matches	Match(sig)	Sequences	Seq(sig)	emPAI	Description
564	1	SwissProt_HUMAN$	2::P55036|PSMD4_HUMAN	236	40711	9	9	9	9	1.84	26S proteasome non-/
565	1	SwissProt_HUMAN$	2::Q08752|PPID_HUMAN	236	40738	8	7	7	6	1.00	Peptidyl-prolyl cis-trar
566	1	SwissProt_HUMAN$	2::P46977|STT3A_HUMAN	236	80477	12	12	10	10	0.80	Dolichyl-diphosphoolic
567	1	SwissProt_HUMAN$	2::Q15717|ELAV1_HUMAN	236	36069	9	8	9	8	1.85	ELAV-like protein 1 OS
568	1	SwissProt_HUMAN$	2::O00303|EIF3F_HUMAN	235	37540	10	10	8	8	1.73	Eukaryotic translation
569	1	SwissProt_HUMAN$	2::O43684|BUB3_HUMAN	235	37131	10	9	9	8	1.76	Mitotic checkpoint pro
570	1	SwissProt_HUMAN$	2::Q13435|SF3B2_HUMAN	235	100165	16	14	13	12	0.77	Splicing factor 3B sub
571	1	SwissProt_HUMAN$	2::P31153|METK2_HUMAN	234	43633	10	9	8	7	1.13	S-adenosylmethionine
572	1	SwissProt_HUMAN$	2::Q8NC51|PAIRB_HUMAN	234	44938	11	9	8	7	1.09	Plasminogen activator
573	1	SwissProt_HUMAN$	2::Q12874|SF3A3_HUMAN	234	58812	12	10	11	9	1.06	Splicing factor 3A sub
574	1	SwissProt_HUMAN$	2::Q8N1G4|LRC47_HUMAN	234	63434	12	11	12	11	1.27	Leucine-rich repeat-cc
575	1	SwissProt_HUMAN$	2::Q9BWF3|RBM4_HUMAN	234	40289	11	8	8	6	1.02	RNA-binding protein 4
576	1	SwissProt_HUMAN$	2::Q15149|PLEC_HUMAN	234	531466	22	17	21	16	0.15	Plectin OS=Homo sap
577	1	SwissProt_HUMAN$	2::P49792|RBP2_HUMAN	233	357974	14	13	14	13	0.19	E3 SUMO-protein ligas
578	1	SwissProt_HUMAN$	2::Q14978|NOLC1_HUMAN	232	73560	10	10	8	8	0.67	Nucleolar and coiled-b
579	1	SwissProt_HUMAN$	2::P61158|ARP3_HUMAN	232	47341	13	13	11	11	2.00	Actin-related protein 3
580	1	SwissProt_HUMAN$	2::P07339|CATD_HUMAN	232	44524	10	10	8	8	1.34	Cathepsin D OS=Hom
581	1	SwissProt_HUMAN$	2::Q12797|ASPH_HUMAN	231	85809	7	5	7	5	0.32	Aspartyl/asparaginyl b
582	1	SwissProt_HUMAN$	2::Q9UQE7|SMC3_HUMAN	230	141454	12	10	12	10	0.40	Structural maintenanc
583	1	SwissProt_HUMAN$	2::Q9Y3B8|ORN_HUMAN	228	26816	9	9	7	7	2.41	Oligoribonuclease, mit
584	1	SwissProt_HUMAN$	2::Q15785|TOM34_HUMAN	227	34538	8	8	8	8	1.98	Mitochondrial import r
585	1	SwissProt_HUMAN$	2::Q8TAT6|NPL4_HUMAN	227	68077	10	9	9	8	0.74	Nuclear protein localiz
586	1	SwissProt_HUMAN$	2::P17931|LEG3_HUMAN	226	26136	11	11	6	6	2.53	Galectin-3 OS=Homo
587	1	SwissProt_HUMAN$	2::P00491|PNPH_HUMAN	226	32097	6	6	5	5	1.08	Purine nucleoside pho
588	1	SwissProt_HUMAN$	2::P30520|PURA2_HUMAN	225	50066	11	9	9	8	1.13	Adenylosuccinate synt
589	1	SwissProt_HUMAN$	2::O00231|PSD11_HUMAN	225	47434	15	13	11	9	1.45	26S proteasome non-/
590	1	SwissProt_HUMAN$	2::P51149|RAB7A_HUMAN	225	23475	9	9	7	7	3.07	Ras-related protein Ra
591	1	SwissProt_HUMAN$	2::P35611|ADDA_HUMAN	225	80905	9	7	9	7	0.51	Alpha-adducin OS=Ho
592	1	SwissProt_HUMAN$	2::P10768|ESTD_HUMAN	224	31442	12	12	9	9	2.86	S-formylglutathione h
593	1	SwissProt_HUMAN$	2::P19224|UD16_HUMAN	224	60711	6	6	5	5	0.48	UDP-glucuronosyltran:
593	2	SwissProt_HUMAN$	2::O60656|UD19_HUMAN	147	59901	3	3	2	2	0.17	UDP-glucuronosyltran:
594	1	SwissProt_HUMAN$	2::P15170|ERF3A_HUMAN	223	55720	16	15	13	12	1.77	Eukaryotic peptide cha
595	1	SwissProt_HUMAN$	2::Q7L014|DDX46_HUMAN	223	117290	11	10	11	10	0.50	Probable ATP-depende
596	1	SwissProt_HUMAN$	2::P11166|GTR1_HUMAN	223	54049	11	11	8	8	1.01	Solute carrier family 2
596	2	SwissProt_HUMAN$	2::P11169|GTR3_HUMAN	159	53889	7	7	4	4	0.69	Solute carrier family 2
597	1	SwissProt_HUMAN$	2::Q9NPH2|INO1_HUMAN	222	61029	10	8	8	6	0.59	Inositol-3-phosphate s
598	1	SwissProt_HUMAN$	2::Q14498|RBM39_HUMAN	222	59343	9	9	8	8	0.89	RNA-binding protein 3
599	1	SwissProt_HUMAN$	2::P09543|CN37_HUMAN	222	47549	13	10	11	8	1.22	2',3'-cyclic-nucleotide
599	2	SwissProt_HUMAN$	2::Q08209|PP2BA_HUMAN	72	58650	6	5	5	4	0.38	Serine/threonine-prot
600	1	SwissProt_HUMAN$	2::Q9NRH3|TBG2_HUMAN	222	51060	9	9	7	7	0.91	Tubulin gamma-2 cha

↑Family	M	DB	Accession	Score	Mass	Matches	Match(sig)	Sequences	Seq(sig)	emPAI	Description
601	1	SwissProt_HUMAN$	2::Q96GQ7|DDX27_HUMAN	222	89779	10	10	9	9	0.61	Probable ATP-depende
602	1	SwissProt_HUMAN$	2::O15230|LAMA5_HUMAN	221	399479	13	12	12	11	0.14	Laminin subunit alpha
603	1	SwissProt_HUMAN$	2::P30048|PRDX3_HUMAN	221	27675	9	8	6	5	1.34	Thioredoxin-depender
604	1	SwissProt_HUMAN$	2::P24539|AT5F1_HUMAN	221	28890	7	7	7	7	2.13	ATP synthase F(0) cor
605	1	SwissProt_HUMAN$	2::Q9NRN7|ADPPT_HUMAN	221	35753	11	9	9	7	1.52	L-aminoadipate-semia
606	1	SwissProt_HUMAN$	2::P51649|SSDH_HUMAN	221	57178	11	10	11	10	1.29	Succinate-semialdehy
607	1	SwissProt_HUMAN$	2::P13861|KAP2_HUMAN	220	45490	10	10	9	9	1.55	cAMP-dependent prote
608	1	SwissProt_HUMAN$	2::A6NDG6|PGP_HUMAN	220	33985	10	10	8	8	2.03	Glycerol-3-phosphate
609	1	SwissProt_HUMAN$	2::Q99459|CDC5L_HUMAN	220	92194	12	11	12	11	0.76	Cell division cycle 5-lil
610	1	SwissProt_HUMAN$	2::P28066|PSA5_HUMAN	219	26394	6	5	5	4	1.04	Proteasome subunit al
611	1	SwissProt_HUMAN$	2::Q5TFE4|NT5D1_HUMAN	219	51812	9	8	8	8	1.07	5'-nucleotidase domai
612	1	SwissProt_HUMAN$	2::Q9NYU2|UGGG1_HUMAN	218	177078	18	15	17	15	0.50	UDP-glucose:glycopro
613	1	SwissProt_HUMAN$	2::P43490|NAMPT_HUMAN	218	55487	9	8	8	7	0.82	Nicotinamide phospho
614	1	SwissProt_HUMAN$	2::P31949|S10AB_HUMAN	218	11733	7	7	5	5	6.17	Protein S100-A11 OS=
615	1	SwissProt_HUMAN$	2::Q14683|SMC1A_HUMAN	217	143144	18	14	15	12	0.49	Structural maintenanc
616	1	SwissProt_HUMAN$	2::Q16543|CDC37_HUMAN	216	44440	10	8	9	8	1.34	Hsp90 co-chaperone C
617	1	SwissProt_HUMAN$	2::Q9UK22|FBX2_HUMAN	216	33307	11	10	8	7	1.69	F-box only protein 2 C
618	1	SwissProt_HUMAN$	2::P00761|TRYP_PIG	215	24394	4	3	3	2	0.47	Trypsin OS=Sus scrofa
619	1	SwissProt_HUMAN$	2::Q8WUM0|NU133_HUMAN	215	128898	10	9	9	8	0.34	Nuclear pore complex
620	1	SwissProt_HUMAN$	2::Q9NXF1|TEX10_HUMAN	215	105608	9	8	8	7	0.37	Testis-expressed prote
621	1	SwissProt_HUMAN$	2::Q9UI26|IPO11_HUMAN	215	112463	9	7	8	6	0.29	Importin-11 OS=Hom
622	1	SwissProt_HUMAN$	2::P17812|PYRG1_HUMAN	215	66648	12	10	11	9	0.90	CTP synthase 1 OS=H
622	2	SwissProt_HUMAN$	2::Q9NRF8|PYRG2_HUMAN	83	65636	4	3	4	3	0.24	CTP synthase 2 OS=H
623	1	SwissProt_HUMAN$	2::Q14739|LBR_HUMAN	214	70658	10	10	8	8	0.71	Delta(14)-sterol reduc
624	1	SwissProt_HUMAN$	2::O15372|EIF3H_HUMAN	214	39905	12	10	11	9	1.90	Eukaryotic translation
625	1	SwissProt_HUMAN$	2::Q93009|UBP7_HUMAN	214	128220	12	11	11	10	0.45	Ubiquitin carboxyl-ter
626	1	SwissProt_HUMAN$	2::P08133|ANXA6_HUMAN	214	75826	20	14	18	13	1.25	Annexin A6 OS=Homo
627	1	SwissProt_HUMAN$	2::P14174|MIF_HUMAN	214	12468	8	8	4	4	5.47	Macrophage migration
628	1	SwissProt_HUMAN$	2::P30043|BLVRB_HUMAN	213	22105	6	6	3	3	0.89	Flavin reductase (NAD
629	1	SwissProt_HUMAN$	2::Q14839|CHD4_HUMAN	213	217867	13	12	11	11	0.27	Chromodomain-helica
630	1	SwissProt_HUMAN$	2::P20645|MPRD_HUMAN	213	30973	6	6	5	5	1.14	Cation-dependent mar
631	1	SwissProt_HUMAN$	2::P49902|5NTC_HUMAN	212	64928	7	7	7	7	0.67	Cytosolic purine 5'-nu
632	1	SwissProt_HUMAN$	2::P42224|STAT1_HUMAN	212	87280	14	13	12	11	0.82	Signal transducer and
633	1	SwissProt_HUMAN$	2::Q9H4A6|GOLP3_HUMAN	212	33790	4	4	4	4	0.75	Golgi phosphoprotein
634	1	SwissProt_HUMAN$	2::Q9UN86|G3BP2_HUMAN	211	54088	7	7	5	5	0.55	Ras GTPase-activating
635	1	SwissProt_HUMAN$	2::Q15181|IPYR_HUMAN	211	32639	12	10	10	9	2.67	Inorganic pyrophospha
635	2	SwissProt_HUMAN$	2::Q9H2U2|IPYR2_HUMAN	45	37896	4	3	2	2	0.28	Inorganic pyrophospha
636	1	SwissProt_HUMAN$	2::P61224|RAP1B_HUMAN	210	20812	7	6	5	4	1.47	Ras-related protein Ra
637	1	SwissProt_HUMAN$	2::P58107|EPIPL_HUMAN	210	555316	4	4	3	3	0.03	Epiplakin OS=Homo sa
638	1	SwissProt_HUMAN$	2::Q9Y512|SAM50_HUMAN	210	51943	11	11	10	10	1.48	Sorting and assembly

↑Family	M	DB	Accession	Score	Mass	Matches	Match(sig)	Sequences	Seq(sig)	emPAI	Description
639	1	SwissProt_HUMAN$	2::O43169|CYB5B_HUMAN	210	16684	11	9	6	5	4.38	Cytochrome b5 type B
640	1	SwissProt_HUMAN$	2::Q15005|SPCS2_HUMAN	209	24987	4	4	3	3	0.76	Signal peptidase comp
641	1	SwissProt_HUMAN$	2::P40616|ARL1_HUMAN	209	20404	5	4	4	3	0.99	ADP-ribosylation facto
642	1	SwissProt_HUMAN$	2::Q03701|CEBPZ_HUMAN	208	120898	14	13	13	12	0.60	CCAAT/enhancer-bind
643	1	SwissProt_HUMAN$	2::P61088|UBE2N_HUMAN	208	17127	11	9	9	8	7.91	Ubiquitin-conjugating
644	1	SwissProt_HUMAN$	2::Q04760|LGUL_HUMAN	208	20764	10	8	6	5	2.09	Lactoylglutathione lya
645	1	SwissProt_HUMAN$	2::Q14011|CIRBP_HUMAN	208	18637	4	4	4	4	1.73	Cold-inducible RNA-bi
646	1	SwissProt_HUMAN$	2::Q13907|IDI1_HUMAN	208	26302	8	8	8	8	3.18	Isopentenyl-diphosph
647	1	SwissProt_HUMAN$	2::P24666|PPAC_HUMAN	207	18031	7	6	6	6	3.76	Low molecular weight
648	1	SwissProt_HUMAN$	2::Q8IY81|SPB1_HUMAN	206	96499	14	11	13	11	0.72	pre-rRNA 2'-O-ribose
649	1	SwissProt_HUMAN$	2::P63208|SKP1_HUMAN	206	18646	9	8	8	7	4.79	S-phase kinase-associ
650	1	SwissProt_HUMAN$	2::Q96KP4|CNDP2_HUMAN	206	52845	9	9	7	7	0.87	Cytosolic non-specific
651	1	SwissProt_HUMAN$	2::O43324|MCA3_HUMAN	206	19798	11	11	8	8	7.44	Eukaryotic translation
652	1	SwissProt_HUMAN$	2::P53992|SC24C_HUMAN	205	118249	12	10	12	10	0.49	Protein transport prot
653	1	SwissProt_HUMAN$	2::Q9H3U1|UN45A_HUMAN	205	103011	10	6	9	5	0.26	Protein unc-45 homol
654	1	SwissProt_HUMAN$	2::P23378|GCSP_HUMAN	205	112657	11	10	10	9	0.46	Glycine dehydrogenas
655	1	SwissProt_HUMAN$	2::Q9UGI8|TES_HUMAN	205	47964	9	9	9	9	1.43	Testin OS=Homo sapi
656	1	SwissProt_HUMAN$	2::Q96CN7|ISOC1_HUMAN	205	32216	5	5	4	4	0.80	Isochorismatase dom
657	1	SwissProt_HUMAN$	2::Q6NVY1|HIBCH_HUMAN	205	43454	8	7	7	7	1.14	3-hydroxyisobutyryl-C
658	1	SwissProt_HUMAN$	2::P28288|ABCD3_HUMAN	204	75428	14	12	13	11	0.99	ATP-binding cassette
659	1	SwissProt_HUMAN$	2::P40429|RL13A_HUMAN	204	23562	15	11	11	9	5.02	60S ribosomal protein
660	1	SwissProt_HUMAN$	2::P46783|RS10_HUMAN	204	18886	11	8	8	6	3.43	40S ribosomal protein
661	1	SwissProt_HUMAN$	2::P49721|PSB2_HUMAN	204	22822	7	7	6	6	2.43	Proteasome subunit b
662	1	SwissProt_HUMAN$	2::P54709|AT1B3_HUMAN	204	31492	6	6	5	5	1.11	Sodium/potassium-tra
663	1	SwissProt_HUMAN$	2::Q93008|USP9X_HUMAN	203	290278	11	9	10	9	0.16	Probable ubiquitin car
663	2	SwissProt_HUMAN$	2::Q96EY7|PTCD3_HUMAN	99	78500	11	8	9	7	0.53	Pentatricopeptide rep
663	3	SwissProt_HUMAN$	2::Q9UPY3|DICER_HUMAN	22	218542	3	2	2	2	0.04	Endoribonuclease Dice
663	4	SwissProt_HUMAN$	2::Q9BQS8|FYCO1_HUMAN	20	166879	4	2	3	2	0.06	FYVE and coiled-coil d
664	1	SwissProt_HUMAN$	2::Q9P289|STK26_HUMAN	203	46500	9	9	9	9	1.50	Serine/threonine-prot
664	2	SwissProt_HUMAN$	2::Q9Y6E0|STK24_HUMAN	131	49277	6	6	6	6	0.78	Serine/threonine-prot
665	1	SwissProt_HUMAN$	2::O14929|HAT1_HUMAN	203	49481	8	7	7	6	0.77	Histone acetyltransfer
666	1	SwissProt_HUMAN$	2::Q00325|MPCP_HUMAN	203	40069	9	7	8	6	1.03	Phosphate carrier prot
667	1	SwissProt_HUMAN$	2::P62841|RS15_HUMAN	203	17029	7	7	5	5	4.21	40S ribosomal protein
668	1	SwissProt_HUMAN$	2::Q02978|M2OM_HUMAN	203	34040	7	7	7	7	1.63	Mitochondrial 2-oxogl
669	1	SwissProt_HUMAN$	2::P82979|SARNP_HUMAN	202	23656	10	9	10	9	4.97	SAP domain-containin
670	1	SwissProt_HUMAN$	2::O60884|DNJA2_HUMAN	201	45717	7	6	6	5	0.68	DnaJ homolog subfam
671	1	SwissProt_HUMAN$	2::Q15631|TSN_HUMAN	201	26167	7	7	6	6	1.94	Translin OS=Homo sa
672	1	SwissProt_HUMAN$	2::Q9P287|BCCIP_HUMAN	201	35957	7	7	5	5	0.93	BRCA2 and CDKN1A-i
673	1	SwissProt_HUMAN$	2::Q13085|ACACA_HUMAN	201	265385	16	14	16	14	0.28	Acetyl-CoA carboxylas
674	1	SwissProt_HUMAN$	2::O60841|IF2P_HUMAN	201	138742	16	12	14	11	0.46	Eukaryotic translation

↑Family	M	DB	Accession	Score	Mass	Matches	Match(sig)	Sequences	Seq(sig)	emPAI	Description
675	1	SwissProt_HUMAN$	2::Q5VYK3|ECM29_HUMAN	201	204160	10	10	8	8	0.20	Proteasome adapter a
676	1	SwissProt_HUMAN$	2::Q9H6S3|ES8L2_HUMAN	200	80570	9	7	8	7	0.51	Epidermal growth fact
677	1	SwissProt_HUMAN$	2::Q5JTZ9|SYAM_HUMAN	200	107273	8	8	7	7	0.36	Alanine--tRNA ligase,
678	1	SwissProt_HUMAN$	2::P49756|RBM25_HUMAN	200	100124	11	9	10	8	0.46	RNA-binding protein 2
679	1	SwissProt_HUMAN$	2::Q8NE71|ABCF1_HUMAN	200	95866	10	8	9	8	0.49	ATP-binding cassette s
680	1	SwissProt_HUMAN$	2::P51114|FXR1_HUMAN	200	69678	8	6	7	5	0.40	Fragile X mental retar
680	2	SwissProt_HUMAN$	2::Q06787|FMR1_HUMAN	112	71131	7	4	7	4	0.31	Synaptic functional re
681	1	SwissProt_HUMAN$	2::P40763|STAT3_HUMAN	200	88011	11	10	8	8	0.54	Signal transducer and
682	1	SwissProt_HUMAN$	2::Q9NTJ3|SMC4_HUMAN	199	147091	12	9	11	8	0.29	Structural maintenanc
683	1	SwissProt_HUMAN$	2::P36915|GNL1_HUMAN	199	68619	10	9	9	8	0.74	Guanine nucleotide-bi
684	1	SwissProt_HUMAN$	2::Q8TCS8|PNPT1_HUMAN	199	85897	11	11	10	10	0.74	Polyribonucleotide nuc
685	1	SwissProt_HUMAN$	2::Q6NXG1|ESRP1_HUMAN	199	75537	8	6	7	6	0.46	Epithelial splicing regu
686	1	SwissProt_HUMAN$	2::O75955|FLOT1_HUMAN	199	47326	11	10	9	8	1.22	Flotillin-1 OS=Homo s
687	1	SwissProt_HUMAN$	2::P41091|IF2G_HUMAN	199	51077	16	13	12	10	1.52	Eukaryotic translation
688	1	SwissProt_HUMAN$	2::P46779|RL28_HUMAN	198	15738	11	10	7	6	9.68	60S ribosomal protein
689	1	SwissProt_HUMAN$	2::P67775|PP2AA_HUMAN	198	35571	10	9	7	6	1.53	Serine/threonine-prot
689	2	SwissProt_HUMAN$	2::P60510|PP4C_HUMAN	84	35057	4	4	3	3	0.71	Serine/threonine-prot
690	1	SwissProt_HUMAN$	2::P14314|GLU2B_HUMAN	198	59388	9	8	8	7	0.75	Glucosidase 2 subunit
691	1	SwissProt_HUMAN$	2::Q9BTE3|MCMBP_HUMAN	198	72934	25	17	9	9	0.79	Mini-chromosome mai
692	1	SwissProt_HUMAN$	2::P39748|FEN1_HUMAN	198	42566	9	9	8	8	1.43	Flap endonuclease 1 C
693	1	SwissProt_HUMAN$	2::O00541|PESC_HUMAN	198	67960	14	12	13	11	1.15	Pescadillo homolog OS
694	1	SwissProt_HUMAN$	2::P30837|AL1B1_HUMAN	197	57170	10	8	8	7	0.78	Aldehyde dehydrogena
695	1	SwissProt_HUMAN$	2::P09936|UCHL1_HUMAN	197	24808	8	8	6	6	2.78	Ubiquitin carboxyl-ter
696	1	SwissProt_HUMAN$	2::P10599|THIO_HUMAN	197	11730	7	7	5	5	9.64	Thioredoxin OS=Homo
697	1	SwissProt_HUMAN$	2::P62910|RL32_HUMAN	196	15850	8	8	5	5	4.84	60S ribosomal protein
698	1	SwissProt_HUMAN$	2::P25325|THTM_HUMAN	196	33158	8	8	5	5	1.03	3-mercaptopyruvate s
699	1	SwissProt_HUMAN$	2::O75844|FACE1_HUMAN	196	54778	8	7	8	7	0.83	CAAX prenyl protease
700	1	SwissProt_HUMAN$	2::Q15392|DHC24_HUMAN	196	60062	7	7	7	7	0.74	Delta(24)-sterol reduc
701	1	SwissProt_HUMAN$	2::P08243|ASNS_HUMAN	195	64329	8	8	6	6	0.55	Asparagine synthetase
702	1	SwissProt_HUMAN$	2::P01857|IGHG1_HUMAN	192	36083	10	10	9	9	2.24	Immunoglobulin heav
702	2	SwissProt_HUMAN$	2::P01859|IGHG2_HUMAN	117	35878	7	7	7	7	1.51	Immunoglobulin heav
703	1	SwissProt_HUMAN$	2::Q9NW13|RBM28_HUMAN	195	85685	10	10	8	8	0.64	RNA-binding protein 2
704	1	SwissProt_HUMAN$	2::P23381|SYWC_HUMAN	195	53132	10	7	8	6	0.71	Tryptophan--tRNA liga
705	1	SwissProt_HUMAN$	2::O43818|U3IP2_HUMAN	194	51809	9	8	8	7	0.89	U3 small nucleolar RN
706	1	SwissProt_HUMAN$	2::Q9Y6G9|DC1L1_HUMAN	194	56544	6	6	5	5	0.52	Cytoplasmic dynein 1
707	1	SwissProt_HUMAN$	2::Q14847|LASP1_HUMAN	193	29698	7	7	5	5	1.21	LIM and SH3 domain p
708	1	SwissProt_HUMAN$	2::Q13011|ECH1_HUMAN	193	35793	12	11	9	8	1.87	Delta(3,5)-Delta(2,4)-
709	1	SwissProt_HUMAN$	2::Q99436|PSB7_HUMAN	193	29946	7	6	6	5	1.19	Proteasome subunit be
710	1	SwissProt_HUMAN$	2::Q6P2E9|EDC4_HUMAN	192	151567	8	7	8	7	0.25	Enhancer of mRNA-de
711	1	SwissProt_HUMAN$	2::P12830|CADH1_HUMAN	191	97396	10	8	8	7	0.41	Cadherin-1 OS=Homo

↑Family	M	DB	Accession	Score	Mass	Matches	Match(sig)	Sequences	Seq(sig)	emPAI	Description
712	1	SwissProt_HUMAN$	2::O14818|PSA7_HUMAN	191	27870	9	8	8	7	2.26	Proteasome subunit al
713	1	SwissProt_HUMAN$	2::O75691|UTP20_HUMAN	191	318182	9	8	9	8	0.13	Small subunit process
714	1	SwissProt_HUMAN$	2::Q16630|CPSF6_HUMAN	191	59173	6	6	5	5	0.49	Cleavage and polyade
715	1	SwissProt_HUMAN$	2::Q9Y6C9|MTCH2_HUMAN	191	33309	9	9	7	7	2.10	Mitochondrial carrier h
716	1	SwissProt_HUMAN$	2::Q8TEQ6|GEMI5_HUMAN	191	168483	12	11	12	11	0.36	Gem-associated prote
717	1	SwissProt_HUMAN$	2::Q96DI7|SNR40_HUMAN	191	39286	2	2	1	1	0.13	U5 small nuclear ribor
718	1	SwissProt_HUMAN$	2::Q8WXX5|DNJC9_HUMAN	191	29891	7	7	6	6	1.58	DnaJ homolog subfam
719	1	SwissProt_HUMAN$	2::O94760|DDAH1_HUMAN	190	31102	9	8	7	6	1.48	N(G),N(G)-dimethylar
719	2	SwissProt_HUMAN$	2::O95865|DDAH2_HUMAN	86	29625	5	5	5	5	1.21	N(G),N(G)-dimethylar
720	1	SwissProt_HUMAN$	2::P61619|S61A1_HUMAN	190	52231	7	5	5	4	0.44	Protein transport prot
721	1	SwissProt_HUMAN$	2::O00203|AP3B1_HUMAN	190	121244	13	11	12	10	0.48	AP-3 complex subunit
722	1	SwissProt_HUMAN$	2::Q9Y2L1|RRP44_HUMAN	190	108934	10	9	9	9	0.48	Exosome complex exo
723	1	SwissProt_HUMAN$	2::P68036|UB2L3_HUMAN	189	17850	14	11	11	10	12.69	Ubiquitin-conjugating
724	1	SwissProt_HUMAN$	2::Q9P258|RCC2_HUMAN	189	56049	14	13	14	13	2.00	Protein RCC2 OS=Hor
725	1	SwissProt_HUMAN$	2::Q13247|SRSF6_HUMAN	189	39563	29	19	9	9	2.30	Serine/arginine-rich s
725	2	SwissProt_HUMAN$	2::Q08170|SRSF4_HUMAN	102	56645	22	13	4	4	0.40	Serine/arginine-rich s
726	1	SwissProt_HUMAN$	2::P54727|RD23B_HUMAN	189	43145	8	6	6	4	0.73	UV excision repair pro
726	2	SwissProt_HUMAN$	2::P54725|RD23A_HUMAN	68	39585	3	3	2	2	0.43	UV excision repair pro
727	1	SwissProt_HUMAN$	2::P18754|RCC1_HUMAN	188	44941	7	7	7	7	1.09	Regulator of chromoso
728	1	SwissProt_HUMAN$	2::Q9H8Y8|GORS2_HUMAN	188	47116	7	7	6	6	0.83	Golgi reassembly-stac
729	1	SwissProt_HUMAN$	2::Q13868|EXOS2_HUMAN	188	32768	5	5	4	4	0.78	Exosome complex con
730	1	SwissProt_HUMAN$	2::P30085|KCY_HUMAN	188	22208	6	5	5	4	1.33	UMP-CMP kinase OS=
731	1	SwissProt_HUMAN$	2::Q9H993|ARMT1_HUMAN	187	51140	6	6	5	5	0.59	Damage-control phos
732	1	SwissProt_HUMAN$	2::P63220|RS21_HUMAN	187	9106	8	6	5	4	11.53	40S ribosomal protein
733	1	SwissProt_HUMAN$	2::O43615|TIM44_HUMAN	187	51323	8	7	8	7	0.91	Mitochondrial import i
734	1	SwissProt_HUMAN$	2::Q99829|CPNE1_HUMAN	186	59022	9	8	9	8	0.90	Copine-1 OS=Homo s
735	1	SwissProt_HUMAN$	2::Q99798|ACON_HUMAN	186	85372	9	7	8	7	0.47	Aconitate hydratase, r
736	1	SwissProt_HUMAN$	2::P62269|RS18_HUMAN	186	17708	15	12	10	8	9.69	40S ribosomal protein
737	1	SwissProt_HUMAN$	2::Q13557|KCC2D_HUMAN	186	56334	9	8	8	7	0.80	Calcium/calmodulin-d
738	1	SwissProt_HUMAN$	2::Q9H0D6|XRN2_HUMAN	186	108513	9	9	8	8	0.42	5'-3' exoribonuclease
739	1	SwissProt_HUMAN$	2::P43487|RANG_HUMAN	186	23296	8	7	6	5	1.74	Ran-specific GTPase-a
740	1	SwissProt_HUMAN$	2::Q01844|EWS_HUMAN	186	68436	5	5	4	4	0.32	RNA-binding protein E
741	1	SwissProt_HUMAN$	2::P53041|PPP5_HUMAN	185	56842	8	6	8	6	0.65	Serine/threonine-prot
742	1	SwissProt_HUMAN$	2::O95433|AHSA1_HUMAN	185	38250	11	9	8	7	1.37	Activator of 90 kDa he
743	1	SwissProt_HUMAN$	2::Q13283|G3BP1_HUMAN	185	52132	12	10	7	5	0.89	Ras GTPase-activating
744	1	SwissProt_HUMAN$	2::Q9BZX2|UCK2_HUMAN	184	29281	6	6	5	5	1.23	Uridine-cytidine kinas
745	1	SwissProt_HUMAN$	2::Q9BUQ8|DDX23_HUMAN	184	95524	10	8	9	7	0.42	Probable ATP-depende
746	1	SwissProt_HUMAN$	2::Q9UBD5|ORC3_HUMAN	184	82201	5	5	4	4	0.26	Origin recognition com
747	1	SwissProt_HUMAN$	2::Q96A33|CCD47_HUMAN	184	55838	8	6	7	5	0.53	Coiled-coil domain-co
748	1	SwissProt_HUMAN$	2::P07942|LAMB1_HUMAN	183	197909	7	6	7	6	0.15	Laminin subunit beta-

↑Family	M	DB	Accession	Score	Mass	Matches	Match(sig)	Sequences	Seq(sig)	emPAI	Description
749	1	SwissProt_HUMAN$	2::Q9Y4R8|TELO2_HUMAN	182	91689	7	5	6	4	0.23	Telomere length regul
750	1	SwissProt_HUMAN$	2::O94979|SC31A_HUMAN	182	132931	14	13	12	11	0.48	Protein transport prot
751	1	SwissProt_HUMAN$	2::P06396|GELS_HUMAN	182	85644	9	6	9	6	0.39	Gelsolin OS=Homo sa
752	1	SwissProt_HUMAN$	2::P37268|FDFT_HUMAN	182	48084	11	9	10	9	1.42	Squalene synthase OS
753	1	SwissProt_HUMAN$	2::O43156|TTI1_HUMAN	182	121992	6	6	6	6	0.26	TELO2-interacting pro
754	1	SwissProt_HUMAN$	2::P61254|RL26_HUMAN	182	17248	13	11	11	10	14.12	60S ribosomal protein
755	1	SwissProt_HUMAN$	2::P11172|UMPS_HUMAN	181	52189	11	10	11	10	1.48	Uridine 5'-monophosp
756	1	SwissProt_HUMAN$	2::P05187|PPB1_HUMAN	181	57917	11	11	11	11	1.45	Alkaline phosphatase,
756	2	SwissProt_HUMAN$	2::P10696|PPBN_HUMAN	143	57341	10	10	10	10	1.28	Alkaline phosphatase,
756	3	SwissProt_HUMAN$	2::P09923|PPBI_HUMAN	85	56776	5	4	5	4	0.40	Intestinal-type alkalin
757	1	SwissProt_HUMAN$	2::P50579|MAP2_HUMAN	180	52858	9	6	8	6	0.71	Methionine aminopept
758	1	SwissProt_HUMAN$	2::P18621|RL17_HUMAN	180	21383	13	10	9	7	4.76	60S ribosomal protein
759	1	SwissProt_HUMAN$	2::Q9NTK5|OLA1_HUMAN	180	44715	10	9	8	7	1.09	Obg-like ATPase 1 OS
760	1	SwissProt_HUMAN$	2::Q8IZL8|PELP1_HUMAN	179	119624	8	8	6	6	0.27	Proline-, glutamic acic
761	1	SwissProt_HUMAN$	2::P05386|RLA1_HUMAN	179	11507	6	6	3	3	2.33	60S acidic ribosomal p
762	1	SwissProt_HUMAN$	2::Q96I99|SUCB2_HUMAN	179	46481	7	7	7	7	1.04	Succinate--CoA ligase
763	1	SwissProt_HUMAN$	2::P22061|PIMT_HUMAN	179	24621	9	8	7	6	2.80	Protein-L-isoaspartate
764	1	SwissProt_HUMAN$	2::P28482|MK01_HUMAN	178	41363	11	11	10	10	2.13	Mitogen-activated pro
764	2	SwissProt_HUMAN$	2::P27361|MK03_HUMAN	144	43108	11	8	10	7	1.15	Mitogen-activated pro
765	1	SwissProt_HUMAN$	2::Q15020|SART3_HUMAN	178	109865	11	9	11	9	0.47	Squamous cell carcinc
766	1	SwissProt_HUMAN$	2::O43290|SNUT1_HUMAN	178	90200	7	4	7	4	0.23	U4/U6.U5 tri-snRNP-a
767	1	SwissProt_HUMAN$	2::P61019|RAB2A_HUMAN	178	23531	8	8	8	8	3.93	Ras-related protein Ra
768	1	SwissProt_HUMAN$	2::Q9Y570|PPME1_HUMAN	178	42288	9	9	8	8	1.73	Protein phosphatase n
769	1	SwissProt_HUMAN$	2::Q14019|COTL1_HUMAN	178	15935	7	7	5	5	3.31	Coactosin-like protein
770	1	SwissProt_HUMAN$	2::Q15274|NADC_HUMAN	178	30826	12	12	9	9	2.95	Nicotinate-nucleotide
771	1	SwissProt_HUMAN$	2::Q9Y6N5|SQOR_HUMAN	178	49929	5	4	4	3	0.33	Sulfide:quinone oxidoı
772	1	SwissProt_HUMAN$	2::Q96FW1|OTUB1_HUMAN	177	31264	9	9	7	7	1.87	Ubiquitin thioesterase
773	1	SwissProt_HUMAN$	2::O60547|GMDS_HUMAN	177	41923	7	7	6	6	0.96	GDP-mannose 4,6 deh
774	1	SwissProt_HUMAN$	2::O15143|ARC1B_HUMAN	177	40923	9	9	6	6	1.00	Actin-related protein 2
774	2	SwissProt_HUMAN$	2::Q92747|ARC1A_HUMAN	69	41543	3	2	3	2	0.26	Actin-related protein 2
775	1	SwissProt_HUMAN$	2::Q9NSD9|SYFB_HUMAN	177	66074	13	12	13	12	1.36	Phenylalanine--tRNA l
776	1	SwissProt_HUMAN$	2::Q9HCE1|MOV10_HUMAN	176	113599	9	8	9	8	0.40	Helicase MOV-10 OS=
777	1	SwissProt_HUMAN$	2::P11279|LAMP1_HUMAN	176	44854	8	7	6	6	0.88	Lysosome-associated
778	1	SwissProt_HUMAN$	2::O60306|AQR_HUMAN	176	171186	9	6	9	6	0.18	RNA helicase aquarius
779	1	SwissProt_HUMAN$	2::Q14444|CAPR1_HUMAN	175	78318	11	8	10	8	0.62	Caprin-1 OS=Homo sa
780	1	SwissProt_HUMAN$	2::Q13561|DCTN2_HUMAN	175	44204	7	7	6	6	0.90	Dynactin subunit 2 OS
781	1	SwissProt_HUMAN$	2::P51858|HDGF_HUMAN	175	26772	7	7	7	7	2.42	Hepatoma-derived grc
782	1	SwissProt_HUMAN$	2::O95292|VAPB_HUMAN	175	27211	8	6	7	5	1.37	Vesicle-associated me
782	2	SwissProt_HUMAN$	2::Q9P0L0|VAPA_HUMAN	42	27875	3	3	3	3	0.66	Vesicle-associated me
783	1	SwissProt_HUMAN$	2::P62316|SMD2_HUMAN	175	13518	8	8	6	6	6.85	Small nuclear ribonucl

↑Family	M	DB	Accession	Score	Mass	Matches	Match(sig)	Sequences	Seq(sig)	emPAI	Description
784	1	SwissProt_HUMAN$	2::Q9Y2B0|CNPY2_HUMAN	175	20639	4	4	4	4	1.48	Protein canopy homolo
785	1	SwissProt_HUMAN$	2::P63151|2ABA_HUMAN	175	51659	7	6	5	5	0.58	Serine/threonine-prote
786	1	SwissProt_HUMAN$	2::O60437|PEPL_HUMAN	174	204623	12	9	10	7	0.18	Periplakin OS=Homo s
787	1	SwissProt_HUMAN$	2::Q15907|RB11B_HUMAN	174	24473	9	9	7	7	2.84	Ras-related protein Ra
788	1	SwissProt_HUMAN$	2::Q9H8H0|NOL11_HUMAN	174	81072	7	6	6	5	0.34	Nucleolar protein 11 C
789	1	SwissProt_HUMAN$	2::Q969V3|NCLN_HUMAN	174	62935	8	8	7	7	0.69	Nicalin OS=Homo sap
790	1	SwissProt_HUMAN$	2::Q14684|RRP1B_HUMAN	174	84375	8	8	8	8	0.57	Ribosomal RNA proces
791	1	SwissProt_HUMAN$	2::Q9Y3T9|NOC2L_HUMAN	173	84866	12	12	12	12	0.95	Nucleolar complex pro
792	1	SwissProt_HUMAN$	2::P61201|CSN2_HUMAN	173	51564	9	7	8	6	0.73	COP9 signalosome cor
793	1	SwissProt_HUMAN$	2::O75146|HIP1R_HUMAN	172	119315	7	6	6	5	0.22	Huntingtin-interacting
794	1	SwissProt_HUMAN$	2::O95239|KIF4A_HUMAN	172	139794	9	6	8	5	0.19	Chromosome-associat
795	1	SwissProt_HUMAN$	2::Q52LJ0|FA98B_HUMAN	172	45519	5	5	5	5	0.68	Protein FAM98B OS=H
795	2	SwissProt_HUMAN$	2::Q8NCA5|FA98A_HUMAN	160	55238	4	4	4	4	0.41	Protein FAM98A OS=H
796	1	SwissProt_HUMAN$	2::Q9H4M9|EHD1_HUMAN	172	60589	8	8	7	7	0.73	EH domain-containing
797	1	SwissProt_HUMAN$	2::Q14244|MAP7_HUMAN	171	84002	10	7	10	7	0.48	Ensconsin OS=Homo s
798	1	SwissProt_HUMAN$	2::Q12864|CAD17_HUMAN	171	92162	14	11	12	10	0.67	Cadherin-17 OS=Hom
799	1	SwissProt_HUMAN$	2::O95816|BAG2_HUMAN	171	23757	10	10	8	8	3.86	BAG family molecular
800	1	SwissProt_HUMAN$	2::Q9Y2W1|TR150_HUMAN	170	108601	9	7	8	6	0.30	Thyroid hormone rece
800	2	SwissProt_HUMAN$	2::Q9NYF8|BCLF1_HUMAN	106	106059	8	7	8	7	0.37	Bcl-2-associated trans
801	1	SwissProt_HUMAN$	2::Q9H444|CHM4B_HUMAN	170	24935	3	3	2	2	0.46	Charged multivesicula
802	1	SwissProt_HUMAN$	2::Q99439|CNN2_HUMAN	170	33675	7	7	4	4	0.75	Calponin-2 OS=Homo
802	2	SwissProt_HUMAN$	2::Q15417|CNN3_HUMAN	55	36391	4	3	4	3	0.47	Calponin-3 OS=Homo
803	1	SwissProt_HUMAN$	2::Q9UHD8|SEPT9_HUMAN	170	65361	12	12	10	10	1.06	Septin-9 OS=Homo sa
804	1	SwissProt_HUMAN$	2::Q29RF7|PDS5A_HUMAN	169	150734	10	8	9	8	0.29	Sister chromatid cohe
804	2	SwissProt_HUMAN$	2::Q9NTI5|PDS5B_HUMAN	155	164563	6	6	6	6	0.19	Sister chromatid cohe
805	1	SwissProt_HUMAN$	2::P51572|BAP31_HUMAN	169	27974	6	6	5	5	1.32	B-cell receptor-associa
806	1	SwissProt_HUMAN$	2::Q9H307|PININ_HUMAN	169	81579	15	10	14	9	0.69	Pinin OS=Homo sapie
807	1	SwissProt_HUMAN$	2::Q15185|TEBP_HUMAN	169	18685	5	5	4	4	1.73	Prostaglandin E syntha
808	1	SwissProt_HUMAN$	2::Q9H2C0|GAN_HUMAN	169	67595	4	4	2	2	0.15	Gigaxonin OS=Homo s
809	1	SwissProt_HUMAN$	2::P83916|CBX1_HUMAN	169	21405	9	7	8	6	2.72	Chromobox protein ho
810	1	SwissProt_HUMAN$	2::P35659|DEK_HUMAN	168	42648	8	6	8	6	0.94	Protein DEK OS=Homo
811	1	SwissProt_HUMAN$	2::Q14166|TTL12_HUMAN	168	74356	10	10	10	10	0.89	Tubulin--tyrosine ligas
812	1	SwissProt_HUMAN$	2::Q07955|SRSF1_HUMAN	168	27728	13	11	10	8	2.88	Serine/arginine-rich sp
813	1	db$	1::P0DTC7|NS7A_SARS2	168	13735	3	3	3	3	1.76	ORF7a protein OS=Se
814	1	SwissProt_HUMAN$	2::O43491|E41L2_HUMAN	168	112519	10	8	9	7	0.34	Band 4.1-like protein
815	1	SwissProt_HUMAN$	2::P23141|EST1_HUMAN	168	62481	10	8	9	8	0.83	Liver carboxylesterase
816	1	SwissProt_HUMAN$	2::O60832|DKC1_HUMAN	167	57638	10	8	9	7	0.78	H/ACA ribonucleoprote
817	1	SwissProt_HUMAN$	2::P11047|LAMC1_HUMAN	167	177489	5	4	4	3	0.08	Laminin subunit gamn
818	1	SwissProt_HUMAN$	2::Q9BS26|ERP44_HUMAN	167	46941	11	11	8	8	1.24	Endoplasmic reticulum
818	2	SwissProt_HUMAN$	2::P43034|LIS1_HUMAN	87	46608	9	8	7	6	0.84	Platelet-activating fact

↑Family	M	DB	Accession	Score	Mass	Matches	Match(sig)	Sequences	Seq(sig)	emPAI	Description
819	1	SwissProt_HUMAN$	2::Q12996|CSTF3_HUMAN	167	82869	8	7	8	7	0.49	Cleavage stimulation f
820	1	SwissProt_HUMAN$	2::P55735|SEC13_HUMAN	167	35518	4	4	4	4	0.70	Protein SEC13 homolo
821	1	SwissProt_HUMAN$	2::Q9BXS5|AP1M1_HUMAN	167	48556	9	9	6	6	0.79	AP-1 complex subunit
821	2	SwissProt_HUMAN$	2::Q9Y6Q5|AP1M2_HUMAN	112	48078	7	6	5	4	0.48	AP-1 complex subunit
822	1	SwissProt_HUMAN$	2::Q9UHD1|CHRD1_HUMAN	167	37466	8	7	8	7	1.41	Cysteine and histidine
823	1	SwissProt_HUMAN$	2::Q15435|PP1R7_HUMAN	166	41539	3	3	2	2	0.26	Protein phosphatase 1
824	1	SwissProt_HUMAN$	2::Q9Y3A5|SBDS_HUMAN	166	28745	6	5	5	4	0.92	Ribosome maturation
825	1	SwissProt_HUMAN$	2::Q9NT62|ATG3_HUMAN	166	35841	6	5	5	4	0.69	Ubiquitin-like-conjuga
826	1	SwissProt_HUMAN$	2::Q9NUU7|DD19A_HUMAN	166	53941	6	6	5	5	0.55	ATP-dependent RNA h
827	1	SwissProt_HUMAN$	2::Q15075|EEA1_HUMAN	166	162367	16	13	14	11	0.38	Early endosome antige
828	1	SwissProt_HUMAN$	2::P40937|RFC5_HUMAN	165	38472	7	6	5	4	0.63	Replication factor C su
829	1	SwissProt_HUMAN$	2::Q9BVI4|NOC4L_HUMAN	165	58431	7	7	7	7	0.76	Nucleolar complex pro
830	1	SwissProt_HUMAN$	2::Q96IR7|HPDL_HUMAN	165	39361	6	6	6	6	1.05	4-hydroxyphenylpyruv
831	1	SwissProt_HUMAN$	2::P21291|CSRP1_HUMAN	165	20554	5	4	4	3	0.98	Cysteine and glycine-r
832	1	SwissProt_HUMAN$	2::O94905|ERLN2_HUMAN	165	37815	5	5	5	5	0.86	Erlin-2 OS=Homo sap
832	2	SwissProt_HUMAN$	2::O75477|ERLN1_HUMAN	157	39146	8	7	8	7	1.32	Erlin-1 OS=Homo sap
833	1	SwissProt_HUMAN$	2::P47756|CAPZB_HUMAN	164	31331	7	7	5	5	1.47	F-actin-capping protei
834	1	SwissProt_HUMAN$	2::Q9Y5L0|TNPO3_HUMAN	164	104136	10	9	7	6	0.31	Transportin-3 OS=Hor
835	1	SwissProt_HUMAN$	2::O75223|GGCT_HUMAN	164	20994	6	6	4	4	1.44	Gamma-glutamylcyclo
836	1	SwissProt_HUMAN$	2::P26196|DDX6_HUMAN	163	54382	9	8	7	6	0.84	Probable ATP-depende
837	1	SwissProt_HUMAN$	2::P42126|ECI1_HUMAN	163	32795	10	7	8	6	1.37	Enoyl-CoA delta isome
838	1	SwissProt_HUMAN$	2::P50213|IDH3A_HUMAN	163	39566	5	5	4	4	0.82	Isocitrate dehydrogen
839	1	SwissProt_HUMAN$	2::Q99567|NUP88_HUMAN	162	83489	7	7	7	7	0.49	Nuclear pore complex
840	1	SwissProt_HUMAN$	2::O14744|ANM5_HUMAN	161	72638	15	14	12	12	1.19	Protein arginine N-me
841	1	SwissProt_HUMAN$	2::P30040|ERP29_HUMAN	161	28975	9	8	7	7	2.67	Endoplasmic reticulum
842	1	SwissProt_HUMAN$	2::P11388|TOP2A_HUMAN	161	174276	13	12	12	11	0.35	DNA topoisomerase 2-
842	2	SwissProt_HUMAN$	2::Q02880|TOP2B_HUMAN	47	183152	5	5	5	5	0.14	DNA topoisomerase 2-
843	1	SwissProt_HUMAN$	2::Q71RC2|LARP4_HUMAN	161	80546	6	6	5	5	0.34	La-related protein 4 O
844	1	SwissProt_HUMAN$	2::Q8N684|CPSF7_HUMAN	160	52018	7	7	6	6	0.72	Cleavage and polyade
845	1	SwissProt_HUMAN$	2::Q7Z7H8|RM10_HUMAN	160	29264	3	3	2	2	0.38	39S ribosomal protein
846	1	SwissProt_HUMAN$	2::O14745|NHRF1_HUMAN	159	38845	7	5	7	5	0.84	Na(+)/H(+) exchange
847	1	SwissProt_HUMAN$	2::P61160|ARP2_HUMAN	159	44732	9	6	7	5	0.70	Actin-related protein 2
848	1	SwissProt_HUMAN$	2::Q9Y606|TRUA_HUMAN	159	47440	5	4	5	4	0.49	tRNA pseudouridine sy
849	1	SwissProt_HUMAN$	2::P55769|NH2L1_HUMAN	159	14165	5	5	4	4	4.15	NHP2-like protein 1 O
850	1	SwissProt_HUMAN$	2::Q8TDD1|DDX54_HUMAN	159	98534	5	5	4	4	0.21	ATP-dependent RNA h
850	2	SwissProt_HUMAN$	2::Q9Y2R4|DDX52_HUMAN	140	67456	7	6	7	6	0.52	Probable ATP-depende
851	1	SwissProt_HUMAN$	2::O43396|TXNL1_HUMAN	159	32231	8	8	7	7	1.79	Thioredoxin-like prote
852	1	SwissProt_HUMAN$	2::Q9BZE4|NOG1_HUMAN	159	73918	8	6	7	6	0.47	Nucleolar GTP-binding
853	1	SwissProt_HUMAN$	2::Q9UBB4|ATX10_HUMAN	159	53455	5	5	5	5	0.56	Ataxin-10 OS=Homo s
854	1	SwissProt_HUMAN$	2::Q9BPX3|CND3_HUMAN	159	114262	9	8	9	8	0.39	Condensin complex su

↑Family	M	DB	Accession	Score	Mass	Matches	Match(sig)	Sequences	Seq(sig)	emPAI	Description
855	1	SwissProt_HUMAN$	2::P62847|RS24_HUMAN	158	15413	8	8	5	5	7.30	40S ribosomal protein
856	1	SwissProt_HUMAN$	2::P09661|RU2A_HUMAN	158	28398	8	7	8	7	2.19	U2 small nuclear ribor
857	1	SwissProt_HUMAN$	2::Q2TAY7|SMU1_HUMAN	158	57507	8	7	7	6	0.64	WD40 repeat-containi
858	1	SwissProt_HUMAN$	2::O00264|PGRC1_HUMAN	158	21658	5	4	4	3	0.92	Membrane-associated
859	1	SwissProt_HUMAN$	2::Q9H6R4|NOL6_HUMAN	156	127513	10	8	10	8	0.35	Nucleolar protein 6 OS
860	1	SwissProt_HUMAN$	2::P01009|A1AT_HUMAN	156	46707	6	5	5	4	0.50	Alpha-1-antitrypsin OS
861	1	SwissProt_HUMAN$	2::P25788|PSA3_HUMAN	156	28415	7	5	7	5	1.29	Proteasome subunit al
862	1	SwissProt_HUMAN$	2::O75439|MPPB_HUMAN	156	54331	10	8	9	8	1.01	Mitochondrial-process
863	1	SwissProt_HUMAN$	2::P10515|ODP2_HUMAN	156	68953	8	8	8	8	0.73	Dihydrolipoyllysine-re
864	1	SwissProt_HUMAN$	2::Q5H9R7|PP6R3_HUMAN	156	97608	10	9	10	9	0.55	Serine/threonine-prot
865	1	SwissProt_HUMAN$	2::P62314|SMD1_HUMAN	154	13273	6	6	4	4	3.04	Small nuclear ribonucl
866	1	SwissProt_HUMAN$	2::O43252|PAPS1_HUMAN	154	70788	7	6	6	5	0.40	Bifunctional 3'-phosph
867	1	SwissProt_HUMAN$	2::P62318|SMD3_HUMAN	154	13907	5	4	4	3	1.74	Small nuclear ribonucl
868	1	SwissProt_HUMAN$	2::Q9UJU6|DBNL_HUMAN	154	48178	6	6	5	5	0.63	Drebrin-like protein O
869	1	SwissProt_HUMAN$	2::Q9UBQ5|EIF3K_HUMAN	154	25043	4	3	4	3	0.75	Eukaryotic translation
870	1	SwissProt_HUMAN$	2::P55084|ECHB_HUMAN	154	51262	7	7	5	5	0.59	Trifunctional enzyme s
871	1	SwissProt_HUMAN$	2::Q15121|PEA15_HUMAN	154	15031	4	4	4	4	2.47	Astrocytic phosphopro
872	1	SwissProt_HUMAN$	2::Q08257|QOR_HUMAN	153	35185	6	6	6	6	1.23	Quinone oxidoreducta
873	1	SwissProt_HUMAN$	2::P07305|H10_HUMAN	153	20850	5	5	4	4	2.07	Histone H1.0 OS=Hon
874	1	SwissProt_HUMAN$	2::Q86Y56|DAAF5_HUMAN	152	93462	9	8	8	7	0.43	Dynein assembly facto
875	1	SwissProt_HUMAN$	2::Q7Z2W4|ZCCHV_HUMAN	152	101367	4	3	4	3	0.15	Zinc finger CCCH-type
876	1	SwissProt_HUMAN$	2::P00492|HPRT_HUMAN	152	24564	6	6	5	5	1.61	Hypoxanthine-guanine
877	1	SwissProt_HUMAN$	2::P52888|THOP1_HUMAN	152	78789	9	8	7	6	0.43	Thimet oligopeptidase
878	1	SwissProt_HUMAN$	2::Q07157|ZO1_HUMAN	152	195340	9	9	9	9	0.24	Tight junction protein
879	1	SwissProt_HUMAN$	2::Q9UNS2|CSN3_HUMAN	152	47842	5	5	5	5	0.64	COP9 signalosome cor
880	1	SwissProt_HUMAN$	2::P02774|VTDB_HUMAN	151	52883	3	3	3	3	0.31	Vitamin D-binding pro
881	1	SwissProt_HUMAN$	2::P13798|ACPH_HUMAN	151	81173	7	6	7	6	0.42	Acylamino-acid-releas
882	1	SwissProt_HUMAN$	2::P49959|MRE11_HUMAN	151	80543	9	9	9	9	0.70	Double-strand break r
883	1	SwissProt_HUMAN$	2::P62266|RS23_HUMAN	151	15798	6	5	5	4	2.27	40S ribosomal protein
884	1	SwissProt_HUMAN$	2::Q15257|PTPA_HUMAN	151	40641	9	9	8	8	1.53	Serine/threonine-prot
885	1	SwissProt_HUMAN$	2::Q14CX7|NAA25_HUMAN	150	112221	7	7	7	7	0.34	N-alpha-acetyltransfer
886	1	SwissProt_HUMAN$	2::O95777|LSM8_HUMAN	150	10396	5	5	4	4	4.88	U6 snRNA-associated
887	1	SwissProt_HUMAN$	2::P62263|RS14_HUMAN	150	16263	9	6	7	4	3.18	40S ribosomal protein
888	1	SwissProt_HUMAN$	2::P48147|PPCE_HUMAN	149	80648	10	10	9	9	0.70	Prolyl endopeptidase (
889	1	SwissProt_HUMAN$	2::P11310|ACADM_HUMAN	149	46559	8	6	6	5	0.66	Medium-chain specific
890	1	SwissProt_HUMAN$	2::Q07021|C1QBP_HUMAN	149	31343	6	6	3	3	0.57	Complement compone
891	1	SwissProt_HUMAN$	2::P55010|IF5_HUMAN	149	49192	8	8	7	7	0.96	Eukaryotic translation
892	1	SwissProt_HUMAN$	2::Q14694|UBP10_HUMAN	149	87080	9	8	8	7	0.46	Ubiquitin carboxyl-ter
893	1	SwissProt_HUMAN$	2::P57740|NU107_HUMAN	149	106307	8	7	8	7	0.37	Nuclear pore complex
894	1	SwissProt_HUMAN$	2::Q2TB90|HKDC1_HUMAN	149	102478	11	10	11	10	0.59	Hexokinase HKDC1 OS

↑Family	M	DB	Accession	Score	Mass	Matches	Match(sig)	Sequences	Seq(sig)	emPAI	Description
894	2	SwissProt_HUMAN$	2::P19367|HXK1_HUMAN	61	102420	3	2	3	2	0.10	Hexokinase-1 OS=Hor
895	1	SwissProt_HUMAN$	2::Q07866|KLC1_HUMAN	148	65269	6	6	6	6	0.55	Kinesin light chain 1 C
896	1	SwissProt_HUMAN$	2::P06703|S10A6_HUMAN	148	10173	4	3	2	2	1.47	Protein S100-A6 OS=I
897	1	SwissProt_HUMAN$	2::Q93034|CUL5_HUMAN	148	90897	9	8	7	6	0.37	Cullin-5 OS=Homo sa
898	1	SwissProt_HUMAN$	2::P09622|DLDH_HUMAN	148	54143	8	8	8	8	1.01	Dihydrolipoyl dehydro
899	1	SwissProt_HUMAN$	2::P52565|GDIR1_HUMAN	148	23193	7	7	5	5	1.75	Rho GDP-dissociation
900	1	SwissProt_HUMAN$	2::P54105|ICLN_HUMAN	148	26199	3	3	3	3	0.71	Methylosome subunit
901	1	SwissProt_HUMAN$	2::Q9BTC8|MTA3_HUMAN	148	67461	9	6	7	5	0.42	Metastasis-associated
902	1	SwissProt_HUMAN$	2::P62857|RS28_HUMAN	147	7836	6	5	4	3	4.72	40S ribosomal protein
903	1	SwissProt_HUMAN$	2::Q99471|PFD5_HUMAN	147	17317	4	4	4	4	1.94	Prefoldin subunit 5 OS
904	1	SwissProt_HUMAN$	2::P13693|TCTP_HUMAN	147	19583	8	8	6	6	4.35	Translationally-control
905	1	SwissProt_HUMAN$	2::P49207|RL34_HUMAN	147	13284	5	5	5	5	4.73	60S ribosomal protein
906	1	SwissProt_HUMAN$	2::Q9UJA5|TRM6_HUMAN	147	55764	5	5	4	4	0.40	tRNA (adenine(58)-N(
907	1	SwissProt_HUMAN$	2::O94874|UFL1_HUMAN	147	89540	8	6	7	5	0.30	E3 UFM1-protein ligas
908	1	SwissProt_HUMAN$	2::O14737|PDCD5_HUMAN	147	14276	7	7	4	4	2.67	Programmed cell deat
909	1	SwissProt_HUMAN$	2::Q9Y266|NUDC_HUMAN	147	38219	7	7	6	6	1.10	Nuclear migration prot
910	1	SwissProt_HUMAN$	2::Q9UI30|TR112_HUMAN	146	14190	3	3	2	2	0.93	Multifunctional methyl
911	1	SwissProt_HUMAN$	2::P52732|KIF11_HUMAN	146	119085	10	8	10	8	0.38	Kinesin-like protein KI
912	1	SwissProt_HUMAN$	2::O14880|MGST3_HUMAN	146	16506	4	4	3	3	1.33	Microsomal glutathion
913	1	SwissProt_HUMAN$	2::Q9BTT6|LRRC1_HUMAN	145	59205	6	6	5	5	0.49	Leucine-rich repeat-cc
913	2	SwissProt_HUMAN$	2::Q14160|SCRIB_HUMAN	74	174778	3	3	2	2	0.06	Protein scribble homol
914	1	SwissProt_HUMAN$	2::P0DP23|CALM1_HUMAN	145	16827	5	4	4	3	1.29	Calmodulin-1 OS=Hor
915	1	SwissProt_HUMAN$	2::Q9Y3Z3|SAMH1_HUMAN	145	72155	7	7	7	7	0.58	Deoxynucleoside triph
916	1	SwissProt_HUMAN$	2::Q13155|AIMP2_HUMAN	145	35326	4	4	4	4	0.71	Aminoacyl tRNA synth
917	1	SwissProt_HUMAN$	2::Q00059|TFAM_HUMAN	145	29078	10	9	8	7	2.10	Transcription factor A,
918	1	SwissProt_HUMAN$	2::Q92538|GBF1_HUMAN	144	206315	11	9	10	9	0.23	Golgi-specific brefeldir
918	2	SwissProt_HUMAN$	2::Q14746|COG2_HUMAN	29	83155	4	2	3	2	0.12	Conserved oligomeric
919	1	SwissProt_HUMAN$	2::Q92896|GSLG1_HUMAN	144	134464	5	5	5	5	0.19	Golgi apparatus protei
920	1	SwissProt_HUMAN$	2::O00154|BACH_HUMAN	144	41769	10	8	8	6	0.97	Cytosolic acyl coenzyr
921	1	SwissProt_HUMAN$	2::P46063|RECQ1_HUMAN	144	73410	5	5	4	4	0.29	ATP-dependent DNA h
922	1	SwissProt_HUMAN$	2::Q96QD8|S38A2_HUMAN	143	55990	3	3	3	3	0.29	Sodium-coupled neutr
923	1	SwissProt_HUMAN$	2::Q96HY6|DDRGK_HUMAN	143	35589	7	6	6	5	0.94	DDRGK domain-conta
924	1	SwissProt_HUMAN$	2::Q9Y617|SERC_HUMAN	143	40397	7	5	7	5	0.79	Phosphoserine aminot
925	1	SwissProt_HUMAN$	2::P46109|CRKL_HUMAN	143	33756	6	6	6	6	1.31	Crk-like protein OS=H
926	1	SwissProt_HUMAN$	2::P78417|GSTO1_HUMAN	143	27548	6	6	6	6	1.79	Glutathione S-transfer
927	1	SwissProt_HUMAN$	2::P61758|PFD3_HUMAN	142	22611	6	6	5	5	1.82	Prefoldin subunit 3 OS
928	1	SwissProt_HUMAN$	2::Q15008|PSMD6_HUMAN	142	45502	15	11	13	10	1.82	26S proteasome non-/
929	1	SwissProt_HUMAN$	2::P35080|PROF2_HUMAN	142	15036	5	5	4	4	3.74	Profilin-2 OS=Homo s
930	1	SwissProt_HUMAN$	2::Q96HR9|REEP6_HUMAN	142	23403	3	3	2	2	0.49	Receptor expression-e
931	1	SwissProt_HUMAN$	2::Q9NVD7|PARVA_HUMAN	142	42217	5	4	4	3	0.40	Alpha-parvin OS=Hom

↑Family	M	DB	Accession	Score	Mass	Matches	Match(sig)	Sequences	Seq(sig)	emPAI	Description
932	1	SwissProt_HUMAN$	2::O14602|IF1AY_HUMAN	142	16432	4	4	4	4	2.12	Eukaryotic translation
933	1	SwissProt_HUMAN$	2::Q16822|PCKGM_HUMAN	142	70654	9	6	8	5	0.40	Phosphoenolpyruvate
934	1	SwissProt_HUMAN$	2::P49407|ARRB1_HUMAN	141	47036	10	10	9	9	1.47	Beta-arrestin-1 OS=H
935	1	SwissProt_HUMAN$	2::Q9NUQ6|SPS2L_HUMAN	141	61691	8	7	8	7	0.71	SPATS2-like protein O
936	1	SwissProt_HUMAN$	2::P62280|RS11_HUMAN	141	18419	11	8	9	6	3.59	40S ribosomal protein
937	1	SwissProt_HUMAN$	2::P78406|RAE1L_HUMAN	141	40942	8	8	8	8	1.51	mRNA export factor O
938	1	SwissProt_HUMAN$	2::P84098|RL19_HUMAN	141	23451	6	5	5	4	1.23	60S ribosomal protein
939	1	SwissProt_HUMAN$	2::P00441|SODC_HUMAN	141	15926	5	5	4	4	2.24	Superoxide dismutase
940	1	SwissProt_HUMAN$	2::Q9P2J5|SYLC_HUMAN	140	134379	7	5	6	5	0.19	Leucine--tRNA ligase,
941	1	SwissProt_HUMAN$	2::Q9NXG2|THUM1_HUMAN	140	39291	5	5	5	5	0.82	THUMP domain-contai
942	1	SwissProt_HUMAN$	2::P51571|SSRD_HUMAN	140	18987	7	7	5	5	2.43	Translocon-associated
943	1	SwissProt_HUMAN$	2::Q53GS9|SNUT2_HUMAN	140	65340	9	6	9	6	0.54	U4/U6.U5 tri-snRNP-a
944	1	SwissProt_HUMAN$	2::Q2NL82|TSR1_HUMAN	139	91752	9	7	9	7	0.44	Pre-rRNA-processing p
945	1	SwissProt_HUMAN$	2::Q9NYK5|RM39_HUMAN	139	38687	6	6	5	5	0.84	39S ribosomal protein
946	1	SwissProt_HUMAN$	2::Q9Y276|BCS1_HUMAN	139	47504	3	3	2	2	0.22	Mitochondrial chapero
947	1	SwissProt_HUMAN$	2::P05198|IF2A_HUMAN	139	36089	11	7	10	6	1.19	Eukaryotic translation
948	1	SwissProt_HUMAN$	2::Q13126|MTAP_HUMAN	139	31216	3	2	3	2	0.35	S-methyl-5'-thioadenc
949	1	SwissProt_HUMAN$	2::P51812|KS6A3_HUMAN	139	83683	13	12	12	11	0.86	Ribosomal protein S6
949	2	SwissProt_HUMAN$	2::Q86UE8|TLK2_HUMAN	27	87606	2	2	2	2	0.11	Serine/threonine-prot
950	1	SwissProt_HUMAN$	2::P28072|PSB6_HUMAN	139	25341	6	5	4	4	1.53	Proteasome subunit b
951	1	SwissProt_HUMAN$	2::O43264|ZW10_HUMAN	138	88773	11	10	11	10	0.71	Centromere/kinetochc
952	1	SwissProt_HUMAN$	2::Q9H6T3|RPAP3_HUMAN	138	75672	4	4	4	4	0.28	RNA polymerase II-as
953	1	SwissProt_HUMAN$	2::Q9Y696|CLIC4_HUMAN	138	28754	3	3	3	3	0.63	Chloride intracellular c
954	1	SwissProt_HUMAN$	2::Q13685|AAMP_HUMAN	138	46721	4	4	4	4	0.50	Angio-associated migr
955	1	SwissProt_HUMAN$	2::P43897|EFTS_HUMAN	138	35368	6	5	6	5	0.95	Elongation factor Ts, r
956	1	SwissProt_HUMAN$	2::P36507|MP2K2_HUMAN	137	44396	6	6	6	6	0.89	Dual specificity mitoge
956	2	SwissProt_HUMAN$	2::Q02750|MP2K1_HUMAN	89	43411	4	4	4	4	0.55	Dual specificity mitoge
957	1	SwissProt_HUMAN$	2::Q9NQW7|XPP1_HUMAN	137	69873	9	6	9	6	0.50	Xaa-Pro aminopeptida
958	1	SwissProt_HUMAN$	2::Q3ZCQ8|TIM50_HUMAN	137	39622	8	6	8	6	1.04	Mitochondrial import i
959	1	SwissProt_HUMAN$	2::Q9BVP2|GNL3_HUMAN	137	61954	5	5	5	5	0.47	Guanine nucleotide-bi
960	1	SwissProt_HUMAN$	2::P46459|NSF_HUMAN	137	82542	8	7	7	6	0.41	Vesicle-fusing ATPase
961	1	SwissProt_HUMAN$	2::Q12800|TFCP2_HUMAN	137	57220	3	3	3	3	0.28	Alpha-globin transcrip
962	1	SwissProt_HUMAN$	2::P22059|OSBP1_HUMAN	136	89365	8	7	8	7	0.45	Oxysterol-binding prot
963	1	SwissProt_HUMAN$	2::Q15642|CIP4_HUMAN	136	68310	6	6	6	6	0.52	Cdc42-interacting prot
964	1	SwissProt_HUMAN$	2::Q07960|RHG01_HUMAN	136	50404	9	6	9	6	0.76	Rho GTPase-activating
965	1	SwissProt_HUMAN$	2::Q99615|DNJC7_HUMAN	136	56405	14	12	11	9	1.13	DnaJ homolog subfam
966	1	SwissProt_HUMAN$	2::Q13895|BYST_HUMAN	136	49570	5	5	5	5	0.61	Bystin OS=Homo sapi
967	1	SwissProt_HUMAN$	2::Q9UKK9|NUDT5_HUMAN	136	24312	7	5	6	5	1.63	ADP-sugar pyrophospl
968	1	SwissProt_HUMAN$	2::P49354|FNTA_HUMAN	136	44381	4	4	4	4	0.53	Protein farnesyltransfe
969	1	SwissProt_HUMAN$	2::Q14137|BOP1_HUMAN	136	83577	8	7	7	6	0.41	Ribosome biogenesis p

↑Family	M	DB	Accession	Score	Mass	Matches	Match(sig)	Sequences	Seq(sig)	emPAI	Description
970	1	SwissProt_HUMAN$	2::P62851|RS25_HUMAN	135	13734	7	7	5	5	4.42	40S ribosomal protein
971	1	SwissProt_HUMAN$	2::P50502|F10A1_HUMAN	135	41305	7	7	7	7	1.23	Hsc70-interacting prot
972	1	SwissProt_HUMAN$	2::Q9NQG5|RPR1B_HUMAN	135	36877	7	7	7	7	1.45	Regulation of nuclear
973	1	SwissProt_HUMAN$	2::Q8WU90|ZC3HF_HUMAN	135	48573	8	6	8	6	0.79	Zinc finger CCCH dom
974	1	SwissProt_HUMAN$	2::Q9HCS7|SYF1_HUMAN	135	99946	4	4	3	3	0.15	Pre-mRNA-splicing fac
975	1	SwissProt_HUMAN$	2::P52907|CAZA1_HUMAN	134	32902	7	7	6	6	1.36	F-actin-capping protei
975	2	SwissProt_HUMAN$	2::P47755|CAZA2_HUMAN	98	32929	7	4	7	4	0.77	F-actin-capping protei
976	1	SwissProt_HUMAN$	2::P20042|IF2B_HUMAN	134	38364	10	9	10	9	2.02	Eukaryotic translation
977	1	SwissProt_HUMAN$	2::Q15942|ZYX_HUMAN	134	61238	6	6	6	6	0.59	Zyxin OS=Homo sapie
978	1	SwissProt_HUMAN$	2::P16455|MGMT_HUMAN	134	21632	3	3	2	2	0.54	Methylated-DNA--prot
979	1	SwissProt_HUMAN$	2::P28070|PSB4_HUMAN	134	29185	7	6	6	5	1.63	Proteasome subunit b
980	1	SwissProt_HUMAN$	2::P62854|RS26_HUMAN	133	13007	5	5	3	3	1.93	40S ribosomal protein
981	1	SwissProt_HUMAN$	2::O00273|DFFA_HUMAN	133	36500	5	5	5	5	0.91	DNA fragmentation fa
982	1	SwissProt_HUMAN$	2::Q9Y4P3|TBL2_HUMAN	133	49766	9	8	9	8	1.14	Transducin beta-like p
983	1	SwissProt_HUMAN$	2::P36543|VATE1_HUMAN	133	26129	4	4	3	3	0.72	V-type proton ATPase
984	1	SwissProt_HUMAN$	2::Q9NR45|SIAS_HUMAN	133	40281	4	3	3	2	0.26	Sialic acid synthase O
985	1	SwissProt_HUMAN$	2::Q9UHX1|PUF60_HUMAN	132	59838	8	8	5	5	0.61	Poly(U)-binding-splicir
986	1	SwissProt_HUMAN$	2::Q9NP79|VTA1_HUMAN	132	33858	4	4	2	2	0.32	Vacuolar protein sortir
987	1	SwissProt_HUMAN$	2::Q9NR19|ACSA_HUMAN	132	78529	8	7	7	6	0.44	Acetyl-coenzyme A sy
988	1	SwissProt_HUMAN$	2::Q8NBT2|SPC24_HUMAN	132	22464	4	4	3	3	0.87	Kinetochore protein Sp
989	1	SwissProt_HUMAN$	2::O15042|SR140_HUMAN	132	118219	12	10	11	9	0.43	U2 snRNP-associated
990	1	SwissProt_HUMAN$	2::Q00577|PURA_HUMAN	132	34889	3	3	3	3	0.50	Transcriptional activat
991	1	SwissProt_HUMAN$	2::Q96H79|ZCCHL_HUMAN	132	32940	6	6	6	6	1.36	Zinc finger CCCH-type
992	1	SwissProt_HUMAN$	2::P51610|HCFC1_HUMAN	131	208602	10	8	8	7	0.17	Host cell factor 1 OS=
993	1	SwissProt_HUMAN$	2::Q7Z739|YTHD3_HUMAN	131	63822	6	6	5	5	0.45	YTH domain-containin
993	2	SwissProt_HUMAN$	2::Q9Y5A9|YTHD2_HUMAN	40	62296	2	2	2	2	0.16	YTH domain-containin
994	1	SwissProt_HUMAN$	2::Q8WY22|BRI3B_HUMAN	131	27818	2	2	1	1	0.18	BRI3-binding protein (
995	1	SwissProt_HUMAN$	2::Q9Y624|JAM1_HUMAN	131	32562	6	5	5	4	0.79	Junctional adhesion m
996	1	SwissProt_HUMAN$	2::Q7Z4H8|PLGT3_HUMAN	131	58535	5	3	5	3	0.27	Protein O-glucosyltran
997	1	SwissProt_HUMAN$	2::Q96EP5|DAZP1_HUMAN	131	43356	4	3	3	2	0.24	DAZ-associated protei
998	1	SwissProt_HUMAN$	2::O75348|VATG1_HUMAN	131	13749	5	4	4	4	2.87	V-type proton ATPase
999	1	SwissProt_HUMAN$	2::O95486|SC24A_HUMAN	130	119674	6	5	6	5	0.22	Protein transport prot
1000	1	SwissProt_HUMAN$	2::P57088|TMM33_HUMAN	130	27960	3	3	3	3	0.66	Transmembrane prote
1001	1	SwissProt_HUMAN$	2::P00966|ASSY_HUMAN	129	46501	9	7	9	7	1.04	Argininosuccinate synt
1002	1	SwissProt_HUMAN$	2::P05556|ITB1_HUMAN	129	88357	7	7	7	7	0.46	Integrin beta-1 OS=H
1003	1	SwissProt_HUMAN$	2::Q99856|ARI3A_HUMAN	129	62850	8	8	6	6	0.57	AT-rich interactive dor
1004	1	SwissProt_HUMAN$	2::Q9NVX2|NLE1_HUMAN	128	53287	6	6	6	6	0.70	Notchless protein hom
1005	1	SwissProt_HUMAN$	2::P14550|AK1A1_HUMAN	128	36550	7	6	6	5	0.91	Aldo-keto reductase fa
1006	1	SwissProt_HUMAN$	2::Q13144|EI2BE_HUMAN	128	80329	8	8	8	8	0.60	Translation initiation f
1007	1	SwissProt_HUMAN$	2::O60231|DHX16_HUMAN	128	119189	8	8	6	6	0.27	Pre-mRNA-splicing fac

↑Family	M	DB	Accession	Score	Mass	Matches	Match(sig)	Sequences	Seq(sig)	emPAI	Description
1008	1	SwissProt_HUMAN$	2::O00499|BIN1_HUMAN	128	64659	5	4	5	4	0.34	Myc box-dependent-ir
1009	1	SwissProt_HUMAN$	2::Q13206|DDX10_HUMAN	128	100825	7	6	7	6	0.33	Probable ATP-depende
1010	1	SwissProt_HUMAN$	2::O15173|PGRC2_HUMAN	128	23804	5	4	5	4	1.21	Membrane-associated
1011	1	SwissProt_HUMAN$	2::Q13404|UB2V1_HUMAN	128	16484	6	5	5	5	3.10	Ubiquitin-conjugating
1012	1	SwissProt_HUMAN$	2::Q14C86|GAPD1_HUMAN	127	164876	2	2	2	2	0.06	GTPase-activating pro
1013	1	SwissProt_HUMAN$	2::Q27J81|INF2_HUMAN	127	135540	4	4	3	3	0.11	Inverted formin-2 OS=
1014	1	SwissProt_HUMAN$	2::Q96TA1|NIBA2_HUMAN	127	84085	7	7	7	7	0.48	Protein Niban 2 OS=H
1015	1	SwissProt_HUMAN$	2::Q16643|DREB_HUMAN	127	71385	5	5	5	5	0.39	Drebrin OS=Homo sap
1016	1	SwissProt_HUMAN$	2::Q96CS3|FAF2_HUMAN	127	52591	9	6	8	5	0.57	FAS-associated factor
1017	1	SwissProt_HUMAN$	2::Q5SRE5|NU188_HUMAN	127	195917	8	8	8	8	0.21	Nucleoporin NUP188 H
1018	1	SwissProt_HUMAN$	2::P36957|ODO2_HUMAN	126	48724	8	8	6	6	0.79	Dihydrolipoyllysine-re
1019	1	SwissProt_HUMAN$	2::P31483|TIA1_HUMAN	126	42936	6	6	6	6	0.93	Nucleolysin TIA-1 isof
1019	2	SwissProt_HUMAN$	2::Q01085|TIAR_HUMAN	116	41564	8	8	8	8	1.48	Nucleolysin TIAR OS=
1020	1	SwissProt_HUMAN$	2::A1L0T0|HACL2_HUMAN	126	67825	5	5	5	5	0.42	2-hydroxyacyl-CoA lya
1021	1	SwissProt_HUMAN$	2::P32322|P5CR1_HUMAN	126	33340	7	6	6	5	1.03	Pyrroline-5-carboxylat
1021	2	SwissProt_HUMAN$	2::Q96C36|P5CR2_HUMAN	115	33616	4	4	2	2	0.32	Pyrroline-5-carboxylat
1022	1	SwissProt_HUMAN$	2::Q15477|SKIV2_HUMAN	126	137668	5	5	5	5	0.19	Helicase SKI2W OS=H
1023	1	SwissProt_HUMAN$	2::Q99661|KIF2C_HUMAN	126	81261	6	6	6	6	0.42	Kinesin-like protein KI
1023	2	SwissProt_HUMAN$	2::O00139|KIF2A_HUMAN	100	79905	4	4	4	4	0.27	Kinesin-like protein KI
1024	1	SwissProt_HUMAN$	2::Q9UHV9|PFD2_HUMAN	126	16638	4	3	2	2	0.75	Prefoldin subunit 2 OS
1025	1	SwissProt_HUMAN$	2::O43847|NRDC_HUMAN	126	131618	8	8	8	8	0.33	Nardilysin OS=Homo s
1026	1	SwissProt_HUMAN$	2::P49589|SYCC_HUMAN	125	85419	8	7	6	6	0.39	Cysteine--tRNA ligase
1027	1	SwissProt_HUMAN$	2::P26358|DNMT1_HUMAN	125	183050	10	10	10	10	0.30	DNA (cytosine-5)-met
1028	1	SwissProt_HUMAN$	2::O43237|DC1L2_HUMAN	125	54066	4	4	4	4	0.42	Cytoplasmic dynein 1
1029	1	SwissProt_HUMAN$	2::Q9BY77|PDIP3_HUMAN	125	46060	7	6	7	6	0.85	Polymerase delta-inte
1030	1	SwissProt_HUMAN$	2::P08621|RU17_HUMAN	125	51526	8	7	7	6	0.73	U1 small nuclear ribor
1031	1	SwissProt_HUMAN$	2::O00422|SAP18_HUMAN	125	17550	3	3	2	2	0.70	Histone deacetylase c
1032	1	SwissProt_HUMAN$	2::P48449|LSS_HUMAN	125	83255	9	8	9	8	0.58	Lanosterol synthase O
1033	1	SwissProt_HUMAN$	2::Q9H223|EHD4_HUMAN	124	61137	7	6	7	6	0.59	EH domain-containing
1034	1	SwissProt_HUMAN$	2::Q5SSJ5|HP1B3_HUMAN	124	61169	12	7	9	5	0.47	Heterochromatin prote
1035	1	SwissProt_HUMAN$	2::P46778|RL21_HUMAN	124	18553	6	5	5	4	1.74	60S ribosomal protein
1036	1	SwissProt_HUMAN$	2::Q9Y333|LSM2_HUMAN	124	10828	2	2	1	1	0.53	U6 snRNA-associated
1037	1	SwissProt_HUMAN$	2::P84103|SRSF3_HUMAN	124	19318	6	5	5	5	2.36	Serine/arginine-rich sp
1038	1	SwissProt_HUMAN$	2::P51398|RT29_HUMAN	124	45538	5	5	5	5	0.68	28S ribosomal protein
1039	1	SwissProt_HUMAN$	2::P60866|RS20_HUMAN	124	13364	6	6	5	5	4.73	40S ribosomal protein
1040	1	SwissProt_HUMAN$	2::P06730|IF4E_HUMAN	124	25082	5	5	4	4	1.12	Eukaryotic translation
1041	1	SwissProt_HUMAN$	2::Q8IUC4|RHPN2_HUMAN	124	76944	5	3	5	3	0.20	Rhophilin-2 OS=Homo
1042	1	SwissProt_HUMAN$	2::O00743|PPP6_HUMAN	124	35121	5	5	3	3	0.50	Serine/threonine-prot
1043	1	SwissProt_HUMAN$	2::P69905|HBA_HUMAN	123	15248	6	6	4	4	3.63	Hemoglobin subunit a
1044	1	SwissProt_HUMAN$	2::O75396|SC22B_HUMAN	123	24578	3	3	3	3	0.78	Vesicle-trafficking pro

↑Family	M	DB	Accession	Score	Mass	Matches	Match(sig)	Sequences	Seq(sig)	emPAI	Description
1045	1	SwissProt_HUMAN$	2::Q8WWM7|ATX2L_HUMAN	123	113304	10	9	10	9	0.46	Ataxin-2-like protein (
1046	1	SwissProt_HUMAN$	2::Q5BKZ1|ZN326_HUMAN	123	65613	8	8	7	7	0.66	DBIRD complex subur
1047	1	SwissProt_HUMAN$	2::Q16625|OCLN_HUMAN	123	59106	4	4	3	3	0.27	Occludin OS=Homo sa
1048	1	SwissProt_HUMAN$	2::Q05048|CSTF1_HUMAN	123	48327	6	6	6	6	0.80	Cleavage stimulation f
1049	1	SwissProt_HUMAN$	2::Q9NQT4|EXOS5_HUMAN	123	25233	2	2	1	1	0.21	Exosome complex con
1050	1	SwissProt_HUMAN$	2::P04424|ARLY_HUMAN	123	51625	4	4	4	4	0.44	Argininosuccinate lyas
1051	1	SwissProt_HUMAN$	2::Q9UKV3|ACINU_HUMAN	123	151771	8	8	8	8	0.28	Apoptotic chromatin c
1052	1	SwissProt_HUMAN$	2::P35251|RFC1_HUMAN	123	128175	3	2	3	2	0.08	Replication factor C su
1053	1	SwissProt_HUMAN$	2::Q5T440|CAF17_HUMAN	123	38131	5	5	4	4	0.64	Putative transferase C
1054	1	SwissProt_HUMAN$	2::Q5JVF3|PCID2_HUMAN	123	46000	4	4	4	4	0.51	PCI domain-containin
1055	1	SwissProt_HUMAN$	2::P21926|CD9_HUMAN	122	25399	5	5	3	3	0.74	CD9 antigen OS=Hom
1056	1	SwissProt_HUMAN$	2::O14545|TRAD1_HUMAN	122	64800	2	1	2	1	0.08	TRAF-type zinc finger
1057	1	SwissProt_HUMAN$	2::P52701|MSH6_HUMAN	122	152689	11	10	10	9	0.32	DNA mismatch repair
1058	1	SwissProt_HUMAN$	2::P62829|RL23_HUMAN	122	14856	5	5	4	4	2.50	60S ribosomal protein
1059	1	SwissProt_HUMAN$	2::O75976|CBPD_HUMAN	121	152835	5	5	4	4	0.13	Carboxypeptidase D C
1060	1	SwissProt_HUMAN$	2::O75821|EIF3G_HUMAN	121	35589	7	6	6	5	0.94	Eukaryotic translation
1061	1	SwissProt_HUMAN$	2::P50914|RL14_HUMAN	121	23417	5	5	4	4	1.23	60S ribosomal protein
1062	1	SwissProt_HUMAN$	2::Q9HCG8|CWC22_HUMAN	121	105402	5	4	5	4	0.20	Pre-mRNA-splicing fac
1063	1	SwissProt_HUMAN$	2::Q14139|UBE4A_HUMAN	120	122482	7	6	7	6	0.26	Ubiquitin conjugation
1064	1	SwissProt_HUMAN$	2::Q8N6H7|ARFG2_HUMAN	120	56685	5	5	5	5	0.52	ADP-ribosylation facto
1065	1	SwissProt_HUMAN$	2::P35613|BASI_HUMAN	120	42174	7	7	5	5	0.75	Basigin OS=Homo sap
1066	1	SwissProt_HUMAN$	2::P42785|PCP_HUMAN	120	55764	5	5	5	5	0.53	Lysosomal Pro-X carb
1067	1	SwissProt_HUMAN$	2::O75608|LYPA1_HUMAN	120	24653	5	5	3	3	0.77	Acyl-protein thioester
1068	1	SwissProt_HUMAN$	2::Q8WYA6|CTBL1_HUMAN	119	65132	5	4	5	4	0.34	Beta-catenin-like prot
1069	1	SwissProt_HUMAN$	2::Q01995|TAGL_HUMAN	119	22596	8	7	8	7	3.27	Transgelin OS=Homo
1070	1	SwissProt_HUMAN$	2::P02765|FETUA_HUMAN	119	39316	4	3	4	3	0.43	Alpha-2-HS-glycoprot
1071	1	SwissProt_HUMAN$	2::Q15428|SF3A2_HUMAN	119	49224	4	3	3	3	0.33	Splicing factor 3A sub
1072	1	SwissProt_HUMAN$	2::P11908|PRPS2_HUMAN	119	34747	7	6	7	6	1.26	Ribose-phosphate pyr
1072	2	SwissProt_HUMAN$	2::P60891|PRPS1_HUMAN	100	34812	5	5	5	5	0.97	Ribose-phosphate pyr
1073	1	SwissProt_HUMAN$	2::P28331|NDUS1_HUMAN	119	79417	4	4	4	4	0.27	NADH-ubiquinone oxic
1074	1	SwissProt_HUMAN$	2::P53007|TXTP_HUMAN	119	33991	10	9	8	7	1.64	Tricarboxylate transpo
1075	1	SwissProt_HUMAN$	2::Q6PGP7|TTC37_HUMAN	118	175375	7	6	7	6	0.18	Tetratricopeptide repe
1076	1	SwissProt_HUMAN$	2::P30044|PRDX5_HUMAN	118	22073	8	6	5	5	1.89	Peroxiredoxin-5, mito
1077	1	SwissProt_HUMAN$	2::O95793|STAU1_HUMAN	118	63143	8	7	8	7	0.69	Double-stranded RNA-
1078	1	SwissProt_HUMAN$	2::Q96S52|PIGS_HUMAN	118	61617	6	5	4	3	0.26	GPI transamidase com
1079	1	SwissProt_HUMAN$	2::Q9NX58|LYAR_HUMAN	118	43588	2	2	2	2	0.24	Cell growth-regulating
1080	1	SwissProt_HUMAN$	2::Q02218|ODO1_HUMAN	118	115861	12	10	11	10	0.51	2-oxoglutarate dehydr
1081	1	SwissProt_HUMAN$	2::Q12849|GRSF1_HUMAN	118	53093	7	7	6	6	0.71	G-rich sequence facto
1082	1	SwissProt_HUMAN$	2::Q16539|MK14_HUMAN	117	41267	4	4	4	4	0.58	Mitogen-activated pro
1082	2	SwissProt_HUMAN$	2::O15264|MK13_HUMAN	47	42063	3	3	3	3	0.40	Mitogen-activated pro

↑Family	M	DB	Accession	Score	Mass	Matches	Match(sig)	Sequences	Seq(sig)	emPAI	Description
1083	1	SwissProt_HUMAN$	2::P61086|UBE2K_HUMAN	117	22393	5	4	4	3	0.87	Ubiquitin-conjugating
1084	1	SwissProt_HUMAN$	2::P14735|IDE_HUMAN	117	117893	8	8	8	8	0.38	Insulin-degrading enz
1085	1	SwissProt_HUMAN$	2::P46926|GNPI1_HUMAN	117	32648	7	6	6	5	1.06	Glucosamine-6-phospl
1086	1	SwissProt_HUMAN$	2::Q04828|AK1C1_HUMAN	117	36765	10	9	9	8	1.79	Aldo-keto reductase fa
1086	2	SwissProt_HUMAN$	2::P52895|AK1C2_HUMAN	52	36712	7	6	6	5	0.90	Aldo-keto reductase fa
1087	1	SwissProt_HUMAN$	2::P09132|SRP19_HUMAN	116	16145	2	2	2	2	0.79	Signal recognition par
1088	1	SwissProt_HUMAN$	2::Q6L8Q7|PDE12_HUMAN	116	67309	8	8	8	8	0.76	2',5'-phosphodiesteras
1089	1	SwissProt_HUMAN$	2::Q9BXW7|HDHD5_HUMAN	116	46292	8	5	7	5	0.67	Haloacid dehalogenase
1090	1	SwissProt_HUMAN$	2::P08579|RU2B_HUMAN	116	25470	6	4	6	4	1.09	U2 small nuclear ribor
1090	2	SwissProt_HUMAN$	2::P09012|SNRPA_HUMAN	87	31259	5	4	4	3	0.57	U1 small nuclear ribor
1091	1	SwissProt_HUMAN$	2::P49585|PCY1A_HUMAN	116	41705	5	4	5	4	0.57	Choline-phosphate cyt
1092	1	SwissProt_HUMAN$	2::Q6DD88|ATLA3_HUMAN	116	60503	8	7	7	6	0.60	Atlastin-3 OS=Homo s
1093	1	SwissProt_HUMAN$	2::Q5T2W1|NHRF3_HUMAN	116	57094	9	7	9	7	0.79	Na(+)/H(+) exchange
1094	1	SwissProt_HUMAN$	2::Q9Y5X3|SNX5_HUMAN	115	46787	6	6	6	6	0.83	Sorting nexin-5 OS=H
1095	1	SwissProt_HUMAN$	2::P42766|RL35_HUMAN	115	14543	10	8	6	5	5.84	60S ribosomal protein
1096	1	SwissProt_HUMAN$	2::Q9NX02|NALP2_HUMAN	115	120437	7	7	6	6	0.27	NACHT, LRR and PYD
1097	1	SwissProt_HUMAN$	2::Q9Y221|NIP7_HUMAN	115	20450	6	4	5	3	0.99	60S ribosome subunit
1098	1	SwissProt_HUMAN$	2::Q8NI27|THOC2_HUMAN	115	182659	9	8	9	8	0.23	THO complex subunit
1099	1	SwissProt_HUMAN$	2::Q9UM00|TMCO1_HUMAN	115	27061	3	3	2	2	0.42	Calcium load-activated
1100	1	SwissProt_HUMAN$	2::P11233|RALA_HUMAN	115	23552	4	4	4	4	1.22	Ras-related protein Ra
1100	2	SwissProt_HUMAN$	2::P11234|RALB_HUMAN	96	23394	4	4	4	4	1.23	Ras-related protein Ra
1101	1	SwissProt_HUMAN$	2::Q92665|RT31_HUMAN	115	45290	4	4	4	4	0.52	28S ribosomal protein
1102	1	SwissProt_HUMAN$	2::P62750|RL23A_HUMAN	115	17684	9	5	8	5	2.76	60S ribosomal protein
1103	1	SwissProt_HUMAN$	2::O00442|RTCA_HUMAN	115	39311	2	2	2	2	0.27	RNA 3'-terminal phosp
1104	1	SwissProt_HUMAN$	2::Q04837|SSBP_HUMAN	114	17249	3	3	3	3	1.26	Single-stranded DNA-l
1105	1	SwissProt_HUMAN$	2::P15374|UCHL3_HUMAN	114	26166	4	4	4	4	1.05	Ubiquitin carboxyl-ter
1106	1	SwissProt_HUMAN$	2::Q9NTX5|ECHD1_HUMAN	114	33677	4	4	3	3	0.52	Ethylmalonyl-CoA dec
1107	1	SwissProt_HUMAN$	2::Q16658|FSCN1_HUMAN	114	54496	4	4	4	4	0.41	Fascin OS=Homo sapi
1108	1	SwissProt_HUMAN$	2::O75436|VP26A_HUMAN	114	38146	5	4	5	4	0.64	Vacuolar protein sortir
1109	1	SwissProt_HUMAN$	2::P02649|APOE_HUMAN	114	36132	7	6	7	6	1.19	Apolipoprotein E OS=I
1110	1	SwissProt_HUMAN$	2::P30533|AMRP_HUMAN	114	41441	5	5	5	5	0.77	Alpha-2-macroglobulir
1111	1	SwissProt_HUMAN$	2::Q7Z4W1|DCXR_HUMAN	113	25897	6	6	5	5	1.48	L-xylulose reductase (
1112	1	SwissProt_HUMAN$	2::Q9NY93|DDX56_HUMAN	113	61551	7	6	7	6	0.59	Probable ATP-depende
1113	1	SwissProt_HUMAN$	2::O15228|GNPAT_HUMAN	113	77138	6	6	6	6	0.45	Dihydroxyacetone pho
1114	1	SwissProt_HUMAN$	2::Q6NUQ4|TM214_HUMAN	113	77101	8	7	8	7	0.54	Transmembrane prote
1115	1	SwissProt_HUMAN$	2::Q15056|IF4H_HUMAN	113	27368	7	7	6	6	2.32	Eukaryotic translation
1116	1	SwissProt_HUMAN$	2::Q9H269|VPS16_HUMAN	113	94634	3	3	3	3	0.16	Vacuolar protein sortir
1117	1	SwissProt_HUMAN$	2::Q96I59|SYNM_HUMAN	113	54056	4	3	4	3	0.30	Probable asparagine--
1118	1	SwissProt_HUMAN$	2::Q92597|NDRG1_HUMAN	113	42808	2	2	2	2	0.25	Protein NDRG1 OS=H
1119	1	SwissProt_HUMAN$	2::Q14914|PTGR1_HUMAN	113	35847	6	5	6	5	0.93	Prostaglandin reducta

↑Family	M	DB	Accession	Score	Mass	Matches	Match(sig)	Sequences	Seq(sig)	emPAI	Description
1120	1	SwissProt_HUMAN$	2::P78316|NOP14_HUMAN	112	97607	7	6	7	6	0.34	Nucleolar protein 14 C
1121	1	SwissProt_HUMAN$	2::Q9GZS3|WDR61_HUMAN	112	33560	3	3	2	2	0.32	WD repeat-containing
1122	1	SwissProt_HUMAN$	2::Q7Z6Z7|HUWE1_HUMAN	112	481589	8	7	7	6	0.06	E3 ubiquitin-protein li
1123	1	SwissProt_HUMAN$	2::P62993|GRB2_HUMAN	112	25190	6	6	5	5	1.54	Growth factor recepto
1124	1	SwissProt_HUMAN$	2::P07099|HYEP_HUMAN	112	52915	5	5	4	4	0.43	Epoxide hydrolase 1 C
1125	1	SwissProt_HUMAN$	2::Q86U42|PABP2_HUMAN	112	32729	5	4	5	4	0.78	Polyadenylate-binding
1126	1	SwissProt_HUMAN$	2::Q96KR1|ZFR_HUMAN	112	116939	3	2	3	2	0.08	Zinc finger RNA-bindir
1127	1	SwissProt_HUMAN$	2::Q9Y3E5|PTH2_HUMAN	112	19181	5	5	4	4	1.65	Peptidyl-tRNA hydrola
1128	1	SwissProt_HUMAN$	2::Q92890|UFD1_HUMAN	112	34478	4	4	4	4	0.73	Ubiquitin recognition f
1129	1	SwissProt_HUMAN$	2::Q16881|TRXR1_HUMAN	112	70862	7	6	7	6	0.49	Thioredoxin reductase
1130	1	SwissProt_HUMAN$	2::P27487|DPP4_HUMAN	112	88222	5	5	5	5	0.31	Dipeptidyl peptidase 4
1131	1	SwissProt_HUMAN$	2::Q13242|SRSF9_HUMAN	111	25526	7	7	6	6	2.02	Serine/arginine-rich s
1132	1	SwissProt_HUMAN$	2::P49247|RPIA_HUMAN	111	33248	4	4	4	4	0.76	Ribose-5-phosphate is
1133	1	SwissProt_HUMAN$	2::Q92979|NEP1_HUMAN	111	26703	5	4	5	4	1.02	Ribosomal RNA small
1134	1	SwissProt_HUMAN$	2::Q9GZZ1|NAA50_HUMAN	111	19386	6	6	6	6	3.25	N-alpha-acetyltransfe
1135	1	SwissProt_HUMAN$	2::Q9BTT0|AN32E_HUMAN	111	30674	4	3	4	3	0.59	Acidic leucine-rich nuc
1136	1	SwissProt_HUMAN$	2::Q8TF05|PP4R1_HUMAN	111	106936	2	2	2	2	0.09	Serine/threonine-prot
1137	1	SwissProt_HUMAN$	2::P55039|DRG2_HUMAN	111	40720	6	5	6	5	0.78	Developmentally-regu
1138	1	SwissProt_HUMAN$	2::P35237|SPB6_HUMAN	111	42594	4	3	4	3	0.39	Serpin B6 OS=Homo s
1139	1	SwissProt_HUMAN$	2::Q92542|NICA_HUMAN	111	78362	4	4	3	3	0.20	Nicastrin OS=Homo sa
1140	1	SwissProt_HUMAN$	2::Q16698|DECR_HUMAN	111	36045	2	2	2	2	0.30	2,4-dienoyl-CoA reduc
1141	1	SwissProt_HUMAN$	2::Q9NPQ8|RIC8A_HUMAN	110	59671	5	4	4	3	0.27	Synembryn-A OS=Hor
1141	2	SwissProt_HUMAN$	2::Q9NVN3|RIC8B_HUMAN	100	58787	4	3	3	2	0.17	Synembryn-B OS=Hor
1142	1	SwissProt_HUMAN$	2::Q99848|EBP2_HUMAN	110	34830	7	6	7	6	1.25	Probable rRNA-proces
1143	1	SwissProt_HUMAN$	2::Q96P48|ARAP1_HUMAN	110	162089	6	6	6	6	0.19	Arf-GAP with Rho-GAF
1144	1	SwissProt_HUMAN$	2::Q96RS6|NUDC1_HUMAN	110	66713	4	3	4	3	0.24	NudC domain-containi
1145	1	SwissProt_HUMAN$	2::Q86YP4|P66A_HUMAN	110	68021	4	4	3	3	0.23	Transcriptional repres
1146	1	SwissProt_HUMAN$	2::Q96RP9|EFGM_HUMAN	109	83418	7	6	6	5	0.33	Elongation factor G, m
1147	1	SwissProt_HUMAN$	2::P08397|HEM3_HUMAN	109	39306	4	4	3	3	0.43	Porphobilinogen deam
1148	1	SwissProt_HUMAN$	2::O60678|ANM3_HUMAN	109	59865	5	5	5	5	0.49	Protein arginine N-me
1149	1	SwissProt_HUMAN$	2::Q7Z417|NUFP2_HUMAN	109	76075	7	5	6	5	0.37	Nuclear fragile X ment
1150	1	SwissProt_HUMAN$	2::Q15041|AR6P1_HUMAN	109	23347	2	2	1	1	0.22	ADP-ribosylation facto
1151	1	SwissProt_HUMAN$	2::O75400|PR40A_HUMAN	109	108737	8	7	6	5	0.24	Pre-mRNA-processing
1152	1	SwissProt_HUMAN$	2::Q99447|PCY2_HUMAN	109	43808	5	5	5	5	0.71	Ethanolamine-phosph
1153	1	SwissProt_HUMAN$	2::Q92667|AKAP1_HUMAN	109	97281	5	3	5	3	0.16	A-kinase anchor prote
1154	1	SwissProt_HUMAN$	2::P10155|RO60_HUMAN	109	60631	5	5	5	5	0.48	60 kDa SS-A/Ro ribon
1155	1	SwissProt_HUMAN$	2::P61604|CH10_HUMAN	109	10925	9	6	7	5	7.39	10 kDa heat shock pro
1156	1	SwissProt_HUMAN$	2::Q9UQB8|BAIP2_HUMAN	109	60830	6	6	6	6	0.59	Brain-specific angioge
1157	1	SwissProt_HUMAN$	2::P51570|GALK1_HUMAN	108	42246	4	4	4	4	0.56	Galactokinase OS=Ho
1158	1	SwissProt_HUMAN$	2::O43809|CPSF5_HUMAN	108	26211	8	8	7	7	2.51	Cleavage and polyade

↑Family	M	DB	Accession	Score	Mass	Matches	Match(sig)	Sequences	Seq(sig)	emPAI	Description
1159	1	SwissProt_HUMAN$	2::Q99638|RAD9A_HUMAN	108	42520	1	1	1	1	0.12	Cell cycle checkpoint c
1160	1	SwissProt_HUMAN$	2::P36551|HEM6_HUMAN	108	50120	6	5	6	5	0.60	Oxygen-dependent co
1161	1	SwissProt_HUMAN$	2::P53990|IST1_HUMAN	108	39725	8	6	6	5	0.81	IST1 homolog OS=Ho
1162	1	SwissProt_HUMAN$	2::Q0VDF9|HSP7E_HUMAN	108	54760	5	5	5	5	0.54	Heat shock 70 kDa pro
1163	1	SwissProt_HUMAN$	2::Q96ER3|SAAL1_HUMAN	108	53524	3	2	2	2	0.19	Protein SAAL1 OS=Ho
1164	1	SwissProt_HUMAN$	2::Q9BSJ2|GCP2_HUMAN	108	102469	9	6	9	6	0.32	Gamma-tubulin compl
1165	1	SwissProt_HUMAN$	2::Q5SY16|NOL9_HUMAN	107	79272	5	5	4	4	0.27	Polynucleotide 5'-hydr
1166	1	SwissProt_HUMAN$	2::P53999|TCP4_HUMAN	107	14386	5	5	3	3	2.64	Activated RNA polyme
1167	1	SwissProt_HUMAN$	2::Q12907|LMAN2_HUMAN	107	40203	5	5	5	5	0.80	Vesicular integral-mer
1168	1	SwissProt_HUMAN$	2::Q15424|SAFB1_HUMAN	106	102580	7	7	7	7	0.38	Scaffold attachment fa
1168	2	SwissProt_HUMAN$	2::Q14151|SAFB2_HUMAN	78	107408	5	5	5	5	0.25	Scaffold attachment fa
1169	1	SwissProt_HUMAN$	2::Q9Y2W2|WBP11_HUMAN	106	69954	4	4	4	4	0.31	WW domain-binding p
1170	1	SwissProt_HUMAN$	2::O43399|TPD54_HUMAN	106	22224	4	4	4	4	1.33	Tumor protein D54 OS
1171	1	SwissProt_HUMAN$	2::Q9NRX1|PNO1_HUMAN	106	27907	6	5	5	4	0.96	RNA-binding protein P
1172	1	SwissProt_HUMAN$	2::Q9Y6A4|CFA20_HUMAN	106	22760	5	5	4	4	1.28	Cilia- and flagella-asso
1173	1	SwissProt_HUMAN$	2::Q9NUQ8|ABCF3_HUMAN	106	79695	9	7	9	7	0.52	ATP-binding cassette s
1174	1	SwissProt_HUMAN$	2::A0AVT1|UBA6_HUMAN	106	117895	9	7	9	7	0.33	Ubiquitin-like modifier
1175	1	SwissProt_HUMAN$	2::P04179|SODM_HUMAN	106	24735	4	4	4	4	1.14	Superoxide dismutase
1176	1	SwissProt_HUMAN$	2::Q9H089|LSG1_HUMAN	105	75178	3	2	3	2	0.13	Large subunit GTPase
1177	1	SwissProt_HUMAN$	2::P08240|SRPRA_HUMAN	105	69767	5	5	5	5	0.40	Signal recognition par
1178	1	SwissProt_HUMAN$	2::Q9BTY7|HGH1_HUMAN	105	42103	3	3	3	3	0.40	Protein HGH1 homolog
1179	1	SwissProt_HUMAN$	2::P0DOX8|IGL1_HUMAN	105	22816	5	5	4	4	1.27	Immunoglobulin lamb
1179	2	SwissProt_HUMAN$	2::P0DOY2|IGLC2_HUMAN	105	11287	5	5	4	4	4.14	Immunoglobulin lamb
1180	1	SwissProt_HUMAN$	2::O60888|CUTA_HUMAN	105	19104	3	3	2	2	0.63	Protein CutA OS=Hom
1181	1	SwissProt_HUMAN$	2::Q8NCW5|NNRE_HUMAN	105	31654	7	6	5	4	0.81	NAD(P)H-hydrate epin
1182	1	SwissProt_HUMAN$	2::P18077|RL35A_HUMAN	105	12530	8	6	5	4	3.40	60S ribosomal protein
1183	1	SwissProt_HUMAN$	2::P33240|CSTF2_HUMAN	104	60920	4	3	3	2	0.17	Cleavage stimulation f
1184	1	SwissProt_HUMAN$	2::Q9BVJ6|UT14A_HUMAN	104	87924	4	4	4	4	0.24	U3 small nucleolar RN
1185	1	SwissProt_HUMAN$	2::P55327|TPD52_HUMAN	104	24312	4	3	4	3	0.79	Tumor protein D52 OS
1186	1	SwissProt_HUMAN$	2::P31350|RIR2_HUMAN	104	44849	3	2	3	2	0.23	Ribonucleoside-diphos
1187	1	SwissProt_HUMAN$	2::P52434|RPAB3_HUMAN	104	17132	5	4	5	4	1.98	DNA-directed RNA pol
1188	1	SwissProt_HUMAN$	2::Q13428|TCOF_HUMAN	104	152015	5	5	4	4	0.13	Treacle protein OS=H
1189	1	SwissProt_HUMAN$	2::P06132|DCUP_HUMAN	104	40761	3	3	3	3	0.42	Uroporphyrinogen dec
1190	1	SwissProt_HUMAN$	2::Q9Y2A7|NCKP1_HUMAN	104	128707	4	4	3	3	0.12	Nck-associated protei
1191	1	SwissProt_HUMAN$	2::Q5T4S7|UBR4_HUMAN	104	573476	6	6	6	6	0.05	E3 ubiquitin-protein li
1192	1	SwissProt_HUMAN$	2::Q9Y2R9|RT07_HUMAN	104	28116	5	4	4	4	0.95	28S ribosomal protein
1193	1	SwissProt_HUMAN$	2::Q9NZ01|TECR_HUMAN	104	36011	6	6	5	5	1.20	Very-long-chain enoyl
1194	1	SwissProt_HUMAN$	2::Q9P2N5|RBM27_HUMAN	104	118645	6	4	5	4	0.17	RNA-binding protein 2
1194	2	SwissProt_HUMAN$	2::Q5T8P6|RBM26_HUMAN	72	113527	2	2	2	2	0.09	RNA-binding protein 2
1195	1	SwissProt_HUMAN$	2::P61077|UB2D3_HUMAN	103	16676	4	4	3	3	1.32	Ubiquitin-conjugating

↑Family	M	DB	Accession	Score	Mass	Matches	Match(sig)	Sequences	Seq(sig)	emPAI	Description
1196	1	SwissProt_HUMAN$	2::P36871|PGM1_HUMAN	103	61411	3	3	3	3	0.26	Phosphoglucomutase-
1197	1	SwissProt_HUMAN$	2::Q7Z2T5|TRM1L_HUMAN	103	81695	3	3	3	3	0.19	TRMT1-like protein OS
1198	1	SwissProt_HUMAN$	2::O75822|EIF3J_HUMAN	103	29045	6	5	6	5	1.25	Eukaryotic translation
1199	1	SwissProt_HUMAN$	2::Q9ULC4|MCTS1_HUMAN	103	20542	4	4	3	3	0.98	Malignant T-cell-ampli
1200	1	SwissProt_HUMAN$	2::P35250|RFC2_HUMAN	103	39132	5	5	5	5	0.83	Replication factor C su
1201	1	SwissProt_HUMAN$	2::Q8IXI1|MIRO2_HUMAN	103	68075	5	5	5	5	0.42	Mitochondrial Rho GTI
1202	1	SwissProt_HUMAN$	2::O96000|NDUBA_HUMAN	103	20763	2	2	2	2	0.57	NADH dehydrogenase
1203	1	SwissProt_HUMAN$	2::Q6P6C2|ALKB5_HUMAN	103	44229	6	6	5	5	0.71	RNA demethylase ALK
1204	1	SwissProt_HUMAN$	2::Q96C23|GALM_HUMAN	103	37742	4	3	4	3	0.46	Galactose mutarotase
1205	1	SwissProt_HUMAN$	2::Q96GW9|SYMM_HUMAN	103	66549	3	3	2	2	0.15	Methionine--tRNA liga
1206	1	SwissProt_HUMAN$	2::P07108|ACBP_HUMAN	103	10038	3	2	3	2	1.52	Acyl-CoA-binding prot
1207	1	SwissProt_HUMAN$	2::P35658|NU214_HUMAN	102	213488	5	4	5	4	0.09	Nuclear pore complex
1208	1	SwissProt_HUMAN$	2::Q9BST9|RTKN_HUMAN	102	62628	2	2	2	2	0.16	Rhotekin OS=Homo sa
1209	1	SwissProt_HUMAN$	2::P50135|HNMT_HUMAN	102	33274	3	3	2	2	0.33	Histamine N-methyltra
1210	1	SwissProt_HUMAN$	2::Q9UKD2|MRT4_HUMAN	102	27543	5	5	5	5	1.35	mRNA turnover protei
1211	1	SwissProt_HUMAN$	2::O00487|PSDE_HUMAN	102	34555	7	7	5	5	0.98	26S proteasome non-/
1212	1	SwissProt_HUMAN$	2::Q9NP81|SYSM_HUMAN	102	58246	5	5	5	5	0.50	Serine--tRNA ligase, n
1213	1	SwissProt_HUMAN$	2::P04183|KITH_HUMAN	102	25452	6	5	5	4	1.09	Thymidine kinase, cyt
1214	1	SwissProt_HUMAN$	2::Q53H82|LACB2_HUMAN	102	32785	7	6	7	6	1.37	Endoribonuclease LAC
1215	1	SwissProt_HUMAN$	2::Q16718|NDUA5_HUMAN	102	13450	2	2	2	2	1.00	NADH dehydrogenase
1216	1	SwissProt_HUMAN$	2::Q9P1Y5|CAMP3_HUMAN	102	134666	3	3	3	3	0.11	Calmodulin-regulated
1217	1	SwissProt_HUMAN$	2::P51648|AL3A2_HUMAN	101	54813	5	5	5	5	0.54	Aldehyde dehydrogena
1218	1	SwissProt_HUMAN$	2::P61513|RL37A_HUMAN	101	10268	4	4	3	3	2.83	60S ribosomal protein
1219	1	SwissProt_HUMAN$	2::Q6DKJ4|NXN_HUMAN	101	48362	2	2	2	2	0.22	Nucleoredoxin OS=Ho
1220	1	SwissProt_HUMAN$	2::Q06124|PTN11_HUMAN	101	67968	8	6	8	6	0.52	Tyrosine-protein phos
1221	1	SwissProt_HUMAN$	2::P63279|UBC9_HUMAN	101	17995	5	4	4	3	1.18	SUMO-conjugating en:
1222	1	SwissProt_HUMAN$	2::P08559|ODPA_HUMAN	101	43268	6	6	5	5	0.73	Pyruvate dehydrogena
1223	1	SwissProt_HUMAN$	2::Q9BTW9|TBCD_HUMAN	101	132515	6	4	6	4	0.15	Tubulin-specific chape
1224	1	SwissProt_HUMAN$	2::Q9GZL7|WDR12_HUMAN	101	47678	5	5	4	4	0.49	Ribosome biogenesis
1225	1	SwissProt_HUMAN$	2::P62899|RL31_HUMAN	100	14454	4	4	4	4	2.64	60S ribosomal protein
1226	1	SwissProt_HUMAN$	2::Q9Y2T2|AP3M1_HUMAN	100	46909	5	5	5	5	0.65	AP-3 complex subunit
1227	1	SwissProt_HUMAN$	2::Q3SY56|SP6_HUMAN	100	39814	2	2	2	2	0.27	Transcription factor S
1228	1	SwissProt_HUMAN$	2::Q4VC31|CCD58_HUMAN	100	16609	4	4	4	4	2.07	Coiled-coil domain-co
1229	1	SwissProt_HUMAN$	2::O15294|OGT1_HUMAN	100	116850	7	7	6	6	0.28	UDP-N-acetylglucosan
1230	1	SwissProt_HUMAN$	2::Q7Z478|DHX29_HUMAN	100	155139	4	4	4	4	0.13	ATP-dependent RNA h
1231	1	SwissProt_HUMAN$	2::Q6NXE6|ARMC6_HUMAN	100	54107	3	3	2	2	0.19	Armadillo repeat-cont
1232	1	SwissProt_HUMAN$	2::P54619|AAKG1_HUMAN	100	37556	2	2	1	1	0.13	5'-AMP-activated prot
1233	1	SwissProt_HUMAN$	2::Q9H488|OFUT1_HUMAN	100	43927	4	4	4	4	0.54	GDP-fucose protein O-
1234	1	SwissProt_HUMAN$	2::P42356|PI4KA_HUMAN	100	236678	3	2	3	2	0.04	Phosphatidylinositol 4-
1235	1	SwissProt_HUMAN$	2::Q96DE0|NUD16_HUMAN	100	21260	2	2	2	2	0.55	U8 snoRNA-decapping

↑Family	M	DB	Accession	Score	Mass	Matches	Match(sig)	Sequences	Seq(sig)	emPAI	Description
1235	2	SwissProt_HUMAN$	2::Q9BRJ7|TIRR_HUMAN	48	23323	2	2	2	2	0.50	Tudor-interacting repa
1236	1	SwissProt_HUMAN$	2::Q6P1J9|CDC73_HUMAN	100	60539	8	6	8	6	0.60	Parafibromin OS=Hon
1237	1	SwissProt_HUMAN$	2::Q13867|BLMH_HUMAN	99	52528	7	6	7	6	0.72	Bleomycin hydrolase (
1238	1	SwissProt_HUMAN$	2::Q13084|RM28_HUMAN	99	30138	6	5	6	5	1.18	39S ribosomal protein
1239	1	SwissProt_HUMAN$	2::P29966|MARCS_HUMAN	99	31536	7	6	5	5	1.11	Myristoylated alanine-
1240	1	SwissProt_HUMAN$	2::Q9BYD6|RM01_HUMAN	99	36885	7	7	7	7	1.45	39S ribosomal protein
1241	1	SwissProt_HUMAN$	2::P49006|MRP_HUMAN	99	19517	2	2	2	2	0.62	MARCKS-related prote
1242	1	SwissProt_HUMAN$	2::Q8WTT2|NOC3L_HUMAN	99	92490	6	5	6	5	0.29	Nucleolar complex prc
1243	1	SwissProt_HUMAN$	2::Q05086|UBE3A_HUMAN	99	100623	2	2	2	2	0.10	Ubiquitin-protein ligas
1244	1	SwissProt_HUMAN$	2::Q9NZJ9|NUDT4_HUMAN	99	20293	5	5	5	5	2.17	Diphosphoinositol poly
1244	2	SwissProt_HUMAN$	2::O95989|NUDT3_HUMAN	35	19459	2	2	2	2	0.62	Diphosphoinositol poly
1245	1	SwissProt_HUMAN$	2::Q16651|PRSS8_HUMAN	99	36408	3	3	2	2	0.30	Prostasin OS=Homo s
1246	1	SwissProt_HUMAN$	2::Q92990|GLMN_HUMAN	98	68165	5	5	4	4	0.32	Glomulin OS=Homo sa
1247	1	SwissProt_HUMAN$	2::Q8IVT2|MISP_HUMAN	98	75311	6	3	6	3	0.21	Mitotic interactor and
1248	1	SwissProt_HUMAN$	2::Q9UHD9|UBQL2_HUMAN	98	65655	1	1	1	1	0.07	Ubiquilin-2 OS=Homo
1249	1	SwissProt_HUMAN$	2::P62070|RRAS2_HUMAN	98	23385	3	3	2	2	0.82	Ras-related protein R-
1250	1	SwissProt_HUMAN$	2::Q13618|CUL3_HUMAN	98	88873	4	4	3	3	0.17	Cullin-3 OS=Homo sa
1251	1	SwissProt_HUMAN$	2::P01024|CO3_HUMAN	98	187030	6	5	6	5	0.14	Complement C3 OS=H
1252	1	SwissProt_HUMAN$	2::P60953|CDC42_HUMAN	98	21245	5	5	5	5	2.02	Cell division control pr
1252	2	SwissProt_HUMAN$	2::P63000|RAC1_HUMAN	86	21436	4	4	4	4	1.40	Ras-related C3 botulir
1252	3	SwissProt_HUMAN$	2::P84095|RHOG_HUMAN	54	21295	3	3	3	3	0.93	Rho-related GTP-bindi
1253	1	SwissProt_HUMAN$	2::Q12959|DLG1_HUMAN	98	100393	6	5	6	5	0.27	Disks large homolog 1
1254	1	SwissProt_HUMAN$	2::Q96G03|PGM2_HUMAN	98	68240	6	6	6	6	0.52	Phosphoglucomutase-
1255	1	SwissProt_HUMAN$	2::Q9UQ35|SRRM2_HUMAN	98	299438	4	4	4	4	0.07	Serine/arginine repeti
1256	1	SwissProt_HUMAN$	2::Q9H2P0|ADNP_HUMAN	98	123485	4	4	4	4	0.17	Activity-dependent ne
1257	1	SwissProt_HUMAN$	2::P50552|VASP_HUMAN	98	39805	4	3	4	3	0.43	Vasodilator-stimulatec
1258	1	SwissProt_HUMAN$	2::Q8N6R0|EFNMT_HUMAN	98	78718	6	4	6	4	0.27	eEF1A lysine and N-te
1259	1	SwissProt_HUMAN$	2::P19525|E2AK2_HUMAN	97	62056	3	3	3	3	0.26	Interferon-induced, dc
1260	1	SwissProt_HUMAN$	2::P48556|PSMD8_HUMAN	97	39587	5	3	5	3	0.43	26S proteasome non-/
1261	1	SwissProt_HUMAN$	2::A5YKK6|CNOT1_HUMAN	97	266768	5	5	5	5	0.09	CCR4-NOT transcriptic
1262	1	SwissProt_HUMAN$	2::Q9P2I0|CPSF2_HUMAN	97	88431	3	3	2	2	0.11	Cleavage and polyade
1263	1	SwissProt_HUMAN$	2::Q9BY32|ITPA_HUMAN	97	21432	6	5	6	5	1.99	Inosine triphosphate p
1264	1	SwissProt_HUMAN$	2::Q8IY67|RAVR1_HUMAN	97	63837	4	3	4	3	0.25	Ribonucleoprotein PTE
1265	1	SwissProt_HUMAN$	2::O14617|AP3D1_HUMAN	97	130076	5	4	5	4	0.16	AP-3 complex subunit
1266	1	SwissProt_HUMAN$	2::P18031|PTN1_HUMAN	96	49935	5	4	5	4	0.46	Tyrosine-protein phos
1267	1	SwissProt_HUMAN$	2::Q86VI3|IQGA3_HUMAN	96	184584	3	3	3	3	0.08	Ras GTPase-activating
1268	1	SwissProt_HUMAN$	2::Q96JG6|VPS50_HUMAN	96	111104	1	1	1	1	0.04	Syndetin OS=Homo sa
1269	1	SwissProt_HUMAN$	2::Q8IWX8|CHERP_HUMAN	96	103637	2	2	2	2	0.10	Calcium homeostasis (
1270	1	SwissProt_HUMAN$	2::O00330|ODPX_HUMAN	96	54089	5	5	4	4	0.42	Pyruvate dehydrogena
1271	1	SwissProt_HUMAN$	2::Q9Y3D6|FIS1_HUMAN	96	16927	4	4	4	4	2.03	Mitochondrial fission 1

↑Family	M	DB	Accession	Score	Mass	Matches	Match(sig)	Sequences	Seq(sig)	emPAI	Description
1272	1	SwissProt_HUMAN$	2::P36542|ATPG_HUMAN	96	32975	5	5	4	4	0.77	ATP synthase subunit
1273	1	SwissProt_HUMAN$	2::Q969U7|PSMG2_HUMAN	95	29377	6	5	5	4	0.90	Proteasome assembly
1274	1	SwissProt_HUMAN$	2::Q9NRG7|D39U1_HUMAN	95	31057	2	2	2	2	0.35	Epimerase family prot
1275	1	SwissProt_HUMAN$	2::P00387|NB5R3_HUMAN	95	34213	6	6	5	5	0.99	NADH-cytochrome b5
1276	1	SwissProt_HUMAN$	2::P04632|CPNS1_HUMAN	95	28298	6	4	5	3	0.65	Calpain small subunit
1277	1	SwissProt_HUMAN$	2::P52594|AGFG1_HUMAN	95	58224	3	3	3	3	0.28	Arf-GAP domain and F
1278	1	SwissProt_HUMAN$	2::O14776|TCRG1_HUMAN	95	123823	9	6	9	6	0.26	Transcription elongati
1279	1	SwissProt_HUMAN$	2::Q16762|THTR_HUMAN	95	33408	5	5	5	5	1.02	Thiosulfate sulfurtrans
1280	1	SwissProt_HUMAN$	2::Q8N1B4|VPS52_HUMAN	95	82170	2	2	2	2	0.12	Vacuolar protein sortir
1281	1	SwissProt_HUMAN$	2::Q9UBQ0|VPS29_HUMAN	95	20493	5	4	5	4	1.49	Vacuolar protein sortir
1282	1	SwissProt_HUMAN$	2::P52735|VAV2_HUMAN	95	101224	6	3	6	3	0.15	Guanine nucleotide ex
1283	1	SwissProt_HUMAN$	2::Q96KA5|CLP1L_HUMAN	95	62189	3	3	3	3	0.26	Cleft lip and palate tra
1284	1	SwissProt_HUMAN$	2::Q14157|UBP2L_HUMAN	95	114465	6	6	6	6	0.28	Ubiquitin-associated p
1285	1	SwissProt_HUMAN$	2::P53794|SC5A3_HUMAN	94	79641	3	3	3	3	0.20	Sodium/myo-inositol c
1286	1	SwissProt_HUMAN$	2::P19823|ITIH2_HUMAN	94	106397	4	3	4	3	0.14	Inter-alpha-trypsin inl
1287	1	SwissProt_HUMAN$	2::O43583|DENR_HUMAN	94	22078	4	4	3	3	0.89	Density-regulated pro
1288	1	SwissProt_HUMAN$	2::Q9H3P2|NELFA_HUMAN	94	57241	3	2	3	2	0.18	Negative elongation fa
1289	1	SwissProt_HUMAN$	2::Q6PIU2|NCEH1_HUMAN	94	45779	3	3	3	3	0.36	Neutral cholesterol est
1290	1	SwissProt_HUMAN$	2::Q86UK7|ZN598_HUMAN	94	98575	2	2	2	2	0.10	E3 ubiquitin-protein lig
1291	1	SwissProt_HUMAN$	2::O15031|PLXB2_HUMAN	94	204997	4	4	3	3	0.07	Plexin-B2 OS=Homo s
1292	1	SwissProt_HUMAN$	2::Q9BZK7|TBL1R_HUMAN	94	55560	3	3	3	3	0.29	F-box-like/WD repeat-
1293	1	SwissProt_HUMAN$	2::Q8IXB1|DJC10_HUMAN	94	91021	6	6	6	6	0.37	DnaJ homolog subfam
1294	1	SwissProt_HUMAN$	2::Q8NBX0|SCPDL_HUMAN	94	47121	5	4	5	4	0.49	Saccharopine dehydro
1295	1	SwissProt_HUMAN$	2::Q8NBQ5|DHB11_HUMAN	94	32915	2	2	1	1	0.15	Estradiol 17-beta-deh
1296	1	SwissProt_HUMAN$	2::Q8WVV4|POF1B_HUMAN	94	68022	5	5	5	5	0.42	Protein POF1B OS=Ho
1297	1	SwissProt_HUMAN$	2::P10321|HLAC_HUMAN	94	40623	3	3	3	3	0.42	HLA class I histocomp
1297	2	SwissProt_HUMAN$	2::P04439|HLAA_HUMAN	58	40815	2	2	2	2	0.26	HLA class I histocomp
1298	1	SwissProt_HUMAN$	2::P49755|TMEDA_HUMAN	93	24960	4	3	3	3	0.76	Transmembrane emp2
1299	1	SwissProt_HUMAN$	2::O75934|SPF27_HUMAN	93	26115	1	1	1	1	0.20	Pre-mRNA-splicing fac
1300	1	SwissProt_HUMAN$	2::P23786|CPT2_HUMAN	93	73730	3	3	3	3	0.21	Carnitine O-palmitoylt
1301	1	SwissProt_HUMAN$	2::Q9Y3P9|RBGP1_HUMAN	93	121660	4	4	4	4	0.17	Rab GTPase-activating
1302	1	SwissProt_HUMAN$	2::P98164|LRP2_HUMAN	93	521616	6	5	5	4	0.04	Low-density lipoprotei
1303	1	SwissProt_HUMAN$	2::Q14155|ARHG7_HUMAN	93	89955	3	3	3	3	0.17	Rho guanine nucleotid
1304	1	SwissProt_HUMAN$	2::Q9H8H3|MET7A_HUMAN	93	28301	2	2	2	2	0.39	Methyltransferase-like
1305	1	SwissProt_HUMAN$	2::Q69YN2|C19L1_HUMAN	93	60581	5	5	4	4	0.37	CWF19-like protein 1
1306	1	SwissProt_HUMAN$	2::Q99598|TSNAX_HUMAN	92	33092	5	4	5	4	0.77	Translin-associated pr
1307	1	SwissProt_HUMAN$	2::O15305|PMM2_HUMAN	92	28064	4	3	3	3	0.65	Phosphomannomutase
1308	1	SwissProt_HUMAN$	2::Q99519|NEUR1_HUMAN	92	45439	2	2	2	2	0.23	Sialidase-1 OS=Homo
1309	1	SwissProt_HUMAN$	2::P68402|PA1B2_HUMAN	92	25553	3	3	2	2	0.45	Platelet-activating fact
1310	1	SwissProt_HUMAN$	2::Q14677|EPN4_HUMAN	92	68216	4	3	4	3	0.23	Clathrin interactor 1 C

↑Family	M	DB	Accession	Score	Mass	Matches	Match(sig)	Sequences	Seq(sig)	emPAI	Description
1311	1	SwissProt_HUMAN$	2::Q13464|ROCK1_HUMAN	92	158076	11	8	8	7	0.23	Rho-associated protei
1312	1	SwissProt_HUMAN$	2::Q8WVJ2|NUDC2_HUMAN	92	17665	2	2	2	2	0.70	NudC domain-containi
1313	1	SwissProt_HUMAN$	2::Q969Q0|RL36L_HUMAN	92	12461	3	3	3	3	2.07	60S ribosomal protein
1313	2	SwissProt_HUMAN$	2::P83881|RL36A_HUMAN	86	12433	3	3	3	3	2.07	60S ribosomal protein
1314	1	SwissProt_HUMAN$	2::Q8NEJ9|NGDN_HUMAN	92	35872	3	3	2	2	0.30	Neuroguidin OS=Hom
1315	1	SwissProt_HUMAN$	2::O00233|PSMD9_HUMAN	91	24667	4	4	4	4	1.14	26S proteasome non-/
1316	1	SwissProt_HUMAN$	2::Q5JTV8|TOIP1_HUMAN	91	66208	5	5	5	5	0.43	Torsin-1A-interacting
1317	1	SwissProt_HUMAN$	2::O76031|CLPX_HUMAN	91	69181	6	5	5	4	0.32	ATP-dependent Clp pr
1318	1	SwissProt_HUMAN$	2::P25685|DNJB1_HUMAN	91	38020	7	6	6	5	0.86	DnaJ homolog subfam
1319	1	SwissProt_HUMAN$	2::Q92797|SYMPK_HUMAN	91	141059	3	3	3	3	0.11	Symplekin OS=Homo
1320	1	SwissProt_HUMAN$	2::Q9NZL4|HPBP1_HUMAN	91	39277	5	5	4	4	0.62	Hsp70-binding protein
1321	1	SwissProt_HUMAN$	2::P60903|S10AA_HUMAN	91	11196	4	3	4	3	2.45	Protein S100-A10 OS=
1322	1	SwissProt_HUMAN$	2::P09417|DHPR_HUMAN	91	25773	5	3	4	2	0.44	Dihydropteridine redu
1323	1	SwissProt_HUMAN$	2::O75676|KS6A4_HUMAN	91	85552	1	1	1	1	0.06	Ribosomal protein S6
1324	1	SwissProt_HUMAN$	2::Q92600|CNOT9_HUMAN	90	33610	4	3	4	3	0.52	CCR4-NOT transcriptic
1325	1	SwissProt_HUMAN$	2::P50613|CDK7_HUMAN	90	39014	2	2	2	2	0.27	Cyclin-dependent kina
1326	1	SwissProt_HUMAN$	2::O95299|NDUAA_HUMAN	90	40725	4	3	3	3	0.42	NADH dehydrogenase
1327	1	SwissProt_HUMAN$	2::O95487|SC24B_HUMAN	90	137331	5	4	5	4	0.15	Protein transport prot
1328	1	SwissProt_HUMAN$	2::Q9UEY8|ADDG_HUMAN	90	79105	5	4	5	4	0.27	Gamma-adducin OS=I
1329	1	SwissProt_HUMAN$	2::Q9UNF0|PACN2_HUMAN	90	55704	8	7	7	6	0.66	Protein kinase C and c
1330	1	SwissProt_HUMAN$	2::Q13409|DC1I2_HUMAN	90	71412	4	3	4	3	0.22	Cytoplasmic dynein 1
1331	1	SwissProt_HUMAN$	2::Q86UP2|KTN1_HUMAN	90	156179	6	5	6	5	0.16	Kinectin OS=Homo sa
1332	1	SwissProt_HUMAN$	2::O14974|MYPT1_HUMAN	90	115211	4	3	4	3	0.13	Protein phosphatase 1
1333	1	SwissProt_HUMAN$	2::Q9C0C9|UBE2O_HUMAN	89	141205	5	4	5	4	0.14	(E3-independent) E2 ι
1334	1	SwissProt_HUMAN$	2::O15144|ARPC2_HUMAN	89	34311	4	3	4	3	0.51	Actin-related protein 2
1335	1	SwissProt_HUMAN$	2::Q9UM54|MYO6_HUMAN	89	149596	4	4	4	4	0.14	Unconventional myosi
1336	1	SwissProt_HUMAN$	2::P50570|DYN2_HUMAN	89	98003	9	4	9	4	0.21	Dynamin-2 OS=Homo
1337	1	SwissProt_HUMAN$	2::P30519|HMOX2_HUMAN	89	36010	4	3	3	2	0.30	Heme oxygenase 2 OS
1338	1	SwissProt_HUMAN$	2::O15127|SCAM2_HUMAN	89	36625	3	2	3	2	0.29	Secretory carrier-asso
1339	1	SwissProt_HUMAN$	2::Q9UBQ7|GRHPR_HUMAN	89	35646	2	2	2	2	0.30	Glyoxylate reductase/
1340	1	SwissProt_HUMAN$	2::Q9Y6D5|BIG2_HUMAN	89	201909	5	4	4	4	0.10	Brefeldin A-inhibited g
1341	1	SwissProt_HUMAN$	2::Q08379|GOGA2_HUMAN	89	113017	5	4	4	3	0.13	Golgin subfamily A me
1342	1	SwissProt_HUMAN$	2::P52789|HXK2_HUMAN	89	102313	6	6	6	6	0.32	Hexokinase-2 OS=Hoı
1343	1	SwissProt_HUMAN$	2::O95049|ZO3_HUMAN	89	101335	3	3	3	3	0.15	Tight junction protein
1344	1	SwissProt_HUMAN$	2::Q7Z4S6|KI21A_HUMAN	88	187063	2	2	2	2	0.05	Kinesin-like protein KI
1345	1	SwissProt_HUMAN$	2::Q08426|ECHP_HUMAN	88	79445	7	5	7	5	0.35	Peroxisomal bifunctior
1346	1	SwissProt_HUMAN$	2::Q14318|FKBP8_HUMAN	88	44534	3	3	3	3	0.37	Peptidyl-prolyl cis-trar
1347	1	SwissProt_HUMAN$	2::P30876|RPB2_HUMAN	88	133811	6	6	6	6	0.24	DNA-directed RNA pol
1348	1	SwissProt_HUMAN$	2::Q14126|DSG2_HUMAN	88	122218	9	5	9	5	0.21	Desmoglein-2 OS=Hoı
1349	1	SwissProt_HUMAN$	2::Q9H0H5|RGAP1_HUMAN	88	70982	5	4	4	3	0.22	Rac GTPase-activating

↑Family	M	DB	Accession	Score	Mass	Matches	Match(sig)	Sequences	Seq(sig)	emPAI	Description
1350	1	SwissProt_HUMAN$	2::Q92734|TFG_HUMAN	88	43421	5	4	5	4	0.55	Protein TFG OS=Homo
1351	1	SwissProt_HUMAN$	2::Q01650|LAT1_HUMAN	88	54974	4	4	3	3	0.29	Large neutral amino a
1352	1	SwissProt_HUMAN$	2::O00151|PDLI1_HUMAN	88	36049	6	6	5	5	0.92	PDZ and LIM domain p
1353	1	SwissProt_HUMAN$	2::Q9UNQ2|DIM1_HUMAN	88	35214	4	4	4	4	0.71	Probable dimethylade
1354	1	SwissProt_HUMAN$	2::Q9Y4C2|TCAF1_HUMAN	87	102061	6	5	6	5	0.26	TRPM8 channel-associ
1355	1	SwissProt_HUMAN$	2::Q8NBJ5|GT251_HUMAN	87	71590	9	7	9	7	0.59	Procollagen galactosyl
1356	1	SwissProt_HUMAN$	2::Q9H845|ACAD9_HUMAN	87	68717	4	4	4	4	0.32	Complex I assembly fa
1357	1	SwissProt_HUMAN$	2::P49257|LMAN1_HUMAN	87	57513	7	6	7	6	0.64	Protein ERGIC-53 OS=
1357	2	SwissProt_HUMAN$	2::Q9Y6Y8|S23IP_HUMAN	69	111007	7	5	7	5	0.24	SEC23-interacting pro
1358	1	SwissProt_HUMAN$	2::Q969Z0|FAKD4_HUMAN	87	70693	6	5	6	5	0.40	FAST kinase domain-c
1359	1	SwissProt_HUMAN$	2::Q9NUQ9|CYRIB_HUMAN	87	36725	4	4	4	4	0.67	CYFIP-related Rac1 in
1360	1	SwissProt_HUMAN$	2::Q9H773|DCTP1_HUMAN	87	18669	1	1	1	1	0.29	dCTP pyrophosphatase
1361	1	SwissProt_HUMAN$	2::Q96FQ6|S10AG_HUMAN	87	11794	6	6	5	5	6.17	Protein S100-A16 OS=
1362	1	SwissProt_HUMAN$	2::Q9ULX3|NOB1_HUMAN	87	46646	5	5	4	4	0.50	RNA-binding protein N
1363	1	SwissProt_HUMAN$	2::Q96T37|RBM15_HUMAN	87	107124	4	3	3	2	0.09	RNA-binding protein 1
1364	1	SwissProt_HUMAN$	2::Q13136|LIPA1_HUMAN	87	135695	3	2	3	2	0.07	Liprin-alpha-1 OS=Ho
1365	1	SwissProt_HUMAN$	2::Q6UWM9|UD2A3_HUMAN	87	60215	7	7	6	6	0.60	UDP-glucuronosyltran
1366	1	SwissProt_HUMAN$	2::P62330|ARF6_HUMAN	86	20069	5	4	4	3	1.54	ADP-ribosylation facto
1367	1	SwissProt_HUMAN$	2::Q9BZE9|ASPC1_HUMAN	86	60146	3	3	3	3	0.27	Tether containing UBX
1368	1	SwissProt_HUMAN$	2::Q9Y4E8|UBP15_HUMAN	86	112348	7	6	7	6	0.29	Ubiquitin carboxyl-ter
1369	1	SwissProt_HUMAN$	2::O75340|PDCD6_HUMAN	86	21855	3	3	3	3	0.90	Programmed cell deat
1370	1	SwissProt_HUMAN$	2::Q9NZ08|ERAP1_HUMAN	86	107166	5	4	5	4	0.19	Endoplasmic reticulum
1371	1	SwissProt_HUMAN$	2::P00167|CYB5_HUMAN	86	15321	1	1	1	1	0.36	Cytochrome b5 OS=H
1372	1	SwissProt_HUMAN$	2::P53597|SUCA_HUMAN	86	36227	4	4	4	4	0.68	Succinate--CoA ligase
1373	1	SwissProt_HUMAN$	2::Q86X29|LSR_HUMAN	86	71394	2	2	2	2	0.14	Lipolysis-stimulated li
1374	1	SwissProt_HUMAN$	2::Q07666|KHDR1_HUMAN	86	48197	6	6	6	6	0.80	KH domain-containing
1374	2	SwissProt_HUMAN$	2::Q6DKI1|RL7L_HUMAN	61	29651	5	4	5	4	0.89	60S ribosomal protein
1375	1	SwissProt_HUMAN$	2::P16278|BGAL_HUMAN	86	76027	4	4	4	4	0.28	Beta-galactosidase OS
1376	1	SwissProt_HUMAN$	2::Q9Y496|KIF3A_HUMAN	85	79991	4	2	3	1	0.06	Kinesin-like protein KI
1377	1	SwissProt_HUMAN$	2::P56134|ATPK_HUMAN	85	10911	4	4	2	2	2.58	ATP synthase subunit
1378	1	SwissProt_HUMAN$	2::O75489|NDUS3_HUMAN	85	30223	4	3	4	3	0.60	NADH dehydrogenase
1379	1	SwissProt_HUMAN$	2::Q16629|SRSF7_HUMAN	85	27350	6	5	6	5	1.37	Serine/arginine-rich sp
1380	1	SwissProt_HUMAN$	2::Q6UB35|C1TM_HUMAN	85	105724	10	7	9	6	0.31	Monofunctional C1-tet
1381	1	SwissProt_HUMAN$	2::P48739|PIPNB_HUMAN	85	31520	5	5	4	4	0.82	Phosphatidylinositol tr
1382	1	SwissProt_HUMAN$	2::Q3LXA3|TKFC_HUMAN	85	58910	6	6	6	6	0.62	Triokinase/FMN cyclas
1383	1	SwissProt_HUMAN$	2::Q9Y5B6|PAXB1_HUMAN	85	104739	3	3	3	3	0.15	PAX3- and PAX7-bindi
1384	1	SwissProt_HUMAN$	2::Q9GZT3|SLIRP_HUMAN	85	12341	4	4	3	3	2.10	SRA stem-loop-interac
1385	1	SwissProt_HUMAN$	2::O14936|CSKP_HUMAN	85	105056	7	5	7	5	0.25	Peripheral plasma me
1386	1	SwissProt_HUMAN$	2::Q92922|SMRC1_HUMAN	85	122790	5	3	5	3	0.12	SWI/SNF complex sub
1386	2	SwissProt_HUMAN$	2::Q8TAQ2|SMRC2_HUMAN	75	132797	5	4	5	4	0.15	SWI/SNF complex sub

↑Family	M	DB	Accession	Score	Mass	Matches	Match(sig)	Sequences	Seq(sig)	emPAI	Description
1387	1	SwissProt_HUMAN$	2::Q15637|SF01_HUMAN	85	68286	5	3	5	3	0.23	Splicing factor 1 OS=H
1388	1	SwissProt_HUMAN$	2::P58546|MTPN_HUMAN	85	12887	1	1	1	1	0.43	Myotrophin OS=Homo
1389	1	SwissProt_HUMAN$	2::Q9HAV7|GRPE1_HUMAN	84	24264	4	3	4	3	0.79	GrpE protein homolog
1390	1	SwissProt_HUMAN$	2::P29317|EPHA2_HUMAN	84	108197	8	6	7	6	0.30	Ephrin type-A recepto
1391	1	SwissProt_HUMAN$	2::Q8WW52|F151A_HUMAN	84	63987	4	3	4	3	0.25	Protein FAM151A OS=
1392	1	SwissProt_HUMAN$	2::Q96HS1|PGAM5_HUMAN	84	31985	6	5	6	5	1.09	Serine/threonine-prot
1393	1	SwissProt_HUMAN$	2::Q9BV86|NTM1A_HUMAN	84	25371	2	2	2	2	0.45	N-terminal Xaa-Pro-Ly
1394	1	SwissProt_HUMAN$	2::Q9H0C8|ILKAP_HUMAN	84	42880	2	2	2	2	0.25	Integrin-linked kinase
1395	1	SwissProt_HUMAN$	2::Q8WX92|NELFB_HUMAN	84	65655	6	5	6	5	0.43	Negative elongation fa
1396	1	SwissProt_HUMAN$	2::P41223|BUD31_HUMAN	84	16989	5	5	5	5	2.96	Protein BUD31 homolo
1397	1	SwissProt_HUMAN$	2::Q5RI15|COX20_HUMAN	84	13283	5	5	3	3	1.85	Cytochrome c oxidase
1398	1	SwissProt_HUMAN$	2::O94888|UBXN7_HUMAN	84	54828	3	3	3	3	0.30	UBX domain-containin
1399	1	SwissProt_HUMAN$	2::Q9UKN8|TF3C4_HUMAN	84	91923	1	1	1	1	0.05	General transcription f
1400	1	SwissProt_HUMAN$	2::Q13596|SNX1_HUMAN	84	59033	4	4	4	4	0.38	Sorting nexin-1 OS=H
1400	2	SwissProt_HUMAN$	2::O60749|SNX2_HUMAN	55	58435	5	5	5	5	0.50	Sorting nexin-2 OS=H
1401	1	SwissProt_HUMAN$	2::Q5VTR2|BRE1A_HUMAN	84	113592	3	3	3	3	0.13	E3 ubiquitin-protein li
1402	1	SwissProt_HUMAN$	2::Q9Y5J1|UTP18_HUMAN	84	61964	3	3	3	3	0.26	U3 small nucleolar RN
1403	1	SwissProt_HUMAN$	2::Q8IXT5|RB12B_HUMAN	83	118030	2	2	2	2	0.08	RNA-binding protein 1
1404	1	SwissProt_HUMAN$	2::Q9P000|COMD9_HUMAN	83	21805	3	3	2	2	0.54	COMM domain-contair
1405	1	SwissProt_HUMAN$	2::Q9UNX4|WDR3_HUMAN	83	106032	6	5	6	5	0.25	WD repeat-containing
1406	1	SwissProt_HUMAN$	2::Q9H490|PIGU_HUMAN	83	50019	2	2	2	2	0.21	Phosphatidylinositol gl
1407	1	SwissProt_HUMAN$	2::Q9Y3C4|TPRKB_HUMAN	83	19648	2	2	2	2	0.61	EKC/KEOPS complex s
1408	1	SwissProt_HUMAN$	2::P04080|CYTB_HUMAN	83	11133	4	4	3	3	2.49	Cystatin-B OS=Homo
1409	1	SwissProt_HUMAN$	2::P61966|AP1S1_HUMAN	83	18721	2	2	2	2	0.65	AP-1 complex subunit
1410	1	SwissProt_HUMAN$	2::P36405|ARL3_HUMAN	83	20443	4	3	4	3	0.99	ADP-ribosylation facto
1411	1	SwissProt_HUMAN$	2::Q9NTJ5|SAC1_HUMAN	83	66924	4	4	4	4	0.33	Phosphatidylinositol-3
1412	1	SwissProt_HUMAN$	2::O14950|ML12B_HUMAN	83	19767	4	4	4	4	1.58	Myosin regulatory ligh
1413	1	SwissProt_HUMAN$	2::O00471|EXOC5_HUMAN	83	81801	6	4	6	4	0.26	Exocyst complex comp
1414	1	SwissProt_HUMAN$	2::Q03154|ACY1_HUMAN	83	45856	4	4	4	4	0.51	Aminoacylase-1 OS=H
1415	1	SwissProt_HUMAN$	2::Q16204|CCDC6_HUMAN	83	53258	6	5	6	5	0.56	Coiled-coil domain-co
1416	1	SwissProt_HUMAN$	2::Q8IYB8|SUV3_HUMAN	83	87935	7	6	7	6	0.38	ATP-dependent RNA h
1417	1	SwissProt_HUMAN$	2::A0MZ66|SHOT1_HUMAN	82	71596	5	3	5	3	0.22	Shootin-1 OS=Homo s
1418	1	SwissProt_HUMAN$	2::P07858|CATB_HUMAN	82	37797	6	4	5	4	0.65	Cathepsin B OS=Hom
1419	1	SwissProt_HUMAN$	2::Q8N8A6|DDX51_HUMAN	82	72413	3	3	3	3	0.22	ATP-dependent RNA h
1420	1	SwissProt_HUMAN$	2::P20962|PTMS_HUMAN	82	11523	3	3	2	2	1.23	Parathymosin OS=Ho
1421	1	SwissProt_HUMAN$	2::Q12765|SCRN1_HUMAN	82	46353	4	4	4	4	0.50	Secernin-1 OS=Homo
1422	1	SwissProt_HUMAN$	2::P49593|PPM1F_HUMAN	81	49800	3	3	3	3	0.33	Protein phosphatase 1
1423	1	SwissProt_HUMAN$	2::Q9HB07|MYG1_HUMAN	81	42450	4	4	4	4	0.56	MYG1 exonuclease OS
1424	1	SwissProt_HUMAN$	2::Q9BRF8|CPPED_HUMAN	81	35526	2	2	2	2	0.30	Serine/threonine-prot
1425	1	SwissProt_HUMAN$	2::Q96HC4|PDLI5_HUMAN	81	63904	4	4	4	4	0.34	PDZ and LIM domain p

↑Family	M	DB	Accession	Score	Mass	Matches	Match(sig)	Sequences	Seq(sig)	emPAI	Description
1426	1	SwissProt_HUMAN$	2::Q9H8S9|MOB1A_HUMAN	81	25064	6	5	4	4	1.12	MOB kinase activator
1427	1	SwissProt_HUMAN$	2::P00568|KAD1_HUMAN	81	21621	3	3	3	3	0.92	Adenylate kinase isoe
1428	1	SwissProt_HUMAN$	2::O60313|OPA1_HUMAN	81	111561	5	5	5	5	0.24	Dynamin-like 120 kDa
1429	1	SwissProt_HUMAN$	2::Q7Z4H3|HDDC2_HUMAN	81	23376	3	2	3	2	0.49	5'-deoxynucleotidase
1430	1	SwissProt_HUMAN$	2::P35249|RFC4_HUMAN	81	39657	6	6	6	6	1.04	Replication factor C su
1431	1	SwissProt_HUMAN$	2::P61956|SUMO2_HUMAN	81	10864	3	3	2	2	1.34	Small ubiquitin-relate
1432	1	SwissProt_HUMAN$	2::Q96QC0|PP1RA_HUMAN	81	98996	3	3	3	3	0.15	Serine/threonine-prot
1433	1	SwissProt_HUMAN$	2::P63165|SUMO1_HUMAN	81	11550	3	3	3	3	2.33	Small ubiquitin-relate
1434	1	SwissProt_HUMAN$	2::Q86X55|CARM1_HUMAN	80	65811	7	6	6	5	0.43	Histone-arginine meth
1435	1	db$	1::P0DTD2|ORF9B_SARS2	80	10790	6	6	5	5	7.57	ORF9b protein OS=Se
1436	1	SwissProt_HUMAN$	2::P48729|KC1A_HUMAN	80	38890	5	4	5	4	0.63	Casein kinase I isofor
1437	1	SwissProt_HUMAN$	2::Q9UJX3|APC7_HUMAN	80	66813	4	2	4	2	0.15	Anaphase-promoting
1438	1	SwissProt_HUMAN$	2::P05026|AT1B1_HUMAN	80	35039	6	6	6	6	1.24	Sodium/potassium-tra
1439	1	SwissProt_HUMAN$	2::O95456|PSMG1_HUMAN	80	32833	2	2	2	2	0.33	Proteasome assembly
1440	1	SwissProt_HUMAN$	2::Q16795|NDUA9_HUMAN	80	42483	4	4	3	3	0.40	NADH dehydrogenase
1441	1	SwissProt_HUMAN$	2::Q9UBN7|HDAC6_HUMAN	80	131336	2	2	2	2	0.07	Histone deacetylase 6
1442	1	SwissProt_HUMAN$	2::Q5VW38|GP107_HUMAN	80	66948	1	1	1	1	0.07	Protein GPR107 OS=H
1443	1	SwissProt_HUMAN$	2::O14964|HGS_HUMAN	80	86138	4	4	4	4	0.25	Hepatocyte growth fa
1444	1	SwissProt_HUMAN$	2::Q9UKS6|PACN3_HUMAN	80	48457	4	4	4	4	0.48	Protein kinase C and
1445	1	SwissProt_HUMAN$	2::O60869|EDF1_HUMAN	79	16359	5	4	5	4	2.14	Endothelial differentia
1446	1	SwissProt_HUMAN$	2::Q7Z4Q2|HEAT3_HUMAN	79	74535	3	3	3	3	0.21	HEAT repeat-containi
1447	1	SwissProt_HUMAN$	2::Q15042|RB3GP_HUMAN	79	110454	4	3	4	3	0.14	Rab3 GTPase-activati
1448	1	SwissProt_HUMAN$	2::Q9HA77|SYCM_HUMAN	79	62185	4	4	4	4	0.36	Probable cysteine--tR
1449	1	SwissProt_HUMAN$	2::Q13573|SNW1_HUMAN	79	61456	3	3	3	3	0.26	SNW domain-containi
1450	1	SwissProt_HUMAN$	2::Q9BV38|WDR18_HUMAN	79	47375	4	3	4	3	0.35	WD repeat-containing
1451	1	SwissProt_HUMAN$	2::P53582|MAP11_HUMAN	79	43187	4	4	4	4	0.55	Methionine aminopept
1452	1	SwissProt_HUMAN$	2::Q9NRV9|HEBP1_HUMAN	79	21084	4	3	3	2	0.56	Heme-binding protein
1453	1	SwissProt_HUMAN$	2::P63173|RL38_HUMAN	79	8213	4	3	3	2	2.06	60S ribosomal protein
1454	1	SwissProt_HUMAN$	2::Q9Y4W6|AFG32_HUMAN	79	88528	4	3	4	3	0.17	AFG3-like protein 2 O
1455	1	SwissProt_HUMAN$	2::Q86W42|THOC6_HUMAN	79	37511	3	2	3	2	0.29	THO complex subunit
1456	1	SwissProt_HUMAN$	2::P14678|RSMB_HUMAN	79	24594	5	4	5	4	1.14	Small nuclear ribonucl
1457	1	SwissProt_HUMAN$	2::Q16186|ADRM1_HUMAN	79	42127	2	2	2	2	0.25	Proteasomal ubiquitin
1458	1	SwissProt_HUMAN$	2::Q9UPN9|TRI33_HUMAN	79	122456	3	3	3	3	0.12	E3 ubiquitin-protein li
1459	1	SwissProt_HUMAN$	2::P40938|RFC3_HUMAN	79	40530	3	3	3	3	0.42	Replication factor C su
1460	1	SwissProt_HUMAN$	2::P41567|EIF1_HUMAN	79	12725	2	2	2	2	1.07	Eukaryotic translation
1461	1	SwissProt_HUMAN$	2::P60468|SC61B_HUMAN	78	9968	1	1	1	1	0.59	Protein transport prot
1462	1	SwissProt_HUMAN$	2::P14635|CCNB1_HUMAN	78	48306	3	3	3	3	0.34	G2/mitotic-specific cy
1463	1	SwissProt_HUMAN$	2::Q8WVC0|LEO1_HUMAN	78	75359	1	1	1	1	0.06	RNA polymerase-asso
1464	1	SwissProt_HUMAN$	2::O95630|STABP_HUMAN	78	48047	6	6	5	5	0.63	STAM-binding protein
1465	1	SwissProt_HUMAN$	2::Q9NV06|DCA13_HUMAN	78	51369	5	3	5	3	0.32	DDB1- and CUL4-asso

↑Family	M	DB	Accession	Score	Mass	Matches	Match(sig)	Sequences	Seq(sig)	emPAI	Description
1466	1	SwissProt_HUMAN$	2::Q9UKZ1|CNO11_HUMAN	78	55180	2	2	2	2	0.19	CCR4-NOT transcriptic
1467	1	SwissProt_HUMAN$	2::Q14257|RCN2_HUMAN	78	36854	3	3	3	3	0.47	Reticulocalbin-2 OS=H
1468	1	SwissProt_HUMAN$	2::Q68E01|INT3_HUMAN	78	117994	3	3	3	3	0.13	Integrator complex su
1469	1	SwissProt_HUMAN$	2::P23610|HAP40_HUMAN	78	39079	2	2	1	1	0.13	40-kDa huntingtin-ass
1470	1	SwissProt_HUMAN$	2::O75165|DJC13_HUMAN	77	254252	2	2	2	2	0.04	DnaJ homolog subfam
1471	1	SwissProt_HUMAN$	2::Q9BPW8|NIPS1_HUMAN	77	33289	5	4	4	4	0.76	Protein NipSnap homo
1471	2	SwissProt_HUMAN$	2::O75323|NIPS2_HUMAN	67	33721	3	3	3	3	0.52	Protein NipSnap homo
1472	1	SwissProt_HUMAN$	2::Q9H9J2|RM44_HUMAN	77	37512	2	1	2	1	0.13	39S ribosomal protein
1473	1	SwissProt_HUMAN$	2::Q9NQ29|LUC7L_HUMAN	77	43701	3	3	3	3	0.38	Putative RNA-binding
1473	2	SwissProt_HUMAN$	2::Q9Y383|LC7L2_HUMAN	74	46486	5	5	4	4	0.50	Putative RNA-binding
1474	1	SwissProt_HUMAN$	2::Q9BVQ7|SPA5L_HUMAN	77	80660	1	1	1	1	0.06	Spermatogenesis-asso
1475	1	SwissProt_HUMAN$	2::Q9UHY7|ENOPH_HUMAN	77	28914	4	4	4	4	0.92	Enolase-phosphatase
1476	1	SwissProt_HUMAN$	2::P61803|DAD1_HUMAN	77	12489	4	3	4	3	2.04	Dolichyl-diphosphoolig
1477	1	SwissProt_HUMAN$	2::P49406|RM19_HUMAN	77	33514	5	5	5	5	1.02	39S ribosomal protein
1478	1	SwissProt_HUMAN$	2::Q9NRG9|AAAS_HUMAN	77	59536	5	4	5	4	0.37	Aladin OS=Homo sapi
1479	1	SwissProt_HUMAN$	2::O15091|MRPP3_HUMAN	77	67272	2	2	2	2	0.15	Mitochondrial ribonucl
1480	1	SwissProt_HUMAN$	2::P62310|LSM3_HUMAN	77	11838	2	2	2	2	1.18	U6 snRNA-associated
1481	1	SwissProt_HUMAN$	2::P26440|IVD_HUMAN	76	46621	3	2	3	2	0.22	Isovaleryl-CoA dehydr
1482	1	SwissProt_HUMAN$	2::Q6PI48|SYDM_HUMAN	76	73516	9	7	9	7	0.57	Aspartate--tRNA ligas
1483	1	SwissProt_HUMAN$	2::Q9NYL9|TMOD3_HUMAN	76	39570	6	5	3	3	0.43	Tropomodulin-3 OS=H
1484	1	SwissProt_HUMAN$	2::P67812|SC11A_HUMAN	76	20612	3	2	2	2	0.57	Signal peptidase comp
1485	1	SwissProt_HUMAN$	2::Q9C0D9|EPT1_HUMAN	76	45200	2	2	2	2	0.23	Ethanolaminephospho
1486	1	SwissProt_HUMAN$	2::Q13823|NOG2_HUMAN	76	83603	6	6	6	6	0.41	Nucleolar GTP-binding
1487	1	SwissProt_HUMAN$	2::Q9NUP9|LIN7C_HUMAN	76	21820	2	2	2	2	0.54	Protein lin-7 homolog
1488	1	SwissProt_HUMAN$	2::Q9Y5S9|RBM8A_HUMAN	76	19877	2	2	2	2	0.60	RNA-binding protein 8
1489	1	SwissProt_HUMAN$	2::Q9Y320|TMX2_HUMAN	76	34016	4	3	3	2	0.32	Thioredoxin-related tr
1490	1	SwissProt_HUMAN$	2::Q6PD62|CTR9_HUMAN	76	133420	6	4	6	4	0.15	RNA polymerase-asso
1491	1	SwissProt_HUMAN$	2::Q8WVM8|SCFD1_HUMAN	76	72334	5	3	5	3	0.22	Sec1 family domain-c
1492	1	SwissProt_HUMAN$	2::Q13416|ORC2_HUMAN	76	65931	3	3	3	3	0.24	Origin recognition com
1493	1	SwissProt_HUMAN$	2::Q92917|GPKOW_HUMAN	76	52197	3	3	3	3	0.31	G-patch domain and K
1494	1	SwissProt_HUMAN$	2::Q9GZR7|DDX24_HUMAN	76	96271	4	4	4	4	0.22	ATP-dependent RNA h
1495	1	SwissProt_HUMAN$	2::Q14254|FLOT2_HUMAN	76	47035	5	3	4	3	0.35	Flotillin-2 OS=Homo s
1496	1	SwissProt_HUMAN$	2::Q9H7D7|WDR26_HUMAN	75	72079	1	1	1	1	0.07	WD repeat-containing
1497	1	SwissProt_HUMAN$	2::Q9Y244|POMP_HUMAN	75	15779	1	1	1	1	0.34	Proteasome maturatio
1498	1	SwissProt_HUMAN$	2::Q8N183|NDUF2_HUMAN	75	19844	2	2	2	2	0.60	NADH dehydrogenase
1499	1	SwissProt_HUMAN$	2::Q9HD45|TM9S3_HUMAN	75	67843	3	3	3	3	0.23	Transmembrane 9 sup
1500	1	SwissProt_HUMAN$	2::P61011|SRP54_HUMAN	75	55668	5	5	5	5	0.53	Signal recognition par
1501	1	SwissProt_HUMAN$	2::O94776|MTA2_HUMAN	75	74976	7	6	5	4	0.29	Metastasis-associated
1502	1	SwissProt_HUMAN$	2::Q15750|TAB1_HUMAN	75	54610	2	2	2	2	0.19	TGF-beta-activated ki
1503	1	SwissProt_HUMAN$	2::Q13637|RAB32_HUMAN	75	24982	2	2	2	2	0.46	Ras-related protein Ra

↑Family	M	DB	Accession	Score	Mass	Matches	Match(sig)	Sequences	Seq(sig)	emPAI	Description
1504	1	SwissProt_HUMAN$	2::Q9UJC3|HOOK1_HUMAN	75	84595	4	3	4	3	0.18	Protein Hook homolog
1505	1	SwissProt_HUMAN$	2::P50443|S26A2_HUMAN	75	81609	3	3	3	3	0.19	Sulfate transporter OS
1506	1	SwissProt_HUMAN$	2::Q9UDY2|ZO2_HUMAN	75	133876	6	4	6	4	0.15	Tight junction protein
1507	1	SwissProt_HUMAN$	2::Q9NR31|SAR1A_HUMAN	75	22353	5	4	4	4	1.32	GTP-binding protein S
1508	1	SwissProt_HUMAN$	2::P33316|DUT_HUMAN	74	26547	3	2	3	2	0.43	Deoxyuridine 5'-tripho
1509	1	SwissProt_HUMAN$	2::Q96PZ0|PUS7_HUMAN	74	74988	6	6	6	6	0.46	Pseudouridylate synth
1510	1	SwissProt_HUMAN$	2::P41240|CSK_HUMAN	74	50672	5	5	5	5	0.59	Tyrosine-protein kinas
1511	1	SwissProt_HUMAN$	2::Q99717|SMAD5_HUMAN	74	52225	3	3	3	3	0.31	Mothers against decap
1512	1	SwissProt_HUMAN$	2::Q9NRP0|OSTC_HUMAN	74	16818	1	1	1	1	0.32	Oligosaccharyltransfer
1513	1	SwissProt_HUMAN$	2::Q8IVM0|CCD50_HUMAN	74	35800	1	1	1	1	0.14	Coiled-coil domain-co
1514	1	SwissProt_HUMAN$	2::Q9NVM6|DJC17_HUMAN	74	34666	2	2	1	1	0.15	DnaJ homolog subfam
1515	1	SwissProt_HUMAN$	2::P51452|DUS3_HUMAN	74	20465	5	5	5	5	2.15	Dual specificity protei
1516	1	SwissProt_HUMAN$	2::P20020|AT2B1_HUMAN	74	134600	4	3	4	3	0.11	Plasma membrane cal
1516	2	SwissProt_HUMAN$	2::P23634|AT2B4_HUMAN	71	137833	4	3	3	3	0.11	Plasma membrane cal
1517	1	SwissProt_HUMAN$	2::Q14997|PSME4_HUMAN	74	211199	4	4	3	3	0.07	Proteasome activator
1518	1	SwissProt_HUMAN$	2::Q5T653|RM02_HUMAN	74	33280	2	2	2	2	0.33	39S ribosomal protein
1519	1	SwissProt_HUMAN$	2::O75947|ATP5H_HUMAN	74	18480	5	5	4	4	1.76	ATP synthase subunit
1520	1	SwissProt_HUMAN$	2::P04818|TYSY_HUMAN	74	35693	3	3	3	3	0.49	Thymidylate synthase
1521	1	SwissProt_HUMAN$	2::Q4G0N4|NAKD2_HUMAN	74	49402	4	4	4	4	0.47	NAD kinase 2, mitoch
1522	1	SwissProt_HUMAN$	2::Q9Y315|DEOC_HUMAN	73	35208	4	3	4	3	0.49	Deoxyribose-phospha
1523	1	SwissProt_HUMAN$	2::Q9NY61|AATF_HUMAN	73	63094	3	3	3	3	0.25	Protein AATF OS=Hon
1524	1	SwissProt_HUMAN$	2::P18615|NELFE_HUMAN	73	43214	2	2	2	2	0.24	Negative elongation fa
1525	1	SwissProt_HUMAN$	2::Q5TBB1|RNH2B_HUMAN	73	35116	2	2	2	2	0.31	Ribonuclease H2 subu
1526	1	SwissProt_HUMAN$	2::O75794|CD123_HUMAN	73	39110	3	3	3	3	0.44	Cell division cycle prot
1527	1	SwissProt_HUMAN$	2::P46734|MP2K3_HUMAN	73	39293	4	3	4	3	0.43	Dual specificity mitoge
1528	1	SwissProt_HUMAN$	2::Q16537|2A5E_HUMAN	73	54664	4	3	4	3	0.30	Serine/threonine-prot
1528	2	SwissProt_HUMAN$	2::Q15172|2A5A_HUMAN	41	56158	3	2	3	2	0.18	Serine/threonine-prot
1529	1	SwissProt_HUMAN$	2::Q9Y4W2|LAS1L_HUMAN	73	83013	3	2	3	2	0.12	Ribosomal biogenesis
1530	1	SwissProt_HUMAN$	2::O76071|CIAO1_HUMAN	73	37816	4	3	4	3	0.45	Probable cytosolic iron
1531	1	SwissProt_HUMAN$	2::O75832|PSD10_HUMAN	73	24412	4	3	4	3	0.78	26S proteasome non-/
1532	1	SwissProt_HUMAN$	2::Q5QJE6|TDIF2_HUMAN	73	84418	2	2	2	2	0.12	Deoxynucleotidyltrans
1533	1	SwissProt_HUMAN$	2::O75312|ZPR1_HUMAN	73	50893	2	2	2	2	0.20	Zinc finger protein ZPI
1534	1	SwissProt_HUMAN$	2::Q13243|SRSF5_HUMAN	73	31245	2	2	2	2	0.35	Serine/arginine-rich s
1535	1	SwissProt_HUMAN$	2::O00255|MEN1_HUMAN	73	67981	1	1	1	1	0.07	Menin OS=Homo sapi
1536	1	SwissProt_HUMAN$	2::P05162|LEG2_HUMAN	73	14635	3	3	2	2	0.89	Galectin-2 OS=Homo
1537	1	SwissProt_HUMAN$	2::Q96C19|EFHD2_HUMAN	73	26680	3	3	3	3	0.70	EF-hand domain-conta
1538	1	SwissProt_HUMAN$	2::Q10471|GALT2_HUMAN	72	64691	4	3	4	3	0.25	Polypeptide N-acetylg
1539	1	SwissProt_HUMAN$	2::Q9H061|T126A_HUMAN	72	21513	2	2	2	2	0.55	Transmembrane prote
1540	1	SwissProt_HUMAN$	2::Q9H2G2|SLK_HUMAN	72	142607	4	3	4	3	0.10	STE20-like serine/thre
1541	1	SwissProt_HUMAN$	2::P82650|RT22_HUMAN	72	41254	3	3	3	3	0.41	28S ribosomal protein

↑Family	M	DB	Accession	Score	Mass	Matches	Match(sig)	Sequences	Seq(sig)	emPAI	Description
1542	1	SwissProt_HUMAN$	2::O75521|ECI2_HUMAN	72	43557	3	2	3	2	0.24	Enoyl-CoA delta isome
1543	1	SwissProt_HUMAN$	2::Q96T51|RUFY1_HUMAN	72	79767	2	1	2	1	0.06	RUN and FYVE domair
1544	1	SwissProt_HUMAN$	2::Q9UN81|LORF1_HUMAN	72	40031	2	2	2	2	0.27	LINE-1 retrotransposa
1545	1	SwissProt_HUMAN$	2::Q32MZ4|LRRF1_HUMAN	72	89199	2	2	2	2	0.11	Leucine-rich repeat fli
1546	1	SwissProt_HUMAN$	2::P05090|APOD_HUMAN	72	21262	4	3	4	3	0.93	Apolipoprotein D OS=
1547	1	SwissProt_HUMAN$	2::Q5EB52|MEST_HUMAN	72	38806	2	2	2	2	0.27	Mesoderm-specific tra
1548	1	SwissProt_HUMAN$	2::Q8NFH3|NUP43_HUMAN	72	42124	2	2	2	2	0.25	Nucleoporin Nup43 OS
1549	1	SwissProt_HUMAN$	2::Q9NW64|RBM22_HUMAN	72	46865	5	4	5	4	0.50	Pre-mRNA-splicing fac
1550	1	SwissProt_HUMAN$	2::O95081|AGFG2_HUMAN	72	48932	3	3	3	3	0.34	Arf-GAP domain and F
1551	1	SwissProt_HUMAN$	2::P52815|RM12_HUMAN	72	21335	3	3	3	3	0.93	39S ribosomal protein
1552	1	SwissProt_HUMAN$	2::Q15125|EBP_HUMAN	72	26336	3	3	2	2	0.43	3-beta-hydroxysteroic
1553	1	SwissProt_HUMAN$	2::Q86TI2|DPP9_HUMAN	72	98201	3	3	2	2	0.10	Dipeptidyl peptidase 9
1554	1	SwissProt_HUMAN$	2::Q03426|KIME_HUMAN	71	42424	4	4	4	4	0.56	Mevalonate kinase OS
1555	1	SwissProt_HUMAN$	2::Q8TC07|TBC15_HUMAN	71	79440	7	7	7	7	0.52	TBC1 domain family m
1556	1	SwissProt_HUMAN$	2::Q9H4G0|E41L1_HUMAN	71	98442	7	5	7	5	0.27	Band 4.1-like protein
1557	1	SwissProt_HUMAN$	2::Q7RTV0|PHF5A_HUMAN	71	12397	2	2	2	2	1.11	PHD finger-like domai
1558	1	SwissProt_HUMAN$	2::P16219|ACADS_HUMAN	71	44269	1	1	1	1	0.11	Short-chain specific ac
1559	1	SwissProt_HUMAN$	2::Q13308|PTK7_HUMAN	71	118317	5	4	5	4	0.17	Inactive tyrosine-prot
1560	1	SwissProt_HUMAN$	2::Q9H9P8|L2HDH_HUMAN	71	50284	3	3	3	3	0.33	L-2-hydroxyglutarate
1561	1	SwissProt_HUMAN$	2::Q99543|DNJC2_HUMAN	71	71952	3	3	3	3	0.22	DnaJ homolog subfam
1562	1	SwissProt_HUMAN$	2::Q9P0S9|TM14C_HUMAN	71	11557	1	1	1	1	0.49	Transmembrane prote
1563	1	SwissProt_HUMAN$	2::Q92544|TM9S4_HUMAN	71	74470	4	2	4	2	0.14	Transmembrane 9 sup
1564	1	SwissProt_HUMAN$	2::Q9H6Z4|RANB3_HUMAN	71	60173	5	5	3	3	0.27	Ran-binding protein 3
1565	1	SwissProt_HUMAN$	2::O43490|PROM1_HUMAN	71	97140	4	4	3	3	0.16	Prominin-1 OS=Homo
1566	1	SwissProt_HUMAN$	2::O14908|GIPC1_HUMAN	71	36027	3	3	3	3	0.48	PDZ domain-containin
1567	1	SwissProt_HUMAN$	2::O15116|LSM1_HUMAN	71	15170	2	2	2	2	0.85	U6 snRNA-associated
1568	1	SwissProt_HUMAN$	2::P62304|RUXE_HUMAN	71	10797	2	2	2	2	1.36	Small nuclear ribonucl
1569	1	SwissProt_HUMAN$	2::Q96A72|MGN2_HUMAN	70	17265	3	3	3	3	1.25	Protein mago nashi hc
1570	1	SwissProt_HUMAN$	2::O43493|TGON2_HUMAN	70	45853	2	2	2	2	0.23	Trans-Golgi network ir
1571	1	SwissProt_HUMAN$	2::Q9Y508|RN114_HUMAN	70	25677	3	3	3	3	0.73	E3 ubiquitin-protein lig
1572	1	SwissProt_HUMAN$	2::Q9BW27|NUP85_HUMAN	70	74971	3	3	3	3	0.21	Nuclear pore complex
1573	1	SwissProt_HUMAN$	2::Q9P265|DIP2B_HUMAN	70	171382	2	2	2	2	0.06	Disco-interacting prot
1574	1	SwissProt_HUMAN$	2::Q8NI60|COQ8A_HUMAN	70	71904	5	5	5	5	0.39	Atypical kinase COQ8/
1575	1	SwissProt_HUMAN$	2::P08574|CY1_HUMAN	70	35399	3	3	2	2	0.31	Cytochrome c1, heme
1576	1	SwissProt_HUMAN$	2::Q96H20|SNF8_HUMAN	70	28846	2	2	2	2	0.39	Vacuolar-sorting prote
1577	1	SwissProt_HUMAN$	2::Q96CW1|AP2M1_HUMAN	70	49623	6	6	5	5	0.61	AP-2 complex subunit
1578	1	SwissProt_HUMAN$	2::P63167|DYL1_HUMAN	70	10359	3	3	3	3	2.83	Dynein light chain 1, c
1579	1	SwissProt_HUMAN$	2::Q9NXE4|NSMA3_HUMAN	70	97747	6	6	6	6	0.34	Sphingomyelin phosph
1580	1	SwissProt_HUMAN$	2::O60502|OGA_HUMAN	70	102849	3	2	3	2	0.10	Protein O-GlcNAcase (
1581	1	SwissProt_HUMAN$	2::Q99816|TS101_HUMAN	70	43916	5	4	5	4	0.54	Tumor susceptibility g

↑Family	M	DB	Accession	Score	Mass	Matches	Match(sig)	Sequences	Seq(sig)	emPAI	Description
1582	1	SwissProt_HUMAN$	2::Q8IYS1|P20D2_HUMAN	70	47746	3	3	3	3	0.35	Peptidase M20 domair
1583	1	SwissProt_HUMAN$	2::Q9NRW7|VPS45_HUMAN	70	65036	3	3	3	3	0.24	Vacuolar protein sortir
1584	1	SwissProt_HUMAN$	2::A8MWD9|RUXGL_HUMAN	70	8538	2	2	1	1	0.71	Putative small nuclear
1585	1	SwissProt_HUMAN$	2::Q15427|SF3B4_HUMAN	70	44357	2	2	2	2	0.24	Splicing factor 3B sub
1586	1	SwissProt_HUMAN$	2::P49790|NU153_HUMAN	70	153843	4	4	4	4	0.13	Nuclear pore complex
1587	1	SwissProt_HUMAN$	2::Q93052|LPP_HUMAN	69	65704	3	3	3	3	0.24	Lipoma-preferred part
1588	1	SwissProt_HUMAN$	2::Q68CZ2|TENS3_HUMAN	69	155169	4	4	4	4	0.13	Tensin-3 OS=Homo sa
1589	1	SwissProt_HUMAN$	2::Q14165|MLEC_HUMAN	69	32214	3	3	3	3	0.55	Malectin OS=Homo sa
1590	1	SwissProt_HUMAN$	2::O14548|COX7R_HUMAN	69	12606	1	1	1	1	0.44	Cytochrome c oxidase
1591	1	SwissProt_HUMAN$	2::Q9BQA1|MEP50_HUMAN	69	36701	3	3	3	3	0.47	Methylosome protein !
1592	1	SwissProt_HUMAN$	2::Q96I25|SPF45_HUMAN	69	44934	5	3	4	2	0.23	Splicing factor 45 OS=
1593	1	SwissProt_HUMAN$	2::Q7Z5K2|WAPL_HUMAN	69	132863	2	2	2	2	0.07	Wings apart-like prote
1594	1	SwissProt_HUMAN$	2::O60341|KDM1A_HUMAN	69	92845	5	5	5	5	0.29	Lysine-specific histone
1595	1	SwissProt_HUMAN$	2::Q14108|SCRB2_HUMAN	69	54255	6	5	5	4	0.42	Lysosome membrane
1596	1	SwissProt_HUMAN$	2::Q9NQ88|TIGAR_HUMAN	69	30043	3	3	3	3	0.60	Fructose-2,6-bisphosp
1597	1	SwissProt_HUMAN$	2::O15382|BCAT2_HUMAN	69	44259	4	3	4	3	0.38	Branched-chain-aminc
1598	1	SwissProt_HUMAN$	2::Q9Y3B4|SF3B6_HUMAN	69	14576	2	2	2	2	0.90	Splicing factor 3B sub
1599	1	SwissProt_HUMAN$	2::O95336|6PGL_HUMAN	69	27530	7	7	7	7	2.31	6-phosphogluconolact
1600	1	SwissProt_HUMAN$	2::Q9H2U1|DHX36_HUMAN	69	114688	7	3	6	3	0.13	ATP-dependent DNA/F
1601	1	SwissProt_HUMAN$	2::Q15102|PA1B3_HUMAN	69	25718	2	2	2	2	0.44	Platelet-activating fact
1602	1	SwissProt_HUMAN$	2::O14641|DVL2_HUMAN	69	78899	1	1	1	1	0.06	Segment polarity prot
1603	1	SwissProt_HUMAN$	2::Q99627|CSN8_HUMAN	69	23211	1	1	1	1	0.22	COP9 signalosome cor
1604	1	SwissProt_HUMAN$	2::P49458|SRP09_HUMAN	69	10105	1	1	1	1	0.58	Signal recognition par
1605	1	SwissProt_HUMAN$	2::Q13564|ULA1_HUMAN	68	60209	2	2	2	2	0.17	NEDD8-activating enz
1606	1	SwissProt_HUMAN$	2::Q8NFV4|ABHDB_HUMAN	68	34668	1	1	1	1	0.15	Protein ABHD11 OS=H
1607	1	SwissProt_HUMAN$	2::O95905|ECD_HUMAN	68	72712	2	2	2	2	0.14	Protein ecdysoneless I
1608	1	SwissProt_HUMAN$	2::Q9BX68|HINT2_HUMAN	68	17151	2	2	2	2	0.72	Histidine triad nucleot
1609	1	SwissProt_HUMAN$	2::P0DMM9|ST1A3_HUMAN	68	34174	2	2	2	2	0.32	Sulfotransferase 1A3 (
1610	1	SwissProt_HUMAN$	2::O75937|DNJC8_HUMAN	68	29823	3	3	3	3	0.60	DnaJ homolog subfam
1611	1	SwissProt_HUMAN$	2::Q6P9B6|MEAK7_HUMAN	68	50961	2	2	2	2	0.20	MTOR-associated prot
1612	1	SwissProt_HUMAN$	2::Q9NVH1|DJC11_HUMAN	68	63239	5	4	5	4	0.35	DnaJ homolog subfam
1613	1	SwissProt_HUMAN$	2::Q92769|HDAC2_HUMAN	68	55329	3	3	3	3	0.29	Histone deacetylase 2
1613	2	SwissProt_HUMAN$	2::Q13547|HDAC1_HUMAN	31	55068	2	2	2	2	0.19	Histone deacetylase 1
1614	1	SwissProt_HUMAN$	2::Q9H074|PAIP1_HUMAN	68	53491	4	3	4	3	0.30	Polyadenylate-binding
1615	1	SwissProt_HUMAN$	2::O96005|CLPT1_HUMAN	68	76048	4	3	4	3	0.21	Cleft lip and palate tra
1616	1	SwissProt_HUMAN$	2::Q8WUX9|CHMP7_HUMAN	68	50879	1	1	1	1	0.10	Charged multivesicula
1617	1	SwissProt_HUMAN$	2::P62306|RUXF_HUMAN	68	9719	1	1	1	1	0.60	Small nuclear ribonucl
1618	1	SwissProt_HUMAN$	2::O00762|UBE2C_HUMAN	68	19640	6	6	5	5	2.29	Ubiquitin-conjugating
1619	1	SwissProt_HUMAN$	2::O00178|GTPB1_HUMAN	67	72408	2	2	2	2	0.14	GTP-binding protein 1
1620	1	SwissProt_HUMAN$	2::Q86TX2|ACOT1_HUMAN	67	46248	5	4	5	4	0.50	Acyl-coenzyme A thioe

↑Family	M	DB	Accession	Score	Mass	Matches	Match(sig)	Sequences	Seq(sig)	emPAI	Description
1621	1	SwissProt_HUMAN$	2::Q5RKV6|EXOS6_HUMAN	67	28218	4	3	4	3	0.65	Exosome complex con
1622	1	SwissProt_HUMAN$	2::Q92905|CSN5_HUMAN	67	37555	3	3	3	3	0.46	COP9 signalosome cor
1623	1	SwissProt_HUMAN$	2::Q9Y5Y2|NUBP2_HUMAN	67	28807	6	6	5	5	1.26	Cytosolic Fe-S cluster
1624	1	SwissProt_HUMAN$	2::Q9Y6W5|WASF2_HUMAN	67	54250	2	2	2	2	0.19	Wiskott-Aldrich syndro
1625	1	SwissProt_HUMAN$	2::O75127|PTCD1_HUMAN	67	78806	1	1	1	1	0.06	Pentatricopeptide repe
1626	1	SwissProt_HUMAN$	2::Q14161|GIT2_HUMAN	67	84490	2	2	2	2	0.12	ARF GTPase-activating
1627	1	SwissProt_HUMAN$	2::P19623|SPEE_HUMAN	67	33803	6	5	6	5	1.01	Spermidine synthase (
1628	1	SwissProt_HUMAN$	2::Q9Y3E0|GOT1B_HUMAN	67	15415	2	2	2	2	0.83	Vesicle transport prote
1629	1	SwissProt_HUMAN$	2::Q9H4L5|OSBL3_HUMAN	67	101160	2	2	2	2	0.10	Oxysterol-binding prot
1630	1	SwissProt_HUMAN$	2::P17301|ITA2_HUMAN	67	129214	5	4	5	4	0.16	Integrin alpha-2 OS=H
1631	1	SwissProt_HUMAN$	2::Q9Y697|NFS1_HUMAN	67	50164	6	5	4	4	0.46	Cysteine desulfurase,
1632	1	db$	1::P0DTC3|AP3A_SARS2	67	31103	3	3	3	3	0.58	ORF3a protein OS=Se
1633	1	SwissProt_HUMAN$	2::P48200|IREB2_HUMAN	66	104992	4	2	4	2	0.09	Iron-responsive eleme
1634	1	SwissProt_HUMAN$	2::Q9H974|QTRT2_HUMAN	66	46683	2	2	2	2	0.22	Queuine tRNA-ribosylt
1635	1	SwissProt_HUMAN$	2::O43813|LANC1_HUMAN	66	45254	4	3	4	3	0.37	Glutathione S-transfer
1636	1	SwissProt_HUMAN$	2::P53004|BIEA_HUMAN	66	33407	3	3	3	3	0.53	Biliverdin reductase A
1637	1	SwissProt_HUMAN$	2::O75879|GATB_HUMAN	66	61825	1	1	1	1	0.08	Glutamyl-tRNA(Gln) a
1638	1	SwissProt_HUMAN$	2::Q96S55|WRIP1_HUMAN	66	72088	6	5	6	5	0.39	ATPase WRNIP1 OS=H
1639	1	SwissProt_HUMAN$	2::O75494|SRS10_HUMAN	66	31282	4	3	3	2	0.35	Serine/arginine-rich sp
1640	1	SwissProt_HUMAN$	2::Q9ULA0|DNPEP_HUMAN	66	53376	3	2	3	2	0.19	Aspartyl aminopeptida
1641	1	SwissProt_HUMAN$	2::Q92552|RT27_HUMAN	66	47581	3	3	3	3	0.35	28S ribosomal protein
1642	1	SwissProt_HUMAN$	2::Q96I24|FUBP3_HUMAN	66	61602	4	4	3	3	0.26	Far upstream element
1643	1	SwissProt_HUMAN$	2::Q96P11|NSUN5_HUMAN	66	46662	2	2	2	2	0.22	28S rRNA (cytosine-C
1644	1	SwissProt_HUMAN$	2::Q8WWC4|MAIP1_HUMAN	66	32524	4	3	4	3	0.54	m-AAA protease-inter
1645	1	SwissProt_HUMAN$	2::Q92572|AP3S1_HUMAN	66	21718	2	2	1	1	0.24	AP-3 complex subunit
1646	1	SwissProt_HUMAN$	2::P62312|LSM6_HUMAN	66	9122	2	1	2	1	0.66	U6 snRNA-associated
1647	1	SwissProt_HUMAN$	2::O15498|YKT6_HUMAN	66	22403	3	3	3	3	0.87	Synaptobrevin homolo
1648	1	SwissProt_HUMAN$	2::Q9H4L7|SMRCD_HUMAN	66	117328	1	1	1	1	0.04	SWI/SNF-related matr
1649	1	SwissProt_HUMAN$	2::Q13098|CSN1_HUMAN	65	55501	5	4	5	4	0.41	COP9 signalosome cor
1650	1	SwissProt_HUMAN$	2::O95861|BPNT1_HUMAN	65	33371	4	4	4	4	0.76	3'(2'),5'-bisphosphate
1651	1	SwissProt_HUMAN$	2::Q14691|PSF1_HUMAN	65	22974	1	1	1	1	0.23	DNA replication compl
1652	1	SwissProt_HUMAN$	2::P51532|SMCA4_HUMAN	65	184530	8	5	7	5	0.14	Transcription activator
1653	1	SwissProt_HUMAN$	2::P16949|STMN1_HUMAN	65	17292	4	4	3	3	1.25	Stathmin OS=Homo s
1654	1	SwissProt_HUMAN$	2::Q96EY1|DNJA3_HUMAN	65	52456	4	3	3	3	0.31	DnaJ homolog subfam
1655	1	SwissProt_HUMAN$	2::P07686|HEXB_HUMAN	65	63071	4	3	4	3	0.25	Beta-hexosaminidase
1656	1	SwissProt_HUMAN$	2::Q9P1F3|ABRAL_HUMAN	65	9051	2	2	2	2	1.75	Costars family protein
1657	1	SwissProt_HUMAN$	2::Q8WVV9|HNRLL_HUMAN	65	60045	2	1	1	1	0.08	Heterogeneous nuclea
1658	1	SwissProt_HUMAN$	2::Q9BW60|ELOV1_HUMAN	64	32641	2	2	2	2	0.33	Elongation of very lon
1659	1	SwissProt_HUMAN$	2::Q9NQ55|SSF1_HUMAN	64	53161	4	3	3	2	0.19	Suppressor of SWI4 1
1660	1	SwissProt_HUMAN$	2::Q13451|FKBP5_HUMAN	64	51180	3	2	2	2	0.20	Peptidyl-prolyl cis-tra

↑Family	M	DB	Accession	Score	Mass	Matches	Match(sig)	Sequences	Seq(sig)	emPAI	Description
1661	1	SwissProt_HUMAN$	2::P15927|RFA2_HUMAN	64	29228	2	2	2	2	0.38	Replication protein A 3
1662	1	SwissProt_HUMAN$	2::Q6FI81|CPIN1_HUMAN	64	33561	3	2	3	2	0.32	Anamorsin OS=Homo
1663	1	SwissProt_HUMAN$	2::O14896|IRF6_HUMAN	64	53096	1	1	1	1	0.09	Interferon regulatory
1664	1	SwissProt_HUMAN$	2::O00469|PLOD2_HUMAN	64	84632	3	3	3	3	0.18	Procollagen-lysine,2-o
1665	1	SwissProt_HUMAN$	2::Q8WXA9|SREK1_HUMAN	64	59345	1	1	1	1	0.08	Splicing regulatory glu
1666	1	SwissProt_HUMAN$	2::Q5VW32|BROX_HUMAN	64	46447	3	3	3	3	0.36	BRO1 domain-contain
1667	1	SwissProt_HUMAN$	2::P57735|RAB25_HUMAN	64	23481	3	3	3	3	0.82	Ras-related protein Ra
1668	1	SwissProt_HUMAN$	2::Q5JRX3|PREP_HUMAN	64	117338	5	5	5	5	0.22	Presequence protease
1669	1	SwissProt_HUMAN$	2::Q92888|ARHG1_HUMAN	64	102371	3	3	3	3	0.15	Rho guanine nucleotid
1670	1	SwissProt_HUMAN$	2::Q5SRD1|TI23B_HUMAN	64	19657	1	1	1	1	0.27	Mitochondrial import i
1671	1	SwissProt_HUMAN$	2::Q969N2|PIGT_HUMAN	64	65658	4	3	3	2	0.15	GPI transamidase com
1672	1	SwissProt_HUMAN$	2::Q68CQ4|DIEXF_HUMAN	63	87001	5	4	5	4	0.24	Digestive organ expar
1673	1	SwissProt_HUMAN$	2::P61923|COPZ1_HUMAN	63	20185	3	3	3	3	1.01	Coatomer subunit zeta
1674	1	SwissProt_HUMAN$	2::Q9UNN5|FAF1_HUMAN	63	73908	3	2	3	2	0.14	FAS-associated factor
1675	1	SwissProt_HUMAN$	2::O60256|KPRB_HUMAN	63	40899	6	5	5	5	0.78	Phosphoribosyl pyroph
1676	1	SwissProt_HUMAN$	2::Q96DV4|RM38_HUMAN	63	44568	3	3	3	3	0.37	39S ribosomal protein
1677	1	SwissProt_HUMAN$	2::Q9UMY1|NOL7_HUMAN	63	29409	3	2	3	2	0.38	Nucleolar protein 7 OS
1678	1	SwissProt_HUMAN$	2::Q13442|HAP28_HUMAN	63	20618	1	1	1	1	0.25	28 kDa heat- and acid
1679	1	SwissProt_HUMAN$	2::P82930|RT34_HUMAN	63	25634	4	4	4	4	1.08	28S ribosomal protein
1680	1	SwissProt_HUMAN$	2::P49821|NDUV1_HUMAN	63	50785	4	4	4	4	0.45	NADH dehydrogenase
1681	1	SwissProt_HUMAN$	2::P21912|SDHB_HUMAN	63	31609	3	3	3	3	0.56	Succinate dehydrogen
1682	1	SwissProt_HUMAN$	2::P52758|RIDA_HUMAN	62	14485	3	3	2	2	0.91	2-iminobutanoate/2-ir
1683	1	SwissProt_HUMAN$	2::P13716|HEM2_HUMAN	62	36271	1	1	1	1	0.14	Delta-aminolevulinic a
1684	1	SwissProt_HUMAN$	2::O95070|YIF1A_HUMAN	62	31991	2	2	1	1	0.16	Protein YIF1A OS=Hor
1685	1	SwissProt_HUMAN$	2::Q9Y3A4|RRP7A_HUMAN	62	32314	2	2	2	2	0.34	Ribosomal RNA-proces
1686	1	SwissProt_HUMAN$	2::P53365|ARFP2_HUMAN	62	37832	4	3	4	3	0.45	Arfaptin-2 OS=Homo
1687	1	SwissProt_HUMAN$	2::O60568|PLOD3_HUMAN	62	84731	4	3	4	3	0.18	Multifunctional procoll
1688	1	SwissProt_HUMAN$	2::Q9H7B2|RPF2_HUMAN	62	35560	3	3	3	3	0.49	Ribosome production
1689	1	SwissProt_HUMAN$	2::Q9BYC9|RM20_HUMAN	62	17432	2	2	1	1	0.31	39S ribosomal protein
1690	1	SwissProt_HUMAN$	2::Q96CT7|CC124_HUMAN	62	25820	2	2	2	2	0.44	Coiled-coil domain-cor
1691	1	SwissProt_HUMAN$	2::Q16222|UAP1_HUMAN	62	58732	5	2	5	2	0.17	UDP-N-acetylhexosam
1691	2	SwissProt_HUMAN$	2::Q3KQV9|UAP1L_HUMAN	42	56994	3	2	3	2	0.18	UDP-N-acetylhexosam
1692	1	SwissProt_HUMAN$	2::O95208|EPN2_HUMAN	62	68439	3	3	2	2	0.15	Epsin-2 OS=Homo sap
1693	1	SwissProt_HUMAN$	2::O00170|AIP_HUMAN	62	37612	7	5	7	5	0.87	AH receptor-interactin
1694	1	SwissProt_HUMAN$	2::Q96HV5|TM41A_HUMAN	62	29646	4	4	4	4	0.89	Transmembrane prote
1695	1	SwissProt_HUMAN$	2::Q9NQZ2|SAS10_HUMAN	62	54525	2	2	2	2	0.19	Something about siler
1696	1	SwissProt_HUMAN$	2::Q9ULW0|TPX2_HUMAN	62	85600	4	4	4	4	0.25	Targeting protein for X
1697	1	SwissProt_HUMAN$	2::Q9UBC2|EP15R_HUMAN	62	94197	2	2	2	2	0.11	Epidermal growth fact
1698	1	SwissProt_HUMAN$	2::Q9BSE5|SPEB_HUMAN	62	37636	5	3	4	3	0.46	Agmatinase, mitochor
1699	1	SwissProt_HUMAN$	2::O14976|GAK_HUMAN	62	143100	1	1	1	1	0.03	Cyclin-G-associated ki

↑Family	M	DB	Accession	Score	Mass	Matches	Match(sig)	Sequences	Seq(sig)	emPAI	Description
1700	1	SwissProt_HUMAN$	2::Q9NRX2|RM17_HUMAN	61	20038	2	2	2	2	0.59	39S ribosomal protein
1701	1	SwissProt_HUMAN$	2::Q5T6V5|QSPP_HUMAN	61	39004	2	2	2	2	0.27	Queuosine salvage pro
1702	1	SwissProt_HUMAN$	2::P61970|NTF2_HUMAN	61	14469	2	2	2	2	0.91	Nuclear transport fact
1703	1	SwissProt_HUMAN$	2::Q969S3|ZN622_HUMAN	61	54237	3	3	3	3	0.30	Zinc finger protein 62
1704	1	SwissProt_HUMAN$	2::Q9P0J0|NDUAD_HUMAN	61	16688	2	2	2	2	0.75	NADH dehydrogenase
1705	1	SwissProt_HUMAN$	2::Q05519|SRS11_HUMAN	61	53510	3	3	2	2	0.19	Serine/arginine-rich s
1706	1	SwissProt_HUMAN$	2::P98175|RBM10_HUMAN	61	103469	3	3	3	3	0.15	RNA-binding protein 1
1707	1	SwissProt_HUMAN$	2::Q9C0B1|FTO_HUMAN	61	58245	3	2	3	2	0.18	Alpha-ketoglutarate-d
1708	1	SwissProt_HUMAN$	2::Q00765|REEP5_HUMAN	61	21479	5	3	5	3	0.92	Receptor expression-e
1709	1	SwissProt_HUMAN$	2::Q5TDH0|DDI2_HUMAN	61	44494	5	3	4	2	0.24	Protein DDI1 homolog
1710	1	SwissProt_HUMAN$	2::Q8NE62|CHDH_HUMAN	61	65317	4	4	4	4	0.34	Choline dehydrogenas
1711	1	SwissProt_HUMAN$	2::Q13123|RED_HUMAN	61	65562	4	3	4	3	0.24	Protein Red OS=Homo
1712	1	SwissProt_HUMAN$	2::Q9NX63|MIC19_HUMAN	61	26136	3	3	3	3	0.72	MICOS complex subur
1713	1	SwissProt_HUMAN$	2::P15428|PGDH_HUMAN	61	28959	1	1	1	1	0.18	15-hydroxyprostaglan
1714	1	SwissProt_HUMAN$	2::O15270|SPTC2_HUMAN	61	62884	1	1	1	1	0.08	Serine palmitoyltransf
1715	1	SwissProt_HUMAN$	2::O95394|AGM1_HUMAN	61	59814	3	3	3	3	0.27	Phosphoacetylglucosa
1716	1	SwissProt_HUMAN$	2::Q9UBR2|CATZ_HUMAN	61	33846	2	2	2	2	0.32	Cathepsin Z OS=Hom
1717	1	SwissProt_HUMAN$	2::Q07812|BAX_HUMAN	60	21171	1	1	1	1	0.25	Apoptosis regulator B
1718	1	SwissProt_HUMAN$	2::Q15006|EMC2_HUMAN	60	34811	1	1	1	1	0.14	ER membrane protein
1719	1	SwissProt_HUMAN$	2::Q8ND24|RN214_HUMAN	60	77619	1	1	1	1	0.06	RING finger protein 2
1720	1	SwissProt_HUMAN$	2::Q9UBU9|NXF1_HUMAN	60	70139	5	5	4	4	0.31	Nuclear RNA export fa
1721	1	SwissProt_HUMAN$	2::Q14790|CASP8_HUMAN	60	55356	3	3	3	3	0.29	Caspase-8 OS=Homo
1722	1	SwissProt_HUMAN$	2::P30049|ATPD_HUMAN	60	17479	1	1	1	1	0.31	ATP synthase subunit
1723	1	SwissProt_HUMAN$	2::Q9BUL8|PDC10_HUMAN	60	24686	5	4	5	4	1.14	Programmed cell deat
1724	1	SwissProt_HUMAN$	2::Q9NPF4|OSGEP_HUMAN	60	36403	2	2	2	2	0.30	Probable tRNA N6-ade
1725	1	SwissProt_HUMAN$	2::P00533|EGFR_HUMAN	60	134190	3	3	3	3	0.11	Epidermal growth fact
1726	1	SwissProt_HUMAN$	2::P61081|UBC12_HUMAN	60	20887	5	4	5	4	1.46	NEDD8-conjugating e
1727	1	SwissProt_HUMAN$	2::Q92685|ALG3_HUMAN	60	50094	2	2	2	2	0.21	Dol-P-Man:Man(5)Glcl
1728	1	SwissProt_HUMAN$	2::O75347|TBCA_HUMAN	60	12847	3	3	2	2	1.06	Tubulin-specific chape
1729	1	SwissProt_HUMAN$	2::O15160|RPAC1_HUMAN	60	39225	3	3	3	3	0.44	DNA-directed RNA pol
1730	1	SwissProt_HUMAN$	2::Q13045|FLII_HUMAN	60	144659	6	5	6	5	0.18	Protein flightless-1 ho
1731	1	SwissProt_HUMAN$	2::Q14692|BMS1_HUMAN	60	145716	5	5	5	5	0.18	Ribosome biogenesis
1732	1	SwissProt_HUMAN$	2::Q9Y6D9|MD1L1_HUMAN	60	83016	4	4	4	4	0.26	Mitotic spindle assemt
1733	1	SwissProt_HUMAN$	2::Q5BJD5|TM41B_HUMAN	60	32492	2	2	1	1	0.16	Transmembrane prote
1734	1	SwissProt_HUMAN$	2::Q6IN85|P4R3A_HUMAN	60	95308	3	3	3	3	0.16	Serine/threonine-prot
1735	1	SwissProt_HUMAN$	2::Q13228|SBP1_HUMAN	59	52358	3	2	3	2	0.20	Methanethiol oxidase
1736	1	SwissProt_HUMAN$	2::P17480|UBF1_HUMAN	59	89350	6	5	6	5	0.30	Nucleolar transcriptior
1737	1	SwissProt_HUMAN$	2::P41236|IPP2_HUMAN	59	23001	2	1	2	1	0.23	Protein phosphatase i
1738	1	SwissProt_HUMAN$	2::Q9NP72|RAB18_HUMAN	59	22963	4	2	4	2	0.50	Ras-related protein Ra
1739	1	SwissProt_HUMAN$	2::Q5C9Z4|NOM1_HUMAN	59	96198	3	3	3	3	0.16	Nucleolar MIF4G doma

↑Family	M	DB	Accession	Score	Mass	Matches	Match(sig)	Sequences	Seq(sig)	emPAI	Description
1740	1	SwissProt_HUMAN$	2::Q9NRZ9|HELLS_HUMAN	59	97012	2	2	2	2	0.10	Lymphoid-specific heli
1741	1	SwissProt_HUMAN$	2::Q9NVM4|ANM7_HUMAN	59	78409	1	1	1	1	0.06	Protein arginine N-me
1742	1	SwissProt_HUMAN$	2::Q86TB9|PATL1_HUMAN	58	86796	2	2	2	2	0.12	Protein PAT1 homolog
1743	1	SwissProt_HUMAN$	2::P01871|IGHM_HUMAN	58	49409	6	3	5	2	0.21	Immunoglobulin heav
1744	1	SwissProt_HUMAN$	2::O95249|GOSR1_HUMAN	58	28595	2	1	2	1	0.18	Golgi SNAP receptor c
1745	1	SwissProt_HUMAN$	2::P47914|RL29_HUMAN	58	17741	3	3	3	3	1.20	60S ribosomal protein
1746	1	SwissProt_HUMAN$	2::Q7Z3B4|NUP54_HUMAN	58	55401	3	3	3	3	0.29	Nucleoporin p54 OS=I
1747	1	SwissProt_HUMAN$	2::Q9UI10|EI2BD_HUMAN	58	57521	3	3	3	3	0.28	Translation initiation f
1748	1	SwissProt_HUMAN$	2::P29992|GNA11_HUMAN	58	42097	4	3	4	3	0.40	Guanine nucleotide-bi
1749	1	SwissProt_HUMAN$	2::O75530|EED_HUMAN	57	50166	3	2	3	2	0.21	Polycomb protein EED
1750	1	SwissProt_HUMAN$	2::P56945|BCAR1_HUMAN	57	93314	3	3	3	3	0.16	Breast cancer anti-est
1751	1	SwissProt_HUMAN$	2::Q7Z5L9|I2BP2_HUMAN	57	60987	1	1	1	1	0.08	Interferon regulatory
1752	1	SwissProt_HUMAN$	2::Q5VV41|ARHGG_HUMAN	57	80055	4	3	4	3	0.19	Rho guanine nucleotid
1753	1	SwissProt_HUMAN$	2::Q9H2M9|RBGPR_HUMAN	57	155886	4	3	4	3	0.10	Rab3 GTPase-activatir
1754	1	SwissProt_HUMAN$	2::P62995|TRA2B_HUMAN	57	33646	6	5	5	4	0.75	Transformer-2 protein
1755	1	SwissProt_HUMAN$	2::Q96EL3|RM53_HUMAN	57	12099	1	1	1	1	0.47	39S ribosomal protein
1756	1	SwissProt_HUMAN$	2::Q9Y3A3|PHOCN_HUMAN	57	26016	1	1	1	1	0.20	MOB-like protein phoc
1757	1	SwissProt_HUMAN$	2::Q9HCM4|E41L5_HUMAN	57	81805	2	2	2	2	0.12	Band 4.1-like protein
1758	1	SwissProt_HUMAN$	2::Q9BTE7|DCNL5_HUMAN	57	27491	2	2	2	2	0.41	DCN1-like protein 5 O
1759	1	SwissProt_HUMAN$	2::Q9H477|RBSK_HUMAN	57	34121	1	1	1	1	0.15	Ribokinase OS=Homo
1760	1	SwissProt_HUMAN$	2::Q9UQ88|CD11A_HUMAN	57	91306	5	5	5	5	0.30	Cyclin-dependent kina
1761	1	SwissProt_HUMAN$	2::P85037|FOXK1_HUMAN	57	75411	3	2	3	2	0.13	Forkhead box protein
1762	1	SwissProt_HUMAN$	2::Q02241|KIF23_HUMAN	57	109990	4	4	4	4	0.19	Kinesin-like protein KI
1763	1	SwissProt_HUMAN$	2::Q14527|HLTF_HUMAN	57	113857	2	2	2	2	0.09	Helicase-like transcrip
1764	1	SwissProt_HUMAN$	2::Q9NUD5|ZCHC3_HUMAN	57	43520	2	2	2	2	0.24	Zinc finger CCHC dom
1765	1	SwissProt_HUMAN$	2::Q6ZXV5|TMTC3_HUMAN	57	103942	2	2	2	2	0.10	Protein O-mannosyl-tr
1766	1	SwissProt_HUMAN$	2::Q9UBB6|NCDN_HUMAN	57	78814	2	2	2	2	0.13	Neurochondrin OS=Hc
1767	1	SwissProt_HUMAN$	2::Q9NRX4|PHP14_HUMAN	56	13824	2	2	1	1	0.40	14 kDa phosphohistidi
1768	1	SwissProt_HUMAN$	2::P16144|ITB4_HUMAN	56	202039	1	1	1	1	0.02	Integrin beta-4 OS=H
1769	1	SwissProt_HUMAN$	2::Q9UJX2|CDC23_HUMAN	56	68790	3	3	3	3	0.23	Cell division cycle prot
1770	1	SwissProt_HUMAN$	2::P23919|KTHY_HUMAN	56	23804	5	4	5	4	1.21	Thymidylate kinase OS
1771	1	SwissProt_HUMAN$	2::P11177|ODPB_HUMAN	56	39208	5	3	4	3	0.44	Pyruvate dehydrogena
1772	1	SwissProt_HUMAN$	2::P02771|FETA_HUMAN	56	68633	2	2	1	1	0.07	Alpha-fetoprotein OS=
1773	1	SwissProt_HUMAN$	2::Q8WUA2|PPIL4_HUMAN	56	57189	3	3	3	3	0.28	Peptidyl-prolyl cis-trar
1774	1	SwissProt_HUMAN$	2::Q9HB40|RISC_HUMAN	56	50798	1	1	1	1	0.10	Retinoid-inducible seri
1775	1	SwissProt_HUMAN$	2::Q9UIV1|CNOT7_HUMAN	56	32724	2	2	2	2	0.33	CCR4-NOT transcriptic
1776	1	SwissProt_HUMAN$	2::Q9NVS9|PNPO_HUMAN	56	29969	3	3	2	2	0.37	Pyridoxine-5'-phospha
1777	1	SwissProt_HUMAN$	2::Q96DA6|TIM14_HUMAN	56	12491	1	1	1	1	0.45	Mitochondrial import i
1778	1	SwissProt_HUMAN$	2::Q9UKF6|CPSF3_HUMAN	56	77436	3	2	3	2	0.13	Cleavage and polyade
1779	1	SwissProt_HUMAN$	2::Q9NQC3|RTN4_HUMAN	55	129851	1	1	1	1	0.04	Reticulon-4 OS=Homc

↑Family	M	DB	Accession	Score	Mass	Matches	Match(sig)	Sequences	Seq(sig)	emPAI	Description
1780	1	SwissProt_HUMAN$	2::Q9Y5K6|CD2AP_HUMAN	55	71407	8	5	7	5	0.39	CD2-associated protei
1781	1	SwissProt_HUMAN$	2::P41235|HNF4A_HUMAN	55	52751	2	2	2	2	0.20	Hepatocyte nuclear fa
1782	1	SwissProt_HUMAN$	2::Q8WUK0|PTPM1_HUMAN	55	22829	3	2	2	1	0.23	Phosphatidylglyceroph
1783	1	SwissProt_HUMAN$	2::P22307|NLTP_HUMAN	55	58956	3	3	3	3	0.27	Non-specific lipid-tran
1784	1	SwissProt_HUMAN$	2::Q96A65|EXOC4_HUMAN	55	110429	3	2	3	2	0.09	Exocyst complex com
1785	1	SwissProt_HUMAN$	2::Q9H3N1|TMX1_HUMAN	55	31771	3	2	3	2	0.35	Thioredoxin-related tr
1786	1	SwissProt_HUMAN$	2::Q9NZ32|ARP10_HUMAN	55	46277	2	2	2	2	0.23	Actin-related protein 1
1787	1	SwissProt_HUMAN$	2::O00625|PIR_HUMAN	55	32093	1	1	1	1	0.16	Pirin OS=Homo sapier
1788	1	SwissProt_HUMAN$	2::Q9Y6B6|SAR1B_HUMAN	55	22396	1	1	1	1	0.23	GTP-binding protein S
1789	1	SwissProt_HUMAN$	2::P30825|SL7A1_HUMAN	55	67594	2	2	2	2	0.15	High affinity cationic a
1790	1	SwissProt_HUMAN$	2::Q92620|PRP16_HUMAN	55	140415	1	1	1	1	0.03	Pre-mRNA-splicing fac
1791	1	SwissProt_HUMAN$	2::Q9Y376|CAB39_HUMAN	55	39844	3	3	3	3	0.43	Calcium-binding prote
1792	1	SwissProt_HUMAN$	2::Q9BYG3|MK67I_HUMAN	55	34201	2	2	2	2	0.32	MKI67 FHA domain-in
1793	1	SwissProt_HUMAN$	2::P02787|TRFE_HUMAN	55	77014	5	4	5	4	0.28	Serotransferrin OS=H
1794	1	SwissProt_HUMAN$	2::P51668|UB2D1_HUMAN	55	16591	2	2	2	2	0.76	Ubiquitin-conjugating
1795	1	SwissProt_HUMAN$	2::Q6WKZ4|RFIP1_HUMAN	55	137083	3	1	3	1	0.04	Rab11 family-interacti
1796	1	SwissProt_HUMAN$	2::Q99808|S29A1_HUMAN	54	50186	2	2	2	2	0.21	Equilibrative nucleosic
1797	1	SwissProt_HUMAN$	2::O75179|ANR17_HUMAN	54	274088	3	2	3	2	0.04	Ankyrin repeat domai
1797	2	SwissProt_HUMAN$	2::Q8IWZ3|ANKH1_HUMAN	36	269291	3	2	3	2	0.04	Ankyrin repeat and KH
1798	1	SwissProt_HUMAN$	2::P53602|MVD1_HUMAN	54	43377	4	3	4	3	0.39	Diphosphomevalonate
1799	1	SwissProt_HUMAN$	2::P82673|RT35_HUMAN	54	36821	3	3	3	3	0.47	28S ribosomal protein
1800	1	SwissProt_HUMAN$	2::O43291|SPIT2_HUMAN	54	28210	1	1	1	1	0.18	Kunitz-type protease i
1801	1	SwissProt_HUMAN$	2::Q8TB22|SPT20_HUMAN	54	87843	4	4	4	4	0.24	Spermatogenesis-assc
1802	1	SwissProt_HUMAN$	2::O75251|NDUS7_HUMAN	54	23548	2	2	2	2	0.49	NADH dehydrogenase
1803	1	SwissProt_HUMAN$	2::Q6IAA8|LTOR1_HUMAN	54	17734	3	2	2	2	0.69	Ragulator complex pro
1804	1	SwissProt_HUMAN$	2::O75964|ATP5L_HUMAN	54	11421	3	3	3	3	2.37	ATP synthase subunit
1805	1	SwissProt_HUMAN$	2::O43172|PRP4_HUMAN	54	58412	3	2	3	2	0.18	U4/U6 small nuclear r
1806	1	SwissProt_HUMAN$	2::Q9H5Q4|TFB2M_HUMAN	54	45320	4	3	4	3	0.37	Dimethyladenosine tra
1807	1	SwissProt_HUMAN$	2::Q15293|RCN1_HUMAN	54	38866	5	3	5	3	0.44	Reticulocalbin-1 OS=H
1808	1	SwissProt_HUMAN$	2::Q13427|PPIG_HUMAN	54	88564	1	1	1	1	0.05	Peptidyl-prolyl cis-trar
1809	1	SwissProt_HUMAN$	2::Q5T0Z8|CF132_HUMAN	54	123958	1	1	1	1	0.04	Uncharacterized prote
1810	1	SwissProt_HUMAN$	2::Q9BSH5|HDHD3_HUMAN	54	27982	3	3	3	3	0.66	Haloacid dehalogenas
1811	1	SwissProt_HUMAN$	2::P13674|P4HA1_HUMAN	54	61011	2	2	2	2	0.17	Prolyl 4-hydroxylase s
1812	1	SwissProt_HUMAN$	2::Q9Y5Z4|HEBP2_HUMAN	54	22861	1	1	1	1	0.23	Heme-binding protein
1813	1	SwissProt_HUMAN$	2::O00560|SDCB1_HUMAN	54	32424	2	2	2	2	0.34	Syntenin-1 OS=Homo
1814	1	SwissProt_HUMAN$	2::Q9Y4F1|FARP1_HUMAN	54	118559	3	3	3	3	0.13	FERM, ARHGEF and pl
1815	1	SwissProt_HUMAN$	2::P67870|CSK2B_HUMAN	54	24926	2	2	2	2	0.46	Casein kinase II subu
1816	1	SwissProt_HUMAN$	2::Q9Y619|ORNT1_HUMAN	54	32715	1	1	1	1	0.15	Mitochondrial ornithin
1817	1	SwissProt_HUMAN$	2::O00767|ACOD_HUMAN	54	41496	3	3	3	3	0.41	Acyl-CoA desaturase (
1818	1	SwissProt_HUMAN$	2::P17706|PTN2_HUMAN	54	48442	2	2	1	1	0.10	Tyrosine-protein phos

↑Family	M	DB	Accession	Score	Mass	Matches	Match(sig)	Sequences	Seq(sig)	emPAI	Description
1819	1	SwissProt_HUMAN$	2::O00411|RPOM_HUMAN	53	138532	4	3	4	3	0.11	DNA-directed RNA pol
1820	1	SwissProt_HUMAN$	2::Q6P1L8|RM14_HUMAN	53	15937	2	2	2	2	0.79	39S ribosomal protein
1821	1	SwissProt_HUMAN$	2::Q9UI12|VATH_HUMAN	53	55847	2	2	2	2	0.18	V-type proton ATPase
1822	1	SwissProt_HUMAN$	2::P98179|RBM3_HUMAN	53	17160	3	3	3	3	1.26	RNA-binding protein 3
1823	1	SwissProt_HUMAN$	2::Q08623|HDHD1_HUMAN	53	25233	3	2	3	2	0.45	Pseudouridine-5'-phos
1824	1	SwissProt_HUMAN$	2::Q9GZZ9|UBA5_HUMAN	53	44834	1	1	1	1	0.11	Ubiquitin-like modifier
1825	1	SwissProt_HUMAN$	2::P78330|SERB_HUMAN	53	24992	3	2	3	2	0.46	Phosphoserine phosph
1826	1	SwissProt_HUMAN$	2::Q6JQN1|ACD10_HUMAN	53	118758	1	1	1	1	0.04	Acyl-CoA dehydrogena
1827	1	SwissProt_HUMAN$	2::Q8WVY7|UBCP1_HUMAN	53	36781	4	3	4	3	0.47	Ubiquitin-like domain-
1828	1	SwissProt_HUMAN$	2::O60784|TOM1_HUMAN	53	53785	1	1	1	1	0.09	Target of Myb protein
1829	1	SwissProt_HUMAN$	2::Q8N0U8|VKORL_HUMAN	53	19823	2	1	2	1	0.27	Vitamin K epoxide red
1830	1	SwissProt_HUMAN$	2::Q9NUJ1|ABHDA_HUMAN	53	33911	3	3	3	3	0.52	Mycophenolic acid acy
1831	1	SwissProt_HUMAN$	2::Q8NFW8|NEUA_HUMAN	53	48349	4	4	3	3	0.34	N-acylneuraminate cy
1832	1	SwissProt_HUMAN$	2::P49023|PAXI_HUMAN	53	64464	3	2	3	2	0.16	Paxillin OS=Homo sap
1833	1	SwissProt_HUMAN$	2::Q9Y450|HBS1L_HUMAN	53	75426	3	3	3	3	0.21	HBS1-like protein OS=
1834	1	SwissProt_HUMAN$	2::P19387|RPB3_HUMAN	53	31422	1	1	1	1	0.16	DNA-directed RNA pol
1835	1	SwissProt_HUMAN$	2::Q9UJC5|SH3L2_HUMAN	53	12318	1	1	1	1	0.46	SH3 domain-binding g
1836	1	SwissProt_HUMAN$	2::P61457|PHS_HUMAN	52	11992	6	5	6	5	5.92	Pterin-4-alpha-carbinc
1837	1	SwissProt_HUMAN$	2::Q6IAN0|DRS7B_HUMAN	52	35097	2	2	1	1	0.14	Dehydrogenase/reduc
1838	1	SwissProt_HUMAN$	2::Q96BP3|PPWD1_HUMAN	52	73528	4	3	4	3	0.21	Peptidylprolyl isomera
1839	1	SwissProt_HUMAN$	2::O60925|PFD1_HUMAN	52	14202	4	4	3	3	1.67	Prefoldin subunit 1 OS
1840	1	SwissProt_HUMAN$	2::P51970|NDUA8_HUMAN	52	20092	2	1	2	1	0.26	NADH dehydrogenase
1841	1	SwissProt_HUMAN$	2::Q8WW12|PCNP_HUMAN	52	18913	2	2	2	2	0.64	PEST proteolytic signa
1842	1	SwissProt_HUMAN$	2::Q8N543|OGFD1_HUMAN	52	63206	4	4	4	4	0.35	Prolyl 3-hydroxylase C
1843	1	SwissProt_HUMAN$	2::Q4KMQ2|ANO6_HUMAN	52	106096	3	2	3	2	0.09	Anoctamin-6 OS=Hom
1844	1	SwissProt_HUMAN$	2::O43823|AKAP8_HUMAN	52	76061	2	2	2	2	0.13	A-kinase anchor prote
1845	1	SwissProt_HUMAN$	2::Q9NZL9|MAT2B_HUMAN	52	37528	2	2	2	2	0.29	Methionine adenosyltr
1846	1	SwissProt_HUMAN$	2::Q9NZW5|MPP6_HUMAN	52	61079	5	3	5	3	0.26	MAGUK p55 subfamily
1847	1	SwissProt_HUMAN$	2::Q16706|MA2A1_HUMAN	52	131057	2	2	2	2	0.08	Alpha-mannosidase 2
1848	1	SwissProt_HUMAN$	2::Q96EE3|SEH1_HUMAN	52	39623	4	3	4	3	0.43	Nucleoporin SEH1 OS=
1849	1	SwissProt_HUMAN$	2::O75663|TIPRL_HUMAN	52	31424	2	1	2	1	0.16	TIP41-like protein OS=
1850	1	SwissProt_HUMAN$	2::P50895|BCAM_HUMAN	52	67363	1	1	1	1	0.07	Basal cell adhesion m
1851	1	SwissProt_HUMAN$	2::O14735|CDIPT_HUMAN	52	23523	1	1	1	1	0.22	CDP-diacylglycerol--in
1852	1	SwissProt_HUMAN$	2::Q9Y3E7|CHMP3_HUMAN	52	25057	2	2	2	2	0.45	Charged multivesicula
1853	1	SwissProt_HUMAN$	2::P46013|KI67_HUMAN	52	358474	5	3	5	3	0.04	Proliferation marker p
1854	1	SwissProt_HUMAN$	2::P51795|CLCN5_HUMAN	52	90726	2	2	2	2	0.11	H(+)/Cl(-) exchange t
1855	1	SwissProt_HUMAN$	2::O00267|SPT5H_HUMAN	52	120925	4	2	4	2	0.08	Transcription elongati
1856	1	SwissProt_HUMAN$	2::Q9HC38|GLOD4_HUMAN	51	34771	1	1	1	1	0.15	Glyoxalase domain-co
1857	1	SwissProt_HUMAN$	2::Q9BRJ6|CG050_HUMAN	51	22070	1	1	1	1	0.24	Uncharacterized prote
1858	1	SwissProt_HUMAN$	2::P53701|CCHL_HUMAN	51	30582	2	2	2	2	0.36	Cytochrome c-type he

↑Family	M	DB	Accession	Score	Mass	Matches	Match(sig)	Sequences	Seq(sig)	emPAI	Description
1859	1	SwissProt_HUMAN$	2::Q15404|RSU1_HUMAN	51	31521	3	3	3	3	0.57	Ras suppressor protei
1860	1	SwissProt_HUMAN$	2::P01876|IGHA1_HUMAN	51	37631	2	2	2	2	0.28	Immunoglobulin heav
1861	1	SwissProt_HUMAN$	2::Q9UJ70|NAGK_HUMAN	51	37352	2	2	2	2	0.29	N-acetyl-D-glucosami
1862	1	SwissProt_HUMAN$	2::P52306|GDS1_HUMAN	51	66275	2	1	2	1	0.07	Rap1 GTPase-GDP dis
1863	1	SwissProt_HUMAN$	2::Q8NHP6|MSPD2_HUMAN	51	59708	1	1	1	1	0.08	Motile sperm domain-
1864	1	SwissProt_HUMAN$	2::Q9HAU5|RENT2_HUMAN	51	147717	4	2	2	2	0.07	Regulator of nonsense
1865	1	SwissProt_HUMAN$	2::Q96QR8|PURB_HUMAN	51	33220	2	1	2	1	0.15	Transcriptional activat
1866	1	SwissProt_HUMAN$	2::P30405|PPIF_HUMAN	51	22026	5	4	5	4	1.35	Peptidyl-prolyl cis-trar
1867	1	SwissProt_HUMAN$	2::O75607|NPM3_HUMAN	51	19331	2	2	2	2	0.62	Nucleoplasmin-3 OS=
1868	1	SwissProt_HUMAN$	2::O43837|IDH3B_HUMAN	51	42157	3	3	3	3	0.40	Isocitrate dehydrogen
1869	1	SwissProt_HUMAN$	2::Q96IX5|ATPMD_HUMAN	51	6453	2	2	2	2	3.10	ATP synthase membra
1870	1	SwissProt_HUMAN$	2::Q6P3X3|TTC27_HUMAN	51	96571	3	3	3	3	0.16	Tetratricopeptide repe
1871	1	SwissProt_HUMAN$	2::Q9HCY8|S10AE_HUMAN	51	11655	1	1	1	1	0.49	Protein S100-A14 OS=
1872	1	SwissProt_HUMAN$	2::P53634|CATC_HUMAN	51	51820	3	3	3	3	0.31	Dipeptidyl peptidase 1
1873	1	SwissProt_HUMAN$	2::Q96C86|DCPS_HUMAN	51	38585	4	2	4	2	0.28	m7GpppX diphosphata
1874	1	SwissProt_HUMAN$	2::Q86W50|MET16_HUMAN	50	63581	1	1	1	1	0.08	RNA N6-adenosine-me
1875	1	SwissProt_HUMAN$	2::Q6P587|FAHD1_HUMAN	50	24827	3	3	2	2	0.46	Acylpyruvase FAHD1,
1876	1	SwissProt_HUMAN$	2::Q9BRX2|PELO_HUMAN	50	43332	4	4	4	4	0.55	Protein pelota homolo
1877	1	SwissProt_HUMAN$	2::O96013|PAK4_HUMAN	50	64032	1	1	1	1	0.08	Serine/threonine-prot
1878	1	SwissProt_HUMAN$	2::P51948|MAT1_HUMAN	50	35800	1	1	1	1	0.14	CDK-activating kinase
1879	1	SwissProt_HUMAN$	2::Q7Z434|MAVS_HUMAN	50	56493	3	3	3	3	0.29	Mitochondrial antiviral
1880	1	SwissProt_HUMAN$	2::O14975|S27A2_HUMAN	50	70267	2	2	2	2	0.14	Very long-chain acyl-C
1881	1	SwissProt_HUMAN$	2::Q9NX18|SDHF2_HUMAN	50	19587	1	1	1	1	0.27	Succinate dehydrogen
1882	1	SwissProt_HUMAN$	2::Q7L5N7|PCAT2_HUMAN	50	60169	2	2	2	2	0.17	Lysophosphatidylcholi
1883	1	SwissProt_HUMAN$	2::Q6Y7W6|GGYF2_HUMAN	50	149978	2	2	2	2	0.07	GRB10-interacting GY
1884	1	SwissProt_HUMAN$	2::Q6GMV2|SMYD5_HUMAN	50	47310	2	2	2	2	0.22	SET and MYND domai
1885	1	SwissProt_HUMAN$	2::Q6PJT7|ZC3HE_HUMAN	50	82823	2	1	2	1	0.06	Zinc finger CCCH dom
1886	1	SwissProt_HUMAN$	2::Q13769|THOC5_HUMAN	50	78458	5	5	4	4	0.27	THO complex subunit
1887	1	SwissProt_HUMAN$	2::O00308|WWP2_HUMAN	50	98850	1	1	1	1	0.05	NEDD4-like E3 ubiquit
1888	1	SwissProt_HUMAN$	2::Q9H936|GHC1_HUMAN	50	34448	4	2	4	2	0.32	Mitochondrial glutama
1889	1	SwissProt_HUMAN$	2::P36639|8ODP_HUMAN	50	22505	1	1	1	1	0.23	7,8-dihydro-8-oxogua
1890	1	SwissProt_HUMAN$	2::Q9NRL3|STRN4_HUMAN	50	80546	2	2	2	2	0.12	Striatin-4 OS=Homo s
1891	1	SwissProt_HUMAN$	2::Q9BWU0|NADAP_HUMAN	50	88759	4	3	4	3	0.17	Kanadaptin OS=Homo
1892	1	SwissProt_HUMAN$	2::Q00796|DHSO_HUMAN	50	38300	7	5	6	5	0.85	Sorbitol dehydrogenas
1893	1	SwissProt_HUMAN$	2::P35790|CHKA_HUMAN	49	52215	2	1	2	1	0.09	Choline kinase alpha (
1894	1	SwissProt_HUMAN$	2::Q9Y6K5|OAS3_HUMAN	49	121093	4	2	4	2	0.08	2'-5'-oligoadenylate sy
1895	1	SwissProt_HUMAN$	2::Q8IUR7|ARMC8_HUMAN	49	75460	2	2	2	2	0.13	Armadillo repeat-conta
1896	1	SwissProt_HUMAN$	2::P49840|GSK3A_HUMAN	49	50949	2	2	2	2	0.20	Glycogen synthase kir
1897	1	SwissProt_HUMAN$	2::P00403|COX2_HUMAN	49	25548	2	2	2	2	0.45	Cytochrome c oxidase
1898	1	SwissProt_HUMAN$	2::P02042|HBD_HUMAN	49	16045	2	2	1	1	0.34	Hemoglobin subunit d

↑Family	M	DB	Accession	Score	Mass	Matches	Match(sig)	Sequences	Seq(sig)	emPAI	Description
1899	1	SwissProt_HUMAN$	2::P56545|CTBP2_HUMAN	49	48914	4	4	4	4	0.47	C-terminal-binding pro
1899	2	SwissProt_HUMAN$	2::Q13363|CTBP1_HUMAN	40	47505	2	2	2	2	0.22	C-terminal-binding pro
1900	1	SwissProt_HUMAN$	2::Q9NSK0|KLC4_HUMAN	49	68598	1	1	1	1	0.07	Kinesin light chain 4 C
1901	1	SwissProt_HUMAN$	2::Q9NX40|OCAD1_HUMAN	49	27609	2	1	2	1	0.19	OCIA domain-containi
1902	1	SwissProt_HUMAN$	2::Q8N5K1|CISD2_HUMAN	49	15268	3	1	3	1	0.36	CDGSH iron-sulfur dor
1903	1	SwissProt_HUMAN$	2::Q9H900|ZWILC_HUMAN	49	67172	2	2	2	2	0.15	Protein zwilch homolo
1904	1	SwissProt_HUMAN$	2::Q6P158|DHX57_HUMAN	49	155507	2	1	2	1	0.03	Putative ATP-depende
1905	1	SwissProt_HUMAN$	2::Q6IA69|NADE_HUMAN	49	79234	3	2	3	2	0.13	Glutamine-dependent
1906	1	SwissProt_HUMAN$	2::Q9BVK6|TMED9_HUMAN	49	27260	4	3	4	3	0.68	Transmembrane emp2
1907	1	SwissProt_HUMAN$	2::Q15386|UBE3C_HUMAN	49	123844	3	2	3	2	0.08	Ubiquitin-protein ligas
1908	1	SwissProt_HUMAN$	2::Q969X6|UTP4_HUMAN	49	76841	2	2	2	2	0.13	U3 small nucleolar RN
1909	1	SwissProt_HUMAN$	2::P55196|AFAD_HUMAN	49	206676	2	2	2	2	0.05	Afadin OS=Homo sapi
1910	1	SwissProt_HUMAN$	2::Q9NWT6|HIF1N_HUMAN	49	40260	1	1	1	1	0.12	Hypoxia-inducible fact
1911	1	SwissProt_HUMAN$	2::Q9UGP8|SEC63_HUMAN	49	87942	2	2	2	2	0.11	Translocation protein :
1912	1	SwissProt_HUMAN$	2::P61353|RL27_HUMAN	49	15788	6	2	5	2	0.81	60S ribosomal protein
1913	1	SwissProt_HUMAN$	2::Q8NHQ9|DDX55_HUMAN	48	68503	2	2	2	2	0.15	ATP-dependent RNA h
1914	1	SwissProt_HUMAN$	2::Q8WVX9|FACR1_HUMAN	48	59319	1	1	1	1	0.08	Fatty acyl-CoA reducta
1915	1	SwissProt_HUMAN$	2::Q96AX1|VP33A_HUMAN	48	67568	2	2	2	2	0.15	Vacuolar protein sortir
1916	1	SwissProt_HUMAN$	2::P17612|KAPCA_HUMAN	48	40564	4	3	4	3	0.42	cAMP-dependent prote
1917	1	SwissProt_HUMAN$	2::A6NNY8|UBP27_HUMAN	48	49598	2	2	1	1	0.10	Ubiquitin carboxyl-ter
1918	1	SwissProt_HUMAN$	2::Q96RD7|PANX1_HUMAN	48	48019	1	1	1	1	0.10	Pannexin-1 OS=Homo
1919	1	SwissProt_HUMAN$	2::O15145|ARPC3_HUMAN	48	20533	1	1	1	1	0.26	Actin-related protein 2
1920	1	SwissProt_HUMAN$	2::Q9NP92|RT30_HUMAN	48	50333	4	3	3	3	0.33	39S ribosomal protein
1921	1	SwissProt_HUMAN$	2::Q7Z4G4|TRM11_HUMAN	48	53387	3	3	3	3	0.30	tRNA (guanine(10)-N2
1922	1	SwissProt_HUMAN$	2::P55011|S12A2_HUMAN	48	131364	3	3	3	3	0.11	Solute carrier family 1
1923	1	SwissProt_HUMAN$	2::O95251|KAT7_HUMAN	48	70598	4	2	3	1	0.07	Histone acetyltransfer
1924	1	SwissProt_HUMAN$	2::Q9UBU8|MO4L1_HUMAN	48	41448	1	1	1	1	0.12	Mortality factor 4-like
1925	1	SwissProt_HUMAN$	2::Q96SB4|SRPK1_HUMAN	48	74278	5	4	5	4	0.29	SRSF protein kinase 1
1925	2	SwissProt_HUMAN$	2::P78362|SRPK2_HUMAN	33	77478	3	3	3	3	0.20	SRSF protein kinase 2
1926	1	SwissProt_HUMAN$	2::Q8IX12|CCAR1_HUMAN	48	132739	6	4	6	4	0.15	Cell division cycle and
1927	1	SwissProt_HUMAN$	2::O95602|RPA1_HUMAN	48	194687	2	1	2	1	0.02	DNA-directed RNA pol
1928	1	SwissProt_HUMAN$	2::Q6PJG6|BRAT1_HUMAN	48	88063	1	1	1	1	0.06	BRCA1-associated ATI
1929	1	SwissProt_HUMAN$	2::Q13112|CAF1B_HUMAN	48	61454	3	3	3	3	0.26	Chromatin assembly fa
1930	1	SwissProt_HUMAN$	2::P48634|PRC2A_HUMAN	48	228724	3	2	3	2	0.04	Protein PRRC2A OS=H
1931	1	SwissProt_HUMAN$	2::Q9NTG7|SIR3_HUMAN	48	43546	1	1	1	1	0.11	NAD-dependent protei
1932	1	SwissProt_HUMAN$	2::Q7L592|NDUF7_HUMAN	48	49206	2	2	2	2	0.21	Protein arginine methy
1933	1	SwissProt_HUMAN$	2::Q9ULR3|PPM1H_HUMAN	48	56413	3	2	2	1	0.09	Protein phosphatase 1
1934	1	SwissProt_HUMAN$	2::Q99426|TBCB_HUMAN	48	27308	2	2	2	2	0.41	Tubulin-folding cofacto
1935	1	SwissProt_HUMAN$	2::Q15061|WDR43_HUMAN	48	74843	3	2	3	2	0.13	WD repeat-containing
1936	1	SwissProt_HUMAN$	2::Q13423|NNTM_HUMAN	48	113823	1	1	1	1	0.04	NAD(P) transhydroger

↑Family	M	DB	Accession	Score	Mass	Matches	Match(sig)	Sequences	Seq(sig)	emPAI	Description
1937	1	SwissProt_HUMAN$	2::P62942|FKB1A_HUMAN	47	11943	1	1	1	1	0.47	Peptidyl-prolyl cis-trar
1938	1	SwissProt_HUMAN$	2::Q9NVJ2|ARL8B_HUMAN	47	21525	2	1	2	1	0.24	ADP-ribosylation facto
1939	1	SwissProt_HUMAN$	2::Q9BT22|ALG1_HUMAN	47	52484	1	1	1	1	0.09	Chitobiosyldiphosphoc
1940	1	SwissProt_HUMAN$	2::P00374|DYR_HUMAN	47	21439	2	2	2	2	0.55	Dihydrofolate reducta:
1941	1	SwissProt_HUMAN$	2::Q02447|SP3_HUMAN	47	81876	2	1	1	1	0.06	Transcription factor Sp
1942	1	SwissProt_HUMAN$	2::P50120|RET2_HUMAN	47	15697	5	4	5	4	2.29	Retinol-binding protei
1943	1	SwissProt_HUMAN$	2::Q9Y305|ACOT9_HUMAN	47	49870	2	2	2	2	0.21	Acyl-coenzyme A thioe
1944	1	SwissProt_HUMAN$	2::Q08AI8|MB214_HUMAN	47	49534	1	1	1	1	0.10	Protein mab-21-like 4
1945	1	SwissProt_HUMAN$	2::Q15843|NEDD8_HUMAN	47	9066	1	1	1	1	0.66	NEDD8 OS=Homo sap
1946	1	SwissProt_HUMAN$	2::Q8N766|EMC1_HUMAN	47	111689	2	2	2	2	0.09	ER membrane protein
1947	1	SwissProt_HUMAN$	2::P32321|DCTD_HUMAN	47	20003	1	1	1	1	0.26	Deoxycytidylate deam
1948	1	SwissProt_HUMAN$	2::Q9UMY4|SNX12_HUMAN	47	18873	7	5	6	5	2.46	Sorting nexin-12 OS=
1948	2	SwissProt_HUMAN$	2::O60493|SNX3_HUMAN	41	18751	6	4	5	4	1.71	Sorting nexin-3 OS=H
1949	1	SwissProt_HUMAN$	2::Q5T6F2|UBAP2_HUMAN	47	117044	3	2	3	2	0.08	Ubiquitin-associated p
1950	1	SwissProt_HUMAN$	2::Q8TDN6|BRX1_HUMAN	47	41375	3	3	3	3	0.41	Ribosome biogenesis p
1951	1	SwissProt_HUMAN$	2::P02788|TRFL_HUMAN	47	78132	2	2	2	2	0.13	Lactotransferrin OS=H
1952	1	SwissProt_HUMAN$	2::Q9BUP3|HTAI2_HUMAN	47	27032	3	1	3	1	0.19	Oxidoreductase HTATI
1953	1	SwissProt_HUMAN$	2::Q7L3T8|SYPM_HUMAN	47	53228	1	1	1	1	0.09	Probable proline--tRN/
1954	1	SwissProt_HUMAN$	2::P08263|GSTA1_HUMAN	47	25615	1	1	1	1	0.20	Glutathione S-transfer
1955	1	SwissProt_HUMAN$	2::Q8WVM7|STAG1_HUMAN	47	144336	4	2	4	2	0.07	Cohesin subunit SA-1
1956	1	SwissProt_HUMAN$	2::Q8TE68|ES8L1_HUMAN	47	80202	3	3	3	3	0.19	Epidermal growth fact
1957	1	SwissProt_HUMAN$	2::Q9HCN4|GPN1_HUMAN	47	41714	1	1	1	1	0.12	GPN-loop GTPase 1 OS
1958	1	SwissProt_HUMAN$	2::Q9NPJ3|ACO13_HUMAN	47	14951	1	1	1	1	0.36	Acyl-coenzyme A thioe
1959	1	SwissProt_HUMAN$	2::O60229|KALRN_HUMAN	47	340047	1	1	1	1	0.01	Kalirin OS=Homo sapi
1960	1	SwissProt_HUMAN$	2::P19174|PLCG1_HUMAN	47	148438	2	1	2	1	0.03	1-phosphatidylinositol
1961	1	SwissProt_HUMAN$	2::P26572|MGAT1_HUMAN	46	50846	1	1	1	1	0.10	Alpha-1,3-mannosyl-g
1962	1	SwissProt_HUMAN$	2::Q92575|UBXN4_HUMAN	46	56743	2	1	2	1	0.09	UBX domain-containin
1963	1	SwissProt_HUMAN$	2::P60033|CD81_HUMAN	46	25792	2	2	2	2	0.44	CD81 antigen OS=Hor
1964	1	SwissProt_HUMAN$	2::Q9UG63|ABCF2_HUMAN	46	71245	2	2	2	2	0.14	ATP-binding cassette s
1965	1	SwissProt_HUMAN$	2::Q9BT09|CNPY3_HUMAN	46	30729	2	2	1	1	0.17	Protein canopy homol
1966	1	SwissProt_HUMAN$	2::O95297|MPZL1_HUMAN	46	29064	2	1	2	1	0.18	Myelin protein zero-lik
1967	1	SwissProt_HUMAN$	2::O43427|FIBP_HUMAN	46	41851	2	2	2	2	0.25	Acidic fibroblast growt
1968	1	SwissProt_HUMAN$	2::Q7LBC6|KDM3B_HUMAN	46	191461	4	3	4	3	0.08	Lysine-specific demetl
1969	1	SwissProt_HUMAN$	2::O60826|CCD22_HUMAN	46	70712	3	3	3	3	0.22	Coiled-coil domain-co
1970	1	SwissProt_HUMAN$	2::Q9H0V9|LMA2L_HUMAN	46	39685	3	3	3	3	0.43	VIP36-like protein OS
1971	1	SwissProt_HUMAN$	2::O43929|ORC4_HUMAN	46	50345	1	1	1	1	0.10	Origin recognition com
1972	1	SwissProt_HUMAN$	2::Q9BQ61|TRIR_HUMAN	46	18408	1	1	1	1	0.29	Telomerase RNA comp
1973	1	SwissProt_HUMAN$	2::Q15043|S39AE_HUMAN	46	54177	2	2	2	2	0.19	Metal cation symporte
1974	1	SwissProt_HUMAN$	2::P21980|TGM2_HUMAN	46	77280	2	2	2	2	0.13	Protein-glutamine gan
1975	1	SwissProt_HUMAN$	2::Q9NZT2|OGFR_HUMAN	46	73280	3	2	3	2	0.14	Opioid growth factor r

↑Family	M	DB	Accession	Score	Mass	Matches	Match(sig)	Sequences	Seq(sig)	emPAI	Description
1976	1	SwissProt_HUMAN$	2::Q8TCT9|HM13_HUMAN	46	41462	2	2	2	2	0.26	Minor histocompatibili
1977	1	SwissProt_HUMAN$	2::Q92522|H1X_HUMAN	46	22474	3	3	3	3	0.87	Histone H1.10 OS=Ho
1978	1	SwissProt_HUMAN$	2::Q4G148|GXLT1_HUMAN	46	50534	2	2	2	2	0.21	Glucoside xylosyltrans
1979	1	SwissProt_HUMAN$	2::P04040|CATA_HUMAN	46	59719	3	3	3	3	0.27	Catalase OS=Homo sa
1980	1	SwissProt_HUMAN$	2::P49441|INPP_HUMAN	46	43970	1	1	1	1	0.11	Inositol polyphosphate
1981	1	SwissProt_HUMAN$	2::Q9GZM8|NDEL1_HUMAN	46	38351	1	1	1	1	0.13	Nuclear distribution pr
1982	1	SwissProt_HUMAN$	2::Q9UNI6|DUS12_HUMAN	46	37663	1	1	1	1	0.13	Dual specificity protei
1983	1	SwissProt_HUMAN$	2::O75190|DNJB6_HUMAN	46	36065	1	1	1	1	0.14	DnaJ homolog subfam
1984	1	SwissProt_HUMAN$	2::Q8NI36|WDR36_HUMAN	46	105255	4	3	4	3	0.14	WD repeat-containing
1985	1	SwissProt_HUMAN$	2::P51690|ARSL_HUMAN	46	65626	2	1	2	1	0.07	Arylsulfatase L OS=Hc
1986	1	SwissProt_HUMAN$	2::Q96T23|RSF1_HUMAN	46	163720	2	2	2	2	0.06	Remodeling and spaci
1987	1	SwissProt_HUMAN$	2::Q7Z3U7|MON2_HUMAN	45	190237	2	2	2	2	0.05	Protein MON2 homolo
1988	1	SwissProt_HUMAN$	2::P20711|DDC_HUMAN	45	53891	1	1	1	1	0.09	Aromatic-L-amino-aci
1989	1	SwissProt_HUMAN$	2::Q9BWE0|REPI1_HUMAN	45	63534	1	1	1	1	0.08	Replication initiator 1
1990	1	SwissProt_HUMAN$	2::Q9Y5K8|VATD_HUMAN	45	28245	1	1	1	1	0.18	V-type proton ATPase
1991	1	SwissProt_HUMAN$	2::Q9NTM9|CUTC_HUMAN	45	29322	1	1	1	1	0.17	Copper homeostasis p
1992	1	SwissProt_HUMAN$	2::P49773|HINT1_HUMAN	45	13793	4	3	4	3	1.76	Histidine triad nucleot
1993	1	SwissProt_HUMAN$	2::Q16850|CP51A_HUMAN	45	56769	2	2	2	2	0.18	Lanosterol 14-alpha d
1994	1	SwissProt_HUMAN$	2::O95674|CDS2_HUMAN	45	51384	1	1	1	1	0.10	Phosphatidate cytidyly
1995	1	SwissProt_HUMAN$	2::O15347|HMGB3_HUMAN	45	22965	3	3	2	2	0.50	High mobility group pi
1996	1	SwissProt_HUMAN$	2::O15258|RER1_HUMAN	45	22943	1	1	1	1	0.23	Protein RER1 OS=Hon
1997	1	SwissProt_HUMAN$	2::Q96D71|REPS1_HUMAN	45	86609	5	2	4	2	0.12	RalBP1-associated Eps
1998	1	SwissProt_HUMAN$	2::Q12979|ABR_HUMAN	45	97536	3	3	3	3	0.16	Active breakpoint clus
1999	1	SwissProt_HUMAN$	2::Q13630|FCL_HUMAN	45	35870	1	1	1	1	0.14	GDP-L-fucose synthas
2000	1	SwissProt_HUMAN$	2::Q13601|KRR1_HUMAN	45	43638	2	2	2	2	0.24	KRR1 small subunit pr
2001	1	SwissProt_HUMAN$	2::Q969E2|SCAM4_HUMAN	45	25711	1	1	1	1	0.20	Secretory carrier-asso
2002	1	SwissProt_HUMAN$	2::Q9NR50|EI2BG_HUMAN	45	50208	3	3	3	3	0.33	Translation initiation f
2003	1	SwissProt_HUMAN$	2::P42695|CNDD3_HUMAN	45	168783	1	1	1	1	0.03	Condensin-2 complex
2004	1	SwissProt_HUMAN$	2::P34949|MPI_HUMAN	45	46626	4	3	3	3	0.36	Mannose-6-phosphate
2005	1	SwissProt_HUMAN$	2::Q92783|STAM1_HUMAN	45	59142	2	2	2	2	0.17	Signal transducing ada
2006	1	SwissProt_HUMAN$	2::P31321|KAP1_HUMAN	45	43046	2	1	2	1	0.12	cAMP-dependent prote
2007	1	SwissProt_HUMAN$	2::P11766|ADHX_HUMAN	45	39698	5	4	4	3	0.43	Alcohol dehydrogenas
2008	1	SwissProt_HUMAN$	2::Q9H0K6|PUS7L_HUMAN	45	80649	2	2	2	2	0.12	Pseudouridylate synth
2009	1	SwissProt_HUMAN$	2::Q16836|HCDH_HUMAN	45	34272	4	2	3	2	0.32	Hydroxyacyl-coenzym
2010	1	SwissProt_HUMAN$	2::Q15397|PUM3_HUMAN	45	73538	5	4	5	4	0.29	Pumilio homolog 3 OS
2011	1	SwissProt_HUMAN$	2::Q9C0C2|TB182_HUMAN	45	181685	2	2	2	2	0.05	182 kDa tankyrase-1-
2012	1	SwissProt_HUMAN$	2::Q96SY0|INT14_HUMAN	44	57434	1	1	1	1	0.09	Integrator complex su
2013	1	SwissProt_HUMAN$	2::Q53EL6|PDCD4_HUMAN	44	51703	3	2	3	2	0.20	Programmed cell deat
2014	1	SwissProt_HUMAN$	2::Q9NVG8|TBC13_HUMAN	44	46524	1	1	1	1	0.11	TBC1 domain family n
2015	1	SwissProt_HUMAN$	2::Q96T88|UHRF1_HUMAN	44	89757	3	3	3	3	0.17	E3 ubiquitin-protein li

↑Family	M	DB	Accession	Score	Mass	Matches	Match(sig)	Sequences	Seq(sig)	emPAI	Description
2016	1	SwissProt_HUMAN$	2::Q9BQ52|RNZ2_HUMAN	44	92160	2	2	2	2	0.11	Zinc phosphodiesteras
2017	1	SwissProt_HUMAN$	2::O95219|SNX4_HUMAN	44	51876	1	1	1	1	0.10	Sorting nexin-4 OS=H
2018	1	SwissProt_HUMAN$	2::O43660|PLRG1_HUMAN	44	57158	2	2	2	2	0.18	Pleiotropic regulator 1
2019	1	SwissProt_HUMAN$	2::Q5TA45|INT11_HUMAN	44	67619	1	1	1	1	0.07	Integrator complex su
2020	1	SwissProt_HUMAN$	2::P41743|KPCI_HUMAN	44	68218	3	3	3	3	0.23	Protein kinase C iota t
2021	1	SwissProt_HUMAN$	2::Q9P2T1|GMPR2_HUMAN	44	37850	2	1	2	1	0.13	GMP reductase 2 OS=
2022	1	SwissProt_HUMAN$	2::Q9BXK5|B2L13_HUMAN	44	52691	1	1	1	1	0.09	Bcl-2-like protein 13 (
2023	1	SwissProt_HUMAN$	2::P82933|RT09_HUMAN	44	45806	2	2	2	2	0.23	28S ribosomal protein
2024	1	SwissProt_HUMAN$	2::Q9BRX8|PXL2A_HUMAN	44	25747	2	2	2	2	0.44	Peroxiredoxin-like 2A
2025	1	SwissProt_HUMAN$	2::P40222|TXLNA_HUMAN	44	61853	3	3	3	3	0.26	Alpha-taxilin OS=Hom
2026	1	SwissProt_HUMAN$	2::O60563|CCNT1_HUMAN	44	80634	2	2	2	2	0.12	Cyclin-T1 OS=Homo s
2027	1	SwissProt_HUMAN$	2::O43819|SCO2_HUMAN	44	29791	2	1	2	1	0.17	Protein SCO2 homolog
2028	1	SwissProt_HUMAN$	2::O15460|P4HA2_HUMAN	44	60864	1	1	1	1	0.08	Prolyl 4-hydroxylase s
2029	1	SwissProt_HUMAN$	2::Q96G23|CERS2_HUMAN	44	44847	2	2	1	1	0.23	Ceramide synthase 2 (
2030	1	SwissProt_HUMAN$	2::Q8N8S7|ENAH_HUMAN	44	66470	4	4	4	4	0.33	Protein enabled homo
2031	1	SwissProt_HUMAN$	2::Q14919|NC2A_HUMAN	44	22336	1	1	1	1	0.23	Dr1-associated corepr
2032	1	SwissProt_HUMAN$	2::Q96RN5|MED15_HUMAN	44	86699	2	1	2	1	0.06	Mediator of RNA polyn
2033	1	SwissProt_HUMAN$	2::Q6YHU6|THADA_HUMAN	44	219465	1	1	1	1	0.02	Thyroid adenoma-ass(
2034	1	SwissProt_HUMAN$	2::Q8IXH7|NELFD_HUMAN	44	66204	4	3	4	3	0.24	Negative elongation fa
2035	1	SwissProt_HUMAN$	2::P46939|UTRO_HUMAN	43	394220	2	2	2	2	0.02	Utrophin OS=Homo sa
2036	1	SwissProt_HUMAN$	2::Q9Y5P6|GMPPB_HUMAN	43	39809	2	2	2	2	0.27	Mannose-1-phosphate
2037	1	SwissProt_HUMAN$	2::Q9BYT8|NEUL_HUMAN	43	80600	2	2	2	2	0.12	Neurolysin, mitochonc
2038	1	SwissProt_HUMAN$	2::Q13523|PRP4B_HUMAN	43	116916	2	1	2	1	0.04	Serine/threonine-prot
2039	1	SwissProt_HUMAN$	2::Q8N201|INT1_HUMAN	43	244143	1	1	1	1	0.02	Integrator complex su
2040	1	SwissProt_HUMAN$	2::O43278|SPIT1_HUMAN	43	58360	3	3	3	3	0.28	Kunitz-type protease i
2041	1	SwissProt_HUMAN$	2::Q9NS69|TOM22_HUMAN	43	15512	2	2	1	1	0.35	Mitochondrial import r
2042	1	SwissProt_HUMAN$	2::Q9NP66|HM20A_HUMAN	43	40119	2	2	2	2	0.27	High mobility group pi
2043	1	SwissProt_HUMAN$	2::P54920|SNAA_HUMAN	43	33211	3	3	3	3	0.53	Alpha-soluble NSF att
2044	1	SwissProt_HUMAN$	2::P98194|AT2C1_HUMAN	43	100512	1	1	1	1	0.05	Calcium-transporting /
2045	1	SwissProt_HUMAN$	2::Q13884|SNTB1_HUMAN	43	58025	1	1	1	1	0.08	Beta-1-syntrophin OS
2046	1	SwissProt_HUMAN$	2::O00165|HAX1_HUMAN	43	31601	1	1	1	1	0.16	HCLS1-associated pro
2047	1	SwissProt_HUMAN$	2::P15529|MCP_HUMAN	43	43719	2	2	1	1	0.11	Membrane cofactor pr
2048	1	SwissProt_HUMAN$	2::Q6UWE0|LRSM1_HUMAN	43	83541	1	1	1	1	0.06	E3 ubiquitin-protein li
2049	1	SwissProt_HUMAN$	2::Q9BXR0|TGT_HUMAN	43	44019	1	1	1	1	0.11	Queuine tRNA-ribosylt
2050	1	SwissProt_HUMAN$	2::P01112|RASH_HUMAN	43	21285	1	1	1	1	0.25	GTPase HRas OS=Hor
2051	1	SwissProt_HUMAN$	2::Q9UBW8|CSN7A_HUMAN	43	30258	1	1	1	1	0.17	COP9 signalosome cor
2052	1	SwissProt_HUMAN$	2::Q9BV57|MTND_HUMAN	43	21485	3	3	3	3	0.92	1,2-dihydroxy-3-keto-
2053	1	SwissProt_HUMAN$	2::Q14320|FA50A_HUMAN	43	40216	2	2	2	2	0.26	Protein FAM50A OS=H
2054	1	SwissProt_HUMAN$	2::Q06265|EXOS9_HUMAN	43	48918	2	2	2	2	0.21	Exosome complex con
2055	1	SwissProt_HUMAN$	2::Q9H9Q4|NHEJ1_HUMAN	43	33316	1	1	1	1	0.15	Non-homologous end-

↑Family	M	DB	Accession	Score	Mass	Matches	Match(sig)	Sequences	Seq(sig)	emPAI	Description
2056	1	SwissProt_HUMAN$	2::Q9Y3C1|NOP16_HUMAN	43	21175	3	3	2	2	0.56	Nucleolar protein 16 C
2057	1	SwissProt_HUMAN$	2::Q8IY37|DHX37_HUMAN	42	129464	3	3	3	3	0.12	Probable ATP-depende
2058	1	SwissProt_HUMAN$	2::Q8NBU5|ATAD1_HUMAN	42	40718	1	1	1	1	0.12	ATPase family AAA do
2059	1	SwissProt_HUMAN$	2::P19838|NFKB1_HUMAN	42	105290	2	2	2	2	0.09	Nuclear factor NF-kap
2060	1	SwissProt_HUMAN$	2::Q9UDR5|AASS_HUMAN	42	102066	2	2	2	2	0.10	Alpha-aminoadipic ser
2061	1	SwissProt_HUMAN$	2::Q8N884|CGAS_HUMAN	42	58777	1	1	1	1	0.08	Cyclic GMP-AMP synth
2062	1	SwissProt_HUMAN$	2::O43488|ARK72_HUMAN	42	39564	3	3	3	3	0.43	Aflatoxin B1 aldehyde
2063	1	SwissProt_HUMAN$	2::Q8N122|RPTOR_HUMAN	42	148942	1	1	1	1	0.03	Regulatory-associated
2064	1	SwissProt_HUMAN$	2::P12259|FA5_HUMAN	42	251546	2	2	1	1	0.02	Coagulation factor V C
2065	1	SwissProt_HUMAN$	2::Q7KZ85|SPT6H_HUMAN	42	198949	3	2	3	2	0.05	Transcription elongati
2066	1	SwissProt_HUMAN$	2::Q86V21|AACS_HUMAN	42	75096	3	3	3	3	0.21	Acetoacetyl-CoA synth
2067	1	SwissProt_HUMAN$	2::P42574|CASP3_HUMAN	42	31587	4	3	4	3	0.56	Caspase-3 OS=Homo
2068	1	SwissProt_HUMAN$	2::Q86WJ1|CHD1L_HUMAN	42	100937	3	3	3	3	0.15	Chromodomain-helica
2069	1	SwissProt_HUMAN$	2::Q9H4Z3|CAPAM_HUMAN	42	80618	3	2	3	2	0.12	mRNA (2'-O-methylad
2070	1	SwissProt_HUMAN$	2::Q9H2W6|RM46_HUMAN	42	31685	2	2	2	2	0.35	39S ribosomal protein
2071	1	SwissProt_HUMAN$	2::P08962|CD63_HUMAN	42	25619	2	2	2	2	0.44	CD63 antigen OS=Hor
2072	1	SwissProt_HUMAN$	2::Q9Y5Y6|ST14_HUMAN	42	94709	2	2	2	2	0.11	Suppressor of tumorig
2073	1	SwissProt_HUMAN$	2::P04792|HSPB1_HUMAN	42	22768	2	1	2	1	0.23	Heat shock protein be
2074	1	SwissProt_HUMAN$	2::Q96GM8|TOE1_HUMAN	42	56512	4	4	3	3	0.29	Target of EGR1 protei
2075	1	SwissProt_HUMAN$	2::P23368|MAOM_HUMAN	42	65402	2	2	2	2	0.16	NAD-dependent malic
2076	1	SwissProt_HUMAN$	2::Q9NPI6|DCP1A_HUMAN	42	63239	1	1	1	1	0.08	mRNA-decapping enzy
2077	1	SwissProt_HUMAN$	2::Q14738|2A5D_HUMAN	42	69947	4	2	4	2	0.15	Serine/threonine-prot
2078	1	SwissProt_HUMAN$	2::Q99442|SEC62_HUMAN	42	45833	2	2	2	2	0.23	Translocation protein
2079	1	SwissProt_HUMAN$	2::Q86UL3|GPAT4_HUMAN	42	52037	1	1	1	1	0.10	Glycerol-3-phosphate
2080	1	SwissProt_HUMAN$	2::Q8WWI1|LMO7_HUMAN	41	192576	3	2	3	2	0.05	LIM domain only prote
2081	1	SwissProt_HUMAN$	2::Q9Y3C6|PPIL1_HUMAN	41	18225	3	3	2	2	0.67	Peptidyl-prolyl cis-trar
2082	1	SwissProt_HUMAN$	2::Q9BWS9|CHID1_HUMAN	41	44912	1	1	1	1	0.11	Chitinase domain-cont
2083	1	SwissProt_HUMAN$	2::Q14118|DAG1_HUMAN	41	97381	1	1	1	1	0.05	Dystroglycan OS=Hon
2084	1	SwissProt_HUMAN$	2::Q9UNH7|SNX6_HUMAN	41	46620	2	2	2	2	0.22	Sorting nexin-6 OS=H
2085	1	SwissProt_HUMAN$	2::Q15050|RRS1_HUMAN	41	41168	5	3	4	2	0.26	Ribosome biogenesis r
2086	1	SwissProt_HUMAN$	2::Q8TBQ9|KISHA_HUMAN	41	8054	1	1	1	1	0.76	Protein kish-A OS=Ho
2087	1	SwissProt_HUMAN$	2::Q5ZPR3|CD276_HUMAN	41	57199	1	1	1	1	0.09	CD276 antigen OS=Ho
2088	1	SwissProt_HUMAN$	2::P13473|LAMP2_HUMAN	41	44932	1	1	1	1	0.11	Lysosome-associated
2089	1	SwissProt_HUMAN$	2::Q96EK5|KBP_HUMAN	41	71768	3	3	3	3	0.22	KIF-binding protein OS
2090	1	SwissProt_HUMAN$	2::Q9BTV4|TMM43_HUMAN	41	44847	2	1	2	1	0.11	Transmembrane prote
2091	1	SwissProt_HUMAN$	2::O94804|STK10_HUMAN	41	112065	1	1	1	1	0.04	Serine/threonine-prot
2092	1	SwissProt_HUMAN$	2::O43719|HTSF1_HUMAN	41	85801	4	4	4	4	0.25	HIV Tat-specific factor
2093	1	SwissProt_HUMAN$	2::Q8WUY1|THEM6_HUMAN	41	23850	2	2	2	2	0.48	Protein THEM6 OS=Ho
2094	1	SwissProt_HUMAN$	2::Q9NW82|WDR70_HUMAN	41	73155	1	1	1	1	0.07	WD repeat-containing
2095	1	SwissProt_HUMAN$	2::Q5VT66|MARC1_HUMAN	41	37476	2	2	2	2	0.29	Mitochondrial amidoxi

↑Family	M	DB	Accession	Score	Mass	Matches	Match(sig)	Sequences	Seq(sig)	emPAI	Description
2096	1	SwissProt_HUMAN$	2::Q9BTC0|DIDO1_HUMAN	41	243723	1	1	1	1	0.02	Death-inducer oblitera
2097	1	SwissProt_HUMAN$	2::Q96T60|PNKP_HUMAN	41	57040	4	4	4	4	0.39	Bifunctional polynucle
2098	1	SwissProt_HUMAN$	2::Q9NPD3|EXOS4_HUMAN	40	26366	2	2	2	2	0.43	Exosome complex con
2099	1	SwissProt_HUMAN$	2::P49643|PRI2_HUMAN	40	58769	2	1	2	1	0.08	DNA primase large sul
2100	1	SwissProt_HUMAN$	2::P18858|DNLI1_HUMAN	40	101673	3	2	3	2	0.10	DNA ligase 1 OS=Hon
2101	1	SwissProt_HUMAN$	2::Q15126|PMVK_HUMAN	40	21981	1	1	1	1	0.24	Phosphomevalonate k
2102	1	SwissProt_HUMAN$	2::O15405|TOX3_HUMAN	40	63302	2	1	2	1	0.08	TOX high mobility gro
2103	1	SwissProt_HUMAN$	2::O00483|NDUA4_HUMAN	40	9364	1	1	1	1	0.64	Cytochrome c oxidase
2104	1	SwissProt_HUMAN$	2::P25098|ARBK1_HUMAN	40	79522	3	3	3	3	0.20	Beta-adrenergic recep
2105	1	SwissProt_HUMAN$	2::Q9NZJ7|MTCH1_HUMAN	40	41517	3	2	3	2	0.26	Mitochondrial carrier h
2106	1	SwissProt_HUMAN$	2::Q8WTX7|CAST1_HUMAN	40	36253	2	2	1	1	0.14	Cytosolic arginine sen
2107	1	SwissProt_HUMAN$	2::O43760|SNG2_HUMAN	40	24794	2	2	2	2	0.46	Synaptogyrin-2 OS=H
2108	1	SwissProt_HUMAN$	2::Q9Y6N9|USH1C_HUMAN	40	62172	1	1	1	1	0.08	Harmonin OS=Homo s
2109	1	SwissProt_HUMAN$	2::Q86SS6|SYT9_HUMAN	40	56153	2	2	2	2	0.18	Synaptotagmin-9 OS=
2110	1	SwissProt_HUMAN$	2::Q86X83|COMD2_HUMAN	40	22731	2	2	2	2	0.51	COMM domain-contair
2111	1	SwissProt_HUMAN$	2::Q9UHA3|RLP24_HUMAN	40	19608	2	1	2	1	0.27	Probable ribosome bio
2112	1	SwissProt_HUMAN$	2::Q6P9B9|INT5_HUMAN	40	107927	1	1	1	1	0.04	Integrator complex su
2113	1	SwissProt_HUMAN$	2::Q7L5N1|CSN6_HUMAN	40	36140	2	2	2	2	0.30	COP9 signalosome cor
2114	1	SwissProt_HUMAN$	2::P54687|BCAT1_HUMAN	40	42939	1	1	1	1	0.12	Branched-chain-aminc
2115	1	SwissProt_HUMAN$	2::P46108|CRK_HUMAN	40	33810	2	2	2	2	0.32	Adapter molecule crk
2116	1	SwissProt_HUMAN$	2::Q9UK61|TASOR_HUMAN	40	188914	2	1	2	1	0.03	Protein TASOR OS=Hc
2117	1	SwissProt_HUMAN$	2::P29350|PTN6_HUMAN	40	67519	1	1	1	1	0.07	Tyrosine-protein phos
2118	1	SwissProt_HUMAN$	2::Q9NP77|SSU72_HUMAN	40	22560	1	1	1	1	0.23	RNA polymerase II su
2119	1	SwissProt_HUMAN$	2::Q96DB5|RMD1_HUMAN	39	35785	2	2	2	2	0.30	Regulator of microtubı
2120	1	SwissProt_HUMAN$	2::P14209|CD99_HUMAN	39	18836	1	1	1	1	0.28	CD99 antigen OS=Hoı
2121	1	SwissProt_HUMAN$	2::Q9NR28|DBLOH_HUMAN	39	27114	2	1	1	1	0.19	Diablo homolog, mitoc
2122	1	SwissProt_HUMAN$	2::Q9H9S4|CB39L_HUMAN	39	39063	5	5	5	5	0.83	Calcium-binding prote
2123	1	SwissProt_HUMAN$	2::Q8TB61|S35B2_HUMAN	39	47484	2	1	2	1	0.10	Adenosine 3'-phospho
2124	1	SwissProt_HUMAN$	2::P62633|CNBP_HUMAN	39	19450	2	1	2	1	0.27	Cellular nucleic acid-b
2125	1	SwissProt_HUMAN$	2::O95400|CD2B2_HUMAN	39	37623	1	1	1	1	0.13	CD2 antigen cytoplasr
2126	1	SwissProt_HUMAN$	2::Q8TBX8|PI42C_HUMAN	39	47270	3	2	3	2	0.22	Phosphatidylinositol 5-
2127	1	SwissProt_HUMAN$	2::O14562|UBFD1_HUMAN	39	33361	1	1	1	1	0.15	Ubiquitin domain-cont
2128	1	SwissProt_HUMAN$	2::Q9BXJ8|TACAN_HUMAN	39	40584	1	1	1	1	0.12	Ion channel TACAN OS
2129	1	SwissProt_HUMAN$	2::Q9H9Q2|CSN7B_HUMAN	39	29603	1	1	1	1	0.17	COP9 signalosome cor
2130	1	SwissProt_HUMAN$	2::Q96IU4|ABHEB_HUMAN	39	22332	3	2	2	2	0.52	Protein ABHD14B OS=
2131	1	SwissProt_HUMAN$	2::Q8NCN5|PDPR_HUMAN	39	99301	4	4	4	4	0.21	Pyruvate dehydrogena
2132	1	SwissProt_HUMAN$	2::Q2T9J0|TYSD1_HUMAN	39	59271	1	1	1	1	0.08	Peroxisomal leader pe
2133	1	SwissProt_HUMAN$	2::Q9NVV4|PAPD1_HUMAN	39	66130	2	2	2	2	0.15	Poly(A) RNA polymera
2134	1	SwissProt_HUMAN$	2::O43598|DNPH1_HUMAN	39	19097	2	2	2	2	0.63	2'-deoxynucleoside 5'-
2135	1	SwissProt_HUMAN$	2::Q9BXY0|MAK16_HUMAN	39	35347	2	1	2	1	0.14	Protein MAK16 homolo

↑Family	M	DB	Accession	Score	Mass	Matches	Match(sig)	Sequences	Seq(sig)	emPAI	Description
2136	1	SwissProt_HUMAN$	2::A0A0B4J2D5|GAL3B_HUMAN	39	28125	2	1	2	1	0.18	Glutamine amidotrans
2137	1	SwissProt_HUMAN$	2::Q9NQP4|PFD4_HUMAN	39	15305	3	2	2	1	0.36	Prefoldin subunit 4 OS
2138	1	SwissProt_HUMAN$	2::Q9Y4Z0|LSM4_HUMAN	39	15340	1	1	1	1	0.36	U6 snRNA-associated
2139	1	SwissProt_HUMAN$	2::Q9UNE7|CHIP_HUMAN	39	34834	2	2	1	1	0.14	E3 ubiquitin-protein li
2140	1	SwissProt_HUMAN$	2::Q5BJF2|SGMR2_HUMAN	39	20834	3	3	3	3	0.96	Sigma intracellular rec
2141	1	SwissProt_HUMAN$	2::A8MXV4|NUD19_HUMAN	38	42207	2	2	2	2	0.25	Nucleoside diphosphat
2142	1	SwissProt_HUMAN$	2::Q86X76|NIT1_HUMAN	38	35873	3	2	3	2	0.30	Deaminated glutathior
2143	1	SwissProt_HUMAN$	2::Q9BSH4|TACO1_HUMAN	38	32457	2	2	2	2	0.34	Translational activator
2144	1	SwissProt_HUMAN$	2::O15066|KIF3B_HUMAN	38	85073	2	2	2	2	0.12	Kinesin-like protein KI
2145	1	SwissProt_HUMAN$	2::Q01780|EXOSX_HUMAN	38	100768	4	4	4	4	0.21	Exosome component :
2146	1	SwissProt_HUMAN$	2::Q15370|ELOB_HUMAN	38	13125	1	1	1	1	0.43	Elongin-B OS=Homo s
2147	1	SwissProt_HUMAN$	2::Q9Y3I1|FBX7_HUMAN	38	58466	2	2	1	1	0.08	F-box only protein 7 C
2148	1	SwissProt_HUMAN$	2::O60645|EXOC3_HUMAN	38	85513	1	1	1	1	0.06	Exocyst complex comp
2149	1	SwissProt_HUMAN$	2::P53801|PTTG_HUMAN	38	20310	2	2	2	2	0.59	Pituitary tumor-transf
2150	1	SwissProt_HUMAN$	2::Q658Y4|F91A1_HUMAN	38	93850	1	1	1	1	0.05	Protein FAM91A1 OS=
2151	1	SwissProt_HUMAN$	2::Q99700|ATX2_HUMAN	38	140196	1	1	1	1	0.03	Ataxin-2 OS=Homo sa
2152	1	SwissProt_HUMAN$	2::Q96A26|F162A_HUMAN	38	17331	2	2	2	2	0.72	Protein FAM162A OS=
2153	1	SwissProt_HUMAN$	2::O15226|NKRF_HUMAN	38	77624	5	3	5	3	0.20	NF-kappa-B-repressin
2154	1	SwissProt_HUMAN$	2::Q8WTW3|COG1_HUMAN	38	108909	2	2	2	2	0.09	Conserved oligomeric
2155	1	SwissProt_HUMAN$	2::P01130|LDLR_HUMAN	38	95314	1	1	1	1	0.05	Low-density lipoprotei
2156	1	SwissProt_HUMAN$	2::Q9NY12|GAR1_HUMAN	38	22334	4	3	4	3	0.88	H/ACA ribonucleoprote
2157	1	SwissProt_HUMAN$	2::Q05682|CALD1_HUMAN	38	93175	2	1	2	1	0.05	Caldesmon OS=Homo
2158	1	SwissProt_HUMAN$	2::Q9NYY8|FAKD2_HUMAN	38	81410	2	2	2	2	0.12	FAST kinase domain-c
2159	1	SwissProt_HUMAN$	2::Q96TA2|YMEL1_HUMAN	38	86401	2	1	2	1	0.06	ATP-dependent zinc m
2160	1	SwissProt_HUMAN$	2::Q9Y399|RT02_HUMAN	38	33228	2	2	2	2	0.33	28S ribosomal protein
2161	1	SwissProt_HUMAN$	2::P02647|APOA1_HUMAN	38	30759	1	1	1	1	0.17	Apolipoprotein A-I OS
2162	1	SwissProt_HUMAN$	2::Q9UID3|VPS51_HUMAN	37	85988	4	4	4	4	0.25	Vacuolar protein sortir
2163	1	SwissProt_HUMAN$	2::Q9H4H8|FA83D_HUMAN	37	64384	1	1	1	1	0.08	Protein FAM83D OS=H
2164	1	SwissProt_HUMAN$	2::O75431|MTX2_HUMAN	37	29744	2	2	2	2	0.37	Metaxin-2 OS=Homo :
2165	1	SwissProt_HUMAN$	2::O00443|P3C2A_HUMAN	37	190559	4	3	4	3	0.08	Phosphatidylinositol 4-
2166	1	SwissProt_HUMAN$	2::P50747|BPL1_HUMAN	37	80709	2	2	2	2	0.12	Biotin--protein ligase (
2167	1	SwissProt_HUMAN$	2::P11182|ODB2_HUMAN	37	53453	2	2	2	2	0.19	Lipoamide acyltransfe
2168	1	SwissProt_HUMAN$	2::Q9Y5I4|PCDC2_HUMAN	37	109383	1	1	1	1	0.04	Protocadherin alpha-C
2169	1	SwissProt_HUMAN$	2::P82932|RT06_HUMAN	37	14218	1	1	1	1	0.39	28S ribosomal protein
2170	1	SwissProt_HUMAN$	2::O75167|PHAR2_HUMAN	37	69658	1	1	1	1	0.07	Phosphatase and actir
2171	1	SwissProt_HUMAN$	2::P23229|ITA6_HUMAN	37	126526	1	1	1	1	0.04	Integrin alpha-6 OS=I
2172	1	SwissProt_HUMAN$	2::Q99614|TTC1_HUMAN	37	33505	3	3	3	3	0.52	Tetratricopeptide repe
2173	1	SwissProt_HUMAN$	2::Q96NC0|ZMAT2_HUMAN	37	23597	1	1	1	1	0.22	Zinc finger matrin-typ
2174	1	SwissProt_HUMAN$	2::Q15345|LRC41_HUMAN	37	88594	1	1	1	1	0.05	Leucine-rich repeat-cc
2175	1	SwissProt_HUMAN$	2::Q9H2J4|PDCL3_HUMAN	37	27597	1	1	1	1	0.19	Phosducin-like protein

↑Family	M	DB	Accession	Score	Mass	Matches	Match(sig)	Sequences	Seq(sig)	emPAI	Description
2176	1	SwissProt_HUMAN$	2::Q9Y287|ITM2B_HUMAN	37	30318	1	1	1	1	0.17	Integral membrane pr
2177	1	SwissProt_HUMAN$	2::P10644|KAP0_HUMAN	37	42955	3	2	3	2	0.25	cAMP-dependent prote
2178	1	SwissProt_HUMAN$	2::O00566|MPP10_HUMAN	37	78816	3	2	3	2	0.13	U3 small nucleolar rib
2179	1	SwissProt_HUMAN$	2::Q14451|GRB7_HUMAN	37	59643	3	2	3	2	0.17	Growth factor recepto
2180	1	SwissProt_HUMAN$	2::Q9P2D3|HTR5B_HUMAN	37	224159	1	1	1	1	0.02	HEAT repeat-containir
2181	1	SwissProt_HUMAN$	2::P42677|RS27_HUMAN	37	9455	5	3	4	2	3.33	40S ribosomal protein
2182	1	SwissProt_HUMAN$	2::Q9UNS1|TIM_HUMAN	37	138572	1	1	1	1	0.03	Protein timeless homo
2183	1	SwissProt_HUMAN$	2::Q9UBS4|DJB11_HUMAN	37	40489	4	3	4	3	0.42	DnaJ homolog subfam
2184	1	SwissProt_HUMAN$	2::O60942|MCE1_HUMAN	37	68513	2	2	2	2	0.15	mRNA-capping enzym
2185	1	SwissProt_HUMAN$	2::Q9NUQ3|TXLNG_HUMAN	37	60548	3	2	3	2	0.17	Gamma-taxilin OS=Hc
2186	1	SwissProt_HUMAN$	2::Q9Y2K7|KDM2A_HUMAN	37	132708	3	3	3	3	0.11	Lysine-specific demetl
2187	1	SwissProt_HUMAN$	2::Q9H008|LHPP_HUMAN	37	29147	1	1	1	1	0.17	Phospholysine phosph
2188	1	SwissProt_HUMAN$	2::Q96A35|RM24_HUMAN	37	24899	1	1	1	1	0.21	39S ribosomal protein
2189	1	SwissProt_HUMAN$	2::Q96J02|ITCH_HUMAN	37	102738	3	2	3	2	0.10	E3 ubiquitin-protein li
2190	1	SwissProt_HUMAN$	2::Q9BX66|SRBS1_HUMAN	37	142426	3	3	3	3	0.11	Sorbin and SH3 doma
2191	1	SwissProt_HUMAN$	2::P49770|EI2BB_HUMAN	37	38965	3	1	3	1	0.13	Translation initiation f
2192	1	SwissProt_HUMAN$	2::Q9UKR5|ERG28_HUMAN	37	15854	2	2	2	2	0.80	Ergosterol biosyntheti
2193	1	SwissProt_HUMAN$	2::Q8IVD9|NUDC3_HUMAN	37	40797	1	1	1	1	0.12	NudC domain-containi
2194	1	SwissProt_HUMAN$	2::Q92974|ARHG2_HUMAN	37	111473	3	3	3	3	0.14	Rho guanine nucleotid
2195	1	SwissProt_HUMAN$	2::Q9NTZ6|RBM12_HUMAN	37	97333	2	2	2	2	0.10	RNA-binding protein 1
2196	1	SwissProt_HUMAN$	2::Q9UKX7|NUP50_HUMAN	37	50113	1	1	1	1	0.10	Nuclear pore complex
2197	1	SwissProt_HUMAN$	2::Q5VZE5|NAA35_HUMAN	37	83584	1	1	1	1	0.06	N-alpha-acetyltransfe
2198	1	SwissProt_HUMAN$	2::P21283|VATC1_HUMAN	37	43914	3	3	3	3	0.38	V-type proton ATPase
2199	1	SwissProt_HUMAN$	2::Q13472|TOP3A_HUMAN	36	112300	1	1	1	1	0.04	DNA topoisomerase 3-
2200	1	SwissProt_HUMAN$	2::O43752|STX6_HUMAN	36	29158	1	1	1	1	0.17	Syntaxin-6 OS=Homo
2201	1	SwissProt_HUMAN$	2::O75940|SPF30_HUMAN	36	26694	2	2	2	2	0.42	Survival of motor neu
2202	1	SwissProt_HUMAN$	2::Q9UHI6|DDX20_HUMAN	36	92182	2	2	2	2	0.11	Probable ATP-depende
2203	1	SwissProt_HUMAN$	2::Q9UBI6|GBG12_HUMAN	36	8001	1	1	1	1	0.77	Guanine nucleotide-bi
2204	1	SwissProt_HUMAN$	2::O43824|GTPB6_HUMAN	36	56862	1	1	1	1	0.09	Putative GTP-binding
2205	1	SwissProt_HUMAN$	2::Q9H2H9|S38A1_HUMAN	36	54012	4	3	3	2	0.19	Sodium-coupled neutr
2206	1	SwissProt_HUMAN$	2::Q9NR56|MBNL1_HUMAN	36	41790	2	1	2	1	0.12	Muscleblind-like prote
2207	1	SwissProt_HUMAN$	2::Q96AT9|RPE_HUMAN	36	24911	1	1	1	1	0.21	Ribulose-phosphate 3-
2208	1	SwissProt_HUMAN$	2::Q5M9N0|CD158_HUMAN	36	127062	1	1	1	1	0.04	Coiled-coil domain-co
2209	1	SwissProt_HUMAN$	2::P07602|SAP_HUMAN	36	58074	1	1	1	1	0.08	Prosaposin OS=Homo
2210	1	SwissProt_HUMAN$	2::Q15363|TMED2_HUMAN	36	22746	1	1	1	1	0.23	Transmembrane emp2
2211	1	SwissProt_HUMAN$	2::Q9UH99|SUN2_HUMAN	36	80262	1	1	1	1	0.06	SUN domain-containir
2212	1	SwissProt_HUMAN$	2::Q92947|GCDH_HUMAN	36	48096	1	1	1	1	0.10	Glutaryl-CoA dehydrog
2213	1	SwissProt_HUMAN$	2::Q9UBB5|MBD2_HUMAN	36	43228	1	1	1	1	0.12	Methyl-CpG-binding d
2214	1	SwissProt_HUMAN$	2::O75027|ABCB7_HUMAN	36	82589	1	1	1	1	0.06	ATP-binding cassette s
2215	1	SwissProt_HUMAN$	2::Q14197|ICT1_HUMAN	36	23615	1	1	1	1	0.22	Peptidyl-tRNA hydrola

↑Family	M	DB	Accession	Score	Mass	Matches	Match(sig)	Sequences	Seq(sig)	emPAI	Description
2216	1	SwissProt_HUMAN$	2::Q8IUR0|TPPC5_HUMAN	36	20770	1	1	1	1	0.25	Trafficking protein par
2217	1	SwissProt_HUMAN$	2::Q9HDC9|APMAP_HUMAN	36	46451	3	2	3	2	0.23	Adipocyte plasma mer
2218	1	SwissProt_HUMAN$	2::Q9Y312|AAR2_HUMAN	36	43444	2	2	2	2	0.24	Protein AAR2 homolog
2219	1	SwissProt_HUMAN$	2::Q86TU7|SETD3_HUMAN	36	67215	2	2	2	2	0.15	Actin-histidine N-meth
2220	1	SwissProt_HUMAN$	2::Q15291|RBBP5_HUMAN	36	59116	1	1	1	1	0.08	Retinoblastoma-bindir
2221	1	SwissProt_HUMAN$	2::O60934|NBN_HUMAN	36	84906	2	2	2	2	0.12	Nibrin OS=Homo sapi
2222	1	SwissProt_HUMAN$	2::Q14657|LAGE3_HUMAN	36	14795	1	1	1	1	0.37	EKC/KEOPS complex s
2223	1	SwissProt_HUMAN$	2::P56182|RRP1_HUMAN	36	52807	3	3	3	3	0.31	Ribosomal RNA proces
2224	1	SwissProt_HUMAN$	2::O15118|NPC1_HUMAN	36	142074	1	1	1	1	0.03	NPC intracellular chole
2225	1	SwissProt_HUMAN$	2::Q9Y2Q9|RT28_HUMAN	36	20830	1	1	1	1	0.25	28S ribosomal protein
2226	1	SwissProt_HUMAN$	2::P37198|NUP62_HUMAN	36	53222	1	1	1	1	0.09	Nuclear pore glycopro
2227	1	SwissProt_HUMAN$	2::O43395|PRPF3_HUMAN	36	77481	4	4	4	4	0.28	U4/U6 small nuclear r
2228	1	SwissProt_HUMAN$	2::Q16890|TPD53_HUMAN	36	22435	1	1	1	1	0.23	Tumor protein D53 OS
2229	1	SwissProt_HUMAN$	2::A6H8Y1|BDP1_HUMAN	36	293705	1	1	1	1	0.02	Transcription factor TF
2230	1	SwissProt_HUMAN$	2::Q96D05|F241B_HUMAN	36	13230	1	1	1	1	0.42	Protein FAM241B OS=
2231	1	SwissProt_HUMAN$	2::Q8NFJ5|RAI3_HUMAN	36	40225	1	1	1	1	0.12	Retinoic acid-induced
2232	1	SwissProt_HUMAN$	2::Q9BTU6|P4K2A_HUMAN	36	53989	1	1	1	1	0.09	Phosphatidylinositol 4
2233	1	SwissProt_HUMAN$	2::Q9NWB6|ARGL1_HUMAN	36	33197	2	1	2	1	0.15	Arginine and glutamat
2234	1	SwissProt_HUMAN$	2::Q13217|DNJC3_HUMAN	36	57544	3	3	3	3	0.28	DnaJ homolog subfam
2235	1	SwissProt_HUMAN$	2::P46937|YAP1_HUMAN	36	54427	1	1	1	1	0.09	Transcriptional coactiv
2236	1	SwissProt_HUMAN$	2::P51553|IDH3G_HUMAN	36	42767	2	2	2	2	0.25	Isocitrate dehydrogen
2237	1	SwissProt_HUMAN$	2::Q9Y2R0|COA3_HUMAN	36	11724	1	1	1	1	0.48	Cytochrome c oxidase
2238	1	SwissProt_HUMAN$	2::Q01130|SRSF2_HUMAN	36	25461	4	3	3	3	0.74	Serine/arginine-rich sp
2239	1	SwissProt_HUMAN$	2::Q7L0Y3|TM10C_HUMAN	35	47317	3	2	3	2	0.22	tRNA methyltransferas
2240	1	SwissProt_HUMAN$	2::Q9HCP0|KC1G1_HUMAN	35	48481	1	1	1	1	0.10	Casein kinase I isoforr
2241	1	SwissProt_HUMAN$	2::Q16775|GLO2_HUMAN	35	33784	3	2	3	2	0.32	Hydroxyacylglutathion
2242	1	SwissProt_HUMAN$	2::O75792|RNH2A_HUMAN	35	33374	3	2	3	2	0.33	Ribonuclease H2 subu
2243	1	SwissProt_HUMAN$	2::Q9BRX5|PSF3_HUMAN	35	24519	1	1	1	1	0.21	DNA replication compl
2244	1	SwissProt_HUMAN$	2::Q9BYD2|RM09_HUMAN	35	30224	2	1	2	1	0.17	39S ribosomal protein
2245	1	SwissProt_HUMAN$	2::Q6ZMG9|CERS6_HUMAN	35	44861	1	1	1	1	0.11	Ceramide synthase 6
2246	1	SwissProt_HUMAN$	2::Q02338|BDH_HUMAN	35	38132	1	1	1	1	0.13	D-beta-hydroxybutyra
2247	1	SwissProt_HUMAN$	2::Q8TC12|RDH11_HUMAN	35	35363	2	2	2	2	0.31	Retinol dehydrogenase
2248	1	SwissProt_HUMAN$	2::P05166|PCCB_HUMAN	35	58179	1	1	1	1	0.08	Propionyl-CoA carboxy
2249	1	SwissProt_HUMAN$	2::O96011|PX11B_HUMAN	35	28413	1	1	1	1	0.18	Peroxisomal membrar
2250	1	SwissProt_HUMAN$	2::Q9Y324|FCF1_HUMAN	35	23354	1	1	1	1	0.22	rRNA-processing prote
2251	1	SwissProt_HUMAN$	2::Q9UPU5|UBP24_HUMAN	35	294178	2	1	1	1	0.02	Ubiquitin carboxyl-ter
2252	1	SwissProt_HUMAN$	2::Q9BW92|SYTM_HUMAN	35	80985	3	3	3	3	0.19	Threonine--tRNA ligas
2253	1	SwissProt_HUMAN$	2::Q8N2K0|ABD12_HUMAN	35	45068	1	1	1	1	0.11	Lysophosphatidylserin
2254	1	SwissProt_HUMAN$	2::Q8NE86|MCU_HUMAN	35	39842	2	2	2	2	0.27	Calcium uniporter prot
2255	1	SwissProt_HUMAN$	2::Q8N668|COMD1_HUMAN	35	21165	1	1	1	1	0.25	COMM domain-contair

↑Family	M	DB	Accession	Score	Mass	Matches	Match(sig)	Sequences	Seq(sig)	emPAI	Description
2256	1	SwissProt_HUMAN$	2::Q9P2W9|STX18_HUMAN	35	38650	1	1	1	1	0.13	Syntaxin-18 OS=Hom
2257	1	SwissProt_HUMAN$	2::P05067|A4_HUMAN	35	86888	2	2	2	2	0.12	Amyloid-beta precurs
2258	1	SwissProt_HUMAN$	2::P84090|ERH_HUMAN	35	12251	2	2	2	2	1.14	Enhancer of rudiment
2259	1	SwissProt_HUMAN$	2::A6NDB9|PALM3_HUMAN	35	71652	3	2	3	2	0.14	Paralemmin-3 OS=Ho
2260	1	SwissProt_HUMAN$	2::Q2TAL8|QRIC1_HUMAN	35	86382	1	1	1	1	0.06	Glutamine-rich protei
2261	1	SwissProt_HUMAN$	2::Q96FV9|THOC1_HUMAN	35	75619	2	2	2	2	0.13	THO complex subunit
2262	1	SwissProt_HUMAN$	2::Q92504|S39A7_HUMAN	35	50087	1	1	1	1	0.10	Zinc transporter SLC3
2263	1	SwissProt_HUMAN$	2::Q9BY42|RTF2_HUMAN	35	33865	2	2	2	2	0.32	Replication terminatio
2264	1	SwissProt_HUMAN$	2::Q7L8L6|FAKD5_HUMAN	35	86519	3	1	3	1	0.06	FAST kinase domain-c
2265	1	SwissProt_HUMAN$	2::Q9BYD3|RM04_HUMAN	35	34897	2	1	2	1	0.14	39S ribosomal protein
2266	1	SwissProt_HUMAN$	2::Q8NBF2|NHLC2_HUMAN	35	79393	6	3	5	3	0.20	NHL repeat-containing
2267	1	SwissProt_HUMAN$	2::Q8N5N7|RM50_HUMAN	35	18313	1	1	1	1	0.29	39S ribosomal protein
2268	1	SwissProt_HUMAN$	2::Q9NVH2|INT7_HUMAN	35	106766	1	1	1	1	0.05	Integrator complex su
2269	1	SwissProt_HUMAN$	2::Q9H0W8|SMG9_HUMAN	35	57614	1	1	1	1	0.09	Protein SMG9 OS=Ho
2270	1	SwissProt_HUMAN$	2::Q9H0A8|COMD4_HUMAN	35	21750	3	2	3	2	0.54	COMM domain-contai
2271	1	SwissProt_HUMAN$	2::Q12768|WASC5_HUMAN	35	134201	1	1	1	1	0.04	WASH complex subun
2272	1	SwissProt_HUMAN$	2::Q8NFF5|FAD1_HUMAN	35	65224	1	1	1	1	0.08	FAD synthase OS=Ho
2273	1	SwissProt_HUMAN$	2::P35625|TIMP3_HUMAN	35	24129	2	2	2	2	0.48	Metalloproteinase inhi
2274	1	SwissProt_HUMAN$	2::O75381|PEX14_HUMAN	35	41212	2	2	2	2	0.26	Peroxisomal membrar
2275	1	SwissProt_HUMAN$	2::Q9BTE6|AASD1_HUMAN	35	45451	1	1	1	1	0.11	Alanyl-tRNA editing pr
2276	1	SwissProt_HUMAN$	2::Q9NRF9|DPOE3_HUMAN	35	16849	1	1	1	1	0.32	DNA polymerase epsil
2277	1	SwissProt_HUMAN$	2::P61927|RL37_HUMAN	35	11071	2	2	1	1	0.52	60S ribosomal protein
2278	1	SwissProt_HUMAN$	2::P21741|MK_HUMAN	34	15575	1	1	1	1	0.35	Midkine OS=Homo sa
2279	1	SwissProt_HUMAN$	2::O94903|PLPHP_HUMAN	34	30325	4	4	4	4	0.86	Pyridoxal phosphate h
2280	1	SwissProt_HUMAN$	2::P08697|A2AP_HUMAN	34	54531	1	1	1	1	0.09	Alpha-2-antiplasmin C
2281	1	SwissProt_HUMAN$	2::Q13158|FADD_HUMAN	34	23265	1	1	1	1	0.22	FAS-associated death
2282	1	SwissProt_HUMAN$	2::Q96CP7|TLCD1_HUMAN	34	28529	1	1	1	1	0.18	TLC domain-containin
2283	1	SwissProt_HUMAN$	2::P61962|DCAF7_HUMAN	34	38901	1	1	1	1	0.13	DDB1- and CUL4-asso
2284	1	SwissProt_HUMAN$	2::Q9HA64|KT3K_HUMAN	34	34390	4	3	3	2	0.32	Ketosamine-3-kinase
2285	1	SwissProt_HUMAN$	2::Q4KWH8|PLCH1_HUMAN	34	189104	1	1	1	1	0.03	1-phosphatidylinositol
2286	1	SwissProt_HUMAN$	2::Q93099|HGD_HUMAN	34	49932	4	2	2	2	0.21	Homogentisate 1,2-di
2287	1	SwissProt_HUMAN$	2::Q9ULR0|ISY1_HUMAN	34	32972	1	1	1	1	0.15	Pre-mRNA-splicing fac
2288	1	SwissProt_HUMAN$	2::O94919|ENDD1_HUMAN	34	54981	4	3	3	2	0.19	Endonuclease domain-
2289	1	SwissProt_HUMAN$	2::P62861|RS30_HUMAN	34	6644	2	2	2	2	2.92	40S ribosomal protein
2290	1	SwissProt_HUMAN$	2::Q8N6M5|ALLC_HUMAN	34	43531	1	1	1	1	0.11	Probable allantoicase
2291	1	SwissProt_HUMAN$	2::P49750|YLPM1_HUMAN	34	241495	3	3	3	3	0.06	YLP motif-containing p
2292	1	SwissProt_HUMAN$	2::O15027|SC16A_HUMAN	34	251738	1	1	1	1	0.02	Protein transport prot
2293	1	SwissProt_HUMAN$	2::Q04941|PLP2_HUMAN	34	16680	2	1	2	1	0.32	Proteolipid protein 2 C
2294	1	SwissProt_HUMAN$	2::Q13418|ILK_HUMAN	34	51386	1	1	1	1	0.10	Integrin-linked protei
2295	1	SwissProt_HUMAN$	2::Q7Z6R9|AP2D_HUMAN	34	49547	1	1	1	1	0.10	Transcription factor AI

↑Family	M	DB	Accession	Score	Mass	Matches	Match(sig)	Sequences	Seq(sig)	emPAI	Description
2296	1	SwissProt_HUMAN$	2::Q9BVG4|PBDC1_HUMAN	34	26041	1	1	1	1	0.20	Protein PBDC1 OS=Hc
2297	1	SwissProt_HUMAN$	2::P49356|FNTB_HUMAN	34	48742	2	2	2	2	0.21	Protein farnesyltransfe
2298	1	SwissProt_HUMAN$	2::P57105|SYJ2B_HUMAN	34	15918	1	1	1	1	0.34	Synaptojanin-2-bindin
2299	1	SwissProt_HUMAN$	2::Q13445|TMED1_HUMAN	34	25190	2	2	2	2	0.45	Transmembrane emp2
2300	1	SwissProt_HUMAN$	2::O43159|RRP8_HUMAN	34	50683	1	1	1	1	0.10	Ribosomal RNA-proces
2301	1	SwissProt_HUMAN$	2::Q6NXR4|TTI2_HUMAN	34	56879	2	2	2	2	0.18	TELO2-interacting pro
2302	1	SwissProt_HUMAN$	2::Q96HW7|INT4_HUMAN	34	108102	1	1	1	1	0.04	Integrator complex su
2303	1	SwissProt_HUMAN$	2::Q9NRK6|ABCBA_HUMAN	34	79098	1	1	1	1	0.06	ATP-binding cassette s
2304	1	SwissProt_HUMAN$	2::Q8IX01|SUGP2_HUMAN	34	120132	4	2	4	2	0.08	SURP and G-patch dor
2305	1	SwissProt_HUMAN$	2::O14493|CLD4_HUMAN	33	22062	2	2	1	1	0.24	Claudin-4 OS=Homo s
2306	1	SwissProt_HUMAN$	2::Q96R06|SPAG5_HUMAN	33	134338	1	1	1	1	0.04	Sperm-associated ant
2307	1	SwissProt_HUMAN$	2::Q9BQ75|CMS1_HUMAN	33	31864	1	1	1	1	0.16	Protein CMSS1 OS=Hc
2308	1	SwissProt_HUMAN$	2::Q6BDS2|URFB1_HUMAN	33	159385	2	1	2	1	0.03	UHRF1-binding proteir
2309	1	SwissProt_HUMAN$	2::Q6ZRP7|QSOX2_HUMAN	33	77480	1	1	1	1	0.06	Sulfhydryl oxidase 2 C
2310	1	SwissProt_HUMAN$	2::Q8N7H5|PAF1_HUMAN	33	59939	2	1	2	1	0.08	RNA polymerase II-as
2311	1	SwissProt_HUMAN$	2::Q9H0E2|TOLIP_HUMAN	33	30262	1	1	1	1	0.17	Toll-interacting protei
2312	1	SwissProt_HUMAN$	2::P06756|ITAV_HUMAN	33	115964	3	2	3	2	0.09	Integrin alpha-V OS=I
2313	1	SwissProt_HUMAN$	2::Q07817|B2CL1_HUMAN	33	26033	1	1	1	1	0.20	Bcl-2-like protein 1 OS
2314	1	SwissProt_HUMAN$	2::Q9UBB9|TFP11_HUMAN	33	96758	3	1	3	1	0.05	Tuftelin-interacting pr
2315	1	SwissProt_HUMAN$	2::Q9NWS0|PIHD1_HUMAN	33	32342	2	2	2	2	0.34	PIH1 domain-containi
2316	1	SwissProt_HUMAN$	2::Q9HAB8|PPCS_HUMAN	33	33984	4	4	4	4	0.74	Phosphopantothenate-
2317	1	SwissProt_HUMAN$	2::Q96SU4|OSBL9_HUMAN	33	83132	2	2	2	2	0.12	Oxysterol-binding prot
2318	1	SwissProt_HUMAN$	2::Q14146|URB2_HUMAN	33	170435	1	1	1	1	0.03	Unhealthy ribosome b
2319	1	SwissProt_HUMAN$	2::Q92643|GPI8_HUMAN	33	45223	2	2	2	2	0.23	GPI-anchor transamid
2320	1	SwissProt_HUMAN$	2::P45985|MP2K4_HUMAN	33	44259	1	1	1	1	0.11	Dual specificity mitoge
2321	1	SwissProt_HUMAN$	2::O60287|NPA1P_HUMAN	33	254227	2	2	2	2	0.04	Nucleolar pre-ribosom
2322	1	SwissProt_HUMAN$	2::O75352|MPU1_HUMAN	33	26620	2	2	1	1	0.19	Mannose-P-dolichol ut
2323	1	SwissProt_HUMAN$	2::Q13042|CDC16_HUMAN	33	71609	2	1	2	1	0.07	Cell division cycle prot
2324	1	SwissProt_HUMAN$	2::O14802|RPC1_HUMAN	33	155542	1	1	1	1	0.03	DNA-directed RNA pol
2325	1	SwissProt_HUMAN$	2::Q9NWT1|PK1IP_HUMAN	33	43936	2	2	2	2	0.24	p21-activated protein
2326	1	SwissProt_HUMAN$	2::Q8NEU8|DP13B_HUMAN	33	74446	3	3	3	3	0.21	DCC-interacting prote
2327	1	SwissProt_HUMAN$	2::Q5BJH7|YIF1B_HUMAN	33	34413	1	1	1	1	0.15	Protein YIF1B OS=Hor
2328	1	SwissProt_HUMAN$	2::Q8NFU1|BEST2_HUMAN	33	57102	1	1	1	1	0.09	Bestrophin-2 OS=Hon
2329	1	SwissProt_HUMAN$	2::Q8NEZ5|FBX22_HUMAN	32	44480	1	1	1	1	0.11	F-box only protein 22
2330	1	SwissProt_HUMAN$	2::Q9UJW0|DCTN4_HUMAN	32	52304	2	1	2	1	0.09	Dynactin subunit 4 OS
2331	1	SwissProt_HUMAN$	2::P29084|T2EB_HUMAN	32	33023	1	1	1	1	0.15	Transcription initiatior
2332	1	SwissProt_HUMAN$	2::P38432|COIL_HUMAN	32	62570	2	2	2	2	0.16	Coilin OS=Homo sapie
2333	1	SwissProt_HUMAN$	2::P56524|HDAC4_HUMAN	32	118966	3	1	1	1	0.04	Histone deacetylase 4
2334	1	SwissProt_HUMAN$	2::Q53FA7|QORX_HUMAN	32	35514	2	2	2	2	0.30	Quinone oxidoreducta
2335	1	SwissProt_HUMAN$	2::O15269|SPTC1_HUMAN	32	52710	2	2	2	2	0.20	Serine palmitoyltransf

↑Family	M	DB	Accession	Score	Mass	Matches	Match(sig)	Sequences	Seq(sig)	emPAI	Description
2336	1	SwissProt_HUMAN$	2::Q96A49|SYAP1_HUMAN	32	39909	2	2	2	2	0.27	Synapse-associated pı
2337	1	SwissProt_HUMAN$	2::O75569|PRKRA_HUMAN	32	34383	2	1	2	1	0.15	Interferon-inducible d
2338	1	SwissProt_HUMAN$	2::P50402|EMD_HUMAN	32	28976	3	2	3	2	0.38	Emerin OS=Homo sap
2339	1	SwissProt_HUMAN$	2::Q969X5|ERGI1_HUMAN	32	32571	2	1	2	1	0.16	Endoplasmic reticulum
2340	1	SwissProt_HUMAN$	2::Q92615|LAR4B_HUMAN	32	80503	2	1	2	1	0.06	La-related protein 4B
2341	1	SwissProt_HUMAN$	2::Q8NHH9|ATLA2_HUMAN	32	66187	1	1	1	1	0.07	Atlastin-2 OS=Homo s
2342	1	SwissProt_HUMAN$	2::P19075|TSN8_HUMAN	32	26026	2	1	1	1	0.20	Tetraspanin-8 OS=Ho
2343	1	SwissProt_HUMAN$	2::Q9Y5Q9|TF3C3_HUMAN	32	101208	2	1	2	1	0.05	General transcription 1
2344	1	SwissProt_HUMAN$	2::Q02809|PLOD1_HUMAN	32	83497	1	1	1	1	0.06	Procollagen-lysine,2-o
2345	1	SwissProt_HUMAN$	2::P35269|T2FA_HUMAN	32	58205	2	2	2	2	0.18	General transcription 1
2346	1	SwissProt_HUMAN$	2::P02751|FINC_HUMAN	32	272150	1	1	1	1	0.02	Fibronectin OS=Homo
2347	1	SwissProt_HUMAN$	2::Q8TB03|CX038_HUMAN	32	36646	1	1	1	1	0.14	Uncharacterized prote
2348	1	SwissProt_HUMAN$	2::Q9H4M3|FBX44_HUMAN	32	29728	1	1	1	1	0.17	F-box only protein 44
2349	1	SwissProt_HUMAN$	2::P09496|CLCA_HUMAN	32	27060	3	3	3	3	0.68	Clathrin light chain A (
2350	1	SwissProt_HUMAN$	2::A6NDU8|CE051_HUMAN	32	33599	1	1	1	1	0.15	UPF0600 protein C5or
2351	1	SwissProt_HUMAN$	2::Q86VM9|ZCH18_HUMAN	32	106316	2	1	2	1	0.05	Zinc finger CCCH dom
2352	1	SwissProt_HUMAN$	2::Q7Z2K6|ERMP1_HUMAN	32	100167	1	1	1	1	0.05	Endoplasmic reticulum
2353	1	SwissProt_HUMAN$	2::Q96Q11|TRNT1_HUMAN	32	50096	2	2	2	2	0.21	CCA tRNA nucleotidylt
2354	1	SwissProt_HUMAN$	2::Q9Y2H0|DLGP4_HUMAN	32	107945	1	1	1	1	0.04	Disks large-associatec
2355	1	SwissProt_HUMAN$	2::Q14554|PDIA5_HUMAN	32	59556	3	3	3	3	0.27	Protein disulfide-isom
2356	1	SwissProt_HUMAN$	2::Q9Y3D0|CIA2B_HUMAN	32	17652	1	1	1	1	0.30	Cytosolic iron-sulfur a
2357	1	SwissProt_HUMAN$	2::O14828|SCAM3_HUMAN	32	38262	1	1	1	1	0.13	Secretory carrier-asso
2358	1	SwissProt_HUMAN$	2::Q96KG9|SCYL1_HUMAN	32	89575	2	1	2	1	0.05	N-terminal kinase-like
2359	1	SwissProt_HUMAN$	2::Q9H7E9|CH033_HUMAN	31	24977	1	1	1	1	0.21	UPF0488 protein C8or
2360	1	SwissProt_HUMAN$	2::Q06203|PUR1_HUMAN	31	57362	1	1	1	1	0.09	Amidophosphoribosylt
2361	1	SwissProt_HUMAN$	2::Q68EM7|RHG17_HUMAN	31	95377	1	1	1	1	0.05	Rho GTPase-activating
2362	1	SwissProt_HUMAN$	2::Q9Y3B3|TMED7_HUMAN	31	25156	4	3	4	3	0.75	Transmembrane emp2
2363	1	SwissProt_HUMAN$	2::Q9BRG1|VPS25_HUMAN	31	20735	2	1	2	1	0.25	Vacuolar protein-sortii
2364	1	SwissProt_HUMAN$	2::Q9Y2V2|CHSP1_HUMAN	31	15882	2	2	2	2	0.80	Calcium-regulated hea
2365	1	SwissProt_HUMAN$	2::Q6XE24|RBMS3_HUMAN	31	47809	1	1	1	1	0.10	RNA-binding motif, sir
2366	1	SwissProt_HUMAN$	2::Q9P015|RM15_HUMAN	31	33399	4	1	4	1	0.15	39S ribosomal protein
2367	1	SwissProt_HUMAN$	2::P0DN79|CBSL_HUMAN	31	60548	1	1	1	1	0.08	Cystathionine beta-sy
2368	1	SwissProt_HUMAN$	2::Q96KB5|TOPK_HUMAN	31	36062	1	1	1	1	0.14	Lymphokine-activated
2369	1	SwissProt_HUMAN$	2::Q8TD19|NEK9_HUMAN	31	107100	2	1	2	1	0.05	Serine/threonine-prot
2370	1	SwissProt_HUMAN$	2::Q15526|SURF1_HUMAN	31	33310	1	1	1	1	0.15	Surfeit locus protein 1
2371	1	SwissProt_HUMAN$	2::P17152|TMM11_HUMAN	31	21527	1	1	1	1	0.24	Transmembrane prote
2372	1	SwissProt_HUMAN$	2::O15213|WDR46_HUMAN	31	68029	2	2	2	2	0.15	WD repeat-containing
2373	1	SwissProt_HUMAN$	2::O94855|SC24D_HUMAN	31	112936	3	3	2	2	0.09	Protein transport prot
2374	1	SwissProt_HUMAN$	2::A6NKL6|T200C_HUMAN	31	63889	4	2	1	1	0.08	Transmembrane prote
2375	1	SwissProt_HUMAN$	2::Q92839|HYAS1_HUMAN	31	64790	1	1	1	1	0.08	Hyaluronan synthase

↑Family	M	DB	Accession	Score	Mass	Matches	Match(sig)	Sequences	Seq(sig)	emPAI	Description
2376	1	SwissProt_HUMAN$	2::Q16513|PKN2_HUMAN	31	111964	5	3	5	3	0.14	Serine/threonine-prot
2377	1	SwissProt_HUMAN$	2::Q9H0U3|MAGT1_HUMAN	31	38011	1	1	1	1	0.13	Magnesium transporte
2378	1	SwissProt_HUMAN$	2::P48506|GSH1_HUMAN	31	72719	2	1	2	1	0.07	Glutamate--cysteine li
2379	1	SwissProt_HUMAN$	2::Q7LBR1|CHM1B_HUMAN	31	22095	1	1	1	1	0.24	Charged multivesicula
2380	1	SwissProt_HUMAN$	2::Q8N983|RM43_HUMAN	31	23416	2	2	2	2	0.49	39S ribosomal protein
2381	1	SwissProt_HUMAN$	2::Q10570|CPSF1_HUMAN	31	160782	1	1	1	1	0.03	Cleavage and polyade
2382	1	SwissProt_HUMAN$	2::Q86U86|PB1_HUMAN	31	192825	2	1	2	1	0.02	Protein polybromo-1 (
2383	1	SwissProt_HUMAN$	2::Q3ZCW2|LEGL_HUMAN	31	18974	2	2	2	2	0.64	Galectin-related prote
2384	1	SwissProt_HUMAN$	2::Q86VR2|RETR3_HUMAN	31	51364	1	1	1	1	0.10	Reticulophagy regulat
2385	1	SwissProt_HUMAN$	2::Q8TBC4|UBA3_HUMAN	31	51819	1	1	1	1	0.10	NEDD8-activating enz
2386	1	SwissProt_HUMAN$	2::Q9P2R3|ANFY1_HUMAN	31	128318	4	2	4	2	0.08	Rabankyrin-5 OS=Hor
2387	1	SwissProt_HUMAN$	2::O43913|ORC5_HUMAN	30	50251	1	1	1	1	0.10	Origin recognition com
2388	1	SwissProt_HUMAN$	2::Q8IYE0|CC146_HUMAN	30	112736	1	1	1	1	0.04	Coiled-coil domain-co
2389	1	SwissProt_HUMAN$	2::Q15554|TERF2_HUMAN	30	59557	2	1	2	1	0.08	Telomeric repeat-bind
2390	1	SwissProt_HUMAN$	2::P23142|FBLN1_HUMAN	30	77162	1	1	1	1	0.06	Fibulin-1 OS=Homo sa
2391	1	SwissProt_HUMAN$	2::Q8IX04|UEVLD_HUMAN	30	52231	1	1	1	1	0.09	Ubiquitin-conjugating
2392	1	SwissProt_HUMAN$	2::Q53H12|AGK_HUMAN	30	47107	5	4	5	4	0.49	Acylglycerol kinase, m
2393	1	SwissProt_HUMAN$	2::P36955|PEDF_HUMAN	30	46283	1	1	1	1	0.11	Pigment epithelium-de
2394	1	SwissProt_HUMAN$	2::Q9NWU5|RM22_HUMAN	30	23626	1	1	1	1	0.22	39S ribosomal protein
2395	1	SwissProt_HUMAN$	2::Q9NX08|COMD8_HUMAN	30	21077	1	1	1	1	0.25	COMM domain-contair
2396	1	SwissProt_HUMAN$	2::O75368|SH3L1_HUMAN	30	12766	1	1	1	1	0.44	SH3 domain-binding g
2397	1	SwissProt_HUMAN$	2::Q9Y679|AUP1_HUMAN	30	45758	1	1	1	1	0.11	Lipid droplet-regulatin
2398	1	SwissProt_HUMAN$	2::Q9NXR7|BABA2_HUMAN	30	43524	2	1	2	1	0.11	BRISC and BRCA1-A c
2399	1	SwissProt_HUMAN$	2::Q7Z7G8|VP13B_HUMAN	30	448378	1	1	1	1	0.01	Vacuolar protein sortir
2400	1	SwissProt_HUMAN$	2::Q9UH65|SWP70_HUMAN	30	68954	2	2	2	2	0.15	Switch-associated pro
2401	1	SwissProt_HUMAN$	2::P09001|RM03_HUMAN	30	38608	1	1	1	1	0.13	39S ribosomal protein
2402	1	SwissProt_HUMAN$	2::P53367|ARFP1_HUMAN	30	41713	1	1	1	1	0.12	Arfaptin-1 OS=Homo
2403	1	SwissProt_HUMAN$	2::Q9Y5K5|UCHL5_HUMAN	30	37583	3	2	3	2	0.28	Ubiquitin carboxyl-ter
2404	1	SwissProt_HUMAN$	2::Q8NCM8|DYHC2_HUMAN	30	492311	1	1	1	1	0.01	Cytoplasmic dynein 2
2405	1	SwissProt_HUMAN$	2::Q96C01|F136A_HUMAN	30	15631	1	1	1	1	0.35	Protein FAM136A OS=
2406	1	SwissProt_HUMAN$	2::O95551|TYDP2_HUMAN	30	40903	1	1	1	1	0.12	Tyrosyl-DNA phospho
2407	1	SwissProt_HUMAN$	2::Q9NX47|MARH5_HUMAN	30	31211	1	1	1	1	0.16	E3 ubiquitin-protein li
2408	1	SwissProt_HUMAN$	2::Q15561|TEAD4_HUMAN	30	48298	1	1	1	1	0.10	Transcriptional enhan
2409	1	SwissProt_HUMAN$	2::Q96GG9|DCNL1_HUMAN	30	30105	2	1	2	1	0.17	DCN1-like protein 1 O
2410	1	SwissProt_HUMAN$	2::Q9NUB1|ACS2L_HUMAN	30	74809	1	1	1	1	0.07	Acetyl-coenzyme A sy
2411	1	SwissProt_HUMAN$	2::O75683|SURF6_HUMAN	30	41426	2	1	2	1	0.12	Surfeit locus protein 6
2412	1	SwissProt_HUMAN$	2::P36404|ARL2_HUMAN	30	20865	2	1	2	1	0.25	ADP-ribosylation facto
2413	1	SwissProt_HUMAN$	2::Q6ZMR3|LDH6A_HUMAN	30	36484	1	1	1	1	0.14	L-lactate dehydrogena
2414	1	SwissProt_HUMAN$	2::O14530|TXND9_HUMAN	30	26517	2	1	2	1	0.19	Thioredoxin domain-c
2415	1	SwissProt_HUMAN$	2::Q8N465|D2HDH_HUMAN	30	56380	1	1	1	1	0.09	D-2-hydroxyglutarate

↑Family	M	DB	Accession	Score	Mass	Matches	Match(sig)	Sequences	Seq(sig)	emPAI	Description
2416	1	SwissProt_HUMAN$	2::O95721|SNP29_HUMAN	30	28953	1	1	1	1	0.18	Synaptosomal-associa
2417	1	SwissProt_HUMAN$	2::Q9GZS1|RPA49_HUMAN	30	47230	1	1	1	1	0.11	DNA-directed RNA pol
2418	1	SwissProt_HUMAN$	2::P46934|NEDD4_HUMAN	30	149022	2	2	2	2	0.07	E3 ubiquitin-protein li
2419	1	SwissProt_HUMAN$	2::P98082|DAB2_HUMAN	30	82397	3	2	2	1	0.06	Disabled homolog 2 O
2420	1	SwissProt_HUMAN$	2::Q99523|SORT_HUMAN	30	92009	1	1	1	1	0.05	Sortilin OS=Homo sap
2421	1	SwissProt_HUMAN$	2::Q99720|SGMR1_HUMAN	29	25112	1	1	1	1	0.21	Sigma non-opioid intr
2422	1	SwissProt_HUMAN$	2::O43447|PPIH_HUMAN	29	19196	4	4	4	4	1.65	Peptidyl-prolyl cis-trar
2423	1	SwissProt_HUMAN$	2::P48735|IDHP_HUMAN	29	50877	2	2	2	2	0.20	Isocitrate dehydrogen
2424	1	SwissProt_HUMAN$	2::P60983|GMFB_HUMAN	29	16702	2	1	2	1	0.32	Glia maturation factor
2425	1	SwissProt_HUMAN$	2::Q9UET6|TRM7_HUMAN	29	36056	1	1	1	1	0.14	Putative tRNA (cytidin
2426	1	SwissProt_HUMAN$	2::Q9ULM6|CNOT6_HUMAN	29	63266	1	1	1	1	0.08	CCR4-NOT transcriptic
2427	1	SwissProt_HUMAN$	2::Q4G176|ACSF3_HUMAN	29	64089	2	1	2	1	0.08	Malonate--CoA ligase
2428	1	SwissProt_HUMAN$	2::Q8IW92|GLBL2_HUMAN	29	72033	2	1	2	1	0.07	Beta-galactosidase-1-
2429	1	SwissProt_HUMAN$	2::Q15269|PWP2_HUMAN	29	102387	1	1	1	1	0.05	Periodic tryptophan pr
2430	1	SwissProt_HUMAN$	2::O75616|ERAL1_HUMAN	29	48319	2	2	2	2	0.22	GTPase Era, mitochon
2431	1	SwissProt_HUMAN$	2::P05543|THBG_HUMAN	29	46295	1	1	1	1	0.11	Thyroxine-binding glo
2432	1	SwissProt_HUMAN$	2::Q5K4L6|S27A3_HUMAN	29	73504	1	1	1	1	0.07	Solute carrier family 2
2433	1	SwissProt_HUMAN$	2::Q96EK6|GNA1_HUMAN	29	20736	1	1	1	1	0.25	Glucosamine 6-phospl
2434	1	SwissProt_HUMAN$	2::Q9UHK6|AMACR_HUMAN	29	42360	1	1	1	1	0.12	Alpha-methylacyl-CoA
2435	1	SwissProt_HUMAN$	2::Q96RQ3|MCCA_HUMAN	29	80422	3	3	3	3	0.19	Methylcrotonoyl-CoA c
2436	1	SwissProt_HUMAN$	2::Q9NV31|IMP3_HUMAN	29	21837	1	1	1	1	0.24	U3 small nucleolar rib
2437	1	SwissProt_HUMAN$	2::Q96M96|FGD4_HUMAN	29	86571	1	1	1	1	0.06	FYVE, RhoGEF and PH
2438	1	SwissProt_HUMAN$	2::A5YM69|ARG35_HUMAN	29	53254	1	1	1	1	0.09	Rho guanine nucleotid
2439	1	SwissProt_HUMAN$	2::Q9UGM6|SYWM_HUMAN	29	40121	1	1	1	1	0.13	Tryptophan--tRNA liga
2440	1	SwissProt_HUMAN$	2::P51003|PAPOA_HUMAN	29	82791	2	1	2	1	0.06	Poly(A) polymerase al
2441	1	SwissProt_HUMAN$	2::P01019|ANGT_HUMAN	29	53121	2	2	1	1	0.09	Angiotensinogen OS=
2442	1	SwissProt_HUMAN$	2::P11532|DMD_HUMAN	29	426484	2	1	2	1	0.01	Dystrophin OS=Homo
2443	1	SwissProt_HUMAN$	2::Q9BYN0|SRXN1_HUMAN	29	14250	1	1	1	1	0.39	Sulfiredoxin-1 OS=Ho
2444	1	SwissProt_HUMAN$	2::Q9BYG5|PAR6B_HUMAN	29	41157	3	2	3	2	0.26	Partitioning defective
2445	1	SwissProt_HUMAN$	2::Q01415|GALK2_HUMAN	29	50346	3	2	3	2	0.21	N-acetylgalactosamine
2446	1	SwissProt_HUMAN$	2::Q9H9A6|LRC40_HUMAN	29	68207	1	1	1	1	0.07	Leucine-rich repeat-cc
2447	1	SwissProt_HUMAN$	2::Q3L8U1|CHD9_HUMAN	29	325819	3	2	3	2	0.03	Chromodomain-helica
2448	1	SwissProt_HUMAN$	2::Q6P5R6|RL22L_HUMAN	29	14598	2	1	2	1	0.37	60S ribosomal protein
2449	1	SwissProt_HUMAN$	2::Q9NXV6|CARF_HUMAN	29	61088	1	1	1	1	0.08	CDKN2A-interacting p
2450	1	SwissProt_HUMAN$	2::Q9UNF1|MAGD2_HUMAN	29	64914	1	1	1	1	0.08	Melanoma-associated
2451	1	SwissProt_HUMAN$	2::O95372|LYPA2_HUMAN	29	24721	1	1	1	1	0.21	Acyl-protein thioester
2452	1	SwissProt_HUMAN$	2::O00214|LEG8_HUMAN	29	35786	2	1	2	1	0.14	Galectin-8 OS=Homo
2453	1	SwissProt_HUMAN$	2::O15164|TIF1A_HUMAN	29	116757	2	1	2	1	0.04	Transcription intermec
2454	1	SwissProt_HUMAN$	2::Q9BW04|SARG_HUMAN	29	63925	1	1	1	1	0.08	Specifically androgen-
2455	1	SwissProt_HUMAN$	2::Q8IWB7|WDFY1_HUMAN	29	46293	1	1	1	1	0.11	WD repeat and FYVE c

↑Family	M	DB	Accession	Score	Mass	Matches	Match(sig)	Sequences	Seq(sig)	emPAI	Description
2456	1	SwissProt_HUMAN$	2::P28676|GRAN_HUMAN	29	23994	1	1	1	1	0.22	Grancalcin OS=Homo
2457	1	SwissProt_HUMAN$	2::O95396|MOCS3_HUMAN	29	49638	1	1	1	1	0.10	Adenylyltransferase ar
2458	1	SwissProt_HUMAN$	2::Q9BZF1|OSBL8_HUMAN	29	101132	2	1	2	1	0.05	Oxysterol-binding prot
2459	1	SwissProt_HUMAN$	2::P49916|DNLI3_HUMAN	29	112835	1	1	1	1	0.04	DNA ligase 3 OS=Hom
2460	1	SwissProt_HUMAN$	2::Q8N5A5|ZGPAT_HUMAN	28	57324	1	1	1	1	0.09	Zinc finger CCCH-type
2461	1	SwissProt_HUMAN$	2::Q9BXB4|OSB11_HUMAN	28	83591	2	2	2	2	0.12	Oxysterol-binding prot
2462	1	SwissProt_HUMAN$	2::Q15022|SUZ12_HUMAN	28	83003	1	1	1	1	0.06	Polycomb protein SUZ
2463	1	SwissProt_HUMAN$	2::P78346|RPP30_HUMAN	28	29303	1	1	1	1	0.17	Ribonuclease P protein
2464	1	SwissProt_HUMAN$	2::Q9NYL4|FKB11_HUMAN	28	22166	2	2	2	2	0.53	Peptidyl-prolyl cis-trar
2465	1	SwissProt_HUMAN$	2::Q8NCM2|KCNH5_HUMAN	28	111805	1	1	1	1	0.04	Potassium voltage-gat
2466	1	SwissProt_HUMAN$	2::P33947|ERD22_HUMAN	28	24406	1	1	1	1	0.21	ER lumen protein-reta
2467	1	SwissProt_HUMAN$	2::Q8IUF8|RIOX2_HUMAN	28	52767	1	1	1	1	0.09	Ribosomal oxygenase
2468	1	SwissProt_HUMAN$	2::O95197|RTN3_HUMAN	28	112541	4	1	3	1	0.04	Reticulon-3 OS=Homo
2469	1	SwissProt_HUMAN$	2::Q9BRR6|ADPGK_HUMAN	28	54055	1	1	1	1	0.09	ADP-dependent glucok
2470	1	SwissProt_HUMAN$	2::Q96CW5|GCP3_HUMAN	28	103506	2	2	2	2	0.10	Gamma-tubulin compl
2471	1	SwissProt_HUMAN$	2::Q13405|RM49_HUMAN	28	19186	1	1	1	1	0.28	39S ribosomal protein
2472	1	SwissProt_HUMAN$	2::Q99836|MYD88_HUMAN	28	33212	1	1	1	1	0.15	Myeloid differentiation
2473	1	SwissProt_HUMAN$	2::Q9P0T7|TMEM9_HUMAN	28	20560	2	2	2	2	0.58	Proton-transporting V-
2474	1	SwissProt_HUMAN$	2::Q8IYB3|SRRM1_HUMAN	28	102274	1	1	1	1	0.05	Serine/arginine repeti
2475	1	SwissProt_HUMAN$	2::Q9Y394|DHRS7_HUMAN	28	38274	3	1	3	1	0.13	Dehydrogenase/reduc
2476	1	SwissProt_HUMAN$	2::Q99541|PLIN2_HUMAN	28	48045	1	1	1	1	0.10	Perilipin-2 OS=Homo
2477	1	SwissProt_HUMAN$	2::Q7Z4V5|HDGR2_HUMAN	28	74272	3	1	2	1	0.07	Hepatoma-derived gro
2478	1	SwissProt_HUMAN$	2::O60443|GSDME_HUMAN	28	54520	2	1	2	1	0.09	Gasdermin-E OS=Hon
2479	1	SwissProt_HUMAN$	2::Q9H9Y6|RPA2_HUMAN	28	128146	2	2	2	2	0.08	DNA-directed RNA pol
2480	1	SwissProt_HUMAN$	2::P04217|A1BG_HUMAN	28	54220	1	1	1	1	0.09	Alpha-1B-glycoprotein
2481	1	SwissProt_HUMAN$	2::Q15334|L2GL1_HUMAN	28	115346	1	1	1	1	0.04	Lethal(2) giant larvae
2482	1	SwissProt_HUMAN$	2::Q9NVE7|PANK4_HUMAN	28	85937	1	1	1	1	0.06	4'-phosphopantethein
2483	1	SwissProt_HUMAN$	2::P29373|RABP2_HUMAN	28	15683	2	1	2	1	0.35	Cellular retinoic acid-b
2484	1	SwissProt_HUMAN$	2::Q2TAP0|GOG7B_HUMAN	28	18323	1	1	1	1	0.29	Golgin subfamily A me
2485	1	SwissProt_HUMAN$	2::Q7Z412|PEX26_HUMAN	28	33877	1	1	1	1	0.15	Peroxisome assembly
2486	1	SwissProt_HUMAN$	2::Q8TCG1|CIP2A_HUMAN	28	102121	1	1	1	1	0.05	Protein CIP2A OS=Ho
2487	1	SwissProt_HUMAN$	2::Q9HCN8|SDF2L_HUMAN	28	23584	2	2	2	2	0.49	Stromal cell-derived fa
2488	1	SwissProt_HUMAN$	2::Q9BU61|NDUF3_HUMAN	28	20337	1	1	1	1	0.26	NADH dehydrogenase
2489	1	SwissProt_HUMAN$	2::O14656|TOR1A_HUMAN	28	37784	1	1	1	1	0.13	Torsin-1A OS=Homo s
2490	1	SwissProt_HUMAN$	2::Q2M3G4|SHRM1_HUMAN	28	90730	1	1	1	1	0.05	Protein Shroom1 OS=
2491	1	SwissProt_HUMAN$	2::Q9Y3D8|KAD6_HUMAN	28	20049	1	1	1	1	0.26	Adenylate kinase isoe
2492	1	SwissProt_HUMAN$	2::Q8IXM3|RM41_HUMAN	28	15373	2	1	2	1	0.36	39S ribosomal protein
2493	1	SwissProt_HUMAN$	2::Q96M27|PRRC1_HUMAN	28	46672	2	2	2	2	0.22	Protein PRRC1 OS=Ho
2494	1	SwissProt_HUMAN$	2::Q8WZB0|CI130_HUMAN	28	14706	2	1	1	1	0.37	Putative uncharacteriz
2495	1	SwissProt_HUMAN$	2::Q9GZT8|NIF3L_HUMAN	28	41942	1	1	1	1	0.12	NIF3-like protein 1 OS

↑Family	M	DB	Accession	Score	Mass	Matches	Match(sig)	Sequences	Seq(sig)	emPAI	Description
2496	1	SwissProt_HUMAN$	2::Q9UHY1|NRBP_HUMAN	28	59807	1	1	1	1	0.08	Nuclear receptor-bindi
2497	1	SwissProt_HUMAN$	2::Q69YN4|VIR_HUMAN	28	201898	1	1	1	1	0.02	Protein virilizer homol
2498	1	SwissProt_HUMAN$	2::Q01658|NC2B_HUMAN	28	19432	1	1	1	1	0.27	Protein Dr1 OS=Homo
2499	1	SwissProt_HUMAN$	2::Q9BSC4|NOL10_HUMAN	27	80251	3	2	3	2	0.13	Nucleolar protein 10 C
2500	1	SwissProt_HUMAN$	2::Q9NYP7|ELOV5_HUMAN	27	35270	1	1	1	1	0.14	Elongation of very lon
2501	1	SwissProt_HUMAN$	2::P46199|IF2M_HUMAN	27	81266	3	1	3	1	0.06	Translation initiation f
2502	1	SwissProt_HUMAN$	2::Q9UBX3|DIC_HUMAN	27	31262	3	2	2	2	0.35	Mitochondrial dicarbox
2503	1	SwissProt_HUMAN$	2::Q86SX6|GLRX5_HUMAN	27	16618	1	1	1	1	0.32	Glutaredoxin-related p
2504	1	SwissProt_HUMAN$	2::P53985|MOT1_HUMAN	27	53909	3	2	2	2	0.19	Monocarboxylate trans
2505	1	SwissProt_HUMAN$	2::Q9UK59|DBR1_HUMAN	27	61516	3	2	3	2	0.17	Lariat debranching en
2506	1	SwissProt_HUMAN$	2::O15511|ARPC5_HUMAN	27	16310	1	1	1	1	0.33	Actin-related protein 2
2507	1	SwissProt_HUMAN$	2::O43681|GET3_HUMAN	27	38767	1	1	1	1	0.13	ATPase GET3 OS=Hor
2508	1	SwissProt_HUMAN$	2::Q9H446|RWDD1_HUMAN	27	27922	1	1	1	1	0.18	RWD domain-containi
2509	1	SwissProt_HUMAN$	2::A6NCS6|CB072_HUMAN	27	30462	3	2	3	2	0.36	Uncharacterized prote
2510	1	SwissProt_HUMAN$	2::B1ANS9|WDR64_HUMAN	27	123553	1	1	1	1	0.04	WD repeat-containing
2511	1	SwissProt_HUMAN$	2::Q13698|CAC1S_HUMAN	27	212212	2	2	1	1	0.02	Voltage-dependent L-t
2512	1	SwissProt_HUMAN$	2::Q15165|PON2_HUMAN	27	39356	4	2	4	2	0.27	Serum paraoxonase/a
2513	1	SwissProt_HUMAN$	2::Q7LGA3|HS2ST_HUMAN	27	41854	1	1	1	1	0.12	Heparan sulfate 2-O-s
2514	1	SwissProt_HUMAN$	2::Q15833|STXB2_HUMAN	27	66410	6	2	4	2	0.15	Syntaxin-binding prot
2515	1	SwissProt_HUMAN$	2::P52657|T2AG_HUMAN	27	12449	1	1	1	1	0.45	Transcription initiatior
2516	1	SwissProt_HUMAN$	2::O95881|TXD12_HUMAN	27	19194	1	1	1	1	0.28	Thioredoxin domain-c
2517	1	SwissProt_HUMAN$	2::Q9HD42|CHM1A_HUMAN	27	21689	1	1	1	1	0.24	Charged multivesicula
2518	1	SwissProt_HUMAN$	2::Q9NW08|RPC2_HUMAN	27	127702	1	1	1	1	0.04	DNA-directed RNA pol
2519	1	SwissProt_HUMAN$	2::Q9HD23|MRS2_HUMAN	27	50285	1	1	1	1	0.10	Magnesium transporte
2520	1	SwissProt_HUMAN$	2::P11498|PYC_HUMAN	27	129551	1	1	1	1	0.04	Pyruvate carboxylase,
2521	1	SwissProt_HUMAN$	2::Q96MW5|COG8_HUMAN	27	68380	1	1	1	1	0.07	Conserved oligomeric
2522	1	SwissProt_HUMAN$	2::Q9P1Q0|VPS54_HUMAN	27	110519	1	1	1	1	0.04	Vacuolar protein sorti
2523	1	SwissProt_HUMAN$	2::P61964|WDR5_HUMAN	27	36565	2	2	2	2	0.29	WD repeat-containing
2524	1	SwissProt_HUMAN$	2::Q8WXD5|GEMI6_HUMAN	27	18812	2	2	1	1	0.28	Gem-associated prote
2525	1	SwissProt_HUMAN$	2::Q8TEM1|PO210_HUMAN	27	204983	3	2	3	2	0.05	Nuclear pore membra
2526	1	SwissProt_HUMAN$	2::P49914|MTHFS_HUMAN	27	23241	1	1	1	1	0.22	5-formyltetrahydrofol
2527	1	SwissProt_HUMAN$	2::O95954|FTCD_HUMAN	27	58889	1	1	1	1	0.08	Formimidoyltransferas
2528	1	SwissProt_HUMAN$	2::Q15024|EXOS7_HUMAN	27	31801	3	2	3	2	0.35	Exosome complex con
2529	1	SwissProt_HUMAN$	2::Q6UW02|CP20A_HUMAN	27	52399	1	1	1	1	0.09	Cytochrome P450 20A
2530	1	SwissProt_HUMAN$	2::P12694|ODBA_HUMAN	27	50439	1	1	1	1	0.10	2-oxoisovalerate dehy
2531	1	SwissProt_HUMAN$	2::P35498|SCN1A_HUMAN	27	228823	1	1	1	1	0.02	Sodium channel prote
2532	1	SwissProt_HUMAN$	2::P43307|SSRA_HUMAN	27	32215	1	1	1	1	0.16	Translocon-associated
2533	1	SwissProt_HUMAN$	2::Q96A22|CK052_HUMAN	27	13912	1	1	1	1	0.40	Uncharacterized prote
2534	1	SwissProt_HUMAN$	2::P10398|ARAF_HUMAN	27	67542	1	1	1	1	0.07	Serine/threonine-prot
2535	1	SwissProt_HUMAN$	2::O43617|TPPC3_HUMAN	27	20261	2	1	2	1	0.26	Trafficking protein par

↑Family	M	DB	Accession	Score	Mass	Matches	Match(sig)	Sequences	Seq(sig)	emPAI	Description
2536	1	SwissProt_HUMAN$	2::Q9H6K4|OPA3_HUMAN	27	19984	1	1	1	1	0.26	Optic atrophy 3 protei
2537	1	SwissProt_HUMAN$	2::Q9H6W3|RIOX1_HUMAN	27	71041	1	1	1	1	0.07	Ribosomal oxygenase
2538	1	SwissProt_HUMAN$	2::Q14353|GAMT_HUMAN	27	26301	1	1	1	1	0.20	Guanidinoacetate N-m
2539	1	SwissProt_HUMAN$	2::Q96RW7|HMCN1_HUMAN	27	613001	3	2	1	1	0.01	Hemicentin-1 OS=Hor
2540	1	SwissProt_HUMAN$	2::Q86U44|MTA70_HUMAN	27	64433	1	1	1	1	0.08	N6-adenosine-methylt
2541	1	SwissProt_HUMAN$	2::O43852|CALU_HUMAN	27	37084	1	1	1	1	0.14	Calumenin OS=Homo
2542	1	SwissProt_HUMAN$	2::Q6ZT62|BGIN_HUMAN	26	73553	1	1	1	1	0.07	Bargin OS=Homo sapi
2543	1	SwissProt_HUMAN$	2::Q9P0U3|SENP1_HUMAN	26	73435	1	1	1	1	0.07	Sentrin-specific protea
2544	1	SwissProt_HUMAN$	2::Q96SZ6|CK5P1_HUMAN	26	67646	1	1	1	1	0.07	Mitochondrial tRNA m
2545	1	SwissProt_HUMAN$	2::Q9UH17|ABC3B_HUMAN	26	45894	1	1	1	1	0.11	DNA dC->dU-editing e
2546	1	SwissProt_HUMAN$	2::P14927|QCR7_HUMAN	26	13522	2	2	2	2	0.99	Cytochrome b-c1 com
2547	1	SwissProt_HUMAN$	2::O95139|NDUB6_HUMAN	26	15479	1	1	1	1	0.35	NADH dehydrogenase
2548	1	SwissProt_HUMAN$	2::Q12999|TSN31_HUMAN	26	23038	1	1	1	1	0.23	Tetraspanin-31 OS=H
2549	1	SwissProt_HUMAN$	2::Q9NRR5|UBQL4_HUMAN	26	63812	1	1	1	1	0.08	Ubiquilin-4 OS=Homo
2550	1	SwissProt_HUMAN$	2::Q99584|S10AD_HUMAN	26	11464	2	2	2	2	1.25	Protein S100-A13 OS=
2551	1	SwissProt_HUMAN$	2::Q9P016|THYN1_HUMAN	26	25681	1	1	1	1	0.20	Thymocyte nuclear pr
2552	1	SwissProt_HUMAN$	2::P11171|EPB41_HUMAN	26	96957	2	1	2	1	0.05	Protein 4.1 OS=Homo
2553	1	SwissProt_HUMAN$	2::Q8TCJ2|STT3B_HUMAN	26	93614	1	1	1	1	0.05	Dolichyl-diphosphoolig
2554	1	SwissProt_HUMAN$	2::P10606|COX5B_HUMAN	26	13687	2	1	1	1	0.41	Cytochrome c oxidase
2555	1	SwissProt_HUMAN$	2::Q96MU7|YTDC1_HUMAN	26	84649	1	1	1	1	0.06	YTH domain-containin
2556	1	SwissProt_HUMAN$	2::P07919|QCR6_HUMAN	26	10732	1	1	1	1	0.54	Cytochrome b-c1 com
2557	1	SwissProt_HUMAN$	2::O75351|VPS4B_HUMAN	26	49271	2	2	2	2	0.21	Vacuolar protein sortir
2558	1	SwissProt_HUMAN$	2::Q13546|RIPK1_HUMAN	26	75883	1	1	1	1	0.06	Receptor-interacting s
2559	1	SwissProt_HUMAN$	2::Q8NC60|NOA1_HUMAN	26	78409	1	1	1	1	0.06	Nitric oxide-associatec
2560	1	SwissProt_HUMAN$	2::P13984|T2FB_HUMAN	26	28363	1	1	1	1	0.18	General transcription f
2561	1	SwissProt_HUMAN$	2::P18583|SON_HUMAN	26	263664	3	1	2	1	0.02	Protein SON OS=Hom
2562	1	SwissProt_HUMAN$	2::Q86WA6|BPHL_HUMAN	26	32522	1	1	1	1	0.16	Valacyclovir hydrolase
2563	1	SwissProt_HUMAN$	2::P09234|RU1C_HUMAN	26	17381	1	1	1	1	0.31	U1 small nuclear ribor
2564	1	SwissProt_HUMAN$	2::Q8TBB5|KLDC4_HUMAN	26	57855	1	1	1	1	0.09	Kelch domain-containi
2565	1	SwissProt_HUMAN$	2::P30260|CDC27_HUMAN	26	91809	1	1	1	1	0.05	Cell division cycle prot
2566	1	SwissProt_HUMAN$	2::Q9BQP7|MGME1_HUMAN	26	39396	1	1	1	1	0.13	Mitochondrial genome
2567	1	SwissProt_HUMAN$	2::Q9BRA2|TXD17_HUMAN	26	13932	2	2	2	2	0.95	Thioredoxin domain-co
2568	1	SwissProt_HUMAN$	2::P36575|ARRC_HUMAN	26	42751	1	1	1	1	0.12	Arrestin-C OS=Homo
2569	1	SwissProt_HUMAN$	2::Q96GC9|VMP1_HUMAN	26	46208	1	1	1	1	0.11	Vacuole membrane pr
2570	1	SwissProt_HUMAN$	2::Q9Y2D5|AKAP2_HUMAN	26	94603	2	1	2	1	0.05	A-kinase anchor prote
2571	1	SwissProt_HUMAN$	2::Q96JM3|CHAP1_HUMAN	26	89043	1	1	1	1	0.05	Chromosome alignme
2572	1	SwissProt_HUMAN$	2::Q16563|SYPL1_HUMAN	26	28547	1	1	1	1	0.18	Synaptophysin-like pr
2573	1	SwissProt_HUMAN$	2::Q96F86|EDC3_HUMAN	26	56042	1	1	1	1	0.09	Enhancer of mRNA-de
2574	1	SwissProt_HUMAN$	2::Q9Y5A7|NUB1_HUMAN	26	70494	1	1	1	1	0.07	NEDD8 ultimate buste
2575	1	SwissProt_HUMAN$	2::P49903|SPS1_HUMAN	26	42883	3	2	3	2	0.25	Selenide, water dikina

↑Family	M	DB	Accession	Score	Mass	Matches	Match(sig)	Sequences	Seq(sig)	emPAI	Description
2576	1	SwissProt_HUMAN$	2::Q9UGV2|NDRG3_HUMAN	26	41382	2	2	2	2	0.26	Protein NDRG3 OS=H
2577	1	SwissProt_HUMAN$	2::P45954|ACDSB_HUMAN	26	47455	3	1	3	1	0.10	Short/branched chain
2578	1	SwissProt_HUMAN$	2::Q9Y241|HIG1A_HUMAN	26	10136	1	1	1	1	0.58	HIG1 domain family m
2579	1	SwissProt_HUMAN$	2::Q9NUL3|STAU2_HUMAN	26	62569	1	1	1	1	0.08	Double-stranded RNA-
2580	1	SwissProt_HUMAN$	2::P04062|GLCM_HUMAN	26	59678	1	1	1	1	0.08	Lysosomal acid glucos
2581	1	SwissProt_HUMAN$	2::Q8WZ82|OVCA2_HUMAN	26	24403	1	1	1	1	0.21	Esterase OVCA2 OS=H
2582	1	SwissProt_HUMAN$	2::Q9H7Z7|PGES2_HUMAN	26	41917	2	1	2	1	0.12	Prostaglandin E synth
2583	1	SwissProt_HUMAN$	2::Q07075|AMPE_HUMAN	25	109176	1	1	1	1	0.04	Glutamyl aminopeptid
2584	1	SwissProt_HUMAN$	2::Q9BT17|MTG1_HUMAN	25	37213	1	1	1	1	0.14	Mitochondrial ribosom
2585	1	SwissProt_HUMAN$	2::Q9H3G5|CPVL_HUMAN	25	54129	1	1	1	1	0.09	Probable serine carbo:
2586	1	SwissProt_HUMAN$	2::Q13033|STRN3_HUMAN	25	87155	2	1	2	1	0.06	Striatin-3 OS=Homo s
2587	1	SwissProt_HUMAN$	2::Q9NX55|HYPK_HUMAN	25	14656	1	1	1	1	0.37	Huntingtin-interacting
2588	1	SwissProt_HUMAN$	2::Q86Y82|STX12_HUMAN	25	31622	1	1	1	1	0.16	Syntaxin-12 OS=Hom
2589	1	db$	1::P0DTC8|NS8_SARS2	25	13822	1	1	1	1	0.40	ORF8 protein OS=Sev
2590	1	SwissProt_HUMAN$	2::Q7L5D6|GET4_HUMAN	25	36481	2	2	2	2	0.29	Golgi to ER traffic prot
2591	1	SwissProt_HUMAN$	2::Q6IBW4|CNDH2_HUMAN	25	68184	1	1	1	1	0.07	Condensin-2 complex
2592	1	SwissProt_HUMAN$	2::Q9BYB4|GNB1L_HUMAN	25	35595	1	1	1	1	0.14	Guanine nucleotide-bi
2593	1	SwissProt_HUMAN$	2::Q8TED0|UTP15_HUMAN	25	58379	2	1	2	1	0.08	U3 small nucleolar RN
2594	1	SwissProt_HUMAN$	2::O14494|PLPP1_HUMAN	25	32135	1	1	1	1	0.16	Phospholipid phosphat
2595	1	SwissProt_HUMAN$	2::Q92530|PSMF1_HUMAN	25	29798	1	1	1	1	0.17	Proteasome inhibitor F
2596	1	SwissProt_HUMAN$	2::Q96GM5|SMRD1_HUMAN	25	58196	2	2	2	2	0.18	SWI/SNF-related matr
2597	1	SwissProt_HUMAN$	2::Q02127|PYRD_HUMAN	25	42841	1	1	1	1	0.12	Dihydroorotate dehyd
2598	1	SwissProt_HUMAN$	2::Q8N357|S35F6_HUMAN	25	40188	2	1	1	1	0.12	Solute carrier family 3
2599	1	SwissProt_HUMAN$	2::Q02509|OC90_HUMAN	25	51694	1	1	1	1	0.10	Otoconin-90 OS=Hom
2600	1	SwissProt_HUMAN$	2::P16383|GCFC2_HUMAN	25	89329	1	1	1	1	0.05	GC-rich sequence DN/
2601	1	SwissProt_HUMAN$	2::P06731|CEAM5_HUMAN	25	76747	1	1	1	1	0.06	Carcinoembryonic ant
2602	1	SwissProt_HUMAN$	2::Q9UGC6|RGS17_HUMAN	25	24344	1	1	1	1	0.21	Regulator of G-protei
2603	1	SwissProt_HUMAN$	2::Q9Y5P4|CERT_HUMAN	25	70791	1	1	1	1	0.07	Ceramide transfer pro
2604	1	SwissProt_HUMAN$	2::Q9NWH9|SLTM_HUMAN	25	117079	2	2	1	1	0.04	SAFB-like transcriptio
2605	1	SwissProt_HUMAN$	2::O95747|OXSR1_HUMAN	25	57986	4	4	4	4	0.39	Serine/threonine-prot
2606	1	SwissProt_HUMAN$	2::A5D8W1|CFA69_HUMAN	25	105816	1	1	1	1	0.05	Cilia- and flagella-ass
2607	1	SwissProt_HUMAN$	2::O96028|NSD2_HUMAN	25	152160	1	1	1	1	0.03	Histone-lysine N-meth
2608	1	SwissProt_HUMAN$	2::Q66K74|MAP1S_HUMAN	25	112142	2	2	2	2	0.09	Microtubule-associate
2609	1	SwissProt_HUMAN$	2::P62273|RS29_HUMAN	25	6672	1	1	1	1	0.98	40S ribosomal protein
2610	1	SwissProt_HUMAN$	2::Q9Y3D9|RT23_HUMAN	25	21757	3	3	3	3	0.91	28S ribosomal protein
2611	1	SwissProt_HUMAN$	2::Q15388|TOM20_HUMAN	25	16288	2	1	2	1	0.33	Mitochondrial import r
2612	1	SwissProt_HUMAN$	2::Q16540|RM23_HUMAN	25	17770	1	1	1	1	0.30	39S ribosomal protein
2613	1	SwissProt_HUMAN$	2::Q7Z2W9|RM21_HUMAN	25	22800	2	2	2	2	0.51	39S ribosomal protein
2614	1	SwissProt_HUMAN$	2::Q96ST3|SIN3A_HUMAN	25	145085	1	1	1	1	0.03	Paired amphipathic he
2615	1	SwissProt_HUMAN$	2::Q8N2F6|ARM10_HUMAN	25	37517	1	1	1	1	0.13	Armadillo repeat-cont

↑Family	M	DB	Accession	Score	Mass	Matches	Match(sig)	Sequences	Seq(sig)	emPAI	Description
2616	1	SwissProt_HUMAN$	2::O14757|CHK1_HUMAN	24	54399	3	2	3	2	0.19	Serine/threonine-prot
2617	1	SwissProt_HUMAN$	2::Q96SQ9|CP2S1_HUMAN	24	55781	2	1	2	1	0.09	Cytochrome P450 2S1
2618	1	SwissProt_HUMAN$	2::Q12965|MYO1E_HUMAN	24	126982	3	3	3	3	0.12	Unconventional myosi
2619	1	SwissProt_HUMAN$	2::Q9HD33|RM47_HUMAN	24	29432	1	1	1	1	0.17	39S ribosomal protein
2620	1	SwissProt_HUMAN$	2::Q12981|SEC20_HUMAN	24	26116	1	1	1	1	0.20	Vesicle transport prote
2621	1	SwissProt_HUMAN$	2::Q9C037|TRIM4_HUMAN	24	57424	1	1	1	1	0.09	E3 ubiquitin-protein li
2622	1	SwissProt_HUMAN$	2::Q01968|OCRL_HUMAN	24	104138	1	1	1	1	0.05	Inositol polyphosphate
2623	1	SwissProt_HUMAN$	2::Q99747|SNAG_HUMAN	24	34724	1	1	1	1	0.15	Gamma-soluble NSF a
2624	1	SwissProt_HUMAN$	2::Q15070|OXA1L_HUMAN	24	48516	1	1	1	1	0.10	Mitochondrial inner m
2625	1	SwissProt_HUMAN$	2::Q08431|MFGM_HUMAN	24	43078	1	1	1	1	0.12	Lactadherin OS=Homo
2626	1	SwissProt_HUMAN$	2::Q13309|SKP2_HUMAN	24	47731	1	1	1	1	0.10	S-phase kinase-associ
2627	1	SwissProt_HUMAN$	2::Q15031|SYLM_HUMAN	24	101911	2	2	2	2	0.10	Probable leucine--tRN
2628	1	SwissProt_HUMAN$	2::Q08AM6|VAC14_HUMAN	24	87917	1	1	1	1	0.06	Protein VAC14 homolo
2629	1	SwissProt_HUMAN$	2::P19388|RPAB1_HUMAN	24	24536	1	1	1	1	0.21	DNA-directed RNA pol
2630	1	SwissProt_HUMAN$	2::Q6NUM9|RETST_HUMAN	24	66777	1	1	1	1	0.07	All-trans-retinol 13,14
2631	1	SwissProt_HUMAN$	2::P22570|ADRO_HUMAN	24	53803	2	1	2	1	0.09	NADPH:adrenodoxin o
2632	1	SwissProt_HUMAN$	2::Q14232|EI2BA_HUMAN	24	33691	1	1	1	1	0.15	Translation initiation f
2633	1	SwissProt_HUMAN$	2::Q63HN8|RN213_HUMAN	24	591030	1	1	1	1	0.01	E3 ubiquitin-protein li
2634	1	SwissProt_HUMAN$	2::Q86YN1|DOPP1_HUMAN	24	27013	1	1	1	1	0.19	Dolichyldiphosphatase
2635	1	SwissProt_HUMAN$	2::O60573|IF4E2_HUMAN	24	28344	1	1	1	1	0.18	Eukaryotic translation
2636	1	SwissProt_HUMAN$	2::Q0WX57|U17LO_HUMAN	24	59673	2	1	2	1	0.08	Ubiquitin carboxyl-ter
2637	1	SwissProt_HUMAN$	2::Q9NX20|RM16_HUMAN	24	28431	2	1	2	1	0.18	39S ribosomal protein
2638	1	SwissProt_HUMAN$	2::P30626|SORCN_HUMAN	24	21662	3	1	3	1	0.24	Sorcin OS=Homo sapi
2639	1	SwissProt_HUMAN$	2::L0R6Q1|S35U4_HUMAN	24	11126	1	1	1	1	0.52	SLC35A4 upstream op
2640	1	SwissProt_HUMAN$	2::Q9P035|HACD3_HUMAN	24	43132	2	1	2	1	0.12	Very-long-chain (3R)-
2641	1	SwissProt_HUMAN$	2::O43670|ZN207_HUMAN	24	50717	2	1	2	1	0.10	BUB3-interacting and
2642	1	SwissProt_HUMAN$	2::Q9HCU5|PREB_HUMAN	24	45440	2	1	2	1	0.11	Prolactin regulatory el
2643	1	SwissProt_HUMAN$	2::Q99569|PKP4_HUMAN	24	131787	1	1	1	1	0.04	Plakophilin-4 OS=Hon
2644	1	SwissProt_HUMAN$	2::P78318|IGBP1_HUMAN	24	39198	1	1	1	1	0.13	Immunoglobulin-bindi
2645	1	SwissProt_HUMAN$	2::P23434|GCSH_HUMAN	24	18873	1	1	1	1	0.28	Glycine cleavage syste
2646	1	SwissProt_HUMAN$	2::Q2NKX8|ERC6L_HUMAN	24	141015	1	1	1	1	0.03	DNA excision repair pr
2647	1	SwissProt_HUMAN$	2::P15559|NQO1_HUMAN	24	30848	1	1	1	1	0.16	NAD(P)H dehydrogena
2648	1	SwissProt_HUMAN$	2::Q6P3W7|SCYL2_HUMAN	24	103642	1	1	1	1	0.05	SCY1-like protein 2 OS
2649	1	SwissProt_HUMAN$	2::Q14653|IRF3_HUMAN	24	47190	1	1	1	1	0.11	Interferon regulatory
2650	1	SwissProt_HUMAN$	2::P30622|CLIP1_HUMAN	24	162147	2	1	2	1	0.03	CAP-Gly domain-conta
2651	1	SwissProt_HUMAN$	2::Q9HCB6|SPON1_HUMAN	24	90914	1	1	1	1	0.05	Spondin-1 OS=Homo
2652	1	SwissProt_HUMAN$	2::Q6WCQ1|MPRIP_HUMAN	24	116461	1	1	1	1	0.04	Myosin phosphatase R
2653	1	SwissProt_HUMAN$	2::P09110|THIK_HUMAN	24	44264	2	2	2	2	0.24	3-ketoacyl-CoA thiolas
2654	1	SwissProt_HUMAN$	2::Q96M32|KAD7_HUMAN	23	82607	1	1	1	1	0.06	Adenylate kinase 7 OS
2655	1	SwissProt_HUMAN$	2::A0FGR8|ESYT2_HUMAN	23	102294	3	2	3	2	0.10	Extended synaptotagr

↑Family	M	DB	Accession	Score	Mass	Matches	Match(sig)	Sequences	Seq(sig)	emPAI	Description
2656	1	SwissProt_HUMAN$	2::Q5M9Q1|NKAPL_HUMAN	23	46284	1	1	1	1	0.11	NKAP-like protein OS=
2657	1	SwissProt_HUMAN$	2::Q13426|XRCC4_HUMAN	23	38263	1	1	1	1	0.13	DNA repair protein XR
2658	1	SwissProt_HUMAN$	2::Q15007|FL2D_HUMAN	23	44217	3	1	2	1	0.11	Pre-mRNA-splicing reg
2659	1	SwissProt_HUMAN$	2::Q14BN4|SLMAP_HUMAN	23	95140	1	1	1	1	0.05	Sarcolemmal membra
2660	1	SwissProt_HUMAN$	2::Q96EK7|F120B_HUMAN	23	103716	1	1	1	1	0.05	Constitutive coactivat
2661	1	SwissProt_HUMAN$	2::Q9NXH9|TRM1_HUMAN	23	72188	1	1	1	1	0.07	tRNA (guanine(26)-N(
2662	1	SwissProt_HUMAN$	2::O15013|ARHGA_HUMAN	23	151516	1	1	1	1	0.03	Rho guanine nucleotid
2663	1	SwissProt_HUMAN$	2::P43652|AFAM_HUMAN	23	69024	1	1	1	1	0.07	Afamin OS=Homo sap
2664	1	SwissProt_HUMAN$	2::P07585|PGS2_HUMAN	23	39722	1	1	1	1	0.13	Decorin OS=Homo sa
2665	1	SwissProt_HUMAN$	2::P53384|NUBP1_HUMAN	23	34512	1	1	1	1	0.15	Cytosolic Fe-S cluster
2666	1	SwissProt_HUMAN$	2::Q99986|VRK1_HUMAN	23	45447	2	1	2	1	0.11	Serine/threonine-prot
2667	1	SwissProt_HUMAN$	2::Q5T9S5|CCD18_HUMAN	23	168857	1	1	1	1	0.03	Coiled-coil domain-co
2668	1	SwissProt_HUMAN$	2::Q96E11|RRFM_HUMAN	23	29259	1	1	1	1	0.17	Ribosome-recycling fa
2669	1	SwissProt_HUMAN$	2::Q6ZRV2|FA83H_HUMAN	23	127044	1	1	1	1	0.04	Protein FAM83H OS=H
2670	1	SwissProt_HUMAN$	2::Q9NXW2|DJB12_HUMAN	23	41834	2	1	2	1	0.12	DnaJ homolog subfam
2671	1	SwissProt_HUMAN$	2::Q712K3|UB2R2_HUMAN	23	27149	1	1	1	1	0.19	Ubiquitin-conjugating
2672	1	SwissProt_HUMAN$	2::O60216|RAD21_HUMAN	23	71645	4	1	3	1	0.07	Double-strand-break r
2673	1	SwissProt_HUMAN$	2::O15162|PLS1_HUMAN	23	35026	1	1	1	1	0.14	Phospholipid scrambla
2674	1	SwissProt_HUMAN$	2::Q8IXU6|S35F2_HUMAN	23	41185	1	1	1	1	0.12	Solute carrier family 3
2675	1	SwissProt_HUMAN$	2::Q13277|STX3_HUMAN	23	33134	1	1	1	1	0.15	Syntaxin-3 OS=Homo
2676	1	SwissProt_HUMAN$	2::Q9NXU5|ARL15_HUMAN	23	22862	1	1	1	1	0.23	ADP-ribosylation facto
2677	1	SwissProt_HUMAN$	2::P56556|NDUA6_HUMAN	23	15127	2	1	2	1	0.36	NADH dehydrogenase
2678	1	SwissProt_HUMAN$	2::Q96S44|PRPK_HUMAN	23	28143	1	1	1	1	0.18	EKC/KEOPS complex s
2679	1	SwissProt_HUMAN$	2::Q9UBV2|SE1L1_HUMAN	23	88698	1	1	1	1	0.05	Protein sel-1 homolog
2680	1	SwissProt_HUMAN$	2::Q8N6M6|AMPO_HUMAN	23	93512	1	1	1	1	0.05	Aminopeptidase O OS
2681	1	SwissProt_HUMAN$	2::Q9UHA4|LTOR3_HUMAN	23	13614	2	1	2	1	0.41	Ragulator complex pro
2682	1	SwissProt_HUMAN$	2::O14949|QCR8_HUMAN	23	9900	1	1	1	1	0.60	Cytochrome b-c1 com
2683	1	SwissProt_HUMAN$	2::Q8N1G2|CMTR1_HUMAN	23	95260	4	2	4	2	0.10	Cap-specific mRNA (n
2684	1	SwissProt_HUMAN$	2::Q15836|VAMP3_HUMAN	23	11302	2	1	2	1	0.51	Vesicle-associated me
2685	1	SwissProt_HUMAN$	2::Q86XX4|FRAS1_HUMAN	23	442928	3	1	1	1	0.01	Extracellular matrix pr
2686	1	SwissProt_HUMAN$	2::Q15800|MSMO1_HUMAN	23	35193	1	1	1	1	0.14	Methylsterol monooxy
2687	1	SwissProt_HUMAN$	2::O95218|ZRAB2_HUMAN	23	37382	2	1	2	1	0.13	Zinc finger Ran-bindin
2688	1	SwissProt_HUMAN$	2::Q01970|PLCB3_HUMAN	23	138713	1	1	1	1	0.03	1-phosphatidylinositol
2689	1	SwissProt_HUMAN$	2::Q9BVL2|NUP58_HUMAN	23	60860	2	2	2	2	0.17	Nucleoporin p58/p45 (
2690	1	SwissProt_HUMAN$	2::P31937|3HIDH_HUMAN	23	35306	1	1	1	1	0.14	3-hydroxyisobutyrate
2691	1	SwissProt_HUMAN$	2::P49366|DHYS_HUMAN	23	40945	1	1	1	1	0.12	Deoxyhypusine syntha
2692	1	SwissProt_HUMAN$	2::P13807|GYS1_HUMAN	23	83732	1	1	1	1	0.06	Glycogen [starch] syn
2693	1	SwissProt_HUMAN$	2::Q68CP9|ARID2_HUMAN	23	197268	1	1	1	1	0.02	AT-rich interactive do
2694	1	SwissProt_HUMAN$	2::Q9BW91|NUDT9_HUMAN	23	39101	2	2	2	2	0.27	ADP-ribose pyrophosp
2695	1	SwissProt_HUMAN$	2::Q9C005|DPY30_HUMAN	23	11243	1	1	1	1	0.51	Protein dpy-30 homolo

↑Family	M	DB	Accession	Score	Mass	Matches	Match(sig)	Sequences	Seq(sig)	emPAI	Description
2696	1	SwissProt_HUMAN$	2::Q9Y530|OARD1_HUMAN	23	17014	1	1	1	1	0.32	ADP-ribose glycohydr
2697	1	SwissProt_HUMAN$	2::Q9C0J8|WDR33_HUMAN	23	145799	2	1	2	1	0.03	pre-mRNA 3' end proc
2698	1	SwissProt_HUMAN$	2::Q8TF09|DLRB2_HUMAN	23	10848	1	1	1	1	0.53	Dynein light chain roa
2699	1	SwissProt_HUMAN$	2::Q9H832|UBE2Z_HUMAN	23	38186	2	2	2	2	0.28	Ubiquitin-conjugating
2700	1	SwissProt_HUMAN$	2::Q9BPX6|MICU1_HUMAN	23	54316	1	1	1	1	0.09	Calcium uptake protei
2701	1	SwissProt_HUMAN$	2::Q01433|AMPD2_HUMAN	23	100624	1	1	1	1	0.05	AMP deaminase 2 OS=
2702	1	SwissProt_HUMAN$	2::P49427|UB2R1_HUMAN	22	26720	2	2	2	2	0.42	Ubiquitin-conjugating
2703	1	SwissProt_HUMAN$	2::Q8WTS6|SETD7_HUMAN	22	40695	1	1	1	1	0.12	Histone-lysine N-meth
2704	1	SwissProt_HUMAN$	2::Q9P0J7|KCMF1_HUMAN	22	41919	1	1	1	1	0.12	E3 ubiquitin-protein li
2705	1	SwissProt_HUMAN$	2::P27105|STOM_HUMAN	22	31711	1	1	1	1	0.16	Stomatin OS=Homo s
2706	1	SwissProt_HUMAN$	2::Q86YL5|TDRP_HUMAN	22	20390	1	1	1	1	0.26	Testis development-re
2707	1	SwissProt_HUMAN$	2::Q5VWZ2|LYPL1_HUMAN	22	26299	1	1	1	1	0.20	Lysophospholipase-lik
2708	1	SwissProt_HUMAN$	2::P63272|SPT4H_HUMAN	22	13185	1	1	1	1	0.42	Transcription elongati
2709	1	SwissProt_HUMAN$	2::Q92759|TF2H4_HUMAN	22	52153	1	1	1	1	0.09	General transcription
2710	1	SwissProt_HUMAN$	2::Q9HCH5|SYTL2_HUMAN	22	104865	1	1	1	1	0.05	Synaptotagmin-like pr
2711	1	SwissProt_HUMAN$	2::Q9Y2Q3|GSTK1_HUMAN	22	25480	1	1	1	1	0.20	Glutathione S-transfer
2712	1	SwissProt_HUMAN$	2::Q9Y6V7|DDX49_HUMAN	22	54192	1	1	1	1	0.09	Probable ATP-depende
2713	1	SwissProt_HUMAN$	2::Q8TF63|DCNP1_HUMAN	22	26687	1	1	1	1	0.19	Dendritic cell nuclear
2714	1	SwissProt_HUMAN$	2::Q96BH1|RNF25_HUMAN	22	51187	1	1	1	1	0.10	E3 ubiquitin-protein li
2715	1	SwissProt_HUMAN$	2::Q14669|TRIPC_HUMAN	22	220296	2	1	2	1	0.02	E3 ubiquitin-protein li
2716	1	SwissProt_HUMAN$	2::Q9H9E3|COG4_HUMAN	22	89026	1	1	1	1	0.05	Conserved oligomeric
2717	1	SwissProt_HUMAN$	2::Q9NR12|PDLI7_HUMAN	22	49813	1	1	1	1	0.10	PDZ and LIM domain
2718	1	SwissProt_HUMAN$	2::P22223|CADH3_HUMAN	22	91362	1	1	1	1	0.05	Cadherin-3 OS=Homo
2719	1	SwissProt_HUMAN$	2::Q969H8|MYDGF_HUMAN	22	18783	3	2	3	2	0.65	Myeloid-derived growt
2720	1	SwissProt_HUMAN$	2::Q9NXA8|SIR5_HUMAN	22	33859	2	2	2	2	0.32	NAD-dependent protei
2721	1	SwissProt_HUMAN$	2::Q9UHR5|S30BP_HUMAN	22	33850	1	1	1	1	0.15	SAP30-binding proteir
2722	1	SwissProt_HUMAN$	2::P82675|RT05_HUMAN	22	47976	1	1	1	1	0.10	28S ribosomal protein
2723	1	SwissProt_HUMAN$	2::Q6P4Q7|CNNM4_HUMAN	22	86552	2	1	2	1	0.06	Metal transporter CNN
2724	1	SwissProt_HUMAN$	2::P29083|T2EA_HUMAN	22	49421	1	1	1	1	0.10	General transcription
2725	1	SwissProt_HUMAN$	2::P09668|CATH_HUMAN	22	37369	1	1	1	1	0.13	Pro-cathepsin H OS=H
2726	1	SwissProt_HUMAN$	2::Q7Z7H5|TMED4_HUMAN	22	25926	3	1	3	1	0.20	Transmembrane emp2
2727	1	SwissProt_HUMAN$	2::P48507|GSH0_HUMAN	22	30708	2	1	2	1	0.17	Glutamate--cysteine li
2728	1	SwissProt_HUMAN$	2::O43283|M3K13_HUMAN	22	108228	2	1	2	1	0.04	Mitogen-activated pro
2729	1	SwissProt_HUMAN$	2::Q8N1Q1|CAH13_HUMAN	22	29425	1	1	1	1	0.17	Carbonic anhydrase 1
2730	1	SwissProt_HUMAN$	2::P49459|UBE2A_HUMAN	22	17305	1	1	1	1	0.31	Ubiquitin-conjugating
2731	1	SwissProt_HUMAN$	2::Q8N806|UBR7_HUMAN	22	47968	1	1	1	1	0.10	Putative E3 ubiquitin-
2732	1	SwissProt_HUMAN$	2::Q9BQ67|GRWD1_HUMAN	22	49388	2	1	2	1	0.10	Glutamate-rich WD re
2733	1	SwissProt_HUMAN$	2::P24468|COT2_HUMAN	22	45542	2	1	2	1	0.11	COUP transcription fa
2734	1	SwissProt_HUMAN$	2::Q86WX3|AROS_HUMAN	22	15424	1	1	1	1	0.35	Active regulator of SIF
2735	1	SwissProt_HUMAN$	2::Q9Y2U8|MAN1_HUMAN	22	99935	2	1	2	1	0.05	Inner nuclear membra

↑Family	M	DB	Accession	Score	Mass	Matches	Match(sig)	Sequences	Seq(sig)	emPAI	Description
2736	1	SwissProt_HUMAN$	2::Q9ULD2|MTUS1_HUMAN	22	141309	1	1	1	1	0.03	Microtubule-associate
2737	1	SwissProt_HUMAN$	2::Q9P1V8|SAM15_HUMAN	22	77103	1	1	1	1	0.06	Sterile alpha motif do
2738	1	SwissProt_HUMAN$	2::Q9Y3B9|RRP15_HUMAN	22	31465	1	1	1	1	0.16	RRP15-like protein OS
2739	1	SwissProt_HUMAN$	2::Q96DX4|RSPRY_HUMAN	22	64139	1	1	1	1	0.08	RING finger and SPRY
2740	1	SwissProt_HUMAN$	2::Q96BR5|COA7_HUMAN	21	25692	1	1	1	1	0.20	Cytochrome c oxidase
2741	1	SwissProt_HUMAN$	2::P49711|CTCF_HUMAN	21	82732	1	1	1	1	0.06	Transcriptional repres
2742	1	SwissProt_HUMAN$	2::Q9BTD8|RBM42_HUMAN	21	50382	1	1	1	1	0.10	RNA-binding protein 4
2743	1	SwissProt_HUMAN$	2::Q13425|SNTB2_HUMAN	21	57913	1	1	1	1	0.09	Beta-2-syntrophin OS
2744	1	SwissProt_HUMAN$	2::P11441|UBL4A_HUMAN	21	17766	2	1	2	1	0.30	Ubiquitin-like protein
2745	1	SwissProt_HUMAN$	2::Q6DHV5|C2D2B_HUMAN	21	166681	2	2	2	2	0.06	Protein CC2D2B OS=H
2746	1	SwissProt_HUMAN$	2::Q15369|ELOC_HUMAN	21	12465	3	3	2	2	1.11	Elongin-C OS=Homo s
2747	1	SwissProt_HUMAN$	2::Q6IQ49|SDE2_HUMAN	21	49711	1	1	1	1	0.10	Replication stress resp
2748	1	SwissProt_HUMAN$	2::P23352|KALM_HUMAN	21	76064	1	1	1	1	0.06	Anosmin-1 OS=Homo
2749	1	SwissProt_HUMAN$	2::Q9H3S7|PTN23_HUMAN	21	178861	1	1	1	1	0.03	Tyrosine-protein phos
2750	1	SwissProt_HUMAN$	2::Q96MW1|CCD43_HUMAN	21	25233	1	1	1	1	0.21	Coiled-coil domain-co
2751	1	SwissProt_HUMAN$	2::Q9HD15|SRA1_HUMAN	21	25657	1	1	1	1	0.20	Steroid receptor RNA
2752	1	SwissProt_HUMAN$	2::O43402|EMC8_HUMAN	21	23758	1	1	1	1	0.22	ER membrane protein
2753	1	SwissProt_HUMAN$	2::Q5VSL9|STRP1_HUMAN	21	95515	2	2	1	1	0.05	Striatin-interacting pr
2754	1	SwissProt_HUMAN$	2::P08174|DAF_HUMAN	21	41374	1	1	1	1	0.12	Complement decay-ac
2755	1	SwissProt_HUMAN$	2::P00747|PLMN_HUMAN	21	90510	1	1	1	1	0.05	Plasminogen OS=Hor
2756	1	SwissProt_HUMAN$	2::Q92989|CLP1_HUMAN	21	47615	2	1	2	1	0.10	Polyribonucleotide 5'-l
2757	1	SwissProt_HUMAN$	2::Q96ST2|IWS1_HUMAN	21	91899	2	1	2	1	0.05	Protein IWS1 homolog
2758	1	SwissProt_HUMAN$	2::Q8WWH5|TRUB1_HUMAN	21	37229	2	2	2	2	0.29	Probable tRNA pseudo
2759	1	SwissProt_HUMAN$	2::Q9ULC3|RAB23_HUMAN	21	26643	1	1	1	1	0.19	Ras-related protein Ra
2760	1	SwissProt_HUMAN$	2::Q15648|MED1_HUMAN	21	168373	2	2	2	2	0.06	Mediator of RNA polyn
2761	1	SwissProt_HUMAN$	2::Q96QE5|TEFM_HUMAN	21	41650	1	1	1	1	0.12	Transcription elongati
2762	1	SwissProt_HUMAN$	2::O95159|ZFPL1_HUMAN	21	34092	1	1	1	1	0.15	Zinc finger protein-like
2763	1	SwissProt_HUMAN$	2::Q8IXI2|MIRO1_HUMAN	21	70739	1	1	1	1	0.07	Mitochondrial Rho GTF
2764	1	SwissProt_HUMAN$	2::Q8WWI5|CTL1_HUMAN	21	73253	1	1	1	1	0.07	Choline transporter-lik
2765	1	SwissProt_HUMAN$	2::Q9H6F5|CCD86_HUMAN	21	40211	1	1	1	1	0.12	Coiled-coil domain-co
2766	1	SwissProt_HUMAN$	2::Q96A59|MALD3_HUMAN	21	44882	1	1	1	1	0.11	MARVEL domain-conta
2767	1	SwissProt_HUMAN$	2::Q99549|MPP8_HUMAN	21	97123	1	1	1	1	0.05	M-phase phosphoprot
2768	1	SwissProt_HUMAN$	2::Q8NFJ9|BBS1_HUMAN	21	65042	2	1	2	1	0.08	Bardet-Biedl syndrom
2769	1	SwissProt_HUMAN$	2::O15121|DEGS1_HUMAN	21	37841	1	1	1	1	0.13	Sphingolipid delta(4)-
2770	1	SwissProt_HUMAN$	2::Q5SNT2|TM201_HUMAN	21	72190	1	1	1	1	0.07	Transmembrane prote
2771	1	SwissProt_HUMAN$	2::O75717|WDHD1_HUMAN	21	125888	2	2	2	2	0.08	WD repeat and HMG-b
2772	1	SwissProt_HUMAN$	2::Q8WUI4|HDAC7_HUMAN	21	102863	2	2	2	2	0.10	Histone deacetylase 7
2773	1	SwissProt_HUMAN$	2::Q6NUQ1|RINT1_HUMAN	21	90574	1	1	1	1	0.05	RAD50-interacting pro
2774	1	SwissProt_HUMAN$	2::Q8IXQ6|PARP9_HUMAN	21	96281	1	1	1	1	0.05	Protein mono-ADP-rib
2775	1	SwissProt_HUMAN$	2::Q8IYM9|TRI22_HUMAN	21	56911	1	1	1	1	0.09	E3 ubiquitin-protein li

↑Family	M	DB	Accession	Score	Mass	Matches	Match(sig)	Sequences	Seq(sig)	emPAI	Description
2776	1	SwissProt_HUMAN$	2::Q9NYH9|UTP6_HUMAN	21	70149	2	1	2	1	0.07	U3 small nucleolar RN
2777	1	SwissProt_HUMAN$	2::Q96RQ1|ERGI2_HUMAN	21	42521	1	1	1	1	0.12	Endoplasmic reticulum
2778	1	SwissProt_HUMAN$	2::Q05D32|CTSL2_HUMAN	21	52966	1	1	1	1	0.09	CTD small phosphatas
2779	1	SwissProt_HUMAN$	2::P14061|DHB1_HUMAN	21	34928	2	1	1	1	0.14	Estradiol 17-beta-deh
2780	1	SwissProt_HUMAN$	2::Q53GQ0|DHB12_HUMAN	21	34302	2	2	2	2	0.32	Very-long-chain 3-oxo
2781	1	SwissProt_HUMAN$	2::Q9UN73|PCDA6_HUMAN	21	102652	1	1	1	1	0.05	Protocadherin alpha-6
2782	1	SwissProt_HUMAN$	2::P57081|WDR4_HUMAN	21	45461	1	1	1	1	0.11	tRNA (guanine-N(7)-)
2783	1	SwissProt_HUMAN$	2::Q92560|BAP1_HUMAN	21	80312	1	1	1	1	0.06	Ubiquitin carboxyl-ter
2784	1	SwissProt_HUMAN$	2::Q13286|CLN3_HUMAN	21	47592	1	1	1	1	0.10	Battenin OS=Homo sa
2785	1	SwissProt_HUMAN$	2::Q96FX8|PERP_HUMAN	21	21372	1	1	1	1	0.24	p53 apoptosis effector
2786	1	SwissProt_HUMAN$	2::Q12934|BFSP1_HUMAN	20	74499	1	1	1	1	0.07	Filensin OS=Homo sa
2787	1	SwissProt_HUMAN$	2::Q14457|BECN1_HUMAN	20	51863	2	1	1	1	0.10	Beclin-1 OS=Homo sa
2788	1	SwissProt_HUMAN$	2::Q8TBF4|ZCRB1_HUMAN	20	24576	1	1	1	1	0.21	Zinc finger CCHC-type
2789	1	SwissProt_HUMAN$	2::Q76NI1|KNDC1_HUMAN	20	191277	1	1	1	1	0.03	Kinase non-catalytic C
2790	1	SwissProt_HUMAN$	2::Q9H6R0|DHX33_HUMAN	20	78824	1	1	1	1	0.06	ATP-dependent RNA h
2791	1	SwissProt_HUMAN$	2::P40121|CAPG_HUMAN	20	38474	1	1	1	1	0.13	Macrophage-capping p
2792	1	SwissProt_HUMAN$	2::Q9H1A4|APC1_HUMAN	20	216361	1	1	1	1	0.02	Anaphase-promoting c
2793	1	SwissProt_HUMAN$	2::Q6P1N9|TATD1_HUMAN	20	33580	1	1	1	1	0.15	Putative deoxyribonuc
2794	1	SwissProt_HUMAN$	2::P52298|NCBP2_HUMAN	20	17990	1	1	1	1	0.30	Nuclear cap-binding p
2795	1	SwissProt_HUMAN$	2::Q9NQT5|EXOS3_HUMAN	20	29553	1	1	1	1	0.17	Exosome complex con
2796	1	SwissProt_HUMAN$	2::Q8N999|CL029_HUMAN	20	37466	1	1	1	1	0.13	Uncharacterized prote
2797	1	SwissProt_HUMAN$	2::O94913|PCF11_HUMAN	20	172944	1	1	1	1	0.03	Pre-mRNA cleavage co
2798	1	SwissProt_HUMAN$	2::P62877|RBX1_HUMAN	20	12266	1	1	1	1	0.46	E3 ubiquitin-protein li
2799	1	SwissProt_HUMAN$	2::Q13951|PEBB_HUMAN	20	21495	2	1	2	1	0.24	Core-binding factor su
2800	1	SwissProt_HUMAN$	2::O14523|C2C2L_HUMAN	20	76134	1	1	1	1	0.06	Phospholipid transfer
2801	1	SwissProt_HUMAN$	2::Q86VD1|MORC1_HUMAN	20	112810	1	1	1	1	0.04	MORC family CW-type
2802	1	SwissProt_HUMAN$	2::Q6YP21|KAT3_HUMAN	20	51368	1	1	1	1	0.10	Kynurenine--oxogluta
2803	1	SwissProt_HUMAN$	2::Q8TDX7|NEK7_HUMAN	20	34528	1	1	1	1	0.15	Serine/threonine-prot
2804	1	SwissProt_HUMAN$	2::P40692|MLH1_HUMAN	20	84548	1	1	1	1	0.06	DNA mismatch repair
2805	1	SwissProt_HUMAN$	2::P0DPD7|EFMT4_HUMAN	20	28288	1	1	1	1	0.18	EEF1A lysine methyltr
2806	1	SwissProt_HUMAN$	2::P21359|NF1_HUMAN	20	319168	1	1	1	1	0.01	Neurofibromin OS=Ho
2807	1	SwissProt_HUMAN$	2::Q9HBH1|DEFM_HUMAN	20	26996	2	1	2	1	0.19	Peptide deformylase,
2808	1	SwissProt_HUMAN$	2::P48651|PTSS1_HUMAN	20	55491	1	1	1	1	0.09	Phosphatidylserine sy
2809	1	SwissProt_HUMAN$	2::Q14807|KIF22_HUMAN	20	73217	1	1	1	1	0.07	Kinesin-like protein KI
2810	1	SwissProt_HUMAN$	2::Q8IZ73|RUSD2_HUMAN	20	61273	1	1	1	1	0.08	RNA pseudouridylate s
2811	1	SwissProt_HUMAN$	2::O75128|COBL_HUMAN	20	135533	1	1	1	1	0.04	Protein cordon-bleu O
2812	1	SwissProt_HUMAN$	2::Q8NC74|RB8NL_HUMAN	20	71388	1	1	1	1	0.07	RBBP8 N-terminal-like
2813	1	SwissProt_HUMAN$	2::Q9NVU7|SDA1_HUMAN	20	79820	1	1	1	1	0.06	Protein SDA1 homolog
2814	1	SwissProt_HUMAN$	2::P47985|UCRI_HUMAN	20	29649	3	1	3	1	0.17	Cytochrome b-c1 com
2815	1	SwissProt_HUMAN$	2::Q12888|TP53B_HUMAN	20	213443	1	1	1	1	0.02	TP53-binding protein

↑Family	M	DB	Accession	Score	Mass	Matches	Match(sig)	Sequences	Seq(sig)	emPAI	Description
2816	1	SwissProt_HUMAN$	2::Q9UP83|COG5_HUMAN	20	92685	3	2	3	2	0.11	Conserved oligomeric
2817	1	SwissProt_HUMAN$	2::Q9UBV8|PEF1_HUMAN	20	30361	1	1	1	1	0.17	Peflin OS=Homo sapie
2818	1	SwissProt_HUMAN$	2::Q9NUI1|DECR2_HUMAN	20	30758	1	1	1	1	0.17	Peroxisomal 2,4-dienc
2819	1	SwissProt_HUMAN$	2::Q9BQL6|FERM1_HUMAN	20	77387	1	1	1	1	0.06	Fermitin family homol
2820	1	SwissProt_HUMAN$	2::O00186|STXB3_HUMAN	20	67721	2	1	2	1	0.07	Syntaxin-binding prot
2821	1	SwissProt_HUMAN$	2::Q9NY15|STAB1_HUMAN	20	275300	2	1	2	1	0.02	Stabilin-1 OS=Homo s
2822	1	SwissProt_HUMAN$	2::Q9NZB2|F120A_HUMAN	20	121811	1	1	1	1	0.04	Constitutive coactivat
2823	1	SwissProt_HUMAN$	2::P41227|NAA10_HUMAN	20	26442	1	1	1	1	0.19	N-alpha-acetyltransfer
2824	1	SwissProt_HUMAN$	2::Q8NEW0|ZNT7_HUMAN	19	41599	1	1	1	1	0.12	Zinc transporter 7 OS
2825	1	SwissProt_HUMAN$	2::Q92614|MY18A_HUMAN	19	232971	1	1	1	1	0.02	Unconventional myosi
2826	1	SwissProt_HUMAN$	2::Q5T0N5|FBP1L_HUMAN	19	70021	2	1	2	1	0.07	Formin-binding protei
2827	1	SwissProt_HUMAN$	2::Q8N6T3|ARFG1_HUMAN	19	44640	1	1	1	1	0.11	ADP-ribosylation facto
2828	1	SwissProt_HUMAN$	2::Q99575|POP1_HUMAN	19	114636	3	1	3	1	0.04	Ribonucleases P/MRP
2829	1	SwissProt_HUMAN$	2::Q6GMV3|PTRD1_HUMAN	19	15795	1	1	1	1	0.34	Putative peptidyl-tRN/
2830	1	SwissProt_HUMAN$	2::P01160|ANF_HUMAN	19	16385	1	1	1	1	0.33	Natriuretic peptides A
2831	1	SwissProt_HUMAN$	2::O95562|SFT2B_HUMAN	19	17767	1	1	1	1	0.30	Vesicle transport prote
2832	1	SwissProt_HUMAN$	2::Q96DG6|CMBL_HUMAN	19	28030	1	1	1	1	0.18	Carboxymethylenebut
2833	1	SwissProt_HUMAN$	2::Q9BUN8|DERL1_HUMAN	19	28782	1	1	1	1	0.18	Derlin-1 OS=Homo sa
2834	1	SwissProt_HUMAN$	2::Q92520|FAM3C_HUMAN	19	24665	2	1	2	1	0.21	Protein FAM3C OS=Hc
2835	1	SwissProt_HUMAN$	2::Q9BXX2|AN30B_HUMAN	19	157950	2	1	2	1	0.03	Ankyrin repeat domaii
2836	1	SwissProt_HUMAN$	2::Q9H6V9|LDAH_HUMAN	19	37294	1	1	1	1	0.13	Lipid droplet-associate
2837	1	SwissProt_HUMAN$	2::O95302|FKBP9_HUMAN	19	63044	1	1	1	1	0.08	Peptidyl-prolyl cis-trar
2838	1	SwissProt_HUMAN$	2::Q6P1N0|C2D1A_HUMAN	19	103998	1	1	1	1	0.05	Coiled-coil and C2 dor
2839	1	SwissProt_HUMAN$	2::Q9BZF9|UACA_HUMAN	19	162404	1	1	1	1	0.03	Uveal autoantigen wit
2840	1	SwissProt_HUMAN$	2::Q9NXH8|TOR4A_HUMAN	19	46885	1	1	1	1	0.11	Torsin-4A OS=Homo s
2841	1	SwissProt_HUMAN$	2::Q8NF37|PCAT1_HUMAN	19	59113	2	2	2	2	0.17	Lysophosphatidylcholi
2842	1	SwissProt_HUMAN$	2::Q9H1I8|ASCC2_HUMAN	19	86306	1	1	1	1	0.06	Activating signal coint
2843	1	SwissProt_HUMAN$	2::Q96ME1|FXL18_HUMAN	19	88284	1	1	1	1	0.06	F-box/LRR-repeat prot
2844	1	SwissProt_HUMAN$	2::P16930|FAAA_HUMAN	19	46344	1	1	1	1	0.11	Fumarylacetoacetase
2845	1	SwissProt_HUMAN$	2::P80303|NUCB2_HUMAN	19	50191	1	1	1	1	0.10	Nucleobindin-2 OS=H
2846	1	SwissProt_HUMAN$	2::O43148|MCES_HUMAN	19	54809	1	1	1	1	0.09	mRNA cap guanine-N7
2847	1	SwissProt_HUMAN$	2::P60520|GBRL2_HUMAN	19	13658	1	1	1	1	0.41	Gamma-aminobutyric
2848	1	SwissProt_HUMAN$	2::Q96FZ2|HMCES_HUMAN	19	40549	2	1	2	1	0.12	Abasic site processing
2849	1	SwissProt_HUMAN$	2::Q16637|SMN_HUMAN	19	31828	1	1	1	1	0.16	Survival motor neuror
2850	1	SwissProt_HUMAN$	2::Q9UIG0|BAZ1B_HUMAN	19	170796	4	3	4	3	0.09	Tyrosine-protein kinas
2851	1	SwissProt_HUMAN$	2::Q9NV70|EXOC1_HUMAN	19	101917	2	2	2	2	0.10	Exocyst complex comp
2852	1	SwissProt_HUMAN$	2::Q2TV78|MST1L_HUMAN	19	79642	1	1	1	1	0.06	Putative macrophage
2853	1	SwissProt_HUMAN$	2::Q96IJ6|GMPPA_HUMAN	19	46262	1	1	1	1	0.11	Mannose-1-phosphate
2854	1	SwissProt_HUMAN$	2::P20936|RASA1_HUMAN	19	116329	1	1	1	1	0.04	Ras GTPase-activating
2855	1	SwissProt_HUMAN$	2::O00635|TRI38_HUMAN	19	53381	1	1	1	1	0.09	E3 ubiquitin-protein li

↑Family	M	DB	Accession	Score	Mass	Matches	Match(sig)	Sequences	Seq(sig)	emPAI	Description
2856	1	SwissProt_HUMAN$	2::Q969T9|WBP2_HUMAN	19	28068	1	1	1	1	0.18	WW domain-binding p
2857	1	SwissProt_HUMAN$	2::Q9Y5Q8|TF3C5_HUMAN	19	59533	3	1	3	1	0.08	General transcription f
2858	1	SwissProt_HUMAN$	2::Q8IV08|PLD3_HUMAN	19	54671	1	1	1	1	0.09	5'-3' exonuclease PLD
2859	1	SwissProt_HUMAN$	2::Q53T59|H1BP3_HUMAN	19	42754	1	1	1	1	0.12	HCLS1-binding proteir
2860	1	SwissProt_HUMAN$	2::Q96BQ3|TRI43_HUMAN	19	52231	1	1	1	1	0.09	Tripartite motif-contai
2861	1	SwissProt_HUMAN$	2::Q8N392|RHG18_HUMAN	19	74930	1	1	1	1	0.07	Rho GTPase-activating
2862	1	SwissProt_HUMAN$	2::Q9UQN3|CHM2B_HUMAN	18	23891	1	1	1	1	0.22	Charged multivesicula
2863	1	SwissProt_HUMAN$	2::P82663|RT25_HUMAN	18	20103	1	1	1	1	0.26	28S ribosomal protein
2864	1	SwissProt_HUMAN$	2::O60271|JIP4_HUMAN	18	146115	3	2	3	2	0.07	C-Jun-amino-terminal
2865	1	SwissProt_HUMAN$	2::A6NCK2|TR43B_HUMAN	18	52276	1	1	1	1	0.09	Tripartite motif-contai
2866	1	SwissProt_HUMAN$	2::Q9UPW0|FOXJ3_HUMAN	18	68917	1	1	1	1	0.07	Forkhead box protein
2867	1	SwissProt_HUMAN$	2::Q96JB5|CK5P3_HUMAN	18	56885	1	1	1	1	0.09	CDK5 regulatory subu
2868	1	SwissProt_HUMAN$	2::O95972|BMP15_HUMAN	18	45026	1	1	1	1	0.11	Bone morphogenetic p
2869	1	SwissProt_HUMAN$	2::Q96JH8|RADIL_HUMAN	18	117381	2	1	1	1	0.04	Ras-associating and d
2870	1	SwissProt_HUMAN$	2::Q7L2J0|MEPCE_HUMAN	18	74310	1	1	1	1	0.07	7SK snRNA methylphc
2871	1	SwissProt_HUMAN$	2::Q9BRP4|PAAF1_HUMAN	18	42163	1	1	1	1	0.12	Proteasomal ATPase-a
2872	1	SwissProt_HUMAN$	2::O14777|NDC80_HUMAN	18	73867	1	1	1	1	0.07	Kinetochore protein N
2873	1	SwissProt_HUMAN$	2::P30038|AL4A1_HUMAN	18	61681	1	1	1	1	0.08	Delta-1-pyrroline-5-ca
2874	1	SwissProt_HUMAN$	2::Q66K66|TM198_HUMAN	18	39449	1	1	1	1	0.13	Transmembrane prote
2875	1	SwissProt_HUMAN$	2::Q5T3F8|CSCL2_HUMAN	18	94896	1	1	1	1	0.05	CSC1-like protein 2 O:
2876	1	SwissProt_HUMAN$	2::P46976|GLYG_HUMAN	18	39359	1	1	1	1	0.13	Glycogenin-1 OS=Hor
2877	1	SwissProt_HUMAN$	2::Q8TB72|PUM2_HUMAN	18	114145	1	1	1	1	0.04	Pumilio homolog 2 OS
2878	1	SwissProt_HUMAN$	2::Q9H0R4|HDHD2_HUMAN	18	28518	1	1	1	1	0.18	Haloacid dehalogenas
2879	1	SwissProt_HUMAN$	2::Q969S9|RRF2M_HUMAN	18	86546	1	1	1	1	0.06	Ribosome-releasing fa
2880	1	SwissProt_HUMAN$	2::P0CG22|DR4L1_HUMAN	18	30588	1	1	1	1	0.17	Putative dehydrogena
2881	1	SwissProt_HUMAN$	2::Q9GZN8|CT027_HUMAN	18	19279	1	1	1	1	0.27	UPF0687 protein C20c
2882	1	SwissProt_HUMAN$	2::P13762|DRB4_HUMAN	18	29922	1	1	1	1	0.17	HLA class II histocom
2883	1	SwissProt_HUMAN$	2::Q3MHD2|LSM12_HUMAN	18	21687	1	1	1	1	0.24	Protein LSM12 homolc
2884	1	SwissProt_HUMAN$	2::O75688|PPM1B_HUMAN	18	52609	1	1	1	1	0.09	Protein phosphatase 1
2885	1	SwissProt_HUMAN$	2::Q9Y673|ALG5_HUMAN	18	36922	3	1	2	1	0.14	Dolichyl-phosphate be
2886	1	SwissProt_HUMAN$	2::Q9BRP1|PDD2L_HUMAN	18	39391	1	1	1	1	0.13	Programmed cell deat
2887	1	SwissProt_HUMAN$	2::Q04323|UBXN1_HUMAN	18	33305	2	2	1	1	0.15	UBX domain-containin
2888	1	SwissProt_HUMAN$	2::Q8TB92|HMGC2_HUMAN	18	39489	2	1	1	1	0.13	3-hydroxy-3-methylgl
2889	1	SwissProt_HUMAN$	2::Q9Y4Y9|LSM5_HUMAN	18	9931	1	1	1	1	0.60	U6 snRNA-associated
2890	1	SwissProt_HUMAN$	2::P09497|CLCB_HUMAN	18	25175	1	1	1	1	0.21	Clathrin light chain B (
2891	1	SwissProt_HUMAN$	2::Q12974|TP4A2_HUMAN	18	19115	1	1	1	1	0.28	Protein tyrosine phosp
2892	1	SwissProt_HUMAN$	2::P60604|UB2G2_HUMAN	18	18554	1	1	1	1	0.29	Ubiquitin-conjugating
2893	1	SwissProt_HUMAN$	2::P01185|NEU2_HUMAN	18	17313	1	1	1	1	0.31	Vasopressin-neurophy
2894	1	SwissProt_HUMAN$	2::P34913|HYES_HUMAN	18	62575	1	1	1	1	0.08	Bifunctional epoxide h
2895	1	SwissProt_HUMAN$	2::Q9HB90|RRAGC_HUMAN	18	44196	1	1	1	1	0.11	Ras-related GTP-bindi

↑Family	M	DB	Accession	Score	Mass	Matches	Match(sig)	Sequences	Seq(sig)	emPAI	Description
2896	1	SwissProt_HUMAN$	2::Q9UK41|VPS28_HUMAN	18	25409	1	1	1	1	0.20	Vacuolar protein sortir
2897	1	SwissProt_HUMAN$	2::P25440|BRD2_HUMAN	17	88006	1	1	1	1	0.06	Bromodomain-contain
2898	1	SwissProt_HUMAN$	2::Q5VWQ8|DAB2P_HUMAN	17	131543	1	1	1	1	0.04	Disabled homolog 2-ir
2899	1	SwissProt_HUMAN$	2::Q9NX46|ADPRS_HUMAN	17	38922	1	1	1	1	0.13	ADP-ribose glycohydrc
2900	1	SwissProt_HUMAN$	2::Q59GN2|R39L5_HUMAN	17	6319	1	1	1	1	1.05	Putative 60S ribosoma
2901	1	SwissProt_HUMAN$	2::Q8ND56|LS14A_HUMAN	17	50499	2	1	2	1	0.10	Protein LSM14 homolc
2902	1	SwissProt_HUMAN$	2::Q4G0J3|LARP7_HUMAN	17	66857	1	1	1	1	0.07	La-related protein 7 O
2903	1	SwissProt_HUMAN$	2::Q9UL45|BL1S6_HUMAN	17	19731	1	1	1	1	0.27	Biogenesis of lysosom
2904	1	SwissProt_HUMAN$	2::Q14728|MFS10_HUMAN	17	48308	1	1	1	1	0.10	Major facilitator super
2905	1	SwissProt_HUMAN$	2::Q9H078|CLPB_HUMAN	17	78680	3	2	3	2	0.13	Caseinolytic peptidase
2906	1	SwissProt_HUMAN$	2::Q9UL63|MKLN1_HUMAN	17	84713	1	1	1	1	0.06	Muskelin OS=Homo sa
2907	1	SwissProt_HUMAN$	2::P10620|MGST1_HUMAN	17	17587	1	1	1	1	0.31	Microsomal glutathion
2908	1	SwissProt_HUMAN$	2::O14618|CCS_HUMAN	17	29022	1	1	1	1	0.18	Copper chaperone for
2909	1	SwissProt_HUMAN$	2::Q9P013|CWC15_HUMAN	17	26608	1	1	1	1	0.19	Spliceosome-associate
2910	1	SwissProt_HUMAN$	2::Q8N6M3|FITM2_HUMAN	17	29835	1	1	1	1	0.17	Fat storage-inducing t
2911	1	SwissProt_HUMAN$	2::Q9UKY7|CDV3_HUMAN	17	27318	1	1	1	1	0.19	Protein CDV3 homolog
2912	1	SwissProt_HUMAN$	2::P27144|KAD4_HUMAN	17	25252	4	1	4	1	0.21	Adenylate kinase 4, m
2913	1	SwissProt_HUMAN$	2::Q92879|CELF1_HUMAN	17	52030	1	1	1	1	0.10	CUGBP Elav-like famil
2914	1	SwissProt_HUMAN$	2::Q68DE3|USF3_HUMAN	16	241503	1	1	1	1	0.02	Basic helix-loop-helix
2915	1	SwissProt_HUMAN$	2::Q969Z3|MARC2_HUMAN	16	37999	1	1	1	1	0.13	Mitochondrial amidoxi
2916	1	SwissProt_HUMAN$	2::Q3YEC7|RABL6_HUMAN	16	79500	1	1	1	1	0.06	Rab-like protein 6 OS=
2917	1	SwissProt_HUMAN$	2::Q9NUL7|DDX28_HUMAN	16	59545	2	2	2	2	0.17	Probable ATP-depende
2918	1	SwissProt_HUMAN$	2::Q9BV81|EMC6_HUMAN	16	12010	1	1	1	1	0.47	ER membrane protein
2919	1	SwissProt_HUMAN$	2::Q6NYC1|JMJD6_HUMAN	16	46433	1	1	1	1	0.11	Bifunctional arginine c
2920	1	SwissProt_HUMAN$	2::P13995|MTDC_HUMAN	16	37871	3	1	3	1	0.13	Bifunctional methylene
2921	1	SwissProt_HUMAN$	2::Q96EN8|MOCOS_HUMAN	16	98058	1	1	1	1	0.05	Molybdenum cofactor
2922	1	SwissProt_HUMAN$	2::P42226|STAT6_HUMAN	16	94075	1	1	1	1	0.05	Signal transducer and
2923	1	SwissProt_HUMAN$	2::Q96GC5|RM48_HUMAN	16	23920	1	1	1	1	0.22	39S ribosomal protein
2924	1	SwissProt_HUMAN$	2::Q9NU22|MDN1_HUMAN	16	632420	4	1	4	1	0.01	Midasin OS=Homo sap
2925	1	SwissProt_HUMAN$	2::Q7Z340|ZN551_HUMAN	16	77465	1	1	1	1	0.06	Zinc finger protein 55
2926	1	SwissProt_HUMAN$	2::Q9H3H5|GPT_HUMAN	16	46060	1	1	1	1	0.11	UDP-N-acetylglucosan
2927	1	SwissProt_HUMAN$	2::Q9NQE9|HINT3_HUMAN	16	20348	1	1	1	1	0.26	Histidine triad nucleot
2928	1	SwissProt_HUMAN$	2::Q9UPT8|ZC3H4_HUMAN	16	140169	1	1	1	1	0.03	Zinc finger CCCH dom
2929	1	SwissProt_HUMAN$	2::Q7L2E3|DHX30_HUMAN	16	133854	4	2	4	2	0.07	ATP-dependent RNA h
2930	1	SwissProt_HUMAN$	2::Q5T8A7|PPR26_HUMAN	16	127274	1	1	1	1	0.04	Protein phosphatase 1
2931	1	SwissProt_HUMAN$	2::Q6ZP82|CC141_HUMAN	16	166157	1	1	1	1	0.03	Coiled-coil domain-co
2932	1	SwissProt_HUMAN$	2::Q9Y6Z4|KIAS1_HUMAN	16	19398	1	1	1	1	0.27	Putative uncharacteriz
2933	1	SwissProt_HUMAN$	2::Q96FC7|PHIPL_HUMAN	16	42459	1	1	1	1	0.12	Phytanoyl-CoA hydrox
2934	1	SwissProt_HUMAN$	2::P18887|XRCC1_HUMAN	16	69434	1	1	1	1	0.07	DNA repair protein XR
2935	1	SwissProt_HUMAN$	2::Q04206|TF65_HUMAN	16	60181	1	1	1	1	0.08	Transcription factor p6

↑Family	M	DB	Accession	Score	Mass	Matches	Match(sig)	Sequences	Seq(sig)	emPAI	Description
2936	1	SwissProt_HUMAN$	2::Q7Z7F7|RM55_HUMAN	16	15119	1	1	1	1	0.36	39S ribosomal protein
2937	1	SwissProt_HUMAN$	2::P14406|CX7A2_HUMAN	16	9390	1	1	1	1	0.63	Cytochrome c oxidase
2938	1	SwissProt_HUMAN$	2::P15121|ALDR_HUMAN	16	35830	1	1	1	1	0.14	Aldo-keto reductase fa
2939	1	SwissProt_HUMAN$	2::Q15262|PTPRK_HUMAN	15	161998	2	1	2	1	0.03	Receptor-type tyrosin
2940	1	SwissProt_HUMAN$	2::Q9H0P0|5NT3A_HUMAN	15	37924	1	1	1	1	0.13	Cytosolic 5'-nucleotida
2941	1	SwissProt_HUMAN$	2::Q9UL26|RB22A_HUMAN	15	21841	1	1	1	1	0.24	Ras-related protein Ra
2942	1	SwissProt_HUMAN$	2::O95996|APCL_HUMAN	15	243799	1	1	1	1	0.02	Adenomatous polypos
2943	1	SwissProt_HUMAN$	2::Q1ED39|KNOP1_HUMAN	15	51557	1	1	1	1	0.10	Lysine-rich nucleolar p
2944	1	SwissProt_HUMAN$	2::Q9P2Z0|THA10_HUMAN	15	28333	1	1	1	1	0.18	THAP domain-containi
2945	1	SwissProt_HUMAN$	2::Q8NHP8|PLBL2_HUMAN	15	65430	2	1	2	1	0.08	Putative phospholipas
2946	1	SwissProt_HUMAN$	2::Q9BYD1|RM13_HUMAN	15	20679	2	1	2	1	0.25	39S ribosomal protein
2947	1	SwissProt_HUMAN$	2::Q04726|TLE3_HUMAN	15	83364	1	1	1	1	0.06	Transducin-like enhan
2948	1	SwissProt_HUMAN$	2::Q96K76|UBP47_HUMAN	15	157212	2	1	2	1	0.03	Ubiquitin carboxyl-ter
2949	1	SwissProt_HUMAN$	2::P17096|HMGA1_HUMAN	15	11669	1	1	1	1	0.49	High mobility group pr
2950	1	SwissProt_HUMAN$	2::Q14242|SELPL_HUMAN	15	43174	1	1	1	1	0.12	P-selectin glycoprotei
2951	1	SwissProt_HUMAN$	2::Q14573|ITPR3_HUMAN	15	303912	2	1	2	1	0.02	Inositol 1,4,5-trisphos
2952	1	SwissProt_HUMAN$	2::Q15643|TRIPB_HUMAN	15	227447	1	1	1	1	0.02	Thyroid receptor-inter
2953	1	SwissProt_HUMAN$	2::Q9Y2P8|RCL1_HUMAN	15	40817	1	1	1	1	0.12	RNA 3'-terminal phosp
2954	1	SwissProt_HUMAN$	2::Q92823|NRCAM_HUMAN	15	143800	1	1	1	1	0.03	Neuronal cell adhesion
2955	1	SwissProt_HUMAN$	2::P78310|CXAR_HUMAN	15	40004	2	1	2	1	0.13	Coxsackievirus and ad
2956	1	SwissProt_HUMAN$	2::O94822|LTN1_HUMAN	14	200423	1	1	1	1	0.02	E3 ubiquitin-protein li
2957	1	SwissProt_HUMAN$	2::Q70CQ2|UBP34_HUMAN	14	403973	1	1	1	1	0.01	Ubiquitin carboxyl-ter
2958	1	SwissProt_HUMAN$	2::Q8IWA5|CTL2_HUMAN	14	80071	1	1	1	1	0.06	Choline transporter-lik
2959	1	SwissProt_HUMAN$	2::Q5VZK9|CARL1_HUMAN	14	151462	1	1	1	1	0.03	F-actin-uncapping pro
2960	1	SwissProt_HUMAN$	2::P35680|HNF1B_HUMAN	14	61285	1	1	1	1	0.08	Hepatocyte nuclear fa
2961	1	SwissProt_HUMAN$	2::Q9C099|LRCC1_HUMAN	14	119522	1	1	1	1	0.04	Leucine-rich repeat ar
2962	1	SwissProt_HUMAN$	2::Q92696|PGTA_HUMAN	14	65030	1	1	1	1	0.08	Geranylgeranyl transf
2963	1	SwissProt_HUMAN$	2::Q9GZR2|REXO4_HUMAN	14	46643	1	1	1	1	0.11	RNA exonuclease 4 OS
2964	1	SwissProt_HUMAN$	2::Q8WXX0|DYH7_HUMAN	14	460862	1	1	1	1	0.01	Dynein heavy chain 7,
2965	1	SwissProt_HUMAN$	2::Q96S21|RB40C_HUMAN	14	31284	1	1	1	1	0.16	Ras-related protein Ra
2966	1	SwissProt_HUMAN$	2::P05165|PCCA_HUMAN	14	80008	2	1	2	1	0.06	Propionyl-CoA carboxy
2967	1	SwissProt_HUMAN$	2::Q9BVG8|KIFC3_HUMAN	14	92718	1	1	1	1	0.05	Kinesin-like protein KI
2968	1	SwissProt_HUMAN$	2::Q9Y691|KCMB2_HUMAN	14	27111	1	1	1	1	0.19	Calcium-activated pot
2969	1	SwissProt_HUMAN$	2::Q969L2|MAL2_HUMAN	14	19113	1	1	1	1	0.28	Protein MAL2 OS=Hon
2970	1	SwissProt_HUMAN$	2::Q16281|CNGA3_HUMAN	14	78789	1	1	1	1	0.06	Cyclic nucleotide-gate
2971	1	SwissProt_HUMAN$	2::Q6ZRS2|SRCAP_HUMAN	14	343343	1	1	1	1	0.01	Helicase SRCAP OS=H
2972	1	SwissProt_HUMAN$	2::Q03431|PTH1R_HUMAN	13	66318	1	1	1	1	0.07	Parathyroid hormone/
2973	1	SwissProt_HUMAN$	2::Q6ZT12|UBR3_HUMAN	13	212296	1	1	1	1	0.02	E3 ubiquitin-protein li
2974	1	SwissProt_HUMAN$	2::Q9NRX5|SERC1_HUMAN	13	50461	2	1	2	1	0.10	Serine incorporator 1
2975	1	SwissProt_HUMAN$	2::Q13137|CACO2_HUMAN	13	52221	1	1	1	1	0.09	Calcium-binding and c

↑Family	M	DB	Accession	Score	Mass	Matches	Match(sig)	Sequences	Seq(sig)	emPAI	Description
2976	1	SwissProt_HUMAN$	2::P42696|RBM34_HUMAN	13	48535	1	1	1	1	0.10	RNA-binding protein 3

Export as CSV

Not what you expected? Try **the select summary**.

Mascot: http://www.matrixscience.com/
