# Supplementary material for: Circulating multimeric immune complexes contribute to immunopathology in COVID-19
Source: Nat Commun. 2022 Sep 26;13:5654. doi: 10.1038/s41467-022-32867-z (PMC9513013; doi:10.1038/s41467-022-32867-z)
Supplement: Supplementary file 3 — Source Data [file 41467_2022_32867_MOESM3_ESM.zip › Source Data/MS_29012021 (whole lane cut).zip!/QX20210214-02_Virologie(AG-Hengel)-20210129_(V5)_-C-_7-5_complete-lane_0k1v20_TRL_db_SARS-CoV-2+SP_H(F229078).pdf]

MASCOT Search Results

User : Wolfgang Bildl
E-mail : wolfgang.bildl@physiologie.uni-freiburg.de
Search title : QX 20210212 (db$ SARS-CoV-2+SwissProt HUMAN$ 5 ppm 20 mmu), QX20210214-02_Virologie(AG-Hengel)-20210129_(V5)_-C-_7-5_complete-lane_0k1v20_TRL_shifted_-2.7.mgf
MS data file : D:\Xcalibur\Data\Wolfgang\Q-Exactive-HF-X_20210212\QX20210214-02_Virologie(AG-Hengel)-20210129_(V5)_-C-_7-5_complete-lane_0k1v20_TRL_shifted_-2.7.mgf
Databases : **1:** db$ 20210114 (14 sequences; 14,319 residues)
2: SwissProt_HUMAN$ 20201007 (20,389 sequences; 11,363,789 residues)
Timestamp : 14 Feb 2021 at 21:10:50 GMT

Not what you expected? Try **the select summary**.

Search parameters

Type of search : MS/MS Ion Search
Enzyme : Trypsin
Variable modifications : **Acetyl (Protein N-term)**, **Carbamidomethyl (C)**, **Gln->pyro-Glu (N-term Q)**, **Oxidation (M)**
Mass values : Monoisotopic
Protein mass : Unrestricted
Peptide mass tolerance : ± 5 ppm
Fragment mass tolerance : ± 20 mmu
Max missed cleavages : 1
Instrument type : ESI-FTICR
Number of queries : 73,276

Score distribution

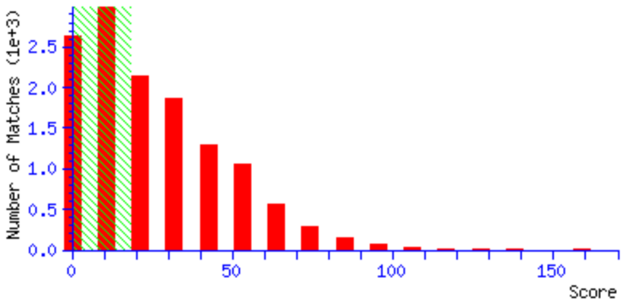

Peptide score distribution. Ions score is $-10\log(P)$, where P is the probability that the observed match is a random event.

There are **6,942** peptide matches above identity threshold and **7,173** matches above homology threshold for **73,276** queries.
On average, individual ions scores **> 18** (beyond green shading) indicate **identity or extensive homology** (p<0.05).

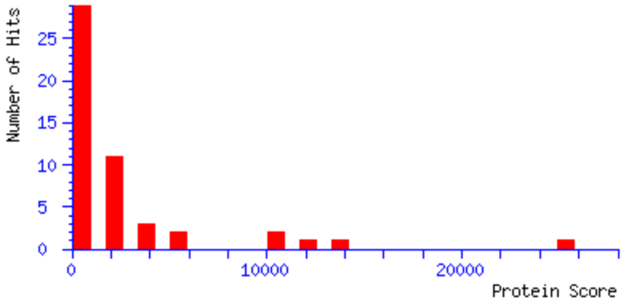

[Deprecated] Protein score distribution. Score distribution for family members in the first 50 proteins. Protein scores are derived from ions scores as a non-probabilistic basis for ranking protein families.

▼Modification statistics

Modification	Site	Above thr.
Carbamidomethyl	C	2800
Oxidation	M	839
Gln->pyro-Glu	N-term	207
Acetyl	Protein N-term	1

▼Legend

Peptide columns and rows

Dupes	...	Expect	Rank	U	1	2	Peptide	
		**0.037**	▶**2**				**GAYSLSLR**	significant
		9	▶1				GFFLFVEGGR	top ranking
		**6.4e-005**	▶**1**				**GSSIFGLAPGK**	significant and top ranking
		**1.3e-006**	▶**1**		■		**SSGTSYPDVLK**	peptide is found in all proteins in family member 1
		**6.2e-007**	▶**1**			■	**VCNYVSWIK**	peptide is found in some but not all proteins in family member 2
		**6.4e-005**	▶**1**	**U**			**GSSIFGLAPGK**	unique
▶**2**		**5.7e-005**	▶**1**				**LNTLETEEWFFK**	peptide has two duplicates
		0.18	▶*1*				*LNTLETEEWFFK*	duplicate peptide

Right-facing triangle (▶) in the Dupes or Rank column indicates content that can be expanded by clicking on it. Down-facing triangle (▼) indicates the content is expanded and can be collapsed. For more details about particular columns, see **results format help**.

Protein quantitation ratios

	Score	...	114/113	115/113
**CFAH_HUMAN**	37559		*0.962*	**1.129**
**FHR2_HUMAN**	1330		**0.859**	1.128

When quantitation method is Reporter (e.g. iTRAQ) or Multiplex (e.g. IPTL), protein ratios are displayed when a family is expanded. Ratios in *italic* indicate that the peptide log-ratios do not appear to come from a normal distribution. **Bold** indicates that if you can assume peptide ratios are normally distributed, the protein ratio is significantly different from 1.0 (at significance level 0.05).

Note that lack of bold or italic can also mean that significance or normality testing has not been performed (for example, if protein ratio type does not support it).

Protein Family Summary

Significance threshold p<	0.05	Max. number of families	AUTO
Ions score or expect cut-off	0.5	Dendrograms cut at	0
Show Percolator scores	☐		
Preferred taxonomy	All entries		

Sensitivity and FDR (reversed protein sequences)

			Target	Decoy	FDR		
PSMs	above	homology	7173	232	3.23%	Adjust to	1%

Decoy results are available in **the decoy report**.

Protein hits (460 proteins)

Filters: (none)

Export as CSV

↑Family	M	DB	Accession	Score	Mass	Matches	Match(sig)	Sequences	Seq(sig)	emPAI	Description
1	1	SwissProt_HUMAN$	2::P02768|ALBU_HUMAN	25327	69321	1031	956	77	75	606.98	Albumin OS=Homo sa
1	2	SwissProt_HUMAN$	2::P02769|ALBU_BOVIN	1785	69248	132	122	9	9	0.98	Albumin OS=Bos taur
2	1	SwissProt_HUMAN$	2::P04114|APOB_HUMAN	13603	515283	547	521	305	293	20.32	Apolipoprotein B-100
3	1	SwissProt_HUMAN$	2::P01024|CO3_HUMAN	11805	187030	515	468	146	137	90.33	Complement C3 OS=I
4	1	SwissProt_HUMAN$	2::P0DOX5|IGG1_HUMAN	11160	49298	656	601	33	32	30.49	Immunoglobulin gami
4	2	SwissProt_HUMAN$	2::P01860|IGHG3_HUMAN	6299	41260	410	383	23	22	20.86	Immunoglobulin heav
4	3	SwissProt_HUMAN$	2::P01859|IGHG2_HUMAN	5357	35878	352	325	22	22	38.65	Immunoglobulin heav
4	4	SwissProt_HUMAN$	2::P01861|IGHG4_HUMAN	3690	35918	259	239	22	22	29.16	Immunoglobulin heav
5	1	SwissProt_HUMAN$	2::P01834|IGKC_HUMAN	10087	11758	418	376	13	12	111.87	Immunoglobulin kapp
5	2	SwissProt_HUMAN$	2::P0DOX7|IGK_HUMAN	9607	23364	379	340	17	16	44.78	Immunoglobulin kapp
5	3	SwissProt_HUMAN$	2::A0A0C4DH67|KV108_HUMAN	397	12529	19	12	4	4	3.39	Immunoglobulin kapp
5	4	SwissProt_HUMAN$	2::A0A075B6S5|KV127_HUMAN	388	12703	19	12	4	4	3.34	Immunoglobulin kapp
5	5	SwissProt_HUMAN$	2::P01599|KV117_HUMAN	248	12770	19	12	6	6	7.87	Immunoglobulin kapp
5	6	SwissProt_HUMAN$	2::A0A0C4DH72|KV106_HUMAN	233	12689	21	12	5	5	5.26	Immunoglobulin kapp
5	7	SwissProt_HUMAN$	2::P01597|KV139_HUMAN	192	12729	19	9	3	3	1.98	Immunoglobulin kapp
5	8	SwissProt_HUMAN$	2::A0A0C4DH73|KV112_HUMAN	179	12637	18	9	3	3	2.01	Immunoglobulin kapp
5	9	SwissProt_HUMAN$	2::P01602|KV105_HUMAN	177	12773	16	8	4	4	3.28	Immunoglobulin kapp
5	10	SwissProt_HUMAN$	2::A0A0C4DH24|KV621_HUMAN	131	12422	12	5	3	3	2.06	Immunoglobulin kapp
5	11	SwissProt_HUMAN$	2::A0A0B4J2D9|KVD13_HUMAN	128	12561	12	5	2	2	1.10	Immunoglobulin kapp
5	12	SwissProt_HUMAN$	2::P01601|KVD16_HUMAN	117	12722	12	5	2	2	1.07	Immunoglobulin kapp
5	13	SwissProt_HUMAN$	2::A0A0A0MT36|KVD21_HUMAN	116	12332	13	6	3	3	2.09	Immunoglobulin kapp
5	14	SwissProt_HUMAN$	2::P04430|KV116_HUMAN	111	12610	12	5	3	3	2.01	Immunoglobulin kapp
6	1	SwissProt_HUMAN$	2::P0C0L5|CO4B_HUMAN	5872	192631	226	219	87	85	9.64	Complement C4-B OS

↑Family	M	DB	Accession	Score	Mass	Matches	Match(sig)	Sequences	Seq(sig)	emPAI	Description
6	2	SwissProt_HUMAN$	2::P0C0L4|CO4A_HUMAN	5732	192664	222	215	85	83	9.12	Complement C4-A OS
7	1	SwissProt_HUMAN$	2::P01023|A2MG_HUMAN	5555	163188	247	227	86	83	18.39	Alpha-2-macroglobuli
7	2	SwissProt_HUMAN$	2::P20742|PZP_HUMAN	643	163760	40	33	12	11	0.46	Pregnancy zone prote
8	1	SwissProt_HUMAN$	2::P01871|IGHM_HUMAN	3719	49409	153	140	35	35	60.03	Immunoglobulin heav
8	2	SwissProt_HUMAN$	2::P0DOX6|IGM_HUMAN	3064	63446	124	112	28	27	9.06	Immunoglobulin mu h
8	3	SwissProt_HUMAN$	2::A0A0C4DH43|HV70D_HUMAN	182	13304	6	6	5	5	4.72	Immunoglobulin heav
8	4	SwissProt_HUMAN$	2::A0A0B4J1V2|HV226_HUMAN	160	13174	6	6	4	4	4.81	Immunoglobulin heav
8	5	SwissProt_HUMAN$	2::P01814|HV270_HUMAN	134	13252	4	4	4	4	3.08	Immunoglobulin heav
9	1	SwissProt_HUMAN$	2::P08603|CFAH_HUMAN	3449	139005	153	139	73	69	12.83	Complement factor H
9	2	SwissProt_HUMAN$	2::Q03591|FHR1_HUMAN	831	37626	31	26	17	16	9.81	Complement factor H-
9	3	SwissProt_HUMAN$	2::P36980|FHR2_HUMAN	305	30631	11	9	7	6	1.51	Complement factor H-
9	4	SwissProt_HUMAN$	2::Q02985|FHR3_HUMAN	164	37299	5	5	4	4	0.66	Complement factor H-
9	5	SwissProt_HUMAN$	2::Q9BXR6|FHR5_HUMAN	138	64377	6	5	6	5	0.44	Complement factor H-
10	1	SwissProt_HUMAN$	2::P02787|TRFE_HUMAN	3220	77014	134	122	60	57	53.25	Serotransferrin OS=H
11	1	SwissProt_HUMAN$	2::P02751|FINC_HUMAN	2977	272150	123	111	81	72	2.83	Fibronectin OS=Homc
12	1	SwissProt_HUMAN$	2::P01876|IGHA1_HUMAN	2790	37631	120	109	23	21	19.22	Immunoglobulin heav
12	2	SwissProt_HUMAN$	2::P0DOX2|IGA2_HUMAN	1065	48903	68	56	21	18	5.25	Immunoglobulin alpha
12	3	SwissProt_HUMAN$	2::P0DP04|HV43D_HUMAN	465	13008	20	17	6	5	16.48	Immunoglobulin heav
12	4	SwissProt_HUMAN$	2::A0A0B4J1X5|HV374_HUMAN	377	12831	20	15	9	7	24.69	Immunoglobulin heav
12	5	SwissProt_HUMAN$	2::P01780|HV307_HUMAN	376	12934	23	16	10	7	16.48	Immunoglobulin heav
12	6	SwissProt_HUMAN$	2::P0DP02|HVC33_HUMAN	367	12980	27	21	8	7	50.12	Immunoglobulin heav
12	7	SwissProt_HUMAN$	2::A0A0B4J1V1|HV321_HUMAN	361	12831	19	14	6	5	7.71	Immunoglobulin heav
12	8	SwissProt_HUMAN$	2::P01764|HV323_HUMAN	356	12574	16	15	7	6	18.30	Immunoglobulin heav
12	9	SwissProt_HUMAN$	2::P01768|HV330_HUMAN	344	12938	19	16	8	7	24.00	Immunoglobulin heav
12	10	SwissProt_HUMAN$	2::P01762|HV311_HUMAN	340	12900	19	14	6	5	7.71	Immunoglobulin heav
12	11	SwissProt_HUMAN$	2::P01763|HV348_HUMAN	339	12804	19	14	6	5	7.87	Immunoglobulin heav
12	12	SwissProt_HUMAN$	2::P01766|HV313_HUMAN	225	12498	11	7	3	2	2.03	Immunoglobulin heav
12	13	SwissProt_HUMAN$	2::A0A0B4J1X8|HV343_HUMAN	213	13068	14	11	6	5	10.97	Immunoglobulin heav
12	14	SwissProt_HUMAN$	2::A0A0C4DH36|HV338_HUMAN	90	12750	7	5	4	2	1.98	Probable non-function
12	15	SwissProt_HUMAN$	2::A0A075B6Q5|HV364_HUMAN	75	12882	8	6	4	3	3.23	Immunoglobulin heav
12	16	SwissProt_HUMAN$	2::A0A0J9YX35|HV64D_HUMAN	52	12814	6	4	5	3	3.28	Immunoglobulin heav
12	17	SwissProt_HUMAN$	2::P0DTE1|HV383_HUMAN	44	12663	5	3	4	2	2.01	Probable non-function
13	1	SwissProt_HUMAN$	2::P0DOY2|IGLC2_HUMAN	2672	11287	100	94	12	12	202.51	Immunoglobulin lamb
13	2	SwissProt_HUMAN$	2::P0DOX8|IGL1_HUMAN	2186	22816	89	81	13	13	20.69	Immunoglobulin lamb
13	3	SwissProt_HUMAN$	2::A0M8Q6|IGLC7_HUMAN	1476	11247	52	48	7	6	16.99	Immunoglobulin lamb
13	4	SwissProt_HUMAN$	2::P01706|LV211_HUMAN	79	12636	4	4	3	3	3.34	Immunoglobulin lamb
13	5	SwissProt_HUMAN$	2::P01709|LV208_HUMAN	71	12374	5	5	3	3	2.09	Immunoglobulin lamb
14	1	SwissProt_HUMAN$	2::P00747|PLMN_HUMAN	2338	90510	99	87	54	50	17.75	Plasminogen OS=Hon
15	1	SwissProt_HUMAN$	2::P00450|CERU_HUMAN	2008	122128	84	79	50	50	7.79	Ceruloplasmin OS=Hc
16	1	SwissProt_HUMAN$	2::P01009|A1AT_HUMAN	1774	46707	98	85	35	33	68.63	Alpha-1-antitrypsin O:

↑Family	M	DB	Accession	Score	Mass	Matches	Match(sig)	Sequences	Seq(sig)	emPAI	Description
17	1	SwissProt_HUMAN$	2::P01031|CO5_HUMAN	1722	188186	89	79	68	63	4.28	Complement C5 OS=I
18	1	SwissProt_HUMAN$	2::P04003|C4BPA_HUMAN	1713	66989	68	64	29	28	9.26	C4b-binding protein a
19	1	SwissProt_HUMAN$	2::P19823|ITIH2_HUMAN	1545	106397	75	62	37	33	4.19	Inter-alpha-trypsin inl
20	1	SwissProt_HUMAN$	2::P19827|ITIH1_HUMAN	1534	101326	61	57	32	31	5.49	Inter-alpha-trypsin inl
21	1	SwissProt_HUMAN$	2::P02647|APOA1_HUMAN	1433	30759	75	68	32	31	210.99	Apolipoprotein A-I OS
22	1	SwissProt_HUMAN$	2::P07357|CO8A_HUMAN	1155	65121	40	37	25	23	6.63	Complement compone
23	1	SwissProt_HUMAN$	2::P02774|VTDB_HUMAN	1146	52883	49	46	27	27	12.37	Vitamin D-binding pro
24	1	SwissProt_HUMAN$	2::P09871|C1S_HUMAN	1123	76635	41	38	24	22	4.30	Complement C1s subc
24	2	SwissProt_HUMAN$	2::P48740|MASP1_HUMAN	69	79195	6	4	5	4	0.27	Mannan-binding lectin
25	1	SwissProt_HUMAN$	2::P06396|GELS_HUMAN	1034	85644	38	35	27	25	3.21	Gelsolin OS=Homo sa
26	1	SwissProt_HUMAN$	2::P10643|CO7_HUMAN	1028	93457	46	43	36	35	5.52	Complement compone
27	1	SwissProt_HUMAN$	2::P00738|HPT_HUMAN	1010	45177	51	47	24	24	21.97	Haptoglobin OS=Hom
27	2	SwissProt_HUMAN$	2::P00739|HPTR_HUMAN	727	39005	38	36	19	19	17.26	Haptoglobin-related p
28	1	SwissProt_HUMAN$	2::P00751|CFAB_HUMAN	994	85479	52	46	36	32	5.22	Complement factor B
29	1	SwissProt_HUMAN$	2::P02749|APOH_HUMAN	972	38273	36	34	20	19	9.37	Beta-2-glycoprotein 1
30	1	SwissProt_HUMAN$	2::P00736|C1R_HUMAN	970	80067	43	41	28	26	4.90	Complement C1r subc
31	1	SwissProt_HUMAN$	2::P13671|CO6_HUMAN	963	104718	48	46	35	34	3.66	Complement compone
32	1	SwissProt_HUMAN$	2::P00734|THRB_HUMAN	876	69992	36	33	21	19	3.73	Prothrombin OS=Hom
33	1	SwissProt_HUMAN$	2::P07358|CO8B_HUMAN	862	67003	36	33	24	22	4.83	Complement compone
34	1	SwissProt_HUMAN$	2::O43866|CD5L_HUMAN	756	38063	32	31	18	17	8.29	CD5 antigen-like OS=
35	1	SwissProt_HUMAN$	2::P07225|PROS_HUMAN	747	75074	33	30	22	21	3.54	Vitamin K-dependent
36	1	SwissProt_HUMAN$	2::P07996|TSP1_HUMAN	728	129300	39	33	33	29	1.90	Thrombospondin-1 OS
37	1	SwissProt_HUMAN$	2::P02649|APOE_HUMAN	675	36132	28	25	22	20	16.55	Apolipoprotein E OS=
38	1	SwissProt_HUMAN$	2::P01008|ANT3_HUMAN	673	52569	31	30	21	20	6.21	Antithrombin-III OS=
39	1	SwissProt_HUMAN$	2::P02748|CO9_HUMAN	672	63133	33	28	23	20	3.82	Complement compone
40	1	SwissProt_HUMAN$	2::P07360|CO8G_HUMAN	636	22264	20	20	11	11	14.36	Complement compone
41	1	SwissProt_HUMAN$	2::P01619|KV320_HUMAN	590	12549	18	16	4	4	3.39	Immunoglobulin kapp
41	2	SwissProt_HUMAN$	2::A0A0C4DH25|KVD20_HUMAN	540	12507	14	13	3	3	2.03	Immunoglobulin kapp
42	1	SwissProt_HUMAN$	2::Q14624|ITIH4_HUMAN	589	103293	29	27	26	24	2.29	Inter-alpha-trypsin inl
43	1	SwissProt_HUMAN$	2::P02766|TTHY_HUMAN	573	15877	18	18	9	9	13.07	Transthyretin OS=Hoı
44	1	SwissProt_HUMAN$	2::P02790|HEMO_HUMAN	571	51643	39	34	25	24	8.86	Hemopexin OS=Homo
45	1	SwissProt_HUMAN$	2::P01019|ANGT_HUMAN	571	53121	18	18	11	11	2.17	Angiotensinogen OS=
46	1	SwissProt_HUMAN$	2::P27169|PON1_HUMAN	542	39706	22	19	16	14	4.93	Serum paraoxonase/a
47	1	SwissProt_HUMAN$	2::P02765|FETUA_HUMAN	541	39316	21	19	11	9	2.74	Alpha-2-HS-glycoprot
48	1	SwissProt_HUMAN$	2::P01042|KNG1_HUMAN	524	71912	21	20	17	16	1.86	Kininogen-1 OS=Hom
49	1	SwissProt_HUMAN$	2::P02753|RET4_HUMAN	520	22995	21	19	11	11	13.21	Retinol-binding protei
50	1	SwissProt_HUMAN$	2::P01011|AACT_HUMAN	503	47621	25	23	20	18	6.27	Alpha-1-antichymotry
51	1	SwissProt_HUMAN$	2::P05155|IC1_HUMAN	490	55119	27	25	19	18	4.09	Plasma protease C1 ir
52	1	SwissProt_HUMAN$	2::P02760|AMBP_HUMAN	472	38974	21	18	13	13	5.14	Protein AMBP OS=Hor
53	1	SwissProt_HUMAN$	2::P02743|SAMP_HUMAN	466	25371	22	18	12	10	5.34	Serum amyloid P-com

↑Family	M	DB	Accession	Score	Mass	Matches	Match(sig)	Sequences	Seq(sig)	emPAI	Description
54	1	SwissProt_HUMAN$	2::P10909|CLUS_HUMAN	454	52461	14	14	10	10	1.46	Clusterin OS=Homo s
55	1	SwissProt_HUMAN$	2::P05156|CFAI_HUMAN	448	65707	22	19	17	14	1.74	Complement factor I (
56	1	SwissProt_HUMAN$	2::P36955|PEDF_HUMAN	448	46283	22	21	17	17	5.94	Pigment epithelium-de
57	1	SwissProt_HUMAN$	2::P04196|HRG_HUMAN	435	59541	27	24	15	15	2.57	Histidine-rich glycopro
58	1	SwissProt_HUMAN$	2::P05160|F13B_HUMAN	421	75461	28	21	19	17	2.09	Coagulation factor XII
59	1	SwissProt_HUMAN$	2::Q08380|LG3BP_HUMAN	397	65289	14	13	11	10	1.06	Galectin-3-binding pro
60	1	SwissProt_HUMAN$	2::P68871|HBB_HUMAN	376	15988	20	19	11	11	32.17	Hemoglobin subunit b
61	1	SwissProt_HUMAN$	2::A0A0C4DH38|HV551_HUMAN	375	12666	15	14	6	5	8.03	Immunoglobulin heav
61	2	SwissProt_HUMAN$	2::A0A0J9YXX1|HV5X1_HUMAN	209	12764	12	11	3	3	5.16	Immunoglobulin heav
62	1	SwissProt_HUMAN$	2::P06331|HV434_HUMAN	355	13806	11	11	5	5	4.42	Immunoglobulin heav
62	2	SwissProt_HUMAN$	2::A0A075B6R2|HV404_HUMAN	283	12840	9	8	6	5	5.07	Immunoglobulin heav
63	1	SwissProt_HUMAN$	2::P0DOX3|IGD_HUMAN	353	56189	18	16	12	11	1.99	Immunoglobulin delta
64	1	SwissProt_HUMAN$	2::P01742|HV169_HUMAN	350	12651	13	11	7	7	12.04	Immunoglobulin heav
64	2	SwissProt_HUMAN$	2::A0A0B4J2H0|HV69D_HUMAN	300	12652	10	8	5	5	5.26	Immunoglobulin heav
64	3	SwissProt_HUMAN$	2::P01743|HV146_HUMAN	193	12924	7	6	3	3	1.95	Immunoglobulin heav
64	4	SwissProt_HUMAN$	2::A0A0C4DH29|HV103_HUMAN	140	12999	6	5	2	2	1.92	Immunoglobulin heav
64	5	SwissProt_HUMAN$	2::P0DP01|HV108_HUMAN	123	12983	5	4	2	2	1.04	Immunoglobulin heav
65	1	SwissProt_HUMAN$	2::A0A0B4J1V0|HV315_HUMAN	346	12917	20	16	10	8	16.91	Immunoglobulin heav
65	2	SwissProt_HUMAN$	2::A0A0B4J1Y9|HV372_HUMAN	311	13194	19	18	10	9	22.71	Immunoglobulin heav
65	3	SwissProt_HUMAN$	2::A0A0A0MS15|HV349_HUMAN	198	13048	11	9	9	8	16.07	Immunoglobulin heav
65	4	SwissProt_HUMAN$	2::A0A0B4J1V6|HV373_HUMAN	197	12849	11	10	7	6	7.71	Immunoglobulin heav
66	1	SwissProt_HUMAN$	2::P05090|APOD_HUMAN	341	21262	12	11	8	8	6.23	Apolipoprotein D OS=
67	1	SwissProt_HUMAN$	2::P05452|TETN_HUMAN	332	22522	11	10	9	9	5.51	Tetranectin OS=Homo
68	1	SwissProt_HUMAN$	2::P29622|KAIN_HUMAN	320	48511	16	14	11	10	1.65	Kallistatin OS=Homo s
69	1	SwissProt_HUMAN$	2::P00748|FA12_HUMAN	320	67748	12	11	10	9	0.88	Coagulation factor XII
70	1	SwissProt_HUMAN$	2::O75636|FCN3_HUMAN	320	32882	16	14	9	9	3.19	Ficolin-3 OS=Homo sa
71	1	SwissProt_HUMAN$	2::P06727|APOA4_HUMAN	310	45344	13	13	10	10	1.83	Apolipoprotein A-IV O
72	1	SwissProt_HUMAN$	2::P03952|KLKB1_HUMAN	308	71323	16	15	15	14	1.70	Plasma kallikrein OS=
73	1	SwissProt_HUMAN$	2::P23142|FBLN1_HUMAN	305	77162	17	15	13	12	1.22	Fibulin-1 OS=Homo sa
74	1	SwissProt_HUMAN$	2::P00761|TRYP_PIG	305	24394	6	6	5	5	1.61	Trypsin OS=Sus scrof
75	1	SwissProt_HUMAN$	2::P08697|A2AP_HUMAN	287	54531	18	16	12	12	2.09	Alpha-2-antiplasmin C
76	1	SwissProt_HUMAN$	2::P02747|C1QC_HUMAN	286	25757	18	15	8	8	5.20	Complement C1q sub
77	1	SwissProt_HUMAN$	2::P04004|VTNC_HUMAN	273	54271	17	15	11	10	2.10	Vitronectin OS=Homo
78	1	SwissProt_HUMAN$	2::P06312|KV401_HUMAN	269	13372	16	15	6	6	7.11	Immunoglobulin kapp
79	1	SwissProt_HUMAN$	2::P35858|ALS_HUMAN	268	65994	12	12	9	9	0.91	Insulin-like growth fac
80	1	SwissProt_HUMAN$	2::A0A075B6R9|KVD24_HUMAN	265	13071	7	7	4	4	3.13	Probable non-function
80	2	SwissProt_HUMAN$	2::A0A087WW87|KV240_HUMAN	256	13302	10	10	4	4	7.11	Immunoglobulin kapp
80	3	SwissProt_HUMAN$	2::A0A075B6P5|KV228_HUMAN	215	12948	9	8	3	3	1.92	Immunoglobulin kapp
80	4	SwissProt_HUMAN$	2::P06310|KV230_HUMAN	154	13177	5	4	2	2	1.02	Immunoglobulin kapp
80	5	SwissProt_HUMAN$	2::A0A075B6S6|KVD30_HUMAN	147	13207	4	4	2	2	1.02	Immunoglobulin kapp

↑Family	M	DB	Accession	Score	Mass	Matches	Match(sig)	Sequences	Seq(sig)	emPAI	Description
81	1	SwissProt_HUMAN$	2::P04275|VWF_HUMAN	261	309058	13	11	13	11	0.18	von Willebrand factor
82	1	SwissProt_HUMAN$	2::Q9Y6R7|FCGBP_HUMAN	253	571639	17	16	17	16	0.14	IgGFc-binding protein
83	1	SwissProt_HUMAN$	2::Q96PD5|PGRP2_HUMAN	248	62178	11	11	9	9	0.98	N-acetylmuramoyl-L-a
84	1	SwissProt_HUMAN$	2::P05546|HEP2_HUMAN	248	57034	16	15	13	13	2.19	Heparin cofactor 2 OS
85	1	SwissProt_HUMAN$	2::O14791|APOL1_HUMAN	245	43947	13	11	10	9	1.63	Apolipoprotein L1 OS=
86	1	SwissProt_HUMAN$	2::P04217|A1BG_HUMAN	238	54220	12	11	8	7	1.01	Alpha-1B-glycoprotein
87	1	SwissProt_HUMAN$	2::P04264|K2C1_HUMAN	235	65999	7	7	6	6	0.54	Keratin, type II cytosl
87	2	SwissProt_HUMAN$	2::P04259|K2C6B_HUMAN	87	60030	5	4	4	4	0.37	Keratin, type II cytosl
88	1	SwissProt_HUMAN$	2::P02652|APOA2_HUMAN	229	11168	9	9	6	6	10.91	Apolipoprotein A-II OS
89	1	SwissProt_HUMAN$	2::P23083|HV102_HUMAN	228	13076	10	10	5	5	7.40	Immunoglobulin heav
89	2	SwissProt_HUMAN$	2::A0A0C4DH31|HV118_HUMAN	204	12812	7	7	3	3	1.98	Immunoglobulin heav
90	1	SwissProt_HUMAN$	2::P01593|KVD33_HUMAN	223	12840	6	3	2	2	1.06	Immunoglobulin kapp
91	1	SwissProt_HUMAN$	2::P35527|K1C9_HUMAN	222	62027	6	6	6	6	0.58	Keratin, type I cytosk
92	1	SwissProt_HUMAN$	2::P02656|APOC3_HUMAN	217	10846	6	4	3	2	1.34	Apolipoprotein C-III C
93	1	SwissProt_HUMAN$	2::P01624|KV315_HUMAN	212	12488	6	6	2	2	1.10	Immunoglobulin kapp
93	2	SwissProt_HUMAN$	2::A0A075B6H7|KV37_HUMAN	95	12775	6	5	3	2	1.07	Probable non-function
94	1	SwissProt_HUMAN$	2::P18428|LBP_HUMAN	212	53350	8	8	7	7	0.86	Lipopolysaccharide-bi
95	1	SwissProt_HUMAN$	2::P43652|AFAM_HUMAN	211	69024	13	13	13	13	1.44	Afamin OS=Homo sap
96	1	SwissProt_HUMAN$	2::P02746|C1QB_HUMAN	210	26704	11	11	6	6	3.08	Complement C1q sub
97	1	SwissProt_HUMAN$	2::P02654|APOC1_HUMAN	210	9326	9	8	5	5	18.35	Apolipoprotein C-I OS
98	1	SwissProt_HUMAN$	2::P80748|LV321_HUMAN	207	12438	7	6	3	3	2.06	Immunoglobulin lamb
99	1	SwissProt_HUMAN$	2::P13645|K1C10_HUMAN	205	58792	11	8	9	7	0.76	Keratin, type I cytosk
99	2	SwissProt_HUMAN$	2::P08727|K1C19_HUMAN	64	44079	3	3	3	3	0.38	Keratin, type I cytosk
99	3	SwissProt_HUMAN$	2::P02533|K1C14_HUMAN	61	51529	3	3	3	3	0.32	Keratin, type I cytosk
100	1	SwissProt_HUMAN$	2::P02763|A1AG1_HUMAN	203	23497	10	10	6	6	2.31	Alpha-1-acid glycopro
100	2	SwissProt_HUMAN$	2::P19652|A1AG2_HUMAN	120	23588	8	8	7	7	3.01	Alpha-1-acid glycopro
101	1	SwissProt_HUMAN$	2::O95445|APOM_HUMAN	202	21239	11	11	8	8	6.31	Apolipoprotein M OS=
102	1	SwissProt_HUMAN$	2::P27918|PROP_HUMAN	202	51242	11	10	10	9	1.29	Properdin OS=Homo s
103	1	SwissProt_HUMAN$	2::P04433|KV311_HUMAN	195	12567	8	8	3	3	2.03	Immunoglobulin kapp
104	1	SwissProt_HUMAN$	2::P69905|HBA_HUMAN	192	15248	11	11	7	7	10.61	Hemoglobin subunit a
105	1	SwissProt_HUMAN$	2::P02776|PLF4_HUMAN	190	10838	9	9	5	5	4.48	Platelet factor 4 OS=H
106	1	SwissProt_HUMAN$	2::A0A0B4J1U7|HV601_HUMAN	189	13473	18	16	5	5	4.64	Immunoglobulin heav
107	1	SwissProt_HUMAN$	2::A0A0C4DH34|HV428_HUMAN	187	13116	7	7	4	4	4.89	Immunoglobulin heav
108	1	SwissProt_HUMAN$	2::P02655|APOC2_HUMAN	181	11277	7	6	5	5	6.73	Apolipoprotein C-II OS
109	1	SwissProt_HUMAN$	2::P35542|SAA4_HUMAN	174	14737	7	7	5	5	3.84	Serum amyloid A-4 pr
110	1	SwissProt_HUMAN$	2::P60709|ACTB_HUMAN	172	41710	6	5	6	5	0.76	Actin, cytoplasmic 1 C
111	1	SwissProt_HUMAN$	2::P01591|IGJ_HUMAN	164	18087	11	11	8	8	6.90	Immunoglobulin J cha
112	1	SwissProt_HUMAN$	2::P20851|C4BPB_HUMAN	157	28338	8	8	5	5	1.29	C4b-binding protein b
113	1	SwissProt_HUMAN$	2::P02745|C1QA_HUMAN	136	26000	7	6	6	5	1.47	Complement C1q sub
114	1	SwissProt_HUMAN$	2::P01700|LV147_HUMAN	130	12276	6	5	3	3	2.09	Immunoglobulin lamb

↑Family	M	DB	Accession	Score	Mass	Matches	Match(sig)	Sequences	Seq(sig)	emPAI	Description
114	2	SwissProt_HUMAN$	2::P01699|LV144_HUMAN	96	12194	4	4	2	2	1.14	Immunoglobulin lamb
115	1	SwissProt_HUMAN$	2::Q06033|ITIH3_HUMAN	129	99787	9	8	7	7	0.46	Inter-alpha-trypsin in
116	1	SwissProt_HUMAN$	2::Q92954|PRG4_HUMAN	123	150968	5	3	4	2	0.06	Proteoglycan 4 OS=H
117	1	SwissProt_HUMAN$	2::P02775|CXCL7_HUMAN	119	13885	3	2	3	2	0.96	Platelet basic protein
118	1	SwissProt_HUMAN$	2::A0A0C4DH33|HV124_HUMAN	116	12816	3	3	2	2	1.07	Immunoglobulin heav
119	1	SwissProt_HUMAN$	2::P05154|IPSP_HUMAN	115	45646	7	6	5	5	0.68	Plasma serine proteas
120	1	SwissProt_HUMAN$	2::P01701|LV151_HUMAN	115	12241	6	3	3	2	1.14	Immunoglobulin lamb
121	1	SwissProt_HUMAN$	2::Q96IY4|CBPB2_HUMAN	113	48393	6	6	6	6	0.80	Carboxypeptidase B2
122	1	SwissProt_HUMAN$	2::A0A0A0MS14|HV145_HUMAN	105	13500	2	2	1	1	0.99	Immunoglobulin heav
123	1	SwissProt_HUMAN$	2::A0A075B6K4|LV310_HUMAN	100	12433	5	4	5	4	3.45	Immunoglobulin lamb
123	2	SwissProt_HUMAN$	2::P01717|LV325_HUMAN	84	12004	5	5	3	3	2.19	Immunoglobulin lamb
123	3	SwissProt_HUMAN$	2::P01718|LV327_HUMAN	54	12157	6	6	3	3	2.13	Immunoglobulin lamb
124	1	SwissProt_HUMAN$	2::P40197|GPV_HUMAN	94	60921	4	4	4	4	0.36	Platelet glycoprotein \
125	1	SwissProt_HUMAN$	2::P22792|CPN2_HUMAN	94	60518	5	5	4	4	0.37	Carboxypeptidase N s
126	1	SwissProt_HUMAN$	2::P05543|THBG_HUMAN	93	46295	4	4	4	4	0.50	Thyroxine-binding glo
127	1	SwissProt_HUMAN$	2::P02750|A2GL_HUMAN	93	38154	6	6	6	6	1.10	Leucine-rich alpha-2-ç
128	1	SwissProt_HUMAN$	2::P02741|CRP_HUMAN	93	25023	3	2	2	2	0.46	C-reactive protein OS
129	1	SwissProt_HUMAN$	2::A0A0B4J1Y8|LV949_HUMAN	91	13016	2	2	2	2	1.04	Immunoglobulin lamb
130	1	SwissProt_HUMAN$	2::P01817|HV205_HUMAN	88	13223	4	4	3	3	1.87	Immunoglobulin heav
131	1	SwissProt_HUMAN$	2::Q9UHG3|PCYOX_HUMAN	88	56604	4	4	4	4	0.40	Prenylcysteine oxidas
132	1	SwissProt_HUMAN$	2::Q15582|BGH3_HUMAN	87	74634	6	4	5	4	0.29	Transforming growth
133	1	SwissProt_HUMAN$	2::P22352|GPX3_HUMAN	87	25537	5	5	5	5	1.51	Glutathione peroxidas
134	1	SwissProt_HUMAN$	2::Q15485|FCN2_HUMAN	86	33980	5	4	3	3	0.74	Ficolin-2 OS=Homo sa
135	1	SwissProt_HUMAN$	2::P00338|LDHA_HUMAN	85	36665	2	2	2	2	0.29	L-lactate dehydrogena
136	1	SwissProt_HUMAN$	2::P25311|ZA2G_HUMAN	84	34237	2	2	2	2	0.32	Zinc-alpha-2-glycopro
137	1	SwissProt_HUMAN$	2::P12259|FA5_HUMAN	80	251546	7	5	7	5	0.10	Coagulation factor V (
138	1	SwissProt_HUMAN$	2::P06681|CO2_HUMAN	79	83214	5	3	5	3	0.19	Complement C2 OS=I
139	1	SwissProt_HUMAN$	2::P04180|LCAT_HUMAN	77	49546	3	3	3	3	0.33	Phosphatidylcholine-s
140	1	SwissProt_HUMAN$	2::P03951|FA11_HUMAN	73	70064	10	7	8	6	0.50	Coagulation factor XI
141	1	SwissProt_HUMAN$	2::P08185|CBG_HUMAN	73	45112	4	3	4	3	0.37	Corticosteroid-binding
142	1	SwissProt_HUMAN$	2::A0A075B6I0|LV861_HUMAN	73	12806	2	2	2	2	1.07	Immunoglobulin lamb
143	1	SwissProt_HUMAN$	2::P11226|MBL2_HUMAN	72	26127	2	2	2	2	0.43	Mannose-binding prot
144	1	SwissProt_HUMAN$	2::P26927|HGFL_HUMAN	72	80268	5	5	5	5	0.34	Hepatocyte growth fa
145	1	SwissProt_HUMAN$	2::P15814|IGLL1_HUMAN	70	22949	2	2	1	1	0.23	Immunoglobulin lamb
146	1	SwissProt_HUMAN$	2::P08519|APOA_HUMAN	70	500995	4	4	4	4	0.04	Apolipoprotein(a) OS=
147	1	SwissProt_HUMAN$	2::P01714|LV319_HUMAN	70	12035	4	4	3	3	2.19	Immunoglobulin lamb
148	1	SwissProt_HUMAN$	2::P38646|GRP75_HUMAN	66	73635	2	2	2	2	0.14	Stress-70 protein, mit
148	2	SwissProt_HUMAN$	2::P11142|HSP7C_HUMAN	49	70854	2	2	2	2	0.14	Heat shock cognate 7
148	3	SwissProt_HUMAN$	2::P11021|BIP_HUMAN	48	72288	2	2	2	2	0.14	Endoplasmic reticulun
149	1	SwissProt_HUMAN$	2::Q04756|HGFA_HUMAN	64	70636	4	3	4	3	0.22	Hepatocyte growth fa

↑Family	M	DB	Accession	Score	Mass	Matches	Match(sig)	Sequences	Seq(sig)	emPAI	Description
150	1	SwissProt_HUMAN$	2::P11597|CETP_HUMAN	63	54721	4	4	4	4	0.41	Cholesteryl ester tran
151	1	SwissProt_HUMAN$	2::A0A087WSZ0|KVD08_HUMAN	62	12828	2	2	1	1	0.43	Immunoglobulin kapp
152	1	SwissProt_HUMAN$	2::P62805|H4_HUMAN	60	11360	4	3	3	2	1.27	Histone H4 OS=Homo
153	1	SwissProt_HUMAN$	2::Q15848|ADIPO_HUMAN	57	26397	2	2	2	2	0.43	Adiponectin OS=Hom
154	1	SwissProt_HUMAN$	2::P04211|LV743_HUMAN	56	12443	3	3	2	2	1.11	Immunoglobulin lamb
154	2	SwissProt_HUMAN$	2::A0A075B6I9|LV746_HUMAN	42	12460	5	3	3	2	1.11	Immunoglobulin lamb
155	1	SwissProt_HUMAN$	2::P15169|CBPN_HUMAN	56	52253	4	3	4	3	0.31	Carboxypeptidase N c
156	1	SwissProt_HUMAN$	2::P51884|LUM_HUMAN	55	38405	4	3	4	3	0.45	Lumican OS=Homo sa
157	1	SwissProt_HUMAN$	2::O00391|QSOX1_HUMAN	55	82526	4	3	4	3	0.19	Sulfhydryl oxidase 1 (
158	1	SwissProt_HUMAN$	2::A0A075B6J9|LV218_HUMAN	54	12404	2	2	1	1	0.45	Immunoglobulin lamb
159	1	SwissProt_HUMAN$	2::P04070|PROC_HUMAN	53	52037	2	2	2	2	0.20	Vitamin K-dependent
160	1	SwissProt_HUMAN$	2::Q12805|FBLN3_HUMAN	53	54604	1	1	1	1	0.09	EGF-containing fibulin
161	1	SwissProt_HUMAN$	2::P61626|LYSC_HUMAN	52	16526	3	3	3	3	1.33	Lysozyme C OS=Hom
162	1	SwissProt_HUMAN$	2::P25705|ATPA_HUMAN	51	59714	3	2	2	2	0.17	ATP synthase subunit
163	1	SwissProt_HUMAN$	2::P01705|LV223_HUMAN	51	11886	3	3	1	1	1.18	Immunoglobulin lamb
164	1	SwissProt_HUMAN$	2::Q8NHQ9|DDX55_HUMAN	50	68503	2	2	1	1	0.07	ATP-dependent RNA h
165	1	SwissProt_HUMAN$	2::Q71DI3|H32_HUMAN	50	15379	2	2	2	2	0.83	Histone H3.2 OS=Hor
166	1	SwissProt_HUMAN$	2::Q14520|HABP2_HUMAN	49	62630	3	3	3	3	0.25	Hyaluronan-binding p
167	1	SwissProt_HUMAN$	2::P08571|CD14_HUMAN	49	40051	3	2	3	2	0.27	Monocyte differentiati
168	1	SwissProt_HUMAN$	2::P00742|FA10_HUMAN	48	54697	5	4	5	4	0.41	Coagulation factor X (
168	2	SwissProt_HUMAN$	2::P00740|FA9_HUMAN	43	51745	5	4	5	4	0.44	Coagulation factor IX
169	1	SwissProt_HUMAN$	2::A0A0B4J1U3|LV136_HUMAN	48	12470	2	1	2	1	0.45	Immunoglobulin lamb
170	1	SwissProt_HUMAN$	2::P08709|FA7_HUMAN	48	51560	1	1	1	1	0.10	Coagulation factor VII
171	1	SwissProt_HUMAN$	2::P02675|FIBB_HUMAN	46	55892	2	1	2	1	0.09	Fibrinogen beta chain
172	1	SwissProt_HUMAN$	2::P04278|SHBG_HUMAN	44	43752	3	3	3	3	0.38	Sex hormone-binding
173	1	SwissProt_HUMAN$	2::A0A075B6I1|LV460_HUMAN	43	12979	1	1	1	1	0.43	Immunoglobulin lamb
174	1	SwissProt_HUMAN$	2::P98160|PGBM_HUMAN	43	468532	1	1	1	1	0.01	Basement membrane-
175	1	SwissProt_HUMAN$	2::P49913|CAMP_HUMAN	42	19289	2	2	2	2	0.62	Cathelicidin antimicro
176	1	SwissProt_HUMAN$	2::Q5T0N5|FBP1L_HUMAN	41	70021	3	3	1	1	0.07	Formin-binding protei
177	1	SwissProt_HUMAN$	2::Q9UK55|ZPI_HUMAN	41	50674	5	4	5	4	0.45	Protein Z-dependent p
178	1	SwissProt_HUMAN$	2::Q13093|PAFA_HUMAN	41	50045	1	1	1	1	0.10	Platelet-activating fac
179	1	SwissProt_HUMAN$	2::Q5VU97|CAHD1_HUMAN	41	142198	2	2	1	1	0.03	VWFA and cache dom
180	1	SwissProt_HUMAN$	2::P00746|CFAD_HUMAN	40	27016	3	3	3	3	0.69	Complement factor D
181	1	SwissProt_HUMAN$	2::P02679|FIBG_HUMAN	40	51479	3	1	2	1	0.10	Fibrinogen gamma ch
182	1	SwissProt_HUMAN$	2::A0A0C4DH35|HV335_HUMAN	39	12802	2	2	2	2	1.07	Probable non-function
183	1	SwissProt_HUMAN$	2::Q5QNW6|H2B2F_HUMAN	39	13912	2	2	2	2	0.96	Histone H2B type 2-F
184	1	SwissProt_HUMAN$	2::Q8N5V2|NGEF_HUMAN	39	82445	1	1	1	1	0.06	Ephexin-1 OS=Homo
185	1	SwissProt_HUMAN$	2::Q6VUC0|AP2E_HUMAN	38	46183	1	1	1	1	0.11	Transcription factor A
186	1	SwissProt_HUMAN$	2::Q8NA31|TERB1_HUMAN	37	83011	1	1	1	1	0.06	Telomere repeats-bin
187	1	SwissProt_HUMAN$	2::P06733|ENOA_HUMAN	37	47139	1	1	1	1	0.11	Alpha-enolase OS=Ho

↑Family	M	DB	Accession	Score	Mass	Matches	Match(sig)	Sequences	Seq(sig)	emPAI	Description
188	1	SwissProt_HUMAN$	2::P01703|LV140_HUMAN	37	12294	2	2	1	1	0.46	Immunoglobulin lamb
189	1	SwissProt_HUMAN$	2::Q9UGM5|FETUB_HUMAN	37	42028	2	2	2	2	0.25	Fetuin-B OS=Homo sa
190	1	SwissProt_HUMAN$	2::P13796|PLSL_HUMAN	36	70244	1	1	1	1	0.07	Plastin-2 OS=Homo s
191	1	SwissProt_HUMAN$	2::P41252|SYIC_HUMAN	36	144406	1	1	1	1	0.03	Isoleucine--tRNA ligas
192	1	SwissProt_HUMAN$	2::P01833|PIGR_HUMAN	36	83232	4	3	4	3	0.19	Polymeric immunoglol
193	1	SwissProt_HUMAN$	2::P55056|APOC4_HUMAN	35	14543	1	1	1	1	0.38	Apolipoprotein C-IV O
194	1	SwissProt_HUMAN$	2::O94887|FARP2_HUMAN	34	119813	1	1	1	1	0.04	FERM, ARHGEF and pl
195	1	SwissProt_HUMAN$	2::Q9ULM0|PKHH1_HUMAN	34	151137	2	1	2	1	0.03	Pleckstrin homology d
196	1	SwissProt_HUMAN$	2::Q96DD0|LRC39_HUMAN	34	38768	1	1	1	1	0.13	Leucine-rich repeat-co
197	1	SwissProt_HUMAN$	2::P20160|CAP7_HUMAN	34	26869	1	1	1	1	0.19	Azurocidin OS=Homo
198	1	SwissProt_HUMAN$	2::Q8NEE6|DRC6_HUMAN	34	83869	1	1	1	1	0.06	Dynein regulatory cor
199	1	SwissProt_HUMAN$	2::Q96EK7|F120B_HUMAN	34	103716	1	1	1	1	0.05	Constitutive coactivat
200	1	SwissProt_HUMAN$	2::Q68DL7|CR063_HUMAN	33	77181	3	3	1	1	0.06	Uncharacterized prote
201	1	SwissProt_HUMAN$	2::P08637|FCG3A_HUMAN	33	29071	2	2	2	2	0.38	Low affinity immunog
202	1	SwissProt_HUMAN$	2::P32249|GP183_HUMAN	33	41196	1	1	1	1	0.12	G-protein coupled rec
203	1	SwissProt_HUMAN$	2::A0A075B6H9|LV469_HUMAN	33	12765	1	1	1	1	0.44	Immunoglobulin lamb
204	1	SwissProt_HUMAN$	2::Q6P087|RUSD3_HUMAN	33	38437	2	1	2	1	0.13	Mitochondrial mRNA p
205	1	SwissProt_HUMAN$	2::P0DOX4|IGE_HUMAN	33	60285	1	1	1	1	0.08	Immunoglobulin epsil
206	1	SwissProt_HUMAN$	2::A0A0G2JS06|LV539_HUMAN	32	13386	3	1	3	1	0.41	Immunoglobulin lamb
207	1	SwissProt_HUMAN$	2::Q9NQ79|CRAC1_HUMAN	31	71376	2	2	2	2	0.14	Cartilage acidic protei
208	1	SwissProt_HUMAN$	2::Q00610|CLH1_HUMAN	31	191493	2	2	1	1	0.03	Clathrin heavy chain 1
209	1	SwissProt_HUMAN$	2::A0A075B6I4|LVX54_HUMAN	31	12387	2	2	1	1	0.45	Immunoglobulin lamb
210	1	SwissProt_HUMAN$	2::Q9BTV5|FSD1_HUMAN	30	55784	2	1	1	1	0.09	Fibronectin type III ar
211	1	SwissProt_HUMAN$	2::Q6DT37|MRCKG_HUMAN	30	172352	2	2	1	1	0.03	Serine/threonine-prot
212	1	SwissProt_HUMAN$	2::Q8IV32|CCD71_HUMAN	30	49618	1	1	1	1	0.10	Coiled-coil domain-co
213	1	SwissProt_HUMAN$	2::Q3SXZ3|ZN718_HUMAN	29	55324	3	3	1	1	0.09	Zinc finger protein 71
214	1	SwissProt_HUMAN$	2::Q9HBU6|EKI1_HUMAN	29	50936	1	1	1	1	0.10	Ethanolamine kinase 1
215	1	SwissProt_HUMAN$	2::Q3L8U1|CHD9_HUMAN	29	325819	1	1	1	1	0.01	Chromodomain-helica
216	1	SwissProt_HUMAN$	2::Q8N163|CCAR2_HUMAN	29	102838	2	1	1	1	0.05	Cell cycle and apoptos
217	1	SwissProt_HUMAN$	2::O43586|PPIP1_HUMAN	29	47562	1	1	1	1	0.10	Proline-serine-threoni
218	1	SwissProt_HUMAN$	2::Q96HU8|DIRA2_HUMAN	29	22471	2	2	1	1	0.23	GTP-binding protein D
219	1	SwissProt_HUMAN$	2::Q99973|TEP1_HUMAN	28	290307	1	1	1	1	0.02	Telomerase protein cc
220	1	SwissProt_HUMAN$	2::O15078|CE290_HUMAN	28	290207	2	1	2	1	0.02	Centrosomal protein c
221	1	SwissProt_HUMAN$	2::Q70Z35|PREX2_HUMAN	28	182505	1	1	1	1	0.03	Phosphatidylinositol 3
222	1	SwissProt_HUMAN$	2::Q99665|I12R2_HUMAN	28	97073	1	1	1	1	0.05	Interleukin-12 recepto
223	1	SwissProt_HUMAN$	2::P56199|ITA1_HUMAN	28	130765	3	1	1	1	0.04	Integrin alpha-1 OS=I
224	1	SwissProt_HUMAN$	2::Q9Y4F1|FARP1_HUMAN	27	118559	1	1	1	1	0.04	FERM, ARHGEF and pl
225	1	SwissProt_HUMAN$	2::P11532|DMD_HUMAN	27	426484	1	1	1	1	0.01	Dystrophin OS=Homo
226	1	SwissProt_HUMAN$	2::P0CG47|UBB_HUMAN	27	25746	1	1	1	1	0.20	Polyubiquitin-B OS=H
227	1	SwissProt_HUMAN$	2::Q12934|BFSP1_HUMAN	27	74499	1	1	1	1	0.07	Filensin OS=Homo sa

↑Family	M	DB	Accession	Score	Mass	Matches	Match(sig)	Sequences	Seq(sig)	emPAI	Description
228	1	SwissProt_HUMAN$	2::Q9NQX4|MYO5C_HUMAN	27	202682	1	1	1	1	0.02	Unconventional myosi
229	1	SwissProt_HUMAN$	2::Q9P2M7|CING_HUMAN	27	136304	1	1	1	1	0.04	Cingulin OS=Homo sa
230	1	SwissProt_HUMAN$	2::Q9NQT8|KI13B_HUMAN	27	202663	1	1	1	1	0.02	Kinesin-like protein KI
231	1	SwissProt_HUMAN$	2::Q92570|NR4A3_HUMAN	27	68186	1	1	1	1	0.07	Nuclear receptor subfa
232	1	SwissProt_HUMAN$	2::P03950|ANGI_HUMAN	26	16539	1	1	1	1	0.33	Angiogenin OS=Homo
233	1	SwissProt_HUMAN$	2::Q9NP80|PLPL8_HUMAN	26	88421	1	1	1	1	0.05	Calcium-independent
234	1	SwissProt_HUMAN$	2::H7C350|CC188_HUMAN	26	43520	1	1	1	1	0.11	Coiled-coil domain-co
235	1	SwissProt_HUMAN$	2::P36957|ODO2_HUMAN	26	48724	1	1	1	1	0.10	Dihydrolipoyllysine-re
236	1	SwissProt_HUMAN$	2::P31939|PUR9_HUMAN	26	64575	1	1	1	1	0.08	Bifunctional purine bi
237	1	SwissProt_HUMAN$	2::Q6ZS17|RIPR1_HUMAN	26	132225	1	1	1	1	0.04	Rho family-interacting
238	1	SwissProt_HUMAN$	2::Q8TAQ9|SUN3_HUMAN	26	40478	1	1	1	1	0.12	SUN domain-containir
239	1	SwissProt_HUMAN$	2::P00488|F13A_HUMAN	26	83215	1	1	1	1	0.06	Coagulation factor XII
240	1	SwissProt_HUMAN$	2::O95757|HS74L_HUMAN	26	94453	1	1	1	1	0.05	Heat shock 70 kDa pr
241	1	SwissProt_HUMAN$	2::Q9Y566|SHAN1_HUMAN	26	224821	3	1	1	1	0.02	SH3 and multiple ank
242	1	SwissProt_HUMAN$	2::Q05513|KPCZ_HUMAN	26	67617	1	1	1	1	0.07	Protein kinase C zeta
243	1	SwissProt_HUMAN$	2::P49643|PRI2_HUMAN	25	58769	2	1	1	1	0.08	DNA primase large su
244	1	SwissProt_HUMAN$	2::Q7Z6Z7|HUWE1_HUMAN	25	481589	1	1	1	1	0.01	E3 ubiquitin-protein li
245	1	SwissProt_HUMAN$	2::Q15113|PCOC1_HUMAN	25	47942	2	2	2	2	0.22	Procollagen C-endope
246	1	SwissProt_HUMAN$	2::Q8NEM7|SP20H_HUMAN	25	85735	1	1	1	1	0.06	Transcription factor S
247	1	SwissProt_HUMAN$	2::O00139|KIF2A_HUMAN	25	79905	1	1	1	1	0.06	Kinesin-like protein KI
248	1	SwissProt_HUMAN$	2::Q8N4P6|LRC71_HUMAN	25	61787	1	1	1	1	0.08	Leucine-rich repeat-co
249	1	SwissProt_HUMAN$	2::Q92750|TAF4B_HUMAN	25	91034	3	3	3	3	0.17	Transcription initiatior
250	1	SwissProt_HUMAN$	2::P78563|RED1_HUMAN	24	80713	2	1	1	1	0.06	Double-stranded RNA
251	1	SwissProt_HUMAN$	2::Q14002|CEAM7_HUMAN	24	29361	1	1	1	1	0.17	Carcinoembryonic ant
252	1	SwissProt_HUMAN$	2::P00966|ASSY_HUMAN	24	46501	1	1	1	1	0.11	Argininosuccinate syn
253	1	SwissProt_HUMAN$	2::O43819|SCO2_HUMAN	24	29791	1	1	1	1	0.17	Protein SCO2 homolo
254	1	SwissProt_HUMAN$	2::Q6ZTR6|ZNFDT_HUMAN	23	17225	1	1	1	1	0.31	Putative uncharacteri
255	1	SwissProt_HUMAN$	2::Q8ND90|PNMA1_HUMAN	23	39736	2	1	1	1	0.13	Paraneoplastic antige
256	1	SwissProt_HUMAN$	2::A2RRD8|ZN320_HUMAN	23	59288	1	1	1	1	0.08	Zinc finger protein 32
257	1	SwissProt_HUMAN$	2::P48039|MTR1A_HUMAN	23	39349	1	1	1	1	0.13	Melatonin receptor ty
258	1	SwissProt_HUMAN$	2::Q58A45|PAN3_HUMAN	23	95552	3	1	2	1	0.05	PAN2-PAN3 deadenyla
259	1	SwissProt_HUMAN$	2::Q13061|TRDN_HUMAN	23	81546	1	1	1	1	0.06	Triadin OS=Homo sap
260	1	SwissProt_HUMAN$	2::Q9Y238|DLEC1_HUMAN	23	195559	1	1	1	1	0.02	Deleted in lung and e
261	1	SwissProt_HUMAN$	2::Q5T2T1|MPP7_HUMAN	23	65483	1	1	1	1	0.07	MAGUK p55 subfamily
262	1	SwissProt_HUMAN$	2::A6NKL6|T200C_HUMAN	23	63889	1	1	1	1	0.08	Transmembrane prote
263	1	SwissProt_HUMAN$	2::Q9Y5K8|VATD_HUMAN	23	28245	1	1	1	1	0.18	V-type proton ATPase
264	1	SwissProt_HUMAN$	2::Q9P2S5|WRP73_HUMAN	22	51555	1	1	1	1	0.10	WD repeat-containing
265	1	SwissProt_HUMAN$	2::O60282|KIF5C_HUMAN	22	109427	4	2	2	1	0.04	Kinesin heavy chain is
266	1	SwissProt_HUMAN$	2::Q7Z5M8|AB12B_HUMAN	22	40750	1	1	1	1	0.12	Protein ABHD12B OS=
267	1	SwissProt_HUMAN$	2::Q7Z4I7|LIMS2_HUMAN	22	38890	1	1	1	1	0.13	LIM and senescent ce

↑Family	M	DB	Accession	Score	Mass	Matches	Match(sig)	Sequences	Seq(sig)	emPAI	Description
268	1	SwissProt_HUMAN$	2::Q8TD08|MK15_HUMAN	22	59795	1	1	1	1	0.08	Mitogen-activated pro
269	1	SwissProt_HUMAN$	2::Q96PP9|GBP4_HUMAN	22	73119	2	1	1	1	0.07	Guanylate-binding prc
270	1	SwissProt_HUMAN$	2::Q92887|MRP2_HUMAN	22	174096	3	1	1	1	0.03	Canalicular multispeci
271	1	SwissProt_HUMAN$	2::Q96JP2|MY15B_HUMAN	22	166983	1	1	1	1	0.03	Unconventional myosi
272	1	SwissProt_HUMAN$	2::P02788|TRFL_HUMAN	22	78132	2	2	2	2	0.13	Lactotransferrin OS=I
273	1	SwissProt_HUMAN$	2::Q8TBE9|NANP_HUMAN	22	27795	1	1	1	1	0.18	N-acylneuraminate-9-
274	1	SwissProt_HUMAN$	2::Q16543|CDC37_HUMAN	22	44440	1	1	1	1	0.11	Hsp90 co-chaperone (
275	1	SwissProt_HUMAN$	2::O75901|RASF9_HUMAN	22	49989	1	1	1	1	0.10	Ras association doma
276	1	SwissProt_HUMAN$	2::Q7Z340|ZN551_HUMAN	21	77465	1	1	1	1	0.06	Zinc finger protein 55
277	1	SwissProt_HUMAN$	2::P54750|PDE1A_HUMAN	21	61213	1	1	1	1	0.08	Calcium/calmodulin-d
278	1	SwissProt_HUMAN$	2::Q86VH2|KIF27_HUMAN	21	160184	1	1	1	1	0.03	Kinesin-like protein KI
279	1	SwissProt_HUMAN$	2::Q96P31|FCRL3_HUMAN	21	80806	1	1	1	1	0.06	Fc receptor-like protei
280	1	SwissProt_HUMAN$	2::Q7Z3F1|GP155_HUMAN	21	96856	1	1	1	1	0.05	Integral membrane pr
281	1	SwissProt_HUMAN$	2::Q7Z418|KCNKI_HUMAN	21	43643	1	1	1	1	0.11	Potassium channel su
282	1	SwissProt_HUMAN$	2::Q8N8K9|K1958_HUMAN	21	79162	1	1	1	1	0.06	Uncharacterized prote
283	1	SwissProt_HUMAN$	2::A0A0A0MT89|KJ01_HUMAN	21	1393	1	1	1	1	14.58	Immunoglobulin kapp
284	1	SwissProt_HUMAN$	2::P06241|FYN_HUMAN	21	60723	1	1	1	1	0.08	Tyrosine-protein kinas
285	1	SwissProt_HUMAN$	2::Q53LP3|SWAHC_HUMAN	21	55638	1	1	1	1	0.09	Ankyrin repeat domai
286	1	SwissProt_HUMAN$	2::P21399|ACOC_HUMAN	21	98337	1	1	1	1	0.05	Cytoplasmic aconitate
287	1	SwissProt_HUMAN$	2::Q9BRX2|PELO_HUMAN	20	43332	1	1	1	1	0.12	Protein pelota homolo
288	1	SwissProt_HUMAN$	2::P56192|SYMC_HUMAN	20	101052	1	1	1	1	0.05	Methionine--tRNA liga
289	1	SwissProt_HUMAN$	2::Q86Y13|DZIP3_HUMAN	20	138516	1	1	1	1	0.03	E3 ubiquitin-protein li
290	1	SwissProt_HUMAN$	2::Q9Y5I4|PCDC2_HUMAN	20	109383	1	1	1	1	0.04	Protocadherin alpha-C
291	1	SwissProt_HUMAN$	2::A0AV02|S12A8_HUMAN	20	78188	1	1	1	1	0.06	Solute carrier family 1
292	1	SwissProt_HUMAN$	2::A0A0B4J1V7|HV781_HUMAN	20	12909	1	1	1	1	0.43	Probable non-function
293	1	SwissProt_HUMAN$	2::O15091|MRPP3_HUMAN	20	67272	1	1	1	1	0.07	Mitochondrial ribonucl
294	1	SwissProt_HUMAN$	2::O75818|RPP40_HUMAN	20	41807	1	1	1	1	0.12	Ribonuclease P protei
295	1	SwissProt_HUMAN$	2::P11309|PIM1_HUMAN	20	35663	2	1	2	1	0.14	Serine/threonine-prot
296	1	SwissProt_HUMAN$	2::Q9NXL9|MCM9_HUMAN	20	127233	1	1	1	1	0.04	DNA helicase MCM9 O
297	1	SwissProt_HUMAN$	2::O15484|CAN5_HUMAN	20	73123	1	1	1	1	0.07	Calpain-5 OS=Homo s
298	1	SwissProt_HUMAN$	2::Q8N7E2|CBLL2_HUMAN	20	48754	1	1	1	1	0.10	E3 ubiquitin-protein li
299	1	SwissProt_HUMAN$	2::Q9NP81|SYSM_HUMAN	20	58246	1	1	1	1	0.08	Serine--tRNA ligase, r
300	1	SwissProt_HUMAN$	2::O15197|EPHB6_HUMAN	20	110630	1	1	1	1	0.04	Ephrin type-B recepto
301	1	SwissProt_HUMAN$	2::Q9NU22|MDN1_HUMAN	20	632420	1	1	1	1	0.01	Midasin OS=Homo sa
302	1	SwissProt_HUMAN$	2::Q8IU68|TMC8_HUMAN	20	81589	2	1	2	1	0.06	Transmembrane chan
303	1	SwissProt_HUMAN$	2::O15417|TNC18_HUMAN	20	314326	1	1	1	1	0.02	Trinucleotide repeat-c
304	1	SwissProt_HUMAN$	2::Q96NL6|SCLT1_HUMAN	20	80861	1	1	1	1	0.06	Sodium channel and c
305	1	SwissProt_HUMAN$	2::Q13474|DRP2_HUMAN	20	107894	1	1	1	1	0.04	Dystrophin-related pr
306	1	SwissProt_HUMAN$	2::O43927|CXL13_HUMAN	19	12656	1	1	1	1	0.44	C-X-C motif chemokir
307	1	SwissProt_HUMAN$	2::Q8TED9|AF1L1_HUMAN	19	86378	1	1	1	1	0.06	Actin filament-associa

↑Family	M	DB	Accession	Score	Mass	Matches	Match(sig)	Sequences	Seq(sig)	emPAI	Description
308	1	SwissProt_HUMAN$	2::Q92878|RAD50_HUMAN	19	153797	1	1	1	1	0.03	DNA repair protein RA
309	1	SwissProt_HUMAN$	2::Q9UFH2|DYH17_HUMAN	19	508987	1	1	1	1	0.01	Dynein heavy chain 1
310	1	SwissProt_HUMAN$	2::P07947|YES_HUMAN	19	60763	1	1	1	1	0.08	Tyrosine-protein kinas
311	1	SwissProt_HUMAN$	2::Q07075|AMPE_HUMAN	19	109176	1	1	1	1	0.04	Glutamyl aminopeptid
312	1	SwissProt_HUMAN$	2::Q9UJX2|CDC23_HUMAN	19	68790	1	1	1	1	0.07	Cell division cycle pro
313	1	SwissProt_HUMAN$	2::P0DJI8|SAA1_HUMAN	19	13524	1	1	1	1	0.41	Serum amyloid A-1 pr
314	1	SwissProt_HUMAN$	2::Q13976|KGP1_HUMAN	19	76316	1	1	1	1	0.06	cGMP-dependent prot
315	1	SwissProt_HUMAN$	2::P78324|SHPS1_HUMAN	19	54933	3	1	2	1	0.09	Tyrosine-protein phos
316	1	SwissProt_HUMAN$	2::Q6ZSZ6|TSH1_HUMAN	19	117843	1	1	1	1	0.04	Teashirt homolog 1 O
317	1	SwissProt_HUMAN$	2::O43896|KIF1C_HUMAN	19	122870	1	1	1	1	0.04	Kinesin-like protein KI
318	1	SwissProt_HUMAN$	2::Q8N7U6|EFHB_HUMAN	19	93743	6	3	1	1	0.05	EF-hand domain-conta
319	1	SwissProt_HUMAN$	2::P49747|COMP_HUMAN	19	82808	1	1	1	1	0.06	Cartilage oligomeric m
320	1	SwissProt_HUMAN$	2::Q9NY97|B3GN2_HUMAN	19	45993	1	1	1	1	0.11	N-acetyllactosaminide
321	1	SwissProt_HUMAN$	2::Q8TEM1|PO210_HUMAN	19	204983	1	1	1	1	0.02	Nuclear pore membra
322	1	SwissProt_HUMAN$	2::Q7Z353|HDX_HUMAN	19	77158	1	1	1	1	0.06	Highly divergent hom
323	1	SwissProt_HUMAN$	2::O00222|GRM8_HUMAN	18	101675	1	1	1	1	0.05	Metabotropic glutama
324	1	SwissProt_HUMAN$	2::O94766|B3GA3_HUMAN	18	37099	1	1	1	1	0.14	Galactosylgalactosylx
325	1	SwissProt_HUMAN$	2::Q96DN5|TBC31_HUMAN	18	124111	1	1	1	1	0.04	TBC1 domain family n
326	1	SwissProt_HUMAN$	2::A9Z1Z3|FR1L4_HUMAN	18	200854	1	1	1	1	0.02	Fer-1-like protein 4 O
327	1	SwissProt_HUMAN$	2::B1AL46|NTM2E_HUMAN	18	93920	1	1	1	1	0.05	NUT family member 2
328	1	SwissProt_HUMAN$	2::Q9BYX4|IFIH1_HUMAN	18	116615	1	1	1	1	0.04	Interferon-induced he
329	1	SwissProt_HUMAN$	2::Q49MG5|MAP9_HUMAN	18	74189	1	1	1	1	0.07	Microtubule-associate
330	1	SwissProt_HUMAN$	2::Q9UBS4|DJB11_HUMAN	18	40489	1	1	1	1	0.12	DnaJ homolog subfam
331	1	SwissProt_HUMAN$	2::Q9UHB6|LIMA1_HUMAN	18	85173	1	1	1	1	0.06	LIM domain and actin
332	1	SwissProt_HUMAN$	2::Q9HCP0|KC1G1_HUMAN	18	48481	1	1	1	1	0.10	Casein kinase I isofor
333	1	SwissProt_HUMAN$	2::Q5JPH6|SYEM_HUMAN	18	58652	1	1	1	1	0.08	Probable glutamate--t
334	1	SwissProt_HUMAN$	2::P13762|DRB4_HUMAN	18	29922	1	1	1	1	0.17	HLA class II histocom
335	1	SwissProt_HUMAN$	2::P20594|ANPRB_HUMAN	17	116948	1	1	1	1	0.04	Atrial natriuretic pepti
336	1	SwissProt_HUMAN$	2::Q6ZS72|PEAK3_HUMAN	17	50478	3	1	1	1	0.10	Protein PEAK3 OS=Ho
337	1	SwissProt_HUMAN$	2::Q93034|CUL5_HUMAN	17	90897	1	1	1	1	0.05	Cullin-5 OS=Homo sa
338	1	SwissProt_HUMAN$	2::P09913|IFIT2_HUMAN	17	54597	1	1	1	1	0.09	Interferon-induced pr
339	1	SwissProt_HUMAN$	2::Q9Y6W5|WASF2_HUMAN	17	54250	1	1	1	1	0.09	Wiskott-Aldrich syndr
340	1	SwissProt_HUMAN$	2::Q8IUH5|ZDH17_HUMAN	17	72592	1	1	1	1	0.07	Palmitoyltransferase Z
341	1	SwissProt_HUMAN$	2::Q7L5N1|CSN6_HUMAN	17	36140	1	1	1	1	0.14	COP9 signalosome co
342	1	SwissProt_HUMAN$	2::Q05586|NMDZ1_HUMAN	17	105306	1	1	1	1	0.05	Glutamate receptor io
343	1	SwissProt_HUMAN$	2::O14746|TERT_HUMAN	17	126916	2	2	1	1	0.04	Telomerase reverse tr
344	1	SwissProt_HUMAN$	2::Q5VXU9|SHOC1_HUMAN	17	165097	1	1	1	1	0.03	Protein shortage in ch
345	1	SwissProt_HUMAN$	2::P51512|MMP16_HUMAN	17	69477	2	1	1	1	0.07	Matrix metalloproteina
346	1	SwissProt_HUMAN$	2::Q9UIX4|KCNG1_HUMAN	17	57876	1	1	1	1	0.09	Potassium voltage-gat
347	1	SwissProt_HUMAN$	2::Q96Q15|SMG1_HUMAN	16	410241	1	1	1	1	0.01	Serine/threonine-prot

↑Family	M	DB	Accession	Score	Mass	Matches	Match(sig)	Sequences	Seq(sig)	emPAI	Description
348	1	SwissProt_HUMAN$	2::Q8IY34|S15A3_HUMAN	16	63518	1	1	1	1	0.08	Solute carrier family 1
349	1	SwissProt_HUMAN$	2::Q9H257|CARD9_HUMAN	16	62202	1	1	1	1	0.08	Caspase recruitment (
350	1	SwissProt_HUMAN$	2::Q96MN2|NALP4_HUMAN	16	113341	1	1	1	1	0.04	NACHT, LRR and PYD
351	1	SwissProt_HUMAN$	2::Q9UKM7|MA1B1_HUMAN	16	79530	1	1	1	1	0.06	Endoplasmic reticulun
352	1	SwissProt_HUMAN$	2::P49716|CEBPD_HUMAN	16	28449	1	1	1	1	0.18	CCAAT/enhancer-bind
353	1	SwissProt_HUMAN$	2::O00478|BT3A3_HUMAN	16	64961	1	1	1	1	0.08	Butyrophilin subfamily
354	1	SwissProt_HUMAN$	2::O14523|C2C2L_HUMAN	16	76134	1	1	1	1	0.06	Phospholipid transfer
355	1	SwissProt_HUMAN$	2::P58512|CU067_HUMAN	16	21294	1	1	1	1	0.25	Uncharacterized prote
356	1	SwissProt_HUMAN$	2::Q8IYD1|ERF3B_HUMAN	16	68840	1	1	1	1	0.07	Eukaryotic peptide ch
357	1	SwissProt_HUMAN$	2::Q9H2V7|SPNS1_HUMAN	16	56594	1	1	1	1	0.09	Protein spinster homo
358	1	SwissProt_HUMAN$	2::A3QJZ7|PRA27_HUMAN	15	55170	1	1	1	1	0.09	PRAME family membe
359	1	SwissProt_HUMAN$	2::Q9BR84|ZN559_HUMAN	15	62277	1	1	1	1	0.08	Zinc finger protein 55
360	1	SwissProt_HUMAN$	2::Q9Y4E5|ZN451_HUMAN	15	121406	1	1	1	1	0.04	E3 SUMO-protein liga
361	1	SwissProt_HUMAN$	2::Q9UIL4|KIF25_HUMAN	15	40661	1	1	1	1	0.12	Kinesin-like protein KI
362	1	SwissProt_HUMAN$	2::Q96NS5|ASB16_HUMAN	15	49605	1	1	1	1	0.10	Ankyrin repeat and SO
363	1	SwissProt_HUMAN$	2::Q8TCY9|URGCP_HUMAN	15	104921	1	1	1	1	0.05	Up-regulator of cell pr
364	1	SwissProt_HUMAN$	2::O94875|SRBS2_HUMAN	15	124030	1	1	1	1	0.04	Sorbin and SH3 doma
365	1	SwissProt_HUMAN$	2::Q86UW6|N4BP2_HUMAN	15	198676	1	1	1	1	0.02	NEDD4-binding protei
366	1	SwissProt_HUMAN$	2::Q86VD1|MORC1_HUMAN	15	112810	1	1	1	1	0.04	MORC family CW-type
367	1	SwissProt_HUMAN$	2::O00187|MASP2_HUMAN	14	75654	1	1	1	1	0.06	Mannan-binding lectin
368	1	SwissProt_HUMAN$	2::P21266|GSTM3_HUMAN	14	26542	1	1	1	1	0.19	Glutathione S-transfer
369	1	SwissProt_HUMAN$	2::Q5VVM6|CCD30_HUMAN	14	91278	1	1	1	1	0.05	Coiled-coil domain-co
370	1	SwissProt_HUMAN$	2::Q8N9Q2|SR1IP_HUMAN	14	18166	1	1	1	1	0.29	Protein SREK1IP1 OS=
371	1	SwissProt_HUMAN$	2::P55786|PSA_HUMAN	14	103211	1	1	1	1	0.05	Puromycin-sensitive a
372	1	SwissProt_HUMAN$	2::Q9H270|VPS11_HUMAN	14	107768	2	1	1	1	0.04	Vacuolar protein sorti
373	1	SwissProt_HUMAN$	2::O75165|DJC13_HUMAN	14	254252	1	1	1	1	0.02	DnaJ homolog subfam
374	1	SwissProt_HUMAN$	2::Q99996|AKAP9_HUMAN	14	452708	2	1	2	1	0.01	A-kinase anchor prote
375	1	SwissProt_HUMAN$	2::Q9BWT1|CDCA7_HUMAN	14	42546	1	1	1	1	0.12	Cell division cycle-ass
376	1	SwissProt_HUMAN$	2::Q96HW7|INT4_HUMAN	14	108102	1	1	1	1	0.04	Integrator complex su
377	1	SwissProt_HUMAN$	2::Q9NQU5|PAK6_HUMAN	13	74822	1	1	1	1	0.07	Serine/threonine-prot
378	1	SwissProt_HUMAN$	2::Q8WW22|DNJA4_HUMAN	13	44769	1	1	1	1	0.11	DnaJ homolog subfam
379	1	SwissProt_HUMAN$	2::Q86VM9|ZCH18_HUMAN	13	106316	1	1	1	1	0.05	Zinc finger CCCH dom
380	1	SwissProt_HUMAN$	2::Q9H8M1|CQ10B_HUMAN	13	27053	1	1	1	1	0.19	Coenzyme Q-binding
381	1	SwissProt_HUMAN$	2::Q9Y257|KCNK6_HUMAN	13	33726	1	1	1	1	0.15	Potassium channel su
382	1	SwissProt_HUMAN$	2::Q0WX57|U17LO_HUMAN	13	59673	2	1	2	1	0.08	Ubiquitin carboxyl-ter
383	1	SwissProt_HUMAN$	2::Q02410|APBA1_HUMAN	13	92807	1	1	1	1	0.05	Amyloid-beta A4 prec
384	1	SwissProt_HUMAN$	2::Q86WK7|AMGO3_HUMAN	13	55214	1	1	1	1	0.09	Amphoterin-induced p

Export as CSV

Not what you expected? Try **the select summary**.

Mascot: http://www.matrixscience.com/
