# Supplementary material for: Circulating multimeric immune complexes contribute to immunopathology in COVID-19
Source: Nat Commun. 2022 Sep 26;13:5654. doi: 10.1038/s41467-022-32867-z (PMC9513013; doi:10.1038/s41467-022-32867-z)
Supplement: Supplementary file 3 — Source Data [file 41467_2022_32867_MOESM3_ESM.zip › Source Data/MS_29012021 (whole lane cut).zip!/QX20210214-03_Virologie(AG-Hengel)-20210129_(V4)_IC-_3-5_complete-lane_0k3v20_TRL_db_SARS-CoV-2+SP_H(F229100).pdf]

MATRIX SCIENCE MASCOT Search Results

User : Wolfgang Bildl
E-mail : wolfgang.bildl@physiologie.uni-freiburg.de
Search title : QX 20210212 (db$ SARS-CoV-2+SwissProt HUMAN$ 5 ppm 20 mmu), QX20210214-03_Virologie(AG-Hengel)-20210129_(V4)_IC-_3-5_complete-lane_0k3v20_TRL_shifted_-3.0.mgf
MS data file : D:\Xcalibur\Data\Wolfgang\Q-Exactive-HF-X_20210212\QX20210214-03_Virologie(AG-Hengel)-20210129_(V4)_IC-_3-5_complete-lane_0k3v20_TRL_shifted_-3.0.mgf
Databases : **1:** db$ 20210114 (14 sequences; 14,319 residues)
2: SwissProt_HUMAN$ 20201007 (20,389 sequences; 11,363,789 residues)
Timestamp : 15 Feb 2021 at 08:23:26 GMT

Not what you expected? Try **the select summary**.

Search parameters

Type of search : MS/MS Ion Search
Enzyme : Trypsin
Variable modifications : **Acetyl (Protein N-term)**, **Carbamidomethyl (C)**, **Gln->pyro-Glu (N-term Q)**, **Oxidation (M)**
Mass values : Monoisotopic
Protein mass : Unrestricted
Peptide mass tolerance : ± 5 ppm
Fragment mass tolerance : ± 20 mmu
Max missed cleavages : 1
Instrument type : ESI-FTICR
Number of queries : 77,338

Score distribution

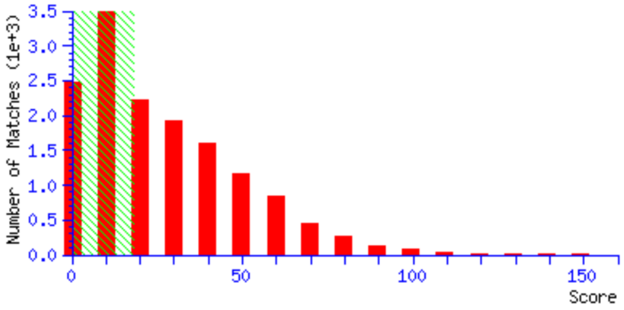

Peptide score distribution. Ions score is $-10\log(P)$, where P is the probability that the observed match is a random event.
There are **7,943** peptide matches above identity threshold and **8,184** matches above homology threshold for **77,338** queries.
On average, individual ions scores **> 18** (beyond green shading) indicate **identity or extensive homology** ($p<0.05$).

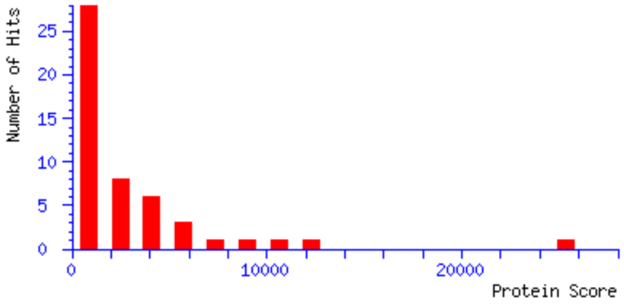

[Deprecated] Protein score distribution. Score distribution for family members in the first 50 proteins. Protein scores are derived from ions scores as a non-probabilistic basis for ranking protein families.

▼Modification statistics

Modification	Site	Above thr.
Carbamidomethyl	C	3188
Oxidation	M	1064
Gln->pyro-Glu	N-term	249
Acetyl	Protein N-term	9

▼Legend

Peptide columns and rows

Dupes	...	Expect	Rank	U	1	2	Peptide	
		**0.037**	▶**2**				**GAYSLSLR**	significant
		9	▶1				GFFLFVEGGR	top ranking
		**6.4e-005**	▶**1**				**GSSIFGLAPGK**	significant and top ranking
		**1.3e-006**	▶**1**		■		**SSGTSYPDVLK**	peptide is found in all proteins in family member 1
		**6.2e-007**	▶**1**			■	**VCNYVSWIK**	peptide is found in some but not all proteins in family member 2
		**6.4e-005**	▶**1**	**U**			**GSSIFGLAPGK**	unique
▶**2**		**5.7e-005**	▶**1**				**LNTLETEEWFFK**	peptide has two duplicates
		0.18	▶*1*				*LNTLETEEWFFK*	duplicate peptide

Right-facing triangle (▶) in the Dupes or Rank column indicates content that can be expanded by clicking on it. Down-facing triangle (▼) indicates the content is expanded and can be collapsed. For more details about particular columns, see **results format help**.

Protein quantitation ratios

	Score	...	114/113	115/113
**CFAH_HUMAN**	37559		*0.962*	**1.129**
**FHR2_HUMAN**	1330		**0.859**	1.128

When quantitation method is Reporter (e.g. iTRAQ) or Multiplex (e.g. IPTL), protein ratios are displayed when a family is expanded. Ratios in *italic* indicate that the peptide log-ratios do not appear to come from a normal distribution. **Bold** indicates that if you can assume peptide ratios are normally distributed, the protein ratio is significantly different from 1.0 (at significance level 0.05).

Note that lack of bold or italic can also mean that significance or normality testing has not been performed (for example, if protein ratio type does not support it).

Protein Family Summary

Significance threshold p<	0.05	Max. number of families	AUTO
Ions score or expect cut-off	0.5	Dendrograms cut at	0
Show Percolator scores	☐		
Preferred taxonomy	All entries		

▼Sensitivity and FDR (reversed protein sequences)

			Target	Decoy	FDR		
PSMs	above	homology	8184	260	3.18%	Adjust to	1%

Decoy results are available in **the decoy report**.

Protein hits (637 proteins)

▶ **Filters: (none)**

Export as CSV

↑Family	M	DB	Accession	Score	Mass	Matches	Match(sig)	Sequences	Seq(sig)	emPAI	Description
1	1	SwissProt_HUMAN$	2::P02768|ALBU_HUMAN	25307	69321	1002	946	79	77	649.89	Albumin OS=Homo sa
1	2	SwissProt_HUMAN$	2::P02769|ALBU_BOVIN	1145	69248	102	92	14	13	1.60	Albumin OS=Bos taur
2	1	SwissProt_HUMAN$	2::P04114|APOB_HUMAN	12815	515283	517	481	288	274	16.57	Apolipoprotein B-100
3	1	SwissProt_HUMAN$	2::P0C0L4|CO4A_HUMAN	10157	192664	384	356	114	106	17.72	Complement C4-A OS
3	2	SwissProt_HUMAN$	2::P0C0L5|CO4B_HUMAN	10009	192631	384	356	114	106	18.22	Complement C4-B OS
4	1	SwissProt_HUMAN$	2::P01024|CO3_HUMAN	9203	187030	399	371	142	137	79.45	Complement C3 OS=I
5	1	SwissProt_HUMAN$	2::P0DOX5|IGG1_HUMAN	7878	49298	429	393	29	29	20.46	Immunoglobulin gamı
5	2	SwissProt_HUMAN$	2::P01860|IGHG3_HUMAN	4705	41260	240	221	24	24	26.47	Immunoglobulin heav
5	3	SwissProt_HUMAN$	2::P01859|IGHG2_HUMAN	3495	35878	213	194	19	19	25.73	Immunoglobulin heav
5	4	SwissProt_HUMAN$	2::P01861|IGHG4_HUMAN	2080	35918	123	109	19	17	11.06	Immunoglobulin heav
6	1	SwissProt_HUMAN$	2::P01834|IGKC_HUMAN	6298	11758	234	219	12	12	111.87	Immunoglobulin kapp
6	2	SwissProt_HUMAN$	2::P0DOX7|IGK_HUMAN	6006	23364	214	201	16	16	44.78	Immunoglobulin kapp
6	3	SwissProt_HUMAN$	2::P01597|KV139_HUMAN	259	12729	10	9	3	3	1.98	Immunoglobulin kapp
6	4	SwissProt_HUMAN$	2::A0A0C4DH72|KV106_HUMAN	245	12689	11	10	4	4	3.34	Immunoglobulin kapp
6	5	SwissProt_HUMAN$	2::A0A0C4DH73|KV112_HUMAN	235	12637	10	9	3	3	2.01	Immunoglobulin kapp
6	6	SwissProt_HUMAN$	2::A0A0C4DH67|KV108_HUMAN	228	12529	8	7	4	4	3.39	Immunoglobulin kapp
6	7	SwissProt_HUMAN$	2::A0A075B6S5|KV127_HUMAN	223	12703	8	7	4	4	3.34	Immunoglobulin kapp
6	8	SwissProt_HUMAN$	2::P01599|KV117_HUMAN	202	12770	8	7	5	5	5.16	Immunoglobulin kapp
6	9	SwissProt_HUMAN$	2::P01602|KV105_HUMAN	166	12773	6	5	3	3	1.98	Immunoglobulin kapp
6	10	SwissProt_HUMAN$	2::P01601|KVD16_HUMAN	111	12722	4	3	2	2	1.07	Immunoglobulin kapp
6	11	SwissProt_HUMAN$	2::A0A0B4J2D9|KVD13_HUMAN	111	12561	4	3	2	2	1.10	Immunoglobulin kapp
6	12	SwissProt_HUMAN$	2::A0A0C4DH24|KV621_HUMAN	97	12422	5	4	3	3	2.06	Immunoglobulin kapp
6	13	SwissProt_HUMAN$	2::P04430|KV116_HUMAN	86	12610	5	4	2	2	1.08	Immunoglobulin kapp

↑Family	M	DB	Accession	Score	Mass	Matches	Match(sig)	Sequences	Seq(sig)	emPAI	Description
6	14	SwissProt_HUMAN$	2::A0A0A0MT36|KVD21_HUMAN	46	12332	5	4	3	3	2.09	Immunoglobulin kapp
7	1	SwissProt_HUMAN$	2::P01871|IGHM_HUMAN	6168	49409	231	220	38	38	107.31	Immunoglobulin heav
7	2	SwissProt_HUMAN$	2::P0DOX6|IGM_HUMAN	5217	63446	189	177	30	29	12.56	Immunoglobulin mu h
7	3	SwissProt_HUMAN$	2::A0A0B4J1V2|HV226_HUMAN	119	13174	5	5	4	4	3.08	Immunoglobulin heav
7	4	SwissProt_HUMAN$	2::P01814|HV270_HUMAN	112	13252	4	4	3	3	3.08	Immunoglobulin heav
8	1	SwissProt_HUMAN$	2::P01023|A2MG_HUMAN	5229	163188	196	184	80	77	15.28	Alpha-2-macroglobuli
8	2	SwissProt_HUMAN$	2::P20742|PZP_HUMAN	506	163760	24	21	10	10	0.42	Pregnancy zone prote
9	1	SwissProt_HUMAN$	2::P02751|FINC_HUMAN	4737	272150	179	170	96	94	4.82	Fibronectin OS=Homc
10	1	SwissProt_HUMAN$	2::P04003|C4BPA_HUMAN	4464	66989	179	164	43	43	30.73	C4b-binding protein a
11	1	SwissProt_HUMAN$	2::P01009|A1AT_HUMAN	4216	46707	206	185	39	38	155.24	Alpha-1-antitrypsin O
12	1	SwissProt_HUMAN$	2::P01031|CO5_HUMAN	4030	188186	160	151	89	86	13.85	Complement C5 OS=I
13	1	SwissProt_HUMAN$	2::P08603|CFAH_HUMAN	3706	139005	166	150	76	70	13.81	Complement factor H
13	2	SwissProt_HUMAN$	2::Q03591|FHR1_HUMAN	850	37626	28	25	17	17	8.53	Complement factor H-
13	3	SwissProt_HUMAN$	2::P36980|FHR2_HUMAN	321	30631	12	11	8	8	2.42	Complement factor H-
13	4	SwissProt_HUMAN$	2::Q9BXR6|FHR5_HUMAN	174	64377	6	6	6	6	0.55	Complement factor H-
13	5	SwissProt_HUMAN$	2::Q02985|FHR3_HUMAN	148	37299	6	6	4	4	0.66	Complement factor H-
14	1	SwissProt_HUMAN$	2::P02787|TRFE_HUMAN	3541	77014	139	132	64	61	68.36	Serotransferrin OS=H
15	1	SwissProt_HUMAN$	2::P00738|HPT_HUMAN	2678	45177	129	125	29	29	37.74	Haptoglobin OS=Hom
15	2	SwissProt_HUMAN$	2::P00739|HPTR_HUMAN	1437	39005	64	63	23	23	28.63	Haptoglobin-related p
16	1	SwissProt_HUMAN$	2::P01876|IGHA1_HUMAN	2313	37631	86	83	21	21	19.22	Immunoglobulin heav
16	2	SwissProt_HUMAN$	2::P0DOX2|IGA2_HUMAN	992	48903	41	40	16	16	4.68	Immunoglobulin alpha
16	3	SwissProt_HUMAN$	2::P0DP02|HVC33_HUMAN	485	12980	18	17	6	6	34.75	Immunoglobulin heav
16	4	SwissProt_HUMAN$	2::P01768|HV330_HUMAN	465	12938	16	15	6	6	24.00	Immunoglobulin heav
16	5	SwissProt_HUMAN$	2::A0A0C4DH42|HV366_HUMAN	396	12690	15	14	5	5	17.82	Immunoglobulin heav
16	6	SwissProt_HUMAN$	2::P01764|HV323_HUMAN	374	12574	14	13	5	5	12.33	Immunoglobulin heav
16	7	SwissProt_HUMAN$	2::A0A0B4J1X5|HV374_HUMAN	343	12831	16	15	7	7	24.69	Immunoglobulin heav
16	8	SwissProt_HUMAN$	2::P01780|HV307_HUMAN	341	12934	17	17	8	8	50.12	Immunoglobulin heav
16	9	SwissProt_HUMAN$	2::A0A0B4J1V1|HV321_HUMAN	326	12831	13	13	5	5	16.91	Immunoglobulin heav
16	10	SwissProt_HUMAN$	2::A0A0C4DH31|HV118_HUMAN	319	12812	9	9	5	5	7.87	Immunoglobulin heav
16	11	SwissProt_HUMAN$	2::P23083|HV102_HUMAN	306	13076	11	9	6	5	10.97	Immunoglobulin heav
16	12	SwissProt_HUMAN$	2::P01782|HV309_HUMAN	270	12936	13	12	4	4	7.55	Immunoglobulin heav
16	13	SwissProt_HUMAN$	2::P01762|HV311_HUMAN	270	12900	11	11	5	5	11.49	Immunoglobulin heav
16	14	SwissProt_HUMAN$	2::P01763|HV348_HUMAN	260	12804	11	11	5	5	11.76	Immunoglobulin heav
16	15	SwissProt_HUMAN$	2::P01742|HV169_HUMAN	234	12651	10	8	5	4	3.34	Immunoglobulin heav
16	16	SwissProt_HUMAN$	2::A0A0C4DH29|HV103_HUMAN	201	12999	8	8	4	4	4.98	Immunoglobulin heav
16	17	SwissProt_HUMAN$	2::A0A0B4J2H0|HV69D_HUMAN	198	12652	7	6	4	3	2.01	Immunoglobulin heav
16	18	SwissProt_HUMAN$	2::P01766|HV313_HUMAN	198	12498	8	8	4	4	5.36	Immunoglobulin heav
16	19	SwissProt_HUMAN$	2::A0A0C4DH33|HV124_HUMAN	184	12816	4	4	3	3	1.98	Immunoglobulin heav
16	20	SwissProt_HUMAN$	2::P01743|HV146_HUMAN	159	12924	8	8	5	5	5.07	Immunoglobulin heav
16	21	SwissProt_HUMAN$	2::A0A075B6Q5|HV364_HUMAN	159	12882	5	4	3	2	1.95	Immunoglobulin heav

↑Family	M	DB	Accession	Score	Mass	Matches	Match(sig)	Sequences	Seq(sig)	emPAI	Description
16	22	SwissProt_HUMAN$	2::P0DP01|HV108_HUMAN	155	12983	7	7	3	3	1.92	Immunoglobulin heav
17	1	SwissProt_HUMAN$	2::P0DOY3|IGLC3_HUMAN	2243	11259	77	69	11	10	92.81	Immunoglobulin lamb
17	2	SwissProt_HUMAN$	2::P0DOX8|IGL1_HUMAN	1976	22816	75	65	13	12	13.39	Immunoglobulin lamb
17	3	SwissProt_HUMAN$	2::A0M8Q6|IGLC7_HUMAN	1129	11247	38	36	6	6	16.99	Immunoglobulin lamb
17	4	SwissProt_HUMAN$	2::P01706|LV211_HUMAN	81	12636	5	5	4	4	5.26	Immunoglobulin lamb
17	5	SwissProt_HUMAN$	2::P01709|LV208_HUMAN	64	12374	5	4	3	3	3.51	Immunoglobulin lamb
18	1	SwissProt_HUMAN$	2::P00450|CERU_HUMAN	2121	122128	81	79	49	48	7.46	Ceruloplasmin OS=Hc
19	1	SwissProt_HUMAN$	2::P01011|AACT_HUMAN	1876	47621	64	58	24	23	16.73	Alpha-1-antichymotry
20	1	SwissProt_HUMAN$	2::P00747|PLMN_HUMAN	1775	90510	84	80	50	49	18.76	Plasminogen OS=Hon
21	1	SwissProt_HUMAN$	2::P00736|C1R_HUMAN	1717	80067	69	65	36	35	10.30	Complement C1r subc
21	2	SwissProt_HUMAN$	2::Q9NZP8|C1RL_HUMAN	134	53464	5	5	4	4	0.42	Complement C1r subc
22	1	SwissProt_HUMAN$	2::P09871|C1S_HUMAN	1680	76635	74	69	33	32	11.60	Complement C1s subc
22	2	SwissProt_HUMAN$	2::P48740|MASP1_HUMAN	178	79195	9	8	8	7	0.52	Mannan-binding lectin
23	1	SwissProt_HUMAN$	2::P02774|VTDB_HUMAN	1555	52883	57	55	29	28	16.49	Vitamin D-binding prc
24	1	SwissProt_HUMAN$	2::P07225|PROS_HUMAN	1514	75074	66	63	33	32	11.46	Vitamin K-dependent
25	1	SwissProt_HUMAN$	2::P02647|APOA1_HUMAN	1298	30759	63	54	32	31	180.91	Apolipoprotein A-I OS
26	1	SwissProt_HUMAN$	2::P00751|CFAB_HUMAN	1289	85479	59	55	37	35	6.35	Complement factor B
27	1	SwissProt_HUMAN$	2::P00734|THRB_HUMAN	1273	69992	49	47	30	29	11.17	Prothrombin OS=Hon
28	1	SwissProt_HUMAN$	2::P04275|VWF_HUMAN	1269	309058	58	53	48	44	1.09	von Willebrand factor
29	1	SwissProt_HUMAN$	2::P05155|IC1_HUMAN	1269	55119	61	60	24	23	12.06	Plasma protease C1 ir
30	1	SwissProt_HUMAN$	2::P19827|ITIH1_HUMAN	1263	101326	46	46	28	28	4.38	Inter-alpha-trypsin inl
31	1	SwissProt_HUMAN$	2::P02741|CRP_HUMAN	1240	25023	86	75	13	11	8.54	C-reactive protein OS
32	1	SwissProt_HUMAN$	2::Q14624|ITIH4_HUMAN	1215	103293	53	52	33	33	4.71	Inter-alpha-trypsin inl
33	1	SwissProt_HUMAN$	2::P10643|CO7_HUMAN	1173	93457	49	44	36	35	5.52	Complement compone
34	1	SwissProt_HUMAN$	2::P02675|FIBB_HUMAN	1168	55892	46	42	31	28	19.93	Fibrinogen beta chain
35	1	SwissProt_HUMAN$	2::P19823|ITIH2_HUMAN	1151	106397	51	46	30	27	2.97	Inter-alpha-trypsin inl
36	1	SwissProt_HUMAN$	2::P02748|CO9_HUMAN	1099	63133	39	36	22	21	4.60	Complement compone
37	1	SwissProt_HUMAN$	2::P07357|CO8A_HUMAN	1094	65121	34	31	20	17	3.94	Complement compone
38	1	SwissProt_HUMAN$	2::P04264|K2C1_HUMAN	1075	65999	36	36	23	23	5.44	Keratin, type II cytosl
38	2	SwissProt_HUMAN$	2::P35908|K22E_HUMAN	354	65393	16	14	14	12	1.38	Keratin, type II cytosl
38	3	SwissProt_HUMAN$	2::P05787|K2C8_HUMAN	242	53671	13	11	11	9	1.21	Keratin, type II cytosl
38	4	SwissProt_HUMAN$	2::P13647|K2C5_HUMAN	165	62340	8	7	6	5	0.46	Keratin, type II cytosl
39	1	SwissProt_HUMAN$	2::P13671|CO6_HUMAN	1075	104718	40	38	29	28	2.72	Complement compone
40	1	SwissProt_HUMAN$	2::P02649|APOE_HUMAN	1044	36132	38	34	22	21	37.34	Apolipoprotein E OS=
41	1	SwissProt_HUMAN$	2::O43866|CD5L_HUMAN	1000	38063	37	33	21	19	10.90	CD5 antigen-like OS=
42	1	SwissProt_HUMAN$	2::P01008|ANT3_HUMAN	952	52569	38	36	26	25	11.36	Antithrombin-III OS=
43	1	SwissProt_HUMAN$	2::P13645|K1C10_HUMAN	940	58792	35	32	21	20	5.88	Keratin, type I cytosk
43	2	SwissProt_HUMAN$	2::P02533|K1C14_HUMAN	192	51529	10	9	8	7	1.08	Keratin, type I cytosk
43	3	SwissProt_HUMAN$	2::P05783|K1C18_HUMAN	75	48029	6	6	6	6	0.80	Keratin, type I cytosk
44	1	SwissProt_HUMAN$	2::P07996|TSP1_HUMAN	933	129300	36	35	29	28	1.90	Thrombospondin-1 OS

↑Family	M	DB	Accession	Score	Mass	Matches	Match(sig)	Sequences	Seq(sig)	emPAI	Description
45	1	SwissProt_HUMAN$	2::P01019|ANGT_HUMAN	900	53121	28	28	12	12	2.79	Angiotensinogen OS=
46	1	SwissProt_HUMAN$	2::P01042|KNG1_HUMAN	897	71912	30	30	21	21	2.98	Kininogen-1 OS=Hom
47	1	SwissProt_HUMAN$	2::P02749|APOH_HUMAN	896	38273	37	36	19	19	9.37	Beta-2-glycoprotein 1
48	1	SwissProt_HUMAN$	2::P06396|GELS_HUMAN	886	85644	34	32	24	22	2.56	Gelsolin OS=Homo sa
49	1	SwissProt_HUMAN$	2::P07358|CO8B_HUMAN	849	67003	30	30	18	18	3.10	Complement compone
50	1	SwissProt_HUMAN$	2::P02746|C1QB_HUMAN	838	26704	41	41	11	11	15.62	Complement C1q sub
51	1	SwissProt_HUMAN$	2::P02790|HEMO_HUMAN	802	51643	49	43	27	26	14.58	Hemopexin OS=Homo
52	1	SwissProt_HUMAN$	2::P35527|K1C9_HUMAN	663	62027	19	19	13	13	2.38	Keratin, type I cytosk
53	1	SwissProt_HUMAN$	2::P07360|CO8G_HUMAN	621	22264	17	17	10	10	9.09	Complement compone
54	1	SwissProt_HUMAN$	2::P02743|SAMP_HUMAN	590	25371	22	22	12	12	8.18	Serum amyloid P-com
55	1	SwissProt_HUMAN$	2::P08519|APOA_HUMAN	590	500995	28	26	20	19	0.21	Apolipoprotein(a) OS=
56	1	SwissProt_HUMAN$	2::P18428|LBP_HUMAN	570	53350	23	22	17	16	3.50	Lipopolysaccharide-bi
57	1	SwissProt_HUMAN$	2::P02747|C1QC_HUMAN	562	25757	28	24	9	9	6.44	Complement C1q sub
58	1	SwissProt_HUMAN$	2::P36955|PEDF_HUMAN	558	46283	27	23	20	17	5.94	Pigment epithelium-d
59	1	SwissProt_HUMAN$	2::P05156|CFAI_HUMAN	553	65707	22	21	17	17	2.65	Complement factor I
60	1	SwissProt_HUMAN$	2::P02679|FIBG_HUMAN	551	51479	26	23	17	15	3.34	Fibrinogen gamma ch
61	1	SwissProt_HUMAN$	2::P06727|APOA4_HUMAN	540	45344	32	28	25	22	8.85	Apolipoprotein A-IV O
62	1	SwissProt_HUMAN$	2::P01619|KV320_HUMAN	537	12549	15	14	4	4	3.39	Immunoglobulin kapp
62	2	SwissProt_HUMAN$	2::A0A0C4DH25|KVD20_HUMAN	456	12507	12	12	3	3	2.03	Immunoglobulin kapp
63	1	SwissProt_HUMAN$	2::P02671|FIBA_HUMAN	513	94914	38	35	25	22	3.04	Fibrinogen alpha chai
64	1	SwissProt_HUMAN$	2::P27169|PON1_HUMAN	513	39706	22	20	17	15	5.68	Serum paraoxonase/a
65	1	SwissProt_HUMAN$	2::P0DJI8|SAA1_HUMAN	508	13524	16	16	8	8	20.97	Serum amyloid A-1 pr
65	2	SwissProt_HUMAN$	2::P0DJI9|SAA2_HUMAN	410	13519	13	13	6	6	10.06	Serum amyloid A-2 pr
66	1	SwissProt_HUMAN$	2::P02763|A1AG1_HUMAN	496	23497	19	18	9	8	5.02	Alpha-1-acid glycopro
66	2	SwissProt_HUMAN$	2::P19652|A1AG2_HUMAN	333	23588	14	13	10	9	7.87	Alpha-1-acid glycopro
67	1	SwissProt_HUMAN$	2::P08697|A2AP_HUMAN	490	54531	18	18	12	12	2.37	Alpha-2-antiplasmin
68	1	SwissProt_HUMAN$	2::Q5QNW6|H2B2F_HUMAN	481	13912	14	13	6	6	19.43	Histone H2B type 2-F
69	1	SwissProt_HUMAN$	2::P02753|RET4_HUMAN	469	22995	19	19	12	12	20.37	Retinol-binding protei
70	1	SwissProt_HUMAN$	2::P02760|AMBP_HUMAN	467	38974	23	22	15	15	5.93	Protein AMBP OS=Ho
71	1	SwissProt_HUMAN$	2::P00761|TRYP_PIG	455	24394	12	12	6	6	2.17	Trypsin OS=Sus scrof
72	1	SwissProt_HUMAN$	2::P04217|A1BG_HUMAN	450	54220	25	23	13	13	2.39	Alpha-1B-glycoprotei
73	1	SwissProt_HUMAN$	2::P02766|TTHY_HUMAN	448	15877	14	13	7	7	6.82	Transthyretin OS=Ho
74	1	SwissProt_HUMAN$	2::P02765|FETUA_HUMAN	428	39316	22	20	11	10	3.76	Alpha-2-HS-glycoprot
75	1	SwissProt_HUMAN$	2::P06331|HV434_HUMAN	422	13806	13	12	5	5	4.42	Immunoglobulin heav
75	2	SwissProt_HUMAN$	2::P0DP08|HVD82_HUMAN	416	13008	12	11	5	5	4.98	Immunoglobulin heav
75	3	SwissProt_HUMAN$	2::A0A075B6R2|HV404_HUMAN	234	12840	10	8	6	5	5.07	Immunoglobulin heav
76	1	SwissProt_HUMAN$	2::P23142|FBLN1_HUMAN	416	77162	16	16	13	13	1.36	Fibulin-1 OS=Homo s
77	1	SwissProt_HUMAN$	2::Q08380|LG3BP_HUMAN	395	65289	11	10	8	7	0.66	Galectin-3-binding pr
78	1	SwissProt_HUMAN$	2::P01833|PIGR_HUMAN	388	83232	15	15	14	14	1.22	Polymeric immunoglo
79	1	SwissProt_HUMAN$	2::P68871|HBB_HUMAN	388	15988	16	15	10	10	23.78	Hemoglobin subunit b

↑Family	M	DB	Accession	Score	Mass	Matches	Match(sig)	Sequences	Seq(sig)	emPAI	Description
80	1	SwissProt_HUMAN$	2::P20851|C4BPB_HUMAN	384	28338	17	17	10	10	7.67	C4b-binding protein b
81	1	SwissProt_HUMAN$	2::P24821|TENA_HUMAN	379	240700	19	18	18	17	0.43	Tenascin OS=Homo s
82	1	SwissProt_HUMAN$	2::O75636|FCN3_HUMAN	370	32882	14	12	9	8	2.63	Ficolin-3 OS=Homo sa
83	1	SwissProt_HUMAN$	2::P12111|CO6A3_HUMAN	368	343457	25	25	23	23	0.37	Collagen alpha-3(VI)
84	1	SwissProt_HUMAN$	2::Q9UK55|ZPI_HUMAN	368	50674	13	13	12	12	2.36	Protein Z-dependent p
85	1	SwissProt_HUMAN$	2::P10909|CLUS_HUMAN	342	52461	14	14	10	10	1.46	Clusterin OS=Homo s
86	1	SwissProt_HUMAN$	2::P62805|H4_HUMAN	339	11360	15	15	10	10	88.83	Histone H4 OS=Homo
87	1	SwissProt_HUMAN$	2::P02745|C1QA_HUMAN	336	26000	15	13	8	7	3.25	Complement C1q sub
88	1	SwissProt_HUMAN$	2::P60709|ACTB_HUMAN	335	41710	18	17	12	12	3.87	Actin, cytoplasmic 1 C
89	1	SwissProt_HUMAN$	2::O14791|APOL1_HUMAN	331	43947	12	12	9	9	1.63	Apolipoprotein L1 OS=
90	1	SwissProt_HUMAN$	2::A0A0C4DH38|HV551_HUMAN	325	12666	14	11	6	4	8.03	Immunoglobulin heav
90	2	SwissProt_HUMAN$	2::A0A0J9YXX1|HV5X1_HUMAN	309	12764	12	12	5	5	11.76	Immunoglobulin heav
91	1	SwissProt_HUMAN$	2::P04004|VTNC_HUMAN	318	54271	23	20	11	11	2.10	Vitronectin OS=Homo
92	1	SwissProt_HUMAN$	2::P02750|A2GL_HUMAN	315	38154	15	14	11	11	2.89	Leucine-rich alpha-2-g
93	1	SwissProt_HUMAN$	2::P03952|KLKB1_HUMAN	310	71323	16	13	15	13	1.37	Plasma kallikrein OS=
94	1	SwissProt_HUMAN$	2::P25311|ZA2G_HUMAN	299	34237	11	10	9	9	2.46	Zinc-alpha-2-glycopro
95	1	SwissProt_HUMAN$	2::P12259|FA5_HUMAN	297	251546	16	15	14	13	0.30	Coagulation factor V C
96	1	SwissProt_HUMAN$	2::P00742|FA10_HUMAN	288	54697	14	11	13	11	1.59	Coagulation factor X C
96	2	SwissProt_HUMAN$	2::P00740|FA9_HUMAN	235	51745	15	13	14	13	2.28	Coagulation factor IX
97	1	SwissProt_HUMAN$	2::P02652|APOA2_HUMAN	287	11168	11	11	7	7	26.19	Apolipoprotein A-II OS
98	1	SwissProt_HUMAN$	2::Q96PD5|PGRP2_HUMAN	280	62178	12	11	10	10	1.14	N-acetylmuramoyl-L-a
99	1	SwissProt_HUMAN$	2::P04433|KV311_HUMAN	279	12567	8	8	3	3	2.03	Immunoglobulin kapp
100	1	SwissProt_HUMAN$	2::P05090|APOD_HUMAN	276	21262	12	10	10	8	6.23	Apolipoprotein D OS=
101	1	SwissProt_HUMAN$	2::P01591|IGJ_HUMAN	271	18087	13	13	8	8	6.90	Immunoglobulin J cha
102	1	SwissProt_HUMAN$	2::Q06033|ITIH3_HUMAN	266	99787	14	14	12	12	0.85	Inter-alpha-trypsin in
103	1	SwissProt_HUMAN$	2::P22792|CPN2_HUMAN	265	60518	11	11	8	8	0.87	Carboxypeptidase N s
104	1	SwissProt_HUMAN$	2::P01714|LV319_HUMAN	260	12035	6	5	4	3	2.19	Immunoglobulin lamb
105	1	SwissProt_HUMAN$	2::P43652|AFAM_HUMAN	258	69024	12	12	12	12	1.28	Afamin OS=Homo sap
106	1	SwissProt_HUMAN$	2::A0A0A0MS15|HV349_HUMAN	244	13048	11	8	7	6	7.40	Immunoglobulin heav
106	2	SwissProt_HUMAN$	2::A0A0B4J1Y9|HV372_HUMAN	238	13194	11	11	7	7	10.73	Immunoglobulin heav
106	3	SwissProt_HUMAN$	2::A0A0B4J1V0|HV315_HUMAN	212	12917	14	13	8	8	24.69	Immunoglobulin heav
107	1	SwissProt_HUMAN$	2::P11226|MBL2_HUMAN	244	26127	7	7	7	7	2.53	Mannose-binding prot
108	1	SwissProt_HUMAN$	2::P04196|HRG_HUMAN	220	59541	15	14	13	13	2.04	Histidine-rich glycopro
109	1	SwissProt_HUMAN$	2::P29622|KAIN_HUMAN	220	48511	15	14	12	12	2.22	Kallistatin OS=Homo
110	1	SwissProt_HUMAN$	2::P00748|FA12_HUMAN	219	67748	10	9	9	8	0.75	Coagulation factor XII
111	1	SwissProt_HUMAN$	2::P62937|PPIA_HUMAN	218	18001	7	7	5	5	3.76	Peptidyl-prolyl cis-tra
112	1	SwissProt_HUMAN$	2::P05546|HEP2_HUMAN	213	57034	14	11	13	11	1.49	Heparin cofactor 2 OS
113	1	SwissProt_HUMAN$	2::P01817|HV205_HUMAN	202	13223	5	5	2	2	1.02	Immunoglobulin heav
114	1	SwissProt_HUMAN$	2::P35858|ALS_HUMAN	199	65994	10	10	8	8	0.77	Insulin-like growth fac
115	1	SwissProt_HUMAN$	2::P00338|LDHA_HUMAN	198	36665	6	5	6	5	0.90	L-lactate dehydrogena

↑Family	M	DB	Accession	Score	Mass	Matches	Match(sig)	Sequences	Seq(sig)	emPAI	Description
115	2	SwissProt_HUMAN$	2::P07195|LDHB_HUMAN	151	36615	6	6	6	6	1.16	L-lactate dehydrogena
115	3	SwissProt_HUMAN$	2::Q6ZMR3|LDH6A_HUMAN	73	36484	2	2	2	2	0.29	L-lactate dehydrogena
116	1	SwissProt_HUMAN$	2::P0DOX3|IGD_HUMAN	196	56189	8	6	8	6	0.66	Immunoglobulin delta
117	1	SwissProt_HUMAN$	2::P27918|PROP_HUMAN	189	51242	9	9	8	8	1.09	Properdin OS=Homo s
118	1	SwissProt_HUMAN$	2::P06312|KV401_HUMAN	189	13372	11	8	6	6	7.11	Immunoglobulin kapp
119	1	SwissProt_HUMAN$	2::P02656|APOC3_HUMAN	177	10846	3	3	2	2	1.34	Apolipoprotein C-III C
120	1	SwissProt_HUMAN$	2::P69905|HBA_HUMAN	171	15248	11	10	7	6	7.54	Hemoglobin subunit a
121	1	SwissProt_HUMAN$	2::P02776|PLF4_HUMAN	170	10838	9	9	4	4	2.58	Platelet factor 4 OS=H
122	1	SwissProt_HUMAN$	2::P23528|COF1_HUMAN	164	18491	6	4	6	4	1.74	Cofilin-1 OS=Homo sa
123	1	SwissProt_HUMAN$	2::P35542|SAA4_HUMAN	162	14737	7	7	5	5	3.84	Serum amyloid A-4 pr
124	1	SwissProt_HUMAN$	2::A0A075B6R9|KVD24_HUMAN	151	13071	5	5	3	3	1.90	Probable non-function
125	1	SwissProt_HUMAN$	2::P08185|CBG_HUMAN	150	45112	8	7	8	7	1.08	Corticosteroid-binding
126	1	SwissProt_HUMAN$	2::A0A075B6P5|KV228_HUMAN	146	12948	5	5	3	3	1.92	Immunoglobulin kapp
126	2	SwissProt_HUMAN$	2::A0A087WW87|KV240_HUMAN	111	13302	5	5	4	4	3.04	Immunoglobulin kapp
126	3	SwissProt_HUMAN$	2::P06310|KV230_HUMAN	96	13177	5	5	3	3	1.87	Immunoglobulin kapp
126	4	SwissProt_HUMAN$	2::A0A075B6S2|KVD29_HUMAN	84	13135	2	2	2	2	1.03	Immunoglobulin kapp
127	1	SwissProt_HUMAN$	2::P08238|HS90B_HUMAN	146	83212	5	5	5	5	0.33	Heat shock protein HS
127	2	SwissProt_HUMAN$	2::P07900|HS90A_HUMAN	110	84607	4	4	4	4	0.25	Heat shock protein HS
127	3	SwissProt_HUMAN$	2::P14625|ENPL_HUMAN	79	92411	2	2	2	2	0.11	Endoplasmin OS=Hon
128	1	SwissProt_HUMAN$	2::P01593|KVD33_HUMAN	142	12840	3	3	2	2	1.06	Immunoglobulin kapp
129	1	SwissProt_HUMAN$	2::P60174|TPIS_HUMAN	142	26653	8	7	8	7	2.44	Triosephosphate isom
130	1	SwissProt_HUMAN$	2::P02654|APOC1_HUMAN	141	9326	9	6	5	4	10.81	Apolipoprotein C-I OS
131	1	SwissProt_HUMAN$	2::Q9Y6R7|FCGBP_HUMAN	138	571639	9	9	9	9	0.08	IgGFc-binding protein
132	1	SwissProt_HUMAN$	2::P51884|LUM_HUMAN	132	38405	9	7	7	6	1.09	Lumican OS=Homo sa
133	1	SwissProt_HUMAN$	2::P01624|KV315_HUMAN	129	12488	4	4	2	2	1.10	Immunoglobulin kapp
134	1	SwissProt_HUMAN$	2::P01700|LV147_HUMAN	129	12276	6	4	4	3	2.09	Immunoglobulin lamb
134	2	SwissProt_HUMAN$	2::P01699|LV144_HUMAN	97	12194	5	3	3	2	1.14	Immunoglobulin lamb
135	1	SwissProt_HUMAN$	2::P63104|1433Z_HUMAN	127	27728	4	4	4	4	0.97	14-3-3 protein zeta/d
135	2	SwissProt_HUMAN$	2::P27348|1433T_HUMAN	99	27747	3	3	3	3	0.66	14-3-3 protein theta (
135	3	SwissProt_HUMAN$	2::P61981|1433G_HUMAN	78	28285	4	4	4	4	0.94	14-3-3 protein gamm
135	4	SwissProt_HUMAN$	2::P31946|1433B_HUMAN	63	28065	2	2	2	2	0.40	14-3-3 protein beta/a
135	5	SwissProt_HUMAN$	2::P62258|1433E_HUMAN	42	29155	2	2	2	2	0.38	14-3-3 protein epsilor
136	1	SwissProt_HUMAN$	2::P08571|CD14_HUMAN	126	40051	6	5	6	5	0.80	Monocyte differentiati
137	1	SwissProt_HUMAN$	2::Q08830|FGL1_HUMAN	126	36357	6	5	6	5	0.91	Fibrinogen-like protei
138	1	SwissProt_HUMAN$	2::P12109|CO6A1_HUMAN	124	108462	3	3	3	3	0.14	Collagen alpha-1(VI) (
139	1	SwissProt_HUMAN$	2::Q71DI3|H32_HUMAN	120	15379	5	5	3	3	1.48	Histone H3.2 OS=Hor
139	2	SwissProt_HUMAN$	2::P84243|H33_HUMAN	74	15318	5	4	3	3	1.49	Histone H3.3 OS=Hor
139	3	SwissProt_HUMAN$	2::P68431|H31_HUMAN	60	15394	5	5	3	3	1.48	Histone H3.1 OS=Hor
140	1	SwissProt_HUMAN$	2::P04070|PROC_HUMAN	118	52037	3	3	3	3	0.31	Vitamin K-dependent
141	1	SwissProt_HUMAN$	2::Q96IY4|CBPB2_HUMAN	118	48393	5	5	5	5	0.63	Carboxypeptidase B2

↑Family	M	DB	Accession	Score	Mass	Matches	Match(sig)	Sequences	Seq(sig)	emPAI	Description
142	1	SwissProt_HUMAN$	2::P61626|LYSC_HUMAN	115	16526	5	5	4	4	3.10	Lysozyme C OS=Hom
143	1	SwissProt_HUMAN$	2::P06681|CO2_HUMAN	112	83214	7	5	7	5	0.33	Complement C2 OS=I
144	1	SwissProt_HUMAN$	2::P22392|NDKB_HUMAN	111	17287	7	7	5	5	4.05	Nucleoside diphospha
145	1	SwissProt_HUMAN$	2::O95445|APOM_HUMAN	109	21239	6	6	5	5	2.02	Apolipoprotein M OS=
146	1	SwissProt_HUMAN$	2::Q6FI13|H2A2A_HUMAN	108	14087	6	5	5	4	2.74	Histone H2A type 2-A
146	2	SwissProt_HUMAN$	2::P04908|H2A1B_HUMAN	67	14127	5	4	4	3	1.69	Histone H2A type 1-B,
147	1	SwissProt_HUMAN$	2::P02655|APOC2_HUMAN	108	11277	7	6	5	5	6.73	Apolipoprotein C-II OS
148	1	SwissProt_HUMAN$	2::P39019|RS19_HUMAN	107	16051	4	4	4	4	2.19	40S ribosomal protein
149	1	SwissProt_HUMAN$	2::P62979|RS27A_HUMAN	107	17953	4	4	3	3	1.18	Ubiquitin-40S ribosom
150	1	SwissProt_HUMAN$	2::P13796|PLSL_HUMAN	106	70244	4	4	4	4	0.31	Plastin-2 OS=Homo s
151	1	SwissProt_HUMAN$	2::P09211|GSTP1_HUMAN	105	23341	6	6	6	6	2.35	Glutathione S-transfe
152	1	SwissProt_HUMAN$	2::P07355|ANXA2_HUMAN	103	38580	5	5	5	5	0.84	Annexin A2 OS=Homo
153	1	SwissProt_HUMAN$	2::A0A0C4DH36|HV338_HUMAN	101	12750	5	5	2	2	1.98	Probable non-function
154	1	SwissProt_HUMAN$	2::P11021|BIP_HUMAN	100	72288	3	3	3	3	0.22	Endoplasmic reticulun
154	2	SwissProt_HUMAN$	2::P11142|HSP7C_HUMAN	55	70854	5	4	5	4	0.31	Heat shock cognate 7
155	1	SwissProt_HUMAN$	2::P62826|RAN_HUMAN	100	24408	4	4	4	4	1.16	GTP-binding nuclear p
156	1	SwissProt_HUMAN$	2::P25705|ATPA_HUMAN	100	59714	3	3	2	2	0.17	ATP synthase subunit
157	1	SwissProt_HUMAN$	2::A0A0C4DH34|HV428_HUMAN	97	13116	3	3	3	3	1.90	Immunoglobulin heav
158	1	SwissProt_HUMAN$	2::A0A075B6K4|LV310_HUMAN	96	12433	6	4	5	4	3.45	Immunoglobulin lamb
158	2	SwissProt_HUMAN$	2::P01717|LV325_HUMAN	87	12004	9	7	4	4	5.91	Immunoglobulin lamb
158	3	SwissProt_HUMAN$	2::A0A075B6K0|LV316_HUMAN	49	12458	4	3	2	2	1.11	Immunoglobulin lamb
158	4	SwissProt_HUMAN$	2::P01718|LV327_HUMAN	48	12157	4	3	2	2	1.14	Immunoglobulin lamb
159	1	SwissProt_HUMAN$	2::P62277|RS13_HUMAN	96	17212	6	6	6	6	4.10	40S ribosomal protein
160	1	SwissProt_HUMAN$	2::A0A0A0MS14|HV145_HUMAN	95	13500	2	2	1	1	0.99	Immunoglobulin heav
161	1	SwissProt_HUMAN$	2::A0A0B4J1U7|HV601_HUMAN	94	13473	11	9	5	4	2.99	Immunoglobulin heav
162	1	SwissProt_HUMAN$	2::Q15582|BGH3_HUMAN	93	74634	8	6	8	6	0.46	Transforming growth
163	1	SwissProt_HUMAN$	2::Q07020|RL18_HUMAN	93	21621	3	3	3	3	0.91	60S ribosomal protein
164	1	SwissProt_HUMAN$	2::P05160|F13B_HUMAN	92	75461	9	6	9	6	0.46	Coagulation factor XII
165	1	SwissProt_HUMAN$	2::Q15848|ADIPO_HUMAN	91	26397	2	2	1	1	0.19	Adiponectin OS=Homo
166	1	SwissProt_HUMAN$	2::Q5VTE0|EF1A3_HUMAN	90	50153	5	5	3	3	0.33	Putative elongation fa
167	1	SwissProt_HUMAN$	2::P06703|S10A6_HUMAN	89	10173	3	3	2	2	1.47	Protein S100-A6 OS=
168	1	SwissProt_HUMAN$	2::P04406|G3P_HUMAN	88	36030	3	3	2	2	0.30	Glyceraldehyde-3-pho
169	1	SwissProt_HUMAN$	2::P22891|PROZ_HUMAN	85	44715	7	7	7	7	1.09	Vitamin K-dependent
170	1	SwissProt_HUMAN$	2::P26022|PTX3_HUMAN	84	41949	3	3	2	2	0.25	Pentraxin-related prot
171	1	SwissProt_HUMAN$	2::P26927|HGFL_HUMAN	83	80268	6	5	6	5	0.34	Hepatocyte growth fa
172	1	SwissProt_HUMAN$	2::P98160|PGBM_HUMAN	82	468532	7	3	7	3	0.03	Basement membrane-
173	1	SwissProt_HUMAN$	2::P62701|RS4X_HUMAN	81	29579	5	3	5	3	0.61	40S ribosomal protein
174	1	SwissProt_HUMAN$	2::Q12805|FBLN3_HUMAN	79	54604	3	3	3	3	0.30	EGF-containing fibulin
175	1	SwissProt_HUMAN$	2::P32119|PRDX2_HUMAN	79	21878	4	4	3	3	1.36	Peroxiredoxin-2 OS=H
175	2	SwissProt_HUMAN$	2::Q06830|PRDX1_HUMAN	59	22096	4	3	3	2	0.89	Peroxiredoxin-1 OS=H

↑Family	M	DB	Accession	Score	Mass	Matches	Match(sig)	Sequences	Seq(sig)	emPAI	Description
176	1	SwissProt_HUMAN$	2::O00187|MASP2_HUMAN	77	75654	3	3	3	3	0.21	Mannan-binding lectin
177	1	SwissProt_HUMAN$	2::P00488|F13A_HUMAN	76	83215	5	5	5	5	0.33	Coagulation factor XII
178	1	SwissProt_HUMAN$	2::P10599|THIO_HUMAN	74	11730	2	2	2	2	1.20	Thioredoxin OS=Hom
179	1	SwissProt_HUMAN$	2::P15169|CBPN_HUMAN	72	52253	3	3	3	3	0.31	Carboxypeptidase N c
180	1	SwissProt_HUMAN$	2::Q14520|HABP2_HUMAN	70	62630	3	3	3	3	0.25	Hyaluronan-binding p
181	1	SwissProt_HUMAN$	2::P49207|RL34_HUMAN	69	13284	4	3	4	3	1.85	60S ribosomal protein
182	1	SwissProt_HUMAN$	2::P61088|UBE2N_HUMAN	69	17127	2	2	2	2	0.73	Ubiquitin-conjugating
183	1	SwissProt_HUMAN$	2::P05452|TETN_HUMAN	69	22522	4	3	4	3	0.87	Tetranectin OS=Homo
184	1	SwissProt_HUMAN$	2::P05154|IPSP_HUMAN	69	45646	4	4	4	4	0.51	Plasma serine proteas
185	1	SwissProt_HUMAN$	2::A0A075B6K5|LV39_HUMAN	68	12324	3	2	2	2	1.12	Immunoglobulin lamb
185	2	SwissProt_HUMAN$	2::P80748|LV321_HUMAN	61	12438	3	2	2	2	1.11	Immunoglobulin lamb
186	1	SwissProt_HUMAN$	2::P07737|PROF1_HUMAN	68	15045	3	2	3	2	0.85	Profilin-1 OS=Homo s
187	1	SwissProt_HUMAN$	2::P35268|RL22_HUMAN	67	14778	2	2	2	2	0.88	60S ribosomal protein
188	1	SwissProt_HUMAN$	2::P61604|CH10_HUMAN	65	10925	2	2	2	2	1.34	10 kDa heat shock pr
189	1	SwissProt_HUMAN$	2::P55259|GP2_HUMAN	65	59442	2	2	2	2	0.17	Pancreatic secretory g
190	1	SwissProt_HUMAN$	2::P61353|RL27_HUMAN	65	15788	2	2	2	2	0.81	60S ribosomal protein
191	1	SwissProt_HUMAN$	2::A0A075B6H9|LV469_HUMAN	65	12765	4	3	2	2	1.07	Immunoglobulin lamb
192	1	SwissProt_HUMAN$	2::P40197|GPV_HUMAN	64	60921	1	1	1	1	0.08	Platelet glycoprotein V
193	1	SwissProt_HUMAN$	2::P62269|RS18_HUMAN	62	17708	4	4	4	4	1.86	40S ribosomal protein
194	1	SwissProt_HUMAN$	2::P26373|RL13_HUMAN	62	24247	3	3	3	3	0.79	60S ribosomal protein
195	1	SwissProt_HUMAN$	2::O00391|QSOX1_HUMAN	61	82526	3	3	3	3	0.19	Sulfhydryl oxidase 1 (
196	1	SwissProt_HUMAN$	2::P07437|TBB5_HUMAN	60	49639	3	3	3	3	0.33	Tubulin beta chain OS
197	1	SwissProt_HUMAN$	2::P38646|GRP75_HUMAN	60	73635	2	1	2	1	0.07	Stress-70 protein, mit
198	1	SwissProt_HUMAN$	2::P62857|RS28_HUMAN	60	7836	1	1	1	1	0.79	40S ribosomal protein
199	1	SwissProt_HUMAN$	2::Q92954|PRG4_HUMAN	60	150968	3	3	3	3	0.10	Proteoglycan 4 OS=H
200	1	SwissProt_HUMAN$	2::P37837|TALDO_HUMAN	59	37516	1	1	1	1	0.13	Transaldolase OS=Ho
201	1	SwissProt_HUMAN$	2::P14174|MIF_HUMAN	59	12468	3	3	2	2	2.06	Macrophage migration
202	1	SwissProt_HUMAN$	2::P05141|ADT2_HUMAN	58	32831	4	3	4	3	0.54	ADP/ATP translocase :
203	1	SwissProt_HUMAN$	2::P18085|ARF4_HUMAN	58	20498	2	2	2	2	0.58	ADP-ribosylation facto
204	1	SwissProt_HUMAN$	2::A0A0B4J1Y8|LV949_HUMAN	58	13016	2	2	2	2	1.04	Immunoglobulin lamb
205	1	SwissProt_HUMAN$	2::P05543|THBG_HUMAN	58	46295	4	4	4	4	0.50	Thyroxine-binding glo
206	1	SwissProt_HUMAN$	2::P31327|CPSM_HUMAN	57	164835	2	2	1	1	0.06	Carbamoyl-phosphate
207	1	SwissProt_HUMAN$	2::A0A0B4J1U3|LV136_HUMAN	57	12470	1	1	1	1	0.45	Immunoglobulin lamb
208	1	SwissProt_HUMAN$	2::P08709|FA7_HUMAN	57	51560	1	1	1	1	0.10	Coagulation factor VII
209	1	SwissProt_HUMAN$	2::P04211|LV743_HUMAN	56	12443	2	2	2	2	1.11	Immunoglobulin lamb
209	2	SwissProt_HUMAN$	2::A0A075B6I9|LV746_HUMAN	42	12460	2	2	2	2	1.11	Immunoglobulin lamb
210	1	SwissProt_HUMAN$	2::P55058|PLTP_HUMAN	56	54705	2	2	2	2	0.19	Phospholipid transfer
211	1	SwissProt_HUMAN$	2::P00746|CFAD_HUMAN	55	27016	3	3	3	3	0.69	Complement factor D
212	1	SwissProt_HUMAN$	2::Q15485|FCN2_HUMAN	55	33980	3	3	2	2	0.32	Ficolin-2 OS=Homo sa
213	1	SwissProt_HUMAN$	2::Q8NHQ9|DDX55_HUMAN	55	68503	3	3	2	2	0.15	ATP-dependent RNA h

↑Family	M	DB	Accession	Score	Mass	Matches	Match(sig)	Sequences	Seq(sig)	emPAI	Description
214	1	SwissProt_HUMAN$	2::Q6IS14|IF5AL_HUMAN	53	16762	2	1	2	1	0.32	Eukaryotic translation
215	1	SwissProt_HUMAN$	2::P23284|PPIB_HUMAN	53	23728	2	2	2	2	0.48	Peptidyl-prolyl cis-tra
216	1	SwissProt_HUMAN$	2::P62263|RS14_HUMAN	52	16263	1	1	1	1	0.33	40S ribosomal protein
217	1	SwissProt_HUMAN$	2::Q16610|ECM1_HUMAN	52	60635	4	3	4	3	0.26	Extracellular matrix p
218	1	SwissProt_HUMAN$	2::P46782|RS5_HUMAN	52	22862	1	1	1	1	0.23	40S ribosomal protein
219	1	SwissProt_HUMAN$	2::P62081|RS7_HUMAN	51	22113	3	3	3	3	0.89	40S ribosomal protein
220	1	SwissProt_HUMAN$	2::P01715|LV301_HUMAN	51	12288	2	2	1	1	0.46	Immunoglobulin lamb
221	1	SwissProt_HUMAN$	2::P46781|RS9_HUMAN	51	22578	4	3	4	3	0.87	40S ribosomal protein
222	1	SwissProt_HUMAN$	2::P46783|RS10_HUMAN	50	18886	2	1	2	1	0.28	40S ribosomal protein
223	1	SwissProt_HUMAN$	2::P15880|RS2_HUMAN	50	31305	2	2	2	2	0.35	40S ribosomal protein
224	1	SwissProt_HUMAN$	2::P62888|RL30_HUMAN	49	12776	4	4	4	4	3.28	60S ribosomal protein
225	1	SwissProt_HUMAN$	2::Q5T0N5|FBP1L_HUMAN	49	70021	3	3	1	1	0.07	Formin-binding protei
226	1	SwissProt_HUMAN$	2::P06748|NPM_HUMAN	49	32555	2	2	2	2	0.34	Nucleophosmin OS=H
227	1	SwissProt_HUMAN$	2::P62316|SMD2_HUMAN	48	13518	1	1	1	1	0.41	Small nuclear ribonuc
228	1	SwissProt_HUMAN$	2::P04278|SHBG_HUMAN	48	43752	2	2	2	2	0.24	Sex hormone-binding
229	1	SwissProt_HUMAN$	2::A0A075B6I0|LV861_HUMAN	48	12806	2	2	2	2	1.07	Immunoglobulin lamb
230	1	SwissProt_HUMAN$	2::P61313|RL15_HUMAN	47	24131	3	3	2	2	0.48	60S ribosomal protein
231	1	SwissProt_HUMAN$	2::Q9NYC9|DYH9_HUMAN	47	511551	3	2	2	1	0.01	Dynein heavy chain 9
232	1	SwissProt_HUMAN$	2::A0A075B6I1|LV460_HUMAN	47	12979	1	1	1	1	0.43	Immunoglobulin lamb
233	1	SwissProt_HUMAN$	2::Q9BQE3|TBA1C_HUMAN	47	49863	3	2	3	2	0.21	Tubulin alpha-1C chai
234	1	SwissProt_HUMAN$	2::P62318|SMD3_HUMAN	46	13907	1	1	1	1	0.40	Small nuclear ribonuc
235	1	SwissProt_HUMAN$	2::P05451|REG1A_HUMAN	45	18719	1	1	1	1	0.28	Lithostathine-1-alpha
236	1	SwissProt_HUMAN$	2::Q5JNZ5|RS26L_HUMAN	45	12994	2	2	2	2	1.04	Putative 40S ribosom
237	1	SwissProt_HUMAN$	2::P32969|RL9_HUMAN	45	21850	2	2	2	2	0.54	60S ribosomal protein
238	1	SwissProt_HUMAN$	2::P62829|RL23_HUMAN	44	14856	3	3	3	3	1.56	60S ribosomal protein
239	1	SwissProt_HUMAN$	2::P62244|RS15A_HUMAN	42	14830	2	2	2	2	0.87	40S ribosomal protein
240	1	SwissProt_HUMAN$	2::Q9UKG4|S13A4_HUMAN	42	69313	1	1	1	1	0.07	Solute carrier family 1
241	1	SwissProt_HUMAN$	2::A0A075B6J1|LV537_HUMAN	42	13268	1	1	1	1	0.42	Immunoglobulin lamb
242	1	SwissProt_HUMAN$	2::P35443|TSP4_HUMAN	42	105802	4	3	4	3	0.14	Thrombospondin-4 OS
242	2	SwissProt_HUMAN$	2::P49747|COMP_HUMAN	21	82808	3	2	3	2	0.12	Cartilage oligomeric m
243	1	SwissProt_HUMAN$	2::P22352|GPX3_HUMAN	42	25537	4	4	4	4	1.09	Glutathione peroxidas
244	1	SwissProt_HUMAN$	2::P62847|RS24_HUMAN	42	15413	1	1	1	1	0.35	40S ribosomal protein
245	1	SwissProt_HUMAN$	2::P61247|RS3A_HUMAN	42	29926	3	3	3	3	0.60	40S ribosomal protein
246	1	SwissProt_HUMAN$	2::O75882|ATRN_HUMAN	41	158432	2	2	2	2	0.06	Attractin OS=Homo s
247	1	SwissProt_HUMAN$	2::O75818|RPP40_HUMAN	41	41807	1	1	1	1	0.12	Ribonuclease P protei
248	1	SwissProt_HUMAN$	2::Q04756|HGFA_HUMAN	41	70636	1	1	1	1	0.07	Hepatocyte growth fa
249	1	SwissProt_HUMAN$	2::Q15365|PCBP1_HUMAN	40	37474	1	1	1	1	0.13	Poly(rC)-binding prote
250	1	SwissProt_HUMAN$	2::P84098|RL19_HUMAN	40	23451	1	1	1	1	0.22	60S ribosomal protein
251	1	SwissProt_HUMAN$	2::P02775|CXCL7_HUMAN	39	13885	1	1	1	1	0.40	Platelet basic protein
252	1	SwissProt_HUMAN$	2::O95994|AGR2_HUMAN	39	19967	2	1	2	1	0.26	Anterior gradient prot

↑Family	M	DB	Accession	Score	Mass	Matches	Match(sig)	Sequences	Seq(sig)	emPAI	Description
253	1	SwissProt_HUMAN$	2::P51649|SSDH_HUMAN	39	57178	1	1	1	1	0.09	Succinate-semialdehy
254	1	SwissProt_HUMAN$	2::P14678|RSMB_HUMAN	39	24594	1	1	1	1	0.21	Small nuclear ribonuc
255	1	SwissProt_HUMAN$	2::Q02543|RL18A_HUMAN	39	20749	1	1	1	1	0.25	60S ribosomal protein
256	1	SwissProt_HUMAN$	2::P60866|RS20_HUMAN	39	13364	2	1	2	1	0.42	40S ribosomal protein
257	1	SwissProt_HUMAN$	2::P36222|CH3L1_HUMAN	39	42598	2	1	2	1	0.12	Chitinase-3-like prote
258	1	SwissProt_HUMAN$	2::P05109|S10A8_HUMAN	38	10828	2	2	2	2	1.34	Protein S100-A8 OS=
259	1	SwissProt_HUMAN$	2::P04075|ALDOA_HUMAN	38	39395	2	1	2	1	0.13	Fructose-bisphosphate
260	1	SwissProt_HUMAN$	2::P42766|RL35_HUMAN	38	14543	2	1	2	1	0.38	60S ribosomal protein
261	1	SwissProt_HUMAN$	2::P62851|RS25_HUMAN	38	13734	1	1	1	1	0.40	40S ribosomal protein
262	1	SwissProt_HUMAN$	2::Q8N4N3|KLH36_HUMAN	38	69852	1	1	1	1	0.07	Kelch-like protein 36 (
263	1	SwissProt_HUMAN$	2::P62917|RL8_HUMAN	37	28007	2	2	2	2	0.40	60S ribosomal protein
264	1	SwissProt_HUMAN$	2::O76031|CLPX_HUMAN	37	69181	1	1	1	1	0.07	ATP-dependent Clp pr
265	1	SwissProt_HUMAN$	2::P18206|VINC_HUMAN	37	123722	1	1	1	1	0.04	Vinculin OS=Homo sa
266	1	SwissProt_HUMAN$	2::Q68DL7|CR063_HUMAN	37	77181	3	3	1	1	0.06	Uncharacterized prote
267	1	SwissProt_HUMAN$	2::O43586|PPIP1_HUMAN	37	47562	1	1	1	1	0.10	Proline-serine-threoni
268	1	SwissProt_HUMAN$	2::P05386|RLA1_HUMAN	36	11507	1	1	1	1	0.49	60S acidic ribosomal p
269	1	SwissProt_HUMAN$	2::P62899|RL31_HUMAN	36	14454	1	1	1	1	0.38	60S ribosomal protein
270	1	SwissProt_HUMAN$	2::P01705|LV223_HUMAN	36	11886	1	1	1	1	0.48	Immunoglobulin lamb
271	1	SwissProt_HUMAN$	2::Q6Q759|SPG17_HUMAN	36	251584	1	1	1	1	0.02	Sperm-associated ant
272	1	SwissProt_HUMAN$	2::Q6ZSZ5|ARHGI_HUMAN	35	151549	1	1	1	1	0.03	Rho guanine nucleotic
273	1	SwissProt_HUMAN$	2::Q96EK7|F120B_HUMAN	35	103716	1	1	1	1	0.05	Constitutive coactivat
274	1	SwissProt_HUMAN$	2::P25398|RS12_HUMAN	35	14505	1	1	1	1	0.38	40S ribosomal protein
275	1	SwissProt_HUMAN$	2::P62249|RS16_HUMAN	35	16435	5	4	5	4	2.12	40S ribosomal protein
276	1	SwissProt_HUMAN$	2::Q6VUC0|AP2E_HUMAN	34	46183	1	1	1	1	0.11	Transcription factor AI
277	1	SwissProt_HUMAN$	2::Q9UGM5|FETUB_HUMAN	34	42028	2	1	2	1	0.12	Fetuin-B OS=Homo sa
278	1	SwissProt_HUMAN$	2::Q96DD0|LRC39_HUMAN	34	38768	1	1	1	1	0.13	Leucine-rich repeat-cc
279	1	SwissProt_HUMAN$	2::Q05513|KPCZ_HUMAN	34	67617	1	1	1	1	0.07	Protein kinase C zeta
280	1	SwissProt_HUMAN$	2::Q9HAW7|UD17_HUMAN	34	59779	2	2	2	2	0.17	UDP-glucuronosyltran
281	1	SwissProt_HUMAN$	2::Q9NRG0|CHRC1_HUMAN	34	14701	1	1	1	1	0.37	Chromatin accessibilit
282	1	SwissProt_HUMAN$	2::Q96EP0|RNF31_HUMAN	33	119575	1	1	1	1	0.04	E3 ubiquitin-protein li
283	1	SwissProt_HUMAN$	2::Q9UHG3|PCYOX_HUMAN	33	56604	4	1	4	1	0.09	Prenylcysteine oxidas
284	1	SwissProt_HUMAN$	2::Q3L8U1|CHD9_HUMAN	33	325819	2	2	2	2	0.03	Chromodomain-helica
285	1	SwissProt_HUMAN$	2::A0A0C4DH35|HV335_HUMAN	32	12802	2	1	2	1	0.44	Probable non-function
286	1	SwissProt_HUMAN$	2::P21399|ACOC_HUMAN	32	98337	1	1	1	1	0.05	Cytoplasmic aconitate
287	1	SwissProt_HUMAN$	2::Q12934|BFSP1_HUMAN	32	74499	2	2	1	1	0.07	Filensin OS=Homo sa
288	1	SwissProt_HUMAN$	2::P18669|PGAM1_HUMAN	32	28786	2	2	2	2	0.39	Phosphoglycerate mut
289	1	SwissProt_HUMAN$	2::P14061|DHB1_HUMAN	31	34928	1	1	1	1	0.14	Estradiol 17-beta-deh
290	1	SwissProt_HUMAN$	2::Q14019|COTL1_HUMAN	31	15935	2	2	2	2	0.79	Coactosin-like protein
291	1	SwissProt_HUMAN$	2::P62314|SMD1_HUMAN	31	13273	2	1	2	1	0.42	Small nuclear ribonuc
292	1	SwissProt_HUMAN$	2::P29401|TKT_HUMAN	31	67835	4	1	3	1	0.07	Transketolase OS=Ho

↑Family	M	DB	Accession	Score	Mass	Matches	Match(sig)	Sequences	Seq(sig)	emPAI	Description
293	1	SwissProt_HUMAN$	2::Q02241|KIF23_HUMAN	31	109990	2	2	2	2	0.09	Kinesin-like protein KI
294	1	SwissProt_HUMAN$	2::Q9H0U4|RAB1B_HUMAN	30	22157	3	2	3	2	0.53	Ras-related protein Ra
295	1	SwissProt_HUMAN$	2::Q3V6T2|GRDN_HUMAN	30	215909	2	1	2	1	0.02	Girdin OS=Homo sapi
296	1	SwissProt_HUMAN$	2::P07741|APT_HUMAN	30	19595	1	1	1	1	0.27	Adenine phosphoribos
297	1	SwissProt_HUMAN$	2::Q8IV32|CCD71_HUMAN	30	49618	1	1	1	1	0.10	Coiled-coil domain-co
298	1	SwissProt_HUMAN$	2::P07988|PSPB_HUMAN	30	42089	2	2	2	2	0.25	Pulmonary surfactant-
299	1	SwissProt_HUMAN$	2::Q8NEE6|DRC6_HUMAN	30	83869	1	1	1	1	0.06	Dynein regulatory cor
300	1	SwissProt_HUMAN$	2::P00966|ASSY_HUMAN	30	46501	1	1	1	1	0.11	Argininosuccinate syn
301	1	SwissProt_HUMAN$	2::Q6XE24|RBMS3_HUMAN	30	47809	1	1	1	1	0.10	RNA-binding motif, sir
302	1	SwissProt_HUMAN$	2::P45880|VDAC2_HUMAN	30	31547	1	1	1	1	0.16	Voltage-dependent ar
303	1	SwissProt_HUMAN$	2::Q9BTV5|FSD1_HUMAN	30	55784	2	1	1	1	0.09	Fibronectin type III ar
304	1	SwissProt_HUMAN$	2::Q9Y566|SHAN1_HUMAN	29	224821	1	1	1	1	0.02	SH3 and multiple ank
305	1	SwissProt_HUMAN$	2::Q6DHV5|C2D2B_HUMAN	29	166681	1	1	1	1	0.03	Protein CC2D2B OS=H
306	1	SwissProt_HUMAN$	2::P43251|BTD_HUMAN	29	61093	1	1	1	1	0.08	Biotinidase OS=Homo
307	1	SwissProt_HUMAN$	2::Q3SXZ3|ZN718_HUMAN	29	55324	4	2	1	1	0.09	Zinc finger protein 71
308	1	SwissProt_HUMAN$	2::Q2M3G4|SHRM1_HUMAN	29	90730	2	1	2	1	0.05	Protein Shroom1 OS=
309	1	SwissProt_HUMAN$	2::Q8IWZ3|ANKH1_HUMAN	29	269291	1	1	1	1	0.02	Ankyrin repeat and KH
310	1	SwissProt_HUMAN$	2::Q9BZI7|REN3B_HUMAN	29	57727	1	1	1	1	0.09	Regulator of nonsense
311	1	SwissProt_HUMAN$	2::Q9UNX3|RL26L_HUMAN	29	17246	1	1	1	1	0.31	60S ribosomal proteir
312	1	SwissProt_HUMAN$	2::P01703|LV140_HUMAN	28	12294	1	1	1	1	0.46	Immunoglobulin lamb
313	1	SwissProt_HUMAN$	2::O14602|IF1AY_HUMAN	28	16432	1	1	1	1	0.33	Eukaryotic translation
314	1	SwissProt_HUMAN$	2::P62266|RS23_HUMAN	28	15798	1	1	1	1	0.34	40S ribosomal proteir
315	1	SwissProt_HUMAN$	2::P08865|RSSA_HUMAN	28	32833	1	1	1	1	0.15	40S ribosomal proteir
316	1	SwissProt_HUMAN$	2::Q3KP66|INAVA_HUMAN	28	72869	1	1	1	1	0.07	Innate immunity activ
317	1	SwissProt_HUMAN$	2::O00429|DNM1L_HUMAN	28	81826	1	1	1	1	0.06	Dynamin-1-like protei
318	1	SwissProt_HUMAN$	2::Q14980|NUMA1_HUMAN	28	238115	1	1	1	1	0.02	Nuclear mitotic appar
319	1	SwissProt_HUMAN$	2::Q96T51|RUFY1_HUMAN	28	79767	2	1	2	1	0.06	RUN and FYVE domair
320	1	SwissProt_HUMAN$	2::P62750|RL23A_HUMAN	28	17684	1	1	1	1	0.30	60S ribosomal proteir
321	1	SwissProt_HUMAN$	2::Q14683|SMC1A_HUMAN	27	143144	1	1	1	1	0.03	Structural maintenanc
322	1	SwissProt_HUMAN$	2::Q8NEF9|SRFB1_HUMAN	27	48604	1	1	1	1	0.10	Serum response facto
323	1	SwissProt_HUMAN$	2::Q96FQ6|S10AG_HUMAN	27	11794	1	1	1	1	0.48	Protein S100-A16 OS=
324	1	SwissProt_HUMAN$	2::Q13093|PAFA_HUMAN	27	50045	1	1	1	1	0.10	Platelet-activating fac
325	1	SwissProt_HUMAN$	2::P18124|RL7_HUMAN	27	29207	3	2	3	2	0.38	60S ribosomal proteir
326	1	SwissProt_HUMAN$	2::Q8N6G1|CA211_HUMAN	27	10497	1	1	1	1	0.55	Putative uncharacteriz
327	1	SwissProt_HUMAN$	2::O43924|PDE6D_HUMAN	27	17409	1	1	1	1	0.31	Retinal rod rhodopsin-
328	1	SwissProt_HUMAN$	2::A0A0J9YX35|HV64D_HUMAN	27	12814	2	1	1	1	0.44	Immunoglobulin heav
329	1	SwissProt_HUMAN$	2::P0DPP9|ETDB_HUMAN	27	6987	1	1	1	1	0.92	Embryonic testis diffe
330	1	SwissProt_HUMAN$	2::P50914|RL14_HUMAN	27	23417	1	1	1	1	0.22	60S ribosomal proteir
331	1	SwissProt_HUMAN$	2::P05164|PERM_HUMAN	26	83815	1	1	1	1	0.06	Myeloperoxidase OS=
332	1	SwissProt_HUMAN$	2::Q9HD64|XAGE1_HUMAN	26	9072	2	1	1	1	0.66	X antigen family mem

↑Family	M	DB	Accession	Score	Mass	Matches	Match(sig)	Sequences	Seq(sig)	emPAI	Description
333	1	SwissProt_HUMAN$	2::Q15276|RABE1_HUMAN	26	99229	1	1	1	1	0.05	Rab GTPase-binding e
334	1	SwissProt_HUMAN$	2::Q8N6F7|GCSAM_HUMAN	26	20992	1	1	1	1	0.25	Germinal center-asso
335	1	SwissProt_HUMAN$	2::Q9NP80|PLPL8_HUMAN	26	88421	1	1	1	1	0.05	Calcium-independent
336	1	SwissProt_HUMAN$	2::Q9ULM0|PKHH1_HUMAN	26	151137	1	1	1	1	0.03	Pleckstrin homology d
337	1	SwissProt_HUMAN$	2::P13804|ETFA_HUMAN	26	35058	1	1	1	1	0.14	Electron transfer flavo
338	1	SwissProt_HUMAN$	2::P28072|PSB6_HUMAN	26	25341	1	1	1	1	0.20	Proteasome subunit b
339	1	SwissProt_HUMAN$	2::P62913|RL11_HUMAN	26	20240	1	1	1	1	0.26	60S ribosomal protein
340	1	SwissProt_HUMAN$	2::P46778|RL21_HUMAN	26	18553	1	1	1	1	0.29	60S ribosomal protein
341	1	SwissProt_HUMAN$	2::Q9NTJ4|MA2C1_HUMAN	26	115762	1	1	1	1	0.04	Alpha-mannosidase 2
342	1	SwissProt_HUMAN$	2::P49643|PRI2_HUMAN	26	58769	3	1	1	1	0.08	DNA primase large su
343	1	SwissProt_HUMAN$	2::Q86VD1|MORC1_HUMAN	26	112810	1	1	1	1	0.04	MORC family CW-type
344	1	SwissProt_HUMAN$	2::O75051|PLXA2_HUMAN	25	210969	1	1	1	1	0.02	Plexin-A2 OS=Homo s
345	1	SwissProt_HUMAN$	2::Q9P0M6|H2AW_HUMAN	25	40033	1	1	1	1	0.12	Core histone macro-H
346	1	SwissProt_HUMAN$	2::P83731|RL24_HUMAN	25	17768	1	1	1	1	0.30	60S ribosomal protein
347	1	SwissProt_HUMAN$	2::Q9BUZ4|TRAF4_HUMAN	25	53507	1	1	1	1	0.09	TNF receptor-associat
348	1	SwissProt_HUMAN$	2::A0A087WSZ0|KVD08_HUMAN	25	12828	1	1	1	1	0.43	Immunoglobulin kapp
349	1	SwissProt_HUMAN$	2::P62753|RS6_HUMAN	25	28663	2	2	1	1	0.18	40S ribosomal protein
350	1	SwissProt_HUMAN$	2::O75197|LRP5_HUMAN	25	179030	1	1	1	1	0.03	Low-density lipoprote
351	1	SwissProt_HUMAN$	2::Q9H329|E41LB_HUMAN	25	99650	1	1	1	1	0.05	Band 4.1-like protein
352	1	SwissProt_HUMAN$	2::Q7Z5M8|AB12B_HUMAN	25	40750	1	1	1	1	0.12	Protein ABHD12B OS=
353	1	SwissProt_HUMAN$	2::P08922|ROS1_HUMAN	25	263747	2	1	2	1	0.02	Proto-oncogene tyrosi
354	1	SwissProt_HUMAN$	2::P06702|S10A9_HUMAN	25	13234	1	1	1	1	0.42	Protein S100-A9 OS=
355	1	SwissProt_HUMAN$	2::Q92496|FHR4_HUMAN	24	65308	1	1	1	1	0.08	Complement factor H-
356	1	SwissProt_HUMAN$	2::O95831|AIFM1_HUMAN	24	66859	1	1	1	1	0.07	Apoptosis-inducing fa
357	1	SwissProt_HUMAN$	2::Q86US8|EST1A_HUMAN	24	160362	1	1	1	1	0.03	Telomerase-binding p
358	1	SwissProt_HUMAN$	2::O94972|TRI37_HUMAN	24	107837	1	1	1	1	0.04	E3 ubiquitin-protein li
359	1	SwissProt_HUMAN$	2::A6NKH3|RL37L_HUMAN	24	10576	1	1	1	1	0.55	Putative 60S ribosoma
360	1	SwissProt_HUMAN$	2::Q6ZTR6|ZNFDT_HUMAN	24	17225	1	1	1	1	0.31	Putative uncharacteriz
361	1	SwissProt_HUMAN$	2::Q9BXX3|AN30A_HUMAN	24	158735	2	1	2	1	0.03	Ankyrin repeat domai
362	1	SwissProt_HUMAN$	2::Q86YJ7|AN13B_HUMAN	24	70162	1	1	1	1	0.07	Ankyrin repeat domai
363	1	SwissProt_HUMAN$	2::Q6P4Q7|CNNM4_HUMAN	24	86552	1	1	1	1	0.06	Metal transporter CNN
364	1	SwissProt_HUMAN$	2::Q8N6S4|AN13C_HUMAN	24	60780	1	1	1	1	0.08	Ankyrin repeat domai
365	1	SwissProt_HUMAN$	2::Q8TD08|MK15_HUMAN	24	59795	1	1	1	1	0.08	Mitogen-activated pro
366	1	SwissProt_HUMAN$	2::Q86YA3|ZGRF1_HUMAN	24	236453	1	1	1	1	0.02	Protein ZGRF1 OS=Ho
367	1	SwissProt_HUMAN$	2::Q15113|PCOC1_HUMAN	24	47942	1	1	1	1	0.10	Procollagen C-endope
368	1	SwissProt_HUMAN$	2::P35222|CTNB1_HUMAN	24	85442	1	1	1	1	0.06	Catenin beta-1 OS=H
369	1	SwissProt_HUMAN$	2::O43819|SCO2_HUMAN	24	29791	1	1	1	1	0.17	Protein SCO2 homolog
370	1	SwissProt_HUMAN$	2::Q07075|AMPE_HUMAN	24	109176	1	1	1	1	0.04	Glutamyl aminopeptid
371	1	SwissProt_HUMAN$	2::Q9ULV5|HSF4_HUMAN	23	52978	1	1	1	1	0.09	Heat shock factor prot
372	1	SwissProt_HUMAN$	2::P51800|CLCKA_HUMAN	23	75236	1	1	1	1	0.06	Chloride channel prot

↑Family	M	DB	Accession	Score	Mass	Matches	Match(sig)	Sequences	Seq(sig)	emPAI	Description
373	1	SwissProt_HUMAN$	2::Q2M385|MPEG1_HUMAN	23	78537	2	1	1	1	0.06	Macrophage-expresse
374	1	SwissProt_HUMAN$	2::Q9C0K3|ARP3C_HUMAN	23	23697	1	1	1	1	0.22	Actin-related protein :
375	1	SwissProt_HUMAN$	2::P38117|ETFB_HUMAN	23	27826	1	1	1	1	0.18	Electron transfer flavc
376	1	SwissProt_HUMAN$	2::Q7Z412|PEX26_HUMAN	23	33877	1	1	1	1	0.15	Peroxisome assembly
377	1	SwissProt_HUMAN$	2::O60673|REV3L_HUMAN	23	352554	3	1	3	1	0.01	DNA polymerase zeta
378	1	SwissProt_HUMAN$	2::Q5VT06|CE350_HUMAN	23	350716	1	1	1	1	0.01	Centrosome-associate
379	1	SwissProt_HUMAN$	2::O00159|MYO1C_HUMAN	23	121606	1	1	1	1	0.04	Unconventional myosi
380	1	SwissProt_HUMAN$	2::Q58A45|PAN3_HUMAN	23	95552	4	1	1	1	0.05	PAN2-PAN3 deadenyla
381	1	SwissProt_HUMAN$	2::O94855|SC24D_HUMAN	23	112936	1	1	1	1	0.04	Protein transport prot
382	1	SwissProt_HUMAN$	2::Q13094|LCP2_HUMAN	23	60151	2	1	1	1	0.08	Lymphocyte cytosolic
383	1	SwissProt_HUMAN$	2::P56199|ITA1_HUMAN	23	130765	1	1	1	1	0.04	Integrin alpha-1 OS=I
384	1	SwissProt_HUMAN$	2::P02788|TRFL_HUMAN	23	78132	3	2	3	2	0.13	Lactotransferrin OS=H
385	1	SwissProt_HUMAN$	2::Q04760|LGUL_HUMAN	23	20764	1	1	1	1	0.25	Lactoylglutathione lya
386	1	SwissProt_HUMAN$	2::Q0WX57|U17LO_HUMAN	23	59673	1	1	1	1	0.08	Ubiquitin carboxyl-ter
387	1	SwissProt_HUMAN$	2::A0A075B6J9|LV218_HUMAN	23	12404	1	1	1	1	0.45	Immunoglobulin lamb
388	1	SwissProt_HUMAN$	2::O60282|KIF5C_HUMAN	23	109427	2	1	2	1	0.04	Kinesin heavy chain is
389	1	SwissProt_HUMAN$	2::O43897|TLL1_HUMAN	23	114635	1	1	1	1	0.04	Tolloid-like protein 1 (
390	1	SwissProt_HUMAN$	2::Q9C0C9|UBE2O_HUMAN	23	141205	2	1	1	1	0.03	(E3-independent) E2 u
391	1	SwissProt_HUMAN$	2::P16383|GCFC2_HUMAN	23	89329	1	1	1	1	0.05	GC-rich sequence DN/
392	1	SwissProt_HUMAN$	2::Q9H254|SPTN4_HUMAN	22	288808	1	1	1	1	0.02	Spectrin beta chain, n
393	1	SwissProt_HUMAN$	2::Q5VUJ9|DRC8_HUMAN	22	29695	3	1	1	1	0.17	Dynein regulatory cor
394	1	SwissProt_HUMAN$	2::P11498|PYC_HUMAN	22	129551	1	1	1	1	0.04	Pyruvate carboxylase,
395	1	SwissProt_HUMAN$	2::P40429|RL13A_HUMAN	22	23562	3	2	3	2	0.49	60S ribosomal proteir
396	1	SwissProt_HUMAN$	2::P23396|RS3_HUMAN	22	26671	2	2	2	2	0.42	40S ribosomal proteir
397	1	SwissProt_HUMAN$	2::Q9P219|DAPLE_HUMAN	22	228091	2	1	2	1	0.02	Protein Daple OS=Hoı
398	1	SwissProt_HUMAN$	2::Q8N228|SCML4_HUMAN	22	44994	1	1	1	1	0.11	Sex comb on midleg-l
399	1	SwissProt_HUMAN$	2::P15311|EZRI_HUMAN	22	69370	1	1	1	1	0.07	Ezrin OS=Homo sapie
400	1	SwissProt_HUMAN$	2::Q8N3K9|CMYA5_HUMAN	22	448935	1	1	1	1	0.01	Cardiomyopathy-asso
401	1	SwissProt_HUMAN$	2::P62841|RS15_HUMAN	22	17029	1	1	1	1	0.32	40S ribosomal proteir
402	1	SwissProt_HUMAN$	2::P0DTE1|HV383_HUMAN	22	12663	1	1	1	1	0.44	Probable non-function
403	1	SwissProt_HUMAN$	2::P01701|LV151_HUMAN	22	12241	2	1	2	1	0.46	Immunoglobulin lamb
404	1	SwissProt_HUMAN$	2::O75821|EIF3G_HUMAN	22	35589	1	1	1	1	0.14	Eukaryotic translation
405	1	SwissProt_HUMAN$	2::P36957|ODO2_HUMAN	22	48724	1	1	1	1	0.10	Dihydrolipoyllysine-re
406	1	SwissProt_HUMAN$	2::P82663|RT25_HUMAN	22	20103	1	1	1	1	0.26	28S ribosomal proteir
407	1	SwissProt_HUMAN$	2::P06733|ENOA_HUMAN	22	47139	2	1	2	1	0.11	Alpha-enolase OS=Hc
408	1	SwissProt_HUMAN$	2::Q5JPH6|SYEM_HUMAN	22	58652	1	1	1	1	0.08	Probable glutamate--t
409	1	SwissProt_HUMAN$	2::Q9Y691|KCMB2_HUMAN	22	27111	1	1	1	1	0.19	Calcium-activated pot
410	1	SwissProt_HUMAN$	2::Q15878|CAC1E_HUMAN	21	261564	1	1	1	1	0.02	Voltage-dependent R-
411	1	SwissProt_HUMAN$	2::P42568|AF9_HUMAN	21	63313	1	1	1	1	0.08	Protein AF-9 OS=Horr
412	1	SwissProt_HUMAN$	2::Q9HCK8|CHD8_HUMAN	21	290338	1	1	1	1	0.02	Chromodomain-helica

↑Family	M	DB	Accession	Score	Mass	Matches	Match(sig)	Sequences	Seq(sig)	emPAI	Description
413	1	SwissProt_HUMAN$	2::P31949|S10AB_HUMAN	21	11733	1	1	1	1	0.48	Protein S100-A11 OS=
414	1	SwissProt_HUMAN$	2::Q13061|TRDN_HUMAN	21	81546	1	1	1	1	0.06	Triadin OS=Homo sap
415	1	SwissProt_HUMAN$	2::Q8NHQ8|RASF8_HUMAN	21	48298	1	1	1	1	0.10	Ras association doma
416	1	SwissProt_HUMAN$	2::Q9UDT6|CLIP2_HUMAN	21	115767	1	1	1	1	0.04	CAP-Gly domain-conta
417	1	SwissProt_HUMAN$	2::Q8ND71|GIMA8_HUMAN	21	74843	1	1	1	1	0.07	GTPase IMAP family m
418	1	SwissProt_HUMAN$	2::P0DKB5|TPBGL_HUMAN	21	40374	1	1	1	1	0.12	Trophoblast glycoprot
419	1	SwissProt_HUMAN$	2::P55056|APOC4_HUMAN	21	14543	1	1	1	1	0.38	Apolipoprotein C-IV O
420	1	SwissProt_HUMAN$	2::P01704|LV214_HUMAN	21	12589	2	1	1	1	0.45	Immunoglobulin lamb
421	1	SwissProt_HUMAN$	2::Q7Z340|ZN551_HUMAN	21	77465	1	1	1	1	0.06	Zinc finger protein 55
422	1	SwissProt_HUMAN$	2::Q16543|CDC37_HUMAN	21	44440	1	1	1	1	0.11	Hsp90 co-chaperone (
423	1	SwissProt_HUMAN$	2::P01034|CYTC_HUMAN	21	15789	1	1	1	1	0.34	Cystatin-C OS=Homo
424	1	SwissProt_HUMAN$	2::P12110|CO6A2_HUMAN	21	108512	3	1	3	1	0.04	Collagen alpha-2(VI) (
425	1	SwissProt_HUMAN$	2::P07148|FABPL_HUMAN	21	14199	1	1	1	1	0.39	Fatty acid-binding pro
426	1	SwissProt_HUMAN$	2::Q9UBX0|HESX1_HUMAN	21	21396	1	1	1	1	0.24	Homeobox expressed
427	1	SwissProt_HUMAN$	2::P60891|PRPS1_HUMAN	21	34812	2	1	2	1	0.14	Ribose-phosphate pyr
428	1	SwissProt_HUMAN$	2::P08637|FCG3A_HUMAN	21	29071	1	1	1	1	0.18	Low affinity immunog
429	1	SwissProt_HUMAN$	2::P55786|PSA_HUMAN	21	103211	1	1	1	1	0.05	Puromycin-sensitive a
430	1	SwissProt_HUMAN$	2::O14523|C2C2L_HUMAN	21	76134	1	1	1	1	0.06	Phospholipid transfer
431	1	SwissProt_HUMAN$	2::P59665|DEF1_HUMAN	21	10194	2	1	2	1	0.57	Neutrophil defensin 1
432	1	SwissProt_HUMAN$	2::Q7Z418|KCNKI_HUMAN	21	43643	1	1	1	1	0.11	Potassium channel su
433	1	SwissProt_HUMAN$	2::Q7Z6Z7|HUWE1_HUMAN	21	481589	1	1	1	1	0.01	E3 ubiquitin-protein li
434	1	SwissProt_HUMAN$	2::Q9ULL4|PLXB3_HUMAN	21	206716	1	1	1	1	0.02	Plexin-B3 OS=Homo s
435	1	SwissProt_HUMAN$	2::P28482|MK01_HUMAN	21	41363	1	1	1	1	0.12	Mitogen-activated pro
436	1	SwissProt_HUMAN$	2::Q8TEP8|CE192_HUMAN	20	278938	1	1	1	1	0.02	Centrosomal protein c
437	1	SwissProt_HUMAN$	2::Q49AJ0|F135B_HUMAN	20	155672	1	1	1	1	0.03	Protein FAM135B OS=
438	1	SwissProt_HUMAN$	2::O14526|FCHO1_HUMAN	20	96801	1	1	1	1	0.05	F-BAR domain only pr
439	1	SwissProt_HUMAN$	2::Q8N6Q8|MET25_HUMAN	20	68169	1	1	1	1	0.07	Methyltransferase-like
440	1	SwissProt_HUMAN$	2::Q63HN8|RN213_HUMAN	20	591030	1	1	1	1	0.01	E3 ubiquitin-protein li
441	1	SwissProt_HUMAN$	2::Q5TGP6|MROH9_HUMAN	20	65008	1	1	1	1	0.08	Maestro heat-like repe
442	1	SwissProt_HUMAN$	2::Q9UHF7|TRPS1_HUMAN	20	141432	1	1	1	1	0.03	Zinc finger transcripti
443	1	SwissProt_HUMAN$	2::B7ZBB8|PP13G_HUMAN	20	37995	1	1	1	1	0.13	Protein phosphatase 1
444	1	SwissProt_HUMAN$	2::O14939|PLD2_HUMAN	20	105920	1	1	1	1	0.05	Phospholipase D2 OS=
445	1	SwissProt_HUMAN$	2::Q9NP81|SYSM_HUMAN	20	58246	1	1	1	1	0.08	Serine--tRNA ligase, r
446	1	SwissProt_HUMAN$	2::O15417|TNC18_HUMAN	20	314326	1	1	1	1	0.02	Trinucleotide repeat-c
447	1	SwissProt_HUMAN$	2::Q6UWR7|ENPP6_HUMAN	20	50208	1	1	1	1	0.10	Glycerophosphocholin
448	1	SwissProt_HUMAN$	2::Q9BVV6|TALD3_HUMAN	20	169201	1	1	1	1	0.03	Protein TALPID3 OS=I
449	1	SwissProt_HUMAN$	2::P0DOX4|IGE_HUMAN	20	60285	1	1	1	1	0.08	Immunoglobulin epsil
450	1	SwissProt_HUMAN$	2::P03951|FA11_HUMAN	20	70064	2	1	2	1	0.07	Coagulation factor XI
451	1	SwissProt_HUMAN$	2::Q8IY82|DRC7_HUMAN	20	103431	1	1	1	1	0.05	Dynein regulatory cor
452	1	SwissProt_HUMAN$	2::Q9UJX2|CDC23_HUMAN	20	68790	1	1	1	1	0.07	Cell division cycle pro

↑Family	M	DB	Accession	Score	Mass	Matches	Match(sig)	Sequences	Seq(sig)	emPAI	Description
453	1	SwissProt_HUMAN$	2::Q2M3V2|SWAHA_HUMAN	20	57407	1	1	1	1	0.09	Ankyrin repeat domai
454	1	SwissProt_HUMAN$	2::Q9P2I0|CPSF2_HUMAN	20	88431	2	2	1	1	0.05	Cleavage and polyade
455	1	SwissProt_HUMAN$	2::Q96FC7|PHIPL_HUMAN	20	42459	1	1	1	1	0.12	Phytanoyl-CoA hydrox
456	1	SwissProt_HUMAN$	2::Q92750|TAF4B_HUMAN	20	91034	2	2	2	2	0.11	Transcription initiatior
457	1	SwissProt_HUMAN$	2::Q86VH2|KIF27_HUMAN	19	160184	1	1	1	1	0.03	Kinesin-like protein KI
458	1	SwissProt_HUMAN$	2::Q13201|MMRN1_HUMAN	19	138023	1	1	1	1	0.03	Multimerin-1 OS=Hon
459	1	SwissProt_HUMAN$	2::P04080|CYTB_HUMAN	19	11133	1	1	1	1	0.52	Cystatin-B OS=Homo
460	1	SwissProt_HUMAN$	2::Q9NRN5|OLFL3_HUMAN	19	45981	1	1	1	1	0.11	Olfactomedin-like prot
461	1	SwissProt_HUMAN$	2::O75564|JERKY_HUMAN	19	61776	1	1	1	1	0.08	Jerky protein homolog
462	1	SwissProt_HUMAN$	2::O75153|CLU_HUMAN	19	146577	1	1	1	1	0.03	Clustered mitochondri
463	1	SwissProt_HUMAN$	2::Q5K651|SAMD9_HUMAN	19	184165	1	1	1	1	0.03	Sterile alpha motif do
464	1	SwissProt_HUMAN$	2::P46779|RL28_HUMAN	19	15738	2	1	2	1	0.34	60S ribosomal proteir
465	1	SwissProt_HUMAN$	2::Q66K66|TM198_HUMAN	19	39449	2	2	2	2	0.27	Transmembrane prote
466	1	SwissProt_HUMAN$	2::Q6DT37|MRCKG_HUMAN	19	172352	2	1	2	1	0.03	Serine/threonine-prot
467	1	SwissProt_HUMAN$	2::Q8NCN4|RN169_HUMAN	19	77147	1	1	1	1	0.06	E3 ubiquitin-protein li
468	1	SwissProt_HUMAN$	2::Q96DN5|TBC31_HUMAN	19	124111	1	1	1	1	0.04	TBC1 domain family n
469	1	SwissProt_HUMAN$	2::P42694|HELZ_HUMAN	19	218833	1	1	1	1	0.02	Probable helicase with
470	1	SwissProt_HUMAN$	2::Q99969|RARR2_HUMAN	19	18606	1	1	1	1	0.29	Retinoic acid receptor
471	1	SwissProt_HUMAN$	2::Q14241|ELOA1_HUMAN	19	89853	1	1	1	1	0.05	Elongin-A OS=Homo s
472	1	SwissProt_HUMAN$	2::Q8TBC4|UBA3_HUMAN	19	51819	1	1	1	1	0.10	NEDD8-activating enz
473	1	SwissProt_HUMAN$	2::A0A087WSX0|LV545_HUMAN	19	13153	1	1	1	1	0.43	Immunoglobulin lamb
474	1	SwissProt_HUMAN$	2::P36507|MP2K2_HUMAN	19	44396	1	1	1	1	0.11	Dual specificity mitog
475	1	SwissProt_HUMAN$	2::Q13976|KGP1_HUMAN	19	76316	1	1	1	1	0.06	cGMP-dependent prot
476	1	SwissProt_HUMAN$	2::P57735|RAB25_HUMAN	19	23481	1	1	1	1	0.22	Ras-related protein Ra
477	1	SwissProt_HUMAN$	2::P11309|PIM1_HUMAN	19	35663	2	1	2	1	0.14	Serine/threonine-prot
478	1	SwissProt_HUMAN$	2::P78527|PRKDC_HUMAN	19	468788	1	1	1	1	0.01	DNA-dependent prote
479	1	SwissProt_HUMAN$	2::Q8IX01|SUGP2_HUMAN	19	120132	1	1	1	1	0.04	SURP and G-patch do
480	1	SwissProt_HUMAN$	2::Q8NA54|IQUB_HUMAN	19	92523	1	1	1	1	0.05	IQ and ubiquitin-like c
481	1	SwissProt_HUMAN$	2::Q00537|CDK17_HUMAN	18	59545	2	1	1	1	0.08	Cyclin-dependent kina
482	1	SwissProt_HUMAN$	2::P84103|SRSF3_HUMAN	18	19318	1	1	1	1	0.27	Serine/arginine-rich s
483	1	SwissProt_HUMAN$	2::Q49MG5|MAP9_HUMAN	18	74189	1	1	1	1	0.07	Microtubule-associate
484	1	SwissProt_HUMAN$	2::A9Z1Z3|FR1L4_HUMAN	18	200854	1	1	1	1	0.02	Fer-1-like protein 4 O
485	1	SwissProt_HUMAN$	2::P49736|MCM2_HUMAN	18	101832	1	1	1	1	0.05	DNA replication licens
486	1	SwissProt_HUMAN$	2::Q92859|NEO1_HUMAN	18	159917	1	1	1	1	0.03	Neogenin OS=Homo s
487	1	SwissProt_HUMAN$	2::Q9NU19|TB22B_HUMAN	18	59044	1	1	1	1	0.08	TBC1 domain family n
488	1	SwissProt_HUMAN$	2::P49720|PSB3_HUMAN	18	22933	1	1	1	1	0.23	Proteasome subunit b
489	1	SwissProt_HUMAN$	2::Q5R3F8|PPR29_HUMAN	18	89630	1	1	1	1	0.05	Protein phosphatase 1
490	1	SwissProt_HUMAN$	2::P35555|FBN1_HUMAN	18	312083	1	1	1	1	0.02	Fibrillin-1 OS=Homo s
491	1	SwissProt_HUMAN$	2::Q9HAU8|RNPL1_HUMAN	18	79949	1	1	1	1	0.06	Aminopeptidase RNPE
492	1	SwissProt_HUMAN$	2::P62906|RL10A_HUMAN	18	24816	1	1	1	1	0.21	60S ribosomal proteir

↑Family	M	DB	Accession	Score	Mass	Matches	Match(sig)	Sequences	Seq(sig)	emPAI	Description
493	1	SwissProt_HUMAN$	2::Q14242|SELPL_HUMAN	18	43174	1	1	1	1	0.12	P-selectin glycoprotei
494	1	SwissProt_HUMAN$	2::Q8NBF2|NHLC2_HUMAN	18	79393	1	1	1	1	0.06	NHL repeat-containing
495	1	SwissProt_HUMAN$	2::Q14678|KANK1_HUMAN	18	147197	1	1	1	1	0.03	KN motif and ankyrin
496	1	SwissProt_HUMAN$	2::Q86WK7|AMGO3_HUMAN	18	55214	1	1	1	1	0.09	Amphoterin-induced p
497	1	SwissProt_HUMAN$	2::Q92570|NR4A3_HUMAN	18	68186	1	1	1	1	0.07	Nuclear receptor subf
498	1	SwissProt_HUMAN$	2::Q8TAV4|STML3_HUMAN	17	32115	1	1	1	1	0.16	Stomatin-like protein
499	1	SwissProt_HUMAN$	2::Q01780|EXOSX_HUMAN	17	100768	2	2	2	2	0.10	Exosome component
500	1	SwissProt_HUMAN$	2::Q15645|PCH2_HUMAN	17	48520	1	1	1	1	0.10	Pachytene checkpoint
501	1	SwissProt_HUMAN$	2::A3KN83|SBNO1_HUMAN	17	154216	1	1	1	1	0.03	Protein strawberry no
502	1	SwissProt_HUMAN$	2::Q9H6S0|YTDC2_HUMAN	17	160147	1	1	1	1	0.03	3'-5' RNA helicase YTI
503	1	SwissProt_HUMAN$	2::Q9NQG7|HPS4_HUMAN	17	76870	1	1	1	1	0.06	Hermansky-Pudlak sy
504	1	SwissProt_HUMAN$	2::Q99996|AKAP9_HUMAN	17	452708	1	1	1	1	0.01	A-kinase anchor prote
505	1	SwissProt_HUMAN$	2::Q9NU22|MDN1_HUMAN	17	632420	1	1	1	1	0.01	Midasin OS=Homo sa
506	1	SwissProt_HUMAN$	2::Q96T25|ZIC5_HUMAN	17	68404	1	1	1	1	0.07	Zinc finger protein ZI
507	1	SwissProt_HUMAN$	2::Q5JPF3|AN36C_HUMAN	16	199626	1	1	1	1	0.02	Ankyrin repeat domai
508	1	SwissProt_HUMAN$	2::Q9HBM0|VEZA_HUMAN	16	88609	1	1	1	1	0.05	Vezatin OS=Homo sa
509	1	SwissProt_HUMAN$	2::Q5SYB0|FRPD1_HUMAN	16	173328	1	1	1	1	0.03	FERM and PDZ domai
510	1	SwissProt_HUMAN$	2::Q9P241|AT10D_HUMAN	16	160171	1	1	1	1	0.03	Probable phospholipid
511	1	SwissProt_HUMAN$	2::P55285|CADH6_HUMAN	16	88254	1	1	1	1	0.06	Cadherin-6 OS=Homo
512	1	SwissProt_HUMAN$	2::Q9BYX4|IFIH1_HUMAN	16	116615	1	1	1	1	0.04	Interferon-induced he
513	1	SwissProt_HUMAN$	2::Q86UW6|N4BP2_HUMAN	15	198676	1	1	1	1	0.02	NEDD4-binding protei
514	1	SwissProt_HUMAN$	2::Q7Z442|PK1L2_HUMAN	15	272340	2	1	1	1	0.02	Polycystic kidney dise
515	1	SwissProt_HUMAN$	2::Q9UIX4|KCNG1_HUMAN	15	57876	1	1	1	1	0.09	Potassium voltage-ga
516	1	SwissProt_HUMAN$	2::P49716|CEBPD_HUMAN	15	28449	1	1	1	1	0.18	CCAAT/enhancer-bind
517	1	SwissProt_HUMAN$	2::A0A0G2JS06|LV539_HUMAN	15	13386	1	1	1	1	0.41	Immunoglobulin lamb
518	1	SwissProt_HUMAN$	2::P53396|ACLY_HUMAN	15	120762	1	1	1	1	0.04	ATP-citrate synthase (
519	1	SwissProt_HUMAN$	2::P55265|DSRAD_HUMAN	15	135981	1	1	1	1	0.04	Double-stranded RNA-
520	1	SwissProt_HUMAN$	2::O60241|AGRB2_HUMAN	15	172544	1	1	1	1	0.03	Adhesion G protein-cc
521	1	SwissProt_HUMAN$	2::Q8NFW9|MYRIP_HUMAN	15	95647	1	1	1	1	0.05	Rab effector MyRIP OS
522	1	SwissProt_HUMAN$	2::P21549|SPYA_HUMAN	15	42982	2	1	2	1	0.12	Serine--pyruvate amii
523	1	SwissProt_HUMAN$	2::Q8IYD1|ERF3B_HUMAN	15	68840	1	1	1	1	0.07	Eukaryotic peptide cha
524	1	SwissProt_HUMAN$	2::A0A1W2PPM1|CPHXL_HUMAN	15	45685	1	1	1	1	0.11	Cytoplasmic polyaden
525	1	SwissProt_HUMAN$	2::Q6ZS17|RIPR1_HUMAN	15	132225	1	1	1	1	0.04	Rho family-interacting
526	1	SwissProt_HUMAN$	2::Q9H765|ASB8_HUMAN	15	31622	1	1	1	1	0.16	Ankyrin repeat and SC
527	1	SwissProt_HUMAN$	2::Q8NA82|MARHA_HUMAN	15	90455	1	1	1	1	0.05	Probable E3 ubiquitin-
528	1	SwissProt_HUMAN$	2::Q5VSL9|STRP1_HUMAN	15	95515	1	1	1	1	0.05	Striatin-interacting pr
529	1	SwissProt_HUMAN$	2::Q9H4I0|RD21L_HUMAN	14	63284	1	1	1	1	0.08	Double-strand-break r
530	1	SwissProt_HUMAN$	2::Q9H4A3|WNK1_HUMAN	14	250640	1	1	1	1	0.02	Serine/threonine-prot
531	1	SwissProt_HUMAN$	2::Q08AI8|MB214_HUMAN	14	49534	1	1	1	1	0.10	Protein mab-21-like 4
532	1	SwissProt_HUMAN$	2::P61769|B2MG_HUMAN	14	13706	2	1	2	1	0.41	Beta-2-microglobulin

↑*Family*	M	DB	Accession	Score	Mass	Matches	Match(sig)	Sequences	Seq(sig)	emPAI	Description
533	1	SwissProt_HUMAN$	2::Q07954|LRP1_HUMAN	14	504276	1	1	1	1	0.01	Prolow-density lipoprc
534	1	SwissProt_HUMAN$	2::Q96L50|LLR1_HUMAN	14	46693	1	1	1	1	0.11	Leucine-rich repeat pr
535	1	SwissProt_HUMAN$	2::Q8IVU3|HERC6_HUMAN	14	115052	1	1	1	1	0.04	Probable E3 ubiquitin-
536	1	SwissProt_HUMAN$	2::P51530|DNA2_HUMAN	14	120337	1	1	1	1	0.04	DNA replication ATP-d
537	1	SwissProt_HUMAN$	2::Q9H257|CARD9_HUMAN	14	62202	1	1	1	1	0.08	Caspase recruitment (
538	1	SwissProt_HUMAN$	2::O76038|SEGN_HUMAN	14	32019	1	1	1	1	0.16	Secretagogin OS=Hor
539	1	SwissProt_HUMAN$	2::Q8WW22|DNJA4_HUMAN	14	44769	1	1	1	1	0.11	DnaJ homolog subfam
540	1	SwissProt_HUMAN$	2::B3KS81|SRRM5_HUMAN	14	80308	1	1	1	1	0.06	Serine/arginine repeti
541	1	SwissProt_HUMAN$	2::Q8ND61|CC020_HUMAN	13	101202	1	1	1	1	0.05	Uncharacterized prote
542	1	SwissProt_HUMAN$	2::Q4KWH8|PLCH1_HUMAN	13	189104	1	1	1	1	0.03	1-phosphatidylinositol
543	1	SwissProt_HUMAN$	2::Q5VVM6|CCD30_HUMAN	13	91278	1	1	1	1	0.05	Coiled-coil domain-co
544	1	SwissProt_HUMAN$	2::O14746|TERT_HUMAN	13	126916	1	1	1	1	0.04	Telomerase reverse tr
545	1	SwissProt_HUMAN$	2::Q9UHV9|PFD2_HUMAN	13	16638	1	1	1	1	0.32	Prefoldin subunit 2 OS
546	1	SwissProt_HUMAN$	2::Q14409|GLPK3_HUMAN	13	60559	1	1	1	1	0.08	Glycerol kinase 3 OS=

Export as CSV

Not what you expected? Try **the select summary**.

Mascot: http://www.matrixscience.com/
