# Supplementary material for: Circulating multimeric immune complexes contribute to immunopathology in COVID-19
Source: Nat Commun. 2022 Sep 26;13:5654. doi: 10.1038/s41467-022-32867-z (PMC9513013; doi:10.1038/s41467-022-32867-z)
Supplement: Supplementary file 3 — Source Data [file 41467_2022_32867_MOESM3_ESM.zip › Source Data/MS_29012021 (whole lane cut).zip!/QX20210214-04_Virologie(AG-Hengel)-20210129_(V3)_IC+_3-5_complete-lane_0k3v20_TRL_db_SARS-CoV-2+SP_H(F229104).pdf]

MATRIX SCIENCE MASCOT Search Results

User : Wolfgang Bildl
E-mail : wolfgang.bildl@physiologie.uni-freiburg.de
Search title : QX 20210212 (db$ SARS-CoV-2+SwissProt HUMAN$ 5 ppm 20 mmu), QX20210214-04_Virologie(AG-Hengel)-20210129_(V3)_IC+_3-5_complete-lane_0k3v20_TRL_shifted_-2.8.mgf
MS data file : D:\Xcalibur\Data\Wolfgang\Q-Exactive-HF-X_20210212\QX20210214-04_Virologie(AG-Hengel)-20210129_(V3)_IC+_3-5_complete-lane_0k3v20_TRL_shifted_-2.8.mgf
Databases : **1:** db$ 20210114 (14 sequences; 14,319 residues)
2: SwissProt_HUMAN$ 20201007 (20,389 sequences; 11,363,789 residues)
Timestamp : 15 Feb 2021 at 08:53:38 GMT

Not what you expected? Try **the select summary**.

Search parameters

**Type of search**	: MS/MS Ion Search
**Enzyme**	: Trypsin
**Variable modifications**	: Acetyl (Protein N-term), Carbamidomethyl (C), Gln->pyro-Glu (N-term Q), Oxidation (M)
**Mass values**	: Monoisotopic
**Protein mass**	: Unrestricted
**Peptide mass tolerance**	: ± 5 ppm
**Fragment mass tolerance**	: ± 20 mmu
**Max missed cleavages**	: 1
**Instrument type**	: ESI-FTICR
**Number of queries**	: 78,920

Score distribution

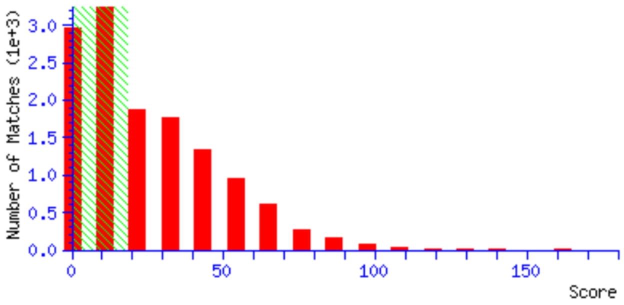

Peptide score distribution. Ions score is $-10\log(P)$, where P is the probability that the observed match is a random event.

There are **6,823** peptide matches above identity threshold and **7,019** matches above homology threshold for **78,920** queries. On average, individual ions scores **> 18** (beyond green shading) indicate **identity or extensive homology** (p<0.05).

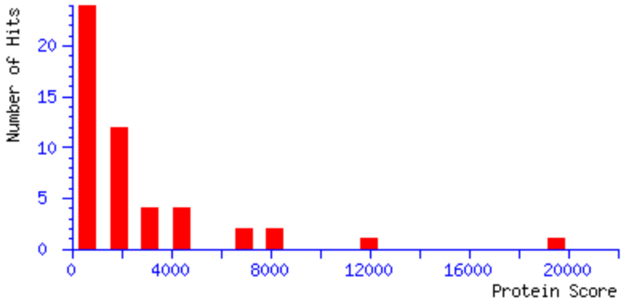

[Deprecated] Protein score distribution. Score distribution for family members in the first 50 proteins. Protein scores are derived from ions scores as a non-probabilistic basis for ranking protein families.

▼Modification statistics

Modification	Site	Above thr.
Carbamidomethyl	C	2632
Oxidation	M	791
Gln->pyro-Glu	N-term	218
Acetyl	Protein N-term	3

▼Legend

Peptide columns and rows

Dupes	...	Expect	Rank	U	1	2	Peptide	
		**0.037**	▶**2**				**GAYSLSLR**	significant
		9	▶1				GFFLFVEGGR	top ranking
		**6.4e-005**	▶**1**				**GSSIFGLAPGK**	significant and top ranking
		**1.3e-006**	▶**1**		■		**SSGTSYPDVLK**	peptide is found in all proteins in family member 1
		**6.2e-007**	▶**1**			■	**VCNYVSWIK**	peptide is found in some but not all proteins in family member 2
		**6.4e-005**	▶**1**	**U**			**GSSIFGLAPGK**	unique
▶**2**		**5.7e-005**	▶**1**				**LNTLETEEWFFK**	peptide has two duplicates
		0.18	▶*1*				*LNTLETEEWFFK*	duplicate peptide

Right-facing triangle (▶) in the Dupes or Rank column indicates content that can be expanded by clicking on it. Down-facing triangle (▼) indicates the content is expanded and can be collapsed. For more details about particular columns, see **results format help**.

Protein quantitation ratios

	Score	...	114/113	115/113
**CFAH_HUMAN**	37559		*0.962*	**1.129**
**FHR2_HUMAN**	1330		**0.859**	1.128

When quantitation method is Reporter (e.g. iTRAQ) or Multiplex (e.g. IPTL), protein ratios are displayed when a family is expanded. Ratios in *italic* indicate that the peptide log-ratios do not appear to come from a normal distribution. **Bold** indicates that if you can assume peptide ratios are normally distributed, the protein ratio is significantly different from 1.0 (at significance level 0.05).

Note that lack of bold or italic can also mean that significance or normality testing has not been performed (for example, if protein ratio type does not support it).

Protein Family Summary

Significance threshold p<	0.05	Max. number of families	AUTO
Ions score or expect cut-off	0.5	Dendrograms cut at	0
Show Percolator scores	☐		
Preferred taxonomy	All entries		

▼Sensitivity and FDR (reversed protein sequences)

			Target	Decoy	FDR		
PSMs	above	homology	7019	238	3.39%	Adjust to	1%

Decoy results are available in **the decoy report**.

Protein hits (533 proteins)

▶Filters: (none)

Export as CSV

↑Family	M	DB	Accession	Score	Mass	Matches	Match(sig)	Sequences	Seq(sig)	emPAI	Description
1	1	SwissProt_HUMAN$	2::P02768|ALBU_HUMAN	19541	69321	777	730	82	79	854.00	Albumin OS=Homo sa
1	2	SwissProt_HUMAN$	2::P02769|ALBU_BOVIN	947	69248	84	70	17	17	2.42	Albumin OS=Bos taur
2	1	SwissProt_HUMAN$	2::P04114|APOB_HUMAN	11942	515283	466	440	280	269	15.17	Apolipoprotein B-100
3	1	SwissProt_HUMAN$	2::P0DOX5|IGG1_HUMAN	8272	49298	478	429	28	28	18.50	Immunoglobulin gam
3	2	SwissProt_HUMAN$	2::P01860|IGHG3_HUMAN	4844	41260	272	257	22	22	20.86	Immunoglobulin heav
3	3	SwissProt_HUMAN$	2::P01859|IGHG2_HUMAN	2850	35878	187	170	20	20	29.48	Immunoglobulin heav
3	4	SwissProt_HUMAN$	2::P01861|IGHG4_HUMAN	2375	35918	144	135	18	18	12.74	Immunoglobulin heav
4	1	SwissProt_HUMAN$	2::P0C0L5|CO4B_HUMAN	8069	192631	281	266	102	96	12.61	Complement C4-B OS
4	2	SwissProt_HUMAN$	2::P0C0L4|CO4A_HUMAN	7981	192664	278	263	101	95	11.94	Complement C4-A OS
5	1	SwissProt_HUMAN$	2::P01024|CO3_HUMAN	7023	187030	304	277	133	126	55.41	Complement C3 OS=
6	1	SwissProt_HUMAN$	2::P01834|IGKC_HUMAN	6641	11758	250	233	13	12	111.87	Immunoglobulin kapp
6	2	SwissProt_HUMAN$	2::P0DOX7|IGK_HUMAN	6125	23364	214	199	16	15	29.61	Immunoglobulin kapp
6	3	SwissProt_HUMAN$	2::A0A0C4DH67|KV108_HUMAN	364	12529	10	8	4	3	2.03	Immunoglobulin kapp
6	4	SwissProt_HUMAN$	2::A0A075B6S5|KV127_HUMAN	348	12703	10	9	4	4	3.34	Immunoglobulin kapp
6	5	SwissProt_HUMAN$	2::P01597|KV139_HUMAN	332	12729	15	14	4	4	3.28	Immunoglobulin kapp
6	6	SwissProt_HUMAN$	2::A0A0C4DH72|KV106_HUMAN	274	12689	16	15	5	5	5.26	Immunoglobulin kapp
6	7	SwissProt_HUMAN$	2::P01599|KV117_HUMAN	192	12770	12	11	6	6	7.87	Immunoglobulin kapp
6	8	SwissProt_HUMAN$	2::A0A0B4J2D9|KVD13_HUMAN	179	12561	8	7	3	3	2.03	Immunoglobulin kapp
6	9	SwissProt_HUMAN$	2::P01602|KV105_HUMAN	166	12773	7	6	3	3	1.98	Immunoglobulin kapp
6	10	SwissProt_HUMAN$	2::A0A0C4DH24|KV621_HUMAN	129	12422	6	5	3	3	2.06	Immunoglobulin kapp
6	11	SwissProt_HUMAN$	2::P01601|KVD16_HUMAN	101	12722	5	4	2	2	1.07	Immunoglobulin kapp
6	12	SwissProt_HUMAN$	2::A0A0A0MT36|KVD21_HUMAN	84	12332	6	5	3	3	2.09	Immunoglobulin kapp
6	13	SwissProt_HUMAN$	2::P04430|KV116_HUMAN	63	12610	5	4	2	2	1.08	Immunoglobulin kapp

↑Family	M	DB	Accession	Score	Mass	Matches	Match(sig)	Sequences	Seq(sig)	emPAI	Description
7	1	SwissProt_HUMAN$	2::P01871|IGHM_HUMAN	4883	49409	182	177	37	36	72.89	Immunoglobulin heav
7	2	SwissProt_HUMAN$	2::P0DOX6|IGM_HUMAN	4002	63446	151	144	30	27	9.84	Immunoglobulin mu h
7	3	SwissProt_HUMAN$	2::A0A0B4J1V2|HV226_HUMAN	146	13174	7	7	5	5	4.81	Immunoglobulin heav
7	4	SwissProt_HUMAN$	2::P01814|HV270_HUMAN	115	13252	4	4	4	4	3.08	Immunoglobulin heav
8	1	SwissProt_HUMAN$	2::P01009|A1AT_HUMAN	4724	46707	217	206	39	38	140.23	Alpha-1-antitrypsin O
9	1	SwissProt_HUMAN$	2::P02751|FINC_HUMAN	4218	272150	153	143	90	85	3.72	Fibronectin OS=Homo
10	1	SwissProt_HUMAN$	2::P04003|C4BPA_HUMAN	3773	66989	139	127	41	39	24.67	C4b-binding protein a
11	1	SwissProt_HUMAN$	2::P08603|CFAH_HUMAN	3651	139005	155	144	70	68	13.31	Complement factor H
11	2	SwissProt_HUMAN$	2::Q03591|FHR1_HUMAN	726	37626	23	22	15	14	6.42	Complement factor H
11	3	SwissProt_HUMAN$	2::P36980|FHR2_HUMAN	266	30631	10	9	7	6	1.51	Complement factor H
11	4	SwissProt_HUMAN$	2::Q9BXR6|FHR5_HUMAN	243	64377	10	9	10	9	0.94	Complement factor H
11	5	SwissProt_HUMAN$	2::Q02985|FHR3_HUMAN	96	37299	6	6	5	5	0.88	Complement factor H
11	6	SwissProt_HUMAN$	2::Q92496|FHR4_HUMAN	27	65308	2	2	2	2	0.16	Complement factor H
12	1	SwissProt_HUMAN$	2::P01023|A2MG_HUMAN	3440	163188	143	132	76	71	10.49	Alpha-2-macroglobuli
12	2	SwissProt_HUMAN$	2::P20742|PZP_HUMAN	401	163760	16	15	10	10	0.38	Pregnancy zone prote
13	1	SwissProt_HUMAN$	2::P02787|TRFE_HUMAN	3007	77014	124	119	57	55	44.12	Serotransferrin OS=H
14	1	SwissProt_HUMAN$	2::P01031|CO5_HUMAN	2909	188186	130	120	81	76	7.74	Complement C5 OS=
15	1	SwissProt_HUMAN$	2::P01876|IGHA1_HUMAN	2485	37631	92	90	22	22	19.22	Immunoglobulin heav
15	2	SwissProt_HUMAN$	2::P0DOX2|IGA2_HUMAN	869	48903	44	39	18	17	4.68	Immunoglobulin alph
15	3	SwissProt_HUMAN$	2::P0DP02|HVC33_HUMAN	520	12980	26	23	8	8	50.12	Immunoglobulin heav
15	4	SwissProt_HUMAN$	2::P01764|HV323_HUMAN	490	12574	18	17	6	5	12.33	Immunoglobulin heav
15	5	SwissProt_HUMAN$	2::A0A0B4J2H0|HV69D_HUMAN	438	12652	14	14	4	4	17.82	Immunoglobulin heav
15	6	SwissProt_HUMAN$	2::P01768|HV330_HUMAN	433	12938	23	20	8	7	24.00	Immunoglobulin heav
15	7	SwissProt_HUMAN$	2::A0A0C4DH42|HV366_HUMAN	359	12690	16	16	5	5	12.04	Immunoglobulin heav
15	8	SwissProt_HUMAN$	2::A0A0C4DH29|HV103_HUMAN	351	12999	12	12	4	4	4.98	Immunoglobulin heav
15	9	SwissProt_HUMAN$	2::P01742|HV169_HUMAN	303	12651	13	13	4	4	12.04	Immunoglobulin heav
15	10	SwissProt_HUMAN$	2::A0A0B4J1X5|HV374_HUMAN	290	12831	15	15	7	7	16.91	Immunoglobulin heav
15	11	SwissProt_HUMAN$	2::P01743|HV146_HUMAN	277	12924	13	11	5	4	3.23	Immunoglobulin heav
15	12	SwissProt_HUMAN$	2::A0A0B4J1V1|HV321_HUMAN	276	12831	12	10	5	5	11.49	Immunoglobulin heav
15	13	SwissProt_HUMAN$	2::P01763|HV348_HUMAN	264	12804	11	9	5	5	7.87	Immunoglobulin heav
15	14	SwissProt_HUMAN$	2::P01780|HV307_HUMAN	257	12934	15	12	9	8	24.00	Immunoglobulin heav
15	15	SwissProt_HUMAN$	2::P01766|HV313_HUMAN	250	12498	10	8	4	4	5.36	Immunoglobulin heav
15	16	SwissProt_HUMAN$	2::P01782|HV309_HUMAN	246	12936	11	8	5	4	7.55	Immunoglobulin heav
15	17	SwissProt_HUMAN$	2::P01762|HV311_HUMAN	235	12900	11	9	5	5	7.71	Immunoglobulin heav
15	18	SwissProt_HUMAN$	2::A0A0C4DH33|HV124_HUMAN	235	12816	7	7	4	4	7.87	Immunoglobulin heav
15	19	SwissProt_HUMAN$	2::A0A0C4DH31|HV118_HUMAN	216	12812	7	7	4	4	3.28	Immunoglobulin heav
15	20	SwissProt_HUMAN$	2::P23083|HV102_HUMAN	212	13076	8	8	4	4	4.89	Immunoglobulin heav
15	21	SwissProt_HUMAN$	2::A0A0C4DH32|HV320_HUMAN	170	12665	9	7	5	5	8.03	Immunoglobulin heav
15	22	SwissProt_HUMAN$	2::P0DP01|HV108_HUMAN	155	12983	8	8	3	3	1.92	Immunoglobulin heav
16	1	SwissProt_HUMAN$	2::P0DOY2|IGLC2_HUMAN	2262	11287	75	73	13	12	202.51	Immunoglobulin lamb

↑Family	M	DB	Accession	Score	Mass	Matches	Match(sig)	Sequences	Seq(sig)	emPAI	Description
16	2	SwissProt_HUMAN$	2::P0DOX8|IGL1_HUMAN	1860	22816	66	63	15	13	20.69	Immunoglobulin lamb
16	3	SwissProt_HUMAN$	2::A0M8Q6|IGLC7_HUMAN	1192	11247	40	40	6	6	16.99	Immunoglobulin lamb
16	4	SwissProt_HUMAN$	2::P01706|LV211_HUMAN	41	12636	3	2	3	2	1.08	Immunoglobulin lamb
16	5	SwissProt_HUMAN$	2::P01709|LV208_HUMAN	28	12374	4	2	3	2	1.12	Immunoglobulin lamb
17	1	SwissProt_HUMAN$	2::P00747|PLMN_HUMAN	1780	90510	76	70	49	46	12.70	Plasminogen OS=Hor
18	1	SwissProt_HUMAN$	2::P00450|CERU_HUMAN	1767	122128	72	67	44	43	5.96	Ceruloplasmin OS=Ho
19	1	SwissProt_HUMAN$	2::P00738|HPT_HUMAN	1686	45177	81	78	28	26	27.31	Haptoglobin OS=Hom
19	2	SwissProt_HUMAN$	2::P00739|HPTR_HUMAN	983	39005	46	45	21	20	17.26	Haptoglobin-related p
20	1	SwissProt_HUMAN$	2::P02649|APOE_HUMAN	1660	36132	53	47	29	27	72.52	Apolipoprotein E OS=
21	1	SwissProt_HUMAN$	2::P13645|K1C10_HUMAN	1637	58792	55	53	29	28	14.36	Keratin, type I cytosk
21	2	SwissProt_HUMAN$	2::P02533|K1C14_HUMAN	340	51529	13	12	12	12	2.01	Keratin, type I cytosk
21	3	SwissProt_HUMAN$	2::P08779|K1C16_HUMAN	265	51236	12	12	12	12	2.02	Keratin, type I cytosk
21	4	SwissProt_HUMAN$	2::P08727|K1C19_HUMAN	111	44079	5	5	5	5	0.71	Keratin, type I cytosk
21	5	SwissProt_HUMAN$	2::P05783|K1C18_HUMAN	94	48029	8	4	8	4	0.48	Keratin, type I cytosk
22	1	SwissProt_HUMAN$	2::P04264|K2C1_HUMAN	1454	65999	44	42	30	29	8.89	Keratin, type II cytos
22	2	SwissProt_HUMAN$	2::P35908|K22E_HUMAN	840	65393	35	29	31	25	5.56	Keratin, type II cytos
22	3	SwissProt_HUMAN$	2::P13647|K2C5_HUMAN	253	62340	15	14	14	13	1.68	Keratin, type II cytos
22	4	SwissProt_HUMAN$	2::P02538|K2C6A_HUMAN	230	60008	14	12	13	11	1.38	Keratin, type II cytos
22	5	SwissProt_HUMAN$	2::P05787|K2C8_HUMAN	203	53671	10	10	9	9	1.21	Keratin, type II cytos
22	6	SwissProt_HUMAN$	2::P08729|K2C7_HUMAN	146	51354	6	6	5	5	0.58	Keratin, type II cytos
23	1	SwissProt_HUMAN$	2::P09871|C1S_HUMAN	1424	76635	59	58	32	31	8.25	Complement C1s sub
23	2	SwissProt_HUMAN$	2::P48740|MASP1_HUMAN	117	79195	7	6	6	5	0.35	Mannan-binding lectir
24	1	SwissProt_HUMAN$	2::P01011|AACT_HUMAN	1421	47621	50	45	24	21	12.17	Alpha-1-antichymotry
25	1	SwissProt_HUMAN$	2::P02774|VTDB_HUMAN	1382	52883	54	51	27	27	14.99	Vitamin D-binding pro
26	1	SwissProt_HUMAN$	2::P07225|PROS_HUMAN	1345	75074	58	55	33	30	9.31	Vitamin K-dependent
27	1	SwissProt_HUMAN$	2::P02647|APOA1_HUMAN	1228	30759	62	55	35	34	334.52	Apolipoprotein A-I OS
28	1	SwissProt_HUMAN$	2::P00751|CFAB_HUMAN	1167	85479	56	53	38	36	6.35	Complement factor B
29	1	SwissProt_HUMAN$	2::P02749|APOH_HUMAN	1167	38273	41	38	20	19	9.37	Beta-2-glycoprotein 1
30	1	SwissProt_HUMAN$	2::P00736|C1R_HUMAN	1108	80067	58	53	33	31	7.41	Complement C1r sub
30	2	SwissProt_HUMAN$	2::Q9NZP8|C1RL_HUMAN	77	53464	4	4	4	4	0.42	Complement C1r sub
31	1	SwissProt_HUMAN$	2::P35527|K1C9_HUMAN	1073	62027	32	30	20	19	3.95	Keratin, type I cytosk
32	1	SwissProt_HUMAN$	2::P10643|CO7_HUMAN	1006	93457	37	34	31	30	3.57	Complement compon
33	1	SwissProt_HUMAN$	2::P19823|ITIH2_HUMAN	967	106397	49	44	31	28	2.80	Inter-alpha-trypsin in
34	1	SwissProt_HUMAN$	2::P05155|IC1_HUMAN	966	55119	47	42	21	18	6.17	Plasma protease C1 i
35	1	SwissProt_HUMAN$	2::P00734|THRB_HUMAN	931	69992	36	31	23	21	4.41	Prothrombin OS=Hon
36	1	SwissProt_HUMAN$	2::P02748|CO9_HUMAN	925	63133	31	28	20	19	3.47	Complement compon
37	1	SwissProt_HUMAN$	2::P07357|CO8A_HUMAN	921	65121	30	29	20	20	4.71	Complement compon
38	1	SwissProt_HUMAN$	2::O43866|CD5L_HUMAN	906	38063	33	31	20	19	10.90	CD5 antigen-like OS=
39	1	SwissProt_HUMAN$	2::P13671|CO6_HUMAN	880	104718	40	37	29	27	2.55	Complement compon
40	1	SwissProt_HUMAN$	2::P02675|FIBB_HUMAN	861	55892	34	31	25	23	6.59	Fibrinogen beta chain

↑Family	M	DB	Accession	Score	Mass	Matches	Match(sig)	Sequences	Seq(sig)	emPAI	Description
41	1	SwissProt_HUMAN$	2::Q14624|ITIH4_HUMAN	855	103293	41	36	28	26	2.96	Inter-alpha-trypsin in
42	1	SwissProt_HUMAN$	2::P07358|CO8B_HUMAN	786	67003	29	28	20	20	3.10	Complement compon
43	1	SwissProt_HUMAN$	2::P01042|KNG1_HUMAN	686	71912	26	26	19	19	2.49	Kininogen-1 OS=Hom
44	1	SwissProt_HUMAN$	2::P06396|GELS_HUMAN	679	85644	25	23	19	18	1.86	Gelsolin OS=Homo sa
45	1	SwissProt_HUMAN$	2::P19827|ITIH1_HUMAN	674	101326	32	30	22	21	2.22	Inter-alpha-trypsin in
46	1	SwissProt_HUMAN$	2::P06727|APOA4_HUMAN	657	45344	36	32	23	22	11.13	Apolipoprotein A-IV C
47	1	SwissProt_HUMAN$	2::P02679|FIBG_HUMAN	634	51479	34	30	20	18	5.26	Fibrinogen gamma ch
48	1	SwissProt_HUMAN$	2::P02746|C1QB_HUMAN	630	26704	35	33	10	10	18.81	Complement C1q sub
49	1	SwissProt_HUMAN$	2::P04275|VWF_HUMAN	626	309058	28	28	26	26	0.49	von Willebrand factor
50	1	SwissProt_HUMAN$	2::P18428|LBP_HUMAN	613	53350	24	23	17	17	3.92	Lipopolysaccharide-bi
51	1	SwissProt_HUMAN$	2::P01008|ANT3_HUMAN	588	52569	32	29	22	19	6.21	Antithrombin-III OS=
52	1	SwissProt_HUMAN$	2::P0DJI8|SAA1_HUMAN	581	13524	22	17	11	8	20.97	Serum amyloid A-1 p
52	2	SwissProt_HUMAN$	2::P0DJI9|SAA2_HUMAN	432	13519	19	14	10	7	14.59	Serum amyloid A-2 p
53	1	SwissProt_HUMAN$	2::P01019|ANGT_HUMAN	575	53121	25	24	13	13	3.14	Angiotensinogen OS=
54	1	SwissProt_HUMAN$	2::P27169|PON1_HUMAN	565	39706	24	21	16	14	4.93	Serum paraoxonase/a
55	1	SwissProt_HUMAN$	2::P02765|FETUA_HUMAN	547	39316	21	20	11	11	4.37	Alpha-2-HS-glycoprot
56	1	SwissProt_HUMAN$	2::P06331|HV434_HUMAN	536	13806	14	13	5	5	4.42	Immunoglobulin heav
56	2	SwissProt_HUMAN$	2::P0DP08|HVD82_HUMAN	489	13008	14	13	5	5	4.98	Immunoglobulin heav
56	3	SwissProt_HUMAN$	2::P01824|HV439_HUMAN	470	13908	13	12	5	5	4.35	Immunoglobulin heav
56	4	SwissProt_HUMAN$	2::A0A0C4DH41|HV461_HUMAN	458	13058	13	12	5	5	4.89	Immunoglobulin heav
56	5	SwissProt_HUMAN$	2::A0A075B6R2|HV404_HUMAN	347	12840	10	9	5	5	5.07	Immunoglobulin heav
57	1	SwissProt_HUMAN$	2::P02741|CRP_HUMAN	530	25023	28	27	11	11	8.54	C-reactive protein OS
58	1	SwissProt_HUMAN$	2::P01833|PIGR_HUMAN	529	83232	20	20	18	18	1.95	Polymeric immunoglo
59	1	SwissProt_HUMAN$	2::P02750|A2GL_HUMAN	514	38154	16	15	10	9	2.04	Leucine-rich alpha-2-
60	1	SwissProt_HUMAN$	2::P68871|HBB_HUMAN	511	15988	15	15	9	9	17.51	Hemoglobin subunit b
61	1	SwissProt_HUMAN$	2::P02790|HEMO_HUMAN	497	51643	34	28	20	18	6.49	Hemopexin OS=Homo
62	1	SwissProt_HUMAN$	2::P05156|CFAI_HUMAN	485	65707	19	17	15	13	1.55	Complement factor I
63	1	SwissProt_HUMAN$	2::P36955|PEDF_HUMAN	475	46283	25	25	18	18	5.94	Pigment epithelium-d
64	1	SwissProt_HUMAN$	2::P07996|TSP1_HUMAN	457	129300	20	19	16	15	0.73	Thrombospondin-1 OS
65	1	SwissProt_HUMAN$	2::P10909|CLUS_HUMAN	452	52461	20	16	13	11	1.69	Clusterin OS=Homo s
66	1	SwissProt_HUMAN$	2::P02747|C1QC_HUMAN	452	25757	22	19	9	8	5.20	Complement C1q sub
67	1	SwissProt_HUMAN$	2::P02766|TTHY_HUMAN	452	15877	16	16	8	8	9.49	Transthyretin OS=Ho
68	1	SwissProt_HUMAN$	2::P07360|CO8G_HUMAN	442	22264	13	13	8	8	5.63	Complement compon
69	1	SwissProt_HUMAN$	2::P02671|FIBA_HUMAN	431	94914	30	26	24	21	2.31	Fibrinogen alpha chai
70	1	SwissProt_HUMAN$	2::P02743|SAMP_HUMAN	427	25371	23	21	12	12	8.18	Serum amyloid P-com
71	1	SwissProt_HUMAN$	2::P12259|FA5_HUMAN	415	251546	24	21	22	19	0.46	Coagulation factor V (
72	1	SwissProt_HUMAN$	2::P00761|TRYP_PIG	413	24394	13	12	6	6	2.84	Trypsin OS=Sus scrof
73	1	SwissProt_HUMAN$	2::P04217|A1BG_HUMAN	392	54220	19	16	11	10	1.61	Alpha-1B-glycoprotei
74	1	SwissProt_HUMAN$	2::P02763|A1AG1_HUMAN	377	23497	19	17	11	9	6.34	Alpha-1-acid glycopro
74	2	SwissProt_HUMAN$	2::P19652|A1AG2_HUMAN	310	23588	18	17	11	10	9.82	Alpha-1-acid glycopro

↑Family	M	DB	Accession	Score	Mass	Matches	Match(sig)	Sequences	Seq(sig)	emPAI	Description
75	1	SwissProt_HUMAN$	2::P01619|KV320_HUMAN	350	12549	9	9	4	4	3.39	Immunoglobulin kapp
75	2	SwissProt_HUMAN$	2::A0A0C4DH25|KVD20_HUMAN	272	12507	5	5	3	3	2.03	Immunoglobulin kapp
76	1	SwissProt_HUMAN$	2::A0A0C4DH38|HV551_HUMAN	346	12666	9	8	5	5	5.26	Immunoglobulin heav
76	2	SwissProt_HUMAN$	2::A0A0J9YXX1|HV5X1_HUMAN	245	12764	8	8	5	5	7.87	Immunoglobulin heav
77	1	SwissProt_HUMAN$	2::P04004|VTNC_HUMAN	343	54271	21	18	10	10	2.10	Vitronectin OS=Homo
78	1	SwissProt_HUMAN$	2::Q08380|LG3BP_HUMAN	333	65289	15	14	12	11	1.22	Galectin-3-binding pr
79	1	SwissProt_HUMAN$	2::Q06033|ITIH3_HUMAN	324	99787	20	18	18	17	1.24	Inter-alpha-trypsin in
80	1	SwissProt_HUMAN$	2::P02760|AMBP_HUMAN	318	38974	15	15	12	12	3.27	Protein AMBP OS=Ho
81	1	SwissProt_HUMAN$	2::A0A0B4J1V0|HV315_HUMAN	300	12917	17	17	7	7	16.91	Immunoglobulin heav
81	2	SwissProt_HUMAN$	2::A0A0A0MS15|HV349_HUMAN	160	13048	7	7	6	6	7.40	Immunoglobulin heav
81	3	SwissProt_HUMAN$	2::A0A0B4J1Y9|HV372_HUMAN	157	13194	9	9	6	6	7.25	Immunoglobulin heav
81	4	SwissProt_HUMAN$	2::A0A0B4J1V6|HV373_HUMAN	100	12849	6	6	4	4	3.23	Immunoglobulin heav
82	1	SwissProt_HUMAN$	2::P60709|ACTB_HUMAN	295	41710	13	11	10	9	2.10	Actin, cytoplasmic 1 (
83	1	SwissProt_HUMAN$	2::P02753|RET4_HUMAN	283	22995	13	11	9	7	4.12	Retinol-binding protei
84	1	SwissProt_HUMAN$	2::P03952|KLKB1_HUMAN	280	71323	11	11	11	11	1.07	Plasma kallikrein OS=
85	1	SwissProt_HUMAN$	2::P01714|LV319_HUMAN	263	12035	7	7	4	4	3.70	Immunoglobulin lamb
86	1	SwissProt_HUMAN$	2::P08697|A2AP_HUMAN	262	54531	15	15	11	11	1.60	Alpha-2-antiplasmin (
87	1	SwissProt_HUMAN$	2::P25311|ZA2G_HUMAN	255	34237	9	8	9	8	2.01	Zinc-alpha-2-glycopro
88	1	SwissProt_HUMAN$	2::P69905|HBA_HUMAN	252	15248	13	12	7	7	10.61	Hemoglobin subunit a
89	1	SwissProt_HUMAN$	2::P02652|APOA2_HUMAN	250	11168	10	10	5	5	10.91	Apolipoprotein A-II O
90	1	SwissProt_HUMAN$	2::P00748|FA12_HUMAN	248	67748	12	11	10	10	1.01	Coagulation factor XI
91	1	SwissProt_HUMAN$	2::P43652|AFAM_HUMAN	246	69024	14	14	13	13	1.44	Afamin OS=Homo sa
92	1	SwissProt_HUMAN$	2::P01591|IGJ_HUMAN	245	18087	12	12	8	8	6.90	Immunoglobulin J cha
93	1	SwissProt_HUMAN$	2::P00740|FA9_HUMAN	245	51745	12	11	12	11	1.73	Coagulation factor IX
94	1	SwissProt_HUMAN$	2::O14791|APOL1_HUMAN	240	43947	10	9	8	7	1.12	Apolipoprotein L1 OS
95	1	SwissProt_HUMAN$	2::P04196|HRG_HUMAN	239	59541	16	13	12	12	1.59	Histidine-rich glycopro
96	1	SwissProt_HUMAN$	2::P06312|KV401_HUMAN	236	13372	10	10	6	6	7.11	Immunoglobulin kapp
97	1	SwissProt_HUMAN$	2::Q5QNW6|H2B2F_HUMAN	231	13912	9	9	6	6	13.61	Histone H2B type 2-F
98	1	SwissProt_HUMAN$	2::P12111|CO6A3_HUMAN	228	343457	13	12	12	11	0.16	Collagen alpha-3(VI)
99	1	SwissProt_HUMAN$	2::P05546|HEP2_HUMAN	227	57034	10	8	10	8	0.94	Heparin cofactor 2 OS
100	1	SwissProt_HUMAN$	2::P02745|C1QA_HUMAN	226	26000	13	12	6	6	2.55	Complement C1q sub
101	1	SwissProt_HUMAN$	2::P01593|KVD33_HUMAN	223	12840	4	4	2	2	1.06	Immunoglobulin kapp
102	1	SwissProt_HUMAN$	2::P02656|APOC3_HUMAN	219	10846	5	4	3	2	1.34	Apolipoprotein C-III (
103	1	SwissProt_HUMAN$	2::P29622|KAIN_HUMAN	217	48511	10	9	9	8	1.18	Kallistatin OS=Homo
104	1	SwissProt_HUMAN$	2::P02655|APOC2_HUMAN	209	11277	9	9	6	6	16.50	Apolipoprotein C-II O
105	1	SwissProt_HUMAN$	2::P04433|KV311_HUMAN	207	12567	6	6	3	3	2.03	Immunoglobulin kapp
106	1	SwissProt_HUMAN$	2::P00742|FA10_HUMAN	207	54697	9	8	9	8	1.00	Coagulation factor X (
107	1	SwissProt_HUMAN$	2::Q96PD5|PGRP2_HUMAN	206	62178	8	8	7	7	0.70	N-acetylmuramoyl-L-
108	1	SwissProt_HUMAN$	2::Q9Y6R7|FCGBP_HUMAN	204	571639	15	14	14	13	0.11	IgGFc-binding proteir
109	1	SwissProt_HUMAN$	2::P04040|CATA_HUMAN	199	59719	5	4	5	4	0.37	Catalase OS=Homo s

↑Family	M	DB	Accession	Score	Mass	Matches	Match(sig)	Sequences	Seq(sig)	emPAI	Description
110	1	SwissProt_HUMAN$	2::P23142|FBLN1_HUMAN	197	77162	10	10	9	9	0.85	Fibulin-1 OS=Homo s
111	1	SwissProt_HUMAN$	2::P20851|C4BPB_HUMAN	196	28338	12	11	8	7	2.20	C4b-binding protein b
112	1	SwissProt_HUMAN$	2::O75636|FCN3_HUMAN	194	32882	12	10	7	7	2.15	Ficolin-3 OS=Homo s
113	1	SwissProt_HUMAN$	2::P01624|KV315_HUMAN	191	12488	6	5	2	2	1.10	Immunoglobulin kapp
114	1	SwissProt_HUMAN$	2::P51884|LUM_HUMAN	184	38405	10	10	7	7	1.36	Lumican OS=Homo sa
115	1	SwissProt_HUMAN$	2::P24821|TENA_HUMAN	181	240700	15	13	14	13	0.29	Tenascin OS=Homo s
116	1	SwissProt_HUMAN$	2::P27918|PROP_HUMAN	181	51242	6	6	5	5	0.58	Properdin OS=Homo
117	1	SwissProt_HUMAN$	2::Q9UK55|ZPI_HUMAN	180	50674	8	7	7	6	0.92	Protein Z-dependent
118	1	SwissProt_HUMAN$	2::P35542|SAA4_HUMAN	164	14737	10	9	8	7	16.10	Serum amyloid A-4 p
119	1	SwissProt_HUMAN$	2::P05160|F13B_HUMAN	162	75461	7	6	7	6	0.46	Coagulation factor XI
120	1	SwissProt_HUMAN$	2::P22792|CPN2_HUMAN	159	60518	9	6	8	5	0.48	Carboxypeptidase N s
121	1	SwissProt_HUMAN$	2::P07988|PSPB_HUMAN	157	42089	8	8	6	6	0.96	Pulmonary surfactant
122	1	SwissProt_HUMAN$	2::P62805|H4_HUMAN	154	11360	7	7	6	6	10.63	Histone H4 OS=Homo
123	1	SwissProt_HUMAN$	2::P11226|MBL2_HUMAN	154	26127	5	4	5	4	1.06	Mannose-binding prot
124	1	SwissProt_HUMAN$	2::P35858|ALS_HUMAN	148	65994	8	8	6	6	0.54	Insulin-like growth fa
125	1	SwissProt_HUMAN$	2::P00338|LDHA_HUMAN	147	36665	5	5	5	5	0.90	L-lactate dehydrogena
125	2	SwissProt_HUMAN$	2::P07195|LDHB_HUMAN	142	36615	5	5	5	5	0.90	L-lactate dehydrogena
126	1	SwissProt_HUMAN$	2::A0A075B6R9|KVD24_HUMAN	143	13071	5	3	4	3	1.90	Probable non-functior
127	1	SwissProt_HUMAN$	2::P08571|CD14_HUMAN	140	40051	7	6	5	5	0.80	Monocyte differentiati
128	1	SwissProt_HUMAN$	2::P04070|PROC_HUMAN	136	52037	4	4	4	4	0.44	Vitamin K-dependent
129	1	SwissProt_HUMAN$	2::P01700|LV147_HUMAN	131	12276	7	6	4	4	3.51	Immunoglobulin lamb
129	2	SwissProt_HUMAN$	2::P01699|LV144_HUMAN	96	12194	5	5	3	3	2.13	Immunoglobulin lamb
130	1	SwissProt_HUMAN$	2::A0A075B6K5|LV39_HUMAN	131	12324	4	4	3	3	2.09	Immunoglobulin lamb
130	2	SwissProt_HUMAN$	2::P80748|LV321_HUMAN	130	12438	5	5	3	3	2.06	Immunoglobulin lamb
131	1	SwissProt_HUMAN$	2::P06310|KV230_HUMAN	128	13177	4	4	3	3	1.87	Immunoglobulin kapp
131	2	SwissProt_HUMAN$	2::A0A075B6S2|KVD29_HUMAN	92	13135	3	2	3	2	1.03	Immunoglobulin kapp
131	3	SwissProt_HUMAN$	2::A0A075B6P5|KV228_HUMAN	89	12948	3	3	3	3	1.92	Immunoglobulin kapp
132	1	SwissProt_HUMAN$	2::P11021|BIP_HUMAN	125	72288	3	3	3	3	0.22	Endoplasmic reticulur
132	2	SwissProt_HUMAN$	2::P11142|HSP7C_HUMAN	113	70854	6	6	6	6	0.49	Heat shock cognate 7
132	3	SwissProt_HUMAN$	2::P38646|GRP75_HUMAN	66	73635	2	2	2	2	0.14	Stress-70 protein, mi
133	1	SwissProt_HUMAN$	2::P08519|APOA_HUMAN	124	500995	6	5	5	5	0.05	Apolipoprotein(a) OS=
134	1	SwissProt_HUMAN$	2::A0A075B6K4|LV310_HUMAN	123	12433	6	6	5	5	5.46	Immunoglobulin lamb
134	2	SwissProt_HUMAN$	2::P01717|LV325_HUMAN	90	12004	6	5	3	2	2.19	Immunoglobulin lamb
135	1	SwissProt_HUMAN$	2::P08238|HS90B_HUMAN	122	83212	5	5	5	5	0.33	Heat shock protein HS
135	2	SwissProt_HUMAN$	2::P07900|HS90A_HUMAN	109	84607	5	4	5	4	0.25	Heat shock protein HS
135	3	SwissProt_HUMAN$	2::P14625|ENPL_HUMAN	62	92411	3	3	3	3	0.17	Endoplasmin OS=Hor
136	1	SwissProt_HUMAN$	2::P08185|CBG_HUMAN	120	45112	7	7	6	6	0.87	Corticosteroid-binding
137	1	SwissProt_HUMAN$	2::O00187|MASP2_HUMAN	112	75654	3	3	3	3	0.21	Mannan-binding lectir
138	1	SwissProt_HUMAN$	2::Q96IY4|CBPB2_HUMAN	111	48393	7	6	6	6	0.80	Carboxypeptidase B2
139	1	SwissProt_HUMAN$	2::P05090|APOD_HUMAN	109	21262	8	5	8	5	2.00	Apolipoprotein D OS=

↑Family	M	DB	Accession	Score	Mass	Matches	Match(sig)	Sequences	Seq(sig)	emPAI	Description
140	1	SwissProt_HUMAN$	2::O00391|QSOX1_HUMAN	106	82526	5	5	5	5	0.33	Sulfhydryl oxidase 1 (
141	1	SwissProt_HUMAN$	2::Q12805|FBLN3_HUMAN	105	54604	4	3	4	3	0.30	EGF-containing fibulir
142	1	SwissProt_HUMAN$	2::P23528|COF1_HUMAN	105	18491	3	3	3	3	1.13	Cofilin-1 OS=Homo s
143	1	SwissProt_HUMAN$	2::Q08830|FGL1_HUMAN	104	36357	6	6	5	5	0.91	Fibrinogen-like protei
144	1	SwissProt_HUMAN$	2::P0DOX3|IGD_HUMAN	104	56189	4	4	3	3	0.29	Immunoglobulin delta
145	1	SwissProt_HUMAN$	2::P06681|CO2_HUMAN	103	83214	6	6	6	6	0.41	Complement C2 OS=
146	1	SwissProt_HUMAN$	2::P05543|THBG_HUMAN	102	46295	8	8	8	8	1.26	Thyroxine-binding glc
147	1	SwissProt_HUMAN$	2::P61626|LYSC_HUMAN	101	16526	6	5	5	5	3.10	Lysozyme C OS=Hom
148	1	SwissProt_HUMAN$	2::A0A0B4J1U7|HV601_HUMAN	100	13473	10	8	4	4	2.99	Immunoglobulin heav
149	1	SwissProt_HUMAN$	2::P13796|PLSL_HUMAN	97	70244	4	4	4	4	0.31	Plastin-2 OS=Homo s
150	1	SwissProt_HUMAN$	2::P26927|HGFL_HUMAN	93	80268	3	3	3	3	0.19	Hepatocyte growth fa
151	1	SwissProt_HUMAN$	2::P02654|APOC1_HUMAN	92	9326	7	6	4	4	10.81	Apolipoprotein C-I OS
152	1	SwissProt_HUMAN$	2::P62937|PPIA_HUMAN	90	18001	3	3	3	3	1.18	Peptidyl-prolyl cis-tra
153	1	SwissProt_HUMAN$	2::P08709|FA7_HUMAN	89	51560	2	2	2	2	0.20	Coagulation factor VI
154	1	SwissProt_HUMAN$	2::Q14520|HABP2_HUMAN	87	62630	4	4	4	4	0.35	Hyaluronan-binding p
155	1	SwissProt_HUMAN$	2::Q9UEW3|MARCO_HUMAN	83	52625	5	5	4	4	0.43	Macrophage receptor
156	1	SwissProt_HUMAN$	2::P01703|LV140_HUMAN	83	12294	2	1	2	1	0.46	Immunoglobulin lamb
157	1	SwissProt_HUMAN$	2::P01715|LV301_HUMAN	82	12288	2	2	2	2	1.12	Immunoglobulin lamb
158	1	SwissProt_HUMAN$	2::P55058|PLTP_HUMAN	81	54705	4	4	3	3	0.41	Phospholipid transfer
159	1	SwissProt_HUMAN$	2::P02776|PLF4_HUMAN	81	10838	4	4	3	3	1.34	Platelet factor 4 OS=I
160	1	SwissProt_HUMAN$	2::A0A0C4DH36|HV338_HUMAN	81	12750	4	3	2	1	1.07	Probable non-functior
161	1	SwissProt_HUMAN$	2::A6NMY6|AXA2L_HUMAN	78	38635	2	2	2	2	0.28	Putative annexin A2-l
162	1	SwissProt_HUMAN$	2::P0C0S8|H2A1_HUMAN	77	14083	5	5	3	3	1.69	Histone H2A type 1 O
163	1	SwissProt_HUMAN$	2::Q92954|PRG4_HUMAN	75	150968	4	4	4	4	0.13	Proteoglycan 4 OS=H
164	1	SwissProt_HUMAN$	2::P12109|CO6A1_HUMAN	73	108462	2	2	2	2	0.09	Collagen alpha-1(VI)
165	1	SwissProt_HUMAN$	2::Q15582|BGH3_HUMAN	72	74634	8	4	8	4	0.29	Transforming growth
166	1	SwissProt_HUMAN$	2::P15814|IGLL1_HUMAN	68	22949	2	2	1	1	0.23	Immunoglobulin lamb
167	1	SwissProt_HUMAN$	2::Q15848|ADIPO_HUMAN	67	26397	1	1	1	1	0.19	Adiponectin OS=Hom
168	1	SwissProt_HUMAN$	2::P62979|RS27A_HUMAN	67	17953	2	2	2	2	0.68	Ubiquitin-40S ribosom
169	1	SwissProt_HUMAN$	2::P25705|ATPA_HUMAN	67	59714	3	2	2	1	0.08	ATP synthase subunit
170	1	SwissProt_HUMAN$	2::P22891|PROZ_HUMAN	67	44715	7	7	7	7	1.09	Vitamin K-dependent
171	1	SwissProt_HUMAN$	2::P00746|CFAD_HUMAN	67	27016	4	3	4	3	0.69	Complement factor D
172	1	SwissProt_HUMAN$	2::Q15485|FCN2_HUMAN	67	33980	4	4	3	3	0.74	Ficolin-2 OS=Homo s
173	1	SwissProt_HUMAN$	2::A0A0C4DH34|HV428_HUMAN	62	13116	5	5	3	3	1.90	Immunoglobulin heav
174	1	SwissProt_HUMAN$	2::A0A0A0MS14|HV145_HUMAN	61	13500	1	1	1	1	0.41	Immunoglobulin heav
175	1	SwissProt_HUMAN$	2::P05154|IPSP_HUMAN	60	45646	2	2	2	2	0.23	Plasma serine proteas
176	1	SwissProt_HUMAN$	2::O95445|APOM_HUMAN	59	21239	4	4	4	4	1.42	Apolipoprotein M OS=
177	1	SwissProt_HUMAN$	2::Q5VTE0|EF1A3_HUMAN	58	50153	4	3	2	2	0.21	Putative elongation fa
178	1	SwissProt_HUMAN$	2::P16070|CD44_HUMAN	56	81487	1	1	1	1	0.06	CD44 antigen OS=Ho
179	1	SwissProt_HUMAN$	2::P31327|CPSM_HUMAN	55	164835	2	2	1	1	0.06	Carbamoyl-phosphate

↑Family	M	DB	Accession	Score	Mass	Matches	Match(sig)	Sequences	Seq(sig)	emPAI	Description
180	1	SwissProt_HUMAN$	2::P02788|TRFL_HUMAN	54	78132	5	2	4	2	0.13	Lactotransferrin OS=l
181	1	SwissProt_HUMAN$	2::P04406|G3P_HUMAN	53	36030	2	1	2	1	0.14	Glyceraldehyde-3-pho
182	1	SwissProt_HUMAN$	2::P15169|CBPN_HUMAN	52	52253	3	3	3	3	0.31	Carboxypeptidase N c
183	1	SwissProt_HUMAN$	2::Q6IS14|IF5AL_HUMAN	52	16762	1	1	1	1	0.32	Eukaryotic translatior
184	1	SwissProt_HUMAN$	2::P0DOX4|IGE_HUMAN	51	60285	1	1	1	1	0.08	Immunoglobulin epsil
185	1	SwissProt_HUMAN$	2::P14174|MIF_HUMAN	51	12468	1	1	1	1	0.45	Macrophage migratioı
186	1	SwissProt_HUMAN$	2::P01817|HV205_HUMAN	50	13223	2	1	2	1	0.42	Immunoglobulin heav
187	1	SwissProt_HUMAN$	2::A0A075B6H9|LV469_HUMAN	50	12765	2	1	2	1	0.44	Immunoglobulin lamt
188	1	SwissProt_HUMAN$	2::A0A075B6I9|LV746_HUMAN	48	12460	4	3	4	3	2.06	Immunoglobulin lamt
188	2	SwissProt_HUMAN$	2::P04211|LV743_HUMAN	42	12443	3	2	3	2	1.11	Immunoglobulin lamt
189	1	SwissProt_HUMAN$	2::Q16610|ECM1_HUMAN	46	60635	2	2	2	2	0.17	Extracellular matrix p
190	1	SwissProt_HUMAN$	2::Q9BQE3|TBA1C_HUMAN	46	49863	1	1	1	1	0.10	Tubulin alpha-1C chai
191	1	SwissProt_HUMAN$	2::P31946|1433B_HUMAN	46	28065	2	2	2	2	0.40	14-3-3 protein beta/a
191	2	SwissProt_HUMAN$	2::P63104|1433Z_HUMAN	34	27728	3	3	3	3	0.66	14-3-3 protein zeta/d
192	1	SwissProt_HUMAN$	2::P98160|PGBM_HUMAN	46	468532	3	1	3	1	0.01	Basement membrane
193	1	SwissProt_HUMAN$	2::P39019|RS19_HUMAN	45	16051	3	2	3	2	0.79	40S ribosomal proteir
194	1	SwissProt_HUMAN$	2::P61353|RL27_HUMAN	45	15788	1	1	1	1	0.34	60S ribosomal proteir
195	1	SwissProt_HUMAN$	2::A0A0B4J1Y8|LV949_HUMAN	44	13016	2	2	2	2	1.04	Immunoglobulin lamt
196	1	SwissProt_HUMAN$	2::P51649|SSDH_HUMAN	43	57178	1	1	1	1	0.09	Succinate-semialdehy
197	1	SwissProt_HUMAN$	2::P37837|TALDO_HUMAN	43	37516	1	1	1	1	0.13	Transaldolase OS=Ho
198	1	SwissProt_HUMAN$	2::P28072|PSB6_HUMAN	43	25341	1	1	1	1	0.20	Proteasome subunit t
199	1	SwissProt_HUMAN$	2::P02786|TFR1_HUMAN	42	84818	1	1	1	1	0.06	Transferrin receptor p
200	1	SwissProt_HUMAN$	2::P03951|FA11_HUMAN	42	70064	2	2	2	2	0.14	Coagulation factor XI
201	1	SwissProt_HUMAN$	2::P04083|ANXA1_HUMAN	42	38690	1	1	1	1	0.13	Annexin A1 OS=Hom
202	1	SwissProt_HUMAN$	2::P62701|RS4X_HUMAN	41	29579	2	2	2	2	0.38	40S ribosomal proteir
203	1	SwissProt_HUMAN$	2::O75882|ATRN_HUMAN	41	158432	2	2	2	2	0.06	Attractin OS=Homo s
204	1	SwissProt_HUMAN$	2::P34096|RNAS4_HUMAN	41	16829	1	1	1	1	0.32	Ribonuclease 4 OS=H
205	1	SwissProt_HUMAN$	2::O75818|RPP40_HUMAN	41	41807	1	1	1	1	0.12	Ribonuclease P protei
206	1	SwissProt_HUMAN$	2::Q13972|RGRF1_HUMAN	40	145142	3	2	2	1	0.03	Ras-specific guanine
207	1	SwissProt_HUMAN$	2::P10599|THIO_HUMAN	39	11730	2	2	2	2	1.20	Thioredoxin OS=Hom
208	1	SwissProt_HUMAN$	2::Q86YZ3|HORN_HUMAN	39	282228	2	2	2	2	0.03	Hornerin OS=Homo s
209	1	SwissProt_HUMAN$	2::P04350|TBB4A_HUMAN	39	49554	1	1	1	1	0.10	Tubulin beta-4A chair
210	1	SwissProt_HUMAN$	2::P05109|S10A8_HUMAN	39	10828	3	2	3	2	1.34	Protein S100-A8 OS=
211	1	SwissProt_HUMAN$	2::Q8N5V2|NGEF_HUMAN	39	82445	1	1	1	1	0.06	Ephexin-1 OS=Homo
212	1	SwissProt_HUMAN$	2::A0A075B6I1|LV460_HUMAN	39	12979	2	1	2	1	0.43	Immunoglobulin lamt
213	1	SwissProt_HUMAN$	2::Q86SS6|SYT9_HUMAN	38	56153	1	1	1	1	0.09	Synaptotagmin-9 OS=
214	1	SwissProt_HUMAN$	2::Q8NGJ3|O52E1_HUMAN	37	34690	3	1	1	1	0.15	Olfactory receptor 52
215	1	SwissProt_HUMAN$	2::P62263|RS14_HUMAN	37	16263	1	1	1	1	0.33	40S ribosomal proteir
216	1	SwissProt_HUMAN$	2::A0A0C4DH35|HV335_HUMAN	37	12802	1	1	1	1	0.44	Probable non-functior
217	1	SwissProt_HUMAN$	2::Q8NGM8|OR6M1_HUMAN	37	35305	2	2	1	1	0.14	Olfactory receptor 6M

↑Family	M	DB	Accession	Score	Mass	Matches	Match(sig)	Sequences	Seq(sig)	emPAI	Description
218	1	SwissProt_HUMAN$	2::Q9UGM5|FETUB_HUMAN	36	42028	3	3	3	3	0.40	Fetuin-B OS=Homo s
219	1	SwissProt_HUMAN$	2::Q13093|PAFA_HUMAN	36	50045	1	1	1	1	0.10	Platelet-activating fac
220	1	SwissProt_HUMAN$	2::Q6Q759|SPG17_HUMAN	35	251584	1	1	1	1	0.02	Sperm-associated ant
221	1	SwissProt_HUMAN$	2::Q9NTJ4|MA2C1_HUMAN	35	115762	1	1	1	1	0.04	Alpha-mannosidase 2
222	1	SwissProt_HUMAN$	2::Q3SXZ3|ZN718_HUMAN	35	55324	3	3	1	1	0.09	Zinc finger protein 71
223	1	SwissProt_HUMAN$	2::P26022|PTX3_HUMAN	35	41949	1	1	1	1	0.12	Pentraxin-related pro
224	1	SwissProt_HUMAN$	2::O00469|PLOD2_HUMAN	35	84632	1	1	1	1	0.06	Procollagen-lysine,2-
225	1	SwissProt_HUMAN$	2::P55056|APOC4_HUMAN	35	14543	2	1	2	1	0.38	Apolipoprotein C-IV C
226	1	SwissProt_HUMAN$	2::P06748|NPM_HUMAN	34	32555	2	2	2	2	0.34	Nucleophosmin OS=H
227	1	SwissProt_HUMAN$	2::P59665|DEF1_HUMAN	34	10194	2	2	2	2	1.47	Neutrophil defensin 1
228	1	SwissProt_HUMAN$	2::Q3L8U1|CHD9_HUMAN	34	325819	2	2	2	2	0.03	Chromodomain-helica
229	1	SwissProt_HUMAN$	2::Q68DL7|CR063_HUMAN	34	77181	2	2	1	1	0.06	Uncharacterized prote
230	1	SwissProt_HUMAN$	2::O76031|CLPX_HUMAN	34	69181	1	1	1	1	0.07	ATP-dependent Clp pı
231	1	SwissProt_HUMAN$	2::Q96DD0|LRC39_HUMAN	34	38768	1	1	1	1	0.13	Leucine-rich repeat-c
232	1	SwissProt_HUMAN$	2::O15078|CE290_HUMAN	33	290207	3	2	3	2	0.03	Centrosomal protein
233	1	SwissProt_HUMAN$	2::Q9NYC9|DYH9_HUMAN	33	511551	2	1	1	1	0.01	Dynein heavy chain 9
234	1	SwissProt_HUMAN$	2::P32119|PRDX2_HUMAN	33	21878	1	1	1	1	0.24	Peroxiredoxin-2 OS=I
235	1	SwissProt_HUMAN$	2::Q8TCB0|IFI44_HUMAN	32	50459	1	1	1	1	0.10	Interferon-induced pr
236	1	SwissProt_HUMAN$	2::Q9HDC9|APMAP_HUMAN	32	46451	1	1	1	1	0.11	Adipocyte plasma me
237	1	SwissProt_HUMAN$	2::O75460|ERN1_HUMAN	32	109666	1	1	1	1	0.04	Serine/threonine-prot
238	1	SwissProt_HUMAN$	2::P15531|NDKA_HUMAN	32	17138	1	1	1	1	0.31	Nucleoside diphospha
239	1	SwissProt_HUMAN$	2::P62249|RS16_HUMAN	32	16435	1	1	1	1	0.33	40S ribosomal proteir
240	1	SwissProt_HUMAN$	2::P04075|ALDOA_HUMAN	32	39395	2	2	2	2	0.27	Fructose-bisphosphat
241	1	SwissProt_HUMAN$	2::Q14980|NUMA1_HUMAN	31	238115	1	1	1	1	0.02	Nuclear mitotic appar
242	1	SwissProt_HUMAN$	2::Q13103|SPP24_HUMAN	31	24322	1	1	1	1	0.21	Secreted phosphopro
243	1	SwissProt_HUMAN$	2::Q12934|BFSP1_HUMAN	31	74499	2	2	1	1	0.07	Filensin OS=Homo sa
244	1	SwissProt_HUMAN$	2::Q9P219|DAPLE_HUMAN	31	228091	1	1	1	1	0.02	Protein Daple OS=Ho
245	1	SwissProt_HUMAN$	2::Q9HAW7|UD17_HUMAN	31	59779	2	2	2	2	0.17	UDP-glucuronosyltrar
246	1	SwissProt_HUMAN$	2::Q14242|SELPL_HUMAN	31	43174	1	1	1	1	0.12	P-selectin glycoprotei
247	1	SwissProt_HUMAN$	2::P01705|LV223_HUMAN	31	11886	3	2	1	1	1.18	Immunoglobulin lamt
248	1	SwissProt_HUMAN$	2::A0A075B6K0|LV316_HUMAN	30	12458	4	2	2	1	0.45	Immunoglobulin lamt
249	1	SwissProt_HUMAN$	2::P02144|MYG_HUMAN	30	17173	1	1	1	1	0.31	Myoglobin OS=Homo
250	1	SwissProt_HUMAN$	2::Q9UKG4|S13A4_HUMAN	30	69313	1	1	1	1	0.07	Solute carrier family
251	1	SwissProt_HUMAN$	2::Q96A32|MLRS_HUMAN	30	19002	1	1	1	1	0.28	Myosin regulatory ligl
252	1	SwissProt_HUMAN$	2::Q5T0N5|FBP1L_HUMAN	30	70021	2	2	1	1	0.07	Formin-binding protei
253	1	SwissProt_HUMAN$	2::Q9UPT5|EXOC7_HUMAN	30	83330	1	1	1	1	0.06	Exocyst complex com
254	1	SwissProt_HUMAN$	2::Q5XXA6|ANO1_HUMAN	30	114005	1	1	1	1	0.04	Anoctamin-1 OS=Hor
255	1	SwissProt_HUMAN$	2::Q9NRG0|CHRC1_HUMAN	30	14701	1	1	1	1	0.37	Chromatin accessibilit
256	1	SwissProt_HUMAN$	2::Q86WI1|PKHL1_HUMAN	30	465439	1	1	1	1	0.01	Fibrocystin-L OS=Hor
257	1	SwissProt_HUMAN$	2::Q8TDN4|CABL1_HUMAN	29	67557	1	1	1	1	0.07	CDK5 and ABL1 enzyı

↑Family	M	DB	Accession	Score	Mass	Matches	Match(sig)	Sequences	Seq(sig)	emPAI	Description
258	1	SwissProt_HUMAN$	2::P14061|DHB1_HUMAN	29	34928	1	1	1	1	0.14	Estradiol 17-beta-deh
259	1	SwissProt_HUMAN$	2::A0A075B6I0|LV861_HUMAN	29	12806	1	1	1	1	0.44	Immunoglobulin lamb
260	1	SwissProt_HUMAN$	2::Q9ULM0|PKHH1_HUMAN	29	151137	1	1	1	1	0.03	Pleckstrin homology c
261	1	SwissProt_HUMAN$	2::Q71DI3|H32_HUMAN	29	15379	3	2	2	2	0.83	Histone H3.2 OS=Hor
262	1	SwissProt_HUMAN$	2::Q00610|CLH1_HUMAN	29	191493	2	2	1	1	0.03	Clathrin heavy chain
263	1	SwissProt_HUMAN$	2::P46778|RL21_HUMAN	28	18553	1	1	1	1	0.29	60S ribosomal protein
264	1	SwissProt_HUMAN$	2::P11498|PYC_HUMAN	28	129551	2	2	1	1	0.04	Pyruvate carboxylase
265	1	SwissProt_HUMAN$	2::Q6DHV5|C2D2B_HUMAN	28	166681	1	1	1	1	0.03	Protein CC2D2B OS=
266	1	SwissProt_HUMAN$	2::Q05513|KPCZ_HUMAN	28	67617	1	1	1	1	0.07	Protein kinase C zeta
267	1	SwissProt_HUMAN$	2::Q8NHQ9|DDX55_HUMAN	28	68503	1	1	1	1	0.07	ATP-dependent RNA h
268	1	SwissProt_HUMAN$	2::P62277|RS13_HUMAN	28	17212	1	1	1	1	0.31	40S ribosomal protein
269	1	SwissProt_HUMAN$	2::P06702|S10A9_HUMAN	28	13234	2	2	2	2	1.02	Protein S100-A9 OS=
270	1	SwissProt_HUMAN$	2::Q07075|AMPE_HUMAN	28	109176	1	1	1	1	0.04	Glutamyl aminopeptic
271	1	SwissProt_HUMAN$	2::Q8NEM7|SP20H_HUMAN	28	85735	1	1	1	1	0.06	Transcription factor S
272	1	SwissProt_HUMAN$	2::P00488|F13A_HUMAN	28	83215	1	1	1	1	0.06	Coagulation factor XI
273	1	SwissProt_HUMAN$	2::P01034|CYTC_HUMAN	28	15789	2	2	2	2	0.81	Cystatin-C OS=Homo
274	1	SwissProt_HUMAN$	2::P61247|RS3A_HUMAN	27	29926	1	1	1	1	0.17	40S ribosomal protein
275	1	SwissProt_HUMAN$	2::Q86VD1|MORC1_HUMAN	27	112810	1	1	1	1	0.04	MORC family CW-type
276	1	SwissProt_HUMAN$	2::P29401|TKT_HUMAN	26	67835	3	2	2	2	0.15	Transketolase OS=Hc
277	1	SwissProt_HUMAN$	2::Q9NP78|ABCB9_HUMAN	27	84421	1	1	1	1	0.06	ATP-binding cassette
278	1	SwissProt_HUMAN$	2::P60174|TPIS_HUMAN	27	26653	2	1	2	1	0.19	Triosephosphate isom
279	1	SwissProt_HUMAN$	2::Q9UHG3|PCYOX_HUMAN	26	56604	2	1	2	1	0.09	Prenylcysteine oxidas
280	1	SwissProt_HUMAN$	2::Q13023|AKAP6_HUMAN	26	256560	1	1	1	1	0.02	A-kinase anchor prote
281	1	SwissProt_HUMAN$	2::P11597|CETP_HUMAN	26	54721	1	1	1	1	0.09	Cholesteryl ester tran
282	1	SwissProt_HUMAN$	2::P61604|CH10_HUMAN	26	10925	1	1	1	1	0.53	10 kDa heat shock pr
283	1	SwissProt_HUMAN$	2::P05164|PERM_HUMAN	26	83815	1	1	1	1	0.06	Myeloperoxidase OS=
284	1	SwissProt_HUMAN$	2::P06703|S10A6_HUMAN	26	10173	1	1	1	1	0.57	Protein S100-A6 OS=
285	1	SwissProt_HUMAN$	2::A0A087WSX0|LV545_HUMAN	26	13153	1	1	1	1	0.43	Immunoglobulin lamb
286	1	SwissProt_HUMAN$	2::P23409|MYF6_HUMAN	26	26935	1	1	1	1	0.19	Myogenic factor 6 OS
287	1	SwissProt_HUMAN$	2::Q9BTT0|AN32E_HUMAN	26	30674	1	1	1	1	0.17	Acidic leucine-rich nu
288	1	SwissProt_HUMAN$	2::P14618|KPYM_HUMAN	26	57900	2	1	2	1	0.09	Pyruvate kinase PKM
289	1	SwissProt_HUMAN$	2::Q15276|RABE1_HUMAN	26	99229	1	1	1	1	0.05	Rab GTPase-binding e
290	1	SwissProt_HUMAN$	2::Q5JNZ5|RS26L_HUMAN	25	12994	1	1	1	1	0.43	Putative 40S ribosom
291	1	SwissProt_HUMAN$	2::Q15113|PCOC1_HUMAN	25	47942	1	1	1	1	0.10	Procollagen C-endope
292	1	SwissProt_HUMAN$	2::O94887|FARP2_HUMAN	25	119813	1	1	1	1	0.04	FERM, ARHGEF and p
293	1	SwissProt_HUMAN$	2::Q4G0X9|CCD40_HUMAN	25	130033	1	1	1	1	0.04	Coiled-coil domain-co
294	1	SwissProt_HUMAN$	2::P04278|SHBG_HUMAN	25	43752	3	3	3	3	0.38	Sex hormone-binding
295	1	SwissProt_HUMAN$	2::P48165|CXA8_HUMAN	25	48199	2	1	1	1	0.10	Gap junction alpha-8
296	1	SwissProt_HUMAN$	2::O43303|CP110_HUMAN	25	113353	1	1	1	1	0.04	Centriolar coiled-coil
297	1	SwissProt_HUMAN$	2::Q9HCG8|CWC22_HUMAN	25	105402	1	1	1	1	0.05	Pre-mRNA-splicing fa

↑Family	M	DB	Accession	Score	Mass	Matches	Match(sig)	Sequences	Seq(sig)	emPAI	Description
298	1	SwissProt_HUMAN$	2::P08922|ROS1_HUMAN	25	263747	2	1	2	1	0.02	Proto-oncogene tyros
299	1	SwissProt_HUMAN$	2::P56182|RRP1_HUMAN	25	52807	1	1	1	1	0.09	Ribosomal RNA proce
300	1	SwissProt_HUMAN$	2::Q8TD08|MK15_HUMAN	25	59795	1	1	1	1	0.08	Mitogen-activated pro
301	1	SwissProt_HUMAN$	2::P05452|TETN_HUMAN	25	22522	1	1	1	1	0.23	Tetranectin OS=Hom
302	1	SwissProt_HUMAN$	2::Q9NP80|PLPL8_HUMAN	25	88421	1	1	1	1	0.05	Calcium-independent
303	1	SwissProt_HUMAN$	2::P21399|ACOC_HUMAN	24	98337	1	1	1	1	0.05	Cytoplasmic aconitate
304	1	SwissProt_HUMAN$	2::P38117|ETFB_HUMAN	24	27826	1	1	1	1	0.18	Electron transfer flav
305	1	SwissProt_HUMAN$	2::Q7Z5H4|VN1R5_HUMAN	24	40752	1	1	1	1	0.12	Vomeronasal type-1 r
306	1	SwissProt_HUMAN$	2::Q86YA3|ZGRF1_HUMAN	24	236453	1	1	1	1	0.02	Protein ZGRF1 OS=H
307	1	SwissProt_HUMAN$	2::Q9Y5I4|PCDC2_HUMAN	24	109383	1	1	1	1	0.04	Protocadherin alpha-(
308	1	SwissProt_HUMAN$	2::Q8NF91|SYNE1_HUMAN	24	1010456	2	1	2	1	0.00	Nesprin-1 OS=Homo
309	1	SwissProt_HUMAN$	2::O94855|SC24D_HUMAN	24	112936	1	1	1	1	0.04	Protein transport prot
310	1	SwissProt_HUMAN$	2::P06733|ENOA_HUMAN	24	47139	1	1	1	1	0.11	Alpha-enolase OS=H
311	1	SwissProt_HUMAN$	2::Q8TB33|CX024_HUMAN	24	10352	1	1	1	1	0.56	Putative uncharacteri
312	1	SwissProt_HUMAN$	2::Q86YJ7|AN13B_HUMAN	24	70162	2	1	1	1	0.07	Ankyrin repeat domai
313	1	SwissProt_HUMAN$	2::Q8N6G1|CA211_HUMAN	23	10497	1	1	1	1	0.55	Putative uncharacteri
314	1	SwissProt_HUMAN$	2::Q8N4N3|KLH36_HUMAN	23	69852	1	1	1	1	0.07	Kelch-like protein 36
315	1	SwissProt_HUMAN$	2::Q7Z418|KCNKI_HUMAN	23	43643	1	1	1	1	0.11	Potassium channel su
316	1	SwissProt_HUMAN$	2::Q14697|GANAB_HUMAN	23	106807	1	1	1	1	0.05	Neutral alpha-glucosi
317	1	SwissProt_HUMAN$	2::P42568|AF9_HUMAN	23	63313	1	1	1	1	0.08	Protein AF-9 OS=Hon
318	1	SwissProt_HUMAN$	2::Q06830|PRDX1_HUMAN	23	22096	2	2	2	2	0.53	Peroxiredoxin-1 OS=I
319	1	SwissProt_HUMAN$	2::O60282|KIF5C_HUMAN	23	109427	3	1	2	1	0.04	Kinesin heavy chain i:
320	1	SwissProt_HUMAN$	2::P22090|RS4Y1_HUMAN	23	29437	1	1	1	1	0.17	40S ribosomal proteir
321	1	SwissProt_HUMAN$	2::P28482|MK01_HUMAN	23	41363	2	2	1	1	0.12	Mitogen-activated pro
322	1	SwissProt_HUMAN$	2::Q16543|CDC37_HUMAN	23	44440	1	1	1	1	0.11	Hsp90 co-chaperone
323	1	SwissProt_HUMAN$	2::Q0WX57|U17LO_HUMAN	23	59673	1	1	1	1	0.08	Ubiquitin carboxyl-ter
324	1	SwissProt_HUMAN$	2::Q01082|SPTB2_HUMAN	23	274439	1	1	1	1	0.02	Spectrin beta chain, r
325	1	SwissProt_HUMAN$	2::P61769|B2MG_HUMAN	23	13706	1	1	1	1	0.41	Beta-2-microglobulin
326	1	SwissProt_HUMAN$	2::P36957|ODO2_HUMAN	22	48724	1	1	1	1	0.10	Dihydrolipoyllysine-re
327	1	SwissProt_HUMAN$	2::Q9NQG7|HPS4_HUMAN	22	76870	1	1	1	1	0.06	Hermansky-Pudlak sy
328	1	SwissProt_HUMAN$	2::Q6P499|NPAL3_HUMAN	22	44712	1	1	1	1	0.11	NIPA-like protein 3 O:
329	1	SwissProt_HUMAN$	2::Q86UW7|CAPS2_HUMAN	22	147641	1	1	1	1	0.03	Calcium-dependent se
330	1	SwissProt_HUMAN$	2::P04180|LCAT_HUMAN	22	49546	2	2	2	2	0.21	Phosphatidylcholine-s
331	1	SwissProt_HUMAN$	2::Q58WW2|DCAF6_HUMAN	22	96232	1	1	1	1	0.05	DDB1- and CUL4-asso
332	1	SwissProt_HUMAN$	2::Q96AA8|JKIP2_HUMAN	22	94875	1	1	1	1	0.05	Janus kinase and mic
333	1	SwissProt_HUMAN$	2::Q9NQ29|LUC7L_HUMAN	22	43701	1	1	1	1	0.11	Putative RNA-binding
334	1	SwissProt_HUMAN$	2::Q92750|TAF4B_HUMAN	22	91034	3	3	3	3	0.17	Transcription initiatio
335	1	SwissProt_HUMAN$	2::Q96K49|TM87B_HUMAN	22	63495	1	1	1	1	0.08	Transmembrane prote
336	1	SwissProt_HUMAN$	2::P14923|PLAK_HUMAN	22	81693	1	1	1	1	0.06	Junction plakoglobin (
337	1	SwissProt_HUMAN$	2::P49910|ZN165_HUMAN	21	55735	1	1	1	1	0.09	Zinc finger protein 16

↑Family	M	DB	Accession	Score	Mass	Matches	Match(sig)	Sequences	Seq(sig)	emPAI	Description
338	1	SwissProt_HUMAN$	2::Q9Y265|RUVB1_HUMAN	21	50196	1	1	1	1	0.10	RuvB-like 1 OS=Hom
339	1	SwissProt_HUMAN$	2::P21108|PRPS3_HUMAN	21	34817	1	1	1	1	0.14	Ribose-phosphate pyı
340	1	SwissProt_HUMAN$	2::Q9NU19|TB22B_HUMAN	21	59044	1	1	1	1	0.08	TBC1 domain family r
341	1	SwissProt_HUMAN$	2::Q15334|L2GL1_HUMAN	21	115346	1	1	1	1	0.04	Lethal(2) giant larvae
342	1	SwissProt_HUMAN$	2::Q8NFZ5|TNIP2_HUMAN	21	48669	1	1	1	1	0.10	TNFAIP3-interacting p
343	1	SwissProt_HUMAN$	2::Q7Z6Z7|HUWE1_HUMAN	21	481589	1	1	1	1	0.01	E3 ubiquitin-protein li
344	1	SwissProt_HUMAN$	2::P46091|GPR1_HUMAN	21	41404	1	1	1	1	0.12	G-protein coupled rec
345	1	SwissProt_HUMAN$	2::Q8WVS4|DC2I1_HUMAN	21	122496	1	1	1	1	0.04	Cytoplasmic dynein 2
346	1	SwissProt_HUMAN$	2::Q9BQS7|HEPH_HUMAN	21	130365	1	1	1	1	0.04	Hephaestin OS=Homo
347	1	SwissProt_HUMAN$	2::P49643|PRI2_HUMAN	21	58769	1	1	1	1	0.08	DNA primase large su
348	1	SwissProt_HUMAN$	2::Q86VH2|KIF27_HUMAN	21	160184	1	1	1	1	0.03	Kinesin-like protein K
349	1	SwissProt_HUMAN$	2::Q8N228|SCML4_HUMAN	21	44994	1	1	1	1	0.11	Sex comb on midleg-
350	1	SwissProt_HUMAN$	2::Q8IUH5|ZDH17_HUMAN	21	72592	1	1	1	1	0.07	Palmitoyltransferase :
351	1	SwissProt_HUMAN$	2::P62244|RS15A_HUMAN	21	14830	1	1	1	1	0.37	40S ribosomal proteir
352	1	SwissProt_HUMAN$	2::P53597|SUCA_HUMAN	20	36227	1	1	1	1	0.14	Succinate--CoA ligase
353	1	SwissProt_HUMAN$	2::Q49MG5|MAP9_HUMAN	20	74189	1	1	1	1	0.07	Microtubule-associate
354	1	SwissProt_HUMAN$	2::Q8IW70|T151B_HUMAN	20	61467	1	1	1	1	0.08	Transmembrane prot
355	1	SwissProt_HUMAN$	2::P31040|SDHA_HUMAN	20	72645	1	1	1	1	0.07	Succinate dehydroger
356	1	SwissProt_HUMAN$	2::P54750|PDE1A_HUMAN	20	61213	1	1	1	1	0.08	Calcium/calmodulin-d
357	1	SwissProt_HUMAN$	2::Q6ZTR6|ZNFDT_HUMAN	20	17225	1	1	1	1	0.31	Putative uncharacteri
358	1	SwissProt_HUMAN$	2::Q9H0L4|CSTFT_HUMAN	20	64396	1	1	1	1	0.08	Cleavage stimulation
359	1	SwissProt_HUMAN$	2::P40763|STAT3_HUMAN	20	88011	2	1	2	1	0.06	Signal transducer anc
360	1	SwissProt_HUMAN$	2::Q9UJX2|CDC23_HUMAN	20	68790	1	1	1	1	0.07	Cell division cycle pro
361	1	SwissProt_HUMAN$	2::P07737|PROF1_HUMAN	20	15045	1	1	1	1	0.36	Profilin-1 OS=Homo s
362	1	SwissProt_HUMAN$	2::P49747|COMP_HUMAN	20	82808	1	1	1	1	0.06	Cartilage oligomeric r
363	1	SwissProt_HUMAN$	2::Q5JPH6|SYEM_HUMAN	20	58652	1	1	1	1	0.08	Probable glutamate--
364	1	SwissProt_HUMAN$	2::Q9Y691|KCMB2_HUMAN	19	27111	1	1	1	1	0.19	Calcium-activated pot
365	1	SwissProt_HUMAN$	2::Q96RU7|TRIB3_HUMAN	19	39553	1	1	1	1	0.13	Tribbles homolog 3 O
366	1	SwissProt_HUMAN$	2::P35222|CTNB1_HUMAN	19	85442	2	1	1	1	0.06	Catenin beta-1 OS=H
367	1	SwissProt_HUMAN$	2::P07237|PDIA1_HUMAN	19	57081	1	1	1	1	0.09	Protein disulfide-isom
368	1	SwissProt_HUMAN$	2::P55786|PSA_HUMAN	19	103211	1	1	1	1	0.05	Puromycin-sensitive a
369	1	SwissProt_HUMAN$	2::O00222|GRM8_HUMAN	19	101675	1	1	1	1	0.05	Metabotropic glutama
370	1	SwissProt_HUMAN$	2::P62857|RS28_HUMAN	19	7836	1	1	1	1	0.79	40S ribosomal proteir
371	1	SwissProt_HUMAN$	2::O60437|PEPL_HUMAN	19	204623	1	1	1	1	0.02	Periplakin OS=Homo
372	1	SwissProt_HUMAN$	2::Q8N4C8|MINK1_HUMAN	19	149729	2	1	2	1	0.03	Misshapen-like kinase
373	1	SwissProt_HUMAN$	2::Q9UKE5|TNIK_HUMAN	19	154848	2	1	2	1	0.03	TRAF2 and NCK-inter
374	1	SwissProt_HUMAN$	2::P61313|RL15_HUMAN	19	24131	1	1	1	1	0.22	60S ribosomal proteir
375	1	SwissProt_HUMAN$	2::P22352|GPX3_HUMAN	19	25537	2	1	2	1	0.20	Glutathione peroxidas
376	1	SwissProt_HUMAN$	2::Q9P2E2|KIF17_HUMAN	19	114997	1	1	1	1	0.04	Kinesin-like protein K
377	1	SwissProt_HUMAN$	2::O43896|KIF1C_HUMAN	19	122870	1	1	1	1	0.04	Kinesin-like protein K

↑Family	M	DB	Accession	Score	Mass	Matches	Match(sig)	Sequences	Seq(sig)	emPAI	Description
378	1	SwissProt_HUMAN$	2::O00429|DNM1L_HUMAN	19	81826	2	1	1	1	0.06	Dynamin-1-like prote
379	1	SwissProt_HUMAN$	2::Q9Y566|SHAN1_HUMAN	18	224821	3	1	2	1	0.02	SH3 and multiple ank
380	1	SwissProt_HUMAN$	2::P01718|LV327_HUMAN	18	12157	2	1	2	1	0.46	Immunoglobulin lamb
381	1	SwissProt_HUMAN$	2::P57735|RAB25_HUMAN	18	23481	1	1	1	1	0.22	Ras-related protein R
382	1	SwissProt_HUMAN$	2::Q66K66|TM198_HUMAN	18	39449	1	1	1	1	0.13	Transmembrane prot
383	1	SwissProt_HUMAN$	2::Q6ZMY3|SPOC1_HUMAN	18	129945	1	1	1	1	0.04	SPOC domain-contair
384	1	SwissProt_HUMAN$	2::Q8NEP3|DAAF1_HUMAN	18	79976	1	1	1	1	0.06	Dynein assembly fact
385	1	SwissProt_HUMAN$	2::Q96HY6|DDRGK_HUMAN	18	35589	1	1	1	1	0.14	DDRGK domain-conta
386	1	SwissProt_HUMAN$	2::Q8N6Q8|MET25_HUMAN	18	68169	1	1	1	1	0.07	Methyltransferase-lik
387	1	SwissProt_HUMAN$	2::Q08AN1|ZN616_HUMAN	18	90215	1	1	1	1	0.05	Zinc finger protein 61
388	1	SwissProt_HUMAN$	2::Q96R06|SPAG5_HUMAN	18	134338	1	1	1	1	0.04	Sperm-associated ant
389	1	SwissProt_HUMAN$	2::P15311|EZRI_HUMAN	18	69370	1	1	1	1	0.07	Ezrin OS=Homo sapie
390	1	SwissProt_HUMAN$	2::Q4KMP7|TB10B_HUMAN	18	87145	1	1	1	1	0.06	TBC1 domain family r
391	1	SwissProt_HUMAN$	2::Q86WK7|AMGO3_HUMAN	18	55214	1	1	1	1	0.09	Amphoterin-induced p
392	1	SwissProt_HUMAN$	2::P08637|FCG3A_HUMAN	18	29071	1	1	1	1	0.18	Low affinity immunog
393	1	SwissProt_HUMAN$	2::O15417|TNC18_HUMAN	18	314326	1	1	1	1	0.02	Trinucleotide repeat-c
394	1	SwissProt_HUMAN$	2::Q93009|UBP7_HUMAN	18	128220	1	1	1	1	0.04	Ubiquitin carboxyl-ter
395	1	SwissProt_HUMAN$	2::Q9NSV4|DIAP3_HUMAN	17	136839	1	1	1	1	0.04	Protein diaphanous h
396	1	SwissProt_HUMAN$	2::Q99996|AKAP9_HUMAN	17	452708	1	1	1	1	0.01	A-kinase anchor prote
397	1	SwissProt_HUMAN$	2::P01701|LV151_HUMAN	17	12241	2	2	2	2	1.14	Immunoglobulin lamb
398	1	SwissProt_HUMAN$	2::P11309|PIM1_HUMAN	17	35663	1	1	1	1	0.14	Serine/threonine-prot
399	1	SwissProt_HUMAN$	2::P0DPP9|ETDB_HUMAN	17	6987	1	1	1	1	0.92	Embryonic testis diffe
400	1	SwissProt_HUMAN$	2::Q6UWY0|ARSK_HUMAN	17	61411	1	1	1	1	0.08	Arylsulfatase K OS=H
401	1	SwissProt_HUMAN$	2::P62906|RL10A_HUMAN	17	24816	1	1	1	1	0.21	60S ribosomal proteir
402	1	SwissProt_HUMAN$	2::Q7Z442|PK1L2_HUMAN	17	272340	2	1	1	1	0.02	Polycystic kidney dise
403	1	SwissProt_HUMAN$	2::Q96NL6|SCLT1_HUMAN	17	80861	2	1	1	1	0.06	Sodium channel and c
404	1	SwissProt_HUMAN$	2::Q9H2K8|TAOK3_HUMAN	17	105340	1	1	1	1	0.05	Serine/threonine-prot
405	1	SwissProt_HUMAN$	2::Q96E66|LRC51_HUMAN	17	22193	1	1	1	1	0.24	Leucine-rich repeat-c
406	1	SwissProt_HUMAN$	2::Q8WZA1|PMGT1_HUMAN	17	75204	1	1	1	1	0.06	Protein O-linked-man
407	1	SwissProt_HUMAN$	2::O94875|SRBS2_HUMAN	16	124030	1	1	1	1	0.04	Sorbin and SH3 doma
408	1	SwissProt_HUMAN$	2::Q9BYX4|IFIH1_HUMAN	16	116615	1	1	1	1	0.04	Interferon-induced he
409	1	SwissProt_HUMAN$	2::Q9BQI3|E2AK1_HUMAN	16	71062	1	1	1	1	0.07	Eukaryotic translatior
410	1	SwissProt_HUMAN$	2::Q96JB2|COG3_HUMAN	16	94036	1	1	1	1	0.05	Conserved oligomeric
411	1	SwissProt_HUMAN$	2::Q15046|SYK_HUMAN	16	68005	1	1	1	1	0.07	Lysine--tRNA ligase C
412	1	SwissProt_HUMAN$	2::Q96MN2|NALP4_HUMAN	16	113341	1	1	1	1	0.04	NACHT, LRR and PYD
413	1	SwissProt_HUMAN$	2::Q14573|ITPR3_HUMAN	16	303912	1	1	1	1	0.02	Inositol 1,4,5-trispho:
414	1	SwissProt_HUMAN$	2::Q15645|PCH2_HUMAN	16	48520	1	1	1	1	0.10	Pachytene checkpoint
415	1	SwissProt_HUMAN$	2::P62917|RL8_HUMAN	16	28007	1	1	1	1	0.18	60S ribosomal proteir
416	1	SwissProt_HUMAN$	2::Q9GZU5|NYX_HUMAN	16	51967	1	1	1	1	0.10	Nyctalopin OS=Homo
417	1	SwissProt_HUMAN$	2::O14939|PLD2_HUMAN	16	105920	1	1	1	1	0.05	Phospholipase D2 OS

↑Family	M	DB	Accession	Score	Mass	Matches	Match(sig)	Sequences	Seq(sig)	emPAI	Description
418	1	SwissProt_HUMAN$	2::Q7Z7L7|ZER1_HUMAN	16	88113	1	1	1	1	0.06	Protein zer-1 homolo
419	1	SwissProt_HUMAN$	2::Q8IYD1|ERF3B_HUMAN	16	68840	1	1	1	1	0.07	Eukaryotic peptide ch
420	1	SwissProt_HUMAN$	2::Q8IVU3|HERC6_HUMAN	16	115052	1	1	1	1	0.04	Probable E3 ubiquitin
421	1	SwissProt_HUMAN$	2::Q9Y2I8|WDR37_HUMAN	16	54632	1	1	1	1	0.09	WD repeat-containin
422	1	SwissProt_HUMAN$	2::Q9UIX4|KCNG1_HUMAN	16	57876	1	1	1	1	0.09	Potassium voltage-ga
423	1	SwissProt_HUMAN$	2::Q9NU22|MDN1_HUMAN	16	632420	1	1	1	1	0.01	Midasin OS=Homo sa
424	1	SwissProt_HUMAN$	2::Q9P2I0|CPSF2_HUMAN	15	88431	1	1	1	1	0.05	Cleavage and polyade
425	1	SwissProt_HUMAN$	2::Q49AJ0|F135B_HUMAN	15	155672	1	1	1	1	0.03	Protein FAM135B OS=
426	1	SwissProt_HUMAN$	2::Q9BXX3|AN30A_HUMAN	15	158735	1	1	1	1	0.03	Ankyrin repeat domai
427	1	SwissProt_HUMAN$	2::Q9Y6Z4|KIAS1_HUMAN	15	19398	1	1	1	1	0.27	Putative uncharacteri
428	1	SwissProt_HUMAN$	2::Q8ND61|CC020_HUMAN	15	101202	1	1	1	1	0.05	Uncharacterized prote
429	1	SwissProt_HUMAN$	2::Q6ZS17|RIPR1_HUMAN	15	132225	1	1	1	1	0.04	Rho family-interactin
430	1	SwissProt_HUMAN$	2::A8MYJ7|TTC34_HUMAN	15	60865	1	1	1	1	0.08	Tetratricopeptide rep
431	1	SwissProt_HUMAN$	2::Q96S42|NODAL_HUMAN	15	39536	1	1	1	1	0.13	Nodal homolog OS=H
432	1	SwissProt_HUMAN$	2::Q5BKX8|CAVN4_HUMAN	15	41874	1	1	1	1	0.12	Caveolae-associated
433	1	SwissProt_HUMAN$	2::Q8NDM7|CFA43_HUMAN	14	191861	1	1	1	1	0.03	Cilia- and flagella-ass
434	1	SwissProt_HUMAN$	2::Q08AI8|MB214_HUMAN	14	49534	1	1	1	1	0.10	Protein mab-21-like 4
435	1	SwissProt_HUMAN$	2::Q9H4A3|WNK1_HUMAN	14	250640	1	1	1	1	0.02	Serine/threonine-prot
436	1	SwissProt_HUMAN$	2::Q9UIF7|MUTYH_HUMAN	14	60031	1	1	1	1	0.08	Adenine DNA glycosy
437	1	SwissProt_HUMAN$	2::O75165|DJC13_HUMAN	14	254252	1	1	1	1	0.02	DnaJ homolog subfan
438	1	SwissProt_HUMAN$	2::Q14571|ITPR2_HUMAN	14	307866	2	1	2	1	0.02	Inositol 1,4,5-trispho
439	1	SwissProt_HUMAN$	2::Q9H7E9|CH033_HUMAN	14	24977	1	1	1	1	0.21	UPF0488 protein C8o
440	1	SwissProt_HUMAN$	2::Q9P2L0|WDR35_HUMAN	14	133461	1	1	1	1	0.04	WD repeat-containin
441	1	SwissProt_HUMAN$	2::Q8IYD8|FANCM_HUMAN	14	232045	1	1	1	1	0.02	Fanconi anemia grou
442	1	SwissProt_HUMAN$	2::Q9H6X5|CS044_HUMAN	14	71300	1	1	1	1	0.07	Uncharacterized prote
443	1	SwissProt_HUMAN$	2::P51530|DNA2_HUMAN	13	120337	1	1	1	1	0.04	DNA replication ATP-
444	1	SwissProt_HUMAN$	2::P49619|DGKG_HUMAN	13	89066	1	1	1	1	0.05	Diacylglycerol kinase
445	1	SwissProt_HUMAN$	2::Q5VSL9|STRP1_HUMAN	13	95515	1	1	1	1	0.05	Striatin-interacting pr
446	1	SwissProt_HUMAN$	2::P28290|ITPI2_HUMAN	13	138300	2	1	2	1	0.03	Protein ITPRID2 OS=
447	1	SwissProt_HUMAN$	2::P60228|EIF3E_HUMAN	13	52187	1	1	1	1	0.09	Eukaryotic translatior

Export as CSV

Not what you expected? Try **the select summary**.

Mascot: http://www.matrixscience.com/
