# Supplementary material for: Circulating multimeric immune complexes contribute to immunopathology in COVID-19
Source: Nat Commun. 2022 Sep 26;13:5654. doi: 10.1038/s41467-022-32867-z (PMC9513013; doi:10.1038/s41467-022-32867-z)
Supplement: Supplementary file 3 — Source Data [file 41467_2022_32867_MOESM3_ESM.zip › Source Data/MS_29012021 (whole lane cut).zip!/QX20210214-05_Virologie(AG-Hengel)-20210129_(V2)_IC-_7-5_complete-lane_0k1v20_TRL_db_SARS-CoV-2+SP_H(F229120).pdf]

MATRIX SCIENCE MASCOT Search Results

User : Wolfgang Bildl
E-mail : wolfgang.bildl@physiologie.uni-freiburg.de
Search title : QX 20210212 (db$ SARS-CoV-2+SwissProt HUMAN$ 5 ppm 20 mmu), QX20210214-05_Virologie(AG-Hengel)-20210129_(V2)_IC-_7-5_complete-lane_0k1v20_TRL_shifted_-2.9.mgf
MS data file : D:\Xcalibur\Data\Wolfgang\Q-Exactive-HF-X_20210212\QX20210214-05_Virologie(AG-Hengel)-20210129_(V2)_IC-_7-5_complete-lane_0k1v20_TRL_shifted_-2.9.mgf
Databases : **1:** db$ 20210114 (14 sequences; 14,319 residues)
2: SwissProt_HUMAN$ 20201007 (20,389 sequences; 11,363,789 residues)
Timestamp : 15 Feb 2021 at 15:59:10 GMT

Not what you expected? Try **the select summary**.

Search parameters

Type of search : MS/MS Ion Search
Enzyme : Trypsin
Variable modifications : **Acetyl (Protein N-term)**, **Carbamidomethyl (C)**, **Gln->pyro-Glu (N-term Q)**, **Oxidation (M)**
Mass values : Monoisotopic
Protein mass : Unrestricted
Peptide mass tolerance : ± 5 ppm
Fragment mass tolerance : ± 20 mmu
Max missed cleavages : 1
Instrument type : ESI-FTICR
Number of queries : 75,974

Score distribution

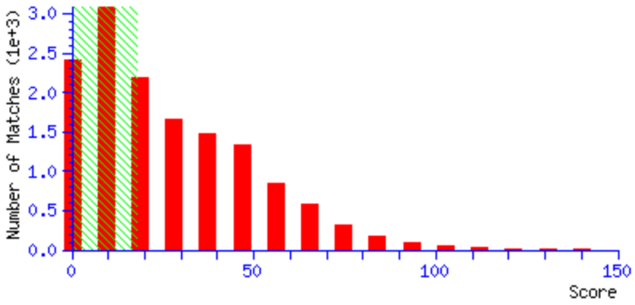

Peptide score distribution. Ions score is $-10\log(P)$, where P is the probability that the observed match is a random event.
There are **7,752** peptide matches above identity threshold and **7,980** matches above homology threshold for **75,974** queries.
On average, individual ions scores **> 18** (beyond green shading) indicate **identity or extensive homology** (p<0.05).

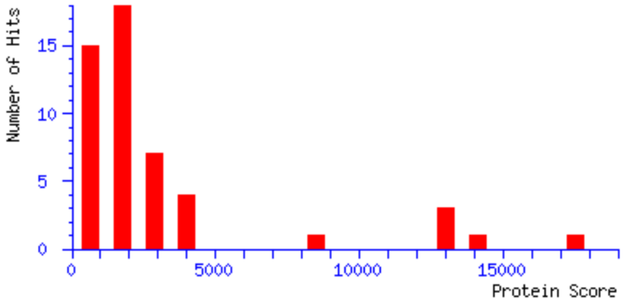

[Deprecated] Protein score distribution. Score distribution for family members in the first 50 proteins. Protein scores are derived from ions scores as a non-probabilistic basis for ranking protein families.

▼Modification statistics

Modification	Site	Above thr.
Carbamidomethyl	C	3163
Oxidation	M	1033
Gln->pyro-Glu	N-term	201
Acetyl	Protein N-term	3

▼Legend

Peptide columns and rows

Dupes	...	Expect	Rank	U	1	2	Peptide	
		**0.037**	▶**2**				**GAYSLSLR**	significant
		9	▶1				GFFLFVEGGR	top ranking
		**6.4e-005**	▶**1**				**GSSIFGLAPGK**	significant and top ranking
		**1.3e-006**	▶**1**		■		**SSGTSYPDVLK**	peptide is found in all proteins in family member 1
		**6.2e-007**	▶**1**			■	**VCNYVSWIK**	peptide is found in some but not all proteins in family member 2
		**6.4e-005**	▶**1**	**U**			**GSSIFGLAPGK**	unique
▶**2**		**5.7e-005**	▶**1**				**LNTLETEEWFFK**	peptide has two duplicates
		0.18	▶*1*				*LNTLETEEWFFK*	duplicate peptide

Right-facing triangle (▶) in the Dupes or Rank column indicates content that can be expanded by clicking on it. Down-facing triangle (▼) indicates the content is expanded and can be collapsed. For more details about particular columns, see **results format help**.

Protein quantitation ratios

	Score	...	114/113	115/113
**CFAH_HUMAN**	37559		*0.962*	**1.129**
**FHR2_HUMAN**	1330		**0.859**	1.128

When quantitation method is Reporter (e.g. iTRAQ) or Multiplex (e.g. IPTL), protein ratios are displayed when a family is expanded. Ratios in *italic* indicate that the peptide log-ratios do not appear to come from a normal distribution. **Bold** indicates that if you can assume peptide ratios are normally distributed, the protein ratio is significantly different from 1.0 (at significance level 0.05).

Note that lack of bold or italic can also mean that significance or normality testing has not been performed (for example, if protein ratio type does not support it).

Protein Family Summary

Significance threshold p<	0.05	Max. number of families	AUTO
Ions score or expect cut-off	0.5	Dendrograms cut at	0
Show Percolator scores	☐		
Preferred taxonomy	All entries		

▼Sensitivity and FDR (reversed protein sequences)

			Target	Decoy	FDR		
PSMs	above	homology	7980	245	3.07%	Adjust to	1%

Decoy results are available in **the decoy report**.

Protein hits (530 proteins)

▶Filters: (none)

Export as CSV

↑Family	M	DB	Accession	Score	Mass	Matches	Match(sig)	Sequences	Seq(sig)	emPAI	Description
1	1	SwissProt_HUMAN$	2::P02768|ALBU_HUMAN	17501	69321	656	615	77	75	431.32	Albumin OS=Homo sa
1	2	SwissProt_HUMAN$	2::P02769|ALBU_BOVIN	687	69248	53	47	12	9	0.85	Albumin OS=Bos taur
2	1	SwissProt_HUMAN$	2::P01024|CO3_HUMAN	13617	187030	576	532	147	141	102.68	Complement C3 OS=I
3	1	SwissProt_HUMAN$	2::P0DOX5|IGG1_HUMAN	13236	49298	677	636	30	30	25.00	Immunoglobulin gami
3	2	SwissProt_HUMAN$	2::P01860|IGHG3_HUMAN	7687	41260	419	377	22	22	20.86	Immunoglobulin heav
3	3	SwissProt_HUMAN$	2::P01859|IGHG2_HUMAN	5799	35878	357	316	22	21	33.76	Immunoglobulin heav
3	4	SwissProt_HUMAN$	2::P01861|IGHG4_HUMAN	4510	35918	261	232	21	20	19.36	Immunoglobulin heav
4	1	SwissProt_HUMAN$	2::P04114|APOB_HUMAN	13151	515283	509	476	300	284	18.80	Apolipoprotein B-100
5	1	SwissProt_HUMAN$	2::P01834|IGKC_HUMAN	12678	11758	430	401	13	13	166.35	Immunoglobulin kapp
5	2	SwissProt_HUMAN$	2::P0DOX7|IGK_HUMAN	12378	23364	389	372	17	17	54.99	Immunoglobulin kapp
5	3	SwissProt_HUMAN$	2::P01597|KV139_HUMAN	521	12729	16	16	5	5	5.16	Immunoglobulin kapp
5	4	SwissProt_HUMAN$	2::A0A075B6S5|KV127_HUMAN	474	12703	12	12	4	4	3.34	Immunoglobulin kapp
5	5	SwissProt_HUMAN$	2::A0A0C4DH73|KV112_HUMAN	418	12637	13	13	4	4	3.34	Immunoglobulin kapp
5	6	SwissProt_HUMAN$	2::A0A0C4DH67|KV108_HUMAN	384	12529	10	10	3	3	2.03	Immunoglobulin kapp
5	7	SwissProt_HUMAN$	2::P01599|KV117_HUMAN	249	12770	11	11	5	5	5.16	Immunoglobulin kapp
5	8	SwissProt_HUMAN$	2::P01602|KV105_HUMAN	206	12773	8	8	3	3	1.98	Immunoglobulin kapp
5	9	SwissProt_HUMAN$	2::A0A0A0MT36|KVD21_HUMAN	159	12332	8	8	3	3	2.09	Immunoglobulin kapp
5	10	SwissProt_HUMAN$	2::A0A0B4J2D9|KVD13_HUMAN	143	12561	7	6	3	3	2.03	Immunoglobulin kapp
5	11	SwissProt_HUMAN$	2::A0A0C4DH24|KV621_HUMAN	142	12422	6	6	3	3	2.06	Immunoglobulin kapp
5	12	SwissProt_HUMAN$	2::A0A075B6S9|KV137_HUMAN	120	12680	8	7	3	2	1.08	Probable non-function
5	13	SwissProt_HUMAN$	2::P04430|KV116_HUMAN	103	12610	5	5	2	2	1.08	Immunoglobulin kapp
6	1	SwissProt_HUMAN$	2::P0C0L5|CO4B_HUMAN	8248	192631	305	284	106	100	15.18	Complement C4-B OS
6	2	SwissProt_HUMAN$	2::P0C0L4|CO4A_HUMAN	8104	192664	303	282	105	99	14.37	Complement C4-A OS

↑Family	M	DB	Accession	Score	Mass	Matches	Match(sig)	Sequences	Seq(sig)	emPAI	Description
7	1	SwissProt_HUMAN$	2::P08603|CFAH_HUMAN	4493	139005	185	168	79	76	18.45	Complement factor H
7	2	SwissProt_HUMAN$	2::Q03591|FHR1_HUMAN	1070	37626	35	31	18	17	11.25	Complement factor H-
7	3	SwissProt_HUMAN$	2::P36980|FHR2_HUMAN	298	30631	13	11	8	7	1.93	Complement factor H-
7	4	SwissProt_HUMAN$	2::Q9BXR6|FHR5_HUMAN	295	64377	11	11	10	10	1.08	Complement factor H-
7	5	SwissProt_HUMAN$	2::Q02985|FHR3_HUMAN	192	37299	7	7	4	4	0.66	Complement factor H-
8	1	SwissProt_HUMAN$	2::P01023|A2MG_HUMAN	4088	163188	179	163	83	79	14.82	Alpha-2-macroglobuli
8	2	SwissProt_HUMAN$	2::P20742|PZP_HUMAN	446	163760	18	16	10	9	0.34	Pregnancy zone prote
9	1	SwissProt_HUMAN$	2::P01031|CO5_HUMAN	3749	188186	142	136	87	83	10.83	Complement C5 OS=H
10	1	SwissProt_HUMAN$	2::P02751|FINC_HUMAN	3609	272150	139	129	84	80	3.56	Fibronectin OS=Homo
11	1	SwissProt_HUMAN$	2::P01871|IGHM_HUMAN	3332	49409	133	126	34	33	40.63	Immunoglobulin heav
11	2	SwissProt_HUMAN$	2::P0DOX6|IGM_HUMAN	2770	63446	104	97	27	25	5.93	Immunoglobulin mu h
11	3	SwissProt_HUMAN$	2::P01814|HV270_HUMAN	250	13252	8	8	5	5	10.73	Immunoglobulin heav
11	4	SwissProt_HUMAN$	2::A0A0C4DH43|HV70D_HUMAN	248	13304	10	10	6	6	15.30	Immunoglobulin heav
11	5	SwissProt_HUMAN$	2::A0A0B4J1V2|HV226_HUMAN	133	13174	5	5	4	4	4.81	Immunoglobulin heav
12	1	SwissProt_HUMAN$	2::P01876|IGHA1_HUMAN	3134	37631	130	122	24	24	32.37	Immunoglobulin heav
12	2	SwissProt_HUMAN$	2::P0DOX2|IGA2_HUMAN	1468	48903	80	71	21	21	7.35	Immunoglobulin alpha
12	3	SwissProt_HUMAN$	2::P01764|HV323_HUMAN	602	12574	26	25	7	7	39.45	Immunoglobulin heav
12	4	SwissProt_HUMAN$	2::P0DP02|HVC33_HUMAN	597	12980	30	28	6	6	24.00	Immunoglobulin heav
12	5	SwissProt_HUMAN$	2::P01768|HV330_HUMAN	593	12938	28	26	7	7	34.75	Immunoglobulin heav
12	6	SwissProt_HUMAN$	2::P01782|HV309_HUMAN	520	12936	23	21	5	5	11.23	Immunoglobulin heav
12	7	SwissProt_HUMAN$	2::P01767|HV353_HUMAN	488	12761	24	23	5	5	11.76	Immunoglobulin heav
12	8	SwissProt_HUMAN$	2::P01780|HV307_HUMAN	459	12934	27	23	8	7	16.48	Immunoglobulin heav
12	9	SwissProt_HUMAN$	2::A0A0B4J2H0|HV69D_HUMAN	436	12652	16	15	5	5	17.82	Immunoglobulin heav
12	10	SwissProt_HUMAN$	2::P01763|HV348_HUMAN	422	12804	22	18	5	5	7.87	Immunoglobulin heav
12	11	SwissProt_HUMAN$	2::A0A0B4J1V1|HV321_HUMAN	420	12831	22	19	5	5	7.71	Immunoglobulin heav
12	12	SwissProt_HUMAN$	2::P01742|HV169_HUMAN	389	12651	18	16	7	7	26.16	Immunoglobulin heav
12	13	SwissProt_HUMAN$	2::A0A0B4J1X5|HV374_HUMAN	374	12831	23	22	5	5	7.71	Immunoglobulin heav
12	14	SwissProt_HUMAN$	2::P23083|HV102_HUMAN	329	13076	12	11	7	7	16.07	Immunoglobulin heav
12	15	SwissProt_HUMAN$	2::A0A0C4DH32|HV320_HUMAN	293	12665	19	17	4	4	5.26	Immunoglobulin heav
12	16	SwissProt_HUMAN$	2::A0A0C4DH31|HV118_HUMAN	281	12812	10	9	5	5	5.16	Immunoglobulin heav
12	17	SwissProt_HUMAN$	2::P01743|HV146_HUMAN	259	12924	11	11	4	4	3.23	Immunoglobulin heav
12	18	SwissProt_HUMAN$	2::P01766|HV313_HUMAN	256	12498	14	12	3	3	3.39	Immunoglobulin heav
12	19	SwissProt_HUMAN$	2::A0A0C4DH29|HV103_HUMAN	217	12999	11	11	4	4	4.98	Immunoglobulin heav
12	20	SwissProt_HUMAN$	2::A0A0B4J1X8|HV343_HUMAN	209	13068	14	12	3	3	3.13	Immunoglobulin heav
12	21	SwissProt_HUMAN$	2::A0A0C4DH33|HV124_HUMAN	204	12816	5	5	4	4	3.28	Immunoglobulin heav
12	22	SwissProt_HUMAN$	2::P0DP01|HV108_HUMAN	176	12983	9	9	3	3	1.92	Immunoglobulin heav
12	23	SwissProt_HUMAN$	2::A0A075B6Q5|HV364_HUMAN	160	12882	7	6	4	3	3.23	Immunoglobulin heav
12	24	SwissProt_HUMAN$	2::A0A0C4DH36|HV338_HUMAN	116	12750	4	4	2	2	1.98	Probable non-function
12	25	SwissProt_HUMAN$	2::P0DTE1|HV383_HUMAN	42	12663	3	3	2	2	2.01	Probable non-function
12	26	SwissProt_HUMAN$	2::A0A0J9YX35|HV64D_HUMAN	41	12814	2	2	2	2	1.07	Immunoglobulin heav

↑Family	M	DB	Accession	Score	Mass	Matches	Match(sig)	Sequences	Seq(sig)	emPAI	Description
13	1	SwissProt_HUMAN$	2::P01009|A1AT_HUMAN	3066	46707	160	139	37	36	114.39	Alpha-1-antitrypsin O
14	1	SwissProt_HUMAN$	2::P02787|TRFE_HUMAN	2800	77014	118	114	59	57	53.25	Serotransferrin OS=H
15	1	SwissProt_HUMAN$	2::P0DOY3|IGLC3_HUMAN	2663	11259	93	85	11	9	61.08	Immunoglobulin lamb
15	2	SwissProt_HUMAN$	2::P0DOX8|IGL1_HUMAN	2460	22816	89	82	13	12	13.39	Immunoglobulin lamb
15	3	SwissProt_HUMAN$	2::A0M8Q6|IGLC7_HUMAN	1432	11247	50	48	6	6	16.99	Immunoglobulin lamb
15	4	SwissProt_HUMAN$	2::P01706|LV211_HUMAN	73	12636	6	5	4	4	5.26	Immunoglobulin lamb
15	5	SwissProt_HUMAN$	2::P01709|LV208_HUMAN	70	12374	6	5	3	3	2.09	Immunoglobulin lamb
16	1	SwissProt_HUMAN$	2::P00450|CERU_HUMAN	2442	122128	95	92	50	49	9.27	Ceruloplasmin OS=Hc
17	1	SwissProt_HUMAN$	2::P00747|PLMN_HUMAN	2339	90510	108	99	56	53	23.37	Plasminogen OS=Hon
18	1	SwissProt_HUMAN$	2::P04003|C4BPA_HUMAN	2232	66989	82	76	32	31	10.82	C4b-binding protein a
19	1	SwissProt_HUMAN$	2::P00738|HPT_HUMAN	2079	45177	101	94	29	28	33.89	Haptoglobin OS=Hom
19	2	SwissProt_HUMAN$	2::P00739|HPTR_HUMAN	1353	39005	54	50	24	23	28.63	Haptoglobin-related p
20	1	SwissProt_HUMAN$	2::P04275|VWF_HUMAN	1732	309058	84	76	64	59	1.63	von Willebrand factor
21	1	SwissProt_HUMAN$	2::P10643|CO7_HUMAN	1600	93457	55	51	38	36	7.40	Complement compone
22	1	SwissProt_HUMAN$	2::P01011|AACT_HUMAN	1585	47621	56	54	22	22	15.06	Alpha-1-antichymotry
23	1	SwissProt_HUMAN$	2::P19827|ITIH1_HUMAN	1572	101326	62	55	33	28	4.91	Inter-alpha-trypsin inl
24	1	SwissProt_HUMAN$	2::P09871|C1S_HUMAN	1528	76635	58	55	32	32	10.13	Complement C1s subc
24	2	SwissProt_HUMAN$	2::P48740|MASP1_HUMAN	132	79195	7	6	7	6	0.43	Mannan-binding lectin
25	1	SwissProt_HUMAN$	2::P00751|CFAB_HUMAN	1486	85479	68	58	44	36	6.35	Complement factor B
26	1	SwissProt_HUMAN$	2::P13671|CO6_HUMAN	1465	104718	54	52	36	35	4.10	Complement compone
27	1	SwissProt_HUMAN$	2::P02774|VTDB_HUMAN	1418	52883	54	52	31	30	21.87	Vitamin D-binding pro
28	1	SwissProt_HUMAN$	2::P02649|APOE_HUMAN	1382	36132	41	38	23	23	37.34	Apolipoprotein E OS=
29	1	SwissProt_HUMAN$	2::P19823|ITIH2_HUMAN	1375	106397	58	51	34	31	3.96	Inter-alpha-trypsin inl
30	1	SwissProt_HUMAN$	2::Q14624|ITIH4_HUMAN	1326	103293	52	51	35	35	5.26	Inter-alpha-trypsin inl
31	1	SwissProt_HUMAN$	2::P07357|CO8A_HUMAN	1320	65121	49	47	29	28	9.20	Complement compone
32	1	SwissProt_HUMAN$	2::P00734|THRB_HUMAN	1280	69992	46	44	30	28	8.94	Prothrombin OS=Hom
33	1	SwissProt_HUMAN$	2::P07358|CO8B_HUMAN	1274	67003	50	48	31	29	8.56	Complement compone
34	1	SwissProt_HUMAN$	2::P02647|APOA1_HUMAN	1258	30759	59	54	32	29	113.94	Apolipoprotein A-I OS
35	1	SwissProt_HUMAN$	2::P02748|CO9_HUMAN	1228	63133	40	37	28	26	6.55	Complement compone
36	1	SwissProt_HUMAN$	2::P00736|C1R_HUMAN	1148	80067	50	47	32	32	8.46	Complement C1r subc
37	1	SwissProt_HUMAN$	2::P06396|GELS_HUMAN	1037	85644	42	40	29	29	4.25	Gelsolin OS=Homo sa
38	1	SwissProt_HUMAN$	2::P02749|APOH_HUMAN	1010	38273	42	37	22	21	14.01	Beta-2-glycoprotein 1
39	1	SwissProt_HUMAN$	2::P07225|PROS_HUMAN	947	75074	42	39	27	25	4.85	Vitamin K-dependent
40	1	SwissProt_HUMAN$	2::P01019|ANGT_HUMAN	932	53121	28	28	13	13	3.95	Angiotensinogen OS=
41	1	SwissProt_HUMAN$	2::P01008|ANT3_HUMAN	885	52569	38	34	20	19	6.89	Antithrombin-III OS=
42	1	SwissProt_HUMAN$	2::P05155|IC1_HUMAN	804	55119	51	46	22	20	9.10	Plasma protease C1 ir
43	1	SwissProt_HUMAN$	2::P01619|KV320_HUMAN	771	12549	25	22	4	3	2.03	Immunoglobulin kapp
43	2	SwissProt_HUMAN$	2::A0A0C4DH25|KVD20_HUMAN	673	12507	19	16	3	2	1.10	Immunoglobulin kapp
44	1	SwissProt_HUMAN$	2::Q9Y6R7|FCGBP_HUMAN	761	571639	33	32	33	32	0.30	IgGFc-binding protein
45	1	SwissProt_HUMAN$	2::P02790|HEMO_HUMAN	711	51643	40	36	24	24	10.84	Hemopexin OS=Homo

↑Family	M	DB	Accession	Score	Mass	Matches	Match(sig)	Sequences	Seq(sig)	emPAI	Description
46	1	SwissProt_HUMAN$	2::P02675|FIBB_HUMAN	676	55892	29	28	21	20	6.59	Fibrinogen beta chain
47	1	SwissProt_HUMAN$	2::P36955|PEDF_HUMAN	670	46283	29	27	20	18	6.69	Pigment epithelium-de
48	1	SwissProt_HUMAN$	2::O43866|CD5L_HUMAN	654	38063	32	30	18	17	7.21	CD5 antigen-like OS=
49	1	SwissProt_HUMAN$	2::P07996|TSP1_HUMAN	647	129300	30	30	24	24	1.50	Thrombospondin-1 OS
50	1	SwissProt_HUMAN$	2::P0DJI8|SAA1_HUMAN	636	13524	17	17	8	8	14.59	Serum amyloid A-1 pr
50	2	SwissProt_HUMAN$	2::P0DJI9|SAA2_HUMAN	461	13519	14	14	7	7	10.06	Serum amyloid A-2 pr
51	1	SwissProt_HUMAN$	2::P06727|APOA4_HUMAN	625	45344	31	28	24	22	8.85	Apolipoprotein A-IV O
52	1	SwissProt_HUMAN$	2::P18428|LBP_HUMAN	618	53350	22	21	14	14	3.12	Lipopolysaccharide-bi
53	1	SwissProt_HUMAN$	2::P13645|K1C10_HUMAN	617	58792	26	24	20	19	4.41	Keratin, type I cytosk
53	2	SwissProt_HUMAN$	2::P35527|K1C9_HUMAN	430	62027	14	11	12	10	1.14	Keratin, type I cytosk
53	3	SwissProt_HUMAN$	2::P08727|K1C19_HUMAN	95	44079	4	4	4	4	0.53	Keratin, type I cytosk
54	1	SwissProt_HUMAN$	2::P05156|CFAI_HUMAN	613	65707	21	20	17	16	2.16	Complement factor I (
55	1	SwissProt_HUMAN$	2::P01042|KNG1_HUMAN	564	71912	24	24	17	17	2.27	Kininogen-1 OS=Hom
56	1	SwissProt_HUMAN$	2::P02741|CRP_HUMAN	559	25023	28	27	11	11	8.54	C-reactive protein OS
57	1	SwissProt_HUMAN$	2::P07360|CO8G_HUMAN	516	22264	20	17	12	10	11.45	Complement compone
58	1	SwissProt_HUMAN$	2::P06331|HV434_HUMAN	512	13806	15	15	5	5	4.42	Immunoglobulin heav
58	2	SwissProt_HUMAN$	2::P0DP08|HVD82_HUMAN	492	13008	14	14	5	5	4.98	Immunoglobulin heav
58	3	SwissProt_HUMAN$	2::A0A075B6R2|HV404_HUMAN	382	12840	13	11	5	4	3.23	Immunoglobulin heav
58	4	SwissProt_HUMAN$	2::A0A087WSY4|HV432_HUMAN	336	13017	10	8	4	2	1.04	Immunoglobulin heav
59	1	SwissProt_HUMAN$	2::P02763|A1AG1_HUMAN	510	23497	20	20	10	10	7.96	Alpha-1-acid glycopro
59	2	SwissProt_HUMAN$	2::P19652|A1AG2_HUMAN	274	23588	15	14	9	9	6.28	Alpha-1-acid glycopro
60	1	SwissProt_HUMAN$	2::P04004|VTNC_HUMAN	509	54271	23	22	13	13	3.03	Vitronectin OS=Homo
61	1	SwissProt_HUMAN$	2::P27169|PON1_HUMAN	501	39706	16	14	12	10	2.28	Serum paraoxonase/a
62	1	SwissProt_HUMAN$	2::P08519|APOA_HUMAN	495	500995	24	19	19	15	0.16	Apolipoprotein(a) OS=
63	1	SwissProt_HUMAN$	2::P02679|FIBG_HUMAN	492	51479	30	26	21	19	5.86	Fibrinogen gamma ch
64	1	SwissProt_HUMAN$	2::P02743|SAMP_HUMAN	487	25371	25	23	12	12	10.04	Serum amyloid P-com
65	1	SwissProt_HUMAN$	2::P02766|TTHY_HUMAN	474	15877	14	14	8	8	9.49	Transthyretin OS=Hor
66	1	SwissProt_HUMAN$	2::O75636|FCN3_HUMAN	472	32882	20	16	13	9	3.19	Ficolin-3 OS=Homo sa
67	1	SwissProt_HUMAN$	2::P02753|RET4_HUMAN	459	22995	22	19	11	10	16.43	Retinol-binding protei
68	1	SwissProt_HUMAN$	2::P04264|K2C1_HUMAN	441	65999	22	15	17	13	1.54	Keratin, type II cytosl
68	2	SwissProt_HUMAN$	2::P35908|K22E_HUMAN	291	65393	18	15	17	14	1.75	Keratin, type II cytosl
68	3	SwissProt_HUMAN$	2::P13647|K2C5_HUMAN	99	62340	6	6	5	5	0.46	Keratin, type II cytosl
68	4	SwissProt_HUMAN$	2::O95678|K2C75_HUMAN	92	59524	5	5	4	4	0.37	Keratin, type II cytosl
69	1	SwissProt_HUMAN$	2::P23142|FBLN1_HUMAN	434	77162	24	20	18	16	2.02	Fibulin-1 OS=Homo sa
70	1	SwissProt_HUMAN$	2::P04217|A1BG_HUMAN	433	54220	19	17	11	11	1.85	Alpha-1B-glycoprotein
71	1	SwissProt_HUMAN$	2::A0A0C4DH38|HV551_HUMAN	431	12666	18	16	7	6	8.03	Immunoglobulin heav
71	2	SwissProt_HUMAN$	2::A0A0J9YXX1|HV5X1_HUMAN	253	12764	15	14	5	5	7.87	Immunoglobulin heav
72	1	SwissProt_HUMAN$	2::Q08380|LG3BP_HUMAN	427	65289	18	17	14	13	1.57	Galectin-3-binding pro
73	1	SwissProt_HUMAN$	2::P02747|C1QC_HUMAN	408	25757	38	36	8	7	4.16	Complement C1q sub
74	1	SwissProt_HUMAN$	2::P12259|FA5_HUMAN	408	251546	20	19	18	17	0.40	Coagulation factor V (

↑Family	M	DB	Accession	Score	Mass	Matches	Match(sig)	Sequences	Seq(sig)	emPAI	Description
75	1	SwissProt_HUMAN$	2::P08697|A2AP_HUMAN	400	54531	17	16	12	12	2.09	Alpha-2-antiplasmin C
76	1	SwissProt_HUMAN$	2::P06681|CO2_HUMAN	393	83214	18	16	16	14	1.48	Complement C2 OS=H
77	1	SwissProt_HUMAN$	2::P10909|CLUS_HUMAN	393	52461	18	17	12	11	1.69	Clusterin OS=Homo s
78	1	SwissProt_HUMAN$	2::P02746|C1QB_HUMAN	367	26704	22	18	10	8	5.90	Complement C1q sub
79	1	SwissProt_HUMAN$	2::P01833|PIGR_HUMAN	366	83232	16	15	16	15	1.35	Polymeric immunoglol
80	1	SwissProt_HUMAN$	2::P05090|APOD_HUMAN	365	21262	14	13	10	9	6.23	Apolipoprotein D OS=
81	1	SwissProt_HUMAN$	2::P02760|AMBP_HUMAN	360	38974	16	16	13	13	4.44	Protein AMBP OS=Hor
82	1	SwissProt_HUMAN$	2::P20851|C4BPB_HUMAN	351	28338	15	15	8	8	5.22	C4b-binding protein b
83	1	SwissProt_HUMAN$	2::A0A0B4J1V0|HV315_HUMAN	346	12917	20	20	9	9	51.84	Immunoglobulin heav
83	2	SwissProt_HUMAN$	2::A0A0B4J1Y9|HV372_HUMAN	257	13194	17	16	9	9	46.92	Immunoglobulin heav
83	3	SwissProt_HUMAN$	2::A0A0B4J1V6|HV373_HUMAN	233	12849	10	10	7	7	11.49	Immunoglobulin heav
83	4	SwissProt_HUMAN$	2::A0A0A0MS15|HV349_HUMAN	219	13048	7	7	6	6	7.40	Immunoglobulin heav
84	1	SwissProt_HUMAN$	2::P02765|FETUA_HUMAN	337	39316	13	12	7	7	1.94	Alpha-2-HS-glycoprot
85	1	SwissProt_HUMAN$	2::P68871|HBB_HUMAN	334	15988	14	13	7	7	9.32	Hemoglobin subunit b
85	2	SwissProt_HUMAN$	2::P02042|HBD_HUMAN	153	16045	7	7	5	5	3.26	Hemoglobin subunit d
86	1	SwissProt_HUMAN$	2::Q06033|ITIH3_HUMAN	327	99787	20	18	18	17	1.35	Inter-alpha-trypsin inl
87	1	SwissProt_HUMAN$	2::P01591|IGJ_HUMAN	317	18087	14	13	8	8	9.23	Immunoglobulin J cha
88	1	SwissProt_HUMAN$	2::A0A075B6K5|LV39_HUMAN	311	12324	8	7	3	3	2.09	Immunoglobulin lamb
88	2	SwissProt_HUMAN$	2::P80748|LV321_HUMAN	300	12438	9	8	3	3	2.06	Immunoglobulin lamb
89	1	SwissProt_HUMAN$	2::P01593|KVD33_HUMAN	310	12840	6	6	2	2	1.06	Immunoglobulin kapp
90	1	SwissProt_HUMAN$	2::P60709|ACTB_HUMAN	300	41710	16	12	12	9	2.10	Actin, cytoplasmic 1 C
91	1	SwissProt_HUMAN$	2::P02652|APOA2_HUMAN	295	11168	12	11	6	6	16.99	Apolipoprotein A-II OS
92	1	SwissProt_HUMAN$	2::P02671|FIBA_HUMAN	286	94914	24	20	20	17	1.58	Fibrinogen alpha chair
93	1	SwissProt_HUMAN$	2::P03952|KLKB1_HUMAN	284	71323	15	14	14	14	1.53	Plasma kallikrein OS=
94	1	SwissProt_HUMAN$	2::P05160|F13B_HUMAN	280	75461	18	12	13	10	0.87	Coagulation factor XII
95	1	SwissProt_HUMAN$	2::P01714|LV319_HUMAN	272	12035	9	8	5	5	5.91	Immunoglobulin lamb
96	1	SwissProt_HUMAN$	2::P04196|HRG_HUMAN	269	59541	17	14	15	13	1.81	Histidine-rich glycopro
97	1	SwissProt_HUMAN$	2::A0A075B6R9|KVD24_HUMAN	265	13071	9	6	4	3	1.90	Probable non-function
98	1	SwissProt_HUMAN$	2::O14791|APOL1_HUMAN	264	43947	14	12	12	11	2.26	Apolipoprotein L1 OS=
99	1	SwissProt_HUMAN$	2::P00761|TRYP_PIG	260	24394	6	6	5	5	1.61	Trypsin OS=Sus scrof
100	1	SwissProt_HUMAN$	2::P04433|KV311_HUMAN	257	12567	10	9	3	3	2.03	Immunoglobulin kapp
101	1	SwissProt_HUMAN$	2::A0A0C4DH34|HV428_HUMAN	255	13116	10	9	4	4	7.40	Immunoglobulin heav
102	1	SwissProt_HUMAN$	2::P00740|FA9_HUMAN	252	51745	12	12	12	12	1.99	Coagulation factor IX
103	1	SwissProt_HUMAN$	2::Q9UK55|ZPI_HUMAN	242	50674	13	12	11	10	1.79	Protein Z-dependent p
104	1	SwissProt_HUMAN$	2::Q15848|ADIPO_HUMAN	239	26397	7	7	4	4	1.04	Adiponectin OS=Hom
105	1	SwissProt_HUMAN$	2::P06312|KV401_HUMAN	235	13372	15	15	6	6	7.11	Immunoglobulin kapp
106	1	SwissProt_HUMAN$	2::P13796|PLSL_HUMAN	224	70244	10	8	8	6	0.50	Plastin-2 OS=Homo s
107	1	SwissProt_HUMAN$	2::P00748|FA12_HUMAN	222	67748	7	7	6	6	0.52	Coagulation factor XII
108	1	SwissProt_HUMAN$	2::A0A087WSY6|KVD15_HUMAN	165	12526	5	5	2	2	1.10	Immunoglobulin kapp
108	2	SwissProt_HUMAN$	2::A0A075B6H7|KV37_HUMAN	96	12775	6	5	2	2	1.07	Probable non-function

↑Family	M	DB	Accession	Score	Mass	Matches	Match(sig)	Sequences	Seq(sig)	emPAI	Description
109	1	SwissProt_HUMAN$	2::P05546|HEP2_HUMAN	215	57034	17	13	13	9	1.29	Heparin cofactor 2 OS
110	1	SwissProt_HUMAN$	2::A0A075B6P5|KV228_HUMAN	211	12948	6	6	3	3	1.92	Immunoglobulin kapp
110	2	SwissProt_HUMAN$	2::A0A087WW87|KV240_HUMAN	201	13302	7	7	4	4	4.72	Immunoglobulin kapp
110	3	SwissProt_HUMAN$	2::A0A075B6S2|KVD29_HUMAN	116	13135	5	3	3	2	1.03	Immunoglobulin kapp
110	4	SwissProt_HUMAN$	2::P06310|KV230_HUMAN	107	13177	6	5	4	3	1.87	Immunoglobulin kapp
111	1	SwissProt_HUMAN$	2::P02750|A2GL_HUMAN	207	38154	10	9	9	8	1.69	Leucine-rich alpha-2-ç
112	1	SwissProt_HUMAN$	2::P11226|MBL2_HUMAN	206	26127	9	8	9	8	3.22	Mannose-binding prot
113	1	SwissProt_HUMAN$	2::P25311|ZA2G_HUMAN	204	34237	8	7	8	7	1.62	Zinc-alpha-2-glycoprc
114	1	SwissProt_HUMAN$	2::P29622|KAIN_HUMAN	204	48511	12	11	10	9	1.40	Kallistatin OS=Homo :
115	1	SwissProt_HUMAN$	2::P05452|TETN_HUMAN	203	22522	5	5	4	4	1.30	Tetranectin OS=Homc
116	1	SwissProt_HUMAN$	2::P0DOX3|IGD_HUMAN	201	56189	9	8	8	7	0.80	Immunoglobulin delta
117	1	SwissProt_HUMAN$	2::P02654|APOC1_HUMAN	199	9326	10	9	5	5	18.35	Apolipoprotein C-I OS
118	1	SwissProt_HUMAN$	2::P00742|FA10_HUMAN	190	54697	9	8	9	8	1.00	Coagulation factor X (
119	1	SwissProt_HUMAN$	2::P26927|HGFL_HUMAN	186	80268	8	8	8	8	0.60	Hepatocyte growth fa
120	1	SwissProt_HUMAN$	2::P02745|C1QA_HUMAN	176	26000	10	9	6	6	1.96	Complement C1q sub
121	1	SwissProt_HUMAN$	2::Q12805|FBLN3_HUMAN	174	54604	5	4	5	4	0.41	EGF-containing fibulin
122	1	SwissProt_HUMAN$	2::Q15582|BGH3_HUMAN	174	74634	11	10	11	10	0.89	Transforming growth
123	1	SwissProt_HUMAN$	2::P22792|CPN2_HUMAN	171	60518	7	7	7	7	0.73	Carboxypeptidase N s
124	1	SwissProt_HUMAN$	2::P35542|SAA4_HUMAN	169	14737	10	10	6	6	11.48	Serum amyloid A-4 pr
125	1	SwissProt_HUMAN$	2::Q96PD5|PGRP2_HUMAN	168	62178	8	7	6	5	0.46	N-acetylmuramoyl-L-a
126	1	SwissProt_HUMAN$	2::P02656|APOC3_HUMAN	165	10846	5	4	2	2	1.34	Apolipoprotein C-III C
127	1	SwissProt_HUMAN$	2::P12111|CO6A3_HUMAN	165	343457	9	9	9	9	0.13	Collagen alpha-3(VI) (
128	1	SwissProt_HUMAN$	2::P69905|HBA_HUMAN	163	15248	10	10	6	6	5.29	Hemoglobin subunit a
129	1	SwissProt_HUMAN$	2::P27918|PROP_HUMAN	161	51242	6	6	6	6	0.74	Properdin OS=Homo s
130	1	SwissProt_HUMAN$	2::O00391|QSOX1_HUMAN	157	82526	6	5	6	5	0.33	Sulfhydryl oxidase 1 (
131	1	SwissProt_HUMAN$	2::P08571|CD14_HUMAN	157	40051	7	5	6	5	0.80	Monocyte differentiati
132	1	SwissProt_HUMAN$	2::Q08830|FGL1_HUMAN	157	36357	7	6	6	5	0.91	Fibrinogen-like proteir
133	1	SwissProt_HUMAN$	2::A0A075B6K4|LV310_HUMAN	155	12433	7	6	5	4	3.45	Immunoglobulin lamb
133	2	SwissProt_HUMAN$	2::P01718|LV327_HUMAN	84	12157	4	4	3	3	2.13	Immunoglobulin lamb
133	3	SwissProt_HUMAN$	2::P01717|LV325_HUMAN	80	12004	6	6	3	3	3.70	Immunoglobulin lamb
133	4	SwissProt_HUMAN$	2::A0A075B6K0|LV316_HUMAN	42	12458	3	3	2	2	1.11	Immunoglobulin lamb
134	1	SwissProt_HUMAN$	2::A0A0B4J1U7|HV601_HUMAN	155	13473	13	11	4	4	2.99	Immunoglobulin heav
135	1	SwissProt_HUMAN$	2::P26022|PTX3_HUMAN	154	41949	5	4	4	3	0.40	Pentraxin-related prot
136	1	SwissProt_HUMAN$	2::P02655|APOC2_HUMAN	154	11277	8	7	6	6	10.63	Apolipoprotein C-II OS
137	1	SwissProt_HUMAN$	2::P43652|AFAM_HUMAN	143	69024	8	7	8	7	0.62	Afamin OS=Homo sap
138	1	SwissProt_HUMAN$	2::P35858|ALS_HUMAN	142	65994	12	10	10	9	0.91	Insulin-like growth fac
139	1	SwissProt_HUMAN$	2::P36222|CH3L1_HUMAN	137	42598	7	5	6	4	0.56	Chitinase-3-like prote
140	1	SwissProt_HUMAN$	2::P24821|TENA_HUMAN	137	240700	9	6	8	6	0.13	Tenascin OS=Homo s
141	1	SwissProt_HUMAN$	2::Q96IY4|CBPB2_HUMAN	137	48393	8	6	7	6	0.80	Carboxypeptidase B2
142	1	SwissProt_HUMAN$	2::O95445|APOM_HUMAN	135	21239	8	8	6	6	3.70	Apolipoprotein M OS=

↑Family	M	DB	Accession	Score	Mass	Matches	Match(sig)	Sequences	Seq(sig)	emPAI	Description
143	1	SwissProt_HUMAN$	2::P01700|LV147_HUMAN	134	12276	7	6	4	4	3.51	Immunoglobulin lamb
143	2	SwissProt_HUMAN$	2::P01699|LV144_HUMAN	99	12194	4	3	3	3	2.13	Immunoglobulin lamb
144	1	SwissProt_HUMAN$	2::P40197|GPV_HUMAN	128	60921	3	3	3	3	0.26	Platelet glycoprotein \
145	1	SwissProt_HUMAN$	2::P03951|FA11_HUMAN	123	70064	12	11	11	11	1.10	Coagulation factor XI
146	1	SwissProt_HUMAN$	2::P12109|CO6A1_HUMAN	122	108462	3	3	2	2	0.09	Collagen alpha-1(VI)
147	1	SwissProt_HUMAN$	2::P18206|VINC_HUMAN	119	123722	6	6	6	6	0.26	Vinculin OS=Homo sa
148	1	SwissProt_HUMAN$	2::A0A075B6H9|LV469_HUMAN	115	12765	4	4	3	3	1.98	Immunoglobulin lamb
149	1	SwissProt_HUMAN$	2::P01715|LV301_HUMAN	113	12288	4	3	3	2	1.12	Immunoglobulin lamb
150	1	SwissProt_HUMAN$	2::P07195|LDHB_HUMAN	111	36615	4	4	4	4	0.67	L-lactate dehydrogena
150	2	SwissProt_HUMAN$	2::P00338|LDHA_HUMAN	90	36665	3	3	3	3	0.47	L-lactate dehydrogena
151	1	SwissProt_HUMAN$	2::A0A0A0MS14|HV145_HUMAN	108	13500	4	4	3	3	2.95	Immunoglobulin heav
152	1	SwissProt_HUMAN$	2::P00746|CFAD_HUMAN	107	27016	8	6	6	6	1.84	Complement factor D
153	1	SwissProt_HUMAN$	2::A0A0G2JMI3|HV692_HUMAN	107	12862	4	4	2	2	1.95	Immunoglobulin heav
154	1	SwissProt_HUMAN$	2::P05154|IPSP_HUMAN	106	45646	5	5	4	4	0.68	Plasma serine proteas
155	1	SwissProt_HUMAN$	2::P04070|PROC_HUMAN	102	52037	4	4	4	4	0.44	Vitamin K-dependent
156	1	SwissProt_HUMAN$	2::P01721|LV657_HUMAN	100	12558	2	2	1	1	0.45	Immunoglobulin lamb
157	1	SwissProt_HUMAN$	2::P04040|CATA_HUMAN	96	59719	6	5	6	5	0.49	Catalase OS=Homo sa
158	1	SwissProt_HUMAN$	2::P11021|BIP_HUMAN	92	72288	5	5	5	5	0.39	Endoplasmic reticulun
159	1	SwissProt_HUMAN$	2::P02776|PLF4_HUMAN	87	10838	5	5	4	4	2.58	Platelet factor 4 OS=I
160	1	SwissProt_HUMAN$	2::P02775|CXCL7_HUMAN	87	13885	4	3	3	3	1.73	Platelet basic protein
161	1	SwissProt_HUMAN$	2::P07900|HS90A_HUMAN	84	84607	2	2	2	2	0.12	Heat shock protein HS
162	1	SwissProt_HUMAN$	2::P01701|LV151_HUMAN	84	12241	4	4	3	3	2.13	Immunoglobulin lamb
163	1	SwissProt_HUMAN$	2::A0A0B4J1Y8|LV949_HUMAN	83	13016	4	4	3	3	1.92	Immunoglobulin lamb
164	1	SwissProt_HUMAN$	2::P04075|ALDOA_HUMAN	82	39395	4	4	4	4	0.61	Fructose-bisphosphate
165	1	SwissProt_HUMAN$	2::P15814|IGLL1_HUMAN	81	22949	2	2	1	1	0.23	Immunoglobulin lamb
166	1	SwissProt_HUMAN$	2::P15169|CBPN_HUMAN	79	52253	3	3	3	3	0.31	Carboxypeptidase N c
167	1	SwissProt_HUMAN$	2::P61626|LYSC_HUMAN	78	16526	7	4	6	3	2.09	Lysozyme C OS=Hom
168	1	SwissProt_HUMAN$	2::Q5QNW6|H2B2F_HUMAN	77	13912	4	4	4	4	2.82	Histone H2B type 2-F
169	1	SwissProt_HUMAN$	2::P25705|ATPA_HUMAN	77	59714	4	4	2	2	0.17	ATP synthase subunit
170	1	SwissProt_HUMAN$	2::P62979|RS27A_HUMAN	77	17953	3	3	3	3	1.18	Ubiquitin-40S ribosom
171	1	SwissProt_HUMAN$	2::P05109|S10A8_HUMAN	76	10828	2	2	2	2	1.34	Protein S100-A8 OS=
172	1	SwissProt_HUMAN$	2::Q15485|FCN2_HUMAN	76	33980	4	2	3	2	0.32	Ficolin-2 OS=Homo sa
173	1	SwissProt_HUMAN$	2::Q04756|HGFA_HUMAN	74	70636	3	3	3	3	0.22	Hepatocyte growth fa
174	1	SwissProt_HUMAN$	2::P51884|LUM_HUMAN	74	38405	6	5	5	5	0.85	Lumican OS=Homo sa
175	1	SwissProt_HUMAN$	2::A0A075B6I1|LV460_HUMAN	73	12979	2	2	2	2	1.04	Immunoglobulin lamb
176	1	SwissProt_HUMAN$	2::P01817|HV205_HUMAN	70	13223	3	2	1	1	0.42	Immunoglobulin heav
177	1	SwissProt_HUMAN$	2::P29401|TKT_HUMAN	69	67835	2	2	2	2	0.15	Transketolase OS=Ho
178	1	SwissProt_HUMAN$	2::A0A087WSZ0|KVD08_HUMAN	68	12828	1	1	1	1	0.43	Immunoglobulin kapp
179	1	SwissProt_HUMAN$	2::A0A0B4J1U3|LV136_HUMAN	66	12470	1	1	1	1	0.45	Immunoglobulin lamb
180	1	SwissProt_HUMAN$	2::A0A075B6I0|LV861_HUMAN	65	12806	3	3	3	3	1.98	Immunoglobulin lamb

↑Family	M	DB	Accession	Score	Mass	Matches	Match(sig)	Sequences	Seq(sig)	emPAI	Description
181	1	SwissProt_HUMAN$	2::Q9UHG3|PCYOX_HUMAN	64	56604	4	3	4	3	0.28	Prenylcysteine oxidas
182	1	SwissProt_HUMAN$	2::A0A075B6I9|LV746_HUMAN	62	12460	3	3	3	3	2.06	Immunoglobulin lamb
182	2	SwissProt_HUMAN$	2::P04211|LV743_HUMAN	53	12443	3	3	3	3	2.06	Immunoglobulin lamb
183	1	SwissProt_HUMAN$	2::P14780|MMP9_HUMAN	61	78408	3	3	3	3	0.20	Matrix metalloproteina
184	1	SwissProt_HUMAN$	2::P38646|GRP75_HUMAN	59	73635	1	1	1	1	0.07	Stress-70 protein, mit
185	1	SwissProt_HUMAN$	2::Q14520|HABP2_HUMAN	59	62630	3	3	3	3	0.25	Hyaluronan-binding p
186	1	SwissProt_HUMAN$	2::P04278|SHBG_HUMAN	58	43752	2	2	2	2	0.24	Sex hormone-binding
187	1	SwissProt_HUMAN$	2::P22352|GPX3_HUMAN	57	25537	5	5	5	5	1.51	Glutathione peroxidas
188	1	SwissProt_HUMAN$	2::P98160|PGBM_HUMAN	57	468532	2	1	2	1	0.01	Basement membrane-
189	1	SwissProt_HUMAN$	2::P59665|DEF1_HUMAN	54	10194	4	4	4	4	5.10	Neutrophil defensin 1
190	1	SwissProt_HUMAN$	2::P49747|COMP_HUMAN	54	82808	3	3	3	3	0.19	Cartilage oligomeric n
191	1	SwissProt_HUMAN$	2::P62805|H4_HUMAN	53	11360	3	2	3	2	1.27	Histone H4 OS=Homo
192	1	SwissProt_HUMAN$	2::P55058|PLTP_HUMAN	53	54705	4	3	3	2	0.30	Phospholipid transfer
193	1	SwissProt_HUMAN$	2::P0DOX4|IGE_HUMAN	52	60285	1	1	1	1	0.08	Immunoglobulin epsil
194	1	SwissProt_HUMAN$	2::P39019|RS19_HUMAN	51	16051	1	1	1	1	0.34	40S ribosomal protein
195	1	SwissProt_HUMAN$	2::P08185|CBG_HUMAN	51	45112	5	4	5	4	0.52	Corticosteroid-binding
196	1	SwissProt_HUMAN$	2::P08709|FA7_HUMAN	49	51560	1	1	1	1	0.10	Coagulation factor VII
197	1	SwissProt_HUMAN$	2::Q92954|PRG4_HUMAN	48	150968	4	3	3	2	0.06	Proteoglycan 4 OS=H
198	1	SwissProt_HUMAN$	2::P00488|F13A_HUMAN	48	83215	2	2	2	2	0.12	Coagulation factor XII
199	1	SwissProt_HUMAN$	2::P01703|LV140_HUMAN	48	12294	3	3	2	2	1.12	Immunoglobulin lamb
200	1	SwissProt_HUMAN$	2::P01705|LV223_HUMAN	46	11886	2	2	1	1	1.18	Immunoglobulin lamb
201	1	SwissProt_HUMAN$	2::Q5T0N5|FBP1L_HUMAN	44	70021	2	2	1	1	0.07	Formin-binding protei
202	1	SwissProt_HUMAN$	2::Q4LDE5|SVEP1_HUMAN	44	389912	1	1	1	1	0.01	Sushi, von Willebrand
203	1	SwissProt_HUMAN$	2::Q8NHQ9|DDX55_HUMAN	44	68503	2	2	1	1	0.07	ATP-dependent RNA h
204	1	SwissProt_HUMAN$	2::P62937|PPIA_HUMAN	43	18001	1	1	1	1	0.30	Peptidyl-prolyl cis-tra
205	1	SwissProt_HUMAN$	2::Q86SS6|SYT9_HUMAN	42	56153	1	1	1	1	0.09	Synaptotagmin-9 OS=
206	1	SwissProt_HUMAN$	2::A0A0C4DH35|HV335_HUMAN	41	12802	2	2	2	2	1.07	Probable non-function
207	1	SwissProt_HUMAN$	2::Q12934|BFSP1_HUMAN	41	74499	2	2	1	1	0.07	Filensin OS=Homo sa
208	1	SwissProt_HUMAN$	2::Q13093|PAFA_HUMAN	41	50045	1	1	1	1	0.10	Platelet-activating fac
209	1	SwissProt_HUMAN$	2::O00187|MASP2_HUMAN	40	75654	5	3	5	3	0.21	Mannan-binding lectin
210	1	SwissProt_HUMAN$	2::P49913|CAMP_HUMAN	40	19289	1	1	1	1	0.27	Cathelicidin antimicrol
211	1	SwissProt_HUMAN$	2::P08637|FCG3A_HUMAN	40	29071	2	2	2	2	0.38	Low affinity immunog
212	1	SwissProt_HUMAN$	2::P49720|PSB3_HUMAN	40	22933	1	1	1	1	0.23	Proteasome subunit b
213	1	SwissProt_HUMAN$	2::Q6VUC0|AP2E_HUMAN	40	46183	1	1	1	1	0.11	Transcription factor A
214	1	SwissProt_HUMAN$	2::H7C350|CC188_HUMAN	39	43520	1	1	1	1	0.11	Coiled-coil domain-co
215	1	SwissProt_HUMAN$	2::P05451|REG1A_HUMAN	39	18719	2	2	2	2	0.65	Lithostathine-1-alpha
216	1	SwissProt_HUMAN$	2::Q9Y279|VSIG4_HUMAN	38	43959	1	1	1	1	0.11	V-set and immunoglol
217	1	SwissProt_HUMAN$	2::Q06141|REG3A_HUMAN	38	19382	2	1	2	1	0.27	Regenerating islet-de
218	1	SwissProt_HUMAN$	2::A0A075B6J9|LV218_HUMAN	38	12404	2	2	2	2	1.11	Immunoglobulin lamb
219	1	SwissProt_HUMAN$	2::P55259|GP2_HUMAN	38	59442	2	1	2	1	0.08	Pancreatic secretory g

↑Family	M	DB	Accession	Score	Mass	Matches	Match(sig)	Sequences	Seq(sig)	emPAI	Description
220	1	SwissProt_HUMAN$	2::P01034|CYTC_HUMAN	37	15789	2	2	2	2	0.81	Cystatin-C OS=Homo
221	1	SwissProt_HUMAN$	2::P02792|FRIL_HUMAN	37	20007	2	2	2	2	0.60	Ferritin light chain OS
222	1	SwissProt_HUMAN$	2::O94887|FARP2_HUMAN	37	119813	1	1	1	1	0.04	FERM, ARHGEF and pl
223	1	SwissProt_HUMAN$	2::P25788|PSA3_HUMAN	36	28415	2	1	2	1	0.18	Proteasome subunit a
224	1	SwissProt_HUMAN$	2::P0C0S8|H2A1_HUMAN	36	14083	2	2	2	2	0.94	Histone H2A type 1 O
225	1	SwissProt_HUMAN$	2::P55056|APOC4_HUMAN	36	14543	2	2	2	2	0.90	Apolipoprotein C-IV O
226	1	SwissProt_HUMAN$	2::Q15113|PCOC1_HUMAN	36	47942	2	2	2	2	0.22	Procollagen C-endope
227	1	SwissProt_HUMAN$	2::Q8N5V2|NGEF_HUMAN	35	82445	1	1	1	1	0.06	Ephexin-1 OS=Homo
228	1	SwissProt_HUMAN$	2::P22891|PROZ_HUMAN	35	44715	2	2	2	2	0.23	Vitamin K-dependent
229	1	SwissProt_HUMAN$	2::Q5TF21|SOGA3_HUMAN	35	103136	2	2	1	1	0.05	Protein SOGA3 OS=H
230	1	SwissProt_HUMAN$	2::P05164|PERM_HUMAN	34	83815	3	3	3	3	0.18	Myeloperoxidase OS=
231	1	SwissProt_HUMAN$	2::P07988|PSPB_HUMAN	34	42089	2	2	2	2	0.25	Pulmonary surfactant-
232	1	SwissProt_HUMAN$	2::Q8N6F7|GCSAM_HUMAN	34	20992	1	1	1	1	0.25	Germinal center-asso
233	1	SwissProt_HUMAN$	2::Q96DD0|LRC39_HUMAN	34	38768	1	1	1	1	0.13	Leucine-rich repeat-c
234	1	SwissProt_HUMAN$	2::Q16610|ECM1_HUMAN	34	60635	2	2	2	2	0.17	Extracellular matrix p
235	1	SwissProt_HUMAN$	2::P03950|ANGI_HUMAN	33	16539	1	1	1	1	0.33	Angiogenin OS=Homo
236	1	SwissProt_HUMAN$	2::Q9UGM5|FETUB_HUMAN	33	42028	2	1	2	1	0.12	Fetuin-B OS=Homo s
237	1	SwissProt_HUMAN$	2::Q68DL7|CR063_HUMAN	33	77181	2	2	1	1	0.06	Uncharacterized prote
238	1	SwissProt_HUMAN$	2::P06733|ENOA_HUMAN	33	47139	1	1	1	1	0.11	Alpha-enolase OS=Ho
239	1	SwissProt_HUMAN$	2::P61769|B2MG_HUMAN	33	13706	1	1	1	1	0.41	Beta-2-microglobulin
240	1	SwissProt_HUMAN$	2::O75460|ERN1_HUMAN	32	109666	1	1	1	1	0.04	Serine/threonine-prot
241	1	SwissProt_HUMAN$	2::O43586|PPIP1_HUMAN	32	47562	1	1	1	1	0.10	Proline-serine-threoni
242	1	SwissProt_HUMAN$	2::P36957|ODO2_HUMAN	32	48724	1	1	1	1	0.10	Dihydrolipoyllysine-re
243	1	SwissProt_HUMAN$	2::Q92496|FHR4_HUMAN	31	65308	1	1	1	1	0.08	Complement factor H-
244	1	SwissProt_HUMAN$	2::Q9NWX5|ASB6_HUMAN	31	47106	1	1	1	1	0.11	Ankyrin repeat and S
245	1	SwissProt_HUMAN$	2::Q05513|KPCZ_HUMAN	31	67617	1	1	1	1	0.07	Protein kinase C zeta
246	1	SwissProt_HUMAN$	2::Q9UK96|FBX10_HUMAN	31	105129	3	1	2	1	0.05	F-box only protein 10
247	1	SwissProt_HUMAN$	2::Q9ULM0|PKHH1_HUMAN	31	151137	2	1	2	1	0.03	Pleckstrin homology d
248	1	SwissProt_HUMAN$	2::Q8TCB0|IFI44_HUMAN	31	50459	1	1	1	1	0.10	Interferon-induced pr
249	1	SwissProt_HUMAN$	2::P49721|PSB2_HUMAN	31	22822	1	1	1	1	0.23	Proteasome subunit b
250	1	SwissProt_HUMAN$	2::P25789|PSA4_HUMAN	30	29465	1	1	1	1	0.17	Proteasome subunit a
251	1	SwissProt_HUMAN$	2::Q3V6T2|GRDN_HUMAN	30	215909	1	1	1	1	0.02	Girdin OS=Homo sapi
252	1	SwissProt_HUMAN$	2::Q9H329|E41LB_HUMAN	30	99650	1	1	1	1	0.05	Band 4.1-like protein
253	1	SwissProt_HUMAN$	2::Q7Z2W7|TRPM8_HUMAN	30	127603	2	2	2	2	0.08	Transient receptor po
254	1	SwissProt_HUMAN$	2::Q5VT06|CE350_HUMAN	30	350716	1	1	1	1	0.01	Centrosome-associate
255	1	SwissProt_HUMAN$	2::Q3L8U1|CHD9_HUMAN	30	325819	2	1	2	1	0.01	Chromodomain-helica
256	1	SwissProt_HUMAN$	2::Q00610|CLH1_HUMAN	30	191493	2	2	1	1	0.03	Clathrin heavy chain 1
257	1	SwissProt_HUMAN$	2::Q92530|PSMF1_HUMAN	30	29798	1	1	1	1	0.17	Proteasome inhibitor I
258	1	SwissProt_HUMAN$	2::Q5XXA6|ANO1_HUMAN	30	114005	1	1	1	1	0.04	Anoctamin-1 OS=Hon
259	1	SwissProt_HUMAN$	2::P10599|THIO_HUMAN	30	11730	1	1	1	1	0.48	Thioredoxin OS=Hom

↑Family	M	DB	Accession	Score	Mass	Matches	Match(sig)	Sequences	Seq(sig)	emPAI	Description
260	1	SwissProt_HUMAN$	2::Q9Y2W3|S45A1_HUMAN	30	84487	1	1	1	1	0.06	Proton-associated sug
261	1	SwissProt_HUMAN$	2::P11309|PIM1_HUMAN	29	35663	2	2	2	2	0.30	Serine/threonine-prot
262	1	SwissProt_HUMAN$	2::Q9HAW4|CLSPN_HUMAN	29	151002	2	1	2	1	0.03	Claspin OS=Homo sap
263	1	SwissProt_HUMAN$	2::Q9HAW7|UD17_HUMAN	29	59779	2	2	2	2	0.17	UDP-glucuronosyltran
264	1	SwissProt_HUMAN$	2::P0DTE7|AMY1B_HUMAN	29	57731	1	1	1	1	0.09	Alpha-amylase 1B OS
265	1	SwissProt_HUMAN$	2::P36575|ARRC_HUMAN	29	42751	1	1	1	1	0.12	Arrestin-C OS=Homo
266	1	SwissProt_HUMAN$	2::P38935|SMBP2_HUMAN	29	109082	1	1	1	1	0.04	DNA-binding protein S
267	1	SwissProt_HUMAN$	2::Q969V4|TEKT1_HUMAN	28	48253	1	1	1	1	0.10	Tektin-1 OS=Homo sa
268	1	SwissProt_HUMAN$	2::O14786|NRP1_HUMAN	28	103069	1	1	1	1	0.05	Neuropilin-1 OS=Hom
269	1	SwissProt_HUMAN$	2::P18065|IBP2_HUMAN	28	34791	1	1	1	1	0.15	Insulin-like growth fac
270	1	SwissProt_HUMAN$	2::P56199|ITA1_HUMAN	28	130765	1	1	1	1	0.04	Integrin alpha-1 OS=I
271	1	SwissProt_HUMAN$	2::Q92738|US6NL_HUMAN	28	94046	1	1	1	1	0.05	USP6 N-terminal-like
272	1	SwissProt_HUMAN$	2::Q8N6G1|CA211_HUMAN	28	10497	1	1	1	1	0.55	Putative uncharacteriz
273	1	SwissProt_HUMAN$	2::Q6DHV5|C2D2B_HUMAN	28	166681	1	1	1	1	0.03	Protein CC2D2B OS=I
274	1	SwissProt_HUMAN$	2::Q9BTV5|FSD1_HUMAN	27	55784	2	1	1	1	0.09	Fibronectin type III ar
275	1	SwissProt_HUMAN$	2::Q9Y566|SHAN1_HUMAN	27	224821	1	1	1	1	0.02	SH3 and multiple ank
276	1	SwissProt_HUMAN$	2::Q8N163|CCAR2_HUMAN	27	102838	2	2	2	2	0.10	Cell cycle and apoptos
277	1	SwissProt_HUMAN$	2::P13804|ETFA_HUMAN	27	35058	1	1	1	1	0.14	Electron transfer flavo
278	1	SwissProt_HUMAN$	2::Q8N4P6|LRC71_HUMAN	27	61787	1	1	1	1	0.08	Leucine-rich repeat-co
279	1	SwissProt_HUMAN$	2::Q7Z5M8|AB12B_HUMAN	27	40750	1	1	1	1	0.12	Protein ABHD12B OS=
280	1	SwissProt_HUMAN$	2::P12107|COBA1_HUMAN	27	180954	1	1	1	1	0.03	Collagen alpha-1(XI)
281	1	SwissProt_HUMAN$	2::Q92570|NR4A3_HUMAN	27	68186	1	1	1	1	0.07	Nuclear receptor subfa
282	1	SwissProt_HUMAN$	2::P34096|RNAS4_HUMAN	26	16829	1	1	1	1	0.32	Ribonuclease 4 OS=H
283	1	SwissProt_HUMAN$	2::Q3SXZ3|ZN718_HUMAN	26	55324	3	3	1	1	0.09	Zinc finger protein 71
284	1	SwissProt_HUMAN$	2::Q5SYB0|FRPD1_HUMAN	26	173328	1	1	1	1	0.03	FERM and PDZ domai
285	1	SwissProt_HUMAN$	2::P21399|ACOC_HUMAN	26	98337	1	1	1	1	0.05	Cytoplasmic aconitate
286	1	SwissProt_HUMAN$	2::Q3MIN7|RGL3_HUMAN	26	78031	1	1	1	1	0.06	Ral guanine nucleotide
287	1	SwissProt_HUMAN$	2::P30531|SC6A1_HUMAN	26	67029	1	1	1	1	0.07	Sodium- and chloride-
288	1	SwissProt_HUMAN$	2::P49643|PRI2_HUMAN	25	58769	2	1	1	1	0.08	DNA primase large su
289	1	SwissProt_HUMAN$	2::Q9HCG8|CWC22_HUMAN	25	105402	1	1	1	1	0.05	Pre-mRNA-splicing fac
290	1	SwissProt_HUMAN$	2::Q66K66|TM198_HUMAN	25	39449	1	1	1	1	0.13	Transmembrane prote
291	1	SwissProt_HUMAN$	2::Q6NXT2|H3C_HUMAN	25	15204	1	1	1	1	0.36	Histone H3.3C OS=Ho
292	1	SwissProt_HUMAN$	2::Q7Z6Z7|HUWE1_HUMAN	25	481589	1	1	1	1	0.01	E3 ubiquitin-protein li
293	1	SwissProt_HUMAN$	2::Q9NP80|PLPL8_HUMAN	25	88421	1	1	1	1	0.05	Calcium-independent
294	1	SwissProt_HUMAN$	2::Q9Y484|WIPI4_HUMAN	25	39842	1	1	1	1	0.13	WD repeat domain ph
295	1	SwissProt_HUMAN$	2::P15121|ALDR_HUMAN	24	35830	1	1	1	1	0.14	Aldo-keto reductase fa
296	1	SwissProt_HUMAN$	2::Q12840|KIF5A_HUMAN	24	117306	2	2	1	1	0.04	Kinesin heavy chain is
297	1	SwissProt_HUMAN$	2::P00966|ASSY_HUMAN	24	46501	1	1	1	1	0.11	Argininosuccinate syn
298	1	SwissProt_HUMAN$	2::Q86VH2|KIF27_HUMAN	24	160184	2	1	1	1	0.03	Kinesin-like protein KI
299	1	SwissProt_HUMAN$	2::O94855|SC24D_HUMAN	24	112936	1	1	1	1	0.04	Protein transport prot

↑Family	M	DB	Accession	Score	Mass	Matches	Match(sig)	Sequences	Seq(sig)	emPAI	Description
300	1	SwissProt_HUMAN$	2::Q6ZR52|ZN493_HUMAN	24	75293	1	1	1	1	0.06	Zinc finger protein 49
301	1	SwissProt_HUMAN$	2::O43303|CP110_HUMAN	24	113353	1	1	1	1	0.04	Centriolar coiled-coil p
302	1	SwissProt_HUMAN$	2::Q6EMB2|TTLL5_HUMAN	24	143488	1	1	1	1	0.03	Tubulin polyglutamyla
303	1	SwissProt_HUMAN$	2::A0A075B6J1|LV537_HUMAN	24	13268	1	1	1	1	0.42	Immunoglobulin lamb
304	1	SwissProt_HUMAN$	2::O14526|FCHO1_HUMAN	24	96801	1	1	1	1	0.05	F-BAR domain only pr
305	1	SwissProt_HUMAN$	2::Q15063|POSTN_HUMAN	24	93255	1	1	1	1	0.05	Periostin OS=Homo sa
306	1	SwissProt_HUMAN$	2::P49910|ZN165_HUMAN	24	55735	1	1	1	1	0.09	Zinc finger protein 16
307	1	SwissProt_HUMAN$	2::P07942|LAMB1_HUMAN	24	197909	2	1	2	1	0.02	Laminin subunit beta-
308	1	SwissProt_HUMAN$	2::Q0WX57|U17LO_HUMAN	24	59673	1	1	1	1	0.08	Ubiquitin carboxyl-ter
309	1	SwissProt_HUMAN$	2::Q5T2T1|MPP7_HUMAN	24	65483	1	1	1	1	0.07	MAGUK p55 subfamily
310	1	SwissProt_HUMAN$	2::O15091|MRPP3_HUMAN	24	67272	1	1	1	1	0.07	Mitochondrial ribonucl
311	1	SwissProt_HUMAN$	2::Q13332|PTPRS_HUMAN	23	216905	2	1	2	1	0.02	Receptor-type tyrosin
312	1	SwissProt_HUMAN$	2::Q58A45|PAN3_HUMAN	23	95552	2	1	1	1	0.05	PAN2-PAN3 deadenyla
313	1	SwissProt_HUMAN$	2::A0A0A0MT89|KJ01_HUMAN	23	1393	1	1	1	1	14.58	Immunoglobulin kapp
314	1	SwissProt_HUMAN$	2::P06702|S10A9_HUMAN	23	13234	1	1	1	1	0.42	Protein S100-A9 OS=
315	1	SwissProt_HUMAN$	2::Q8WZ74|CTTB2_HUMAN	23	180938	2	1	2	1	0.03	Cortactin-binding prot
316	1	SwissProt_HUMAN$	2::Q16629|SRSF7_HUMAN	22	27350	1	1	1	1	0.19	Serine/arginine-rich s
317	1	SwissProt_HUMAN$	2::P60174|TPIS_HUMAN	22	26653	2	2	2	2	0.42	Triosephosphate isom
318	1	SwissProt_HUMAN$	2::Q5JPB2|ZN831_HUMAN	22	177839	1	1	1	1	0.03	Zinc finger protein 83
319	1	SwissProt_HUMAN$	2::P07478|TRY2_HUMAN	22	26471	1	1	1	1	0.19	Trypsin-2 OS=Homo s
320	1	SwissProt_HUMAN$	2::P02788|TRFL_HUMAN	22	78132	1	1	1	1	0.06	Lactotransferrin OS=H
321	1	SwissProt_HUMAN$	2::Q2TBA0|KLH40_HUMAN	22	69213	1	1	1	1	0.07	Kelch-like protein 40 (
322	1	SwissProt_HUMAN$	2::Q8TCY9|URGCP_HUMAN	22	104921	1	1	1	1	0.05	Up-regulator of cell pr
323	1	SwissProt_HUMAN$	2::Q9H254|SPTN4_HUMAN	22	288808	1	1	1	1	0.02	Spectrin beta chain, n
324	1	SwissProt_HUMAN$	2::Q13459|MYO9B_HUMAN	22	243249	1	1	1	1	0.02	Unconventional myosi
325	1	SwissProt_HUMAN$	2::Q96E66|LRC51_HUMAN	22	22193	1	1	1	1	0.24	Leucine-rich repeat-co
326	1	SwissProt_HUMAN$	2::Q6P499|NPAL3_HUMAN	22	44712	2	1	1	1	0.11	NIPA-like protein 3 OS
327	1	SwissProt_HUMAN$	2::P38117|ETFB_HUMAN	22	27826	1	1	1	1	0.18	Electron transfer flavo
328	1	SwissProt_HUMAN$	2::Q9UH65|SWP70_HUMAN	22	68954	1	1	1	1	0.07	Switch-associated pro
329	1	SwissProt_HUMAN$	2::Q03468|ERCC6_HUMAN	22	168311	1	1	1	1	0.03	DNA excision repair pr
330	1	SwissProt_HUMAN$	2::P51572|BAP31_HUMAN	22	27974	1	1	1	1	0.18	B-cell receptor-associa
331	1	SwissProt_HUMAN$	2::Q68E01|INT3_HUMAN	21	117994	1	1	1	1	0.04	Integrator complex su
332	1	SwissProt_HUMAN$	2::P48039|MTR1A_HUMAN	21	39349	1	1	1	1	0.13	Melatonin receptor typ
333	1	SwissProt_HUMAN$	2::P07237|PDIA1_HUMAN	21	57081	1	1	1	1	0.09	Protein disulfide-isom
334	1	SwissProt_HUMAN$	2::Q8N6Q8|MET25_HUMAN	21	68169	1	1	1	1	0.07	Methyltransferase-like
335	1	SwissProt_HUMAN$	2::O76031|CLPX_HUMAN	21	69181	1	1	1	1	0.07	ATP-dependent Clp pr
336	1	SwissProt_HUMAN$	2::Q9Y6K9|NEMO_HUMAN	21	48167	1	1	1	1	0.10	NF-kappa-B essential
337	1	SwissProt_HUMAN$	2::Q8NEF9|SRFB1_HUMAN	21	48604	1	1	1	1	0.10	Serum response facto
338	1	SwissProt_HUMAN$	2::Q7Z418|KCNKI_HUMAN	21	43643	1	1	1	1	0.11	Potassium channel su
339	1	SwissProt_HUMAN$	2::Q6ZTR6|ZNFDT_HUMAN	21	17225	1	1	1	1	0.31	Putative uncharacteriz

↑Family	M	DB	Accession	Score	Mass	Matches	Match(sig)	Sequences	Seq(sig)	emPAI	Description
340	1	SwissProt_HUMAN$	2::Q00537|CDK17_HUMAN	21	59545	1	1	1	1	0.08	Cyclin-dependent kina
341	1	SwissProt_HUMAN$	2::P42331|RHG25_HUMAN	21	73389	1	1	1	1	0.07	Rho GTPase-activating
342	1	SwissProt_HUMAN$	2::Q6WRI0|IGS10_HUMAN	21	290655	1	1	1	1	0.02	Immunoglobulin supe
343	1	SwissProt_HUMAN$	2::P35228|NOS2_HUMAN	21	131033	1	1	1	1	0.04	Nitric oxide synthase,
344	1	SwissProt_HUMAN$	2::Q9NP81|SYSM_HUMAN	21	58246	1	1	1	1	0.08	Serine--tRNA ligase, r
345	1	SwissProt_HUMAN$	2::Q92616|GCN1_HUMAN	21	292572	1	1	1	1	0.02	eIF-2-alpha kinase ac
346	1	SwissProt_HUMAN$	2::O43526|KCNQ2_HUMAN	20	95787	1	1	1	1	0.05	Potassium voltage-gat
347	1	SwissProt_HUMAN$	2::Q9UKT9|IKZF3_HUMAN	20	57986	2	1	2	1	0.09	Zinc finger protein Aic
348	1	SwissProt_HUMAN$	2::O43897|TLL1_HUMAN	20	114635	1	1	1	1	0.04	Tolloid-like protein 1 (
349	1	SwissProt_HUMAN$	2::O75882|ATRN_HUMAN	20	158432	1	1	1	1	0.03	Attractin OS=Homo sa
350	1	SwissProt_HUMAN$	2::Q9P2S5|WRP73_HUMAN	20	51555	1	1	1	1	0.10	WD repeat-containing
351	1	SwissProt_HUMAN$	2::Q9Y2I7|FYV1_HUMAN	20	236986	1	1	1	1	0.02	1-phosphatidylinositol
352	1	SwissProt_HUMAN$	2::Q86WK7|AMGO3_HUMAN	20	55214	1	1	1	1	0.09	Amphoterin-induced p
353	1	SwissProt_HUMAN$	2::Q15878|CAC1E_HUMAN	20	261564	1	1	1	1	0.02	Voltage-dependent R-
354	1	SwissProt_HUMAN$	2::P82930|RT34_HUMAN	20	25634	1	1	1	1	0.20	28S ribosomal proteir
355	1	SwissProt_HUMAN$	2::Q96GD3|SCMH1_HUMAN	20	73307	1	1	1	1	0.07	Polycomb protein SCM
356	1	SwissProt_HUMAN$	2::P78559|MAP1A_HUMAN	20	305298	2	1	2	1	0.02	Microtubule-associate
357	1	SwissProt_HUMAN$	2::Q9UJX2|CDC23_HUMAN	20	68790	1	1	1	1	0.07	Cell division cycle pro
358	1	SwissProt_HUMAN$	2::Q6ZMR5|TM11A_HUMAN	20	47538	1	1	1	1	0.10	Transmembrane prote
359	1	SwissProt_HUMAN$	2::Q92887|MRP2_HUMAN	20	174096	1	1	1	1	0.03	Canalicular multispeci
360	1	SwissProt_HUMAN$	2::Q9P2W1|HOP2_HUMAN	20	24891	1	1	1	1	0.21	Homologous-pairing p
361	1	SwissProt_HUMAN$	2::Q9NYK5|RM39_HUMAN	20	38687	1	1	1	1	0.13	39S ribosomal proteir
362	1	SwissProt_HUMAN$	2::Q86XI8|ZSWM9_HUMAN	20	70029	1	1	1	1	0.07	Uncharacterized prote
363	1	SwissProt_HUMAN$	2::P17041|ZNF32_HUMAN	20	31009	1	1	1	1	0.16	Zinc finger protein 32
364	1	SwissProt_HUMAN$	2::P05543|THBG_HUMAN	20	46295	1	1	1	1	0.11	Thyroxine-binding glo
365	1	SwissProt_HUMAN$	2::Q96NL6|SCLT1_HUMAN	20	80861	1	1	1	1	0.06	Sodium channel and c
366	1	SwissProt_HUMAN$	2::Q07075|AMPE_HUMAN	20	109176	1	1	1	1	0.04	Glutamyl aminopeptid
367	1	SwissProt_HUMAN$	2::Q9BWP8|COL11_HUMAN	19	28647	1	1	1	1	0.18	Collectin-11 OS=Hom
368	1	SwissProt_HUMAN$	2::Q8TBF8|FA81A_HUMAN	19	42365	1	1	1	1	0.12	Protein FAM81A OS=H
369	1	SwissProt_HUMAN$	2::P52333|JAK3_HUMAN	19	125019	1	1	1	1	0.04	Tyrosine-protein kinas
370	1	SwissProt_HUMAN$	2::A0A075B6I4|LVX54_HUMAN	19	12387	2	2	1	1	0.45	Immunoglobulin lamb
371	1	SwissProt_HUMAN$	2::A4FU49|SH321_HUMAN	19	70476	1	1	1	1	0.07	SH3 domain-containir
372	1	SwissProt_HUMAN$	2::P01704|LV214_HUMAN	19	12589	2	1	1	1	0.45	Immunoglobulin lamb
373	1	SwissProt_HUMAN$	2::Q9Y388|RBMX2_HUMAN	19	37313	1	1	1	1	0.13	RNA-binding motif prc
374	1	SwissProt_HUMAN$	2::Q9UFH2|DYH17_HUMAN	19	508987	1	1	1	1	0.01	Dynein heavy chain 1
375	1	SwissProt_HUMAN$	2::Q92750|TAF4B_HUMAN	19	91034	2	2	2	2	0.11	Transcription initiatior
376	1	SwissProt_HUMAN$	2::Q8TBC4|UBA3_HUMAN	19	51819	1	1	1	1	0.10	NEDD8-activating enz
377	1	SwissProt_HUMAN$	2::Q9NYC9|DYH9_HUMAN	19	511551	2	1	1	1	0.01	Dynein heavy chain 9
378	1	SwissProt_HUMAN$	2::O95196|CSPG5_HUMAN	19	59979	1	1	1	1	0.08	Chondroitin sulfate pr
379	1	SwissProt_HUMAN$	2::Q6J272|F166A_HUMAN	19	36142	1	1	1	1	0.14	Protein FAM166A OS=

↑Family	M	DB	Accession	Score	Mass	Matches	Match(sig)	Sequences	Seq(sig)	emPAI	Description
380	1	SwissProt_HUMAN$	2::Q96JP2|MY15B_HUMAN	18	166983	1	1	1	1	0.03	Unconventional myosi
381	1	SwissProt_HUMAN$	2::Q5JPF3|AN36C_HUMAN	18	199626	1	1	1	1	0.02	Ankyrin repeat domai
382	1	SwissProt_HUMAN$	2::Q96DN5|TBC31_HUMAN	18	124111	2	1	1	1	0.04	TBC1 domain family n
383	1	SwissProt_HUMAN$	2::Q96P31|FCRL3_HUMAN	18	80806	1	1	1	1	0.06	Fc receptor-like protei
384	1	SwissProt_HUMAN$	2::Q5VWZ2|LYPL1_HUMAN	18	26299	1	1	1	1	0.20	Lysophospholipase-lik
385	1	SwissProt_HUMAN$	2::Q9BQS8|FYCO1_HUMAN	18	166879	1	1	1	1	0.03	FYVE and coiled-coil d
386	1	SwissProt_HUMAN$	2::P04180|LCAT_HUMAN	18	49546	1	1	1	1	0.10	Phosphatidylcholine-s
387	1	SwissProt_HUMAN$	2::Q8N1B4|VPS52_HUMAN	18	82170	1	1	1	1	0.06	Vacuolar protein sorti
388	1	SwissProt_HUMAN$	2::P13985|HRES1_HUMAN	18	24328	1	1	1	1	0.21	Putative HTLV-1-relat
389	1	SwissProt_HUMAN$	2::Q14679|TTLL4_HUMAN	18	133294	2	1	2	1	0.04	Tubulin polyglutamyla
390	1	SwissProt_HUMAN$	2::A0A087WSX0|LV545_HUMAN	18	13153	1	1	1	1	0.43	Immunoglobulin lamb
391	1	SwissProt_HUMAN$	2::Q96Q15|SMG1_HUMAN	18	410241	1	1	1	1	0.01	Serine/threonine-prot
392	1	SwissProt_HUMAN$	2::O43896|KIF1C_HUMAN	18	122870	1	1	1	1	0.04	Kinesin-like protein KI
393	1	SwissProt_HUMAN$	2::O60673|REV3L_HUMAN	18	352554	2	1	2	1	0.01	DNA polymerase zeta
394	1	SwissProt_HUMAN$	2::A9Z1Z3|FR1L4_HUMAN	18	200854	1	1	1	1	0.02	Fer-1-like protein 4 O
395	1	SwissProt_HUMAN$	2::Q9UIL4|KIF25_HUMAN	17	40661	3	1	1	1	0.12	Kinesin-like protein KI
396	1	SwissProt_HUMAN$	2::Q99996|AKAP9_HUMAN	17	452708	1	1	1	1	0.01	A-kinase anchor prote
397	1	SwissProt_HUMAN$	2::Q9BSG1|ZNF2_HUMAN	17	48676	1	1	1	1	0.10	Zinc finger protein 2 (
398	1	SwissProt_HUMAN$	2::O94972|TRI37_HUMAN	17	107837	1	1	1	1	0.04	E3 ubiquitin-protein li
399	1	SwissProt_HUMAN$	2::Q9BXX3|AN30A_HUMAN	17	158735	1	1	1	1	0.03	Ankyrin repeat domai
400	1	SwissProt_HUMAN$	2::Q96EN8|MOCOS_HUMAN	17	98058	1	1	1	1	0.05	Molybdenum cofactor
401	1	SwissProt_HUMAN$	2::Q9NU22|MDN1_HUMAN	17	632420	1	1	1	1	0.01	Midasin OS=Homo sa
402	1	SwissProt_HUMAN$	2::Q9BQI3|E2AK1_HUMAN	17	71062	1	1	1	1	0.07	Eukaryotic translation
403	1	SwissProt_HUMAN$	2::Q53RD9|FBLN7_HUMAN	17	47345	1	1	1	1	0.10	Fibulin-7 OS=Homo s
404	1	SwissProt_HUMAN$	2::Q9H5I5|PIEZ2_HUMAN	17	317858	1	1	1	1	0.02	Piezo-type mechanose
405	1	SwissProt_HUMAN$	2::Q13349|ITAD_HUMAN	17	126678	1	1	1	1	0.04	Integrin alpha-D OS=
406	1	SwissProt_HUMAN$	2::Q8IYD1|ERF3B_HUMAN	17	68840	1	1	1	1	0.07	Eukaryotic peptide ch
407	1	SwissProt_HUMAN$	2::Q9BYX4|IFIH1_HUMAN	16	116615	2	1	2	1	0.04	Interferon-induced he
408	1	SwissProt_HUMAN$	2::P55285|CADH6_HUMAN	16	88254	1	1	1	1	0.06	Cadherin-6 OS=Homo
409	1	SwissProt_HUMAN$	2::P25786|PSA1_HUMAN	16	29537	1	1	1	1	0.17	Proteasome subunit a
410	1	SwissProt_HUMAN$	2::P51512|MMP16_HUMAN	16	69477	2	1	1	1	0.07	Matrix metalloproteina
411	1	SwissProt_HUMAN$	2::O15417|TNC18_HUMAN	16	314326	1	1	1	1	0.02	Trinucleotide repeat-c
412	1	SwissProt_HUMAN$	2::Q5VXU9|SHOC1_HUMAN	16	165097	1	1	1	1	0.03	Protein shortage in ch
413	1	SwissProt_HUMAN$	2::Q8N9Q2|SR1IP_HUMAN	16	18166	1	1	1	1	0.29	Protein SREK1IP1 OS=
414	1	SwissProt_HUMAN$	2::Q9UIX4|KCNG1_HUMAN	16	57876	1	1	1	1	0.09	Potassium voltage-gat
415	1	SwissProt_HUMAN$	2::O00461|GOLI4_HUMAN	16	81831	1	1	1	1	0.06	Golgi integral membra
416	1	SwissProt_HUMAN$	2::Q9NXD2|MTMRA_HUMAN	16	88217	1	1	1	1	0.06	Myotubularin-related
417	1	SwissProt_HUMAN$	2::P62258|1433E_HUMAN	15	29155	2	1	2	1	0.17	14-3-3 protein epsilor
418	1	SwissProt_HUMAN$	2::Q15046|SYK_HUMAN	15	68005	1	1	1	1	0.07	Lysine--tRNA ligase O
419	1	SwissProt_HUMAN$	2::Q9P2D7|DYH1_HUMAN	15	487166	1	1	1	1	0.01	Dynein heavy chain 1

↑Family	M	DB	Accession	Score	Mass	Matches	Match(sig)	Sequences	Seq(sig)	emPAI	Description
420	1	SwissProt_HUMAN$	2::Q6ZS17|RIPR1_HUMAN	15	132225	1	1	1	1	0.04	Rho family-interacting
421	1	SwissProt_HUMAN$	2::Q9H4A3|WNK1_HUMAN	15	250640	1	1	1	1	0.02	Serine/threonine-prot
422	1	SwissProt_HUMAN$	2::Q96NS5|ASB16_HUMAN	15	49605	1	1	1	1	0.10	Ankyrin repeat and S(
423	1	SwissProt_HUMAN$	2::P19367|HXK1_HUMAN	15	102420	1	1	1	1	0.05	Hexokinase-1 OS=Ho
424	1	SwissProt_HUMAN$	2::Q16851|UGPA_HUMAN	14	56905	2	1	2	1	0.09	UTP--glucose-1-phosp
425	1	SwissProt_HUMAN$	2::Q8N697|S15A4_HUMAN	14	61993	2	1	2	1	0.08	Solute carrier family 1
426	1	SwissProt_HUMAN$	2::A0A1W2PPM1|CPHXL_HUMAN	14	45685	1	1	1	1	0.11	Cytoplasmic polyaden
427	1	SwissProt_HUMAN$	2::Q9NSI6|BRWD1_HUMAN	14	262772	1	1	1	1	0.02	Bromodomain and WD
428	1	SwissProt_HUMAN$	2::A6NMY6|AXA2L_HUMAN	14	38635	1	1	1	1	0.13	Putative annexin A2-li
429	1	SwissProt_HUMAN$	2::Q96RD6|PANX2_HUMAN	14	74400	1	1	1	1	0.07	Pannexin-2 OS=Homo
430	1	SwissProt_HUMAN$	2::O75602|SPAG6_HUMAN	14	55441	1	1	1	1	0.09	Sperm-associated ant
431	1	SwissProt_HUMAN$	2::Q6UB99|ANR11_HUMAN	14	297731	1	1	1	1	0.02	Ankyrin repeat domai
432	1	SwissProt_HUMAN$	2::Q6ZSZ6|TSH1_HUMAN	14	117843	1	1	1	1	0.04	Teashirt homolog 1 O:
433	1	SwissProt_HUMAN$	2::Q7L5N1|CSN6_HUMAN	14	36140	1	1	1	1	0.14	COP9 signalosome co
434	1	SwissProt_HUMAN$	2::Q9NQW1|SC31B_HUMAN	14	128615	1	1	1	1	0.04	Protein transport prot
435	1	SwissProt_HUMAN$	2::Q86VD1|MORC1_HUMAN	14	112810	1	1	1	1	0.04	MORC family CW-type
436	1	SwissProt_HUMAN$	2::Q8WW22|DNJA4_HUMAN	14	44769	1	1	1	1	0.11	DnaJ homolog subfam
437	1	SwissProt_HUMAN$	2::P35241|RADI_HUMAN	14	68521	1	1	1	1	0.07	Radixin OS=Homo sa
438	1	SwissProt_HUMAN$	2::O14827|RGRF2_HUMAN	14	140675	2	1	2	1	0.03	Ras-specific guanine r
439	1	SwissProt_HUMAN$	2::Q9UKA4|AKA11_HUMAN	14	210380	2	1	2	1	0.02	A-kinase anchor prote
440	1	SwissProt_HUMAN$	2::Q96AC6|KIFC2_HUMAN	14	90092	1	1	1	1	0.05	Kinesin-like protein KI
441	1	SwissProt_HUMAN$	2::Q9H257|CARD9_HUMAN	14	62202	1	1	1	1	0.08	Caspase recruitment (
442	1	SwissProt_HUMAN$	2::Q9P2L0|WDR35_HUMAN	14	133461	2	1	1	1	0.04	WD repeat-containing
443	1	SwissProt_HUMAN$	2::Q5VSL9|STRP1_HUMAN	13	95515	1	1	1	1	0.05	Striatin-interacting pr
444	1	SwissProt_HUMAN$	2::Q9H270|VPS11_HUMAN	13	107768	1	1	1	1	0.04	Vacuolar protein sorti
445	1	SwissProt_HUMAN$	2::Q4KWH8|PLCH1_HUMAN	13	189104	1	1	1	1	0.03	1-phosphatidylinositol
446	1	SwissProt_HUMAN$	2::Q8TAA3|PSMA8_HUMAN	13	28512	1	1	1	1	0.18	Proteasome subunit a

Export as CSV

Not what you expected? Try **the select summary**.

Mascot: http://www.matrixscience.com/
