# Supplementary material for: Circulating multimeric immune complexes contribute to immunopathology in COVID-19
Source: Nat Commun. 2022 Sep 26;13:5654. doi: 10.1038/s41467-022-32867-z (PMC9513013; doi:10.1038/s41467-022-32867-z)
Supplement: Supplementary file 3 — Source Data [file 41467_2022_32867_MOESM3_ESM.zip › Source Data/MS_29012021 (whole lane cut).zip!/QX20210214-06_Virologie(AG-Hengel)-20210129_(V1)_IC+_7-5_complete-lane_0k1v20_TRL_db_SARS-CoV-2+SP_H(F229151).pdf]

MATRIX SCIENCE MASCOT Search Results

User : Wolfgang Bildl
E-mail : wolfgang.bildl@physiologie.uni-freiburg.de
Search title : QX 20210212 (db$ SARS-CoV-2+SwissProt HUMAN$ 5 ppm 20 mmu), QX20210214-06_Virologie(AG-Hengel)-20210129_(V1)_IC+_7-5_complete-lane_0k1v20_TRL_shifted_-3.0.mgf
MS data file : D:\Xcalibur\Data\Wolfgang\Q-Exactive-HF-X_20210212\QX20210214-06_Virologie(AG-Hengel)-20210129_(V1)_IC+_7-5_complete-lane_0k1v20_TRL_shifted_-3.0.mgf
Databases : **1:** db$ 20210114 (14 sequences; 14,319 residues)
2: SwissProt_HUMAN$ 20201007 (20,389 sequences; 11,363,789 residues)
Timestamp : 16 Feb 2021 at 08:59:00 GMT

Not what you expected? Try **the select summary**.

Search parameters

**Type of search**	: MS/MS Ion Search
**Enzyme**	: Trypsin
**Variable modifications**	: **Acetyl (Protein N-term)**, **Carbamidomethyl (C)**, **Gln->pyro-Glu (N-term Q)**, **Oxidation (M)**
**Mass values**	: Monoisotopic
**Protein mass**	: Unrestricted
**Peptide mass tolerance**	: ± 5 ppm
**Fragment mass tolerance**	: ± 20 mmu
**Max missed cleavages**	: 1
**Instrument type**	: ESI-FTICR
**Number of queries**	: 73,748

Score distribution

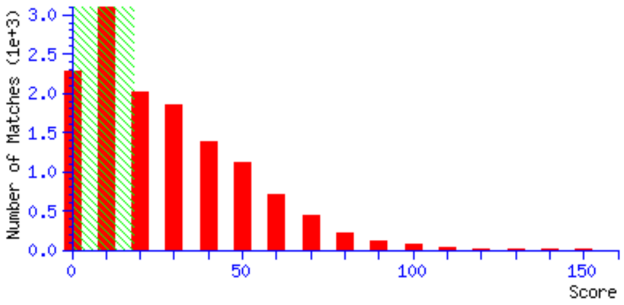

Peptide score distribution. Ions score is $-10\log(P)$, where P is the probability that the observed match is a random event.
There are **7,258** peptide matches above identity threshold and **7,486** matches above homology threshold for **73,748** queries.
On average, individual ions scores **> 18** (beyond green shading) indicate **identity or extensive homology** (p<0.05).

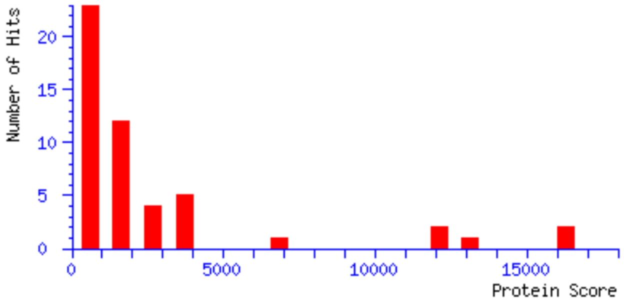

[Deprecated] Protein score distribution. Score distribution for family members in the first 50 proteins. Protein scores are derived from ions scores as a non-probabilistic basis for ranking protein families.

▼Modification statistics

Modification	Site	Above thr.
Carbamidomethyl	C	2651
Oxidation	M	836
Gln->pyro-Glu	N-term	186
Acetyl	Protein N-term	2

▼Legend

Peptide columns and rows

Dupes	...	Expect	Rank	U	1	2	Peptide	
		**0.037**	▶**2**				**GAYSLSLR**	significant
		9	▶1				GFFLFVEGGR	top ranking
		**6.4e-005**	▶**1**				**GSSIFGLAPGK**	significant and top ranking
		**1.3e-006**	▶**1**		■		**SSGTSYPDVLK**	peptide is found in all proteins in family member 1
		**6.2e-007**	▶**1**			■	**VCNYVSWIK**	peptide is found in some but not all proteins in family member 2
		**6.4e-005**	▶**1**	**U**			**GSSIFGLAPGK**	unique
▶**2**		**5.7e-005**	▶**1**				**LNTLETEEWFFK**	peptide has two duplicates
		0.18	▶*1*				*LNTLETEEWFFK*	duplicate peptide

Right-facing triangle (▶) in the Dupes or Rank column indicates content that can be expanded by clicking on it. Down-facing triangle (▼) indicates the content is expanded and can be collapsed. For more details about particular columns, see **results format help**.

Protein quantitation ratios

	Score	...	114/113	115/113
**CFAH_HUMAN**	37559		*0.962*	**1.129**
**FHR2_HUMAN**	1330		**0.859**	1.128

When quantitation method is Reporter (e.g. iTRAQ) or Multiplex (e.g. IPTL), protein ratios are displayed when a family is expanded. Ratios in *italic* indicate that the peptide log-ratios do not appear to come from a normal distribution. **Bold** indicates that if you can assume peptide ratios are normally distributed, the protein ratio is significantly different from 1.0 (at significance level 0.05).

Note that lack of bold or italic can also mean that significance or normality testing has not been performed (for example, if protein ratio type does not support it).

Protein Family Summary

Significance threshold p<	0.05	Max. number of families	AUTO
Ions score or expect cut-off	0.5	Dendrograms cut at	0
Show Percolator scores	☐		
Preferred taxonomy	All entries		

Sensitivity and FDR (reversed protein sequences)

			Target	Decoy	FDR		
PSMs	above	homology	7486	222	2.97%	Adjust to	1%

Decoy results are available in **the decoy report**.

Protein hits (487 proteins)

Filters: (none)

Export as CSV

↑Family	M	DB	Accession	Score	Mass	Matches	Match(sig)	Sequences	Seq(sig)	emPAI	Description
1	1	SwissProt_HUMAN$	2::P01834|IGKC_HUMAN	16280	11758	485	461	13	12	111.87	Immunoglobulin kapp
1	2	SwissProt_HUMAN$	2::P0DOX7|IGK_HUMAN	15932	23364	465	436	17	16	44.78	Immunoglobulin kapp
1	3	SwissProt_HUMAN$	2::P01597|KV139_HUMAN	639	12729	26	22	4	4	3.28	Immunoglobulin kapp
1	4	SwissProt_HUMAN$	2::A0A0C4DH72|KV106_HUMAN	584	12689	25	21	5	5	5.26	Immunoglobulin kapp
1	5	SwissProt_HUMAN$	2::A0A0C4DH73|KV112_HUMAN	560	12637	23	19	3	3	2.01	Immunoglobulin kapp
1	6	SwissProt_HUMAN$	2::A0A075B6S5|KV127_HUMAN	478	12703	21	17	4	4	3.34	Immunoglobulin kapp
1	7	SwissProt_HUMAN$	2::A0A0C4DH67|KV108_HUMAN	460	12529	20	16	4	4	3.39	Immunoglobulin kapp
1	8	SwissProt_HUMAN$	2::A0A0B4J2D9|KVD13_HUMAN	345	12561	17	13	3	3	2.03	Immunoglobulin kapp
1	9	SwissProt_HUMAN$	2::P01602|KV105_HUMAN	335	12773	20	16	4	4	3.28	Immunoglobulin kapp
1	10	SwissProt_HUMAN$	2::P01599|KV117_HUMAN	277	12770	19	15	6	6	7.87	Immunoglobulin kapp
1	11	SwissProt_HUMAN$	2::P04430|KV116_HUMAN	186	12610	15	10	3	2	1.08	Immunoglobulin kapp
1	12	SwissProt_HUMAN$	2::P01601|KVD16_HUMAN	138	12722	13	9	2	2	1.07	Immunoglobulin kapp
1	13	SwissProt_HUMAN$	2::A0A0C4DH24|KV621_HUMAN	131	12422	13	9	2	2	1.11	Immunoglobulin kapp
1	14	SwissProt_HUMAN$	2::A0A0A0MT36|KVD21_HUMAN	118	12332	14	10	2	2	1.12	Immunoglobulin kapp
1	15	SwissProt_HUMAN$	2::A0A075B6S9|KV137_HUMAN	112	12680	14	10	2	2	1.08	Probable non-function
2	1	SwissProt_HUMAN$	2::P0DOX5|IGG1_HUMAN	16157	49298	824	773	34	34	54.96	Immunoglobulin gamı
2	2	SwissProt_HUMAN$	2::P01860|IGHG3_HUMAN	8563	41260	520	476	25	25	37.69	Immunoglobulin heav
2	3	SwissProt_HUMAN$	2::P01859|IGHG2_HUMAN	5824	35878	389	351	21	21	44.21	Immunoglobulin heav
2	4	SwissProt_HUMAN$	2::P01861|IGHG4_HUMAN	5125	35918	324	300	23	22	29.16	Immunoglobulin heav
3	1	SwissProt_HUMAN$	2::P04114|APOB_HUMAN	13153	515283	515	488	286	279	17.91	Apolipoprotein B-100
4	1	SwissProt_HUMAN$	2::P02768|ALBU_HUMAN	11945	69321	447	425	73	72	328.11	Albumin OS=Homo sa
5	1	SwissProt_HUMAN$	2::P01024|CO3_HUMAN	11707	187030	520	476	156	154	136.04	Complement C3 OS=I
6	1	SwissProt_HUMAN$	2::P0C0L4|CO4A_HUMAN	6903	192664	269	243	98	95	12.59	Complement C4-A OS

↑*Family*	M	DB	Accession	Score	Mass	Matches	Match(sig)	Sequences	Seq(sig)	emPAI	Description
6	2	SwissProt_HUMAN$	2::P0C0L5|CO4B_HUMAN	6869	192631	268	242	97	94	12.61	Complement C4-B OS
7	1	SwissProt_HUMAN$	2::P01023|A2MG_HUMAN	3772	163188	151	143	76	74	12.29	Alpha-2-macroglobuli
8	1	SwissProt_HUMAN$	2::P01876|IGHA1_HUMAN	3622	37631	157	139	23	22	21.92	Immunoglobulin heav
8	2	SwissProt_HUMAN$	2::P0DOX2|IGA2_HUMAN	1161	48903	75	63	19	19	5.89	Immunoglobulin alpha
8	3	SwissProt_HUMAN$	2::P0DP02|HVC33_HUMAN	718	12980	36	34	9	9	72.10	Immunoglobulin heav
8	4	SwissProt_HUMAN$	2::P01772|HV333_HUMAN	685	13066	33	31	9	9	69.54	Immunoglobulin heav
8	5	SwissProt_HUMAN$	2::P01768|HV330_HUMAN	679	12938	31	29	9	9	50.12	Immunoglobulin heav
8	6	SwissProt_HUMAN$	2::P01764|HV323_HUMAN	637	12574	27	25	8	7	26.94	Immunoglobulin heav
8	7	SwissProt_HUMAN$	2::A0A0B4J1X5|HV374_HUMAN	596	12831	33	30	11	11	107.71	Immunoglobulin heav
8	8	SwissProt_HUMAN$	2::A0A0C4DH42|HV366_HUMAN	557	12690	28	27	6	6	26.16	Immunoglobulin heav
8	9	SwissProt_HUMAN$	2::A0A0B4J1V0|HV315_HUMAN	555	12917	22	20	9	9	35.84	Immunoglobulin heav
8	10	SwissProt_HUMAN$	2::A0A0B4J2H0|HV69D_HUMAN	536	12652	18	16	5	5	17.82	Immunoglobulin heav
8	11	SwissProt_HUMAN$	2::P01780|HV307_HUMAN	498	12934	28	27	12	12	148.47	Immunoglobulin heav
8	12	SwissProt_HUMAN$	2::P01742|HV169_HUMAN	494	12651	18	17	7	7	38.19	Immunoglobulin heav
8	13	SwissProt_HUMAN$	2::A0A0B4J1V1|HV321_HUMAN	436	12831	20	19	7	7	35.84	Immunoglobulin heav
8	14	SwissProt_HUMAN$	2::P01766|HV313_HUMAN	428	12498	16	15	5	5	8.21	Immunoglobulin heav
8	15	SwissProt_HUMAN$	2::A0A0C4DH33|HV124_HUMAN	402	12816	13	12	5	5	11.76	Immunoglobulin heav
8	16	SwissProt_HUMAN$	2::P01763|HV348_HUMAN	377	12804	18	17	7	7	25.41	Immunoglobulin heav
8	17	SwissProt_HUMAN$	2::P01762|HV311_HUMAN	365	12900	18	17	7	7	24.69	Immunoglobulin heav
8	18	SwissProt_HUMAN$	2::A0A0A0MS15|HV349_HUMAN	354	13048	13	12	9	9	23.34	Immunoglobulin heav
8	19	SwissProt_HUMAN$	2::P01782|HV309_HUMAN	348	12936	16	16	8	8	34.75	Immunoglobulin heav
8	20	SwissProt_HUMAN$	2::P01743|HV146_HUMAN	347	12924	11	11	5	5	5.07	Immunoglobulin heav
8	21	SwissProt_HUMAN$	2::P0DP04|HV43D_HUMAN	285	13008	13	13	6	6	16.48	Immunoglobulin heav
8	22	SwissProt_HUMAN$	2::P23083|HV102_HUMAN	268	13076	11	11	7	7	16.07	Immunoglobulin heav
8	23	SwissProt_HUMAN$	2::A0A0B4J1Y9|HV372_HUMAN	247	13194	14	13	7	7	10.73	Immunoglobulin heav
8	24	SwissProt_HUMAN$	2::A0A0C4DH32|HV320_HUMAN	226	12665	10	10	6	6	12.04	Immunoglobulin heav
8	25	SwissProt_HUMAN$	2::A0A0C4DH29|HV103_HUMAN	224	12999	12	12	6	6	16.48	Immunoglobulin heav
8	26	SwissProt_HUMAN$	2::A0A0B4J1V6|HV373_HUMAN	198	12849	11	10	6	6	7.71	Immunoglobulin heav
8	27	SwissProt_HUMAN$	2::P0DP01|HV108_HUMAN	164	12983	8	7	3	3	1.92	Immunoglobulin heav
8	28	SwissProt_HUMAN$	2::A0A0C4DH31|HV118_HUMAN	153	12812	7	7	5	5	5.16	Immunoglobulin heav
8	29	SwissProt_HUMAN$	2::A0A075B6Q5|HV364_HUMAN	153	12882	6	6	3	3	1.95	Immunoglobulin heav
8	30	SwissProt_HUMAN$	2::A0A0J9YVY3|HV741_HUMAN	147	12840	7	7	3	3	1.95	Immunoglobulin heav
8	31	SwissProt_HUMAN$	2::A0A0B4J1X8|HV343_HUMAN	132	13068	8	8	4	4	4.89	Immunoglobulin heav
8	32	SwissProt_HUMAN$	2::A0A0G2JMI3|HV692_HUMAN	115	12862	7	5	2	2	1.95	Immunoglobulin heav
8	33	SwissProt_HUMAN$	2::A0A0C4DH36|HV338_HUMAN	67	12750	5	5	3	3	5.16	Probable non-function
8	34	SwissProt_HUMAN$	2::A0A0J9YX35|HV64D_HUMAN	44	12814	3	3	2	2	1.07	Immunoglobulin heav
8	35	SwissProt_HUMAN$	2::P0DTE1|HV383_HUMAN	23	12663	2	2	2	2	1.08	Probable non-function
9	1	SwissProt_HUMAN$	2::P08603|CFAH_HUMAN	3443	139005	163	154	78	76	17.80	Complement factor H
9	2	SwissProt_HUMAN$	2::Q03591|FHR1_HUMAN	888	37626	34	31	18	18	12.88	Complement factor H-
9	3	SwissProt_HUMAN$	2::P36980|FHR2_HUMAN	436	30631	16	15	10	10	3.65	Complement factor H-

↑Family	M	DB	Accession	Score	Mass	Matches	Match(sig)	Sequences	Seq(sig)	emPAI	Description
9	4	SwissProt_HUMAN$	2::Q9BXR6|FHR5_HUMAN	207	64377	10	10	10	10	1.08	Complement factor H-
9	5	SwissProt_HUMAN$	2::Q02985|FHR3_HUMAN	137	37299	8	7	5	5	0.88	Complement factor H-
9	6	SwissProt_HUMAN$	2::Q92496|FHR4_HUMAN	31	65308	2	2	2	2	0.16	Complement factor H-
10	1	SwissProt_HUMAN$	2::P02751|FINC_HUMAN	3422	272150	142	127	87	81	3.64	Fibronectin OS=Homc
11	1	SwissProt_HUMAN$	2::P01871|IGHM_HUMAN	3411	49409	145	133	38	36	66.15	Immunoglobulin heav
11	2	SwissProt_HUMAN$	2::P0DOX6|IGM_HUMAN	2758	63446	114	104	30	28	9.84	Immunoglobulin mu h
11	3	SwissProt_HUMAN$	2::A0A0C4DH43|HV70D_HUMAN	318	13304	8	8	6	6	10.50	Immunoglobulin heav
11	4	SwissProt_HUMAN$	2::P01814|HV270_HUMAN	289	13252	6	6	5	5	7.25	Immunoglobulin heav
11	5	SwissProt_HUMAN$	2::A0A0B4J1V2|HV226_HUMAN	177	13174	7	7	6	6	7.25	Immunoglobulin heav
12	1	SwissProt_HUMAN$	2::P01009|A1AT_HUMAN	3119	46707	141	138	34	33	93.28	Alpha-1-antitrypsin O
13	1	SwissProt_HUMAN$	2::P01031|CO5_HUMAN	2965	188186	135	128	87	86	10.83	Complement C5 OS=I
14	1	SwissProt_HUMAN$	2::P0DOY3|IGLC3_HUMAN	2771	11259	108	92	13	12	213.21	Immunoglobulin lamb
14	2	SwissProt_HUMAN$	2::P0DOX8|IGL1_HUMAN	2364	22816	106	89	15	14	25.62	Immunoglobulin lamb
14	3	SwissProt_HUMAN$	2::A0M8Q6|IGLC7_HUMAN	1352	11247	56	46	7	6	16.99	Immunoglobulin lamb
14	4	SwissProt_HUMAN$	2::P01706|LV211_HUMAN	129	12636	8	8	5	5	8.03	Immunoglobulin lamb
14	5	SwissProt_HUMAN$	2::P01709|LV208_HUMAN	105	12374	11	8	3	3	3.51	Immunoglobulin lamb
15	1	SwissProt_HUMAN$	2::P02787|TRFE_HUMAN	2243	77014	106	98	55	52	34.29	Serotransferrin OS=H
16	1	SwissProt_HUMAN$	2::P00450|CERU_HUMAN	2148	122128	78	74	49	49	7.79	Ceruloplasmin OS=Hc
17	1	SwissProt_HUMAN$	2::P00747|PLMN_HUMAN	2077	90510	90	79	51	50	17.75	Plasminogen OS=Hom
18	1	SwissProt_HUMAN$	2::P04003|C4BPA_HUMAN	2011	66989	76	72	29	29	8.56	C4b-binding protein a
19	1	SwissProt_HUMAN$	2::P09871|C1S_HUMAN	1477	76635	53	52	29	29	7.70	Complement C1s subc
19	2	SwissProt_HUMAN$	2::P48740|MASP1_HUMAN	160	79195	9	9	7	7	0.52	Mannan-binding lectir
20	1	SwissProt_HUMAN$	2::P02649|APOE_HUMAN	1462	36132	50	48	28	28	107.66	Apolipoprotein E OS=
21	1	SwissProt_HUMAN$	2::P00738|HPT_HUMAN	1362	45177	62	57	26	25	19.70	Haptoglobin OS=Hom
21	2	SwissProt_HUMAN$	2::P00739|HPTR_HUMAN	747	39005	37	35	22	21	15.18	Haptoglobin-related p
22	1	SwissProt_HUMAN$	2::P19827|ITIH1_HUMAN	1237	101326	49	48	30	29	4.13	Inter-alpha-trypsin inl
23	1	SwissProt_HUMAN$	2::P02647|APOA1_HUMAN	1227	30759	57	54	31	29	132.94	Apolipoprotein A-I OS
24	1	SwissProt_HUMAN$	2::P01011|AACT_HUMAN	1221	47621	49	47	23	22	15.06	Alpha-1-antichymotry
25	1	SwissProt_HUMAN$	2::P19823|ITIH2_HUMAN	1204	106397	52	48	31	30	3.54	Inter-alpha-trypsin inl
26	1	SwissProt_HUMAN$	2::P10643|CO7_HUMAN	1196	93457	51	45	41	37	6.22	Complement compone
27	1	SwissProt_HUMAN$	2::P00751|CFAB_HUMAN	1190	85479	70	65	42	39	8.69	Complement factor B
28	1	SwissProt_HUMAN$	2::P07357|CO8A_HUMAN	1081	65121	34	32	24	22	5.60	Complement compone
29	1	SwissProt_HUMAN$	2::P13671|CO6_HUMAN	1044	104718	46	43	32	29	3.07	Complement compone
30	1	SwissProt_HUMAN$	2::P02749|APOH_HUMAN	1007	38273	46	42	24	22	15.97	Beta-2-glycoprotein 1
31	1	SwissProt_HUMAN$	2::P04275|VWF_HUMAN	1005	309058	47	45	39	38	0.85	von Willebrand factor
32	1	SwissProt_HUMAN$	2::Q14624|ITIH4_HUMAN	988	103293	39	37	30	30	3.54	Inter-alpha-trypsin inl
33	1	SwissProt_HUMAN$	2::P00736|C1R_HUMAN	942	80067	46	42	28	27	5.26	Complement C1r subc
34	1	SwissProt_HUMAN$	2::P07358|CO8B_HUMAN	896	67003	35	33	23	23	5.26	Complement compone
35	1	SwissProt_HUMAN$	2::P00734|THRB_HUMAN	877	69992	33	33	23	23	5.20	Prothrombin OS=Hom
36	1	SwissProt_HUMAN$	2::P07225|PROS_HUMAN	877	75074	36	35	24	23	3.84	Vitamin K-dependent

↑Family	M	DB	Accession	Score	Mass	Matches	Match(sig)	Sequences	Seq(sig)	emPAI	Description
37	1	SwissProt_HUMAN$	2::P02748|CO9_HUMAN	869	63133	41	37	28	26	6.55	Complement compone
38	1	SwissProt_HUMAN$	2::P02774|VTDB_HUMAN	856	52883	46	40	28	26	12.37	Vitamin D-binding pro
39	1	SwissProt_HUMAN$	2::Q9Y6R7|FCGBP_HUMAN	855	571639	38	37	33	33	0.32	IgGFc-binding protein
40	1	SwissProt_HUMAN$	2::P05155|IC1_HUMAN	812	55119	40	36	21	20	5.58	Plasma protease C1 ir
41	1	SwissProt_HUMAN$	2::P0DJI8|SAA1_HUMAN	797	13524	28	25	9	8	20.97	Serum amyloid A-1 pr
41	2	SwissProt_HUMAN$	2::P0DJI9|SAA2_HUMAN	610	13519	22	19	9	8	20.97	Serum amyloid A-2 pr
42	1	SwissProt_HUMAN$	2::P01008|ANT3_HUMAN	791	52569	33	30	24	22	6.89	Antithrombin-III OS=
43	1	SwissProt_HUMAN$	2::P06396|GELS_HUMAN	772	85644	32	32	22	22	2.56	Gelsolin OS=Homo sa
44	1	SwissProt_HUMAN$	2::P01619|KV320_HUMAN	722	12549	16	16	4	4	3.39	Immunoglobulin kapp
44	2	SwissProt_HUMAN$	2::A0A0C4DH25|KVD20_HUMAN	625	12507	14	14	3	3	2.03	Immunoglobulin kapp
45	1	SwissProt_HUMAN$	2::P01019|ANGT_HUMAN	705	53121	24	24	15	15	3.53	Angiotensinogen OS=
46	1	SwissProt_HUMAN$	2::P07360|CO8G_HUMAN	654	22264	18	17	11	11	14.36	Complement compone
47	1	SwissProt_HUMAN$	2::P18428|LBP_HUMAN	601	53350	21	21	15	15	3.12	Lipopolysaccharide-bi
48	1	SwissProt_HUMAN$	2::P36955|PEDF_HUMAN	592	46283	22	21	17	16	5.27	Pigment epithelium-de
49	1	SwissProt_HUMAN$	2::P06727|APOA4_HUMAN	585	45344	27	27	23	23	9.93	Apolipoprotein A-IV O
50	1	SwissProt_HUMAN$	2::P02675|FIBB_HUMAN	582	55892	25	21	21	18	3.58	Fibrinogen beta chain
51	1	SwissProt_HUMAN$	2::P0DP08|HVD82_HUMAN	574	13008	17	16	5	5	4.98	Immunoglobulin heav
51	2	SwissProt_HUMAN$	2::P06331|HV434_HUMAN	574	13806	17	16	5	5	4.42	Immunoglobulin heav
51	3	SwissProt_HUMAN$	2::A0A0C4DH41|HV461_HUMAN	568	13058	17	16	5	5	4.89	Immunoglobulin heav
51	4	SwissProt_HUMAN$	2::A0A075B6R2|HV404_HUMAN	426	12840	11	10	5	4	3.23	Immunoglobulin heav
52	1	SwissProt_HUMAN$	2::O43866|CD5L_HUMAN	568	38063	26	22	16	14	5.41	CD5 antigen-like OS=
53	1	SwissProt_HUMAN$	2::P68871|HBB_HUMAN	553	15988	21	20	12	12	43.42	Hemoglobin subunit b
53	2	SwissProt_HUMAN$	2::P02042|HBD_HUMAN	213	16045	10	10	7	7	6.61	Hemoglobin subunit d
54	1	SwissProt_HUMAN$	2::P02790|HEMO_HUMAN	549	51643	30	29	20	20	6.49	Hemopexin OS=Homo
55	1	SwissProt_HUMAN$	2::P13645|K1C10_HUMAN	526	58792	22	20	17	16	2.92	Keratin, type I cytosk
55	2	SwissProt_HUMAN$	2::P35527|K1C9_HUMAN	233	62027	9	8	8	7	0.70	Keratin, type I cytosk
55	3	SwissProt_HUMAN$	2::P08727|K1C19_HUMAN	43	44079	5	5	5	5	0.71	Keratin, type I cytosk
56	1	SwissProt_HUMAN$	2::P02679|FIBG_HUMAN	466	51479	23	22	17	17	4.71	Fibrinogen gamma ch
57	1	SwissProt_HUMAN$	2::P04264|K2C1_HUMAN	465	65999	19	18	18	17	2.38	Keratin, type II cytosk
57	2	SwissProt_HUMAN$	2::P35908|K22E_HUMAN	196	65393	11	8	10	8	0.78	Keratin, type II cytosk
57	3	SwissProt_HUMAN$	2::P13647|K2C5_HUMAN	46	62340	4	2	3	2	0.16	Keratin, type II cytosk
58	1	SwissProt_HUMAN$	2::P02743|SAMP_HUMAN	464	25371	19	18	12	11	6.63	Serum amyloid P-com
59	1	SwissProt_HUMAN$	2::P02753|RET4_HUMAN	446	22995	23	20	13	12	16.43	Retinol-binding protei
60	1	SwissProt_HUMAN$	2::P02765|FETUA_HUMAN	436	39316	14	14	8	8	2.32	Alpha-2-HS-glycoprot
61	1	SwissProt_HUMAN$	2::O75636|FCN3_HUMAN	432	32882	21	20	12	11	5.44	Ficolin-3 OS=Homo sa
62	1	SwissProt_HUMAN$	2::P05156|CFAI_HUMAN	424	65707	21	19	18	17	2.65	Complement factor I (
63	1	SwissProt_HUMAN$	2::P01833|PIGR_HUMAN	420	83232	20	17	20	17	1.63	Polymeric immunoglol
64	1	SwissProt_HUMAN$	2::P27169|PON1_HUMAN	415	39706	20	18	14	12	3.68	Serum paraoxonase/a
65	1	SwissProt_HUMAN$	2::P01042|KNG1_HUMAN	414	71912	17	16	14	13	1.51	Kininogen-1 OS=Hom
66	1	SwissProt_HUMAN$	2::P02741|CRP_HUMAN	414	25023	19	17	10	9	5.55	C-reactive protein OS

↑Family	M	DB	Accession	Score	Mass	Matches	Match(sig)	Sequences	Seq(sig)	emPAI	Description
67	1	SwissProt_HUMAN$	2::P02750|A2GL_HUMAN	370	38154	15	15	10	10	2.44	Leucine-rich alpha-2-(
68	1	SwissProt_HUMAN$	2::P02656|APOC3_HUMAN	364	10846	10	8	6	5	7.38	Apolipoprotein C-III C
69	1	SwissProt_HUMAN$	2::P02763|A1AG1_HUMAN	355	23497	16	16	9	9	5.02	Alpha-1-acid glycopro
69	2	SwissProt_HUMAN$	2::P19652|A1AG2_HUMAN	270	23588	17	16	10	10	6.28	Alpha-1-acid glycopro
70	1	SwissProt_HUMAN$	2::P12259|FA5_HUMAN	351	251546	19	17	17	15	0.35	Coagulation factor V (
71	1	SwissProt_HUMAN$	2::P04040|CATA_HUMAN	346	59719	10	10	10	10	1.21	Catalase OS=Homo sa
72	1	SwissProt_HUMAN$	2::O14791|APOL1_HUMAN	344	43947	15	13	11	10	1.93	Apolipoprotein L1 OS=
73	1	SwissProt_HUMAN$	2::P04004|VTNC_HUMAN	337	54271	18	15	9	8	1.19	Vitronectin OS=Homo
74	1	SwissProt_HUMAN$	2::A0A0C4DH38|HV551_HUMAN	337	12666	12	11	5	5	5.26	Immunoglobulin heav
74	2	SwissProt_HUMAN$	2::A0A0J9YXX1|HV5X1_HUMAN	325	12764	16	15	6	5	7.87	Immunoglobulin heav
75	1	SwissProt_HUMAN$	2::P02746|C1QB_HUMAN	331	26704	16	16	9	9	7.23	Complement C1q sub(
76	1	SwissProt_HUMAN$	2::P02766|TTHY_HUMAN	327	15877	10	10	8	8	9.49	Transthyretin OS=Hor
77	1	SwissProt_HUMAN$	2::P02747|C1QC_HUMAN	313	25757	15	15	8	8	4.16	Complement C1q sub(
78	1	SwissProt_HUMAN$	2::Q06033|ITIH3_HUMAN	309	99787	16	13	16	13	0.85	Inter-alpha-trypsin inl
79	1	SwissProt_HUMAN$	2::P01624|KV315_HUMAN	309	12488	12	10	2	2	1.10	Immunoglobulin kapp
79	2	SwissProt_HUMAN$	2::A0A087WSY6|KVD15_HUMAN	204	12526	6	6	2	2	1.10	Immunoglobulin kapp
80	1	SwissProt_HUMAN$	2::P04217|A1BG_HUMAN	296	54220	14	11	10	8	1.19	Alpha-1B-glycoproteir
81	1	SwissProt_HUMAN$	2::P04196|HRG_HUMAN	289	59541	15	15	14	14	2.04	Histidine-rich glycopr(
82	1	SwissProt_HUMAN$	2::P35542|SAA4_HUMAN	288	14737	12	12	6	6	16.10	Serum amyloid A-4 pr
83	1	SwissProt_HUMAN$	2::P01714|LV319_HUMAN	286	12035	10	10	5	5	5.91	Immunoglobulin lamb
84	1	SwissProt_HUMAN$	2::Q08380|LG3BP_HUMAN	285	65289	15	13	11	10	1.06	Galectin-3-binding pr(
85	1	SwissProt_HUMAN$	2::P10909|CLUS_HUMAN	284	52461	15	13	11	9	1.25	Clusterin OS=Homo s
86	1	SwissProt_HUMAN$	2::P60709|ACTB_HUMAN	277	41710	16	15	13	12	3.35	Actin, cytoplasmic 1 (
87	1	SwissProt_HUMAN$	2::P03952|KLKB1_HUMAN	270	71323	14	12	12	11	1.07	Plasma kallikrein OS=
88	1	SwissProt_HUMAN$	2::P08571|CD14_HUMAN	265	40051	7	7	6	6	1.03	Monocyte differentiati
89	1	SwissProt_HUMAN$	2::P00761|TRYP_PIG	262	24394	6	6	4	4	1.61	Trypsin OS=Sus scrof
90	1	SwissProt_HUMAN$	2::P04433|KV311_HUMAN	259	12567	12	11	3	3	2.03	Immunoglobulin kapp
91	1	SwissProt_HUMAN$	2::P05546|HEP2_HUMAN	251	57034	13	12	9	8	1.11	Heparin cofactor 2 OS
92	1	SwissProt_HUMAN$	2::A0A087WW87|KV240_HUMAN	246	13302	7	7	5	5	4.72	Immunoglobulin kapp
92	2	SwissProt_HUMAN$	2::A0A075B6P5|KV228_HUMAN	211	12948	7	7	4	4	3.18	Immunoglobulin kapp
92	3	SwissProt_HUMAN$	2::P06310|KV230_HUMAN	165	13177	9	9	5	5	4.81	Immunoglobulin kapp
92	4	SwissProt_HUMAN$	2::A0A075B6S2|KVD29_HUMAN	147	13135	6	5	4	3	1.90	Immunoglobulin kapp
93	1	SwissProt_HUMAN$	2::P05090|APOD_HUMAN	244	21262	14	12	11	11	10.23	Apolipoprotein D OS=
94	1	SwissProt_HUMAN$	2::P07996|TSP1_HUMAN	240	129300	10	9	10	9	0.39	Thrombospondin-1 OS
95	1	SwissProt_HUMAN$	2::P01591|IGJ_HUMAN	228	18087	12	11	7	7	5.10	Immunoglobulin J cha
96	1	SwissProt_HUMAN$	2::P23142|FBLN1_HUMAN	226	77162	15	14	11	11	1.09	Fibulin-1 OS=Homo sa
97	1	SwissProt_HUMAN$	2::P05160|F13B_HUMAN	223	75461	14	11	11	9	0.76	Coagulation factor XII
98	1	SwissProt_HUMAN$	2::P02760|AMBP_HUMAN	223	38974	14	11	10	8	1.97	Protein AMBP OS=Hor
99	1	SwissProt_HUMAN$	2::P02671|FIBA_HUMAN	221	94914	19	18	17	16	1.46	Fibrinogen alpha chair
100	1	SwissProt_HUMAN$	2::P06312|KV401_HUMAN	214	13372	15	15	5	5	4.72	Immunoglobulin kapp

↑Family	M	DB	Accession	Score	Mass	Matches	Match(sig)	Sequences	Seq(sig)	emPAI	Description
101	1	SwissProt_HUMAN$	2::P02655|APOC2_HUMAN	214	11277	10	9	6	5	16.50	Apolipoprotein C-II OS
102	1	SwissProt_HUMAN$	2::P00748|FA12_HUMAN	214	67748	9	8	9	8	0.75	Coagulation factor XII
103	1	SwissProt_HUMAN$	2::Q15582|BGH3_HUMAN	214	74634	12	10	12	10	0.89	Transforming growth
104	1	SwissProt_HUMAN$	2::P11226|MBL2_HUMAN	207	26127	8	8	8	8	3.22	Mannose-binding prot
105	1	SwissProt_HUMAN$	2::Q96IY4|CBPB2_HUMAN	203	48393	8	8	8	8	1.18	Carboxypeptidase B2
106	1	SwissProt_HUMAN$	2::Q96PD5|PGRP2_HUMAN	200	62178	9	8	7	6	0.58	N-acetylmuramoyl-L-a
107	1	SwissProt_HUMAN$	2::P0DOX3|IGD_HUMAN	196	56189	9	8	7	7	0.96	Immunoglobulin delta
108	1	SwissProt_HUMAN$	2::P02652|APOA2_HUMAN	191	11168	9	7	6	5	6.88	Apolipoprotein A-II OS
109	1	SwissProt_HUMAN$	2::P02654|APOC1_HUMAN	188	9326	11	9	5	5	18.35	Apolipoprotein C-I OS
110	1	SwissProt_HUMAN$	2::P01593|KVD33_HUMAN	186	12840	5	5	3	3	1.95	Immunoglobulin kapp
111	1	SwissProt_HUMAN$	2::Q92954|PRG4_HUMAN	186	150968	8	8	6	6	0.21	Proteoglycan 4 OS=H
112	1	SwissProt_HUMAN$	2::A0A0C4DH34|HV428_HUMAN	177	13116	13	9	4	3	4.89	Immunoglobulin heav
113	1	SwissProt_HUMAN$	2::A0A075B6R9|KVD24_HUMAN	169	13071	4	4	2	2	1.03	Probable non-function
114	1	SwissProt_HUMAN$	2::P29622|KAIN_HUMAN	168	48511	9	9	8	8	1.18	Kallistatin OS=Homo
115	1	SwissProt_HUMAN$	2::P06681|CO2_HUMAN	168	83214	11	9	10	8	0.67	Complement C2 OS=H
116	1	SwissProt_HUMAN$	2::P69905|HBA_HUMAN	165	15248	11	10	7	7	7.54	Hemoglobin subunit a
117	1	SwissProt_HUMAN$	2::P20851|C4BPB_HUMAN	164	28338	10	8	8	6	1.71	C4b-binding protein b
118	1	SwissProt_HUMAN$	2::A0A075B6K5|LV39_HUMAN	164	12324	3	3	2	2	1.12	Immunoglobulin lamb
119	1	SwissProt_HUMAN$	2::P01817|HV205_HUMAN	162	13223	5	5	3	3	1.87	Immunoglobulin heav
120	1	SwissProt_HUMAN$	2::P43652|AFAM_HUMAN	161	69024	11	11	11	11	1.12	Afamin OS=Homo sap
121	1	SwissProt_HUMAN$	2::P13796|PLSL_HUMAN	158	70244	8	8	7	7	0.60	Plastin-2 OS=Homo s
122	1	SwissProt_HUMAN$	2::O95445|APOM_HUMAN	156	21239	9	8	7	7	3.70	Apolipoprotein M OS=
123	1	SwissProt_HUMAN$	2::P01700|LV147_HUMAN	153	12276	8	7	4	4	3.51	Immunoglobulin lamb
123	2	SwissProt_HUMAN$	2::P01699|LV144_HUMAN	100	12194	7	5	3	3	2.13	Immunoglobulin lamb
124	1	SwissProt_HUMAN$	2::A0A0B4J1U7|HV601_HUMAN	150	13473	11	10	5	5	4.64	Immunoglobulin heav
125	1	SwissProt_HUMAN$	2::O00187|MASP2_HUMAN	146	75654	6	6	5	5	0.37	Mannan-binding lectin
126	1	SwissProt_HUMAN$	2::Q15485|FCN2_HUMAN	143	33980	6	5	5	4	1.00	Ficolin-2 OS=Homo sa
127	1	SwissProt_HUMAN$	2::P05452|TETN_HUMAN	142	22522	6	5	5	4	1.30	Tetranectin OS=Homo
128	1	SwissProt_HUMAN$	2::P02745|C1QA_HUMAN	141	26000	10	8	6	6	1.96	Complement C1q sub
129	1	SwissProt_HUMAN$	2::P35858|ALS_HUMAN	140	65994	7	7	6	6	0.54	Insulin-like growth fa
130	1	SwissProt_HUMAN$	2::P08697|A2AP_HUMAN	140	54531	13	11	12	11	1.60	Alpha-2-antiplasmin C
131	1	SwissProt_HUMAN$	2::A0A075B6K4|LV310_HUMAN	139	12433	8	6	6	4	3.45	Immunoglobulin lamb
131	2	SwissProt_HUMAN$	2::P01717|LV325_HUMAN	103	12004	8	6	4	3	3.70	Immunoglobulin lamb
131	3	SwissProt_HUMAN$	2::P01718|LV327_HUMAN	49	12157	4	3	3	3	2.13	Immunoglobulin lamb
131	4	SwissProt_HUMAN$	2::A0A075B6K0|LV316_HUMAN	42	12458	3	2	2	2	1.11	Immunoglobulin lamb
132	1	SwissProt_HUMAN$	2::P22792|CPN2_HUMAN	130	60518	6	6	5	5	0.48	Carboxypeptidase N s
133	1	SwissProt_HUMAN$	2::P11021|BIP_HUMAN	123	72288	5	5	5	5	0.39	Endoplasmic reticulun
134	1	SwissProt_HUMAN$	2::P01701|LV151_HUMAN	122	12241	6	5	3	2	1.14	Immunoglobulin lamb
135	1	SwissProt_HUMAN$	2::P05154|IPSP_HUMAN	119	45646	6	6	6	6	0.86	Plasma serine proteas
136	1	SwissProt_HUMAN$	2::P61626|LYSC_HUMAN	118	16526	8	7	6	5	4.44	Lysozyme C OS=Hom

↑Family	M	DB	Accession	Score	Mass	Matches	Match(sig)	Sequences	Seq(sig)	emPAI	Description
137	1	SwissProt_HUMAN$	2::P02775|CXCL7_HUMAN	116	13885	4	3	3	2	0.96	Platelet basic protein
138	1	SwissProt_HUMAN$	2::P07988|PSPB_HUMAN	115	42089	4	4	3	3	0.40	Pulmonary surfactant-
139	1	SwissProt_HUMAN$	2::P26927|HGFL_HUMAN	110	80268	4	4	4	4	0.27	Hepatocyte growth fa
140	1	SwissProt_HUMAN$	2::Q9UK55|ZPI_HUMAN	110	50674	7	7	7	7	0.92	Protein Z-dependent p
141	1	SwissProt_HUMAN$	2::P00742|FA10_HUMAN	108	54697	5	4	4	3	0.30	Coagulation factor X (
142	1	SwissProt_HUMAN$	2::P00740|FA9_HUMAN	107	51745	7	7	6	6	0.73	Coagulation factor IX
143	1	SwissProt_HUMAN$	2::P07195|LDHB_HUMAN	102	36615	5	5	5	5	0.90	L-lactate dehydrogena
143	2	SwissProt_HUMAN$	2::P00338|LDHA_HUMAN	83	36665	3	3	3	3	0.47	L-lactate dehydrogena
144	1	SwissProt_HUMAN$	2::P01721|LV657_HUMAN	101	12558	2	1	1	1	0.45	Immunoglobulin lamb
145	1	SwissProt_HUMAN$	2::P27918|PROP_HUMAN	101	51242	4	4	4	4	0.45	Properdin OS=Homo s
146	1	SwissProt_HUMAN$	2::P15814|IGLL1_HUMAN	97	22949	2	2	1	1	0.23	Immunoglobulin lamb
147	1	SwissProt_HUMAN$	2::P03951|FA11_HUMAN	94	70064	7	6	7	6	0.50	Coagulation factor XI
148	1	SwissProt_HUMAN$	2::P24821|TENA_HUMAN	94	240700	6	6	6	6	0.13	Tenascin OS=Homo s
149	1	SwissProt_HUMAN$	2::Q12805|FBLN3_HUMAN	90	54604	3	3	3	3	0.30	EGF-containing fibulin
150	1	SwissProt_HUMAN$	2::P02776|PLF4_HUMAN	84	10838	5	4	5	4	4.48	Platelet factor 4 OS=H
151	1	SwissProt_HUMAN$	2::A0A075B6I1|LV460_HUMAN	83	12979	2	2	2	2	1.04	Immunoglobulin lamb
152	1	SwissProt_HUMAN$	2::P15169|CBPN_HUMAN	81	52253	4	4	4	4	0.44	Carboxypeptidase N c
153	1	SwissProt_HUMAN$	2::O00391|QSOX1_HUMAN	79	82526	6	6	5	5	0.33	Sulfhydryl oxidase 1 (
154	1	SwissProt_HUMAN$	2::P04070|PROC_HUMAN	76	52037	3	3	3	3	0.31	Vitamin K-dependent
155	1	SwissProt_HUMAN$	2::P29401|TKT_HUMAN	75	67835	4	3	4	3	0.23	Transketolase OS=Ho
156	1	SwissProt_HUMAN$	2::P18206|VINC_HUMAN	73	123722	4	4	4	4	0.17	Vinculin OS=Homo sa
157	1	SwissProt_HUMAN$	2::P51884|LUM_HUMAN	72	38405	4	3	4	3	0.45	Lumican OS=Homo sa
158	1	SwissProt_HUMAN$	2::P01715|LV301_HUMAN	71	12288	4	3	3	2	1.12	Immunoglobulin lamb
159	1	SwissProt_HUMAN$	2::P05109|S10A8_HUMAN	67	10828	3	3	3	3	2.58	Protein S100-A8 OS=
160	1	SwissProt_HUMAN$	2::P28072|PSB6_HUMAN	66	25341	2	2	2	2	0.45	Proteasome subunit b
161	1	SwissProt_HUMAN$	2::Q08830|FGL1_HUMAN	65	36357	4	4	4	4	0.68	Fibrinogen-like protei
162	1	SwissProt_HUMAN$	2::P05164|PERM_HUMAN	64	83815	3	2	3	2	0.12	Myeloperoxidase OS=
163	1	SwissProt_HUMAN$	2::A0A075B6J1|LV537_HUMAN	64	13268	1	1	1	1	0.42	Immunoglobulin lamb
164	1	SwissProt_HUMAN$	2::P04211|LV743_HUMAN	64	12443	4	3	3	3	2.06	Immunoglobulin lamb
164	2	SwissProt_HUMAN$	2::A0A075B6I9|LV746_HUMAN	62	12460	5	3	4	3	2.06	Immunoglobulin lamb
165	1	SwissProt_HUMAN$	2::Q9Y279|VSIG4_HUMAN	63	43959	4	3	4	3	0.38	V-set and immunoglol
166	1	SwissProt_HUMAN$	2::P25311|ZA2G_HUMAN	63	34237	8	5	7	4	0.99	Zinc-alpha-2-glycoprc
167	1	SwissProt_HUMAN$	2::P08519|APOA_HUMAN	62	500995	3	3	3	3	0.03	Apolipoprotein(a) OS=
168	1	SwissProt_HUMAN$	2::P38646|GRP75_HUMAN	61	73635	1	1	1	1	0.07	Stress-70 protein, mit
169	1	SwissProt_HUMAN$	2::Q5T0N5|FBP1L_HUMAN	61	70021	3	3	1	1	0.07	Formin-binding protei
170	1	SwissProt_HUMAN$	2::A0A0B4J1U3|LV136_HUMAN	61	12470	2	1	2	1	0.45	Immunoglobulin lamb
171	1	SwissProt_HUMAN$	2::P0DOX4|IGE_HUMAN	61	60285	2	2	1	1	0.17	Immunoglobulin epsil
172	1	SwissProt_HUMAN$	2::Q71DI3|H32_HUMAN	60	15379	2	1	2	1	0.35	Histone H3.2 OS=Hor
173	1	SwissProt_HUMAN$	2::P08709|FA7_HUMAN	60	51560	1	1	1	1	0.10	Coagulation factor VII
174	1	SwissProt_HUMAN$	2::P36222|CH3L1_HUMAN	60	42598	5	4	5	4	0.56	Chitinase-3-like prote

↑Family	M	DB	Accession	Score	Mass	Matches	Match(sig)	Sequences	Seq(sig)	emPAI	Description
175	1	SwissProt_HUMAN$	2::Q8NHM4|TRY6_HUMAN	59	26520	1	1	1	1	0.19	Putative trypsin-6 OS
176	1	SwissProt_HUMAN$	2::A0A0A0MS14|HV145_HUMAN	58	13500	1	1	1	1	0.41	Immunoglobulin heav
177	1	SwissProt_HUMAN$	2::A0A075B6I0|LV861_HUMAN	56	12806	3	3	3	3	1.98	Immunoglobulin lamb
178	1	SwissProt_HUMAN$	2::A0A0C4DH35|HV335_HUMAN	56	12802	3	2	2	2	1.07	Probable non-function
179	1	SwissProt_HUMAN$	2::P05543|THBG_HUMAN	56	46295	3	3	3	3	0.36	Thyroxine-binding glo
180	1	SwissProt_HUMAN$	2::Q6ZRR7|LRRC9_HUMAN	54	166805	2	2	1	1	0.03	Leucine-rich repeat-co
181	1	SwissProt_HUMAN$	2::A0A075B6H9|LV469_HUMAN	54	12765	4	3	3	3	1.98	Immunoglobulin lamb
182	1	SwissProt_HUMAN$	2::Q06141|REG3A_HUMAN	52	19382	2	2	2	2	0.62	Regenerating islet-der
183	1	SwissProt_HUMAN$	2::Q9UGM5|FETUB_HUMAN	51	42028	2	2	2	2	0.25	Fetuin-B OS=Homo sa
184	1	SwissProt_HUMAN$	2::P55058|PLTP_HUMAN	51	54705	3	3	3	3	0.30	Phospholipid transfer
185	1	SwissProt_HUMAN$	2::Q8NGJ3|O52E1_HUMAN	49	34690	11	2	1	1	0.15	Olfactory receptor 52E
186	1	SwissProt_HUMAN$	2::P30531|SC6A1_HUMAN	49	67029	2	2	1	1	0.07	Sodium- and chloride-
187	1	SwissProt_HUMAN$	2::P02792|FRIL_HUMAN	48	20007	1	1	1	1	0.26	Ferritin light chain OS
188	1	SwissProt_HUMAN$	2::P08185|CBG_HUMAN	47	45112	3	3	3	3	0.37	Corticosteroid-binding
189	1	SwissProt_HUMAN$	2::P12111|CO6A3_HUMAN	47	343457	4	4	4	4	0.06	Collagen alpha-3(VI)
190	1	SwissProt_HUMAN$	2::Q5QNW6|H2B2F_HUMAN	47	13912	4	3	3	3	1.73	Histone H2B type 2-F
191	1	SwissProt_HUMAN$	2::P02788|TRFL_HUMAN	46	78132	2	2	2	2	0.13	Lactotransferrin OS=H
192	1	SwissProt_HUMAN$	2::P01705|LV223_HUMAN	46	11886	2	2	1	1	1.18	Immunoglobulin lamb
193	1	SwissProt_HUMAN$	2::P01034|CYTC_HUMAN	46	15789	3	2	2	2	0.81	Cystatin-C OS=Homo
194	1	SwissProt_HUMAN$	2::P00746|CFAD_HUMAN	43	27016	4	2	3	2	0.42	Complement factor D
195	1	SwissProt_HUMAN$	2::Q86SS6|SYT9_HUMAN	43	56153	1	1	1	1	0.09	Synaptotagmin-9 OS=
196	1	SwissProt_HUMAN$	2::P22352|GPX3_HUMAN	42	25537	4	3	4	3	0.74	Glutathione peroxidas
197	1	SwissProt_HUMAN$	2::P61769|B2MG_HUMAN	41	13706	1	1	1	1	0.41	Beta-2-microglobulin
198	1	SwissProt_HUMAN$	2::P04278|SHBG_HUMAN	41	43752	2	2	2	2	0.24	Sex hormone-binding
199	1	SwissProt_HUMAN$	2::P05062|ALDOB_HUMAN	41	39448	2	2	2	2	0.27	Fructose-bisphosphate
199	2	SwissProt_HUMAN$	2::P04075|ALDOA_HUMAN	40	39395	3	3	3	3	0.43	Fructose-bisphosphate
200	1	SwissProt_HUMAN$	2::P59665|DEF1_HUMAN	41	10194	3	1	3	1	0.57	Neutrophil defensin 1
201	1	SwissProt_HUMAN$	2::P10599|THIO_HUMAN	40	11730	1	1	1	1	0.48	Thioredoxin OS=Homo
202	1	SwissProt_HUMAN$	2::Q8N5V2|NGEF_HUMAN	40	82445	1	1	1	1	0.06	Ephexin-1 OS=Homo
203	1	SwissProt_HUMAN$	2::P25788|PSA3_HUMAN	39	28415	3	1	3	1	0.18	Proteasome subunit a
204	1	SwissProt_HUMAN$	2::P0C0S8|H2A1_HUMAN	39	14083	1	1	1	1	0.39	Histone H2A type 1 O
205	1	SwissProt_HUMAN$	2::P05451|REG1A_HUMAN	38	18719	2	2	2	2	0.65	Lithostathine-1-alpha
206	1	SwissProt_HUMAN$	2::A0A0B4J1Y8|LV949_HUMAN	38	13016	3	1	3	1	0.43	Immunoglobulin lamb
207	1	SwissProt_HUMAN$	2::P08637|FCG3A_HUMAN	37	29071	2	2	2	2	0.38	Low affinity immunog
208	1	SwissProt_HUMAN$	2::Q66K66|TM198_HUMAN	37	39449	1	1	1	1	0.13	Transmembrane prote
209	1	SwissProt_HUMAN$	2::Q2NKX8|ERC6L_HUMAN	37	141015	1	1	1	1	0.03	DNA excision repair p
210	1	SwissProt_HUMAN$	2::Q12934|BFSP1_HUMAN	37	74499	2	2	1	1	0.07	Filensin OS=Homo sa
211	1	SwissProt_HUMAN$	2::Q5VU97|CAHD1_HUMAN	37	142198	2	2	1	1	0.03	VWFA and cache dom
212	1	SwissProt_HUMAN$	2::Q8N6G1|CA211_HUMAN	36	10497	1	1	1	1	0.55	Putative uncharacteri
213	1	SwissProt_HUMAN$	2::Q15113|PCOC1_HUMAN	35	47942	1	1	1	1	0.10	Procollagen C-endope

↑Family	M	DB	Accession	Score	Mass	Matches	Match(sig)	Sequences	Seq(sig)	emPAI	Description
214	1	SwissProt_HUMAN$	2::Q6VUC0|AP2E_HUMAN	34	46183	1	1	1	1	0.11	Transcription factor AI
215	1	SwissProt_HUMAN$	2::Q9NWX5|ASB6_HUMAN	34	47106	1	1	1	1	0.11	Ankyrin repeat and SO
216	1	SwissProt_HUMAN$	2::P62805|H4_HUMAN	34	11360	2	2	2	2	1.27	Histone H4 OS=Homo
217	1	SwissProt_HUMAN$	2::P00915|CAH1_HUMAN	34	28852	1	1	1	1	0.18	Carbonic anhydrase 1
218	1	SwissProt_HUMAN$	2::P49913|CAMP_HUMAN	33	19289	1	1	1	1	0.27	Cathelicidin antimicro
219	1	SwissProt_HUMAN$	2::Q5SYB0|FRPD1_HUMAN	33	173328	1	1	1	1	0.03	FERM and PDZ domai
220	1	SwissProt_HUMAN$	2::Q8NHQ9|DDX55_HUMAN	32	68503	1	1	1	1	0.07	ATP-dependent RNA h
221	1	SwissProt_HUMAN$	2::P55774|CCL18_HUMAN	32	9842	1	1	1	1	0.60	C-C motif chemokine
222	1	SwissProt_HUMAN$	2::O75460|ERN1_HUMAN	32	109666	1	1	1	1	0.04	Serine/threonine-prot
223	1	SwissProt_HUMAN$	2::P11908|PRPS2_HUMAN	31	34747	2	1	1	1	0.15	Ribose-phosphate pyr
224	1	SwissProt_HUMAN$	2::P25705|ATPA_HUMAN	31	59714	1	1	1	1	0.08	ATP synthase subunit
225	1	SwissProt_HUMAN$	2::Q6DHV5|C2D2B_HUMAN	31	166681	2	2	1	1	0.03	Protein CC2D2B OS=H
226	1	SwissProt_HUMAN$	2::Q14204|DYHC1_HUMAN	31	532072	1	1	1	1	0.01	Cytoplasmic dynein 1
227	1	SwissProt_HUMAN$	2::P62701|RS4X_HUMAN	30	29579	1	1	1	1	0.17	40S ribosomal protein
228	1	SwissProt_HUMAN$	2::Q92530|PSMF1_HUMAN	30	29798	1	1	1	1	0.17	Proteasome inhibitor I
229	1	SwissProt_HUMAN$	2::Q9BTV5|FSD1_HUMAN	30	55784	2	1	1	1	0.09	Fibronectin type III ar
230	1	SwissProt_HUMAN$	2::A0A075B6J9|LV218_HUMAN	30	12404	2	1	2	1	0.45	Immunoglobulin lamb
231	1	SwissProt_HUMAN$	2::Q8IWZ3|ANKH1_HUMAN	30	269291	3	1	2	1	0.02	Ankyrin repeat and KH
232	1	SwissProt_HUMAN$	2::Q5XXA6|ANO1_HUMAN	30	114005	1	1	1	1	0.04	Anoctamin-1 OS=Hon
233	1	SwissProt_HUMAN$	2::P78563|RED1_HUMAN	30	80713	1	1	1	1	0.06	Double-stranded RNA-
234	1	SwissProt_HUMAN$	2::A0A075B6I4|LVX54_HUMAN	29	12387	1	1	1	1	0.45	Immunoglobulin lamb
235	1	SwissProt_HUMAN$	2::Q9ULM0|PKHH1_HUMAN	29	151137	2	1	2	1	0.03	Pleckstrin homology d
236	1	SwissProt_HUMAN$	2::P49643|PRI2_HUMAN	29	58769	1	1	1	1	0.08	DNA primase large su
237	1	SwissProt_HUMAN$	2::P01703|LV140_HUMAN	29	12294	1	1	1	1	0.46	Immunoglobulin lamb
238	1	SwissProt_HUMAN$	2::Q96AY4|TTC28_HUMAN	29	270715	1	1	1	1	0.02	Tetratricopeptide repe
239	1	SwissProt_HUMAN$	2::Q8N163|CCAR2_HUMAN	29	102838	1	1	1	1	0.05	Cell cycle and apoptos
240	1	SwissProt_HUMAN$	2::O95757|HS74L_HUMAN	29	94453	1	1	1	1	0.05	Heat shock 70 kDa pr
241	1	SwissProt_HUMAN$	2::Q00610|CLH1_HUMAN	29	191493	2	2	1	1	0.03	Clathrin heavy chain 1
242	1	SwissProt_HUMAN$	2::Q8WYA0|IFT81_HUMAN	29	79696	1	1	1	1	0.06	Intraflagellar transpor
243	1	SwissProt_HUMAN$	2::P98160|PGBM_HUMAN	29	468532	1	1	1	1	0.01	Basement membrane-
244	1	SwissProt_HUMAN$	2::Q69YQ0|CYTSA_HUMAN	29	124525	1	1	1	1	0.04	Cytospin-A OS=Homo
245	1	SwissProt_HUMAN$	2::P18065|IBP2_HUMAN	28	34791	1	1	1	1	0.15	Insulin-like growth fac
246	1	SwissProt_HUMAN$	2::O60229|KALRN_HUMAN	28	340047	1	1	1	1	0.01	Kalirin OS=Homo sapi
247	1	SwissProt_HUMAN$	2::P55056|APOC4_HUMAN	28	14543	2	2	2	2	0.90	Apolipoprotein C-IV O
248	1	SwissProt_HUMAN$	2::Q0WX57|U17LO_HUMAN	28	59673	1	1	1	1	0.08	Ubiquitin carboxyl-ter
249	1	SwissProt_HUMAN$	2::P28066|PSA5_HUMAN	28	26394	1	1	1	1	0.19	Proteasome subunit a
250	1	SwissProt_HUMAN$	2::Q6V702|CF299_HUMAN	28	26853	1	1	1	1	0.19	Cilia- and flagella-ass
251	1	SwissProt_HUMAN$	2::Q99832|TCPH_HUMAN	28	59329	1	1	1	1	0.08	T-complex protein 1 s
252	1	SwissProt_HUMAN$	2::P22891|PROZ_HUMAN	28	44715	2	1	2	1	0.11	Vitamin K-dependent
253	1	SwissProt_HUMAN$	2::O15417|TNC18_HUMAN	27	314326	1	1	1	1	0.02	Trinucleotide repeat-c

↑Family	M	DB	Accession	Score	Mass	Matches	Match(sig)	Sequences	Seq(sig)	emPAI	Description
254	1	SwissProt_HUMAN$	2::P39060|COIA1_HUMAN	27	178077	1	1	1	1	0.03	Collagen alpha-1(XVII
255	1	SwissProt_HUMAN$	2::Q9NP78|ABCB9_HUMAN	27	84421	1	1	1	1	0.06	ATP-binding cassette
256	1	SwissProt_HUMAN$	2::Q68DL7|CR063_HUMAN	27	77181	2	2	1	1	0.06	Uncharacterized prote
257	1	SwissProt_HUMAN$	2::Q9UKM7|MA1B1_HUMAN	27	79530	1	1	1	1	0.06	Endoplasmic reticulun
258	1	SwissProt_HUMAN$	2::Q8NEM7|SP20H_HUMAN	27	85735	1	1	1	1	0.06	Transcription factor S
259	1	SwissProt_HUMAN$	2::Q9UHG3|PCYOX_HUMAN	27	56604	2	1	2	1	0.09	Prenylcysteine oxidas
260	1	SwissProt_HUMAN$	2::Q1XH10|SKDA1_HUMAN	27	98087	3	2	1	1	0.05	SKI/DACH domain-co
261	1	SwissProt_HUMAN$	2::Q9NZ08|ERAP1_HUMAN	27	107166	1	1	1	1	0.05	Endoplasmic reticulun
262	1	SwissProt_HUMAN$	2::Q9Y566|SHAN1_HUMAN	26	224821	2	1	1	1	0.02	SH3 and multiple ank
263	1	SwissProt_HUMAN$	2::O43819|SCO2_HUMAN	26	29791	1	1	1	1	0.17	Protein SCO2 homolo
264	1	SwissProt_HUMAN$	2::A4D1U4|DEN11_HUMAN	26	51413	1	1	1	1	0.10	DENN domain-contain
265	1	SwissProt_HUMAN$	2::Q07075|AMPE_HUMAN	26	109176	1	1	1	1	0.04	Glutamyl aminopeptid
266	1	SwissProt_HUMAN$	2::Q6ZS17|RIPR1_HUMAN	26	132225	1	1	1	1	0.04	Rho family-interacting
267	1	SwissProt_HUMAN$	2::Q92570|NR4A3_HUMAN	26	68186	1	1	1	1	0.07	Nuclear receptor subf
268	1	SwissProt_HUMAN$	2::Q9NP80|PLPL8_HUMAN	26	88421	1	1	1	1	0.05	Calcium-independent
269	1	SwissProt_HUMAN$	2::Q7Z6Z7|HUWE1_HUMAN	25	481589	1	1	1	1	0.01	E3 ubiquitin-protein li
270	1	SwissProt_HUMAN$	2::P36957|ODO2_HUMAN	25	48724	3	1	2	1	0.10	Dihydrolipoyllysine-re
271	1	SwissProt_HUMAN$	2::P36575|ARRC_HUMAN	25	42751	1	1	1	1	0.12	Arrestin-C OS=Homo
272	1	SwissProt_HUMAN$	2::Q7KZ85|SPT6H_HUMAN	25	198949	1	1	1	1	0.02	Transcription elongati
273	1	SwissProt_HUMAN$	2::Q14520|HABP2_HUMAN	25	62630	2	2	2	2	0.16	Hyaluronan-binding p
274	1	SwissProt_HUMAN$	2::Q9H1Z8|AUGN_HUMAN	25	17173	1	1	1	1	0.31	Augurin OS=Homo sa
275	1	SwissProt_HUMAN$	2::O43586|PPIP1_HUMAN	25	47562	1	1	1	1	0.10	Proline-serine-threoni
276	1	SwissProt_HUMAN$	2::Q8TD08|MK15_HUMAN	25	59795	1	1	1	1	0.08	Mitogen-activated pro
277	1	SwissProt_HUMAN$	2::P32119|PRDX2_HUMAN	24	21878	2	1	2	1	0.24	Peroxiredoxin-2 OS=H
278	1	SwissProt_HUMAN$	2::Q9ULL4|PLXB3_HUMAN	24	206716	2	1	2	1	0.02	Plexin-B3 OS=Homo s
279	1	SwissProt_HUMAN$	2::Q16740|CLPP_HUMAN	24	30161	1	1	1	1	0.17	ATP-dependent Clp pr
280	1	SwissProt_HUMAN$	2::Q9UDT6|CLIP2_HUMAN	24	115767	1	1	1	1	0.04	CAP-Gly domain-conta
281	1	SwissProt_HUMAN$	2::Q9Y5I4|PCDC2_HUMAN	24	109383	1	1	1	1	0.04	Protocadherin alpha-C
282	1	SwissProt_HUMAN$	2::Q86T24|KAISO_HUMAN	24	74438	1	1	1	1	0.07	Transcriptional regula
283	1	SwissProt_HUMAN$	2::Q08211|DHX9_HUMAN	24	140869	4	1	2	1	0.03	ATP-dependent RNA h
284	1	SwissProt_HUMAN$	2::Q567U6|CCD93_HUMAN	24	73152	1	1	1	1	0.07	Coiled-coil domain-co
285	1	SwissProt_HUMAN$	2::P35228|NOS2_HUMAN	24	131033	1	1	1	1	0.04	Nitric oxide synthase,
286	1	SwissProt_HUMAN$	2::Q9UH65|SWP70_HUMAN	24	68954	1	1	1	1	0.07	Switch-associated pro
287	1	SwissProt_HUMAN$	2::Q96S86|HPLN3_HUMAN	24	40868	1	1	1	1	0.12	Hyaluronan and prote
288	1	SwissProt_HUMAN$	2::P15121|ALDR_HUMAN	24	35830	1	1	1	1	0.14	Aldo-keto reductase f
289	1	SwissProt_HUMAN$	2::Q9H254|SPTN4_HUMAN	24	288808	1	1	1	1	0.02	Spectrin beta chain, n
290	1	SwissProt_HUMAN$	2::Q8TAY7|F110D_HUMAN	24	28693	2	1	1	1	0.18	Protein FAM110D OS=
291	1	SwissProt_HUMAN$	2::Q6ZTR6|ZNFDT_HUMAN	24	17225	1	1	1	1	0.31	Putative uncharacteri
292	1	SwissProt_HUMAN$	2::Q9P2S5|WRP73_HUMAN	24	51555	1	1	1	1	0.10	WD repeat-containing
293	1	SwissProt_HUMAN$	2::P54750|PDE1A_HUMAN	24	61213	1	1	1	1	0.08	Calcium/calmodulin-d

↑Family	M	DB	Accession	Score	Mass	Matches	Match(sig)	Sequences	Seq(sig)	emPAI	Description
294	1	SwissProt_HUMAN$	2::P46778|RL21_HUMAN	23	18553	1	1	1	1	0.29	60S ribosomal protein
295	1	SwissProt_HUMAN$	2::Q5VT06|CE350_HUMAN	23	350716	3	1	3	1	0.01	Centrosome-associate
296	1	SwissProt_HUMAN$	2::O75821|EIF3G_HUMAN	23	35589	1	1	1	1	0.14	Eukaryotic translation
297	1	SwissProt_HUMAN$	2::Q05513|KPCZ_HUMAN	23	67617	1	1	1	1	0.07	Protein kinase C zeta
298	1	SwissProt_HUMAN$	2::P08294|SODE_HUMAN	23	25835	1	1	1	1	0.20	Extracellular superoxi
299	1	SwissProt_HUMAN$	2::A0A087WSX0|LV545_HUMAN	23	13153	2	2	2	2	1.03	Immunoglobulin lamb
300	1	SwissProt_HUMAN$	2::P0CG47|UBB_HUMAN	23	25746	1	1	1	1	0.20	Polyubiquitin-B OS=H
301	1	SwissProt_HUMAN$	2::Q96RW7|HMCN1_HUMAN	22	613001	1	1	1	1	0.01	Hemicentin-1 OS=Hor
302	1	SwissProt_HUMAN$	2::Q01082|SPTB2_HUMAN	22	274439	1	1	1	1	0.02	Spectrin beta chain, n
303	1	SwissProt_HUMAN$	2::Q9C0C9|UBE2O_HUMAN	22	141205	2	1	1	1	0.03	(E3-independent) E2
304	1	SwissProt_HUMAN$	2::Q5T2T1|MPP7_HUMAN	22	65483	1	1	1	1	0.07	MAGUK p55 subfamily
305	1	SwissProt_HUMAN$	2::Q3SXZ3|ZN718_HUMAN	22	55324	2	2	1	1	0.09	Zinc finger protein 71
306	1	SwissProt_HUMAN$	2::Q9NXL9|MCM9_HUMAN	22	127233	1	1	1	1	0.04	DNA helicase MCM9 O
307	1	SwissProt_HUMAN$	2::Q9NU22|MDN1_HUMAN	22	632420	1	1	1	1	0.01	Midasin OS=Homo sa
308	1	SwissProt_HUMAN$	2::Q12840|KIF5A_HUMAN	21	117306	2	1	1	1	0.04	Kinesin heavy chain is
309	1	SwissProt_HUMAN$	2::P56199|ITA1_HUMAN	21	130765	2	1	1	1	0.04	Integrin alpha-1 OS=
310	1	SwissProt_HUMAN$	2::Q92878|RAD50_HUMAN	21	153797	2	1	2	1	0.03	DNA repair protein RA
311	1	SwissProt_HUMAN$	2::P31327|CPSM_HUMAN	21	164835	1	1	1	1	0.03	Carbamoyl-phosphate
312	1	SwissProt_HUMAN$	2::Q86VH2|KIF27_HUMAN	21	160184	1	1	1	1	0.03	Kinesin-like protein KI
313	1	SwissProt_HUMAN$	2::Q9UFH2|DYH17_HUMAN	21	508987	1	1	1	1	0.01	Dynein heavy chain 1
314	1	SwissProt_HUMAN$	2::Q9H329|E41LB_HUMAN	21	99650	1	1	1	1	0.05	Band 4.1-like protein
315	1	SwissProt_HUMAN$	2::Q99459|CDC5L_HUMAN	21	92194	1	1	1	1	0.05	Cell division cycle 5-li
316	1	SwissProt_HUMAN$	2::Q7Z340|ZN551_HUMAN	21	77465	2	1	1	1	0.06	Zinc finger protein 55
317	1	SwissProt_HUMAN$	2::Q9BTA9|WAC_HUMAN	21	70681	1	1	1	1	0.07	WW domain-containin
318	1	SwissProt_HUMAN$	2::Q9ULG6|CCPG1_HUMAN	21	87286	1	1	1	1	0.06	Cell cycle progression
319	1	SwissProt_HUMAN$	2::Q9NP81|SYSM_HUMAN	21	58246	1	1	1	1	0.08	Serine--tRNA ligase, r
320	1	SwissProt_HUMAN$	2::Q6PRD1|GP179_HUMAN	20	257202	1	1	1	1	0.02	Probable G-protein co
321	1	SwissProt_HUMAN$	2::Q9H2D6|TARA_HUMAN	20	261217	1	1	1	1	0.02	TRIO and F-actin-binc
322	1	SwissProt_HUMAN$	2::P01704|LV214_HUMAN	20	12589	2	2	1	1	0.45	Immunoglobulin lamb
323	1	SwissProt_HUMAN$	2::Q13093|PAFA_HUMAN	20	50045	1	1	1	1	0.10	Platelet-activating fac
324	1	SwissProt_HUMAN$	2::O14786|NRP1_HUMAN	20	103069	1	1	1	1	0.05	Neuropilin-1 OS=Hom
325	1	SwissProt_HUMAN$	2::O00221|IKBE_HUMAN	20	52831	1	1	1	1	0.09	NF-kappa-B inhibitor
326	1	SwissProt_HUMAN$	2::P15531|NDKA_HUMAN	20	17138	1	1	1	1	0.31	Nucleoside diphospha
327	1	SwissProt_HUMAN$	2::Q9H257|CARD9_HUMAN	20	62202	1	1	1	1	0.08	Caspase recruitment
328	1	SwissProt_HUMAN$	2::Q58A45|PAN3_HUMAN	20	95552	2	1	1	1	0.05	PAN2-PAN3 deadenyla
329	1	SwissProt_HUMAN$	2::Q96GD3|SCMH1_HUMAN	20	73307	1	1	1	1	0.07	Polycomb protein SCM
330	1	SwissProt_HUMAN$	2::Q8TED9|AF1L1_HUMAN	20	86378	1	1	1	1	0.06	Actin filament-associa
331	1	SwissProt_HUMAN$	2::O15091|MRPP3_HUMAN	20	67272	1	1	1	1	0.07	Mitochondrial ribonucl
332	1	SwissProt_HUMAN$	2::Q9UJX2|CDC23_HUMAN	20	68790	1	1	1	1	0.07	Cell division cycle pro
333	1	SwissProt_HUMAN$	2::Q9H4D5|NXF3_HUMAN	20	60063	1	1	1	1	0.08	Nuclear RNA export fa

↑Family	M	DB	Accession	Score	Mass	Matches	Match(sig)	Sequences	Seq(sig)	emPAI	Description
334	1	SwissProt_HUMAN$	2::A2RRD8|ZN320_HUMAN	20	59288	1	1	1	1	0.08	Zinc finger protein 32
335	1	SwissProt_HUMAN$	2::Q9H4L7|SMRCD_HUMAN	20	117328	1	1	1	1	0.04	SWI/SNF-related matr
336	1	SwissProt_HUMAN$	2::Q8N7U6|EFHB_HUMAN	20	93743	4	3	1	1	0.05	EF-hand domain-conta
337	1	SwissProt_HUMAN$	2::P07237|PDIA1_HUMAN	20	57081	1	1	1	1	0.09	Protein disulfide-isom
338	1	SwissProt_HUMAN$	2::Q96DN5|TBC31_HUMAN	20	124111	2	1	1	1	0.04	TBC1 domain family n
339	1	SwissProt_HUMAN$	2::Q16851|UGPA_HUMAN	19	56905	2	1	2	1	0.09	UTP--glucose-1-phosp
340	1	SwissProt_HUMAN$	2::P04350|TBB4A_HUMAN	19	49554	1	1	1	1	0.10	Tubulin beta-4A chain
341	1	SwissProt_HUMAN$	2::Q9NYC9|DYH9_HUMAN	19	511551	2	1	2	1	0.01	Dynein heavy chain 9
342	1	SwissProt_HUMAN$	2::P82930|RT34_HUMAN	19	25634	1	1	1	1	0.20	28S ribosomal proteir
343	1	SwissProt_HUMAN$	2::Q96P31|FCRL3_HUMAN	19	80806	1	1	1	1	0.06	Fc receptor-like protei
344	1	SwissProt_HUMAN$	2::Q13867|BLMH_HUMAN	19	52528	1	1	1	1	0.09	Bleomycin hydrolase (
345	1	SwissProt_HUMAN$	2::Q14103|HNRPD_HUMAN	19	38410	1	1	1	1	0.13	Heterogeneous nuclea
346	1	SwissProt_HUMAN$	2::Q11203|SIAT6_HUMAN	19	42144	1	1	1	1	0.12	CMP-N-acetylneurami
347	1	SwissProt_HUMAN$	2::P11309|PIM1_HUMAN	19	35663	1	1	1	1	0.14	Serine/threonine-prot
348	1	SwissProt_HUMAN$	2::Q8NI27|THOC2_HUMAN	19	182659	1	1	1	1	0.03	THO complex subunit
349	1	SwissProt_HUMAN$	2::P51572|BAP31_HUMAN	19	27974	1	1	1	1	0.18	B-cell receptor-associ
350	1	SwissProt_HUMAN$	2::P0C221|CC175_HUMAN	19	93567	1	1	1	1	0.05	Coiled-coil domain-co
351	1	SwissProt_HUMAN$	2::Q8IYD8|FANCM_HUMAN	19	232045	1	1	1	1	0.02	Fanconi anemia group
352	1	SwissProt_HUMAN$	2::Q02809|PLOD1_HUMAN	19	83497	1	1	1	1	0.06	Procollagen-lysine,2-c
353	1	SwissProt_HUMAN$	2::Q7Z2Q7|LRR70_HUMAN	19	70257	1	1	1	1	0.07	Leucine-rich repeat-cc
354	1	SwissProt_HUMAN$	2::O00222|GRM8_HUMAN	19	101675	1	1	1	1	0.05	Metabotropic glutama
355	1	SwissProt_HUMAN$	2::P12081|HARS1_HUMAN	19	57374	1	1	1	1	0.09	Histidine--tRNA ligase
356	1	SwissProt_HUMAN$	2::P55786|PSA_HUMAN	18	103211	1	1	1	1	0.05	Puromycin-sensitive a
357	1	SwissProt_HUMAN$	2::P25786|PSA1_HUMAN	18	29537	1	1	1	1	0.17	Proteasome subunit a
358	1	SwissProt_HUMAN$	2::Q96MN2|NALP4_HUMAN	17	113341	3	1	1	1	0.04	NACHT, LRR and PYD
359	1	SwissProt_HUMAN$	2::Q8WTW3|COG1_HUMAN	17	108909	1	1	1	1	0.04	Conserved oligomeric
360	1	SwissProt_HUMAN$	2::P09913|IFIT2_HUMAN	17	54597	1	1	1	1	0.09	Interferon-induced pr
361	1	SwissProt_HUMAN$	2::P06702|S10A9_HUMAN	17	13234	1	1	1	1	0.42	Protein S100-A9 OS=
362	1	SwissProt_HUMAN$	2::P20618|PSB1_HUMAN	17	26472	1	1	1	1	0.19	Proteasome subunit b
363	1	SwissProt_HUMAN$	2::Q13619|CUL4A_HUMAN	17	87624	1	1	1	1	0.06	Cullin-4A OS=Homo s
364	1	SwissProt_HUMAN$	2::P14780|MMP9_HUMAN	17	78408	2	2	2	2	0.13	Matrix metalloproteina
365	1	SwissProt_HUMAN$	2::O15355|PPM1G_HUMAN	17	59235	1	1	1	1	0.08	Protein phosphatase 1
366	1	SwissProt_HUMAN$	2::Q92823|NRCAM_HUMAN	17	143800	1	1	1	1	0.03	Neuronal cell adhesior
367	1	SwissProt_HUMAN$	2::Q9UIX4|KCNG1_HUMAN	17	57876	1	1	1	1	0.09	Potassium voltage-gat
368	1	SwissProt_HUMAN$	2::O00635|TRI38_HUMAN	16	53381	1	1	1	1	0.09	E3 ubiquitin-protein li
369	1	SwissProt_HUMAN$	2::Q92616|GCN1_HUMAN	16	292572	1	1	1	1	0.02	eIF-2-alpha kinase ac
370	1	SwissProt_HUMAN$	2::Q13976|KGP1_HUMAN	16	76316	1	1	1	1	0.06	cGMP-dependent prot
371	1	SwissProt_HUMAN$	2::Q99996|AKAP9_HUMAN	16	452708	1	1	1	1	0.01	A-kinase anchor prote
372	1	SwissProt_HUMAN$	2::Q8TCY9|URGCP_HUMAN	16	104921	1	1	1	1	0.05	Up-regulator of cell pr
373	1	SwissProt_HUMAN$	2::Q9H9A7|RMI1_HUMAN	16	70100	1	1	1	1	0.07	RecQ-mediated genor

↑*Family*	M	DB	Accession	Score	Mass	Matches	Match(sig)	Sequences	Seq(sig)	emPAI	Description
374	1	SwissProt_HUMAN$	2::Q8WW22|DNJA4_HUMAN	16	44769	1	1	1	1	0.11	DnaJ homolog subfam
375	1	SwissProt_HUMAN$	2::P38117|ETFB_HUMAN	16	27826	1	1	1	1	0.18	Electron transfer flavo
376	1	SwissProt_HUMAN$	2::Q8IYD1|ERF3B_HUMAN	15	68840	1	1	1	1	0.07	Eukaryotic peptide ch
377	1	SwissProt_HUMAN$	2::Q9H2V7|SPNS1_HUMAN	15	56594	1	1	1	1	0.09	Protein spinster homo
378	1	SwissProt_HUMAN$	2::Q9H8M1|CQ10B_HUMAN	15	27053	1	1	1	1	0.19	Coenzyme Q-binding
379	1	SwissProt_HUMAN$	2::O95153|RIMB1_HUMAN	15	199928	2	1	2	1	0.02	Peripheral-type benzo
380	1	SwissProt_HUMAN$	2::Q9P2L0|WDR35_HUMAN	15	133461	3	1	1	1	0.04	WD repeat-containing
381	1	SwissProt_HUMAN$	2::P49720|PSB3_HUMAN	15	22933	2	1	2	1	0.23	Proteasome subunit b
382	1	SwissProt_HUMAN$	2::O15085|ARHGB_HUMAN	15	167601	1	1	1	1	0.03	Rho guanine nucleotic
383	1	SwissProt_HUMAN$	2::A0A0B4J1V7|HV781_HUMAN	15	12909	1	1	1	1	0.43	Probable non-function
384	1	SwissProt_HUMAN$	2::Q92750|TAF4B_HUMAN	15	91034	1	1	1	1	0.05	Transcription initiatior
385	1	SwissProt_HUMAN$	2::Q5T4S7|UBR4_HUMAN	15	573476	1	1	1	1	0.01	E3 ubiquitin-protein li
386	1	SwissProt_HUMAN$	2::Q9Y2K6|UBP20_HUMAN	15	101938	1	1	1	1	0.05	Ubiquitin carboxyl-ter
387	1	SwissProt_HUMAN$	2::Q9NVH1|DJC11_HUMAN	15	63239	1	1	1	1	0.08	DnaJ homolog subfam
388	1	SwissProt_HUMAN$	2::Q9BWP8|COL11_HUMAN	14	28647	1	1	1	1	0.18	Collectin-11 OS=Hom
389	1	SwissProt_HUMAN$	2::Q7Z406|MYH14_HUMAN	14	227732	1	1	1	1	0.02	Myosin-14 OS=Homo
390	1	SwissProt_HUMAN$	2::Q07954|LRP1_HUMAN	14	504276	1	1	1	1	0.01	Prolow-density lipoprc
391	1	SwissProt_HUMAN$	2::O14939|PLD2_HUMAN	14	105920	1	1	1	1	0.05	Phospholipase D2 OS=
392	1	SwissProt_HUMAN$	2::P42694|HELZ_HUMAN	14	218833	1	1	1	1	0.02	Probable helicase with
393	1	SwissProt_HUMAN$	2::Q86WK7|AMGO3_HUMAN	13	55214	1	1	1	1	0.09	Amphoterin-induced p
394	1	SwissProt_HUMAN$	2::Q9H5L6|THAP9_HUMAN	13	103345	1	1	1	1	0.05	DNA transposase THA
395	1	SwissProt_HUMAN$	2::Q9UNU6|CP8B1_HUMAN	13	58031	1	1	1	1	0.08	7-alpha-hydroxychole
396	1	SwissProt_HUMAN$	2::Q9UBW5|BIN2_HUMAN	13	61837	1	1	1	1	0.08	Bridging integrator 2
397	1	SwissProt_HUMAN$	2::P60509|ERB1_HUMAN	13	58483	1	1	1	1	0.08	Endogenous retroviru

Export as CSV

Not what you expected? Try **the select summary**.

Mascot: http://www.matrixscience.com/
